# Purity control of simulated moving bed based on advanced fuzzy controller

**DOI:** 10.1038/s41598-024-59847-1

**Published:** 2024-04-20

**Authors:** Chao-Fan Xie, Xiong Chen, Hong Zhang

**Affiliations:** 1 School of big data and artificial intelligence, Fujian Polytechnic Normal University, Fuzhou, FuJian China; 2 School of Business and Trade Management, Fujian Polytechnic of Information Technology, Fuzhou, FuJian China; 3 Key Laboratory of Nondestructive Testing, Fujian Ploytechnic Normal University, Fujian Province University, Fuzhou, FuJian China

**Keywords:** SMB, Advanced fuzzy, Chromatographic, Control, Chemical engineering, Mathematics and computing, Applied mathematics

## Abstract

Simulated moving bed (SMB) technology is considered one of the most successful techniques in chromatographic separation. However, due to the nonlinearity caused by discrete events and sensitivity to numerous separation performance parameters, purity control in SMB systems has been a challenging issue. Fuzzy controllers are increasingly popular in industrial environments due to their simplicity and effectiveness in handling nonlinearity. However, traditional fuzzy controllers used in industry often overlook considerations of error acceleration, resulting in slight deviations from target values under steady-state conditions and oscillatory behavior when system parameters change. This study proposes an advanced fuzzy controller, where in a series of experiments, the purity control targets for component B are set at 94% and 96%, and for component A are set at 96% and 96%, respectively. Experimental results indicate that the advanced fuzzy controller achieves higher precision, with an average deviation of around 0.1%, for both components B and A. Importantly, under variations in adsorbent parameter(from 0.01 to 0.03), feed concentration(from 4.5 to 5.2), and switching time(from 178 to 182), the experimental results demonstrate smoother control with the advanced controller, particularly when oscillations occur with conventional fuzzy controllers due to switching time variations, indicating robust control with the advanced fuzzy controller.

## Introduction

The SMB system consists of a series of fixed-bed towers, where a circulating pump facilitates solvent flow. The feed mixture enters the system at the inlet of one tower and the purified product is extracted at different positions within the loop. The desorbent is introduced into the loop to clean the adsorbent beds. The effective countercurrent movement of the liquid and solid phases as well as the separation of components is achieved through cyclic switching of the inlet (feed and desorbent) and outlet (raffinate and extract) in the direction of liquid flow. The switching speed of the ports is chosen between the speeds of the strongly and weakly adsorbed components, such that a component migrates upstream relative to the port and is withdrawn at the raffinate port, while another component migrates downstream and is withdrawn at the extract port^1,2^.

Due to the complexity and coupling of the simulated moving bed (SMB) process, there are numerous parameters that can affect its separation efficiency. Many researchers have mathematically modeled the process to provide reference models for control and optimization, which are then applied to practical SMB simulations^3–5^. The simplest and most practical model is known as the Equilibrium Dispersive Model (EDM). In particular, recent advancements in computer technology have made it possible to achieve online prediction and control of SMB systems, leading to a flourishing development in SMB research^6–8^.

In reference^9^, a numerical simulation was performed using parameters such as isotherms, mass transfer resistance, cycle switch time, number of columns in series, column type, length, diameter, volume, feed concentration, and recycle flow rate to establish a model for column movement. This work laid the foundation for the optimization control of the column. Majeed et al. investigated the accuracy and efficiency of solving partial differential equations in separation engineering using the orthogonal collocation method, where the numerical solutions provided were nearly as accurate as the actual machine precision^10^. In the study conducted by Andrade Neto et al., they employed the finite element method to simulate and control the separation of enantiomers of quinidine in a simulated moving bed system. They achieved excellent results in practical predictive control based on their simulation approach^11^. Wei et al. proposed a control method based on piecewise affine models in order to optimize the performance of the control system in SMB chromatographic separation processes. Simulation results demonstrate the feasibility of this method, as the actual yield-production curve of the target compound and impurities can be fitted to achieve a desired target yield rate^12^. Heinonen et al. presented a model-based optimization control scheme for SMB chromatographic separation processes and its application in the separation of fructose and glucose. The study discusses the design of the control scheme and demonstrates its successful application in achieving efficient separation of fructose and glucose using SMB chromatography^13^. Mun and Wang proposed an optimization method for separation control, focusing on the design of column configurations in SMB systems. The research suggests that under the optimized design of the SMB system structure, the usage of desorbent can be reduced by 25%^14^. Reference^15^ utilizes the principle of near-input and near-output linearization to estimate non-linear states and designs an SMB control architecture based on the Real Moving Bed (RMB) model. On the other hand, reference^16^ utilizes a simple P controller with the difference in ultraviolet spectroscopy waveforms as the control parameter for online control of SMB. In the study by I-Chun et al., ultraviolet sensors were used to monitor the concentration of substances, and an NN model was employed to make real-time predictions of the substance concentration^17^. These predicted concentrations can be utilized for real-time control of the moving bed system. Other controller research on SMB can found in literature^18–23^.

This paper presents a digital simulation of the simulated moving bed (SMB) process using computer simulation techniques. The purity separation in SMB is controlled using an advanced type fuzzy controller, and its performance is compared with a traditional fuzzy controller through a comprehensive analysis. Additionally, the performance of both controllers under parameter perturbations is observed.

## SMB mathematical model

For SMB, the mass balance of mobile and solid phase is:

1 $$\frac{{\partial C_{ij} }}{\partial t} = D_{i} \frac{{\partial^{2} C_{ij} }}{{\partial x^{2} }} - v_{j}^{*} \frac{{\partial C_{ij} }}{\partial x} - \frac{1 - \varepsilon }{\varepsilon }k_{i} (q_{ij}^{*} - q_{ij} ),$$

2 $$\frac{{\partial q_{ij} }}{\partial t} = k_{i} (q_{ij}^{*} - q_{ij} ).$$

The parameters meaning is shown in Table1.

**Table 1:** Parameters of SMB system.

Parameter	Describe
$x(cm)$	Axial distance
$k(gL^{ - 1} )$	Comprehensive mass transfer constant
$v^{*} (cm\min^{ - 1} )$	Effect velocity of body
$u_{s} (cm\min^{ - 1} )$	Solid flow rate
$C(gL^{ - 1} )$	Mobile phase concentration
$q(gL^{ - 1} )$	Solid phase concentration
$q^{*} (gL^{ - 1} )$	Solid phase concentration at equilibrium between solid phase and mobile phase
$Q(cm^{3} \min^{ - 1} )$	Volume flow rate
$t(\sec ond)$	Time
$D(cm^{2} \min^{ - 1} )$	Effective dispersion coefficient
$\varepsilon$	Bulk void fraction
$i$	Material index: A or B
$j$	Column number:1, 2, 3, 4, 5, 6, 7, 8

Formula (2) substituted into (1) , it get as follows:

3 $$\frac{{\partial C_{ij} }}{\partial t} = D_{i} \frac{{\partial^{2} C_{ij} }}{{\partial x^{2} }} - v_{{_{j} }}^{*} \frac{{\partial C_{ij} }}{\partial x} - \frac{1 - \varepsilon }{\varepsilon }\frac{{\partial q_{ij} }}{\partial t}.$$

The adsorption equilibrium of the two enantiomers is expressed by linear isotherms.

4 $$q_{ij} = H_{i} C_{ij}$$

Purity formulas are:

5 $$\mathop C\limits^{ - }_{E,B} = \frac{{C_{E,B} }}{{C_{E,A} + C_{E,B} }},$$

6 $$\mathop C\limits^{ - }_{R,A} = \frac{{C_{R,A} }}{{C_{R,A} + C_{R,B} }},$$

$\mathop C\limits^{ - }_{E,B}$ represents extract material *B* of purity, $\mathop C\limits^{ - }_{R,A}$ represents raffinate material *A* of purity, $C_{E,B}$ represents extract material *B* of concentration, and $C_{R,A}$ represents raffinate material *A* of concentration.

## Simulation

To validate the inference correctness of the aforementioned SMB discrete system, a simulated experiment was conducted using an 8-column SMB model with a 2–2-2–2 configuration, where each region consists of two columns. The relationships between these two variables can be expressed formula (10), $r$ is the radius of the column.

7 $$v_{j} = \frac{{Q_{j} }}{{\varepsilon \pi r^{2} }}.$$

The time step is set to 0.1 s, and the length of each column in the spatial domain is divided into 100 equal parts. All digital calculations were performed on a PC machine equipped with an Intel Core i7-3770 k 3.53 GHz processor and 16 GB of memory. The initial parameters for SMB are shown in Table 2.

**Table 2:** The initial parameters for SMB.

Parameter	Value	Parameter	Value
$L(cm)$	25	$C_{f,i} (gL^{ - 1} )$	5
$d(cm)$	0.46	$\theta (\min )$	3
$H_{A}$	0.001	$Q_{I} (cm^{{3}} \min^{ - 1} )$	6.75
$H_{B}$	0.45	$Q_{II} (cm^{{3}} \min^{ - 1} )$	6.6
$D_{A} (cm^{2} \min^{ - 1} )$	0.2	$Q_{III} (cm^{{3}} \min^{ - 1} )$	7
$D_{B} (cm^{2} \min^{ - 1} )$	1.265	$Q_{IV} (cm^{{3}} \min^{ - 1} )$	2
$\varepsilon$	0.8	Spatial number	50

### Advance fuzzy type controller design

The traditional fuzzy controller used in industry often does not consider the error acceleration, which is a little less than the target value in steady state. In this paper, advanced fuzzy type controller is used to control the purity of the simulated moving bed. Several common formulas are as follows:

8 $$e(k) = desired - y(k),$$

9 $$\Delta e(k) = e(k) - e(k - 1),$$

10 $$\Delta e(k{ - 1}) = e(k{ - 1}) - e(k - {2}),$$

11 $$\Delta^{2} e(k) = \Delta e(k) - \Delta e(k - 1).$$

In order to construct a rule base with acceleration as a consideration, an example is given to illustrate the following:

When $$\Delta^{2} e(k) > 0$$, $$\Delta e(k) < 0$$ and $$e(k) < 0$$, from $$\Delta^{2} e(k) > 0$$$$\Delta e(k) < 0$$ and above formula, can get $$\Delta e(k{ - 1}) < 0$$, From $$\Delta^{2} e(k) > 0$$$$\Delta e(k) < 0$$ and above formula, can set $$e(k{ - 1}) = 0$$, $$\Delta e(k{ - 1}) < 0$$,$$e(k{ - 1}) = 0$$ and above formula, can get $$e(k{ - 2}) > 0$$.

**Figure 1:**
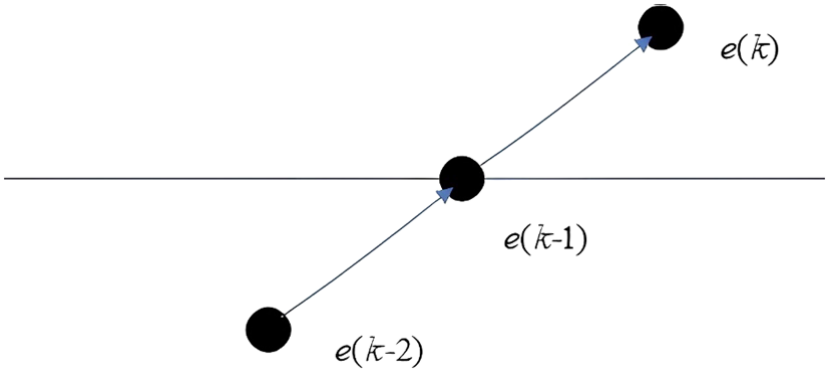
$$\Delta^{2} e(k) > 0$$, $$\Delta e(k) < 0$$** 且 **$$e(k) < 0$$.

Expressing physical meaning by $e(k)$,$e(k{ - 1})$ and $e(k{ - 2})$ as follow Fig. 1:

Usually, a completed fuzzy system is mainly composed of four parts: fuzzifier, fuzzy rule base, fuzzy inference engine and defuzzifier^24^. In the fuzzy controller, the input variables are selected as error, first-order error difference. The formula is as follows:

12 $$e_{1} = desired \, B - C_{E,B} ,$$

13 $$e_{2} = desired \, A - C_{R,A} ,$$

14 $$e_{3} = e_{1} + e_{2} ,$$

15 $$\Delta e_{1} = e_{1} (k) - e_{1} (k - 1),$$

16 $$\Delta e_{2} = e_{2} (k) - e_{2} (k - 1),$$

17 $$\Delta e_{3} = \Delta e_{1} + \Delta e_{2} ,$$

18 $$\Delta^{{2}} e_{1} = \Delta e_{1} (k) - \Delta e_{1} (k - 1),$$

19 $$\Delta^{{2}} e_{2} = \Delta e_{{2}} (k) - \Delta e_{{2}} (k - 1),$$

20 $$\Delta^{{2}} e_{{3}} = \Delta^{{2}} e_{{1}} { + }\Delta^{{2}} e_{{2}} ,$$

where $e_{1}$ and $\Delta e_{1}$ are the input of the zone I controller, $e_{2}$ and $\Delta e_{2}$ are the input of the zone II controller, $e_{3}$ and $\Delta e_{3}$ are the input of the zone III controller.

**Figure 2:**
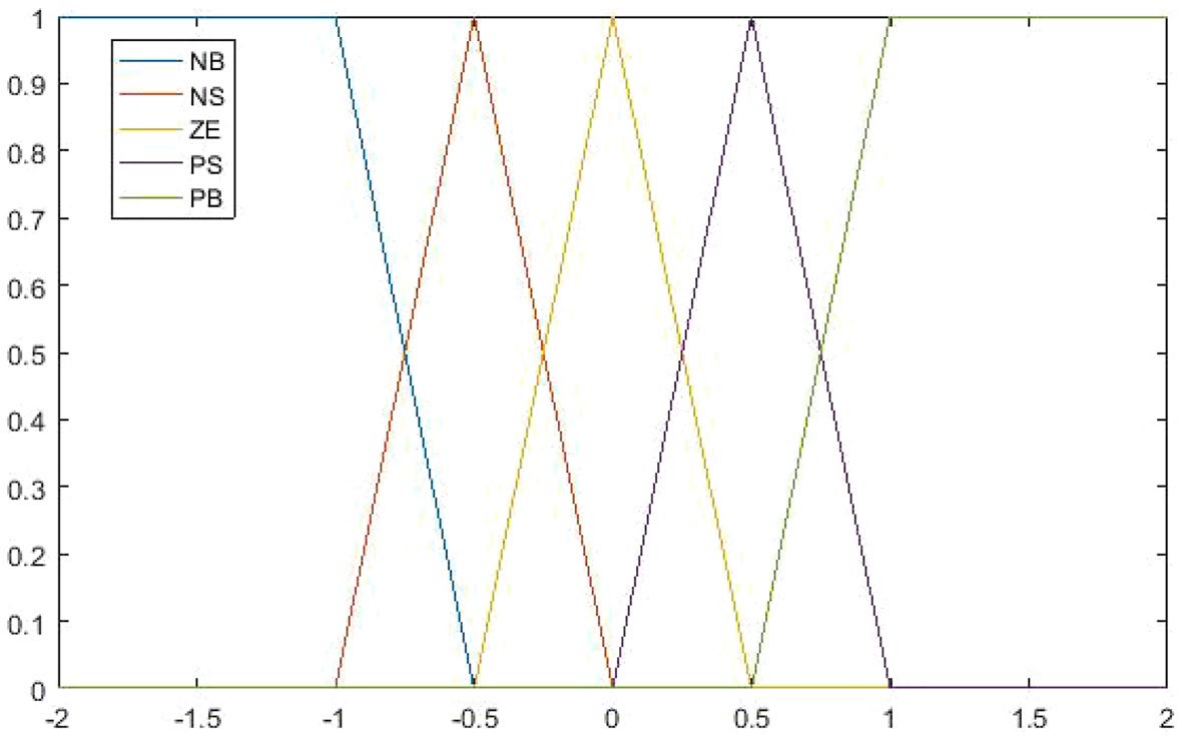
The memberships function for $e_{i}$ and $\Delta e_{i}$.

**Figure 3:**
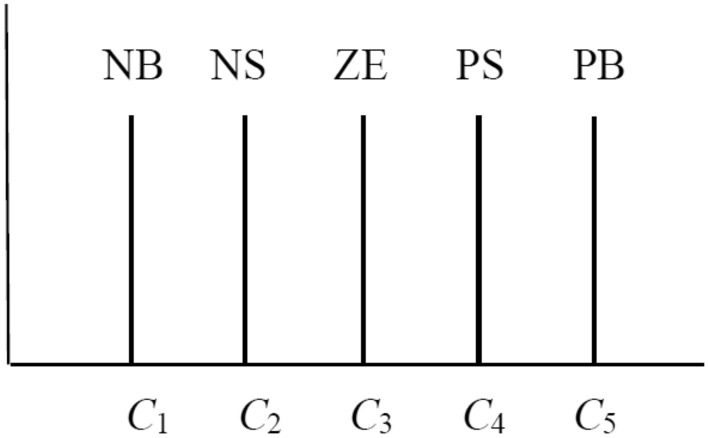
The unipolar fuzzy form of $\Delta Q_{i} ,i = 1,2,3$.

The fuzzy system define five linguistic variable values on errors: *NB, NS, ZE, PS, PB* and five linguistic for the first order difference of errors: *NB, NS, ZE, PS, PB.* The membership function is shown in the below Fig. 2. Then defines the control parameter flow rate $\Delta {\text{Q}}_{{\text{I}}} ,\Delta {\text{Q}}_{{{\text{II}}}} ,\Delta {\text{Q}}_{{{\text{III}}}}$ which are chosen as three independent defuzzification output variables denoted as $\Delta Q_{i}$ $(i = 1,2,3)$ respectively. The membership function of $\Delta Q_{i}$ is shown in Fig. 3, the values of (C1, C2, C3, C4, C5) are set as {0.15, 0.1, 0, − 0.1, − 0.15}, {0.006, 0.004, 0, − 0.004, − 0.006} and {0.08, 0.05, 0, − 0.05, − 0.08}.

Then, we draw on the kinematics principle to formulate the corresponding fuzzy rule table shown in Tables 3, 4 and 5:

**Table 3:** Fuzzy rule for $\Delta Q_{i}$ when $\Delta^{2} e(k) > 0$.

∆*e*	*e*				
	*NB*	*NS*	*ZE*	*PS*	*PB*
*NB*	*NB*	*NB*	*NB*	*NS*	*ZE*
*NS*	*NB*	*NB*	*NS*	*ZE*	*PS*
*ZE*	*NB*	*NS*	*ZE*	*PS*	*PB*
*PS*	*NS*	*ZE*	*PS*	*PB*	*PB*
*PB*	*ZE*	*PS*	*PB*	*PB*	*PB*

**Table 4:** Fuzzy rule for $\Delta Q_{i}$ when $\Delta^{2} e(k){ = }0$.

∆*e*	*e*				
	*NB*	*NS*	*ZE*	*PS*	*PB*
*NB*	*NB*	*NB*	*NS*	*ZE*	*PS*
*NS*	*NB*	*NB*	*NS*	*PS*	*PB*
*ZE*	*NB*	*NS*	*ZE*	*PS*	*PB*
*PS*	*ZE*	*PS*	*PB*	*PB*	*PB*
*PB*	*PS*	*PB*	*PB*	*PB*	*PB*

**Table 5:** Fuzzy rule for $\Delta Q_{i}$ when $\Delta^{2} e(k) < 0$.

∆*e*	*e*				
	*NB*	*NS*	*ZE*	*PS*	*PB*
*NB*	*NB*	*NB*	*NB*	*NB*	*NS*
*NS*	*NB*	*NB*	*NB*	*NS*	*ZE*
*ZE*	*NB*	*NS*	*ZE*	*PS*	*PB*
*PS*	*NB*	*NS*	*PS*	*PB*	*PB*
*PB*	*NS*	*ZE*	*PB*	*PB*	*PB*

Fuzzy inference engine adopts product form, that is the strength of the If part multiply the output value of the Then part of the fuzzy rule^16^. The last step is defuzzifer, where the center-average value is used to solve the defuzzifer. According to the fuzzy rule base and membership function of input variables, each input will cause four rules to be activated. The formula is shown in formula (21), (22) and (23)

21 $$V = T(u_{1} ,u_{3} ) \times y_{1} + T(u_{1} ,u_{{4}} ) \times y_{2} { + }T(u_{2} ,u_{3} ) \times y_{3} + T(u_{2} ,u_{{4}} ) \times y_{4} ,$$

22 $$W = T(u_{1} ,u_{3} ) + T(u_{1} ,u_{{4}} ){ + }T(u_{2} ,u_{3} ) + T(u_{2} ,u_{{4}} ),$$

23 $$U = \frac{V}{W}.$$

$u_{1} ,u_{2}$ are the fuzzy error values,$u_{{3}} ,u_{{4}}$ are the fuzzy error velocity, that is, the first-order difference.$y_{1} \cdots y_{{4}}$ correspond to the unipolar value of the four start rules, that is the fuzzy single pole value of the Then part.$T$ represents the product* T*-norm, and the final output value of defuzzifier is $U$.

### Experiment results

**Figure 4:**
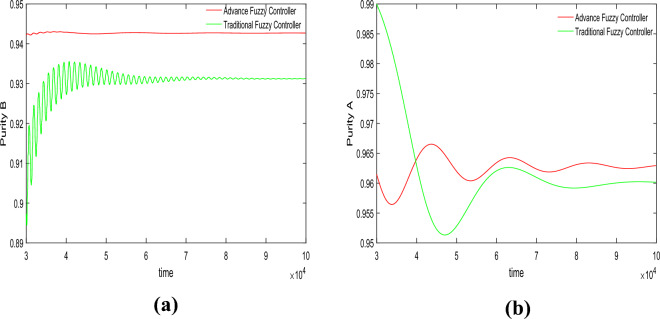
Desired *B* = 94%, desired *A* = 96%, Switch time = 180 s.

**Figure 5:**
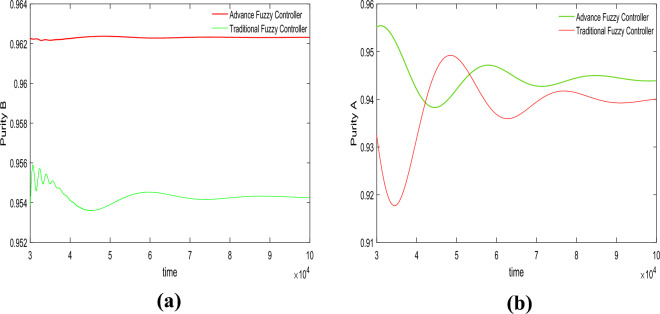
Desired *B* = 96%, desired *A* = 94%, Switch time = 180 s.

In this section, we conducted purity control experiments on the SMB system using an advanced fuzzy controller and compared it with a traditional fuzzy controller. The results of the control experiments are shown in Figs. 4 and 5. The effects of variations in adsorbent parameters, feed concentration, and switching time on the controller performance were observed. Each set of experiments was further divided into five trials, targeting different purity levels for component B (92%, 93%, 94%, 95%, and 96%) and component A (93%, 94%, 95%, 96%, and 97%).

In the first set of experiments shown in Fig. 4, the switching time parameter was set to 180 s. The target control purity for component A was 96%, and for component B, it was 94%. For the advanced fuzzy controller, the actual control purity achieved for component A was 96.14%, and for component B, it was 94.27%. For the traditional fuzzy controller, the actual control purity achieved for component A was 96.02%, and for component B, it was 93.13%.

In the second group of experiments shown in Fig. 5, the switching time parameter was set to 180 s. The target control purity for material A was 94%, and for material B, it was 96%. For the advanced fuzzy controller, the actual control purity achieved for material A was 94.11%, and for material B, it was 96.23%.For the traditional fuzzy controller, the actual control purity achieved for material A was 94.01%, and for material B, it was 95.43%.

**Figure 6:**
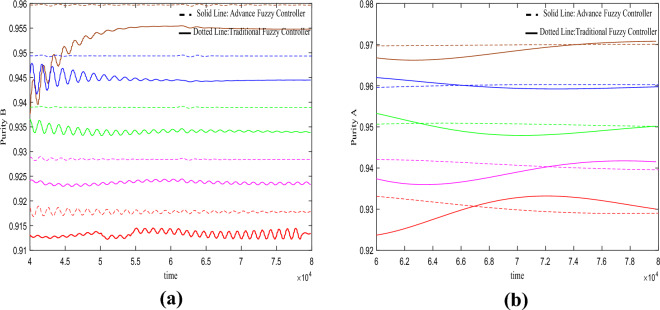
Under the change of adsorbent parameters $H_{A} = 0.01 \to 0.03$.

In the third set of experiments shown in Fig. 6, it can be observed that the purity of material B and material A remains stable under the variations in adsorbent parameters. However, the traditional fuzzy controller exhibits small sawtooth oscillations in the purity control of material B, while the advanced fuzzy controller, although devoid of oscillations, consistently exhibits a steady-state error larger than the target. Furthermore, the advanced fuzzy controller demonstrates larger fluctuations in the purity control of material A.

**Figure 7:**
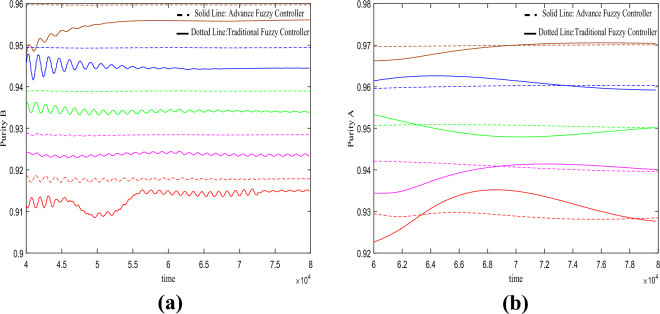
Under the change of feed port concentration $C_{{_{f} }} = 4.5 \to 5.2$.

In the fourth set of experiments shown in Fig. 7, it is observed that under the variations in feed concentration, the results are similar to those seen in the experiments depicted in Fig. 6.

**Figure 8:**
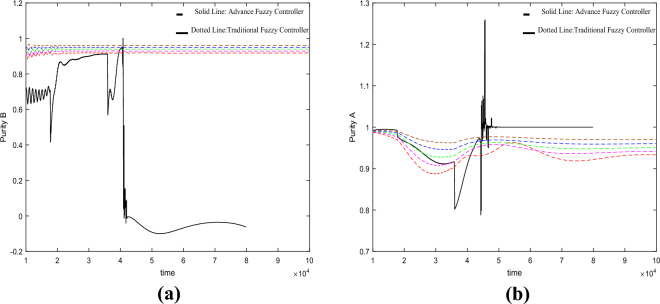
Under the change of switch time $\theta = 178s \to 182s$.

From the results of the fifth set of experiments shown in Fig. 8, it can be observed that the traditional fuzzy controller exhibits ill-conditioned characteristics under variations in the switching time. On the other hand, although the advanced fuzzy controller does not exhibit ill-conditioned behavior, it demonstrates higher fluctuations in the control results.

Overall, the traditional fuzzy controller exhibits a smaller steady-state error compared to the target, while the advanced fuzzy controller shows a slightly larger error. In the case of the SMB system, the advanced fuzzy controller proves to be more stable than the traditional fuzzy controller.

## Conclusion

This study employed an advanced fuzzy controller to regulate the separation purity of SMB. The traditional fuzzy controller exhibited a steady-state error smaller than the target value, while the advanced fuzzy controller’s control results were slightly larger than the target value. Experimental results indicated that the traditional fuzzy controller exhibited oscillatory behavior under variations in adsorbent parameters and feed concentration, whereas the advanced fuzzy controller did not. Regarding changes in the switching time, the traditional fuzzy controller exhibited oscillations, while the advanced fuzzy controller did not. However, the advanced fuzzy controller showed higher fluctuations in the control results.

## Supplementary Information

Supplementary Information 1.

Supplementary Information 2.

Supplementary Information 3.

Supplementary Information 4.

Supplementary Information 5.

Supplementary Information 6.

Supplementary Information 7.

Supplementary Information 8.

Supplementary Information 9.

## Data Availability

All data generated or analyzed during this study are included in this published article [and its Supplementary Information files].
