# Supplementary material for: Purity control of simulated moving bed based on advanced fuzzy controller
Source: Sci Rep. 2024 Apr 20;14:9083. doi: 10.1038/s41598-024-59847-1 (PMC11576947; doi:10.1038/s41598-024-59847-1)
Supplement: Supplementary file 1 — Supplementary Information 1. [file 41598_2024_59847_MOESM1_ESM.docx]

**Figure 4(a):**

1 1.468380e-80 3.616623e-76

2 1.468380e-80 3.616623e-76

3 1.468380e-80 3.616623e-76

4 1.468380e-80 3.616623e-76

5 1.468380e-80 3.616623e-76

6 1.468380e-80 3.616623e-76

7 1.468380e-80 3.616623e-76

8 1.468380e-80 3.616623e-76

9 1.468380e-80 3.616623e-76

10 1.468380e-80 3.616623e-76

11 1.468380e-80 3.616623e-76

12 1.468380e-80 3.616623e-76

13 1.468380e-80 3.616623e-76

14 1.468380e-80 3.616623e-76

15 1.468380e-80 3.616623e-76

16 1.468380e-80 3.616623e-76

17 1.468380e-80 3.616623e-76

18 1.468380e-80 3.616623e-76

19 1.468380e-80 3.616623e-76

20 1.468380e-80 3.616623e-76

21 1.468380e-80 3.616623e-76

22 1.468380e-80 3.616623e-76

23 1.468380e-80 3.616623e-76

24 1.468380e-80 3.616623e-76

25 1.468380e-80 3.616623e-76

26 1.468380e-80 3.616623e-76

27 1.468380e-80 3.616623e-76

28 1.468380e-80 3.616623e-76

29 1.468380e-80 3.616623e-76

30 1.468380e-80 3.616623e-76

31 1.468380e-80 3.616623e-76

32 1.468380e-80 3.616623e-76

33 1.468380e-80 3.616623e-76

34 1.468380e-80 3.616623e-76

35 1.468380e-80 3.616623e-76

36 1.468380e-80 3.616623e-76

37 1.468380e-80 3.616623e-76

38 1.468380e-80 3.616623e-76

39 1.468380e-80 3.616623e-76

40 1.468380e-80 3.616623e-76

41 1.468380e-80 3.616623e-76

42 1.468380e-80 3.616623e-76

43 1.468380e-80 3.616623e-76

44 1.468380e-80 3.616624e-76

45 1.468380e-80 3.616624e-76

46 1.468380e-80 3.616624e-76

47 1.468380e-80 3.616624e-76

48 1.468380e-80 3.616624e-76

49 1.468380e-80 3.616624e-76

50 1.468380e-80 3.616624e-76

51 1.468380e-80 3.616624e-76

52 1.468380e-80 3.616626e-76

53 1.468381e-80 3.616628e-76

54 1.468382e-80 3.616632e-76

55 1.468384e-80 3.616640e-76

56 1.468389e-80 3.616658e-76

57 1.468398e-80 3.616692e-76

58 1.468417e-80 3.616760e-76

59 1.468454e-80 3.616893e-76

60 1.468527e-80 3.617155e-76

61 1.468670e-80 3.617666e-76

62 1.468949e-80 3.618654e-76

63 1.469492e-80 3.620559e-76

64 1.470541e-80 3.624208e-76

65 1.472560e-80 3.631160e-76

66 1.476425e-80 3.644334e-76

67 1.483786e-80 3.669165e-76

68 1.497744e-80 3.715727e-76

69 1.524084e-80 3.802595e-76

70 1.573568e-80 3.964914e-76

71 1.666132e-80 4.268721e-76

72 1.838561e-80 4.836404e-76

73 2.158505e-80 5.893886e-76

74 2.749958e-80 7.856697e-76

75 3.839530e-80 1.148650e-75

76 5.840261e-80 1.817516e-75

77 9.503213e-80 3.045908e-75

78 1.619124e-79 5.294908e-75

79 2.837282e-79 9.400892e-75

80 5.051214e-79 1.687818e-74

81 9.067185e-79 3.046415e-74

82 1.633979e-78 5.510049e-74

83 2.949093e-78 9.969802e-74

84 5.324317e-78 1.803095e-73

85 9.609777e-78 3.258310e-73

86 1.733509e-77 5.882415e-73

87 3.125142e-77 1.060987e-72

88 5.630534e-77 1.911968e-72

89 1.013881e-76 3.442707e-72

90 1.824772e-76 6.194391e-72

91 3.282752e-76 1.113782e-71

92 5.903306e-76 2.001339e-71

93 1.061192e-75 3.593901e-71

94 1.906958e-75 6.449618e-71

95 3.425627e-75 1.156682e-70

96 6.151599e-75 2.072948e-70

97 1.104281e-74 3.712234e-70

98 1.981554e-74 6.642447e-70

99 3.550950e-74 1.187513e-69

100 6.350934e-74 2.120979e-69

101 1.133854e-73 3.784368e-69

102 2.021365e-73 6.744995e-69

103 3.599372e-73 1.200814e-68

104 6.403139e-73 2.135273e-68

105 1.138154e-72 3.792232e-68

106 2.021541e-72 6.726411e-68

107 3.588000e-72 1.191531e-67

108 6.363744e-72 2.107896e-67

109 1.127865e-71 3.733030e-67

110 1.997436e-71 6.628160e-67

111 3.534628e-71 1.179326e-66

112 6.249543e-71 2.100745e-66

113 1.103984e-70 3.743108e-66

114 1.948319e-70 6.666880e-66

115 3.434875e-70 1.186428e-65

116 6.048996e-70 2.108868e-65

117 1.064005e-69 3.743270e-65

118 1.869204e-69 6.634039e-65

119 3.279335e-69 1.173754e-64

120 5.745050e-69 2.073030e-64

121 1.004948e-68 3.654476e-64

122 1.755081e-68 6.429846e-64

123 3.059976e-68 1.129012e-63

124 5.325620e-68 1.978273e-63

125 9.251651e-68 3.458860e-63

126 1.604102e-67 6.034043e-63

127 2.775733e-67 1.050228e-62

128 4.793217e-67 1.823608e-62

129 8.259499e-67 3.158830e-62

130 1.420146e-66 5.458132e-62

131 2.436369e-66 9.407253e-62

132 4.170272e-66 1.617196e-61

133 7.121628e-66 2.772841e-61

134 1.213313e-65 4.741697e-61

135 2.062206e-65 8.086760e-61

136 3.496598e-65 1.375416e-60

137 5.914319e-65 2.332929e-60

138 9.979336e-65 3.946086e-60

139 1.679696e-64 6.656117e-60

140 2.820249e-64 1.119586e-59

141 4.723560e-64 1.877890e-59

142 7.891779e-64 3.140903e-59

143 1.315237e-63 5.238507e-59

144 2.186541e-63 8.712184e-59

145 3.626087e-63 1.444816e-58

146 5.998589e-63 2.389271e-58

147 9.899084e-63 3.939924e-58

148 1.629601e-62 6.489743e-58

149 2.676175e-62 1.069142e-57

150 4.384318e-62 1.761330e-57

151 7.165592e-62 2.899936e-57

152 1.168352e-61 4.768863e-57

153 1.900536e-61 7.828996e-57

154 3.084393e-61 1.282649e-56

155 4.994193e-61 2.096608e-56

156 8.068150e-61 3.418771e-56

157 1.300490e-60 5.560701e-56

158 2.091590e-60 9.021485e-56

159 3.356559e-60 1.459856e-55

160 5.374925e-60 2.356290e-55

161 8.588616e-60 3.793515e-55

162 1.369490e-59 6.092008e-55

163 2.179172e-59 9.758831e-55

164 3.460458e-59 1.559438e-54

165 5.483994e-59 2.485914e-54

166 8.673489e-59 3.953364e-54

167 1.369102e-58 6.272281e-54

168 2.156924e-58 9.928350e-54

169 3.391580e-58 1.567965e-53

170 5.322908e-58 2.470688e-53

171 8.338474e-58 3.884510e-53

172 1.303845e-57 6.094039e-53

173 2.035060e-57 9.539790e-53

174 3.170672e-57 1.490220e-52

175 4.931264e-57 2.323020e-52

176 7.656108e-57 3.613761e-52

177 1.186617e-56 5.610247e-52

178 1.836010e-56 8.692256e-52

179 2.836032e-56 1.344071e-51

180 4.373488e-56 2.074255e-51

181 6.733398e-56 3.194937e-51

182 6.733398e-56 3.194937e-51

183 6.733398e-56 3.194937e-51

184 6.733398e-56 3.194937e-51

185 6.733398e-56 3.194937e-51

186 6.733398e-56 3.194937e-51

187 6.733398e-56 3.194937e-51

188 6.733398e-56 3.194937e-51

189 6.733398e-56 3.194937e-51

190 6.733398e-56 3.194937e-51

191 6.733398e-56 3.194937e-51

192 6.733398e-56 3.194937e-51

193 6.733398e-56 3.194937e-51

194 6.733398e-56 3.194937e-51

195 6.733398e-56 3.194937e-51

196 6.733398e-56 3.194937e-51

197 6.733398e-56 3.194937e-51

198 6.733398e-56 3.194937e-51

199 6.733398e-56 3.194937e-51

200 6.733398e-56 3.194937e-51

201 6.733398e-56 3.194937e-51

202 6.733398e-56 3.194937e-51

203 6.733398e-56 3.194937e-51

204 6.733398e-56 3.194937e-51

205 6.733398e-56 3.194937e-51

206 6.733398e-56 3.194937e-51

207 6.733398e-56 3.194937e-51

208 6.733398e-56 3.194937e-51

209 6.733398e-56 3.194937e-51

210 6.733398e-56 3.194937e-51

211 6.733398e-56 3.194937e-51

212 6.733398e-56 3.194937e-51

213 6.733398e-56 3.194937e-51

214 6.733398e-56 3.194937e-51

215 6.733398e-56 3.194937e-51

216 6.733398e-56 3.194937e-51

217 6.733398e-56 3.194937e-51

218 6.733398e-56 3.194937e-51

219 6.733398e-56 3.194937e-51

220 6.733398e-56 3.194937e-51

221 6.733398e-56 3.194937e-51

222 6.733398e-56 3.194937e-51

223 6.733398e-56 3.194937e-51

224 6.733398e-56 3.194937e-51

225 6.733398e-56 3.194937e-51

226 6.733398e-56 3.194937e-51

227 6.733398e-56 3.194937e-51

228 6.733398e-56 3.194937e-51

229 6.733398e-56 3.194937e-51

230 6.733398e-56 3.194937e-51

231 6.733398e-56 3.194937e-51

232 6.733398e-56 3.194937e-51

233 6.733398e-56 3.194937e-51

234 6.733398e-56 3.194937e-51

235 6.733398e-56 3.194937e-51

236 6.733398e-56 3.194937e-51

237 6.733398e-56 3.194937e-51

238 6.733399e-56 3.194937e-51

239 6.733399e-56 3.194937e-51

240 6.733400e-56 3.194938e-51

241 6.733400e-56 3.194939e-51

242 6.733402e-56 3.194940e-51

243 6.733404e-56 3.194942e-51

244 6.733407e-56 3.194944e-51

245 6.733412e-56 3.194949e-51

246 6.733419e-56 3.194955e-51

247 6.733429e-56 3.194964e-51

248 6.733445e-56 3.194979e-51

249 6.733469e-56 3.195000e-51

250 6.733505e-56 3.195032e-51

251 6.733559e-56 3.195081e-51

252 6.733640e-56 3.195154e-51

253 6.733762e-56 3.195262e-51

254 6.733943e-56 3.195425e-51

255 6.734213e-56 3.195667e-51

256 6.734616e-56 3.196028e-51

257 6.735215e-56 3.196564e-51

258 6.736106e-56 3.197360e-51

259 6.737429e-56 3.198540e-51

260 6.739391e-56 3.200286e-51

261 6.742297e-56 3.202865e-51

262 6.746595e-56 3.206669e-51

263 6.752944e-56 3.212270e-51

264 6.762309e-56 3.220526e-51

265 6.776104e-56 3.232719e-51

266 6.796398e-56 3.250735e-51

267 6.826209e-56 3.277336e-51

268 6.869936e-56 3.316571e-51

269 6.933980e-56 3.374353e-51

270 7.027641e-56 3.459304e-51

271 7.164402e-56 3.583968e-51

272 7.363783e-56 3.766555e-51

273 7.653989e-56 4.033439e-51

274 8.075703e-56 4.422734e-51

275 8.687498e-56 4.989393e-51

276 9.573541e-56 5.812464e-51

277 1.085456e-55 7.005389e-51

278 1.270337e-55 8.730593e-51

279 1.536690e-55 1.122008e-50

280 1.919729e-55 1.480448e-50

281 2.469570e-55 1.995386e-50

282 3.257400e-55 2.733494e-50

283 4.384145e-55 3.789124e-50

284 5.992606e-55 5.295482e-50

285 8.284445e-55 7.440207e-50

286 1.154390e-54 1.048701e-49

287 1.617085e-54 1.480567e-49

288 2.272678e-54 2.091359e-49

289 3.199863e-54 2.953313e-49

290 4.508740e-54 4.167064e-49

291 6.353063e-54 5.872522e-49

292 8.947189e-54 8.263801e-49

293 1.258946e-53 1.160969e-48

294 1.769441e-53 1.628165e-48

295 2.483717e-53 2.279211e-48

296 3.481442e-53 3.184664e-48

297 4.872814e-53 4.441510e-48

298 6.810058e-53 6.182846e-48

299 9.503172e-53 8.591026e-48

300 1.324150e-52 1.191552e-47

301 1.842325e-52 1.649713e-47

302 2.559586e-52 2.281401e-47

303 3.551120e-52 3.153425e-47

304 4.920091e-52 4.357523e-47

305 6.807938e-52 6.019549e-47

306 9.408421e-52 8.312007e-47

307 1.298680e-51 1.147114e-46

308 1.790602e-51 1.582018e-46

309 2.466234e-51 2.180111e-46

310 3.393413e-51 3.001774e-46

311 4.664819e-51 4.129453e-46

312 6.407040e-51 5.675648e-46

313 8.792929e-51 7.793788e-46

314 1.205846e-50 1.069302e-45

315 1.652573e-50 1.465832e-45

316 2.263431e-50 2.007785e-45

317 3.098410e-50 2.748012e-45

318 4.239368e-50 3.758456e-45

319 5.798008e-50 5.137020e-45

320 7.926749e-50 7.016904e-45

321 1.083358e-49 9.579294e-45

322 1.480232e-49 1.307060e-44

323 2.022027e-49 1.782589e-44

324 2.761599e-49 2.430067e-44

325 3.771082e-49 3.311416e-44

326 5.148919e-49 4.510790e-44

327 7.029472e-49 6.142545e-44

328 9.596120e-49 8.362052e-44

329 1.309915e-48 1.138037e-43

330 1.788016e-48 1.548416e-43

331 2.440539e-48 2.106269e-43

332 3.331115e-48 2.864444e-43

333 4.546588e-48 3.894678e-43

334 6.205474e-48 5.294323e-43

335 8.469500e-48 7.195466e-43

336 1.155936e-47 9.777276e-43

337 1.577616e-47 1.328273e-42

338 2.153074e-47 1.804125e-42

339 2.938354e-47 2.449932e-42

340 4.009908e-47 3.327954e-42

341 5.472010e-47 4.524717e-42

342 7.466872e-47 6.158447e-42

343 1.018842e-46 8.390680e-42

344 1.390106e-46 1.144210e-41

345 1.896529e-46 1.561414e-41

346 2.587248e-46 2.131842e-41

347 3.529238e-46 2.911689e-41

348 4.813768e-46 3.977648e-41

349 6.565201e-46 5.434349e-41

350 8.952960e-46 7.424495e-41

351 1.220782e-45 1.014264e-40

352 1.664407e-45 1.385391e-40

353 2.268964e-45 1.891952e-40

354 3.092712e-45 2.583131e-40

355 4.214939e-45 3.525876e-40

356 5.743538e-45 4.811277e-40

357 7.825277e-45 6.563204e-40

358 1.065976e-44 8.950036e-40

359 1.451838e-44 1.220053e-39

360 1.976995e-44 1.662531e-39

361 2.691562e-44 2.264596e-39

362 3.135913e-06 4.903528e-03

363 5.909657e-06 9.653368e-03

364 8.361252e-06 1.422861e-02

365 1.052650e-05 1.860761e-02

366 1.243740e-05 2.276839e-02

367 1.412253e-05 2.668920e-02

368 1.560743e-05 3.034918e-02

369 1.691485e-05 3.372915e-02

370 1.806509e-05 3.681248e-02

371 1.907621e-05 3.958600e-02

372 1.996432e-05 4.204086e-02

373 2.074371e-05 4.417336e-02

374 2.142712e-05 4.598549e-02

375 2.202585e-05 4.748506e-02

376 2.254991e-05 4.868562e-02

377 2.300825e-05 4.960628e-02

378 2.340879e-05 5.027089e-02

379 2.375856e-05 5.070695e-02

380 2.406375e-05 5.094429e-02

381 2.432981e-05 5.101364e-02

382 2.456157e-05 5.094539e-02

383 2.476328e-05 5.076835e-02

384 2.493868e-05 5.050897e-02

385 2.509105e-05 5.019068e-02

386 2.522330e-05 4.983366e-02

387 2.533796e-05 4.945472e-02

388 2.543729e-05 4.906745e-02

389 2.552322e-05 4.868253e-02

390 2.559750e-05 4.830802e-02

391 2.566163e-05 4.794975e-02

392 2.571693e-05 4.761171e-02

393 2.576456e-05 4.729638e-02

394 2.580553e-05 4.700506e-02

395 2.584072e-05 4.673814e-02

396 2.587091e-05 4.649533e-02

397 2.589677e-05 4.627584e-02

398 2.591889e-05 4.607854e-02

399 2.593777e-05 4.590208e-02

400 2.595387e-05 4.574499e-02

401 2.596757e-05 4.560574e-02

402 2.597920e-05 4.548280e-02

403 2.598906e-05 4.537467e-02

404 2.599740e-05 4.527991e-02

405 2.600444e-05 4.519716e-02

406 2.601036e-05 4.512515e-02

407 2.601533e-05 4.506270e-02

408 2.601949e-05 4.500875e-02

409 2.602296e-05 4.496232e-02

410 2.602585e-05 4.492253e-02

411 2.602824e-05 4.488857e-02

412 2.603021e-05 4.485973e-02

413 2.603183e-05 4.483534e-02

414 2.603315e-05 4.481482e-02

415 2.603422e-05 4.479765e-02

416 2.603508e-05 4.478337e-02

417 2.603577e-05 4.477156e-02

418 2.603632e-05 4.476187e-02

419 2.603674e-05 4.475398e-02

420 2.603707e-05 4.474762e-02

421 2.603732e-05 4.474254e-02

422 2.603751e-05 4.473854e-02

423 2.603764e-05 4.473543e-02

424 2.603772e-05 4.473307e-02

425 2.603778e-05 4.473132e-02

426 2.603781e-05 4.473007e-02

427 2.603781e-05 4.472922e-02

428 2.603781e-05 4.472869e-02

429 2.603779e-05 4.472842e-02

430 2.603776e-05 4.472835e-02

431 2.603772e-05 4.472842e-02

432 2.603768e-05 4.472861e-02

433 2.603764e-05 4.472889e-02

434 2.603760e-05 4.472921e-02

435 2.603756e-05 4.472958e-02

436 2.603752e-05 4.472996e-02

437 2.603748e-05 4.473035e-02

438 2.603745e-05 4.473073e-02

439 2.603741e-05 4.473111e-02

440 2.603738e-05 4.473146e-02

441 2.603735e-05 4.473180e-02

442 2.603732e-05 4.473212e-02

443 2.603730e-05 4.473242e-02

444 2.603727e-05 4.473269e-02

445 2.603725e-05 4.473293e-02

446 2.603723e-05 4.473316e-02

447 2.603722e-05 4.473336e-02

448 2.603720e-05 4.473354e-02

449 2.603719e-05 4.473370e-02

450 2.603717e-05 4.473384e-02

451 2.603716e-05 4.473397e-02

452 2.603715e-05 4.473408e-02

453 2.603715e-05 4.473417e-02

454 2.603714e-05 4.473425e-02

455 2.603713e-05 4.473433e-02

456 2.603713e-05 4.473439e-02

457 2.603712e-05 4.473444e-02

458 2.603712e-05 4.473448e-02

459 2.603711e-05 4.473452e-02

460 2.603711e-05 4.473455e-02

461 2.603711e-05 4.473458e-02

462 2.603710e-05 4.473460e-02

463 2.603710e-05 4.473462e-02

464 2.603710e-05 4.473463e-02

465 2.603710e-05 4.473464e-02

466 2.603710e-05 4.473465e-02

467 2.603710e-05 4.473466e-02

468 2.603710e-05 4.473466e-02

469 2.603710e-05 4.473467e-02

470 2.603709e-05 4.473467e-02

471 2.603709e-05 4.473467e-02

472 2.603709e-05 4.473467e-02

473 2.603709e-05 4.473467e-02

474 2.603709e-05 4.473467e-02

475 2.603709e-05 4.473467e-02

476 2.603709e-05 4.473467e-02

477 2.603709e-05 4.473467e-02

478 2.603709e-05 4.473467e-02

479 2.603709e-05 4.473467e-02

480 2.603709e-05 4.473467e-02

481 2.603709e-05 4.473467e-02

482 2.603709e-05 4.473467e-02

483 2.603709e-05 4.473467e-02

484 2.603709e-05 4.473467e-02

485 2.603709e-05 4.473466e-02

486 2.603709e-05 4.473466e-02

487 2.603709e-05 4.473466e-02

488 2.603709e-05 4.473466e-02

489 2.603709e-05 4.473466e-02

490 2.603709e-05 4.473466e-02

491 2.603709e-05 4.473466e-02

492 2.603709e-05 4.473466e-02

493 2.603709e-05 4.473466e-02

494 2.603709e-05 4.473466e-02

495 2.603709e-05 4.473466e-02

496 2.603709e-05 4.473466e-02

497 2.603709e-05 4.473466e-02

498 2.603709e-05 4.473466e-02

499 2.603709e-05 4.473466e-02

500 2.603709e-05 4.473466e-02

501 2.603709e-05 4.473466e-02

502 2.603709e-05 4.473466e-02

503 2.603709e-05 4.473466e-02

504 2.603709e-05 4.473466e-02

505 2.603709e-05 4.473466e-02

506 2.603709e-05 4.473466e-02

507 2.603709e-05 4.473466e-02

508 2.603709e-05 4.473466e-02

509 2.603709e-05 4.473466e-02

510 2.603709e-05 4.473466e-02

511 2.603709e-05 4.473466e-02

512 2.603709e-05 4.473466e-02

513 2.603709e-05 4.473466e-02

514 2.603709e-05 4.473466e-02

515 2.603709e-05 4.473466e-02

516 2.603709e-05 4.473466e-02

517 2.603709e-05 4.473466e-02

518 2.603709e-05 4.473466e-02

519 2.603709e-05 4.473466e-02

520 2.603709e-05 4.473466e-02

521 2.603709e-05 4.473466e-02

522 2.603709e-05 4.473466e-02

523 2.603709e-05 4.473466e-02

524 2.603709e-05 4.473466e-02

525 2.603709e-05 4.473466e-02

526 2.603709e-05 4.473466e-02

527 2.603709e-05 4.473466e-02

528 2.603709e-05 4.473466e-02

529 2.603709e-05 4.473466e-02

530 2.603709e-05 4.473466e-02

531 2.603709e-05 4.473466e-02

532 2.603709e-05 4.473466e-02

533 2.603709e-05 4.473466e-02

534 2.603709e-05 4.473466e-02

535 2.603709e-05 4.473466e-02

536 2.603709e-05 4.473466e-02

537 2.603709e-05 4.473466e-02

538 2.603709e-05 4.473466e-02

539 2.603709e-05 4.473466e-02

540 2.603709e-05 4.473466e-02

541 2.603709e-05 4.473466e-02

542 9.896490e-04 4.538569e-02

543 1.903071e-03 4.619026e-02

544 2.768194e-03 4.716929e-02

545 3.586928e-03 4.834441e-02

546 4.361185e-03 4.973761e-02

547 5.092849e-03 5.137064e-02

548 5.783773e-03 5.326436e-02

549 6.435775e-03 5.543795e-02

550 7.050635e-03 5.790805e-02

551 7.630088e-03 6.068783e-02

552 8.175825e-03 6.378612e-02

553 8.689489e-03 6.720663e-02

554 9.172673e-03 7.094738e-02

555 9.626917e-03 7.500055e-02

556 1.005371e-02 7.935256e-02

557 1.045449e-02 8.398432e-02

558 1.083064e-02 8.887195e-02

559 1.118347e-02 9.398795e-02

560 1.151428e-02 9.930246e-02

561 1.182427e-02 1.047847e-01

562 1.211462e-02 1.104044e-01

563 1.238644e-02 1.161325e-01

564 1.264079e-02 1.219427e-01

565 1.287869e-02 1.278114e-01

566 1.310111e-02 1.337185e-01

567 1.330895e-02 1.396471e-01

568 1.350310e-02 1.455835e-01

569 1.368438e-02 1.515170e-01

570 1.385358e-02 1.574395e-01

571 1.401144e-02 1.633451e-01

572 1.415866e-02 1.692297e-01

573 1.429590e-02 1.750907e-01

574 1.442381e-02 1.809268e-01

575 1.454295e-02 1.867374e-01

576 1.465391e-02 1.925228e-01

577 1.475720e-02 1.982835e-01

578 1.485332e-02 2.040206e-01

579 1.494273e-02 2.097352e-01

580 1.502589e-02 2.154287e-01

581 1.510319e-02 2.211024e-01

582 1.517503e-02 2.267576e-01

583 1.524178e-02 2.323956e-01

584 1.530376e-02 2.380176e-01

585 1.536132e-02 2.436247e-01

586 1.541474e-02 2.492178e-01

587 1.546431e-02 2.547979e-01

588 1.551029e-02 2.603656e-01

589 1.555293e-02 2.659214e-01

590 1.559246e-02 2.714660e-01

591 1.562909e-02 2.769995e-01

592 1.566304e-02 2.825222e-01

593 1.569448e-02 2.880341e-01

594 1.572359e-02 2.935353e-01

595 1.575054e-02 2.990256e-01

596 1.577548e-02 3.045045e-01

597 1.579856e-02 3.099717e-01

598 1.581991e-02 3.154264e-01

599 1.583964e-02 3.208681e-01

600 1.585789e-02 3.262957e-01

601 1.587475e-02 3.317081e-01

602 1.589034e-02 3.371041e-01

603 1.590473e-02 3.424823e-01

604 1.591802e-02 3.478411e-01

605 1.593029e-02 3.531786e-01

606 1.594161e-02 3.584929e-01

607 1.595206e-02 3.637816e-01

608 1.596170e-02 3.690423e-01

609 1.597059e-02 3.742721e-01

610 1.597879e-02 3.794682e-01

611 1.598634e-02 3.846272e-01

612 1.599330e-02 3.897455e-01

613 1.599971e-02 3.948192e-01

614 1.600562e-02 3.998441e-01

615 1.601106e-02 4.048157e-01

616 1.601607e-02 4.097291e-01

617 1.602067e-02 4.145792e-01

618 1.602491e-02 4.193605e-01

619 1.602881e-02 4.240674e-01

620 1.603240e-02 4.286937e-01

621 1.603569e-02 4.332333e-01

622 1.603872e-02 4.376796e-01

623 1.604151e-02 4.420257e-01

624 1.604407e-02 4.462646e-01

625 1.604642e-02 4.503892e-01

626 1.604857e-02 4.543926e-01

627 1.605055e-02 4.582679e-01

628 1.605237e-02 4.620087e-01

629 1.605404e-02 4.656090e-01

630 1.605556e-02 4.690633e-01

631 1.605697e-02 4.723666e-01

632 1.605825e-02 4.755152e-01

633 1.605943e-02 4.785058e-01

634 1.606051e-02 4.813365e-01

635 1.606150e-02 4.840061e-01

636 1.606240e-02 4.865148e-01

637 1.606323e-02 4.888638e-01

638 1.606399e-02 4.910553e-01

639 1.606469e-02 4.930926e-01

640 1.606532e-02 4.949800e-01

641 1.606590e-02 4.967226e-01

642 1.606643e-02 4.983260e-01

643 1.606692e-02 4.997968e-01

644 1.606736e-02 5.011418e-01

645 1.606777e-02 5.023681e-01

646 1.606814e-02 5.034832e-01

647 1.606848e-02 5.044944e-01

648 1.606879e-02 5.054092e-01

649 1.606907e-02 5.062346e-01

650 1.606933e-02 5.069777e-01

651 1.606956e-02 5.076449e-01

652 1.606978e-02 5.082426e-01

653 1.606997e-02 5.087770e-01

654 1.607015e-02 5.092538e-01

655 1.607031e-02 5.096784e-01

656 1.607046e-02 5.100558e-01

657 1.607059e-02 5.103907e-01

658 1.607072e-02 5.106875e-01

659 1.607083e-02 5.109500e-01

660 1.607093e-02 5.111820e-01

661 1.607102e-02 5.113866e-01

662 1.607111e-02 5.115670e-01

663 1.607118e-02 5.117257e-01

664 1.607125e-02 5.118653e-01

665 1.607132e-02 5.119878e-01

666 1.607137e-02 5.120953e-01

667 1.607143e-02 5.121896e-01

668 1.607147e-02 5.122721e-01

669 1.607152e-02 5.123443e-01

670 1.607156e-02 5.124074e-01

671 1.607159e-02 5.124625e-01

672 1.607162e-02 5.125107e-01

673 1.607165e-02 5.125526e-01

674 1.607168e-02 5.125891e-01

675 1.607170e-02 5.126210e-01

676 1.607172e-02 5.126486e-01

677 1.607174e-02 5.126727e-01

678 1.607176e-02 5.126935e-01

679 1.607178e-02 5.127116e-01

680 1.607179e-02 5.127273e-01

681 1.607181e-02 5.127408e-01

682 1.607182e-02 5.127525e-01

683 1.607183e-02 5.127626e-01

684 1.607184e-02 5.127713e-01

685 1.607185e-02 5.127787e-01

686 1.607186e-02 5.127852e-01

687 1.607186e-02 5.127907e-01

688 1.607187e-02 5.127954e-01

689 1.607188e-02 5.127994e-01

690 1.607188e-02 5.128029e-01

691 1.607189e-02 5.128058e-01

692 1.607189e-02 5.128084e-01

693 1.607189e-02 5.128105e-01

694 1.607190e-02 5.128123e-01

695 1.607190e-02 5.128139e-01

696 1.607190e-02 5.128152e-01

697 1.607191e-02 5.128163e-01

698 1.607191e-02 5.128172e-01

699 1.607191e-02 5.128180e-01

700 1.607191e-02 5.128186e-01

701 1.607191e-02 5.128192e-01

702 1.607192e-02 5.128196e-01

703 1.607192e-02 5.128200e-01

704 1.607192e-02 5.128203e-01

705 1.607192e-02 5.128206e-01

706 1.607192e-02 5.128208e-01

707 1.607192e-02 5.128210e-01

708 1.607192e-02 5.128211e-01

709 1.607192e-02 5.128212e-01

710 1.607192e-02 5.128213e-01

711 1.607192e-02 5.128214e-01

712 1.607192e-02 5.128215e-01

713 1.607192e-02 5.128215e-01

714 1.607193e-02 5.128215e-01

715 1.607193e-02 5.128216e-01

716 1.607193e-02 5.128216e-01

717 1.607193e-02 5.128216e-01

718 1.607193e-02 5.128216e-01

719 1.607193e-02 5.128216e-01

720 1.607193e-02 5.128216e-01

721 1.607193e-02 5.128216e-01

722 1.905264e-02 5.128226e-01

723 2.203018e-02 5.128236e-01

724 2.500130e-02 5.128248e-01

725 2.796273e-02 5.128262e-01

726 3.091125e-02 5.128277e-01

727 3.384370e-02 5.128293e-01

728 3.675699e-02 5.128311e-01

729 3.964810e-02 5.128330e-01

730 4.251411e-02 5.128350e-01

731 4.535219e-02 5.128372e-01

732 4.815964e-02 5.128394e-01

733 5.093382e-02 5.128419e-01

734 5.367227e-02 5.128444e-01

735 5.637260e-02 5.128471e-01

736 5.903259e-02 5.128500e-01

737 6.165014e-02 5.128530e-01

738 6.422328e-02 5.128562e-01

739 6.675021e-02 5.128595e-01

740 6.922925e-02 5.128630e-01

741 7.165887e-02 5.128668e-01

742 7.403772e-02 5.128708e-01

743 7.636456e-02 5.128751e-01

744 7.863833e-02 5.128796e-01

745 8.085812e-02 5.128845e-01

746 8.302314e-02 5.128898e-01

747 8.513279e-02 5.128954e-01

748 8.718659e-02 5.129016e-01

749 8.918420e-02 5.129082e-01

750 9.112544e-02 5.129154e-01

751 9.301023e-02 5.129233e-01

752 9.483866e-02 5.129320e-01

753 9.661091e-02 5.129414e-01

754 9.832730e-02 5.129518e-01

755 9.998826e-02 5.129632e-01

756 1.015943e-01 5.129757e-01

757 1.031461e-01 5.129895e-01

758 1.046444e-01 5.130047e-01

759 1.060899e-01 5.130215e-01

760 1.074835e-01 5.130400e-01

761 1.088263e-01 5.130604e-01

762 1.101191e-01 5.130829e-01

763 1.113630e-01 5.131078e-01

764 1.125590e-01 5.131352e-01

765 1.137083e-01 5.131655e-01

766 1.148119e-01 5.131989e-01

767 1.158710e-01 5.132358e-01

768 1.168869e-01 5.132765e-01

769 1.178606e-01 5.133214e-01

770 1.187933e-01 5.133709e-01

771 1.196863e-01 5.134255e-01

772 1.205408e-01 5.134856e-01

773 1.213579e-01 5.135520e-01

774 1.221390e-01 5.136251e-01

775 1.228851e-01 5.137058e-01

776 1.235975e-01 5.137947e-01

777 1.242773e-01 5.138927e-01

778 1.249257e-01 5.140009e-01

779 1.255439e-01 5.141202e-01

780 1.261329e-01 5.142520e-01

781 1.266940e-01 5.143973e-01

782 1.272281e-01 5.145578e-01

783 1.277364e-01 5.147350e-01

784 1.282199e-01 5.149306e-01

785 1.286795e-01 5.151466e-01

786 1.291164e-01 5.153851e-01

787 1.295314e-01 5.156485e-01

788 1.299255e-01 5.159392e-01

789 1.302996e-01 5.162601e-01

790 1.306546e-01 5.166142e-01

791 1.309913e-01 5.170049e-01

792 1.313106e-01 5.174357e-01

793 1.316133e-01 5.179105e-01

794 1.319000e-01 5.184335e-01

795 1.321717e-01 5.190093e-01

796 1.324289e-01 5.196426e-01

797 1.326724e-01 5.203385e-01

798 1.329028e-01 5.211025e-01

799 1.331208e-01 5.219403e-01

800 1.333269e-01 5.228577e-01

801 1.335218e-01 5.238610e-01

802 1.337061e-01 5.249567e-01

803 1.338801e-01 5.261515e-01

804 1.340446e-01 5.274525e-01

805 1.341999e-01 5.288665e-01

806 1.343465e-01 5.304004e-01

807 1.344849e-01 5.320610e-01

808 1.346155e-01 5.338544e-01

809 1.347387e-01 5.357868e-01

810 1.348550e-01 5.378634e-01

811 1.349645e-01 5.400888e-01

812 1.350678e-01 5.424670e-01

813 1.351652e-01 5.450007e-01

814 1.352570e-01 5.476918e-01

815 1.353434e-01 5.505412e-01

816 1.354248e-01 5.535485e-01

817 1.355015e-01 5.567123e-01

818 1.355736e-01 5.600301e-01

819 1.356416e-01 5.634981e-01

820 1.357055e-01 5.671120e-01

821 1.357656e-01 5.708663e-01

822 1.358222e-01 5.747547e-01

823 1.358754e-01 5.787705e-01

824 1.359254e-01 5.829064e-01

825 1.359724e-01 5.871548e-01

826 1.360166e-01 5.915077e-01

827 1.360582e-01 5.959572e-01

828 1.360972e-01 6.004951e-01

829 1.361338e-01 6.051138e-01

830 1.361683e-01 6.098055e-01

831 1.362006e-01 6.145630e-01

832 1.362309e-01 6.193787e-01

833 1.362594e-01 6.242459e-01

834 1.362861e-01 6.291576e-01

835 1.363112e-01 6.341072e-01

836 1.363347e-01 6.390887e-01

837 1.363568e-01 6.440959e-01

838 1.363774e-01 6.491231e-01

839 1.363968e-01 6.541649e-01

840 1.364150e-01 6.592159e-01

841 1.364320e-01 6.642711e-01

842 1.364480e-01 6.693256e-01

843 1.364630e-01 6.743746e-01

844 1.364770e-01 6.794136e-01

845 1.364901e-01 6.844380e-01

846 1.365024e-01 6.894434e-01

847 1.365139e-01 6.944253e-01

848 1.365247e-01 6.993795e-01

849 1.365347e-01 7.043015e-01

850 1.365442e-01 7.091869e-01

851 1.365530e-01 7.140315e-01

852 1.365612e-01 7.188307e-01

853 1.365690e-01 7.235802e-01

854 1.365762e-01 7.282753e-01

855 1.365829e-01 7.329115e-01

856 1.365892e-01 7.374839e-01

857 1.365951e-01 7.419875e-01

858 1.366006e-01 7.464173e-01

859 1.366058e-01 7.507684e-01

860 1.366106e-01 7.550358e-01

861 1.366151e-01 7.592144e-01

862 1.366193e-01 7.632993e-01

863 1.366232e-01 7.672858e-01

864 1.366268e-01 7.711693e-01

865 1.366303e-01 7.749452e-01

866 1.366334e-01 7.786095e-01

867 1.366364e-01 7.821582e-01

868 1.366392e-01 7.855880e-01

869 1.366418e-01 7.888957e-01

870 1.366442e-01 7.920789e-01

871 1.366464e-01 7.951353e-01

872 1.366485e-01 7.980634e-01

873 1.366505e-01 8.008624e-01

874 1.366523e-01 8.035317e-01

875 1.366540e-01 8.060717e-01

876 1.366556e-01 8.084831e-01

877 1.366570e-01 8.107672e-01

878 1.366584e-01 8.129259e-01

879 1.366597e-01 8.149618e-01

880 1.366609e-01 8.168776e-01

881 1.366620e-01 8.186767e-01

882 1.366630e-01 8.203626e-01

883 1.366640e-01 8.219388e-01

884 1.366649e-01 8.234094e-01

885 1.366657e-01 8.247784e-01

886 1.366665e-01 8.260504e-01

887 1.366672e-01 8.272299e-01

888 1.366678e-01 8.283215e-01

889 1.366685e-01 8.293301e-01

890 1.366690e-01 8.302604e-01

891 1.366696e-01 8.311170e-01

892 1.366701e-01 8.319046e-01

893 1.366705e-01 8.326278e-01

894 1.366710e-01 8.332908e-01

895 1.366714e-01 8.338980e-01

896 1.366717e-01 8.344534e-01

897 1.366721e-01 8.349608e-01

898 1.366724e-01 8.354239e-01

899 1.366727e-01 8.358462e-01

900 1.366730e-01 8.362309e-01

901 1.366732e-01 8.365810e-01

902 1.378301e-01 8.365811e-01

903 1.390257e-01 8.365811e-01

904 1.402607e-01 8.365812e-01

905 1.415359e-01 8.365812e-01

906 1.428521e-01 8.365812e-01

907 1.442097e-01 8.365813e-01

908 1.456095e-01 8.365813e-01

909 1.470520e-01 8.365814e-01

910 1.485376e-01 8.365814e-01

911 1.500666e-01 8.365815e-01

912 1.516395e-01 8.365816e-01

913 1.532564e-01 8.365816e-01

914 1.549175e-01 8.365817e-01

915 1.566228e-01 8.365818e-01

916 1.583723e-01 8.365819e-01

917 1.601658e-01 8.365820e-01

918 1.620031e-01 8.365821e-01

919 1.638838e-01 8.365822e-01

920 1.658074e-01 8.365823e-01

921 1.677733e-01 8.365824e-01

922 1.697808e-01 8.365825e-01

923 1.718291e-01 8.365827e-01

924 1.739173e-01 8.365829e-01

925 1.760442e-01 8.365830e-01

926 1.782087e-01 8.365832e-01

927 1.804094e-01 8.365834e-01

928 1.826451e-01 8.365837e-01

929 1.849140e-01 8.365839e-01

930 1.872147e-01 8.365842e-01

931 1.895453e-01 8.365845e-01

932 1.919040e-01 8.365848e-01

933 1.942888e-01 8.365851e-01

934 1.966977e-01 8.365855e-01

935 1.991286e-01 8.365858e-01

936 2.015793e-01 8.365863e-01

937 2.040475e-01 8.365867e-01

938 2.065310e-01 8.365872e-01

939 2.090272e-01 8.365877e-01

940 2.115338e-01 8.365883e-01

941 2.140484e-01 8.365889e-01

942 2.165685e-01 8.365896e-01

943 2.190917e-01 8.365903e-01

944 2.216155e-01 8.365910e-01

945 2.241374e-01 8.365918e-01

946 2.266551e-01 8.365927e-01

947 2.291661e-01 8.365936e-01

948 2.316680e-01 8.365946e-01

949 2.341585e-01 8.365957e-01

950 2.366353e-01 8.365969e-01

951 2.390962e-01 8.365981e-01

952 2.415389e-01 8.365994e-01

953 2.439614e-01 8.366009e-01

954 2.463614e-01 8.366024e-01

955 2.487371e-01 8.366040e-01

956 2.510866e-01 8.366058e-01

957 2.534079e-01 8.366077e-01

958 2.556993e-01 8.366097e-01

959 2.579592e-01 8.366119e-01

960 2.601860e-01 8.366142e-01

961 2.623782e-01 8.366167e-01

962 2.645344e-01 8.366194e-01

963 2.666535e-01 8.366223e-01

964 2.687342e-01 8.366255e-01

965 2.707754e-01 8.366288e-01

966 2.727763e-01 8.366325e-01

967 2.747360e-01 8.366364e-01

968 2.766537e-01 8.366406e-01

969 2.785288e-01 8.366451e-01

970 2.803609e-01 8.366500e-01

971 2.821494e-01 8.366552e-01

972 2.838941e-01 8.366609e-01

973 2.855947e-01 8.366670e-01

974 2.872511e-01 8.366736e-01

975 2.888634e-01 8.366807e-01

976 2.904315e-01 8.366884e-01

977 2.919556e-01 8.366967e-01

978 2.934359e-01 8.367056e-01

979 2.948728e-01 8.367152e-01

980 2.962666e-01 8.367256e-01

981 2.976178e-01 8.367368e-01

982 2.989268e-01 8.367489e-01

983 3.001944e-01 8.367620e-01

984 3.014210e-01 8.367762e-01

985 3.026075e-01 8.367915e-01

986 3.037544e-01 8.368080e-01

987 3.048627e-01 8.368259e-01

988 3.059330e-01 8.368453e-01

989 3.069662e-01 8.368662e-01

990 3.079630e-01 8.368889e-01

991 3.089244e-01 8.369135e-01

992 3.098512e-01 8.369401e-01

993 3.107442e-01 8.369689e-01

994 3.116042e-01 8.370001e-01

995 3.124320e-01 8.370340e-01

996 3.132287e-01 8.370706e-01

997 3.139949e-01 8.371104e-01

998 3.147315e-01 8.371535e-01

999 3.154394e-01 8.372002e-01

1000 3.161195e-01 8.372508e-01

1001 3.167725e-01 8.373057e-01

1002 3.173992e-01 8.373653e-01

1003 3.180005e-01 8.374298e-01

1004 3.185773e-01 8.374999e-01

1005 3.191302e-01 8.375758e-01

1006 3.196601e-01 8.376581e-01

1007 3.201678e-01 8.377473e-01

1008 3.206540e-01 8.378441e-01

1009 3.211194e-01 8.379490e-01

1010 3.215649e-01 8.380629e-01

1011 3.219910e-01 8.381865e-01

1012 3.223986e-01 8.383207e-01

1013 3.227882e-01 8.384663e-01

1014 3.231606e-01 8.386244e-01

1015 3.235165e-01 8.387961e-01

1016 3.238563e-01 8.389826e-01

1017 3.241809e-01 8.391850e-01

1018 3.244907e-01 8.394049e-01

1019 3.247864e-01 8.396436e-01

1020 3.250685e-01 8.399028e-01

1021 3.253376e-01 8.401841e-01

1022 3.255942e-01 8.404895e-01

1023 3.258388e-01 8.408208e-01

1024 3.260719e-01 8.411802e-01

1025 3.262941e-01 8.415700e-01

1026 3.265057e-01 8.419925e-01

1027 3.267072e-01 8.424503e-01

1028 3.268991e-01 8.429461e-01

1029 3.270818e-01 8.434827e-01

1030 3.272556e-01 8.440633e-01

1031 3.274210e-01 8.446908e-01

1032 3.275784e-01 8.453687e-01

1033 3.277280e-01 8.461003e-01

1034 3.278703e-01 8.468893e-01

1035 3.280056e-01 8.477395e-01

1036 3.281342e-01 8.486549e-01

1037 3.282564e-01 8.496398e-01

1038 3.283725e-01 8.506986e-01

1039 3.284828e-01 8.518354e-01

1040 3.285876e-01 8.530549e-01

1041 3.286870e-01 8.543613e-01

1042 3.287815e-01 8.557590e-01

1043 3.288711e-01 8.572523e-01

1044 3.289562e-01 8.588452e-01

1045 3.290369e-01 8.605417e-01

1046 3.291134e-01 8.623452e-01

1047 3.291861e-01 8.642592e-01

1048 3.292550e-01 8.662866e-01

1049 3.293203e-01 8.684297e-01

1050 3.293822e-01 8.706907e-01

1051 3.294409e-01 8.730708e-01

1052 3.294965e-01 8.755711e-01

1053 3.295493e-01 8.781918e-01

1054 3.295992e-01 8.809326e-01

1055 3.296465e-01 8.837925e-01

1056 3.296913e-01 8.867699e-01

1057 3.297338e-01 8.898626e-01

1058 3.297740e-01 8.930677e-01

1059 3.298120e-01 8.963818e-01

1060 3.298480e-01 8.998010e-01

1061 3.298821e-01 9.033206e-01

1062 3.299143e-01 9.069361e-01

1063 3.299449e-01 9.106425e-01

1064 3.299737e-01 9.144345e-01

1065 3.300010e-01 9.183063e-01

1066 3.300268e-01 9.222521e-01

1067 3.300513e-01 9.262655e-01

1068 3.300743e-01 9.303400e-01

1069 3.300962e-01 9.344692e-01

1070 3.301168e-01 9.386462e-01

1071 3.301363e-01 9.428643e-01

1072 3.301547e-01 9.471166e-01

1073 3.301721e-01 9.513963e-01

1074 3.301885e-01 9.556967e-01

1075 3.302041e-01 9.600109e-01

1076 3.302187e-01 9.643322e-01

1077 3.302326e-01 9.686542e-01

1078 3.302456e-01 9.729702e-01

1079 3.302580e-01 9.772739e-01

1080 3.302696e-01 9.815590e-01

1081 3.302806e-01 9.858193e-01

1082 3.305503e-01 9.858194e-01

1083 3.308301e-01 9.858194e-01

1084 3.311205e-01 9.858195e-01

1085 3.314220e-01 9.858195e-01

1086 3.317347e-01 9.858196e-01

1087 3.320592e-01 9.858196e-01

1088 3.323960e-01 9.858196e-01

1089 3.327453e-01 9.858197e-01

1090 3.331077e-01 9.858197e-01

1091 3.334835e-01 9.858198e-01

1092 3.338734e-01 9.858198e-01

1093 3.342777e-01 9.858199e-01

1094 3.346970e-01 9.858199e-01

1095 3.351318e-01 9.858199e-01

1096 3.355825e-01 9.858200e-01

1097 3.360497e-01 9.858200e-01

1098 3.365341e-01 9.858200e-01

1099 3.370360e-01 9.858201e-01

1100 3.375561e-01 9.858201e-01

1101 3.380950e-01 9.858201e-01

1102 3.386533e-01 9.858202e-01

1103 3.392315e-01 9.858202e-01

1104 3.398303e-01 9.858202e-01

1105 3.404502e-01 9.858202e-01

1106 3.410920e-01 9.858203e-01

1107 3.417561e-01 9.858203e-01

1108 3.424434e-01 9.858203e-01

1109 3.431544e-01 9.858204e-01

1110 3.438896e-01 9.858204e-01

1111 3.446499e-01 9.858204e-01

1112 3.454358e-01 9.858205e-01

1113 3.462479e-01 9.858205e-01

1114 3.470869e-01 9.858206e-01

1115 3.479533e-01 9.858206e-01

1116 3.488479e-01 9.858207e-01

1117 3.497711e-01 9.858207e-01

1118 3.507236e-01 9.858208e-01

1119 3.517059e-01 9.858208e-01

1120 3.527186e-01 9.858209e-01

1121 3.537622e-01 9.858210e-01

1122 3.548372e-01 9.858210e-01

1123 3.559439e-01 9.858211e-01

1124 3.570829e-01 9.858212e-01

1125 3.582546e-01 9.858213e-01

1126 3.594592e-01 9.858214e-01

1127 3.606971e-01 9.858215e-01

1128 3.619685e-01 9.858216e-01

1129 3.632736e-01 9.858217e-01

1130 3.646126e-01 9.858218e-01

1131 3.659856e-01 9.858219e-01

1132 3.673925e-01 9.858221e-01

1133 3.688334e-01 9.858222e-01

1134 3.703081e-01 9.858224e-01

1135 3.718164e-01 9.858226e-01

1136 3.733581e-01 9.858227e-01

1137 3.749329e-01 9.858229e-01

1138 3.765403e-01 9.858231e-01

1139 3.781799e-01 9.858234e-01

1140 3.798510e-01 9.858236e-01

1141 3.815531e-01 9.858238e-01

1142 3.832853e-01 9.858241e-01

1143 3.850469e-01 9.858244e-01

1144 3.868369e-01 9.858247e-01

1145 3.886543e-01 9.858250e-01

1146 3.904981e-01 9.858253e-01

1147 3.923671e-01 9.858257e-01

1148 3.942601e-01 9.858260e-01

1149 3.961756e-01 9.858265e-01

1150 3.981124e-01 9.858269e-01

1151 4.000690e-01 9.858273e-01

1152 4.020437e-01 9.858278e-01

1153 4.040351e-01 9.858284e-01

1154 4.060415e-01 9.858289e-01

1155 4.080610e-01 9.858295e-01

1156 4.100921e-01 9.858301e-01

1157 4.121328e-01 9.858308e-01

1158 4.141813e-01 9.858315e-01

1159 4.162358e-01 9.858323e-01

1160 4.182942e-01 9.858331e-01

1161 4.203548e-01 9.858340e-01

1162 4.224155e-01 9.858349e-01

1163 4.244744e-01 9.858359e-01

1164 4.265296e-01 9.858370e-01

1165 4.285792e-01 9.858381e-01

1166 4.306211e-01 9.858393e-01

1167 4.326536e-01 9.858406e-01

1168 4.346747e-01 9.858420e-01

1169 4.366827e-01 9.858434e-01

1170 4.386758e-01 9.858450e-01

1171 4.406522e-01 9.858466e-01

1172 4.426105e-01 9.858484e-01

1173 4.445490e-01 9.858503e-01

1174 4.464662e-01 9.858523e-01

1175 4.483607e-01 9.858545e-01

1176 4.502311e-01 9.858568e-01

1177 4.520762e-01 9.858593e-01

1178 4.538949e-01 9.858619e-01

1179 4.556859e-01 9.858647e-01

1180 4.574482e-01 9.858677e-01

1181 4.591809e-01 9.858709e-01

1182 4.608831e-01 9.858743e-01

1183 4.625541e-01 9.858779e-01

1184 4.641931e-01 9.858818e-01

1185 4.657995e-01 9.858859e-01

1186 4.673727e-01 9.858904e-01

1187 4.689124e-01 9.858951e-01

1188 4.704181e-01 9.859002e-01

1189 4.718895e-01 9.859056e-01

1190 4.733264e-01 9.859114e-01

1191 4.747286e-01 9.859176e-01

1192 4.760962e-01 9.859242e-01

1193 4.774290e-01 9.859313e-01

1194 4.787271e-01 9.859389e-01

1195 4.799907e-01 9.859471e-01

1196 4.812199e-01 9.859558e-01

1197 4.824150e-01 9.859651e-01

1198 4.835762e-01 9.859751e-01

1199 4.847040e-01 9.859858e-01

1200 4.857987e-01 9.859973e-01

1201 4.868607e-01 9.860096e-01

1202 4.878905e-01 9.860228e-01

1203 4.888887e-01 9.860369e-01

1204 4.898558e-01 9.860520e-01

1205 4.907924e-01 9.860683e-01

1206 4.916990e-01 9.860856e-01

1207 4.925763e-01 9.861043e-01

1208 4.934250e-01 9.861243e-01

1209 4.942457e-01 9.861457e-01

1210 4.950392e-01 9.861686e-01

1211 4.958059e-01 9.861932e-01

1212 4.965468e-01 9.862197e-01

1213 4.972623e-01 9.862480e-01

1214 4.979532e-01 9.862784e-01

1215 4.986202e-01 9.863111e-01

1216 4.992637e-01 9.863462e-01

1217 4.998845e-01 9.863839e-01

1218 5.004832e-01 9.864244e-01

1219 5.010604e-01 9.864680e-01

1220 5.016167e-01 9.865147e-01

1221 5.021527e-01 9.865650e-01

1222 5.026689e-01 9.866191e-01

1223 5.031660e-01 9.866772e-01

1224 5.036445e-01 9.867397e-01

1225 5.041049e-01 9.868069e-01

1226 5.045479e-01 9.868791e-01

1227 5.049739e-01 9.869568e-01

1228 5.053835e-01 9.870403e-01

1229 5.057773e-01 9.871300e-01

1230 5.061556e-01 9.872265e-01

1231 5.065191e-01 9.873303e-01

1232 5.068682e-01 9.874418e-01

1233 5.072033e-01 9.875616e-01

1234 5.075251e-01 9.876904e-01

1235 5.078338e-01 9.878289e-01

1236 5.081301e-01 9.879776e-01

1237 5.084142e-01 9.881374e-01

1238 5.086867e-01 9.883092e-01

1239 5.089479e-01 9.884938e-01

1240 5.091983e-01 9.886923e-01

1241 5.094382e-01 9.889057e-01

1242 5.096681e-01 9.891350e-01

1243 5.098883e-01 9.893817e-01

1244 5.100992e-01 9.896468e-01

1245 5.103010e-01 9.899318e-01

1246 5.104943e-01 9.902382e-01

1247 5.106792e-01 9.905674e-01

1248 5.108562e-01 9.909211e-01

1249 5.110255e-01 9.913010e-01

1250 5.111874e-01 9.917090e-01

1251 5.113423e-01 9.921469e-01

1252 5.114903e-01 9.926168e-01

1253 5.116318e-01 9.931208e-01

1254 5.117671e-01 9.936610e-01

1255 5.118963e-01 9.942398e-01

1256 5.120198e-01 9.948597e-01

1257 5.121378e-01 9.955229e-01

1258 5.122504e-01 9.962322e-01

1259 5.123580e-01 9.969902e-01

1260 5.124607e-01 9.977995e-01

1261 5.125588e-01 9.986630e-01

1262 5.126340e-01 9.986630e-01

1263 5.127122e-01 9.986630e-01

1264 5.127933e-01 9.986630e-01

1265 5.128775e-01 9.986630e-01

1266 5.129648e-01 9.986630e-01

1267 5.130554e-01 9.986630e-01

1268 5.131494e-01 9.986630e-01

1269 5.132469e-01 9.986630e-01

1270 5.133480e-01 9.986630e-01

1271 5.134528e-01 9.986630e-01

1272 5.135615e-01 9.986630e-01

1273 5.136742e-01 9.986630e-01

1274 5.137911e-01 9.986630e-01

1275 5.139122e-01 9.986630e-01

1276 5.140377e-01 9.986630e-01

1277 5.141678e-01 9.986630e-01

1278 5.143027e-01 9.986631e-01

1279 5.144424e-01 9.986631e-01

1280 5.145872e-01 9.986631e-01

1281 5.147373e-01 9.986631e-01

1282 5.148928e-01 9.986631e-01

1283 5.150539e-01 9.986631e-01

1284 5.152208e-01 9.986631e-01

1285 5.153937e-01 9.986631e-01

1286 5.155729e-01 9.986632e-01

1287 5.157585e-01 9.986632e-01

1288 5.159507e-01 9.986632e-01

1289 5.161499e-01 9.986632e-01

1290 5.163562e-01 9.986632e-01

1291 5.165699e-01 9.986632e-01

1292 5.167912e-01 9.986632e-01

1293 5.170205e-01 9.986633e-01

1294 5.172579e-01 9.986633e-01

1295 5.175038e-01 9.986633e-01

1296 5.177585e-01 9.986633e-01

1297 5.180222e-01 9.986633e-01

1298 5.182952e-01 9.986633e-01

1299 5.185780e-01 9.986634e-01

1300 5.188707e-01 9.986634e-01

1301 5.191738e-01 9.986634e-01

1302 5.194875e-01 9.986634e-01

1303 5.198123e-01 9.986634e-01

1304 5.201484e-01 9.986635e-01

1305 5.204964e-01 9.986635e-01

1306 5.208564e-01 9.986635e-01

1307 5.212291e-01 9.986635e-01

1308 5.216146e-01 9.986635e-01

1309 5.220134e-01 9.986636e-01

1310 5.224260e-01 9.986636e-01

1311 5.228528e-01 9.986636e-01

1312 5.232941e-01 9.986636e-01

1313 5.237505e-01 9.986637e-01

1314 5.242224e-01 9.986637e-01

1315 5.247101e-01 9.986637e-01

1316 5.252142e-01 9.986638e-01

1317 5.257352e-01 9.986638e-01

1318 5.262734e-01 9.986639e-01

1319 5.268293e-01 9.986639e-01

1320 5.274035e-01 9.986639e-01

1321 5.279963e-01 9.986640e-01

1322 5.286083e-01 9.986640e-01

1323 5.292399e-01 9.986641e-01

1324 5.298915e-01 9.986641e-01

1325 5.305637e-01 9.986642e-01

1326 5.312568e-01 9.986642e-01

1327 5.319713e-01 9.986643e-01

1328 5.327077e-01 9.986644e-01

1329 5.334664e-01 9.986644e-01

1330 5.342477e-01 9.986645e-01

1331 5.350522e-01 9.986646e-01

1332 5.358801e-01 9.986647e-01

1333 5.367318e-01 9.986648e-01

1334 5.376077e-01 9.986649e-01

1335 5.385080e-01 9.986650e-01

1336 5.394332e-01 9.986651e-01

1337 5.403834e-01 9.986652e-01

1338 5.413588e-01 9.986653e-01

1339 5.423598e-01 9.986654e-01

1340 5.433864e-01 9.986655e-01

1341 5.444387e-01 9.986657e-01

1342 5.455169e-01 9.986658e-01

1343 5.466210e-01 9.986660e-01

1344 5.477510e-01 9.986661e-01

1345 5.489069e-01 9.986663e-01

1346 5.500884e-01 9.986665e-01

1347 5.512956e-01 9.986667e-01

1348 5.525282e-01 9.986669e-01

1349 5.537859e-01 9.986671e-01

1350 5.550684e-01 9.986673e-01

1351 5.563754e-01 9.986676e-01

1352 5.577063e-01 9.986678e-01

1353 5.590607e-01 9.986681e-01

1354 5.604381e-01 9.986684e-01

1355 5.618377e-01 9.986687e-01

1356 5.632590e-01 9.986690e-01

1357 5.647011e-01 9.986694e-01

1358 5.661633e-01 9.986697e-01

1359 5.676446e-01 9.986701e-01

1360 5.691442e-01 9.986705e-01

1361 5.706611e-01 9.986710e-01

1362 5.721941e-01 9.986715e-01

1363 5.737422e-01 9.986720e-01

1364 5.753042e-01 9.986725e-01

1365 5.768790e-01 9.986730e-01

1366 5.784653e-01 9.986736e-01

1367 5.800617e-01 9.986743e-01

1368 5.816669e-01 9.986749e-01

1369 5.832796e-01 9.986756e-01

1370 5.848984e-01 9.986764e-01

1371 5.865218e-01 9.986772e-01

1372 5.881484e-01 9.986780e-01

1373 5.897768e-01 9.986789e-01

1374 5.914054e-01 9.986799e-01

1375 5.930327e-01 9.986809e-01

1376 5.946573e-01 9.986820e-01

1377 5.962778e-01 9.986831e-01

1378 5.978926e-01 9.986843e-01

1379 5.995002e-01 9.986856e-01

1380 6.010993e-01 9.986870e-01

1381 6.026884e-01 9.986885e-01

1382 6.042662e-01 9.986900e-01

1383 6.058313e-01 9.986916e-01

1384 6.073824e-01 9.986934e-01

1385 6.089182e-01 9.986952e-01

1386 6.104375e-01 9.986972e-01

1387 6.119391e-01 9.986993e-01

1388 6.134220e-01 9.987015e-01

1389 6.148850e-01 9.987039e-01

1390 6.163271e-01 9.987064e-01

1391 6.177475e-01 9.987090e-01

1392 6.191453e-01 9.987119e-01

1393 6.205196e-01 9.987149e-01

1394 6.218699e-01 9.987180e-01

1395 6.231954e-01 9.987214e-01

1396 6.244958e-01 9.987250e-01

1397 6.257704e-01 9.987288e-01

1398 6.270189e-01 9.987329e-01

1399 6.282410e-01 9.987372e-01

1400 6.294365e-01 9.987418e-01

1401 6.306050e-01 9.987466e-01

1402 6.317466e-01 9.987518e-01

1403 6.328610e-01 9.987573e-01

1404 6.339484e-01 9.987631e-01

1405 6.350087e-01 9.987693e-01

1406 6.360420e-01 9.987759e-01

1407 6.370485e-01 9.987829e-01

1408 6.380283e-01 9.987904e-01

1409 6.389816e-01 9.987983e-01

1410 6.399088e-01 9.988068e-01

1411 6.408101e-01 9.988158e-01

1412 6.416859e-01 9.988254e-01

1413 6.425364e-01 9.988357e-01

1414 6.433622e-01 9.988466e-01

1415 6.441635e-01 9.988582e-01

1416 6.449410e-01 9.988706e-01

1417 6.456949e-01 9.988839e-01

1418 6.464259e-01 9.988979e-01

1419 6.471344e-01 9.989130e-01

1420 6.478208e-01 9.989290e-01

1421 6.484858e-01 9.989461e-01

1422 6.491298e-01 9.989643e-01

1423 6.497534e-01 9.989837e-01

1424 6.503571e-01 9.990044e-01

1425 6.509414e-01 9.990265e-01

1426 6.515069e-01 9.990501e-01

1427 6.520541e-01 9.990752e-01

1428 6.525834e-01 9.991020e-01

1429 6.530954e-01 9.991306e-01

1430 6.535904e-01 9.991610e-01

1431 6.540689e-01 9.991936e-01

1432 6.545314e-01 9.992283e-01

1433 6.549783e-01 9.992655e-01

1434 6.554101e-01 9.993051e-01

1435 6.558270e-01 9.993475e-01

1436 6.562296e-01 9.993927e-01

1437 6.566183e-01 9.994411e-01

1438 6.569934e-01 9.994928e-01

1439 6.573553e-01 9.995481e-01

1440 6.577044e-01 9.996072e-01

1441 6.580411e-01 9.996705e-01

1442 6.580688e-01 9.996704e-01

1443 6.580974e-01 9.996704e-01

1444 6.581270e-01 9.996704e-01

1445 6.581577e-01 9.996704e-01

1446 6.581895e-01 9.996704e-01

1447 6.582223e-01 9.996704e-01

1448 6.582564e-01 9.996704e-01

1449 6.582916e-01 9.996703e-01

1450 6.583280e-01 9.996703e-01

1451 6.583657e-01 9.996703e-01

1452 6.584047e-01 9.996703e-01

1453 6.584451e-01 9.996703e-01

1454 6.584869e-01 9.996703e-01

1455 6.585301e-01 9.996703e-01

1456 6.585749e-01 9.996703e-01

1457 6.586212e-01 9.996703e-01

1458 6.586691e-01 9.996703e-01

1459 6.587186e-01 9.996703e-01

1460 6.587699e-01 9.996703e-01

1461 6.588229e-01 9.996703e-01

1462 6.588778e-01 9.996703e-01

1463 6.589345e-01 9.996703e-01

1464 6.589932e-01 9.996703e-01

1465 6.590540e-01 9.996703e-01

1466 6.591169e-01 9.996703e-01

1467 6.591819e-01 9.996703e-01

1468 6.592491e-01 9.996703e-01

1469 6.593187e-01 9.996703e-01

1470 6.593907e-01 9.996703e-01

1471 6.594652e-01 9.996703e-01

1472 6.595422e-01 9.996703e-01

1473 6.596219e-01 9.996703e-01

1474 6.597044e-01 9.996703e-01

1475 6.597897e-01 9.996703e-01

1476 6.598779e-01 9.996703e-01

1477 6.599692e-01 9.996703e-01

1478 6.600636e-01 9.996703e-01

1479 6.601613e-01 9.996703e-01

1480 6.602624e-01 9.996703e-01

1481 6.603670e-01 9.996703e-01

1482 6.604752e-01 9.996703e-01

1483 6.605871e-01 9.996703e-01

1484 6.607029e-01 9.996703e-01

1485 6.608227e-01 9.996703e-01

1486 6.609466e-01 9.996704e-01

1487 6.610748e-01 9.996704e-01

1488 6.612075e-01 9.996704e-01

1489 6.613447e-01 9.996704e-01

1490 6.614867e-01 9.996704e-01

1491 6.616336e-01 9.996704e-01

1492 6.617855e-01 9.996704e-01

1493 6.619427e-01 9.996704e-01

1494 6.621054e-01 9.996704e-01

1495 6.622736e-01 9.996704e-01

1496 6.624477e-01 9.996704e-01

1497 6.626277e-01 9.996705e-01

1498 6.628140e-01 9.996705e-01

1499 6.630066e-01 9.996705e-01

1500 6.632059e-01 9.996705e-01

1501 6.634120e-01 9.996705e-01

1502 6.636252e-01 9.996705e-01

1503 6.638457e-01 9.996705e-01

1504 6.640737e-01 9.996705e-01

1505 6.643096e-01 9.996706e-01

1506 6.645534e-01 9.996706e-01

1507 6.648056e-01 9.996706e-01

1508 6.650663e-01 9.996706e-01

1509 6.653359e-01 9.996706e-01

1510 6.656146e-01 9.996707e-01

1511 6.659026e-01 9.996707e-01

1512 6.662004e-01 9.996707e-01

1513 6.665081e-01 9.996707e-01

1514 6.668262e-01 9.996708e-01

1515 6.671548e-01 9.996708e-01

1516 6.674943e-01 9.996708e-01

1517 6.678450e-01 9.996708e-01

1518 6.682073e-01 9.996709e-01

1519 6.685815e-01 9.996709e-01

1520 6.689678e-01 9.996709e-01

1521 6.693667e-01 9.996710e-01

1522 6.697784e-01 9.996710e-01

1523 6.702034e-01 9.996711e-01

1524 6.706419e-01 9.996711e-01

1525 6.710943e-01 9.996711e-01

1526 6.715610e-01 9.996712e-01

1527 6.720422e-01 9.996712e-01

1528 6.725384e-01 9.996713e-01

1529 6.730499e-01 9.996714e-01

1530 6.735769e-01 9.996714e-01

1531 6.741199e-01 9.996715e-01

1532 6.746792e-01 9.996716e-01

1533 6.752550e-01 9.996716e-01

1534 6.758478e-01 9.996717e-01

1535 6.764578e-01 9.996718e-01

1536 6.770853e-01 9.996719e-01

1537 6.777307e-01 9.996720e-01

1538 6.783941e-01 9.996720e-01

1539 6.790758e-01 9.996721e-01

1540 6.797762e-01 9.996723e-01

1541 6.804953e-01 9.996724e-01

1542 6.812335e-01 9.996725e-01

1543 6.819909e-01 9.996726e-01

1544 6.827677e-01 9.996727e-01

1545 6.835640e-01 9.996729e-01

1546 6.843799e-01 9.996730e-01

1547 6.852156e-01 9.996732e-01

1548 6.860710e-01 9.996734e-01

1549 6.869462e-01 9.996735e-01

1550 6.878412e-01 9.996737e-01

1551 6.887560e-01 9.996739e-01

1552 6.896905e-01 9.996741e-01

1553 6.906445e-01 9.996744e-01

1554 6.916179e-01 9.996746e-01

1555 6.926105e-01 9.996749e-01

1556 6.936221e-01 9.996751e-01

1557 6.946524e-01 9.996754e-01

1558 6.957011e-01 9.996757e-01

1559 6.967678e-01 9.996760e-01

1560 6.978520e-01 9.996764e-01

1561 6.989534e-01 9.996767e-01

1562 7.000714e-01 9.996771e-01

1563 7.012055e-01 9.996775e-01

1564 7.023550e-01 9.996779e-01

1565 7.035194e-01 9.996784e-01

1566 7.046979e-01 9.996789e-01

1567 7.058898e-01 9.996794e-01

1568 7.070944e-01 9.996799e-01

1569 7.083107e-01 9.996805e-01

1570 7.095381e-01 9.996811e-01

1571 7.107755e-01 9.996818e-01

1572 7.120221e-01 9.996825e-01

1573 7.132769e-01 9.996832e-01

1574 7.145390e-01 9.996840e-01

1575 7.158072e-01 9.996848e-01

1576 7.170805e-01 9.996857e-01

1577 7.183580e-01 9.996866e-01

1578 7.196385e-01 9.996876e-01

1579 7.209209e-01 9.996886e-01

1580 7.222041e-01 9.996897e-01

1581 7.234870e-01 9.996909e-01

1582 7.247686e-01 9.996922e-01

1583 7.260476e-01 9.996935e-01

1584 7.273230e-01 9.996949e-01

1585 7.285937e-01 9.996964e-01

1586 7.298586e-01 9.996980e-01

1587 7.311166e-01 9.996996e-01

1588 7.323667e-01 9.997014e-01

1589 7.336079e-01 9.997033e-01

1590 7.348390e-01 9.997053e-01

1591 7.360592e-01 9.997074e-01

1592 7.372676e-01 9.997097e-01

1593 7.384632e-01 9.997121e-01

1594 7.396451e-01 9.997146e-01

1595 7.408125e-01 9.997173e-01

1596 7.419646e-01 9.997202e-01

1597 7.431007e-01 9.997233e-01

1598 7.442201e-01 9.997265e-01

1599 7.453221e-01 9.997299e-01

1600 7.464062e-01 9.997336e-01

1601 7.474717e-01 9.997374e-01

1602 7.485181e-01 9.997415e-01

1603 7.495451e-01 9.997459e-01

1604 7.505522e-01 9.997505e-01

1605 7.515390e-01 9.997554e-01

1606 7.525052e-01 9.997606e-01

1607 7.534507e-01 9.997661e-01

1608 7.543753e-01 9.997720e-01

1609 7.552789e-01 9.997782e-01

1610 7.561614e-01 9.997848e-01

1611 7.570229e-01 9.997918e-01

1612 7.578633e-01 9.997992e-01

1613 7.586828e-01 9.998071e-01

1614 7.594815e-01 9.998155e-01

1615 7.602595e-01 9.998244e-01

1616 7.610171e-01 9.998338e-01

1617 7.617545e-01 9.998438e-01

1618 7.624719e-01 9.998544e-01

1619 7.631696e-01 9.998657e-01

1620 7.638479e-01 9.998777e-01

1621 7.645072e-01 9.998904e-01

1622 7.645217e-01 9.998904e-01

1623 7.645366e-01 9.998904e-01

1624 7.645519e-01 9.998903e-01

1625 7.645676e-01 9.998903e-01

1626 7.645838e-01 9.998903e-01

1627 7.646003e-01 9.998903e-01

1628 7.646174e-01 9.998903e-01

1629 7.646348e-01 9.998903e-01

1630 7.646528e-01 9.998903e-01

1631 7.646712e-01 9.998903e-01

1632 7.646901e-01 9.998903e-01

1633 7.647096e-01 9.998903e-01

1634 7.647295e-01 9.998903e-01

1635 7.647500e-01 9.998903e-01

1636 7.647711e-01 9.998903e-01

1637 7.647927e-01 9.998903e-01

1638 7.648150e-01 9.998902e-01

1639 7.648378e-01 9.998902e-01

1640 7.648612e-01 9.998902e-01

1641 7.648853e-01 9.998902e-01

1642 7.649101e-01 9.998902e-01

1643 7.649355e-01 9.998902e-01

1644 7.649617e-01 9.998902e-01

1645 7.649885e-01 9.998902e-01

1646 7.650162e-01 9.998902e-01

1647 7.650445e-01 9.998902e-01

1648 7.650737e-01 9.998902e-01

1649 7.651037e-01 9.998902e-01

1650 7.651345e-01 9.998902e-01

1651 7.651662e-01 9.998902e-01

1652 7.651988e-01 9.998902e-01

1653 7.652323e-01 9.998902e-01

1654 7.652668e-01 9.998902e-01

1655 7.653023e-01 9.998902e-01

1656 7.653387e-01 9.998902e-01

1657 7.653762e-01 9.998902e-01

1658 7.654148e-01 9.998902e-01

1659 7.654545e-01 9.998902e-01

1660 7.654953e-01 9.998902e-01

1661 7.655373e-01 9.998902e-01

1662 7.655806e-01 9.998902e-01

1663 7.656251e-01 9.998902e-01

1664 7.656709e-01 9.998902e-01

1665 7.657180e-01 9.998902e-01

1666 7.657665e-01 9.998902e-01

1667 7.658164e-01 9.998903e-01

1668 7.658678e-01 9.998903e-01

1669 7.659207e-01 9.998903e-01

1670 7.659752e-01 9.998903e-01

1671 7.660313e-01 9.998903e-01

1672 7.660891e-01 9.998903e-01

1673 7.661486e-01 9.998903e-01

1674 7.662098e-01 9.998903e-01

1675 7.662729e-01 9.998903e-01

1676 7.663379e-01 9.998903e-01

1677 7.664048e-01 9.998903e-01

1678 7.664738e-01 9.998903e-01

1679 7.665448e-01 9.998903e-01

1680 7.666180e-01 9.998903e-01

1681 7.666933e-01 9.998903e-01

1682 7.667710e-01 9.998903e-01

1683 7.668510e-01 9.998903e-01

1684 7.669334e-01 9.998903e-01

1685 7.670184e-01 9.998903e-01

1686 7.671059e-01 9.998903e-01

1687 7.671961e-01 9.998903e-01

1688 7.672890e-01 9.998904e-01

1689 7.673848e-01 9.998904e-01

1690 7.674835e-01 9.998904e-01

1691 7.675852e-01 9.998904e-01

1692 7.676900e-01 9.998904e-01

1693 7.677980e-01 9.998904e-01

1694 7.679093e-01 9.998904e-01

1695 7.680240e-01 9.998904e-01

1696 7.681423e-01 9.998904e-01

1697 7.682641e-01 9.998904e-01

1698 7.683897e-01 9.998904e-01

1699 7.685191e-01 9.998905e-01

1700 7.686524e-01 9.998905e-01

1701 7.687898e-01 9.998905e-01

1702 7.689315e-01 9.998905e-01

1703 7.690774e-01 9.998905e-01

1704 7.692277e-01 9.998905e-01

1705 7.693826e-01 9.998905e-01

1706 7.695423e-01 9.998906e-01

1707 7.697067e-01 9.998906e-01

1708 7.698761e-01 9.998906e-01

1709 7.700506e-01 9.998906e-01

1710 7.702304e-01 9.998906e-01

1711 7.704155e-01 9.998907e-01

1712 7.706062e-01 9.998907e-01

1713 7.708026e-01 9.998907e-01

1714 7.710047e-01 9.998907e-01

1715 7.712129e-01 9.998907e-01

1716 7.714272e-01 9.998908e-01

1717 7.716478e-01 9.998908e-01

1718 7.718748e-01 9.998908e-01

1719 7.721084e-01 9.998909e-01

1720 7.723487e-01 9.998909e-01

1721 7.725960e-01 9.998909e-01

1722 7.728503e-01 9.998910e-01

1723 7.731118e-01 9.998910e-01

1724 7.733807e-01 9.998910e-01

1725 7.736572e-01 9.998911e-01

1726 7.739413e-01 9.998911e-01

1727 7.742332e-01 9.998912e-01

1728 7.745331e-01 9.998912e-01

1729 7.748411e-01 9.998913e-01

1730 7.751574e-01 9.998913e-01

1731 7.754821e-01 9.998914e-01

1732 7.758153e-01 9.998914e-01

1733 7.761572e-01 9.998915e-01

1734 7.765078e-01 9.998916e-01

1735 7.768673e-01 9.998916e-01

1736 7.772358e-01 9.998917e-01

1737 7.776135e-01 9.998918e-01

1738 7.780003e-01 9.998919e-01

1739 7.783963e-01 9.998920e-01

1740 7.788017e-01 9.998921e-01

1741 7.792165e-01 9.998922e-01

1742 7.796407e-01 9.998923e-01

1743 7.800744e-01 9.998924e-01

1744 7.805176e-01 9.998925e-01

1745 7.809703e-01 9.998926e-01

1746 7.814325e-01 9.998927e-01

1747 7.819041e-01 9.998929e-01

1748 7.823852e-01 9.998930e-01

1749 7.828756e-01 9.998931e-01

1750 7.833752e-01 9.998933e-01

1751 7.838841e-01 9.998935e-01

1752 7.844020e-01 9.998936e-01

1753 7.849289e-01 9.998938e-01

1754 7.854645e-01 9.998940e-01

1755 7.860087e-01 9.998942e-01

1756 7.865614e-01 9.998944e-01

1757 7.871221e-01 9.998947e-01

1758 7.876908e-01 9.998949e-01

1759 7.882672e-01 9.998952e-01

1760 7.888509e-01 9.998954e-01

1761 7.894417e-01 9.998957e-01

1762 7.900392e-01 9.998960e-01

1763 7.906431e-01 9.998963e-01

1764 7.912529e-01 9.998966e-01

1765 7.918684e-01 9.998970e-01

1766 7.924891e-01 9.998974e-01

1767 7.931145e-01 9.998978e-01

1768 7.937442e-01 9.998982e-01

1769 7.943777e-01 9.998986e-01

1770 7.950146e-01 9.998991e-01

1771 7.956544e-01 9.998996e-01

1772 7.962965e-01 9.999001e-01

1773 7.969404e-01 9.999006e-01

1774 7.975856e-01 9.999012e-01

1775 7.982315e-01 9.999018e-01

1776 7.988777e-01 9.999025e-01

1777 7.995235e-01 9.999031e-01

1778 8.001683e-01 9.999039e-01

1779 8.008117e-01 9.999046e-01

1780 8.014531e-01 9.999054e-01

1781 8.020919e-01 9.999063e-01

1782 8.027276e-01 9.999072e-01

1783 8.033597e-01 9.999081e-01

1784 8.039875e-01 9.999091e-01

1785 8.046107e-01 9.999102e-01

1786 8.052286e-01 9.999113e-01

1787 8.058408e-01 9.999124e-01

1788 8.064468e-01 9.999137e-01

1789 8.070462e-01 9.999150e-01

1790 8.076385e-01 9.999164e-01

1791 8.082232e-01 9.999179e-01

1792 8.088001e-01 9.999194e-01

1793 8.093686e-01 9.999210e-01

1794 8.099285e-01 9.999228e-01

1795 8.104794e-01 9.999246e-01

1796 8.110210e-01 9.999265e-01

1797 8.115530e-01 9.999286e-01

1798 8.120752e-01 9.999307e-01

1799 8.125873e-01 9.999330e-01

1800 8.130891e-01 9.999354e-01

1801 8.135805e-01 9.999379e-01

1802 8.135866e-01 9.999379e-01

1803 8.135925e-01 9.999379e-01

1804 8.135983e-01 9.999379e-01

1805 8.136039e-01 9.999380e-01

1806 8.136094e-01 9.999380e-01

1807 8.136148e-01 9.999380e-01

1808 8.136200e-01 9.999380e-01

1809 8.136250e-01 9.999380e-01

1810 8.136300e-01 9.999380e-01

1811 8.136348e-01 9.999380e-01

1812 8.136394e-01 9.999380e-01

1813 8.136440e-01 9.999380e-01

1814 8.136484e-01 9.999380e-01

1815 8.136527e-01 9.999380e-01

1816 8.136568e-01 9.999380e-01

1817 8.136609e-01 9.999380e-01

1818 8.136648e-01 9.999380e-01

1819 8.136686e-01 9.999380e-01

1820 8.136723e-01 9.999380e-01

1821 8.136758e-01 9.999380e-01

1822 8.136793e-01 9.999380e-01

1823 8.136826e-01 9.999380e-01

1824 8.136858e-01 9.999380e-01

1825 8.136888e-01 9.999380e-01

1826 8.136918e-01 9.999380e-01

1827 8.136946e-01 9.999380e-01

1828 8.136973e-01 9.999380e-01

1829 8.136998e-01 9.999380e-01

1830 8.137022e-01 9.999380e-01

1831 8.137045e-01 9.999380e-01

1832 8.137067e-01 9.999380e-01

1833 8.137087e-01 9.999380e-01

1834 8.137105e-01 9.999380e-01

1835 8.137122e-01 9.999380e-01

1836 8.137137e-01 9.999380e-01

1837 8.137151e-01 9.999380e-01

1838 8.137163e-01 9.999380e-01

1839 8.137173e-01 9.999380e-01

1840 8.137182e-01 9.999380e-01

1841 8.137189e-01 9.999380e-01

1842 8.137193e-01 9.999380e-01

1843 8.137196e-01 9.999380e-01

1844 8.137197e-01 9.999380e-01

1845 8.137196e-01 9.999380e-01

1846 8.137192e-01 9.999380e-01

1847 8.137186e-01 9.999380e-01

1848 8.137178e-01 9.999380e-01

1849 8.137167e-01 9.999380e-01

1850 8.137153e-01 9.999380e-01

1851 8.137137e-01 9.999380e-01

1852 8.137118e-01 9.999380e-01

1853 8.137096e-01 9.999380e-01

1854 8.137071e-01 9.999380e-01

1855 8.137042e-01 9.999380e-01

1856 8.137010e-01 9.999380e-01

1857 8.136975e-01 9.999380e-01

1858 8.136936e-01 9.999380e-01

1859 8.136893e-01 9.999380e-01

1860 8.136846e-01 9.999380e-01

1861 8.136795e-01 9.999380e-01

1862 8.136739e-01 9.999380e-01

1863 8.136679e-01 9.999380e-01

1864 8.136614e-01 9.999380e-01

1865 8.136544e-01 9.999380e-01

1866 8.136469e-01 9.999380e-01

1867 8.136388e-01 9.999380e-01

1868 8.136302e-01 9.999380e-01

1869 8.136210e-01 9.999380e-01

1870 8.136111e-01 9.999380e-01

1871 8.136006e-01 9.999380e-01

1872 8.135894e-01 9.999380e-01

1873 8.135775e-01 9.999380e-01

1874 8.135648e-01 9.999380e-01

1875 8.135514e-01 9.999380e-01

1876 8.135371e-01 9.999380e-01

1877 8.135220e-01 9.999380e-01

1878 8.135061e-01 9.999380e-01

1879 8.134892e-01 9.999380e-01

1880 8.134713e-01 9.999380e-01

1881 8.134525e-01 9.999380e-01

1882 8.134326e-01 9.999380e-01

1883 8.134116e-01 9.999380e-01

1884 8.133894e-01 9.999380e-01

1885 8.133661e-01 9.999380e-01

1886 8.133416e-01 9.999380e-01

1887 8.133158e-01 9.999380e-01

1888 8.132886e-01 9.999380e-01

1889 8.132600e-01 9.999380e-01

1890 8.132300e-01 9.999380e-01

1891 8.131985e-01 9.999380e-01

1892 8.131654e-01 9.999380e-01

1893 8.131307e-01 9.999380e-01

1894 8.130943e-01 9.999380e-01

1895 8.130562e-01 9.999380e-01

1896 8.130162e-01 9.999380e-01

1897 8.129743e-01 9.999380e-01

1898 8.129304e-01 9.999380e-01

1899 8.128845e-01 9.999380e-01

1900 8.128365e-01 9.999380e-01

1901 8.127862e-01 9.999380e-01

1902 8.127337e-01 9.999380e-01

1903 8.126788e-01 9.999380e-01

1904 8.126215e-01 9.999380e-01

1905 8.125617e-01 9.999380e-01

1906 8.124992e-01 9.999380e-01

1907 8.124340e-01 9.999380e-01

1908 8.123661e-01 9.999380e-01

1909 8.122952e-01 9.999380e-01

1910 8.122214e-01 9.999380e-01

1911 8.121445e-01 9.999380e-01

1912 8.120644e-01 9.999380e-01

1913 8.119811e-01 9.999380e-01

1914 8.118944e-01 9.999380e-01

1915 8.118043e-01 9.999380e-01

1916 8.117106e-01 9.999380e-01

1917 8.116132e-01 9.999380e-01

1918 8.115122e-01 9.999380e-01

1919 8.114073e-01 9.999380e-01

1920 8.112984e-01 9.999380e-01

1921 8.111855e-01 9.999380e-01

1922 8.110686e-01 9.999380e-01

1923 8.109474e-01 9.999380e-01

1924 8.108219e-01 9.999380e-01

1925 8.106920e-01 9.999380e-01

1926 8.105577e-01 9.999380e-01

1927 8.104189e-01 9.999380e-01

1928 8.102755e-01 9.999380e-01

1929 8.101274e-01 9.999380e-01

1930 8.099745e-01 9.999380e-01

1931 8.098169e-01 9.999380e-01

1932 8.096544e-01 9.999380e-01

1933 8.094871e-01 9.999380e-01

1934 8.093148e-01 9.999380e-01

1935 8.091376e-01 9.999380e-01

1936 8.089553e-01 9.999380e-01

1937 8.087682e-01 9.999380e-01

1938 8.085760e-01 9.999380e-01

1939 8.083788e-01 9.999380e-01

1940 8.081767e-01 9.999380e-01

1941 8.079697e-01 9.999380e-01

1942 8.077577e-01 9.999380e-01

1943 8.075409e-01 9.999380e-01

1944 8.073193e-01 9.999380e-01

1945 8.070930e-01 9.999380e-01

1946 8.068621e-01 9.999380e-01

1947 8.066266e-01 9.999380e-01

1948 8.063867e-01 9.999380e-01

1949 8.061425e-01 9.999380e-01

1950 8.058941e-01 9.999380e-01

1951 8.056417e-01 9.999380e-01

1952 8.053855e-01 9.999380e-01

1953 8.051256e-01 9.999380e-01

1954 8.048621e-01 9.999380e-01

1955 8.045954e-01 9.999380e-01

1956 8.043255e-01 9.999380e-01

1957 8.040527e-01 9.999380e-01

1958 8.037773e-01 9.999380e-01

1959 8.034994e-01 9.999380e-01

1960 8.032193e-01 9.999379e-01

1961 8.029373e-01 9.999379e-01

1962 8.026536e-01 9.999379e-01

1963 8.023684e-01 9.999379e-01

1964 8.020821e-01 9.999379e-01

1965 8.017949e-01 9.999379e-01

1966 8.015071e-01 9.999379e-01

1967 8.012189e-01 9.999379e-01

1968 8.009306e-01 9.999378e-01

1969 8.006426e-01 9.999378e-01

1970 8.003550e-01 9.999378e-01

1971 8.000681e-01 9.999378e-01

1972 7.997823e-01 9.999377e-01

1973 7.994977e-01 9.999377e-01

1974 7.992147e-01 9.999377e-01

1975 7.989334e-01 9.999377e-01

1976 7.986541e-01 9.999376e-01

1977 7.983771e-01 9.999376e-01

1978 7.981026e-01 9.999375e-01

1979 7.978308e-01 9.999375e-01

1980 7.975618e-01 9.999374e-01

1981 7.972960e-01 9.999374e-01

1982 7.972838e-01 9.999374e-01

1983 7.972708e-01 9.999374e-01

1984 7.972571e-01 9.999374e-01

1985 7.972425e-01 9.999374e-01

1986 7.972271e-01 9.999375e-01

1987 7.972109e-01 9.999375e-01

1988 7.971940e-01 9.999375e-01

1989 7.971761e-01 9.999375e-01

1990 7.971575e-01 9.999375e-01

1991 7.971380e-01 9.999375e-01

1992 7.971177e-01 9.999375e-01

1993 7.970964e-01 9.999375e-01

1994 7.970743e-01 9.999375e-01

1995 7.970513e-01 9.999375e-01

1996 7.970274e-01 9.999375e-01

1997 7.970025e-01 9.999375e-01

1998 7.969767e-01 9.999375e-01

1999 7.969499e-01 9.999375e-01

2000 7.969221e-01 9.999375e-01

2001 7.968933e-01 9.999375e-01

2002 7.968635e-01 9.999375e-01

2003 7.968326e-01 9.999375e-01

2004 7.968005e-01 9.999375e-01

2005 7.967674e-01 9.999375e-01

2006 7.967331e-01 9.999375e-01

2007 7.966976e-01 9.999375e-01

2008 7.966609e-01 9.999375e-01

2009 7.966230e-01 9.999375e-01

2010 7.965838e-01 9.999375e-01

2011 7.965432e-01 9.999375e-01

2012 7.965013e-01 9.999375e-01

2013 7.964580e-01 9.999375e-01

2014 7.964133e-01 9.999375e-01

2015 7.963671e-01 9.999375e-01

2016 7.963193e-01 9.999375e-01

2017 7.962700e-01 9.999375e-01

2018 7.962190e-01 9.999375e-01

2019 7.961664e-01 9.999375e-01

2020 7.961121e-01 9.999375e-01

2021 7.960559e-01 9.999375e-01

2022 7.959980e-01 9.999375e-01

2023 7.959381e-01 9.999375e-01

2024 7.958763e-01 9.999375e-01

2025 7.958125e-01 9.999375e-01

2026 7.957466e-01 9.999375e-01

2027 7.956785e-01 9.999375e-01

2028 7.956082e-01 9.999375e-01

2029 7.955356e-01 9.999375e-01

2030 7.954607e-01 9.999375e-01

2031 7.953833e-01 9.999375e-01

2032 7.953034e-01 9.999375e-01

2033 7.952209e-01 9.999375e-01

2034 7.951356e-01 9.999374e-01

2035 7.950476e-01 9.999374e-01

2036 7.949567e-01 9.999374e-01

2037 7.948629e-01 9.999374e-01

2038 7.947659e-01 9.999374e-01

2039 7.946658e-01 9.999374e-01

2040 7.945624e-01 9.999374e-01

2041 7.944557e-01 9.999374e-01

2042 7.943454e-01 9.999374e-01

2043 7.942315e-01 9.999374e-01

2044 7.941139e-01 9.999374e-01

2045 7.939924e-01 9.999374e-01

2046 7.938669e-01 9.999374e-01

2047 7.937373e-01 9.999374e-01

2048 7.936035e-01 9.999374e-01

2049 7.934653e-01 9.999374e-01

2050 7.933226e-01 9.999374e-01

2051 7.931752e-01 9.999374e-01

2052 7.930229e-01 9.999374e-01

2053 7.928657e-01 9.999373e-01

2054 7.927033e-01 9.999373e-01

2055 7.925357e-01 9.999373e-01

2056 7.923626e-01 9.999373e-01

2057 7.921838e-01 9.999373e-01

2058 7.919992e-01 9.999373e-01

2059 7.918086e-01 9.999373e-01

2060 7.916118e-01 9.999373e-01

2061 7.914087e-01 9.999373e-01

2062 7.911990e-01 9.999373e-01

2063 7.909825e-01 9.999372e-01

2064 7.907591e-01 9.999372e-01

2065 7.905285e-01 9.999372e-01

2066 7.902906e-01 9.999372e-01

2067 7.900450e-01 9.999372e-01

2068 7.897917e-01 9.999372e-01

2069 7.895303e-01 9.999371e-01

2070 7.892607e-01 9.999371e-01

2071 7.889826e-01 9.999371e-01

2072 7.886959e-01 9.999371e-01

2073 7.884002e-01 9.999371e-01

2074 7.880954e-01 9.999371e-01

2075 7.877811e-01 9.999370e-01

2076 7.874573e-01 9.999370e-01

2077 7.871236e-01 9.999370e-01

2078 7.867799e-01 9.999370e-01

2079 7.864258e-01 9.999369e-01

2080 7.860612e-01 9.999369e-01

2081 7.856859e-01 9.999369e-01

2082 7.852995e-01 9.999368e-01

2083 7.849019e-01 9.999368e-01

2084 7.844929e-01 9.999368e-01

2085 7.840722e-01 9.999367e-01

2086 7.836397e-01 9.999367e-01

2087 7.831951e-01 9.999367e-01

2088 7.827382e-01 9.999366e-01

2089 7.822689e-01 9.999366e-01

2090 7.817870e-01 9.999365e-01

2091 7.812923e-01 9.999365e-01

2092 7.807847e-01 9.999364e-01

2093 7.802640e-01 9.999364e-01

2094 7.797301e-01 9.999363e-01

2095 7.791829e-01 9.999363e-01

2096 7.786223e-01 9.999362e-01

2097 7.780482e-01 9.999361e-01

2098 7.774606e-01 9.999361e-01

2099 7.768595e-01 9.999360e-01

2100 7.762448e-01 9.999359e-01

2101 7.756165e-01 9.999358e-01

2102 7.749748e-01 9.999358e-01

2103 7.743195e-01 9.999357e-01

2104 7.736509e-01 9.999356e-01

2105 7.729691e-01 9.999355e-01

2106 7.722742e-01 9.999354e-01

2107 7.715663e-01 9.999353e-01

2108 7.708458e-01 9.999352e-01

2109 7.701128e-01 9.999350e-01

2110 7.693675e-01 9.999349e-01

2111 7.686104e-01 9.999348e-01

2112 7.678417e-01 9.999346e-01

2113 7.670619e-01 9.999345e-01

2114 7.662713e-01 9.999343e-01

2115 7.654703e-01 9.999342e-01

2116 7.646595e-01 9.999340e-01

2117 7.638393e-01 9.999338e-01

2118 7.630103e-01 9.999336e-01

2119 7.621730e-01 9.999334e-01

2120 7.613281e-01 9.999332e-01

2121 7.604762e-01 9.999330e-01

2122 7.596178e-01 9.999327e-01

2123 7.587538e-01 9.999325e-01

2124 7.578848e-01 9.999322e-01

2125 7.570116e-01 9.999319e-01

2126 7.561348e-01 9.999316e-01

2127 7.552552e-01 9.999313e-01

2128 7.543737e-01 9.999310e-01

2129 7.534910e-01 9.999306e-01

2130 7.526079e-01 9.999303e-01

2131 7.517252e-01 9.999299e-01

2132 7.508437e-01 9.999295e-01

2133 7.499643e-01 9.999290e-01

2134 7.490877e-01 9.999286e-01

2135 7.482147e-01 9.999281e-01

2136 7.473462e-01 9.999276e-01

2137 7.464829e-01 9.999271e-01

2138 7.456255e-01 9.999265e-01

2139 7.447749e-01 9.999259e-01

2140 7.439318e-01 9.999253e-01

2141 7.430968e-01 9.999246e-01

2142 7.422707e-01 9.999239e-01

2143 7.414540e-01 9.999232e-01

2144 7.406476e-01 9.999224e-01

2145 7.398518e-01 9.999216e-01

2146 7.390673e-01 9.999207e-01

2147 7.382947e-01 9.999198e-01

2148 7.375344e-01 9.999188e-01

2149 7.367868e-01 9.999178e-01

2150 7.360525e-01 9.999167e-01

2151 7.353317e-01 9.999155e-01

2152 7.346249e-01 9.999143e-01

2153 7.339323e-01 9.999131e-01

2154 7.332542e-01 9.999117e-01

2155 7.325908e-01 9.999103e-01

2156 7.319424e-01 9.999088e-01

2157 7.313090e-01 9.999072e-01

2158 7.306909e-01 9.999055e-01

2159 7.300880e-01 9.999038e-01

2160 7.295005e-01 9.999019e-01

2161 7.289283e-01 9.998999e-01

2162 7.288821e-01 9.998999e-01

2163 7.288346e-01 9.998999e-01

2164 7.287857e-01 9.998999e-01

2165 7.287353e-01 9.998999e-01

2166 7.286836e-01 9.998999e-01

2167 7.286304e-01 9.998999e-01

2168 7.285758e-01 9.998999e-01

2169 7.285196e-01 9.998999e-01

2170 7.284619e-01 9.998999e-01

2171 7.284026e-01 9.998999e-01

2172 7.283417e-01 9.998999e-01

2173 7.282792e-01 9.998999e-01

2174 7.282150e-01 9.998999e-01

2175 7.281490e-01 9.998999e-01

2176 7.280813e-01 9.998999e-01

2177 7.280117e-01 9.998999e-01

2178 7.279403e-01 9.998999e-01

2179 7.278670e-01 9.998999e-01

2180 7.277917e-01 9.998999e-01

2181 7.277145e-01 9.998999e-01

2182 7.276351e-01 9.998999e-01

2183 7.275536e-01 9.998999e-01

2184 7.274700e-01 9.998999e-01

2185 7.273841e-01 9.998999e-01

2186 7.272960e-01 9.998999e-01

2187 7.272054e-01 9.998999e-01

2188 7.271125e-01 9.998999e-01

2189 7.270171e-01 9.998999e-01

2190 7.269191e-01 9.998999e-01

2191 7.268185e-01 9.998999e-01

2192 7.267152e-01 9.998999e-01

2193 7.266092e-01 9.998999e-01

2194 7.265002e-01 9.998999e-01

2195 7.263884e-01 9.998999e-01

2196 7.262736e-01 9.998999e-01

2197 7.261556e-01 9.998999e-01

2198 7.260345e-01 9.998999e-01

2199 7.259102e-01 9.998999e-01

2200 7.257825e-01 9.998998e-01

2201 7.256513e-01 9.998998e-01

2202 7.255166e-01 9.998998e-01

2203 7.253783e-01 9.998998e-01

2204 7.252362e-01 9.998998e-01

2205 7.250903e-01 9.998998e-01

2206 7.249405e-01 9.998998e-01

2207 7.247866e-01 9.998998e-01

2208 7.246285e-01 9.998998e-01

2209 7.244662e-01 9.998998e-01

2210 7.242995e-01 9.998998e-01

2211 7.241283e-01 9.998998e-01

2212 7.239524e-01 9.998998e-01

2213 7.237718e-01 9.998997e-01

2214 7.235864e-01 9.998997e-01

2215 7.233959e-01 9.998997e-01

2216 7.232003e-01 9.998997e-01

2217 7.229995e-01 9.998997e-01

2218 7.227932e-01 9.998997e-01

2219 7.225814e-01 9.998997e-01

2220 7.223639e-01 9.998997e-01

2221 7.221406e-01 9.998997e-01

2222 7.219113e-01 9.998996e-01

2223 7.216759e-01 9.998996e-01

2224 7.214343e-01 9.998996e-01

2225 7.211862e-01 9.998996e-01

2226 7.209315e-01 9.998996e-01

2227 7.206702e-01 9.998996e-01

2228 7.204019e-01 9.998995e-01

2229 7.201266e-01 9.998995e-01

2230 7.198441e-01 9.998995e-01

2231 7.195543e-01 9.998995e-01

2232 7.192569e-01 9.998995e-01

2233 7.189519e-01 9.998994e-01

2234 7.186390e-01 9.998994e-01

2235 7.183181e-01 9.998994e-01

2236 7.179891e-01 9.998994e-01

2237 7.176517e-01 9.998993e-01

2238 7.173059e-01 9.998993e-01

2239 7.169514e-01 9.998993e-01

2240 7.165882e-01 9.998992e-01

2241 7.162160e-01 9.998992e-01

2242 7.158347e-01 9.998992e-01

2243 7.154442e-01 9.998991e-01

2244 7.150444e-01 9.998991e-01

2245 7.146350e-01 9.998991e-01

2246 7.142159e-01 9.998990e-01

2247 7.137871e-01 9.998990e-01

2248 7.133485e-01 9.998989e-01

2249 7.128998e-01 9.998989e-01

2250 7.124410e-01 9.998988e-01

2251 7.119720e-01 9.998988e-01

2252 7.114928e-01 9.998987e-01

2253 7.110032e-01 9.998987e-01

2254 7.105032e-01 9.998986e-01

2255 7.099927e-01 9.998986e-01

2256 7.094717e-01 9.998985e-01

2257 7.089402e-01 9.998984e-01

2258 7.083981e-01 9.998984e-01

2259 7.078455e-01 9.998983e-01

2260 7.072823e-01 9.998982e-01

2261 7.067087e-01 9.998981e-01

2262 7.061247e-01 9.998980e-01

2263 7.055303e-01 9.998979e-01

2264 7.049256e-01 9.998978e-01

2265 7.043109e-01 9.998977e-01

2266 7.036861e-01 9.998976e-01

2267 7.030514e-01 9.998975e-01

2268 7.024071e-01 9.998974e-01

2269 7.017533e-01 9.998973e-01

2270 7.010902e-01 9.998972e-01

2271 7.004182e-01 9.998970e-01

2272 6.997374e-01 9.998969e-01

2273 6.990482e-01 9.998967e-01

2274 6.983509e-01 9.998966e-01

2275 6.976459e-01 9.998964e-01

2276 6.969335e-01 9.998963e-01

2277 6.962141e-01 9.998961e-01

2278 6.954881e-01 9.998959e-01

2279 6.947560e-01 9.998957e-01

2280 6.940183e-01 9.998955e-01

2281 6.932754e-01 9.998953e-01

2282 6.925279e-01 9.998950e-01

2283 6.917762e-01 9.998948e-01

2284 6.910210e-01 9.998945e-01

2285 6.902628e-01 9.998943e-01

2286 6.895022e-01 9.998940e-01

2287 6.887398e-01 9.998937e-01

2288 6.879762e-01 9.998934e-01

2289 6.872120e-01 9.998930e-01

2290 6.864480e-01 9.998927e-01

2291 6.856846e-01 9.998923e-01

2292 6.849227e-01 9.998919e-01

2293 6.841628e-01 9.998915e-01

2294 6.834056e-01 9.998911e-01

2295 6.826517e-01 9.998906e-01

2296 6.819018e-01 9.998902e-01

2297 6.811566e-01 9.998897e-01

2298 6.804167e-01 9.998891e-01

2299 6.796827e-01 9.998886e-01

2300 6.789553e-01 9.998880e-01

2301 6.782350e-01 9.998874e-01

2302 6.775224e-01 9.998867e-01

2303 6.768182e-01 9.998861e-01

2304 6.761228e-01 9.998854e-01

2305 6.754367e-01 9.998846e-01

2306 6.747606e-01 9.998838e-01

2307 6.740947e-01 9.998830e-01

2308 6.734397e-01 9.998821e-01

2309 6.727959e-01 9.998812e-01

2310 6.721637e-01 9.998802e-01

2311 6.715435e-01 9.998792e-01

2312 6.709356e-01 9.998781e-01

2313 6.703402e-01 9.998769e-01

2314 6.697578e-01 9.998757e-01

2315 6.691884e-01 9.998745e-01

2316 6.686322e-01 9.998731e-01

2317 6.680896e-01 9.998717e-01

2318 6.675605e-01 9.998702e-01

2319 6.670451e-01 9.998687e-01

2320 6.665435e-01 9.998670e-01

2321 6.660557e-01 9.998653e-01

2322 6.655818e-01 9.998635e-01

2323 6.651216e-01 9.998616e-01

2324 6.646752e-01 9.998595e-01

2325 6.642425e-01 9.998574e-01

2326 6.638235e-01 9.998552e-01

2327 6.634179e-01 9.998528e-01

2328 6.630257e-01 9.998503e-01

2329 6.626467e-01 9.998477e-01

2330 6.622808e-01 9.998449e-01

2331 6.619277e-01 9.998420e-01

2332 6.615872e-01 9.998389e-01

2333 6.612591e-01 9.998357e-01

2334 6.609432e-01 9.998323e-01

2335 6.606393e-01 9.998287e-01

2336 6.603470e-01 9.998249e-01

2337 6.600661e-01 9.998209e-01

2338 6.597963e-01 9.998166e-01

2339 6.595374e-01 9.998122e-01

2340 6.592891e-01 9.998075e-01

2341 6.590510e-01 9.998026e-01

2342 6.589957e-01 9.998026e-01

2343 6.589393e-01 9.998026e-01

2344 6.588819e-01 9.998026e-01

2345 6.588235e-01 9.998026e-01

2346 6.587641e-01 9.998026e-01

2347 6.587036e-01 9.998026e-01

2348 6.586421e-01 9.998026e-01

2349 6.585796e-01 9.998026e-01

2350 6.585160e-01 9.998026e-01

2351 6.584513e-01 9.998026e-01

2352 6.583855e-01 9.998026e-01

2353 6.583187e-01 9.998026e-01

2354 6.582508e-01 9.998025e-01

2355 6.581818e-01 9.998025e-01

2356 6.581116e-01 9.998025e-01

2357 6.580403e-01 9.998025e-01

2358 6.579679e-01 9.998025e-01

2359 6.578944e-01 9.998025e-01

2360 6.578197e-01 9.998025e-01

2361 6.577439e-01 9.998025e-01

2362 6.576668e-01 9.998025e-01

2363 6.575886e-01 9.998025e-01

2364 6.575092e-01 9.998025e-01

2365 6.574286e-01 9.998025e-01

2366 6.573468e-01 9.998025e-01

2367 6.572638e-01 9.998025e-01

2368 6.571795e-01 9.998025e-01

2369 6.570941e-01 9.998025e-01

2370 6.570073e-01 9.998024e-01

2371 6.569194e-01 9.998024e-01

2372 6.568302e-01 9.998024e-01

2373 6.567397e-01 9.998024e-01

2374 6.566480e-01 9.998024e-01

2375 6.565551e-01 9.998024e-01

2376 6.564608e-01 9.998024e-01

2377 6.563653e-01 9.998024e-01

2378 6.562686e-01 9.998024e-01

2379 6.561706e-01 9.998024e-01

2380 6.560713e-01 9.998023e-01

2381 6.559708e-01 9.998023e-01

2382 6.558690e-01 9.998023e-01

2383 6.557660e-01 9.998023e-01

2384 6.556618e-01 9.998023e-01

2385 6.555564e-01 9.998023e-01

2386 6.554497e-01 9.998023e-01

2387 6.553419e-01 9.998022e-01

2388 6.552328e-01 9.998022e-01

2389 6.551226e-01 9.998022e-01

2390 6.550113e-01 9.998022e-01

2391 6.548989e-01 9.998022e-01

2392 6.547853e-01 9.998022e-01

2393 6.546707e-01 9.998021e-01

2394 6.545550e-01 9.998021e-01

2395 6.544384e-01 9.998021e-01

2396 6.543208e-01 9.998021e-01

2397 6.542022e-01 9.998020e-01

2398 6.540828e-01 9.998020e-01

2399 6.539625e-01 9.998020e-01

2400 6.538414e-01 9.998020e-01

2401 6.537195e-01 9.998019e-01

2402 6.535970e-01 9.998019e-01

2403 6.534738e-01 9.998019e-01

2404 6.533501e-01 9.998019e-01

2405 6.532258e-01 9.998018e-01

2406 6.531011e-01 9.998018e-01

2407 6.529761e-01 9.998017e-01

2408 6.528507e-01 9.998017e-01

2409 6.527252e-01 9.998017e-01

2410 6.525995e-01 9.998016e-01

2411 6.524738e-01 9.998016e-01

2412 6.523482e-01 9.998015e-01

2413 6.522228e-01 9.998015e-01

2414 6.520976e-01 9.998014e-01

2415 6.519728e-01 9.998014e-01

2416 6.518486e-01 9.998013e-01

2417 6.517250e-01 9.998013e-01

2418 6.516021e-01 9.998012e-01

2419 6.514801e-01 9.998012e-01

2420 6.513592e-01 9.998011e-01

2421 6.512394e-01 9.998010e-01

2422 6.511210e-01 9.998010e-01

2423 6.510040e-01 9.998009e-01

2424 6.508886e-01 9.998008e-01

2425 6.507750e-01 9.998008e-01

2426 6.506634e-01 9.998007e-01

2427 6.505540e-01 9.998006e-01

2428 6.504468e-01 9.998005e-01

2429 6.503421e-01 9.998004e-01

2430 6.502401e-01 9.998003e-01

2431 6.501410e-01 9.998002e-01

2432 6.500449e-01 9.998001e-01

2433 6.499521e-01 9.998000e-01

2434 6.498628e-01 9.997999e-01

2435 6.497771e-01 9.997998e-01

2436 6.496953e-01 9.997996e-01

2437 6.496176e-01 9.997995e-01

2438 6.495441e-01 9.997994e-01

2439 6.494752e-01 9.997992e-01

2440 6.494109e-01 9.997991e-01

2441 6.493516e-01 9.997989e-01

2442 6.492974e-01 9.997987e-01

2443 6.492485e-01 9.997986e-01

2444 6.492052e-01 9.997984e-01

2445 6.491676e-01 9.997982e-01

2446 6.491360e-01 9.997980e-01

2447 6.491105e-01 9.997978e-01

2448 6.490913e-01 9.997975e-01

2449 6.490787e-01 9.997973e-01

2450 6.490727e-01 9.997971e-01

2451 6.490736e-01 9.997968e-01

2452 6.490816e-01 9.997965e-01

2453 6.490967e-01 9.997963e-01

2454 6.491192e-01 9.997960e-01

2455 6.491491e-01 9.997957e-01

2456 6.491866e-01 9.997953e-01

2457 6.492318e-01 9.997950e-01

2458 6.492848e-01 9.997947e-01

2459 6.493457e-01 9.997943e-01

2460 6.494145e-01 9.997939e-01

2461 6.494913e-01 9.997935e-01

2462 6.495762e-01 9.997931e-01

2463 6.496692e-01 9.997926e-01

2464 6.497702e-01 9.997921e-01

2465 6.498793e-01 9.997916e-01

2466 6.499965e-01 9.997911e-01

2467 6.501217e-01 9.997906e-01

2468 6.502548e-01 9.997900e-01

2469 6.503959e-01 9.997894e-01

2470 6.505447e-01 9.997888e-01

2471 6.507012e-01 9.997881e-01

2472 6.508652e-01 9.997875e-01

2473 6.510367e-01 9.997867e-01

2474 6.512154e-01 9.997860e-01

2475 6.514013e-01 9.997852e-01

2476 6.515940e-01 9.997844e-01

2477 6.517934e-01 9.997835e-01

2478 6.519992e-01 9.997826e-01

2479 6.522113e-01 9.997816e-01

2480 6.524295e-01 9.997806e-01

2481 6.526533e-01 9.997796e-01

2482 6.528827e-01 9.997785e-01

2483 6.531172e-01 9.997773e-01

2484 6.533567e-01 9.997761e-01

2485 6.536008e-01 9.997748e-01

2486 6.538492e-01 9.997735e-01

2487 6.541017e-01 9.997721e-01

2488 6.543579e-01 9.997706e-01

2489 6.546175e-01 9.997691e-01

2490 6.548803e-01 9.997675e-01

2491 6.551458e-01 9.997658e-01

2492 6.554138e-01 9.997640e-01

2493 6.556840e-01 9.997621e-01

2494 6.559560e-01 9.997602e-01

2495 6.562296e-01 9.997581e-01

2496 6.565044e-01 9.997560e-01

2497 6.567802e-01 9.997537e-01

2498 6.570567e-01 9.997514e-01

2499 6.573335e-01 9.997489e-01

2500 6.576104e-01 9.997463e-01

2501 6.578871e-01 9.997436e-01

2502 6.581634e-01 9.997407e-01

2503 6.584390e-01 9.997377e-01

2504 6.587137e-01 9.997346e-01

2505 6.589872e-01 9.997313e-01

2506 6.592593e-01 9.997278e-01

2507 6.595298e-01 9.997242e-01

2508 6.597985e-01 9.997204e-01

2509 6.600652e-01 9.997164e-01

2510 6.603297e-01 9.997122e-01

2511 6.605919e-01 9.997079e-01

2512 6.608515e-01 9.997033e-01

2513 6.611085e-01 9.996985e-01

2514 6.613627e-01 9.996934e-01

2515 6.616140e-01 9.996881e-01

2516 6.618623e-01 9.996826e-01

2517 6.621075e-01 9.996768e-01

2518 6.623494e-01 9.996707e-01

2519 6.625880e-01 9.996643e-01

2520 6.628232e-01 9.996576e-01

2521 6.630550e-01 9.996506e-01

2522 6.630699e-01 9.996506e-01

2523 6.630856e-01 9.996506e-01

2524 6.631020e-01 9.996506e-01

2525 6.631191e-01 9.996506e-01

2526 6.631370e-01 9.996506e-01

2527 6.631558e-01 9.996505e-01

2528 6.631754e-01 9.996505e-01

2529 6.631959e-01 9.996505e-01

2530 6.632172e-01 9.996505e-01

2531 6.632395e-01 9.996505e-01

2532 6.632627e-01 9.996505e-01

2533 6.632869e-01 9.996505e-01

2534 6.633122e-01 9.996505e-01

2535 6.633384e-01 9.996505e-01

2536 6.633658e-01 9.996505e-01

2537 6.633943e-01 9.996505e-01

2538 6.634239e-01 9.996505e-01

2539 6.634547e-01 9.996505e-01

2540 6.634868e-01 9.996505e-01

2541 6.635201e-01 9.996504e-01

2542 6.635548e-01 9.996504e-01

2543 6.635908e-01 9.996504e-01

2544 6.636282e-01 9.996504e-01

2545 6.636671e-01 9.996504e-01

2546 6.637075e-01 9.996504e-01

2547 6.637495e-01 9.996504e-01

2548 6.637930e-01 9.996504e-01

2549 6.638383e-01 9.996504e-01

2550 6.638852e-01 9.996504e-01

2551 6.639340e-01 9.996503e-01

2552 6.639845e-01 9.996503e-01

2553 6.640370e-01 9.996503e-01

2554 6.640914e-01 9.996503e-01

2555 6.641479e-01 9.996503e-01

2556 6.642064e-01 9.996503e-01

2557 6.642671e-01 9.996503e-01

2558 6.643300e-01 9.996502e-01

2559 6.643953e-01 9.996502e-01

2560 6.644629e-01 9.996502e-01

2561 6.645330e-01 9.996502e-01

2562 6.646056e-01 9.996502e-01

2563 6.646808e-01 9.996502e-01

2564 6.647588e-01 9.996501e-01

2565 6.648395e-01 9.996501e-01

2566 6.649231e-01 9.996501e-01

2567 6.650097e-01 9.996501e-01

2568 6.650993e-01 9.996501e-01

2569 6.651921e-01 9.996500e-01

2570 6.652881e-01 9.996500e-01

2571 6.653875e-01 9.996500e-01

2572 6.654904e-01 9.996500e-01

2573 6.655968e-01 9.996499e-01

2574 6.657069e-01 9.996499e-01

2575 6.658208e-01 9.996499e-01

2576 6.659386e-01 9.996498e-01

2577 6.660605e-01 9.996498e-01

2578 6.661864e-01 9.996498e-01

2579 6.663166e-01 9.996498e-01

2580 6.664512e-01 9.996497e-01

2581 6.665903e-01 9.996497e-01

2582 6.667341e-01 9.996496e-01

2583 6.668826e-01 9.996496e-01

2584 6.670361e-01 9.996496e-01

2585 6.671945e-01 9.996495e-01

2586 6.673582e-01 9.996495e-01

2587 6.675272e-01 9.996494e-01

2588 6.677017e-01 9.996494e-01

2589 6.678818e-01 9.996493e-01

2590 6.680677e-01 9.996493e-01

2591 6.682595e-01 9.996492e-01

2592 6.684573e-01 9.996492e-01

2593 6.686614e-01 9.996491e-01

2594 6.688719e-01 9.996491e-01

2595 6.690890e-01 9.996490e-01

2596 6.693127e-01 9.996490e-01

2597 6.695433e-01 9.996489e-01

2598 6.697810e-01 9.996488e-01

2599 6.700258e-01 9.996488e-01

2600 6.702781e-01 9.996487e-01

2601 6.705378e-01 9.996486e-01

2602 6.708052e-01 9.996485e-01

2603 6.710805e-01 9.996484e-01

2604 6.713638e-01 9.996484e-01

2605 6.716553e-01 9.996483e-01

2606 6.719552e-01 9.996482e-01

2607 6.722635e-01 9.996481e-01

2608 6.725806e-01 9.996480e-01

2609 6.729064e-01 9.996479e-01

2610 6.732413e-01 9.996478e-01

2611 6.735853e-01 9.996477e-01

2612 6.739385e-01 9.996476e-01

2613 6.743013e-01 9.996474e-01

2614 6.746736e-01 9.996473e-01

2615 6.750556e-01 9.996472e-01

2616 6.754475e-01 9.996471e-01

2617 6.758494e-01 9.996469e-01

2618 6.762614e-01 9.996468e-01

2619 6.766836e-01 9.996466e-01

2620 6.771161e-01 9.996465e-01

2621 6.775591e-01 9.996463e-01

2622 6.780127e-01 9.996461e-01

2623 6.784768e-01 9.996459e-01

2624 6.789516e-01 9.996458e-01

2625 6.794373e-01 9.996456e-01

2626 6.799337e-01 9.996454e-01

2627 6.804410e-01 9.996452e-01

2628 6.809592e-01 9.996450e-01

2629 6.814883e-01 9.996447e-01

2630 6.820283e-01 9.996445e-01

2631 6.825793e-01 9.996443e-01

2632 6.831413e-01 9.996440e-01

2633 6.837141e-01 9.996437e-01

2634 6.842977e-01 9.996435e-01

2635 6.848922e-01 9.996432e-01

2636 6.854974e-01 9.996429e-01

2637 6.861132e-01 9.996426e-01

2638 6.867395e-01 9.996423e-01

2639 6.873763e-01 9.996420e-01

2640 6.880233e-01 9.996416e-01

2641 6.886804e-01 9.996413e-01

2642 6.893475e-01 9.996409e-01

2643 6.900244e-01 9.996405e-01

2644 6.907109e-01 9.996401e-01

2645 6.914067e-01 9.996397e-01

2646 6.921116e-01 9.996393e-01

2647 6.928253e-01 9.996388e-01

2648 6.935477e-01 9.996384e-01

2649 6.942783e-01 9.996379e-01

2650 6.950170e-01 9.996374e-01

2651 6.957634e-01 9.996369e-01

2652 6.965171e-01 9.996364e-01

2653 6.972778e-01 9.996358e-01

2654 6.980451e-01 9.996352e-01

2655 6.988188e-01 9.996346e-01

2656 6.995983e-01 9.996340e-01

2657 7.003833e-01 9.996334e-01

2658 7.011733e-01 9.996327e-01

2659 7.019680e-01 9.996321e-01

2660 7.027670e-01 9.996313e-01

2661 7.035697e-01 9.996306e-01

2662 7.043757e-01 9.996299e-01

2663 7.051846e-01 9.996291e-01

2664 7.059959e-01 9.996283e-01

2665 7.068091e-01 9.996274e-01

2666 7.076238e-01 9.996265e-01

2667 7.084394e-01 9.996256e-01

2668 7.092556e-01 9.996247e-01

2669 7.100718e-01 9.996237e-01

2670 7.108876e-01 9.996228e-01

2671 7.117025e-01 9.996217e-01

2672 7.125159e-01 9.996207e-01

2673 7.133275e-01 9.996196e-01

2674 7.141368e-01 9.996184e-01

2675 7.149432e-01 9.996173e-01

2676 7.157464e-01 9.996161e-01

2677 7.165460e-01 9.996148e-01

2678 7.173414e-01 9.996135e-01

2679 7.181323e-01 9.996122e-01

2680 7.189182e-01 9.996109e-01

2681 7.196987e-01 9.996095e-01

2682 7.204734e-01 9.996080e-01

2683 7.212421e-01 9.996065e-01

2684 7.220042e-01 9.996050e-01

2685 7.227594e-01 9.996034e-01

2686 7.235074e-01 9.996018e-01

2687 7.242480e-01 9.996002e-01

2688 7.249806e-01 9.995985e-01

2689 7.257052e-01 9.995967e-01

2690 7.264214e-01 9.995950e-01

2691 7.271289e-01 9.995931e-01

2692 7.278275e-01 9.995913e-01

2693 7.285170e-01 9.995893e-01

2694 7.291971e-01 9.995874e-01

2695 7.298678e-01 9.995854e-01

2696 7.305287e-01 9.995834e-01

2697 7.311798e-01 9.995813e-01

2698 7.318208e-01 9.995792e-01

2699 7.324518e-01 9.995770e-01

2700 7.330725e-01 9.995748e-01

2701 7.336829e-01 9.995726e-01

2702 7.337233e-01 9.995726e-01

2703 7.337647e-01 9.995726e-01

2704 7.338070e-01 9.995726e-01

2705 7.338502e-01 9.995726e-01

2706 7.338943e-01 9.995726e-01

2707 7.339395e-01 9.995726e-01

2708 7.339855e-01 9.995726e-01

2709 7.340326e-01 9.995726e-01

2710 7.340807e-01 9.995726e-01

2711 7.341298e-01 9.995726e-01

2712 7.341799e-01 9.995725e-01

2713 7.342311e-01 9.995725e-01

2714 7.342834e-01 9.995725e-01

2715 7.343368e-01 9.995725e-01

2716 7.343914e-01 9.995725e-01

2717 7.344471e-01 9.995725e-01

2718 7.345039e-01 9.995725e-01

2719 7.345620e-01 9.995725e-01

2720 7.346213e-01 9.995725e-01

2721 7.346818e-01 9.995725e-01

2722 7.347436e-01 9.995725e-01

2723 7.348068e-01 9.995725e-01

2724 7.348712e-01 9.995725e-01

2725 7.349371e-01 9.995725e-01

2726 7.350043e-01 9.995725e-01

2727 7.350729e-01 9.995725e-01

2728 7.351430e-01 9.995725e-01

2729 7.352146e-01 9.995725e-01

2730 7.352877e-01 9.995724e-01

2731 7.353624e-01 9.995724e-01

2732 7.354386e-01 9.995724e-01

2733 7.355165e-01 9.995724e-01

2734 7.355960e-01 9.995724e-01

2735 7.356772e-01 9.995724e-01

2736 7.357602e-01 9.995724e-01

2737 7.358449e-01 9.995724e-01

2738 7.359314e-01 9.995724e-01

2739 7.360197e-01 9.995724e-01

2740 7.361100e-01 9.995724e-01

2741 7.362021e-01 9.995724e-01

2742 7.362963e-01 9.995723e-01

2743 7.363924e-01 9.995723e-01

2744 7.364906e-01 9.995723e-01

2745 7.365909e-01 9.995723e-01

2746 7.366933e-01 9.995723e-01

2747 7.367979e-01 9.995723e-01

2748 7.369048e-01 9.995723e-01

2749 7.370139e-01 9.995723e-01

2750 7.371254e-01 9.995723e-01

2751 7.372393e-01 9.995723e-01

2752 7.373555e-01 9.995722e-01

2753 7.374743e-01 9.995722e-01

2754 7.375956e-01 9.995722e-01

2755 7.377195e-01 9.995722e-01

2756 7.378461e-01 9.995722e-01

2757 7.379753e-01 9.995722e-01

2758 7.381073e-01 9.995722e-01

2759 7.382421e-01 9.995722e-01

2760 7.383798e-01 9.995722e-01

2761 7.385204e-01 9.995721e-01

2762 7.386640e-01 9.995721e-01

2763 7.388107e-01 9.995721e-01

2764 7.389604e-01 9.995721e-01

2765 7.391133e-01 9.995721e-01

2766 7.392695e-01 9.995721e-01

2767 7.394290e-01 9.995721e-01

2768 7.395918e-01 9.995720e-01

2769 7.397580e-01 9.995720e-01

2770 7.399278e-01 9.995720e-01

2771 7.401011e-01 9.995720e-01

2772 7.402780e-01 9.995720e-01

2773 7.404586e-01 9.995720e-01

2774 7.406430e-01 9.995720e-01

2775 7.408313e-01 9.995720e-01

2776 7.410234e-01 9.995719e-01

2777 7.412195e-01 9.995719e-01

2778 7.414196e-01 9.995719e-01

2779 7.416239e-01 9.995719e-01

2780 7.418323e-01 9.995719e-01

2781 7.420450e-01 9.995719e-01

2782 7.422620e-01 9.995719e-01

2783 7.424834e-01 9.995718e-01

2784 7.427092e-01 9.995718e-01

2785 7.429396e-01 9.995718e-01

2786 7.431746e-01 9.995718e-01

2787 7.434143e-01 9.995718e-01

2788 7.436587e-01 9.995718e-01

2789 7.439079e-01 9.995718e-01

2790 7.441619e-01 9.995718e-01

2791 7.444210e-01 9.995718e-01

2792 7.446850e-01 9.995717e-01

2793 7.449541e-01 9.995717e-01

2794 7.452283e-01 9.995717e-01

2795 7.455077e-01 9.995717e-01

2796 7.457924e-01 9.995717e-01

2797 7.460824e-01 9.995717e-01

2798 7.463777e-01 9.995717e-01

2799 7.466785e-01 9.995717e-01

2800 7.469847e-01 9.995717e-01

2801 7.472965e-01 9.995717e-01

2802 7.476138e-01 9.995717e-01

2803 7.479367e-01 9.995717e-01

2804 7.482653e-01 9.995718e-01

2805 7.485995e-01 9.995718e-01

2806 7.489394e-01 9.995718e-01

2807 7.492851e-01 9.995718e-01

2808 7.496365e-01 9.995718e-01

2809 7.499937e-01 9.995719e-01

2810 7.503568e-01 9.995719e-01

2811 7.507256e-01 9.995719e-01

2812 7.511002e-01 9.995720e-01

2813 7.514806e-01 9.995720e-01

2814 7.518669e-01 9.995721e-01

2815 7.522589e-01 9.995721e-01

2816 7.526567e-01 9.995722e-01

2817 7.530603e-01 9.995723e-01

2818 7.534696e-01 9.995724e-01

2819 7.538846e-01 9.995724e-01

2820 7.543052e-01 9.995725e-01

2821 7.547314e-01 9.995726e-01

2822 7.551632e-01 9.995727e-01

2823 7.556004e-01 9.995729e-01

2824 7.560430e-01 9.995730e-01

2825 7.564910e-01 9.995732e-01

2826 7.569442e-01 9.995733e-01

2827 7.574025e-01 9.995735e-01

2828 7.578659e-01 9.995737e-01

2829 7.583342e-01 9.995739e-01

2830 7.588073e-01 9.995741e-01

2831 7.592851e-01 9.995743e-01

2832 7.597674e-01 9.995746e-01

2833 7.602541e-01 9.995748e-01

2834 7.607451e-01 9.995751e-01

2835 7.612401e-01 9.995755e-01

2836 7.617391e-01 9.995758e-01

2837 7.622418e-01 9.995762e-01

2838 7.627480e-01 9.995766e-01

2839 7.632577e-01 9.995770e-01

2840 7.637705e-01 9.995774e-01

2841 7.642862e-01 9.995779e-01

2842 7.648047e-01 9.995784e-01

2843 7.653258e-01 9.995790e-01

2844 7.658491e-01 9.995796e-01

2845 7.663745e-01 9.995802e-01

2846 7.669017e-01 9.995809e-01

2847 7.674305e-01 9.995816e-01

2848 7.679606e-01 9.995824e-01

2849 7.684918e-01 9.995832e-01

2850 7.690238e-01 9.995841e-01

2851 7.695564e-01 9.995850e-01

2852 7.700892e-01 9.995860e-01

2853 7.706221e-01 9.995871e-01

2854 7.711548e-01 9.995882e-01

2855 7.716869e-01 9.995894e-01

2856 7.722182e-01 9.995907e-01

2857 7.727485e-01 9.995921e-01

2858 7.732774e-01 9.995936e-01

2859 7.738047e-01 9.995951e-01

2860 7.743301e-01 9.995968e-01

2861 7.748534e-01 9.995985e-01

2862 7.753742e-01 9.996004e-01

2863 7.758924e-01 9.996024e-01

2864 7.764076e-01 9.996045e-01

2865 7.769196e-01 9.996068e-01

2866 7.774281e-01 9.996092e-01

2867 7.779330e-01 9.996117e-01

2868 7.784339e-01 9.996144e-01

2869 7.789306e-01 9.996173e-01

2870 7.794229e-01 9.996203e-01

2871 7.799106e-01 9.996235e-01

2872 7.803934e-01 9.996270e-01

2873 7.808712e-01 9.996306e-01

2874 7.813437e-01 9.996344e-01

2875 7.818108e-01 9.996385e-01

2876 7.822722e-01 9.996428e-01

2877 7.827279e-01 9.996474e-01

2878 7.831775e-01 9.996523e-01

2879 7.836211e-01 9.996574e-01

2880 7.840585e-01 9.996628e-01

2881 7.844894e-01 9.996686e-01

2882 7.845025e-01 9.996686e-01

2883 7.845157e-01 9.996686e-01

2884 7.845291e-01 9.996686e-01

2885 7.845427e-01 9.996686e-01

2886 7.845564e-01 9.996686e-01

2887 7.845704e-01 9.996686e-01

2888 7.845845e-01 9.996686e-01

2889 7.845988e-01 9.996686e-01

2890 7.846133e-01 9.996686e-01

2891 7.846280e-01 9.996686e-01

2892 7.846428e-01 9.996686e-01

2893 7.846578e-01 9.996686e-01

2894 7.846730e-01 9.996686e-01

2895 7.846884e-01 9.996686e-01

2896 7.847040e-01 9.996686e-01

2897 7.847198e-01 9.996686e-01

2898 7.847357e-01 9.996686e-01

2899 7.847518e-01 9.996686e-01

2900 7.847682e-01 9.996686e-01

2901 7.847847e-01 9.996686e-01

2902 7.848014e-01 9.996686e-01

2903 7.848182e-01 9.996686e-01

2904 7.848353e-01 9.996686e-01

2905 7.848526e-01 9.996686e-01

2906 7.848701e-01 9.996687e-01

2907 7.848877e-01 9.996687e-01

2908 7.849056e-01 9.996687e-01

2909 7.849236e-01 9.996687e-01

2910 7.849418e-01 9.996687e-01

2911 7.849602e-01 9.996687e-01

2912 7.849789e-01 9.996687e-01

2913 7.849977e-01 9.996687e-01

2914 7.850167e-01 9.996687e-01

2915 7.850358e-01 9.996687e-01

2916 7.850552e-01 9.996687e-01

2917 7.850748e-01 9.996687e-01

2918 7.850945e-01 9.996687e-01

2919 7.851144e-01 9.996687e-01

2920 7.851346e-01 9.996687e-01

2921 7.851549e-01 9.996687e-01

2922 7.851753e-01 9.996687e-01

2923 7.851960e-01 9.996688e-01

2924 7.852168e-01 9.996688e-01

2925 7.852378e-01 9.996688e-01

2926 7.852590e-01 9.996688e-01

2927 7.852803e-01 9.996688e-01

2928 7.853018e-01 9.996688e-01

2929 7.853235e-01 9.996688e-01

2930 7.853453e-01 9.996688e-01

2931 7.853673e-01 9.996688e-01

2932 7.853894e-01 9.996689e-01

2933 7.854116e-01 9.996689e-01

2934 7.854340e-01 9.996689e-01

2935 7.854565e-01 9.996689e-01

2936 7.854791e-01 9.996689e-01

2937 7.855019e-01 9.996689e-01

2938 7.855247e-01 9.996690e-01

2939 7.855477e-01 9.996690e-01

2940 7.855707e-01 9.996690e-01

2941 7.855938e-01 9.996690e-01

2942 7.856170e-01 9.996690e-01

2943 7.856402e-01 9.996691e-01

2944 7.856635e-01 9.996691e-01

2945 7.856869e-01 9.996691e-01

2946 7.857102e-01 9.996692e-01

2947 7.857336e-01 9.996692e-01

2948 7.857570e-01 9.996692e-01

2949 7.857804e-01 9.996692e-01

2950 7.858037e-01 9.996693e-01

2951 7.858270e-01 9.996693e-01

2952 7.858503e-01 9.996694e-01

2953 7.858735e-01 9.996694e-01

2954 7.858966e-01 9.996694e-01

2955 7.859196e-01 9.996695e-01

2956 7.859425e-01 9.996695e-01

2957 7.859652e-01 9.996696e-01

2958 7.859878e-01 9.996696e-01

2959 7.860102e-01 9.996697e-01

2960 7.860324e-01 9.996697e-01

2961 7.860544e-01 9.996698e-01

2962 7.860761e-01 9.996699e-01

2963 7.860975e-01 9.996699e-01

2964 7.861187e-01 9.996700e-01

2965 7.861395e-01 9.996701e-01

2966 7.861600e-01 9.996701e-01

2967 7.861801e-01 9.996702e-01

2968 7.861998e-01 9.996703e-01

2969 7.862191e-01 9.996704e-01

2970 7.862379e-01 9.996705e-01

2971 7.862562e-01 9.996706e-01

2972 7.862739e-01 9.996707e-01

2973 7.862911e-01 9.996708e-01

2974 7.863078e-01 9.996709e-01

2975 7.863237e-01 9.996710e-01

2976 7.863390e-01 9.996711e-01

2977 7.863536e-01 9.996713e-01

2978 7.863675e-01 9.996714e-01

2979 7.863806e-01 9.996715e-01

2980 7.863928e-01 9.996717e-01

2981 7.864042e-01 9.996718e-01

2982 7.864147e-01 9.996720e-01

2983 7.864242e-01 9.996722e-01

2984 7.864327e-01 9.996724e-01

2985 7.864402e-01 9.996726e-01

2986 7.864466e-01 9.996728e-01

2987 7.864518e-01 9.996730e-01

2988 7.864559e-01 9.996732e-01

2989 7.864588e-01 9.996734e-01

2990 7.864604e-01 9.996737e-01

2991 7.864606e-01 9.996739e-01

2992 7.864595e-01 9.996742e-01

2993 7.864570e-01 9.996745e-01

2994 7.864530e-01 9.996748e-01

2995 7.864474e-01 9.996751e-01

2996 7.864403e-01 9.996754e-01

2997 7.864315e-01 9.996757e-01

2998 7.864211e-01 9.996761e-01

2999 7.864089e-01 9.996765e-01

3000 7.863950e-01 9.996768e-01

3001 7.863792e-01 9.996773e-01

3002 7.863615e-01 9.996777e-01

3003 7.863419e-01 9.996781e-01

3004 7.863203e-01 9.996786e-01

3005 7.862967e-01 9.996791e-01

3006 7.862710e-01 9.996796e-01

3007 7.862431e-01 9.996802e-01

3008 7.862131e-01 9.996807e-01

3009 7.861809e-01 9.996813e-01

3010 7.861464e-01 9.996820e-01

3011 7.861095e-01 9.996826e-01

3012 7.860704e-01 9.996833e-01

3013 7.860288e-01 9.996840e-01

3014 7.859848e-01 9.996848e-01

3015 7.859383e-01 9.996856e-01

3016 7.858894e-01 9.996864e-01

3017 7.858379e-01 9.996873e-01

3018 7.857839e-01 9.996882e-01

3019 7.857273e-01 9.996892e-01

3020 7.856682e-01 9.996902e-01

3021 7.856064e-01 9.996912e-01

3022 7.855419e-01 9.996923e-01

3023 7.854749e-01 9.996935e-01

3024 7.854051e-01 9.996947e-01

3025 7.853328e-01 9.996960e-01

3026 7.852577e-01 9.996973e-01

3027 7.851801e-01 9.996987e-01

3028 7.850997e-01 9.997002e-01

3029 7.850168e-01 9.997017e-01

3030 7.849312e-01 9.997033e-01

3031 7.848430e-01 9.997050e-01

3032 7.847523e-01 9.997068e-01

3033 7.846590e-01 9.997086e-01

3034 7.845631e-01 9.997106e-01

3035 7.844648e-01 9.997126e-01

3036 7.843641e-01 9.997147e-01

3037 7.842609e-01 9.997169e-01

3038 7.841555e-01 9.997193e-01

3039 7.840477e-01 9.997217e-01

3040 7.839377e-01 9.997243e-01

3041 7.838255e-01 9.997270e-01

3042 7.837112e-01 9.997298e-01

3043 7.835948e-01 9.997327e-01

3044 7.834765e-01 9.997358e-01

3045 7.833563e-01 9.997390e-01

3046 7.832343e-01 9.997424e-01

3047 7.831105e-01 9.997459e-01

3048 7.829851e-01 9.997496e-01

3049 7.828581e-01 9.997535e-01

3050 7.827297e-01 9.997576e-01

3051 7.825998e-01 9.997618e-01

3052 7.824687e-01 9.997662e-01

3053 7.823364e-01 9.997709e-01

3054 7.822030e-01 9.997757e-01

3055 7.820686e-01 9.997808e-01

3056 7.819333e-01 9.997861e-01

3057 7.817972e-01 9.997917e-01

3058 7.816604e-01 9.997975e-01

3059 7.815230e-01 9.998035e-01

3060 7.813851e-01 9.998099e-01

3061 7.812468e-01 9.998165e-01

3062 7.812277e-01 9.998165e-01

3063 7.812081e-01 9.998165e-01

3064 7.811879e-01 9.998165e-01

3065 7.811671e-01 9.998165e-01

3066 7.811457e-01 9.998165e-01

3067 7.811236e-01 9.998165e-01

3068 7.811009e-01 9.998165e-01

3069 7.810776e-01 9.998165e-01

3070 7.810536e-01 9.998165e-01

3071 7.810289e-01 9.998165e-01

3072 7.810034e-01 9.998165e-01

3073 7.809773e-01 9.998165e-01

3074 7.809504e-01 9.998165e-01

3075 7.809227e-01 9.998165e-01

3076 7.808942e-01 9.998166e-01

3077 7.808649e-01 9.998166e-01

3078 7.808347e-01 9.998166e-01

3079 7.808037e-01 9.998166e-01

3080 7.807717e-01 9.998166e-01

3081 7.807389e-01 9.998166e-01

3082 7.807051e-01 9.998166e-01

3083 7.806703e-01 9.998166e-01

3084 7.806346e-01 9.998166e-01

3085 7.805978e-01 9.998166e-01

3086 7.805599e-01 9.998166e-01

3087 7.805210e-01 9.998166e-01

3088 7.804809e-01 9.998166e-01

3089 7.804397e-01 9.998166e-01

3090 7.803973e-01 9.998167e-01

3091 7.803537e-01 9.998167e-01

3092 7.803088e-01 9.998167e-01

3093 7.802626e-01 9.998167e-01

3094 7.802152e-01 9.998167e-01

3095 7.801663e-01 9.998167e-01

3096 7.801160e-01 9.998167e-01

3097 7.800643e-01 9.998167e-01

3098 7.800112e-01 9.998167e-01

3099 7.799565e-01 9.998167e-01

3100 7.799002e-01 9.998168e-01

3101 7.798423e-01 9.998168e-01

3102 7.797827e-01 9.998168e-01

3103 7.797214e-01 9.998168e-01

3104 7.796584e-01 9.998168e-01

3105 7.795936e-01 9.998168e-01

3106 7.795269e-01 9.998168e-01

3107 7.794583e-01 9.998169e-01

3108 7.793877e-01 9.998169e-01

3109 7.793151e-01 9.998169e-01

3110 7.792404e-01 9.998169e-01

3111 7.791636e-01 9.998169e-01

3112 7.790846e-01 9.998169e-01

3113 7.790033e-01 9.998170e-01

3114 7.789198e-01 9.998170e-01

3115 7.788338e-01 9.998170e-01

3116 7.787454e-01 9.998170e-01

3117 7.786544e-01 9.998170e-01

3118 7.785609e-01 9.998171e-01

3119 7.784647e-01 9.998171e-01

3120 7.783658e-01 9.998171e-01

3121 7.782641e-01 9.998171e-01

3122 7.781595e-01 9.998172e-01

3123 7.780519e-01 9.998172e-01

3124 7.779414e-01 9.998172e-01

3125 7.778276e-01 9.998172e-01

3126 7.777107e-01 9.998173e-01

3127 7.775905e-01 9.998173e-01

3128 7.774670e-01 9.998173e-01

3129 7.773400e-01 9.998174e-01

3130 7.772094e-01 9.998174e-01

3131 7.770752e-01 9.998174e-01

3132 7.769372e-01 9.998175e-01

3133 7.767955e-01 9.998175e-01

3134 7.766498e-01 9.998175e-01

3135 7.765001e-01 9.998176e-01

3136 7.763463e-01 9.998176e-01

3137 7.761882e-01 9.998177e-01

3138 7.760259e-01 9.998177e-01

3139 7.758591e-01 9.998178e-01

3140 7.756878e-01 9.998178e-01

3141 7.755119e-01 9.998179e-01

3142 7.753312e-01 9.998179e-01

3143 7.751457e-01 9.998180e-01

3144 7.749553e-01 9.998180e-01

3145 7.747597e-01 9.998181e-01

3146 7.745590e-01 9.998181e-01

3147 7.743531e-01 9.998182e-01

3148 7.741417e-01 9.998183e-01

3149 7.739248e-01 9.998183e-01

3150 7.737023e-01 9.998184e-01

3151 7.734741e-01 9.998185e-01

3152 7.732400e-01 9.998186e-01

3153 7.730000e-01 9.998187e-01

3154 7.727539e-01 9.998187e-01

3155 7.725017e-01 9.998188e-01

3156 7.722431e-01 9.998189e-01

3157 7.719782e-01 9.998190e-01

3158 7.717068e-01 9.998191e-01

3159 7.714287e-01 9.998192e-01

3160 7.711440e-01 9.998193e-01

3161 7.708525e-01 9.998194e-01

3162 7.705540e-01 9.998195e-01

3163 7.702486e-01 9.998196e-01

3164 7.699361e-01 9.998198e-01

3165 7.696164e-01 9.998199e-01

3166 7.692894e-01 9.998200e-01

3167 7.689551e-01 9.998202e-01

3168 7.686134e-01 9.998203e-01

3169 7.682642e-01 9.998205e-01

3170 7.679074e-01 9.998206e-01

3171 7.675430e-01 9.998208e-01

3172 7.671709e-01 9.998209e-01

3173 7.667911e-01 9.998211e-01

3174 7.664036e-01 9.998213e-01

3175 7.660082e-01 9.998215e-01

3176 7.656051e-01 9.998217e-01

3177 7.651941e-01 9.998219e-01

3178 7.647752e-01 9.998221e-01

3179 7.643485e-01 9.998223e-01

3180 7.639140e-01 9.998225e-01

3181 7.634716e-01 9.998228e-01

3182 7.630214e-01 9.998230e-01

3183 7.625635e-01 9.998233e-01

3184 7.620978e-01 9.998235e-01

3185 7.616246e-01 9.998238e-01

3186 7.611437e-01 9.998241e-01

3187 7.606553e-01 9.998244e-01

3188 7.601595e-01 9.998247e-01

3189 7.596565e-01 9.998250e-01

3190 7.591462e-01 9.998254e-01

3191 7.586289e-01 9.998257e-01

3192 7.581047e-01 9.998261e-01

3193 7.575738e-01 9.998265e-01

3194 7.570362e-01 9.998269e-01

3195 7.564923e-01 9.998273e-01

3196 7.559422e-01 9.998277e-01

3197 7.553860e-01 9.998282e-01

3198 7.548241e-01 9.998286e-01

3199 7.542567e-01 9.998291e-01

3200 7.536839e-01 9.998296e-01

3201 7.531061e-01 9.998301e-01

3202 7.525235e-01 9.998307e-01

3203 7.519364e-01 9.998312e-01

3204 7.513451e-01 9.998318e-01

3205 7.507499e-01 9.998324e-01

3206 7.501511e-01 9.998330e-01

3207 7.495490e-01 9.998337e-01

3208 7.489439e-01 9.998344e-01

3209 7.483362e-01 9.998351e-01

3210 7.477263e-01 9.998358e-01

3211 7.471144e-01 9.998366e-01

3212 7.465010e-01 9.998374e-01

3213 7.458863e-01 9.998382e-01

3214 7.452708e-01 9.998390e-01

3215 7.446548e-01 9.998399e-01

3216 7.440386e-01 9.998409e-01

3217 7.434228e-01 9.998418e-01

3218 7.428076e-01 9.998428e-01

3219 7.421934e-01 9.998439e-01

3220 7.415805e-01 9.998450e-01

3221 7.409694e-01 9.998461e-01

3222 7.403604e-01 9.998473e-01

3223 7.397539e-01 9.998485e-01

3224 7.391503e-01 9.998497e-01

3225 7.385497e-01 9.998510e-01

3226 7.379528e-01 9.998524e-01

3227 7.373596e-01 9.998538e-01

3228 7.367707e-01 9.998553e-01

3229 7.361863e-01 9.998568e-01

3230 7.356067e-01 9.998584e-01

3231 7.350322e-01 9.998601e-01

3232 7.344632e-01 9.998618e-01

3233 7.338998e-01 9.998637e-01

3234 7.333424e-01 9.998655e-01

3235 7.327912e-01 9.998675e-01

3236 7.322464e-01 9.998696e-01

3237 7.317084e-01 9.998717e-01

3238 7.311772e-01 9.998739e-01

3239 7.306531e-01 9.998763e-01

3240 7.301363e-01 9.998787e-01

3241 7.296269e-01 9.998812e-01

3242 7.295671e-01 9.998812e-01

3243 7.295059e-01 9.998812e-01

3244 7.294432e-01 9.998813e-01

3245 7.293791e-01 9.998813e-01

3246 7.293134e-01 9.998813e-01

3247 7.292461e-01 9.998813e-01

3248 7.291773e-01 9.998813e-01

3249 7.291068e-01 9.998813e-01

3250 7.290347e-01 9.998813e-01

3251 7.289608e-01 9.998813e-01

3252 7.288852e-01 9.998813e-01

3253 7.288079e-01 9.998813e-01

3254 7.287287e-01 9.998813e-01

3255 7.286476e-01 9.998813e-01

3256 7.285646e-01 9.998813e-01

3257 7.284797e-01 9.998813e-01

3258 7.283928e-01 9.998813e-01

3259 7.283038e-01 9.998813e-01

3260 7.282126e-01 9.998813e-01

3261 7.281194e-01 9.998813e-01

3262 7.280239e-01 9.998813e-01

3263 7.279262e-01 9.998813e-01

3264 7.278261e-01 9.998813e-01

3265 7.277237e-01 9.998813e-01

3266 7.276189e-01 9.998813e-01

3267 7.275115e-01 9.998813e-01

3268 7.274016e-01 9.998813e-01

3269 7.272891e-01 9.998813e-01

3270 7.271739e-01 9.998813e-01

3271 7.270560e-01 9.998813e-01

3272 7.269353e-01 9.998813e-01

3273 7.268117e-01 9.998813e-01

3274 7.266852e-01 9.998813e-01

3275 7.265557e-01 9.998814e-01

3276 7.264231e-01 9.998814e-01

3277 7.262873e-01 9.998814e-01

3278 7.261483e-01 9.998814e-01

3279 7.260060e-01 9.998814e-01

3280 7.258604e-01 9.998814e-01

3281 7.257112e-01 9.998814e-01

3282 7.255586e-01 9.998814e-01

3283 7.254023e-01 9.998814e-01

3284 7.252423e-01 9.998814e-01

3285 7.250785e-01 9.998814e-01

3286 7.249109e-01 9.998814e-01

3287 7.247392e-01 9.998814e-01

3288 7.245636e-01 9.998814e-01

3289 7.243838e-01 9.998814e-01

3290 7.241997e-01 9.998815e-01

3291 7.240113e-01 9.998815e-01

3292 7.238185e-01 9.998815e-01

3293 7.236212e-01 9.998815e-01

3294 7.234193e-01 9.998815e-01

3295 7.232127e-01 9.998815e-01

3296 7.230012e-01 9.998815e-01

3297 7.227848e-01 9.998815e-01

3298 7.225634e-01 9.998815e-01

3299 7.223369e-01 9.998816e-01

3300 7.221052e-01 9.998816e-01

3301 7.218681e-01 9.998816e-01

3302 7.216256e-01 9.998816e-01

3303 7.213775e-01 9.998816e-01

3304 7.211238e-01 9.998816e-01

3305 7.208644e-01 9.998816e-01

3306 7.205991e-01 9.998817e-01

3307 7.203278e-01 9.998817e-01

3308 7.200504e-01 9.998817e-01

3309 7.197668e-01 9.998817e-01

3310 7.194770e-01 9.998817e-01

3311 7.191807e-01 9.998817e-01

3312 7.188779e-01 9.998818e-01

3313 7.185686e-01 9.998818e-01

3314 7.182524e-01 9.998818e-01

3315 7.179295e-01 9.998818e-01

3316 7.175996e-01 9.998818e-01

3317 7.172627e-01 9.998819e-01

3318 7.169186e-01 9.998819e-01

3319 7.165674e-01 9.998819e-01

3320 7.162087e-01 9.998819e-01

3321 7.158427e-01 9.998820e-01

3322 7.154692e-01 9.998820e-01

3323 7.150880e-01 9.998820e-01

3324 7.146992e-01 9.998821e-01

3325 7.143027e-01 9.998821e-01

3326 7.138982e-01 9.998821e-01

3327 7.134859e-01 9.998822e-01

3328 7.130656e-01 9.998822e-01

3329 7.126373e-01 9.998822e-01

3330 7.122009e-01 9.998823e-01

3331 7.117563e-01 9.998823e-01

3332 7.113035e-01 9.998824e-01

3333 7.108426e-01 9.998824e-01

3334 7.103733e-01 9.998825e-01

3335 7.098958e-01 9.998825e-01

3336 7.094100e-01 9.998826e-01

3337 7.089159e-01 9.998826e-01

3338 7.084135e-01 9.998827e-01

3339 7.079028e-01 9.998827e-01

3340 7.073838e-01 9.998828e-01

3341 7.068566e-01 9.998828e-01

3342 7.063212e-01 9.998829e-01

3343 7.057777e-01 9.998830e-01

3344 7.052260e-01 9.998830e-01

3345 7.046664e-01 9.998831e-01

3346 7.040987e-01 9.998832e-01

3347 7.035233e-01 9.998832e-01

3348 7.029401e-01 9.998833e-01

3349 7.023493e-01 9.998834e-01

3350 7.017510e-01 9.998835e-01

3351 7.011454e-01 9.998836e-01

3352 7.005325e-01 9.998837e-01

3353 6.999127e-01 9.998838e-01

3354 6.992860e-01 9.998839e-01

3355 6.986527e-01 9.998840e-01

3356 6.980130e-01 9.998841e-01

3357 6.973671e-01 9.998842e-01

3358 6.967152e-01 9.998843e-01

3359 6.960575e-01 9.998844e-01

3360 6.953944e-01 9.998846e-01

3361 6.947262e-01 9.998847e-01

3362 6.940530e-01 9.998848e-01

3363 6.933752e-01 9.998850e-01

3364 6.926932e-01 9.998851e-01

3365 6.920071e-01 9.998853e-01

3366 6.913175e-01 9.998855e-01

3367 6.906245e-01 9.998856e-01

3368 6.899287e-01 9.998858e-01

3369 6.892302e-01 9.998860e-01

3370 6.885295e-01 9.998862e-01

3371 6.878271e-01 9.998864e-01

3372 6.871231e-01 9.998866e-01

3373 6.864182e-01 9.998868e-01

3374 6.857126e-01 9.998870e-01

3375 6.850067e-01 9.998873e-01

3376 6.843010e-01 9.998875e-01

3377 6.835959e-01 9.998878e-01

3378 6.828918e-01 9.998880e-01

3379 6.821890e-01 9.998883e-01

3380 6.814881e-01 9.998886e-01

3381 6.807894e-01 9.998889e-01

3382 6.800934e-01 9.998892e-01

3383 6.794004e-01 9.998895e-01

3384 6.787108e-01 9.998899e-01

3385 6.780251e-01 9.998902e-01

3386 6.773436e-01 9.998906e-01

3387 6.766667e-01 9.998910e-01

3388 6.759949e-01 9.998913e-01

3389 6.753284e-01 9.998918e-01

3390 6.746676e-01 9.998922e-01

3391 6.740130e-01 9.998926e-01

3392 6.733647e-01 9.998931e-01

3393 6.727232e-01 9.998936e-01

3394 6.720888e-01 9.998941e-01

3395 6.714617e-01 9.998946e-01

3396 6.708423e-01 9.998951e-01

3397 6.702308e-01 9.998957e-01

3398 6.696274e-01 9.998963e-01

3399 6.690326e-01 9.998969e-01

3400 6.684463e-01 9.998975e-01

3401 6.678689e-01 9.998982e-01

3402 6.673006e-01 9.998989e-01

3403 6.667416e-01 9.998996e-01

3404 6.661919e-01 9.999003e-01

3405 6.656518e-01 9.999011e-01

3406 6.651214e-01 9.999019e-01

3407 6.646007e-01 9.999027e-01

3408 6.640900e-01 9.999036e-01

3409 6.635892e-01 9.999045e-01

3410 6.630985e-01 9.999055e-01

3411 6.626179e-01 9.999064e-01

3412 6.621475e-01 9.999075e-01

3413 6.616872e-01 9.999085e-01

3414 6.612370e-01 9.999096e-01

3415 6.607971e-01 9.999108e-01

3416 6.603672e-01 9.999120e-01

3417 6.599475e-01 9.999132e-01

3418 6.595379e-01 9.999145e-01

3419 6.591382e-01 9.999158e-01

3420 6.587486e-01 9.999172e-01

3421 6.583687e-01 9.999187e-01

3422 6.582812e-01 9.999187e-01

3423 6.581920e-01 9.999187e-01

3424 6.581011e-01 9.999187e-01

3425 6.580087e-01 9.999187e-01

3426 6.579145e-01 9.999187e-01

3427 6.578187e-01 9.999187e-01

3428 6.577212e-01 9.999187e-01

3429 6.576219e-01 9.999187e-01

3430 6.575209e-01 9.999187e-01

3431 6.574181e-01 9.999187e-01

3432 6.573136e-01 9.999187e-01

3433 6.572072e-01 9.999187e-01

3434 6.570989e-01 9.999187e-01

3435 6.569888e-01 9.999187e-01

3436 6.568768e-01 9.999187e-01

3437 6.567629e-01 9.999187e-01

3438 6.566470e-01 9.999187e-01

3439 6.565291e-01 9.999187e-01

3440 6.564093e-01 9.999187e-01

3441 6.562874e-01 9.999187e-01

3442 6.561634e-01 9.999187e-01

3443 6.560374e-01 9.999187e-01

3444 6.559092e-01 9.999187e-01

3445 6.557789e-01 9.999187e-01

3446 6.556464e-01 9.999187e-01

3447 6.555118e-01 9.999187e-01

3448 6.553748e-01 9.999188e-01

3449 6.552356e-01 9.999188e-01

3450 6.550942e-01 9.999188e-01

3451 6.549504e-01 9.999188e-01

3452 6.548042e-01 9.999188e-01

3453 6.546557e-01 9.999188e-01

3454 6.545048e-01 9.999188e-01

3455 6.543515e-01 9.999188e-01

3456 6.541956e-01 9.999188e-01

3457 6.540373e-01 9.999188e-01

3458 6.538765e-01 9.999188e-01

3459 6.537132e-01 9.999188e-01

3460 6.535473e-01 9.999188e-01

3461 6.533788e-01 9.999188e-01

3462 6.532077e-01 9.999188e-01

3463 6.530339e-01 9.999188e-01

3464 6.528575e-01 9.999188e-01

3465 6.526784e-01 9.999188e-01

3466 6.524966e-01 9.999188e-01

3467 6.523121e-01 9.999188e-01

3468 6.521248e-01 9.999188e-01

3469 6.519348e-01 9.999188e-01

3470 6.517420e-01 9.999188e-01

3471 6.515464e-01 9.999188e-01

3472 6.513480e-01 9.999189e-01

3473 6.511467e-01 9.999189e-01

3474 6.509427e-01 9.999189e-01

3475 6.507358e-01 9.999189e-01

3476 6.505260e-01 9.999189e-01

3477 6.503134e-01 9.999189e-01

3478 6.500980e-01 9.999189e-01

3479 6.498796e-01 9.999189e-01

3480 6.496585e-01 9.999189e-01

3481 6.494344e-01 9.999189e-01

3482 6.492075e-01 9.999189e-01

3483 6.489778e-01 9.999189e-01

3484 6.487452e-01 9.999189e-01

3485 6.485098e-01 9.999189e-01

3486 6.482715e-01 9.999190e-01

3487 6.480305e-01 9.999190e-01

3488 6.477867e-01 9.999190e-01

3489 6.475402e-01 9.999190e-01

3490 6.472909e-01 9.999190e-01

3491 6.470389e-01 9.999190e-01

3492 6.467843e-01 9.999190e-01

3493 6.465270e-01 9.999190e-01

3494 6.462672e-01 9.999190e-01

3495 6.460048e-01 9.999191e-01

3496 6.457400e-01 9.999191e-01

3497 6.454727e-01 9.999191e-01

3498 6.452030e-01 9.999191e-01

3499 6.449310e-01 9.999191e-01

3500 6.446567e-01 9.999191e-01

3501 6.443803e-01 9.999191e-01

3502 6.441017e-01 9.999192e-01

3503 6.438211e-01 9.999192e-01

3504 6.435385e-01 9.999192e-01

3505 6.432541e-01 9.999192e-01

3506 6.429678e-01 9.999192e-01

3507 6.426799e-01 9.999192e-01

3508 6.423904e-01 9.999193e-01

3509 6.420994e-01 9.999193e-01

3510 6.418071e-01 9.999193e-01

3511 6.415134e-01 9.999193e-01

3512 6.412187e-01 9.999193e-01

3513 6.409229e-01 9.999194e-01

3514 6.406262e-01 9.999194e-01

3515 6.403287e-01 9.999194e-01

3516 6.400306e-01 9.999194e-01

3517 6.397321e-01 9.999195e-01

3518 6.394331e-01 9.999195e-01

3519 6.391340e-01 9.999195e-01

3520 6.388348e-01 9.999196e-01

3521 6.385358e-01 9.999196e-01

3522 6.382369e-01 9.999196e-01

3523 6.379386e-01 9.999196e-01

3524 6.376407e-01 9.999197e-01

3525 6.373437e-01 9.999197e-01

3526 6.370475e-01 9.999197e-01

3527 6.367525e-01 9.999198e-01

3528 6.364587e-01 9.999198e-01

3529 6.361663e-01 9.999199e-01

3530 6.358756e-01 9.999199e-01

3531 6.355866e-01 9.999200e-01

3532 6.352996e-01 9.999200e-01

3533 6.350147e-01 9.999200e-01

3534 6.347321e-01 9.999201e-01

3535 6.344521e-01 9.999201e-01

3536 6.341746e-01 9.999202e-01

3537 6.339000e-01 9.999202e-01

3538 6.336285e-01 9.999203e-01

3539 6.333600e-01 9.999204e-01

3540 6.330950e-01 9.999204e-01

3541 6.328334e-01 9.999205e-01

3542 6.325755e-01 9.999205e-01

3543 6.323213e-01 9.999206e-01

3544 6.320712e-01 9.999207e-01

3545 6.318252e-01 9.999208e-01

3546 6.315834e-01 9.999208e-01

3547 6.313460e-01 9.999209e-01

3548 6.311131e-01 9.999210e-01

3549 6.308848e-01 9.999211e-01

3550 6.306613e-01 9.999212e-01

3551 6.304427e-01 9.999213e-01

3552 6.302290e-01 9.999213e-01

3553 6.300204e-01 9.999214e-01

3554 6.298170e-01 9.999215e-01

3555 6.296187e-01 9.999216e-01

3556 6.294258e-01 9.999218e-01

3557 6.292383e-01 9.999219e-01

3558 6.290562e-01 9.999220e-01

3559 6.288795e-01 9.999221e-01

3560 6.287084e-01 9.999222e-01

3561 6.285428e-01 9.999224e-01

3562 6.283828e-01 9.999225e-01

3563 6.282284e-01 9.999226e-01

3564 6.280796e-01 9.999228e-01

3565 6.279364e-01 9.999229e-01

3566 6.277988e-01 9.999231e-01

3567 6.276667e-01 9.999233e-01

3568 6.275402e-01 9.999234e-01

3569 6.274192e-01 9.999236e-01

3570 6.273037e-01 9.999238e-01

3571 6.271936e-01 9.999240e-01

3572 6.270889e-01 9.999242e-01

3573 6.269895e-01 9.999244e-01

3574 6.268954e-01 9.999246e-01

3575 6.268064e-01 9.999248e-01

3576 6.267226e-01 9.999250e-01

3577 6.266438e-01 9.999253e-01

3578 6.265699e-01 9.999255e-01

3579 6.265009e-01 9.999258e-01

3580 6.264366e-01 9.999260e-01

3581 6.263770e-01 9.999263e-01

3582 6.263219e-01 9.999266e-01

3583 6.262713e-01 9.999269e-01

3584 6.262250e-01 9.999272e-01

3585 6.261830e-01 9.999275e-01

3586 6.261450e-01 9.999278e-01

3587 6.261111e-01 9.999281e-01

3588 6.260811e-01 9.999285e-01

3589 6.260548e-01 9.999289e-01

3590 6.260322e-01 9.999292e-01

3591 6.260131e-01 9.999296e-01

3592 6.259975e-01 9.999300e-01

3593 6.259851e-01 9.999304e-01

3594 6.259760e-01 9.999309e-01

3595 6.259699e-01 9.999313e-01

3596 6.259668e-01 9.999318e-01

3597 6.259665e-01 9.999323e-01

3598 6.259689e-01 9.999328e-01

3599 6.259740e-01 9.999333e-01

3600 6.259816e-01 9.999338e-01

3601 6.259916e-01 9.999344e-01

3602 6.259712e-01 9.999344e-01

3603 6.259511e-01 9.999344e-01

3604 6.259312e-01 9.999344e-01

3605 6.259115e-01 9.999344e-01

3606 6.258922e-01 9.999344e-01

3607 6.258731e-01 9.999344e-01

3608 6.258543e-01 9.999344e-01

3609 6.258359e-01 9.999344e-01

3610 6.258179e-01 9.999344e-01

3611 6.258002e-01 9.999344e-01

3612 6.257830e-01 9.999344e-01

3613 6.257662e-01 9.999344e-01

3614 6.257499e-01 9.999344e-01

3615 6.257341e-01 9.999344e-01

3616 6.257189e-01 9.999344e-01

3617 6.257042e-01 9.999344e-01

3618 6.256901e-01 9.999344e-01

3619 6.256766e-01 9.999344e-01

3620 6.256638e-01 9.999344e-01

3621 6.256517e-01 9.999344e-01

3622 6.256404e-01 9.999344e-01

3623 6.256298e-01 9.999344e-01

3624 6.256200e-01 9.999344e-01

3625 6.256111e-01 9.999344e-01

3626 6.256031e-01 9.999344e-01

3627 6.255961e-01 9.999344e-01

3628 6.255900e-01 9.999344e-01

3629 6.255849e-01 9.999344e-01

3630 6.255809e-01 9.999344e-01

3631 6.255781e-01 9.999344e-01

3632 6.255764e-01 9.999344e-01

3633 6.255759e-01 9.999344e-01

3634 6.255767e-01 9.999344e-01

3635 6.255788e-01 9.999344e-01

3636 6.255824e-01 9.999344e-01

3637 6.255873e-01 9.999344e-01

3638 6.255938e-01 9.999344e-01

3639 6.256018e-01 9.999344e-01

3640 6.256114e-01 9.999344e-01

3641 6.256228e-01 9.999344e-01

3642 6.256358e-01 9.999344e-01

3643 6.256507e-01 9.999344e-01

3644 6.256675e-01 9.999344e-01

3645 6.256862e-01 9.999344e-01

3646 6.257069e-01 9.999344e-01

3647 6.257298e-01 9.999344e-01

3648 6.257548e-01 9.999344e-01

3649 6.257820e-01 9.999344e-01

3650 6.258116e-01 9.999344e-01

3651 6.258436e-01 9.999344e-01

3652 6.258780e-01 9.999344e-01

3653 6.259151e-01 9.999344e-01

3654 6.259548e-01 9.999344e-01

3655 6.259972e-01 9.999344e-01

3656 6.260425e-01 9.999344e-01

3657 6.260906e-01 9.999344e-01

3658 6.261418e-01 9.999344e-01

3659 6.261961e-01 9.999344e-01

3660 6.262536e-01 9.999344e-01

3661 6.263144e-01 9.999344e-01

3662 6.263785e-01 9.999344e-01

3663 6.264462e-01 9.999344e-01

3664 6.265174e-01 9.999344e-01

3665 6.265923e-01 9.999344e-01

3666 6.266710e-01 9.999344e-01

3667 6.267536e-01 9.999344e-01

3668 6.268402e-01 9.999344e-01

3669 6.269308e-01 9.999345e-01

3670 6.270257e-01 9.999345e-01

3671 6.271249e-01 9.999345e-01

3672 6.272285e-01 9.999345e-01

3673 6.273366e-01 9.999345e-01

3674 6.274493e-01 9.999345e-01

3675 6.275668e-01 9.999345e-01

3676 6.276891e-01 9.999345e-01

3677 6.278164e-01 9.999345e-01

3678 6.279487e-01 9.999345e-01

3679 6.280862e-01 9.999345e-01

3680 6.282289e-01 9.999345e-01

3681 6.283770e-01 9.999345e-01

3682 6.285305e-01 9.999345e-01

3683 6.286897e-01 9.999345e-01

3684 6.288545e-01 9.999345e-01

3685 6.290251e-01 9.999345e-01

3686 6.292015e-01 9.999345e-01

3687 6.293839e-01 9.999345e-01

3688 6.295724e-01 9.999345e-01

3689 6.297670e-01 9.999345e-01

3690 6.299678e-01 9.999345e-01

3691 6.301749e-01 9.999345e-01

3692 6.303885e-01 9.999345e-01

3693 6.306085e-01 9.999345e-01

3694 6.308350e-01 9.999345e-01

3695 6.310682e-01 9.999345e-01

3696 6.313080e-01 9.999345e-01

3697 6.315546e-01 9.999345e-01

3698 6.318079e-01 9.999345e-01

3699 6.320681e-01 9.999345e-01

3700 6.323352e-01 9.999345e-01

3701 6.326092e-01 9.999345e-01

3702 6.328902e-01 9.999345e-01

3703 6.331781e-01 9.999345e-01

3704 6.334731e-01 9.999345e-01

3705 6.337750e-01 9.999345e-01

3706 6.340840e-01 9.999345e-01

3707 6.344000e-01 9.999345e-01

3708 6.347230e-01 9.999345e-01

3709 6.350530e-01 9.999345e-01

3710 6.353900e-01 9.999345e-01

3711 6.357339e-01 9.999345e-01

3712 6.360847e-01 9.999345e-01

3713 6.364424e-01 9.999345e-01

3714 6.368069e-01 9.999345e-01

3715 6.371781e-01 9.999345e-01

3716 6.375560e-01 9.999345e-01

3717 6.379405e-01 9.999345e-01

3718 6.383314e-01 9.999345e-01

3719 6.387288e-01 9.999345e-01

3720 6.391325e-01 9.999345e-01

3721 6.395423e-01 9.999346e-01

3722 6.399582e-01 9.999346e-01

3723 6.403800e-01 9.999346e-01

3724 6.408077e-01 9.999346e-01

3725 6.412409e-01 9.999346e-01

3726 6.416797e-01 9.999346e-01

3727 6.421237e-01 9.999346e-01

3728 6.425729e-01 9.999346e-01

3729 6.430271e-01 9.999346e-01

3730 6.434861e-01 9.999346e-01

3731 6.439496e-01 9.999346e-01

3732 6.444176e-01 9.999346e-01

3733 6.448897e-01 9.999346e-01

3734 6.453659e-01 9.999346e-01

3735 6.458457e-01 9.999346e-01

3736 6.463292e-01 9.999346e-01

3737 6.468159e-01 9.999346e-01

3738 6.473057e-01 9.999345e-01

3739 6.477984e-01 9.999345e-01

3740 6.482937e-01 9.999345e-01

3741 6.487913e-01 9.999345e-01

3742 6.492911e-01 9.999345e-01

3743 6.497927e-01 9.999345e-01

3744 6.502960e-01 9.999345e-01

3745 6.508006e-01 9.999345e-01

3746 6.513064e-01 9.999345e-01

3747 6.518130e-01 9.999345e-01

3748 6.523203e-01 9.999345e-01

3749 6.528279e-01 9.999345e-01

3750 6.533357e-01 9.999345e-01

3751 6.538433e-01 9.999345e-01

3752 6.543506e-01 9.999344e-01

3753 6.548572e-01 9.999344e-01

3754 6.553630e-01 9.999344e-01

3755 6.558677e-01 9.999344e-01

3756 6.563711e-01 9.999344e-01

3757 6.568729e-01 9.999344e-01

3758 6.573730e-01 9.999343e-01

3759 6.578710e-01 9.999343e-01

3760 6.583669e-01 9.999343e-01

3761 6.588603e-01 9.999343e-01

3762 6.593510e-01 9.999342e-01

3763 6.598390e-01 9.999342e-01

3764 6.603239e-01 9.999342e-01

3765 6.608057e-01 9.999341e-01

3766 6.612840e-01 9.999341e-01

3767 6.617588e-01 9.999340e-01

3768 6.622299e-01 9.999340e-01

3769 6.626971e-01 9.999339e-01

3770 6.631602e-01 9.999339e-01

3771 6.636192e-01 9.999338e-01

3772 6.640739e-01 9.999337e-01

3773 6.645242e-01 9.999337e-01

3774 6.649699e-01 9.999336e-01

3775 6.654109e-01 9.999335e-01

3776 6.658472e-01 9.999334e-01

3777 6.662786e-01 9.999333e-01

3778 6.667051e-01 9.999332e-01

3779 6.671265e-01 9.999331e-01

3780 6.675428e-01 9.999330e-01

3781 6.679539e-01 9.999329e-01

3782 6.680218e-01 9.999329e-01

3783 6.680910e-01 9.999329e-01

3784 6.681618e-01 9.999329e-01

3785 6.682340e-01 9.999329e-01

3786 6.683077e-01 9.999329e-01

3787 6.683829e-01 9.999329e-01

3788 6.684597e-01 9.999329e-01

3789 6.685381e-01 9.999329e-01

3790 6.686180e-01 9.999329e-01

3791 6.686996e-01 9.999329e-01

3792 6.687829e-01 9.999329e-01

3793 6.688678e-01 9.999329e-01

3794 6.689545e-01 9.999329e-01

3795 6.690429e-01 9.999329e-01

3796 6.691331e-01 9.999329e-01

3797 6.692251e-01 9.999329e-01

3798 6.693190e-01 9.999329e-01

3799 6.694148e-01 9.999329e-01

3800 6.695125e-01 9.999329e-01

3801 6.696122e-01 9.999329e-01

3802 6.697139e-01 9.999329e-01

3803 6.698176e-01 9.999329e-01

3804 6.699235e-01 9.999329e-01

3805 6.700314e-01 9.999329e-01

3806 6.701416e-01 9.999328e-01

3807 6.702540e-01 9.999328e-01

3808 6.703686e-01 9.999328e-01

3809 6.704855e-01 9.999328e-01

3810 6.706049e-01 9.999328e-01

3811 6.707266e-01 9.999328e-01

3812 6.708507e-01 9.999328e-01

3813 6.709774e-01 9.999328e-01

3814 6.711066e-01 9.999328e-01

3815 6.712384e-01 9.999328e-01

3816 6.713729e-01 9.999328e-01

3817 6.715101e-01 9.999328e-01

3818 6.716500e-01 9.999328e-01

3819 6.717928e-01 9.999328e-01

3820 6.719384e-01 9.999328e-01

3821 6.720870e-01 9.999328e-01

3822 6.722385e-01 9.999328e-01

3823 6.723931e-01 9.999328e-01

3824 6.725507e-01 9.999328e-01

3825 6.727115e-01 9.999328e-01

3826 6.728755e-01 9.999328e-01

3827 6.730428e-01 9.999328e-01

3828 6.732135e-01 9.999328e-01

3829 6.733875e-01 9.999328e-01

3830 6.735650e-01 9.999328e-01

3831 6.737460e-01 9.999328e-01

3832 6.739306e-01 9.999328e-01

3833 6.741188e-01 9.999328e-01

3834 6.743108e-01 9.999328e-01

3835 6.745065e-01 9.999328e-01

3836 6.747061e-01 9.999328e-01

3837 6.749096e-01 9.999328e-01

3838 6.751171e-01 9.999328e-01

3839 6.753286e-01 9.999328e-01

3840 6.755443e-01 9.999327e-01

3841 6.757641e-01 9.999327e-01

3842 6.759882e-01 9.999327e-01

3843 6.762166e-01 9.999327e-01

3844 6.764494e-01 9.999327e-01

3845 6.766867e-01 9.999327e-01

3846 6.769285e-01 9.999327e-01

3847 6.771749e-01 9.999327e-01

3848 6.774259e-01 9.999327e-01

3849 6.776817e-01 9.999327e-01

3850 6.779423e-01 9.999327e-01

3851 6.782078e-01 9.999327e-01

3852 6.784783e-01 9.999327e-01

3853 6.787537e-01 9.999327e-01

3854 6.790342e-01 9.999326e-01

3855 6.793199e-01 9.999326e-01

3856 6.796108e-01 9.999326e-01

3857 6.799070e-01 9.999326e-01

3858 6.802085e-01 9.999326e-01

3859 6.805154e-01 9.999326e-01

3860 6.808278e-01 9.999326e-01

3861 6.811457e-01 9.999326e-01

3862 6.814692e-01 9.999326e-01

3863 6.817983e-01 9.999325e-01

3864 6.821332e-01 9.999325e-01

3865 6.824738e-01 9.999325e-01

3866 6.828202e-01 9.999325e-01

3867 6.831725e-01 9.999325e-01

3868 6.835307e-01 9.999325e-01

3869 6.838949e-01 9.999325e-01

3870 6.842650e-01 9.999324e-01

3871 6.846412e-01 9.999324e-01

3872 6.850235e-01 9.999324e-01

3873 6.854119e-01 9.999324e-01

3874 6.858064e-01 9.999324e-01

3875 6.862071e-01 9.999323e-01

3876 6.866141e-01 9.999323e-01

3877 6.870272e-01 9.999323e-01

3878 6.874466e-01 9.999323e-01

3879 6.878722e-01 9.999323e-01

3880 6.883041e-01 9.999322e-01

3881 6.887423e-01 9.999322e-01

3882 6.891868e-01 9.999322e-01

3883 6.896375e-01 9.999321e-01

3884 6.900945e-01 9.999321e-01

3885 6.905577e-01 9.999321e-01

3886 6.910272e-01 9.999321e-01

3887 6.915029e-01 9.999320e-01

3888 6.919848e-01 9.999320e-01

3889 6.924728e-01 9.999320e-01

3890 6.929669e-01 9.999319e-01

3891 6.934672e-01 9.999319e-01

3892 6.939734e-01 9.999318e-01

3893 6.944856e-01 9.999318e-01

3894 6.950037e-01 9.999317e-01

3895 6.955277e-01 9.999317e-01

3896 6.960574e-01 9.999317e-01

3897 6.965928e-01 9.999316e-01

3898 6.971337e-01 9.999315e-01

3899 6.976802e-01 9.999315e-01

3900 6.982321e-01 9.999314e-01

3901 6.987893e-01 9.999314e-01

3902 6.993517e-01 9.999313e-01

3903 6.999192e-01 9.999312e-01

3904 7.004916e-01 9.999312e-01

3905 7.010688e-01 9.999311e-01

3906 7.016508e-01 9.999310e-01

3907 7.022372e-01 9.999310e-01

3908 7.028280e-01 9.999309e-01

3909 7.034231e-01 9.999308e-01

3910 7.040222e-01 9.999307e-01

3911 7.046253e-01 9.999306e-01

3912 7.052321e-01 9.999305e-01

3913 7.058424e-01 9.999304e-01

3914 7.064561e-01 9.999303e-01

3915 7.070730e-01 9.999302e-01

3916 7.076928e-01 9.999301e-01

3917 7.083155e-01 9.999300e-01

3918 7.089407e-01 9.999298e-01

3919 7.095683e-01 9.999297e-01

3920 7.101980e-01 9.999296e-01

3921 7.108297e-01 9.999294e-01

3922 7.114632e-01 9.999293e-01

3923 7.120981e-01 9.999291e-01

3924 7.127343e-01 9.999290e-01

3925 7.133715e-01 9.999288e-01

3926 7.140096e-01 9.999286e-01

3927 7.146482e-01 9.999284e-01

3928 7.152872e-01 9.999282e-01

3929 7.159263e-01 9.999280e-01

3930 7.165652e-01 9.999278e-01

3931 7.172038e-01 9.999276e-01

3932 7.178418e-01 9.999273e-01

3933 7.184789e-01 9.999271e-01

3934 7.191150e-01 9.999268e-01

3935 7.197497e-01 9.999265e-01

3936 7.203829e-01 9.999263e-01

3937 7.210142e-01 9.999260e-01

3938 7.216436e-01 9.999256e-01

3939 7.222707e-01 9.999253e-01

3940 7.228952e-01 9.999250e-01

3941 7.235171e-01 9.999246e-01

3942 7.241361e-01 9.999242e-01

3943 7.247519e-01 9.999238e-01

3944 7.253643e-01 9.999234e-01

3945 7.259731e-01 9.999229e-01

3946 7.265782e-01 9.999225e-01

3947 7.271793e-01 9.999220e-01

3948 7.277762e-01 9.999215e-01

3949 7.283687e-01 9.999209e-01

3950 7.289566e-01 9.999203e-01

3951 7.295398e-01 9.999197e-01

3952 7.301181e-01 9.999191e-01

3953 7.306914e-01 9.999184e-01

3954 7.312593e-01 9.999177e-01

3955 7.318219e-01 9.999170e-01

3956 7.323790e-01 9.999162e-01

3957 7.329303e-01 9.999154e-01

3958 7.334759e-01 9.999146e-01

3959 7.340155e-01 9.999137e-01

3960 7.345490e-01 9.999127e-01

3961 7.350763e-01 9.999117e-01

3962 7.351358e-01 9.999117e-01

3963 7.351961e-01 9.999117e-01

3964 7.352570e-01 9.999117e-01

3965 7.353188e-01 9.999117e-01

3966 7.353813e-01 9.999117e-01

3967 7.354447e-01 9.999117e-01

3968 7.355088e-01 9.999117e-01

3969 7.355738e-01 9.999117e-01

3970 7.356396e-01 9.999117e-01

3971 7.357062e-01 9.999117e-01

3972 7.357737e-01 9.999117e-01

3973 7.358421e-01 9.999117e-01

3974 7.359115e-01 9.999117e-01

3975 7.359817e-01 9.999117e-01

3976 7.360528e-01 9.999117e-01

3977 7.361250e-01 9.999117e-01

3978 7.361981e-01 9.999117e-01

3979 7.362721e-01 9.999116e-01

3980 7.363472e-01 9.999116e-01

3981 7.364233e-01 9.999116e-01

3982 7.365005e-01 9.999116e-01

3983 7.365787e-01 9.999116e-01

3984 7.366580e-01 9.999116e-01

3985 7.367383e-01 9.999116e-01

3986 7.368198e-01 9.999116e-01

3987 7.369025e-01 9.999116e-01

3988 7.369863e-01 9.999116e-01

3989 7.370712e-01 9.999116e-01

3990 7.371574e-01 9.999116e-01

3991 7.372448e-01 9.999116e-01

3992 7.373334e-01 9.999116e-01

3993 7.374233e-01 9.999116e-01

3994 7.375145e-01 9.999116e-01

3995 7.376069e-01 9.999116e-01

3996 7.377007e-01 9.999116e-01

3997 7.377958e-01 9.999116e-01

3998 7.378923e-01 9.999116e-01

3999 7.379902e-01 9.999116e-01

4000 7.380895e-01 9.999116e-01

4001 7.381902e-01 9.999115e-01

4002 7.382923e-01 9.999115e-01

4003 7.383960e-01 9.999115e-01

4004 7.385011e-01 9.999115e-01

4005 7.386077e-01 9.999115e-01

4006 7.387159e-01 9.999115e-01

4007 7.388257e-01 9.999115e-01

4008 7.389370e-01 9.999115e-01

4009 7.390500e-01 9.999115e-01

4010 7.391646e-01 9.999115e-01

4011 7.392808e-01 9.999115e-01

4012 7.393987e-01 9.999115e-01

4013 7.395183e-01 9.999114e-01

4014 7.396397e-01 9.999114e-01

4015 7.397627e-01 9.999114e-01

4016 7.398876e-01 9.999114e-01

4017 7.400143e-01 9.999114e-01

4018 7.401427e-01 9.999114e-01

4019 7.402730e-01 9.999114e-01

4020 7.404052e-01 9.999114e-01

4021 7.405392e-01 9.999113e-01

4022 7.406752e-01 9.999113e-01

4023 7.408131e-01 9.999113e-01

4024 7.409529e-01 9.999113e-01

4025 7.410948e-01 9.999113e-01

4026 7.412386e-01 9.999113e-01

4027 7.413844e-01 9.999113e-01

4028 7.415323e-01 9.999112e-01

4029 7.416822e-01 9.999112e-01

4030 7.418343e-01 9.999112e-01

4031 7.419884e-01 9.999112e-01

4032 7.421447e-01 9.999112e-01

4033 7.423031e-01 9.999111e-01

4034 7.424636e-01 9.999111e-01

4035 7.426264e-01 9.999111e-01

4036 7.427914e-01 9.999111e-01

4037 7.429586e-01 9.999110e-01

4038 7.431280e-01 9.999110e-01

4039 7.432997e-01 9.999110e-01

4040 7.434736e-01 9.999110e-01

4041 7.436499e-01 9.999109e-01

4042 7.438285e-01 9.999109e-01

4043 7.440093e-01 9.999109e-01

4044 7.441926e-01 9.999108e-01

4045 7.443781e-01 9.999108e-01

4046 7.445661e-01 9.999108e-01

4047 7.447564e-01 9.999107e-01

4048 7.449491e-01 9.999107e-01

4049 7.451441e-01 9.999107e-01

4050 7.453416e-01 9.999106e-01

4051 7.455415e-01 9.999106e-01

4052 7.457438e-01 9.999105e-01

4053 7.459486e-01 9.999105e-01

4054 7.461557e-01 9.999104e-01

4055 7.463653e-01 9.999104e-01

4056 7.465773e-01 9.999103e-01

4057 7.467918e-01 9.999103e-01

4058 7.470086e-01 9.999102e-01

4059 7.472279e-01 9.999102e-01

4060 7.474496e-01 9.999101e-01

4061 7.476737e-01 9.999101e-01

4062 7.479003e-01 9.999100e-01

4063 7.481292e-01 9.999099e-01

4064 7.483605e-01 9.999099e-01

4065 7.485942e-01 9.999098e-01

4066 7.488302e-01 9.999097e-01

4067 7.490685e-01 9.999096e-01

4068 7.493092e-01 9.999095e-01

4069 7.495522e-01 9.999095e-01

4070 7.497974e-01 9.999094e-01

4071 7.500449e-01 9.999093e-01

4072 7.502946e-01 9.999092e-01

4073 7.505465e-01 9.999091e-01

4074 7.508006e-01 9.999090e-01

4075 7.510568e-01 9.999089e-01

4076 7.513150e-01 9.999087e-01

4077 7.515753e-01 9.999086e-01

4078 7.518377e-01 9.999085e-01

4079 7.521019e-01 9.999084e-01

4080 7.523681e-01 9.999082e-01

4081 7.526361e-01 9.999081e-01

4082 7.529060e-01 9.999079e-01

4083 7.531776e-01 9.999078e-01

4084 7.534509e-01 9.999076e-01

4085 7.537259e-01 9.999074e-01

4086 7.540024e-01 9.999072e-01

4087 7.542804e-01 9.999070e-01

4088 7.545599e-01 9.999068e-01

4089 7.548408e-01 9.999066e-01

4090 7.551230e-01 9.999064e-01

4091 7.554064e-01 9.999062e-01

4092 7.556909e-01 9.999059e-01

4093 7.559765e-01 9.999057e-01

4094 7.562632e-01 9.999054e-01

4095 7.565507e-01 9.999051e-01

4096 7.568391e-01 9.999048e-01

4097 7.571282e-01 9.999045e-01

4098 7.574179e-01 9.999042e-01

4099 7.577082e-01 9.999039e-01

4100 7.579990e-01 9.999035e-01

4101 7.582901e-01 9.999032e-01

4102 7.585815e-01 9.999028e-01

4103 7.588731e-01 9.999023e-01

4104 7.591647e-01 9.999019e-01

4105 7.594563e-01 9.999015e-01

4106 7.597478e-01 9.999010e-01

4107 7.600390e-01 9.999005e-01

4108 7.603299e-01 9.998999e-01

4109 7.606204e-01 9.998994e-01

4110 7.609103e-01 9.998988e-01

4111 7.611995e-01 9.998982e-01

4112 7.614880e-01 9.998975e-01

4113 7.617756e-01 9.998968e-01

4114 7.620623e-01 9.998961e-01

4115 7.623478e-01 9.998953e-01

4116 7.626322e-01 9.998945e-01

4117 7.629152e-01 9.998937e-01

4118 7.631969e-01 9.998928e-01

4119 7.634771e-01 9.998918e-01

4120 7.637556e-01 9.998908e-01

4121 7.640325e-01 9.998898e-01

4122 7.643075e-01 9.998887e-01

4123 7.645806e-01 9.998875e-01

4124 7.648517e-01 9.998862e-01

4125 7.651207e-01 9.998849e-01

4126 7.653875e-01 9.998835e-01

4127 7.656521e-01 9.998820e-01

4128 7.659142e-01 9.998805e-01

4129 7.661739e-01 9.998788e-01

4130 7.664310e-01 9.998771e-01

4131 7.666855e-01 9.998753e-01

4132 7.669373e-01 9.998733e-01

4133 7.671863e-01 9.998712e-01

4134 7.674324e-01 9.998691e-01

4135 7.676756e-01 9.998667e-01

4136 7.679158e-01 9.998643e-01

4137 7.681529e-01 9.998617e-01

4138 7.683869e-01 9.998590e-01

4139 7.686177e-01 9.998560e-01

4140 7.688453e-01 9.998529e-01

4141 7.690696e-01 9.998497e-01

4142 7.690978e-01 9.998497e-01

4143 7.691259e-01 9.998497e-01

4144 7.691538e-01 9.998497e-01

4145 7.691816e-01 9.998497e-01

4146 7.692091e-01 9.998497e-01

4147 7.692365e-01 9.998497e-01

4148 7.692636e-01 9.998497e-01

4149 7.692906e-01 9.998497e-01

4150 7.693173e-01 9.998496e-01

4151 7.693438e-01 9.998496e-01

4152 7.693701e-01 9.998496e-01

4153 7.693962e-01 9.998496e-01

4154 7.694220e-01 9.998496e-01

4155 7.694476e-01 9.998496e-01

4156 7.694729e-01 9.998496e-01

4157 7.694980e-01 9.998496e-01

4158 7.695228e-01 9.998496e-01

4159 7.695474e-01 9.998496e-01

4160 7.695716e-01 9.998496e-01

4161 7.695956e-01 9.998496e-01

4162 7.696193e-01 9.998496e-01

4163 7.696427e-01 9.998496e-01

4164 7.696658e-01 9.998496e-01

4165 7.696886e-01 9.998496e-01

4166 7.697111e-01 9.998496e-01

4167 7.697333e-01 9.998496e-01

4168 7.697552e-01 9.998495e-01

4169 7.697767e-01 9.998495e-01

4170 7.697978e-01 9.998495e-01

4171 7.698187e-01 9.998495e-01

4172 7.698391e-01 9.998495e-01

4173 7.698592e-01 9.998495e-01

4174 7.698790e-01 9.998495e-01

4175 7.698983e-01 9.998495e-01

4176 7.699173e-01 9.998495e-01

4177 7.699358e-01 9.998495e-01

4178 7.699540e-01 9.998495e-01

4179 7.699717e-01 9.998495e-01

4180 7.699891e-01 9.998494e-01

4181 7.700059e-01 9.998494e-01

4182 7.700224e-01 9.998494e-01

4183 7.700384e-01 9.998494e-01

4184 7.700539e-01 9.998494e-01

4185 7.700689e-01 9.998494e-01

4186 7.700834e-01 9.998494e-01

4187 7.700974e-01 9.998494e-01

4188 7.701109e-01 9.998493e-01

4189 7.701239e-01 9.998493e-01

4190 7.701363e-01 9.998493e-01

4191 7.701482e-01 9.998493e-01

4192 7.701595e-01 9.998493e-01

4193 7.701702e-01 9.998493e-01

4194 7.701803e-01 9.998492e-01

4195 7.701897e-01 9.998492e-01

4196 7.701985e-01 9.998492e-01

4197 7.702067e-01 9.998492e-01

4198 7.702141e-01 9.998492e-01

4199 7.702209e-01 9.998491e-01

4200 7.702269e-01 9.998491e-01

4201 7.702322e-01 9.998491e-01

4202 7.702368e-01 9.998491e-01

4203 7.702405e-01 9.998491e-01

4204 7.702435e-01 9.998490e-01

4205 7.702456e-01 9.998490e-01

4206 7.702468e-01 9.998490e-01

4207 7.702472e-01 9.998489e-01

4208 7.702467e-01 9.998489e-01

4209 7.702452e-01 9.998489e-01

4210 7.702428e-01 9.998489e-01

4211 7.702394e-01 9.998488e-01

4212 7.702350e-01 9.998488e-01

4213 7.702296e-01 9.998488e-01

4214 7.702231e-01 9.998487e-01

4215 7.702155e-01 9.998487e-01

4216 7.702067e-01 9.998486e-01

4217 7.701968e-01 9.998486e-01

4218 7.701857e-01 9.998486e-01

4219 7.701734e-01 9.998485e-01

4220 7.701598e-01 9.998485e-01

4221 7.701449e-01 9.998484e-01

4222 7.701287e-01 9.998484e-01

4223 7.701111e-01 9.998483e-01

4224 7.700921e-01 9.998483e-01

4225 7.700717e-01 9.998482e-01

4226 7.700498e-01 9.998481e-01

4227 7.700264e-01 9.998481e-01

4228 7.700014e-01 9.998480e-01

4229 7.699748e-01 9.998479e-01

4230 7.699466e-01 9.998479e-01

4231 7.699168e-01 9.998478e-01

4232 7.698852e-01 9.998477e-01

4233 7.698519e-01 9.998476e-01

4234 7.698167e-01 9.998476e-01

4235 7.697798e-01 9.998475e-01

4236 7.697409e-01 9.998474e-01

4237 7.697002e-01 9.998473e-01

4238 7.696574e-01 9.998472e-01

4239 7.696127e-01 9.998471e-01

4240 7.695659e-01 9.998470e-01

4241 7.695170e-01 9.998469e-01

4242 7.694660e-01 9.998467e-01

4243 7.694129e-01 9.998466e-01

4244 7.693574e-01 9.998465e-01

4245 7.692998e-01 9.998463e-01

4246 7.692398e-01 9.998462e-01

4247 7.691775e-01 9.998460e-01

4248 7.691127e-01 9.998459e-01

4249 7.690456e-01 9.998457e-01

4250 7.689760e-01 9.998455e-01

4251 7.689038e-01 9.998453e-01

4252 7.688291e-01 9.998452e-01

4253 7.687518e-01 9.998450e-01

4254 7.686719e-01 9.998447e-01

4255 7.685893e-01 9.998445e-01

4256 7.685041e-01 9.998443e-01

4257 7.684160e-01 9.998440e-01

4258 7.683252e-01 9.998438e-01

4259 7.682316e-01 9.998435e-01

4260 7.681352e-01 9.998432e-01

4261 7.680359e-01 9.998429e-01

4262 7.679337e-01 9.998426e-01

4263 7.678286e-01 9.998422e-01

4264 7.677205e-01 9.998419e-01

4265 7.676095e-01 9.998415e-01

4266 7.674955e-01 9.998411e-01

4267 7.673784e-01 9.998407e-01

4268 7.672584e-01 9.998402e-01

4269 7.671353e-01 9.998397e-01

4270 7.670092e-01 9.998393e-01

4271 7.668800e-01 9.998387e-01

4272 7.667478e-01 9.998382e-01

4273 7.666125e-01 9.998376e-01

4274 7.664741e-01 9.998370e-01

4275 7.663327e-01 9.998363e-01

4276 7.661882e-01 9.998356e-01

4277 7.660407e-01 9.998349e-01

4278 7.658901e-01 9.998341e-01

4279 7.657365e-01 9.998333e-01

4280 7.655800e-01 9.998324e-01

4281 7.654204e-01 9.998315e-01

4282 7.652579e-01 9.998306e-01

4283 7.650924e-01 9.998295e-01

4284 7.649241e-01 9.998285e-01

4285 7.647529e-01 9.998273e-01

4286 7.645789e-01 9.998261e-01

4287 7.644021e-01 9.998248e-01

4288 7.642226e-01 9.998234e-01

4289 7.640404e-01 9.998220e-01

4290 7.638555e-01 9.998205e-01

4291 7.636681e-01 9.998188e-01

4292 7.634782e-01 9.998171e-01

4293 7.632859e-01 9.998153e-01

4294 7.630911e-01 9.998134e-01

4295 7.628941e-01 9.998113e-01

4296 7.626948e-01 9.998091e-01

4297 7.624934e-01 9.998068e-01

4298 7.622899e-01 9.998044e-01

4299 7.620844e-01 9.998018e-01

4300 7.618770e-01 9.997991e-01

4301 7.616678e-01 9.997961e-01

4302 7.614568e-01 9.997930e-01

4303 7.612442e-01 9.997898e-01

4304 7.610301e-01 9.997863e-01

4305 7.608145e-01 9.997826e-01

4306 7.605976e-01 9.997787e-01

4307 7.603794e-01 9.997745e-01

4308 7.601601e-01 9.997701e-01

4309 7.599397e-01 9.997654e-01

4310 7.597184e-01 9.997605e-01

4311 7.594963e-01 9.997552e-01

4312 7.592735e-01 9.997496e-01

4313 7.590500e-01 9.997437e-01

4314 7.588261e-01 9.997374e-01

4315 7.586017e-01 9.997308e-01

4316 7.583771e-01 9.997237e-01

4317 7.581523e-01 9.997162e-01

4318 7.579274e-01 9.997082e-01

4319 7.577025e-01 9.996998e-01

4320 7.574777e-01 9.996908e-01

4321 7.572532e-01 9.996813e-01

4322 7.572406e-01 9.996813e-01

4323 7.572270e-01 9.996813e-01

4324 7.572126e-01 9.996813e-01

4325 7.571972e-01 9.996813e-01

4326 7.571808e-01 9.996813e-01

4327 7.571635e-01 9.996813e-01

4328 7.571451e-01 9.996813e-01

4329 7.571257e-01 9.996813e-01

4330 7.571052e-01 9.996813e-01

4331 7.570835e-01 9.996813e-01

4332 7.570607e-01 9.996813e-01

4333 7.570368e-01 9.996813e-01

4334 7.570116e-01 9.996813e-01

4335 7.569851e-01 9.996813e-01

4336 7.569574e-01 9.996812e-01

4337 7.569283e-01 9.996812e-01

4338 7.568979e-01 9.996812e-01

4339 7.568661e-01 9.996812e-01

4340 7.568328e-01 9.996812e-01

4341 7.567981e-01 9.996812e-01

4342 7.567619e-01 9.996812e-01

4343 7.567242e-01 9.996812e-01

4344 7.566849e-01 9.996812e-01

4345 7.566439e-01 9.996812e-01

4346 7.566014e-01 9.996812e-01

4347 7.565571e-01 9.996812e-01

4348 7.565111e-01 9.996812e-01

4349 7.564633e-01 9.996812e-01

4350 7.564137e-01 9.996811e-01

4351 7.563623e-01 9.996811e-01

4352 7.563089e-01 9.996811e-01

4353 7.562537e-01 9.996811e-01

4354 7.561964e-01 9.996811e-01

4355 7.561372e-01 9.996811e-01

4356 7.560758e-01 9.996811e-01

4357 7.560124e-01 9.996811e-01

4358 7.559468e-01 9.996811e-01

4359 7.558790e-01 9.996810e-01

4360 7.558090e-01 9.996810e-01

4361 7.557366e-01 9.996810e-01

4362 7.556619e-01 9.996810e-01

4363 7.555849e-01 9.996810e-01

4364 7.555054e-01 9.996810e-01

4365 7.554233e-01 9.996810e-01

4366 7.553388e-01 9.996809e-01

4367 7.552517e-01 9.996809e-01

4368 7.551619e-01 9.996809e-01

4369 7.550694e-01 9.996809e-01

4370 7.549741e-01 9.996809e-01

4371 7.548761e-01 9.996808e-01

4372 7.547752e-01 9.996808e-01

4373 7.546713e-01 9.996808e-01

4374 7.545645e-01 9.996808e-01

4375 7.544547e-01 9.996808e-01

4376 7.543417e-01 9.996807e-01

4377 7.542256e-01 9.996807e-01

4378 7.541063e-01 9.996807e-01

4379 7.539837e-01 9.996807e-01

4380 7.538578e-01 9.996806e-01

4381 7.537285e-01 9.996806e-01

4382 7.535957e-01 9.996806e-01

4383 7.534593e-01 9.996805e-01

4384 7.533194e-01 9.996805e-01

4385 7.531758e-01 9.996805e-01

4386 7.530285e-01 9.996804e-01

4387 7.528774e-01 9.996804e-01

4388 7.527225e-01 9.996804e-01

4389 7.525636e-01 9.996803e-01

4390 7.524007e-01 9.996803e-01

4391 7.522337e-01 9.996802e-01

4392 7.520626e-01 9.996802e-01

4393 7.518873e-01 9.996801e-01

4394 7.517077e-01 9.996801e-01

4395 7.515238e-01 9.996800e-01

4396 7.513355e-01 9.996800e-01

4397 7.511427e-01 9.996799e-01

4398 7.509453e-01 9.996798e-01

4399 7.507433e-01 9.996798e-01

4400 7.505366e-01 9.996797e-01

4401 7.503251e-01 9.996796e-01

4402 7.501088e-01 9.996796e-01

4403 7.498876e-01 9.996795e-01

4404 7.496614e-01 9.996794e-01

4405 7.494301e-01 9.996793e-01

4406 7.491938e-01 9.996792e-01

4407 7.489523e-01 9.996791e-01

4408 7.487055e-01 9.996790e-01

4409 7.484535e-01 9.996789e-01

4410 7.481960e-01 9.996788e-01

4411 7.479332e-01 9.996787e-01

4412 7.476649e-01 9.996785e-01

4413 7.473910e-01 9.996784e-01

4414 7.471115e-01 9.996783e-01

4415 7.468263e-01 9.996781e-01

4416 7.465355e-01 9.996780e-01

4417 7.462389e-01 9.996778e-01

4418 7.459365e-01 9.996776e-01

4419 7.456283e-01 9.996775e-01

4420 7.453141e-01 9.996773e-01

4421 7.449941e-01 9.996771e-01

4422 7.446680e-01 9.996769e-01

4423 7.443360e-01 9.996766e-01

4424 7.439980e-01 9.996764e-01

4425 7.436539e-01 9.996761e-01

4426 7.433038e-01 9.996759e-01

4427 7.429476e-01 9.996756e-01

4428 7.425853e-01 9.996753e-01

4429 7.422170e-01 9.996750e-01

4430 7.418425e-01 9.996747e-01

4431 7.414619e-01 9.996743e-01

4432 7.410753e-01 9.996740e-01

4433 7.406826e-01 9.996736e-01

4434 7.402838e-01 9.996731e-01

4435 7.398791e-01 9.996727e-01

4436 7.394683e-01 9.996722e-01

4437 7.390516e-01 9.996718e-01

4438 7.386290e-01 9.996712e-01

4439 7.382005e-01 9.996707e-01

4440 7.377662e-01 9.996701e-01

4441 7.373262e-01 9.996695e-01

4442 7.368805e-01 9.996688e-01

4443 7.364292e-01 9.996682e-01

4444 7.359724e-01 9.996674e-01

4445 7.355102e-01 9.996667e-01

4446 7.350427e-01 9.996658e-01

4447 7.345700e-01 9.996650e-01

4448 7.340922e-01 9.996640e-01

4449 7.336094e-01 9.996631e-01

4450 7.331217e-01 9.996620e-01

4451 7.326293e-01 9.996609e-01

4452 7.321324e-01 9.996598e-01

4453 7.316310e-01 9.996586e-01

4454 7.311253e-01 9.996573e-01

4455 7.306155e-01 9.996559e-01

4456 7.301018e-01 9.996544e-01

4457 7.295843e-01 9.996529e-01

4458 7.290632e-01 9.996512e-01

4459 7.285387e-01 9.996495e-01

4460 7.280110e-01 9.996476e-01

4461 7.274803e-01 9.996457e-01

4462 7.269468e-01 9.996436e-01

4463 7.264107e-01 9.996414e-01

4464 7.258723e-01 9.996391e-01

4465 7.253316e-01 9.996367e-01

4466 7.247891e-01 9.996341e-01

4467 7.242448e-01 9.996313e-01

4468 7.236991e-01 9.996284e-01

4469 7.231521e-01 9.996253e-01

4470 7.226042e-01 9.996220e-01

4471 7.220555e-01 9.996186e-01

4472 7.215062e-01 9.996149e-01

4473 7.209567e-01 9.996110e-01

4474 7.204072e-01 9.996069e-01

4475 7.198579e-01 9.996025e-01

4476 7.193091e-01 9.995979e-01

4477 7.187610e-01 9.995931e-01

4478 7.182139e-01 9.995879e-01

4479 7.176679e-01 9.995824e-01

4480 7.171235e-01 9.995767e-01

4481 7.165807e-01 9.995706e-01

4482 7.160398e-01 9.995641e-01

4483 7.155012e-01 9.995573e-01

4484 7.149649e-01 9.995500e-01

4485 7.144312e-01 9.995424e-01

4486 7.139004e-01 9.995343e-01

4487 7.133727e-01 9.995258e-01

4488 7.128483e-01 9.995168e-01

4489 7.123274e-01 9.995072e-01

4490 7.118102e-01 9.994972e-01

4491 7.112968e-01 9.994865e-01

4492 7.107876e-01 9.994753e-01

4493 7.102827e-01 9.994634e-01

4494 7.097822e-01 9.994509e-01

4495 7.092864e-01 9.994377e-01

4496 7.087954e-01 9.994237e-01

4497 7.083093e-01 9.994089e-01

4498 7.078283e-01 9.993934e-01

4499 7.073526e-01 9.993770e-01

4500 7.068823e-01 9.993596e-01

4501 7.064174e-01 9.993414e-01

4502 7.063562e-01 9.993414e-01

4503 7.062931e-01 9.993414e-01

4504 7.062282e-01 9.993414e-01

4505 7.061615e-01 9.993413e-01

4506 7.060930e-01 9.993413e-01

4507 7.060224e-01 9.993413e-01

4508 7.059500e-01 9.993413e-01

4509 7.058755e-01 9.993413e-01

4510 7.057990e-01 9.993413e-01

4511 7.057204e-01 9.993413e-01

4512 7.056397e-01 9.993413e-01

4513 7.055569e-01 9.993413e-01

4514 7.054718e-01 9.993413e-01

4515 7.053844e-01 9.993413e-01

4516 7.052948e-01 9.993413e-01

4517 7.052028e-01 9.993413e-01

4518 7.051084e-01 9.993413e-01

4519 7.050115e-01 9.993413e-01

4520 7.049121e-01 9.993413e-01

4521 7.048102e-01 9.993413e-01

4522 7.047057e-01 9.993413e-01

4523 7.045986e-01 9.993413e-01

4524 7.044887e-01 9.993413e-01

4525 7.043760e-01 9.993413e-01

4526 7.042606e-01 9.993413e-01

4527 7.041423e-01 9.993412e-01

4528 7.040210e-01 9.993412e-01

4529 7.038968e-01 9.993412e-01

4530 7.037695e-01 9.993412e-01

4531 7.036392e-01 9.993412e-01

4532 7.035057e-01 9.993412e-01

4533 7.033689e-01 9.993412e-01

4534 7.032289e-01 9.993412e-01

4535 7.030856e-01 9.993412e-01

4536 7.029389e-01 9.993412e-01

4537 7.027888e-01 9.993412e-01

4538 7.026352e-01 9.993412e-01

4539 7.024779e-01 9.993411e-01

4540 7.023171e-01 9.993411e-01

4541 7.021526e-01 9.993411e-01

4542 7.019843e-01 9.993411e-01

4543 7.018122e-01 9.993411e-01

4544 7.016362e-01 9.993411e-01

4545 7.014563e-01 9.993411e-01

4546 7.012724e-01 9.993410e-01

4547 7.010845e-01 9.993410e-01

4548 7.008924e-01 9.993410e-01

4549 7.006961e-01 9.993410e-01

4550 7.004956e-01 9.993410e-01

4551 7.002907e-01 9.993410e-01

4552 7.000815e-01 9.993409e-01

4553 6.998678e-01 9.993409e-01

4554 6.996497e-01 9.993409e-01

4555 6.994269e-01 9.993409e-01

4556 6.991996e-01 9.993408e-01

4557 6.989675e-01 9.993408e-01

4558 6.987307e-01 9.993408e-01

4559 6.984891e-01 9.993408e-01

4560 6.982426e-01 9.993407e-01

4561 6.979912e-01 9.993407e-01

4562 6.977348e-01 9.993407e-01

4563 6.974734e-01 9.993406e-01

4564 6.972068e-01 9.993406e-01

4565 6.969351e-01 9.993406e-01

4566 6.966581e-01 9.993405e-01

4567 6.963759e-01 9.993405e-01

4568 6.960884e-01 9.993404e-01

4569 6.957955e-01 9.993404e-01

4570 6.954972e-01 9.993403e-01

4571 6.951934e-01 9.993403e-01

4572 6.948841e-01 9.993402e-01

4573 6.945692e-01 9.993401e-01

4574 6.942487e-01 9.993401e-01

4575 6.939226e-01 9.993400e-01

4576 6.935909e-01 9.993399e-01

4577 6.932534e-01 9.993399e-01

4578 6.929102e-01 9.993398e-01

4579 6.925612e-01 9.993397e-01

4580 6.922064e-01 9.993396e-01

4581 6.918458e-01 9.993395e-01

4582 6.914794e-01 9.993394e-01

4583 6.911071e-01 9.993393e-01

4584 6.907290e-01 9.993392e-01

4585 6.903450e-01 9.993391e-01

4586 6.899551e-01 9.993390e-01

4587 6.895594e-01 9.993388e-01

4588 6.891578e-01 9.993387e-01

4589 6.887503e-01 9.993386e-01

4590 6.883370e-01 9.993384e-01

4591 6.879179e-01 9.993383e-01

4592 6.874930e-01 9.993381e-01

4593 6.870623e-01 9.993379e-01

4594 6.866259e-01 9.993377e-01

4595 6.861838e-01 9.993375e-01

4596 6.857360e-01 9.993373e-01

4597 6.852827e-01 9.993371e-01

4598 6.848238e-01 9.993368e-01

4599 6.843594e-01 9.993366e-01

4600 6.838896e-01 9.993363e-01

4601 6.834144e-01 9.993360e-01

4602 6.829341e-01 9.993357e-01

4603 6.824485e-01 9.993354e-01

4604 6.819579e-01 9.993351e-01

4605 6.814623e-01 9.993347e-01

4606 6.809619e-01 9.993343e-01

4607 6.804567e-01 9.993339e-01

4608 6.799469e-01 9.993335e-01

4609 6.794326e-01 9.993331e-01

4610 6.789139e-01 9.993326e-01

4611 6.783910e-01 9.993321e-01

4612 6.778640e-01 9.993316e-01

4613 6.773331e-01 9.993310e-01

4614 6.767984e-01 9.993304e-01

4615 6.762602e-01 9.993298e-01

4616 6.757185e-01 9.993291e-01

4617 6.751736e-01 9.993284e-01

4618 6.746256e-01 9.993277e-01

4619 6.740748e-01 9.993269e-01

4620 6.735213e-01 9.993261e-01

4621 6.729653e-01 9.993252e-01

4622 6.724070e-01 9.993243e-01

4623 6.718468e-01 9.993233e-01

4624 6.712847e-01 9.993223e-01

4625 6.707210e-01 9.993212e-01

4626 6.701559e-01 9.993201e-01

4627 6.695896e-01 9.993189e-01

4628 6.690225e-01 9.993176e-01

4629 6.684546e-01 9.993162e-01

4630 6.678863e-01 9.993148e-01

4631 6.673179e-01 9.993133e-01

4632 6.667494e-01 9.993117e-01

4633 6.661813e-01 9.993100e-01

4634 6.656137e-01 9.993082e-01

4635 6.650468e-01 9.993064e-01

4636 6.644810e-01 9.993044e-01

4637 6.639165e-01 9.993023e-01

4638 6.633535e-01 9.993001e-01

4639 6.627923e-01 9.992978e-01

4640 6.622331e-01 9.992954e-01

4641 6.616761e-01 9.992928e-01

4642 6.611217e-01 9.992901e-01

4643 6.605699e-01 9.992872e-01

4644 6.600212e-01 9.992842e-01

4645 6.594757e-01 9.992810e-01

4646 6.589336e-01 9.992777e-01

4647 6.583951e-01 9.992742e-01

4648 6.578605e-01 9.992704e-01

4649 6.573301e-01 9.992665e-01

4650 6.568039e-01 9.992624e-01

4651 6.562822e-01 9.992581e-01

4652 6.557652e-01 9.992536e-01

4653 6.552531e-01 9.992488e-01

4654 6.547460e-01 9.992438e-01

4655 6.542442e-01 9.992385e-01

4656 6.537478e-01 9.992329e-01

4657 6.532570e-01 9.992271e-01

4658 6.527719e-01 9.992210e-01

4659 6.522926e-01 9.992145e-01

4660 6.518194e-01 9.992078e-01

4661 6.513523e-01 9.992007e-01

4662 6.508915e-01 9.991932e-01

4663 6.504370e-01 9.991854e-01

4664 6.499890e-01 9.991772e-01

4665 6.495476e-01 9.991686e-01

4666 6.491128e-01 9.991596e-01

4667 6.486848e-01 9.991501e-01

4668 6.482635e-01 9.991402e-01

4669 6.478492e-01 9.991298e-01

4670 6.474417e-01 9.991189e-01

4671 6.470413e-01 9.991075e-01

4672 6.466478e-01 9.990955e-01

4673 6.462614e-01 9.990830e-01

4674 6.458820e-01 9.990699e-01

4675 6.455097e-01 9.990562e-01

4676 6.451445e-01 9.990419e-01

4677 6.447864e-01 9.990269e-01

4678 6.444354e-01 9.990112e-01

4679 6.440914e-01 9.989948e-01

4680 6.437544e-01 9.989777e-01

4681 6.434245e-01 9.989598e-01

4682 6.433516e-01 9.989598e-01

4683 6.432771e-01 9.989598e-01

4684 6.432011e-01 9.989598e-01

4685 6.431235e-01 9.989598e-01

4686 6.430443e-01 9.989598e-01

4687 6.429636e-01 9.989598e-01

4688 6.428812e-01 9.989598e-01

4689 6.427972e-01 9.989598e-01

4690 6.427116e-01 9.989598e-01

4691 6.426242e-01 9.989598e-01

4692 6.425352e-01 9.989598e-01

4693 6.424445e-01 9.989598e-01

4694 6.423521e-01 9.989598e-01

4695 6.422580e-01 9.989598e-01

4696 6.421620e-01 9.989598e-01

4697 6.420643e-01 9.989598e-01

4698 6.419648e-01 9.989598e-01

4699 6.418635e-01 9.989598e-01

4700 6.417603e-01 9.989598e-01

4701 6.416552e-01 9.989598e-01

4702 6.415483e-01 9.989598e-01

4703 6.414394e-01 9.989598e-01

4704 6.413286e-01 9.989598e-01

4705 6.412159e-01 9.989598e-01

4706 6.411012e-01 9.989598e-01

4707 6.409845e-01 9.989598e-01

4708 6.408658e-01 9.989598e-01

4709 6.407451e-01 9.989598e-01

4710 6.406223e-01 9.989598e-01

4711 6.404975e-01 9.989598e-01

4712 6.403706e-01 9.989598e-01

4713 6.402416e-01 9.989598e-01

4714 6.401104e-01 9.989598e-01

4715 6.399772e-01 9.989597e-01

4716 6.398418e-01 9.989597e-01

4717 6.397043e-01 9.989597e-01

4718 6.395646e-01 9.989597e-01

4719 6.394227e-01 9.989597e-01

4720 6.392786e-01 9.989597e-01

4721 6.391323e-01 9.989597e-01

4722 6.389839e-01 9.989597e-01

4723 6.388332e-01 9.989597e-01

4724 6.386802e-01 9.989597e-01

4725 6.385251e-01 9.989597e-01

4726 6.383677e-01 9.989597e-01

4727 6.382081e-01 9.989597e-01

4728 6.380463e-01 9.989596e-01

4729 6.378822e-01 9.989596e-01

4730 6.377159e-01 9.989596e-01

4731 6.375474e-01 9.989596e-01

4732 6.373766e-01 9.989596e-01

4733 6.372037e-01 9.989596e-01

4734 6.370285e-01 9.989596e-01

4735 6.368511e-01 9.989595e-01

4736 6.366715e-01 9.989595e-01

4737 6.364898e-01 9.989595e-01

4738 6.363059e-01 9.989595e-01

4739 6.361199e-01 9.989595e-01

4740 6.359318e-01 9.989594e-01

4741 6.357415e-01 9.989594e-01

4742 6.355492e-01 9.989594e-01

4743 6.353549e-01 9.989594e-01

4744 6.351585e-01 9.989593e-01

4745 6.349601e-01 9.989593e-01

4746 6.347598e-01 9.989593e-01

4747 6.345575e-01 9.989592e-01

4748 6.343534e-01 9.989592e-01

4749 6.341474e-01 9.989591e-01

4750 6.339396e-01 9.989591e-01

4751 6.337301e-01 9.989591e-01

4752 6.335188e-01 9.989590e-01

4753 6.333059e-01 9.989590e-01

4754 6.330913e-01 9.989589e-01

4755 6.328752e-01 9.989588e-01

4756 6.326576e-01 9.989588e-01

4757 6.324385e-01 9.989587e-01

4758 6.322181e-01 9.989587e-01

4759 6.319964e-01 9.989586e-01

4760 6.317733e-01 9.989585e-01

4761 6.315491e-01 9.989584e-01

4762 6.313238e-01 9.989583e-01

4763 6.310975e-01 9.989582e-01

4764 6.308702e-01 9.989581e-01

4765 6.306420e-01 9.989580e-01

4766 6.304130e-01 9.989579e-01

4767 6.301833e-01 9.989578e-01

4768 6.299529e-01 9.989577e-01

4769 6.297221e-01 9.989576e-01

4770 6.294907e-01 9.989574e-01

4771 6.292590e-01 9.989573e-01

4772 6.290270e-01 9.989571e-01

4773 6.287949e-01 9.989570e-01

4774 6.285627e-01 9.989568e-01

4775 6.283305e-01 9.989566e-01

4776 6.280984e-01 9.989565e-01

4777 6.278666e-01 9.989563e-01

4778 6.276351e-01 9.989561e-01

4779 6.274041e-01 9.989558e-01

4780 6.271736e-01 9.989556e-01

4781 6.269438e-01 9.989554e-01

4782 6.267147e-01 9.989551e-01

4783 6.264865e-01 9.989548e-01

4784 6.262593e-01 9.989545e-01

4785 6.260332e-01 9.989542e-01

4786 6.258083e-01 9.989539e-01

4787 6.255848e-01 9.989536e-01

4788 6.253626e-01 9.989532e-01

4789 6.251421e-01 9.989529e-01

4790 6.249231e-01 9.989525e-01

4791 6.247060e-01 9.989521e-01

4792 6.244907e-01 9.989517e-01

4793 6.242774e-01 9.989512e-01

4794 6.240662e-01 9.989507e-01

4795 6.238572e-01 9.989502e-01

4796 6.236505e-01 9.989497e-01

4797 6.234463e-01 9.989492e-01

4798 6.232445e-01 9.989486e-01

4799 6.230453e-01 9.989480e-01

4800 6.228488e-01 9.989473e-01

4801 6.226552e-01 9.989466e-01

4802 6.224644e-01 9.989459e-01

4803 6.222766e-01 9.989452e-01

4804 6.220919e-01 9.989444e-01

4805 6.219103e-01 9.989436e-01

4806 6.217319e-01 9.989427e-01

4807 6.215568e-01 9.989418e-01

4808 6.213851e-01 9.989409e-01

4809 6.212169e-01 9.989399e-01

4810 6.210521e-01 9.989389e-01

4811 6.208910e-01 9.989378e-01

4812 6.207334e-01 9.989367e-01

4813 6.205795e-01 9.989355e-01

4814 6.204294e-01 9.989342e-01

4815 6.202830e-01 9.989329e-01

4816 6.201404e-01 9.989316e-01

4817 6.200017e-01 9.989301e-01

4818 6.198668e-01 9.989286e-01

4819 6.197359e-01 9.989271e-01

4820 6.196088e-01 9.989254e-01

4821 6.194857e-01 9.989237e-01

4822 6.193666e-01 9.989220e-01

4823 6.192514e-01 9.989201e-01

4824 6.191401e-01 9.989182e-01

4825 6.190329e-01 9.989162e-01

4826 6.189296e-01 9.989140e-01

4827 6.188302e-01 9.989118e-01

4828 6.187347e-01 9.989096e-01

4829 6.186432e-01 9.989072e-01

4830 6.185556e-01 9.989047e-01

4831 6.184718e-01 9.989021e-01

4832 6.183919e-01 9.988994e-01

4833 6.183158e-01 9.988966e-01

4834 6.182434e-01 9.988937e-01

4835 6.181748e-01 9.988907e-01

4836 6.181098e-01 9.988875e-01

4837 6.180485e-01 9.988843e-01

4838 6.179908e-01 9.988809e-01

4839 6.179367e-01 9.988774e-01

4840 6.178860e-01 9.988737e-01

4841 6.178388e-01 9.988700e-01

4842 6.177949e-01 9.988660e-01

4843 6.177543e-01 9.988620e-01

4844 6.177170e-01 9.988578e-01

4845 6.176829e-01 9.988535e-01

4846 6.176519e-01 9.988490e-01

4847 6.176239e-01 9.988444e-01

4848 6.175989e-01 9.988396e-01

4849 6.175769e-01 9.988347e-01

4850 6.175577e-01 9.988296e-01

4851 6.175412e-01 9.988243e-01

4852 6.175275e-01 9.988189e-01

4853 6.175164e-01 9.988134e-01

4854 6.175078e-01 9.988076e-01

4855 6.175018e-01 9.988018e-01

4856 6.174981e-01 9.987957e-01

4857 6.174968e-01 9.987895e-01

4858 6.174978e-01 9.987832e-01

4859 6.175010e-01 9.987767e-01

4860 6.175063e-01 9.987700e-01

4861 6.175137e-01 9.987632e-01

4862 6.175404e-01 9.987632e-01

4863 6.175681e-01 9.987632e-01

4864 6.175969e-01 9.987632e-01

4865 6.176266e-01 9.987632e-01

4866 6.176573e-01 9.987632e-01

4867 6.176890e-01 9.987632e-01

4868 6.177218e-01 9.987632e-01

4869 6.177556e-01 9.987632e-01

4870 6.177906e-01 9.987632e-01

4871 6.178266e-01 9.987632e-01

4872 6.178637e-01 9.987632e-01

4873 6.179020e-01 9.987632e-01

4874 6.179414e-01 9.987632e-01

4875 6.179819e-01 9.987632e-01

4876 6.180237e-01 9.987632e-01

4877 6.180667e-01 9.987632e-01

4878 6.181109e-01 9.987632e-01

4879 6.181563e-01 9.987632e-01

4880 6.182030e-01 9.987632e-01

4881 6.182510e-01 9.987632e-01

4882 6.183003e-01 9.987632e-01

4883 6.183509e-01 9.987632e-01

4884 6.184029e-01 9.987632e-01

4885 6.184563e-01 9.987632e-01

4886 6.185110e-01 9.987632e-01

4887 6.185672e-01 9.987632e-01

4888 6.186248e-01 9.987632e-01

4889 6.186839e-01 9.987632e-01

4890 6.187445e-01 9.987632e-01

4891 6.188066e-01 9.987632e-01

4892 6.188703e-01 9.987632e-01

4893 6.189355e-01 9.987632e-01

4894 6.190023e-01 9.987632e-01

4895 6.190708e-01 9.987632e-01

4896 6.191409e-01 9.987632e-01

4897 6.192127e-01 9.987632e-01

4898 6.192862e-01 9.987631e-01

4899 6.193615e-01 9.987631e-01

4900 6.194385e-01 9.987631e-01

4901 6.195174e-01 9.987631e-01

4902 6.195981e-01 9.987631e-01

4903 6.196807e-01 9.987631e-01

4904 6.197653e-01 9.987631e-01

4905 6.198518e-01 9.987631e-01

4906 6.199403e-01 9.987631e-01

4907 6.200308e-01 9.987631e-01

4908 6.201234e-01 9.987631e-01

4909 6.202181e-01 9.987630e-01

4910 6.203150e-01 9.987630e-01

4911 6.204141e-01 9.987630e-01

4912 6.205154e-01 9.987630e-01

4913 6.206190e-01 9.987630e-01

4914 6.207249e-01 9.987630e-01

4915 6.208333e-01 9.987629e-01

4916 6.209440e-01 9.987629e-01

4917 6.210572e-01 9.987629e-01

4918 6.211730e-01 9.987629e-01

4919 6.212913e-01 9.987628e-01

4920 6.214123e-01 9.987628e-01

4921 6.215359e-01 9.987628e-01

4922 6.216622e-01 9.987627e-01

4923 6.217913e-01 9.987627e-01

4924 6.219233e-01 9.987626e-01

4925 6.220581e-01 9.987626e-01

4926 6.221959e-01 9.987625e-01

4927 6.223366e-01 9.987625e-01

4928 6.224804e-01 9.987624e-01

4929 6.226273e-01 9.987624e-01

4930 6.227774e-01 9.987623e-01

4931 6.229306e-01 9.987623e-01

4932 6.230871e-01 9.987622e-01

4933 6.232469e-01 9.987621e-01

4934 6.234101e-01 9.987620e-01

4935 6.235767e-01 9.987619e-01

4936 6.237468e-01 9.987618e-01

4937 6.239204e-01 9.987618e-01

4938 6.240975e-01 9.987617e-01

4939 6.242784e-01 9.987615e-01

4940 6.244628e-01 9.987614e-01

4941 6.246510e-01 9.987613e-01

4942 6.248430e-01 9.987612e-01

4943 6.250388e-01 9.987610e-01

4944 6.252385e-01 9.987609e-01

4945 6.254421e-01 9.987607e-01

4946 6.256497e-01 9.987606e-01

4947 6.258612e-01 9.987604e-01

4948 6.260768e-01 9.987602e-01

4949 6.262965e-01 9.987600e-01

4950 6.265203e-01 9.987598e-01

4951 6.267482e-01 9.987596e-01

4952 6.269803e-01 9.987593e-01

4953 6.272166e-01 9.987591e-01

4954 6.274571e-01 9.987588e-01

4955 6.277019e-01 9.987585e-01

4956 6.279509e-01 9.987582e-01

4957 6.282043e-01 9.987579e-01

4958 6.284619e-01 9.987576e-01

4959 6.287239e-01 9.987572e-01

4960 6.289902e-01 9.987569e-01

4961 6.292608e-01 9.987565e-01

4962 6.295357e-01 9.987560e-01

4963 6.298150e-01 9.987556e-01

4964 6.300986e-01 9.987551e-01

4965 6.303865e-01 9.987546e-01

4966 6.306788e-01 9.987541e-01

4967 6.309753e-01 9.987536e-01

4968 6.312760e-01 9.987530e-01

4969 6.315810e-01 9.987524e-01

4970 6.318902e-01 9.987517e-01

4971 6.322036e-01 9.987510e-01

4972 6.325211e-01 9.987503e-01

4973 6.328426e-01 9.987495e-01

4974 6.331683e-01 9.987487e-01

4975 6.334979e-01 9.987479e-01

4976 6.338314e-01 9.987470e-01

4977 6.341688e-01 9.987460e-01

4978 6.345101e-01 9.987450e-01

4979 6.348550e-01 9.987439e-01

4980 6.352037e-01 9.987428e-01

4981 6.355559e-01 9.987416e-01

4982 6.359116e-01 9.987404e-01

4983 6.362708e-01 9.987390e-01

4984 6.366333e-01 9.987377e-01

4985 6.369990e-01 9.987362e-01

4986 6.373678e-01 9.987346e-01

4987 6.377397e-01 9.987330e-01

4988 6.381146e-01 9.987313e-01

4989 6.384922e-01 9.987295e-01

4990 6.388726e-01 9.987276e-01

4991 6.392556e-01 9.987255e-01

4992 6.396410e-01 9.987234e-01

4993 6.400288e-01 9.987212e-01

4994 6.404188e-01 9.987188e-01

4995 6.408109e-01 9.987163e-01

4996 6.412050e-01 9.987137e-01

4997 6.416009e-01 9.987110e-01

4998 6.419986e-01 9.987081e-01

4999 6.423977e-01 9.987050e-01

5000 6.427983e-01 9.987018e-01

5001 6.432002e-01 9.986984e-01

5002 6.436033e-01 9.986949e-01

5003 6.440073e-01 9.986912e-01

5004 6.444122e-01 9.986872e-01

5005 6.448178e-01 9.986831e-01

5006 6.452240e-01 9.986788e-01

5007 6.456305e-01 9.986742e-01

5008 6.460374e-01 9.986694e-01

5009 6.464444e-01 9.986644e-01

5010 6.468513e-01 9.986591e-01

5011 6.472581e-01 9.986535e-01

5012 6.476646e-01 9.986477e-01

5013 6.480706e-01 9.986416e-01

5014 6.484761e-01 9.986351e-01

5015 6.488808e-01 9.986284e-01

5016 6.492846e-01 9.986213e-01

5017 6.496875e-01 9.986139e-01

5018 6.500891e-01 9.986061e-01

5019 6.504895e-01 9.985979e-01

5020 6.508885e-01 9.985893e-01

5021 6.512860e-01 9.985803e-01

5022 6.516818e-01 9.985709e-01

5023 6.520758e-01 9.985610e-01

5024 6.524679e-01 9.985507e-01

5025 6.528580e-01 9.985398e-01

5026 6.532459e-01 9.985285e-01

5027 6.536317e-01 9.985166e-01

5028 6.540150e-01 9.985041e-01

5029 6.543959e-01 9.984911e-01

5030 6.547743e-01 9.984774e-01

5031 6.551500e-01 9.984631e-01

5032 6.555229e-01 9.984482e-01

5033 6.558931e-01 9.984325e-01

5034 6.562603e-01 9.984161e-01

5035 6.566245e-01 9.983990e-01

5036 6.569857e-01 9.983811e-01

5037 6.573437e-01 9.983624e-01

5038 6.576985e-01 9.983428e-01

5039 6.580501e-01 9.983224e-01

5040 6.583983e-01 9.983010e-01

5041 6.587431e-01 9.982787e-01

5042 6.588523e-01 9.982787e-01

5043 6.589643e-01 9.982787e-01

5044 6.590790e-01 9.982787e-01

5045 6.591965e-01 9.982787e-01

5046 6.593167e-01 9.982787e-01

5047 6.594398e-01 9.982787e-01

5048 6.595657e-01 9.982787e-01

5049 6.596945e-01 9.982787e-01

5050 6.598263e-01 9.982787e-01

5051 6.599610e-01 9.982787e-01

5052 6.600987e-01 9.982787e-01

5053 6.602394e-01 9.982786e-01

5054 6.603832e-01 9.982786e-01

5055 6.605302e-01 9.982786e-01

5056 6.606802e-01 9.982786e-01

5057 6.608335e-01 9.982786e-01

5058 6.609899e-01 9.982786e-01

5059 6.611496e-01 9.982786e-01

5060 6.613126e-01 9.982786e-01

5061 6.614789e-01 9.982786e-01

5062 6.616486e-01 9.982786e-01

5063 6.618217e-01 9.982785e-01

5064 6.619982e-01 9.982785e-01

5065 6.621782e-01 9.982785e-01

5066 6.623616e-01 9.982785e-01

5067 6.625487e-01 9.982785e-01

5068 6.627392e-01 9.982785e-01

5069 6.629334e-01 9.982784e-01

5070 6.631312e-01 9.982784e-01

5071 6.633327e-01 9.982784e-01

5072 6.635379e-01 9.982784e-01

5073 6.637468e-01 9.982784e-01

5074 6.639594e-01 9.982783e-01

5075 6.641758e-01 9.982783e-01

5076 6.643961e-01 9.982783e-01

5077 6.646202e-01 9.982783e-01

5078 6.648481e-01 9.982782e-01

5079 6.650800e-01 9.982782e-01

5080 6.653157e-01 9.982782e-01

5081 6.655555e-01 9.982781e-01

5082 6.657991e-01 9.982781e-01

5083 6.660468e-01 9.982781e-01

5084 6.662985e-01 9.982780e-01

5085 6.665542e-01 9.982780e-01

5086 6.668140e-01 9.982779e-01

5087 6.670778e-01 9.982779e-01

5088 6.673458e-01 9.982778e-01

5089 6.676179e-01 9.982778e-01

5090 6.678940e-01 9.982777e-01

5091 6.681744e-01 9.982777e-01

5092 6.684589e-01 9.982776e-01

5093 6.687476e-01 9.982776e-01

5094 6.690405e-01 9.982775e-01

5095 6.693376e-01 9.982774e-01

5096 6.696389e-01 9.982774e-01

5097 6.699444e-01 9.982773e-01

5098 6.702542e-01 9.982772e-01

5099 6.705683e-01 9.982771e-01

5100 6.708866e-01 9.982770e-01

5101 6.712092e-01 9.982769e-01

5102 6.715360e-01 9.982768e-01

5103 6.718672e-01 9.982767e-01

5104 6.722027e-01 9.982766e-01

5105 6.725425e-01 9.982765e-01

5106 6.728865e-01 9.982764e-01

5107 6.732349e-01 9.982762e-01

5108 6.735876e-01 9.982761e-01

5109 6.739447e-01 9.982759e-01

5110 6.743060e-01 9.982758e-01

5111 6.746717e-01 9.982756e-01

5112 6.750417e-01 9.982755e-01

5113 6.754160e-01 9.982753e-01

5114 6.757946e-01 9.982751e-01

5115 6.761776e-01 9.982749e-01

5116 6.765648e-01 9.982747e-01

5117 6.769564e-01 9.982745e-01

5118 6.773522e-01 9.982743e-01

5119 6.777524e-01 9.982740e-01

5120 6.781568e-01 9.982738e-01

5121 6.785654e-01 9.982735e-01

5122 6.789784e-01 9.982732e-01

5123 6.793955e-01 9.982729e-01

5124 6.798169e-01 9.982726e-01

5125 6.802426e-01 9.982723e-01

5126 6.806724e-01 9.982720e-01

5127 6.811063e-01 9.982716e-01

5128 6.815445e-01 9.982712e-01

5129 6.819867e-01 9.982709e-01

5130 6.824331e-01 9.982704e-01

5131 6.828835e-01 9.982700e-01

5132 6.833380e-01 9.982696e-01

5133 6.837965e-01 9.982691e-01

5134 6.842590e-01 9.982686e-01

5135 6.847254e-01 9.982680e-01

5136 6.851958e-01 9.982675e-01

5137 6.856700e-01 9.982669e-01

5138 6.861481e-01 9.982663e-01

5139 6.866300e-01 9.982657e-01

5140 6.871156e-01 9.982650e-01

5141 6.876049e-01 9.982643e-01

5142 6.880979e-01 9.982636e-01

5143 6.885944e-01 9.982628e-01

5144 6.890945e-01 9.982620e-01

5145 6.895981e-01 9.982611e-01

5146 6.901052e-01 9.982602e-01

5147 6.906155e-01 9.982593e-01

5148 6.911292e-01 9.982583e-01

5149 6.916461e-01 9.982573e-01

5150 6.921662e-01 9.982562e-01

5151 6.926893e-01 9.982551e-01

5152 6.932155e-01 9.982539e-01

5153 6.937446e-01 9.982527e-01

5154 6.942765e-01 9.982514e-01

5155 6.948112e-01 9.982500e-01

5156 6.953486e-01 9.982486e-01

5157 6.958885e-01 9.982471e-01

5158 6.964310e-01 9.982455e-01

5159 6.969758e-01 9.982439e-01

5160 6.975229e-01 9.982422e-01

5161 6.980722e-01 9.982404e-01

5162 6.986236e-01 9.982385e-01

5163 6.991770e-01 9.982365e-01

5164 6.997322e-01 9.982345e-01

5165 7.002892e-01 9.982323e-01

5166 7.008478e-01 9.982301e-01

5167 7.014080e-01 9.982277e-01

5168 7.019696e-01 9.982252e-01

5169 7.025324e-01 9.982226e-01

5170 7.030964e-01 9.982199e-01

5171 7.036614e-01 9.982171e-01

5172 7.042274e-01 9.982142e-01

5173 7.047941e-01 9.982111e-01

5174 7.053614e-01 9.982079e-01

5175 7.059292e-01 9.982045e-01

5176 7.064974e-01 9.982010e-01

5177 7.070658e-01 9.981973e-01

5178 7.076344e-01 9.981934e-01

5179 7.082028e-01 9.981894e-01

5180 7.087711e-01 9.981852e-01

5181 7.093390e-01 9.981809e-01

5182 7.099065e-01 9.981763e-01

5183 7.104733e-01 9.981715e-01

5184 7.110393e-01 9.981665e-01

5185 7.116045e-01 9.981613e-01

5186 7.121686e-01 9.981559e-01

5187 7.127314e-01 9.981503e-01

5188 7.132930e-01 9.981444e-01

5189 7.138530e-01 9.981382e-01

5190 7.144114e-01 9.981318e-01

5191 7.149680e-01 9.981252e-01

5192 7.155227e-01 9.981182e-01

5193 7.160753e-01 9.981109e-01

5194 7.166257e-01 9.981034e-01

5195 7.171738e-01 9.980955e-01

5196 7.177194e-01 9.980873e-01

5197 7.182623e-01 9.980788e-01

5198 7.188025e-01 9.980699e-01

5199 7.193398e-01 9.980607e-01

5200 7.198741e-01 9.980511e-01

5201 7.204053e-01 9.980411e-01

5202 7.209331e-01 9.980307e-01

5203 7.214576e-01 9.980199e-01

5204 7.219785e-01 9.980086e-01

5205 7.224959e-01 9.979970e-01

5206 7.230094e-01 9.979848e-01

5207 7.235191e-01 9.979722e-01

5208 7.240248e-01 9.979591e-01

5209 7.245265e-01 9.979455e-01

5210 7.250240e-01 9.979314e-01

5211 7.255171e-01 9.979168e-01

5212 7.260060e-01 9.979016e-01

5213 7.264903e-01 9.978858e-01

5214 7.269701e-01 9.978695e-01

5215 7.274453e-01 9.978525e-01

5216 7.279157e-01 9.978349e-01

5217 7.283814e-01 9.978167e-01

5218 7.288422e-01 9.977978e-01

5219 7.292981e-01 9.977783e-01

5220 7.297490e-01 9.977581e-01

5221 7.301948e-01 9.977371e-01

5222 7.302626e-01 9.977371e-01

5223 7.303320e-01 9.977371e-01

5224 7.304032e-01 9.977371e-01

5225 7.304761e-01 9.977371e-01

5226 7.305507e-01 9.977371e-01

5227 7.306271e-01 9.977371e-01

5228 7.307054e-01 9.977370e-01

5229 7.307855e-01 9.977370e-01

5230 7.308675e-01 9.977370e-01

5231 7.309515e-01 9.977370e-01

5232 7.310374e-01 9.977370e-01

5233 7.311253e-01 9.977370e-01

5234 7.312152e-01 9.977370e-01

5235 7.313072e-01 9.977370e-01

5236 7.314014e-01 9.977369e-01

5237 7.314976e-01 9.977369e-01

5238 7.315961e-01 9.977369e-01

5239 7.316968e-01 9.977369e-01

5240 7.317997e-01 9.977369e-01

5241 7.319050e-01 9.977369e-01

5242 7.320125e-01 9.977368e-01

5243 7.321225e-01 9.977368e-01

5244 7.322349e-01 9.977368e-01

5245 7.323497e-01 9.977368e-01

5246 7.324670e-01 9.977368e-01

5247 7.325869e-01 9.977367e-01

5248 7.327093e-01 9.977367e-01

5249 7.328343e-01 9.977367e-01

5250 7.329619e-01 9.977366e-01

5251 7.330923e-01 9.977366e-01

5252 7.332253e-01 9.977366e-01

5253 7.333611e-01 9.977365e-01

5254 7.334997e-01 9.977365e-01

5255 7.336411e-01 9.977365e-01

5256 7.337854e-01 9.977364e-01

5257 7.339325e-01 9.977364e-01

5258 7.340826e-01 9.977363e-01

5259 7.342356e-01 9.977363e-01

5260 7.343917e-01 9.977363e-01

5261 7.345507e-01 9.977362e-01

5262 7.347128e-01 9.977361e-01

5263 7.348779e-01 9.977361e-01

5264 7.350462e-01 9.977360e-01

5265 7.352176e-01 9.977360e-01

5266 7.353921e-01 9.977359e-01

5267 7.355698e-01 9.977358e-01

5268 7.357507e-01 9.977358e-01

5269 7.359349e-01 9.977357e-01

5270 7.361222e-01 9.977356e-01

5271 7.363129e-01 9.977355e-01

5272 7.365068e-01 9.977354e-01

5273 7.367040e-01 9.977353e-01

5274 7.369046e-01 9.977352e-01

5275 7.371084e-01 9.977351e-01

5276 7.373156e-01 9.977350e-01

5277 7.375261e-01 9.977349e-01

5278 7.377400e-01 9.977348e-01

5279 7.379572e-01 9.977347e-01

5280 7.381778e-01 9.977345e-01

5281 7.384018e-01 9.977344e-01

5282 7.386291e-01 9.977343e-01

5283 7.388598e-01 9.977341e-01

5284 7.390938e-01 9.977339e-01

5285 7.393312e-01 9.977338e-01

5286 7.395719e-01 9.977336e-01

5287 7.398159e-01 9.977334e-01

5288 7.400633e-01 9.977332e-01

5289 7.403140e-01 9.977330e-01

5290 7.405680e-01 9.977328e-01

5291 7.408252e-01 9.977326e-01

5292 7.410857e-01 9.977323e-01

5293 7.413494e-01 9.977321e-01

5294 7.416164e-01 9.977318e-01

5295 7.418865e-01 9.977316e-01

5296 7.421598e-01 9.977313e-01

5297 7.424361e-01 9.977310e-01

5298 7.427156e-01 9.977307e-01

5299 7.429981e-01 9.977303e-01

5300 7.432837e-01 9.977300e-01

5301 7.435722e-01 9.977296e-01

5302 7.438636e-01 9.977293e-01

5303 7.441579e-01 9.977289e-01

5304 7.444551e-01 9.977285e-01

5305 7.447551e-01 9.977280e-01

5306 7.450578e-01 9.977276e-01

5307 7.453632e-01 9.977271e-01

5308 7.456712e-01 9.977266e-01

5309 7.459818e-01 9.977261e-01

5310 7.462950e-01 9.977255e-01

5311 7.466106e-01 9.977249e-01

5312 7.469286e-01 9.977243e-01

5313 7.472489e-01 9.977237e-01

5314 7.475716e-01 9.977231e-01

5315 7.478964e-01 9.977224e-01

5316 7.482234e-01 9.977217e-01

5317 7.485524e-01 9.977209e-01

5318 7.488834e-01 9.977201e-01

5319 7.492164e-01 9.977193e-01

5320 7.495512e-01 9.977184e-01

5321 7.498877e-01 9.977175e-01

5322 7.502260e-01 9.977166e-01

5323 7.505659e-01 9.977156e-01

5324 7.509073e-01 9.977145e-01

5325 7.512501e-01 9.977135e-01

5326 7.515943e-01 9.977123e-01

5327 7.519398e-01 9.977112e-01

5328 7.522865e-01 9.977099e-01

5329 7.526343e-01 9.977086e-01

5330 7.529831e-01 9.977073e-01

5331 7.533329e-01 9.977059e-01

5332 7.536834e-01 9.977044e-01

5333 7.540348e-01 9.977028e-01

5334 7.543868e-01 9.977012e-01

5335 7.547393e-01 9.976995e-01

5336 7.550924e-01 9.976978e-01

5337 7.554458e-01 9.976959e-01

5338 7.557995e-01 9.976940e-01

5339 7.561534e-01 9.976920e-01

5340 7.565074e-01 9.976899e-01

5341 7.568614e-01 9.976877e-01

5342 7.572153e-01 9.976854e-01

5343 7.575690e-01 9.976830e-01

5344 7.579224e-01 9.976805e-01

5345 7.582755e-01 9.976779e-01

5346 7.586280e-01 9.976752e-01

5347 7.589800e-01 9.976723e-01

5348 7.593313e-01 9.976694e-01

5349 7.596819e-01 9.976663e-01

5350 7.600316e-01 9.976630e-01

5351 7.603804e-01 9.976597e-01

5352 7.607281e-01 9.976562e-01

5353 7.610746e-01 9.976525e-01

5354 7.614199e-01 9.976487e-01

5355 7.617639e-01 9.976447e-01

5356 7.621065e-01 9.976405e-01

5357 7.624475e-01 9.976362e-01

5358 7.627870e-01 9.976317e-01

5359 7.631247e-01 9.976269e-01

5360 7.634607e-01 9.976220e-01

5361 7.637948e-01 9.976169e-01

5362 7.641269e-01 9.976116e-01

5363 7.644570e-01 9.976060e-01

5364 7.647850e-01 9.976002e-01

5365 7.651107e-01 9.975942e-01

5366 7.654342e-01 9.975879e-01

5367 7.657553e-01 9.975814e-01

5368 7.660739e-01 9.975746e-01

5369 7.663900e-01 9.975675e-01

5370 7.667035e-01 9.975601e-01

5371 7.670143e-01 9.975525e-01

5372 7.673224e-01 9.975445e-01

5373 7.676276e-01 9.975362e-01

5374 7.679300e-01 9.975276e-01

5375 7.682294e-01 9.975186e-01

5376 7.685258e-01 9.975093e-01

5377 7.688191e-01 9.974996e-01

5378 7.691093e-01 9.974895e-01

5379 7.693962e-01 9.974790e-01

5380 7.696800e-01 9.974682e-01

5381 7.699604e-01 9.974569e-01

5382 7.702375e-01 9.974451e-01

5383 7.705111e-01 9.974330e-01

5384 7.707813e-01 9.974203e-01

5385 7.710481e-01 9.974072e-01

5386 7.713113e-01 9.973936e-01

5387 7.715709e-01 9.973795e-01

5388 7.718269e-01 9.973648e-01

5389 7.720793e-01 9.973496e-01

5390 7.723280e-01 9.973339e-01

5391 7.725730e-01 9.973176e-01

5392 7.728143e-01 9.973006e-01

5393 7.730518e-01 9.972831e-01

5394 7.732856e-01 9.972649e-01

5395 7.735156e-01 9.972461e-01

5396 7.737418e-01 9.972266e-01

5397 7.739641e-01 9.972064e-01

5398 7.741827e-01 9.971855e-01

5399 7.743974e-01 9.971639e-01

5400 7.746083e-01 9.971415e-01

5401 7.748154e-01 9.971184e-01

5402 7.748386e-01 9.971184e-01

5403 7.748623e-01 9.971184e-01

5404 7.748864e-01 9.971183e-01

5405 7.749111e-01 9.971183e-01

5406 7.749363e-01 9.971183e-01

5407 7.749620e-01 9.971183e-01

5408 7.749882e-01 9.971183e-01

5409 7.750149e-01 9.971183e-01

5410 7.750422e-01 9.971183e-01

5411 7.750700e-01 9.971182e-01

5412 7.750983e-01 9.971182e-01

5413 7.751272e-01 9.971182e-01

5414 7.751567e-01 9.971182e-01

5415 7.751867e-01 9.971182e-01

5416 7.752174e-01 9.971181e-01

5417 7.752485e-01 9.971181e-01

5418 7.752803e-01 9.971181e-01

5419 7.753127e-01 9.971180e-01

5420 7.753457e-01 9.971180e-01

5421 7.753793e-01 9.971180e-01

5422 7.754135e-01 9.971180e-01

5423 7.754484e-01 9.971179e-01

5424 7.754839e-01 9.971179e-01

5425 7.755200e-01 9.971178e-01

5426 7.755568e-01 9.971178e-01

5427 7.755942e-01 9.971178e-01

5428 7.756323e-01 9.971177e-01

5429 7.756710e-01 9.971177e-01

5430 7.757104e-01 9.971176e-01

5431 7.757505e-01 9.971176e-01

5432 7.757913e-01 9.971175e-01

5433 7.758327e-01 9.971175e-01

5434 7.758749e-01 9.971174e-01

5435 7.759177e-01 9.971174e-01

5436 7.759612e-01 9.971173e-01

5437 7.760054e-01 9.971172e-01

5438 7.760503e-01 9.971171e-01

5439 7.760959e-01 9.971171e-01

5440 7.761422e-01 9.971170e-01

5441 7.761891e-01 9.971169e-01

5442 7.762368e-01 9.971168e-01

5443 7.762852e-01 9.971167e-01

5444 7.763342e-01 9.971166e-01

5445 7.763839e-01 9.971165e-01

5446 7.764344e-01 9.971164e-01

5447 7.764854e-01 9.971163e-01

5448 7.765372e-01 9.971162e-01

5449 7.765896e-01 9.971161e-01

5450 7.766426e-01 9.971160e-01

5451 7.766963e-01 9.971158e-01

5452 7.767506e-01 9.971157e-01

5453 7.768056e-01 9.971155e-01

5454 7.768611e-01 9.971154e-01

5455 7.769172e-01 9.971152e-01

5456 7.769739e-01 9.971151e-01

5457 7.770311e-01 9.971149e-01

5458 7.770889e-01 9.971147e-01

5459 7.771472e-01 9.971145e-01

5460 7.772059e-01 9.971143e-01

5461 7.772652e-01 9.971141e-01

5462 7.773248e-01 9.971138e-01

5463 7.773849e-01 9.971136e-01

5464 7.774453e-01 9.971134e-01

5465 7.775061e-01 9.971131e-01

5466 7.775672e-01 9.971128e-01

5467 7.776286e-01 9.971126e-01

5468 7.776902e-01 9.971123e-01

5469 7.777521e-01 9.971119e-01

5470 7.778141e-01 9.971116e-01

5471 7.778762e-01 9.971113e-01

5472 7.779384e-01 9.971109e-01

5473 7.780006e-01 9.971106e-01

5474 7.780628e-01 9.971102e-01

5475 7.781249e-01 9.971098e-01

5476 7.781869e-01 9.971093e-01

5477 7.782488e-01 9.971089e-01

5478 7.783104e-01 9.971084e-01

5479 7.783717e-01 9.971079e-01

5480 7.784326e-01 9.971074e-01

5481 7.784932e-01 9.971069e-01

5482 7.785532e-01 9.971063e-01

5483 7.786128e-01 9.971057e-01

5484 7.786717e-01 9.971051e-01

5485 7.787299e-01 9.971044e-01

5486 7.787874e-01 9.971038e-01

5487 7.788440e-01 9.971031e-01

5488 7.788998e-01 9.971023e-01

5489 7.789545e-01 9.971016e-01

5490 7.790083e-01 9.971007e-01

5491 7.790608e-01 9.970999e-01

5492 7.791122e-01 9.970990e-01

5493 7.791623e-01 9.970981e-01

5494 7.792110e-01 9.970971e-01

5495 7.792582e-01 9.970961e-01

5496 7.793039e-01 9.970951e-01

5497 7.793479e-01 9.970940e-01

5498 7.793903e-01 9.970928e-01

5499 7.794308e-01 9.970916e-01

5500 7.794694e-01 9.970904e-01

5501 7.795060e-01 9.970890e-01

5502 7.795406e-01 9.970877e-01

5503 7.795730e-01 9.970862e-01

5504 7.796032e-01 9.970847e-01

5505 7.796310e-01 9.970832e-01

5506 7.796564e-01 9.970815e-01

5507 7.796793e-01 9.970798e-01

5508 7.796996e-01 9.970780e-01

5509 7.797172e-01 9.970762e-01

5510 7.797320e-01 9.970742e-01

5511 7.797440e-01 9.970722e-01

5512 7.797530e-01 9.970701e-01

5513 7.797590e-01 9.970679e-01

5514 7.797620e-01 9.970655e-01

5515 7.797618e-01 9.970631e-01

5516 7.797583e-01 9.970606e-01

5517 7.797515e-01 9.970580e-01

5518 7.797413e-01 9.970552e-01

5519 7.797277e-01 9.970524e-01

5520 7.797105e-01 9.970494e-01

5521 7.796897e-01 9.970463e-01

5522 7.796653e-01 9.970430e-01

5523 7.796372e-01 9.970396e-01

5524 7.796054e-01 9.970361e-01

5525 7.795697e-01 9.970324e-01

5526 7.795302e-01 9.970285e-01

5527 7.794867e-01 9.970245e-01

5528 7.794394e-01 9.970203e-01

5529 7.793881e-01 9.970159e-01

5530 7.793327e-01 9.970114e-01

5531 7.792733e-01 9.970066e-01

5532 7.792099e-01 9.970017e-01

5533 7.791424e-01 9.969965e-01

5534 7.790708e-01 9.969911e-01

5535 7.789951e-01 9.969855e-01

5536 7.789153e-01 9.969796e-01

5537 7.788314e-01 9.969735e-01

5538 7.787434e-01 9.969672e-01

5539 7.786513e-01 9.969606e-01

5540 7.785551e-01 9.969537e-01

5541 7.784548e-01 9.969465e-01

5542 7.783505e-01 9.969390e-01

5543 7.782422e-01 9.969312e-01

5544 7.781298e-01 9.969231e-01

5545 7.780136e-01 9.969147e-01

5546 7.778934e-01 9.969059e-01

5547 7.777693e-01 9.968967e-01

5548 7.776414e-01 9.968872e-01

5549 7.775097e-01 9.968772e-01

5550 7.773743e-01 9.968669e-01

5551 7.772352e-01 9.968562e-01

5552 7.770926e-01 9.968450e-01

5553 7.769464e-01 9.968333e-01

5554 7.767968e-01 9.968212e-01

5555 7.766438e-01 9.968086e-01

5556 7.764876e-01 9.967955e-01

5557 7.763281e-01 9.967819e-01

5558 7.761655e-01 9.967677e-01

5559 7.759999e-01 9.967530e-01

5560 7.758313e-01 9.967377e-01

5561 7.756599e-01 9.967218e-01

5562 7.754857e-01 9.967052e-01

5563 7.753089e-01 9.966880e-01

5564 7.751295e-01 9.966701e-01

5565 7.749477e-01 9.966515e-01

5566 7.747636e-01 9.966322e-01

5567 7.745773e-01 9.966121e-01

5568 7.743888e-01 9.965912e-01

5569 7.741984e-01 9.965696e-01

5570 7.740060e-01 9.965471e-01

5571 7.738119e-01 9.965237e-01

5572 7.736161e-01 9.964994e-01

5573 7.734187e-01 9.964741e-01

5574 7.732200e-01 9.964479e-01

5575 7.730199e-01 9.964207e-01

5576 7.728185e-01 9.963924e-01

5577 7.726161e-01 9.963631e-01

5578 7.724127e-01 9.963326e-01

5579 7.722085e-01 9.963010e-01

5580 7.720034e-01 9.962681e-01

5581 7.717978e-01 9.962340e-01

5582 7.717927e-01 9.962340e-01

5583 7.717873e-01 9.962340e-01

5584 7.717815e-01 9.962340e-01

5585 7.717754e-01 9.962339e-01

5586 7.717689e-01 9.962339e-01

5587 7.717620e-01 9.962339e-01

5588 7.717547e-01 9.962339e-01

5589 7.717469e-01 9.962338e-01

5590 7.717387e-01 9.962338e-01

5591 7.717300e-01 9.962338e-01

5592 7.717208e-01 9.962337e-01

5593 7.717111e-01 9.962337e-01

5594 7.717008e-01 9.962336e-01

5595 7.716900e-01 9.962336e-01

5596 7.716785e-01 9.962336e-01

5597 7.716665e-01 9.962335e-01

5598 7.716538e-01 9.962335e-01

5599 7.716404e-01 9.962334e-01

5600 7.716263e-01 9.962334e-01

5601 7.716115e-01 9.962333e-01

5602 7.715959e-01 9.962333e-01

5603 7.715795e-01 9.962332e-01

5604 7.715623e-01 9.962331e-01

5605 7.715442e-01 9.962331e-01

5606 7.715252e-01 9.962330e-01

5607 7.715053e-01 9.962329e-01

5608 7.714844e-01 9.962328e-01

5609 7.714625e-01 9.962328e-01

5610 7.714395e-01 9.962327e-01

5611 7.714154e-01 9.962326e-01

5612 7.713902e-01 9.962325e-01

5613 7.713638e-01 9.962324e-01

5614 7.713362e-01 9.962323e-01

5615 7.713072e-01 9.962322e-01

5616 7.712770e-01 9.962321e-01

5617 7.712454e-01 9.962320e-01

5618 7.712123e-01 9.962318e-01

5619 7.711778e-01 9.962317e-01

5620 7.711417e-01 9.962316e-01

5621 7.711040e-01 9.962314e-01

5622 7.710647e-01 9.962313e-01

5623 7.710237e-01 9.962311e-01

5624 7.709809e-01 9.962310e-01

5625 7.709362e-01 9.962308e-01

5626 7.708897e-01 9.962306e-01

5627 7.708412e-01 9.962304e-01

5628 7.707906e-01 9.962302e-01

5629 7.707380e-01 9.962300e-01

5630 7.706831e-01 9.962298e-01

5631 7.706261e-01 9.962296e-01

5632 7.705667e-01 9.962293e-01

5633 7.705049e-01 9.962291e-01

5634 7.704406e-01 9.962288e-01

5635 7.703738e-01 9.962286e-01

5636 7.703043e-01 9.962283e-01

5637 7.702321e-01 9.962280e-01

5638 7.701571e-01 9.962277e-01

5639 7.700792e-01 9.962273e-01

5640 7.699983e-01 9.962270e-01

5641 7.699144e-01 9.962266e-01

5642 7.698273e-01 9.962263e-01

5643 7.697369e-01 9.962259e-01

5644 7.696432e-01 9.962255e-01

5645 7.695461e-01 9.962250e-01

5646 7.694454e-01 9.962246e-01

5647 7.693411e-01 9.962241e-01

5648 7.692330e-01 9.962236e-01

5649 7.691211e-01 9.962231e-01

5650 7.690053e-01 9.962226e-01

5651 7.688854e-01 9.962220e-01

5652 7.687613e-01 9.962214e-01

5653 7.686331e-01 9.962208e-01

5654 7.685005e-01 9.962202e-01

5655 7.683634e-01 9.962195e-01

5656 7.682217e-01 9.962188e-01

5657 7.680754e-01 9.962181e-01

5658 7.679243e-01 9.962173e-01

5659 7.677684e-01 9.962165e-01

5660 7.676074e-01 9.962157e-01

5661 7.674414e-01 9.962148e-01

5662 7.672702e-01 9.962139e-01

5663 7.670937e-01 9.962129e-01

5664 7.669118e-01 9.962119e-01

5665 7.667243e-01 9.962109e-01

5666 7.665313e-01 9.962098e-01

5667 7.663326e-01 9.962086e-01

5668 7.661281e-01 9.962074e-01

5669 7.659177e-01 9.962062e-01

5670 7.657013e-01 9.962049e-01

5671 7.654789e-01 9.962035e-01

5672 7.652503e-01 9.962021e-01

5673 7.650154e-01 9.962006e-01

5674 7.647742e-01 9.961990e-01

5675 7.645267e-01 9.961974e-01

5676 7.642726e-01 9.961957e-01

5677 7.640120e-01 9.961940e-01

5678 7.637448e-01 9.961921e-01

5679 7.634709e-01 9.961902e-01

5680 7.631902e-01 9.961882e-01

5681 7.629028e-01 9.961861e-01

5682 7.626086e-01 9.961839e-01

5683 7.623074e-01 9.961816e-01

5684 7.619994e-01 9.961792e-01

5685 7.616844e-01 9.961767e-01

5686 7.613624e-01 9.961741e-01

5687 7.610335e-01 9.961713e-01

5688 7.606975e-01 9.961685e-01

5689 7.603545e-01 9.961655e-01

5690 7.600046e-01 9.961624e-01

5691 7.596476e-01 9.961592e-01

5692 7.592837e-01 9.961558e-01

5693 7.589128e-01 9.961523e-01

5694 7.585349e-01 9.961486e-01

5695 7.581502e-01 9.961448e-01

5696 7.577586e-01 9.961408e-01

5697 7.573602e-01 9.961366e-01

5698 7.569551e-01 9.961322e-01

5699 7.565433e-01 9.961277e-01

5700 7.561250e-01 9.961230e-01

5701 7.557001e-01 9.961180e-01

5702 7.552688e-01 9.961128e-01

5703 7.548312e-01 9.961074e-01

5704 7.543875e-01 9.961018e-01

5705 7.539376e-01 9.960960e-01

5706 7.534818e-01 9.960898e-01

5707 7.530201e-01 9.960835e-01

5708 7.525528e-01 9.960768e-01

5709 7.520799e-01 9.960698e-01

5710 7.516016e-01 9.960626e-01

5711 7.511182e-01 9.960550e-01

5712 7.506296e-01 9.960472e-01

5713 7.501362e-01 9.960389e-01

5714 7.496381e-01 9.960304e-01

5715 7.491356e-01 9.960214e-01

5716 7.486287e-01 9.960121e-01

5717 7.481177e-01 9.960024e-01

5718 7.476028e-01 9.959922e-01

5719 7.470842e-01 9.959816e-01

5720 7.465622e-01 9.959706e-01

5721 7.460369e-01 9.959591e-01

5722 7.455086e-01 9.959471e-01

5723 7.449775e-01 9.959346e-01

5724 7.444439e-01 9.959215e-01

5725 7.439079e-01 9.959079e-01

5726 7.433699e-01 9.958938e-01

5727 7.428300e-01 9.958790e-01

5728 7.422886e-01 9.958636e-01

5729 7.417458e-01 9.958475e-01

5730 7.412019e-01 9.958307e-01

5731 7.406572e-01 9.958133e-01

5732 7.401118e-01 9.957951e-01

5733 7.395661e-01 9.957761e-01

5734 7.390203e-01 9.957563e-01

5735 7.384746e-01 9.957357e-01

5736 7.379293e-01 9.957142e-01

5737 7.373846e-01 9.956918e-01

5738 7.368408e-01 9.956684e-01

5739 7.362980e-01 9.956441e-01

5740 7.357566e-01 9.956187e-01

5741 7.352168e-01 9.955922e-01

5742 7.346788e-01 9.955646e-01

5743 7.341427e-01 9.955359e-01

5744 7.336089e-01 9.955059e-01

5745 7.330776e-01 9.954747e-01

5746 7.325489e-01 9.954421e-01

5747 7.320230e-01 9.954082e-01

5748 7.315002e-01 9.953729e-01

5749 7.309807e-01 9.953360e-01

5750 7.304646e-01 9.952976e-01

5751 7.299521e-01 9.952576e-01

5752 7.294434e-01 9.952158e-01

5753 7.289386e-01 9.951724e-01

5754 7.284380e-01 9.951270e-01

5755 7.279416e-01 9.950798e-01

5756 7.274497e-01 9.950306e-01

5757 7.269623e-01 9.949793e-01

5758 7.264796e-01 9.949259e-01

5759 7.260017e-01 9.948702e-01

5760 7.255288e-01 9.948122e-01

5761 7.250609e-01 9.947518e-01

5762 7.250274e-01 9.947517e-01

5763 7.249926e-01 9.947517e-01

5764 7.249566e-01 9.947516e-01

5765 7.249193e-01 9.947516e-01

5766 7.248806e-01 9.947515e-01

5767 7.248406e-01 9.947515e-01

5768 7.247992e-01 9.947514e-01

5769 7.247563e-01 9.947514e-01

5770 7.247119e-01 9.947513e-01

5771 7.246659e-01 9.947513e-01

5772 7.246183e-01 9.947512e-01

5773 7.245691e-01 9.947511e-01

5774 7.245182e-01 9.947511e-01

5775 7.244655e-01 9.947510e-01

5776 7.244110e-01 9.947509e-01

5777 7.243546e-01 9.947508e-01

5778 7.242962e-01 9.947507e-01

5779 7.242359e-01 9.947506e-01

5780 7.241735e-01 9.947505e-01

5781 7.241090e-01 9.947504e-01

5782 7.240423e-01 9.947503e-01

5783 7.239733e-01 9.947502e-01

5784 7.239021e-01 9.947501e-01

5785 7.238284e-01 9.947500e-01

5786 7.237522e-01 9.947499e-01

5787 7.236735e-01 9.947497e-01

5788 7.235922e-01 9.947496e-01

5789 7.235082e-01 9.947495e-01

5790 7.234214e-01 9.947493e-01

5791 7.233318e-01 9.947491e-01

5792 7.232392e-01 9.947490e-01

5793 7.231436e-01 9.947488e-01

5794 7.230449e-01 9.947486e-01

5795 7.229430e-01 9.947484e-01

5796 7.228378e-01 9.947482e-01

5797 7.227293e-01 9.947480e-01

5798 7.226172e-01 9.947478e-01

5799 7.225016e-01 9.947476e-01

5800 7.223823e-01 9.947474e-01

5801 7.222593e-01 9.947471e-01

5802 7.221325e-01 9.947468e-01

5803 7.220016e-01 9.947466e-01

5804 7.218668e-01 9.947463e-01

5805 7.217277e-01 9.947460e-01

5806 7.215844e-01 9.947457e-01

5807 7.214368e-01 9.947454e-01

5808 7.212846e-01 9.947450e-01

5809 7.211279e-01 9.947447e-01

5810 7.209665e-01 9.947443e-01

5811 7.208003e-01 9.947439e-01

5812 7.206293e-01 9.947435e-01

5813 7.204532e-01 9.947431e-01

5814 7.202720e-01 9.947426e-01

5815 7.200855e-01 9.947422e-01

5816 7.198937e-01 9.947417e-01

5817 7.196965e-01 9.947412e-01

5818 7.194937e-01 9.947406e-01

5819 7.192852e-01 9.947401e-01

5820 7.190710e-01 9.947395e-01

5821 7.188509e-01 9.947389e-01

5822 7.186248e-01 9.947383e-01

5823 7.183926e-01 9.947376e-01

5824 7.181542e-01 9.947369e-01

5825 7.179095e-01 9.947362e-01

5826 7.176583e-01 9.947354e-01

5827 7.174007e-01 9.947347e-01

5828 7.171365e-01 9.947338e-01

5829 7.168655e-01 9.947330e-01

5830 7.165878e-01 9.947321e-01

5831 7.163032e-01 9.947311e-01

5832 7.160116e-01 9.947302e-01

5833 7.157130e-01 9.947291e-01

5834 7.154072e-01 9.947281e-01

5835 7.150943e-01 9.947269e-01

5836 7.147740e-01 9.947258e-01

5837 7.144465e-01 9.947246e-01

5838 7.141115e-01 9.947233e-01

5839 7.137690e-01 9.947220e-01

5840 7.134191e-01 9.947206e-01

5841 7.130615e-01 9.947191e-01

5842 7.126964e-01 9.947176e-01

5843 7.123237e-01 9.947160e-01

5844 7.119433e-01 9.947144e-01

5845 7.115552e-01 9.947127e-01

5846 7.111594e-01 9.947109e-01

5847 7.107559e-01 9.947090e-01

5848 7.103447e-01 9.947070e-01

5849 7.099259e-01 9.947050e-01

5850 7.094993e-01 9.947028e-01

5851 7.090651e-01 9.947006e-01

5852 7.086233e-01 9.946983e-01

5853 7.081739e-01 9.946958e-01

5854 7.077170e-01 9.946933e-01

5855 7.072526e-01 9.946906e-01

5856 7.067808e-01 9.946879e-01

5857 7.063017e-01 9.946850e-01

5858 7.058153e-01 9.946820e-01

5859 7.053218e-01 9.946788e-01

5860 7.048213e-01 9.946755e-01

5861 7.043138e-01 9.946721e-01

5862 7.037996e-01 9.946685e-01

5863 7.032786e-01 9.946648e-01

5864 7.027512e-01 9.946609e-01

5865 7.022173e-01 9.946568e-01

5866 7.016772e-01 9.946525e-01

5867 7.011311e-01 9.946481e-01

5868 7.005791e-01 9.946434e-01

5869 7.000214e-01 9.946386e-01

5870 6.994582e-01 9.946335e-01

5871 6.988897e-01 9.946283e-01

5872 6.983161e-01 9.946227e-01

5873 6.977376e-01 9.946170e-01

5874 6.971545e-01 9.946110e-01

5875 6.965670e-01 9.946047e-01

5876 6.959754e-01 9.945981e-01

5877 6.953798e-01 9.945913e-01

5878 6.947805e-01 9.945841e-01

5879 6.941779e-01 9.945766e-01

5880 6.935720e-01 9.945688e-01

5881 6.929633e-01 9.945607e-01

5882 6.923520e-01 9.945522e-01

5883 6.917384e-01 9.945433e-01

5884 6.911227e-01 9.945340e-01

5885 6.905052e-01 9.945243e-01

5886 6.898863e-01 9.945142e-01

5887 6.892662e-01 9.945036e-01

5888 6.886451e-01 9.944925e-01

5889 6.880235e-01 9.944810e-01

5890 6.874015e-01 9.944689e-01

5891 6.867795e-01 9.944563e-01

5892 6.861578e-01 9.944431e-01

5893 6.855366e-01 9.944293e-01

5894 6.849163e-01 9.944150e-01

5895 6.842971e-01 9.943999e-01

5896 6.836793e-01 9.943842e-01

5897 6.830632e-01 9.943678e-01

5898 6.824491e-01 9.943507e-01

5899 6.818372e-01 9.943328e-01

5900 6.812278e-01 9.943141e-01

5901 6.806212e-01 9.942945e-01

5902 6.800176e-01 9.942741e-01

5903 6.794174e-01 9.942527e-01

5904 6.788207e-01 9.942304e-01

5905 6.782277e-01 9.942071e-01

5906 6.776388e-01 9.941828e-01

5907 6.770542e-01 9.941573e-01

5908 6.764740e-01 9.941307e-01

5909 6.758985e-01 9.941029e-01

5910 6.753278e-01 9.940739e-01

5911 6.747623e-01 9.940435e-01

5912 6.742021e-01 9.940118e-01

5913 6.736473e-01 9.939786e-01

5914 6.730981e-01 9.939440e-01

5915 6.725547e-01 9.939078e-01

5916 6.720173e-01 9.938700e-01

5917 6.714860e-01 9.938305e-01

5918 6.709610e-01 9.937892e-01

5919 6.704423e-01 9.937461e-01

5920 6.699301e-01 9.937010e-01

5921 6.694245e-01 9.936539e-01

5922 6.689255e-01 9.936047e-01

5923 6.684334e-01 9.935534e-01

5924 6.679482e-01 9.934997e-01

5925 6.674700e-01 9.934436e-01

5926 6.669987e-01 9.933850e-01

5927 6.665346e-01 9.933239e-01

5928 6.660776e-01 9.932600e-01

5929 6.656278e-01 9.931933e-01

5930 6.651852e-01 9.931236e-01

5931 6.647498e-01 9.930509e-01

5932 6.643217e-01 9.929750e-01

5933 6.639009e-01 9.928957e-01

5934 6.634874e-01 9.928130e-01

5935 6.630812e-01 9.927267e-01

5936 6.626822e-01 9.926366e-01

5937 6.622906e-01 9.925426e-01

5938 6.619061e-01 9.924445e-01

5939 6.615289e-01 9.923423e-01

5940 6.611589e-01 9.922356e-01

5941 6.607960e-01 9.921243e-01

5942 6.607464e-01 9.921243e-01

5943 6.606954e-01 9.921242e-01

5944 6.606429e-01 9.921241e-01

5945 6.605890e-01 9.921240e-01

5946 6.605337e-01 9.921239e-01

5947 6.604768e-01 9.921238e-01

5948 6.604184e-01 9.921237e-01

5949 6.603585e-01 9.921236e-01

5950 6.602970e-01 9.921235e-01

5951 6.602338e-01 9.921234e-01

5952 6.601690e-01 9.921233e-01

5953 6.601025e-01 9.921232e-01

5954 6.600343e-01 9.921231e-01

5955 6.599644e-01 9.921229e-01

5956 6.598927e-01 9.921228e-01

5957 6.598191e-01 9.921226e-01

5958 6.597437e-01 9.921225e-01

5959 6.596664e-01 9.921223e-01

5960 6.595872e-01 9.921222e-01

5961 6.595060e-01 9.921220e-01

5962 6.594229e-01 9.921218e-01

5963 6.593377e-01 9.921216e-01

5964 6.592504e-01 9.921214e-01

5965 6.591610e-01 9.921212e-01

5966 6.590695e-01 9.921210e-01

5967 6.589758e-01 9.921208e-01

5968 6.588799e-01 9.921206e-01

5969 6.587817e-01 9.921203e-01

5970 6.586813e-01 9.921201e-01

5971 6.585785e-01 9.921198e-01

5972 6.584733e-01 9.921195e-01

5973 6.583658e-01 9.921192e-01

5974 6.582558e-01 9.921189e-01

5975 6.581433e-01 9.921186e-01

5976 6.580283e-01 9.921183e-01

5977 6.579108e-01 9.921179e-01

5978 6.577907e-01 9.921176e-01

5979 6.576681e-01 9.921172e-01

5980 6.575427e-01 9.921168e-01

5981 6.574147e-01 9.921164e-01

5982 6.572840e-01 9.921160e-01

5983 6.571506e-01 9.921155e-01

5984 6.570145e-01 9.921150e-01

5985 6.568755e-01 9.921146e-01

5986 6.567338e-01 9.921140e-01

5987 6.565892e-01 9.921135e-01

5988 6.564417e-01 9.921130e-01

5989 6.562914e-01 9.921124e-01

5990 6.561382e-01 9.921118e-01

5991 6.559821e-01 9.921111e-01

5992 6.558231e-01 9.921105e-01

5993 6.556611e-01 9.921098e-01

5994 6.554962e-01 9.921091e-01

5995 6.553283e-01 9.921083e-01

5996 6.551574e-01 9.921075e-01

5997 6.549836e-01 9.921067e-01

5998 6.548069e-01 9.921059e-01

5999 6.546271e-01 9.921050e-01

6000 6.544444e-01 9.921040e-01

6001 6.542587e-01 9.921031e-01

6002 6.540701e-01 9.921021e-01

6003 6.538785e-01 9.921010e-01

6004 6.536840e-01 9.920999e-01

6005 6.534866e-01 9.920987e-01

6006 6.532863e-01 9.920975e-01

6007 6.530831e-01 9.920963e-01

6008 6.528771e-01 9.920950e-01

6009 6.526683e-01 9.920936e-01

6010 6.524568e-01 9.920922e-01

6011 6.522425e-01 9.920907e-01

6012 6.520255e-01 9.920891e-01

6013 6.518058e-01 9.920875e-01

6014 6.515836e-01 9.920858e-01

6015 6.513588e-01 9.920840e-01

6016 6.511315e-01 9.920822e-01

6017 6.509018e-01 9.920802e-01

6018 6.506697e-01 9.920782e-01

6019 6.504353e-01 9.920761e-01

6020 6.501986e-01 9.920739e-01

6021 6.499598e-01 9.920716e-01

6022 6.497190e-01 9.920692e-01

6023 6.494761e-01 9.920667e-01

6024 6.492313e-01 9.920641e-01

6025 6.489846e-01 9.920614e-01

6026 6.487363e-01 9.920586e-01

6027 6.484863e-01 9.920556e-01

6028 6.482347e-01 9.920525e-01

6029 6.479817e-01 9.920492e-01

6030 6.477274e-01 9.920459e-01

6031 6.474718e-01 9.920423e-01

6032 6.472151e-01 9.920386e-01

6033 6.469574e-01 9.920348e-01

6034 6.466988e-01 9.920307e-01

6035 6.464394e-01 9.920265e-01

6036 6.461794e-01 9.920221e-01

6037 6.459188e-01 9.920176e-01

6038 6.456578e-01 9.920128e-01

6039 6.453966e-01 9.920077e-01

6040 6.451351e-01 9.920025e-01

6041 6.448736e-01 9.919970e-01

6042 6.446123e-01 9.919913e-01

6043 6.443511e-01 9.919853e-01

6044 6.440903e-01 9.919791e-01

6045 6.438300e-01 9.919726e-01

6046 6.435702e-01 9.919657e-01

6047 6.433113e-01 9.919586e-01

6048 6.430532e-01 9.919511e-01

6049 6.427961e-01 9.919433e-01

6050 6.425401e-01 9.919352e-01

6051 6.422854e-01 9.919266e-01

6052 6.420321e-01 9.919177e-01

6053 6.417803e-01 9.919084e-01

6054 6.415301e-01 9.918986e-01

6055 6.412817e-01 9.918884e-01

6056 6.410351e-01 9.918778e-01

6057 6.407906e-01 9.918666e-01

6058 6.405481e-01 9.918549e-01

6059 6.403079e-01 9.918427e-01

6060 6.400700e-01 9.918299e-01

6061 6.398345e-01 9.918165e-01

6062 6.396015e-01 9.918025e-01

6063 6.393712e-01 9.917879e-01

6064 6.391436e-01 9.917726e-01

6065 6.389189e-01 9.917565e-01

6066 6.386970e-01 9.917397e-01

6067 6.384781e-01 9.917221e-01

6068 6.382624e-01 9.917038e-01

6069 6.380497e-01 9.916845e-01

6070 6.378403e-01 9.916644e-01

6071 6.376341e-01 9.916433e-01

6072 6.374313e-01 9.916212e-01

6073 6.372319e-01 9.915981e-01

6074 6.370360e-01 9.915739e-01

6075 6.368436e-01 9.915486e-01

6076 6.366547e-01 9.915221e-01

6077 6.364695e-01 9.914944e-01

6078 6.362878e-01 9.914654e-01

6079 6.361098e-01 9.914351e-01

6080 6.359355e-01 9.914033e-01

6081 6.357650e-01 9.913700e-01

6082 6.355981e-01 9.913352e-01

6083 6.354350e-01 9.912988e-01

6084 6.352756e-01 9.912607e-01

6085 6.351200e-01 9.912208e-01

6086 6.349682e-01 9.911791e-01

6087 6.348201e-01 9.911355e-01

6088 6.346758e-01 9.910898e-01

6089 6.345352e-01 9.910420e-01

6090 6.343984e-01 9.909921e-01

6091 6.342652e-01 9.909398e-01

6092 6.341358e-01 9.908852e-01

6093 6.340100e-01 9.908280e-01

6094 6.338879e-01 9.907682e-01

6095 6.337694e-01 9.907057e-01

6096 6.336545e-01 9.906404e-01

6097 6.335431e-01 9.905721e-01

6098 6.334352e-01 9.905008e-01

6099 6.333308e-01 9.904262e-01

6100 6.332299e-01 9.903482e-01

6101 6.331323e-01 9.902668e-01

6102 6.330381e-01 9.901818e-01

6103 6.329472e-01 9.900929e-01

6104 6.328595e-01 9.900002e-01

6105 6.327751e-01 9.899033e-01

6106 6.326938e-01 9.898022e-01

6107 6.326156e-01 9.896967e-01

6108 6.325404e-01 9.895866e-01

6109 6.324682e-01 9.894717e-01

6110 6.323990e-01 9.893519e-01

6111 6.323327e-01 9.892270e-01

6112 6.322692e-01 9.890967e-01

6113 6.322084e-01 9.889610e-01

6114 6.321504e-01 9.888195e-01

6115 6.320950e-01 9.886721e-01

6116 6.320423e-01 9.885186e-01

6117 6.319921e-01 9.883587e-01

6118 6.319443e-01 9.881924e-01

6119 6.318990e-01 9.880192e-01

6120 6.318561e-01 9.878391e-01

6121 6.318155e-01 9.876518e-01

6122 6.318212e-01 9.876517e-01

6123 6.318275e-01 9.876516e-01

6124 6.318344e-01 9.876515e-01

6125 6.318421e-01 9.876513e-01

6126 6.318504e-01 9.876512e-01

6127 6.318595e-01 9.876510e-01

6128 6.318694e-01 9.876509e-01

6129 6.318801e-01 9.876507e-01

6130 6.318917e-01 9.876506e-01

6131 6.319041e-01 9.876504e-01

6132 6.319175e-01 9.876502e-01

6133 6.319319e-01 9.876500e-01

6134 6.319472e-01 9.876498e-01

6135 6.319636e-01 9.876496e-01

6136 6.319811e-01 9.876494e-01

6137 6.319998e-01 9.876492e-01

6138 6.320196e-01 9.876489e-01

6139 6.320407e-01 9.876487e-01

6140 6.320630e-01 9.876485e-01

6141 6.320867e-01 9.876482e-01

6142 6.321118e-01 9.876479e-01

6143 6.321383e-01 9.876476e-01

6144 6.321663e-01 9.876473e-01

6145 6.321958e-01 9.876470e-01

6146 6.322270e-01 9.876467e-01

6147 6.322598e-01 9.876464e-01

6148 6.322943e-01 9.876460e-01

6149 6.323307e-01 9.876456e-01

6150 6.323688e-01 9.876452e-01

6151 6.324089e-01 9.876448e-01

6152 6.324510e-01 9.876444e-01

6153 6.324951e-01 9.876440e-01

6154 6.325414e-01 9.876435e-01

6155 6.325898e-01 9.876431e-01

6156 6.326404e-01 9.876426e-01

6157 6.326934e-01 9.876421e-01

6158 6.327488e-01 9.876415e-01

6159 6.328067e-01 9.876410e-01

6160 6.328672e-01 9.876404e-01

6161 6.329302e-01 9.876398e-01

6162 6.329960e-01 9.876391e-01

6163 6.330646e-01 9.876385e-01

6164 6.331361e-01 9.876378e-01

6165 6.332105e-01 9.876371e-01

6166 6.332879e-01 9.876363e-01

6167 6.333685e-01 9.876355e-01

6168 6.334523e-01 9.876347e-01

6169 6.335394e-01 9.876339e-01

6170 6.336298e-01 9.876330e-01

6171 6.337238e-01 9.876321e-01

6172 6.338212e-01 9.876311e-01

6173 6.339224e-01 9.876301e-01

6174 6.340273e-01 9.876290e-01

6175 6.341360e-01 9.876279e-01

6176 6.342486e-01 9.876268e-01

6177 6.343652e-01 9.876256e-01

6178 6.344859e-01 9.876244e-01

6179 6.346108e-01 9.876231e-01

6180 6.347400e-01 9.876217e-01

6181 6.348735e-01 9.876203e-01

6182 6.350115e-01 9.876188e-01

6183 6.351540e-01 9.876173e-01

6184 6.353012e-01 9.876157e-01

6185 6.354530e-01 9.876140e-01

6186 6.356096e-01 9.876122e-01

6187 6.357711e-01 9.876104e-01

6188 6.359375e-01 9.876085e-01

6189 6.361089e-01 9.876065e-01

6190 6.362855e-01 9.876044e-01

6191 6.364672e-01 9.876023e-01

6192 6.366541e-01 9.876000e-01

6193 6.368464e-01 9.875976e-01

6194 6.370440e-01 9.875951e-01

6195 6.372470e-01 9.875926e-01

6196 6.374556e-01 9.875899e-01

6197 6.376697e-01 9.875870e-01

6198 6.378894e-01 9.875841e-01

6199 6.381147e-01 9.875810e-01

6200 6.383457e-01 9.875778e-01

6201 6.385825e-01 9.875745e-01

6202 6.388250e-01 9.875710e-01

6203 6.390732e-01 9.875673e-01

6204 6.393273e-01 9.875635e-01

6205 6.395872e-01 9.875595e-01

6206 6.398530e-01 9.875553e-01

6207 6.401246e-01 9.875509e-01

6208 6.404021e-01 9.875463e-01

6209 6.406854e-01 9.875415e-01

6210 6.409745e-01 9.875366e-01

6211 6.412695e-01 9.875313e-01

6212 6.415702e-01 9.875259e-01

6213 6.418768e-01 9.875201e-01

6214 6.421890e-01 9.875142e-01

6215 6.425070e-01 9.875079e-01

6216 6.428306e-01 9.875014e-01

6217 6.431599e-01 9.874945e-01

6218 6.434946e-01 9.874874e-01

6219 6.438349e-01 9.874799e-01

6220 6.441805e-01 9.874721e-01

6221 6.445315e-01 9.874639e-01

6222 6.448876e-01 9.874553e-01

6223 6.452490e-01 9.874463e-01

6224 6.456153e-01 9.874369e-01

6225 6.459866e-01 9.874270e-01

6226 6.463628e-01 9.874167e-01

6227 6.467436e-01 9.874060e-01

6228 6.471291e-01 9.873947e-01

6229 6.475189e-01 9.873829e-01

6230 6.479132e-01 9.873705e-01

6231 6.483116e-01 9.873575e-01

6232 6.487140e-01 9.873440e-01

6233 6.491203e-01 9.873297e-01

6234 6.495304e-01 9.873149e-01

6235 6.499440e-01 9.872993e-01

6236 6.503611e-01 9.872830e-01

6237 6.507814e-01 9.872659e-01

6238 6.512048e-01 9.872480e-01

6239 6.516310e-01 9.872292e-01

6240 6.520600e-01 9.872096e-01

6241 6.524916e-01 9.871890e-01

6242 6.529255e-01 9.871675e-01

6243 6.533616e-01 9.871449e-01

6244 6.537997e-01 9.871213e-01

6245 6.542396e-01 9.870965e-01

6246 6.546811e-01 9.870706e-01

6247 6.551240e-01 9.870435e-01

6248 6.555682e-01 9.870151e-01

6249 6.560134e-01 9.869853e-01

6250 6.564594e-01 9.869542e-01

6251 6.569061e-01 9.869216e-01

6252 6.573533e-01 9.868874e-01

6253 6.578008e-01 9.868517e-01

6254 6.582483e-01 9.868143e-01

6255 6.586958e-01 9.867751e-01

6256 6.591429e-01 9.867341e-01

6257 6.595896e-01 9.866912e-01

6258 6.600357e-01 9.866463e-01

6259 6.604809e-01 9.865994e-01

6260 6.609252e-01 9.865503e-01

6261 6.613682e-01 9.864989e-01

6262 6.618100e-01 9.864452e-01

6263 6.622502e-01 9.863890e-01

6264 6.626888e-01 9.863303e-01

6265 6.631255e-01 9.862688e-01

6266 6.635603e-01 9.862047e-01

6267 6.639929e-01 9.861376e-01

6268 6.644233e-01 9.860675e-01

6269 6.648512e-01 9.859942e-01

6270 6.652766e-01 9.859178e-01

6271 6.656993e-01 9.858379e-01

6272 6.661192e-01 9.857545e-01

6273 6.665362e-01 9.856674e-01

6274 6.669501e-01 9.855765e-01

6275 6.673609e-01 9.854817e-01

6276 6.677684e-01 9.853827e-01

6277 6.681725e-01 9.852796e-01

6278 6.685732e-01 9.851720e-01

6279 6.689703e-01 9.850598e-01

6280 6.693638e-01 9.849429e-01

6281 6.697536e-01 9.848211e-01

6282 6.701395e-01 9.846942e-01

6283 6.705216e-01 9.845621e-01

6284 6.708997e-01 9.844245e-01

6285 6.712739e-01 9.842813e-01

6286 6.716440e-01 9.841324e-01

6287 6.720099e-01 9.839774e-01

6288 6.723718e-01 9.838163e-01

6289 6.727294e-01 9.836489e-01

6290 6.730828e-01 9.834749e-01

6291 6.734320e-01 9.832942e-01

6292 6.737768e-01 9.831066e-01

6293 6.741174e-01 9.829119e-01

6294 6.744536e-01 9.827099e-01

6295 6.747854e-01 9.825004e-01

6296 6.751129e-01 9.822832e-01

6297 6.754360e-01 9.820582e-01

6298 6.757548e-01 9.818252e-01

6299 6.760691e-01 9.815839e-01

6300 6.763791e-01 9.813343e-01

6301 6.766848e-01 9.810761e-01

6302 6.767428e-01 9.810759e-01

6303 6.768026e-01 9.810758e-01

6304 6.768644e-01 9.810756e-01

6305 6.769281e-01 9.810754e-01

6306 6.769937e-01 9.810752e-01

6307 6.770614e-01 9.810750e-01

6308 6.771312e-01 9.810748e-01

6309 6.772031e-01 9.810746e-01

6310 6.772772e-01 9.810744e-01

6311 6.773536e-01 9.810742e-01

6312 6.774322e-01 9.810739e-01

6313 6.775133e-01 9.810737e-01

6314 6.775967e-01 9.810734e-01

6315 6.776826e-01 9.810732e-01

6316 6.777711e-01 9.810729e-01

6317 6.778622e-01 9.810726e-01

6318 6.779559e-01 9.810723e-01

6319 6.780525e-01 9.810720e-01

6320 6.781518e-01 9.810717e-01

6321 6.782540e-01 9.810713e-01

6322 6.783592e-01 9.810710e-01

6323 6.784674e-01 9.810706e-01

6324 6.785787e-01 9.810702e-01

6325 6.786932e-01 9.810698e-01

6326 6.788110e-01 9.810694e-01

6327 6.789321e-01 9.810690e-01

6328 6.790566e-01 9.810685e-01

6329 6.791847e-01 9.810680e-01

6330 6.793164e-01 9.810675e-01

6331 6.794517e-01 9.810670e-01

6332 6.795908e-01 9.810665e-01

6333 6.797337e-01 9.810659e-01

6334 6.798806e-01 9.810654e-01

6335 6.800315e-01 9.810648e-01

6336 6.801866e-01 9.810641e-01

6337 6.803459e-01 9.810635e-01

6338 6.805094e-01 9.810628e-01

6339 6.806774e-01 9.810621e-01

6340 6.808499e-01 9.810613e-01

6341 6.810270e-01 9.810606e-01

6342 6.812087e-01 9.810598e-01

6343 6.813953e-01 9.810589e-01

6344 6.815867e-01 9.810581e-01

6345 6.817832e-01 9.810571e-01

6346 6.819847e-01 9.810562e-01

6347 6.821914e-01 9.810552e-01

6348 6.824034e-01 9.810542e-01

6349 6.826208e-01 9.810531e-01

6350 6.828436e-01 9.810520e-01

6351 6.830721e-01 9.810508e-01

6352 6.833063e-01 9.810496e-01

6353 6.835462e-01 9.810483e-01

6354 6.837920e-01 9.810470e-01

6355 6.840439e-01 9.810456e-01

6356 6.843018e-01 9.810441e-01

6357 6.845659e-01 9.810426e-01

6358 6.848363e-01 9.810410e-01

6359 6.851131e-01 9.810394e-01

6360 6.853964e-01 9.810377e-01

6361 6.856863e-01 9.810359e-01

6362 6.859828e-01 9.810340e-01

6363 6.862861e-01 9.810320e-01

6364 6.865963e-01 9.810300e-01

6365 6.869134e-01 9.810278e-01

6366 6.872376e-01 9.810256e-01

6367 6.875689e-01 9.810233e-01

6368 6.879074e-01 9.810208e-01

6369 6.882531e-01 9.810183e-01

6370 6.886063e-01 9.810156e-01

6371 6.889668e-01 9.810129e-01

6372 6.893349e-01 9.810100e-01

6373 6.897106e-01 9.810069e-01

6374 6.900939e-01 9.810038e-01

6375 6.904849e-01 9.810004e-01

6376 6.908836e-01 9.809970e-01

6377 6.912902e-01 9.809934e-01

6378 6.917047e-01 9.809896e-01

6379 6.921270e-01 9.809856e-01

6380 6.925573e-01 9.809815e-01

6381 6.929955e-01 9.809771e-01

6382 6.934418e-01 9.809726e-01

6383 6.938961e-01 9.809679e-01

6384 6.943584e-01 9.809629e-01

6385 6.948288e-01 9.809578e-01

6386 6.953072e-01 9.809523e-01

6387 6.957937e-01 9.809467e-01

6388 6.962883e-01 9.809408e-01

6389 6.967908e-01 9.809345e-01

6390 6.973014e-01 9.809281e-01

6391 6.978200e-01 9.809213e-01

6392 6.983465e-01 9.809142e-01

6393 6.988810e-01 9.809067e-01

6394 6.994232e-01 9.808989e-01

6395 6.999733e-01 9.808908e-01

6396 7.005311e-01 9.808823e-01

6397 7.010965e-01 9.808733e-01

6398 7.016696e-01 9.808640e-01

6399 7.022500e-01 9.808542e-01

6400 7.028379e-01 9.808440e-01

6401 7.034330e-01 9.808333e-01

6402 7.040353e-01 9.808221e-01

6403 7.046446e-01 9.808103e-01

6404 7.052609e-01 9.807980e-01

6405 7.058838e-01 9.807852e-01

6406 7.065134e-01 9.807717e-01

6407 7.071494e-01 9.807576e-01

6408 7.077917e-01 9.807428e-01

6409 7.084402e-01 9.807274e-01

6410 7.090945e-01 9.807112e-01

6411 7.097546e-01 9.806942e-01

6412 7.104202e-01 9.806765e-01

6413 7.110912e-01 9.806579e-01

6414 7.117673e-01 9.806385e-01

6415 7.124483e-01 9.806182e-01

6416 7.131339e-01 9.805969e-01

6417 7.138241e-01 9.805746e-01

6418 7.145184e-01 9.805512e-01

6419 7.152167e-01 9.805268e-01

6420 7.159187e-01 9.805013e-01

6421 7.166242e-01 9.804745e-01

6422 7.173329e-01 9.804465e-01

6423 7.180445e-01 9.804173e-01

6424 7.187587e-01 9.803866e-01

6425 7.194754e-01 9.803546e-01

6426 7.201942e-01 9.803211e-01

6427 7.209148e-01 9.802860e-01

6428 7.216369e-01 9.802494e-01

6429 7.223603e-01 9.802111e-01

6430 7.230847e-01 9.801710e-01

6431 7.238098e-01 9.801292e-01

6432 7.245353e-01 9.800854e-01

6433 7.252608e-01 9.800397e-01

6434 7.259862e-01 9.799920e-01

6435 7.267112e-01 9.799421e-01

6436 7.274354e-01 9.798900e-01

6437 7.281585e-01 9.798356e-01

6438 7.288803e-01 9.797788e-01

6439 7.296005e-01 9.797195e-01

6440 7.303187e-01 9.796576e-01

6441 7.310348e-01 9.795931e-01

6442 7.317485e-01 9.795258e-01

6443 7.324594e-01 9.794556e-01

6444 7.331674e-01 9.793823e-01

6445 7.338721e-01 9.793060e-01

6446 7.345733e-01 9.792265e-01

6447 7.352707e-01 9.791436e-01

6448 7.359642e-01 9.790573e-01

6449 7.366534e-01 9.789675e-01

6450 7.373381e-01 9.788739e-01

6451 7.380181e-01 9.787765e-01

6452 7.386932e-01 9.786752e-01

6453 7.393631e-01 9.785699e-01

6454 7.400277e-01 9.784603e-01

6455 7.406868e-01 9.783464e-01

6456 7.413401e-01 9.782281e-01

6457 7.419875e-01 9.781052e-01

6458 7.426288e-01 9.779775e-01

6459 7.432639e-01 9.778450e-01

6460 7.438925e-01 9.777075e-01

6461 7.445146e-01 9.775649e-01

6462 7.451299e-01 9.774171e-01

6463 7.457384e-01 9.772638e-01

6464 7.463399e-01 9.771051e-01

6465 7.469343e-01 9.769406e-01

6466 7.475215e-01 9.767704e-01

6467 7.481014e-01 9.765943e-01

6468 7.486739e-01 9.764122e-01

6469 7.492389e-01 9.762239e-01

6470 7.497964e-01 9.760294e-01

6471 7.503462e-01 9.758285e-01

6472 7.508884e-01 9.756211e-01

6473 7.514228e-01 9.754072e-01

6474 7.519494e-01 9.751866e-01

6475 7.524681e-01 9.749592e-01

6476 7.529790e-01 9.747250e-01

6477 7.534821e-01 9.744839e-01

6478 7.539772e-01 9.742359e-01

6479 7.544644e-01 9.739808e-01

6480 7.549437e-01 9.737187e-01

6481 7.554151e-01 9.734495e-01

6482 7.554498e-01 9.734493e-01

6483 7.554854e-01 9.734492e-01

6484 7.555221e-01 9.734490e-01

6485 7.555597e-01 9.734488e-01

6486 7.555984e-01 9.734486e-01

6487 7.556382e-01 9.734484e-01

6488 7.556790e-01 9.734482e-01

6489 7.557210e-01 9.734479e-01

6490 7.557641e-01 9.734477e-01

6491 7.558083e-01 9.734475e-01

6492 7.558538e-01 9.734472e-01

6493 7.559005e-01 9.734469e-01

6494 7.559485e-01 9.734467e-01

6495 7.559978e-01 9.734464e-01

6496 7.560484e-01 9.734461e-01

6497 7.561003e-01 9.734458e-01

6498 7.561537e-01 9.734455e-01

6499 7.562085e-01 9.734451e-01

6500 7.562648e-01 9.734448e-01

6501 7.563225e-01 9.734444e-01

6502 7.563818e-01 9.734440e-01

6503 7.564427e-01 9.734436e-01

6504 7.565052e-01 9.734432e-01

6505 7.565694e-01 9.734428e-01

6506 7.566352e-01 9.734424e-01

6507 7.567028e-01 9.734419e-01

6508 7.567722e-01 9.734414e-01

6509 7.568435e-01 9.734409e-01

6510 7.569166e-01 9.734404e-01

6511 7.569916e-01 9.734399e-01

6512 7.570686e-01 9.734393e-01

6513 7.571476e-01 9.734388e-01

6514 7.572287e-01 9.734381e-01

6515 7.573118e-01 9.734375e-01

6516 7.573972e-01 9.734369e-01

6517 7.574847e-01 9.734362e-01

6518 7.575745e-01 9.734354e-01

6519 7.576666e-01 9.734347e-01

6520 7.577611e-01 9.734339e-01

6521 7.578579e-01 9.734331e-01

6522 7.579573e-01 9.734323e-01

6523 7.580592e-01 9.734314e-01

6524 7.581636e-01 9.734305e-01

6525 7.582707e-01 9.734295e-01

6526 7.583805e-01 9.734285e-01

6527 7.584930e-01 9.734275e-01

6528 7.586083e-01 9.734264e-01

6529 7.587265e-01 9.734252e-01

6530 7.588475e-01 9.734241e-01

6531 7.589716e-01 9.734228e-01

6532 7.590987e-01 9.734215e-01

6533 7.592289e-01 9.734202e-01

6534 7.593622e-01 9.734188e-01

6535 7.594987e-01 9.734173e-01

6536 7.596385e-01 9.734158e-01

6537 7.597816e-01 9.734142e-01

6538 7.599281e-01 9.734125e-01

6539 7.600780e-01 9.734108e-01

6540 7.602315e-01 9.734090e-01

6541 7.603884e-01 9.734071e-01

6542 7.605490e-01 9.734051e-01

6543 7.607133e-01 9.734030e-01

6544 7.608813e-01 9.734009e-01

6545 7.610530e-01 9.733986e-01

6546 7.612286e-01 9.733963e-01

6547 7.614081e-01 9.733938e-01

6548 7.615915e-01 9.733912e-01

6549 7.617789e-01 9.733885e-01

6550 7.619704e-01 9.733857e-01

6551 7.621659e-01 9.733828e-01

6552 7.623656e-01 9.733797e-01

6553 7.625695e-01 9.733765e-01

6554 7.627775e-01 9.733732e-01

6555 7.629899e-01 9.733697e-01

6556 7.632066e-01 9.733660e-01

6557 7.634276e-01 9.733622e-01

6558 7.636530e-01 9.733582e-01

6559 7.638828e-01 9.733540e-01

6560 7.641171e-01 9.733497e-01

6561 7.643558e-01 9.733451e-01

6562 7.645991e-01 9.733403e-01

6563 7.648469e-01 9.733353e-01

6564 7.650992e-01 9.733301e-01

6565 7.653561e-01 9.733247e-01

6566 7.656176e-01 9.733190e-01

6567 7.658836e-01 9.733130e-01

6568 7.661542e-01 9.733068e-01

6569 7.664294e-01 9.733002e-01

6570 7.667093e-01 9.732934e-01

6571 7.669936e-01 9.732863e-01

6572 7.672826e-01 9.732789e-01

6573 7.675761e-01 9.732711e-01

6574 7.678741e-01 9.732629e-01

6575 7.681767e-01 9.732544e-01

6576 7.684837e-01 9.732455e-01

6577 7.687952e-01 9.732362e-01

6578 7.691110e-01 9.732264e-01

6579 7.694313e-01 9.732163e-01

6580 7.697558e-01 9.732056e-01

6581 7.700846e-01 9.731945e-01

6582 7.704175e-01 9.731829e-01

6583 7.707546e-01 9.731707e-01

6584 7.710957e-01 9.731580e-01

6585 7.714408e-01 9.731447e-01

6586 7.717898e-01 9.731308e-01

6587 7.721425e-01 9.731163e-01

6588 7.724990e-01 9.731011e-01

6589 7.728590e-01 9.730853e-01

6590 7.732225e-01 9.730687e-01

6591 7.735894e-01 9.730514e-01

6592 7.739596e-01 9.730333e-01

6593 7.743328e-01 9.730144e-01

6594 7.747091e-01 9.729947e-01

6595 7.750882e-01 9.729740e-01

6596 7.754700e-01 9.729525e-01

6597 7.758544e-01 9.729300e-01

6598 7.762411e-01 9.729065e-01

6599 7.766302e-01 9.728820e-01

6600 7.770213e-01 9.728564e-01

6601 7.774143e-01 9.728296e-01

6602 7.778091e-01 9.728017e-01

6603 7.782054e-01 9.727726e-01

6604 7.786032e-01 9.727422e-01

6605 7.790021e-01 9.727105e-01

6606 7.794021e-01 9.726775e-01

6607 7.798028e-01 9.726430e-01

6608 7.802042e-01 9.726071e-01

6609 7.806061e-01 9.725696e-01

6610 7.810081e-01 9.725305e-01

6611 7.814102e-01 9.724898e-01

6612 7.818121e-01 9.724474e-01

6613 7.822136e-01 9.724033e-01

6614 7.826146e-01 9.723573e-01

6615 7.830147e-01 9.723094e-01

6616 7.834139e-01 9.722596e-01

6617 7.838118e-01 9.722077e-01

6618 7.842083e-01 9.721538e-01

6619 7.846032e-01 9.720977e-01

6620 7.849963e-01 9.720393e-01

6621 7.853874e-01 9.719787e-01

6622 7.857762e-01 9.719157e-01

6623 7.861626e-01 9.718502e-01

6624 7.865465e-01 9.717823e-01

6625 7.869274e-01 9.717117e-01

6626 7.873054e-01 9.716385e-01

6627 7.876803e-01 9.715625e-01

6628 7.880517e-01 9.714837e-01

6629 7.884197e-01 9.714020e-01

6630 7.887839e-01 9.713174e-01

6631 7.891442e-01 9.712297e-01

6632 7.895006e-01 9.711389e-01

6633 7.898527e-01 9.710449e-01

6634 7.902005e-01 9.709476e-01

6635 7.905439e-01 9.708470e-01

6636 7.908826e-01 9.707429e-01

6637 7.912166e-01 9.706354e-01

6638 7.915458e-01 9.705244e-01

6639 7.918700e-01 9.704097e-01

6640 7.921891e-01 9.702914e-01

6641 7.925030e-01 9.701693e-01

6642 7.928117e-01 9.700435e-01

6643 7.931150e-01 9.699138e-01

6644 7.934129e-01 9.697802e-01

6645 7.937053e-01 9.696426e-01

6646 7.939921e-01 9.695011e-01

6647 7.942732e-01 9.693556e-01

6648 7.945487e-01 9.692060e-01

6649 7.948185e-01 9.690524e-01

6650 7.950826e-01 9.688947e-01

6651 7.953408e-01 9.687329e-01

6652 7.955933e-01 9.685669e-01

6653 7.958399e-01 9.683969e-01

6654 7.960807e-01 9.682228e-01

6655 7.963157e-01 9.680445e-01

6656 7.965449e-01 9.678623e-01

6657 7.967683e-01 9.676760e-01

6658 7.969859e-01 9.674857e-01

6659 7.971977e-01 9.672915e-01

6660 7.974038e-01 9.670934e-01

6661 7.976042e-01 9.668915e-01

6662 7.976125e-01 9.668913e-01

6663 7.976210e-01 9.668912e-01

6664 7.976296e-01 9.668910e-01

6665 7.976384e-01 9.668908e-01

6666 7.976473e-01 9.668907e-01

6667 7.976564e-01 9.668905e-01

6668 7.976657e-01 9.668903e-01

6669 7.976751e-01 9.668901e-01

6670 7.976847e-01 9.668899e-01

6671 7.976944e-01 9.668897e-01

6672 7.977043e-01 9.668895e-01

6673 7.977144e-01 9.668893e-01

6674 7.977247e-01 9.668890e-01

6675 7.977351e-01 9.668888e-01

6676 7.977457e-01 9.668885e-01

6677 7.977565e-01 9.668883e-01

6678 7.977675e-01 9.668880e-01

6679 7.977786e-01 9.668877e-01

6680 7.977899e-01 9.668874e-01

6681 7.978014e-01 9.668871e-01

6682 7.978131e-01 9.668868e-01

6683 7.978250e-01 9.668865e-01

6684 7.978370e-01 9.668861e-01

6685 7.978493e-01 9.668857e-01

6686 7.978617e-01 9.668854e-01

6687 7.978743e-01 9.668850e-01

6688 7.978871e-01 9.668846e-01

6689 7.979001e-01 9.668842e-01

6690 7.979133e-01 9.668837e-01

6691 7.979266e-01 9.668833e-01

6692 7.979402e-01 9.668828e-01

6693 7.979539e-01 9.668823e-01

6694 7.979678e-01 9.668818e-01

6695 7.979819e-01 9.668812e-01

6696 7.979961e-01 9.668807e-01

6697 7.980106e-01 9.668801e-01

6698 7.980252e-01 9.668795e-01

6699 7.980400e-01 9.668788e-01

6700 7.980549e-01 9.668782e-01

6701 7.980700e-01 9.668775e-01

6702 7.980853e-01 9.668768e-01

6703 7.981007e-01 9.668760e-01

6704 7.981162e-01 9.668752e-01

6705 7.981319e-01 9.668744e-01

6706 7.981477e-01 9.668736e-01

6707 7.981637e-01 9.668727e-01

6708 7.981797e-01 9.668718e-01

6709 7.981959e-01 9.668708e-01

6710 7.982122e-01 9.668698e-01

6711 7.982285e-01 9.668688e-01

6712 7.982449e-01 9.668677e-01

6713 7.982614e-01 9.668666e-01

6714 7.982779e-01 9.668654e-01

6715 7.982944e-01 9.668641e-01

6716 7.983110e-01 9.668629e-01

6717 7.983276e-01 9.668615e-01

6718 7.983441e-01 9.668601e-01

6719 7.983606e-01 9.668586e-01

6720 7.983770e-01 9.668571e-01

6721 7.983933e-01 9.668555e-01

6722 7.984096e-01 9.668539e-01

6723 7.984257e-01 9.668521e-01

6724 7.984416e-01 9.668503e-01

6725 7.984574e-01 9.668484e-01

6726 7.984729e-01 9.668465e-01

6727 7.984882e-01 9.668444e-01

6728 7.985032e-01 9.668423e-01

6729 7.985179e-01 9.668400e-01

6730 7.985322e-01 9.668377e-01

6731 7.985462e-01 9.668353e-01

6732 7.985597e-01 9.668327e-01

6733 7.985727e-01 9.668301e-01

6734 7.985852e-01 9.668273e-01

6735 7.985972e-01 9.668244e-01

6736 7.986085e-01 9.668214e-01

6737 7.986192e-01 9.668182e-01

6738 7.986291e-01 9.668150e-01

6739 7.986383e-01 9.668115e-01

6740 7.986467e-01 9.668079e-01

6741 7.986541e-01 9.668042e-01

6742 7.986606e-01 9.668003e-01

6743 7.986661e-01 9.667962e-01

6744 7.986705e-01 9.667920e-01

6745 7.986737e-01 9.667876e-01

6746 7.986757e-01 9.667829e-01

6747 7.986765e-01 9.667781e-01

6748 7.986758e-01 9.667731e-01

6749 7.986737e-01 9.667678e-01

6750 7.986700e-01 9.667624e-01

6751 7.986647e-01 9.667566e-01

6752 7.986577e-01 9.667507e-01

6753 7.986489e-01 9.667445e-01

6754 7.986382e-01 9.667380e-01

6755 7.986255e-01 9.667312e-01

6756 7.986108e-01 9.667242e-01

6757 7.985938e-01 9.667168e-01

6758 7.985746e-01 9.667091e-01

6759 7.985531e-01 9.667011e-01

6760 7.985290e-01 9.666928e-01

6761 7.985023e-01 9.666841e-01

6762 7.984730e-01 9.666750e-01

6763 7.984409e-01 9.666656e-01

6764 7.984058e-01 9.666557e-01

6765 7.983678e-01 9.666455e-01

6766 7.983266e-01 9.666348e-01

6767 7.982822e-01 9.666236e-01

6768 7.982344e-01 9.666120e-01

6769 7.981832e-01 9.665999e-01

6770 7.981284e-01 9.665873e-01

6771 7.980700e-01 9.665742e-01

6772 7.980077e-01 9.665606e-01

6773 7.979416e-01 9.665464e-01

6774 7.978715e-01 9.665316e-01

6775 7.977972e-01 9.665162e-01

6776 7.977188e-01 9.665001e-01

6777 7.976360e-01 9.664834e-01

6778 7.975489e-01 9.664661e-01

6779 7.974572e-01 9.664480e-01

6780 7.973610e-01 9.664292e-01

6781 7.972600e-01 9.664097e-01

6782 7.971543e-01 9.663894e-01

6783 7.970438e-01 9.663683e-01

6784 7.969283e-01 9.663463e-01

6785 7.968078e-01 9.663235e-01

6786 7.966823e-01 9.662998e-01

6787 7.965517e-01 9.662752e-01

6788 7.964159e-01 9.662497e-01

6789 7.962749e-01 9.662231e-01

6790 7.961286e-01 9.661956e-01

6791 7.959770e-01 9.661670e-01

6792 7.958201e-01 9.661374e-01

6793 7.956578e-01 9.661066e-01

6794 7.954902e-01 9.660747e-01

6795 7.953173e-01 9.660417e-01

6796 7.951390e-01 9.660075e-01

6797 7.949553e-01 9.659720e-01

6798 7.947664e-01 9.659353e-01

6799 7.945721e-01 9.658972e-01

6800 7.943726e-01 9.658579e-01

6801 7.941678e-01 9.658172e-01

6802 7.939579e-01 9.657751e-01

6803 7.937430e-01 9.657316e-01

6804 7.935230e-01 9.656866e-01

6805 7.932980e-01 9.656402e-01

6806 7.930682e-01 9.655923e-01

6807 7.928336e-01 9.655428e-01

6808 7.925944e-01 9.654918e-01

6809 7.923506e-01 9.654392e-01

6810 7.921024e-01 9.653849e-01

6811 7.918499e-01 9.653291e-01

6812 7.915932e-01 9.652716e-01

6813 7.913326e-01 9.652124e-01

6814 7.910680e-01 9.651515e-01

6815 7.907997e-01 9.650889e-01

6816 7.905278e-01 9.650245e-01

6817 7.902525e-01 9.649585e-01

6818 7.899740e-01 9.648907e-01

6819 7.896924e-01 9.648211e-01

6820 7.894079e-01 9.647498e-01

6821 7.891207e-01 9.646767e-01

6822 7.888310e-01 9.646019e-01

6823 7.885389e-01 9.645253e-01

6824 7.882447e-01 9.644469e-01

6825 7.879486e-01 9.643669e-01

6826 7.876506e-01 9.642851e-01

6827 7.873511e-01 9.642016e-01

6828 7.870502e-01 9.641164e-01

6829 7.867480e-01 9.640296e-01

6830 7.864449e-01 9.639412e-01

6831 7.861410e-01 9.638511e-01

6832 7.858364e-01 9.637596e-01

6833 7.855314e-01 9.636665e-01

6834 7.852261e-01 9.635720e-01

6835 7.849208e-01 9.634761e-01

6836 7.846155e-01 9.633788e-01

6837 7.843105e-01 9.632803e-01

6838 7.840060e-01 9.631805e-01

6839 7.837020e-01 9.630796e-01

6840 7.833989e-01 9.629777e-01

6841 7.830966e-01 9.628747e-01

6842 7.830856e-01 9.628746e-01

6843 7.830742e-01 9.628745e-01

6844 7.830622e-01 9.628744e-01

6845 7.830498e-01 9.628742e-01

6846 7.830369e-01 9.628741e-01

6847 7.830234e-01 9.628740e-01

6848 7.830093e-01 9.628738e-01

6849 7.829947e-01 9.628737e-01

6850 7.829794e-01 9.628735e-01

6851 7.829636e-01 9.628734e-01

6852 7.829471e-01 9.628732e-01

6853 7.829299e-01 9.628731e-01

6854 7.829120e-01 9.628729e-01

6855 7.828934e-01 9.628727e-01

6856 7.828740e-01 9.628725e-01

6857 7.828539e-01 9.628723e-01

6858 7.828329e-01 9.628721e-01

6859 7.828111e-01 9.628719e-01

6860 7.827884e-01 9.628717e-01

6861 7.827649e-01 9.628715e-01

6862 7.827403e-01 9.628712e-01

6863 7.827148e-01 9.628710e-01

6864 7.826883e-01 9.628707e-01

6865 7.826607e-01 9.628705e-01

6866 7.826320e-01 9.628702e-01

6867 7.826022e-01 9.628699e-01

6868 7.825712e-01 9.628696e-01

6869 7.825390e-01 9.628693e-01

6870 7.825055e-01 9.628690e-01

6871 7.824707e-01 9.628686e-01

6872 7.824345e-01 9.628683e-01

6873 7.823969e-01 9.628679e-01

6874 7.823579e-01 9.628675e-01

6875 7.823173e-01 9.628671e-01

6876 7.822751e-01 9.628667e-01

6877 7.822312e-01 9.628663e-01

6878 7.821857e-01 9.628659e-01

6879 7.821384e-01 9.628654e-01

6880 7.820893e-01 9.628649e-01

6881 7.820382e-01 9.628644e-01

6882 7.819852e-01 9.628639e-01

6883 7.819302e-01 9.628633e-01

6884 7.818730e-01 9.628627e-01

6885 7.818136e-01 9.628622e-01

6886 7.817520e-01 9.628615e-01

6887 7.816880e-01 9.628609e-01

6888 7.816216e-01 9.628602e-01

6889 7.815527e-01 9.628595e-01

6890 7.814811e-01 9.628588e-01

6891 7.814068e-01 9.628580e-01

6892 7.813298e-01 9.628572e-01

6893 7.812498e-01 9.628564e-01

6894 7.811668e-01 9.628556e-01

6895 7.810808e-01 9.628547e-01

6896 7.809915e-01 9.628537e-01

6897 7.808989e-01 9.628528e-01

6898 7.808029e-01 9.628518e-01

6899 7.807033e-01 9.628507e-01

6900 7.806000e-01 9.628496e-01

6901 7.804930e-01 9.628485e-01

6902 7.803821e-01 9.628473e-01

6903 7.802671e-01 9.628460e-01

6904 7.801479e-01 9.628448e-01

6905 7.800244e-01 9.628434e-01

6906 7.798965e-01 9.628420e-01

6907 7.797640e-01 9.628406e-01

6908 7.796268e-01 9.628390e-01

6909 7.794847e-01 9.628375e-01

6910 7.793376e-01 9.628358e-01

6911 7.791853e-01 9.628341e-01

6912 7.790277e-01 9.628323e-01

6913 7.788646e-01 9.628305e-01

6914 7.786959e-01 9.628285e-01

6915 7.785214e-01 9.628265e-01

6916 7.783409e-01 9.628244e-01

6917 7.781543e-01 9.628222e-01

6918 7.779614e-01 9.628200e-01

6919 7.777620e-01 9.628176e-01

6920 7.775560e-01 9.628151e-01

6921 7.773432e-01 9.628126e-01

6922 7.771234e-01 9.628099e-01

6923 7.768965e-01 9.628072e-01

6924 7.766623e-01 9.628043e-01

6925 7.764205e-01 9.628013e-01

6926 7.761711e-01 9.627982e-01

6927 7.759139e-01 9.627949e-01

6928 7.756487e-01 9.627915e-01

6929 7.753753e-01 9.627880e-01

6930 7.750936e-01 9.627844e-01

6931 7.748033e-01 9.627806e-01

6932 7.745045e-01 9.627767e-01

6933 7.741968e-01 9.627726e-01

6934 7.738801e-01 9.627683e-01

6935 7.735543e-01 9.627639e-01

6936 7.732193e-01 9.627593e-01

6937 7.728749e-01 9.627545e-01

6938 7.725210e-01 9.627495e-01

6939 7.721574e-01 9.627444e-01

6940 7.717840e-01 9.627391e-01

6941 7.714008e-01 9.627335e-01

6942 7.710076e-01 9.627277e-01

6943 7.706043e-01 9.627218e-01

6944 7.701909e-01 9.627155e-01

6945 7.697673e-01 9.627091e-01

6946 7.693334e-01 9.627024e-01

6947 7.688891e-01 9.626955e-01

6948 7.684345e-01 9.626883e-01

6949 7.679696e-01 9.626809e-01

6950 7.674942e-01 9.626731e-01

6951 7.670085e-01 9.626651e-01

6952 7.665124e-01 9.626568e-01

6953 7.660060e-01 9.626482e-01

6954 7.654893e-01 9.626393e-01

6955 7.649624e-01 9.626301e-01

6956 7.644253e-01 9.626206e-01

6957 7.638783e-01 9.626107e-01

6958 7.633213e-01 9.626005e-01

6959 7.627545e-01 9.625899e-01

6960 7.621781e-01 9.625790e-01

6961 7.615923e-01 9.625677e-01

6962 7.609972e-01 9.625560e-01

6963 7.603930e-01 9.625439e-01

6964 7.597799e-01 9.625314e-01

6965 7.591582e-01 9.625185e-01

6966 7.585282e-01 9.625052e-01

6967 7.578900e-01 9.624914e-01

6968 7.572441e-01 9.624772e-01

6969 7.565906e-01 9.624626e-01

6970 7.559300e-01 9.624474e-01

6971 7.552625e-01 9.624318e-01

6972 7.545885e-01 9.624158e-01

6973 7.539083e-01 9.623992e-01

6974 7.532224e-01 9.623821e-01

6975 7.525310e-01 9.623645e-01

6976 7.518347e-01 9.623464e-01

6977 7.511338e-01 9.623277e-01

6978 7.504287e-01 9.623085e-01

6979 7.497198e-01 9.622888e-01

6980 7.490076e-01 9.622685e-01

6981 7.482925e-01 9.622477e-01

6982 7.475750e-01 9.622263e-01

6983 7.468555e-01 9.622043e-01

6984 7.461344e-01 9.621818e-01

6985 7.454122e-01 9.621587e-01

6986 7.446894e-01 9.621350e-01

6987 7.439663e-01 9.621108e-01

6988 7.432435e-01 9.620860e-01

6989 7.425214e-01 9.620606e-01

6990 7.418004e-01 9.620347e-01

6991 7.410809e-01 9.620082e-01

6992 7.403635e-01 9.619812e-01

6993 7.396484e-01 9.619536e-01

6994 7.389362e-01 9.619255e-01

6995 7.382272e-01 9.618968e-01

6996 7.375218e-01 9.618676e-01

6997 7.368204e-01 9.618380e-01

6998 7.361234e-01 9.618078e-01

6999 7.354312e-01 9.617772e-01

7000 7.347441e-01 9.617461e-01

7001 7.340623e-01 9.617146e-01

7002 7.333864e-01 9.616828e-01

7003 7.327165e-01 9.616505e-01

7004 7.320529e-01 9.616179e-01

7005 7.313960e-01 9.615849e-01

7006 7.307459e-01 9.615517e-01

7007 7.301030e-01 9.615182e-01

7008 7.294675e-01 9.614844e-01

7009 7.288395e-01 9.614505e-01

7010 7.282194e-01 9.614164e-01

7011 7.276072e-01 9.613823e-01

7012 7.270032e-01 9.613480e-01

7013 7.264075e-01 9.613138e-01

7014 7.258202e-01 9.612795e-01

7015 7.252414e-01 9.612453e-01

7016 7.246714e-01 9.612113e-01

7017 7.241101e-01 9.611774e-01

7018 7.235577e-01 9.611437e-01

7019 7.230142e-01 9.611103e-01

7020 7.224796e-01 9.610772e-01

7021 7.219541e-01 9.610445e-01

7022 7.219174e-01 9.610444e-01

7023 7.218795e-01 9.610444e-01

7024 7.218403e-01 9.610443e-01

7025 7.217997e-01 9.610442e-01

7026 7.217578e-01 9.610440e-01

7027 7.217145e-01 9.610439e-01

7028 7.216697e-01 9.610438e-01

7029 7.216235e-01 9.610437e-01

7030 7.215757e-01 9.610436e-01

7031 7.215263e-01 9.610434e-01

7032 7.214753e-01 9.610433e-01

7033 7.214226e-01 9.610432e-01

7034 7.213682e-01 9.610430e-01

7035 7.213120e-01 9.610429e-01

7036 7.212540e-01 9.610427e-01

7037 7.211940e-01 9.610426e-01

7038 7.211321e-01 9.610424e-01

7039 7.210682e-01 9.610422e-01

7040 7.210021e-01 9.610420e-01

7041 7.209339e-01 9.610418e-01

7042 7.208635e-01 9.610416e-01

7043 7.207908e-01 9.610414e-01

7044 7.207157e-01 9.610412e-01

7045 7.206382e-01 9.610410e-01

7046 7.205582e-01 9.610407e-01

7047 7.204756e-01 9.610405e-01

7048 7.203903e-01 9.610402e-01

7049 7.203023e-01 9.610399e-01

7050 7.202114e-01 9.610397e-01

7051 7.201176e-01 9.610394e-01

7052 7.200208e-01 9.610391e-01

7053 7.199208e-01 9.610387e-01

7054 7.198177e-01 9.610384e-01

7055 7.197113e-01 9.610381e-01

7056 7.196015e-01 9.610377e-01

7057 7.194882e-01 9.610373e-01

7058 7.193712e-01 9.610369e-01

7059 7.192506e-01 9.610365e-01

7060 7.191262e-01 9.610361e-01

7061 7.189979e-01 9.610357e-01

7062 7.188655e-01 9.610352e-01

7063 7.187290e-01 9.610347e-01

7064 7.185882e-01 9.610342e-01

7065 7.184430e-01 9.610337e-01

7066 7.182933e-01 9.610332e-01

7067 7.181390e-01 9.610326e-01

7068 7.179799e-01 9.610320e-01

7069 7.178160e-01 9.610314e-01

7070 7.176470e-01 9.610308e-01

7071 7.174729e-01 9.610301e-01

7072 7.172935e-01 9.610294e-01

7073 7.171087e-01 9.610287e-01

7074 7.169184e-01 9.610280e-01

7075 7.167223e-01 9.610272e-01

7076 7.165204e-01 9.610264e-01

7077 7.163126e-01 9.610256e-01

7078 7.160986e-01 9.610247e-01

7079 7.158784e-01 9.610238e-01

7080 7.156517e-01 9.610228e-01

7081 7.154185e-01 9.610218e-01

7082 7.151787e-01 9.610208e-01

7083 7.149319e-01 9.610198e-01

7084 7.146782e-01 9.610186e-01

7085 7.144174e-01 9.610175e-01

7086 7.141493e-01 9.610163e-01

7087 7.138738e-01 9.610150e-01

7088 7.135907e-01 9.610137e-01

7089 7.132999e-01 9.610124e-01

7090 7.130013e-01 9.610110e-01

7091 7.126947e-01 9.610095e-01

7092 7.123800e-01 9.610080e-01

7093 7.120571e-01 9.610064e-01

7094 7.117258e-01 9.610048e-01

7095 7.113861e-01 9.610031e-01

7096 7.110377e-01 9.610013e-01

7097 7.106807e-01 9.609995e-01

7098 7.103148e-01 9.609976e-01

7099 7.099400e-01 9.609956e-01

7100 7.095562e-01 9.609935e-01

7101 7.091633e-01 9.609914e-01

7102 7.087613e-01 9.609892e-01

7103 7.083500e-01 9.609868e-01

7104 7.079293e-01 9.609844e-01

7105 7.074994e-01 9.609820e-01

7106 7.070600e-01 9.609794e-01

7107 7.066112e-01 9.609767e-01

7108 7.061530e-01 9.609739e-01

7109 7.056852e-01 9.609710e-01

7110 7.052081e-01 9.609680e-01

7111 7.047214e-01 9.609649e-01

7112 7.042254e-01 9.609617e-01

7113 7.037200e-01 9.609583e-01

7114 7.032052e-01 9.609549e-01

7115 7.026812e-01 9.609513e-01

7116 7.021480e-01 9.609476e-01

7117 7.016058e-01 9.609437e-01

7118 7.010546e-01 9.609397e-01

7119 7.004946e-01 9.609356e-01

7120 6.999258e-01 9.609313e-01

7121 6.993486e-01 9.609268e-01

7122 6.987630e-01 9.609222e-01

7123 6.981693e-01 9.609175e-01

7124 6.975677e-01 9.609126e-01

7125 6.969583e-01 9.609075e-01

7126 6.963415e-01 9.609022e-01

7127 6.957174e-01 9.608968e-01

7128 6.950865e-01 9.608911e-01

7129 6.944489e-01 9.608853e-01

7130 6.938049e-01 9.608793e-01

7131 6.931550e-01 9.608731e-01

7132 6.924993e-01 9.608666e-01

7133 6.918384e-01 9.608600e-01

7134 6.911724e-01 9.608532e-01

7135 6.905019e-01 9.608461e-01

7136 6.898271e-01 9.608388e-01

7137 6.891486e-01 9.608313e-01

7138 6.884666e-01 9.608236e-01

7139 6.877816e-01 9.608156e-01

7140 6.870940e-01 9.608073e-01

7141 6.864043e-01 9.607989e-01

7142 6.857129e-01 9.607901e-01

7143 6.850201e-01 9.607811e-01

7144 6.843266e-01 9.607719e-01

7145 6.836326e-01 9.607623e-01

7146 6.829387e-01 9.607525e-01

7147 6.822454e-01 9.607424e-01

7148 6.815529e-01 9.607321e-01

7149 6.808618e-01 9.607214e-01

7150 6.801726e-01 9.607105e-01

7151 6.794856e-01 9.606992e-01

7152 6.788014e-01 9.606876e-01

7153 6.781202e-01 9.606758e-01

7154 6.774425e-01 9.606636e-01

7155 6.767688e-01 9.606511e-01

7156 6.760994e-01 9.606383e-01

7157 6.754348e-01 9.606251e-01

7158 6.747752e-01 9.606117e-01

7159 6.741211e-01 9.605979e-01

7160 6.734727e-01 9.605838e-01

7161 6.728306e-01 9.605694e-01

7162 6.721948e-01 9.605547e-01

7163 6.715659e-01 9.605396e-01

7164 6.709439e-01 9.605242e-01

7165 6.703294e-01 9.605085e-01

7166 6.697224e-01 9.604925e-01

7167 6.691232e-01 9.604762e-01

7168 6.685321e-01 9.604595e-01

7169 6.679493e-01 9.604426e-01

7170 6.673749e-01 9.604254e-01

7171 6.668092e-01 9.604079e-01

7172 6.662523e-01 9.603901e-01

7173 6.657044e-01 9.603720e-01

7174 6.651655e-01 9.603537e-01

7175 6.646358e-01 9.603352e-01

7176 6.641154e-01 9.603164e-01

7177 6.636043e-01 9.602974e-01

7178 6.631027e-01 9.602782e-01

7179 6.626105e-01 9.602589e-01

7180 6.621279e-01 9.602394e-01

7181 6.616548e-01 9.602197e-01

7182 6.611913e-01 9.601999e-01

7183 6.607373e-01 9.601800e-01

7184 6.602928e-01 9.601600e-01

7185 6.598579e-01 9.601400e-01

7186 6.594324e-01 9.601200e-01

7187 6.590163e-01 9.600999e-01

7188 6.586096e-01 9.600799e-01

7189 6.582121e-01 9.600599e-01

7190 6.578239e-01 9.600400e-01

7191 6.574448e-01 9.600203e-01

7192 6.570748e-01 9.600007e-01

7193 6.567137e-01 9.599813e-01

7194 6.563614e-01 9.599621e-01

7195 6.560178e-01 9.599431e-01

7196 6.556829e-01 9.599244e-01

7197 6.553564e-01 9.599061e-01

7198 6.550382e-01 9.598881e-01

7199 6.547283e-01 9.598705e-01

7200 6.544265e-01 9.598534e-01

7201 6.541326e-01 9.598367e-01

7202 6.540887e-01 9.598366e-01

7203 6.540438e-01 9.598365e-01

7204 6.539977e-01 9.598363e-01

7205 6.539506e-01 9.598362e-01

7206 6.539023e-01 9.598361e-01

7207 6.538529e-01 9.598359e-01

7208 6.538023e-01 9.598358e-01

7209 6.537506e-01 9.598356e-01

7210 6.536976e-01 9.598355e-01

7211 6.536434e-01 9.598353e-01

7212 6.535881e-01 9.598351e-01

7213 6.535314e-01 9.598349e-01

7214 6.534735e-01 9.598347e-01

7215 6.534144e-01 9.598345e-01

7216 6.533539e-01 9.598343e-01

7217 6.532921e-01 9.598341e-01

7218 6.532289e-01 9.598339e-01

7219 6.531645e-01 9.598336e-01

7220 6.530986e-01 9.598334e-01

7221 6.530313e-01 9.598331e-01

7222 6.529626e-01 9.598328e-01

7223 6.528925e-01 9.598325e-01

7224 6.528210e-01 9.598322e-01

7225 6.527479e-01 9.598319e-01

7226 6.526734e-01 9.598316e-01

7227 6.525974e-01 9.598313e-01

7228 6.525199e-01 9.598309e-01

7229 6.524408e-01 9.598305e-01

7230 6.523602e-01 9.598302e-01

7231 6.522780e-01 9.598298e-01

7232 6.521943e-01 9.598293e-01

7233 6.521089e-01 9.598289e-01

7234 6.520220e-01 9.598284e-01

7235 6.519334e-01 9.598280e-01

7236 6.518432e-01 9.598275e-01

7237 6.517514e-01 9.598270e-01

7238 6.516579e-01 9.598264e-01

7239 6.515628e-01 9.598259e-01

7240 6.514660e-01 9.598253e-01

7241 6.513676e-01 9.598247e-01

7242 6.512675e-01 9.598240e-01

7243 6.511658e-01 9.598234e-01

7244 6.510623e-01 9.598227e-01

7245 6.509573e-01 9.598220e-01

7246 6.508505e-01 9.598212e-01

7247 6.507422e-01 9.598205e-01

7248 6.506322e-01 9.598196e-01

7249 6.505205e-01 9.598188e-01

7250 6.504073e-01 9.598179e-01

7251 6.502924e-01 9.598170e-01

7252 6.501760e-01 9.598160e-01

7253 6.500580e-01 9.598151e-01

7254 6.499385e-01 9.598140e-01

7255 6.498174e-01 9.598129e-01

7256 6.496949e-01 9.598118e-01

7257 6.495709e-01 9.598106e-01

7258 6.494456e-01 9.598094e-01

7259 6.493188e-01 9.598081e-01

7260 6.491907e-01 9.598068e-01

7261 6.490613e-01 9.598054e-01

7262 6.489307e-01 9.598040e-01

7263 6.487989e-01 9.598025e-01

7264 6.486659e-01 9.598010e-01

7265 6.485319e-01 9.597994e-01

7266 6.483968e-01 9.597977e-01

7267 6.482608e-01 9.597959e-01

7268 6.481240e-01 9.597941e-01

7269 6.479863e-01 9.597922e-01

7270 6.478479e-01 9.597902e-01

7271 6.477088e-01 9.597882e-01

7272 6.475692e-01 9.597860e-01

7273 6.474291e-01 9.597838e-01

7274 6.472886e-01 9.597815e-01

7275 6.471479e-01 9.597791e-01

7276 6.470069e-01 9.597766e-01

7277 6.468659e-01 9.597740e-01

7278 6.467249e-01 9.597713e-01

7279 6.465840e-01 9.597685e-01

7280 6.464434e-01 9.597656e-01

7281 6.463032e-01 9.597625e-01

7282 6.461634e-01 9.597594e-01

7283 6.460243e-01 9.597561e-01

7284 6.458859e-01 9.597527e-01

7285 6.457483e-01 9.597492e-01

7286 6.456118e-01 9.597455e-01

7287 6.454764e-01 9.597417e-01

7288 6.453422e-01 9.597377e-01

7289 6.452095e-01 9.597336e-01

7290 6.450783e-01 9.597293e-01

7291 6.449487e-01 9.597248e-01

7292 6.448210e-01 9.597202e-01

7293 6.446952e-01 9.597154e-01

7294 6.445715e-01 9.597104e-01

7295 6.444501e-01 9.597053e-01

7296 6.443310e-01 9.596999e-01

7297 6.442144e-01 9.596944e-01

7298 6.441004e-01 9.596886e-01

7299 6.439892e-01 9.596826e-01

7300 6.438810e-01 9.596764e-01

7301 6.437757e-01 9.596699e-01

7302 6.436736e-01 9.596633e-01

7303 6.435748e-01 9.596563e-01

7304 6.434794e-01 9.596492e-01

7305 6.433875e-01 9.596417e-01

7306 6.432992e-01 9.596340e-01

7307 6.432146e-01 9.596260e-01

7308 6.431338e-01 9.596178e-01

7309 6.430569e-01 9.596092e-01

7310 6.429841e-01 9.596003e-01

7311 6.429153e-01 9.595911e-01

7312 6.428506e-01 9.595816e-01

7313 6.427902e-01 9.595718e-01

7314 6.427341e-01 9.595616e-01

7315 6.426822e-01 9.595510e-01

7316 6.426348e-01 9.595401e-01

7317 6.425917e-01 9.595289e-01

7318 6.425531e-01 9.595172e-01

7319 6.425190e-01 9.595051e-01

7320 6.424893e-01 9.594927e-01

7321 6.424641e-01 9.594798e-01

7322 6.424433e-01 9.594665e-01

7323 6.424271e-01 9.594528e-01

7324 6.424152e-01 9.594386e-01

7325 6.424079e-01 9.594239e-01

7326 6.424048e-01 9.594088e-01

7327 6.424062e-01 9.593932e-01

7328 6.424118e-01 9.593771e-01

7329 6.424217e-01 9.593605e-01

7330 6.424358e-01 9.593433e-01

7331 6.424540e-01 9.593257e-01

7332 6.424762e-01 9.593075e-01

7333 6.425024e-01 9.592887e-01

7334 6.425325e-01 9.592694e-01

7335 6.425664e-01 9.592495e-01

7336 6.426040e-01 9.592291e-01

7337 6.426451e-01 9.592080e-01

7338 6.426898e-01 9.591863e-01

7339 6.427378e-01 9.591640e-01

7340 6.427891e-01 9.591411e-01

7341 6.428436e-01 9.591175e-01

7342 6.429011e-01 9.590933e-01

7343 6.429615e-01 9.590685e-01

7344 6.430248e-01 9.590430e-01

7345 6.430907e-01 9.590168e-01

7346 6.431592e-01 9.589899e-01

7347 6.432301e-01 9.589624e-01

7348 6.433034e-01 9.589342e-01

7349 6.433788e-01 9.589053e-01

7350 6.434563e-01 9.588757e-01

7351 6.435358e-01 9.588454e-01

7352 6.436170e-01 9.588144e-01

7353 6.437000e-01 9.587827e-01

7354 6.437846e-01 9.587504e-01

7355 6.438706e-01 9.587173e-01

7356 6.439580e-01 9.586836e-01

7357 6.440466e-01 9.586491e-01

7358 6.441364e-01 9.586140e-01

7359 6.442272e-01 9.585782e-01

7360 6.443189e-01 9.585418e-01

7361 6.444114e-01 9.585046e-01

7362 6.445047e-01 9.584669e-01

7363 6.445985e-01 9.584285e-01

7364 6.446929e-01 9.583895e-01

7365 6.447878e-01 9.583498e-01

7366 6.448830e-01 9.583096e-01

7367 6.449785e-01 9.582688e-01

7368 6.450741e-01 9.582275e-01

7369 6.451699e-01 9.581856e-01

7370 6.452658e-01 9.581432e-01

7371 6.453616e-01 9.581003e-01

7372 6.454573e-01 9.580569e-01

7373 6.455529e-01 9.580131e-01

7374 6.456483e-01 9.579689e-01

7375 6.457434e-01 9.579243e-01

7376 6.458382e-01 9.578793e-01

7377 6.459326e-01 9.578341e-01

7378 6.460267e-01 9.577885e-01

7379 6.461203e-01 9.577426e-01

7380 6.462134e-01 9.576966e-01

7381 6.463060e-01 9.576503e-01

7382 6.463269e-01 9.576501e-01

7383 6.463488e-01 9.576499e-01

7384 6.463717e-01 9.576497e-01

7385 6.463955e-01 9.576495e-01

7386 6.464205e-01 9.576492e-01

7387 6.464465e-01 9.576490e-01

7388 6.464736e-01 9.576487e-01

7389 6.465019e-01 9.576484e-01

7390 6.465314e-01 9.576482e-01

7391 6.465621e-01 9.576479e-01

7392 6.465940e-01 9.576476e-01

7393 6.466273e-01 9.576472e-01

7394 6.466620e-01 9.576469e-01

7395 6.466981e-01 9.576465e-01

7396 6.467356e-01 9.576462e-01

7397 6.467746e-01 9.576458e-01

7398 6.468152e-01 9.576454e-01

7399 6.468574e-01 9.576450e-01

7400 6.469013e-01 9.576446e-01

7401 6.469469e-01 9.576441e-01

7402 6.469942e-01 9.576436e-01

7403 6.470435e-01 9.576432e-01

7404 6.470946e-01 9.576427e-01

7405 6.471477e-01 9.576421e-01

7406 6.472029e-01 9.576416e-01

7407 6.472601e-01 9.576410e-01

7408 6.473195e-01 9.576404e-01

7409 6.473812e-01 9.576398e-01

7410 6.474453e-01 9.576391e-01

7411 6.475117e-01 9.576384e-01

7412 6.475806e-01 9.576377e-01

7413 6.476521e-01 9.576370e-01

7414 6.477262e-01 9.576362e-01

7415 6.478030e-01 9.576354e-01

7416 6.478827e-01 9.576346e-01

7417 6.479653e-01 9.576337e-01

7418 6.480509e-01 9.576328e-01

7419 6.481396e-01 9.576318e-01

7420 6.482316e-01 9.576308e-01

7421 6.483268e-01 9.576298e-01

7422 6.484254e-01 9.576287e-01

7423 6.485275e-01 9.576276e-01

7424 6.486332e-01 9.576265e-01

7425 6.487427e-01 9.576252e-01

7426 6.488560e-01 9.576240e-01

7427 6.489732e-01 9.576227e-01

7428 6.490945e-01 9.576213e-01

7429 6.492200e-01 9.576199e-01

7430 6.493498e-01 9.576184e-01

7431 6.494840e-01 9.576168e-01

7432 6.496228e-01 9.576152e-01

7433 6.497662e-01 9.576135e-01

7434 6.499144e-01 9.576118e-01

7435 6.500676e-01 9.576100e-01

7436 6.502259e-01 9.576081e-01

7437 6.503893e-01 9.576061e-01

7438 6.505581e-01 9.576040e-01

7439 6.507324e-01 9.576019e-01

7440 6.509123e-01 9.575996e-01

7441 6.510980e-01 9.575973e-01

7442 6.512895e-01 9.575949e-01

7443 6.514871e-01 9.575924e-01

7444 6.516909e-01 9.575898e-01

7445 6.519010e-01 9.575870e-01

7446 6.521176e-01 9.575842e-01

7447 6.523408e-01 9.575812e-01

7448 6.525707e-01 9.575782e-01

7449 6.528076e-01 9.575750e-01

7450 6.530515e-01 9.575716e-01

7451 6.533025e-01 9.575682e-01

7452 6.535610e-01 9.575646e-01

7453 6.538268e-01 9.575608e-01

7454 6.541003e-01 9.575569e-01

7455 6.543815e-01 9.575529e-01

7456 6.546706e-01 9.575487e-01

7457 6.549677e-01 9.575443e-01

7458 6.552730e-01 9.575397e-01

7459 6.555865e-01 9.575350e-01

7460 6.559083e-01 9.575301e-01

7461 6.562387e-01 9.575249e-01

7462 6.565776e-01 9.575196e-01

7463 6.569253e-01 9.575141e-01

7464 6.572817e-01 9.575083e-01

7465 6.576471e-01 9.575023e-01

7466 6.580214e-01 9.574961e-01

7467 6.584048e-01 9.574896e-01

7468 6.587974e-01 9.574829e-01

7469 6.591992e-01 9.574759e-01

7470 6.596102e-01 9.574687e-01

7471 6.600305e-01 9.574612e-01

7472 6.604602e-01 9.574533e-01

7473 6.608993e-01 9.574452e-01

7474 6.613478e-01 9.574368e-01

7475 6.618057e-01 9.574280e-01

7476 6.622730e-01 9.574189e-01

7477 6.627498e-01 9.574095e-01

7478 6.632359e-01 9.573997e-01

7479 6.637313e-01 9.573895e-01

7480 6.642361e-01 9.573789e-01

7481 6.647502e-01 9.573679e-01

7482 6.652734e-01 9.573566e-01

7483 6.658057e-01 9.573448e-01

7484 6.663470e-01 9.573325e-01

7485 6.668972e-01 9.573198e-01

7486 6.674562e-01 9.573066e-01

7487 6.680238e-01 9.572930e-01

7488 6.685999e-01 9.572788e-01

7489 6.691843e-01 9.572641e-01

7490 6.697769e-01 9.572489e-01

7491 6.703775e-01 9.572331e-01

7492 6.709858e-01 9.572167e-01

7493 6.716017e-01 9.571998e-01

7494 6.722250e-01 9.571822e-01

7495 6.728553e-01 9.571640e-01

7496 6.734925e-01 9.571452e-01

7497 6.741363e-01 9.571257e-01

7498 6.747863e-01 9.571055e-01

7499 6.754424e-01 9.570846e-01

7500 6.761043e-01 9.570629e-01

7501 6.767716e-01 9.570405e-01

7502 6.774440e-01 9.570174e-01

7503 6.781211e-01 9.569934e-01

7504 6.788028e-01 9.569686e-01

7505 6.794886e-01 9.569430e-01

7506 6.801782e-01 9.569165e-01

7507 6.808712e-01 9.568891e-01

7508 6.815673e-01 9.568608e-01

7509 6.822661e-01 9.568316e-01

7510 6.829672e-01 9.568014e-01

7511 6.836703e-01 9.567702e-01

7512 6.843751e-01 9.567380e-01

7513 6.850811e-01 9.567048e-01

7514 6.857879e-01 9.566706e-01

7515 6.864953e-01 9.566352e-01

7516 6.872027e-01 9.565988e-01

7517 6.879100e-01 9.565612e-01

7518 6.886165e-01 9.565225e-01

7519 6.893222e-01 9.564826e-01

7520 6.900264e-01 9.564416e-01

7521 6.907289e-01 9.563993e-01

7522 6.914294e-01 9.563558e-01

7523 6.921275e-01 9.563110e-01

7524 6.928228e-01 9.562649e-01

7525 6.935150e-01 9.562176e-01

7526 6.942038e-01 9.561689e-01

7527 6.948889e-01 9.561189e-01

7528 6.955699e-01 9.560676e-01

7529 6.962466e-01 9.560148e-01

7530 6.969187e-01 9.559607e-01

7531 6.975858e-01 9.559052e-01

7532 6.982478e-01 9.558483e-01

7533 6.989043e-01 9.557900e-01

7534 6.995551e-01 9.557302e-01

7535 7.002000e-01 9.556689e-01

7536 7.008387e-01 9.556063e-01

7537 7.014710e-01 9.555421e-01

7538 7.020968e-01 9.554765e-01

7539 7.027157e-01 9.554094e-01

7540 7.033277e-01 9.553409e-01

7541 7.039326e-01 9.552709e-01

7542 7.045302e-01 9.551994e-01

7543 7.051204e-01 9.551264e-01

7544 7.057031e-01 9.550520e-01

7545 7.062780e-01 9.549761e-01

7546 7.068452e-01 9.548988e-01

7547 7.074045e-01 9.548201e-01

7548 7.079558e-01 9.547399e-01

7549 7.084991e-01 9.546584e-01

7550 7.090342e-01 9.545754e-01

7551 7.095612e-01 9.544911e-01

7552 7.100800e-01 9.544054e-01

7553 7.105905e-01 9.543185e-01

7554 7.110928e-01 9.542302e-01

7555 7.115868e-01 9.541407e-01

7556 7.120725e-01 9.540499e-01

7557 7.125499e-01 9.539580e-01

7558 7.130190e-01 9.538649e-01

7559 7.134799e-01 9.537707e-01

7560 7.139325e-01 9.536754e-01

7561 7.143769e-01 9.535790e-01

7562 7.144232e-01 9.535787e-01

7563 7.144707e-01 9.535783e-01

7564 7.145196e-01 9.535779e-01

7565 7.145699e-01 9.535775e-01

7566 7.146215e-01 9.535771e-01

7567 7.146747e-01 9.535767e-01

7568 7.147293e-01 9.535763e-01

7569 7.147854e-01 9.535758e-01

7570 7.148430e-01 9.535753e-01

7571 7.149022e-01 9.535748e-01

7572 7.149631e-01 9.535743e-01

7573 7.150256e-01 9.535738e-01

7574 7.150899e-01 9.535732e-01

7575 7.151559e-01 9.535726e-01

7576 7.152236e-01 9.535720e-01

7577 7.152932e-01 9.535713e-01

7578 7.153647e-01 9.535707e-01

7579 7.154382e-01 9.535700e-01

7580 7.155136e-01 9.535693e-01

7581 7.155910e-01 9.535685e-01

7582 7.156706e-01 9.535677e-01

7583 7.157522e-01 9.535669e-01

7584 7.158361e-01 9.535661e-01

7585 7.159222e-01 9.535652e-01

7586 7.160105e-01 9.535643e-01

7587 7.161013e-01 9.535633e-01

7588 7.161944e-01 9.535623e-01

7589 7.162901e-01 9.535613e-01

7590 7.163883e-01 9.535602e-01

7591 7.164890e-01 9.535591e-01

7592 7.165925e-01 9.535579e-01

7593 7.166986e-01 9.535567e-01

7594 7.168076e-01 9.535555e-01

7595 7.169194e-01 9.535541e-01

7596 7.170342e-01 9.535528e-01

7597 7.171519e-01 9.535513e-01

7598 7.172728e-01 9.535499e-01

7599 7.173968e-01 9.535483e-01

7600 7.175240e-01 9.535467e-01

7601 7.176545e-01 9.535450e-01

7602 7.177883e-01 9.535433e-01

7603 7.179257e-01 9.535415e-01

7604 7.180665e-01 9.535396e-01

7605 7.182110e-01 9.535376e-01

7606 7.183592e-01 9.535356e-01

7607 7.185111e-01 9.535335e-01

7608 7.186670e-01 9.535313e-01

7609 7.188267e-01 9.535290e-01

7610 7.189906e-01 9.535266e-01

7611 7.191585e-01 9.535241e-01

7612 7.193306e-01 9.535215e-01

7613 7.195071e-01 9.535188e-01

7614 7.196880e-01 9.535160e-01

7615 7.198733e-01 9.535131e-01

7616 7.200633e-01 9.535101e-01

7617 7.202579e-01 9.535069e-01

7618 7.204573e-01 9.535036e-01

7619 7.206616e-01 9.535002e-01

7620 7.208708e-01 9.534967e-01

7621 7.210851e-01 9.534930e-01

7622 7.213046e-01 9.534892e-01

7623 7.215294e-01 9.534852e-01

7624 7.217595e-01 9.534811e-01

7625 7.219951e-01 9.534768e-01

7626 7.222362e-01 9.534723e-01

7627 7.224830e-01 9.534676e-01

7628 7.227356e-01 9.534628e-01

7629 7.229941e-01 9.534578e-01

7630 7.232585e-01 9.534526e-01

7631 7.235290e-01 9.534471e-01

7632 7.238057e-01 9.534415e-01

7633 7.240886e-01 9.534357e-01

7634 7.243780e-01 9.534296e-01

7635 7.246737e-01 9.534232e-01

7636 7.249761e-01 9.534167e-01

7637 7.252851e-01 9.534098e-01

7638 7.256008e-01 9.534028e-01

7639 7.259234e-01 9.533954e-01

7640 7.262529e-01 9.533877e-01

7641 7.265895e-01 9.533798e-01

7642 7.269332e-01 9.533715e-01

7643 7.272840e-01 9.533630e-01

7644 7.276421e-01 9.533541e-01

7645 7.280076e-01 9.533448e-01

7646 7.283805e-01 9.533352e-01

7647 7.287609e-01 9.533253e-01

7648 7.291489e-01 9.533149e-01

7649 7.295444e-01 9.533042e-01

7650 7.299477e-01 9.532931e-01

7651 7.303587e-01 9.532815e-01

7652 7.307775e-01 9.532695e-01

7653 7.312042e-01 9.532571e-01

7654 7.316387e-01 9.532441e-01

7655 7.320811e-01 9.532307e-01

7656 7.325315e-01 9.532168e-01

7657 7.329898e-01 9.532024e-01

7658 7.334561e-01 9.531875e-01

7659 7.339303e-01 9.531720e-01

7660 7.344126e-01 9.531559e-01

7661 7.349028e-01 9.531392e-01

7662 7.354010e-01 9.531219e-01

7663 7.359071e-01 9.531040e-01

7664 7.364211e-01 9.530854e-01

7665 7.369430e-01 9.530662e-01

7666 7.374727e-01 9.530462e-01

7667 7.380101e-01 9.530255e-01

7668 7.385553e-01 9.530041e-01

7669 7.391081e-01 9.529819e-01

7670 7.396684e-01 9.529589e-01

7671 7.402361e-01 9.529351e-01

7672 7.408112e-01 9.529105e-01

7673 7.413935e-01 9.528850e-01

7674 7.419829e-01 9.528586e-01

7675 7.425793e-01 9.528312e-01

7676 7.431825e-01 9.528029e-01

7677 7.437923e-01 9.527736e-01

7678 7.444086e-01 9.527434e-01

7679 7.450312e-01 9.527120e-01

7680 7.456599e-01 9.526796e-01

7681 7.462946e-01 9.526461e-01

7682 7.469350e-01 9.526115e-01

7683 7.475808e-01 9.525757e-01

7684 7.482320e-01 9.525386e-01

7685 7.488882e-01 9.525004e-01

7686 7.495491e-01 9.524609e-01

7687 7.502146e-01 9.524201e-01

7688 7.508843e-01 9.523780e-01

7689 7.515581e-01 9.523345e-01

7690 7.522355e-01 9.522897e-01

7691 7.529164e-01 9.522434e-01

7692 7.536003e-01 9.521956e-01

7693 7.542871e-01 9.521464e-01

7694 7.549764e-01 9.520956e-01

7695 7.556679e-01 9.520433e-01

7696 7.563612e-01 9.519894e-01

7697 7.570561e-01 9.519339e-01

7698 7.577521e-01 9.518767e-01

7699 7.584491e-01 9.518179e-01

7700 7.591465e-01 9.517573e-01

7701 7.598442e-01 9.516950e-01

7702 7.605417e-01 9.516309e-01

7703 7.612387e-01 9.515650e-01

7704 7.619349e-01 9.514973e-01

7705 7.626299e-01 9.514277e-01

7706 7.633233e-01 9.513563e-01

7707 7.640149e-01 9.512829e-01

7708 7.647043e-01 9.512076e-01

7709 7.653911e-01 9.511303e-01

7710 7.660751e-01 9.510510e-01

7711 7.667559e-01 9.509698e-01

7712 7.674331e-01 9.508865e-01

7713 7.681065e-01 9.508011e-01

7714 7.687757e-01 9.507137e-01

7715 7.694405e-01 9.506243e-01

7716 7.701005e-01 9.505327e-01

7717 7.707555e-01 9.504390e-01

7718 7.714051e-01 9.503433e-01

7719 7.720491e-01 9.502454e-01

7720 7.726873e-01 9.501453e-01

7721 7.733193e-01 9.500432e-01

7722 7.739450e-01 9.499389e-01

7723 7.745640e-01 9.498325e-01

7724 7.751763e-01 9.497240e-01

7725 7.757814e-01 9.496133e-01

7726 7.763794e-01 9.495006e-01

7727 7.769699e-01 9.493857e-01

7728 7.775528e-01 9.492688e-01

7729 7.781279e-01 9.491497e-01

7730 7.786951e-01 9.490287e-01

7731 7.792542e-01 9.489056e-01

7732 7.798051e-01 9.487805e-01

7733 7.803476e-01 9.486534e-01

7734 7.808818e-01 9.485244e-01

7735 7.814073e-01 9.483935e-01

7736 7.819243e-01 9.482607e-01

7737 7.824325e-01 9.481260e-01

7738 7.829320e-01 9.479896e-01

7739 7.834227e-01 9.478514e-01

7740 7.839045e-01 9.477116e-01

7741 7.843774e-01 9.475701e-01

7742 7.843983e-01 9.475695e-01

7743 7.844197e-01 9.475689e-01

7744 7.844417e-01 9.475684e-01

7745 7.844641e-01 9.475678e-01

7746 7.844872e-01 9.475671e-01

7747 7.845108e-01 9.475665e-01

7748 7.845349e-01 9.475658e-01

7749 7.845597e-01 9.475651e-01

7750 7.845850e-01 9.475644e-01

7751 7.846109e-01 9.475636e-01

7752 7.846375e-01 9.475628e-01

7753 7.846647e-01 9.475620e-01

7754 7.846925e-01 9.475611e-01

7755 7.847211e-01 9.475602e-01

7756 7.847502e-01 9.475593e-01

7757 7.847801e-01 9.475583e-01

7758 7.848107e-01 9.475573e-01

7759 7.848420e-01 9.475563e-01

7760 7.848741e-01 9.475552e-01

7761 7.849069e-01 9.475541e-01

7762 7.849405e-01 9.475529e-01

7763 7.849749e-01 9.475517e-01

7764 7.850101e-01 9.475505e-01

7765 7.850461e-01 9.475492e-01

7766 7.850830e-01 9.475478e-01

7767 7.851208e-01 9.475464e-01

7768 7.851594e-01 9.475449e-01

7769 7.851989e-01 9.475434e-01

7770 7.852394e-01 9.475418e-01

7771 7.852808e-01 9.475402e-01

7772 7.853231e-01 9.475385e-01

7773 7.853665e-01 9.475367e-01

7774 7.854108e-01 9.475349e-01

7775 7.854562e-01 9.475329e-01

7776 7.855026e-01 9.475310e-01

7777 7.855501e-01 9.475289e-01

7778 7.855987e-01 9.475268e-01

7779 7.856483e-01 9.475245e-01

7780 7.856992e-01 9.475222e-01

7781 7.857512e-01 9.475198e-01

7782 7.858043e-01 9.475173e-01

7783 7.858587e-01 9.475147e-01

7784 7.859143e-01 9.475121e-01

7785 7.859711e-01 9.475093e-01

7786 7.860292e-01 9.475064e-01

7787 7.860886e-01 9.475034e-01

7788 7.861494e-01 9.475002e-01

7789 7.862114e-01 9.474970e-01

7790 7.862749e-01 9.474936e-01

7791 7.863397e-01 9.474901e-01

7792 7.864059e-01 9.474865e-01

7793 7.864736e-01 9.474828e-01

7794 7.865427e-01 9.474789e-01

7795 7.866134e-01 9.474748e-01

7796 7.866855e-01 9.474706e-01

7797 7.867592e-01 9.474662e-01

7798 7.868344e-01 9.474617e-01

7799 7.869112e-01 9.474570e-01

7800 7.869896e-01 9.474521e-01

7801 7.870696e-01 9.474470e-01

7802 7.871512e-01 9.474418e-01

7803 7.872346e-01 9.474363e-01

7804 7.873196e-01 9.474307e-01

7805 7.874063e-01 9.474248e-01

7806 7.874948e-01 9.474187e-01

7807 7.875850e-01 9.474124e-01

7808 7.876769e-01 9.474058e-01

7809 7.877707e-01 9.473990e-01

7810 7.878662e-01 9.473920e-01

7811 7.879636e-01 9.473847e-01

7812 7.880628e-01 9.473771e-01

7813 7.881639e-01 9.473692e-01

7814 7.882668e-01 9.473611e-01

7815 7.883716e-01 9.473526e-01

7816 7.884783e-01 9.473438e-01

7817 7.885869e-01 9.473347e-01

7818 7.886974e-01 9.473253e-01

7819 7.888098e-01 9.473155e-01

7820 7.889242e-01 9.473054e-01

7821 7.890405e-01 9.472949e-01

7822 7.891587e-01 9.472840e-01

7823 7.892788e-01 9.472727e-01

7824 7.894009e-01 9.472611e-01

7825 7.895249e-01 9.472489e-01

7826 7.896509e-01 9.472364e-01

7827 7.897788e-01 9.472234e-01

7828 7.899086e-01 9.472099e-01

7829 7.900403e-01 9.471960e-01

7830 7.901739e-01 9.471815e-01

7831 7.903094e-01 9.471665e-01

7832 7.904467e-01 9.471510e-01

7833 7.905859e-01 9.471350e-01

7834 7.907269e-01 9.471184e-01

7835 7.908698e-01 9.471011e-01

7836 7.910143e-01 9.470833e-01

7837 7.911607e-01 9.470649e-01

7838 7.913087e-01 9.470457e-01

7839 7.914584e-01 9.470260e-01

7840 7.916098e-01 9.470055e-01

7841 7.917627e-01 9.469843e-01

7842 7.919172e-01 9.469624e-01

7843 7.920732e-01 9.469397e-01

7844 7.922306e-01 9.469163e-01

7845 7.923894e-01 9.468920e-01

7846 7.925496e-01 9.468669e-01

7847 7.927110e-01 9.468410e-01

7848 7.928736e-01 9.468141e-01

7849 7.930373e-01 9.467864e-01

7850 7.932021e-01 9.467577e-01

7851 7.933679e-01 9.467280e-01

7852 7.935346e-01 9.466974e-01

7853 7.937021e-01 9.466657e-01

7854 7.938704e-01 9.466330e-01

7855 7.940393e-01 9.465991e-01

7856 7.942087e-01 9.465642e-01

7857 7.943786e-01 9.465281e-01

7858 7.945488e-01 9.464909e-01

7859 7.947193e-01 9.464524e-01

7860 7.948900e-01 9.464126e-01

7861 7.950607e-01 9.463716e-01

7862 7.952313e-01 9.463293e-01

7863 7.954017e-01 9.462857e-01

7864 7.955718e-01 9.462406e-01

7865 7.957415e-01 9.461942e-01

7866 7.959106e-01 9.461463e-01

7867 7.960791e-01 9.460969e-01

7868 7.962468e-01 9.460460e-01

7869 7.964135e-01 9.459935e-01

7870 7.965792e-01 9.459395e-01

7871 7.967438e-01 9.458838e-01

7872 7.969070e-01 9.458265e-01

7873 7.970688e-01 9.457675e-01

7874 7.972291e-01 9.457068e-01

7875 7.973876e-01 9.456443e-01

7876 7.975444e-01 9.455801e-01

7877 7.976991e-01 9.455140e-01

7878 7.978519e-01 9.454460e-01

7879 7.980024e-01 9.453762e-01

7880 7.981506e-01 9.453044e-01

7881 7.982963e-01 9.452307e-01

7882 7.984395e-01 9.451550e-01

7883 7.985800e-01 9.450773e-01

7884 7.987177e-01 9.449975e-01

7885 7.988525e-01 9.449157e-01

7886 7.989843e-01 9.448318e-01

7887 7.991130e-01 9.447457e-01

7888 7.992385e-01 9.446575e-01

7889 7.993607e-01 9.445670e-01

7890 7.994794e-01 9.444744e-01

7891 7.995947e-01 9.443796e-01

7892 7.997065e-01 9.442825e-01

7893 7.998146e-01 9.441831e-01

7894 7.999190e-01 9.440814e-01

7895 8.000196e-01 9.439775e-01

7896 8.001164e-01 9.438712e-01

7897 8.002094e-01 9.437626e-01

7898 8.002984e-01 9.436516e-01

7899 8.003835e-01 9.435383e-01

7900 8.004646e-01 9.434226e-01

7901 8.005416e-01 9.433045e-01

7902 8.006147e-01 9.431841e-01

7903 8.006837e-01 9.430614e-01

7904 8.007487e-01 9.429362e-01

7905 8.008096e-01 9.428087e-01

7906 8.008665e-01 9.426788e-01

7907 8.009193e-01 9.425466e-01

7908 8.009682e-01 9.424121e-01

7909 8.010130e-01 9.422753e-01

7910 8.010539e-01 9.421361e-01

7911 8.010909e-01 9.419947e-01

7912 8.011240e-01 9.418511e-01

7913 8.011533e-01 9.417052e-01

7914 8.011788e-01 9.415572e-01

7915 8.012005e-01 9.414070e-01

7916 8.012186e-01 9.412547e-01

7917 8.012331e-01 9.411003e-01

7918 8.012440e-01 9.409438e-01

7919 8.012515e-01 9.407854e-01

7920 8.012556e-01 9.406251e-01

7921 8.012564e-01 9.404629e-01

7922 8.012560e-01 9.404621e-01

7923 8.012555e-01 9.404614e-01

7924 8.012549e-01 9.404606e-01

7925 8.012541e-01 9.404598e-01

7926 8.012532e-01 9.404590e-01

7927 8.012521e-01 9.404581e-01

7928 8.012509e-01 9.404572e-01

7929 8.012494e-01 9.404563e-01

7930 8.012478e-01 9.404553e-01

7931 8.012460e-01 9.404543e-01

7932 8.012440e-01 9.404533e-01

7933 8.012418e-01 9.404522e-01

7934 8.012394e-01 9.404511e-01

7935 8.012367e-01 9.404500e-01

7936 8.012338e-01 9.404488e-01

7937 8.012307e-01 9.404475e-01

7938 8.012272e-01 9.404462e-01

7939 8.012235e-01 9.404449e-01

7940 8.012195e-01 9.404435e-01

7941 8.012152e-01 9.404421e-01

7942 8.012106e-01 9.404406e-01

7943 8.012056e-01 9.404391e-01

7944 8.012003e-01 9.404375e-01

7945 8.011946e-01 9.404359e-01

7946 8.011885e-01 9.404342e-01

7947 8.011820e-01 9.404324e-01

7948 8.011751e-01 9.404306e-01

7949 8.011677e-01 9.404287e-01

7950 8.011599e-01 9.404267e-01

7951 8.011516e-01 9.404247e-01

7952 8.011428e-01 9.404226e-01

7953 8.011334e-01 9.404204e-01

7954 8.011236e-01 9.404182e-01

7955 8.011131e-01 9.404158e-01

7956 8.011020e-01 9.404134e-01

7957 8.010903e-01 9.404109e-01

7958 8.010780e-01 9.404083e-01

7959 8.010649e-01 9.404056e-01

7960 8.010512e-01 9.404028e-01

7961 8.010367e-01 9.404000e-01

7962 8.010214e-01 9.403970e-01

7963 8.010053e-01 9.403939e-01

7964 8.009884e-01 9.403907e-01

7965 8.009706e-01 9.403874e-01

7966 8.009518e-01 9.403839e-01

7967 8.009321e-01 9.403804e-01

7968 8.009114e-01 9.403767e-01

7969 8.008897e-01 9.403729e-01

7970 8.008668e-01 9.403689e-01

7971 8.008429e-01 9.403649e-01

7972 8.008177e-01 9.403606e-01

7973 8.007913e-01 9.403563e-01

7974 8.007637e-01 9.403517e-01

7975 8.007347e-01 9.403471e-01

7976 8.007043e-01 9.403422e-01

7977 8.006724e-01 9.403372e-01

7978 8.006391e-01 9.403320e-01

7979 8.006042e-01 9.403266e-01

7980 8.005676e-01 9.403211e-01

7981 8.005294e-01 9.403153e-01

7982 8.004894e-01 9.403094e-01

7983 8.004475e-01 9.403032e-01

7984 8.004038e-01 9.402969e-01

7985 8.003581e-01 9.402903e-01

7986 8.003103e-01 9.402835e-01

7987 8.002604e-01 9.402764e-01

7988 8.002083e-01 9.402691e-01

7989 8.001539e-01 9.402616e-01

7990 8.000971e-01 9.402538e-01

7991 8.000378e-01 9.402457e-01

7992 7.999759e-01 9.402374e-01

7993 7.999114e-01 9.402287e-01

7994 7.998441e-01 9.402198e-01

7995 7.997740e-01 9.402106e-01

7996 7.997009e-01 9.402011e-01

7997 7.996247e-01 9.401912e-01

7998 7.995454e-01 9.401810e-01

7999 7.994628e-01 9.401705e-01

8000 7.993768e-01 9.401596e-01

8001 7.992872e-01 9.401483e-01

8002 7.991941e-01 9.401367e-01

8003 7.990971e-01 9.401247e-01

8004 7.989963e-01 9.401123e-01

8005 7.988915e-01 9.400994e-01

8006 7.987826e-01 9.400862e-01

8007 7.986694e-01 9.400725e-01

8008 7.985519e-01 9.400583e-01

8009 7.984298e-01 9.400437e-01

8010 7.983031e-01 9.400286e-01

8011 7.981715e-01 9.400130e-01

8012 7.980351e-01 9.399968e-01

8013 7.978935e-01 9.399802e-01

8014 7.977468e-01 9.399630e-01

8015 7.975947e-01 9.399452e-01

8016 7.974371e-01 9.399269e-01

8017 7.972739e-01 9.399080e-01

8018 7.971049e-01 9.398884e-01

8019 7.969300e-01 9.398683e-01

8020 7.967490e-01 9.398474e-01

8021 7.965618e-01 9.398259e-01

8022 7.963683e-01 9.398037e-01

8023 7.961682e-01 9.397809e-01

8024 7.959616e-01 9.397572e-01

8025 7.957482e-01 9.397328e-01

8026 7.955280e-01 9.397077e-01

8027 7.953007e-01 9.396817e-01

8028 7.950663e-01 9.396550e-01

8029 7.948246e-01 9.396274e-01

8030 7.945756e-01 9.395989e-01

8031 7.943191e-01 9.395695e-01

8032 7.940550e-01 9.395392e-01

8033 7.937832e-01 9.395080e-01

8034 7.935036e-01 9.394758e-01

8035 7.932161e-01 9.394426e-01

8036 7.929208e-01 9.394084e-01

8037 7.926173e-01 9.393732e-01

8038 7.923058e-01 9.393368e-01

8039 7.919862e-01 9.392994e-01

8040 7.916584e-01 9.392609e-01

8041 7.913224e-01 9.392212e-01

8042 7.909781e-01 9.391803e-01

8043 7.906256e-01 9.391382e-01

8044 7.902649e-01 9.390948e-01

8045 7.898959e-01 9.390502e-01

8046 7.895187e-01 9.390043e-01

8047 7.891334e-01 9.389571e-01

8048 7.887399e-01 9.389085e-01

8049 7.883383e-01 9.388586e-01

8050 7.879288e-01 9.388072e-01

8051 7.875114e-01 9.387544e-01

8052 7.870862e-01 9.387001e-01

8053 7.866533e-01 9.386443e-01

8054 7.862128e-01 9.385869e-01

8055 7.857650e-01 9.385280e-01

8056 7.853099e-01 9.384675e-01

8057 7.848478e-01 9.384054e-01

8058 7.843789e-01 9.383417e-01

8059 7.839032e-01 9.382762e-01

8060 7.834211e-01 9.382091e-01

8061 7.829328e-01 9.381402e-01

8062 7.824386e-01 9.380696e-01

8063 7.819386e-01 9.379972e-01

8064 7.814332e-01 9.379229e-01

8065 7.809226e-01 9.378469e-01

8066 7.804071e-01 9.377689e-01

8067 7.798871e-01 9.376891e-01

8068 7.793628e-01 9.376073e-01

8069 7.788346e-01 9.375236e-01

8070 7.783028e-01 9.374379e-01

8071 7.777676e-01 9.373503e-01

8072 7.772296e-01 9.372606e-01

8073 7.766889e-01 9.371690e-01

8074 7.761461e-01 9.370753e-01

8075 7.756013e-01 9.369795e-01

8076 7.750550e-01 9.368816e-01

8077 7.745076e-01 9.367817e-01

8078 7.739593e-01 9.366797e-01

8079 7.734106e-01 9.365756e-01

8080 7.728619e-01 9.364693e-01

8081 7.723134e-01 9.363610e-01

8082 7.717655e-01 9.362505e-01

8083 7.712187e-01 9.361379e-01

8084 7.706731e-01 9.360232e-01

8085 7.701293e-01 9.359063e-01

8086 7.695874e-01 9.357874e-01

8087 7.690479e-01 9.356664e-01

8088 7.685110e-01 9.355432e-01

8089 7.679771e-01 9.354180e-01

8090 7.674465e-01 9.352908e-01

8091 7.669194e-01 9.351615e-01

8092 7.663961e-01 9.350301e-01

8093 7.658770e-01 9.348968e-01

8094 7.653622e-01 9.347616e-01

8095 7.648521e-01 9.346244e-01

8096 7.643468e-01 9.344853e-01

8097 7.638466e-01 9.343444e-01

8098 7.633516e-01 9.342017e-01

8099 7.628622e-01 9.340572e-01

8100 7.623784e-01 9.339110e-01

8101 7.619005e-01 9.337632e-01

8102 7.618781e-01 9.337624e-01

8103 7.618549e-01 9.337616e-01

8104 7.618309e-01 9.337607e-01

8105 7.618060e-01 9.337599e-01

8106 7.617802e-01 9.337590e-01

8107 7.617534e-01 9.337580e-01

8108 7.617257e-01 9.337571e-01

8109 7.616969e-01 9.337561e-01

8110 7.616672e-01 9.337551e-01

8111 7.616363e-01 9.337540e-01

8112 7.616044e-01 9.337529e-01

8113 7.615713e-01 9.337518e-01

8114 7.615370e-01 9.337506e-01

8115 7.615015e-01 9.337494e-01

8116 7.614648e-01 9.337482e-01

8117 7.614267e-01 9.337469e-01

8118 7.613873e-01 9.337456e-01

8119 7.613465e-01 9.337442e-01

8120 7.613042e-01 9.337428e-01

8121 7.612604e-01 9.337414e-01

8122 7.612151e-01 9.337399e-01

8123 7.611681e-01 9.337383e-01

8124 7.611195e-01 9.337367e-01

8125 7.610692e-01 9.337351e-01

8126 7.610171e-01 9.337334e-01

8127 7.609632e-01 9.337316e-01

8128 7.609073e-01 9.337298e-01

8129 7.608496e-01 9.337279e-01

8130 7.607897e-01 9.337260e-01

8131 7.607278e-01 9.337240e-01

8132 7.606637e-01 9.337220e-01

8133 7.605973e-01 9.337199e-01

8134 7.605287e-01 9.337177e-01

8135 7.604576e-01 9.337154e-01

8136 7.603840e-01 9.337131e-01

8137 7.603079e-01 9.337107e-01

8138 7.602291e-01 9.337082e-01

8139 7.601476e-01 9.337057e-01

8140 7.600633e-01 9.337030e-01

8141 7.599760e-01 9.337003e-01

8142 7.598857e-01 9.336975e-01

8143 7.597923e-01 9.336946e-01

8144 7.596957e-01 9.336917e-01

8145 7.595958e-01 9.336886e-01

8146 7.594924e-01 9.336854e-01

8147 7.593855e-01 9.336822e-01

8148 7.592749e-01 9.336788e-01

8149 7.591606e-01 9.336753e-01

8150 7.590423e-01 9.336717e-01

8151 7.589201e-01 9.336681e-01

8152 7.587937e-01 9.336643e-01

8153 7.586630e-01 9.336603e-01

8154 7.585280e-01 9.336563e-01

8155 7.583884e-01 9.336521e-01

8156 7.582441e-01 9.336479e-01

8157 7.580950e-01 9.336434e-01

8158 7.579410e-01 9.336389e-01

8159 7.577818e-01 9.336342e-01

8160 7.576174e-01 9.336293e-01

8161 7.574475e-01 9.336244e-01

8162 7.572721e-01 9.336192e-01

8163 7.570910e-01 9.336139e-01

8164 7.569040e-01 9.336085e-01

8165 7.567109e-01 9.336029e-01

8166 7.565116e-01 9.335971e-01

8167 7.563059e-01 9.335911e-01

8168 7.560936e-01 9.335850e-01

8169 7.558746e-01 9.335787e-01

8170 7.556486e-01 9.335722e-01

8171 7.554156e-01 9.335655e-01

8172 7.551753e-01 9.335586e-01

8173 7.549275e-01 9.335515e-01

8174 7.546721e-01 9.335442e-01

8175 7.544088e-01 9.335366e-01

8176 7.541376e-01 9.335289e-01

8177 7.538581e-01 9.335209e-01

8178 7.535703e-01 9.335127e-01

8179 7.532739e-01 9.335042e-01

8180 7.529687e-01 9.334955e-01

8181 7.526547e-01 9.334865e-01

8182 7.523315e-01 9.334773e-01

8183 7.519990e-01 9.334678e-01

8184 7.516570e-01 9.334580e-01

8185 7.513054e-01 9.334480e-01

8186 7.509440e-01 9.334376e-01

8187 7.505726e-01 9.334270e-01

8188 7.501911e-01 9.334160e-01

8189 7.497992e-01 9.334047e-01

8190 7.493969e-01 9.333931e-01

8191 7.489841e-01 9.333812e-01

8192 7.485604e-01 9.333689e-01

8193 7.481260e-01 9.333563e-01

8194 7.476805e-01 9.333433e-01

8195 7.472240e-01 9.333299e-01

8196 7.467563e-01 9.333162e-01

8197 7.462773e-01 9.333020e-01

8198 7.457869e-01 9.332875e-01

8199 7.452851e-01 9.332725e-01

8200 7.447718e-01 9.332571e-01

8201 7.442470e-01 9.332413e-01

8202 7.437106e-01 9.332250e-01

8203 7.431626e-01 9.332083e-01

8204 7.426031e-01 9.331911e-01

8205 7.420321e-01 9.331734e-01

8206 7.414495e-01 9.331552e-01

8207 7.408555e-01 9.331364e-01

8208 7.402501e-01 9.331172e-01

8209 7.396334e-01 9.330974e-01

8210 7.390056e-01 9.330771e-01

8211 7.383666e-01 9.330562e-01

8212 7.377168e-01 9.330347e-01

8213 7.370563e-01 9.330126e-01

8214 7.363852e-01 9.329899e-01

8215 7.357037e-01 9.329666e-01

8216 7.350122e-01 9.329426e-01

8217 7.343108e-01 9.329180e-01

8218 7.335999e-01 9.328927e-01

8219 7.328797e-01 9.328667e-01

8220 7.321505e-01 9.328399e-01

8221 7.314127e-01 9.328125e-01

8222 7.306666e-01 9.327843e-01

8223 7.299126e-01 9.327553e-01

8224 7.291511e-01 9.327255e-01

8225 7.283825e-01 9.326950e-01

8226 7.276073e-01 9.326636e-01

8227 7.268259e-01 9.326314e-01

8228 7.260387e-01 9.325983e-01

8229 7.252462e-01 9.325643e-01

8230 7.244489e-01 9.325294e-01

8231 7.236474e-01 9.324937e-01

8232 7.228421e-01 9.324570e-01

8233 7.220335e-01 9.324193e-01

8234 7.212223e-01 9.323806e-01

8235 7.204089e-01 9.323410e-01

8236 7.195938e-01 9.323003e-01

8237 7.187777e-01 9.322586e-01

8238 7.179611e-01 9.322159e-01

8239 7.171445e-01 9.321720e-01

8240 7.163285e-01 9.321271e-01

8241 7.155136e-01 9.320811e-01

8242 7.147005e-01 9.320339e-01

8243 7.138896e-01 9.319856e-01

8244 7.130814e-01 9.319361e-01

8245 7.122767e-01 9.318854e-01

8246 7.114757e-01 9.318334e-01

8247 7.106791e-01 9.317803e-01

8248 7.098874e-01 9.317259e-01

8249 7.091010e-01 9.316703e-01

8250 7.083204e-01 9.316134e-01

8251 7.075461e-01 9.315551e-01

8252 7.067786e-01 9.314956e-01

8253 7.060182e-01 9.314348e-01

8254 7.052653e-01 9.313726e-01

8255 7.045205e-01 9.313091e-01

8256 7.037839e-01 9.312442e-01

8257 7.030561e-01 9.311780e-01

8258 7.023372e-01 9.311103e-01

8259 7.016277e-01 9.310414e-01

8260 7.009277e-01 9.309710e-01

8261 7.002377e-01 9.308993e-01

8262 6.995578e-01 9.308261e-01

8263 6.988882e-01 9.307516e-01

8264 6.982292e-01 9.306757e-01

8265 6.975810e-01 9.305985e-01

8266 6.969436e-01 9.305198e-01

8267 6.963173e-01 9.304398e-01

8268 6.957022e-01 9.303585e-01

8269 6.950983e-01 9.302759e-01

8270 6.945058e-01 9.301919e-01

8271 6.939247e-01 9.301066e-01

8272 6.933550e-01 9.300201e-01

8273 6.927969e-01 9.299323e-01

8274 6.922502e-01 9.298433e-01

8275 6.917151e-01 9.297531e-01

8276 6.911914e-01 9.296617e-01

8277 6.906791e-01 9.295692e-01

8278 6.901782e-01 9.294756e-01

8279 6.896887e-01 9.293810e-01

8280 6.892103e-01 9.292854e-01

8281 6.887432e-01 9.291888e-01

8282 6.886950e-01 9.291881e-01

8283 6.886453e-01 9.291874e-01

8284 6.885941e-01 9.291867e-01

8285 6.885415e-01 9.291860e-01

8286 6.884872e-01 9.291853e-01

8287 6.884314e-01 9.291845e-01

8288 6.883740e-01 9.291838e-01

8289 6.883149e-01 9.291830e-01

8290 6.882541e-01 9.291821e-01

8291 6.881915e-01 9.291813e-01

8292 6.881272e-01 9.291804e-01

8293 6.880610e-01 9.291795e-01

8294 6.879930e-01 9.291786e-01

8295 6.879230e-01 9.291777e-01

8296 6.878510e-01 9.291767e-01

8297 6.877770e-01 9.291758e-01

8298 6.877009e-01 9.291747e-01

8299 6.876227e-01 9.291737e-01

8300 6.875423e-01 9.291726e-01

8301 6.874596e-01 9.291715e-01

8302 6.873747e-01 9.291704e-01

8303 6.872873e-01 9.291693e-01

8304 6.871976e-01 9.291681e-01

8305 6.871054e-01 9.291669e-01

8306 6.870106e-01 9.291656e-01

8307 6.869132e-01 9.291644e-01

8308 6.868131e-01 9.291631e-01

8309 6.867103e-01 9.291617e-01

8310 6.866047e-01 9.291603e-01

8311 6.864962e-01 9.291589e-01

8312 6.863847e-01 9.291575e-01

8313 6.862703e-01 9.291560e-01

8314 6.861527e-01 9.291545e-01

8315 6.860320e-01 9.291529e-01

8316 6.859080e-01 9.291514e-01

8317 6.857807e-01 9.291497e-01

8318 6.856501e-01 9.291480e-01

8319 6.855160e-01 9.291463e-01

8320 6.853783e-01 9.291446e-01

8321 6.852370e-01 9.291428e-01

8322 6.850920e-01 9.291409e-01

8323 6.849432e-01 9.291391e-01

8324 6.847905e-01 9.291371e-01

8325 6.846339e-01 9.291351e-01

8326 6.844733e-01 9.291331e-01

8327 6.843085e-01 9.291310e-01

8328 6.841396e-01 9.291289e-01

8329 6.839664e-01 9.291268e-01

8330 6.837888e-01 9.291245e-01

8331 6.836068e-01 9.291223e-01

8332 6.834202e-01 9.291199e-01

8333 6.832290e-01 9.291176e-01

8334 6.830332e-01 9.291151e-01

8335 6.828326e-01 9.291126e-01

8336 6.826271e-01 9.291101e-01

8337 6.824167e-01 9.291075e-01

8338 6.822012e-01 9.291048e-01

8339 6.819807e-01 9.291021e-01

8340 6.817550e-01 9.290993e-01

8341 6.815241e-01 9.290965e-01

8342 6.812879e-01 9.290936e-01

8343 6.810463e-01 9.290906e-01

8344 6.807992e-01 9.290876e-01

8345 6.805466e-01 9.290845e-01

8346 6.802885e-01 9.290814e-01

8347 6.800247e-01 9.290781e-01

8348 6.797552e-01 9.290748e-01

8349 6.794799e-01 9.290715e-01

8350 6.791989e-01 9.290680e-01

8351 6.789120e-01 9.290645e-01

8352 6.786193e-01 9.290609e-01

8353 6.783206e-01 9.290573e-01

8354 6.780160e-01 9.290536e-01

8355 6.777055e-01 9.290498e-01

8356 6.773890e-01 9.290459e-01

8357 6.770664e-01 9.290419e-01

8358 6.767379e-01 9.290379e-01

8359 6.764034e-01 9.290337e-01

8360 6.760629e-01 9.290295e-01

8361 6.757164e-01 9.290253e-01

8362 6.753640e-01 9.290209e-01

8363 6.750057e-01 9.290164e-01

8364 6.746415e-01 9.290119e-01

8365 6.742715e-01 9.290073e-01

8366 6.738958e-01 9.290025e-01

8367 6.735143e-01 9.289977e-01

8368 6.731273e-01 9.289928e-01

8369 6.727347e-01 9.289878e-01

8370 6.723367e-01 9.289828e-01

8371 6.719334e-01 9.289776e-01

8372 6.715249e-01 9.289723e-01

8373 6.711113e-01 9.289669e-01

8374 6.706927e-01 9.289614e-01

8375 6.702694e-01 9.289559e-01

8376 6.698415e-01 9.289502e-01

8377 6.694091e-01 9.289444e-01

8378 6.689724e-01 9.289385e-01

8379 6.685317e-01 9.289325e-01

8380 6.680871e-01 9.289264e-01

8381 6.676388e-01 9.289201e-01

8382 6.671871e-01 9.289138e-01

8383 6.667322e-01 9.289073e-01

8384 6.662743e-01 9.289008e-01

8385 6.658137e-01 9.288941e-01

8386 6.653507e-01 9.288873e-01

8387 6.648855e-01 9.288803e-01

8388 6.644183e-01 9.288733e-01

8389 6.639495e-01 9.288661e-01

8390 6.634794e-01 9.288587e-01

8391 6.630083e-01 9.288513e-01

8392 6.625363e-01 9.288437e-01

8393 6.620640e-01 9.288359e-01

8394 6.615915e-01 9.288281e-01

8395 6.611193e-01 9.288200e-01

8396 6.606475e-01 9.288119e-01

8397 6.601765e-01 9.288035e-01

8398 6.597067e-01 9.287950e-01

8399 6.592383e-01 9.287864e-01

8400 6.587717e-01 9.287776e-01

8401 6.583072e-01 9.287686e-01

8402 6.578450e-01 9.287595e-01

8403 6.573856e-01 9.287501e-01

8404 6.569292e-01 9.287406e-01

8405 6.564761e-01 9.287310e-01

8406 6.560266e-01 9.287211e-01

8407 6.555810e-01 9.287110e-01

8408 6.551395e-01 9.287007e-01

8409 6.547025e-01 9.286903e-01

8410 6.542702e-01 9.286796e-01

8411 6.538429e-01 9.286687e-01

8412 6.534208e-01 9.286576e-01

8413 6.530041e-01 9.286462e-01

8414 6.525930e-01 9.286346e-01

8415 6.521878e-01 9.286228e-01

8416 6.517887e-01 9.286108e-01

8417 6.513958e-01 9.285985e-01

8418 6.510093e-01 9.285859e-01

8419 6.506295e-01 9.285731e-01

8420 6.502563e-01 9.285600e-01

8421 6.498900e-01 9.285467e-01

8422 6.495306e-01 9.285331e-01

8423 6.491784e-01 9.285192e-01

8424 6.488333e-01 9.285050e-01

8425 6.484954e-01 9.284906e-01

8426 6.481649e-01 9.284758e-01

8427 6.478418e-01 9.284608e-01

8428 6.475260e-01 9.284454e-01

8429 6.472177e-01 9.284298e-01

8430 6.469169e-01 9.284138e-01

8431 6.466235e-01 9.283976e-01

8432 6.463375e-01 9.283810e-01

8433 6.460591e-01 9.283641e-01

8434 6.457880e-01 9.283469e-01

8435 6.455243e-01 9.283294e-01

8436 6.452679e-01 9.283116e-01

8437 6.450187e-01 9.282934e-01

8438 6.447768e-01 9.282750e-01

8439 6.445420e-01 9.282562e-01

8440 6.443143e-01 9.282372e-01

8441 6.440935e-01 9.282178e-01

8442 6.438796e-01 9.281981e-01

8443 6.436724e-01 9.281782e-01

8444 6.434720e-01 9.281580e-01

8445 6.432780e-01 9.281375e-01

8446 6.430905e-01 9.281167e-01

8447 6.429094e-01 9.280957e-01

8448 6.427344e-01 9.280744e-01

8449 6.425655e-01 9.280529e-01

8450 6.424026e-01 9.280312e-01

8451 6.422455e-01 9.280093e-01

8452 6.420940e-01 9.279871e-01

8453 6.419482e-01 9.279649e-01

8454 6.418077e-01 9.279424e-01

8455 6.416726e-01 9.279198e-01

8456 6.415426e-01 9.278971e-01

8457 6.414177e-01 9.278743e-01

8458 6.412976e-01 9.278514e-01

8459 6.411823e-01 9.278285e-01

8460 6.410717e-01 9.278056e-01

8461 6.409655e-01 9.277826e-01

8462 6.409460e-01 9.277822e-01

8463 6.409263e-01 9.277818e-01

8464 6.409065e-01 9.277814e-01

8465 6.408865e-01 9.277810e-01

8466 6.408664e-01 9.277806e-01

8467 6.408461e-01 9.277802e-01

8468 6.408258e-01 9.277797e-01

8469 6.408053e-01 9.277793e-01

8470 6.407848e-01 9.277789e-01

8471 6.407642e-01 9.277784e-01

8472 6.407435e-01 9.277780e-01

8473 6.407228e-01 9.277775e-01

8474 6.407021e-01 9.277770e-01

8475 6.406814e-01 9.277765e-01

8476 6.406608e-01 9.277761e-01

8477 6.406402e-01 9.277756e-01

8478 6.406196e-01 9.277751e-01

8479 6.405992e-01 9.277746e-01

8480 6.405788e-01 9.277741e-01

8481 6.405586e-01 9.277735e-01

8482 6.405386e-01 9.277730e-01

8483 6.405188e-01 9.277725e-01

8484 6.404992e-01 9.277719e-01

8485 6.404799e-01 9.277714e-01

8486 6.404609e-01 9.277709e-01

8487 6.404422e-01 9.277703e-01

8488 6.404238e-01 9.277697e-01

8489 6.404059e-01 9.277692e-01

8490 6.403884e-01 9.277686e-01

8491 6.403714e-01 9.277680e-01

8492 6.403550e-01 9.277675e-01

8493 6.403391e-01 9.277669e-01

8494 6.403238e-01 9.277663e-01

8495 6.403092e-01 9.277657e-01

8496 6.402953e-01 9.277651e-01

8497 6.402822e-01 9.277645e-01

8498 6.402700e-01 9.277639e-01

8499 6.402586e-01 9.277634e-01

8500 6.402482e-01 9.277628e-01

8501 6.402387e-01 9.277622e-01

8502 6.402304e-01 9.277616e-01

8503 6.402232e-01 9.277610e-01

8504 6.402173e-01 9.277604e-01

8505 6.402126e-01 9.277598e-01

8506 6.402093e-01 9.277592e-01

8507 6.402074e-01 9.277586e-01

8508 6.402071e-01 9.277581e-01

8509 6.402084e-01 9.277575e-01

8510 6.402113e-01 9.277569e-01

8511 6.402161e-01 9.277564e-01

8512 6.402227e-01 9.277558e-01

8513 6.402314e-01 9.277553e-01

8514 6.402420e-01 9.277548e-01

8515 6.402549e-01 9.277542e-01

8516 6.402701e-01 9.277537e-01

8517 6.402876e-01 9.277533e-01

8518 6.403076e-01 9.277528e-01

8519 6.403303e-01 9.277523e-01

8520 6.403556e-01 9.277519e-01

8521 6.403838e-01 9.277515e-01

8522 6.404150e-01 9.277511e-01

8523 6.404492e-01 9.277507e-01

8524 6.404867e-01 9.277504e-01

8525 6.405275e-01 9.277501e-01

8526 6.405717e-01 9.277498e-01

8527 6.406196e-01 9.277495e-01

8528 6.406712e-01 9.277493e-01

8529 6.407267e-01 9.277491e-01

8530 6.407862e-01 9.277489e-01

8531 6.408498e-01 9.277488e-01

8532 6.409177e-01 9.277487e-01

8533 6.409901e-01 9.277487e-01

8534 6.410670e-01 9.277487e-01

8535 6.411486e-01 9.277488e-01

8536 6.412352e-01 9.277489e-01

8537 6.413267e-01 9.277491e-01

8538 6.414233e-01 9.277493e-01

8539 6.415253e-01 9.277496e-01

8540 6.416327e-01 9.277500e-01

8541 6.417457e-01 9.277504e-01

8542 6.418644e-01 9.277509e-01

8543 6.419890e-01 9.277515e-01

8544 6.421195e-01 9.277521e-01

8545 6.422562e-01 9.277529e-01

8546 6.423992e-01 9.277537e-01

8547 6.425485e-01 9.277546e-01

8548 6.427044e-01 9.277556e-01

8549 6.428669e-01 9.277567e-01

8550 6.430361e-01 9.277579e-01

8551 6.432122e-01 9.277592e-01

8552 6.433952e-01 9.277606e-01

8553 6.435853e-01 9.277622e-01

8554 6.437826e-01 9.277639e-01

8555 6.439870e-01 9.277657e-01

8556 6.441988e-01 9.277676e-01

8557 6.444180e-01 9.277697e-01

8558 6.446446e-01 9.277719e-01

8559 6.448787e-01 9.277742e-01

8560 6.451204e-01 9.277768e-01

8561 6.453696e-01 9.277794e-01

8562 6.456264e-01 9.277823e-01

8563 6.458908e-01 9.277853e-01

8564 6.461628e-01 9.277885e-01

8565 6.464425e-01 9.277919e-01

8566 6.467297e-01 9.277955e-01

8567 6.470244e-01 9.277994e-01

8568 6.473267e-01 9.278034e-01

8569 6.476364e-01 9.278076e-01

8570 6.479536e-01 9.278121e-01

8571 6.482780e-01 9.278168e-01

8572 6.486097e-01 9.278217e-01

8573 6.489485e-01 9.278269e-01

8574 6.492942e-01 9.278324e-01

8575 6.496469e-01 9.278381e-01

8576 6.500063e-01 9.278441e-01

8577 6.503723e-01 9.278504e-01

8578 6.507448e-01 9.278569e-01

8579 6.511235e-01 9.278638e-01

8580 6.515082e-01 9.278710e-01

8581 6.518989e-01 9.278785e-01

8582 6.522952e-01 9.278864e-01

8583 6.526970e-01 9.278946e-01

8584 6.531041e-01 9.279031e-01

8585 6.535161e-01 9.279120e-01

8586 6.539329e-01 9.279213e-01

8587 6.543542e-01 9.279309e-01

8588 6.547797e-01 9.279409e-01

8589 6.552093e-01 9.279513e-01

8590 6.556426e-01 9.279621e-01

8591 6.560793e-01 9.279734e-01

8592 6.565192e-01 9.279850e-01

8593 6.569619e-01 9.279971e-01

8594 6.574073e-01 9.280096e-01

8595 6.578550e-01 9.280226e-01

8596 6.583047e-01 9.280361e-01

8597 6.587561e-01 9.280500e-01

8598 6.592089e-01 9.280644e-01

8599 6.596629e-01 9.280793e-01

8600 6.601177e-01 9.280947e-01

8601 6.605730e-01 9.281106e-01

8602 6.610286e-01 9.281271e-01

8603 6.614842e-01 9.281441e-01

8604 6.619395e-01 9.281616e-01

8605 6.623942e-01 9.281797e-01

8606 6.628481e-01 9.281984e-01

8607 6.633008e-01 9.282177e-01

8608 6.637521e-01 9.282375e-01

8609 6.642018e-01 9.282580e-01

8610 6.646496e-01 9.282791e-01

8611 6.650953e-01 9.283008e-01

8612 6.655386e-01 9.283231e-01

8613 6.659793e-01 9.283461e-01

8614 6.664172e-01 9.283698e-01

8615 6.668521e-01 9.283942e-01

8616 6.672838e-01 9.284192e-01

8617 6.677120e-01 9.284449e-01

8618 6.681367e-01 9.284714e-01

8619 6.685576e-01 9.284985e-01

8620 6.689745e-01 9.285264e-01

8621 6.693874e-01 9.285550e-01

8622 6.697960e-01 9.285844e-01

8623 6.702002e-01 9.286146e-01

8624 6.706000e-01 9.286455e-01

8625 6.709951e-01 9.286772e-01

8626 6.713856e-01 9.287096e-01

8627 6.717712e-01 9.287429e-01

8628 6.721519e-01 9.287770e-01

8629 6.725276e-01 9.288119e-01

8630 6.728982e-01 9.288476e-01

8631 6.732638e-01 9.288841e-01

8632 6.736241e-01 9.289215e-01

8633 6.739793e-01 9.289597e-01

8634 6.743292e-01 9.289987e-01

8635 6.746738e-01 9.290386e-01

8636 6.750130e-01 9.290793e-01

8637 6.753470e-01 9.291208e-01

8638 6.756756e-01 9.291632e-01

8639 6.759989e-01 9.292065e-01

8640 6.763168e-01 9.292505e-01

8641 6.766294e-01 9.292955e-01

8642 6.766768e-01 9.292953e-01

8643 6.767256e-01 9.292952e-01

8644 6.767759e-01 9.292951e-01

8645 6.768278e-01 9.292949e-01

8646 6.768812e-01 9.292948e-01

8647 6.769362e-01 9.292947e-01

8648 6.769928e-01 9.292945e-01

8649 6.770511e-01 9.292944e-01

8650 6.771110e-01 9.292942e-01

8651 6.771727e-01 9.292941e-01

8652 6.772362e-01 9.292939e-01

8653 6.773015e-01 9.292938e-01

8654 6.773687e-01 9.292936e-01

8655 6.774377e-01 9.292934e-01

8656 6.775088e-01 9.292932e-01

8657 6.775818e-01 9.292930e-01

8658 6.776569e-01 9.292928e-01

8659 6.777340e-01 9.292926e-01

8660 6.778134e-01 9.292924e-01

8661 6.778950e-01 9.292921e-01

8662 6.779788e-01 9.292919e-01

8663 6.780650e-01 9.292916e-01

8664 6.781535e-01 9.292913e-01

8665 6.782445e-01 9.292910e-01

8666 6.783380e-01 9.292907e-01

8667 6.784341e-01 9.292903e-01

8668 6.785328e-01 9.292900e-01

8669 6.786343e-01 9.292896e-01

8670 6.787385e-01 9.292892e-01

8671 6.788455e-01 9.292888e-01

8672 6.789555e-01 9.292883e-01

8673 6.790684e-01 9.292879e-01

8674 6.791845e-01 9.292874e-01

8675 6.793036e-01 9.292869e-01

8676 6.794260e-01 9.292863e-01

8677 6.795517e-01 9.292857e-01

8678 6.796807e-01 9.292851e-01

8679 6.798132e-01 9.292845e-01

8680 6.799493e-01 9.292838e-01

8681 6.800890e-01 9.292830e-01

8682 6.802324e-01 9.292823e-01

8683 6.803796e-01 9.292815e-01

8684 6.805307e-01 9.292806e-01

8685 6.806858e-01 9.292797e-01

8686 6.808450e-01 9.292787e-01

8687 6.810083e-01 9.292777e-01

8688 6.811760e-01 9.292767e-01

8689 6.813480e-01 9.292756e-01

8690 6.815244e-01 9.292744e-01

8691 6.817055e-01 9.292731e-01

8692 6.818912e-01 9.292718e-01

8693 6.820817e-01 9.292704e-01

8694 6.822770e-01 9.292689e-01

8695 6.824774e-01 9.292674e-01

8696 6.826829e-01 9.292657e-01

8697 6.828935e-01 9.292640e-01

8698 6.831095e-01 9.292622e-01

8699 6.833309e-01 9.292603e-01

8700 6.835578e-01 9.292582e-01

8701 6.837904e-01 9.292561e-01

8702 6.840288e-01 9.292538e-01

8703 6.842730e-01 9.292514e-01

8704 6.845232e-01 9.292489e-01

8705 6.847796e-01 9.292463e-01

8706 6.850421e-01 9.292435e-01

8707 6.853110e-01 9.292405e-01

8708 6.855863e-01 9.292374e-01

8709 6.858682e-01 9.292342e-01

8710 6.861568e-01 9.292307e-01

8711 6.864521e-01 9.292271e-01

8712 6.867544e-01 9.292232e-01

8713 6.870637e-01 9.292192e-01

8714 6.873801e-01 9.292149e-01

8715 6.877037e-01 9.292104e-01

8716 6.880347e-01 9.292057e-01

8717 6.883732e-01 9.292007e-01

8718 6.887192e-01 9.291954e-01

8719 6.890728e-01 9.291899e-01

8720 6.894343e-01 9.291841e-01

8721 6.898035e-01 9.291779e-01

8722 6.901808e-01 9.291715e-01

8723 6.905660e-01 9.291647e-01

8724 6.909594e-01 9.291575e-01

8725 6.913610e-01 9.291499e-01

8726 6.917709e-01 9.291420e-01

8727 6.921892e-01 9.291336e-01

8728 6.926158e-01 9.291248e-01

8729 6.930510e-01 9.291156e-01

8730 6.934947e-01 9.291058e-01

8731 6.939470e-01 9.290956e-01

8732 6.944080e-01 9.290848e-01

8733 6.948776e-01 9.290735e-01

8734 6.953560e-01 9.290615e-01

8735 6.958431e-01 9.290490e-01

8736 6.963389e-01 9.290358e-01

8737 6.968436e-01 9.290220e-01

8738 6.973570e-01 9.290074e-01

8739 6.978791e-01 9.289921e-01

8740 6.984100e-01 9.289760e-01

8741 6.989497e-01 9.289591e-01

8742 6.994980e-01 9.289414e-01

8743 7.000549e-01 9.289227e-01

8744 7.006204e-01 9.289032e-01

8745 7.011945e-01 9.288826e-01

8746 7.017769e-01 9.288611e-01

8747 7.023678e-01 9.288384e-01

8748 7.029668e-01 9.288147e-01

8749 7.035741e-01 9.287898e-01

8750 7.041893e-01 9.287637e-01

8751 7.048124e-01 9.287363e-01

8752 7.054433e-01 9.287076e-01

8753 7.060818e-01 9.286775e-01

8754 7.067277e-01 9.286460e-01

8755 7.073809e-01 9.286130e-01

8756 7.080411e-01 9.285784e-01

8757 7.087082e-01 9.285422e-01

8758 7.093819e-01 9.285044e-01

8759 7.100620e-01 9.284647e-01

8760 7.107483e-01 9.284233e-01

8761 7.114406e-01 9.283799e-01

8762 7.121385e-01 9.283346e-01

8763 7.128419e-01 9.282872e-01

8764 7.135504e-01 9.282377e-01

8765 7.142637e-01 9.281861e-01

8766 7.149816e-01 9.281321e-01

8767 7.157038e-01 9.280759e-01

8768 7.164299e-01 9.280171e-01

8769 7.171596e-01 9.279559e-01

8770 7.178926e-01 9.278921e-01

8771 7.186286e-01 9.278257e-01

8772 7.193672e-01 9.277564e-01

8773 7.201080e-01 9.276844e-01

8774 7.208507e-01 9.276094e-01

8775 7.215951e-01 9.275314e-01

8776 7.223406e-01 9.274503e-01

8777 7.230869e-01 9.273660e-01

8778 7.238337e-01 9.272785e-01

8779 7.245806e-01 9.271876e-01

8780 7.253272e-01 9.270933e-01

8781 7.260732e-01 9.269955e-01

8782 7.268182e-01 9.268941e-01

8783 7.275618e-01 9.267890e-01

8784 7.283037e-01 9.266802e-01

8785 7.290434e-01 9.265675e-01

8786 7.297808e-01 9.264509e-01

8787 7.305153e-01 9.263304e-01

8788 7.312466e-01 9.262058e-01

8789 7.319744e-01 9.260771e-01

8790 7.326984e-01 9.259443e-01

8791 7.334183e-01 9.258072e-01

8792 7.341336e-01 9.256659e-01

8793 7.348441e-01 9.255202e-01

8794 7.355495e-01 9.253702e-01

8795 7.362494e-01 9.252157e-01

8796 7.369437e-01 9.250568e-01

8797 7.376319e-01 9.248934e-01

8798 7.383139e-01 9.247255e-01

8799 7.389894e-01 9.245531e-01

8800 7.396582e-01 9.243762e-01

8801 7.403199e-01 9.241948e-01

8802 7.409744e-01 9.240089e-01

8803 7.416214e-01 9.238184e-01

8804 7.422608e-01 9.236235e-01

8805 7.428924e-01 9.234241e-01

8806 7.435160e-01 9.232202e-01

8807 7.441314e-01 9.230121e-01

8808 7.447385e-01 9.227995e-01

8809 7.453371e-01 9.225827e-01

8810 7.459272e-01 9.223618e-01

8811 7.465085e-01 9.221367e-01

8812 7.470810e-01 9.219075e-01

8813 7.476446e-01 9.216745e-01

8814 7.481993e-01 9.214376e-01

8815 7.487448e-01 9.211969e-01

8816 7.492813e-01 9.209527e-01

8817 7.498087e-01 9.207050e-01

8818 7.503268e-01 9.204539e-01

8819 7.508357e-01 9.201996e-01

8820 7.513354e-01 9.199423e-01

8821 7.518260e-01 9.196820e-01

8822 7.518587e-01 9.196807e-01

8823 7.518924e-01 9.196793e-01

8824 7.519269e-01 9.196779e-01

8825 7.519623e-01 9.196764e-01

8826 7.519986e-01 9.196749e-01

8827 7.520358e-01 9.196733e-01

8828 7.520739e-01 9.196717e-01

8829 7.521131e-01 9.196700e-01

8830 7.521532e-01 9.196683e-01

8831 7.521944e-01 9.196665e-01

8832 7.522366e-01 9.196647e-01

8833 7.522799e-01 9.196628e-01

8834 7.523243e-01 9.196608e-01

8835 7.523698e-01 9.196588e-01

8836 7.524164e-01 9.196567e-01

8837 7.524642e-01 9.196545e-01

8838 7.525132e-01 9.196523e-01

8839 7.525635e-01 9.196500e-01

8840 7.526150e-01 9.196477e-01

8841 7.526678e-01 9.196453e-01

8842 7.527220e-01 9.196427e-01

8843 7.527774e-01 9.196402e-01

8844 7.528343e-01 9.196375e-01

8845 7.528926e-01 9.196348e-01

8846 7.529523e-01 9.196319e-01

8847 7.530135e-01 9.196290e-01

8848 7.530763e-01 9.196260e-01

8849 7.531406e-01 9.196229e-01

8850 7.532064e-01 9.196197e-01

8851 7.532739e-01 9.196164e-01

8852 7.533431e-01 9.196130e-01

8853 7.534140e-01 9.196095e-01

8854 7.534866e-01 9.196059e-01

8855 7.535609e-01 9.196022e-01

8856 7.536371e-01 9.195983e-01

8857 7.537152e-01 9.195944e-01

8858 7.537951e-01 9.195903e-01

8859 7.538770e-01 9.195861e-01

8860 7.539609e-01 9.195817e-01

8861 7.540468e-01 9.195772e-01

8862 7.541347e-01 9.195726e-01

8863 7.542248e-01 9.195678e-01

8864 7.543170e-01 9.195628e-01

8865 7.544114e-01 9.195578e-01

8866 7.545080e-01 9.195525e-01

8867 7.546070e-01 9.195471e-01

8868 7.547082e-01 9.195415e-01

8869 7.548119e-01 9.195357e-01

8870 7.549180e-01 9.195297e-01

8871 7.550266e-01 9.195235e-01

8872 7.551377e-01 9.195172e-01

8873 7.552513e-01 9.195106e-01

8874 7.553676e-01 9.195038e-01

8875 7.554866e-01 9.194968e-01

8876 7.556083e-01 9.194895e-01

8877 7.557328e-01 9.194821e-01

8878 7.558600e-01 9.194743e-01

8879 7.559902e-01 9.194663e-01

8880 7.561233e-01 9.194581e-01

8881 7.562594e-01 9.194496e-01

8882 7.563985e-01 9.194408e-01

8883 7.565407e-01 9.194317e-01

8884 7.566860e-01 9.194223e-01

8885 7.568345e-01 9.194126e-01

8886 7.569862e-01 9.194025e-01

8887 7.571412e-01 9.193921e-01

8888 7.572996e-01 9.193814e-01

8889 7.574613e-01 9.193703e-01

8890 7.576265e-01 9.193589e-01

8891 7.577952e-01 9.193470e-01

8892 7.579674e-01 9.193348e-01

8893 7.581431e-01 9.193221e-01

8894 7.583226e-01 9.193090e-01

8895 7.585057e-01 9.192955e-01

8896 7.586925e-01 9.192815e-01

8897 7.588831e-01 9.192671e-01

8898 7.590775e-01 9.192521e-01

8899 7.592758e-01 9.192366e-01

8900 7.594780e-01 9.192207e-01

8901 7.596842e-01 9.192041e-01

8902 7.598943e-01 9.191870e-01

8903 7.601085e-01 9.191694e-01

8904 7.603267e-01 9.191511e-01

8905 7.605490e-01 9.191322e-01

8906 7.607754e-01 9.191127e-01

8907 7.610060e-01 9.190925e-01

8908 7.612408e-01 9.190716e-01

8909 7.614798e-01 9.190500e-01

8910 7.617231e-01 9.190277e-01

8911 7.619706e-01 9.190046e-01

8912 7.622224e-01 9.189807e-01

8913 7.624785e-01 9.189561e-01

8914 7.627389e-01 9.189306e-01

8915 7.630036e-01 9.189042e-01

8916 7.632727e-01 9.188770e-01

8917 7.635461e-01 9.188488e-01

8918 7.638238e-01 9.188197e-01

8919 7.641058e-01 9.187896e-01

8920 7.643922e-01 9.187586e-01

8921 7.646828e-01 9.187265e-01

8922 7.649777e-01 9.186933e-01

8923 7.652769e-01 9.186590e-01

8924 7.655804e-01 9.186237e-01

8925 7.658880e-01 9.185871e-01

8926 7.661999e-01 9.185494e-01

8927 7.665158e-01 9.185104e-01

8928 7.668358e-01 9.184702e-01

8929 7.671599e-01 9.184287e-01

8930 7.674880e-01 9.183859e-01

8931 7.678199e-01 9.183417e-01

8932 7.681558e-01 9.182961e-01

8933 7.684954e-01 9.182491e-01

8934 7.688387e-01 9.182006e-01

8935 7.691856e-01 9.181507e-01

8936 7.695360e-01 9.180992e-01

8937 7.698899e-01 9.180462e-01

8938 7.702471e-01 9.179915e-01

8939 7.706076e-01 9.179353e-01

8940 7.709711e-01 9.178774e-01

8941 7.713376e-01 9.178178e-01

8942 7.717070e-01 9.177566e-01

8943 7.720791e-01 9.176936e-01

8944 7.724538e-01 9.176288e-01

8945 7.728310e-01 9.175622e-01

8946 7.732104e-01 9.174939e-01

8947 7.735920e-01 9.174237e-01

8948 7.739756e-01 9.173517e-01

8949 7.743610e-01 9.172778e-01

8950 7.747481e-01 9.172020e-01

8951 7.751367e-01 9.171243e-01

8952 7.755265e-01 9.170447e-01

8953 7.759175e-01 9.169632e-01

8954 7.763095e-01 9.168798e-01

8955 7.767021e-01 9.167944e-01

8956 7.770954e-01 9.167071e-01

8957 7.774889e-01 9.166179e-01

8958 7.778827e-01 9.165267e-01

8959 7.782764e-01 9.164336e-01

8960 7.786698e-01 9.163386e-01

8961 7.790628e-01 9.162417e-01

8962 7.794552e-01 9.161429e-01

8963 7.798466e-01 9.160422e-01

8964 7.802370e-01 9.159397e-01

8965 7.806262e-01 9.158354e-01

8966 7.810138e-01 9.157293e-01

8967 7.813997e-01 9.156214e-01

8968 7.817838e-01 9.155118e-01

8969 7.821657e-01 9.154005e-01

8970 7.825454e-01 9.152876e-01

8971 7.829225e-01 9.151732e-01

8972 7.832970e-01 9.150572e-01

8973 7.836686e-01 9.149398e-01

8974 7.840371e-01 9.148209e-01

8975 7.844023e-01 9.147008e-01

8976 7.847642e-01 9.145794e-01

8977 7.851224e-01 9.144568e-01

8978 7.854768e-01 9.143331e-01

8979 7.858273e-01 9.142083e-01

8980 7.861737e-01 9.140827e-01

8981 7.865158e-01 9.139562e-01

8982 7.868536e-01 9.138290e-01

8983 7.871869e-01 9.137011e-01

8984 7.875154e-01 9.135727e-01

8985 7.878392e-01 9.134438e-01

8986 7.881582e-01 9.133146e-01

8987 7.884721e-01 9.131852e-01

8988 7.887809e-01 9.130557e-01

8989 7.890845e-01 9.129261e-01

8990 7.893828e-01 9.127967e-01

8991 7.896758e-01 9.126675e-01

8992 7.899634e-01 9.125387e-01

8993 7.902454e-01 9.124103e-01

8994 7.905220e-01 9.122825e-01

8995 7.907929e-01 9.121554e-01

8996 7.910582e-01 9.120291e-01

8997 7.913179e-01 9.119038e-01

8998 7.915719e-01 9.117795e-01

8999 7.918202e-01 9.116563e-01

9000 7.920628e-01 9.115345e-01

9001 7.922997e-01 9.114140e-01

9002 7.923086e-01 9.114130e-01

9003 7.923178e-01 9.114119e-01

9004 7.923270e-01 9.114108e-01

9005 7.923364e-01 9.114097e-01

9006 7.923460e-01 9.114086e-01

9007 7.923558e-01 9.114074e-01

9008 7.923657e-01 9.114062e-01

9009 7.923758e-01 9.114050e-01

9010 7.923860e-01 9.114037e-01

9011 7.923964e-01 9.114024e-01

9012 7.924069e-01 9.114011e-01

9013 7.924177e-01 9.113997e-01

9014 7.924286e-01 9.113983e-01

9015 7.924396e-01 9.113969e-01

9016 7.924509e-01 9.113954e-01

9017 7.924623e-01 9.113939e-01

9018 7.924738e-01 9.113923e-01

9019 7.924856e-01 9.113907e-01

9020 7.924975e-01 9.113891e-01

9021 7.925096e-01 9.113874e-01

9022 7.925218e-01 9.113856e-01

9023 7.925342e-01 9.113839e-01

9024 7.925468e-01 9.113820e-01

9025 7.925596e-01 9.113802e-01

9026 7.925725e-01 9.113782e-01

9027 7.925856e-01 9.113763e-01

9028 7.925989e-01 9.113742e-01

9029 7.926123e-01 9.113722e-01

9030 7.926259e-01 9.113700e-01

9031 7.926396e-01 9.113678e-01

9032 7.926536e-01 9.113656e-01

9033 7.926676e-01 9.113633e-01

9034 7.926819e-01 9.113609e-01

9035 7.926962e-01 9.113585e-01

9036 7.927108e-01 9.113560e-01

9037 7.927254e-01 9.113534e-01

9038 7.927402e-01 9.113508e-01

9039 7.927552e-01 9.113481e-01

9040 7.927702e-01 9.113453e-01

9041 7.927854e-01 9.113425e-01

9042 7.928007e-01 9.113395e-01

9043 7.928161e-01 9.113365e-01

9044 7.928316e-01 9.113335e-01

9045 7.928473e-01 9.113303e-01

9046 7.928629e-01 9.113271e-01

9047 7.928787e-01 9.113237e-01

9048 7.928945e-01 9.113203e-01

9049 7.929104e-01 9.113168e-01

9050 7.929263e-01 9.113132e-01

9051 7.929423e-01 9.113095e-01

9052 7.929582e-01 9.113057e-01

9053 7.929742e-01 9.113019e-01

9054 7.929901e-01 9.112979e-01

9055 7.930060e-01 9.112938e-01

9056 7.930219e-01 9.112896e-01

9057 7.930377e-01 9.112853e-01

9058 7.930534e-01 9.112809e-01

9059 7.930690e-01 9.112764e-01

9060 7.930844e-01 9.112717e-01

9061 7.930997e-01 9.112670e-01

9062 7.931149e-01 9.112621e-01

9063 7.931298e-01 9.112571e-01

9064 7.931445e-01 9.112519e-01

9065 7.931589e-01 9.112467e-01

9066 7.931731e-01 9.112413e-01

9067 7.931869e-01 9.112358e-01

9068 7.932004e-01 9.112301e-01

9069 7.932135e-01 9.112243e-01

9070 7.932261e-01 9.112184e-01

9071 7.932383e-01 9.112123e-01

9072 7.932501e-01 9.112061e-01

9073 7.932612e-01 9.111997e-01

9074 7.932718e-01 9.111932e-01

9075 7.932818e-01 9.111865e-01

9076 7.932911e-01 9.111797e-01

9077 7.932997e-01 9.111727e-01

9078 7.933075e-01 9.111655e-01

9079 7.933145e-01 9.111582e-01

9080 7.933206e-01 9.111507e-01

9081 7.933257e-01 9.111430e-01

9082 7.933299e-01 9.111352e-01

9083 7.933331e-01 9.111272e-01

9084 7.933351e-01 9.111191e-01

9085 7.933360e-01 9.111107e-01

9086 7.933356e-01 9.111022e-01

9087 7.933340e-01 9.110935e-01

9088 7.933309e-01 9.110847e-01

9089 7.933264e-01 9.110757e-01

9090 7.933204e-01 9.110664e-01

9091 7.933128e-01 9.110571e-01

9092 7.933035e-01 9.110475e-01

9093 7.932925e-01 9.110377e-01

9094 7.932797e-01 9.110278e-01

9095 7.932649e-01 9.110178e-01

9096 7.932481e-01 9.110075e-01

9097 7.932293e-01 9.109971e-01

9098 7.932083e-01 9.109865e-01

9099 7.931850e-01 9.109757e-01

9100 7.931594e-01 9.109648e-01

9101 7.931313e-01 9.109538e-01

9102 7.931007e-01 9.109426e-01

9103 7.930675e-01 9.109312e-01

9104 7.930316e-01 9.109197e-01

9105 7.929928e-01 9.109081e-01

9106 7.929512e-01 9.108964e-01

9107 7.929065e-01 9.108846e-01

9108 7.928588e-01 9.108726e-01

9109 7.928078e-01 9.108606e-01

9110 7.927535e-01 9.108485e-01

9111 7.926959e-01 9.108363e-01

9112 7.926348e-01 9.108241e-01

9113 7.925701e-01 9.108119e-01

9114 7.925017e-01 9.107996e-01

9115 7.924296e-01 9.107873e-01

9116 7.923536e-01 9.107751e-01

9117 7.922737e-01 9.107628e-01

9118 7.921898e-01 9.107507e-01

9119 7.921017e-01 9.107386e-01

9120 7.920095e-01 9.107267e-01

9121 7.919130e-01 9.107148e-01

9122 7.918123e-01 9.107032e-01

9123 7.917071e-01 9.106917e-01

9124 7.915974e-01 9.106804e-01

9125 7.914832e-01 9.106694e-01

9126 7.913645e-01 9.106587e-01

9127 7.912411e-01 9.106483e-01

9128 7.911130e-01 9.106383e-01

9129 7.909802e-01 9.106287e-01

9130 7.908427e-01 9.106195e-01

9131 7.907004e-01 9.106108e-01

9132 7.905534e-01 9.106026e-01

9133 7.904015e-01 9.105950e-01

9134 7.902448e-01 9.105880e-01

9135 7.900832e-01 9.105817e-01

9136 7.899169e-01 9.105762e-01

9137 7.897458e-01 9.105714e-01

9138 7.895699e-01 9.105674e-01

9139 7.893893e-01 9.105643e-01

9140 7.892040e-01 9.105621e-01

9141 7.890140e-01 9.105610e-01

9142 7.888194e-01 9.105609e-01

9143 7.886203e-01 9.105619e-01

9144 7.884168e-01 9.105641e-01

9145 7.882088e-01 9.105675e-01

9146 7.879966e-01 9.105723e-01

9147 7.877801e-01 9.105784e-01

9148 7.875596e-01 9.105860e-01

9149 7.873350e-01 9.105950e-01

9150 7.871066e-01 9.106056e-01

9151 7.868745e-01 9.106179e-01

9152 7.866387e-01 9.106318e-01

9153 7.863995e-01 9.106475e-01

9154 7.861569e-01 9.106651e-01

9155 7.859112e-01 9.106845e-01

9156 7.856625e-01 9.107058e-01

9157 7.854108e-01 9.107292e-01

9158 7.851565e-01 9.107547e-01

9159 7.848997e-01 9.107822e-01

9160 7.846405e-01 9.108120e-01

9161 7.843792e-01 9.108440e-01

9162 7.841159e-01 9.108783e-01

9163 7.838508e-01 9.109150e-01

9164 7.835840e-01 9.109540e-01

9165 7.833159e-01 9.109955e-01

9166 7.830465e-01 9.110394e-01

9167 7.827760e-01 9.110859e-01

9168 7.825047e-01 9.111349e-01

9169 7.822327e-01 9.111865e-01

9170 7.819602e-01 9.112407e-01

9171 7.816875e-01 9.112976e-01

9172 7.814146e-01 9.113570e-01

9173 7.811418e-01 9.114192e-01

9174 7.808692e-01 9.114840e-01

9175 7.805970e-01 9.115515e-01

9176 7.803255e-01 9.116216e-01

9177 7.800547e-01 9.116940e-01

9178 7.797848e-01 9.117685e-01

9179 7.795160e-01 9.118450e-01

9180 7.792485e-01 9.119231e-01

9181 7.789824e-01 9.120028e-01

9182 7.789717e-01 9.120025e-01

9183 7.789606e-01 9.120023e-01

9184 7.789490e-01 9.120020e-01

9185 7.789369e-01 9.120017e-01

9186 7.789244e-01 9.120013e-01

9187 7.789113e-01 9.120010e-01

9188 7.788976e-01 9.120007e-01

9189 7.788834e-01 9.120003e-01

9190 7.788686e-01 9.120000e-01

9191 7.788532e-01 9.119996e-01

9192 7.788371e-01 9.119992e-01

9193 7.788204e-01 9.119988e-01

9194 7.788031e-01 9.119983e-01

9195 7.787850e-01 9.119979e-01

9196 7.787662e-01 9.119974e-01

9197 7.787466e-01 9.119969e-01

9198 7.787263e-01 9.119964e-01

9199 7.787051e-01 9.119958e-01

9200 7.786831e-01 9.119953e-01

9201 7.786603e-01 9.119947e-01

9202 7.786365e-01 9.119941e-01

9203 7.786118e-01 9.119934e-01

9204 7.785861e-01 9.119927e-01

9205 7.785594e-01 9.119920e-01

9206 7.785317e-01 9.119913e-01

9207 7.785029e-01 9.119905e-01

9208 7.784729e-01 9.119897e-01

9209 7.784418e-01 9.119888e-01

9210 7.784095e-01 9.119880e-01

9211 7.783760e-01 9.119870e-01

9212 7.783411e-01 9.119861e-01

9213 7.783049e-01 9.119850e-01

9214 7.782673e-01 9.119840e-01

9215 7.782283e-01 9.119829e-01

9216 7.781878e-01 9.119817e-01

9217 7.781457e-01 9.119805e-01

9218 7.781020e-01 9.119792e-01

9219 7.780567e-01 9.119779e-01

9220 7.780097e-01 9.119765e-01

9221 7.779608e-01 9.119751e-01

9222 7.779102e-01 9.119736e-01

9223 7.778576e-01 9.119720e-01

9224 7.778031e-01 9.119704e-01

9225 7.777465e-01 9.119687e-01

9226 7.776878e-01 9.119669e-01

9227 7.776269e-01 9.119650e-01

9228 7.775637e-01 9.119630e-01

9229 7.774982e-01 9.119610e-01

9230 7.774303e-01 9.119588e-01

9231 7.773599e-01 9.119566e-01

9232 7.772869e-01 9.119543e-01

9233 7.772112e-01 9.119519e-01

9234 7.771328e-01 9.119493e-01

9235 7.770515e-01 9.119467e-01

9236 7.769672e-01 9.119439e-01

9237 7.768799e-01 9.119410e-01

9238 7.767895e-01 9.119380e-01

9239 7.766958e-01 9.119349e-01

9240 7.765987e-01 9.119316e-01

9241 7.764981e-01 9.119282e-01

9242 7.763940e-01 9.119247e-01

9243 7.762862e-01 9.119209e-01

9244 7.761745e-01 9.119171e-01

9245 7.760590e-01 9.119130e-01

9246 7.759394e-01 9.119088e-01

9247 7.758156e-01 9.119045e-01

9248 7.756874e-01 9.118999e-01

9249 7.755549e-01 9.118951e-01

9250 7.754178e-01 9.118902e-01

9251 7.752760e-01 9.118850e-01

9252 7.751293e-01 9.118796e-01

9253 7.749777e-01 9.118740e-01

9254 7.748209e-01 9.118682e-01

9255 7.746589e-01 9.118621e-01

9256 7.744914e-01 9.118558e-01

9257 7.743184e-01 9.118492e-01

9258 7.741397e-01 9.118423e-01

9259 7.739551e-01 9.118352e-01

9260 7.737645e-01 9.118277e-01

9261 7.735677e-01 9.118200e-01

9262 7.733646e-01 9.118120e-01

9263 7.731550e-01 9.118036e-01

9264 7.729388e-01 9.117949e-01

9265 7.727157e-01 9.117858e-01

9266 7.724857e-01 9.117764e-01

9267 7.722486e-01 9.117666e-01

9268 7.720042e-01 9.117565e-01

9269 7.717524e-01 9.117459e-01

9270 7.714930e-01 9.117349e-01

9271 7.712258e-01 9.117235e-01

9272 7.709508e-01 9.117117e-01

9273 7.706677e-01 9.116994e-01

9274 7.703764e-01 9.116866e-01

9275 7.700768e-01 9.116734e-01

9276 7.697688e-01 9.116596e-01

9277 7.694521e-01 9.116453e-01

9278 7.691267e-01 9.116305e-01

9279 7.687925e-01 9.116151e-01

9280 7.684493e-01 9.115992e-01

9281 7.680969e-01 9.115827e-01

9282 7.677354e-01 9.115656e-01

9283 7.673646e-01 9.115478e-01

9284 7.669844e-01 9.115294e-01

9285 7.665947e-01 9.115104e-01

9286 7.661955e-01 9.114906e-01

9287 7.657867e-01 9.114702e-01

9288 7.653682e-01 9.114490e-01

9289 7.649401e-01 9.114272e-01

9290 7.645022e-01 9.114045e-01

9291 7.640545e-01 9.113811e-01

9292 7.635971e-01 9.113569e-01

9293 7.631300e-01 9.113319e-01

9294 7.626531e-01 9.113060e-01

9295 7.621665e-01 9.112793e-01

9296 7.616703e-01 9.112517e-01

9297 7.611646e-01 9.112233e-01

9298 7.606493e-01 9.111939e-01

9299 7.601246e-01 9.111636e-01

9300 7.595907e-01 9.111324e-01

9301 7.590476e-01 9.111002e-01

9302 7.584955e-01 9.110671e-01

9303 7.579346e-01 9.110329e-01

9304 7.573650e-01 9.109978e-01

9305 7.567870e-01 9.109616e-01

9306 7.562007e-01 9.109244e-01

9307 7.556064e-01 9.108862e-01

9308 7.550043e-01 9.108469e-01

9309 7.543947e-01 9.108065e-01

9310 7.537779e-01 9.107650e-01

9311 7.531542e-01 9.107225e-01

9312 7.525238e-01 9.106788e-01

9313 7.518872e-01 9.106341e-01

9314 7.512446e-01 9.105882e-01

9315 7.505965e-01 9.105412e-01

9316 7.499431e-01 9.104931e-01

9317 7.492849e-01 9.104439e-01

9318 7.486223e-01 9.103935e-01

9319 7.479556e-01 9.103420e-01

9320 7.472853e-01 9.102894e-01

9321 7.466118e-01 9.102356e-01

9322 7.459355e-01 9.101807e-01

9323 7.452569e-01 9.101247e-01

9324 7.445764e-01 9.100676e-01

9325 7.438945e-01 9.100094e-01

9326 7.432115e-01 9.099501e-01

9327 7.425281e-01 9.098897e-01

9328 7.418445e-01 9.098282e-01

9329 7.411613e-01 9.097657e-01

9330 7.404789e-01 9.097022e-01

9331 7.397977e-01 9.096376e-01

9332 7.391183e-01 9.095720e-01

9333 7.384410e-01 9.095054e-01

9334 7.377662e-01 9.094379e-01

9335 7.370945e-01 9.093695e-01

9336 7.364262e-01 9.093001e-01

9337 7.357617e-01 9.092299e-01

9338 7.351015e-01 9.091588e-01

9339 7.344458e-01 9.090869e-01

9340 7.337951e-01 9.090142e-01

9341 7.331498e-01 9.089408e-01

9342 7.325101e-01 9.088666e-01

9343 7.318765e-01 9.087917e-01

9344 7.312492e-01 9.087162e-01

9345 7.306286e-01 9.086401e-01

9346 7.300149e-01 9.085634e-01

9347 7.294084e-01 9.084861e-01

9348 7.288094e-01 9.084084e-01

9349 7.282181e-01 9.083302e-01

9350 7.276346e-01 9.082516e-01

9351 7.270593e-01 9.081725e-01

9352 7.264924e-01 9.080932e-01

9353 7.259339e-01 9.080135e-01

9354 7.253840e-01 9.079336e-01

9355 7.248430e-01 9.078535e-01

9356 7.243108e-01 9.077733e-01

9357 7.237876e-01 9.076933e-01

9358 7.232735e-01 9.076138e-01

9359 7.227686e-01 9.075349e-01

9360 7.222729e-01 9.074569e-01

9361 7.217865e-01 9.073801e-01

9362 7.217518e-01 9.073787e-01

9363 7.217160e-01 9.073774e-01

9364 7.216789e-01 9.073759e-01

9365 7.216407e-01 9.073745e-01

9366 7.216012e-01 9.073730e-01

9367 7.215604e-01 9.073715e-01

9368 7.215183e-01 9.073699e-01

9369 7.214748e-01 9.073683e-01

9370 7.214299e-01 9.073666e-01

9371 7.213835e-01 9.073649e-01

9372 7.213357e-01 9.073632e-01

9373 7.212864e-01 9.073614e-01

9374 7.212354e-01 9.073595e-01

9375 7.211829e-01 9.073576e-01

9376 7.211286e-01 9.073557e-01

9377 7.210727e-01 9.073537e-01

9378 7.210150e-01 9.073516e-01

9379 7.209554e-01 9.073495e-01

9380 7.208940e-01 9.073474e-01

9381 7.208306e-01 9.073452e-01

9382 7.207652e-01 9.073429e-01

9383 7.206978e-01 9.073406e-01

9384 7.206282e-01 9.073382e-01

9385 7.205565e-01 9.073358e-01

9386 7.204825e-01 9.073333e-01

9387 7.204062e-01 9.073307e-01

9388 7.203276e-01 9.073281e-01

9389 7.202464e-01 9.073254e-01

9390 7.201628e-01 9.073226e-01

9391 7.200765e-01 9.073198e-01

9392 7.199876e-01 9.073169e-01

9393 7.198959e-01 9.073139e-01

9394 7.198014e-01 9.073109e-01

9395 7.197040e-01 9.073078e-01

9396 7.196036e-01 9.073046e-01

9397 7.195001e-01 9.073013e-01

9398 7.193934e-01 9.072980e-01

9399 7.192835e-01 9.072945e-01

9400 7.191702e-01 9.072910e-01

9401 7.190535e-01 9.072874e-01

9402 7.189333e-01 9.072837e-01

9403 7.188094e-01 9.072800e-01

9404 7.186818e-01 9.072761e-01

9405 7.185504e-01 9.072722e-01

9406 7.184150e-01 9.072681e-01

9407 7.182756e-01 9.072640e-01

9408 7.181320e-01 9.072598e-01

9409 7.179842e-01 9.072555e-01

9410 7.178320e-01 9.072510e-01

9411 7.176754e-01 9.072465e-01

9412 7.175141e-01 9.072419e-01

9413 7.173482e-01 9.072372e-01

9414 7.171774e-01 9.072323e-01

9415 7.170018e-01 9.072274e-01

9416 7.168210e-01 9.072224e-01

9417 7.166351e-01 9.072172e-01

9418 7.164438e-01 9.072120e-01

9419 7.162472e-01 9.072066e-01

9420 7.160450e-01 9.072012e-01

9421 7.158372e-01 9.071956e-01

9422 7.156235e-01 9.071899e-01

9423 7.154040e-01 9.071841e-01

9424 7.151784e-01 9.071782e-01

9425 7.149466e-01 9.071722e-01

9426 7.147085e-01 9.071660e-01

9427 7.144640e-01 9.071598e-01

9428 7.142130e-01 9.071534e-01

9429 7.139554e-01 9.071470e-01

9430 7.136909e-01 9.071404e-01

9431 7.134195e-01 9.071337e-01

9432 7.131411e-01 9.071269e-01

9433 7.128556e-01 9.071200e-01

9434 7.125628e-01 9.071130e-01

9435 7.122627e-01 9.071059e-01

9436 7.119550e-01 9.070987e-01

9437 7.116398e-01 9.070914e-01

9438 7.113169e-01 9.070839e-01

9439 7.109863e-01 9.070765e-01

9440 7.106478e-01 9.070689e-01

9441 7.103013e-01 9.070612e-01

9442 7.099467e-01 9.070534e-01

9443 7.095841e-01 9.070456e-01

9444 7.092133e-01 9.070377e-01

9445 7.088342e-01 9.070298e-01

9446 7.084469e-01 9.070218e-01

9447 7.080512e-01 9.070137e-01

9448 7.076471e-01 9.070056e-01

9449 7.072346e-01 9.069975e-01

9450 7.068137e-01 9.069894e-01

9451 7.063843e-01 9.069812e-01

9452 7.059464e-01 9.069731e-01

9453 7.055002e-01 9.069649e-01

9454 7.050455e-01 9.069568e-01

9455 7.045824e-01 9.069487e-01

9456 7.041111e-01 9.069407e-01

9457 7.036314e-01 9.069327e-01

9458 7.031435e-01 9.069248e-01

9459 7.026475e-01 9.069170e-01

9460 7.021435e-01 9.069094e-01

9461 7.016315e-01 9.069019e-01

9462 7.011118e-01 9.068945e-01

9463 7.005844e-01 9.068873e-01

9464 7.000495e-01 9.068803e-01

9465 6.995073e-01 9.068736e-01

9466 6.989579e-01 9.068671e-01

9467 6.984016e-01 9.068609e-01

9468 6.978386e-01 9.068550e-01

9469 6.972690e-01 9.068495e-01

9470 6.966932e-01 9.068443e-01

9471 6.961114e-01 9.068395e-01

9472 6.955238e-01 9.068352e-01

9473 6.949308e-01 9.068313e-01

9474 6.943326e-01 9.068279e-01

9475 6.937296e-01 9.068252e-01

9476 6.931221e-01 9.068229e-01

9477 6.925104e-01 9.068214e-01

9478 6.918948e-01 9.068205e-01

9479 6.912758e-01 9.068203e-01

9480 6.906537e-01 9.068209e-01

9481 6.900288e-01 9.068224e-01

9482 6.894016e-01 9.068246e-01

9483 6.887724e-01 9.068279e-01

9484 6.881417e-01 9.068320e-01

9485 6.875099e-01 9.068372e-01

9486 6.868773e-01 9.068435e-01

9487 6.862444e-01 9.068510e-01

9488 6.856116e-01 9.068596e-01

9489 6.849794e-01 9.068695e-01

9490 6.843480e-01 9.068807e-01

9491 6.837180e-01 9.068932e-01

9492 6.830898e-01 9.069073e-01

9493 6.824638e-01 9.069228e-01

9494 6.818404e-01 9.069398e-01

9495 6.812200e-01 9.069585e-01

9496 6.806029e-01 9.069789e-01

9497 6.799897e-01 9.070011e-01

9498 6.793806e-01 9.070251e-01

9499 6.787761e-01 9.070509e-01

9500 6.781765e-01 9.070788e-01

9501 6.775821e-01 9.071086e-01

9502 6.769934e-01 9.071406e-01

9503 6.764106e-01 9.071746e-01

9504 6.758340e-01 9.072109e-01

9505 6.752640e-01 9.072495e-01

9506 6.747008e-01 9.072904e-01

9507 6.741447e-01 9.073337e-01

9508 6.735960e-01 9.073795e-01

9509 6.730548e-01 9.074277e-01

9510 6.725215e-01 9.074786e-01

9511 6.719963e-01 9.075321e-01

9512 6.714792e-01 9.075882e-01

9513 6.709706e-01 9.076471e-01

9514 6.704706e-01 9.077087e-01

9515 6.699792e-01 9.077732e-01

9516 6.694967e-01 9.078405e-01

9517 6.690231e-01 9.079107e-01

9518 6.685585e-01 9.079838e-01

9519 6.681031e-01 9.080599e-01

9520 6.676568e-01 9.081389e-01

9521 6.672198e-01 9.082210e-01

9522 6.667920e-01 9.083061e-01

9523 6.663735e-01 9.083942e-01

9524 6.659642e-01 9.084853e-01

9525 6.655643e-01 9.085796e-01

9526 6.651735e-01 9.086768e-01

9527 6.647920e-01 9.087771e-01

9528 6.644197e-01 9.088805e-01

9529 6.640565e-01 9.089869e-01

9530 6.637024e-01 9.090963e-01

9531 6.633572e-01 9.092086e-01

9532 6.630209e-01 9.093239e-01

9533 6.626934e-01 9.094417e-01

9534 6.623746e-01 9.095619e-01

9535 6.620644e-01 9.096843e-01

9536 6.617626e-01 9.098086e-01

9537 6.614692e-01 9.099347e-01

9538 6.611840e-01 9.100623e-01

9539 6.609070e-01 9.101913e-01

9540 6.606378e-01 9.103215e-01

9541 6.603765e-01 9.104527e-01

9542 6.603388e-01 9.104525e-01

9543 6.603002e-01 9.104522e-01

9544 6.602607e-01 9.104519e-01

9545 6.602203e-01 9.104516e-01

9546 6.601789e-01 9.104513e-01

9547 6.601366e-01 9.104510e-01

9548 6.600933e-01 9.104507e-01

9549 6.600491e-01 9.104504e-01

9550 6.600038e-01 9.104500e-01

9551 6.599576e-01 9.104496e-01

9552 6.599104e-01 9.104493e-01

9553 6.598621e-01 9.104489e-01

9554 6.598128e-01 9.104485e-01

9555 6.597624e-01 9.104480e-01

9556 6.597110e-01 9.104476e-01

9557 6.596585e-01 9.104471e-01

9558 6.596049e-01 9.104466e-01

9559 6.595502e-01 9.104461e-01

9560 6.594944e-01 9.104456e-01

9561 6.594375e-01 9.104451e-01

9562 6.593794e-01 9.104445e-01

9563 6.593201e-01 9.104439e-01

9564 6.592597e-01 9.104433e-01

9565 6.591980e-01 9.104427e-01

9566 6.591352e-01 9.104420e-01

9567 6.590712e-01 9.104413e-01

9568 6.590059e-01 9.104406e-01

9569 6.589394e-01 9.104399e-01

9570 6.588717e-01 9.104391e-01

9571 6.588027e-01 9.104383e-01

9572 6.587324e-01 9.104375e-01

9573 6.586609e-01 9.104366e-01

9574 6.585881e-01 9.104357e-01

9575 6.585140e-01 9.104348e-01

9576 6.584386e-01 9.104338e-01

9577 6.583619e-01 9.104328e-01

9578 6.582839e-01 9.104317e-01

9579 6.582046e-01 9.104306e-01

9580 6.581240e-01 9.104295e-01

9581 6.580420e-01 9.104284e-01

9582 6.579588e-01 9.104272e-01

9583 6.578742e-01 9.104259e-01

9584 6.577884e-01 9.104246e-01

9585 6.577012e-01 9.104233e-01

9586 6.576127e-01 9.104219e-01

9587 6.575230e-01 9.104204e-01

9588 6.574319e-01 9.104189e-01

9589 6.573396e-01 9.104174e-01

9590 6.572461e-01 9.104158e-01

9591 6.571513e-01 9.104141e-01

9592 6.570552e-01 9.104124e-01

9593 6.569580e-01 9.104106e-01

9594 6.568596e-01 9.104088e-01

9595 6.567600e-01 9.104069e-01

9596 6.566593e-01 9.104050e-01

9597 6.565574e-01 9.104029e-01

9598 6.564545e-01 9.104008e-01

9599 6.563506e-01 9.103987e-01

9600 6.562456e-01 9.103965e-01

9601 6.561397e-01 9.103941e-01

9602 6.560328e-01 9.103918e-01

9603 6.559251e-01 9.103893e-01

9604 6.558165e-01 9.103868e-01

9605 6.557071e-01 9.103842e-01

9606 6.555970e-01 9.103815e-01

9607 6.554862e-01 9.103787e-01

9608 6.553748e-01 9.103758e-01

9609 6.552629e-01 9.103729e-01

9610 6.551504e-01 9.103698e-01

9611 6.550375e-01 9.103667e-01

9612 6.549243e-01 9.103635e-01

9613 6.548108e-01 9.103601e-01

9614 6.546971e-01 9.103567e-01

9615 6.545833e-01 9.103532e-01

9616 6.544695e-01 9.103496e-01

9617 6.543557e-01 9.103459e-01

9618 6.542421e-01 9.103420e-01

9619 6.541287e-01 9.103381e-01

9620 6.540157e-01 9.103340e-01

9621 6.539032e-01 9.103299e-01

9622 6.537911e-01 9.103256e-01

9623 6.536798e-01 9.103212e-01

9624 6.535692e-01 9.103167e-01

9625 6.534595e-01 9.103121e-01

9626 6.533508e-01 9.103073e-01

9627 6.532432e-01 9.103025e-01

9628 6.531368e-01 9.102975e-01

9629 6.530318e-01 9.102923e-01

9630 6.529283e-01 9.102871e-01

9631 6.528263e-01 9.102817e-01

9632 6.527261e-01 9.102762e-01

9633 6.526278e-01 9.102706e-01

9634 6.525314e-01 9.102648e-01

9635 6.524371e-01 9.102589e-01

9636 6.523451e-01 9.102528e-01

9637 6.522554e-01 9.102466e-01

9638 6.521682e-01 9.102403e-01

9639 6.520837e-01 9.102338e-01

9640 6.520019e-01 9.102272e-01

9641 6.519229e-01 9.102204e-01

9642 6.518469e-01 9.102135e-01

9643 6.517741e-01 9.102064e-01

9644 6.517044e-01 9.101992e-01

9645 6.516381e-01 9.101919e-01

9646 6.515753e-01 9.101844e-01

9647 6.515160e-01 9.101767e-01

9648 6.514603e-01 9.101689e-01

9649 6.514084e-01 9.101610e-01

9650 6.513604e-01 9.101529e-01

9651 6.513163e-01 9.101446e-01

9652 6.512762e-01 9.101362e-01

9653 6.512402e-01 9.101277e-01

9654 6.512083e-01 9.101190e-01

9655 6.511807e-01 9.101101e-01

9656 6.511574e-01 9.101011e-01

9657 6.511384e-01 9.100919e-01

9658 6.511238e-01 9.100826e-01

9659 6.511136e-01 9.100731e-01

9660 6.511079e-01 9.100635e-01

9661 6.511066e-01 9.100537e-01

9662 6.511098e-01 9.100438e-01

9663 6.511175e-01 9.100337e-01

9664 6.511297e-01 9.100235e-01

9665 6.511463e-01 9.100132e-01

9666 6.511674e-01 9.100026e-01

9667 6.511930e-01 9.099920e-01

9668 6.512229e-01 9.099812e-01

9669 6.512572e-01 9.099702e-01

9670 6.512958e-01 9.099591e-01

9671 6.513386e-01 9.099479e-01

9672 6.513856e-01 9.099365e-01

9673 6.514368e-01 9.099250e-01

9674 6.514920e-01 9.099133e-01

9675 6.515511e-01 9.099015e-01

9676 6.516141e-01 9.098895e-01

9677 6.516809e-01 9.098774e-01

9678 6.517513e-01 9.098651e-01

9679 6.518253e-01 9.098527e-01

9680 6.519028e-01 9.098402e-01

9681 6.519836e-01 9.098275e-01

9682 6.520676e-01 9.098146e-01

9683 6.521547e-01 9.098017e-01

9684 6.522448e-01 9.097885e-01

9685 6.523377e-01 9.097752e-01

9686 6.524334e-01 9.097618e-01

9687 6.525316e-01 9.097482e-01

9688 6.526323e-01 9.097344e-01

9689 6.527353e-01 9.097205e-01

9690 6.528404e-01 9.097064e-01

9691 6.529476e-01 9.096922e-01

9692 6.530567e-01 9.096778e-01

9693 6.531676e-01 9.096632e-01

9694 6.532801e-01 9.096485e-01

9695 6.533941e-01 9.096336e-01

9696 6.535094e-01 9.096185e-01

9697 6.536260e-01 9.096032e-01

9698 6.537437e-01 9.095877e-01

9699 6.538624e-01 9.095720e-01

9700 6.539820e-01 9.095562e-01

9701 6.541023e-01 9.095401e-01

9702 6.542232e-01 9.095239e-01

9703 6.543446e-01 9.095074e-01

9704 6.544664e-01 9.094907e-01

9705 6.545885e-01 9.094738e-01

9706 6.547107e-01 9.094567e-01

9707 6.548331e-01 9.094394e-01

9708 6.549554e-01 9.094218e-01

9709 6.550777e-01 9.094040e-01

9710 6.551997e-01 9.093859e-01

9711 6.553214e-01 9.093677e-01

9712 6.554428e-01 9.093492e-01

9713 6.555638e-01 9.093309e-01

9714 6.556843e-01 9.093128e-01

9715 6.558041e-01 9.092952e-01

9716 6.559234e-01 9.092781e-01

9717 6.560419e-01 9.092618e-01

9718 6.561597e-01 9.092464e-01

9719 6.562767e-01 9.092320e-01

9720 6.563928e-01 9.092187e-01

9721 6.565080e-01 9.092066e-01

9722 6.565294e-01 9.092053e-01

9723 6.565517e-01 9.092040e-01

9724 6.565748e-01 9.092027e-01

9725 6.565989e-01 9.092014e-01

9726 6.566240e-01 9.092000e-01

9727 6.566501e-01 9.091986e-01

9728 6.566772e-01 9.091971e-01

9729 6.567054e-01 9.091956e-01

9730 6.567346e-01 9.091941e-01

9731 6.567650e-01 9.091926e-01

9732 6.567966e-01 9.091910e-01

9733 6.568294e-01 9.091894e-01

9734 6.568634e-01 9.091878e-01

9735 6.568987e-01 9.091861e-01

9736 6.569353e-01 9.091844e-01

9737 6.569734e-01 9.091826e-01

9738 6.570128e-01 9.091809e-01

9739 6.570537e-01 9.091790e-01

9740 6.570961e-01 9.091772e-01

9741 6.571401e-01 9.091753e-01

9742 6.571857e-01 9.091734e-01

9743 6.572330e-01 9.091715e-01

9744 6.572819e-01 9.091695e-01

9745 6.573327e-01 9.091674e-01

9746 6.573853e-01 9.091654e-01

9747 6.574398e-01 9.091633e-01

9748 6.574962e-01 9.091612e-01

9749 6.575547e-01 9.091590e-01

9750 6.576153e-01 9.091568e-01

9751 6.576780e-01 9.091546e-01

9752 6.577429e-01 9.091523e-01

9753 6.578101e-01 9.091500e-01

9754 6.578797e-01 9.091476e-01

9755 6.579517e-01 9.091453e-01

9756 6.580262e-01 9.091428e-01

9757 6.581033e-01 9.091404e-01

9758 6.581830e-01 9.091379e-01

9759 6.582656e-01 9.091354e-01

9760 6.583509e-01 9.091328e-01

9761 6.584392e-01 9.091303e-01

9762 6.585304e-01 9.091277e-01

9763 6.586248e-01 9.091250e-01

9764 6.587224e-01 9.091223e-01

9765 6.588232e-01 9.091196e-01

9766 6.589274e-01 9.091169e-01

9767 6.590351e-01 9.091142e-01

9768 6.591464e-01 9.091114e-01

9769 6.592613e-01 9.091086e-01

9770 6.593801e-01 9.091058e-01

9771 6.595027e-01 9.091030e-01

9772 6.596293e-01 9.091001e-01

9773 6.597600e-01 9.090973e-01

9774 6.598950e-01 9.090944e-01

9775 6.600343e-01 9.090915e-01

9776 6.601780e-01 9.090886e-01

9777 6.603264e-01 9.090857e-01

9778 6.604794e-01 9.090828e-01

9779 6.606372e-01 9.090800e-01

9780 6.607999e-01 9.090771e-01

9781 6.609677e-01 9.090742e-01

9782 6.611407e-01 9.090714e-01

9783 6.613191e-01 9.090685e-01

9784 6.615028e-01 9.090657e-01

9785 6.616921e-01 9.090630e-01

9786 6.618872e-01 9.090602e-01

9787 6.620880e-01 9.090576e-01

9788 6.622948e-01 9.090549e-01

9789 6.625078e-01 9.090523e-01

9790 6.627269e-01 9.090498e-01

9791 6.629524e-01 9.090474e-01

9792 6.631844e-01 9.090450e-01

9793 6.634230e-01 9.090427e-01

9794 6.636684e-01 9.090405e-01

9795 6.639206e-01 9.090384e-01

9796 6.641799e-01 9.090364e-01

9797 6.644463e-01 9.090346e-01

9798 6.647200e-01 9.090329e-01

9799 6.650010e-01 9.090313e-01

9800 6.652896e-01 9.090299e-01

9801 6.655858e-01 9.090286e-01

9802 6.658898e-01 9.090275e-01

9803 6.662016e-01 9.090267e-01

9804 6.665213e-01 9.090260e-01

9805 6.668492e-01 9.090256e-01

9806 6.671852e-01 9.090254e-01

9807 6.675295e-01 9.090254e-01

9808 6.678821e-01 9.090257e-01

9809 6.682432e-01 9.090264e-01

9810 6.686128e-01 9.090273e-01

9811 6.689910e-01 9.090286e-01

9812 6.693778e-01 9.090302e-01

9813 6.697734e-01 9.090321e-01

9814 6.701777e-01 9.090345e-01

9815 6.705909e-01 9.090373e-01

9816 6.710129e-01 9.090405e-01

9817 6.714437e-01 9.090442e-01

9818 6.718835e-01 9.090484e-01

9819 6.723322e-01 9.090531e-01

9820 6.727898e-01 9.090584e-01

9821 6.732562e-01 9.090642e-01

9822 6.737315e-01 9.090706e-01

9823 6.742157e-01 9.090777e-01

9824 6.747087e-01 9.090854e-01

9825 6.752103e-01 9.090938e-01

9826 6.757207e-01 9.091030e-01

9827 6.762396e-01 9.091129e-01

9828 6.767671e-01 9.091236e-01

9829 6.773030e-01 9.091352e-01

9830 6.778471e-01 9.091477e-01

9831 6.783994e-01 9.091611e-01

9832 6.789597e-01 9.091754e-01

9833 6.795279e-01 9.091907e-01

9834 6.801038e-01 9.092071e-01

9835 6.806872e-01 9.092246e-01

9836 6.812780e-01 9.092432e-01

9837 6.818759e-01 9.092630e-01

9838 6.824808e-01 9.092840e-01

9839 6.830923e-01 9.093063e-01

9840 6.837104e-01 9.093299e-01

9841 6.843346e-01 9.093549e-01

9842 6.849648e-01 9.093813e-01

9843 6.856008e-01 9.094091e-01

9844 6.862421e-01 9.094385e-01

9845 6.868886e-01 9.094694e-01

9846 6.875399e-01 9.095020e-01

9847 6.881958e-01 9.095363e-01

9848 6.888558e-01 9.095722e-01

9849 6.895198e-01 9.096100e-01

9850 6.901874e-01 9.096496e-01

9851 6.908581e-01 9.096911e-01

9852 6.915318e-01 9.097346e-01

9853 6.922081e-01 9.097800e-01

9854 6.928865e-01 9.098275e-01

9855 6.935668e-01 9.098772e-01

9856 6.942486e-01 9.099290e-01

9857 6.949315e-01 9.099830e-01

9858 6.956152e-01 9.100393e-01

9859 6.962993e-01 9.100979e-01

9860 6.969835e-01 9.101590e-01

9861 6.976674e-01 9.102224e-01

9862 6.983506e-01 9.102884e-01

9863 6.990329e-01 9.103569e-01

9864 6.997137e-01 9.104280e-01

9865 7.003929e-01 9.105018e-01

9866 7.010700e-01 9.105782e-01

9867 7.017447e-01 9.106574e-01

9868 7.024167e-01 9.107394e-01

9869 7.030856e-01 9.108242e-01

9870 7.037512e-01 9.109119e-01

9871 7.044131e-01 9.110024e-01

9872 7.050710e-01 9.110960e-01

9873 7.057247e-01 9.111925e-01

9874 7.063738e-01 9.112921e-01

9875 7.070180e-01 9.113947e-01

9876 7.076571e-01 9.115004e-01

9877 7.082909e-01 9.116091e-01

9878 7.089191e-01 9.117211e-01

9879 7.095414e-01 9.118361e-01

9880 7.101577e-01 9.119544e-01

9881 7.107677e-01 9.120758e-01

9882 7.113712e-01 9.122004e-01

9883 7.119680e-01 9.123282e-01

9884 7.125580e-01 9.124592e-01

9885 7.131409e-01 9.125934e-01

9886 7.137167e-01 9.127308e-01

9887 7.142852e-01 9.128715e-01

9888 7.148463e-01 9.130152e-01

9889 7.153997e-01 9.131622e-01

9890 7.159455e-01 9.133122e-01

9891 7.164835e-01 9.134650e-01

9892 7.170137e-01 9.136204e-01

9893 7.175360e-01 9.137783e-01

9894 7.180502e-01 9.139383e-01

9895 7.185564e-01 9.141004e-01

9896 7.190545e-01 9.142644e-01

9897 7.195444e-01 9.144300e-01

9898 7.200262e-01 9.145971e-01

9899 7.204999e-01 9.147656e-01

9900 7.209653e-01 9.149354e-01

9901 7.214226e-01 9.151061e-01

9902 7.214663e-01 9.151062e-01

9903 7.215111e-01 9.151062e-01

9904 7.215573e-01 9.151062e-01

9905 7.216047e-01 9.151062e-01

9906 7.216534e-01 9.151062e-01

9907 7.217035e-01 9.151063e-01

9908 7.217549e-01 9.151063e-01

9909 7.218077e-01 9.151063e-01

9910 7.218620e-01 9.151063e-01

9911 7.219178e-01 9.151064e-01

9912 7.219750e-01 9.151064e-01

9913 7.220338e-01 9.151064e-01

9914 7.220941e-01 9.151064e-01

9915 7.221560e-01 9.151064e-01

9916 7.222196e-01 9.151064e-01

9917 7.222849e-01 9.151064e-01

9918 7.223519e-01 9.151065e-01

9919 7.224207e-01 9.151065e-01

9920 7.224912e-01 9.151065e-01

9921 7.225637e-01 9.151065e-01

9922 7.226380e-01 9.151065e-01

9923 7.227142e-01 9.151065e-01

9924 7.227925e-01 9.151065e-01

9925 7.228728e-01 9.151065e-01

9926 7.229551e-01 9.151065e-01

9927 7.230396e-01 9.151065e-01

9928 7.231263e-01 9.151065e-01

9929 7.232152e-01 9.151064e-01

9930 7.233064e-01 9.151064e-01

9931 7.233999e-01 9.151064e-01

9932 7.234958e-01 9.151064e-01

9933 7.235942e-01 9.151064e-01

9934 7.236950e-01 9.151064e-01

9935 7.237985e-01 9.151063e-01

9936 7.239045e-01 9.151063e-01

9937 7.240133e-01 9.151063e-01

9938 7.241248e-01 9.151063e-01

9939 7.242391e-01 9.151062e-01

9940 7.243562e-01 9.151062e-01

9941 7.244763e-01 9.151062e-01

9942 7.245994e-01 9.151062e-01

9943 7.247255e-01 9.151061e-01

9944 7.248548e-01 9.151061e-01

9945 7.249873e-01 9.151061e-01

9946 7.251230e-01 9.151060e-01

9947 7.252621e-01 9.151060e-01

9948 7.254046e-01 9.151060e-01

9949 7.255506e-01 9.151059e-01

9950 7.257001e-01 9.151059e-01

9951 7.258533e-01 9.151059e-01

9952 7.260101e-01 9.151059e-01

9953 7.261707e-01 9.151058e-01

9954 7.263352e-01 9.151058e-01

9955 7.265036e-01 9.151058e-01

9956 7.266760e-01 9.151058e-01

9957 7.268525e-01 9.151058e-01

9958 7.270332e-01 9.151058e-01

9959 7.272181e-01 9.151057e-01

9960 7.274074e-01 9.151057e-01

9961 7.276010e-01 9.151058e-01

9962 7.277992e-01 9.151058e-01

9963 7.280019e-01 9.151058e-01

9964 7.282092e-01 9.151058e-01

9965 7.284214e-01 9.151058e-01

9966 7.286383e-01 9.151059e-01

9967 7.288601e-01 9.151059e-01

9968 7.290869e-01 9.151060e-01

9969 7.293188e-01 9.151060e-01

9970 7.295558e-01 9.151061e-01

9971 7.297981e-01 9.151062e-01

9972 7.300457e-01 9.151063e-01

9973 7.302987e-01 9.151064e-01

9974 7.305572e-01 9.151065e-01

9975 7.308212e-01 9.151067e-01

9976 7.310909e-01 9.151068e-01

9977 7.313663e-01 9.151070e-01

9978 7.316475e-01 9.151072e-01

9979 7.319345e-01 9.151074e-01

9980 7.322275e-01 9.151076e-01

9981 7.325266e-01 9.151079e-01

9982 7.328317e-01 9.151081e-01

9983 7.331430e-01 9.151084e-01

9984 7.334605e-01 9.151087e-01

9985 7.337843e-01 9.151090e-01

9986 7.341145e-01 9.151094e-01

9987 7.344511e-01 9.151097e-01

9988 7.347942e-01 9.151101e-01

9989 7.351438e-01 9.151106e-01

9990 7.354999e-01 9.151110e-01

9991 7.358627e-01 9.151115e-01

9992 7.362322e-01 9.151120e-01

9993 7.366084e-01 9.151125e-01

9994 7.369913e-01 9.151131e-01

9995 7.373810e-01 9.151136e-01

9996 7.377775e-01 9.151143e-01

9997 7.381809e-01 9.151149e-01

9998 7.385910e-01 9.151156e-01

9999 7.390080e-01 9.151163e-01

10000 7.394319e-01 9.151170e-01

10001 7.398626e-01 9.151178e-01

10002 7.403001e-01 9.151186e-01

10003 7.407445e-01 9.151194e-01

10004 7.411957e-01 9.151203e-01

10005 7.416536e-01 9.151212e-01

10006 7.421183e-01 9.151221e-01

10007 7.425897e-01 9.151230e-01

10008 7.430677e-01 9.151240e-01

10009 7.435524e-01 9.151250e-01

10010 7.440435e-01 9.151261e-01

10011 7.445411e-01 9.151271e-01

10012 7.450451e-01 9.151282e-01

10013 7.455553e-01 9.151293e-01

10014 7.460717e-01 9.151305e-01

10015 7.465942e-01 9.151316e-01

10016 7.471227e-01 9.151328e-01

10017 7.476570e-01 9.151340e-01

10018 7.481970e-01 9.151352e-01

10019 7.487425e-01 9.151364e-01

10020 7.492934e-01 9.151376e-01

10021 7.498496e-01 9.151388e-01

10022 7.504109e-01 9.151400e-01

10023 7.509770e-01 9.151413e-01

10024 7.515479e-01 9.151425e-01

10025 7.521232e-01 9.151437e-01

10026 7.527029e-01 9.151449e-01

10027 7.532867e-01 9.151460e-01

10028 7.538743e-01 9.151472e-01

10029 7.544656e-01 9.151483e-01

10030 7.550603e-01 9.151494e-01

10031 7.556582e-01 9.151504e-01

10032 7.562590e-01 9.151514e-01

10033 7.568625e-01 9.151523e-01

10034 7.574683e-01 9.151532e-01

10035 7.580764e-01 9.151540e-01

10036 7.586862e-01 9.151548e-01

10037 7.592977e-01 9.151555e-01

10038 7.599104e-01 9.151561e-01

10039 7.605242e-01 9.151566e-01

10040 7.611387e-01 9.151570e-01

10041 7.617536e-01 9.151573e-01

10042 7.623686e-01 9.151576e-01

10043 7.629835e-01 9.151576e-01

10044 7.635979e-01 9.151576e-01

10045 7.642115e-01 9.151575e-01

10046 7.648241e-01 9.151572e-01

10047 7.654353e-01 9.151567e-01

10048 7.660449e-01 9.151561e-01

10049 7.666524e-01 9.151554e-01

10050 7.672578e-01 9.151544e-01

10051 7.678605e-01 9.151533e-01

10052 7.684605e-01 9.151521e-01

10053 7.690573e-01 9.151506e-01

10054 7.696507e-01 9.151489e-01

10055 7.702404e-01 9.151471e-01

10056 7.708262e-01 9.151450e-01

10057 7.714077e-01 9.151427e-01

10058 7.719848e-01 9.151402e-01

10059 7.725571e-01 9.151375e-01

10060 7.731245e-01 9.151345e-01

10061 7.736867e-01 9.151313e-01

10062 7.742434e-01 9.151278e-01

10063 7.747945e-01 9.151241e-01

10064 7.753397e-01 9.151202e-01

10065 7.758789e-01 9.151159e-01

10066 7.764117e-01 9.151114e-01

10067 7.769382e-01 9.151067e-01

10068 7.774581e-01 9.151016e-01

10069 7.779711e-01 9.150963e-01

10070 7.784773e-01 9.150908e-01

10071 7.789764e-01 9.150853e-01

10072 7.794682e-01 9.150801e-01

10073 7.799528e-01 9.150751e-01

10074 7.804300e-01 9.150707e-01

10075 7.808996e-01 9.150670e-01

10076 7.813616e-01 9.150641e-01

10077 7.818159e-01 9.150621e-01

10078 7.822624e-01 9.150611e-01

10079 7.827011e-01 9.150613e-01

10080 7.831319e-01 9.150627e-01

10081 7.835548e-01 9.150654e-01

10082 7.835737e-01 9.150642e-01

10083 7.835929e-01 9.150629e-01

10084 7.836127e-01 9.150616e-01

10085 7.836329e-01 9.150603e-01

10086 7.836536e-01 9.150590e-01

10087 7.836748e-01 9.150576e-01

10088 7.836965e-01 9.150562e-01

10089 7.837188e-01 9.150548e-01

10090 7.837415e-01 9.150534e-01

10091 7.837648e-01 9.150519e-01

10092 7.837887e-01 9.150504e-01

10093 7.838131e-01 9.150489e-01

10094 7.838380e-01 9.150474e-01

10095 7.838636e-01 9.150458e-01

10096 7.838898e-01 9.150442e-01

10097 7.839165e-01 9.150426e-01

10098 7.839439e-01 9.150410e-01

10099 7.839720e-01 9.150393e-01

10100 7.840006e-01 9.150376e-01

10101 7.840300e-01 9.150359e-01

10102 7.840600e-01 9.150342e-01

10103 7.840907e-01 9.150324e-01

10104 7.841221e-01 9.150306e-01

10105 7.841542e-01 9.150288e-01

10106 7.841871e-01 9.150269e-01

10107 7.842207e-01 9.150251e-01

10108 7.842551e-01 9.150232e-01

10109 7.842903e-01 9.150213e-01

10110 7.843262e-01 9.150193e-01

10111 7.843630e-01 9.150174e-01

10112 7.844006e-01 9.150154e-01

10113 7.844390e-01 9.150134e-01

10114 7.844783e-01 9.150113e-01

10115 7.845184e-01 9.150093e-01

10116 7.845595e-01 9.150072e-01

10117 7.846014e-01 9.150052e-01

10118 7.846443e-01 9.150030e-01

10119 7.846881e-01 9.150009e-01

10120 7.847329e-01 9.149988e-01

10121 7.847786e-01 9.149966e-01

10122 7.848253e-01 9.149945e-01

10123 7.848730e-01 9.149923e-01

10124 7.849218e-01 9.149901e-01

10125 7.849716e-01 9.149879e-01

10126 7.850224e-01 9.149857e-01

10127 7.850743e-01 9.149835e-01

10128 7.851273e-01 9.149813e-01

10129 7.851814e-01 9.149791e-01

10130 7.852366e-01 9.149769e-01

10131 7.852929e-01 9.149746e-01

10132 7.853504e-01 9.149724e-01

10133 7.854090e-01 9.149702e-01

10134 7.854688e-01 9.149680e-01

10135 7.855298e-01 9.149658e-01

10136 7.855920e-01 9.149636e-01

10137 7.856554e-01 9.149615e-01

10138 7.857201e-01 9.149593e-01

10139 7.857860e-01 9.149572e-01

10140 7.858531e-01 9.149551e-01

10141 7.859215e-01 9.149531e-01

10142 7.859912e-01 9.149511e-01

10143 7.860622e-01 9.149491e-01

10144 7.861345e-01 9.149471e-01

10145 7.862080e-01 9.149453e-01

10146 7.862829e-01 9.149434e-01

10147 7.863591e-01 9.149416e-01

10148 7.864367e-01 9.149399e-01

10149 7.865156e-01 9.149383e-01

10150 7.865958e-01 9.149367e-01

10151 7.866773e-01 9.149352e-01

10152 7.867602e-01 9.149338e-01

10153 7.868445e-01 9.149325e-01

10154 7.869301e-01 9.149313e-01

10155 7.870170e-01 9.149302e-01

10156 7.871053e-01 9.149292e-01

10157 7.871949e-01 9.149283e-01

10158 7.872858e-01 9.149276e-01

10159 7.873781e-01 9.149270e-01

10160 7.874717e-01 9.149266e-01

10161 7.875666e-01 9.149263e-01

10162 7.876627e-01 9.149262e-01

10163 7.877602e-01 9.149263e-01

10164 7.878590e-01 9.149265e-01

10165 7.879589e-01 9.149270e-01

10166 7.880602e-01 9.149277e-01

10167 7.881626e-01 9.149286e-01

10168 7.882662e-01 9.149298e-01

10169 7.883710e-01 9.149312e-01

10170 7.884769e-01 9.149329e-01

10171 7.885839e-01 9.149349e-01

10172 7.886920e-01 9.149372e-01

10173 7.888011e-01 9.149398e-01

10174 7.889113e-01 9.149427e-01

10175 7.890224e-01 9.149460e-01

10176 7.891344e-01 9.149497e-01

10177 7.892473e-01 9.149537e-01

10178 7.893611e-01 9.149581e-01

10179 7.894756e-01 9.149630e-01

10180 7.895908e-01 9.149684e-01

10181 7.897068e-01 9.149742e-01

10182 7.898233e-01 9.149804e-01

10183 7.899404e-01 9.149873e-01

10184 7.900580e-01 9.149946e-01

10185 7.901761e-01 9.150025e-01

10186 7.902945e-01 9.150110e-01

10187 7.904132e-01 9.150202e-01

10188 7.905322e-01 9.150299e-01

10189 7.906513e-01 9.150404e-01

10190 7.907705e-01 9.150515e-01

10191 7.908896e-01 9.150634e-01

10192 7.910087e-01 9.150761e-01

10193 7.911276e-01 9.150895e-01

10194 7.912463e-01 9.151038e-01

10195 7.913646e-01 9.151190e-01

10196 7.914824e-01 9.151350e-01

10197 7.915997e-01 9.151520e-01

10198 7.917164e-01 9.151699e-01

10199 7.918324e-01 9.151888e-01

10200 7.919475e-01 9.152088e-01

10201 7.920618e-01 9.152299e-01

10202 7.921750e-01 9.152520e-01

10203 7.922870e-01 9.152754e-01

10204 7.923979e-01 9.152999e-01

10205 7.925074e-01 9.153257e-01

10206 7.926154e-01 9.153527e-01

10207 7.927220e-01 9.153811e-01

10208 7.928269e-01 9.154108e-01

10209 7.929300e-01 9.154420e-01

10210 7.930313e-01 9.154746e-01

10211 7.931306e-01 9.155087e-01

10212 7.932279e-01 9.155444e-01

10213 7.933230e-01 9.155816e-01

10214 7.934158e-01 9.156205e-01

10215 7.935063e-01 9.156610e-01

10216 7.935943e-01 9.157033e-01

10217 7.936798e-01 9.157474e-01

10218 7.937627e-01 9.157933e-01

10219 7.938428e-01 9.158411e-01

10220 7.939201e-01 9.158907e-01

10221 7.939946e-01 9.159424e-01

10222 7.940660e-01 9.159961e-01

10223 7.941344e-01 9.160518e-01

10224 7.941997e-01 9.161096e-01

10225 7.942618e-01 9.161696e-01

10226 7.943207e-01 9.162318e-01

10227 7.943763e-01 9.162962e-01

10228 7.944285e-01 9.163629e-01

10229 7.944773e-01 9.164319e-01

10230 7.945226e-01 9.165034e-01

10231 7.945645e-01 9.165772e-01

10232 7.946029e-01 9.166535e-01

10233 7.946377e-01 9.167323e-01

10234 7.946689e-01 9.168136e-01

10235 7.946966e-01 9.168975e-01

10236 7.947207e-01 9.169840e-01

10237 7.947412e-01 9.170731e-01

10238 7.947582e-01 9.171649e-01

10239 7.947715e-01 9.172594e-01

10240 7.947813e-01 9.173566e-01

10241 7.947875e-01 9.174566e-01

10242 7.947903e-01 9.175593e-01

10243 7.947895e-01 9.176649e-01

10244 7.947853e-01 9.177732e-01

10245 7.947776e-01 9.178844e-01

10246 7.947666e-01 9.179984e-01

10247 7.947523e-01 9.181153e-01

10248 7.947347e-01 9.182350e-01

10249 7.947140e-01 9.183575e-01

10250 7.946901e-01 9.184829e-01

10251 7.946631e-01 9.186112e-01

10252 7.946331e-01 9.187423e-01

10253 7.946002e-01 9.188762e-01

10254 7.945644e-01 9.190126e-01

10255 7.945259e-01 9.191514e-01

10256 7.944847e-01 9.192923e-01

10257 7.944410e-01 9.194353e-01

10258 7.943947e-01 9.195801e-01

10259 7.943460e-01 9.197266e-01

10260 7.942949e-01 9.198746e-01

10261 7.942417e-01 9.200241e-01

10262 7.942399e-01 9.200240e-01

10263 7.942379e-01 9.200240e-01

10264 7.942357e-01 9.200240e-01

10265 7.942334e-01 9.200240e-01

10266 7.942308e-01 9.200239e-01

10267 7.942281e-01 9.200239e-01

10268 7.942251e-01 9.200239e-01

10269 7.942219e-01 9.200238e-01

10270 7.942184e-01 9.200238e-01

10271 7.942148e-01 9.200238e-01

10272 7.942108e-01 9.200237e-01

10273 7.942066e-01 9.200237e-01

10274 7.942021e-01 9.200236e-01

10275 7.941973e-01 9.200236e-01

10276 7.941922e-01 9.200235e-01

10277 7.941867e-01 9.200235e-01

10278 7.941810e-01 9.200234e-01

10279 7.941748e-01 9.200233e-01

10280 7.941683e-01 9.200233e-01

10281 7.941615e-01 9.200232e-01

10282 7.941542e-01 9.200231e-01

10283 7.941465e-01 9.200231e-01

10284 7.941383e-01 9.200230e-01

10285 7.941297e-01 9.200229e-01

10286 7.941206e-01 9.200228e-01

10287 7.941110e-01 9.200227e-01

10288 7.941009e-01 9.200226e-01

10289 7.940902e-01 9.200224e-01

10290 7.940790e-01 9.200223e-01

10291 7.940672e-01 9.200222e-01

10292 7.940547e-01 9.200220e-01

10293 7.940417e-01 9.200219e-01

10294 7.940279e-01 9.200217e-01

10295 7.940134e-01 9.200216e-01

10296 7.939982e-01 9.200214e-01

10297 7.939823e-01 9.200212e-01

10298 7.939655e-01 9.200210e-01

10299 7.939480e-01 9.200209e-01

10300 7.939295e-01 9.200206e-01

10301 7.939102e-01 9.200204e-01

10302 7.938899e-01 9.200202e-01

10303 7.938687e-01 9.200200e-01

10304 7.938464e-01 9.200197e-01

10305 7.938231e-01 9.200194e-01

10306 7.937986e-01 9.200192e-01

10307 7.937731e-01 9.200189e-01

10308 7.937463e-01 9.200186e-01

10309 7.937183e-01 9.200183e-01

10310 7.936890e-01 9.200179e-01

10311 7.936584e-01 9.200176e-01

10312 7.936264e-01 9.200172e-01

10313 7.935929e-01 9.200169e-01

10314 7.935579e-01 9.200165e-01

10315 7.935213e-01 9.200160e-01

10316 7.934831e-01 9.200156e-01

10317 7.934433e-01 9.200152e-01

10318 7.934016e-01 9.200147e-01

10319 7.933581e-01 9.200142e-01

10320 7.933128e-01 9.200137e-01

10321 7.932654e-01 9.200132e-01

10322 7.932161e-01 9.200126e-01

10323 7.931645e-01 9.200120e-01

10324 7.931108e-01 9.200114e-01

10325 7.930549e-01 9.200108e-01

10326 7.929965e-01 9.200101e-01

10327 7.929357e-01 9.200094e-01

10328 7.928723e-01 9.200087e-01

10329 7.928063e-01 9.200079e-01

10330 7.927376e-01 9.200071e-01

10331 7.926661e-01 9.200063e-01

10332 7.925916e-01 9.200054e-01

10333 7.925141e-01 9.200045e-01

10334 7.924335e-01 9.200036e-01

10335 7.923496e-01 9.200026e-01

10336 7.922624e-01 9.200016e-01

10337 7.921717e-01 9.200005e-01

10338 7.920774e-01 9.199994e-01

10339 7.919795e-01 9.199982e-01

10340 7.918777e-01 9.199970e-01

10341 7.917720e-01 9.199958e-01

10342 7.916622e-01 9.199944e-01

10343 7.915483e-01 9.199931e-01

10344 7.914300e-01 9.199916e-01

10345 7.913073e-01 9.199901e-01

10346 7.911800e-01 9.199885e-01

10347 7.910480e-01 9.199869e-01

10348 7.909112e-01 9.199852e-01

10349 7.907694e-01 9.199834e-01

10350 7.906224e-01 9.199815e-01

10351 7.904702e-01 9.199796e-01

10352 7.903126e-01 9.199775e-01

10353 7.901495e-01 9.199754e-01

10354 7.899807e-01 9.199732e-01

10355 7.898061e-01 9.199709e-01

10356 7.896255e-01 9.199685e-01

10357 7.894388e-01 9.199660e-01

10358 7.892459e-01 9.199634e-01

10359 7.890466e-01 9.199606e-01

10360 7.888408e-01 9.199578e-01

10361 7.886283e-01 9.199548e-01

10362 7.884091e-01 9.199517e-01

10363 7.881829e-01 9.199485e-01

10364 7.879497e-01 9.199451e-01

10365 7.877093e-01 9.199416e-01

10366 7.874617e-01 9.199379e-01

10367 7.872066e-01 9.199341e-01

10368 7.869441e-01 9.199301e-01

10369 7.866739e-01 9.199260e-01

10370 7.863960e-01 9.199217e-01

10371 7.861102e-01 9.199172e-01

10372 7.858166e-01 9.199125e-01

10373 7.855150e-01 9.199076e-01

10374 7.852054e-01 9.199025e-01

10375 7.848876e-01 9.198972e-01

10376 7.845617e-01 9.198917e-01

10377 7.842275e-01 9.198860e-01

10378 7.838851e-01 9.198801e-01

10379 7.835344e-01 9.198739e-01

10380 7.831754e-01 9.198675e-01

10381 7.828080e-01 9.198608e-01

10382 7.824324e-01 9.198538e-01

10383 7.820485e-01 9.198466e-01

10384 7.816564e-01 9.198391e-01

10385 7.812561e-01 9.198314e-01

10386 7.808476e-01 9.198233e-01

10387 7.804310e-01 9.198149e-01

10388 7.800065e-01 9.198063e-01

10389 7.795740e-01 9.197973e-01

10390 7.791338e-01 9.197880e-01

10391 7.786859e-01 9.197783e-01

10392 7.782305e-01 9.197683e-01

10393 7.777677e-01 9.197579e-01

10394 7.772978e-01 9.197472e-01

10395 7.768208e-01 9.197361e-01

10396 7.763370e-01 9.197247e-01

10397 7.758467e-01 9.197128e-01

10398 7.753499e-01 9.197006e-01

10399 7.748470e-01 9.196879e-01

10400 7.743382e-01 9.196749e-01

10401 7.738238e-01 9.196614e-01

10402 7.733041e-01 9.196475e-01

10403 7.727793e-01 9.196331e-01

10404 7.722497e-01 9.196183e-01

10405 7.717157e-01 9.196031e-01

10406 7.711776e-01 9.195873e-01

10407 7.706356e-01 9.195712e-01

10408 7.700902e-01 9.195545e-01

10409 7.695417e-01 9.195374e-01

10410 7.689904e-01 9.195197e-01

10411 7.684366e-01 9.195016e-01

10412 7.678808e-01 9.194830e-01

10413 7.673233e-01 9.194639e-01

10414 7.667645e-01 9.194443e-01

10415 7.662047e-01 9.194241e-01

10416 7.656443e-01 9.194034e-01

10417 7.650836e-01 9.193822e-01

10418 7.645232e-01 9.193605e-01

10419 7.639632e-01 9.193383e-01

10420 7.634041e-01 9.193155e-01

10421 7.628462e-01 9.192922e-01

10422 7.622899e-01 9.192683e-01

10423 7.617356e-01 9.192439e-01

10424 7.611835e-01 9.192190e-01

10425 7.606340e-01 9.191935e-01

10426 7.600875e-01 9.191675e-01

10427 7.595442e-01 9.191409e-01

10428 7.590045e-01 9.191138e-01

10429 7.584687e-01 9.190862e-01

10430 7.579370e-01 9.190580e-01

10431 7.574097e-01 9.190293e-01

10432 7.568872e-01 9.190000e-01

10433 7.563695e-01 9.189703e-01

10434 7.558571e-01 9.189404e-01

10435 7.553501e-01 9.189104e-01

10436 7.548487e-01 9.188805e-01

10437 7.543532e-01 9.188508e-01

10438 7.538637e-01 9.188216e-01

10439 7.533804e-01 9.187928e-01

10440 7.529034e-01 9.187647e-01

10441 7.524330e-01 9.187373e-01

10442 7.524094e-01 9.187358e-01

10443 7.523850e-01 9.187342e-01

10444 7.523596e-01 9.187325e-01

10445 7.523334e-01 9.187308e-01

10446 7.523062e-01 9.187291e-01

10447 7.522780e-01 9.187274e-01

10448 7.522488e-01 9.187256e-01

10449 7.522186e-01 9.187238e-01

10450 7.521873e-01 9.187220e-01

10451 7.521549e-01 9.187201e-01

10452 7.521213e-01 9.187182e-01

10453 7.520866e-01 9.187162e-01

10454 7.520506e-01 9.187142e-01

10455 7.520134e-01 9.187122e-01

10456 7.519749e-01 9.187101e-01

10457 7.519350e-01 9.187080e-01

10458 7.518937e-01 9.187059e-01

10459 7.518510e-01 9.187037e-01

10460 7.518067e-01 9.187015e-01

10461 7.517609e-01 9.186992e-01

10462 7.517136e-01 9.186969e-01

10463 7.516645e-01 9.186946e-01

10464 7.516138e-01 9.186922e-01

10465 7.515613e-01 9.186898e-01

10466 7.515070e-01 9.186873e-01

10467 7.514508e-01 9.186848e-01

10468 7.513926e-01 9.186823e-01

10469 7.513325e-01 9.186797e-01

10470 7.512702e-01 9.186770e-01

10471 7.512058e-01 9.186744e-01

10472 7.511392e-01 9.186716e-01

10473 7.510703e-01 9.186689e-01

10474 7.509991e-01 9.186661e-01

10475 7.509254e-01 9.186632e-01

10476 7.508491e-01 9.186603e-01

10477 7.507703e-01 9.186574e-01

10478 7.506887e-01 9.186544e-01

10479 7.506044e-01 9.186514e-01

10480 7.505172e-01 9.186483e-01

10481 7.504271e-01 9.186452e-01

10482 7.503339e-01 9.186421e-01

10483 7.502375e-01 9.186389e-01

10484 7.501379e-01 9.186356e-01

10485 7.500349e-01 9.186323e-01

10486 7.499285e-01 9.186290e-01

10487 7.498185e-01 9.186256e-01

10488 7.497049e-01 9.186222e-01

10489 7.495874e-01 9.186187e-01

10490 7.494661e-01 9.186152e-01

10491 7.493407e-01 9.186117e-01

10492 7.492112e-01 9.186081e-01

10493 7.490774e-01 9.186044e-01

10494 7.489392e-01 9.186007e-01

10495 7.487965e-01 9.185970e-01

10496 7.486491e-01 9.185933e-01

10497 7.484970e-01 9.185895e-01

10498 7.483399e-01 9.185857e-01

10499 7.481777e-01 9.185818e-01

10500 7.480103e-01 9.185779e-01

10501 7.478376e-01 9.185739e-01

10502 7.476594e-01 9.185700e-01

10503 7.474755e-01 9.185660e-01

10504 7.472858e-01 9.185620e-01

10505 7.470901e-01 9.185579e-01

10506 7.468883e-01 9.185538e-01

10507 7.466803e-01 9.185497e-01

10508 7.464658e-01 9.185456e-01

10509 7.462447e-01 9.185415e-01

10510 7.460169e-01 9.185373e-01

10511 7.457821e-01 9.185331e-01

10512 7.455402e-01 9.185289e-01

10513 7.452911e-01 9.185248e-01

10514 7.450346e-01 9.185206e-01

10515 7.447705e-01 9.185164e-01

10516 7.444986e-01 9.185122e-01

10517 7.442188e-01 9.185080e-01

10518 7.439310e-01 9.185038e-01

10519 7.436349e-01 9.184997e-01

10520 7.433304e-01 9.184956e-01

10521 7.430173e-01 9.184915e-01

10522 7.426955e-01 9.184874e-01

10523 7.423649e-01 9.184834e-01

10524 7.420252e-01 9.184794e-01

10525 7.416763e-01 9.184754e-01

10526 7.413182e-01 9.184715e-01

10527 7.409505e-01 9.184677e-01

10528 7.405733e-01 9.184640e-01

10529 7.401864e-01 9.184603e-01

10530 7.397896e-01 9.184567e-01

10531 7.393828e-01 9.184532e-01

10532 7.389661e-01 9.184499e-01

10533 7.385391e-01 9.184466e-01

10534 7.381019e-01 9.184435e-01

10535 7.376544e-01 9.184405e-01

10536 7.371965e-01 9.184376e-01

10537 7.367282e-01 9.184349e-01

10538 7.362493e-01 9.184324e-01

10539 7.357599e-01 9.184300e-01

10540 7.352600e-01 9.184279e-01

10541 7.347495e-01 9.184259e-01

10542 7.342284e-01 9.184242e-01

10543 7.336968e-01 9.184227e-01

10544 7.331547e-01 9.184215e-01

10545 7.326021e-01 9.184205e-01

10546 7.320391e-01 9.184199e-01

10547 7.314658e-01 9.184195e-01

10548 7.308824e-01 9.184195e-01

10549 7.302888e-01 9.184198e-01

10550 7.296852e-01 9.184204e-01

10551 7.290719e-01 9.184215e-01

10552 7.284489e-01 9.184229e-01

10553 7.278165e-01 9.184248e-01

10554 7.271748e-01 9.184271e-01

10555 7.265242e-01 9.184299e-01

10556 7.258648e-01 9.184332e-01

10557 7.251969e-01 9.184370e-01

10558 7.245207e-01 9.184414e-01

10559 7.238367e-01 9.184463e-01

10560 7.231451e-01 9.184519e-01

10561 7.224462e-01 9.184581e-01

10562 7.217404e-01 9.184649e-01

10563 7.210281e-01 9.184724e-01

10564 7.203096e-01 9.184807e-01

10565 7.195854e-01 9.184896e-01

10566 7.188559e-01 9.184994e-01

10567 7.181215e-01 9.185100e-01

10568 7.173827e-01 9.185214e-01

10569 7.166399e-01 9.185337e-01

10570 7.158935e-01 9.185470e-01

10571 7.151441e-01 9.185611e-01

10572 7.143921e-01 9.185763e-01

10573 7.136381e-01 9.185925e-01

10574 7.128825e-01 9.186097e-01

10575 7.121258e-01 9.186281e-01

10576 7.113685e-01 9.186475e-01

10577 7.106112e-01 9.186682e-01

10578 7.098543e-01 9.186900e-01

10579 7.090984e-01 9.187131e-01

10580 7.083440e-01 9.187375e-01

10581 7.075916e-01 9.187632e-01

10582 7.068416e-01 9.187903e-01

10583 7.060945e-01 9.188188e-01

10584 7.053509e-01 9.188488e-01

10585 7.046113e-01 9.188802e-01

10586 7.038760e-01 9.189131e-01

10587 7.031455e-01 9.189476e-01

10588 7.024204e-01 9.189837e-01

10589 7.017010e-01 9.190215e-01

10590 7.009877e-01 9.190609e-01

10591 7.002810e-01 9.191020e-01

10592 6.995811e-01 9.191449e-01

10593 6.988886e-01 9.191896e-01

10594 6.982037e-01 9.192361e-01

10595 6.975269e-01 9.192845e-01

10596 6.968583e-01 9.193347e-01

10597 6.961983e-01 9.193869e-01

10598 6.955472e-01 9.194410e-01

10599 6.949052e-01 9.194972e-01

10600 6.942727e-01 9.195553e-01

10601 6.936497e-01 9.196155e-01

10602 6.930365e-01 9.196778e-01

10603 6.924333e-01 9.197422e-01

10604 6.918403e-01 9.198087e-01

10605 6.912575e-01 9.198773e-01

10606 6.906852e-01 9.199481e-01

10607 6.901234e-01 9.200211e-01

10608 6.895722e-01 9.200962e-01

10609 6.890316e-01 9.201736e-01

10610 6.885018e-01 9.202532e-01

10611 6.879828e-01 9.203350e-01

10612 6.874746e-01 9.204191e-01

10613 6.869771e-01 9.205054e-01

10614 6.864904e-01 9.205939e-01

10615 6.860145e-01 9.206847e-01

10616 6.855493e-01 9.207777e-01

10617 6.850947e-01 9.208730e-01

10618 6.846507e-01 9.209704e-01

10619 6.842173e-01 9.210697e-01

10620 6.837942e-01 9.211708e-01

10621 6.833815e-01 9.212735e-01

10622 6.833372e-01 9.212731e-01

10623 6.832915e-01 9.212728e-01

10624 6.832446e-01 9.212724e-01

10625 6.831962e-01 9.212720e-01

10626 6.831465e-01 9.212716e-01

10627 6.830954e-01 9.212712e-01

10628 6.830428e-01 9.212708e-01

10629 6.829887e-01 9.212704e-01

10630 6.829332e-01 9.212700e-01

10631 6.828760e-01 9.212696e-01

10632 6.828173e-01 9.212691e-01

10633 6.827570e-01 9.212687e-01

10634 6.826950e-01 9.212682e-01

10635 6.826313e-01 9.212677e-01

10636 6.825659e-01 9.212672e-01

10637 6.824986e-01 9.212667e-01

10638 6.824296e-01 9.212661e-01

10639 6.823587e-01 9.212656e-01

10640 6.822858e-01 9.212650e-01

10641 6.822111e-01 9.212644e-01

10642 6.821343e-01 9.212638e-01

10643 6.820554e-01 9.212631e-01

10644 6.819744e-01 9.212625e-01

10645 6.818913e-01 9.212618e-01

10646 6.818060e-01 9.212611e-01

10647 6.817185e-01 9.212603e-01

10648 6.816286e-01 9.212596e-01

10649 6.815363e-01 9.212588e-01

10650 6.814417e-01 9.212580e-01

10651 6.813446e-01 9.212571e-01

10652 6.812450e-01 9.212562e-01

10653 6.811427e-01 9.212553e-01

10654 6.810379e-01 9.212544e-01

10655 6.809304e-01 9.212534e-01

10656 6.808201e-01 9.212524e-01

10657 6.807070e-01 9.212513e-01

10658 6.805910e-01 9.212503e-01

10659 6.804721e-01 9.212491e-01

10660 6.803503e-01 9.212480e-01

10661 6.802253e-01 9.212468e-01

10662 6.800973e-01 9.212455e-01

10663 6.799661e-01 9.212442e-01

10664 6.798317e-01 9.212429e-01

10665 6.796940e-01 9.212415e-01

10666 6.795530e-01 9.212401e-01

10667 6.794085e-01 9.212386e-01

10668 6.792606e-01 9.212370e-01

10669 6.791092e-01 9.212355e-01

10670 6.789541e-01 9.212338e-01

10671 6.787955e-01 9.212321e-01

10672 6.786331e-01 9.212303e-01

10673 6.784669e-01 9.212285e-01

10674 6.782970e-01 9.212266e-01

10675 6.781231e-01 9.212247e-01

10676 6.779454e-01 9.212226e-01

10677 6.777636e-01 9.212205e-01

10678 6.775778e-01 9.212184e-01

10679 6.773879e-01 9.212161e-01

10680 6.771939e-01 9.212138e-01

10681 6.769957e-01 9.212114e-01

10682 6.767933e-01 9.212089e-01

10683 6.765866e-01 9.212063e-01

10684 6.763756e-01 9.212037e-01

10685 6.761603e-01 9.212009e-01

10686 6.759406e-01 9.211981e-01

10687 6.757164e-01 9.211951e-01

10688 6.754879e-01 9.211921e-01

10689 6.752548e-01 9.211889e-01

10690 6.750173e-01 9.211857e-01

10691 6.747753e-01 9.211823e-01

10692 6.745287e-01 9.211788e-01

10693 6.742776e-01 9.211752e-01

10694 6.740220e-01 9.211714e-01

10695 6.737619e-01 9.211676e-01

10696 6.734973e-01 9.211636e-01

10697 6.732281e-01 9.211594e-01

10698 6.729544e-01 9.211552e-01

10699 6.726763e-01 9.211508e-01

10700 6.723938e-01 9.211462e-01

10701 6.721068e-01 9.211415e-01

10702 6.718155e-01 9.211366e-01

10703 6.715199e-01 9.211315e-01

10704 6.712200e-01 9.211263e-01

10705 6.709159e-01 9.211209e-01

10706 6.706077e-01 9.211153e-01

10707 6.702954e-01 9.211096e-01

10708 6.699792e-01 9.211036e-01

10709 6.696591e-01 9.210974e-01

10710 6.693352e-01 9.210911e-01

10711 6.690077e-01 9.210845e-01

10712 6.686766e-01 9.210777e-01

10713 6.683421e-01 9.210707e-01

10714 6.680043e-01 9.210634e-01

10715 6.676633e-01 9.210559e-01

10716 6.673193e-01 9.210481e-01

10717 6.669725e-01 9.210401e-01

10718 6.666230e-01 9.210319e-01

10719 6.662710e-01 9.210233e-01

10720 6.659166e-01 9.210145e-01

10721 6.655601e-01 9.210054e-01

10722 6.652016e-01 9.209960e-01

10723 6.648414e-01 9.209862e-01

10724 6.644796e-01 9.209762e-01

10725 6.641164e-01 9.209658e-01

10726 6.637521e-01 9.209551e-01

10727 6.633869e-01 9.209441e-01

10728 6.630210e-01 9.209327e-01

10729 6.626547e-01 9.209209e-01

10730 6.622881e-01 9.209088e-01

10731 6.619216e-01 9.208963e-01

10732 6.615553e-01 9.208834e-01

10733 6.611896e-01 9.208700e-01

10734 6.608245e-01 9.208563e-01

10735 6.604605e-01 9.208421e-01

10736 6.600978e-01 9.208275e-01

10737 6.597365e-01 9.208125e-01

10738 6.593770e-01 9.207969e-01

10739 6.590195e-01 9.207809e-01

10740 6.586642e-01 9.207644e-01

10741 6.583113e-01 9.207475e-01

10742 6.579612e-01 9.207300e-01

10743 6.576140e-01 9.207120e-01

10744 6.572700e-01 9.206934e-01

10745 6.569293e-01 9.206743e-01

10746 6.565923e-01 9.206547e-01

10747 6.562591e-01 9.206345e-01

10748 6.559299e-01 9.206137e-01

10749 6.556050e-01 9.205923e-01

10750 6.552844e-01 9.205703e-01

10751 6.549684e-01 9.205477e-01

10752 6.546572e-01 9.205244e-01

10753 6.543509e-01 9.205005e-01

10754 6.540497e-01 9.204760e-01

10755 6.537537e-01 9.204508e-01

10756 6.534630e-01 9.204249e-01

10757 6.531778e-01 9.203983e-01

10758 6.528982e-01 9.203710e-01

10759 6.526243e-01 9.203430e-01

10760 6.523561e-01 9.203143e-01

10761 6.520938e-01 9.202848e-01

10762 6.518374e-01 9.202546e-01

10763 6.515869e-01 9.202236e-01

10764 6.513425e-01 9.201919e-01

10765 6.511042e-01 9.201593e-01

10766 6.508719e-01 9.201260e-01

10767 6.506458e-01 9.200919e-01

10768 6.504257e-01 9.200569e-01

10769 6.502118e-01 9.200211e-01

10770 6.500040e-01 9.199845e-01

10771 6.498022e-01 9.199471e-01

10772 6.496065e-01 9.199088e-01

10773 6.494168e-01 9.198696e-01

10774 6.492331e-01 9.198296e-01

10775 6.490553e-01 9.197887e-01

10776 6.488833e-01 9.197469e-01

10777 6.487172e-01 9.197042e-01

10778 6.485567e-01 9.196607e-01

10779 6.484019e-01 9.196162e-01

10780 6.482526e-01 9.195708e-01

10781 6.481089e-01 9.195246e-01

10782 6.479705e-01 9.194774e-01

10783 6.478373e-01 9.194294e-01

10784 6.477094e-01 9.193804e-01

10785 6.475865e-01 9.193305e-01

10786 6.474686e-01 9.192797e-01

10787 6.473556e-01 9.192279e-01

10788 6.472473e-01 9.191753e-01

10789 6.471437e-01 9.191218e-01

10790 6.470446e-01 9.190674e-01

10791 6.469499e-01 9.190120e-01

10792 6.468595e-01 9.189558e-01

10793 6.467734e-01 9.188987e-01

10794 6.466913e-01 9.188406e-01

10795 6.466131e-01 9.187818e-01

10796 6.465388e-01 9.187220e-01

10797 6.464683e-01 9.186614e-01

10798 6.464013e-01 9.186000e-01

10799 6.463379e-01 9.185381e-01

10800 6.462779e-01 9.184757e-01

10801 6.462211e-01 9.184132e-01

10802 6.462122e-01 9.184111e-01

10803 6.462034e-01 9.184089e-01

10804 6.461948e-01 9.184068e-01

10805 6.461862e-01 9.184045e-01

10806 6.461778e-01 9.184022e-01

10807 6.461695e-01 9.183999e-01

10808 6.461614e-01 9.183975e-01

10809 6.461535e-01 9.183950e-01

10810 6.461458e-01 9.183925e-01

10811 6.461384e-01 9.183900e-01

10812 6.461312e-01 9.183874e-01

10813 6.461243e-01 9.183847e-01

10814 6.461177e-01 9.183820e-01

10815 6.461114e-01 9.183793e-01

10816 6.461055e-01 9.183765e-01

10817 6.460999e-01 9.183736e-01

10818 6.460948e-01 9.183707e-01

10819 6.460901e-01 9.183677e-01

10820 6.460858e-01 9.183646e-01

10821 6.460821e-01 9.183615e-01

10822 6.460789e-01 9.183584e-01

10823 6.460763e-01 9.183552e-01

10824 6.460742e-01 9.183519e-01

10825 6.460728e-01 9.183486e-01

10826 6.460721e-01 9.183452e-01

10827 6.460721e-01 9.183417e-01

10828 6.460729e-01 9.183382e-01

10829 6.460744e-01 9.183346e-01

10830 6.460769e-01 9.183310e-01

10831 6.460802e-01 9.183272e-01

10832 6.460844e-01 9.183235e-01

10833 6.460897e-01 9.183196e-01

10834 6.460960e-01 9.183157e-01

10835 6.461034e-01 9.183117e-01

10836 6.461120e-01 9.183077e-01

10837 6.461218e-01 9.183035e-01

10838 6.461328e-01 9.182993e-01

10839 6.461452e-01 9.182951e-01

10840 6.461591e-01 9.182907e-01

10841 6.461744e-01 9.182863e-01

10842 6.461912e-01 9.182818e-01

10843 6.462097e-01 9.182773e-01

10844 6.462299e-01 9.182726e-01

10845 6.462518e-01 9.182679e-01

10846 6.462756e-01 9.182631e-01

10847 6.463013e-01 9.182582e-01

10848 6.463291e-01 9.182533e-01

10849 6.463589e-01 9.182483e-01

10850 6.463910e-01 9.182432e-01

10851 6.464253e-01 9.182380e-01

10852 6.464620e-01 9.182327e-01

10853 6.465012e-01 9.182274e-01

10854 6.465430e-01 9.182219e-01

10855 6.465875e-01 9.182164e-01

10856 6.466347e-01 9.182109e-01

10857 6.466849e-01 9.182052e-01

10858 6.467380e-01 9.181995e-01

10859 6.467942e-01 9.181936e-01

10860 6.468537e-01 9.181877e-01

10861 6.469166e-01 9.181817e-01

10862 6.469828e-01 9.181757e-01

10863 6.470527e-01 9.181695e-01

10864 6.471263e-01 9.181633e-01

10865 6.472037e-01 9.181570e-01

10866 6.472851e-01 9.181506e-01

10867 6.473706e-01 9.181442e-01

10868 6.474603e-01 9.181377e-01

10869 6.475543e-01 9.181311e-01

10870 6.476528e-01 9.181244e-01

10871 6.477560e-01 9.181176e-01

10872 6.478638e-01 9.181108e-01

10873 6.479766e-01 9.181039e-01

10874 6.480944e-01 9.180970e-01

10875 6.482174e-01 9.180900e-01

10876 6.483456e-01 9.180829e-01

10877 6.484793e-01 9.180757e-01

10878 6.486185e-01 9.180686e-01

10879 6.487635e-01 9.180613e-01

10880 6.489142e-01 9.180540e-01

10881 6.490709e-01 9.180467e-01

10882 6.492338e-01 9.180393e-01

10883 6.494028e-01 9.180318e-01

10884 6.495782e-01 9.180244e-01

10885 6.497600e-01 9.180169e-01

10886 6.499484e-01 9.180094e-01

10887 6.501435e-01 9.180018e-01

10888 6.503454e-01 9.179943e-01

10889 6.505543e-01 9.179867e-01

10890 6.507701e-01 9.179791e-01

10891 6.509931e-01 9.179715e-01

10892 6.512232e-01 9.179640e-01

10893 6.514606e-01 9.179564e-01

10894 6.517054e-01 9.179489e-01

10895 6.519577e-01 9.179414e-01

10896 6.522174e-01 9.179339e-01

10897 6.524846e-01 9.179265e-01

10898 6.527595e-01 9.179192e-01

10899 6.530420e-01 9.179119e-01

10900 6.533321e-01 9.179047e-01

10901 6.536300e-01 9.178976e-01

10902 6.539355e-01 9.178906e-01

10903 6.542487e-01 9.178837e-01

10904 6.545696e-01 9.178769e-01

10905 6.548982e-01 9.178702e-01

10906 6.552344e-01 9.178637e-01

10907 6.555782e-01 9.178574e-01

10908 6.559295e-01 9.178512e-01

10909 6.562882e-01 9.178453e-01

10910 6.566544e-01 9.178395e-01

10911 6.570279e-01 9.178340e-01

10912 6.574085e-01 9.178287e-01

10913 6.577963e-01 9.178237e-01

10914 6.581910e-01 9.178190e-01

10915 6.585925e-01 9.178145e-01

10916 6.590007e-01 9.178104e-01

10917 6.594155e-01 9.178066e-01

10918 6.598365e-01 9.178032e-01

10919 6.602638e-01 9.178002e-01

10920 6.606970e-01 9.177976e-01

10921 6.611360e-01 9.177954e-01

10922 6.615806e-01 9.177937e-01

10923 6.620304e-01 9.177924e-01

10924 6.624854e-01 9.177917e-01

10925 6.629453e-01 9.177915e-01

10926 6.634098e-01 9.177918e-01

10927 6.638786e-01 9.177927e-01

10928 6.643515e-01 9.177943e-01

10929 6.648283e-01 9.177965e-01

10930 6.653086e-01 9.177993e-01

10931 6.657922e-01 9.178029e-01

10932 6.662787e-01 9.178072e-01

10933 6.667679e-01 9.178122e-01

10934 6.672595e-01 9.178181e-01

10935 6.677532e-01 9.178248e-01

10936 6.682487e-01 9.178323e-01

10937 6.687457e-01 9.178407e-01

10938 6.692439e-01 9.178501e-01

10939 6.697430e-01 9.178604e-01

10940 6.702426e-01 9.178716e-01

10941 6.707426e-01 9.178840e-01

10942 6.712425e-01 9.178973e-01

10943 6.717421e-01 9.179118e-01

10944 6.722411e-01 9.179273e-01

10945 6.727393e-01 9.179441e-01

10946 6.732363e-01 9.179620e-01

10947 6.737318e-01 9.179811e-01

10948 6.742256e-01 9.180015e-01

10949 6.747175e-01 9.180232e-01

10950 6.752070e-01 9.180462e-01

10951 6.756941e-01 9.180706e-01

10952 6.761785e-01 9.180964e-01

10953 6.766599e-01 9.181236e-01

10954 6.771381e-01 9.181522e-01

10955 6.776128e-01 9.181823e-01

10956 6.780839e-01 9.182140e-01

10957 6.785512e-01 9.182471e-01

10958 6.790145e-01 9.182819e-01

10959 6.794735e-01 9.183182e-01

10960 6.799282e-01 9.183562e-01

10961 6.803782e-01 9.183958e-01

10962 6.808236e-01 9.184371e-01

10963 6.812642e-01 9.184801e-01

10964 6.816997e-01 9.185249e-01

10965 6.821302e-01 9.185713e-01

10966 6.825554e-01 9.186195e-01

10967 6.829753e-01 9.186695e-01

10968 6.833897e-01 9.187213e-01

10969 6.837987e-01 9.187749e-01

10970 6.842020e-01 9.188304e-01

10971 6.845997e-01 9.188876e-01

10972 6.849917e-01 9.189467e-01

10973 6.853779e-01 9.190077e-01

10974 6.857583e-01 9.190705e-01

10975 6.861328e-01 9.191352e-01

10976 6.865015e-01 9.192018e-01

10977 6.868643e-01 9.192702e-01

10978 6.872211e-01 9.193404e-01

10979 6.875721e-01 9.194126e-01

10980 6.879172e-01 9.194865e-01

10981 6.882563e-01 9.195619e-01

10982 6.883014e-01 9.195613e-01

10983 6.883478e-01 9.195608e-01

10984 6.883956e-01 9.195602e-01

10985 6.884448e-01 9.195596e-01

10986 6.884956e-01 9.195590e-01

10987 6.885478e-01 9.195584e-01

10988 6.886015e-01 9.195578e-01

10989 6.886568e-01 9.195572e-01

10990 6.887138e-01 9.195565e-01

10991 6.887723e-01 9.195558e-01

10992 6.888326e-01 9.195552e-01

10993 6.888945e-01 9.195545e-01

10994 6.889583e-01 9.195538e-01

10995 6.890238e-01 9.195530e-01

10996 6.890912e-01 9.195523e-01

10997 6.891605e-01 9.195515e-01

10998 6.892317e-01 9.195507e-01

10999 6.893049e-01 9.195499e-01

11000 6.893802e-01 9.195490e-01

11001 6.894576e-01 9.195482e-01

11002 6.895371e-01 9.195473e-01

11003 6.896188e-01 9.195463e-01

11004 6.897028e-01 9.195454e-01

11005 6.897891e-01 9.195444e-01

11006 6.898778e-01 9.195434e-01

11007 6.899689e-01 9.195423e-01

11008 6.900625e-01 9.195412e-01

11009 6.901587e-01 9.195401e-01

11010 6.902574e-01 9.195390e-01

11011 6.903589e-01 9.195378e-01

11012 6.904632e-01 9.195366e-01

11013 6.905702e-01 9.195353e-01

11014 6.906802e-01 9.195340e-01

11015 6.907931e-01 9.195326e-01

11016 6.909090e-01 9.195313e-01

11017 6.910281e-01 9.195298e-01

11018 6.911504e-01 9.195284e-01

11019 6.912759e-01 9.195268e-01

11020 6.914048e-01 9.195253e-01

11021 6.915370e-01 9.195237e-01

11022 6.916728e-01 9.195220e-01

11023 6.918122e-01 9.195203e-01

11024 6.919552e-01 9.195185e-01

11025 6.921020e-01 9.195167e-01

11026 6.922527e-01 9.195148e-01

11027 6.924073e-01 9.195128e-01

11028 6.925659e-01 9.195108e-01

11029 6.927286e-01 9.195088e-01

11030 6.928955e-01 9.195066e-01

11031 6.930667e-01 9.195044e-01

11032 6.932423e-01 9.195022e-01

11033 6.934224e-01 9.194998e-01

11034 6.936071e-01 9.194974e-01

11035 6.937965e-01 9.194949e-01

11036 6.939906e-01 9.194924e-01

11037 6.941897e-01 9.194897e-01

11038 6.943937e-01 9.194870e-01

11039 6.946028e-01 9.194842e-01

11040 6.948171e-01 9.194813e-01

11041 6.950368e-01 9.194783e-01

11042 6.952618e-01 9.194753e-01

11043 6.954923e-01 9.194721e-01

11044 6.957285e-01 9.194688e-01

11045 6.959703e-01 9.194655e-01

11046 6.962180e-01 9.194620e-01

11047 6.964717e-01 9.194584e-01

11048 6.967314e-01 9.194547e-01

11049 6.969972e-01 9.194509e-01

11050 6.972693e-01 9.194470e-01

11051 6.975477e-01 9.194430e-01

11052 6.978326e-01 9.194388e-01

11053 6.981241e-01 9.194345e-01

11054 6.984223e-01 9.194301e-01

11055 6.987273e-01 9.194256e-01

11056 6.990391e-01 9.194209e-01

11057 6.993579e-01 9.194161e-01

11058 6.996838e-01 9.194111e-01

11059 7.000169e-01 9.194060e-01

11060 7.003572e-01 9.194007e-01

11061 7.007049e-01 9.193952e-01

11062 7.010601e-01 9.193896e-01

11063 7.014228e-01 9.193838e-01

11064 7.017931e-01 9.193779e-01

11065 7.021712e-01 9.193717e-01

11066 7.025570e-01 9.193654e-01

11067 7.029506e-01 9.193589e-01

11068 7.033522e-01 9.193522e-01

11069 7.037617e-01 9.193453e-01

11070 7.041793e-01 9.193381e-01

11071 7.046050e-01 9.193308e-01

11072 7.050388e-01 9.193232e-01

11073 7.054808e-01 9.193154e-01

11074 7.059310e-01 9.193074e-01

11075 7.063895e-01 9.192991e-01

11076 7.068562e-01 9.192906e-01

11077 7.073312e-01 9.192818e-01

11078 7.078144e-01 9.192727e-01

11079 7.083060e-01 9.192634e-01

11080 7.088059e-01 9.192538e-01

11081 7.093140e-01 9.192439e-01

11082 7.098303e-01 9.192337e-01

11083 7.103548e-01 9.192232e-01

11084 7.108875e-01 9.192124e-01

11085 7.114283e-01 9.192012e-01

11086 7.119771e-01 9.191898e-01

11087 7.125340e-01 9.191779e-01

11088 7.130986e-01 9.191658e-01

11089 7.136711e-01 9.191532e-01

11090 7.142513e-01 9.191403e-01

11091 7.148390e-01 9.191270e-01

11092 7.154342e-01 9.191133e-01

11093 7.160368e-01 9.190992e-01

11094 7.166464e-01 9.190847e-01

11095 7.172631e-01 9.190698e-01

11096 7.178867e-01 9.190544e-01

11097 7.185169e-01 9.190386e-01

11098 7.191536e-01 9.190223e-01

11099 7.197965e-01 9.190055e-01

11100 7.204455e-01 9.189883e-01

11101 7.211004e-01 9.189706e-01

11102 7.217609e-01 9.189523e-01

11103 7.224267e-01 9.189335e-01

11104 7.230977e-01 9.189142e-01

11105 7.237735e-01 9.188944e-01

11106 7.244539e-01 9.188740e-01

11107 7.251386e-01 9.188530e-01

11108 7.258273e-01 9.188315e-01

11109 7.265197e-01 9.188093e-01

11110 7.272155e-01 9.187865e-01

11111 7.279145e-01 9.187631e-01

11112 7.286162e-01 9.187391e-01

11113 7.293204e-01 9.187144e-01

11114 7.300267e-01 9.186890e-01

11115 7.307349e-01 9.186630e-01

11116 7.314445e-01 9.186362e-01

11117 7.321552e-01 9.186088e-01

11118 7.328666e-01 9.185806e-01

11119 7.335785e-01 9.185517e-01

11120 7.342905e-01 9.185221e-01

11121 7.350022e-01 9.184916e-01

11122 7.357133e-01 9.184604e-01

11123 7.364234e-01 9.184284e-01

11124 7.371321e-01 9.183956e-01

11125 7.378392e-01 9.183620e-01

11126 7.385443e-01 9.183276e-01

11127 7.392470e-01 9.182923e-01

11128 7.399470e-01 9.182561e-01

11129 7.406439e-01 9.182191e-01

11130 7.413375e-01 9.181812e-01

11131 7.420274e-01 9.181424e-01

11132 7.427133e-01 9.181027e-01

11133 7.433949e-01 9.180620e-01

11134 7.440718e-01 9.180205e-01

11135 7.447438e-01 9.179780e-01

11136 7.454106e-01 9.179345e-01

11137 7.460720e-01 9.178901e-01

11138 7.467275e-01 9.178447e-01

11139 7.473771e-01 9.177984e-01

11140 7.480204e-01 9.177510e-01

11141 7.486571e-01 9.177027e-01

11142 7.492872e-01 9.176534e-01

11143 7.499103e-01 9.176030e-01

11144 7.505263e-01 9.175517e-01

11145 7.511349e-01 9.174993e-01

11146 7.517359e-01 9.174460e-01

11147 7.523293e-01 9.173916e-01

11148 7.529148e-01 9.173362e-01

11149 7.534923e-01 9.172797e-01

11150 7.540617e-01 9.172223e-01

11151 7.546228e-01 9.171638e-01

11152 7.551755e-01 9.171043e-01

11153 7.557197e-01 9.170438e-01

11154 7.562553e-01 9.169822e-01

11155 7.567823e-01 9.169197e-01

11156 7.573006e-01 9.168561e-01

11157 7.578101e-01 9.167916e-01

11158 7.583108e-01 9.167261e-01

11159 7.588027e-01 9.166595e-01

11160 7.592856e-01 9.165921e-01

11161 7.597597e-01 9.165241e-01

11162 7.597878e-01 9.165218e-01

11163 7.598166e-01 9.165194e-01

11164 7.598462e-01 9.165170e-01

11165 7.598765e-01 9.165145e-01

11166 7.599076e-01 9.165120e-01

11167 7.599395e-01 9.165094e-01

11168 7.599723e-01 9.165068e-01

11169 7.600059e-01 9.165041e-01

11170 7.600403e-01 9.165014e-01

11171 7.600757e-01 9.164986e-01

11172 7.601119e-01 9.164957e-01

11173 7.601491e-01 9.164929e-01

11174 7.601872e-01 9.164899e-01

11175 7.602262e-01 9.164869e-01

11176 7.602663e-01 9.164839e-01

11177 7.603074e-01 9.164808e-01

11178 7.603495e-01 9.164776e-01

11179 7.603927e-01 9.164744e-01

11180 7.604370e-01 9.164712e-01

11181 7.604824e-01 9.164679e-01

11182 7.605290e-01 9.164645e-01

11183 7.605767e-01 9.164611e-01

11184 7.606256e-01 9.164576e-01

11185 7.606758e-01 9.164541e-01

11186 7.607272e-01 9.164505e-01

11187 7.607799e-01 9.164468e-01

11188 7.608339e-01 9.164431e-01

11189 7.608892e-01 9.164394e-01

11190 7.609459e-01 9.164356e-01

11191 7.610040e-01 9.164317e-01

11192 7.610636e-01 9.164278e-01

11193 7.611246e-01 9.164238e-01

11194 7.611872e-01 9.164197e-01

11195 7.612512e-01 9.164156e-01

11196 7.613169e-01 9.164115e-01

11197 7.613841e-01 9.164072e-01

11198 7.614530e-01 9.164029e-01

11199 7.615235e-01 9.163986e-01

11200 7.615958e-01 9.163942e-01

11201 7.616698e-01 9.163897e-01

11202 7.617456e-01 9.163852e-01

11203 7.618232e-01 9.163806e-01

11204 7.619027e-01 9.163760e-01

11205 7.619841e-01 9.163713e-01

11206 7.620674e-01 9.163665e-01

11207 7.621527e-01 9.163617e-01

11208 7.622400e-01 9.163568e-01

11209 7.623293e-01 9.163519e-01

11210 7.624208e-01 9.163469e-01

11211 7.625144e-01 9.163418e-01

11212 7.626101e-01 9.163367e-01

11213 7.627081e-01 9.163315e-01

11214 7.628083e-01 9.163263e-01

11215 7.629108e-01 9.163210e-01

11216 7.630157e-01 9.163157e-01

11217 7.631229e-01 9.163103e-01

11218 7.632326e-01 9.163049e-01

11219 7.633448e-01 9.162994e-01

11220 7.634594e-01 9.162938e-01

11221 7.635766e-01 9.162883e-01

11222 7.636964e-01 9.162826e-01

11223 7.638188e-01 9.162769e-01

11224 7.639439e-01 9.162712e-01

11225 7.640717e-01 9.162655e-01

11226 7.642023e-01 9.162597e-01

11227 7.643357e-01 9.162538e-01

11228 7.644719e-01 9.162479e-01

11229 7.646110e-01 9.162420e-01

11230 7.647530e-01 9.162361e-01

11231 7.648980e-01 9.162301e-01

11232 7.650460e-01 9.162242e-01

11233 7.651970e-01 9.162182e-01

11234 7.653511e-01 9.162121e-01

11235 7.655083e-01 9.162061e-01

11236 7.656687e-01 9.162001e-01

11237 7.658322e-01 9.161940e-01

11238 7.659989e-01 9.161880e-01

11239 7.661689e-01 9.161819e-01

11240 7.663422e-01 9.161759e-01

11241 7.665188e-01 9.161699e-01

11242 7.666987e-01 9.161639e-01

11243 7.668820e-01 9.161579e-01

11244 7.670686e-01 9.161519e-01

11245 7.672587e-01 9.161460e-01

11246 7.674522e-01 9.161402e-01

11247 7.676492e-01 9.161344e-01

11248 7.678496e-01 9.161286e-01

11249 7.680535e-01 9.161229e-01

11250 7.682610e-01 9.161173e-01

11251 7.684719e-01 9.161118e-01

11252 7.686863e-01 9.161063e-01

11253 7.689042e-01 9.161010e-01

11254 7.691257e-01 9.160958e-01

11255 7.693507e-01 9.160906e-01

11256 7.695792e-01 9.160856e-01

11257 7.698112e-01 9.160808e-01

11258 7.700467e-01 9.160761e-01

11259 7.702857e-01 9.160716e-01

11260 7.705281e-01 9.160672e-01

11261 7.707740e-01 9.160630e-01

11262 7.710233e-01 9.160591e-01

11263 7.712760e-01 9.160553e-01

11264 7.715321e-01 9.160518e-01

11265 7.717914e-01 9.160485e-01

11266 7.720540e-01 9.160455e-01

11267 7.723199e-01 9.160427e-01

11268 7.725889e-01 9.160403e-01

11269 7.728611e-01 9.160381e-01

11270 7.731363e-01 9.160363e-01

11271 7.734145e-01 9.160348e-01

11272 7.736956e-01 9.160337e-01

11273 7.739795e-01 9.160329e-01

11274 7.742663e-01 9.160326e-01

11275 7.745557e-01 9.160326e-01

11276 7.748477e-01 9.160332e-01

11277 7.751422e-01 9.160341e-01

11278 7.754390e-01 9.160356e-01

11279 7.757382e-01 9.160376e-01

11280 7.760396e-01 9.160401e-01

11281 7.763430e-01 9.160432e-01

11282 7.766484e-01 9.160468e-01

11283 7.769556e-01 9.160510e-01

11284 7.772646e-01 9.160559e-01

11285 7.775750e-01 9.160615e-01

11286 7.778869e-01 9.160677e-01

11287 7.782001e-01 9.160746e-01

11288 7.785144e-01 9.160823e-01

11289 7.788298e-01 9.160907e-01

11290 7.791459e-01 9.160999e-01

11291 7.794627e-01 9.161100e-01

11292 7.797801e-01 9.161209e-01

11293 7.800978e-01 9.161327e-01

11294 7.804157e-01 9.161454e-01

11295 7.807336e-01 9.161590e-01

11296 7.810514e-01 9.161736e-01

11297 7.813689e-01 9.161892e-01

11298 7.816858e-01 9.162059e-01

11299 7.820021e-01 9.162236e-01

11300 7.823176e-01 9.162424e-01

11301 7.826320e-01 9.162624e-01

11302 7.829453e-01 9.162835e-01

11303 7.832572e-01 9.163058e-01

11304 7.835675e-01 9.163293e-01

11305 7.838761e-01 9.163541e-01

11306 7.841828e-01 9.163802e-01

11307 7.844875e-01 9.164076e-01

11308 7.847900e-01 9.164364e-01

11309 7.850900e-01 9.164665e-01

11310 7.853875e-01 9.164981e-01

11311 7.856822e-01 9.165310e-01

11312 7.859741e-01 9.165655e-01

11313 7.862630e-01 9.166015e-01

11314 7.865487e-01 9.166390e-01

11315 7.868311e-01 9.166781e-01

11316 7.871100e-01 9.167187e-01

11317 7.873853e-01 9.167610e-01

11318 7.876569e-01 9.168049e-01

11319 7.879247e-01 9.168505e-01

11320 7.881885e-01 9.168978e-01

11321 7.884482e-01 9.169468e-01

11322 7.887038e-01 9.169975e-01

11323 7.889551e-01 9.170500e-01

11324 7.892021e-01 9.171043e-01

11325 7.894446e-01 9.171603e-01

11326 7.896826e-01 9.172182e-01

11327 7.899160e-01 9.172779e-01

11328 7.901447e-01 9.173395e-01

11329 7.903688e-01 9.174029e-01

11330 7.905880e-01 9.174682e-01

11331 7.908025e-01 9.175354e-01

11332 7.910122e-01 9.176045e-01

11333 7.912170e-01 9.176755e-01

11334 7.914169e-01 9.177483e-01

11335 7.916119e-01 9.178231e-01

11336 7.918020e-01 9.178998e-01

11337 7.919872e-01 9.179783e-01

11338 7.921674e-01 9.180585e-01

11339 7.923428e-01 9.181402e-01

11340 7.925133e-01 9.182231e-01

11341 7.926790e-01 9.183073e-01

11342 7.926849e-01 9.183070e-01

11343 7.926909e-01 9.183067e-01

11344 7.926970e-01 9.183064e-01

11345 7.927031e-01 9.183062e-01

11346 7.927093e-01 9.183059e-01

11347 7.927156e-01 9.183056e-01

11348 7.927220e-01 9.183053e-01

11349 7.927284e-01 9.183050e-01

11350 7.927349e-01 9.183047e-01

11351 7.927414e-01 9.183044e-01

11352 7.927481e-01 9.183041e-01

11353 7.927547e-01 9.183038e-01

11354 7.927615e-01 9.183035e-01

11355 7.927683e-01 9.183032e-01

11356 7.927751e-01 9.183029e-01

11357 7.927820e-01 9.183025e-01

11358 7.927890e-01 9.183022e-01

11359 7.927960e-01 9.183018e-01

11360 7.928030e-01 9.183015e-01

11361 7.928101e-01 9.183011e-01

11362 7.928172e-01 9.183007e-01

11363 7.928243e-01 9.183003e-01

11364 7.928315e-01 9.182999e-01

11365 7.928387e-01 9.182995e-01

11366 7.928459e-01 9.182991e-01

11367 7.928531e-01 9.182986e-01

11368 7.928604e-01 9.182982e-01

11369 7.928676e-01 9.182977e-01

11370 7.928748e-01 9.182972e-01

11371 7.928821e-01 9.182967e-01

11372 7.928893e-01 9.182962e-01

11373 7.928964e-01 9.182957e-01

11374 7.929036e-01 9.182952e-01

11375 7.929107e-01 9.182946e-01

11376 7.929177e-01 9.182941e-01

11377 7.929247e-01 9.182935e-01

11378 7.929316e-01 9.182929e-01

11379 7.929384e-01 9.182923e-01

11380 7.929452e-01 9.182916e-01

11381 7.929518e-01 9.182910e-01

11382 7.929583e-01 9.182903e-01

11383 7.929646e-01 9.182896e-01

11384 7.929708e-01 9.182889e-01

11385 7.929769e-01 9.182882e-01

11386 7.929828e-01 9.182874e-01

11387 7.929884e-01 9.182867e-01

11388 7.929939e-01 9.182859e-01

11389 7.929991e-01 9.182851e-01

11390 7.930041e-01 9.182842e-01

11391 7.930087e-01 9.182834e-01

11392 7.930131e-01 9.182825e-01

11393 7.930172e-01 9.182816e-01

11394 7.930209e-01 9.182806e-01

11395 7.930242e-01 9.182797e-01

11396 7.930272e-01 9.182787e-01

11397 7.930297e-01 9.182777e-01

11398 7.930317e-01 9.182766e-01

11399 7.930333e-01 9.182755e-01

11400 7.930343e-01 9.182744e-01

11401 7.930348e-01 9.182733e-01

11402 7.930347e-01 9.182721e-01

11403 7.930340e-01 9.182709e-01

11404 7.930326e-01 9.182697e-01

11405 7.930305e-01 9.182684e-01

11406 7.930277e-01 9.182671e-01

11407 7.930241e-01 9.182657e-01

11408 7.930196e-01 9.182643e-01

11409 7.930143e-01 9.182629e-01

11410 7.930080e-01 9.182614e-01

11411 7.930007e-01 9.182599e-01

11412 7.929924e-01 9.182583e-01

11413 7.929831e-01 9.182567e-01

11414 7.929725e-01 9.182550e-01

11415 7.929608e-01 9.182533e-01

11416 7.929478e-01 9.182515e-01

11417 7.929335e-01 9.182497e-01

11418 7.929178e-01 9.182478e-01

11419 7.929007e-01 9.182459e-01

11420 7.928820e-01 9.182439e-01

11421 7.928617e-01 9.182418e-01

11422 7.928398e-01 9.182397e-01

11423 7.928161e-01 9.182375e-01

11424 7.927906e-01 9.182352e-01

11425 7.927632e-01 9.182329e-01

11426 7.927338e-01 9.182305e-01

11427 7.927024e-01 9.182280e-01

11428 7.926688e-01 9.182255e-01

11429 7.926330e-01 9.182228e-01

11430 7.925948e-01 9.182201e-01

11431 7.925542e-01 9.182173e-01

11432 7.925112e-01 9.182144e-01

11433 7.924655e-01 9.182114e-01

11434 7.924171e-01 9.182082e-01

11435 7.923659e-01 9.182050e-01

11436 7.923119e-01 9.182017e-01

11437 7.922548e-01 9.181983e-01

11438 7.921946e-01 9.181947e-01

11439 7.921313e-01 9.181911e-01

11440 7.920646e-01 9.181873e-01

11441 7.919946e-01 9.181834e-01

11442 7.919210e-01 9.181793e-01

11443 7.918438e-01 9.181751e-01

11444 7.917629e-01 9.181708e-01

11445 7.916781e-01 9.181663e-01

11446 7.915895e-01 9.181617e-01

11447 7.914968e-01 9.181569e-01

11448 7.913999e-01 9.181519e-01

11449 7.912988e-01 9.181468e-01

11450 7.911934e-01 9.181415e-01

11451 7.910836e-01 9.181359e-01

11452 7.909692e-01 9.181302e-01

11453 7.908502e-01 9.181243e-01

11454 7.907264e-01 9.181182e-01

11455 7.905979e-01 9.181119e-01

11456 7.904644e-01 9.181054e-01

11457 7.903260e-01 9.180986e-01

11458 7.901825e-01 9.180916e-01

11459 7.900339e-01 9.180843e-01

11460 7.898800e-01 9.180768e-01

11461 7.897209e-01 9.180690e-01

11462 7.895565e-01 9.180610e-01

11463 7.893867e-01 9.180527e-01

11464 7.892115e-01 9.180440e-01

11465 7.890308e-01 9.180351e-01

11466 7.888446e-01 9.180259e-01

11467 7.886528e-01 9.180164e-01

11468 7.884555e-01 9.180065e-01

11469 7.882527e-01 9.179963e-01

11470 7.880443e-01 9.179857e-01

11471 7.878303e-01 9.179748e-01

11472 7.876107e-01 9.179635e-01

11473 7.873857e-01 9.179519e-01

11474 7.871551e-01 9.179398e-01

11475 7.869191e-01 9.179273e-01

11476 7.866776e-01 9.179145e-01

11477 7.864308e-01 9.179012e-01

11478 7.861788e-01 9.178875e-01

11479 7.859215e-01 9.178733e-01

11480 7.856591e-01 9.178587e-01

11481 7.853916e-01 9.178436e-01

11482 7.851193e-01 9.178280e-01

11483 7.848421e-01 9.178119e-01

11484 7.845603e-01 9.177954e-01

11485 7.842739e-01 9.177783e-01

11486 7.839831e-01 9.177607e-01

11487 7.836880e-01 9.177426e-01

11488 7.833888e-01 9.177239e-01

11489 7.830858e-01 9.177047e-01

11490 7.827789e-01 9.176849e-01

11491 7.824685e-01 9.176645e-01

11492 7.821547e-01 9.176436e-01

11493 7.818377e-01 9.176220e-01

11494 7.815178e-01 9.175999e-01

11495 7.811950e-01 9.175771e-01

11496 7.808697e-01 9.175537e-01

11497 7.805420e-01 9.175297e-01

11498 7.802122e-01 9.175051e-01

11499 7.798805e-01 9.174798e-01

11500 7.795471e-01 9.174538e-01

11501 7.792123e-01 9.174272e-01

11502 7.788762e-01 9.174000e-01

11503 7.785391e-01 9.173720e-01

11504 7.782013e-01 9.173434e-01

11505 7.778629e-01 9.173141e-01

11506 7.775242e-01 9.172841e-01

11507 7.771855e-01 9.172535e-01

11508 7.768468e-01 9.172221e-01

11509 7.765086e-01 9.171900e-01

11510 7.761709e-01 9.171573e-01

11511 7.758341e-01 9.171238e-01

11512 7.754982e-01 9.170897e-01

11513 7.751636e-01 9.170549e-01

11514 7.748304e-01 9.170194e-01

11515 7.744988e-01 9.169831e-01

11516 7.741690e-01 9.169462e-01

11517 7.738412e-01 9.169087e-01

11518 7.735156e-01 9.168708e-01

11519 7.731923e-01 9.168326e-01

11520 7.728715e-01 9.167944e-01

11521 7.725534e-01 9.167562e-01

11522 7.725403e-01 9.167543e-01

11523 7.725267e-01 9.167524e-01

11524 7.725125e-01 9.167504e-01

11525 7.724978e-01 9.167484e-01

11526 7.724825e-01 9.167464e-01

11527 7.724665e-01 9.167444e-01

11528 7.724500e-01 9.167423e-01

11529 7.724328e-01 9.167402e-01

11530 7.724149e-01 9.167381e-01

11531 7.723963e-01 9.167359e-01

11532 7.723770e-01 9.167337e-01

11533 7.723569e-01 9.167315e-01

11534 7.723361e-01 9.167293e-01

11535 7.723145e-01 9.167270e-01

11536 7.722920e-01 9.167247e-01

11537 7.722687e-01 9.167224e-01

11538 7.722445e-01 9.167200e-01

11539 7.722193e-01 9.167176e-01

11540 7.721932e-01 9.167152e-01

11541 7.721661e-01 9.167128e-01

11542 7.721380e-01 9.167104e-01

11543 7.721088e-01 9.167079e-01

11544 7.720784e-01 9.167054e-01

11545 7.720470e-01 9.167029e-01

11546 7.720143e-01 9.167003e-01

11547 7.719805e-01 9.166978e-01

11548 7.719453e-01 9.166952e-01

11549 7.719088e-01 9.166926e-01

11550 7.718710e-01 9.166900e-01

11551 7.718317e-01 9.166873e-01

11552 7.717910e-01 9.166846e-01

11553 7.717487e-01 9.166820e-01

11554 7.717049e-01 9.166793e-01

11555 7.716595e-01 9.166765e-01

11556 7.716123e-01 9.166738e-01

11557 7.715634e-01 9.166711e-01

11558 7.715127e-01 9.166683e-01

11559 7.714602e-01 9.166655e-01

11560 7.714057e-01 9.166628e-01

11561 7.713492e-01 9.166600e-01

11562 7.712906e-01 9.166572e-01

11563 7.712298e-01 9.166544e-01

11564 7.711669e-01 9.166516e-01

11565 7.711016e-01 9.166487e-01

11566 7.710340e-01 9.166459e-01

11567 7.709639e-01 9.166431e-01

11568 7.708913e-01 9.166403e-01

11569 7.708161e-01 9.166375e-01

11570 7.707381e-01 9.166347e-01

11571 7.706574e-01 9.166319e-01

11572 7.705737e-01 9.166291e-01

11573 7.704871e-01 9.166263e-01

11574 7.703973e-01 9.166235e-01

11575 7.703044e-01 9.166208e-01

11576 7.702081e-01 9.166180e-01

11577 7.701085e-01 9.166153e-01

11578 7.700054e-01 9.166126e-01

11579 7.698986e-01 9.166100e-01

11580 7.697881e-01 9.166073e-01

11581 7.696737e-01 9.166047e-01

11582 7.695553e-01 9.166022e-01

11583 7.694328e-01 9.165997e-01

11584 7.693061e-01 9.165972e-01

11585 7.691751e-01 9.165948e-01

11586 7.690395e-01 9.165924e-01

11587 7.688994e-01 9.165901e-01

11588 7.687544e-01 9.165879e-01

11589 7.686046e-01 9.165857e-01

11590 7.684497e-01 9.165836e-01

11591 7.682897e-01 9.165815e-01

11592 7.681243e-01 9.165796e-01

11593 7.679534e-01 9.165777e-01

11594 7.677769e-01 9.165760e-01

11595 7.675946e-01 9.165743e-01

11596 7.674063e-01 9.165727e-01

11597 7.672120e-01 9.165713e-01

11598 7.670114e-01 9.165700e-01

11599 7.668044e-01 9.165688e-01

11600 7.665908e-01 9.165677e-01

11601 7.663705e-01 9.165668e-01

11602 7.661433e-01 9.165660e-01

11603 7.659090e-01 9.165654e-01

11604 7.656675e-01 9.165650e-01

11605 7.654187e-01 9.165647e-01

11606 7.651623e-01 9.165646e-01

11607 7.648982e-01 9.165647e-01

11608 7.646262e-01 9.165650e-01

11609 7.643463e-01 9.165656e-01

11610 7.640581e-01 9.165663e-01

11611 7.637617e-01 9.165673e-01

11612 7.634568e-01 9.165686e-01

11613 7.631433e-01 9.165701e-01

11614 7.628210e-01 9.165719e-01

11615 7.624898e-01 9.165740e-01

11616 7.621497e-01 9.165763e-01

11617 7.618004e-01 9.165790e-01

11618 7.614418e-01 9.165821e-01

11619 7.610739e-01 9.165854e-01

11620 7.606964e-01 9.165891e-01

11621 7.603094e-01 9.165932e-01

11622 7.599128e-01 9.165977e-01

11623 7.595064e-01 9.166026e-01

11624 7.590902e-01 9.166080e-01

11625 7.586641e-01 9.166137e-01

11626 7.582281e-01 9.166200e-01

11627 7.577822e-01 9.166267e-01

11628 7.573263e-01 9.166339e-01

11629 7.568604e-01 9.166416e-01

11630 7.563845e-01 9.166499e-01

11631 7.558987e-01 9.166588e-01

11632 7.554029e-01 9.166682e-01

11633 7.548972e-01 9.166782e-01

11634 7.543817e-01 9.166889e-01

11635 7.538565e-01 9.167002e-01

11636 7.533216e-01 9.167122e-01

11637 7.527772e-01 9.167249e-01

11638 7.522233e-01 9.167383e-01

11639 7.516602e-01 9.167525e-01

11640 7.510879e-01 9.167675e-01

11641 7.505068e-01 9.167832e-01

11642 7.499168e-01 9.167998e-01

11643 7.493184e-01 9.168173e-01

11644 7.487117e-01 9.168356e-01

11645 7.480970e-01 9.168549e-01

11646 7.474745e-01 9.168751e-01

11647 7.468445e-01 9.168962e-01

11648 7.462074e-01 9.169184e-01

11649 7.455634e-01 9.169416e-01

11650 7.449128e-01 9.169659e-01

11651 7.442561e-01 9.169913e-01

11652 7.435936e-01 9.170178e-01

11653 7.429256e-01 9.170454e-01

11654 7.422526e-01 9.170743e-01

11655 7.415750e-01 9.171044e-01

11656 7.408931e-01 9.171357e-01

11657 7.402075e-01 9.171684e-01

11658 7.395185e-01 9.172023e-01

11659 7.388265e-01 9.172377e-01

11660 7.381321e-01 9.172744e-01

11661 7.374357e-01 9.173125e-01

11662 7.367378e-01 9.173521e-01

11663 7.360388e-01 9.173932e-01

11664 7.353391e-01 9.174358e-01

11665 7.346393e-01 9.174800e-01

11666 7.339399e-01 9.175258e-01

11667 7.332412e-01 9.175732e-01

11668 7.325438e-01 9.176222e-01

11669 7.318481e-01 9.176730e-01

11670 7.311546e-01 9.177255e-01

11671 7.304638e-01 9.177797e-01

11672 7.297760e-01 9.178358e-01

11673 7.290917e-01 9.178936e-01

11674 7.284114e-01 9.179533e-01

11675 7.277354e-01 9.180149e-01

11676 7.270641e-01 9.180785e-01

11677 7.263981e-01 9.181439e-01

11678 7.257375e-01 9.182113e-01

11679 7.250829e-01 9.182807e-01

11680 7.244345e-01 9.183522e-01

11681 7.237926e-01 9.184257e-01

11682 7.231577e-01 9.185012e-01

11683 7.225300e-01 9.185789e-01

11684 7.219097e-01 9.186587e-01

11685 7.212972e-01 9.187406e-01

11686 7.206927e-01 9.188247e-01

11687 7.200964e-01 9.189109e-01

11688 7.195086e-01 9.189993e-01

11689 7.189294e-01 9.190900e-01

11690 7.183591e-01 9.191828e-01

11691 7.177977e-01 9.192779e-01

11692 7.172455e-01 9.193752e-01

11693 7.167026e-01 9.194748e-01

11694 7.161690e-01 9.195765e-01

11695 7.156449e-01 9.196803e-01

11696 7.151303e-01 9.197859e-01

11697 7.146254e-01 9.198932e-01

11698 7.141301e-01 9.200022e-01

11699 7.136444e-01 9.201126e-01

11700 7.131685e-01 9.202243e-01

11701 7.127023e-01 9.203373e-01

11702 7.126665e-01 9.203377e-01

11703 7.126295e-01 9.203380e-01

11704 7.125913e-01 9.203384e-01

11705 7.125519e-01 9.203388e-01

11706 7.125112e-01 9.203392e-01

11707 7.124693e-01 9.203397e-01

11708 7.124260e-01 9.203401e-01

11709 7.123813e-01 9.203406e-01

11710 7.123352e-01 9.203410e-01

11711 7.122877e-01 9.203415e-01

11712 7.122387e-01 9.203420e-01

11713 7.121882e-01 9.203425e-01

11714 7.121361e-01 9.203430e-01

11715 7.120824e-01 9.203435e-01

11716 7.120270e-01 9.203440e-01

11717 7.119699e-01 9.203446e-01

11718 7.119110e-01 9.203451e-01

11719 7.118503e-01 9.203457e-01

11720 7.117878e-01 9.203463e-01

11721 7.117233e-01 9.203468e-01

11722 7.116568e-01 9.203474e-01

11723 7.115883e-01 9.203481e-01

11724 7.115178e-01 9.203487e-01

11725 7.114450e-01 9.203493e-01

11726 7.113701e-01 9.203500e-01

11727 7.112929e-01 9.203506e-01

11728 7.112133e-01 9.203513e-01

11729 7.111313e-01 9.203520e-01

11730 7.110468e-01 9.203527e-01

11731 7.109598e-01 9.203534e-01

11732 7.108702e-01 9.203541e-01

11733 7.107779e-01 9.203549e-01

11734 7.106828e-01 9.203556e-01

11735 7.105848e-01 9.203564e-01

11736 7.104839e-01 9.203572e-01

11737 7.103801e-01 9.203580e-01

11738 7.102731e-01 9.203588e-01

11739 7.101630e-01 9.203596e-01

11740 7.100496e-01 9.203605e-01

11741 7.099329e-01 9.203613e-01

11742 7.098127e-01 9.203622e-01

11743 7.096891e-01 9.203631e-01

11744 7.095618e-01 9.203640e-01

11745 7.094308e-01 9.203649e-01

11746 7.092961e-01 9.203659e-01

11747 7.091574e-01 9.203668e-01

11748 7.090147e-01 9.203678e-01

11749 7.088680e-01 9.203688e-01

11750 7.087171e-01 9.203698e-01

11751 7.085619e-01 9.203708e-01

11752 7.084022e-01 9.203718e-01

11753 7.082381e-01 9.203729e-01

11754 7.080694e-01 9.203740e-01

11755 7.078960e-01 9.203751e-01

11756 7.077178e-01 9.203762e-01

11757 7.075347e-01 9.203773e-01

11758 7.073465e-01 9.203785e-01

11759 7.071532e-01 9.203796e-01

11760 7.069547e-01 9.203808e-01

11761 7.067508e-01 9.203820e-01

11762 7.065414e-01 9.203832e-01

11763 7.063265e-01 9.203845e-01

11764 7.061059e-01 9.203857e-01

11765 7.058796e-01 9.203870e-01

11766 7.056473e-01 9.203883e-01

11767 7.054090e-01 9.203896e-01

11768 7.051647e-01 9.203909e-01

11769 7.049141e-01 9.203923e-01

11770 7.046573e-01 9.203936e-01

11771 7.043940e-01 9.203950e-01

11772 7.041242e-01 9.203964e-01

11773 7.038479e-01 9.203978e-01

11774 7.035649e-01 9.203992e-01

11775 7.032751e-01 9.204006e-01

11776 7.029784e-01 9.204021e-01

11777 7.026748e-01 9.204036e-01

11778 7.023643e-01 9.204050e-01

11779 7.020466e-01 9.204065e-01

11780 7.017218e-01 9.204080e-01

11781 7.013898e-01 9.204096e-01

11782 7.010505e-01 9.204111e-01

11783 7.007039e-01 9.204126e-01

11784 7.003500e-01 9.204142e-01

11785 6.999887e-01 9.204157e-01

11786 6.996200e-01 9.204173e-01

11787 6.992439e-01 9.204188e-01

11788 6.988604e-01 9.204204e-01

11789 6.984694e-01 9.204220e-01

11790 6.980711e-01 9.204235e-01

11791 6.976653e-01 9.204251e-01

11792 6.972521e-01 9.204267e-01

11793 6.968316e-01 9.204282e-01

11794 6.964039e-01 9.204298e-01

11795 6.959689e-01 9.204313e-01

11796 6.955268e-01 9.204329e-01

11797 6.950776e-01 9.204344e-01

11798 6.946214e-01 9.204359e-01

11799 6.941584e-01 9.204374e-01

11800 6.936887e-01 9.204389e-01

11801 6.932123e-01 9.204403e-01

11802 6.927296e-01 9.204418e-01

11803 6.922405e-01 9.204432e-01

11804 6.917453e-01 9.204446e-01

11805 6.912442e-01 9.204459e-01

11806 6.907374e-01 9.204472e-01

11807 6.902250e-01 9.204485e-01

11808 6.897074e-01 9.204497e-01

11809 6.891847e-01 9.204508e-01

11810 6.886572e-01 9.204520e-01

11811 6.881252e-01 9.204530e-01

11812 6.875890e-01 9.204540e-01

11813 6.870488e-01 9.204549e-01

11814 6.865049e-01 9.204558e-01

11815 6.859576e-01 9.204566e-01

11816 6.854073e-01 9.204573e-01

11817 6.848542e-01 9.204579e-01

11818 6.842988e-01 9.204584e-01

11819 6.837414e-01 9.204589e-01

11820 6.831822e-01 9.204592e-01

11821 6.826217e-01 9.204594e-01

11822 6.820603e-01 9.204595e-01

11823 6.814982e-01 9.204595e-01

11824 6.809359e-01 9.204594e-01

11825 6.803738e-01 9.204591e-01

11826 6.798121e-01 9.204587e-01

11827 6.792514e-01 9.204581e-01

11828 6.786919e-01 9.204574e-01

11829 6.781340e-01 9.204565e-01

11830 6.775782e-01 9.204555e-01

11831 6.770247e-01 9.204543e-01

11832 6.764740e-01 9.204529e-01

11833 6.759263e-01 9.204513e-01

11834 6.753822e-01 9.204495e-01

11835 6.748418e-01 9.204476e-01

11836 6.743055e-01 9.204454e-01

11837 6.737737e-01 9.204430e-01

11838 6.732467e-01 9.204403e-01

11839 6.727247e-01 9.204375e-01

11840 6.722082e-01 9.204344e-01

11841 6.716973e-01 9.204310e-01

11842 6.711923e-01 9.204274e-01

11843 6.706936e-01 9.204235e-01

11844 6.702013e-01 9.204194e-01

11845 6.697157e-01 9.204150e-01

11846 6.692369e-01 9.204103e-01

11847 6.687653e-01 9.204053e-01

11848 6.683010e-01 9.204000e-01

11849 6.678441e-01 9.203945e-01

11850 6.673949e-01 9.203886e-01

11851 6.669534e-01 9.203824e-01

11852 6.665199e-01 9.203758e-01

11853 6.660943e-01 9.203690e-01

11854 6.656769e-01 9.203618e-01

11855 6.652676e-01 9.203542e-01

11856 6.648666e-01 9.203463e-01

11857 6.644740e-01 9.203381e-01

11858 6.640897e-01 9.203295e-01

11859 6.637138e-01 9.203206e-01

11860 6.633463e-01 9.203112e-01

11861 6.629872e-01 9.203015e-01

11862 6.626366e-01 9.202915e-01

11863 6.622943e-01 9.202811e-01

11864 6.619604e-01 9.202702e-01

11865 6.616348e-01 9.202590e-01

11866 6.613174e-01 9.202475e-01

11867 6.610083e-01 9.202355e-01

11868 6.607073e-01 9.202231e-01

11869 6.604143e-01 9.202104e-01

11870 6.601293e-01 9.201972e-01

11871 6.598522e-01 9.201837e-01

11872 6.595828e-01 9.201698e-01

11873 6.593211e-01 9.201555e-01

11874 6.590669e-01 9.201409e-01

11875 6.588202e-01 9.201261e-01

11876 6.585808e-01 9.201114e-01

11877 6.583485e-01 9.200968e-01

11878 6.581233e-01 9.200825e-01

11879 6.579051e-01 9.200686e-01

11880 6.576936e-01 9.200552e-01

11881 6.574887e-01 9.200425e-01

11882 6.574586e-01 9.200410e-01

11883 6.574278e-01 9.200395e-01

11884 6.573964e-01 9.200381e-01

11885 6.573643e-01 9.200365e-01

11886 6.573315e-01 9.200350e-01

11887 6.572981e-01 9.200335e-01

11888 6.572640e-01 9.200319e-01

11889 6.572292e-01 9.200303e-01

11890 6.571938e-01 9.200287e-01

11891 6.571577e-01 9.200271e-01

11892 6.571209e-01 9.200255e-01

11893 6.570834e-01 9.200239e-01

11894 6.570452e-01 9.200222e-01

11895 6.570063e-01 9.200205e-01

11896 6.569667e-01 9.200189e-01

11897 6.569264e-01 9.200172e-01

11898 6.568855e-01 9.200155e-01

11899 6.568438e-01 9.200138e-01

11900 6.568014e-01 9.200120e-01

11901 6.567582e-01 9.200103e-01

11902 6.567144e-01 9.200086e-01

11903 6.566699e-01 9.200068e-01

11904 6.566246e-01 9.200051e-01

11905 6.565786e-01 9.200033e-01

11906 6.565320e-01 9.200015e-01

11907 6.564846e-01 9.199998e-01

11908 6.564365e-01 9.199980e-01

11909 6.563877e-01 9.199962e-01

11910 6.563382e-01 9.199944e-01

11911 6.562880e-01 9.199926e-01

11912 6.562371e-01 9.199908e-01

11913 6.561855e-01 9.199890e-01

11914 6.561333e-01 9.199872e-01

11915 6.560804e-01 9.199854e-01

11916 6.560268e-01 9.199837e-01

11917 6.559726e-01 9.199819e-01

11918 6.559178e-01 9.199801e-01

11919 6.558624e-01 9.199783e-01

11920 6.558064e-01 9.199766e-01

11921 6.557498e-01 9.199748e-01

11922 6.556926e-01 9.199731e-01

11923 6.556349e-01 9.199713e-01

11924 6.555767e-01 9.199696e-01

11925 6.555180e-01 9.199679e-01

11926 6.554588e-01 9.199662e-01

11927 6.553992e-01 9.199646e-01

11928 6.553392e-01 9.199629e-01

11929 6.552789e-01 9.199613e-01

11930 6.552181e-01 9.199597e-01

11931 6.551571e-01 9.199581e-01

11932 6.550958e-01 9.199566e-01

11933 6.550343e-01 9.199551e-01

11934 6.549727e-01 9.199536e-01

11935 6.549108e-01 9.199522e-01

11936 6.548490e-01 9.199508e-01

11937 6.547870e-01 9.199494e-01

11938 6.547251e-01 9.199481e-01

11939 6.546633e-01 9.199469e-01

11940 6.546016e-01 9.199457e-01

11941 6.545401e-01 9.199445e-01

11942 6.544788e-01 9.199434e-01

11943 6.544179e-01 9.199424e-01

11944 6.543574e-01 9.199415e-01

11945 6.542973e-01 9.199406e-01

11946 6.542378e-01 9.199398e-01

11947 6.541789e-01 9.199390e-01

11948 6.541207e-01 9.199384e-01

11949 6.540633e-01 9.199378e-01

11950 6.540068e-01 9.199374e-01

11951 6.539512e-01 9.199370e-01

11952 6.538967e-01 9.199367e-01

11953 6.538433e-01 9.199366e-01

11954 6.537912e-01 9.199365e-01

11955 6.537404e-01 9.199366e-01

11956 6.536910e-01 9.199368e-01

11957 6.536432e-01 9.199372e-01

11958 6.535971e-01 9.199377e-01

11959 6.535527e-01 9.199383e-01

11960 6.535102e-01 9.199391e-01

11961 6.534697e-01 9.199401e-01

11962 6.534313e-01 9.199412e-01

11963 6.533950e-01 9.199425e-01

11964 6.533612e-01 9.199440e-01

11965 6.533297e-01 9.199457e-01

11966 6.533009e-01 9.199476e-01

11967 6.532747e-01 9.199497e-01

11968 6.532513e-01 9.199520e-01

11969 6.532309e-01 9.199546e-01

11970 6.532134e-01 9.199574e-01

11971 6.531992e-01 9.199604e-01

11972 6.531882e-01 9.199638e-01

11973 6.531806e-01 9.199674e-01

11974 6.531766e-01 9.199713e-01

11975 6.531761e-01 9.199755e-01

11976 6.531794e-01 9.199800e-01

11977 6.531865e-01 9.199848e-01

11978 6.531976e-01 9.199900e-01

11979 6.532127e-01 9.199956e-01

11980 6.532320e-01 9.200015e-01

11981 6.532555e-01 9.200078e-01

11982 6.532834e-01 9.200146e-01

11983 6.533156e-01 9.200217e-01

11984 6.533524e-01 9.200293e-01

11985 6.533938e-01 9.200373e-01

11986 6.534398e-01 9.200458e-01

11987 6.534905e-01 9.200548e-01

11988 6.535459e-01 9.200643e-01

11989 6.536062e-01 9.200743e-01

11990 6.536714e-01 9.200849e-01

11991 6.537414e-01 9.200961e-01

11992 6.538164e-01 9.201078e-01

11993 6.538964e-01 9.201202e-01

11994 6.539812e-01 9.201332e-01

11995 6.540711e-01 9.201468e-01

11996 6.541659e-01 9.201611e-01

11997 6.542657e-01 9.201762e-01

11998 6.543704e-01 9.201919e-01

11999 6.544800e-01 9.202085e-01

12000 6.545944e-01 9.202257e-01

12001 6.547137e-01 9.202438e-01

12002 6.548377e-01 9.202628e-01

12003 6.549665e-01 9.202826e-01

12004 6.550998e-01 9.203032e-01

12005 6.552378e-01 9.203248e-01

12006 6.553801e-01 9.203473e-01

12007 6.555269e-01 9.203708e-01

12008 6.556779e-01 9.203953e-01

12009 6.558330e-01 9.204208e-01

12010 6.559922e-01 9.204474e-01

12011 6.561553e-01 9.204751e-01

12012 6.563222e-01 9.205039e-01

12013 6.564927e-01 9.205338e-01

12014 6.566667e-01 9.205649e-01

12015 6.568441e-01 9.205973e-01

12016 6.570246e-01 9.206309e-01

12017 6.572083e-01 9.206657e-01

12018 6.573947e-01 9.207019e-01

12019 6.575840e-01 9.207395e-01

12020 6.577757e-01 9.207784e-01

12021 6.579699e-01 9.208187e-01

12022 6.581662e-01 9.208605e-01

12023 6.583646e-01 9.209038e-01

12024 6.585649e-01 9.209486e-01

12025 6.587668e-01 9.209949e-01

12026 6.589703e-01 9.210429e-01

12027 6.591751e-01 9.210924e-01

12028 6.593811e-01 9.211436e-01

12029 6.595881e-01 9.211965e-01

12030 6.597959e-01 9.212512e-01

12031 6.600044e-01 9.213076e-01

12032 6.602133e-01 9.213657e-01

12033 6.604226e-01 9.214257e-01

12034 6.606321e-01 9.214876e-01

12035 6.608416e-01 9.215513e-01

12036 6.610510e-01 9.216170e-01

12037 6.612601e-01 9.216846e-01

12038 6.614688e-01 9.217542e-01

12039 6.616769e-01 9.218258e-01

12040 6.618843e-01 9.218994e-01

12041 6.620909e-01 9.219751e-01

12042 6.622965e-01 9.220529e-01

12043 6.625011e-01 9.221328e-01

12044 6.627045e-01 9.222148e-01

12045 6.629067e-01 9.222991e-01

12046 6.631074e-01 9.223855e-01

12047 6.633066e-01 9.224741e-01

12048 6.635043e-01 9.225649e-01

12049 6.637003e-01 9.226580e-01

12050 6.638946e-01 9.227534e-01

12051 6.640870e-01 9.228510e-01

12052 6.642776e-01 9.229510e-01

12053 6.644662e-01 9.230532e-01

12054 6.646528e-01 9.231578e-01

12055 6.648374e-01 9.232646e-01

12056 6.650198e-01 9.233735e-01

12057 6.652001e-01 9.234844e-01

12058 6.653782e-01 9.235972e-01

12059 6.655541e-01 9.237116e-01

12060 6.657277e-01 9.238277e-01

12061 6.658990e-01 9.239453e-01

12062 6.659268e-01 9.239458e-01

12063 6.659557e-01 9.239464e-01

12064 6.659856e-01 9.239470e-01

12065 6.660165e-01 9.239476e-01

12066 6.660486e-01 9.239482e-01

12067 6.660817e-01 9.239489e-01

12068 6.661160e-01 9.239495e-01

12069 6.661515e-01 9.239502e-01

12070 6.661882e-01 9.239508e-01

12071 6.662262e-01 9.239515e-01

12072 6.662654e-01 9.239522e-01

12073 6.663059e-01 9.239529e-01

12074 6.663478e-01 9.239536e-01

12075 6.663911e-01 9.239544e-01

12076 6.664358e-01 9.239551e-01

12077 6.664820e-01 9.239558e-01

12078 6.665297e-01 9.239566e-01

12079 6.665790e-01 9.239574e-01

12080 6.666298e-01 9.239582e-01

12081 6.666824e-01 9.239590e-01

12082 6.667366e-01 9.239598e-01

12083 6.667926e-01 9.239606e-01

12084 6.668504e-01 9.239614e-01

12085 6.669100e-01 9.239622e-01

12086 6.669716e-01 9.239631e-01

12087 6.670351e-01 9.239639e-01

12088 6.671007e-01 9.239648e-01

12089 6.671683e-01 9.239657e-01

12090 6.672381e-01 9.239666e-01

12091 6.673101e-01 9.239675e-01

12092 6.673844e-01 9.239684e-01

12093 6.674610e-01 9.239693e-01

12094 6.675401e-01 9.239702e-01

12095 6.676216e-01 9.239711e-01

12096 6.677056e-01 9.239721e-01

12097 6.677923e-01 9.239731e-01

12098 6.678817e-01 9.239740e-01

12099 6.679738e-01 9.239750e-01

12100 6.680688e-01 9.239760e-01

12101 6.681667e-01 9.239770e-01

12102 6.682677e-01 9.239780e-01

12103 6.683717e-01 9.239790e-01

12104 6.684789e-01 9.239800e-01

12105 6.685894e-01 9.239810e-01

12106 6.687032e-01 9.239821e-01

12107 6.688205e-01 9.239831e-01

12108 6.689413e-01 9.239842e-01

12109 6.690657e-01 9.239852e-01

12110 6.691939e-01 9.239863e-01

12111 6.693259e-01 9.239874e-01

12112 6.694618e-01 9.239884e-01

12113 6.696017e-01 9.239895e-01

12114 6.697458e-01 9.239906e-01

12115 6.698940e-01 9.239917e-01

12116 6.700467e-01 9.239928e-01

12117 6.702037e-01 9.239939e-01

12118 6.703654e-01 9.239950e-01

12119 6.705316e-01 9.239962e-01

12120 6.707027e-01 9.239973e-01

12121 6.708786e-01 9.239984e-01

12122 6.710596e-01 9.239995e-01

12123 6.712456e-01 9.240006e-01

12124 6.714369e-01 9.240017e-01

12125 6.716335e-01 9.240029e-01

12126 6.718356e-01 9.240040e-01

12127 6.720432e-01 9.240051e-01

12128 6.722566e-01 9.240062e-01

12129 6.724758e-01 9.240073e-01

12130 6.727009e-01 9.240084e-01

12131 6.729320e-01 9.240095e-01

12132 6.731693e-01 9.240106e-01

12133 6.734129e-01 9.240117e-01

12134 6.736629e-01 9.240127e-01

12135 6.739194e-01 9.240138e-01

12136 6.741825e-01 9.240148e-01

12137 6.744524e-01 9.240159e-01

12138 6.747291e-01 9.240169e-01

12139 6.750127e-01 9.240179e-01

12140 6.753035e-01 9.240189e-01

12141 6.756013e-01 9.240198e-01

12142 6.759065e-01 9.240208e-01

12143 6.762190e-01 9.240217e-01

12144 6.765390e-01 9.240226e-01

12145 6.768665e-01 9.240234e-01

12146 6.772017e-01 9.240243e-01

12147 6.775445e-01 9.240251e-01

12148 6.778952e-01 9.240259e-01

12149 6.782537e-01 9.240266e-01

12150 6.786202e-01 9.240273e-01

12151 6.789946e-01 9.240279e-01

12152 6.793771e-01 9.240286e-01

12153 6.797676e-01 9.240291e-01

12154 6.801663e-01 9.240296e-01

12155 6.805732e-01 9.240301e-01

12156 6.809882e-01 9.240305e-01

12157 6.814114e-01 9.240309e-01

12158 6.818429e-01 9.240312e-01

12159 6.822826e-01 9.240314e-01

12160 6.827304e-01 9.240316e-01

12161 6.831865e-01 9.240317e-01

12162 6.836507e-01 9.240317e-01

12163 6.841230e-01 9.240317e-01

12164 6.846033e-01 9.240315e-01

12165 6.850917e-01 9.240313e-01

12166 6.855881e-01 9.240310e-01

12167 6.860922e-01 9.240306e-01

12168 6.866042e-01 9.240301e-01

12169 6.871238e-01 9.240295e-01

12170 6.876510e-01 9.240288e-01

12171 6.881856e-01 9.240280e-01

12172 6.887274e-01 9.240271e-01

12173 6.892764e-01 9.240261e-01

12174 6.898324e-01 9.240249e-01

12175 6.903952e-01 9.240236e-01

12176 6.909646e-01 9.240222e-01

12177 6.915405e-01 9.240206e-01

12178 6.921225e-01 9.240189e-01

12179 6.927106e-01 9.240170e-01

12180 6.933045e-01 9.240150e-01

12181 6.939039e-01 9.240129e-01

12182 6.945086e-01 9.240105e-01

12183 6.951183e-01 9.240080e-01

12184 6.957328e-01 9.240053e-01

12185 6.963518e-01 9.240025e-01

12186 6.969750e-01 9.239994e-01

12187 6.976021e-01 9.239962e-01

12188 6.982329e-01 9.239927e-01

12189 6.988669e-01 9.239891e-01

12190 6.995040e-01 9.239852e-01

12191 7.001437e-01 9.239812e-01

12192 7.007858e-01 9.239769e-01

12193 7.014299e-01 9.239724e-01

12194 7.020757e-01 9.239676e-01

12195 7.027228e-01 9.239626e-01

12196 7.033710e-01 9.239574e-01

12197 7.040198e-01 9.239519e-01

12198 7.046690e-01 9.239462e-01

12199 7.053181e-01 9.239402e-01

12200 7.059669e-01 9.239339e-01

12201 7.066150e-01 9.239274e-01

12202 7.072621e-01 9.239206e-01

12203 7.079078e-01 9.239135e-01

12204 7.085518e-01 9.239061e-01

12205 7.091938e-01 9.238985e-01

12206 7.098334e-01 9.238905e-01

12207 7.104703e-01 9.238823e-01

12208 7.111043e-01 9.238737e-01

12209 7.117349e-01 9.238648e-01

12210 7.123620e-01 9.238557e-01

12211 7.129852e-01 9.238462e-01

12212 7.136041e-01 9.238364e-01

12213 7.142186e-01 9.238262e-01

12214 7.148284e-01 9.238158e-01

12215 7.154332e-01 9.238050e-01

12216 7.160328e-01 9.237939e-01

12217 7.166269e-01 9.237825e-01

12218 7.172153e-01 9.237707e-01

12219 7.177978e-01 9.237586e-01

12220 7.183741e-01 9.237462e-01

12221 7.189441e-01 9.237334e-01

12222 7.195076e-01 9.237203e-01

12223 7.200645e-01 9.237068e-01

12224 7.206144e-01 9.236930e-01

12225 7.211574e-01 9.236789e-01

12226 7.216932e-01 9.236644e-01

12227 7.222218e-01 9.236496e-01

12228 7.227430e-01 9.236345e-01

12229 7.232567e-01 9.236190e-01

12230 7.237628e-01 9.236032e-01

12231 7.242612e-01 9.235871e-01

12232 7.247519e-01 9.235707e-01

12233 7.252347e-01 9.235539e-01

12234 7.257097e-01 9.235368e-01

12235 7.261768e-01 9.235194e-01

12236 7.266360e-01 9.235020e-01

12237 7.270872e-01 9.234846e-01

12238 7.275305e-01 9.234675e-01

12239 7.279657e-01 9.234506e-01

12240 7.283930e-01 9.234342e-01

12241 7.288123e-01 9.234184e-01

12242 7.288475e-01 9.234168e-01

12243 7.288836e-01 9.234151e-01

12244 7.289207e-01 9.234135e-01

12245 7.289589e-01 9.234118e-01

12246 7.289981e-01 9.234101e-01

12247 7.290384e-01 9.234083e-01

12248 7.290798e-01 9.234066e-01

12249 7.291223e-01 9.234048e-01

12250 7.291660e-01 9.234029e-01

12251 7.292109e-01 9.234011e-01

12252 7.292570e-01 9.233992e-01

12253 7.293043e-01 9.233973e-01

12254 7.293529e-01 9.233954e-01

12255 7.294028e-01 9.233935e-01

12256 7.294541e-01 9.233915e-01

12257 7.295067e-01 9.233895e-01

12258 7.295608e-01 9.233875e-01

12259 7.296163e-01 9.233855e-01

12260 7.296733e-01 9.233835e-01

12261 7.297318e-01 9.233814e-01

12262 7.297918e-01 9.233793e-01

12263 7.298535e-01 9.233772e-01

12264 7.299167e-01 9.233750e-01

12265 7.299817e-01 9.233729e-01

12266 7.300484e-01 9.233707e-01

12267 7.301168e-01 9.233685e-01

12268 7.301870e-01 9.233662e-01

12269 7.302591e-01 9.233640e-01

12270 7.303330e-01 9.233617e-01

12271 7.304089e-01 9.233594e-01

12272 7.304868e-01 9.233571e-01

12273 7.305667e-01 9.233548e-01

12274 7.306487e-01 9.233524e-01

12275 7.307328e-01 9.233501e-01

12276 7.308191e-01 9.233477e-01

12277 7.309076e-01 9.233453e-01

12278 7.309984e-01 9.233428e-01

12279 7.310915e-01 9.233404e-01

12280 7.311870e-01 9.233379e-01

12281 7.312849e-01 9.233355e-01

12282 7.313854e-01 9.233330e-01

12283 7.314883e-01 9.233305e-01

12284 7.315939e-01 9.233280e-01

12285 7.317022e-01 9.233254e-01

12286 7.318132e-01 9.233229e-01

12287 7.319270e-01 9.233203e-01

12288 7.320436e-01 9.233178e-01

12289 7.321631e-01 9.233152e-01

12290 7.322856e-01 9.233126e-01

12291 7.324111e-01 9.233101e-01

12292 7.325398e-01 9.233075e-01

12293 7.326715e-01 9.233049e-01

12294 7.328066e-01 9.233023e-01

12295 7.329449e-01 9.232997e-01

12296 7.330865e-01 9.232971e-01

12297 7.332316e-01 9.232945e-01

12298 7.333802e-01 9.232919e-01

12299 7.335324e-01 9.232893e-01

12300 7.336882e-01 9.232867e-01

12301 7.338476e-01 9.232842e-01

12302 7.340109e-01 9.232816e-01

12303 7.341780e-01 9.232791e-01

12304 7.343490e-01 9.232766e-01

12305 7.345240e-01 9.232741e-01

12306 7.347031e-01 9.232716e-01

12307 7.348863e-01 9.232691e-01

12308 7.350736e-01 9.232667e-01

12309 7.352653e-01 9.232643e-01

12310 7.354613e-01 9.232619e-01

12311 7.356617e-01 9.232596e-01

12312 7.358666e-01 9.232573e-01

12313 7.360760e-01 9.232551e-01

12314 7.362900e-01 9.232529e-01

12315 7.365088e-01 9.232508e-01

12316 7.367323e-01 9.232487e-01

12317 7.369606e-01 9.232467e-01

12318 7.371938e-01 9.232447e-01

12319 7.374320e-01 9.232428e-01

12320 7.376752e-01 9.232411e-01

12321 7.379235e-01 9.232393e-01

12322 7.381769e-01 9.232377e-01

12323 7.384355e-01 9.232362e-01

12324 7.386994e-01 9.232347e-01

12325 7.389686e-01 9.232334e-01

12326 7.392432e-01 9.232322e-01

12327 7.395232e-01 9.232311e-01

12328 7.398086e-01 9.232302e-01

12329 7.400996e-01 9.232293e-01

12330 7.403961e-01 9.232286e-01

12331 7.406982e-01 9.232281e-01

12332 7.410060e-01 9.232277e-01

12333 7.413194e-01 9.232276e-01

12334 7.416385e-01 9.232275e-01

12335 7.419634e-01 9.232277e-01

12336 7.422939e-01 9.232281e-01

12337 7.426302e-01 9.232287e-01

12338 7.429723e-01 9.232295e-01

12339 7.433202e-01 9.232305e-01

12340 7.436738e-01 9.232318e-01

12341 7.440332e-01 9.232333e-01

12342 7.443983e-01 9.232351e-01

12343 7.447692e-01 9.232372e-01

12344 7.451458e-01 9.232396e-01

12345 7.455281e-01 9.232423e-01

12346 7.459160e-01 9.232453e-01

12347 7.463096e-01 9.232487e-01

12348 7.467088e-01 9.232524e-01

12349 7.471134e-01 9.232565e-01

12350 7.475236e-01 9.232610e-01

12351 7.479391e-01 9.232659e-01

12352 7.483599e-01 9.232712e-01

12353 7.487860e-01 9.232769e-01

12354 7.492172e-01 9.232831e-01

12355 7.496534e-01 9.232898e-01

12356 7.500946e-01 9.232970e-01

12357 7.505406e-01 9.233047e-01

12358 7.509913e-01 9.233130e-01

12359 7.514466e-01 9.233218e-01

12360 7.519063e-01 9.233312e-01

12361 7.523703e-01 9.233413e-01

12362 7.528385e-01 9.233519e-01

12363 7.533106e-01 9.233632e-01

12364 7.537866e-01 9.233752e-01

12365 7.542662e-01 9.233879e-01

12366 7.547492e-01 9.234013e-01

12367 7.552355e-01 9.234155e-01

12368 7.557249e-01 9.234305e-01

12369 7.562171e-01 9.234462e-01

12370 7.567120e-01 9.234628e-01

12371 7.572093e-01 9.234803e-01

12372 7.577089e-01 9.234987e-01

12373 7.582104e-01 9.235179e-01

12374 7.587137e-01 9.235382e-01

12375 7.592186e-01 9.235594e-01

12376 7.597246e-01 9.235816e-01

12377 7.602318e-01 9.236049e-01

12378 7.607397e-01 9.236292e-01

12379 7.612481e-01 9.236546e-01

12380 7.617568e-01 9.236812e-01

12381 7.622656e-01 9.237089e-01

12382 7.627741e-01 9.237379e-01

12383 7.632820e-01 9.237680e-01

12384 7.637893e-01 9.237995e-01

12385 7.642955e-01 9.238322e-01

12386 7.648004e-01 9.238662e-01

12387 7.653037e-01 9.239016e-01

12388 7.658053e-01 9.239384e-01

12389 7.663048e-01 9.239766e-01

12390 7.668019e-01 9.240162e-01

12391 7.672966e-01 9.240574e-01

12392 7.677884e-01 9.241001e-01

12393 7.682771e-01 9.241443e-01

12394 7.687626e-01 9.241901e-01

12395 7.692445e-01 9.242375e-01

12396 7.697227e-01 9.242866e-01

12397 7.701969e-01 9.243373e-01

12398 7.706669e-01 9.243898e-01

12399 7.711325e-01 9.244440e-01

12400 7.715935e-01 9.244999e-01

12401 7.720497e-01 9.245577e-01

12402 7.725009e-01 9.246173e-01

12403 7.729470e-01 9.246787e-01

12404 7.733877e-01 9.247420e-01

12405 7.738229e-01 9.248072e-01

12406 7.742525e-01 9.248743e-01

12407 7.746764e-01 9.249434e-01

12408 7.750943e-01 9.250145e-01

12409 7.755061e-01 9.250876e-01

12410 7.759118e-01 9.251627e-01

12411 7.763112e-01 9.252398e-01

12412 7.767043e-01 9.253190e-01

12413 7.770909e-01 9.254002e-01

12414 7.774710e-01 9.254836e-01

12415 7.778445e-01 9.255690e-01

12416 7.782113e-01 9.256566e-01

12417 7.785715e-01 9.257463e-01

12418 7.789249e-01 9.258381e-01

12419 7.792714e-01 9.259320e-01

12420 7.796112e-01 9.260278e-01

12421 7.799442e-01 9.261255e-01

12422 7.799587e-01 9.261257e-01

12423 7.799735e-01 9.261259e-01

12424 7.799887e-01 9.261262e-01

12425 7.800042e-01 9.261264e-01

12426 7.800201e-01 9.261267e-01

12427 7.800364e-01 9.261270e-01

12428 7.800530e-01 9.261272e-01

12429 7.800700e-01 9.261275e-01

12430 7.800874e-01 9.261278e-01

12431 7.801053e-01 9.261280e-01

12432 7.801235e-01 9.261283e-01

12433 7.801421e-01 9.261286e-01

12434 7.801612e-01 9.261289e-01

12435 7.801807e-01 9.261291e-01

12436 7.802006e-01 9.261294e-01

12437 7.802210e-01 9.261297e-01

12438 7.802419e-01 9.261299e-01

12439 7.802632e-01 9.261302e-01

12440 7.802850e-01 9.261304e-01

12441 7.803073e-01 9.261307e-01

12442 7.803301e-01 9.261309e-01

12443 7.803534e-01 9.261311e-01

12444 7.803772e-01 9.261313e-01

12445 7.804015e-01 9.261316e-01

12446 7.804264e-01 9.261318e-01

12447 7.804518e-01 9.261320e-01

12448 7.804777e-01 9.261321e-01

12449 7.805043e-01 9.261323e-01

12450 7.805314e-01 9.261325e-01

12451 7.805590e-01 9.261326e-01

12452 7.805873e-01 9.261328e-01

12453 7.806162e-01 9.261329e-01

12454 7.806457e-01 9.261330e-01

12455 7.806758e-01 9.261331e-01

12456 7.807066e-01 9.261332e-01

12457 7.807380e-01 9.261332e-01
[truncated: 2,882,978 more chars]
