# Supplementary material for: Purity control of simulated moving bed based on advanced fuzzy controller
Source: Sci Rep. 2024 Apr 20;14:9083. doi: 10.1038/s41598-024-59847-1 (PMC11576947; doi:10.1038/s41598-024-59847-1)
Supplement: Supplementary file 2 — Supplementary Information 2. [file 41598_2024_59847_MOESM2_ESM.docx]

**Figure 4 (b):**

1 8.604928e-06 1.158060e-04

2 8.604928e-06 1.158060e-04

3 8.604928e-06 1.158060e-04

4 8.604928e-06 1.158060e-04

5 8.604928e-06 1.158060e-04

6 8.604928e-06 1.158060e-04

7 8.604928e-06 1.158060e-04

8 8.604928e-06 1.158060e-04

9 8.604928e-06 1.158060e-04

10 8.604928e-06 1.158060e-04

11 8.604928e-06 1.158060e-04

12 8.604928e-06 1.158060e-04

13 8.604928e-06 1.158060e-04

14 8.604928e-06 1.158060e-04

15 8.604928e-06 1.158060e-04

16 8.604928e-06 1.158060e-04

17 8.604928e-06 1.158060e-04

18 8.604928e-06 1.158060e-04

19 8.604928e-06 1.158060e-04

20 8.604928e-06 1.158060e-04

21 8.604928e-06 1.158060e-04

22 8.604928e-06 1.158060e-04

23 8.604928e-06 1.158060e-04

24 8.604928e-06 1.158060e-04

25 8.604928e-06 1.158060e-04

26 8.604928e-06 1.158060e-04

27 8.604928e-06 1.158060e-04

28 8.604928e-06 1.158060e-04

29 8.604928e-06 1.158060e-04

30 8.604928e-06 1.158060e-04

31 8.604928e-06 1.158060e-04

32 8.604928e-06 1.158060e-04

33 8.604928e-06 1.158060e-04

34 8.604928e-06 1.158060e-04

35 8.604928e-06 1.158060e-04

36 8.604928e-06 1.158060e-04

37 8.604928e-06 1.158060e-04

38 8.604928e-06 1.158060e-04

39 8.604928e-06 1.158060e-04

40 8.604928e-06 1.158060e-04

41 8.604928e-06 1.158060e-04

42 8.604928e-06 1.158060e-04

43 8.604928e-06 1.158060e-04

44 8.604928e-06 1.158060e-04

45 8.604928e-06 1.158060e-04

46 8.604928e-06 1.158060e-04

47 8.604928e-06 1.158060e-04

48 8.604928e-06 1.158060e-04

49 8.604928e-06 1.158060e-04

50 8.604928e-06 1.158060e-04

51 8.604928e-06 1.158060e-04

52 8.604928e-06 1.158060e-04

53 8.604928e-06 1.158060e-04

54 8.604928e-06 1.158061e-04

55 8.604929e-06 1.158061e-04

56 8.604929e-06 1.158061e-04

57 8.604929e-06 1.158061e-04

58 8.604930e-06 1.158061e-04

59 8.604930e-06 1.158061e-04

60 8.604931e-06 1.158061e-04

61 8.604932e-06 1.158061e-04

62 8.604933e-06 1.158061e-04

63 8.604934e-06 1.158061e-04

64 8.604936e-06 1.158062e-04

65 8.604938e-06 1.158062e-04

66 8.604941e-06 1.158063e-04

67 8.604945e-06 1.158063e-04

68 8.604950e-06 1.158064e-04

69 8.604956e-06 1.158065e-04

70 8.604964e-06 1.158066e-04

71 8.604973e-06 1.158068e-04

72 8.604986e-06 1.158070e-04

73 8.605001e-06 1.158072e-04

74 8.605020e-06 1.158075e-04

75 8.605045e-06 1.158079e-04

76 8.605075e-06 1.158084e-04

77 8.605114e-06 1.158090e-04

78 8.605161e-06 1.158098e-04

79 8.605221e-06 1.158107e-04

80 8.605296e-06 1.158119e-04

81 8.605389e-06 1.158135e-04

82 8.605504e-06 1.158153e-04

83 8.605647e-06 1.158177e-04

84 8.605825e-06 1.158206e-04

85 8.606045e-06 1.158242e-04

86 8.606318e-06 1.158286e-04

87 8.606654e-06 1.158341e-04

88 8.607068e-06 1.158409e-04

89 8.607579e-06 1.158493e-04

90 8.608207e-06 1.158597e-04

91 8.608978e-06 1.158725e-04

92 8.609924e-06 1.158881e-04

93 8.611082e-06 1.159074e-04

94 8.612499e-06 1.159310e-04

95 8.614231e-06 1.159599e-04

96 8.616344e-06 1.159952e-04

97 8.618918e-06 1.160383e-04

98 8.622052e-06 1.160909e-04

99 8.625860e-06 1.161549e-04

100 8.630484e-06 1.162329e-04

101 8.636089e-06 1.163275e-04

102 8.642876e-06 1.164424e-04

103 8.651085e-06 1.165816e-04

104 8.661000e-06 1.167502e-04

105 8.672962e-06 1.169539e-04

106 8.687376e-06 1.171999e-04

107 8.704722e-06 1.174966e-04

108 8.725573e-06 1.178539e-04

109 8.750606e-06 1.182838e-04

110 8.780625e-06 1.188005e-04

111 8.816580e-06 1.194206e-04

112 8.859594e-06 1.201640e-04

113 8.910993e-06 1.210541e-04

114 8.972339e-06 1.221187e-04

115 9.045474e-06 1.233905e-04

116 9.132562e-06 1.249081e-04

117 9.236146e-06 1.267168e-04

118 9.359212e-06 1.288700e-04

119 9.505258e-06 1.314304e-04

120 9.678379e-06 1.344714e-04

121 9.883365e-06 1.380792e-04

122 1.012581e-05 1.423545e-04

123 1.041225e-05 1.474150e-04

124 1.075028e-05 1.533979e-04

125 1.114876e-05 1.604635e-04

126 1.161800e-05 1.687978e-04

127 1.216995e-05 1.786175e-04

128 1.281850e-05 1.901738e-04

129 1.357973e-05 2.037579e-04

130 1.447225e-05 2.197067e-04

131 1.551761e-05 2.384097e-04

132 1.674067e-05 2.603160e-04

133 1.817014e-05 2.859427e-04

134 1.983908e-05 3.158845e-04

135 2.178556e-05 3.508236e-04

136 2.405338e-05 3.915412e-04

137 2.669283e-05 4.389299e-04

138 2.976161e-05 4.940075e-04

139 3.332586e-05 5.579314e-04

140 3.746130e-05 6.320146e-04

141 4.225450e-05 7.177421e-04

142 4.780437e-05 8.167885e-04

143 5.422373e-05 9.310356e-04

144 6.164115e-05 1.062591e-03

145 7.020296e-05 1.213804e-03

146 8.007554e-05 1.387285e-03

147 9.144777e-05 1.585916e-03

148 1.045339e-04 1.812867e-03

149 1.195765e-04 2.071602e-03

150 1.368500e-04 2.365880e-03

151 1.566645e-04 2.699755e-03

152 1.793695e-04 3.077563e-03

153 2.053591e-04 3.503902e-03

154 2.350762e-04 3.983602e-03

155 2.690186e-04 4.521680e-03

156 3.077440e-04 5.123290e-03

157 3.518771e-04 5.793657e-03

158 4.021158e-04 6.537997e-03

159 4.592387e-04 7.361434e-03

160 5.241123e-04 8.268905e-03

161 5.976997e-04 9.265062e-03

162 6.810684e-04 1.035417e-02

163 7.753990e-04 1.154004e-02

164 8.819945e-04 1.282588e-02

165 1.002289e-03 1.421430e-02

166 1.137855e-03 1.570719e-02

167 1.290415e-03 1.730575e-02

168 1.461848e-03 1.901039e-02

169 1.654194e-03 2.082081e-02

170 1.869664e-03 2.273598e-02

171 2.110643e-03 2.475420e-02

172 2.379692e-03 2.687315e-02

173 2.679550e-03 2.908996e-02

174 3.013129e-03 3.140127e-02

175 3.383511e-03 3.380335e-02

176 3.793937e-03 3.629209e-02

177 4.247795e-03 3.886318e-02

178 4.748599e-03 4.151207e-02

179 5.299971e-03 4.423408e-02

180 5.905609e-03 4.702444e-02

181 6.569256e-03 4.987828e-02

182 6.569256e-03 4.987828e-02

183 6.569256e-03 4.987828e-02

184 6.569256e-03 4.987828e-02

185 6.569256e-03 4.987828e-02

186 6.569256e-03 4.987828e-02

187 6.569256e-03 4.987828e-02

188 6.569256e-03 4.987828e-02

189 6.569256e-03 4.987828e-02

190 6.569256e-03 4.987828e-02

191 6.569256e-03 4.987828e-02

192 6.569256e-03 4.987828e-02

193 6.569256e-03 4.987828e-02

194 6.569256e-03 4.987828e-02

195 6.569256e-03 4.987828e-02

196 6.569256e-03 4.987828e-02

197 6.569256e-03 4.987828e-02

198 6.569256e-03 4.987828e-02

199 6.569256e-03 4.987828e-02

200 6.569256e-03 4.987829e-02

201 6.569256e-03 4.987829e-02

202 6.569256e-03 4.987829e-02

203 6.569256e-03 4.987829e-02

204 6.569256e-03 4.987829e-02

205 6.569256e-03 4.987829e-02

206 6.569256e-03 4.987829e-02

207 6.569257e-03 4.987829e-02

208 6.569257e-03 4.987829e-02

209 6.569257e-03 4.987829e-02

210 6.569257e-03 4.987830e-02

211 6.569257e-03 4.987830e-02

212 6.569257e-03 4.987830e-02

213 6.569257e-03 4.987830e-02

214 6.569257e-03 4.987831e-02

215 6.569257e-03 4.987831e-02

216 6.569258e-03 4.987832e-02

217 6.569258e-03 4.987833e-02

218 6.569258e-03 4.987833e-02

219 6.569259e-03 4.987834e-02

220 6.569259e-03 4.987835e-02

221 6.569260e-03 4.987837e-02

222 6.569261e-03 4.987838e-02

223 6.569261e-03 4.987840e-02

224 6.569262e-03 4.987843e-02

225 6.569263e-03 4.987845e-02

226 6.569265e-03 4.987848e-02

227 6.569266e-03 4.987852e-02

228 6.569268e-03 4.987856e-02

229 6.569270e-03 4.987862e-02

230 6.569273e-03 4.987868e-02

231 6.569276e-03 4.987875e-02

232 6.569279e-03 4.987883e-02

233 6.569284e-03 4.987893e-02

234 6.569288e-03 4.987904e-02

235 6.569294e-03 4.987918e-02

236 6.569301e-03 4.987934e-02

237 6.569308e-03 4.987952e-02

238 6.569317e-03 4.987974e-02

239 6.569328e-03 4.987999e-02

240 6.569340e-03 4.988029e-02

241 6.569354e-03 4.988063e-02

242 6.569371e-03 4.988103e-02

243 6.569390e-03 4.988150e-02

244 6.569413e-03 4.988205e-02

245 6.569439e-03 4.988268e-02

246 6.569469e-03 4.988342e-02

247 6.569504e-03 4.988427e-02

248 6.569544e-03 4.988527e-02

249 6.569591e-03 4.988642e-02

250 6.569646e-03 4.988775e-02

251 6.569708e-03 4.988930e-02

252 6.569781e-03 4.989109e-02

253 6.569865e-03 4.989315e-02

254 6.569961e-03 4.989554e-02

255 6.570072e-03 4.989830e-02

256 6.570200e-03 4.990148e-02

257 6.570348e-03 4.990514e-02

258 6.570517e-03 4.990937e-02

259 6.570712e-03 4.991423e-02

260 6.570936e-03 4.991982e-02

261 6.571192e-03 4.992624e-02

262 6.571487e-03 4.993362e-02

263 6.571824e-03 4.994209e-02

264 6.572210e-03 4.995180e-02

265 6.572652e-03 4.996292e-02

266 6.573157e-03 4.997566e-02

267 6.573735e-03 4.999023e-02

268 6.574394e-03 5.000689e-02

269 6.575146e-03 5.002592e-02

270 6.576004e-03 5.004764e-02

271 6.576981e-03 5.007241e-02

272 6.578094e-03 5.010065e-02

273 6.579360e-03 5.013279e-02

274 6.580800e-03 5.016936e-02

275 6.582436e-03 5.021094e-02

276 6.584294e-03 5.025816e-02

277 6.586402e-03 5.031174e-02

278 6.588792e-03 5.037249e-02

279 6.591501e-03 5.044130e-02

280 6.594569e-03 5.051916e-02

281 6.598040e-03 5.060719e-02

282 6.601965e-03 5.070660e-02

283 6.606402e-03 5.081876e-02

284 6.611411e-03 5.094515e-02

285 6.617065e-03 5.108743e-02

286 6.623441e-03 5.124739e-02

287 6.630627e-03 5.142702e-02

288 6.638721e-03 5.162847e-02

289 6.647829e-03 5.185409e-02

290 6.658073e-03 5.210642e-02

291 6.669587e-03 5.238820e-02

292 6.682519e-03 5.270238e-02

293 6.697033e-03 5.305210e-02

294 6.713312e-03 5.344072e-02

295 6.731557e-03 5.387177e-02

296 6.751992e-03 5.434898e-02

297 6.774863e-03 5.487624e-02

298 6.800443e-03 5.545757e-02

299 6.829031e-03 5.609710e-02

300 6.860958e-03 5.679904e-02

301 6.896588e-03 5.756764e-02

302 6.936321e-03 5.840712e-02

303 6.980597e-03 5.932164e-02

304 7.029896e-03 6.031522e-02

305 7.084747e-03 6.139173e-02

306 7.145727e-03 6.255473e-02

307 7.213466e-03 6.380753e-02

308 7.288653e-03 6.515302e-02

309 7.372037e-03 6.659367e-02

310 7.464433e-03 6.813147e-02

311 7.566727e-03 6.976786e-02

312 7.679879e-03 7.150376e-02

313 7.804927e-03 7.333943e-02

314 7.942993e-03 7.527457e-02

315 8.095286e-03 7.730825e-02

316 8.263105e-03 7.943892e-02

317 8.447846e-03 8.166443e-02

318 8.651001e-03 8.398205e-02

319 8.874164e-03 8.638851e-02

320 9.119029e-03 8.887999e-02

321 9.387396e-03 9.145219e-02

322 9.681169e-03 9.410038e-02

323 1.000235e-02 9.681937e-02

324 1.035305e-02 9.960361e-02

325 1.073546e-02 1.024472e-01

326 1.115188e-02 1.053438e-01

327 1.160468e-02 1.082871e-01

328 1.209630e-02 1.112700e-01

329 1.262926e-02 1.142856e-01

330 1.320609e-02 1.173266e-01

331 1.382938e-02 1.203855e-01

332 1.450172e-02 1.234546e-01

333 1.522567e-02 1.265261e-01

334 1.600377e-02 1.295921e-01

335 1.683849e-02 1.326447e-01

336 1.773219e-02 1.356759e-01

337 1.868714e-02 1.386779e-01

338 1.970540e-02 1.416429e-01

339 2.078888e-02 1.445636e-01

340 2.193924e-02 1.474327e-01

341 2.315790e-02 1.502436e-01

342 2.444596e-02 1.529900e-01

343 2.580421e-02 1.556663e-01

344 2.723309e-02 1.582676e-01

345 2.873264e-02 1.607897e-01

346 3.030251e-02 1.632292e-01

347 3.194190e-02 1.655835e-01

348 3.364960e-02 1.678509e-01

349 3.542391e-02 1.700305e-01

350 3.726269e-02 1.721221e-01

351 3.916332e-02 1.741263e-01

352 4.112275e-02 1.760443e-01

353 4.313745e-02 1.778780e-01

354 4.520348e-02 1.796297e-01

355 4.731647e-02 1.813022e-01

356 4.947170e-02 1.828986e-01

357 5.166406e-02 1.844222e-01

358 5.388816e-02 1.858764e-01

359 5.613832e-02 1.872649e-01

360 5.840867e-02 1.885913e-01

361 6.069316e-02 1.898592e-01

362 6.069316e-02 1.898592e-01

363 6.069316e-02 1.898593e-01

364 6.069316e-02 1.898593e-01

365 6.069317e-02 1.898594e-01

366 6.069317e-02 1.898594e-01

367 6.069317e-02 1.898595e-01

368 6.069317e-02 1.898596e-01

369 6.069317e-02 1.898597e-01

370 6.069318e-02 1.898598e-01

371 6.069318e-02 1.898599e-01

372 6.069318e-02 1.898600e-01

373 6.069319e-02 1.898601e-01

374 6.069319e-02 1.898603e-01

375 6.069320e-02 1.898605e-01

376 6.069320e-02 1.898607e-01

377 6.069321e-02 1.898610e-01

378 6.069322e-02 1.898613e-01

379 6.069323e-02 1.898616e-01

380 6.069324e-02 1.898620e-01

381 6.069325e-02 1.898624e-01

382 6.069326e-02 1.898630e-01

383 6.069328e-02 1.898635e-01

384 6.069330e-02 1.898642e-01

385 6.069332e-02 1.898649e-01

386 6.069334e-02 1.898658e-01

387 6.069337e-02 1.898667e-01

388 6.069340e-02 1.898678e-01

389 6.069343e-02 1.898691e-01

390 6.069347e-02 1.898705e-01

391 6.069351e-02 1.898721e-01

392 6.069356e-02 1.898740e-01

393 6.069362e-02 1.898760e-01

394 6.069368e-02 1.898784e-01

395 6.069375e-02 1.898811e-01

396 6.069383e-02 1.898841e-01

397 6.069392e-02 1.898876e-01

398 6.069403e-02 1.898914e-01

399 6.069414e-02 1.898958e-01

400 6.069427e-02 1.899008e-01

401 6.069442e-02 1.899064e-01

402 6.069459e-02 1.899128e-01

403 6.069478e-02 1.899200e-01

404 6.069499e-02 1.899280e-01

405 6.069523e-02 1.899372e-01

406 6.069551e-02 1.899474e-01

407 6.069581e-02 1.899590e-01

408 6.069615e-02 1.899720e-01

409 6.069654e-02 1.899867e-01

410 6.069697e-02 1.900031e-01

411 6.069745e-02 1.900216e-01

412 6.069800e-02 1.900424e-01

413 6.069861e-02 1.900657e-01

414 6.069929e-02 1.900919e-01

415 6.070006e-02 1.901212e-01

416 6.070092e-02 1.901541e-01

417 6.070188e-02 1.901909e-01

418 6.070295e-02 1.902321e-01

419 6.070416e-02 1.902781e-01

420 6.070550e-02 1.903296e-01

421 6.070700e-02 1.903871e-01

422 6.070867e-02 1.904513e-01

423 6.071054e-02 1.905228e-01

424 6.071263e-02 1.906026e-01

425 6.071495e-02 1.906915e-01

426 6.071754e-02 1.907903e-01

427 6.072042e-02 1.909003e-01

428 6.072362e-02 1.910225e-01

429 6.072719e-02 1.911582e-01

430 6.073116e-02 1.913087e-01

431 6.073556e-02 1.914755e-01

432 6.074046e-02 1.916603e-01

433 6.074589e-02 1.918646e-01

434 6.075192e-02 1.920905e-01

435 6.075861e-02 1.923399e-01

436 6.076602e-02 1.926150e-01

437 6.077423e-02 1.929180e-01

438 6.078332e-02 1.932514e-01

439 6.079338e-02 1.936178e-01

440 6.080451e-02 1.940199e-01

441 6.081681e-02 1.944607e-01

442 6.083040e-02 1.949430e-01

443 6.084541e-02 1.954702e-01

444 6.086198e-02 1.960453e-01

445 6.088026e-02 1.966718e-01

446 6.090042e-02 1.973532e-01

447 6.092263e-02 1.980927e-01

448 6.094709e-02 1.988940e-01

449 6.097403e-02 1.997605e-01

450 6.100366e-02 2.006956e-01

451 6.103625e-02 2.017026e-01

452 6.107206e-02 2.027847e-01

453 6.111139e-02 2.039450e-01

454 6.115457e-02 2.051862e-01

455 6.120195e-02 2.065108e-01

456 6.125390e-02 2.079210e-01

457 6.131083e-02 2.094186e-01

458 6.137317e-02 2.110050e-01

459 6.144141e-02 2.126812e-01

460 6.151606e-02 2.144476e-01

461 6.159766e-02 2.163043e-01

462 6.168680e-02 2.182505e-01

463 6.178412e-02 2.202854e-01

464 6.189030e-02 2.224070e-01

465 6.200606e-02 2.246133e-01

466 6.213218e-02 2.269014e-01

467 6.226949e-02 2.292680e-01

468 6.241887e-02 2.317092e-01

469 6.258126e-02 2.342207e-01

470 6.275765e-02 2.367976e-01

471 6.294910e-02 2.394346e-01

472 6.315673e-02 2.421260e-01

473 6.338171e-02 2.448657e-01

474 6.362528e-02 2.476472e-01

475 6.388873e-02 2.504637e-01

476 6.417343e-02 2.533084e-01

477 6.448079e-02 2.561740e-01

478 6.481229e-02 2.590532e-01

479 6.516945e-02 2.619384e-01

480 6.555386e-02 2.648222e-01

481 6.596713e-02 2.676970e-01

482 6.641094e-02 2.705555e-01

483 6.688697e-02 2.733904e-01

484 6.739694e-02 2.761945e-01

485 6.794258e-02 2.789612e-01

486 6.852563e-02 2.816839e-01

487 6.914780e-02 2.843566e-01

488 6.981080e-02 2.869736e-01

489 7.051628e-02 2.895299e-01

490 7.126585e-02 2.920210e-01

491 7.206103e-02 2.944428e-01

492 7.290325e-02 2.967922e-01

493 7.379384e-02 2.990664e-01

494 7.473399e-02 3.012634e-01

495 7.572472e-02 3.033818e-01

496 7.676691e-02 3.054208e-01

497 7.786119e-02 3.073803e-01

498 7.900802e-02 3.092605e-01

499 8.020761e-02 3.110624e-01

500 8.145989e-02 3.127872e-01

501 8.276456e-02 3.144367e-01

502 8.412100e-02 3.160128e-01

503 8.552829e-02 3.175179e-01

504 8.698523e-02 3.189545e-01

505 8.849028e-02 3.203254e-01

506 9.004160e-02 3.216334e-01

507 9.163703e-02 3.228813e-01

508 9.327409e-02 3.240722e-01

509 9.495002e-02 3.252091e-01

510 9.666176e-02 3.262947e-01

511 9.840599e-02 3.273321e-01

512 1.001791e-01 3.283240e-01

513 1.019774e-01 3.292730e-01

514 1.037969e-01 3.301819e-01

515 1.056334e-01 3.310532e-01

516 1.074827e-01 3.318890e-01

517 1.093405e-01 3.326918e-01

518 1.112024e-01 3.334637e-01

519 1.130640e-01 3.342066e-01

520 1.149211e-01 3.349224e-01

521 1.167693e-01 3.356129e-01

522 1.186045e-01 3.362797e-01

523 1.204229e-01 3.369244e-01

524 1.222206e-01 3.375483e-01

525 1.239940e-01 3.381528e-01

526 1.257399e-01 3.387392e-01

527 1.274552e-01 3.393086e-01

528 1.291372e-01 3.398620e-01

529 1.307834e-01 3.404004e-01

530 1.323919e-01 3.409247e-01

531 1.339606e-01 3.414357e-01

532 1.354882e-01 3.419343e-01

533 1.369734e-01 3.424212e-01

534 1.384154e-01 3.428969e-01

535 1.398137e-01 3.433622e-01

536 1.411677e-01 3.438176e-01

537 1.424776e-01 3.442635e-01

538 1.437434e-01 3.447006e-01

539 1.449655e-01 3.451292e-01

540 1.461445e-01 3.455497e-01

541 1.472812e-01 3.459626e-01

542 1.472812e-01 3.459666e-01

543 1.472813e-01 3.459710e-01

544 1.472814e-01 3.459760e-01

545 1.472815e-01 3.459815e-01

546 1.472816e-01 3.459877e-01

547 1.472817e-01 3.459946e-01

548 1.472818e-01 3.460023e-01

549 1.472820e-01 3.460109e-01

550 1.472821e-01 3.460205e-01

551 1.472823e-01 3.460311e-01

552 1.472825e-01 3.460429e-01

553 1.472827e-01 3.460561e-01

554 1.472829e-01 3.460707e-01

555 1.472832e-01 3.460870e-01

556 1.472835e-01 3.461051e-01

557 1.472838e-01 3.461251e-01

558 1.472842e-01 3.461474e-01

559 1.472846e-01 3.461721e-01

560 1.472850e-01 3.461994e-01

561 1.472855e-01 3.462298e-01

562 1.472861e-01 3.462634e-01

563 1.472867e-01 3.463006e-01

564 1.472874e-01 3.463417e-01

565 1.472881e-01 3.463872e-01

566 1.472889e-01 3.464376e-01

567 1.472898e-01 3.464932e-01

568 1.472908e-01 3.465546e-01

569 1.472920e-01 3.466224e-01

570 1.472932e-01 3.466972e-01

571 1.472945e-01 3.467796e-01

572 1.472960e-01 3.468705e-01

573 1.472977e-01 3.469705e-01

574 1.472995e-01 3.470807e-01

575 1.473015e-01 3.472018e-01

576 1.473036e-01 3.473349e-01

577 1.473061e-01 3.474812e-01

578 1.473087e-01 3.476417e-01

579 1.473116e-01 3.478178e-01

580 1.473148e-01 3.480108e-01

581 1.473184e-01 3.482222e-01

582 1.473222e-01 3.484536e-01

583 1.473265e-01 3.487065e-01

584 1.473312e-01 3.489829e-01

585 1.473363e-01 3.492846e-01

586 1.473419e-01 3.496136e-01

587 1.473480e-01 3.499720e-01

588 1.473548e-01 3.503621e-01

589 1.473622e-01 3.507861e-01

590 1.473702e-01 3.512466e-01

591 1.473791e-01 3.517460e-01

592 1.473888e-01 3.522870e-01

593 1.473993e-01 3.528724e-01

594 1.474109e-01 3.535048e-01

595 1.474235e-01 3.541871e-01

596 1.474373e-01 3.549221e-01

597 1.474524e-01 3.557128e-01

598 1.474688e-01 3.565620e-01

599 1.474867e-01 3.574725e-01

600 1.475062e-01 3.584471e-01

601 1.475275e-01 3.594884e-01

602 1.475507e-01 3.605990e-01

603 1.475760e-01 3.617811e-01

604 1.476034e-01 3.630371e-01

605 1.476333e-01 3.643687e-01

606 1.476658e-01 3.657776e-01

607 1.477011e-01 3.672651e-01

608 1.477395e-01 3.688323e-01

609 1.477811e-01 3.704797e-01

610 1.478263e-01 3.722075e-01

611 1.478753e-01 3.740155e-01

612 1.479285e-01 3.759029e-01

613 1.479861e-01 3.778687e-01

614 1.480486e-01 3.799111e-01

615 1.481161e-01 3.820281e-01

616 1.481893e-01 3.842171e-01

617 1.482684e-01 3.864748e-01

618 1.483539e-01 3.887978e-01

619 1.484463e-01 3.911821e-01

620 1.485460e-01 3.936231e-01

621 1.486537e-01 3.961160e-01

622 1.487699e-01 3.986555e-01

623 1.488951e-01 4.012361e-01

624 1.490300e-01 4.038519e-01

625 1.491752e-01 4.064966e-01

626 1.493315e-01 4.091641e-01

627 1.494996e-01 4.118476e-01

628 1.496803e-01 4.145406e-01

629 1.498743e-01 4.172364e-01

630 1.500825e-01 4.199282e-01

631 1.503058e-01 4.226095e-01

632 1.505451e-01 4.252737e-01

633 1.508014e-01 4.279144e-01

634 1.510756e-01 4.305254e-01

635 1.513688e-01 4.331009e-01

636 1.516820e-01 4.356354e-01

637 1.520163e-01 4.381236e-01

638 1.523728e-01 4.405608e-01

639 1.527527e-01 4.429428e-01

640 1.531571e-01 4.452655e-01

641 1.535871e-01 4.475258e-01

642 1.540439e-01 4.497208e-01

643 1.545288e-01 4.518482e-01

644 1.550428e-01 4.539062e-01

645 1.555871e-01 4.558935e-01

646 1.561628e-01 4.578094e-01

647 1.567711e-01 4.596535e-01

648 1.574129e-01 4.614258e-01

649 1.580894e-01 4.631271e-01

650 1.588013e-01 4.647580e-01

651 1.595497e-01 4.663199e-01

652 1.603352e-01 4.678143e-01

653 1.611585e-01 4.692429e-01

654 1.620202e-01 4.706076e-01

655 1.629207e-01 4.719106e-01

656 1.638604e-01 4.731541e-01

657 1.648393e-01 4.743406e-01

658 1.658575e-01 4.754724e-01

659 1.669148e-01 4.765520e-01

660 1.680108e-01 4.775818e-01

661 1.691449e-01 4.785644e-01

662 1.703164e-01 4.795020e-01

663 1.715243e-01 4.803972e-01

664 1.727675e-01 4.812522e-01

665 1.740446e-01 4.820693e-01

666 1.753541e-01 4.828507e-01

667 1.766941e-01 4.835984e-01

668 1.780627e-01 4.843144e-01

669 1.794577e-01 4.850006e-01

670 1.808769e-01 4.856589e-01

671 1.823178e-01 4.862909e-01

672 1.837777e-01 4.868983e-01

673 1.852538e-01 4.874825e-01

674 1.867434e-01 4.880451e-01

675 1.882435e-01 4.885874e-01

676 1.897511e-01 4.891106e-01

677 1.912632e-01 4.896159e-01

678 1.927767e-01 4.901045e-01

679 1.942886e-01 4.905774e-01

680 1.957959e-01 4.910355e-01

681 1.972957e-01 4.914798e-01

682 1.987851e-01 4.919110e-01

683 2.002614e-01 4.923301e-01

684 2.017221e-01 4.927376e-01

685 2.031646e-01 4.931344e-01

686 2.045866e-01 4.935209e-01

687 2.059860e-01 4.938979e-01

688 2.073610e-01 4.942659e-01

689 2.087096e-01 4.946253e-01

690 2.100304e-01 4.949766e-01

691 2.113220e-01 4.953204e-01

692 2.125832e-01 4.956569e-01

693 2.138132e-01 4.959866e-01

694 2.150110e-01 4.963098e-01

695 2.161762e-01 4.966269e-01

696 2.173084e-01 4.969381e-01

697 2.184073e-01 4.972438e-01

698 2.194728e-01 4.975441e-01

699 2.205050e-01 4.978395e-01

700 2.215042e-01 4.981300e-01

701 2.224706e-01 4.984159e-01

702 2.234047e-01 4.986974e-01

703 2.243070e-01 4.989747e-01

704 2.251783e-01 4.992479e-01

705 2.260191e-01 4.995173e-01

706 2.268302e-01 4.997829e-01

707 2.276125e-01 5.000449e-01

708 2.283667e-01 5.003035e-01

709 2.290939e-01 5.005587e-01

710 2.297948e-01 5.008108e-01

711 2.304705e-01 5.010597e-01

712 2.311218e-01 5.013056e-01

713 2.317497e-01 5.015486e-01

714 2.323551e-01 5.017888e-01

715 2.329389e-01 5.020263e-01

716 2.335021e-01 5.022611e-01

717 2.340455e-01 5.024933e-01

718 2.345700e-01 5.027230e-01

719 2.350765e-01 5.029503e-01

720 2.355657e-01 5.031752e-01

721 2.360386e-01 5.033978e-01

722 2.360396e-01 5.034979e-01

723 2.360406e-01 5.036073e-01

724 2.360418e-01 5.037267e-01

725 2.360431e-01 5.038569e-01

726 2.360445e-01 5.039988e-01

727 2.360460e-01 5.041535e-01

728 2.360477e-01 5.043219e-01

729 2.360495e-01 5.045052e-01

730 2.360515e-01 5.047045e-01

731 2.360537e-01 5.049212e-01

732 2.360560e-01 5.051565e-01

733 2.360586e-01 5.054120e-01

734 2.360614e-01 5.056890e-01

735 2.360645e-01 5.059893e-01

736 2.360678e-01 5.063144e-01

737 2.360714e-01 5.066662e-01

738 2.360754e-01 5.070465e-01

739 2.360797e-01 5.074573e-01

740 2.360843e-01 5.079006e-01

741 2.360894e-01 5.083784e-01

742 2.360949e-01 5.088930e-01

743 2.361009e-01 5.094466e-01

744 2.361074e-01 5.100415e-01

745 2.361144e-01 5.106800e-01

746 2.361221e-01 5.113644e-01

747 2.361304e-01 5.120972e-01

748 2.361394e-01 5.128808e-01

749 2.361491e-01 5.137175e-01

750 2.361597e-01 5.146097e-01

751 2.361711e-01 5.155595e-01

752 2.361835e-01 5.165693e-01

753 2.361969e-01 5.176411e-01

754 2.362114e-01 5.187768e-01

755 2.362271e-01 5.199783e-01

756 2.362441e-01 5.212470e-01

757 2.362624e-01 5.225845e-01

758 2.362823e-01 5.239917e-01

759 2.363037e-01 5.254696e-01

760 2.363268e-01 5.270187e-01

761 2.363517e-01 5.286392e-01

762 2.363786e-01 5.303309e-01

763 2.364077e-01 5.320934e-01

764 2.364389e-01 5.339258e-01

765 2.364727e-01 5.358267e-01

766 2.365090e-01 5.377945e-01

767 2.365481e-01 5.398271e-01

768 2.365902e-01 5.419218e-01

769 2.366355e-01 5.440759e-01

770 2.366843e-01 5.462859e-01

771 2.367367e-01 5.485482e-01

772 2.367930e-01 5.508586e-01

773 2.368535e-01 5.532128e-01

774 2.369184e-01 5.556059e-01

775 2.369882e-01 5.580331e-01

776 2.370630e-01 5.604890e-01

777 2.371432e-01 5.629681e-01

778 2.372292e-01 5.654649e-01

779 2.373213e-01 5.679735e-01

780 2.374200e-01 5.704882e-01

781 2.375256e-01 5.730031e-01

782 2.376387e-01 5.755122e-01

783 2.377595e-01 5.780100e-01

784 2.378887e-01 5.804906e-01

785 2.380267e-01 5.829487e-01

786 2.381740e-01 5.853789e-01

787 2.383313e-01 5.877761e-01

788 2.384990e-01 5.901357e-01

789 2.386777e-01 5.924531e-01

790 2.388682e-01 5.947244e-01

791 2.390709e-01 5.969458e-01

792 2.392866e-01 5.991140e-01

793 2.395160e-01 6.012261e-01

794 2.397598e-01 6.032798e-01

795 2.400186e-01 6.052728e-01

796 2.402933e-01 6.072036e-01

797 2.405846e-01 6.090710e-01

798 2.408933e-01 6.108743e-01

799 2.412201e-01 6.126128e-01

800 2.415659e-01 6.142867e-01

801 2.419314e-01 6.158961e-01

802 2.423175e-01 6.174416e-01

803 2.427250e-01 6.189241e-01

804 2.431547e-01 6.203447e-01

805 2.436073e-01 6.217047e-01

806 2.440836e-01 6.230057e-01

807 2.445845e-01 6.242492e-01

808 2.451106e-01 6.254371e-01

809 2.456626e-01 6.265713e-01

810 2.462412e-01 6.276538e-01

811 2.468470e-01 6.286866e-01

812 2.474805e-01 6.296718e-01

813 2.481423e-01 6.306114e-01

814 2.488328e-01 6.315076e-01

815 2.495524e-01 6.323624e-01

816 2.503012e-01 6.331778e-01

817 2.510796e-01 6.339559e-01

818 2.518875e-01 6.346985e-01

819 2.527250e-01 6.354075e-01

820 2.535919e-01 6.360848e-01

821 2.544880e-01 6.367322e-01

822 2.554129e-01 6.373512e-01

823 2.563661e-01 6.379436e-01

824 2.573469e-01 6.385108e-01

825 2.583547e-01 6.390543e-01

826 2.593884e-01 6.395755e-01

827 2.604471e-01 6.400758e-01

828 2.615296e-01 6.405563e-01

829 2.626346e-01 6.410182e-01

830 2.637607e-01 6.414628e-01

831 2.649064e-01 6.418909e-01

832 2.660699e-01 6.423036e-01

833 2.672496e-01 6.427019e-01

834 2.684436e-01 6.430865e-01

835 2.696499e-01 6.434584e-01

836 2.708666e-01 6.438182e-01

837 2.720916e-01 6.441667e-01

838 2.733228e-01 6.445046e-01

839 2.745580e-01 6.448325e-01

840 2.757952e-01 6.451509e-01

841 2.770322e-01 6.454605e-01

842 2.782668e-01 6.457617e-01

843 2.794971e-01 6.460551e-01

844 2.807208e-01 6.463410e-01

845 2.819361e-01 6.466199e-01

846 2.831411e-01 6.468922e-01

847 2.843338e-01 6.471582e-01

848 2.855127e-01 6.474183e-01

849 2.866759e-01 6.476729e-01

850 2.878222e-01 6.479221e-01

851 2.889499e-01 6.481663e-01

852 2.900580e-01 6.484057e-01

853 2.911452e-01 6.486407e-01

854 2.922104e-01 6.488713e-01

855 2.932530e-01 6.490979e-01

856 2.942720e-01 6.493205e-01

857 2.952668e-01 6.495395e-01

858 2.962370e-01 6.497550e-01

859 2.971821e-01 6.499671e-01

860 2.981020e-01 6.501759e-01

861 2.989964e-01 6.503817e-01

862 2.998652e-01 6.505846e-01

863 3.007086e-01 6.507847e-01

864 3.015266e-01 6.509820e-01

865 3.023194e-01 6.511768e-01

866 3.030874e-01 6.513690e-01

867 3.038309e-01 6.515589e-01

868 3.045503e-01 6.517465e-01

869 3.052460e-01 6.519319e-01

870 3.059186e-01 6.521151e-01

871 3.065686e-01 6.522963e-01

872 3.071966e-01 6.524755e-01

873 3.078032e-01 6.526527e-01

874 3.083890e-01 6.528281e-01

875 3.089547e-01 6.530017e-01

876 3.095009e-01 6.531735e-01

877 3.100283e-01 6.533437e-01

878 3.105375e-01 6.535122e-01

879 3.110293e-01 6.536790e-01

880 3.115041e-01 6.538444e-01

881 3.119628e-01 6.540082e-01

882 3.124059e-01 6.541705e-01

883 3.128341e-01 6.543314e-01

884 3.132480e-01 6.544909e-01

885 3.136482e-01 6.546491e-01

886 3.140352e-01 6.548059e-01

887 3.144098e-01 6.549613e-01

888 3.147723e-01 6.551156e-01

889 3.151233e-01 6.552685e-01

890 3.154634e-01 6.554202e-01

891 3.157931e-01 6.555708e-01

892 3.161128e-01 6.557201e-01

893 3.164230e-01 6.558683e-01

894 3.167242e-01 6.560154e-01

895 3.170167e-01 6.561614e-01

896 3.173010e-01 6.563062e-01

897 3.175775e-01 6.564500e-01

898 3.178465e-01 6.565928e-01

899 3.181084e-01 6.567345e-01

900 3.183635e-01 6.568752e-01

901 3.186122e-01 6.570149e-01

902 3.186181e-01 6.578643e-01

903 3.186244e-01 6.587651e-01

904 3.186312e-01 6.597193e-01

905 3.186385e-01 6.607285e-01

906 3.186464e-01 6.617944e-01

907 3.186550e-01 6.629185e-01

908 3.186641e-01 6.641022e-01

909 3.186740e-01 6.653468e-01

910 3.186846e-01 6.666531e-01

911 3.186960e-01 6.680221e-01

912 3.187083e-01 6.694542e-01

913 3.187214e-01 6.709498e-01

914 3.187356e-01 6.725089e-01

915 3.187508e-01 6.741312e-01

916 3.187672e-01 6.758162e-01

917 3.187847e-01 6.775628e-01

918 3.188035e-01 6.793699e-01

919 3.188237e-01 6.812359e-01

920 3.188454e-01 6.831588e-01

921 3.188686e-01 6.851364e-01

922 3.188934e-01 6.871661e-01

923 3.189201e-01 6.892448e-01

924 3.189487e-01 6.913694e-01

925 3.189792e-01 6.935362e-01

926 3.190120e-01 6.957413e-01

927 3.190470e-01 6.979808e-01

928 3.190845e-01 7.002501e-01

929 3.191245e-01 7.025447e-01

930 3.191673e-01 7.048599e-01

931 3.192131e-01 7.071908e-01

932 3.192620e-01 7.095324e-01

933 3.193142e-01 7.118795e-01

934 3.193699e-01 7.142272e-01

935 3.194294e-01 7.165704e-01

936 3.194928e-01 7.189040e-01

937 3.195604e-01 7.212231e-01

938 3.196325e-01 7.235230e-01

939 3.197093e-01 7.257990e-01

940 3.197912e-01 7.280466e-01

941 3.198783e-01 7.302617e-01

942 3.199710e-01 7.324403e-01

943 3.200696e-01 7.345788e-01

944 3.201744e-01 7.366740e-01

945 3.202858e-01 7.387226e-01

946 3.204042e-01 7.407221e-01

947 3.205300e-01 7.426702e-01

948 3.206634e-01 7.445648e-01

949 3.208050e-01 7.464044e-01

950 3.209551e-01 7.481876e-01

951 3.211142e-01 7.499134e-01

952 3.212828e-01 7.515813e-01

953 3.214612e-01 7.531909e-01

954 3.216501e-01 7.547421e-01

955 3.218498e-01 7.562353e-01

956 3.220609e-01 7.576708e-01

957 3.222840e-01 7.590494e-01

958 3.225195e-01 7.603719e-01

959 3.227680e-01 7.616396e-01

960 3.230300e-01 7.628536e-01

961 3.233062e-01 7.640152e-01

962 3.235970e-01 7.651261e-01

963 3.239030e-01 7.661877e-01

964 3.242248e-01 7.672018e-01

965 3.245631e-01 7.681700e-01

966 3.249182e-01 7.690941e-01

967 3.252909e-01 7.699758e-01

968 3.256816e-01 7.708169e-01

969 3.260909e-01 7.716192e-01

970 3.265194e-01 7.723845e-01

971 3.269674e-01 7.731144e-01

972 3.274356e-01 7.738107e-01

973 3.279243e-01 7.744749e-01

974 3.284340e-01 7.751088e-01

975 3.289650e-01 7.757139e-01

976 3.295178e-01 7.762916e-01

977 3.300925e-01 7.768435e-01

978 3.306895e-01 7.773709e-01

979 3.313090e-01 7.778751e-01

980 3.319510e-01 7.783575e-01

981 3.326156e-01 7.788193e-01

982 3.333028e-01 7.792616e-01

983 3.340126e-01 7.796854e-01

984 3.347448e-01 7.800920e-01

985 3.354992e-01 7.804822e-01

986 3.362754e-01 7.808571e-01

987 3.370730e-01 7.812174e-01

988 3.378916e-01 7.815641e-01

989 3.387305e-01 7.818979e-01

990 3.395892e-01 7.822196e-01

991 3.404668e-01 7.825298e-01

992 3.413624e-01 7.828294e-01

993 3.422752e-01 7.831187e-01

994 3.432041e-01 7.833986e-01

995 3.441481e-01 7.836694e-01

996 3.451059e-01 7.839318e-01

997 3.460763e-01 7.841862e-01

998 3.470581e-01 7.844331e-01

999 3.480497e-01 7.846729e-01

1000 3.490499e-01 7.849059e-01

1001 3.500571e-01 7.851327e-01

1002 3.510698e-01 7.853535e-01

1003 3.520866e-01 7.855686e-01

1004 3.531059e-01 7.857785e-01

1005 3.541262e-01 7.859833e-01

1006 3.551459e-01 7.861833e-01

1007 3.561634e-01 7.863789e-01

1008 3.571774e-01 7.865701e-01

1009 3.581863e-01 7.867574e-01

1010 3.591887e-01 7.869408e-01

1011 3.601832e-01 7.871206e-01

1012 3.611685e-01 7.872969e-01

1013 3.621433e-01 7.874700e-01

1014 3.631064e-01 7.876400e-01

1015 3.640567e-01 7.878070e-01

1016 3.649932e-01 7.879712e-01

1017 3.659148e-01 7.881327e-01

1018 3.668208e-01 7.882917e-01

1019 3.677103e-01 7.884482e-01

1020 3.685826e-01 7.886024e-01

1021 3.694371e-01 7.887544e-01

1022 3.702733e-01 7.889043e-01

1023 3.710906e-01 7.890521e-01

1024 3.718888e-01 7.891981e-01

1025 3.726676e-01 7.893421e-01

1026 3.734267e-01 7.894844e-01

1027 3.741661e-01 7.896249e-01

1028 3.748856e-01 7.897639e-01

1029 3.755853e-01 7.899012e-01

1030 3.762652e-01 7.900370e-01

1031 3.769254e-01 7.901714e-01

1032 3.775662e-01 7.903043e-01

1033 3.781878e-01 7.904359e-01

1034 3.787903e-01 7.905662e-01

1035 3.793742e-01 7.906953e-01

1036 3.799398e-01 7.908230e-01

1037 3.804873e-01 7.909497e-01

1038 3.810174e-01 7.910751e-01

1039 3.815302e-01 7.911995e-01

1040 3.820264e-01 7.913228e-01

1041 3.825062e-01 7.914450e-01

1042 3.829703e-01 7.915662e-01

1043 3.834191e-01 7.916865e-01

1044 3.838530e-01 7.918057e-01

1045 3.842725e-01 7.919241e-01

1046 3.846781e-01 7.920415e-01

1047 3.850704e-01 7.921581e-01

1048 3.854497e-01 7.922738e-01

1049 3.858165e-01 7.923886e-01

1050 3.861713e-01 7.925027e-01

1051 3.865146e-01 7.926159e-01

1052 3.868468e-01 7.927283e-01

1053 3.871684e-01 7.928400e-01

1054 3.874797e-01 7.929509e-01

1055 3.877813e-01 7.930611e-01

1056 3.880734e-01 7.931706e-01

1057 3.883566e-01 7.932794e-01

1058 3.886312e-01 7.933874e-01

1059 3.888975e-01 7.934948e-01

1060 3.891559e-01 7.936016e-01

1061 3.894068e-01 7.937076e-01

1062 3.896505e-01 7.938131e-01

1063 3.898873e-01 7.939178e-01

1064 3.901175e-01 7.940220e-01

1065 3.903415e-01 7.941256e-01

1066 3.905595e-01 7.942285e-01

1067 3.907718e-01 7.943309e-01

1068 3.909786e-01 7.944327e-01

1069 3.911803e-01 7.945339e-01

1070 3.913769e-01 7.946345e-01

1071 3.915689e-01 7.947346e-01

1072 3.917563e-01 7.948341e-01

1073 3.919395e-01 7.949331e-01

1074 3.921185e-01 7.950316e-01

1075 3.922937e-01 7.951295e-01

1076 3.924651e-01 7.952269e-01

1077 3.926330e-01 7.953237e-01

1078 3.927975e-01 7.954201e-01

1079 3.929588e-01 7.955160e-01

1080 3.931170e-01 7.956113e-01

1081 3.932723e-01 7.957062e-01

1082 3.932923e-01 7.978246e-01

1083 3.933135e-01 7.999659e-01

1084 3.933362e-01 8.021260e-01

1085 3.933604e-01 8.043007e-01

1086 3.933862e-01 8.064857e-01

1087 3.934136e-01 8.086765e-01

1088 3.934428e-01 8.108689e-01

1089 3.934739e-01 8.130583e-01

1090 3.935071e-01 8.152405e-01

1091 3.935423e-01 8.174110e-01

1092 3.935798e-01 8.195658e-01

1093 3.936196e-01 8.217007e-01

1094 3.936620e-01 8.238119e-01

1095 3.937070e-01 8.258955e-01

1096 3.937549e-01 8.279482e-01

1097 3.938057e-01 8.299666e-01

1098 3.938596e-01 8.319476e-01

1099 3.939169e-01 8.338885e-01

1100 3.939777e-01 8.357868e-01

1101 3.940421e-01 8.376402e-01

1102 3.941104e-01 8.394468e-01

1103 3.941829e-01 8.412050e-01

1104 3.942596e-01 8.429133e-01

1105 3.943409e-01 8.445708e-01

1106 3.944270e-01 8.461766e-01

1107 3.945181e-01 8.477301e-01

1108 3.946145e-01 8.492311e-01

1109 3.947164e-01 8.506795e-01

1110 3.948242e-01 8.520754e-01

1111 3.949381e-01 8.534193e-01

1112 3.950584e-01 8.547117e-01

1113 3.951855e-01 8.559534e-01

1114 3.953196e-01 8.571451e-01

1115 3.954611e-01 8.582880e-01

1116 3.956103e-01 8.593831e-01

1117 3.957675e-01 8.604318e-01

1118 3.959332e-01 8.614352e-01

1119 3.961077e-01 8.623949e-01

1120 3.962913e-01 8.633121e-01

1121 3.964845e-01 8.641885e-01

1122 3.966877e-01 8.650254e-01

1123 3.969011e-01 8.658245e-01

1124 3.971253e-01 8.665871e-01

1125 3.973607e-01 8.673149e-01

1126 3.976076e-01 8.680093e-01

1127 3.978665e-01 8.686718e-01

1128 3.981378e-01 8.693039e-01

1129 3.984219e-01 8.699069e-01

1130 3.987192e-01 8.704823e-01

1131 3.990301e-01 8.710314e-01

1132 3.993550e-01 8.715554e-01

1133 3.996943e-01 8.720558e-01

1134 4.000485e-01 8.725336e-01

1135 4.004178e-01 8.729900e-01

1136 4.008026e-01 8.734262e-01

1137 4.012033e-01 8.738433e-01

1138 4.016202e-01 8.742422e-01

1139 4.020535e-01 8.746239e-01

1140 4.025036e-01 8.749894e-01

1141 4.029707e-01 8.753395e-01

1142 4.034549e-01 8.756752e-01

1143 4.039565e-01 8.759971e-01

1144 4.044756e-01 8.763062e-01

1145 4.050122e-01 8.766030e-01

1146 4.055665e-01 8.768882e-01

1147 4.061384e-01 8.771626e-01

1148 4.067279e-01 8.774268e-01

1149 4.073348e-01 8.776812e-01

1150 4.079591e-01 8.779265e-01

1151 4.086004e-01 8.781631e-01

1152 4.092586e-01 8.783916e-01

1153 4.099333e-01 8.786124e-01

1154 4.106241e-01 8.788259e-01

1155 4.113305e-01 8.790326e-01

1156 4.120520e-01 8.792328e-01

1157 4.127881e-01 8.794269e-01

1158 4.135381e-01 8.796152e-01

1159 4.143013e-01 8.797981e-01

1160 4.150769e-01 8.799758e-01

1161 4.158643e-01 8.801486e-01

1162 4.166623e-01 8.803169e-01

1163 4.174703e-01 8.804807e-01

1164 4.182872e-01 8.806405e-01

1165 4.191120e-01 8.807964e-01

1166 4.199436e-01 8.809485e-01

1167 4.207811e-01 8.810972e-01

1168 4.216233e-01 8.812425e-01

1169 4.224692e-01 8.813848e-01

1170 4.233175e-01 8.815240e-01

1171 4.241671e-01 8.816604e-01

1172 4.250170e-01 8.817942e-01

1173 4.258660e-01 8.819254e-01

1174 4.267130e-01 8.820541e-01

1175 4.275568e-01 8.821806e-01

1176 4.283965e-01 8.823049e-01

1177 4.292308e-01 8.824271e-01

1178 4.300589e-01 8.825473e-01

1179 4.308798e-01 8.826657e-01

1180 4.316924e-01 8.827822e-01

1181 4.324960e-01 8.828971e-01

1182 4.332896e-01 8.830102e-01

1183 4.340726e-01 8.831219e-01

1184 4.348441e-01 8.832320e-01

1185 4.356034e-01 8.833407e-01

1186 4.363501e-01 8.834481e-01

1187 4.370834e-01 8.835541e-01

1188 4.378029e-01 8.836589e-01

1189 4.385083e-01 8.837625e-01

1190 4.391990e-01 8.838649e-01

1191 4.398748e-01 8.839663e-01

1192 4.405354e-01 8.840665e-01

1193 4.411807e-01 8.841658e-01

1194 4.418104e-01 8.842640e-01

1195 4.424244e-01 8.843614e-01

1196 4.430228e-01 8.844578e-01

1197 4.436055e-01 8.845533e-01

1198 4.441726e-01 8.846480e-01

1199 4.447241e-01 8.847419e-01

1200 4.452601e-01 8.848350e-01

1201 4.457808e-01 8.849273e-01

1202 4.462864e-01 8.850189e-01

1203 4.467771e-01 8.851098e-01

1204 4.472532e-01 8.852000e-01

1205 4.477148e-01 8.852895e-01

1206 4.481623e-01 8.853783e-01

1207 4.485960e-01 8.854666e-01

1208 4.490161e-01 8.855542e-01

1209 4.494231e-01 8.856412e-01

1210 4.498172e-01 8.857277e-01

1211 4.501989e-01 8.858136e-01

1212 4.505683e-01 8.858989e-01

1213 4.509260e-01 8.859837e-01

1214 4.512722e-01 8.860680e-01

1215 4.516073e-01 8.861518e-01

1216 4.519317e-01 8.862351e-01

1217 4.522458e-01 8.863179e-01

1218 4.525498e-01 8.864003e-01

1219 4.528441e-01 8.864821e-01

1220 4.531292e-01 8.865636e-01

1221 4.534052e-01 8.866446e-01

1222 4.536726e-01 8.867251e-01

1223 4.539317e-01 8.868052e-01

1224 4.541828e-01 8.868850e-01

1225 4.544262e-01 8.869643e-01

1226 4.546623e-01 8.870432e-01

1227 4.548912e-01 8.871217e-01

1228 4.551134e-01 8.871999e-01

1229 4.553290e-01 8.872776e-01

1230 4.555384e-01 8.873550e-01

1231 4.557418e-01 8.874321e-01

1232 4.559395e-01 8.875087e-01

1233 4.561318e-01 8.875851e-01

1234 4.563187e-01 8.876610e-01

1235 4.565007e-01 8.877367e-01

1236 4.566778e-01 8.878120e-01

1237 4.568504e-01 8.878869e-01

1238 4.570186e-01 8.879616e-01

1239 4.571826e-01 8.880359e-01

1240 4.573426e-01 8.881099e-01

1241 4.574987e-01 8.881836e-01

1242 4.576513e-01 8.882570e-01

1243 4.578003e-01 8.883301e-01

1244 4.579460e-01 8.884028e-01

1245 4.580886e-01 8.884753e-01

1246 4.582281e-01 8.885475e-01

1247 4.583647e-01 8.886194e-01

1248 4.584986e-01 8.886910e-01

1249 4.586298e-01 8.887623e-01

1250 4.587586e-01 8.888334e-01

1251 4.588849e-01 8.889042e-01

1252 4.590090e-01 8.889747e-01

1253 4.591309e-01 8.890449e-01

1254 4.592508e-01 8.891148e-01

1255 4.593686e-01 8.891845e-01

1256 4.594846e-01 8.892540e-01

1257 4.595988e-01 8.893231e-01

1258 4.597113e-01 8.893920e-01

1259 4.598222e-01 8.894607e-01

1260 4.599315e-01 8.895291e-01

1261 4.600394e-01 8.895972e-01

1262 4.600864e-01 8.910922e-01

1263 4.601361e-01 8.925400e-01

1264 4.601886e-01 8.939405e-01

1265 4.602441e-01 8.952935e-01

1266 4.603027e-01 8.965993e-01

1267 4.603646e-01 8.978580e-01

1268 4.604299e-01 8.990702e-01

1269 4.604989e-01 9.002364e-01

1270 4.605716e-01 9.013573e-01

1271 4.606484e-01 9.024339e-01

1272 4.607293e-01 9.034670e-01

1273 4.608145e-01 9.044576e-01

1274 4.609044e-01 9.054069e-01

1275 4.609990e-01 9.063160e-01

1276 4.610987e-01 9.071861e-01

1277 4.612035e-01 9.080185e-01

1278 4.613139e-01 9.088144e-01

1279 4.614299e-01 9.095751e-01

1280 4.615519e-01 9.103020e-01

1281 4.616801e-01 9.109962e-01

1282 4.618148e-01 9.116592e-01

1283 4.619562e-01 9.122922e-01

1284 4.621046e-01 9.128964e-01

1285 4.622603e-01 9.134732e-01

1286 4.624236e-01 9.140237e-01

1287 4.625948e-01 9.145491e-01

1288 4.627741e-01 9.150506e-01

1289 4.629619e-01 9.155294e-01

1290 4.631585e-01 9.159864e-01

1291 4.633641e-01 9.164228e-01

1292 4.635791e-01 9.168396e-01

1293 4.638038e-01 9.172377e-01

1294 4.640385e-01 9.176182e-01

1295 4.642835e-01 9.179818e-01

1296 4.645392e-01 9.183295e-01

1297 4.648057e-01 9.186621e-01

1298 4.650834e-01 9.189804e-01

1299 4.653726e-01 9.192851e-01

1300 4.656737e-01 9.195769e-01

1301 4.659867e-01 9.198566e-01

1302 4.663121e-01 9.201248e-01

1303 4.666501e-01 9.203821e-01

1304 4.670009e-01 9.206290e-01

1305 4.673647e-01 9.208663e-01

1306 4.677418e-01 9.210942e-01

1307 4.681322e-01 9.213135e-01

1308 4.685363e-01 9.215245e-01

1309 4.689541e-01 9.217277e-01

1310 4.693857e-01 9.219235e-01

1311 4.698312e-01 9.221124e-01

1312 4.702906e-01 9.222946e-01

1313 4.707641e-01 9.224706e-01

1314 4.712515e-01 9.226407e-01

1315 4.717529e-01 9.228052e-01

1316 4.722680e-01 9.229645e-01

1317 4.727969e-01 9.231187e-01

1318 4.733393e-01 9.232683e-01

1319 4.738951e-01 9.234133e-01

1320 4.744639e-01 9.235541e-01

1321 4.750455e-01 9.236909e-01

1322 4.756396e-01 9.238240e-01

1323 4.762457e-01 9.239534e-01

1324 4.768635e-01 9.240794e-01

1325 4.774925e-01 9.242022e-01

1326 4.781321e-01 9.243220e-01

1327 4.787819e-01 9.244389e-01

1328 4.794412e-01 9.245530e-01

1329 4.801094e-01 9.246645e-01

1330 4.807858e-01 9.247736e-01

1331 4.814698e-01 9.248803e-01

1332 4.821606e-01 9.249848e-01

1333 4.828574e-01 9.250872e-01

1334 4.835595e-01 9.251875e-01

1335 4.842661e-01 9.252860e-01

1336 4.849763e-01 9.253827e-01

1337 4.856893e-01 9.254776e-01

1338 4.864042e-01 9.255709e-01

1339 4.871203e-01 9.256626e-01

1340 4.878366e-01 9.257529e-01

1341 4.885523e-01 9.258417e-01

1342 4.892665e-01 9.259292e-01

1343 4.899785e-01 9.260154e-01

1344 4.906873e-01 9.261004e-01

1345 4.913923e-01 9.261842e-01

1346 4.920925e-01 9.262669e-01

1347 4.927873e-01 9.263485e-01

1348 4.934760e-01 9.264291e-01

1349 4.941577e-01 9.265088e-01

1350 4.948320e-01 9.265875e-01

1351 4.954981e-01 9.266653e-01

1352 4.961554e-01 9.267422e-01

1353 4.968035e-01 9.268183e-01

1354 4.974418e-01 9.268937e-01

1355 4.980698e-01 9.269683e-01

1356 4.986871e-01 9.270421e-01

1357 4.992934e-01 9.271153e-01

1358 4.998883e-01 9.271878e-01

1359 5.004714e-01 9.272597e-01

1360 5.010427e-01 9.273309e-01

1361 5.016018e-01 9.274016e-01

1362 5.021486e-01 9.274717e-01

1363 5.026829e-01 9.275412e-01

1364 5.032047e-01 9.276102e-01

1365 5.037139e-01 9.276787e-01

1366 5.042104e-01 9.277467e-01

1367 5.046943e-01 9.278142e-01

1368 5.051657e-01 9.278813e-01

1369 5.056245e-01 9.279479e-01

1370 5.060710e-01 9.280140e-01

1371 5.065051e-01 9.280798e-01

1372 5.069271e-01 9.281452e-01

1373 5.073371e-01 9.282101e-01

1374 5.077352e-01 9.282747e-01

1375 5.081218e-01 9.283389e-01

1376 5.084969e-01 9.284028e-01

1377 5.088609e-01 9.284663e-01

1378 5.092140e-01 9.285295e-01

1379 5.095563e-01 9.285923e-01

1380 5.098882e-01 9.286548e-01

1381 5.102099e-01 9.287170e-01

1382 5.105217e-01 9.287789e-01

1383 5.108238e-01 9.288406e-01

1384 5.111166e-01 9.289019e-01

1385 5.114002e-01 9.289629e-01

1386 5.116751e-01 9.290237e-01

1387 5.119413e-01 9.290842e-01

1388 5.121993e-01 9.291444e-01

1389 5.124493e-01 9.292044e-01

1390 5.126916e-01 9.292642e-01

1391 5.129263e-01 9.293237e-01

1392 5.131539e-01 9.293829e-01

1393 5.133744e-01 9.294419e-01

1394 5.135883e-01 9.295007e-01

1395 5.137957e-01 9.295593e-01

1396 5.139969e-01 9.296176e-01

1397 5.141920e-01 9.296758e-01

1398 5.143815e-01 9.297337e-01

1399 5.145654e-01 9.297914e-01

1400 5.147439e-01 9.298489e-01

1401 5.149174e-01 9.299062e-01

1402 5.150860e-01 9.299633e-01

1403 5.152498e-01 9.300203e-01

1404 5.154092e-01 9.300770e-01

1405 5.155642e-01 9.301335e-01

1406 5.157150e-01 9.301899e-01

1407 5.158619e-01 9.302461e-01

1408 5.160050e-01 9.303021e-01

1409 5.161445e-01 9.303579e-01

1410 5.162805e-01 9.304135e-01

1411 5.164131e-01 9.304690e-01

1412 5.165426e-01 9.305243e-01

1413 5.166690e-01 9.305795e-01

1414 5.167924e-01 9.306345e-01

1415 5.169131e-01 9.306893e-01

1416 5.170311e-01 9.307439e-01

1417 5.171466e-01 9.307984e-01

1418 5.172597e-01 9.308528e-01

1419 5.173704e-01 9.309070e-01

1420 5.174789e-01 9.309610e-01

1421 5.175853e-01 9.310149e-01

1422 5.176896e-01 9.310686e-01

1423 5.177921e-01 9.311222e-01

1424 5.178927e-01 9.311757e-01

1425 5.179915e-01 9.312290e-01

1426 5.180887e-01 9.312822e-01

1427 5.181842e-01 9.313352e-01

1428 5.182783e-01 9.313881e-01

1429 5.183709e-01 9.314408e-01

1430 5.184621e-01 9.314934e-01

1431 5.185520e-01 9.315459e-01

1432 5.186406e-01 9.315982e-01

1433 5.187281e-01 9.316504e-01

1434 5.188144e-01 9.317025e-01

1435 5.188996e-01 9.317545e-01

1436 5.189838e-01 9.318063e-01

1437 5.190671e-01 9.318579e-01

1438 5.191494e-01 9.319095e-01

1439 5.192308e-01 9.319609e-01

1440 5.193114e-01 9.320122e-01

1441 5.193911e-01 9.320634e-01

1442 5.194772e-01 9.326144e-01

1443 5.195676e-01 9.331407e-01

1444 5.196624e-01 9.336434e-01

1445 5.197618e-01 9.341235e-01

1446 5.198660e-01 9.345820e-01

1447 5.199752e-01 9.350198e-01

1448 5.200896e-01 9.354380e-01

1449 5.202095e-01 9.358374e-01

1450 5.203349e-01 9.362189e-01

1451 5.204662e-01 9.365834e-01

1452 5.206035e-01 9.369317e-01

1453 5.207470e-01 9.372646e-01

1454 5.208971e-01 9.375828e-01

1455 5.210538e-01 9.378870e-01

1456 5.212176e-01 9.381781e-01

1457 5.213884e-01 9.384565e-01

1458 5.215667e-01 9.387231e-01

1459 5.217527e-01 9.389783e-01

1460 5.219465e-01 9.392228e-01

1461 5.221484e-01 9.394570e-01

1462 5.223587e-01 9.396816e-01

1463 5.225775e-01 9.398971e-01

1464 5.228052e-01 9.401038e-01

1465 5.230419e-01 9.403024e-01

1466 5.232879e-01 9.404931e-01

1467 5.235434e-01 9.406764e-01

1468 5.238085e-01 9.408527e-01

1469 5.240836e-01 9.410224e-01

1470 5.243687e-01 9.411857e-01

1471 5.246641e-01 9.413432e-01

1472 5.249700e-01 9.414949e-01

1473 5.252865e-01 9.416413e-01

1474 5.256137e-01 9.417827e-01

1475 5.259519e-01 9.419193e-01

1476 5.263010e-01 9.420513e-01

1477 5.266613e-01 9.421789e-01

1478 5.270328e-01 9.423026e-01

1479 5.274155e-01 9.424223e-01

1480 5.278095e-01 9.425383e-01

1481 5.282148e-01 9.426509e-01

1482 5.286313e-01 9.427602e-01

1483 5.290591e-01 9.428664e-01

1484 5.294981e-01 9.429696e-01

1485 5.299482e-01 9.430700e-01

1486 5.304093e-01 9.431677e-01

1487 5.308811e-01 9.432629e-01

1488 5.313636e-01 9.433557e-01

1489 5.318565e-01 9.434462e-01

1490 5.323596e-01 9.435345e-01

1491 5.328726e-01 9.436208e-01

1492 5.333952e-01 9.437052e-01

1493 5.339269e-01 9.437876e-01

1494 5.344676e-01 9.438684e-01

1495 5.350167e-01 9.439474e-01

1496 5.355739e-01 9.440249e-01

1497 5.361387e-01 9.441008e-01

1498 5.367105e-01 9.441754e-01

1499 5.372889e-01 9.442485e-01

1500 5.378733e-01 9.443203e-01

1501 5.384631e-01 9.443909e-01

1502 5.390579e-01 9.444604e-01

1503 5.396569e-01 9.445287e-01

1504 5.402596e-01 9.445959e-01

1505 5.408653e-01 9.446621e-01

1506 5.414734e-01 9.447273e-01

1507 5.420832e-01 9.447916e-01

1508 5.426941e-01 9.448550e-01

1509 5.433054e-01 9.449176e-01

1510 5.439165e-01 9.449794e-01

1511 5.445266e-01 9.450404e-01

1512 5.451353e-01 9.451006e-01

1513 5.457417e-01 9.451602e-01

1514 5.463453e-01 9.452190e-01

1515 5.469455e-01 9.452773e-01

1516 5.475417e-01 9.453349e-01

1517 5.481332e-01 9.453919e-01

1518 5.487196e-01 9.454484e-01

1519 5.493003e-01 9.455043e-01

1520 5.498748e-01 9.455597e-01

1521 5.504426e-01 9.456146e-01

1522 5.510033e-01 9.456690e-01

1523 5.515563e-01 9.457230e-01

1524 5.521015e-01 9.457765e-01

1525 5.526382e-01 9.458296e-01

1526 5.531663e-01 9.458823e-01

1527 5.536855e-01 9.459346e-01

1528 5.541954e-01 9.459866e-01

1529 5.546958e-01 9.460381e-01

1530 5.551865e-01 9.460894e-01

1531 5.556674e-01 9.461403e-01

1532 5.561382e-01 9.461908e-01

1533 5.565989e-01 9.462411e-01

1534 5.570494e-01 9.462910e-01

1535 5.574896e-01 9.463407e-01

1536 5.579195e-01 9.463901e-01

1537 5.583390e-01 9.464392e-01

1538 5.587482e-01 9.464881e-01

1539 5.591471e-01 9.465367e-01

1540 5.595357e-01 9.465850e-01

1541 5.599142e-01 9.466331e-01

1542 5.602826e-01 9.466810e-01

1543 5.606410e-01 9.467287e-01

1544 5.609896e-01 9.467762e-01

1545 5.613285e-01 9.468234e-01

1546 5.616578e-01 9.468705e-01

1547 5.619778e-01 9.469173e-01

1548 5.622885e-01 9.469640e-01

1549 5.625902e-01 9.470105e-01

1550 5.628830e-01 9.470568e-01

1551 5.631671e-01 9.471029e-01

1552 5.634428e-01 9.471489e-01

1553 5.637103e-01 9.471947e-01

1554 5.639697e-01 9.472403e-01

1555 5.642213e-01 9.472858e-01

1556 5.644652e-01 9.473312e-01

1557 5.647018e-01 9.473763e-01

1558 5.649311e-01 9.474214e-01

1559 5.651535e-01 9.474663e-01

1560 5.653691e-01 9.475111e-01

1561 5.655781e-01 9.475557e-01

1562 5.657808e-01 9.476002e-01

1563 5.659774e-01 9.476446e-01

1564 5.661680e-01 9.476888e-01

1565 5.663529e-01 9.477329e-01

1566 5.665323e-01 9.477769e-01

1567 5.667063e-01 9.478208e-01

1568 5.668752e-01 9.478646e-01

1569 5.670391e-01 9.479083e-01

1570 5.671982e-01 9.479519e-01

1571 5.673527e-01 9.479953e-01

1572 5.675028e-01 9.480387e-01

1573 5.676486e-01 9.480819e-01

1574 5.677903e-01 9.481250e-01

1575 5.679281e-01 9.481681e-01

1576 5.680621e-01 9.482110e-01

1577 5.681924e-01 9.482539e-01

1578 5.683193e-01 9.482967e-01

1579 5.684428e-01 9.483393e-01

1580 5.685631e-01 9.483819e-01

1581 5.686803e-01 9.484244e-01

1582 5.687945e-01 9.484668e-01

1583 5.689059e-01 9.485091e-01

1584 5.690146e-01 9.485514e-01

1585 5.691207e-01 9.485935e-01

1586 5.692243e-01 9.486356e-01

1587 5.693255e-01 9.486776e-01

1588 5.694244e-01 9.487195e-01

1589 5.695211e-01 9.487614e-01

1590 5.696158e-01 9.488031e-01

1591 5.697084e-01 9.488448e-01

1592 5.697992e-01 9.488864e-01

1593 5.698881e-01 9.489280e-01

1594 5.699752e-01 9.489694e-01

1595 5.700607e-01 9.490108e-01

1596 5.701446e-01 9.490521e-01

1597 5.702270e-01 9.490934e-01

1598 5.703079e-01 9.491346e-01

1599 5.703875e-01 9.491757e-01

1600 5.704657e-01 9.492167e-01

1601 5.705426e-01 9.492577e-01

1602 5.706184e-01 9.492986e-01

1603 5.706930e-01 9.493395e-01

1604 5.707665e-01 9.493803e-01

1605 5.708390e-01 9.494210e-01

1606 5.709104e-01 9.494617e-01

1607 5.709810e-01 9.495023e-01

1608 5.710506e-01 9.495428e-01

1609 5.711194e-01 9.495833e-01

1610 5.711873e-01 9.496237e-01

1611 5.712545e-01 9.496640e-01

1612 5.713210e-01 9.497043e-01

1613 5.713868e-01 9.497446e-01

1614 5.714519e-01 9.497847e-01

1615 5.715163e-01 9.498249e-01

1616 5.715802e-01 9.498649e-01

1617 5.716435e-01 9.499049e-01

1618 5.717063e-01 9.499449e-01

1619 5.717686e-01 9.499847e-01

1620 5.718304e-01 9.500246e-01

1621 5.718917e-01 9.500643e-01

1622 5.720237e-01 9.502610e-01

1623 5.721613e-01 9.504495e-01

1624 5.723046e-01 9.506304e-01

1625 5.724538e-01 9.508039e-01

1626 5.726092e-01 9.509706e-01

1627 5.727709e-01 9.511307e-01

1628 5.729391e-01 9.512845e-01

1629 5.731140e-01 9.514324e-01

1630 5.732957e-01 9.515747e-01

1631 5.734845e-01 9.517117e-01

1632 5.736804e-01 9.518436e-01

1633 5.738838e-01 9.519707e-01

1634 5.740948e-01 9.520932e-01

1635 5.743135e-01 9.522114e-01

1636 5.745402e-01 9.523255e-01

1637 5.747749e-01 9.524357e-01

1638 5.750178e-01 9.525421e-01

1639 5.752692e-01 9.526451e-01

1640 5.755290e-01 9.527447e-01

1641 5.757975e-01 9.528412e-01

1642 5.760748e-01 9.529347e-01

1643 5.763610e-01 9.530253e-01

1644 5.766562e-01 9.531132e-01

1645 5.769604e-01 9.531985e-01

1646 5.772738e-01 9.532814e-01

1647 5.775964e-01 9.533619e-01

1648 5.779283e-01 9.534402e-01

1649 5.782695e-01 9.535165e-01

1650 5.786199e-01 9.535908e-01

1651 5.789796e-01 9.536631e-01

1652 5.793486e-01 9.537337e-01

1653 5.797268e-01 9.538026e-01

1654 5.801141e-01 9.538698e-01

1655 5.805105e-01 9.539355e-01

1656 5.809158e-01 9.539997e-01

1657 5.813299e-01 9.540625e-01

1658 5.817526e-01 9.541240e-01

1659 5.821838e-01 9.541842e-01

1660 5.826233e-01 9.542433e-01

1661 5.830708e-01 9.543011e-01

1662 5.835260e-01 9.543579e-01

1663 5.839887e-01 9.544137e-01

1664 5.844586e-01 9.544684e-01

1665 5.849354e-01 9.545222e-01

1666 5.854187e-01 9.545751e-01

1667 5.859082e-01 9.546272e-01

1668 5.864034e-01 9.546784e-01

1669 5.869040e-01 9.547289e-01

1670 5.874095e-01 9.547786e-01

1671 5.879194e-01 9.548276e-01

1672 5.884335e-01 9.548759e-01

1673 5.889510e-01 9.549236e-01

1674 5.894716e-01 9.549707e-01

1675 5.899948e-01 9.550172e-01

1676 5.905201e-01 9.550631e-01

1677 5.910469e-01 9.551085e-01

1678 5.915748e-01 9.551533e-01

1679 5.921032e-01 9.551977e-01

1680 5.926316e-01 9.552416e-01

1681 5.931594e-01 9.552851e-01

1682 5.936862e-01 9.553281e-01

1683 5.942115e-01 9.553708e-01

1684 5.947348e-01 9.554130e-01

1685 5.952555e-01 9.554548e-01

1686 5.957732e-01 9.554963e-01

1687 5.962874e-01 9.555375e-01

1688 5.967976e-01 9.555783e-01

1689 5.973035e-01 9.556188e-01

1690 5.978046e-01 9.556590e-01

1691 5.983005e-01 9.556989e-01

1692 5.987908e-01 9.557385e-01

1693 5.992751e-01 9.557778e-01

1694 5.997532e-01 9.558169e-01

1695 6.002247e-01 9.558558e-01

1696 6.006893e-01 9.558944e-01

1697 6.011467e-01 9.559328e-01

1698 6.015967e-01 9.559709e-01

1699 6.020391e-01 9.560089e-01

1700 6.024737e-01 9.560466e-01

1701 6.029002e-01 9.560842e-01

1702 6.033187e-01 9.561215e-01

1703 6.037288e-01 9.561587e-01

1704 6.041305e-01 9.561957e-01

1705 6.045237e-01 9.562326e-01

1706 6.049084e-01 9.562692e-01

1707 6.052846e-01 9.563057e-01

1708 6.056521e-01 9.563421e-01

1709 6.060110e-01 9.563783e-01

1710 6.063613e-01 9.564144e-01

1711 6.067030e-01 9.564504e-01

1712 6.070362e-01 9.564862e-01

1713 6.073609e-01 9.565219e-01

1714 6.076772e-01 9.565574e-01

1715 6.079853e-01 9.565929e-01

1716 6.082851e-01 9.566282e-01

1717 6.085768e-01 9.566634e-01

1718 6.088605e-01 9.566986e-01

1719 6.091363e-01 9.567336e-01

1720 6.094044e-01 9.567685e-01

1721 6.096650e-01 9.568033e-01

1722 6.099181e-01 9.568380e-01

1723 6.101639e-01 9.568727e-01

1724 6.104026e-01 9.569072e-01

1725 6.106343e-01 9.569417e-01

1726 6.108593e-01 9.569761e-01

1727 6.110776e-01 9.570104e-01

1728 6.112894e-01 9.570446e-01

1729 6.114950e-01 9.570787e-01

1730 6.116944e-01 9.571128e-01

1731 6.118879e-01 9.571468e-01

1732 6.120756e-01 9.571807e-01

1733 6.122577e-01 9.572146e-01

1734 6.124343e-01 9.572484e-01

1735 6.126057e-01 9.572822e-01

1736 6.127720e-01 9.573158e-01

1737 6.129333e-01 9.573495e-01

1738 6.130898e-01 9.573830e-01

1739 6.132418e-01 9.574165e-01

1740 6.133892e-01 9.574500e-01

1741 6.135323e-01 9.574834e-01

1742 6.136713e-01 9.575167e-01

1743 6.138062e-01 9.575500e-01

1744 6.139372e-01 9.575832e-01

1745 6.140645e-01 9.576164e-01

1746 6.141882e-01 9.576496e-01

1747 6.143085e-01 9.576827e-01

1748 6.144254e-01 9.577158e-01

1749 6.145390e-01 9.577488e-01

1750 6.146496e-01 9.577817e-01

1751 6.147572e-01 9.578147e-01

1752 6.148620e-01 9.578476e-01

1753 6.149640e-01 9.578804e-01

1754 6.150633e-01 9.579132e-01

1755 6.151602e-01 9.579460e-01

1756 6.152546e-01 9.579787e-01

1757 6.153466e-01 9.580114e-01

1758 6.154364e-01 9.580441e-01

1759 6.155241e-01 9.580767e-01

1760 6.156097e-01 9.581093e-01

1761 6.156933e-01 9.581419e-01

1762 6.157751e-01 9.581744e-01

1763 6.158550e-01 9.582069e-01

1764 6.159332e-01 9.582394e-01

1765 6.160097e-01 9.582719e-01

1766 6.160846e-01 9.583043e-01

1767 6.161580e-01 9.583366e-01

1768 6.162300e-01 9.583690e-01

1769 6.163005e-01 9.584013e-01

1770 6.163697e-01 9.584336e-01

1771 6.164377e-01 9.584659e-01

1772 6.165044e-01 9.584981e-01

1773 6.165700e-01 9.585304e-01

1774 6.166345e-01 9.585625e-01

1775 6.166979e-01 9.585947e-01

1776 6.167603e-01 9.586268e-01

1777 6.168217e-01 9.586590e-01

1778 6.168822e-01 9.586910e-01

1779 6.169418e-01 9.587231e-01

1780 6.170006e-01 9.587552e-01

1781 6.170586e-01 9.587872e-01

1782 6.171159e-01 9.588192e-01

1783 6.171724e-01 9.588511e-01

1784 6.172283e-01 9.588831e-01

1785 6.172834e-01 9.589150e-01

1786 6.173380e-01 9.589469e-01

1787 6.173920e-01 9.589788e-01

1788 6.174455e-01 9.590107e-01

1789 6.174984e-01 9.590425e-01

1790 6.175508e-01 9.590743e-01

1791 6.176027e-01 9.591061e-01

1792 6.176542e-01 9.591379e-01

1793 6.177053e-01 9.591696e-01

1794 6.177560e-01 9.592013e-01

1795 6.178063e-01 9.592331e-01

1796 6.178562e-01 9.592647e-01

1797 6.179058e-01 9.592964e-01

1798 6.179551e-01 9.593280e-01

1799 6.180042e-01 9.593597e-01

1800 6.180529e-01 9.593913e-01

1801 6.181014e-01 9.594228e-01

1802 6.182787e-01 9.595061e-01

1803 6.184624e-01 9.595867e-01

1804 6.186525e-01 9.596647e-01

1805 6.188492e-01 9.597402e-01

1806 6.190526e-01 9.598134e-01

1807 6.192628e-01 9.598844e-01

1808 6.194800e-01 9.599533e-01

1809 6.197043e-01 9.600201e-01

1810 6.199358e-01 9.600851e-01

1811 6.201746e-01 9.601482e-01

1812 6.204207e-01 9.602096e-01

1813 6.206743e-01 9.602694e-01

1814 6.209355e-01 9.603276e-01

1815 6.212042e-01 9.603843e-01

1816 6.214807e-01 9.604396e-01

1817 6.217648e-01 9.604935e-01

1818 6.220567e-01 9.605462e-01

1819 6.223564e-01 9.605976e-01

1820 6.226638e-01 9.606479e-01

1821 6.229790e-01 9.606970e-01

1822 6.233019e-01 9.607451e-01

1823 6.236326e-01 9.607922e-01

1824 6.239708e-01 9.608383e-01

1825 6.243167e-01 9.608835e-01

1826 6.246701e-01 9.609279e-01

1827 6.250308e-01 9.609714e-01

1828 6.253988e-01 9.610141e-01

1829 6.257739e-01 9.610561e-01

1830 6.261560e-01 9.610973e-01

1831 6.265449e-01 9.611379e-01

1832 6.269404e-01 9.611778e-01

1833 6.273422e-01 9.612171e-01

1834 6.277502e-01 9.612558e-01

1835 6.281641e-01 9.612939e-01

1836 6.285836e-01 9.613315e-01

1837 6.290085e-01 9.613685e-01

1838 6.294384e-01 9.614051e-01

1839 6.298730e-01 9.614412e-01

1840 6.303121e-01 9.614768e-01

1841 6.307552e-01 9.615120e-01

1842 6.312020e-01 9.615468e-01

1843 6.316521e-01 9.615812e-01

1844 6.321051e-01 9.616152e-01

1845 6.325608e-01 9.616489e-01

1846 6.330185e-01 9.616822e-01

1847 6.334781e-01 9.617152e-01

1848 6.339389e-01 9.617478e-01

1849 6.344007e-01 9.617802e-01

1850 6.348631e-01 9.618123e-01

1851 6.353255e-01 9.618441e-01

1852 6.357875e-01 9.618756e-01

1853 6.362489e-01 9.619069e-01

1854 6.367091e-01 9.619380e-01

1855 6.371677e-01 9.619688e-01

1856 6.376243e-01 9.619994e-01

1857 6.380786e-01 9.620298e-01

1858 6.385302e-01 9.620600e-01

1859 6.389787e-01 9.620900e-01

1860 6.394236e-01 9.621198e-01

1861 6.398648e-01 9.621494e-01

1862 6.403018e-01 9.621789e-01

1863 6.407343e-01 9.622081e-01

1864 6.411621e-01 9.622373e-01

1865 6.415848e-01 9.622662e-01

1866 6.420021e-01 9.622951e-01

1867 6.424138e-01 9.623238e-01

1868 6.428197e-01 9.623523e-01

1869 6.432196e-01 9.623808e-01

1870 6.436132e-01 9.624091e-01

1871 6.440004e-01 9.624373e-01

1872 6.443809e-01 9.624654e-01

1873 6.447548e-01 9.624933e-01

1874 6.451217e-01 9.625212e-01

1875 6.454817e-01 9.625490e-01

1876 6.458346e-01 9.625766e-01

1877 6.461804e-01 9.626042e-01

1878 6.465189e-01 9.626317e-01

1879 6.468502e-01 9.626591e-01

1880 6.471742e-01 9.626864e-01

1881 6.474910e-01 9.627137e-01

1882 6.478004e-01 9.627408e-01

1883 6.481026e-01 9.627679e-01

1884 6.483975e-01 9.627950e-01

1885 6.486852e-01 9.628219e-01

1886 6.489658e-01 9.628488e-01

1887 6.492393e-01 9.628757e-01

1888 6.495057e-01 9.629024e-01

1889 6.497653e-01 9.629291e-01

1890 6.500179e-01 9.629558e-01

1891 6.502638e-01 9.629824e-01

1892 6.505031e-01 9.630090e-01

1893 6.507358e-01 9.630355e-01

1894 6.509621e-01 9.630619e-01

1895 6.511820e-01 9.630884e-01

1896 6.513958e-01 9.631147e-01

1897 6.516035e-01 9.631411e-01

1898 6.518053e-01 9.631674e-01

1899 6.520013e-01 9.631936e-01

1900 6.521917e-01 9.632198e-01

1901 6.523765e-01 9.632460e-01

1902 6.525559e-01 9.632722e-01

1903 6.527301e-01 9.632983e-01

1904 6.528992e-01 9.633244e-01

1905 6.530633e-01 9.633504e-01

1906 6.532226e-01 9.633764e-01

1907 6.533772e-01 9.634024e-01

1908 6.535272e-01 9.634284e-01

1909 6.536728e-01 9.634544e-01

1910 6.538141e-01 9.634803e-01

1911 6.539513e-01 9.635062e-01

1912 6.540845e-01 9.635320e-01

1913 6.542137e-01 9.635579e-01

1914 6.543392e-01 9.635837e-01

1915 6.544611e-01 9.636095e-01

1916 6.545794e-01 9.636353e-01

1917 6.546943e-01 9.636611e-01

1918 6.548060e-01 9.636869e-01

1919 6.549144e-01 9.637126e-01

1920 6.550199e-01 9.637383e-01

1921 6.551223e-01 9.637641e-01

1922 6.552219e-01 9.637898e-01

1923 6.553188e-01 9.638154e-01

1924 6.554130e-01 9.638411e-01

1925 6.555047e-01 9.638668e-01

1926 6.555940e-01 9.638924e-01

1927 6.556809e-01 9.639181e-01

1928 6.557655e-01 9.639437e-01

1929 6.558479e-01 9.639693e-01

1930 6.559283e-01 9.639949e-01

1931 6.560066e-01 9.640205e-01

1932 6.560830e-01 9.640461e-01

1933 6.561576e-01 9.640717e-01

1934 6.562303e-01 9.640972e-01

1935 6.563014e-01 9.641228e-01

1936 6.563708e-01 9.641483e-01

1937 6.564386e-01 9.641739e-01

1938 6.565049e-01 9.641994e-01

1939 6.565698e-01 9.642250e-01

1940 6.566333e-01 9.642505e-01

1941 6.566954e-01 9.642760e-01

1942 6.567563e-01 9.643015e-01

1943 6.568160e-01 9.643271e-01

1944 6.568744e-01 9.643526e-01

1945 6.569318e-01 9.643781e-01

1946 6.569881e-01 9.644036e-01

1947 6.570434e-01 9.644291e-01

1948 6.570977e-01 9.644546e-01

1949 6.571511e-01 9.644801e-01

1950 6.572036e-01 9.645056e-01

1951 6.572552e-01 9.645310e-01

1952 6.573060e-01 9.645565e-01

1953 6.573560e-01 9.645820e-01

1954 6.574053e-01 9.646075e-01

1955 6.574539e-01 9.646330e-01

1956 6.575019e-01 9.646584e-01

1957 6.575492e-01 9.646839e-01

1958 6.575958e-01 9.647094e-01

1959 6.576419e-01 9.647348e-01

1960 6.576875e-01 9.647603e-01

1961 6.577325e-01 9.647858e-01

1962 6.577771e-01 9.648112e-01

1963 6.578211e-01 9.648367e-01

1964 6.578647e-01 9.648621e-01

1965 6.579079e-01 9.648876e-01

1966 6.579507e-01 9.649130e-01

1967 6.579932e-01 9.649385e-01

1968 6.580352e-01 9.649639e-01

1969 6.580769e-01 9.649894e-01

1970 6.581184e-01 9.650148e-01

1971 6.581595e-01 9.650403e-01

1972 6.582003e-01 9.650657e-01

1973 6.582409e-01 9.650911e-01

1974 6.582812e-01 9.651166e-01

1975 6.583214e-01 9.651420e-01

1976 6.583613e-01 9.651674e-01

1977 6.584010e-01 9.651928e-01

1978 6.584405e-01 9.652183e-01

1979 6.584798e-01 9.652437e-01

1980 6.585190e-01 9.652691e-01

1981 6.585581e-01 9.652945e-01

1982 6.587738e-01 9.653368e-01

1983 6.589959e-01 9.653781e-01

1984 6.592243e-01 9.654184e-01

1985 6.594592e-01 9.654578e-01

1986 6.597005e-01 9.654963e-01

1987 6.599484e-01 9.655340e-01

1988 6.602029e-01 9.655709e-01

1989 6.604639e-01 9.656070e-01

1990 6.607315e-01 9.656424e-01

1991 6.610057e-01 9.656772e-01

1992 6.612865e-01 9.657112e-01

1993 6.615739e-01 9.657446e-01

1994 6.618677e-01 9.657775e-01

1995 6.621681e-01 9.658097e-01

1996 6.624749e-01 9.658414e-01

1997 6.627881e-01 9.658726e-01

1998 6.631075e-01 9.659033e-01

1999 6.634331e-01 9.659335e-01

2000 6.637647e-01 9.659633e-01

2001 6.641023e-01 9.659926e-01

2002 6.644456e-01 9.660215e-01

2003 6.647946e-01 9.660500e-01

2004 6.651490e-01 9.660781e-01

2005 6.655087e-01 9.661059e-01

2006 6.658735e-01 9.661333e-01

2007 6.662431e-01 9.661604e-01

2008 6.666174e-01 9.661872e-01

2009 6.669960e-01 9.662137e-01

2010 6.673788e-01 9.662399e-01

2011 6.677654e-01 9.662658e-01

2012 6.681557e-01 9.662914e-01

2013 6.685492e-01 9.663168e-01

2014 6.689458e-01 9.663420e-01

2015 6.693451e-01 9.663669e-01

2016 6.697467e-01 9.663916e-01

2017 6.701505e-01 9.664161e-01

2018 6.705560e-01 9.664404e-01

2019 6.709629e-01 9.664645e-01

2020 6.713708e-01 9.664884e-01

2021 6.717795e-01 9.665122e-01

2022 6.721886e-01 9.665357e-01

2023 6.725978e-01 9.665591e-01

2024 6.730066e-01 9.665824e-01

2025 6.734148e-01 9.666055e-01

2026 6.738221e-01 9.666285e-01

2027 6.742280e-01 9.666513e-01

2028 6.746323e-01 9.666740e-01

2029 6.750347e-01 9.666966e-01

2030 6.754347e-01 9.667190e-01

2031 6.758322e-01 9.667414e-01

2032 6.762268e-01 9.667636e-01

2033 6.766183e-01 9.667858e-01

2034 6.770063e-01 9.668078e-01

2035 6.773905e-01 9.668298e-01

2036 6.777708e-01 9.668516e-01

2037 6.781468e-01 9.668734e-01

2038 6.785184e-01 9.668951e-01

2039 6.788854e-01 9.669167e-01

2040 6.792474e-01 9.669382e-01

2041 6.796044e-01 9.669596e-01

2042 6.799561e-01 9.669810e-01

2043 6.803025e-01 9.670023e-01

2044 6.806433e-01 9.670236e-01

2045 6.809784e-01 9.670448e-01

2046 6.813077e-01 9.670659e-01

2047 6.816311e-01 9.670870e-01

2048 6.819485e-01 9.671081e-01

2049 6.822599e-01 9.671290e-01

2050 6.825651e-01 9.671500e-01

2051 6.828641e-01 9.671709e-01

2052 6.831570e-01 9.671917e-01

2053 6.834436e-01 9.672125e-01

2054 6.837239e-01 9.672333e-01

2055 6.839980e-01 9.672540e-01

2056 6.842658e-01 9.672747e-01

2057 6.845274e-01 9.672953e-01

2058 6.847828e-01 9.673160e-01

2059 6.850320e-01 9.673366e-01

2060 6.852752e-01 9.673571e-01

2061 6.855123e-01 9.673777e-01

2062 6.857434e-01 9.673982e-01

2063 6.859685e-01 9.674187e-01

2064 6.861878e-01 9.674391e-01

2065 6.864014e-01 9.674596e-01

2066 6.866093e-01 9.674800e-01

2067 6.868116e-01 9.675004e-01

2068 6.870084e-01 9.675208e-01

2069 6.871998e-01 9.675411e-01

2070 6.873859e-01 9.675615e-01

2071 6.875668e-01 9.675818e-01

2072 6.877427e-01 9.676021e-01

2073 6.879136e-01 9.676224e-01

2074 6.880796e-01 9.676427e-01

2075 6.882409e-01 9.676630e-01

2076 6.883976e-01 9.676833e-01

2077 6.885498e-01 9.677035e-01

2078 6.886975e-01 9.677238e-01

2079 6.888410e-01 9.677440e-01

2080 6.889804e-01 9.677643e-01

2081 6.891156e-01 9.677845e-01

2082 6.892470e-01 9.678047e-01

2083 6.893745e-01 9.678249e-01

2084 6.894983e-01 9.678452e-01

2085 6.896185e-01 9.678654e-01

2086 6.897352e-01 9.678856e-01

2087 6.898485e-01 9.679058e-01

2088 6.899585e-01 9.679260e-01

2089 6.900653e-01 9.679462e-01

2090 6.901691e-01 9.679664e-01

2091 6.902699e-01 9.679866e-01

2092 6.903678e-01 9.680068e-01

2093 6.904629e-01 9.680270e-01

2094 6.905554e-01 9.680472e-01

2095 6.906452e-01 9.680674e-01

2096 6.907325e-01 9.680876e-01

2097 6.908175e-01 9.681078e-01

2098 6.909000e-01 9.681280e-01

2099 6.909804e-01 9.681482e-01

2100 6.910585e-01 9.681684e-01

2101 6.911346e-01 9.681886e-01

2102 6.912087e-01 9.682089e-01

2103 6.912808e-01 9.682291e-01

2104 6.913510e-01 9.682493e-01

2105 6.914195e-01 9.682695e-01

2106 6.914862e-01 9.682898e-01

2107 6.915512e-01 9.683100e-01

2108 6.916147e-01 9.683303e-01

2109 6.916766e-01 9.683505e-01

2110 6.917370e-01 9.683708e-01

2111 6.917961e-01 9.683911e-01

2112 6.918537e-01 9.684114e-01

2113 6.919101e-01 9.684316e-01

2114 6.919651e-01 9.684519e-01

2115 6.920190e-01 9.684722e-01

2116 6.920717e-01 9.684925e-01

2117 6.921234e-01 9.685128e-01

2118 6.921739e-01 9.685332e-01

2119 6.922234e-01 9.685535e-01

2120 6.922720e-01 9.685738e-01

2121 6.923196e-01 9.685942e-01

2122 6.923663e-01 9.686145e-01

2123 6.924122e-01 9.686349e-01

2124 6.924572e-01 9.686553e-01

2125 6.925015e-01 9.686756e-01

2126 6.925450e-01 9.686960e-01

2127 6.925878e-01 9.687164e-01

2128 6.926299e-01 9.687368e-01

2129 6.926714e-01 9.687572e-01

2130 6.927122e-01 9.687777e-01

2131 6.927525e-01 9.687981e-01

2132 6.927922e-01 9.688185e-01

2133 6.928313e-01 9.688390e-01

2134 6.928700e-01 9.688594e-01

2135 6.929081e-01 9.688799e-01

2136 6.929458e-01 9.689004e-01

2137 6.929830e-01 9.689208e-01

2138 6.930198e-01 9.689413e-01

2139 6.930562e-01 9.689618e-01

2140 6.930923e-01 9.689823e-01

2141 6.931280e-01 9.690028e-01

2142 6.931633e-01 9.690234e-01

2143 6.931984e-01 9.690439e-01

2144 6.932331e-01 9.690644e-01

2145 6.932675e-01 9.690850e-01

2146 6.933017e-01 9.691055e-01

2147 6.933356e-01 9.691261e-01

2148 6.933693e-01 9.691466e-01

2149 6.934028e-01 9.691672e-01

2150 6.934361e-01 9.691878e-01

2151 6.934692e-01 9.692084e-01

2152 6.935021e-01 9.692290e-01

2153 6.935348e-01 9.692496e-01

2154 6.935674e-01 9.692702e-01

2155 6.935998e-01 9.692908e-01

2156 6.936321e-01 9.693114e-01

2157 6.936643e-01 9.693320e-01

2158 6.936964e-01 9.693526e-01

2159 6.937284e-01 9.693733e-01

2160 6.937603e-01 9.693939e-01

2161 6.937921e-01 9.694145e-01

2162 6.940353e-01 9.694393e-01

2163 6.942842e-01 9.694637e-01

2164 6.945387e-01 9.694877e-01

2165 6.947988e-01 9.695114e-01

2166 6.950646e-01 9.695346e-01

2167 6.953359e-01 9.695576e-01

2168 6.956127e-01 9.695802e-01

2169 6.958949e-01 9.696024e-01

2170 6.961825e-01 9.696244e-01

2171 6.964754e-01 9.696461e-01

2172 6.967734e-01 9.696675e-01

2173 6.970765e-01 9.696887e-01

2174 6.973846e-01 9.697096e-01

2175 6.976974e-01 9.697303e-01

2176 6.980150e-01 9.697507e-01

2177 6.983370e-01 9.697709e-01

2178 6.986634e-01 9.697909e-01

2179 6.989939e-01 9.698107e-01

2180 6.993285e-01 9.698304e-01

2181 6.996668e-01 9.698498e-01

2182 7.000087e-01 9.698691e-01

2183 7.003539e-01 9.698881e-01

2184 7.007023e-01 9.699071e-01

2185 7.010535e-01 9.699259e-01

2186 7.014074e-01 9.699445e-01

2187 7.017637e-01 9.699630e-01

2188 7.021221e-01 9.699813e-01

2189 7.024824e-01 9.699996e-01

2190 7.028442e-01 9.700177e-01

2191 7.032074e-01 9.700357e-01

2192 7.035717e-01 9.700535e-01

2193 7.039367e-01 9.700713e-01

2194 7.043021e-01 9.700890e-01

2195 7.046678e-01 9.701066e-01

2196 7.050334e-01 9.701240e-01

2197 7.053986e-01 9.701414e-01

2198 7.057631e-01 9.701588e-01

2199 7.061267e-01 9.701760e-01

2200 7.064890e-01 9.701931e-01

2201 7.068499e-01 9.702102e-01

2202 7.072090e-01 9.702272e-01

2203 7.075661e-01 9.702442e-01

2204 7.079209e-01 9.702610e-01

2205 7.082732e-01 9.702778e-01

2206 7.086227e-01 9.702946e-01

2207 7.089692e-01 9.703113e-01

2208 7.093125e-01 9.703279e-01

2209 7.096523e-01 9.703445e-01

2210 7.099885e-01 9.703611e-01

2211 7.103208e-01 9.703776e-01

2212 7.106491e-01 9.703941e-01

2213 7.109732e-01 9.704105e-01

2214 7.112929e-01 9.704269e-01

2215 7.116082e-01 9.704432e-01

2216 7.119188e-01 9.704595e-01

2217 7.122246e-01 9.704758e-01

2218 7.125255e-01 9.704921e-01

2219 7.128214e-01 9.705083e-01

2220 7.131123e-01 9.705245e-01

2221 7.133979e-01 9.705406e-01

2222 7.136784e-01 9.705568e-01

2223 7.139535e-01 9.705729e-01

2224 7.142233e-01 9.705890e-01

2225 7.144877e-01 9.706051e-01

2226 7.147467e-01 9.706211e-01

2227 7.150003e-01 9.706372e-01

2228 7.152485e-01 9.706532e-01

2229 7.154912e-01 9.706692e-01

2230 7.157285e-01 9.706852e-01

2231 7.159604e-01 9.707012e-01

2232 7.161869e-01 9.707171e-01

2233 7.164081e-01 9.707331e-01

2234 7.166239e-01 9.707490e-01

2235 7.168345e-01 9.707650e-01

2236 7.170399e-01 9.707809e-01

2237 7.172401e-01 9.707968e-01

2238 7.174353e-01 9.708127e-01

2239 7.176254e-01 9.708287e-01

2240 7.178106e-01 9.708446e-01

2241 7.179909e-01 9.708605e-01

2242 7.181664e-01 9.708764e-01

2243 7.183373e-01 9.708923e-01

2244 7.185034e-01 9.709082e-01

2245 7.186651e-01 9.709240e-01

2246 7.188223e-01 9.709399e-01

2247 7.189752e-01 9.709558e-01

2248 7.191237e-01 9.709717e-01

2249 7.192682e-01 9.709876e-01

2250 7.194085e-01 9.710035e-01

2251 7.195449e-01 9.710194e-01

2252 7.196774e-01 9.710353e-01

2253 7.198061e-01 9.710512e-01

2254 7.199311e-01 9.710671e-01

2255 7.200526e-01 9.710830e-01

2256 7.201705e-01 9.710990e-01

2257 7.202851e-01 9.711149e-01

2258 7.203963e-01 9.711308e-01

2259 7.205043e-01 9.711467e-01

2260 7.206092e-01 9.711627e-01

2261 7.207111e-01 9.711786e-01

2262 7.208101e-01 9.711946e-01

2263 7.209062e-01 9.712106e-01

2264 7.209995e-01 9.712265e-01

2265 7.210901e-01 9.712425e-01

2266 7.211782e-01 9.712585e-01

2267 7.212638e-01 9.712745e-01

2268 7.213469e-01 9.712905e-01

2269 7.214277e-01 9.713065e-01

2270 7.215062e-01 9.713226e-01

2271 7.215825e-01 9.713386e-01

2272 7.216567e-01 9.713546e-01

2273 7.217288e-01 9.713707e-01

2274 7.217990e-01 9.713868e-01

2275 7.218673e-01 9.714028e-01

2276 7.219337e-01 9.714189e-01

2277 7.219983e-01 9.714350e-01

2278 7.220612e-01 9.714512e-01

2279 7.221225e-01 9.714673e-01

2280 7.221822e-01 9.714834e-01

2281 7.222403e-01 9.714996e-01

2282 7.222970e-01 9.715157e-01

2283 7.223522e-01 9.715319e-01

2284 7.224061e-01 9.715481e-01

2285 7.224587e-01 9.715643e-01

2286 7.225099e-01 9.715805e-01

2287 7.225600e-01 9.715967e-01

2288 7.226089e-01 9.716130e-01

2289 7.226567e-01 9.716292e-01

2290 7.227034e-01 9.716455e-01

2291 7.227490e-01 9.716618e-01

2292 7.227937e-01 9.716781e-01

2293 7.228374e-01 9.716944e-01

2294 7.228802e-01 9.717107e-01

2295 7.229221e-01 9.717270e-01

2296 7.229631e-01 9.717434e-01

2297 7.230033e-01 9.717597e-01

2298 7.230428e-01 9.717761e-01

2299 7.230815e-01 9.717925e-01

2300 7.231195e-01 9.718089e-01

2301 7.231568e-01 9.718253e-01

2302 7.231935e-01 9.718417e-01

2303 7.232295e-01 9.718582e-01

2304 7.232650e-01 9.718746e-01

2305 7.232999e-01 9.718911e-01

2306 7.233342e-01 9.719076e-01

2307 7.233680e-01 9.719241e-01

2308 7.234013e-01 9.719406e-01

2309 7.234342e-01 9.719571e-01

2310 7.234665e-01 9.719737e-01

2311 7.234985e-01 9.719902e-01

2312 7.235300e-01 9.720068e-01

2313 7.235612e-01 9.720233e-01

2314 7.235920e-01 9.720399e-01

2315 7.236224e-01 9.720565e-01

2316 7.236525e-01 9.720731e-01

2317 7.236823e-01 9.720898e-01

2318 7.237118e-01 9.721064e-01

2319 7.237410e-01 9.721230e-01

2320 7.237699e-01 9.721397e-01

2321 7.237986e-01 9.721564e-01

2322 7.238270e-01 9.721730e-01

2323 7.238552e-01 9.721897e-01

2324 7.238832e-01 9.722064e-01

2325 7.239110e-01 9.722232e-01

2326 7.239386e-01 9.722399e-01

2327 7.239661e-01 9.722566e-01

2328 7.239933e-01 9.722734e-01

2329 7.240205e-01 9.722901e-01

2330 7.240474e-01 9.723069e-01

2331 7.240743e-01 9.723236e-01

2332 7.241010e-01 9.723404e-01

2333 7.241277e-01 9.723572e-01

2334 7.241542e-01 9.723740e-01

2335 7.241806e-01 9.723908e-01

2336 7.242070e-01 9.724076e-01

2337 7.242333e-01 9.724244e-01

2338 7.242595e-01 9.724412e-01

2339 7.242856e-01 9.724580e-01

2340 7.243117e-01 9.724749e-01

2341 7.243378e-01 9.724917e-01

2342 7.245964e-01 9.725079e-01

2343 7.248596e-01 9.725238e-01

2344 7.251272e-01 9.725397e-01

2345 7.253994e-01 9.725553e-01

2346 7.256758e-01 9.725708e-01

2347 7.259565e-01 9.725861e-01

2348 7.262413e-01 9.726013e-01

2349 7.265301e-01 9.726164e-01

2350 7.268228e-01 9.726313e-01

2351 7.271191e-01 9.726461e-01

2352 7.274190e-01 9.726608e-01

2353 7.277222e-01 9.726753e-01

2354 7.280287e-01 9.726898e-01

2355 7.283382e-01 9.727041e-01

2356 7.286505e-01 9.727184e-01

2357 7.289655e-01 9.727325e-01

2358 7.292830e-01 9.727466e-01

2359 7.296027e-01 9.727605e-01

2360 7.299244e-01 9.727744e-01

2361 7.302479e-01 9.727882e-01

2362 7.305730e-01 9.728020e-01

2363 7.308995e-01 9.728156e-01

2364 7.312271e-01 9.728292e-01

2365 7.315555e-01 9.728427e-01

2366 7.318846e-01 9.728562e-01

2367 7.322142e-01 9.728696e-01

2368 7.325439e-01 9.728829e-01

2369 7.328735e-01 9.728962e-01

2370 7.332028e-01 9.729094e-01

2371 7.335316e-01 9.729226e-01

2372 7.338596e-01 9.729358e-01

2373 7.341865e-01 9.729489e-01

2374 7.345122e-01 9.729619e-01

2375 7.348364e-01 9.729749e-01

2376 7.351589e-01 9.729879e-01

2377 7.354795e-01 9.730008e-01

2378 7.357980e-01 9.730138e-01

2379 7.361140e-01 9.730266e-01

2380 7.364276e-01 9.730395e-01

2381 7.367384e-01 9.730523e-01

2382 7.370463e-01 9.730651e-01

2383 7.373511e-01 9.730778e-01

2384 7.376526e-01 9.730906e-01

2385 7.379506e-01 9.731033e-01

2386 7.382451e-01 9.731160e-01

2387 7.385359e-01 9.731287e-01

2388 7.388228e-01 9.731413e-01

2389 7.391057e-01 9.731540e-01

2390 7.393845e-01 9.731666e-01

2391 7.396591e-01 9.731792e-01

2392 7.399294e-01 9.731918e-01

2393 7.401952e-01 9.732044e-01

2394 7.404567e-01 9.732169e-01

2395 7.407136e-01 9.732295e-01

2396 7.409658e-01 9.732420e-01

2397 7.412135e-01 9.732546e-01

2398 7.414564e-01 9.732671e-01

2399 7.416947e-01 9.732797e-01

2400 7.419282e-01 9.732922e-01

2401 7.421569e-01 9.733047e-01

2402 7.423808e-01 9.733172e-01

2403 7.426000e-01 9.733297e-01

2404 7.428144e-01 9.733422e-01

2405 7.430241e-01 9.733547e-01

2406 7.432290e-01 9.733673e-01

2407 7.434293e-01 9.733798e-01

2408 7.436248e-01 9.733923e-01

2409 7.438157e-01 9.734048e-01

2410 7.440021e-01 9.734173e-01

2411 7.441838e-01 9.734298e-01

2412 7.443611e-01 9.734423e-01

2413 7.445339e-01 9.734548e-01

2414 7.447024e-01 9.734673e-01

2415 7.448665e-01 9.734799e-01

2416 7.450264e-01 9.734924e-01

2417 7.451821e-01 9.735049e-01

2418 7.453336e-01 9.735175e-01

2419 7.454811e-01 9.735300e-01

2420 7.456247e-01 9.735426e-01

2421 7.457644e-01 9.735551e-01

2422 7.459002e-01 9.735677e-01

2423 7.460323e-01 9.735802e-01

2424 7.461608e-01 9.735928e-01

2425 7.462857e-01 9.736054e-01

2426 7.464071e-01 9.736180e-01

2427 7.465250e-01 9.736306e-01

2428 7.466397e-01 9.736432e-01

2429 7.467511e-01 9.736559e-01

2430 7.468594e-01 9.736685e-01

2431 7.469645e-01 9.736811e-01

2432 7.470667e-01 9.736938e-01

2433 7.471659e-01 9.737065e-01

2434 7.472623e-01 9.737192e-01

2435 7.473560e-01 9.737318e-01

2436 7.474469e-01 9.737445e-01

2437 7.475353e-01 9.737573e-01

2438 7.476211e-01 9.737700e-01

2439 7.477044e-01 9.737827e-01

2440 7.477854e-01 9.737955e-01

2441 7.478641e-01 9.738082e-01

2442 7.479405e-01 9.738210e-01

2443 7.480147e-01 9.738338e-01

2444 7.480869e-01 9.738466e-01

2445 7.481570e-01 9.738594e-01

2446 7.482252e-01 9.738723e-01

2447 7.482914e-01 9.738851e-01

2448 7.483558e-01 9.738980e-01

2449 7.484184e-01 9.739108e-01

2450 7.484793e-01 9.739237e-01

2451 7.485386e-01 9.739366e-01

2452 7.485962e-01 9.739495e-01

2453 7.486523e-01 9.739625e-01

2454 7.487068e-01 9.739754e-01

2455 7.487600e-01 9.739884e-01

2456 7.488117e-01 9.740013e-01

2457 7.488621e-01 9.740143e-01

2458 7.489112e-01 9.740273e-01

2459 7.489590e-01 9.740404e-01

2460 7.490057e-01 9.740534e-01

2461 7.490512e-01 9.740664e-01

2462 7.490955e-01 9.740795e-01

2463 7.491388e-01 9.740926e-01

2464 7.491811e-01 9.741057e-01

2465 7.492223e-01 9.741188e-01

2466 7.492626e-01 9.741319e-01

2467 7.493020e-01 9.741451e-01

2468 7.493405e-01 9.741582e-01

2469 7.493782e-01 9.741714e-01

2470 7.494150e-01 9.741846e-01

2471 7.494510e-01 9.741978e-01

2472 7.494863e-01 9.742110e-01

2473 7.495209e-01 9.742242e-01

2474 7.495547e-01 9.742375e-01

2475 7.495879e-01 9.742507e-01

2476 7.496205e-01 9.742640e-01

2477 7.496524e-01 9.742773e-01

2478 7.496838e-01 9.742906e-01

2479 7.497146e-01 9.743039e-01

2480 7.497448e-01 9.743173e-01

2481 7.497746e-01 9.743306e-01

2482 7.498038e-01 9.743440e-01

2483 7.498326e-01 9.743573e-01

2484 7.498609e-01 9.743707e-01

2485 7.498888e-01 9.743841e-01

2486 7.499163e-01 9.743976e-01

2487 7.499434e-01 9.744110e-01

2488 7.499701e-01 9.744244e-01

2489 7.499965e-01 9.744379e-01

2490 7.500225e-01 9.744514e-01

2491 7.500482e-01 9.744649e-01

2492 7.500736e-01 9.744784e-01

2493 7.500987e-01 9.744919e-01

2494 7.501235e-01 9.745054e-01

2495 7.501480e-01 9.745189e-01

2496 7.501723e-01 9.745325e-01

2497 7.501964e-01 9.745460e-01

2498 7.502202e-01 9.745596e-01

2499 7.502439e-01 9.745732e-01

2500 7.502673e-01 9.745868e-01

2501 7.502905e-01 9.746004e-01

2502 7.503136e-01 9.746140e-01

2503 7.503365e-01 9.746276e-01

2504 7.503592e-01 9.746412e-01

2505 7.503818e-01 9.746549e-01

2506 7.504043e-01 9.746685e-01

2507 7.504266e-01 9.746822e-01

2508 7.504489e-01 9.746959e-01

2509 7.504710e-01 9.747095e-01

2510 7.504930e-01 9.747232e-01

2511 7.505149e-01 9.747369e-01

2512 7.505367e-01 9.747506e-01

2513 7.505585e-01 9.747643e-01

2514 7.505802e-01 9.747780e-01

2515 7.506018e-01 9.747917e-01

2516 7.506234e-01 9.748054e-01

2517 7.506449e-01 9.748191e-01

2518 7.506664e-01 9.748328e-01

2519 7.506878e-01 9.748465e-01

2520 7.507092e-01 9.748602e-01

2521 7.507306e-01 9.748739e-01

2522 7.509936e-01 9.748853e-01

2523 7.512599e-01 9.748965e-01

2524 7.515295e-01 9.749077e-01

2525 7.518021e-01 9.749188e-01

2526 7.520778e-01 9.749298e-01

2527 7.523562e-01 9.749407e-01

2528 7.526374e-01 9.749516e-01

2529 7.529211e-01 9.749624e-01

2530 7.532071e-01 9.749732e-01

2531 7.534953e-01 9.749839e-01

2532 7.537855e-01 9.749945e-01

2533 7.540775e-01 9.750051e-01

2534 7.543711e-01 9.750157e-01

2535 7.546662e-01 9.750262e-01

2536 7.549626e-01 9.750367e-01

2537 7.552600e-01 9.750471e-01

2538 7.555582e-01 9.750575e-01

2539 7.558571e-01 9.750678e-01

2540 7.561565e-01 9.750781e-01

2541 7.564561e-01 9.750884e-01

2542 7.567557e-01 9.750986e-01

2543 7.570551e-01 9.751088e-01

2544 7.573542e-01 9.751190e-01

2545 7.576527e-01 9.751292e-01

2546 7.579504e-01 9.751393e-01

2547 7.582472e-01 9.751494e-01

2548 7.585428e-01 9.751595e-01

2549 7.588369e-01 9.751696e-01

2550 7.591296e-01 9.751796e-01

2551 7.594204e-01 9.751896e-01

2552 7.597094e-01 9.751996e-01

2553 7.599962e-01 9.752096e-01

2554 7.602808e-01 9.752196e-01

2555 7.605629e-01 9.752296e-01

2556 7.608424e-01 9.752395e-01

2557 7.611192e-01 9.752494e-01

2558 7.613931e-01 9.752594e-01

2559 7.616639e-01 9.752693e-01

2560 7.619315e-01 9.752792e-01

2561 7.621959e-01 9.752891e-01

2562 7.624569e-01 9.752990e-01

2563 7.627143e-01 9.753089e-01

2564 7.629681e-01 9.753188e-01

2565 7.632183e-01 9.753286e-01

2566 7.634646e-01 9.753385e-01

2567 7.637070e-01 9.753484e-01

2568 7.639455e-01 9.753582e-01

2569 7.641800e-01 9.753681e-01

2570 7.644105e-01 9.753780e-01

2571 7.646369e-01 9.753878e-01

2572 7.648591e-01 9.753977e-01

2573 7.650771e-01 9.754076e-01

2574 7.652910e-01 9.754174e-01

2575 7.655006e-01 9.754273e-01

2576 7.657060e-01 9.754372e-01

2577 7.659072e-01 9.754470e-01

2578 7.661042e-01 9.754569e-01

2579 7.662969e-01 9.754668e-01

2580 7.664854e-01 9.754767e-01

2581 7.666698e-01 9.754866e-01

2582 7.668500e-01 9.754965e-01

2583 7.670260e-01 9.755064e-01

2584 7.671979e-01 9.755163e-01

2585 7.673658e-01 9.755262e-01

2586 7.675296e-01 9.755361e-01

2587 7.676895e-01 9.755460e-01

2588 7.678454e-01 9.755560e-01

2589 7.679974e-01 9.755659e-01

2590 7.681456e-01 9.755759e-01

2591 7.682900e-01 9.755858e-01

2592 7.684307e-01 9.755958e-01

2593 7.685677e-01 9.756058e-01

2594 7.687012e-01 9.756158e-01

2595 7.688311e-01 9.756258e-01

2596 7.689575e-01 9.756358e-01

2597 7.690806e-01 9.756458e-01

2598 7.692003e-01 9.756558e-01

2599 7.693168e-01 9.756658e-01

2600 7.694300e-01 9.756759e-01

2601 7.695402e-01 9.756860e-01

2602 7.696473e-01 9.756960e-01

2603 7.697514e-01 9.757061e-01

2604 7.698526e-01 9.757162e-01

2605 7.699510e-01 9.757263e-01

2606 7.700465e-01 9.757364e-01

2607 7.701394e-01 9.757466e-01

2608 7.702297e-01 9.757567e-01

2609 7.703174e-01 9.757669e-01

2610 7.704026e-01 9.757770e-01

2611 7.704854e-01 9.757872e-01

2612 7.705658e-01 9.757974e-01

2613 7.706439e-01 9.758076e-01

2614 7.707198e-01 9.758179e-01

2615 7.707936e-01 9.758281e-01

2616 7.708652e-01 9.758384e-01

2617 7.709348e-01 9.758486e-01

2618 7.710024e-01 9.758589e-01

2619 7.710681e-01 9.758692e-01

2620 7.711320e-01 9.758795e-01

2621 7.711940e-01 9.758898e-01

2622 7.712543e-01 9.759002e-01

2623 7.713129e-01 9.759105e-01

2624 7.713699e-01 9.759209e-01

2625 7.714253e-01 9.759313e-01

2626 7.714792e-01 9.759417e-01

2627 7.715315e-01 9.759521e-01

2628 7.715825e-01 9.759625e-01

2629 7.716320e-01 9.759730e-01

2630 7.716803e-01 9.759834e-01

2631 7.717272e-01 9.759939e-01

2632 7.717729e-01 9.760044e-01

2633 7.718174e-01 9.760149e-01

2634 7.718607e-01 9.760254e-01

2635 7.719029e-01 9.760359e-01

2636 7.719440e-01 9.760465e-01

2637 7.719840e-01 9.760570e-01

2638 7.720231e-01 9.760676e-01

2639 7.720612e-01 9.760782e-01

2640 7.720983e-01 9.760888e-01

2641 7.721346e-01 9.760994e-01

2642 7.721699e-01 9.761101e-01

2643 7.722045e-01 9.761207e-01

2644 7.722382e-01 9.761314e-01

2645 7.722712e-01 9.761421e-01

2646 7.723034e-01 9.761527e-01

2647 7.723349e-01 9.761635e-01

2648 7.723657e-01 9.761742e-01

2649 7.723959e-01 9.761849e-01

2650 7.724254e-01 9.761957e-01

2651 7.724543e-01 9.762064e-01

2652 7.724826e-01 9.762172e-01

2653 7.725104e-01 9.762280e-01

2654 7.725376e-01 9.762388e-01

2655 7.725643e-01 9.762496e-01

2656 7.725905e-01 9.762605e-01

2657 7.726162e-01 9.762713e-01

2658 7.726415e-01 9.762822e-01

2659 7.726663e-01 9.762930e-01

2660 7.726907e-01 9.763039e-01

2661 7.727147e-01 9.763148e-01

2662 7.727383e-01 9.763257e-01

2663 7.727616e-01 9.763366e-01

2664 7.727845e-01 9.763476e-01

2665 7.728071e-01 9.763585e-01

2666 7.728293e-01 9.763695e-01

2667 7.728512e-01 9.763804e-01

2668 7.728729e-01 9.763914e-01

2669 7.728943e-01 9.764024e-01

2670 7.729154e-01 9.764134e-01

2671 7.729362e-01 9.764244e-01

2672 7.729568e-01 9.764354e-01

2673 7.729772e-01 9.764464e-01

2674 7.729974e-01 9.764575e-01

2675 7.730174e-01 9.764685e-01

2676 7.730372e-01 9.764796e-01

2677 7.730567e-01 9.764906e-01

2678 7.730762e-01 9.765017e-01

2679 7.730954e-01 9.765128e-01

2680 7.731145e-01 9.765238e-01

2681 7.731335e-01 9.765349e-01

2682 7.731523e-01 9.765460e-01

2683 7.731710e-01 9.765571e-01

2684 7.731896e-01 9.765682e-01

2685 7.732080e-01 9.765793e-01

2686 7.732264e-01 9.765904e-01

2687 7.732447e-01 9.766016e-01

2688 7.732628e-01 9.766127e-01

2689 7.732809e-01 9.766238e-01

2690 7.732989e-01 9.766349e-01

2691 7.733169e-01 9.766460e-01

2692 7.733348e-01 9.766572e-01

2693 7.733526e-01 9.766683e-01

2694 7.733704e-01 9.766794e-01

2695 7.733881e-01 9.766905e-01

2696 7.734058e-01 9.767016e-01

2697 7.734235e-01 9.767128e-01

2698 7.734411e-01 9.767239e-01

2699 7.734587e-01 9.767350e-01

2700 7.734763e-01 9.767461e-01

2701 7.734939e-01 9.767572e-01

2702 7.737525e-01 9.767655e-01

2703 7.740132e-01 9.767738e-01

2704 7.742760e-01 9.767821e-01

2705 7.745406e-01 9.767903e-01

2706 7.748069e-01 9.767985e-01

2707 7.750748e-01 9.768067e-01

2708 7.753441e-01 9.768148e-01

2709 7.756146e-01 9.768230e-01

2710 7.758861e-01 9.768310e-01

2711 7.761586e-01 9.768391e-01

2712 7.764317e-01 9.768472e-01

2713 7.767054e-01 9.768552e-01

2714 7.769795e-01 9.768632e-01

2715 7.772537e-01 9.768712e-01

2716 7.775279e-01 9.768791e-01

2717 7.778020e-01 9.768871e-01

2718 7.780757e-01 9.768950e-01

2719 7.783488e-01 9.769029e-01

2720 7.786213e-01 9.769108e-01

2721 7.788929e-01 9.769187e-01

2722 7.791635e-01 9.769266e-01

2723 7.794328e-01 9.769345e-01

2724 7.797008e-01 9.769423e-01

2725 7.799672e-01 9.769502e-01

2726 7.802319e-01 9.769580e-01

2727 7.804948e-01 9.769659e-01

2728 7.807557e-01 9.769737e-01

2729 7.810144e-01 9.769815e-01

2730 7.812709e-01 9.769894e-01

2731 7.815249e-01 9.769972e-01

2732 7.817764e-01 9.770050e-01

2733 7.820252e-01 9.770128e-01

2734 7.822713e-01 9.770206e-01

2735 7.825144e-01 9.770284e-01

2736 7.827546e-01 9.770362e-01

2737 7.829916e-01 9.770440e-01

2738 7.832255e-01 9.770518e-01

2739 7.834561e-01 9.770596e-01

2740 7.836833e-01 9.770674e-01

2741 7.839072e-01 9.770753e-01

2742 7.841275e-01 9.770831e-01

2743 7.843443e-01 9.770909e-01

2744 7.845576e-01 9.770987e-01

2745 7.847672e-01 9.771065e-01

2746 7.849731e-01 9.771143e-01

2747 7.851753e-01 9.771222e-01

2748 7.853738e-01 9.771300e-01

2749 7.855685e-01 9.771378e-01

2750 7.857595e-01 9.771457e-01

2751 7.859467e-01 9.771535e-01

2752 7.861301e-01 9.771614e-01

2753 7.863097e-01 9.771693e-01

2754 7.864856e-01 9.771771e-01

2755 7.866577e-01 9.771850e-01

2756 7.868260e-01 9.771929e-01

2757 7.869906e-01 9.772008e-01

2758 7.871515e-01 9.772087e-01

2759 7.873088e-01 9.772166e-01

2760 7.874624e-01 9.772245e-01

2761 7.876124e-01 9.772324e-01

2762 7.877588e-01 9.772404e-01

2763 7.879017e-01 9.772483e-01

2764 7.880410e-01 9.772563e-01

2765 7.881770e-01 9.772642e-01

2766 7.883095e-01 9.772722e-01

2767 7.884388e-01 9.772802e-01

2768 7.885647e-01 9.772882e-01

2769 7.886873e-01 9.772962e-01

2770 7.888068e-01 9.773042e-01

2771 7.889232e-01 9.773122e-01

2772 7.890365e-01 9.773203e-01

2773 7.891468e-01 9.773283e-01

2774 7.892541e-01 9.773364e-01

2775 7.893585e-01 9.773444e-01

2776 7.894601e-01 9.773525e-01

2777 7.895589e-01 9.773606e-01

2778 7.896551e-01 9.773687e-01

2779 7.897485e-01 9.773769e-01

2780 7.898394e-01 9.773850e-01

2781 7.899278e-01 9.773931e-01

2782 7.900137e-01 9.774013e-01

2783 7.900971e-01 9.774095e-01

2784 7.901783e-01 9.774176e-01

2785 7.902571e-01 9.774258e-01

2786 7.903338e-01 9.774340e-01

2787 7.904082e-01 9.774423e-01

2788 7.904806e-01 9.774505e-01

2789 7.905509e-01 9.774587e-01

2790 7.906192e-01 9.774670e-01

2791 7.906855e-01 9.774753e-01

2792 7.907500e-01 9.774836e-01

2793 7.908127e-01 9.774919e-01

2794 7.908735e-01 9.775002e-01

2795 7.909327e-01 9.775085e-01

2796 7.909901e-01 9.775168e-01

2797 7.910460e-01 9.775252e-01

2798 7.911003e-01 9.775336e-01

2799 7.911530e-01 9.775419e-01

2800 7.912043e-01 9.775503e-01

2801 7.912541e-01 9.775588e-01

2802 7.913026e-01 9.775672e-01

2803 7.913497e-01 9.775756e-01

2804 7.913955e-01 9.775841e-01

2805 7.914400e-01 9.775925e-01

2806 7.914834e-01 9.776010e-01

2807 7.915255e-01 9.776095e-01

2808 7.915665e-01 9.776180e-01

2809 7.916065e-01 9.776265e-01

2810 7.916453e-01 9.776350e-01

2811 7.916831e-01 9.776436e-01

2812 7.917200e-01 9.776521e-01

2813 7.917559e-01 9.776607e-01

2814 7.917908e-01 9.776693e-01

2815 7.918249e-01 9.776779e-01

2816 7.918581e-01 9.776865e-01

2817 7.918905e-01 9.776951e-01

2818 7.919220e-01 9.777038e-01

2819 7.919528e-01 9.777124e-01

2820 7.919829e-01 9.777211e-01

2821 7.920122e-01 9.777297e-01

2822 7.920409e-01 9.777384e-01

2823 7.920688e-01 9.777471e-01

2824 7.920962e-01 9.777558e-01

2825 7.921229e-01 9.777645e-01

2826 7.921490e-01 9.777733e-01

2827 7.921746e-01 9.777820e-01

2828 7.921996e-01 9.777908e-01

2829 7.922241e-01 9.777995e-01

2830 7.922481e-01 9.778083e-01

2831 7.922716e-01 9.778171e-01

2832 7.922946e-01 9.778259e-01

2833 7.923172e-01 9.778347e-01

2834 7.923393e-01 9.778435e-01

2835 7.923610e-01 9.778524e-01

2836 7.923823e-01 9.778612e-01

2837 7.924033e-01 9.778701e-01

2838 7.924239e-01 9.778789e-01

2839 7.924441e-01 9.778878e-01

2840 7.924640e-01 9.778967e-01

2841 7.924836e-01 9.779055e-01

2842 7.925028e-01 9.779144e-01

2843 7.925218e-01 9.779233e-01

2844 7.925405e-01 9.779322e-01

2845 7.925589e-01 9.779412e-01

2846 7.925771e-01 9.779501e-01

2847 7.925950e-01 9.779590e-01

2848 7.926127e-01 9.779680e-01

2849 7.926302e-01 9.779769e-01

2850 7.926474e-01 9.779858e-01

2851 7.926645e-01 9.779948e-01

2852 7.926813e-01 9.780038e-01

2853 7.926980e-01 9.780127e-01

2854 7.927145e-01 9.780217e-01

2855 7.927309e-01 9.780307e-01

2856 7.927470e-01 9.780396e-01

2857 7.927631e-01 9.780486e-01

2858 7.927790e-01 9.780576e-01

2859 7.927948e-01 9.780666e-01

2860 7.928104e-01 9.780755e-01

2861 7.928259e-01 9.780845e-01

2862 7.928414e-01 9.780935e-01

2863 7.928567e-01 9.781025e-01

2864 7.928719e-01 9.781115e-01

2865 7.928871e-01 9.781204e-01

2866 7.929021e-01 9.781294e-01

2867 7.929171e-01 9.781384e-01

2868 7.929320e-01 9.781474e-01

2869 7.929468e-01 9.781563e-01

2870 7.929616e-01 9.781653e-01

2871 7.929764e-01 9.781742e-01

2872 7.929910e-01 9.781832e-01

2873 7.930057e-01 9.781921e-01

2874 7.930203e-01 9.782011e-01

2875 7.930348e-01 9.782100e-01

2876 7.930494e-01 9.782189e-01

2877 7.930639e-01 9.782278e-01

2878 7.930783e-01 9.782367e-01

2879 7.930928e-01 9.782456e-01

2880 7.931073e-01 9.782545e-01

2881 7.931217e-01 9.782634e-01

2882 7.933698e-01 9.782697e-01

2883 7.936189e-01 9.782761e-01

2884 7.938690e-01 9.782824e-01

2885 7.941199e-01 9.782887e-01

2886 7.943715e-01 9.782950e-01

2887 7.946235e-01 9.783013e-01

2888 7.948759e-01 9.783076e-01

2889 7.951285e-01 9.783138e-01

2890 7.953811e-01 9.783201e-01

2891 7.956336e-01 9.783264e-01

2892 7.958858e-01 9.783326e-01

2893 7.961376e-01 9.783388e-01

2894 7.963888e-01 9.783451e-01

2895 7.966392e-01 9.783513e-01

2896 7.968888e-01 9.783575e-01

2897 7.971374e-01 9.783637e-01

2898 7.973847e-01 9.783699e-01

2899 7.976308e-01 9.783761e-01

2900 7.978753e-01 9.783823e-01

2901 7.981183e-01 9.783886e-01

2902 7.983596e-01 9.783948e-01

2903 7.985990e-01 9.784010e-01

2904 7.988364e-01 9.784072e-01

2905 7.990717e-01 9.784134e-01

2906 7.993048e-01 9.784196e-01

2907 7.995356e-01 9.784258e-01

2908 7.997639e-01 9.784320e-01

2909 7.999897e-01 9.784382e-01

2910 8.002129e-01 9.784444e-01

2911 8.004333e-01 9.784506e-01

2912 8.006510e-01 9.784568e-01

2913 8.008658e-01 9.784630e-01

2914 8.010776e-01 9.784692e-01

2915 8.012864e-01 9.784754e-01

2916 8.014921e-01 9.784817e-01

2917 8.016947e-01 9.784879e-01

2918 8.018941e-01 9.784941e-01

2919 8.020903e-01 9.785004e-01

2920 8.022832e-01 9.785066e-01

2921 8.024728e-01 9.785129e-01

2922 8.026591e-01 9.785191e-01

2923 8.028420e-01 9.785254e-01

2924 8.030215e-01 9.785317e-01

2925 8.031977e-01 9.785380e-01

2926 8.033704e-01 9.785442e-01

2927 8.035397e-01 9.785505e-01

2928 8.037057e-01 9.785568e-01

2929 8.038682e-01 9.785632e-01

2930 8.040273e-01 9.785695e-01

2931 8.041831e-01 9.785758e-01

2932 8.043355e-01 9.785821e-01

2933 8.044845e-01 9.785885e-01

2934 8.046303e-01 9.785948e-01

2935 8.047727e-01 9.786012e-01

2936 8.049119e-01 9.786075e-01

2937 8.050478e-01 9.786139e-01

2938 8.051805e-01 9.786203e-01

2939 8.053101e-01 9.786267e-01

2940 8.054365e-01 9.786331e-01

2941 8.055598e-01 9.786395e-01

2942 8.056801e-01 9.786460e-01

2943 8.057974e-01 9.786524e-01

2944 8.059118e-01 9.786589e-01

2945 8.060232e-01 9.786653e-01

2946 8.061318e-01 9.786718e-01

2947 8.062376e-01 9.786783e-01

2948 8.063406e-01 9.786847e-01

2949 8.064409e-01 9.786912e-01

2950 8.065386e-01 9.786978e-01

2951 8.066336e-01 9.787043e-01

2952 8.067261e-01 9.787108e-01

2953 8.068162e-01 9.787174e-01

2954 8.069037e-01 9.787239e-01

2955 8.069889e-01 9.787305e-01

2956 8.070718e-01 9.787371e-01

2957 8.071524e-01 9.787437e-01

2958 8.072307e-01 9.787503e-01

2959 8.073069e-01 9.787569e-01

2960 8.073809e-01 9.787635e-01

2961 8.074529e-01 9.787701e-01

2962 8.075229e-01 9.787768e-01

2963 8.075909e-01 9.787834e-01

2964 8.076570e-01 9.787901e-01

2965 8.077212e-01 9.787968e-01

2966 8.077836e-01 9.788035e-01

2967 8.078443e-01 9.788102e-01

2968 8.079032e-01 9.788169e-01

2969 8.079605e-01 9.788236e-01

2970 8.080161e-01 9.788304e-01

2971 8.080702e-01 9.788371e-01

2972 8.081227e-01 9.788439e-01

2973 8.081738e-01 9.788507e-01

2974 8.082234e-01 9.788575e-01

2975 8.082716e-01 9.788643e-01

2976 8.083185e-01 9.788711e-01

2977 8.083640e-01 9.788779e-01

2978 8.084083e-01 9.788847e-01

2979 8.084513e-01 9.788916e-01

2980 8.084932e-01 9.788985e-01

2981 8.085338e-01 9.789053e-01

2982 8.085734e-01 9.789122e-01

2983 8.086118e-01 9.789191e-01

2984 8.086493e-01 9.789260e-01

2985 8.086857e-01 9.789329e-01

2986 8.087211e-01 9.789398e-01

2987 8.087555e-01 9.789468e-01

2988 8.087890e-01 9.789537e-01

2989 8.088217e-01 9.789607e-01

2990 8.088535e-01 9.789677e-01

2991 8.088844e-01 9.789746e-01

2992 8.089146e-01 9.789816e-01

2993 8.089439e-01 9.789886e-01

2994 8.089726e-01 9.789956e-01

2995 8.090004e-01 9.790027e-01

2996 8.090276e-01 9.790097e-01

2997 8.090542e-01 9.790167e-01

2998 8.090800e-01 9.790238e-01

2999 8.091053e-01 9.790308e-01

3000 8.091299e-01 9.790379e-01

3001 8.091540e-01 9.790450e-01

3002 8.091775e-01 9.790521e-01

3003 8.092004e-01 9.790592e-01

3004 8.092229e-01 9.790663e-01

3005 8.092448e-01 9.790734e-01

3006 8.092663e-01 9.790805e-01

3007 8.092873e-01 9.790876e-01

3008 8.093078e-01 9.790948e-01

3009 8.093279e-01 9.791019e-01

3010 8.093476e-01 9.791090e-01

3011 8.093669e-01 9.791162e-01

3012 8.093858e-01 9.791234e-01

3013 8.094044e-01 9.791305e-01

3014 8.094226e-01 9.791377e-01

3015 8.094404e-01 9.791449e-01

3016 8.094579e-01 9.791521e-01

3017 8.094752e-01 9.791593e-01

3018 8.094921e-01 9.791665e-01

3019 8.095087e-01 9.791737e-01

3020 8.095251e-01 9.791809e-01

3021 8.095412e-01 9.791881e-01

3022 8.095570e-01 9.791953e-01

3023 8.095726e-01 9.792025e-01

3024 8.095880e-01 9.792097e-01

3025 8.096031e-01 9.792169e-01

3026 8.096181e-01 9.792241e-01

3027 8.096328e-01 9.792314e-01

3028 8.096474e-01 9.792386e-01

3029 8.096617e-01 9.792458e-01

3030 8.096759e-01 9.792530e-01

3031 8.096900e-01 9.792603e-01

3032 8.097038e-01 9.792675e-01

3033 8.097175e-01 9.792747e-01

3034 8.097311e-01 9.792819e-01

3035 8.097445e-01 9.792891e-01

3036 8.097578e-01 9.792963e-01

3037 8.097710e-01 9.793035e-01

3038 8.097841e-01 9.793108e-01

3039 8.097971e-01 9.793180e-01

3040 8.098099e-01 9.793252e-01

3041 8.098227e-01 9.793323e-01

3042 8.098354e-01 9.793395e-01

3043 8.098479e-01 9.793467e-01

3044 8.098604e-01 9.793539e-01

3045 8.098729e-01 9.793610e-01

3046 8.098852e-01 9.793682e-01

3047 8.098975e-01 9.793753e-01

3048 8.099097e-01 9.793825e-01

3049 8.099219e-01 9.793896e-01

3050 8.099340e-01 9.793967e-01

3051 8.099461e-01 9.794038e-01

3052 8.099581e-01 9.794109e-01

3053 8.099701e-01 9.794179e-01

3054 8.099820e-01 9.794250e-01

3055 8.099940e-01 9.794320e-01

3056 8.100058e-01 9.794390e-01

3057 8.100177e-01 9.794461e-01

3058 8.100295e-01 9.794530e-01

3059 8.100413e-01 9.794600e-01

3060 8.100531e-01 9.794670e-01

3061 8.100649e-01 9.794739e-01

3062 8.102986e-01 9.794788e-01

3063 8.105325e-01 9.794838e-01

3064 8.107666e-01 9.794888e-01

3065 8.110006e-01 9.794937e-01

3066 8.112344e-01 9.794987e-01

3067 8.114679e-01 9.795036e-01

3068 8.117010e-01 9.795086e-01

3069 8.119334e-01 9.795135e-01

3070 8.121652e-01 9.795185e-01

3071 8.123961e-01 9.795234e-01

3072 8.126260e-01 9.795284e-01

3073 8.128548e-01 9.795333e-01

3074 8.130824e-01 9.795382e-01

3075 8.133086e-01 9.795432e-01

3076 8.135334e-01 9.795481e-01

3077 8.137566e-01 9.795531e-01

3078 8.139780e-01 9.795580e-01

3079 8.141977e-01 9.795630e-01

3080 8.144154e-01 9.795679e-01

3081 8.146312e-01 9.795729e-01

3082 8.148448e-01 9.795778e-01

3083 8.150562e-01 9.795828e-01

3084 8.152653e-01 9.795878e-01

3085 8.154721e-01 9.795927e-01

3086 8.156763e-01 9.795977e-01

3087 8.158781e-01 9.796027e-01

3088 8.160772e-01 9.796077e-01

3089 8.162737e-01 9.796127e-01

3090 8.164675e-01 9.796176e-01

3091 8.166585e-01 9.796226e-01

3092 8.168466e-01 9.796276e-01

3093 8.170319e-01 9.796327e-01

3094 8.172142e-01 9.796377e-01

3095 8.173936e-01 9.796427e-01

3096 8.175700e-01 9.796477e-01

3097 8.177434e-01 9.796527e-01

3098 8.179137e-01 9.796578e-01

3099 8.180810e-01 9.796628e-01

3100 8.182453e-01 9.796679e-01

3101 8.184064e-01 9.796729e-01

3102 8.185645e-01 9.796780e-01

3103 8.187195e-01 9.796831e-01

3104 8.188713e-01 9.796882e-01

3105 8.190202e-01 9.796933e-01

3106 8.191659e-01 9.796984e-01

3107 8.193086e-01 9.797035e-01

3108 8.194482e-01 9.797086e-01

3109 8.195848e-01 9.797137e-01

3110 8.197184e-01 9.797188e-01

3111 8.198490e-01 9.797240e-01

3112 8.199767e-01 9.797291e-01

3113 8.201014e-01 9.797343e-01

3114 8.202232e-01 9.797394e-01

3115 8.203422e-01 9.797446e-01

3116 8.204583e-01 9.797498e-01

3117 8.205717e-01 9.797550e-01

3118 8.206823e-01 9.797602e-01

3119 8.207901e-01 9.797654e-01

3120 8.208953e-01 9.797706e-01

3121 8.209979e-01 9.797759e-01

3122 8.210979e-01 9.797811e-01

3123 8.211953e-01 9.797864e-01

3124 8.212902e-01 9.797916e-01

3125 8.213826e-01 9.797969e-01

3126 8.214727e-01 9.798022e-01

3127 8.215604e-01 9.798074e-01

3128 8.216457e-01 9.798127e-01

3129 8.217288e-01 9.798180e-01

3130 8.218097e-01 9.798234e-01

3131 8.218883e-01 9.798287e-01

3132 8.219649e-01 9.798340e-01

3133 8.220394e-01 9.798394e-01

3134 8.221118e-01 9.798447e-01

3135 8.221822e-01 9.798501e-01

3136 8.222507e-01 9.798555e-01

3137 8.223174e-01 9.798609e-01

3138 8.223821e-01 9.798663e-01

3139 8.224451e-01 9.798717e-01

3140 8.225063e-01 9.798771e-01

3141 8.225658e-01 9.798825e-01

3142 8.226236e-01 9.798879e-01

3143 8.226798e-01 9.798934e-01

3144 8.227344e-01 9.798988e-01

3145 8.227875e-01 9.799043e-01

3146 8.228391e-01 9.799098e-01

3147 8.228892e-01 9.799153e-01

3148 8.229379e-01 9.799208e-01

3149 8.229852e-01 9.799263e-01

3150 8.230312e-01 9.799318e-01

3151 8.230759e-01 9.799373e-01

3152 8.231194e-01 9.799428e-01

3153 8.231616e-01 9.799484e-01

3154 8.232026e-01 9.799539e-01

3155 8.232425e-01 9.799595e-01

3156 8.232812e-01 9.799650e-01

3157 8.233189e-01 9.799706e-01

3158 8.233555e-01 9.799762e-01

3159 8.233912e-01 9.799818e-01

3160 8.234258e-01 9.799874e-01

3161 8.234595e-01 9.799930e-01

3162 8.234922e-01 9.799986e-01

3163 8.235240e-01 9.800043e-01

3164 8.235550e-01 9.800099e-01

3165 8.235852e-01 9.800155e-01

3166 8.236145e-01 9.800212e-01

3167 8.236430e-01 9.800268e-01

3168 8.236708e-01 9.800325e-01

3169 8.236979e-01 9.800382e-01

3170 8.237242e-01 9.800439e-01

3171 8.237499e-01 9.800496e-01

3172 8.237749e-01 9.800552e-01

3173 8.237992e-01 9.800609e-01

3174 8.238229e-01 9.800667e-01

3175 8.238461e-01 9.800724e-01

3176 8.238686e-01 9.800781e-01

3177 8.238906e-01 9.800838e-01

3178 8.239121e-01 9.800896e-01

3179 8.239330e-01 9.800953e-01

3180 8.239535e-01 9.801010e-01

3181 8.239734e-01 9.801068e-01

3182 8.239929e-01 9.801125e-01

3183 8.240120e-01 9.801183e-01

3184 8.240306e-01 9.801241e-01

3185 8.240488e-01 9.801298e-01

3186 8.240665e-01 9.801356e-01

3187 8.240840e-01 9.801414e-01

3188 8.241010e-01 9.801471e-01

3189 8.241177e-01 9.801529e-01

3190 8.241340e-01 9.801587e-01

3191 8.241500e-01 9.801645e-01

3192 8.241657e-01 9.801703e-01

3193 8.241810e-01 9.801760e-01

3194 8.241961e-01 9.801818e-01

3195 8.242109e-01 9.801876e-01

3196 8.242254e-01 9.801934e-01

3197 8.242397e-01 9.801992e-01

3198 8.242537e-01 9.802050e-01

3199 8.242674e-01 9.802108e-01

3200 8.242809e-01 9.802166e-01

3201 8.242942e-01 9.802223e-01

3202 8.243073e-01 9.802281e-01

3203 8.243202e-01 9.802339e-01

3204 8.243329e-01 9.802397e-01

3205 8.243454e-01 9.802454e-01

3206 8.243577e-01 9.802512e-01

3207 8.243699e-01 9.802570e-01

3208 8.243818e-01 9.802627e-01

3209 8.243936e-01 9.802685e-01

3210 8.244053e-01 9.802742e-01

3211 8.244168e-01 9.802800e-01

3212 8.244282e-01 9.802857e-01

3213 8.244394e-01 9.802915e-01

3214 8.244505e-01 9.802972e-01

3215 8.244615e-01 9.803029e-01

3216 8.244724e-01 9.803086e-01

3217 8.244832e-01 9.803143e-01

3218 8.244938e-01 9.803200e-01

3219 8.245044e-01 9.803256e-01

3220 8.245148e-01 9.803313e-01

3221 8.245251e-01 9.803369e-01

3222 8.245354e-01 9.803426e-01

3223 8.245456e-01 9.803482e-01

3224 8.245556e-01 9.803538e-01

3225 8.245656e-01 9.803594e-01

3226 8.245756e-01 9.803650e-01

3227 8.245854e-01 9.803705e-01

3228 8.245952e-01 9.803761e-01

3229 8.246049e-01 9.803816e-01

3230 8.246145e-01 9.803871e-01

3231 8.246240e-01 9.803926e-01

3232 8.246335e-01 9.803981e-01

3233 8.246429e-01 9.804035e-01

3234 8.246523e-01 9.804089e-01

3235 8.246616e-01 9.804143e-01

3236 8.246708e-01 9.804197e-01

3237 8.246800e-01 9.804250e-01

3238 8.246890e-01 9.804304e-01

3239 8.246981e-01 9.804356e-01

3240 8.247070e-01 9.804409e-01

3241 8.247159e-01 9.804462e-01

3242 8.249333e-01 9.804501e-01

3243 8.251503e-01 9.804541e-01

3244 8.253667e-01 9.804580e-01

3245 8.255825e-01 9.804620e-01

3246 8.257975e-01 9.804659e-01

3247 8.260116e-01 9.804699e-01

3248 8.262246e-01 9.804739e-01

3249 8.264366e-01 9.804778e-01

3250 8.266473e-01 9.804818e-01

3251 8.268567e-01 9.804857e-01

3252 8.270646e-01 9.804897e-01

3253 8.272710e-01 9.804937e-01

3254 8.274757e-01 9.804977e-01

3255 8.276788e-01 9.805016e-01

3256 8.278800e-01 9.805056e-01

3257 8.280792e-01 9.805096e-01

3258 8.282765e-01 9.805136e-01

3259 8.284717e-01 9.805176e-01

3260 8.286648e-01 9.805216e-01

3261 8.288557e-01 9.805256e-01

3262 8.290442e-01 9.805296e-01

3263 8.292304e-01 9.805336e-01

3264 8.294142e-01 9.805376e-01

3265 8.295956e-01 9.805417e-01

3266 8.297744e-01 9.805457e-01

3267 8.299507e-01 9.805497e-01

3268 8.301243e-01 9.805538e-01

3269 8.302953e-01 9.805578e-01

3270 8.304637e-01 9.805618e-01

3271 8.306293e-01 9.805659e-01

3272 8.307922e-01 9.805700e-01

3273 8.309523e-01 9.805740e-01

3274 8.311096e-01 9.805781e-01

3275 8.312642e-01 9.805822e-01

3276 8.314159e-01 9.805863e-01

3277 8.315649e-01 9.805904e-01

3278 8.317110e-01 9.805945e-01

3279 8.318543e-01 9.805986e-01

3280 8.319948e-01 9.806027e-01

3281 8.321325e-01 9.806068e-01

3282 8.322674e-01 9.806110e-01

3283 8.323995e-01 9.806151e-01

3284 8.325288e-01 9.806192e-01

3285 8.326553e-01 9.806234e-01

3286 8.327792e-01 9.806276e-01

3287 8.329003e-01 9.806317e-01

3288 8.330187e-01 9.806359e-01

3289 8.331344e-01 9.806401e-01

3290 8.332475e-01 9.806443e-01

3291 8.333579e-01 9.806485e-01

3292 8.334658e-01 9.806527e-01

3293 8.335711e-01 9.806569e-01

3294 8.336739e-01 9.806611e-01

3295 8.337743e-01 9.806653e-01

3296 8.338721e-01 9.806696e-01

3297 8.339676e-01 9.806738e-01

3298 8.340607e-01 9.806781e-01

3299 8.341514e-01 9.806823e-01

3300 8.342399e-01 9.806866e-01

3301 8.343261e-01 9.806909e-01

3302 8.344101e-01 9.806952e-01

3303 8.344919e-01 9.806995e-01

3304 8.345716e-01 9.807038e-01

3305 8.346492e-01 9.807081e-01

3306 8.347247e-01 9.807124e-01

3307 8.347983e-01 9.807167e-01

3308 8.348698e-01 9.807211e-01

3309 8.349395e-01 9.807254e-01

3310 8.350073e-01 9.807297e-01

3311 8.350732e-01 9.807341e-01

3312 8.351373e-01 9.807385e-01

3313 8.351997e-01 9.807428e-01

3314 8.352604e-01 9.807472e-01

3315 8.353194e-01 9.807516e-01

3316 8.353768e-01 9.807560e-01

3317 8.354325e-01 9.807604e-01

3318 8.354868e-01 9.807648e-01

3319 8.355395e-01 9.807692e-01

3320 8.355907e-01 9.807737e-01

3321 8.356405e-01 9.807781e-01

3322 8.356889e-01 9.807825e-01

3323 8.357360e-01 9.807870e-01

3324 8.357817e-01 9.807914e-01

3325 8.358262e-01 9.807959e-01

3326 8.358694e-01 9.808004e-01

3327 8.359113e-01 9.808049e-01

3328 8.359521e-01 9.808093e-01

3329 8.359918e-01 9.808138e-01

3330 8.360303e-01 9.808183e-01

3331 8.360677e-01 9.808228e-01

3332 8.361041e-01 9.808273e-01

3333 8.361395e-01 9.808319e-01

3334 8.361738e-01 9.808364e-01

3335 8.362073e-01 9.808409e-01

3336 8.362397e-01 9.808455e-01

3337 8.362713e-01 9.808500e-01

3338 8.363020e-01 9.808545e-01

3339 8.363318e-01 9.808591e-01

3340 8.363609e-01 9.808636e-01

3341 8.363891e-01 9.808682e-01

3342 8.364165e-01 9.808728e-01

3343 8.364432e-01 9.808774e-01

3344 8.364692e-01 9.808819e-01

3345 8.364944e-01 9.808865e-01

3346 8.365190e-01 9.808911e-01

3347 8.365429e-01 9.808957e-01

3348 8.365662e-01 9.809003e-01

3349 8.365889e-01 9.809049e-01

3350 8.366110e-01 9.809095e-01

3351 8.366325e-01 9.809141e-01

3352 8.366534e-01 9.809187e-01

3353 8.366738e-01 9.809233e-01

3354 8.366937e-01 9.809279e-01

3355 8.367130e-01 9.809325e-01

3356 8.367319e-01 9.809372e-01

3357 8.367503e-01 9.809418e-01

3358 8.367683e-01 9.809464e-01

3359 8.367858e-01 9.809510e-01

3360 8.368029e-01 9.809556e-01

3361 8.368196e-01 9.809603e-01

3362 8.368358e-01 9.809649e-01

3363 8.368517e-01 9.809695e-01

3364 8.368673e-01 9.809741e-01

3365 8.368824e-01 9.809788e-01

3366 8.368972e-01 9.809834e-01

3367 8.369117e-01 9.809880e-01

3368 8.369259e-01 9.809926e-01

3369 8.369398e-01 9.809972e-01

3370 8.369533e-01 9.810019e-01

3371 8.369666e-01 9.810065e-01

3372 8.369796e-01 9.810111e-01

3373 8.369923e-01 9.810157e-01

3374 8.370048e-01 9.810203e-01

3375 8.370170e-01 9.810249e-01

3376 8.370289e-01 9.810295e-01

3377 8.370406e-01 9.810341e-01

3378 8.370521e-01 9.810386e-01

3379 8.370634e-01 9.810432e-01

3380 8.370745e-01 9.810478e-01

3381 8.370853e-01 9.810524e-01

3382 8.370960e-01 9.810569e-01

3383 8.371064e-01 9.810615e-01

3384 8.371167e-01 9.810660e-01

3385 8.371268e-01 9.810705e-01

3386 8.371367e-01 9.810751e-01

3387 8.371465e-01 9.810796e-01

3388 8.371560e-01 9.810841e-01

3389 8.371655e-01 9.810886e-01

3390 8.371747e-01 9.810930e-01

3391 8.371838e-01 9.810975e-01

3392 8.371927e-01 9.811020e-01

3393 8.372015e-01 9.811064e-01

3394 8.372102e-01 9.811108e-01

3395 8.372187e-01 9.811152e-01

3396 8.372270e-01 9.811196e-01

3397 8.372353e-01 9.811240e-01

3398 8.372433e-01 9.811284e-01

3399 8.372513e-01 9.811327e-01

3400 8.372591e-01 9.811371e-01

3401 8.372668e-01 9.811414e-01

3402 8.372743e-01 9.811457e-01

3403 8.372817e-01 9.811499e-01

3404 8.372889e-01 9.811542e-01

3405 8.372960e-01 9.811584e-01

3406 8.373030e-01 9.811626e-01

3407 8.373099e-01 9.811668e-01

3408 8.373166e-01 9.811709e-01

3409 8.373231e-01 9.811751e-01

3410 8.373295e-01 9.811792e-01

3411 8.373357e-01 9.811833e-01

3412 8.373418e-01 9.811873e-01

3413 8.373477e-01 9.811913e-01

3414 8.373535e-01 9.811953e-01

3415 8.373591e-01 9.811993e-01

3416 8.373645e-01 9.812032e-01

3417 8.373697e-01 9.812071e-01

3418 8.373748e-01 9.812110e-01

3419 8.373796e-01 9.812149e-01

3420 8.373842e-01 9.812187e-01

3421 8.373886e-01 9.812224e-01

3422 8.375891e-01 9.812256e-01

3423 8.377887e-01 9.812288e-01

3424 8.379873e-01 9.812320e-01

3425 8.381848e-01 9.812352e-01

3426 8.383811e-01 9.812384e-01

3427 8.385760e-01 9.812416e-01

3428 8.387696e-01 9.812448e-01

3429 8.389617e-01 9.812480e-01

3430 8.391523e-01 9.812512e-01

3431 8.393412e-01 9.812544e-01

3432 8.395284e-01 9.812576e-01

3433 8.397138e-01 9.812609e-01

3434 8.398973e-01 9.812641e-01

3435 8.400789e-01 9.812673e-01

3436 8.402585e-01 9.812705e-01

3437 8.404360e-01 9.812738e-01

3438 8.406114e-01 9.812770e-01

3439 8.407847e-01 9.812803e-01

3440 8.409557e-01 9.812835e-01

3441 8.411244e-01 9.812868e-01

3442 8.412908e-01 9.812901e-01

3443 8.414548e-01 9.812933e-01

3444 8.416164e-01 9.812966e-01

3445 8.417756e-01 9.812999e-01

3446 8.419323e-01 9.813032e-01

3447 8.420865e-01 9.813064e-01

3448 8.422382e-01 9.813097e-01

3449 8.423874e-01 9.813130e-01

3450 8.425341e-01 9.813163e-01

3451 8.426781e-01 9.813197e-01

3452 8.428196e-01 9.813230e-01

3453 8.429585e-01 9.813263e-01

3454 8.430949e-01 9.813296e-01

3455 8.432286e-01 9.813330e-01

3456 8.433598e-01 9.813363e-01

3457 8.434884e-01 9.813397e-01

3458 8.436144e-01 9.813430e-01

3459 8.437378e-01 9.813464e-01

3460 8.438588e-01 9.813497e-01

3461 8.439771e-01 9.813531e-01

3462 8.440930e-01 9.813565e-01

3463 8.442064e-01 9.813599e-01

3464 8.443172e-01 9.813633e-01

3465 8.444257e-01 9.813667e-01

3466 8.445317e-01 9.813701e-01

3467 8.446352e-01 9.813735e-01

3468 8.447365e-01 9.813769e-01

3469 8.448353e-01 9.813803e-01

3470 8.449319e-01 9.813837e-01

3471 8.450261e-01 9.813872e-01

3472 8.451181e-01 9.813906e-01

3473 8.452078e-01 9.813941e-01

3474 8.452954e-01 9.813975e-01

3475 8.453808e-01 9.814010e-01

3476 8.454641e-01 9.814044e-01

3477 8.455453e-01 9.814079e-01

3478 8.456245e-01 9.814114e-01

3479 8.457016e-01 9.814149e-01

3480 8.457768e-01 9.814184e-01

3481 8.458500e-01 9.814219e-01

3482 8.459213e-01 9.814254e-01

3483 8.459907e-01 9.814289e-01

3484 8.460584e-01 9.814324e-01

3485 8.461242e-01 9.814359e-01

3486 8.461883e-01 9.814394e-01

3487 8.462507e-01 9.814430e-01

3488 8.463114e-01 9.814465e-01

3489 8.463704e-01 9.814501e-01

3490 8.464279e-01 9.814536e-01

3491 8.464838e-01 9.814572e-01

3492 8.465381e-01 9.814607e-01

3493 8.465910e-01 9.814643e-01

3494 8.466424e-01 9.814679e-01

3495 8.466924e-01 9.814715e-01

3496 8.467410e-01 9.814750e-01

3497 8.467883e-01 9.814786e-01

3498 8.468342e-01 9.814822e-01

3499 8.468789e-01 9.814858e-01

3500 8.469223e-01 9.814894e-01

3501 8.469645e-01 9.814931e-01

3502 8.470055e-01 9.814967e-01

3503 8.470454e-01 9.815003e-01

3504 8.470841e-01 9.815039e-01

3505 8.471218e-01 9.815075e-01

3506 8.471584e-01 9.815112e-01

3507 8.471939e-01 9.815148e-01

3508 8.472285e-01 9.815184e-01

3509 8.472621e-01 9.815221e-01

3510 8.472947e-01 9.815257e-01

3511 8.473264e-01 9.815294e-01

3512 8.473572e-01 9.815330e-01

3513 8.473872e-01 9.815367e-01

3514 8.474163e-01 9.815404e-01

3515 8.474446e-01 9.815440e-01

3516 8.474721e-01 9.815477e-01

3517 8.474988e-01 9.815514e-01

3518 8.475248e-01 9.815550e-01

3519 8.475501e-01 9.815587e-01

3520 8.475746e-01 9.815624e-01

3521 8.475985e-01 9.815661e-01

3522 8.476217e-01 9.815697e-01

3523 8.476443e-01 9.815734e-01

3524 8.476662e-01 9.815771e-01

3525 8.476876e-01 9.815808e-01

3526 8.477083e-01 9.815845e-01

3527 8.477285e-01 9.815882e-01

3528 8.477482e-01 9.815918e-01

3529 8.477673e-01 9.815955e-01

3530 8.477859e-01 9.815992e-01

3531 8.478040e-01 9.816029e-01

3532 8.478216e-01 9.816066e-01

3533 8.478388e-01 9.816103e-01

3534 8.478554e-01 9.816139e-01

3535 8.478717e-01 9.816176e-01

3536 8.478875e-01 9.816213e-01

3537 8.479029e-01 9.816250e-01

3538 8.479179e-01 9.816286e-01

3539 8.479326e-01 9.816323e-01

3540 8.479468e-01 9.816360e-01

3541 8.479607e-01 9.816396e-01

3542 8.479742e-01 9.816433e-01

3543 8.479874e-01 9.816469e-01

3544 8.480002e-01 9.816506e-01

3545 8.480127e-01 9.816542e-01

3546 8.480249e-01 9.816579e-01

3547 8.480368e-01 9.816615e-01

3548 8.480484e-01 9.816651e-01

3549 8.480597e-01 9.816688e-01

3550 8.480707e-01 9.816724e-01

3551 8.480815e-01 9.816760e-01

3552 8.480919e-01 9.816796e-01

3553 8.481021e-01 9.816832e-01

3554 8.481121e-01 9.816867e-01

3555 8.481218e-01 9.816903e-01

3556 8.481313e-01 9.816939e-01

3557 8.481405e-01 9.816974e-01

3558 8.481495e-01 9.817010e-01

3559 8.481582e-01 9.817045e-01

3560 8.481667e-01 9.817080e-01

3561 8.481751e-01 9.817115e-01

3562 8.481831e-01 9.817150e-01

3563 8.481910e-01 9.817185e-01

3564 8.481987e-01 9.817220e-01

3565 8.482061e-01 9.817254e-01

3566 8.482133e-01 9.817289e-01

3567 8.482203e-01 9.817323e-01

3568 8.482271e-01 9.817357e-01

3569 8.482338e-01 9.817391e-01

3570 8.482401e-01 9.817425e-01

3571 8.482463e-01 9.817458e-01

3572 8.482523e-01 9.817491e-01

3573 8.482581e-01 9.817525e-01

3574 8.482636e-01 9.817558e-01

3575 8.482690e-01 9.817590e-01

3576 8.482741e-01 9.817623e-01

3577 8.482790e-01 9.817655e-01

3578 8.482837e-01 9.817687e-01

3579 8.482881e-01 9.817719e-01

3580 8.482924e-01 9.817751e-01

3581 8.482964e-01 9.817782e-01

3582 8.483001e-01 9.817814e-01

3583 8.483036e-01 9.817845e-01

3584 8.483069e-01 9.817875e-01

3585 8.483099e-01 9.817905e-01

3586 8.483127e-01 9.817936e-01

3587 8.483151e-01 9.817965e-01

3588 8.483173e-01 9.817995e-01

3589 8.483192e-01 9.818024e-01

3590 8.483208e-01 9.818053e-01

3591 8.483221e-01 9.818081e-01

3592 8.483231e-01 9.818109e-01

3593 8.483238e-01 9.818137e-01

3594 8.483241e-01 9.818165e-01

3595 8.483240e-01 9.818192e-01

3596 8.483236e-01 9.818219e-01

3597 8.483228e-01 9.818245e-01

3598 8.483216e-01 9.818271e-01

3599 8.483200e-01 9.818297e-01

3600 8.483180e-01 9.818322e-01

3601 8.483156e-01 9.818346e-01

3602 8.484994e-01 9.818372e-01

3603 8.486820e-01 9.818398e-01

3604 8.488634e-01 9.818424e-01

3605 8.490433e-01 9.818450e-01

3606 8.492217e-01 9.818476e-01

3607 8.493986e-01 9.818502e-01

3608 8.495739e-01 9.818528e-01

3609 8.497475e-01 9.818555e-01

3610 8.499194e-01 9.818581e-01

3611 8.500895e-01 9.818607e-01

3612 8.502576e-01 9.818633e-01

3613 8.504239e-01 9.818659e-01

3614 8.505882e-01 9.818686e-01

3615 8.507505e-01 9.818712e-01

3616 8.509107e-01 9.818739e-01

3617 8.510688e-01 9.818765e-01

3618 8.512248e-01 9.818791e-01

3619 8.513786e-01 9.818818e-01

3620 8.515301e-01 9.818845e-01

3621 8.516794e-01 9.818871e-01

3622 8.518265e-01 9.818898e-01

3623 8.519712e-01 9.818925e-01

3624 8.521136e-01 9.818951e-01

3625 8.522537e-01 9.818978e-01

3626 8.523914e-01 9.819005e-01

3627 8.525268e-01 9.819032e-01

3628 8.526598e-01 9.819059e-01

3629 8.527904e-01 9.819086e-01

3630 8.529187e-01 9.819113e-01

3631 8.530445e-01 9.819140e-01

3632 8.531680e-01 9.819167e-01

3633 8.532891e-01 9.819194e-01

3634 8.534078e-01 9.819222e-01

3635 8.535242e-01 9.819249e-01

3636 8.536382e-01 9.819276e-01

3637 8.537498e-01 9.819304e-01

3638 8.538592e-01 9.819331e-01

3639 8.539662e-01 9.819358e-01

3640 8.540710e-01 9.819386e-01

3641 8.541734e-01 9.819414e-01

3642 8.542737e-01 9.819441e-01

3643 8.543717e-01 9.819469e-01

3644 8.544674e-01 9.819497e-01

3645 8.545610e-01 9.819524e-01

3646 8.546525e-01 9.819552e-01

3647 8.547418e-01 9.819580e-01

3648 8.548291e-01 9.819608e-01

3649 8.549142e-01 9.819636e-01

3650 8.549973e-01 9.819664e-01

3651 8.550784e-01 9.819692e-01

3652 8.551576e-01 9.819720e-01

3653 8.552348e-01 9.819748e-01

3654 8.553100e-01 9.819777e-01

3655 8.553834e-01 9.819805e-01

3656 8.554550e-01 9.819833e-01

3657 8.555247e-01 9.819861e-01

3658 8.555927e-01 9.819890e-01

3659 8.556589e-01 9.819918e-01

3660 8.557234e-01 9.819947e-01

3661 8.557862e-01 9.819975e-01

3662 8.558474e-01 9.820004e-01

3663 8.559069e-01 9.820032e-01

3664 8.559649e-01 9.820061e-01

3665 8.560214e-01 9.820090e-01

3666 8.560763e-01 9.820118e-01

3667 8.561297e-01 9.820147e-01

3668 8.561818e-01 9.820176e-01

3669 8.562324e-01 9.820205e-01

3670 8.562816e-01 9.820234e-01

3671 8.563295e-01 9.820262e-01

3672 8.563761e-01 9.820291e-01

3673 8.564213e-01 9.820320e-01

3674 8.564654e-01 9.820349e-01

3675 8.565082e-01 9.820378e-01

3676 8.565498e-01 9.820407e-01

3677 8.565903e-01 9.820436e-01

3678 8.566296e-01 9.820465e-01

3679 8.566679e-01 9.820495e-01

3680 8.567051e-01 9.820524e-01

3681 8.567412e-01 9.820553e-01

3682 8.567763e-01 9.820582e-01

3683 8.568104e-01 9.820611e-01

3684 8.568436e-01 9.820640e-01

3685 8.568758e-01 9.820670e-01

3686 8.569071e-01 9.820699e-01

3687 8.569375e-01 9.820728e-01

3688 8.569671e-01 9.820757e-01

3689 8.569958e-01 9.820787e-01

3690 8.570237e-01 9.820816e-01

3691 8.570508e-01 9.820845e-01

3692 8.570771e-01 9.820874e-01

3693 8.571027e-01 9.820904e-01

3694 8.571275e-01 9.820933e-01

3695 8.571517e-01 9.820962e-01

3696 8.571751e-01 9.820992e-01

3697 8.571979e-01 9.821021e-01

3698 8.572201e-01 9.821050e-01

3699 8.572416e-01 9.821079e-01

3700 8.572625e-01 9.821109e-01

3701 8.572828e-01 9.821138e-01

3702 8.573025e-01 9.821167e-01

3703 8.573216e-01 9.821196e-01

3704 8.573402e-01 9.821225e-01

3705 8.573583e-01 9.821254e-01

3706 8.573758e-01 9.821283e-01

3707 8.573929e-01 9.821313e-01

3708 8.574095e-01 9.821342e-01

3709 8.574256e-01 9.821371e-01

3710 8.574412e-01 9.821400e-01

3711 8.574564e-01 9.821428e-01

3712 8.574711e-01 9.821457e-01

3713 8.574854e-01 9.821486e-01

3714 8.574993e-01 9.821515e-01

3715 8.575128e-01 9.821544e-01

3716 8.575259e-01 9.821572e-01

3717 8.575386e-01 9.821601e-01

3718 8.575509e-01 9.821629e-01

3719 8.575629e-01 9.821658e-01

3720 8.575745e-01 9.821686e-01

3721 8.575858e-01 9.821714e-01

3722 8.575967e-01 9.821743e-01

3723 8.576073e-01 9.821771e-01

3724 8.576176e-01 9.821799e-01

3725 8.576275e-01 9.821827e-01

3726 8.576372e-01 9.821854e-01

3727 8.576465e-01 9.821882e-01

3728 8.576555e-01 9.821910e-01

3729 8.576642e-01 9.821937e-01

3730 8.576727e-01 9.821965e-01

3731 8.576808e-01 9.821992e-01

3732 8.576887e-01 9.822019e-01

3733 8.576962e-01 9.822046e-01

3734 8.577035e-01 9.822073e-01

3735 8.577105e-01 9.822100e-01

3736 8.577173e-01 9.822127e-01

3737 8.577237e-01 9.822153e-01

3738 8.577299e-01 9.822179e-01

3739 8.577359e-01 9.822205e-01

3740 8.577415e-01 9.822231e-01

3741 8.577469e-01 9.822257e-01

3742 8.577520e-01 9.822283e-01

3743 8.577569e-01 9.822308e-01

3744 8.577614e-01 9.822334e-01

3745 8.577657e-01 9.822359e-01

3746 8.577697e-01 9.822384e-01

3747 8.577735e-01 9.822408e-01

3748 8.577769e-01 9.822433e-01

3749 8.577801e-01 9.822457e-01

3750 8.577830e-01 9.822481e-01

3751 8.577856e-01 9.822505e-01

3752 8.577879e-01 9.822529e-01

3753 8.577899e-01 9.822552e-01

3754 8.577916e-01 9.822575e-01

3755 8.577930e-01 9.822598e-01

3756 8.577941e-01 9.822620e-01

3757 8.577948e-01 9.822643e-01

3758 8.577952e-01 9.822665e-01

3759 8.577953e-01 9.822686e-01

3760 8.577950e-01 9.822708e-01

3761 8.577944e-01 9.822729e-01

3762 8.577934e-01 9.822750e-01

3763 8.577920e-01 9.822770e-01

3764 8.577902e-01 9.822790e-01

3765 8.577880e-01 9.822810e-01

3766 8.577855e-01 9.822830e-01

3767 8.577825e-01 9.822849e-01

3768 8.577791e-01 9.822868e-01

3769 8.577752e-01 9.822886e-01

3770 8.577709e-01 9.822904e-01

3771 8.577661e-01 9.822922e-01

3772 8.577609e-01 9.822939e-01

3773 8.577551e-01 9.822956e-01

3774 8.577488e-01 9.822972e-01

3775 8.577420e-01 9.822988e-01

3776 8.577347e-01 9.823004e-01

3777 8.577268e-01 9.823019e-01

3778 8.577183e-01 9.823034e-01

3779 8.577092e-01 9.823048e-01

3780 8.576995e-01 9.823062e-01

3781 8.576892e-01 9.823075e-01

3782 8.578573e-01 9.823096e-01

3783 8.580239e-01 9.823118e-01

3784 8.581890e-01 9.823139e-01

3785 8.583526e-01 9.823160e-01

3786 8.585145e-01 9.823181e-01

3787 8.586747e-01 9.823203e-01

3788 8.588332e-01 9.823224e-01

3789 8.589899e-01 9.823245e-01

3790 8.591448e-01 9.823267e-01

3791 8.592978e-01 9.823288e-01

3792 8.594489e-01 9.823310e-01

3793 8.595981e-01 9.823331e-01

3794 8.597452e-01 9.823353e-01

3795 8.598904e-01 9.823374e-01

3796 8.600334e-01 9.823396e-01

3797 8.601744e-01 9.823417e-01

3798 8.603133e-01 9.823439e-01

3799 8.604501e-01 9.823461e-01

3800 8.605847e-01 9.823482e-01

3801 8.607172e-01 9.823504e-01

3802 8.608475e-01 9.823526e-01

3803 8.609756e-01 9.823548e-01

3804 8.611015e-01 9.823570e-01

3805 8.612252e-01 9.823592e-01

3806 8.613467e-01 9.823614e-01

3807 8.614660e-01 9.823636e-01

3808 8.615831e-01 9.823658e-01

3809 8.616980e-01 9.823680e-01

3810 8.618107e-01 9.823702e-01

3811 8.619212e-01 9.823724e-01

3812 8.620296e-01 9.823746e-01

3813 8.621357e-01 9.823768e-01

3814 8.622397e-01 9.823791e-01

3815 8.623415e-01 9.823813e-01

3816 8.624413e-01 9.823835e-01

3817 8.625388e-01 9.823858e-01

3818 8.626344e-01 9.823880e-01

3819 8.627278e-01 9.823902e-01

3820 8.628192e-01 9.823925e-01

3821 8.629085e-01 9.823947e-01

3822 8.629958e-01 9.823970e-01

3823 8.630812e-01 9.823992e-01

3824 8.631646e-01 9.824015e-01

3825 8.632460e-01 9.824038e-01

3826 8.633256e-01 9.824060e-01

3827 8.634032e-01 9.824083e-01

3828 8.634790e-01 9.824106e-01

3829 8.635530e-01 9.824128e-01

3830 8.636252e-01 9.824151e-01

3831 8.636956e-01 9.824174e-01

3832 8.637643e-01 9.824197e-01

3833 8.638313e-01 9.824220e-01

3834 8.638966e-01 9.824243e-01

3835 8.639602e-01 9.824265e-01

3836 8.640223e-01 9.824288e-01

3837 8.640827e-01 9.824311e-01

3838 8.641416e-01 9.824334e-01

3839 8.641990e-01 9.824357e-01

3840 8.642549e-01 9.824380e-01

3841 8.643093e-01 9.824403e-01

3842 8.643623e-01 9.824427e-01

3843 8.644139e-01 9.824450e-01

3844 8.644641e-01 9.824473e-01

3845 8.645130e-01 9.824496e-01

3846 8.645606e-01 9.824519e-01

3847 8.646069e-01 9.824542e-01

3848 8.646519e-01 9.824565e-01

3849 8.646957e-01 9.824589e-01

3850 8.647384e-01 9.824612e-01

3851 8.647798e-01 9.824635e-01

3852 8.648201e-01 9.824658e-01

3853 8.648593e-01 9.824681e-01

3854 8.648974e-01 9.824705e-01

3855 8.649345e-01 9.824728e-01

3856 8.649705e-01 9.824751e-01

3857 8.650055e-01 9.824774e-01

3858 8.650395e-01 9.824797e-01

3859 8.650726e-01 9.824821e-01

3860 8.651048e-01 9.824844e-01

3861 8.651360e-01 9.824867e-01

3862 8.651663e-01 9.824890e-01

3863 8.651958e-01 9.824913e-01

3864 8.652245e-01 9.824937e-01

3865 8.652523e-01 9.824960e-01

3866 8.652793e-01 9.824983e-01

3867 8.653056e-01 9.825006e-01

3868 8.653311e-01 9.825029e-01

3869 8.653558e-01 9.825052e-01

3870 8.653799e-01 9.825075e-01

3871 8.654032e-01 9.825098e-01

3872 8.654259e-01 9.825121e-01

3873 8.654479e-01 9.825144e-01

3874 8.654693e-01 9.825167e-01

3875 8.654900e-01 9.825190e-01

3876 8.655101e-01 9.825213e-01

3877 8.655297e-01 9.825236e-01

3878 8.655486e-01 9.825259e-01

3879 8.655670e-01 9.825282e-01

3880 8.655848e-01 9.825304e-01

3881 8.656021e-01 9.825327e-01

3882 8.656189e-01 9.825350e-01

3883 8.656352e-01 9.825372e-01

3884 8.656509e-01 9.825395e-01

3885 8.656662e-01 9.825417e-01

3886 8.656810e-01 9.825440e-01

3887 8.656954e-01 9.825462e-01

3888 8.657093e-01 9.825484e-01

3889 8.657227e-01 9.825506e-01

3890 8.657358e-01 9.825528e-01

3891 8.657484e-01 9.825550e-01

3892 8.657606e-01 9.825572e-01

3893 8.657723e-01 9.825594e-01

3894 8.657837e-01 9.825616e-01

3895 8.657948e-01 9.825637e-01

3896 8.658054e-01 9.825659e-01

3897 8.658156e-01 9.825680e-01

3898 8.658255e-01 9.825702e-01

3899 8.658351e-01 9.825723e-01

3900 8.658442e-01 9.825744e-01

3901 8.658531e-01 9.825765e-01

3902 8.658616e-01 9.825786e-01

3903 8.658697e-01 9.825807e-01

3904 8.658775e-01 9.825827e-01

3905 8.658850e-01 9.825848e-01

3906 8.658921e-01 9.825868e-01

3907 8.658990e-01 9.825888e-01

3908 8.659055e-01 9.825908e-01

3909 8.659117e-01 9.825928e-01

3910 8.659175e-01 9.825948e-01

3911 8.659231e-01 9.825968e-01

3912 8.659283e-01 9.825987e-01

3913 8.659333e-01 9.826006e-01

3914 8.659379e-01 9.826025e-01

3915 8.659422e-01 9.826044e-01

3916 8.659461e-01 9.826063e-01

3917 8.659498e-01 9.826081e-01

3918 8.659532e-01 9.826100e-01

3919 8.659562e-01 9.826118e-01

3920 8.659589e-01 9.826136e-01

3921 8.659613e-01 9.826153e-01

3922 8.659634e-01 9.826171e-01

3923 8.659651e-01 9.826188e-01

3924 8.659666e-01 9.826205e-01

3925 8.659677e-01 9.826222e-01

3926 8.659684e-01 9.826238e-01

3927 8.659688e-01 9.826254e-01

3928 8.659689e-01 9.826270e-01

3929 8.659686e-01 9.826286e-01

3930 8.659679e-01 9.826302e-01

3931 8.659669e-01 9.826317e-01

3932 8.659656e-01 9.826332e-01

3933 8.659638e-01 9.826346e-01

3934 8.659617e-01 9.826360e-01

3935 8.659592e-01 9.826374e-01

3936 8.659563e-01 9.826388e-01

3937 8.659530e-01 9.826401e-01

3938 8.659493e-01 9.826414e-01

3939 8.659451e-01 9.826427e-01

3940 8.659406e-01 9.826439e-01

3941 8.659356e-01 9.826451e-01

3942 8.659302e-01 9.826463e-01

3943 8.659243e-01 9.826474e-01

3944 8.659179e-01 9.826485e-01

3945 8.659111e-01 9.826496e-01

3946 8.659038e-01 9.826506e-01

3947 8.658961e-01 9.826515e-01

3948 8.658878e-01 9.826525e-01

3949 8.658790e-01 9.826534e-01

3950 8.658697e-01 9.826542e-01

3951 8.658598e-01 9.826550e-01

3952 8.658495e-01 9.826558e-01

3953 8.658386e-01 9.826565e-01

3954 8.658271e-01 9.826571e-01

3955 8.658150e-01 9.826578e-01

3956 8.658024e-01 9.826583e-01

3957 8.657892e-01 9.826589e-01

3958 8.657754e-01 9.826593e-01

3959 8.657609e-01 9.826598e-01

3960 8.657459e-01 9.826601e-01

3961 8.657302e-01 9.826604e-01

3962 8.658836e-01 9.826622e-01

3963 8.660354e-01 9.826639e-01

3964 8.661856e-01 9.826656e-01

3965 8.663342e-01 9.826674e-01

3966 8.664810e-01 9.826691e-01

3967 8.666261e-01 9.826708e-01

3968 8.667695e-01 9.826726e-01

3969 8.669110e-01 9.826743e-01

3970 8.670507e-01 9.826761e-01

3971 8.671885e-01 9.826778e-01

3972 8.673244e-01 9.826796e-01

3973 8.674584e-01 9.826813e-01

3974 8.675905e-01 9.826831e-01

3975 8.677205e-01 9.826848e-01

3976 8.678486e-01 9.826866e-01

3977 8.679747e-01 9.826884e-01

3978 8.680988e-01 9.826901e-01

3979 8.682208e-01 9.826919e-01

3980 8.683408e-01 9.826937e-01

3981 8.684587e-01 9.826955e-01

3982 8.685746e-01 9.826972e-01

3983 8.686885e-01 9.826990e-01

3984 8.688003e-01 9.827008e-01

3985 8.689101e-01 9.827026e-01

3986 8.690178e-01 9.827044e-01

3987 8.691234e-01 9.827062e-01

3988 8.692271e-01 9.827080e-01

3989 8.693287e-01 9.827098e-01

3990 8.694283e-01 9.827116e-01

3991 8.695259e-01 9.827134e-01

3992 8.696215e-01 9.827152e-01

3993 8.697152e-01 9.827170e-01

3994 8.698068e-01 9.827188e-01

3995 8.698966e-01 9.827206e-01

3996 8.699844e-01 9.827224e-01

3997 8.700703e-01 9.827242e-01

3998 8.701544e-01 9.827260e-01

3999 8.702365e-01 9.827278e-01

4000 8.703169e-01 9.827297e-01

4001 8.703954e-01 9.827315e-01

4002 8.704721e-01 9.827333e-01

4003 8.705470e-01 9.827351e-01

4004 8.706203e-01 9.827370e-01

4005 8.706917e-01 9.827388e-01

4006 8.707615e-01 9.827406e-01

4007 8.708297e-01 9.827424e-01

4008 8.708962e-01 9.827443e-01

4009 8.709611e-01 9.827461e-01

4010 8.710243e-01 9.827479e-01

4011 8.710861e-01 9.827498e-01

4012 8.711463e-01 9.827516e-01

4013 8.712050e-01 9.827534e-01

4014 8.712622e-01 9.827553e-01

4015 8.713180e-01 9.827571e-01

4016 8.713724e-01 9.827590e-01

4017 8.714253e-01 9.827608e-01

4018 8.714770e-01 9.827626e-01

4019 8.715272e-01 9.827645e-01

4020 8.715762e-01 9.827663e-01

4021 8.716239e-01 9.827682e-01

4022 8.716703e-01 9.827700e-01

4023 8.717155e-01 9.827718e-01

4024 8.717595e-01 9.827737e-01

4025 8.718024e-01 9.827755e-01

4026 8.718440e-01 9.827773e-01

4027 8.718846e-01 9.827792e-01

4028 8.719241e-01 9.827810e-01

4029 8.719625e-01 9.827828e-01

4030 8.719998e-01 9.827847e-01

4031 8.720362e-01 9.827865e-01

4032 8.720715e-01 9.827883e-01

4033 8.721058e-01 9.827902e-01

4034 8.721393e-01 9.827920e-01

4035 8.721717e-01 9.827938e-01

4036 8.722033e-01 9.827956e-01

4037 8.722340e-01 9.827975e-01

4038 8.722638e-01 9.827993e-01

4039 8.722928e-01 9.828011e-01

4040 8.723210e-01 9.828029e-01

4041 8.723484e-01 9.828047e-01

4042 8.723749e-01 9.828065e-01

4043 8.724008e-01 9.828083e-01

4044 8.724259e-01 9.828101e-01

4045 8.724502e-01 9.828119e-01

4046 8.724739e-01 9.828137e-01

4047 8.724969e-01 9.828155e-01

4048 8.725192e-01 9.828172e-01

4049 8.725408e-01 9.828190e-01

4050 8.725618e-01 9.828208e-01

4051 8.725822e-01 9.828225e-01

4052 8.726020e-01 9.828243e-01

4053 8.726212e-01 9.828261e-01

4054 8.726398e-01 9.828278e-01

4055 8.726578e-01 9.828295e-01

4056 8.726753e-01 9.828313e-01

4057 8.726923e-01 9.828330e-01

4058 8.727087e-01 9.828347e-01

4059 8.727246e-01 9.828364e-01

4060 8.727400e-01 9.828381e-01

4061 8.727549e-01 9.828398e-01

4062 8.727694e-01 9.828415e-01

4063 8.727834e-01 9.828432e-01

4064 8.727969e-01 9.828448e-01

4065 8.728099e-01 9.828465e-01

4066 8.728226e-01 9.828481e-01

4067 8.728348e-01 9.828498e-01

4068 8.728465e-01 9.828514e-01

4069 8.728579e-01 9.828530e-01

4070 8.728688e-01 9.828546e-01

4071 8.728794e-01 9.828562e-01

4072 8.728895e-01 9.828578e-01

4073 8.728993e-01 9.828594e-01

4074 8.729087e-01 9.828609e-01

4075 8.729177e-01 9.828625e-01

4076 8.729264e-01 9.828640e-01

4077 8.729346e-01 9.828655e-01

4078 8.729426e-01 9.828670e-01

4079 8.729501e-01 9.828685e-01

4080 8.729573e-01 9.828700e-01

4081 8.729642e-01 9.828714e-01

4082 8.729707e-01 9.828729e-01

4083 8.729769e-01 9.828743e-01

4084 8.729827e-01 9.828757e-01

4085 8.729882e-01 9.828771e-01

4086 8.729934e-01 9.828784e-01

4087 8.729982e-01 9.828798e-01

4088 8.730027e-01 9.828811e-01

4089 8.730069e-01 9.828824e-01

4090 8.730108e-01 9.828837e-01

4091 8.730143e-01 9.828850e-01

4092 8.730175e-01 9.828862e-01

4093 8.730203e-01 9.828875e-01

4094 8.730228e-01 9.828887e-01

4095 8.730250e-01 9.828899e-01

4096 8.730269e-01 9.828910e-01

4097 8.730284e-01 9.828922e-01

4098 8.730296e-01 9.828933e-01

4099 8.730305e-01 9.828944e-01

4100 8.730310e-01 9.828954e-01

4101 8.730312e-01 9.828965e-01

4102 8.730310e-01 9.828975e-01

4103 8.730305e-01 9.828985e-01

4104 8.730297e-01 9.828994e-01

4105 8.730285e-01 9.829003e-01

4106 8.730269e-01 9.829012e-01

4107 8.730250e-01 9.829021e-01

4108 8.730227e-01 9.829030e-01

4109 8.730201e-01 9.829038e-01

4110 8.730171e-01 9.829045e-01

4111 8.730137e-01 9.829053e-01

4112 8.730100e-01 9.829060e-01

4113 8.730059e-01 9.829067e-01

4114 8.730014e-01 9.829073e-01

4115 8.729965e-01 9.829079e-01

4116 8.729912e-01 9.829085e-01

4117 8.729856e-01 9.829090e-01

4118 8.729795e-01 9.829095e-01

4119 8.729730e-01 9.829100e-01

4120 8.729661e-01 9.829104e-01

4121 8.729588e-01 9.829107e-01

4122 8.729511e-01 9.829111e-01

4123 8.729430e-01 9.829114e-01

4124 8.729345e-01 9.829116e-01

4125 8.729255e-01 9.829118e-01

4126 8.729161e-01 9.829120e-01

4127 8.729063e-01 9.829121e-01

4128 8.728960e-01 9.829122e-01

4129 8.728853e-01 9.829122e-01

4130 8.728742e-01 9.829122e-01

4131 8.728626e-01 9.829121e-01

4132 8.728506e-01 9.829120e-01

4133 8.728382e-01 9.829119e-01

4134 8.728252e-01 9.829116e-01

4135 8.728119e-01 9.829114e-01

4136 8.727981e-01 9.829111e-01

4137 8.727838e-01 9.829107e-01

4138 8.727692e-01 9.829102e-01

4139 8.727540e-01 9.829098e-01

4140 8.727385e-01 9.829092e-01

4141 8.727224e-01 9.829086e-01

4142 8.728624e-01 9.829100e-01

4143 8.730008e-01 9.829114e-01

4144 8.731376e-01 9.829129e-01

4145 8.732727e-01 9.829143e-01

4146 8.734060e-01 9.829157e-01

4147 8.735376e-01 9.829171e-01

4148 8.736675e-01 9.829185e-01

4149 8.737956e-01 9.829199e-01

4150 8.739219e-01 9.829214e-01

4151 8.740463e-01 9.829228e-01

4152 8.741689e-01 9.829242e-01

4153 8.742896e-01 9.829256e-01

4154 8.744085e-01 9.829271e-01

4155 8.745255e-01 9.829285e-01

4156 8.746406e-01 9.829299e-01

4157 8.747538e-01 9.829314e-01

4158 8.748651e-01 9.829328e-01

4159 8.749744e-01 9.829342e-01

4160 8.750819e-01 9.829357e-01

4161 8.751875e-01 9.829371e-01

4162 8.752911e-01 9.829385e-01

4163 8.753929e-01 9.829400e-01

4164 8.754927e-01 9.829414e-01

4165 8.755907e-01 9.829429e-01

4166 8.756868e-01 9.829443e-01

4167 8.757810e-01 9.829458e-01

4168 8.758734e-01 9.829472e-01

4169 8.759639e-01 9.829487e-01

4170 8.760526e-01 9.829501e-01

4171 8.761394e-01 9.829516e-01

4172 8.762245e-01 9.829530e-01

4173 8.763078e-01 9.829545e-01

4174 8.763893e-01 9.829559e-01

4175 8.764690e-01 9.829574e-01

4176 8.765471e-01 9.829588e-01

4177 8.766234e-01 9.829603e-01

4178 8.766980e-01 9.829617e-01

4179 8.767710e-01 9.829632e-01

4180 8.768423e-01 9.829646e-01

4181 8.769120e-01 9.829661e-01

4182 8.769801e-01 9.829675e-01

4183 8.770466e-01 9.829690e-01

4184 8.771116e-01 9.829705e-01

4185 8.771750e-01 9.829719e-01

4186 8.772370e-01 9.829734e-01

4187 8.772974e-01 9.829748e-01

4188 8.773564e-01 9.829763e-01

4189 8.774140e-01 9.829777e-01

4190 8.774702e-01 9.829792e-01

4191 8.775250e-01 9.829806e-01

4192 8.775785e-01 9.829821e-01

4193 8.776306e-01 9.829835e-01

4194 8.776815e-01 9.829850e-01

4195 8.777310e-01 9.829864e-01

4196 8.777793e-01 9.829879e-01

4197 8.778264e-01 9.829893e-01

4198 8.778723e-01 9.829907e-01

4199 8.779170e-01 9.829922e-01

4200 8.779606e-01 9.829936e-01

4201 8.780030e-01 9.829951e-01

4202 8.780443e-01 9.829965e-01

4203 8.780846e-01 9.829979e-01

4204 8.781238e-01 9.829994e-01

4205 8.781620e-01 9.830008e-01

4206 8.781991e-01 9.830022e-01

4207 8.782353e-01 9.830036e-01

4208 8.782705e-01 9.830050e-01

4209 8.783048e-01 9.830064e-01

4210 8.783381e-01 9.830079e-01

4211 8.783706e-01 9.830093e-01

4212 8.784022e-01 9.830107e-01

4213 8.784329e-01 9.830121e-01

4214 8.784628e-01 9.830135e-01

4215 8.784918e-01 9.830148e-01

4216 8.785201e-01 9.830162e-01

4217 8.785476e-01 9.830176e-01

4218 8.785743e-01 9.830190e-01

4219 8.786003e-01 9.830203e-01

4220 8.786255e-01 9.830217e-01

4221 8.786501e-01 9.830231e-01

4222 8.786739e-01 9.830244e-01

4223 8.786971e-01 9.830258e-01

4224 8.787197e-01 9.830271e-01

4225 8.787415e-01 9.830284e-01

4226 8.787628e-01 9.830297e-01

4227 8.787834e-01 9.830311e-01

4228 8.788035e-01 9.830324e-01

4229 8.788229e-01 9.830337e-01

4230 8.788418e-01 9.830350e-01

4231 8.788602e-01 9.830362e-01

4232 8.788779e-01 9.830375e-01

4233 8.788952e-01 9.830388e-01

4234 8.789119e-01 9.830400e-01

4235 8.789281e-01 9.830413e-01

4236 8.789439e-01 9.830425e-01

4237 8.789591e-01 9.830438e-01

4238 8.789739e-01 9.830450e-01

4239 8.789882e-01 9.830462e-01

4240 8.790020e-01 9.830474e-01

4241 8.790154e-01 9.830486e-01

4242 8.790283e-01 9.830497e-01

4243 8.790408e-01 9.830509e-01

4244 8.790529e-01 9.830520e-01

4245 8.790646e-01 9.830532e-01

4246 8.790759e-01 9.830543e-01

4247 8.790867e-01 9.830554e-01

4248 8.790972e-01 9.830565e-01

4249 8.791073e-01 9.830576e-01

4250 8.791170e-01 9.830586e-01

4251 8.791263e-01 9.830597e-01

4252 8.791353e-01 9.830607e-01

4253 8.791439e-01 9.830617e-01

4254 8.791521e-01 9.830627e-01

4255 8.791600e-01 9.830637e-01

4256 8.791675e-01 9.830647e-01

4257 8.791747e-01 9.830657e-01

4258 8.791816e-01 9.830666e-01

4259 8.791881e-01 9.830675e-01

4260 8.791942e-01 9.830684e-01

4261 8.792001e-01 9.830693e-01

4262 8.792056e-01 9.830701e-01

4263 8.792107e-01 9.830710e-01

4264 8.792156e-01 9.830718e-01

4265 8.792201e-01 9.830726e-01

4266 8.792243e-01 9.830733e-01

4267 8.792282e-01 9.830741e-01

4268 8.792318e-01 9.830748e-01

4269 8.792350e-01 9.830755e-01

4270 8.792380e-01 9.830762e-01

4271 8.792406e-01 9.830769e-01

4272 8.792429e-01 9.830775e-01

4273 8.792449e-01 9.830781e-01

4274 8.792466e-01 9.830787e-01

4275 8.792479e-01 9.830792e-01

4276 8.792490e-01 9.830797e-01

4277 8.792497e-01 9.830802e-01

4278 8.792501e-01 9.830807e-01

4279 8.792502e-01 9.830811e-01

4280 8.792500e-01 9.830816e-01

4281 8.792495e-01 9.830819e-01

4282 8.792487e-01 9.830823e-01

4283 8.792476e-01 9.830826e-01

4284 8.792461e-01 9.830829e-01

4285 8.792443e-01 9.830831e-01

4286 8.792422e-01 9.830833e-01

4287 8.792398e-01 9.830835e-01

4288 8.792371e-01 9.830837e-01

4289 8.792340e-01 9.830838e-01

4290 8.792307e-01 9.830839e-01

4291 8.792270e-01 9.830839e-01

4292 8.792230e-01 9.830839e-01

4293 8.792187e-01 9.830839e-01

4294 8.792141e-01 9.830838e-01

4295 8.792091e-01 9.830837e-01

4296 8.792038e-01 9.830835e-01

4297 8.791982e-01 9.830833e-01

4298 8.791923e-01 9.830831e-01

4299 8.791861e-01 9.830828e-01

4300 8.791796e-01 9.830825e-01

4301 8.791727e-01 9.830821e-01

4302 8.791656e-01 9.830817e-01

4303 8.791581e-01 9.830812e-01

4304 8.791503e-01 9.830807e-01

4305 8.791422e-01 9.830802e-01

4306 8.791338e-01 9.830796e-01

4307 8.791251e-01 9.830789e-01

4308 8.791161e-01 9.830782e-01

4309 8.791068e-01 9.830775e-01

4310 8.790972e-01 9.830767e-01

4311 8.790874e-01 9.830758e-01

4312 8.790772e-01 9.830749e-01

4313 8.790668e-01 9.830739e-01

4314 8.790561e-01 9.830729e-01

4315 8.790451e-01 9.830718e-01

4316 8.790338e-01 9.830707e-01

4317 8.790224e-01 9.830695e-01

4318 8.790106e-01 9.830683e-01

4319 8.789986e-01 9.830669e-01

4320 8.789864e-01 9.830656e-01

4321 8.789740e-01 9.830641e-01

4322 8.791021e-01 9.830653e-01

4323 8.792285e-01 9.830664e-01

4324 8.793534e-01 9.830676e-01

4325 8.794765e-01 9.830687e-01

4326 8.795980e-01 9.830699e-01

4327 8.797178e-01 9.830710e-01

4328 8.798359e-01 9.830721e-01

4329 8.799523e-01 9.830733e-01

4330 8.800670e-01 9.830744e-01

4331 8.801798e-01 9.830756e-01

4332 8.802910e-01 9.830767e-01

4333 8.804003e-01 9.830779e-01

4334 8.805079e-01 9.830790e-01

4335 8.806138e-01 9.830802e-01

4336 8.807178e-01 9.830813e-01

4337 8.808201e-01 9.830825e-01

4338 8.809206e-01 9.830836e-01

4339 8.810193e-01 9.830848e-01

4340 8.811162e-01 9.830859e-01

4341 8.812114e-01 9.830871e-01

4342 8.813049e-01 9.830882e-01

4343 8.813966e-01 9.830894e-01

4344 8.814865e-01 9.830905e-01

4345 8.815747e-01 9.830917e-01

4346 8.816612e-01 9.830928e-01

4347 8.817460e-01 9.830940e-01

4348 8.818291e-01 9.830951e-01

4349 8.819106e-01 9.830963e-01

4350 8.819903e-01 9.830974e-01

4351 8.820685e-01 9.830986e-01

4352 8.821450e-01 9.830997e-01

4353 8.822199e-01 9.831009e-01

4354 8.822932e-01 9.831020e-01

4355 8.823649e-01 9.831032e-01

4356 8.824351e-01 9.831043e-01

4357 8.825037e-01 9.831054e-01

4358 8.825709e-01 9.831066e-01

4359 8.826365e-01 9.831077e-01

4360 8.827007e-01 9.831089e-01

4361 8.827634e-01 9.831100e-01

4362 8.828248e-01 9.831111e-01

4363 8.828847e-01 9.831123e-01

4364 8.829432e-01 9.831134e-01

4365 8.830004e-01 9.831145e-01

4366 8.830563e-01 9.831157e-01

4367 8.831108e-01 9.831168e-01

4368 8.831641e-01 9.831179e-01

4369 8.832161e-01 9.831190e-01

4370 8.832668e-01 9.831202e-01

4371 8.833164e-01 9.831213e-01

4372 8.833647e-01 9.831224e-01

4373 8.834119e-01 9.831235e-01

4374 8.834580e-01 9.831246e-01

4375 8.835029e-01 9.831257e-01

4376 8.835467e-01 9.831268e-01

4377 8.835894e-01 9.831279e-01

4378 8.836311e-01 9.831290e-01

4379 8.836717e-01 9.831301e-01

4380 8.837114e-01 9.831312e-01

4381 8.837500e-01 9.831323e-01

4382 8.837877e-01 9.831333e-01

4383 8.838244e-01 9.831344e-01

4384 8.838602e-01 9.831355e-01

4385 8.838951e-01 9.831365e-01

4386 8.839291e-01 9.831376e-01

4387 8.839622e-01 9.831386e-01

4388 8.839945e-01 9.831397e-01

4389 8.840259e-01 9.831407e-01

4390 8.840566e-01 9.831418e-01

4391 8.840864e-01 9.831428e-01

4392 8.841155e-01 9.831438e-01

4393 8.841438e-01 9.831448e-01

4394 8.841714e-01 9.831458e-01

4395 8.841982e-01 9.831468e-01

4396 8.842244e-01 9.831478e-01

4397 8.842499e-01 9.831488e-01

4398 8.842747e-01 9.831498e-01

4399 8.842988e-01 9.831508e-01

4400 8.843223e-01 9.831517e-01

4401 8.843451e-01 9.831527e-01

4402 8.843674e-01 9.831536e-01

4403 8.843891e-01 9.831546e-01

4404 8.844101e-01 9.831555e-01

4405 8.844306e-01 9.831564e-01

4406 8.844506e-01 9.831573e-01

4407 8.844699e-01 9.831582e-01

4408 8.844888e-01 9.831591e-01

4409 8.845071e-01 9.831600e-01

4410 8.845249e-01 9.831609e-01

4411 8.845423e-01 9.831617e-01

4412 8.845591e-01 9.831626e-01

4413 8.845755e-01 9.831634e-01

4414 8.845913e-01 9.831642e-01

4415 8.846068e-01 9.831650e-01

4416 8.846217e-01 9.831658e-01

4417 8.846363e-01 9.831666e-01

4418 8.846504e-01 9.831674e-01

4419 8.846641e-01 9.831681e-01

4420 8.846773e-01 9.831689e-01

4421 8.846902e-01 9.831696e-01

4422 8.847026e-01 9.831703e-01

4423 8.847147e-01 9.831710e-01

4424 8.847264e-01 9.831717e-01

4425 8.847377e-01 9.831724e-01

4426 8.847486e-01 9.831730e-01

4427 8.847592e-01 9.831736e-01

4428 8.847694e-01 9.831742e-01

4429 8.847792e-01 9.831748e-01

4430 8.847887e-01 9.831754e-01

4431 8.847979e-01 9.831760e-01

4432 8.848067e-01 9.831765e-01

4433 8.848152e-01 9.831770e-01

4434 8.848233e-01 9.831775e-01

4435 8.848311e-01 9.831780e-01

4436 8.848386e-01 9.831785e-01

4437 8.848458e-01 9.831789e-01

4438 8.848526e-01 9.831793e-01

4439 8.848592e-01 9.831797e-01

4440 8.848654e-01 9.831801e-01

4441 8.848714e-01 9.831805e-01

4442 8.848770e-01 9.831808e-01

4443 8.848823e-01 9.831811e-01

4444 8.848873e-01 9.831814e-01

4445 8.848921e-01 9.831816e-01

4446 8.848965e-01 9.831819e-01

4447 8.849007e-01 9.831821e-01

4448 8.849045e-01 9.831822e-01

4449 8.849081e-01 9.831824e-01

4450 8.849114e-01 9.831825e-01

4451 8.849144e-01 9.831826e-01

4452 8.849171e-01 9.831827e-01

4453 8.849196e-01 9.831827e-01

4454 8.849217e-01 9.831827e-01

4455 8.849236e-01 9.831827e-01

4456 8.849252e-01 9.831826e-01

4457 8.849266e-01 9.831825e-01

4458 8.849276e-01 9.831824e-01

4459 8.849284e-01 9.831822e-01

4460 8.849289e-01 9.831821e-01

4461 8.849292e-01 9.831818e-01

4462 8.849292e-01 9.831816e-01

4463 8.849289e-01 9.831813e-01

4464 8.849283e-01 9.831809e-01

4465 8.849275e-01 9.831806e-01

4466 8.849265e-01 9.831802e-01

4467 8.849251e-01 9.831797e-01

4468 8.849235e-01 9.831792e-01

4469 8.849217e-01 9.831787e-01

4470 8.849196e-01 9.831782e-01

4471 8.849172e-01 9.831776e-01

4472 8.849146e-01 9.831769e-01

4473 8.849118e-01 9.831762e-01

4474 8.849087e-01 9.831755e-01

4475 8.849054e-01 9.831747e-01

4476 8.849018e-01 9.831739e-01

4477 8.848980e-01 9.831730e-01

4478 8.848939e-01 9.831721e-01

4479 8.848896e-01 9.831712e-01

4480 8.848851e-01 9.831701e-01

4481 8.848804e-01 9.831691e-01

4482 8.848754e-01 9.831680e-01

4483 8.848702e-01 9.831668e-01

4484 8.848648e-01 9.831656e-01

4485 8.848592e-01 9.831644e-01

4486 8.848534e-01 9.831630e-01

4487 8.848474e-01 9.831617e-01

4488 8.848412e-01 9.831603e-01

4489 8.848348e-01 9.831588e-01

4490 8.848282e-01 9.831572e-01

4491 8.848215e-01 9.831557e-01

4492 8.848146e-01 9.831540e-01

4493 8.848075e-01 9.831523e-01

4494 8.848002e-01 9.831505e-01

4495 8.847928e-01 9.831487e-01

4496 8.847852e-01 9.831468e-01

4497 8.847775e-01 9.831449e-01

4498 8.847697e-01 9.831429e-01

4499 8.847617e-01 9.831408e-01

4500 8.847536e-01 9.831387e-01

4501 8.847454e-01 9.831365e-01

4502 8.848631e-01 9.831374e-01

4503 8.849792e-01 9.831383e-01

4504 8.850938e-01 9.831392e-01

4505 8.852068e-01 9.831401e-01

4506 8.853181e-01 9.831410e-01

4507 8.854279e-01 9.831419e-01

4508 8.855360e-01 9.831428e-01

4509 8.856425e-01 9.831437e-01

4510 8.857473e-01 9.831446e-01

4511 8.858505e-01 9.831456e-01

4512 8.859521e-01 9.831465e-01

4513 8.860520e-01 9.831474e-01

4514 8.861502e-01 9.831483e-01

4515 8.862468e-01 9.831492e-01

4516 8.863418e-01 9.831501e-01

4517 8.864351e-01 9.831510e-01

4518 8.865267e-01 9.831519e-01

4519 8.866168e-01 9.831528e-01

4520 8.867052e-01 9.831537e-01

4521 8.867920e-01 9.831546e-01

4522 8.868772e-01 9.831555e-01

4523 8.869608e-01 9.831564e-01

4524 8.870428e-01 9.831573e-01

4525 8.871233e-01 9.831582e-01

4526 8.872021e-01 9.831591e-01

4527 8.872795e-01 9.831600e-01

4528 8.873553e-01 9.831609e-01

4529 8.874296e-01 9.831618e-01

4530 8.875024e-01 9.831627e-01

4531 8.875738e-01 9.831635e-01

4532 8.876437e-01 9.831644e-01

4533 8.877121e-01 9.831653e-01

4534 8.877791e-01 9.831662e-01

4535 8.878447e-01 9.831671e-01

4536 8.879089e-01 9.831679e-01

4537 8.879718e-01 9.831688e-01

4538 8.880333e-01 9.831697e-01

4539 8.880935e-01 9.831705e-01

4540 8.881523e-01 9.831714e-01

4541 8.882099e-01 9.831723e-01

4542 8.882663e-01 9.831731e-01

4543 8.883214e-01 9.831740e-01

4544 8.883752e-01 9.831748e-01

4545 8.884279e-01 9.831757e-01

4546 8.884794e-01 9.831765e-01

4547 8.885297e-01 9.831773e-01

4548 8.885789e-01 9.831782e-01

4549 8.886270e-01 9.831790e-01

4550 8.886739e-01 9.831798e-01

4551 8.887198e-01 9.831807e-01

4552 8.887647e-01 9.831815e-01

4553 8.888085e-01 9.831823e-01

4554 8.888513e-01 9.831831e-01

4555 8.888931e-01 9.831839e-01

4556 8.889340e-01 9.831847e-01

4557 8.889739e-01 9.831855e-01

4558 8.890128e-01 9.831863e-01

4559 8.890509e-01 9.831870e-01

4560 8.890880e-01 9.831878e-01

4561 8.891243e-01 9.831886e-01

4562 8.891597e-01 9.831893e-01

4563 8.891943e-01 9.831901e-01

4564 8.892281e-01 9.831908e-01

4565 8.892610e-01 9.831916e-01

4566 8.892932e-01 9.831923e-01

4567 8.893246e-01 9.831930e-01

4568 8.893553e-01 9.831937e-01

4569 8.893852e-01 9.831945e-01

4570 8.894144e-01 9.831952e-01

4571 8.894429e-01 9.831958e-01

4572 8.894708e-01 9.831965e-01

4573 8.894979e-01 9.831972e-01

4574 8.895244e-01 9.831979e-01

4575 8.895503e-01 9.831985e-01

4576 8.895755e-01 9.831992e-01

4577 8.896002e-01 9.831998e-01

4578 8.896242e-01 9.832004e-01

4579 8.896476e-01 9.832010e-01

4580 8.896705e-01 9.832017e-01

4581 8.896928e-01 9.832022e-01

4582 8.897146e-01 9.832028e-01

4583 8.897358e-01 9.832034e-01

4584 8.897565e-01 9.832040e-01

4585 8.897767e-01 9.832045e-01

4586 8.897964e-01 9.832050e-01

4587 8.898156e-01 9.832056e-01

4588 8.898343e-01 9.832061e-01

4589 8.898525e-01 9.832066e-01

4590 8.898703e-01 9.832071e-01

4591 8.898877e-01 9.832075e-01

4592 8.899045e-01 9.832080e-01

4593 8.899210e-01 9.832084e-01

4594 8.899370e-01 9.832088e-01

4595 8.899527e-01 9.832093e-01

4596 8.899679e-01 9.832096e-01

4597 8.899827e-01 9.832100e-01

4598 8.899971e-01 9.832104e-01

4599 8.900111e-01 9.832107e-01

4600 8.900248e-01 9.832111e-01

4601 8.900380e-01 9.832114e-01

4602 8.900509e-01 9.832117e-01

4603 8.900635e-01 9.832119e-01

4604 8.900757e-01 9.832122e-01

4605 8.900875e-01 9.832124e-01

4606 8.900990e-01 9.832126e-01

4607 8.901102e-01 9.832128e-01

4608 8.901210e-01 9.832130e-01

4609 8.901315e-01 9.832132e-01

4610 8.901417e-01 9.832133e-01

4611 8.901516e-01 9.832134e-01

4612 8.901611e-01 9.832135e-01

4613 8.901704e-01 9.832136e-01

4614 8.901793e-01 9.832136e-01

4615 8.901879e-01 9.832136e-01

4616 8.901962e-01 9.832136e-01

4617 8.902043e-01 9.832136e-01

4618 8.902120e-01 9.832135e-01

4619 8.902195e-01 9.832135e-01

4620 8.902266e-01 9.832134e-01

4621 8.902335e-01 9.832132e-01

4622 8.902401e-01 9.832131e-01

4623 8.902464e-01 9.832129e-01

4624 8.902525e-01 9.832127e-01

4625 8.902582e-01 9.832124e-01

4626 8.902637e-01 9.832122e-01

4627 8.902690e-01 9.832118e-01

4628 8.902740e-01 9.832115e-01

4629 8.902787e-01 9.832112e-01

4630 8.902831e-01 9.832108e-01

4631 8.902873e-01 9.832103e-01

4632 8.902912e-01 9.832099e-01

4633 8.902949e-01 9.832094e-01

4634 8.902983e-01 9.832089e-01

4635 8.903015e-01 9.832083e-01

4636 8.903044e-01 9.832077e-01

4637 8.903071e-01 9.832071e-01

4638 8.903095e-01 9.832064e-01

4639 8.903117e-01 9.832057e-01

4640 8.903137e-01 9.832049e-01

4641 8.903154e-01 9.832042e-01

4642 8.903168e-01 9.832033e-01

4643 8.903181e-01 9.832025e-01

4644 8.903191e-01 9.832016e-01

4645 8.903199e-01 9.832006e-01

4646 8.903204e-01 9.831997e-01

4647 8.903207e-01 9.831986e-01

4648 8.903208e-01 9.831976e-01

4649 8.903207e-01 9.831964e-01

4650 8.903204e-01 9.831953e-01

4651 8.903198e-01 9.831941e-01

4652 8.903190e-01 9.831928e-01

4653 8.903181e-01 9.831915e-01

4654 8.903169e-01 9.831902e-01

4655 8.903155e-01 9.831888e-01

4656 8.903139e-01 9.831874e-01

4657 8.903121e-01 9.831859e-01

4658 8.903101e-01 9.831843e-01

4659 8.903079e-01 9.831827e-01

4660 8.903056e-01 9.831811e-01

4661 8.903030e-01 9.831794e-01

4662 8.903003e-01 9.831776e-01

4663 8.902973e-01 9.831758e-01

4664 8.902942e-01 9.831740e-01

4665 8.902910e-01 9.831721e-01

4666 8.902875e-01 9.831701e-01

4667 8.902839e-01 9.831681e-01

4668 8.902802e-01 9.831660e-01

4669 8.902763e-01 9.831638e-01

4670 8.902722e-01 9.831616e-01

4671 8.902680e-01 9.831593e-01

4672 8.902636e-01 9.831570e-01

4673 8.902591e-01 9.831546e-01

4674 8.902545e-01 9.831521e-01

4675 8.902497e-01 9.831496e-01

4676 8.902448e-01 9.831470e-01

4677 8.902398e-01 9.831444e-01

4678 8.902347e-01 9.831416e-01

4679 8.902295e-01 9.831388e-01

4680 8.902241e-01 9.831360e-01

4681 8.902186e-01 9.831330e-01

4682 8.903277e-01 9.831337e-01

4683 8.904353e-01 9.831345e-01

4684 8.905414e-01 9.831352e-01

4685 8.906460e-01 9.831359e-01

4686 8.907490e-01 9.831366e-01

4687 8.908506e-01 9.831373e-01

4688 8.909506e-01 9.831380e-01

4689 8.910491e-01 9.831387e-01

4690 8.911460e-01 9.831394e-01

4691 8.912414e-01 9.831401e-01

4692 8.913353e-01 9.831408e-01

4693 8.914277e-01 9.831415e-01

4694 8.915186e-01 9.831422e-01

4695 8.916079e-01 9.831429e-01

4696 8.916957e-01 9.831436e-01

4697 8.917820e-01 9.831443e-01

4698 8.918668e-01 9.831450e-01

4699 8.919501e-01 9.831457e-01

4700 8.920320e-01 9.831463e-01

4701 8.921124e-01 9.831470e-01

4702 8.921913e-01 9.831477e-01

4703 8.922688e-01 9.831484e-01

4704 8.923448e-01 9.831491e-01

4705 8.924194e-01 9.831497e-01

4706 8.924927e-01 9.831504e-01

4707 8.925645e-01 9.831511e-01

4708 8.926349e-01 9.831517e-01

4709 8.927040e-01 9.831524e-01

4710 8.927718e-01 9.831530e-01

4711 8.928382e-01 9.831537e-01

4712 8.929034e-01 9.831544e-01

4713 8.929672e-01 9.831550e-01

4714 8.930298e-01 9.831556e-01

4715 8.930911e-01 9.831563e-01

4716 8.931511e-01 9.831569e-01

4717 8.932100e-01 9.831575e-01

4718 8.932676e-01 9.831582e-01

4719 8.933241e-01 9.831588e-01

4720 8.933794e-01 9.831594e-01

4721 8.934336e-01 9.831600e-01

4722 8.934866e-01 9.831606e-01

4723 8.935386e-01 9.831612e-01

4724 8.935894e-01 9.831618e-01

4725 8.936392e-01 9.831624e-01

4726 8.936879e-01 9.831630e-01

4727 8.937357e-01 9.831636e-01

4728 8.937824e-01 9.831642e-01

4729 8.938281e-01 9.831647e-01

4730 8.938728e-01 9.831653e-01

4731 8.939166e-01 9.831658e-01

4732 8.939594e-01 9.831664e-01

4733 8.940014e-01 9.831669e-01

4734 8.940424e-01 9.831675e-01

4735 8.940826e-01 9.831680e-01

4736 8.941219e-01 9.831685e-01

4737 8.941603e-01 9.831690e-01

4738 8.941979e-01 9.831695e-01

4739 8.942347e-01 9.831700e-01

4740 8.942708e-01 9.831705e-01

4741 8.943060e-01 9.831710e-01

4742 8.943405e-01 9.831715e-01

4743 8.943742e-01 9.831719e-01

4744 8.944072e-01 9.831724e-01

4745 8.944395e-01 9.831728e-01

4746 8.944711e-01 9.831733e-01

4747 8.945020e-01 9.831737e-01

4748 8.945322e-01 9.831741e-01

4749 8.945618e-01 9.831745e-01

4750 8.945907e-01 9.831749e-01

4751 8.946190e-01 9.831753e-01

4752 8.946467e-01 9.831757e-01

4753 8.946737e-01 9.831761e-01

4754 8.947002e-01 9.831764e-01

4755 8.947261e-01 9.831767e-01

4756 8.947515e-01 9.831771e-01

4757 8.947763e-01 9.831774e-01

4758 8.948005e-01 9.831777e-01

4759 8.948243e-01 9.831780e-01

4760 8.948475e-01 9.831783e-01

4761 8.948702e-01 9.831785e-01

4762 8.948923e-01 9.831788e-01

4763 8.949140e-01 9.831790e-01

4764 8.949353e-01 9.831792e-01

4765 8.949560e-01 9.831794e-01

4766 8.949763e-01 9.831796e-01

4767 8.949962e-01 9.831798e-01

4768 8.950155e-01 9.831800e-01

4769 8.950345e-01 9.831801e-01

4770 8.950530e-01 9.831803e-01

4771 8.950712e-01 9.831804e-01

4772 8.950889e-01 9.831805e-01

4773 8.951062e-01 9.831805e-01

4774 8.951231e-01 9.831806e-01

4775 8.951396e-01 9.831806e-01

4776 8.951557e-01 9.831807e-01

4777 8.951715e-01 9.831807e-01

4778 8.951868e-01 9.831807e-01

4779 8.952019e-01 9.831806e-01

4780 8.952165e-01 9.831806e-01

4781 8.952308e-01 9.831805e-01

4782 8.952448e-01 9.831804e-01

4783 8.952584e-01 9.831803e-01

4784 8.952717e-01 9.831802e-01

4785 8.952846e-01 9.831800e-01

4786 8.952972e-01 9.831798e-01

4787 8.953095e-01 9.831796e-01

4788 8.953214e-01 9.831794e-01

4789 8.953331e-01 9.831791e-01

4790 8.953444e-01 9.831788e-01

4791 8.953555e-01 9.831785e-01

4792 8.953662e-01 9.831782e-01

4793 8.953766e-01 9.831779e-01

4794 8.953867e-01 9.831775e-01

4795 8.953965e-01 9.831771e-01

4796 8.954061e-01 9.831766e-01

4797 8.954153e-01 9.831762e-01

4798 8.954243e-01 9.831757e-01

4799 8.954330e-01 9.831752e-01

4800 8.954414e-01 9.831746e-01

4801 8.954495e-01 9.831740e-01

4802 8.954573e-01 9.831734e-01

4803 8.954649e-01 9.831728e-01

4804 8.954722e-01 9.831721e-01

4805 8.954792e-01 9.831714e-01

4806 8.954860e-01 9.831707e-01

4807 8.954925e-01 9.831699e-01

4808 8.954988e-01 9.831691e-01

4809 8.955048e-01 9.831682e-01

4810 8.955105e-01 9.831674e-01

4811 8.955160e-01 9.831665e-01

4812 8.955212e-01 9.831655e-01

4813 8.955262e-01 9.831645e-01

4814 8.955309e-01 9.831635e-01

4815 8.955354e-01 9.831624e-01

4816 8.955396e-01 9.831613e-01

4817 8.955436e-01 9.831602e-01

4818 8.955474e-01 9.831590e-01

4819 8.955509e-01 9.831578e-01

4820 8.955542e-01 9.831565e-01

4821 8.955572e-01 9.831552e-01

4822 8.955600e-01 9.831539e-01

4823 8.955626e-01 9.831525e-01

4824 8.955650e-01 9.831511e-01

4825 8.955671e-01 9.831496e-01

4826 8.955690e-01 9.831481e-01

4827 8.955707e-01 9.831465e-01

4828 8.955722e-01 9.831449e-01

4829 8.955735e-01 9.831432e-01

4830 8.955745e-01 9.831415e-01

4831 8.955754e-01 9.831397e-01

4832 8.955760e-01 9.831379e-01

4833 8.955765e-01 9.831360e-01

4834 8.955767e-01 9.831341e-01

4835 8.955767e-01 9.831321e-01

4836 8.955766e-01 9.831301e-01

4837 8.955762e-01 9.831280e-01

4838 8.955756e-01 9.831259e-01

4839 8.955749e-01 9.831237e-01

4840 8.955740e-01 9.831215e-01

4841 8.955729e-01 9.831192e-01

4842 8.955716e-01 9.831168e-01

4843 8.955701e-01 9.831144e-01

4844 8.955685e-01 9.831119e-01

4845 8.955667e-01 9.831094e-01

4846 8.955647e-01 9.831068e-01

4847 8.955626e-01 9.831041e-01

4848 8.955603e-01 9.831014e-01

4849 8.955579e-01 9.830986e-01

4850 8.955553e-01 9.830957e-01

4851 8.955525e-01 9.830928e-01

4852 8.955496e-01 9.830898e-01

4853 8.955466e-01 9.830867e-01

4854 8.955434e-01 9.830836e-01

4855 8.955401e-01 9.830804e-01

4856 8.955367e-01 9.830772e-01

4857 8.955331e-01 9.830738e-01

4858 8.955294e-01 9.830704e-01

4859 8.955256e-01 9.830669e-01

4860 8.955217e-01 9.830634e-01

4861 8.955176e-01 9.830598e-01

4862 8.956199e-01 9.830603e-01

4863 8.957208e-01 9.830608e-01

4864 8.958203e-01 9.830614e-01

4865 8.959183e-01 9.830619e-01

4866 8.960150e-01 9.830624e-01

4867 8.961102e-01 9.830630e-01

4868 8.962040e-01 9.830635e-01

4869 8.962964e-01 9.830640e-01

4870 8.963874e-01 9.830645e-01

4871 8.964770e-01 9.830651e-01

4872 8.965652e-01 9.830656e-01

4873 8.966519e-01 9.830661e-01

4874 8.967373e-01 9.830666e-01

4875 8.968213e-01 9.830671e-01

4876 8.969039e-01 9.830676e-01

4877 8.969852e-01 9.830681e-01

4878 8.970651e-01 9.830686e-01

4879 8.971436e-01 9.830691e-01

4880 8.972208e-01 9.830696e-01

4881 8.972966e-01 9.830701e-01

4882 8.973712e-01 9.830706e-01

4883 8.974444e-01 9.830710e-01

4884 8.975163e-01 9.830715e-01

4885 8.975870e-01 9.830720e-01

4886 8.976564e-01 9.830725e-01

4887 8.977245e-01 9.830729e-01

4888 8.977914e-01 9.830734e-01

4889 8.978571e-01 9.830738e-01

4890 8.979216e-01 9.830743e-01

4891 8.979849e-01 9.830747e-01

4892 8.980470e-01 9.830752e-01

4893 8.981080e-01 9.830756e-01

4894 8.981678e-01 9.830760e-01

4895 8.982265e-01 9.830765e-01

4896 8.982840e-01 9.830769e-01

4897 8.983405e-01 9.830773e-01

4898 8.983959e-01 9.830777e-01

4899 8.984503e-01 9.830781e-01

4900 8.985036e-01 9.830785e-01

4901 8.985559e-01 9.830789e-01

4902 8.986072e-01 9.830793e-01

4903 8.986575e-01 9.830796e-01

4904 8.987068e-01 9.830800e-01

4905 8.987552e-01 9.830804e-01

4906 8.988026e-01 9.830807e-01

4907 8.988491e-01 9.830811e-01

4908 8.988947e-01 9.830814e-01

4909 8.989394e-01 9.830817e-01

4910 8.989833e-01 9.830821e-01

4911 8.990263e-01 9.830824e-01

4912 8.990684e-01 9.830827e-01

4913 8.991097e-01 9.830830e-01

4914 8.991502e-01 9.830833e-01

4915 8.991899e-01 9.830835e-01

4916 8.992289e-01 9.830838e-01

4917 8.992670e-01 9.830841e-01

4918 8.993045e-01 9.830843e-01

4919 8.993411e-01 9.830846e-01

4920 8.993771e-01 9.830848e-01

4921 8.994124e-01 9.830850e-01

4922 8.994469e-01 9.830852e-01

4923 8.994808e-01 9.830854e-01

4924 8.995140e-01 9.830856e-01

4925 8.995466e-01 9.830858e-01

4926 8.995785e-01 9.830860e-01

4927 8.996098e-01 9.830861e-01

4928 8.996405e-01 9.830863e-01

4929 8.996706e-01 9.830864e-01

4930 8.997001e-01 9.830865e-01

4931 8.997290e-01 9.830866e-01

4932 8.997573e-01 9.830867e-01

4933 8.997851e-01 9.830868e-01

4934 8.998123e-01 9.830869e-01

4935 8.998390e-01 9.830869e-01

4936 8.998652e-01 9.830869e-01

4937 8.998908e-01 9.830870e-01

4938 8.999159e-01 9.830870e-01

4939 8.999406e-01 9.830870e-01

4940 8.999647e-01 9.830869e-01

4941 8.999884e-01 9.830869e-01

4942 9.000116e-01 9.830868e-01

4943 9.000343e-01 9.830868e-01

4944 9.000566e-01 9.830867e-01

4945 9.000784e-01 9.830866e-01

4946 9.000998e-01 9.830865e-01

4947 9.001207e-01 9.830863e-01

4948 9.001412e-01 9.830862e-01

4949 9.001613e-01 9.830860e-01

4950 9.001810e-01 9.830858e-01

4951 9.002003e-01 9.830856e-01

4952 9.002191e-01 9.830853e-01

4953 9.002376e-01 9.830851e-01

4954 9.002557e-01 9.830848e-01

4955 9.002734e-01 9.830845e-01

4956 9.002907e-01 9.830842e-01

4957 9.003077e-01 9.830838e-01

4958 9.003243e-01 9.830835e-01

4959 9.003405e-01 9.830831e-01

4960 9.003563e-01 9.830827e-01

4961 9.003718e-01 9.830822e-01

4962 9.003870e-01 9.830818e-01

4963 9.004018e-01 9.830813e-01

4964 9.004163e-01 9.830808e-01

4965 9.004304e-01 9.830803e-01

4966 9.004442e-01 9.830797e-01

4967 9.004577e-01 9.830791e-01

4968 9.004708e-01 9.830785e-01

4969 9.004837e-01 9.830779e-01

4970 9.004962e-01 9.830772e-01

4971 9.005084e-01 9.830765e-01

4972 9.005202e-01 9.830758e-01

4973 9.005318e-01 9.830751e-01

4974 9.005431e-01 9.830743e-01

4975 9.005540e-01 9.830735e-01

4976 9.005647e-01 9.830726e-01

4977 9.005750e-01 9.830718e-01

4978 9.005851e-01 9.830709e-01

4979 9.005949e-01 9.830699e-01

4980 9.006044e-01 9.830690e-01

4981 9.006136e-01 9.830680e-01

4982 9.006225e-01 9.830669e-01

4983 9.006311e-01 9.830658e-01

4984 9.006395e-01 9.830647e-01

4985 9.006475e-01 9.830636e-01

4986 9.006553e-01 9.830624e-01

4987 9.006629e-01 9.830612e-01

4988 9.006701e-01 9.830600e-01

4989 9.006771e-01 9.830587e-01

4990 9.006838e-01 9.830573e-01

4991 9.006903e-01 9.830560e-01

4992 9.006965e-01 9.830546e-01

4993 9.007024e-01 9.830531e-01

4994 9.007081e-01 9.830516e-01

4995 9.007136e-01 9.830501e-01

4996 9.007188e-01 9.830485e-01

4997 9.007237e-01 9.830469e-01

4998 9.007284e-01 9.830452e-01

4999 9.007328e-01 9.830435e-01

5000 9.007370e-01 9.830418e-01

5001 9.007410e-01 9.830400e-01

5002 9.007448e-01 9.830381e-01

5003 9.007483e-01 9.830362e-01

5004 9.007515e-01 9.830343e-01

5005 9.007546e-01 9.830323e-01

5006 9.007574e-01 9.830303e-01

5007 9.007600e-01 9.830282e-01

5008 9.007624e-01 9.830260e-01

5009 9.007646e-01 9.830238e-01

5010 9.007665e-01 9.830216e-01

5011 9.007683e-01 9.830193e-01

5012 9.007698e-01 9.830169e-01

5013 9.007712e-01 9.830145e-01

5014 9.007723e-01 9.830120e-01

5015 9.007732e-01 9.830095e-01

5016 9.007740e-01 9.830069e-01

5017 9.007746e-01 9.830043e-01

5018 9.007749e-01 9.830016e-01

5019 9.007751e-01 9.829988e-01

5020 9.007752e-01 9.829960e-01

5021 9.007750e-01 9.829931e-01

5022 9.007747e-01 9.829902e-01

5023 9.007742e-01 9.829872e-01

5024 9.007735e-01 9.829841e-01

5025 9.007727e-01 9.829810e-01

5026 9.007718e-01 9.829778e-01

5027 9.007706e-01 9.829745e-01

5028 9.007694e-01 9.829712e-01

5029 9.007680e-01 9.829678e-01

5030 9.007664e-01 9.829643e-01

5031 9.007648e-01 9.829608e-01

5032 9.007630e-01 9.829571e-01

5033 9.007610e-01 9.829535e-01

5034 9.007590e-01 9.829497e-01

5035 9.007568e-01 9.829459e-01

5036 9.007545e-01 9.829419e-01

5037 9.007522e-01 9.829380e-01

5038 9.007497e-01 9.829339e-01

5039 9.007471e-01 9.829298e-01

5040 9.007444e-01 9.829255e-01

5041 9.007417e-01 9.829212e-01

5042 9.008390e-01 9.829216e-01

5043 9.009350e-01 9.829220e-01

5044 9.010297e-01 9.829224e-01

5045 9.011231e-01 9.829228e-01

5046 9.012152e-01 9.829231e-01

5047 9.013060e-01 9.829235e-01

5048 9.013955e-01 9.829239e-01

5049 9.014837e-01 9.829242e-01

5050 9.015706e-01 9.829246e-01

5051 9.016562e-01 9.829249e-01

5052 9.017405e-01 9.829253e-01

5053 9.018235e-01 9.829256e-01

5054 9.019053e-01 9.829260e-01

5055 9.019857e-01 9.829263e-01

5056 9.020650e-01 9.829267e-01

5057 9.021429e-01 9.829270e-01

5058 9.022197e-01 9.829273e-01

5059 9.022952e-01 9.829276e-01

5060 9.023695e-01 9.829280e-01

5061 9.024425e-01 9.829283e-01

5062 9.025144e-01 9.829286e-01

5063 9.025851e-01 9.829289e-01

5064 9.026546e-01 9.829292e-01

5065 9.027230e-01 9.829295e-01

5066 9.027902e-01 9.829297e-01

5067 9.028562e-01 9.829300e-01

5068 9.029212e-01 9.829303e-01

5069 9.029850e-01 9.829306e-01

5070 9.030478e-01 9.829308e-01

5071 9.031095e-01 9.829311e-01

5072 9.031701e-01 9.829313e-01

5073 9.032296e-01 9.829316e-01

5074 9.032882e-01 9.829318e-01

5075 9.033457e-01 9.829320e-01

5076 9.034022e-01 9.829323e-01

5077 9.034577e-01 9.829325e-01

5078 9.035122e-01 9.829327e-01

5079 9.035658e-01 9.829329e-01

5080 9.036184e-01 9.829331e-01

5081 9.036701e-01 9.829332e-01

5082 9.037208e-01 9.829334e-01

5083 9.037707e-01 9.829336e-01

5084 9.038197e-01 9.829337e-01

5085 9.038678e-01 9.829339e-01

5086 9.039151e-01 9.829340e-01

5087 9.039615e-01 9.829342e-01

5088 9.040070e-01 9.829343e-01

5089 9.040518e-01 9.829344e-01

5090 9.040958e-01 9.829345e-01

5091 9.041389e-01 9.829346e-01

5092 9.041813e-01 9.829347e-01

5093 9.042229e-01 9.829348e-01

5094 9.042638e-01 9.829348e-01

5095 9.043040e-01 9.829349e-01

5096 9.043434e-01 9.829349e-01

5097 9.043821e-01 9.829349e-01

5098 9.044201e-01 9.829350e-01

5099 9.044574e-01 9.829350e-01

5100 9.044941e-01 9.829350e-01

5101 9.045301e-01 9.829349e-01

5102 9.045654e-01 9.829349e-01

5103 9.046001e-01 9.829349e-01

5104 9.046342e-01 9.829348e-01

5105 9.046677e-01 9.829347e-01

5106 9.047005e-01 9.829347e-01

5107 9.047328e-01 9.829346e-01

5108 9.047644e-01 9.829344e-01

5109 9.047955e-01 9.829343e-01

5110 9.048260e-01 9.829342e-01

5111 9.048560e-01 9.829340e-01

5112 9.048854e-01 9.829338e-01

5113 9.049143e-01 9.829337e-01

5114 9.049426e-01 9.829335e-01

5115 9.049704e-01 9.829332e-01

5116 9.049977e-01 9.829330e-01

5117 9.050245e-01 9.829327e-01

5118 9.050508e-01 9.829325e-01

5119 9.050766e-01 9.829322e-01

5120 9.051019e-01 9.829319e-01

5121 9.051268e-01 9.829316e-01

5122 9.051512e-01 9.829312e-01

5123 9.051751e-01 9.829309e-01

5124 9.051985e-01 9.829305e-01

5125 9.052215e-01 9.829301e-01

5126 9.052441e-01 9.829297e-01

5127 9.052662e-01 9.829292e-01

5128 9.052879e-01 9.829288e-01

5129 9.053092e-01 9.829283e-01

5130 9.053301e-01 9.829278e-01

5131 9.053505e-01 9.829272e-01

5132 9.053705e-01 9.829267e-01

5133 9.053901e-01 9.829261e-01

5134 9.054094e-01 9.829255e-01

5135 9.054282e-01 9.829249e-01

5136 9.054466e-01 9.829243e-01

5137 9.054647e-01 9.829236e-01

5138 9.054824e-01 9.829229e-01

5139 9.054997e-01 9.829222e-01

5140 9.055166e-01 9.829214e-01

5141 9.055331e-01 9.829207e-01

5142 9.055493e-01 9.829199e-01

5143 9.055652e-01 9.829190e-01

5144 9.055807e-01 9.829182e-01

5145 9.055958e-01 9.829173e-01

5146 9.056106e-01 9.829164e-01

5147 9.056250e-01 9.829155e-01

5148 9.056391e-01 9.829145e-01

5149 9.056528e-01 9.829135e-01

5150 9.056663e-01 9.829125e-01

5151 9.056793e-01 9.829114e-01

5152 9.056921e-01 9.829103e-01

5153 9.057045e-01 9.829092e-01

5154 9.057166e-01 9.829080e-01

5155 9.057284e-01 9.829069e-01

5156 9.057399e-01 9.829056e-01

5157 9.057511e-01 9.829044e-01

5158 9.057620e-01 9.829031e-01

5159 9.057725e-01 9.829017e-01

5160 9.057828e-01 9.829004e-01

5161 9.057927e-01 9.828990e-01

5162 9.058024e-01 9.828975e-01

5163 9.058117e-01 9.828961e-01

5164 9.058208e-01 9.828945e-01

5165 9.058296e-01 9.828930e-01

5166 9.058381e-01 9.828914e-01

5167 9.058463e-01 9.828898e-01

5168 9.058543e-01 9.828881e-01

5169 9.058619e-01 9.828864e-01

5170 9.058693e-01 9.828846e-01

5171 9.058765e-01 9.828828e-01

5172 9.058833e-01 9.828809e-01

5173 9.058899e-01 9.828791e-01

5174 9.058963e-01 9.828771e-01

5175 9.059024e-01 9.828751e-01

5176 9.059082e-01 9.828731e-01

5177 9.059138e-01 9.828710e-01

5178 9.059192e-01 9.828689e-01

5179 9.059243e-01 9.828667e-01

5180 9.059292e-01 9.828645e-01

5181 9.059338e-01 9.828622e-01

5182 9.059383e-01 9.828599e-01

5183 9.059424e-01 9.828575e-01

5184 9.059464e-01 9.828551e-01

5185 9.059502e-01 9.828526e-01

5186 9.059537e-01 9.828501e-01

5187 9.059571e-01 9.828475e-01

5188 9.059602e-01 9.828449e-01

5189 9.059632e-01 9.828422e-01

5190 9.059659e-01 9.828394e-01

5191 9.059685e-01 9.828366e-01

5192 9.059709e-01 9.828337e-01

5193 9.059730e-01 9.828308e-01

5194 9.059751e-01 9.828278e-01

5195 9.059769e-01 9.828247e-01

5196 9.059786e-01 9.828216e-01

5197 9.059801e-01 9.828184e-01

5198 9.059815e-01 9.828152e-01

5199 9.059827e-01 9.828119e-01

5200 9.059838e-01 9.828085e-01

5201 9.059847e-01 9.828051e-01

5202 9.059855e-01 9.828016e-01

5203 9.059861e-01 9.827980e-01

5204 9.059867e-01 9.827944e-01

5205 9.059871e-01 9.827906e-01

5206 9.059874e-01 9.827869e-01

5207 9.059876e-01 9.827830e-01

5208 9.059877e-01 9.827791e-01

5209 9.059877e-01 9.827751e-01

5210 9.059876e-01 9.827710e-01

5211 9.059874e-01 9.827669e-01

5212 9.059871e-01 9.827626e-01

5213 9.059868e-01 9.827583e-01

5214 9.059864e-01 9.827540e-01

5215 9.059860e-01 9.827495e-01

5216 9.059855e-01 9.827450e-01

5217 9.059849e-01 9.827404e-01

5218 9.059843e-01 9.827357e-01

5219 9.059837e-01 9.827309e-01

5220 9.059830e-01 9.827260e-01

5221 9.059823e-01 9.827211e-01

5222 9.060764e-01 9.827213e-01

5223 9.061693e-01 9.827216e-01

5224 9.062610e-01 9.827218e-01

5225 9.063515e-01 9.827220e-01

5226 9.064408e-01 9.827223e-01

5227 9.065289e-01 9.827225e-01

5228 9.066157e-01 9.827227e-01

5229 9.067014e-01 9.827229e-01

5230 9.067859e-01 9.827231e-01

5231 9.068692e-01 9.827233e-01

5232 9.069512e-01 9.827235e-01

5233 9.070322e-01 9.827237e-01

5234 9.071119e-01 9.827239e-01

5235 9.071905e-01 9.827241e-01

5236 9.072679e-01 9.827243e-01

5237 9.073442e-01 9.827245e-01

5238 9.074193e-01 9.827246e-01

5239 9.074933e-01 9.827248e-01

5240 9.075662e-01 9.827250e-01

5241 9.076379e-01 9.827251e-01

5242 9.077086e-01 9.827252e-01

5243 9.077782e-01 9.827254e-01

5244 9.078467e-01 9.827255e-01

5245 9.079141e-01 9.827256e-01

5246 9.079805e-01 9.827258e-01

5247 9.080458e-01 9.827259e-01

5248 9.081101e-01 9.827260e-01

5249 9.081734e-01 9.827261e-01

5250 9.082356e-01 9.827262e-01

5251 9.082969e-01 9.827262e-01

5252 9.083572e-01 9.827263e-01

5253 9.084165e-01 9.827264e-01

5254 9.084749e-01 9.827264e-01

5255 9.085323e-01 9.827265e-01

5256 9.085887e-01 9.827265e-01

5257 9.086443e-01 9.827265e-01

5258 9.086990e-01 9.827266e-01

5259 9.087527e-01 9.827266e-01

5260 9.088056e-01 9.827266e-01

5261 9.088576e-01 9.827266e-01

5262 9.089087e-01 9.827266e-01

5263 9.089590e-01 9.827265e-01

5264 9.090085e-01 9.827265e-01

5265 9.090571e-01 9.827264e-01

5266 9.091050e-01 9.827264e-01

5267 9.091520e-01 9.827263e-01

5268 9.091983e-01 9.827262e-01

5269 9.092437e-01 9.827262e-01

5270 9.092885e-01 9.827261e-01

5271 9.093324e-01 9.827259e-01

5272 9.093757e-01 9.827258e-01

5273 9.094182e-01 9.827257e-01

5274 9.094600e-01 9.827255e-01

5275 9.095010e-01 9.827254e-01

5276 9.095414e-01 9.827252e-01

5277 9.095811e-01 9.827250e-01

5278 9.096202e-01 9.827248e-01

5279 9.096585e-01 9.827246e-01

5280 9.096962e-01 9.827243e-01

5281 9.097333e-01 9.827241e-01

5282 9.097697e-01 9.827238e-01

5283 9.098055e-01 9.827236e-01

5284 9.098407e-01 9.827233e-01

5285 9.098753e-01 9.827230e-01

5286 9.099092e-01 9.827227e-01

5287 9.099426e-01 9.827223e-01

5288 9.099754e-01 9.827220e-01

5289 9.100077e-01 9.827216e-01

5290 9.100393e-01 9.827212e-01

5291 9.100704e-01 9.827208e-01

5292 9.101010e-01 9.827204e-01

5293 9.101310e-01 9.827200e-01

5294 9.101605e-01 9.827195e-01

5295 9.101894e-01 9.827190e-01

5296 9.102179e-01 9.827185e-01

5297 9.102458e-01 9.827180e-01

5298 9.102732e-01 9.827175e-01

5299 9.103001e-01 9.827169e-01

5300 9.103265e-01 9.827164e-01

5301 9.103524e-01 9.827158e-01

5302 9.103779e-01 9.827152e-01

5303 9.104028e-01 9.827145e-01

5304 9.104273e-01 9.827139e-01

5305 9.104514e-01 9.827132e-01

5306 9.104749e-01 9.827125e-01

5307 9.104980e-01 9.827118e-01

5308 9.105207e-01 9.827110e-01

5309 9.105429e-01 9.827102e-01

5310 9.105647e-01 9.827094e-01

5311 9.105861e-01 9.827086e-01

5312 9.106070e-01 9.827078e-01

5313 9.106275e-01 9.827069e-01

5314 9.106476e-01 9.827060e-01

5315 9.106673e-01 9.827051e-01

5316 9.106866e-01 9.827041e-01

5317 9.107055e-01 9.827032e-01

5318 9.107239e-01 9.827022e-01

5319 9.107420e-01 9.827011e-01

5320 9.107597e-01 9.827001e-01

5321 9.107770e-01 9.826990e-01

5322 9.107939e-01 9.826978e-01

5323 9.108105e-01 9.826967e-01

5324 9.108266e-01 9.826955e-01

5325 9.108424e-01 9.826943e-01

5326 9.108579e-01 9.826931e-01

5327 9.108729e-01 9.826918e-01

5328 9.108876e-01 9.826905e-01

5329 9.109020e-01 9.826891e-01

5330 9.109160e-01 9.826877e-01

5331 9.109297e-01 9.826863e-01

5332 9.109430e-01 9.826849e-01

5333 9.109560e-01 9.826834e-01

5334 9.109687e-01 9.826819e-01

5335 9.109811e-01 9.826803e-01

5336 9.109931e-01 9.826787e-01

5337 9.110048e-01 9.826771e-01

5338 9.110162e-01 9.826754e-01

5339 9.110272e-01 9.826737e-01

5340 9.110380e-01 9.826720e-01

5341 9.110485e-01 9.826702e-01

5342 9.110586e-01 9.826684e-01

5343 9.110685e-01 9.826665e-01

5344 9.110781e-01 9.826646e-01

5345 9.110874e-01 9.826626e-01

5346 9.110964e-01 9.826606e-01

5347 9.111052e-01 9.826586e-01

5348 9.111136e-01 9.826565e-01

5349 9.111218e-01 9.826544e-01

5350 9.111298e-01 9.826522e-01

5351 9.111375e-01 9.826500e-01

5352 9.111449e-01 9.826477e-01

5353 9.111521e-01 9.826454e-01

5354 9.111591e-01 9.826430e-01

5355 9.111658e-01 9.826406e-01

5356 9.111723e-01 9.826381e-01

5357 9.111785e-01 9.826356e-01

5358 9.111845e-01 9.826330e-01

5359 9.111903e-01 9.826304e-01

5360 9.111959e-01 9.826277e-01

5361 9.112013e-01 9.826250e-01

5362 9.112065e-01 9.826222e-01

5363 9.112115e-01 9.826194e-01

5364 9.112163e-01 9.826165e-01

5365 9.112210e-01 9.826135e-01

5366 9.112254e-01 9.826105e-01

5367 9.112297e-01 9.826074e-01

5368 9.112338e-01 9.826043e-01

5369 9.112377e-01 9.826011e-01

5370 9.112415e-01 9.825979e-01

5371 9.112452e-01 9.825946e-01

5372 9.112486e-01 9.825912e-01

5373 9.112520e-01 9.825877e-01

5374 9.112552e-01 9.825842e-01

5375 9.112583e-01 9.825807e-01

5376 9.112613e-01 9.825770e-01

5377 9.112642e-01 9.825733e-01

5378 9.112669e-01 9.825696e-01

5379 9.112696e-01 9.825657e-01

5380 9.112721e-01 9.825618e-01

5381 9.112746e-01 9.825578e-01

5382 9.112770e-01 9.825538e-01

5383 9.112793e-01 9.825497e-01

5384 9.112816e-01 9.825455e-01

5385 9.112837e-01 9.825412e-01

5386 9.112859e-01 9.825368e-01

5387 9.112880e-01 9.825324e-01

5388 9.112900e-01 9.825279e-01

5389 9.112920e-01 9.825234e-01

5390 9.112940e-01 9.825187e-01

5391 9.112959e-01 9.825140e-01

5392 9.112978e-01 9.825092e-01

5393 9.112998e-01 9.825043e-01

5394 9.113017e-01 9.824993e-01

5395 9.113036e-01 9.824942e-01

5396 9.113055e-01 9.824891e-01

5397 9.113075e-01 9.824839e-01

5398 9.113094e-01 9.824785e-01

5399 9.113114e-01 9.824731e-01

5400 9.113135e-01 9.824676e-01

5401 9.113156e-01 9.824621e-01

5402 9.114080e-01 9.824622e-01

5403 9.114994e-01 9.824623e-01

5404 9.115896e-01 9.824624e-01

5405 9.116787e-01 9.824625e-01

5406 9.117667e-01 9.824626e-01

5407 9.118535e-01 9.824627e-01

5408 9.119392e-01 9.824628e-01

5409 9.120238e-01 9.824629e-01

5410 9.121073e-01 9.824629e-01

5411 9.121897e-01 9.824630e-01

5412 9.122709e-01 9.824631e-01

5413 9.123511e-01 9.824631e-01

5414 9.124301e-01 9.824632e-01

5415 9.125081e-01 9.824632e-01

5416 9.125850e-01 9.824632e-01

5417 9.126608e-01 9.824633e-01

5418 9.127355e-01 9.824633e-01

5419 9.128092e-01 9.824633e-01

5420 9.128819e-01 9.824633e-01

5421 9.129535e-01 9.824633e-01

5422 9.130240e-01 9.824633e-01

5423 9.130936e-01 9.824633e-01

5424 9.131621e-01 9.824633e-01

5425 9.132296e-01 9.824632e-01

5426 9.132962e-01 9.824632e-01

5427 9.133617e-01 9.824632e-01

5428 9.134263e-01 9.824631e-01

5429 9.134899e-01 9.824630e-01

5430 9.135525e-01 9.824630e-01

5431 9.136142e-01 9.824629e-01

5432 9.136750e-01 9.824628e-01

5433 9.137348e-01 9.824627e-01

5434 9.137938e-01 9.824626e-01

5435 9.138518e-01 9.824625e-01

5436 9.139090e-01 9.824623e-01

5437 9.139652e-01 9.824622e-01

5438 9.140206e-01 9.824620e-01

5439 9.140751e-01 9.824619e-01

5440 9.141288e-01 9.824617e-01

5441 9.141817e-01 9.824615e-01

5442 9.142337e-01 9.824613e-01

5443 9.142849e-01 9.824611e-01

5444 9.143352e-01 9.824609e-01

5445 9.143848e-01 9.824607e-01

5446 9.144336e-01 9.824604e-01

5447 9.144817e-01 9.824602e-01

5448 9.145289e-01 9.824599e-01

5449 9.145754e-01 9.824596e-01

5450 9.146212e-01 9.824593e-01

5451 9.146662e-01 9.824590e-01

5452 9.147104e-01 9.824587e-01

5453 9.147540e-01 9.824583e-01

5454 9.147969e-01 9.824580e-01

5455 9.148390e-01 9.824576e-01

5456 9.148805e-01 9.824573e-01

5457 9.149212e-01 9.824569e-01

5458 9.149613e-01 9.824564e-01

5459 9.150008e-01 9.824560e-01

5460 9.150395e-01 9.824556e-01

5461 9.150777e-01 9.824551e-01

5462 9.151151e-01 9.824546e-01

5463 9.151520e-01 9.824542e-01

5464 9.151882e-01 9.824536e-01

5465 9.152238e-01 9.824531e-01

5466 9.152588e-01 9.824526e-01

5467 9.152932e-01 9.824520e-01

5468 9.153270e-01 9.824514e-01

5469 9.153602e-01 9.824508e-01

5470 9.153928e-01 9.824502e-01

5471 9.154249e-01 9.824496e-01

5472 9.154564e-01 9.824489e-01

5473 9.154873e-01 9.824483e-01

5474 9.155177e-01 9.824476e-01

5475 9.155475e-01 9.824468e-01

5476 9.155768e-01 9.824461e-01

5477 9.156056e-01 9.824453e-01

5478 9.156338e-01 9.824446e-01

5479 9.156615e-01 9.824438e-01

5480 9.156887e-01 9.824429e-01

5481 9.157154e-01 9.824421e-01

5482 9.157416e-01 9.824412e-01

5483 9.157672e-01 9.824403e-01

5484 9.157924e-01 9.824394e-01

5485 9.158172e-01 9.824384e-01

5486 9.158414e-01 9.824375e-01

5487 9.158651e-01 9.824365e-01

5488 9.158884e-01 9.824355e-01

5489 9.159113e-01 9.824344e-01

5490 9.159336e-01 9.824333e-01

5491 9.159556e-01 9.824322e-01

5492 9.159770e-01 9.824311e-01

5493 9.159981e-01 9.824299e-01

5494 9.160187e-01 9.824288e-01

5495 9.160388e-01 9.824275e-01

5496 9.160586e-01 9.824263e-01

5497 9.160779e-01 9.824250e-01

5498 9.160968e-01 9.824237e-01

5499 9.161153e-01 9.824224e-01

5500 9.161334e-01 9.824210e-01

5501 9.161511e-01 9.824196e-01

5502 9.161684e-01 9.824182e-01

5503 9.161854e-01 9.824167e-01

5504 9.162019e-01 9.824152e-01

5505 9.162181e-01 9.824137e-01

5506 9.162339e-01 9.824121e-01

5507 9.162493e-01 9.824105e-01

5508 9.162644e-01 9.824088e-01

5509 9.162791e-01 9.824072e-01

5510 9.162934e-01 9.824055e-01

5511 9.163074e-01 9.824037e-01

5512 9.163211e-01 9.824019e-01

5513 9.163344e-01 9.824001e-01

5514 9.163474e-01 9.823982e-01

5515 9.163601e-01 9.823963e-01

5516 9.163725e-01 9.823944e-01

5517 9.163846e-01 9.823924e-01

5518 9.163963e-01 9.823903e-01

5519 9.164077e-01 9.823883e-01

5520 9.164189e-01 9.823862e-01

5521 9.164298e-01 9.823840e-01

5522 9.164403e-01 9.823818e-01

5523 9.164506e-01 9.823795e-01

5524 9.164606e-01 9.823773e-01

5525 9.164704e-01 9.823749e-01

5526 9.164799e-01 9.823725e-01

5527 9.164891e-01 9.823701e-01

5528 9.164981e-01 9.823676e-01

5529 9.165069e-01 9.823651e-01

5530 9.165154e-01 9.823625e-01

5531 9.165237e-01 9.823599e-01

5532 9.165317e-01 9.823572e-01

5533 9.165395e-01 9.823544e-01

5534 9.165471e-01 9.823517e-01

5535 9.165546e-01 9.823488e-01

5536 9.165618e-01 9.823459e-01

5537 9.165688e-01 9.823430e-01

5538 9.165756e-01 9.823400e-01

5539 9.165823e-01 9.823369e-01

5540 9.165887e-01 9.823338e-01

5541 9.165950e-01 9.823306e-01

5542 9.166012e-01 9.823274e-01

5543 9.166071e-01 9.823241e-01

5544 9.166130e-01 9.823207e-01

5545 9.166186e-01 9.823173e-01

5546 9.166242e-01 9.823138e-01

5547 9.166296e-01 9.823103e-01

5548 9.166349e-01 9.823067e-01

5549 9.166401e-01 9.823030e-01

5550 9.166451e-01 9.822993e-01

5551 9.166501e-01 9.822955e-01

5552 9.166550e-01 9.822916e-01

5553 9.166597e-01 9.822877e-01

5554 9.166644e-01 9.822837e-01

5555 9.166690e-01 9.822796e-01

5556 9.166735e-01 9.822755e-01

5557 9.166780e-01 9.822712e-01

5558 9.166824e-01 9.822670e-01

5559 9.166868e-01 9.822626e-01

5560 9.166911e-01 9.822582e-01

5561 9.166953e-01 9.822537e-01

5562 9.166996e-01 9.822491e-01

5563 9.167038e-01 9.822444e-01

5564 9.167079e-01 9.822397e-01

5565 9.167121e-01 9.822349e-01

5566 9.167163e-01 9.822300e-01

5567 9.167204e-01 9.822250e-01

5568 9.167246e-01 9.822199e-01

5569 9.167287e-01 9.822148e-01

5570 9.167329e-01 9.822096e-01

5571 9.167371e-01 9.822043e-01

5572 9.167414e-01 9.821989e-01

5573 9.167456e-01 9.821934e-01

5574 9.167499e-01 9.821878e-01

5575 9.167543e-01 9.821822e-01

5576 9.167587e-01 9.821764e-01

5577 9.167631e-01 9.821706e-01

5578 9.167676e-01 9.821647e-01

5579 9.167722e-01 9.821587e-01

5580 9.167769e-01 9.821526e-01

5581 9.167816e-01 9.821464e-01

5582 9.168738e-01 9.821464e-01

5583 9.169648e-01 9.821464e-01

5584 9.170548e-01 9.821463e-01

5585 9.171437e-01 9.821463e-01

5586 9.172315e-01 9.821463e-01

5587 9.173183e-01 9.821463e-01

5588 9.174040e-01 9.821462e-01

5589 9.174886e-01 9.821462e-01

5590 9.175722e-01 9.821461e-01

5591 9.176547e-01 9.821461e-01

5592 9.177361e-01 9.821460e-01

5593 9.178165e-01 9.821459e-01

5594 9.178959e-01 9.821458e-01

5595 9.179742e-01 9.821457e-01

5596 9.180514e-01 9.821456e-01

5597 9.181277e-01 9.821455e-01

5598 9.182029e-01 9.821454e-01

5599 9.182771e-01 9.821453e-01

5600 9.183502e-01 9.821452e-01

5601 9.184224e-01 9.821450e-01

5602 9.184936e-01 9.821449e-01

5603 9.185638e-01 9.821447e-01

5604 9.186330e-01 9.821446e-01

5605 9.187013e-01 9.821444e-01

5606 9.187685e-01 9.821442e-01

5607 9.188348e-01 9.821440e-01

5608 9.189002e-01 9.821438e-01

5609 9.189646e-01 9.821436e-01

5610 9.190281e-01 9.821434e-01

5611 9.190907e-01 9.821431e-01

5612 9.191524e-01 9.821429e-01

5613 9.192131e-01 9.821426e-01

5614 9.192730e-01 9.821423e-01

5615 9.193319e-01 9.821421e-01

5616 9.193900e-01 9.821418e-01

5617 9.194472e-01 9.821415e-01

5618 9.195036e-01 9.821411e-01

5619 9.195591e-01 9.821408e-01

5620 9.196137e-01 9.821405e-01

5621 9.196675e-01 9.821401e-01

5622 9.197205e-01 9.821398e-01

5623 9.197727e-01 9.821394e-01

5624 9.198241e-01 9.821390e-01

5625 9.198746e-01 9.821386e-01

5626 9.199244e-01 9.821382e-01

5627 9.199734e-01 9.821377e-01

5628 9.200216e-01 9.821373e-01

5629 9.200690e-01 9.821368e-01

5630 9.201157e-01 9.821363e-01

5631 9.201616e-01 9.821358e-01

5632 9.202068e-01 9.821353e-01

5633 9.202513e-01 9.821348e-01

5634 9.202950e-01 9.821343e-01

5635 9.203380e-01 9.821337e-01

5636 9.203803e-01 9.821331e-01

5637 9.204220e-01 9.821325e-01

5638 9.204629e-01 9.821319e-01

5639 9.205031e-01 9.821313e-01

5640 9.205427e-01 9.821307e-01

5641 9.205815e-01 9.821300e-01

5642 9.206198e-01 9.821293e-01

5643 9.206573e-01 9.821286e-01

5644 9.206942e-01 9.821279e-01

5645 9.207305e-01 9.821272e-01

5646 9.207662e-01 9.821264e-01

5647 9.208012e-01 9.821256e-01

5648 9.208356e-01 9.821248e-01

5649 9.208694e-01 9.821240e-01

5650 9.209026e-01 9.821232e-01

5651 9.209352e-01 9.821223e-01

5652 9.209672e-01 9.821214e-01

5653 9.209986e-01 9.821205e-01

5654 9.210294e-01 9.821196e-01

5655 9.210597e-01 9.821186e-01

5656 9.210894e-01 9.821177e-01

5657 9.211185e-01 9.821167e-01

5658 9.211472e-01 9.821157e-01

5659 9.211752e-01 9.821146e-01

5660 9.212027e-01 9.821135e-01

5661 9.212297e-01 9.821124e-01

5662 9.212562e-01 9.821113e-01

5663 9.212822e-01 9.821102e-01

5664 9.213076e-01 9.821090e-01

5665 9.213326e-01 9.821078e-01

5666 9.213570e-01 9.821066e-01

5667 9.213810e-01 9.821053e-01

5668 9.214045e-01 9.821040e-01

5669 9.214275e-01 9.821027e-01

5670 9.214500e-01 9.821013e-01

5671 9.214721e-01 9.821000e-01

5672 9.214937e-01 9.820986e-01

5673 9.215148e-01 9.820971e-01

5674 9.215355e-01 9.820957e-01

5675 9.215558e-01 9.820942e-01

5676 9.215756e-01 9.820926e-01

5677 9.215950e-01 9.820911e-01

5678 9.216140e-01 9.820895e-01

5679 9.216326e-01 9.820878e-01

5680 9.216508e-01 9.820862e-01

5681 9.216686e-01 9.820845e-01

5682 9.216859e-01 9.820827e-01

5683 9.217029e-01 9.820809e-01

5684 9.217195e-01 9.820791e-01

5685 9.217358e-01 9.820773e-01

5686 9.217516e-01 9.820754e-01

5687 9.217671e-01 9.820735e-01

5688 9.217823e-01 9.820715e-01

5689 9.217971e-01 9.820695e-01

5690 9.218116e-01 9.820675e-01

5691 9.218257e-01 9.820654e-01

5692 9.218395e-01 9.820633e-01

5693 9.218530e-01 9.820611e-01

5694 9.218661e-01 9.820589e-01

5695 9.218790e-01 9.820566e-01

5696 9.218915e-01 9.820543e-01

5697 9.219038e-01 9.820520e-01

5698 9.219157e-01 9.820496e-01

5699 9.219274e-01 9.820472e-01

5700 9.219388e-01 9.820447e-01

5701 9.219500e-01 9.820422e-01

5702 9.219608e-01 9.820396e-01

5703 9.219714e-01 9.820370e-01

5704 9.219818e-01 9.820343e-01

5705 9.219919e-01 9.820316e-01

5706 9.220018e-01 9.820289e-01

5707 9.220115e-01 9.820260e-01

5708 9.220210e-01 9.820232e-01

5709 9.220302e-01 9.820202e-01

5710 9.220392e-01 9.820173e-01

5711 9.220480e-01 9.820142e-01

5712 9.220566e-01 9.820111e-01

5713 9.220651e-01 9.820080e-01

5714 9.220733e-01 9.820048e-01

5715 9.220814e-01 9.820015e-01

5716 9.220893e-01 9.819982e-01

5717 9.220971e-01 9.819949e-01

5718 9.221047e-01 9.819914e-01

5719 9.221121e-01 9.819879e-01

5720 9.221194e-01 9.819844e-01

5721 9.221266e-01 9.819808e-01

5722 9.221336e-01 9.819771e-01

5723 9.221406e-01 9.819733e-01

5724 9.221474e-01 9.819695e-01

5725 9.221541e-01 9.819657e-01

5726 9.221606e-01 9.819617e-01

5727 9.221671e-01 9.819577e-01

5728 9.221735e-01 9.819537e-01

5729 9.221799e-01 9.819495e-01

5730 9.221861e-01 9.819453e-01

5731 9.221923e-01 9.819410e-01

5732 9.221984e-01 9.819367e-01

5733 9.222044e-01 9.819323e-01

5734 9.222104e-01 9.819278e-01

5735 9.222164e-01 9.819232e-01

5736 9.222223e-01 9.819186e-01

5737 9.222281e-01 9.819138e-01

5738 9.222340e-01 9.819090e-01

5739 9.222398e-01 9.819042e-01

5740 9.222456e-01 9.818992e-01

5741 9.222513e-01 9.818942e-01

5742 9.222571e-01 9.818891e-01

5743 9.222629e-01 9.818839e-01

5744 9.222686e-01 9.818786e-01

5745 9.222744e-01 9.818733e-01

5746 9.222802e-01 9.818678e-01

5747 9.222860e-01 9.818623e-01

5748 9.222918e-01 9.818567e-01

5749 9.222977e-01 9.818510e-01

5750 9.223036e-01 9.818452e-01

5751 9.223095e-01 9.818393e-01

5752 9.223154e-01 9.818334e-01

5753 9.223214e-01 9.818273e-01

5754 9.223275e-01 9.818212e-01

5755 9.223336e-01 9.818149e-01

5756 9.223398e-01 9.818086e-01

5757 9.223460e-01 9.818022e-01

5758 9.223523e-01 9.817957e-01

5759 9.223586e-01 9.817891e-01

5760 9.223651e-01 9.817824e-01

5761 9.223716e-01 9.817755e-01

5762 9.224643e-01 9.817754e-01

5763 9.225559e-01 9.817753e-01

5764 9.226465e-01 9.817752e-01

5765 9.227361e-01 9.817750e-01

5766 9.228246e-01 9.817749e-01

5767 9.229120e-01 9.817748e-01

5768 9.229984e-01 9.817746e-01

5769 9.230838e-01 9.817744e-01

5770 9.231681e-01 9.817743e-01

5771 9.232514e-01 9.817741e-01

5772 9.233336e-01 9.817739e-01

5773 9.234148e-01 9.817737e-01

5774 9.234950e-01 9.817735e-01

5775 9.235742e-01 9.817733e-01

5776 9.236523e-01 9.817730e-01

5777 9.237294e-01 9.817728e-01

5778 9.238055e-01 9.817726e-01

5779 9.238806e-01 9.817723e-01

5780 9.239546e-01 9.817720e-01

5781 9.240277e-01 9.817718e-01

5782 9.240998e-01 9.817715e-01

5783 9.241709e-01 9.817712e-01

5784 9.242410e-01 9.817709e-01

5785 9.243102e-01 9.817706e-01

5786 9.243784e-01 9.817702e-01

5787 9.244456e-01 9.817699e-01

5788 9.245118e-01 9.817696e-01

5789 9.245772e-01 9.817692e-01

5790 9.246416e-01 9.817688e-01

5791 9.247050e-01 9.817684e-01

5792 9.247675e-01 9.817680e-01

5793 9.248291e-01 9.817676e-01

5794 9.248898e-01 9.817672e-01

5795 9.249496e-01 9.817668e-01

5796 9.250086e-01 9.817663e-01

5797 9.250666e-01 9.817659e-01

5798 9.251237e-01 9.817654e-01

5799 9.251800e-01 9.817649e-01

5800 9.252354e-01 9.817644e-01

5801 9.252899e-01 9.817639e-01

5802 9.253436e-01 9.817634e-01

5803 9.253965e-01 9.817628e-01

5804 9.254485e-01 9.817623e-01

5805 9.254997e-01 9.817617e-01

5806 9.255501e-01 9.817611e-01

5807 9.255997e-01 9.817605e-01

5808 9.256485e-01 9.817599e-01

5809 9.256965e-01 9.817592e-01

5810 9.257437e-01 9.817586e-01

5811 9.257901e-01 9.817579e-01

5812 9.258358e-01 9.817572e-01

5813 9.258807e-01 9.817565e-01

5814 9.259248e-01 9.817558e-01

5815 9.259682e-01 9.817550e-01

5816 9.260109e-01 9.817543e-01

5817 9.260529e-01 9.817535e-01

5818 9.260941e-01 9.817527e-01

5819 9.261346e-01 9.817519e-01

5820 9.261744e-01 9.817510e-01

5821 9.262135e-01 9.817502e-01

5822 9.262519e-01 9.817493e-01

5823 9.262896e-01 9.817484e-01

5824 9.263267e-01 9.817475e-01

5825 9.263631e-01 9.817465e-01

5826 9.263988e-01 9.817456e-01

5827 9.264339e-01 9.817446e-01

5828 9.264683e-01 9.817436e-01

5829 9.265021e-01 9.817425e-01

5830 9.265353e-01 9.817415e-01

5831 9.265679e-01 9.817404e-01

5832 9.265998e-01 9.817393e-01

5833 9.266312e-01 9.817382e-01

5834 9.266619e-01 9.817370e-01

5835 9.266921e-01 9.817358e-01

5836 9.267216e-01 9.817346e-01

5837 9.267506e-01 9.817334e-01

5838 9.267791e-01 9.817322e-01

5839 9.268069e-01 9.817309e-01

5840 9.268342e-01 9.817296e-01

5841 9.268610e-01 9.817282e-01

5842 9.268873e-01 9.817269e-01

5843 9.269130e-01 9.817255e-01

5844 9.269382e-01 9.817240e-01

5845 9.269628e-01 9.817226e-01

5846 9.269870e-01 9.817211e-01

5847 9.270107e-01 9.817196e-01

5848 9.270339e-01 9.817180e-01

5849 9.270566e-01 9.817165e-01

5850 9.270788e-01 9.817148e-01

5851 9.271005e-01 9.817132e-01

5852 9.271218e-01 9.817115e-01

5853 9.271427e-01 9.817098e-01

5854 9.271630e-01 9.817081e-01

5855 9.271830e-01 9.817063e-01

5856 9.272025e-01 9.817045e-01

5857 9.272216e-01 9.817026e-01

5858 9.272403e-01 9.817007e-01

5859 9.272586e-01 9.816988e-01

5860 9.272765e-01 9.816969e-01

5861 9.272940e-01 9.816949e-01

5862 9.273111e-01 9.816928e-01

5863 9.273278e-01 9.816908e-01

5864 9.273441e-01 9.816886e-01

5865 9.273601e-01 9.816865e-01

5866 9.273758e-01 9.816843e-01

5867 9.273910e-01 9.816821e-01

5868 9.274060e-01 9.816798e-01

5869 9.274206e-01 9.816775e-01

5870 9.274349e-01 9.816751e-01

5871 9.274489e-01 9.816727e-01

5872 9.274625e-01 9.816702e-01

5873 9.274759e-01 9.816677e-01

5874 9.274889e-01 9.816652e-01

5875 9.275017e-01 9.816626e-01

5876 9.275142e-01 9.816600e-01

5877 9.275264e-01 9.816573e-01

5878 9.275384e-01 9.816546e-01

5879 9.275501e-01 9.816518e-01

5880 9.275615e-01 9.816489e-01

5881 9.275727e-01 9.816461e-01

5882 9.275837e-01 9.816431e-01

5883 9.275944e-01 9.816401e-01

5884 9.276049e-01 9.816371e-01

5885 9.276152e-01 9.816340e-01

5886 9.276253e-01 9.816309e-01

5887 9.276352e-01 9.816277e-01

5888 9.276448e-01 9.816244e-01

5889 9.276543e-01 9.816211e-01

5890 9.276636e-01 9.816177e-01

5891 9.276728e-01 9.816143e-01

5892 9.276817e-01 9.816108e-01

5893 9.276905e-01 9.816073e-01

5894 9.276992e-01 9.816037e-01

5895 9.277077e-01 9.816000e-01

5896 9.277160e-01 9.815963e-01

5897 9.277242e-01 9.815925e-01

5898 9.277323e-01 9.815886e-01

5899 9.277403e-01 9.815847e-01

5900 9.277481e-01 9.815808e-01

5901 9.277559e-01 9.815767e-01

5902 9.277635e-01 9.815726e-01

5903 9.277710e-01 9.815684e-01

5904 9.277785e-01 9.815642e-01

5905 9.277858e-01 9.815598e-01

5906 9.277931e-01 9.815555e-01

5907 9.278003e-01 9.815510e-01

5908 9.278074e-01 9.815465e-01

5909 9.278144e-01 9.815419e-01

5910 9.278214e-01 9.815372e-01

5911 9.278283e-01 9.815324e-01

5912 9.278352e-01 9.815276e-01

5913 9.278421e-01 9.815227e-01

5914 9.278489e-01 9.815177e-01

5915 9.278556e-01 9.815127e-01

5916 9.278623e-01 9.815075e-01

5917 9.278690e-01 9.815023e-01

5918 9.278757e-01 9.814970e-01

5919 9.278824e-01 9.814916e-01

5920 9.278890e-01 9.814862e-01

5921 9.278957e-01 9.814806e-01

5922 9.279023e-01 9.814750e-01

5923 9.279089e-01 9.814693e-01

5924 9.279156e-01 9.814635e-01

5925 9.279222e-01 9.814576e-01

5926 9.279289e-01 9.814516e-01

5927 9.279355e-01 9.814456e-01

5928 9.279422e-01 9.814394e-01

5929 9.279489e-01 9.814332e-01

5930 9.279557e-01 9.814268e-01

5931 9.279624e-01 9.814204e-01

5932 9.279692e-01 9.814139e-01

5933 9.279761e-01 9.814073e-01

5934 9.279829e-01 9.814006e-01

5935 9.279898e-01 9.813938e-01

5936 9.279968e-01 9.813869e-01

5937 9.280038e-01 9.813799e-01

5938 9.280108e-01 9.813728e-01

5939 9.280179e-01 9.813656e-01

5940 9.280250e-01 9.813583e-01

5941 9.280322e-01 9.813509e-01

5942 9.281259e-01 9.813506e-01

5943 9.282186e-01 9.813504e-01

5944 9.283103e-01 9.813502e-01

5945 9.284009e-01 9.813499e-01

5946 9.284904e-01 9.813497e-01

5947 9.285788e-01 9.813494e-01

5948 9.286662e-01 9.813491e-01

5949 9.287526e-01 9.813489e-01

5950 9.288379e-01 9.813486e-01

5951 9.289221e-01 9.813483e-01

5952 9.290053e-01 9.813480e-01

5953 9.290874e-01 9.813476e-01

5954 9.291685e-01 9.813473e-01

5955 9.292486e-01 9.813470e-01

5956 9.293276e-01 9.813466e-01

5957 9.294055e-01 9.813463e-01

5958 9.294825e-01 9.813459e-01

5959 9.295584e-01 9.813455e-01

5960 9.296332e-01 9.813451e-01

5961 9.297071e-01 9.813447e-01

5962 9.297799e-01 9.813443e-01

5963 9.298517e-01 9.813439e-01

5964 9.299226e-01 9.813435e-01

5965 9.299924e-01 9.813430e-01

5966 9.300612e-01 9.813426e-01

5967 9.301290e-01 9.813421e-01

5968 9.301959e-01 9.813416e-01

5969 9.302618e-01 9.813411e-01

5970 9.303267e-01 9.813406e-01

5971 9.303906e-01 9.813401e-01

5972 9.304536e-01 9.813395e-01

5973 9.305156e-01 9.813390e-01

5974 9.305767e-01 9.813384e-01

5975 9.306369e-01 9.813378e-01

5976 9.306961e-01 9.813372e-01

5977 9.307544e-01 9.813366e-01

5978 9.308118e-01 9.813360e-01

5979 9.308683e-01 9.813354e-01

5980 9.309239e-01 9.813347e-01

5981 9.309786e-01 9.813340e-01

5982 9.310324e-01 9.813333e-01

5983 9.310854e-01 9.813326e-01

5984 9.311375e-01 9.813319e-01

5985 9.311887e-01 9.813312e-01

5986 9.312391e-01 9.813304e-01

5987 9.312886e-01 9.813296e-01

5988 9.313373e-01 9.813289e-01

5989 9.313852e-01 9.813280e-01

5990 9.314322e-01 9.813272e-01

5991 9.314785e-01 9.813264e-01

5992 9.315239e-01 9.813255e-01

5993 9.315686e-01 9.813246e-01

5994 9.316124e-01 9.813237e-01

5995 9.316555e-01 9.813228e-01

5996 9.316979e-01 9.813218e-01

5997 9.317394e-01 9.813209e-01

5998 9.317802e-01 9.813199e-01

5999 9.318203e-01 9.813189e-01

6000 9.318597e-01 9.813179e-01

6001 9.318983e-01 9.813168e-01

6002 9.319362e-01 9.813157e-01

6003 9.319734e-01 9.813146e-01

6004 9.320099e-01 9.813135e-01

6005 9.320457e-01 9.813124e-01

6006 9.320809e-01 9.813112e-01

6007 9.321153e-01 9.813100e-01

6008 9.321491e-01 9.813088e-01

6009 9.321823e-01 9.813076e-01

6010 9.322148e-01 9.813063e-01

6011 9.322466e-01 9.813050e-01

6012 9.322779e-01 9.813037e-01

6013 9.323085e-01 9.813023e-01

6014 9.323385e-01 9.813010e-01

6015 9.323679e-01 9.812996e-01

6016 9.323967e-01 9.812981e-01

6017 9.324249e-01 9.812967e-01

6018 9.324526e-01 9.812952e-01

6019 9.324797e-01 9.812937e-01

6020 9.325062e-01 9.812921e-01

6021 9.325322e-01 9.812905e-01

6022 9.325576e-01 9.812889e-01

6023 9.325825e-01 9.812873e-01

6024 9.326069e-01 9.812856e-01

6025 9.326308e-01 9.812839e-01

6026 9.326542e-01 9.812822e-01

6027 9.326771e-01 9.812804e-01

6028 9.326994e-01 9.812786e-01

6029 9.327213e-01 9.812768e-01

6030 9.327428e-01 9.812749e-01

6031 9.327638e-01 9.812730e-01

6032 9.327843e-01 9.812711e-01

6033 9.328043e-01 9.812691e-01

6034 9.328240e-01 9.812671e-01

6035 9.328432e-01 9.812650e-01

6036 9.328620e-01 9.812629e-01

6037 9.328804e-01 9.812608e-01

6038 9.328984e-01 9.812586e-01

6039 9.329159e-01 9.812564e-01

6040 9.329331e-01 9.812542e-01

6041 9.329500e-01 9.812519e-01

6042 9.329664e-01 9.812496e-01

6043 9.329825e-01 9.812472e-01

6044 9.329982e-01 9.812448e-01

6045 9.330136e-01 9.812423e-01

6046 9.330287e-01 9.812398e-01

6047 9.330434e-01 9.812373e-01

6048 9.330578e-01 9.812347e-01

6049 9.330719e-01 9.812321e-01

6050 9.330857e-01 9.812294e-01

6051 9.330992e-01 9.812267e-01

6052 9.331124e-01 9.812239e-01

6053 9.331254e-01 9.812211e-01

6054 9.331380e-01 9.812182e-01

6055 9.331504e-01 9.812153e-01

6056 9.331625e-01 9.812123e-01

6057 9.331744e-01 9.812093e-01

6058 9.331860e-01 9.812062e-01

6059 9.331974e-01 9.812030e-01

6060 9.332086e-01 9.811999e-01

6061 9.332195e-01 9.811966e-01

6062 9.332303e-01 9.811933e-01

6063 9.332408e-01 9.811900e-01

6064 9.332511e-01 9.811866e-01

6065 9.332612e-01 9.811831e-01

6066 9.332712e-01 9.811796e-01

6067 9.332809e-01 9.811760e-01

6068 9.332905e-01 9.811724e-01

6069 9.332999e-01 9.811687e-01

6070 9.333091e-01 9.811650e-01

6071 9.333182e-01 9.811611e-01

6072 9.333272e-01 9.811573e-01

6073 9.333360e-01 9.811533e-01

6074 9.333446e-01 9.811493e-01

6075 9.333531e-01 9.811452e-01

6076 9.333615e-01 9.811411e-01

6077 9.333698e-01 9.811369e-01

6078 9.333780e-01 9.811326e-01

6079 9.333860e-01 9.811283e-01

6080 9.333940e-01 9.811239e-01

6081 9.334019e-01 9.811194e-01

6082 9.334096e-01 9.811149e-01

6083 9.334173e-01 9.811103e-01

6084 9.334249e-01 9.811056e-01

6085 9.334324e-01 9.811008e-01

6086 9.334398e-01 9.810960e-01

6087 9.334472e-01 9.810911e-01

6088 9.334545e-01 9.810861e-01

6089 9.334617e-01 9.810810e-01

6090 9.334689e-01 9.810759e-01

6091 9.334760e-01 9.810707e-01

6092 9.334831e-01 9.810654e-01

6093 9.334902e-01 9.810600e-01

6094 9.334972e-01 9.810545e-01

6095 9.335042e-01 9.810490e-01

6096 9.335111e-01 9.810434e-01

6097 9.335180e-01 9.810377e-01

6098 9.335249e-01 9.810319e-01

6099 9.335318e-01 9.810260e-01

6100 9.335386e-01 9.810200e-01

6101 9.335454e-01 9.810140e-01

6102 9.335523e-01 9.810078e-01

6103 9.335591e-01 9.810016e-01

6104 9.335659e-01 9.809953e-01

6105 9.335727e-01 9.809889e-01

6106 9.335795e-01 9.809824e-01

6107 9.335863e-01 9.809758e-01

6108 9.335931e-01 9.809691e-01

6109 9.336000e-01 9.809623e-01

6110 9.336068e-01 9.809555e-01

6111 9.336136e-01 9.809485e-01

6112 9.336205e-01 9.809414e-01

6113 9.336274e-01 9.809343e-01

6114 9.336343e-01 9.809270e-01

6115 9.336412e-01 9.809197e-01

6116 9.336481e-01 9.809122e-01

6117 9.336551e-01 9.809046e-01

6118 9.336621e-01 9.808970e-01

6119 9.336691e-01 9.808892e-01

6120 9.336762e-01 9.808813e-01

6121 9.336832e-01 9.808733e-01

6122 9.337780e-01 9.808730e-01

6123 9.338717e-01 9.808727e-01

6124 9.339643e-01 9.808723e-01

6125 9.340558e-01 9.808720e-01

6126 9.341462e-01 9.808716e-01

6127 9.342354e-01 9.808713e-01

6128 9.343236e-01 9.808709e-01

6129 9.344107e-01 9.808705e-01

6130 9.344967e-01 9.808701e-01

6131 9.345816e-01 9.808697e-01

6132 9.346654e-01 9.808693e-01

6133 9.347481e-01 9.808688e-01

6134 9.348297e-01 9.808684e-01

6135 9.349103e-01 9.808679e-01

6136 9.349897e-01 9.808675e-01

6137 9.350680e-01 9.808670e-01

6138 9.351453e-01 9.808665e-01

6139 9.352215e-01 9.808660e-01

6140 9.352966e-01 9.808655e-01

6141 9.353707e-01 9.808650e-01

6142 9.354437e-01 9.808644e-01

6143 9.355156e-01 9.808639e-01

6144 9.355865e-01 9.808633e-01

6145 9.356564e-01 9.808627e-01

6146 9.357251e-01 9.808622e-01

6147 9.357929e-01 9.808615e-01

6148 9.358596e-01 9.808609e-01

6149 9.359254e-01 9.808603e-01

6150 9.359901e-01 9.808596e-01

6151 9.360537e-01 9.808590e-01

6152 9.361164e-01 9.808583e-01

6153 9.361781e-01 9.808576e-01

6154 9.362388e-01 9.808569e-01

6155 9.362986e-01 9.808562e-01

6156 9.363573e-01 9.808554e-01

6157 9.364151e-01 9.808547e-01

6158 9.364720e-01 9.808539e-01

6159 9.365279e-01 9.808531e-01

6160 9.365828e-01 9.808523e-01

6161 9.366368e-01 9.808515e-01

6162 9.366899e-01 9.808506e-01

6163 9.367421e-01 9.808498e-01

6164 9.367934e-01 9.808489e-01

6165 9.368438e-01 9.808480e-01

6166 9.368933e-01 9.808471e-01

6167 9.369420e-01 9.808461e-01

6168 9.369897e-01 9.808452e-01

6169 9.370367e-01 9.808442e-01

6170 9.370827e-01 9.808432e-01

6171 9.371279e-01 9.808422e-01

6172 9.371723e-01 9.808412e-01

6173 9.372159e-01 9.808401e-01

6174 9.372587e-01 9.808390e-01

6175 9.373007e-01 9.808379e-01

6176 9.373418e-01 9.808368e-01

6177 9.373822e-01 9.808357e-01

6178 9.374219e-01 9.808345e-01

6179 9.374607e-01 9.808333e-01

6180 9.374988e-01 9.808321e-01

6181 9.375362e-01 9.808308e-01

6182 9.375728e-01 9.808296e-01

6183 9.376088e-01 9.808283e-01

6184 9.376440e-01 9.808270e-01

6185 9.376785e-01 9.808256e-01

6186 9.377123e-01 9.808243e-01

6187 9.377454e-01 9.808229e-01

6188 9.377779e-01 9.808214e-01

6189 9.378097e-01 9.808200e-01

6190 9.378408e-01 9.808185e-01

6191 9.378714e-01 9.808170e-01

6192 9.379012e-01 9.808155e-01

6193 9.379305e-01 9.808139e-01

6194 9.379591e-01 9.808123e-01

6195 9.379872e-01 9.808107e-01

6196 9.380146e-01 9.808091e-01

6197 9.380415e-01 9.808074e-01

6198 9.380678e-01 9.808057e-01

6199 9.380936e-01 9.808039e-01

6200 9.381188e-01 9.808021e-01

6201 9.381434e-01 9.808003e-01

6202 9.381675e-01 9.807985e-01

6203 9.381911e-01 9.807966e-01

6204 9.382142e-01 9.807947e-01

6205 9.382368e-01 9.807927e-01

6206 9.382589e-01 9.807908e-01

6207 9.382805e-01 9.807887e-01

6208 9.383016e-01 9.807867e-01

6209 9.383223e-01 9.807846e-01

6210 9.383425e-01 9.807825e-01

6211 9.383623e-01 9.807803e-01

6212 9.383816e-01 9.807781e-01

6213 9.384005e-01 9.807759e-01

6214 9.384190e-01 9.807736e-01

6215 9.384371e-01 9.807713e-01

6216 9.384548e-01 9.807689e-01

6217 9.384720e-01 9.807665e-01

6218 9.384889e-01 9.807640e-01

6219 9.385055e-01 9.807616e-01

6220 9.385216e-01 9.807590e-01

6221 9.385375e-01 9.807564e-01

6222 9.385529e-01 9.807538e-01

6223 9.385681e-01 9.807512e-01

6224 9.385828e-01 9.807484e-01

6225 9.385973e-01 9.807457e-01

6226 9.386115e-01 9.807429e-01

6227 9.386253e-01 9.807400e-01

6228 9.386389e-01 9.807371e-01

6229 9.386522e-01 9.807342e-01

6230 9.386652e-01 9.807312e-01

6231 9.386779e-01 9.807281e-01

6232 9.386903e-01 9.807250e-01

6233 9.387025e-01 9.807219e-01

6234 9.387144e-01 9.807187e-01

6235 9.387261e-01 9.807154e-01

6236 9.387376e-01 9.807121e-01

6237 9.387488e-01 9.807088e-01

6238 9.387598e-01 9.807053e-01

6239 9.387706e-01 9.807019e-01

6240 9.387812e-01 9.806983e-01

6241 9.387915e-01 9.806947e-01

6242 9.388017e-01 9.806911e-01

6243 9.388117e-01 9.806874e-01

6244 9.388215e-01 9.806836e-01

6245 9.388312e-01 9.806798e-01

6246 9.388406e-01 9.806759e-01

6247 9.388499e-01 9.806720e-01

6248 9.388591e-01 9.806680e-01

6249 9.388681e-01 9.806639e-01

6250 9.388769e-01 9.806597e-01

6251 9.388856e-01 9.806555e-01

6252 9.388942e-01 9.806513e-01

6253 9.389026e-01 9.806469e-01

6254 9.389109e-01 9.806425e-01

6255 9.389191e-01 9.806381e-01

6256 9.389272e-01 9.806335e-01

6257 9.389352e-01 9.806289e-01

6258 9.389430e-01 9.806242e-01

6259 9.389508e-01 9.806195e-01

6260 9.389585e-01 9.806147e-01

6261 9.389661e-01 9.806098e-01

6262 9.389736e-01 9.806048e-01

6263 9.389810e-01 9.805997e-01

6264 9.389883e-01 9.805946e-01

6265 9.389956e-01 9.805894e-01

6266 9.390028e-01 9.805841e-01

6267 9.390099e-01 9.805788e-01

6268 9.390170e-01 9.805733e-01

6269 9.390240e-01 9.805678e-01

6270 9.390310e-01 9.805622e-01

6271 9.390379e-01 9.805566e-01

6272 9.390447e-01 9.805508e-01

6273 9.390516e-01 9.805450e-01

6274 9.390583e-01 9.805390e-01

6275 9.390651e-01 9.805330e-01

6276 9.390718e-01 9.805269e-01

6277 9.390785e-01 9.805207e-01

6278 9.390851e-01 9.805145e-01

6279 9.390918e-01 9.805081e-01

6280 9.390984e-01 9.805016e-01

6281 9.391050e-01 9.804951e-01

6282 9.391115e-01 9.804885e-01

6283 9.391181e-01 9.804817e-01

6284 9.391246e-01 9.804749e-01

6285 9.391312e-01 9.804680e-01

6286 9.391377e-01 9.804610e-01

6287 9.391442e-01 9.804539e-01

6288 9.391507e-01 9.804467e-01

6289 9.391573e-01 9.804394e-01

6290 9.391638e-01 9.804320e-01

6291 9.391703e-01 9.804245e-01

6292 9.391768e-01 9.804169e-01

6293 9.391834e-01 9.804092e-01

6294 9.391899e-01 9.804014e-01

6295 9.391964e-01 9.803935e-01

6296 9.392030e-01 9.803855e-01

6297 9.392096e-01 9.803774e-01

6298 9.392162e-01 9.803692e-01

6299 9.392227e-01 9.803609e-01

6300 9.392294e-01 9.803525e-01

6301 9.392360e-01 9.803439e-01

6302 9.393313e-01 9.803435e-01

6303 9.394254e-01 9.803431e-01

6304 9.395183e-01 9.803426e-01

6305 9.396101e-01 9.803422e-01

6306 9.397008e-01 9.803417e-01

6307 9.397902e-01 9.803412e-01

6308 9.398785e-01 9.803408e-01

6309 9.399657e-01 9.803403e-01

6310 9.400516e-01 9.803398e-01

6311 9.401364e-01 9.803392e-01

6312 9.402201e-01 9.803387e-01

6313 9.403026e-01 9.803382e-01

6314 9.403839e-01 9.803376e-01

6315 9.404641e-01 9.803370e-01

6316 9.405431e-01 9.803365e-01

6317 9.406210e-01 9.803359e-01

6318 9.406977e-01 9.803353e-01

6319 9.407733e-01 9.803346e-01

6320 9.408478e-01 9.803340e-01

6321 9.409211e-01 9.803334e-01

6322 9.409933e-01 9.803327e-01

6323 9.410645e-01 9.803320e-01

6324 9.411345e-01 9.803313e-01

6325 9.412034e-01 9.803306e-01

6326 9.412712e-01 9.803299e-01

6327 9.413379e-01 9.803292e-01

6328 9.414035e-01 9.803284e-01

6329 9.414681e-01 9.803277e-01

6330 9.415316e-01 9.803269e-01

6331 9.415941e-01 9.803261e-01

6332 9.416555e-01 9.803253e-01

6333 9.417158e-01 9.803244e-01

6334 9.417752e-01 9.803236e-01

6335 9.418335e-01 9.803227e-01

6336 9.418908e-01 9.803218e-01

6337 9.419471e-01 9.803209e-01

6338 9.420024e-01 9.803200e-01

6339 9.420567e-01 9.803191e-01

6340 9.421101e-01 9.803181e-01

6341 9.421625e-01 9.803171e-01

6342 9.422139e-01 9.803161e-01

6343 9.422644e-01 9.803151e-01

6344 9.423140e-01 9.803141e-01

6345 9.423627e-01 9.803130e-01

6346 9.424104e-01 9.803120e-01

6347 9.424572e-01 9.803109e-01

6348 9.425032e-01 9.803097e-01

6349 9.425482e-01 9.803086e-01

6350 9.425925e-01 9.803074e-01

6351 9.426358e-01 9.803063e-01

6352 9.426783e-01 9.803051e-01

6353 9.427200e-01 9.803038e-01

6354 9.427608e-01 9.803026e-01

6355 9.428008e-01 9.803013e-01

6356 9.428401e-01 9.803000e-01

6357 9.428785e-01 9.802987e-01

6358 9.429162e-01 9.802973e-01

6359 9.429531e-01 9.802959e-01

6360 9.429892e-01 9.802945e-01

6361 9.430246e-01 9.802931e-01

6362 9.430593e-01 9.802916e-01

6363 9.430933e-01 9.802902e-01

6364 9.431265e-01 9.802886e-01

6365 9.431590e-01 9.802871e-01

6366 9.431909e-01 9.802855e-01

6367 9.432221e-01 9.802840e-01

6368 9.432526e-01 9.802823e-01

6369 9.432825e-01 9.802807e-01

6370 9.433117e-01 9.802790e-01

6371 9.433403e-01 9.802773e-01

6372 9.433682e-01 9.802755e-01

6373 9.433956e-01 9.802738e-01

6374 9.434224e-01 9.802720e-01

6375 9.434485e-01 9.802701e-01

6376 9.434741e-01 9.802682e-01

6377 9.434992e-01 9.802663e-01

6378 9.435237e-01 9.802644e-01

6379 9.435476e-01 9.802624e-01

6380 9.435710e-01 9.802604e-01

6381 9.435939e-01 9.802584e-01

6382 9.436163e-01 9.802563e-01

6383 9.436382e-01 9.802542e-01

6384 9.436596e-01 9.802520e-01

6385 9.436805e-01 9.802498e-01

6386 9.437009e-01 9.802476e-01

6387 9.437209e-01 9.802453e-01

6388 9.437404e-01 9.802430e-01

6389 9.437595e-01 9.802407e-01

6390 9.437782e-01 9.802383e-01

6391 9.437964e-01 9.802359e-01

6392 9.438142e-01 9.802334e-01

6393 9.438317e-01 9.802309e-01

6394 9.438487e-01 9.802284e-01

6395 9.438654e-01 9.802258e-01

6396 9.438816e-01 9.802231e-01

6397 9.438976e-01 9.802205e-01

6398 9.439131e-01 9.802177e-01

6399 9.439283e-01 9.802150e-01

6400 9.439432e-01 9.802121e-01

6401 9.439578e-01 9.802093e-01

6402 9.439720e-01 9.802064e-01

6403 9.439859e-01 9.802034e-01

6404 9.439995e-01 9.802004e-01

6405 9.440128e-01 9.801973e-01

6406 9.440259e-01 9.801942e-01

6407 9.440386e-01 9.801911e-01

6408 9.440511e-01 9.801879e-01

6409 9.440633e-01 9.801846e-01

6410 9.440753e-01 9.801813e-01

6411 9.440870e-01 9.801779e-01

6412 9.440984e-01 9.801745e-01

6413 9.441097e-01 9.801710e-01

6414 9.441207e-01 9.801675e-01

6415 9.441314e-01 9.801639e-01

6416 9.441420e-01 9.801603e-01

6417 9.441524e-01 9.801566e-01

6418 9.441625e-01 9.801528e-01

6419 9.441725e-01 9.801490e-01

6420 9.441823e-01 9.801451e-01

6421 9.441919e-01 9.801412e-01

6422 9.442013e-01 9.801372e-01

6423 9.442105e-01 9.801331e-01

6424 9.442196e-01 9.801290e-01

6425 9.442286e-01 9.801248e-01

6426 9.442373e-01 9.801206e-01

6427 9.442460e-01 9.801162e-01

6428 9.442544e-01 9.801119e-01

6429 9.442628e-01 9.801074e-01

6430 9.442710e-01 9.801029e-01

6431 9.442791e-01 9.800983e-01

6432 9.442871e-01 9.800936e-01

6433 9.442950e-01 9.800889e-01

6434 9.443027e-01 9.800841e-01

6435 9.443103e-01 9.800793e-01

6436 9.443179e-01 9.800743e-01

6437 9.443253e-01 9.800693e-01

6438 9.443327e-01 9.800642e-01

6439 9.443399e-01 9.800590e-01

6440 9.443471e-01 9.800538e-01

6441 9.443542e-01 9.800485e-01

6442 9.443612e-01 9.800431e-01

6443 9.443682e-01 9.800376e-01

6444 9.443751e-01 9.800321e-01

6445 9.443819e-01 9.800265e-01

6446 9.443886e-01 9.800207e-01

6447 9.443953e-01 9.800150e-01

6448 9.444020e-01 9.800091e-01

6449 9.444085e-01 9.800031e-01

6450 9.444151e-01 9.799971e-01

6451 9.444216e-01 9.799910e-01

6452 9.444280e-01 9.799847e-01

6453 9.444344e-01 9.799784e-01

6454 9.444408e-01 9.799721e-01

6455 9.444471e-01 9.799656e-01

6456 9.444534e-01 9.799590e-01

6457 9.444597e-01 9.799524e-01

6458 9.444660e-01 9.799456e-01

6459 9.444722e-01 9.799388e-01

6460 9.444784e-01 9.799318e-01

6461 9.444846e-01 9.799248e-01

6462 9.444908e-01 9.799177e-01

6463 9.444970e-01 9.799105e-01

6464 9.445031e-01 9.799032e-01

6465 9.445092e-01 9.798958e-01

6466 9.445154e-01 9.798883e-01

6467 9.445215e-01 9.798807e-01

6468 9.445276e-01 9.798730e-01

6469 9.445338e-01 9.798652e-01

6470 9.445399e-01 9.798573e-01

6471 9.445460e-01 9.798492e-01

6472 9.445521e-01 9.798411e-01

6473 9.445583e-01 9.798329e-01

6474 9.445644e-01 9.798246e-01

6475 9.445706e-01 9.798162e-01

6476 9.445767e-01 9.798077e-01

6477 9.445829e-01 9.797990e-01

6478 9.445891e-01 9.797903e-01

6479 9.445953e-01 9.797815e-01

6480 9.446015e-01 9.797725e-01

6481 9.446078e-01 9.797634e-01

6482 9.447026e-01 9.797629e-01

6483 9.447962e-01 9.797624e-01

6484 9.448885e-01 9.797618e-01

6485 9.449796e-01 9.797613e-01

6486 9.450694e-01 9.797607e-01

6487 9.451580e-01 9.797602e-01

6488 9.452453e-01 9.797596e-01

6489 9.453314e-01 9.797590e-01

6490 9.454163e-01 9.797583e-01

6491 9.454999e-01 9.797577e-01

6492 9.455823e-01 9.797571e-01

6493 9.456634e-01 9.797564e-01

6494 9.457434e-01 9.797558e-01

6495 9.458221e-01 9.797551e-01

6496 9.458996e-01 9.797544e-01

6497 9.459758e-01 9.797537e-01

6498 9.460509e-01 9.797530e-01

6499 9.461248e-01 9.797522e-01

6500 9.461975e-01 9.797515e-01

6501 9.462690e-01 9.797507e-01

6502 9.463393e-01 9.797499e-01

6503 9.464085e-01 9.797491e-01

6504 9.464765e-01 9.797483e-01

6505 9.465434e-01 9.797475e-01

6506 9.466091e-01 9.797466e-01

6507 9.466737e-01 9.797458e-01

6508 9.467371e-01 9.797449e-01

6509 9.467995e-01 9.797440e-01

6510 9.468607e-01 9.797431e-01

6511 9.469209e-01 9.797421e-01

6512 9.469800e-01 9.797412e-01

6513 9.470380e-01 9.797402e-01

6514 9.470949e-01 9.797392e-01

6515 9.471508e-01 9.797382e-01

6516 9.472056e-01 9.797372e-01

6517 9.472595e-01 9.797362e-01

6518 9.473123e-01 9.797351e-01

6519 9.473641e-01 9.797340e-01

6520 9.474149e-01 9.797329e-01

6521 9.474647e-01 9.797318e-01

6522 9.475136e-01 9.797306e-01

6523 9.475615e-01 9.797295e-01

6524 9.476085e-01 9.797283e-01

6525 9.476545e-01 9.797271e-01

6526 9.476996e-01 9.797258e-01

6527 9.477439e-01 9.797246e-01

6528 9.477872e-01 9.797233e-01

6529 9.478296e-01 9.797220e-01

6530 9.478712e-01 9.797207e-01

6531 9.479119e-01 9.797193e-01

6532 9.479518e-01 9.797179e-01

6533 9.479908e-01 9.797165e-01

6534 9.480291e-01 9.797151e-01

6535 9.480665e-01 9.797136e-01

6536 9.481031e-01 9.797122e-01

6537 9.481390e-01 9.797107e-01

6538 9.481741e-01 9.797091e-01

6539 9.482084e-01 9.797076e-01

6540 9.482420e-01 9.797060e-01

6541 9.482749e-01 9.797044e-01

6542 9.483070e-01 9.797027e-01

6543 9.483385e-01 9.797010e-01

6544 9.483692e-01 9.796993e-01

6545 9.483993e-01 9.796976e-01

6546 9.484287e-01 9.796958e-01

6547 9.484575e-01 9.796940e-01

6548 9.484856e-01 9.796922e-01

6549 9.485131e-01 9.796904e-01

6550 9.485400e-01 9.796885e-01

6551 9.485663e-01 9.796866e-01

6552 9.485919e-01 9.796846e-01

6553 9.486170e-01 9.796826e-01

6554 9.486416e-01 9.796806e-01

6555 9.486655e-01 9.796785e-01

6556 9.486890e-01 9.796764e-01

6557 9.487118e-01 9.796743e-01

6558 9.487342e-01 9.796721e-01

6559 9.487561e-01 9.796699e-01

6560 9.487774e-01 9.796677e-01

6561 9.487983e-01 9.796654e-01

6562 9.488187e-01 9.796631e-01

6563 9.488386e-01 9.796608e-01

6564 9.488580e-01 9.796584e-01

6565 9.488770e-01 9.796559e-01

6566 9.488956e-01 9.796535e-01

6567 9.489138e-01 9.796510e-01

6568 9.489315e-01 9.796484e-01

6569 9.489488e-01 9.796458e-01

6570 9.489657e-01 9.796432e-01

6571 9.489823e-01 9.796405e-01

6572 9.489984e-01 9.796377e-01

6573 9.490142e-01 9.796350e-01

6574 9.490296e-01 9.796322e-01

6575 9.490447e-01 9.796293e-01

6576 9.490595e-01 9.796264e-01

6577 9.490739e-01 9.796234e-01

6578 9.490879e-01 9.796204e-01

6579 9.491017e-01 9.796174e-01

6580 9.491152e-01 9.796143e-01

6581 9.491283e-01 9.796111e-01

6582 9.491412e-01 9.796079e-01

6583 9.491538e-01 9.796047e-01

6584 9.491661e-01 9.796014e-01

6585 9.491782e-01 9.795980e-01

6586 9.491899e-01 9.795946e-01

6587 9.492015e-01 9.795911e-01

6588 9.492128e-01 9.795876e-01

6589 9.492238e-01 9.795841e-01

6590 9.492347e-01 9.795804e-01

6591 9.492453e-01 9.795768e-01

6592 9.492557e-01 9.795730e-01

6593 9.492658e-01 9.795692e-01

6594 9.492758e-01 9.795654e-01

6595 9.492856e-01 9.795614e-01

6596 9.492952e-01 9.795575e-01

6597 9.493046e-01 9.795534e-01

6598 9.493138e-01 9.795493e-01

6599 9.493229e-01 9.795452e-01

6600 9.493318e-01 9.795410e-01

6601 9.493405e-01 9.795367e-01

6602 9.493491e-01 9.795323e-01

6603 9.493576e-01 9.795279e-01

6604 9.493658e-01 9.795234e-01

6605 9.493740e-01 9.795189e-01

6606 9.493820e-01 9.795143e-01

6607 9.493899e-01 9.795096e-01

6608 9.493977e-01 9.795048e-01

6609 9.494053e-01 9.795000e-01

6610 9.494129e-01 9.794951e-01

6611 9.494203e-01 9.794901e-01

6612 9.494276e-01 9.794851e-01

6613 9.494348e-01 9.794800e-01

6614 9.494420e-01 9.794748e-01

6615 9.494490e-01 9.794696e-01

6616 9.494559e-01 9.794642e-01

6617 9.494628e-01 9.794588e-01

6618 9.494696e-01 9.794533e-01

6619 9.494763e-01 9.794478e-01

6620 9.494829e-01 9.794421e-01

6621 9.494895e-01 9.794364e-01

6622 9.494960e-01 9.794306e-01

6623 9.495025e-01 9.794247e-01

6624 9.495088e-01 9.794187e-01

6625 9.495152e-01 9.794127e-01

6626 9.495214e-01 9.794065e-01

6627 9.495277e-01 9.794003e-01

6628 9.495339e-01 9.793940e-01

6629 9.495400e-01 9.793876e-01

6630 9.495461e-01 9.793811e-01

6631 9.495522e-01 9.793746e-01

6632 9.495582e-01 9.793679e-01

6633 9.495642e-01 9.793612e-01

6634 9.495702e-01 9.793543e-01

6635 9.495761e-01 9.793474e-01

6636 9.495821e-01 9.793404e-01

6637 9.495880e-01 9.793333e-01

6638 9.495939e-01 9.793261e-01

6639 9.495997e-01 9.793188e-01

6640 9.496056e-01 9.793114e-01

6641 9.496115e-01 9.793039e-01

6642 9.496173e-01 9.792963e-01

6643 9.496231e-01 9.792886e-01

6644 9.496290e-01 9.792808e-01

6645 9.496348e-01 9.792729e-01

6646 9.496406e-01 9.792649e-01

6647 9.496465e-01 9.792569e-01

6648 9.496523e-01 9.792487e-01

6649 9.496581e-01 9.792404e-01

6650 9.496640e-01 9.792320e-01

6651 9.496698e-01 9.792235e-01

6652 9.496757e-01 9.792149e-01

6653 9.496816e-01 9.792062e-01

6654 9.496875e-01 9.791974e-01

6655 9.496934e-01 9.791884e-01

6656 9.496993e-01 9.791794e-01

6657 9.497053e-01 9.791703e-01

6658 9.497113e-01 9.791610e-01

6659 9.497173e-01 9.791517e-01

6660 9.497233e-01 9.791422e-01

6661 9.497293e-01 9.791326e-01

6662 9.498224e-01 9.791320e-01

6663 9.499142e-01 9.791314e-01

6664 9.500046e-01 9.791307e-01

6665 9.500937e-01 9.791301e-01

6666 9.501814e-01 9.791294e-01

6667 9.502678e-01 9.791287e-01

6668 9.503529e-01 9.791281e-01

6669 9.504367e-01 9.791273e-01

6670 9.505192e-01 9.791266e-01

6671 9.506004e-01 9.791259e-01

6672 9.506802e-01 9.791251e-01

6673 9.507588e-01 9.791244e-01

6674 9.508361e-01 9.791236e-01

6675 9.509121e-01 9.791228e-01

6676 9.509869e-01 9.791220e-01

6677 9.510603e-01 9.791212e-01

6678 9.511326e-01 9.791204e-01

6679 9.512035e-01 9.791195e-01

6680 9.512733e-01 9.791186e-01

6681 9.513418e-01 9.791177e-01

6682 9.514091e-01 9.791168e-01

6683 9.514752e-01 9.791159e-01

6684 9.515401e-01 9.791150e-01

6685 9.516038e-01 9.791140e-01

6686 9.516664e-01 9.791131e-01

6687 9.517278e-01 9.791121e-01

6688 9.517880e-01 9.791111e-01

6689 9.518471e-01 9.791100e-01

6690 9.519051e-01 9.791090e-01

6691 9.519620e-01 9.791079e-01

6692 9.520178e-01 9.791068e-01

6693 9.520725e-01 9.791057e-01

6694 9.521261e-01 9.791046e-01

6695 9.521787e-01 9.791035e-01

6696 9.522302e-01 9.791023e-01

6697 9.522807e-01 9.791011e-01

6698 9.523302e-01 9.790999e-01

6699 9.523787e-01 9.790987e-01

6700 9.524262e-01 9.790974e-01

6701 9.524727e-01 9.790961e-01

6702 9.525182e-01 9.790948e-01

6703 9.525628e-01 9.790935e-01

6704 9.526065e-01 9.790922e-01

6705 9.526493e-01 9.790908e-01

6706 9.526911e-01 9.790894e-01

6707 9.527321e-01 9.790880e-01

6708 9.527722e-01 9.790865e-01

6709 9.528114e-01 9.790851e-01

6710 9.528498e-01 9.790836e-01

6711 9.528873e-01 9.790821e-01

6712 9.529241e-01 9.790805e-01

6713 9.529600e-01 9.790789e-01

6714 9.529951e-01 9.790773e-01

6715 9.530295e-01 9.790757e-01

6716 9.530631e-01 9.790741e-01

6717 9.530959e-01 9.790724e-01

6718 9.531280e-01 9.790707e-01

6719 9.531594e-01 9.790689e-01

6720 9.531901e-01 9.790671e-01

6721 9.532201e-01 9.790653e-01

6722 9.532494e-01 9.790635e-01

6723 9.532780e-01 9.790616e-01

6724 9.533060e-01 9.790597e-01

6725 9.533333e-01 9.790578e-01

6726 9.533600e-01 9.790559e-01

6727 9.533861e-01 9.790539e-01

6728 9.534116e-01 9.790518e-01

6729 9.534365e-01 9.790498e-01

6730 9.534608e-01 9.790477e-01

6731 9.534845e-01 9.790455e-01

6732 9.535077e-01 9.790434e-01

6733 9.535304e-01 9.790412e-01

6734 9.535525e-01 9.790389e-01

6735 9.535741e-01 9.790367e-01

6736 9.535952e-01 9.790343e-01

6737 9.536158e-01 9.790320e-01

6738 9.536359e-01 9.790296e-01

6739 9.536556e-01 9.790272e-01

6740 9.536748e-01 9.790247e-01

6741 9.536935e-01 9.790222e-01

6742 9.537118e-01 9.790196e-01

6743 9.537297e-01 9.790171e-01

6744 9.537471e-01 9.790144e-01

6745 9.537642e-01 9.790118e-01

6746 9.537808e-01 9.790090e-01

6747 9.537970e-01 9.790063e-01

6748 9.538129e-01 9.790035e-01

6749 9.538284e-01 9.790006e-01

6750 9.538436e-01 9.789977e-01

6751 9.538584e-01 9.789948e-01

6752 9.538728e-01 9.789918e-01

6753 9.538870e-01 9.789888e-01

6754 9.539008e-01 9.789857e-01

6755 9.539143e-01 9.789825e-01

6756 9.539274e-01 9.789794e-01

6757 9.539403e-01 9.789761e-01

6758 9.539529e-01 9.789729e-01

6759 9.539652e-01 9.789695e-01

6760 9.539773e-01 9.789661e-01

6761 9.539891e-01 9.789627e-01

6762 9.540006e-01 9.789592e-01

6763 9.540119e-01 9.789557e-01

6764 9.540229e-01 9.789521e-01

6765 9.540337e-01 9.789484e-01

6766 9.540442e-01 9.789447e-01

6767 9.540546e-01 9.789410e-01

6768 9.540647e-01 9.789371e-01

6769 9.540747e-01 9.789333e-01

6770 9.540844e-01 9.789293e-01

6771 9.540939e-01 9.789253e-01

6772 9.541033e-01 9.789213e-01

6773 9.541124e-01 9.789172e-01

6774 9.541214e-01 9.789130e-01

6775 9.541302e-01 9.789087e-01

6776 9.541389e-01 9.789044e-01

6777 9.541473e-01 9.789001e-01

6778 9.541557e-01 9.788956e-01

6779 9.541639e-01 9.788911e-01

6780 9.541719e-01 9.788866e-01

6781 9.541798e-01 9.788820e-01

6782 9.541876e-01 9.788773e-01

6783 9.541953e-01 9.788725e-01

6784 9.542028e-01 9.788677e-01

6785 9.542102e-01 9.788628e-01

6786 9.542175e-01 9.788578e-01

6787 9.542247e-01 9.788527e-01

6788 9.542318e-01 9.788476e-01

6789 9.542388e-01 9.788424e-01

6790 9.542457e-01 9.788372e-01

6791 9.542525e-01 9.788318e-01

6792 9.542592e-01 9.788264e-01

6793 9.542658e-01 9.788209e-01

6794 9.542724e-01 9.788154e-01

6795 9.542788e-01 9.788097e-01

6796 9.542853e-01 9.788040e-01

6797 9.542916e-01 9.787982e-01

6798 9.542979e-01 9.787923e-01

6799 9.543041e-01 9.787864e-01

6800 9.543102e-01 9.787803e-01

6801 9.543163e-01 9.787742e-01

6802 9.543224e-01 9.787680e-01

6803 9.543284e-01 9.787617e-01

6804 9.543343e-01 9.787553e-01

6805 9.543403e-01 9.787488e-01

6806 9.543461e-01 9.787423e-01

6807 9.543520e-01 9.787357e-01

6808 9.543578e-01 9.787289e-01

6809 9.543635e-01 9.787221e-01

6810 9.543693e-01 9.787152e-01

6811 9.543750e-01 9.787082e-01

6812 9.543807e-01 9.787011e-01

6813 9.543864e-01 9.786939e-01

6814 9.543921e-01 9.786867e-01

6815 9.543977e-01 9.786793e-01

6816 9.544033e-01 9.786718e-01

6817 9.544090e-01 9.786643e-01

6818 9.544146e-01 9.786566e-01

6819 9.544202e-01 9.786489e-01

6820 9.544258e-01 9.786410e-01

6821 9.544314e-01 9.786331e-01

6822 9.544370e-01 9.786250e-01

6823 9.544426e-01 9.786169e-01

6824 9.544482e-01 9.786086e-01

6825 9.544539e-01 9.786003e-01

6826 9.544595e-01 9.785918e-01

6827 9.544651e-01 9.785833e-01

6828 9.544708e-01 9.785746e-01

6829 9.544764e-01 9.785659e-01

6830 9.544821e-01 9.785570e-01

6831 9.544878e-01 9.785480e-01

6832 9.544935e-01 9.785389e-01

6833 9.544993e-01 9.785297e-01

6834 9.545050e-01 9.785205e-01

6835 9.545108e-01 9.785110e-01

6836 9.545166e-01 9.785015e-01

6837 9.545224e-01 9.784919e-01

6838 9.545283e-01 9.784822e-01

6839 9.545342e-01 9.784723e-01

6840 9.545401e-01 9.784624e-01

6841 9.545461e-01 9.784523e-01

6842 9.546360e-01 9.784516e-01

6843 9.547246e-01 9.784509e-01

6844 9.548117e-01 9.784501e-01

6845 9.548975e-01 9.784494e-01

6846 9.549818e-01 9.784486e-01

6847 9.550648e-01 9.784478e-01

6848 9.551463e-01 9.784470e-01

6849 9.552265e-01 9.784462e-01

6850 9.553054e-01 9.784454e-01

6851 9.553828e-01 9.784446e-01

6852 9.554590e-01 9.784437e-01

6853 9.555337e-01 9.784428e-01

6854 9.556072e-01 9.784420e-01

6855 9.556794e-01 9.784411e-01

6856 9.557502e-01 9.784401e-01

6857 9.558198e-01 9.784392e-01

6858 9.558880e-01 9.784382e-01

6859 9.559550e-01 9.784373e-01

6860 9.560208e-01 9.784363e-01

6861 9.560853e-01 9.784353e-01

6862 9.561486e-01 9.784343e-01

6863 9.562106e-01 9.784332e-01

6864 9.562715e-01 9.784322e-01

6865 9.563312e-01 9.784311e-01

6866 9.563897e-01 9.784300e-01

6867 9.564470e-01 9.784289e-01

6868 9.565032e-01 9.784277e-01

6869 9.565583e-01 9.784266e-01

6870 9.566122e-01 9.784254e-01

6871 9.566651e-01 9.784242e-01

6872 9.567168e-01 9.784230e-01

6873 9.567675e-01 9.784217e-01

6874 9.568171e-01 9.784205e-01

6875 9.568657e-01 9.784192e-01

6876 9.569133e-01 9.784179e-01

6877 9.569599e-01 9.784165e-01

6878 9.570054e-01 9.784152e-01

6879 9.570500e-01 9.784138e-01

6880 9.570937e-01 9.784124e-01

6881 9.571364e-01 9.784110e-01

6882 9.571781e-01 9.784095e-01

6883 9.572189e-01 9.784081e-01

6884 9.572589e-01 9.784066e-01

6885 9.572979e-01 9.784050e-01

6886 9.573361e-01 9.784035e-01

6887 9.573735e-01 9.784019e-01

6888 9.574100e-01 9.784003e-01

6889 9.574456e-01 9.783987e-01

6890 9.574805e-01 9.783970e-01

6891 9.575146e-01 9.783953e-01

6892 9.575479e-01 9.783936e-01

6893 9.575804e-01 9.783919e-01

6894 9.576122e-01 9.783901e-01

6895 9.576432e-01 9.783883e-01

6896 9.576736e-01 9.783864e-01

6897 9.577032e-01 9.783846e-01

6898 9.577321e-01 9.783827e-01

6899 9.577604e-01 9.783808e-01

6900 9.577880e-01 9.783788e-01

6901 9.578150e-01 9.783768e-01

6902 9.578413e-01 9.783748e-01

6903 9.578670e-01 9.783727e-01

6904 9.578921e-01 9.783706e-01

6905 9.579166e-01 9.783685e-01

6906 9.579405e-01 9.783664e-01

6907 9.579638e-01 9.783642e-01

6908 9.579866e-01 9.783619e-01

6909 9.580089e-01 9.783597e-01

6910 9.580306e-01 9.783574e-01

6911 9.580518e-01 9.783550e-01

6912 9.580725e-01 9.783526e-01

6913 9.580927e-01 9.783502e-01

6914 9.581124e-01 9.783478e-01

6915 9.581317e-01 9.783453e-01

6916 9.581504e-01 9.783427e-01

6917 9.581688e-01 9.783402e-01

6918 9.581867e-01 9.783376e-01

6919 9.582042e-01 9.783349e-01

6920 9.582212e-01 9.783322e-01

6921 9.582379e-01 9.783295e-01

6922 9.582541e-01 9.783267e-01

6923 9.582700e-01 9.783238e-01

6924 9.582855e-01 9.783210e-01

6925 9.583006e-01 9.783181e-01

6926 9.583154e-01 9.783151e-01

6927 9.583298e-01 9.783121e-01

6928 9.583439e-01 9.783090e-01

6929 9.583577e-01 9.783059e-01

6930 9.583712e-01 9.783028e-01

6931 9.583843e-01 9.782996e-01

6932 9.583971e-01 9.782963e-01

6933 9.584097e-01 9.782930e-01

6934 9.584219e-01 9.782897e-01

6935 9.584339e-01 9.782863e-01

6936 9.584456e-01 9.782828e-01

6937 9.584571e-01 9.782793e-01

6938 9.584683e-01 9.782758e-01

6939 9.584793e-01 9.782722e-01

6940 9.584900e-01 9.782685e-01

6941 9.585005e-01 9.782648e-01

6942 9.585107e-01 9.782610e-01

6943 9.585208e-01 9.782572e-01

6944 9.585306e-01 9.782533e-01

6945 9.585402e-01 9.782494e-01

6946 9.585497e-01 9.782454e-01

6947 9.585589e-01 9.782413e-01

6948 9.585680e-01 9.782372e-01

6949 9.585769e-01 9.782330e-01

6950 9.585856e-01 9.782287e-01

6951 9.585941e-01 9.782244e-01

6952 9.586025e-01 9.782201e-01

6953 9.586107e-01 9.782156e-01

6954 9.586188e-01 9.782111e-01

6955 9.586267e-01 9.782066e-01

6956 9.586345e-01 9.782019e-01

6957 9.586422e-01 9.781973e-01

6958 9.586497e-01 9.781925e-01

6959 9.586571e-01 9.781877e-01

6960 9.586644e-01 9.781828e-01

6961 9.586716e-01 9.781778e-01

6962 9.586786e-01 9.781728e-01

6963 9.586856e-01 9.781677e-01

6964 9.586924e-01 9.781625e-01

6965 9.586992e-01 9.781572e-01

6966 9.587058e-01 9.781519e-01

6967 9.587124e-01 9.781465e-01

6968 9.587189e-01 9.781410e-01

6969 9.587253e-01 9.781355e-01

6970 9.587316e-01 9.781298e-01

6971 9.587379e-01 9.781241e-01

6972 9.587440e-01 9.781184e-01

6973 9.587502e-01 9.781125e-01

6974 9.587562e-01 9.781066e-01

6975 9.587622e-01 9.781005e-01

6976 9.587681e-01 9.780944e-01

6977 9.587740e-01 9.780883e-01

6978 9.587798e-01 9.780820e-01

6979 9.587856e-01 9.780756e-01

6980 9.587914e-01 9.780692e-01

6981 9.587970e-01 9.780627e-01

6982 9.588027e-01 9.780561e-01

6983 9.588083e-01 9.780494e-01

6984 9.588139e-01 9.780426e-01

6985 9.588195e-01 9.780357e-01

6986 9.588250e-01 9.780288e-01

6987 9.588305e-01 9.780217e-01

6988 9.588360e-01 9.780146e-01

6989 9.588414e-01 9.780074e-01

6990 9.588469e-01 9.780001e-01

6991 9.588523e-01 9.779926e-01

6992 9.588577e-01 9.779851e-01

6993 9.588631e-01 9.779775e-01

6994 9.588685e-01 9.779698e-01

6995 9.588739e-01 9.779620e-01

6996 9.588792e-01 9.779541e-01

6997 9.588846e-01 9.779461e-01

6998 9.588900e-01 9.779381e-01

6999 9.588953e-01 9.779299e-01

7000 9.589007e-01 9.779216e-01

7001 9.589061e-01 9.779132e-01

7002 9.589115e-01 9.779047e-01

7003 9.589169e-01 9.778961e-01

7004 9.589223e-01 9.778874e-01

7005 9.589277e-01 9.778786e-01

7006 9.589331e-01 9.778697e-01

7007 9.589385e-01 9.778607e-01

7008 9.589440e-01 9.778516e-01

7009 9.589494e-01 9.778424e-01

7010 9.589549e-01 9.778330e-01

7011 9.589604e-01 9.778236e-01

7012 9.589660e-01 9.778141e-01

7013 9.589715e-01 9.778044e-01

7014 9.589771e-01 9.777946e-01

7015 9.589827e-01 9.777848e-01

7016 9.589883e-01 9.777748e-01

7017 9.589939e-01 9.777647e-01

7018 9.589996e-01 9.777545e-01

7019 9.590053e-01 9.777442e-01

7020 9.590111e-01 9.777337e-01

7021 9.590168e-01 9.777232e-01

7022 9.591023e-01 9.777224e-01

7023 9.591864e-01 9.777215e-01

7024 9.592690e-01 9.777207e-01

7025 9.593502e-01 9.777199e-01

7026 9.594299e-01 9.777190e-01

7027 9.595082e-01 9.777181e-01

7028 9.595851e-01 9.777172e-01

7029 9.596606e-01 9.777163e-01

7030 9.597347e-01 9.777154e-01

7031 9.598074e-01 9.777144e-01

7032 9.598788e-01 9.777135e-01

7033 9.599488e-01 9.777125e-01

7034 9.600175e-01 9.777115e-01

7035 9.600848e-01 9.777105e-01

7036 9.601509e-01 9.777095e-01

7037 9.602156e-01 9.777084e-01

7038 9.602791e-01 9.777073e-01

7039 9.603413e-01 9.777063e-01

7040 9.604023e-01 9.777052e-01

7041 9.604620e-01 9.777040e-01

7042 9.605206e-01 9.777029e-01

7043 9.605779e-01 9.777017e-01

7044 9.606340e-01 9.777005e-01

7045 9.606890e-01 9.776993e-01

7046 9.607428e-01 9.776981e-01

7047 9.607955e-01 9.776969e-01

7048 9.608471e-01 9.776956e-01

7049 9.608976e-01 9.776943e-01

7050 9.609469e-01 9.776930e-01

7051 9.609953e-01 9.776917e-01

7052 9.610426e-01 9.776903e-01

7053 9.610888e-01 9.776889e-01

7054 9.611340e-01 9.776875e-01

7055 9.611783e-01 9.776861e-01

7056 9.612215e-01 9.776847e-01

7057 9.612638e-01 9.776832e-01

7058 9.613052e-01 9.776817e-01

7059 9.613456e-01 9.776802e-01

7060 9.613851e-01 9.776786e-01

7061 9.614237e-01 9.776770e-01

7062 9.614614e-01 9.776754e-01

7063 9.614982e-01 9.776738e-01

7064 9.615343e-01 9.776721e-01

7065 9.615694e-01 9.776705e-01

7066 9.616038e-01 9.776687e-01

7067 9.616374e-01 9.776670e-01

7068 9.616701e-01 9.776652e-01

7069 9.617021e-01 9.776634e-01

7070 9.617334e-01 9.776616e-01

7071 9.617639e-01 9.776598e-01

7072 9.617937e-01 9.776579e-01

7073 9.618228e-01 9.776560e-01

7074 9.618512e-01 9.776540e-01

7075 9.618790e-01 9.776520e-01

7076 9.619060e-01 9.776500e-01

7077 9.619324e-01 9.776480e-01

7078 9.619582e-01 9.776459e-01

7079 9.619834e-01 9.776438e-01

7080 9.620079e-01 9.776416e-01

7081 9.620319e-01 9.776395e-01

7082 9.620553e-01 9.776373e-01

7083 9.620781e-01 9.776350e-01

7084 9.621004e-01 9.776327e-01

7085 9.621221e-01 9.776304e-01

7086 9.621433e-01 9.776280e-01

7087 9.621640e-01 9.776256e-01

7088 9.621842e-01 9.776232e-01

7089 9.622039e-01 9.776207e-01

7090 9.622231e-01 9.776182e-01

7091 9.622419e-01 9.776157e-01

7092 9.622602e-01 9.776131e-01

7093 9.622780e-01 9.776105e-01

7094 9.622955e-01 9.776078e-01

7095 9.623125e-01 9.776051e-01

7096 9.623291e-01 9.776023e-01

7097 9.623453e-01 9.775995e-01

7098 9.623611e-01 9.775967e-01

7099 9.623765e-01 9.775938e-01

7100 9.623916e-01 9.775909e-01

7101 9.624063e-01 9.775879e-01

7102 9.624207e-01 9.775849e-01

7103 9.624347e-01 9.775818e-01

7104 9.624484e-01 9.775787e-01

7105 9.624617e-01 9.775756e-01

7106 9.624748e-01 9.775724e-01

7107 9.624875e-01 9.775691e-01

7108 9.625000e-01 9.775658e-01

7109 9.625121e-01 9.775625e-01

7110 9.625240e-01 9.775590e-01

7111 9.625356e-01 9.775556e-01

7112 9.625470e-01 9.775521e-01

7113 9.625581e-01 9.775485e-01

7114 9.625689e-01 9.775449e-01

7115 9.625796e-01 9.775413e-01

7116 9.625899e-01 9.775375e-01

7117 9.626001e-01 9.775338e-01

7118 9.626100e-01 9.775299e-01

7119 9.626198e-01 9.775260e-01

7120 9.626293e-01 9.775221e-01

7121 9.626386e-01 9.775181e-01

7122 9.626477e-01 9.775141e-01

7123 9.626567e-01 9.775099e-01

7124 9.626654e-01 9.775058e-01

7125 9.626740e-01 9.775015e-01

7126 9.626824e-01 9.774972e-01

7127 9.626907e-01 9.774929e-01

7128 9.626988e-01 9.774884e-01

7129 9.627067e-01 9.774840e-01

7130 9.627145e-01 9.774794e-01

7131 9.627222e-01 9.774748e-01

7132 9.627297e-01 9.774701e-01

7133 9.627371e-01 9.774654e-01

7134 9.627443e-01 9.774606e-01

7135 9.627515e-01 9.774557e-01

7136 9.627585e-01 9.774507e-01

7137 9.627654e-01 9.774457e-01

7138 9.627722e-01 9.774406e-01

7139 9.627789e-01 9.774355e-01

7140 9.627855e-01 9.774303e-01

7141 9.627920e-01 9.774250e-01

7142 9.627984e-01 9.774196e-01

7143 9.628047e-01 9.774141e-01

7144 9.628110e-01 9.774086e-01

7145 9.628171e-01 9.774030e-01

7146 9.628232e-01 9.773973e-01

7147 9.628292e-01 9.773916e-01

7148 9.628352e-01 9.773858e-01

7149 9.628410e-01 9.773799e-01

7150 9.628468e-01 9.773739e-01

7151 9.628526e-01 9.773678e-01

7152 9.628583e-01 9.773617e-01

7153 9.628639e-01 9.773555e-01

7154 9.628695e-01 9.773492e-01

7155 9.628750e-01 9.773428e-01

7156 9.628805e-01 9.773363e-01

7157 9.628859e-01 9.773297e-01

7158 9.628913e-01 9.773231e-01

7159 9.628967e-01 9.773164e-01

7160 9.629020e-01 9.773096e-01

7161 9.629073e-01 9.773026e-01

7162 9.629126e-01 9.772957e-01

7163 9.629178e-01 9.772886e-01

7164 9.629231e-01 9.772814e-01

7165 9.629283e-01 9.772741e-01

7166 9.629334e-01 9.772668e-01

7167 9.629386e-01 9.772594e-01

7168 9.629437e-01 9.772518e-01

7169 9.629488e-01 9.772442e-01

7170 9.629539e-01 9.772365e-01

7171 9.629590e-01 9.772286e-01

7172 9.629641e-01 9.772207e-01

7173 9.629692e-01 9.772127e-01

7174 9.629743e-01 9.772046e-01

7175 9.629793e-01 9.771964e-01

7176 9.629844e-01 9.771881e-01

7177 9.629894e-01 9.771797e-01

7178 9.629945e-01 9.771712e-01

7179 9.629996e-01 9.771625e-01

7180 9.630046e-01 9.771538e-01

7181 9.630097e-01 9.771450e-01

7182 9.630148e-01 9.771361e-01

7183 9.630199e-01 9.771271e-01

7184 9.630250e-01 9.771179e-01

7185 9.630301e-01 9.771087e-01

7186 9.630352e-01 9.770994e-01

7187 9.630403e-01 9.770899e-01

7188 9.630454e-01 9.770804e-01

7189 9.630506e-01 9.770707e-01

7190 9.630558e-01 9.770609e-01

7191 9.630609e-01 9.770511e-01

7192 9.630661e-01 9.770411e-01

7193 9.630714e-01 9.770310e-01

7194 9.630766e-01 9.770208e-01

7195 9.630819e-01 9.770104e-01

7196 9.630871e-01 9.770000e-01

7197 9.630924e-01 9.769894e-01

7198 9.630978e-01 9.769788e-01

7199 9.631031e-01 9.769680e-01

7200 9.631085e-01 9.769571e-01

7201 9.631139e-01 9.769461e-01

7202 9.631939e-01 9.769452e-01

7203 9.632724e-01 9.769443e-01

7204 9.633495e-01 9.769434e-01

7205 9.634251e-01 9.769424e-01

7206 9.634993e-01 9.769414e-01

7207 9.635721e-01 9.769405e-01

7208 9.636434e-01 9.769395e-01

7209 9.637134e-01 9.769384e-01

7210 9.637819e-01 9.769374e-01

7211 9.638491e-01 9.769364e-01

7212 9.639150e-01 9.769353e-01

7213 9.639795e-01 9.769342e-01

7214 9.640427e-01 9.769331e-01

7215 9.641047e-01 9.769320e-01

7216 9.641653e-01 9.769308e-01

7217 9.642247e-01 9.769297e-01

7218 9.642828e-01 9.769285e-01

7219 9.643397e-01 9.769273e-01

7220 9.643954e-01 9.769261e-01

7221 9.644499e-01 9.769248e-01

7222 9.645033e-01 9.769235e-01

7223 9.645554e-01 9.769223e-01

7224 9.646065e-01 9.769210e-01

7225 9.646564e-01 9.769196e-01

7226 9.647052e-01 9.769183e-01

7227 9.647530e-01 9.769169e-01

7228 9.647997e-01 9.769155e-01

7229 9.648453e-01 9.769141e-01

7230 9.648899e-01 9.769126e-01

7231 9.649335e-01 9.769112e-01

7232 9.649761e-01 9.769097e-01

7233 9.650177e-01 9.769082e-01

7234 9.650584e-01 9.769066e-01

7235 9.650982e-01 9.769051e-01

7236 9.651370e-01 9.769035e-01

7237 9.651749e-01 9.769018e-01

7238 9.652120e-01 9.769002e-01

7239 9.652482e-01 9.768985e-01

7240 9.652835e-01 9.768968e-01

7241 9.653180e-01 9.768951e-01

7242 9.653517e-01 9.768933e-01

7243 9.653845e-01 9.768916e-01

7244 9.654166e-01 9.768897e-01

7245 9.654480e-01 9.768879e-01

7246 9.654785e-01 9.768860e-01

7247 9.655084e-01 9.768841e-01

7248 9.655375e-01 9.768822e-01

7249 9.655659e-01 9.768802e-01

7250 9.655937e-01 9.768783e-01

7251 9.656207e-01 9.768762e-01

7252 9.656472e-01 9.768742e-01

7253 9.656729e-01 9.768721e-01

7254 9.656981e-01 9.768700e-01

7255 9.657226e-01 9.768678e-01

7256 9.657465e-01 9.768656e-01

7257 9.657699e-01 9.768634e-01

7258 9.657926e-01 9.768611e-01

7259 9.658148e-01 9.768588e-01

7260 9.658365e-01 9.768565e-01

7261 9.658577e-01 9.768541e-01

7262 9.658783e-01 9.768517e-01

7263 9.658984e-01 9.768493e-01

7264 9.659180e-01 9.768468e-01

7265 9.659371e-01 9.768443e-01

7266 9.659558e-01 9.768418e-01

7267 9.659740e-01 9.768392e-01

7268 9.659918e-01 9.768365e-01

7269 9.660091e-01 9.768338e-01

7270 9.660260e-01 9.768311e-01

7271 9.660425e-01 9.768284e-01

7272 9.660586e-01 9.768256e-01

7273 9.660743e-01 9.768227e-01

7274 9.660896e-01 9.768198e-01

7275 9.661046e-01 9.768169e-01

7276 9.661192e-01 9.768139e-01

7277 9.661334e-01 9.768109e-01

7278 9.661473e-01 9.768079e-01

7279 9.661609e-01 9.768048e-01

7280 9.661741e-01 9.768016e-01

7281 9.661871e-01 9.767984e-01

7282 9.661997e-01 9.767951e-01

7283 9.662120e-01 9.767918e-01

7284 9.662241e-01 9.767885e-01

7285 9.662358e-01 9.767851e-01

7286 9.662473e-01 9.767817e-01

7287 9.662585e-01 9.767782e-01

7288 9.662695e-01 9.767746e-01

7289 9.662802e-01 9.767710e-01

7290 9.662907e-01 9.767673e-01

7291 9.663010e-01 9.767636e-01

7292 9.663110e-01 9.767599e-01

7293 9.663208e-01 9.767561e-01

7294 9.663304e-01 9.767522e-01

7295 9.663398e-01 9.767483e-01

7296 9.663489e-01 9.767443e-01

7297 9.663579e-01 9.767402e-01

7298 9.663667e-01 9.767361e-01

7299 9.663753e-01 9.767320e-01

7300 9.663838e-01 9.767278e-01

7301 9.663920e-01 9.767235e-01

7302 9.664001e-01 9.767191e-01

7303 9.664081e-01 9.767147e-01

7304 9.664159e-01 9.767103e-01

7305 9.664235e-01 9.767058e-01

7306 9.664310e-01 9.767012e-01

7307 9.664384e-01 9.766965e-01

7308 9.664456e-01 9.766918e-01

7309 9.664527e-01 9.766870e-01

7310 9.664597e-01 9.766822e-01

7311 9.664666e-01 9.766773e-01

7312 9.664733e-01 9.766723e-01

7313 9.664799e-01 9.766672e-01

7314 9.664864e-01 9.766621e-01

7315 9.664929e-01 9.766569e-01

7316 9.664992e-01 9.766516e-01

7317 9.665054e-01 9.766463e-01

7318 9.665115e-01 9.766409e-01

7319 9.665176e-01 9.766354e-01

7320 9.665235e-01 9.766299e-01

7321 9.665294e-01 9.766243e-01

7322 9.665352e-01 9.766185e-01

7323 9.665410e-01 9.766128e-01

7324 9.665466e-01 9.766069e-01

7325 9.665522e-01 9.766010e-01

7326 9.665577e-01 9.765950e-01

7327 9.665632e-01 9.765889e-01

7328 9.665686e-01 9.765827e-01

7329 9.665739e-01 9.765765e-01

7330 9.665792e-01 9.765701e-01

7331 9.665845e-01 9.765637e-01

7332 9.665897e-01 9.765572e-01

7333 9.665948e-01 9.765506e-01

7334 9.666000e-01 9.765440e-01

7335 9.666050e-01 9.765372e-01

7336 9.666101e-01 9.765304e-01

7337 9.666150e-01 9.765235e-01

7338 9.666200e-01 9.765165e-01

7339 9.666249e-01 9.765094e-01

7340 9.666298e-01 9.765022e-01

7341 9.666347e-01 9.764949e-01

7342 9.666396e-01 9.764875e-01

7343 9.666444e-01 9.764801e-01

7344 9.666492e-01 9.764725e-01

7345 9.666540e-01 9.764649e-01

7346 9.666587e-01 9.764572e-01

7347 9.666635e-01 9.764493e-01

7348 9.666682e-01 9.764414e-01

7349 9.666730e-01 9.764334e-01

7350 9.666777e-01 9.764253e-01

7351 9.666824e-01 9.764170e-01

7352 9.666871e-01 9.764087e-01

7353 9.666917e-01 9.764003e-01

7354 9.666964e-01 9.763918e-01

7355 9.667011e-01 9.763832e-01

7356 9.667058e-01 9.763745e-01

7357 9.667104e-01 9.763657e-01

7358 9.667151e-01 9.763567e-01

7359 9.667198e-01 9.763477e-01

7360 9.667244e-01 9.763386e-01

7361 9.667291e-01 9.763294e-01

7362 9.667338e-01 9.763200e-01

7363 9.667384e-01 9.763106e-01

7364 9.667431e-01 9.763011e-01

7365 9.667478e-01 9.762914e-01

7366 9.667525e-01 9.762816e-01

7367 9.667572e-01 9.762718e-01

7368 9.667619e-01 9.762618e-01

7369 9.667666e-01 9.762517e-01

7370 9.667714e-01 9.762415e-01

7371 9.667761e-01 9.762312e-01

7372 9.667809e-01 9.762208e-01

7373 9.667856e-01 9.762103e-01

7374 9.667904e-01 9.761996e-01

7375 9.667952e-01 9.761889e-01

7376 9.668000e-01 9.761780e-01

7377 9.668048e-01 9.761670e-01

7378 9.668097e-01 9.761559e-01

7379 9.668145e-01 9.761447e-01

7380 9.668194e-01 9.761334e-01

7381 9.668242e-01 9.761219e-01

7382 9.668980e-01 9.761209e-01

7383 9.669703e-01 9.761199e-01

7384 9.670412e-01 9.761189e-01

7385 9.671106e-01 9.761178e-01

7386 9.671787e-01 9.761168e-01

7387 9.672453e-01 9.761157e-01

7388 9.673105e-01 9.761146e-01

7389 9.673744e-01 9.761135e-01

7390 9.674370e-01 9.761123e-01

7391 9.674983e-01 9.761112e-01

7392 9.675582e-01 9.761100e-01

7393 9.676169e-01 9.761088e-01

7394 9.676743e-01 9.761076e-01

7395 9.677304e-01 9.761063e-01

7396 9.677853e-01 9.761051e-01

7397 9.678391e-01 9.761038e-01

7398 9.678916e-01 9.761025e-01

7399 9.679430e-01 9.761012e-01

7400 9.679932e-01 9.760998e-01

7401 9.680423e-01 9.760985e-01

7402 9.680903e-01 9.760971e-01

7403 9.681372e-01 9.760957e-01

7404 9.681831e-01 9.760942e-01

7405 9.682279e-01 9.760928e-01

7406 9.682717e-01 9.760913e-01

7407 9.683144e-01 9.760898e-01

7408 9.683562e-01 9.760883e-01

7409 9.683970e-01 9.760867e-01

7410 9.684368e-01 9.760851e-01

7411 9.684757e-01 9.760835e-01

7412 9.685137e-01 9.760819e-01

7413 9.685508e-01 9.760803e-01

7414 9.685870e-01 9.760786e-01

7415 9.686223e-01 9.760769e-01

7416 9.686568e-01 9.760751e-01

7417 9.686905e-01 9.760734e-01

7418 9.687234e-01 9.760716e-01

7419 9.687554e-01 9.760698e-01

7420 9.687867e-01 9.760679e-01

7421 9.688173e-01 9.760660e-01

7422 9.688471e-01 9.760641e-01

7423 9.688761e-01 9.760622e-01

7424 9.689045e-01 9.760602e-01

7425 9.689322e-01 9.760582e-01

7426 9.689591e-01 9.760562e-01

7427 9.689855e-01 9.760541e-01

7428 9.690111e-01 9.760520e-01

7429 9.690362e-01 9.760499e-01

7430 9.690606e-01 9.760478e-01

7431 9.690844e-01 9.760456e-01

7432 9.691077e-01 9.760433e-01

7433 9.691303e-01 9.760411e-01

7434 9.691524e-01 9.760388e-01

7435 9.691740e-01 9.760364e-01

7436 9.691950e-01 9.760341e-01

7437 9.692155e-01 9.760317e-01

7438 9.692355e-01 9.760292e-01

7439 9.692549e-01 9.760268e-01

7440 9.692739e-01 9.760242e-01

7441 9.692925e-01 9.760217e-01

7442 9.693106e-01 9.760191e-01

7443 9.693282e-01 9.760165e-01

7444 9.693454e-01 9.760138e-01

7445 9.693622e-01 9.760111e-01

7446 9.693785e-01 9.760083e-01

7447 9.693945e-01 9.760055e-01

7448 9.694100e-01 9.760027e-01

7449 9.694252e-01 9.759998e-01

7450 9.694400e-01 9.759969e-01

7451 9.694545e-01 9.759939e-01

7452 9.694686e-01 9.759909e-01

7453 9.694823e-01 9.759879e-01

7454 9.694957e-01 9.759848e-01

7455 9.695088e-01 9.759816e-01

7456 9.695216e-01 9.759784e-01

7457 9.695341e-01 9.759752e-01

7458 9.695463e-01 9.759719e-01

7459 9.695582e-01 9.759686e-01

7460 9.695698e-01 9.759652e-01

7461 9.695811e-01 9.759618e-01

7462 9.695922e-01 9.759583e-01

7463 9.696030e-01 9.759547e-01

7464 9.696136e-01 9.759512e-01

7465 9.696239e-01 9.759475e-01

7466 9.696340e-01 9.759438e-01

7467 9.696439e-01 9.759401e-01

7468 9.696535e-01 9.759363e-01

7469 9.696629e-01 9.759324e-01

7470 9.696721e-01 9.759285e-01

7471 9.696812e-01 9.759246e-01

7472 9.696900e-01 9.759206e-01

7473 9.696986e-01 9.759165e-01

7474 9.697071e-01 9.759124e-01

7475 9.697154e-01 9.759082e-01

7476 9.697235e-01 9.759039e-01

7477 9.697314e-01 9.758996e-01

7478 9.697392e-01 9.758953e-01

7479 9.697468e-01 9.758908e-01

7480 9.697543e-01 9.758863e-01

7481 9.697616e-01 9.758818e-01

7482 9.697688e-01 9.758772e-01

7483 9.697758e-01 9.758725e-01

7484 9.697827e-01 9.758678e-01

7485 9.697895e-01 9.758629e-01

7486 9.697962e-01 9.758581e-01

7487 9.698027e-01 9.758531e-01

7488 9.698092e-01 9.758481e-01

7489 9.698155e-01 9.758431e-01

7490 9.698217e-01 9.758379e-01

7491 9.698278e-01 9.758327e-01

7492 9.698338e-01 9.758274e-01

7493 9.698398e-01 9.758221e-01

7494 9.698456e-01 9.758166e-01

7495 9.698513e-01 9.758111e-01

7496 9.698570e-01 9.758056e-01

7497 9.698626e-01 9.757999e-01

7498 9.698681e-01 9.757942e-01

7499 9.698735e-01 9.757884e-01

7500 9.698789e-01 9.757825e-01

7501 9.698842e-01 9.757766e-01

7502 9.698894e-01 9.757706e-01

7503 9.698945e-01 9.757645e-01

7504 9.698996e-01 9.757583e-01

7505 9.699047e-01 9.757520e-01

7506 9.699097e-01 9.757457e-01

7507 9.699146e-01 9.757393e-01

7508 9.699195e-01 9.757328e-01

7509 9.699243e-01 9.757262e-01

7510 9.699291e-01 9.757195e-01

7511 9.699338e-01 9.757128e-01

7512 9.699385e-01 9.757059e-01

7513 9.699432e-01 9.756990e-01

7514 9.699478e-01 9.756920e-01

7515 9.699524e-01 9.756849e-01

7516 9.699570e-01 9.756777e-01

7517 9.699615e-01 9.756704e-01

7518 9.699660e-01 9.756631e-01

7519 9.699705e-01 9.756556e-01

7520 9.699750e-01 9.756481e-01

7521 9.699794e-01 9.756404e-01

7522 9.699838e-01 9.756327e-01

7523 9.699882e-01 9.756249e-01

7524 9.699925e-01 9.756169e-01

7525 9.699969e-01 9.756089e-01

7526 9.700012e-01 9.756008e-01

7527 9.700055e-01 9.755926e-01

7528 9.700098e-01 9.755843e-01

7529 9.700141e-01 9.755759e-01

7530 9.700184e-01 9.755674e-01

7531 9.700226e-01 9.755588e-01

7532 9.700269e-01 9.755501e-01

7533 9.700311e-01 9.755413e-01

7534 9.700354e-01 9.755324e-01

7535 9.700396e-01 9.755234e-01

7536 9.700438e-01 9.755143e-01

7537 9.700481e-01 9.755051e-01

7538 9.700523e-01 9.754958e-01

7539 9.700565e-01 9.754864e-01

7540 9.700607e-01 9.754769e-01

7541 9.700649e-01 9.754673e-01

7542 9.700692e-01 9.754576e-01

7543 9.700734e-01 9.754477e-01

7544 9.700776e-01 9.754378e-01

7545 9.700819e-01 9.754277e-01

7546 9.700861e-01 9.754176e-01

7547 9.700903e-01 9.754073e-01

7548 9.700946e-01 9.753969e-01

7549 9.700988e-01 9.753864e-01

7550 9.701031e-01 9.753758e-01

7551 9.701073e-01 9.753651e-01

7552 9.701116e-01 9.753543e-01

7553 9.701159e-01 9.753434e-01

7554 9.701202e-01 9.753323e-01

7555 9.701245e-01 9.753211e-01

7556 9.701288e-01 9.753099e-01

7557 9.701331e-01 9.752985e-01

7558 9.701374e-01 9.752870e-01

7559 9.701417e-01 9.752753e-01

7560 9.701461e-01 9.752636e-01

7561 9.701504e-01 9.752518e-01

7562 9.702175e-01 9.752507e-01

7563 9.702832e-01 9.752496e-01

7564 9.703476e-01 9.752484e-01

7565 9.704105e-01 9.752473e-01

7566 9.704721e-01 9.752461e-01

7567 9.705324e-01 9.752449e-01

7568 9.705913e-01 9.752437e-01

7569 9.706490e-01 9.752425e-01

7570 9.707054e-01 9.752412e-01

7571 9.707605e-01 9.752400e-01

7572 9.708144e-01 9.752387e-01

7573 9.708671e-01 9.752374e-01

7574 9.709186e-01 9.752361e-01

7575 9.709690e-01 9.752347e-01

7576 9.710182e-01 9.752333e-01

7577 9.710663e-01 9.752319e-01

7578 9.711133e-01 9.752305e-01

7579 9.711591e-01 9.752291e-01

7580 9.712040e-01 9.752276e-01

7581 9.712477e-01 9.752261e-01

7582 9.712905e-01 9.752246e-01

7583 9.713323e-01 9.752231e-01

7584 9.713730e-01 9.752215e-01

7585 9.714128e-01 9.752200e-01

7586 9.714517e-01 9.752184e-01

7587 9.714896e-01 9.752167e-01

7588 9.715266e-01 9.752151e-01

7589 9.715627e-01 9.752134e-01

7590 9.715980e-01 9.752117e-01

7591 9.716324e-01 9.752099e-01

7592 9.716660e-01 9.752082e-01

7593 9.716987e-01 9.752064e-01

7594 9.717307e-01 9.752045e-01

7595 9.717619e-01 9.752027e-01

7596 9.717923e-01 9.752008e-01

7597 9.718219e-01 9.751989e-01

7598 9.718509e-01 9.751970e-01

7599 9.718791e-01 9.751950e-01

7600 9.719066e-01 9.751930e-01

7601 9.719334e-01 9.751910e-01

7602 9.719596e-01 9.751889e-01

7603 9.719851e-01 9.751868e-01

7604 9.720100e-01 9.751847e-01

7605 9.720343e-01 9.751825e-01

7606 9.720580e-01 9.751804e-01

7607 9.720810e-01 9.751781e-01

7608 9.721035e-01 9.751759e-01

7609 9.721255e-01 9.751736e-01

7610 9.721468e-01 9.751713e-01

7611 9.721677e-01 9.751689e-01

7612 9.721880e-01 9.751665e-01

7613 9.722078e-01 9.751641e-01

7614 9.722272e-01 9.751616e-01

7615 9.722460e-01 9.751591e-01

7616 9.722644e-01 9.751565e-01

7617 9.722823e-01 9.751540e-01

7618 9.722997e-01 9.751513e-01

7619 9.723168e-01 9.751487e-01

7620 9.723334e-01 9.751460e-01

7621 9.723496e-01 9.751432e-01

7622 9.723653e-01 9.751405e-01

7623 9.723807e-01 9.751376e-01

7624 9.723957e-01 9.751348e-01

7625 9.724104e-01 9.751319e-01

7626 9.724247e-01 9.751289e-01

7627 9.724386e-01 9.751259e-01

7628 9.724522e-01 9.751229e-01

7629 9.724654e-01 9.751198e-01

7630 9.724784e-01 9.751167e-01

7631 9.724910e-01 9.751135e-01

7632 9.725033e-01 9.751103e-01

7633 9.725153e-01 9.751070e-01

7634 9.725270e-01 9.751037e-01

7635 9.725385e-01 9.751003e-01

7636 9.725497e-01 9.750969e-01

7637 9.725606e-01 9.750935e-01

7638 9.725712e-01 9.750900e-01

7639 9.725816e-01 9.750864e-01

7640 9.725918e-01 9.750828e-01

7641 9.726017e-01 9.750792e-01

7642 9.726114e-01 9.750754e-01

7643 9.726209e-01 9.750717e-01

7644 9.726301e-01 9.750679e-01

7645 9.726392e-01 9.750640e-01

7646 9.726480e-01 9.750601e-01

7647 9.726567e-01 9.750561e-01

7648 9.726651e-01 9.750520e-01

7649 9.726734e-01 9.750480e-01

7650 9.726815e-01 9.750438e-01

7651 9.726894e-01 9.750396e-01

7652 9.726972e-01 9.750353e-01

7653 9.727048e-01 9.750310e-01

7654 9.727122e-01 9.750266e-01

7655 9.727195e-01 9.750222e-01

7656 9.727266e-01 9.750177e-01

7657 9.727336e-01 9.750131e-01

7658 9.727405e-01 9.750085e-01

7659 9.727472e-01 9.750038e-01

7660 9.727538e-01 9.749990e-01

7661 9.727603e-01 9.749942e-01

7662 9.727666e-01 9.749893e-01

7663 9.727729e-01 9.749844e-01

7664 9.727790e-01 9.749794e-01

7665 9.727850e-01 9.749743e-01

7666 9.727909e-01 9.749691e-01

7667 9.727967e-01 9.749639e-01

7668 9.728024e-01 9.749586e-01

7669 9.728080e-01 9.749532e-01

7670 9.728136e-01 9.749478e-01

7671 9.728190e-01 9.749423e-01

7672 9.728244e-01 9.749367e-01

7673 9.728296e-01 9.749311e-01

7674 9.728348e-01 9.749254e-01

7675 9.728399e-01 9.749196e-01

7676 9.728450e-01 9.749137e-01

7677 9.728500e-01 9.749077e-01

7678 9.728549e-01 9.749017e-01

7679 9.728597e-01 9.748956e-01

7680 9.728645e-01 9.748894e-01

7681 9.728692e-01 9.748832e-01

7682 9.728739e-01 9.748768e-01

7683 9.728785e-01 9.748704e-01

7684 9.728831e-01 9.748639e-01

7685 9.728876e-01 9.748574e-01

7686 9.728921e-01 9.748507e-01

7687 9.728965e-01 9.748439e-01

7688 9.729009e-01 9.748371e-01

7689 9.729052e-01 9.748302e-01

7690 9.729095e-01 9.748232e-01

7691 9.729138e-01 9.748161e-01

7692 9.729180e-01 9.748089e-01

7693 9.729222e-01 9.748017e-01

7694 9.729264e-01 9.747943e-01

7695 9.729305e-01 9.747869e-01

7696 9.729346e-01 9.747794e-01

7697 9.729387e-01 9.747718e-01

7698 9.729427e-01 9.747640e-01

7699 9.729467e-01 9.747562e-01

7700 9.729507e-01 9.747484e-01

7701 9.729547e-01 9.747404e-01

7702 9.729587e-01 9.747323e-01

7703 9.729626e-01 9.747241e-01

7704 9.729666e-01 9.747158e-01

7705 9.729705e-01 9.747075e-01

7706 9.729744e-01 9.746990e-01

7707 9.729783e-01 9.746904e-01

7708 9.729821e-01 9.746818e-01

7709 9.729860e-01 9.746730e-01

7710 9.729898e-01 9.746642e-01

7711 9.729937e-01 9.746552e-01

7712 9.729975e-01 9.746462e-01

7713 9.730014e-01 9.746370e-01

7714 9.730052e-01 9.746277e-01

7715 9.730090e-01 9.746184e-01

7716 9.730128e-01 9.746089e-01

7717 9.730166e-01 9.745993e-01

7718 9.730204e-01 9.745897e-01

7719 9.730242e-01 9.745799e-01

7720 9.730280e-01 9.745700e-01

7721 9.730318e-01 9.745600e-01

7722 9.730357e-01 9.745499e-01

7723 9.730395e-01 9.745397e-01

7724 9.730433e-01 9.745294e-01

7725 9.730471e-01 9.745189e-01

7726 9.730509e-01 9.745084e-01

7727 9.730547e-01 9.744978e-01

7728 9.730586e-01 9.744870e-01

7729 9.730624e-01 9.744761e-01

7730 9.730662e-01 9.744652e-01

7731 9.730701e-01 9.744541e-01

7732 9.730739e-01 9.744429e-01

7733 9.730778e-01 9.744316e-01

7734 9.730817e-01 9.744201e-01

7735 9.730855e-01 9.744086e-01

7736 9.730894e-01 9.743969e-01

7737 9.730933e-01 9.743852e-01

7738 9.730972e-01 9.743733e-01

7739 9.731012e-01 9.743613e-01

7740 9.731051e-01 9.743492e-01

7741 9.731090e-01 9.743370e-01

7742 9.731694e-01 9.743358e-01

7743 9.732284e-01 9.743346e-01

7744 9.732862e-01 9.743333e-01

7745 9.733426e-01 9.743321e-01

7746 9.733978e-01 9.743308e-01

7747 9.734517e-01 9.743295e-01

7748 9.735044e-01 9.743282e-01

7749 9.735559e-01 9.743269e-01

7750 9.736062e-01 9.743255e-01

7751 9.736554e-01 9.743242e-01

7752 9.737034e-01 9.743228e-01

7753 9.737503e-01 9.743214e-01

7754 9.737961e-01 9.743199e-01

7755 9.738408e-01 9.743185e-01

7756 9.738845e-01 9.743170e-01

7757 9.739271e-01 9.743155e-01

7758 9.739687e-01 9.743139e-01

7759 9.740094e-01 9.743124e-01

7760 9.740490e-01 9.743108e-01

7761 9.740877e-01 9.743092e-01

7762 9.741255e-01 9.743075e-01

7763 9.741624e-01 9.743059e-01

7764 9.741983e-01 9.743042e-01

7765 9.742334e-01 9.743025e-01

7766 9.742676e-01 9.743008e-01

7767 9.743010e-01 9.742990e-01

7768 9.743336e-01 9.742972e-01

7769 9.743654e-01 9.742954e-01

7770 9.743964e-01 9.742935e-01

7771 9.744266e-01 9.742917e-01

7772 9.744561e-01 9.742898e-01

7773 9.744848e-01 9.742878e-01

7774 9.745128e-01 9.742859e-01

7775 9.745401e-01 9.742839e-01

7776 9.745668e-01 9.742819e-01

7777 9.745928e-01 9.742798e-01

7778 9.746181e-01 9.742777e-01

7779 9.746428e-01 9.742756e-01

7780 9.746668e-01 9.742735e-01

7781 9.746903e-01 9.742713e-01

7782 9.747132e-01 9.742691e-01

7783 9.747355e-01 9.742668e-01

7784 9.747572e-01 9.742645e-01

7785 9.747784e-01 9.742622e-01

7786 9.747991e-01 9.742599e-01

7787 9.748192e-01 9.742575e-01

7788 9.748388e-01 9.742551e-01

7789 9.748580e-01 9.742526e-01

7790 9.748766e-01 9.742501e-01

7791 9.748948e-01 9.742476e-01

7792 9.749125e-01 9.742450e-01

7793 9.749298e-01 9.742424e-01

7794 9.749466e-01 9.742398e-01

7795 9.749630e-01 9.742371e-01

7796 9.749790e-01 9.742344e-01

7797 9.749946e-01 9.742316e-01

7798 9.750099e-01 9.742288e-01

7799 9.750247e-01 9.742260e-01

7800 9.750392e-01 9.742231e-01

7801 9.750533e-01 9.742202e-01

7802 9.750670e-01 9.742172e-01

7803 9.750804e-01 9.742142e-01

7804 9.750935e-01 9.742111e-01

7805 9.751062e-01 9.742080e-01

7806 9.751187e-01 9.742049e-01

7807 9.751308e-01 9.742017e-01

7808 9.751427e-01 9.741985e-01

7809 9.751542e-01 9.741952e-01

7810 9.751655e-01 9.741918e-01

7811 9.751765e-01 9.741885e-01

7812 9.751872e-01 9.741850e-01

7813 9.751977e-01 9.741816e-01

7814 9.752079e-01 9.741781e-01

7815 9.752179e-01 9.741745e-01

7816 9.752277e-01 9.741709e-01

7817 9.752372e-01 9.741672e-01

7818 9.752465e-01 9.741635e-01

7819 9.752556e-01 9.741597e-01

7820 9.752644e-01 9.741559e-01

7821 9.752731e-01 9.741520e-01

7822 9.752816e-01 9.741480e-01

7823 9.752899e-01 9.741440e-01

7824 9.752980e-01 9.741400e-01

7825 9.753059e-01 9.741359e-01

7826 9.753137e-01 9.741317e-01

7827 9.753212e-01 9.741275e-01

7828 9.753287e-01 9.741233e-01

7829 9.753359e-01 9.741189e-01

7830 9.753430e-01 9.741145e-01

7831 9.753500e-01 9.741101e-01

7832 9.753568e-01 9.741056e-01

7833 9.753635e-01 9.741010e-01

7834 9.753700e-01 9.740964e-01

7835 9.753764e-01 9.740917e-01

7836 9.753827e-01 9.740869e-01

7837 9.753888e-01 9.740821e-01

7838 9.753949e-01 9.740772e-01

7839 9.754008e-01 9.740722e-01

7840 9.754066e-01 9.740672e-01

7841 9.754123e-01 9.740621e-01

7842 9.754179e-01 9.740570e-01

7843 9.754234e-01 9.740518e-01

7844 9.754288e-01 9.740465e-01

7845 9.754342e-01 9.740411e-01

7846 9.754394e-01 9.740357e-01

7847 9.754445e-01 9.740302e-01

7848 9.754496e-01 9.740246e-01

7849 9.754546e-01 9.740190e-01

7850 9.754595e-01 9.740133e-01

7851 9.754643e-01 9.740075e-01

7852 9.754690e-01 9.740016e-01

7853 9.754737e-01 9.739957e-01

7854 9.754783e-01 9.739897e-01

7855 9.754829e-01 9.739836e-01

7856 9.754874e-01 9.739774e-01

7857 9.754918e-01 9.739712e-01

7858 9.754962e-01 9.739649e-01

7859 9.755005e-01 9.739585e-01

7860 9.755048e-01 9.739520e-01

7861 9.755090e-01 9.739455e-01

7862 9.755132e-01 9.739388e-01

7863 9.755173e-01 9.739321e-01

7864 9.755214e-01 9.739253e-01

7865 9.755254e-01 9.739184e-01

7866 9.755294e-01 9.739114e-01

7867 9.755334e-01 9.739044e-01

7868 9.755373e-01 9.738972e-01

7869 9.755412e-01 9.738900e-01

7870 9.755451e-01 9.738827e-01

7871 9.755489e-01 9.738753e-01

7872 9.755527e-01 9.738678e-01

7873 9.755565e-01 9.738602e-01

7874 9.755602e-01 9.738525e-01

7875 9.755639e-01 9.738448e-01

7876 9.755676e-01 9.738369e-01

7877 9.755713e-01 9.738290e-01

7878 9.755749e-01 9.738210e-01

7879 9.755786e-01 9.738128e-01

7880 9.755822e-01 9.738046e-01

7881 9.755858e-01 9.737963e-01

7882 9.755893e-01 9.737879e-01

7883 9.755929e-01 9.737794e-01

7884 9.755964e-01 9.737708e-01

7885 9.756000e-01 9.737621e-01

7886 9.756035e-01 9.737533e-01

7887 9.756070e-01 9.737444e-01

7888 9.756105e-01 9.737354e-01

7889 9.756140e-01 9.737263e-01

7890 9.756175e-01 9.737171e-01

7891 9.756210e-01 9.737078e-01

7892 9.756245e-01 9.736984e-01

7893 9.756280e-01 9.736889e-01

7894 9.756315e-01 9.736793e-01

7895 9.756349e-01 9.736696e-01

7896 9.756384e-01 9.736598e-01

7897 9.756419e-01 9.736499e-01

7898 9.756453e-01 9.736398e-01

7899 9.756488e-01 9.736297e-01

7900 9.756523e-01 9.736195e-01

7901 9.756558e-01 9.736091e-01

7902 9.756592e-01 9.735987e-01

7903 9.756627e-01 9.735881e-01

7904 9.756662e-01 9.735775e-01

7905 9.756697e-01 9.735667e-01

7906 9.756732e-01 9.735558e-01

7907 9.756767e-01 9.735449e-01

7908 9.756802e-01 9.735338e-01

7909 9.756838e-01 9.735226e-01

7910 9.756873e-01 9.735112e-01

7911 9.756909e-01 9.734998e-01

7912 9.756944e-01 9.734883e-01

7913 9.756980e-01 9.734766e-01

7914 9.757016e-01 9.734649e-01

7915 9.757051e-01 9.734530e-01

7916 9.757088e-01 9.734410e-01

7917 9.757124e-01 9.734289e-01

7918 9.757160e-01 9.734167e-01

7919 9.757196e-01 9.734044e-01

7920 9.757233e-01 9.733920e-01

7921 9.757270e-01 9.733794e-01

7922 9.757808e-01 9.733781e-01

7923 9.758333e-01 9.733768e-01

7924 9.758847e-01 9.733755e-01

7925 9.759348e-01 9.733741e-01

7926 9.759838e-01 9.733728e-01

7927 9.760316e-01 9.733714e-01

7928 9.760783e-01 9.733699e-01

7929 9.761239e-01 9.733685e-01

7930 9.761685e-01 9.733671e-01

7931 9.762119e-01 9.733656e-01

7932 9.762544e-01 9.733641e-01

7933 9.762958e-01 9.733625e-01

7934 9.763362e-01 9.733610e-01

7935 9.763756e-01 9.733594e-01

7936 9.764141e-01 9.733578e-01

7937 9.764516e-01 9.733562e-01

7938 9.764883e-01 9.733545e-01

7939 9.765240e-01 9.733529e-01

7940 9.765588e-01 9.733512e-01

7941 9.765928e-01 9.733494e-01

7942 9.766260e-01 9.733477e-01

7943 9.766583e-01 9.733459e-01

7944 9.766899e-01 9.733441e-01

7945 9.767206e-01 9.733422e-01

7946 9.767506e-01 9.733404e-01

7947 9.767799e-01 9.733385e-01

7948 9.768084e-01 9.733366e-01

7949 9.768362e-01 9.733346e-01

7950 9.768633e-01 9.733326e-01

7951 9.768897e-01 9.733306e-01

7952 9.769154e-01 9.733286e-01

7953 9.769405e-01 9.733265e-01

7954 9.769650e-01 9.733244e-01

7955 9.769888e-01 9.733223e-01

7956 9.770121e-01 9.733201e-01

7957 9.770347e-01 9.733179e-01

7958 9.770568e-01 9.733157e-01

7959 9.770783e-01 9.733134e-01

7960 9.770993e-01 9.733111e-01

7961 9.771198e-01 9.733088e-01

7962 9.771397e-01 9.733064e-01

7963 9.771591e-01 9.733040e-01

7964 9.771780e-01 9.733016e-01

7965 9.771965e-01 9.732991e-01

7966 9.772144e-01 9.732966e-01

7967 9.772320e-01 9.732941e-01

7968 9.772490e-01 9.732915e-01

7969 9.772657e-01 9.732889e-01

7970 9.772819e-01 9.732862e-01

7971 9.772977e-01 9.732835e-01

7972 9.773131e-01 9.732808e-01

7973 9.773282e-01 9.732780e-01

7974 9.773428e-01 9.732752e-01

7975 9.773571e-01 9.732723e-01

7976 9.773710e-01 9.732695e-01

7977 9.773846e-01 9.732665e-01

7978 9.773978e-01 9.732635e-01

7979 9.774107e-01 9.732605e-01

7980 9.774233e-01 9.732574e-01

7981 9.774355e-01 9.732543e-01

7982 9.774475e-01 9.732512e-01

7983 9.774592e-01 9.732480e-01

7984 9.774706e-01 9.732447e-01

7985 9.774817e-01 9.732414e-01

7986 9.774925e-01 9.732381e-01

7987 9.775031e-01 9.732347e-01

7988 9.775134e-01 9.732313e-01

7989 9.775234e-01 9.732278e-01

7990 9.775332e-01 9.732243e-01

7991 9.775428e-01 9.732207e-01

7992 9.775522e-01 9.732171e-01

7993 9.775613e-01 9.732134e-01

7994 9.775702e-01 9.732097e-01

7995 9.775789e-01 9.732059e-01

7996 9.775875e-01 9.732021e-01

7997 9.775958e-01 9.731982e-01

7998 9.776039e-01 9.731942e-01

7999 9.776118e-01 9.731902e-01

8000 9.776196e-01 9.731862e-01

8001 9.776272e-01 9.731821e-01

8002 9.776346e-01 9.731779e-01

8003 9.776418e-01 9.731737e-01

8004 9.776489e-01 9.731695e-01

8005 9.776559e-01 9.731651e-01

8006 9.776626e-01 9.731607e-01

8007 9.776693e-01 9.731563e-01

8008 9.776758e-01 9.731518e-01

8009 9.776822e-01 9.731472e-01

8010 9.776884e-01 9.731426e-01

8011 9.776945e-01 9.731379e-01

8012 9.777005e-01 9.731332e-01

8013 9.777064e-01 9.731283e-01

8014 9.777121e-01 9.731235e-01

8015 9.777177e-01 9.731185e-01

8016 9.777233e-01 9.731135e-01

8017 9.777287e-01 9.731084e-01

8018 9.777340e-01 9.731033e-01

8019 9.777393e-01 9.730981e-01

8020 9.777444e-01 9.730928e-01

8021 9.777494e-01 9.730875e-01

8022 9.777544e-01 9.730821e-01

8023 9.777593e-01 9.730766e-01

8024 9.777640e-01 9.730711e-01

8025 9.777688e-01 9.730654e-01

8026 9.777734e-01 9.730598e-01

8027 9.777779e-01 9.730540e-01

8028 9.777824e-01 9.730482e-01

8029 9.777869e-01 9.730422e-01

8030 9.777912e-01 9.730363e-01

8031 9.777955e-01 9.730302e-01

8032 9.777997e-01 9.730241e-01

8033 9.778039e-01 9.730179e-01

8034 9.778080e-01 9.730116e-01

8035 9.778121e-01 9.730052e-01

8036 9.778161e-01 9.729988e-01

8037 9.778200e-01 9.729922e-01

8038 9.778240e-01 9.729856e-01

8039 9.778278e-01 9.729790e-01

8040 9.778316e-01 9.729722e-01

8041 9.778354e-01 9.729653e-01

8042 9.778392e-01 9.729584e-01

8043 9.778429e-01 9.729514e-01

8044 9.778465e-01 9.729443e-01

8045 9.778502e-01 9.729371e-01

8046 9.778538e-01 9.729299e-01

8047 9.778573e-01 9.729225e-01

8048 9.778609e-01 9.729151e-01

8049 9.778644e-01 9.729075e-01

8050 9.778678e-01 9.728999e-01

8051 9.778713e-01 9.728922e-01

8052 9.778747e-01 9.728844e-01

8053 9.778781e-01 9.728766e-01

8054 9.778815e-01 9.728686e-01

8055 9.778849e-01 9.728605e-01

8056 9.778882e-01 9.728524e-01

8057 9.778916e-01 9.728441e-01

8058 9.778949e-01 9.728358e-01

8059 9.778982e-01 9.728273e-01

8060 9.779015e-01 9.728188e-01

8061 9.779048e-01 9.728102e-01

8062 9.779080e-01 9.728015e-01

8063 9.779113e-01 9.727926e-01

8064 9.779145e-01 9.727837e-01

8065 9.779178e-01 9.727747e-01

8066 9.779210e-01 9.727656e-01

8067 9.779242e-01 9.727564e-01

8068 9.779274e-01 9.727471e-01

8069 9.779306e-01 9.727377e-01

8070 9.779338e-01 9.727282e-01

8071 9.779370e-01 9.727186e-01

8072 9.779403e-01 9.727088e-01

8073 9.779435e-01 9.726990e-01

8074 9.779467e-01 9.726891e-01

8075 9.779499e-01 9.726791e-01

8076 9.779531e-01 9.726690e-01

8077 9.779563e-01 9.726588e-01

8078 9.779595e-01 9.726484e-01

8079 9.779627e-01 9.726380e-01

8080 9.779659e-01 9.726275e-01

8081 9.779692e-01 9.726168e-01

8082 9.779724e-01 9.726061e-01

8083 9.779756e-01 9.725952e-01

8084 9.779789e-01 9.725842e-01

8085 9.779822e-01 9.725732e-01

8086 9.779854e-01 9.725620e-01

8087 9.779887e-01 9.725507e-01

8088 9.779920e-01 9.725393e-01

8089 9.779953e-01 9.725278e-01

8090 9.779986e-01 9.725162e-01

8091 9.780019e-01 9.725045e-01

8092 9.780053e-01 9.724926e-01

8093 9.780086e-01 9.724807e-01

8094 9.780120e-01 9.724687e-01

8095 9.780154e-01 9.724565e-01

8096 9.780188e-01 9.724442e-01

8097 9.780222e-01 9.724318e-01

8098 9.780256e-01 9.724194e-01

8099 9.780291e-01 9.724068e-01

8100 9.780325e-01 9.723941e-01

8101 9.780360e-01 9.723812e-01

8102 9.780836e-01 9.723798e-01

8103 9.781300e-01 9.723784e-01

8104 9.781753e-01 9.723770e-01

8105 9.782196e-01 9.723756e-01

8106 9.782628e-01 9.723741e-01

8107 9.783049e-01 9.723726e-01

8108 9.783460e-01 9.723711e-01

8109 9.783862e-01 9.723695e-01

8110 9.784253e-01 9.723680e-01

8111 9.784635e-01 9.723664e-01

8112 9.785008e-01 9.723648e-01

8113 9.785371e-01 9.723631e-01

8114 9.785726e-01 9.723614e-01

8115 9.786071e-01 9.723598e-01

8116 9.786409e-01 9.723580e-01

8117 9.786737e-01 9.723563e-01

8118 9.787058e-01 9.723545e-01

8119 9.787371e-01 9.723527e-01

8120 9.787676e-01 9.723509e-01

8121 9.787973e-01 9.723491e-01

8122 9.788263e-01 9.723472e-01

8123 9.788545e-01 9.723453e-01

8124 9.788820e-01 9.723434e-01

8125 9.789089e-01 9.723414e-01

8126 9.789350e-01 9.723394e-01

8127 9.789605e-01 9.723374e-01

8128 9.789854e-01 9.723353e-01

8129 9.790096e-01 9.723332e-01

8130 9.790332e-01 9.723311e-01

8131 9.790562e-01 9.723290e-01

8132 9.790787e-01 9.723268e-01

8133 9.791005e-01 9.723246e-01

8134 9.791218e-01 9.723224e-01

8135 9.791426e-01 9.723201e-01

8136 9.791628e-01 9.723178e-01

8137 9.791825e-01 9.723154e-01

8138 9.792017e-01 9.723131e-01

8139 9.792204e-01 9.723107e-01

8140 9.792387e-01 9.723082e-01

8141 9.792564e-01 9.723057e-01

8142 9.792738e-01 9.723032e-01

8143 9.792906e-01 9.723007e-01

8144 9.793071e-01 9.722981e-01

8145 9.793231e-01 9.722955e-01

8146 9.793387e-01 9.722928e-01

8147 9.793540e-01 9.722901e-01

8148 9.793688e-01 9.722873e-01

8149 9.793833e-01 9.722846e-01

8150 9.793974e-01 9.722817e-01

8151 9.794111e-01 9.722789e-01

8152 9.794245e-01 9.722760e-01

8153 9.794376e-01 9.722730e-01

8154 9.794503e-01 9.722701e-01

8155 9.794627e-01 9.722670e-01

8156 9.794748e-01 9.722640e-01

8157 9.794866e-01 9.722609e-01

8158 9.794981e-01 9.722577e-01

8159 9.795093e-01 9.722545e-01

8160 9.795203e-01 9.722512e-01

8161 9.795309e-01 9.722480e-01

8162 9.795413e-01 9.722446e-01

8163 9.795515e-01 9.722412e-01

8164 9.795614e-01 9.722378e-01

8165 9.795711e-01 9.722343e-01

8166 9.795805e-01 9.722308e-01

8167 9.795897e-01 9.722272e-01

8168 9.795987e-01 9.722236e-01

8169 9.796074e-01 9.722199e-01

8170 9.796160e-01 9.722162e-01

8171 9.796244e-01 9.722124e-01

8172 9.796325e-01 9.722086e-01

8173 9.796405e-01 9.722047e-01

8174 9.796483e-01 9.722008e-01

8175 9.796559e-01 9.721968e-01

8176 9.796633e-01 9.721928e-01

8177 9.796706e-01 9.721887e-01

8178 9.796777e-01 9.721845e-01

8179 9.796846e-01 9.721803e-01

8180 9.796914e-01 9.721761e-01

8181 9.796981e-01 9.721718e-01

8182 9.797046e-01 9.721674e-01

8183 9.797109e-01 9.721630e-01

8184 9.797171e-01 9.721585e-01

8185 9.797232e-01 9.721539e-01

8186 9.797292e-01 9.721493e-01

8187 9.797350e-01 9.721446e-01

8188 9.797407e-01 9.721399e-01

8189 9.797463e-01 9.721351e-01

8190 9.797518e-01 9.721303e-01

8191 9.797572e-01 9.721253e-01

8192 9.797625e-01 9.721204e-01

8193 9.797676e-01 9.721153e-01

8194 9.797727e-01 9.721102e-01

8195 9.797777e-01 9.721050e-01

8196 9.797826e-01 9.720998e-01

8197 9.797874e-01 9.720945e-01

8198 9.797921e-01 9.720891e-01

8199 9.797967e-01 9.720837e-01

8200 9.798012e-01 9.720781e-01

8201 9.798057e-01 9.720726e-01

8202 9.798101e-01 9.720669e-01

8203 9.798144e-01 9.720612e-01

8204 9.798187e-01 9.720554e-01

8205 9.798229e-01 9.720495e-01

8206 9.798270e-01 9.720436e-01

8207 9.798311e-01 9.720375e-01

8208 9.798351e-01 9.720315e-01

8209 9.798390e-01 9.720253e-01

8210 9.798429e-01 9.720190e-01

8211 9.798467e-01 9.720127e-01

8212 9.798505e-01 9.720063e-01

8213 9.798542e-01 9.719999e-01

8214 9.798579e-01 9.719933e-01

8215 9.798616e-01 9.719867e-01

8216 9.798652e-01 9.719800e-01

8217 9.798687e-01 9.719732e-01

8218 9.798722e-01 9.719663e-01

8219 9.798757e-01 9.719594e-01

8220 9.798792e-01 9.719523e-01

8221 9.798826e-01 9.719452e-01

8222 9.798860e-01 9.719380e-01

8223 9.798893e-01 9.719307e-01

8224 9.798926e-01 9.719234e-01

8225 9.798959e-01 9.719159e-01

8226 9.798992e-01 9.719084e-01

8227 9.799024e-01 9.719007e-01

8228 9.799056e-01 9.718930e-01

8229 9.799088e-01 9.718852e-01

8230 9.799120e-01 9.718773e-01

8231 9.799151e-01 9.718693e-01

8232 9.799182e-01 9.718613e-01

8233 9.799213e-01 9.718531e-01

8234 9.799244e-01 9.718449e-01

8235 9.799275e-01 9.718365e-01

8236 9.799306e-01 9.718281e-01

8237 9.799336e-01 9.718195e-01

8238 9.799367e-01 9.718109e-01

8239 9.799397e-01 9.718022e-01

8240 9.799427e-01 9.717934e-01

8241 9.799457e-01 9.717845e-01

8242 9.799487e-01 9.717755e-01

8243 9.799517e-01 9.717664e-01

8244 9.799547e-01 9.717572e-01

8245 9.799577e-01 9.717479e-01

8246 9.799607e-01 9.717385e-01

8247 9.799636e-01 9.717290e-01

8248 9.799666e-01 9.717194e-01

8249 9.799696e-01 9.717097e-01

8250 9.799725e-01 9.717000e-01

8251 9.799755e-01 9.716901e-01

8252 9.799785e-01 9.716801e-01

8253 9.799815e-01 9.716700e-01

8254 9.799844e-01 9.716598e-01

8255 9.799874e-01 9.716495e-01

8256 9.799904e-01 9.716391e-01

8257 9.799934e-01 9.716286e-01

8258 9.799964e-01 9.716180e-01

8259 9.799994e-01 9.716073e-01

8260 9.800024e-01 9.715965e-01

8261 9.800054e-01 9.715856e-01

8262 9.800084e-01 9.715746e-01

8263 9.800115e-01 9.715635e-01

8264 9.800145e-01 9.715523e-01

8265 9.800176e-01 9.715409e-01

8266 9.800206e-01 9.715295e-01

8267 9.800237e-01 9.715179e-01

8268 9.800268e-01 9.715063e-01

8269 9.800299e-01 9.714945e-01

8270 9.800330e-01 9.714827e-01

8271 9.800361e-01 9.714707e-01

8272 9.800392e-01 9.714587e-01

8273 9.800424e-01 9.714465e-01

8274 9.800455e-01 9.714342e-01

8275 9.800487e-01 9.714218e-01

8276 9.800519e-01 9.714093e-01

8277 9.800551e-01 9.713967e-01

8278 9.800583e-01 9.713840e-01

8279 9.800615e-01 9.713712e-01

8280 9.800648e-01 9.713582e-01

8281 9.800681e-01 9.713452e-01

8282 9.801099e-01 9.713437e-01

8283 9.801507e-01 9.713422e-01

8284 9.801905e-01 9.713407e-01

8285 9.802293e-01 9.713391e-01

8286 9.802672e-01 9.713376e-01

8287 9.803041e-01 9.713360e-01

8288 9.803401e-01 9.713343e-01

8289 9.803753e-01 9.713327e-01

8290 9.804095e-01 9.713310e-01

8291 9.804429e-01 9.713293e-01

8292 9.804755e-01 9.713276e-01

8293 9.805073e-01 9.713259e-01

8294 9.805383e-01 9.713241e-01

8295 9.805685e-01 9.713223e-01

8296 9.805979e-01 9.713204e-01

8297 9.806266e-01 9.713186e-01

8298 9.806546e-01 9.713167e-01

8299 9.806818e-01 9.713148e-01

8300 9.807084e-01 9.713129e-01

8301 9.807343e-01 9.713109e-01

8302 9.807596e-01 9.713089e-01

8303 9.807842e-01 9.713069e-01

8304 9.808081e-01 9.713048e-01

8305 9.808315e-01 9.713027e-01

8306 9.808543e-01 9.713006e-01

8307 9.808765e-01 9.712984e-01

8308 9.808981e-01 9.712963e-01

8309 9.809192e-01 9.712941e-01

8310 9.809397e-01 9.712918e-01

8311 9.809597e-01 9.712895e-01

8312 9.809792e-01 9.712872e-01

8313 9.809982e-01 9.712849e-01

8314 9.810167e-01 9.712825e-01

8315 9.810347e-01 9.712801e-01

8316 9.810523e-01 9.712776e-01

8317 9.810694e-01 9.712752e-01

8318 9.810861e-01 9.712726e-01

8319 9.811024e-01 9.712701e-01

8320 9.811182e-01 9.712675e-01

8321 9.811337e-01 9.712649e-01

8322 9.811487e-01 9.712622e-01

8323 9.811634e-01 9.712595e-01

8324 9.811777e-01 9.712568e-01

8325 9.811916e-01 9.712540e-01

8326 9.812052e-01 9.712512e-01

8327 9.812184e-01 9.712483e-01

8328 9.812313e-01 9.712454e-01

8329 9.812438e-01 9.712425e-01

8330 9.812561e-01 9.712395e-01

8331 9.812680e-01 9.712365e-01

8332 9.812797e-01 9.712334e-01

8333 9.812910e-01 9.712303e-01

8334 9.813021e-01 9.712272e-01

8335 9.813129e-01 9.712240e-01

8336 9.813234e-01 9.712207e-01

8337 9.813337e-01 9.712174e-01

8338 9.813437e-01 9.712141e-01

8339 9.813535e-01 9.712107e-01

8340 9.813630e-01 9.712073e-01

8341 9.813723e-01 9.712039e-01

8342 9.813813e-01 9.712003e-01

8343 9.813902e-01 9.711968e-01

8344 9.813988e-01 9.711932e-01

8345 9.814073e-01 9.711895e-01

8346 9.814155e-01 9.711858e-01

8347 9.814235e-01 9.711820e-01

8348 9.814314e-01 9.711782e-01

8349 9.814390e-01 9.711744e-01

8350 9.814465e-01 9.711705e-01

8351 9.814538e-01 9.711665e-01

8352 9.814609e-01 9.711625e-01

8353 9.814679e-01 9.711584e-01

8354 9.814747e-01 9.711543e-01

8355 9.814814e-01 9.711501e-01

8356 9.814879e-01 9.711459e-01

8357 9.814942e-01 9.711416e-01

8358 9.815005e-01 9.711372e-01

8359 9.815066e-01 9.711328e-01

8360 9.815125e-01 9.711283e-01

8361 9.815183e-01 9.711238e-01

8362 9.815241e-01 9.711192e-01

8363 9.815296e-01 9.711146e-01

8364 9.815351e-01 9.711099e-01

8365 9.815405e-01 9.711051e-01

8366 9.815457e-01 9.711003e-01

8367 9.815509e-01 9.710954e-01

8368 9.815559e-01 9.710905e-01

8369 9.815608e-01 9.710855e-01

8370 9.815657e-01 9.710804e-01

8371 9.815704e-01 9.710753e-01

8372 9.815751e-01 9.710701e-01

8373 9.815797e-01 9.710648e-01

8374 9.815842e-01 9.710595e-01

8375 9.815886e-01 9.710541e-01

8376 9.815929e-01 9.710486e-01

8377 9.815972e-01 9.710431e-01

8378 9.816013e-01 9.710374e-01

8379 9.816055e-01 9.710318e-01

8380 9.816095e-01 9.710260e-01

8381 9.816135e-01 9.710202e-01

8382 9.816174e-01 9.710143e-01

8383 9.816213e-01 9.710084e-01

8384 9.816251e-01 9.710023e-01

8385 9.816288e-01 9.709962e-01

8386 9.816325e-01 9.709900e-01

8387 9.816361e-01 9.709838e-01

8388 9.816397e-01 9.709775e-01

8389 9.816432e-01 9.709710e-01

8390 9.816467e-01 9.709646e-01

8391 9.816502e-01 9.709580e-01

8392 9.816536e-01 9.709514e-01

8393 9.816569e-01 9.709447e-01

8394 9.816602e-01 9.709379e-01

8395 9.816635e-01 9.709310e-01

8396 9.816668e-01 9.709240e-01

8397 9.816700e-01 9.709170e-01

8398 9.816732e-01 9.709099e-01

8399 9.816763e-01 9.709027e-01

8400 9.816794e-01 9.708954e-01

8401 9.816825e-01 9.708880e-01

8402 9.816856e-01 9.708806e-01

8403 9.816886e-01 9.708730e-01

8404 9.816916e-01 9.708654e-01

8405 9.816946e-01 9.708577e-01

8406 9.816976e-01 9.708499e-01

8407 9.817005e-01 9.708420e-01

8408 9.817034e-01 9.708341e-01

8409 9.817063e-01 9.708260e-01

8410 9.817092e-01 9.708179e-01

8411 9.817121e-01 9.708096e-01

8412 9.817150e-01 9.708013e-01

8413 9.817178e-01 9.707929e-01

8414 9.817206e-01 9.707844e-01

8415 9.817235e-01 9.707758e-01

8416 9.817263e-01 9.707671e-01

8417 9.817291e-01 9.707583e-01

8418 9.817319e-01 9.707495e-01

8419 9.817346e-01 9.707405e-01

8420 9.817374e-01 9.707314e-01

8421 9.817402e-01 9.707223e-01

8422 9.817429e-01 9.707130e-01

8423 9.817457e-01 9.707037e-01

8424 9.817484e-01 9.706943e-01

8425 9.817512e-01 9.706847e-01

8426 9.817539e-01 9.706751e-01

8427 9.817567e-01 9.706654e-01

8428 9.817594e-01 9.706555e-01

8429 9.817622e-01 9.706456e-01

8430 9.817649e-01 9.706356e-01

8431 9.817677e-01 9.706254e-01

8432 9.817704e-01 9.706152e-01

8433 9.817732e-01 9.706049e-01

8434 9.817759e-01 9.705945e-01

8435 9.817787e-01 9.705840e-01

8436 9.817814e-01 9.705733e-01

8437 9.817842e-01 9.705626e-01

8438 9.817870e-01 9.705518e-01

8439 9.817898e-01 9.705409e-01

8440 9.817925e-01 9.705298e-01

8441 9.817953e-01 9.705187e-01

8442 9.817981e-01 9.705075e-01

8443 9.818009e-01 9.704961e-01

8444 9.818037e-01 9.704847e-01

8445 9.818066e-01 9.704732e-01

8446 9.818094e-01 9.704615e-01

8447 9.818122e-01 9.704498e-01

8448 9.818151e-01 9.704379e-01

8449 9.818179e-01 9.704260e-01

8450 9.818208e-01 9.704139e-01

8451 9.818237e-01 9.704018e-01

8452 9.818266e-01 9.703895e-01

8453 9.818295e-01 9.703772e-01

8454 9.818324e-01 9.703647e-01

8455 9.818353e-01 9.703521e-01

8456 9.818383e-01 9.703395e-01

8457 9.818412e-01 9.703267e-01

8458 9.818442e-01 9.703138e-01

8459 9.818472e-01 9.703008e-01

8460 9.818502e-01 9.702878e-01

8461 9.818532e-01 9.702746e-01

8462 9.818898e-01 9.702730e-01

8463 9.819255e-01 9.702714e-01

8464 9.819603e-01 9.702698e-01

8465 9.819942e-01 9.702681e-01

8466 9.820273e-01 9.702664e-01

8467 9.820595e-01 9.702647e-01

8468 9.820910e-01 9.702630e-01

8469 9.821217e-01 9.702612e-01

8470 9.821516e-01 9.702595e-01

8471 9.821807e-01 9.702577e-01

8472 9.822091e-01 9.702558e-01

8473 9.822368e-01 9.702540e-01

8474 9.822638e-01 9.702521e-01

8475 9.822901e-01 9.702502e-01

8476 9.823157e-01 9.702482e-01

8477 9.823407e-01 9.702463e-01

8478 9.823651e-01 9.702443e-01

8479 9.823888e-01 9.702422e-01

8480 9.824119e-01 9.702402e-01

8481 9.824344e-01 9.702381e-01

8482 9.824564e-01 9.702360e-01

8483 9.824778e-01 9.702338e-01

8484 9.824986e-01 9.702317e-01

8485 9.825189e-01 9.702294e-01

8486 9.825387e-01 9.702272e-01

8487 9.825580e-01 9.702249e-01

8488 9.825768e-01 9.702226e-01

8489 9.825951e-01 9.702203e-01

8490 9.826130e-01 9.702179e-01

8491 9.826303e-01 9.702155e-01

8492 9.826473e-01 9.702131e-01

8493 9.826638e-01 9.702106e-01

8494 9.826799e-01 9.702081e-01

8495 9.826955e-01 9.702055e-01

8496 9.827108e-01 9.702029e-01

8497 9.827257e-01 9.702003e-01

8498 9.827402e-01 9.701977e-01

8499 9.827543e-01 9.701950e-01

8500 9.827681e-01 9.701922e-01

8501 9.827815e-01 9.701895e-01

8502 9.827946e-01 9.701867e-01

8503 9.828073e-01 9.701838e-01

8504 9.828197e-01 9.701809e-01

8505 9.828318e-01 9.701780e-01

8506 9.828436e-01 9.701750e-01

8507 9.828551e-01 9.701720e-01

8508 9.828663e-01 9.701690e-01

8509 9.828773e-01 9.701659e-01

8510 9.828879e-01 9.701627e-01

8511 9.828983e-01 9.701596e-01

8512 9.829085e-01 9.701563e-01

8513 9.829183e-01 9.701531e-01

8514 9.829280e-01 9.701498e-01

8515 9.829374e-01 9.701464e-01

8516 9.829466e-01 9.701430e-01

8517 9.829555e-01 9.701396e-01

8518 9.829642e-01 9.701361e-01

8519 9.829727e-01 9.701325e-01

8520 9.829810e-01 9.701289e-01

8521 9.829892e-01 9.701253e-01

8522 9.829971e-01 9.701216e-01

8523 9.830048e-01 9.701179e-01

8524 9.830123e-01 9.701141e-01

8525 9.830197e-01 9.701103e-01

8526 9.830269e-01 9.701064e-01

8527 9.830339e-01 9.701024e-01

8528 9.830408e-01 9.700984e-01

8529 9.830475e-01 9.700944e-01

8530 9.830540e-01 9.700903e-01

8531 9.830604e-01 9.700862e-01

8532 9.830667e-01 9.700819e-01

8533 9.830728e-01 9.700777e-01

8534 9.830788e-01 9.700734e-01

8535 9.830846e-01 9.700690e-01

8536 9.830903e-01 9.700646e-01

8537 9.830959e-01 9.700601e-01

8538 9.831014e-01 9.700556e-01

8539 9.831068e-01 9.700510e-01

8540 9.831120e-01 9.700463e-01

8541 9.831171e-01 9.700416e-01

8542 9.831222e-01 9.700368e-01

8543 9.831271e-01 9.700320e-01

8544 9.831319e-01 9.700271e-01

8545 9.831366e-01 9.700221e-01

8546 9.831413e-01 9.700171e-01

8547 9.831458e-01 9.700120e-01

8548 9.831503e-01 9.700069e-01

8549 9.831547e-01 9.700016e-01

8550 9.831590e-01 9.699964e-01

8551 9.831632e-01 9.699910e-01

8552 9.831673e-01 9.699856e-01

8553 9.831714e-01 9.699801e-01

8554 9.831754e-01 9.699746e-01

8555 9.831793e-01 9.699690e-01

8556 9.831831e-01 9.699633e-01

8557 9.831869e-01 9.699575e-01

8558 9.831907e-01 9.699517e-01

8559 9.831943e-01 9.699458e-01

8560 9.831979e-01 9.699399e-01

8561 9.832015e-01 9.699338e-01

8562 9.832050e-01 9.699277e-01

8563 9.832084e-01 9.699216e-01

8564 9.832118e-01 9.699153e-01

8565 9.832152e-01 9.699090e-01

8566 9.832185e-01 9.699026e-01

8567 9.832218e-01 9.698961e-01

8568 9.832250e-01 9.698895e-01

8569 9.832281e-01 9.698829e-01

8570 9.832313e-01 9.698762e-01

8571 9.832344e-01 9.698694e-01

8572 9.832374e-01 9.698626e-01

8573 9.832405e-01 9.698556e-01

8574 9.832435e-01 9.698486e-01

8575 9.832464e-01 9.698415e-01

8576 9.832494e-01 9.698343e-01

8577 9.832523e-01 9.698271e-01

8578 9.832551e-01 9.698197e-01

8579 9.832580e-01 9.698123e-01

8580 9.832608e-01 9.698048e-01

8581 9.832636e-01 9.697972e-01

8582 9.832664e-01 9.697895e-01

8583 9.832691e-01 9.697818e-01

8584 9.832719e-01 9.697739e-01

8585 9.832746e-01 9.697660e-01

8586 9.832773e-01 9.697580e-01

8587 9.832800e-01 9.697499e-01

8588 9.832826e-01 9.697417e-01

8589 9.832853e-01 9.697334e-01

8590 9.832879e-01 9.697251e-01

8591 9.832905e-01 9.697166e-01

8592 9.832931e-01 9.697081e-01

8593 9.832957e-01 9.696994e-01

8594 9.832983e-01 9.696907e-01

8595 9.833009e-01 9.696819e-01

8596 9.833035e-01 9.696730e-01

8597 9.833060e-01 9.696640e-01

8598 9.833086e-01 9.696549e-01

8599 9.833111e-01 9.696457e-01

8600 9.833136e-01 9.696365e-01

8601 9.833162e-01 9.696271e-01

8602 9.833187e-01 9.696176e-01

8603 9.833212e-01 9.696081e-01

8604 9.833237e-01 9.695984e-01

8605 9.833263e-01 9.695887e-01

8606 9.833288e-01 9.695789e-01

8607 9.833313e-01 9.695689e-01

8608 9.833338e-01 9.695589e-01

8609 9.833363e-01 9.695488e-01

8610 9.833388e-01 9.695385e-01

8611 9.833413e-01 9.695282e-01

8612 9.833439e-01 9.695178e-01

8613 9.833464e-01 9.695073e-01

8614 9.833489e-01 9.694967e-01

8615 9.833514e-01 9.694860e-01

8616 9.833539e-01 9.694752e-01

8617 9.833565e-01 9.694643e-01

8618 9.833590e-01 9.694533e-01

8619 9.833616e-01 9.694422e-01

8620 9.833641e-01 9.694310e-01

8621 9.833666e-01 9.694197e-01

8622 9.833692e-01 9.694083e-01

8623 9.833718e-01 9.693968e-01

8624 9.833743e-01 9.693852e-01

8625 9.833769e-01 9.693735e-01

8626 9.833795e-01 9.693617e-01

8627 9.833821e-01 9.693498e-01

8628 9.833847e-01 9.693378e-01

8629 9.833873e-01 9.693257e-01

8630 9.833899e-01 9.693135e-01

8631 9.833925e-01 9.693012e-01

8632 9.833951e-01 9.692888e-01

8633 9.833978e-01 9.692763e-01

8634 9.834004e-01 9.692638e-01

8635 9.834030e-01 9.692511e-01

8636 9.834057e-01 9.692383e-01

8637 9.834084e-01 9.692254e-01

8638 9.834111e-01 9.692124e-01

8639 9.834138e-01 9.691993e-01

8640 9.834165e-01 9.691862e-01

8641 9.834192e-01 9.691729e-01

8642 9.834511e-01 9.691712e-01

8643 9.834822e-01 9.691695e-01

8644 9.835126e-01 9.691678e-01

8645 9.835422e-01 9.691660e-01

8646 9.835710e-01 9.691643e-01

8647 9.835991e-01 9.691625e-01

8648 9.836265e-01 9.691606e-01

8649 9.836533e-01 9.691588e-01

8650 9.836793e-01 9.691569e-01

8651 9.837047e-01 9.691550e-01

8652 9.837294e-01 9.691530e-01

8653 9.837535e-01 9.691511e-01

8654 9.837770e-01 9.691491e-01

8655 9.837998e-01 9.691471e-01

8656 9.838221e-01 9.691450e-01

8657 9.838439e-01 9.691429e-01

8658 9.838650e-01 9.691408e-01

8659 9.838857e-01 9.691387e-01

8660 9.839058e-01 9.691365e-01

8661 9.839253e-01 9.691343e-01

8662 9.839444e-01 9.691321e-01

8663 9.839630e-01 9.691298e-01

8664 9.839811e-01 9.691275e-01

8665 9.839988e-01 9.691252e-01

8666 9.840160e-01 9.691228e-01

8667 9.840327e-01 9.691204e-01

8668 9.840491e-01 9.691180e-01

8669 9.840650e-01 9.691155e-01

8670 9.840805e-01 9.691130e-01

8671 9.840956e-01 9.691105e-01

8672 9.841103e-01 9.691079e-01

8673 9.841246e-01 9.691053e-01

8674 9.841386e-01 9.691027e-01

8675 9.841522e-01 9.691000e-01

8676 9.841655e-01 9.690973e-01

8677 9.841784e-01 9.690945e-01

8678 9.841911e-01 9.690917e-01

8679 9.842033e-01 9.690889e-01

8680 9.842153e-01 9.690860e-01

8681 9.842270e-01 9.690831e-01

8682 9.842384e-01 9.690802e-01

8683 9.842494e-01 9.690772e-01

8684 9.842603e-01 9.690741e-01

8685 9.842708e-01 9.690711e-01

8686 9.842811e-01 9.690679e-01

8687 9.842911e-01 9.690648e-01

8688 9.843009e-01 9.690616e-01

8689 9.843104e-01 9.690584e-01

8690 9.843197e-01 9.690551e-01

8691 9.843287e-01 9.690517e-01

8692 9.843376e-01 9.690484e-01

8693 9.843462e-01 9.690449e-01

8694 9.843546e-01 9.690415e-01

8695 9.843628e-01 9.690380e-01

8696 9.843708e-01 9.690344e-01

8697 9.843786e-01 9.690308e-01

8698 9.843862e-01 9.690272e-01

8699 9.843937e-01 9.690234e-01

8700 9.844009e-01 9.690197e-01

8701 9.844080e-01 9.690159e-01

8702 9.844150e-01 9.690120e-01

8703 9.844217e-01 9.690081e-01

8704 9.844283e-01 9.690042e-01

8705 9.844348e-01 9.690002e-01

8706 9.844411e-01 9.689961e-01

8707 9.844472e-01 9.689920e-01

8708 9.844532e-01 9.689879e-01

8709 9.844591e-01 9.689837e-01

8710 9.844649e-01 9.689794e-01

8711 9.844705e-01 9.689751e-01

8712 9.844760e-01 9.689707e-01

8713 9.844814e-01 9.689663e-01

8714 9.844866e-01 9.689618e-01

8715 9.844918e-01 9.689572e-01

8716 9.844968e-01 9.689526e-01

8717 9.845017e-01 9.689480e-01

8718 9.845066e-01 9.689432e-01

8719 9.845113e-01 9.689385e-01

8720 9.845159e-01 9.689336e-01

8721 9.845205e-01 9.689287e-01

8722 9.845249e-01 9.689238e-01

8723 9.845293e-01 9.689187e-01

8724 9.845335e-01 9.689136e-01

8725 9.845377e-01 9.689085e-01

8726 9.845418e-01 9.689033e-01

8727 9.845459e-01 9.688980e-01

8728 9.845498e-01 9.688927e-01

8729 9.845537e-01 9.688873e-01

8730 9.845575e-01 9.688818e-01

8731 9.845612e-01 9.688763e-01

8732 9.845649e-01 9.688707e-01

8733 9.845685e-01 9.688650e-01

8734 9.845721e-01 9.688592e-01

8735 9.845756e-01 9.688534e-01

8736 9.845790e-01 9.688476e-01

8737 9.845824e-01 9.688416e-01

8738 9.845857e-01 9.688356e-01

8739 9.845890e-01 9.688295e-01

8740 9.845922e-01 9.688234e-01

8741 9.845954e-01 9.688171e-01

8742 9.845986e-01 9.688108e-01

8743 9.846016e-01 9.688044e-01

8744 9.846047e-01 9.687980e-01

8745 9.846077e-01 9.687915e-01

8746 9.846107e-01 9.687849e-01

8747 9.846136e-01 9.687782e-01

8748 9.846165e-01 9.687715e-01

8749 9.846193e-01 9.687646e-01

8750 9.846222e-01 9.687577e-01

8751 9.846249e-01 9.687507e-01

8752 9.846277e-01 9.687437e-01

8753 9.846304e-01 9.687366e-01

8754 9.846331e-01 9.687293e-01

8755 9.846358e-01 9.687220e-01

8756 9.846385e-01 9.687147e-01

8757 9.846411e-01 9.687072e-01

8758 9.846437e-01 9.686997e-01

8759 9.846462e-01 9.686920e-01

8760 9.846488e-01 9.686843e-01

8761 9.846513e-01 9.686766e-01

8762 9.846538e-01 9.686687e-01

8763 9.846563e-01 9.686607e-01

8764 9.846588e-01 9.686527e-01

8765 9.846613e-01 9.686446e-01

8766 9.846637e-01 9.686364e-01

8767 9.846662e-01 9.686281e-01

8768 9.846686e-01 9.686197e-01

8769 9.846710e-01 9.686112e-01

8770 9.846734e-01 9.686027e-01

8771 9.846757e-01 9.685940e-01

8772 9.846781e-01 9.685853e-01

8773 9.846805e-01 9.685765e-01

8774 9.846828e-01 9.685676e-01

8775 9.846851e-01 9.685586e-01

8776 9.846875e-01 9.685495e-01

8777 9.846898e-01 9.685403e-01

8778 9.846921e-01 9.685310e-01

8779 9.846944e-01 9.685217e-01

8780 9.846967e-01 9.685122e-01

8781 9.846990e-01 9.685027e-01

8782 9.847013e-01 9.684931e-01

8783 9.847036e-01 9.684833e-01

8784 9.847059e-01 9.684735e-01

8785 9.847082e-01 9.684636e-01

8786 9.847105e-01 9.684536e-01

8787 9.847128e-01 9.684435e-01

8788 9.847151e-01 9.684333e-01

8789 9.847173e-01 9.684231e-01

8790 9.847196e-01 9.684127e-01

8791 9.847219e-01 9.684022e-01

8792 9.847242e-01 9.683916e-01

8793 9.847265e-01 9.683810e-01

8794 9.847288e-01 9.683702e-01

8795 9.847311e-01 9.683594e-01

8796 9.847334e-01 9.683484e-01

8797 9.847356e-01 9.683374e-01

8798 9.847379e-01 9.683263e-01

8799 9.847402e-01 9.683150e-01

8800 9.847426e-01 9.683037e-01

8801 9.847449e-01 9.682923e-01

8802 9.847472e-01 9.682808e-01

8803 9.847495e-01 9.682692e-01

8804 9.847518e-01 9.682574e-01

8805 9.847542e-01 9.682456e-01

8806 9.847565e-01 9.682337e-01

8807 9.847588e-01 9.682217e-01

8808 9.847612e-01 9.682097e-01

8809 9.847635e-01 9.681975e-01

8810 9.847659e-01 9.681852e-01

8811 9.847683e-01 9.681728e-01

8812 9.847706e-01 9.681603e-01

8813 9.847730e-01 9.681478e-01

8814 9.847754e-01 9.681351e-01

8815 9.847778e-01 9.681224e-01

8816 9.847802e-01 9.681095e-01

8817 9.847826e-01 9.680966e-01

8818 9.847851e-01 9.680835e-01

8819 9.847875e-01 9.680704e-01

8820 9.847899e-01 9.680572e-01

8821 9.847924e-01 9.680439e-01

8822 9.848202e-01 9.680421e-01

8823 9.848473e-01 9.680403e-01

8824 9.848738e-01 9.680385e-01

8825 9.848995e-01 9.680366e-01

8826 9.849246e-01 9.680348e-01

8827 9.849491e-01 9.680329e-01

8828 9.849729e-01 9.680309e-01

8829 9.849962e-01 9.680290e-01

8830 9.850188e-01 9.680270e-01

8831 9.850409e-01 9.680250e-01

8832 9.850624e-01 9.680229e-01

8833 9.850833e-01 9.680209e-01

8834 9.851038e-01 9.680188e-01

8835 9.851237e-01 9.680166e-01

8836 9.851430e-01 9.680145e-01

8837 9.851619e-01 9.680123e-01

8838 9.851803e-01 9.680101e-01

8839 9.851983e-01 9.680078e-01

8840 9.852157e-01 9.680055e-01

8841 9.852328e-01 9.680032e-01

8842 9.852494e-01 9.680009e-01

8843 9.852655e-01 9.679985e-01

8844 9.852813e-01 9.679961e-01

8845 9.852966e-01 9.679936e-01

8846 9.853116e-01 9.679911e-01

8847 9.853261e-01 9.679886e-01

8848 9.853403e-01 9.679861e-01

8849 9.853542e-01 9.679835e-01

8850 9.853677e-01 9.679809e-01

8851 9.853808e-01 9.679782e-01

8852 9.853936e-01 9.679755e-01

8853 9.854061e-01 9.679728e-01

8854 9.854182e-01 9.679700e-01

8855 9.854301e-01 9.679672e-01

8856 9.854416e-01 9.679644e-01

8857 9.854529e-01 9.679615e-01

8858 9.854639e-01 9.679586e-01

8859 9.854746e-01 9.679556e-01

8860 9.854850e-01 9.679526e-01

8861 9.854952e-01 9.679496e-01

8862 9.855051e-01 9.679465e-01

8863 9.855148e-01 9.679433e-01

8864 9.855242e-01 9.679402e-01

8865 9.855334e-01 9.679370e-01

8866 9.855423e-01 9.679337e-01

8867 9.855511e-01 9.679304e-01

8868 9.855596e-01 9.679271e-01

8869 9.855679e-01 9.679237e-01

8870 9.855760e-01 9.679203e-01

8871 9.855839e-01 9.679168e-01

8872 9.855916e-01 9.679133e-01

8873 9.855992e-01 9.679097e-01

8874 9.856065e-01 9.679061e-01

8875 9.856137e-01 9.679024e-01

8876 9.856207e-01 9.678987e-01

8877 9.856275e-01 9.678950e-01

8878 9.856342e-01 9.678912e-01

8879 9.856407e-01 9.678873e-01

8880 9.856471e-01 9.678834e-01

8881 9.856533e-01 9.678795e-01

8882 9.856594e-01 9.678755e-01

8883 9.856653e-01 9.678714e-01

8884 9.856711e-01 9.678673e-01

8885 9.856768e-01 9.678631e-01

8886 9.856823e-01 9.678589e-01

8887 9.856877e-01 9.678547e-01

8888 9.856930e-01 9.678503e-01

8889 9.856982e-01 9.678460e-01

8890 9.857033e-01 9.678415e-01

8891 9.857082e-01 9.678371e-01

8892 9.857130e-01 9.678325e-01

8893 9.857178e-01 9.678279e-01

8894 9.857224e-01 9.678233e-01

8895 9.857270e-01 9.678186e-01

8896 9.857314e-01 9.678138e-01

8897 9.857358e-01 9.678090e-01

8898 9.857400e-01 9.678041e-01

8899 9.857442e-01 9.677991e-01

8900 9.857483e-01 9.677941e-01

8901 9.857523e-01 9.677890e-01

8902 9.857563e-01 9.677839e-01

8903 9.857601e-01 9.677787e-01

8904 9.857639e-01 9.677735e-01

8905 9.857676e-01 9.677681e-01

8906 9.857713e-01 9.677628e-01

8907 9.857748e-01 9.677573e-01

8908 9.857784e-01 9.677518e-01

8909 9.857818e-01 9.677462e-01

8910 9.857852e-01 9.677406e-01

8911 9.857885e-01 9.677349e-01

8912 9.857918e-01 9.677291e-01

8913 9.857950e-01 9.677233e-01

8914 9.857982e-01 9.677173e-01

8915 9.858013e-01 9.677114e-01

8916 9.858044e-01 9.677053e-01

8917 9.858074e-01 9.676992e-01

8918 9.858104e-01 9.676930e-01

8919 9.858133e-01 9.676867e-01

8920 9.858162e-01 9.676804e-01

8921 9.858190e-01 9.676740e-01

8922 9.858218e-01 9.676675e-01

8923 9.858246e-01 9.676610e-01

8924 9.858273e-01 9.676543e-01

8925 9.858300e-01 9.676477e-01

8926 9.858327e-01 9.676409e-01

8927 9.858353e-01 9.676340e-01

8928 9.858379e-01 9.676271e-01

8929 9.858405e-01 9.676201e-01

8930 9.858430e-01 9.676130e-01

8931 9.858455e-01 9.676059e-01

8932 9.858480e-01 9.675987e-01

8933 9.858505e-01 9.675913e-01

8934 9.858529e-01 9.675840e-01

8935 9.858553e-01 9.675765e-01

8936 9.858577e-01 9.675689e-01

8937 9.858601e-01 9.675613e-01

8938 9.858624e-01 9.675536e-01

8939 9.858647e-01 9.675458e-01

8940 9.858670e-01 9.675380e-01

8941 9.858693e-01 9.675300e-01

8942 9.858716e-01 9.675220e-01

8943 9.858739e-01 9.675139e-01

8944 9.858761e-01 9.675057e-01

8945 9.858783e-01 9.674974e-01

8946 9.858805e-01 9.674890e-01

8947 9.858827e-01 9.674806e-01

8948 9.858849e-01 9.674720e-01

8949 9.858871e-01 9.674634e-01

8950 9.858893e-01 9.674547e-01

8951 9.858914e-01 9.674459e-01

8952 9.858936e-01 9.674370e-01

8953 9.858957e-01 9.674281e-01

8954 9.858978e-01 9.674190e-01

8955 9.859000e-01 9.674099e-01

8956 9.859021e-01 9.674006e-01

8957 9.859042e-01 9.673913e-01

8958 9.859063e-01 9.673819e-01

8959 9.859084e-01 9.673724e-01

8960 9.859105e-01 9.673628e-01

8961 9.859126e-01 9.673532e-01

8962 9.859146e-01 9.673434e-01

8963 9.859167e-01 9.673335e-01

8964 9.859188e-01 9.673236e-01

8965 9.859209e-01 9.673136e-01

8966 9.859230e-01 9.673034e-01

8967 9.859250e-01 9.672932e-01

8968 9.859271e-01 9.672829e-01

8969 9.859292e-01 9.672725e-01

8970 9.859313e-01 9.672620e-01

8971 9.859334e-01 9.672515e-01

8972 9.859354e-01 9.672408e-01

8973 9.859375e-01 9.672300e-01

8974 9.859396e-01 9.672192e-01

8975 9.859417e-01 9.672082e-01

8976 9.859438e-01 9.671972e-01

8977 9.859459e-01 9.671861e-01

8978 9.859480e-01 9.671749e-01

8979 9.859500e-01 9.671636e-01

8980 9.859522e-01 9.671521e-01

8981 9.859543e-01 9.671407e-01

8982 9.859564e-01 9.671291e-01

8983 9.859585e-01 9.671174e-01

8984 9.859606e-01 9.671056e-01

8985 9.859627e-01 9.670938e-01

8986 9.859649e-01 9.670818e-01

8987 9.859670e-01 9.670698e-01

8988 9.859692e-01 9.670576e-01

8989 9.859713e-01 9.670454e-01

8990 9.859735e-01 9.670331e-01

8991 9.859756e-01 9.670207e-01

8992 9.859778e-01 9.670081e-01

8993 9.859800e-01 9.669956e-01

8994 9.859822e-01 9.669829e-01

8995 9.859844e-01 9.669701e-01

8996 9.859866e-01 9.669572e-01

8997 9.859888e-01 9.669443e-01

8998 9.859911e-01 9.669312e-01

8999 9.859933e-01 9.669181e-01

9000 9.859956e-01 9.669049e-01

9001 9.859978e-01 9.668916e-01

9002 9.860220e-01 9.668897e-01

9003 9.860456e-01 9.668878e-01

9004 9.860686e-01 9.668859e-01

9005 9.860911e-01 9.668840e-01

9006 9.861129e-01 9.668820e-01

9007 9.861342e-01 9.668800e-01

9008 9.861549e-01 9.668780e-01

9009 9.861752e-01 9.668759e-01

9010 9.861949e-01 9.668739e-01

9011 9.862140e-01 9.668718e-01

9012 9.862327e-01 9.668696e-01

9013 9.862510e-01 9.668674e-01

9014 9.862687e-01 9.668652e-01

9015 9.862860e-01 9.668630e-01

9016 9.863029e-01 9.668607e-01

9017 9.863193e-01 9.668585e-01

9018 9.863353e-01 9.668561e-01

9019 9.863509e-01 9.668538e-01

9020 9.863661e-01 9.668514e-01

9021 9.863809e-01 9.668490e-01

9022 9.863954e-01 9.668465e-01

9023 9.864094e-01 9.668440e-01

9024 9.864231e-01 9.668415e-01

9025 9.864365e-01 9.668389e-01

9026 9.864495e-01 9.668363e-01

9027 9.864622e-01 9.668337e-01

9028 9.864746e-01 9.668310e-01

9029 9.864866e-01 9.668283e-01

9030 9.864984e-01 9.668256e-01

9031 9.865098e-01 9.668228e-01

9032 9.865209e-01 9.668200e-01

9033 9.865318e-01 9.668172e-01

9034 9.865424e-01 9.668143e-01

9035 9.865527e-01 9.668113e-01

9036 9.865628e-01 9.668084e-01

9037 9.865726e-01 9.668054e-01

9038 9.865822e-01 9.668023e-01

9039 9.865915e-01 9.667992e-01

9040 9.866006e-01 9.667961e-01

9041 9.866095e-01 9.667929e-01

9042 9.866182e-01 9.667897e-01

9043 9.866266e-01 9.667865e-01

9044 9.866348e-01 9.667832e-01

9045 9.866429e-01 9.667798e-01

9046 9.866507e-01 9.667765e-01

9047 9.866583e-01 9.667730e-01

9048 9.866658e-01 9.667696e-01

9049 9.866731e-01 9.667660e-01

9050 9.866802e-01 9.667625e-01

9051 9.866871e-01 9.667589e-01

9052 9.866939e-01 9.667552e-01

9053 9.867005e-01 9.667515e-01

9054 9.867069e-01 9.667478e-01

9055 9.867132e-01 9.667440e-01

9056 9.867193e-01 9.667401e-01

9057 9.867253e-01 9.667362e-01

9058 9.867312e-01 9.667323e-01

9059 9.867369e-01 9.667283e-01

9060 9.867425e-01 9.667243e-01

9061 9.867480e-01 9.667202e-01

9062 9.867533e-01 9.667160e-01

9063 9.867585e-01 9.667118e-01

9064 9.867636e-01 9.667076e-01

9065 9.867686e-01 9.667033e-01

9066 9.867735e-01 9.666989e-01

9067 9.867782e-01 9.666945e-01

9068 9.867829e-01 9.666900e-01

9069 9.867875e-01 9.666855e-01

9070 9.867919e-01 9.666809e-01

9071 9.867963e-01 9.666763e-01

9072 9.868006e-01 9.666716e-01

9073 9.868047e-01 9.666669e-01

9074 9.868088e-01 9.666621e-01

9075 9.868129e-01 9.666572e-01

9076 9.868168e-01 9.666523e-01

9077 9.868206e-01 9.666473e-01

9078 9.868244e-01 9.666423e-01

9079 9.868281e-01 9.666372e-01

9080 9.868318e-01 9.666320e-01

9081 9.868353e-01 9.666268e-01

9082 9.868388e-01 9.666215e-01

9083 9.868422e-01 9.666162e-01

9084 9.868456e-01 9.666108e-01

9085 9.868489e-01 9.666053e-01

9086 9.868521e-01 9.665997e-01

9087 9.868553e-01 9.665941e-01

9088 9.868585e-01 9.665885e-01

9089 9.868615e-01 9.665828e-01

9090 9.868646e-01 9.665770e-01

9091 9.868675e-01 9.665711e-01

9092 9.868704e-01 9.665652e-01

9093 9.868733e-01 9.665592e-01

9094 9.868762e-01 9.665531e-01

9095 9.868789e-01 9.665470e-01

9096 9.868817e-01 9.665408e-01

9097 9.868844e-01 9.665345e-01

9098 9.868870e-01 9.665282e-01

9099 9.868897e-01 9.665218e-01

9100 9.868923e-01 9.665153e-01

9101 9.868948e-01 9.665087e-01

9102 9.868973e-01 9.665021e-01

9103 9.868998e-01 9.664954e-01

9104 9.869023e-01 9.664886e-01

9105 9.869047e-01 9.664818e-01

9106 9.869071e-01 9.664749e-01

9107 9.869094e-01 9.664679e-01

9108 9.869118e-01 9.664608e-01

9109 9.869141e-01 9.664536e-01

9110 9.869164e-01 9.664464e-01

9111 9.869186e-01 9.664391e-01

9112 9.869209e-01 9.664318e-01

9113 9.869231e-01 9.664243e-01

9114 9.869253e-01 9.664168e-01

9115 9.869275e-01 9.664092e-01

9116 9.869296e-01 9.664015e-01

9117 9.869317e-01 9.663937e-01

9118 9.869339e-01 9.663859e-01

9119 9.869360e-01 9.663780e-01

9120 9.869381e-01 9.663700e-01

9121 9.869401e-01 9.663619e-01

9122 9.869422e-01 9.663537e-01

9123 9.869442e-01 9.663455e-01

9124 9.869463e-01 9.663372e-01

9125 9.869483e-01 9.663287e-01

9126 9.869503e-01 9.663203e-01

9127 9.869523e-01 9.663117e-01

9128 9.869543e-01 9.663030e-01

9129 9.869562e-01 9.662943e-01

9130 9.869582e-01 9.662855e-01

9131 9.869602e-01 9.662766e-01

9132 9.869621e-01 9.662676e-01

9133 9.869641e-01 9.662585e-01

9134 9.869660e-01 9.662493e-01

9135 9.869679e-01 9.662401e-01

9136 9.869699e-01 9.662307e-01

9137 9.869718e-01 9.662213e-01

9138 9.869737e-01 9.662118e-01

9139 9.869756e-01 9.662022e-01

9140 9.869775e-01 9.661926e-01

9141 9.869795e-01 9.661828e-01

9142 9.869814e-01 9.661729e-01

9143 9.869833e-01 9.661630e-01

9144 9.869852e-01 9.661530e-01

9145 9.869871e-01 9.661429e-01

9146 9.869890e-01 9.661327e-01

9147 9.869909e-01 9.661224e-01

9148 9.869928e-01 9.661120e-01

9149 9.869947e-01 9.661015e-01

9150 9.869966e-01 9.660910e-01

9151 9.869985e-01 9.660803e-01

9152 9.870004e-01 9.660696e-01

9153 9.870023e-01 9.660588e-01

9154 9.870043e-01 9.660479e-01

9155 9.870062e-01 9.660369e-01

9156 9.870081e-01 9.660258e-01

9157 9.870100e-01 9.660147e-01

9158 9.870120e-01 9.660034e-01

9159 9.870139e-01 9.659921e-01

9160 9.870159e-01 9.659806e-01

9161 9.870178e-01 9.659691e-01

9162 9.870198e-01 9.659575e-01

9163 9.870217e-01 9.659458e-01

9164 9.870237e-01 9.659340e-01

9165 9.870257e-01 9.659221e-01

9166 9.870277e-01 9.659102e-01

9167 9.870297e-01 9.658981e-01

9168 9.870317e-01 9.658860e-01

9169 9.870337e-01 9.658737e-01

9170 9.870357e-01 9.658614e-01

9171 9.870377e-01 9.658490e-01

9172 9.870398e-01 9.658365e-01

9173 9.870418e-01 9.658240e-01

9174 9.870439e-01 9.658113e-01

9175 9.870459e-01 9.657986e-01

9176 9.870480e-01 9.657857e-01

9177 9.870501e-01 9.657728e-01

9178 9.870522e-01 9.657598e-01

9179 9.870543e-01 9.657467e-01

9180 9.870564e-01 9.657336e-01

9181 9.870586e-01 9.657203e-01

9182 9.870796e-01 9.657184e-01

9183 9.871002e-01 9.657164e-01

9184 9.871202e-01 9.657144e-01

9185 9.871397e-01 9.657124e-01

9186 9.871587e-01 9.657103e-01

9187 9.871772e-01 9.657082e-01

9188 9.871953e-01 9.657061e-01

9189 9.872129e-01 9.657039e-01

9190 9.872300e-01 9.657018e-01

9191 9.872467e-01 9.656996e-01

9192 9.872630e-01 9.656973e-01

9193 9.872789e-01 9.656951e-01

9194 9.872943e-01 9.656928e-01

9195 9.873094e-01 9.656904e-01

9196 9.873241e-01 9.656881e-01

9197 9.873384e-01 9.656857e-01

9198 9.873523e-01 9.656833e-01

9199 9.873659e-01 9.656808e-01

9200 9.873791e-01 9.656783e-01

9201 9.873920e-01 9.656758e-01

9202 9.874046e-01 9.656732e-01

9203 9.874169e-01 9.656706e-01

9204 9.874288e-01 9.656680e-01

9205 9.874405e-01 9.656653e-01

9206 9.874518e-01 9.656626e-01

9207 9.874629e-01 9.656599e-01

9208 9.874736e-01 9.656571e-01

9209 9.874841e-01 9.656543e-01

9210 9.874944e-01 9.656515e-01

9211 9.875044e-01 9.656486e-01

9212 9.875141e-01 9.656457e-01

9213 9.875236e-01 9.656427e-01

9214 9.875328e-01 9.656397e-01

9215 9.875419e-01 9.656367e-01

9216 9.875507e-01 9.656336e-01

9217 9.875592e-01 9.656305e-01

9218 9.875676e-01 9.656273e-01

9219 9.875758e-01 9.656241e-01

9220 9.875837e-01 9.656209e-01

9221 9.875915e-01 9.656176e-01

9222 9.875990e-01 9.656142e-01

9223 9.876064e-01 9.656109e-01

9224 9.876136e-01 9.656075e-01

9225 9.876207e-01 9.656040e-01

9226 9.876275e-01 9.656005e-01

9227 9.876342e-01 9.655970e-01

9228 9.876408e-01 9.655934e-01

9229 9.876471e-01 9.655897e-01

9230 9.876534e-01 9.655860e-01

9231 9.876594e-01 9.655823e-01

9232 9.876654e-01 9.655785e-01

9233 9.876712e-01 9.655747e-01

9234 9.876768e-01 9.655708e-01

9235 9.876824e-01 9.655669e-01

9236 9.876878e-01 9.655629e-01

9237 9.876930e-01 9.655589e-01

9238 9.876982e-01 9.655548e-01

9239 9.877032e-01 9.655507e-01

9240 9.877081e-01 9.655466e-01

9241 9.877129e-01 9.655423e-01

9242 9.877176e-01 9.655381e-01

9243 9.877222e-01 9.655337e-01

9244 9.877267e-01 9.655294e-01

9245 9.877311e-01 9.655249e-01

9246 9.877354e-01 9.655204e-01

9247 9.877397e-01 9.655159e-01

9248 9.877438e-01 9.655113e-01

9249 9.877478e-01 9.655066e-01

9250 9.877518e-01 9.655019e-01

9251 9.877556e-01 9.654972e-01

9252 9.877594e-01 9.654924e-01

9253 9.877631e-01 9.654875e-01

9254 9.877667e-01 9.654825e-01

9255 9.877703e-01 9.654776e-01

9256 9.877738e-01 9.654725e-01

9257 9.877772e-01 9.654674e-01

9258 9.877806e-01 9.654622e-01

9259 9.877839e-01 9.654570e-01

9260 9.877871e-01 9.654517e-01

9261 9.877902e-01 9.654463e-01

9262 9.877934e-01 9.654409e-01

9263 9.877964e-01 9.654355e-01

9264 9.877994e-01 9.654299e-01

9265 9.878023e-01 9.654243e-01

9266 9.878052e-01 9.654187e-01

9267 9.878081e-01 9.654129e-01

9268 9.878109e-01 9.654071e-01

9269 9.878136e-01 9.654013e-01

9270 9.878163e-01 9.653953e-01

9271 9.878190e-01 9.653894e-01

9272 9.878216e-01 9.653833e-01

9273 9.878242e-01 9.653772e-01

9274 9.878267e-01 9.653710e-01

9275 9.878292e-01 9.653647e-01

9276 9.878316e-01 9.653584e-01

9277 9.878341e-01 9.653520e-01

9278 9.878365e-01 9.653455e-01

9279 9.878388e-01 9.653390e-01

9280 9.878411e-01 9.653324e-01

9281 9.878434e-01 9.653257e-01

9282 9.878457e-01 9.653190e-01

9283 9.878479e-01 9.653121e-01

9284 9.878501e-01 9.653053e-01

9285 9.878523e-01 9.652983e-01

9286 9.878545e-01 9.652913e-01

9287 9.878566e-01 9.652841e-01

9288 9.878587e-01 9.652770e-01

9289 9.878608e-01 9.652697e-01

9290 9.878629e-01 9.652624e-01

9291 9.878649e-01 9.652550e-01

9292 9.878670e-01 9.652475e-01

9293 9.878690e-01 9.652399e-01

9294 9.878710e-01 9.652323e-01

9295 9.878729e-01 9.652246e-01

9296 9.878749e-01 9.652168e-01

9297 9.878768e-01 9.652089e-01

9298 9.878788e-01 9.652010e-01

9299 9.878807e-01 9.651930e-01

9300 9.878826e-01 9.651849e-01

9301 9.878844e-01 9.651767e-01

9302 9.878863e-01 9.651684e-01

9303 9.878882e-01 9.651601e-01

9304 9.878900e-01 9.651517e-01

9305 9.878919e-01 9.651432e-01

9306 9.878937e-01 9.651346e-01

9307 9.878955e-01 9.651259e-01

9308 9.878974e-01 9.651172e-01

9309 9.878992e-01 9.651084e-01

9310 9.879010e-01 9.650995e-01

9311 9.879028e-01 9.650905e-01

9312 9.879046e-01 9.650814e-01

9313 9.879063e-01 9.650723e-01

9314 9.879081e-01 9.650630e-01

9315 9.879099e-01 9.650537e-01

9316 9.879117e-01 9.650443e-01

9317 9.879134e-01 9.650348e-01

9318 9.879152e-01 9.650253e-01

9319 9.879170e-01 9.650156e-01

9320 9.879187e-01 9.650059e-01

9321 9.879205e-01 9.649961e-01

9322 9.879223e-01 9.649862e-01

9323 9.879240e-01 9.649762e-01

9324 9.879258e-01 9.649661e-01

9325 9.879276e-01 9.649560e-01

9326 9.879293e-01 9.649458e-01

9327 9.879311e-01 9.649354e-01

9328 9.879329e-01 9.649250e-01

9329 9.879346e-01 9.649145e-01

9330 9.879364e-01 9.649040e-01

9331 9.879382e-01 9.648933e-01

9332 9.879400e-01 9.648826e-01

9333 9.879417e-01 9.648717e-01

9334 9.879435e-01 9.648608e-01

9335 9.879453e-01 9.648498e-01

9336 9.879471e-01 9.648387e-01

9337 9.879489e-01 9.648276e-01

9338 9.879507e-01 9.648163e-01

9339 9.879525e-01 9.648050e-01

9340 9.879544e-01 9.647935e-01

9341 9.879562e-01 9.647820e-01

9342 9.879580e-01 9.647704e-01

9343 9.879599e-01 9.647588e-01

9344 9.879617e-01 9.647470e-01

9345 9.879636e-01 9.647352e-01

9346 9.879655e-01 9.647232e-01

9347 9.879673e-01 9.647112e-01

9348 9.879692e-01 9.646991e-01

9349 9.879711e-01 9.646870e-01

9350 9.879730e-01 9.646747e-01

9351 9.879749e-01 9.646623e-01

9352 9.879769e-01 9.646499e-01

9353 9.879788e-01 9.646374e-01

9354 9.879807e-01 9.646248e-01

9355 9.879827e-01 9.646121e-01

9356 9.879847e-01 9.645994e-01

9357 9.879866e-01 9.645866e-01

9358 9.879886e-01 9.645736e-01

9359 9.879906e-01 9.645606e-01

9360 9.879926e-01 9.645476e-01

9361 9.879947e-01 9.645344e-01

9362 9.880130e-01 9.645324e-01

9363 9.880309e-01 9.645303e-01

9364 9.880484e-01 9.645282e-01

9365 9.880653e-01 9.645261e-01

9366 9.880819e-01 9.645240e-01

9367 9.880980e-01 9.645218e-01

9368 9.881138e-01 9.645196e-01

9369 9.881291e-01 9.645174e-01

9370 9.881440e-01 9.645151e-01

9371 9.881586e-01 9.645128e-01

9372 9.881728e-01 9.645105e-01

9373 9.881866e-01 9.645081e-01

9374 9.882001e-01 9.645058e-01

9375 9.882132e-01 9.645033e-01

9376 9.882260e-01 9.645009e-01

9377 9.882385e-01 9.644984e-01

9378 9.882506e-01 9.644959e-01

9379 9.882625e-01 9.644933e-01

9380 9.882740e-01 9.644907e-01

9381 9.882853e-01 9.644881e-01

9382 9.882962e-01 9.644855e-01

9383 9.883069e-01 9.644828e-01

9384 9.883173e-01 9.644800e-01

9385 9.883275e-01 9.644773e-01

9386 9.883374e-01 9.644745e-01

9387 9.883471e-01 9.644716e-01

9388 9.883565e-01 9.644688e-01

9389 9.883657e-01 9.644659e-01

9390 9.883746e-01 9.644629e-01

9391 9.883834e-01 9.644599e-01

9392 9.883919e-01 9.644569e-01

9393 9.884002e-01 9.644538e-01

9394 9.884083e-01 9.644507e-01

9395 9.884162e-01 9.644476e-01

9396 9.884239e-01 9.644444e-01

9397 9.884314e-01 9.644412e-01

9398 9.884387e-01 9.644379e-01

9399 9.884458e-01 9.644346e-01

9400 9.884528e-01 9.644313e-01

9401 9.884596e-01 9.644279e-01

9402 9.884663e-01 9.644244e-01

9403 9.884727e-01 9.644210e-01

9404 9.884791e-01 9.644174e-01

9405 9.884852e-01 9.644139e-01

9406 9.884912e-01 9.644103e-01

9407 9.884971e-01 9.644066e-01

9408 9.885029e-01 9.644029e-01

9409 9.885085e-01 9.643992e-01

9410 9.885139e-01 9.643954e-01

9411 9.885193e-01 9.643915e-01

9412 9.885245e-01 9.643876e-01

9413 9.885296e-01 9.643837e-01

9414 9.885346e-01 9.643797e-01

9415 9.885395e-01 9.643757e-01

9416 9.885442e-01 9.643716e-01

9417 9.885489e-01 9.643675e-01

9418 9.885534e-01 9.643633e-01

9419 9.885579e-01 9.643591e-01

9420 9.885622e-01 9.643548e-01

9421 9.885665e-01 9.643504e-01

9422 9.885706e-01 9.643460e-01

9423 9.885747e-01 9.643416e-01

9424 9.885787e-01 9.643371e-01

9425 9.885826e-01 9.643326e-01

9426 9.885864e-01 9.643280e-01

9427 9.885901e-01 9.643233e-01

9428 9.885937e-01 9.643186e-01

9429 9.885973e-01 9.643138e-01

9430 9.886008e-01 9.643090e-01

9431 9.886042e-01 9.643041e-01

9432 9.886076e-01 9.642992e-01

9433 9.886109e-01 9.642942e-01

9434 9.886141e-01 9.642892e-01

9435 9.886173e-01 9.642841e-01

9436 9.886204e-01 9.642789e-01

9437 9.886234e-01 9.642737e-01

9438 9.886264e-01 9.642684e-01

9439 9.886294e-01 9.642631e-01

9440 9.886322e-01 9.642577e-01

9441 9.886351e-01 9.642522e-01

9442 9.886378e-01 9.642467e-01

9443 9.886406e-01 9.642411e-01

9444 9.886432e-01 9.642354e-01

9445 9.886459e-01 9.642297e-01

9446 9.886485e-01 9.642240e-01

9447 9.886510e-01 9.642181e-01

9448 9.886535e-01 9.642122e-01

9449 9.886560e-01 9.642063e-01

9450 9.886584e-01 9.642002e-01

9451 9.886608e-01 9.641941e-01

9452 9.886631e-01 9.641880e-01

9453 9.886654e-01 9.641817e-01

9454 9.886677e-01 9.641754e-01

9455 9.886700e-01 9.641691e-01

9456 9.886722e-01 9.641627e-01

9457 9.886744e-01 9.641562e-01

9458 9.886765e-01 9.641496e-01

9459 9.886786e-01 9.641430e-01

9460 9.886807e-01 9.641363e-01

9461 9.886828e-01 9.641295e-01

9462 9.886849e-01 9.641226e-01

9463 9.886869e-01 9.641157e-01

9464 9.886889e-01 9.641088e-01

9465 9.886909e-01 9.641017e-01

9466 9.886928e-01 9.640946e-01

9467 9.886948e-01 9.640874e-01

9468 9.886967e-01 9.640801e-01

9469 9.886986e-01 9.640728e-01

9470 9.887004e-01 9.640653e-01

9471 9.887023e-01 9.640579e-01

9472 9.887041e-01 9.640503e-01

9473 9.887060e-01 9.640427e-01

9474 9.887078e-01 9.640349e-01

9475 9.887096e-01 9.640272e-01

9476 9.887114e-01 9.640193e-01

9477 9.887131e-01 9.640114e-01

9478 9.887149e-01 9.640033e-01

9479 9.887167e-01 9.639953e-01

9480 9.887184e-01 9.639871e-01

9481 9.887201e-01 9.639789e-01

9482 9.887218e-01 9.639705e-01

9483 9.887235e-01 9.639621e-01

9484 9.887252e-01 9.639537e-01

9485 9.887269e-01 9.639451e-01

9486 9.887286e-01 9.639365e-01

9487 9.887303e-01 9.639278e-01

9488 9.887320e-01 9.639190e-01

9489 9.887336e-01 9.639101e-01

9490 9.887353e-01 9.639012e-01

9491 9.887370e-01 9.638921e-01

9492 9.887386e-01 9.638830e-01

9493 9.887403e-01 9.638739e-01

9494 9.887419e-01 9.638646e-01

9495 9.887436e-01 9.638553e-01

9496 9.887452e-01 9.638458e-01

9497 9.887468e-01 9.638363e-01

9498 9.887485e-01 9.638267e-01

9499 9.887501e-01 9.638171e-01

9500 9.887517e-01 9.638073e-01

9501 9.887534e-01 9.637975e-01

9502 9.887550e-01 9.637876e-01

9503 9.887567e-01 9.637776e-01

9504 9.887583e-01 9.637675e-01

9505 9.887599e-01 9.637574e-01

9506 9.887616e-01 9.637472e-01

9507 9.887632e-01 9.637368e-01

9508 9.887649e-01 9.637264e-01

9509 9.887665e-01 9.637160e-01

9510 9.887682e-01 9.637054e-01

9511 9.887698e-01 9.636948e-01

9512 9.887715e-01 9.636840e-01

9513 9.887732e-01 9.636732e-01

9514 9.887748e-01 9.636624e-01

9515 9.887765e-01 9.636514e-01

9516 9.887782e-01 9.636404e-01

9517 9.887799e-01 9.636292e-01

9518 9.887816e-01 9.636180e-01

9519 9.887833e-01 9.636067e-01

9520 9.887850e-01 9.635954e-01

9521 9.887867e-01 9.635839e-01

9522 9.887884e-01 9.635724e-01

9523 9.887902e-01 9.635608e-01

9524 9.887919e-01 9.635491e-01

9525 9.887936e-01 9.635373e-01

9526 9.887954e-01 9.635254e-01

9527 9.887972e-01 9.635135e-01

9528 9.887989e-01 9.635015e-01

9529 9.888007e-01 9.634894e-01

9530 9.888025e-01 9.634772e-01

9531 9.888043e-01 9.634650e-01

9532 9.888061e-01 9.634527e-01

9533 9.888079e-01 9.634403e-01

9534 9.888097e-01 9.634278e-01

9535 9.888116e-01 9.634152e-01

9536 9.888134e-01 9.634026e-01

9537 9.888153e-01 9.633899e-01

9538 9.888172e-01 9.633771e-01

9539 9.888190e-01 9.633642e-01

9540 9.888209e-01 9.633513e-01

9541 9.888228e-01 9.633383e-01

9542 9.888388e-01 9.633362e-01

9543 9.888544e-01 9.633340e-01

9544 9.888696e-01 9.633319e-01

9545 9.888844e-01 9.633297e-01

9546 9.888989e-01 9.633275e-01

9547 9.889129e-01 9.633252e-01

9548 9.889267e-01 9.633229e-01

9549 9.889400e-01 9.633206e-01

9550 9.889531e-01 9.633183e-01

9551 9.889658e-01 9.633159e-01

9552 9.889781e-01 9.633135e-01

9553 9.889902e-01 9.633111e-01

9554 9.890020e-01 9.633086e-01

9555 9.890134e-01 9.633061e-01

9556 9.890246e-01 9.633035e-01

9557 9.890355e-01 9.633010e-01

9558 9.890461e-01 9.632984e-01

9559 9.890565e-01 9.632957e-01

9560 9.890666e-01 9.632931e-01

9561 9.890764e-01 9.632903e-01

9562 9.890860e-01 9.632876e-01

9563 9.890953e-01 9.632848e-01

9564 9.891045e-01 9.632820e-01

9565 9.891133e-01 9.632792e-01

9566 9.891220e-01 9.632763e-01

9567 9.891305e-01 9.632733e-01

9568 9.891387e-01 9.632704e-01

9569 9.891468e-01 9.632674e-01

9570 9.891546e-01 9.632643e-01

9571 9.891622e-01 9.632613e-01

9572 9.891697e-01 9.632582e-01

9573 9.891770e-01 9.632550e-01

9574 9.891841e-01 9.632518e-01

9575 9.891910e-01 9.632486e-01

9576 9.891978e-01 9.632453e-01

9577 9.892043e-01 9.632420e-01

9578 9.892108e-01 9.632386e-01

9579 9.892171e-01 9.632352e-01

9580 9.892232e-01 9.632318e-01

9581 9.892292e-01 9.632283e-01

9582 9.892350e-01 9.632248e-01

9583 9.892407e-01 9.632212e-01

9584 9.892463e-01 9.632176e-01

9585 9.892517e-01 9.632139e-01

9586 9.892570e-01 9.632102e-01

9587 9.892622e-01 9.632064e-01

9588 9.892672e-01 9.632026e-01

9589 9.892722e-01 9.631988e-01

9590 9.892770e-01 9.631949e-01

9591 9.892817e-01 9.631910e-01

9592 9.892863e-01 9.631870e-01

9593 9.892908e-01 9.631830e-01

9594 9.892952e-01 9.631789e-01

9595 9.892995e-01 9.631747e-01

9596 9.893037e-01 9.631706e-01

9597 9.893079e-01 9.631663e-01

9598 9.893119e-01 9.631621e-01

9599 9.893158e-01 9.631577e-01

9600 9.893197e-01 9.631534e-01

9601 9.893234e-01 9.631489e-01

9602 9.893271e-01 9.631444e-01

9603 9.893307e-01 9.631399e-01

9604 9.893342e-01 9.631353e-01

9605 9.893377e-01 9.631307e-01

9606 9.893411e-01 9.631260e-01

9607 9.893444e-01 9.631212e-01

9608 9.893476e-01 9.631164e-01

9609 9.893508e-01 9.631116e-01

9610 9.893539e-01 9.631067e-01

9611 9.893570e-01 9.631017e-01

9612 9.893600e-01 9.630967e-01

9613 9.893629e-01 9.630916e-01

9614 9.893658e-01 9.630864e-01

9615 9.893686e-01 9.630813e-01

9616 9.893714e-01 9.630760e-01

9617 9.893741e-01 9.630707e-01

9618 9.893768e-01 9.630653e-01

9619 9.893794e-01 9.630599e-01

9620 9.893820e-01 9.630544e-01

9621 9.893845e-01 9.630488e-01

9622 9.893870e-01 9.630432e-01

9623 9.893894e-01 9.630376e-01

9624 9.893919e-01 9.630318e-01

9625 9.893942e-01 9.630260e-01

9626 9.893965e-01 9.630202e-01

9627 9.893988e-01 9.630143e-01

9628 9.894011e-01 9.630083e-01

9629 9.894033e-01 9.630022e-01

9630 9.894055e-01 9.629961e-01

9631 9.894076e-01 9.629899e-01

9632 9.894097e-01 9.629837e-01

9633 9.894118e-01 9.629774e-01

9634 9.894139e-01 9.629710e-01

9635 9.894159e-01 9.629646e-01

9636 9.894179e-01 9.629581e-01

9637 9.894199e-01 9.629515e-01

9638 9.894218e-01 9.629449e-01

9639 9.894238e-01 9.629382e-01

9640 9.894257e-01 9.629314e-01

9641 9.894275e-01 9.629246e-01

9642 9.894294e-01 9.629177e-01

9643 9.894312e-01 9.629107e-01

9644 9.894331e-01 9.629037e-01

9645 9.894349e-01 9.628966e-01

9646 9.894366e-01 9.628894e-01

9647 9.894384e-01 9.628821e-01

9648 9.894401e-01 9.628748e-01

9649 9.894419e-01 9.628674e-01

9650 9.894436e-01 9.628599e-01

9651 9.894453e-01 9.628524e-01

9652 9.894470e-01 9.628448e-01

9653 9.894486e-01 9.628371e-01

9654 9.894503e-01 9.628293e-01

9655 9.894520e-01 9.628215e-01

9656 9.894536e-01 9.628136e-01

9657 9.894552e-01 9.628056e-01

9658 9.894568e-01 9.627976e-01

9659 9.894584e-01 9.627895e-01

9660 9.894600e-01 9.627813e-01

9661 9.894616e-01 9.627730e-01

9662 9.894632e-01 9.627647e-01

9663 9.894648e-01 9.627562e-01

9664 9.894663e-01 9.627477e-01

9665 9.894679e-01 9.627392e-01

9666 9.894694e-01 9.627305e-01

9667 9.894710e-01 9.627218e-01

9668 9.894725e-01 9.627130e-01

9669 9.894741e-01 9.627041e-01

9670 9.894756e-01 9.626952e-01

9671 9.894771e-01 9.626861e-01

9672 9.894787e-01 9.626770e-01

9673 9.894802e-01 9.626679e-01

9674 9.894817e-01 9.626586e-01

9675 9.894832e-01 9.626493e-01

9676 9.894848e-01 9.626399e-01

9677 9.894863e-01 9.626304e-01

9678 9.894878e-01 9.626208e-01

9679 9.894893e-01 9.626112e-01

9680 9.894908e-01 9.626014e-01

9681 9.894924e-01 9.625916e-01

9682 9.894939e-01 9.625818e-01

9683 9.894954e-01 9.625718e-01

9684 9.894969e-01 9.625618e-01

9685 9.894984e-01 9.625517e-01

9686 9.895000e-01 9.625415e-01

9687 9.895015e-01 9.625312e-01

9688 9.895030e-01 9.625209e-01

9689 9.895046e-01 9.625104e-01

9690 9.895061e-01 9.624999e-01

9691 9.895076e-01 9.624894e-01

9692 9.895092e-01 9.624787e-01

9693 9.895107e-01 9.624680e-01

9694 9.895123e-01 9.624572e-01

9695 9.895139e-01 9.624463e-01

9696 9.895154e-01 9.624353e-01

9697 9.895170e-01 9.624243e-01

9698 9.895186e-01 9.624131e-01

9699 9.895201e-01 9.624019e-01

9700 9.895217e-01 9.623907e-01

9701 9.895233e-01 9.623793e-01

9702 9.895249e-01 9.623679e-01

9703 9.895265e-01 9.623564e-01

9704 9.895281e-01 9.623448e-01

9705 9.895298e-01 9.623332e-01

9706 9.895314e-01 9.623214e-01

9707 9.895330e-01 9.623096e-01

9708 9.895347e-01 9.622977e-01

9709 9.895363e-01 9.622858e-01

9710 9.895380e-01 9.622738e-01

9711 9.895396e-01 9.622616e-01

9712 9.895413e-01 9.622495e-01

9713 9.895430e-01 9.622372e-01

9714 9.895447e-01 9.622249e-01

9715 9.895464e-01 9.622125e-01

9716 9.895481e-01 9.622000e-01

9717 9.895498e-01 9.621875e-01

9718 9.895515e-01 9.621749e-01

9719 9.895533e-01 9.621622e-01

9720 9.895550e-01 9.621494e-01

9721 9.895568e-01 9.621366e-01

9722 9.895707e-01 9.621344e-01

9723 9.895843e-01 9.621322e-01

9724 9.895976e-01 9.621300e-01

9725 9.896106e-01 9.621277e-01

9726 9.896232e-01 9.621254e-01

9727 9.896355e-01 9.621231e-01

9728 9.896474e-01 9.621208e-01

9729 9.896591e-01 9.621184e-01

9730 9.896705e-01 9.621159e-01

9731 9.896816e-01 9.621135e-01

9732 9.896924e-01 9.621110e-01

9733 9.897030e-01 9.621085e-01

9734 9.897133e-01 9.621060e-01

9735 9.897233e-01 9.621034e-01

9736 9.897331e-01 9.621008e-01

9737 9.897426e-01 9.620981e-01

9738 9.897519e-01 9.620954e-01

9739 9.897610e-01 9.620927e-01

9740 9.897698e-01 9.620900e-01

9741 9.897784e-01 9.620872e-01

9742 9.897868e-01 9.620844e-01

9743 9.897950e-01 9.620815e-01

9744 9.898030e-01 9.620786e-01

9745 9.898108e-01 9.620757e-01

9746 9.898184e-01 9.620727e-01

9747 9.898258e-01 9.620697e-01

9748 9.898331e-01 9.620667e-01

9749 9.898401e-01 9.620636e-01

9750 9.898470e-01 9.620605e-01

9751 9.898537e-01 9.620573e-01

9752 9.898603e-01 9.620541e-01

9753 9.898667e-01 9.620509e-01

9754 9.898729e-01 9.620476e-01

9755 9.898790e-01 9.620443e-01

9756 9.898850e-01 9.620409e-01

9757 9.898908e-01 9.620375e-01

9758 9.898964e-01 9.620341e-01

9759 9.899019e-01 9.620306e-01

9760 9.899073e-01 9.620271e-01

9761 9.899126e-01 9.620235e-01

9762 9.899178e-01 9.620199e-01

9763 9.899228e-01 9.620163e-01

9764 9.899277e-01 9.620126e-01

9765 9.899325e-01 9.620088e-01

9766 9.899372e-01 9.620050e-01

9767 9.899417e-01 9.620012e-01

9768 9.899462e-01 9.619973e-01

9769 9.899506e-01 9.619934e-01

9770 9.899549e-01 9.619894e-01

9771 9.899590e-01 9.619854e-01

9772 9.899631e-01 9.619814e-01

9773 9.899671e-01 9.619772e-01

9774 9.899710e-01 9.619731e-01

9775 9.899748e-01 9.619689e-01

9776 9.899785e-01 9.619646e-01

9777 9.899822e-01 9.619603e-01

9778 9.899858e-01 9.619560e-01

9779 9.899893e-01 9.619515e-01

9780 9.899927e-01 9.619471e-01

9781 9.899960e-01 9.619426e-01

9782 9.899993e-01 9.619380e-01

9783 9.900025e-01 9.619334e-01

9784 9.900056e-01 9.619287e-01

9785 9.900087e-01 9.619240e-01

9786 9.900117e-01 9.619193e-01

9787 9.900147e-01 9.619144e-01

9788 9.900176e-01 9.619096e-01

9789 9.900204e-01 9.619046e-01

9790 9.900232e-01 9.618996e-01

9791 9.900259e-01 9.618946e-01

9792 9.900286e-01 9.618895e-01

9793 9.900312e-01 9.618844e-01

9794 9.900338e-01 9.618791e-01

9795 9.900363e-01 9.618739e-01

9796 9.900388e-01 9.618686e-01

9797 9.900413e-01 9.618632e-01

9798 9.900437e-01 9.618577e-01

9799 9.900460e-01 9.618522e-01

9800 9.900483e-01 9.618467e-01

9801 9.900506e-01 9.618411e-01

9802 9.900528e-01 9.618354e-01

9803 9.900550e-01 9.618297e-01

9804 9.900572e-01 9.618239e-01

9805 9.900593e-01 9.618180e-01

9806 9.900614e-01 9.618121e-01

9807 9.900635e-01 9.618061e-01

9808 9.900655e-01 9.618001e-01

9809 9.900675e-01 9.617940e-01

9810 9.900695e-01 9.617878e-01

9811 9.900714e-01 9.617816e-01

9812 9.900733e-01 9.617753e-01

9813 9.900752e-01 9.617690e-01

9814 9.900771e-01 9.617625e-01

9815 9.900789e-01 9.617561e-01

9816 9.900807e-01 9.617495e-01

9817 9.900825e-01 9.617429e-01

9818 9.900843e-01 9.617362e-01

9819 9.900861e-01 9.617295e-01

9820 9.900878e-01 9.617227e-01

9821 9.900895e-01 9.617158e-01

9822 9.900912e-01 9.617089e-01

9823 9.900929e-01 9.617018e-01

9824 9.900945e-01 9.616948e-01

9825 9.900962e-01 9.616876e-01

9826 9.900978e-01 9.616804e-01

9827 9.900994e-01 9.616731e-01

9828 9.901010e-01 9.616658e-01

9829 9.901026e-01 9.616584e-01

9830 9.901041e-01 9.616509e-01

9831 9.901057e-01 9.616433e-01

9832 9.901072e-01 9.616357e-01

9833 9.901087e-01 9.616280e-01

9834 9.901103e-01 9.616202e-01

9835 9.901118e-01 9.616124e-01

9836 9.901133e-01 9.616045e-01

9837 9.901148e-01 9.615965e-01

9838 9.901162e-01 9.615885e-01

9839 9.901177e-01 9.615803e-01

9840 9.901192e-01 9.615722e-01

9841 9.901206e-01 9.615639e-01

9842 9.901221e-01 9.615555e-01

9843 9.901235e-01 9.615471e-01

9844 9.901250e-01 9.615387e-01

9845 9.901264e-01 9.615301e-01

9846 9.901278e-01 9.615215e-01

9847 9.901293e-01 9.615128e-01

9848 9.901307e-01 9.615040e-01

9849 9.901321e-01 9.614952e-01

9850 9.901335e-01 9.614862e-01

9851 9.901349e-01 9.614772e-01

9852 9.901363e-01 9.614682e-01

9853 9.901378e-01 9.614590e-01

9854 9.901392e-01 9.614498e-01

9855 9.901406e-01 9.614405e-01

9856 9.901420e-01 9.614312e-01

9857 9.901434e-01 9.614217e-01

9858 9.901448e-01 9.614122e-01

9859 9.901462e-01 9.614026e-01

9860 9.901476e-01 9.613930e-01

9861 9.901490e-01 9.613832e-01

9862 9.901504e-01 9.613734e-01

9863 9.901518e-01 9.613636e-01

9864 9.901532e-01 9.613536e-01

9865 9.901546e-01 9.613436e-01

9866 9.901560e-01 9.613335e-01

9867 9.901574e-01 9.613233e-01

9868 9.901588e-01 9.613130e-01

9869 9.901602e-01 9.613027e-01

9870 9.901617e-01 9.612923e-01

9871 9.901631e-01 9.612818e-01

9872 9.901645e-01 9.612713e-01

9873 9.901659e-01 9.612607e-01

9874 9.901674e-01 9.612500e-01

9875 9.901688e-01 9.612392e-01

9876 9.901703e-01 9.612283e-01

9877 9.901717e-01 9.612174e-01

9878 9.901732e-01 9.612064e-01

9879 9.901746e-01 9.611954e-01

9880 9.901761e-01 9.611842e-01

9881 9.901775e-01 9.611730e-01

9882 9.901790e-01 9.611618e-01

9883 9.901805e-01 9.611504e-01

9884 9.901820e-01 9.611390e-01

9885 9.901835e-01 9.611275e-01

9886 9.901850e-01 9.611159e-01

9887 9.901865e-01 9.611043e-01

9888 9.901880e-01 9.610926e-01

9889 9.901895e-01 9.610808e-01

9890 9.901910e-01 9.610690e-01

9891 9.901925e-01 9.610570e-01

9892 9.901941e-01 9.610451e-01

9893 9.901956e-01 9.610330e-01

9894 9.901972e-01 9.610209e-01

9895 9.901987e-01 9.610087e-01

9896 9.902003e-01 9.609964e-01

9897 9.902019e-01 9.609841e-01

9898 9.902034e-01 9.609717e-01

9899 9.902050e-01 9.609593e-01

9900 9.902066e-01 9.609467e-01

9901 9.902082e-01 9.609342e-01

9902 9.902204e-01 9.609319e-01

9903 9.902324e-01 9.609297e-01

9904 9.902440e-01 9.609274e-01

9905 9.902553e-01 9.609250e-01

9906 9.902663e-01 9.609227e-01

9907 9.902771e-01 9.609203e-01

9908 9.902876e-01 9.609179e-01

9909 9.902978e-01 9.609154e-01

9910 9.903078e-01 9.609129e-01

9911 9.903175e-01 9.609104e-01

9912 9.903270e-01 9.609079e-01

9913 9.903362e-01 9.609053e-01

9914 9.903452e-01 9.609027e-01

9915 9.903540e-01 9.609000e-01

9916 9.903626e-01 9.608974e-01

9917 9.903710e-01 9.608946e-01

9918 9.903791e-01 9.608919e-01

9919 9.903871e-01 9.608891e-01

9920 9.903948e-01 9.608863e-01

9921 9.904024e-01 9.608834e-01

9922 9.904098e-01 9.608806e-01

9923 9.904170e-01 9.608776e-01

9924 9.904240e-01 9.608747e-01

9925 9.904308e-01 9.608717e-01

9926 9.904375e-01 9.608687e-01

9927 9.904441e-01 9.608656e-01

9928 9.904504e-01 9.608625e-01

9929 9.904566e-01 9.608593e-01

9930 9.904627e-01 9.608561e-01

9931 9.904686e-01 9.608529e-01

9932 9.904744e-01 9.608497e-01

9933 9.904800e-01 9.608464e-01

9934 9.904855e-01 9.608430e-01

9935 9.904909e-01 9.608396e-01

9936 9.904961e-01 9.608362e-01

9937 9.905013e-01 9.608327e-01

9938 9.905063e-01 9.608292e-01

9939 9.905111e-01 9.608257e-01

9940 9.905159e-01 9.608221e-01

9941 9.905206e-01 9.608185e-01

9942 9.905251e-01 9.608148e-01

9943 9.905296e-01 9.608111e-01

9944 9.905339e-01 9.608073e-01

9945 9.905381e-01 9.608035e-01

9946 9.905423e-01 9.607996e-01

9947 9.905463e-01 9.607957e-01

9948 9.905503e-01 9.607918e-01

9949 9.905542e-01 9.607878e-01

9950 9.905580e-01 9.607838e-01

9951 9.905617e-01 9.607797e-01

9952 9.905653e-01 9.607756e-01

9953 9.905688e-01 9.607714e-01

9954 9.905723e-01 9.607672e-01

9955 9.905757e-01 9.607629e-01

9956 9.905790e-01 9.607586e-01

9957 9.905823e-01 9.607542e-01

9958 9.905854e-01 9.607498e-01

9959 9.905885e-01 9.607453e-01

9960 9.905916e-01 9.607408e-01

9961 9.905946e-01 9.607363e-01

9962 9.905975e-01 9.607316e-01

9963 9.906004e-01 9.607270e-01

9964 9.906032e-01 9.607223e-01

9965 9.906059e-01 9.607175e-01

9966 9.906086e-01 9.607127e-01

9967 9.906112e-01 9.607078e-01

9968 9.906138e-01 9.607029e-01

9969 9.906164e-01 9.606979e-01

9970 9.906189e-01 9.606928e-01

9971 9.906213e-01 9.606877e-01

9972 9.906237e-01 9.606826e-01

9973 9.906261e-01 9.606774e-01

9974 9.906284e-01 9.606721e-01

9975 9.906306e-01 9.606668e-01

9976 9.906329e-01 9.606615e-01

9977 9.906351e-01 9.606560e-01

9978 9.906372e-01 9.606506e-01

9979 9.906393e-01 9.606450e-01

9980 9.906414e-01 9.606394e-01

9981 9.906435e-01 9.606338e-01

9982 9.906455e-01 9.606281e-01

9983 9.906475e-01 9.606223e-01

9984 9.906494e-01 9.606165e-01

9985 9.906513e-01 9.606106e-01

9986 9.906532e-01 9.606046e-01

9987 9.906551e-01 9.605986e-01

9988 9.906569e-01 9.605926e-01

9989 9.906587e-01 9.605864e-01

9990 9.906605e-01 9.605802e-01

9991 9.906623e-01 9.605740e-01

9992 9.906640e-01 9.605677e-01

9993 9.906657e-01 9.605613e-01

9994 9.906674e-01 9.605549e-01

9995 9.906691e-01 9.605484e-01

9996 9.906707e-01 9.605418e-01

9997 9.906724e-01 9.605352e-01

9998 9.906740e-01 9.605285e-01

9999 9.906756e-01 9.605217e-01

10000 9.906771e-01 9.605149e-01

10001 9.906787e-01 9.605080e-01

10002 9.906802e-01 9.605011e-01

10003 9.906818e-01 9.604941e-01

10004 9.906833e-01 9.604870e-01

10005 9.906848e-01 9.604799e-01

10006 9.906862e-01 9.604727e-01

10007 9.906877e-01 9.604654e-01

10008 9.906892e-01 9.604580e-01

10009 9.906906e-01 9.604506e-01

10010 9.906920e-01 9.604432e-01

10011 9.906935e-01 9.604356e-01

10012 9.906949e-01 9.604280e-01

10013 9.906963e-01 9.604203e-01

10014 9.906977e-01 9.604126e-01

10015 9.906990e-01 9.604048e-01

10016 9.907004e-01 9.603969e-01

10017 9.907018e-01 9.603890e-01

10018 9.907031e-01 9.603809e-01

10019 9.907045e-01 9.603729e-01

10020 9.907058e-01 9.603647e-01

10021 9.907072e-01 9.603565e-01

10022 9.907085e-01 9.603482e-01

10023 9.907098e-01 9.603398e-01

10024 9.907112e-01 9.603314e-01

10025 9.907125e-01 9.603229e-01

10026 9.907138e-01 9.603143e-01

10027 9.907151e-01 9.603057e-01

10028 9.907164e-01 9.602970e-01

10029 9.907177e-01 9.602882e-01

10030 9.907190e-01 9.602793e-01

10031 9.907203e-01 9.602704e-01

10032 9.907216e-01 9.602614e-01

10033 9.907229e-01 9.602524e-01

10034 9.907242e-01 9.602432e-01

10035 9.907255e-01 9.602340e-01

10036 9.907268e-01 9.602248e-01

10037 9.907281e-01 9.602154e-01

10038 9.907293e-01 9.602060e-01

10039 9.907306e-01 9.601965e-01

10040 9.907319e-01 9.601870e-01

10041 9.907332e-01 9.601773e-01

10042 9.907345e-01 9.601676e-01

10043 9.907358e-01 9.601579e-01

10044 9.907371e-01 9.601480e-01

10045 9.907384e-01 9.601381e-01

10046 9.907397e-01 9.601282e-01

10047 9.907410e-01 9.601181e-01

10048 9.907423e-01 9.601080e-01

10049 9.907436e-01 9.600978e-01

10050 9.907449e-01 9.600875e-01

10051 9.907462e-01 9.600772e-01

10052 9.907475e-01 9.600668e-01

10053 9.907488e-01 9.600563e-01

10054 9.907502e-01 9.600458e-01

10055 9.907515e-01 9.600352e-01

10056 9.907528e-01 9.600245e-01

10057 9.907541e-01 9.600138e-01

10058 9.907555e-01 9.600030e-01

10059 9.907568e-01 9.599921e-01

10060 9.907582e-01 9.599811e-01

10061 9.907595e-01 9.599701e-01

10062 9.907609e-01 9.599590e-01

10063 9.907622e-01 9.599479e-01

10064 9.907636e-01 9.599367e-01

10065 9.907650e-01 9.599254e-01

10066 9.907663e-01 9.599140e-01

10067 9.907677e-01 9.599026e-01

10068 9.907691e-01 9.598911e-01

10069 9.907705e-01 9.598796e-01

10070 9.907719e-01 9.598680e-01

10071 9.907733e-01 9.598563e-01

10072 9.907747e-01 9.598446e-01

10073 9.907762e-01 9.598328e-01

10074 9.907776e-01 9.598209e-01

10075 9.907790e-01 9.598090e-01

10076 9.907805e-01 9.597970e-01

10077 9.907819e-01 9.597849e-01

10078 9.907834e-01 9.597728e-01

10079 9.907848e-01 9.597606e-01

10080 9.907863e-01 9.597484e-01

10081 9.907878e-01 9.597361e-01

10082 9.907985e-01 9.597338e-01

10083 9.908089e-01 9.597315e-01

10084 9.908191e-01 9.597291e-01

10085 9.908290e-01 9.597268e-01

10086 9.908387e-01 9.597243e-01

10087 9.908481e-01 9.597219e-01

10088 9.908573e-01 9.597194e-01

10089 9.908663e-01 9.597169e-01

10090 9.908751e-01 9.597144e-01

10091 9.908836e-01 9.597118e-01

10092 9.908919e-01 9.597092e-01

10093 9.909000e-01 9.597066e-01

10094 9.909080e-01 9.597039e-01

10095 9.909157e-01 9.597012e-01

10096 9.909232e-01 9.596985e-01

10097 9.909306e-01 9.596957e-01

10098 9.909377e-01 9.596929e-01

10099 9.909447e-01 9.596901e-01

10100 9.909516e-01 9.596872e-01

10101 9.909582e-01 9.596843e-01

10102 9.909647e-01 9.596814e-01

10103 9.909711e-01 9.596784e-01

10104 9.909773e-01 9.596754e-01

10105 9.909833e-01 9.596723e-01

10106 9.909892e-01 9.596692e-01

10107 9.909949e-01 9.596661e-01

10108 9.910006e-01 9.596630e-01

10109 9.910060e-01 9.596598e-01

10110 9.910114e-01 9.596565e-01

10111 9.910166e-01 9.596532e-01

10112 9.910217e-01 9.596499e-01

10113 9.910267e-01 9.596466e-01

10114 9.910316e-01 9.596432e-01

10115 9.910363e-01 9.596397e-01

10116 9.910409e-01 9.596363e-01

10117 9.910455e-01 9.596327e-01

10118 9.910499e-01 9.596292e-01

10119 9.910542e-01 9.596256e-01

10120 9.910584e-01 9.596220e-01

10121 9.910626e-01 9.596183e-01

10122 9.910666e-01 9.596145e-01

10123 9.910705e-01 9.596108e-01

10124 9.910744e-01 9.596070e-01

10125 9.910782e-01 9.596031e-01

10126 9.910818e-01 9.595992e-01

10127 9.910854e-01 9.595953e-01

10128 9.910890e-01 9.595913e-01

10129 9.910924e-01 9.595872e-01

10130 9.910958e-01 9.595832e-01

10131 9.910991e-01 9.595790e-01

10132 9.911023e-01 9.595749e-01

10133 9.911054e-01 9.595707e-01

10134 9.911085e-01 9.595664e-01

10135 9.911116e-01 9.595621e-01

10136 9.911145e-01 9.595577e-01

10137 9.911174e-01 9.595533e-01

10138 9.911202e-01 9.595489e-01

10139 9.911230e-01 9.595443e-01

10140 9.911257e-01 9.595398e-01

10141 9.911284e-01 9.595352e-01

10142 9.911310e-01 9.595305e-01

10143 9.911336e-01 9.595258e-01

10144 9.911361e-01 9.595211e-01

10145 9.911386e-01 9.595163e-01

10146 9.911410e-01 9.595114e-01

10147 9.911433e-01 9.595065e-01

10148 9.911457e-01 9.595016e-01

10149 9.911479e-01 9.594965e-01

10150 9.911502e-01 9.594915e-01

10151 9.911524e-01 9.594864e-01

10152 9.911545e-01 9.594812e-01

10153 9.911566e-01 9.594760e-01

10154 9.911587e-01 9.594707e-01

10155 9.911608e-01 9.594654e-01

10156 9.911628e-01 9.594600e-01

10157 9.911647e-01 9.594545e-01

10158 9.911667e-01 9.594490e-01

10159 9.911686e-01 9.594435e-01

10160 9.911705e-01 9.594379e-01

10161 9.911723e-01 9.594322e-01

10162 9.911741e-01 9.594265e-01

10163 9.911759e-01 9.594207e-01

10164 9.911777e-01 9.594149e-01

10165 9.911794e-01 9.594090e-01

10166 9.911811e-01 9.594030e-01

10167 9.911828e-01 9.593970e-01

10168 9.911845e-01 9.593909e-01

10169 9.911861e-01 9.593848e-01

10170 9.911877e-01 9.593786e-01

10171 9.911893e-01 9.593724e-01

10172 9.911909e-01 9.593661e-01

10173 9.911925e-01 9.593597e-01

10174 9.911940e-01 9.593533e-01

10175 9.911955e-01 9.593468e-01

10176 9.911970e-01 9.593402e-01

10177 9.911985e-01 9.593336e-01

10178 9.912000e-01 9.593270e-01

10179 9.912014e-01 9.593202e-01

10180 9.912028e-01 9.593134e-01

10181 9.912043e-01 9.593066e-01

10182 9.912057e-01 9.592997e-01

10183 9.912071e-01 9.592927e-01

10184 9.912084e-01 9.592856e-01

10185 9.912098e-01 9.592785e-01

10186 9.912112e-01 9.592714e-01

10187 9.912125e-01 9.592641e-01

10188 9.912138e-01 9.592568e-01

10189 9.912151e-01 9.592495e-01

10190 9.912165e-01 9.592420e-01

10191 9.912177e-01 9.592345e-01

10192 9.912190e-01 9.592270e-01

10193 9.912203e-01 9.592194e-01

10194 9.912216e-01 9.592117e-01

10195 9.912229e-01 9.592039e-01

10196 9.912241e-01 9.591961e-01

10197 9.912254e-01 9.591882e-01

10198 9.912266e-01 9.591803e-01

10199 9.912279e-01 9.591723e-01

10200 9.912291e-01 9.591642e-01

10201 9.912303e-01 9.591561e-01

10202 9.912315e-01 9.591479e-01

10203 9.912328e-01 9.591396e-01

10204 9.912340e-01 9.591312e-01

10205 9.912352e-01 9.591228e-01

10206 9.912364e-01 9.591143e-01

10207 9.912376e-01 9.591058e-01

10208 9.912388e-01 9.590972e-01

10209 9.912400e-01 9.590885e-01

10210 9.912412e-01 9.590798e-01

10211 9.912424e-01 9.590710e-01

10212 9.912436e-01 9.590621e-01

10213 9.912448e-01 9.590532e-01

10214 9.912460e-01 9.590442e-01

10215 9.912471e-01 9.590351e-01

10216 9.912483e-01 9.590259e-01

10217 9.912495e-01 9.590167e-01

10218 9.912507e-01 9.590074e-01

10219 9.912519e-01 9.589981e-01

10220 9.912531e-01 9.589887e-01

10221 9.912543e-01 9.589792e-01

10222 9.912555e-01 9.589697e-01

10223 9.912567e-01 9.589601e-01

10224 9.912579e-01 9.589504e-01

10225 9.912591e-01 9.589407e-01

10226 9.912603e-01 9.589309e-01

10227 9.912615e-01 9.589210e-01

10228 9.912627e-01 9.589110e-01

10229 9.912639e-01 9.589010e-01

10230 9.912651e-01 9.588910e-01

10231 9.912663e-01 9.588808e-01

10232 9.912675e-01 9.588706e-01

10233 9.912687e-01 9.588604e-01

10234 9.912699e-01 9.588500e-01

10235 9.912712e-01 9.588396e-01

10236 9.912724e-01 9.588292e-01

10237 9.912736e-01 9.588187e-01

10238 9.912749e-01 9.588081e-01

10239 9.912761e-01 9.587974e-01

10240 9.912774e-01 9.587867e-01

10241 9.912786e-01 9.587759e-01

10242 9.912799e-01 9.587651e-01

10243 9.912812e-01 9.587542e-01

10244 9.912824e-01 9.587432e-01

10245 9.912837e-01 9.587322e-01

10246 9.912850e-01 9.587211e-01

10247 9.912863e-01 9.587100e-01

10248 9.912876e-01 9.586988e-01

10249 9.912889e-01 9.586875e-01

10250 9.912902e-01 9.586762e-01

10251 9.912915e-01 9.586648e-01

10252 9.912928e-01 9.586534e-01

10253 9.912942e-01 9.586419e-01

10254 9.912955e-01 9.586303e-01

10255 9.912969e-01 9.586187e-01

10256 9.912982e-01 9.586070e-01

10257 9.912996e-01 9.585953e-01

10258 9.913009e-01 9.585835e-01

10259 9.913023e-01 9.585717e-01

10260 9.913037e-01 9.585598e-01

10261 9.913051e-01 9.585478e-01

10262 9.913145e-01 9.585455e-01

10263 9.913237e-01 9.585431e-01

10264 9.913326e-01 9.585407e-01

10265 9.913413e-01 9.585383e-01

10266 9.913498e-01 9.585358e-01

10267 9.913581e-01 9.585333e-01

10268 9.913662e-01 9.585308e-01

10269 9.913741e-01 9.585283e-01

10270 9.913818e-01 9.585257e-01

10271 9.913893e-01 9.585231e-01

10272 9.913966e-01 9.585204e-01

10273 9.914038e-01 9.585178e-01

10274 9.914107e-01 9.585151e-01

10275 9.914175e-01 9.585123e-01

10276 9.914242e-01 9.585095e-01

10277 9.914307e-01 9.585067e-01

10278 9.914370e-01 9.585039e-01

10279 9.914431e-01 9.585010e-01

10280 9.914492e-01 9.584981e-01

10281 9.914550e-01 9.584952e-01

10282 9.914608e-01 9.584922e-01

10283 9.914664e-01 9.584892e-01

10284 9.914718e-01 9.584861e-01

10285 9.914772e-01 9.584830e-01

10286 9.914824e-01 9.584799e-01

10287 9.914875e-01 9.584767e-01

10288 9.914924e-01 9.584735e-01

10289 9.914973e-01 9.584703e-01

10290 9.915020e-01 9.584670e-01

10291 9.915066e-01 9.584637e-01

10292 9.915111e-01 9.584603e-01

10293 9.915156e-01 9.584569e-01

10294 9.915199e-01 9.584535e-01

10295 9.915241e-01 9.584500e-01

10296 9.915282e-01 9.584465e-01

10297 9.915322e-01 9.584430e-01

10298 9.915361e-01 9.584394e-01

10299 9.915400e-01 9.584357e-01

10300 9.915437e-01 9.584321e-01

10301 9.915474e-01 9.584284e-01

10302 9.915510e-01 9.584246e-01

10303 9.915545e-01 9.584208e-01

10304 9.915579e-01 9.584170e-01

10305 9.915613e-01 9.584131e-01

10306 9.915645e-01 9.584091e-01

10307 9.915677e-01 9.584052e-01

10308 9.915709e-01 9.584012e-01

10309 9.915739e-01 9.583971e-01

10310 9.915770e-01 9.583930e-01

10311 9.915799e-01 9.583888e-01

10312 9.915828e-01 9.583846e-01

10313 9.915856e-01 9.583804e-01

10314 9.915883e-01 9.583761e-01

10315 9.915910e-01 9.583718e-01

10316 9.915937e-01 9.583674e-01

10317 9.915963e-01 9.583630e-01

10318 9.915988e-01 9.583585e-01

10319 9.916013e-01 9.583540e-01

10320 9.916037e-01 9.583494e-01

10321 9.916061e-01 9.583448e-01

10322 9.916085e-01 9.583401e-01

10323 9.916108e-01 9.583354e-01

10324 9.916130e-01 9.583306e-01

10325 9.916152e-01 9.583258e-01

10326 9.916174e-01 9.583210e-01

10327 9.916195e-01 9.583160e-01

10328 9.916216e-01 9.583111e-01

10329 9.916236e-01 9.583061e-01

10330 9.916257e-01 9.583010e-01

10331 9.916276e-01 9.582959e-01

10332 9.916296e-01 9.582907e-01

10333 9.916315e-01 9.582855e-01

10334 9.916334e-01 9.582802e-01

10335 9.916352e-01 9.582749e-01

10336 9.916370e-01 9.582695e-01

10337 9.916388e-01 9.582640e-01

10338 9.916405e-01 9.582586e-01

10339 9.916423e-01 9.582530e-01

10340 9.916440e-01 9.582474e-01

10341 9.916456e-01 9.582418e-01

10342 9.916473e-01 9.582361e-01

10343 9.916489e-01 9.582303e-01

10344 9.916505e-01 9.582245e-01

10345 9.916521e-01 9.582186e-01

10346 9.916536e-01 9.582127e-01

10347 9.916552e-01 9.582067e-01

10348 9.916567e-01 9.582006e-01

10349 9.916582e-01 9.581945e-01

10350 9.916596e-01 9.581884e-01

10351 9.916611e-01 9.581822e-01

10352 9.916625e-01 9.581759e-01

10353 9.916639e-01 9.581696e-01

10354 9.916653e-01 9.581632e-01

10355 9.916667e-01 9.581567e-01

10356 9.916681e-01 9.581502e-01

10357 9.916694e-01 9.581437e-01

10358 9.916707e-01 9.581371e-01

10359 9.916721e-01 9.581304e-01

10360 9.916734e-01 9.581236e-01

10361 9.916747e-01 9.581168e-01

10362 9.916759e-01 9.581100e-01

10363 9.916772e-01 9.581031e-01

10364 9.916785e-01 9.580961e-01

10365 9.916797e-01 9.580890e-01

10366 9.916810e-01 9.580819e-01

10367 9.916822e-01 9.580748e-01

10368 9.916834e-01 9.580675e-01

10369 9.916846e-01 9.580603e-01

10370 9.916858e-01 9.580529e-01

10371 9.916870e-01 9.580455e-01

10372 9.916882e-01 9.580380e-01

10373 9.916894e-01 9.580305e-01

10374 9.916905e-01 9.580229e-01

10375 9.916917e-01 9.580153e-01

10376 9.916929e-01 9.580075e-01

10377 9.916940e-01 9.579998e-01

10378 9.916951e-01 9.579919e-01

10379 9.916963e-01 9.579840e-01

10380 9.916974e-01 9.579761e-01

10381 9.916986e-01 9.579680e-01

10382 9.916997e-01 9.579599e-01

10383 9.917008e-01 9.579518e-01

10384 9.917019e-01 9.579436e-01

10385 9.917030e-01 9.579353e-01

10386 9.917042e-01 9.579270e-01

10387 9.917053e-01 9.579185e-01

10388 9.917064e-01 9.579101e-01

10389 9.917075e-01 9.579015e-01

10390 9.917086e-01 9.578930e-01

10391 9.917097e-01 9.578843e-01

10392 9.917108e-01 9.578756e-01

10393 9.917119e-01 9.578668e-01

10394 9.917130e-01 9.578580e-01

10395 9.917141e-01 9.578490e-01

10396 9.917152e-01 9.578401e-01

10397 9.917163e-01 9.578310e-01

10398 9.917174e-01 9.578219e-01

10399 9.917185e-01 9.578128e-01

10400 9.917196e-01 9.578036e-01

10401 9.917207e-01 9.577943e-01

10402 9.917218e-01 9.577849e-01

10403 9.917229e-01 9.577755e-01

10404 9.917241e-01 9.577661e-01

10405 9.917252e-01 9.577565e-01

10406 9.917263e-01 9.577469e-01

10407 9.917274e-01 9.577373e-01

10408 9.917285e-01 9.577276e-01

10409 9.917297e-01 9.577178e-01

10410 9.917308e-01 9.577080e-01

10411 9.917319e-01 9.576981e-01

10412 9.917331e-01 9.576881e-01

10413 9.917342e-01 9.576781e-01

10414 9.917353e-01 9.576680e-01

10415 9.917365e-01 9.576579e-01

10416 9.917376e-01 9.576477e-01

10417 9.917388e-01 9.576374e-01

10418 9.917400e-01 9.576271e-01

10419 9.917411e-01 9.576167e-01

10420 9.917423e-01 9.576063e-01

10421 9.917435e-01 9.575958e-01

10422 9.917447e-01 9.575853e-01

10423 9.917459e-01 9.575747e-01

10424 9.917471e-01 9.575640e-01

10425 9.917483e-01 9.575533e-01

10426 9.917495e-01 9.575425e-01

10427 9.917507e-01 9.575317e-01

10428 9.917519e-01 9.575208e-01

10429 9.917532e-01 9.575099e-01

10430 9.917544e-01 9.574989e-01

10431 9.917557e-01 9.574879e-01

10432 9.917569e-01 9.574768e-01

10433 9.917582e-01 9.574656e-01

10434 9.917595e-01 9.574544e-01

10435 9.917607e-01 9.574432e-01

10436 9.917620e-01 9.574319e-01

10437 9.917633e-01 9.574205e-01

10438 9.917646e-01 9.574091e-01

10439 9.917659e-01 9.573977e-01

10440 9.917673e-01 9.573862e-01

10441 9.917686e-01 9.573746e-01

10442 9.917769e-01 9.573723e-01

10443 9.917849e-01 9.573699e-01

10444 9.917928e-01 9.573675e-01

10445 9.918005e-01 9.573650e-01

10446 9.918080e-01 9.573625e-01

10447 9.918153e-01 9.573600e-01

10448 9.918224e-01 9.573574e-01

10449 9.918293e-01 9.573549e-01

10450 9.918361e-01 9.573522e-01

10451 9.918427e-01 9.573496e-01

10452 9.918492e-01 9.573469e-01

10453 9.918555e-01 9.573442e-01

10454 9.918617e-01 9.573415e-01

10455 9.918677e-01 9.573387e-01

10456 9.918735e-01 9.573359e-01

10457 9.918793e-01 9.573331e-01

10458 9.918848e-01 9.573302e-01

10459 9.918903e-01 9.573273e-01

10460 9.918956e-01 9.573243e-01

10461 9.919008e-01 9.573214e-01

10462 9.919059e-01 9.573184e-01

10463 9.919108e-01 9.573153e-01

10464 9.919157e-01 9.573122e-01

10465 9.919204e-01 9.573091e-01

10466 9.919250e-01 9.573060e-01

10467 9.919295e-01 9.573028e-01

10468 9.919339e-01 9.572995e-01

10469 9.919382e-01 9.572963e-01

10470 9.919424e-01 9.572930e-01

10471 9.919465e-01 9.572896e-01

10472 9.919505e-01 9.572863e-01

10473 9.919544e-01 9.572829e-01

10474 9.919583e-01 9.572794e-01

10475 9.919620e-01 9.572759e-01

10476 9.919657e-01 9.572724e-01

10477 9.919692e-01 9.572688e-01

10478 9.919727e-01 9.572652e-01

10479 9.919761e-01 9.572615e-01

10480 9.919795e-01 9.572579e-01

10481 9.919827e-01 9.572541e-01

10482 9.919859e-01 9.572504e-01

10483 9.919891e-01 9.572465e-01

10484 9.919921e-01 9.572427e-01

10485 9.919951e-01 9.572388e-01

10486 9.919980e-01 9.572348e-01

10487 9.920009e-01 9.572309e-01

10488 9.920037e-01 9.572268e-01

10489 9.920064e-01 9.572228e-01

10490 9.920091e-01 9.572187e-01

10491 9.920118e-01 9.572145e-01

10492 9.920143e-01 9.572103e-01

10493 9.920168e-01 9.572061e-01

10494 9.920193e-01 9.572018e-01

10495 9.920217e-01 9.571974e-01

10496 9.920241e-01 9.571930e-01

10497 9.920264e-01 9.571886e-01

10498 9.920287e-01 9.571841e-01

10499 9.920309e-01 9.571796e-01

10500 9.920331e-01 9.571751e-01

10501 9.920353e-01 9.571704e-01

10502 9.920374e-01 9.571658e-01

10503 9.920394e-01 9.571611e-01

10504 9.920415e-01 9.571563e-01

10505 9.920434e-01 9.571515e-01

10506 9.920454e-01 9.571467e-01

10507 9.920473e-01 9.571418e-01

10508 9.920492e-01 9.571368e-01

10509 9.920510e-01 9.571318e-01

10510 9.920529e-01 9.571268e-01

10511 9.920546e-01 9.571217e-01

10512 9.920564e-01 9.571165e-01

10513 9.920581e-01 9.571113e-01

10514 9.920598e-01 9.571061e-01

10515 9.920615e-01 9.571008e-01

10516 9.920631e-01 9.570954e-01

10517 9.920647e-01 9.570900e-01

10518 9.920663e-01 9.570846e-01

10519 9.920679e-01 9.570791e-01

10520 9.920694e-01 9.570735e-01

10521 9.920709e-01 9.570679e-01

10522 9.920724e-01 9.570622e-01

10523 9.920739e-01 9.570565e-01

10524 9.920753e-01 9.570507e-01

10525 9.920768e-01 9.570449e-01

10526 9.920782e-01 9.570390e-01

10527 9.920796e-01 9.570331e-01

10528 9.920809e-01 9.570271e-01

10529 9.920823e-01 9.570211e-01

10530 9.920836e-01 9.570150e-01

10531 9.920849e-01 9.570088e-01

10532 9.920862e-01 9.570026e-01

10533 9.920875e-01 9.569963e-01

10534 9.920888e-01 9.569900e-01

10535 9.920901e-01 9.569837e-01

10536 9.920913e-01 9.569772e-01

10537 9.920926e-01 9.569707e-01

10538 9.920938e-01 9.569642e-01

10539 9.920950e-01 9.569576e-01

10540 9.920962e-01 9.569509e-01

10541 9.920974e-01 9.569442e-01

10542 9.920985e-01 9.569375e-01

10543 9.920997e-01 9.569306e-01

10544 9.921009e-01 9.569238e-01

10545 9.921020e-01 9.569168e-01

10546 9.921031e-01 9.569098e-01

10547 9.921043e-01 9.569028e-01

10548 9.921054e-01 9.568956e-01

10549 9.921065e-01 9.568885e-01

10550 9.921076e-01 9.568812e-01

10551 9.921087e-01 9.568740e-01

10552 9.921098e-01 9.568666e-01

10553 9.921109e-01 9.568592e-01

10554 9.921119e-01 9.568517e-01

10555 9.921130e-01 9.568442e-01

10556 9.921141e-01 9.568366e-01

10557 9.921151e-01 9.568290e-01

10558 9.921162e-01 9.568213e-01

10559 9.921173e-01 9.568135e-01

10560 9.921183e-01 9.568057e-01

10561 9.921194e-01 9.567978e-01

10562 9.921204e-01 9.567899e-01

10563 9.921214e-01 9.567819e-01

10564 9.921225e-01 9.567739e-01

10565 9.921235e-01 9.567658e-01

10566 9.921245e-01 9.567576e-01

10567 9.921256e-01 9.567494e-01

10568 9.921266e-01 9.567411e-01

10569 9.921276e-01 9.567327e-01

10570 9.921287e-01 9.567243e-01

10571 9.921297e-01 9.567159e-01

10572 9.921307e-01 9.567073e-01

10573 9.921318e-01 9.566987e-01

10574 9.921328e-01 9.566901e-01

10575 9.921338e-01 9.566814e-01

10576 9.921348e-01 9.566727e-01

10577 9.921359e-01 9.566638e-01

10578 9.921369e-01 9.566550e-01

10579 9.921379e-01 9.566460e-01

10580 9.921390e-01 9.566370e-01

10581 9.921400e-01 9.566280e-01

10582 9.921410e-01 9.566189e-01

10583 9.921421e-01 9.566097e-01

10584 9.921431e-01 9.566005e-01

10585 9.921442e-01 9.565912e-01

10586 9.921452e-01 9.565819e-01

10587 9.921463e-01 9.565725e-01

10588 9.921473e-01 9.565631e-01

10589 9.921484e-01 9.565536e-01

10590 9.921494e-01 9.565440e-01

10591 9.921505e-01 9.565344e-01

10592 9.921516e-01 9.565247e-01

10593 9.921527e-01 9.565150e-01

10594 9.921537e-01 9.565052e-01

10595 9.921548e-01 9.564954e-01

10596 9.921559e-01 9.564855e-01

10597 9.921570e-01 9.564756e-01

10598 9.921581e-01 9.564656e-01

10599 9.921592e-01 9.564556e-01

10600 9.921603e-01 9.564455e-01

10601 9.921614e-01 9.564353e-01

10602 9.921626e-01 9.564251e-01

10603 9.921637e-01 9.564149e-01

10604 9.921648e-01 9.564046e-01

10605 9.921660e-01 9.563942e-01

10606 9.921671e-01 9.563838e-01

10607 9.921683e-01 9.563734e-01

10608 9.921694e-01 9.563629e-01

10609 9.921706e-01 9.563523e-01

10610 9.921718e-01 9.563417e-01

10611 9.921730e-01 9.563311e-01

10612 9.921742e-01 9.563204e-01

10613 9.921754e-01 9.563096e-01

10614 9.921766e-01 9.562989e-01

10615 9.921778e-01 9.562880e-01

10616 9.921790e-01 9.562772e-01

10617 9.921802e-01 9.562663e-01

10618 9.921815e-01 9.562553e-01

10619 9.921827e-01 9.562443e-01

10620 9.921840e-01 9.562333e-01

10621 9.921852e-01 9.562222e-01

10622 9.921925e-01 9.562198e-01

10623 9.921996e-01 9.562174e-01

10624 9.922066e-01 9.562149e-01

10625 9.922133e-01 9.562124e-01

10626 9.922200e-01 9.562099e-01

10627 9.922264e-01 9.562074e-01

10628 9.922327e-01 9.562048e-01

10629 9.922388e-01 9.562022e-01

10630 9.922448e-01 9.561996e-01

10631 9.922507e-01 9.561969e-01

10632 9.922564e-01 9.561942e-01

10633 9.922620e-01 9.561915e-01

10634 9.922674e-01 9.561887e-01

10635 9.922727e-01 9.561860e-01

10636 9.922779e-01 9.561831e-01

10637 9.922830e-01 9.561803e-01

10638 9.922879e-01 9.561774e-01

10639 9.922928e-01 9.561745e-01

10640 9.922975e-01 9.561715e-01

10641 9.923021e-01 9.561685e-01

10642 9.923066e-01 9.561655e-01

10643 9.923110e-01 9.561624e-01

10644 9.923153e-01 9.561594e-01

10645 9.923195e-01 9.561562e-01

10646 9.923236e-01 9.561531e-01

10647 9.923276e-01 9.561499e-01

10648 9.923315e-01 9.561466e-01

10649 9.923353e-01 9.561434e-01

10650 9.923390e-01 9.561401e-01

10651 9.923427e-01 9.561367e-01

10652 9.923462e-01 9.561333e-01

10653 9.923497e-01 9.561299e-01

10654 9.923531e-01 9.561265e-01

10655 9.923565e-01 9.561230e-01

10656 9.923597e-01 9.561194e-01

10657 9.923629e-01 9.561159e-01

10658 9.923660e-01 9.561122e-01

10659 9.923691e-01 9.561086e-01

10660 9.923720e-01 9.561049e-01

10661 9.923750e-01 9.561012e-01

10662 9.923778e-01 9.560974e-01

10663 9.923806e-01 9.560936e-01

10664 9.923833e-01 9.560897e-01

10665 9.923860e-01 9.560859e-01

10666 9.923886e-01 9.560819e-01

10667 9.923912e-01 9.560780e-01

10668 9.923937e-01 9.560739e-01

10669 9.923962e-01 9.560699e-01

10670 9.923986e-01 9.560658e-01

10671 9.924009e-01 9.560616e-01

10672 9.924032e-01 9.560575e-01

10673 9.924055e-01 9.560532e-01

10674 9.924077e-01 9.560490e-01

10675 9.924099e-01 9.560446e-01

10676 9.924120e-01 9.560403e-01

10677 9.924141e-01 9.560359e-01

10678 9.924161e-01 9.560314e-01

10679 9.924181e-01 9.560269e-01

10680 9.924201e-01 9.560224e-01

10681 9.924220e-01 9.560178e-01

10682 9.924239e-01 9.560132e-01

10683 9.924258e-01 9.560085e-01

10684 9.924276e-01 9.560038e-01

10685 9.924294e-01 9.559990e-01

10686 9.924312e-01 9.559942e-01

10687 9.924329e-01 9.559893e-01

10688 9.924346e-01 9.559844e-01

10689 9.924363e-01 9.559795e-01

10690 9.924379e-01 9.559745e-01

10691 9.924395e-01 9.559694e-01

10692 9.924411e-01 9.559643e-01

10693 9.924427e-01 9.559592e-01

10694 9.924442e-01 9.559540e-01

10695 9.924457e-01 9.559487e-01

10696 9.924472e-01 9.559434e-01

10697 9.924487e-01 9.559381e-01

10698 9.924501e-01 9.559327e-01

10699 9.924515e-01 9.559273e-01

10700 9.924529e-01 9.559218e-01

10701 9.924543e-01 9.559162e-01

10702 9.924557e-01 9.559106e-01

10703 9.924570e-01 9.559050e-01

10704 9.924583e-01 9.558993e-01

10705 9.924596e-01 9.558935e-01

10706 9.924609e-01 9.558877e-01

10707 9.924622e-01 9.558819e-01

10708 9.924634e-01 9.558760e-01

10709 9.924647e-01 9.558700e-01

10710 9.924659e-01 9.558640e-01

10711 9.924671e-01 9.558580e-01

10712 9.924683e-01 9.558519e-01

10713 9.924695e-01 9.558457e-01

10714 9.924706e-01 9.558395e-01

10715 9.924718e-01 9.558332e-01

10716 9.924729e-01 9.558269e-01

10717 9.924741e-01 9.558205e-01

10718 9.924752e-01 9.558141e-01

10719 9.924763e-01 9.558076e-01

10720 9.924774e-01 9.558011e-01

10721 9.924785e-01 9.557945e-01

10722 9.924795e-01 9.557878e-01

10723 9.924806e-01 9.557811e-01

10724 9.924817e-01 9.557744e-01

10725 9.924827e-01 9.557676e-01

10726 9.924838e-01 9.557607e-01

10727 9.924848e-01 9.557538e-01

10728 9.924859e-01 9.557468e-01

10729 9.924869e-01 9.557398e-01

10730 9.924879e-01 9.557327e-01

10731 9.924889e-01 9.557256e-01

10732 9.924899e-01 9.557184e-01

10733 9.924909e-01 9.557111e-01

10734 9.924919e-01 9.557039e-01

10735 9.924929e-01 9.556965e-01

10736 9.924939e-01 9.556891e-01

10737 9.924949e-01 9.556816e-01

10738 9.924959e-01 9.556741e-01

10739 9.924969e-01 9.556665e-01

10740 9.924978e-01 9.556589e-01

10741 9.924988e-01 9.556512e-01

10742 9.924998e-01 9.556435e-01

10743 9.925007e-01 9.556357e-01

10744 9.925017e-01 9.556278e-01

10745 9.925027e-01 9.556199e-01

10746 9.925036e-01 9.556120e-01

10747 9.925046e-01 9.556040e-01

10748 9.925056e-01 9.555959e-01

10749 9.925065e-01 9.555878e-01

10750 9.925075e-01 9.555796e-01

10751 9.925084e-01 9.555714e-01

10752 9.925094e-01 9.555631e-01

10753 9.925103e-01 9.555548e-01

10754 9.925113e-01 9.555464e-01

10755 9.925123e-01 9.555379e-01

10756 9.925132e-01 9.555294e-01

10757 9.925142e-01 9.555209e-01

10758 9.925152e-01 9.555123e-01

10759 9.925161e-01 9.555036e-01

10760 9.925171e-01 9.554949e-01

10761 9.925181e-01 9.554861e-01

10762 9.925190e-01 9.554773e-01

10763 9.925200e-01 9.554685e-01

10764 9.925210e-01 9.554595e-01

10765 9.925220e-01 9.554506e-01

10766 9.925230e-01 9.554415e-01

10767 9.925239e-01 9.554325e-01

10768 9.925249e-01 9.554233e-01

10769 9.925259e-01 9.554142e-01

10770 9.925269e-01 9.554049e-01

10771 9.925279e-01 9.553957e-01

10772 9.925289e-01 9.553863e-01

10773 9.925299e-01 9.553770e-01

10774 9.925310e-01 9.553675e-01

10775 9.925320e-01 9.553581e-01

10776 9.925330e-01 9.553485e-01

10777 9.925340e-01 9.553390e-01

10778 9.925351e-01 9.553293e-01

10779 9.925361e-01 9.553197e-01

10780 9.925372e-01 9.553100e-01

10781 9.925382e-01 9.553002e-01

10782 9.925393e-01 9.552904e-01

10783 9.925403e-01 9.552806e-01

10784 9.925414e-01 9.552707e-01

10785 9.925425e-01 9.552607e-01

10786 9.925435e-01 9.552508e-01

10787 9.925446e-01 9.552407e-01

10788 9.925457e-01 9.552307e-01

10789 9.925468e-01 9.552206e-01

10790 9.925479e-01 9.552104e-01

10791 9.925490e-01 9.552002e-01

10792 9.925502e-01 9.551900e-01

10793 9.925513e-01 9.551797e-01

10794 9.925524e-01 9.551694e-01

10795 9.925536e-01 9.551591e-01

10796 9.925547e-01 9.551487e-01

10797 9.925559e-01 9.551383e-01

10798 9.925570e-01 9.551278e-01

10799 9.925582e-01 9.551173e-01

10800 9.925594e-01 9.551068e-01

10801 9.925606e-01 9.550962e-01

10802 9.925670e-01 9.550938e-01

10803 9.925733e-01 9.550914e-01

10804 9.925794e-01 9.550889e-01

10805 9.925854e-01 9.550865e-01

10806 9.925912e-01 9.550839e-01

10807 9.925969e-01 9.550814e-01

10808 9.926025e-01 9.550788e-01

10809 9.926079e-01 9.550762e-01

10810 9.926133e-01 9.550736e-01

10811 9.926184e-01 9.550709e-01

10812 9.926235e-01 9.550682e-01

10813 9.926284e-01 9.550655e-01

10814 9.926333e-01 9.550627e-01

10815 9.926380e-01 9.550599e-01

10816 9.926426e-01 9.550571e-01

10817 9.926471e-01 9.550542e-01

10818 9.926515e-01 9.550514e-01

10819 9.926558e-01 9.550484e-01

10820 9.926599e-01 9.550455e-01

10821 9.926640e-01 9.550425e-01

10822 9.926680e-01 9.550395e-01

10823 9.926719e-01 9.550364e-01

10824 9.926758e-01 9.550333e-01

10825 9.926795e-01 9.550302e-01

10826 9.926831e-01 9.550270e-01

10827 9.926867e-01 9.550239e-01

10828 9.926902e-01 9.550206e-01

10829 9.926936e-01 9.550174e-01

10830 9.926969e-01 9.550141e-01

10831 9.927002e-01 9.550107e-01

10832 9.927034e-01 9.550074e-01

10833 9.927065e-01 9.550040e-01

10834 9.927095e-01 9.550005e-01

10835 9.927125e-01 9.549970e-01

10836 9.927154e-01 9.549935e-01

10837 9.927182e-01 9.549900e-01

10838 9.927210e-01 9.549864e-01

10839 9.927237e-01 9.549827e-01

10840 9.927264e-01 9.549791e-01

10841 9.927290e-01 9.549754e-01

10842 9.927316e-01 9.549716e-01

10843 9.927341e-01 9.549678e-01

10844 9.927365e-01 9.549640e-01

10845 9.927389e-01 9.549601e-01

10846 9.927413e-01 9.549562e-01

10847 9.927436e-01 9.549523e-01

10848 9.927458e-01 9.549483e-01

10849 9.927480e-01 9.549443e-01

10850 9.927502e-01 9.549402e-01

10851 9.927523e-01 9.549361e-01

10852 9.927544e-01 9.549320e-01

10853 9.927564e-01 9.549278e-01

10854 9.927584e-01 9.549236e-01

10855 9.927604e-01 9.549193e-01

10856 9.927623e-01 9.549150e-01

10857 9.927642e-01 9.549106e-01

10858 9.927660e-01 9.549062e-01

10859 9.927678e-01 9.549018e-01

10860 9.927696e-01 9.548973e-01

10861 9.927713e-01 9.548927e-01

10862 9.927730e-01 9.548882e-01

10863 9.927747e-01 9.548836e-01

10864 9.927764e-01 9.548789e-01

10865 9.927780e-01 9.548742e-01

10866 9.927796e-01 9.548694e-01

10867 9.927812e-01 9.548646e-01

10868 9.927827e-01 9.548598e-01

10869 9.927842e-01 9.548549e-01

10870 9.927857e-01 9.548500e-01

10871 9.927872e-01 9.548450e-01

10872 9.927886e-01 9.548400e-01

10873 9.927900e-01 9.548349e-01

10874 9.927914e-01 9.548298e-01

10875 9.927928e-01 9.548246e-01

10876 9.927941e-01 9.548194e-01

10877 9.927955e-01 9.548141e-01

10878 9.927968e-01 9.548088e-01

10879 9.927981e-01 9.548035e-01

10880 9.927993e-01 9.547981e-01

10881 9.928006e-01 9.547926e-01

10882 9.928018e-01 9.547871e-01

10883 9.928031e-01 9.547816e-01

10884 9.928043e-01 9.547760e-01

10885 9.928055e-01 9.547704e-01

10886 9.928066e-01 9.547647e-01

10887 9.928078e-01 9.547589e-01

10888 9.928089e-01 9.547531e-01

10889 9.928101e-01 9.547473e-01

10890 9.928112e-01 9.547414e-01

10891 9.928123e-01 9.547355e-01

10892 9.928134e-01 9.547295e-01

10893 9.928145e-01 9.547235e-01

10894 9.928155e-01 9.547174e-01

10895 9.928166e-01 9.547112e-01

10896 9.928176e-01 9.547051e-01

10897 9.928187e-01 9.546988e-01

10898 9.928197e-01 9.546925e-01

10899 9.928207e-01 9.546862e-01

10900 9.928218e-01 9.546798e-01

10901 9.928228e-01 9.546734e-01

10902 9.928238e-01 9.546669e-01

10903 9.928247e-01 9.546604e-01

10904 9.928257e-01 9.546538e-01

10905 9.928267e-01 9.546471e-01

10906 9.928277e-01 9.546405e-01

10907 9.928286e-01 9.546337e-01

10908 9.928296e-01 9.546269e-01

10909 9.928305e-01 9.546201e-01

10910 9.928315e-01 9.546132e-01

10911 9.928324e-01 9.546062e-01

10912 9.928333e-01 9.545993e-01

10913 9.928343e-01 9.545922e-01

10914 9.928352e-01 9.545851e-01

10915 9.928361e-01 9.545780e-01

10916 9.928370e-01 9.545708e-01

10917 9.928379e-01 9.545635e-01

10918 9.928389e-01 9.545562e-01

10919 9.928398e-01 9.545489e-01

10920 9.928407e-01 9.545415e-01

10921 9.928416e-01 9.545340e-01

10922 9.928425e-01 9.545265e-01

10923 9.928434e-01 9.545190e-01

10924 9.928443e-01 9.545114e-01

10925 9.928452e-01 9.545037e-01

10926 9.928461e-01 9.544960e-01

10927 9.928470e-01 9.544882e-01

10928 9.928479e-01 9.544804e-01

10929 9.928488e-01 9.544726e-01

10930 9.928497e-01 9.544647e-01

10931 9.928505e-01 9.544567e-01

10932 9.928514e-01 9.544487e-01

10933 9.928523e-01 9.544407e-01

10934 9.928532e-01 9.544326e-01

10935 9.928541e-01 9.544244e-01

10936 9.928550e-01 9.544162e-01

10937 9.928559e-01 9.544080e-01

10938 9.928568e-01 9.543997e-01

10939 9.928577e-01 9.543913e-01

10940 9.928586e-01 9.543830e-01

10941 9.928595e-01 9.543745e-01

10942 9.928605e-01 9.543660e-01

10943 9.928614e-01 9.543575e-01

10944 9.928623e-01 9.543489e-01

10945 9.928632e-01 9.543403e-01

10946 9.928641e-01 9.543316e-01

10947 9.928650e-01 9.543229e-01

10948 9.928660e-01 9.543142e-01

10949 9.928669e-01 9.543054e-01

10950 9.928678e-01 9.542965e-01

10951 9.928688e-01 9.542876e-01

10952 9.928697e-01 9.542787e-01

10953 9.928706e-01 9.542697e-01

10954 9.928716e-01 9.542607e-01

10955 9.928725e-01 9.542516e-01

10956 9.928735e-01 9.542425e-01

10957 9.928744e-01 9.542333e-01

10958 9.928754e-01 9.542241e-01

10959 9.928764e-01 9.542149e-01

10960 9.928774e-01 9.542056e-01

10961 9.928783e-01 9.541963e-01

10962 9.928793e-01 9.541870e-01

10963 9.928803e-01 9.541776e-01

10964 9.928813e-01 9.541681e-01

10965 9.928823e-01 9.541587e-01

10966 9.928833e-01 9.541492e-01

10967 9.928843e-01 9.541396e-01

10968 9.928853e-01 9.541300e-01

10969 9.928863e-01 9.541204e-01

10970 9.928874e-01 9.541108e-01

10971 9.928884e-01 9.541011e-01

10972 9.928894e-01 9.540914e-01

10973 9.928905e-01 9.540816e-01

10974 9.928915e-01 9.540719e-01

10975 9.928926e-01 9.540620e-01

10976 9.928937e-01 9.540522e-01

10977 9.928947e-01 9.540423e-01

10978 9.928958e-01 9.540324e-01

10979 9.928969e-01 9.540225e-01

10980 9.928980e-01 9.540125e-01

10981 9.928991e-01 9.540026e-01

10982 9.929048e-01 9.540002e-01

10983 9.929103e-01 9.539977e-01

10984 9.929158e-01 9.539953e-01

10985 9.929211e-01 9.539928e-01

10986 9.929262e-01 9.539903e-01

10987 9.929313e-01 9.539877e-01

10988 9.929362e-01 9.539852e-01

10989 9.929411e-01 9.539826e-01

10990 9.929458e-01 9.539799e-01

10991 9.929504e-01 9.539773e-01

10992 9.929549e-01 9.539746e-01

10993 9.929593e-01 9.539719e-01

10994 9.929635e-01 9.539691e-01

10995 9.929677e-01 9.539663e-01

10996 9.929718e-01 9.539635e-01

10997 9.929758e-01 9.539607e-01

10998 9.929797e-01 9.539578e-01

10999 9.929836e-01 9.539549e-01

11000 9.929873e-01 9.539520e-01

11001 9.929909e-01 9.539490e-01

11002 9.929945e-01 9.539460e-01

11003 9.929980e-01 9.539429e-01

11004 9.930014e-01 9.539399e-01

11005 9.930047e-01 9.539368e-01

11006 9.930080e-01 9.539336e-01

11007 9.930111e-01 9.539305e-01

11008 9.930142e-01 9.539273e-01

11009 9.930173e-01 9.539240e-01

11010 9.930203e-01 9.539208e-01

11011 9.930232e-01 9.539175e-01

11012 9.930260e-01 9.539141e-01

11013 9.930288e-01 9.539107e-01

11014 9.930315e-01 9.539073e-01

11015 9.930342e-01 9.539039e-01

11016 9.930368e-01 9.539004e-01

11017 9.930393e-01 9.538969e-01

11018 9.930418e-01 9.538933e-01

11019 9.930443e-01 9.538897e-01

11020 9.930467e-01 9.538861e-01

11021 9.930490e-01 9.538824e-01

11022 9.930513e-01 9.538787e-01

11023 9.930536e-01 9.538750e-01

11024 9.930558e-01 9.538712e-01

11025 9.930579e-01 9.538674e-01

11026 9.930600e-01 9.538635e-01

11027 9.930621e-01 9.538596e-01

11028 9.930641e-01 9.538557e-01

11029 9.930661e-01 9.538517e-01

11030 9.930680e-01 9.538477e-01

11031 9.930699e-01 9.538437e-01

11032 9.930718e-01 9.538396e-01

11033 9.930737e-01 9.538355e-01

11034 9.930755e-01 9.538313e-01

11035 9.930772e-01 9.538271e-01

11036 9.930789e-01 9.538228e-01

11037 9.930806e-01 9.538186e-01

11038 9.930823e-01 9.538142e-01

11039 9.930840e-01 9.538098e-01

11040 9.930856e-01 9.538054e-01

11041 9.930871e-01 9.538010e-01

11042 9.930887e-01 9.537965e-01

11043 9.930902e-01 9.537919e-01

11044 9.930917e-01 9.537874e-01

11045 9.930932e-01 9.537827e-01

11046 9.930946e-01 9.537781e-01

11047 9.930960e-01 9.537734e-01

11048 9.930974e-01 9.537686e-01

11049 9.930988e-01 9.537638e-01

11050 9.931002e-01 9.537590e-01

11051 9.931015e-01 9.537541e-01

11052 9.931028e-01 9.537492e-01

11053 9.931041e-01 9.537442e-01

11054 9.931054e-01 9.537392e-01

11055 9.931066e-01 9.537341e-01

11056 9.931078e-01 9.537290e-01

11057 9.931091e-01 9.537239e-01

11058 9.931103e-01 9.537187e-01

11059 9.931114e-01 9.537135e-01

11060 9.931126e-01 9.537082e-01

11061 9.931137e-01 9.537029e-01

11062 9.931149e-01 9.536975e-01

11063 9.931160e-01 9.536921e-01

11064 9.931171e-01 9.536866e-01

11065 9.931182e-01 9.536811e-01

11066 9.931193e-01 9.536756e-01

11067 9.931203e-01 9.536700e-01

11068 9.931214e-01 9.536643e-01

11069 9.931224e-01 9.536586e-01

11070 9.931234e-01 9.536529e-01

11071 9.931245e-01 9.536471e-01

11072 9.931255e-01 9.536413e-01

11073 9.931265e-01 9.536354e-01

11074 9.931274e-01 9.536295e-01

11075 9.931284e-01 9.536235e-01

11076 9.931294e-01 9.536175e-01

11077 9.931303e-01 9.536115e-01

11078 9.931313e-01 9.536053e-01

11079 9.931322e-01 9.535992e-01

11080 9.931332e-01 9.535930e-01

11081 9.931341e-01 9.535867e-01

11082 9.931350e-01 9.535805e-01

11083 9.931359e-01 9.535741e-01

11084 9.931368e-01 9.535677e-01

11085 9.931377e-01 9.535613e-01

11086 9.931386e-01 9.535548e-01

11087 9.931395e-01 9.535483e-01

11088 9.931404e-01 9.535417e-01

11089 9.931413e-01 9.535351e-01

11090 9.931421e-01 9.535284e-01

11091 9.931430e-01 9.535217e-01

11092 9.931439e-01 9.535149e-01

11093 9.931447e-01 9.535081e-01

11094 9.931456e-01 9.535013e-01

11095 9.931464e-01 9.534944e-01

11096 9.931473e-01 9.534874e-01

11097 9.931481e-01 9.534804e-01

11098 9.931490e-01 9.534734e-01

11099 9.931498e-01 9.534663e-01

11100 9.931507e-01 9.534591e-01

11101 9.931515e-01 9.534520e-01

11102 9.931523e-01 9.534447e-01

11103 9.931532e-01 9.534375e-01

11104 9.931540e-01 9.534301e-01

11105 9.931549e-01 9.534228e-01

11106 9.931557e-01 9.534154e-01

11107 9.931565e-01 9.534079e-01

11108 9.931573e-01 9.534004e-01

11109 9.931582e-01 9.533929e-01

11110 9.931590e-01 9.533853e-01

11111 9.931598e-01 9.533776e-01

11112 9.931607e-01 9.533700e-01

11113 9.931615e-01 9.533622e-01

11114 9.931623e-01 9.533545e-01

11115 9.931632e-01 9.533467e-01

11116 9.931640e-01 9.533388e-01

11117 9.931648e-01 9.533309e-01

11118 9.931657e-01 9.533230e-01

11119 9.931665e-01 9.533150e-01

11120 9.931674e-01 9.533070e-01

11121 9.931682e-01 9.532989e-01

11122 9.931690e-01 9.532908e-01

11123 9.931699e-01 9.532826e-01

11124 9.931707e-01 9.532745e-01

11125 9.931716e-01 9.532662e-01

11126 9.931724e-01 9.532580e-01

11127 9.931733e-01 9.532496e-01

11128 9.931742e-01 9.532413e-01

11129 9.931750e-01 9.532329e-01

11130 9.931759e-01 9.532245e-01

11131 9.931768e-01 9.532160e-01

11132 9.931776e-01 9.532075e-01

11133 9.931785e-01 9.531990e-01

11134 9.931794e-01 9.531904e-01

11135 9.931803e-01 9.531818e-01

11136 9.931812e-01 9.531731e-01

11137 9.931820e-01 9.531644e-01

11138 9.931829e-01 9.531557e-01

11139 9.931838e-01 9.531470e-01

11140 9.931847e-01 9.531382e-01

11141 9.931856e-01 9.531293e-01

11142 9.931866e-01 9.531205e-01

11143 9.931875e-01 9.531116e-01

11144 9.931884e-01 9.531027e-01

11145 9.931893e-01 9.530937e-01

11146 9.931902e-01 9.530847e-01

11147 9.931912e-01 9.530757e-01

11148 9.931921e-01 9.530667e-01

11149 9.931931e-01 9.530576e-01

11150 9.931940e-01 9.530485e-01

11151 9.931950e-01 9.530394e-01

11152 9.931959e-01 9.530302e-01

11153 9.931969e-01 9.530211e-01

11154 9.931979e-01 9.530119e-01

11155 9.931988e-01 9.530026e-01

11156 9.931998e-01 9.529934e-01

11157 9.932008e-01 9.529841e-01

11158 9.932018e-01 9.529748e-01

11159 9.932028e-01 9.529655e-01

11160 9.932038e-01 9.529562e-01

11161 9.932048e-01 9.529468e-01

11162 9.932099e-01 9.529444e-01

11163 9.932148e-01 9.529420e-01

11164 9.932196e-01 9.529396e-01

11165 9.932244e-01 9.529371e-01

11166 9.932290e-01 9.529346e-01

11167 9.932335e-01 9.529321e-01

11168 9.932378e-01 9.529295e-01

11169 9.932421e-01 9.529269e-01

11170 9.932463e-01 9.529243e-01

11171 9.932504e-01 9.529217e-01

11172 9.932544e-01 9.529190e-01

11173 9.932583e-01 9.529163e-01

11174 9.932621e-01 9.529136e-01

11175 9.932659e-01 9.529109e-01

11176 9.932695e-01 9.529081e-01

11177 9.932731e-01 9.529053e-01

11178 9.932766e-01 9.529024e-01

11179 9.932800e-01 9.528995e-01

11180 9.932833e-01 9.528966e-01

11181 9.932866e-01 9.528937e-01

11182 9.932897e-01 9.528907e-01

11183 9.932929e-01 9.528877e-01

11184 9.932959e-01 9.528847e-01

11185 9.932989e-01 9.528816e-01

11186 9.933018e-01 9.528785e-01

11187 9.933046e-01 9.528754e-01

11188 9.933074e-01 9.528722e-01

11189 9.933101e-01 9.528690e-01

11190 9.933128e-01 9.528658e-01

11191 9.933154e-01 9.528626e-01

11192 9.933180e-01 9.528593e-01

11193 9.933205e-01 9.528559e-01

11194 9.933229e-01 9.528526e-01

11195 9.933253e-01 9.528492e-01

11196 9.933276e-01 9.528457e-01

11197 9.933299e-01 9.528423e-01

11198 9.933322e-01 9.528388e-01

11199 9.933343e-01 9.528352e-01

11200 9.933365e-01 9.528317e-01

11201 9.933386e-01 9.528281e-01

11202 9.933407e-01 9.528244e-01

11203 9.933427e-01 9.528207e-01

11204 9.933447e-01 9.528170e-01

11205 9.933466e-01 9.528133e-01

11206 9.933485e-01 9.528095e-01

11207 9.933504e-01 9.528057e-01

11208 9.933522e-01 9.528018e-01

11209 9.933540e-01 9.527979e-01

11210 9.933558e-01 9.527940e-01

11211 9.933575e-01 9.527900e-01

11212 9.933592e-01 9.527860e-01

11213 9.933608e-01 9.527820e-01

11214 9.933624e-01 9.527779e-01

11215 9.933640e-01 9.527738e-01

11216 9.933656e-01 9.527696e-01

11217 9.933671e-01 9.527654e-01

11218 9.933687e-01 9.527612e-01

11219 9.933701e-01 9.527569e-01

11220 9.933716e-01 9.527526e-01

11221 9.933730e-01 9.527482e-01

11222 9.933744e-01 9.527438e-01

11223 9.933758e-01 9.527394e-01

11224 9.933772e-01 9.527349e-01

11225 9.933785e-01 9.527304e-01

11226 9.933798e-01 9.527258e-01

11227 9.933811e-01 9.527212e-01

11228 9.933824e-01 9.527166e-01

11229 9.933836e-01 9.527119e-01

11230 9.933849e-01 9.527072e-01

11231 9.933861e-01 9.527025e-01

11232 9.933873e-01 9.526977e-01

11233 9.933885e-01 9.526928e-01

11234 9.933896e-01 9.526879e-01

11235 9.933908e-01 9.526830e-01

11236 9.933919e-01 9.526781e-01

11237 9.933930e-01 9.526731e-01

11238 9.933941e-01 9.526680e-01

11239 9.933952e-01 9.526629e-01

11240 9.933962e-01 9.526578e-01

11241 9.933973e-01 9.526526e-01

11242 9.933983e-01 9.526474e-01

11243 9.933993e-01 9.526421e-01

11244 9.934003e-01 9.526368e-01

11245 9.934013e-01 9.526315e-01

11246 9.934023e-01 9.526261e-01

11247 9.934033e-01 9.526207e-01

11248 9.934043e-01 9.526152e-01

11249 9.934052e-01 9.526097e-01

11250 9.934062e-01 9.526042e-01

11251 9.934071e-01 9.525986e-01

11252 9.934080e-01 9.525929e-01

11253 9.934089e-01 9.525872e-01

11254 9.934098e-01 9.525815e-01

11255 9.934107e-01 9.525758e-01

11256 9.934116e-01 9.525699e-01

11257 9.934125e-01 9.525641e-01

11258 9.934134e-01 9.525582e-01

11259 9.934143e-01 9.525523e-01

11260 9.934151e-01 9.525463e-01

11261 9.934160e-01 9.525402e-01

11262 9.934168e-01 9.525342e-01

11263 9.934177e-01 9.525281e-01

11264 9.934185e-01 9.525219e-01

11265 9.934193e-01 9.525157e-01

11266 9.934201e-01 9.525095e-01

11267 9.934210e-01 9.525032e-01

11268 9.934218e-01 9.524969e-01

11269 9.934226e-01 9.524905e-01

11270 9.934234e-01 9.524841e-01

11271 9.934242e-01 9.524776e-01

11272 9.934250e-01 9.524711e-01

11273 9.934258e-01 9.524646e-01

11274 9.934266e-01 9.524580e-01

11275 9.934274e-01 9.524514e-01

11276 9.934282e-01 9.524447e-01

11277 9.934290e-01 9.524380e-01

11278 9.934297e-01 9.524313e-01

11279 9.934305e-01 9.524245e-01

11280 9.934313e-01 9.524176e-01

11281 9.934321e-01 9.524108e-01

11282 9.934329e-01 9.524038e-01

11283 9.934336e-01 9.523969e-01

11284 9.934344e-01 9.523899e-01

11285 9.934352e-01 9.523829e-01

11286 9.934359e-01 9.523758e-01

11287 9.934367e-01 9.523687e-01

11288 9.934375e-01 9.523615e-01

11289 9.934383e-01 9.523543e-01

11290 9.934390e-01 9.523471e-01

11291 9.934398e-01 9.523398e-01

11292 9.934406e-01 9.523325e-01

11293 9.934414e-01 9.523251e-01

11294 9.934421e-01 9.523177e-01

11295 9.934429e-01 9.523103e-01

11296 9.934437e-01 9.523028e-01

11297 9.934444e-01 9.522953e-01

11298 9.934452e-01 9.522878e-01

11299 9.934460e-01 9.522802e-01

11300 9.934468e-01 9.522726e-01

11301 9.934476e-01 9.522649e-01

11302 9.934484e-01 9.522573e-01

11303 9.934491e-01 9.522495e-01

11304 9.934499e-01 9.522418e-01

11305 9.934507e-01 9.522340e-01

11306 9.934515e-01 9.522261e-01

11307 9.934523e-01 9.522183e-01

11308 9.934531e-01 9.522104e-01

11309 9.934539e-01 9.522025e-01

11310 9.934547e-01 9.521945e-01

11311 9.934555e-01 9.521865e-01

11312 9.934563e-01 9.521785e-01

11313 9.934571e-01 9.521704e-01

11314 9.934580e-01 9.521623e-01

11315 9.934588e-01 9.521542e-01

11316 9.934596e-01 9.521461e-01

11317 9.934604e-01 9.521379e-01

11318 9.934613e-01 9.521297e-01

11319 9.934621e-01 9.521215e-01

11320 9.934629e-01 9.521132e-01

11321 9.934638e-01 9.521049e-01

11322 9.934646e-01 9.520966e-01

11323 9.934655e-01 9.520883e-01

11324 9.934663e-01 9.520799e-01

11325 9.934672e-01 9.520715e-01

11326 9.934681e-01 9.520631e-01

11327 9.934689e-01 9.520547e-01

11328 9.934698e-01 9.520462e-01

11329 9.934707e-01 9.520377e-01

11330 9.934716e-01 9.520292e-01

11331 9.934725e-01 9.520207e-01

11332 9.934734e-01 9.520122e-01

11333 9.934743e-01 9.520036e-01

11334 9.934752e-01 9.519950e-01

11335 9.934761e-01 9.519864e-01

11336 9.934770e-01 9.519778e-01

11337 9.934780e-01 9.519692e-01

11338 9.934789e-01 9.519605e-01

11339 9.934798e-01 9.519518e-01

11340 9.934808e-01 9.519432e-01

11341 9.934817e-01 9.519345e-01

11342 9.934862e-01 9.519321e-01

11343 9.934906e-01 9.519297e-01

11344 9.934949e-01 9.519273e-01

11345 9.934991e-01 9.519249e-01

11346 9.935032e-01 9.519224e-01

11347 9.935072e-01 9.519199e-01

11348 9.935111e-01 9.519174e-01

11349 9.935149e-01 9.519149e-01

11350 9.935187e-01 9.519123e-01

11351 9.935223e-01 9.519097e-01

11352 9.935259e-01 9.519071e-01

11353 9.935294e-01 9.519044e-01

11354 9.935328e-01 9.519017e-01

11355 9.935361e-01 9.518990e-01

11356 9.935394e-01 9.518963e-01

11357 9.935426e-01 9.518935e-01

11358 9.935457e-01 9.518907e-01

11359 9.935487e-01 9.518879e-01

11360 9.935517e-01 9.518850e-01

11361 9.935546e-01 9.518821e-01

11362 9.935575e-01 9.518792e-01

11363 9.935602e-01 9.518763e-01

11364 9.935630e-01 9.518733e-01

11365 9.935656e-01 9.518703e-01

11366 9.935682e-01 9.518672e-01

11367 9.935708e-01 9.518642e-01

11368 9.935733e-01 9.518610e-01

11369 9.935757e-01 9.518579e-01

11370 9.935781e-01 9.518547e-01

11371 9.935805e-01 9.518516e-01

11372 9.935828e-01 9.518483e-01

11373 9.935850e-01 9.518451e-01

11374 9.935872e-01 9.518418e-01

11375 9.935893e-01 9.518384e-01

11376 9.935914e-01 9.518351e-01

11377 9.935935e-01 9.518317e-01

11378 9.935955e-01 9.518283e-01

11379 9.935975e-01 9.518248e-01

11380 9.935994e-01 9.518213e-01

11381 9.936013e-01 9.518178e-01

11382 9.936032e-01 9.518142e-01

11383 9.936050e-01 9.518106e-01

11384 9.936068e-01 9.518070e-01

11385 9.936086e-01 9.518033e-01

11386 9.936103e-01 9.517997e-01

11387 9.936120e-01 9.517959e-01

11388 9.936136e-01 9.517922e-01

11389 9.936152e-01 9.517884e-01

11390 9.936168e-01 9.517845e-01

11391 9.936184e-01 9.517807e-01

11392 9.936199e-01 9.517767e-01

11393 9.936214e-01 9.517728e-01

11394 9.936229e-01 9.517688e-01

11395 9.936243e-01 9.517648e-01

11396 9.936258e-01 9.517608e-01

11397 9.936271e-01 9.517567e-01

11398 9.936285e-01 9.517526e-01

11399 9.936299e-01 9.517484e-01

11400 9.936312e-01 9.517442e-01

11401 9.936325e-01 9.517400e-01

11402 9.936338e-01 9.517357e-01

11403 9.936350e-01 9.517314e-01

11404 9.936363e-01 9.517271e-01

11405 9.936375e-01 9.517227e-01

11406 9.936387e-01 9.517183e-01

11407 9.936399e-01 9.517138e-01

11408 9.936410e-01 9.517093e-01

11409 9.936422e-01 9.517048e-01

11410 9.936433e-01 9.517002e-01

11411 9.936444e-01 9.516956e-01

11412 9.936455e-01 9.516910e-01

11413 9.936465e-01 9.516863e-01

11414 9.936476e-01 9.516815e-01

11415 9.936486e-01 9.516768e-01

11416 9.936497e-01 9.516720e-01

11417 9.936507e-01 9.516672e-01

11418 9.936517e-01 9.516623e-01

11419 9.936527e-01 9.516574e-01

11420 9.936536e-01 9.516524e-01

11421 9.936546e-01 9.516474e-01

11422 9.936556e-01 9.516424e-01

11423 9.936565e-01 9.516373e-01

11424 9.936574e-01 9.516322e-01

11425 9.936583e-01 9.516271e-01

11426 9.936592e-01 9.516219e-01

11427 9.936601e-01 9.516167e-01

11428 9.936610e-01 9.516114e-01

11429 9.936619e-01 9.516061e-01

11430 9.936628e-01 9.516007e-01

11431 9.936636e-01 9.515954e-01

11432 9.936645e-01 9.515899e-01

11433 9.936653e-01 9.515845e-01

11434 9.936662e-01 9.515790e-01

11435 9.936670e-01 9.515734e-01

11436 9.936678e-01 9.515679e-01

11437 9.936686e-01 9.515623e-01

11438 9.936694e-01 9.515566e-01

11439 9.936702e-01 9.515509e-01

11440 9.936710e-01 9.515452e-01

11441 9.936718e-01 9.515394e-01

11442 9.936726e-01 9.515336e-01

11443 9.936734e-01 9.515277e-01

11444 9.936741e-01 9.515218e-01

11445 9.936749e-01 9.515159e-01

11446 9.936757e-01 9.515100e-01

11447 9.936764e-01 9.515039e-01

11448 9.936772e-01 9.514979e-01

11449 9.936779e-01 9.514918e-01

11450 9.936787e-01 9.514857e-01

11451 9.936794e-01 9.514796e-01

11452 9.936802e-01 9.514734e-01

11453 9.936809e-01 9.514671e-01

11454 9.936816e-01 9.514609e-01

11455 9.936824e-01 9.514545e-01

11456 9.936831e-01 9.514482e-01

11457 9.936838e-01 9.514418e-01

11458 9.936845e-01 9.514354e-01

11459 9.936853e-01 9.514289e-01

11460 9.936860e-01 9.514225e-01

11461 9.936867e-01 9.514159e-01

11462 9.936874e-01 9.514094e-01

11463 9.936882e-01 9.514028e-01

11464 9.936889e-01 9.513961e-01

11465 9.936896e-01 9.513895e-01

11466 9.936903e-01 9.513827e-01

11467 9.936910e-01 9.513760e-01

11468 9.936917e-01 9.513692e-01

11469 9.936925e-01 9.513624e-01

11470 9.936932e-01 9.513556e-01

11471 9.936939e-01 9.513487e-01

11472 9.936946e-01 9.513418e-01

11473 9.936953e-01 9.513348e-01

11474 9.936961e-01 9.513279e-01

11475 9.936968e-01 9.513208e-01

11476 9.936975e-01 9.513138e-01

11477 9.936982e-01 9.513067e-01

11478 9.936989e-01 9.512996e-01

11479 9.936997e-01 9.512925e-01

11480 9.937004e-01 9.512853e-01

11481 9.937011e-01 9.512781e-01

11482 9.937019e-01 9.512709e-01

11483 9.937026e-01 9.512636e-01

11484 9.937033e-01 9.512563e-01

11485 9.937041e-01 9.512490e-01

11486 9.937048e-01 9.512417e-01

11487 9.937056e-01 9.512343e-01

11488 9.937063e-01 9.512269e-01

11489 9.937071e-01 9.512195e-01

11490 9.937078e-01 9.512120e-01

11491 9.937086e-01 9.512045e-01

11492 9.937093e-01 9.511970e-01

11493 9.937101e-01 9.511895e-01

11494 9.937109e-01 9.511820e-01

11495 9.937116e-01 9.511744e-01

11496 9.937124e-01 9.511668e-01

11497 9.937132e-01 9.511592e-01

11498 9.937140e-01 9.511515e-01

11499 9.937148e-01 9.511438e-01

11500 9.937155e-01 9.511361e-01

11501 9.937163e-01 9.511284e-01

11502 9.937171e-01 9.511207e-01

11503 9.937179e-01 9.511130e-01

11504 9.937188e-01 9.511052e-01

11505 9.937196e-01 9.510974e-01

11506 9.937204e-01 9.510896e-01

11507 9.937212e-01 9.510818e-01

11508 9.937220e-01 9.510739e-01

11509 9.937229e-01 9.510661e-01

11510 9.937237e-01 9.510582e-01

11511 9.937246e-01 9.510503e-01

11512 9.937254e-01 9.510424e-01

11513 9.937263e-01 9.510345e-01

11514 9.937271e-01 9.510266e-01

11515 9.937280e-01 9.510187e-01

11516 9.937289e-01 9.510107e-01

11517 9.937298e-01 9.510028e-01

11518 9.937307e-01 9.509948e-01

11519 9.937316e-01 9.509868e-01

11520 9.937325e-01 9.509788e-01

11521 9.937334e-01 9.509708e-01

11522 9.937374e-01 9.509685e-01

11523 9.937413e-01 9.509662e-01

11524 9.937451e-01 9.509638e-01

11525 9.937489e-01 9.509614e-01

11526 9.937525e-01 9.509590e-01

11527 9.937561e-01 9.509566e-01

11528 9.937596e-01 9.509541e-01

11529 9.937630e-01 9.509516e-01

11530 9.937663e-01 9.509491e-01

11531 9.937696e-01 9.509465e-01

11532 9.937728e-01 9.509440e-01

11533 9.937759e-01 9.509414e-01

11534 9.937790e-01 9.509387e-01

11535 9.937819e-01 9.509361e-01

11536 9.937849e-01 9.509334e-01

11537 9.937877e-01 9.509307e-01

11538 9.937905e-01 9.509279e-01

11539 9.937932e-01 9.509252e-01

11540 9.937959e-01 9.509224e-01

11541 9.937985e-01 9.509195e-01

11542 9.938011e-01 9.509167e-01

11543 9.938036e-01 9.509138e-01

11544 9.938060e-01 9.509109e-01

11545 9.938084e-01 9.509080e-01

11546 9.938107e-01 9.509050e-01

11547 9.938130e-01 9.509020e-01

11548 9.938153e-01 9.508990e-01

11549 9.938175e-01 9.508959e-01

11550 9.938196e-01 9.508928e-01

11551 9.938217e-01 9.508897e-01

11552 9.938238e-01 9.508866e-01

11553 9.938258e-01 9.508834e-01

11554 9.938278e-01 9.508802e-01

11555 9.938297e-01 9.508769e-01

11556 9.938316e-01 9.508737e-01

11557 9.938335e-01 9.508704e-01

11558 9.938353e-01 9.508670e-01

11559 9.938371e-01 9.508637e-01

11560 9.938388e-01 9.508603e-01

11561 9.938405e-01 9.508569e-01

11562 9.938422e-01 9.508534e-01

11563 9.938439e-01 9.508499e-01

11564 9.938455e-01 9.508464e-01

11565 9.938471e-01 9.508428e-01

11566 9.938486e-01 9.508393e-01

11567 9.938501e-01 9.508356e-01

11568 9.938516e-01 9.508320e-01

11569 9.938531e-01 9.508283e-01

11570 9.938546e-01 9.508246e-01

11571 9.938560e-01 9.508208e-01

11572 9.938574e-01 9.508171e-01

11573 9.938587e-01 9.508132e-01

11574 9.938601e-01 9.508094e-01

11575 9.938614e-01 9.508055e-01

11576 9.938627e-01 9.508016e-01

11577 9.938639e-01 9.507977e-01

11578 9.938652e-01 9.507937e-01

11579 9.938664e-01 9.507897e-01

11580 9.938676e-01 9.507856e-01

11581 9.938688e-01 9.507815e-01

11582 9.938699e-01 9.507774e-01

11583 9.938711e-01 9.507732e-01

11584 9.938722e-01 9.507691e-01

11585 9.938733e-01 9.507648e-01

11586 9.938744e-01 9.507606e-01

11587 9.938755e-01 9.507563e-01

11588 9.938766e-01 9.507520e-01

11589 9.938776e-01 9.507476e-01

11590 9.938786e-01 9.507432e-01

11591 9.938796e-01 9.507388e-01

11592 9.938806e-01 9.507343e-01

11593 9.938816e-01 9.507298e-01

11594 9.938826e-01 9.507253e-01

11595 9.938835e-01 9.507207e-01

11596 9.938845e-01 9.507161e-01

11597 9.938854e-01 9.507114e-01

11598 9.938863e-01 9.507068e-01

11599 9.938872e-01 9.507021e-01

11600 9.938881e-01 9.506973e-01

11601 9.938890e-01 9.506925e-01

11602 9.938899e-01 9.506877e-01

11603 9.938907e-01 9.506828e-01

11604 9.938916e-01 9.506780e-01

11605 9.938924e-01 9.506730e-01

11606 9.938933e-01 9.506681e-01

11607 9.938941e-01 9.506631e-01

11608 9.938949e-01 9.506580e-01

11609 9.938957e-01 9.506530e-01

11610 9.938965e-01 9.506479e-01

11611 9.938973e-01 9.506427e-01

11612 9.938981e-01 9.506376e-01

11613 9.938989e-01 9.506324e-01

11614 9.938996e-01 9.506271e-01

11615 9.939004e-01 9.506218e-01

11616 9.939011e-01 9.506165e-01

11617 9.939019e-01 9.506112e-01

11618 9.939026e-01 9.506058e-01

11619 9.939034e-01 9.506004e-01

11620 9.939041e-01 9.505949e-01

11621 9.939048e-01 9.505894e-01

11622 9.939056e-01 9.505839e-01

11623 9.939063e-01 9.505784e-01

11624 9.939070e-01 9.505728e-01

11625 9.939077e-01 9.505672e-01

11626 9.939084e-01 9.505615e-01

11627 9.939091e-01 9.505558e-01

11628 9.939098e-01 9.505501e-01

11629 9.939105e-01 9.505443e-01

11630 9.939112e-01 9.505385e-01

11631 9.939119e-01 9.505327e-01

11632 9.939126e-01 9.505268e-01

11633 9.939133e-01 9.505210e-01

11634 9.939139e-01 9.505150e-01

11635 9.939146e-01 9.505091e-01

11636 9.939153e-01 9.505031e-01

11637 9.939160e-01 9.504971e-01

11638 9.939167e-01 9.504910e-01

11639 9.939173e-01 9.504849e-01

11640 9.939180e-01 9.504788e-01

11641 9.939187e-01 9.504727e-01

11642 9.939194e-01 9.504665e-01

11643 9.939200e-01 9.504603e-01

11644 9.939207e-01 9.504540e-01

11645 9.939214e-01 9.504478e-01

11646 9.939220e-01 9.504415e-01

11647 9.939227e-01 9.504351e-01

11648 9.939234e-01 9.504288e-01

11649 9.939240e-01 9.504224e-01

11650 9.939247e-01 9.504160e-01

11651 9.939254e-01 9.504095e-01

11652 9.939261e-01 9.504030e-01

11653 9.939267e-01 9.503965e-01

11654 9.939274e-01 9.503900e-01

11655 9.939281e-01 9.503835e-01

11656 9.939288e-01 9.503769e-01

11657 9.939294e-01 9.503703e-01

11658 9.939301e-01 9.503636e-01

11659 9.939308e-01 9.503570e-01

11660 9.939315e-01 9.503503e-01

11661 9.939322e-01 9.503436e-01

11662 9.939329e-01 9.503368e-01

11663 9.939336e-01 9.503301e-01

11664 9.939342e-01 9.503233e-01

11665 9.939349e-01 9.503165e-01

11666 9.939356e-01 9.503097e-01

11667 9.939363e-01 9.503028e-01

11668 9.939371e-01 9.502960e-01

11669 9.939378e-01 9.502891e-01

11670 9.939385e-01 9.502822e-01

11671 9.939392e-01 9.502752e-01

11672 9.939399e-01 9.502683e-01

11673 9.939406e-01 9.502613e-01

11674 9.939414e-01 9.502543e-01

11675 9.939421e-01 9.502473e-01

11676 9.939428e-01 9.502403e-01

11677 9.939436e-01 9.502333e-01

11678 9.939443e-01 9.502262e-01

11679 9.939450e-01 9.502191e-01

11680 9.939458e-01 9.502120e-01

11681 9.939466e-01 9.502049e-01

11682 9.939473e-01 9.501978e-01

11683 9.939481e-01 9.501907e-01

11684 9.939488e-01 9.501835e-01

11685 9.939496e-01 9.501764e-01

11686 9.939504e-01 9.501692e-01

11687 9.939512e-01 9.501620e-01

11688 9.939520e-01 9.501549e-01

11689 9.939528e-01 9.501477e-01

11690 9.939536e-01 9.501405e-01

11691 9.939544e-01 9.501332e-01

11692 9.939552e-01 9.501260e-01

11693 9.939560e-01 9.501188e-01

11694 9.939568e-01 9.501116e-01

11695 9.939577e-01 9.501043e-01

11696 9.939585e-01 9.500971e-01

11697 9.939594e-01 9.500898e-01

11698 9.939602e-01 9.500826e-01

11699 9.939611e-01 9.500753e-01

11700 9.939619e-01 9.500681e-01

11701 9.939628e-01 9.500608e-01

11702 9.939664e-01 9.500585e-01

11703 9.939699e-01 9.500563e-01

11704 9.939733e-01 9.500539e-01

11705 9.939766e-01 9.500516e-01

11706 9.939799e-01 9.500493e-01

11707 9.939831e-01 9.500469e-01

11708 9.939862e-01 9.500445e-01

11709 9.939893e-01 9.500420e-01

11710 9.939923e-01 9.500396e-01

11711 9.939952e-01 9.500371e-01

11712 9.939981e-01 9.500346e-01

11713 9.940009e-01 9.500321e-01

11714 9.940036e-01 9.500295e-01

11715 9.940063e-01 9.500269e-01

11716 9.940089e-01 9.500243e-01

11717 9.940114e-01 9.500217e-01

11718 9.940139e-01 9.500190e-01

11719 9.940164e-01 9.500163e-01

11720 9.940188e-01 9.500136e-01

11721 9.940211e-01 9.500109e-01

11722 9.940234e-01 9.500081e-01

11723 9.940257e-01 9.500053e-01

11724 9.940279e-01 9.500025e-01

11725 9.940300e-01 9.499996e-01

11726 9.940321e-01 9.499967e-01

11727 9.940342e-01 9.499938e-01

11728 9.940362e-01 9.499909e-01

11729 9.940382e-01 9.499879e-01

11730 9.940401e-01 9.499849e-01

11731 9.940420e-01 9.499819e-01

11732 9.940439e-01 9.499789e-01

11733 9.940457e-01 9.499758e-01

11734 9.940475e-01 9.499727e-01

11735 9.940493e-01 9.499696e-01

11736 9.940510e-01 9.499664e-01

11737 9.940526e-01 9.499632e-01

11738 9.940543e-01 9.499600e-01

11739 9.940559e-01 9.499568e-01

11740 9.940575e-01 9.499535e-01

11741 9.940590e-01 9.499502e-01

11742 9.940606e-01 9.499468e-01

11743 9.940620e-01 9.499435e-01

11744 9.940635e-01 9.499401e-01

11745 9.940649e-01 9.499367e-01

11746 9.940664e-01 9.499332e-01

11747 9.940677e-01 9.499297e-01

11748 9.940691e-01 9.499262e-01

11749 9.940704e-01 9.499227e-01

11750 9.940717e-01 9.499191e-01

11751 9.940730e-01 9.499155e-01

11752 9.940743e-01 9.499118e-01

11753 9.940755e-01 9.499082e-01

11754 9.940767e-01 9.499045e-01

11755 9.940779e-01 9.499007e-01

11756 9.940791e-01 9.498970e-01

11757 9.940803e-01 9.498932e-01

11758 9.940814e-01 9.498894e-01

11759 9.940825e-01 9.498855e-01

11760 9.940836e-01 9.498816e-01

11761 9.940847e-01 9.498777e-01

11762 9.940857e-01 9.498738e-01

11763 9.940868e-01 9.498698e-01

11764 9.940878e-01 9.498658e-01

11765 9.940888e-01 9.498618e-01

11766 9.940898e-01 9.498577e-01

11767 9.940908e-01 9.498536e-01

11768 9.940918e-01 9.498494e-01

11769 9.940927e-01 9.498453e-01

11770 9.940937e-01 9.498411e-01

11771 9.940946e-01 9.498368e-01

11772 9.940955e-01 9.498326e-01

11773 9.940964e-01 9.498283e-01

11774 9.940973e-01 9.498240e-01

11775 9.940982e-01 9.498196e-01

11776 9.940990e-01 9.498152e-01

11777 9.940999e-01 9.498108e-01

11778 9.941007e-01 9.498063e-01

11779 9.941016e-01 9.498019e-01

11780 9.941024e-01 9.497973e-01

11781 9.941032e-01 9.497928e-01

11782 9.941040e-01 9.497882e-01

11783 9.941048e-01 9.497836e-01

11784 9.941056e-01 9.497790e-01

11785 9.941063e-01 9.497743e-01

11786 9.941071e-01 9.497696e-01

11787 9.941079e-01 9.497648e-01

11788 9.941086e-01 9.497601e-01

11789 9.941094e-01 9.497553e-01

11790 9.941101e-01 9.497504e-01

11791 9.941108e-01 9.497456e-01

11792 9.941116e-01 9.497407e-01

11793 9.941123e-01 9.497358e-01

11794 9.941130e-01 9.497308e-01

11795 9.941137e-01 9.497258e-01

11796 9.941144e-01 9.497208e-01

11797 9.941151e-01 9.497157e-01

11798 9.941158e-01 9.497107e-01

11799 9.941165e-01 9.497055e-01

11800 9.941171e-01 9.497004e-01

11801 9.941178e-01 9.496952e-01

11802 9.941185e-01 9.496900e-01

11803 9.941192e-01 9.496848e-01

11804 9.941198e-01 9.496795e-01

11805 9.941205e-01 9.496743e-01

11806 9.941211e-01 9.496689e-01

11807 9.941218e-01 9.496636e-01

11808 9.941224e-01 9.496582e-01

11809 9.941231e-01 9.496528e-01

11810 9.941237e-01 9.496474e-01

11811 9.941244e-01 9.496419e-01

11812 9.941250e-01 9.496364e-01

11813 9.941257e-01 9.496309e-01

11814 9.941263e-01 9.496253e-01

11815 9.941269e-01 9.496198e-01

11816 9.941276e-01 9.496142e-01

11817 9.941282e-01 9.496085e-01

11818 9.941288e-01 9.496029e-01

11819 9.941295e-01 9.495972e-01

11820 9.941301e-01 9.495915e-01

11821 9.941307e-01 9.495857e-01

11822 9.941313e-01 9.495800e-01

11823 9.941320e-01 9.495742e-01

11824 9.941326e-01 9.495684e-01

11825 9.941332e-01 9.495625e-01

11826 9.941339e-01 9.495567e-01

11827 9.941345e-01 9.495508e-01

11828 9.941351e-01 9.495449e-01

11829 9.941357e-01 9.495389e-01

11830 9.941364e-01 9.495330e-01

11831 9.941370e-01 9.495270e-01

11832 9.941376e-01 9.495210e-01

11833 9.941383e-01 9.495150e-01

11834 9.941389e-01 9.495089e-01

11835 9.941395e-01 9.495029e-01

11836 9.941402e-01 9.494968e-01

11837 9.941408e-01 9.494907e-01

11838 9.941415e-01 9.494845e-01

11839 9.941421e-01 9.494784e-01

11840 9.941428e-01 9.494722e-01

11841 9.941434e-01 9.494660e-01

11842 9.941441e-01 9.494598e-01

11843 9.941447e-01 9.494536e-01

11844 9.941454e-01 9.494474e-01

11845 9.941460e-01 9.494411e-01

11846 9.941467e-01 9.494348e-01

11847 9.941473e-01 9.494285e-01

11848 9.941480e-01 9.494222e-01

11849 9.941487e-01 9.494159e-01

11850 9.941494e-01 9.494096e-01

11851 9.941500e-01 9.494032e-01

11852 9.941507e-01 9.493968e-01

11853 9.941514e-01 9.493905e-01

11854 9.941521e-01 9.493841e-01

11855 9.941528e-01 9.493777e-01

11856 9.941535e-01 9.493712e-01

11857 9.941542e-01 9.493648e-01

11858 9.941549e-01 9.493584e-01

11859 9.941556e-01 9.493519e-01

11860 9.941563e-01 9.493455e-01

11861 9.941570e-01 9.493390e-01

11862 9.941577e-01 9.493325e-01

11863 9.941585e-01 9.493260e-01

11864 9.941592e-01 9.493196e-01

11865 9.941599e-01 9.493131e-01

11866 9.941607e-01 9.493066e-01

11867 9.941614e-01 9.493001e-01

11868 9.941622e-01 9.492935e-01

11869 9.941629e-01 9.492870e-01

11870 9.941637e-01 9.492805e-01

11871 9.941644e-01 9.492740e-01

11872 9.941652e-01 9.492675e-01

11873 9.941660e-01 9.492609e-01

11874 9.941668e-01 9.492544e-01

11875 9.941676e-01 9.492479e-01

11876 9.941684e-01 9.492414e-01

11877 9.941692e-01 9.492348e-01

11878 9.941700e-01 9.492283e-01

11879 9.941708e-01 9.492218e-01

11880 9.941716e-01 9.492153e-01

11881 9.941724e-01 9.492088e-01

11882 9.941756e-01 9.492066e-01

11883 9.941788e-01 9.492044e-01

11884 9.941818e-01 9.492021e-01

11885 9.941848e-01 9.491999e-01

11886 9.941877e-01 9.491976e-01

11887 9.941906e-01 9.491953e-01

11888 9.941934e-01 9.491929e-01

11889 9.941962e-01 9.491906e-01

11890 9.941988e-01 9.491882e-01

11891 9.942015e-01 9.491858e-01

11892 9.942040e-01 9.491834e-01

11893 9.942065e-01 9.491809e-01

11894 9.942090e-01 9.491785e-01

11895 9.942114e-01 9.491760e-01

11896 9.942137e-01 9.491734e-01

11897 9.942160e-01 9.491709e-01

11898 9.942183e-01 9.491683e-01

11899 9.942205e-01 9.491657e-01

11900 9.942226e-01 9.491631e-01

11901 9.942248e-01 9.491605e-01

11902 9.942268e-01 9.491578e-01

11903 9.942289e-01 9.491551e-01

11904 9.942308e-01 9.491524e-01

11905 9.942328e-01 9.491496e-01

11906 9.942347e-01 9.491469e-01

11907 9.942365e-01 9.491441e-01

11908 9.942384e-01 9.491412e-01

11909 9.942401e-01 9.491384e-01

11910 9.942419e-01 9.491355e-01

11911 9.942436e-01 9.491326e-01

11912 9.942453e-01 9.491297e-01

11913 9.942469e-01 9.491267e-01

11914 9.942486e-01 9.491238e-01

11915 9.942501e-01 9.491207e-01

11916 9.942517e-01 9.491177e-01

11917 9.942532e-01 9.491147e-01

11918 9.942547e-01 9.491116e-01

11919 9.942562e-01 9.491085e-01

11920 9.942576e-01 9.491053e-01

11921 9.942590e-01 9.491021e-01

11922 9.942604e-01 9.490990e-01

11923 9.942617e-01 9.490957e-01

11924 9.942631e-01 9.490925e-01

11925 9.942644e-01 9.490892e-01

11926 9.942656e-01 9.490859e-01

11927 9.942669e-01 9.490826e-01

11928 9.942681e-01 9.490792e-01

11929 9.942693e-01 9.490758e-01

11930 9.942705e-01 9.490724e-01

11931 9.942717e-01 9.490690e-01

11932 9.942728e-01 9.490655e-01

11933 9.942740e-01 9.490620e-01

11934 9.942751e-01 9.490585e-01

11935 9.942762e-01 9.490549e-01

11936 9.942772e-01 9.490513e-01

11937 9.942783e-01 9.490477e-01

11938 9.942793e-01 9.490441e-01

11939 9.942803e-01 9.490404e-01

11940 9.942814e-01 9.490367e-01

11941 9.942823e-01 9.490330e-01

11942 9.942833e-01 9.490293e-01

11943 9.942843e-01 9.490255e-01

11944 9.942852e-01 9.490217e-01

11945 9.942861e-01 9.490178e-01

11946 9.942870e-01 9.490140e-01

11947 9.942879e-01 9.490101e-01

11948 9.942888e-01 9.490062e-01

11949 9.942897e-01 9.490022e-01

11950 9.942906e-01 9.489982e-01

11951 9.942914e-01 9.489942e-01

11952 9.942923e-01 9.489902e-01

11953 9.942931e-01 9.489861e-01

11954 9.942939e-01 9.489820e-01

11955 9.942947e-01 9.489779e-01

11956 9.942955e-01 9.489738e-01

11957 9.942963e-01 9.489696e-01

11958 9.942971e-01 9.489654e-01

11959 9.942978e-01 9.489612e-01

11960 9.942986e-01 9.489569e-01

11961 9.942993e-01 9.489526e-01

11962 9.943001e-01 9.489483e-01

11963 9.943008e-01 9.489440e-01

11964 9.943015e-01 9.489396e-01

11965 9.943022e-01 9.489352e-01

11966 9.943029e-01 9.489308e-01

11967 9.943036e-01 9.489263e-01

11968 9.943043e-01 9.489218e-01

11969 9.943050e-01 9.489173e-01

11970 9.943057e-01 9.489128e-01

11971 9.943064e-01 9.489082e-01

11972 9.943071e-01 9.489036e-01

11973 9.943077e-01 9.488990e-01

11974 9.943084e-01 9.488944e-01

11975 9.943090e-01 9.488897e-01

11976 9.943097e-01 9.488850e-01

11977 9.943103e-01 9.488803e-01

11978 9.943110e-01 9.488755e-01

11979 9.943116e-01 9.488707e-01

11980 9.943122e-01 9.488659e-01

11981 9.943129e-01 9.488611e-01

11982 9.943135e-01 9.488563e-01

11983 9.943141e-01 9.488514e-01

11984 9.943147e-01 9.488465e-01

11985 9.943153e-01 9.488416e-01

11986 9.943160e-01 9.488366e-01

11987 9.943166e-01 9.488316e-01

11988 9.943172e-01 9.488266e-01

11989 9.943178e-01 9.488216e-01

11990 9.943184e-01 9.488165e-01

11991 9.943190e-01 9.488115e-01

11992 9.943196e-01 9.488064e-01

11993 9.943202e-01 9.488012e-01

11994 9.943208e-01 9.487961e-01

11995 9.943214e-01 9.487909e-01

11996 9.943220e-01 9.487857e-01

11997 9.943225e-01 9.487805e-01

11998 9.943231e-01 9.487753e-01

11999 9.943237e-01 9.487700e-01

12000 9.943243e-01 9.487648e-01

12001 9.943249e-01 9.487595e-01

12002 9.943255e-01 9.487542e-01

12003 9.943261e-01 9.487488e-01

12004 9.943267e-01 9.487435e-01

12005 9.943273e-01 9.487381e-01

12006 9.943279e-01 9.487327e-01

12007 9.943284e-01 9.487273e-01

12008 9.943290e-01 9.487218e-01

12009 9.943296e-01 9.487164e-01

12010 9.943302e-01 9.487109e-01

12011 9.943308e-01 9.487054e-01

12012 9.943314e-01 9.486999e-01

12013 9.943320e-01 9.486944e-01

12014 9.943326e-01 9.486889e-01

12015 9.943332e-01 9.486833e-01

12016 9.943338e-01 9.486777e-01

12017 9.943344e-01 9.486722e-01

12018 9.943350e-01 9.486666e-01

12019 9.943356e-01 9.486609e-01

12020 9.943362e-01 9.486553e-01

12021 9.943368e-01 9.486497e-01

12022 9.943374e-01 9.486440e-01

12023 9.943381e-01 9.486383e-01

12024 9.943387e-01 9.486327e-01

12025 9.943393e-01 9.486270e-01

12026 9.943399e-01 9.486213e-01

12027 9.943405e-01 9.486156e-01

12028 9.943412e-01 9.486098e-01

12029 9.943418e-01 9.486041e-01

12030 9.943424e-01 9.485984e-01

12031 9.943431e-01 9.485926e-01

12032 9.943437e-01 9.485868e-01

12033 9.943444e-01 9.485811e-01

12034 9.943450e-01 9.485753e-01

12035 9.943457e-01 9.485695e-01

12036 9.943463e-01 9.485637e-01

12037 9.943470e-01 9.485579e-01

12038 9.943476e-01 9.485521e-01

12039 9.943483e-01 9.485463e-01

12040 9.943490e-01 9.485405e-01

12041 9.943496e-01 9.485347e-01

12042 9.943503e-01 9.485289e-01

12043 9.943510e-01 9.485231e-01

12044 9.943517e-01 9.485173e-01

12045 9.943524e-01 9.485115e-01

12046 9.943531e-01 9.485056e-01

12047 9.943538e-01 9.484998e-01

12048 9.943545e-01 9.484940e-01

12049 9.943552e-01 9.484882e-01

12050 9.943559e-01 9.484824e-01

12051 9.943566e-01 9.484766e-01

12052 9.943573e-01 9.484707e-01

12053 9.943581e-01 9.484649e-01

12054 9.943588e-01 9.484591e-01

12055 9.943595e-01 9.484533e-01

12056 9.943603e-01 9.484476e-01

12057 9.943610e-01 9.484418e-01

12058 9.943618e-01 9.484360e-01

12059 9.943625e-01 9.484302e-01

12060 9.943633e-01 9.484245e-01

12061 9.943641e-01 9.484187e-01

12062 9.943669e-01 9.484166e-01

12063 9.943698e-01 9.484145e-01

12064 9.943725e-01 9.484123e-01

12065 9.943752e-01 9.484101e-01

12066 9.943778e-01 9.484079e-01

12067 9.943804e-01 9.484057e-01

12068 9.943829e-01 9.484035e-01

12069 9.943854e-01 9.484012e-01

12070 9.943878e-01 9.483989e-01

12071 9.943901e-01 9.483966e-01

12072 9.943924e-01 9.483943e-01

12073 9.943947e-01 9.483919e-01

12074 9.943969e-01 9.483896e-01

12075 9.943991e-01 9.483872e-01

12076 9.944012e-01 9.483847e-01

12077 9.944033e-01 9.483823e-01

12078 9.944053e-01 9.483798e-01

12079 9.944073e-01 9.483774e-01

12080 9.944092e-01 9.483749e-01

12081 9.944111e-01 9.483723e-01

12082 9.944130e-01 9.483698e-01

12083 9.944148e-01 9.483672e-01

12084 9.944166e-01 9.483646e-01

12085 9.944184e-01 9.483620e-01

12086 9.944201e-01 9.483593e-01

12087 9.944218e-01 9.483566e-01

12088 9.944234e-01 9.483539e-01

12089 9.944250e-01 9.483512e-01

12090 9.944266e-01 9.483485e-01

12091 9.944281e-01 9.483457e-01

12092 9.944297e-01 9.483429e-01

12093 9.944312e-01 9.483401e-01

12094 9.944326e-01 9.483373e-01

12095 9.944341e-01 9.483344e-01

12096 9.944355e-01 9.483315e-01

12097 9.944368e-01 9.483286e-01

12098 9.944382e-01 9.483257e-01

12099 9.944395e-01 9.483227e-01

12100 9.944408e-01 9.483197e-01

12101 9.944421e-01 9.483167e-01

12102 9.944434e-01 9.483137e-01

12103 9.944446e-01 9.483106e-01

12104 9.944458e-01 9.483075e-01

12105 9.944470e-01 9.483044e-01

12106 9.944481e-01 9.483013e-01

12107 9.944493e-01 9.482981e-01

12108 9.944504e-01 9.482950e-01

12109 9.944515e-01 9.482917e-01

12110 9.944526e-01 9.482885e-01

12111 9.944537e-01 9.482853e-01

12112 9.944547e-01 9.482820e-01

12113 9.944557e-01 9.482787e-01

12114 9.944568e-01 9.482753e-01

12115 9.944577e-01 9.482720e-01

12116 9.944587e-01 9.482686e-01

12117 9.944597e-01 9.482652e-01

12118 9.944606e-01 9.482617e-01

12119 9.944616e-01 9.482583e-01

12120 9.944625e-01 9.482548e-01

12121 9.944634e-01 9.482513e-01

12122 9.944643e-01 9.482478e-01

12123 9.944652e-01 9.482442e-01

12124 9.944660e-01 9.482406e-01

12125 9.944669e-01 9.482370e-01

12126 9.944677e-01 9.482334e-01

12127 9.944685e-01 9.482297e-01

12128 9.944693e-01 9.482260e-01

12129 9.944701e-01 9.482223e-01

12130 9.944709e-01 9.482186e-01

12131 9.944717e-01 9.482148e-01

12132 9.944725e-01 9.482110e-01

12133 9.944732e-01 9.482072e-01

12134 9.944740e-01 9.482034e-01

12135 9.944747e-01 9.481995e-01

12136 9.944755e-01 9.481957e-01

12137 9.944762e-01 9.481918e-01

12138 9.944769e-01 9.481878e-01

12139 9.944776e-01 9.481839e-01

12140 9.944783e-01 9.481799e-01

12141 9.944790e-01 9.481759e-01

12142 9.944797e-01 9.481719e-01

12143 9.944804e-01 9.481678e-01

12144 9.944810e-01 9.481637e-01

12145 9.944817e-01 9.481596e-01

12146 9.944823e-01 9.481555e-01

12147 9.944830e-01 9.481514e-01

12148 9.944836e-01 9.481472e-01

12149 9.944843e-01 9.481430e-01

12150 9.944849e-01 9.481388e-01

12151 9.944855e-01 9.481345e-01

12152 9.944862e-01 9.481303e-01

12153 9.944868e-01 9.481260e-01

12154 9.944874e-01 9.481217e-01

12155 9.944880e-01 9.481174e-01

12156 9.944886e-01 9.481130e-01

12157 9.944892e-01 9.481086e-01

12158 9.944898e-01 9.481042e-01

12159 9.944904e-01 9.480998e-01

12160 9.944910e-01 9.480954e-01

12161 9.944916e-01 9.480909e-01

12162 9.944921e-01 9.480864e-01

12163 9.944927e-01 9.480819e-01

12164 9.944933e-01 9.480774e-01

12165 9.944939e-01 9.480729e-01

12166 9.944944e-01 9.480683e-01

12167 9.944950e-01 9.480637e-01

12168 9.944956e-01 9.480591e-01

12169 9.944961e-01 9.480545e-01

12170 9.944967e-01 9.480499e-01

12171 9.944973e-01 9.480452e-01

12172 9.944978e-01 9.480405e-01

12173 9.944984e-01 9.480358e-01

12174 9.944989e-01 9.480311e-01

12175 9.944995e-01 9.480264e-01

12176 9.945000e-01 9.480216e-01

12177 9.945006e-01 9.480169e-01

12178 9.945011e-01 9.480121e-01

12179 9.945017e-01 9.480073e-01

12180 9.945022e-01 9.480025e-01

12181 9.945028e-01 9.479976e-01

12182 9.945034e-01 9.479928e-01

12183 9.945039e-01 9.479879e-01

12184 9.945045e-01 9.479830e-01

12185 9.945050e-01 9.479781e-01

12186 9.945056e-01 9.479732e-01

12187 9.945061e-01 9.479683e-01

12188 9.945067e-01 9.479634e-01

12189 9.945072e-01 9.479584e-01

12190 9.945078e-01 9.479535e-01

12191 9.945083e-01 9.479485e-01

12192 9.945089e-01 9.479435e-01

12193 9.945094e-01 9.479385e-01

12194 9.945100e-01 9.479335e-01

12195 9.945106e-01 9.479285e-01

12196 9.945111e-01 9.479234e-01

12197 9.945117e-01 9.479184e-01

12198 9.945123e-01 9.479133e-01

12199 9.945128e-01 9.479083e-01

12200 9.945134e-01 9.479032e-01

12201 9.945140e-01 9.478981e-01

12202 9.945145e-01 9.478931e-01

12203 9.945151e-01 9.478880e-01

12204 9.945157e-01 9.478829e-01

12205 9.945163e-01 9.478778e-01

12206 9.945169e-01 9.478727e-01

12207 9.945174e-01 9.478675e-01

12208 9.945180e-01 9.478624e-01

12209 9.945186e-01 9.478573e-01

12210 9.945192e-01 9.478522e-01

12211 9.945198e-01 9.478470e-01

12212 9.945204e-01 9.478419e-01

12213 9.945210e-01 9.478368e-01

12214 9.945216e-01 9.478316e-01

12215 9.945222e-01 9.478265e-01

12216 9.945228e-01 9.478213e-01

12217 9.945234e-01 9.478162e-01

12218 9.945241e-01 9.478110e-01

12219 9.945247e-01 9.478059e-01

12220 9.945253e-01 9.478008e-01

12221 9.945259e-01 9.477956e-01

12222 9.945266e-01 9.477905e-01

12223 9.945272e-01 9.477853e-01

12224 9.945278e-01 9.477802e-01

12225 9.945285e-01 9.477751e-01

12226 9.945291e-01 9.477699e-01

12227 9.945298e-01 9.477648e-01

12228 9.945304e-01 9.477597e-01

12229 9.945311e-01 9.477546e-01

12230 9.945318e-01 9.477495e-01

12231 9.945324e-01 9.477444e-01

12232 9.945331e-01 9.477393e-01

12233 9.945338e-01 9.477342e-01

12234 9.945345e-01 9.477292e-01

12235 9.945351e-01 9.477241e-01

12236 9.945358e-01 9.477191e-01

12237 9.945365e-01 9.477140e-01

12238 9.945372e-01 9.477090e-01

12239 9.945379e-01 9.477040e-01

12240 9.945386e-01 9.476990e-01

12241 9.945393e-01 9.476940e-01

12242 9.945419e-01 9.476920e-01

12243 9.945444e-01 9.476899e-01

12244 9.945469e-01 9.476879e-01

12245 9.945493e-01 9.476858e-01

12246 9.945517e-01 9.476837e-01

12247 9.945540e-01 9.476816e-01

12248 9.945563e-01 9.476794e-01

12249 9.945585e-01 9.476773e-01

12250 9.945607e-01 9.476751e-01

12251 9.945628e-01 9.476729e-01

12252 9.945649e-01 9.476707e-01

12253 9.945669e-01 9.476685e-01

12254 9.945689e-01 9.476662e-01

12255 9.945708e-01 9.476639e-01

12256 9.945727e-01 9.476616e-01

12257 9.945746e-01 9.476593e-01

12258 9.945764e-01 9.476570e-01

12259 9.945782e-01 9.476546e-01

12260 9.945800e-01 9.476522e-01

12261 9.945817e-01 9.476498e-01

12262 9.945834e-01 9.476474e-01

12263 9.945851e-01 9.476449e-01

12264 9.945867e-01 9.476425e-01

12265 9.945883e-01 9.476400e-01

12266 9.945898e-01 9.476375e-01

12267 9.945913e-01 9.476349e-01

12268 9.945928e-01 9.476324e-01

12269 9.945943e-01 9.476298e-01

12270 9.945957e-01 9.476272e-01

12271 9.945971e-01 9.476246e-01

12272 9.945985e-01 9.476220e-01

12273 9.945999e-01 9.476193e-01

12274 9.946012e-01 9.476166e-01

12275 9.946025e-01 9.476139e-01

12276 9.946038e-01 9.476112e-01

12277 9.946050e-01 9.476084e-01

12278 9.946063e-01 9.476057e-01

12279 9.946075e-01 9.476029e-01

12280 9.946086e-01 9.476001e-01

12281 9.946098e-01 9.475972e-01

12282 9.946109e-01 9.475944e-01

12283 9.946121e-01 9.475915e-01

12284 9.946132e-01 9.475886e-01

12285 9.946142e-01 9.475857e-01

12286 9.946153e-01 9.475827e-01

12287 9.946164e-01 9.475798e-01

12288 9.946174e-01 9.475768e-01

12289 9.946184e-01 9.475738e-01

12290 9.946194e-01 9.475707e-01

12291 9.946203e-01 9.475677e-01

12292 9.946213e-01 9.475646e-01

12293 9.946222e-01 9.475615e-01

12294 9.946232e-01 9.475584e-01

12295 9.946241e-01 9.475552e-01

12296 9.946250e-01 9.475521e-01

12297 9.946259e-01 9.475489e-01

12298 9.946267e-01 9.475457e-01

12299 9.946276e-01 9.475424e-01

12300 9.946284e-01 9.475392e-01

12301 9.946293e-01 9.475359e-01

12302 9.946301e-01 9.475326e-01

12303 9.946309e-01 9.475293e-01

12304 9.946317e-01 9.475259e-01

12305 9.946324e-01 9.475226e-01

12306 9.946332e-01 9.475192e-01

12307 9.946340e-01 9.475158e-01

12308 9.946347e-01 9.475123e-01

12309 9.946355e-01 9.475089e-01

12310 9.946362e-01 9.475054e-01

12311 9.946369e-01 9.475019e-01

12312 9.946376e-01 9.474984e-01

12313 9.946383e-01 9.474949e-01

12314 9.946390e-01 9.474913e-01

12315 9.946397e-01 9.474878e-01

12316 9.946404e-01 9.474842e-01

12317 9.946410e-01 9.474805e-01

12318 9.946417e-01 9.474769e-01

12319 9.946423e-01 9.474732e-01

12320 9.946430e-01 9.474696e-01

12321 9.946436e-01 9.474659e-01

12322 9.946443e-01 9.474621e-01

12323 9.946449e-01 9.474584e-01

12324 9.946455e-01 9.474546e-01

12325 9.946461e-01 9.474509e-01

12326 9.946467e-01 9.474471e-01

12327 9.946473e-01 9.474432e-01

12328 9.946479e-01 9.474394e-01

12329 9.946485e-01 9.474355e-01

12330 9.946491e-01 9.474317e-01

12331 9.946497e-01 9.474278e-01

12332 9.946503e-01 9.474239e-01

12333 9.946508e-01 9.474199e-01

12334 9.946514e-01 9.474160e-01

12335 9.946520e-01 9.474120e-01

12336 9.946525e-01 9.474080e-01

12337 9.946531e-01 9.474040e-01

12338 9.946536e-01 9.474000e-01

12339 9.946542e-01 9.473959e-01

12340 9.946547e-01 9.473919e-01

12341 9.946553e-01 9.473878e-01

12342 9.946558e-01 9.473837e-01

12343 9.946564e-01 9.473796e-01

12344 9.946569e-01 9.473755e-01

12345 9.946574e-01 9.473714e-01

12346 9.946580e-01 9.473672e-01

12347 9.946585e-01 9.473630e-01

12348 9.946590e-01 9.473589e-01

12349 9.946595e-01 9.473547e-01

12350 9.946601e-01 9.473505e-01

12351 9.946606e-01 9.473462e-01

12352 9.946611e-01 9.473420e-01

12353 9.946616e-01 9.473377e-01

12354 9.946621e-01 9.473335e-01

12355 9.946627e-01 9.473292e-01

12356 9.946632e-01 9.473249e-01

12357 9.946637e-01 9.473206e-01

12358 9.946642e-01 9.473163e-01

12359 9.946647e-01 9.473119e-01

12360 9.946652e-01 9.473076e-01

12361 9.946658e-01 9.473032e-01

12362 9.946663e-01 9.472989e-01

12363 9.946668e-01 9.472945e-01

12364 9.946673e-01 9.472901e-01

12365 9.946678e-01 9.472857e-01

12366 9.946683e-01 9.472813e-01

12367 9.946688e-01 9.472769e-01

12368 9.946694e-01 9.472725e-01

12369 9.946699e-01 9.472681e-01

12370 9.946704e-01 9.472636e-01

12371 9.946709e-01 9.472592e-01

12372 9.946714e-01 9.472547e-01

12373 9.946720e-01 9.472503e-01

12374 9.946725e-01 9.472458e-01

12375 9.946730e-01 9.472413e-01

12376 9.946735e-01 9.472369e-01

12377 9.946741e-01 9.472324e-01

12378 9.946746e-01 9.472279e-01

12379 9.946751e-01 9.472234e-01

12380 9.946756e-01 9.472189e-01

12381 9.946762e-01 9.472144e-01

12382 9.946767e-01 9.472099e-01

12383 9.946773e-01 9.472054e-01

12384 9.946778e-01 9.472009e-01

12385 9.946783e-01 9.471964e-01

12386 9.946789e-01 9.471919e-01

12387 9.946794e-01 9.471874e-01

12388 9.946800e-01 9.471829e-01

12389 9.946805e-01 9.471784e-01

12390 9.946811e-01 9.471739e-01

12391 9.946816e-01 9.471694e-01

12392 9.946822e-01 9.471649e-01

12393 9.946828e-01 9.471604e-01

12394 9.946833e-01 9.471559e-01

12395 9.946839e-01 9.471514e-01

12396 9.946845e-01 9.471469e-01

12397 9.946850e-01 9.471424e-01

12398 9.946856e-01 9.471379e-01

12399 9.946862e-01 9.471335e-01

12400 9.946868e-01 9.471290e-01

12401 9.946873e-01 9.471245e-01

12402 9.946879e-01 9.471201e-01

12403 9.946885e-01 9.471156e-01

12404 9.946891e-01 9.471112e-01

12405 9.946897e-01 9.471068e-01

12406 9.946903e-01 9.471023e-01

12407 9.946909e-01 9.470979e-01

12408 9.946915e-01 9.470935e-01

12409 9.946922e-01 9.470891e-01

12410 9.946928e-01 9.470847e-01

12411 9.946934e-01 9.470804e-01

12412 9.946940e-01 9.470760e-01

12413 9.946946e-01 9.470717e-01

12414 9.946953e-01 9.470673e-01

12415 9.946959e-01 9.470630e-01

12416 9.946966e-01 9.470587e-01

12417 9.946972e-01 9.470544e-01

12418 9.946979e-01 9.470502e-01

12419 9.946985e-01 9.470459e-01

12420 9.946992e-01 9.470417e-01

12421 9.946998e-01 9.470374e-01

12422 9.947022e-01 9.470355e-01

12423 9.947044e-01 9.470336e-01

12424 9.947066e-01 9.470316e-01

12425 9.947088e-01 9.470297e-01

12426 9.947110e-01 9.470277e-01

12427 9.947130e-01 9.470257e-01

12428 9.947151e-01 9.470237e-01

12429 9.947171e-01 9.470216e-01

12430 9.947190e-01 9.470196e-01

12431 9.947209e-01 9.470175e-01

12432 9.947228e-01 9.470154e-01

12433 9.947247e-01 9.470133e-01

12434 9.947265e-01 9.470112e-01

12435 9.947282e-01 9.470090e-01

12436 9.947299e-01 9.470068e-01

12437 9.947316e-01 9.470047e-01

12438 9.947333e-01 9.470025e-01

12439 9.947349e-01 9.470002e-01

12440 9.947365e-01 9.469980e-01

12441 9.947381e-01 9.469957e-01

12442 9.947396e-01 9.469934e-01

12443 9.947411e-01 9.469911e-01

12444 9.947426e-01 9.469888e-01

12445 9.947440e-01 9.469865e-01

12446 9.947454e-01 9.469841e-01

12447 9.947468e-01 9.469818e-01

12448 9.947481e-01 9.469794e-01

12449 9.947495e-01 9.469769e-01

12450 9.947508e-01 9.469745e-01

12451 9.947520e-01 9.469721e-01

12452 9.947533e-01 9.469696e-01

12453 9.947545e-01 9.469671e-01

12454 9.947557e-01 9.469646e-01

12455 9.947569e-01 9.469621e-01

12456 9.947581e-01 9.469595e-01

12457 9.947592e-01 9.469569e-01
[truncated: 2,882,978 more chars]
