# Supplementary material for: Purity control of simulated moving bed based on advanced fuzzy controller
Source: Sci Rep. 2024 Apr 20;14:9083. doi: 10.1038/s41598-024-59847-1 (PMC11576947; doi:10.1038/s41598-024-59847-1)
Supplement: Supplementary file 3 — Supplementary Information 3. [file 41598_2024_59847_MOESM3_ESM.docx]

**Figure 5 (a):**

1 1.463816e-80 3.996838e-76

2 1.463816e-80 3.996838e-76

3 1.463816e-80 3.996838e-76

4 1.463816e-80 3.996838e-76

5 1.463816e-80 3.996838e-76

6 1.463816e-80 3.996838e-76

7 1.463816e-80 3.996838e-76

8 1.463816e-80 3.996838e-76

9 1.463816e-80 3.996838e-76

10 1.463816e-80 3.996838e-76

11 1.463816e-80 3.996838e-76

12 1.463816e-80 3.996838e-76

13 1.463816e-80 3.996838e-76

14 1.463816e-80 3.996838e-76

15 1.463816e-80 3.996838e-76

16 1.463816e-80 3.996838e-76

17 1.463816e-80 3.996838e-76

18 1.463816e-80 3.996838e-76

19 1.463816e-80 3.996838e-76

20 1.463816e-80 3.996838e-76

21 1.463816e-80 3.996838e-76

22 1.463816e-80 3.996838e-76

23 1.463816e-80 3.996838e-76

24 1.463816e-80 3.996838e-76

25 1.463816e-80 3.996838e-76

26 1.463816e-80 3.996838e-76

27 1.463816e-80 3.996838e-76

28 1.463816e-80 3.996838e-76

29 1.463816e-80 3.996838e-76

30 1.463816e-80 3.996838e-76

31 1.463816e-80 3.996838e-76

32 1.463816e-80 3.996838e-76

33 1.463816e-80 3.996838e-76

34 1.463816e-80 3.996838e-76

35 1.463816e-80 3.996838e-76

36 1.463816e-80 3.996838e-76

37 1.463816e-80 3.996838e-76

38 1.463816e-80 3.996838e-76

39 1.463816e-80 3.996838e-76

40 1.463816e-80 3.996838e-76

41 1.463816e-80 3.996838e-76

42 1.463816e-80 3.996838e-76

43 1.463816e-80 3.996838e-76

44 1.463816e-80 3.996838e-76

45 1.463816e-80 3.996838e-76

46 1.463816e-80 3.996838e-76

47 1.463816e-80 3.996838e-76

48 1.463816e-80 3.996838e-76

49 1.463816e-80 3.996838e-76

50 1.463816e-80 3.996838e-76

51 1.463816e-80 3.996839e-76

52 1.463817e-80 3.996840e-76

53 1.463817e-80 3.996842e-76

54 1.463818e-80 3.996847e-76

55 1.463821e-80 3.996856e-76

56 1.463825e-80 3.996874e-76

57 1.463835e-80 3.996910e-76

58 1.463853e-80 3.996982e-76

59 1.463890e-80 3.997122e-76

60 1.463962e-80 3.997396e-76

61 1.464105e-80 3.997927e-76

62 1.464382e-80 3.998956e-76

63 1.464922e-80 4.000932e-76

64 1.465966e-80 4.004736e-76

65 1.467973e-80 4.012071e-76

66 1.471816e-80 4.026177e-76

67 1.479137e-80 4.053196e-76

68 1.493018e-80 4.104710e-76

69 1.519214e-80 4.202447e-76

70 1.568429e-80 4.386987e-76

71 1.660493e-80 4.733776e-76

72 1.831999e-80 5.382497e-76

73 2.150243e-80 6.590746e-76

74 2.738585e-80 8.831858e-76

75 3.822483e-80 1.297269e-75

76 5.812913e-80 2.059599e-75

77 9.457236e-80 3.458377e-75

78 1.611170e-79 6.017114e-75

79 2.823306e-79 1.068472e-74

80 5.026467e-79 1.917797e-74

81 9.023237e-79 3.459782e-74

82 1.626172e-78 6.253752e-74

83 2.935241e-78 1.130735e-73

84 5.299785e-78 2.043391e-73

85 9.566423e-78 3.689362e-73

86 1.725866e-77 6.654281e-73

87 3.111697e-77 1.198927e-72

88 5.606939e-77 2.157939e-72

89 1.009750e-76 3.880256e-72

90 1.817555e-76 6.970664e-72

91 3.270171e-76 1.251100e-71

92 5.881422e-76 2.243461e-71

93 1.057393e-75 4.019303e-71

94 1.900380e-75 7.194155e-71

95 3.414258e-75 1.286446e-70

96 6.131999e-75 2.298104e-70

97 1.100909e-74 4.101053e-70

98 1.975770e-74 7.310564e-70

99 3.544427e-74 1.301726e-69

100 6.355788e-74 2.315190e-69

101 1.139185e-73 4.112834e-69

102 2.040837e-73 7.315294e-69

103 3.654257e-73 1.304690e-68

104 6.539640e-73 2.332173e-68

105 1.169660e-72 4.174361e-68

106 2.090752e-72 7.475338e-68

107 3.734807e-72 1.338483e-67

108 6.660891e-72 2.395261e-67

109 1.185309e-71 4.282849e-67

110 2.104883e-71 7.650287e-67

111 3.731238e-71 1.365020e-66

112 6.604199e-71 2.432671e-66

113 1.167365e-70 4.329964e-66

114 2.060888e-70 7.696946e-66

115 3.633950e-70 1.366352e-65

116 6.399954e-70 2.422100e-65

117 1.125724e-69 4.287266e-65

118 1.977512e-69 7.577019e-65

119 3.469032e-69 1.336950e-64

120 6.076680e-69 2.355036e-64

121 1.062815e-68 4.141057e-64

122 1.855865e-68 7.268103e-64

123 3.235164e-68 1.273184e-63

124 5.629525e-68 2.225795e-63

125 9.777738e-68 3.883001e-63

126 1.694974e-67 6.759324e-63

127 2.932341e-67 1.173973e-62

128 5.062478e-67 2.034224e-62

129 8.721308e-67 3.516362e-62

130 1.499149e-66 6.063347e-62

131 2.571165e-66 1.042862e-61

132 4.399635e-66 1.788998e-61

133 7.510814e-66 3.060814e-61

134 1.279161e-65 5.222590e-61

135 2.173293e-65 8.886614e-61

136 3.683455e-65 1.507893e-60

137 6.227701e-65 2.551370e-60

138 1.050338e-64 4.304599e-60

139 1.767074e-64 7.241666e-60

140 2.965530e-64 1.216989e-59

141 4.964450e-64 2.045656e-59

142 8.290146e-64 3.438481e-59

143 1.380949e-63 5.775573e-59

144 2.294676e-63 9.687865e-59

145 3.803627e-63 1.621948e-58

146 6.289456e-63 2.709320e-58

147 1.037465e-62 4.514312e-58

148 1.707205e-62 7.501800e-58

149 2.802582e-62 1.243212e-57

150 4.589858e-62 2.054535e-57

151 7.499237e-62 3.385827e-57

152 1.222421e-61 5.564194e-57

153 1.988015e-61 9.118730e-57

154 3.225695e-61 1.490296e-56

155 5.222052e-61 2.429014e-56

156 8.434968e-61 3.948409e-56

157 1.359440e-60 6.401243e-56

158 2.186157e-60 1.035077e-55

159 3.507985e-60 1.669411e-55

160 5.616941e-60 2.685669e-55

161 8.974671e-60 4.309796e-55

162 1.430950e-59 6.899096e-55

163 2.276820e-59 1.101730e-54

164 3.615284e-59 1.755178e-54

165 5.728967e-59 2.789630e-54

166 9.060283e-59 4.423503e-54

167 1.430045e-58 6.998353e-54

168 2.252739e-58 1.104714e-53

169 3.541903e-58 1.739974e-53

170 5.558243e-58 2.734565e-53

171 8.706109e-58 4.288443e-53

172 1.361153e-57 6.711041e-53

173 2.124205e-57 1.048023e-52

174 3.309043e-57 1.633254e-52

175 5.145586e-57 2.540092e-52

176 7.987362e-57 3.942465e-52

177 1.237706e-56 6.106863e-52

178 1.914635e-56 9.452339e-52

179 2.956774e-56 1.463489e-51

180 4.558504e-56 2.266635e-51

181 7.016284e-56 3.510462e-51

182 7.016284e-56 3.510462e-51

183 7.016284e-56 3.510462e-51

184 7.016284e-56 3.510462e-51

185 7.016284e-56 3.510462e-51

186 7.016284e-56 3.510462e-51

187 7.016284e-56 3.510462e-51

188 7.016284e-56 3.510462e-51

189 7.016284e-56 3.510462e-51

190 7.016284e-56 3.510462e-51

191 7.016284e-56 3.510462e-51

192 7.016284e-56 3.510462e-51

193 7.016284e-56 3.510462e-51

194 7.016284e-56 3.510462e-51

195 7.016284e-56 3.510462e-51

196 7.016284e-56 3.510462e-51

197 7.016284e-56 3.510462e-51

198 7.016284e-56 3.510462e-51

199 7.016284e-56 3.510462e-51

200 7.016284e-56 3.510462e-51

201 7.016284e-56 3.510462e-51

202 7.016284e-56 3.510462e-51

203 7.016284e-56 3.510462e-51

204 7.016284e-56 3.510462e-51

205 7.016284e-56 3.510462e-51

206 7.016284e-56 3.510462e-51

207 7.016284e-56 3.510462e-51

208 7.016284e-56 3.510462e-51

209 7.016284e-56 3.510462e-51

210 7.016284e-56 3.510462e-51

211 7.016284e-56 3.510462e-51

212 7.016284e-56 3.510462e-51

213 7.016284e-56 3.510462e-51

214 7.016284e-56 3.510462e-51

215 7.016284e-56 3.510462e-51

216 7.016284e-56 3.510462e-51

217 7.016284e-56 3.510462e-51

218 7.016284e-56 3.510462e-51

219 7.016284e-56 3.510462e-51

220 7.016284e-56 3.510462e-51

221 7.016284e-56 3.510462e-51

222 7.016284e-56 3.510462e-51

223 7.016284e-56 3.510462e-51

224 7.016284e-56 3.510462e-51

225 7.016284e-56 3.510462e-51

226 7.016284e-56 3.510462e-51

227 7.016284e-56 3.510462e-51

228 7.016284e-56 3.510462e-51

229 7.016284e-56 3.510462e-51

230 7.016284e-56 3.510462e-51

231 7.016284e-56 3.510462e-51

232 7.016284e-56 3.510462e-51

233 7.016284e-56 3.510462e-51

234 7.016284e-56 3.510462e-51

235 7.016284e-56 3.510462e-51

236 7.016284e-56 3.510462e-51

237 7.016284e-56 3.510462e-51

238 7.016285e-56 3.510463e-51

239 7.016285e-56 3.510463e-51

240 7.016286e-56 3.510464e-51

241 7.016287e-56 3.510465e-51

242 7.016288e-56 3.510466e-51

243 7.016290e-56 3.510468e-51

244 7.016293e-56 3.510471e-51

245 7.016298e-56 3.510476e-51

246 7.016305e-56 3.510483e-51

247 7.016316e-56 3.510494e-51

248 7.016333e-56 3.510510e-51

249 7.016357e-56 3.510535e-51

250 7.016394e-56 3.510571e-51

251 7.016450e-56 3.510626e-51

252 7.016533e-56 3.510708e-51

253 7.016657e-56 3.510830e-51

254 7.016842e-56 3.511013e-51

255 7.017118e-56 3.511286e-51

256 7.017529e-56 3.511696e-51

257 7.018140e-56 3.512310e-51

258 7.019049e-56 3.513229e-51

259 7.020396e-56 3.514603e-51

260 7.022393e-56 3.516652e-51

261 7.025349e-56 3.519705e-51

262 7.029717e-56 3.524247e-51

263 7.036164e-56 3.530991e-51

264 7.045666e-56 3.540986e-51

265 7.059654e-56 3.555774e-51

266 7.080216e-56 3.577612e-51

267 7.110398e-56 3.609801e-51

268 7.154638e-56 3.657158e-51

269 7.219387e-56 3.726695e-51

270 7.314013e-56 3.828604e-51

271 7.452088e-56 3.977661e-51

272 7.653250e-56 4.195244e-51

273 7.945858e-56 4.512214e-51

274 8.370792e-56 4.973032e-51

275 8.986876e-56 5.641594e-51

276 9.878596e-56 6.609536e-51

277 1.116707e-55 8.007973e-51

278 1.302559e-55 1.002408e-50

279 1.570166e-55 1.292446e-50

280 1.954805e-55 1.708797e-50

281 2.506663e-55 2.305178e-50

282 3.296998e-55 3.157573e-50

283 4.426794e-55 4.373227e-50

284 6.038883e-55 6.103156e-50

285 8.334900e-55 8.559533e-50

286 1.159895e-54 1.203980e-49

287 1.623057e-54 1.695999e-49

288 2.279062e-54 2.390077e-49

289 3.206494e-54 3.367082e-49

290 4.515265e-54 4.739403e-49

291 6.358830e-54 6.662908e-49

292 8.951073e-54 9.359420e-49

293 1.258960e-53 1.314312e-48

294 1.768787e-53 1.845125e-48

295 2.481933e-53 2.589126e-48

296 3.477823e-53 3.630565e-48

297 4.866299e-53 5.086089e-48

298 6.799065e-53 7.116936e-48

299 9.485368e-53 9.945613e-48

300 1.321348e-52 1.387871e-47

301 1.838006e-52 1.933804e-47

302 2.553040e-52 2.690315e-47

303 3.541327e-52 3.736922e-47

304 4.905600e-52 5.182600e-47

305 6.786693e-52 7.176518e-47

306 9.377525e-52 9.922647e-47

307 1.294219e-51 1.369963e-46

308 1.784198e-51 1.888775e-46

309 2.457095e-51 2.600566e-46

310 3.380436e-51 3.576010e-46

311 4.646476e-51 4.911359e-46

312 6.381224e-51 6.737617e-46

313 8.756736e-51 9.233000e-46

314 1.200790e-50 1.263983e-45

315 1.645535e-50 1.728750e-45

316 2.253665e-50 2.362353e-45

317 3.084899e-50 3.225583e-45

318 4.220729e-50 4.400992e-45

319 5.772363e-50 6.000657e-45

320 7.891556e-50 8.176707e-45

321 1.078540e-49 1.113560e-44

322 1.473652e-49 1.515747e-44

323 2.013062e-49 2.062236e-44

324 2.749410e-49 2.804578e-44

325 3.754544e-49 3.812686e-44

326 5.126528e-49 5.181361e-44

327 6.999219e-49 7.039130e-44

328 9.555325e-49 9.560215e-44

329 1.304425e-48 1.298073e-43

330 1.780642e-48 1.762984e-43

331 2.430655e-48 2.396499e-43

332 3.317893e-48 3.261124e-43

333 4.528935e-48 4.442273e-43

334 6.181951e-48 6.056659e-43

335 8.438224e-48 8.263739e-43

336 1.151786e-47 1.128133e-42

337 1.572122e-47 1.540691e-42

338 2.145816e-47 2.104662e-42

339 2.928791e-47 2.875484e-42

340 3.997337e-47 3.928806e-42

341 5.455531e-47 5.367829e-42

342 7.445329e-47 7.333293e-42

343 1.016034e-46 1.001708e-41

344 1.386459e-46 1.368070e-41

345 1.891807e-46 1.868049e-41

346 2.581159e-46 2.550179e-41

347 3.521419e-46 3.480548e-41

348 4.803776e-46 4.749116e-41

349 6.552499e-46 6.478300e-41

350 8.936910e-46 8.834632e-41

351 1.218768e-45 1.204458e-40

352 1.661900e-45 1.641601e-40

353 2.265873e-45 2.236729e-40

354 3.088944e-45 3.046679e-40

355 4.210412e-45 4.148630e-40

356 5.738194e-45 5.647344e-40

357 7.819119e-45 7.684968e-40

358 1.065290e-44 1.045429e-39

359 1.451108e-44 1.421663e-39

360 1.976280e-44 1.932610e-39

361 2.690963e-44 2.626218e-39

362 3.065712e-06 4.698384e-03

363 5.777382e-06 9.207459e-03

364 8.174132e-06 1.350387e-02

365 1.029096e-05 1.756419e-02

366 1.215914e-05 2.136560e-02

367 1.380662e-05 2.488671e-02

368 1.525835e-05 2.810825e-02

369 1.653658e-05 3.101397e-02

370 1.766114e-05 3.359181e-02

371 1.864971e-05 3.583486e-02

372 1.951801e-05 3.774214e-02

373 2.028004e-05 3.931909e-02

374 2.094822e-05 4.057773e-02

375 2.153361e-05 4.153633e-02

376 2.204601e-05 4.221871e-02

377 2.249412e-05 4.265313e-02

378 2.288563e-05 4.287087e-02

379 2.322737e-05 4.290475e-02

380 2.352539e-05 4.278762e-02

381 2.378501e-05 4.255103e-02

382 2.401095e-05 4.222417e-02

383 2.420739e-05 4.183313e-02

384 2.437798e-05 4.140047e-02

385 2.452597e-05 4.094510e-02

386 2.465420e-05 4.048236e-02

387 2.476519e-05 4.002428e-02

388 2.486114e-05 3.957994e-02

389 2.494398e-05 3.915590e-02

390 2.501540e-05 3.875659e-02

391 2.507691e-05 3.838473e-02

392 2.512981e-05 3.804168e-02

393 2.517523e-05 3.772776e-02

394 2.521419e-05 3.744250e-02

395 2.524755e-05 3.718488e-02

396 2.527608e-05 3.695349e-02

397 2.530045e-05 3.674669e-02

398 2.532122e-05 3.656269e-02

399 2.533889e-05 3.639971e-02

400 2.535391e-05 3.625597e-02

401 2.536664e-05 3.612973e-02

402 2.537741e-05 3.601931e-02

403 2.538651e-05 3.592311e-02

404 2.539417e-05 3.583961e-02

405 2.540061e-05 3.576742e-02

406 2.540601e-05 3.570525e-02

407 2.541052e-05 3.565192e-02

408 2.541427e-05 3.560636e-02

409 2.541739e-05 3.556760e-02

410 2.541996e-05 3.553478e-02

411 2.542208e-05 3.550711e-02

412 2.542382e-05 3.548391e-02

413 2.542523e-05 3.546456e-02

414 2.542638e-05 3.544852e-02

415 2.542730e-05 3.543530e-02

416 2.542804e-05 3.542450e-02

417 2.542861e-05 3.541575e-02

418 2.542906e-05 3.540873e-02

419 2.542941e-05 3.540315e-02

420 2.542967e-05 3.539880e-02

421 2.542986e-05 3.539544e-02

422 2.543000e-05 3.539292e-02

423 2.543009e-05 3.539108e-02

424 2.543014e-05 3.538980e-02

425 2.543016e-05 3.538896e-02

426 2.543017e-05 3.538847e-02

427 2.543016e-05 3.538826e-02

428 2.543013e-05 3.538827e-02

429 2.543010e-05 3.538844e-02

430 2.543006e-05 3.538872e-02

431 2.543002e-05 3.538910e-02

432 2.542997e-05 3.538952e-02

433 2.542993e-05 3.538999e-02

434 2.542988e-05 3.539047e-02

435 2.542984e-05 3.539095e-02

436 2.542980e-05 3.539142e-02

437 2.542976e-05 3.539187e-02

438 2.542972e-05 3.539231e-02

439 2.542968e-05 3.539271e-02

440 2.542965e-05 3.539309e-02

441 2.542962e-05 3.539344e-02

442 2.542959e-05 3.539376e-02

443 2.542957e-05 3.539406e-02

444 2.542955e-05 3.539432e-02

445 2.542953e-05 3.539455e-02

446 2.542951e-05 3.539476e-02

447 2.542949e-05 3.539495e-02

448 2.542948e-05 3.539512e-02

449 2.542946e-05 3.539526e-02

450 2.542945e-05 3.539539e-02

451 2.542944e-05 3.539549e-02

452 2.542943e-05 3.539559e-02

453 2.542942e-05 3.539567e-02

454 2.542942e-05 3.539574e-02

455 2.542941e-05 3.539580e-02

456 2.542941e-05 3.539585e-02

457 2.542940e-05 3.539589e-02

458 2.542940e-05 3.539593e-02

459 2.542939e-05 3.539595e-02

460 2.542939e-05 3.539598e-02

461 2.542939e-05 3.539600e-02

462 2.542939e-05 3.539601e-02

463 2.542938e-05 3.539603e-02

464 2.542938e-05 3.539604e-02

465 2.542938e-05 3.539604e-02

466 2.542938e-05 3.539605e-02

467 2.542938e-05 3.539605e-02

468 2.542938e-05 3.539606e-02

469 2.542938e-05 3.539606e-02

470 2.542938e-05 3.539606e-02

471 2.542938e-05 3.539606e-02

472 2.542938e-05 3.539606e-02

473 2.542938e-05 3.539606e-02

474 2.542938e-05 3.539606e-02

475 2.542938e-05 3.539606e-02

476 2.542938e-05 3.539606e-02

477 2.542938e-05 3.539606e-02

478 2.542938e-05 3.539605e-02

479 2.542938e-05 3.539605e-02

480 2.542938e-05 3.539605e-02

481 2.542938e-05 3.539605e-02

482 2.542938e-05 3.539605e-02

483 2.542938e-05 3.539605e-02

484 2.542938e-05 3.539605e-02

485 2.542938e-05 3.539605e-02

486 2.542938e-05 3.539605e-02

487 2.542938e-05 3.539605e-02

488 2.542938e-05 3.539605e-02

489 2.542938e-05 3.539604e-02

490 2.542938e-05 3.539604e-02

491 2.542938e-05 3.539604e-02

492 2.542938e-05 3.539604e-02

493 2.542938e-05 3.539604e-02

494 2.542938e-05 3.539604e-02

495 2.542938e-05 3.539604e-02

496 2.542938e-05 3.539604e-02

497 2.542938e-05 3.539604e-02

498 2.542938e-05 3.539604e-02

499 2.542938e-05 3.539604e-02

500 2.542938e-05 3.539604e-02

501 2.542938e-05 3.539604e-02

502 2.542938e-05 3.539604e-02

503 2.542938e-05 3.539604e-02

504 2.542938e-05 3.539604e-02

505 2.542938e-05 3.539604e-02

506 2.542938e-05 3.539604e-02

507 2.542938e-05 3.539604e-02

508 2.542938e-05 3.539604e-02

509 2.542938e-05 3.539604e-02

510 2.542938e-05 3.539604e-02

511 2.542938e-05 3.539604e-02

512 2.542938e-05 3.539604e-02

513 2.542938e-05 3.539604e-02

514 2.542938e-05 3.539604e-02

515 2.542938e-05 3.539604e-02

516 2.542938e-05 3.539604e-02

517 2.542938e-05 3.539604e-02

518 2.542938e-05 3.539604e-02

519 2.542938e-05 3.539604e-02

520 2.542938e-05 3.539604e-02

521 2.542938e-05 3.539604e-02

522 2.542938e-05 3.539604e-02

523 2.542938e-05 3.539604e-02

524 2.542938e-05 3.539604e-02

525 2.542938e-05 3.539604e-02

526 2.542938e-05 3.539604e-02

527 2.542938e-05 3.539604e-02

528 2.542938e-05 3.539604e-02

529 2.542938e-05 3.539604e-02

530 2.542938e-05 3.539604e-02

531 2.542938e-05 3.539604e-02

532 2.542938e-05 3.539604e-02

533 2.542938e-05 3.539604e-02

534 2.542938e-05 3.539604e-02

535 2.542938e-05 3.539604e-02

536 2.542938e-05 3.539604e-02

537 2.542938e-05 3.539604e-02

538 2.542938e-05 3.539604e-02

539 2.542938e-05 3.539604e-02

540 2.542938e-05 3.539604e-02

541 2.542938e-05 3.539604e-02

542 9.634980e-04 3.625204e-02

543 1.852423e-03 3.729719e-02

544 2.694088e-03 3.855485e-02

545 3.490398e-03 4.004846e-02

546 4.243250e-03 4.180083e-02

547 4.954513e-03 4.383336e-02

548 5.626023e-03 4.616531e-02

549 6.259575e-03 4.881295e-02

550 6.856925e-03 5.178832e-02

551 7.419783e-03 5.509835e-02

552 7.949811e-03 5.874400e-02

553 8.448623e-03 6.271983e-02

554 8.917781e-03 6.701382e-02

555 9.358795e-03 7.160770e-02

556 9.773122e-03 7.647764e-02

557 1.016217e-02 8.159536e-02

558 1.052728e-02 8.692957e-02

559 1.086975e-02 9.244743e-02

560 1.119083e-02 9.811607e-02

561 1.149170e-02 1.039039e-01

562 1.177350e-02 1.097818e-01

563 1.203731e-02 1.157235e-01

564 1.228418e-02 1.217064e-01

565 1.251508e-02 1.277117e-01

566 1.273097e-02 1.337239e-01

567 1.293271e-02 1.397309e-01

568 1.312118e-02 1.457237e-01

569 1.329716e-02 1.516954e-01

570 1.346143e-02 1.576418e-01

571 1.361469e-02 1.635599e-01

572 1.375764e-02 1.694483e-01

573 1.389091e-02 1.753066e-01

574 1.401512e-02 1.811351e-01

575 1.413085e-02 1.869348e-01

576 1.423862e-02 1.927068e-01

577 1.433897e-02 1.984528e-01

578 1.443235e-02 2.041743e-01

579 1.451924e-02 2.098729e-01

580 1.460005e-02 2.155503e-01

581 1.467518e-02 2.212079e-01

582 1.474502e-02 2.268472e-01

583 1.480990e-02 2.324695e-01

584 1.487018e-02 2.380761e-01

585 1.492615e-02 2.436681e-01

586 1.497811e-02 2.492464e-01

587 1.502633e-02 2.548118e-01

588 1.507106e-02 2.603650e-01

589 1.511256e-02 2.659065e-01

590 1.515103e-02 2.714366e-01

591 1.518669e-02 2.769555e-01

592 1.521974e-02 2.824634e-01

593 1.525035e-02 2.879601e-01

594 1.527871e-02 2.934454e-01

595 1.530496e-02 2.989189e-01

596 1.532926e-02 3.043801e-01

597 1.535175e-02 3.098283e-01

598 1.537255e-02 3.152628e-01

599 1.539180e-02 3.206825e-01

600 1.540959e-02 3.260862e-01

601 1.542603e-02 3.314727e-01

602 1.544123e-02 3.368403e-01

603 1.545527e-02 3.421875e-01

604 1.546824e-02 3.475122e-01

605 1.548022e-02 3.528123e-01

606 1.549127e-02 3.580855e-01

607 1.550148e-02 3.633292e-01

608 1.551089e-02 3.685403e-01

609 1.551957e-02 3.737159e-01

610 1.552758e-02 3.788525e-01

611 1.553496e-02 3.839464e-01

612 1.554177e-02 3.889935e-01

613 1.554804e-02 3.939895e-01

614 1.555382e-02 3.989296e-01

615 1.555914e-02 4.038087e-01

616 1.556403e-02 4.086212e-01

617 1.556854e-02 4.133613e-01

618 1.557269e-02 4.180226e-01

619 1.557651e-02 4.225987e-01

620 1.558002e-02 4.270828e-01

621 1.558325e-02 4.314680e-01

622 1.558622e-02 4.357472e-01

623 1.558895e-02 4.399132e-01

624 1.559146e-02 4.439591e-01

625 1.559376e-02 4.478779e-01

626 1.559588e-02 4.516630e-01

627 1.559782e-02 4.553082e-01

628 1.559960e-02 4.588079e-01

629 1.560124e-02 4.621572e-01

630 1.560274e-02 4.653518e-01

631 1.560412e-02 4.683885e-01

632 1.560538e-02 4.712650e-01

633 1.560654e-02 4.739801e-01

634 1.560760e-02 4.765337e-01

635 1.560857e-02 4.789268e-01

636 1.560946e-02 4.811616e-01

637 1.561028e-02 4.832410e-01

638 1.561103e-02 4.851692e-01

639 1.561171e-02 4.869512e-01

640 1.561233e-02 4.885926e-01

641 1.561291e-02 4.900995e-01

642 1.561343e-02 4.914783e-01

643 1.561391e-02 4.927359e-01

644 1.561435e-02 4.938791e-01

645 1.561475e-02 4.949155e-01

646 1.561511e-02 4.958522e-01

647 1.561545e-02 4.966967e-01

648 1.561575e-02 4.974560e-01

649 1.561603e-02 4.981371e-01

650 1.561629e-02 4.987466e-01

651 1.561652e-02 4.992910e-01

652 1.561673e-02 4.997761e-01

653 1.561692e-02 5.002078e-01

654 1.561710e-02 5.005911e-01

655 1.561726e-02 5.009309e-01

656 1.561741e-02 5.012318e-01

657 1.561754e-02 5.014977e-01

658 1.561766e-02 5.017325e-01

659 1.561777e-02 5.019395e-01

660 1.561787e-02 5.021218e-01

661 1.561796e-02 5.022821e-01

662 1.561805e-02 5.024230e-01

663 1.561812e-02 5.025467e-01

664 1.561819e-02 5.026552e-01

665 1.561825e-02 5.027502e-01

666 1.561831e-02 5.028334e-01

667 1.561836e-02 5.029062e-01

668 1.561841e-02 5.029698e-01

669 1.561845e-02 5.030253e-01

670 1.561849e-02 5.030737e-01

671 1.561853e-02 5.031158e-01

672 1.561856e-02 5.031524e-01

673 1.561859e-02 5.031842e-01

674 1.561862e-02 5.032118e-01

675 1.561864e-02 5.032358e-01

676 1.561866e-02 5.032565e-01

677 1.561868e-02 5.032744e-01

678 1.561870e-02 5.032899e-01

679 1.561871e-02 5.033032e-01

680 1.561873e-02 5.033147e-01

681 1.561874e-02 5.033246e-01

682 1.561875e-02 5.033331e-01

683 1.561877e-02 5.033404e-01

684 1.561878e-02 5.033466e-01

685 1.561878e-02 5.033520e-01

686 1.561879e-02 5.033566e-01

687 1.561880e-02 5.033605e-01

688 1.561881e-02 5.033638e-01

689 1.561881e-02 5.033666e-01

690 1.561882e-02 5.033690e-01

691 1.561882e-02 5.033711e-01

692 1.561883e-02 5.033728e-01

693 1.561883e-02 5.033743e-01

694 1.561883e-02 5.033755e-01

695 1.561884e-02 5.033766e-01

696 1.561884e-02 5.033774e-01

697 1.561884e-02 5.033782e-01

698 1.561884e-02 5.033788e-01

699 1.561885e-02 5.033793e-01

700 1.561885e-02 5.033797e-01

701 1.561885e-02 5.033801e-01

702 1.561885e-02 5.033803e-01

703 1.561885e-02 5.033806e-01

704 1.561885e-02 5.033808e-01

705 1.561886e-02 5.033809e-01

706 1.561886e-02 5.033811e-01

707 1.561886e-02 5.033812e-01

708 1.561886e-02 5.033812e-01

709 1.561886e-02 5.033813e-01

710 1.561886e-02 5.033813e-01

711 1.561886e-02 5.033814e-01

712 1.561886e-02 5.033814e-01

713 1.561886e-02 5.033814e-01

714 1.561886e-02 5.033814e-01

715 1.561886e-02 5.033814e-01

716 1.561886e-02 5.033814e-01

717 1.561886e-02 5.033815e-01

718 1.561886e-02 5.033815e-01

719 1.561886e-02 5.033814e-01

720 1.561886e-02 5.033814e-01

721 1.561886e-02 5.033814e-01

722 1.861753e-02 5.033825e-01

723 2.161206e-02 5.033838e-01

724 2.459922e-02 5.033852e-01

725 2.757577e-02 5.033867e-01

726 3.053852e-02 5.033884e-01

727 3.348435e-02 5.033902e-01

728 3.641021e-02 5.033922e-01

729 3.931314e-02 5.033943e-01

730 4.219028e-02 5.033965e-01

731 4.503884e-02 5.033989e-01

732 4.785618e-02 5.034014e-01

733 5.063975e-02 5.034041e-01

734 5.338713e-02 5.034069e-01

735 5.609602e-02 5.034098e-01

736 5.876425e-02 5.034129e-01

737 6.138980e-02 5.034161e-01

738 6.397078e-02 5.034196e-01

739 6.650543e-02 5.034232e-01

740 6.899216e-02 5.034271e-01

741 7.142951e-02 5.034312e-01

742 7.381617e-02 5.034356e-01

743 7.615099e-02 5.034402e-01

744 7.843294e-02 5.034452e-01

745 8.066118e-02 5.034506e-01

746 8.283498e-02 5.034565e-01

747 8.495376e-02 5.034628e-01

748 8.701710e-02 5.034696e-01

749 8.902470e-02 5.034771e-01

750 9.097640e-02 5.034852e-01

751 9.287217e-02 5.034941e-01

752 9.471210e-02 5.035039e-01

753 9.649640e-02 5.035146e-01

754 9.822540e-02 5.035264e-01

755 9.989950e-02 5.035394e-01

756 1.015192e-01 5.035537e-01

757 1.030851e-01 5.035694e-01

758 1.045978e-01 5.035868e-01

759 1.060581e-01 5.036060e-01

760 1.074667e-01 5.036272e-01

761 1.088245e-01 5.036505e-01

762 1.101324e-01 5.036763e-01

763 1.113914e-01 5.037048e-01

764 1.126025e-01 5.037362e-01

765 1.137667e-01 5.037708e-01

766 1.148851e-01 5.038091e-01

767 1.159587e-01 5.038512e-01

768 1.169888e-01 5.038978e-01

769 1.179765e-01 5.039491e-01

770 1.189230e-01 5.040058e-01

771 1.198294e-01 5.040684e-01

772 1.206969e-01 5.041374e-01

773 1.215267e-01 5.042137e-01

774 1.223201e-01 5.042979e-01

775 1.230781e-01 5.043909e-01

776 1.238020e-01 5.044936e-01

777 1.244930e-01 5.046071e-01

778 1.251523e-01 5.047324e-01

779 1.257809e-01 5.048709e-01

780 1.263799e-01 5.050238e-01

781 1.269507e-01 5.051928e-01

782 1.274941e-01 5.053795e-01

783 1.280113e-01 5.055858e-01

784 1.285033e-01 5.058136e-01

785 1.289712e-01 5.060653e-01

786 1.294159e-01 5.063432e-01

787 1.298384e-01 5.066500e-01

788 1.302397e-01 5.069886e-01

789 1.306207e-01 5.073622e-01

790 1.309823e-01 5.077743e-01

791 1.313253e-01 5.082284e-01

792 1.316506e-01 5.087287e-01

793 1.319590e-01 5.092795e-01

794 1.322512e-01 5.098855e-01

795 1.325280e-01 5.105517e-01

796 1.327902e-01 5.112838e-01

797 1.330384e-01 5.120876e-01

798 1.332732e-01 5.129692e-01

799 1.334955e-01 5.139351e-01

800 1.337057e-01 5.149920e-01

801 1.339044e-01 5.161467e-01

802 1.340923e-01 5.174062e-01

803 1.342698e-01 5.187775e-01

804 1.344376e-01 5.202676e-01

805 1.345960e-01 5.218832e-01

806 1.347455e-01 5.236309e-01

807 1.348867e-01 5.255167e-01

808 1.350200e-01 5.275460e-01

809 1.351457e-01 5.297236e-01

810 1.352643e-01 5.320537e-01

811 1.353762e-01 5.345391e-01

812 1.354816e-01 5.371820e-01

813 1.355810e-01 5.399833e-01

814 1.356746e-01 5.429430e-01

815 1.357629e-01 5.460596e-01

816 1.358460e-01 5.493308e-01

817 1.359243e-01 5.527532e-01

818 1.359979e-01 5.563223e-01

819 1.360673e-01 5.600328e-01

820 1.361326e-01 5.638785e-01

821 1.361940e-01 5.678530e-01

822 1.362518e-01 5.719492e-01

823 1.363061e-01 5.761600e-01

824 1.363572e-01 5.804776e-01

825 1.364053e-01 5.848942e-01

826 1.364504e-01 5.894016e-01

827 1.364928e-01 5.939918e-01

828 1.365327e-01 5.986569e-01

829 1.365701e-01 6.033891e-01

830 1.366053e-01 6.081807e-01

831 1.366383e-01 6.130244e-01

832 1.366693e-01 6.179131e-01

833 1.366984e-01 6.228400e-01

834 1.367258e-01 6.277984e-01

835 1.367514e-01 6.327823e-01

836 1.367754e-01 6.377857e-01

837 1.367980e-01 6.428028e-01

838 1.368191e-01 6.478282e-01

839 1.368390e-01 6.528564e-01

840 1.368575e-01 6.578824e-01

841 1.368750e-01 6.629010e-01

842 1.368913e-01 6.679074e-01

843 1.369066e-01 6.728969e-01

844 1.369209e-01 6.778646e-01

845 1.369343e-01 6.828058e-01

846 1.369469e-01 6.877159e-01

847 1.369587e-01 6.925902e-01

848 1.369697e-01 6.974239e-01

849 1.369800e-01 7.022123e-01

850 1.369896e-01 7.069507e-01

851 1.369987e-01 7.116342e-01

852 1.370071e-01 7.162579e-01

853 1.370150e-01 7.208170e-01

854 1.370224e-01 7.253065e-01

855 1.370293e-01 7.297214e-01

856 1.370357e-01 7.340567e-01

857 1.370418e-01 7.383076e-01

858 1.370474e-01 7.424692e-01

859 1.370527e-01 7.465367e-01

860 1.370576e-01 7.505056e-01

861 1.370622e-01 7.543714e-01

862 1.370665e-01 7.581299e-01

863 1.370705e-01 7.617772e-01

864 1.370742e-01 7.653096e-01

865 1.370777e-01 7.687233e-01

866 1.370810e-01 7.720151e-01

867 1.370840e-01 7.751821e-01

868 1.370869e-01 7.782221e-01

869 1.370895e-01 7.811333e-01

870 1.370920e-01 7.839146e-01

871 1.370943e-01 7.865653e-01

872 1.370964e-01 7.890855e-01

873 1.370984e-01 7.914758e-01

874 1.371003e-01 7.937376e-01

875 1.371020e-01 7.958727e-01

876 1.371037e-01 7.978835e-01

877 1.371052e-01 7.997729e-01

878 1.371066e-01 8.015441e-01

879 1.371079e-01 8.032011e-01

880 1.371091e-01 8.047480e-01

881 1.371102e-01 8.061890e-01

882 1.371113e-01 8.075288e-01

883 1.371123e-01 8.087723e-01

884 1.371132e-01 8.099242e-01

885 1.371140e-01 8.109896e-01

886 1.371148e-01 8.119733e-01

887 1.371156e-01 8.128803e-01

888 1.371162e-01 8.137153e-01

889 1.371169e-01 8.144830e-01

890 1.371175e-01 8.151879e-01

891 1.371180e-01 8.158344e-01

892 1.371185e-01 8.164265e-01

893 1.371190e-01 8.169679e-01

894 1.371194e-01 8.174624e-01

895 1.371199e-01 8.179135e-01

896 1.371202e-01 8.183246e-01

897 1.371206e-01 8.186987e-01

898 1.371209e-01 8.190390e-01

899 1.371212e-01 8.193481e-01

900 1.371215e-01 8.196287e-01

901 1.371218e-01 8.198832e-01

902 1.382842e-01 8.198833e-01

903 1.394852e-01 8.198833e-01

904 1.407256e-01 8.198833e-01

905 1.420061e-01 8.198834e-01

906 1.433273e-01 8.198834e-01

907 1.446898e-01 8.198835e-01

908 1.460944e-01 8.198835e-01

909 1.475413e-01 8.198836e-01

910 1.490311e-01 8.198836e-01

911 1.505642e-01 8.198837e-01

912 1.521408e-01 8.198838e-01

913 1.537611e-01 8.198839e-01

914 1.554253e-01 8.198840e-01

915 1.571333e-01 8.198840e-01

916 1.588851e-01 8.198841e-01

917 1.606805e-01 8.198843e-01

918 1.625193e-01 8.198844e-01

919 1.644010e-01 8.198845e-01

920 1.663251e-01 8.198846e-01

921 1.682911e-01 8.198848e-01

922 1.702982e-01 8.198850e-01

923 1.723456e-01 8.198851e-01

924 1.744323e-01 8.198853e-01

925 1.765573e-01 8.198855e-01

926 1.787194e-01 8.198858e-01

927 1.809173e-01 8.198860e-01

928 1.831495e-01 8.198863e-01

929 1.854146e-01 8.198866e-01

930 1.877110e-01 8.198869e-01

931 1.900369e-01 8.198872e-01

932 1.923906e-01 8.198876e-01

933 1.947701e-01 8.198880e-01

934 1.971734e-01 8.198884e-01

935 1.995985e-01 8.198888e-01

936 2.020433e-01 8.198893e-01

937 2.045057e-01 8.198899e-01

938 2.069834e-01 8.198904e-01

939 2.094743e-01 8.198911e-01

940 2.119760e-01 8.198917e-01

941 2.144863e-01 8.198925e-01

942 2.170028e-01 8.198932e-01

943 2.195232e-01 8.198941e-01

944 2.220453e-01 8.198950e-01

945 2.245666e-01 8.198959e-01

946 2.270848e-01 8.198970e-01

947 2.295976e-01 8.198981e-01

948 2.321027e-01 8.198993e-01

949 2.345979e-01 8.199006e-01

950 2.370810e-01 8.199020e-01

951 2.395497e-01 8.199034e-01

952 2.420019e-01 8.199050e-01

953 2.444356e-01 8.199068e-01

954 2.468487e-01 8.199086e-01

955 2.492392e-01 8.199106e-01

956 2.516053e-01 8.199127e-01

957 2.539452e-01 8.199150e-01

958 2.562571e-01 8.199175e-01

959 2.585394e-01 8.199201e-01

960 2.607905e-01 8.199230e-01

961 2.630090e-01 8.199261e-01

962 2.651935e-01 8.199294e-01

963 2.673427e-01 8.199329e-01

964 2.694555e-01 8.199367e-01

965 2.715307e-01 8.199408e-01

966 2.735674e-01 8.199452e-01

967 2.755647e-01 8.199500e-01

968 2.775218e-01 8.199551e-01

969 2.794382e-01 8.199607e-01

970 2.813130e-01 8.199667e-01

971 2.831460e-01 8.199731e-01

972 2.849368e-01 8.199801e-01

973 2.866850e-01 8.199876e-01

974 2.883904e-01 8.199957e-01

975 2.900530e-01 8.200045e-01

976 2.916727e-01 8.200139e-01

977 2.932497e-01 8.200242e-01

978 2.947840e-01 8.200353e-01

979 2.962758e-01 8.200472e-01

980 2.977256e-01 8.200602e-01

981 2.991335e-01 8.200742e-01

982 3.005001e-01 8.200893e-01

983 3.018257e-01 8.201057e-01

984 3.031107e-01 8.201234e-01

985 3.043559e-01 8.201426e-01

986 3.055615e-01 8.201633e-01

987 3.067284e-01 8.201857e-01

988 3.078571e-01 8.202100e-01

989 3.089482e-01 8.202363e-01

990 3.100024e-01 8.202648e-01

991 3.110205e-01 8.202956e-01

992 3.120032e-01 8.203290e-01

993 3.129511e-01 8.203651e-01

994 3.138652e-01 8.204043e-01

995 3.147461e-01 8.204469e-01

996 3.155947e-01 8.204930e-01

997 3.164118e-01 8.205430e-01

998 3.171981e-01 8.205973e-01

999 3.179544e-01 8.206562e-01

1000 3.186817e-01 8.207201e-01

1001 3.193807e-01 8.207895e-01

1002 3.200521e-01 8.208649e-01

1003 3.206969e-01 8.209467e-01

1004 3.213159e-01 8.210355e-01

1005 3.219097e-01 8.211320e-01

1006 3.224793e-01 8.212368e-01

1007 3.230254e-01 8.213506e-01

1008 3.235488e-01 8.214742e-01

1009 3.240502e-01 8.216084e-01

1010 3.245303e-01 8.217542e-01

1011 3.249900e-01 8.219124e-01

1012 3.254300e-01 8.220842e-01

1013 3.258509e-01 8.222708e-01

1014 3.262534e-01 8.224732e-01

1015 3.266383e-01 8.226930e-01

1016 3.270062e-01 8.229314e-01

1017 3.273576e-01 8.231900e-01

1018 3.276934e-01 8.234707e-01

1019 3.280140e-01 8.237752e-01

1020 3.283200e-01 8.241056e-01

1021 3.286121e-01 8.244640e-01

1022 3.288908e-01 8.248529e-01

1023 3.291566e-01 8.252745e-01

1024 3.294101e-01 8.257317e-01

1025 3.296518e-01 8.262272e-01

1026 3.298821e-01 8.267639e-01

1027 3.301016e-01 8.273451e-01

1028 3.303107e-01 8.279740e-01

1029 3.305099e-01 8.286542e-01

1030 3.306995e-01 8.293893e-01

1031 3.308801e-01 8.301830e-01

1032 3.310519e-01 8.310394e-01

1033 3.312154e-01 8.319624e-01

1034 3.313709e-01 8.329561e-01

1035 3.315189e-01 8.340249e-01

1036 3.316596e-01 8.351729e-01

1037 3.317933e-01 8.364044e-01

1038 3.319205e-01 8.377235e-01

1039 3.320414e-01 8.391345e-01

1040 3.321562e-01 8.406413e-01

1041 3.322653e-01 8.422477e-01

1042 3.323689e-01 8.439573e-01

1043 3.324673e-01 8.457735e-01

1044 3.325607e-01 8.476995e-01

1045 3.326494e-01 8.497382e-01

1046 3.327336e-01 8.518925e-01

1047 3.328135e-01 8.541645e-01

1048 3.328893e-01 8.565558e-01

1049 3.329612e-01 8.590674e-01

1050 3.330294e-01 8.616999e-01

1051 3.330941e-01 8.644528e-01

1052 3.331554e-01 8.673255e-01

1053 3.332136e-01 8.703162e-01

1054 3.332687e-01 8.734228e-01

1055 3.333209e-01 8.766424e-01

1056 3.333704e-01 8.799716e-01

1057 3.334173e-01 8.834062e-01

1058 3.334617e-01 8.869417e-01

1059 3.335038e-01 8.905731e-01

1060 3.335436e-01 8.942949e-01

1061 3.335814e-01 8.981011e-01

1062 3.336171e-01 9.019857e-01

1063 3.336509e-01 9.059422e-01

1064 3.336829e-01 9.099639e-01

1065 3.337131e-01 9.140442e-01

1066 3.337418e-01 9.181761e-01

1067 3.337689e-01 9.223526e-01

1068 3.337945e-01 9.265669e-01

1069 3.338187e-01 9.308119e-01

1070 3.338417e-01 9.350808e-01

1071 3.338633e-01 9.393667e-01

1072 3.338838e-01 9.436632e-01

1073 3.339032e-01 9.479635e-01

1074 3.339215e-01 9.522613e-01

1075 3.339388e-01 9.565502e-01

1076 3.339551e-01 9.608236e-01

1077 3.339705e-01 9.650751e-01

1078 3.339851e-01 9.692984e-01

1079 3.339989e-01 9.734871e-01

1080 3.340119e-01 9.776351e-01

1081 3.340242e-01 9.817363e-01

1082 3.342999e-01 9.817364e-01

1083 3.345862e-01 9.817364e-01

1084 3.348833e-01 9.817365e-01

1085 3.351916e-01 9.817365e-01

1086 3.355116e-01 9.817365e-01

1087 3.358436e-01 9.817366e-01

1088 3.361880e-01 9.817366e-01

1089 3.365454e-01 9.817367e-01

1090 3.369160e-01 9.817367e-01

1091 3.373005e-01 9.817367e-01

1092 3.376992e-01 9.817368e-01

1093 3.381127e-01 9.817368e-01

1094 3.385415e-01 9.817368e-01

1095 3.389860e-01 9.817369e-01

1096 3.394467e-01 9.817369e-01

1097 3.399243e-01 9.817369e-01

1098 3.404193e-01 9.817370e-01

1099 3.409321e-01 9.817370e-01

1100 3.414635e-01 9.817370e-01

1101 3.420139e-01 9.817371e-01

1102 3.425840e-01 9.817371e-01

1103 3.431743e-01 9.817371e-01

1104 3.437855e-01 9.817372e-01

1105 3.444181e-01 9.817372e-01

1106 3.450728e-01 9.817372e-01

1107 3.457502e-01 9.817373e-01

1108 3.464510e-01 9.817373e-01

1109 3.471757e-01 9.817373e-01

1110 3.479250e-01 9.817374e-01

1111 3.486995e-01 9.817374e-01

1112 3.494999e-01 9.817374e-01

1113 3.503268e-01 9.817375e-01

1114 3.511807e-01 9.817375e-01

1115 3.520624e-01 9.817376e-01

1116 3.529724e-01 9.817377e-01

1117 3.539113e-01 9.817377e-01

1118 3.548797e-01 9.817378e-01

1119 3.558781e-01 9.817379e-01

1120 3.569071e-01 9.817379e-01

1121 3.579671e-01 9.817380e-01

1122 3.590587e-01 9.817381e-01

1123 3.601824e-01 9.817382e-01

1124 3.613385e-01 9.817383e-01

1125 3.625274e-01 9.817384e-01

1126 3.637495e-01 9.817385e-01

1127 3.650051e-01 9.817386e-01

1128 3.662945e-01 9.817388e-01

1129 3.676179e-01 9.817389e-01

1130 3.689754e-01 9.817391e-01

1131 3.703671e-01 9.817392e-01

1132 3.717932e-01 9.817394e-01

1133 3.732535e-01 9.817396e-01

1134 3.747481e-01 9.817397e-01

1135 3.762767e-01 9.817399e-01

1136 3.778392e-01 9.817402e-01

1137 3.794353e-01 9.817404e-01

1138 3.810646e-01 9.817406e-01

1139 3.827268e-01 9.817409e-01

1140 3.844212e-01 9.817412e-01

1141 3.861473e-01 9.817415e-01

1142 3.879045e-01 9.817418e-01

1143 3.896920e-01 9.817421e-01

1144 3.915089e-01 9.817425e-01

1145 3.933545e-01 9.817429e-01

1146 3.952276e-01 9.817433e-01

1147 3.971273e-01 9.817437e-01

1148 3.990524e-01 9.817441e-01

1149 4.010016e-01 9.817446e-01

1150 4.029738e-01 9.817452e-01

1151 4.049676e-01 9.817457e-01

1152 4.069814e-01 9.817463e-01

1153 4.090140e-01 9.817469e-01

1154 4.110637e-01 9.817476e-01

1155 4.131289e-01 9.817483e-01

1156 4.152079e-01 9.817491e-01

1157 4.172992e-01 9.817499e-01

1158 4.194009e-01 9.817508e-01

1159 4.215113e-01 9.817517e-01

1160 4.236287e-01 9.817527e-01

1161 4.257511e-01 9.817537e-01

1162 4.278768e-01 9.817549e-01

1163 4.300040e-01 9.817561e-01

1164 4.321309e-01 9.817573e-01

1165 4.342557e-01 9.817587e-01

1166 4.363767e-01 9.817601e-01

1167 4.384921e-01 9.817617e-01

1168 4.406002e-01 9.817633e-01

1169 4.426992e-01 9.817651e-01

1170 4.447875e-01 9.817670e-01

1171 4.468634e-01 9.817690e-01

1172 4.489253e-01 9.817711e-01

1173 4.509718e-01 9.817734e-01

1174 4.530012e-01 9.817759e-01

1175 4.550121e-01 9.817785e-01

1176 4.570031e-01 9.817812e-01

1177 4.589729e-01 9.817842e-01

1178 4.609201e-01 9.817874e-01

1179 4.628435e-01 9.817907e-01

1180 4.647420e-01 9.817944e-01

1181 4.666145e-01 9.817982e-01

1182 4.684599e-01 9.818024e-01

1183 4.702774e-01 9.818068e-01

1184 4.720659e-01 9.818115e-01

1185 4.738247e-01 9.818166e-01

1186 4.755531e-01 9.818220e-01

1187 4.772503e-01 9.818278e-01

1188 4.789159e-01 9.818340e-01

1189 4.805493e-01 9.818407e-01

1190 4.821500e-01 9.818478e-01

1191 4.837176e-01 9.818554e-01

1192 4.852520e-01 9.818636e-01

1193 4.867528e-01 9.818723e-01

1194 4.882199e-01 9.818817e-01

1195 4.896531e-01 9.818917e-01

1196 4.910526e-01 9.819025e-01

1197 4.924181e-01 9.819140e-01

1198 4.937500e-01 9.819264e-01

1199 4.950482e-01 9.819396e-01

1200 4.963130e-01 9.819538e-01

1201 4.975446e-01 9.819690e-01

1202 4.987434e-01 9.819853e-01

1203 4.999096e-01 9.820028e-01

1204 5.010436e-01 9.820216e-01

1205 5.021458e-01 9.820417e-01

1206 5.032166e-01 9.820633e-01

1207 5.042563e-01 9.820865e-01

1208 5.052655e-01 9.821115e-01

1209 5.062445e-01 9.821382e-01

1210 5.071939e-01 9.821670e-01

1211 5.081141e-01 9.821979e-01

1212 5.090057e-01 9.822311e-01

1213 5.098691e-01 9.822668e-01

1214 5.107049e-01 9.823052e-01

1215 5.115137e-01 9.823465e-01

1216 5.122959e-01 9.823909e-01

1217 5.130522e-01 9.824387e-01

1218 5.137831e-01 9.824900e-01

1219 5.144892e-01 9.825452e-01

1220 5.151710e-01 9.826046e-01

1221 5.158292e-01 9.826685e-01

1222 5.164644e-01 9.827372e-01

1223 5.170770e-01 9.828110e-01

1224 5.176678e-01 9.828905e-01

1225 5.182372e-01 9.829759e-01

1226 5.187859e-01 9.830677e-01

1227 5.193144e-01 9.831664e-01

1228 5.198233e-01 9.832725e-01

1229 5.203132e-01 9.833866e-01

1230 5.207846e-01 9.835093e-01

1231 5.212381e-01 9.836413e-01

1232 5.216743e-01 9.837833e-01

1233 5.220936e-01 9.839360e-01

1234 5.224966e-01 9.841003e-01

1235 5.228838e-01 9.842772e-01

1236 5.232558e-01 9.844675e-01

1237 5.236130e-01 9.846722e-01

1238 5.239560e-01 9.848926e-01

1239 5.242852e-01 9.851297e-01

1240 5.246010e-01 9.853847e-01

1241 5.249041e-01 9.856591e-01

1242 5.251947e-01 9.859542e-01

1243 5.254733e-01 9.862715e-01

1244 5.257405e-01 9.866126e-01

1245 5.259965e-01 9.869791e-01

1246 5.262418e-01 9.873729e-01

1247 5.264768e-01 9.877957e-01

1248 5.267019e-01 9.882495e-01

1249 5.269174e-01 9.887364e-01

1250 5.271237e-01 9.892585e-01

1251 5.273212e-01 9.898180e-01

1252 5.275102e-01 9.904172e-01

1253 5.276910e-01 9.910586e-01

1254 5.278639e-01 9.917445e-01

1255 5.280293e-01 9.924776e-01

1256 5.281875e-01 9.932606e-01

1257 5.283388e-01 9.940964e-01

1258 5.284833e-01 9.949880e-01

1259 5.286214e-01 9.959386e-01

1260 5.287534e-01 9.969511e-01

1261 5.288796e-01 9.980287e-01

1262 5.289557e-01 9.980287e-01

1263 5.290348e-01 9.980287e-01

1264 5.291171e-01 9.980287e-01

1265 5.292026e-01 9.980287e-01

1266 5.292914e-01 9.980287e-01

1267 5.293836e-01 9.980287e-01

1268 5.294793e-01 9.980287e-01

1269 5.295786e-01 9.980287e-01

1270 5.296817e-01 9.980287e-01

1271 5.297887e-01 9.980288e-01

1272 5.298997e-01 9.980288e-01

1273 5.300147e-01 9.980288e-01

1274 5.301341e-01 9.980288e-01

1275 5.302578e-01 9.980288e-01

1276 5.303860e-01 9.980288e-01

1277 5.305189e-01 9.980288e-01

1278 5.306567e-01 9.980288e-01

1279 5.307994e-01 9.980289e-01

1280 5.309473e-01 9.980289e-01

1281 5.311006e-01 9.980289e-01

1282 5.312593e-01 9.980289e-01

1283 5.314237e-01 9.980289e-01

1284 5.315940e-01 9.980289e-01

1285 5.317704e-01 9.980289e-01

1286 5.319531e-01 9.980289e-01

1287 5.321423e-01 9.980290e-01

1288 5.323381e-01 9.980290e-01

1289 5.325409e-01 9.980290e-01

1290 5.327509e-01 9.980290e-01

1291 5.329682e-01 9.980290e-01

1292 5.331933e-01 9.980290e-01

1293 5.334262e-01 9.980290e-01

1294 5.336673e-01 9.980291e-01

1295 5.339168e-01 9.980291e-01

1296 5.341751e-01 9.980291e-01

1297 5.344424e-01 9.980291e-01

1298 5.347190e-01 9.980291e-01

1299 5.350052e-01 9.980291e-01

1300 5.353013e-01 9.980292e-01

1301 5.356077e-01 9.980292e-01

1302 5.359246e-01 9.980292e-01

1303 5.362525e-01 9.980292e-01

1304 5.365916e-01 9.980293e-01

1305 5.369424e-01 9.980293e-01

1306 5.373052e-01 9.980293e-01

1307 5.376803e-01 9.980293e-01

1308 5.380682e-01 9.980294e-01

1309 5.384693e-01 9.980294e-01

1310 5.388838e-01 9.980294e-01

1311 5.393124e-01 9.980295e-01

1312 5.397552e-01 9.980295e-01

1313 5.402129e-01 9.980295e-01

1314 5.406858e-01 9.980296e-01

1315 5.411743e-01 9.980296e-01

1316 5.416790e-01 9.980297e-01

1317 5.422001e-01 9.980297e-01

1318 5.427383e-01 9.980298e-01

1319 5.432939e-01 9.980298e-01

1320 5.438673e-01 9.980299e-01

1321 5.444592e-01 9.980299e-01

1322 5.450699e-01 9.980300e-01

1323 5.456998e-01 9.980300e-01

1324 5.463495e-01 9.980301e-01

1325 5.470194e-01 9.980302e-01

1326 5.477100e-01 9.980303e-01

1327 5.484217e-01 9.980304e-01

1328 5.491549e-01 9.980304e-01

1329 5.499102e-01 9.980305e-01

1330 5.506879e-01 9.980306e-01

1331 5.514885e-01 9.980307e-01

1332 5.523123e-01 9.980308e-01

1333 5.531599e-01 9.980310e-01

1334 5.540314e-01 9.980311e-01

1335 5.549274e-01 9.980312e-01

1336 5.558482e-01 9.980314e-01

1337 5.567941e-01 9.980315e-01

1338 5.577653e-01 9.980317e-01

1339 5.587622e-01 9.980318e-01

1340 5.597850e-01 9.980320e-01

1341 5.608340e-01 9.980322e-01

1342 5.619093e-01 9.980324e-01

1343 5.630110e-01 9.980326e-01

1344 5.641393e-01 9.980329e-01

1345 5.652942e-01 9.980331e-01

1346 5.664759e-01 9.980333e-01

1347 5.676842e-01 9.980336e-01

1348 5.689191e-01 9.980339e-01

1349 5.701805e-01 9.980342e-01

1350 5.714683e-01 9.980345e-01

1351 5.727822e-01 9.980349e-01

1352 5.741220e-01 9.980353e-01

1353 5.754874e-01 9.980356e-01

1354 5.768780e-01 9.980361e-01

1355 5.782934e-01 9.980365e-01

1356 5.797332e-01 9.980370e-01

1357 5.811967e-01 9.980375e-01

1358 5.826833e-01 9.980380e-01

1359 5.841925e-01 9.980386e-01

1360 5.857236e-01 9.980392e-01

1361 5.872756e-01 9.980398e-01

1362 5.888479e-01 9.980405e-01

1363 5.904395e-01 9.980412e-01

1364 5.920495e-01 9.980420e-01

1365 5.936769e-01 9.980428e-01

1366 5.953206e-01 9.980436e-01

1367 5.969796e-01 9.980446e-01

1368 5.986527e-01 9.980455e-01

1369 6.003388e-01 9.980466e-01

1370 6.020365e-01 9.980477e-01

1371 6.037447e-01 9.980488e-01

1372 6.054620e-01 9.980501e-01

1373 6.071872e-01 9.980514e-01

1374 6.089188e-01 9.980528e-01

1375 6.106555e-01 9.980543e-01

1376 6.123958e-01 9.980559e-01

1377 6.141384e-01 9.980576e-01

1378 6.158818e-01 9.980594e-01

1379 6.176246e-01 9.980613e-01

1380 6.193654e-01 9.980634e-01

1381 6.211027e-01 9.980655e-01

1382 6.228351e-01 9.980678e-01

1383 6.245612e-01 9.980703e-01

1384 6.262796e-01 9.980729e-01

1385 6.279890e-01 9.980757e-01

1386 6.296882e-01 9.980786e-01

1387 6.313757e-01 9.980817e-01

1388 6.330505e-01 9.980851e-01

1389 6.347114e-01 9.980886e-01

1390 6.363572e-01 9.980924e-01

1391 6.379869e-01 9.980964e-01

1392 6.395994e-01 9.981006e-01

1393 6.411937e-01 9.981052e-01

1394 6.427690e-01 9.981100e-01

1395 6.443244e-01 9.981151e-01

1396 6.458590e-01 9.981205e-01

1397 6.473721e-01 9.981263e-01

1398 6.488629e-01 9.981325e-01

1399 6.503308e-01 9.981391e-01

1400 6.517753e-01 9.981460e-01

1401 6.531958e-01 9.981535e-01

1402 6.545917e-01 9.981614e-01

1403 6.559627e-01 9.981698e-01

1404 6.573084e-01 9.981788e-01

1405 6.586284e-01 9.981884e-01

1406 6.599226e-01 9.981986e-01

1407 6.611906e-01 9.982095e-01

1408 6.624324e-01 9.982211e-01

1409 6.636478e-01 9.982334e-01

1410 6.648367e-01 9.982467e-01

1411 6.659991e-01 9.982607e-01

1412 6.671351e-01 9.982758e-01

1413 6.682448e-01 9.982918e-01

1414 6.693281e-01 9.983089e-01

1415 6.703854e-01 9.983272e-01

1416 6.714167e-01 9.983467e-01

1417 6.724223e-01 9.983675e-01

1418 6.734024e-01 9.983897e-01

1419 6.743573e-01 9.984134e-01

1420 6.752874e-01 9.984387e-01

1421 6.761930e-01 9.984657e-01

1422 6.770743e-01 9.984945e-01

1423 6.779318e-01 9.985253e-01

1424 6.787658e-01 9.985581e-01

1425 6.795766e-01 9.985931e-01

1426 6.803646e-01 9.986305e-01

1427 6.811302e-01 9.986704e-01

1428 6.818737e-01 9.987130e-01

1429 6.825955e-01 9.987585e-01

1430 6.832960e-01 9.988072e-01

1431 6.839755e-01 9.988592e-01

1432 6.846346e-01 9.989148e-01

1433 6.852736e-01 9.989742e-01

1434 6.858928e-01 9.990377e-01

1435 6.864927e-01 9.991057e-01

1436 6.870737e-01 9.991783e-01

1437 6.876362e-01 9.992560e-01

1438 6.881807e-01 9.993390e-01

1439 6.887075e-01 9.994279e-01

1440 6.892170e-01 9.995228e-01

1441 6.897097e-01 9.996244e-01

1442 6.897355e-01 9.996244e-01

1443 6.897624e-01 9.996244e-01

1444 6.897905e-01 9.996244e-01

1445 6.898197e-01 9.996243e-01

1446 6.898501e-01 9.996243e-01

1447 6.898817e-01 9.996243e-01

1448 6.899146e-01 9.996243e-01

1449 6.899488e-01 9.996243e-01

1450 6.899843e-01 9.996243e-01

1451 6.900212e-01 9.996243e-01

1452 6.900595e-01 9.996243e-01

1453 6.900992e-01 9.996243e-01

1454 6.901405e-01 9.996243e-01

1455 6.901833e-01 9.996243e-01

1456 6.902276e-01 9.996243e-01

1457 6.902736e-01 9.996243e-01

1458 6.903213e-01 9.996243e-01

1459 6.903707e-01 9.996243e-01

1460 6.904219e-01 9.996243e-01

1461 6.904750e-01 9.996243e-01

1462 6.905299e-01 9.996243e-01

1463 6.905867e-01 9.996243e-01

1464 6.906456e-01 9.996243e-01

1465 6.907066e-01 9.996243e-01

1466 6.907696e-01 9.996243e-01

1467 6.908349e-01 9.996243e-01

1468 6.909025e-01 9.996243e-01

1469 6.909724e-01 9.996243e-01

1470 6.910447e-01 9.996243e-01

1471 6.911195e-01 9.996243e-01

1472 6.911969e-01 9.996243e-01

1473 6.912770e-01 9.996243e-01

1474 6.913598e-01 9.996243e-01

1475 6.914454e-01 9.996243e-01

1476 6.915340e-01 9.996243e-01

1477 6.916256e-01 9.996244e-01

1478 6.917203e-01 9.996244e-01

1479 6.918182e-01 9.996244e-01

1480 6.919194e-01 9.996244e-01

1481 6.920241e-01 9.996244e-01

1482 6.921323e-01 9.996244e-01

1483 6.922442e-01 9.996244e-01

1484 6.923598e-01 9.996244e-01

1485 6.924794e-01 9.996244e-01

1486 6.926030e-01 9.996244e-01

1487 6.927308e-01 9.996244e-01

1488 6.928629e-01 9.996245e-01

1489 6.929994e-01 9.996245e-01

1490 6.931406e-01 9.996245e-01

1491 6.932865e-01 9.996245e-01

1492 6.934372e-01 9.996245e-01

1493 6.935931e-01 9.996245e-01

1494 6.937542e-01 9.996245e-01

1495 6.939207e-01 9.996245e-01

1496 6.940928e-01 9.996246e-01

1497 6.942706e-01 9.996246e-01

1498 6.944544e-01 9.996246e-01

1499 6.946443e-01 9.996246e-01

1500 6.948406e-01 9.996246e-01

1501 6.950435e-01 9.996247e-01

1502 6.952531e-01 9.996247e-01

1503 6.954697e-01 9.996247e-01

1504 6.956935e-01 9.996247e-01

1505 6.959248e-01 9.996247e-01

1506 6.961637e-01 9.996248e-01

1507 6.964105e-01 9.996248e-01

1508 6.966655e-01 9.996248e-01

1509 6.969290e-01 9.996248e-01

1510 6.972011e-01 9.996249e-01

1511 6.974822e-01 9.996249e-01

1512 6.977725e-01 9.996249e-01

1513 6.980723e-01 9.996250e-01

1514 6.983819e-01 9.996250e-01

1515 6.987016e-01 9.996250e-01

1516 6.990318e-01 9.996251e-01

1517 6.993726e-01 9.996251e-01

1518 6.997244e-01 9.996252e-01

1519 7.000876e-01 9.996252e-01

1520 7.004625e-01 9.996253e-01

1521 7.008493e-01 9.996253e-01

1522 7.012485e-01 9.996254e-01

1523 7.016603e-01 9.996254e-01

1524 7.020851e-01 9.996255e-01

1525 7.025234e-01 9.996256e-01

1526 7.029753e-01 9.996256e-01

1527 7.034413e-01 9.996257e-01

1528 7.039217e-01 9.996258e-01

1529 7.044169e-01 9.996259e-01

1530 7.049272e-01 9.996260e-01

1531 7.054531e-01 9.996260e-01

1532 7.059948e-01 9.996261e-01

1533 7.065528e-01 9.996262e-01

1534 7.071273e-01 9.996264e-01

1535 7.077189e-01 9.996265e-01

1536 7.083277e-01 9.996266e-01

1537 7.089542e-01 9.996267e-01

1538 7.095988e-01 9.996268e-01

1539 7.102616e-01 9.996270e-01

1540 7.109432e-01 9.996271e-01

1541 7.116438e-01 9.996273e-01

1542 7.123637e-01 9.996274e-01

1543 7.131032e-01 9.996276e-01

1544 7.138627e-01 9.996278e-01

1545 7.146423e-01 9.996280e-01

1546 7.154424e-01 9.996282e-01

1547 7.162632e-01 9.996284e-01

1548 7.171050e-01 9.996287e-01

1549 7.179678e-01 9.996289e-01

1550 7.188520e-01 9.996292e-01

1551 7.197576e-01 9.996294e-01

1552 7.206849e-01 9.996297e-01

1553 7.216338e-01 9.996300e-01

1554 7.226046e-01 9.996303e-01

1555 7.235971e-01 9.996307e-01

1556 7.246115e-01 9.996310e-01

1557 7.256478e-01 9.996314e-01

1558 7.267058e-01 9.996318e-01

1559 7.277856e-01 9.996323e-01

1560 7.288869e-01 9.996327e-01

1561 7.300096e-01 9.996332e-01

1562 7.311534e-01 9.996337e-01

1563 7.323183e-01 9.996342e-01

1564 7.335038e-01 9.996348e-01

1565 7.347097e-01 9.996354e-01

1566 7.359355e-01 9.996360e-01

1567 7.371808e-01 9.996367e-01

1568 7.384452e-01 9.996374e-01

1569 7.397282e-01 9.996381e-01

1570 7.410292e-01 9.996389e-01

1571 7.423476e-01 9.996398e-01

1572 7.436828e-01 9.996407e-01

1573 7.450341e-01 9.996416e-01

1574 7.464008e-01 9.996426e-01

1575 7.477820e-01 9.996437e-01

1576 7.491771e-01 9.996448e-01

1577 7.505850e-01 9.996460e-01

1578 7.520051e-01 9.996472e-01

1579 7.534362e-01 9.996485e-01

1580 7.548776e-01 9.996499e-01

1581 7.563281e-01 9.996514e-01

1582 7.577868e-01 9.996530e-01

1583 7.592527e-01 9.996546e-01

1584 7.607247e-01 9.996564e-01

1585 7.622016e-01 9.996583e-01

1586 7.636824e-01 9.996602e-01

1587 7.651661e-01 9.996623e-01

1588 7.666513e-01 9.996645e-01

1589 7.681371e-01 9.996668e-01

1590 7.696223e-01 9.996693e-01

1591 7.711058e-01 9.996719e-01

1592 7.725863e-01 9.996747e-01

1593 7.740629e-01 9.996776e-01

1594 7.755343e-01 9.996807e-01

1595 7.769994e-01 9.996840e-01

1596 7.784573e-01 9.996874e-01

1597 7.799067e-01 9.996911e-01

1598 7.813466e-01 9.996950e-01

1599 7.827761e-01 9.996991e-01

1600 7.841941e-01 9.997035e-01

1601 7.855997e-01 9.997081e-01

1602 7.869919e-01 9.997130e-01

1603 7.883699e-01 9.997181e-01

1604 7.897329e-01 9.997236e-01

1605 7.910801e-01 9.997295e-01

1606 7.924109e-01 9.997356e-01

1607 7.937245e-01 9.997422e-01

1608 7.950204e-01 9.997491e-01

1609 7.962980e-01 9.997565e-01

1610 7.975568e-01 9.997644e-01

1611 7.987963e-01 9.997727e-01

1612 8.000161e-01 9.997815e-01

1613 8.012159e-01 9.997909e-01

1614 8.023952e-01 9.998008e-01

1615 8.035539e-01 9.998114e-01

1616 8.046917e-01 9.998226e-01

1617 8.058084e-01 9.998345e-01

1618 8.069037e-01 9.998472e-01

1619 8.079777e-01 9.998606e-01

1620 8.090303e-01 9.998749e-01

1621 8.100613e-01 9.998900e-01

1622 8.100733e-01 9.998900e-01

1623 8.100858e-01 9.998900e-01

1624 8.100990e-01 9.998900e-01

1625 8.101126e-01 9.998900e-01

1626 8.101269e-01 9.998900e-01

1627 8.101418e-01 9.998900e-01

1628 8.101572e-01 9.998900e-01

1629 8.101732e-01 9.998899e-01

1630 8.101899e-01 9.998899e-01

1631 8.102072e-01 9.998899e-01

1632 8.102251e-01 9.998899e-01

1633 8.102437e-01 9.998899e-01

1634 8.102629e-01 9.998899e-01

1635 8.102828e-01 9.998899e-01

1636 8.103034e-01 9.998899e-01

1637 8.103247e-01 9.998899e-01

1638 8.103467e-01 9.998899e-01

1639 8.103694e-01 9.998899e-01

1640 8.103929e-01 9.998899e-01

1641 8.104172e-01 9.998899e-01

1642 8.104422e-01 9.998899e-01

1643 8.104681e-01 9.998899e-01

1644 8.104948e-01 9.998899e-01

1645 8.105223e-01 9.998899e-01

1646 8.105507e-01 9.998899e-01

1647 8.105800e-01 9.998899e-01

1648 8.106102e-01 9.998899e-01

1649 8.106413e-01 9.998899e-01

1650 8.106734e-01 9.998899e-01

1651 8.107064e-01 9.998899e-01

1652 8.107405e-01 9.998899e-01

1653 8.107756e-01 9.998899e-01

1654 8.108118e-01 9.998899e-01

1655 8.108490e-01 9.998899e-01

1656 8.108874e-01 9.998899e-01

1657 8.109270e-01 9.998899e-01

1658 8.109677e-01 9.998899e-01

1659 8.110097e-01 9.998899e-01

1660 8.110529e-01 9.998899e-01

1661 8.110974e-01 9.998899e-01

1662 8.111432e-01 9.998899e-01

1663 8.111904e-01 9.998899e-01

1664 8.112390e-01 9.998899e-01

1665 8.112890e-01 9.998899e-01

1666 8.113405e-01 9.998899e-01

1667 8.113936e-01 9.998899e-01

1668 8.114483e-01 9.998899e-01

1669 8.115045e-01 9.998899e-01

1670 8.115625e-01 9.998899e-01

1671 8.116221e-01 9.998899e-01

1672 8.116836e-01 9.998899e-01

1673 8.117469e-01 9.998899e-01

1674 8.118120e-01 9.998899e-01

1675 8.118791e-01 9.998899e-01

1676 8.119482e-01 9.998899e-01

1677 8.120194e-01 9.998900e-01

1678 8.120926e-01 9.998900e-01

1679 8.121681e-01 9.998900e-01

1680 8.122458e-01 9.998900e-01

1681 8.123259e-01 9.998900e-01

1682 8.124083e-01 9.998900e-01

1683 8.124932e-01 9.998900e-01

1684 8.125807e-01 9.998900e-01

1685 8.126708e-01 9.998900e-01

1686 8.127635e-01 9.998900e-01

1687 8.128591e-01 9.998900e-01

1688 8.129575e-01 9.998900e-01

1689 8.130589e-01 9.998901e-01

1690 8.131634e-01 9.998901e-01

1691 8.132710e-01 9.998901e-01

1692 8.133818e-01 9.998901e-01

1693 8.134960e-01 9.998901e-01

1694 8.136136e-01 9.998901e-01

1695 8.137347e-01 9.998901e-01

1696 8.138595e-01 9.998902e-01

1697 8.139881e-01 9.998902e-01

1698 8.141206e-01 9.998902e-01

1699 8.142570e-01 9.998902e-01

1700 8.143976e-01 9.998902e-01

1701 8.145424e-01 9.998902e-01

1702 8.146916e-01 9.998903e-01

1703 8.148453e-01 9.998903e-01

1704 8.150036e-01 9.998903e-01

1705 8.151667e-01 9.998903e-01

1706 8.153347e-01 9.998904e-01

1707 8.155078e-01 9.998904e-01

1708 8.156861e-01 9.998904e-01

1709 8.158697e-01 9.998904e-01

1710 8.160589e-01 9.998905e-01

1711 8.162537e-01 9.998905e-01

1712 8.164544e-01 9.998905e-01

1713 8.166611e-01 9.998906e-01

1714 8.168740e-01 9.998906e-01

1715 8.170932e-01 9.998906e-01

1716 8.173189e-01 9.998907e-01

1717 8.175514e-01 9.998907e-01

1718 8.177908e-01 9.998908e-01

1719 8.180372e-01 9.998908e-01

1720 8.182909e-01 9.998909e-01

1721 8.185521e-01 9.998909e-01

1722 8.188210e-01 9.998910e-01

1723 8.190977e-01 9.998910e-01

1724 8.193825e-01 9.998911e-01

1725 8.196756e-01 9.998911e-01

1726 8.199771e-01 9.998912e-01

1727 8.202874e-01 9.998913e-01

1728 8.206065e-01 9.998913e-01

1729 8.209348e-01 9.998914e-01

1730 8.212724e-01 9.998915e-01

1731 8.216196e-01 9.998916e-01

1732 8.219766e-01 9.998917e-01

1733 8.223435e-01 9.998918e-01

1734 8.227206e-01 9.998919e-01

1735 8.231082e-01 9.998920e-01

1736 8.235064e-01 9.998921e-01

1737 8.239155e-01 9.998922e-01

1738 8.243356e-01 9.998923e-01

1739 8.247670e-01 9.998924e-01

1740 8.252099e-01 9.998926e-01

1741 8.256645e-01 9.998927e-01

1742 8.261309e-01 9.998929e-01

1743 8.266095e-01 9.998930e-01

1744 8.271003e-01 9.998932e-01

1745 8.276036e-01 9.998934e-01

1746 8.281195e-01 9.998935e-01

1747 8.286482e-01 9.998937e-01

1748 8.291898e-01 9.998939e-01

1749 8.297446e-01 9.998942e-01

1750 8.303125e-01 9.998944e-01

1751 8.308939e-01 9.998946e-01

1752 8.314887e-01 9.998949e-01

1753 8.320971e-01 9.998951e-01

1754 8.327192e-01 9.998954e-01

1755 8.333550e-01 9.998957e-01

1756 8.340045e-01 9.998960e-01

1757 8.346679e-01 9.998964e-01

1758 8.353452e-01 9.998967e-01

1759 8.360363e-01 9.998971e-01

1760 8.367412e-01 9.998975e-01

1761 8.374598e-01 9.998979e-01

1762 8.381922e-01 9.998983e-01

1763 8.389382e-01 9.998988e-01

1764 8.396977e-01 9.998992e-01

1765 8.404706e-01 9.998997e-01

1766 8.412567e-01 9.999003e-01

1767 8.420558e-01 9.999008e-01

1768 8.428677e-01 9.999014e-01

1769 8.436922e-01 9.999021e-01

1770 8.445290e-01 9.999027e-01

1771 8.453778e-01 9.999034e-01

1772 8.462383e-01 9.999042e-01

1773 8.471102e-01 9.999049e-01

1774 8.479930e-01 9.999057e-01

1775 8.488864e-01 9.999066e-01

1776 8.497899e-01 9.999075e-01

1777 8.507030e-01 9.999085e-01

1778 8.516254e-01 9.999095e-01

1779 8.525564e-01 9.999106e-01

1780 8.534955e-01 9.999117e-01

1781 8.544422e-01 9.999129e-01

1782 8.553959e-01 9.999141e-01

1783 8.563560e-01 9.999154e-01

1784 8.573218e-01 9.999168e-01

1785 8.582927e-01 9.999183e-01

1786 8.592681e-01 9.999199e-01

1787 8.602472e-01 9.999215e-01

1788 8.612295e-01 9.999232e-01

1789 8.622140e-01 9.999251e-01

1790 8.632003e-01 9.999270e-01

1791 8.641875e-01 9.999290e-01

1792 8.651748e-01 9.999311e-01

1793 8.661616e-01 9.999334e-01

1794 8.671472e-01 9.999358e-01

1795 8.681308e-01 9.999383e-01

1796 8.691116e-01 9.999409e-01

1797 8.700889e-01 9.999437e-01

1798 8.710621e-01 9.999467e-01

1799 8.720303e-01 9.999498e-01

1800 8.729929e-01 9.999531e-01

1801 8.739493e-01 9.999565e-01

1802 8.739580e-01 9.999565e-01

1803 8.739669e-01 9.999565e-01

1804 8.739758e-01 9.999565e-01

1805 8.739847e-01 9.999565e-01

1806 8.739938e-01 9.999565e-01

1807 8.740029e-01 9.999565e-01

1808 8.740121e-01 9.999565e-01

1809 8.740214e-01 9.999565e-01

1810 8.740308e-01 9.999564e-01

1811 8.740403e-01 9.999564e-01

1812 8.740499e-01 9.999564e-01

1813 8.740596e-01 9.999564e-01

1814 8.740694e-01 9.999564e-01

1815 8.740794e-01 9.999564e-01

1816 8.740895e-01 9.999564e-01

1817 8.740997e-01 9.999564e-01

1818 8.741101e-01 9.999564e-01

1819 8.741206e-01 9.999564e-01

1820 8.741313e-01 9.999564e-01

1821 8.741421e-01 9.999564e-01

1822 8.741531e-01 9.999564e-01

1823 8.741643e-01 9.999564e-01

1824 8.741756e-01 9.999564e-01

1825 8.741872e-01 9.999564e-01

1826 8.741989e-01 9.999564e-01

1827 8.742108e-01 9.999564e-01

1828 8.742229e-01 9.999564e-01

1829 8.742352e-01 9.999564e-01

1830 8.742477e-01 9.999564e-01

1831 8.742605e-01 9.999564e-01

1832 8.742734e-01 9.999564e-01

1833 8.742866e-01 9.999564e-01

1834 8.743001e-01 9.999564e-01

1835 8.743137e-01 9.999564e-01

1836 8.743277e-01 9.999564e-01

1837 8.743419e-01 9.999564e-01

1838 8.743563e-01 9.999564e-01

1839 8.743711e-01 9.999564e-01

1840 8.743861e-01 9.999564e-01

1841 8.744014e-01 9.999564e-01

1842 8.744170e-01 9.999564e-01

1843 8.744329e-01 9.999564e-01

1844 8.744491e-01 9.999564e-01

1845 8.744657e-01 9.999564e-01

1846 8.744826e-01 9.999564e-01

1847 8.744998e-01 9.999564e-01

1848 8.745174e-01 9.999564e-01

1849 8.745353e-01 9.999564e-01

1850 8.745536e-01 9.999564e-01

1851 8.745723e-01 9.999564e-01

1852 8.745914e-01 9.999564e-01

1853 8.746109e-01 9.999564e-01

1854 8.746308e-01 9.999564e-01

1855 8.746511e-01 9.999564e-01

1856 8.746719e-01 9.999564e-01

1857 8.746931e-01 9.999564e-01

1858 8.747147e-01 9.999564e-01

1859 8.747369e-01 9.999564e-01

1860 8.747595e-01 9.999564e-01

1861 8.747826e-01 9.999564e-01

1862 8.748062e-01 9.999564e-01

1863 8.748303e-01 9.999564e-01

1864 8.748550e-01 9.999564e-01

1865 8.748802e-01 9.999564e-01

1866 8.749060e-01 9.999564e-01

1867 8.749323e-01 9.999564e-01

1868 8.749593e-01 9.999564e-01

1869 8.749868e-01 9.999564e-01

1870 8.750150e-01 9.999564e-01

1871 8.750438e-01 9.999565e-01

1872 8.750733e-01 9.999565e-01

1873 8.751034e-01 9.999565e-01

1874 8.751342e-01 9.999565e-01

1875 8.751658e-01 9.999565e-01

1876 8.751980e-01 9.999565e-01

1877 8.752310e-01 9.999565e-01

1878 8.752648e-01 9.999565e-01

1879 8.752993e-01 9.999565e-01

1880 8.753347e-01 9.999565e-01

1881 8.753708e-01 9.999565e-01

1882 8.754078e-01 9.999565e-01

1883 8.754457e-01 9.999565e-01

1884 8.754844e-01 9.999565e-01

1885 8.755241e-01 9.999565e-01

1886 8.755646e-01 9.999565e-01

1887 8.756061e-01 9.999566e-01

1888 8.756486e-01 9.999566e-01

1889 8.756921e-01 9.999566e-01

1890 8.757366e-01 9.999566e-01

1891 8.757821e-01 9.999566e-01

1892 8.758287e-01 9.999566e-01

1893 8.758764e-01 9.999566e-01

1894 8.759251e-01 9.999566e-01

1895 8.759751e-01 9.999566e-01

1896 8.760262e-01 9.999566e-01

1897 8.760785e-01 9.999567e-01

1898 8.761320e-01 9.999567e-01

1899 8.761867e-01 9.999567e-01

1900 8.762428e-01 9.999567e-01

1901 8.763001e-01 9.999567e-01

1902 8.763587e-01 9.999567e-01

1903 8.764188e-01 9.999568e-01

1904 8.764802e-01 9.999568e-01

1905 8.765430e-01 9.999568e-01

1906 8.766072e-01 9.999568e-01

1907 8.766730e-01 9.999568e-01

1908 8.767402e-01 9.999569e-01

1909 8.768090e-01 9.999569e-01

1910 8.768793e-01 9.999569e-01

1911 8.769512e-01 9.999569e-01

1912 8.770247e-01 9.999569e-01

1913 8.770999e-01 9.999570e-01

1914 8.771768e-01 9.999570e-01

1915 8.772553e-01 9.999570e-01

1916 8.773356e-01 9.999571e-01

1917 8.774177e-01 9.999571e-01

1918 8.775016e-01 9.999571e-01

1919 8.775872e-01 9.999572e-01

1920 8.776748e-01 9.999572e-01

1921 8.777641e-01 9.999572e-01

1922 8.778554e-01 9.999573e-01

1923 8.779486e-01 9.999573e-01

1924 8.780438e-01 9.999574e-01

1925 8.781409e-01 9.999574e-01

1926 8.782400e-01 9.999575e-01

1927 8.783411e-01 9.999575e-01

1928 8.784443e-01 9.999576e-01

1929 8.785495e-01 9.999576e-01

1930 8.786567e-01 9.999577e-01

1931 8.787661e-01 9.999577e-01

1932 8.788775e-01 9.999578e-01

1933 8.789910e-01 9.999579e-01

1934 8.791066e-01 9.999580e-01

1935 8.792244e-01 9.999580e-01

1936 8.793442e-01 9.999581e-01

1937 8.794662e-01 9.999582e-01

1938 8.795903e-01 9.999583e-01

1939 8.797165e-01 9.999584e-01

1940 8.798448e-01 9.999585e-01

1941 8.799752e-01 9.999586e-01

1942 8.801076e-01 9.999587e-01

1943 8.802422e-01 9.999588e-01

1944 8.803787e-01 9.999589e-01

1945 8.805173e-01 9.999590e-01

1946 8.806579e-01 9.999592e-01

1947 8.808004e-01 9.999593e-01

1948 8.809449e-01 9.999594e-01

1949 8.810912e-01 9.999596e-01

1950 8.812393e-01 9.999597e-01

1951 8.813892e-01 9.999599e-01

1952 8.815409e-01 9.999601e-01

1953 8.816942e-01 9.999603e-01

1954 8.818491e-01 9.999605e-01

1955 8.820056e-01 9.999607e-01

1956 8.821635e-01 9.999609e-01

1957 8.823228e-01 9.999611e-01

1958 8.824834e-01 9.999613e-01

1959 8.826452e-01 9.999616e-01

1960 8.828082e-01 9.999618e-01

1961 8.829721e-01 9.999621e-01

1962 8.831370e-01 9.999624e-01

1963 8.833027e-01 9.999627e-01

1964 8.834692e-01 9.999630e-01

1965 8.836362e-01 9.999633e-01

1966 8.838038e-01 9.999637e-01

1967 8.839717e-01 9.999641e-01

1968 8.841398e-01 9.999645e-01

1969 8.843081e-01 9.999649e-01

1970 8.844764e-01 9.999653e-01

1971 8.846445e-01 9.999657e-01

1972 8.848124e-01 9.999662e-01

1973 8.849799e-01 9.999667e-01

1974 8.851468e-01 9.999672e-01

1975 8.853131e-01 9.999678e-01

1976 8.854785e-01 9.999683e-01

1977 8.856430e-01 9.999689e-01

1978 8.858064e-01 9.999696e-01

1979 8.859687e-01 9.999702e-01

1980 8.861295e-01 9.999709e-01

1981 8.862889e-01 9.999717e-01

1982 8.862957e-01 9.999717e-01

1983 8.863019e-01 9.999717e-01

1984 8.863076e-01 9.999717e-01

1985 8.863129e-01 9.999717e-01

1986 8.863177e-01 9.999718e-01

1987 8.863220e-01 9.999718e-01

1988 8.863259e-01 9.999718e-01

1989 8.863293e-01 9.999718e-01

1990 8.863323e-01 9.999718e-01

1991 8.863348e-01 9.999718e-01

1992 8.863369e-01 9.999718e-01

1993 8.863386e-01 9.999718e-01

1994 8.863398e-01 9.999718e-01

1995 8.863406e-01 9.999718e-01

1996 8.863410e-01 9.999718e-01

1997 8.863410e-01 9.999718e-01

1998 8.863406e-01 9.999718e-01

1999 8.863397e-01 9.999718e-01

2000 8.863385e-01 9.999718e-01

2001 8.863368e-01 9.999718e-01

2002 8.863348e-01 9.999718e-01

2003 8.863323e-01 9.999718e-01

2004 8.863294e-01 9.999718e-01

2005 8.863261e-01 9.999718e-01

2006 8.863224e-01 9.999718e-01

2007 8.863183e-01 9.999718e-01

2008 8.863137e-01 9.999718e-01

2009 8.863088e-01 9.999718e-01

2010 8.863034e-01 9.999718e-01

2011 8.862976e-01 9.999718e-01

2012 8.862914e-01 9.999718e-01

2013 8.862848e-01 9.999718e-01

2014 8.862777e-01 9.999718e-01

2015 8.862702e-01 9.999718e-01

2016 8.862622e-01 9.999718e-01

2017 8.862538e-01 9.999718e-01

2018 8.862449e-01 9.999718e-01

2019 8.862356e-01 9.999718e-01

2020 8.862258e-01 9.999718e-01

2021 8.862155e-01 9.999718e-01

2022 8.862047e-01 9.999718e-01

2023 8.861934e-01 9.999718e-01

2024 8.861815e-01 9.999718e-01

2025 8.861692e-01 9.999718e-01

2026 8.861563e-01 9.999718e-01

2027 8.861428e-01 9.999718e-01

2028 8.861288e-01 9.999718e-01

2029 8.861142e-01 9.999718e-01

2030 8.860990e-01 9.999718e-01

2031 8.860832e-01 9.999718e-01

2032 8.860668e-01 9.999718e-01

2033 8.860497e-01 9.999718e-01

2034 8.860320e-01 9.999718e-01

2035 8.860135e-01 9.999718e-01

2036 8.859944e-01 9.999718e-01

2037 8.859745e-01 9.999718e-01

2038 8.859540e-01 9.999718e-01

2039 8.859326e-01 9.999718e-01

2040 8.859104e-01 9.999718e-01

2041 8.858875e-01 9.999718e-01

2042 8.858636e-01 9.999718e-01

2043 8.858390e-01 9.999718e-01

2044 8.858134e-01 9.999718e-01

2045 8.857869e-01 9.999718e-01

2046 8.857594e-01 9.999718e-01

2047 8.857310e-01 9.999718e-01

2048 8.857015e-01 9.999718e-01

2049 8.856710e-01 9.999718e-01

2050 8.856394e-01 9.999718e-01

2051 8.856067e-01 9.999718e-01

2052 8.855728e-01 9.999718e-01

2053 8.855377e-01 9.999718e-01

2054 8.855014e-01 9.999718e-01

2055 8.854638e-01 9.999718e-01

2056 8.854249e-01 9.999718e-01

2057 8.853846e-01 9.999718e-01

2058 8.853429e-01 9.999718e-01

2059 8.852998e-01 9.999718e-01

2060 8.852551e-01 9.999718e-01

2061 8.852089e-01 9.999718e-01

2062 8.851610e-01 9.999718e-01

2063 8.851115e-01 9.999718e-01

2064 8.850603e-01 9.999718e-01

2065 8.850072e-01 9.999718e-01

2066 8.849523e-01 9.999718e-01

2067 8.848955e-01 9.999718e-01

2068 8.848368e-01 9.999718e-01

2069 8.847760e-01 9.999718e-01

2070 8.847130e-01 9.999718e-01

2071 8.846479e-01 9.999718e-01

2072 8.845806e-01 9.999718e-01

2073 8.845109e-01 9.999718e-01

2074 8.844388e-01 9.999718e-01

2075 8.843642e-01 9.999718e-01

2076 8.842870e-01 9.999718e-01

2077 8.842072e-01 9.999718e-01

2078 8.841247e-01 9.999718e-01

2079 8.840393e-01 9.999718e-01

2080 8.839509e-01 9.999718e-01

2081 8.838596e-01 9.999718e-01

2082 8.837651e-01 9.999718e-01

2083 8.836673e-01 9.999718e-01

2084 8.835663e-01 9.999718e-01

2085 8.834618e-01 9.999718e-01

2086 8.833538e-01 9.999718e-01

2087 8.832421e-01 9.999718e-01

2088 8.831267e-01 9.999718e-01

2089 8.830073e-01 9.999718e-01

2090 8.828840e-01 9.999718e-01

2091 8.827565e-01 9.999718e-01

2092 8.826248e-01 9.999718e-01

2093 8.824886e-01 9.999718e-01

2094 8.823480e-01 9.999718e-01

2095 8.822027e-01 9.999718e-01

2096 8.820527e-01 9.999718e-01

2097 8.818977e-01 9.999718e-01

2098 8.817376e-01 9.999718e-01

2099 8.815724e-01 9.999718e-01

2100 8.814018e-01 9.999718e-01

2101 8.812258e-01 9.999718e-01

2102 8.810441e-01 9.999717e-01

2103 8.808567e-01 9.999717e-01

2104 8.806633e-01 9.999717e-01

2105 8.804638e-01 9.999717e-01

2106 8.802582e-01 9.999717e-01

2107 8.800461e-01 9.999717e-01

2108 8.798276e-01 9.999717e-01

2109 8.796024e-01 9.999717e-01

2110 8.793703e-01 9.999717e-01

2111 8.791314e-01 9.999717e-01

2112 8.788853e-01 9.999717e-01

2113 8.786319e-01 9.999717e-01

2114 8.783712e-01 9.999717e-01

2115 8.781030e-01 9.999716e-01

2116 8.778271e-01 9.999716e-01

2117 8.775434e-01 9.999716e-01

2118 8.772519e-01 9.999716e-01

2119 8.769523e-01 9.999716e-01

2120 8.766446e-01 9.999716e-01

2121 8.763286e-01 9.999716e-01

2122 8.760044e-01 9.999715e-01

2123 8.756717e-01 9.999715e-01

2124 8.753305e-01 9.999715e-01

2125 8.749807e-01 9.999715e-01

2126 8.746223e-01 9.999715e-01

2127 8.742552e-01 9.999714e-01

2128 8.738794e-01 9.999714e-01

2129 8.734949e-01 9.999714e-01

2130 8.731016e-01 9.999714e-01

2131 8.726996e-01 9.999713e-01

2132 8.722888e-01 9.999713e-01

2133 8.718694e-01 9.999713e-01

2134 8.714413e-01 9.999712e-01

2135 8.710046e-01 9.999712e-01

2136 8.705594e-01 9.999712e-01

2137 8.701058e-01 9.999711e-01

2138 8.696440e-01 9.999711e-01

2139 8.691739e-01 9.999711e-01

2140 8.686959e-01 9.999710e-01

2141 8.682101e-01 9.999710e-01

2142 8.677167e-01 9.999709e-01

2143 8.672159e-01 9.999709e-01

2144 8.667079e-01 9.999708e-01

2145 8.661930e-01 9.999707e-01

2146 8.656714e-01 9.999707e-01

2147 8.651435e-01 9.999706e-01

2148 8.646096e-01 9.999705e-01

2149 8.640699e-01 9.999705e-01

2150 8.635249e-01 9.999704e-01

2151 8.629749e-01 9.999703e-01

2152 8.624202e-01 9.999702e-01

2153 8.618613e-01 9.999701e-01

2154 8.612986e-01 9.999700e-01

2155 8.607325e-01 9.999699e-01

2156 8.601634e-01 9.999698e-01

2157 8.595918e-01 9.999697e-01

2158 8.590181e-01 9.999695e-01

2159 8.584428e-01 9.999694e-01

2160 8.578664e-01 9.999693e-01

2161 8.572893e-01 9.999691e-01

2162 8.572800e-01 9.999691e-01

2163 8.572699e-01 9.999691e-01

2164 8.572591e-01 9.999692e-01

2165 8.572475e-01 9.999692e-01

2166 8.572351e-01 9.999692e-01

2167 8.572219e-01 9.999692e-01

2168 8.572079e-01 9.999692e-01

2169 8.571932e-01 9.999692e-01

2170 8.571776e-01 9.999692e-01

2171 8.571613e-01 9.999692e-01

2172 8.571441e-01 9.999692e-01

2173 8.571261e-01 9.999692e-01

2174 8.571073e-01 9.999693e-01

2175 8.570876e-01 9.999693e-01

2176 8.570672e-01 9.999693e-01

2177 8.570458e-01 9.999693e-01

2178 8.570236e-01 9.999693e-01

2179 8.570004e-01 9.999693e-01

2180 8.569764e-01 9.999693e-01

2181 8.569515e-01 9.999693e-01

2182 8.569256e-01 9.999693e-01

2183 8.568988e-01 9.999693e-01

2184 8.568710e-01 9.999693e-01

2185 8.568422e-01 9.999693e-01

2186 8.568124e-01 9.999693e-01

2187 8.567815e-01 9.999693e-01

2188 8.567496e-01 9.999693e-01

2189 8.567167e-01 9.999693e-01

2190 8.566826e-01 9.999693e-01

2191 8.566473e-01 9.999693e-01

2192 8.566110e-01 9.999693e-01

2193 8.565734e-01 9.999693e-01

2194 8.565346e-01 9.999693e-01

2195 8.564945e-01 9.999693e-01

2196 8.564531e-01 9.999693e-01

2197 8.564105e-01 9.999693e-01

2198 8.563664e-01 9.999693e-01

2199 8.563210e-01 9.999693e-01

2200 8.562741e-01 9.999693e-01

2201 8.562257e-01 9.999693e-01

2202 8.561758e-01 9.999693e-01

2203 8.561244e-01 9.999693e-01

2204 8.560713e-01 9.999693e-01

2205 8.560166e-01 9.999693e-01

2206 8.559602e-01 9.999693e-01

2207 8.559020e-01 9.999693e-01

2208 8.558420e-01 9.999693e-01

2209 8.557801e-01 9.999693e-01

2210 8.557163e-01 9.999693e-01

2211 8.556505e-01 9.999693e-01

2212 8.555827e-01 9.999693e-01

2213 8.555127e-01 9.999693e-01

2214 8.554406e-01 9.999692e-01

2215 8.553663e-01 9.999692e-01

2216 8.552896e-01 9.999692e-01

2217 8.552106e-01 9.999692e-01

2218 8.551290e-01 9.999692e-01

2219 8.550450e-01 9.999692e-01

2220 8.549583e-01 9.999692e-01

2221 8.548689e-01 9.999692e-01

2222 8.547768e-01 9.999692e-01

2223 8.546818e-01 9.999692e-01

2224 8.545838e-01 9.999692e-01

2225 8.544827e-01 9.999692e-01

2226 8.543785e-01 9.999692e-01

2227 8.542710e-01 9.999692e-01

2228 8.541602e-01 9.999692e-01

2229 8.540459e-01 9.999692e-01

2230 8.539280e-01 9.999692e-01

2231 8.538064e-01 9.999692e-01

2232 8.536810e-01 9.999692e-01

2233 8.535517e-01 9.999692e-01

2234 8.534183e-01 9.999692e-01

2235 8.532807e-01 9.999692e-01

2236 8.531389e-01 9.999692e-01

2237 8.529925e-01 9.999691e-01

2238 8.528416e-01 9.999691e-01

2239 8.526860e-01 9.999691e-01

2240 8.525254e-01 9.999691e-01

2241 8.523598e-01 9.999691e-01

2242 8.521891e-01 9.999691e-01

2243 8.520130e-01 9.999691e-01

2244 8.518313e-01 9.999691e-01

2245 8.516440e-01 9.999691e-01

2246 8.514509e-01 9.999691e-01

2247 8.512517e-01 9.999691e-01

2248 8.510463e-01 9.999691e-01

2249 8.508345e-01 9.999690e-01

2250 8.506161e-01 9.999690e-01

2251 8.503909e-01 9.999690e-01

2252 8.501587e-01 9.999690e-01

2253 8.499194e-01 9.999690e-01

2254 8.496727e-01 9.999690e-01

2255 8.494183e-01 9.999690e-01

2256 8.491562e-01 9.999689e-01

2257 8.488860e-01 9.999689e-01

2258 8.486076e-01 9.999689e-01

2259 8.483206e-01 9.999689e-01

2260 8.480250e-01 9.999689e-01

2261 8.477204e-01 9.999689e-01

2262 8.474067e-01 9.999688e-01

2263 8.470836e-01 9.999688e-01

2264 8.467508e-01 9.999688e-01

2265 8.464081e-01 9.999688e-01

2266 8.460552e-01 9.999687e-01

2267 8.456920e-01 9.999687e-01

2268 8.453182e-01 9.999687e-01

2269 8.449335e-01 9.999687e-01

2270 8.445377e-01 9.999686e-01

2271 8.441306e-01 9.999686e-01

2272 8.437118e-01 9.999686e-01

2273 8.432812e-01 9.999685e-01

2274 8.428386e-01 9.999685e-01

2275 8.423836e-01 9.999685e-01

2276 8.419161e-01 9.999684e-01

2277 8.414359e-01 9.999684e-01

2278 8.409426e-01 9.999683e-01

2279 8.404362e-01 9.999683e-01

2280 8.399163e-01 9.999683e-01

2281 8.393829e-01 9.999682e-01

2282 8.388357e-01 9.999681e-01

2283 8.382745e-01 9.999681e-01

2284 8.376993e-01 9.999680e-01

2285 8.371097e-01 9.999680e-01

2286 8.365057e-01 9.999679e-01

2287 8.358873e-01 9.999678e-01

2288 8.352541e-01 9.999678e-01

2289 8.346063e-01 9.999677e-01

2290 8.339436e-01 9.999676e-01

2291 8.332662e-01 9.999676e-01

2292 8.325738e-01 9.999675e-01

2293 8.318666e-01 9.999674e-01

2294 8.311445e-01 9.999673e-01

2295 8.304076e-01 9.999672e-01

2296 8.296560e-01 9.999671e-01

2297 8.288897e-01 9.999670e-01

2298 8.281090e-01 9.999669e-01

2299 8.273138e-01 9.999667e-01

2300 8.265045e-01 9.999666e-01

2301 8.256812e-01 9.999665e-01

2302 8.248441e-01 9.999663e-01

2303 8.239937e-01 9.999662e-01

2304 8.231301e-01 9.999660e-01

2305 8.222537e-01 9.999659e-01

2306 8.213649e-01 9.999657e-01

2307 8.204641e-01 9.999655e-01

2308 8.195517e-01 9.999654e-01

2309 8.186283e-01 9.999652e-01

2310 8.176942e-01 9.999649e-01

2311 8.167502e-01 9.999647e-01

2312 8.157967e-01 9.999645e-01

2313 8.148343e-01 9.999643e-01

2314 8.138637e-01 9.999640e-01

2315 8.128855e-01 9.999637e-01

2316 8.119004e-01 9.999635e-01

2317 8.109091e-01 9.999632e-01

2318 8.099124e-01 9.999628e-01

2319 8.089110e-01 9.999625e-01

2320 8.079057e-01 9.999622e-01

2321 8.068973e-01 9.999618e-01

2322 8.058865e-01 9.999614e-01

2323 8.048742e-01 9.999610e-01

2324 8.038612e-01 9.999606e-01

2325 8.028484e-01 9.999602e-01

2326 8.018366e-01 9.999597e-01

2327 8.008265e-01 9.999592e-01

2328 7.998191e-01 9.999587e-01

2329 7.988152e-01 9.999581e-01

2330 7.978156e-01 9.999576e-01

2331 7.968210e-01 9.999570e-01

2332 7.958324e-01 9.999563e-01

2333 7.948505e-01 9.999557e-01

2334 7.938761e-01 9.999549e-01

2335 7.929099e-01 9.999542e-01

2336 7.919526e-01 9.999534e-01

2337 7.910049e-01 9.999526e-01

2338 7.900675e-01 9.999517e-01

2339 7.891411e-01 9.999508e-01

2340 7.882262e-01 9.999499e-01

2341 7.873235e-01 9.999489e-01

2342 7.872847e-01 9.999489e-01

2343 7.872448e-01 9.999489e-01

2344 7.872037e-01 9.999489e-01

2345 7.871614e-01 9.999489e-01

2346 7.871179e-01 9.999489e-01

2347 7.870731e-01 9.999489e-01

2348 7.870270e-01 9.999489e-01

2349 7.869797e-01 9.999489e-01

2350 7.869309e-01 9.999489e-01

2351 7.868808e-01 9.999489e-01

2352 7.868294e-01 9.999489e-01

2353 7.867764e-01 9.999489e-01

2354 7.867220e-01 9.999489e-01

2355 7.866661e-01 9.999489e-01

2356 7.866087e-01 9.999489e-01

2357 7.865497e-01 9.999489e-01

2358 7.864890e-01 9.999489e-01

2359 7.864267e-01 9.999489e-01

2360 7.863627e-01 9.999489e-01

2361 7.862969e-01 9.999489e-01

2362 7.862293e-01 9.999489e-01

2363 7.861599e-01 9.999489e-01

2364 7.860886e-01 9.999489e-01

2365 7.860153e-01 9.999489e-01

2366 7.859401e-01 9.999489e-01

2367 7.858628e-01 9.999489e-01

2368 7.857833e-01 9.999489e-01

2369 7.857017e-01 9.999489e-01

2370 7.856179e-01 9.999489e-01

2371 7.855318e-01 9.999489e-01

2372 7.854433e-01 9.999489e-01

2373 7.853524e-01 9.999488e-01

2374 7.852590e-01 9.999488e-01

2375 7.851630e-01 9.999488e-01

2376 7.850644e-01 9.999488e-01

2377 7.849631e-01 9.999488e-01

2378 7.848590e-01 9.999488e-01

2379 7.847520e-01 9.999488e-01

2380 7.846421e-01 9.999488e-01

2381 7.845291e-01 9.999488e-01

2382 7.844130e-01 9.999488e-01

2383 7.842937e-01 9.999488e-01

2384 7.841711e-01 9.999488e-01

2385 7.840452e-01 9.999488e-01

2386 7.839157e-01 9.999488e-01

2387 7.837826e-01 9.999488e-01

2388 7.836458e-01 9.999488e-01

2389 7.835053e-01 9.999488e-01

2390 7.833608e-01 9.999488e-01

2391 7.832123e-01 9.999488e-01

2392 7.830597e-01 9.999488e-01

2393 7.829028e-01 9.999488e-01

2394 7.827416e-01 9.999487e-01

2395 7.825759e-01 9.999487e-01

2396 7.824055e-01 9.999487e-01

2397 7.822304e-01 9.999487e-01

2398 7.820504e-01 9.999487e-01

2399 7.818654e-01 9.999487e-01

2400 7.816753e-01 9.999487e-01

2401 7.814799e-01 9.999487e-01

2402 7.812790e-01 9.999487e-01

2403 7.810725e-01 9.999487e-01

2404 7.808603e-01 9.999487e-01

2405 7.806422e-01 9.999486e-01

2406 7.804180e-01 9.999486e-01

2407 7.801876e-01 9.999486e-01

2408 7.799509e-01 9.999486e-01

2409 7.797076e-01 9.999486e-01

2410 7.794575e-01 9.999486e-01

2411 7.792006e-01 9.999486e-01

2412 7.789366e-01 9.999486e-01

2413 7.786654e-01 9.999485e-01

2414 7.783867e-01 9.999485e-01

2415 7.781005e-01 9.999485e-01

2416 7.778064e-01 9.999485e-01

2417 7.775043e-01 9.999485e-01

2418 7.771940e-01 9.999484e-01

2419 7.768754e-01 9.999484e-01

2420 7.765482e-01 9.999484e-01

2421 7.762122e-01 9.999484e-01

2422 7.758673e-01 9.999484e-01

2423 7.755132e-01 9.999483e-01

2424 7.751497e-01 9.999483e-01

2425 7.747766e-01 9.999483e-01

2426 7.743938e-01 9.999483e-01

2427 7.740011e-01 9.999482e-01

2428 7.735982e-01 9.999482e-01

2429 7.731849e-01 9.999482e-01

2430 7.727610e-01 9.999481e-01

2431 7.723264e-01 9.999481e-01

2432 7.718809e-01 9.999481e-01

2433 7.714243e-01 9.999480e-01

2434 7.709563e-01 9.999480e-01

2435 7.704769e-01 9.999480e-01

2436 7.699858e-01 9.999479e-01

2437 7.694828e-01 9.999479e-01

2438 7.689679e-01 9.999478e-01

2439 7.684409e-01 9.999478e-01

2440 7.679015e-01 9.999477e-01

2441 7.673498e-01 9.999477e-01

2442 7.667855e-01 9.999476e-01

2443 7.662085e-01 9.999476e-01

2444 7.656188e-01 9.999475e-01

2445 7.650162e-01 9.999475e-01

2446 7.644007e-01 9.999474e-01

2447 7.637722e-01 9.999473e-01

2448 7.631306e-01 9.999472e-01

2449 7.624760e-01 9.999472e-01

2450 7.618083e-01 9.999471e-01

2451 7.611276e-01 9.999470e-01

2452 7.604337e-01 9.999469e-01

2453 7.597269e-01 9.999468e-01

2454 7.590071e-01 9.999467e-01

2455 7.582744e-01 9.999466e-01

2456 7.575290e-01 9.999465e-01

2457 7.567710e-01 9.999464e-01

2458 7.560005e-01 9.999463e-01

2459 7.552178e-01 9.999462e-01

2460 7.544230e-01 9.999461e-01

2461 7.536164e-01 9.999460e-01

2462 7.527982e-01 9.999458e-01

2463 7.519688e-01 9.999457e-01

2464 7.511284e-01 9.999455e-01

2465 7.502774e-01 9.999454e-01

2466 7.494161e-01 9.999452e-01

2467 7.485451e-01 9.999450e-01

2468 7.476646e-01 9.999448e-01

2469 7.467752e-01 9.999447e-01

2470 7.458774e-01 9.999445e-01

2471 7.449716e-01 9.999442e-01

2472 7.440584e-01 9.999440e-01

2473 7.431384e-01 9.999438e-01

2474 7.422121e-01 9.999435e-01

2475 7.412802e-01 9.999433e-01

2476 7.403433e-01 9.999430e-01

2477 7.394020e-01 9.999427e-01

2478 7.384571e-01 9.999424e-01

2479 7.375092e-01 9.999421e-01

2480 7.365589e-01 9.999418e-01

2481 7.356072e-01 9.999415e-01

2482 7.346545e-01 9.999411e-01

2483 7.337018e-01 9.999407e-01

2484 7.327497e-01 9.999403e-01

2485 7.317991e-01 9.999399e-01

2486 7.308505e-01 9.999395e-01

2487 7.299049e-01 9.999390e-01

2488 7.289630e-01 9.999385e-01

2489 7.280254e-01 9.999380e-01

2490 7.270930e-01 9.999375e-01

2491 7.261665e-01 9.999370e-01

2492 7.252465e-01 9.999364e-01

2493 7.243339e-01 9.999358e-01

2494 7.234293e-01 9.999351e-01

2495 7.225333e-01 9.999344e-01

2496 7.216466e-01 9.999337e-01

2497 7.207698e-01 9.999330e-01

2498 7.199036e-01 9.999322e-01

2499 7.190485e-01 9.999314e-01

2500 7.182051e-01 9.999305e-01

2501 7.173738e-01 9.999296e-01

2502 7.165552e-01 9.999286e-01

2503 7.157497e-01 9.999276e-01

2504 7.149577e-01 9.999266e-01

2505 7.141797e-01 9.999255e-01

2506 7.134159e-01 9.999243e-01

2507 7.126668e-01 9.999231e-01

2508 7.119326e-01 9.999218e-01

2509 7.112135e-01 9.999205e-01

2510 7.105098e-01 9.999190e-01

2511 7.098217e-01 9.999176e-01

2512 7.091493e-01 9.999160e-01

2513 7.084927e-01 9.999144e-01

2514 7.078520e-01 9.999126e-01

2515 7.072274e-01 9.999108e-01

2516 7.066187e-01 9.999089e-01

2517 7.060260e-01 9.999069e-01

2518 7.054493e-01 9.999048e-01

2519 7.048886e-01 9.999026e-01

2520 7.043436e-01 9.999003e-01

2521 7.038144e-01 9.998978e-01

2522 7.037531e-01 9.998978e-01

2523 7.036906e-01 9.998978e-01

2524 7.036268e-01 9.998978e-01

2525 7.035616e-01 9.998978e-01

2526 7.034951e-01 9.998978e-01

2527 7.034273e-01 9.998978e-01

2528 7.033581e-01 9.998978e-01

2529 7.032874e-01 9.998978e-01

2530 7.032154e-01 9.998978e-01

2531 7.031419e-01 9.998978e-01

2532 7.030669e-01 9.998978e-01

2533 7.029904e-01 9.998978e-01

2534 7.029124e-01 9.998978e-01

2535 7.028328e-01 9.998978e-01

2536 7.027517e-01 9.998978e-01

2537 7.026689e-01 9.998978e-01

2538 7.025845e-01 9.998978e-01

2539 7.024985e-01 9.998978e-01

2540 7.024107e-01 9.998978e-01

2541 7.023212e-01 9.998978e-01

2542 7.022300e-01 9.998978e-01

2543 7.021369e-01 9.998978e-01

2544 7.020421e-01 9.998978e-01

2545 7.019453e-01 9.998978e-01

2546 7.018467e-01 9.998978e-01

2547 7.017462e-01 9.998978e-01

2548 7.016436e-01 9.998977e-01

2549 7.015391e-01 9.998977e-01

2550 7.014326e-01 9.998977e-01

2551 7.013240e-01 9.998977e-01

2552 7.012133e-01 9.998977e-01

2553 7.011004e-01 9.998977e-01

2554 7.009854e-01 9.998977e-01

2555 7.008682e-01 9.998977e-01

2556 7.007487e-01 9.998977e-01

2557 7.006269e-01 9.998977e-01

2558 7.005029e-01 9.998977e-01

2559 7.003764e-01 9.998977e-01

2560 7.002476e-01 9.998977e-01

2561 7.001163e-01 9.998977e-01

2562 6.999825e-01 9.998976e-01

2563 6.998462e-01 9.998976e-01

2564 6.997074e-01 9.998976e-01

2565 6.995660e-01 9.998976e-01

2566 6.994220e-01 9.998976e-01

2567 6.992753e-01 9.998976e-01

2568 6.991259e-01 9.998976e-01

2569 6.989738e-01 9.998976e-01

2570 6.988190e-01 9.998976e-01

2571 6.986613e-01 9.998975e-01

2572 6.985008e-01 9.998975e-01

2573 6.983374e-01 9.998975e-01

2574 6.981711e-01 9.998975e-01

2575 6.980019e-01 9.998975e-01

2576 6.978297e-01 9.998975e-01

2577 6.976546e-01 9.998975e-01

2578 6.974764e-01 9.998974e-01

2579 6.972952e-01 9.998974e-01

2580 6.971109e-01 9.998974e-01

2581 6.969235e-01 9.998974e-01

2582 6.967330e-01 9.998974e-01

2583 6.965393e-01 9.998973e-01

2584 6.963425e-01 9.998973e-01

2585 6.961425e-01 9.998973e-01

2586 6.959393e-01 9.998973e-01

2587 6.957330e-01 9.998972e-01

2588 6.955234e-01 9.998972e-01

2589 6.953106e-01 9.998972e-01

2590 6.950946e-01 9.998972e-01

2591 6.948754e-01 9.998971e-01

2592 6.946530e-01 9.998971e-01

2593 6.944274e-01 9.998971e-01

2594 6.941986e-01 9.998971e-01

2595 6.939666e-01 9.998970e-01

2596 6.937315e-01 9.998970e-01

2597 6.934932e-01 9.998969e-01

2598 6.932518e-01 9.998969e-01

2599 6.930074e-01 9.998969e-01

2600 6.927599e-01 9.998968e-01

2601 6.925094e-01 9.998968e-01

2602 6.922560e-01 9.998967e-01

2603 6.919997e-01 9.998967e-01

2604 6.917405e-01 9.998967e-01

2605 6.914786e-01 9.998966e-01

2606 6.912140e-01 9.998965e-01

2607 6.909467e-01 9.998965e-01

2608 6.906770e-01 9.998964e-01

2609 6.904047e-01 9.998964e-01

2610 6.901302e-01 9.998963e-01

2611 6.898533e-01 9.998963e-01

2612 6.895744e-01 9.998962e-01

2613 6.892934e-01 9.998961e-01

2614 6.890105e-01 9.998960e-01

2615 6.887259e-01 9.998960e-01

2616 6.884396e-01 9.998959e-01

2617 6.881518e-01 9.998958e-01

2618 6.878628e-01 9.998957e-01

2619 6.875726e-01 9.998956e-01

2620 6.872813e-01 9.998955e-01

2621 6.869893e-01 9.998954e-01

2622 6.866966e-01 9.998953e-01

2623 6.864035e-01 9.998952e-01

2624 6.861102e-01 9.998951e-01

2625 6.858168e-01 9.998950e-01

2626 6.855235e-01 9.998949e-01

2627 6.852307e-01 9.998947e-01

2628 6.849384e-01 9.998946e-01

2629 6.846470e-01 9.998945e-01

2630 6.843567e-01 9.998943e-01

2631 6.840676e-01 9.998942e-01

2632 6.837801e-01 9.998940e-01

2633 6.834944e-01 9.998939e-01

2634 6.832108e-01 9.998937e-01

2635 6.829294e-01 9.998935e-01

2636 6.826505e-01 9.998933e-01

2637 6.823745e-01 9.998931e-01

2638 6.821015e-01 9.998929e-01

2639 6.818317e-01 9.998927e-01

2640 6.815656e-01 9.998925e-01

2641 6.813032e-01 9.998922e-01

2642 6.810450e-01 9.998920e-01

2643 6.807910e-01 9.998917e-01

2644 6.805415e-01 9.998914e-01

2645 6.802969e-01 9.998912e-01

2646 6.800573e-01 9.998909e-01

2647 6.798229e-01 9.998905e-01

2648 6.795939e-01 9.998902e-01

2649 6.793707e-01 9.998899e-01

2650 6.791533e-01 9.998895e-01

2651 6.789420e-01 9.998892e-01

2652 6.787369e-01 9.998888e-01

2653 6.785383e-01 9.998884e-01

2654 6.783462e-01 9.998879e-01

2655 6.781609e-01 9.998875e-01

2656 6.779824e-01 9.998870e-01

2657 6.778109e-01 9.998865e-01

2658 6.776466e-01 9.998860e-01

2659 6.774894e-01 9.998855e-01

2660 6.773395e-01 9.998849e-01

2661 6.771969e-01 9.998844e-01

2662 6.770617e-01 9.998837e-01

2663 6.769340e-01 9.998831e-01

2664 6.768137e-01 9.998824e-01

2665 6.767008e-01 9.998817e-01

2666 6.765954e-01 9.998810e-01

2667 6.764975e-01 9.998802e-01

2668 6.764069e-01 9.998794e-01

2669 6.763237e-01 9.998786e-01

2670 6.762478e-01 9.998777e-01

2671 6.761790e-01 9.998768e-01

2672 6.761174e-01 9.998759e-01

2673 6.760629e-01 9.998749e-01

2674 6.760152e-01 9.998738e-01

2675 6.759743e-01 9.998727e-01

2676 6.759400e-01 9.998716e-01

2677 6.759123e-01 9.998704e-01

2678 6.758909e-01 9.998691e-01

2679 6.758757e-01 9.998678e-01

2680 6.758665e-01 9.998665e-01

2681 6.758632e-01 9.998650e-01

2682 6.758655e-01 9.998635e-01

2683 6.758733e-01 9.998620e-01

2684 6.758864e-01 9.998603e-01

2685 6.759047e-01 9.998586e-01

2686 6.759278e-01 9.998568e-01

2687 6.759557e-01 9.998549e-01

2688 6.759880e-01 9.998530e-01

2689 6.760247e-01 9.998509e-01

2690 6.760655e-01 9.998488e-01

2691 6.761102e-01 9.998465e-01

2692 6.761586e-01 9.998442e-01

2693 6.762105e-01 9.998417e-01

2694 6.762658e-01 9.998392e-01

2695 6.763242e-01 9.998365e-01

2696 6.763855e-01 9.998337e-01

2697 6.764496e-01 9.998307e-01

2698 6.765163e-01 9.998276e-01

2699 6.765854e-01 9.998244e-01

2700 6.766567e-01 9.998211e-01

2701 6.767300e-01 9.998176e-01

2702 6.767194e-01 9.998176e-01

2703 6.767090e-01 9.998176e-01

2704 6.766988e-01 9.998176e-01

2705 6.766889e-01 9.998176e-01

2706 6.766794e-01 9.998176e-01

2707 6.766701e-01 9.998175e-01

2708 6.766612e-01 9.998175e-01

2709 6.766527e-01 9.998175e-01

2710 6.766445e-01 9.998175e-01

2711 6.766367e-01 9.998175e-01

2712 6.766294e-01 9.998175e-01

2713 6.766225e-01 9.998175e-01

2714 6.766161e-01 9.998175e-01

2715 6.766102e-01 9.998175e-01

2716 6.766049e-01 9.998175e-01

2717 6.766001e-01 9.998175e-01

2718 6.765959e-01 9.998175e-01

2719 6.765923e-01 9.998175e-01

2720 6.765894e-01 9.998175e-01

2721 6.765871e-01 9.998175e-01

2722 6.765856e-01 9.998175e-01

2723 6.765849e-01 9.998175e-01

2724 6.765849e-01 9.998175e-01

2725 6.765858e-01 9.998174e-01

2726 6.765876e-01 9.998174e-01

2727 6.765903e-01 9.998174e-01

2728 6.765940e-01 9.998174e-01

2729 6.765986e-01 9.998174e-01

2730 6.766044e-01 9.998174e-01

2731 6.766112e-01 9.998174e-01

2732 6.766192e-01 9.998174e-01

2733 6.766284e-01 9.998174e-01

2734 6.766388e-01 9.998174e-01

2735 6.766506e-01 9.998174e-01

2736 6.766637e-01 9.998174e-01

2737 6.766783e-01 9.998173e-01

2738 6.766943e-01 9.998173e-01

2739 6.767119e-01 9.998173e-01

2740 6.767312e-01 9.998173e-01

2741 6.767521e-01 9.998173e-01

2742 6.767748e-01 9.998173e-01

2743 6.767993e-01 9.998173e-01

2744 6.768257e-01 9.998173e-01

2745 6.768541e-01 9.998172e-01

2746 6.768846e-01 9.998172e-01

2747 6.769172e-01 9.998172e-01

2748 6.769520e-01 9.998172e-01

2749 6.769892e-01 9.998172e-01

2750 6.770287e-01 9.998172e-01

2751 6.770707e-01 9.998172e-01

2752 6.771154e-01 9.998171e-01

2753 6.771627e-01 9.998171e-01

2754 6.772128e-01 9.998171e-01

2755 6.772658e-01 9.998171e-01

2756 6.773218e-01 9.998171e-01

2757 6.773809e-01 9.998170e-01

2758 6.774432e-01 9.998170e-01

2759 6.775088e-01 9.998170e-01

2760 6.775779e-01 9.998170e-01

2761 6.776506e-01 9.998169e-01

2762 6.777269e-01 9.998169e-01

2763 6.778071e-01 9.998169e-01

2764 6.778912e-01 9.998169e-01

2765 6.779794e-01 9.998168e-01

2766 6.780718e-01 9.998168e-01

2767 6.781686e-01 9.998168e-01

2768 6.782699e-01 9.998168e-01

2769 6.783758e-01 9.998167e-01

2770 6.784865e-01 9.998167e-01

2771 6.786022e-01 9.998167e-01

2772 6.787229e-01 9.998166e-01

2773 6.788489e-01 9.998166e-01

2774 6.789803e-01 9.998165e-01

2775 6.791172e-01 9.998165e-01

2776 6.792599e-01 9.998165e-01

2777 6.794085e-01 9.998164e-01

2778 6.795631e-01 9.998164e-01

2779 6.797240e-01 9.998163e-01

2780 6.798913e-01 9.998163e-01

2781 6.800651e-01 9.998162e-01

2782 6.802457e-01 9.998162e-01

2783 6.804333e-01 9.998161e-01

2784 6.806279e-01 9.998161e-01

2785 6.808298e-01 9.998160e-01

2786 6.810392e-01 9.998160e-01

2787 6.812562e-01 9.998159e-01

2788 6.814810e-01 9.998159e-01

2789 6.817139e-01 9.998158e-01

2790 6.819549e-01 9.998157e-01

2791 6.822042e-01 9.998157e-01

2792 6.824621e-01 9.998156e-01

2793 6.827287e-01 9.998155e-01

2794 6.830042e-01 9.998154e-01

2795 6.832887e-01 9.998154e-01

2796 6.835824e-01 9.998153e-01

2797 6.838855e-01 9.998152e-01

2798 6.841982e-01 9.998151e-01

2799 6.845205e-01 9.998150e-01

2800 6.848527e-01 9.998149e-01

2801 6.851949e-01 9.998148e-01

2802 6.855473e-01 9.998147e-01

2803 6.859099e-01 9.998146e-01

2804 6.862829e-01 9.998145e-01

2805 6.866664e-01 9.998144e-01

2806 6.870606e-01 9.998142e-01

2807 6.874655e-01 9.998141e-01

2808 6.878813e-01 9.998140e-01

2809 6.883080e-01 9.998139e-01

2810 6.887457e-01 9.998137e-01

2811 6.891945e-01 9.998136e-01

2812 6.896544e-01 9.998134e-01

2813 6.901255e-01 9.998133e-01

2814 6.906078e-01 9.998131e-01

2815 6.911013e-01 9.998129e-01

2816 6.916061e-01 9.998128e-01

2817 6.921221e-01 9.998126e-01

2818 6.926493e-01 9.998124e-01

2819 6.931877e-01 9.998122e-01

2820 6.937373e-01 9.998120e-01

2821 6.942979e-01 9.998118e-01

2822 6.948695e-01 9.998116e-01

2823 6.954520e-01 9.998113e-01

2824 6.960452e-01 9.998111e-01

2825 6.966492e-01 9.998109e-01

2826 6.972636e-01 9.998106e-01

2827 6.978885e-01 9.998104e-01

2828 6.985235e-01 9.998101e-01

2829 6.991685e-01 9.998098e-01

2830 6.998233e-01 9.998095e-01

2831 7.004876e-01 9.998092e-01

2832 7.011613e-01 9.998089e-01

2833 7.018441e-01 9.998086e-01

2834 7.025356e-01 9.998083e-01

2835 7.032357e-01 9.998079e-01

2836 7.039439e-01 9.998076e-01

2837 7.046601e-01 9.998072e-01

2838 7.053839e-01 9.998068e-01

2839 7.061148e-01 9.998064e-01

2840 7.068527e-01 9.998060e-01

2841 7.075970e-01 9.998056e-01

2842 7.083475e-01 9.998052e-01

2843 7.091037e-01 9.998047e-01

2844 7.098652e-01 9.998042e-01

2845 7.106317e-01 9.998038e-01

2846 7.114027e-01 9.998033e-01

2847 7.121778e-01 9.998028e-01

2848 7.129565e-01 9.998022e-01

2849 7.137385e-01 9.998017e-01

2850 7.145232e-01 9.998011e-01

2851 7.153103e-01 9.998005e-01

2852 7.160992e-01 9.997999e-01

2853 7.168897e-01 9.997993e-01

2854 7.176811e-01 9.997986e-01

2855 7.184730e-01 9.997980e-01

2856 7.192651e-01 9.997973e-01

2857 7.200568e-01 9.997966e-01

2858 7.208478e-01 9.997959e-01

2859 7.216375e-01 9.997951e-01

2860 7.224256e-01 9.997943e-01

2861 7.232116e-01 9.997935e-01

2862 7.239952e-01 9.997927e-01

2863 7.247758e-01 9.997919e-01

2864 7.255531e-01 9.997910e-01

2865 7.263268e-01 9.997901e-01

2866 7.270963e-01 9.997892e-01

2867 7.278615e-01 9.997882e-01

2868 7.286218e-01 9.997872e-01

2869 7.293769e-01 9.997862e-01

2870 7.301266e-01 9.997852e-01

2871 7.308704e-01 9.997841e-01

2872 7.316082e-01 9.997830e-01

2873 7.323395e-01 9.997819e-01

2874 7.330641e-01 9.997808e-01

2875 7.337817e-01 9.997796e-01

2876 7.344921e-01 9.997784e-01

2877 7.351951e-01 9.997772e-01

2878 7.358903e-01 9.997759e-01

2879 7.365777e-01 9.997746e-01

2880 7.372569e-01 9.997733e-01

2881 7.379279e-01 9.997719e-01

2882 7.379742e-01 9.997719e-01

2883 7.380216e-01 9.997719e-01

2884 7.380701e-01 9.997719e-01

2885 7.381198e-01 9.997719e-01

2886 7.381708e-01 9.997719e-01

2887 7.382229e-01 9.997719e-01

2888 7.382762e-01 9.997719e-01

2889 7.383309e-01 9.997719e-01

2890 7.383868e-01 9.997719e-01

2891 7.384440e-01 9.997719e-01

2892 7.385026e-01 9.997719e-01

2893 7.385625e-01 9.997719e-01

2894 7.386238e-01 9.997719e-01

2895 7.386866e-01 9.997719e-01

2896 7.387508e-01 9.997719e-01

2897 7.388165e-01 9.997719e-01

2898 7.388838e-01 9.997719e-01

2899 7.389526e-01 9.997719e-01

2900 7.390229e-01 9.997719e-01

2901 7.390950e-01 9.997719e-01

2902 7.391687e-01 9.997719e-01

2903 7.392441e-01 9.997719e-01

2904 7.393212e-01 9.997718e-01

2905 7.394002e-01 9.997718e-01

2906 7.394809e-01 9.997718e-01

2907 7.395636e-01 9.997718e-01

2908 7.396482e-01 9.997718e-01

2909 7.397347e-01 9.997718e-01

2910 7.398232e-01 9.997718e-01

2911 7.399138e-01 9.997718e-01

2912 7.400065e-01 9.997718e-01

2913 7.401014e-01 9.997718e-01

2914 7.401984e-01 9.997718e-01

2915 7.402977e-01 9.997718e-01

2916 7.403994e-01 9.997718e-01

2917 7.405033e-01 9.997718e-01

2918 7.406097e-01 9.997718e-01

2919 7.407186e-01 9.997718e-01

2920 7.408300e-01 9.997718e-01

2921 7.409440e-01 9.997718e-01

2922 7.410607e-01 9.997718e-01

2923 7.411801e-01 9.997717e-01

2924 7.413022e-01 9.997717e-01

2925 7.414272e-01 9.997717e-01

2926 7.415551e-01 9.997717e-01

2927 7.416859e-01 9.997717e-01

2928 7.418199e-01 9.997717e-01

2929 7.419569e-01 9.997717e-01

2930 7.420971e-01 9.997717e-01

2931 7.422405e-01 9.997717e-01

2932 7.423873e-01 9.997717e-01

2933 7.425375e-01 9.997717e-01

2934 7.426912e-01 9.997717e-01

2935 7.428485e-01 9.997717e-01

2936 7.430094e-01 9.997717e-01

2937 7.431740e-01 9.997716e-01

2938 7.433424e-01 9.997716e-01

2939 7.435147e-01 9.997716e-01

2940 7.436910e-01 9.997716e-01

2941 7.438714e-01 9.997716e-01

2942 7.440559e-01 9.997716e-01

2943 7.442447e-01 9.997716e-01

2944 7.444378e-01 9.997716e-01

2945 7.446353e-01 9.997716e-01

2946 7.448374e-01 9.997716e-01

2947 7.450441e-01 9.997716e-01

2948 7.452555e-01 9.997715e-01

2949 7.454718e-01 9.997715e-01

2950 7.456929e-01 9.997715e-01

2951 7.459191e-01 9.997715e-01

2952 7.461504e-01 9.997715e-01

2953 7.463870e-01 9.997715e-01

2954 7.466289e-01 9.997715e-01

2955 7.468762e-01 9.997715e-01

2956 7.471291e-01 9.997715e-01

2957 7.473877e-01 9.997715e-01

2958 7.476521e-01 9.997715e-01

2959 7.479223e-01 9.997714e-01

2960 7.481985e-01 9.997714e-01

2961 7.484809e-01 9.997714e-01

2962 7.487694e-01 9.997714e-01

2963 7.490644e-01 9.997714e-01

2964 7.493657e-01 9.997714e-01

2965 7.496737e-01 9.997714e-01

2966 7.499883e-01 9.997714e-01

2967 7.503098e-01 9.997714e-01

2968 7.506382e-01 9.997714e-01

2969 7.509736e-01 9.997714e-01

2970 7.513161e-01 9.997714e-01

2971 7.516660e-01 9.997713e-01

2972 7.520232e-01 9.997713e-01

2973 7.523879e-01 9.997713e-01

2974 7.527603e-01 9.997713e-01

2975 7.531403e-01 9.997713e-01

2976 7.535282e-01 9.997713e-01

2977 7.539241e-01 9.997713e-01

2978 7.543280e-01 9.997713e-01

2979 7.547401e-01 9.997713e-01

2980 7.551605e-01 9.997713e-01

2981 7.555892e-01 9.997713e-01

2982 7.560265e-01 9.997713e-01

2983 7.564723e-01 9.997713e-01

2984 7.569267e-01 9.997713e-01

2985 7.573900e-01 9.997713e-01

2986 7.578621e-01 9.997713e-01

2987 7.583431e-01 9.997713e-01

2988 7.588331e-01 9.997713e-01

2989 7.593323e-01 9.997714e-01

2990 7.598406e-01 9.997714e-01

2991 7.603582e-01 9.997714e-01

2992 7.608851e-01 9.997714e-01

2993 7.614213e-01 9.997714e-01

2994 7.619669e-01 9.997715e-01

2995 7.625220e-01 9.997715e-01

2996 7.630866e-01 9.997715e-01

2997 7.636608e-01 9.997716e-01

2998 7.642445e-01 9.997716e-01

2999 7.648377e-01 9.997716e-01

3000 7.654406e-01 9.997717e-01

3001 7.660531e-01 9.997717e-01

3002 7.666751e-01 9.997718e-01

3003 7.673068e-01 9.997718e-01

3004 7.679480e-01 9.997719e-01

3005 7.685987e-01 9.997720e-01

3006 7.692589e-01 9.997721e-01

3007 7.699286e-01 9.997721e-01

3008 7.706076e-01 9.997722e-01

3009 7.712960e-01 9.997723e-01

3010 7.719936e-01 9.997724e-01

3011 7.727003e-01 9.997726e-01

3012 7.734162e-01 9.997727e-01

3013 7.741409e-01 9.997728e-01

3014 7.748745e-01 9.997730e-01

3015 7.756168e-01 9.997731e-01

3016 7.763676e-01 9.997733e-01

3017 7.771269e-01 9.997735e-01

3018 7.778943e-01 9.997737e-01

3019 7.786699e-01 9.997739e-01

3020 7.794533e-01 9.997741e-01

3021 7.802444e-01 9.997743e-01

3022 7.810429e-01 9.997746e-01

3023 7.818488e-01 9.997749e-01

3024 7.826616e-01 9.997751e-01

3025 7.834812e-01 9.997755e-01

3026 7.843073e-01 9.997758e-01

3027 7.851397e-01 9.997761e-01

3028 7.859781e-01 9.997765e-01

3029 7.868222e-01 9.997769e-01

3030 7.876717e-01 9.997773e-01

3031 7.885263e-01 9.997778e-01

3032 7.893858e-01 9.997783e-01

3033 7.902497e-01 9.997788e-01

3034 7.911177e-01 9.997793e-01

3035 7.919896e-01 9.997799e-01

3036 7.928650e-01 9.997805e-01

3037 7.937435e-01 9.997812e-01

3038 7.946247e-01 9.997819e-01

3039 7.955084e-01 9.997826e-01

3040 7.963941e-01 9.997834e-01

3041 7.972815e-01 9.997842e-01

3042 7.981702e-01 9.997851e-01

3043 7.990598e-01 9.997861e-01

3044 7.999500e-01 9.997871e-01

3045 8.008403e-01 9.997881e-01

3046 8.017304e-01 9.997893e-01

3047 8.026198e-01 9.997904e-01

3048 8.035083e-01 9.997917e-01

3049 8.043954e-01 9.997930e-01

3050 8.052807e-01 9.997944e-01

3051 8.061638e-01 9.997959e-01

3052 8.070445e-01 9.997975e-01

3053 8.079222e-01 9.997992e-01

3054 8.087967e-01 9.998010e-01

3055 8.096675e-01 9.998028e-01

3056 8.105343e-01 9.998048e-01

3057 8.113968e-01 9.998069e-01

3058 8.122545e-01 9.998091e-01

3059 8.131072e-01 9.998115e-01

3060 8.139546e-01 9.998140e-01

3061 8.147962e-01 9.998166e-01

3062 8.148289e-01 9.998166e-01

3063 8.148623e-01 9.998166e-01

3064 8.148963e-01 9.998166e-01

3065 8.149309e-01 9.998166e-01

3066 8.149662e-01 9.998166e-01

3067 8.150021e-01 9.998166e-01

3068 8.150386e-01 9.998166e-01

3069 8.150759e-01 9.998166e-01

3070 8.151138e-01 9.998166e-01

3071 8.151525e-01 9.998166e-01

3072 8.151918e-01 9.998166e-01

3073 8.152319e-01 9.998166e-01

3074 8.152727e-01 9.998166e-01

3075 8.153143e-01 9.998166e-01

3076 8.153567e-01 9.998166e-01

3077 8.153999e-01 9.998166e-01

3078 8.154439e-01 9.998166e-01

3079 8.154887e-01 9.998166e-01

3080 8.155343e-01 9.998166e-01

3081 8.155808e-01 9.998166e-01

3082 8.156282e-01 9.998166e-01

3083 8.156765e-01 9.998166e-01

3084 8.157257e-01 9.998166e-01

3085 8.157759e-01 9.998166e-01

3086 8.158270e-01 9.998166e-01

3087 8.158791e-01 9.998166e-01

3088 8.159322e-01 9.998166e-01

3089 8.159863e-01 9.998166e-01

3090 8.160414e-01 9.998166e-01

3091 8.160976e-01 9.998166e-01

3092 8.161549e-01 9.998166e-01

3093 8.162132e-01 9.998166e-01

3094 8.162727e-01 9.998166e-01

3095 8.163334e-01 9.998166e-01

3096 8.163952e-01 9.998166e-01

3097 8.164582e-01 9.998167e-01

3098 8.165225e-01 9.998167e-01

3099 8.165880e-01 9.998167e-01

3100 8.166547e-01 9.998167e-01

3101 8.167228e-01 9.998167e-01

3102 8.167922e-01 9.998167e-01

3103 8.168629e-01 9.998167e-01

3104 8.169350e-01 9.998167e-01

3105 8.170085e-01 9.998167e-01

3106 8.170835e-01 9.998167e-01

3107 8.171599e-01 9.998167e-01

3108 8.172378e-01 9.998167e-01

3109 8.173172e-01 9.998167e-01

3110 8.173982e-01 9.998167e-01

3111 8.174808e-01 9.998167e-01

3112 8.175650e-01 9.998168e-01

3113 8.176508e-01 9.998168e-01

3114 8.177383e-01 9.998168e-01

3115 8.178275e-01 9.998168e-01

3116 8.179185e-01 9.998168e-01

3117 8.180112e-01 9.998168e-01

3118 8.181058e-01 9.998168e-01

3119 8.182022e-01 9.998168e-01

3120 8.183005e-01 9.998169e-01

3121 8.184008e-01 9.998169e-01

3122 8.185030e-01 9.998169e-01

3123 8.186072e-01 9.998169e-01

3124 8.187134e-01 9.998169e-01

3125 8.188217e-01 9.998169e-01

3126 8.189322e-01 9.998170e-01

3127 8.190447e-01 9.998170e-01

3128 8.191595e-01 9.998170e-01

3129 8.192766e-01 9.998170e-01

3130 8.193959e-01 9.998170e-01

3131 8.195175e-01 9.998171e-01

3132 8.196415e-01 9.998171e-01

3133 8.197679e-01 9.998171e-01

3134 8.198967e-01 9.998171e-01

3135 8.200281e-01 9.998172e-01

3136 8.201620e-01 9.998172e-01

3137 8.202985e-01 9.998172e-01

3138 8.204376e-01 9.998173e-01

3139 8.205795e-01 9.998173e-01

3140 8.207240e-01 9.998173e-01

3141 8.208713e-01 9.998174e-01

3142 8.210215e-01 9.998174e-01

3143 8.211745e-01 9.998174e-01

3144 8.213305e-01 9.998175e-01

3145 8.214894e-01 9.998175e-01

3146 8.216514e-01 9.998176e-01

3147 8.218164e-01 9.998176e-01

3148 8.219845e-01 9.998177e-01

3149 8.221559e-01 9.998177e-01

3150 8.223305e-01 9.998178e-01

3151 8.225083e-01 9.998178e-01

3152 8.226895e-01 9.998179e-01

3153 8.228740e-01 9.998180e-01

3154 8.230620e-01 9.998180e-01

3155 8.232535e-01 9.998181e-01

3156 8.234486e-01 9.998182e-01

3157 8.236472e-01 9.998183e-01

3158 8.238494e-01 9.998184e-01

3159 8.240554e-01 9.998184e-01

3160 8.242651e-01 9.998185e-01

3161 8.244786e-01 9.998186e-01

3162 8.246960e-01 9.998187e-01

3163 8.249173e-01 9.998188e-01

3164 8.251425e-01 9.998189e-01

3165 8.253717e-01 9.998190e-01

3166 8.256049e-01 9.998192e-01

3167 8.258423e-01 9.998193e-01

3168 8.260838e-01 9.998194e-01

3169 8.263295e-01 9.998196e-01

3170 8.265795e-01 9.998197e-01

3171 8.268337e-01 9.998198e-01

3172 8.270923e-01 9.998200e-01

3173 8.273552e-01 9.998202e-01

3174 8.276225e-01 9.998203e-01

3175 8.278943e-01 9.998205e-01

3176 8.281706e-01 9.998207e-01

3177 8.284514e-01 9.998209e-01

3178 8.287368e-01 9.998211e-01

3179 8.290267e-01 9.998213e-01

3180 8.293213e-01 9.998216e-01

3181 8.296205e-01 9.998218e-01

3182 8.299244e-01 9.998220e-01

3183 8.302330e-01 9.998223e-01

3184 8.305463e-01 9.998226e-01

3185 8.308644e-01 9.998228e-01

3186 8.311872e-01 9.998231e-01

3187 8.315148e-01 9.998235e-01

3188 8.318472e-01 9.998238e-01

3189 8.321843e-01 9.998241e-01

3190 8.325263e-01 9.998245e-01

3191 8.328730e-01 9.998248e-01

3192 8.332245e-01 9.998252e-01

3193 8.335807e-01 9.998256e-01

3194 8.339418e-01 9.998261e-01

3195 8.343075e-01 9.998265e-01

3196 8.346780e-01 9.998270e-01

3197 8.350532e-01 9.998274e-01

3198 8.354330e-01 9.998280e-01

3199 8.358175e-01 9.998285e-01

3200 8.362066e-01 9.998290e-01

3201 8.366002e-01 9.998296e-01

3202 8.369983e-01 9.998302e-01

3203 8.374008e-01 9.998309e-01

3204 8.378078e-01 9.998315e-01

3205 8.382190e-01 9.998322e-01

3206 8.386345e-01 9.998329e-01

3207 8.390542e-01 9.998337e-01

3208 8.394780e-01 9.998345e-01

3209 8.399057e-01 9.998353e-01

3210 8.403374e-01 9.998362e-01

3211 8.407729e-01 9.998371e-01

3212 8.412121e-01 9.998381e-01

3213 8.416549e-01 9.998390e-01

3214 8.421012e-01 9.998401e-01

3215 8.425509e-01 9.998412e-01

3216 8.430038e-01 9.998423e-01

3217 8.434598e-01 9.998435e-01

3218 8.439188e-01 9.998447e-01

3219 8.443806e-01 9.998460e-01

3220 8.448452e-01 9.998474e-01

3221 8.453122e-01 9.998488e-01

3222 8.457816e-01 9.998502e-01

3223 8.462533e-01 9.998518e-01

3224 8.467269e-01 9.998534e-01

3225 8.472025e-01 9.998551e-01

3226 8.476797e-01 9.998568e-01

3227 8.481585e-01 9.998587e-01

3228 8.486386e-01 9.998606e-01

3229 8.491199e-01 9.998626e-01

3230 8.496021e-01 9.998647e-01

3231 8.500850e-01 9.998668e-01

3232 8.505686e-01 9.998691e-01

3233 8.510525e-01 9.998715e-01

3234 8.515366e-01 9.998739e-01

3235 8.520206e-01 9.998765e-01

3236 8.525044e-01 9.998792e-01

3237 8.529878e-01 9.998820e-01

3238 8.534705e-01 9.998849e-01

3239 8.539523e-01 9.998879e-01

3240 8.544331e-01 9.998911e-01

3241 8.549126e-01 9.998944e-01

3242 8.549254e-01 9.998944e-01

3243 8.549384e-01 9.998944e-01

3244 8.549514e-01 9.998944e-01

3245 8.549646e-01 9.998944e-01

3246 8.549779e-01 9.998944e-01

3247 8.549913e-01 9.998944e-01

3248 8.550048e-01 9.998944e-01

3249 8.550184e-01 9.998944e-01

3250 8.550321e-01 9.998945e-01

3251 8.550460e-01 9.998945e-01

3252 8.550600e-01 9.998945e-01

3253 8.550741e-01 9.998945e-01

3254 8.550884e-01 9.998945e-01

3255 8.551028e-01 9.998945e-01

3256 8.551173e-01 9.998945e-01

3257 8.551320e-01 9.998945e-01

3258 8.551468e-01 9.998945e-01

3259 8.551617e-01 9.998945e-01

3260 8.551768e-01 9.998945e-01

3261 8.551921e-01 9.998945e-01

3262 8.552075e-01 9.998945e-01

3263 8.552231e-01 9.998945e-01

3264 8.552388e-01 9.998945e-01

3265 8.552547e-01 9.998945e-01

3266 8.552708e-01 9.998945e-01

3267 8.552870e-01 9.998945e-01

3268 8.553034e-01 9.998945e-01

3269 8.553200e-01 9.998945e-01

3270 8.553367e-01 9.998946e-01

3271 8.553537e-01 9.998946e-01

3272 8.553708e-01 9.998946e-01

3273 8.553881e-01 9.998946e-01

3274 8.554056e-01 9.998946e-01

3275 8.554233e-01 9.998946e-01

3276 8.554412e-01 9.998946e-01

3277 8.554593e-01 9.998946e-01

3278 8.554776e-01 9.998946e-01

3279 8.554961e-01 9.998946e-01

3280 8.555148e-01 9.998946e-01

3281 8.555337e-01 9.998946e-01

3282 8.555528e-01 9.998946e-01

3283 8.555722e-01 9.998947e-01

3284 8.555918e-01 9.998947e-01

3285 8.556116e-01 9.998947e-01

3286 8.556316e-01 9.998947e-01

3287 8.556518e-01 9.998947e-01

3288 8.556723e-01 9.998947e-01

3289 8.556930e-01 9.998947e-01

3290 8.557140e-01 9.998947e-01

3291 8.557352e-01 9.998947e-01

3292 8.557566e-01 9.998947e-01

3293 8.557783e-01 9.998948e-01

3294 8.558002e-01 9.998948e-01

3295 8.558224e-01 9.998948e-01

3296 8.558448e-01 9.998948e-01

3297 8.558675e-01 9.998948e-01

3298 8.558904e-01 9.998948e-01

3299 8.559136e-01 9.998948e-01

3300 8.559370e-01 9.998949e-01

3301 8.559607e-01 9.998949e-01

3302 8.559846e-01 9.998949e-01

3303 8.560088e-01 9.998949e-01

3304 8.560333e-01 9.998949e-01

3305 8.560581e-01 9.998950e-01

3306 8.560831e-01 9.998950e-01

3307 8.561083e-01 9.998950e-01

3308 8.561339e-01 9.998950e-01

3309 8.561597e-01 9.998950e-01

3310 8.561857e-01 9.998951e-01

3311 8.562120e-01 9.998951e-01

3312 8.562386e-01 9.998951e-01

3313 8.562655e-01 9.998951e-01

3314 8.562926e-01 9.998952e-01

3315 8.563200e-01 9.998952e-01

3316 8.563477e-01 9.998952e-01

3317 8.563756e-01 9.998952e-01

3318 8.564037e-01 9.998953e-01

3319 8.564322e-01 9.998953e-01

3320 8.564608e-01 9.998953e-01

3321 8.564898e-01 9.998954e-01

3322 8.565190e-01 9.998954e-01

3323 8.565484e-01 9.998954e-01

3324 8.565781e-01 9.998955e-01

3325 8.566080e-01 9.998955e-01

3326 8.566381e-01 9.998956e-01

3327 8.566685e-01 9.998956e-01

3328 8.566991e-01 9.998956e-01

3329 8.567299e-01 9.998957e-01

3330 8.567610e-01 9.998957e-01

3331 8.567922e-01 9.998958e-01

3332 8.568237e-01 9.998958e-01

3333 8.568554e-01 9.998959e-01

3334 8.568872e-01 9.998959e-01

3335 8.569192e-01 9.998960e-01

3336 8.569514e-01 9.998960e-01

3337 8.569838e-01 9.998961e-01

3338 8.570163e-01 9.998962e-01

3339 8.570490e-01 9.998962e-01

3340 8.570818e-01 9.998963e-01

3341 8.571147e-01 9.998964e-01

3342 8.571478e-01 9.998964e-01

3343 8.571809e-01 9.998965e-01

3344 8.572142e-01 9.998966e-01

3345 8.572475e-01 9.998967e-01

3346 8.572809e-01 9.998967e-01

3347 8.573143e-01 9.998968e-01

3348 8.573478e-01 9.998969e-01

3349 8.573812e-01 9.998970e-01

3350 8.574147e-01 9.998971e-01

3351 8.574482e-01 9.998972e-01

3352 8.574816e-01 9.998973e-01

3353 8.575150e-01 9.998974e-01

3354 8.575483e-01 9.998975e-01

3355 8.575815e-01 9.998976e-01

3356 8.576146e-01 9.998977e-01

3357 8.576476e-01 9.998979e-01

3358 8.576804e-01 9.998980e-01

3359 8.577131e-01 9.998981e-01

3360 8.577455e-01 9.998983e-01

3361 8.577777e-01 9.998984e-01

3362 8.578097e-01 9.998985e-01

3363 8.578414e-01 9.998987e-01

3364 8.578728e-01 9.998989e-01

3365 8.579038e-01 9.998990e-01

3366 8.579345e-01 9.998992e-01

3367 8.579648e-01 9.998994e-01

3368 8.579947e-01 9.998995e-01

3369 8.580242e-01 9.998997e-01

3370 8.580532e-01 9.998999e-01

3371 8.580816e-01 9.999001e-01

3372 8.581095e-01 9.999004e-01

3373 8.581369e-01 9.999006e-01

3374 8.581636e-01 9.999008e-01

3375 8.581897e-01 9.999010e-01

3376 8.582152e-01 9.999013e-01

3377 8.582399e-01 9.999015e-01

3378 8.582639e-01 9.999018e-01

3379 8.582870e-01 9.999021e-01

3380 8.583094e-01 9.999024e-01

3381 8.583309e-01 9.999027e-01

3382 8.583515e-01 9.999030e-01

3383 8.583712e-01 9.999033e-01

3384 8.583899e-01 9.999036e-01

3385 8.584077e-01 9.999040e-01

3386 8.584243e-01 9.999043e-01

3387 8.584399e-01 9.999047e-01

3388 8.584544e-01 9.999051e-01

3389 8.584677e-01 9.999055e-01

3390 8.584798e-01 9.999059e-01

3391 8.584907e-01 9.999063e-01

3392 8.585003e-01 9.999068e-01

3393 8.585086e-01 9.999072e-01

3394 8.585155e-01 9.999077e-01

3395 8.585211e-01 9.999082e-01

3396 8.585253e-01 9.999087e-01

3397 8.585280e-01 9.999093e-01

3398 8.585293e-01 9.999098e-01

3399 8.585290e-01 9.999104e-01

3400 8.585272e-01 9.999110e-01

3401 8.585238e-01 9.999116e-01

3402 8.585188e-01 9.999123e-01

3403 8.585122e-01 9.999129e-01

3404 8.585039e-01 9.999136e-01

3405 8.584940e-01 9.999143e-01

3406 8.584823e-01 9.999151e-01

3407 8.584690e-01 9.999159e-01

3408 8.584539e-01 9.999167e-01

3409 8.584370e-01 9.999175e-01

3410 8.584183e-01 9.999184e-01

3411 8.583979e-01 9.999193e-01

3412 8.583756e-01 9.999203e-01

3413 8.583515e-01 9.999213e-01

3414 8.583256e-01 9.999223e-01

3415 8.582979e-01 9.999234e-01

3416 8.582684e-01 9.999245e-01

3417 8.582370e-01 9.999256e-01

3418 8.582038e-01 9.999269e-01

3419 8.581688e-01 9.999281e-01

3420 8.581319e-01 9.999295e-01

3421 8.580932e-01 9.999308e-01

3422 8.580885e-01 9.999308e-01

3423 8.580835e-01 9.999308e-01

3424 8.580783e-01 9.999308e-01

3425 8.580727e-01 9.999308e-01

3426 8.580669e-01 9.999308e-01

3427 8.580607e-01 9.999308e-01

3428 8.580542e-01 9.999308e-01

3429 8.580474e-01 9.999308e-01

3430 8.580403e-01 9.999308e-01

3431 8.580329e-01 9.999308e-01

3432 8.580251e-01 9.999308e-01

3433 8.580169e-01 9.999308e-01

3434 8.580084e-01 9.999308e-01

3435 8.579995e-01 9.999308e-01

3436 8.579903e-01 9.999308e-01

3437 8.579806e-01 9.999309e-01

3438 8.579706e-01 9.999309e-01

3439 8.579601e-01 9.999309e-01

3440 8.579493e-01 9.999309e-01

3441 8.579380e-01 9.999309e-01

3442 8.579262e-01 9.999309e-01

3443 8.579140e-01 9.999309e-01

3444 8.579014e-01 9.999309e-01

3445 8.578883e-01 9.999309e-01

3446 8.578747e-01 9.999309e-01

3447 8.578605e-01 9.999309e-01

3448 8.578459e-01 9.999309e-01

3449 8.578308e-01 9.999309e-01

3450 8.578151e-01 9.999309e-01

3451 8.577988e-01 9.999309e-01

3452 8.577820e-01 9.999309e-01

3453 8.577647e-01 9.999309e-01

3454 8.577467e-01 9.999309e-01

3455 8.577281e-01 9.999309e-01

3456 8.577089e-01 9.999309e-01

3457 8.576890e-01 9.999309e-01

3458 8.576685e-01 9.999309e-01

3459 8.576473e-01 9.999309e-01

3460 8.576254e-01 9.999309e-01

3461 8.576028e-01 9.999309e-01

3462 8.575794e-01 9.999309e-01

3463 8.575553e-01 9.999309e-01

3464 8.575304e-01 9.999310e-01

3465 8.575048e-01 9.999310e-01

3466 8.574783e-01 9.999310e-01

3467 8.574510e-01 9.999310e-01

3468 8.574228e-01 9.999310e-01

3469 8.573938e-01 9.999310e-01

3470 8.573638e-01 9.999310e-01

3471 8.573330e-01 9.999310e-01

3472 8.573011e-01 9.999310e-01

3473 8.572683e-01 9.999310e-01

3474 8.572345e-01 9.999310e-01

3475 8.571997e-01 9.999310e-01

3476 8.571638e-01 9.999310e-01

3477 8.571268e-01 9.999310e-01

3478 8.570887e-01 9.999311e-01

3479 8.570494e-01 9.999311e-01

3480 8.570090e-01 9.999311e-01

3481 8.569674e-01 9.999311e-01

3482 8.569245e-01 9.999311e-01

3483 8.568804e-01 9.999311e-01

3484 8.568349e-01 9.999311e-01

3485 8.567881e-01 9.999311e-01

3486 8.567399e-01 9.999311e-01

3487 8.566904e-01 9.999311e-01

3488 8.566393e-01 9.999312e-01

3489 8.565868e-01 9.999312e-01

3490 8.565327e-01 9.999312e-01

3491 8.564771e-01 9.999312e-01

3492 8.564198e-01 9.999312e-01

3493 8.563609e-01 9.999312e-01

3494 8.563003e-01 9.999312e-01

3495 8.562379e-01 9.999313e-01

3496 8.561738e-01 9.999313e-01

3497 8.561078e-01 9.999313e-01

3498 8.560399e-01 9.999313e-01

3499 8.559701e-01 9.999313e-01

3500 8.558983e-01 9.999313e-01

3501 8.558245e-01 9.999314e-01

3502 8.557486e-01 9.999314e-01

3503 8.556706e-01 9.999314e-01

3504 8.555903e-01 9.999314e-01

3505 8.555078e-01 9.999315e-01

3506 8.554230e-01 9.999315e-01

3507 8.553358e-01 9.999315e-01

3508 8.552462e-01 9.999315e-01

3509 8.551541e-01 9.999316e-01

3510 8.550594e-01 9.999316e-01

3511 8.549622e-01 9.999316e-01

3512 8.548622e-01 9.999316e-01

3513 8.547596e-01 9.999317e-01

3514 8.546541e-01 9.999317e-01

3515 8.545457e-01 9.999317e-01

3516 8.544344e-01 9.999318e-01

3517 8.543201e-01 9.999318e-01

3518 8.542027e-01 9.999318e-01

3519 8.540822e-01 9.999319e-01

3520 8.539584e-01 9.999319e-01

3521 8.538313e-01 9.999319e-01

3522 8.537009e-01 9.999320e-01

3523 8.535670e-01 9.999320e-01

3524 8.534295e-01 9.999321e-01

3525 8.532885e-01 9.999321e-01

3526 8.531438e-01 9.999322e-01

3527 8.529954e-01 9.999322e-01

3528 8.528431e-01 9.999323e-01

3529 8.526869e-01 9.999323e-01

3530 8.525267e-01 9.999324e-01

3531 8.523624e-01 9.999324e-01

3532 8.521940e-01 9.999325e-01

3533 8.520213e-01 9.999326e-01

3534 8.518443e-01 9.999326e-01

3535 8.516630e-01 9.999327e-01

3536 8.514771e-01 9.999328e-01

3537 8.512867e-01 9.999328e-01

3538 8.510917e-01 9.999329e-01

3539 8.508919e-01 9.999330e-01

3540 8.506874e-01 9.999331e-01

3541 8.504780e-01 9.999332e-01

3542 8.502637e-01 9.999333e-01

3543 8.500443e-01 9.999333e-01

3544 8.498198e-01 9.999334e-01

3545 8.495902e-01 9.999335e-01

3546 8.493554e-01 9.999336e-01

3547 8.491152e-01 9.999337e-01

3548 8.488697e-01 9.999339e-01

3549 8.486187e-01 9.999340e-01

3550 8.483623e-01 9.999341e-01

3551 8.481003e-01 9.999342e-01

3552 8.478327e-01 9.999343e-01

3553 8.475594e-01 9.999345e-01

3554 8.472804e-01 9.999346e-01

3555 8.469956e-01 9.999348e-01

3556 8.467051e-01 9.999349e-01

3557 8.464087e-01 9.999351e-01

3558 8.461064e-01 9.999352e-01

3559 8.457983e-01 9.999354e-01

3560 8.454842e-01 9.999356e-01

3561 8.451642e-01 9.999357e-01

3562 8.448382e-01 9.999359e-01

3563 8.445062e-01 9.999361e-01

3564 8.441683e-01 9.999363e-01

3565 8.438245e-01 9.999365e-01

3566 8.434746e-01 9.999368e-01

3567 8.431189e-01 9.999370e-01

3568 8.427572e-01 9.999372e-01

3569 8.423897e-01 9.999375e-01

3570 8.420163e-01 9.999377e-01

3571 8.416371e-01 9.999380e-01

3572 8.412521e-01 9.999382e-01

3573 8.408615e-01 9.999385e-01

3574 8.404652e-01 9.999388e-01

3575 8.400634e-01 9.999391e-01

3576 8.396562e-01 9.999394e-01

3577 8.392436e-01 9.999398e-01

3578 8.388257e-01 9.999401e-01

3579 8.384026e-01 9.999405e-01

3580 8.379745e-01 9.999408e-01

3581 8.375415e-01 9.999412e-01

3582 8.371036e-01 9.999416e-01

3583 8.366612e-01 9.999420e-01

3584 8.362142e-01 9.999425e-01

3585 8.357629e-01 9.999429e-01

3586 8.353074e-01 9.999434e-01

3587 8.348479e-01 9.999439e-01

3588 8.343845e-01 9.999443e-01

3589 8.339175e-01 9.999449e-01

3590 8.334471e-01 9.999454e-01

3591 8.329734e-01 9.999460e-01

3592 8.324966e-01 9.999465e-01

3593 8.320170e-01 9.999471e-01

3594 8.315348e-01 9.999478e-01

3595 8.310502e-01 9.999484e-01

3596 8.305634e-01 9.999491e-01

3597 8.300747e-01 9.999498e-01

3598 8.295842e-01 9.999505e-01

3599 8.290923e-01 9.999512e-01

3600 8.285992e-01 9.999520e-01

3601 8.281051e-01 9.999528e-01

3602 8.280774e-01 9.999528e-01

3603 8.280490e-01 9.999528e-01

3604 8.280198e-01 9.999528e-01

3605 8.279898e-01 9.999528e-01

3606 8.279589e-01 9.999528e-01

3607 8.279273e-01 9.999528e-01

3608 8.278948e-01 9.999528e-01

3609 8.278615e-01 9.999528e-01

3610 8.278272e-01 9.999528e-01

3611 8.277921e-01 9.999528e-01

3612 8.277560e-01 9.999528e-01

3613 8.277190e-01 9.999528e-01

3614 8.276810e-01 9.999528e-01

3615 8.276420e-01 9.999528e-01

3616 8.276020e-01 9.999529e-01

3617 8.275609e-01 9.999529e-01

3618 8.275187e-01 9.999529e-01

3619 8.274755e-01 9.999529e-01

3620 8.274311e-01 9.999529e-01

3621 8.273855e-01 9.999529e-01

3622 8.273388e-01 9.999529e-01

3623 8.272908e-01 9.999529e-01

3624 8.272416e-01 9.999529e-01

3625 8.271912e-01 9.999529e-01

3626 8.271394e-01 9.999529e-01

3627 8.270862e-01 9.999529e-01

3628 8.270317e-01 9.999529e-01

3629 8.269758e-01 9.999529e-01

3630 8.269185e-01 9.999529e-01

3631 8.268596e-01 9.999529e-01

3632 8.267993e-01 9.999529e-01

3633 8.267373e-01 9.999529e-01

3634 8.266738e-01 9.999529e-01

3635 8.266087e-01 9.999529e-01

3636 8.265419e-01 9.999529e-01

3637 8.264733e-01 9.999529e-01

3638 8.264030e-01 9.999529e-01

3639 8.263309e-01 9.999529e-01

3640 8.262570e-01 9.999529e-01

3641 8.261811e-01 9.999529e-01

3642 8.261033e-01 9.999529e-01

3643 8.260236e-01 9.999529e-01

3644 8.259417e-01 9.999529e-01

3645 8.258578e-01 9.999529e-01

3646 8.257718e-01 9.999529e-01

3647 8.256836e-01 9.999529e-01

3648 8.255931e-01 9.999529e-01

3649 8.255003e-01 9.999529e-01

3650 8.254051e-01 9.999529e-01

3651 8.253076e-01 9.999530e-01

3652 8.252075e-01 9.999530e-01

3653 8.251049e-01 9.999530e-01

3654 8.249997e-01 9.999530e-01

3655 8.248919e-01 9.999530e-01

3656 8.247813e-01 9.999530e-01

3657 8.246679e-01 9.999530e-01

3658 8.245517e-01 9.999530e-01

3659 8.244325e-01 9.999530e-01

3660 8.243103e-01 9.999530e-01

3661 8.241851e-01 9.999530e-01

3662 8.240567e-01 9.999530e-01

3663 8.239251e-01 9.999530e-01

3664 8.237901e-01 9.999530e-01

3665 8.236518e-01 9.999530e-01

3666 8.235101e-01 9.999530e-01

3667 8.233648e-01 9.999530e-01

3668 8.232159e-01 9.999531e-01

3669 8.230632e-01 9.999531e-01

3670 8.229068e-01 9.999531e-01

3671 8.227465e-01 9.999531e-01

3672 8.225822e-01 9.999531e-01

3673 8.224139e-01 9.999531e-01

3674 8.222414e-01 9.999531e-01

3675 8.220647e-01 9.999531e-01

3676 8.218837e-01 9.999531e-01

3677 8.216982e-01 9.999531e-01

3678 8.215081e-01 9.999531e-01

3679 8.213135e-01 9.999531e-01

3680 8.211141e-01 9.999532e-01

3681 8.209099e-01 9.999532e-01

3682 8.207007e-01 9.999532e-01

3683 8.204865e-01 9.999532e-01

3684 8.202672e-01 9.999532e-01

3685 8.200426e-01 9.999532e-01

3686 8.198126e-01 9.999532e-01

3687 8.195772e-01 9.999532e-01

3688 8.193362e-01 9.999533e-01

3689 8.190895e-01 9.999533e-01

3690 8.188371e-01 9.999533e-01

3691 8.185787e-01 9.999533e-01

3692 8.183143e-01 9.999533e-01

3693 8.180438e-01 9.999533e-01

3694 8.177670e-01 9.999534e-01

3695 8.174839e-01 9.999534e-01

3696 8.171944e-01 9.999534e-01

3697 8.168982e-01 9.999534e-01

3698 8.165954e-01 9.999534e-01

3699 8.162858e-01 9.999534e-01

3700 8.159693e-01 9.999535e-01

3701 8.156458e-01 9.999535e-01

3702 8.153151e-01 9.999535e-01

3703 8.149773e-01 9.999535e-01

3704 8.146321e-01 9.999536e-01

3705 8.142794e-01 9.999536e-01

3706 8.139193e-01 9.999536e-01

3707 8.135515e-01 9.999536e-01

3708 8.131760e-01 9.999537e-01

3709 8.127926e-01 9.999537e-01

3710 8.124014e-01 9.999537e-01

3711 8.120022e-01 9.999537e-01

3712 8.115949e-01 9.999538e-01

3713 8.111794e-01 9.999538e-01

3714 8.107558e-01 9.999538e-01

3715 8.103238e-01 9.999539e-01

3716 8.098835e-01 9.999539e-01

3717 8.094347e-01 9.999539e-01

3718 8.089775e-01 9.999540e-01

3719 8.085117e-01 9.999540e-01

3720 8.080374e-01 9.999540e-01

3721 8.075546e-01 9.999541e-01

3722 8.070630e-01 9.999541e-01

3723 8.065629e-01 9.999542e-01

3724 8.060541e-01 9.999542e-01

3725 8.055366e-01 9.999543e-01

3726 8.050105e-01 9.999543e-01

3727 8.044758e-01 9.999544e-01

3728 8.039324e-01 9.999544e-01

3729 8.033805e-01 9.999545e-01

3730 8.028200e-01 9.999545e-01

3731 8.022510e-01 9.999546e-01

3732 8.016736e-01 9.999546e-01

3733 8.010879e-01 9.999547e-01

3734 8.004939e-01 9.999548e-01

3735 7.998917e-01 9.999548e-01

3736 7.992814e-01 9.999549e-01

3737 7.986632e-01 9.999550e-01

3738 7.980372e-01 9.999550e-01

3739 7.974034e-01 9.999551e-01

3740 7.967622e-01 9.999552e-01

3741 7.961136e-01 9.999553e-01

3742 7.954578e-01 9.999553e-01

3743 7.947950e-01 9.999554e-01

3744 7.941254e-01 9.999555e-01

3745 7.934493e-01 9.999556e-01

3746 7.927668e-01 9.999557e-01

3747 7.920781e-01 9.999558e-01

3748 7.913836e-01 9.999559e-01

3749 7.906835e-01 9.999560e-01

3750 7.899781e-01 9.999561e-01

3751 7.892676e-01 9.999562e-01

3752 7.885523e-01 9.999563e-01

3753 7.878325e-01 9.999565e-01

3754 7.871086e-01 9.999566e-01

3755 7.863809e-01 9.999567e-01

3756 7.856497e-01 9.999569e-01

3757 7.849153e-01 9.999570e-01

3758 7.841781e-01 9.999571e-01

3759 7.834385e-01 9.999573e-01

3760 7.826967e-01 9.999574e-01

3761 7.819532e-01 9.999576e-01

3762 7.812083e-01 9.999577e-01

3763 7.804624e-01 9.999579e-01

3764 7.797159e-01 9.999581e-01

3765 7.789692e-01 9.999583e-01

3766 7.782226e-01 9.999584e-01

3767 7.774765e-01 9.999586e-01

3768 7.767314e-01 9.999588e-01

3769 7.759875e-01 9.999590e-01

3770 7.752453e-01 9.999592e-01

3771 7.745052e-01 9.999595e-01

3772 7.737674e-01 9.999597e-01

3773 7.730325e-01 9.999599e-01

3774 7.723008e-01 9.999602e-01

3775 7.715725e-01 9.999604e-01

3776 7.708482e-01 9.999607e-01

3777 7.701281e-01 9.999609e-01

3778 7.694126e-01 9.999612e-01

3779 7.687020e-01 9.999615e-01

3780 7.679967e-01 9.999618e-01

3781 7.672969e-01 9.999621e-01

3782 7.672365e-01 9.999621e-01

3783 7.671747e-01 9.999621e-01

3784 7.671116e-01 9.999621e-01

3785 7.670469e-01 9.999621e-01

3786 7.669808e-01 9.999621e-01

3787 7.669132e-01 9.999621e-01

3788 7.668440e-01 9.999621e-01

3789 7.667733e-01 9.999621e-01

3790 7.667009e-01 9.999621e-01

3791 7.666269e-01 9.999621e-01

3792 7.665512e-01 9.999621e-01

3793 7.664738e-01 9.999621e-01

3794 7.663947e-01 9.999621e-01

3795 7.663138e-01 9.999621e-01

3796 7.662310e-01 9.999621e-01

3797 7.661464e-01 9.999621e-01

3798 7.660599e-01 9.999621e-01

3799 7.659714e-01 9.999621e-01

3800 7.658809e-01 9.999621e-01

3801 7.657883e-01 9.999621e-01

3802 7.656937e-01 9.999621e-01

3803 7.655970e-01 9.999621e-01

3804 7.654980e-01 9.999621e-01

3805 7.653969e-01 9.999621e-01

3806 7.652934e-01 9.999621e-01

3807 7.651877e-01 9.999621e-01

3808 7.650795e-01 9.999621e-01

3809 7.649689e-01 9.999621e-01

3810 7.648558e-01 9.999621e-01

3811 7.647402e-01 9.999621e-01

3812 7.646220e-01 9.999621e-01

3813 7.645011e-01 9.999621e-01

3814 7.643776e-01 9.999621e-01

3815 7.642512e-01 9.999621e-01

3816 7.641220e-01 9.999621e-01

3817 7.639899e-01 9.999621e-01

3818 7.638549e-01 9.999621e-01

3819 7.637168e-01 9.999621e-01

3820 7.635757e-01 9.999621e-01

3821 7.634314e-01 9.999621e-01

3822 7.632839e-01 9.999621e-01

3823 7.631331e-01 9.999621e-01

3824 7.629789e-01 9.999621e-01

3825 7.628213e-01 9.999621e-01

3826 7.626602e-01 9.999621e-01

3827 7.624956e-01 9.999621e-01

3828 7.623273e-01 9.999621e-01

3829 7.621553e-01 9.999621e-01

3830 7.619794e-01 9.999621e-01

3831 7.617997e-01 9.999621e-01

3832 7.616161e-01 9.999621e-01

3833 7.614284e-01 9.999621e-01

3834 7.612366e-01 9.999621e-01

3835 7.610407e-01 9.999621e-01

3836 7.608404e-01 9.999621e-01

3837 7.606358e-01 9.999621e-01

3838 7.604267e-01 9.999621e-01

3839 7.602132e-01 9.999621e-01

3840 7.599950e-01 9.999621e-01

3841 7.597721e-01 9.999621e-01

3842 7.595444e-01 9.999621e-01

3843 7.593118e-01 9.999621e-01

3844 7.590743e-01 9.999621e-01

3845 7.588317e-01 9.999621e-01

3846 7.585840e-01 9.999621e-01

3847 7.583311e-01 9.999621e-01

3848 7.580728e-01 9.999621e-01

3849 7.578091e-01 9.999621e-01

3850 7.575399e-01 9.999621e-01

3851 7.572652e-01 9.999621e-01

3852 7.569847e-01 9.999621e-01

3853 7.566985e-01 9.999621e-01

3854 7.564064e-01 9.999622e-01

3855 7.561084e-01 9.999622e-01

3856 7.558043e-01 9.999622e-01

3857 7.554941e-01 9.999622e-01

3858 7.551776e-01 9.999622e-01

3859 7.548549e-01 9.999622e-01

3860 7.545258e-01 9.999622e-01

3861 7.541902e-01 9.999622e-01

3862 7.538481e-01 9.999622e-01

3863 7.534994e-01 9.999622e-01

3864 7.531439e-01 9.999622e-01

3865 7.527816e-01 9.999622e-01

3866 7.524125e-01 9.999622e-01

3867 7.520365e-01 9.999622e-01

3868 7.516535e-01 9.999622e-01

3869 7.512634e-01 9.999622e-01

3870 7.508661e-01 9.999622e-01

3871 7.504617e-01 9.999622e-01

3872 7.500501e-01 9.999622e-01

3873 7.496311e-01 9.999622e-01

3874 7.492048e-01 9.999622e-01

3875 7.487712e-01 9.999622e-01

3876 7.483301e-01 9.999622e-01

3877 7.478816e-01 9.999622e-01

3878 7.474256e-01 9.999622e-01

3879 7.469621e-01 9.999622e-01

3880 7.464910e-01 9.999622e-01

3881 7.460125e-01 9.999622e-01

3882 7.455264e-01 9.999622e-01

3883 7.450328e-01 9.999622e-01

3884 7.445317e-01 9.999622e-01

3885 7.440231e-01 9.999622e-01

3886 7.435070e-01 9.999622e-01

3887 7.429834e-01 9.999622e-01

3888 7.424525e-01 9.999622e-01

3889 7.419142e-01 9.999622e-01

3890 7.413686e-01 9.999622e-01

3891 7.408158e-01 9.999622e-01

3892 7.402558e-01 9.999622e-01

3893 7.396888e-01 9.999622e-01

3894 7.391148e-01 9.999622e-01

3895 7.385339e-01 9.999622e-01

3896 7.379462e-01 9.999622e-01

3897 7.373520e-01 9.999622e-01

3898 7.367512e-01 9.999622e-01

3899 7.361440e-01 9.999623e-01

3900 7.355306e-01 9.999623e-01

3901 7.349112e-01 9.999623e-01

3902 7.342859e-01 9.999623e-01

3903 7.336549e-01 9.999623e-01

3904 7.330185e-01 9.999623e-01

3905 7.323767e-01 9.999623e-01

3906 7.317298e-01 9.999623e-01

3907 7.310781e-01 9.999623e-01

3908 7.304218e-01 9.999623e-01

3909 7.297611e-01 9.999623e-01

3910 7.290962e-01 9.999623e-01

3911 7.284275e-01 9.999623e-01

3912 7.277552e-01 9.999623e-01

3913 7.270796e-01 9.999623e-01

3914 7.264009e-01 9.999623e-01

3915 7.257195e-01 9.999623e-01

3916 7.250357e-01 9.999623e-01

3917 7.243497e-01 9.999622e-01

3918 7.236619e-01 9.999622e-01

3919 7.229726e-01 9.999622e-01

3920 7.222822e-01 9.999622e-01

3921 7.215909e-01 9.999622e-01

3922 7.208991e-01 9.999622e-01

3923 7.202071e-01 9.999622e-01

3924 7.195154e-01 9.999622e-01

3925 7.188241e-01 9.999622e-01

3926 7.181337e-01 9.999622e-01

3927 7.174445e-01 9.999622e-01

3928 7.167568e-01 9.999622e-01

3929 7.160711e-01 9.999622e-01

3930 7.153876e-01 9.999621e-01

3931 7.147066e-01 9.999621e-01

3932 7.140286e-01 9.999621e-01

3933 7.133539e-01 9.999621e-01

3934 7.126827e-01 9.999621e-01

3935 7.120154e-01 9.999621e-01

3936 7.113524e-01 9.999620e-01

3937 7.106939e-01 9.999620e-01

3938 7.100403e-01 9.999620e-01

3939 7.093918e-01 9.999620e-01

3940 7.087488e-01 9.999619e-01

3941 7.081115e-01 9.999619e-01

3942 7.074803e-01 9.999619e-01

3943 7.068553e-01 9.999618e-01

3944 7.062368e-01 9.999618e-01

3945 7.056251e-01 9.999617e-01

3946 7.050204e-01 9.999617e-01

3947 7.044230e-01 9.999616e-01

3948 7.038330e-01 9.999616e-01

3949 7.032506e-01 9.999615e-01

3950 7.026761e-01 9.999615e-01

3951 7.021096e-01 9.999614e-01

3952 7.015512e-01 9.999613e-01

3953 7.010012e-01 9.999612e-01

3954 7.004597e-01 9.999611e-01

3955 6.999267e-01 9.999611e-01

3956 6.994025e-01 9.999610e-01

3957 6.988871e-01 9.999609e-01

3958 6.983805e-01 9.999607e-01

3959 6.978830e-01 9.999606e-01

3960 6.973945e-01 9.999605e-01

3961 6.969151e-01 9.999604e-01

3962 6.968375e-01 9.999604e-01

3963 6.967585e-01 9.999604e-01

3964 6.966780e-01 9.999604e-01

3965 6.965961e-01 9.999604e-01

3966 6.965127e-01 9.999604e-01

3967 6.964279e-01 9.999604e-01

3968 6.963415e-01 9.999604e-01

3969 6.962535e-01 9.999604e-01

3970 6.961641e-01 9.999604e-01

3971 6.960730e-01 9.999604e-01

3972 6.959804e-01 9.999604e-01

3973 6.958862e-01 9.999604e-01

3974 6.957903e-01 9.999604e-01

3975 6.956927e-01 9.999604e-01

3976 6.955935e-01 9.999604e-01

3977 6.954926e-01 9.999604e-01

3978 6.953899e-01 9.999604e-01

3979 6.952855e-01 9.999604e-01

3980 6.951793e-01 9.999604e-01

3981 6.950713e-01 9.999604e-01

3982 6.949615e-01 9.999604e-01

3983 6.948499e-01 9.999604e-01

3984 6.947363e-01 9.999603e-01

3985 6.946209e-01 9.999603e-01

3986 6.945035e-01 9.999603e-01

3987 6.943842e-01 9.999603e-01

3988 6.942629e-01 9.999603e-01

3989 6.941396e-01 9.999603e-01

3990 6.940143e-01 9.999603e-01

3991 6.938869e-01 9.999603e-01

3992 6.937575e-01 9.999603e-01

3993 6.936259e-01 9.999603e-01

3994 6.934923e-01 9.999603e-01

3995 6.933564e-01 9.999603e-01

3996 6.932184e-01 9.999603e-01

3997 6.930782e-01 9.999603e-01

3998 6.929358e-01 9.999603e-01

3999 6.927911e-01 9.999603e-01

4000 6.926441e-01 9.999603e-01

4001 6.924949e-01 9.999603e-01

4002 6.923433e-01 9.999603e-01

4003 6.921894e-01 9.999603e-01

4004 6.920331e-01 9.999603e-01

4005 6.918744e-01 9.999603e-01

4006 6.917134e-01 9.999603e-01

4007 6.915499e-01 9.999603e-01

4008 6.913840e-01 9.999603e-01

4009 6.912156e-01 9.999603e-01

4010 6.910447e-01 9.999603e-01

4011 6.908713e-01 9.999603e-01

4012 6.906955e-01 9.999603e-01

4013 6.905170e-01 9.999603e-01

4014 6.903361e-01 9.999603e-01

4015 6.901526e-01 9.999603e-01

4016 6.899665e-01 9.999603e-01

4017 6.897778e-01 9.999603e-01

4018 6.895866e-01 9.999603e-01

4019 6.893927e-01 9.999603e-01

4020 6.891963e-01 9.999603e-01

4021 6.889972e-01 9.999603e-01

4022 6.887956e-01 9.999603e-01

4023 6.885913e-01 9.999603e-01

4024 6.883844e-01 9.999603e-01

4025 6.881749e-01 9.999603e-01

4026 6.879627e-01 9.999603e-01

4027 6.877480e-01 9.999603e-01

4028 6.875307e-01 9.999603e-01

4029 6.873108e-01 9.999603e-01

4030 6.870882e-01 9.999603e-01

4031 6.868632e-01 9.999602e-01

4032 6.866355e-01 9.999602e-01

4033 6.864054e-01 9.999602e-01

4034 6.861727e-01 9.999602e-01

4035 6.859375e-01 9.999602e-01

4036 6.856999e-01 9.999602e-01

4037 6.854598e-01 9.999602e-01

4038 6.852174e-01 9.999602e-01

4039 6.849725e-01 9.999602e-01

4040 6.847253e-01 9.999602e-01

4041 6.844758e-01 9.999602e-01

4042 6.842241e-01 9.999602e-01

4043 6.839702e-01 9.999602e-01

4044 6.837140e-01 9.999602e-01

4045 6.834558e-01 9.999602e-01

4046 6.831956e-01 9.999601e-01

4047 6.829333e-01 9.999601e-01

4048 6.826691e-01 9.999601e-01

4049 6.824031e-01 9.999601e-01

4050 6.821352e-01 9.999601e-01

4051 6.818657e-01 9.999601e-01

4052 6.815945e-01 9.999601e-01

4053 6.813217e-01 9.999601e-01

4054 6.810474e-01 9.999601e-01

4055 6.807718e-01 9.999600e-01

4056 6.804948e-01 9.999600e-01

4057 6.802166e-01 9.999600e-01

4058 6.799373e-01 9.999600e-01

4059 6.796570e-01 9.999600e-01

4060 6.793758e-01 9.999600e-01

4061 6.790938e-01 9.999599e-01

4062 6.788111e-01 9.999599e-01

4063 6.785278e-01 9.999599e-01

4064 6.782440e-01 9.999599e-01

4065 6.779599e-01 9.999599e-01

4066 6.776756e-01 9.999598e-01

4067 6.773911e-01 9.999598e-01

4068 6.771068e-01 9.999598e-01

4069 6.768225e-01 9.999598e-01

4070 6.765386e-01 9.999598e-01

4071 6.762551e-01 9.999597e-01

4072 6.759722e-01 9.999597e-01

4073 6.756900e-01 9.999597e-01

4074 6.754086e-01 9.999596e-01

4075 6.751282e-01 9.999596e-01

4076 6.748490e-01 9.999596e-01

4077 6.745710e-01 9.999595e-01

4078 6.742945e-01 9.999595e-01

4079 6.740195e-01 9.999595e-01

4080 6.737462e-01 9.999594e-01

4081 6.734748e-01 9.999594e-01

4082 6.732054e-01 9.999593e-01

4083 6.729382e-01 9.999593e-01

4084 6.726732e-01 9.999592e-01

4085 6.724106e-01 9.999592e-01

4086 6.721507e-01 9.999591e-01

4087 6.718934e-01 9.999591e-01

4088 6.716390e-01 9.999590e-01

4089 6.713876e-01 9.999590e-01

4090 6.711392e-01 9.999589e-01

4091 6.708942e-01 9.999588e-01

4092 6.706525e-01 9.999588e-01

4093 6.704142e-01 9.999587e-01

4094 6.701796e-01 9.999586e-01

4095 6.699487e-01 9.999585e-01

4096 6.697217e-01 9.999584e-01

4097 6.694985e-01 9.999584e-01

4098 6.692795e-01 9.999583e-01

4099 6.690645e-01 9.999582e-01

4100 6.688538e-01 9.999581e-01

4101 6.686474e-01 9.999580e-01

4102 6.684454e-01 9.999578e-01

4103 6.682478e-01 9.999577e-01

4104 6.680548e-01 9.999576e-01

4105 6.678664e-01 9.999575e-01

4106 6.676826e-01 9.999573e-01

4107 6.675036e-01 9.999572e-01

4108 6.673293e-01 9.999570e-01

4109 6.671598e-01 9.999569e-01

4110 6.669951e-01 9.999567e-01

4111 6.668353e-01 9.999565e-01

4112 6.666804e-01 9.999563e-01

4113 6.665303e-01 9.999561e-01

4114 6.663852e-01 9.999559e-01

4115 6.662450e-01 9.999557e-01

4116 6.661096e-01 9.999555e-01

4117 6.659792e-01 9.999552e-01

4118 6.658536e-01 9.999550e-01

4119 6.657329e-01 9.999547e-01

4120 6.656170e-01 9.999544e-01

4121 6.655059e-01 9.999541e-01

4122 6.653996e-01 9.999538e-01

4123 6.652980e-01 9.999535e-01

4124 6.652011e-01 9.999532e-01

4125 6.651088e-01 9.999528e-01

4126 6.650212e-01 9.999524e-01

4127 6.649380e-01 9.999520e-01

4128 6.648593e-01 9.999516e-01

4129 6.647850e-01 9.999511e-01

4130 6.647150e-01 9.999506e-01

4131 6.646493e-01 9.999501e-01

4132 6.645878e-01 9.999496e-01

4133 6.645303e-01 9.999490e-01

4134 6.644770e-01 9.999484e-01

4135 6.644275e-01 9.999478e-01

4136 6.643819e-01 9.999471e-01

4137 6.643401e-01 9.999464e-01

4138 6.643020e-01 9.999457e-01

4139 6.642675e-01 9.999449e-01

4140 6.642366e-01 9.999441e-01

4141 6.642090e-01 9.999432e-01

4142 6.641941e-01 9.999432e-01

4143 6.641793e-01 9.999432e-01

4144 6.641646e-01 9.999432e-01

4145 6.641501e-01 9.999432e-01

4146 6.641358e-01 9.999432e-01

4147 6.641217e-01 9.999432e-01

4148 6.641078e-01 9.999432e-01

4149 6.640941e-01 9.999432e-01

4150 6.640807e-01 9.999432e-01

4151 6.640675e-01 9.999432e-01

4152 6.640547e-01 9.999432e-01

4153 6.640421e-01 9.999432e-01

4154 6.640299e-01 9.999432e-01

4155 6.640180e-01 9.999432e-01

4156 6.640065e-01 9.999432e-01

4157 6.639954e-01 9.999432e-01

4158 6.639847e-01 9.999432e-01

4159 6.639744e-01 9.999432e-01

4160 6.639647e-01 9.999432e-01

4161 6.639554e-01 9.999432e-01

4162 6.639466e-01 9.999432e-01

4163 6.639383e-01 9.999432e-01

4164 6.639307e-01 9.999432e-01

4165 6.639236e-01 9.999432e-01

4166 6.639172e-01 9.999432e-01

4167 6.639115e-01 9.999431e-01

4168 6.639064e-01 9.999431e-01

4169 6.639021e-01 9.999431e-01

4170 6.638985e-01 9.999431e-01

4171 6.638958e-01 9.999431e-01

4172 6.638939e-01 9.999431e-01

4173 6.638928e-01 9.999431e-01

4174 6.638927e-01 9.999431e-01

4175 6.638935e-01 9.999431e-01

4176 6.638953e-01 9.999431e-01

4177 6.638982e-01 9.999431e-01

4178 6.639021e-01 9.999431e-01

4179 6.639072e-01 9.999431e-01

4180 6.639134e-01 9.999431e-01

4181 6.639209e-01 9.999431e-01

4182 6.639296e-01 9.999431e-01

4183 6.639396e-01 9.999431e-01

4184 6.639510e-01 9.999431e-01

4185 6.639638e-01 9.999431e-01

4186 6.639781e-01 9.999431e-01

4187 6.639939e-01 9.999431e-01

4188 6.640113e-01 9.999431e-01

4189 6.640303e-01 9.999431e-01

4190 6.640511e-01 9.999430e-01

4191 6.640735e-01 9.999430e-01

4192 6.640979e-01 9.999430e-01

4193 6.641240e-01 9.999430e-01

4194 6.641522e-01 9.999430e-01

4195 6.641823e-01 9.999430e-01

4196 6.642146e-01 9.999430e-01

4197 6.642489e-01 9.999430e-01

4198 6.642855e-01 9.999430e-01

4199 6.643244e-01 9.999430e-01

4200 6.643656e-01 9.999430e-01

4201 6.644093e-01 9.999430e-01

4202 6.644555e-01 9.999429e-01

4203 6.645042e-01 9.999429e-01

4204 6.645557e-01 9.999429e-01

4205 6.646098e-01 9.999429e-01

4206 6.646668e-01 9.999429e-01

4207 6.647266e-01 9.999429e-01

4208 6.647895e-01 9.999429e-01

4209 6.648554e-01 9.999429e-01

4210 6.649244e-01 9.999429e-01

4211 6.649967e-01 9.999428e-01

4212 6.650723e-01 9.999428e-01

4213 6.651513e-01 9.999428e-01

4214 6.652337e-01 9.999428e-01

4215 6.653197e-01 9.999428e-01

4216 6.654094e-01 9.999428e-01

4217 6.655029e-01 9.999427e-01

4218 6.656001e-01 9.999427e-01

4219 6.657013e-01 9.999427e-01

4220 6.658065e-01 9.999427e-01

4221 6.659158e-01 9.999427e-01

4222 6.660292e-01 9.999426e-01

4223 6.661470e-01 9.999426e-01

4224 6.662691e-01 9.999426e-01

4225 6.663956e-01 9.999426e-01

4226 6.665267e-01 9.999425e-01

4227 6.666624e-01 9.999425e-01

4228 6.668028e-01 9.999425e-01

4229 6.669480e-01 9.999425e-01

4230 6.670981e-01 9.999424e-01

4231 6.672531e-01 9.999424e-01

4232 6.674132e-01 9.999424e-01

4233 6.675784e-01 9.999423e-01

4234 6.677487e-01 9.999423e-01

4235 6.679244e-01 9.999423e-01

4236 6.681054e-01 9.999422e-01

4237 6.682917e-01 9.999422e-01

4238 6.684836e-01 9.999421e-01

4239 6.686810e-01 9.999421e-01

4240 6.688840e-01 9.999420e-01

4241 6.690927e-01 9.999420e-01

4242 6.693071e-01 9.999419e-01

4243 6.695273e-01 9.999419e-01

4244 6.697534e-01 9.999418e-01

4245 6.699853e-01 9.999418e-01

4246 6.702231e-01 9.999417e-01

4247 6.704668e-01 9.999416e-01

4248 6.707166e-01 9.999416e-01

4249 6.709723e-01 9.999415e-01

4250 6.712341e-01 9.999414e-01

4251 6.715020e-01 9.999414e-01

4252 6.717760e-01 9.999413e-01

4253 6.720560e-01 9.999412e-01

4254 6.723421e-01 9.999411e-01

4255 6.726343e-01 9.999410e-01

4256 6.729326e-01 9.999409e-01

4257 6.732370e-01 9.999408e-01

4258 6.735474e-01 9.999407e-01

4259 6.738638e-01 9.999406e-01

4260 6.741863e-01 9.999405e-01

4261 6.745147e-01 9.999404e-01

4262 6.748490e-01 9.999402e-01

4263 6.751892e-01 9.999401e-01

4264 6.755352e-01 9.999400e-01

4265 6.758870e-01 9.999398e-01

4266 6.762445e-01 9.999396e-01

4267 6.766076e-01 9.999395e-01

4268 6.769762e-01 9.999393e-01

4269 6.773502e-01 9.999391e-01

4270 6.777297e-01 9.999389e-01

4271 6.781143e-01 9.999387e-01

4272 6.785042e-01 9.999385e-01

4273 6.788990e-01 9.999383e-01

4274 6.792988e-01 9.999381e-01

4275 6.797034e-01 9.999378e-01

4276 6.801127e-01 9.999376e-01

4277 6.805264e-01 9.999373e-01

4278 6.809446e-01 9.999370e-01

4279 6.813671e-01 9.999367e-01

4280 6.817936e-01 9.999364e-01

4281 6.822241e-01 9.999360e-01

4282 6.826583e-01 9.999357e-01

4283 6.830962e-01 9.999353e-01

4284 6.835375e-01 9.999349e-01

4285 6.839821e-01 9.999345e-01

4286 6.844298e-01 9.999340e-01

4287 6.848805e-01 9.999335e-01

4288 6.853338e-01 9.999330e-01

4289 6.857897e-01 9.999325e-01

4290 6.862480e-01 9.999320e-01

4291 6.867084e-01 9.999314e-01

4292 6.871709e-01 9.999307e-01

4293 6.876351e-01 9.999301e-01

4294 6.881009e-01 9.999294e-01

4295 6.885681e-01 9.999286e-01

4296 6.890364e-01 9.999279e-01

4297 6.895058e-01 9.999270e-01

4298 6.899760e-01 9.999261e-01

4299 6.904468e-01 9.999252e-01

4300 6.909180e-01 9.999242e-01

4301 6.913894e-01 9.999232e-01

4302 6.918608e-01 9.999221e-01

4303 6.923320e-01 9.999209e-01

4304 6.928029e-01 9.999197e-01

4305 6.932732e-01 9.999183e-01

4306 6.937428e-01 9.999169e-01

4307 6.942115e-01 9.999154e-01

4308 6.946790e-01 9.999139e-01

4309 6.951453e-01 9.999122e-01

4310 6.956102e-01 9.999104e-01

4311 6.960734e-01 9.999085e-01

4312 6.965348e-01 9.999065e-01

4313 6.969943e-01 9.999044e-01

4314 6.974516e-01 9.999022e-01

4315 6.979067e-01 9.998998e-01

4316 6.983594e-01 9.998972e-01

4317 6.988096e-01 9.998945e-01

4318 6.992571e-01 9.998916e-01

4319 6.997017e-01 9.998886e-01

4320 7.001434e-01 9.998854e-01

4321 7.005820e-01 9.998819e-01

4322 7.006544e-01 9.998819e-01

4323 7.007282e-01 9.998819e-01

4324 7.008034e-01 9.998819e-01

4325 7.008802e-01 9.998819e-01

4326 7.009584e-01 9.998819e-01

4327 7.010382e-01 9.998819e-01

4328 7.011195e-01 9.998819e-01

4329 7.012023e-01 9.998819e-01

4330 7.012867e-01 9.998819e-01

4331 7.013728e-01 9.998819e-01

4332 7.014605e-01 9.998819e-01

4333 7.015498e-01 9.998819e-01

4334 7.016408e-01 9.998819e-01

4335 7.017335e-01 9.998819e-01

4336 7.018280e-01 9.998819e-01

4337 7.019243e-01 9.998819e-01

4338 7.020223e-01 9.998819e-01

4339 7.021222e-01 9.998819e-01

4340 7.022240e-01 9.998819e-01

4341 7.023276e-01 9.998818e-01

4342 7.024332e-01 9.998818e-01

4343 7.025408e-01 9.998818e-01

4344 7.026504e-01 9.998818e-01

4345 7.027620e-01 9.998818e-01

4346 7.028758e-01 9.998818e-01

4347 7.029916e-01 9.998818e-01

4348 7.031096e-01 9.998818e-01

4349 7.032298e-01 9.998818e-01

4350 7.033522e-01 9.998818e-01

4351 7.034769e-01 9.998818e-01

4352 7.036040e-01 9.998818e-01

4353 7.037334e-01 9.998818e-01

4354 7.038652e-01 9.998818e-01

4355 7.039994e-01 9.998818e-01

4356 7.041362e-01 9.998818e-01

4357 7.042755e-01 9.998817e-01

4358 7.044174e-01 9.998817e-01

4359 7.045619e-01 9.998817e-01

4360 7.047091e-01 9.998817e-01

4361 7.048591e-01 9.998817e-01

4362 7.050118e-01 9.998817e-01

4363 7.051674e-01 9.998817e-01

4364 7.053258e-01 9.998817e-01

4365 7.054872e-01 9.998817e-01

4366 7.056516e-01 9.998817e-01

4367 7.058190e-01 9.998816e-01

4368 7.059895e-01 9.998816e-01

4369 7.061631e-01 9.998816e-01

4370 7.063400e-01 9.998816e-01

4371 7.065201e-01 9.998816e-01

4372 7.067036e-01 9.998816e-01

4373 7.068904e-01 9.998816e-01

4374 7.070806e-01 9.998816e-01

4375 7.072744e-01 9.998815e-01

4376 7.074717e-01 9.998815e-01

4377 7.076726e-01 9.998815e-01

4378 7.078771e-01 9.998815e-01

4379 7.080854e-01 9.998815e-01

4380 7.082975e-01 9.998815e-01

4381 7.085135e-01 9.998814e-01

4382 7.087334e-01 9.998814e-01

4383 7.089573e-01 9.998814e-01

4384 7.091852e-01 9.998814e-01

4385 7.094172e-01 9.998813e-01

4386 7.096534e-01 9.998813e-01

4387 7.098938e-01 9.998813e-01

4388 7.101386e-01 9.998813e-01

4389 7.103877e-01 9.998812e-01

4390 7.106412e-01 9.998812e-01

4391 7.108993e-01 9.998812e-01

4392 7.111619e-01 9.998812e-01

4393 7.114291e-01 9.998811e-01

4394 7.117011e-01 9.998811e-01

4395 7.119778e-01 9.998811e-01

4396 7.122593e-01 9.998810e-01

4397 7.125457e-01 9.998810e-01

4398 7.128371e-01 9.998809e-01

4399 7.131335e-01 9.998809e-01

4400 7.134349e-01 9.998808e-01

4401 7.137416e-01 9.998808e-01

4402 7.140534e-01 9.998808e-01

4403 7.143705e-01 9.998807e-01

4404 7.146929e-01 9.998807e-01

4405 7.150207e-01 9.998806e-01

4406 7.153540e-01 9.998805e-01

4407 7.156927e-01 9.998805e-01

4408 7.160371e-01 9.998804e-01

4409 7.163870e-01 9.998804e-01

4410 7.167426e-01 9.998803e-01

4411 7.171040e-01 9.998802e-01

4412 7.174711e-01 9.998801e-01

4413 7.178441e-01 9.998801e-01

4414 7.182229e-01 9.998800e-01

4415 7.186076e-01 9.998799e-01

4416 7.189983e-01 9.998798e-01

4417 7.193950e-01 9.998797e-01

4418 7.197978e-01 9.998796e-01

4419 7.202066e-01 9.998795e-01

4420 7.206215e-01 9.998794e-01

4421 7.210426e-01 9.998793e-01

4422 7.214698e-01 9.998791e-01

4423 7.219032e-01 9.998790e-01

4424 7.223429e-01 9.998789e-01

4425 7.227888e-01 9.998787e-01

4426 7.232409e-01 9.998786e-01

4427 7.236993e-01 9.998784e-01

4428 7.241640e-01 9.998782e-01

4429 7.246349e-01 9.998781e-01

4430 7.251122e-01 9.998779e-01

4431 7.255957e-01 9.998777e-01

4432 7.260854e-01 9.998775e-01

4433 7.265815e-01 9.998772e-01

4434 7.270838e-01 9.998770e-01

4435 7.275923e-01 9.998768e-01

4436 7.281070e-01 9.998765e-01

4437 7.286278e-01 9.998762e-01

4438 7.291549e-01 9.998759e-01

4439 7.296880e-01 9.998756e-01

4440 7.302272e-01 9.998753e-01

4441 7.307723e-01 9.998749e-01

4442 7.313235e-01 9.998746e-01

4443 7.318805e-01 9.998742e-01

4444 7.324434e-01 9.998738e-01

4445 7.330120e-01 9.998734e-01

4446 7.335864e-01 9.998729e-01

4447 7.341663e-01 9.998724e-01

4448 7.347518e-01 9.998719e-01

4449 7.353427e-01 9.998714e-01

4450 7.359390e-01 9.998708e-01

4451 7.365405e-01 9.998702e-01

4452 7.371471e-01 9.998695e-01

4453 7.377588e-01 9.998688e-01

4454 7.383754e-01 9.998681e-01

4455 7.389968e-01 9.998673e-01

4456 7.396228e-01 9.998665e-01

4457 7.402534e-01 9.998657e-01

4458 7.408884e-01 9.998647e-01

4459 7.415276e-01 9.998638e-01

4460 7.421709e-01 9.998627e-01

4461 7.428182e-01 9.998616e-01

4462 7.434693e-01 9.998605e-01

4463 7.441240e-01 9.998592e-01

4464 7.447821e-01 9.998579e-01

4465 7.454436e-01 9.998565e-01

4466 7.461082e-01 9.998550e-01

4467 7.467757e-01 9.998534e-01

4468 7.474460e-01 9.998518e-01

4469 7.481189e-01 9.998500e-01

4470 7.487942e-01 9.998481e-01

4471 7.494716e-01 9.998461e-01

4472 7.501511e-01 9.998440e-01

4473 7.508323e-01 9.998417e-01

4474 7.515152e-01 9.998393e-01

4475 7.521994e-01 9.998367e-01

4476 7.528848e-01 9.998340e-01

4477 7.535712e-01 9.998311e-01

4478 7.542584e-01 9.998280e-01

4479 7.549461e-01 9.998248e-01

4480 7.556342e-01 9.998213e-01

4481 7.563224e-01 9.998176e-01

4482 7.570106e-01 9.998137e-01

4483 7.576984e-01 9.998095e-01

4484 7.583857e-01 9.998051e-01

4485 7.590723e-01 9.998004e-01

4486 7.597580e-01 9.997954e-01

4487 7.604426e-01 9.997901e-01

4488 7.611258e-01 9.997845e-01

4489 7.618074e-01 9.997785e-01

4490 7.624872e-01 9.997721e-01

4491 7.631651e-01 9.997654e-01

4492 7.638408e-01 9.997582e-01

4493 7.645141e-01 9.997506e-01

4494 7.651848e-01 9.997425e-01

4495 7.658527e-01 9.997339e-01

4496 7.665177e-01 9.997247e-01

4497 7.671795e-01 9.997150e-01

4498 7.678379e-01 9.997047e-01

4499 7.684929e-01 9.996938e-01

4500 7.691441e-01 9.996822e-01

4501 7.697914e-01 9.996698e-01

4502 7.698671e-01 9.996698e-01

4503 7.699438e-01 9.996698e-01

4504 7.700217e-01 9.996698e-01

4505 7.701006e-01 9.996698e-01

4506 7.701807e-01 9.996698e-01

4507 7.702620e-01 9.996698e-01

4508 7.703444e-01 9.996698e-01

4509 7.704281e-01 9.996698e-01

4510 7.705129e-01 9.996698e-01

4511 7.705990e-01 9.996698e-01

4512 7.706863e-01 9.996698e-01

4513 7.707750e-01 9.996698e-01

4514 7.708649e-01 9.996698e-01

4515 7.709562e-01 9.996698e-01

4516 7.710488e-01 9.996698e-01

4517 7.711427e-01 9.996697e-01

4518 7.712381e-01 9.996697e-01

4519 7.713349e-01 9.996697e-01

4520 7.714331e-01 9.996697e-01

4521 7.715327e-01 9.996697e-01

4522 7.716339e-01 9.996697e-01

4523 7.717366e-01 9.996697e-01

4524 7.718408e-01 9.996697e-01

4525 7.719465e-01 9.996697e-01

4526 7.720539e-01 9.996697e-01

4527 7.721628e-01 9.996697e-01

4528 7.722734e-01 9.996697e-01

4529 7.723857e-01 9.996697e-01

4530 7.724996e-01 9.996696e-01

4531 7.726153e-01 9.996696e-01

4532 7.727327e-01 9.996696e-01

4533 7.728519e-01 9.996696e-01

4534 7.729729e-01 9.996696e-01

4535 7.730957e-01 9.996696e-01

4536 7.732204e-01 9.996696e-01

4537 7.733469e-01 9.996696e-01

4538 7.734754e-01 9.996695e-01

4539 7.736058e-01 9.996695e-01

4540 7.737382e-01 9.996695e-01

4541 7.738725e-01 9.996695e-01

4542 7.740090e-01 9.996695e-01

4543 7.741474e-01 9.996695e-01

4544 7.742880e-01 9.996695e-01

4545 7.744307e-01 9.996694e-01

4546 7.745756e-01 9.996694e-01

4547 7.747226e-01 9.996694e-01

4548 7.748719e-01 9.996694e-01

4549 7.750234e-01 9.996694e-01

4550 7.751772e-01 9.996693e-01

4551 7.753333e-01 9.996693e-01

4552 7.754918e-01 9.996693e-01

4553 7.756526e-01 9.996693e-01

4554 7.758159e-01 9.996692e-01

4555 7.759817e-01 9.996692e-01

4556 7.761499e-01 9.996692e-01

4557 7.763206e-01 9.996691e-01

4558 7.764939e-01 9.996691e-01

4559 7.766698e-01 9.996691e-01

4560 7.768484e-01 9.996690e-01

4561 7.770296e-01 9.996690e-01

4562 7.772134e-01 9.996690e-01

4563 7.774001e-01 9.996689e-01

4564 7.775895e-01 9.996689e-01

4565 7.777817e-01 9.996688e-01

4566 7.779767e-01 9.996688e-01

4567 7.781747e-01 9.996688e-01

4568 7.783755e-01 9.996687e-01

4569 7.785793e-01 9.996686e-01

4570 7.787861e-01 9.996686e-01

4571 7.789960e-01 9.996685e-01

4572 7.792089e-01 9.996685e-01

4573 7.794249e-01 9.996684e-01

4574 7.796440e-01 9.996683e-01

4575 7.798664e-01 9.996683e-01

4576 7.800919e-01 9.996682e-01

4577 7.803207e-01 9.996681e-01

4578 7.805528e-01 9.996680e-01

4579 7.807883e-01 9.996679e-01

4580 7.810271e-01 9.996678e-01

4581 7.812693e-01 9.996677e-01

4582 7.815149e-01 9.996676e-01

4583 7.817641e-01 9.996675e-01

4584 7.820167e-01 9.996674e-01

4585 7.822729e-01 9.996673e-01

4586 7.825327e-01 9.996672e-01

4587 7.827961e-01 9.996670e-01

4588 7.830631e-01 9.996669e-01

4589 7.833339e-01 9.996667e-01

4590 7.836084e-01 9.996666e-01

4591 7.838866e-01 9.996664e-01

4592 7.841686e-01 9.996662e-01

4593 7.844545e-01 9.996661e-01

4594 7.847442e-01 9.996659e-01

4595 7.850378e-01 9.996657e-01

4596 7.853354e-01 9.996654e-01

4597 7.856368e-01 9.996652e-01

4598 7.859423e-01 9.996650e-01

4599 7.862518e-01 9.996647e-01

4600 7.865653e-01 9.996644e-01

4601 7.868828e-01 9.996641e-01

4602 7.872045e-01 9.996638e-01

4603 7.875302e-01 9.996635e-01

4604 7.878601e-01 9.996632e-01

4605 7.881941e-01 9.996628e-01

4606 7.885323e-01 9.996624e-01

4607 7.888747e-01 9.996620e-01

4608 7.892212e-01 9.996616e-01

4609 7.895720e-01 9.996611e-01

4610 7.899271e-01 9.996606e-01

4611 7.902863e-01 9.996601e-01

4612 7.906499e-01 9.996596e-01

4613 7.910177e-01 9.996590e-01

4614 7.913897e-01 9.996584e-01

4615 7.917660e-01 9.996577e-01

4616 7.921466e-01 9.996570e-01

4617 7.925315e-01 9.996563e-01

4618 7.929206e-01 9.996555e-01

4619 7.933141e-01 9.996547e-01

4620 7.937117e-01 9.996538e-01

4621 7.941136e-01 9.996529e-01

4622 7.945198e-01 9.996519e-01

4623 7.949301e-01 9.996508e-01

4624 7.953447e-01 9.996497e-01

4625 7.957634e-01 9.996486e-01

4626 7.961863e-01 9.996473e-01

4627 7.966133e-01 9.996460e-01

4628 7.970445e-01 9.996446e-01

4629 7.974797e-01 9.996431e-01

4630 7.979189e-01 9.996415e-01

4631 7.983621e-01 9.996399e-01

4632 7.988093e-01 9.996381e-01

4633 7.992604e-01 9.996362e-01

4634 7.997154e-01 9.996342e-01

4635 8.001742e-01 9.996321e-01

4636 8.006367e-01 9.996298e-01

4637 8.011029e-01 9.996275e-01

4638 8.015728e-01 9.996249e-01

4639 8.020462e-01 9.996222e-01

4640 8.025231e-01 9.996194e-01

4641 8.030035e-01 9.996164e-01

4642 8.034872e-01 9.996132e-01

4643 8.039742e-01 9.996098e-01

4644 8.044644e-01 9.996062e-01

4645 8.049577e-01 9.996024e-01

4646 8.054540e-01 9.995984e-01

4647 8.059533e-01 9.995941e-01

4648 8.064553e-01 9.995896e-01

4649 8.069601e-01 9.995848e-01

4650 8.074676e-01 9.995797e-01

4651 8.079775e-01 9.995743e-01

4652 8.084899e-01 9.995686e-01

4653 8.090045e-01 9.995625e-01

4654 8.095214e-01 9.995561e-01

4655 8.100403e-01 9.995494e-01

4656 8.105611e-01 9.995422e-01

4657 8.110838e-01 9.995346e-01

4658 8.116081e-01 9.995265e-01

4659 8.121340e-01 9.995180e-01

4660 8.126613e-01 9.995090e-01

4661 8.131899e-01 9.994995e-01

4662 8.137197e-01 9.994894e-01

4663 8.142505e-01 9.994787e-01

4664 8.147821e-01 9.994674e-01

4665 8.153145e-01 9.994555e-01

4666 8.158475e-01 9.994429e-01

4667 8.163809e-01 9.994296e-01

4668 8.169145e-01 9.994155e-01

4669 8.174484e-01 9.994006e-01

4670 8.179821e-01 9.993848e-01

4671 8.185158e-01 9.993682e-01

4672 8.190491e-01 9.993506e-01

4673 8.195819e-01 9.993320e-01

4674 8.201141e-01 9.993124e-01

4675 8.206455e-01 9.992917e-01

4676 8.211760e-01 9.992699e-01

4677 8.217053e-01 9.992468e-01

4678 8.222335e-01 9.992225e-01

4679 8.227602e-01 9.991968e-01

4680 8.232854e-01 9.991697e-01

4681 8.238089e-01 9.991411e-01

4682 8.238640e-01 9.991411e-01

4683 8.239199e-01 9.991411e-01

4684 8.239765e-01 9.991411e-01

4685 8.240338e-01 9.991411e-01

4686 8.240919e-01 9.991411e-01

4687 8.241508e-01 9.991411e-01

4688 8.242103e-01 9.991411e-01

4689 8.242706e-01 9.991411e-01

4690 8.243317e-01 9.991411e-01

4691 8.243934e-01 9.991411e-01

4692 8.244559e-01 9.991411e-01

4693 8.245191e-01 9.991411e-01

4694 8.245831e-01 9.991411e-01

4695 8.246478e-01 9.991411e-01

4696 8.247132e-01 9.991410e-01

4697 8.247793e-01 9.991410e-01

4698 8.248461e-01 9.991410e-01

4699 8.249137e-01 9.991410e-01

4700 8.249820e-01 9.991410e-01

4701 8.250511e-01 9.991410e-01

4702 8.251209e-01 9.991410e-01

4703 8.251914e-01 9.991410e-01

4704 8.252626e-01 9.991410e-01

4705 8.253346e-01 9.991410e-01

4706 8.254074e-01 9.991410e-01

4707 8.254808e-01 9.991409e-01

4708 8.255550e-01 9.991409e-01

4709 8.256300e-01 9.991409e-01

4710 8.257057e-01 9.991409e-01

4711 8.257822e-01 9.991409e-01

4712 8.258595e-01 9.991409e-01

4713 8.259375e-01 9.991409e-01

4714 8.260163e-01 9.991408e-01

4715 8.260958e-01 9.991408e-01

4716 8.261761e-01 9.991408e-01

4717 8.262573e-01 9.991408e-01

4718 8.263392e-01 9.991408e-01

4719 8.264219e-01 9.991407e-01

4720 8.265054e-01 9.991407e-01

4721 8.265898e-01 9.991407e-01

4722 8.266749e-01 9.991407e-01

4723 8.267609e-01 9.991407e-01

4724 8.268477e-01 9.991406e-01

4725 8.269354e-01 9.991406e-01

4726 8.270239e-01 9.991406e-01

4727 8.271133e-01 9.991405e-01

4728 8.272035e-01 9.991405e-01

4729 8.272946e-01 9.991405e-01

4730 8.273866e-01 9.991404e-01

4731 8.274795e-01 9.991404e-01

4732 8.275733e-01 9.991404e-01

4733 8.276680e-01 9.991403e-01

4734 8.277636e-01 9.991403e-01

4735 8.278602e-01 9.991402e-01

4736 8.279577e-01 9.991402e-01

4737 8.280561e-01 9.991401e-01

4738 8.281555e-01 9.991401e-01

4739 8.282559e-01 9.991400e-01

4740 8.283573e-01 9.991400e-01

4741 8.284596e-01 9.991399e-01

4742 8.285630e-01 9.991399e-01

4743 8.286674e-01 9.991398e-01

4744 8.287728e-01 9.991397e-01

4745 8.288792e-01 9.991396e-01

4746 8.289867e-01 9.991396e-01

4747 8.290952e-01 9.991395e-01

4748 8.292048e-01 9.991394e-01

4749 8.293154e-01 9.991393e-01

4750 8.294272e-01 9.991392e-01

4751 8.295400e-01 9.991391e-01

4752 8.296540e-01 9.991390e-01

4753 8.297690e-01 9.991389e-01

4754 8.298852e-01 9.991387e-01

4755 8.300026e-01 9.991386e-01

4756 8.301210e-01 9.991385e-01

4757 8.302407e-01 9.991383e-01

4758 8.303615e-01 9.991382e-01

4759 8.304834e-01 9.991380e-01

4760 8.306066e-01 9.991378e-01

4761 8.307309e-01 9.991377e-01

4762 8.308565e-01 9.991375e-01

4763 8.309832e-01 9.991373e-01

4764 8.311112e-01 9.991371e-01

4765 8.312404e-01 9.991368e-01

4766 8.313708e-01 9.991366e-01

4767 8.315025e-01 9.991363e-01

4768 8.316354e-01 9.991361e-01

4769 8.317696e-01 9.991358e-01

4770 8.319050e-01 9.991355e-01

4771 8.320417e-01 9.991352e-01

4772 8.321796e-01 9.991349e-01

4773 8.323189e-01 9.991345e-01

4774 8.324594e-01 9.991341e-01

4775 8.326012e-01 9.991337e-01

4776 8.327442e-01 9.991333e-01

4777 8.328886e-01 9.991329e-01

4778 8.330342e-01 9.991324e-01

4779 8.331812e-01 9.991319e-01

4780 8.333294e-01 9.991314e-01

4781 8.334789e-01 9.991309e-01

4782 8.336298e-01 9.991303e-01

4783 8.337819e-01 9.991297e-01

4784 8.339353e-01 9.991291e-01

4785 8.340900e-01 9.991284e-01

4786 8.342459e-01 9.991276e-01

4787 8.344032e-01 9.991269e-01

4788 8.345617e-01 9.991261e-01

4789 8.347216e-01 9.991252e-01

4790 8.348826e-01 9.991243e-01

4791 8.350450e-01 9.991234e-01

4792 8.352086e-01 9.991224e-01

4793 8.353734e-01 9.991213e-01

4794 8.355395e-01 9.991202e-01

4795 8.357068e-01 9.991190e-01

4796 8.358753e-01 9.991177e-01

4797 8.360450e-01 9.991164e-01

4798 8.362160e-01 9.991150e-01

4799 8.363880e-01 9.991135e-01

4800 8.365613e-01 9.991120e-01

4801 8.367357e-01 9.991103e-01

4802 8.369112e-01 9.991086e-01

4803 8.370879e-01 9.991067e-01

4804 8.372656e-01 9.991048e-01

4805 8.374444e-01 9.991027e-01

4806 8.376243e-01 9.991006e-01

4807 8.378052e-01 9.990983e-01

4808 8.379871e-01 9.990959e-01

4809 8.381700e-01 9.990933e-01

4810 8.383538e-01 9.990906e-01

4811 8.385386e-01 9.990878e-01

4812 8.387242e-01 9.990848e-01

4813 8.389108e-01 9.990817e-01

4814 8.390982e-01 9.990783e-01

4815 8.392864e-01 9.990748e-01

4816 8.394754e-01 9.990711e-01

4817 8.396651e-01 9.990672e-01

4818 8.398556e-01 9.990631e-01

4819 8.400467e-01 9.990588e-01

4820 8.402385e-01 9.990542e-01

4821 8.404308e-01 9.990494e-01

4822 8.406237e-01 9.990444e-01

4823 8.408172e-01 9.990390e-01

4824 8.410111e-01 9.990334e-01

4825 8.412055e-01 9.990275e-01

4826 8.414003e-01 9.990213e-01

4827 8.415954e-01 9.990148e-01

4828 8.417908e-01 9.990079e-01

4829 8.419865e-01 9.990006e-01

4830 8.421823e-01 9.989930e-01

4831 8.423784e-01 9.989850e-01

4832 8.425745e-01 9.989766e-01

4833 8.427707e-01 9.989678e-01

4834 8.429670e-01 9.989585e-01

4835 8.431631e-01 9.989487e-01

4836 8.433592e-01 9.989384e-01

4837 8.435551e-01 9.989276e-01

4838 8.437509e-01 9.989163e-01

4839 8.439463e-01 9.989044e-01

4840 8.441415e-01 9.988920e-01

4841 8.443362e-01 9.988789e-01

4842 8.445306e-01 9.988652e-01

4843 8.447244e-01 9.988508e-01

4844 8.449178e-01 9.988357e-01

4845 8.451105e-01 9.988198e-01

4846 8.453025e-01 9.988032e-01

4847 8.454939e-01 9.987858e-01

4848 8.456845e-01 9.987676e-01

4849 8.458742e-01 9.987485e-01

4850 8.460630e-01 9.987285e-01

4851 8.462509e-01 9.987076e-01

4852 8.464378e-01 9.986857e-01

4853 8.466237e-01 9.986628e-01

4854 8.468084e-01 9.986388e-01

4855 8.469919e-01 9.986137e-01

4856 8.471742e-01 9.985875e-01

4857 8.473552e-01 9.985601e-01

4858 8.475349e-01 9.985314e-01

4859 8.477131e-01 9.985015e-01

4860 8.478899e-01 9.984702e-01

4861 8.480652e-01 9.984376e-01

4862 8.480873e-01 9.984376e-01

4863 8.481099e-01 9.984376e-01

4864 8.481330e-01 9.984376e-01

4865 8.481566e-01 9.984375e-01

4866 8.481807e-01 9.984375e-01

4867 8.482054e-01 9.984375e-01

4868 8.482306e-01 9.984375e-01

4869 8.482564e-01 9.984375e-01

4870 8.482827e-01 9.984375e-01

4871 8.483095e-01 9.984375e-01

4872 8.483368e-01 9.984375e-01

4873 8.483646e-01 9.984375e-01

4874 8.483929e-01 9.984375e-01

4875 8.484217e-01 9.984375e-01

4876 8.484511e-01 9.984375e-01

4877 8.484809e-01 9.984375e-01

4878 8.485112e-01 9.984375e-01

4879 8.485419e-01 9.984375e-01

4880 8.485731e-01 9.984375e-01

4881 8.486048e-01 9.984374e-01

4882 8.486369e-01 9.984374e-01

4883 8.486695e-01 9.984374e-01

4884 8.487024e-01 9.984374e-01

4885 8.487358e-01 9.984374e-01

4886 8.487695e-01 9.984374e-01

4887 8.488036e-01 9.984374e-01

4888 8.488381e-01 9.984374e-01

4889 8.488730e-01 9.984374e-01

4890 8.489081e-01 9.984373e-01

4891 8.489436e-01 9.984373e-01

4892 8.489793e-01 9.984373e-01

4893 8.490154e-01 9.984373e-01

4894 8.490517e-01 9.984373e-01

4895 8.490882e-01 9.984373e-01

4896 8.491250e-01 9.984372e-01

4897 8.491619e-01 9.984372e-01

4898 8.491990e-01 9.984372e-01

4899 8.492363e-01 9.984372e-01

4900 8.492737e-01 9.984372e-01

4901 8.493112e-01 9.984371e-01

4902 8.493489e-01 9.984371e-01

4903 8.493865e-01 9.984371e-01

4904 8.494243e-01 9.984370e-01

4905 8.494620e-01 9.984370e-01

4906 8.494998e-01 9.984370e-01

4907 8.495375e-01 9.984369e-01

4908 8.495751e-01 9.984369e-01

4909 8.496127e-01 9.984369e-01

4910 8.496502e-01 9.984368e-01

4911 8.496876e-01 9.984368e-01

4912 8.497247e-01 9.984367e-01

4913 8.497618e-01 9.984367e-01

4914 8.497986e-01 9.984366e-01

4915 8.498352e-01 9.984366e-01

4916 8.498715e-01 9.984365e-01

4917 8.499075e-01 9.984365e-01

4918 8.499432e-01 9.984364e-01

4919 8.499786e-01 9.984363e-01

4920 8.500136e-01 9.984363e-01

4921 8.500483e-01 9.984362e-01

4922 8.500825e-01 9.984361e-01

4923 8.501162e-01 9.984360e-01

4924 8.501495e-01 9.984360e-01

4925 8.501823e-01 9.984359e-01

4926 8.502146e-01 9.984358e-01

4927 8.502463e-01 9.984357e-01

4928 8.502774e-01 9.984356e-01

4929 8.503079e-01 9.984355e-01

4930 8.503377e-01 9.984354e-01

4931 8.503669e-01 9.984352e-01

4932 8.503954e-01 9.984351e-01

4933 8.504232e-01 9.984350e-01

4934 8.504502e-01 9.984348e-01

4935 8.504765e-01 9.984347e-01

4936 8.505019e-01 9.984345e-01

4937 8.505265e-01 9.984343e-01

4938 8.505502e-01 9.984342e-01

4939 8.505731e-01 9.984340e-01

4940 8.505950e-01 9.984338e-01

4941 8.506159e-01 9.984336e-01

4942 8.506359e-01 9.984334e-01

4943 8.506548e-01 9.984331e-01

4944 8.506727e-01 9.984329e-01

4945 8.506895e-01 9.984327e-01

4946 8.507053e-01 9.984324e-01

4947 8.507198e-01 9.984321e-01

4948 8.507333e-01 9.984318e-01

4949 8.507455e-01 9.984315e-01

4950 8.507564e-01 9.984312e-01

4951 8.507662e-01 9.984308e-01

4952 8.507746e-01 9.984305e-01

4953 8.507817e-01 9.984301e-01

4954 8.507874e-01 9.984297e-01

4955 8.507917e-01 9.984293e-01

4956 8.507946e-01 9.984289e-01

4957 8.507961e-01 9.984284e-01

4958 8.507960e-01 9.984279e-01

4959 8.507944e-01 9.984274e-01

4960 8.507913e-01 9.984269e-01

4961 8.507865e-01 9.984263e-01

4962 8.507801e-01 9.984257e-01

4963 8.507721e-01 9.984251e-01

4964 8.507623e-01 9.984245e-01

4965 8.507508e-01 9.984238e-01

4966 8.507375e-01 9.984231e-01

4967 8.507223e-01 9.984223e-01

4968 8.507054e-01 9.984215e-01

4969 8.506865e-01 9.984207e-01

4970 8.506657e-01 9.984198e-01

4971 8.506429e-01 9.984189e-01

4972 8.506182e-01 9.984180e-01

4973 8.505913e-01 9.984170e-01

4974 8.505625e-01 9.984159e-01

4975 8.505314e-01 9.984148e-01

4976 8.504983e-01 9.984136e-01

4977 8.504629e-01 9.984124e-01

4978 8.504253e-01 9.984112e-01

4979 8.503854e-01 9.984098e-01

4980 8.503432e-01 9.984085e-01

4981 8.502986e-01 9.984070e-01

4982 8.502517e-01 9.984055e-01

4983 8.502023e-01 9.984039e-01

4984 8.501505e-01 9.984022e-01

4985 8.500962e-01 9.984005e-01

4986 8.500393e-01 9.983986e-01

4987 8.499799e-01 9.983967e-01

4988 8.499178e-01 9.983947e-01

4989 8.498531e-01 9.983926e-01

4990 8.497857e-01 9.983904e-01

4991 8.497157e-01 9.983882e-01

4992 8.496428e-01 9.983858e-01

4993 8.495672e-01 9.983833e-01

4994 8.494888e-01 9.983807e-01

4995 8.494075e-01 9.983780e-01

4996 8.493234e-01 9.983751e-01

4997 8.492363e-01 9.983722e-01

4998 8.491464e-01 9.983691e-01

4999 8.490535e-01 9.983659e-01

5000 8.489576e-01 9.983625e-01

5001 8.488587e-01 9.983590e-01

5002 8.487568e-01 9.983554e-01

5003 8.486518e-01 9.983516e-01

5004 8.485438e-01 9.983476e-01

5005 8.484327e-01 9.983435e-01

5006 8.483185e-01 9.983393e-01

5007 8.482012e-01 9.983348e-01

5008 8.480808e-01 9.983302e-01

5009 8.479572e-01 9.983254e-01

5010 8.478306e-01 9.983204e-01

5011 8.477007e-01 9.983152e-01

5012 8.475678e-01 9.983098e-01

5013 8.474317e-01 9.983042e-01

5014 8.472925e-01 9.982984e-01

5015 8.471501e-01 9.982924e-01

5016 8.470046e-01 9.982861e-01

5017 8.468560e-01 9.982796e-01

5018 8.467043e-01 9.982729e-01

5019 8.465495e-01 9.982660e-01

5020 8.463917e-01 9.982588e-01

5021 8.462308e-01 9.982514e-01

5022 8.460668e-01 9.982437e-01

5023 8.458999e-01 9.982358e-01

5024 8.457300e-01 9.982275e-01

5025 8.455571e-01 9.982191e-01

5026 8.453814e-01 9.982103e-01

5027 8.452027e-01 9.982013e-01

5028 8.450213e-01 9.981920e-01

5029 8.448371e-01 9.981824e-01

5030 8.446501e-01 9.981726e-01

5031 8.444605e-01 9.981624e-01

5032 8.442682e-01 9.981520e-01

5033 8.440733e-01 9.981412e-01

5034 8.438759e-01 9.981302e-01

5035 8.436761e-01 9.981189e-01

5036 8.434738e-01 9.981073e-01

5037 8.432693e-01 9.980954e-01

5038 8.430624e-01 9.980832e-01

5039 8.428534e-01 9.980707e-01

5040 8.426422e-01 9.980579e-01

5041 8.424290e-01 9.980447e-01

5042 8.424291e-01 9.980447e-01

5043 8.424292e-01 9.980447e-01

5044 8.424292e-01 9.980447e-01

5045 8.424292e-01 9.980447e-01

5046 8.424291e-01 9.980447e-01

5047 8.424289e-01 9.980447e-01

5048 8.424286e-01 9.980447e-01

5049 8.424282e-01 9.980447e-01

5050 8.424277e-01 9.980447e-01

5051 8.424271e-01 9.980447e-01

5052 8.424264e-01 9.980447e-01

5053 8.424255e-01 9.980447e-01

5054 8.424245e-01 9.980447e-01

5055 8.424233e-01 9.980446e-01

5056 8.424219e-01 9.980446e-01

5057 8.424203e-01 9.980446e-01

5058 8.424185e-01 9.980446e-01

5059 8.424165e-01 9.980446e-01

5060 8.424143e-01 9.980446e-01

5061 8.424118e-01 9.980446e-01

5062 8.424090e-01 9.980446e-01

5063 8.424059e-01 9.980446e-01

5064 8.424025e-01 9.980446e-01

5065 8.423988e-01 9.980446e-01

5066 8.423947e-01 9.980446e-01

5067 8.423902e-01 9.980446e-01

5068 8.423853e-01 9.980445e-01

5069 8.423799e-01 9.980445e-01

5070 8.423741e-01 9.980445e-01

5071 8.423678e-01 9.980445e-01

5072 8.423610e-01 9.980445e-01

5073 8.423536e-01 9.980445e-01

5074 8.423456e-01 9.980445e-01

5075 8.423370e-01 9.980444e-01

5076 8.423278e-01 9.980444e-01

5077 8.423178e-01 9.980444e-01

5078 8.423072e-01 9.980444e-01

5079 8.422958e-01 9.980444e-01

5080 8.422835e-01 9.980443e-01

5081 8.422705e-01 9.980443e-01

5082 8.422565e-01 9.980443e-01

5083 8.422416e-01 9.980443e-01

5084 8.422257e-01 9.980442e-01

5085 8.422089e-01 9.980442e-01

5086 8.421909e-01 9.980442e-01

5087 8.421719e-01 9.980441e-01

5088 8.421517e-01 9.980441e-01

5089 8.421302e-01 9.980440e-01

5090 8.421075e-01 9.980440e-01

5091 8.420835e-01 9.980440e-01

5092 8.420582e-01 9.980439e-01

5093 8.420314e-01 9.980438e-01

5094 8.420031e-01 9.980438e-01

5095 8.419733e-01 9.980437e-01

5096 8.419419e-01 9.980437e-01

5097 8.419089e-01 9.980436e-01

5098 8.418741e-01 9.980435e-01

5099 8.418376e-01 9.980435e-01

5100 8.417992e-01 9.980434e-01

5101 8.417590e-01 9.980433e-01

5102 8.417168e-01 9.980432e-01

5103 8.416725e-01 9.980431e-01

5104 8.416262e-01 9.980430e-01

5105 8.415778e-01 9.980429e-01

5106 8.415271e-01 9.980428e-01

5107 8.414741e-01 9.980427e-01

5108 8.414188e-01 9.980426e-01

5109 8.413611e-01 9.980424e-01

5110 8.413008e-01 9.980423e-01

5111 8.412380e-01 9.980422e-01

5112 8.411726e-01 9.980420e-01

5113 8.411045e-01 9.980419e-01

5114 8.410336e-01 9.980417e-01

5115 8.409599e-01 9.980415e-01

5116 8.408833e-01 9.980413e-01

5117 8.408037e-01 9.980411e-01

5118 8.407210e-01 9.980409e-01

5119 8.406352e-01 9.980407e-01

5120 8.405462e-01 9.980404e-01

5121 8.404539e-01 9.980402e-01

5122 8.403583e-01 9.980399e-01

5123 8.402592e-01 9.980397e-01

5124 8.401566e-01 9.980394e-01

5125 8.400505e-01 9.980391e-01

5126 8.399407e-01 9.980387e-01

5127 8.398272e-01 9.980384e-01

5128 8.397099e-01 9.980380e-01

5129 8.395888e-01 9.980377e-01

5130 8.394637e-01 9.980373e-01

5131 8.393345e-01 9.980368e-01

5132 8.392013e-01 9.980364e-01

5133 8.390639e-01 9.980359e-01

5134 8.389223e-01 9.980354e-01

5135 8.387764e-01 9.980349e-01

5136 8.386261e-01 9.980344e-01

5137 8.384713e-01 9.980338e-01

5138 8.383120e-01 9.980332e-01

5139 8.381481e-01 9.980325e-01

5140 8.379795e-01 9.980319e-01

5141 8.378062e-01 9.980311e-01

5142 8.376281e-01 9.980304e-01

5143 8.374451e-01 9.980296e-01

5144 8.372571e-01 9.980288e-01

5145 8.370642e-01 9.980279e-01

5146 8.368661e-01 9.980270e-01

5147 8.366630e-01 9.980260e-01

5148 8.364546e-01 9.980250e-01

5149 8.362409e-01 9.980240e-01

5150 8.360220e-01 9.980228e-01

5151 8.357976e-01 9.980217e-01

5152 8.355679e-01 9.980204e-01

5153 8.353326e-01 9.980191e-01

5154 8.350918e-01 9.980177e-01

5155 8.348453e-01 9.980163e-01

5156 8.345933e-01 9.980148e-01

5157 8.343355e-01 9.980132e-01

5158 8.340720e-01 9.980115e-01

5159 8.338028e-01 9.980098e-01

5160 8.335277e-01 9.980079e-01

5161 8.332467e-01 9.980060e-01

5162 8.329599e-01 9.980040e-01

5163 8.326672e-01 9.980018e-01

5164 8.323685e-01 9.979996e-01

5165 8.320639e-01 9.979972e-01

5166 8.317532e-01 9.979947e-01

5167 8.314366e-01 9.979921e-01

5168 8.311139e-01 9.979894e-01

5169 8.307853e-01 9.979866e-01

5170 8.304506e-01 9.979836e-01

5171 8.301099e-01 9.979804e-01

5172 8.297631e-01 9.979771e-01

5173 8.294103e-01 9.979736e-01

5174 8.290516e-01 9.979700e-01

5175 8.286868e-01 9.979662e-01

5176 8.283161e-01 9.979622e-01

5177 8.279395e-01 9.979580e-01

5178 8.275569e-01 9.979536e-01

5179 8.271685e-01 9.979490e-01

5180 8.267742e-01 9.979442e-01

5181 8.263742e-01 9.979392e-01

5182 8.259685e-01 9.979339e-01

5183 8.255571e-01 9.979283e-01

5184 8.251401e-01 9.979226e-01

5185 8.247176e-01 9.979165e-01

5186 8.242896e-01 9.979101e-01

5187 8.238563e-01 9.979035e-01

5188 8.234177e-01 9.978965e-01

5189 8.229739e-01 9.978893e-01

5190 8.225250e-01 9.978817e-01

5191 8.220711e-01 9.978737e-01

5192 8.216124e-01 9.978654e-01

5193 8.211489e-01 9.978567e-01

5194 8.206807e-01 9.978476e-01

5195 8.202081e-01 9.978381e-01

5196 8.197311e-01 9.978282e-01

5197 8.192499e-01 9.978179e-01

5198 8.187645e-01 9.978070e-01

5199 8.182752e-01 9.977957e-01

5200 8.177822e-01 9.977840e-01

5201 8.172855e-01 9.977716e-01

5202 8.167854e-01 9.977588e-01

5203 8.162819e-01 9.977454e-01

5204 8.157753e-01 9.977314e-01

5205 8.152658e-01 9.977168e-01

5206 8.147535e-01 9.977016e-01

5207 8.142387e-01 9.976858e-01

5208 8.137214e-01 9.976692e-01

5209 8.132020e-01 9.976520e-01

5210 8.126805e-01 9.976340e-01

5211 8.121572e-01 9.976154e-01

5212 8.116324e-01 9.975959e-01

5213 8.111061e-01 9.975756e-01

5214 8.105786e-01 9.975545e-01

5215 8.100501e-01 9.975325e-01

5216 8.095207e-01 9.975097e-01

5217 8.089909e-01 9.974859e-01

5218 8.084606e-01 9.974611e-01

5219 8.079301e-01 9.974354e-01

5220 8.073997e-01 9.974088e-01

5221 8.068695e-01 9.973811e-01

5222 8.068524e-01 9.973811e-01

5223 8.068346e-01 9.973811e-01

5224 8.068162e-01 9.973811e-01

5225 8.067970e-01 9.973811e-01

5226 8.067770e-01 9.973811e-01

5227 8.067563e-01 9.973811e-01

5228 8.067348e-01 9.973811e-01

5229 8.067124e-01 9.973810e-01

5230 8.066892e-01 9.973810e-01

5231 8.066650e-01 9.973810e-01

5232 8.066400e-01 9.973810e-01

5233 8.066139e-01 9.973810e-01

5234 8.065869e-01 9.973810e-01

5235 8.065589e-01 9.973810e-01

5236 8.065297e-01 9.973809e-01

5237 8.064995e-01 9.973809e-01

5238 8.064681e-01 9.973809e-01

5239 8.064355e-01 9.973809e-01

5240 8.064017e-01 9.973809e-01

5241 8.063667e-01 9.973809e-01

5242 8.063303e-01 9.973808e-01

5243 8.062925e-01 9.973808e-01

5244 8.062533e-01 9.973808e-01

5245 8.062127e-01 9.973808e-01

5246 8.061706e-01 9.973807e-01

5247 8.061269e-01 9.973807e-01

5248 8.060817e-01 9.973807e-01

5249 8.060347e-01 9.973806e-01

5250 8.059861e-01 9.973806e-01

5251 8.059356e-01 9.973806e-01

5252 8.058834e-01 9.973805e-01

5253 8.058293e-01 9.973805e-01

5254 8.057732e-01 9.973805e-01

5255 8.057151e-01 9.973804e-01

5256 8.056550e-01 9.973804e-01

5257 8.055927e-01 9.973803e-01

5258 8.055283e-01 9.973803e-01

5259 8.054616e-01 9.973802e-01

5260 8.053925e-01 9.973802e-01

5261 8.053211e-01 9.973801e-01

5262 8.052472e-01 9.973801e-01

5263 8.051707e-01 9.973800e-01

5264 8.050917e-01 9.973799e-01

5265 8.050100e-01 9.973799e-01

5266 8.049255e-01 9.973798e-01

5267 8.048382e-01 9.973797e-01

5268 8.047479e-01 9.973796e-01

5269 8.046547e-01 9.973795e-01

5270 8.045584e-01 9.973795e-01

5271 8.044590e-01 9.973794e-01

5272 8.043563e-01 9.973793e-01

5273 8.042504e-01 9.973792e-01

5274 8.041410e-01 9.973791e-01

5275 8.040282e-01 9.973789e-01

5276 8.039118e-01 9.973788e-01

5277 8.037917e-01 9.973787e-01

5278 8.036679e-01 9.973786e-01

5279 8.035403e-01 9.973784e-01

5280 8.034087e-01 9.973783e-01

5281 8.032732e-01 9.973781e-01

5282 8.031335e-01 9.973780e-01

5283 8.029897e-01 9.973778e-01

5284 8.028416e-01 9.973776e-01

5285 8.026891e-01 9.973775e-01

5286 8.025321e-01 9.973773e-01

5287 8.023707e-01 9.973771e-01

5288 8.022045e-01 9.973769e-01

5289 8.020336e-01 9.973767e-01

5290 8.018579e-01 9.973764e-01

5291 8.016773e-01 9.973762e-01

5292 8.014917e-01 9.973759e-01

5293 8.013009e-01 9.973757e-01

5294 8.011050e-01 9.973754e-01

5295 8.009037e-01 9.973751e-01

5296 8.006971e-01 9.973748e-01

5297 8.004850e-01 9.973745e-01

5298 8.002673e-01 9.973742e-01

5299 8.000440e-01 9.973738e-01

5300 7.998150e-01 9.973735e-01

5301 7.995801e-01 9.973731e-01

5302 7.993392e-01 9.973727e-01

5303 7.990924e-01 9.973723e-01

5304 7.988395e-01 9.973718e-01

5305 7.985804e-01 9.973714e-01

5306 7.983151e-01 9.973709e-01

5307 7.980434e-01 9.973704e-01

5308 7.977653e-01 9.973699e-01

5309 7.974808e-01 9.973694e-01

5310 7.971896e-01 9.973688e-01

5311 7.968919e-01 9.973682e-01

5312 7.965874e-01 9.973676e-01

5313 7.962761e-01 9.973669e-01

5314 7.959580e-01 9.973663e-01

5315 7.956330e-01 9.973655e-01

5316 7.953011e-01 9.973648e-01

5317 7.949621e-01 9.973640e-01

5318 7.946161e-01 9.973632e-01

5319 7.942629e-01 9.973624e-01

5320 7.939026e-01 9.973615e-01

5321 7.935350e-01 9.973606e-01

5322 7.931602e-01 9.973597e-01

5323 7.927782e-01 9.973587e-01

5324 7.923888e-01 9.973576e-01

5325 7.919920e-01 9.973565e-01

5326 7.915880e-01 9.973554e-01

5327 7.911765e-01 9.973542e-01

5328 7.907576e-01 9.973530e-01

5329 7.903313e-01 9.973517e-01

5330 7.898977e-01 9.973504e-01

5331 7.894566e-01 9.973490e-01

5332 7.890082e-01 9.973475e-01

5333 7.885523e-01 9.973460e-01

5334 7.880892e-01 9.973445e-01

5335 7.876186e-01 9.973428e-01

5336 7.871408e-01 9.973411e-01

5337 7.866558e-01 9.973393e-01

5338 7.861635e-01 9.973375e-01

5339 7.856640e-01 9.973356e-01

5340 7.851575e-01 9.973336e-01

5341 7.846439e-01 9.973315e-01

5342 7.841234e-01 9.973293e-01

5343 7.835959e-01 9.973271e-01

5344 7.830617e-01 9.973247e-01

5345 7.825207e-01 9.973223e-01

5346 7.819732e-01 9.973197e-01

5347 7.814191e-01 9.973171e-01

5348 7.808586e-01 9.973144e-01

5349 7.802919e-01 9.973115e-01

5350 7.797191e-01 9.973086e-01

5351 7.791402e-01 9.973055e-01

5352 7.785555e-01 9.973023e-01

5353 7.779651e-01 9.972990e-01

5354 7.773691e-01 9.972956e-01

5355 7.767677e-01 9.972920e-01

5356 7.761611e-01 9.972883e-01

5357 7.755495e-01 9.972845e-01

5358 7.749331e-01 9.972805e-01

5359 7.743119e-01 9.972764e-01

5360 7.736863e-01 9.972721e-01

5361 7.730565e-01 9.972677e-01

5362 7.724226e-01 9.972631e-01

5363 7.717849e-01 9.972583e-01

5364 7.711436e-01 9.972534e-01

5365 7.704989e-01 9.972483e-01

5366 7.698510e-01 9.972431e-01

5367 7.692002e-01 9.972376e-01

5368 7.685468e-01 9.972320e-01

5369 7.678910e-01 9.972261e-01

5370 7.672329e-01 9.972201e-01

5371 7.665730e-01 9.972139e-01

5372 7.659113e-01 9.972074e-01

5373 7.652483e-01 9.972008e-01

5374 7.645841e-01 9.971939e-01

5375 7.639191e-01 9.971868e-01

5376 7.632534e-01 9.971795e-01

5377 7.625874e-01 9.971719e-01

5378 7.619212e-01 9.971641e-01

5379 7.612553e-01 9.971561e-01

5380 7.605898e-01 9.971479e-01

5381 7.599251e-01 9.971393e-01

5382 7.592613e-01 9.971306e-01

5383 7.585987e-01 9.971216e-01

5384 7.579377e-01 9.971123e-01

5385 7.572784e-01 9.971027e-01

5386 7.566212e-01 9.970929e-01

5387 7.559662e-01 9.970828e-01

5388 7.553138e-01 9.970725e-01

5389 7.546641e-01 9.970619e-01

5390 7.540175e-01 9.970510e-01

5391 7.533741e-01 9.970398e-01

5392 7.527342e-01 9.970284e-01

5393 7.520981e-01 9.970166e-01

5394 7.514659e-01 9.970046e-01

5395 7.508379e-01 9.969923e-01

5396 7.502143e-01 9.969798e-01

5397 7.495952e-01 9.969669e-01

5398 7.489809e-01 9.969538e-01

5399 7.483717e-01 9.969404e-01

5400 7.477676e-01 9.969268e-01

5401 7.471688e-01 9.969129e-01

5402 7.471317e-01 9.969128e-01

5403 7.470933e-01 9.969128e-01

5404 7.470536e-01 9.969128e-01

5405 7.470125e-01 9.969128e-01

5406 7.469701e-01 9.969128e-01

5407 7.469262e-01 9.969128e-01

5408 7.468808e-01 9.969128e-01

5409 7.468340e-01 9.969128e-01

5410 7.467855e-01 9.969127e-01

5411 7.467355e-01 9.969127e-01

5412 7.466838e-01 9.969127e-01

5413 7.466304e-01 9.969127e-01

5414 7.465753e-01 9.969127e-01

5415 7.465184e-01 9.969127e-01

5416 7.464597e-01 9.969126e-01

5417 7.463990e-01 9.969126e-01

5418 7.463364e-01 9.969126e-01

5419 7.462719e-01 9.969126e-01

5420 7.462052e-01 9.969126e-01

5421 7.461365e-01 9.969125e-01

5422 7.460657e-01 9.969125e-01

5423 7.459926e-01 9.969125e-01

5424 7.459172e-01 9.969124e-01

5425 7.458395e-01 9.969124e-01

5426 7.457594e-01 9.969124e-01

5427 7.456769e-01 9.969124e-01

5428 7.455918e-01 9.969123e-01

5429 7.455042e-01 9.969123e-01

5430 7.454140e-01 9.969122e-01

5431 7.453210e-01 9.969122e-01

5432 7.452253e-01 9.969122e-01

5433 7.451268e-01 9.969121e-01

5434 7.450253e-01 9.969121e-01

5435 7.449210e-01 9.969120e-01

5436 7.448135e-01 9.969120e-01

5437 7.447030e-01 9.969119e-01

5438 7.445893e-01 9.969119e-01

5439 7.444724e-01 9.969118e-01

5440 7.443522e-01 9.969117e-01

5441 7.442286e-01 9.969117e-01

5442 7.441016e-01 9.969116e-01

5443 7.439710e-01 9.969115e-01

5444 7.438369e-01 9.969115e-01

5445 7.436991e-01 9.969114e-01

5446 7.435576e-01 9.969113e-01

5447 7.434123e-01 9.969112e-01

5448 7.432631e-01 9.969111e-01

5449 7.431100e-01 9.969110e-01

5450 7.429529e-01 9.969109e-01

5451 7.427917e-01 9.969108e-01

5452 7.426263e-01 9.969107e-01

5453 7.424567e-01 9.969106e-01

5454 7.422829e-01 9.969105e-01

5455 7.421046e-01 9.969104e-01

5456 7.419219e-01 9.969103e-01

5457 7.417347e-01 9.969101e-01

5458 7.415429e-01 9.969100e-01

5459 7.413465e-01 9.969098e-01

5460 7.411454e-01 9.969097e-01

5461 7.409395e-01 9.969095e-01

5462 7.407287e-01 9.969094e-01

5463 7.405130e-01 9.969092e-01

5464 7.402924e-01 9.969090e-01

5465 7.400667e-01 9.969088e-01

5466 7.398360e-01 9.969086e-01

5467 7.396000e-01 9.969084e-01

5468 7.393589e-01 9.969082e-01

5469 7.391125e-01 9.969080e-01

5470 7.388608e-01 9.969077e-01

5471 7.386037e-01 9.969075e-01

5472 7.383412e-01 9.969072e-01

5473 7.380732e-01 9.969070e-01

5474 7.377997e-01 9.969067e-01

5475 7.375206e-01 9.969064e-01

5476 7.372360e-01 9.969061e-01

5477 7.369457e-01 9.969058e-01

5478 7.366497e-01 9.969055e-01

5479 7.363480e-01 9.969051e-01

5480 7.360406e-01 9.969048e-01

5481 7.357274e-01 9.969044e-01

5482 7.354085e-01 9.969040e-01

5483 7.350838e-01 9.969036e-01

5484 7.347532e-01 9.969032e-01

5485 7.344168e-01 9.969027e-01

5486 7.340746e-01 9.969023e-01

5487 7.337266e-01 9.969018e-01

5488 7.333727e-01 9.969013e-01

5489 7.330130e-01 9.969008e-01

5490 7.326475e-01 9.969002e-01

5491 7.322762e-01 9.968997e-01

5492 7.318991e-01 9.968991e-01

5493 7.315163e-01 9.968985e-01

5494 7.311277e-01 9.968978e-01

5495 7.307334e-01 9.968972e-01

5496 7.303335e-01 9.968965e-01

5497 7.299279e-01 9.968958e-01

5498 7.295168e-01 9.968950e-01

5499 7.291002e-01 9.968942e-01

5500 7.286781e-01 9.968934e-01

5501 7.282507e-01 9.968926e-01

5502 7.278179e-01 9.968917e-01

5503 7.273799e-01 9.968908e-01

5504 7.269368e-01 9.968899e-01

5505 7.264885e-01 9.968889e-01

5506 7.260354e-01 9.968879e-01

5507 7.255773e-01 9.968868e-01

5508 7.251145e-01 9.968857e-01

5509 7.246470e-01 9.968846e-01

5510 7.241750e-01 9.968834e-01

5511 7.236986e-01 9.968822e-01

5512 7.232178e-01 9.968809e-01

5513 7.227330e-01 9.968796e-01

5514 7.222441e-01 9.968782e-01

5515 7.217513e-01 9.968768e-01

5516 7.212548e-01 9.968753e-01

5517 7.207548e-01 9.968738e-01

5518 7.202513e-01 9.968722e-01

5519 7.197446e-01 9.968706e-01

5520 7.192349e-01 9.968689e-01

5521 7.187222e-01 9.968671e-01

5522 7.182068e-01 9.968653e-01

5523 7.176889e-01 9.968634e-01

5524 7.171686e-01 9.968614e-01

5525 7.166462e-01 9.968594e-01

5526 7.161217e-01 9.968573e-01

5527 7.155956e-01 9.968551e-01

5528 7.150678e-01 9.968529e-01

5529 7.145387e-01 9.968506e-01

5530 7.140084e-01 9.968482e-01

5531 7.134772e-01 9.968457e-01

5532 7.129452e-01 9.968431e-01

5533 7.124127e-01 9.968404e-01

5534 7.118799e-01 9.968377e-01

5535 7.113470e-01 9.968348e-01

5536 7.108142e-01 9.968319e-01

5537 7.102817e-01 9.968289e-01

5538 7.097498e-01 9.968257e-01

5539 7.092187e-01 9.968225e-01

5540 7.086885e-01 9.968191e-01

5541 7.081595e-01 9.968157e-01

5542 7.076319e-01 9.968121e-01

5543 7.071060e-01 9.968085e-01

5544 7.065818e-01 9.968047e-01

5545 7.060597e-01 9.968008e-01

5546 7.055399e-01 9.967967e-01

5547 7.050224e-01 9.967926e-01

5548 7.045077e-01 9.967883e-01

5549 7.039958e-01 9.967839e-01

5550 7.034869e-01 9.967794e-01

5551 7.029812e-01 9.967747e-01

5552 7.024789e-01 9.967700e-01

5553 7.019802e-01 9.967650e-01

5554 7.014853e-01 9.967600e-01

5555 7.009944e-01 9.967548e-01

5556 7.005075e-01 9.967494e-01

5557 7.000249e-01 9.967439e-01

5558 6.995467e-01 9.967383e-01

5559 6.990730e-01 9.967325e-01

5560 6.986041e-01 9.967266e-01

5561 6.981400e-01 9.967205e-01

5562 6.976809e-01 9.967143e-01

5563 6.972269e-01 9.967079e-01

5564 6.967781e-01 9.967013e-01

5565 6.963347e-01 9.966947e-01

5566 6.958967e-01 9.966878e-01

5567 6.954642e-01 9.966808e-01

5568 6.950374e-01 9.966737e-01

5569 6.946162e-01 9.966664e-01

5570 6.942009e-01 9.966590e-01

5571 6.937915e-01 9.966514e-01

5572 6.933879e-01 9.966437e-01

5573 6.929904e-01 9.966358e-01

5574 6.925989e-01 9.966278e-01

5575 6.922135e-01 9.966196e-01

5576 6.918343e-01 9.966113e-01

5577 6.914612e-01 9.966029e-01

5578 6.910944e-01 9.965943e-01

5579 6.907337e-01 9.965856e-01

5580 6.903793e-01 9.965769e-01

5581 6.900312e-01 9.965680e-01

5582 6.899986e-01 9.965679e-01

5583 6.899652e-01 9.965679e-01

5584 6.899311e-01 9.965679e-01

5585 6.898961e-01 9.965679e-01

5586 6.898604e-01 9.965679e-01

5587 6.898239e-01 9.965679e-01

5588 6.897866e-01 9.965678e-01

5589 6.897484e-01 9.965678e-01

5590 6.897094e-01 9.965678e-01

5591 6.896696e-01 9.965678e-01

5592 6.896289e-01 9.965678e-01

5593 6.895874e-01 9.965677e-01

5594 6.895450e-01 9.965677e-01

5595 6.895018e-01 9.965677e-01

5596 6.894576e-01 9.965677e-01

5597 6.894126e-01 9.965676e-01

5598 6.893667e-01 9.965676e-01

5599 6.893199e-01 9.965676e-01

5600 6.892722e-01 9.965676e-01

5601 6.892235e-01 9.965675e-01

5602 6.891739e-01 9.965675e-01

5603 6.891234e-01 9.965674e-01

5604 6.890720e-01 9.965674e-01

5605 6.890196e-01 9.965674e-01

5606 6.889663e-01 9.965673e-01

5607 6.889119e-01 9.965673e-01

5608 6.888567e-01 9.965672e-01

5609 6.888004e-01 9.965672e-01

5610 6.887432e-01 9.965671e-01

5611 6.886850e-01 9.965671e-01

5612 6.886258e-01 9.965670e-01

5613 6.885656e-01 9.965670e-01

5614 6.885044e-01 9.965669e-01

5615 6.884422e-01 9.965668e-01

5616 6.883790e-01 9.965668e-01

5617 6.883147e-01 9.965667e-01

5618 6.882495e-01 9.965666e-01

5619 6.881832e-01 9.965666e-01

5620 6.881160e-01 9.965665e-01

5621 6.880477e-01 9.965664e-01

5622 6.879784e-01 9.965663e-01

5623 6.879080e-01 9.965662e-01

5624 6.878366e-01 9.965661e-01

5625 6.877642e-01 9.965660e-01

5626 6.876908e-01 9.965659e-01

5627 6.876163e-01 9.965658e-01

5628 6.875409e-01 9.965657e-01

5629 6.874643e-01 9.965656e-01

5630 6.873868e-01 9.965654e-01

5631 6.873082e-01 9.965653e-01

5632 6.872287e-01 9.965652e-01

5633 6.871481e-01 9.965650e-01

5634 6.870665e-01 9.965649e-01

5635 6.869838e-01 9.965647e-01

5636 6.869002e-01 9.965646e-01

5637 6.868156e-01 9.965644e-01

5638 6.867300e-01 9.965642e-01

5639 6.866434e-01 9.965640e-01

5640 6.865558e-01 9.965638e-01

5641 6.864673e-01 9.965636e-01

5642 6.863778e-01 9.965634e-01

5643 6.862873e-01 9.965632e-01

5644 6.861959e-01 9.965629e-01

5645 6.861036e-01 9.965627e-01

5646 6.860104e-01 9.965624e-01

5647 6.859163e-01 9.965622e-01

5648 6.858212e-01 9.965619e-01

5649 6.857254e-01 9.965616e-01

5650 6.856286e-01 9.965613e-01

5651 6.855310e-01 9.965610e-01

5652 6.854327e-01 9.965607e-01

5653 6.853335e-01 9.965603e-01

5654 6.852335e-01 9.965600e-01

5655 6.851328e-01 9.965596e-01

5656 6.850314e-01 9.965592e-01

5657 6.849292e-01 9.965588e-01

5658 6.848264e-01 9.965584e-01

5659 6.847230e-01 9.965579e-01

5660 6.846189e-01 9.965574e-01

5661 6.845143e-01 9.965570e-01

5662 6.844091e-01 9.965565e-01

5663 6.843034e-01 9.965559e-01

5664 6.841972e-01 9.965554e-01

5665 6.840905e-01 9.965548e-01

5666 6.839835e-01 9.965542e-01

5667 6.838761e-01 9.965536e-01

5668 6.837683e-01 9.965530e-01

5669 6.836603e-01 9.965523e-01

5670 6.835521e-01 9.965516e-01

5671 6.834437e-01 9.965509e-01

5672 6.833351e-01 9.965501e-01

5673 6.832264e-01 9.965493e-01

5674 6.831178e-01 9.965485e-01

5675 6.830091e-01 9.965477e-01

5676 6.829005e-01 9.965468e-01

5677 6.827920e-01 9.965458e-01

5678 6.826837e-01 9.965449e-01

5679 6.825757e-01 9.965439e-01

5680 6.824679e-01 9.965428e-01

5681 6.823605e-01 9.965418e-01

5682 6.822535e-01 9.965406e-01

5683 6.821470e-01 9.965395e-01

5684 6.820411e-01 9.965382e-01

5685 6.819357e-01 9.965370e-01

5686 6.818311e-01 9.965356e-01

5687 6.817271e-01 9.965343e-01

5688 6.816240e-01 9.965329e-01

5689 6.815217e-01 9.965314e-01

5690 6.814204e-01 9.965298e-01

5691 6.813201e-01 9.965282e-01

5692 6.812209e-01 9.965266e-01

5693 6.811228e-01 9.965248e-01

5694 6.810258e-01 9.965230e-01

5695 6.809302e-01 9.965212e-01

5696 6.808359e-01 9.965193e-01

5697 6.807430e-01 9.965172e-01

5698 6.806516e-01 9.965152e-01

5699 6.805617e-01 9.965130e-01

5700 6.804734e-01 9.965107e-01

5701 6.803867e-01 9.965084e-01

5702 6.803017e-01 9.965060e-01

5703 6.802186e-01 9.965035e-01

5704 6.801372e-01 9.965009e-01

5705 6.800577e-01 9.964982e-01

5706 6.799802e-01 9.964954e-01

5707 6.799047e-01 9.964925e-01

5708 6.798312e-01 9.964894e-01

5709 6.797598e-01 9.964863e-01

5710 6.796906e-01 9.964831e-01

5711 6.796235e-01 9.964797e-01

5712 6.795587e-01 9.964762e-01

5713 6.794961e-01 9.964726e-01

5714 6.794359e-01 9.964689e-01

5715 6.793779e-01 9.964650e-01

5716 6.793224e-01 9.964610e-01

5717 6.792692e-01 9.964568e-01

5718 6.792185e-01 9.964525e-01

5719 6.791703e-01 9.964480e-01

5720 6.791245e-01 9.964434e-01

5721 6.790812e-01 9.964386e-01

5722 6.790404e-01 9.964337e-01

5723 6.790021e-01 9.964286e-01

5724 6.789664e-01 9.964232e-01

5725 6.789332e-01 9.964178e-01

5726 6.789026e-01 9.964121e-01

5727 6.788745e-01 9.964062e-01

5728 6.788490e-01 9.964001e-01

5729 6.788260e-01 9.963938e-01

5730 6.788055e-01 9.963873e-01

5731 6.787876e-01 9.963806e-01

5732 6.787722e-01 9.963737e-01

5733 6.787593e-01 9.963665e-01

5734 6.787489e-01 9.963590e-01

5735 6.787409e-01 9.963514e-01

5736 6.787354e-01 9.963435e-01

5737 6.787324e-01 9.963353e-01

5738 6.787317e-01 9.963268e-01

5739 6.787334e-01 9.963181e-01

5740 6.787374e-01 9.963091e-01

5741 6.787437e-01 9.962998e-01

5742 6.787523e-01 9.962902e-01

5743 6.787632e-01 9.962803e-01

5744 6.787762e-01 9.962700e-01

5745 6.787914e-01 9.962595e-01

5746 6.788088e-01 9.962486e-01

5747 6.788282e-01 9.962374e-01

5748 6.788496e-01 9.962258e-01

5749 6.788731e-01 9.962139e-01

5750 6.788985e-01 9.962016e-01

5751 6.789258e-01 9.961889e-01

5752 6.789550e-01 9.961758e-01

5753 6.789860e-01 9.961624e-01

5754 6.790188e-01 9.961485e-01

5755 6.790533e-01 9.961342e-01

5756 6.790894e-01 9.961195e-01

5757 6.791272e-01 9.961043e-01

5758 6.791666e-01 9.960887e-01

5759 6.792075e-01 9.960727e-01

5760 6.792498e-01 9.960561e-01

5761 6.792937e-01 9.960391e-01

5762 6.793162e-01 9.960391e-01

5763 6.793399e-01 9.960391e-01

5764 6.793647e-01 9.960390e-01

5765 6.793906e-01 9.960390e-01

5766 6.794178e-01 9.960390e-01

5767 6.794463e-01 9.960390e-01

5768 6.794760e-01 9.960389e-01

5769 6.795071e-01 9.960389e-01

5770 6.795396e-01 9.960389e-01

5771 6.795735e-01 9.960388e-01

5772 6.796089e-01 9.960388e-01

5773 6.796458e-01 9.960388e-01

5774 6.796844e-01 9.960387e-01

5775 6.797245e-01 9.960387e-01

5776 6.797664e-01 9.960386e-01

5777 6.798100e-01 9.960386e-01

5778 6.798554e-01 9.960385e-01

5779 6.799027e-01 9.960385e-01

5780 6.799519e-01 9.960384e-01

5781 6.800030e-01 9.960384e-01

5782 6.800562e-01 9.960383e-01

5783 6.801116e-01 9.960383e-01

5784 6.801690e-01 9.960382e-01

5785 6.802287e-01 9.960381e-01

5786 6.802907e-01 9.960381e-01

5787 6.803551e-01 9.960380e-01

5788 6.804218e-01 9.960379e-01

5789 6.804911e-01 9.960378e-01

5790 6.805629e-01 9.960377e-01

5791 6.806373e-01 9.960376e-01

5792 6.807144e-01 9.960376e-01

5793 6.807943e-01 9.960375e-01

5794 6.808771e-01 9.960374e-01

5795 6.809627e-01 9.960372e-01

5796 6.810513e-01 9.960371e-01

5797 6.811430e-01 9.960370e-01

5798 6.812378e-01 9.960369e-01

5799 6.813358e-01 9.960368e-01

5800 6.814370e-01 9.960366e-01

5801 6.815416e-01 9.960365e-01

5802 6.816496e-01 9.960363e-01

5803 6.817611e-01 9.960362e-01

5804 6.818762e-01 9.960360e-01

5805 6.819949e-01 9.960358e-01

5806 6.821173e-01 9.960357e-01

5807 6.822434e-01 9.960355e-01

5808 6.823735e-01 9.960353e-01

5809 6.825074e-01 9.960351e-01

5810 6.826454e-01 9.960349e-01

5811 6.827874e-01 9.960346e-01

5812 6.829335e-01 9.960344e-01

5813 6.830839e-01 9.960342e-01

5814 6.832385e-01 9.960339e-01

5815 6.833975e-01 9.960337e-01

5816 6.835608e-01 9.960334e-01

5817 6.837287e-01 9.960331e-01

5818 6.839010e-01 9.960328e-01

5819 6.840780e-01 9.960325e-01

5820 6.842596e-01 9.960321e-01

5821 6.844460e-01 9.960318e-01

5822 6.846371e-01 9.960314e-01

5823 6.848330e-01 9.960310e-01

5824 6.850338e-01 9.960306e-01

5825 6.852395e-01 9.960302e-01

5826 6.854503e-01 9.960298e-01

5827 6.856660e-01 9.960294e-01

5828 6.858868e-01 9.960289e-01

5829 6.861128e-01 9.960284e-01

5830 6.863439e-01 9.960279e-01

5831 6.865802e-01 9.960273e-01

5832 6.868217e-01 9.960268e-01

5833 6.870685e-01 9.960262e-01

5834 6.873206e-01 9.960256e-01

5835 6.875780e-01 9.960250e-01

5836 6.878407e-01 9.960243e-01

5837 6.881088e-01 9.960236e-01

5838 6.883823e-01 9.960229e-01

5839 6.886612e-01 9.960221e-01

5840 6.889455e-01 9.960214e-01

5841 6.892353e-01 9.960205e-01

5842 6.895304e-01 9.960197e-01

5843 6.898310e-01 9.960188e-01

5844 6.901370e-01 9.960179e-01

5845 6.904485e-01 9.960169e-01

5846 6.907654e-01 9.960159e-01

5847 6.910876e-01 9.960148e-01

5848 6.914153e-01 9.960137e-01

5849 6.917483e-01 9.960126e-01

5850 6.920867e-01 9.960114e-01

5851 6.924305e-01 9.960101e-01

5852 6.927795e-01 9.960089e-01

5853 6.931338e-01 9.960075e-01

5854 6.934934e-01 9.960061e-01

5855 6.938581e-01 9.960046e-01

5856 6.942280e-01 9.960031e-01

5857 6.946030e-01 9.960015e-01

5858 6.949831e-01 9.959998e-01

5859 6.953682e-01 9.959981e-01

5860 6.957583e-01 9.959963e-01

5861 6.961532e-01 9.959944e-01

5862 6.965530e-01 9.959925e-01

5863 6.969575e-01 9.959905e-01

5864 6.973667e-01 9.959884e-01

5865 6.977806e-01 9.959861e-01

5866 6.981990e-01 9.959839e-01

5867 6.986219e-01 9.959815e-01

5868 6.990491e-01 9.959790e-01

5869 6.994807e-01 9.959764e-01

5870 6.999165e-01 9.959737e-01

5871 7.003564e-01 9.959709e-01

5872 7.008003e-01 9.959680e-01

5873 7.012482e-01 9.959650e-01

5874 7.016998e-01 9.959618e-01

5875 7.021553e-01 9.959585e-01

5876 7.026143e-01 9.959551e-01

5877 7.030768e-01 9.959516e-01

5878 7.035428e-01 9.959479e-01

5879 7.040120e-01 9.959440e-01

5880 7.044844e-01 9.959400e-01

5881 7.049598e-01 9.959359e-01

5882 7.054382e-01 9.959315e-01

5883 7.059194e-01 9.959270e-01

5884 7.064033e-01 9.959224e-01

5885 7.068897e-01 9.959175e-01

5886 7.073785e-01 9.959125e-01

5887 7.078697e-01 9.959072e-01

5888 7.083630e-01 9.959018e-01

5889 7.088584e-01 9.958961e-01

5890 7.093557e-01 9.958902e-01

5891 7.098547e-01 9.958841e-01

5892 7.103554e-01 9.958777e-01

5893 7.108575e-01 9.958711e-01

5894 7.113610e-01 9.958642e-01

5895 7.118657e-01 9.958571e-01

5896 7.123715e-01 9.958497e-01

5897 7.128782e-01 9.958420e-01

5898 7.133857e-01 9.958340e-01

5899 7.138939e-01 9.958256e-01

5900 7.144026e-01 9.958170e-01

5901 7.149116e-01 9.958080e-01

5902 7.154208e-01 9.957987e-01

5903 7.159302e-01 9.957890e-01

5904 7.164395e-01 9.957790e-01

5905 7.169485e-01 9.957686e-01

5906 7.174573e-01 9.957577e-01

5907 7.179655e-01 9.957465e-01

5908 7.184732e-01 9.957348e-01

5909 7.189801e-01 9.957227e-01

5910 7.194861e-01 9.957101e-01

5911 7.199911e-01 9.956971e-01

5912 7.204949e-01 9.956835e-01

5913 7.209974e-01 9.956695e-01

5914 7.214985e-01 9.956549e-01

5915 7.219981e-01 9.956397e-01

5916 7.224960e-01 9.956240e-01

5917 7.229921e-01 9.956077e-01

5918 7.234863e-01 9.955908e-01

5919 7.239784e-01 9.955732e-01

5920 7.244684e-01 9.955550e-01

5921 7.249561e-01 9.955361e-01

5922 7.254415e-01 9.955165e-01

5923 7.259243e-01 9.954962e-01

5924 7.264045e-01 9.954751e-01

5925 7.268821e-01 9.954532e-01

5926 7.273568e-01 9.954305e-01

5927 7.278286e-01 9.954070e-01

5928 7.282974e-01 9.953826e-01

5929 7.287632e-01 9.953573e-01

5930 7.292257e-01 9.953310e-01

5931 7.296850e-01 9.953038e-01

5932 7.301409e-01 9.952755e-01

5933 7.305934e-01 9.952462e-01

5934 7.310424e-01 9.952159e-01

5935 7.314878e-01 9.951844e-01

5936 7.319296e-01 9.951517e-01

5937 7.323676e-01 9.951178e-01

5938 7.328019e-01 9.950827e-01

5939 7.332323e-01 9.950463e-01

5940 7.336589e-01 9.950086e-01

5941 7.340815e-01 9.949695e-01

5942 7.341277e-01 9.949694e-01

5943 7.341756e-01 9.949694e-01

5944 7.342250e-01 9.949693e-01

5945 7.342760e-01 9.949693e-01

5946 7.343286e-01 9.949692e-01

5947 7.343830e-01 9.949691e-01

5948 7.344391e-01 9.949691e-01

5949 7.344970e-01 9.949690e-01

5950 7.345568e-01 9.949690e-01

5951 7.346184e-01 9.949689e-01

5952 7.346820e-01 9.949688e-01

5953 7.347476e-01 9.949687e-01

5954 7.348153e-01 9.949687e-01

5955 7.348851e-01 9.949686e-01

5956 7.349570e-01 9.949685e-01

5957 7.350312e-01 9.949684e-01

5958 7.351078e-01 9.949683e-01

5959 7.351866e-01 9.949682e-01

5960 7.352679e-01 9.949681e-01

5961 7.353517e-01 9.949680e-01

5962 7.354381e-01 9.949679e-01

5963 7.355271e-01 9.949678e-01

5964 7.356188e-01 9.949676e-01

5965 7.357133e-01 9.949675e-01

5966 7.358106e-01 9.949674e-01

5967 7.359109e-01 9.949672e-01

5968 7.360142e-01 9.949671e-01

5969 7.361205e-01 9.949669e-01

5970 7.362300e-01 9.949667e-01

5971 7.363428e-01 9.949666e-01

5972 7.364589e-01 9.949664e-01

5973 7.365784e-01 9.949662e-01

5974 7.367013e-01 9.949660e-01

5975 7.368279e-01 9.949658e-01

5976 7.369581e-01 9.949656e-01

5977 7.370921e-01 9.949654e-01

5978 7.372299e-01 9.949651e-01

5979 7.373717e-01 9.949649e-01

5980 7.375175e-01 9.949646e-01

5981 7.376674e-01 9.949644e-01

5982 7.378215e-01 9.949641e-01

5983 7.379799e-01 9.949638e-01

5984 7.381427e-01 9.949635e-01

5985 7.383101e-01 9.949631e-01

5986 7.384820e-01 9.949628e-01

5987 7.386586e-01 9.949625e-01

5988 7.388400e-01 9.949621e-01

5989 7.390263e-01 9.949617e-01

5990 7.392176e-01 9.949613e-01

5991 7.394140e-01 9.949609e-01

5992 7.396156e-01 9.949605e-01

5993 7.398224e-01 9.949600e-01

5994 7.400347e-01 9.949595e-01

5995 7.402524e-01 9.949590e-01

5996 7.404757e-01 9.949585e-01

5997 7.407047e-01 9.949580e-01

5998 7.409395e-01 9.949574e-01

5999 7.411802e-01 9.949568e-01

6000 7.414268e-01 9.949562e-01

6001 7.416796e-01 9.949555e-01

6002 7.419385e-01 9.949549e-01

6003 7.422037e-01 9.949542e-01

6004 7.424752e-01 9.949534e-01

6005 7.427532e-01 9.949527e-01

6006 7.430378e-01 9.949519e-01

6007 7.433290e-01 9.949510e-01

6008 7.436269e-01 9.949502e-01

6009 7.439316e-01 9.949493e-01

6010 7.442432e-01 9.949483e-01

6011 7.445619e-01 9.949473e-01

6012 7.448875e-01 9.949463e-01

6013 7.452203e-01 9.949452e-01

6014 7.455603e-01 9.949441e-01

6015 7.459076e-01 9.949429e-01

6016 7.462622e-01 9.949417e-01

6017 7.466243e-01 9.949404e-01

6018 7.469938e-01 9.949391e-01

6019 7.473709e-01 9.949377e-01

6020 7.477555e-01 9.949363e-01

6021 7.481478e-01 9.949348e-01

6022 7.485478e-01 9.949332e-01

6023 7.489555e-01 9.949316e-01

6024 7.493709e-01 9.949299e-01

6025 7.497942e-01 9.949281e-01

6026 7.502253e-01 9.949263e-01

6027 7.506643e-01 9.949244e-01

6028 7.511111e-01 9.949223e-01

6029 7.515659e-01 9.949203e-01

6030 7.520285e-01 9.949181e-01

6031 7.524990e-01 9.949158e-01

6032 7.529775e-01 9.949134e-01

6033 7.534638e-01 9.949110e-01

6034 7.539580e-01 9.949084e-01

6035 7.544601e-01 9.949057e-01

6036 7.549701e-01 9.949030e-01

6037 7.554878e-01 9.949001e-01

6038 7.560134e-01 9.948970e-01

6039 7.565466e-01 9.948939e-01

6040 7.570876e-01 9.948906e-01

6041 7.576362e-01 9.948872e-01

6042 7.581924e-01 9.948837e-01

6043 7.587560e-01 9.948800e-01

6044 7.593271e-01 9.948761e-01

6045 7.599056e-01 9.948721e-01

6046 7.604913e-01 9.948679e-01

6047 7.610842e-01 9.948636e-01

6048 7.616842e-01 9.948591e-01

6049 7.622912e-01 9.948544e-01

6050 7.629050e-01 9.948495e-01

6051 7.635255e-01 9.948443e-01

6052 7.641527e-01 9.948390e-01

6053 7.647864e-01 9.948335e-01

6054 7.654264e-01 9.948278e-01

6055 7.660726e-01 9.948218e-01

6056 7.667249e-01 9.948155e-01

6057 7.673831e-01 9.948090e-01

6058 7.680471e-01 9.948023e-01

6059 7.687166e-01 9.947953e-01

6060 7.693916e-01 9.947879e-01

6061 7.700718e-01 9.947803e-01

6062 7.707571e-01 9.947724e-01

6063 7.714472e-01 9.947642e-01

6064 7.721420e-01 9.947556e-01

6065 7.728414e-01 9.947467e-01

6066 7.735450e-01 9.947374e-01

6067 7.742527e-01 9.947277e-01

6068 7.749644e-01 9.947177e-01

6069 7.756797e-01 9.947072e-01

6070 7.763984e-01 9.946963e-01

6071 7.771204e-01 9.946850e-01

6072 7.778455e-01 9.946732e-01

6073 7.785733e-01 9.946609e-01

6074 7.793038e-01 9.946481e-01

6075 7.800366e-01 9.946349e-01

6076 7.807715e-01 9.946210e-01

6077 7.815084e-01 9.946067e-01

6078 7.822469e-01 9.945917e-01

6079 7.829868e-01 9.945761e-01

6080 7.837280e-01 9.945599e-01

6081 7.844701e-01 9.945431e-01

6082 7.852130e-01 9.945256e-01

6083 7.859563e-01 9.945073e-01

6084 7.867000e-01 9.944884e-01

6085 7.874436e-01 9.944687e-01

6086 7.881871e-01 9.944481e-01

6087 7.889301e-01 9.944268e-01

6088 7.896725e-01 9.944046e-01

6089 7.904140e-01 9.943815e-01

6090 7.911544e-01 9.943574e-01

6091 7.918934e-01 9.943324e-01

6092 7.926309e-01 9.943064e-01

6093 7.933666e-01 9.942794e-01

6094 7.941003e-01 9.942512e-01

6095 7.948317e-01 9.942219e-01

6096 7.955608e-01 9.941915e-01

6097 7.962871e-01 9.941598e-01

6098 7.970107e-01 9.941269e-01

6099 7.977312e-01 9.940926e-01

6100 7.984484e-01 9.940570e-01

6101 7.991622e-01 9.940199e-01

6102 7.998724e-01 9.939813e-01

6103 8.005788e-01 9.939412e-01

6104 8.012811e-01 9.938995e-01

6105 8.019793e-01 9.938562e-01

6106 8.026731e-01 9.938110e-01

6107 8.033625e-01 9.937641e-01

6108 8.040471e-01 9.937153e-01

6109 8.047270e-01 9.936646e-01

6110 8.054018e-01 9.936119e-01

6111 8.060715e-01 9.935570e-01

6112 8.067360e-01 9.934999e-01

6113 8.073950e-01 9.934406e-01

6114 8.080485e-01 9.933789e-01

6115 8.086964e-01 9.933148e-01

6116 8.093384e-01 9.932482e-01

6117 8.099746e-01 9.931788e-01

6118 8.106048e-01 9.931068e-01

6119 8.112289e-01 9.930319e-01

6120 8.118467e-01 9.929541e-01

6121 8.124583e-01 9.928731e-01

6122 8.124837e-01 9.928731e-01

6123 8.125097e-01 9.928730e-01

6124 8.125365e-01 9.928729e-01

6125 8.125641e-01 9.928727e-01

6126 8.125925e-01 9.928726e-01

6127 8.126217e-01 9.928725e-01

6128 8.126517e-01 9.928724e-01

6129 8.126825e-01 9.928723e-01

6130 8.127142e-01 9.928721e-01

6131 8.127469e-01 9.928720e-01

6132 8.127804e-01 9.928719e-01

6133 8.128149e-01 9.928717e-01

6134 8.128504e-01 9.928716e-01

6135 8.128869e-01 9.928714e-01

6136 8.129244e-01 9.928712e-01

6137 8.129630e-01 9.928710e-01

6138 8.130027e-01 9.928709e-01

6139 8.130435e-01 9.928707e-01

6140 8.130855e-01 9.928705e-01

6141 8.131287e-01 9.928702e-01

6142 8.131731e-01 9.928700e-01

6143 8.132187e-01 9.928698e-01

6144 8.132657e-01 9.928695e-01

6145 8.133140e-01 9.928693e-01

6146 8.133636e-01 9.928690e-01

6147 8.134147e-01 9.928687e-01

6148 8.134672e-01 9.928685e-01

6149 8.135212e-01 9.928682e-01

6150 8.135767e-01 9.928678e-01

6151 8.136338e-01 9.928675e-01

6152 8.136925e-01 9.928672e-01

6153 8.137528e-01 9.928668e-01

6154 8.138149e-01 9.928664e-01

6155 8.138787e-01 9.928661e-01

6156 8.139443e-01 9.928656e-01

6157 8.140118e-01 9.928652e-01

6158 8.140812e-01 9.928648e-01

6159 8.141525e-01 9.928643e-01

6160 8.142258e-01 9.928638e-01

6161 8.143011e-01 9.928633e-01

6162 8.143786e-01 9.928628e-01

6163 8.144582e-01 9.928623e-01

6164 8.145400e-01 9.928617e-01

6165 8.146241e-01 9.928611e-01

6166 8.147105e-01 9.928605e-01

6167 8.147993e-01 9.928598e-01

6168 8.148906e-01 9.928592e-01

6169 8.149844e-01 9.928584e-01

6170 8.150807e-01 9.928577e-01

6171 8.151797e-01 9.928569e-01

6172 8.152814e-01 9.928562e-01

6173 8.153858e-01 9.928553e-01

6174 8.154931e-01 9.928545e-01

6175 8.156033e-01 9.928535e-01

6176 8.157164e-01 9.928526e-01

6177 8.158326e-01 9.928516e-01

6178 8.159518e-01 9.928506e-01

6179 8.160743e-01 9.928495e-01

6180 8.162000e-01 9.928484e-01

6181 8.163290e-01 9.928473e-01

6182 8.164614e-01 9.928461e-01

6183 8.165973e-01 9.928448e-01

6184 8.167367e-01 9.928435e-01

6185 8.168797e-01 9.928421e-01

6186 8.170264e-01 9.928407e-01

6187 8.171768e-01 9.928392e-01

6188 8.173311e-01 9.928377e-01

6189 8.174893e-01 9.928360e-01

6190 8.176515e-01 9.928344e-01

6191 8.178178e-01 9.928326e-01

6192 8.179882e-01 9.928308e-01

6193 8.181628e-01 9.928289e-01

6194 8.183418e-01 9.928269e-01

6195 8.185251e-01 9.928249e-01

6196 8.187128e-01 9.928227e-01

6197 8.189051e-01 9.928205e-01

6198 8.191019e-01 9.928182e-01

6199 8.193035e-01 9.928158e-01

6200 8.195098e-01 9.928132e-01

6201 8.197209e-01 9.928106e-01

6202 8.199369e-01 9.928079e-01

6203 8.201578e-01 9.928050e-01

6204 8.203838e-01 9.928021e-01

6205 8.206148e-01 9.927990e-01

6206 8.208511e-01 9.927958e-01

6207 8.210925e-01 9.927924e-01

6208 8.213393e-01 9.927889e-01

6209 8.215914e-01 9.927853e-01

6210 8.218489e-01 9.927815e-01

6211 8.221119e-01 9.927776e-01

6212 8.223804e-01 9.927735e-01

6213 8.226544e-01 9.927693e-01

6214 8.229341e-01 9.927648e-01

6215 8.232194e-01 9.927602e-01

6216 8.235105e-01 9.927554e-01

6217 8.238072e-01 9.927504e-01

6218 8.241098e-01 9.927452e-01

6219 8.244182e-01 9.927397e-01

6220 8.247324e-01 9.927341e-01

6221 8.250524e-01 9.927282e-01

6222 8.253784e-01 9.927221e-01

6223 8.257102e-01 9.927157e-01

6224 8.260480e-01 9.927091e-01

6225 8.263916e-01 9.927022e-01

6226 8.267412e-01 9.926950e-01

6227 8.270967e-01 9.926875e-01

6228 8.274581e-01 9.926797e-01

6229 8.278253e-01 9.926715e-01

6230 8.281985e-01 9.926631e-01

6231 8.285774e-01 9.926543e-01

6232 8.289622e-01 9.926451e-01

6233 8.293528e-01 9.926355e-01

6234 8.297491e-01 9.926256e-01

6235 8.301511e-01 9.926152e-01

6236 8.305587e-01 9.926044e-01

6237 8.309719e-01 9.925932e-01

6238 8.313906e-01 9.925815e-01

6239 8.318147e-01 9.925693e-01

6240 8.322442e-01 9.925566e-01

6241 8.326789e-01 9.925434e-01

6242 8.331188e-01 9.925296e-01

6243 8.335638e-01 9.925153e-01

6244 8.340137e-01 9.925003e-01

6245 8.344685e-01 9.924847e-01

6246 8.349281e-01 9.924685e-01

6247 8.353923e-01 9.924516e-01

6248 8.358609e-01 9.924340e-01

6249 8.363339e-01 9.924157e-01

6250 8.368112e-01 9.923965e-01

6251 8.372925e-01 9.923766e-01

6252 8.377777e-01 9.923559e-01

6253 8.382666e-01 9.923343e-01

6254 8.387591e-01 9.923117e-01

6255 8.392551e-01 9.922883e-01

6256 8.397543e-01 9.922638e-01

6257 8.402565e-01 9.922383e-01

6258 8.407616e-01 9.922118e-01

6259 8.412694e-01 9.921841e-01

6260 8.417797e-01 9.921553e-01

6261 8.422923e-01 9.921252e-01

6262 8.428070e-01 9.920939e-01

6263 8.433236e-01 9.920613e-01

6264 8.438418e-01 9.920273e-01

6265 8.443616e-01 9.919919e-01

6266 8.448825e-01 9.919550e-01

6267 8.454046e-01 9.919165e-01

6268 8.459274e-01 9.918765e-01

6269 8.464509e-01 9.918347e-01

6270 8.469747e-01 9.917912e-01

6271 8.474987e-01 9.917459e-01

6272 8.480227e-01 9.916986e-01

6273 8.485463e-01 9.916494e-01

6274 8.490695e-01 9.915981e-01

6275 8.495920e-01 9.915447e-01

6276 8.501135e-01 9.914891e-01

6277 8.506338e-01 9.914311e-01

6278 8.511528e-01 9.913707e-01

6279 8.516702e-01 9.913077e-01

6280 8.521857e-01 9.912422e-01

6281 8.526993e-01 9.911739e-01

6282 8.532106e-01 9.911028e-01

6283 8.537195e-01 9.910288e-01

6284 8.542258e-01 9.909516e-01

6285 8.547292e-01 9.908713e-01

6286 8.552297e-01 9.907877e-01

6287 8.557269e-01 9.907006e-01

6288 8.562207e-01 9.906100e-01

6289 8.567109e-01 9.905156e-01

6290 8.571974e-01 9.904174e-01

6291 8.576799e-01 9.903152e-01

6292 8.581583e-01 9.902088e-01

6293 8.586326e-01 9.900981e-01

6294 8.591024e-01 9.899830e-01

6295 8.595676e-01 9.898632e-01

6296 8.600282e-01 9.897386e-01

6297 8.604839e-01 9.896090e-01

6298 8.609346e-01 9.894742e-01

6299 8.613803e-01 9.893342e-01

6300 8.618208e-01 9.891885e-01

6301 8.622560e-01 9.890372e-01

6302 8.622651e-01 9.890370e-01

6303 8.622744e-01 9.890369e-01

6304 8.622840e-01 9.890367e-01

6305 8.622937e-01 9.890365e-01

6306 8.623037e-01 9.890363e-01

6307 8.623140e-01 9.890361e-01

6308 8.623245e-01 9.890359e-01

6309 8.623352e-01 9.890356e-01

6310 8.623461e-01 9.890354e-01

6311 8.623574e-01 9.890351e-01

6312 8.623689e-01 9.890349e-01

6313 8.623806e-01 9.890346e-01

6314 8.623927e-01 9.890343e-01

6315 8.624050e-01 9.890341e-01

6316 8.624176e-01 9.890338e-01

6317 8.624305e-01 9.890334e-01

6318 8.624437e-01 9.890331e-01

6319 8.624573e-01 9.890328e-01

6320 8.624711e-01 9.890324e-01

6321 8.624853e-01 9.890320e-01

6322 8.624999e-01 9.890317e-01

6323 8.625148e-01 9.890312e-01

6324 8.625300e-01 9.890308e-01

6325 8.625456e-01 9.890304e-01

6326 8.625616e-01 9.890299e-01

6327 8.625780e-01 9.890295e-01

6328 8.625947e-01 9.890290e-01

6329 8.626119e-01 9.890285e-01

6330 8.626295e-01 9.890279e-01

6331 8.626475e-01 9.890274e-01

6332 8.626660e-01 9.890268e-01

6333 8.626849e-01 9.890262e-01

6334 8.627043e-01 9.890256e-01

6335 8.627241e-01 9.890249e-01

6336 8.627445e-01 9.890242e-01

6337 8.627653e-01 9.890235e-01

6338 8.627867e-01 9.890228e-01

6339 8.628085e-01 9.890220e-01

6340 8.628309e-01 9.890212e-01

6341 8.628539e-01 9.890204e-01

6342 8.628774e-01 9.890195e-01

6343 8.629015e-01 9.890186e-01

6344 8.629262e-01 9.890177e-01

6345 8.629515e-01 9.890167e-01

6346 8.629774e-01 9.890157e-01

6347 8.630040e-01 9.890146e-01

6348 8.630312e-01 9.890135e-01

6349 8.630591e-01 9.890124e-01

6350 8.630876e-01 9.890112e-01

6351 8.631169e-01 9.890099e-01

6352 8.631469e-01 9.890086e-01

6353 8.631776e-01 9.890073e-01

6354 8.632091e-01 9.890059e-01

6355 8.632413e-01 9.890044e-01

6356 8.632743e-01 9.890029e-01

6357 8.633081e-01 9.890014e-01

6358 8.633427e-01 9.889997e-01

6359 8.633782e-01 9.889980e-01

6360 8.634145e-01 9.889962e-01

6361 8.634517e-01 9.889944e-01

6362 8.634898e-01 9.889925e-01

6363 8.635287e-01 9.889905e-01

6364 8.635687e-01 9.889884e-01

6365 8.636095e-01 9.889862e-01

6366 8.636513e-01 9.889840e-01

6367 8.636941e-01 9.889817e-01

6368 8.637379e-01 9.889792e-01

6369 8.637827e-01 9.889767e-01

6370 8.638286e-01 9.889740e-01

6371 8.638755e-01 9.889713e-01

6372 8.639235e-01 9.889684e-01

6373 8.639726e-01 9.889655e-01

6374 8.640228e-01 9.889624e-01

6375 8.640741e-01 9.889592e-01

6376 8.641265e-01 9.889558e-01

6377 8.641802e-01 9.889523e-01

6378 8.642350e-01 9.889487e-01

6379 8.642909e-01 9.889449e-01

6380 8.643482e-01 9.889410e-01

6381 8.644066e-01 9.889369e-01

6382 8.644663e-01 9.889327e-01

6383 8.645272e-01 9.889282e-01

6384 8.645894e-01 9.889236e-01

6385 8.646529e-01 9.889188e-01

6386 8.647176e-01 9.889138e-01

6387 8.647837e-01 9.889087e-01

6388 8.648511e-01 9.889032e-01

6389 8.649198e-01 9.888976e-01

6390 8.649899e-01 9.888917e-01

6391 8.650613e-01 9.888856e-01

6392 8.651340e-01 9.888793e-01

6393 8.652081e-01 9.888727e-01

6394 8.652836e-01 9.888658e-01

6395 8.653604e-01 9.888586e-01

6396 8.654386e-01 9.888511e-01

6397 8.655181e-01 9.888433e-01

6398 8.655990e-01 9.888352e-01

6399 8.656812e-01 9.888268e-01

6400 8.657648e-01 9.888180e-01

6401 8.658497e-01 9.888088e-01

6402 8.659360e-01 9.887993e-01

6403 8.660235e-01 9.887894e-01

6404 8.661124e-01 9.887790e-01

6405 8.662026e-01 9.887682e-01

6406 8.662940e-01 9.887569e-01

6407 8.663866e-01 9.887452e-01

6408 8.664805e-01 9.887330e-01

6409 8.665755e-01 9.887203e-01

6410 8.666718e-01 9.887070e-01

6411 8.667691e-01 9.886932e-01

6412 8.668676e-01 9.886787e-01

6413 8.669671e-01 9.886637e-01

6414 8.670676e-01 9.886480e-01

6415 8.671691e-01 9.886317e-01

6416 8.672716e-01 9.886146e-01

6417 8.673749e-01 9.885969e-01

6418 8.674791e-01 9.885783e-01

6419 8.675840e-01 9.885590e-01

6420 8.676896e-01 9.885388e-01

6421 8.677959e-01 9.885178e-01

6422 8.679028e-01 9.884959e-01

6423 8.680102e-01 9.884730e-01

6424 8.681180e-01 9.884492e-01

6425 8.682262e-01 9.884243e-01

6426 8.683347e-01 9.883983e-01

6427 8.684435e-01 9.883712e-01

6428 8.685523e-01 9.883430e-01

6429 8.686612e-01 9.883135e-01

6430 8.687701e-01 9.882828e-01

6431 8.688788e-01 9.882507e-01

6432 8.689872e-01 9.882172e-01

6433 8.690954e-01 9.881823e-01

6434 8.692031e-01 9.881459e-01

6435 8.693103e-01 9.881079e-01

6436 8.694168e-01 9.880683e-01

6437 8.695226e-01 9.880270e-01

6438 8.696276e-01 9.879838e-01

6439 8.697316e-01 9.879389e-01

6440 8.698345e-01 9.878919e-01

6441 8.699362e-01 9.878430e-01

6442 8.700367e-01 9.877919e-01

6443 8.701357e-01 9.877387e-01

6444 8.702332e-01 9.876832e-01

6445 8.703291e-01 9.876253e-01

6446 8.704232e-01 9.875649e-01

6447 8.705154e-01 9.875019e-01

6448 8.706056e-01 9.874363e-01

6449 8.706937e-01 9.873678e-01

6450 8.707797e-01 9.872964e-01

6451 8.708633e-01 9.872221e-01

6452 8.709444e-01 9.871445e-01

6453 8.710231e-01 9.870637e-01

6454 8.710991e-01 9.869795e-01

6455 8.711723e-01 9.868918e-01

6456 8.712427e-01 9.868003e-01

6457 8.713101e-01 9.867051e-01

6458 8.713745e-01 9.866059e-01

6459 8.714358e-01 9.865026e-01

6460 8.714939e-01 9.863950e-01

6461 8.715486e-01 9.862830e-01

6462 8.716000e-01 9.861664e-01

6463 8.716479e-01 9.860450e-01

6464 8.716923e-01 9.859188e-01

6465 8.717332e-01 9.857874e-01

6466 8.717703e-01 9.856507e-01

6467 8.718038e-01 9.855085e-01

6468 8.718336e-01 9.853607e-01

6469 8.718595e-01 9.852071e-01

6470 8.718816e-01 9.850474e-01

6471 8.718999e-01 9.848814e-01

6472 8.719142e-01 9.847091e-01

6473 8.719247e-01 9.845300e-01

6474 8.719312e-01 9.843441e-01

6475 8.719338e-01 9.841512e-01

6476 8.719324e-01 9.839509e-01

6477 8.719271e-01 9.837432e-01

6478 8.719179e-01 9.835278e-01

6479 8.719048e-01 9.833044e-01

6480 8.718877e-01 9.830729e-01

6481 8.718668e-01 9.828331e-01

6482 8.718668e-01 9.828328e-01

6483 8.718667e-01 9.828326e-01

6484 8.718665e-01 9.828323e-01

6485 8.718663e-01 9.828320e-01

6486 8.718661e-01 9.828317e-01

6487 8.718657e-01 9.828314e-01

6488 8.718653e-01 9.828311e-01

6489 8.718648e-01 9.828308e-01

6490 8.718643e-01 9.828304e-01

6491 8.718636e-01 9.828301e-01

6492 8.718629e-01 9.828297e-01

6493 8.718621e-01 9.828293e-01

6494 8.718612e-01 9.828289e-01

6495 8.718601e-01 9.828285e-01

6496 8.718590e-01 9.828280e-01

6497 8.718578e-01 9.828276e-01

6498 8.718564e-01 9.828271e-01

6499 8.718549e-01 9.828266e-01

6500 8.718533e-01 9.828261e-01

6501 8.718515e-01 9.828256e-01

6502 8.718496e-01 9.828251e-01

6503 8.718476e-01 9.828245e-01

6504 8.718453e-01 9.828239e-01

6505 8.718429e-01 9.828233e-01

6506 8.718404e-01 9.828227e-01

6507 8.718376e-01 9.828220e-01

6508 8.718347e-01 9.828213e-01

6509 8.718315e-01 9.828206e-01

6510 8.718282e-01 9.828199e-01

6511 8.718246e-01 9.828191e-01

6512 8.718207e-01 9.828183e-01

6513 8.718167e-01 9.828175e-01

6514 8.718123e-01 9.828166e-01

6515 8.718077e-01 9.828157e-01

6516 8.718029e-01 9.828148e-01

6517 8.717977e-01 9.828139e-01

6518 8.717922e-01 9.828129e-01

6519 8.717864e-01 9.828118e-01

6520 8.717803e-01 9.828107e-01

6521 8.717738e-01 9.828096e-01

6522 8.717670e-01 9.828085e-01

6523 8.717597e-01 9.828073e-01

6524 8.717521e-01 9.828060e-01

6525 8.717440e-01 9.828047e-01

6526 8.717356e-01 9.828033e-01

6527 8.717266e-01 9.828019e-01

6528 8.717172e-01 9.828005e-01

6529 8.717073e-01 9.827990e-01

6530 8.716969e-01 9.827974e-01

6531 8.716859e-01 9.827957e-01

6532 8.716744e-01 9.827940e-01

6533 8.716623e-01 9.827923e-01

6534 8.716496e-01 9.827904e-01

6535 8.716363e-01 9.827885e-01

6536 8.716223e-01 9.827865e-01

6537 8.716076e-01 9.827845e-01

6538 8.715921e-01 9.827823e-01

6539 8.715760e-01 9.827801e-01

6540 8.715590e-01 9.827778e-01

6541 8.715413e-01 9.827754e-01

6542 8.715227e-01 9.827729e-01

6543 8.715032e-01 9.827702e-01

6544 8.714828e-01 9.827675e-01

6545 8.714614e-01 9.827647e-01

6546 8.714390e-01 9.827618e-01

6547 8.714156e-01 9.827587e-01

6548 8.713912e-01 9.827556e-01

6549 8.713656e-01 9.827523e-01

6550 8.713388e-01 9.827489e-01

6551 8.713108e-01 9.827453e-01

6552 8.712816e-01 9.827416e-01

6553 8.712511e-01 9.827377e-01

6554 8.712192e-01 9.827337e-01

6555 8.711859e-01 9.827295e-01

6556 8.711511e-01 9.827252e-01

6557 8.711148e-01 9.827207e-01

6558 8.710769e-01 9.827160e-01

6559 8.710373e-01 9.827111e-01

6560 8.709961e-01 9.827060e-01

6561 8.709530e-01 9.827007e-01

6562 8.709082e-01 9.826951e-01

6563 8.708614e-01 9.826894e-01

6564 8.708127e-01 9.826834e-01

6565 8.707619e-01 9.826771e-01

6566 8.707089e-01 9.826706e-01

6567 8.706538e-01 9.826639e-01

6568 8.705964e-01 9.826568e-01

6569 8.705366e-01 9.826495e-01

6570 8.704744e-01 9.826418e-01

6571 8.704096e-01 9.826339e-01

6572 8.703422e-01 9.826256e-01

6573 8.702721e-01 9.826169e-01

6574 8.701992e-01 9.826079e-01

6575 8.701233e-01 9.825985e-01

6576 8.700445e-01 9.825887e-01

6577 8.699626e-01 9.825785e-01

6578 8.698775e-01 9.825679e-01

6579 8.697890e-01 9.825568e-01

6580 8.696972e-01 9.825452e-01

6581 8.696019e-01 9.825331e-01

6582 8.695029e-01 9.825206e-01

6583 8.694002e-01 9.825074e-01

6584 8.692936e-01 9.824938e-01

6585 8.691831e-01 9.824795e-01

6586 8.690685e-01 9.824646e-01

6587 8.689497e-01 9.824491e-01

6588 8.688265e-01 9.824329e-01

6589 8.686990e-01 9.824160e-01

6590 8.685669e-01 9.823984e-01

6591 8.684301e-01 9.823800e-01

6592 8.682885e-01 9.823608e-01

6593 8.681419e-01 9.823408e-01

6594 8.679904e-01 9.823199e-01

6595 8.678336e-01 9.822981e-01

6596 8.676716e-01 9.822753e-01

6597 8.675041e-01 9.822516e-01

6598 8.673312e-01 9.822268e-01

6599 8.671525e-01 9.822009e-01

6600 8.669681e-01 9.821739e-01

6601 8.667778e-01 9.821458e-01

6602 8.665815e-01 9.821164e-01

6603 8.663791e-01 9.820857e-01

6604 8.661704e-01 9.820537e-01

6605 8.659554e-01 9.820203e-01

6606 8.657340e-01 9.819854e-01

6607 8.655060e-01 9.819490e-01

6608 8.652714e-01 9.819111e-01

6609 8.650301e-01 9.818715e-01

6610 8.647819e-01 9.818302e-01

6611 8.645269e-01 9.817871e-01

6612 8.642649e-01 9.817421e-01

6613 8.639958e-01 9.816952e-01

6614 8.637197e-01 9.816463e-01

6615 8.634363e-01 9.815953e-01

6616 8.631458e-01 9.815420e-01

6617 8.628481e-01 9.814866e-01

6618 8.625430e-01 9.814287e-01

6619 8.622306e-01 9.813684e-01

6620 8.619110e-01 9.813055e-01

6621 8.615840e-01 9.812400e-01

6622 8.612496e-01 9.811717e-01

6623 8.609080e-01 9.811006e-01

6624 8.605590e-01 9.810265e-01

6625 8.602028e-01 9.809493e-01

6626 8.598394e-01 9.808688e-01

6627 8.594688e-01 9.807851e-01

6628 8.590911e-01 9.806979e-01

6629 8.587063e-01 9.806072e-01

6630 8.583146e-01 9.805127e-01

6631 8.579161e-01 9.804144e-01

6632 8.575108e-01 9.803122e-01

6633 8.570989e-01 9.802059e-01

6634 8.566805e-01 9.800953e-01

6635 8.562557e-01 9.799803e-01

6636 8.558247e-01 9.798608e-01

6637 8.553877e-01 9.797367e-01

6638 8.549447e-01 9.796077e-01

6639 8.544961e-01 9.794737e-01

6640 8.540419e-01 9.793346e-01

6641 8.535824e-01 9.791903e-01

6642 8.531178e-01 9.790405e-01

6643 8.526483e-01 9.788851e-01

6644 8.521741e-01 9.787239e-01

6645 8.516955e-01 9.785569e-01

6646 8.512127e-01 9.783837e-01

6647 8.507259e-01 9.782044e-01

6648 8.502353e-01 9.780187e-01

6649 8.497413e-01 9.778264e-01

6650 8.492442e-01 9.776274e-01

6651 8.487440e-01 9.774216e-01

6652 8.482412e-01 9.772088e-01

6653 8.477360e-01 9.769889e-01

6654 8.472287e-01 9.767617e-01

6655 8.467195e-01 9.765271e-01

6656 8.462087e-01 9.762849e-01

6657 8.456967e-01 9.760351e-01

6658 8.451836e-01 9.757775e-01

6659 8.446697e-01 9.755119e-01

6660 8.441554e-01 9.752384e-01

6661 8.436408e-01 9.749568e-01

6662 8.436311e-01 9.749565e-01

6663 8.436210e-01 9.749562e-01

6664 8.436105e-01 9.749559e-01

6665 8.435997e-01 9.749556e-01

6666 8.435884e-01 9.749553e-01

6667 8.435767e-01 9.749549e-01

6668 8.435645e-01 9.749546e-01

6669 8.435519e-01 9.749542e-01

6670 8.435388e-01 9.749539e-01

6671 8.435252e-01 9.749535e-01

6672 8.435110e-01 9.749531e-01

6673 8.434964e-01 9.749527e-01

6674 8.434811e-01 9.749523e-01

6675 8.434654e-01 9.749518e-01

6676 8.434490e-01 9.749514e-01

6677 8.434319e-01 9.749509e-01

6678 8.434143e-01 9.749504e-01

6679 8.433959e-01 9.749499e-01

6680 8.433769e-01 9.749494e-01

6681 8.433572e-01 9.749489e-01

6682 8.433367e-01 9.749483e-01

6683 8.433154e-01 9.749477e-01

6684 8.432933e-01 9.749471e-01

6685 8.432704e-01 9.749465e-01

6686 8.432466e-01 9.749459e-01

6687 8.432219e-01 9.749452e-01

6688 8.431963e-01 9.749445e-01

6689 8.431697e-01 9.749438e-01

6690 8.431421e-01 9.749430e-01

6691 8.431135e-01 9.749423e-01

6692 8.430838e-01 9.749415e-01

6693 8.430530e-01 9.749406e-01

6694 8.430210e-01 9.749398e-01

6695 8.429878e-01 9.749389e-01

6696 8.429533e-01 9.749380e-01

6697 8.429175e-01 9.749370e-01

6698 8.428805e-01 9.749360e-01

6699 8.428420e-01 9.749350e-01

6700 8.428020e-01 9.749339e-01

6701 8.427606e-01 9.749328e-01

6702 8.427176e-01 9.749317e-01

6703 8.426730e-01 9.749305e-01

6704 8.426267e-01 9.749293e-01

6705 8.425787e-01 9.749280e-01

6706 8.425289e-01 9.749267e-01

6707 8.424773e-01 9.749253e-01

6708 8.424237e-01 9.749238e-01

6709 8.423681e-01 9.749224e-01

6710 8.423105e-01 9.749208e-01

6711 8.422507e-01 9.749192e-01

6712 8.421887e-01 9.749176e-01

6713 8.421244e-01 9.749158e-01

6714 8.420578e-01 9.749140e-01

6715 8.419887e-01 9.749122e-01

6716 8.419170e-01 9.749103e-01

6717 8.418427e-01 9.749083e-01

6718 8.417656e-01 9.749062e-01

6719 8.416858e-01 9.749040e-01

6720 8.416030e-01 9.749018e-01

6721 8.415172e-01 9.748994e-01

6722 8.414283e-01 9.748970e-01

6723 8.413361e-01 9.748945e-01

6724 8.412406e-01 9.748918e-01

6725 8.411417e-01 9.748891e-01

6726 8.410391e-01 9.748863e-01

6727 8.409329e-01 9.748833e-01

6728 8.408229e-01 9.748803e-01

6729 8.407089e-01 9.748771e-01

6730 8.405909e-01 9.748738e-01

6731 8.404687e-01 9.748703e-01

6732 8.403421e-01 9.748667e-01

6733 8.402110e-01 9.748630e-01

6734 8.400754e-01 9.748591e-01

6735 8.399350e-01 9.748550e-01

6736 8.397896e-01 9.748508e-01

6737 8.396392e-01 9.748465e-01

6738 8.394836e-01 9.748419e-01

6739 8.393227e-01 9.748372e-01

6740 8.391561e-01 9.748322e-01

6741 8.389839e-01 9.748271e-01

6742 8.388058e-01 9.748217e-01

6743 8.386217e-01 9.748161e-01

6744 8.384314e-01 9.748103e-01

6745 8.382347e-01 9.748042e-01

6746 8.380314e-01 9.747979e-01

6747 8.378213e-01 9.747914e-01

6748 8.376044e-01 9.747845e-01

6749 8.373803e-01 9.747774e-01

6750 8.371490e-01 9.747700e-01

6751 8.369102e-01 9.747622e-01

6752 8.366637e-01 9.747541e-01

6753 8.364093e-01 9.747457e-01

6754 8.361469e-01 9.747369e-01

6755 8.358763e-01 9.747278e-01

6756 8.355972e-01 9.747183e-01

6757 8.353095e-01 9.747083e-01

6758 8.350130e-01 9.746980e-01

6759 8.347076e-01 9.746872e-01

6760 8.343929e-01 9.746759e-01

6761 8.340689e-01 9.746642e-01

6762 8.337354e-01 9.746519e-01

6763 8.333921e-01 9.746391e-01

6764 8.330390e-01 9.746258e-01

6765 8.326758e-01 9.746119e-01

6766 8.323024e-01 9.745974e-01

6767 8.319186e-01 9.745823e-01

6768 8.315242e-01 9.745666e-01

6769 8.311192e-01 9.745502e-01

6770 8.307034e-01 9.745330e-01

6771 8.302766e-01 9.745152e-01

6772 8.298387e-01 9.744965e-01

6773 8.293896e-01 9.744771e-01

6774 8.289293e-01 9.744568e-01

6775 8.284576e-01 9.744357e-01

6776 8.279744e-01 9.744137e-01

6777 8.274796e-01 9.743907e-01

6778 8.269733e-01 9.743668e-01

6779 8.264553e-01 9.743418e-01

6780 8.259257e-01 9.743158e-01

6781 8.253844e-01 9.742887e-01

6782 8.248314e-01 9.742604e-01

6783 8.242667e-01 9.742309e-01

6784 8.236904e-01 9.742002e-01

6785 8.231025e-01 9.741682e-01

6786 8.225030e-01 9.741349e-01

6787 8.218922e-01 9.741002e-01

6788 8.212699e-01 9.740640e-01

6789 8.206365e-01 9.740263e-01

6790 8.199919e-01 9.739871e-01

6791 8.193365e-01 9.739463e-01

6792 8.186703e-01 9.739038e-01

6793 8.179935e-01 9.738595e-01

6794 8.173063e-01 9.738135e-01

6795 8.166091e-01 9.737656e-01

6796 8.159019e-01 9.737158e-01

6797 8.151852e-01 9.736640e-01

6798 8.144592e-01 9.736102e-01

6799 8.137241e-01 9.735542e-01

6800 8.129804e-01 9.734960e-01

6801 8.122283e-01 9.734355e-01

6802 8.114683e-01 9.733727e-01

6803 8.107006e-01 9.733075e-01

6804 8.099257e-01 9.732398e-01

6805 8.091440e-01 9.731695e-01

6806 8.083559e-01 9.730966e-01

6807 8.075618e-01 9.730209e-01

6808 8.067621e-01 9.729425e-01

6809 8.059574e-01 9.728611e-01

6810 8.051480e-01 9.727768e-01

6811 8.043345e-01 9.726895e-01

6812 8.035173e-01 9.725990e-01

6813 8.026969e-01 9.725054e-01

6814 8.018737e-01 9.724084e-01

6815 8.010484e-01 9.723081e-01

6816 8.002213e-01 9.722044e-01

6817 7.993930e-01 9.720972e-01

6818 7.985639e-01 9.719864e-01

6819 7.977345e-01 9.718719e-01

6820 7.969054e-01 9.717537e-01

6821 7.960771e-01 9.716317e-01

6822 7.952499e-01 9.715059e-01

6823 7.944244e-01 9.713761e-01

6824 7.936011e-01 9.712424e-01

6825 7.927804e-01 9.711046e-01

6826 7.919628e-01 9.709627e-01

6827 7.911486e-01 9.708167e-01

6828 7.903385e-01 9.706665e-01

6829 7.895326e-01 9.705120e-01

6830 7.887316e-01 9.703533e-01

6831 7.879357e-01 9.701904e-01

6832 7.871454e-01 9.700231e-01

6833 7.863609e-01 9.698515e-01

6834 7.855828e-01 9.696755e-01

6835 7.848112e-01 9.694953e-01

6836 7.840466e-01 9.693107e-01

6837 7.832892e-01 9.691218e-01

6838 7.825393e-01 9.689286e-01

6839 7.817972e-01 9.687312e-01

6840 7.810632e-01 9.685295e-01

6841 7.803374e-01 9.683237e-01

6842 7.803109e-01 9.683236e-01

6843 7.802834e-01 9.683234e-01

6844 7.802550e-01 9.683232e-01

6845 7.802256e-01 9.683230e-01

6846 7.801952e-01 9.683227e-01

6847 7.801638e-01 9.683225e-01

6848 7.801313e-01 9.683223e-01

6849 7.800977e-01 9.683221e-01

6850 7.800630e-01 9.683218e-01

6851 7.800270e-01 9.683216e-01

6852 7.799899e-01 9.683213e-01

6853 7.799514e-01 9.683210e-01

6854 7.799117e-01 9.683208e-01

6855 7.798707e-01 9.683205e-01

6856 7.798282e-01 9.683202e-01

6857 7.797843e-01 9.683199e-01

6858 7.797389e-01 9.683196e-01

6859 7.796920e-01 9.683193e-01

6860 7.796435e-01 9.683189e-01

6861 7.795934e-01 9.683186e-01

6862 7.795416e-01 9.683183e-01

6863 7.794880e-01 9.683179e-01

6864 7.794326e-01 9.683175e-01

6865 7.793754e-01 9.683171e-01

6866 7.793162e-01 9.683167e-01

6867 7.792550e-01 9.683163e-01

6868 7.791918e-01 9.683159e-01

6869 7.791265e-01 9.683155e-01

6870 7.790589e-01 9.683150e-01

6871 7.789891e-01 9.683145e-01

6872 7.789170e-01 9.683141e-01

6873 7.788424e-01 9.683136e-01

6874 7.787654e-01 9.683131e-01

6875 7.786858e-01 9.683125e-01

6876 7.786035e-01 9.683120e-01

6877 7.785185e-01 9.683114e-01

6878 7.784306e-01 9.683108e-01

6879 7.783399e-01 9.683102e-01

6880 7.782461e-01 9.683096e-01

6881 7.781492e-01 9.683089e-01

6882 7.780491e-01 9.683082e-01

6883 7.779457e-01 9.683075e-01

6884 7.778389e-01 9.683068e-01

6885 7.777286e-01 9.683061e-01

6886 7.776146e-01 9.683053e-01

6887 7.774970e-01 9.683045e-01

6888 7.773754e-01 9.683037e-01

6889 7.772500e-01 9.683028e-01

6890 7.771204e-01 9.683019e-01

6891 7.769866e-01 9.683010e-01

6892 7.768485e-01 9.683000e-01

6893 7.767060e-01 9.682990e-01

6894 7.765588e-01 9.682980e-01

6895 7.764070e-01 9.682970e-01

6896 7.762503e-01 9.682959e-01

6897 7.760886e-01 9.682947e-01

6898 7.759217e-01 9.682935e-01

6899 7.757496e-01 9.682923e-01

6900 7.755721e-01 9.682910e-01

6901 7.753890e-01 9.682897e-01

6902 7.752001e-01 9.682883e-01

6903 7.750054e-01 9.682869e-01

6904 7.748047e-01 9.682854e-01

6905 7.745977e-01 9.682838e-01

6906 7.743845e-01 9.682822e-01

6907 7.741647e-01 9.682806e-01

6908 7.739382e-01 9.682789e-01

6909 7.737049e-01 9.682771e-01

6910 7.734647e-01 9.682752e-01

6911 7.732172e-01 9.682733e-01

6912 7.729624e-01 9.682713e-01

6913 7.727001e-01 9.682692e-01

6914 7.724302e-01 9.682670e-01

6915 7.721524e-01 9.682647e-01

6916 7.718666e-01 9.682624e-01

6917 7.715727e-01 9.682600e-01

6918 7.712704e-01 9.682574e-01

6919 7.709596e-01 9.682548e-01

6920 7.706401e-01 9.682521e-01

6921 7.703119e-01 9.682492e-01

6922 7.699746e-01 9.682463e-01

6923 7.696282e-01 9.682432e-01

6924 7.692726e-01 9.682400e-01

6925 7.689075e-01 9.682366e-01

6926 7.685328e-01 9.682332e-01

6927 7.681484e-01 9.682296e-01

6928 7.677542e-01 9.682258e-01

6929 7.673500e-01 9.682219e-01

6930 7.669357e-01 9.682179e-01

6931 7.665112e-01 9.682137e-01

6932 7.660763e-01 9.682093e-01

6933 7.656311e-01 9.682047e-01

6934 7.651754e-01 9.682000e-01

6935 7.647091e-01 9.681951e-01

6936 7.642322e-01 9.681899e-01

6937 7.637446e-01 9.681846e-01

6938 7.632462e-01 9.681790e-01

6939 7.627371e-01 9.681733e-01

6940 7.622172e-01 9.681673e-01

6941 7.616864e-01 9.681610e-01

6942 7.611449e-01 9.681545e-01

6943 7.605926e-01 9.681478e-01

6944 7.600296e-01 9.681408e-01

6945 7.594559e-01 9.681335e-01

6946 7.588715e-01 9.681259e-01

6947 7.582766e-01 9.681180e-01

6948 7.576713e-01 9.681098e-01

6949 7.570557e-01 9.681013e-01

6950 7.564299e-01 9.680924e-01

6951 7.557941e-01 9.680832e-01

6952 7.551484e-01 9.680737e-01

6953 7.544930e-01 9.680638e-01

6954 7.538282e-01 9.680535e-01

6955 7.531542e-01 9.680428e-01

6956 7.524711e-01 9.680316e-01

6957 7.517793e-01 9.680201e-01

6958 7.510791e-01 9.680081e-01

6959 7.503707e-01 9.679957e-01

6960 7.496545e-01 9.679828e-01

6961 7.489307e-01 9.679694e-01

6962 7.481998e-01 9.679556e-01

6963 7.474621e-01 9.679412e-01

6964 7.467179e-01 9.679263e-01

6965 7.459678e-01 9.679108e-01

6966 7.452120e-01 9.678948e-01

6967 7.444510e-01 9.678782e-01

6968 7.436852e-01 9.678610e-01

6969 7.429151e-01 9.678432e-01

6970 7.421411e-01 9.678248e-01

6971 7.413637e-01 9.678058e-01

6972 7.405833e-01 9.677861e-01

6973 7.398005e-01 9.677657e-01

6974 7.390156e-01 9.677446e-01

6975 7.382293e-01 9.677229e-01

6976 7.374420e-01 9.677004e-01

6977 7.366541e-01 9.676772e-01

6978 7.358662e-01 9.676532e-01

6979 7.350787e-01 9.676285e-01

6980 7.342922e-01 9.676030e-01

6981 7.335071e-01 9.675767e-01

6982 7.327239e-01 9.675496e-01

6983 7.319431e-01 9.675218e-01

6984 7.311651e-01 9.674931e-01

6985 7.303905e-01 9.674636e-01

6986 7.296196e-01 9.674332e-01

6987 7.288529e-01 9.674020e-01

6988 7.280909e-01 9.673700e-01

6989 7.273340e-01 9.673371e-01

6990 7.265824e-01 9.673034e-01

6991 7.258368e-01 9.672688e-01

6992 7.250974e-01 9.672334e-01

6993 7.243646e-01 9.671971e-01

6994 7.236387e-01 9.671599e-01

6995 7.229201e-01 9.671220e-01

6996 7.222091e-01 9.670832e-01

6997 7.215060e-01 9.670436e-01

6998 7.208111e-01 9.670031e-01

6999 7.201246e-01 9.669619e-01

7000 7.194468e-01 9.669199e-01

7001 7.187779e-01 9.668772e-01

7002 7.181182e-01 9.668337e-01

7003 7.174677e-01 9.667896e-01

7004 7.168268e-01 9.667447e-01

7005 7.161955e-01 9.666992e-01

7006 7.155740e-01 9.666532e-01

7007 7.149624e-01 9.666065e-01

7008 7.143608e-01 9.665593e-01

7009 7.137694e-01 9.665117e-01

7010 7.131881e-01 9.664636e-01

7011 7.126171e-01 9.664151e-01

7012 7.120564e-01 9.663663e-01

7013 7.115061e-01 9.663173e-01

7014 7.109661e-01 9.662680e-01

7015 7.104364e-01 9.662186e-01

7016 7.099172e-01 9.661691e-01

7017 7.094082e-01 9.661196e-01

7018 7.089095e-01 9.660701e-01

7019 7.084211e-01 9.660204e-01

7020 7.079429e-01 9.659706e-01

7021 7.074749e-01 9.659204e-01

7022 7.074362e-01 9.659204e-01

7023 7.073965e-01 9.659203e-01

7024 7.073557e-01 9.659203e-01

7025 7.073137e-01 9.659202e-01

7026 7.072706e-01 9.659202e-01

7027 7.072264e-01 9.659201e-01

7028 7.071810e-01 9.659201e-01

7029 7.071343e-01 9.659200e-01

7030 7.070864e-01 9.659200e-01

7031 7.070372e-01 9.659199e-01

7032 7.069868e-01 9.659199e-01

7033 7.069350e-01 9.659198e-01

7034 7.068818e-01 9.659197e-01

7035 7.068273e-01 9.659197e-01

7036 7.067713e-01 9.659196e-01

7037 7.067139e-01 9.659195e-01

7038 7.066550e-01 9.659194e-01

7039 7.065946e-01 9.659193e-01

7040 7.065326e-01 9.659192e-01

7041 7.064691e-01 9.659191e-01

7042 7.064039e-01 9.659190e-01

7043 7.063371e-01 9.659189e-01

7044 7.062686e-01 9.659188e-01

7045 7.061984e-01 9.659186e-01

7046 7.061263e-01 9.659185e-01

7047 7.060525e-01 9.659184e-01

7048 7.059769e-01 9.659182e-01

7049 7.058994e-01 9.659180e-01

7050 7.058199e-01 9.659179e-01

7051 7.057385e-01 9.659177e-01

7052 7.056551e-01 9.659175e-01

7053 7.055697e-01 9.659173e-01

7054 7.054822e-01 9.659170e-01

7055 7.053926e-01 9.659168e-01

7056 7.053008e-01 9.659165e-01

7057 7.052068e-01 9.659163e-01

7058 7.051106e-01 9.659160e-01

7059 7.050121e-01 9.659157e-01

7060 7.049113e-01 9.659154e-01

7061 7.048081e-01 9.659150e-01

7062 7.047026e-01 9.659147e-01

7063 7.045946e-01 9.659143e-01

7064 7.044841e-01 9.659139e-01

7065 7.043711e-01 9.659134e-01

7066 7.042555e-01 9.659130e-01

7067 7.041374e-01 9.659125e-01

7068 7.040166e-01 9.659120e-01

7069 7.038931e-01 9.659114e-01

7070 7.037669e-01 9.659109e-01

7071 7.036380e-01 9.659102e-01

7072 7.035063e-01 9.659096e-01

7073 7.033718e-01 9.659089e-01

7074 7.032345e-01 9.659082e-01

7075 7.030942e-01 9.659074e-01

7076 7.029511e-01 9.659066e-01

7077 7.028050e-01 9.659057e-01

7078 7.026560e-01 9.659048e-01

7079 7.025040e-01 9.659039e-01

7080 7.023489e-01 9.659028e-01

7081 7.021908e-01 9.659018e-01

7082 7.020297e-01 9.659006e-01

7083 7.018655e-01 9.658994e-01

7084 7.016983e-01 9.658981e-01

7085 7.015279e-01 9.658968e-01

7086 7.013544e-01 9.658954e-01

7087 7.011778e-01 9.658938e-01

7088 7.009982e-01 9.658923e-01

7089 7.008154e-01 9.658906e-01

7090 7.006294e-01 9.658888e-01

7091 7.004404e-01 9.658869e-01

7092 7.002483e-01 9.658849e-01

7093 7.000531e-01 9.658828e-01

7094 6.998549e-01 9.658806e-01

7095 6.996536e-01 9.658783e-01

7096 6.994493e-01 9.658758e-01

7097 6.992420e-01 9.658732e-01

7098 6.990318e-01 9.658704e-01

7099 6.988187e-01 9.658675e-01

7100 6.986027e-01 9.658645e-01

7101 6.983840e-01 9.658613e-01

7102 6.981624e-01 9.658579e-01

7103 6.979382e-01 9.658543e-01

7104 6.977114e-01 9.658505e-01

7105 6.974820e-01 9.658465e-01

7106 6.972501e-01 9.658423e-01

7107 6.970158e-01 9.658379e-01

7108 6.967792e-01 9.658332e-01

7109 6.965404e-01 9.658283e-01

7110 6.962995e-01 9.658231e-01

7111 6.960566e-01 9.658176e-01

7112 6.958118e-01 9.658119e-01

7113 6.955651e-01 9.658058e-01

7114 6.953168e-01 9.657995e-01

7115 6.950670e-01 9.657928e-01

7116 6.948158e-01 9.657857e-01

7117 6.945632e-01 9.657783e-01

7118 6.943095e-01 9.657705e-01

7119 6.940549e-01 9.657623e-01

7120 6.937993e-01 9.657536e-01

7121 6.935431e-01 9.657446e-01

7122 6.932864e-01 9.657350e-01

7123 6.930292e-01 9.657250e-01

7124 6.927719e-01 9.657144e-01

7125 6.925145e-01 9.657034e-01

7126 6.922573e-01 9.656917e-01

7127 6.920003e-01 9.656795e-01

7128 6.917438e-01 9.656667e-01

7129 6.914880e-01 9.656532e-01

7130 6.912330e-01 9.656390e-01

7131 6.909789e-01 9.656242e-01

7132 6.907261e-01 9.656086e-01

7133 6.904746e-01 9.655922e-01

7134 6.902247e-01 9.655751e-01

7135 6.899765e-01 9.655571e-01

7136 6.897301e-01 9.655382e-01

7137 6.894858e-01 9.655184e-01

7138 6.892438e-01 9.654977e-01

7139 6.890041e-01 9.654760e-01

7140 6.887670e-01 9.654532e-01

7141 6.885326e-01 9.654294e-01

7142 6.883012e-01 9.654044e-01

7143 6.880727e-01 9.653783e-01

7144 6.878474e-01 9.653509e-01

7145 6.876254e-01 9.653223e-01

7146 6.874069e-01 9.652924e-01

7147 6.871920e-01 9.652611e-01

7148 6.869808e-01 9.652284e-01

7149 6.867734e-01 9.651942e-01

7150 6.865699e-01 9.651585e-01

7151 6.863705e-01 9.651212e-01

7152 6.861752e-01 9.650823e-01

7153 6.859841e-01 9.650416e-01

7154 6.857974e-01 9.649992e-01

7155 6.856150e-01 9.649549e-01

7156 6.854370e-01 9.649088e-01

7157 6.852635e-01 9.648607e-01

7158 6.850946e-01 9.648105e-01

7159 6.849303e-01 9.647583e-01

7160 6.847705e-01 9.647039e-01

7161 6.846154e-01 9.646473e-01

7162 6.844650e-01 9.645884e-01

7163 6.843192e-01 9.645272e-01

7164 6.841781e-01 9.644635e-01

7165 6.840417e-01 9.643973e-01

7166 6.839099e-01 9.643285e-01

7167 6.837828e-01 9.642571e-01

7168 6.836602e-01 9.641829e-01

7169 6.835423e-01 9.641060e-01

7170 6.834289e-01 9.640262e-01

7171 6.833200e-01 9.639435e-01

7172 6.832156e-01 9.638578e-01

7173 6.831155e-01 9.637690e-01

7174 6.830199e-01 9.636771e-01

7175 6.829285e-01 9.635820e-01

7176 6.828413e-01 9.634836e-01

7177 6.827582e-01 9.633819e-01

7178 6.826793e-01 9.632768e-01

7179 6.826044e-01 9.631682e-01

7180 6.825333e-01 9.630562e-01

7181 6.824662e-01 9.629406e-01

7182 6.824028e-01 9.628214e-01

7183 6.823430e-01 9.626985e-01

7184 6.822869e-01 9.625719e-01

7185 6.822343e-01 9.624416e-01

7186 6.821851e-01 9.623074e-01

7187 6.821392e-01 9.621695e-01

7188 6.820966e-01 9.620277e-01

7189 6.820571e-01 9.618821e-01

7190 6.820207e-01 9.617326e-01

7191 6.819873e-01 9.615791e-01

7192 6.819567e-01 9.614218e-01

7193 6.819290e-01 9.612606e-01

7194 6.819039e-01 9.610954e-01

7195 6.818815e-01 9.609264e-01

7196 6.818616e-01 9.607535e-01

7197 6.818442e-01 9.605767e-01

7198 6.818292e-01 9.603962e-01

7199 6.818164e-01 9.602122e-01

7200 6.818059e-01 9.600249e-01

7201 6.817975e-01 9.598345e-01

7202 6.817983e-01 9.598343e-01

7203 6.817994e-01 9.598340e-01

7204 6.818010e-01 9.598337e-01

7205 6.818029e-01 9.598334e-01

7206 6.818052e-01 9.598331e-01

7207 6.818080e-01 9.598328e-01

7208 6.818112e-01 9.598325e-01

7209 6.818149e-01 9.598321e-01

7210 6.818191e-01 9.598318e-01

7211 6.818239e-01 9.598314e-01

7212 6.818292e-01 9.598310e-01

7213 6.818352e-01 9.598306e-01

7214 6.818417e-01 9.598302e-01

7215 6.818489e-01 9.598298e-01

7216 6.818568e-01 9.598294e-01

7217 6.818654e-01 9.598289e-01

7218 6.818747e-01 9.598284e-01

7219 6.818849e-01 9.598279e-01

7220 6.818958e-01 9.598274e-01

7221 6.819077e-01 9.598269e-01

7222 6.819204e-01 9.598264e-01

7223 6.819341e-01 9.598258e-01

7224 6.819487e-01 9.598252e-01

7225 6.819644e-01 9.598246e-01

7226 6.819812e-01 9.598240e-01

7227 6.819991e-01 9.598233e-01

7228 6.820182e-01 9.598227e-01

7229 6.820385e-01 9.598220e-01

7230 6.820601e-01 9.598212e-01

7231 6.820830e-01 9.598205e-01

7232 6.821074e-01 9.598197e-01

7233 6.821331e-01 9.598189e-01

7234 6.821604e-01 9.598180e-01

7235 6.821893e-01 9.598172e-01

7236 6.822198e-01 9.598163e-01

7237 6.822520e-01 9.598153e-01

7238 6.822860e-01 9.598143e-01

7239 6.823219e-01 9.598133e-01

7240 6.823596e-01 9.598123e-01

7241 6.823994e-01 9.598112e-01

7242 6.824413e-01 9.598101e-01

7243 6.824853e-01 9.598089e-01

7244 6.825315e-01 9.598077e-01

7245 6.825801e-01 9.598064e-01

7246 6.826311e-01 9.598051e-01

7247 6.826846e-01 9.598037e-01

7248 6.827407e-01 9.598023e-01

7249 6.827995e-01 9.598008e-01

7250 6.828611e-01 9.597993e-01

7251 6.829256e-01 9.597977e-01

7252 6.829931e-01 9.597961e-01

7253 6.830637e-01 9.597944e-01

7254 6.831375e-01 9.597926e-01

7255 6.832146e-01 9.597907e-01

7256 6.832952e-01 9.597888e-01

7257 6.833793e-01 9.597868e-01

7258 6.834671e-01 9.597848e-01

7259 6.835587e-01 9.597826e-01

7260 6.836542e-01 9.597804e-01

7261 6.837537e-01 9.597781e-01

7262 6.838575e-01 9.597757e-01

7263 6.839655e-01 9.597732e-01

7264 6.840779e-01 9.597706e-01

7265 6.841950e-01 9.597679e-01

7266 6.843167e-01 9.597651e-01

7267 6.844432e-01 9.597622e-01

7268 6.845748e-01 9.597592e-01

7269 6.847115e-01 9.597561e-01

7270 6.848534e-01 9.597529e-01

7271 6.850007e-01 9.597495e-01

7272 6.851536e-01 9.597460e-01

7273 6.853122e-01 9.597424e-01

7274 6.854767e-01 9.597386e-01

7275 6.856471e-01 9.597347e-01

7276 6.858237e-01 9.597306e-01

7277 6.860066e-01 9.597264e-01

7278 6.861959e-01 9.597220e-01

7279 6.863918e-01 9.597175e-01

7280 6.865944e-01 9.597127e-01

7281 6.868038e-01 9.597078e-01

7282 6.870203e-01 9.597028e-01

7283 6.872440e-01 9.596975e-01

7284 6.874749e-01 9.596920e-01

7285 6.877133e-01 9.596864e-01

7286 6.879593e-01 9.596805e-01

7287 6.882129e-01 9.596744e-01

7288 6.884743e-01 9.596680e-01

7289 6.887437e-01 9.596615e-01

7290 6.890212e-01 9.596547e-01

7291 6.893068e-01 9.596476e-01

7292 6.896008e-01 9.596403e-01

7293 6.899031e-01 9.596327e-01

7294 6.902139e-01 9.596249e-01

7295 6.905332e-01 9.596167e-01

7296 6.908613e-01 9.596083e-01

7297 6.911980e-01 9.595995e-01

7298 6.915436e-01 9.595905e-01

7299 6.918980e-01 9.595811e-01

7300 6.922614e-01 9.595714e-01

7301 6.926337e-01 9.595614e-01

7302 6.930150e-01 9.595510e-01

7303 6.934053e-01 9.595402e-01

7304 6.938047e-01 9.595291e-01

7305 6.942131e-01 9.595176e-01

7306 6.946305e-01 9.595057e-01

7307 6.950569e-01 9.594934e-01

7308 6.954923e-01 9.594807e-01

7309 6.959366e-01 9.594676e-01

7310 6.963898e-01 9.594540e-01

7311 6.968519e-01 9.594400e-01

7312 6.973227e-01 9.594256e-01

7313 6.978021e-01 9.594106e-01

7314 6.982901e-01 9.593952e-01

7315 6.987865e-01 9.593793e-01

7316 6.992912e-01 9.593629e-01

7317 6.998041e-01 9.593460e-01

7318 7.003250e-01 9.593286e-01

7319 7.008537e-01 9.593107e-01

7320 7.013902e-01 9.592922e-01

7321 7.019341e-01 9.592732e-01

7322 7.024853e-01 9.592536e-01

7323 7.030436e-01 9.592334e-01

7324 7.036087e-01 9.592127e-01

7325 7.041805e-01 9.591914e-01

7326 7.047586e-01 9.591695e-01

7327 7.053428e-01 9.591470e-01

7328 7.059329e-01 9.591239e-01

7329 7.065286e-01 9.591003e-01

7330 7.071296e-01 9.590760e-01

7331 7.077356e-01 9.590511e-01

7332 7.083463e-01 9.590255e-01

7333 7.089614e-01 9.589994e-01

7334 7.095806e-01 9.589727e-01

7335 7.102036e-01 9.589453e-01

7336 7.108300e-01 9.589173e-01

7337 7.114596e-01 9.588887e-01

7338 7.120920e-01 9.588595e-01

7339 7.127268e-01 9.588297e-01

7340 7.133638e-01 9.587993e-01

7341 7.140026e-01 9.587683e-01

7342 7.146428e-01 9.587368e-01

7343 7.152841e-01 9.587047e-01

7344 7.159263e-01 9.586720e-01

7345 7.165688e-01 9.586388e-01

7346 7.172115e-01 9.586051e-01

7347 7.178540e-01 9.585709e-01

7348 7.184960e-01 9.585362e-01

7349 7.191370e-01 9.585011e-01

7350 7.197769e-01 9.584655e-01

7351 7.204153e-01 9.584296e-01

7352 7.210518e-01 9.583933e-01

7353 7.216863e-01 9.583567e-01

7354 7.223183e-01 9.583197e-01

7355 7.229477e-01 9.582826e-01

7356 7.235740e-01 9.582452e-01

7357 7.241971e-01 9.582076e-01

7358 7.248167e-01 9.581699e-01

7359 7.254325e-01 9.581321e-01

7360 7.260443e-01 9.580944e-01

7361 7.266518e-01 9.580566e-01

7362 7.272549e-01 9.580189e-01

7363 7.278532e-01 9.579814e-01

7364 7.284466e-01 9.579441e-01

7365 7.290349e-01 9.579071e-01

7366 7.296179e-01 9.578704e-01

7367 7.301954e-01 9.578341e-01

7368 7.307673e-01 9.577983e-01

7369 7.313333e-01 9.577631e-01

7370 7.318934e-01 9.577285e-01

7371 7.324474e-01 9.576946e-01

7372 7.329952e-01 9.576615e-01

7373 7.335367e-01 9.576292e-01

7374 7.340717e-01 9.575980e-01

7375 7.346001e-01 9.575677e-01

7376 7.351220e-01 9.575386e-01

7377 7.356371e-01 9.575107e-01

7378 7.361455e-01 9.574841e-01

7379 7.366471e-01 9.574589e-01

7380 7.371417e-01 9.574350e-01

7381 7.376293e-01 9.574124e-01

7382 7.376682e-01 9.574123e-01

7383 7.377083e-01 9.574121e-01

7384 7.377496e-01 9.574120e-01

7385 7.377921e-01 9.574118e-01

7386 7.378359e-01 9.574117e-01

7387 7.378810e-01 9.574115e-01

7388 7.379275e-01 9.574114e-01

7389 7.379753e-01 9.574112e-01

7390 7.380246e-01 9.574110e-01

7391 7.380753e-01 9.574108e-01

7392 7.381274e-01 9.574106e-01

7393 7.381811e-01 9.574104e-01

7394 7.382363e-01 9.574102e-01

7395 7.382932e-01 9.574099e-01

7396 7.383516e-01 9.574097e-01

7397 7.384118e-01 9.574094e-01

7398 7.384737e-01 9.574092e-01

7399 7.385373e-01 9.574089e-01

7400 7.386028e-01 9.574086e-01

7401 7.386702e-01 9.574083e-01

7402 7.387394e-01 9.574079e-01

7403 7.388107e-01 9.574076e-01

7404 7.388839e-01 9.574072e-01

7405 7.389592e-01 9.574068e-01

7406 7.390367e-01 9.574064e-01

7407 7.391163e-01 9.574060e-01

7408 7.391982e-01 9.574055e-01

7409 7.392824e-01 9.574050e-01

7410 7.393689e-01 9.574045e-01

7411 7.394579e-01 9.574040e-01

7412 7.395494e-01 9.574035e-01

7413 7.396434e-01 9.574029e-01

7414 7.397400e-01 9.574023e-01

7415 7.398393e-01 9.574017e-01

7416 7.399414e-01 9.574010e-01

7417 7.400463e-01 9.574003e-01

7418 7.401542e-01 9.573995e-01

7419 7.402649e-01 9.573988e-01

7420 7.403788e-01 9.573979e-01

7421 7.404957e-01 9.573971e-01

7422 7.406159e-01 9.573962e-01

7423 7.407394e-01 9.573952e-01

7424 7.408662e-01 9.573943e-01

7425 7.409965e-01 9.573932e-01

7426 7.411303e-01 9.573921e-01

7427 7.412678e-01 9.573910e-01

7428 7.414089e-01 9.573898e-01

7429 7.415539e-01 9.573885e-01

7430 7.417028e-01 9.573872e-01

7431 7.418556e-01 9.573858e-01

7432 7.420126e-01 9.573844e-01

7433 7.421737e-01 9.573828e-01

7434 7.423391e-01 9.573812e-01

7435 7.425089e-01 9.573796e-01

7436 7.426832e-01 9.573778e-01

7437 7.428620e-01 9.573760e-01

7438 7.430456e-01 9.573740e-01

7439 7.432339e-01 9.573720e-01

7440 7.434272e-01 9.573699e-01

7441 7.436254e-01 9.573677e-01

7442 7.438288e-01 9.573653e-01

7443 7.440374e-01 9.573629e-01

7444 7.442514e-01 9.573604e-01

7445 7.444708e-01 9.573577e-01

7446 7.446959e-01 9.573549e-01

7447 7.449266e-01 9.573519e-01

7448 7.451631e-01 9.573489e-01

7449 7.454055e-01 9.573456e-01

7450 7.456540e-01 9.573423e-01

7451 7.459087e-01 9.573388e-01

7452 7.461697e-01 9.573351e-01

7453 7.464371e-01 9.573312e-01

7454 7.467110e-01 9.573272e-01

7455 7.469916e-01 9.573229e-01

7456 7.472789e-01 9.573185e-01

7457 7.475731e-01 9.573139e-01

7458 7.478744e-01 9.573090e-01

7459 7.481828e-01 9.573039e-01

7460 7.484985e-01 9.572986e-01

7461 7.488216e-01 9.572931e-01

7462 7.491521e-01 9.572873e-01

7463 7.494903e-01 9.572812e-01

7464 7.498362e-01 9.572749e-01

7465 7.501900e-01 9.572682e-01

7466 7.505518e-01 9.572613e-01

7467 7.509216e-01 9.572541e-01

7468 7.512996e-01 9.572465e-01

7469 7.516859e-01 9.572386e-01

7470 7.520807e-01 9.572303e-01

7471 7.524839e-01 9.572217e-01

7472 7.528957e-01 9.572127e-01

7473 7.533163e-01 9.572032e-01

7474 7.537456e-01 9.571934e-01

7475 7.541838e-01 9.571831e-01

7476 7.546310e-01 9.571724e-01

7477 7.550872e-01 9.571612e-01

7478 7.555525e-01 9.571495e-01

7479 7.560270e-01 9.571373e-01

7480 7.565108e-01 9.571246e-01

7481 7.570039e-01 9.571113e-01

7482 7.575063e-01 9.570974e-01

7483 7.580181e-01 9.570829e-01

7484 7.585394e-01 9.570679e-01

7485 7.590702e-01 9.570521e-01

7486 7.596104e-01 9.570357e-01

7487 7.601601e-01 9.570186e-01

7488 7.607194e-01 9.570008e-01

7489 7.612881e-01 9.569822e-01

7490 7.618664e-01 9.569629e-01

7491 7.624541e-01 9.569427e-01

7492 7.630514e-01 9.569217e-01

7493 7.636580e-01 9.568999e-01

7494 7.642740e-01 9.568771e-01

7495 7.648993e-01 9.568534e-01

7496 7.655338e-01 9.568287e-01

7497 7.661775e-01 9.568030e-01

7498 7.668303e-01 9.567763e-01

7499 7.674921e-01 9.567486e-01

7500 7.681627e-01 9.567197e-01

7501 7.688421e-01 9.566897e-01

7502 7.695301e-01 9.566584e-01

7503 7.702266e-01 9.566260e-01

7504 7.709314e-01 9.565923e-01

7505 7.716443e-01 9.565574e-01

7506 7.723651e-01 9.565210e-01

7507 7.730938e-01 9.564833e-01

7508 7.738300e-01 9.564442e-01

7509 7.745736e-01 9.564037e-01

7510 7.753243e-01 9.563616e-01

7511 7.760819e-01 9.563180e-01

7512 7.768462e-01 9.562728e-01

7513 7.776169e-01 9.562259e-01

7514 7.783937e-01 9.561774e-01

7515 7.791764e-01 9.561272e-01

7516 7.799646e-01 9.560753e-01

7517 7.807581e-01 9.560215e-01

7518 7.815566e-01 9.559659e-01

7519 7.823598e-01 9.559084e-01

7520 7.831673e-01 9.558490e-01

7521 7.839788e-01 9.557876e-01

7522 7.847940e-01 9.557243e-01

7523 7.856125e-01 9.556589e-01

7524 7.864340e-01 9.555914e-01

7525 7.872581e-01 9.555218e-01

7526 7.880845e-01 9.554500e-01

7527 7.889128e-01 9.553761e-01

7528 7.897427e-01 9.552999e-01

7529 7.905737e-01 9.552215e-01

7530 7.914055e-01 9.551408e-01

7531 7.922378e-01 9.550578e-01

7532 7.930701e-01 9.549724e-01

7533 7.939021e-01 9.548847e-01

7534 7.947334e-01 9.547946e-01

7535 7.955636e-01 9.547020e-01

7536 7.963924e-01 9.546071e-01

7537 7.972194e-01 9.545097e-01

7538 7.980441e-01 9.544099e-01

7539 7.988664e-01 9.543076e-01

7540 7.996857e-01 9.542028e-01

7541 8.005018e-01 9.540956e-01

7542 8.013142e-01 9.539859e-01

7543 8.021228e-01 9.538737e-01

7544 8.029270e-01 9.537591e-01

7545 8.037266e-01 9.536420e-01

7546 8.045213e-01 9.535225e-01

7547 8.053107e-01 9.534006e-01

7548 8.060945e-01 9.532762e-01

7549 8.068725e-01 9.531496e-01

7550 8.076444e-01 9.530205e-01

7551 8.084098e-01 9.528892e-01

7552 8.091686e-01 9.527556e-01

7553 8.099204e-01 9.526198e-01

7554 8.106651e-01 9.524818e-01

7555 8.114023e-01 9.523417e-01

7556 8.121319e-01 9.521995e-01

7557 8.128536e-01 9.520553e-01

7558 8.135673e-01 9.519091e-01

7559 8.142727e-01 9.517611e-01

7560 8.149698e-01 9.516114e-01

7561 8.156583e-01 9.514602e-01

7562 8.156809e-01 9.514598e-01

7563 8.157041e-01 9.514593e-01

7564 8.157279e-01 9.514588e-01

7565 8.157524e-01 9.514583e-01

7566 8.157775e-01 9.514578e-01

7567 8.158032e-01 9.514572e-01

7568 8.158296e-01 9.514567e-01

7569 8.158567e-01 9.514561e-01

7570 8.158845e-01 9.514555e-01

7571 8.159131e-01 9.514549e-01

7572 8.159424e-01 9.514542e-01

7573 8.159724e-01 9.514535e-01

7574 8.160033e-01 9.514528e-01

7575 8.160349e-01 9.514521e-01

7576 8.160674e-01 9.514513e-01

7577 8.161008e-01 9.514505e-01

7578 8.161350e-01 9.514497e-01

7579 8.161701e-01 9.514489e-01

7580 8.162061e-01 9.514480e-01

7581 8.162431e-01 9.514471e-01

7582 8.162811e-01 9.514461e-01

7583 8.163201e-01 9.514452e-01

7584 8.163601e-01 9.514442e-01

7585 8.164011e-01 9.514431e-01

7586 8.164433e-01 9.514420e-01

7587 8.164865e-01 9.514409e-01

7588 8.165309e-01 9.514397e-01

7589 8.165764e-01 9.514385e-01

7590 8.166232e-01 9.514372e-01

7591 8.166712e-01 9.514359e-01

7592 8.167204e-01 9.514346e-01

7593 8.167709e-01 9.514332e-01

7594 8.168228e-01 9.514317e-01

7595 8.168760e-01 9.514302e-01

7596 8.169306e-01 9.514287e-01

7597 8.169867e-01 9.514271e-01

7598 8.170442e-01 9.514254e-01

7599 8.171033e-01 9.514237e-01

7600 8.171638e-01 9.514219e-01

7601 8.172260e-01 9.514200e-01

7602 8.172898e-01 9.514181e-01

7603 8.173552e-01 9.514161e-01

7604 8.174224e-01 9.514141e-01

7605 8.174913e-01 9.514119e-01

7606 8.175620e-01 9.514097e-01

7607 8.176345e-01 9.514074e-01

7608 8.177089e-01 9.514051e-01

7609 8.177852e-01 9.514026e-01

7610 8.178635e-01 9.514001e-01

7611 8.179438e-01 9.513974e-01

7612 8.180262e-01 9.513947e-01

7613 8.181107e-01 9.513919e-01

7614 8.181974e-01 9.513890e-01

7615 8.182862e-01 9.513860e-01

7616 8.183774e-01 9.513829e-01

7617 8.184709e-01 9.513796e-01

7618 8.185667e-01 9.513763e-01

7619 8.186650e-01 9.513729e-01

7620 8.187657e-01 9.513693e-01

7621 8.188690e-01 9.513656e-01

7622 8.189749e-01 9.513618e-01

7623 8.190835e-01 9.513578e-01

7624 8.191948e-01 9.513538e-01

7625 8.193088e-01 9.513496e-01

7626 8.194257e-01 9.513452e-01

7627 8.195455e-01 9.513407e-01

7628 8.196682e-01 9.513360e-01

7629 8.197940e-01 9.513312e-01

7630 8.199229e-01 9.513263e-01

7631 8.200549e-01 9.513212e-01

7632 8.201901e-01 9.513159e-01

7633 8.203286e-01 9.513104e-01

7634 8.204704e-01 9.513048e-01

7635 8.206157e-01 9.512990e-01

7636 8.207645e-01 9.512929e-01

7637 8.209168e-01 9.512867e-01

7638 8.210727e-01 9.512804e-01

7639 8.212323e-01 9.512738e-01

7640 8.213957e-01 9.512670e-01

7641 8.215628e-01 9.512600e-01

7642 8.217339e-01 9.512527e-01

7643 8.219090e-01 9.512453e-01

7644 8.220881e-01 9.512376e-01

7645 8.222713e-01 9.512297e-01

7646 8.224587e-01 9.512216e-01

7647 8.226503e-01 9.512132e-01

7648 8.228463e-01 9.512045e-01

7649 8.230467e-01 9.511957e-01

7650 8.232515e-01 9.511865e-01

7651 8.234608e-01 9.511771e-01

7652 8.236747e-01 9.511675e-01

7653 8.238933e-01 9.511575e-01

7654 8.241167e-01 9.511473e-01

7655 8.243448e-01 9.511368e-01

7656 8.245778e-01 9.511260e-01

7657 8.248157e-01 9.511150e-01

7658 8.250586e-01 9.511036e-01

7659 8.253066e-01 9.510919e-01

7660 8.255597e-01 9.510800e-01

7661 8.258179e-01 9.510677e-01

7662 8.260814e-01 9.510551e-01

7663 8.263502e-01 9.510423e-01

7664 8.266243e-01 9.510291e-01

7665 8.269037e-01 9.510156e-01

7666 8.271886e-01 9.510017e-01

7667 8.274790e-01 9.509876e-01

7668 8.277749e-01 9.509731e-01

7669 8.280763e-01 9.509583e-01

7670 8.283834e-01 9.509433e-01

7671 8.286960e-01 9.509278e-01

7672 8.290143e-01 9.509121e-01

7673 8.293383e-01 9.508961e-01

7674 8.296680e-01 9.508797e-01

7675 8.300034e-01 9.508631e-01

7676 8.303445e-01 9.508461e-01

7677 8.306914e-01 9.508289e-01

7678 8.310439e-01 9.508113e-01

7679 8.314023e-01 9.507935e-01

7680 8.317663e-01 9.507755e-01

7681 8.321360e-01 9.507571e-01

7682 8.325115e-01 9.507385e-01

7683 8.328926e-01 9.507197e-01

7684 8.332793e-01 9.507006e-01

7685 8.336717e-01 9.506814e-01

7686 8.340696e-01 9.506619e-01

7687 8.344730e-01 9.506423e-01

7688 8.348819e-01 9.506225e-01

7689 8.352961e-01 9.506026e-01

7690 8.357157e-01 9.505826e-01

7691 8.361405e-01 9.505625e-01

7692 8.365705e-01 9.505423e-01

7693 8.370056e-01 9.505221e-01

7694 8.374457e-01 9.505019e-01

7695 8.378906e-01 9.504817e-01

7696 8.383403e-01 9.504616e-01

7697 8.387946e-01 9.504415e-01

7698 8.392534e-01 9.504216e-01

7699 8.397166e-01 9.504018e-01

7700 8.401841e-01 9.503823e-01

7701 8.406556e-01 9.503630e-01

7702 8.411311e-01 9.503440e-01

7703 8.416103e-01 9.503253e-01

7704 8.420931e-01 9.503070e-01

7705 8.425794e-01 9.502892e-01

7706 8.430689e-01 9.502718e-01

7707 8.435615e-01 9.502550e-01

7708 8.440569e-01 9.502388e-01

7709 8.445549e-01 9.502232e-01

7710 8.450554e-01 9.502083e-01

7711 8.455581e-01 9.501943e-01

7712 8.460629e-01 9.501810e-01

7713 8.465694e-01 9.501687e-01

7714 8.470775e-01 9.501573e-01

7715 8.475869e-01 9.501469e-01

7716 8.480974e-01 9.501377e-01

7717 8.486088e-01 9.501296e-01

7718 8.491207e-01 9.501228e-01

7719 8.496330e-01 9.501173e-01

7720 8.501454e-01 9.501132e-01

7721 8.506577e-01 9.501106e-01

7722 8.511696e-01 9.501095e-01

7723 8.516809e-01 9.501100e-01

7724 8.521913e-01 9.501122e-01

7725 8.527005e-01 9.501162e-01

7726 8.532083e-01 9.501220e-01

7727 8.537145e-01 9.501297e-01

7728 8.542188e-01 9.501394e-01

7729 8.547209e-01 9.501512e-01

7730 8.552207e-01 9.501652e-01

7731 8.557179e-01 9.501813e-01

7732 8.562121e-01 9.501997e-01

7733 8.567033e-01 9.502205e-01

7734 8.571912e-01 9.502437e-01

7735 8.576755e-01 9.502693e-01

7736 8.581561e-01 9.502975e-01

7737 8.586326e-01 9.503283e-01

7738 8.591050e-01 9.503618e-01

7739 8.595730e-01 9.503979e-01

7740 8.600364e-01 9.504364e-01

7741 8.604951e-01 9.504774e-01

7742 8.605036e-01 9.504771e-01

7743 8.605123e-01 9.504769e-01

7744 8.605212e-01 9.504766e-01

7745 8.605303e-01 9.504764e-01

7746 8.605396e-01 9.504761e-01

7747 8.605490e-01 9.504758e-01

7748 8.605587e-01 9.504755e-01

7749 8.605685e-01 9.504752e-01

7750 8.605786e-01 9.504749e-01

7751 8.605888e-01 9.504746e-01

7752 8.605993e-01 9.504742e-01

7753 8.606099e-01 9.504739e-01

7754 8.606208e-01 9.504735e-01

7755 8.606319e-01 9.504731e-01

7756 8.606433e-01 9.504727e-01

7757 8.606549e-01 9.504722e-01

7758 8.606667e-01 9.504718e-01

7759 8.606787e-01 9.504713e-01

7760 8.606910e-01 9.504708e-01

7761 8.607036e-01 9.504703e-01

7762 8.607164e-01 9.504697e-01

7763 8.607295e-01 9.504692e-01

7764 8.607429e-01 9.504686e-01

7765 8.607566e-01 9.504680e-01

7766 8.607705e-01 9.504673e-01

7767 8.607847e-01 9.504667e-01

7768 8.607993e-01 9.504660e-01

7769 8.608141e-01 9.504652e-01

7770 8.608292e-01 9.504644e-01

7771 8.608447e-01 9.504636e-01

7772 8.608605e-01 9.504628e-01

7773 8.608766e-01 9.504619e-01

7774 8.608931e-01 9.504610e-01

7775 8.609099e-01 9.504601e-01

7776 8.609271e-01 9.504591e-01

7777 8.609446e-01 9.504580e-01

7778 8.609625e-01 9.504570e-01

7779 8.609808e-01 9.504558e-01

7780 8.609995e-01 9.504546e-01

7781 8.610186e-01 9.504534e-01

7782 8.610380e-01 9.504521e-01

7783 8.610579e-01 9.504508e-01

7784 8.610782e-01 9.504494e-01

7785 8.610990e-01 9.504479e-01

7786 8.611201e-01 9.504464e-01

7787 8.611417e-01 9.504449e-01

7788 8.611638e-01 9.504432e-01

7789 8.611864e-01 9.504415e-01

7790 8.612094e-01 9.504397e-01

7791 8.612328e-01 9.504378e-01

7792 8.612568e-01 9.504359e-01

7793 8.612813e-01 9.504339e-01

7794 8.613063e-01 9.504318e-01

7795 8.613318e-01 9.504296e-01

7796 8.613578e-01 9.504273e-01

7797 8.613844e-01 9.504249e-01

7798 8.614115e-01 9.504225e-01

7799 8.614391e-01 9.504199e-01

7800 8.614673e-01 9.504172e-01

7801 8.614961e-01 9.504144e-01

7802 8.615255e-01 9.504115e-01

7803 8.615554e-01 9.504085e-01

7804 8.615860e-01 9.504054e-01

7805 8.616172e-01 9.504021e-01

7806 8.616489e-01 9.503987e-01

7807 8.616813e-01 9.503952e-01

7808 8.617143e-01 9.503915e-01

7809 8.617480e-01 9.503877e-01

7810 8.617823e-01 9.503838e-01

7811 8.618172e-01 9.503797e-01

7812 8.618529e-01 9.503754e-01

7813 8.618891e-01 9.503709e-01

7814 8.619261e-01 9.503663e-01

7815 8.619637e-01 9.503615e-01

7816 8.620020e-01 9.503566e-01

7817 8.620410e-01 9.503514e-01

7818 8.620808e-01 9.503460e-01

7819 8.621212e-01 9.503405e-01

7820 8.621623e-01 9.503347e-01

7821 8.622041e-01 9.503287e-01

7822 8.622466e-01 9.503225e-01

7823 8.622899e-01 9.503160e-01

7824 8.623339e-01 9.503093e-01

7825 8.623785e-01 9.503023e-01

7826 8.624240e-01 9.502951e-01

7827 8.624701e-01 9.502877e-01

7828 8.625169e-01 9.502799e-01

7829 8.625645e-01 9.502719e-01

7830 8.626128e-01 9.502636e-01

7831 8.626618e-01 9.502549e-01

7832 8.627115e-01 9.502460e-01

7833 8.627619e-01 9.502367e-01

7834 8.628130e-01 9.502272e-01

7835 8.628648e-01 9.502172e-01

7836 8.629173e-01 9.502069e-01

7837 8.629705e-01 9.501963e-01

7838 8.630243e-01 9.501853e-01

7839 8.630787e-01 9.501739e-01

7840 8.631339e-01 9.501621e-01

7841 8.631896e-01 9.501499e-01

7842 8.632459e-01 9.501372e-01

7843 8.633029e-01 9.501242e-01

7844 8.633604e-01 9.501107e-01

7845 8.634184e-01 9.500967e-01

7846 8.634770e-01 9.500823e-01

7847 8.635361e-01 9.500673e-01

7848 8.635957e-01 9.500519e-01

7849 8.636558e-01 9.500360e-01

7850 8.637162e-01 9.500196e-01

7851 8.637771e-01 9.500026e-01

7852 8.638383e-01 9.499850e-01

7853 8.638999e-01 9.499670e-01

7854 8.639618e-01 9.499483e-01

7855 8.640239e-01 9.499290e-01

7856 8.640862e-01 9.499092e-01

7857 8.641487e-01 9.498887e-01

7858 8.642114e-01 9.498676e-01

7859 8.642741e-01 9.498458e-01

7860 8.643369e-01 9.498234e-01

7861 8.643997e-01 9.498003e-01

7862 8.644624e-01 9.497765e-01

7863 8.645250e-01 9.497520e-01

7864 8.645874e-01 9.497268e-01

7865 8.646497e-01 9.497009e-01

7866 8.647116e-01 9.496742e-01

7867 8.647732e-01 9.496467e-01

7868 8.648343e-01 9.496185e-01

7869 8.648950e-01 9.495896e-01

7870 8.649552e-01 9.495598e-01

7871 8.650147e-01 9.495292e-01

7872 8.650736e-01 9.494978e-01

7873 8.651317e-01 9.494656e-01

7874 8.651889e-01 9.494325e-01

7875 8.652453e-01 9.493986e-01

7876 8.653007e-01 9.493638e-01

7877 8.653551e-01 9.493282e-01

7878 8.654083e-01 9.492916e-01

7879 8.654603e-01 9.492542e-01

7880 8.655110e-01 9.492159e-01

7881 8.655604e-01 9.491767e-01

7882 8.656083e-01 9.491365e-01

7883 8.656546e-01 9.490955e-01

7884 8.656994e-01 9.490535e-01

7885 8.657425e-01 9.490106e-01

7886 8.657838e-01 9.489668e-01

7887 8.658232e-01 9.489220e-01

7888 8.658607e-01 9.488762e-01

7889 8.658962e-01 9.488296e-01

7890 8.659296e-01 9.487820e-01

7891 8.659608e-01 9.487334e-01

7892 8.659898e-01 9.486839e-01

7893 8.660165e-01 9.486335e-01

7894 8.660408e-01 9.485821e-01

7895 8.660627e-01 9.485297e-01

7896 8.660820e-01 9.484765e-01

7897 8.660988e-01 9.484223e-01

7898 8.661129e-01 9.483671e-01

7899 8.661243e-01 9.483110e-01

7900 8.661330e-01 9.482541e-01

7901 8.661388e-01 9.481962e-01

7902 8.661419e-01 9.481374e-01

7903 8.661420e-01 9.480777e-01

7904 8.661391e-01 9.480171e-01

7905 8.661333e-01 9.479557e-01

7906 8.661246e-01 9.478933e-01

7907 8.661127e-01 9.478302e-01

7908 8.660979e-01 9.477661e-01

7909 8.660799e-01 9.477013e-01

7910 8.660589e-01 9.476356e-01

7911 8.660348e-01 9.475692e-01

7912 8.660076e-01 9.475019e-01

7913 8.659773e-01 9.474339e-01

7914 8.659439e-01 9.473651e-01

7915 8.659075e-01 9.472956e-01

7916 8.658680e-01 9.472254e-01

7917 8.658254e-01 9.471544e-01

7918 8.657799e-01 9.470828e-01

7919 8.657314e-01 9.470106e-01

7920 8.656799e-01 9.469380e-01

7921 8.656255e-01 9.468652e-01

7922 8.656246e-01 9.468646e-01

7923 8.656236e-01 9.468640e-01

7924 8.656225e-01 9.468633e-01

7925 8.656213e-01 9.468626e-01

7926 8.656200e-01 9.468619e-01

7927 8.656186e-01 9.468612e-01

7928 8.656170e-01 9.468605e-01

7929 8.656154e-01 9.468597e-01

7930 8.656135e-01 9.468589e-01

7931 8.656116e-01 9.468580e-01

7932 8.656095e-01 9.468572e-01

7933 8.656072e-01 9.468563e-01

7934 8.656048e-01 9.468554e-01

7935 8.656022e-01 9.468544e-01

7936 8.655995e-01 9.468535e-01

7937 8.655965e-01 9.468525e-01

7938 8.655934e-01 9.468514e-01

7939 8.655901e-01 9.468504e-01

7940 8.655865e-01 9.468492e-01

7941 8.655828e-01 9.468481e-01

7942 8.655788e-01 9.468469e-01

7943 8.655746e-01 9.468457e-01

7944 8.655701e-01 9.468445e-01

7945 8.655654e-01 9.468432e-01

7946 8.655604e-01 9.468418e-01

7947 8.655551e-01 9.468404e-01

7948 8.655496e-01 9.468390e-01

7949 8.655437e-01 9.468376e-01

7950 8.655376e-01 9.468361e-01

7951 8.655311e-01 9.468345e-01

7952 8.655242e-01 9.468329e-01

7953 8.655171e-01 9.468313e-01

7954 8.655095e-01 9.468296e-01

7955 8.655016e-01 9.468278e-01

7956 8.654932e-01 9.468260e-01

7957 8.654845e-01 9.468242e-01

7958 8.654753e-01 9.468222e-01

7959 8.654657e-01 9.468203e-01

7960 8.654556e-01 9.468182e-01

7961 8.654450e-01 9.468162e-01

7962 8.654339e-01 9.468140e-01

7963 8.654223e-01 9.468118e-01

7964 8.654101e-01 9.468095e-01

7965 8.653974e-01 9.468072e-01

7966 8.653841e-01 9.468048e-01

7967 8.653702e-01 9.468023e-01

7968 8.653556e-01 9.467998e-01

7969 8.653404e-01 9.467972e-01

7970 8.653245e-01 9.467945e-01

7971 8.653079e-01 9.467918e-01

7972 8.652906e-01 9.467889e-01

7973 8.652724e-01 9.467860e-01

7974 8.652535e-01 9.467831e-01

7975 8.652338e-01 9.467800e-01

7976 8.652132e-01 9.467769e-01

7977 8.651917e-01 9.467736e-01

7978 8.651693e-01 9.467703e-01

7979 8.651459e-01 9.467670e-01

7980 8.651215e-01 9.467635e-01

7981 8.650961e-01 9.467599e-01

7982 8.650696e-01 9.467563e-01

7983 8.650420e-01 9.467526e-01

7984 8.650132e-01 9.467487e-01

7985 8.649832e-01 9.467448e-01

7986 8.649520e-01 9.467408e-01

7987 8.649195e-01 9.467367e-01

7988 8.648857e-01 9.467325e-01

7989 8.648505e-01 9.467282e-01

7990 8.648138e-01 9.467239e-01

7991 8.647757e-01 9.467194e-01

7992 8.647360e-01 9.467148e-01

7993 8.646947e-01 9.467102e-01

7994 8.646517e-01 9.467054e-01

7995 8.646070e-01 9.467006e-01

7996 8.645606e-01 9.466956e-01

7997 8.645123e-01 9.466906e-01

7998 8.644621e-01 9.466855e-01

7999 8.644099e-01 9.466803e-01

8000 8.643556e-01 9.466749e-01

8001 8.642993e-01 9.466695e-01

8002 8.642408e-01 9.466641e-01

8003 8.641800e-01 9.466585e-01

8004 8.641168e-01 9.466528e-01

8005 8.640512e-01 9.466471e-01

8006 8.639832e-01 9.466413e-01

8007 8.639125e-01 9.466354e-01

8008 8.638392e-01 9.466294e-01

8009 8.637631e-01 9.466234e-01

8010 8.636841e-01 9.466173e-01

8011 8.636022e-01 9.466112e-01

8012 8.635173e-01 9.466050e-01

8013 8.634292e-01 9.465987e-01

8014 8.633379e-01 9.465924e-01

8015 8.632432e-01 9.465861e-01

8016 8.631451e-01 9.465797e-01

8017 8.630435e-01 9.465734e-01

8018 8.629382e-01 9.465670e-01

8019 8.628291e-01 9.465606e-01

8020 8.627162e-01 9.465542e-01

8021 8.625992e-01 9.465478e-01

8022 8.624782e-01 9.465415e-01

8023 8.623529e-01 9.465352e-01

8024 8.622233e-01 9.465290e-01

8025 8.620892e-01 9.465228e-01

8026 8.619506e-01 9.465167e-01

8027 8.618072e-01 9.465107e-01

8028 8.616590e-01 9.465048e-01

8029 8.615058e-01 9.464991e-01

8030 8.613476e-01 9.464935e-01

8031 8.611842e-01 9.464880e-01

8032 8.610154e-01 9.464828e-01

8033 8.608412e-01 9.464777e-01

8034 8.606613e-01 9.464729e-01

8035 8.604758e-01 9.464683e-01

8036 8.602844e-01 9.464640e-01

8037 8.600871e-01 9.464600e-01

8038 8.598837e-01 9.464563e-01

8039 8.596741e-01 9.464530e-01

8040 8.594581e-01 9.464501e-01

8041 8.592357e-01 9.464476e-01

8042 8.590068e-01 9.464455e-01

8043 8.587711e-01 9.464439e-01

8044 8.585287e-01 9.464427e-01

8045 8.582794e-01 9.464422e-01

8046 8.580231e-01 9.464422e-01

8047 8.577598e-01 9.464428e-01

8048 8.574892e-01 9.464441e-01

8049 8.572114e-01 9.464461e-01

8050 8.569262e-01 9.464488e-01

8051 8.566336e-01 9.464523e-01

8052 8.563335e-01 9.464566e-01

8053 8.560258e-01 9.464618e-01

8054 8.557106e-01 9.464679e-01

8055 8.553876e-01 9.464749e-01

8056 8.550570e-01 9.464829e-01

8057 8.547187e-01 9.464920e-01

8058 8.543726e-01 9.465022e-01

8059 8.540188e-01 9.465136e-01

8060 8.536572e-01 9.465262e-01

8061 8.532878e-01 9.465400e-01

8062 8.529107e-01 9.465551e-01

8063 8.525260e-01 9.465716e-01

8064 8.521336e-01 9.465895e-01

8065 8.517336e-01 9.466089e-01

8066 8.513260e-01 9.466298e-01

8067 8.509111e-01 9.466524e-01

8068 8.504887e-01 9.466765e-01

8069 8.500592e-01 9.467024e-01

8070 8.496224e-01 9.467300e-01

8071 8.491787e-01 9.467595e-01

8072 8.487281e-01 9.467908e-01

8073 8.482708e-01 9.468240e-01

8074 8.478070e-01 9.468593e-01

8075 8.473368e-01 9.468965e-01

8076 8.468605e-01 9.469359e-01

8077 8.463782e-01 9.469774e-01

8078 8.458901e-01 9.470212e-01

8079 8.453965e-01 9.470672e-01

8080 8.448977e-01 9.471155e-01

8081 8.443938e-01 9.471661e-01

8082 8.438851e-01 9.472192e-01

8083 8.433719e-01 9.472747e-01

8084 8.428546e-01 9.473328e-01

8085 8.423333e-01 9.473934e-01

8086 8.418083e-01 9.474565e-01

8087 8.412800e-01 9.475223e-01

8088 8.407487e-01 9.475908e-01

8089 8.402147e-01 9.476619e-01

8090 8.396782e-01 9.477358e-01

8091 8.391397e-01 9.478124e-01

8092 8.385995e-01 9.478918e-01

8093 8.380578e-01 9.479740e-01

8094 8.375150e-01 9.480590e-01

8095 8.369715e-01 9.481469e-01

8096 8.364276e-01 9.482375e-01

8097 8.358835e-01 9.483307e-01

8098 8.353397e-01 9.484264e-01

8099 8.347965e-01 9.485243e-01

8100 8.342541e-01 9.486244e-01

8101 8.337130e-01 9.487264e-01

8102 8.337009e-01 9.487262e-01

8103 8.336884e-01 9.487260e-01

8104 8.336755e-01 9.487258e-01

8105 8.336620e-01 9.487255e-01

8106 8.336481e-01 9.487253e-01

8107 8.336337e-01 9.487250e-01

8108 8.336187e-01 9.487248e-01

8109 8.336032e-01 9.487245e-01

8110 8.335871e-01 9.487242e-01

8111 8.335704e-01 9.487240e-01

8112 8.335531e-01 9.487237e-01

8113 8.335352e-01 9.487233e-01

8114 8.335166e-01 9.487230e-01

8115 8.334974e-01 9.487227e-01

8116 8.334775e-01 9.487223e-01

8117 8.334568e-01 9.487220e-01

8118 8.334354e-01 9.487216e-01

8119 8.334132e-01 9.487212e-01

8120 8.333902e-01 9.487208e-01

8121 8.333664e-01 9.487204e-01

8122 8.333417e-01 9.487200e-01

8123 8.333161e-01 9.487195e-01

8124 8.332896e-01 9.487191e-01

8125 8.332621e-01 9.487186e-01

8126 8.332337e-01 9.487181e-01

8127 8.332042e-01 9.487176e-01

8128 8.331737e-01 9.487170e-01

8129 8.331421e-01 9.487165e-01

8130 8.331093e-01 9.487159e-01

8131 8.330754e-01 9.487153e-01

8132 8.330402e-01 9.487146e-01

8133 8.330038e-01 9.487140e-01

8134 8.329661e-01 9.487133e-01

8135 8.329270e-01 9.487126e-01

8136 8.328865e-01 9.487119e-01

8137 8.328446e-01 9.487112e-01

8138 8.328012e-01 9.487104e-01

8139 8.327563e-01 9.487096e-01

8140 8.327098e-01 9.487088e-01

8141 8.326616e-01 9.487079e-01

8142 8.326117e-01 9.487070e-01

8143 8.325600e-01 9.487061e-01

8144 8.325065e-01 9.487051e-01

8145 8.324511e-01 9.487041e-01

8146 8.323938e-01 9.487031e-01

8147 8.323345e-01 9.487021e-01

8148 8.322730e-01 9.487010e-01

8149 8.322094e-01 9.486998e-01

8150 8.321436e-01 9.486987e-01

8151 8.320755e-01 9.486975e-01

8152 8.320049e-01 9.486962e-01

8153 8.319320e-01 9.486949e-01

8154 8.318565e-01 9.486936e-01

8155 8.317783e-01 9.486922e-01

8156 8.316975e-01 9.486908e-01

8157 8.316138e-01 9.486893e-01

8158 8.315272e-01 9.486878e-01

8159 8.314377e-01 9.486862e-01

8160 8.313451e-01 9.486846e-01

8161 8.312493e-01 9.486830e-01

8162 8.311502e-01 9.486812e-01

8163 8.310477e-01 9.486795e-01

8164 8.309417e-01 9.486776e-01

8165 8.308321e-01 9.486757e-01

8166 8.307188e-01 9.486738e-01

8167 8.306016e-01 9.486718e-01

8168 8.304805e-01 9.486697e-01

8169 8.303553e-01 9.486676e-01

8170 8.302258e-01 9.486654e-01

8171 8.300921e-01 9.486631e-01

8172 8.299538e-01 9.486608e-01

8173 8.298110e-01 9.486584e-01

8174 8.296634e-01 9.486559e-01

8175 8.295110e-01 9.486534e-01

8176 8.293535e-01 9.486508e-01

8177 8.291909e-01 9.486481e-01

8178 8.290229e-01 9.486453e-01

8179 8.288494e-01 9.486425e-01

8180 8.286704e-01 9.486396e-01

8181 8.284855e-01 9.486366e-01

8182 8.282947e-01 9.486335e-01

8183 8.280978e-01 9.486303e-01

8184 8.278946e-01 9.486270e-01

8185 8.276850e-01 9.486237e-01

8186 8.274688e-01 9.486202e-01

8187 8.272458e-01 9.486167e-01

8188 8.270159e-01 9.486131e-01

8189 8.267788e-01 9.486093e-01

8190 8.265344e-01 9.486055e-01

8191 8.262825e-01 9.486016e-01

8192 8.260230e-01 9.485976e-01

8193 8.257557e-01 9.485934e-01

8194 8.254803e-01 9.485892e-01

8195 8.251968e-01 9.485848e-01

8196 8.249048e-01 9.485804e-01

8197 8.246043e-01 9.485758e-01

8198 8.242951e-01 9.485712e-01

8199 8.239770e-01 9.485664e-01

8200 8.236498e-01 9.485614e-01

8201 8.233134e-01 9.485564e-01

8202 8.229675e-01 9.485513e-01

8203 8.226120e-01 9.485460e-01

8204 8.222468e-01 9.485406e-01

8205 8.218717e-01 9.485351e-01

8206 8.214865e-01 9.485294e-01

8207 8.210911e-01 9.485236e-01

8208 8.206854e-01 9.485177e-01

8209 8.202691e-01 9.485116e-01

8210 8.198423e-01 9.485054e-01

8211 8.194047e-01 9.484990e-01

8212 8.189562e-01 9.484925e-01

8213 8.184968e-01 9.484859e-01

8214 8.180263e-01 9.484791e-01

8215 8.175446e-01 9.484721e-01

8216 8.170518e-01 9.484650e-01

8217 8.165477e-01 9.484577e-01

8218 8.160322e-01 9.484503e-01

8219 8.155054e-01 9.484427e-01

8220 8.149672e-01 9.484349e-01

8221 8.144175e-01 9.484269e-01

8222 8.138565e-01 9.484188e-01

8223 8.132842e-01 9.484104e-01

8224 8.127004e-01 9.484019e-01

8225 8.121054e-01 9.483932e-01

8226 8.114992e-01 9.483843e-01

8227 8.108819e-01 9.483752e-01

8228 8.102536e-01 9.483658e-01

8229 8.096143e-01 9.483563e-01

8230 8.089643e-01 9.483465e-01

8231 8.083038e-01 9.483365e-01

8232 8.076328e-01 9.483263e-01

8233 8.069516e-01 9.483158e-01

8234 8.062605e-01 9.483051e-01

8235 8.055596e-01 9.482941e-01

8236 8.048493e-01 9.482828e-01

8237 8.041297e-01 9.482713e-01

8238 8.034013e-01 9.482595e-01

8239 8.026643e-01 9.482474e-01

8240 8.019190e-01 9.482350e-01

8241 8.011659e-01 9.482223e-01

8242 8.004052e-01 9.482093e-01

8243 7.996375e-01 9.481959e-01

8244 7.988630e-01 9.481822e-01

8245 7.980822e-01 9.481681e-01

8246 7.972956e-01 9.481536e-01

8247 7.965036e-01 9.481388e-01

8248 7.957066e-01 9.481236e-01

8249 7.949052e-01 9.481079e-01

8250 7.940998e-01 9.480918e-01

8251 7.932909e-01 9.480753e-01

8252 7.924790e-01 9.480583e-01

8253 7.916647e-01 9.480408e-01

8254 7.908484e-01 9.480228e-01

8255 7.900307e-01 9.480043e-01

8256 7.892120e-01 9.479853e-01

8257 7.883930e-01 9.479657e-01

8258 7.875742e-01 9.479455e-01

8259 7.867560e-01 9.479248e-01

8260 7.859390e-01 9.479034e-01

8261 7.851238e-01 9.478814e-01

8262 7.843107e-01 9.478587e-01

8263 7.835004e-01 9.478353e-01

8264 7.826934e-01 9.478112e-01

8265 7.818900e-01 9.477864e-01

8266 7.810909e-01 9.477609e-01

8267 7.802965e-01 9.477345e-01

8268 7.795072e-01 9.477073e-01

8269 7.787234e-01 9.476793e-01

8270 7.779457e-01 9.476505e-01

8271 7.771744e-01 9.476207e-01

8272 7.764099e-01 9.475900e-01

8273 7.756527e-01 9.475584e-01

8274 7.749030e-01 9.475259e-01

8275 7.741612e-01 9.474923e-01

8276 7.734276e-01 9.474578e-01

8277 7.727026e-01 9.474226e-01

8278 7.719865e-01 9.473867e-01

8279 7.712795e-01 9.473502e-01

8280 7.705818e-01 9.473134e-01

8281 7.698938e-01 9.472763e-01

8282 7.698643e-01 9.472757e-01

8283 7.698338e-01 9.472750e-01

8284 7.698023e-01 9.472743e-01

8285 7.697699e-01 9.472736e-01

8286 7.697363e-01 9.472729e-01

8287 7.697017e-01 9.472721e-01

8288 7.696660e-01 9.472713e-01

8289 7.696291e-01 9.472705e-01

8290 7.695910e-01 9.472697e-01

8291 7.695518e-01 9.472689e-01

8292 7.695112e-01 9.472680e-01

8293 7.694694e-01 9.472671e-01

8294 7.694263e-01 9.472662e-01

8295 7.693818e-01 9.472653e-01

8296 7.693359e-01 9.472643e-01

8297 7.692885e-01 9.472634e-01

8298 7.692396e-01 9.472624e-01

8299 7.691892e-01 9.472613e-01

8300 7.691372e-01 9.472603e-01

8301 7.690835e-01 9.472592e-01

8302 7.690282e-01 9.472581e-01

8303 7.689711e-01 9.472570e-01

8304 7.689122e-01 9.472558e-01

8305 7.688515e-01 9.472546e-01

8306 7.687888e-01 9.472534e-01

8307 7.687242e-01 9.472522e-01

8308 7.686576e-01 9.472509e-01

8309 7.685889e-01 9.472496e-01

8310 7.685180e-01 9.472483e-01

8311 7.684450e-01 9.472469e-01

8312 7.683696e-01 9.472455e-01

8313 7.682919e-01 9.472441e-01

8314 7.682118e-01 9.472427e-01

8315 7.681292e-01 9.472412e-01

8316 7.680441e-01 9.472397e-01

8317 7.679563e-01 9.472382e-01

8318 7.678658e-01 9.472366e-01

8319 7.677725e-01 9.472350e-01

8320 7.676763e-01 9.472333e-01

8321 7.675771e-01 9.472317e-01

8322 7.674750e-01 9.472300e-01

8323 7.673697e-01 9.472282e-01

8324 7.672611e-01 9.472265e-01

8325 7.671493e-01 9.472247e-01

8326 7.670340e-01 9.472229e-01

8327 7.669153e-01 9.472210e-01

8328 7.667930e-01 9.472191e-01

8329 7.666669e-01 9.472172e-01

8330 7.665371e-01 9.472152e-01

8331 7.664033e-01 9.472133e-01

8332 7.662656e-01 9.472112e-01

8333 7.661237e-01 9.472092e-01

8334 7.659776e-01 9.472071e-01

8335 7.658271e-01 9.472050e-01

8336 7.656722e-01 9.472029e-01

8337 7.655127e-01 9.472008e-01

8338 7.653486e-01 9.471986e-01

8339 7.651796e-01 9.471964e-01

8340 7.650057e-01 9.471941e-01

8341 7.648267e-01 9.471919e-01

8342 7.646425e-01 9.471896e-01

8343 7.644531e-01 9.471873e-01

8344 7.642581e-01 9.471850e-01

8345 7.640577e-01 9.471827e-01

8346 7.638515e-01 9.471803e-01

8347 7.636395e-01 9.471780e-01

8348 7.634215e-01 9.471756e-01

8349 7.631974e-01 9.471732e-01

8350 7.629671e-01 9.471708e-01

8351 7.627304e-01 9.471685e-01

8352 7.624872e-01 9.471661e-01

8353 7.622374e-01 9.471637e-01

8354 7.619808e-01 9.471613e-01

8355 7.617172e-01 9.471589e-01

8356 7.614467e-01 9.471566e-01

8357 7.611689e-01 9.471543e-01

8358 7.608838e-01 9.471519e-01

8359 7.605913e-01 9.471496e-01

8360 7.602911e-01 9.471474e-01

8361 7.599833e-01 9.471452e-01

8362 7.596676e-01 9.471430e-01

8363 7.593440e-01 9.471408e-01

8364 7.590123e-01 9.471387e-01

8365 7.586723e-01 9.471367e-01

8366 7.583241e-01 9.471347e-01

8367 7.579675e-01 9.471328e-01

8368 7.576023e-01 9.471310e-01

8369 7.572285e-01 9.471293e-01

8370 7.568460e-01 9.471277e-01

8371 7.564546e-01 9.471261e-01

8372 7.560544e-01 9.471247e-01

8373 7.556453e-01 9.471234e-01

8374 7.552271e-01 9.471222e-01

8375 7.547998e-01 9.471212e-01

8376 7.543634e-01 9.471203e-01

8377 7.539178e-01 9.471195e-01

8378 7.534630e-01 9.471190e-01

8379 7.529989e-01 9.471186e-01

8380 7.525257e-01 9.471184e-01

8381 7.520431e-01 9.471185e-01

8382 7.515514e-01 9.471187e-01

8383 7.510505e-01 9.471192e-01

8384 7.505404e-01 9.471200e-01

8385 7.500211e-01 9.471210e-01

8386 7.494929e-01 9.471223e-01

8387 7.489556e-01 9.471239e-01

8388 7.484095e-01 9.471258e-01

8389 7.478546e-01 9.471281e-01

8390 7.472910e-01 9.471307e-01

8391 7.467189e-01 9.471337e-01

8392 7.461385e-01 9.471370e-01

8393 7.455498e-01 9.471408e-01

8394 7.449531e-01 9.471450e-01

8395 7.443486e-01 9.471497e-01

8396 7.437365e-01 9.471549e-01

8397 7.431170e-01 9.471605e-01

8398 7.424904e-01 9.471667e-01

8399 7.418569e-01 9.471734e-01

8400 7.412168e-01 9.471807e-01

8401 7.405704e-01 9.471886e-01

8402 7.399181e-01 9.471971e-01

8403 7.392600e-01 9.472063e-01

8404 7.385966e-01 9.472161e-01

8405 7.379283e-01 9.472267e-01

8406 7.372553e-01 9.472380e-01

8407 7.365780e-01 9.472501e-01

8408 7.358969e-01 9.472629e-01

8409 7.352123e-01 9.472766e-01

8410 7.345247e-01 9.472912e-01

8411 7.338344e-01 9.473066e-01

8412 7.331418e-01 9.473229e-01

8413 7.324475e-01 9.473402e-01

8414 7.317518e-01 9.473585e-01

8415 7.310552e-01 9.473778e-01

8416 7.303581e-01 9.473982e-01

8417 7.296610e-01 9.474197e-01

8418 7.289643e-01 9.474422e-01

8419 7.282685e-01 9.474660e-01

8420 7.275739e-01 9.474909e-01

8421 7.268812e-01 9.475171e-01

8422 7.261906e-01 9.475445e-01

8423 7.255028e-01 9.475732e-01

8424 7.248179e-01 9.476033e-01

8425 7.241366e-01 9.476347e-01

8426 7.234592e-01 9.476676e-01

8427 7.227862e-01 9.477019e-01

8428 7.221179e-01 9.477376e-01

8429 7.214547e-01 9.477749e-01

8430 7.207970e-01 9.478138e-01

8431 7.201452e-01 9.478542e-01

8432 7.194997e-01 9.478963e-01

8433 7.188607e-01 9.479400e-01

8434 7.182285e-01 9.479854e-01

8435 7.176036e-01 9.480325e-01

8436 7.169862e-01 9.480814e-01

8437 7.163766e-01 9.481321e-01

8438 7.157750e-01 9.481846e-01

8439 7.151817e-01 9.482389e-01

8440 7.145969e-01 9.482952e-01

8441 7.140208e-01 9.483533e-01

8442 7.134536e-01 9.484134e-01

8443 7.128955e-01 9.484754e-01

8444 7.123466e-01 9.485395e-01

8445 7.118072e-01 9.486055e-01

8446 7.112772e-01 9.486736e-01

8447 7.107569e-01 9.487437e-01

8448 7.102463e-01 9.488159e-01

8449 7.097455e-01 9.488903e-01

8450 7.092545e-01 9.489667e-01

8451 7.087734e-01 9.490452e-01

8452 7.083022e-01 9.491259e-01

8453 7.078410e-01 9.492087e-01

8454 7.073897e-01 9.492936e-01

8455 7.069483e-01 9.493808e-01

8456 7.065168e-01 9.494700e-01

8457 7.060951e-01 9.495614e-01

8458 7.056833e-01 9.496547e-01

8459 7.052812e-01 9.497496e-01

8460 7.048888e-01 9.498462e-01

8461 7.045060e-01 9.499442e-01

8462 7.044714e-01 9.499440e-01

8463 7.044359e-01 9.499439e-01

8464 7.043995e-01 9.499437e-01

8465 7.043622e-01 9.499436e-01

8466 7.043240e-01 9.499434e-01

8467 7.042849e-01 9.499433e-01

8468 7.042448e-01 9.499431e-01

8469 7.042038e-01 9.499429e-01

8470 7.041618e-01 9.499427e-01

8471 7.041188e-01 9.499426e-01

8472 7.040748e-01 9.499424e-01

8473 7.040298e-01 9.499422e-01

8474 7.039837e-01 9.499420e-01

8475 7.039365e-01 9.499418e-01

8476 7.038882e-01 9.499416e-01

8477 7.038389e-01 9.499414e-01

8478 7.037884e-01 9.499412e-01

8479 7.037367e-01 9.499410e-01

8480 7.036839e-01 9.499407e-01

8481 7.036299e-01 9.499405e-01

8482 7.035747e-01 9.499402e-01

8483 7.035183e-01 9.499400e-01

8484 7.034606e-01 9.499397e-01

8485 7.034016e-01 9.499395e-01

8486 7.033413e-01 9.499392e-01

8487 7.032797e-01 9.499389e-01

8488 7.032168e-01 9.499387e-01

8489 7.031525e-01 9.499384e-01

8490 7.030869e-01 9.499381e-01

8491 7.030198e-01 9.499377e-01

8492 7.029513e-01 9.499374e-01

8493 7.028814e-01 9.499371e-01

8494 7.028100e-01 9.499368e-01

8495 7.027371e-01 9.499364e-01

8496 7.026627e-01 9.499361e-01

8497 7.025868e-01 9.499357e-01

8498 7.025093e-01 9.499353e-01

8499 7.024303e-01 9.499349e-01

8500 7.023497e-01 9.499345e-01

8501 7.022674e-01 9.499341e-01

8502 7.021836e-01 9.499337e-01

8503 7.020981e-01 9.499333e-01

8504 7.020110e-01 9.499328e-01

8505 7.019222e-01 9.499324e-01

8506 7.018317e-01 9.499319e-01

8507 7.017395e-01 9.499314e-01

8508 7.016456e-01 9.499309e-01

8509 7.015499e-01 9.499304e-01

8510 7.014526e-01 9.499299e-01

8511 7.013534e-01 9.499294e-01

8512 7.012526e-01 9.499288e-01

8513 7.011499e-01 9.499283e-01

8514 7.010455e-01 9.499277e-01

8515 7.009393e-01 9.499271e-01

8516 7.008313e-01 9.499265e-01

8517 7.007216e-01 9.499259e-01

8518 7.006100e-01 9.499252e-01

8519 7.004967e-01 9.499246e-01

8520 7.003816e-01 9.499239e-01

8521 7.002647e-01 9.499232e-01

8522 7.001460e-01 9.499225e-01

8523 7.000256e-01 9.499218e-01

8524 6.999034e-01 9.499210e-01

8525 6.997795e-01 9.499202e-01

8526 6.996539e-01 9.499194e-01

8527 6.995266e-01 9.499186e-01

8528 6.993976e-01 9.499178e-01

8529 6.992670e-01 9.499169e-01

8530 6.991347e-01 9.499160e-01

8531 6.990008e-01 9.499151e-01

8532 6.988654e-01 9.499141e-01

8533 6.987284e-01 9.499132e-01

8534 6.985900e-01 9.499122e-01

8535 6.984501e-01 9.499112e-01

8536 6.983088e-01 9.499101e-01

8537 6.981661e-01 9.499090e-01

8538 6.980222e-01 9.499079e-01

8539 6.978770e-01 9.499067e-01

8540 6.977306e-01 9.499055e-01

8541 6.975831e-01 9.499043e-01

8542 6.974345e-01 9.499030e-01

8543 6.972850e-01 9.499017e-01

8544 6.971345e-01 9.499004e-01

8545 6.969832e-01 9.498990e-01

8546 6.968312e-01 9.498975e-01

8547 6.966785e-01 9.498960e-01

8548 6.965252e-01 9.498945e-01

8549 6.963715e-01 9.498929e-01

8550 6.962173e-01 9.498912e-01

8551 6.960629e-01 9.498895e-01

8552 6.959083e-01 9.498877e-01

8553 6.957536e-01 9.498859e-01

8554 6.955990e-01 9.498840e-01

8555 6.954445e-01 9.498820e-01

8556 6.952903e-01 9.498799e-01

8557 6.951365e-01 9.498778e-01

8558 6.949832e-01 9.498756e-01

8559 6.948306e-01 9.498733e-01

8560 6.946787e-01 9.498709e-01

8561 6.945278e-01 9.498684e-01

8562 6.943778e-01 9.498657e-01

8563 6.942291e-01 9.498630e-01

8564 6.940816e-01 9.498602e-01

8565 6.939356e-01 9.498572e-01

8566 6.937912e-01 9.498542e-01

8567 6.936485e-01 9.498509e-01

8568 6.935077e-01 9.498476e-01

8569 6.933689e-01 9.498441e-01

8570 6.932322e-01 9.498404e-01

8571 6.930978e-01 9.498365e-01

8572 6.929658e-01 9.498325e-01

8573 6.928364e-01 9.498283e-01

8574 6.927096e-01 9.498239e-01

8575 6.925857e-01 9.498193e-01

8576 6.924647e-01 9.498145e-01

8577 6.923467e-01 9.498094e-01

8578 6.922320e-01 9.498041e-01

8579 6.921206e-01 9.497986e-01

8580 6.920126e-01 9.497928e-01

8581 6.919081e-01 9.497866e-01

8582 6.918073e-01 9.497802e-01

8583 6.917102e-01 9.497735e-01

8584 6.916170e-01 9.497665e-01

8585 6.915277e-01 9.497590e-01

8586 6.914424e-01 9.497513e-01

8587 6.913611e-01 9.497431e-01

8588 6.912840e-01 9.497346e-01

8589 6.912112e-01 9.497256e-01

8590 6.911425e-01 9.497162e-01

8591 6.910782e-01 9.497063e-01

8592 6.910183e-01 9.496959e-01

8593 6.909627e-01 9.496850e-01

8594 6.909116e-01 9.496736e-01

8595 6.908649e-01 9.496616e-01

8596 6.908226e-01 9.496490e-01

8597 6.907847e-01 9.496359e-01

8598 6.907513e-01 9.496220e-01

8599 6.907224e-01 9.496076e-01

8600 6.906978e-01 9.495924e-01

8601 6.906776e-01 9.495765e-01

8602 6.906617e-01 9.495598e-01

8603 6.906501e-01 9.495424e-01

8604 6.906428e-01 9.495241e-01

8605 6.906397e-01 9.495050e-01

8606 6.906407e-01 9.494850e-01

8607 6.906458e-01 9.494641e-01

8608 6.906548e-01 9.494423e-01

8609 6.906678e-01 9.494194e-01

8610 6.906846e-01 9.493955e-01

8611 6.907052e-01 9.493706e-01

8612 6.907294e-01 9.493446e-01

8613 6.907571e-01 9.493174e-01

8614 6.907883e-01 9.492891e-01

8615 6.908229e-01 9.492595e-01

8616 6.908607e-01 9.492287e-01

8617 6.909016e-01 9.491966e-01

8618 6.909455e-01 9.491632e-01

8619 6.909924e-01 9.491285e-01

8620 6.910421e-01 9.490923e-01

8621 6.910944e-01 9.490546e-01

8622 6.911493e-01 9.490155e-01

8623 6.912067e-01 9.489749e-01

8624 6.912663e-01 9.489327e-01

8625 6.913282e-01 9.488889e-01

8626 6.913922e-01 9.488435e-01

8627 6.914582e-01 9.487964e-01

8628 6.915261e-01 9.487476e-01

8629 6.915957e-01 9.486970e-01

8630 6.916670e-01 9.486447e-01

8631 6.917399e-01 9.485905e-01

8632 6.918141e-01 9.485346e-01

8633 6.918897e-01 9.484767e-01

8634 6.919666e-01 9.484169e-01

8635 6.920445e-01 9.483552e-01

8636 6.921235e-01 9.482916e-01

8637 6.922035e-01 9.482260e-01

8638 6.922843e-01 9.481587e-01

8639 6.923658e-01 9.480898e-01

8640 6.924481e-01 9.480194e-01

8641 6.925309e-01 9.479477e-01

8642 6.925408e-01 9.479470e-01

8643 6.925513e-01 9.479462e-01

8644 6.925623e-01 9.479454e-01

8645 6.925740e-01 9.479446e-01

8646 6.925863e-01 9.479437e-01

8647 6.925992e-01 9.479429e-01

8648 6.926127e-01 9.479420e-01

8649 6.926270e-01 9.479411e-01

8650 6.926420e-01 9.479402e-01

8651 6.926577e-01 9.479393e-01

8652 6.926742e-01 9.479383e-01

8653 6.926915e-01 9.479373e-01

8654 6.927096e-01 9.479363e-01

8655 6.927286e-01 9.479353e-01

8656 6.927484e-01 9.479342e-01

8657 6.927692e-01 9.479331e-01

8658 6.927910e-01 9.479320e-01

8659 6.928138e-01 9.479309e-01

8660 6.928375e-01 9.479297e-01

8661 6.928624e-01 9.479286e-01

8662 6.928884e-01 9.479274e-01

8663 6.929155e-01 9.479261e-01

8664 6.929438e-01 9.479249e-01

8665 6.929734e-01 9.479236e-01

8666 6.930042e-01 9.479223e-01

8667 6.930364e-01 9.479210e-01

8668 6.930700e-01 9.479196e-01

8669 6.931049e-01 9.479182e-01

8670 6.931414e-01 9.479168e-01

8671 6.931794e-01 9.479154e-01

8672 6.932189e-01 9.479139e-01

8673 6.932601e-01 9.479124e-01

8674 6.933031e-01 9.479109e-01

8675 6.933477e-01 9.479093e-01

8676 6.933942e-01 9.479077e-01

8677 6.934426e-01 9.479061e-01

8678 6.934929e-01 9.479045e-01

8679 6.935452e-01 9.479028e-01

8680 6.935996e-01 9.479011e-01

8681 6.936561e-01 9.478993e-01

8682 6.937149e-01 9.478976e-01

8683 6.937760e-01 9.478958e-01

8684 6.938394e-01 9.478940e-01

8685 6.939053e-01 9.478921e-01

8686 6.939737e-01 9.478902e-01

8687 6.940448e-01 9.478883e-01

8688 6.941185e-01 9.478863e-01

8689 6.941951e-01 9.478844e-01

8690 6.942745e-01 9.478824e-01

8691 6.943568e-01 9.478803e-01

8692 6.944423e-01 9.478782e-01

8693 6.945308e-01 9.478761e-01

8694 6.946227e-01 9.478740e-01

8695 6.947179e-01 9.478718e-01

8696 6.948165e-01 9.478696e-01

8697 6.949187e-01 9.478674e-01

8698 6.950246e-01 9.478652e-01

8699 6.951343e-01 9.478629e-01

8700 6.952478e-01 9.478606e-01

8701 6.953654e-01 9.478582e-01

8702 6.954870e-01 9.478559e-01

8703 6.956129e-01 9.478535e-01

8704 6.957431e-01 9.478511e-01

8705 6.958778e-01 9.478486e-01

8706 6.960171e-01 9.478461e-01

8707 6.961612e-01 9.478436e-01

8708 6.963100e-01 9.478411e-01

8709 6.964639e-01 9.478386e-01

8710 6.966228e-01 9.478360e-01

8711 6.967870e-01 9.478334e-01

8712 6.969565e-01 9.478308e-01

8713 6.971316e-01 9.478282e-01

8714 6.973122e-01 9.478255e-01

8715 6.974987e-01 9.478229e-01

8716 6.976910e-01 9.478202e-01

8717 6.978894e-01 9.478175e-01

8718 6.980939e-01 9.478148e-01

8719 6.983048e-01 9.478121e-01

8720 6.985221e-01 9.478094e-01

8721 6.987460e-01 9.478066e-01

8722 6.989766e-01 9.478039e-01

8723 6.992140e-01 9.478012e-01

8724 6.994584e-01 9.477984e-01

8725 6.997100e-01 9.477957e-01

8726 6.999688e-01 9.477930e-01

8727 7.002350e-01 9.477903e-01

8728 7.005087e-01 9.477876e-01

8729 7.007900e-01 9.477849e-01

8730 7.010791e-01 9.477822e-01

8731 7.013760e-01 9.477795e-01

8732 7.016809e-01 9.477769e-01

8733 7.019939e-01 9.477743e-01

8734 7.023151e-01 9.477717e-01

8735 7.026446e-01 9.477691e-01

8736 7.029825e-01 9.477666e-01

8737 7.033289e-01 9.477642e-01

8738 7.036838e-01 9.477617e-01

8739 7.040474e-01 9.477594e-01

8740 7.044198e-01 9.477571e-01

8741 7.048009e-01 9.477548e-01

8742 7.051909e-01 9.477526e-01

8743 7.055898e-01 9.477505e-01

8744 7.059976e-01 9.477484e-01

8745 7.064144e-01 9.477465e-01

8746 7.068402e-01 9.477446e-01

8747 7.072751e-01 9.477428e-01

8748 7.077190e-01 9.477412e-01

8749 7.081719e-01 9.477396e-01

8750 7.086338e-01 9.477382e-01

8751 7.091047e-01 9.477369e-01

8752 7.095846e-01 9.477357e-01

8753 7.100735e-01 9.477346e-01

8754 7.105711e-01 9.477337e-01

8755 7.110776e-01 9.477330e-01

8756 7.115928e-01 9.477324e-01

8757 7.121167e-01 9.477320e-01

8758 7.126490e-01 9.477318e-01

8759 7.131898e-01 9.477318e-01

8760 7.137389e-01 9.477320e-01

8761 7.142961e-01 9.477324e-01

8762 7.148613e-01 9.477330e-01

8763 7.154344e-01 9.477339e-01

8764 7.160152e-01 9.477350e-01

8765 7.166034e-01 9.477364e-01

8766 7.171989e-01 9.477381e-01

8767 7.178015e-01 9.477400e-01

8768 7.184109e-01 9.477423e-01

8769 7.190270e-01 9.477448e-01

8770 7.196495e-01 9.477477e-01

8771 7.202781e-01 9.477510e-01

8772 7.209126e-01 9.477546e-01

8773 7.215527e-01 9.477586e-01

8774 7.221981e-01 9.477629e-01

8775 7.228485e-01 9.477677e-01

8776 7.235037e-01 9.477729e-01

8777 7.241633e-01 9.477785e-01

8778 7.248270e-01 9.477846e-01

8779 7.254946e-01 9.477911e-01

8780 7.261656e-01 9.477982e-01

8781 7.268397e-01 9.478057e-01

8782 7.275167e-01 9.478138e-01

8783 7.281962e-01 9.478224e-01

8784 7.288778e-01 9.478316e-01

8785 7.295612e-01 9.478414e-01

8786 7.302460e-01 9.478518e-01

8787 7.309320e-01 9.478628e-01

8788 7.316187e-01 9.478744e-01

8789 7.323058e-01 9.478867e-01

8790 7.329930e-01 9.478997e-01

8791 7.336799e-01 9.479134e-01

8792 7.343662e-01 9.479279e-01

8793 7.350515e-01 9.479431e-01

8794 7.357356e-01 9.479590e-01

8795 7.364180e-01 9.479758e-01

8796 7.370984e-01 9.479934e-01

8797 7.377766e-01 9.480118e-01

8798 7.384522e-01 9.480311e-01

8799 7.391249e-01 9.480513e-01

8800 7.397944e-01 9.480724e-01

8801 7.404604e-01 9.480945e-01

8802 7.411227e-01 9.481175e-01

8803 7.417808e-01 9.481414e-01

8804 7.424347e-01 9.481664e-01

8805 7.430840e-01 9.481925e-01

8806 7.437285e-01 9.482195e-01

8807 7.443679e-01 9.482477e-01

8808 7.450020e-01 9.482770e-01

8809 7.456305e-01 9.483073e-01

8810 7.462534e-01 9.483388e-01

8811 7.468703e-01 9.483715e-01

8812 7.474811e-01 9.484054e-01

8813 7.480856e-01 9.484404e-01

8814 7.486837e-01 9.484767e-01

8815 7.492752e-01 9.485143e-01

8816 7.498599e-01 9.485530e-01

8817 7.504377e-01 9.485931e-01

8818 7.510084e-01 9.486345e-01

8819 7.515721e-01 9.486771e-01

8820 7.521285e-01 9.487211e-01

8821 7.526776e-01 9.487664e-01

8822 7.527155e-01 9.487662e-01

8823 7.527546e-01 9.487659e-01

8824 7.527947e-01 9.487657e-01

8825 7.528360e-01 9.487654e-01

8826 7.528785e-01 9.487652e-01

8827 7.529222e-01 9.487649e-01

8828 7.529672e-01 9.487647e-01

8829 7.530134e-01 9.487644e-01

8830 7.530609e-01 9.487642e-01

8831 7.531097e-01 9.487639e-01

8832 7.531599e-01 9.487636e-01

8833 7.532115e-01 9.487634e-01

8834 7.532645e-01 9.487631e-01

8835 7.533190e-01 9.487628e-01

8836 7.533749e-01 9.487625e-01

8837 7.534324e-01 9.487622e-01

8838 7.534915e-01 9.487618e-01

8839 7.535522e-01 9.487615e-01

8840 7.536146e-01 9.487612e-01

8841 7.536786e-01 9.487608e-01

8842 7.537444e-01 9.487605e-01

8843 7.538119e-01 9.487601e-01

8844 7.538813e-01 9.487598e-01

8845 7.539526e-01 9.487594e-01

8846 7.540257e-01 9.487590e-01

8847 7.541009e-01 9.487586e-01

8848 7.541780e-01 9.487581e-01

8849 7.542573e-01 9.487577e-01

8850 7.543386e-01 9.487572e-01

8851 7.544221e-01 9.487568e-01

8852 7.545078e-01 9.487563e-01

8853 7.545958e-01 9.487558e-01

8854 7.546862e-01 9.487553e-01

8855 7.547789e-01 9.487548e-01

8856 7.548741e-01 9.487542e-01

8857 7.549718e-01 9.487537e-01

8858 7.550721e-01 9.487531e-01

8859 7.551749e-01 9.487525e-01

8860 7.552805e-01 9.487519e-01

8861 7.553889e-01 9.487512e-01

8862 7.555000e-01 9.487506e-01

8863 7.556141e-01 9.487499e-01

8864 7.557311e-01 9.487492e-01

8865 7.558512e-01 9.487485e-01

8866 7.559743e-01 9.487477e-01

8867 7.561007e-01 9.487469e-01

8868 7.562302e-01 9.487461e-01

8869 7.563631e-01 9.487453e-01

8870 7.564994e-01 9.487444e-01

8871 7.566392e-01 9.487435e-01

8872 7.567826e-01 9.487426e-01

8873 7.569295e-01 9.487417e-01

8874 7.570802e-01 9.487407e-01

8875 7.572347e-01 9.487396e-01

8876 7.573931e-01 9.487386e-01

8877 7.575554e-01 9.487375e-01

8878 7.577218e-01 9.487363e-01

8879 7.578923e-01 9.487351e-01

8880 7.580671e-01 9.487339e-01

8881 7.582462e-01 9.487326e-01

8882 7.584297e-01 9.487313e-01

8883 7.586177e-01 9.487299e-01

8884 7.588104e-01 9.487284e-01

8885 7.590077e-01 9.487270e-01

8886 7.592098e-01 9.487254e-01

8887 7.594168e-01 9.487238e-01

8888 7.596288e-01 9.487221e-01

8889 7.598459e-01 9.487204e-01

8890 7.600682e-01 9.487186e-01

8891 7.602958e-01 9.487167e-01

8892 7.605288e-01 9.487147e-01

8893 7.607672e-01 9.487127e-01

8894 7.610113e-01 9.487105e-01

8895 7.612611e-01 9.487083e-01

8896 7.615166e-01 9.487060e-01

8897 7.617781e-01 9.487036e-01

8898 7.620456e-01 9.487011e-01

8899 7.623192e-01 9.486985e-01

8900 7.625991e-01 9.486958e-01

8901 7.628852e-01 9.486930e-01

8902 7.631778e-01 9.486900e-01

8903 7.634770e-01 9.486870e-01

8904 7.637828e-01 9.486837e-01

8905 7.640953e-01 9.486804e-01

8906 7.644147e-01 9.486769e-01

8907 7.647410e-01 9.486733e-01

8908 7.650743e-01 9.486695e-01

8909 7.654148e-01 9.486655e-01

8910 7.657626e-01 9.486614e-01

8911 7.661177e-01 9.486570e-01

8912 7.664802e-01 9.486525e-01

8913 7.668503e-01 9.486478e-01

8914 7.672279e-01 9.486429e-01

8915 7.676133e-01 9.486377e-01

8916 7.680065e-01 9.486324e-01

8917 7.684075e-01 9.486268e-01

8918 7.688164e-01 9.486209e-01

8919 7.692334e-01 9.486148e-01

8920 7.696585e-01 9.486084e-01

8921 7.700918e-01 9.486017e-01

8922 7.705332e-01 9.485947e-01

8923 7.709830e-01 9.485875e-01

8924 7.714410e-01 9.485798e-01

8925 7.719075e-01 9.485719e-01

8926 7.723824e-01 9.485636e-01

8927 7.728658e-01 9.485549e-01

8928 7.733576e-01 9.485458e-01

8929 7.738580e-01 9.485363e-01

8930 7.743670e-01 9.485264e-01

8931 7.748844e-01 9.485160e-01

8932 7.754105e-01 9.485052e-01

8933 7.759451e-01 9.484939e-01

8934 7.764883e-01 9.484821e-01

8935 7.770401e-01 9.484698e-01

8936 7.776003e-01 9.484570e-01

8937 7.781690e-01 9.484435e-01

8938 7.787462e-01 9.484295e-01

8939 7.793318e-01 9.484148e-01

8940 7.799257e-01 9.483996e-01

8941 7.805279e-01 9.483836e-01

8942 7.811384e-01 9.483670e-01

8943 7.817569e-01 9.483496e-01

8944 7.823834e-01 9.483315e-01

8945 7.830179e-01 9.483126e-01

8946 7.836602e-01 9.482929e-01

8947 7.843101e-01 9.482724e-01

8948 7.849676e-01 9.482510e-01

8949 7.856325e-01 9.482287e-01

8950 7.863047e-01 9.482055e-01

8951 7.869839e-01 9.481813e-01

8952 7.876701e-01 9.481561e-01

8953 7.883629e-01 9.481299e-01

8954 7.890624e-01 9.481026e-01

8955 7.897681e-01 9.480743e-01

8956 7.904800e-01 9.480447e-01

8957 7.911978e-01 9.480140e-01

8958 7.919212e-01 9.479821e-01

8959 7.926500e-01 9.479489e-01

8960 7.933841e-01 9.479145e-01

8961 7.941230e-01 9.478787e-01

8962 7.948666e-01 9.478415e-01

8963 7.956145e-01 9.478030e-01

8964 7.963666e-01 9.477629e-01

8965 7.971224e-01 9.477214e-01

8966 7.978818e-01 9.476784e-01

8967 7.986443e-01 9.476338e-01

8968 7.994097e-01 9.475875e-01

8969 8.001777e-01 9.475396e-01

8970 8.009479e-01 9.474901e-01

8971 8.017201e-01 9.474387e-01

8972 8.024938e-01 9.473856e-01

8973 8.032688e-01 9.473307e-01

8974 8.040447e-01 9.472739e-01

8975 8.048213e-01 9.472153e-01

8976 8.055980e-01 9.471547e-01

8977 8.063747e-01 9.470921e-01

8978 8.071509e-01 9.470275e-01

8979 8.079263e-01 9.469609e-01

8980 8.087006e-01 9.468921e-01

8981 8.094734e-01 9.468213e-01

8982 8.102444e-01 9.467483e-01

8983 8.110133e-01 9.466731e-01

8984 8.117797e-01 9.465957e-01

8985 8.125433e-01 9.465161e-01

8986 8.133037e-01 9.464342e-01

8987 8.140607e-01 9.463500e-01

8988 8.148139e-01 9.462635e-01

8989 8.155631e-01 9.461746e-01

8990 8.163078e-01 9.460834e-01

8991 8.170479e-01 9.459898e-01

8992 8.177830e-01 9.458938e-01

8993 8.185128e-01 9.457954e-01

8994 8.192371e-01 9.456946e-01

8995 8.199556e-01 9.455913e-01

8996 8.206680e-01 9.454857e-01

8997 8.213741e-01 9.453775e-01

8998 8.220737e-01 9.452670e-01

8999 8.227665e-01 9.451540e-01

9000 8.234523e-01 9.450387e-01

9001 8.241310e-01 9.449210e-01

9002 8.241522e-01 9.449200e-01

9003 8.241739e-01 9.449190e-01

9004 8.241962e-01 9.449180e-01

9005 8.242190e-01 9.449170e-01

9006 8.242424e-01 9.449159e-01

9007 8.242664e-01 9.449148e-01

9008 8.242911e-01 9.449137e-01

9009 8.243163e-01 9.449126e-01

9010 8.243422e-01 9.449114e-01

9011 8.243687e-01 9.449102e-01

9012 8.243959e-01 9.449090e-01

9013 8.244239e-01 9.449077e-01

9014 8.244525e-01 9.449064e-01

9015 8.244818e-01 9.449051e-01

9016 8.245119e-01 9.449037e-01

9017 8.245427e-01 9.449024e-01

9018 8.245743e-01 9.449010e-01

9019 8.246068e-01 9.448995e-01

9020 8.246400e-01 9.448980e-01

9021 8.246741e-01 9.448965e-01

9022 8.247090e-01 9.448950e-01

9023 8.247449e-01 9.448934e-01

9024 8.247816e-01 9.448918e-01

9025 8.248193e-01 9.448901e-01

9026 8.248579e-01 9.448885e-01

9027 8.248976e-01 9.448867e-01

9028 8.249382e-01 9.448850e-01

9029 8.249798e-01 9.448832e-01

9030 8.250225e-01 9.448813e-01

9031 8.250663e-01 9.448795e-01

9032 8.251111e-01 9.448775e-01

9033 8.251572e-01 9.448756e-01

9034 8.252043e-01 9.448736e-01

9035 8.252527e-01 9.448715e-01

9036 8.253022e-01 9.448695e-01

9037 8.253531e-01 9.448673e-01

9038 8.254052e-01 9.448652e-01

9039 8.254586e-01 9.448629e-01

9040 8.255133e-01 9.448607e-01

9041 8.255694e-01 9.448584e-01

9042 8.256269e-01 9.448560e-01

9043 8.256858e-01 9.448536e-01

9044 8.257463e-01 9.448511e-01

9045 8.258082e-01 9.448486e-01

9046 8.258716e-01 9.448460e-01

9047 8.259366e-01 9.448434e-01

9048 8.260033e-01 9.448407e-01

9049 8.260715e-01 9.448380e-01

9050 8.261415e-01 9.448352e-01

9051 8.262131e-01 9.448323e-01

9052 8.262866e-01 9.448294e-01

9053 8.263618e-01 9.448265e-01

9054 8.264389e-01 9.448234e-01

9055 8.265178e-01 9.448203e-01

9056 8.265987e-01 9.448172e-01

9057 8.266815e-01 9.448140e-01

9058 8.267663e-01 9.448107e-01

9059 8.268532e-01 9.448073e-01

9060 8.269421e-01 9.448039e-01

9061 8.270332e-01 9.448004e-01

9062 8.271264e-01 9.447968e-01

9063 8.272219e-01 9.447932e-01

9064 8.273197e-01 9.447895e-01

9065 8.274197e-01 9.447857e-01

9066 8.275221e-01 9.447819e-01

9067 8.276270e-01 9.447779e-01

9068 8.277342e-01 9.447739e-01

9069 8.278440e-01 9.447698e-01

9070 8.279563e-01 9.447656e-01

9071 8.280713e-01 9.447614e-01

9072 8.281888e-01 9.447570e-01

9073 8.283091e-01 9.447526e-01

9074 8.284321e-01 9.447481e-01

9075 8.285579e-01 9.447435e-01

9076 8.286866e-01 9.447388e-01

9077 8.288182e-01 9.447340e-01

9078 8.289527e-01 9.447291e-01

9079 8.290902e-01 9.447242e-01

9080 8.292308e-01 9.447191e-01

9081 8.293745e-01 9.447139e-01

9082 8.295213e-01 9.447087e-01

9083 8.296714e-01 9.447033e-01

9084 8.298247e-01 9.446978e-01

9085 8.299814e-01 9.446923e-01

9086 8.301414e-01 9.446866e-01

9087 8.303048e-01 9.446808e-01

9088 8.304716e-01 9.446749e-01

9089 8.306420e-01 9.446690e-01

9090 8.308160e-01 9.446629e-01

9091 8.309936e-01 9.446566e-01

9092 8.311748e-01 9.446503e-01

9093 8.313598e-01 9.446439e-01

9094 8.315485e-01 9.446374e-01

9095 8.317411e-01 9.446307e-01

9096 8.319375e-01 9.446239e-01

9097 8.321378e-01 9.446171e-01

9098 8.323420e-01 9.446101e-01

9099 8.325503e-01 9.446030e-01

9100 8.327625e-01 9.445957e-01

9101 8.329789e-01 9.445884e-01

9102 8.331993e-01 9.445809e-01

9103 8.334239e-01 9.445733e-01

9104 8.336527e-01 9.445657e-01

9105 8.338857e-01 9.445579e-01

9106 8.341229e-01 9.445499e-01

9107 8.343645e-01 9.445419e-01

9108 8.346103e-01 9.445337e-01

9109 8.348605e-01 9.445255e-01

9110 8.351150e-01 9.445171e-01

9111 8.353739e-01 9.445086e-01

9112 8.356371e-01 9.445001e-01

9113 8.359048e-01 9.444914e-01

9114 8.361769e-01 9.444826e-01

9115 8.364535e-01 9.444737e-01

9116 8.367344e-01 9.444647e-01

9117 8.370198e-01 9.444557e-01

9118 8.373096e-01 9.444465e-01

9119 8.376039e-01 9.444373e-01

9120 8.379025e-01 9.444280e-01

9121 8.382056e-01 9.444186e-01

9122 8.385131e-01 9.444091e-01

9123 8.388249e-01 9.443996e-01

9124 8.391410e-01 9.443901e-01

9125 8.394615e-01 9.443805e-01

9126 8.397862e-01 9.443708e-01

9127 8.401151e-01 9.443612e-01

9128 8.404482e-01 9.443515e-01

9129 8.407855e-01 9.443418e-01

9130 8.411268e-01 9.443322e-01

9131 8.414722e-01 9.443225e-01

9132 8.418215e-01 9.443128e-01

9133 8.421747e-01 9.443032e-01

9134 8.425317e-01 9.442937e-01

9135 8.428924e-01 9.442842e-01

9136 8.432567e-01 9.442748e-01

9137 8.436246e-01 9.442655e-01

9138 8.439960e-01 9.442563e-01

9139 8.443706e-01 9.442472e-01

9140 8.447485e-01 9.442383e-01

9141 8.451296e-01 9.442295e-01

9142 8.455136e-01 9.442209e-01

9143 8.459005e-01 9.442126e-01

9144 8.462901e-01 9.442044e-01

9145 8.466823e-01 9.441965e-01

9146 8.470770e-01 9.441888e-01

9147 8.474740e-01 9.441814e-01

9148 8.478732e-01 9.441744e-01

9149 8.482743e-01 9.441676e-01

9150 8.486774e-01 9.441612e-01

9151 8.490820e-01 9.441552e-01

9152 8.494883e-01 9.441497e-01

9153 8.498958e-01 9.441445e-01

9154 8.503045e-01 9.441398e-01

9155 8.507142e-01 9.441356e-01

9156 8.511247e-01 9.441319e-01

9157 8.515358e-01 9.441288e-01

9158 8.519473e-01 9.441263e-01

9159 8.523591e-01 9.441243e-01

9160 8.527709e-01 9.441230e-01

9161 8.531825e-01 9.441224e-01

9162 8.535938e-01 9.441224e-01

9163 8.540045e-01 9.441232e-01

9164 8.544145e-01 9.441248e-01

9165 8.548235e-01 9.441272e-01

9166 8.552314e-01 9.441304e-01

9167 8.556379e-01 9.441344e-01

9168 8.560429e-01 9.441394e-01

9169 8.564462e-01 9.441453e-01

9170 8.568475e-01 9.441521e-01

9171 8.572467e-01 9.441599e-01

9172 8.576437e-01 9.441688e-01

9173 8.580381e-01 9.441787e-01

9174 8.584298e-01 9.441897e-01

9175 8.588187e-01 9.442018e-01

9176 8.592045e-01 9.442151e-01

9177 8.595871e-01 9.442295e-01

9178 8.599663e-01 9.442451e-01

9179 8.603420e-01 9.442620e-01

9180 8.607140e-01 9.442801e-01

9181 8.610822e-01 9.442994e-01

9182 8.610896e-01 9.442991e-01

9183 8.610972e-01 9.442987e-01

9184 8.611049e-01 9.442984e-01

9185 8.611128e-01 9.442981e-01

9186 8.611208e-01 9.442978e-01

9187 8.611289e-01 9.442974e-01

9188 8.611372e-01 9.442971e-01

9189 8.611457e-01 9.442967e-01

9190 8.611543e-01 9.442964e-01

9191 8.611630e-01 9.442960e-01

9192 8.611719e-01 9.442956e-01

9193 8.611810e-01 9.442952e-01

9194 8.611903e-01 9.442948e-01

9195 8.611997e-01 9.442944e-01

9196 8.612093e-01 9.442940e-01

9197 8.612191e-01 9.442936e-01

9198 8.612290e-01 9.442931e-01

9199 8.612392e-01 9.442927e-01

9200 8.612495e-01 9.442922e-01

9201 8.612600e-01 9.442917e-01

9202 8.612707e-01 9.442912e-01

9203 8.612816e-01 9.442907e-01

9204 8.612927e-01 9.442901e-01

9205 8.613040e-01 9.442896e-01

9206 8.613155e-01 9.442890e-01

9207 8.613272e-01 9.442884e-01

9208 8.613391e-01 9.442878e-01

9209 8.613513e-01 9.442872e-01

9210 8.613636e-01 9.442865e-01

9211 8.613762e-01 9.442858e-01

9212 8.613890e-01 9.442852e-01

9213 8.614021e-01 9.442844e-01

9214 8.614154e-01 9.442837e-01

9215 8.614289e-01 9.442829e-01

9216 8.614426e-01 9.442821e-01

9217 8.614567e-01 9.442813e-01

9218 8.614709e-01 9.442804e-01

9219 8.614854e-01 9.442795e-01

9220 8.615002e-01 9.442786e-01

9221 8.615152e-01 9.442777e-01

9222 8.615305e-01 9.442767e-01

9223 8.615461e-01 9.442757e-01

9224 8.615620e-01 9.442746e-01

9225 8.615781e-01 9.442735e-01

9226 8.615945e-01 9.442724e-01

9227 8.616111e-01 9.442712e-01

9228 8.616281e-01 9.442700e-01

9229 8.616453e-01 9.442687e-01

9230 8.616629e-01 9.442674e-01

9231 8.616807e-01 9.442660e-01

9232 8.616989e-01 9.442646e-01

9233 8.617173e-01 9.442631e-01

9234 8.617360e-01 9.442616e-01

9235 8.617550e-01 9.442600e-01

9236 8.617744e-01 9.442584e-01

9237 8.617940e-01 9.442567e-01

9238 8.618140e-01 9.442549e-01

9239 8.618342e-01 9.442531e-01

9240 8.618548e-01 9.442512e-01

9241 8.618757e-01 9.442492e-01

9242 8.618969e-01 9.442471e-01

9243 8.619184e-01 9.442450e-01

9244 8.619402e-01 9.442428e-01

9245 8.619624e-01 9.442405e-01

9246 8.619848e-01 9.442381e-01

9247 8.620076e-01 9.442356e-01

9248 8.620306e-01 9.442330e-01

9249 8.620540e-01 9.442303e-01

9250 8.620777e-01 9.442275e-01

9251 8.621016e-01 9.442246e-01

9252 8.621259e-01 9.442216e-01

9253 8.621504e-01 9.442184e-01

9254 8.621753e-01 9.442152e-01

9255 8.622004e-01 9.442118e-01

9256 8.622258e-01 9.442082e-01

9257 8.622515e-01 9.442046e-01

9258 8.622774e-01 9.442008e-01

9259 8.623035e-01 9.441968e-01

9260 8.623300e-01 9.441927e-01

9261 8.623566e-01 9.441884e-01

9262 8.623835e-01 9.441839e-01

9263 8.624106e-01 9.441793e-01

9264 8.624379e-01 9.441744e-01

9265 8.624653e-01 9.441694e-01

9266 8.624930e-01 9.441642e-01

9267 8.625208e-01 9.441587e-01

9268 8.625488e-01 9.441531e-01

9269 8.625768e-01 9.441472e-01

9270 8.626050e-01 9.441411e-01

9271 8.626333e-01 9.441347e-01

9272 8.626616e-01 9.441281e-01

9273 8.626900e-01 9.441212e-01

9274 8.627184e-01 9.441140e-01

9275 8.627468e-01 9.441066e-01

9276 8.627752e-01 9.440988e-01

9277 8.628035e-01 9.440908e-01

9278 8.628318e-01 9.440824e-01

9279 8.628599e-01 9.440737e-01

9280 8.628879e-01 9.440646e-01

9281 8.629157e-01 9.440552e-01

9282 8.629433e-01 9.440454e-01

9283 8.629707e-01 9.440352e-01

9284 8.629978e-01 9.440246e-01

9285 8.630246e-01 9.440135e-01

9286 8.630510e-01 9.440021e-01

9287 8.630770e-01 9.439902e-01

9288 8.631026e-01 9.439778e-01

9289 8.631277e-01 9.439649e-01

9290 8.631522e-01 9.439515e-01

9291 8.631762e-01 9.439376e-01

9292 8.631996e-01 9.439232e-01

9293 8.632223e-01 9.439082e-01

9294 8.632442e-01 9.438926e-01

9295 8.632654e-01 9.438764e-01

9296 8.632857e-01 9.438595e-01

9297 8.633051e-01 9.438421e-01

9298 8.633236e-01 9.438239e-01

9299 8.633410e-01 9.438051e-01

9300 8.633574e-01 9.437856e-01

9301 8.633726e-01 9.437653e-01

9302 8.633866e-01 9.437442e-01

9303 8.633994e-01 9.437224e-01

9304 8.634108e-01 9.436998e-01

9305 8.634208e-01 9.436763e-01

9306 8.634294e-01 9.436520e-01

9307 8.634364e-01 9.436267e-01

9308 8.634418e-01 9.436006e-01

9309 8.634455e-01 9.435735e-01

9310 8.634474e-01 9.435455e-01

9311 8.634475e-01 9.435164e-01

9312 8.634458e-01 9.434863e-01

9313 8.634420e-01 9.434552e-01

9314 8.634362e-01 9.434230e-01

9315 8.634283e-01 9.433897e-01

9316 8.634181e-01 9.433552e-01

9317 8.634058e-01 9.433195e-01

9318 8.633910e-01 9.432827e-01

9319 8.633739e-01 9.432446e-01

9320 8.633543e-01 9.432052e-01

9321 8.633321e-01 9.431646e-01

9322 8.633073e-01 9.431226e-01

9323 8.632799e-01 9.430793e-01

9324 8.632497e-01 9.430346e-01

9325 8.632166e-01 9.429884e-01

9326 8.631807e-01 9.429409e-01

9327 8.631419e-01 9.428918e-01

9328 8.631001e-01 9.428412e-01

9329 8.630552e-01 9.427892e-01

9330 8.630073e-01 9.427355e-01

9331 8.629562e-01 9.426803e-01

9332 8.629019e-01 9.426234e-01

9333 8.628444e-01 9.425649e-01

9334 8.627837e-01 9.425047e-01

9335 8.627196e-01 9.424428e-01

9336 8.626523e-01 9.423792e-01

9337 8.625816e-01 9.423139e-01

9338 8.625075e-01 9.422468e-01

9339 8.624301e-01 9.421778e-01

9340 8.623492e-01 9.421071e-01

9341 8.622650e-01 9.420346e-01

9342 8.621774e-01 9.419602e-01

9343 8.620863e-01 9.418839e-01

9344 8.619920e-01 9.418057e-01

9345 8.618942e-01 9.417257e-01

9346 8.617931e-01 9.416437e-01

9347 8.616887e-01 9.415599e-01

9348 8.615809e-01 9.414741e-01

9349 8.614700e-01 9.413863e-01

9350 8.613558e-01 9.412967e-01

9351 8.612384e-01 9.412050e-01

9352 8.611180e-01 9.411115e-01

9353 8.609945e-01 9.410160e-01

9354 8.608679e-01 9.409185e-01

9355 8.607385e-01 9.408192e-01

9356 8.606062e-01 9.407179e-01

9357 8.604711e-01 9.406147e-01

9358 8.603333e-01 9.405095e-01

9359 8.601928e-01 9.404025e-01

9360 8.600499e-01 9.402937e-01

9361 8.599045e-01 9.401830e-01

9362 8.599022e-01 9.401820e-01

9363 8.598998e-01 9.401810e-01

9364 8.598973e-01 9.401799e-01

9365 8.598946e-01 9.401788e-01

9366 8.598917e-01 9.401776e-01

9367 8.598887e-01 9.401765e-01

9368 8.598855e-01 9.401753e-01

9369 8.598821e-01 9.401740e-01

9370 8.598785e-01 9.401728e-01

9371 8.598748e-01 9.401715e-01

9372 8.598708e-01 9.401702e-01

9373 8.598666e-01 9.401689e-01

9374 8.598622e-01 9.401675e-01

9375 8.598576e-01 9.401661e-01

9376 8.598527e-01 9.401647e-01

9377 8.598475e-01 9.401632e-01

9378 8.598421e-01 9.401617e-01

9379 8.598364e-01 9.401602e-01

9380 8.598305e-01 9.401586e-01

9381 8.598242e-01 9.401570e-01

9382 8.598176e-01 9.401554e-01

9383 8.598107e-01 9.401537e-01

9384 8.598035e-01 9.401521e-01

9385 8.597959e-01 9.401503e-01

9386 8.597880e-01 9.401486e-01

9387 8.597796e-01 9.401468e-01

9388 8.597709e-01 9.401449e-01

9389 8.597618e-01 9.401430e-01

9390 8.597522e-01 9.401411e-01

9391 8.597422e-01 9.401392e-01

9392 8.597318e-01 9.401372e-01

9393 8.597208e-01 9.401352e-01

9394 8.597094e-01 9.401331e-01

9395 8.596975e-01 9.401310e-01

9396 8.596850e-01 9.401288e-01

9397 8.596720e-01 9.401266e-01

9398 8.596584e-01 9.401244e-01

9399 8.596442e-01 9.401221e-01

9400 8.596293e-01 9.401197e-01

9401 8.596138e-01 9.401174e-01

9402 8.595977e-01 9.401149e-01

9403 8.595809e-01 9.401124e-01

9404 8.595633e-01 9.401099e-01

9405 8.595450e-01 9.401073e-01

9406 8.595259e-01 9.401047e-01

9407 8.595060e-01 9.401020e-01

9408 8.594852e-01 9.400993e-01

9409 8.594636e-01 9.400965e-01

9410 8.594411e-01 9.400937e-01

9411 8.594177e-01 9.400908e-01

9412 8.593932e-01 9.400879e-01

9413 8.593678e-01 9.400849e-01

9414 8.593413e-01 9.400818e-01

9415 8.593138e-01 9.400787e-01

9416 8.592851e-01 9.400755e-01

9417 8.592553e-01 9.400723e-01

9418 8.592243e-01 9.400690e-01

9419 8.591920e-01 9.400657e-01

9420 8.591584e-01 9.400623e-01

9421 8.591235e-01 9.400588e-01

9422 8.590872e-01 9.400553e-01

9423 8.590494e-01 9.400517e-01

9424 8.590102e-01 9.400480e-01

9425 8.589694e-01 9.400443e-01

9426 8.589270e-01 9.400405e-01

9427 8.588830e-01 9.400367e-01

9428 8.588373e-01 9.400327e-01

9429 8.587898e-01 9.400288e-01

9430 8.587404e-01 9.400247e-01

9431 8.586892e-01 9.400206e-01

9432 8.586360e-01 9.400164e-01

9433 8.585807e-01 9.400122e-01

9434 8.585234e-01 9.400079e-01

9435 8.584639e-01 9.400035e-01

9436 8.584022e-01 9.399991e-01

9437 8.583381e-01 9.399946e-01

9438 8.582716e-01 9.399900e-01

9439 8.582027e-01 9.399854e-01

9440 8.581312e-01 9.399807e-01

9441 8.580570e-01 9.399759e-01

9442 8.579802e-01 9.399711e-01

9443 8.579005e-01 9.399663e-01

9444 8.578179e-01 9.399613e-01

9445 8.577323e-01 9.399563e-01

9446 8.576436e-01 9.399513e-01

9447 8.575517e-01 9.399461e-01

9448 8.574565e-01 9.399410e-01

9449 8.573579e-01 9.399358e-01

9450 8.572558e-01 9.399305e-01

9451 8.571501e-01 9.399252e-01

9452 8.570407e-01 9.399198e-01

9453 8.569275e-01 9.399144e-01

9454 8.568103e-01 9.399090e-01

9455 8.566891e-01 9.399035e-01

9456 8.565637e-01 9.398980e-01

9457 8.564340e-01 9.398925e-01

9458 8.562999e-01 9.398869e-01

9459 8.561613e-01 9.398813e-01

9460 8.560181e-01 9.398757e-01

9461 8.558700e-01 9.398701e-01

9462 8.557171e-01 9.398645e-01

9463 8.555591e-01 9.398589e-01

9464 8.553960e-01 9.398533e-01

9465 8.552276e-01 9.398478e-01

9466 8.550537e-01 9.398422e-01

9467 8.548743e-01 9.398367e-01

9468 8.546893e-01 9.398312e-01

9469 8.544984e-01 9.398258e-01

9470 8.543016e-01 9.398204e-01

9471 8.540987e-01 9.398151e-01

9472 8.538896e-01 9.398099e-01

9473 8.536741e-01 9.398048e-01

9474 8.534522e-01 9.397998e-01

9475 8.532237e-01 9.397949e-01

9476 8.529885e-01 9.397901e-01

9477 8.527465e-01 9.397854e-01

9478 8.524975e-01 9.397810e-01

9479 8.522414e-01 9.397767e-01

9480 8.519781e-01 9.397725e-01

9481 8.517075e-01 9.397686e-01

9482 8.514295e-01 9.397649e-01

9483 8.511440e-01 9.397615e-01

9484 8.508509e-01 9.397583e-01

9485 8.505500e-01 9.397554e-01

9486 8.502414e-01 9.397528e-01

9487 8.499249e-01 9.397505e-01

9488 8.496004e-01 9.397485e-01

9489 8.492679e-01 9.397469e-01

9490 8.489273e-01 9.397458e-01

9491 8.485786e-01 9.397450e-01

9492 8.482217e-01 9.397446e-01

9493 8.478566e-01 9.397448e-01

9494 8.474833e-01 9.397454e-01

9495 8.471016e-01 9.397466e-01

9496 8.467117e-01 9.397483e-01

9497 8.463136e-01 9.397505e-01

9498 8.459072e-01 9.397534e-01

9499 8.454925e-01 9.397570e-01

9500 8.450697e-01 9.397612e-01

9501 8.446388e-01 9.397661e-01

9502 8.441997e-01 9.397718e-01

9503 8.437527e-01 9.397782e-01

9504 8.432977e-01 9.397854e-01

9505 8.428349e-01 9.397935e-01

9506 8.423644e-01 9.398025e-01

9507 8.418863e-01 9.398124e-01

9508 8.414008e-01 9.398233e-01

9509 8.409080e-01 9.398351e-01

9510 8.404080e-01 9.398480e-01

9511 8.399012e-01 9.398620e-01

9512 8.393875e-01 9.398770e-01

9513 8.388673e-01 9.398933e-01

9514 8.383408e-01 9.399107e-01

9515 8.378081e-01 9.399293e-01

9516 8.372697e-01 9.399493e-01

9517 8.367256e-01 9.399705e-01

9518 8.361761e-01 9.399931e-01

9519 8.356216e-01 9.400171e-01

9520 8.350623e-01 9.400425e-01

9521 8.344986e-01 9.400694e-01

9522 8.339306e-01 9.400978e-01

9523 8.333588e-01 9.401278e-01

9524 8.327835e-01 9.401594e-01

9525 8.322049e-01 9.401926e-01

9526 8.316235e-01 9.402274e-01

9527 8.310395e-01 9.402640e-01

9528 8.304534e-01 9.403023e-01

9529 8.298654e-01 9.403424e-01

9530 8.292759e-01 9.403843e-01

9531 8.286853e-01 9.404280e-01

9532 8.280939e-01 9.404736e-01

9533 8.275021e-01 9.405211e-01

9534 8.269103e-01 9.405706e-01

9535 8.263188e-01 9.406219e-01

9536 8.257279e-01 9.406749e-01

9537 8.251380e-01 9.407295e-01

9538 8.245494e-01 9.407856e-01

9539 8.239626e-01 9.408429e-01

9540 8.233778e-01 9.409015e-01

9541 8.227953e-01 9.409611e-01

9542 8.227809e-01 9.409610e-01

9543 8.227660e-01 9.409609e-01

9544 8.227506e-01 9.409607e-01

9545 8.227346e-01 9.409606e-01

9546 8.227180e-01 9.409604e-01

9547 8.227008e-01 9.409603e-01

9548 8.226830e-01 9.409601e-01

9549 8.226646e-01 9.409599e-01

9550 8.226455e-01 9.409598e-01

9551 8.226257e-01 9.409596e-01

9552 8.226052e-01 9.409594e-01

9553 8.225839e-01 9.409592e-01

9554 8.225619e-01 9.409591e-01

9555 8.225391e-01 9.409589e-01

9556 8.225155e-01 9.409587e-01

9557 8.224911e-01 9.409585e-01

9558 8.224658e-01 9.409582e-01

9559 8.224395e-01 9.409580e-01

9560 8.224124e-01 9.409578e-01

9561 8.223843e-01 9.409576e-01

9562 8.223551e-01 9.409573e-01

9563 8.223250e-01 9.409571e-01

9564 8.222937e-01 9.409568e-01

9565 8.222614e-01 9.409565e-01

9566 8.222279e-01 9.409563e-01

9567 8.221933e-01 9.409560e-01

9568 8.221574e-01 9.409557e-01

9569 8.221202e-01 9.409554e-01

9570 8.220818e-01 9.409550e-01

9571 8.220420e-01 9.409547e-01

9572 8.220007e-01 9.409543e-01

9573 8.219581e-01 9.409540e-01

9574 8.219139e-01 9.409536e-01

9575 8.218682e-01 9.409532e-01

9576 8.218209e-01 9.409528e-01

9577 8.217719e-01 9.409524e-01

9578 8.217212e-01 9.409520e-01

9579 8.216688e-01 9.409515e-01

9580 8.216145e-01 9.409510e-01

9581 8.215584e-01 9.409505e-01

9582 8.215002e-01 9.409500e-01

9583 8.214401e-01 9.409495e-01

9584 8.213779e-01 9.409490e-01

9585 8.213136e-01 9.409484e-01

9586 8.212470e-01 9.409478e-01

9587 8.211781e-01 9.409472e-01

9588 8.211069e-01 9.409465e-01

9589 8.210332e-01 9.409459e-01

9590 8.209570e-01 9.409452e-01

9591 8.208781e-01 9.409445e-01

9592 8.207966e-01 9.409437e-01

9593 8.207123e-01 9.409429e-01

9594 8.206252e-01 9.409421e-01

9595 8.205351e-01 9.409413e-01

9596 8.204419e-01 9.409404e-01

9597 8.203456e-01 9.409395e-01

9598 8.202461e-01 9.409386e-01

9599 8.201432e-01 9.409376e-01

9600 8.200368e-01 9.409366e-01

9601 8.199269e-01 9.409356e-01

9602 8.198133e-01 9.409345e-01

9603 8.196960e-01 9.409333e-01

9604 8.195747e-01 9.409321e-01

9605 8.194495e-01 9.409309e-01

9606 8.193201e-01 9.409296e-01

9607 8.191864e-01 9.409283e-01

9608 8.190484e-01 9.409269e-01

9609 8.189059e-01 9.409255e-01

9610 8.187587e-01 9.409240e-01

9611 8.186067e-01 9.409224e-01

9612 8.184499e-01 9.409208e-01

9613 8.182880e-01 9.409192e-01

9614 8.181209e-01 9.409174e-01

9615 8.179484e-01 9.409156e-01

9616 8.177705e-01 9.409137e-01

9617 8.175870e-01 9.409118e-01

9618 8.173977e-01 9.409098e-01

9619 8.172024e-01 9.409077e-01

9620 8.170011e-01 9.409055e-01

9621 8.167934e-01 9.409032e-01

9622 8.165794e-01 9.409008e-01

9623 8.163589e-01 9.408984e-01

9624 8.161315e-01 9.408958e-01

9625 8.158973e-01 9.408932e-01

9626 8.156560e-01 9.408904e-01

9627 8.154075e-01 9.408876e-01

9628 8.151516e-01 9.408846e-01

9629 8.148881e-01 9.408815e-01

9630 8.146169e-01 9.408783e-01

9631 8.143378e-01 9.408750e-01

9632 8.140506e-01 9.408715e-01

9633 8.137551e-01 9.408679e-01

9634 8.134513e-01 9.408642e-01

9635 8.131388e-01 9.408603e-01

9636 8.128177e-01 9.408563e-01

9637 8.124876e-01 9.408521e-01

9638 8.121484e-01 9.408478e-01

9639 8.118001e-01 9.408433e-01

9640 8.114424e-01 9.408386e-01

9641 8.110751e-01 9.408338e-01

9642 8.106982e-01 9.408287e-01

9643 8.103115e-01 9.408235e-01

9644 8.099148e-01 9.408180e-01

9645 8.095080e-01 9.408124e-01

9646 8.090911e-01 9.408065e-01

9647 8.086638e-01 9.408005e-01

9648 8.082261e-01 9.407942e-01

9649 8.077779e-01 9.407876e-01

9650 8.073190e-01 9.407808e-01

9651 8.068495e-01 9.407738e-01

9652 8.063691e-01 9.407665e-01

9653 8.058779e-01 9.407589e-01

9654 8.053759e-01 9.407510e-01

9655 8.048629e-01 9.407429e-01

9656 8.043389e-01 9.407344e-01

9657 8.038039e-01 9.407256e-01

9658 8.032580e-01 9.407165e-01

9659 8.027011e-01 9.407071e-01

9660 8.021333e-01 9.406973e-01

9661 8.015545e-01 9.406872e-01

9662 8.009650e-01 9.406767e-01

9663 8.003646e-01 9.406658e-01

9664 7.997536e-01 9.406546e-01

9665 7.991320e-01 9.406429e-01

9666 7.985000e-01 9.406309e-01

9667 7.978577e-01 9.406184e-01

9668 7.972052e-01 9.406054e-01

9669 7.965428e-01 9.405921e-01

9670 7.958706e-01 9.405782e-01

9671 7.951889e-01 9.405639e-01

9672 7.944978e-01 9.405491e-01

9673 7.937977e-01 9.405338e-01

9674 7.930888e-01 9.405179e-01

9675 7.923714e-01 9.405016e-01

9676 7.916458e-01 9.404846e-01

9677 7.909123e-01 9.404672e-01

9678 7.901712e-01 9.404491e-01

9679 7.894230e-01 9.404305e-01

9680 7.886680e-01 9.404113e-01

9681 7.879066e-01 9.403914e-01

9682 7.871392e-01 9.403710e-01

9683 7.863661e-01 9.403499e-01

9684 7.855879e-01 9.403281e-01

9685 7.848050e-01 9.403056e-01

9686 7.840179e-01 9.402825e-01

9687 7.832269e-01 9.402587e-01

9688 7.824326e-01 9.402341e-01

9689 7.816354e-01 9.402089e-01

9690 7.808359e-01 9.401828e-01

9691 7.800345e-01 9.401561e-01

9692 7.792318e-01 9.401285e-01

9693 7.784282e-01 9.401002e-01

9694 7.776243e-01 9.400711e-01

9695 7.768205e-01 9.400412e-01

9696 7.760174e-01 9.400104e-01

9697 7.752154e-01 9.399789e-01

9698 7.744152e-01 9.399465e-01

9699 7.736170e-01 9.399132e-01

9700 7.728215e-01 9.398791e-01

9701 7.720292e-01 9.398441e-01

9702 7.712405e-01 9.398082e-01

9703 7.704558e-01 9.397715e-01

9704 7.696757e-01 9.397338e-01

9705 7.689006e-01 9.396953e-01

9706 7.681309e-01 9.396558e-01

9707 7.673670e-01 9.396155e-01

9708 7.666094e-01 9.395742e-01

9709 7.658584e-01 9.395320e-01

9710 7.651144e-01 9.394888e-01

9711 7.643778e-01 9.394447e-01

9712 7.636489e-01 9.393998e-01

9713 7.629281e-01 9.393538e-01

9714 7.622156e-01 9.393070e-01

9715 7.615117e-01 9.392593e-01

9716 7.608168e-01 9.392109e-01

9717 7.601311e-01 9.391621e-01

9718 7.594547e-01 9.391129e-01

9719 7.587880e-01 9.390636e-01

9720 7.581311e-01 9.390142e-01

9721 7.574842e-01 9.389649e-01

9722 7.574525e-01 9.389640e-01

9723 7.574198e-01 9.389632e-01

9724 7.573861e-01 9.389624e-01

9725 7.573513e-01 9.389615e-01

9726 7.573154e-01 9.389606e-01

9727 7.572784e-01 9.389597e-01

9728 7.572402e-01 9.389588e-01

9729 7.572009e-01 9.389579e-01

9730 7.571603e-01 9.389569e-01

9731 7.571184e-01 9.389560e-01

9732 7.570752e-01 9.389550e-01

9733 7.570307e-01 9.389540e-01

9734 7.569848e-01 9.389530e-01

9735 7.569376e-01 9.389519e-01

9736 7.568888e-01 9.389509e-01

9737 7.568386e-01 9.389498e-01

9738 7.567868e-01 9.389487e-01

9739 7.567334e-01 9.389476e-01

9740 7.566784e-01 9.389465e-01

9741 7.566217e-01 9.389454e-01

9742 7.565633e-01 9.389443e-01

9743 7.565031e-01 9.389431e-01

9744 7.564410e-01 9.389419e-01

9745 7.563771e-01 9.389407e-01

9746 7.563112e-01 9.389395e-01

9747 7.562434e-01 9.389383e-01

9748 7.561735e-01 9.389370e-01

9749 7.561014e-01 9.389358e-01

9750 7.560272e-01 9.389345e-01

9751 7.559508e-01 9.389332e-01

9752 7.558721e-01 9.389319e-01

9753 7.557910e-01 9.389306e-01

9754 7.557075e-01 9.389292e-01

9755 7.556214e-01 9.389279e-01

9756 7.555329e-01 9.389265e-01

9757 7.554416e-01 9.389251e-01

9758 7.553477e-01 9.389237e-01

9759 7.552510e-01 9.389223e-01

9760 7.551514e-01 9.389209e-01

9761 7.550489e-01 9.389195e-01

9762 7.549434e-01 9.389180e-01

9763 7.548348e-01 9.389166e-01

9764 7.547229e-01 9.389151e-01

9765 7.546079e-01 9.389136e-01

9766 7.544894e-01 9.389121e-01

9767 7.543676e-01 9.389106e-01

9768 7.542422e-01 9.389091e-01

9769 7.541132e-01 9.389076e-01

9770 7.539804e-01 9.389061e-01

9771 7.538439e-01 9.389045e-01

9772 7.537035e-01 9.389030e-01

9773 7.535591e-01 9.389015e-01

9774 7.534106e-01 9.388999e-01

9775 7.532578e-01 9.388984e-01

9776 7.531008e-01 9.388968e-01

9777 7.529394e-01 9.388953e-01

9778 7.527735e-01 9.388938e-01

9779 7.526030e-01 9.388922e-01

9780 7.524278e-01 9.388907e-01

9781 7.522477e-01 9.388892e-01

9782 7.520627e-01 9.388877e-01

9783 7.518727e-01 9.388862e-01

9784 7.516776e-01 9.388847e-01

9785 7.514771e-01 9.388833e-01

9786 7.512714e-01 9.388818e-01

9787 7.510601e-01 9.388804e-01

9788 7.508433e-01 9.388790e-01

9789 7.506207e-01 9.388777e-01

9790 7.503924e-01 9.388764e-01

9791 7.501581e-01 9.388751e-01

9792 7.499178e-01 9.388738e-01

9793 7.496714e-01 9.388726e-01

9794 7.494187e-01 9.388715e-01

9795 7.491597e-01 9.388704e-01

9796 7.488942e-01 9.388693e-01

9797 7.486222e-01 9.388683e-01

9798 7.483435e-01 9.388674e-01

9799 7.480580e-01 9.388665e-01

9800 7.477657e-01 9.388658e-01

9801 7.474665e-01 9.388651e-01

9802 7.471602e-01 9.388644e-01

9803 7.468467e-01 9.388639e-01

9804 7.465261e-01 9.388635e-01

9805 7.461982e-01 9.388632e-01

9806 7.458629e-01 9.388630e-01

9807 7.455202e-01 9.388630e-01

9808 7.451700e-01 9.388630e-01

9809 7.448122e-01 9.388632e-01

9810 7.444468e-01 9.388636e-01

9811 7.440738e-01 9.388641e-01

9812 7.436931e-01 9.388648e-01

9813 7.433047e-01 9.388656e-01

9814 7.429085e-01 9.388667e-01

9815 7.425045e-01 9.388679e-01

9816 7.420928e-01 9.388694e-01

9817 7.416733e-01 9.388711e-01

9818 7.412460e-01 9.388730e-01

9819 7.408110e-01 9.388751e-01

9820 7.403683e-01 9.388776e-01

9821 7.399179e-01 9.388803e-01

9822 7.394599e-01 9.388833e-01

9823 7.389943e-01 9.388866e-01

9824 7.385212e-01 9.388902e-01

9825 7.380408e-01 9.388942e-01

9826 7.375530e-01 9.388985e-01

9827 7.370580e-01 9.389032e-01

9828 7.365560e-01 9.389083e-01

9829 7.360470e-01 9.389138e-01

9830 7.355312e-01 9.389198e-01

9831 7.350088e-01 9.389262e-01

9832 7.344799e-01 9.389331e-01

9833 7.339448e-01 9.389405e-01

9834 7.334035e-01 9.389484e-01

9835 7.328564e-01 9.389569e-01

9836 7.323036e-01 9.389660e-01

9837 7.317454e-01 9.389756e-01

9838 7.311821e-01 9.389859e-01

9839 7.306138e-01 9.389968e-01

9840 7.300410e-01 9.390084e-01

9841 7.294637e-01 9.390208e-01

9842 7.288824e-01 9.390339e-01

9843 7.282974e-01 9.390477e-01

9844 7.277089e-01 9.390624e-01

9845 7.271172e-01 9.390779e-01

9846 7.265228e-01 9.390943e-01

9847 7.259260e-01 9.391116e-01

9848 7.253270e-01 9.391298e-01

9849 7.247264e-01 9.391490e-01

9850 7.241243e-01 9.391692e-01

9851 7.235212e-01 9.391905e-01

9852 7.229175e-01 9.392129e-01

9853 7.223135e-01 9.392364e-01

9854 7.217096e-01 9.392611e-01

9855 7.211061e-01 9.392870e-01

9856 7.205036e-01 9.393142e-01

9857 7.199022e-01 9.393426e-01

9858 7.193025e-01 9.393725e-01

9859 7.187047e-01 9.394037e-01

9860 7.181094e-01 9.394363e-01

9861 7.175167e-01 9.394704e-01

9862 7.169271e-01 9.395061e-01

9863 7.163409e-01 9.395433e-01

9864 7.157586e-01 9.395822e-01

9865 7.151803e-01 9.396227e-01

9866 7.146066e-01 9.396649e-01

9867 7.140376e-01 9.397089e-01

9868 7.134737e-01 9.397547e-01

9869 7.129152e-01 9.398024e-01

9870 7.123624e-01 9.398520e-01

9871 7.118155e-01 9.399036e-01

9872 7.112749e-01 9.399571e-01

9873 7.107408e-01 9.400128e-01

9874 7.102135e-01 9.400705e-01

9875 7.096931e-01 9.401304e-01

9876 7.091799e-01 9.401925e-01

9877 7.086741e-01 9.402569e-01

9878 7.081758e-01 9.403236e-01

9879 7.076853e-01 9.403926e-01

9880 7.072026e-01 9.404640e-01

9881 7.067280e-01 9.405379e-01

9882 7.062616e-01 9.406143e-01

9883 7.058035e-01 9.406932e-01

9884 7.053537e-01 9.407746e-01

9885 7.049124e-01 9.408587e-01

9886 7.044796e-01 9.409455e-01

9887 7.040554e-01 9.410349e-01

9888 7.036399e-01 9.411271e-01

9889 7.032330e-01 9.412219e-01

9890 7.028348e-01 9.413193e-01

9891 7.024454e-01 9.414192e-01

9892 7.020646e-01 9.415215e-01

9893 7.016924e-01 9.416260e-01

9894 7.013290e-01 9.417326e-01

9895 7.009741e-01 9.418413e-01

9896 7.006278e-01 9.419520e-01

9897 7.002900e-01 9.420645e-01

9898 6.999607e-01 9.421789e-01

9899 6.996397e-01 9.422950e-01

9900 6.993270e-01 9.424127e-01

9901 6.990225e-01 9.425320e-01

9902 6.989935e-01 9.425321e-01

9903 6.989637e-01 9.425323e-01

9904 6.989333e-01 9.425325e-01

9905 6.989023e-01 9.425327e-01

9906 6.988705e-01 9.425329e-01

9907 6.988381e-01 9.425331e-01

9908 6.988049e-01 9.425333e-01

9909 6.987710e-01 9.425335e-01

9910 6.987364e-01 9.425337e-01

9911 6.987011e-01 9.425339e-01

9912 6.986650e-01 9.425341e-01

9913 6.986282e-01 9.425343e-01

9914 6.985907e-01 9.425346e-01

9915 6.985523e-01 9.425348e-01

9916 6.985132e-01 9.425350e-01

9917 6.984733e-01 9.425353e-01

9918 6.984326e-01 9.425355e-01

9919 6.983911e-01 9.425358e-01

9920 6.983488e-01 9.425360e-01

9921 6.983056e-01 9.425363e-01

9922 6.982616e-01 9.425366e-01

9923 6.982168e-01 9.425369e-01

9924 6.981712e-01 9.425371e-01

9925 6.981246e-01 9.425374e-01

9926 6.980772e-01 9.425377e-01

9927 6.980290e-01 9.425380e-01

9928 6.979798e-01 9.425383e-01

9929 6.979298e-01 9.425386e-01

9930 6.978788e-01 9.425390e-01

9931 6.978270e-01 9.425393e-01

9932 6.977743e-01 9.425396e-01

9933 6.977206e-01 9.425399e-01

9934 6.976660e-01 9.425403e-01

9935 6.976105e-01 9.425406e-01

9936 6.975541e-01 9.425410e-01

9937 6.974968e-01 9.425414e-01

9938 6.974385e-01 9.425417e-01

9939 6.973793e-01 9.425421e-01

9940 6.973191e-01 9.425425e-01

9941 6.972580e-01 9.425429e-01

9942 6.971960e-01 9.425433e-01

9943 6.971331e-01 9.425437e-01

9944 6.970693e-01 9.425441e-01

9945 6.970045e-01 9.425445e-01

9946 6.969388e-01 9.425450e-01

9947 6.968722e-01 9.425454e-01

9948 6.968048e-01 9.425458e-01

9949 6.967364e-01 9.425463e-01

9950 6.966671e-01 9.425468e-01

9951 6.965970e-01 9.425472e-01

9952 6.965261e-01 9.425477e-01

9953 6.964543e-01 9.425482e-01

9954 6.963817e-01 9.425487e-01

9955 6.963082e-01 9.425492e-01

9956 6.962340e-01 9.425497e-01

9957 6.961591e-01 9.425503e-01

9958 6.960834e-01 9.425508e-01

9959 6.960070e-01 9.425513e-01

9960 6.959300e-01 9.425519e-01

9961 6.958523e-01 9.425525e-01

9962 6.957739e-01 9.425530e-01

9963 6.956950e-01 9.425536e-01

9964 6.956156e-01 9.425542e-01

9965 6.955356e-01 9.425548e-01

9966 6.954552e-01 9.425554e-01

9967 6.953743e-01 9.425561e-01

9968 6.952931e-01 9.425567e-01

9969 6.952116e-01 9.425573e-01

9970 6.951298e-01 9.425580e-01

9971 6.950477e-01 9.425587e-01

9972 6.949656e-01 9.425594e-01

9973 6.948833e-01 9.425600e-01

9974 6.948009e-01 9.425607e-01

9975 6.947186e-01 9.425614e-01

9976 6.946364e-01 9.425622e-01

9977 6.945543e-01 9.425629e-01

9978 6.944725e-01 9.425636e-01

9979 6.943909e-01 9.425644e-01

9980 6.943098e-01 9.425652e-01

9981 6.942291e-01 9.425659e-01

9982 6.941490e-01 9.425667e-01

9983 6.940695e-01 9.425675e-01

9984 6.939907e-01 9.425683e-01

9985 6.939128e-01 9.425691e-01

9986 6.938358e-01 9.425699e-01

9987 6.937597e-01 9.425708e-01

9988 6.936848e-01 9.425716e-01

9989 6.936111e-01 9.425725e-01

9990 6.935387e-01 9.425733e-01

9991 6.934677e-01 9.425742e-01

9992 6.933982e-01 9.425750e-01

9993 6.933303e-01 9.425759e-01

9994 6.932642e-01 9.425768e-01

9995 6.931999e-01 9.425777e-01

9996 6.931376e-01 9.425786e-01

9997 6.930773e-01 9.425795e-01

9998 6.930193e-01 9.425804e-01

9999 6.929635e-01 9.425813e-01

10000 6.929101e-01 9.425822e-01

10001 6.928592e-01 9.425831e-01

10002 6.928110e-01 9.425840e-01

10003 6.927655e-01 9.425849e-01

10004 6.927229e-01 9.425858e-01

10005 6.926832e-01 9.425867e-01

10006 6.926466e-01 9.425876e-01

10007 6.926132e-01 9.425885e-01

10008 6.925831e-01 9.425894e-01

10009 6.925564e-01 9.425903e-01

10010 6.925331e-01 9.425912e-01

10011 6.925135e-01 9.425920e-01

10012 6.924975e-01 9.425929e-01

10013 6.924852e-01 9.425937e-01

10014 6.924768e-01 9.425945e-01

10015 6.924724e-01 9.425953e-01

10016 6.924719e-01 9.425961e-01

10017 6.924756e-01 9.425968e-01

10018 6.924833e-01 9.425975e-01

10019 6.924953e-01 9.425982e-01

10020 6.925115e-01 9.425989e-01

10021 6.925321e-01 9.425995e-01

10022 6.925569e-01 9.426001e-01

10023 6.925862e-01 9.426007e-01

10024 6.926198e-01 9.426012e-01

10025 6.926579e-01 9.426016e-01

10026 6.927005e-01 9.426020e-01

10027 6.927475e-01 9.426024e-01

10028 6.927989e-01 9.426027e-01

10029 6.928548e-01 9.426030e-01

10030 6.929152e-01 9.426032e-01

10031 6.929799e-01 9.426033e-01

10032 6.930491e-01 9.426033e-01

10033 6.931226e-01 9.426033e-01

10034 6.932004e-01 9.426032e-01

10035 6.932824e-01 9.426030e-01

10036 6.933687e-01 9.426028e-01

10037 6.934591e-01 9.426024e-01

10038 6.935535e-01 9.426020e-01

10039 6.936520e-01 9.426014e-01

10040 6.937544e-01 9.426008e-01

10041 6.938606e-01 9.426000e-01

10042 6.939705e-01 9.425991e-01

10043 6.940840e-01 9.425981e-01

10044 6.942011e-01 9.425970e-01

10045 6.943216e-01 9.425958e-01

10046 6.944453e-01 9.425944e-01

10047 6.945723e-01 9.425929e-01

10048 6.947023e-01 9.425913e-01

10049 6.948352e-01 9.425895e-01

10050 6.949709e-01 9.425876e-01

10051 6.951093e-01 9.425855e-01

10052 6.952503e-01 9.425833e-01

10053 6.953936e-01 9.425808e-01

10054 6.955392e-01 9.425783e-01

10055 6.956869e-01 9.425755e-01

10056 6.958367e-01 9.425726e-01

10057 6.959882e-01 9.425695e-01

10058 6.961415e-01 9.425662e-01

10059 6.962963e-01 9.425627e-01

10060 6.964526e-01 9.425590e-01

10061 6.966102e-01 9.425552e-01

10062 6.967689e-01 9.425511e-01

10063 6.969286e-01 9.425468e-01

10064 6.970893e-01 9.425423e-01

10065 6.972507e-01 9.425376e-01

10066 6.974128e-01 9.425326e-01

10067 6.975753e-01 9.425275e-01

10068 6.977383e-01 9.425221e-01

10069 6.979015e-01 9.425166e-01

10070 6.980649e-01 9.425111e-01

10071 6.982283e-01 9.425057e-01

10072 6.983916e-01 9.425005e-01

10073 6.985548e-01 9.424958e-01

10074 6.987177e-01 9.424916e-01

10075 6.988803e-01 9.424880e-01

10076 6.990424e-01 9.424851e-01

10077 6.992040e-01 9.424831e-01

10078 6.993649e-01 9.424820e-01

10079 6.995251e-01 9.424819e-01

10080 6.996845e-01 9.424830e-01

10081 6.998431e-01 9.424851e-01

10082 6.998614e-01 9.424845e-01

10083 6.998806e-01 9.424839e-01

10084 6.999005e-01 9.424833e-01

10085 6.999213e-01 9.424827e-01

10086 6.999428e-01 9.424820e-01

10087 6.999652e-01 9.424813e-01

10088 6.999885e-01 9.424806e-01

10089 7.000127e-01 9.424799e-01

10090 7.000379e-01 9.424792e-01

10091 7.000640e-01 9.424784e-01

10092 7.000911e-01 9.424777e-01

10093 7.001192e-01 9.424769e-01

10094 7.001484e-01 9.424761e-01

10095 7.001787e-01 9.424752e-01

10096 7.002101e-01 9.424744e-01

10097 7.002427e-01 9.424735e-01

10098 7.002765e-01 9.424726e-01

10099 7.003115e-01 9.424716e-01

10100 7.003478e-01 9.424706e-01

10101 7.003854e-01 9.424696e-01

10102 7.004244e-01 9.424686e-01

10103 7.004648e-01 9.424675e-01

10104 7.005066e-01 9.424664e-01

10105 7.005500e-01 9.424653e-01

10106 7.005949e-01 9.424641e-01

10107 7.006413e-01 9.424629e-01

10108 7.006895e-01 9.424616e-01

10109 7.007393e-01 9.424603e-01

10110 7.007908e-01 9.424590e-01

10111 7.008442e-01 9.424576e-01

10112 7.008994e-01 9.424562e-01

10113 7.009566e-01 9.424547e-01

10114 7.010157e-01 9.424532e-01

10115 7.010769e-01 9.424517e-01

10116 7.011401e-01 9.424501e-01

10117 7.012055e-01 9.424484e-01

10118 7.012732e-01 9.424467e-01

10119 7.013431e-01 9.424449e-01

10120 7.014154e-01 9.424430e-01

10121 7.014901e-01 9.424411e-01

10122 7.015674e-01 9.424392e-01

10123 7.016472e-01 9.424372e-01

10124 7.017297e-01 9.424351e-01

10125 7.018149e-01 9.424329e-01

10126 7.019029e-01 9.424307e-01

10127 7.019939e-01 9.424284e-01

10128 7.020878e-01 9.424260e-01

10129 7.021848e-01 9.424235e-01

10130 7.022849e-01 9.424210e-01

10131 7.023883e-01 9.424184e-01

10132 7.024950e-01 9.424157e-01

10133 7.026051e-01 9.424128e-01

10134 7.027187e-01 9.424100e-01

10135 7.028360e-01 9.424070e-01

10136 7.029570e-01 9.424039e-01

10137 7.030818e-01 9.424007e-01

10138 7.032105e-01 9.423974e-01

10139 7.033432e-01 9.423940e-01

10140 7.034800e-01 9.423904e-01

10141 7.036211e-01 9.423868e-01

10142 7.037665e-01 9.423830e-01

10143 7.039164e-01 9.423791e-01

10144 7.040709e-01 9.423751e-01

10145 7.042300e-01 9.423710e-01

10146 7.043939e-01 9.423667e-01

10147 7.045627e-01 9.423622e-01

10148 7.047365e-01 9.423577e-01

10149 7.049155e-01 9.423529e-01

10150 7.050998e-01 9.423480e-01

10151 7.052894e-01 9.423430e-01

10152 7.054845e-01 9.423377e-01

10153 7.056853e-01 9.423323e-01

10154 7.058918e-01 9.423267e-01

10155 7.061042e-01 9.423210e-01

10156 7.063226e-01 9.423150e-01

10157 7.065471e-01 9.423088e-01

10158 7.067778e-01 9.423024e-01

10159 7.070149e-01 9.422959e-01

10160 7.072585e-01 9.422891e-01

10161 7.075088e-01 9.422820e-01

10162 7.077657e-01 9.422747e-01

10163 7.080295e-01 9.422672e-01

10164 7.083003e-01 9.422595e-01

10165 7.085782e-01 9.422514e-01

10166 7.088632e-01 9.422431e-01

10167 7.091556e-01 9.422346e-01

10168 7.094555e-01 9.422257e-01

10169 7.097628e-01 9.422166e-01

10170 7.100778e-01 9.422071e-01

10171 7.104006e-01 9.421973e-01

10172 7.107312e-01 9.421873e-01

10173 7.110697e-01 9.421768e-01

10174 7.114163e-01 9.421660e-01

10175 7.117710e-01 9.421549e-01

10176 7.121339e-01 9.421434e-01

10177 7.125051e-01 9.421315e-01

10178 7.128847e-01 9.421193e-01

10179 7.132726e-01 9.421066e-01

10180 7.136691e-01 9.420935e-01

10181 7.140741e-01 9.420800e-01

10182 7.144876e-01 9.420660e-01

10183 7.149098e-01 9.420516e-01

10184 7.153406e-01 9.420367e-01

10185 7.157801e-01 9.420213e-01

10186 7.162283e-01 9.420054e-01

10187 7.166852e-01 9.419891e-01

10188 7.171508e-01 9.419721e-01

10189 7.176251e-01 9.419547e-01

10190 7.181080e-01 9.419366e-01

10191 7.185997e-01 9.419180e-01

10192 7.190999e-01 9.418988e-01

10193 7.196086e-01 9.418790e-01

10194 7.201259e-01 9.418586e-01

10195 7.206516e-01 9.418375e-01

10196 7.211857e-01 9.418158e-01

10197 7.217280e-01 9.417934e-01

10198 7.222785e-01 9.417703e-01

10199 7.228370e-01 9.417465e-01

10200 7.234034e-01 9.417219e-01

10201 7.239777e-01 9.416966e-01

10202 7.245596e-01 9.416705e-01

10203 7.251490e-01 9.416437e-01

10204 7.257458e-01 9.416160e-01

10205 7.263497e-01 9.415875e-01

10206 7.269605e-01 9.415582e-01

10207 7.275781e-01 9.415280e-01

10208 7.282023e-01 9.414969e-01

10209 7.288328e-01 9.414649e-01

10210 7.294695e-01 9.414320e-01

10211 7.301120e-01 9.413981e-01

10212 7.307601e-01 9.413633e-01

10213 7.314136e-01 9.413275e-01

10214 7.320722e-01 9.412907e-01

10215 7.327356e-01 9.412529e-01

10216 7.334036e-01 9.412140e-01

10217 7.340758e-01 9.411741e-01

10218 7.347520e-01 9.411331e-01

10219 7.354318e-01 9.410910e-01

10220 7.361150e-01 9.410477e-01

10221 7.368012e-01 9.410034e-01

10222 7.374901e-01 9.409579e-01

10223 7.381814e-01 9.409112e-01

10224 7.388748e-01 9.408633e-01

10225 7.395700e-01 9.408143e-01

10226 7.402665e-01 9.407640e-01

10227 7.409641e-01 9.407125e-01

10228 7.416625e-01 9.406597e-01

10229 7.423613e-01 9.406056e-01

10230 7.430601e-01 9.405503e-01

10231 7.437587e-01 9.404937e-01

10232 7.444567e-01 9.404358e-01

10233 7.451538e-01 9.403766e-01

10234 7.458496e-01 9.403160e-01

10235 7.465439e-01 9.402541e-01

10236 7.472362e-01 9.401909e-01

10237 7.479264e-01 9.401263e-01

10238 7.486141e-01 9.400604e-01

10239 7.492989e-01 9.399931e-01

10240 7.499807e-01 9.399244e-01

10241 7.506590e-01 9.398544e-01

10242 7.513336e-01 9.397830e-01

10243 7.520043e-01 9.397102e-01

10244 7.526708e-01 9.396361e-01

10245 7.533327e-01 9.395606e-01

10246 7.539899e-01 9.394838e-01

10247 7.546422e-01 9.394056e-01

10248 7.552892e-01 9.393261e-01

10249 7.559307e-01 9.392452e-01

10250 7.565666e-01 9.391630e-01

10251 7.571967e-01 9.390795e-01

10252 7.578206e-01 9.389947e-01

10253 7.584384e-01 9.389087e-01

10254 7.590497e-01 9.388213e-01

10255 7.596544e-01 9.387328e-01

10256 7.602524e-01 9.386430e-01

10257 7.608435e-01 9.385520e-01

10258 7.614277e-01 9.384598e-01

10259 7.620046e-01 9.383665e-01

10260 7.625744e-01 9.382721e-01

10261 7.631368e-01 9.381767e-01

10262 7.631715e-01 9.381753e-01

10263 7.632073e-01 9.381738e-01

10264 7.632441e-01 9.381723e-01

10265 7.632819e-01 9.381707e-01

10266 7.633209e-01 9.381691e-01

10267 7.633609e-01 9.381675e-01

10268 7.634021e-01 9.381658e-01

10269 7.634444e-01 9.381641e-01

10270 7.634879e-01 9.381623e-01

10271 7.635326e-01 9.381605e-01

10272 7.635786e-01 9.381587e-01

10273 7.636259e-01 9.381568e-01

10274 7.636745e-01 9.381549e-01

10275 7.637244e-01 9.381530e-01

10276 7.637757e-01 9.381510e-01

10277 7.638284e-01 9.381489e-01

10278 7.638826e-01 9.381468e-01

10279 7.639382e-01 9.381447e-01

10280 7.639954e-01 9.381425e-01

10281 7.640542e-01 9.381403e-01

10282 7.641145e-01 9.381380e-01

10283 7.641765e-01 9.381357e-01

10284 7.642402e-01 9.381334e-01

10285 7.643056e-01 9.381310e-01

10286 7.643728e-01 9.381285e-01

10287 7.644418e-01 9.381260e-01

10288 7.645127e-01 9.381235e-01

10289 7.645855e-01 9.381209e-01

10290 7.646602e-01 9.381182e-01

10291 7.647370e-01 9.381155e-01

10292 7.648158e-01 9.381128e-01

10293 7.648967e-01 9.381099e-01

10294 7.649798e-01 9.381071e-01

10295 7.650651e-01 9.381042e-01

10296 7.651526e-01 9.381012e-01

10297 7.652425e-01 9.380981e-01

10298 7.653348e-01 9.380950e-01

10299 7.654295e-01 9.380919e-01

10300 7.655267e-01 9.380887e-01

10301 7.656265e-01 9.380854e-01

10302 7.657289e-01 9.380821e-01

10303 7.658339e-01 9.380787e-01

10304 7.659417e-01 9.380752e-01

10305 7.660523e-01 9.380717e-01

10306 7.661658e-01 9.380682e-01

10307 7.662822e-01 9.380645e-01

10308 7.664017e-01 9.380608e-01

10309 7.665242e-01 9.380570e-01

10310 7.666498e-01 9.380532e-01

10311 7.667787e-01 9.380493e-01

10312 7.669108e-01 9.380453e-01

10313 7.670464e-01 9.380413e-01

10314 7.671854e-01 9.380372e-01

10315 7.673278e-01 9.380330e-01

10316 7.674739e-01 9.380288e-01

10317 7.676237e-01 9.380244e-01

10318 7.677772e-01 9.380201e-01

10319 7.679346e-01 9.380156e-01

10320 7.680958e-01 9.380111e-01

10321 7.682611e-01 9.380065e-01

10322 7.684304e-01 9.380018e-01

10323 7.686039e-01 9.379971e-01

10324 7.687817e-01 9.379923e-01

10325 7.689638e-01 9.379874e-01

10326 7.691504e-01 9.379825e-01

10327 7.693414e-01 9.379775e-01

10328 7.695371e-01 9.379724e-01

10329 7.697375e-01 9.379673e-01

10330 7.699427e-01 9.379621e-01

10331 7.701527e-01 9.379568e-01

10332 7.703677e-01 9.379514e-01

10333 7.705878e-01 9.379460e-01

10334 7.708131e-01 9.379405e-01

10335 7.710436e-01 9.379350e-01

10336 7.712795e-01 9.379294e-01

10337 7.715209e-01 9.379238e-01

10338 7.717677e-01 9.379180e-01

10339 7.720202e-01 9.379123e-01

10340 7.722785e-01 9.379064e-01

10341 7.725426e-01 9.379006e-01

10342 7.728126e-01 9.378946e-01

10343 7.730886e-01 9.378887e-01

10344 7.733708e-01 9.378827e-01

10345 7.736592e-01 9.378766e-01

10346 7.739538e-01 9.378705e-01

10347 7.742549e-01 9.378644e-01

10348 7.745624e-01 9.378583e-01

10349 7.748766e-01 9.378521e-01

10350 7.751974e-01 9.378459e-01

10351 7.755249e-01 9.378397e-01

10352 7.758593e-01 9.378335e-01

10353 7.762007e-01 9.378273e-01

10354 7.765490e-01 9.378210e-01

10355 7.769044e-01 9.378148e-01

10356 7.772670e-01 9.378086e-01

10357 7.776369e-01 9.378025e-01

10358 7.780140e-01 9.377963e-01

10359 7.783986e-01 9.377902e-01

10360 7.787906e-01 9.377842e-01

10361 7.791901e-01 9.377782e-01

10362 7.795972e-01 9.377723e-01

10363 7.800119e-01 9.377664e-01

10364 7.804343e-01 9.377607e-01

10365 7.808644e-01 9.377550e-01

10366 7.813023e-01 9.377495e-01

10367 7.817480e-01 9.377440e-01

10368 7.822016e-01 9.377388e-01

10369 7.826630e-01 9.377336e-01

10370 7.831323e-01 9.377287e-01

10371 7.836095e-01 9.377239e-01

10372 7.840946e-01 9.377193e-01

10373 7.845877e-01 9.377149e-01

10374 7.850886e-01 9.377108e-01

10375 7.855975e-01 9.377069e-01

10376 7.861143e-01 9.377033e-01

10377 7.866389e-01 9.377000e-01

10378 7.871713e-01 9.376970e-01

10379 7.877116e-01 9.376944e-01

10380 7.882596e-01 9.376921e-01

10381 7.888153e-01 9.376901e-01

10382 7.893786e-01 9.376886e-01

10383 7.899495e-01 9.376876e-01

10384 7.905278e-01 9.376869e-01

10385 7.911136e-01 9.376868e-01

10386 7.917066e-01 9.376872e-01

10387 7.923068e-01 9.376882e-01

10388 7.929141e-01 9.376897e-01

10389 7.935284e-01 9.376918e-01

10390 7.941494e-01 9.376946e-01

10391 7.947771e-01 9.376980e-01

10392 7.954114e-01 9.377022e-01

10393 7.960520e-01 9.377071e-01

10394 7.966987e-01 9.377128e-01

10395 7.973515e-01 9.377194e-01

10396 7.980101e-01 9.377267e-01

10397 7.986743e-01 9.377350e-01

10398 7.993439e-01 9.377443e-01

10399 8.000187e-01 9.377545e-01

10400 8.006985e-01 9.377657e-01

10401 8.013831e-01 9.377781e-01

10402 8.020721e-01 9.377915e-01

10403 8.027654e-01 9.378061e-01

10404 8.034627e-01 9.378219e-01

10405 8.041637e-01 9.378390e-01

10406 8.048682e-01 9.378574e-01

10407 8.055760e-01 9.378771e-01

10408 8.062866e-01 9.378982e-01

10409 8.069998e-01 9.379208e-01

10410 8.077154e-01 9.379449e-01

10411 8.084331e-01 9.379705e-01

10412 8.091524e-01 9.379978e-01

10413 8.098733e-01 9.380267e-01

10414 8.105952e-01 9.380573e-01

10415 8.113180e-01 9.380896e-01

10416 8.120412e-01 9.381238e-01

10417 8.127647e-01 9.381599e-01

10418 8.134880e-01 9.381979e-01

10419 8.142109e-01 9.382378e-01

10420 8.149330e-01 9.382798e-01

10421 8.156540e-01 9.383238e-01

10422 8.163737e-01 9.383700e-01

10423 8.170916e-01 9.384184e-01

10424 8.178075e-01 9.384690e-01

10425 8.185211e-01 9.385218e-01

10426 8.192320e-01 9.385771e-01

10427 8.199400e-01 9.386346e-01

10428 8.206448e-01 9.386947e-01

10429 8.213460e-01 9.387571e-01

10430 8.220435e-01 9.388221e-01

10431 8.227368e-01 9.388896e-01

10432 8.234257e-01 9.389595e-01

10433 8.241100e-01 9.390317e-01

10434 8.247894e-01 9.391060e-01

10435 8.254636e-01 9.391824e-01

10436 8.261324e-01 9.392609e-01

10437 8.267955e-01 9.393412e-01

10438 8.274528e-01 9.394234e-01

10439 8.281039e-01 9.395075e-01

10440 8.287487e-01 9.395932e-01

10441 8.293870e-01 9.396806e-01

10442 8.294056e-01 9.396804e-01

10443 8.294246e-01 9.396802e-01

10444 8.294441e-01 9.396800e-01

10445 8.294641e-01 9.396798e-01

10446 8.294847e-01 9.396796e-01

10447 8.295057e-01 9.396794e-01

10448 8.295273e-01 9.396792e-01

10449 8.295494e-01 9.396790e-01

10450 8.295721e-01 9.396788e-01

10451 8.295954e-01 9.396785e-01

10452 8.296192e-01 9.396783e-01

10453 8.296437e-01 9.396781e-01

10454 8.296688e-01 9.396778e-01

10455 8.296945e-01 9.396775e-01

10456 8.297209e-01 9.396773e-01

10457 8.297479e-01 9.396770e-01

10458 8.297756e-01 9.396767e-01

10459 8.298040e-01 9.396764e-01

10460 8.298332e-01 9.396761e-01

10461 8.298630e-01 9.396758e-01

10462 8.298937e-01 9.396755e-01

10463 8.299251e-01 9.396751e-01

10464 8.299573e-01 9.396748e-01

10465 8.299904e-01 9.396744e-01

10466 8.300242e-01 9.396741e-01

10467 8.300589e-01 9.396737e-01

10468 8.300946e-01 9.396733e-01

10469 8.301311e-01 9.396729e-01

10470 8.301685e-01 9.396725e-01

10471 8.302069e-01 9.396720e-01

10472 8.302462e-01 9.396716e-01

10473 8.302866e-01 9.396711e-01

10474 8.303280e-01 9.396707e-01

10475 8.303704e-01 9.396702e-01

10476 8.304139e-01 9.396697e-01

10477 8.304584e-01 9.396692e-01

10478 8.305041e-01 9.396686e-01

10479 8.305510e-01 9.396681e-01

10480 8.305990e-01 9.396675e-01

10481 8.306483e-01 9.396670e-01

10482 8.306987e-01 9.396664e-01

10483 8.307505e-01 9.396658e-01

10484 8.308035e-01 9.396651e-01

10485 8.308578e-01 9.396645e-01

10486 8.309136e-01 9.396638e-01

10487 8.309706e-01 9.396631e-01

10488 8.310291e-01 9.396624e-01

10489 8.310891e-01 9.396617e-01

10490 8.311505e-01 9.396610e-01

10491 8.312135e-01 9.396602e-01

10492 8.312780e-01 9.396594e-01

10493 8.313441e-01 9.396586e-01

10494 8.314118e-01 9.396578e-01

10495 8.314811e-01 9.396569e-01

10496 8.315521e-01 9.396561e-01

10497 8.316249e-01 9.396552e-01

10498 8.316994e-01 9.396542e-01

10499 8.317758e-01 9.396533e-01

10500 8.318539e-01 9.396523e-01

10501 8.319340e-01 9.396513e-01

10502 8.320160e-01 9.396503e-01

10503 8.320999e-01 9.396492e-01

10504 8.321858e-01 9.396482e-01

10505 8.322737e-01 9.396471e-01

10506 8.323638e-01 9.396459e-01

10507 8.324559e-01 9.396448e-01

10508 8.325502e-01 9.396436e-01

10509 8.326467e-01 9.396423e-01

10510 8.327454e-01 9.396411e-01

10511 8.328465e-01 9.396398e-01

10512 8.329498e-01 9.396385e-01

10513 8.330555e-01 9.396372e-01

10514 8.331637e-01 9.396358e-01

10515 8.332742e-01 9.396344e-01

10516 8.333873e-01 9.396329e-01

10517 8.335030e-01 9.396314e-01

10518 8.336212e-01 9.396299e-01

10519 8.337420e-01 9.396284e-01

10520 8.338656e-01 9.396268e-01

10521 8.339918e-01 9.396252e-01

10522 8.341208e-01 9.396235e-01

10523 8.342526e-01 9.396218e-01

10524 8.343873e-01 9.396201e-01

10525 8.345248e-01 9.396183e-01

10526 8.346653e-01 9.396165e-01

10527 8.348088e-01 9.396146e-01

10528 8.349553e-01 9.396127e-01

10529 8.351048e-01 9.396108e-01

10530 8.352575e-01 9.396088e-01

10531 8.354133e-01 9.396068e-01

10532 8.355723e-01 9.396048e-01

10533 8.357345e-01 9.396027e-01

10534 8.358999e-01 9.396006e-01

10535 8.360687e-01 9.395984e-01

10536 8.362408e-01 9.395962e-01

10537 8.364163e-01 9.395939e-01

10538 8.365952e-01 9.395916e-01

10539 8.367775e-01 9.395892e-01

10540 8.369633e-01 9.395868e-01

10541 8.371526e-01 9.395844e-01

10542 8.373455e-01 9.395819e-01

10543 8.375419e-01 9.395794e-01

10544 8.377419e-01 9.395768e-01

10545 8.379454e-01 9.395742e-01

10546 8.381527e-01 9.395716e-01

10547 8.383636e-01 9.395689e-01

10548 8.385781e-01 9.395661e-01

10549 8.387963e-01 9.395634e-01

10550 8.390183e-01 9.395606e-01

10551 8.392439e-01 9.395577e-01

10552 8.394733e-01 9.395548e-01

10553 8.397064e-01 9.395519e-01

10554 8.399432e-01 9.395489e-01

10555 8.401837e-01 9.395459e-01

10556 8.404280e-01 9.395429e-01

10557 8.406760e-01 9.395398e-01

10558 8.409277e-01 9.395367e-01

10559 8.411830e-01 9.395336e-01

10560 8.414421e-01 9.395304e-01

10561 8.417048e-01 9.395272e-01

10562 8.419711e-01 9.395240e-01

10563 8.422411e-01 9.395208e-01

10564 8.425146e-01 9.395175e-01

10565 8.427917e-01 9.395143e-01

10566 8.430722e-01 9.395110e-01

10567 8.433562e-01 9.395077e-01

10568 8.436436e-01 9.395044e-01

10569 8.439343e-01 9.395011e-01

10570 8.442284e-01 9.394978e-01

10571 8.445256e-01 9.394946e-01

10572 8.448260e-01 9.394913e-01

10573 8.451295e-01 9.394880e-01

10574 8.454361e-01 9.394848e-01

10575 8.457455e-01 9.394816e-01

10576 8.460578e-01 9.394784e-01

10577 8.463729e-01 9.394752e-01

10578 8.466906e-01 9.394721e-01

10579 8.470108e-01 9.394691e-01

10580 8.473336e-01 9.394660e-01

10581 8.476586e-01 9.394631e-01

10582 8.479859e-01 9.394602e-01

10583 8.483154e-01 9.394574e-01

10584 8.486468e-01 9.394546e-01

10585 8.489801e-01 9.394520e-01

10586 8.493151e-01 9.394494e-01

10587 8.496517e-01 9.394470e-01

10588 8.499898e-01 9.394447e-01

10589 8.503292e-01 9.394424e-01

10590 8.506697e-01 9.394404e-01

10591 8.510113e-01 9.394384e-01

10592 8.513538e-01 9.394366e-01

10593 8.516970e-01 9.394350e-01

10594 8.520407e-01 9.394335e-01

10595 8.523848e-01 9.394322e-01

10596 8.527292e-01 9.394311e-01

10597 8.530736e-01 9.394302e-01

10598 8.534180e-01 9.394295e-01

10599 8.537621e-01 9.394290e-01

10600 8.541057e-01 9.394287e-01

10601 8.544488e-01 9.394287e-01

10602 8.547910e-01 9.394289e-01

10603 8.551324e-01 9.394294e-01

10604 8.554726e-01 9.394302e-01

10605 8.558115e-01 9.394313e-01

10606 8.561490e-01 9.394326e-01

10607 8.564849e-01 9.394343e-01

10608 8.568189e-01 9.394362e-01

10609 8.571511e-01 9.394385e-01

10610 8.574811e-01 9.394412e-01

10611 8.578088e-01 9.394442e-01

10612 8.581341e-01 9.394479e-01

10613 8.584569e-01 9.394523e-01

10614 8.587769e-01 9.394575e-01

10615 8.590940e-01 9.394638e-01

10616 8.594081e-01 9.394712e-01

10617 8.597190e-01 9.394799e-01

10618 8.600267e-01 9.394900e-01

10619 8.603309e-01 9.395014e-01

10620 8.606316e-01 9.395142e-01

10621 8.609285e-01 9.395283e-01

10622 8.609345e-01 9.395274e-01

10623 8.609406e-01 9.395265e-01

10624 8.609468e-01 9.395256e-01

10625 8.609531e-01 9.395247e-01

10626 8.609595e-01 9.395238e-01

10627 8.609660e-01 9.395228e-01

10628 8.609726e-01 9.395218e-01

10629 8.609793e-01 9.395208e-01

10630 8.609861e-01 9.395198e-01

10631 8.609931e-01 9.395187e-01

10632 8.610001e-01 9.395176e-01

10633 8.610073e-01 9.395165e-01

10634 8.610146e-01 9.395153e-01

10635 8.610220e-01 9.395142e-01

10636 8.610295e-01 9.395130e-01

10637 8.610372e-01 9.395117e-01

10638 8.610449e-01 9.395104e-01

10639 8.610528e-01 9.395091e-01

10640 8.610608e-01 9.395078e-01

10641 8.610690e-01 9.395064e-01

10642 8.610773e-01 9.395049e-01

10643 8.610857e-01 9.395035e-01

10644 8.610942e-01 9.395020e-01

10645 8.611029e-01 9.395004e-01

10646 8.611117e-01 9.394988e-01

10647 8.611206e-01 9.394972e-01

10648 8.611297e-01 9.394955e-01

10649 8.611389e-01 9.394938e-01

10650 8.611483e-01 9.394920e-01

10651 8.611578e-01 9.394902e-01

10652 8.611675e-01 9.394883e-01

10653 8.611773e-01 9.394864e-01

10654 8.611872e-01 9.394844e-01

10655 8.611973e-01 9.394823e-01

10656 8.612075e-01 9.394802e-01

10657 8.612179e-01 9.394781e-01

10658 8.612284e-01 9.394758e-01

10659 8.612391e-01 9.394736e-01

10660 8.612500e-01 9.394712e-01

10661 8.612610e-01 9.394688e-01

10662 8.612721e-01 9.394663e-01

10663 8.612834e-01 9.394638e-01

10664 8.612949e-01 9.394611e-01

10665 8.613065e-01 9.394584e-01

10666 8.613182e-01 9.394557e-01

10667 8.613301e-01 9.394528e-01

10668 8.613422e-01 9.394499e-01

10669 8.613544e-01 9.394469e-01

10670 8.613668e-01 9.394438e-01

10671 8.613793e-01 9.394406e-01

10672 8.613919e-01 9.394373e-01

10673 8.614047e-01 9.394340e-01

10674 8.614177e-01 9.394305e-01

10675 8.614308e-01 9.394269e-01

10676 8.614440e-01 9.394233e-01

10677 8.614574e-01 9.394195e-01

10678 8.614709e-01 9.394156e-01

10679 8.614845e-01 9.394117e-01

10680 8.614983e-01 9.394076e-01

10681 8.615122e-01 9.394034e-01

10682 8.615261e-01 9.393991e-01

10683 8.615402e-01 9.393946e-01

10684 8.615545e-01 9.393901e-01

10685 8.615688e-01 9.393854e-01

10686 8.615832e-01 9.393806e-01

10687 8.615976e-01 9.393756e-01

10688 8.616122e-01 9.393705e-01

10689 8.616268e-01 9.393653e-01

10690 8.616415e-01 9.393599e-01

10691 8.616562e-01 9.393544e-01

10692 8.616710e-01 9.393487e-01

10693 8.616858e-01 9.393429e-01

10694 8.617006e-01 9.393369e-01

10695 8.617154e-01 9.393307e-01

10696 8.617302e-01 9.393244e-01

10697 8.617450e-01 9.393179e-01

10698 8.617597e-01 9.393112e-01

10699 8.617744e-01 9.393044e-01

10700 8.617890e-01 9.392973e-01

10701 8.618035e-01 9.392901e-01

10702 8.618179e-01 9.392826e-01

10703 8.618322e-01 9.392750e-01

10704 8.618463e-01 9.392672e-01

10705 8.618602e-01 9.392591e-01

10706 8.618740e-01 9.392509e-01

10707 8.618875e-01 9.392424e-01

10708 8.619008e-01 9.392337e-01

10709 8.619138e-01 9.392247e-01

10710 8.619265e-01 9.392155e-01

10711 8.619389e-01 9.392061e-01

10712 8.619509e-01 9.391965e-01

10713 8.619625e-01 9.391865e-01

10714 8.619737e-01 9.391764e-01

10715 8.619844e-01 9.391659e-01

10716 8.619946e-01 9.391552e-01

10717 8.620043e-01 9.391442e-01

10718 8.620134e-01 9.391330e-01

10719 8.620219e-01 9.391214e-01

10720 8.620298e-01 9.391095e-01

10721 8.620369e-01 9.390974e-01

10722 8.620433e-01 9.390849e-01

10723 8.620489e-01 9.390722e-01

10724 8.620537e-01 9.390591e-01

10725 8.620576e-01 9.390457e-01

10726 8.620605e-01 9.390319e-01

10727 8.620624e-01 9.390178e-01

10728 8.620633e-01 9.390034e-01

10729 8.620631e-01 9.389887e-01

10730 8.620617e-01 9.389735e-01

10731 8.620591e-01 9.389581e-01

10732 8.620552e-01 9.389422e-01

10733 8.620500e-01 9.389260e-01

10734 8.620434e-01 9.389094e-01

10735 8.620353e-01 9.388924e-01

10736 8.620256e-01 9.388751e-01

10737 8.620143e-01 9.388573e-01

10738 8.620014e-01 9.388392e-01

10739 8.619867e-01 9.388206e-01

10740 8.619702e-01 9.388017e-01

10741 8.619518e-01 9.387823e-01

10742 8.619315e-01 9.387626e-01

10743 8.619091e-01 9.387424e-01

10744 8.618846e-01 9.387217e-01

10745 8.618579e-01 9.387007e-01

10746 8.618289e-01 9.386792e-01

10747 8.617976e-01 9.386573e-01

10748 8.617639e-01 9.386350e-01

10749 8.617277e-01 9.386122e-01

10750 8.616890e-01 9.385889e-01

10751 8.616476e-01 9.385653e-01

10752 8.616034e-01 9.385411e-01

10753 8.615565e-01 9.385166e-01

10754 8.615067e-01 9.384915e-01

10755 8.614540e-01 9.384660e-01

10756 8.613983e-01 9.384401e-01

10757 8.613395e-01 9.384137e-01

10758 8.612775e-01 9.383869e-01

10759 8.612123e-01 9.383596e-01

10760 8.611438e-01 9.383318e-01

10761 8.610720e-01 9.383036e-01

10762 8.609968e-01 9.382750e-01

10763 8.609181e-01 9.382458e-01

10764 8.608359e-01 9.382163e-01

10765 8.607502e-01 9.381863e-01

10766 8.606608e-01 9.381558e-01

10767 8.605677e-01 9.381249e-01

10768 8.604710e-01 9.380935e-01

10769 8.603705e-01 9.380618e-01

10770 8.602662e-01 9.380295e-01

10771 8.601582e-01 9.379969e-01

10772 8.600463e-01 9.379638e-01

10773 8.599306e-01 9.379303e-01

10774 8.598111e-01 9.378964e-01

10775 8.596877e-01 9.378621e-01

10776 8.595604e-01 9.378274e-01

10777 8.594293e-01 9.377923e-01

10778 8.592944e-01 9.377568e-01

10779 8.591556e-01 9.377209e-01

10780 8.590130e-01 9.376847e-01

10781 8.588666e-01 9.376481e-01

10782 8.587165e-01 9.376111e-01

10783 8.585627e-01 9.375738e-01

10784 8.584052e-01 9.375362e-01

10785 8.582441e-01 9.374982e-01

10786 8.580794e-01 9.374599e-01

10787 8.579113e-01 9.374213e-01

10788 8.577397e-01 9.373824e-01

10789 8.575648e-01 9.373432e-01

10790 8.573866e-01 9.373037e-01

10791 8.572052e-01 9.372640e-01

10792 8.570208e-01 9.372240e-01

10793 8.568333e-01 9.371838e-01

10794 8.566430e-01 9.371433e-01

10795 8.564498e-01 9.371026e-01

10796 8.562540e-01 9.370617e-01

10797 8.560556e-01 9.370205e-01

10798 8.558548e-01 9.369792e-01

10799 8.556516e-01 9.369378e-01

10800 8.554463e-01 9.368965e-01

10801 8.552388e-01 9.368554e-01

10802 8.552353e-01 9.368539e-01

10803 8.552317e-01 9.368523e-01

10804 8.552278e-01 9.368507e-01

10805 8.552238e-01 9.368491e-01

10806 8.552196e-01 9.368474e-01

10807 8.552151e-01 9.368457e-01

10808 8.552105e-01 9.368440e-01

10809 8.552056e-01 9.368422e-01

10810 8.552005e-01 9.368404e-01

10811 8.551951e-01 9.368386e-01

10812 8.551895e-01 9.368367e-01

10813 8.551836e-01 9.368349e-01

10814 8.551775e-01 9.368329e-01

10815 8.551710e-01 9.368310e-01

10816 8.551643e-01 9.368290e-01

10817 8.551573e-01 9.368270e-01

10818 8.551499e-01 9.368249e-01

10819 8.551422e-01 9.368228e-01

10820 8.551342e-01 9.368207e-01

10821 8.551258e-01 9.368186e-01

10822 8.551170e-01 9.368164e-01

10823 8.551079e-01 9.368142e-01

10824 8.550983e-01 9.368119e-01

10825 8.550883e-01 9.368097e-01

10826 8.550779e-01 9.368073e-01

10827 8.550670e-01 9.368050e-01

10828 8.550557e-01 9.368026e-01

10829 8.550439e-01 9.368002e-01

10830 8.550315e-01 9.367978e-01

10831 8.550186e-01 9.367953e-01

10832 8.550052e-01 9.367928e-01

10833 8.549912e-01 9.367903e-01

10834 8.549767e-01 9.367878e-01

10835 8.549615e-01 9.367852e-01

10836 8.549456e-01 9.367826e-01

10837 8.549291e-01 9.367799e-01

10838 8.549120e-01 9.367772e-01

10839 8.548941e-01 9.367745e-01

10840 8.548755e-01 9.367718e-01

10841 8.548561e-01 9.367691e-01

10842 8.548359e-01 9.367663e-01

10843 8.548149e-01 9.367635e-01

10844 8.547930e-01 9.367606e-01

10845 8.547703e-01 9.367578e-01

10846 8.547466e-01 9.367549e-01

10847 8.547220e-01 9.367520e-01

10848 8.546964e-01 9.367491e-01

10849 8.546697e-01 9.367462e-01

10850 8.546420e-01 9.367432e-01

10851 8.546132e-01 9.367402e-01

10852 8.545833e-01 9.367373e-01

10853 8.545522e-01 9.367343e-01

10854 8.545198e-01 9.367313e-01

10855 8.544862e-01 9.367282e-01

10856 8.544513e-01 9.367252e-01

10857 8.544150e-01 9.367222e-01

10858 8.543773e-01 9.367192e-01

10859 8.543381e-01 9.367162e-01

10860 8.542974e-01 9.367131e-01

10861 8.542552e-01 9.367101e-01

10862 8.542113e-01 9.367071e-01

10863 8.541658e-01 9.367041e-01

10864 8.541185e-01 9.367011e-01

10865 8.540694e-01 9.366982e-01

10866 8.540185e-01 9.366953e-01

10867 8.539656e-01 9.366924e-01

10868 8.539108e-01 9.366895e-01

10869 8.538539e-01 9.366866e-01

10870 8.537948e-01 9.366838e-01

10871 8.537336e-01 9.366811e-01

10872 8.536701e-01 9.366784e-01

10873 8.536042e-01 9.366758e-01

10874 8.535359e-01 9.366732e-01

10875 8.534651e-01 9.366707e-01

10876 8.533917e-01 9.366682e-01

10877 8.533156e-01 9.366659e-01

10878 8.532368e-01 9.366636e-01

10879 8.531551e-01 9.366614e-01

10880 8.530704e-01 9.366594e-01

10881 8.529828e-01 9.366574e-01

10882 8.528919e-01 9.366556e-01

10883 8.527979e-01 9.366539e-01

10884 8.527005e-01 9.366523e-01

10885 8.525996e-01 9.366509e-01

10886 8.524952e-01 9.366496e-01

10887 8.523872e-01 9.366485e-01

10888 8.522754e-01 9.366476e-01

10889 8.521597e-01 9.366469e-01

10890 8.520400e-01 9.366464e-01

10891 8.519162e-01 9.366461e-01

10892 8.517882e-01 9.366461e-01

10893 8.516558e-01 9.366462e-01

10894 8.515190e-01 9.366467e-01

10895 8.513775e-01 9.366474e-01

10896 8.512314e-01 9.366484e-01

10897 8.510803e-01 9.366497e-01

10898 8.509243e-01 9.366513e-01

10899 8.507632e-01 9.366533e-01

10900 8.505969e-01 9.366556e-01

10901 8.504251e-01 9.366583e-01

10902 8.502479e-01 9.366614e-01

10903 8.500650e-01 9.366649e-01

10904 8.498763e-01 9.366689e-01

10905 8.496817e-01 9.366733e-01

10906 8.494810e-01 9.366782e-01

10907 8.492741e-01 9.366836e-01

10908 8.490609e-01 9.366895e-01

10909 8.488412e-01 9.366960e-01

10910 8.486150e-01 9.367031e-01

10911 8.483820e-01 9.367108e-01

10912 8.481421e-01 9.367191e-01

10913 8.478952e-01 9.367281e-01

10914 8.476411e-01 9.367378e-01

10915 8.473799e-01 9.367482e-01

10916 8.471112e-01 9.367593e-01

10917 8.468350e-01 9.367713e-01

10918 8.465512e-01 9.367841e-01

10919 8.462597e-01 9.367977e-01

10920 8.459603e-01 9.368122e-01

10921 8.456530e-01 9.368276e-01

10922 8.453377e-01 9.368440e-01

10923 8.450142e-01 9.368614e-01

10924 8.446824e-01 9.368798e-01

10925 8.443424e-01 9.368993e-01

10926 8.439940e-01 9.369200e-01

10927 8.436371e-01 9.369417e-01

10928 8.432718e-01 9.369647e-01

10929 8.428979e-01 9.369889e-01

10930 8.425154e-01 9.370144e-01

10931 8.421243e-01 9.370413e-01

10932 8.417245e-01 9.370695e-01

10933 8.413162e-01 9.370991e-01

10934 8.408991e-01 9.371302e-01

10935 8.404734e-01 9.371628e-01

10936 8.400392e-01 9.371970e-01

10937 8.395963e-01 9.372327e-01

10938 8.391449e-01 9.372702e-01

10939 8.386851e-01 9.373093e-01

10940 8.382169e-01 9.373502e-01

10941 8.377404e-01 9.373929e-01

10942 8.372556e-01 9.374375e-01

10943 8.367628e-01 9.374840e-01

10944 8.362621e-01 9.375324e-01

10945 8.357535e-01 9.375829e-01

10946 8.352373e-01 9.376354e-01

10947 8.347136e-01 9.376901e-01

10948 8.341826e-01 9.377469e-01

10949 8.336445e-01 9.378060e-01

10950 8.330995e-01 9.378673e-01

10951 8.325478e-01 9.379310e-01

10952 8.319898e-01 9.379970e-01

10953 8.314255e-01 9.380655e-01

10954 8.308554e-01 9.381364e-01

10955 8.302796e-01 9.382099e-01

10956 8.296985e-01 9.382860e-01

10957 8.291123e-01 9.383647e-01

10958 8.285214e-01 9.384461e-01

10959 8.279261e-01 9.385302e-01

10960 8.273268e-01 9.386171e-01

10961 8.267236e-01 9.387069e-01

10962 8.261171e-01 9.387995e-01

10963 8.255076e-01 9.388950e-01

10964 8.248953e-01 9.389934e-01

10965 8.242807e-01 9.390947e-01

10966 8.236642e-01 9.391986e-01

10967 8.230461e-01 9.393053e-01

10968 8.224267e-01 9.394146e-01

10969 8.218066e-01 9.395264e-01

10970 8.211859e-01 9.396407e-01

10971 8.205652e-01 9.397575e-01

10972 8.199448e-01 9.398766e-01

10973 8.193251e-01 9.399980e-01

10974 8.187063e-01 9.401218e-01

10975 8.180890e-01 9.402477e-01

10976 8.174735e-01 9.403758e-01

10977 8.168601e-01 9.405061e-01

10978 8.162491e-01 9.406385e-01

10979 8.156410e-01 9.407729e-01

10980 8.150360e-01 9.409093e-01

10981 8.144345e-01 9.410477e-01

10982 8.144185e-01 9.410479e-01

10983 8.144020e-01 9.410482e-01

10984 8.143850e-01 9.410484e-01

10985 8.143673e-01 9.410487e-01

10986 8.143489e-01 9.410490e-01

10987 8.143299e-01 9.410493e-01

10988 8.143103e-01 9.410496e-01

10989 8.142899e-01 9.410499e-01

10990 8.142688e-01 9.410502e-01

10991 8.142470e-01 9.410505e-01

10992 8.142244e-01 9.410508e-01

10993 8.142010e-01 9.410512e-01

10994 8.141768e-01 9.410515e-01

10995 8.141517e-01 9.410519e-01

10996 8.141258e-01 9.410523e-01

10997 8.140989e-01 9.410526e-01

10998 8.140711e-01 9.410530e-01

10999 8.140423e-01 9.410534e-01

11000 8.140125e-01 9.410539e-01

11001 8.139816e-01 9.410543e-01

11002 8.139497e-01 9.410548e-01

11003 8.139167e-01 9.410552e-01

11004 8.138825e-01 9.410557e-01

11005 8.138471e-01 9.410562e-01

11006 8.138105e-01 9.410567e-01

11007 8.137725e-01 9.410572e-01

11008 8.137333e-01 9.410578e-01

11009 8.136927e-01 9.410584e-01

11010 8.136507e-01 9.410589e-01

11011 8.136073e-01 9.410595e-01

11012 8.135623e-01 9.410602e-01

11013 8.135157e-01 9.410608e-01

11014 8.134676e-01 9.410615e-01

11015 8.134178e-01 9.410622e-01

11016 8.133663e-01 9.410629e-01

11017 8.133130e-01 9.410636e-01

11018 8.132578e-01 9.410644e-01

11019 8.132008e-01 9.410652e-01

11020 8.131418e-01 9.410660e-01

11021 8.130807e-01 9.410668e-01

11022 8.130176e-01 9.410677e-01

11023 8.129524e-01 9.410686e-01

11024 8.128848e-01 9.410695e-01

11025 8.128150e-01 9.410705e-01

11026 8.127428e-01 9.410715e-01

11027 8.126682e-01 9.410725e-01

11028 8.125910e-01 9.410736e-01

11029 8.125112e-01 9.410747e-01

11030 8.124287e-01 9.410759e-01

11031 8.123435e-01 9.410771e-01

11032 8.122553e-01 9.410783e-01

11033 8.121642e-01 9.410796e-01

11034 8.120701e-01 9.410809e-01

11035 8.119727e-01 9.410823e-01

11036 8.118722e-01 9.410837e-01

11037 8.117683e-01 9.410852e-01

11038 8.116609e-01 9.410867e-01

11039 8.115500e-01 9.410883e-01

11040 8.114354e-01 9.410900e-01

11041 8.113170e-01 9.410917e-01

11042 8.111947e-01 9.410934e-01

11043 8.110685e-01 9.410953e-01

11044 8.109381e-01 9.410972e-01

11045 8.108034e-01 9.410991e-01

11046 8.106644e-01 9.411012e-01

11047 8.105209e-01 9.411033e-01

11048 8.103728e-01 9.411055e-01

11049 8.102199e-01 9.411078e-01

11050 8.100621e-01 9.411102e-01

11051 8.098992e-01 9.411126e-01

11052 8.097312e-01 9.411152e-01

11053 8.095579e-01 9.411178e-01

11054 8.093792e-01 9.411206e-01

11055 8.091948e-01 9.411234e-01

11056 8.090047e-01 9.411263e-01

11057 8.088086e-01 9.411294e-01

11058 8.086066e-01 9.411326e-01

11059 8.083983e-01 9.411359e-01

11060 8.081836e-01 9.411393e-01

11061 8.079624e-01 9.411428e-01

11062 8.077345e-01 9.411465e-01

11063 8.074998e-01 9.411503e-01

11064 8.072581e-01 9.411542e-01

11065 8.070092e-01 9.411583e-01

11066 8.067529e-01 9.411625e-01

11067 8.064892e-01 9.411669e-01

11068 8.062178e-01 9.411715e-01

11069 8.059386e-01 9.411762e-01

11070 8.056514e-01 9.411811e-01

11071 8.053560e-01 9.411862e-01

11072 8.050524e-01 9.411914e-01

11073 8.047402e-01 9.411969e-01

11074 8.044195e-01 9.412025e-01

11075 8.040899e-01 9.412084e-01

11076 8.037514e-01 9.412144e-01

11077 8.034039e-01 9.412207e-01

11078 8.030471e-01 9.412272e-01

11079 8.026809e-01 9.412339e-01

11080 8.023052e-01 9.412409e-01

11081 8.019198e-01 9.412481e-01

11082 8.015247e-01 9.412556e-01

11083 8.011197e-01 9.412633e-01

11084 8.007047e-01 9.412713e-01

11085 8.002795e-01 9.412796e-01

11086 7.998441e-01 9.412882e-01

11087 7.993984e-01 9.412971e-01

11088 7.989424e-01 9.413062e-01

11089 7.984758e-01 9.413157e-01

11090 7.979987e-01 9.413255e-01

11091 7.975110e-01 9.413357e-01

11092 7.970127e-01 9.413462e-01

11093 7.965038e-01 9.413570e-01

11094 7.959841e-01 9.413682e-01

11095 7.954538e-01 9.413798e-01

11096 7.949128e-01 9.413917e-01

11097 7.943612e-01 9.414041e-01

11098 7.937989e-01 9.414168e-01

11099 7.932261e-01 9.414300e-01

11100 7.926428e-01 9.414435e-01

11101 7.920491e-01 9.414575e-01

11102 7.914450e-01 9.414720e-01

11103 7.908308e-01 9.414869e-01

11104 7.902065e-01 9.415022e-01

11105 7.895724e-01 9.415181e-01

11106 7.889284e-01 9.415344e-01

11107 7.882750e-01 9.415512e-01

11108 7.876122e-01 9.415685e-01

11109 7.869402e-01 9.415864e-01

11110 7.862594e-01 9.416047e-01

11111 7.855700e-01 9.416236e-01

11112 7.848722e-01 9.416431e-01

11113 7.841664e-01 9.416631e-01

11114 7.834528e-01 9.416837e-01

11115 7.827318e-01 9.417049e-01

11116 7.820037e-01 9.417267e-01

11117 7.812689e-01 9.417490e-01

11118 7.805278e-01 9.417720e-01

11119 7.797807e-01 9.417957e-01

11120 7.790281e-01 9.418199e-01

11121 7.782704e-01 9.418448e-01

11122 7.775079e-01 9.418704e-01

11123 7.767412e-01 9.418966e-01

11124 7.759707e-01 9.419236e-01

11125 7.751968e-01 9.419512e-01

11126 7.744201e-01 9.419795e-01

11127 7.736410e-01 9.420085e-01

11128 7.728599e-01 9.420382e-01

11129 7.720774e-01 9.420687e-01

11130 7.712939e-01 9.420999e-01

11131 7.705100e-01 9.421318e-01

11132 7.697262e-01 9.421645e-01

11133 7.689429e-01 9.421980e-01

11134 7.681606e-01 9.422322e-01

11135 7.673798e-01 9.422672e-01

11136 7.666011e-01 9.423030e-01

11137 7.658248e-01 9.423395e-01

11138 7.650515e-01 9.423769e-01

11139 7.642816e-01 9.424150e-01

11140 7.635156e-01 9.424540e-01

11141 7.627540e-01 9.424938e-01

11142 7.619971e-01 9.425343e-01

11143 7.612454e-01 9.425757e-01

11144 7.604994e-01 9.426180e-01

11145 7.597594e-01 9.426613e-01

11146 7.590258e-01 9.427057e-01

11147 7.582990e-01 9.427513e-01

11148 7.575793e-01 9.427982e-01

11149 7.568671e-01 9.428466e-01

11150 7.561627e-01 9.428965e-01

11151 7.554665e-01 9.429480e-01

11152 7.547786e-01 9.430012e-01

11153 7.540994e-01 9.430561e-01

11154 7.534292e-01 9.431128e-01

11155 7.527681e-01 9.431712e-01

11156 7.521164e-01 9.432312e-01

11157 7.514743e-01 9.432928e-01

11158 7.508420e-01 9.433558e-01

11159 7.502196e-01 9.434204e-01

11160 7.496073e-01 9.434863e-01

11161 7.490052e-01 9.435535e-01

11162 7.489732e-01 9.435531e-01

11163 7.489402e-01 9.435527e-01

11164 7.489062e-01 9.435522e-01

11165 7.488711e-01 9.435518e-01

11166 7.488350e-01 9.435514e-01

11167 7.487977e-01 9.435509e-01

11168 7.487593e-01 9.435504e-01

11169 7.487197e-01 9.435500e-01

11170 7.486790e-01 9.435495e-01

11171 7.486370e-01 9.435490e-01

11172 7.485937e-01 9.435485e-01

11173 7.485491e-01 9.435479e-01

11174 7.485032e-01 9.435474e-01

11175 7.484558e-01 9.435469e-01

11176 7.484071e-01 9.435463e-01

11177 7.483569e-01 9.435457e-01

11178 7.483052e-01 9.435452e-01

11179 7.482520e-01 9.435446e-01

11180 7.481972e-01 9.435439e-01

11181 7.481407e-01 9.435433e-01

11182 7.480826e-01 9.435427e-01

11183 7.480227e-01 9.435420e-01

11184 7.479611e-01 9.435414e-01

11185 7.478976e-01 9.435407e-01

11186 7.478323e-01 9.435400e-01

11187 7.477650e-01 9.435392e-01

11188 7.476958e-01 9.435385e-01

11189 7.476245e-01 9.435378e-01

11190 7.475512e-01 9.435370e-01

11191 7.474757e-01 9.435362e-01

11192 7.473980e-01 9.435354e-01

11193 7.473180e-01 9.435346e-01

11194 7.472357e-01 9.435337e-01

11195 7.471510e-01 9.435328e-01

11196 7.470639e-01 9.435319e-01

11197 7.469742e-01 9.435310e-01

11198 7.468820e-01 9.435301e-01

11199 7.467871e-01 9.435292e-01

11200 7.466895e-01 9.435282e-01

11201 7.465891e-01 9.435272e-01

11202 7.464858e-01 9.435262e-01

11203 7.463796e-01 9.435251e-01

11204 7.462704e-01 9.435241e-01

11205 7.461581e-01 9.435230e-01

11206 7.460426e-01 9.435219e-01

11207 7.459239e-01 9.435207e-01

11208 7.458018e-01 9.435195e-01

11209 7.456764e-01 9.435184e-01

11210 7.455474e-01 9.435171e-01

11211 7.454149e-01 9.435159e-01

11212 7.452788e-01 9.435146e-01

11213 7.451389e-01 9.435133e-01

11214 7.449951e-01 9.435120e-01

11215 7.448475e-01 9.435106e-01

11216 7.446958e-01 9.435092e-01

11217 7.445401e-01 9.435078e-01

11218 7.443801e-01 9.435063e-01

11219 7.442159e-01 9.435048e-01

11220 7.440474e-01 9.435033e-01

11221 7.438743e-01 9.435018e-01

11222 7.436967e-01 9.435002e-01

11223 7.435145e-01 9.434986e-01

11224 7.433275e-01 9.434969e-01

11225 7.431358e-01 9.434952e-01

11226 7.429390e-01 9.434935e-01

11227 7.427373e-01 9.434917e-01

11228 7.425305e-01 9.434899e-01

11229 7.423184e-01 9.434881e-01

11230 7.421011e-01 9.434862e-01

11231 7.418784e-01 9.434843e-01

11232 7.416502e-01 9.434823e-01

11233 7.414165e-01 9.434803e-01

11234 7.411771e-01 9.434783e-01

11235 7.409320e-01 9.434762e-01

11236 7.406811e-01 9.434741e-01

11237 7.404243e-01 9.434719e-01

11238 7.401616e-01 9.434697e-01

11239 7.398928e-01 9.434674e-01

11240 7.396178e-01 9.434651e-01

11241 7.393367e-01 9.434628e-01

11242 7.390494e-01 9.434604e-01

11243 7.387557e-01 9.434579e-01

11244 7.384557e-01 9.434555e-01

11245 7.381493e-01 9.434529e-01

11246 7.378364e-01 9.434503e-01

11247 7.375170e-01 9.434477e-01

11248 7.371910e-01 9.434450e-01

11249 7.368584e-01 9.434423e-01

11250 7.365193e-01 9.434395e-01

11251 7.361735e-01 9.434366e-01

11252 7.358211e-01 9.434337e-01

11253 7.354621e-01 9.434308e-01

11254 7.350964e-01 9.434277e-01

11255 7.347241e-01 9.434247e-01

11256 7.343452e-01 9.434215e-01

11257 7.339597e-01 9.434184e-01

11258 7.335676e-01 9.434151e-01

11259 7.331691e-01 9.434118e-01

11260 7.327641e-01 9.434084e-01

11261 7.323527e-01 9.434050e-01

11262 7.319350e-01 9.434015e-01

11263 7.315111e-01 9.433979e-01

11264 7.310810e-01 9.433943e-01

11265 7.306449e-01 9.433906e-01

11266 7.302029e-01 9.433869e-01

11267 7.297551e-01 9.433830e-01

11268 7.293016e-01 9.433791e-01

11269 7.288427e-01 9.433752e-01

11270 7.283783e-01 9.433711e-01

11271 7.279088e-01 9.433670e-01

11272 7.274343e-01 9.433628e-01

11273 7.269550e-01 9.433586e-01

11274 7.264710e-01 9.433542e-01

11275 7.259827e-01 9.433498e-01

11276 7.254901e-01 9.433453e-01

11277 7.249937e-01 9.433407e-01

11278 7.244935e-01 9.433361e-01

11279 7.239898e-01 9.433313e-01

11280 7.234830e-01 9.433265e-01

11281 7.229732e-01 9.433216e-01

11282 7.224608e-01 9.433166e-01

11283 7.219461e-01 9.433115e-01

11284 7.214292e-01 9.433063e-01

11285 7.209106e-01 9.433010e-01

11286 7.203905e-01 9.432956e-01

11287 7.198692e-01 9.432901e-01

11288 7.193471e-01 9.432846e-01

11289 7.188245e-01 9.432789e-01

11290 7.183017e-01 9.432731e-01

11291 7.177789e-01 9.432672e-01

11292 7.172567e-01 9.432612e-01

11293 7.167351e-01 9.432551e-01

11294 7.162147e-01 9.432489e-01

11295 7.156957e-01 9.432426e-01

11296 7.151785e-01 9.432361e-01

11297 7.146633e-01 9.432296e-01

11298 7.141505e-01 9.432229e-01

11299 7.136404e-01 9.432161e-01

11300 7.131333e-01 9.432091e-01

11301 7.126295e-01 9.432021e-01

11302 7.121293e-01 9.431949e-01

11303 7.116330e-01 9.431875e-01

11304 7.111409e-01 9.431801e-01

11305 7.106532e-01 9.431724e-01

11306 7.101703e-01 9.431647e-01

11307 7.096923e-01 9.431568e-01

11308 7.092196e-01 9.431487e-01

11309 7.087523e-01 9.431406e-01

11310 7.082907e-01 9.431322e-01

11311 7.078350e-01 9.431237e-01

11312 7.073854e-01 9.431150e-01

11313 7.069421e-01 9.431062e-01

11314 7.065053e-01 9.430972e-01

11315 7.060751e-01 9.430881e-01

11316 7.056516e-01 9.430787e-01

11317 7.052351e-01 9.430692e-01

11318 7.048257e-01 9.430596e-01

11319 7.044234e-01 9.430497e-01

11320 7.040283e-01 9.430397e-01

11321 7.036406e-01 9.430295e-01

11322 7.032604e-01 9.430191e-01

11323 7.028876e-01 9.430085e-01

11324 7.025224e-01 9.429977e-01

11325 7.021648e-01 9.429868e-01

11326 7.018148e-01 9.429756e-01

11327 7.014725e-01 9.429643e-01

11328 7.011378e-01 9.429528e-01

11329 7.008107e-01 9.429410e-01

11330 7.004913e-01 9.429291e-01

11331 7.001795e-01 9.429170e-01

11332 6.998753e-01 9.429047e-01

11333 6.995786e-01 9.428921e-01

11334 6.992894e-01 9.428795e-01

11335 6.990076e-01 9.428668e-01

11336 6.987332e-01 9.428543e-01

11337 6.984661e-01 9.428420e-01

11338 6.982062e-01 9.428301e-01

11339 6.979535e-01 9.428187e-01

11340 6.977078e-01 9.428078e-01

11341 6.974691e-01 9.427976e-01

11342 6.974460e-01 9.427964e-01

11343 6.974225e-01 9.427951e-01

11344 6.973986e-01 9.427937e-01

11345 6.973741e-01 9.427924e-01

11346 6.973492e-01 9.427910e-01

11347 6.973238e-01 9.427896e-01

11348 6.972979e-01 9.427882e-01

11349 6.972715e-01 9.427868e-01

11350 6.972447e-01 9.427854e-01

11351 6.972174e-01 9.427839e-01

11352 6.971896e-01 9.427825e-01

11353 6.971613e-01 9.427810e-01

11354 6.971325e-01 9.427795e-01

11355 6.971032e-01 9.427780e-01

11356 6.970734e-01 9.427764e-01

11357 6.970431e-01 9.427749e-01

11358 6.970124e-01 9.427733e-01

11359 6.969811e-01 9.427717e-01

11360 6.969493e-01 9.427701e-01

11361 6.969170e-01 9.427685e-01

11362 6.968842e-01 9.427669e-01

11363 6.968510e-01 9.427653e-01

11364 6.968172e-01 9.427636e-01

11365 6.967829e-01 9.427620e-01

11366 6.967482e-01 9.427603e-01

11367 6.967129e-01 9.427587e-01

11368 6.966772e-01 9.427570e-01

11369 6.966409e-01 9.427553e-01

11370 6.966042e-01 9.427536e-01

11371 6.965670e-01 9.427519e-01

11372 6.965294e-01 9.427502e-01

11373 6.964913e-01 9.427485e-01

11374 6.964527e-01 9.427467e-01

11375 6.964136e-01 9.427450e-01

11376 6.963742e-01 9.427433e-01

11377 6.963343e-01 9.427415e-01

11378 6.962940e-01 9.427398e-01

11379 6.962533e-01 9.427381e-01

11380 6.962122e-01 9.427364e-01

11381 6.961707e-01 9.427346e-01

11382 6.961288e-01 9.427329e-01

11383 6.960866e-01 9.427312e-01

11384 6.960441e-01 9.427295e-01

11385 6.960013e-01 9.427278e-01

11386 6.959582e-01 9.427262e-01

11387 6.959148e-01 9.427245e-01

11388 6.958712e-01 9.427228e-01

11389 6.958274e-01 9.427212e-01

11390 6.957833e-01 9.427196e-01

11391 6.957392e-01 9.427180e-01

11392 6.956948e-01 9.427165e-01

11393 6.956504e-01 9.427149e-01

11394 6.956060e-01 9.427134e-01

11395 6.955615e-01 9.427119e-01

11396 6.955170e-01 9.427105e-01

11397 6.954725e-01 9.427091e-01

11398 6.954282e-01 9.427077e-01

11399 6.953839e-01 9.427064e-01

11400 6.953399e-01 9.427052e-01

11401 6.952961e-01 9.427039e-01

11402 6.952525e-01 9.427028e-01

11403 6.952093e-01 9.427017e-01

11404 6.951664e-01 9.427006e-01

11405 6.951240e-01 9.426997e-01

11406 6.950821e-01 9.426987e-01

11407 6.950408e-01 9.426979e-01

11408 6.950000e-01 9.426972e-01

11409 6.949600e-01 9.426965e-01

11410 6.949207e-01 9.426959e-01

11411 6.948822e-01 9.426954e-01

11412 6.948447e-01 9.426951e-01

11413 6.948080e-01 9.426948e-01

11414 6.947725e-01 9.426946e-01

11415 6.947381e-01 9.426946e-01

11416 6.947048e-01 9.426947e-01

11417 6.946729e-01 9.426949e-01

11418 6.946424e-01 9.426952e-01

11419 6.946133e-01 9.426957e-01

11420 6.945857e-01 9.426964e-01

11421 6.945599e-01 9.426972e-01

11422 6.945357e-01 9.426982e-01

11423 6.945135e-01 9.426993e-01

11424 6.944931e-01 9.427007e-01

11425 6.944748e-01 9.427022e-01

11426 6.944586e-01 9.427039e-01

11427 6.944447e-01 9.427059e-01

11428 6.944331e-01 9.427081e-01

11429 6.944240e-01 9.427105e-01

11430 6.944174e-01 9.427131e-01

11431 6.944135e-01 9.427160e-01

11432 6.944124e-01 9.427192e-01

11433 6.944141e-01 9.427226e-01

11434 6.944188e-01 9.427264e-01

11435 6.944267e-01 9.427304e-01

11436 6.944377e-01 9.427348e-01

11437 6.944520e-01 9.427395e-01

11438 6.944697e-01 9.427445e-01

11439 6.944909e-01 9.427499e-01

11440 6.945158e-01 9.427557e-01

11441 6.945444e-01 9.427618e-01

11442 6.945768e-01 9.427684e-01

11443 6.946130e-01 9.427754e-01

11444 6.946534e-01 9.427828e-01

11445 6.946978e-01 9.427907e-01

11446 6.947463e-01 9.427991e-01

11447 6.947992e-01 9.428079e-01

11448 6.948564e-01 9.428173e-01

11449 6.949180e-01 9.428272e-01

11450 6.949842e-01 9.428376e-01

11451 6.950548e-01 9.428486e-01

11452 6.951302e-01 9.428602e-01

11453 6.952102e-01 9.428725e-01

11454 6.952949e-01 9.428853e-01

11455 6.953844e-01 9.428988e-01

11456 6.954787e-01 9.429130e-01

11457 6.955779e-01 9.429280e-01

11458 6.956820e-01 9.429436e-01

11459 6.957909e-01 9.429600e-01

11460 6.959048e-01 9.429772e-01

11461 6.960235e-01 9.429952e-01

11462 6.961472e-01 9.430140e-01

11463 6.962758e-01 9.430338e-01

11464 6.964092e-01 9.430543e-01

11465 6.965475e-01 9.430759e-01

11466 6.966906e-01 9.430983e-01

11467 6.968385e-01 9.431218e-01

11468 6.969911e-01 9.431463e-01

11469 6.971484e-01 9.431718e-01

11470 6.973103e-01 9.431984e-01

11471 6.974767e-01 9.432261e-01

11472 6.976476e-01 9.432549e-01

11473 6.978228e-01 9.432849e-01

11474 6.980024e-01 9.433162e-01

11475 6.981860e-01 9.433486e-01

11476 6.983738e-01 9.433824e-01

11477 6.985655e-01 9.434175e-01

11478 6.987610e-01 9.434539e-01

11479 6.989603e-01 9.434917e-01

11480 6.991631e-01 9.435309e-01

11481 6.993693e-01 9.435716e-01

11482 6.995788e-01 9.436138e-01

11483 6.997915e-01 9.436576e-01

11484 7.000071e-01 9.437029e-01

11485 7.002256e-01 9.437498e-01

11486 7.004468e-01 9.437984e-01

11487 7.006705e-01 9.438487e-01

11488 7.008965e-01 9.439007e-01

11489 7.011248e-01 9.439546e-01

11490 7.013550e-01 9.440102e-01

11491 7.015871e-01 9.440677e-01

11492 7.018209e-01 9.441271e-01

11493 7.020562e-01 9.441884e-01

11494 7.022929e-01 9.442517e-01

11495 7.025307e-01 9.443170e-01

11496 7.027696e-01 9.443844e-01

11497 7.030092e-01 9.444539e-01

11498 7.032496e-01 9.445255e-01

11499 7.034905e-01 9.445994e-01

11500 7.037317e-01 9.446754e-01

11501 7.039731e-01 9.447537e-01

11502 7.042146e-01 9.448343e-01

11503 7.044559e-01 9.449173e-01

11504 7.046970e-01 9.450026e-01

11505 7.049377e-01 9.450904e-01

11506 7.051779e-01 9.451806e-01

11507 7.054174e-01 9.452733e-01

11508 7.056560e-01 9.453683e-01

11509 7.058938e-01 9.454656e-01

11510 7.061304e-01 9.455651e-01

11511 7.063659e-01 9.456669e-01

11512 7.066001e-01 9.457707e-01

11513 7.068329e-01 9.458766e-01

11514 7.070643e-01 9.459845e-01

11515 7.072940e-01 9.460944e-01

11516 7.075220e-01 9.462062e-01

11517 7.077483e-01 9.463199e-01

11518 7.079727e-01 9.464354e-01

11519 7.081952e-01 9.465527e-01

11520 7.084157e-01 9.466718e-01

11521 7.086341e-01 9.467926e-01

11522 7.086569e-01 9.467930e-01

11523 7.086806e-01 9.467934e-01

11524 7.087051e-01 9.467937e-01

11525 7.087305e-01 9.467941e-01

11526 7.087568e-01 9.467945e-01

11527 7.087840e-01 9.467949e-01

11528 7.088122e-01 9.467954e-01

11529 7.088415e-01 9.467958e-01

11530 7.088717e-01 9.467962e-01

11531 7.089030e-01 9.467967e-01

11532 7.089354e-01 9.467972e-01

11533 7.089689e-01 9.467976e-01

11534 7.090035e-01 9.467981e-01

11535 7.090394e-01 9.467986e-01

11536 7.090764e-01 9.467991e-01

11537 7.091147e-01 9.467997e-01

11538 7.091543e-01 9.468002e-01

11539 7.091953e-01 9.468008e-01

11540 7.092376e-01 9.468013e-01

11541 7.092813e-01 9.468019e-01

11542 7.093264e-01 9.468025e-01

11543 7.093731e-01 9.468031e-01

11544 7.094213e-01 9.468038e-01

11545 7.094711e-01 9.468044e-01

11546 7.095225e-01 9.468051e-01

11547 7.095757e-01 9.468057e-01

11548 7.096305e-01 9.468064e-01

11549 7.096871e-01 9.468071e-01

11550 7.097456e-01 9.468079e-01

11551 7.098060e-01 9.468086e-01

11552 7.098683e-01 9.468094e-01

11553 7.099326e-01 9.468102e-01

11554 7.099989e-01 9.468110e-01

11555 7.100674e-01 9.468118e-01

11556 7.101381e-01 9.468127e-01

11557 7.102110e-01 9.468136e-01

11558 7.102863e-01 9.468145e-01

11559 7.103639e-01 9.468154e-01

11560 7.104439e-01 9.468163e-01

11561 7.105265e-01 9.468173e-01

11562 7.106117e-01 9.468183e-01

11563 7.106995e-01 9.468193e-01

11564 7.107900e-01 9.468204e-01

11565 7.108833e-01 9.468214e-01

11566 7.109796e-01 9.468226e-01

11567 7.110787e-01 9.468237e-01

11568 7.111810e-01 9.468249e-01

11569 7.112863e-01 9.468261e-01

11570 7.113949e-01 9.468273e-01

11571 7.115068e-01 9.468286e-01

11572 7.116220e-01 9.468299e-01

11573 7.117407e-01 9.468312e-01

11574 7.118630e-01 9.468326e-01

11575 7.119890e-01 9.468340e-01

11576 7.121187e-01 9.468354e-01

11577 7.122522e-01 9.468369e-01

11578 7.123897e-01 9.468385e-01

11579 7.125312e-01 9.468400e-01

11580 7.126769e-01 9.468416e-01

11581 7.128269e-01 9.468433e-01

11582 7.129812e-01 9.468450e-01

11583 7.131399e-01 9.468468e-01

11584 7.133032e-01 9.468486e-01

11585 7.134712e-01 9.468504e-01

11586 7.136440e-01 9.468523e-01

11587 7.138216e-01 9.468543e-01

11588 7.140043e-01 9.468563e-01

11589 7.141921e-01 9.468583e-01

11590 7.143851e-01 9.468604e-01

11591 7.145835e-01 9.468626e-01

11592 7.147874e-01 9.468648e-01

11593 7.149968e-01 9.468671e-01

11594 7.152119e-01 9.468695e-01

11595 7.154328e-01 9.468719e-01

11596 7.156597e-01 9.468744e-01

11597 7.158926e-01 9.468770e-01

11598 7.161316e-01 9.468796e-01

11599 7.163770e-01 9.468823e-01

11600 7.166287e-01 9.468850e-01

11601 7.168870e-01 9.468879e-01

11602 7.171519e-01 9.468908e-01

11603 7.174235e-01 9.468938e-01

11604 7.177020e-01 9.468969e-01

11605 7.179875e-01 9.469000e-01

11606 7.182800e-01 9.469032e-01

11607 7.185797e-01 9.469066e-01

11608 7.188867e-01 9.469100e-01

11609 7.192011e-01 9.469135e-01

11610 7.195230e-01 9.469171e-01

11611 7.198525e-01 9.469208e-01

11612 7.201896e-01 9.469245e-01

11613 7.205346e-01 9.469284e-01

11614 7.208874e-01 9.469324e-01

11615 7.212481e-01 9.469365e-01

11616 7.216169e-01 9.469407e-01

11617 7.219937e-01 9.469450e-01

11618 7.223787e-01 9.469494e-01

11619 7.227719e-01 9.469539e-01

11620 7.231734e-01 9.469585e-01

11621 7.235833e-01 9.469633e-01

11622 7.240015e-01 9.469681e-01

11623 7.244281e-01 9.469731e-01

11624 7.248632e-01 9.469782e-01

11625 7.253067e-01 9.469835e-01

11626 7.257587e-01 9.469888e-01

11627 7.262192e-01 9.469943e-01

11628 7.266882e-01 9.469999e-01

11629 7.271657e-01 9.470057e-01

11630 7.276516e-01 9.470116e-01

11631 7.281460e-01 9.470176e-01

11632 7.286488e-01 9.470238e-01

11633 7.291599e-01 9.470302e-01

11634 7.296793e-01 9.470366e-01

11635 7.302070e-01 9.470433e-01

11636 7.307428e-01 9.470500e-01

11637 7.312866e-01 9.470570e-01

11638 7.318385e-01 9.470641e-01

11639 7.323982e-01 9.470713e-01

11640 7.329657e-01 9.470787e-01

11641 7.335408e-01 9.470863e-01

11642 7.341234e-01 9.470940e-01

11643 7.347133e-01 9.471019e-01

11644 7.353104e-01 9.471100e-01

11645 7.359146e-01 9.471182e-01

11646 7.365255e-01 9.471266e-01

11647 7.371431e-01 9.471352e-01

11648 7.377671e-01 9.471440e-01

11649 7.383973e-01 9.471530e-01

11650 7.390335e-01 9.471621e-01

11651 7.396755e-01 9.471714e-01

11652 7.403231e-01 9.471809e-01

11653 7.409759e-01 9.471906e-01

11654 7.416337e-01 9.472005e-01

11655 7.422963e-01 9.472106e-01

11656 7.429634e-01 9.472209e-01

11657 7.436346e-01 9.472313e-01

11658 7.443098e-01 9.472420e-01

11659 7.449886e-01 9.472529e-01

11660 7.456707e-01 9.472639e-01

11661 7.463559e-01 9.472752e-01

11662 7.470437e-01 9.472867e-01

11663 7.477339e-01 9.472984e-01

11664 7.484261e-01 9.473103e-01

11665 7.491201e-01 9.473224e-01

11666 7.498155e-01 9.473347e-01

11667 7.505120e-01 9.473472e-01

11668 7.512093e-01 9.473599e-01

11669 7.519069e-01 9.473729e-01

11670 7.526047e-01 9.473861e-01

11671 7.533022e-01 9.473995e-01

11672 7.539992e-01 9.474131e-01

11673 7.546953e-01 9.474269e-01

11674 7.553901e-01 9.474410e-01

11675 7.560834e-01 9.474552e-01

11676 7.567749e-01 9.474697e-01

11677 7.574642e-01 9.474845e-01

11678 7.581510e-01 9.474994e-01

11679 7.588350e-01 9.475146e-01

11680 7.595159e-01 9.475300e-01

11681 7.601935e-01 9.475456e-01

11682 7.608673e-01 9.475615e-01

11683 7.615373e-01 9.475776e-01

11684 7.622030e-01 9.475939e-01

11685 7.628643e-01 9.476105e-01

11686 7.635208e-01 9.476273e-01

11687 7.641724e-01 9.476443e-01

11688 7.648187e-01 9.476618e-01

11689 7.654596e-01 9.476798e-01

11690 7.660948e-01 9.476983e-01

11691 7.667242e-01 9.477176e-01

11692 7.673475e-01 9.477376e-01

11693 7.679645e-01 9.477585e-01

11694 7.685751e-01 9.477804e-01

11695 7.691791e-01 9.478033e-01

11696 7.697763e-01 9.478272e-01

11697 7.703666e-01 9.478523e-01

11698 7.709498e-01 9.478787e-01

11699 7.715259e-01 9.479063e-01

11700 7.720947e-01 9.479352e-01

11701 7.726561e-01 9.479652e-01

11702 7.726866e-01 9.479645e-01

11703 7.727180e-01 9.479638e-01

11704 7.727504e-01 9.479631e-01

11705 7.727836e-01 9.479624e-01

11706 7.728178e-01 9.479616e-01

11707 7.728529e-01 9.479608e-01

11708 7.728890e-01 9.479601e-01

11709 7.729262e-01 9.479593e-01

11710 7.729644e-01 9.479585e-01

11711 7.730037e-01 9.479576e-01

11712 7.730440e-01 9.479568e-01

11713 7.730855e-01 9.479559e-01

11714 7.731282e-01 9.479550e-01

11715 7.731720e-01 9.479541e-01

11716 7.732171e-01 9.479532e-01

11717 7.732634e-01 9.479523e-01

11718 7.733110e-01 9.479513e-01

11719 7.733599e-01 9.479503e-01

11720 7.734102e-01 9.479493e-01

11721 7.734618e-01 9.479482e-01

11722 7.735149e-01 9.479472e-01

11723 7.735694e-01 9.479461e-01

11724 7.736255e-01 9.479449e-01

11725 7.736830e-01 9.479438e-01

11726 7.737422e-01 9.479426e-01

11727 7.738029e-01 9.479414e-01

11728 7.738654e-01 9.479402e-01

11729 7.739295e-01 9.479389e-01

11730 7.739954e-01 9.479376e-01

11731 7.740630e-01 9.479362e-01

11732 7.741325e-01 9.479349e-01

11733 7.742039e-01 9.479335e-01

11734 7.742772e-01 9.479320e-01

11735 7.743525e-01 9.479305e-01

11736 7.744298e-01 9.479290e-01

11737 7.745092e-01 9.479274e-01

11738 7.745908e-01 9.479258e-01

11739 7.746745e-01 9.479242e-01

11740 7.747604e-01 9.479225e-01

11741 7.748486e-01 9.479208e-01

11742 7.749392e-01 9.479190e-01

11743 7.750322e-01 9.479171e-01

11744 7.751276e-01 9.479153e-01

11745 7.752256e-01 9.479133e-01

11746 7.753261e-01 9.479114e-01

11747 7.754293e-01 9.479093e-01

11748 7.755352e-01 9.479073e-01

11749 7.756439e-01 9.479051e-01

11750 7.757553e-01 9.479029e-01

11751 7.758697e-01 9.479007e-01

11752 7.759871e-01 9.478984e-01

11753 7.761075e-01 9.478960e-01

11754 7.762310e-01 9.478936e-01

11755 7.763576e-01 9.478911e-01

11756 7.764875e-01 9.478885e-01

11757 7.766207e-01 9.478858e-01

11758 7.767574e-01 9.478831e-01

11759 7.768974e-01 9.478803e-01

11760 7.770411e-01 9.478775e-01

11761 7.771883e-01 9.478746e-01

11762 7.773392e-01 9.478715e-01

11763 7.774940e-01 9.478684e-01

11764 7.776525e-01 9.478653e-01

11765 7.778150e-01 9.478620e-01

11766 7.779816e-01 9.478586e-01

11767 7.781522e-01 9.478552e-01

11768 7.783270e-01 9.478516e-01

11769 7.785061e-01 9.478480e-01

11770 7.786895e-01 9.478443e-01

11771 7.788774e-01 9.478404e-01

11772 7.790698e-01 9.478365e-01

11773 7.792669e-01 9.478324e-01

11774 7.794686e-01 9.478283e-01

11775 7.796751e-01 9.478240e-01

11776 7.798865e-01 9.478196e-01

11777 7.801029e-01 9.478151e-01

11778 7.803244e-01 9.478104e-01

11779 7.805510e-01 9.478056e-01

11780 7.807828e-01 9.478007e-01

11781 7.810200e-01 9.477957e-01

11782 7.812626e-01 9.477905e-01

11783 7.815107e-01 9.477852e-01

11784 7.817644e-01 9.477797e-01

11785 7.820238e-01 9.477741e-01

11786 7.822890e-01 9.477683e-01

11787 7.825601e-01 9.477624e-01

11788 7.828371e-01 9.477563e-01

11789 7.831202e-01 9.477500e-01

11790 7.834094e-01 9.477436e-01

11791 7.837048e-01 9.477369e-01

11792 7.840066e-01 9.477301e-01

11793 7.843147e-01 9.477231e-01

11794 7.846293e-01 9.477160e-01

11795 7.849504e-01 9.477086e-01

11796 7.852781e-01 9.477010e-01

11797 7.856126e-01 9.476932e-01

11798 7.859538e-01 9.476852e-01

11799 7.863018e-01 9.476769e-01

11800 7.866568e-01 9.476684e-01

11801 7.870187e-01 9.476597e-01

11802 7.873876e-01 9.476508e-01

11803 7.877637e-01 9.476416e-01

11804 7.881468e-01 9.476322e-01

11805 7.885372e-01 9.476225e-01

11806 7.889348e-01 9.476125e-01

11807 7.893397e-01 9.476022e-01

11808 7.897519e-01 9.475917e-01

11809 7.901714e-01 9.475809e-01

11810 7.905983e-01 9.475698e-01

11811 7.910326e-01 9.475584e-01

11812 7.914743e-01 9.475466e-01

11813 7.919235e-01 9.475346e-01

11814 7.923801e-01 9.475222e-01

11815 7.928441e-01 9.475095e-01

11816 7.933155e-01 9.474965e-01

11817 7.937943e-01 9.474831e-01

11818 7.942805e-01 9.474694e-01

11819 7.947741e-01 9.474553e-01

11820 7.952750e-01 9.474408e-01

11821 7.957831e-01 9.474259e-01

11822 7.962985e-01 9.474107e-01

11823 7.968211e-01 9.473950e-01

11824 7.973508e-01 9.473790e-01

11825 7.978875e-01 9.473625e-01

11826 7.984311e-01 9.473456e-01

11827 7.989816e-01 9.473283e-01

11828 7.995388e-01 9.473106e-01

11829 8.001027e-01 9.472923e-01

11830 8.006732e-01 9.472737e-01

11831 8.012500e-01 9.472545e-01

11832 8.018331e-01 9.472349e-01

11833 8.024223e-01 9.472148e-01

11834 8.030175e-01 9.471942e-01

11835 8.036185e-01 9.471731e-01

11836 8.042252e-01 9.471515e-01

11837 8.048373e-01 9.471294e-01

11838 8.054547e-01 9.471067e-01

11839 8.060772e-01 9.470835e-01

11840 8.067045e-01 9.470598e-01

11841 8.073366e-01 9.470355e-01

11842 8.079730e-01 9.470106e-01

11843 8.086137e-01 9.469852e-01

11844 8.092584e-01 9.469592e-01

11845 8.099068e-01 9.469326e-01

11846 8.105588e-01 9.469054e-01

11847 8.112139e-01 9.468776e-01

11848 8.118721e-01 9.468491e-01

11849 8.125329e-01 9.468201e-01

11850 8.131962e-01 9.467904e-01

11851 8.138617e-01 9.467601e-01

11852 8.145290e-01 9.467291e-01

11853 8.151979e-01 9.466975e-01

11854 8.158682e-01 9.466653e-01

11855 8.165394e-01 9.466324e-01

11856 8.172114e-01 9.465988e-01

11857 8.178837e-01 9.465645e-01

11858 8.185562e-01 9.465296e-01

11859 8.192285e-01 9.464940e-01

11860 8.199003e-01 9.464577e-01

11861 8.205713e-01 9.464206e-01

11862 8.212413e-01 9.463829e-01

11863 8.219098e-01 9.463445e-01

11864 8.225766e-01 9.463054e-01

11865 8.232415e-01 9.462656e-01

11866 8.239041e-01 9.462251e-01

11867 8.245640e-01 9.461839e-01

11868 8.252212e-01 9.461419e-01

11869 8.258752e-01 9.460993e-01

11870 8.265257e-01 9.460559e-01

11871 8.271726e-01 9.460118e-01

11872 8.278155e-01 9.459670e-01

11873 8.284542e-01 9.459215e-01

11874 8.290884e-01 9.458753e-01

11875 8.297178e-01 9.458284e-01

11876 8.303423e-01 9.457807e-01

11877 8.309616e-01 9.457324e-01

11878 8.315754e-01 9.456833e-01

11879 8.321836e-01 9.456336e-01

11880 8.327860e-01 9.455832e-01

11881 8.333823e-01 9.455323e-01

11882 8.333984e-01 9.455304e-01

11883 8.334149e-01 9.455285e-01

11884 8.334318e-01 9.455265e-01

11885 8.334492e-01 9.455245e-01

11886 8.334670e-01 9.455224e-01

11887 8.334852e-01 9.455203e-01

11888 8.335039e-01 9.455182e-01

11889 8.335231e-01 9.455160e-01

11890 8.335428e-01 9.455138e-01

11891 8.335629e-01 9.455116e-01

11892 8.335836e-01 9.455093e-01

11893 8.336047e-01 9.455070e-01

11894 8.336264e-01 9.455046e-01

11895 8.336487e-01 9.455022e-01

11896 8.336715e-01 9.454998e-01

11897 8.336949e-01 9.454973e-01

11898 8.337189e-01 9.454948e-01

11899 8.337434e-01 9.454923e-01

11900 8.337686e-01 9.454897e-01

11901 8.337945e-01 9.454871e-01

11902 8.338209e-01 9.454845e-01

11903 8.338481e-01 9.454818e-01

11904 8.338759e-01 9.454791e-01

11905 8.339044e-01 9.454763e-01

11906 8.339337e-01 9.454735e-01

11907 8.339637e-01 9.454707e-01

11908 8.339944e-01 9.454678e-01

11909 8.340259e-01 9.454649e-01

11910 8.340582e-01 9.454619e-01

11911 8.340913e-01 9.454589e-01

11912 8.341253e-01 9.454559e-01

11913 8.341601e-01 9.454529e-01

11914 8.341958e-01 9.454498e-01

11915 8.342324e-01 9.454466e-01

11916 8.342699e-01 9.454434e-01

11917 8.343083e-01 9.454402e-01

11918 8.343477e-01 9.454370e-01

11919 8.343881e-01 9.454337e-01

11920 8.344295e-01 9.454303e-01

11921 8.344719e-01 9.454270e-01

11922 8.345154e-01 9.454236e-01

11923 8.345600e-01 9.454201e-01

11924 8.346057e-01 9.454166e-01

11925 8.346525e-01 9.454131e-01

11926 8.347005e-01 9.454095e-01

11927 8.347496e-01 9.454059e-01

11928 8.348000e-01 9.454023e-01

11929 8.348517e-01 9.453986e-01

11930 8.349046e-01 9.453949e-01

11931 8.349588e-01 9.453912e-01

11932 8.350143e-01 9.453874e-01

11933 8.350712e-01 9.453836e-01

11934 8.351295e-01 9.453797e-01

11935 8.351892e-01 9.453759e-01

11936 8.352504e-01 9.453720e-01

11937 8.353130e-01 9.453680e-01

11938 8.353772e-01 9.453640e-01

11939 8.354429e-01 9.453600e-01

11940 8.355101e-01 9.453560e-01

11941 8.355790e-01 9.453519e-01

11942 8.356496e-01 9.453478e-01

11943 8.357218e-01 9.453437e-01

11944 8.357957e-01 9.453396e-01

11945 8.358714e-01 9.453354e-01

11946 8.359489e-01 9.453312e-01

11947 8.360281e-01 9.453270e-01

11948 8.361093e-01 9.453228e-01

11949 8.361923e-01 9.453186e-01

11950 8.362772e-01 9.453143e-01

11951 8.363641e-01 9.453100e-01

11952 8.364530e-01 9.453057e-01

11953 8.365440e-01 9.453014e-01

11954 8.366370e-01 9.452971e-01

11955 8.367321e-01 9.452928e-01

11956 8.368293e-01 9.452885e-01

11957 8.369288e-01 9.452842e-01

11958 8.370304e-01 9.452799e-01

11959 8.371343e-01 9.452756e-01

11960 8.372405e-01 9.452713e-01

11961 8.373490e-01 9.452671e-01

11962 8.374599e-01 9.452628e-01

11963 8.375732e-01 9.452586e-01

11964 8.376890e-01 9.452544e-01

11965 8.378072e-01 9.452502e-01

11966 8.379279e-01 9.452460e-01

11967 8.380512e-01 9.452419e-01

11968 8.381770e-01 9.452378e-01

11969 8.383055e-01 9.452338e-01

11970 8.384366e-01 9.452298e-01

11971 8.385704e-01 9.452259e-01

11972 8.387069e-01 9.452220e-01

11973 8.388461e-01 9.452183e-01

11974 8.389882e-01 9.452145e-01

11975 8.391330e-01 9.452109e-01

11976 8.392807e-01 9.452074e-01

11977 8.394312e-01 9.452039e-01

11978 8.395847e-01 9.452006e-01

11979 8.397411e-01 9.451973e-01

11980 8.399004e-01 9.451942e-01

11981 8.400627e-01 9.451912e-01

11982 8.402280e-01 9.451883e-01

11983 8.403962e-01 9.451856e-01

11984 8.405676e-01 9.451830e-01

11985 8.407419e-01 9.451806e-01

11986 8.409194e-01 9.451784e-01

11987 8.410999e-01 9.451763e-01

11988 8.412835e-01 9.451745e-01

11989 8.414702e-01 9.451728e-01

11990 8.416600e-01 9.451714e-01

11991 8.418529e-01 9.451702e-01

11992 8.420490e-01 9.451692e-01

11993 8.422481e-01 9.451685e-01

11994 8.424503e-01 9.451681e-01

11995 8.426557e-01 9.451680e-01

11996 8.428642e-01 9.451681e-01

11997 8.430757e-01 9.451686e-01

11998 8.432903e-01 9.451694e-01

11999 8.435080e-01 9.451705e-01

12000 8.437286e-01 9.451720e-01

12001 8.439523e-01 9.451739e-01

12002 8.441790e-01 9.451761e-01

12003 8.444086e-01 9.451788e-01

12004 8.446412e-01 9.451819e-01

12005 8.448766e-01 9.451855e-01

12006 8.451148e-01 9.451896e-01

12007 8.453559e-01 9.451941e-01

12008 8.455996e-01 9.451992e-01

12009 8.458461e-01 9.452048e-01

12010 8.460952e-01 9.452109e-01

12011 8.463468e-01 9.452176e-01

12012 8.466010e-01 9.452250e-01

12013 8.468575e-01 9.452329e-01

12014 8.471164e-01 9.452416e-01

12015 8.473776e-01 9.452509e-01

12016 8.476410e-01 9.452608e-01

12017 8.479065e-01 9.452716e-01

12018 8.481740e-01 9.452830e-01

12019 8.484434e-01 9.452953e-01

12020 8.487147e-01 9.453084e-01

12021 8.489877e-01 9.453223e-01

12022 8.492622e-01 9.453370e-01

12023 8.495383e-01 9.453527e-01

12024 8.498158e-01 9.453693e-01

12025 8.500945e-01 9.453868e-01

12026 8.503744e-01 9.454053e-01

12027 8.506554e-01 9.454249e-01

12028 8.509372e-01 9.454454e-01

12029 8.512198e-01 9.454671e-01

12030 8.515030e-01 9.454898e-01

12031 8.517867e-01 9.455137e-01

12032 8.520708e-01 9.455388e-01

12033 8.523551e-01 9.455650e-01

12034 8.526395e-01 9.455925e-01

12035 8.529239e-01 9.456213e-01

12036 8.532080e-01 9.456514e-01

12037 8.534918e-01 9.456828e-01

12038 8.537751e-01 9.457155e-01

12039 8.540577e-01 9.457497e-01

12040 8.543395e-01 9.457853e-01

12041 8.546204e-01 9.458223e-01

12042 8.549002e-01 9.458609e-01

12043 8.551787e-01 9.459010e-01

12044 8.554559e-01 9.459427e-01

12045 8.557315e-01 9.459859e-01

12046 8.560055e-01 9.460308e-01

12047 8.562776e-01 9.460774e-01

12048 8.565478e-01 9.461256e-01

12049 8.568159e-01 9.461756e-01

12050 8.570817e-01 9.462274e-01

12051 8.573452e-01 9.462809e-01

12052 8.576061e-01 9.463363e-01

12053 8.578645e-01 9.463935e-01

12054 8.581201e-01 9.464525e-01

12055 8.583728e-01 9.465133e-01

12056 8.586226e-01 9.465757e-01

12057 8.588692e-01 9.466398e-01

12058 8.591127e-01 9.467053e-01

12059 8.593528e-01 9.467723e-01

12060 8.595895e-01 9.468406e-01

12061 8.598228e-01 9.469104e-01

12062 8.598274e-01 9.469102e-01

12063 8.598321e-01 9.469101e-01

12064 8.598369e-01 9.469099e-01

12065 8.598418e-01 9.469098e-01

12066 8.598467e-01 9.469096e-01

12067 8.598516e-01 9.469095e-01

12068 8.598567e-01 9.469093e-01

12069 8.598618e-01 9.469092e-01

12070 8.598670e-01 9.469090e-01

12071 8.598722e-01 9.469089e-01

12072 8.598775e-01 9.469087e-01

12073 8.598829e-01 9.469085e-01

12074 8.598883e-01 9.469084e-01

12075 8.598938e-01 9.469082e-01

12076 8.598994e-01 9.469080e-01

12077 8.599051e-01 9.469078e-01

12078 8.599108e-01 9.469077e-01

12079 8.599166e-01 9.469075e-01

12080 8.599224e-01 9.469073e-01

12081 8.599283e-01 9.469071e-01

12082 8.599343e-01 9.469069e-01

12083 8.599403e-01 9.469066e-01

12084 8.599465e-01 9.469064e-01

12085 8.599526e-01 9.469062e-01

12086 8.599589e-01 9.469060e-01

12087 8.599652e-01 9.469057e-01

12088 8.599716e-01 9.469055e-01

12089 8.599780e-01 9.469052e-01

12090 8.599845e-01 9.469049e-01

12091 8.599911e-01 9.469047e-01

12092 8.599977e-01 9.469044e-01

12093 8.600044e-01 9.469041e-01

12094 8.600111e-01 9.469038e-01

12095 8.600179e-01 9.469035e-01

12096 8.600247e-01 9.469032e-01

12097 8.600316e-01 9.469029e-01

12098 8.600386e-01 9.469026e-01

12099 8.600456e-01 9.469022e-01

12100 8.600527e-01 9.469019e-01

12101 8.600598e-01 9.469015e-01

12102 8.600669e-01 9.469011e-01

12103 8.600741e-01 9.469008e-01

12104 8.600813e-01 9.469004e-01

12105 8.600885e-01 9.469000e-01

12106 8.600958e-01 9.468996e-01

12107 8.601031e-01 9.468991e-01

12108 8.601104e-01 9.468987e-01

12109 8.601177e-01 9.468983e-01

12110 8.601251e-01 9.468978e-01

12111 8.601324e-01 9.468973e-01

12112 8.601398e-01 9.468969e-01

12113 8.601471e-01 9.468964e-01

12114 8.601544e-01 9.468959e-01

12115 8.601618e-01 9.468954e-01

12116 8.601690e-01 9.468948e-01

12117 8.601763e-01 9.468943e-01

12118 8.601835e-01 9.468937e-01

12119 8.601907e-01 9.468931e-01

12120 8.601977e-01 9.468925e-01

12121 8.602048e-01 9.468919e-01

12122 8.602117e-01 9.468913e-01

12123 8.602186e-01 9.468907e-01

12124 8.602253e-01 9.468900e-01

12125 8.602320e-01 9.468894e-01

12126 8.602385e-01 9.468887e-01

12127 8.602449e-01 9.468880e-01

12128 8.602511e-01 9.468872e-01

12129 8.602572e-01 9.468865e-01

12130 8.602630e-01 9.468857e-01

12131 8.602687e-01 9.468850e-01

12132 8.602742e-01 9.468842e-01

12133 8.602794e-01 9.468833e-01

12134 8.602843e-01 9.468825e-01

12135 8.602890e-01 9.468816e-01

12136 8.602934e-01 9.468808e-01

12137 8.602975e-01 9.468799e-01

12138 8.603013e-01 9.468789e-01

12139 8.603046e-01 9.468780e-01

12140 8.603076e-01 9.468770e-01

12141 8.603102e-01 9.468760e-01

12142 8.603123e-01 9.468750e-01

12143 8.603140e-01 9.468739e-01

12144 8.603152e-01 9.468729e-01

12145 8.603158e-01 9.468718e-01

12146 8.603159e-01 9.468707e-01

12147 8.603154e-01 9.468695e-01

12148 8.603143e-01 9.468683e-01

12149 8.603125e-01 9.468671e-01

12150 8.603100e-01 9.468659e-01

12151 8.603067e-01 9.468646e-01

12152 8.603027e-01 9.468633e-01

12153 8.602978e-01 9.468620e-01

12154 8.602921e-01 9.468607e-01

12155 8.602855e-01 9.468593e-01

12156 8.602780e-01 9.468579e-01

12157 8.602694e-01 9.468564e-01

12158 8.602598e-01 9.468549e-01

12159 8.602490e-01 9.468534e-01

12160 8.602372e-01 9.468518e-01

12161 8.602241e-01 9.468502e-01

12162 8.602098e-01 9.468486e-01

12163 8.601941e-01 9.468469e-01

12164 8.601771e-01 9.468452e-01

12165 8.601586e-01 9.468435e-01

12166 8.601386e-01 9.468417e-01

12167 8.601171e-01 9.468399e-01

12168 8.600939e-01 9.468380e-01

12169 8.600691e-01 9.468361e-01

12170 8.600425e-01 9.468341e-01

12171 8.600140e-01 9.468321e-01

12172 8.599837e-01 9.468301e-01

12173 8.599514e-01 9.468280e-01

12174 8.599171e-01 9.468258e-01

12175 8.598806e-01 9.468236e-01

12176 8.598420e-01 9.468214e-01

12177 8.598011e-01 9.468191e-01

12178 8.597579e-01 9.468168e-01

12179 8.597122e-01 9.468144e-01

12180 8.596641e-01 9.468119e-01

12181 8.596134e-01 9.468094e-01

12182 8.595600e-01 9.468069e-01

12183 8.595039e-01 9.468043e-01

12184 8.594450e-01 9.468016e-01

12185 8.593832e-01 9.467989e-01

12186 8.593184e-01 9.467961e-01

12187 8.592506e-01 9.467933e-01

12188 8.591797e-01 9.467904e-01

12189 8.591055e-01 9.467874e-01

12190 8.590281e-01 9.467844e-01

12191 8.589473e-01 9.467813e-01

12192 8.588631e-01 9.467781e-01

12193 8.587754e-01 9.467749e-01

12194 8.586842e-01 9.467716e-01

12195 8.585893e-01 9.467682e-01

12196 8.584906e-01 9.467648e-01

12197 8.583883e-01 9.467613e-01

12198 8.582820e-01 9.467578e-01

12199 8.581719e-01 9.467541e-01

12200 8.580579e-01 9.467504e-01

12201 8.579398e-01 9.467466e-01

12202 8.578177e-01 9.467428e-01

12203 8.576915e-01 9.467388e-01

12204 8.575612e-01 9.467348e-01

12205 8.574267e-01 9.467307e-01

12206 8.572881e-01 9.467265e-01

12207 8.571452e-01 9.467223e-01

12208 8.569980e-01 9.467180e-01

12209 8.568466e-01 9.467136e-01

12210 8.566910e-01 9.467091e-01

12211 8.565310e-01 9.467045e-01

12212 8.563668e-01 9.466998e-01

12213 8.561984e-01 9.466951e-01

12214 8.560257e-01 9.466902e-01

12215 8.558488e-01 9.466853e-01

12216 8.556677e-01 9.466803e-01

12217 8.554824e-01 9.466752e-01

12218 8.552930e-01 9.466700e-01

12219 8.550995e-01 9.466647e-01

12220 8.549020e-01 9.466594e-01

12221 8.547006e-01 9.466539e-01

12222 8.544952e-01 9.466483e-01

12223 8.542861e-01 9.466427e-01

12224 8.540732e-01 9.466369e-01

12225 8.538567e-01 9.466311e-01

12226 8.536366e-01 9.466251e-01

12227 8.534130e-01 9.466191e-01

12228 8.531861e-01 9.466129e-01

12229 8.529560e-01 9.466067e-01

12230 8.527228e-01 9.466003e-01

12231 8.524865e-01 9.465939e-01

12232 8.522474e-01 9.465873e-01

12233 8.520056e-01 9.465806e-01

12234 8.517612e-01 9.465739e-01

12235 8.515143e-01 9.465672e-01

12236 8.512651e-01 9.465607e-01

12237 8.510137e-01 9.465545e-01

12238 8.507604e-01 9.465487e-01

12239 8.505052e-01 9.465433e-01

12240 8.502483e-01 9.465384e-01

12241 8.499898e-01 9.465341e-01

12242 8.499851e-01 9.465329e-01

12243 8.499802e-01 9.465316e-01

12244 8.499750e-01 9.465303e-01

12245 8.499697e-01 9.465289e-01

12246 8.499641e-01 9.465276e-01

12247 8.499582e-01 9.465262e-01

12248 8.499521e-01 9.465249e-01

12249 8.499457e-01 9.465235e-01

12250 8.499390e-01 9.465221e-01

12251 8.499321e-01 9.465207e-01

12252 8.499248e-01 9.465193e-01

12253 8.499172e-01 9.465179e-01

12254 8.499094e-01 9.465164e-01

12255 8.499011e-01 9.465150e-01

12256 8.498926e-01 9.465135e-01

12257 8.498836e-01 9.465121e-01

12258 8.498743e-01 9.465106e-01

12259 8.498646e-01 9.465091e-01

12260 8.498545e-01 9.465077e-01

12261 8.498439e-01 9.465062e-01

12262 8.498330e-01 9.465047e-01

12263 8.498215e-01 9.465032e-01

12264 8.498096e-01 9.465017e-01

12265 8.497972e-01 9.465001e-01

12266 8.497843e-01 9.464986e-01

12267 8.497709e-01 9.464971e-01

12268 8.497569e-01 9.464956e-01

12269 8.497424e-01 9.464941e-01

12270 8.497272e-01 9.464925e-01

12271 8.497115e-01 9.464910e-01

12272 8.496951e-01 9.464895e-01

12273 8.496781e-01 9.464880e-01

12274 8.496603e-01 9.464864e-01

12275 8.496419e-01 9.464849e-01

12276 8.496227e-01 9.464834e-01

12277 8.496028e-01 9.464819e-01

12278 8.495821e-01 9.464804e-01

12279 8.495605e-01 9.464789e-01

12280 8.495381e-01 9.464774e-01

12281 8.495148e-01 9.464760e-01

12282 8.494906e-01 9.464745e-01

12283 8.494655e-01 9.464730e-01

12284 8.494394e-01 9.464716e-01

12285 8.494122e-01 9.464702e-01

12286 8.493841e-01 9.464688e-01

12287 8.493548e-01 9.464674e-01

12288 8.493244e-01 9.464660e-01

12289 8.492928e-01 9.464647e-01

12290 8.492600e-01 9.464634e-01

12291 8.492259e-01 9.464621e-01

12292 8.491906e-01 9.464608e-01

12293 8.491539e-01 9.464596e-01

12294 8.491158e-01 9.464584e-01

12295 8.490763e-01 9.464573e-01

12296 8.490352e-01 9.464561e-01

12297 8.489927e-01 9.464550e-01

12298 8.489485e-01 9.464540e-01

12299 8.489027e-01 9.464530e-01

12300 8.488551e-01 9.464521e-01

12301 8.488058e-01 9.464512e-01

12302 8.487546e-01 9.464503e-01

12303 8.487016e-01 9.464495e-01

12304 8.486466e-01 9.464488e-01

12305 8.485895e-01 9.464482e-01

12306 8.485304e-01 9.464476e-01

12307 8.484691e-01 9.464470e-01

12308 8.484055e-01 9.464466e-01

12309 8.483397e-01 9.464462e-01

12310 8.482714e-01 9.464460e-01

12311 8.482007e-01 9.464458e-01

12312 8.481274e-01 9.464457e-01

12313 8.480515e-01 9.464457e-01

12314 8.479728e-01 9.464458e-01

12315 8.478913e-01 9.464460e-01

12316 8.478070e-01 9.464463e-01

12317 8.477196e-01 9.464468e-01

12318 8.476292e-01 9.464473e-01

12319 8.475355e-01 9.464480e-01

12320 8.474386e-01 9.464488e-01

12321 8.473383e-01 9.464498e-01

12322 8.472345e-01 9.464509e-01

12323 8.471271e-01 9.464522e-01

12324 8.470160e-01 9.464537e-01

12325 8.469010e-01 9.464553e-01

12326 8.467821e-01 9.464571e-01

12327 8.466592e-01 9.464591e-01

12328 8.465321e-01 9.464612e-01

12329 8.464007e-01 9.464636e-01

12330 8.462649e-01 9.464662e-01

12331 8.461246e-01 9.464690e-01

12332 8.459796e-01 9.464721e-01

12333 8.458298e-01 9.464753e-01

12334 8.456751e-01 9.464789e-01

12335 8.455153e-01 9.464827e-01

12336 8.453503e-01 9.464867e-01

12337 8.451801e-01 9.464911e-01

12338 8.450043e-01 9.464957e-01

12339 8.448230e-01 9.465006e-01

12340 8.446359e-01 9.465059e-01

12341 8.444430e-01 9.465115e-01

12342 8.442440e-01 9.465174e-01

12343 8.440390e-01 9.465237e-01

12344 8.438276e-01 9.465303e-01

12345 8.436098e-01 9.465374e-01

12346 8.433854e-01 9.465448e-01

12347 8.431543e-01 9.465526e-01

12348 8.429163e-01 9.465609e-01

12349 8.426714e-01 9.465696e-01

12350 8.424194e-01 9.465788e-01

12351 8.421601e-01 9.465884e-01

12352 8.418934e-01 9.465986e-01

12353 8.416192e-01 9.466092e-01

12354 8.413374e-01 9.466204e-01

12355 8.410479e-01 9.466321e-01

12356 8.407504e-01 9.466444e-01

12357 8.404450e-01 9.466573e-01

12358 8.401315e-01 9.466708e-01

12359 8.398098e-01 9.466849e-01

12360 8.394798e-01 9.466996e-01

12361 8.391414e-01 9.467150e-01

12362 8.387945e-01 9.467311e-01

12363 8.384391e-01 9.467479e-01

12364 8.380750e-01 9.467655e-01

12365 8.377023e-01 9.467838e-01

12366 8.373209e-01 9.468028e-01

12367 8.369306e-01 9.468227e-01

12368 8.365316e-01 9.468434e-01

12369 8.361237e-01 9.468649e-01

12370 8.357069e-01 9.468873e-01

12371 8.352813e-01 9.469106e-01

12372 8.348468e-01 9.469348e-01

12373 8.344036e-01 9.469600e-01

12374 8.339515e-01 9.469862e-01

12375 8.334906e-01 9.470134e-01

12376 8.330211e-01 9.470415e-01

12377 8.325429e-01 9.470708e-01

12378 8.320562e-01 9.471011e-01

12379 8.315611e-01 9.471326e-01

12380 8.310576e-01 9.471652e-01

12381 8.305460e-01 9.471990e-01

12382 8.300263e-01 9.472340e-01

12383 8.294986e-01 9.472702e-01

12384 8.289633e-01 9.473077e-01

12385 8.284204e-01 9.473464e-01

12386 8.278701e-01 9.473865e-01

12387 8.273127e-01 9.474280e-01

12388 8.267485e-01 9.474708e-01

12389 8.261775e-01 9.475151e-01

12390 8.256001e-01 9.475608e-01

12391 8.250166e-01 9.476080e-01

12392 8.244273e-01 9.476567e-01

12393 8.238324e-01 9.477069e-01

12394 8.232322e-01 9.477587e-01

12395 8.226271e-01 9.478121e-01

12396 8.220174e-01 9.478672e-01

12397 8.214034e-01 9.479239e-01

12398 8.207854e-01 9.479823e-01

12399 8.201639e-01 9.480425e-01

12400 8.195392e-01 9.481044e-01

12401 8.189116e-01 9.481681e-01

12402 8.182816e-01 9.482336e-01

12403 8.176495e-01 9.483010e-01

12404 8.170157e-01 9.483702e-01

12405 8.163806e-01 9.484414e-01

12406 8.157445e-01 9.485146e-01

12407 8.151080e-01 9.485896e-01

12408 8.144712e-01 9.486667e-01

12409 8.138348e-01 9.487459e-01

12410 8.131989e-01 9.488270e-01

12411 8.125641e-01 9.489101e-01

12412 8.119307e-01 9.489950e-01

12413 8.112991e-01 9.490817e-01

12414 8.106696e-01 9.491702e-01

12415 8.100427e-01 9.492604e-01

12416 8.094186e-01 9.493522e-01

12417 8.087978e-01 9.494456e-01

12418 8.081805e-01 9.495405e-01

12419 8.075672e-01 9.496370e-01

12420 8.069581e-01 9.497349e-01

12421 8.063535e-01 9.498342e-01

12422 8.063361e-01 9.498347e-01

12423 8.063181e-01 9.498352e-01

12424 8.062995e-01 9.498357e-01

12425 8.062802e-01 9.498363e-01

12426 8.062602e-01 9.498368e-01

12427 8.062396e-01 9.498374e-01

12428 8.062182e-01 9.498380e-01

12429 8.061961e-01 9.498386e-01

12430 8.061732e-01 9.498392e-01

12431 8.061496e-01 9.498398e-01

12432 8.061251e-01 9.498405e-01

12433 8.060998e-01 9.498411e-01

12434 8.060735e-01 9.498418e-01

12435 8.060464e-01 9.498424e-01

12436 8.060184e-01 9.498431e-01

12437 8.059894e-01 9.498438e-01

12438 8.059594e-01 9.498445e-01

12439 8.059283e-01 9.498452e-01

12440 8.058962e-01 9.498460e-01

12441 8.058630e-01 9.498467e-01

12442 8.058286e-01 9.498475e-01

12443 8.057931e-01 9.498482e-01

12444 8.057564e-01 9.498490e-01

12445 8.057184e-01 9.498498e-01

12446 8.056790e-01 9.498506e-01

12447 8.056384e-01 9.498515e-01

12448 8.055964e-01 9.498523e-01

12449 8.055529e-01 9.498532e-01

12450 8.055080e-01 9.498541e-01

12451 8.054615e-01 9.498550e-01

12452 8.054134e-01 9.498559e-01

12453 8.053637e-01 9.498568e-01

12454 8.053124e-01 9.498578e-01

12455 8.052593e-01 9.498587e-01

12456 8.052043e-01 9.498597e-01

12457 8.051476e-01 9.498607e-01
[truncated: 2,882,978 more chars]
