# Supplementary material for: Purity control of simulated moving bed based on advanced fuzzy controller
Source: Sci Rep. 2024 Apr 20;14:9083. doi: 10.1038/s41598-024-59847-1 (PMC11576947; doi:10.1038/s41598-024-59847-1)
Supplement: Supplementary file 4 — Supplementary Information 4. [file 41598_2024_59847_MOESM4_ESM.docx]

**Figure 5(b):**

1 8.622241e-06 1.176603e-04

2 8.622241e-06 1.176603e-04

3 8.622241e-06 1.176603e-04

4 8.622241e-06 1.176603e-04

5 8.622241e-06 1.176603e-04

6 8.622241e-06 1.176603e-04

7 8.622241e-06 1.176603e-04

8 8.622241e-06 1.176603e-04

9 8.622241e-06 1.176603e-04

10 8.622241e-06 1.176603e-04

11 8.622241e-06 1.176603e-04

12 8.622241e-06 1.176603e-04

13 8.622241e-06 1.176603e-04

14 8.622241e-06 1.176603e-04

15 8.622241e-06 1.176603e-04

16 8.622241e-06 1.176603e-04

17 8.622241e-06 1.176603e-04

18 8.622241e-06 1.176603e-04

19 8.622241e-06 1.176603e-04

20 8.622241e-06 1.176603e-04

21 8.622241e-06 1.176603e-04

22 8.622241e-06 1.176603e-04

23 8.622241e-06 1.176603e-04

24 8.622241e-06 1.176603e-04

25 8.622241e-06 1.176603e-04

26 8.622241e-06 1.176603e-04

27 8.622241e-06 1.176603e-04

28 8.622241e-06 1.176603e-04

29 8.622241e-06 1.176603e-04

30 8.622241e-06 1.176603e-04

31 8.622241e-06 1.176603e-04

32 8.622241e-06 1.176603e-04

33 8.622241e-06 1.176603e-04

34 8.622241e-06 1.176603e-04

35 8.622241e-06 1.176603e-04

36 8.622241e-06 1.176603e-04

37 8.622241e-06 1.176603e-04

38 8.622241e-06 1.176603e-04

39 8.622241e-06 1.176603e-04

40 8.622241e-06 1.176603e-04

41 8.622241e-06 1.176603e-04

42 8.622241e-06 1.176603e-04

43 8.622241e-06 1.176603e-04

44 8.622241e-06 1.176603e-04

45 8.622241e-06 1.176603e-04

46 8.622241e-06 1.176603e-04

47 8.622241e-06 1.176603e-04

48 8.622241e-06 1.176603e-04

49 8.622241e-06 1.176603e-04

50 8.622241e-06 1.176603e-04

51 8.622242e-06 1.176603e-04

52 8.622242e-06 1.176603e-04

53 8.622242e-06 1.176603e-04

54 8.622242e-06 1.176603e-04

55 8.622242e-06 1.176603e-04

56 8.622242e-06 1.176603e-04

57 8.622243e-06 1.176603e-04

58 8.622243e-06 1.176603e-04

59 8.622244e-06 1.176603e-04

60 8.622244e-06 1.176603e-04

61 8.622245e-06 1.176603e-04

62 8.622246e-06 1.176604e-04

63 8.622248e-06 1.176604e-04

64 8.622250e-06 1.176604e-04

65 8.622252e-06 1.176605e-04

66 8.622255e-06 1.176605e-04

67 8.622259e-06 1.176606e-04

68 8.622263e-06 1.176606e-04

69 8.622269e-06 1.176607e-04

70 8.622277e-06 1.176609e-04

71 8.622287e-06 1.176610e-04

72 8.622299e-06 1.176612e-04

73 8.622315e-06 1.176615e-04

74 8.622334e-06 1.176618e-04

75 8.622359e-06 1.176622e-04

76 8.622389e-06 1.176627e-04

77 8.622428e-06 1.176633e-04

78 8.622476e-06 1.176641e-04

79 8.622536e-06 1.176651e-04

80 8.622611e-06 1.176664e-04

81 8.622704e-06 1.176680e-04

82 8.622820e-06 1.176699e-04

83 8.622964e-06 1.176723e-04

84 8.623143e-06 1.176753e-04

85 8.623364e-06 1.176791e-04

86 8.623637e-06 1.176837e-04

87 8.623975e-06 1.176894e-04

88 8.624392e-06 1.176965e-04

89 8.624904e-06 1.177052e-04

90 8.625535e-06 1.177159e-04

91 8.626310e-06 1.177292e-04

92 8.627260e-06 1.177454e-04

93 8.628424e-06 1.177654e-04

94 8.629848e-06 1.177899e-04

95 8.631588e-06 1.178198e-04

96 8.633710e-06 1.178565e-04

97 8.636297e-06 1.179012e-04

98 8.639445e-06 1.179558e-04

99 8.643272e-06 1.180222e-04

100 8.647917e-06 1.181031e-04

101 8.653549e-06 1.182013e-04

102 8.660368e-06 1.183206e-04

103 8.668616e-06 1.184651e-04

104 8.678578e-06 1.186400e-04

105 8.690597e-06 1.188514e-04

106 8.705078e-06 1.191068e-04

107 8.722507e-06 1.194148e-04

108 8.743457e-06 1.197857e-04

109 8.768610e-06 1.202321e-04

110 8.798772e-06 1.207685e-04

111 8.834899e-06 1.214123e-04

112 8.878118e-06 1.221841e-04

113 8.929763e-06 1.231084e-04

114 8.991403e-06 1.242138e-04

115 9.064889e-06 1.255344e-04

116 9.152395e-06 1.271103e-04

117 9.256477e-06 1.289885e-04

118 9.380135e-06 1.312246e-04

119 9.526883e-06 1.338835e-04

120 9.700837e-06 1.370417e-04

121 9.906812e-06 1.407886e-04

122 1.015043e-05 1.452288e-04

123 1.043824e-05 1.504846e-04

124 1.077791e-05 1.566985e-04

125 1.117832e-05 1.640369e-04

126 1.164983e-05 1.726932e-04

127 1.220445e-05 1.828922e-04

128 1.285614e-05 1.948949e-04

129 1.362105e-05 2.090037e-04

130 1.451790e-05 2.255683e-04

131 1.556833e-05 2.449931e-04

132 1.679732e-05 2.677443e-04

133 1.823372e-05 2.943586e-04

134 1.991076e-05 3.254531e-04

135 2.186670e-05 3.617354e-04

136 2.414553e-05 4.040159e-04

137 2.679779e-05 4.532200e-04

138 2.988148e-05 5.104026e-04

139 3.346305e-05 5.767629e-04

140 3.761858e-05 6.536606e-04

141 4.243507e-05 7.426329e-04

142 4.801190e-05 8.454118e-04

143 5.446244e-05 9.639430e-04

144 6.191588e-05 1.100403e-03

145 7.051927e-05 1.257217e-03

146 8.043977e-05 1.437075e-03

147 9.186718e-05 1.642946e-03

148 1.050167e-04 1.878090e-03

149 1.201322e-04 2.146064e-03

150 1.374894e-04 2.450721e-03

151 1.573997e-04 2.796210e-03

152 1.802145e-04 3.186959e-03

153 2.063296e-04 3.627652e-03

154 2.361900e-04 4.123193e-03

155 2.702958e-04 4.678667e-03

156 3.092074e-04 5.299276e-03

157 3.535523e-04 5.990272e-03

158 4.040316e-04 6.756876e-03

159 4.614273e-04 7.604192e-03

160 5.266101e-04 8.537107e-03

161 6.005471e-04 9.560193e-03

162 6.843105e-04 1.067761e-02

163 7.790862e-04 1.189302e-02

164 8.861826e-04 1.320948e-02

165 1.007039e-03 1.462940e-02

166 1.143237e-03 1.615449e-02

167 1.296504e-03 1.778571e-02

168 1.468725e-03 1.952328e-02

169 1.661950e-03 2.136666e-02

170 1.878398e-03 2.331462e-02

171 2.120462e-03 2.536526e-02

172 2.390711e-03 2.751608e-02

173 2.691893e-03 2.976406e-02

174 3.026930e-03 3.210570e-02

175 3.398912e-03 3.453714e-02

176 3.811088e-03 3.705420e-02

177 4.266854e-03 3.965249e-02

178 4.769733e-03 4.232741e-02

179 5.323350e-03 4.507426e-02

180 5.931412e-03 4.788824e-02

181 6.597664e-03 5.076449e-02

182 6.597664e-03 5.076449e-02

183 6.597664e-03 5.076449e-02

184 6.597664e-03 5.076449e-02

185 6.597664e-03 5.076449e-02

186 6.597664e-03 5.076449e-02

187 6.597664e-03 5.076449e-02

188 6.597664e-03 5.076449e-02

189 6.597664e-03 5.076449e-02

190 6.597664e-03 5.076449e-02

191 6.597664e-03 5.076449e-02

192 6.597664e-03 5.076449e-02

193 6.597664e-03 5.076449e-02

194 6.597664e-03 5.076449e-02

195 6.597664e-03 5.076449e-02

196 6.597664e-03 5.076449e-02

197 6.597664e-03 5.076449e-02

198 6.597664e-03 5.076449e-02

199 6.597664e-03 5.076449e-02

200 6.597664e-03 5.076449e-02

201 6.597664e-03 5.076449e-02

202 6.597664e-03 5.076449e-02

203 6.597664e-03 5.076449e-02

204 6.597664e-03 5.076449e-02

205 6.597664e-03 5.076450e-02

206 6.597664e-03 5.076450e-02

207 6.597664e-03 5.076450e-02

208 6.597664e-03 5.076450e-02

209 6.597664e-03 5.076450e-02

210 6.597664e-03 5.076450e-02

211 6.597664e-03 5.076451e-02

212 6.597664e-03 5.076451e-02

213 6.597665e-03 5.076451e-02

214 6.597665e-03 5.076452e-02

215 6.597665e-03 5.076452e-02

216 6.597665e-03 5.076453e-02

217 6.597665e-03 5.076454e-02

218 6.597666e-03 5.076454e-02

219 6.597666e-03 5.076455e-02

220 6.597667e-03 5.076457e-02

221 6.597667e-03 5.076458e-02

222 6.597668e-03 5.076460e-02

223 6.597669e-03 5.076462e-02

224 6.597670e-03 5.076464e-02

225 6.597671e-03 5.076467e-02

226 6.597672e-03 5.076470e-02

227 6.597674e-03 5.076474e-02

228 6.597676e-03 5.076479e-02

229 6.597678e-03 5.076484e-02

230 6.597681e-03 5.076491e-02

231 6.597684e-03 5.076498e-02

232 6.597687e-03 5.076507e-02

233 6.597691e-03 5.076518e-02

234 6.597696e-03 5.076530e-02

235 6.597702e-03 5.076544e-02

236 6.597708e-03 5.076561e-02

237 6.597716e-03 5.076581e-02

238 6.597725e-03 5.076604e-02

239 6.597736e-03 5.076631e-02

240 6.597748e-03 5.076662e-02

241 6.597763e-03 5.076699e-02

242 6.597779e-03 5.076742e-02

243 6.597799e-03 5.076792e-02

244 6.597821e-03 5.076850e-02

245 6.597848e-03 5.076917e-02

246 6.597878e-03 5.076996e-02

247 6.597913e-03 5.077087e-02

248 6.597954e-03 5.077192e-02

249 6.598002e-03 5.077315e-02

250 6.598057e-03 5.077457e-02

251 6.598120e-03 5.077621e-02

252 6.598193e-03 5.077812e-02

253 6.598277e-03 5.078032e-02

254 6.598375e-03 5.078286e-02

255 6.598487e-03 5.078579e-02

256 6.598616e-03 5.078917e-02

257 6.598765e-03 5.079307e-02

258 6.598936e-03 5.079757e-02

259 6.599132e-03 5.080274e-02

260 6.599358e-03 5.080868e-02

261 6.599617e-03 5.081551e-02

262 6.599914e-03 5.082336e-02

263 6.600254e-03 5.083237e-02

264 6.600644e-03 5.084269e-02

265 6.601090e-03 5.085452e-02

266 6.601600e-03 5.086806e-02

267 6.602182e-03 5.088356e-02

268 6.602847e-03 5.090127e-02

269 6.603606e-03 5.092150e-02

270 6.604471e-03 5.094458e-02

271 6.605457e-03 5.097091e-02

272 6.606580e-03 5.100091e-02

273 6.607858e-03 5.103507e-02

274 6.609310e-03 5.107392e-02

275 6.610961e-03 5.111809e-02

276 6.612835e-03 5.116824e-02

277 6.614962e-03 5.122514e-02

278 6.617373e-03 5.128964e-02

279 6.620106e-03 5.136269e-02

280 6.623201e-03 5.144533e-02

281 6.626703e-03 5.153874e-02

282 6.630663e-03 5.164420e-02

283 6.635138e-03 5.176315e-02

284 6.640192e-03 5.189716e-02

285 6.645896e-03 5.204796e-02

286 6.652329e-03 5.221745e-02

287 6.659578e-03 5.240770e-02

288 6.667743e-03 5.262098e-02

289 6.676931e-03 5.285974e-02

290 6.687266e-03 5.312664e-02

291 6.698881e-03 5.342452e-02

292 6.711927e-03 5.375646e-02

293 6.726568e-03 5.412572e-02

294 6.742990e-03 5.453578e-02

295 6.761395e-03 5.499028e-02

296 6.782009e-03 5.549306e-02

297 6.805080e-03 5.604809e-02

298 6.830883e-03 5.665948e-02

299 6.859721e-03 5.733144e-02

300 6.891926e-03 5.806822e-02

301 6.927866e-03 5.887409e-02

302 6.967944e-03 5.975327e-02

303 7.012604e-03 6.070988e-02

304 7.062330e-03 6.174790e-02

305 7.117654e-03 6.287106e-02

306 7.179159e-03 6.408283e-02

307 7.247481e-03 6.538633e-02

308 7.323312e-03 6.678424e-02

309 7.407408e-03 6.827883e-02

310 7.500592e-03 6.987181e-02

311 7.603755e-03 7.156436e-02

312 7.717864e-03 7.335707e-02

313 7.843967e-03 7.524993e-02

314 7.983192e-03 7.724231e-02

315 8.136758e-03 7.933296e-02

316 8.305973e-03 8.152003e-02

317 8.492242e-03 8.380107e-02

318 8.697069e-03 8.617307e-02

319 8.922056e-03 8.863249e-02

320 9.168911e-03 9.117528e-02

321 9.439443e-03 9.379690e-02

322 9.735567e-03 9.649243e-02

323 1.005930e-02 9.925649e-02

324 1.041276e-02 1.020834e-01

325 1.079815e-02 1.049671e-01

326 1.121778e-02 1.079012e-01

327 1.167404e-02 1.108790e-01

328 1.216937e-02 1.138938e-01

329 1.270629e-02 1.169382e-01

330 1.328735e-02 1.200051e-01

331 1.391516e-02 1.230868e-01

332 1.459228e-02 1.261756e-01

333 1.532130e-02 1.292637e-01

334 1.610476e-02 1.323433e-01

335 1.694511e-02 1.354063e-01

336 1.784473e-02 1.384448e-01

337 1.880585e-02 1.414512e-01

338 1.983056e-02 1.444176e-01

339 2.092073e-02 1.473367e-01

340 2.207802e-02 1.502016e-01

341 2.330381e-02 1.530056e-01

342 2.459920e-02 1.557428e-01

343 2.596495e-02 1.584076e-01

344 2.740146e-02 1.609955e-01

345 2.890876e-02 1.635024e-01

346 3.048646e-02 1.659252e-01

347 3.213373e-02 1.682617e-01

348 3.384931e-02 1.705103e-01

349 3.563149e-02 1.726703e-01

350 3.747807e-02 1.747419e-01

351 3.938641e-02 1.767259e-01

352 4.135341e-02 1.786238e-01

353 4.337552e-02 1.804376e-01

354 4.544875e-02 1.821697e-01

355 4.756871e-02 1.838231e-01

356 4.973064e-02 1.854010e-01

357 5.192941e-02 1.869069e-01

358 5.415959e-02 1.883442e-01

359 5.641549e-02 1.897165e-01

360 5.869122e-02 1.910277e-01

361 6.098070e-02 1.922812e-01

362 6.098071e-02 1.922812e-01

363 6.098071e-02 1.922812e-01

364 6.098071e-02 1.922813e-01

365 6.098071e-02 1.922813e-01

366 6.098071e-02 1.922814e-01

367 6.098071e-02 1.922815e-01

368 6.098071e-02 1.922816e-01

369 6.098072e-02 1.922817e-01

370 6.098072e-02 1.922818e-01

371 6.098072e-02 1.922819e-01

372 6.098073e-02 1.922820e-01

373 6.098073e-02 1.922822e-01

374 6.098074e-02 1.922824e-01

375 6.098074e-02 1.922826e-01

376 6.098075e-02 1.922828e-01

377 6.098075e-02 1.922831e-01

378 6.098076e-02 1.922834e-01

379 6.098077e-02 1.922838e-01

380 6.098078e-02 1.922842e-01

381 6.098079e-02 1.922847e-01

382 6.098081e-02 1.922852e-01

383 6.098082e-02 1.922859e-01

384 6.098084e-02 1.922866e-01

385 6.098086e-02 1.922874e-01

386 6.098089e-02 1.922883e-01

387 6.098091e-02 1.922894e-01

388 6.098094e-02 1.922906e-01

389 6.098098e-02 1.922919e-01

390 6.098102e-02 1.922935e-01

391 6.098106e-02 1.922952e-01

392 6.098111e-02 1.922972e-01

393 6.098117e-02 1.922995e-01

394 6.098123e-02 1.923021e-01

395 6.098130e-02 1.923050e-01

396 6.098138e-02 1.923083e-01

397 6.098148e-02 1.923120e-01

398 6.098158e-02 1.923163e-01

399 6.098170e-02 1.923211e-01

400 6.098183e-02 1.923265e-01

401 6.098199e-02 1.923326e-01

402 6.098216e-02 1.923395e-01

403 6.098235e-02 1.923473e-01

404 6.098256e-02 1.923561e-01

405 6.098281e-02 1.923660e-01

406 6.098308e-02 1.923771e-01

407 6.098339e-02 1.923897e-01

408 6.098374e-02 1.924038e-01

409 6.098413e-02 1.924197e-01

410 6.098457e-02 1.924376e-01

411 6.098506e-02 1.924577e-01

412 6.098561e-02 1.924803e-01

413 6.098623e-02 1.925056e-01

414 6.098692e-02 1.925340e-01

415 6.098770e-02 1.925658e-01

416 6.098857e-02 1.926015e-01

417 6.098955e-02 1.926414e-01

418 6.099064e-02 1.926861e-01

419 6.099186e-02 1.927360e-01

420 6.099322e-02 1.927918e-01

421 6.099474e-02 1.928541e-01

422 6.099644e-02 1.929237e-01

423 6.099833e-02 1.930012e-01

424 6.100045e-02 1.930876e-01

425 6.100280e-02 1.931837e-01

426 6.100543e-02 1.932908e-01

427 6.100835e-02 1.934097e-01

428 6.101160e-02 1.935419e-01

429 6.101522e-02 1.936885e-01

430 6.101924e-02 1.938512e-01

431 6.102371e-02 1.940314e-01

432 6.102868e-02 1.942308e-01

433 6.103419e-02 1.944513e-01

434 6.104030e-02 1.946950e-01

435 6.104708e-02 1.949638e-01

436 6.105460e-02 1.952600e-01

437 6.106292e-02 1.955862e-01

438 6.107214e-02 1.959448e-01

439 6.108235e-02 1.963386e-01

440 6.109363e-02 1.967705e-01

441 6.110611e-02 1.972434e-01

442 6.111989e-02 1.977605e-01

443 6.113511e-02 1.983251e-01

444 6.115192e-02 1.989404e-01

445 6.117045e-02 1.996100e-01

446 6.119089e-02 2.003372e-01

447 6.121342e-02 2.011256e-01

448 6.123823e-02 2.019788e-01

449 6.126554e-02 2.029001e-01

450 6.129558e-02 2.038929e-01

451 6.132863e-02 2.049605e-01

452 6.136494e-02 2.061060e-01

453 6.140482e-02 2.073323e-01

454 6.144861e-02 2.086418e-01

455 6.149664e-02 2.100370e-01

456 6.154931e-02 2.115197e-01

457 6.160703e-02 2.130915e-01

458 6.167024e-02 2.147534e-01

459 6.173942e-02 2.165061e-01

460 6.181509e-02 2.183497e-01

461 6.189781e-02 2.202837e-01

462 6.198818e-02 2.223071e-01

463 6.208683e-02 2.244186e-01

464 6.219445e-02 2.266158e-01

465 6.231178e-02 2.288963e-01

466 6.243961e-02 2.312568e-01

467 6.257877e-02 2.336936e-01

468 6.273016e-02 2.362024e-01

469 6.289472e-02 2.387786e-01

470 6.307347e-02 2.414169e-01

471 6.326746e-02 2.441117e-01

472 6.347783e-02 2.468571e-01

473 6.370577e-02 2.496466e-01

474 6.395252e-02 2.524737e-01

475 6.421939e-02 2.553313e-01

476 6.450777e-02 2.582124e-01

477 6.481907e-02 2.611096e-01

478 6.515478e-02 2.640154e-01

479 6.551646e-02 2.669224e-01

480 6.590568e-02 2.698230e-01

481 6.632408e-02 2.727097e-01

482 6.677334e-02 2.755753e-01

483 6.725516e-02 2.784124e-01

484 6.777127e-02 2.812142e-01

485 6.832340e-02 2.839740e-01

486 6.891330e-02 2.866856e-01

487 6.954269e-02 2.893432e-01

488 7.021327e-02 2.919415e-01

489 7.092670e-02 2.944756e-01

490 7.168457e-02 2.969414e-01

491 7.248842e-02 2.993353e-01

492 7.333967e-02 3.016543e-01

493 7.423962e-02 3.038963e-01

494 7.518946e-02 3.060594e-01

495 7.619019e-02 3.081427e-01

496 7.724265e-02 3.101458e-01

497 7.834748e-02 3.120687e-01

498 7.950508e-02 3.139123e-01

499 8.071564e-02 3.156775e-01

500 8.197907e-02 3.173661e-01

501 8.329501e-02 3.189798e-01

502 8.466280e-02 3.205209e-01

503 8.608150e-02 3.219920e-01

504 8.754984e-02 3.233957e-01

505 8.906625e-02 3.247348e-01

506 9.062884e-02 3.260123e-01

507 9.223539e-02 3.272310e-01

508 9.388339e-02 3.283941e-01

509 9.557002e-02 3.295044e-01

510 9.729218e-02 3.305649e-01

511 9.904650e-02 3.315785e-01

512 1.008294e-01 3.325479e-01

513 1.026370e-01 3.334758e-01

514 1.044654e-01 3.343648e-01

515 1.063103e-01 3.352172e-01

516 1.081675e-01 3.360355e-01

517 1.100327e-01 3.368218e-01

518 1.119015e-01 3.375781e-01

519 1.137694e-01 3.383065e-01

520 1.156322e-01 3.390087e-01

521 1.174857e-01 3.396864e-01

522 1.193256e-01 3.403412e-01

523 1.211480e-01 3.409746e-01

524 1.229493e-01 3.415880e-01

525 1.247258e-01 3.421827e-01

526 1.264742e-01 3.427598e-01

527 1.281916e-01 3.433204e-01

528 1.298753e-01 3.438656e-01

529 1.315227e-01 3.443963e-01

530 1.331320e-01 3.449133e-01

531 1.347012e-01 3.454175e-01

532 1.362289e-01 3.459096e-01

533 1.377140e-01 3.463903e-01

534 1.391557e-01 3.468602e-01

535 1.405532e-01 3.473200e-01

536 1.419065e-01 3.477701e-01

537 1.432153e-01 3.482110e-01

538 1.444800e-01 3.486433e-01

539 1.457009e-01 3.490674e-01

540 1.468785e-01 3.494836e-01

541 1.480137e-01 3.498923e-01

542 1.480138e-01 3.498967e-01

543 1.480138e-01 3.499016e-01

544 1.480139e-01 3.499072e-01

545 1.480140e-01 3.499133e-01

546 1.480141e-01 3.499202e-01

547 1.480142e-01 3.499278e-01

548 1.480144e-01 3.499363e-01

549 1.480145e-01 3.499458e-01

550 1.480147e-01 3.499564e-01

551 1.480148e-01 3.499682e-01

552 1.480150e-01 3.499813e-01

553 1.480153e-01 3.499958e-01

554 1.480155e-01 3.500120e-01

555 1.480158e-01 3.500300e-01

556 1.480161e-01 3.500500e-01

557 1.480164e-01 3.500721e-01

558 1.480168e-01 3.500967e-01

559 1.480172e-01 3.501240e-01

560 1.480177e-01 3.501542e-01

561 1.480182e-01 3.501877e-01

562 1.480187e-01 3.502248e-01

563 1.480193e-01 3.502658e-01

564 1.480200e-01 3.503112e-01

565 1.480208e-01 3.503614e-01

566 1.480216e-01 3.504169e-01

567 1.480226e-01 3.504782e-01

568 1.480236e-01 3.505459e-01

569 1.480247e-01 3.506206e-01

570 1.480260e-01 3.507029e-01

571 1.480273e-01 3.507937e-01

572 1.480289e-01 3.508937e-01

573 1.480305e-01 3.510037e-01

574 1.480324e-01 3.511248e-01

575 1.480344e-01 3.512579e-01

576 1.480367e-01 3.514042e-01

577 1.480391e-01 3.515647e-01

578 1.480418e-01 3.517409e-01

579 1.480448e-01 3.519341e-01

580 1.480481e-01 3.521457e-01

581 1.480517e-01 3.523773e-01

582 1.480556e-01 3.526306e-01

583 1.480600e-01 3.529075e-01

584 1.480647e-01 3.532097e-01

585 1.480700e-01 3.535393e-01

586 1.480757e-01 3.538985e-01

587 1.480820e-01 3.542895e-01

588 1.480889e-01 3.547146e-01

589 1.480964e-01 3.551763e-01

590 1.481047e-01 3.556772e-01

591 1.481137e-01 3.562199e-01

592 1.481236e-01 3.568071e-01

593 1.481344e-01 3.574417e-01

594 1.481462e-01 3.581265e-01

595 1.481591e-01 3.588643e-01

596 1.481732e-01 3.596581e-01

597 1.481885e-01 3.605108e-01

598 1.482053e-01 3.614252e-01

599 1.482236e-01 3.624040e-01

600 1.482435e-01 3.634500e-01

601 1.482653e-01 3.645658e-01

602 1.482889e-01 3.657536e-01

603 1.483147e-01 3.670157e-01

604 1.483427e-01 3.683540e-01

605 1.483733e-01 3.697702e-01

606 1.484064e-01 3.712656e-01

607 1.484425e-01 3.728413e-01

608 1.484816e-01 3.744977e-01

609 1.485241e-01 3.762352e-01

610 1.485703e-01 3.780536e-01

611 1.486203e-01 3.799520e-01

612 1.486746e-01 3.819295e-01

613 1.487334e-01 3.839843e-01

614 1.487971e-01 3.861143e-01

615 1.488661e-01 3.883170e-01

616 1.489408e-01 3.905891e-01

617 1.490215e-01 3.929273e-01

618 1.491087e-01 3.953273e-01

619 1.492030e-01 3.977848e-01

620 1.493048e-01 4.002949e-01

621 1.494147e-01 4.028524e-01

622 1.495332e-01 4.054516e-01

623 1.496609e-01 4.080866e-01

624 1.497986e-01 4.107513e-01

625 1.499467e-01 4.134393e-01

626 1.501062e-01 4.161441e-01

627 1.502776e-01 4.188589e-01

628 1.504619e-01 4.215771e-01

629 1.506597e-01 4.242918e-01

630 1.508720e-01 4.269965e-01

631 1.510997e-01 4.296845e-01

632 1.513437e-01 4.323494e-01

633 1.516049e-01 4.349850e-01

634 1.518844e-01 4.375853e-01

635 1.521832e-01 4.401448e-01

636 1.525024e-01 4.426581e-01

637 1.528431e-01 4.451204e-01

638 1.532063e-01 4.475274e-01

639 1.535933e-01 4.498751e-01

640 1.540051e-01 4.521600e-01

641 1.544430e-01 4.543794e-01

642 1.549081e-01 4.565307e-01

643 1.554017e-01 4.586122e-01

644 1.559248e-01 4.606225e-01

645 1.564787e-01 4.625607e-01

646 1.570644e-01 4.644264e-01

647 1.576831e-01 4.662197e-01

648 1.583358e-01 4.679412e-01

649 1.590236e-01 4.695916e-01

650 1.597472e-01 4.711721e-01

651 1.605077e-01 4.726842e-01

652 1.613056e-01 4.741297e-01

653 1.621418e-01 4.755105e-01

654 1.630167e-01 4.768288e-01

655 1.639308e-01 4.780868e-01

656 1.648843e-01 4.792868e-01

657 1.658773e-01 4.804314e-01

658 1.669099e-01 4.815231e-01

659 1.679816e-01 4.825642e-01

660 1.690922e-01 4.835573e-01

661 1.702411e-01 4.845049e-01

662 1.714274e-01 4.854093e-01

663 1.726501e-01 4.862730e-01

664 1.739081e-01 4.870981e-01

665 1.751998e-01 4.878869e-01

666 1.765237e-01 4.886415e-01

667 1.778780e-01 4.893640e-01

668 1.792607e-01 4.900562e-01

669 1.806695e-01 4.907201e-01

670 1.821020e-01 4.913572e-01

671 1.835558e-01 4.919694e-01

672 1.850282e-01 4.925581e-01

673 1.865164e-01 4.931248e-01

674 1.880174e-01 4.936708e-01

675 1.895284e-01 4.941976e-01

676 1.910464e-01 4.947061e-01

677 1.925681e-01 4.951977e-01

678 1.940907e-01 4.956734e-01

679 1.956110e-01 4.961341e-01

680 1.971260e-01 4.965807e-01

681 1.986329e-01 4.970141e-01

682 2.001287e-01 4.974352e-01

683 2.016109e-01 4.978446e-01

684 2.030766e-01 4.982431e-01

685 2.045237e-01 4.986313e-01

686 2.059496e-01 4.990097e-01

687 2.073524e-01 4.993790e-01

688 2.087301e-01 4.997397e-01

689 2.100810e-01 5.000922e-01

690 2.114036e-01 5.004370e-01

691 2.126965e-01 5.007745e-01

692 2.139586e-01 5.011051e-01

693 2.151891e-01 5.014291e-01

694 2.163871e-01 5.017470e-01

695 2.175521e-01 5.020589e-01

696 2.186839e-01 5.023652e-01

697 2.197820e-01 5.026661e-01

698 2.208466e-01 5.029620e-01

699 2.218777e-01 5.032530e-01

700 2.228756e-01 5.035393e-01

701 2.238406e-01 5.038212e-01

702 2.247732e-01 5.040989e-01

703 2.256739e-01 5.043724e-01

704 2.265435e-01 5.046421e-01

705 2.273825e-01 5.049079e-01

706 2.281919e-01 5.051702e-01

707 2.289723e-01 5.054290e-01

708 2.297248e-01 5.056844e-01

709 2.304502e-01 5.059365e-01

710 2.311493e-01 5.061856e-01

711 2.318232e-01 5.064316e-01

712 2.324728e-01 5.066747e-01

713 2.330990e-01 5.069149e-01

714 2.337028e-01 5.071524e-01

715 2.342850e-01 5.073872e-01

716 2.348466e-01 5.076194e-01

717 2.353886e-01 5.078491e-01

718 2.359116e-01 5.080763e-01

719 2.364167e-01 5.083011e-01

720 2.369046e-01 5.085236e-01

721 2.373762e-01 5.087439e-01

722 2.373772e-01 5.088554e-01

723 2.373783e-01 5.089772e-01

724 2.373795e-01 5.091100e-01

725 2.373808e-01 5.092548e-01

726 2.373823e-01 5.094125e-01

727 2.373839e-01 5.095844e-01

728 2.373856e-01 5.097714e-01

729 2.373875e-01 5.099748e-01

730 2.373895e-01 5.101958e-01

731 2.373917e-01 5.104359e-01

732 2.373942e-01 5.106966e-01

733 2.373968e-01 5.109793e-01

734 2.373997e-01 5.112856e-01

735 2.374029e-01 5.116174e-01

736 2.374063e-01 5.119763e-01

737 2.374100e-01 5.123644e-01

738 2.374141e-01 5.127835e-01

739 2.374185e-01 5.132357e-01

740 2.374233e-01 5.137232e-01

741 2.374285e-01 5.142481e-01

742 2.374342e-01 5.148128e-01

743 2.374404e-01 5.154195e-01

744 2.374471e-01 5.160706e-01

745 2.374543e-01 5.167685e-01

746 2.374622e-01 5.175156e-01

747 2.374707e-01 5.183143e-01

748 2.374800e-01 5.191670e-01

749 2.374900e-01 5.200761e-01

750 2.375009e-01 5.210438e-01

751 2.375127e-01 5.220722e-01

752 2.375255e-01 5.231636e-01

753 2.375393e-01 5.243198e-01

754 2.375542e-01 5.255426e-01

755 2.375704e-01 5.268335e-01

756 2.375879e-01 5.281939e-01

757 2.376068e-01 5.296248e-01

758 2.376272e-01 5.311271e-01

759 2.376492e-01 5.327013e-01

760 2.376730e-01 5.343474e-01

761 2.376987e-01 5.360653e-01

762 2.377264e-01 5.378544e-01

763 2.377563e-01 5.397138e-01

764 2.377885e-01 5.416419e-01

765 2.378232e-01 5.436372e-01

766 2.378606e-01 5.456972e-01

767 2.379009e-01 5.478195e-01

768 2.379443e-01 5.500011e-01

769 2.379909e-01 5.522383e-01

770 2.380411e-01 5.545276e-01

771 2.380950e-01 5.568647e-01

772 2.381530e-01 5.592451e-01

773 2.382153e-01 5.616639e-01

774 2.382821e-01 5.641162e-01

775 2.383539e-01 5.665965e-01

776 2.384309e-01 5.690993e-01

777 2.385135e-01 5.716190e-01

778 2.386020e-01 5.741497e-01

779 2.386968e-01 5.766855e-01

780 2.387983e-01 5.792206e-01

781 2.389070e-01 5.817490e-01

782 2.390233e-01 5.842650e-01

783 2.391476e-01 5.867629e-01

784 2.392805e-01 5.892372e-01

785 2.394224e-01 5.916826e-01

786 2.395740e-01 5.940941e-01

787 2.397357e-01 5.964669e-01

788 2.399081e-01 5.987965e-01

789 2.400919e-01 6.010790e-01

790 2.402877e-01 6.033107e-01

791 2.404961e-01 6.054883e-01

792 2.407179e-01 6.076089e-01

793 2.409536e-01 6.096701e-01

794 2.412041e-01 6.116699e-01

795 2.414700e-01 6.136068e-01

796 2.417522e-01 6.154795e-01

797 2.420514e-01 6.172873e-01

798 2.423684e-01 6.190299e-01

799 2.427039e-01 6.207071e-01

800 2.430589e-01 6.223194e-01

801 2.434340e-01 6.238672e-01

802 2.438302e-01 6.253517e-01

803 2.442483e-01 6.267737e-01

804 2.446889e-01 6.281349e-01

805 2.451531e-01 6.294366e-01

806 2.456414e-01 6.306806e-01

807 2.461547e-01 6.318687e-01

808 2.466938e-01 6.330029e-01

809 2.472592e-01 6.340852e-01

810 2.478517e-01 6.351175e-01

811 2.484718e-01 6.361022e-01

812 2.491202e-01 6.370411e-01

813 2.497972e-01 6.379365e-01

814 2.505034e-01 6.387904e-01

815 2.512390e-01 6.396049e-01

816 2.520042e-01 6.403819e-01

817 2.527993e-01 6.411235e-01

818 2.536243e-01 6.418314e-01

819 2.544791e-01 6.425076e-01

820 2.553636e-01 6.431538e-01

821 2.562774e-01 6.437717e-01

822 2.572201e-01 6.443629e-01

823 2.581913e-01 6.449289e-01

824 2.591901e-01 6.454713e-01

825 2.602159e-01 6.459914e-01

826 2.612676e-01 6.464905e-01

827 2.623441e-01 6.469699e-01

828 2.634443e-01 6.474308e-01

829 2.645668e-01 6.478742e-01

830 2.657100e-01 6.483013e-01

831 2.668725e-01 6.487129e-01

832 2.680525e-01 6.491102e-01

833 2.692482e-01 6.494938e-01

834 2.704577e-01 6.498646e-01

835 2.716790e-01 6.502235e-01

836 2.729102e-01 6.505710e-01

837 2.741490e-01 6.509080e-01

838 2.753934e-01 6.512349e-01

839 2.766412e-01 6.515525e-01

840 2.778902e-01 6.518612e-01

841 2.791384e-01 6.521615e-01

842 2.803835e-01 6.524540e-01

843 2.816234e-01 6.527391e-01

844 2.828561e-01 6.530172e-01

845 2.840797e-01 6.532886e-01

846 2.852922e-01 6.535539e-01

847 2.864917e-01 6.538132e-01

848 2.876767e-01 6.540670e-01

849 2.888454e-01 6.543154e-01

850 2.899965e-01 6.545589e-01

851 2.911284e-01 6.547976e-01

852 2.922401e-01 6.550318e-01

853 2.933302e-01 6.552617e-01

854 2.943980e-01 6.554875e-01

855 2.954425e-01 6.557095e-01

856 2.964630e-01 6.559278e-01

857 2.974590e-01 6.561425e-01

858 2.984298e-01 6.563539e-01

859 2.993753e-01 6.565621e-01

860 3.002952e-01 6.567673e-01

861 3.011892e-01 6.569695e-01

862 3.020575e-01 6.571689e-01

863 3.029001e-01 6.573655e-01

864 3.037171e-01 6.575597e-01

865 3.045088e-01 6.577513e-01

866 3.052755e-01 6.579405e-01

867 3.060175e-01 6.581275e-01

868 3.067353e-01 6.583122e-01

869 3.074294e-01 6.584948e-01

870 3.081003e-01 6.586753e-01

871 3.087486e-01 6.588539e-01

872 3.093748e-01 6.590305e-01

873 3.099796e-01 6.592053e-01

874 3.105636e-01 6.593782e-01

875 3.111275e-01 6.595494e-01

876 3.116719e-01 6.597189e-01

877 3.121975e-01 6.598868e-01

878 3.127050e-01 6.600531e-01

879 3.131950e-01 6.602178e-01

880 3.136682e-01 6.603810e-01

881 3.141253e-01 6.605427e-01

882 3.145668e-01 6.607030e-01

883 3.149935e-01 6.608619e-01

884 3.154059e-01 6.610194e-01

885 3.158046e-01 6.611756e-01

886 3.161903e-01 6.613304e-01

887 3.165635e-01 6.614840e-01

888 3.169247e-01 6.616364e-01

889 3.172746e-01 6.617875e-01

890 3.176135e-01 6.619374e-01

891 3.179421e-01 6.620862e-01

892 3.182607e-01 6.622337e-01

893 3.185699e-01 6.623802e-01

894 3.188701e-01 6.625255e-01

895 3.191616e-01 6.626698e-01

896 3.194450e-01 6.628130e-01

897 3.197207e-01 6.629551e-01

898 3.199889e-01 6.630962e-01

899 3.202500e-01 6.632363e-01

900 3.205044e-01 6.633753e-01

901 3.207524e-01 6.635134e-01

902 3.207585e-01 6.644411e-01

903 3.207650e-01 6.654232e-01

904 3.207721e-01 6.664614e-01

905 3.207798e-01 6.675575e-01

906 3.207880e-01 6.687128e-01

907 3.207969e-01 6.699287e-01

908 3.208064e-01 6.712064e-01

909 3.208167e-01 6.725467e-01

910 3.208277e-01 6.739504e-01

911 3.208396e-01 6.754179e-01

912 3.208524e-01 6.769495e-01

913 3.208661e-01 6.785451e-01

914 3.208808e-01 6.802042e-01

915 3.208967e-01 6.819261e-01

916 3.209137e-01 6.837098e-01

917 3.209319e-01 6.855539e-01

918 3.209515e-01 6.874566e-01

919 3.209725e-01 6.894159e-01

920 3.209951e-01 6.914294e-01

921 3.210192e-01 6.934942e-01

922 3.210451e-01 6.956074e-01

923 3.210728e-01 6.977653e-01

924 3.211026e-01 6.999645e-01

925 3.211344e-01 7.022008e-01

926 3.211684e-01 7.044699e-01

927 3.212049e-01 7.067675e-01

928 3.212439e-01 7.090888e-01

929 3.212855e-01 7.114291e-01

930 3.213301e-01 7.137832e-01

931 3.213777e-01 7.161462e-01

932 3.214286e-01 7.185130e-01

933 3.214829e-01 7.208784e-01

934 3.215408e-01 7.232374e-01

935 3.216027e-01 7.255848e-01

936 3.216687e-01 7.279159e-01

937 3.217390e-01 7.302258e-01

938 3.218140e-01 7.325099e-01

939 3.218938e-01 7.347639e-01

940 3.219789e-01 7.369836e-01

941 3.220695e-01 7.391651e-01

942 3.221658e-01 7.413049e-01

943 3.222684e-01 7.433998e-01

944 3.223773e-01 7.454468e-01

945 3.224932e-01 7.474433e-01

946 3.226162e-01 7.493872e-01

947 3.227468e-01 7.512765e-01

948 3.228855e-01 7.531097e-01

949 3.230326e-01 7.548857e-01

950 3.231885e-01 7.566035e-01

951 3.233538e-01 7.582627e-01

952 3.235288e-01 7.598630e-01

953 3.237142e-01 7.614046e-01

954 3.239102e-01 7.628876e-01

955 3.241176e-01 7.643127e-01

956 3.243367e-01 7.656807e-01

957 3.245682e-01 7.669926e-01

958 3.248125e-01 7.682494e-01

959 3.250703e-01 7.694526e-01

960 3.253420e-01 7.706035e-01

961 3.256284e-01 7.717038e-01

962 3.259298e-01 7.727549e-01

963 3.262470e-01 7.737587e-01

964 3.265805e-01 7.747169e-01

965 3.269308e-01 7.756311e-01

966 3.272987e-01 7.765033e-01

967 3.276845e-01 7.773352e-01

968 3.280889e-01 7.781285e-01

969 3.285124e-01 7.788851e-01

970 3.289556e-01 7.796067e-01

971 3.294189e-01 7.802949e-01

972 3.299028e-01 7.809515e-01

973 3.304077e-01 7.815780e-01

974 3.309342e-01 7.821759e-01

975 3.314825e-01 7.827469e-01

976 3.320530e-01 7.832923e-01

977 3.326459e-01 7.838135e-01

978 3.332615e-01 7.843119e-01

979 3.338999e-01 7.847886e-01

980 3.345613e-01 7.852450e-01

981 3.352456e-01 7.856822e-01

982 3.359529e-01 7.861012e-01

983 3.366831e-01 7.865032e-01

984 3.374358e-01 7.868890e-01

985 3.382109e-01 7.872596e-01

986 3.390080e-01 7.876160e-01

987 3.398267e-01 7.879589e-01

988 3.406663e-01 7.882890e-01

989 3.415263e-01 7.886073e-01

990 3.424060e-01 7.889143e-01

991 3.433045e-01 7.892107e-01

992 3.442208e-01 7.894971e-01

993 3.451542e-01 7.897741e-01

994 3.461034e-01 7.900422e-01

995 3.470673e-01 7.903020e-01

996 3.480446e-01 7.905540e-01

997 3.490342e-01 7.907985e-01

998 3.500345e-01 7.910360e-01

999 3.510443e-01 7.912669e-01

1000 3.520620e-01 7.914916e-01

1001 3.530862e-01 7.917104e-01

1002 3.541153e-01 7.919237e-01

1003 3.551477e-01 7.921317e-01

1004 3.561819e-01 7.923348e-01

1005 3.572164e-01 7.925331e-01

1006 3.582495e-01 7.927271e-01

1007 3.592798e-01 7.929168e-01

1008 3.603057e-01 7.931025e-01

1009 3.613258e-01 7.932845e-01

1010 3.623385e-01 7.934628e-01

1011 3.633427e-01 7.936378e-01

1012 3.643368e-01 7.938096e-01

1013 3.653197e-01 7.939783e-01

1014 3.662902e-01 7.941440e-01

1015 3.672472e-01 7.943070e-01

1016 3.681897e-01 7.944674e-01

1017 3.691167e-01 7.946252e-01

1018 3.700274e-01 7.947807e-01

1019 3.709210e-01 7.949338e-01

1020 3.717968e-01 7.950847e-01

1021 3.726543e-01 7.952336e-01

1022 3.734929e-01 7.953804e-01

1023 3.743123e-01 7.955254e-01

1024 3.751121e-01 7.956685e-01

1025 3.758920e-01 7.958098e-01

1026 3.766520e-01 7.959494e-01

1027 3.773918e-01 7.960874e-01

1028 3.781114e-01 7.962239e-01

1029 3.788110e-01 7.963588e-01

1030 3.794905e-01 7.964923e-01

1031 3.801502e-01 7.966244e-01

1032 3.807902e-01 7.967552e-01

1033 3.814108e-01 7.968846e-01

1034 3.820123e-01 7.970128e-01

1035 3.825949e-01 7.971398e-01

1036 3.831592e-01 7.972656e-01

1037 3.837053e-01 7.973903e-01

1038 3.842339e-01 7.975139e-01

1039 3.847452e-01 7.976364e-01

1040 3.852398e-01 7.977579e-01

1041 3.857181e-01 7.978783e-01

1042 3.861806e-01 7.979978e-01

1043 3.866277e-01 7.981163e-01

1044 3.870600e-01 7.982340e-01

1045 3.874780e-01 7.983507e-01

1046 3.878821e-01 7.984665e-01

1047 3.882728e-01 7.985814e-01

1048 3.886505e-01 7.986956e-01

1049 3.890159e-01 7.988089e-01

1050 3.893693e-01 7.989214e-01

1051 3.897112e-01 7.990331e-01

1052 3.900421e-01 7.991441e-01

1053 3.903623e-01 7.992543e-01

1054 3.906724e-01 7.993638e-01

1055 3.909728e-01 7.994726e-01

1056 3.912637e-01 7.995806e-01

1057 3.915458e-01 7.996880e-01

1058 3.918193e-01 7.997947e-01

1059 3.920845e-01 7.999008e-01

1060 3.923420e-01 8.000061e-01

1061 3.925920e-01 8.001109e-01

1062 3.928348e-01 8.002150e-01

1063 3.930707e-01 8.003185e-01

1064 3.933002e-01 8.004213e-01

1065 3.935234e-01 8.005236e-01

1066 3.937406e-01 8.006253e-01

1067 3.939522e-01 8.007264e-01

1068 3.941584e-01 8.008269e-01

1069 3.943594e-01 8.009269e-01

1070 3.945555e-01 8.010262e-01

1071 3.947468e-01 8.011251e-01

1072 3.949337e-01 8.012234e-01

1073 3.951164e-01 8.013212e-01

1074 3.952950e-01 8.014184e-01

1075 3.954696e-01 8.015151e-01

1076 3.956406e-01 8.016113e-01

1077 3.958081e-01 8.017069e-01

1078 3.959722e-01 8.018021e-01

1079 3.961331e-01 8.018968e-01

1080 3.962910e-01 8.019910e-01

1081 3.964460e-01 8.020846e-01

1082 3.964670e-01 8.042557e-01

1083 3.964894e-01 8.064432e-01

1084 3.965133e-01 8.086430e-01

1085 3.965388e-01 8.108507e-01

1086 3.965660e-01 8.130618e-01

1087 3.965949e-01 8.152718e-01

1088 3.966257e-01 8.174764e-01

1089 3.966585e-01 8.196711e-01

1090 3.966934e-01 8.218517e-01

1091 3.967305e-01 8.240140e-01

1092 3.967700e-01 8.261541e-01

1093 3.968120e-01 8.282680e-01

1094 3.968567e-01 8.303521e-01

1095 3.969041e-01 8.324030e-01

1096 3.969545e-01 8.344175e-01

1097 3.970080e-01 8.363927e-01

1098 3.970648e-01 8.383260e-01

1099 3.971252e-01 8.402149e-01

1100 3.971891e-01 8.420574e-01

1101 3.972570e-01 8.438517e-01

1102 3.973290e-01 8.455962e-01

1103 3.974052e-01 8.472899e-01

1104 3.974860e-01 8.489316e-01

1105 3.975716e-01 8.505208e-01

1106 3.976622e-01 8.520571e-01

1107 3.977581e-01 8.535403e-01

1108 3.978596e-01 8.549704e-01

1109 3.979668e-01 8.563478e-01

1110 3.980802e-01 8.576730e-01

1111 3.982001e-01 8.589465e-01

1112 3.983267e-01 8.601693e-01

1113 3.984603e-01 8.613423e-01

1114 3.986013e-01 8.624666e-01

1115 3.987501e-01 8.635434e-01

1116 3.989069e-01 8.645740e-01

1117 3.990722e-01 8.655597e-01

1118 3.992464e-01 8.665021e-01

1119 3.994297e-01 8.674025e-01

1120 3.996226e-01 8.682624e-01

1121 3.998255e-01 8.690834e-01

1122 4.000388e-01 8.698671e-01

1123 4.002629e-01 8.706149e-01

1124 4.004983e-01 8.713283e-01

1125 4.007452e-01 8.720089e-01

1126 4.010043e-01 8.726582e-01

1127 4.012758e-01 8.732775e-01

1128 4.015602e-01 8.738683e-01

1129 4.018579e-01 8.744320e-01

1130 4.021694e-01 8.749699e-01

1131 4.024951e-01 8.754834e-01

1132 4.028353e-01 8.759735e-01

1133 4.031905e-01 8.764416e-01

1134 4.035611e-01 8.768888e-01

1135 4.039474e-01 8.773162e-01

1136 4.043497e-01 8.777249e-01

1137 4.047684e-01 8.781158e-01

1138 4.052039e-01 8.784900e-01

1139 4.056563e-01 8.788483e-01

1140 4.061261e-01 8.791916e-01

1141 4.066132e-01 8.795208e-01

1142 4.071181e-01 8.798366e-01

1143 4.076408e-01 8.801398e-01

1144 4.081814e-01 8.804310e-01

1145 4.087399e-01 8.807110e-01

1146 4.093165e-01 8.809804e-01

1147 4.099111e-01 8.812398e-01

1148 4.105235e-01 8.814897e-01

1149 4.111537e-01 8.817307e-01

1150 4.118015e-01 8.819632e-01

1151 4.124665e-01 8.821878e-01

1152 4.131485e-01 8.824049e-01

1153 4.138471e-01 8.826150e-01

1154 4.145619e-01 8.828183e-01

1155 4.152923e-01 8.830153e-01

1156 4.160378e-01 8.832064e-01

1157 4.167977e-01 8.833918e-01

1158 4.175714e-01 8.835719e-01

1159 4.183581e-01 8.837470e-01

1160 4.191570e-01 8.839174e-01

1161 4.199672e-01 8.840832e-01

1162 4.207878e-01 8.842448e-01

1163 4.216178e-01 8.844024e-01

1164 4.224563e-01 8.845561e-01

1165 4.233023e-01 8.847063e-01

1166 4.241545e-01 8.848530e-01

1167 4.250119e-01 8.849965e-01

1168 4.258735e-01 8.851370e-01

1169 4.267379e-01 8.852745e-01

1170 4.276042e-01 8.854093e-01

1171 4.284711e-01 8.855414e-01

1172 4.293374e-01 8.856711e-01

1173 4.302021e-01 8.857984e-01

1174 4.310640e-01 8.859235e-01

1175 4.319219e-01 8.860464e-01

1176 4.327748e-01 8.861673e-01

1177 4.336217e-01 8.862862e-01

1178 4.344615e-01 8.864033e-01

1179 4.352933e-01 8.865186e-01

1180 4.361161e-01 8.866323e-01

1181 4.369290e-01 8.867443e-01

1182 4.377313e-01 8.868548e-01

1183 4.385221e-01 8.869639e-01

1184 4.393008e-01 8.870715e-01

1185 4.400666e-01 8.871778e-01

1186 4.408191e-01 8.872829e-01

1187 4.415577e-01 8.873867e-01

1188 4.422819e-01 8.874893e-01

1189 4.429913e-01 8.875908e-01

1190 4.436856e-01 8.876912e-01

1191 4.443644e-01 8.877905e-01

1192 4.450276e-01 8.878889e-01

1193 4.456750e-01 8.879863e-01

1194 4.463065e-01 8.880827e-01

1195 4.469220e-01 8.881783e-01

1196 4.475214e-01 8.882730e-01

1197 4.481048e-01 8.883668e-01

1198 4.486723e-01 8.884599e-01

1199 4.492240e-01 8.885522e-01

1200 4.497600e-01 8.886437e-01

1201 4.502805e-01 8.887345e-01

1202 4.507857e-01 8.888246e-01

1203 4.512758e-01 8.889140e-01

1204 4.517512e-01 8.890027e-01

1205 4.522120e-01 8.890909e-01

1206 4.526586e-01 8.891783e-01

1207 4.530912e-01 8.892652e-01

1208 4.535103e-01 8.893515e-01

1209 4.539162e-01 8.894372e-01

1210 4.543091e-01 8.895224e-01

1211 4.546895e-01 8.896070e-01

1212 4.550578e-01 8.896911e-01

1213 4.554142e-01 8.897746e-01

1214 4.557592e-01 8.898577e-01

1215 4.560931e-01 8.899403e-01

1216 4.564162e-01 8.900224e-01

1217 4.567291e-01 8.901040e-01

1218 4.570319e-01 8.901852e-01

1219 4.573251e-01 8.902659e-01

1220 4.576090e-01 8.903462e-01

1221 4.578839e-01 8.904260e-01

1222 4.581503e-01 8.905054e-01

1223 4.584084e-01 8.905845e-01

1224 4.586585e-01 8.906631e-01

1225 4.589009e-01 8.907413e-01

1226 4.591360e-01 8.908191e-01

1227 4.593641e-01 8.908965e-01

1228 4.595854e-01 8.909736e-01

1229 4.598003e-01 8.910503e-01

1230 4.600089e-01 8.911266e-01

1231 4.602116e-01 8.912026e-01

1232 4.604086e-01 8.912782e-01

1233 4.606001e-01 8.913535e-01

1234 4.607865e-01 8.914284e-01

1235 4.609678e-01 8.915030e-01

1236 4.611444e-01 8.915772e-01

1237 4.613165e-01 8.916511e-01

1238 4.614841e-01 8.917247e-01

1239 4.616477e-01 8.917980e-01

1240 4.618072e-01 8.918710e-01

1241 4.619630e-01 8.919436e-01

1242 4.621151e-01 8.920160e-01

1243 4.622638e-01 8.920880e-01

1244 4.624091e-01 8.921598e-01

1245 4.625513e-01 8.922312e-01

1246 4.626906e-01 8.923024e-01

1247 4.628269e-01 8.923732e-01

1248 4.629605e-01 8.924438e-01

1249 4.630915e-01 8.925141e-01

1250 4.632200e-01 8.925841e-01

1251 4.633461e-01 8.926539e-01

1252 4.634700e-01 8.927233e-01

1253 4.635917e-01 8.927925e-01

1254 4.637113e-01 8.928614e-01

1255 4.638290e-01 8.929301e-01

1256 4.639449e-01 8.929984e-01

1257 4.640589e-01 8.930666e-01

1258 4.641712e-01 8.931344e-01

1259 4.642820e-01 8.932020e-01

1260 4.643912e-01 8.932693e-01

1261 4.644989e-01 8.933364e-01

1262 4.645491e-01 8.947615e-01

1263 4.646022e-01 8.961387e-01

1264 4.646584e-01 8.974680e-01

1265 4.647177e-01 8.987496e-01

1266 4.647804e-01 8.999840e-01

1267 4.648465e-01 9.011717e-01

1268 4.649164e-01 9.023135e-01

1269 4.649900e-01 9.034102e-01

1270 4.650678e-01 9.044628e-01

1271 4.651498e-01 9.054721e-01

1272 4.652362e-01 9.064394e-01

1273 4.653273e-01 9.073657e-01

1274 4.654232e-01 9.082524e-01

1275 4.655243e-01 9.091006e-01

1276 4.656307e-01 9.099116e-01

1277 4.657426e-01 9.106868e-01

1278 4.658604e-01 9.114274e-01

1279 4.659842e-01 9.121348e-01

1280 4.661144e-01 9.128103e-01

1281 4.662512e-01 9.134551e-01

1282 4.663948e-01 9.140707e-01

1283 4.665456e-01 9.146581e-01

1284 4.667038e-01 9.152188e-01

1285 4.668698e-01 9.157538e-01

1286 4.670438e-01 9.162644e-01

1287 4.672262e-01 9.167518e-01

1288 4.674172e-01 9.172170e-01

1289 4.676171e-01 9.176611e-01

1290 4.678264e-01 9.180851e-01

1291 4.680452e-01 9.184901e-01

1292 4.682739e-01 9.188770e-01

1293 4.685129e-01 9.192467e-01

1294 4.687624e-01 9.196001e-01

1295 4.690228e-01 9.199381e-01

1296 4.692943e-01 9.202615e-01

1297 4.695774e-01 9.205709e-01

1298 4.698722e-01 9.208673e-01

1299 4.701791e-01 9.211512e-01

1300 4.704983e-01 9.214234e-01

1301 4.708302e-01 9.216844e-01

1302 4.711750e-01 9.219348e-01

1303 4.715330e-01 9.221753e-01

1304 4.719043e-01 9.224064e-01

1305 4.722892e-01 9.226285e-01

1306 4.726879e-01 9.228422e-01

1307 4.731006e-01 9.230480e-01

1308 4.735273e-01 9.232462e-01

1309 4.739683e-01 9.234372e-01

1310 4.744236e-01 9.236215e-01

1311 4.748932e-01 9.237995e-01

1312 4.753772e-01 9.239714e-01

1313 4.758757e-01 9.241376e-01

1314 4.763884e-01 9.242984e-01

1315 4.769154e-01 9.244541e-01

1316 4.774566e-01 9.246050e-01

1317 4.780117e-01 9.247513e-01

1318 4.785806e-01 9.248933e-01

1319 4.791629e-01 9.250313e-01

1320 4.797585e-01 9.251653e-01

1321 4.803670e-01 9.252957e-01

1322 4.809879e-01 9.254226e-01

1323 4.816209e-01 9.255462e-01

1324 4.822655e-01 9.256667e-01

1325 4.829211e-01 9.257842e-01

1326 4.835873e-01 9.258990e-01

1327 4.842633e-01 9.260111e-01

1328 4.849486e-01 9.261206e-01

1329 4.856424e-01 9.262278e-01

1330 4.863441e-01 9.263327e-01

1331 4.870530e-01 9.264355e-01

1332 4.877681e-01 9.265362e-01

1333 4.884888e-01 9.266350e-01

1334 4.892141e-01 9.267319e-01

1335 4.899433e-01 9.268271e-01

1336 4.906756e-01 9.269206e-01

1337 4.914099e-01 9.270125e-01

1338 4.921455e-01 9.271028e-01

1339 4.928814e-01 9.271918e-01

1340 4.936168e-01 9.272794e-01

1341 4.943509e-01 9.273656e-01

1342 4.950827e-01 9.274506e-01

1343 4.958114e-01 9.275345e-01

1344 4.965361e-01 9.276171e-01

1345 4.972562e-01 9.276987e-01

1346 4.979707e-01 9.277793e-01

1347 4.986790e-01 9.278588e-01

1348 4.993803e-01 9.279375e-01

1349 5.000739e-01 9.280152e-01

1350 5.007593e-01 9.280920e-01

1351 5.014356e-01 9.281680e-01

1352 5.021026e-01 9.282432e-01

1353 5.027594e-01 9.283176e-01

1354 5.034058e-01 9.283913e-01

1355 5.040413e-01 9.284643e-01

1356 5.046654e-01 9.285366e-01

1357 5.052778e-01 9.286082e-01

1358 5.058783e-01 9.286792e-01

1359 5.064665e-01 9.287496e-01

1360 5.070422e-01 9.288195e-01

1361 5.076053e-01 9.288887e-01

1362 5.081556e-01 9.289575e-01

1363 5.086929e-01 9.290257e-01

1364 5.092174e-01 9.290934e-01

1365 5.097288e-01 9.291606e-01

1366 5.102272e-01 9.292273e-01

1367 5.107128e-01 9.292936e-01

1368 5.111854e-01 9.293594e-01

1369 5.116453e-01 9.294249e-01

1370 5.120925e-01 9.294899e-01

1371 5.125271e-01 9.295545e-01

1372 5.129494e-01 9.296187e-01

1373 5.133596e-01 9.296825e-01

1374 5.137578e-01 9.297460e-01

1375 5.141442e-01 9.298091e-01

1376 5.145191e-01 9.298719e-01

1377 5.148827e-01 9.299343e-01

1378 5.152352e-01 9.299964e-01

1379 5.155770e-01 9.300582e-01

1380 5.159083e-01 9.301197e-01

1381 5.162294e-01 9.301809e-01

1382 5.165405e-01 9.302418e-01

1383 5.168419e-01 9.303024e-01

1384 5.171339e-01 9.303627e-01

1385 5.174168e-01 9.304227e-01

1386 5.176908e-01 9.304825e-01

1387 5.179563e-01 9.305420e-01

1388 5.182136e-01 9.306012e-01

1389 5.184628e-01 9.306602e-01

1390 5.187042e-01 9.307190e-01

1391 5.189383e-01 9.307775e-01

1392 5.191651e-01 9.308358e-01

1393 5.193849e-01 9.308938e-01

1394 5.195981e-01 9.309517e-01

1395 5.198049e-01 9.310092e-01

1396 5.200054e-01 9.310666e-01

1397 5.202000e-01 9.311238e-01

1398 5.203888e-01 9.311807e-01

1399 5.205722e-01 9.312375e-01

1400 5.207502e-01 9.312940e-01

1401 5.209232e-01 9.313504e-01

1402 5.210913e-01 9.314065e-01

1403 5.212547e-01 9.314624e-01

1404 5.214136e-01 9.315182e-01

1405 5.215683e-01 9.315737e-01

1406 5.217188e-01 9.316291e-01

1407 5.218653e-01 9.316843e-01

1408 5.220081e-01 9.317393e-01

1409 5.221473e-01 9.317942e-01

1410 5.222830e-01 9.318488e-01

1411 5.224154e-01 9.319033e-01

1412 5.225446e-01 9.319576e-01

1413 5.226708e-01 9.320117e-01

1414 5.227941e-01 9.320657e-01

1415 5.229146e-01 9.321195e-01

1416 5.230325e-01 9.321732e-01

1417 5.231478e-01 9.322266e-01

1418 5.232607e-01 9.322800e-01

1419 5.233713e-01 9.323331e-01

1420 5.234797e-01 9.323861e-01

1421 5.235860e-01 9.324390e-01

1422 5.236902e-01 9.324916e-01

1423 5.237926e-01 9.325442e-01

1424 5.238931e-01 9.325966e-01

1425 5.239919e-01 9.326488e-01

1426 5.240890e-01 9.327009e-01

1427 5.241845e-01 9.327528e-01

1428 5.242785e-01 9.328046e-01

1429 5.243710e-01 9.328562e-01

1430 5.244622e-01 9.329077e-01

1431 5.245521e-01 9.329591e-01

1432 5.246407e-01 9.330103e-01

1433 5.247281e-01 9.330614e-01

1434 5.248143e-01 9.331123e-01

1435 5.248995e-01 9.331631e-01

1436 5.249837e-01 9.332137e-01

1437 5.250669e-01 9.332642e-01

1438 5.251491e-01 9.333146e-01

1439 5.252305e-01 9.333648e-01

1440 5.253110e-01 9.334149e-01

1441 5.253907e-01 9.334649e-01

1442 5.254842e-01 9.339723e-01

1443 5.255824e-01 9.344568e-01

1444 5.256853e-01 9.349195e-01

1445 5.257932e-01 9.353612e-01

1446 5.259064e-01 9.357831e-01

1447 5.260249e-01 9.361860e-01

1448 5.261490e-01 9.365707e-01

1449 5.262790e-01 9.369383e-01

1450 5.264150e-01 9.372894e-01

1451 5.265573e-01 9.376249e-01

1452 5.267062e-01 9.379456e-01

1453 5.268617e-01 9.382522e-01

1454 5.270243e-01 9.385455e-01

1455 5.271940e-01 9.388260e-01

1456 5.273713e-01 9.390944e-01

1457 5.275562e-01 9.393514e-01

1458 5.277491e-01 9.395975e-01

1459 5.279502e-01 9.398333e-01

1460 5.281597e-01 9.400594e-01

1461 5.283779e-01 9.402762e-01

1462 5.286051e-01 9.404842e-01

1463 5.288414e-01 9.406838e-01

1464 5.290871e-01 9.408756e-01

1465 5.293425e-01 9.410599e-01

1466 5.296077e-01 9.412372e-01

1467 5.298830e-01 9.414077e-01

1468 5.301686e-01 9.415718e-01

1469 5.304648e-01 9.417300e-01

1470 5.307716e-01 9.418824e-01

1471 5.310893e-01 9.420294e-01

1472 5.314180e-01 9.421713e-01

1473 5.317579e-01 9.423084e-01

1474 5.321092e-01 9.424408e-01

1475 5.324719e-01 9.425689e-01

1476 5.328461e-01 9.426929e-01

1477 5.332320e-01 9.428130e-01

1478 5.336296e-01 9.429293e-01

1479 5.340389e-01 9.430422e-01

1480 5.344600e-01 9.431517e-01

1481 5.348927e-01 9.432580e-01

1482 5.353372e-01 9.433614e-01

1483 5.357933e-01 9.434619e-01

1484 5.362608e-01 9.435597e-01

1485 5.367398e-01 9.436550e-01

1486 5.372300e-01 9.437478e-01

1487 5.377312e-01 9.438384e-01

1488 5.382431e-01 9.439267e-01

1489 5.387657e-01 9.440130e-01

1490 5.392984e-01 9.440973e-01

1491 5.398411e-01 9.441797e-01

1492 5.403934e-01 9.442604e-01

1493 5.409548e-01 9.443393e-01

1494 5.415250e-01 9.444167e-01

1495 5.421034e-01 9.444925e-01

1496 5.426897e-01 9.445669e-01

1497 5.432832e-01 9.446399e-01

1498 5.438835e-01 9.447115e-01

1499 5.444900e-01 9.447819e-01

1500 5.451020e-01 9.448512e-01

1501 5.457191e-01 9.449192e-01

1502 5.463405e-01 9.449862e-01

1503 5.469657e-01 9.450522e-01

1504 5.475939e-01 9.451172e-01

1505 5.482244e-01 9.451812e-01

1506 5.488567e-01 9.452444e-01

1507 5.494900e-01 9.453067e-01

1508 5.501237e-01 9.453681e-01

1509 5.507570e-01 9.454288e-01

1510 5.513892e-01 9.454888e-01

1511 5.520198e-01 9.455480e-01

1512 5.526480e-01 9.456065e-01

1513 5.532732e-01 9.456644e-01

1514 5.538948e-01 9.457217e-01

1515 5.545121e-01 9.457784e-01

1516 5.551245e-01 9.458345e-01

1517 5.557315e-01 9.458900e-01

1518 5.563325e-01 9.459450e-01

1519 5.569270e-01 9.459996e-01

1520 5.575144e-01 9.460536e-01

1521 5.580944e-01 9.461071e-01

1522 5.586665e-01 9.461603e-01

1523 5.592302e-01 9.462129e-01

1524 5.597852e-01 9.462652e-01

1525 5.603313e-01 9.463171e-01

1526 5.608679e-01 9.463686e-01

1527 5.613949e-01 9.464197e-01

1528 5.619121e-01 9.464705e-01

1529 5.624192e-01 9.465209e-01

1530 5.629160e-01 9.465710e-01

1531 5.634024e-01 9.466208e-01

1532 5.638782e-01 9.466702e-01

1533 5.643435e-01 9.467194e-01

1534 5.647981e-01 9.467683e-01

1535 5.652419e-01 9.468169e-01

1536 5.656750e-01 9.468653e-01

1537 5.660974e-01 9.469133e-01

1538 5.665092e-01 9.469612e-01

1539 5.669103e-01 9.470088e-01

1540 5.673009e-01 9.470561e-01

1541 5.676810e-01 9.471032e-01

1542 5.680509e-01 9.471502e-01

1543 5.684105e-01 9.471968e-01

1544 5.687601e-01 9.472433e-01

1545 5.690998e-01 9.472896e-01

1546 5.694298e-01 9.473357e-01

1547 5.697502e-01 9.473816e-01

1548 5.700613e-01 9.474273e-01

1549 5.703632e-01 9.474728e-01

1550 5.706562e-01 9.475182e-01

1551 5.709404e-01 9.475633e-01

1552 5.712161e-01 9.476084e-01

1553 5.714834e-01 9.476532e-01

1554 5.717427e-01 9.476979e-01

1555 5.719941e-01 9.477424e-01

1556 5.722378e-01 9.477868e-01

1557 5.724741e-01 9.478310e-01

1558 5.727032e-01 9.478751e-01

1559 5.729253e-01 9.479191e-01

1560 5.731406e-01 9.479629e-01

1561 5.733493e-01 9.480066e-01

1562 5.735517e-01 9.480501e-01

1563 5.737480e-01 9.480935e-01

1564 5.739383e-01 9.481368e-01

1565 5.741229e-01 9.481800e-01

1566 5.743020e-01 9.482230e-01

1567 5.744758e-01 9.482659e-01

1568 5.746444e-01 9.483087e-01

1569 5.748080e-01 9.483514e-01

1570 5.749669e-01 9.483940e-01

1571 5.751212e-01 9.484364e-01

1572 5.752711e-01 9.484788e-01

1573 5.754168e-01 9.485210e-01

1574 5.755583e-01 9.485632e-01

1575 5.756960e-01 9.486052e-01

1576 5.758298e-01 9.486471e-01

1577 5.759601e-01 9.486890e-01

1578 5.760868e-01 9.487307e-01

1579 5.762103e-01 9.487723e-01

1580 5.763305e-01 9.488139e-01

1581 5.764477e-01 9.488553e-01

1582 5.765619e-01 9.488966e-01

1583 5.766733e-01 9.489379e-01

1584 5.767820e-01 9.489790e-01

1585 5.768881e-01 9.490201e-01

1586 5.769917e-01 9.490611e-01

1587 5.770930e-01 9.491020e-01

1588 5.771919e-01 9.491428e-01

1589 5.772887e-01 9.491835e-01

1590 5.773835e-01 9.492241e-01

1591 5.774762e-01 9.492647e-01

1592 5.775670e-01 9.493051e-01

1593 5.776560e-01 9.493455e-01

1594 5.777432e-01 9.493858e-01

1595 5.778288e-01 9.494260e-01

1596 5.779128e-01 9.494661e-01

1597 5.779953e-01 9.495062e-01

1598 5.780763e-01 9.495461e-01

1599 5.781560e-01 9.495860e-01

1600 5.782343e-01 9.496258e-01

1601 5.783114e-01 9.496656e-01

1602 5.783872e-01 9.497052e-01

1603 5.784619e-01 9.497448e-01

1604 5.785356e-01 9.497843e-01

1605 5.786081e-01 9.498237e-01

1606 5.786797e-01 9.498631e-01

1607 5.787503e-01 9.499023e-01

1608 5.788201e-01 9.499415e-01

1609 5.788889e-01 9.499807e-01

1610 5.789570e-01 9.500197e-01

1611 5.790242e-01 9.500587e-01

1612 5.790908e-01 9.500976e-01

1613 5.791566e-01 9.501364e-01

1614 5.792218e-01 9.501751e-01

1615 5.792863e-01 9.502138e-01

1616 5.793502e-01 9.502524e-01

1617 5.794136e-01 9.502910e-01

1618 5.794764e-01 9.503294e-01

1619 5.795387e-01 9.503678e-01

1620 5.796006e-01 9.504061e-01

1621 5.796619e-01 9.504443e-01

1622 5.798076e-01 9.506243e-01

1623 5.799595e-01 9.507970e-01

1624 5.801176e-01 9.509628e-01

1625 5.802822e-01 9.511221e-01

1626 5.804536e-01 9.512751e-01

1627 5.806318e-01 9.514222e-01

1628 5.808170e-01 9.515637e-01

1629 5.810096e-01 9.516999e-01

1630 5.812096e-01 9.518311e-01

1631 5.814173e-01 9.519574e-01

1632 5.816328e-01 9.520792e-01

1633 5.818563e-01 9.521967e-01

1634 5.820880e-01 9.523101e-01

1635 5.823281e-01 9.524197e-01

1636 5.825767e-01 9.525255e-01

1637 5.828341e-01 9.526278e-01

1638 5.831003e-01 9.527268e-01

1639 5.833755e-01 9.528227e-01

1640 5.836599e-01 9.529155e-01

1641 5.839535e-01 9.530055e-01

1642 5.842565e-01 9.530928e-01

1643 5.845690e-01 9.531775e-01

1644 5.848910e-01 9.532598e-01

1645 5.852227e-01 9.533398e-01

1646 5.855641e-01 9.534176e-01

1647 5.859152e-01 9.534933e-01

1648 5.862761e-01 9.535670e-01

1649 5.866467e-01 9.536389e-01

1650 5.870271e-01 9.537089e-01

1651 5.874171e-01 9.537772e-01

1652 5.878169e-01 9.538439e-01

1653 5.882262e-01 9.539091e-01

1654 5.886449e-01 9.539728e-01

1655 5.890730e-01 9.540351e-01

1656 5.895102e-01 9.540961e-01

1657 5.899565e-01 9.541558e-01

1658 5.904115e-01 9.542143e-01

1659 5.908752e-01 9.542717e-01

1660 5.913471e-01 9.543280e-01

1661 5.918271e-01 9.543832e-01

1662 5.923148e-01 9.544374e-01

1663 5.928099e-01 9.544907e-01

1664 5.933121e-01 9.545432e-01

1665 5.938210e-01 9.545947e-01

1666 5.943361e-01 9.546454e-01

1667 5.948571e-01 9.546954e-01

1668 5.953836e-01 9.547446e-01

1669 5.959150e-01 9.547931e-01

1670 5.964509e-01 9.548409e-01

1671 5.969909e-01 9.548881e-01

1672 5.975343e-01 9.549347e-01

1673 5.980808e-01 9.549807e-01

1674 5.986297e-01 9.550261e-01

1675 5.991806e-01 9.550709e-01

1676 5.997328e-01 9.551153e-01

1677 6.002859e-01 9.551591e-01

1678 6.008394e-01 9.552025e-01

1679 6.013925e-01 9.552455e-01

1680 6.019449e-01 9.552880e-01

1681 6.024960e-01 9.553301e-01

1682 6.030452e-01 9.553718e-01

1683 6.035921e-01 9.554131e-01

1684 6.041360e-01 9.554540e-01

1685 6.046766e-01 9.554946e-01

1686 6.052133e-01 9.555349e-01

1687 6.057456e-01 9.555749e-01

1688 6.062732e-01 9.556145e-01

1689 6.067956e-01 9.556539e-01

1690 6.073123e-01 9.556929e-01

1691 6.078230e-01 9.557317e-01

1692 6.083273e-01 9.557702e-01

1693 6.088249e-01 9.558085e-01

1694 6.093154e-01 9.558465e-01

1695 6.097985e-01 9.558843e-01

1696 6.102741e-01 9.559219e-01

1697 6.107418e-01 9.559593e-01

1698 6.112013e-01 9.559964e-01

1699 6.116526e-01 9.560333e-01

1700 6.120955e-01 9.560701e-01

1701 6.125297e-01 9.561067e-01

1702 6.129552e-01 9.561431e-01

1703 6.133718e-01 9.561793e-01

1704 6.137796e-01 9.562153e-01

1705 6.141783e-01 9.562512e-01

1706 6.145681e-01 9.562869e-01

1707 6.149489e-01 9.563225e-01

1708 6.153206e-01 9.563579e-01

1709 6.156833e-01 9.563932e-01

1710 6.160371e-01 9.564284e-01

1711 6.163819e-01 9.564634e-01

1712 6.167179e-01 9.564983e-01

1713 6.170452e-01 9.565331e-01

1714 6.173638e-01 9.565677e-01

1715 6.176738e-01 9.566022e-01

1716 6.179755e-01 9.566367e-01

1717 6.182688e-01 9.566710e-01

1718 6.185539e-01 9.567052e-01

1719 6.188310e-01 9.567393e-01

1720 6.191002e-01 9.567733e-01

1721 6.193617e-01 9.568072e-01

1722 6.196157e-01 9.568410e-01

1723 6.198622e-01 9.568747e-01

1724 6.201016e-01 9.569083e-01

1725 6.203338e-01 9.569418e-01

1726 6.205593e-01 9.569753e-01

1727 6.207780e-01 9.570087e-01

1728 6.209902e-01 9.570419e-01

1729 6.211960e-01 9.570751e-01

1730 6.213957e-01 9.571083e-01

1731 6.215894e-01 9.571413e-01

1732 6.217773e-01 9.571743e-01

1733 6.219596e-01 9.572072e-01

1734 6.221364e-01 9.572401e-01

1735 6.223079e-01 9.572728e-01

1736 6.224744e-01 9.573056e-01

1737 6.226358e-01 9.573382e-01

1738 6.227925e-01 9.573708e-01

1739 6.229445e-01 9.574033e-01

1740 6.230921e-01 9.574357e-01

1741 6.232353e-01 9.574681e-01

1742 6.233744e-01 9.575005e-01

1743 6.235095e-01 9.575328e-01

1744 6.236407e-01 9.575650e-01

1745 6.237682e-01 9.575971e-01

1746 6.238920e-01 9.576293e-01

1747 6.240125e-01 9.576613e-01

1748 6.241295e-01 9.576933e-01

1749 6.242434e-01 9.577253e-01

1750 6.243542e-01 9.577572e-01

1751 6.244620e-01 9.577890e-01

1752 6.245670e-01 9.578208e-01

1753 6.246693e-01 9.578526e-01

1754 6.247689e-01 9.578843e-01

1755 6.248660e-01 9.579160e-01

1756 6.249606e-01 9.579476e-01

1757 6.250529e-01 9.579791e-01

1758 6.251430e-01 9.580107e-01

1759 6.252310e-01 9.580421e-01

1760 6.253169e-01 9.580736e-01

1761 6.254008e-01 9.581050e-01

1762 6.254829e-01 9.581363e-01

1763 6.255631e-01 9.581676e-01

1764 6.256416e-01 9.581989e-01

1765 6.257184e-01 9.582301e-01

1766 6.257937e-01 9.582613e-01

1767 6.258674e-01 9.582924e-01

1768 6.259397e-01 9.583235e-01

1769 6.260105e-01 9.583546e-01

1770 6.260801e-01 9.583856e-01

1771 6.261484e-01 9.584166e-01

1772 6.262154e-01 9.584475e-01

1773 6.262813e-01 9.584784e-01

1774 6.263461e-01 9.585093e-01

1775 6.264098e-01 9.585401e-01

1776 6.264725e-01 9.585709e-01

1777 6.265342e-01 9.586017e-01

1778 6.265950e-01 9.586324e-01

1779 6.266550e-01 9.586630e-01

1780 6.267141e-01 9.586937e-01

1781 6.267724e-01 9.587243e-01

1782 6.268299e-01 9.587548e-01

1783 6.268867e-01 9.587853e-01

1784 6.269428e-01 9.588158e-01

1785 6.269983e-01 9.588463e-01

1786 6.270531e-01 9.588767e-01

1787 6.271073e-01 9.589070e-01

1788 6.271610e-01 9.589374e-01

1789 6.272142e-01 9.589676e-01

1790 6.272668e-01 9.589979e-01

1791 6.273190e-01 9.590281e-01

1792 6.273707e-01 9.590583e-01

1793 6.274219e-01 9.590884e-01

1794 6.274728e-01 9.591185e-01

1795 6.275233e-01 9.591486e-01

1796 6.275734e-01 9.591786e-01

1797 6.276232e-01 9.592086e-01

1798 6.276727e-01 9.592385e-01

1799 6.277218e-01 9.592684e-01

1800 6.277707e-01 9.592983e-01

1801 6.278194e-01 9.593281e-01

1802 6.280183e-01 9.594045e-01

1803 6.282242e-01 9.594785e-01

1804 6.284372e-01 9.595503e-01

1805 6.286574e-01 9.596199e-01

1806 6.288850e-01 9.596874e-01

1807 6.291201e-01 9.597529e-01

1808 6.293628e-01 9.598166e-01

1809 6.296132e-01 9.598785e-01

1810 6.298714e-01 9.599387e-01

1811 6.301376e-01 9.599973e-01

1812 6.304117e-01 9.600544e-01

1813 6.306939e-01 9.601100e-01

1814 6.309843e-01 9.601643e-01

1815 6.312828e-01 9.602172e-01

1816 6.315896e-01 9.602688e-01

1817 6.319046e-01 9.603193e-01

1818 6.322278e-01 9.603686e-01

1819 6.325594e-01 9.604169e-01

1820 6.328992e-01 9.604641e-01

1821 6.332471e-01 9.605103e-01

1822 6.336033e-01 9.605555e-01

1823 6.339675e-01 9.605999e-01

1824 6.343397e-01 9.606434e-01

1825 6.347197e-01 9.606861e-01

1826 6.351076e-01 9.607281e-01

1827 6.355030e-01 9.607693e-01

1828 6.359059e-01 9.608098e-01

1829 6.363160e-01 9.608496e-01

1830 6.367332e-01 9.608887e-01

1831 6.371573e-01 9.609273e-01

1832 6.375879e-01 9.609653e-01

1833 6.380249e-01 9.610027e-01

1834 6.384679e-01 9.610395e-01

1835 6.389167e-01 9.610759e-01

1836 6.393709e-01 9.611118e-01

1837 6.398302e-01 9.611472e-01

1838 6.402943e-01 9.611822e-01

1839 6.407627e-01 9.612167e-01

1840 6.412352e-01 9.612508e-01

1841 6.417113e-01 9.612846e-01

1842 6.421906e-01 9.613180e-01

1843 6.426727e-01 9.613510e-01

1844 6.431572e-01 9.613837e-01

1845 6.436437e-01 9.614160e-01

1846 6.441316e-01 9.614480e-01

1847 6.446207e-01 9.614798e-01

1848 6.451104e-01 9.615112e-01

1849 6.456003e-01 9.615424e-01

1850 6.460899e-01 9.615734e-01

1851 6.465789e-01 9.616040e-01

1852 6.470667e-01 9.616345e-01

1853 6.475529e-01 9.616646e-01

1854 6.480371e-01 9.616946e-01

1855 6.485190e-01 9.617244e-01

1856 6.489980e-01 9.617540e-01

1857 6.494738e-01 9.617833e-01

1858 6.499460e-01 9.618125e-01

1859 6.504142e-01 9.618415e-01

1860 6.508781e-01 9.618703e-01

1861 6.513373e-01 9.618990e-01

1862 6.517915e-01 9.619275e-01

1863 6.522404e-01 9.619558e-01

1864 6.526836e-01 9.619840e-01

1865 6.531210e-01 9.620121e-01

1866 6.535523e-01 9.620400e-01

1867 6.539772e-01 9.620678e-01

1868 6.543955e-01 9.620955e-01

1869 6.548071e-01 9.621230e-01

1870 6.552117e-01 9.621505e-01

1871 6.556092e-01 9.621778e-01

1872 6.559994e-01 9.622050e-01

1873 6.563823e-01 9.622321e-01

1874 6.567577e-01 9.622591e-01

1875 6.571255e-01 9.622860e-01

1876 6.574857e-01 9.623128e-01

1877 6.578383e-01 9.623396e-01

1878 6.581831e-01 9.623662e-01

1879 6.585202e-01 9.623928e-01

1880 6.588497e-01 9.624192e-01

1881 6.591714e-01 9.624457e-01

1882 6.594854e-01 9.624720e-01

1883 6.597918e-01 9.624982e-01

1884 6.600906e-01 9.625244e-01

1885 6.603819e-01 9.625505e-01

1886 6.606657e-01 9.625766e-01

1887 6.609422e-01 9.626026e-01

1888 6.612114e-01 9.626285e-01

1889 6.614734e-01 9.626544e-01

1890 6.617284e-01 9.626802e-01

1891 6.619764e-01 9.627060e-01

1892 6.622176e-01 9.627317e-01

1893 6.624520e-01 9.627573e-01

1894 6.626799e-01 9.627829e-01

1895 6.629013e-01 9.628085e-01

1896 6.631165e-01 9.628340e-01

1897 6.633254e-01 9.628595e-01

1898 6.635283e-01 9.628849e-01

1899 6.637254e-01 9.629103e-01

1900 6.639167e-01 9.629356e-01

1901 6.641024e-01 9.629609e-01

1902 6.642826e-01 9.629862e-01

1903 6.644576e-01 9.630114e-01

1904 6.646274e-01 9.630366e-01

1905 6.647922e-01 9.630617e-01

1906 6.649521e-01 9.630868e-01

1907 6.651074e-01 9.631119e-01

1908 6.652580e-01 9.631370e-01

1909 6.654042e-01 9.631620e-01

1910 6.655461e-01 9.631870e-01

1911 6.656838e-01 9.632119e-01

1912 6.658175e-01 9.632369e-01

1913 6.659473e-01 9.632618e-01

1914 6.660733e-01 9.632866e-01

1915 6.661956e-01 9.633115e-01

1916 6.663145e-01 9.633363e-01

1917 6.664299e-01 9.633611e-01

1918 6.665421e-01 9.633859e-01

1919 6.666511e-01 9.634106e-01

1920 6.667570e-01 9.634353e-01

1921 6.668600e-01 9.634600e-01

1922 6.669601e-01 9.634847e-01

1923 6.670575e-01 9.635094e-01

1924 6.671523e-01 9.635340e-01

1925 6.672445e-01 9.635586e-01

1926 6.673343e-01 9.635832e-01

1927 6.674217e-01 9.636077e-01

1928 6.675069e-01 9.636323e-01

1929 6.675899e-01 9.636568e-01

1930 6.676708e-01 9.636813e-01

1931 6.677497e-01 9.637058e-01

1932 6.678266e-01 9.637303e-01

1933 6.679017e-01 9.637547e-01

1934 6.679751e-01 9.637792e-01

1935 6.680467e-01 9.638036e-01

1936 6.681167e-01 9.638280e-01

1937 6.681850e-01 9.638524e-01

1938 6.682519e-01 9.638767e-01

1939 6.683174e-01 9.639011e-01

1940 6.683814e-01 9.639254e-01

1941 6.684441e-01 9.639497e-01

1942 6.685056e-01 9.639740e-01

1943 6.685658e-01 9.639983e-01

1944 6.686248e-01 9.640225e-01

1945 6.686828e-01 9.640468e-01

1946 6.687396e-01 9.640710e-01

1947 6.687954e-01 9.640952e-01

1948 6.688503e-01 9.641194e-01

1949 6.689042e-01 9.641436e-01

1950 6.689572e-01 9.641677e-01

1951 6.690094e-01 9.641919e-01

1952 6.690607e-01 9.642160e-01

1953 6.691112e-01 9.642401e-01

1954 6.691610e-01 9.642642e-01

1955 6.692101e-01 9.642883e-01

1956 6.692586e-01 9.643123e-01

1957 6.693063e-01 9.643364e-01

1958 6.693535e-01 9.643604e-01

1959 6.694000e-01 9.643844e-01

1960 6.694460e-01 9.644084e-01

1961 6.694915e-01 9.644324e-01

1962 6.695365e-01 9.644563e-01

1963 6.695810e-01 9.644803e-01

1964 6.696250e-01 9.645042e-01

1965 6.696687e-01 9.645281e-01

1966 6.697119e-01 9.645520e-01

1967 6.697547e-01 9.645758e-01

1968 6.697971e-01 9.645997e-01

1969 6.698392e-01 9.646235e-01

1970 6.698810e-01 9.646473e-01

1971 6.699225e-01 9.646711e-01

1972 6.699637e-01 9.646949e-01

1973 6.700046e-01 9.647186e-01

1974 6.700453e-01 9.647424e-01

1975 6.700857e-01 9.647661e-01

1976 6.701259e-01 9.647898e-01

1977 6.701659e-01 9.648134e-01

1978 6.702057e-01 9.648371e-01

1979 6.702454e-01 9.648607e-01

1980 6.702848e-01 9.648843e-01

1981 6.703242e-01 9.649079e-01

1982 6.705697e-01 9.649469e-01

1983 6.708221e-01 9.649851e-01

1984 6.710815e-01 9.650224e-01

1985 6.713480e-01 9.650589e-01

1986 6.716215e-01 9.650947e-01

1987 6.719021e-01 9.651297e-01

1988 6.721898e-01 9.651641e-01

1989 6.724847e-01 9.651978e-01

1990 6.727866e-01 9.652308e-01

1991 6.730956e-01 9.652633e-01

1992 6.734116e-01 9.652951e-01

1993 6.737346e-01 9.653265e-01

1994 6.740646e-01 9.653573e-01

1995 6.744013e-01 9.653876e-01

1996 6.747448e-01 9.654174e-01

1997 6.750949e-01 9.654467e-01

1998 6.754515e-01 9.654756e-01

1999 6.758145e-01 9.655042e-01

2000 6.761837e-01 9.655323e-01

2001 6.765589e-01 9.655600e-01

2002 6.769399e-01 9.655873e-01

2003 6.773266e-01 9.656143e-01

2004 6.777188e-01 9.656410e-01

2005 6.781161e-01 9.656674e-01

2006 6.785183e-01 9.656934e-01

2007 6.789252e-01 9.657192e-01

2008 6.793366e-01 9.657447e-01

2009 6.797520e-01 9.657699e-01

2010 6.801713e-01 9.657948e-01

2011 6.805941e-01 9.658196e-01

2012 6.810201e-01 9.658440e-01

2013 6.814489e-01 9.658683e-01

2014 6.818802e-01 9.658923e-01

2015 6.823137e-01 9.659162e-01

2016 6.827491e-01 9.659398e-01

2017 6.831858e-01 9.659633e-01

2018 6.836237e-01 9.659866e-01

2019 6.840623e-01 9.660097e-01

2020 6.845012e-01 9.660326e-01

2021 6.849402e-01 9.660554e-01

2022 6.853787e-01 9.660780e-01

2023 6.858165e-01 9.661005e-01

2024 6.862532e-01 9.661228e-01

2025 6.866884e-01 9.661450e-01

2026 6.871218e-01 9.661671e-01

2027 6.875531e-01 9.661891e-01

2028 6.879818e-01 9.662109e-01

2029 6.884077e-01 9.662326e-01

2030 6.888304e-01 9.662543e-01

2031 6.892497e-01 9.662758e-01

2032 6.896652e-01 9.662972e-01

2033 6.900767e-01 9.663185e-01

2034 6.904839e-01 9.663398e-01

2035 6.908864e-01 9.663609e-01

2036 6.912842e-01 9.663820e-01

2037 6.916769e-01 9.664029e-01

2038 6.920643e-01 9.664238e-01

2039 6.924463e-01 9.664447e-01

2040 6.928226e-01 9.664654e-01

2041 6.931931e-01 9.664861e-01

2042 6.935576e-01 9.665067e-01

2043 6.939160e-01 9.665273e-01

2044 6.942682e-01 9.665478e-01

2045 6.946140e-01 9.665682e-01

2046 6.949534e-01 9.665886e-01

2047 6.952863e-01 9.666090e-01

2048 6.956126e-01 9.666292e-01

2049 6.959322e-01 9.666495e-01

2050 6.962452e-01 9.666697e-01

2051 6.965515e-01 9.666898e-01

2052 6.968512e-01 9.667099e-01

2053 6.971441e-01 9.667299e-01

2054 6.974303e-01 9.667500e-01

2055 6.977099e-01 9.667699e-01

2056 6.979828e-01 9.667899e-01

2057 6.982492e-01 9.668098e-01

2058 6.985090e-01 9.668297e-01

2059 6.987624e-01 9.668495e-01

2060 6.990093e-01 9.668693e-01

2061 6.992499e-01 9.668891e-01

2062 6.994843e-01 9.669088e-01

2063 6.997125e-01 9.669286e-01

2064 6.999347e-01 9.669483e-01

2065 7.001509e-01 9.669679e-01

2066 7.003612e-01 9.669876e-01

2067 7.005658e-01 9.670072e-01

2068 7.007647e-01 9.670268e-01

2069 7.009581e-01 9.670464e-01

2070 7.011460e-01 9.670659e-01

2071 7.013287e-01 9.670855e-01

2072 7.015062e-01 9.671050e-01

2073 7.016786e-01 9.671245e-01

2074 7.018461e-01 9.671440e-01

2075 7.020087e-01 9.671635e-01

2076 7.021667e-01 9.671829e-01

2077 7.023201e-01 9.672024e-01

2078 7.024690e-01 9.672218e-01

2079 7.026137e-01 9.672412e-01

2080 7.027541e-01 9.672606e-01

2081 7.028904e-01 9.672800e-01

2082 7.030227e-01 9.672994e-01

2083 7.031512e-01 9.673187e-01

2084 7.032760e-01 9.673381e-01

2085 7.033971e-01 9.673574e-01

2086 7.035147e-01 9.673767e-01

2087 7.036289e-01 9.673961e-01

2088 7.037398e-01 9.674154e-01

2089 7.038475e-01 9.674347e-01

2090 7.039521e-01 9.674540e-01

2091 7.040538e-01 9.674733e-01

2092 7.041526e-01 9.674925e-01

2093 7.042485e-01 9.675118e-01

2094 7.043418e-01 9.675311e-01

2095 7.044325e-01 9.675503e-01

2096 7.045207e-01 9.675696e-01

2097 7.046064e-01 9.675888e-01

2098 7.046898e-01 9.676081e-01

2099 7.047710e-01 9.676273e-01

2100 7.048500e-01 9.676465e-01

2101 7.049269e-01 9.676657e-01

2102 7.050018e-01 9.676850e-01

2103 7.050747e-01 9.677042e-01

2104 7.051458e-01 9.677234e-01

2105 7.052151e-01 9.677426e-01

2106 7.052826e-01 9.677618e-01

2107 7.053485e-01 9.677810e-01

2108 7.054128e-01 9.678002e-01

2109 7.054755e-01 9.678193e-01

2110 7.055368e-01 9.678385e-01

2111 7.055966e-01 9.678577e-01

2112 7.056551e-01 9.678769e-01

2113 7.057122e-01 9.678960e-01

2114 7.057681e-01 9.679152e-01

2115 7.058228e-01 9.679344e-01

2116 7.058763e-01 9.679535e-01

2117 7.059287e-01 9.679727e-01

2118 7.059801e-01 9.679918e-01

2119 7.060304e-01 9.680110e-01

2120 7.060797e-01 9.680301e-01

2121 7.061281e-01 9.680492e-01

2122 7.061756e-01 9.680684e-01

2123 7.062222e-01 9.680875e-01

2124 7.062680e-01 9.681066e-01

2125 7.063130e-01 9.681257e-01

2126 7.063573e-01 9.681448e-01

2127 7.064008e-01 9.681640e-01

2128 7.064436e-01 9.681831e-01

2129 7.064858e-01 9.682022e-01

2130 7.065273e-01 9.682213e-01

2131 7.065683e-01 9.682404e-01

2132 7.066086e-01 9.682594e-01

2133 7.066484e-01 9.682785e-01

2134 7.066877e-01 9.682976e-01

2135 7.067265e-01 9.683167e-01

2136 7.067648e-01 9.683357e-01

2137 7.068027e-01 9.683548e-01

2138 7.068401e-01 9.683738e-01

2139 7.068771e-01 9.683929e-01

2140 7.069138e-01 9.684119e-01

2141 7.069500e-01 9.684310e-01

2142 7.069859e-01 9.684500e-01

2143 7.070215e-01 9.684690e-01

2144 7.070568e-01 9.684880e-01

2145 7.070918e-01 9.685070e-01

2146 7.071264e-01 9.685260e-01

2147 7.071609e-01 9.685450e-01

2148 7.071951e-01 9.685639e-01

2149 7.072290e-01 9.685829e-01

2150 7.072627e-01 9.686019e-01

2151 7.072962e-01 9.686208e-01

2152 7.073296e-01 9.686397e-01

2153 7.073627e-01 9.686587e-01

2154 7.073957e-01 9.686776e-01

2155 7.074285e-01 9.686965e-01

2156 7.074612e-01 9.687153e-01

2157 7.074938e-01 9.687342e-01

2158 7.075262e-01 9.687531e-01

2159 7.075585e-01 9.687719e-01

2160 7.075907e-01 9.687907e-01

2161 7.076228e-01 9.688096e-01

2162 7.079029e-01 9.688326e-01

2163 7.081890e-01 9.688553e-01

2164 7.084813e-01 9.688777e-01

2165 7.087796e-01 9.688998e-01

2166 7.090839e-01 9.689215e-01

2167 7.093940e-01 9.689430e-01

2168 7.097100e-01 9.689642e-01

2169 7.100317e-01 9.689851e-01

2170 7.103589e-01 9.690057e-01

2171 7.106916e-01 9.690261e-01

2172 7.110297e-01 9.690463e-01

2173 7.113728e-01 9.690662e-01

2174 7.117210e-01 9.690859e-01

2175 7.120740e-01 9.691054e-01

2176 7.124317e-01 9.691247e-01

2177 7.127937e-01 9.691439e-01

2178 7.131600e-01 9.691628e-01

2179 7.135303e-01 9.691816e-01

2180 7.139043e-01 9.692002e-01

2181 7.142818e-01 9.692186e-01

2182 7.146626e-01 9.692369e-01

2183 7.150464e-01 9.692550e-01

2184 7.154329e-01 9.692730e-01

2185 7.158218e-01 9.692909e-01

2186 7.162129e-01 9.693087e-01

2187 7.166058e-01 9.693263e-01

2188 7.170003e-01 9.693438e-01

2189 7.173960e-01 9.693612e-01

2190 7.177927e-01 9.693784e-01

2191 7.181900e-01 9.693956e-01

2192 7.185877e-01 9.694127e-01

2193 7.189853e-01 9.694297e-01

2194 7.193827e-01 9.694466e-01

2195 7.197795e-01 9.694634e-01

2196 7.201753e-01 9.694801e-01

2197 7.205699e-01 9.694968e-01

2198 7.209631e-01 9.695133e-01

2199 7.213544e-01 9.695298e-01

2200 7.217436e-01 9.695463e-01

2201 7.221304e-01 9.695626e-01

2202 7.225146e-01 9.695789e-01

2203 7.228959e-01 9.695952e-01

2204 7.232740e-01 9.696114e-01

2205 7.236487e-01 9.696275e-01

2206 7.240197e-01 9.696436e-01

2207 7.243868e-01 9.696596e-01

2208 7.247499e-01 9.696756e-01

2209 7.251086e-01 9.696915e-01

2210 7.254629e-01 9.697074e-01

2211 7.258125e-01 9.697232e-01

2212 7.261572e-01 9.697390e-01

2213 7.264969e-01 9.697548e-01

2214 7.268316e-01 9.697705e-01

2215 7.271609e-01 9.697862e-01

2216 7.274849e-01 9.698019e-01

2217 7.278034e-01 9.698175e-01

2218 7.281163e-01 9.698331e-01

2219 7.284236e-01 9.698487e-01

2220 7.287251e-01 9.698642e-01

2221 7.290209e-01 9.698797e-01

2222 7.293108e-01 9.698952e-01

2223 7.295949e-01 9.699107e-01

2224 7.298732e-01 9.699262e-01

2225 7.301455e-01 9.699416e-01

2226 7.304119e-01 9.699570e-01

2227 7.306725e-01 9.699724e-01

2228 7.309272e-01 9.699877e-01

2229 7.311760e-01 9.700031e-01

2230 7.314191e-01 9.700184e-01

2231 7.316563e-01 9.700337e-01

2232 7.318879e-01 9.700491e-01

2233 7.321138e-01 9.700643e-01

2234 7.323340e-01 9.700796e-01

2235 7.325488e-01 9.700949e-01

2236 7.327580e-01 9.701102e-01

2237 7.329619e-01 9.701254e-01

2238 7.331604e-01 9.701406e-01

2239 7.333538e-01 9.701559e-01

2240 7.335419e-01 9.701711e-01

2241 7.337251e-01 9.701863e-01

2242 7.339032e-01 9.702015e-01

2243 7.340765e-01 9.702167e-01

2244 7.342451e-01 9.702319e-01

2245 7.344089e-01 9.702471e-01

2246 7.345683e-01 9.702623e-01

2247 7.347231e-01 9.702774e-01

2248 7.348736e-01 9.702926e-01

2249 7.350198e-01 9.703078e-01

2250 7.351619e-01 9.703229e-01

2251 7.353000e-01 9.703381e-01

2252 7.354340e-01 9.703533e-01

2253 7.355643e-01 9.703684e-01

2254 7.356908e-01 9.703836e-01

2255 7.358137e-01 9.703987e-01

2256 7.359330e-01 9.704139e-01

2257 7.360489e-01 9.704290e-01

2258 7.361615e-01 9.704442e-01

2259 7.362708e-01 9.704594e-01

2260 7.363770e-01 9.704745e-01

2261 7.364801e-01 9.704897e-01

2262 7.365803e-01 9.705048e-01

2263 7.366776e-01 9.705200e-01

2264 7.367721e-01 9.705351e-01

2265 7.368639e-01 9.705503e-01

2266 7.369532e-01 9.705654e-01

2267 7.370399e-01 9.705806e-01

2268 7.371241e-01 9.705958e-01

2269 7.372060e-01 9.706109e-01

2270 7.372857e-01 9.706261e-01

2271 7.373631e-01 9.706413e-01

2272 7.374384e-01 9.706564e-01

2273 7.375117e-01 9.706716e-01

2274 7.375829e-01 9.706868e-01

2275 7.376523e-01 9.707020e-01

2276 7.377198e-01 9.707171e-01

2277 7.377855e-01 9.707323e-01

2278 7.378495e-01 9.707475e-01

2279 7.379119e-01 9.707627e-01

2280 7.379726e-01 9.707779e-01

2281 7.380318e-01 9.707931e-01

2282 7.380896e-01 9.708083e-01

2283 7.381459e-01 9.708235e-01

2284 7.382008e-01 9.708387e-01

2285 7.382544e-01 9.708539e-01

2286 7.383067e-01 9.708691e-01

2287 7.383579e-01 9.708843e-01

2288 7.384078e-01 9.708995e-01

2289 7.384566e-01 9.709148e-01

2290 7.385043e-01 9.709300e-01

2291 7.385509e-01 9.709452e-01

2292 7.385966e-01 9.709604e-01

2293 7.386413e-01 9.709757e-01

2294 7.386850e-01 9.709909e-01

2295 7.387279e-01 9.710061e-01

2296 7.387699e-01 9.710214e-01

2297 7.388111e-01 9.710366e-01

2298 7.388515e-01 9.710519e-01

2299 7.388911e-01 9.710671e-01

2300 7.389300e-01 9.710824e-01

2301 7.389682e-01 9.710976e-01

2302 7.390058e-01 9.711129e-01

2303 7.390427e-01 9.711281e-01

2304 7.390790e-01 9.711434e-01

2305 7.391147e-01 9.711586e-01

2306 7.391499e-01 9.711739e-01

2307 7.391845e-01 9.711892e-01

2308 7.392186e-01 9.712044e-01

2309 7.392522e-01 9.712197e-01

2310 7.392854e-01 9.712349e-01

2311 7.393181e-01 9.712502e-01

2312 7.393504e-01 9.712654e-01

2313 7.393822e-01 9.712807e-01

2314 7.394137e-01 9.712960e-01

2315 7.394448e-01 9.713112e-01

2316 7.394756e-01 9.713265e-01

2317 7.395060e-01 9.713417e-01

2318 7.395361e-01 9.713570e-01

2319 7.395659e-01 9.713722e-01

2320 7.395954e-01 9.713875e-01

2321 7.396246e-01 9.714027e-01

2322 7.396536e-01 9.714179e-01

2323 7.396823e-01 9.714332e-01

2324 7.397108e-01 9.714484e-01

2325 7.397391e-01 9.714636e-01

2326 7.397671e-01 9.714789e-01

2327 7.397950e-01 9.714941e-01

2328 7.398227e-01 9.715093e-01

2329 7.398501e-01 9.715245e-01

2330 7.398775e-01 9.715397e-01

2331 7.399046e-01 9.715549e-01

2332 7.399316e-01 9.715700e-01

2333 7.399585e-01 9.715852e-01

2334 7.399852e-01 9.716004e-01

2335 7.400118e-01 9.716155e-01

2336 7.400383e-01 9.716307e-01

2337 7.400647e-01 9.716458e-01

2338 7.400910e-01 9.716609e-01

2339 7.401172e-01 9.716760e-01

2340 7.401433e-01 9.716911e-01

2341 7.401693e-01 9.717062e-01

2342 7.404698e-01 9.717213e-01

2343 7.407751e-01 9.717363e-01

2344 7.410850e-01 9.717511e-01

2345 7.413995e-01 9.717658e-01

2346 7.417184e-01 9.717804e-01

2347 7.420416e-01 9.717948e-01

2348 7.423688e-01 9.718091e-01

2349 7.427000e-01 9.718233e-01

2350 7.430348e-01 9.718374e-01

2351 7.433733e-01 9.718514e-01

2352 7.437150e-01 9.718652e-01

2353 7.440599e-01 9.718790e-01

2354 7.444077e-01 9.718927e-01

2355 7.447582e-01 9.719062e-01

2356 7.451112e-01 9.719197e-01

2357 7.454664e-01 9.719332e-01

2358 7.458235e-01 9.719465e-01

2359 7.461824e-01 9.719598e-01

2360 7.465428e-01 9.719729e-01

2361 7.469043e-01 9.719861e-01

2362 7.472669e-01 9.719991e-01

2363 7.476301e-01 9.720121e-01

2364 7.479937e-01 9.720251e-01

2365 7.483575e-01 9.720379e-01

2366 7.487212e-01 9.720507e-01

2367 7.490845e-01 9.720635e-01

2368 7.494472e-01 9.720762e-01

2369 7.498090e-01 9.720889e-01

2370 7.501696e-01 9.721015e-01

2371 7.505289e-01 9.721141e-01

2372 7.508864e-01 9.721267e-01

2373 7.512420e-01 9.721392e-01

2374 7.515955e-01 9.721516e-01

2375 7.519466e-01 9.721641e-01

2376 7.522951e-01 9.721765e-01

2377 7.526408e-01 9.721888e-01

2378 7.529834e-01 9.722012e-01

2379 7.533228e-01 9.722135e-01

2380 7.536588e-01 9.722257e-01

2381 7.539911e-01 9.722380e-01

2382 7.543196e-01 9.722502e-01

2383 7.546441e-01 9.722624e-01

2384 7.549646e-01 9.722746e-01

2385 7.552807e-01 9.722868e-01

2386 7.555925e-01 9.722989e-01

2387 7.558997e-01 9.723110e-01

2388 7.562023e-01 9.723231e-01

2389 7.565002e-01 9.723352e-01

2390 7.567932e-01 9.723473e-01

2391 7.570813e-01 9.723593e-01

2392 7.573644e-01 9.723714e-01

2393 7.576424e-01 9.723834e-01

2394 7.579153e-01 9.723954e-01

2395 7.581831e-01 9.724074e-01

2396 7.584457e-01 9.724194e-01

2397 7.587031e-01 9.724314e-01

2398 7.589552e-01 9.724434e-01

2399 7.592021e-01 9.724554e-01

2400 7.594438e-01 9.724673e-01

2401 7.596802e-01 9.724793e-01

2402 7.599114e-01 9.724912e-01

2403 7.601374e-01 9.725032e-01

2404 7.603583e-01 9.725151e-01

2405 7.605740e-01 9.725271e-01

2406 7.607847e-01 9.725390e-01

2407 7.609903e-01 9.725509e-01

2408 7.611909e-01 9.725629e-01

2409 7.613866e-01 9.725748e-01

2410 7.615774e-01 9.725867e-01

2411 7.617634e-01 9.725986e-01

2412 7.619447e-01 9.726105e-01

2413 7.621212e-01 9.726225e-01

2414 7.622932e-01 9.726344e-01

2415 7.624607e-01 9.726463e-01

2416 7.626238e-01 9.726582e-01

2417 7.627825e-01 9.726702e-01

2418 7.629369e-01 9.726821e-01

2419 7.630871e-01 9.726940e-01

2420 7.632332e-01 9.727059e-01

2421 7.633754e-01 9.727179e-01

2422 7.635136e-01 9.727298e-01

2423 7.636479e-01 9.727417e-01

2424 7.637786e-01 9.727537e-01

2425 7.639055e-01 9.727656e-01

2426 7.640289e-01 9.727776e-01

2427 7.641488e-01 9.727895e-01

2428 7.642653e-01 9.728015e-01

2429 7.643786e-01 9.728134e-01

2430 7.644886e-01 9.728254e-01

2431 7.645954e-01 9.728374e-01

2432 7.646993e-01 9.728493e-01

2433 7.648001e-01 9.728613e-01

2434 7.648981e-01 9.728733e-01

2435 7.649933e-01 9.728853e-01

2436 7.650858e-01 9.728973e-01

2437 7.651756e-01 9.729093e-01

2438 7.652629e-01 9.729213e-01

2439 7.653478e-01 9.729333e-01

2440 7.654302e-01 9.729453e-01

2441 7.655103e-01 9.729573e-01

2442 7.655881e-01 9.729694e-01

2443 7.656638e-01 9.729814e-01

2444 7.657373e-01 9.729934e-01

2445 7.658088e-01 9.730055e-01

2446 7.658783e-01 9.730175e-01

2447 7.659459e-01 9.730296e-01

2448 7.660116e-01 9.730417e-01

2449 7.660756e-01 9.730537e-01

2450 7.661378e-01 9.730658e-01

2451 7.661983e-01 9.730779e-01

2452 7.662572e-01 9.730900e-01

2453 7.663146e-01 9.731021e-01

2454 7.663705e-01 9.731142e-01

2455 7.664249e-01 9.731263e-01

2456 7.664779e-01 9.731385e-01

2457 7.665295e-01 9.731506e-01

2458 7.665799e-01 9.731627e-01

2459 7.666290e-01 9.731749e-01

2460 7.666768e-01 9.731870e-01

2461 7.667235e-01 9.731992e-01

2462 7.667691e-01 9.732113e-01

2463 7.668136e-01 9.732235e-01

2464 7.668570e-01 9.732357e-01

2465 7.668994e-01 9.732478e-01

2466 7.669409e-01 9.732600e-01

2467 7.669814e-01 9.732722e-01

2468 7.670210e-01 9.732844e-01

2469 7.670598e-01 9.732966e-01

2470 7.670977e-01 9.733088e-01

2471 7.671348e-01 9.733210e-01

2472 7.671711e-01 9.733332e-01

2473 7.672067e-01 9.733454e-01

2474 7.672416e-01 9.733577e-01

2475 7.672758e-01 9.733699e-01

2476 7.673094e-01 9.733821e-01

2477 7.673423e-01 9.733944e-01

2478 7.673746e-01 9.734066e-01

2479 7.674063e-01 9.734189e-01

2480 7.674375e-01 9.734311e-01

2481 7.674681e-01 9.734434e-01

2482 7.674982e-01 9.734556e-01

2483 7.675278e-01 9.734679e-01

2484 7.675569e-01 9.734801e-01

2485 7.675856e-01 9.734924e-01

2486 7.676138e-01 9.735047e-01

2487 7.676417e-01 9.735169e-01

2488 7.676691e-01 9.735292e-01

2489 7.676961e-01 9.735415e-01

2490 7.677228e-01 9.735537e-01

2491 7.677491e-01 9.735660e-01

2492 7.677750e-01 9.735783e-01

2493 7.678007e-01 9.735906e-01

2494 7.678260e-01 9.736028e-01

2495 7.678510e-01 9.736151e-01

2496 7.678757e-01 9.736274e-01

2497 7.679002e-01 9.736396e-01

2498 7.679244e-01 9.736519e-01

2499 7.679484e-01 9.736642e-01

2500 7.679721e-01 9.736764e-01

2501 7.679956e-01 9.736887e-01

2502 7.680188e-01 9.737009e-01

2503 7.680419e-01 9.737132e-01

2504 7.680647e-01 9.737254e-01

2505 7.680874e-01 9.737377e-01

2506 7.681098e-01 9.737499e-01

2507 7.681321e-01 9.737621e-01

2508 7.681542e-01 9.737744e-01

2509 7.681762e-01 9.737866e-01

2510 7.681980e-01 9.737988e-01

2511 7.682196e-01 9.738110e-01

2512 7.682411e-01 9.738232e-01

2513 7.682625e-01 9.738353e-01

2514 7.682837e-01 9.738475e-01

2515 7.683048e-01 9.738597e-01

2516 7.683257e-01 9.738718e-01

2517 7.683466e-01 9.738839e-01

2518 7.683673e-01 9.738961e-01

2519 7.683879e-01 9.739082e-01

2520 7.684084e-01 9.739203e-01

2521 7.684288e-01 9.739324e-01

2522 7.687363e-01 9.739430e-01

2523 7.690470e-01 9.739536e-01

2524 7.693608e-01 9.739641e-01

2525 7.696775e-01 9.739745e-01

2526 7.699970e-01 9.739849e-01

2527 7.703189e-01 9.739953e-01

2528 7.706432e-01 9.740055e-01

2529 7.709696e-01 9.740158e-01

2530 7.712979e-01 9.740259e-01

2531 7.716279e-01 9.740361e-01

2532 7.719594e-01 9.740462e-01

2533 7.722922e-01 9.740562e-01

2534 7.726260e-01 9.740662e-01

2535 7.729606e-01 9.740762e-01

2536 7.732958e-01 9.740861e-01

2537 7.736314e-01 9.740960e-01

2538 7.739671e-01 9.741059e-01

2539 7.743027e-01 9.741157e-01

2540 7.746379e-01 9.741255e-01

2541 7.749726e-01 9.741352e-01

2542 7.753064e-01 9.741450e-01

2543 7.756393e-01 9.741547e-01

2544 7.759709e-01 9.741644e-01

2545 7.763011e-01 9.741740e-01

2546 7.766296e-01 9.741837e-01

2547 7.769562e-01 9.741933e-01

2548 7.772807e-01 9.742029e-01

2549 7.776029e-01 9.742125e-01

2550 7.779227e-01 9.742221e-01

2551 7.782398e-01 9.742316e-01

2552 7.785540e-01 9.742411e-01

2553 7.788652e-01 9.742507e-01

2554 7.791733e-01 9.742602e-01

2555 7.794780e-01 9.742697e-01

2556 7.797792e-01 9.742792e-01

2557 7.800768e-01 9.742886e-01

2558 7.803707e-01 9.742981e-01

2559 7.806606e-01 9.743075e-01

2560 7.809466e-01 9.743170e-01

2561 7.812285e-01 9.743264e-01

2562 7.815061e-01 9.743359e-01

2563 7.817795e-01 9.743453e-01

2564 7.820486e-01 9.743547e-01

2565 7.823132e-01 9.743641e-01

2566 7.825733e-01 9.743735e-01

2567 7.828288e-01 9.743829e-01

2568 7.830798e-01 9.743923e-01

2569 7.833261e-01 9.744017e-01

2570 7.835677e-01 9.744111e-01

2571 7.838047e-01 9.744205e-01

2572 7.840370e-01 9.744299e-01

2573 7.842646e-01 9.744393e-01

2574 7.844874e-01 9.744487e-01

2575 7.847056e-01 9.744581e-01

2576 7.849191e-01 9.744674e-01

2577 7.851279e-01 9.744768e-01

2578 7.853321e-01 9.744862e-01

2579 7.855316e-01 9.744956e-01

2580 7.857266e-01 9.745050e-01

2581 7.859170e-01 9.745144e-01

2582 7.861029e-01 9.745238e-01

2583 7.862844e-01 9.745332e-01

2584 7.864615e-01 9.745426e-01

2585 7.866342e-01 9.745520e-01

2586 7.868026e-01 9.745614e-01

2587 7.869668e-01 9.745709e-01

2588 7.871269e-01 9.745803e-01

2589 7.872828e-01 9.745897e-01

2590 7.874347e-01 9.745991e-01

2591 7.875827e-01 9.746086e-01

2592 7.877267e-01 9.746180e-01

2593 7.878670e-01 9.746274e-01

2594 7.880035e-01 9.746369e-01

2595 7.881363e-01 9.746464e-01

2596 7.882655e-01 9.746558e-01

2597 7.883913e-01 9.746653e-01

2598 7.885136e-01 9.746748e-01

2599 7.886325e-01 9.746842e-01

2600 7.887481e-01 9.746937e-01

2601 7.888606e-01 9.747032e-01

2602 7.889699e-01 9.747127e-01

2603 7.890762e-01 9.747222e-01

2604 7.891795e-01 9.747317e-01

2605 7.892799e-01 9.747413e-01

2606 7.893774e-01 9.747508e-01

2607 7.894723e-01 9.747603e-01

2608 7.895644e-01 9.747699e-01

2609 7.896540e-01 9.747794e-01

2610 7.897410e-01 9.747890e-01

2611 7.898255e-01 9.747985e-01

2612 7.899077e-01 9.748081e-01

2613 7.899876e-01 9.748177e-01

2614 7.900652e-01 9.748273e-01

2615 7.901406e-01 9.748369e-01

2616 7.902138e-01 9.748465e-01

2617 7.902851e-01 9.748561e-01

2618 7.903543e-01 9.748657e-01

2619 7.904216e-01 9.748754e-01

2620 7.904870e-01 9.748850e-01

2621 7.905506e-01 9.748946e-01

2622 7.906124e-01 9.749043e-01

2623 7.906726e-01 9.749140e-01

2624 7.907310e-01 9.749236e-01

2625 7.907879e-01 9.749333e-01

2626 7.908433e-01 9.749430e-01

2627 7.908971e-01 9.749527e-01

2628 7.909495e-01 9.749624e-01

2629 7.910005e-01 9.749721e-01

2630 7.910501e-01 9.749818e-01

2631 7.910985e-01 9.749915e-01

2632 7.911455e-01 9.750012e-01

2633 7.911914e-01 9.750110e-01

2634 7.912361e-01 9.750207e-01

2635 7.912796e-01 9.750305e-01

2636 7.913220e-01 9.750402e-01

2637 7.913634e-01 9.750500e-01

2638 7.914038e-01 9.750598e-01

2639 7.914431e-01 9.750696e-01

2640 7.914815e-01 9.750793e-01

2641 7.915190e-01 9.750891e-01

2642 7.915556e-01 9.750989e-01

2643 7.915914e-01 9.751087e-01

2644 7.916263e-01 9.751185e-01

2645 7.916604e-01 9.751284e-01

2646 7.916937e-01 9.751382e-01

2647 7.917263e-01 9.751480e-01

2648 7.917582e-01 9.751578e-01

2649 7.917894e-01 9.751677e-01

2650 7.918200e-01 9.751775e-01

2651 7.918499e-01 9.751874e-01

2652 7.918792e-01 9.751972e-01

2653 7.919079e-01 9.752071e-01

2654 7.919360e-01 9.752169e-01

2655 7.919636e-01 9.752268e-01

2656 7.919906e-01 9.752367e-01

2657 7.920171e-01 9.752465e-01

2658 7.920432e-01 9.752564e-01

2659 7.920687e-01 9.752663e-01

2660 7.920938e-01 9.752762e-01

2661 7.921185e-01 9.752860e-01

2662 7.921428e-01 9.752959e-01

2663 7.921666e-01 9.753058e-01

2664 7.921900e-01 9.753157e-01

2665 7.922131e-01 9.753256e-01

2666 7.922358e-01 9.753355e-01

2667 7.922582e-01 9.753453e-01

2668 7.922802e-01 9.753552e-01

2669 7.923019e-01 9.753651e-01

2670 7.923232e-01 9.753750e-01

2671 7.923443e-01 9.753849e-01

2672 7.923651e-01 9.753947e-01

2673 7.923856e-01 9.754046e-01

2674 7.924058e-01 9.754145e-01

2675 7.924257e-01 9.754244e-01

2676 7.924454e-01 9.754342e-01

2677 7.924649e-01 9.754441e-01

2678 7.924841e-01 9.754540e-01

2679 7.925031e-01 9.754638e-01

2680 7.925219e-01 9.754737e-01

2681 7.925404e-01 9.754835e-01

2682 7.925588e-01 9.754933e-01

2683 7.925769e-01 9.755032e-01

2684 7.925949e-01 9.755130e-01

2685 7.926126e-01 9.755228e-01

2686 7.926302e-01 9.755326e-01

2687 7.926476e-01 9.755424e-01

2688 7.926648e-01 9.755522e-01

2689 7.926819e-01 9.755620e-01

2690 7.926987e-01 9.755717e-01

2691 7.927155e-01 9.755815e-01

2692 7.927320e-01 9.755912e-01

2693 7.927484e-01 9.756009e-01

2694 7.927646e-01 9.756106e-01

2695 7.927807e-01 9.756203e-01

2696 7.927967e-01 9.756300e-01

2697 7.928125e-01 9.756397e-01

2698 7.928281e-01 9.756493e-01

2699 7.928436e-01 9.756589e-01

2700 7.928589e-01 9.756686e-01

2701 7.928742e-01 9.756781e-01

2702 7.931776e-01 9.756860e-01

2703 7.934829e-01 9.756938e-01

2704 7.937897e-01 9.757016e-01

2705 7.940978e-01 9.757094e-01

2706 7.944071e-01 9.757172e-01

2707 7.947173e-01 9.757249e-01

2708 7.950284e-01 9.757326e-01

2709 7.953399e-01 9.757403e-01

2710 7.956519e-01 9.757479e-01

2711 7.959640e-01 9.757556e-01

2712 7.962761e-01 9.757632e-01

2713 7.965880e-01 9.757708e-01

2714 7.968994e-01 9.757784e-01

2715 7.972101e-01 9.757860e-01

2716 7.975200e-01 9.757935e-01

2717 7.978289e-01 9.758011e-01

2718 7.981366e-01 9.758086e-01

2719 7.984428e-01 9.758161e-01

2720 7.987474e-01 9.758236e-01

2721 7.990502e-01 9.758311e-01

2722 7.993511e-01 9.758386e-01

2723 7.996498e-01 9.758461e-01

2724 7.999463e-01 9.758536e-01

2725 8.002402e-01 9.758610e-01

2726 8.005316e-01 9.758685e-01

2727 8.008201e-01 9.758760e-01

2728 8.011058e-01 9.758834e-01

2729 8.013884e-01 9.758908e-01

2730 8.016678e-01 9.758983e-01

2731 8.019439e-01 9.759057e-01

2732 8.022166e-01 9.759131e-01

2733 8.024858e-01 9.759206e-01

2734 8.027513e-01 9.759280e-01

2735 8.030132e-01 9.759354e-01

2736 8.032712e-01 9.759428e-01

2737 8.035254e-01 9.759502e-01

2738 8.037756e-01 9.759577e-01

2739 8.040218e-01 9.759651e-01

2740 8.042639e-01 9.759725e-01

2741 8.045020e-01 9.759799e-01

2742 8.047358e-01 9.759873e-01

2743 8.049655e-01 9.759948e-01

2744 8.051910e-01 9.760022e-01

2745 8.054122e-01 9.760096e-01

2746 8.056291e-01 9.760170e-01

2747 8.058418e-01 9.760245e-01

2748 8.060503e-01 9.760319e-01

2749 8.062544e-01 9.760393e-01

2750 8.064544e-01 9.760468e-01

2751 8.066500e-01 9.760542e-01

2752 8.068415e-01 9.760617e-01

2753 8.070287e-01 9.760691e-01

2754 8.072118e-01 9.760766e-01

2755 8.073908e-01 9.760840e-01

2756 8.075656e-01 9.760915e-01

2757 8.077364e-01 9.760990e-01

2758 8.079031e-01 9.761064e-01

2759 8.080659e-01 9.761139e-01

2760 8.082247e-01 9.761214e-01

2761 8.083797e-01 9.761289e-01

2762 8.085309e-01 9.761364e-01

2763 8.086782e-01 9.761439e-01

2764 8.088219e-01 9.761514e-01

2765 8.089619e-01 9.761589e-01

2766 8.090984e-01 9.761665e-01

2767 8.092313e-01 9.761740e-01

2768 8.093607e-01 9.761815e-01

2769 8.094868e-01 9.761891e-01

2770 8.096095e-01 9.761966e-01

2771 8.097290e-01 9.762042e-01

2772 8.098453e-01 9.762117e-01

2773 8.099584e-01 9.762193e-01

2774 8.100685e-01 9.762269e-01

2775 8.101757e-01 9.762345e-01

2776 8.102798e-01 9.762421e-01

2777 8.103812e-01 9.762497e-01

2778 8.104797e-01 9.762573e-01

2779 8.105755e-01 9.762649e-01

2780 8.106687e-01 9.762725e-01

2781 8.107593e-01 9.762802e-01

2782 8.108474e-01 9.762878e-01

2783 8.109330e-01 9.762955e-01

2784 8.110162e-01 9.763031e-01

2785 8.110971e-01 9.763108e-01

2786 8.111757e-01 9.763185e-01

2787 8.112521e-01 9.763262e-01

2788 8.113264e-01 9.763338e-01

2789 8.113986e-01 9.763415e-01

2790 8.114688e-01 9.763492e-01

2791 8.115370e-01 9.763570e-01

2792 8.116032e-01 9.763647e-01

2793 8.116677e-01 9.763724e-01

2794 8.117303e-01 9.763801e-01

2795 8.117912e-01 9.763879e-01

2796 8.118504e-01 9.763956e-01

2797 8.119079e-01 9.764034e-01

2798 8.119639e-01 9.764112e-01

2799 8.120183e-01 9.764190e-01

2800 8.120712e-01 9.764267e-01

2801 8.121227e-01 9.764345e-01

2802 8.121727e-01 9.764423e-01

2803 8.122214e-01 9.764501e-01

2804 8.122688e-01 9.764579e-01

2805 8.123149e-01 9.764658e-01

2806 8.123597e-01 9.764736e-01

2807 8.124034e-01 9.764814e-01

2808 8.124459e-01 9.764893e-01

2809 8.124873e-01 9.764971e-01

2810 8.125276e-01 9.765050e-01

2811 8.125668e-01 9.765128e-01

2812 8.126051e-01 9.765207e-01

2813 8.126423e-01 9.765286e-01

2814 8.126786e-01 9.765364e-01

2815 8.127140e-01 9.765443e-01

2816 8.127485e-01 9.765522e-01

2817 8.127822e-01 9.765601e-01

2818 8.128150e-01 9.765680e-01

2819 8.128470e-01 9.765759e-01

2820 8.128783e-01 9.765838e-01

2821 8.129088e-01 9.765917e-01

2822 8.129385e-01 9.765997e-01

2823 8.129676e-01 9.766076e-01

2824 8.129960e-01 9.766155e-01

2825 8.130238e-01 9.766234e-01

2826 8.130509e-01 9.766314e-01

2827 8.130774e-01 9.766393e-01

2828 8.131034e-01 9.766473e-01

2829 8.131287e-01 9.766552e-01

2830 8.131536e-01 9.766631e-01

2831 8.131779e-01 9.766711e-01

2832 8.132016e-01 9.766790e-01

2833 8.132249e-01 9.766870e-01

2834 8.132478e-01 9.766949e-01

2835 8.132701e-01 9.767029e-01

2836 8.132920e-01 9.767109e-01

2837 8.133135e-01 9.767188e-01

2838 8.133346e-01 9.767268e-01

2839 8.133552e-01 9.767347e-01

2840 8.133755e-01 9.767427e-01

2841 8.133954e-01 9.767506e-01

2842 8.134150e-01 9.767586e-01

2843 8.134341e-01 9.767665e-01

2844 8.134530e-01 9.767745e-01

2845 8.134715e-01 9.767824e-01

2846 8.134897e-01 9.767904e-01

2847 8.135076e-01 9.767983e-01

2848 8.135252e-01 9.768062e-01

2849 8.135425e-01 9.768142e-01

2850 8.135595e-01 9.768221e-01

2851 8.135763e-01 9.768300e-01

2852 8.135928e-01 9.768379e-01

2853 8.136090e-01 9.768458e-01

2854 8.136250e-01 9.768537e-01

2855 8.136408e-01 9.768616e-01

2856 8.136563e-01 9.768695e-01

2857 8.136716e-01 9.768774e-01

2858 8.136866e-01 9.768853e-01

2859 8.137015e-01 9.768931e-01

2860 8.137161e-01 9.769010e-01

2861 8.137306e-01 9.769088e-01

2862 8.137448e-01 9.769166e-01

2863 8.137588e-01 9.769245e-01

2864 8.137727e-01 9.769323e-01

2865 8.137863e-01 9.769400e-01

2866 8.137998e-01 9.769478e-01

2867 8.138131e-01 9.769556e-01

2868 8.138262e-01 9.769633e-01

2869 8.138391e-01 9.769710e-01

2870 8.138519e-01 9.769788e-01

2871 8.138645e-01 9.769864e-01

2872 8.138769e-01 9.769941e-01

2873 8.138892e-01 9.770018e-01

2874 8.139013e-01 9.770094e-01

2875 8.139133e-01 9.770170e-01

2876 8.139251e-01 9.770246e-01

2877 8.139367e-01 9.770322e-01

2878 8.139482e-01 9.770397e-01

2879 8.139595e-01 9.770473e-01

2880 8.139707e-01 9.770547e-01

2881 8.139817e-01 9.770622e-01

2882 8.142732e-01 9.770682e-01

2883 8.145651e-01 9.770742e-01

2884 8.148573e-01 9.770802e-01

2885 8.151495e-01 9.770862e-01

2886 8.154417e-01 9.770921e-01

2887 8.157335e-01 9.770981e-01

2888 8.160249e-01 9.771040e-01

2889 8.163156e-01 9.771100e-01

2890 8.166055e-01 9.771159e-01

2891 8.168943e-01 9.771218e-01

2892 8.171821e-01 9.771278e-01

2893 8.174685e-01 9.771337e-01

2894 8.177534e-01 9.771396e-01

2895 8.180366e-01 9.771455e-01

2896 8.183180e-01 9.771514e-01

2897 8.185975e-01 9.771573e-01

2898 8.188748e-01 9.771632e-01

2899 8.191499e-01 9.771691e-01

2900 8.194226e-01 9.771749e-01

2901 8.196927e-01 9.771808e-01

2902 8.199602e-01 9.771867e-01

2903 8.202249e-01 9.771926e-01

2904 8.204867e-01 9.771985e-01

2905 8.207455e-01 9.772044e-01

2906 8.210013e-01 9.772103e-01

2907 8.212538e-01 9.772161e-01

2908 8.215030e-01 9.772220e-01

2909 8.217489e-01 9.772279e-01

2910 8.219913e-01 9.772338e-01

2911 8.222301e-01 9.772397e-01

2912 8.224654e-01 9.772456e-01

2913 8.226971e-01 9.772515e-01

2914 8.229250e-01 9.772574e-01

2915 8.231493e-01 9.772633e-01

2916 8.233697e-01 9.772692e-01

2917 8.235863e-01 9.772751e-01

2918 8.237991e-01 9.772810e-01

2919 8.240080e-01 9.772869e-01

2920 8.242130e-01 9.772928e-01

2921 8.244142e-01 9.772987e-01

2922 8.246114e-01 9.773046e-01

2923 8.248048e-01 9.773106e-01

2924 8.249943e-01 9.773165e-01

2925 8.251798e-01 9.773225e-01

2926 8.253616e-01 9.773284e-01

2927 8.255394e-01 9.773343e-01

2928 8.257134e-01 9.773403e-01

2929 8.258837e-01 9.773463e-01

2930 8.260501e-01 9.773522e-01

2931 8.262128e-01 9.773582e-01

2932 8.263718e-01 9.773642e-01

2933 8.265271e-01 9.773702e-01

2934 8.266788e-01 9.773762e-01

2935 8.268269e-01 9.773821e-01

2936 8.269715e-01 9.773881e-01

2937 8.271125e-01 9.773942e-01

2938 8.272501e-01 9.774002e-01

2939 8.273844e-01 9.774062e-01

2940 8.275153e-01 9.774122e-01

2941 8.276429e-01 9.774183e-01

2942 8.277672e-01 9.774243e-01

2943 8.278884e-01 9.774304e-01

2944 8.280065e-01 9.774364e-01

2945 8.281215e-01 9.774425e-01

2946 8.282336e-01 9.774485e-01

2947 8.283427e-01 9.774546e-01

2948 8.284489e-01 9.774607e-01

2949 8.285523e-01 9.774668e-01

2950 8.286529e-01 9.774729e-01

2951 8.287508e-01 9.774790e-01

2952 8.288461e-01 9.774851e-01

2953 8.289389e-01 9.774913e-01

2954 8.290291e-01 9.774974e-01

2955 8.291168e-01 9.775035e-01

2956 8.292021e-01 9.775097e-01

2957 8.292851e-01 9.775158e-01

2958 8.293659e-01 9.775220e-01

2959 8.294444e-01 9.775281e-01

2960 8.295207e-01 9.775343e-01

2961 8.295949e-01 9.775405e-01

2962 8.296671e-01 9.775467e-01

2963 8.297372e-01 9.775529e-01

2964 8.298054e-01 9.775591e-01

2965 8.298717e-01 9.775653e-01

2966 8.299362e-01 9.775715e-01

2967 8.299988e-01 9.775777e-01

2968 8.300598e-01 9.775840e-01

2969 8.301190e-01 9.775902e-01

2970 8.301766e-01 9.775964e-01

2971 8.302325e-01 9.776027e-01

2972 8.302870e-01 9.776089e-01

2973 8.303399e-01 9.776152e-01

2974 8.303913e-01 9.776215e-01

2975 8.304413e-01 9.776277e-01

2976 8.304900e-01 9.776340e-01

2977 8.305373e-01 9.776403e-01

2978 8.305833e-01 9.776466e-01

2979 8.306281e-01 9.776529e-01

2980 8.306716e-01 9.776592e-01

2981 8.307140e-01 9.776655e-01

2982 8.307552e-01 9.776718e-01

2983 8.307953e-01 9.776782e-01

2984 8.308343e-01 9.776845e-01

2985 8.308723e-01 9.776908e-01

2986 8.309092e-01 9.776972e-01

2987 8.309452e-01 9.777035e-01

2988 8.309802e-01 9.777099e-01

2989 8.310144e-01 9.777162e-01

2990 8.310476e-01 9.777226e-01

2991 8.310800e-01 9.777289e-01

2992 8.311115e-01 9.777353e-01

2993 8.311422e-01 9.777417e-01

2994 8.311722e-01 9.777480e-01

2995 8.312014e-01 9.777544e-01

2996 8.312298e-01 9.777608e-01

2997 8.312576e-01 9.777672e-01

2998 8.312847e-01 9.777735e-01

2999 8.313111e-01 9.777799e-01

3000 8.313368e-01 9.777863e-01

3001 8.313620e-01 9.777927e-01

3002 8.313865e-01 9.777991e-01

3003 8.314105e-01 9.778055e-01

3004 8.314339e-01 9.778119e-01

3005 8.314568e-01 9.778183e-01

3006 8.314791e-01 9.778247e-01

3007 8.315009e-01 9.778310e-01

3008 8.315223e-01 9.778374e-01

3009 8.315431e-01 9.778438e-01

3010 8.315635e-01 9.778502e-01

3011 8.315835e-01 9.778566e-01

3012 8.316030e-01 9.778630e-01

3013 8.316221e-01 9.778694e-01

3014 8.316408e-01 9.778758e-01

3015 8.316591e-01 9.778821e-01

3016 8.316770e-01 9.778885e-01

3017 8.316945e-01 9.778949e-01

3018 8.317117e-01 9.779013e-01

3019 8.317286e-01 9.779076e-01

3020 8.317451e-01 9.779140e-01

3021 8.317613e-01 9.779203e-01

3022 8.317772e-01 9.779267e-01

3023 8.317928e-01 9.779330e-01

3024 8.318081e-01 9.779394e-01

3025 8.318231e-01 9.779457e-01

3026 8.318378e-01 9.779520e-01

3027 8.318522e-01 9.779583e-01

3028 8.318664e-01 9.779646e-01

3029 8.318804e-01 9.779709e-01

3030 8.318940e-01 9.779772e-01

3031 8.319075e-01 9.779835e-01

3032 8.319207e-01 9.779897e-01

3033 8.319337e-01 9.779960e-01

3034 8.319465e-01 9.780022e-01

3035 8.319590e-01 9.780085e-01

3036 8.319714e-01 9.780147e-01

3037 8.319835e-01 9.780209e-01

3038 8.319955e-01 9.780271e-01

3039 8.320073e-01 9.780332e-01

3040 8.320188e-01 9.780394e-01

3041 8.320302e-01 9.780455e-01

3042 8.320414e-01 9.780516e-01

3043 8.320525e-01 9.780577e-01

3044 8.320634e-01 9.780638e-01

3045 8.320741e-01 9.780699e-01

3046 8.320846e-01 9.780759e-01

3047 8.320950e-01 9.780819e-01

3048 8.321053e-01 9.780879e-01

3049 8.321154e-01 9.780939e-01

3050 8.321253e-01 9.780999e-01

3051 8.321351e-01 9.781058e-01

3052 8.321448e-01 9.781117e-01

3053 8.321543e-01 9.781176e-01

3054 8.321637e-01 9.781234e-01

3055 8.321730e-01 9.781292e-01

3056 8.321821e-01 9.781350e-01

3057 8.321911e-01 9.781408e-01

3058 8.322000e-01 9.781465e-01

3059 8.322088e-01 9.781522e-01

3060 8.322174e-01 9.781579e-01

3061 8.322260e-01 9.781635e-01

3062 8.325004e-01 9.781682e-01

3063 8.327743e-01 9.781729e-01

3064 8.330473e-01 9.781776e-01

3065 8.333195e-01 9.781823e-01

3066 8.335905e-01 9.781870e-01

3067 8.338604e-01 9.781917e-01

3068 8.341288e-01 9.781964e-01

3069 8.343958e-01 9.782011e-01

3070 8.346611e-01 9.782058e-01

3071 8.349246e-01 9.782104e-01

3072 8.351862e-01 9.782151e-01

3073 8.354457e-01 9.782198e-01

3074 8.357031e-01 9.782245e-01

3075 8.359581e-01 9.782292e-01

3076 8.362108e-01 9.782339e-01

3077 8.364609e-01 9.782385e-01

3078 8.367084e-01 9.782432e-01

3079 8.369532e-01 9.782479e-01

3080 8.371951e-01 9.782526e-01

3081 8.374341e-01 9.782573e-01

3082 8.376702e-01 9.782620e-01

3083 8.379032e-01 9.782667e-01

3084 8.381330e-01 9.782714e-01

3085 8.383596e-01 9.782761e-01

3086 8.385830e-01 9.782808e-01

3087 8.388031e-01 9.782855e-01

3088 8.390197e-01 9.782902e-01

3089 8.392330e-01 9.782950e-01

3090 8.394428e-01 9.782997e-01

3091 8.396491e-01 9.783044e-01

3092 8.398519e-01 9.783091e-01

3093 8.400512e-01 9.783139e-01

3094 8.402469e-01 9.783186e-01

3095 8.404390e-01 9.783234e-01

3096 8.406276e-01 9.783281e-01

3097 8.408126e-01 9.783329e-01

3098 8.409940e-01 9.783376e-01

3099 8.411718e-01 9.783424e-01

3100 8.413460e-01 9.783472e-01

3101 8.415167e-01 9.783519e-01

3102 8.416839e-01 9.783567e-01

3103 8.418475e-01 9.783615e-01

3104 8.420076e-01 9.783663e-01

3105 8.421643e-01 9.783711e-01

3106 8.423175e-01 9.783759e-01

3107 8.424672e-01 9.783807e-01

3108 8.426136e-01 9.783855e-01

3109 8.427567e-01 9.783903e-01

3110 8.428964e-01 9.783952e-01

3111 8.430329e-01 9.784000e-01

3112 8.431662e-01 9.784048e-01

3113 8.432963e-01 9.784097e-01

3114 8.434232e-01 9.784145e-01

3115 8.435471e-01 9.784194e-01

3116 8.436679e-01 9.784242e-01

3117 8.437857e-01 9.784291e-01

3118 8.439006e-01 9.784340e-01

3119 8.440126e-01 9.784389e-01

3120 8.441218e-01 9.784438e-01

3121 8.442282e-01 9.784487e-01

3122 8.443318e-01 9.784536e-01

3123 8.444328e-01 9.784585e-01

3124 8.445311e-01 9.784634e-01

3125 8.446269e-01 9.784683e-01

3126 8.447202e-01 9.784732e-01

3127 8.448110e-01 9.784782e-01

3128 8.448994e-01 9.784831e-01

3129 8.449854e-01 9.784880e-01

3130 8.450691e-01 9.784930e-01

3131 8.451506e-01 9.784980e-01

3132 8.452299e-01 9.785029e-01

3133 8.453070e-01 9.785079e-01

3134 8.453821e-01 9.785129e-01

3135 8.454551e-01 9.785179e-01

3136 8.455261e-01 9.785228e-01

3137 8.455952e-01 9.785278e-01

3138 8.456624e-01 9.785328e-01

3139 8.457277e-01 9.785378e-01

3140 8.457913e-01 9.785429e-01

3141 8.458531e-01 9.785479e-01

3142 8.459132e-01 9.785529e-01

3143 8.459716e-01 9.785579e-01

3144 8.460285e-01 9.785630e-01

3145 8.460837e-01 9.785680e-01

3146 8.461375e-01 9.785730e-01

3147 8.461897e-01 9.785781e-01

3148 8.462406e-01 9.785831e-01

3149 8.462900e-01 9.785882e-01

3150 8.463380e-01 9.785933e-01

3151 8.463848e-01 9.785983e-01

3152 8.464302e-01 9.786034e-01

3153 8.464744e-01 9.786085e-01

3154 8.465174e-01 9.786136e-01

3155 8.465593e-01 9.786187e-01

3156 8.465999e-01 9.786237e-01

3157 8.466395e-01 9.786288e-01

3158 8.466780e-01 9.786339e-01

3159 8.467155e-01 9.786390e-01

3160 8.467519e-01 9.786441e-01

3161 8.467874e-01 9.786492e-01

3162 8.468219e-01 9.786544e-01

3163 8.468555e-01 9.786595e-01

3164 8.468882e-01 9.786646e-01

3165 8.469201e-01 9.786697e-01

3166 8.469511e-01 9.786748e-01

3167 8.469813e-01 9.786799e-01

3168 8.470106e-01 9.786851e-01

3169 8.470393e-01 9.786902e-01

3170 8.470672e-01 9.786953e-01

3171 8.470943e-01 9.787004e-01

3172 8.471208e-01 9.787056e-01

3173 8.471466e-01 9.787107e-01

3174 8.471718e-01 9.787158e-01

3175 8.471963e-01 9.787209e-01

3176 8.472202e-01 9.787261e-01

3177 8.472435e-01 9.787312e-01

3178 8.472663e-01 9.787363e-01

3179 8.472885e-01 9.787414e-01

3180 8.473101e-01 9.787466e-01

3181 8.473312e-01 9.787517e-01

3182 8.473519e-01 9.787568e-01

3183 8.473720e-01 9.787619e-01

3184 8.473916e-01 9.787670e-01

3185 8.474108e-01 9.787721e-01

3186 8.474296e-01 9.787772e-01

3187 8.474479e-01 9.787823e-01

3188 8.474658e-01 9.787874e-01

3189 8.474833e-01 9.787925e-01

3190 8.475004e-01 9.787976e-01

3191 8.475172e-01 9.788027e-01

3192 8.475336e-01 9.788078e-01

3193 8.475496e-01 9.788128e-01

3194 8.475652e-01 9.788179e-01

3195 8.475806e-01 9.788229e-01

3196 8.475956e-01 9.788280e-01

3197 8.476103e-01 9.788330e-01

3198 8.476247e-01 9.788381e-01

3199 8.476388e-01 9.788431e-01

3200 8.476526e-01 9.788481e-01

3201 8.476661e-01 9.788531e-01

3202 8.476794e-01 9.788581e-01

3203 8.476924e-01 9.788631e-01

3204 8.477052e-01 9.788680e-01

3205 8.477177e-01 9.788730e-01

3206 8.477300e-01 9.788779e-01

3207 8.477421e-01 9.788828e-01

3208 8.477539e-01 9.788878e-01

3209 8.477656e-01 9.788927e-01

3210 8.477770e-01 9.788975e-01

3211 8.477882e-01 9.789024e-01

3212 8.477992e-01 9.789072e-01

3213 8.478101e-01 9.789121e-01

3214 8.478207e-01 9.789169e-01

3215 8.478312e-01 9.789217e-01

3216 8.478415e-01 9.789265e-01

3217 8.478516e-01 9.789312e-01

3218 8.478616e-01 9.789360e-01

3219 8.478714e-01 9.789407e-01

3220 8.478811e-01 9.789454e-01

3221 8.478906e-01 9.789500e-01

3222 8.479000e-01 9.789547e-01

3223 8.479092e-01 9.789593e-01

3224 8.479183e-01 9.789639e-01

3225 8.479273e-01 9.789685e-01

3226 8.479361e-01 9.789730e-01

3227 8.479448e-01 9.789775e-01

3228 8.479534e-01 9.789820e-01

3229 8.479619e-01 9.789864e-01

3230 8.479703e-01 9.789909e-01

3231 8.479786e-01 9.789953e-01

3232 8.479867e-01 9.789996e-01

3233 8.479948e-01 9.790039e-01

3234 8.480027e-01 9.790082e-01

3235 8.480106e-01 9.790125e-01

3236 8.480183e-01 9.790167e-01

3237 8.480260e-01 9.790209e-01

3238 8.480336e-01 9.790250e-01

3239 8.480411e-01 9.790291e-01

3240 8.480485e-01 9.790332e-01

3241 8.480558e-01 9.790372e-01

3242 8.483106e-01 9.790409e-01

3243 8.485639e-01 9.790447e-01

3244 8.488158e-01 9.790484e-01

3245 8.490660e-01 9.790522e-01

3246 8.493145e-01 9.790559e-01

3247 8.495612e-01 9.790597e-01

3248 8.498059e-01 9.790634e-01

3249 8.500485e-01 9.790672e-01

3250 8.502889e-01 9.790709e-01

3251 8.505270e-01 9.790747e-01

3252 8.507628e-01 9.790784e-01

3253 8.509960e-01 9.790822e-01

3254 8.512268e-01 9.790860e-01

3255 8.514548e-01 9.790897e-01

3256 8.516802e-01 9.790935e-01

3257 8.519028e-01 9.790973e-01

3258 8.521224e-01 9.791010e-01

3259 8.523392e-01 9.791048e-01

3260 8.525530e-01 9.791086e-01

3261 8.527638e-01 9.791124e-01

3262 8.529714e-01 9.791162e-01

3263 8.531760e-01 9.791200e-01

3264 8.533773e-01 9.791238e-01

3265 8.535755e-01 9.791276e-01

3266 8.537705e-01 9.791314e-01

3267 8.539622e-01 9.791352e-01

3268 8.541506e-01 9.791390e-01

3269 8.543357e-01 9.791428e-01

3270 8.545175e-01 9.791466e-01

3271 8.546960e-01 9.791505e-01

3272 8.548712e-01 9.791543e-01

3273 8.550431e-01 9.791581e-01

3274 8.552117e-01 9.791620e-01

3275 8.553769e-01 9.791658e-01

3276 8.555389e-01 9.791697e-01

3277 8.556976e-01 9.791735e-01

3278 8.558530e-01 9.791774e-01

3279 8.560052e-01 9.791812e-01

3280 8.561542e-01 9.791851e-01

3281 8.563000e-01 9.791890e-01

3282 8.564426e-01 9.791929e-01

3283 8.565820e-01 9.791968e-01

3284 8.567184e-01 9.792007e-01

3285 8.568517e-01 9.792046e-01

3286 8.569820e-01 9.792085e-01

3287 8.571092e-01 9.792124e-01

3288 8.572335e-01 9.792163e-01

3289 8.573549e-01 9.792202e-01

3290 8.574734e-01 9.792241e-01

3291 8.575890e-01 9.792280e-01

3292 8.577019e-01 9.792320e-01

3293 8.578120e-01 9.792359e-01

3294 8.579195e-01 9.792399e-01

3295 8.580242e-01 9.792438e-01

3296 8.581264e-01 9.792478e-01

3297 8.582260e-01 9.792517e-01

3298 8.583231e-01 9.792557e-01

3299 8.584177e-01 9.792597e-01

3300 8.585100e-01 9.792636e-01

3301 8.585998e-01 9.792676e-01

3302 8.586873e-01 9.792716e-01

3303 8.587726e-01 9.792756e-01

3304 8.588557e-01 9.792796e-01

3305 8.589365e-01 9.792836e-01

3306 8.590153e-01 9.792876e-01

3307 8.590919e-01 9.792916e-01

3308 8.591666e-01 9.792956e-01

3309 8.592392e-01 9.792996e-01

3310 8.593100e-01 9.793037e-01

3311 8.593788e-01 9.793077e-01

3312 8.594458e-01 9.793117e-01

3313 8.595110e-01 9.793158e-01

3314 8.595744e-01 9.793198e-01

3315 8.596361e-01 9.793239e-01

3316 8.596962e-01 9.793279e-01

3317 8.597546e-01 9.793320e-01

3318 8.598114e-01 9.793360e-01

3319 8.598667e-01 9.793401e-01

3320 8.599205e-01 9.793442e-01

3321 8.599729e-01 9.793482e-01

3322 8.600238e-01 9.793523e-01

3323 8.600733e-01 9.793564e-01

3324 8.601215e-01 9.793604e-01

3325 8.601683e-01 9.793645e-01

3326 8.602139e-01 9.793686e-01

3327 8.602583e-01 9.793727e-01

3328 8.603014e-01 9.793768e-01

3329 8.603434e-01 9.793809e-01

3330 8.603842e-01 9.793850e-01

3331 8.604240e-01 9.793891e-01

3332 8.604626e-01 9.793932e-01

3333 8.605002e-01 9.793973e-01

3334 8.605369e-01 9.794014e-01

3335 8.605725e-01 9.794055e-01

3336 8.606071e-01 9.794096e-01

3337 8.606409e-01 9.794137e-01

3338 8.606737e-01 9.794178e-01

3339 8.607057e-01 9.794219e-01

3340 8.607368e-01 9.794260e-01

3341 8.607671e-01 9.794301e-01

3342 8.607966e-01 9.794342e-01

3343 8.608253e-01 9.794383e-01

3344 8.608533e-01 9.794424e-01

3345 8.608805e-01 9.794465e-01

3346 8.609070e-01 9.794506e-01

3347 8.609329e-01 9.794547e-01

3348 8.609581e-01 9.794588e-01

3349 8.609826e-01 9.794629e-01

3350 8.610065e-01 9.794670e-01

3351 8.610298e-01 9.794711e-01

3352 8.610525e-01 9.794752e-01

3353 8.610747e-01 9.794793e-01

3354 8.610963e-01 9.794833e-01

3355 8.611174e-01 9.794874e-01

3356 8.611379e-01 9.794915e-01

3357 8.611579e-01 9.794956e-01

3358 8.611775e-01 9.794996e-01

3359 8.611966e-01 9.795037e-01

3360 8.612152e-01 9.795077e-01

3361 8.612334e-01 9.795118e-01

3362 8.612512e-01 9.795158e-01

3363 8.612685e-01 9.795199e-01

3364 8.612854e-01 9.795239e-01

3365 8.613020e-01 9.795279e-01

3366 8.613181e-01 9.795319e-01

3367 8.613340e-01 9.795359e-01

3368 8.613494e-01 9.795399e-01

3369 8.613645e-01 9.795439e-01

3370 8.613793e-01 9.795479e-01

3371 8.613937e-01 9.795518e-01

3372 8.614078e-01 9.795558e-01

3373 8.614217e-01 9.795597e-01

3374 8.614352e-01 9.795637e-01

3375 8.614484e-01 9.795676e-01

3376 8.614614e-01 9.795715e-01

3377 8.614741e-01 9.795754e-01

3378 8.614865e-01 9.795793e-01

3379 8.614987e-01 9.795831e-01

3380 8.615107e-01 9.795870e-01

3381 8.615224e-01 9.795908e-01

3382 8.615338e-01 9.795946e-01

3383 8.615451e-01 9.795984e-01

3384 8.615561e-01 9.796022e-01

3385 8.615669e-01 9.796060e-01

3386 8.615776e-01 9.796097e-01

3387 8.615880e-01 9.796134e-01

3388 8.615982e-01 9.796172e-01

3389 8.616082e-01 9.796208e-01

3390 8.616181e-01 9.796245e-01

3391 8.616277e-01 9.796282e-01

3392 8.616372e-01 9.796318e-01

3393 8.616466e-01 9.796354e-01

3394 8.616557e-01 9.796390e-01

3395 8.616647e-01 9.796425e-01

3396 8.616736e-01 9.796460e-01

3397 8.616823e-01 9.796495e-01

3398 8.616908e-01 9.796530e-01

3399 8.616992e-01 9.796564e-01

3400 8.617075e-01 9.796599e-01

3401 8.617156e-01 9.796632e-01

3402 8.617236e-01 9.796666e-01

3403 8.617314e-01 9.796699e-01

3404 8.617391e-01 9.796732e-01

3405 8.617467e-01 9.796765e-01

3406 8.617541e-01 9.796797e-01

3407 8.617614e-01 9.796829e-01

3408 8.617686e-01 9.796860e-01

3409 8.617756e-01 9.796891e-01

3410 8.617825e-01 9.796922e-01

3411 8.617893e-01 9.796953e-01

3412 8.617960e-01 9.796983e-01

3413 8.618025e-01 9.797012e-01

3414 8.618089e-01 9.797041e-01

3415 8.618152e-01 9.797070e-01

3416 8.618214e-01 9.797098e-01

3417 8.618274e-01 9.797126e-01

3418 8.618332e-01 9.797154e-01

3419 8.618390e-01 9.797180e-01

3420 8.618446e-01 9.797207e-01

3421 8.618500e-01 9.797233e-01

3422 8.620842e-01 9.797263e-01

3423 8.623165e-01 9.797293e-01

3424 8.625468e-01 9.797323e-01

3425 8.627750e-01 9.797354e-01

3426 8.630011e-01 9.797384e-01

3427 8.632250e-01 9.797414e-01

3428 8.634465e-01 9.797444e-01

3429 8.636656e-01 9.797475e-01

3430 8.638823e-01 9.797505e-01

3431 8.640964e-01 9.797536e-01

3432 8.643079e-01 9.797566e-01

3433 8.645167e-01 9.797596e-01

3434 8.647228e-01 9.797627e-01

3435 8.649261e-01 9.797657e-01

3436 8.651266e-01 9.797688e-01

3437 8.653242e-01 9.797719e-01

3438 8.655189e-01 9.797749e-01

3439 8.657107e-01 9.797780e-01

3440 8.658994e-01 9.797811e-01

3441 8.660852e-01 9.797841e-01

3442 8.662680e-01 9.797872e-01

3443 8.664477e-01 9.797903e-01

3444 8.666243e-01 9.797934e-01

3445 8.667979e-01 9.797965e-01

3446 8.669684e-01 9.797996e-01

3447 8.671358e-01 9.798027e-01

3448 8.673001e-01 9.798058e-01

3449 8.674614e-01 9.798089e-01

3450 8.676195e-01 9.798120e-01

3451 8.677747e-01 9.798151e-01

3452 8.679267e-01 9.798182e-01

3453 8.680757e-01 9.798213e-01

3454 8.682217e-01 9.798245e-01

3455 8.683647e-01 9.798276e-01

3456 8.685047e-01 9.798307e-01

3457 8.686418e-01 9.798339e-01

3458 8.687759e-01 9.798370e-01

3459 8.689071e-01 9.798402e-01

3460 8.690355e-01 9.798433e-01

3461 8.691610e-01 9.798465e-01

3462 8.692836e-01 9.798496e-01

3463 8.694036e-01 9.798528e-01

3464 8.695208e-01 9.798560e-01

3465 8.696352e-01 9.798591e-01

3466 8.697471e-01 9.798623e-01

3467 8.698563e-01 9.798655e-01

3468 8.699629e-01 9.798687e-01

3469 8.700669e-01 9.798719e-01

3470 8.701685e-01 9.798751e-01

3471 8.702676e-01 9.798783e-01

3472 8.703643e-01 9.798815e-01

3473 8.704587e-01 9.798847e-01

3474 8.705507e-01 9.798879e-01

3475 8.706404e-01 9.798911e-01

3476 8.707278e-01 9.798943e-01

3477 8.708131e-01 9.798975e-01

3478 8.708962e-01 9.799008e-01

3479 8.709773e-01 9.799040e-01

3480 8.710562e-01 9.799072e-01

3481 8.711331e-01 9.799105e-01

3482 8.712081e-01 9.799137e-01

3483 8.712811e-01 9.799169e-01

3484 8.713522e-01 9.799202e-01

3485 8.714215e-01 9.799234e-01

3486 8.714889e-01 9.799267e-01

3487 8.715546e-01 9.799299e-01

3488 8.716186e-01 9.799332e-01

3489 8.716809e-01 9.799364e-01

3490 8.717415e-01 9.799397e-01

3491 8.718006e-01 9.799430e-01

3492 8.718581e-01 9.799462e-01

3493 8.719140e-01 9.799495e-01

3494 8.719685e-01 9.799528e-01

3495 8.720215e-01 9.799560e-01

3496 8.720731e-01 9.799593e-01

3497 8.721233e-01 9.799626e-01

3498 8.721722e-01 9.799659e-01

3499 8.722198e-01 9.799691e-01

3500 8.722661e-01 9.799724e-01

3501 8.723112e-01 9.799757e-01

3502 8.723551e-01 9.799790e-01

3503 8.723978e-01 9.799823e-01

3504 8.724393e-01 9.799855e-01

3505 8.724798e-01 9.799888e-01

3506 8.725191e-01 9.799921e-01

3507 8.725574e-01 9.799954e-01

3508 8.725947e-01 9.799987e-01

3509 8.726310e-01 9.800020e-01

3510 8.726664e-01 9.800052e-01

3511 8.727008e-01 9.800085e-01

3512 8.727343e-01 9.800118e-01

3513 8.727669e-01 9.800151e-01

3514 8.727986e-01 9.800183e-01

3515 8.728295e-01 9.800216e-01

3516 8.728596e-01 9.800249e-01

3517 8.728889e-01 9.800282e-01

3518 8.729175e-01 9.800314e-01

3519 8.729453e-01 9.800347e-01

3520 8.729723e-01 9.800380e-01

3521 8.729987e-01 9.800412e-01

3522 8.730244e-01 9.800445e-01

3523 8.730494e-01 9.800477e-01

3524 8.730738e-01 9.800510e-01

3525 8.730976e-01 9.800542e-01

3526 8.731208e-01 9.800575e-01

3527 8.731433e-01 9.800607e-01

3528 8.731653e-01 9.800639e-01

3529 8.731868e-01 9.800671e-01

3530 8.732077e-01 9.800704e-01

3531 8.732281e-01 9.800736e-01

3532 8.732480e-01 9.800768e-01

3533 8.732674e-01 9.800800e-01

3534 8.732863e-01 9.800831e-01

3535 8.733047e-01 9.800863e-01

3536 8.733227e-01 9.800895e-01

3537 8.733403e-01 9.800927e-01

3538 8.733574e-01 9.800958e-01

3539 8.733742e-01 9.800990e-01

3540 8.733905e-01 9.801021e-01

3541 8.734065e-01 9.801052e-01

3542 8.734220e-01 9.801083e-01

3543 8.734372e-01 9.801114e-01

3544 8.734521e-01 9.801145e-01

3545 8.734666e-01 9.801176e-01

3546 8.734807e-01 9.801207e-01

3547 8.734946e-01 9.801237e-01

3548 8.735081e-01 9.801268e-01

3549 8.735213e-01 9.801298e-01

3550 8.735342e-01 9.801328e-01

3551 8.735468e-01 9.801358e-01

3552 8.735591e-01 9.801388e-01

3553 8.735711e-01 9.801418e-01

3554 8.735829e-01 9.801447e-01

3555 8.735944e-01 9.801477e-01

3556 8.736057e-01 9.801506e-01

3557 8.736166e-01 9.801535e-01

3558 8.736274e-01 9.801564e-01

3559 8.736379e-01 9.801592e-01

3560 8.736481e-01 9.801621e-01

3561 8.736582e-01 9.801649e-01

3562 8.736680e-01 9.801677e-01

3563 8.736775e-01 9.801704e-01

3564 8.736869e-01 9.801732e-01

3565 8.736960e-01 9.801759e-01

3566 8.737049e-01 9.801786e-01

3567 8.737137e-01 9.801813e-01

3568 8.737222e-01 9.801840e-01

3569 8.737304e-01 9.801866e-01

3570 8.737385e-01 9.801892e-01

3571 8.737464e-01 9.801918e-01

3572 8.737541e-01 9.801943e-01

3573 8.737616e-01 9.801968e-01

3574 8.737688e-01 9.801993e-01

3575 8.737759e-01 9.802018e-01

3576 8.737828e-01 9.802042e-01

3577 8.737895e-01 9.802066e-01

3578 8.737959e-01 9.802090e-01

3579 8.738022e-01 9.802113e-01

3580 8.738083e-01 9.802136e-01

3581 8.738141e-01 9.802158e-01

3582 8.738197e-01 9.802181e-01

3583 8.738252e-01 9.802203e-01

3584 8.738304e-01 9.802224e-01

3585 8.738354e-01 9.802245e-01

3586 8.738401e-01 9.802266e-01

3587 8.738446e-01 9.802286e-01

3588 8.738489e-01 9.802306e-01

3589 8.738530e-01 9.802325e-01

3590 8.738568e-01 9.802344e-01

3591 8.738604e-01 9.802362e-01

3592 8.738636e-01 9.802380e-01

3593 8.738667e-01 9.802398e-01

3594 8.738694e-01 9.802415e-01

3595 8.738719e-01 9.802431e-01

3596 8.738740e-01 9.802447e-01

3597 8.738759e-01 9.802463e-01

3598 8.738774e-01 9.802478e-01

3599 8.738786e-01 9.802492e-01

3600 8.738795e-01 9.802506e-01

3601 8.738800e-01 9.802520e-01

3602 8.740940e-01 9.802544e-01

3603 8.743058e-01 9.802569e-01

3604 8.745154e-01 9.802593e-01

3605 8.747226e-01 9.802618e-01

3606 8.749275e-01 9.802642e-01

3607 8.751299e-01 9.802667e-01

3608 8.753298e-01 9.802692e-01

3609 8.755272e-01 9.802716e-01

3610 8.757219e-01 9.802741e-01

3611 8.759141e-01 9.802766e-01

3612 8.761035e-01 9.802790e-01

3613 8.762902e-01 9.802815e-01

3614 8.764742e-01 9.802840e-01

3615 8.766554e-01 9.802865e-01

3616 8.768338e-01 9.802890e-01

3617 8.770094e-01 9.802915e-01

3618 8.771821e-01 9.802940e-01

3619 8.773520e-01 9.802965e-01

3620 8.775190e-01 9.802990e-01

3621 8.776832e-01 9.803015e-01

3622 8.778444e-01 9.803040e-01

3623 8.780028e-01 9.803065e-01

3624 8.781583e-01 9.803090e-01

3625 8.783109e-01 9.803115e-01

3626 8.784606e-01 9.803141e-01

3627 8.786075e-01 9.803166e-01

3628 8.787516e-01 9.803191e-01

3629 8.788928e-01 9.803216e-01

3630 8.790311e-01 9.803242e-01

3631 8.791667e-01 9.803267e-01

3632 8.792995e-01 9.803293e-01

3633 8.794296e-01 9.803318e-01

3634 8.795569e-01 9.803343e-01

3635 8.796816e-01 9.803369e-01

3636 8.798035e-01 9.803394e-01

3637 8.799228e-01 9.803420e-01

3638 8.800395e-01 9.803446e-01

3639 8.801536e-01 9.803471e-01

3640 8.802652e-01 9.803497e-01

3641 8.803742e-01 9.803523e-01

3642 8.804808e-01 9.803548e-01

3643 8.805849e-01 9.803574e-01

3644 8.806866e-01 9.803600e-01

3645 8.807859e-01 9.803626e-01

3646 8.808829e-01 9.803652e-01

3647 8.809776e-01 9.803677e-01

3648 8.810701e-01 9.803703e-01

3649 8.811603e-01 9.803729e-01

3650 8.812484e-01 9.803755e-01

3651 8.813343e-01 9.803781e-01

3652 8.814181e-01 9.803807e-01

3653 8.814999e-01 9.803833e-01

3654 8.815797e-01 9.803859e-01

3655 8.816574e-01 9.803885e-01

3656 8.817333e-01 9.803911e-01

3657 8.818072e-01 9.803937e-01

3658 8.818793e-01 9.803963e-01

3659 8.819496e-01 9.803989e-01

3660 8.820181e-01 9.804016e-01

3661 8.820848e-01 9.804042e-01

3662 8.821499e-01 9.804068e-01

3663 8.822133e-01 9.804094e-01

3664 8.822750e-01 9.804120e-01

3665 8.823352e-01 9.804146e-01

3666 8.823938e-01 9.804173e-01

3667 8.824509e-01 9.804199e-01

3668 8.825065e-01 9.804225e-01

3669 8.825607e-01 9.804251e-01

3670 8.826135e-01 9.804277e-01

3671 8.826649e-01 9.804304e-01

3672 8.827150e-01 9.804330e-01

3673 8.827637e-01 9.804356e-01

3674 8.828112e-01 9.804382e-01

3675 8.828575e-01 9.804409e-01

3676 8.829025e-01 9.804435e-01

3677 8.829463e-01 9.804461e-01

3678 8.829890e-01 9.804487e-01

3679 8.830306e-01 9.804513e-01

3680 8.830711e-01 9.804539e-01

3681 8.831105e-01 9.804566e-01

3682 8.831489e-01 9.804592e-01

3683 8.831863e-01 9.804618e-01

3684 8.832227e-01 9.804644e-01

3685 8.832582e-01 9.804670e-01

3686 8.832927e-01 9.804696e-01

3687 8.833263e-01 9.804722e-01

3688 8.833591e-01 9.804748e-01

3689 8.833910e-01 9.804774e-01

3690 8.834220e-01 9.804800e-01

3691 8.834523e-01 9.804826e-01

3692 8.834817e-01 9.804851e-01

3693 8.835104e-01 9.804877e-01

3694 8.835383e-01 9.804903e-01

3695 8.835656e-01 9.804929e-01

3696 8.835921e-01 9.804954e-01

3697 8.836179e-01 9.804980e-01

3698 8.836431e-01 9.805005e-01

3699 8.836676e-01 9.805031e-01

3700 8.836915e-01 9.805056e-01

3701 8.837148e-01 9.805081e-01

3702 8.837374e-01 9.805107e-01

3703 8.837595e-01 9.805132e-01

3704 8.837811e-01 9.805157e-01

3705 8.838020e-01 9.805182e-01

3706 8.838225e-01 9.805207e-01

3707 8.838424e-01 9.805232e-01

3708 8.838618e-01 9.805257e-01

3709 8.838807e-01 9.805281e-01

3710 8.838992e-01 9.805306e-01

3711 8.839171e-01 9.805330e-01

3712 8.839347e-01 9.805355e-01

3713 8.839517e-01 9.805379e-01

3714 8.839684e-01 9.805403e-01

3715 8.839846e-01 9.805427e-01

3716 8.840004e-01 9.805451e-01

3717 8.840158e-01 9.805475e-01

3718 8.840308e-01 9.805499e-01

3719 8.840454e-01 9.805522e-01

3720 8.840597e-01 9.805545e-01

3721 8.840736e-01 9.805569e-01

3722 8.840871e-01 9.805592e-01

3723 8.841003e-01 9.805615e-01

3724 8.841131e-01 9.805638e-01

3725 8.841256e-01 9.805660e-01

3726 8.841378e-01 9.805683e-01

3727 8.841496e-01 9.805705e-01

3728 8.841611e-01 9.805727e-01

3729 8.841723e-01 9.805749e-01

3730 8.841832e-01 9.805771e-01

3731 8.841938e-01 9.805792e-01

3732 8.842041e-01 9.805814e-01

3733 8.842141e-01 9.805835e-01

3734 8.842238e-01 9.805856e-01

3735 8.842332e-01 9.805876e-01

3736 8.842423e-01 9.805897e-01

3737 8.842512e-01 9.805917e-01

3738 8.842598e-01 9.805937e-01

3739 8.842680e-01 9.805957e-01

3740 8.842760e-01 9.805976e-01

3741 8.842837e-01 9.805996e-01

3742 8.842912e-01 9.806015e-01

3743 8.842983e-01 9.806033e-01

3744 8.843052e-01 9.806052e-01

3745 8.843118e-01 9.806070e-01

3746 8.843181e-01 9.806088e-01

3747 8.843242e-01 9.806106e-01

3748 8.843299e-01 9.806123e-01

3749 8.843354e-01 9.806140e-01

3750 8.843405e-01 9.806156e-01

3751 8.843454e-01 9.806173e-01

3752 8.843500e-01 9.806189e-01

3753 8.843543e-01 9.806204e-01

3754 8.843582e-01 9.806220e-01

3755 8.843619e-01 9.806235e-01

3756 8.843652e-01 9.806249e-01

3757 8.843682e-01 9.806263e-01

3758 8.843708e-01 9.806277e-01

3759 8.843732e-01 9.806291e-01

3760 8.843751e-01 9.806304e-01

3761 8.843768e-01 9.806316e-01

3762 8.843780e-01 9.806328e-01

3763 8.843789e-01 9.806340e-01

3764 8.843794e-01 9.806351e-01

3765 8.843795e-01 9.806362e-01

3766 8.843791e-01 9.806373e-01

3767 8.843784e-01 9.806382e-01

3768 8.843772e-01 9.806392e-01

3769 8.843756e-01 9.806401e-01

3770 8.843735e-01 9.806409e-01

3771 8.843710e-01 9.806417e-01

3772 8.843680e-01 9.806424e-01

3773 8.843644e-01 9.806431e-01

3774 8.843604e-01 9.806438e-01

3775 8.843558e-01 9.806443e-01

3776 8.843506e-01 9.806449e-01

3777 8.843449e-01 9.806453e-01

3778 8.843385e-01 9.806457e-01

3779 8.843316e-01 9.806461e-01

3780 8.843240e-01 9.806463e-01

3781 8.843158e-01 9.806466e-01

3782 8.845108e-01 9.806486e-01

3783 8.847034e-01 9.806506e-01

3784 8.848937e-01 9.806526e-01

3785 8.850815e-01 9.806546e-01

3786 8.852669e-01 9.806566e-01

3787 8.854497e-01 9.806586e-01

3788 8.856300e-01 9.806606e-01

3789 8.858077e-01 9.806626e-01

3790 8.859829e-01 9.806646e-01

3791 8.861553e-01 9.806666e-01

3792 8.863252e-01 9.806686e-01

3793 8.864924e-01 9.806707e-01

3794 8.866569e-01 9.806727e-01

3795 8.868187e-01 9.806747e-01

3796 8.869778e-01 9.806767e-01

3797 8.871342e-01 9.806788e-01

3798 8.872879e-01 9.806808e-01

3799 8.874389e-01 9.806829e-01

3800 8.875872e-01 9.806849e-01

3801 8.877328e-01 9.806869e-01

3802 8.878757e-01 9.806890e-01

3803 8.880159e-01 9.806910e-01

3804 8.881535e-01 9.806931e-01

3805 8.882884e-01 9.806951e-01

3806 8.884206e-01 9.806972e-01

3807 8.885503e-01 9.806992e-01

3808 8.886773e-01 9.807013e-01

3809 8.888017e-01 9.807033e-01

3810 8.889236e-01 9.807054e-01

3811 8.890430e-01 9.807075e-01

3812 8.891599e-01 9.807095e-01

3813 8.892742e-01 9.807116e-01

3814 8.893862e-01 9.807137e-01

3815 8.894957e-01 9.807157e-01

3816 8.896028e-01 9.807178e-01

3817 8.897075e-01 9.807199e-01

3818 8.898099e-01 9.807220e-01

3819 8.899101e-01 9.807240e-01

3820 8.900079e-01 9.807261e-01

3821 8.901036e-01 9.807282e-01

3822 8.901970e-01 9.807303e-01

3823 8.902883e-01 9.807324e-01

3824 8.903775e-01 9.807345e-01

3825 8.904646e-01 9.807365e-01

3826 8.905496e-01 9.807386e-01

3827 8.906326e-01 9.807407e-01

3828 8.907137e-01 9.807428e-01

3829 8.907928e-01 9.807449e-01

3830 8.908700e-01 9.807470e-01

3831 8.909453e-01 9.807491e-01

3832 8.910188e-01 9.807512e-01

3833 8.910905e-01 9.807533e-01

3834 8.911605e-01 9.807554e-01

3835 8.912287e-01 9.807574e-01

3836 8.912953e-01 9.807595e-01

3837 8.913602e-01 9.807616e-01

3838 8.914235e-01 9.807637e-01

3839 8.914852e-01 9.807658e-01

3840 8.915453e-01 9.807679e-01

3841 8.916040e-01 9.807700e-01

3842 8.916612e-01 9.807721e-01

3843 8.917169e-01 9.807742e-01

3844 8.917712e-01 9.807763e-01

3845 8.918241e-01 9.807783e-01

3846 8.918757e-01 9.807804e-01

3847 8.919260e-01 9.807825e-01

3848 8.919750e-01 9.807846e-01

3849 8.920228e-01 9.807867e-01

3850 8.920693e-01 9.807888e-01

3851 8.921146e-01 9.807908e-01

3852 8.921588e-01 9.807929e-01

3853 8.922018e-01 9.807950e-01

3854 8.922438e-01 9.807970e-01

3855 8.922846e-01 9.807991e-01

3856 8.923244e-01 9.808012e-01

3857 8.923632e-01 9.808032e-01

3858 8.924010e-01 9.808053e-01

3859 8.924377e-01 9.808073e-01

3860 8.924736e-01 9.808094e-01

3861 8.925085e-01 9.808114e-01

3862 8.925425e-01 9.808135e-01

3863 8.925756e-01 9.808155e-01

3864 8.926079e-01 9.808175e-01

3865 8.926393e-01 9.808195e-01

3866 8.926699e-01 9.808216e-01

3867 8.926998e-01 9.808236e-01

3868 8.927288e-01 9.808256e-01

3869 8.927571e-01 9.808276e-01

3870 8.927847e-01 9.808296e-01

3871 8.928115e-01 9.808316e-01

3872 8.928376e-01 9.808335e-01

3873 8.928631e-01 9.808355e-01

3874 8.928879e-01 9.808375e-01

3875 8.929120e-01 9.808394e-01

3876 8.929356e-01 9.808414e-01

3877 8.929585e-01 9.808433e-01

3878 8.929808e-01 9.808452e-01

3879 8.930025e-01 9.808472e-01

3880 8.930236e-01 9.808491e-01

3881 8.930442e-01 9.808510e-01

3882 8.930642e-01 9.808529e-01

3883 8.930837e-01 9.808548e-01

3884 8.931027e-01 9.808566e-01

3885 8.931212e-01 9.808585e-01

3886 8.931392e-01 9.808603e-01

3887 8.931566e-01 9.808622e-01

3888 8.931737e-01 9.808640e-01

3889 8.931902e-01 9.808658e-01

3890 8.932063e-01 9.808676e-01

3891 8.932219e-01 9.808694e-01

3892 8.932371e-01 9.808711e-01

3893 8.932519e-01 9.808729e-01

3894 8.932663e-01 9.808746e-01

3895 8.932802e-01 9.808764e-01

3896 8.932937e-01 9.808781e-01

3897 8.933068e-01 9.808798e-01

3898 8.933196e-01 9.808814e-01

3899 8.933319e-01 9.808831e-01

3900 8.933439e-01 9.808847e-01

3901 8.933554e-01 9.808864e-01

3902 8.933666e-01 9.808880e-01

3903 8.933775e-01 9.808895e-01

3904 8.933879e-01 9.808911e-01

3905 8.933980e-01 9.808927e-01

3906 8.934078e-01 9.808942e-01

3907 8.934171e-01 9.808957e-01

3908 8.934261e-01 9.808972e-01

3909 8.934348e-01 9.808986e-01

3910 8.934431e-01 9.809000e-01

3911 8.934511e-01 9.809015e-01

3912 8.934587e-01 9.809028e-01

3913 8.934659e-01 9.809042e-01

3914 8.934728e-01 9.809055e-01

3915 8.934794e-01 9.809068e-01

3916 8.934855e-01 9.809081e-01

3917 8.934914e-01 9.809094e-01

3918 8.934968e-01 9.809106e-01

3919 8.935019e-01 9.809118e-01

3920 8.935067e-01 9.809130e-01

3921 8.935110e-01 9.809141e-01

3922 8.935150e-01 9.809152e-01

3923 8.935187e-01 9.809163e-01

3924 8.935219e-01 9.809173e-01

3925 8.935247e-01 9.809183e-01

3926 8.935272e-01 9.809193e-01

3927 8.935292e-01 9.809203e-01

3928 8.935309e-01 9.809212e-01

3929 8.935321e-01 9.809220e-01

3930 8.935329e-01 9.809229e-01

3931 8.935333e-01 9.809237e-01

3932 8.935333e-01 9.809244e-01

3933 8.935328e-01 9.809251e-01

3934 8.935318e-01 9.809258e-01

3935 8.935304e-01 9.809264e-01

3936 8.935285e-01 9.809270e-01

3937 8.935261e-01 9.809276e-01

3938 8.935232e-01 9.809281e-01

3939 8.935198e-01 9.809286e-01

3940 8.935159e-01 9.809290e-01

3941 8.935115e-01 9.809293e-01

3942 8.935065e-01 9.809297e-01

3943 8.935009e-01 9.809299e-01

3944 8.934948e-01 9.809302e-01

3945 8.934880e-01 9.809303e-01

3946 8.934807e-01 9.809305e-01

3947 8.934728e-01 9.809305e-01

3948 8.934642e-01 9.809306e-01

3949 8.934549e-01 9.809305e-01

3950 8.934450e-01 9.809304e-01

3951 8.934344e-01 9.809303e-01

3952 8.934231e-01 9.809301e-01

3953 8.934111e-01 9.809298e-01

3954 8.933983e-01 9.809295e-01

3955 8.933848e-01 9.809291e-01

3956 8.933705e-01 9.809287e-01

3957 8.933554e-01 9.809282e-01

3958 8.933395e-01 9.809276e-01

3959 8.933228e-01 9.809270e-01

3960 8.933052e-01 9.809263e-01

3961 8.932868e-01 9.809255e-01

3962 8.934643e-01 9.809272e-01

3963 8.936394e-01 9.809288e-01

3964 8.938121e-01 9.809304e-01

3965 8.939823e-01 9.809321e-01

3966 8.941501e-01 9.809337e-01

3967 8.943154e-01 9.809353e-01

3968 8.944782e-01 9.809370e-01

3969 8.946385e-01 9.809386e-01

3970 8.947962e-01 9.809402e-01

3971 8.949514e-01 9.809419e-01

3972 8.951040e-01 9.809435e-01

3973 8.952541e-01 9.809452e-01

3974 8.954016e-01 9.809468e-01

3975 8.955466e-01 9.809485e-01

3976 8.956890e-01 9.809501e-01

3977 8.958289e-01 9.809518e-01

3978 8.959663e-01 9.809534e-01

3979 8.961011e-01 9.809551e-01

3980 8.962334e-01 9.809567e-01

3981 8.963632e-01 9.809584e-01

3982 8.964905e-01 9.809600e-01

3983 8.966154e-01 9.809617e-01

3984 8.967377e-01 9.809634e-01

3985 8.968577e-01 9.809650e-01

3986 8.969753e-01 9.809667e-01

3987 8.970904e-01 9.809683e-01

3988 8.972032e-01 9.809700e-01

3989 8.973137e-01 9.809717e-01

3990 8.974218e-01 9.809733e-01

3991 8.975277e-01 9.809750e-01

3992 8.976313e-01 9.809767e-01

3993 8.977327e-01 9.809783e-01

3994 8.978319e-01 9.809800e-01

3995 8.979289e-01 9.809817e-01

3996 8.980238e-01 9.809833e-01

3997 8.981165e-01 9.809850e-01

3998 8.982072e-01 9.809867e-01

3999 8.982959e-01 9.809883e-01

4000 8.983825e-01 9.809900e-01

4001 8.984672e-01 9.809917e-01

4002 8.985500e-01 9.809934e-01

4003 8.986308e-01 9.809950e-01

4004 8.987097e-01 9.809967e-01

4005 8.987868e-01 9.809984e-01

4006 8.988621e-01 9.810000e-01

4007 8.989357e-01 9.810017e-01

4008 8.990074e-01 9.810034e-01

4009 8.990775e-01 9.810050e-01

4010 8.991459e-01 9.810067e-01

4011 8.992127e-01 9.810083e-01

4012 8.992778e-01 9.810100e-01

4013 8.993414e-01 9.810117e-01

4014 8.994035e-01 9.810133e-01

4015 8.994640e-01 9.810150e-01

4016 8.995230e-01 9.810166e-01

4017 8.995806e-01 9.810183e-01

4018 8.996368e-01 9.810199e-01

4019 8.996916e-01 9.810216e-01

4020 8.997450e-01 9.810232e-01

4021 8.997972e-01 9.810249e-01

4022 8.998480e-01 9.810265e-01

4023 8.998975e-01 9.810281e-01

4024 8.999459e-01 9.810298e-01

4025 8.999930e-01 9.810314e-01

4026 9.000389e-01 9.810330e-01

4027 9.000837e-01 9.810346e-01

4028 9.001273e-01 9.810363e-01

4029 9.001699e-01 9.810379e-01

4030 9.002113e-01 9.810395e-01

4031 9.002517e-01 9.810411e-01

4032 9.002911e-01 9.810427e-01

4033 9.003295e-01 9.810443e-01

4034 9.003669e-01 9.810459e-01

4035 9.004034e-01 9.810474e-01

4036 9.004389e-01 9.810490e-01

4037 9.004735e-01 9.810506e-01

4038 9.005072e-01 9.810522e-01

4039 9.005401e-01 9.810537e-01

4040 9.005721e-01 9.810553e-01

4041 9.006033e-01 9.810568e-01

4042 9.006337e-01 9.810584e-01

4043 9.006632e-01 9.810599e-01

4044 9.006921e-01 9.810614e-01

4045 9.007201e-01 9.810629e-01

4046 9.007475e-01 9.810644e-01

4047 9.007741e-01 9.810659e-01

4048 9.008000e-01 9.810674e-01

4049 9.008252e-01 9.810689e-01

4050 9.008498e-01 9.810704e-01

4051 9.008737e-01 9.810719e-01

4052 9.008969e-01 9.810733e-01

4053 9.009196e-01 9.810747e-01

4054 9.009416e-01 9.810762e-01

4055 9.009630e-01 9.810776e-01

4056 9.009838e-01 9.810790e-01

4057 9.010041e-01 9.810804e-01

4058 9.010238e-01 9.810818e-01

4059 9.010429e-01 9.810832e-01

4060 9.010615e-01 9.810845e-01

4061 9.010796e-01 9.810859e-01

4062 9.010972e-01 9.810872e-01

4063 9.011142e-01 9.810885e-01

4064 9.011307e-01 9.810898e-01

4065 9.011468e-01 9.810911e-01

4066 9.011623e-01 9.810924e-01

4067 9.011774e-01 9.810937e-01

4068 9.011920e-01 9.810949e-01

4069 9.012061e-01 9.810961e-01

4070 9.012198e-01 9.810974e-01

4071 9.012330e-01 9.810986e-01

4072 9.012458e-01 9.810997e-01

4073 9.012581e-01 9.811009e-01

4074 9.012700e-01 9.811020e-01

4075 9.012815e-01 9.811032e-01

4076 9.012925e-01 9.811043e-01

4077 9.013031e-01 9.811053e-01

4078 9.013133e-01 9.811064e-01

4079 9.013231e-01 9.811074e-01

4080 9.013324e-01 9.811085e-01

4081 9.013414e-01 9.811095e-01

4082 9.013499e-01 9.811104e-01

4083 9.013580e-01 9.811114e-01

4084 9.013656e-01 9.811123e-01

4085 9.013729e-01 9.811132e-01

4086 9.013798e-01 9.811141e-01

4087 9.013862e-01 9.811149e-01

4088 9.013922e-01 9.811158e-01

4089 9.013978e-01 9.811166e-01

4090 9.014030e-01 9.811173e-01

4091 9.014078e-01 9.811181e-01

4092 9.014121e-01 9.811188e-01

4093 9.014160e-01 9.811195e-01

4094 9.014195e-01 9.811202e-01

4095 9.014225e-01 9.811208e-01

4096 9.014251e-01 9.811214e-01

4097 9.014273e-01 9.811219e-01

4098 9.014290e-01 9.811225e-01

4099 9.014303e-01 9.811230e-01

4100 9.014311e-01 9.811234e-01

4101 9.014314e-01 9.811239e-01

4102 9.014313e-01 9.811243e-01

4103 9.014307e-01 9.811246e-01

4104 9.014296e-01 9.811250e-01

4105 9.014280e-01 9.811252e-01

4106 9.014259e-01 9.811255e-01

4107 9.014233e-01 9.811257e-01

4108 9.014202e-01 9.811259e-01

4109 9.014165e-01 9.811260e-01

4110 9.014124e-01 9.811261e-01

4111 9.014076e-01 9.811261e-01

4112 9.014024e-01 9.811261e-01

4113 9.013965e-01 9.811261e-01

4114 9.013901e-01 9.811260e-01

4115 9.013831e-01 9.811259e-01

4116 9.013756e-01 9.811257e-01

4117 9.013674e-01 9.811255e-01

4118 9.013586e-01 9.811252e-01

4119 9.013492e-01 9.811249e-01

4120 9.013391e-01 9.811245e-01

4121 9.013284e-01 9.811241e-01

4122 9.013171e-01 9.811236e-01

4123 9.013050e-01 9.811231e-01

4124 9.012923e-01 9.811225e-01

4125 9.012789e-01 9.811219e-01

4126 9.012648e-01 9.811212e-01

4127 9.012500e-01 9.811205e-01

4128 9.012345e-01 9.811197e-01

4129 9.012182e-01 9.811188e-01

4130 9.012012e-01 9.811179e-01

4131 9.011835e-01 9.811169e-01

4132 9.011649e-01 9.811159e-01

4133 9.011456e-01 9.811148e-01

4134 9.011256e-01 9.811136e-01

4135 9.011047e-01 9.811124e-01

4136 9.010830e-01 9.811111e-01

4137 9.010605e-01 9.811098e-01

4138 9.010372e-01 9.811083e-01

4139 9.010131e-01 9.811068e-01

4140 9.009881e-01 9.811053e-01

4141 9.009623e-01 9.811037e-01

4142 9.011240e-01 9.811050e-01

4143 9.012834e-01 9.811063e-01

4144 9.014403e-01 9.811076e-01

4145 9.015949e-01 9.811089e-01

4146 9.017470e-01 9.811103e-01

4147 9.018968e-01 9.811116e-01

4148 9.020441e-01 9.811129e-01

4149 9.021890e-01 9.811142e-01

4150 9.023315e-01 9.811156e-01

4151 9.024716e-01 9.811169e-01

4152 9.026093e-01 9.811182e-01

4153 9.027445e-01 9.811196e-01

4154 9.028774e-01 9.811209e-01

4155 9.030078e-01 9.811222e-01

4156 9.031359e-01 9.811235e-01

4157 9.032616e-01 9.811249e-01

4158 9.033850e-01 9.811262e-01

4159 9.035060e-01 9.811275e-01

4160 9.036247e-01 9.811289e-01

4161 9.037411e-01 9.811302e-01

4162 9.038552e-01 9.811315e-01

4163 9.039670e-01 9.811329e-01

4164 9.040766e-01 9.811342e-01

4165 9.041840e-01 9.811355e-01

4166 9.042892e-01 9.811369e-01

4167 9.043923e-01 9.811382e-01

4168 9.044932e-01 9.811395e-01

4169 9.045919e-01 9.811408e-01

4170 9.046886e-01 9.811422e-01

4171 9.047833e-01 9.811435e-01

4172 9.048759e-01 9.811448e-01

4173 9.049665e-01 9.811462e-01

4174 9.050551e-01 9.811475e-01

4175 9.051418e-01 9.811488e-01

4176 9.052266e-01 9.811501e-01

4177 9.053095e-01 9.811515e-01

4178 9.053906e-01 9.811528e-01

4179 9.054698e-01 9.811541e-01

4180 9.055472e-01 9.811554e-01

4181 9.056229e-01 9.811567e-01

4182 9.056969e-01 9.811580e-01

4183 9.057691e-01 9.811594e-01

4184 9.058397e-01 9.811607e-01

4185 9.059087e-01 9.811620e-01

4186 9.059761e-01 9.811633e-01

4187 9.060419e-01 9.811646e-01

4188 9.061061e-01 9.811659e-01

4189 9.061688e-01 9.811672e-01

4190 9.062301e-01 9.811685e-01

4191 9.062899e-01 9.811698e-01

4192 9.063483e-01 9.811710e-01

4193 9.064052e-01 9.811723e-01

4194 9.064608e-01 9.811736e-01

4195 9.065151e-01 9.811749e-01

4196 9.065681e-01 9.811762e-01

4197 9.066198e-01 9.811774e-01

4198 9.066702e-01 9.811787e-01

4199 9.067194e-01 9.811800e-01

4200 9.067674e-01 9.811812e-01

4201 9.068143e-01 9.811825e-01

4202 9.068599e-01 9.811837e-01

4203 9.069045e-01 9.811850e-01

4204 9.069480e-01 9.811862e-01

4205 9.069903e-01 9.811874e-01

4206 9.070317e-01 9.811886e-01

4207 9.070720e-01 9.811899e-01

4208 9.071113e-01 9.811911e-01

4209 9.071496e-01 9.811923e-01

4210 9.071870e-01 9.811935e-01

4211 9.072234e-01 9.811947e-01

4212 9.072589e-01 9.811959e-01

4213 9.072935e-01 9.811970e-01

4214 9.073272e-01 9.811982e-01

4215 9.073601e-01 9.811994e-01

4216 9.073921e-01 9.812005e-01

4217 9.074233e-01 9.812017e-01

4218 9.074537e-01 9.812028e-01

4219 9.074833e-01 9.812039e-01

4220 9.075121e-01 9.812051e-01

4221 9.075402e-01 9.812062e-01

4222 9.075676e-01 9.812073e-01

4223 9.075942e-01 9.812084e-01

4224 9.076202e-01 9.812094e-01

4225 9.076454e-01 9.812105e-01

4226 9.076699e-01 9.812116e-01

4227 9.076938e-01 9.812126e-01

4228 9.077171e-01 9.812136e-01

4229 9.077397e-01 9.812147e-01

4230 9.077617e-01 9.812157e-01

4231 9.077830e-01 9.812167e-01

4232 9.078038e-01 9.812177e-01

4233 9.078239e-01 9.812186e-01

4234 9.078435e-01 9.812196e-01

4235 9.078625e-01 9.812206e-01

4236 9.078810e-01 9.812215e-01

4237 9.078989e-01 9.812224e-01

4238 9.079163e-01 9.812233e-01

4239 9.079331e-01 9.812242e-01

4240 9.079494e-01 9.812251e-01

4241 9.079651e-01 9.812259e-01

4242 9.079804e-01 9.812268e-01

4243 9.079951e-01 9.812276e-01

4244 9.080094e-01 9.812284e-01

4245 9.080231e-01 9.812292e-01

4246 9.080364e-01 9.812299e-01

4247 9.080491e-01 9.812307e-01

4248 9.080614e-01 9.812314e-01

4249 9.080733e-01 9.812321e-01

4250 9.080846e-01 9.812328e-01

4251 9.080955e-01 9.812335e-01

4252 9.081059e-01 9.812341e-01

4253 9.081158e-01 9.812348e-01

4254 9.081253e-01 9.812354e-01

4255 9.081343e-01 9.812360e-01

4256 9.081429e-01 9.812365e-01

4257 9.081510e-01 9.812371e-01

4258 9.081586e-01 9.812376e-01

4259 9.081658e-01 9.812381e-01

4260 9.081725e-01 9.812385e-01

4261 9.081788e-01 9.812390e-01

4262 9.081846e-01 9.812394e-01

4263 9.081900e-01 9.812398e-01

4264 9.081949e-01 9.812401e-01

4265 9.081994e-01 9.812404e-01

4266 9.082034e-01 9.812407e-01

4267 9.082069e-01 9.812410e-01

4268 9.082099e-01 9.812413e-01

4269 9.082125e-01 9.812415e-01

4270 9.082146e-01 9.812417e-01

4271 9.082163e-01 9.812418e-01

4272 9.082175e-01 9.812419e-01

4273 9.082181e-01 9.812420e-01

4274 9.082184e-01 9.812421e-01

4275 9.082181e-01 9.812421e-01

4276 9.082173e-01 9.812421e-01

4277 9.082160e-01 9.812420e-01

4278 9.082142e-01 9.812419e-01

4279 9.082119e-01 9.812418e-01

4280 9.082092e-01 9.812416e-01

4281 9.082058e-01 9.812414e-01

4282 9.082020e-01 9.812412e-01

4283 9.081977e-01 9.812409e-01

4284 9.081928e-01 9.812406e-01

4285 9.081873e-01 9.812402e-01

4286 9.081814e-01 9.812398e-01

4287 9.081749e-01 9.812394e-01

4288 9.081678e-01 9.812389e-01

4289 9.081602e-01 9.812384e-01

4290 9.081520e-01 9.812378e-01

4291 9.081432e-01 9.812371e-01

4292 9.081339e-01 9.812365e-01

4293 9.081240e-01 9.812358e-01

4294 9.081135e-01 9.812350e-01

4295 9.081024e-01 9.812342e-01

4296 9.080907e-01 9.812333e-01

4297 9.080785e-01 9.812324e-01

4298 9.080656e-01 9.812314e-01

4299 9.080521e-01 9.812304e-01

4300 9.080380e-01 9.812293e-01

4301 9.080234e-01 9.812282e-01

4302 9.080081e-01 9.812270e-01

4303 9.079922e-01 9.812258e-01

4304 9.079756e-01 9.812245e-01

4305 9.079585e-01 9.812231e-01

4306 9.079407e-01 9.812217e-01

4307 9.079223e-01 9.812202e-01

4308 9.079034e-01 9.812187e-01

4309 9.078837e-01 9.812171e-01

4310 9.078635e-01 9.812155e-01

4311 9.078427e-01 9.812137e-01

4312 9.078213e-01 9.812120e-01

4313 9.077992e-01 9.812101e-01

4314 9.077766e-01 9.812082e-01

4315 9.077533e-01 9.812062e-01

4316 9.077295e-01 9.812042e-01

4317 9.077051e-01 9.812021e-01

4318 9.076801e-01 9.811999e-01

4319 9.076546e-01 9.811976e-01

4320 9.076285e-01 9.811953e-01

4321 9.076019e-01 9.811929e-01

4322 9.077495e-01 9.811940e-01

4323 9.078949e-01 9.811950e-01

4324 9.080379e-01 9.811961e-01

4325 9.081786e-01 9.811971e-01

4326 9.083171e-01 9.811982e-01

4327 9.084532e-01 9.811993e-01

4328 9.085870e-01 9.812003e-01

4329 9.087186e-01 9.812014e-01

4330 9.088479e-01 9.812025e-01

4331 9.089749e-01 9.812035e-01

4332 9.090996e-01 9.812046e-01

4333 9.092221e-01 9.812056e-01

4334 9.093424e-01 9.812067e-01

4335 9.094604e-01 9.812078e-01

4336 9.095762e-01 9.812088e-01

4337 9.096899e-01 9.812099e-01

4338 9.098014e-01 9.812109e-01

4339 9.099107e-01 9.812120e-01

4340 9.100179e-01 9.812130e-01

4341 9.101230e-01 9.812141e-01

4342 9.102260e-01 9.812151e-01

4343 9.103269e-01 9.812162e-01

4344 9.104258e-01 9.812172e-01

4345 9.105227e-01 9.812183e-01

4346 9.106177e-01 9.812193e-01

4347 9.107106e-01 9.812204e-01

4348 9.108016e-01 9.812214e-01

4349 9.108907e-01 9.812225e-01

4350 9.109779e-01 9.812235e-01

4351 9.110633e-01 9.812245e-01

4352 9.111469e-01 9.812256e-01

4353 9.112286e-01 9.812266e-01

4354 9.113086e-01 9.812276e-01

4355 9.113868e-01 9.812287e-01

4356 9.114634e-01 9.812297e-01

4357 9.115382e-01 9.812307e-01

4358 9.116114e-01 9.812317e-01

4359 9.116829e-01 9.812327e-01

4360 9.117529e-01 9.812338e-01

4361 9.118213e-01 9.812348e-01

4362 9.118881e-01 9.812358e-01

4363 9.119535e-01 9.812368e-01

4364 9.120173e-01 9.812378e-01

4365 9.120797e-01 9.812388e-01

4366 9.121407e-01 9.812398e-01

4367 9.122003e-01 9.812407e-01

4368 9.122585e-01 9.812417e-01

4369 9.123153e-01 9.812427e-01

4370 9.123709e-01 9.812437e-01

4371 9.124251e-01 9.812446e-01

4372 9.124781e-01 9.812456e-01

4373 9.125298e-01 9.812466e-01

4374 9.125803e-01 9.812475e-01

4375 9.126296e-01 9.812485e-01

4376 9.126777e-01 9.812494e-01

4377 9.127247e-01 9.812503e-01

4378 9.127706e-01 9.812513e-01

4379 9.128154e-01 9.812522e-01

4380 9.128591e-01 9.812531e-01

4381 9.129018e-01 9.812540e-01

4382 9.129434e-01 9.812549e-01

4383 9.129840e-01 9.812558e-01

4384 9.130237e-01 9.812567e-01

4385 9.130624e-01 9.812576e-01

4386 9.131001e-01 9.812584e-01

4387 9.131369e-01 9.812593e-01

4388 9.131728e-01 9.812601e-01

4389 9.132078e-01 9.812610e-01

4390 9.132419e-01 9.812618e-01

4391 9.132752e-01 9.812627e-01

4392 9.133077e-01 9.812635e-01

4393 9.133393e-01 9.812643e-01

4394 9.133702e-01 9.812651e-01

4395 9.134002e-01 9.812659e-01

4396 9.134295e-01 9.812667e-01

4397 9.134580e-01 9.812674e-01

4398 9.134858e-01 9.812682e-01

4399 9.135129e-01 9.812689e-01

4400 9.135393e-01 9.812697e-01

4401 9.135649e-01 9.812704e-01

4402 9.135899e-01 9.812711e-01

4403 9.136142e-01 9.812718e-01

4404 9.136379e-01 9.812725e-01

4405 9.136609e-01 9.812731e-01

4406 9.136833e-01 9.812738e-01

4407 9.137050e-01 9.812744e-01

4408 9.137261e-01 9.812751e-01

4409 9.137467e-01 9.812757e-01

4410 9.137666e-01 9.812763e-01

4411 9.137859e-01 9.812769e-01

4412 9.138047e-01 9.812774e-01

4413 9.138229e-01 9.812780e-01

4414 9.138405e-01 9.812785e-01

4415 9.138576e-01 9.812791e-01

4416 9.138742e-01 9.812796e-01

4417 9.138902e-01 9.812801e-01

4418 9.139056e-01 9.812805e-01

4419 9.139206e-01 9.812810e-01

4420 9.139350e-01 9.812814e-01

4421 9.139489e-01 9.812818e-01

4422 9.139623e-01 9.812822e-01

4423 9.139752e-01 9.812826e-01

4424 9.139876e-01 9.812829e-01

4425 9.139994e-01 9.812833e-01

4426 9.140108e-01 9.812836e-01

4427 9.140217e-01 9.812839e-01

4428 9.140322e-01 9.812841e-01

4429 9.140421e-01 9.812844e-01

4430 9.140516e-01 9.812846e-01

4431 9.140605e-01 9.812848e-01

4432 9.140690e-01 9.812850e-01

4433 9.140770e-01 9.812851e-01

4434 9.140846e-01 9.812852e-01

4435 9.140917e-01 9.812853e-01

4436 9.140983e-01 9.812854e-01

4437 9.141044e-01 9.812855e-01

4438 9.141101e-01 9.812855e-01

4439 9.141152e-01 9.812855e-01

4440 9.141200e-01 9.812854e-01

4441 9.141242e-01 9.812854e-01

4442 9.141280e-01 9.812853e-01

4443 9.141313e-01 9.812851e-01

4444 9.141341e-01 9.812850e-01

4445 9.141365e-01 9.812848e-01

4446 9.141384e-01 9.812846e-01

4447 9.141398e-01 9.812843e-01

4448 9.141408e-01 9.812840e-01

4449 9.141413e-01 9.812837e-01

4450 9.141413e-01 9.812834e-01

4451 9.141408e-01 9.812830e-01

4452 9.141398e-01 9.812825e-01

4453 9.141384e-01 9.812821e-01

4454 9.141365e-01 9.812816e-01

4455 9.141341e-01 9.812811e-01

4456 9.141312e-01 9.812805e-01

4457 9.141279e-01 9.812799e-01

4458 9.141240e-01 9.812792e-01

4459 9.141197e-01 9.812785e-01

4460 9.141149e-01 9.812778e-01

4461 9.141096e-01 9.812770e-01

4462 9.141038e-01 9.812762e-01

4463 9.140976e-01 9.812753e-01

4464 9.140908e-01 9.812744e-01

4465 9.140836e-01 9.812735e-01

4466 9.140759e-01 9.812725e-01

4467 9.140677e-01 9.812715e-01

4468 9.140590e-01 9.812704e-01

4469 9.140499e-01 9.812692e-01

4470 9.140402e-01 9.812681e-01

4471 9.140301e-01 9.812668e-01

4472 9.140196e-01 9.812655e-01

4473 9.140085e-01 9.812642e-01

4474 9.139970e-01 9.812628e-01

4475 9.139850e-01 9.812614e-01

4476 9.139726e-01 9.812599e-01

4477 9.139597e-01 9.812584e-01

4478 9.139464e-01 9.812568e-01

4479 9.139326e-01 9.812551e-01

4480 9.139184e-01 9.812534e-01

4481 9.139037e-01 9.812516e-01

4482 9.138887e-01 9.812498e-01

4483 9.138732e-01 9.812479e-01

4484 9.138573e-01 9.812460e-01

4485 9.138411e-01 9.812440e-01

4486 9.138244e-01 9.812419e-01

4487 9.138074e-01 9.812398e-01

4488 9.137900e-01 9.812376e-01

4489 9.137723e-01 9.812353e-01

4490 9.137542e-01 9.812330e-01

4491 9.137358e-01 9.812306e-01

4492 9.137171e-01 9.812282e-01

4493 9.136981e-01 9.812257e-01

4494 9.136789e-01 9.812231e-01

4495 9.136593e-01 9.812204e-01

4496 9.136395e-01 9.812177e-01

4497 9.136195e-01 9.812149e-01

4498 9.135993e-01 9.812120e-01

4499 9.135789e-01 9.812091e-01

4500 9.135583e-01 9.812061e-01

4501 9.135376e-01 9.812030e-01

4502 9.136729e-01 9.812038e-01

4503 9.138060e-01 9.812047e-01

4504 9.139369e-01 9.812055e-01

4505 9.140657e-01 9.812064e-01

4506 9.141922e-01 9.812072e-01

4507 9.143167e-01 9.812080e-01

4508 9.144389e-01 9.812089e-01

4509 9.145591e-01 9.812097e-01

4510 9.146771e-01 9.812105e-01

4511 9.147930e-01 9.812114e-01

4512 9.149068e-01 9.812122e-01

4513 9.150185e-01 9.812130e-01

4514 9.151281e-01 9.812139e-01

4515 9.152357e-01 9.812147e-01

4516 9.153413e-01 9.812155e-01

4517 9.154449e-01 9.812163e-01

4518 9.155465e-01 9.812171e-01

4519 9.156461e-01 9.812180e-01

4520 9.157437e-01 9.812188e-01

4521 9.158395e-01 9.812196e-01

4522 9.159333e-01 9.812204e-01

4523 9.160253e-01 9.812212e-01

4524 9.161155e-01 9.812220e-01

4525 9.162038e-01 9.812228e-01

4526 9.162903e-01 9.812236e-01

4527 9.163750e-01 9.812244e-01

4528 9.164580e-01 9.812252e-01

4529 9.165393e-01 9.812260e-01

4530 9.166189e-01 9.812268e-01

4531 9.166968e-01 9.812275e-01

4532 9.167730e-01 9.812283e-01

4533 9.168477e-01 9.812291e-01

4534 9.169207e-01 9.812299e-01

4535 9.169922e-01 9.812306e-01

4536 9.170622e-01 9.812314e-01

4537 9.171307e-01 9.812322e-01

4538 9.171976e-01 9.812329e-01

4539 9.172631e-01 9.812337e-01

4540 9.173272e-01 9.812344e-01

4541 9.173899e-01 9.812351e-01

4542 9.174512e-01 9.812359e-01

4543 9.175111e-01 9.812366e-01

4544 9.175697e-01 9.812373e-01

4545 9.176271e-01 9.812380e-01

4546 9.176831e-01 9.812388e-01

4547 9.177379e-01 9.812395e-01

4548 9.177914e-01 9.812402e-01

4549 9.178438e-01 9.812408e-01

4550 9.178949e-01 9.812415e-01

4551 9.179449e-01 9.812422e-01

4552 9.179938e-01 9.812429e-01

4553 9.180415e-01 9.812436e-01

4554 9.180882e-01 9.812442e-01

4555 9.181338e-01 9.812449e-01

4556 9.181783e-01 9.812455e-01

4557 9.182218e-01 9.812461e-01

4558 9.182643e-01 9.812468e-01

4559 9.183058e-01 9.812474e-01

4560 9.183464e-01 9.812480e-01

4561 9.183860e-01 9.812486e-01

4562 9.184246e-01 9.812492e-01

4563 9.184624e-01 9.812498e-01

4564 9.184992e-01 9.812504e-01

4565 9.185352e-01 9.812509e-01

4566 9.185703e-01 9.812515e-01

4567 9.186046e-01 9.812520e-01

4568 9.186381e-01 9.812526e-01

4569 9.186707e-01 9.812531e-01

4570 9.187026e-01 9.812536e-01

4571 9.187337e-01 9.812541e-01

4572 9.187640e-01 9.812546e-01

4573 9.187935e-01 9.812551e-01

4574 9.188223e-01 9.812555e-01

4575 9.188504e-01 9.812560e-01

4576 9.188778e-01 9.812564e-01

4577 9.189045e-01 9.812568e-01

4578 9.189305e-01 9.812573e-01

4579 9.189558e-01 9.812577e-01

4580 9.189805e-01 9.812581e-01

4581 9.190045e-01 9.812584e-01

4582 9.190279e-01 9.812588e-01

4583 9.190507e-01 9.812591e-01

4584 9.190728e-01 9.812595e-01

4585 9.190943e-01 9.812598e-01

4586 9.191152e-01 9.812601e-01

4587 9.191355e-01 9.812603e-01

4588 9.191553e-01 9.812606e-01

4589 9.191744e-01 9.812608e-01

4590 9.191930e-01 9.812611e-01

4591 9.192110e-01 9.812613e-01

4592 9.192285e-01 9.812615e-01

4593 9.192454e-01 9.812617e-01

4594 9.192618e-01 9.812618e-01

4595 9.192777e-01 9.812619e-01

4596 9.192930e-01 9.812621e-01

4597 9.193078e-01 9.812622e-01

4598 9.193221e-01 9.812622e-01

4599 9.193359e-01 9.812623e-01

4600 9.193491e-01 9.812623e-01

4601 9.193619e-01 9.812623e-01

4602 9.193742e-01 9.812623e-01

4603 9.193860e-01 9.812623e-01

4604 9.193972e-01 9.812622e-01

4605 9.194080e-01 9.812622e-01

4606 9.194183e-01 9.812620e-01

4607 9.194282e-01 9.812619e-01

4608 9.194375e-01 9.812618e-01

4609 9.194464e-01 9.812616e-01

4610 9.194549e-01 9.812614e-01

4611 9.194628e-01 9.812611e-01

4612 9.194703e-01 9.812609e-01

4613 9.194773e-01 9.812606e-01

4614 9.194839e-01 9.812603e-01

4615 9.194900e-01 9.812599e-01

4616 9.194957e-01 9.812596e-01

4617 9.195009e-01 9.812592e-01

4618 9.195056e-01 9.812587e-01

4619 9.195099e-01 9.812583e-01

4620 9.195138e-01 9.812578e-01

4621 9.195172e-01 9.812572e-01

4622 9.195202e-01 9.812567e-01

4623 9.195227e-01 9.812561e-01

4624 9.195248e-01 9.812555e-01

4625 9.195265e-01 9.812548e-01

4626 9.195278e-01 9.812541e-01

4627 9.195286e-01 9.812534e-01

4628 9.195290e-01 9.812526e-01

4629 9.195289e-01 9.812518e-01

4630 9.195285e-01 9.812510e-01

4631 9.195276e-01 9.812501e-01

4632 9.195263e-01 9.812492e-01

4633 9.195246e-01 9.812482e-01

4634 9.195225e-01 9.812472e-01

4635 9.195199e-01 9.812462e-01

4636 9.195170e-01 9.812451e-01

4637 9.195137e-01 9.812440e-01

4638 9.195100e-01 9.812428e-01

4639 9.195059e-01 9.812416e-01

4640 9.195015e-01 9.812403e-01

4641 9.194966e-01 9.812390e-01

4642 9.194914e-01 9.812377e-01

4643 9.194858e-01 9.812363e-01

4644 9.194799e-01 9.812349e-01

4645 9.194736e-01 9.812334e-01

4646 9.194669e-01 9.812319e-01

4647 9.194600e-01 9.812303e-01

4648 9.194527e-01 9.812287e-01

4649 9.194450e-01 9.812270e-01

4650 9.194371e-01 9.812252e-01

4651 9.194289e-01 9.812235e-01

4652 9.194203e-01 9.812216e-01

4653 9.194115e-01 9.812197e-01

4654 9.194024e-01 9.812178e-01

4655 9.193931e-01 9.812158e-01

4656 9.193834e-01 9.812137e-01

4657 9.193736e-01 9.812116e-01

4658 9.193635e-01 9.812094e-01

4659 9.193532e-01 9.812072e-01

4660 9.193427e-01 9.812049e-01

4661 9.193320e-01 9.812026e-01

4662 9.193211e-01 9.812002e-01

4663 9.193101e-01 9.811977e-01

4664 9.192989e-01 9.811952e-01

4665 9.192875e-01 9.811926e-01

4666 9.192761e-01 9.811899e-01

4667 9.192645e-01 9.811872e-01

4668 9.192529e-01 9.811844e-01

4669 9.192411e-01 9.811816e-01

4670 9.192294e-01 9.811786e-01

4671 9.192175e-01 9.811757e-01

4672 9.192057e-01 9.811726e-01

4673 9.191938e-01 9.811695e-01

4674 9.191820e-01 9.811663e-01

4675 9.191701e-01 9.811630e-01

4676 9.191583e-01 9.811597e-01

4677 9.191466e-01 9.811562e-01

4678 9.191350e-01 9.811528e-01

4679 9.191234e-01 9.811492e-01

4680 9.191120e-01 9.811456e-01

4681 9.191007e-01 9.811419e-01

4682 9.192255e-01 9.811425e-01

4683 9.193483e-01 9.811432e-01

4684 9.194690e-01 9.811438e-01

4685 9.195877e-01 9.811444e-01

4686 9.197043e-01 9.811451e-01

4687 9.198190e-01 9.811457e-01

4688 9.199316e-01 9.811464e-01

4689 9.200423e-01 9.811470e-01

4690 9.201510e-01 9.811476e-01

4691 9.202577e-01 9.811483e-01

4692 9.203625e-01 9.811489e-01

4693 9.204654e-01 9.811495e-01

4694 9.205664e-01 9.811501e-01

4695 9.206655e-01 9.811508e-01

4696 9.207627e-01 9.811514e-01

4697 9.208582e-01 9.811520e-01

4698 9.209518e-01 9.811526e-01

4699 9.210436e-01 9.811532e-01

4700 9.211337e-01 9.811538e-01

4701 9.212220e-01 9.811544e-01

4702 9.213086e-01 9.811550e-01

4703 9.213935e-01 9.811556e-01

4704 9.214767e-01 9.811562e-01

4705 9.215583e-01 9.811568e-01

4706 9.216382e-01 9.811573e-01

4707 9.217165e-01 9.811579e-01

4708 9.217933e-01 9.811585e-01

4709 9.218685e-01 9.811591e-01

4710 9.219422e-01 9.811596e-01

4711 9.220144e-01 9.811602e-01

4712 9.220851e-01 9.811607e-01

4713 9.221543e-01 9.811613e-01

4714 9.222221e-01 9.811618e-01

4715 9.222885e-01 9.811623e-01

4716 9.223536e-01 9.811629e-01

4717 9.224172e-01 9.811634e-01

4718 9.224796e-01 9.811639e-01

4719 9.225406e-01 9.811644e-01

4720 9.226003e-01 9.811649e-01

4721 9.226588e-01 9.811654e-01

4722 9.227160e-01 9.811659e-01

4723 9.227721e-01 9.811664e-01

4724 9.228269e-01 9.811669e-01

4725 9.228805e-01 9.811673e-01

4726 9.229330e-01 9.811678e-01

4727 9.229844e-01 9.811683e-01

4728 9.230347e-01 9.811687e-01

4729 9.230838e-01 9.811692e-01

4730 9.231319e-01 9.811696e-01

4731 9.231790e-01 9.811700e-01

4732 9.232250e-01 9.811704e-01

4733 9.232700e-01 9.811708e-01

4734 9.233141e-01 9.811712e-01

4735 9.233571e-01 9.811716e-01

4736 9.233992e-01 9.811720e-01

4737 9.234404e-01 9.811724e-01

4738 9.234807e-01 9.811727e-01

4739 9.235200e-01 9.811731e-01

4740 9.235585e-01 9.811734e-01

4741 9.235961e-01 9.811737e-01

4742 9.236329e-01 9.811740e-01

4743 9.236688e-01 9.811743e-01

4744 9.237039e-01 9.811746e-01

4745 9.237382e-01 9.811749e-01

4746 9.237718e-01 9.811752e-01

4747 9.238045e-01 9.811754e-01

4748 9.238365e-01 9.811757e-01

4749 9.238677e-01 9.811759e-01

4750 9.238982e-01 9.811761e-01

4751 9.239280e-01 9.811763e-01

4752 9.239571e-01 9.811765e-01

4753 9.239854e-01 9.811767e-01

4754 9.240131e-01 9.811769e-01

4755 9.240401e-01 9.811770e-01

4756 9.240665e-01 9.811772e-01

4757 9.240922e-01 9.811773e-01

4758 9.241172e-01 9.811774e-01

4759 9.241416e-01 9.811775e-01

4760 9.241654e-01 9.811775e-01

4761 9.241886e-01 9.811776e-01

4762 9.242112e-01 9.811776e-01

4763 9.242332e-01 9.811776e-01

4764 9.242546e-01 9.811776e-01

4765 9.242754e-01 9.811776e-01

4766 9.242956e-01 9.811776e-01

4767 9.243153e-01 9.811775e-01

4768 9.243345e-01 9.811775e-01

4769 9.243530e-01 9.811774e-01

4770 9.243711e-01 9.811773e-01

4771 9.243886e-01 9.811771e-01

4772 9.244056e-01 9.811770e-01

4773 9.244220e-01 9.811768e-01

4774 9.244380e-01 9.811766e-01

4775 9.244534e-01 9.811764e-01

4776 9.244683e-01 9.811762e-01

4777 9.244828e-01 9.811759e-01

4778 9.244967e-01 9.811756e-01

4779 9.245102e-01 9.811753e-01

4780 9.245231e-01 9.811750e-01

4781 9.245356e-01 9.811746e-01

4782 9.245477e-01 9.811742e-01

4783 9.245592e-01 9.811738e-01

4784 9.245703e-01 9.811734e-01

4785 9.245810e-01 9.811729e-01

4786 9.245912e-01 9.811725e-01

4787 9.246009e-01 9.811719e-01

4788 9.246102e-01 9.811714e-01

4789 9.246191e-01 9.811708e-01

4790 9.246275e-01 9.811702e-01

4791 9.246355e-01 9.811696e-01

4792 9.246431e-01 9.811689e-01

4793 9.246502e-01 9.811683e-01

4794 9.246570e-01 9.811675e-01

4795 9.246633e-01 9.811668e-01

4796 9.246692e-01 9.811660e-01

4797 9.246748e-01 9.811652e-01

4798 9.246799e-01 9.811643e-01

4799 9.246846e-01 9.811635e-01

4800 9.246890e-01 9.811626e-01

4801 9.246930e-01 9.811616e-01

4802 9.246966e-01 9.811606e-01

4803 9.246998e-01 9.811596e-01

4804 9.247027e-01 9.811585e-01

4805 9.247052e-01 9.811574e-01

4806 9.247073e-01 9.811563e-01

4807 9.247091e-01 9.811551e-01

4808 9.247106e-01 9.811539e-01

4809 9.247117e-01 9.811527e-01

4810 9.247125e-01 9.811514e-01

4811 9.247130e-01 9.811500e-01

4812 9.247132e-01 9.811487e-01

4813 9.247131e-01 9.811473e-01

4814 9.247126e-01 9.811458e-01

4815 9.247119e-01 9.811443e-01

4816 9.247109e-01 9.811428e-01

4817 9.247097e-01 9.811412e-01

4818 9.247081e-01 9.811395e-01

4819 9.247063e-01 9.811378e-01

4820 9.247043e-01 9.811361e-01

4821 9.247020e-01 9.811343e-01

4822 9.246995e-01 9.811325e-01

4823 9.246967e-01 9.811306e-01

4824 9.246938e-01 9.811287e-01

4825 9.246907e-01 9.811267e-01

4826 9.246873e-01 9.811247e-01

4827 9.246838e-01 9.811226e-01

4828 9.246801e-01 9.811205e-01

4829 9.246763e-01 9.811183e-01

4830 9.246723e-01 9.811161e-01

4831 9.246682e-01 9.811138e-01

4832 9.246640e-01 9.811114e-01

4833 9.246597e-01 9.811090e-01

4834 9.246552e-01 9.811066e-01

4835 9.246507e-01 9.811041e-01

4836 9.246461e-01 9.811015e-01

4837 9.246414e-01 9.810989e-01

4838 9.246367e-01 9.810962e-01

4839 9.246320e-01 9.810934e-01

4840 9.246272e-01 9.810906e-01

4841 9.246224e-01 9.810877e-01

4842 9.246177e-01 9.810848e-01

4843 9.246129e-01 9.810817e-01

4844 9.246082e-01 9.810787e-01

4845 9.246036e-01 9.810755e-01

4846 9.245989e-01 9.810723e-01

4847 9.245944e-01 9.810691e-01

4848 9.245900e-01 9.810657e-01

4849 9.245856e-01 9.810623e-01

4850 9.245814e-01 9.810588e-01

4851 9.245772e-01 9.810553e-01

4852 9.245733e-01 9.810517e-01

4853 9.245694e-01 9.810480e-01

4854 9.245658e-01 9.810442e-01

4855 9.245623e-01 9.810404e-01

4856 9.245590e-01 9.810365e-01

4857 9.245560e-01 9.810325e-01

4858 9.245531e-01 9.810285e-01

4859 9.245505e-01 9.810243e-01

4860 9.245481e-01 9.810201e-01

4861 9.245459e-01 9.810158e-01

4862 9.246622e-01 9.810163e-01

4863 9.247766e-01 9.810168e-01

4864 9.248890e-01 9.810173e-01

4865 9.249996e-01 9.810177e-01

4866 9.251083e-01 9.810182e-01

4867 9.252151e-01 9.810187e-01

4868 9.253201e-01 9.810191e-01

4869 9.254232e-01 9.810196e-01

4870 9.255246e-01 9.810200e-01

4871 9.256241e-01 9.810205e-01

4872 9.257219e-01 9.810209e-01

4873 9.258179e-01 9.810214e-01

4874 9.259122e-01 9.810218e-01

4875 9.260047e-01 9.810223e-01

4876 9.260956e-01 9.810227e-01

4877 9.261848e-01 9.810231e-01

4878 9.262723e-01 9.810236e-01

4879 9.263582e-01 9.810240e-01

4880 9.264425e-01 9.810244e-01

4881 9.265253e-01 9.810248e-01

4882 9.266064e-01 9.810252e-01

4883 9.266860e-01 9.810256e-01

4884 9.267641e-01 9.810260e-01

4885 9.268407e-01 9.810264e-01

4886 9.269158e-01 9.810268e-01

4887 9.269895e-01 9.810271e-01

4888 9.270618e-01 9.810275e-01

4889 9.271326e-01 9.810279e-01

4890 9.272020e-01 9.810282e-01

4891 9.272701e-01 9.810286e-01

4892 9.273369e-01 9.810289e-01

4893 9.274023e-01 9.810293e-01

4894 9.274665e-01 9.810296e-01

4895 9.275293e-01 9.810299e-01

4896 9.275909e-01 9.810303e-01

4897 9.276513e-01 9.810306e-01

4898 9.277105e-01 9.810309e-01

4899 9.277685e-01 9.810312e-01

4900 9.278253e-01 9.810315e-01

4901 9.278810e-01 9.810317e-01

4902 9.279355e-01 9.810320e-01

4903 9.279890e-01 9.810323e-01

4904 9.280413e-01 9.810325e-01

4905 9.280926e-01 9.810328e-01

4906 9.281428e-01 9.810330e-01

4907 9.281921e-01 9.810332e-01

4908 9.282403e-01 9.810335e-01

4909 9.282875e-01 9.810337e-01

4910 9.283337e-01 9.810339e-01

4911 9.283789e-01 9.810340e-01

4912 9.284233e-01 9.810342e-01

4913 9.284667e-01 9.810344e-01

4914 9.285092e-01 9.810345e-01

4915 9.285508e-01 9.810347e-01

4916 9.285915e-01 9.810348e-01

4917 9.286314e-01 9.810350e-01

4918 9.286704e-01 9.810351e-01

4919 9.287086e-01 9.810352e-01

4920 9.287460e-01 9.810353e-01

4921 9.287825e-01 9.810353e-01

4922 9.288183e-01 9.810354e-01

4923 9.288533e-01 9.810354e-01

4924 9.288876e-01 9.810355e-01

4925 9.289211e-01 9.810355e-01

4926 9.289538e-01 9.810355e-01

4927 9.289859e-01 9.810355e-01

4928 9.290172e-01 9.810355e-01

4929 9.290478e-01 9.810354e-01

4930 9.290778e-01 9.810354e-01

4931 9.291070e-01 9.810353e-01

4932 9.291356e-01 9.810352e-01

4933 9.291636e-01 9.810351e-01

4934 9.291908e-01 9.810350e-01

4935 9.292175e-01 9.810349e-01

4936 9.292435e-01 9.810347e-01

4937 9.292689e-01 9.810346e-01

4938 9.292937e-01 9.810344e-01

4939 9.293179e-01 9.810342e-01

4940 9.293415e-01 9.810340e-01

4941 9.293646e-01 9.810337e-01

4942 9.293870e-01 9.810335e-01

4943 9.294089e-01 9.810332e-01

4944 9.294302e-01 9.810329e-01

4945 9.294510e-01 9.810326e-01

4946 9.294712e-01 9.810322e-01

4947 9.294910e-01 9.810319e-01

4948 9.295101e-01 9.810315e-01

4949 9.295288e-01 9.810311e-01

4950 9.295469e-01 9.810307e-01

4951 9.295646e-01 9.810302e-01

4952 9.295817e-01 9.810298e-01

4953 9.295984e-01 9.810293e-01

4954 9.296145e-01 9.810288e-01

4955 9.296302e-01 9.810282e-01

4956 9.296455e-01 9.810276e-01

4957 9.296602e-01 9.810271e-01

4958 9.296745e-01 9.810264e-01

4959 9.296883e-01 9.810258e-01

4960 9.297017e-01 9.810251e-01

4961 9.297147e-01 9.810244e-01

4962 9.297272e-01 9.810237e-01

4963 9.297393e-01 9.810230e-01

4964 9.297510e-01 9.810222e-01

4965 9.297622e-01 9.810214e-01

4966 9.297731e-01 9.810205e-01

4967 9.297835e-01 9.810197e-01

4968 9.297936e-01 9.810188e-01

4969 9.298033e-01 9.810178e-01

4970 9.298125e-01 9.810169e-01

4971 9.298214e-01 9.810159e-01

4972 9.298300e-01 9.810148e-01

4973 9.298382e-01 9.810138e-01

4974 9.298460e-01 9.810127e-01

4975 9.298535e-01 9.810116e-01

4976 9.298606e-01 9.810104e-01

4977 9.298674e-01 9.810092e-01

4978 9.298739e-01 9.810080e-01

4979 9.298800e-01 9.810067e-01

4980 9.298859e-01 9.810054e-01

4981 9.298914e-01 9.810041e-01

4982 9.298966e-01 9.810027e-01

4983 9.299016e-01 9.810013e-01

4984 9.299062e-01 9.809998e-01

4985 9.299106e-01 9.809983e-01

4986 9.299148e-01 9.809968e-01

4987 9.299187e-01 9.809952e-01

4988 9.299223e-01 9.809936e-01

4989 9.299257e-01 9.809919e-01

4990 9.299288e-01 9.809902e-01

4991 9.299318e-01 9.809884e-01

4992 9.299345e-01 9.809866e-01

4993 9.299370e-01 9.809848e-01

4994 9.299393e-01 9.809829e-01

4995 9.299415e-01 9.809810e-01

4996 9.299435e-01 9.809790e-01

4997 9.299453e-01 9.809770e-01

4998 9.299469e-01 9.809749e-01

4999 9.299484e-01 9.809728e-01

5000 9.299498e-01 9.809706e-01

5001 9.299510e-01 9.809684e-01

5002 9.299522e-01 9.809661e-01

5003 9.299532e-01 9.809638e-01

5004 9.299541e-01 9.809614e-01

5005 9.299550e-01 9.809590e-01

5006 9.299557e-01 9.809565e-01

5007 9.299564e-01 9.809539e-01

5008 9.299571e-01 9.809513e-01

5009 9.299577e-01 9.809487e-01

5010 9.299583e-01 9.809460e-01

5011 9.299589e-01 9.809432e-01

5012 9.299594e-01 9.809404e-01

5013 9.299600e-01 9.809375e-01

5014 9.299605e-01 9.809346e-01

5015 9.299611e-01 9.809316e-01

5016 9.299617e-01 9.809285e-01

5017 9.299624e-01 9.809254e-01

5018 9.299631e-01 9.809222e-01

5019 9.299639e-01 9.809190e-01

5020 9.299647e-01 9.809156e-01

5021 9.299657e-01 9.809123e-01

5022 9.299667e-01 9.809088e-01

5023 9.299678e-01 9.809053e-01

5024 9.299691e-01 9.809017e-01

5025 9.299705e-01 9.808981e-01

5026 9.299720e-01 9.808944e-01

5027 9.299736e-01 9.808906e-01

5028 9.299754e-01 9.808867e-01

5029 9.299774e-01 9.808828e-01

5030 9.299795e-01 9.808788e-01

5031 9.299818e-01 9.808747e-01

5032 9.299843e-01 9.808706e-01

5033 9.299870e-01 9.808664e-01

5034 9.299899e-01 9.808621e-01

5035 9.299930e-01 9.808577e-01

5036 9.299964e-01 9.808533e-01

5037 9.299999e-01 9.808488e-01

5038 9.300037e-01 9.808442e-01

5039 9.300078e-01 9.808395e-01

5040 9.300121e-01 9.808348e-01

5041 9.300166e-01 9.808299e-01

5042 9.301263e-01 9.808303e-01

5043 9.302342e-01 9.808306e-01

5044 9.303403e-01 9.808309e-01

5045 9.304447e-01 9.808312e-01

5046 9.305474e-01 9.808316e-01

5047 9.306483e-01 9.808319e-01

5048 9.307475e-01 9.808322e-01

5049 9.308451e-01 9.808325e-01

5050 9.309409e-01 9.808328e-01

5051 9.310352e-01 9.808331e-01

5052 9.311278e-01 9.808333e-01

5053 9.312187e-01 9.808336e-01

5054 9.313081e-01 9.808339e-01

5055 9.313959e-01 9.808342e-01

5056 9.314822e-01 9.808344e-01

5057 9.315669e-01 9.808347e-01

5058 9.316501e-01 9.808350e-01

5059 9.317318e-01 9.808352e-01

5060 9.318121e-01 9.808355e-01

5061 9.318909e-01 9.808357e-01

5062 9.319682e-01 9.808359e-01

5063 9.320442e-01 9.808361e-01

5064 9.321188e-01 9.808364e-01

5065 9.321920e-01 9.808366e-01

5066 9.322638e-01 9.808368e-01

5067 9.323343e-01 9.808370e-01

5068 9.324035e-01 9.808372e-01

5069 9.324714e-01 9.808374e-01

5070 9.325381e-01 9.808375e-01

5071 9.326035e-01 9.808377e-01

5072 9.326677e-01 9.808379e-01

5073 9.327307e-01 9.808380e-01

5074 9.327924e-01 9.808382e-01

5075 9.328530e-01 9.808383e-01

5076 9.329125e-01 9.808384e-01

5077 9.329708e-01 9.808385e-01

5078 9.330280e-01 9.808386e-01

5079 9.330842e-01 9.808387e-01

5080 9.331392e-01 9.808388e-01

5081 9.331932e-01 9.808389e-01

5082 9.332461e-01 9.808390e-01

5083 9.332981e-01 9.808390e-01

5084 9.333490e-01 9.808391e-01

5085 9.333989e-01 9.808391e-01

5086 9.334478e-01 9.808392e-01

5087 9.334958e-01 9.808392e-01

5088 9.335429e-01 9.808392e-01

5089 9.335890e-01 9.808392e-01

5090 9.336342e-01 9.808392e-01

5091 9.336785e-01 9.808392e-01

5092 9.337220e-01 9.808391e-01

5093 9.337645e-01 9.808391e-01

5094 9.338063e-01 9.808390e-01

5095 9.338471e-01 9.808389e-01

5096 9.338872e-01 9.808388e-01

5097 9.339264e-01 9.808387e-01

5098 9.339649e-01 9.808386e-01

5099 9.340026e-01 9.808385e-01

5100 9.340395e-01 9.808383e-01

5101 9.340756e-01 9.808382e-01

5102 9.341110e-01 9.808380e-01

5103 9.341456e-01 9.808378e-01

5104 9.341796e-01 9.808376e-01

5105 9.342128e-01 9.808374e-01

5106 9.342453e-01 9.808372e-01

5107 9.342771e-01 9.808369e-01

5108 9.343082e-01 9.808367e-01

5109 9.343387e-01 9.808364e-01

5110 9.343685e-01 9.808361e-01

5111 9.343977e-01 9.808358e-01

5112 9.344262e-01 9.808354e-01

5113 9.344541e-01 9.808351e-01

5114 9.344814e-01 9.808347e-01

5115 9.345081e-01 9.808343e-01

5116 9.345341e-01 9.808339e-01

5117 9.345596e-01 9.808335e-01

5118 9.345845e-01 9.808330e-01

5119 9.346088e-01 9.808325e-01

5120 9.346326e-01 9.808321e-01

5121 9.346558e-01 9.808315e-01

5122 9.346784e-01 9.808310e-01

5123 9.347005e-01 9.808305e-01

5124 9.347221e-01 9.808299e-01

5125 9.347431e-01 9.808293e-01

5126 9.347636e-01 9.808287e-01

5127 9.347837e-01 9.808280e-01

5128 9.348032e-01 9.808274e-01

5129 9.348222e-01 9.808267e-01

5130 9.348408e-01 9.808259e-01

5131 9.348588e-01 9.808252e-01

5132 9.348764e-01 9.808244e-01

5133 9.348936e-01 9.808236e-01

5134 9.349103e-01 9.808228e-01

5135 9.349265e-01 9.808220e-01

5136 9.349423e-01 9.808211e-01

5137 9.349577e-01 9.808202e-01

5138 9.349726e-01 9.808193e-01

5139 9.349871e-01 9.808183e-01

5140 9.350012e-01 9.808173e-01

5141 9.350150e-01 9.808163e-01

5142 9.350283e-01 9.808153e-01

5143 9.350412e-01 9.808142e-01

5144 9.350538e-01 9.808131e-01

5145 9.350660e-01 9.808120e-01

5146 9.350778e-01 9.808108e-01

5147 9.350893e-01 9.808096e-01

5148 9.351004e-01 9.808084e-01

5149 9.351112e-01 9.808071e-01

5150 9.351217e-01 9.808058e-01

5151 9.351318e-01 9.808045e-01

5152 9.351417e-01 9.808031e-01

5153 9.351512e-01 9.808017e-01

5154 9.351604e-01 9.808002e-01

5155 9.351694e-01 9.807988e-01

5156 9.351780e-01 9.807972e-01

5157 9.351864e-01 9.807957e-01

5158 9.351945e-01 9.807941e-01

5159 9.352024e-01 9.807925e-01

5160 9.352100e-01 9.807908e-01

5161 9.352174e-01 9.807891e-01

5162 9.352246e-01 9.807873e-01

5163 9.352315e-01 9.807855e-01

5164 9.352382e-01 9.807837e-01

5165 9.352447e-01 9.807818e-01

5166 9.352511e-01 9.807799e-01

5167 9.352572e-01 9.807779e-01

5168 9.352632e-01 9.807759e-01

5169 9.352690e-01 9.807739e-01

5170 9.352746e-01 9.807718e-01

5171 9.352801e-01 9.807696e-01

5172 9.352854e-01 9.807674e-01

5173 9.352907e-01 9.807652e-01

5174 9.352958e-01 9.807629e-01

5175 9.353008e-01 9.807606e-01

5176 9.353056e-01 9.807582e-01

5177 9.353104e-01 9.807558e-01

5178 9.353151e-01 9.807533e-01

5179 9.353198e-01 9.807507e-01

5180 9.353243e-01 9.807481e-01

5181 9.353288e-01 9.807455e-01

5182 9.353333e-01 9.807428e-01

5183 9.353377e-01 9.807401e-01

5184 9.353421e-01 9.807372e-01

5185 9.353465e-01 9.807344e-01

5186 9.353508e-01 9.807315e-01

5187 9.353552e-01 9.807285e-01

5188 9.353595e-01 9.807255e-01

5189 9.353639e-01 9.807224e-01

5190 9.353682e-01 9.807192e-01

5191 9.353727e-01 9.807160e-01

5192 9.353771e-01 9.807127e-01

5193 9.353816e-01 9.807094e-01

5194 9.353861e-01 9.807060e-01

5195 9.353907e-01 9.807025e-01

5196 9.353954e-01 9.806990e-01

5197 9.354002e-01 9.806954e-01

5198 9.354050e-01 9.806918e-01

5199 9.354099e-01 9.806881e-01

5200 9.354149e-01 9.806843e-01

5201 9.354201e-01 9.806804e-01

5202 9.354253e-01 9.806765e-01

5203 9.354306e-01 9.806725e-01

5204 9.354361e-01 9.806685e-01

5205 9.354417e-01 9.806644e-01

5206 9.354475e-01 9.806602e-01

5207 9.354534e-01 9.806559e-01

5208 9.354594e-01 9.806515e-01

5209 9.354656e-01 9.806471e-01

5210 9.354719e-01 9.806426e-01

5211 9.354785e-01 9.806381e-01

5212 9.354851e-01 9.806334e-01

5213 9.354920e-01 9.806287e-01

5214 9.354990e-01 9.806239e-01

5215 9.355062e-01 9.806191e-01

5216 9.355136e-01 9.806141e-01

5217 9.355212e-01 9.806091e-01

5218 9.355290e-01 9.806040e-01

5219 9.355370e-01 9.805988e-01

5220 9.355452e-01 9.805935e-01

5221 9.355536e-01 9.805882e-01

5222 9.356585e-01 9.805884e-01

5223 9.357617e-01 9.805885e-01

5224 9.358633e-01 9.805887e-01

5225 9.359633e-01 9.805889e-01

5226 9.360616e-01 9.805891e-01

5227 9.361583e-01 9.805893e-01

5228 9.362535e-01 9.805894e-01

5229 9.363470e-01 9.805896e-01

5230 9.364391e-01 9.805897e-01

5231 9.365296e-01 9.805899e-01

5232 9.366186e-01 9.805900e-01

5233 9.367060e-01 9.805902e-01

5234 9.367920e-01 9.805903e-01

5235 9.368766e-01 9.805904e-01

5236 9.369597e-01 9.805905e-01

5237 9.370414e-01 9.805906e-01

5238 9.371216e-01 9.805907e-01

5239 9.372005e-01 9.805908e-01

5240 9.372780e-01 9.805909e-01

5241 9.373542e-01 9.805910e-01

5242 9.374290e-01 9.805911e-01

5243 9.375025e-01 9.805911e-01

5244 9.375748e-01 9.805912e-01

5245 9.376457e-01 9.805912e-01

5246 9.377154e-01 9.805913e-01

5247 9.377839e-01 9.805913e-01

5248 9.378511e-01 9.805913e-01

5249 9.379171e-01 9.805913e-01

5250 9.379820e-01 9.805914e-01

5251 9.380456e-01 9.805914e-01

5252 9.381082e-01 9.805913e-01

5253 9.381695e-01 9.805913e-01

5254 9.382298e-01 9.805913e-01

5255 9.382890e-01 9.805912e-01

5256 9.383471e-01 9.805912e-01

5257 9.384041e-01 9.805911e-01

5258 9.384601e-01 9.805911e-01

5259 9.385150e-01 9.805910e-01

5260 9.385689e-01 9.805909e-01

5261 9.386218e-01 9.805908e-01

5262 9.386738e-01 9.805907e-01

5263 9.387247e-01 9.805906e-01

5264 9.387747e-01 9.805904e-01

5265 9.388238e-01 9.805903e-01

5266 9.388719e-01 9.805901e-01

5267 9.389191e-01 9.805899e-01

5268 9.389654e-01 9.805897e-01

5269 9.390109e-01 9.805895e-01

5270 9.390554e-01 9.805893e-01

5271 9.390991e-01 9.805891e-01

5272 9.391420e-01 9.805889e-01

5273 9.391840e-01 9.805886e-01

5274 9.392252e-01 9.805883e-01

5275 9.392656e-01 9.805881e-01

5276 9.393052e-01 9.805878e-01

5277 9.393440e-01 9.805875e-01

5278 9.393820e-01 9.805871e-01

5279 9.394193e-01 9.805868e-01

5280 9.394558e-01 9.805864e-01

5281 9.394916e-01 9.805860e-01

5282 9.395267e-01 9.805857e-01

5283 9.395610e-01 9.805852e-01

5284 9.395947e-01 9.805848e-01

5285 9.396276e-01 9.805844e-01

5286 9.396599e-01 9.805839e-01

5287 9.396915e-01 9.805834e-01

5288 9.397225e-01 9.805829e-01

5289 9.397528e-01 9.805824e-01

5290 9.397824e-01 9.805819e-01

5291 9.398114e-01 9.805813e-01

5292 9.398398e-01 9.805808e-01

5293 9.398676e-01 9.805802e-01

5294 9.398948e-01 9.805796e-01

5295 9.399214e-01 9.805789e-01

5296 9.399474e-01 9.805783e-01

5297 9.399728e-01 9.805776e-01

5298 9.399977e-01 9.805769e-01

5299 9.400220e-01 9.805762e-01

5300 9.400458e-01 9.805754e-01

5301 9.400690e-01 9.805747e-01

5302 9.400917e-01 9.805739e-01

5303 9.401139e-01 9.805731e-01

5304 9.401355e-01 9.805722e-01

5305 9.401567e-01 9.805714e-01

5306 9.401774e-01 9.805705e-01

5307 9.401975e-01 9.805696e-01

5308 9.402172e-01 9.805686e-01

5309 9.402365e-01 9.805677e-01

5310 9.402553e-01 9.805667e-01

5311 9.402736e-01 9.805657e-01

5312 9.402915e-01 9.805646e-01

5313 9.403090e-01 9.805635e-01

5314 9.403260e-01 9.805624e-01

5315 9.403426e-01 9.805613e-01

5316 9.403588e-01 9.805602e-01

5317 9.403746e-01 9.805590e-01

5318 9.403901e-01 9.805577e-01

5319 9.404051e-01 9.805565e-01

5320 9.404198e-01 9.805552e-01

5321 9.404341e-01 9.805539e-01

5322 9.404480e-01 9.805525e-01

5323 9.404617e-01 9.805512e-01

5324 9.404749e-01 9.805498e-01

5325 9.404879e-01 9.805483e-01

5326 9.405005e-01 9.805468e-01

5327 9.405128e-01 9.805453e-01

5328 9.405248e-01 9.805438e-01

5329 9.405365e-01 9.805422e-01

5330 9.405480e-01 9.805406e-01

5331 9.405591e-01 9.805389e-01

5332 9.405700e-01 9.805372e-01

5333 9.405806e-01 9.805355e-01

5334 9.405910e-01 9.805337e-01

5335 9.406011e-01 9.805319e-01

5336 9.406110e-01 9.805300e-01

5337 9.406207e-01 9.805281e-01

5338 9.406302e-01 9.805262e-01

5339 9.406394e-01 9.805242e-01

5340 9.406485e-01 9.805222e-01

5341 9.406573e-01 9.805202e-01

5342 9.406660e-01 9.805181e-01

5343 9.406745e-01 9.805159e-01

5344 9.406829e-01 9.805137e-01

5345 9.406911e-01 9.805115e-01

5346 9.406991e-01 9.805092e-01

5347 9.407070e-01 9.805069e-01

5348 9.407148e-01 9.805045e-01

5349 9.407224e-01 9.805021e-01

5350 9.407300e-01 9.804996e-01

5351 9.407374e-01 9.804971e-01

5352 9.407447e-01 9.804945e-01

5353 9.407520e-01 9.804919e-01

5354 9.407592e-01 9.804892e-01

5355 9.407662e-01 9.804865e-01

5356 9.407733e-01 9.804837e-01

5357 9.407802e-01 9.804809e-01

5358 9.407872e-01 9.804780e-01

5359 9.407940e-01 9.804751e-01

5360 9.408009e-01 9.804721e-01

5361 9.408077e-01 9.804691e-01

5362 9.408144e-01 9.804660e-01

5363 9.408212e-01 9.804628e-01

5364 9.408280e-01 9.804596e-01

5365 9.408347e-01 9.804563e-01

5366 9.408415e-01 9.804530e-01

5367 9.408483e-01 9.804496e-01

5368 9.408551e-01 9.804462e-01

5369 9.408619e-01 9.804426e-01

5370 9.408688e-01 9.804391e-01

5371 9.408757e-01 9.804354e-01

5372 9.408826e-01 9.804317e-01

5373 9.408896e-01 9.804280e-01

5374 9.408966e-01 9.804241e-01

5375 9.409037e-01 9.804203e-01

5376 9.409109e-01 9.804163e-01

5377 9.409182e-01 9.804123e-01

5378 9.409255e-01 9.804082e-01

5379 9.409329e-01 9.804040e-01

5380 9.409403e-01 9.803998e-01

5381 9.409479e-01 9.803955e-01

5382 9.409556e-01 9.803911e-01

5383 9.409634e-01 9.803867e-01

5384 9.409712e-01 9.803821e-01

5385 9.409792e-01 9.803775e-01

5386 9.409873e-01 9.803729e-01

5387 9.409955e-01 9.803681e-01

5388 9.410038e-01 9.803633e-01

5389 9.410122e-01 9.803584e-01

5390 9.410208e-01 9.803535e-01

5391 9.410295e-01 9.803484e-01

5392 9.410383e-01 9.803433e-01

5393 9.410473e-01 9.803381e-01

5394 9.410563e-01 9.803328e-01

5395 9.410656e-01 9.803275e-01

5396 9.410749e-01 9.803220e-01

5397 9.410844e-01 9.803165e-01

5398 9.410941e-01 9.803109e-01

5399 9.411039e-01 9.803052e-01

5400 9.411138e-01 9.802994e-01

5401 9.411239e-01 9.802936e-01

5402 9.412254e-01 9.802936e-01

5403 9.413253e-01 9.802937e-01

5404 9.414236e-01 9.802938e-01

5405 9.415204e-01 9.802938e-01

5406 9.416157e-01 9.802939e-01

5407 9.417094e-01 9.802939e-01

5408 9.418016e-01 9.802939e-01

5409 9.418924e-01 9.802940e-01

5410 9.419817e-01 9.802940e-01

5411 9.420695e-01 9.802940e-01

5412 9.421559e-01 9.802940e-01

5413 9.422409e-01 9.802940e-01

5414 9.423245e-01 9.802940e-01

5415 9.424067e-01 9.802940e-01

5416 9.424875e-01 9.802939e-01

5417 9.425670e-01 9.802939e-01

5418 9.426451e-01 9.802939e-01

5419 9.427219e-01 9.802938e-01

5420 9.427975e-01 9.802938e-01

5421 9.428717e-01 9.802937e-01

5422 9.429446e-01 9.802936e-01

5423 9.430164e-01 9.802935e-01

5424 9.430868e-01 9.802934e-01

5425 9.431561e-01 9.802933e-01

5426 9.432241e-01 9.802932e-01

5427 9.432910e-01 9.802931e-01

5428 9.433567e-01 9.802930e-01

5429 9.434212e-01 9.802928e-01

5430 9.434846e-01 9.802927e-01

5431 9.435468e-01 9.802925e-01

5432 9.436080e-01 9.802924e-01

5433 9.436681e-01 9.802922e-01

5434 9.437270e-01 9.802920e-01

5435 9.437850e-01 9.802918e-01

5436 9.438418e-01 9.802916e-01

5437 9.438977e-01 9.802913e-01

5438 9.439525e-01 9.802911e-01

5439 9.440063e-01 9.802909e-01

5440 9.440591e-01 9.802906e-01

5441 9.441110e-01 9.802903e-01

5442 9.441619e-01 9.802900e-01

5443 9.442118e-01 9.802897e-01

5444 9.442608e-01 9.802894e-01

5445 9.443089e-01 9.802891e-01

5446 9.443560e-01 9.802887e-01

5447 9.444023e-01 9.802884e-01

5448 9.444477e-01 9.802880e-01

5449 9.444922e-01 9.802876e-01

5450 9.445359e-01 9.802872e-01

5451 9.445787e-01 9.802868e-01

5452 9.446207e-01 9.802864e-01

5453 9.446619e-01 9.802859e-01

5454 9.447023e-01 9.802855e-01

5455 9.447418e-01 9.802850e-01

5456 9.447806e-01 9.802845e-01

5457 9.448187e-01 9.802840e-01

5458 9.448559e-01 9.802835e-01

5459 9.448924e-01 9.802829e-01

5460 9.449282e-01 9.802824e-01

5461 9.449632e-01 9.802818e-01

5462 9.449976e-01 9.802812e-01

5463 9.450312e-01 9.802806e-01

5464 9.450641e-01 9.802800e-01

5465 9.450964e-01 9.802793e-01

5466 9.451280e-01 9.802786e-01

5467 9.451589e-01 9.802779e-01

5468 9.451892e-01 9.802772e-01

5469 9.452188e-01 9.802765e-01

5470 9.452478e-01 9.802757e-01

5471 9.452762e-01 9.802750e-01

5472 9.453040e-01 9.802742e-01

5473 9.453312e-01 9.802733e-01

5474 9.453578e-01 9.802725e-01

5475 9.453838e-01 9.802716e-01

5476 9.454092e-01 9.802707e-01

5477 9.454341e-01 9.802698e-01

5478 9.454584e-01 9.802689e-01

5479 9.454823e-01 9.802679e-01

5480 9.455055e-01 9.802670e-01

5481 9.455283e-01 9.802660e-01

5482 9.455505e-01 9.802649e-01

5483 9.455723e-01 9.802639e-01

5484 9.455936e-01 9.802628e-01

5485 9.456143e-01 9.802617e-01

5486 9.456346e-01 9.802605e-01

5487 9.456545e-01 9.802594e-01

5488 9.456739e-01 9.802582e-01

5489 9.456929e-01 9.802569e-01

5490 9.457114e-01 9.802557e-01

5491 9.457295e-01 9.802544e-01

5492 9.457472e-01 9.802531e-01

5493 9.457645e-01 9.802518e-01

5494 9.457814e-01 9.802504e-01

5495 9.457979e-01 9.802490e-01

5496 9.458141e-01 9.802476e-01

5497 9.458298e-01 9.802461e-01

5498 9.458452e-01 9.802446e-01

5499 9.458603e-01 9.802431e-01

5500 9.458750e-01 9.802415e-01

5501 9.458895e-01 9.802399e-01

5502 9.459035e-01 9.802383e-01

5503 9.459173e-01 9.802366e-01

5504 9.459308e-01 9.802349e-01

5505 9.459440e-01 9.802331e-01

5506 9.459569e-01 9.802314e-01

5507 9.459695e-01 9.802296e-01

5508 9.459818e-01 9.802277e-01

5509 9.459939e-01 9.802258e-01

5510 9.460058e-01 9.802239e-01

5511 9.460174e-01 9.802219e-01

5512 9.460288e-01 9.802199e-01

5513 9.460399e-01 9.802179e-01

5514 9.460508e-01 9.802158e-01

5515 9.460616e-01 9.802136e-01

5516 9.460721e-01 9.802115e-01

5517 9.460825e-01 9.802093e-01

5518 9.460926e-01 9.802070e-01

5519 9.461026e-01 9.802047e-01

5520 9.461124e-01 9.802024e-01

5521 9.461221e-01 9.802000e-01

5522 9.461316e-01 9.801975e-01

5523 9.461410e-01 9.801950e-01

5524 9.461503e-01 9.801925e-01

5525 9.461594e-01 9.801899e-01

5526 9.461684e-01 9.801873e-01

5527 9.461773e-01 9.801846e-01

5528 9.461861e-01 9.801819e-01

5529 9.461947e-01 9.801791e-01

5530 9.462033e-01 9.801763e-01

5531 9.462119e-01 9.801734e-01

5532 9.462203e-01 9.801705e-01

5533 9.462287e-01 9.801675e-01

5534 9.462370e-01 9.801645e-01

5535 9.462452e-01 9.801614e-01

5536 9.462534e-01 9.801582e-01

5537 9.462616e-01 9.801550e-01

5538 9.462697e-01 9.801518e-01

5539 9.462778e-01 9.801485e-01

5540 9.462858e-01 9.801451e-01

5541 9.462939e-01 9.801417e-01

5542 9.463019e-01 9.801382e-01

5543 9.463099e-01 9.801346e-01

5544 9.463179e-01 9.801310e-01

5545 9.463260e-01 9.801274e-01

5546 9.463340e-01 9.801236e-01

5547 9.463420e-01 9.801199e-01

5548 9.463501e-01 9.801160e-01

5549 9.463581e-01 9.801121e-01

5550 9.463662e-01 9.801081e-01

5551 9.463744e-01 9.801040e-01

5552 9.463825e-01 9.800999e-01

5553 9.463907e-01 9.800957e-01

5554 9.463990e-01 9.800915e-01

5555 9.464073e-01 9.800872e-01

5556 9.464156e-01 9.800828e-01

5557 9.464240e-01 9.800783e-01

5558 9.464325e-01 9.800738e-01

5559 9.464410e-01 9.800692e-01

5560 9.464495e-01 9.800645e-01

5561 9.464582e-01 9.800598e-01

5562 9.464669e-01 9.800550e-01

5563 9.464757e-01 9.800501e-01

5564 9.464846e-01 9.800451e-01

5565 9.464935e-01 9.800401e-01

5566 9.465025e-01 9.800350e-01

5567 9.465116e-01 9.800298e-01

5568 9.465208e-01 9.800245e-01

5569 9.465301e-01 9.800191e-01

5570 9.465395e-01 9.800137e-01

5571 9.465490e-01 9.800082e-01

5572 9.465585e-01 9.800026e-01

5573 9.465682e-01 9.799969e-01

5574 9.465779e-01 9.799912e-01

5575 9.465878e-01 9.799853e-01

5576 9.465977e-01 9.799794e-01

5577 9.466078e-01 9.799734e-01

5578 9.466179e-01 9.799673e-01

5579 9.466282e-01 9.799611e-01

5580 9.466386e-01 9.799548e-01

5581 9.466490e-01 9.799485e-01

5582 9.467478e-01 9.799484e-01

5583 9.468450e-01 9.799484e-01

5584 9.469407e-01 9.799483e-01

5585 9.470349e-01 9.799483e-01

5586 9.471277e-01 9.799482e-01

5587 9.472189e-01 9.799481e-01

5588 9.473087e-01 9.799480e-01

5589 9.473971e-01 9.799479e-01

5590 9.474840e-01 9.799478e-01

5591 9.475696e-01 9.799477e-01

5592 9.476537e-01 9.799476e-01

5593 9.477365e-01 9.799474e-01

5594 9.478179e-01 9.799473e-01

5595 9.478979e-01 9.799472e-01

5596 9.479766e-01 9.799470e-01

5597 9.480540e-01 9.799468e-01

5598 9.481301e-01 9.799467e-01

5599 9.482049e-01 9.799465e-01

5600 9.482784e-01 9.799463e-01

5601 9.483507e-01 9.799461e-01

5602 9.484217e-01 9.799459e-01

5603 9.484915e-01 9.799457e-01

5604 9.485601e-01 9.799454e-01

5605 9.486275e-01 9.799452e-01

5606 9.486937e-01 9.799449e-01

5607 9.487587e-01 9.799447e-01

5608 9.488226e-01 9.799444e-01

5609 9.488853e-01 9.799441e-01

5610 9.489469e-01 9.799438e-01

5611 9.490074e-01 9.799435e-01

5612 9.490669e-01 9.799432e-01

5613 9.491252e-01 9.799428e-01

5614 9.491825e-01 9.799425e-01

5615 9.492387e-01 9.799421e-01

5616 9.492939e-01 9.799418e-01

5617 9.493480e-01 9.799414e-01

5618 9.494012e-01 9.799410e-01

5619 9.494533e-01 9.799406e-01

5620 9.495045e-01 9.799401e-01

5621 9.495547e-01 9.799397e-01

5622 9.496039e-01 9.799393e-01

5623 9.496522e-01 9.799388e-01

5624 9.496996e-01 9.799383e-01

5625 9.497461e-01 9.799378e-01

5626 9.497916e-01 9.799373e-01

5627 9.498363e-01 9.799368e-01

5628 9.498801e-01 9.799362e-01

5629 9.499230e-01 9.799357e-01

5630 9.499651e-01 9.799351e-01

5631 9.500064e-01 9.799345e-01

5632 9.500468e-01 9.799339e-01

5633 9.500864e-01 9.799333e-01

5634 9.501252e-01 9.799326e-01

5635 9.501632e-01 9.799320e-01

5636 9.502005e-01 9.799313e-01

5637 9.502370e-01 9.799306e-01

5638 9.502727e-01 9.799299e-01

5639 9.503077e-01 9.799291e-01

5640 9.503420e-01 9.799284e-01

5641 9.503756e-01 9.799276e-01

5642 9.504084e-01 9.799268e-01

5643 9.504406e-01 9.799260e-01

5644 9.504721e-01 9.799252e-01

5645 9.505029e-01 9.799243e-01

5646 9.505330e-01 9.799234e-01

5647 9.505626e-01 9.799225e-01

5648 9.505914e-01 9.799216e-01

5649 9.506197e-01 9.799207e-01

5650 9.506473e-01 9.799197e-01

5651 9.506744e-01 9.799187e-01

5652 9.507008e-01 9.799177e-01

5653 9.507267e-01 9.799167e-01

5654 9.507520e-01 9.799156e-01

5655 9.507768e-01 9.799145e-01

5656 9.508010e-01 9.799134e-01

5657 9.508247e-01 9.799123e-01

5658 9.508478e-01 9.799111e-01

5659 9.508705e-01 9.799100e-01

5660 9.508926e-01 9.799087e-01

5661 9.509143e-01 9.799075e-01

5662 9.509354e-01 9.799062e-01

5663 9.509561e-01 9.799049e-01

5664 9.509764e-01 9.799036e-01

5665 9.509961e-01 9.799023e-01

5666 9.510155e-01 9.799009e-01

5667 9.510344e-01 9.798995e-01

5668 9.510529e-01 9.798980e-01

5669 9.510710e-01 9.798966e-01

5670 9.510887e-01 9.798951e-01

5671 9.511060e-01 9.798935e-01

5672 9.511229e-01 9.798920e-01

5673 9.511394e-01 9.798904e-01

5674 9.511556e-01 9.798887e-01

5675 9.511715e-01 9.798871e-01

5676 9.511870e-01 9.798854e-01

5677 9.512021e-01 9.798837e-01

5678 9.512170e-01 9.798819e-01

5679 9.512315e-01 9.798801e-01

5680 9.512457e-01 9.798783e-01

5681 9.512597e-01 9.798764e-01

5682 9.512733e-01 9.798745e-01

5683 9.512867e-01 9.798725e-01

5684 9.512998e-01 9.798705e-01

5685 9.513126e-01 9.798685e-01

5686 9.513252e-01 9.798665e-01

5687 9.513376e-01 9.798644e-01

5688 9.513497e-01 9.798622e-01

5689 9.513616e-01 9.798600e-01

5690 9.513733e-01 9.798578e-01

5691 9.513847e-01 9.798555e-01

5692 9.513960e-01 9.798532e-01

5693 9.514071e-01 9.798509e-01

5694 9.514180e-01 9.798485e-01

5695 9.514287e-01 9.798461e-01

5696 9.514392e-01 9.798436e-01

5697 9.514496e-01 9.798410e-01

5698 9.514598e-01 9.798385e-01

5699 9.514699e-01 9.798359e-01

5700 9.514799e-01 9.798332e-01

5701 9.514897e-01 9.798305e-01

5702 9.514994e-01 9.798277e-01

5703 9.515089e-01 9.798249e-01

5704 9.515184e-01 9.798220e-01

5705 9.515277e-01 9.798191e-01

5706 9.515370e-01 9.798162e-01

5707 9.515461e-01 9.798132e-01

5708 9.515552e-01 9.798101e-01

5709 9.515642e-01 9.798070e-01

5710 9.515731e-01 9.798038e-01

5711 9.515819e-01 9.798006e-01

5712 9.515907e-01 9.797973e-01

5713 9.515994e-01 9.797940e-01

5714 9.516080e-01 9.797906e-01

5715 9.516167e-01 9.797871e-01

5716 9.516252e-01 9.797836e-01

5717 9.516338e-01 9.797801e-01

5718 9.516423e-01 9.797764e-01

5719 9.516507e-01 9.797727e-01

5720 9.516592e-01 9.797690e-01

5721 9.516676e-01 9.797652e-01

5722 9.516761e-01 9.797613e-01

5723 9.516845e-01 9.797574e-01

5724 9.516929e-01 9.797534e-01

5725 9.517013e-01 9.797494e-01

5726 9.517097e-01 9.797452e-01

5727 9.517181e-01 9.797411e-01

5728 9.517265e-01 9.797368e-01

5729 9.517349e-01 9.797325e-01

5730 9.517434e-01 9.797281e-01

5731 9.517519e-01 9.797237e-01

5732 9.517604e-01 9.797191e-01

5733 9.517689e-01 9.797145e-01

5734 9.517775e-01 9.797099e-01

5735 9.517860e-01 9.797052e-01

5736 9.517947e-01 9.797004e-01

5737 9.518033e-01 9.796955e-01

5738 9.518120e-01 9.796905e-01

5739 9.518208e-01 9.796855e-01

5740 9.518296e-01 9.796804e-01

5741 9.518384e-01 9.796752e-01

5742 9.518473e-01 9.796700e-01

5743 9.518563e-01 9.796646e-01

5744 9.518653e-01 9.796592e-01

5745 9.518743e-01 9.796537e-01

5746 9.518835e-01 9.796482e-01

5747 9.518926e-01 9.796425e-01

5748 9.519019e-01 9.796368e-01

5749 9.519112e-01 9.796310e-01

5750 9.519206e-01 9.796251e-01

5751 9.519300e-01 9.796191e-01

5752 9.519395e-01 9.796131e-01

5753 9.519491e-01 9.796069e-01

5754 9.519587e-01 9.796007e-01

5755 9.519685e-01 9.795944e-01

5756 9.519783e-01 9.795880e-01

5757 9.519881e-01 9.795815e-01

5758 9.519981e-01 9.795749e-01

5759 9.520081e-01 9.795682e-01

5760 9.520182e-01 9.795615e-01

5761 9.520284e-01 9.795546e-01

5762 9.521243e-01 9.795545e-01

5763 9.522187e-01 9.795543e-01

5764 9.523117e-01 9.795541e-01

5765 9.524031e-01 9.795540e-01

5766 9.524931e-01 9.795538e-01

5767 9.525816e-01 9.795536e-01

5768 9.526687e-01 9.795534e-01

5769 9.527543e-01 9.795531e-01

5770 9.528385e-01 9.795529e-01

5771 9.529214e-01 9.795527e-01

5772 9.530028e-01 9.795525e-01

5773 9.530829e-01 9.795522e-01

5774 9.531616e-01 9.795519e-01

5775 9.532389e-01 9.795517e-01

5776 9.533150e-01 9.795514e-01

5777 9.533897e-01 9.795511e-01

5778 9.534631e-01 9.795508e-01

5779 9.535352e-01 9.795505e-01

5780 9.536061e-01 9.795502e-01

5781 9.536757e-01 9.795498e-01

5782 9.537441e-01 9.795495e-01

5783 9.538112e-01 9.795491e-01

5784 9.538772e-01 9.795488e-01

5785 9.539419e-01 9.795484e-01

5786 9.540055e-01 9.795480e-01

5787 9.540679e-01 9.795476e-01

5788 9.541291e-01 9.795472e-01

5789 9.541892e-01 9.795468e-01

5790 9.542482e-01 9.795463e-01

5791 9.543061e-01 9.795459e-01

5792 9.543629e-01 9.795454e-01

5793 9.544186e-01 9.795449e-01

5794 9.544733e-01 9.795445e-01

5795 9.545269e-01 9.795439e-01

5796 9.545794e-01 9.795434e-01

5797 9.546310e-01 9.795429e-01

5798 9.546815e-01 9.795423e-01

5799 9.547311e-01 9.795418e-01

5800 9.547797e-01 9.795412e-01

5801 9.548273e-01 9.795406e-01

5802 9.548740e-01 9.795400e-01

5803 9.549197e-01 9.795394e-01

5804 9.549645e-01 9.795387e-01

5805 9.550085e-01 9.795381e-01

5806 9.550515e-01 9.795374e-01

5807 9.550936e-01 9.795367e-01

5808 9.551349e-01 9.795360e-01

5809 9.551753e-01 9.795353e-01

5810 9.552149e-01 9.795345e-01

5811 9.552537e-01 9.795338e-01

5812 9.552917e-01 9.795330e-01

5813 9.553288e-01 9.795322e-01

5814 9.553652e-01 9.795314e-01

5815 9.554008e-01 9.795305e-01

5816 9.554357e-01 9.795297e-01

5817 9.554698e-01 9.795288e-01

5818 9.555032e-01 9.795279e-01

5819 9.555358e-01 9.795270e-01

5820 9.555678e-01 9.795261e-01

5821 9.555991e-01 9.795251e-01

5822 9.556296e-01 9.795241e-01

5823 9.556596e-01 9.795231e-01

5824 9.556888e-01 9.795221e-01

5825 9.557175e-01 9.795210e-01

5826 9.557454e-01 9.795200e-01

5827 9.557728e-01 9.795189e-01

5828 9.557996e-01 9.795177e-01

5829 9.558258e-01 9.795166e-01

5830 9.558514e-01 9.795154e-01

5831 9.558764e-01 9.795142e-01

5832 9.559009e-01 9.795130e-01

5833 9.559248e-01 9.795118e-01

5834 9.559482e-01 9.795105e-01

5835 9.559711e-01 9.795092e-01

5836 9.559934e-01 9.795079e-01

5837 9.560153e-01 9.795066e-01

5838 9.560367e-01 9.795052e-01

5839 9.560575e-01 9.795038e-01

5840 9.560780e-01 9.795023e-01

5841 9.560980e-01 9.795009e-01

5842 9.561175e-01 9.794994e-01

5843 9.561366e-01 9.794979e-01

5844 9.561553e-01 9.794963e-01

5845 9.561735e-01 9.794947e-01

5846 9.561914e-01 9.794931e-01

5847 9.562088e-01 9.794915e-01

5848 9.562259e-01 9.794898e-01

5849 9.562427e-01 9.794881e-01

5850 9.562590e-01 9.794863e-01

5851 9.562750e-01 9.794846e-01

5852 9.562907e-01 9.794827e-01

5853 9.563060e-01 9.794809e-01

5854 9.563210e-01 9.794790e-01

5855 9.563357e-01 9.794771e-01

5856 9.563501e-01 9.794752e-01

5857 9.563642e-01 9.794732e-01

5858 9.563780e-01 9.794711e-01

5859 9.563916e-01 9.794691e-01

5860 9.564048e-01 9.794670e-01

5861 9.564179e-01 9.794648e-01

5862 9.564306e-01 9.794627e-01

5863 9.564431e-01 9.794604e-01

5864 9.564554e-01 9.794582e-01

5865 9.564675e-01 9.794559e-01

5866 9.564793e-01 9.794535e-01

5867 9.564910e-01 9.794512e-01

5868 9.565024e-01 9.794487e-01

5869 9.565136e-01 9.794463e-01

5870 9.565247e-01 9.794438e-01

5871 9.565356e-01 9.794412e-01

5872 9.565463e-01 9.794386e-01

5873 9.565568e-01 9.794360e-01

5874 9.565672e-01 9.794333e-01

5875 9.565774e-01 9.794305e-01

5876 9.565875e-01 9.794278e-01

5877 9.565974e-01 9.794249e-01

5878 9.566073e-01 9.794220e-01

5879 9.566169e-01 9.794191e-01

5880 9.566265e-01 9.794161e-01

5881 9.566360e-01 9.794131e-01

5882 9.566453e-01 9.794100e-01

5883 9.566546e-01 9.794069e-01

5884 9.566637e-01 9.794037e-01

5885 9.566728e-01 9.794005e-01

5886 9.566818e-01 9.793972e-01

5887 9.566907e-01 9.793938e-01

5888 9.566995e-01 9.793904e-01

5889 9.567083e-01 9.793870e-01

5890 9.567170e-01 9.793835e-01

5891 9.567256e-01 9.793799e-01

5892 9.567342e-01 9.793763e-01

5893 9.567427e-01 9.793726e-01

5894 9.567512e-01 9.793689e-01

5895 9.567597e-01 9.793651e-01

5896 9.567681e-01 9.793612e-01

5897 9.567764e-01 9.793573e-01

5898 9.567848e-01 9.793533e-01

5899 9.567931e-01 9.793493e-01

5900 9.568014e-01 9.793451e-01

5901 9.568097e-01 9.793410e-01

5902 9.568180e-01 9.793367e-01

5903 9.568263e-01 9.793324e-01

5904 9.568345e-01 9.793281e-01

5905 9.568428e-01 9.793236e-01

5906 9.568510e-01 9.793191e-01

5907 9.568593e-01 9.793146e-01

5908 9.568676e-01 9.793099e-01

5909 9.568758e-01 9.793052e-01

5910 9.568841e-01 9.793004e-01

5911 9.568924e-01 9.792956e-01

5912 9.569008e-01 9.792907e-01

5913 9.569091e-01 9.792857e-01

5914 9.569175e-01 9.792806e-01

5915 9.569258e-01 9.792754e-01

5916 9.569343e-01 9.792702e-01

5917 9.569427e-01 9.792649e-01

5918 9.569512e-01 9.792596e-01

5919 9.569597e-01 9.792541e-01

5920 9.569682e-01 9.792486e-01

5921 9.569768e-01 9.792430e-01

5922 9.569854e-01 9.792373e-01

5923 9.569941e-01 9.792315e-01

5924 9.570028e-01 9.792257e-01

5925 9.570115e-01 9.792198e-01

5926 9.570203e-01 9.792138e-01

5927 9.570292e-01 9.792077e-01

5928 9.570380e-01 9.792015e-01

5929 9.570470e-01 9.791952e-01

5930 9.570560e-01 9.791889e-01

5931 9.570650e-01 9.791825e-01

5932 9.570741e-01 9.791759e-01

5933 9.570832e-01 9.791693e-01

5934 9.570925e-01 9.791626e-01

5935 9.571017e-01 9.791559e-01

5936 9.571110e-01 9.791490e-01

5937 9.571204e-01 9.791420e-01

5938 9.571299e-01 9.791350e-01

5939 9.571394e-01 9.791278e-01

5940 9.571489e-01 9.791206e-01

5941 9.571585e-01 9.791132e-01

5942 9.572508e-01 9.791130e-01

5943 9.573416e-01 9.791127e-01

5944 9.574309e-01 9.791124e-01

5945 9.575187e-01 9.791121e-01

5946 9.576050e-01 9.791118e-01

5947 9.576898e-01 9.791115e-01

5948 9.577732e-01 9.791112e-01

5949 9.578551e-01 9.791109e-01

5950 9.579356e-01 9.791106e-01

5951 9.580147e-01 9.791102e-01

5952 9.580924e-01 9.791099e-01

5953 9.581688e-01 9.791095e-01

5954 9.582437e-01 9.791091e-01

5955 9.583174e-01 9.791087e-01

5956 9.583896e-01 9.791083e-01

5957 9.584606e-01 9.791079e-01

5958 9.585303e-01 9.791075e-01

5959 9.585986e-01 9.791071e-01

5960 9.586657e-01 9.791066e-01

5961 9.587316e-01 9.791062e-01

5962 9.587962e-01 9.791057e-01

5963 9.588596e-01 9.791052e-01

5964 9.589218e-01 9.791047e-01

5965 9.589828e-01 9.791042e-01

5966 9.590426e-01 9.791037e-01

5967 9.591012e-01 9.791032e-01

5968 9.591587e-01 9.791026e-01

5969 9.592151e-01 9.791021e-01

5970 9.592704e-01 9.791015e-01

5971 9.593246e-01 9.791009e-01

5972 9.593776e-01 9.791003e-01

5973 9.594297e-01 9.790997e-01

5974 9.594806e-01 9.790991e-01

5975 9.595306e-01 9.790984e-01

5976 9.595795e-01 9.790978e-01

5977 9.596274e-01 9.790971e-01

5978 9.596744e-01 9.790964e-01

5979 9.597203e-01 9.790957e-01

5980 9.597653e-01 9.790950e-01

5981 9.598094e-01 9.790942e-01

5982 9.598525e-01 9.790935e-01

5983 9.598948e-01 9.790927e-01

5984 9.599361e-01 9.790919e-01

5985 9.599766e-01 9.790911e-01

5986 9.600161e-01 9.790902e-01

5987 9.600549e-01 9.790894e-01

5988 9.600928e-01 9.790885e-01

5989 9.601299e-01 9.790876e-01

5990 9.601661e-01 9.790867e-01

5991 9.602016e-01 9.790858e-01

5992 9.602363e-01 9.790848e-01

5993 9.602703e-01 9.790839e-01

5994 9.603035e-01 9.790829e-01

5995 9.603359e-01 9.790819e-01

5996 9.603677e-01 9.790809e-01

5997 9.603987e-01 9.790798e-01

5998 9.604290e-01 9.790787e-01

5999 9.604587e-01 9.790776e-01

6000 9.604877e-01 9.790765e-01

6001 9.605160e-01 9.790754e-01

6002 9.605437e-01 9.790742e-01

6003 9.605708e-01 9.790730e-01

6004 9.605973e-01 9.790718e-01

6005 9.606232e-01 9.790706e-01

6006 9.606484e-01 9.790693e-01

6007 9.606731e-01 9.790680e-01

6008 9.606973e-01 9.790667e-01

6009 9.607209e-01 9.790654e-01

6010 9.607440e-01 9.790640e-01

6011 9.607665e-01 9.790626e-01

6012 9.607885e-01 9.790612e-01

6013 9.608101e-01 9.790598e-01

6014 9.608311e-01 9.790583e-01

6015 9.608517e-01 9.790568e-01

6016 9.608718e-01 9.790553e-01

6017 9.608914e-01 9.790537e-01

6018 9.609106e-01 9.790521e-01

6019 9.609294e-01 9.790505e-01

6020 9.609478e-01 9.790488e-01

6021 9.609657e-01 9.790472e-01

6022 9.609833e-01 9.790454e-01

6023 9.610004e-01 9.790437e-01

6024 9.610172e-01 9.790419e-01

6025 9.610337e-01 9.790401e-01

6026 9.610497e-01 9.790383e-01

6027 9.610654e-01 9.790364e-01

6028 9.610808e-01 9.790345e-01

6029 9.610959e-01 9.790325e-01

6030 9.611106e-01 9.790306e-01

6031 9.611250e-01 9.790286e-01

6032 9.611392e-01 9.790265e-01

6033 9.611530e-01 9.790244e-01

6034 9.611666e-01 9.790223e-01

6035 9.611799e-01 9.790201e-01

6036 9.611929e-01 9.790179e-01

6037 9.612057e-01 9.790157e-01

6038 9.612182e-01 9.790134e-01

6039 9.612305e-01 9.790111e-01

6040 9.612425e-01 9.790087e-01

6041 9.612544e-01 9.790063e-01

6042 9.612660e-01 9.790039e-01

6043 9.612774e-01 9.790014e-01

6044 9.612886e-01 9.789989e-01

6045 9.612996e-01 9.789963e-01

6046 9.613104e-01 9.789937e-01

6047 9.613211e-01 9.789910e-01

6048 9.613316e-01 9.789883e-01

6049 9.613419e-01 9.789856e-01

6050 9.613521e-01 9.789828e-01

6051 9.613621e-01 9.789800e-01

6052 9.613720e-01 9.789771e-01

6053 9.613817e-01 9.789741e-01

6054 9.613913e-01 9.789711e-01

6055 9.614007e-01 9.789681e-01

6056 9.614101e-01 9.789650e-01

6057 9.614193e-01 9.789619e-01

6058 9.614285e-01 9.789587e-01

6059 9.614375e-01 9.789555e-01

6060 9.614464e-01 9.789522e-01

6061 9.614552e-01 9.789489e-01

6062 9.614640e-01 9.789455e-01

6063 9.614726e-01 9.789420e-01

6064 9.614812e-01 9.789385e-01

6065 9.614897e-01 9.789350e-01

6066 9.614981e-01 9.789313e-01

6067 9.615065e-01 9.789277e-01

6068 9.615148e-01 9.789239e-01

6069 9.615231e-01 9.789202e-01

6070 9.615312e-01 9.789163e-01

6071 9.615394e-01 9.789124e-01

6072 9.615475e-01 9.789084e-01

6073 9.615556e-01 9.789044e-01

6074 9.615636e-01 9.789003e-01

6075 9.615716e-01 9.788962e-01

6076 9.615796e-01 9.788920e-01

6077 9.615875e-01 9.788877e-01

6078 9.615954e-01 9.788833e-01

6079 9.616033e-01 9.788789e-01

6080 9.616112e-01 9.788745e-01

6081 9.616191e-01 9.788699e-01

6082 9.616269e-01 9.788653e-01

6083 9.616348e-01 9.788607e-01

6084 9.616426e-01 9.788559e-01

6085 9.616504e-01 9.788511e-01

6086 9.616583e-01 9.788462e-01

6087 9.616661e-01 9.788413e-01

6088 9.616740e-01 9.788362e-01

6089 9.616818e-01 9.788311e-01

6090 9.616897e-01 9.788260e-01

6091 9.616976e-01 9.788207e-01

6092 9.617055e-01 9.788154e-01

6093 9.617134e-01 9.788100e-01

6094 9.617213e-01 9.788045e-01

6095 9.617293e-01 9.787990e-01

6096 9.617373e-01 9.787934e-01

6097 9.617453e-01 9.787877e-01

6098 9.617533e-01 9.787819e-01

6099 9.617614e-01 9.787760e-01

6100 9.617695e-01 9.787701e-01

6101 9.617776e-01 9.787640e-01

6102 9.617857e-01 9.787579e-01

6103 9.617939e-01 9.787517e-01

6104 9.618021e-01 9.787455e-01

6105 9.618104e-01 9.787391e-01

6106 9.618187e-01 9.787327e-01

6107 9.618270e-01 9.787261e-01

6108 9.618354e-01 9.787195e-01

6109 9.618439e-01 9.787128e-01

6110 9.618523e-01 9.787060e-01

6111 9.618609e-01 9.786991e-01

6112 9.618694e-01 9.786922e-01

6113 9.618780e-01 9.786851e-01

6114 9.618867e-01 9.786780e-01

6115 9.618954e-01 9.786707e-01

6116 9.619042e-01 9.786634e-01

6117 9.619130e-01 9.786560e-01

6118 9.619218e-01 9.786484e-01

6119 9.619308e-01 9.786408e-01

6120 9.619397e-01 9.786331e-01

6121 9.619488e-01 9.786253e-01

6122 9.620361e-01 9.786250e-01

6123 9.621220e-01 9.786246e-01

6124 9.622063e-01 9.786242e-01

6125 9.622891e-01 9.786238e-01

6126 9.623704e-01 9.786234e-01

6127 9.624502e-01 9.786230e-01

6128 9.625286e-01 9.786226e-01

6129 9.626055e-01 9.786222e-01

6130 9.626810e-01 9.786217e-01

6131 9.627551e-01 9.786213e-01

6132 9.628278e-01 9.786208e-01

6133 9.628991e-01 9.786203e-01

6134 9.629690e-01 9.786198e-01

6135 9.630377e-01 9.786193e-01

6136 9.631049e-01 9.786188e-01

6137 9.631709e-01 9.786183e-01

6138 9.632356e-01 9.786178e-01

6139 9.632990e-01 9.786172e-01

6140 9.633611e-01 9.786167e-01

6141 9.634221e-01 9.786161e-01

6142 9.634818e-01 9.786155e-01

6143 9.635402e-01 9.786149e-01

6144 9.635975e-01 9.786143e-01

6145 9.636537e-01 9.786136e-01

6146 9.637086e-01 9.786130e-01

6147 9.637625e-01 9.786123e-01

6148 9.638152e-01 9.786117e-01

6149 9.638668e-01 9.786110e-01

6150 9.639174e-01 9.786103e-01

6151 9.639668e-01 9.786096e-01

6152 9.640153e-01 9.786088e-01

6153 9.640627e-01 9.786081e-01

6154 9.641090e-01 9.786073e-01

6155 9.641544e-01 9.786065e-01

6156 9.641988e-01 9.786057e-01

6157 9.642423e-01 9.786049e-01

6158 9.642848e-01 9.786041e-01

6159 9.643263e-01 9.786032e-01

6160 9.643670e-01 9.786023e-01

6161 9.644067e-01 9.786014e-01

6162 9.644456e-01 9.786005e-01

6163 9.644836e-01 9.785996e-01

6164 9.645208e-01 9.785987e-01

6165 9.645571e-01 9.785977e-01

6166 9.645926e-01 9.785967e-01

6167 9.646273e-01 9.785957e-01

6168 9.646613e-01 9.785947e-01

6169 9.646944e-01 9.785936e-01

6170 9.647268e-01 9.785926e-01

6171 9.647585e-01 9.785915e-01

6172 9.647894e-01 9.785904e-01

6173 9.648197e-01 9.785893e-01

6174 9.648492e-01 9.785881e-01

6175 9.648781e-01 9.785869e-01

6176 9.649063e-01 9.785857e-01

6177 9.649338e-01 9.785845e-01

6178 9.649607e-01 9.785833e-01

6179 9.649870e-01 9.785820e-01

6180 9.650127e-01 9.785807e-01

6181 9.650378e-01 9.785794e-01

6182 9.650623e-01 9.785780e-01

6183 9.650862e-01 9.785767e-01

6184 9.651096e-01 9.785753e-01

6185 9.651324e-01 9.785739e-01

6186 9.651548e-01 9.785724e-01

6187 9.651766e-01 9.785709e-01

6188 9.651978e-01 9.785694e-01

6189 9.652186e-01 9.785679e-01

6190 9.652390e-01 9.785663e-01

6191 9.652588e-01 9.785648e-01

6192 9.652782e-01 9.785631e-01

6193 9.652972e-01 9.785615e-01

6194 9.653157e-01 9.785598e-01

6195 9.653338e-01 9.785581e-01

6196 9.653515e-01 9.785564e-01

6197 9.653688e-01 9.785546e-01

6198 9.653857e-01 9.785528e-01

6199 9.654022e-01 9.785510e-01

6200 9.654184e-01 9.785491e-01

6201 9.654342e-01 9.785472e-01

6202 9.654497e-01 9.785453e-01

6203 9.654648e-01 9.785433e-01

6204 9.654796e-01 9.785413e-01

6205 9.654941e-01 9.785393e-01

6206 9.655082e-01 9.785372e-01

6207 9.655221e-01 9.785351e-01

6208 9.655357e-01 9.785330e-01

6209 9.655490e-01 9.785308e-01

6210 9.655620e-01 9.785286e-01

6211 9.655748e-01 9.785264e-01

6212 9.655873e-01 9.785241e-01

6213 9.655995e-01 9.785217e-01

6214 9.656116e-01 9.785194e-01

6215 9.656234e-01 9.785170e-01

6216 9.656349e-01 9.785145e-01

6217 9.656463e-01 9.785120e-01

6218 9.656574e-01 9.785095e-01

6219 9.656684e-01 9.785069e-01

6220 9.656791e-01 9.785043e-01

6221 9.656897e-01 9.785017e-01

6222 9.657001e-01 9.784989e-01

6223 9.657103e-01 9.784962e-01

6224 9.657203e-01 9.784934e-01

6225 9.657302e-01 9.784906e-01

6226 9.657400e-01 9.784877e-01

6227 9.657495e-01 9.784848e-01

6228 9.657590e-01 9.784818e-01

6229 9.657683e-01 9.784788e-01

6230 9.657775e-01 9.784757e-01

6231 9.657865e-01 9.784726e-01

6232 9.657955e-01 9.784694e-01

6233 9.658043e-01 9.784662e-01

6234 9.658130e-01 9.784629e-01

6235 9.658216e-01 9.784596e-01

6236 9.658301e-01 9.784562e-01

6237 9.658385e-01 9.784528e-01

6238 9.658468e-01 9.784493e-01

6239 9.658551e-01 9.784457e-01

6240 9.658632e-01 9.784421e-01

6241 9.658713e-01 9.784385e-01

6242 9.658793e-01 9.784348e-01

6243 9.658873e-01 9.784310e-01

6244 9.658951e-01 9.784272e-01

6245 9.659030e-01 9.784233e-01

6246 9.659107e-01 9.784194e-01

6247 9.659184e-01 9.784154e-01

6248 9.659261e-01 9.784113e-01

6249 9.659337e-01 9.784072e-01

6250 9.659413e-01 9.784030e-01

6251 9.659489e-01 9.783988e-01

6252 9.659564e-01 9.783945e-01

6253 9.659638e-01 9.783901e-01

6254 9.659713e-01 9.783857e-01

6255 9.659787e-01 9.783812e-01

6256 9.659861e-01 9.783766e-01

6257 9.659935e-01 9.783720e-01

6258 9.660009e-01 9.783673e-01

6259 9.660082e-01 9.783626e-01

6260 9.660156e-01 9.783577e-01

6261 9.660229e-01 9.783528e-01

6262 9.660302e-01 9.783479e-01

6263 9.660376e-01 9.783428e-01

6264 9.660449e-01 9.783377e-01

6265 9.660522e-01 9.783325e-01

6266 9.660595e-01 9.783273e-01

6267 9.660669e-01 9.783219e-01

6268 9.660742e-01 9.783165e-01

6269 9.660816e-01 9.783110e-01

6270 9.660889e-01 9.783055e-01

6271 9.660963e-01 9.782998e-01

6272 9.661037e-01 9.782941e-01

6273 9.661111e-01 9.782883e-01

6274 9.661185e-01 9.782825e-01

6275 9.661260e-01 9.782765e-01

6276 9.661335e-01 9.782705e-01

6277 9.661409e-01 9.782644e-01

6278 9.661485e-01 9.782582e-01

6279 9.661560e-01 9.782519e-01

6280 9.661636e-01 9.782456e-01

6281 9.661712e-01 9.782391e-01

6282 9.661789e-01 9.782326e-01

6283 9.661865e-01 9.782260e-01

6284 9.661942e-01 9.782193e-01

6285 9.662020e-01 9.782125e-01

6286 9.662098e-01 9.782057e-01

6287 9.662176e-01 9.781987e-01

6288 9.662254e-01 9.781917e-01

6289 9.662333e-01 9.781845e-01

6290 9.662413e-01 9.781773e-01

6291 9.662493e-01 9.781700e-01

6292 9.662573e-01 9.781626e-01

6293 9.662654e-01 9.781551e-01

6294 9.662735e-01 9.781475e-01

6295 9.662817e-01 9.781398e-01

6296 9.662899e-01 9.781320e-01

6297 9.662981e-01 9.781242e-01

6298 9.663064e-01 9.781162e-01

6299 9.663148e-01 9.781082e-01

6300 9.663232e-01 9.781000e-01

6301 9.663317e-01 9.780917e-01

6302 9.664127e-01 9.780913e-01

6303 9.664922e-01 9.780908e-01

6304 9.665701e-01 9.780903e-01

6305 9.666466e-01 9.780899e-01

6306 9.667216e-01 9.780894e-01

6307 9.667951e-01 9.780888e-01

6308 9.668671e-01 9.780883e-01

6309 9.669378e-01 9.780878e-01

6310 9.670070e-01 9.780872e-01

6311 9.670749e-01 9.780867e-01

6312 9.671414e-01 9.780861e-01

6313 9.672065e-01 9.780855e-01

6314 9.672703e-01 9.780849e-01

6315 9.673328e-01 9.780843e-01

6316 9.673940e-01 9.780837e-01

6317 9.674539e-01 9.780831e-01

6318 9.675126e-01 9.780824e-01

6319 9.675700e-01 9.780817e-01

6320 9.676263e-01 9.780811e-01

6321 9.676813e-01 9.780804e-01

6322 9.677352e-01 9.780797e-01

6323 9.677879e-01 9.780789e-01

6324 9.678394e-01 9.780782e-01

6325 9.678899e-01 9.780775e-01

6326 9.679392e-01 9.780767e-01

6327 9.679875e-01 9.780759e-01

6328 9.680347e-01 9.780751e-01

6329 9.680808e-01 9.780743e-01

6330 9.681260e-01 9.780735e-01

6331 9.681701e-01 9.780726e-01

6332 9.682132e-01 9.780717e-01

6333 9.682554e-01 9.780709e-01

6334 9.682966e-01 9.780700e-01

6335 9.683369e-01 9.780690e-01

6336 9.683763e-01 9.780681e-01

6337 9.684148e-01 9.780672e-01

6338 9.684524e-01 9.780662e-01

6339 9.684891e-01 9.780652e-01

6340 9.685250e-01 9.780642e-01

6341 9.685600e-01 9.780631e-01

6342 9.685943e-01 9.780621e-01

6343 9.686277e-01 9.780610e-01

6344 9.686604e-01 9.780599e-01

6345 9.686923e-01 9.780588e-01

6346 9.687234e-01 9.780577e-01

6347 9.687539e-01 9.780565e-01

6348 9.687836e-01 9.780553e-01

6349 9.688126e-01 9.780541e-01

6350 9.688409e-01 9.780529e-01

6351 9.688686e-01 9.780517e-01

6352 9.688956e-01 9.780504e-01

6353 9.689220e-01 9.780491e-01

6354 9.689477e-01 9.780478e-01

6355 9.689729e-01 9.780465e-01

6356 9.689974e-01 9.780451e-01

6357 9.690214e-01 9.780437e-01

6358 9.690448e-01 9.780423e-01

6359 9.690676e-01 9.780408e-01

6360 9.690900e-01 9.780394e-01

6361 9.691117e-01 9.780379e-01

6362 9.691330e-01 9.780364e-01

6363 9.691538e-01 9.780348e-01

6364 9.691740e-01 9.780332e-01

6365 9.691939e-01 9.780316e-01

6366 9.692132e-01 9.780300e-01

6367 9.692321e-01 9.780283e-01

6368 9.692505e-01 9.780267e-01

6369 9.692686e-01 9.780249e-01

6370 9.692862e-01 9.780232e-01

6371 9.693034e-01 9.780214e-01

6372 9.693202e-01 9.780196e-01

6373 9.693366e-01 9.780178e-01

6374 9.693526e-01 9.780159e-01

6375 9.693683e-01 9.780140e-01

6376 9.693837e-01 9.780120e-01

6377 9.693987e-01 9.780101e-01

6378 9.694133e-01 9.780081e-01

6379 9.694277e-01 9.780060e-01

6380 9.694417e-01 9.780040e-01

6381 9.694554e-01 9.780018e-01

6382 9.694688e-01 9.779997e-01

6383 9.694820e-01 9.779975e-01

6384 9.694949e-01 9.779953e-01

6385 9.695074e-01 9.779930e-01

6386 9.695198e-01 9.779908e-01

6387 9.695319e-01 9.779884e-01

6388 9.695437e-01 9.779861e-01

6389 9.695553e-01 9.779837e-01

6390 9.695667e-01 9.779812e-01

6391 9.695779e-01 9.779787e-01

6392 9.695888e-01 9.779762e-01

6393 9.695995e-01 9.779736e-01

6394 9.696101e-01 9.779710e-01

6395 9.696204e-01 9.779684e-01

6396 9.696306e-01 9.779657e-01

6397 9.696406e-01 9.779629e-01

6398 9.696504e-01 9.779602e-01

6399 9.696600e-01 9.779573e-01

6400 9.696695e-01 9.779545e-01

6401 9.696789e-01 9.779515e-01

6402 9.696881e-01 9.779486e-01

6403 9.696971e-01 9.779456e-01

6404 9.697060e-01 9.779425e-01

6405 9.697148e-01 9.779394e-01

6406 9.697234e-01 9.779362e-01

6407 9.697320e-01 9.779330e-01

6408 9.697404e-01 9.779298e-01

6409 9.697487e-01 9.779265e-01

6410 9.697569e-01 9.779231e-01

6411 9.697650e-01 9.779197e-01

6412 9.697730e-01 9.779163e-01

6413 9.697809e-01 9.779128e-01

6414 9.697887e-01 9.779092e-01

6415 9.697965e-01 9.779056e-01

6416 9.698041e-01 9.779019e-01

6417 9.698117e-01 9.778982e-01

6418 9.698192e-01 9.778944e-01

6419 9.698267e-01 9.778906e-01

6420 9.698341e-01 9.778867e-01

6421 9.698414e-01 9.778827e-01

6422 9.698487e-01 9.778787e-01

6423 9.698559e-01 9.778746e-01

6424 9.698631e-01 9.778705e-01

6425 9.698702e-01 9.778663e-01

6426 9.698773e-01 9.778620e-01

6427 9.698843e-01 9.778577e-01

6428 9.698913e-01 9.778533e-01

6429 9.698983e-01 9.778489e-01

6430 9.699052e-01 9.778444e-01

6431 9.699121e-01 9.778398e-01

6432 9.699190e-01 9.778352e-01

6433 9.699259e-01 9.778305e-01

6434 9.699328e-01 9.778257e-01

6435 9.699396e-01 9.778208e-01

6436 9.699464e-01 9.778159e-01

6437 9.699532e-01 9.778110e-01

6438 9.699600e-01 9.778059e-01

6439 9.699668e-01 9.778008e-01

6440 9.699736e-01 9.777956e-01

6441 9.699804e-01 9.777904e-01

6442 9.699871e-01 9.777850e-01

6443 9.699939e-01 9.777796e-01

6444 9.700007e-01 9.777741e-01

6445 9.700075e-01 9.777686e-01

6446 9.700143e-01 9.777630e-01

6447 9.700211e-01 9.777573e-01

6448 9.700279e-01 9.777515e-01

6449 9.700347e-01 9.777456e-01

6450 9.700416e-01 9.777397e-01

6451 9.700484e-01 9.777337e-01

6452 9.700553e-01 9.777276e-01

6453 9.700622e-01 9.777214e-01

6454 9.700691e-01 9.777151e-01

6455 9.700760e-01 9.777088e-01

6456 9.700830e-01 9.777024e-01

6457 9.700900e-01 9.776959e-01

6458 9.700970e-01 9.776893e-01

6459 9.701040e-01 9.776826e-01

6460 9.701111e-01 9.776759e-01

6461 9.701182e-01 9.776690e-01

6462 9.701253e-01 9.776621e-01

6463 9.701325e-01 9.776551e-01

6464 9.701397e-01 9.776480e-01

6465 9.701469e-01 9.776408e-01

6466 9.701541e-01 9.776335e-01

6467 9.701614e-01 9.776261e-01

6468 9.701688e-01 9.776187e-01

6469 9.701761e-01 9.776111e-01

6470 9.701836e-01 9.776035e-01

6471 9.701910e-01 9.775958e-01

6472 9.701985e-01 9.775879e-01

6473 9.702060e-01 9.775800e-01

6474 9.702136e-01 9.775720e-01

6475 9.702212e-01 9.775639e-01

6476 9.702289e-01 9.775557e-01

6477 9.702366e-01 9.775474e-01

6478 9.702444e-01 9.775390e-01

6479 9.702522e-01 9.775306e-01

6480 9.702601e-01 9.775220e-01

6481 9.702680e-01 9.775133e-01

6482 9.703414e-01 9.775128e-01

6483 9.704134e-01 9.775122e-01

6484 9.704839e-01 9.775116e-01

6485 9.705530e-01 9.775110e-01

6486 9.706206e-01 9.775104e-01

6487 9.706868e-01 9.775098e-01

6488 9.707516e-01 9.775092e-01

6489 9.708150e-01 9.775086e-01

6490 9.708771e-01 9.775079e-01

6491 9.709379e-01 9.775073e-01

6492 9.709974e-01 9.775066e-01

6493 9.710555e-01 9.775059e-01

6494 9.711124e-01 9.775052e-01

6495 9.711681e-01 9.775045e-01

6496 9.712225e-01 9.775037e-01

6497 9.712758e-01 9.775030e-01

6498 9.713278e-01 9.775022e-01

6499 9.713787e-01 9.775015e-01

6500 9.714285e-01 9.775007e-01

6501 9.714771e-01 9.774999e-01

6502 9.715247e-01 9.774990e-01

6503 9.715711e-01 9.774982e-01

6504 9.716165e-01 9.774973e-01

6505 9.716609e-01 9.774965e-01

6506 9.717042e-01 9.774956e-01

6507 9.717466e-01 9.774947e-01

6508 9.717879e-01 9.774937e-01

6509 9.718284e-01 9.774928e-01

6510 9.718678e-01 9.774918e-01

6511 9.719064e-01 9.774909e-01

6512 9.719440e-01 9.774899e-01

6513 9.719808e-01 9.774889e-01

6514 9.720167e-01 9.774878e-01

6515 9.720517e-01 9.774868e-01

6516 9.720859e-01 9.774857e-01

6517 9.721193e-01 9.774846e-01

6518 9.721519e-01 9.774835e-01

6519 9.721838e-01 9.774824e-01

6520 9.722148e-01 9.774812e-01

6521 9.722452e-01 9.774800e-01

6522 9.722748e-01 9.774788e-01

6523 9.723037e-01 9.774776e-01

6524 9.723319e-01 9.774764e-01

6525 9.723594e-01 9.774751e-01

6526 9.723862e-01 9.774738e-01

6527 9.724124e-01 9.774725e-01

6528 9.724380e-01 9.774712e-01

6529 9.724630e-01 9.774698e-01

6530 9.724873e-01 9.774685e-01

6531 9.725111e-01 9.774671e-01

6532 9.725343e-01 9.774656e-01

6533 9.725570e-01 9.774642e-01

6534 9.725791e-01 9.774627e-01

6535 9.726006e-01 9.774612e-01

6536 9.726217e-01 9.774597e-01

6537 9.726422e-01 9.774581e-01

6538 9.726623e-01 9.774565e-01

6539 9.726818e-01 9.774549e-01

6540 9.727010e-01 9.774533e-01

6541 9.727196e-01 9.774516e-01

6542 9.727378e-01 9.774499e-01

6543 9.727556e-01 9.774482e-01

6544 9.727730e-01 9.774464e-01

6545 9.727899e-01 9.774446e-01

6546 9.728065e-01 9.774428e-01

6547 9.728227e-01 9.774410e-01

6548 9.728385e-01 9.774391e-01

6549 9.728539e-01 9.774372e-01

6550 9.728690e-01 9.774353e-01

6551 9.728837e-01 9.774333e-01

6552 9.728981e-01 9.774313e-01

6553 9.729122e-01 9.774293e-01

6554 9.729260e-01 9.774272e-01

6555 9.729394e-01 9.774251e-01

6556 9.729526e-01 9.774229e-01

6557 9.729655e-01 9.774208e-01

6558 9.729781e-01 9.774185e-01

6559 9.729904e-01 9.774163e-01

6560 9.730025e-01 9.774140e-01

6561 9.730143e-01 9.774117e-01

6562 9.730258e-01 9.774093e-01

6563 9.730372e-01 9.774069e-01

6564 9.730483e-01 9.774045e-01

6565 9.730592e-01 9.774020e-01

6566 9.730698e-01 9.773995e-01

6567 9.730803e-01 9.773970e-01

6568 9.730905e-01 9.773944e-01

6569 9.731006e-01 9.773917e-01

6570 9.731105e-01 9.773890e-01

6571 9.731201e-01 9.773863e-01

6572 9.731297e-01 9.773836e-01

6573 9.731390e-01 9.773807e-01

6574 9.731482e-01 9.773779e-01

6575 9.731572e-01 9.773750e-01

6576 9.731661e-01 9.773721e-01

6577 9.731748e-01 9.773691e-01

6578 9.731834e-01 9.773660e-01

6579 9.731919e-01 9.773630e-01

6580 9.732002e-01 9.773598e-01

6581 9.732084e-01 9.773567e-01

6582 9.732165e-01 9.773534e-01

6583 9.732245e-01 9.773502e-01

6584 9.732323e-01 9.773469e-01

6585 9.732401e-01 9.773435e-01

6586 9.732477e-01 9.773401e-01

6587 9.732553e-01 9.773366e-01

6588 9.732627e-01 9.773331e-01

6589 9.732701e-01 9.773295e-01

6590 9.732774e-01 9.773258e-01

6591 9.732846e-01 9.773222e-01

6592 9.732918e-01 9.773184e-01

6593 9.732988e-01 9.773146e-01

6594 9.733058e-01 9.773108e-01

6595 9.733127e-01 9.773069e-01

6596 9.733196e-01 9.773029e-01

6597 9.733264e-01 9.772989e-01

6598 9.733332e-01 9.772948e-01

6599 9.733399e-01 9.772906e-01

6600 9.733465e-01 9.772864e-01

6601 9.733532e-01 9.772822e-01

6602 9.733597e-01 9.772779e-01

6603 9.733662e-01 9.772735e-01

6604 9.733727e-01 9.772690e-01

6605 9.733792e-01 9.772645e-01

6606 9.733856e-01 9.772600e-01

6607 9.733920e-01 9.772553e-01

6608 9.733984e-01 9.772506e-01

6609 9.734047e-01 9.772458e-01

6610 9.734111e-01 9.772410e-01

6611 9.734174e-01 9.772361e-01

6612 9.734237e-01 9.772311e-01

6613 9.734299e-01 9.772261e-01

6614 9.734362e-01 9.772210e-01

6615 9.734425e-01 9.772158e-01

6616 9.734487e-01 9.772106e-01

6617 9.734549e-01 9.772052e-01

6618 9.734612e-01 9.771999e-01

6619 9.734674e-01 9.771944e-01

6620 9.734736e-01 9.771889e-01

6621 9.734799e-01 9.771832e-01

6622 9.734861e-01 9.771776e-01

6623 9.734923e-01 9.771718e-01

6624 9.734986e-01 9.771660e-01

6625 9.735048e-01 9.771600e-01

6626 9.735111e-01 9.771541e-01

6627 9.735174e-01 9.771480e-01

6628 9.735237e-01 9.771418e-01

6629 9.735300e-01 9.771356e-01

6630 9.735363e-01 9.771293e-01

6631 9.735426e-01 9.771229e-01

6632 9.735490e-01 9.771164e-01

6633 9.735554e-01 9.771099e-01

6634 9.735617e-01 9.771033e-01

6635 9.735682e-01 9.770965e-01

6636 9.735746e-01 9.770897e-01

6637 9.735811e-01 9.770829e-01

6638 9.735875e-01 9.770759e-01

6639 9.735941e-01 9.770688e-01

6640 9.736006e-01 9.770617e-01

6641 9.736072e-01 9.770545e-01

6642 9.736138e-01 9.770472e-01

6643 9.736204e-01 9.770398e-01

6644 9.736270e-01 9.770323e-01

6645 9.736337e-01 9.770247e-01

6646 9.736405e-01 9.770170e-01

6647 9.736472e-01 9.770092e-01

6648 9.736540e-01 9.770014e-01

6649 9.736608e-01 9.769935e-01

6650 9.736677e-01 9.769854e-01

6651 9.736746e-01 9.769773e-01

6652 9.736815e-01 9.769691e-01

6653 9.736885e-01 9.769608e-01

6654 9.736955e-01 9.769523e-01

6655 9.737026e-01 9.769438e-01

6656 9.737097e-01 9.769352e-01

6657 9.737168e-01 9.769266e-01

6658 9.737240e-01 9.769178e-01

6659 9.737312e-01 9.769089e-01

6660 9.737385e-01 9.768999e-01

6661 9.737458e-01 9.768908e-01

6662 9.738110e-01 9.768902e-01

6663 9.738749e-01 9.768895e-01

6664 9.739373e-01 9.768889e-01

6665 9.739983e-01 9.768882e-01

6666 9.740581e-01 9.768875e-01

6667 9.741164e-01 9.768868e-01

6668 9.741735e-01 9.768861e-01

6669 9.742293e-01 9.768853e-01

6670 9.742839e-01 9.768846e-01

6671 9.743372e-01 9.768838e-01

6672 9.743893e-01 9.768830e-01

6673 9.744402e-01 9.768822e-01

6674 9.744899e-01 9.768814e-01

6675 9.745385e-01 9.768806e-01

6676 9.745860e-01 9.768798e-01

6677 9.746323e-01 9.768789e-01

6678 9.746776e-01 9.768780e-01

6679 9.747219e-01 9.768771e-01

6680 9.747651e-01 9.768762e-01

6681 9.748072e-01 9.768753e-01

6682 9.748484e-01 9.768744e-01

6683 9.748886e-01 9.768734e-01

6684 9.749279e-01 9.768724e-01

6685 9.749662e-01 9.768714e-01

6686 9.750036e-01 9.768704e-01

6687 9.750401e-01 9.768694e-01

6688 9.750757e-01 9.768684e-01

6689 9.751104e-01 9.768673e-01

6690 9.751444e-01 9.768662e-01

6691 9.751775e-01 9.768651e-01

6692 9.752098e-01 9.768640e-01

6693 9.752413e-01 9.768628e-01

6694 9.752720e-01 9.768617e-01

6695 9.753021e-01 9.768605e-01

6696 9.753313e-01 9.768593e-01

6697 9.753599e-01 9.768581e-01

6698 9.753877e-01 9.768568e-01

6699 9.754149e-01 9.768555e-01

6700 9.754414e-01 9.768542e-01

6701 9.754673e-01 9.768529e-01

6702 9.754925e-01 9.768516e-01

6703 9.755171e-01 9.768502e-01

6704 9.755411e-01 9.768488e-01

6705 9.755646e-01 9.768474e-01

6706 9.755874e-01 9.768460e-01

6707 9.756097e-01 9.768445e-01

6708 9.756314e-01 9.768431e-01

6709 9.756526e-01 9.768416e-01

6710 9.756733e-01 9.768400e-01

6711 9.756935e-01 9.768385e-01

6712 9.757132e-01 9.768369e-01

6713 9.757324e-01 9.768353e-01

6714 9.757512e-01 9.768336e-01

6715 9.757695e-01 9.768319e-01

6716 9.757874e-01 9.768303e-01

6717 9.758048e-01 9.768285e-01

6718 9.758218e-01 9.768268e-01

6719 9.758384e-01 9.768250e-01

6720 9.758546e-01 9.768232e-01

6721 9.758705e-01 9.768213e-01

6722 9.758859e-01 9.768195e-01

6723 9.759010e-01 9.768176e-01

6724 9.759158e-01 9.768156e-01

6725 9.759302e-01 9.768137e-01

6726 9.759442e-01 9.768117e-01

6727 9.759580e-01 9.768096e-01

6728 9.759714e-01 9.768076e-01

6729 9.759846e-01 9.768055e-01

6730 9.759974e-01 9.768033e-01

6731 9.760099e-01 9.768012e-01

6732 9.760222e-01 9.767990e-01

6733 9.760342e-01 9.767967e-01

6734 9.760460e-01 9.767945e-01

6735 9.760574e-01 9.767922e-01

6736 9.760687e-01 9.767898e-01

6737 9.760797e-01 9.767874e-01

6738 9.760905e-01 9.767850e-01

6739 9.761010e-01 9.767826e-01

6740 9.761113e-01 9.767801e-01

6741 9.761215e-01 9.767775e-01

6742 9.761314e-01 9.767750e-01

6743 9.761411e-01 9.767723e-01

6744 9.761507e-01 9.767697e-01

6745 9.761600e-01 9.767670e-01

6746 9.761692e-01 9.767643e-01

6747 9.761782e-01 9.767615e-01

6748 9.761871e-01 9.767587e-01

6749 9.761958e-01 9.767558e-01

6750 9.762043e-01 9.767529e-01

6751 9.762127e-01 9.767499e-01

6752 9.762210e-01 9.767469e-01

6753 9.762291e-01 9.767439e-01

6754 9.762371e-01 9.767408e-01

6755 9.762449e-01 9.767377e-01

6756 9.762526e-01 9.767345e-01

6757 9.762603e-01 9.767312e-01

6758 9.762678e-01 9.767280e-01

6759 9.762752e-01 9.767246e-01

6760 9.762824e-01 9.767212e-01

6761 9.762896e-01 9.767178e-01

6762 9.762967e-01 9.767143e-01

6763 9.763037e-01 9.767108e-01

6764 9.763107e-01 9.767072e-01

6765 9.763175e-01 9.767036e-01

6766 9.763242e-01 9.766999e-01

6767 9.763309e-01 9.766961e-01

6768 9.763375e-01 9.766923e-01

6769 9.763441e-01 9.766885e-01

6770 9.763505e-01 9.766846e-01

6771 9.763569e-01 9.766806e-01

6772 9.763633e-01 9.766766e-01

6773 9.763696e-01 9.766725e-01

6774 9.763758e-01 9.766684e-01

6775 9.763820e-01 9.766642e-01

6776 9.763882e-01 9.766599e-01

6777 9.763943e-01 9.766556e-01

6778 9.764003e-01 9.766512e-01

6779 9.764063e-01 9.766468e-01

6780 9.764123e-01 9.766423e-01

6781 9.764182e-01 9.766377e-01

6782 9.764242e-01 9.766331e-01

6783 9.764300e-01 9.766284e-01

6784 9.764359e-01 9.766237e-01

6785 9.764417e-01 9.766188e-01

6786 9.764475e-01 9.766140e-01

6787 9.764533e-01 9.766090e-01

6788 9.764591e-01 9.766040e-01

6789 9.764648e-01 9.765989e-01

6790 9.764706e-01 9.765937e-01

6791 9.764763e-01 9.765885e-01

6792 9.764820e-01 9.765832e-01

6793 9.764877e-01 9.765779e-01

6794 9.764934e-01 9.765724e-01

6795 9.764991e-01 9.765669e-01

6796 9.765048e-01 9.765613e-01

6797 9.765105e-01 9.765557e-01

6798 9.765162e-01 9.765499e-01

6799 9.765219e-01 9.765441e-01

6800 9.765275e-01 9.765383e-01

6801 9.765332e-01 9.765323e-01

6802 9.765389e-01 9.765263e-01

6803 9.765446e-01 9.765202e-01

6804 9.765504e-01 9.765140e-01

6805 9.765561e-01 9.765077e-01

6806 9.765618e-01 9.765014e-01

6807 9.765676e-01 9.764950e-01

6808 9.765733e-01 9.764885e-01

6809 9.765791e-01 9.764819e-01

6810 9.765849e-01 9.764752e-01

6811 9.765907e-01 9.764685e-01

6812 9.765965e-01 9.764616e-01

6813 9.766023e-01 9.764547e-01

6814 9.766082e-01 9.764477e-01

6815 9.766141e-01 9.764407e-01

6816 9.766200e-01 9.764335e-01

6817 9.766259e-01 9.764262e-01

6818 9.766318e-01 9.764189e-01

6819 9.766378e-01 9.764115e-01

6820 9.766438e-01 9.764040e-01

6821 9.766498e-01 9.763964e-01

6822 9.766559e-01 9.763887e-01

6823 9.766620e-01 9.763809e-01

6824 9.766681e-01 9.763730e-01

6825 9.766742e-01 9.763651e-01

6826 9.766804e-01 9.763570e-01

6827 9.766865e-01 9.763489e-01

6828 9.766928e-01 9.763407e-01

6829 9.766990e-01 9.763323e-01

6830 9.767053e-01 9.763239e-01

6831 9.767116e-01 9.763154e-01

6832 9.767180e-01 9.763068e-01

6833 9.767243e-01 9.762981e-01

6834 9.767308e-01 9.762894e-01

6835 9.767372e-01 9.762805e-01

6836 9.767437e-01 9.762715e-01

6837 9.767502e-01 9.762624e-01

6838 9.767568e-01 9.762533e-01

6839 9.767634e-01 9.762440e-01

6840 9.767700e-01 9.762346e-01

6841 9.767766e-01 9.762252e-01

6842 9.768335e-01 9.762245e-01

6843 9.768890e-01 9.762237e-01

6844 9.769432e-01 9.762229e-01

6845 9.769962e-01 9.762222e-01

6846 9.770480e-01 9.762214e-01

6847 9.770986e-01 9.762206e-01

6848 9.771479e-01 9.762197e-01

6849 9.771961e-01 9.762189e-01

6850 9.772432e-01 9.762181e-01

6851 9.772892e-01 9.762172e-01

6852 9.773340e-01 9.762163e-01

6853 9.773778e-01 9.762154e-01

6854 9.774206e-01 9.762145e-01

6855 9.774623e-01 9.762136e-01

6856 9.775030e-01 9.762126e-01

6857 9.775428e-01 9.762116e-01

6858 9.775816e-01 9.762107e-01

6859 9.776194e-01 9.762097e-01

6860 9.776563e-01 9.762086e-01

6861 9.776923e-01 9.762076e-01

6862 9.777275e-01 9.762066e-01

6863 9.777617e-01 9.762055e-01

6864 9.777952e-01 9.762044e-01

6865 9.778278e-01 9.762033e-01

6866 9.778596e-01 9.762021e-01

6867 9.778906e-01 9.762010e-01

6868 9.779209e-01 9.761998e-01

6869 9.779504e-01 9.761986e-01

6870 9.779792e-01 9.761974e-01

6871 9.780072e-01 9.761962e-01

6872 9.780346e-01 9.761949e-01

6873 9.780613e-01 9.761937e-01

6874 9.780873e-01 9.761924e-01

6875 9.781127e-01 9.761911e-01

6876 9.781375e-01 9.761897e-01

6877 9.781616e-01 9.761884e-01

6878 9.781852e-01 9.761870e-01

6879 9.782081e-01 9.761856e-01

6880 9.782305e-01 9.761841e-01

6881 9.782523e-01 9.761827e-01

6882 9.782736e-01 9.761812e-01

6883 9.782944e-01 9.761797e-01

6884 9.783146e-01 9.761781e-01

6885 9.783344e-01 9.761766e-01

6886 9.783536e-01 9.761750e-01

6887 9.783724e-01 9.761734e-01

6888 9.783908e-01 9.761718e-01

6889 9.784086e-01 9.761701e-01

6890 9.784261e-01 9.761684e-01

6891 9.784431e-01 9.761667e-01

6892 9.784597e-01 9.761650e-01

6893 9.784759e-01 9.761632e-01

6894 9.784917e-01 9.761614e-01

6895 9.785071e-01 9.761595e-01

6896 9.785222e-01 9.761577e-01

6897 9.785369e-01 9.761558e-01

6898 9.785513e-01 9.761539e-01

6899 9.785653e-01 9.761519e-01

6900 9.785790e-01 9.761499e-01

6901 9.785923e-01 9.761479e-01

6902 9.786054e-01 9.761459e-01

6903 9.786182e-01 9.761438e-01

6904 9.786306e-01 9.761417e-01

6905 9.786428e-01 9.761395e-01

6906 9.786547e-01 9.761373e-01

6907 9.786664e-01 9.761351e-01

6908 9.786777e-01 9.761329e-01

6909 9.786889e-01 9.761306e-01

6910 9.786998e-01 9.761283e-01

6911 9.787104e-01 9.761259e-01

6912 9.787208e-01 9.761235e-01

6913 9.787310e-01 9.761211e-01

6914 9.787410e-01 9.761186e-01

6915 9.787508e-01 9.761161e-01

6916 9.787604e-01 9.761136e-01

6917 9.787698e-01 9.761110e-01

6918 9.787790e-01 9.761083e-01

6919 9.787880e-01 9.761057e-01

6920 9.787968e-01 9.761030e-01

6921 9.788055e-01 9.761002e-01

6922 9.788140e-01 9.760974e-01

6923 9.788223e-01 9.760946e-01

6924 9.788305e-01 9.760917e-01

6925 9.788386e-01 9.760888e-01

6926 9.788465e-01 9.760859e-01

6927 9.788542e-01 9.760829e-01

6928 9.788619e-01 9.760798e-01

6929 9.788694e-01 9.760767e-01

6930 9.788768e-01 9.760736e-01

6931 9.788840e-01 9.760704e-01

6932 9.788912e-01 9.760671e-01

6933 9.788982e-01 9.760639e-01

6934 9.789051e-01 9.760605e-01

6935 9.789120e-01 9.760571e-01

6936 9.789187e-01 9.760537e-01

6937 9.789253e-01 9.760502e-01

6938 9.789319e-01 9.760467e-01

6939 9.789384e-01 9.760431e-01

6940 9.789447e-01 9.760395e-01

6941 9.789510e-01 9.760358e-01

6942 9.789573e-01 9.760321e-01

6943 9.789634e-01 9.760283e-01

6944 9.789695e-01 9.760244e-01

6945 9.789755e-01 9.760205e-01

6946 9.789815e-01 9.760166e-01

6947 9.789874e-01 9.760126e-01

6948 9.789932e-01 9.760085e-01

6949 9.789990e-01 9.760044e-01

6950 9.790047e-01 9.760002e-01

6951 9.790104e-01 9.759960e-01

6952 9.790160e-01 9.759917e-01

6953 9.790216e-01 9.759873e-01

6954 9.790272e-01 9.759829e-01

6955 9.790327e-01 9.759784e-01

6956 9.790382e-01 9.759739e-01

6957 9.790436e-01 9.759693e-01

6958 9.790490e-01 9.759646e-01

6959 9.790544e-01 9.759599e-01

6960 9.790597e-01 9.759551e-01

6961 9.790651e-01 9.759502e-01

6962 9.790704e-01 9.759453e-01

6963 9.790756e-01 9.759403e-01

6964 9.790809e-01 9.759352e-01

6965 9.790861e-01 9.759301e-01

6966 9.790914e-01 9.759249e-01

6967 9.790966e-01 9.759197e-01

6968 9.791018e-01 9.759143e-01

6969 9.791069e-01 9.759089e-01

6970 9.791121e-01 9.759035e-01

6971 9.791173e-01 9.758979e-01

6972 9.791224e-01 9.758923e-01

6973 9.791276e-01 9.758866e-01

6974 9.791328e-01 9.758809e-01

6975 9.791379e-01 9.758750e-01

6976 9.791430e-01 9.758691e-01

6977 9.791482e-01 9.758631e-01

6978 9.791533e-01 9.758571e-01

6979 9.791585e-01 9.758509e-01

6980 9.791637e-01 9.758447e-01

6981 9.791688e-01 9.758385e-01

6982 9.791740e-01 9.758321e-01

6983 9.791792e-01 9.758256e-01

6984 9.791843e-01 9.758191e-01

6985 9.791895e-01 9.758125e-01

6986 9.791947e-01 9.758058e-01

6987 9.791999e-01 9.757991e-01

6988 9.792052e-01 9.757922e-01

6989 9.792104e-01 9.757853e-01

6990 9.792157e-01 9.757783e-01

6991 9.792209e-01 9.757712e-01

6992 9.792262e-01 9.757640e-01

6993 9.792315e-01 9.757568e-01

6994 9.792368e-01 9.757494e-01

6995 9.792422e-01 9.757420e-01

6996 9.792475e-01 9.757345e-01

6997 9.792529e-01 9.757269e-01

6998 9.792583e-01 9.757192e-01

6999 9.792637e-01 9.757114e-01

7000 9.792691e-01 9.757035e-01

7001 9.792746e-01 9.756956e-01

7002 9.792801e-01 9.756875e-01

7003 9.792856e-01 9.756794e-01

7004 9.792911e-01 9.756712e-01

7005 9.792967e-01 9.756629e-01

7006 9.793023e-01 9.756544e-01

7007 9.793079e-01 9.756459e-01

7008 9.793135e-01 9.756374e-01

7009 9.793191e-01 9.756287e-01

7010 9.793248e-01 9.756199e-01

7011 9.793305e-01 9.756110e-01

7012 9.793363e-01 9.756021e-01

7013 9.793420e-01 9.755930e-01

7014 9.793478e-01 9.755839e-01

7015 9.793536e-01 9.755746e-01

7016 9.793595e-01 9.755653e-01

7017 9.793654e-01 9.755559e-01

7018 9.793713e-01 9.755463e-01

7019 9.793772e-01 9.755367e-01

7020 9.793832e-01 9.755270e-01

7021 9.793892e-01 9.755172e-01

7022 9.794379e-01 9.755163e-01

7023 9.794855e-01 9.755155e-01

7024 9.795319e-01 9.755146e-01

7025 9.795772e-01 9.755138e-01

7026 9.796214e-01 9.755129e-01

7027 9.796645e-01 9.755120e-01

7028 9.797066e-01 9.755111e-01

7029 9.797477e-01 9.755101e-01

7030 9.797877e-01 9.755092e-01

7031 9.798268e-01 9.755082e-01

7032 9.798649e-01 9.755072e-01

7033 9.799021e-01 9.755062e-01

7034 9.799384e-01 9.755052e-01

7035 9.799737e-01 9.755041e-01

7036 9.800082e-01 9.755031e-01

7037 9.800419e-01 9.755020e-01

7038 9.800747e-01 9.755009e-01

7039 9.801066e-01 9.754998e-01

7040 9.801378e-01 9.754987e-01

7041 9.801682e-01 9.754975e-01

7042 9.801979e-01 9.754964e-01

7043 9.802268e-01 9.754952e-01

7044 9.802550e-01 9.754940e-01

7045 9.802824e-01 9.754927e-01

7046 9.803092e-01 9.754915e-01

7047 9.803353e-01 9.754902e-01

7048 9.803608e-01 9.754889e-01

7049 9.803856e-01 9.754876e-01

7050 9.804098e-01 9.754863e-01

7051 9.804334e-01 9.754849e-01

7052 9.804564e-01 9.754835e-01

7053 9.804788e-01 9.754821e-01

7054 9.805006e-01 9.754807e-01

7055 9.805219e-01 9.754792e-01

7056 9.805427e-01 9.754778e-01

7057 9.805630e-01 9.754763e-01

7058 9.805827e-01 9.754747e-01

7059 9.806020e-01 9.754732e-01

7060 9.806207e-01 9.754716e-01

7061 9.806390e-01 9.754700e-01

7062 9.806569e-01 9.754684e-01

7063 9.806743e-01 9.754667e-01

7064 9.806913e-01 9.754651e-01

7065 9.807079e-01 9.754634e-01

7066 9.807240e-01 9.754616e-01

7067 9.807398e-01 9.754599e-01

7068 9.807551e-01 9.754581e-01

7069 9.807701e-01 9.754563e-01

7070 9.807848e-01 9.754544e-01

7071 9.807991e-01 9.754525e-01

7072 9.808130e-01 9.754506e-01

7073 9.808266e-01 9.754487e-01

7074 9.808399e-01 9.754467e-01

7075 9.808529e-01 9.754447e-01

7076 9.808655e-01 9.754427e-01

7077 9.808779e-01 9.754407e-01

7078 9.808900e-01 9.754386e-01

7079 9.809018e-01 9.754364e-01

7080 9.809133e-01 9.754343e-01

7081 9.809246e-01 9.754321e-01

7082 9.809356e-01 9.754299e-01

7083 9.809463e-01 9.754276e-01

7084 9.809568e-01 9.754253e-01

7085 9.809671e-01 9.754230e-01

7086 9.809772e-01 9.754206e-01

7087 9.809870e-01 9.754182e-01

7088 9.809967e-01 9.754158e-01

7089 9.810061e-01 9.754133e-01

7090 9.810153e-01 9.754108e-01

7091 9.810244e-01 9.754082e-01

7092 9.810332e-01 9.754056e-01

7093 9.810419e-01 9.754030e-01

7094 9.810504e-01 9.754003e-01

7095 9.810587e-01 9.753976e-01

7096 9.810669e-01 9.753949e-01

7097 9.810749e-01 9.753921e-01

7098 9.810827e-01 9.753893e-01

7099 9.810904e-01 9.753864e-01

7100 9.810980e-01 9.753835e-01

7101 9.811054e-01 9.753805e-01

7102 9.811127e-01 9.753775e-01

7103 9.811198e-01 9.753745e-01

7104 9.811269e-01 9.753714e-01

7105 9.811338e-01 9.753682e-01

7106 9.811406e-01 9.753651e-01

7107 9.811473e-01 9.753618e-01

7108 9.811539e-01 9.753585e-01

7109 9.811604e-01 9.753552e-01

7110 9.811667e-01 9.753518e-01

7111 9.811730e-01 9.753484e-01

7112 9.811792e-01 9.753450e-01

7113 9.811853e-01 9.753414e-01

7114 9.811913e-01 9.753379e-01

7115 9.811973e-01 9.753343e-01

7116 9.812031e-01 9.753306e-01

7117 9.812089e-01 9.753269e-01

7118 9.812146e-01 9.753231e-01

7119 9.812203e-01 9.753193e-01

7120 9.812259e-01 9.753154e-01

7121 9.812314e-01 9.753114e-01

7122 9.812368e-01 9.753074e-01

7123 9.812422e-01 9.753034e-01

7124 9.812476e-01 9.752993e-01

7125 9.812529e-01 9.752951e-01

7126 9.812581e-01 9.752909e-01

7127 9.812633e-01 9.752866e-01

7128 9.812685e-01 9.752823e-01

7129 9.812736e-01 9.752779e-01

7130 9.812786e-01 9.752735e-01

7131 9.812837e-01 9.752689e-01

7132 9.812886e-01 9.752644e-01

7133 9.812936e-01 9.752597e-01

7134 9.812985e-01 9.752550e-01

7135 9.813034e-01 9.752503e-01

7136 9.813083e-01 9.752455e-01

7137 9.813131e-01 9.752406e-01

7138 9.813179e-01 9.752356e-01

7139 9.813227e-01 9.752306e-01

7140 9.813275e-01 9.752255e-01

7141 9.813322e-01 9.752204e-01

7142 9.813370e-01 9.752151e-01

7143 9.813417e-01 9.752099e-01

7144 9.813464e-01 9.752045e-01

7145 9.813511e-01 9.751991e-01

7146 9.813558e-01 9.751936e-01

7147 9.813604e-01 9.751880e-01

7148 9.813651e-01 9.751824e-01

7149 9.813697e-01 9.751767e-01

7150 9.813744e-01 9.751709e-01

7151 9.813790e-01 9.751651e-01

7152 9.813836e-01 9.751591e-01

7153 9.813883e-01 9.751531e-01

7154 9.813929e-01 9.751471e-01

7155 9.813975e-01 9.751409e-01

7156 9.814021e-01 9.751347e-01

7157 9.814068e-01 9.751284e-01

7158 9.814114e-01 9.751220e-01

7159 9.814160e-01 9.751156e-01

7160 9.814207e-01 9.751090e-01

7161 9.814253e-01 9.751024e-01

7162 9.814299e-01 9.750957e-01

7163 9.814346e-01 9.750890e-01

7164 9.814393e-01 9.750821e-01

7165 9.814439e-01 9.750752e-01

7166 9.814486e-01 9.750682e-01

7167 9.814533e-01 9.750611e-01

7168 9.814580e-01 9.750539e-01

7169 9.814627e-01 9.750466e-01

7170 9.814675e-01 9.750393e-01

7171 9.814722e-01 9.750319e-01

7172 9.814769e-01 9.750244e-01

7173 9.814817e-01 9.750168e-01

7174 9.814865e-01 9.750091e-01

7175 9.814913e-01 9.750013e-01

7176 9.814961e-01 9.749934e-01

7177 9.815009e-01 9.749855e-01

7178 9.815058e-01 9.749775e-01

7179 9.815107e-01 9.749693e-01

7180 9.815155e-01 9.749611e-01

7181 9.815204e-01 9.749528e-01

7182 9.815254e-01 9.749444e-01

7183 9.815303e-01 9.749359e-01

7184 9.815353e-01 9.749274e-01

7185 9.815403e-01 9.749187e-01

7186 9.815453e-01 9.749100e-01

7187 9.815503e-01 9.749011e-01

7188 9.815553e-01 9.748922e-01

7189 9.815604e-01 9.748831e-01

7190 9.815655e-01 9.748740e-01

7191 9.815706e-01 9.748648e-01

7192 9.815757e-01 9.748555e-01

7193 9.815809e-01 9.748461e-01

7194 9.815861e-01 9.748366e-01

7195 9.815913e-01 9.748270e-01

7196 9.815965e-01 9.748173e-01

7197 9.816018e-01 9.748075e-01

7198 9.816070e-01 9.747976e-01

7199 9.816123e-01 9.747877e-01

7200 9.816177e-01 9.747776e-01

7201 9.816230e-01 9.747674e-01

7202 9.816643e-01 9.747665e-01

7203 9.817045e-01 9.747656e-01

7204 9.817437e-01 9.747646e-01

7205 9.817820e-01 9.747636e-01

7206 9.818193e-01 9.747626e-01

7207 9.818557e-01 9.747616e-01

7208 9.818912e-01 9.747606e-01

7209 9.819258e-01 9.747596e-01

7210 9.819595e-01 9.747585e-01

7211 9.819924e-01 9.747575e-01

7212 9.820245e-01 9.747564e-01

7213 9.820557e-01 9.747553e-01

7214 9.820862e-01 9.747541e-01

7215 9.821159e-01 9.747530e-01

7216 9.821448e-01 9.747518e-01

7217 9.821731e-01 9.747506e-01

7218 9.822006e-01 9.747494e-01

7219 9.822274e-01 9.747482e-01

7220 9.822535e-01 9.747470e-01

7221 9.822789e-01 9.747457e-01

7222 9.823038e-01 9.747444e-01

7223 9.823280e-01 9.747431e-01

7224 9.823515e-01 9.747418e-01

7225 9.823745e-01 9.747404e-01

7226 9.823969e-01 9.747390e-01

7227 9.824187e-01 9.747377e-01

7228 9.824400e-01 9.747362e-01

7229 9.824608e-01 9.747348e-01

7230 9.824810e-01 9.747333e-01

7231 9.825007e-01 9.747318e-01

7232 9.825199e-01 9.747303e-01

7233 9.825386e-01 9.747288e-01

7234 9.825569e-01 9.747272e-01

7235 9.825747e-01 9.747257e-01

7236 9.825920e-01 9.747240e-01

7237 9.826089e-01 9.747224e-01

7238 9.826254e-01 9.747207e-01

7239 9.826415e-01 9.747191e-01

7240 9.826572e-01 9.747173e-01

7241 9.826725e-01 9.747156e-01

7242 9.826874e-01 9.747138e-01

7243 9.827020e-01 9.747120e-01

7244 9.827162e-01 9.747102e-01

7245 9.827300e-01 9.747083e-01

7246 9.827435e-01 9.747065e-01

7247 9.827567e-01 9.747046e-01

7248 9.827696e-01 9.747026e-01

7249 9.827822e-01 9.747006e-01

7250 9.827944e-01 9.746986e-01

7251 9.828064e-01 9.746966e-01

7252 9.828181e-01 9.746945e-01

7253 9.828295e-01 9.746924e-01

7254 9.828406e-01 9.746903e-01

7255 9.828515e-01 9.746881e-01

7256 9.828622e-01 9.746860e-01

7257 9.828725e-01 9.746837e-01

7258 9.828827e-01 9.746815e-01

7259 9.828926e-01 9.746792e-01

7260 9.829023e-01 9.746768e-01

7261 9.829118e-01 9.746745e-01

7262 9.829211e-01 9.746721e-01

7263 9.829302e-01 9.746696e-01

7264 9.829391e-01 9.746671e-01

7265 9.829477e-01 9.746646e-01

7266 9.829562e-01 9.746621e-01

7267 9.829646e-01 9.746595e-01

7268 9.829727e-01 9.746569e-01

7269 9.829807e-01 9.746542e-01

7270 9.829885e-01 9.746515e-01

7271 9.829962e-01 9.746488e-01

7272 9.830037e-01 9.746460e-01

7273 9.830111e-01 9.746431e-01

7274 9.830183e-01 9.746403e-01

7275 9.830254e-01 9.746374e-01

7276 9.830324e-01 9.746344e-01

7277 9.830392e-01 9.746314e-01

7278 9.830459e-01 9.746284e-01

7279 9.830525e-01 9.746253e-01

7280 9.830590e-01 9.746222e-01

7281 9.830653e-01 9.746190e-01

7282 9.830716e-01 9.746158e-01

7283 9.830777e-01 9.746125e-01

7284 9.830838e-01 9.746092e-01

7285 9.830897e-01 9.746059e-01

7286 9.830956e-01 9.746025e-01

7287 9.831014e-01 9.745990e-01

7288 9.831070e-01 9.745955e-01

7289 9.831126e-01 9.745919e-01

7290 9.831182e-01 9.745883e-01

7291 9.831236e-01 9.745847e-01

7292 9.831290e-01 9.745810e-01

7293 9.831343e-01 9.745772e-01

7294 9.831395e-01 9.745734e-01

7295 9.831447e-01 9.745696e-01

7296 9.831498e-01 9.745657e-01

7297 9.831548e-01 9.745617e-01

7298 9.831598e-01 9.745577e-01

7299 9.831647e-01 9.745536e-01

7300 9.831696e-01 9.745495e-01

7301 9.831744e-01 9.745453e-01

7302 9.831792e-01 9.745410e-01

7303 9.831839e-01 9.745367e-01

7304 9.831886e-01 9.745324e-01

7305 9.831933e-01 9.745280e-01

7306 9.831979e-01 9.745235e-01

7307 9.832025e-01 9.745189e-01

7308 9.832070e-01 9.745144e-01

7309 9.832115e-01 9.745097e-01

7310 9.832160e-01 9.745050e-01

7311 9.832204e-01 9.745002e-01

7312 9.832248e-01 9.744953e-01

7313 9.832292e-01 9.744904e-01

7314 9.832335e-01 9.744855e-01

7315 9.832379e-01 9.744804e-01

7316 9.832422e-01 9.744753e-01

7317 9.832465e-01 9.744702e-01

7318 9.832507e-01 9.744649e-01

7319 9.832550e-01 9.744596e-01

7320 9.832592e-01 9.744543e-01

7321 9.832635e-01 9.744488e-01

7322 9.832677e-01 9.744433e-01

7323 9.832719e-01 9.744377e-01

7324 9.832760e-01 9.744321e-01

7325 9.832802e-01 9.744264e-01

7326 9.832844e-01 9.744206e-01

7327 9.832885e-01 9.744147e-01

7328 9.832927e-01 9.744088e-01

7329 9.832968e-01 9.744028e-01

7330 9.833010e-01 9.743967e-01

7331 9.833051e-01 9.743906e-01

7332 9.833092e-01 9.743843e-01

7333 9.833134e-01 9.743780e-01

7334 9.833175e-01 9.743717e-01

7335 9.833216e-01 9.743652e-01

7336 9.833258e-01 9.743587e-01

7337 9.833299e-01 9.743521e-01

7338 9.833341e-01 9.743454e-01

7339 9.833382e-01 9.743386e-01

7340 9.833423e-01 9.743318e-01

7341 9.833465e-01 9.743248e-01

7342 9.833506e-01 9.743178e-01

7343 9.833548e-01 9.743107e-01

7344 9.833590e-01 9.743036e-01

7345 9.833632e-01 9.742963e-01

7346 9.833673e-01 9.742890e-01

7347 9.833715e-01 9.742816e-01

7348 9.833758e-01 9.742741e-01

7349 9.833800e-01 9.742665e-01

7350 9.833842e-01 9.742588e-01

7351 9.833884e-01 9.742510e-01

7352 9.833927e-01 9.742432e-01

7353 9.833970e-01 9.742353e-01

7354 9.834012e-01 9.742273e-01

7355 9.834055e-01 9.742192e-01

7356 9.834098e-01 9.742110e-01

7357 9.834141e-01 9.742027e-01

7358 9.834185e-01 9.741943e-01

7359 9.834228e-01 9.741859e-01

7360 9.834272e-01 9.741773e-01

7361 9.834316e-01 9.741687e-01

7362 9.834360e-01 9.741599e-01

7363 9.834404e-01 9.741511e-01

7364 9.834448e-01 9.741422e-01

7365 9.834493e-01 9.741332e-01

7366 9.834537e-01 9.741241e-01

7367 9.834582e-01 9.741149e-01

7368 9.834627e-01 9.741056e-01

7369 9.834672e-01 9.740963e-01

7370 9.834718e-01 9.740868e-01

7371 9.834763e-01 9.740772e-01

7372 9.834809e-01 9.740676e-01

7373 9.834855e-01 9.740578e-01

7374 9.834901e-01 9.740480e-01

7375 9.834948e-01 9.740380e-01

7376 9.834994e-01 9.740280e-01

7377 9.835041e-01 9.740179e-01

7378 9.835088e-01 9.740076e-01

7379 9.835135e-01 9.739973e-01

7380 9.835183e-01 9.739869e-01

7381 9.835230e-01 9.739764e-01

7382 9.835576e-01 9.739753e-01

7383 9.835914e-01 9.739743e-01

7384 9.836243e-01 9.739733e-01

7385 9.836563e-01 9.739722e-01

7386 9.836876e-01 9.739711e-01

7387 9.837180e-01 9.739700e-01

7388 9.837477e-01 9.739689e-01

7389 9.837767e-01 9.739677e-01

7390 9.838048e-01 9.739666e-01

7391 9.838323e-01 9.739654e-01

7392 9.838591e-01 9.739642e-01

7393 9.838852e-01 9.739630e-01

7394 9.839106e-01 9.739618e-01

7395 9.839354e-01 9.739605e-01

7396 9.839595e-01 9.739592e-01

7397 9.839831e-01 9.739579e-01

7398 9.840060e-01 9.739566e-01

7399 9.840283e-01 9.739553e-01

7400 9.840501e-01 9.739539e-01

7401 9.840713e-01 9.739525e-01

7402 9.840920e-01 9.739511e-01

7403 9.841122e-01 9.739497e-01

7404 9.841318e-01 9.739483e-01

7405 9.841509e-01 9.739468e-01

7406 9.841696e-01 9.739453e-01

7407 9.841878e-01 9.739438e-01

7408 9.842055e-01 9.739422e-01

7409 9.842228e-01 9.739407e-01

7410 9.842396e-01 9.739391e-01

7411 9.842560e-01 9.739375e-01

7412 9.842720e-01 9.739358e-01

7413 9.842876e-01 9.739342e-01

7414 9.843029e-01 9.739325e-01

7415 9.843177e-01 9.739307e-01

7416 9.843322e-01 9.739290e-01

7417 9.843463e-01 9.739272e-01

7418 9.843600e-01 9.739254e-01

7419 9.843734e-01 9.739236e-01

7420 9.843865e-01 9.739217e-01

7421 9.843993e-01 9.739198e-01

7422 9.844117e-01 9.739179e-01

7423 9.844239e-01 9.739160e-01

7424 9.844358e-01 9.739140e-01

7425 9.844473e-01 9.739120e-01

7426 9.844586e-01 9.739100e-01

7427 9.844697e-01 9.739079e-01

7428 9.844804e-01 9.739058e-01

7429 9.844909e-01 9.739037e-01

7430 9.845012e-01 9.739015e-01

7431 9.845112e-01 9.738993e-01

7432 9.845210e-01 9.738971e-01

7433 9.845306e-01 9.738948e-01

7434 9.845400e-01 9.738925e-01

7435 9.845491e-01 9.738902e-01

7436 9.845580e-01 9.738879e-01

7437 9.845668e-01 9.738855e-01

7438 9.845753e-01 9.738830e-01

7439 9.845837e-01 9.738806e-01

7440 9.845919e-01 9.738780e-01

7441 9.845999e-01 9.738755e-01

7442 9.846077e-01 9.738729e-01

7443 9.846154e-01 9.738703e-01

7444 9.846229e-01 9.738676e-01

7445 9.846302e-01 9.738650e-01

7446 9.846374e-01 9.738622e-01

7447 9.846445e-01 9.738594e-01

7448 9.846514e-01 9.738566e-01

7449 9.846582e-01 9.738538e-01

7450 9.846648e-01 9.738509e-01

7451 9.846713e-01 9.738479e-01

7452 9.846777e-01 9.738450e-01

7453 9.846840e-01 9.738419e-01

7454 9.846902e-01 9.738389e-01

7455 9.846962e-01 9.738358e-01

7456 9.847022e-01 9.738326e-01

7457 9.847080e-01 9.738294e-01

7458 9.847137e-01 9.738262e-01

7459 9.847194e-01 9.738229e-01

7460 9.847249e-01 9.738195e-01

7461 9.847304e-01 9.738162e-01

7462 9.847358e-01 9.738127e-01

7463 9.847411e-01 9.738092e-01

7464 9.847463e-01 9.738057e-01

7465 9.847514e-01 9.738021e-01

7466 9.847564e-01 9.737985e-01

7467 9.847614e-01 9.737948e-01

7468 9.847663e-01 9.737911e-01

7469 9.847712e-01 9.737873e-01

7470 9.847760e-01 9.737835e-01

7471 9.847807e-01 9.737796e-01

7472 9.847853e-01 9.737757e-01

7473 9.847899e-01 9.737717e-01

7474 9.847945e-01 9.737677e-01

7475 9.847990e-01 9.737636e-01

7476 9.848034e-01 9.737594e-01

7477 9.848078e-01 9.737552e-01

7478 9.848122e-01 9.737509e-01

7479 9.848165e-01 9.737466e-01

7480 9.848207e-01 9.737423e-01

7481 9.848249e-01 9.737378e-01

7482 9.848291e-01 9.737333e-01

7483 9.848333e-01 9.737288e-01

7484 9.848374e-01 9.737242e-01

7485 9.848415e-01 9.737195e-01

7486 9.848455e-01 9.737148e-01

7487 9.848495e-01 9.737100e-01

7488 9.848535e-01 9.737051e-01

7489 9.848575e-01 9.737002e-01

7490 9.848614e-01 9.736952e-01

7491 9.848653e-01 9.736902e-01

7492 9.848692e-01 9.736850e-01

7493 9.848731e-01 9.736799e-01

7494 9.848769e-01 9.736746e-01

7495 9.848807e-01 9.736693e-01

7496 9.848846e-01 9.736639e-01

7497 9.848884e-01 9.736585e-01

7498 9.848921e-01 9.736530e-01

7499 9.848959e-01 9.736474e-01

7500 9.848997e-01 9.736418e-01

7501 9.849034e-01 9.736360e-01

7502 9.849071e-01 9.736303e-01

7503 9.849109e-01 9.736244e-01

7504 9.849146e-01 9.736185e-01

7505 9.849183e-01 9.736125e-01

7506 9.849220e-01 9.736064e-01

7507 9.849257e-01 9.736002e-01

7508 9.849294e-01 9.735940e-01

7509 9.849331e-01 9.735877e-01

7510 9.849367e-01 9.735813e-01

7511 9.849404e-01 9.735749e-01

7512 9.849441e-01 9.735684e-01

7513 9.849478e-01 9.735618e-01

7514 9.849515e-01 9.735551e-01

7515 9.849551e-01 9.735483e-01

7516 9.849588e-01 9.735415e-01

7517 9.849625e-01 9.735346e-01

7518 9.849662e-01 9.735276e-01

7519 9.849699e-01 9.735205e-01

7520 9.849736e-01 9.735133e-01

7521 9.849773e-01 9.735061e-01

7522 9.849810e-01 9.734988e-01

7523 9.849847e-01 9.734914e-01

7524 9.849884e-01 9.734839e-01

7525 9.849922e-01 9.734763e-01

7526 9.849959e-01 9.734687e-01

7527 9.849997e-01 9.734609e-01

7528 9.850034e-01 9.734531e-01

7529 9.850072e-01 9.734452e-01

7530 9.850110e-01 9.734372e-01

7531 9.850147e-01 9.734291e-01

7532 9.850185e-01 9.734210e-01

7533 9.850223e-01 9.734127e-01

7534 9.850262e-01 9.734044e-01

7535 9.850300e-01 9.733960e-01

7536 9.850338e-01 9.733874e-01

7537 9.850377e-01 9.733788e-01

7538 9.850416e-01 9.733701e-01

7539 9.850455e-01 9.733613e-01

7540 9.850494e-01 9.733525e-01

7541 9.850533e-01 9.733435e-01

7542 9.850572e-01 9.733344e-01

7543 9.850611e-01 9.733253e-01

7544 9.850651e-01 9.733160e-01

7545 9.850691e-01 9.733067e-01

7546 9.850731e-01 9.732972e-01

7547 9.850771e-01 9.732877e-01

7548 9.850811e-01 9.732781e-01

7549 9.850851e-01 9.732684e-01

7550 9.850892e-01 9.732586e-01

7551 9.850933e-01 9.732487e-01

7552 9.850974e-01 9.732387e-01

7553 9.851015e-01 9.732286e-01

7554 9.851056e-01 9.732184e-01

7555 9.851098e-01 9.732081e-01

7556 9.851139e-01 9.731977e-01

7557 9.851181e-01 9.731872e-01

7558 9.851223e-01 9.731766e-01

7559 9.851266e-01 9.731660e-01

7560 9.851308e-01 9.731552e-01

7561 9.851351e-01 9.731443e-01

7562 9.851639e-01 9.731432e-01

7563 9.851921e-01 9.731421e-01

7564 9.852195e-01 9.731409e-01

7565 9.852462e-01 9.731398e-01

7566 9.852722e-01 9.731386e-01

7567 9.852975e-01 9.731374e-01

7568 9.853222e-01 9.731362e-01

7569 9.853463e-01 9.731349e-01

7570 9.853698e-01 9.731337e-01

7571 9.853926e-01 9.731324e-01

7572 9.854149e-01 9.731311e-01

7573 9.854366e-01 9.731298e-01

7574 9.854577e-01 9.731284e-01

7575 9.854783e-01 9.731271e-01

7576 9.854984e-01 9.731257e-01

7577 9.855180e-01 9.731243e-01

7578 9.855370e-01 9.731228e-01

7579 9.855556e-01 9.731214e-01

7580 9.855737e-01 9.731199e-01

7581 9.855913e-01 9.731184e-01

7582 9.856085e-01 9.731169e-01

7583 9.856253e-01 9.731153e-01

7584 9.856416e-01 9.731138e-01

7585 9.856575e-01 9.731122e-01

7586 9.856730e-01 9.731105e-01

7587 9.856881e-01 9.731089e-01

7588 9.857029e-01 9.731072e-01

7589 9.857172e-01 9.731055e-01

7590 9.857313e-01 9.731038e-01

7591 9.857449e-01 9.731021e-01

7592 9.857582e-01 9.731003e-01

7593 9.857712e-01 9.730985e-01

7594 9.857839e-01 9.730967e-01

7595 9.857963e-01 9.730948e-01

7596 9.858083e-01 9.730929e-01

7597 9.858201e-01 9.730910e-01

7598 9.858315e-01 9.730891e-01

7599 9.858427e-01 9.730871e-01

7600 9.858537e-01 9.730851e-01

7601 9.858643e-01 9.730831e-01

7602 9.858747e-01 9.730810e-01

7603 9.858849e-01 9.730789e-01

7604 9.858948e-01 9.730768e-01

7605 9.859045e-01 9.730746e-01

7606 9.859139e-01 9.730724e-01

7607 9.859232e-01 9.730702e-01

7608 9.859322e-01 9.730680e-01

7609 9.859410e-01 9.730657e-01

7610 9.859496e-01 9.730634e-01

7611 9.859580e-01 9.730610e-01

7612 9.859663e-01 9.730586e-01

7613 9.859743e-01 9.730562e-01

7614 9.859822e-01 9.730537e-01

7615 9.859899e-01 9.730512e-01

7616 9.859974e-01 9.730487e-01

7617 9.860047e-01 9.730461e-01

7618 9.860120e-01 9.730435e-01

7619 9.860190e-01 9.730409e-01

7620 9.860259e-01 9.730382e-01

7621 9.860327e-01 9.730355e-01

7622 9.860393e-01 9.730327e-01

7623 9.860458e-01 9.730299e-01

7624 9.860521e-01 9.730271e-01

7625 9.860584e-01 9.730242e-01

7626 9.860645e-01 9.730213e-01

7627 9.860705e-01 9.730183e-01

7628 9.860763e-01 9.730153e-01

7629 9.860821e-01 9.730123e-01

7630 9.860878e-01 9.730092e-01

7631 9.860933e-01 9.730061e-01

7632 9.860988e-01 9.730029e-01

7633 9.861041e-01 9.729997e-01

7634 9.861094e-01 9.729964e-01

7635 9.861146e-01 9.729931e-01

7636 9.861197e-01 9.729897e-01

7637 9.861247e-01 9.729863e-01

7638 9.861296e-01 9.729829e-01

7639 9.861344e-01 9.729794e-01

7640 9.861392e-01 9.729758e-01

7641 9.861439e-01 9.729723e-01

7642 9.861485e-01 9.729686e-01

7643 9.861531e-01 9.729649e-01

7644 9.861576e-01 9.729612e-01

7645 9.861620e-01 9.729574e-01

7646 9.861663e-01 9.729535e-01

7647 9.861707e-01 9.729496e-01

7648 9.861749e-01 9.729457e-01

7649 9.861791e-01 9.729417e-01

7650 9.861832e-01 9.729376e-01

7651 9.861873e-01 9.729335e-01

7652 9.861914e-01 9.729294e-01

7653 9.861954e-01 9.729251e-01

7654 9.861993e-01 9.729209e-01

7655 9.862033e-01 9.729165e-01

7656 9.862071e-01 9.729121e-01

7657 9.862110e-01 9.729077e-01

7658 9.862148e-01 9.729032e-01

7659 9.862185e-01 9.728986e-01

7660 9.862222e-01 9.728940e-01

7661 9.862259e-01 9.728893e-01

7662 9.862296e-01 9.728846e-01

7663 9.862332e-01 9.728798e-01

7664 9.862368e-01 9.728749e-01

7665 9.862404e-01 9.728700e-01

7666 9.862440e-01 9.728650e-01

7667 9.862475e-01 9.728600e-01

7668 9.862510e-01 9.728549e-01

7669 9.862545e-01 9.728497e-01

7670 9.862580e-01 9.728444e-01

7671 9.862614e-01 9.728391e-01

7672 9.862648e-01 9.728337e-01

7673 9.862683e-01 9.728283e-01

7674 9.862717e-01 9.728228e-01

7675 9.862750e-01 9.728172e-01

7676 9.862784e-01 9.728116e-01

7677 9.862818e-01 9.728059e-01

7678 9.862851e-01 9.728001e-01

7679 9.862885e-01 9.727942e-01

7680 9.862918e-01 9.727883e-01

7681 9.862951e-01 9.727823e-01

7682 9.862984e-01 9.727762e-01

7683 9.863017e-01 9.727701e-01

7684 9.863050e-01 9.727639e-01

7685 9.863083e-01 9.727576e-01

7686 9.863116e-01 9.727512e-01

7687 9.863149e-01 9.727448e-01

7688 9.863182e-01 9.727383e-01

7689 9.863214e-01 9.727317e-01

7690 9.863247e-01 9.727250e-01

7691 9.863280e-01 9.727183e-01

7692 9.863313e-01 9.727115e-01

7693 9.863346e-01 9.727046e-01

7694 9.863378e-01 9.726976e-01

7695 9.863411e-01 9.726905e-01

7696 9.863444e-01 9.726834e-01

7697 9.863477e-01 9.726762e-01

7698 9.863510e-01 9.726689e-01

7699 9.863543e-01 9.726615e-01

7700 9.863576e-01 9.726540e-01

7701 9.863609e-01 9.726465e-01

7702 9.863642e-01 9.726389e-01

7703 9.863675e-01 9.726312e-01

7704 9.863709e-01 9.726234e-01

7705 9.863742e-01 9.726155e-01

7706 9.863775e-01 9.726075e-01

7707 9.863809e-01 9.725995e-01

7708 9.863843e-01 9.725913e-01

7709 9.863876e-01 9.725831e-01

7710 9.863910e-01 9.725748e-01

7711 9.863944e-01 9.725664e-01

7712 9.863978e-01 9.725579e-01

7713 9.864012e-01 9.725493e-01

7714 9.864046e-01 9.725407e-01

7715 9.864081e-01 9.725319e-01

7716 9.864115e-01 9.725231e-01

7717 9.864150e-01 9.725141e-01

7718 9.864185e-01 9.725051e-01

7719 9.864220e-01 9.724960e-01

7720 9.864255e-01 9.724868e-01

7721 9.864290e-01 9.724775e-01

7722 9.864325e-01 9.724681e-01

7723 9.864360e-01 9.724586e-01

7724 9.864396e-01 9.724490e-01

7725 9.864432e-01 9.724393e-01

7726 9.864468e-01 9.724296e-01

7727 9.864504e-01 9.724197e-01

7728 9.864540e-01 9.724097e-01

7729 9.864577e-01 9.723997e-01

7730 9.864613e-01 9.723895e-01

7731 9.864650e-01 9.723793e-01

7732 9.864687e-01 9.723690e-01

7733 9.864724e-01 9.723585e-01

7734 9.864761e-01 9.723480e-01

7735 9.864799e-01 9.723374e-01

7736 9.864836e-01 9.723266e-01

7737 9.864874e-01 9.723158e-01

7738 9.864912e-01 9.723049e-01

7739 9.864950e-01 9.722939e-01

7740 9.864989e-01 9.722827e-01

7741 9.865027e-01 9.722715e-01

7742 9.865267e-01 9.722703e-01

7743 9.865501e-01 9.722691e-01

7744 9.865728e-01 9.722678e-01

7745 9.865950e-01 9.722666e-01

7746 9.866166e-01 9.722653e-01

7747 9.866376e-01 9.722640e-01

7748 9.866581e-01 9.722627e-01

7749 9.866781e-01 9.722613e-01

7750 9.866976e-01 9.722600e-01

7751 9.867166e-01 9.722586e-01

7752 9.867350e-01 9.722572e-01

7753 9.867530e-01 9.722557e-01

7754 9.867706e-01 9.722543e-01

7755 9.867877e-01 9.722528e-01

7756 9.868043e-01 9.722513e-01

7757 9.868206e-01 9.722498e-01

7758 9.868364e-01 9.722482e-01

7759 9.868518e-01 9.722467e-01

7760 9.868669e-01 9.722451e-01

7761 9.868815e-01 9.722434e-01

7762 9.868958e-01 9.722418e-01

7763 9.869097e-01 9.722401e-01

7764 9.869233e-01 9.722384e-01

7765 9.869365e-01 9.722367e-01

7766 9.869494e-01 9.722350e-01

7767 9.869620e-01 9.722332e-01

7768 9.869742e-01 9.722314e-01

7769 9.869862e-01 9.722296e-01

7770 9.869979e-01 9.722277e-01

7771 9.870092e-01 9.722259e-01

7772 9.870203e-01 9.722240e-01

7773 9.870312e-01 9.722220e-01

7774 9.870417e-01 9.722201e-01

7775 9.870520e-01 9.722181e-01

7776 9.870621e-01 9.722160e-01

7777 9.870719e-01 9.722140e-01

7778 9.870815e-01 9.722119e-01

7779 9.870908e-01 9.722098e-01

7780 9.871000e-01 9.722076e-01

7781 9.871089e-01 9.722055e-01

7782 9.871176e-01 9.722032e-01

7783 9.871261e-01 9.722010e-01

7784 9.871344e-01 9.721987e-01

7785 9.871425e-01 9.721964e-01

7786 9.871504e-01 9.721941e-01

7787 9.871582e-01 9.721917e-01

7788 9.871658e-01 9.721893e-01

7789 9.871732e-01 9.721869e-01

7790 9.871804e-01 9.721844e-01

7791 9.871875e-01 9.721819e-01

7792 9.871944e-01 9.721793e-01

7793 9.872012e-01 9.721767e-01

7794 9.872078e-01 9.721741e-01

7795 9.872143e-01 9.721714e-01

7796 9.872207e-01 9.721687e-01

7797 9.872269e-01 9.721660e-01

7798 9.872330e-01 9.721632e-01

7799 9.872389e-01 9.721604e-01

7800 9.872448e-01 9.721575e-01

7801 9.872505e-01 9.721546e-01

7802 9.872561e-01 9.721517e-01

7803 9.872616e-01 9.721487e-01

7804 9.872670e-01 9.721457e-01

7805 9.872723e-01 9.721426e-01

7806 9.872775e-01 9.721395e-01

7807 9.872826e-01 9.721364e-01

7808 9.872877e-01 9.721332e-01

7809 9.872926e-01 9.721300e-01

7810 9.872974e-01 9.721267e-01

7811 9.873022e-01 9.721234e-01

7812 9.873068e-01 9.721200e-01

7813 9.873114e-01 9.721166e-01

7814 9.873159e-01 9.721131e-01

7815 9.873204e-01 9.721096e-01

7816 9.873247e-01 9.721060e-01

7817 9.873290e-01 9.721024e-01

7818 9.873332e-01 9.720988e-01

7819 9.873374e-01 9.720951e-01

7820 9.873415e-01 9.720913e-01

7821 9.873456e-01 9.720875e-01

7822 9.873496e-01 9.720837e-01

7823 9.873535e-01 9.720798e-01

7824 9.873574e-01 9.720758e-01

7825 9.873612e-01 9.720718e-01

7826 9.873650e-01 9.720677e-01

7827 9.873687e-01 9.720636e-01

7828 9.873724e-01 9.720594e-01

7829 9.873760e-01 9.720552e-01

7830 9.873796e-01 9.720509e-01

7831 9.873832e-01 9.720466e-01

7832 9.873867e-01 9.720422e-01

7833 9.873902e-01 9.720378e-01

7834 9.873937e-01 9.720333e-01

7835 9.873971e-01 9.720287e-01

7836 9.874005e-01 9.720241e-01

7837 9.874038e-01 9.720194e-01

7838 9.874071e-01 9.720146e-01

7839 9.874104e-01 9.720098e-01

7840 9.874137e-01 9.720050e-01

7841 9.874169e-01 9.720001e-01

7842 9.874202e-01 9.719951e-01

7843 9.874233e-01 9.719900e-01

7844 9.874265e-01 9.719849e-01

7845 9.874297e-01 9.719797e-01

7846 9.874328e-01 9.719745e-01

7847 9.874359e-01 9.719692e-01

7848 9.874390e-01 9.719638e-01

7849 9.874421e-01 9.719584e-01

7850 9.874451e-01 9.719529e-01

7851 9.874482e-01 9.719473e-01

7852 9.874512e-01 9.719417e-01

7853 9.874542e-01 9.719360e-01

7854 9.874572e-01 9.719302e-01

7855 9.874602e-01 9.719244e-01

7856 9.874632e-01 9.719185e-01

7857 9.874662e-01 9.719125e-01

7858 9.874692e-01 9.719064e-01

7859 9.874721e-01 9.719003e-01

7860 9.874751e-01 9.718941e-01

7861 9.874780e-01 9.718878e-01

7862 9.874810e-01 9.718815e-01

7863 9.874839e-01 9.718751e-01

7864 9.874869e-01 9.718686e-01

7865 9.874898e-01 9.718620e-01

7866 9.874927e-01 9.718553e-01

7867 9.874957e-01 9.718486e-01

7868 9.874986e-01 9.718418e-01

7869 9.875015e-01 9.718350e-01

7870 9.875045e-01 9.718280e-01

7871 9.875074e-01 9.718210e-01

7872 9.875103e-01 9.718139e-01

7873 9.875132e-01 9.718067e-01

7874 9.875162e-01 9.717994e-01

7875 9.875191e-01 9.717920e-01

7876 9.875221e-01 9.717846e-01

7877 9.875250e-01 9.717771e-01

7878 9.875280e-01 9.717695e-01

7879 9.875309e-01 9.717618e-01

7880 9.875339e-01 9.717541e-01

7881 9.875368e-01 9.717462e-01

7882 9.875398e-01 9.717383e-01

7883 9.875428e-01 9.717303e-01

7884 9.875458e-01 9.717222e-01

7885 9.875488e-01 9.717140e-01

7886 9.875518e-01 9.717057e-01

7887 9.875548e-01 9.716973e-01

7888 9.875578e-01 9.716889e-01

7889 9.875609e-01 9.716803e-01

7890 9.875639e-01 9.716717e-01

7891 9.875670e-01 9.716630e-01

7892 9.875700e-01 9.716542e-01

7893 9.875731e-01 9.716453e-01

7894 9.875762e-01 9.716363e-01

7895 9.875793e-01 9.716273e-01

7896 9.875824e-01 9.716181e-01

7897 9.875856e-01 9.716088e-01

7898 9.875887e-01 9.715995e-01

7899 9.875918e-01 9.715900e-01

7900 9.875950e-01 9.715805e-01

7901 9.875982e-01 9.715709e-01

7902 9.876014e-01 9.715612e-01

7903 9.876046e-01 9.715513e-01

7904 9.876078e-01 9.715414e-01

7905 9.876111e-01 9.715314e-01

7906 9.876143e-01 9.715213e-01

7907 9.876176e-01 9.715111e-01

7908 9.876209e-01 9.715008e-01

7909 9.876242e-01 9.714905e-01

7910 9.876275e-01 9.714800e-01

7911 9.876309e-01 9.714694e-01

7912 9.876342e-01 9.714587e-01

7913 9.876376e-01 9.714479e-01

7914 9.876410e-01 9.714371e-01

7915 9.876444e-01 9.714261e-01

7916 9.876478e-01 9.714150e-01

7917 9.876513e-01 9.714039e-01

7918 9.876547e-01 9.713926e-01

7919 9.876582e-01 9.713813e-01

7920 9.876617e-01 9.713698e-01

7921 9.876653e-01 9.713583e-01

7922 9.876851e-01 9.713570e-01

7923 9.877045e-01 9.713556e-01

7924 9.877234e-01 9.713543e-01

7925 9.877418e-01 9.713529e-01

7926 9.877597e-01 9.713515e-01

7927 9.877771e-01 9.713501e-01

7928 9.877941e-01 9.713487e-01

7929 9.878107e-01 9.713472e-01

7930 9.878268e-01 9.713458e-01

7931 9.878426e-01 9.713443e-01

7932 9.878579e-01 9.713428e-01

7933 9.878728e-01 9.713412e-01

7934 9.878874e-01 9.713397e-01

7935 9.879016e-01 9.713381e-01

7936 9.879154e-01 9.713365e-01

7937 9.879289e-01 9.713348e-01

7938 9.879421e-01 9.713332e-01

7939 9.879549e-01 9.713315e-01

7940 9.879674e-01 9.713298e-01

7941 9.879795e-01 9.713280e-01

7942 9.879914e-01 9.713263e-01

7943 9.880030e-01 9.713245e-01

7944 9.880143e-01 9.713226e-01

7945 9.880253e-01 9.713208e-01

7946 9.880360e-01 9.713189e-01

7947 9.880465e-01 9.713170e-01

7948 9.880567e-01 9.713151e-01

7949 9.880667e-01 9.713132e-01

7950 9.880764e-01 9.713112e-01

7951 9.880859e-01 9.713092e-01

7952 9.880952e-01 9.713071e-01

7953 9.881042e-01 9.713050e-01

7954 9.881130e-01 9.713029e-01

7955 9.881217e-01 9.713008e-01

7956 9.881301e-01 9.712986e-01

7957 9.881383e-01 9.712965e-01

7958 9.881463e-01 9.712942e-01

7959 9.881541e-01 9.712920e-01

7960 9.881618e-01 9.712897e-01

7961 9.881693e-01 9.712874e-01

7962 9.881766e-01 9.712850e-01

7963 9.881837e-01 9.712826e-01

7964 9.881907e-01 9.712802e-01

7965 9.881975e-01 9.712777e-01

7966 9.882042e-01 9.712752e-01

7967 9.882107e-01 9.712727e-01

7968 9.882171e-01 9.712701e-01

7969 9.882233e-01 9.712675e-01

7970 9.882294e-01 9.712649e-01

7971 9.882354e-01 9.712622e-01

7972 9.882412e-01 9.712595e-01

7973 9.882470e-01 9.712567e-01

7974 9.882526e-01 9.712539e-01

7975 9.882581e-01 9.712511e-01

7976 9.882635e-01 9.712482e-01

7977 9.882687e-01 9.712453e-01

7978 9.882739e-01 9.712424e-01

7979 9.882790e-01 9.712394e-01

7980 9.882839e-01 9.712364e-01

7981 9.882888e-01 9.712333e-01

7982 9.882936e-01 9.712302e-01

7983 9.882983e-01 9.712270e-01

7984 9.883029e-01 9.712238e-01

7985 9.883074e-01 9.712206e-01

7986 9.883118e-01 9.712173e-01

7987 9.883162e-01 9.712139e-01

7988 9.883205e-01 9.712106e-01

7989 9.883247e-01 9.712071e-01

7990 9.883288e-01 9.712037e-01

7991 9.883329e-01 9.712001e-01

7992 9.883369e-01 9.711966e-01

7993 9.883408e-01 9.711930e-01

7994 9.883447e-01 9.711893e-01

7995 9.883485e-01 9.711856e-01

7996 9.883523e-01 9.711818e-01

7997 9.883560e-01 9.711780e-01

7998 9.883597e-01 9.711742e-01

7999 9.883633e-01 9.711703e-01

8000 9.883668e-01 9.711663e-01

8001 9.883703e-01 9.711623e-01

8002 9.883738e-01 9.711582e-01

8003 9.883772e-01 9.711541e-01

8004 9.883805e-01 9.711499e-01

8005 9.883839e-01 9.711457e-01

8006 9.883872e-01 9.711414e-01

8007 9.883904e-01 9.711371e-01

8008 9.883936e-01 9.711327e-01

8009 9.883968e-01 9.711282e-01

8010 9.883999e-01 9.711237e-01

8011 9.884030e-01 9.711192e-01

8012 9.884061e-01 9.711146e-01

8013 9.884092e-01 9.711099e-01

8014 9.884122e-01 9.711052e-01

8015 9.884152e-01 9.711004e-01

8016 9.884181e-01 9.710955e-01

8017 9.884211e-01 9.710906e-01

8018 9.884240e-01 9.710856e-01

8019 9.884269e-01 9.710806e-01

8020 9.884298e-01 9.710755e-01

8021 9.884326e-01 9.710703e-01

8022 9.884355e-01 9.710651e-01

8023 9.884383e-01 9.710598e-01

8024 9.884411e-01 9.710544e-01

8025 9.884438e-01 9.710490e-01

8026 9.884466e-01 9.710435e-01

8027 9.884494e-01 9.710380e-01

8028 9.884521e-01 9.710323e-01

8029 9.884548e-01 9.710266e-01

8030 9.884575e-01 9.710209e-01

8031 9.884602e-01 9.710151e-01

8032 9.884629e-01 9.710092e-01

8033 9.884656e-01 9.710032e-01

8034 9.884683e-01 9.709972e-01

8035 9.884709e-01 9.709911e-01

8036 9.884736e-01 9.709849e-01

8037 9.884763e-01 9.709786e-01

8038 9.884789e-01 9.709723e-01

8039 9.884815e-01 9.709659e-01

8040 9.884842e-01 9.709594e-01

8041 9.884868e-01 9.709529e-01

8042 9.884894e-01 9.709463e-01

8043 9.884921e-01 9.709396e-01

8044 9.884947e-01 9.709328e-01

8045 9.884973e-01 9.709260e-01

8046 9.884999e-01 9.709190e-01

8047 9.885026e-01 9.709120e-01

8048 9.885052e-01 9.709050e-01

8049 9.885078e-01 9.708978e-01

8050 9.885104e-01 9.708906e-01

8051 9.885131e-01 9.708832e-01

8052 9.885157e-01 9.708758e-01

8053 9.885183e-01 9.708684e-01

8054 9.885210e-01 9.708608e-01

8055 9.885236e-01 9.708532e-01

8056 9.885263e-01 9.708454e-01

8057 9.885289e-01 9.708376e-01

8058 9.885316e-01 9.708297e-01

8059 9.885342e-01 9.708218e-01

8060 9.885369e-01 9.708137e-01

8061 9.885396e-01 9.708055e-01

8062 9.885423e-01 9.707973e-01

8063 9.885450e-01 9.707890e-01

8064 9.885477e-01 9.707806e-01

8065 9.885504e-01 9.707721e-01

8066 9.885531e-01 9.707635e-01

8067 9.885558e-01 9.707548e-01

8068 9.885586e-01 9.707461e-01

8069 9.885613e-01 9.707372e-01

8070 9.885641e-01 9.707283e-01

8071 9.885668e-01 9.707193e-01

8072 9.885696e-01 9.707102e-01

8073 9.885724e-01 9.707010e-01

8074 9.885752e-01 9.706917e-01

8075 9.885780e-01 9.706823e-01

8076 9.885808e-01 9.706728e-01

8077 9.885837e-01 9.706632e-01

8078 9.885865e-01 9.706535e-01

8079 9.885894e-01 9.706438e-01

8080 9.885923e-01 9.706339e-01

8081 9.885952e-01 9.706240e-01

8082 9.885981e-01 9.706139e-01

8083 9.886010e-01 9.706038e-01

8084 9.886039e-01 9.705936e-01

8085 9.886069e-01 9.705833e-01

8086 9.886098e-01 9.705728e-01

8087 9.886128e-01 9.705623e-01

8088 9.886158e-01 9.705517e-01

8089 9.886188e-01 9.705410e-01

8090 9.886219e-01 9.705302e-01

8091 9.886249e-01 9.705193e-01

8092 9.886280e-01 9.705083e-01

8093 9.886311e-01 9.704972e-01

8094 9.886341e-01 9.704860e-01

8095 9.886373e-01 9.704747e-01

8096 9.886404e-01 9.704633e-01

8097 9.886435e-01 9.704518e-01

8098 9.886467e-01 9.704403e-01

8099 9.886499e-01 9.704286e-01

8100 9.886531e-01 9.704168e-01

8101 9.886563e-01 9.704049e-01

8102 9.886728e-01 9.704035e-01

8103 9.886889e-01 9.704021e-01

8104 9.887045e-01 9.704006e-01

8105 9.887197e-01 9.703992e-01

8106 9.887346e-01 9.703977e-01

8107 9.887491e-01 9.703962e-01

8108 9.887632e-01 9.703946e-01

8109 9.887769e-01 9.703931e-01

8110 9.887903e-01 9.703915e-01

8111 9.888034e-01 9.703899e-01

8112 9.888161e-01 9.703883e-01

8113 9.888286e-01 9.703866e-01

8114 9.888407e-01 9.703849e-01

8115 9.888525e-01 9.703832e-01

8116 9.888640e-01 9.703815e-01

8117 9.888752e-01 9.703798e-01

8118 9.888861e-01 9.703780e-01

8119 9.888968e-01 9.703762e-01

8120 9.889072e-01 9.703743e-01

8121 9.889173e-01 9.703725e-01

8122 9.889272e-01 9.703706e-01

8123 9.889369e-01 9.703687e-01

8124 9.889463e-01 9.703667e-01

8125 9.889555e-01 9.703648e-01

8126 9.889644e-01 9.703628e-01

8127 9.889732e-01 9.703608e-01

8128 9.889817e-01 9.703587e-01

8129 9.889900e-01 9.703566e-01

8130 9.889982e-01 9.703545e-01

8131 9.890061e-01 9.703523e-01

8132 9.890139e-01 9.703502e-01

8133 9.890214e-01 9.703480e-01

8134 9.890288e-01 9.703457e-01

8135 9.890361e-01 9.703434e-01

8136 9.890431e-01 9.703411e-01

8137 9.890500e-01 9.703388e-01

8138 9.890567e-01 9.703364e-01

8139 9.890633e-01 9.703340e-01

8140 9.890698e-01 9.703316e-01

8141 9.890760e-01 9.703291e-01

8142 9.890822e-01 9.703266e-01

8143 9.890882e-01 9.703241e-01

8144 9.890941e-01 9.703215e-01

8145 9.890998e-01 9.703189e-01

8146 9.891055e-01 9.703162e-01

8147 9.891110e-01 9.703135e-01

8148 9.891164e-01 9.703108e-01

8149 9.891216e-01 9.703081e-01

8150 9.891268e-01 9.703053e-01

8151 9.891319e-01 9.703024e-01

8152 9.891368e-01 9.702995e-01

8153 9.891417e-01 9.702966e-01

8154 9.891465e-01 9.702937e-01

8155 9.891511e-01 9.702907e-01

8156 9.891557e-01 9.702876e-01

8157 9.891602e-01 9.702845e-01

8158 9.891646e-01 9.702814e-01

8159 9.891689e-01 9.702782e-01

8160 9.891731e-01 9.702750e-01

8161 9.891773e-01 9.702718e-01

8162 9.891814e-01 9.702685e-01

8163 9.891854e-01 9.702652e-01

8164 9.891893e-01 9.702618e-01

8165 9.891932e-01 9.702583e-01

8166 9.891970e-01 9.702549e-01

8167 9.892008e-01 9.702513e-01

8168 9.892044e-01 9.702478e-01

8169 9.892081e-01 9.702442e-01

8170 9.892116e-01 9.702405e-01

8171 9.892151e-01 9.702368e-01

8172 9.892186e-01 9.702330e-01

8173 9.892220e-01 9.702292e-01

8174 9.892253e-01 9.702254e-01

8175 9.892286e-01 9.702214e-01

8176 9.892319e-01 9.702175e-01

8177 9.892351e-01 9.702135e-01

8178 9.892383e-01 9.702094e-01

8179 9.892414e-01 9.702053e-01

8180 9.892445e-01 9.702011e-01

8181 9.892475e-01 9.701969e-01

8182 9.892505e-01 9.701926e-01

8183 9.892535e-01 9.701883e-01

8184 9.892564e-01 9.701839e-01

8185 9.892593e-01 9.701795e-01

8186 9.892622e-01 9.701750e-01

8187 9.892650e-01 9.701704e-01

8188 9.892678e-01 9.701658e-01

8189 9.892706e-01 9.701611e-01

8190 9.892734e-01 9.701564e-01

8191 9.892761e-01 9.701516e-01

8192 9.892788e-01 9.701468e-01

8193 9.892815e-01 9.701419e-01

8194 9.892841e-01 9.701369e-01

8195 9.892867e-01 9.701319e-01

8196 9.892893e-01 9.701268e-01

8197 9.892919e-01 9.701216e-01

8198 9.892945e-01 9.701164e-01

8199 9.892971e-01 9.701112e-01

8200 9.892996e-01 9.701058e-01

8201 9.893021e-01 9.701004e-01

8202 9.893046e-01 9.700949e-01

8203 9.893071e-01 9.700894e-01

8204 9.893096e-01 9.700838e-01

8205 9.893121e-01 9.700781e-01

8206 9.893145e-01 9.700724e-01

8207 9.893170e-01 9.700666e-01

8208 9.893194e-01 9.700607e-01

8209 9.893218e-01 9.700548e-01

8210 9.893242e-01 9.700488e-01

8211 9.893266e-01 9.700427e-01

8212 9.893290e-01 9.700365e-01

8213 9.893314e-01 9.700303e-01

8214 9.893338e-01 9.700240e-01

8215 9.893362e-01 9.700176e-01

8216 9.893386e-01 9.700112e-01

8217 9.893409e-01 9.700047e-01

8218 9.893433e-01 9.699981e-01

8219 9.893457e-01 9.699914e-01

8220 9.893480e-01 9.699847e-01

8221 9.893504e-01 9.699779e-01

8222 9.893527e-01 9.699710e-01

8223 9.893551e-01 9.699640e-01

8224 9.893574e-01 9.699570e-01

8225 9.893598e-01 9.699498e-01

8226 9.893622e-01 9.699426e-01

8227 9.893645e-01 9.699354e-01

8228 9.893669e-01 9.699280e-01

8229 9.893692e-01 9.699206e-01

8230 9.893716e-01 9.699130e-01

8231 9.893740e-01 9.699054e-01

8232 9.893763e-01 9.698978e-01

8233 9.893787e-01 9.698900e-01

8234 9.893811e-01 9.698821e-01

8235 9.893835e-01 9.698742e-01

8236 9.893859e-01 9.698662e-01

8237 9.893883e-01 9.698581e-01

8238 9.893907e-01 9.698499e-01

8239 9.893931e-01 9.698416e-01

8240 9.893955e-01 9.698333e-01

8241 9.893979e-01 9.698248e-01

8242 9.894003e-01 9.698163e-01

8243 9.894028e-01 9.698077e-01

8244 9.894052e-01 9.697990e-01

8245 9.894077e-01 9.697902e-01

8246 9.894101e-01 9.697813e-01

8247 9.894126e-01 9.697724e-01

8248 9.894151e-01 9.697633e-01

8249 9.894176e-01 9.697541e-01

8250 9.894201e-01 9.697449e-01

8251 9.894226e-01 9.697356e-01

8252 9.894251e-01 9.697262e-01

8253 9.894276e-01 9.697166e-01

8254 9.894302e-01 9.697070e-01

8255 9.894327e-01 9.696974e-01

8256 9.894353e-01 9.696876e-01

8257 9.894379e-01 9.696777e-01

8258 9.894405e-01 9.696677e-01

8259 9.894431e-01 9.696576e-01

8260 9.894457e-01 9.696475e-01

8261 9.894483e-01 9.696372e-01

8262 9.894510e-01 9.696269e-01

8263 9.894536e-01 9.696164e-01

8264 9.894563e-01 9.696059e-01

8265 9.894590e-01 9.695953e-01

8266 9.894617e-01 9.695845e-01

8267 9.894644e-01 9.695737e-01

8268 9.894671e-01 9.695628e-01

8269 9.894698e-01 9.695518e-01

8270 9.894726e-01 9.695407e-01

8271 9.894754e-01 9.695295e-01

8272 9.894781e-01 9.695182e-01

8273 9.894809e-01 9.695068e-01

8274 9.894838e-01 9.694953e-01

8275 9.894866e-01 9.694837e-01

8276 9.894894e-01 9.694720e-01

8277 9.894923e-01 9.694602e-01

8278 9.894952e-01 9.694483e-01

8279 9.894981e-01 9.694363e-01

8280 9.895010e-01 9.694242e-01

8281 9.895039e-01 9.694121e-01

8282 9.895176e-01 9.694106e-01

8283 9.895309e-01 9.694090e-01

8284 9.895439e-01 9.694075e-01

8285 9.895566e-01 9.694059e-01

8286 9.895689e-01 9.694043e-01

8287 9.895809e-01 9.694027e-01

8288 9.895927e-01 9.694011e-01

8289 9.896041e-01 9.693994e-01

8290 9.896153e-01 9.693977e-01

8291 9.896261e-01 9.693960e-01

8292 9.896367e-01 9.693943e-01

8293 9.896471e-01 9.693925e-01

8294 9.896571e-01 9.693907e-01

8295 9.896670e-01 9.693889e-01

8296 9.896765e-01 9.693870e-01

8297 9.896859e-01 9.693852e-01

8298 9.896950e-01 9.693833e-01

8299 9.897039e-01 9.693814e-01

8300 9.897126e-01 9.693794e-01

8301 9.897211e-01 9.693774e-01

8302 9.897293e-01 9.693754e-01

8303 9.897374e-01 9.693734e-01

8304 9.897453e-01 9.693713e-01

8305 9.897530e-01 9.693692e-01

8306 9.897605e-01 9.693671e-01

8307 9.897678e-01 9.693649e-01

8308 9.897750e-01 9.693627e-01

8309 9.897819e-01 9.693605e-01

8310 9.897888e-01 9.693583e-01

8311 9.897954e-01 9.693560e-01

8312 9.898019e-01 9.693537e-01

8313 9.898083e-01 9.693513e-01

8314 9.898145e-01 9.693489e-01

8315 9.898206e-01 9.693465e-01

8316 9.898265e-01 9.693441e-01

8317 9.898323e-01 9.693416e-01

8318 9.898380e-01 9.693391e-01

8319 9.898436e-01 9.693365e-01

8320 9.898490e-01 9.693340e-01

8321 9.898543e-01 9.693313e-01

8322 9.898595e-01 9.693287e-01

8323 9.898646e-01 9.693260e-01

8324 9.898696e-01 9.693233e-01

8325 9.898745e-01 9.693205e-01

8326 9.898792e-01 9.693177e-01

8327 9.898839e-01 9.693148e-01

8328 9.898885e-01 9.693119e-01

8329 9.898930e-01 9.693090e-01

8330 9.898974e-01 9.693061e-01

8331 9.899017e-01 9.693031e-01

8332 9.899059e-01 9.693000e-01

8333 9.899100e-01 9.692969e-01

8334 9.899141e-01 9.692938e-01

8335 9.899181e-01 9.692906e-01

8336 9.899220e-01 9.692874e-01

8337 9.899258e-01 9.692842e-01

8338 9.899296e-01 9.692809e-01

8339 9.899333e-01 9.692775e-01

8340 9.899369e-01 9.692742e-01

8341 9.899405e-01 9.692707e-01

8342 9.899440e-01 9.692672e-01

8343 9.899474e-01 9.692637e-01

8344 9.899508e-01 9.692602e-01

8345 9.899541e-01 9.692565e-01

8346 9.899574e-01 9.692529e-01

8347 9.899606e-01 9.692492e-01

8348 9.899638e-01 9.692454e-01

8349 9.899669e-01 9.692416e-01

8350 9.899700e-01 9.692378e-01

8351 9.899731e-01 9.692339e-01

8352 9.899760e-01 9.692299e-01

8353 9.899790e-01 9.692259e-01

8354 9.899819e-01 9.692218e-01

8355 9.899848e-01 9.692177e-01

8356 9.899876e-01 9.692136e-01

8357 9.899904e-01 9.692094e-01

8358 9.899931e-01 9.692051e-01

8359 9.899958e-01 9.692008e-01

8360 9.899985e-01 9.691964e-01

8361 9.900012e-01 9.691920e-01

8362 9.900038e-01 9.691875e-01

8363 9.900064e-01 9.691829e-01

8364 9.900090e-01 9.691783e-01

8365 9.900115e-01 9.691737e-01

8366 9.900140e-01 9.691690e-01

8367 9.900165e-01 9.691642e-01

8368 9.900190e-01 9.691594e-01

8369 9.900214e-01 9.691545e-01

8370 9.900238e-01 9.691495e-01

8371 9.900262e-01 9.691445e-01

8372 9.900286e-01 9.691394e-01

8373 9.900310e-01 9.691343e-01

8374 9.900333e-01 9.691291e-01

8375 9.900356e-01 9.691239e-01

8376 9.900379e-01 9.691185e-01

8377 9.900402e-01 9.691132e-01

8378 9.900425e-01 9.691077e-01

8379 9.900448e-01 9.691022e-01

8380 9.900470e-01 9.690966e-01

8381 9.900493e-01 9.690910e-01

8382 9.900515e-01 9.690853e-01

8383 9.900537e-01 9.690795e-01

8384 9.900559e-01 9.690736e-01

8385 9.900581e-01 9.690677e-01

8386 9.900603e-01 9.690617e-01

8387 9.900624e-01 9.690557e-01

8388 9.900646e-01 9.690496e-01

8389 9.900668e-01 9.690434e-01

8390 9.900689e-01 9.690371e-01

8391 9.900711e-01 9.690308e-01

8392 9.900732e-01 9.690244e-01

8393 9.900754e-01 9.690179e-01

8394 9.900775e-01 9.690113e-01

8395 9.900796e-01 9.690047e-01

8396 9.900817e-01 9.689980e-01

8397 9.900839e-01 9.689912e-01

8398 9.900860e-01 9.689844e-01

8399 9.900881e-01 9.689775e-01

8400 9.900902e-01 9.689705e-01

8401 9.900923e-01 9.689634e-01

8402 9.900945e-01 9.689562e-01

8403 9.900966e-01 9.689490e-01

8404 9.900987e-01 9.689417e-01

8405 9.901008e-01 9.689343e-01

8406 9.901029e-01 9.689268e-01

8407 9.901051e-01 9.689192e-01

8408 9.901072e-01 9.689116e-01

8409 9.901093e-01 9.689039e-01

8410 9.901114e-01 9.688961e-01

8411 9.901136e-01 9.688882e-01

8412 9.901157e-01 9.688803e-01

8413 9.901179e-01 9.688722e-01

8414 9.901200e-01 9.688641e-01

8415 9.901222e-01 9.688559e-01

8416 9.901243e-01 9.688476e-01

8417 9.901265e-01 9.688392e-01

8418 9.901286e-01 9.688307e-01

8419 9.901308e-01 9.688222e-01

8420 9.901330e-01 9.688135e-01

8421 9.901352e-01 9.688048e-01

8422 9.901374e-01 9.687960e-01

8423 9.901396e-01 9.687871e-01

8424 9.901418e-01 9.687781e-01

8425 9.901440e-01 9.687690e-01

8426 9.901462e-01 9.687599e-01

8427 9.901485e-01 9.687506e-01

8428 9.901507e-01 9.687413e-01

8429 9.901530e-01 9.687318e-01

8430 9.901552e-01 9.687223e-01

8431 9.901575e-01 9.687127e-01

8432 9.901598e-01 9.687030e-01

8433 9.901621e-01 9.686932e-01

8434 9.901644e-01 9.686833e-01

8435 9.901667e-01 9.686733e-01

8436 9.901690e-01 9.686632e-01

8437 9.901714e-01 9.686530e-01

8438 9.901737e-01 9.686428e-01

8439 9.901761e-01 9.686324e-01

8440 9.901784e-01 9.686220e-01

8441 9.901808e-01 9.686114e-01

8442 9.901832e-01 9.686008e-01

8443 9.901856e-01 9.685901e-01

8444 9.901880e-01 9.685792e-01

8445 9.901904e-01 9.685683e-01

8446 9.901929e-01 9.685573e-01

8447 9.901953e-01 9.685462e-01

8448 9.901978e-01 9.685350e-01

8449 9.902003e-01 9.685237e-01

8450 9.902028e-01 9.685123e-01

8451 9.902053e-01 9.685008e-01

8452 9.902078e-01 9.684892e-01

8453 9.902103e-01 9.684776e-01

8454 9.902128e-01 9.684658e-01

8455 9.902154e-01 9.684539e-01

8456 9.902180e-01 9.684420e-01

8457 9.902206e-01 9.684299e-01

8458 9.902232e-01 9.684178e-01

8459 9.902258e-01 9.684055e-01

8460 9.902284e-01 9.683932e-01

8461 9.902310e-01 9.683807e-01

8462 9.902424e-01 9.683791e-01

8463 9.902535e-01 9.683775e-01

8464 9.902643e-01 9.683758e-01

8465 9.902748e-01 9.683742e-01

8466 9.902851e-01 9.683725e-01

8467 9.902951e-01 9.683707e-01

8468 9.903049e-01 9.683690e-01

8469 9.903144e-01 9.683672e-01

8470 9.903237e-01 9.683654e-01

8471 9.903328e-01 9.683636e-01

8472 9.903417e-01 9.683617e-01

8473 9.903503e-01 9.683599e-01

8474 9.903587e-01 9.683580e-01

8475 9.903669e-01 9.683560e-01

8476 9.903749e-01 9.683541e-01

8477 9.903828e-01 9.683521e-01

8478 9.903904e-01 9.683501e-01

8479 9.903978e-01 9.683480e-01

8480 9.904051e-01 9.683459e-01

8481 9.904122e-01 9.683438e-01

8482 9.904191e-01 9.683417e-01

8483 9.904259e-01 9.683395e-01

8484 9.904325e-01 9.683373e-01

8485 9.904390e-01 9.683351e-01

8486 9.904453e-01 9.683329e-01

8487 9.904514e-01 9.683306e-01

8488 9.904575e-01 9.683283e-01

8489 9.904633e-01 9.683259e-01

8490 9.904691e-01 9.683235e-01

8491 9.904747e-01 9.683211e-01

8492 9.904802e-01 9.683186e-01

8493 9.904856e-01 9.683162e-01

8494 9.904908e-01 9.683136e-01

8495 9.904959e-01 9.683111e-01

8496 9.905010e-01 9.683085e-01

8497 9.905059e-01 9.683059e-01

8498 9.905107e-01 9.683032e-01

8499 9.905154e-01 9.683005e-01

8500 9.905200e-01 9.682978e-01

8501 9.905245e-01 9.682950e-01

8502 9.905289e-01 9.682922e-01

8503 9.905332e-01 9.682894e-01

8504 9.905374e-01 9.682865e-01

8505 9.905416e-01 9.682836e-01

8506 9.905457e-01 9.682806e-01

8507 9.905496e-01 9.682776e-01

8508 9.905535e-01 9.682745e-01

8509 9.905574e-01 9.682715e-01

8510 9.905611e-01 9.682683e-01

8511 9.905648e-01 9.682652e-01

8512 9.905684e-01 9.682620e-01

8513 9.905719e-01 9.682587e-01

8514 9.905754e-01 9.682554e-01

8515 9.905788e-01 9.682521e-01

8516 9.905822e-01 9.682487e-01

8517 9.905855e-01 9.682453e-01

8518 9.905887e-01 9.682418e-01

8519 9.905919e-01 9.682383e-01

8520 9.905950e-01 9.682347e-01

8521 9.905981e-01 9.682311e-01

8522 9.906011e-01 9.682275e-01

8523 9.906041e-01 9.682238e-01

8524 9.906070e-01 9.682200e-01

8525 9.906099e-01 9.682162e-01

8526 9.906127e-01 9.682124e-01

8527 9.906155e-01 9.682085e-01

8528 9.906183e-01 9.682045e-01

8529 9.906210e-01 9.682005e-01

8530 9.906237e-01 9.681965e-01

8531 9.906263e-01 9.681924e-01

8532 9.906289e-01 9.681882e-01

8533 9.906315e-01 9.681840e-01

8534 9.906340e-01 9.681798e-01

8535 9.906365e-01 9.681755e-01

8536 9.906389e-01 9.681711e-01

8537 9.906414e-01 9.681667e-01

8538 9.906438e-01 9.681622e-01

8539 9.906462e-01 9.681577e-01

8540 9.906485e-01 9.681531e-01

8541 9.906508e-01 9.681485e-01

8542 9.906531e-01 9.681438e-01

8543 9.906554e-01 9.681390e-01

8544 9.906577e-01 9.681342e-01

8545 9.906599e-01 9.681294e-01

8546 9.906621e-01 9.681244e-01

8547 9.906643e-01 9.681194e-01

8548 9.906665e-01 9.681144e-01

8549 9.906686e-01 9.681093e-01

8550 9.906708e-01 9.681041e-01

8551 9.906729e-01 9.680989e-01

8552 9.906750e-01 9.680936e-01

8553 9.906771e-01 9.680882e-01

8554 9.906791e-01 9.680828e-01

8555 9.906812e-01 9.680773e-01

8556 9.906832e-01 9.680718e-01

8557 9.906853e-01 9.680662e-01

8558 9.906873e-01 9.680605e-01

8559 9.906893e-01 9.680547e-01

8560 9.906913e-01 9.680489e-01

8561 9.906933e-01 9.680430e-01

8562 9.906953e-01 9.680371e-01

8563 9.906972e-01 9.680311e-01

8564 9.906992e-01 9.680250e-01

8565 9.907012e-01 9.680188e-01

8566 9.907031e-01 9.680126e-01

8567 9.907050e-01 9.680063e-01

8568 9.907070e-01 9.679999e-01

8569 9.907089e-01 9.679935e-01

8570 9.907108e-01 9.679870e-01

8571 9.907128e-01 9.679804e-01

8572 9.907147e-01 9.679738e-01

8573 9.907166e-01 9.679670e-01

8574 9.907185e-01 9.679602e-01

8575 9.907204e-01 9.679533e-01

8576 9.907223e-01 9.679464e-01

8577 9.907242e-01 9.679394e-01

8578 9.907261e-01 9.679323e-01

8579 9.907280e-01 9.679251e-01

8580 9.907299e-01 9.679178e-01

8581 9.907318e-01 9.679105e-01

8582 9.907337e-01 9.679030e-01

8583 9.907356e-01 9.678955e-01

8584 9.907375e-01 9.678880e-01

8585 9.907394e-01 9.678803e-01

8586 9.907413e-01 9.678726e-01

8587 9.907432e-01 9.678648e-01

8588 9.907452e-01 9.678569e-01

8589 9.907471e-01 9.678489e-01

8590 9.907490e-01 9.678408e-01

8591 9.907509e-01 9.678327e-01

8592 9.907528e-01 9.678244e-01

8593 9.907548e-01 9.678161e-01

8594 9.907567e-01 9.678077e-01

8595 9.907586e-01 9.677992e-01

8596 9.907606e-01 9.677907e-01

8597 9.907625e-01 9.677820e-01

8598 9.907645e-01 9.677733e-01

8599 9.907664e-01 9.677645e-01

8600 9.907684e-01 9.677555e-01

8601 9.907704e-01 9.677466e-01

8602 9.907724e-01 9.677375e-01

8603 9.907743e-01 9.677283e-01

8604 9.907763e-01 9.677190e-01

8605 9.907783e-01 9.677097e-01

8606 9.907803e-01 9.677002e-01

8607 9.907824e-01 9.676907e-01

8608 9.907844e-01 9.676811e-01

8609 9.907864e-01 9.676714e-01

8610 9.907885e-01 9.676616e-01

8611 9.907905e-01 9.676517e-01

8612 9.907926e-01 9.676417e-01

8613 9.907946e-01 9.676316e-01

8614 9.907967e-01 9.676215e-01

8615 9.907988e-01 9.676112e-01

8616 9.908009e-01 9.676009e-01

8617 9.908030e-01 9.675904e-01

8618 9.908051e-01 9.675799e-01

8619 9.908072e-01 9.675693e-01

8620 9.908093e-01 9.675586e-01

8621 9.908115e-01 9.675478e-01

8622 9.908136e-01 9.675369e-01

8623 9.908158e-01 9.675259e-01

8624 9.908180e-01 9.675148e-01

8625 9.908202e-01 9.675036e-01

8626 9.908223e-01 9.674924e-01

8627 9.908246e-01 9.674810e-01

8628 9.908268e-01 9.674695e-01

8629 9.908290e-01 9.674580e-01

8630 9.908312e-01 9.674463e-01

8631 9.908335e-01 9.674346e-01

8632 9.908357e-01 9.674228e-01

8633 9.908380e-01 9.674109e-01

8634 9.908403e-01 9.673988e-01

8635 9.908426e-01 9.673867e-01

8636 9.908449e-01 9.673745e-01

8637 9.908472e-01 9.673622e-01

8638 9.908495e-01 9.673498e-01

8639 9.908519e-01 9.673374e-01

8640 9.908542e-01 9.673248e-01

8641 9.908566e-01 9.673121e-01

8642 9.908660e-01 9.673104e-01

8643 9.908753e-01 9.673087e-01

8644 9.908843e-01 9.673069e-01

8645 9.908931e-01 9.673052e-01

8646 9.909017e-01 9.673034e-01

8647 9.909101e-01 9.673015e-01

8648 9.909182e-01 9.672997e-01

8649 9.909262e-01 9.672978e-01

8650 9.909340e-01 9.672959e-01

8651 9.909416e-01 9.672939e-01

8652 9.909490e-01 9.672920e-01

8653 9.909562e-01 9.672900e-01

8654 9.909633e-01 9.672879e-01

8655 9.909702e-01 9.672859e-01

8656 9.909769e-01 9.672838e-01

8657 9.909835e-01 9.672817e-01

8658 9.909899e-01 9.672796e-01

8659 9.909961e-01 9.672774e-01

8660 9.910022e-01 9.672752e-01

8661 9.910082e-01 9.672730e-01

8662 9.910141e-01 9.672707e-01

8663 9.910198e-01 9.672684e-01

8664 9.910253e-01 9.672661e-01

8665 9.910308e-01 9.672638e-01

8666 9.910361e-01 9.672614e-01

8667 9.910413e-01 9.672589e-01

8668 9.910464e-01 9.672565e-01

8669 9.910513e-01 9.672540e-01

8670 9.910562e-01 9.672515e-01

8671 9.910609e-01 9.672489e-01

8672 9.910656e-01 9.672463e-01

8673 9.910701e-01 9.672437e-01

8674 9.910746e-01 9.672411e-01

8675 9.910789e-01 9.672384e-01

8676 9.910832e-01 9.672356e-01

8677 9.910874e-01 9.672329e-01

8678 9.910915e-01 9.672301e-01

8679 9.910955e-01 9.672272e-01

8680 9.910994e-01 9.672243e-01

8681 9.911032e-01 9.672214e-01

8682 9.911070e-01 9.672184e-01

8683 9.911107e-01 9.672154e-01

8684 9.911143e-01 9.672124e-01

8685 9.911178e-01 9.672093e-01

8686 9.911213e-01 9.672062e-01

8687 9.911247e-01 9.672030e-01

8688 9.911280e-01 9.671998e-01

8689 9.911313e-01 9.671966e-01

8690 9.911345e-01 9.671933e-01

8691 9.911377e-01 9.671900e-01

8692 9.911408e-01 9.671866e-01

8693 9.911438e-01 9.671832e-01

8694 9.911468e-01 9.671797e-01

8695 9.911498e-01 9.671762e-01

8696 9.911527e-01 9.671727e-01

8697 9.911555e-01 9.671691e-01

8698 9.911583e-01 9.671654e-01

8699 9.911610e-01 9.671617e-01

8700 9.911637e-01 9.671580e-01

8701 9.911664e-01 9.671542e-01

8702 9.911690e-01 9.671504e-01

8703 9.911716e-01 9.671465e-01

8704 9.911741e-01 9.671426e-01

8705 9.911766e-01 9.671386e-01

8706 9.911791e-01 9.671346e-01

8707 9.911815e-01 9.671305e-01

8708 9.911839e-01 9.671263e-01

8709 9.911863e-01 9.671222e-01

8710 9.911886e-01 9.671179e-01

8711 9.911909e-01 9.671136e-01

8712 9.911931e-01 9.671093e-01

8713 9.911954e-01 9.671049e-01

8714 9.911976e-01 9.671004e-01

8715 9.911998e-01 9.670959e-01

8716 9.912019e-01 9.670914e-01

8717 9.912041e-01 9.670868e-01

8718 9.912062e-01 9.670821e-01

8719 9.912083e-01 9.670774e-01

8720 9.912103e-01 9.670726e-01

8721 9.912124e-01 9.670677e-01

8722 9.912144e-01 9.670628e-01

8723 9.912164e-01 9.670579e-01

8724 9.912184e-01 9.670529e-01

8725 9.912204e-01 9.670478e-01

8726 9.912223e-01 9.670426e-01

8727 9.912242e-01 9.670374e-01

8728 9.912262e-01 9.670322e-01

8729 9.912281e-01 9.670268e-01

8730 9.912299e-01 9.670215e-01

8731 9.912318e-01 9.670160e-01

8732 9.912337e-01 9.670105e-01

8733 9.912355e-01 9.670049e-01

8734 9.912374e-01 9.669993e-01

8735 9.912392e-01 9.669936e-01

8736 9.912410e-01 9.669878e-01

8737 9.912428e-01 9.669819e-01

8738 9.912446e-01 9.669760e-01

8739 9.912464e-01 9.669701e-01

8740 9.912482e-01 9.669640e-01

8741 9.912499e-01 9.669579e-01

8742 9.912517e-01 9.669517e-01

8743 9.912535e-01 9.669455e-01

8744 9.912552e-01 9.669391e-01

8745 9.912570e-01 9.669328e-01

8746 9.912587e-01 9.669263e-01

8747 9.912604e-01 9.669198e-01

8748 9.912621e-01 9.669132e-01

8749 9.912639e-01 9.669065e-01

8750 9.912656e-01 9.668997e-01

8751 9.912673e-01 9.668929e-01

8752 9.912690e-01 9.668860e-01

8753 9.912707e-01 9.668790e-01

8754 9.912724e-01 9.668720e-01

8755 9.912742e-01 9.668649e-01

8756 9.912759e-01 9.668577e-01

8757 9.912776e-01 9.668504e-01

8758 9.912793e-01 9.668430e-01

8759 9.912810e-01 9.668356e-01

8760 9.912827e-01 9.668281e-01

8761 9.912844e-01 9.668205e-01

8762 9.912861e-01 9.668128e-01

8763 9.912878e-01 9.668051e-01

8764 9.912895e-01 9.667972e-01

8765 9.912912e-01 9.667893e-01

8766 9.912929e-01 9.667813e-01

8767 9.912947e-01 9.667733e-01

8768 9.912964e-01 9.667651e-01

8769 9.912981e-01 9.667569e-01

8770 9.912998e-01 9.667486e-01

8771 9.913015e-01 9.667402e-01

8772 9.913033e-01 9.667317e-01

8773 9.913050e-01 9.667231e-01

8774 9.913067e-01 9.667145e-01

8775 9.913085e-01 9.667057e-01

8776 9.913102e-01 9.666969e-01

8777 9.913120e-01 9.666880e-01

8778 9.913137e-01 9.666790e-01

8779 9.913155e-01 9.666699e-01

8780 9.913173e-01 9.666608e-01

8781 9.913190e-01 9.666515e-01

8782 9.913208e-01 9.666422e-01

8783 9.913226e-01 9.666327e-01

8784 9.913244e-01 9.666232e-01

8785 9.913262e-01 9.666136e-01

8786 9.913280e-01 9.666039e-01

8787 9.913298e-01 9.665942e-01

8788 9.913316e-01 9.665843e-01

8789 9.913334e-01 9.665743e-01

8790 9.913353e-01 9.665643e-01

8791 9.913371e-01 9.665541e-01

8792 9.913390e-01 9.665439e-01

8793 9.913408e-01 9.665336e-01

8794 9.913427e-01 9.665232e-01

8795 9.913446e-01 9.665127e-01

8796 9.913464e-01 9.665021e-01

8797 9.913483e-01 9.664914e-01

8798 9.913502e-01 9.664807e-01

8799 9.913521e-01 9.664698e-01

8800 9.913540e-01 9.664589e-01

8801 9.913559e-01 9.664478e-01

8802 9.913579e-01 9.664367e-01

8803 9.913598e-01 9.664255e-01

8804 9.913618e-01 9.664141e-01

8805 9.913637e-01 9.664027e-01

8806 9.913657e-01 9.663912e-01

8807 9.913677e-01 9.663797e-01

8808 9.913696e-01 9.663680e-01

8809 9.913716e-01 9.663562e-01

8810 9.913736e-01 9.663444e-01

8811 9.913756e-01 9.663324e-01

8812 9.913777e-01 9.663204e-01

8813 9.913797e-01 9.663082e-01

8814 9.913817e-01 9.662960e-01

8815 9.913838e-01 9.662837e-01

8816 9.913858e-01 9.662713e-01

8817 9.913879e-01 9.662588e-01

8818 9.913900e-01 9.662462e-01

8819 9.913921e-01 9.662336e-01

8820 9.913942e-01 9.662208e-01

8821 9.913963e-01 9.662079e-01

8822 9.914042e-01 9.662061e-01

8823 9.914119e-01 9.662043e-01

8824 9.914195e-01 9.662024e-01

8825 9.914269e-01 9.662006e-01

8826 9.914341e-01 9.661986e-01

8827 9.914411e-01 9.661967e-01

8828 9.914479e-01 9.661947e-01

8829 9.914546e-01 9.661927e-01

8830 9.914612e-01 9.661907e-01

8831 9.914675e-01 9.661887e-01

8832 9.914738e-01 9.661866e-01

8833 9.914798e-01 9.661845e-01

8834 9.914858e-01 9.661823e-01

8835 9.914916e-01 9.661802e-01

8836 9.914972e-01 9.661780e-01

8837 9.915028e-01 9.661757e-01

8838 9.915082e-01 9.661735e-01

8839 9.915134e-01 9.661712e-01

8840 9.915186e-01 9.661689e-01

8841 9.915237e-01 9.661665e-01

8842 9.915286e-01 9.661641e-01

8843 9.915334e-01 9.661617e-01

8844 9.915381e-01 9.661592e-01

8845 9.915427e-01 9.661568e-01

8846 9.915472e-01 9.661542e-01

8847 9.915516e-01 9.661517e-01

8848 9.915559e-01 9.661491e-01

8849 9.915602e-01 9.661465e-01

8850 9.915643e-01 9.661438e-01

8851 9.915683e-01 9.661411e-01

8852 9.915723e-01 9.661384e-01

8853 9.915761e-01 9.661356e-01

8854 9.915799e-01 9.661328e-01

8855 9.915836e-01 9.661300e-01

8856 9.915873e-01 9.661271e-01

8857 9.915908e-01 9.661242e-01

8858 9.915943e-01 9.661213e-01

8859 9.915977e-01 9.661183e-01

8860 9.916011e-01 9.661152e-01

8861 9.916044e-01 9.661122e-01

8862 9.916076e-01 9.661091e-01

8863 9.916107e-01 9.661059e-01

8864 9.916138e-01 9.661027e-01

8865 9.916169e-01 9.660995e-01

8866 9.916199e-01 9.660962e-01

8867 9.916228e-01 9.660929e-01

8868 9.916256e-01 9.660895e-01

8869 9.916285e-01 9.660861e-01

8870 9.916312e-01 9.660827e-01

8871 9.916340e-01 9.660792e-01

8872 9.916366e-01 9.660756e-01

8873 9.916393e-01 9.660720e-01

8874 9.916418e-01 9.660684e-01

8875 9.916444e-01 9.660647e-01

8876 9.916469e-01 9.660610e-01

8877 9.916493e-01 9.660572e-01

8878 9.916518e-01 9.660534e-01

8879 9.916541e-01 9.660496e-01

8880 9.916565e-01 9.660456e-01

8881 9.916588e-01 9.660417e-01

8882 9.916611e-01 9.660377e-01

8883 9.916633e-01 9.660336e-01

8884 9.916655e-01 9.660295e-01

8885 9.916677e-01 9.660253e-01

8886 9.916698e-01 9.660211e-01

8887 9.916719e-01 9.660169e-01

8888 9.916740e-01 9.660125e-01

8889 9.916761e-01 9.660082e-01

8890 9.916781e-01 9.660037e-01

8891 9.916801e-01 9.659993e-01

8892 9.916821e-01 9.659947e-01

8893 9.916841e-01 9.659901e-01

8894 9.916860e-01 9.659855e-01

8895 9.916879e-01 9.659808e-01

8896 9.916898e-01 9.659760e-01

8897 9.916917e-01 9.659712e-01

8898 9.916936e-01 9.659664e-01

8899 9.916954e-01 9.659614e-01

8900 9.916972e-01 9.659564e-01

8901 9.916990e-01 9.659514e-01

8902 9.917008e-01 9.659463e-01

8903 9.917026e-01 9.659411e-01

8904 9.917043e-01 9.659359e-01

8905 9.917061e-01 9.659306e-01

8906 9.917078e-01 9.659253e-01

8907 9.917095e-01 9.659198e-01

8908 9.917112e-01 9.659144e-01

8909 9.917129e-01 9.659088e-01

8910 9.917145e-01 9.659032e-01

8911 9.917162e-01 9.658976e-01

8912 9.917179e-01 9.658918e-01

8913 9.917195e-01 9.658860e-01

8914 9.917211e-01 9.658802e-01

8915 9.917227e-01 9.658742e-01

8916 9.917244e-01 9.658683e-01

8917 9.917260e-01 9.658622e-01

8918 9.917276e-01 9.658561e-01

8919 9.917292e-01 9.658499e-01

8920 9.917307e-01 9.658436e-01

8921 9.917323e-01 9.658373e-01

8922 9.917339e-01 9.658309e-01

8923 9.917354e-01 9.658244e-01

8924 9.917370e-01 9.658178e-01

8925 9.917386e-01 9.658112e-01

8926 9.917401e-01 9.658045e-01

8927 9.917417e-01 9.657978e-01

8928 9.917432e-01 9.657909e-01

8929 9.917447e-01 9.657840e-01

8930 9.917463e-01 9.657770e-01

8931 9.917478e-01 9.657700e-01

8932 9.917494e-01 9.657628e-01

8933 9.917509e-01 9.657556e-01

8934 9.917524e-01 9.657483e-01

8935 9.917539e-01 9.657410e-01

8936 9.917555e-01 9.657335e-01

8937 9.917570e-01 9.657260e-01

8938 9.917585e-01 9.657184e-01

8939 9.917601e-01 9.657108e-01

8940 9.917616e-01 9.657030e-01

8941 9.917631e-01 9.656952e-01

8942 9.917646e-01 9.656873e-01

8943 9.917662e-01 9.656793e-01

8944 9.917677e-01 9.656712e-01

8945 9.917692e-01 9.656631e-01

8946 9.917708e-01 9.656549e-01

8947 9.917723e-01 9.656466e-01

8948 9.917739e-01 9.656382e-01

8949 9.917754e-01 9.656297e-01

8950 9.917769e-01 9.656211e-01

8951 9.917785e-01 9.656125e-01

8952 9.917800e-01 9.656038e-01

8953 9.917816e-01 9.655950e-01

8954 9.917832e-01 9.655861e-01

8955 9.917847e-01 9.655771e-01

8956 9.917863e-01 9.655681e-01

8957 9.917879e-01 9.655589e-01

8958 9.917894e-01 9.655497e-01

8959 9.917910e-01 9.655404e-01

8960 9.917926e-01 9.655310e-01

8961 9.917942e-01 9.655215e-01

8962 9.917958e-01 9.655119e-01

8963 9.917974e-01 9.655022e-01

8964 9.917990e-01 9.654925e-01

8965 9.918006e-01 9.654827e-01

8966 9.918022e-01 9.654728e-01

8967 9.918038e-01 9.654627e-01

8968 9.918055e-01 9.654526e-01

8969 9.918071e-01 9.654425e-01

8970 9.918088e-01 9.654322e-01

8971 9.918104e-01 9.654218e-01

8972 9.918121e-01 9.654114e-01

8973 9.918137e-01 9.654008e-01

8974 9.918154e-01 9.653902e-01

8975 9.918171e-01 9.653795e-01

8976 9.918188e-01 9.653687e-01

8977 9.918204e-01 9.653578e-01

8978 9.918221e-01 9.653468e-01

8979 9.918239e-01 9.653358e-01

8980 9.918256e-01 9.653246e-01

8981 9.918273e-01 9.653134e-01

8982 9.918290e-01 9.653020e-01

8983 9.918307e-01 9.652906e-01

8984 9.918325e-01 9.652791e-01

8985 9.918342e-01 9.652675e-01

8986 9.918360e-01 9.652558e-01

8987 9.918378e-01 9.652440e-01

8988 9.918395e-01 9.652322e-01

8989 9.918413e-01 9.652202e-01

8990 9.918431e-01 9.652082e-01

8991 9.918449e-01 9.651960e-01

8992 9.918467e-01 9.651838e-01

8993 9.918486e-01 9.651715e-01

8994 9.918504e-01 9.651591e-01

8995 9.918522e-01 9.651466e-01

8996 9.918541e-01 9.651341e-01

8997 9.918559e-01 9.651214e-01

8998 9.918578e-01 9.651087e-01

8999 9.918596e-01 9.650958e-01

9000 9.918615e-01 9.650829e-01

9001 9.918634e-01 9.650699e-01

9002 9.918701e-01 9.650680e-01

9003 9.918766e-01 9.650661e-01

9004 9.918829e-01 9.650641e-01

9005 9.918891e-01 9.650621e-01

9006 9.918952e-01 9.650601e-01

9007 9.919011e-01 9.650580e-01

9008 9.919068e-01 9.650560e-01

9009 9.919125e-01 9.650539e-01

9010 9.919180e-01 9.650517e-01

9011 9.919234e-01 9.650495e-01

9012 9.919286e-01 9.650474e-01

9013 9.919337e-01 9.650451e-01

9014 9.919387e-01 9.650429e-01

9015 9.919436e-01 9.650406e-01

9016 9.919484e-01 9.650383e-01

9017 9.919531e-01 9.650359e-01

9018 9.919577e-01 9.650335e-01

9019 9.919622e-01 9.650311e-01

9020 9.919665e-01 9.650287e-01

9021 9.919708e-01 9.650262e-01

9022 9.919750e-01 9.650237e-01

9023 9.919791e-01 9.650211e-01

9024 9.919831e-01 9.650185e-01

9025 9.919870e-01 9.650159e-01

9026 9.919908e-01 9.650133e-01

9027 9.919946e-01 9.650106e-01

9028 9.919982e-01 9.650079e-01

9029 9.920018e-01 9.650051e-01

9030 9.920054e-01 9.650023e-01

9031 9.920088e-01 9.649995e-01

9032 9.920122e-01 9.649966e-01

9033 9.920155e-01 9.649937e-01

9034 9.920187e-01 9.649908e-01

9035 9.920219e-01 9.649878e-01

9036 9.920250e-01 9.649848e-01

9037 9.920280e-01 9.649817e-01

9038 9.920310e-01 9.649786e-01

9039 9.920340e-01 9.649755e-01

9040 9.920368e-01 9.649723e-01

9041 9.920397e-01 9.649691e-01

9042 9.920424e-01 9.649658e-01

9043 9.920451e-01 9.649625e-01

9044 9.920478e-01 9.649592e-01

9045 9.920504e-01 9.649558e-01

9046 9.920530e-01 9.649523e-01

9047 9.920555e-01 9.649489e-01

9048 9.920580e-01 9.649453e-01

9049 9.920604e-01 9.649418e-01

9050 9.920628e-01 9.649382e-01

9051 9.920652e-01 9.649345e-01

9052 9.920675e-01 9.649308e-01

9053 9.920698e-01 9.649270e-01

9054 9.920720e-01 9.649232e-01

9055 9.920742e-01 9.649194e-01

9056 9.920764e-01 9.649155e-01

9057 9.920785e-01 9.649116e-01

9058 9.920806e-01 9.649076e-01

9059 9.920827e-01 9.649035e-01

9060 9.920847e-01 9.648995e-01

9061 9.920867e-01 9.648953e-01

9062 9.920887e-01 9.648911e-01

9063 9.920907e-01 9.648869e-01

9064 9.920926e-01 9.648826e-01

9065 9.920945e-01 9.648783e-01

9066 9.920964e-01 9.648739e-01

9067 9.920982e-01 9.648694e-01

9068 9.921000e-01 9.648649e-01

9069 9.921018e-01 9.648604e-01

9070 9.921036e-01 9.648557e-01

9071 9.921054e-01 9.648511e-01

9072 9.921071e-01 9.648463e-01

9073 9.921089e-01 9.648416e-01

9074 9.921106e-01 9.648367e-01

9075 9.921122e-01 9.648318e-01

9076 9.921139e-01 9.648269e-01

9077 9.921156e-01 9.648219e-01

9078 9.921172e-01 9.648168e-01

9079 9.921188e-01 9.648117e-01

9080 9.921204e-01 9.648065e-01

9081 9.921220e-01 9.648013e-01

9082 9.921236e-01 9.647960e-01

9083 9.921251e-01 9.647906e-01

9084 9.921267e-01 9.647852e-01

9085 9.921282e-01 9.647797e-01

9086 9.921298e-01 9.647741e-01

9087 9.921313e-01 9.647685e-01

9088 9.921328e-01 9.647628e-01

9089 9.921343e-01 9.647571e-01

9090 9.921358e-01 9.647513e-01

9091 9.921372e-01 9.647454e-01

9092 9.921387e-01 9.647395e-01

9093 9.921402e-01 9.647334e-01

9094 9.921416e-01 9.647274e-01

9095 9.921430e-01 9.647212e-01

9096 9.921445e-01 9.647150e-01

9097 9.921459e-01 9.647087e-01

9098 9.921473e-01 9.647024e-01

9099 9.921487e-01 9.646960e-01

9100 9.921502e-01 9.646895e-01

9101 9.921516e-01 9.646830e-01

9102 9.921530e-01 9.646763e-01

9103 9.921544e-01 9.646696e-01

9104 9.921557e-01 9.646629e-01

9105 9.921571e-01 9.646560e-01

9106 9.921585e-01 9.646491e-01

9107 9.921599e-01 9.646421e-01

9108 9.921613e-01 9.646351e-01

9109 9.921627e-01 9.646280e-01

9110 9.921640e-01 9.646207e-01

9111 9.921654e-01 9.646135e-01

9112 9.921668e-01 9.646061e-01

9113 9.921681e-01 9.645987e-01

9114 9.921695e-01 9.645912e-01

9115 9.921709e-01 9.645836e-01

9116 9.921723e-01 9.645759e-01

9117 9.921736e-01 9.645682e-01

9118 9.921750e-01 9.645604e-01

9119 9.921764e-01 9.645525e-01

9120 9.921777e-01 9.645445e-01

9121 9.921791e-01 9.645365e-01

9122 9.921805e-01 9.645283e-01

9123 9.921818e-01 9.645201e-01

9124 9.921832e-01 9.645118e-01

9125 9.921846e-01 9.645035e-01

9126 9.921860e-01 9.644950e-01

9127 9.921873e-01 9.644865e-01

9128 9.921887e-01 9.644779e-01

9129 9.921901e-01 9.644692e-01

9130 9.921915e-01 9.644604e-01

9131 9.921929e-01 9.644516e-01

9132 9.921943e-01 9.644426e-01

9133 9.921957e-01 9.644336e-01

9134 9.921971e-01 9.644245e-01

9135 9.921985e-01 9.644153e-01

9136 9.921999e-01 9.644060e-01

9137 9.922013e-01 9.643967e-01

9138 9.922027e-01 9.643872e-01

9139 9.922041e-01 9.643777e-01

9140 9.922056e-01 9.643681e-01

9141 9.922070e-01 9.643584e-01

9142 9.922084e-01 9.643486e-01

9143 9.922099e-01 9.643387e-01

9144 9.922113e-01 9.643288e-01

9145 9.922127e-01 9.643187e-01

9146 9.922142e-01 9.643086e-01

9147 9.922157e-01 9.642984e-01

9148 9.922171e-01 9.642881e-01

9149 9.922186e-01 9.642777e-01

9150 9.922201e-01 9.642672e-01

9151 9.922216e-01 9.642567e-01

9152 9.922230e-01 9.642460e-01

9153 9.922245e-01 9.642353e-01

9154 9.922260e-01 9.642245e-01

9155 9.922275e-01 9.642136e-01

9156 9.922291e-01 9.642026e-01

9157 9.922306e-01 9.641915e-01

9158 9.922321e-01 9.641804e-01

9159 9.922336e-01 9.641691e-01

9160 9.922352e-01 9.641578e-01

9161 9.922367e-01 9.641464e-01

9162 9.922383e-01 9.641349e-01

9163 9.922398e-01 9.641233e-01

9164 9.922414e-01 9.641116e-01

9165 9.922430e-01 9.640998e-01

9166 9.922446e-01 9.640880e-01

9167 9.922462e-01 9.640760e-01

9168 9.922478e-01 9.640640e-01

9169 9.922494e-01 9.640519e-01

9170 9.922510e-01 9.640397e-01

9171 9.922526e-01 9.640274e-01

9172 9.922542e-01 9.640150e-01

9173 9.922559e-01 9.640026e-01

9174 9.922575e-01 9.639901e-01

9175 9.922592e-01 9.639774e-01

9176 9.922608e-01 9.639647e-01

9177 9.922625e-01 9.639520e-01

9178 9.922642e-01 9.639391e-01

9179 9.922659e-01 9.639262e-01

9180 9.922676e-01 9.639131e-01

9181 9.922693e-01 9.639000e-01

9182 9.922749e-01 9.638980e-01

9183 9.922804e-01 9.638959e-01

9184 9.922857e-01 9.638939e-01

9185 9.922909e-01 9.638918e-01

9186 9.922961e-01 9.638896e-01

9187 9.923010e-01 9.638875e-01

9188 9.923059e-01 9.638853e-01

9189 9.923107e-01 9.638831e-01

9190 9.923153e-01 9.638808e-01

9191 9.923199e-01 9.638785e-01

9192 9.923243e-01 9.638762e-01

9193 9.923287e-01 9.638739e-01

9194 9.923329e-01 9.638715e-01

9195 9.923371e-01 9.638691e-01

9196 9.923412e-01 9.638666e-01

9197 9.923451e-01 9.638642e-01

9198 9.923490e-01 9.638617e-01

9199 9.923528e-01 9.638591e-01

9200 9.923565e-01 9.638566e-01

9201 9.923602e-01 9.638539e-01

9202 9.923637e-01 9.638513e-01

9203 9.923672e-01 9.638486e-01

9204 9.923706e-01 9.638459e-01

9205 9.923740e-01 9.638432e-01

9206 9.923772e-01 9.638404e-01

9207 9.923804e-01 9.638376e-01

9208 9.923836e-01 9.638347e-01

9209 9.923867e-01 9.638318e-01

9210 9.923897e-01 9.638289e-01

9211 9.923926e-01 9.638259e-01

9212 9.923955e-01 9.638229e-01

9213 9.923983e-01 9.638199e-01

9214 9.924011e-01 9.638168e-01

9215 9.924038e-01 9.638137e-01

9216 9.924065e-01 9.638105e-01

9217 9.924091e-01 9.638073e-01

9218 9.924117e-01 9.638040e-01

9219 9.924142e-01 9.638008e-01

9220 9.924167e-01 9.637974e-01

9221 9.924191e-01 9.637941e-01

9222 9.924215e-01 9.637906e-01

9223 9.924239e-01 9.637872e-01

9224 9.924262e-01 9.637837e-01

9225 9.924284e-01 9.637801e-01

9226 9.924306e-01 9.637765e-01

9227 9.924328e-01 9.637729e-01

9228 9.924350e-01 9.637692e-01

9229 9.924371e-01 9.637655e-01

9230 9.924392e-01 9.637617e-01

9231 9.924412e-01 9.637579e-01

9232 9.924432e-01 9.637541e-01

9233 9.924452e-01 9.637501e-01

9234 9.924471e-01 9.637462e-01

9235 9.924491e-01 9.637422e-01

9236 9.924509e-01 9.637381e-01

9237 9.924528e-01 9.637340e-01

9238 9.924546e-01 9.637298e-01

9239 9.924564e-01 9.637256e-01

9240 9.924582e-01 9.637214e-01

9241 9.924600e-01 9.637171e-01

9242 9.924617e-01 9.637127e-01

9243 9.924634e-01 9.637083e-01

9244 9.924651e-01 9.637038e-01

9245 9.924668e-01 9.636993e-01

9246 9.924684e-01 9.636947e-01

9247 9.924700e-01 9.636901e-01

9248 9.924716e-01 9.636854e-01

9249 9.924732e-01 9.636807e-01

9250 9.924748e-01 9.636759e-01

9251 9.924763e-01 9.636710e-01

9252 9.924779e-01 9.636661e-01

9253 9.924794e-01 9.636612e-01

9254 9.924809e-01 9.636561e-01

9255 9.924824e-01 9.636511e-01

9256 9.924839e-01 9.636459e-01

9257 9.924853e-01 9.636407e-01

9258 9.924868e-01 9.636355e-01

9259 9.924882e-01 9.636302e-01

9260 9.924896e-01 9.636248e-01

9261 9.924910e-01 9.636193e-01

9262 9.924924e-01 9.636138e-01

9263 9.924938e-01 9.636083e-01

9264 9.924952e-01 9.636027e-01

9265 9.924965e-01 9.635970e-01

9266 9.924979e-01 9.635912e-01

9267 9.924992e-01 9.635854e-01

9268 9.925005e-01 9.635795e-01

9269 9.925019e-01 9.635736e-01

9270 9.925032e-01 9.635676e-01

9271 9.925045e-01 9.635615e-01

9272 9.925058e-01 9.635554e-01

9273 9.925071e-01 9.635491e-01

9274 9.925084e-01 9.635429e-01

9275 9.925097e-01 9.635365e-01

9276 9.925110e-01 9.635301e-01

9277 9.925122e-01 9.635236e-01

9278 9.925135e-01 9.635171e-01

9279 9.925148e-01 9.635105e-01

9280 9.925160e-01 9.635038e-01

9281 9.925173e-01 9.634970e-01

9282 9.925185e-01 9.634902e-01

9283 9.925198e-01 9.634833e-01

9284 9.925210e-01 9.634763e-01

9285 9.925223e-01 9.634693e-01

9286 9.925235e-01 9.634622e-01

9287 9.925247e-01 9.634550e-01

9288 9.925260e-01 9.634477e-01

9289 9.925272e-01 9.634404e-01

9290 9.925284e-01 9.634330e-01

9291 9.925297e-01 9.634255e-01

9292 9.925309e-01 9.634179e-01

9293 9.925321e-01 9.634103e-01

9294 9.925333e-01 9.634026e-01

9295 9.925346e-01 9.633948e-01

9296 9.925358e-01 9.633869e-01

9297 9.925370e-01 9.633790e-01

9298 9.925382e-01 9.633709e-01

9299 9.925395e-01 9.633628e-01

9300 9.925407e-01 9.633547e-01

9301 9.925419e-01 9.633464e-01

9302 9.925432e-01 9.633381e-01

9303 9.925444e-01 9.633297e-01

9304 9.925456e-01 9.633212e-01

9305 9.925469e-01 9.633126e-01

9306 9.925481e-01 9.633040e-01

9307 9.925493e-01 9.632952e-01

9308 9.925506e-01 9.632864e-01

9309 9.925518e-01 9.632775e-01

9310 9.925531e-01 9.632685e-01

9311 9.925543e-01 9.632595e-01

9312 9.925556e-01 9.632503e-01

9313 9.925568e-01 9.632411e-01

9314 9.925581e-01 9.632318e-01

9315 9.925593e-01 9.632224e-01

9316 9.925606e-01 9.632130e-01

9317 9.925619e-01 9.632034e-01

9318 9.925632e-01 9.631938e-01

9319 9.925644e-01 9.631841e-01

9320 9.925657e-01 9.631743e-01

9321 9.925670e-01 9.631644e-01

9322 9.925683e-01 9.631544e-01

9323 9.925696e-01 9.631444e-01

9324 9.925709e-01 9.631342e-01

9325 9.925722e-01 9.631240e-01

9326 9.925735e-01 9.631137e-01

9327 9.925748e-01 9.631033e-01

9328 9.925761e-01 9.630928e-01

9329 9.925775e-01 9.630823e-01

9330 9.925788e-01 9.630716e-01

9331 9.925801e-01 9.630609e-01

9332 9.925815e-01 9.630501e-01

9333 9.925828e-01 9.630392e-01

9334 9.925842e-01 9.630282e-01

9335 9.925855e-01 9.630172e-01

9336 9.925869e-01 9.630060e-01

9337 9.925883e-01 9.629948e-01

9338 9.925897e-01 9.629835e-01

9339 9.925910e-01 9.629721e-01

9340 9.925924e-01 9.629606e-01

9341 9.925938e-01 9.629490e-01

9342 9.925952e-01 9.629373e-01

9343 9.925967e-01 9.629256e-01

9344 9.925981e-01 9.629138e-01

9345 9.925995e-01 9.629019e-01

9346 9.926009e-01 9.628899e-01

9347 9.926024e-01 9.628778e-01

9348 9.926038e-01 9.628657e-01

9349 9.926053e-01 9.628535e-01

9350 9.926067e-01 9.628411e-01

9351 9.926082e-01 9.628287e-01

9352 9.926097e-01 9.628163e-01

9353 9.926112e-01 9.628037e-01

9354 9.926127e-01 9.627911e-01

9355 9.926142e-01 9.627783e-01

9356 9.926157e-01 9.627655e-01

9357 9.926172e-01 9.627527e-01

9358 9.926187e-01 9.627397e-01

9359 9.926202e-01 9.627267e-01

9360 9.926218e-01 9.627136e-01

9361 9.926233e-01 9.627004e-01

9362 9.926281e-01 9.626982e-01

9363 9.926327e-01 9.626961e-01

9364 9.926372e-01 9.626939e-01

9365 9.926417e-01 9.626917e-01

9366 9.926460e-01 9.626894e-01

9367 9.926502e-01 9.626872e-01

9368 9.926544e-01 9.626849e-01

9369 9.926584e-01 9.626825e-01

9370 9.926624e-01 9.626802e-01

9371 9.926662e-01 9.626778e-01

9372 9.926700e-01 9.626753e-01

9373 9.926737e-01 9.626729e-01

9374 9.926773e-01 9.626704e-01

9375 9.926809e-01 9.626678e-01

9376 9.926843e-01 9.626653e-01

9377 9.926877e-01 9.626627e-01

9378 9.926910e-01 9.626601e-01

9379 9.926943e-01 9.626574e-01

9380 9.926974e-01 9.626547e-01

9381 9.927006e-01 9.626520e-01

9382 9.927036e-01 9.626492e-01

9383 9.927066e-01 9.626464e-01

9384 9.927095e-01 9.626436e-01

9385 9.927124e-01 9.626407e-01

9386 9.927152e-01 9.626378e-01

9387 9.927179e-01 9.626348e-01

9388 9.927206e-01 9.626318e-01

9389 9.927232e-01 9.626288e-01

9390 9.927258e-01 9.626257e-01

9391 9.927284e-01 9.626226e-01

9392 9.927308e-01 9.626195e-01

9393 9.927333e-01 9.626163e-01

9394 9.927357e-01 9.626131e-01

9395 9.927380e-01 9.626098e-01

9396 9.927403e-01 9.626065e-01

9397 9.927426e-01 9.626031e-01

9398 9.927448e-01 9.625998e-01

9399 9.927470e-01 9.625963e-01

9400 9.927491e-01 9.625928e-01

9401 9.927512e-01 9.625893e-01

9402 9.927533e-01 9.625858e-01

9403 9.927553e-01 9.625822e-01

9404 9.927573e-01 9.625785e-01

9405 9.927593e-01 9.625748e-01

9406 9.927612e-01 9.625711e-01

9407 9.927631e-01 9.625673e-01

9408 9.927650e-01 9.625634e-01

9409 9.927668e-01 9.625596e-01

9410 9.927686e-01 9.625556e-01

9411 9.927704e-01 9.625517e-01

9412 9.927721e-01 9.625476e-01

9413 9.927739e-01 9.625436e-01

9414 9.927756e-01 9.625394e-01

9415 9.927772e-01 9.625353e-01

9416 9.927789e-01 9.625310e-01

9417 9.927805e-01 9.625268e-01

9418 9.927821e-01 9.625224e-01

9419 9.927837e-01 9.625181e-01

9420 9.927853e-01 9.625136e-01

9421 9.927868e-01 9.625092e-01

9422 9.927883e-01 9.625046e-01

9423 9.927898e-01 9.625000e-01

9424 9.927913e-01 9.624954e-01

9425 9.927928e-01 9.624907e-01

9426 9.927942e-01 9.624860e-01

9427 9.927956e-01 9.624812e-01

9428 9.927970e-01 9.624763e-01

9429 9.927984e-01 9.624714e-01

9430 9.927998e-01 9.624664e-01

9431 9.928012e-01 9.624614e-01

9432 9.928025e-01 9.624563e-01

9433 9.928039e-01 9.624512e-01

9434 9.928052e-01 9.624460e-01

9435 9.928065e-01 9.624407e-01

9436 9.928078e-01 9.624354e-01

9437 9.928091e-01 9.624300e-01

9438 9.928104e-01 9.624246e-01

9439 9.928116e-01 9.624191e-01

9440 9.928129e-01 9.624135e-01

9441 9.928141e-01 9.624079e-01

9442 9.928154e-01 9.624022e-01

9443 9.928166e-01 9.623964e-01

9444 9.928178e-01 9.623906e-01

9445 9.928190e-01 9.623848e-01

9446 9.928202e-01 9.623788e-01

9447 9.928214e-01 9.623728e-01

9448 9.928226e-01 9.623667e-01

9449 9.928238e-01 9.623606e-01

9450 9.928250e-01 9.623544e-01

9451 9.928261e-01 9.623481e-01

9452 9.928273e-01 9.623418e-01

9453 9.928285e-01 9.623354e-01

9454 9.928296e-01 9.623289e-01

9455 9.928308e-01 9.623224e-01

9456 9.928319e-01 9.623158e-01

9457 9.928330e-01 9.623091e-01

9458 9.928342e-01 9.623024e-01

9459 9.928353e-01 9.622956e-01

9460 9.928364e-01 9.622887e-01

9461 9.928375e-01 9.622818e-01

9462 9.928387e-01 9.622747e-01

9463 9.928398e-01 9.622676e-01

9464 9.928409e-01 9.622605e-01

9465 9.928420e-01 9.622532e-01

9466 9.928431e-01 9.622459e-01

9467 9.928442e-01 9.622385e-01

9468 9.928453e-01 9.622311e-01

9469 9.928464e-01 9.622236e-01

9470 9.928475e-01 9.622160e-01

9471 9.928486e-01 9.622083e-01

9472 9.928497e-01 9.622005e-01

9473 9.928508e-01 9.621927e-01

9474 9.928519e-01 9.621848e-01

9475 9.928530e-01 9.621768e-01

9476 9.928541e-01 9.621688e-01

9477 9.928552e-01 9.621606e-01

9478 9.928563e-01 9.621524e-01

9479 9.928575e-01 9.621441e-01

9480 9.928586e-01 9.621358e-01

9481 9.928597e-01 9.621273e-01

9482 9.928608e-01 9.621188e-01

9483 9.928619e-01 9.621102e-01

9484 9.928630e-01 9.621015e-01

9485 9.928641e-01 9.620928e-01

9486 9.928652e-01 9.620839e-01

9487 9.928663e-01 9.620750e-01

9488 9.928674e-01 9.620660e-01

9489 9.928686e-01 9.620569e-01

9490 9.928697e-01 9.620478e-01

9491 9.928708e-01 9.620385e-01

9492 9.928719e-01 9.620292e-01

9493 9.928731e-01 9.620198e-01

9494 9.928742e-01 9.620104e-01

9495 9.928754e-01 9.620008e-01

9496 9.928765e-01 9.619912e-01

9497 9.928776e-01 9.619814e-01

9498 9.928788e-01 9.619716e-01

9499 9.928799e-01 9.619618e-01

9500 9.928811e-01 9.619518e-01

9501 9.928823e-01 9.619417e-01

9502 9.928834e-01 9.619316e-01

9503 9.928846e-01 9.619214e-01

9504 9.928858e-01 9.619111e-01

9505 9.928870e-01 9.619007e-01

9506 9.928881e-01 9.618903e-01

9507 9.928893e-01 9.618797e-01

9508 9.928905e-01 9.618691e-01

9509 9.928917e-01 9.618584e-01

9510 9.928929e-01 9.618476e-01

9511 9.928941e-01 9.618367e-01

9512 9.928954e-01 9.618258e-01

9513 9.928966e-01 9.618147e-01

9514 9.928978e-01 9.618036e-01

9515 9.928990e-01 9.617924e-01

9516 9.929003e-01 9.617811e-01

9517 9.929015e-01 9.617698e-01

9518 9.929028e-01 9.617583e-01

9519 9.929040e-01 9.617468e-01

9520 9.929053e-01 9.617352e-01

9521 9.929065e-01 9.617235e-01

9522 9.929078e-01 9.617117e-01

9523 9.929091e-01 9.616999e-01

9524 9.929104e-01 9.616880e-01

9525 9.929117e-01 9.616760e-01

9526 9.929130e-01 9.616639e-01

9527 9.929143e-01 9.616517e-01

9528 9.929156e-01 9.616394e-01

9529 9.929169e-01 9.616271e-01

9530 9.929182e-01 9.616147e-01

9531 9.929196e-01 9.616022e-01

9532 9.929209e-01 9.615896e-01

9533 9.929223e-01 9.615770e-01

9534 9.929236e-01 9.615643e-01

9535 9.929250e-01 9.615515e-01

9536 9.929263e-01 9.615386e-01

9537 9.929277e-01 9.615257e-01

9538 9.929291e-01 9.615126e-01

9539 9.929305e-01 9.614995e-01

9540 9.929319e-01 9.614863e-01

9541 9.929333e-01 9.614731e-01

9542 9.929373e-01 9.614709e-01

9543 9.929412e-01 9.614686e-01

9544 9.929451e-01 9.614663e-01

9545 9.929489e-01 9.614640e-01

9546 9.929525e-01 9.614616e-01

9547 9.929561e-01 9.614592e-01

9548 9.929597e-01 9.614568e-01

9549 9.929631e-01 9.614544e-01

9550 9.929665e-01 9.614519e-01

9551 9.929698e-01 9.614494e-01

9552 9.929730e-01 9.614468e-01

9553 9.929761e-01 9.614443e-01

9554 9.929792e-01 9.614417e-01

9555 9.929823e-01 9.614390e-01

9556 9.929852e-01 9.614363e-01

9557 9.929881e-01 9.614336e-01

9558 9.929910e-01 9.614309e-01

9559 9.929937e-01 9.614281e-01

9560 9.929965e-01 9.614253e-01

9561 9.929991e-01 9.614224e-01

9562 9.930017e-01 9.614195e-01

9563 9.930043e-01 9.614166e-01

9564 9.930068e-01 9.614136e-01

9565 9.930093e-01 9.614106e-01

9566 9.930117e-01 9.614075e-01

9567 9.930140e-01 9.614045e-01

9568 9.930164e-01 9.614013e-01

9569 9.930186e-01 9.613982e-01

9570 9.930209e-01 9.613950e-01

9571 9.930231e-01 9.613917e-01

9572 9.930252e-01 9.613885e-01

9573 9.930273e-01 9.613851e-01

9574 9.930294e-01 9.613818e-01

9575 9.930314e-01 9.613784e-01

9576 9.930334e-01 9.613749e-01

9577 9.930354e-01 9.613714e-01

9578 9.930373e-01 9.613679e-01

9579 9.930392e-01 9.613643e-01

9580 9.930410e-01 9.613607e-01

9581 9.930428e-01 9.613570e-01

9582 9.930446e-01 9.613533e-01

9583 9.930464e-01 9.613496e-01

9584 9.930481e-01 9.613458e-01

9585 9.930498e-01 9.613419e-01

9586 9.930515e-01 9.613380e-01

9587 9.930532e-01 9.613341e-01

9588 9.930548e-01 9.613301e-01

9589 9.930564e-01 9.613261e-01

9590 9.930580e-01 9.613220e-01

9591 9.930595e-01 9.613178e-01

9592 9.930611e-01 9.613137e-01

9593 9.930626e-01 9.613094e-01

9594 9.930641e-01 9.613052e-01

9595 9.930655e-01 9.613008e-01

9596 9.930670e-01 9.612964e-01

9597 9.930684e-01 9.612920e-01

9598 9.930698e-01 9.612875e-01

9599 9.930712e-01 9.612830e-01

9600 9.930726e-01 9.612784e-01

9601 9.930739e-01 9.612738e-01

9602 9.930752e-01 9.612691e-01

9603 9.930766e-01 9.612643e-01

9604 9.930779e-01 9.612595e-01

9605 9.930792e-01 9.612546e-01

9606 9.930804e-01 9.612497e-01

9607 9.930817e-01 9.612448e-01

9608 9.930829e-01 9.612397e-01

9609 9.930842e-01 9.612346e-01

9610 9.930854e-01 9.612295e-01

9611 9.930866e-01 9.612243e-01

9612 9.930878e-01 9.612191e-01

9613 9.930890e-01 9.612137e-01

9614 9.930902e-01 9.612084e-01

9615 9.930913e-01 9.612029e-01

9616 9.930925e-01 9.611974e-01

9617 9.930936e-01 9.611919e-01

9618 9.930947e-01 9.611863e-01

9619 9.930959e-01 9.611806e-01

9620 9.930970e-01 9.611749e-01

9621 9.930981e-01 9.611691e-01

9622 9.930992e-01 9.611632e-01

9623 9.931003e-01 9.611573e-01

9624 9.931014e-01 9.611513e-01

9625 9.931024e-01 9.611452e-01

9626 9.931035e-01 9.611391e-01

9627 9.931046e-01 9.611329e-01

9628 9.931056e-01 9.611267e-01

9629 9.931067e-01 9.611204e-01

9630 9.931077e-01 9.611140e-01

9631 9.931088e-01 9.611076e-01

9632 9.931098e-01 9.611010e-01

9633 9.931109e-01 9.610945e-01

9634 9.931119e-01 9.610878e-01

9635 9.931129e-01 9.610811e-01

9636 9.931139e-01 9.610743e-01

9637 9.931149e-01 9.610675e-01

9638 9.931160e-01 9.610606e-01

9639 9.931170e-01 9.610536e-01

9640 9.931180e-01 9.610465e-01

9641 9.931190e-01 9.610394e-01

9642 9.931200e-01 9.610322e-01

9643 9.931210e-01 9.610249e-01

9644 9.931220e-01 9.610176e-01

9645 9.931230e-01 9.610101e-01

9646 9.931240e-01 9.610027e-01

9647 9.931250e-01 9.609951e-01

9648 9.931260e-01 9.609875e-01

9649 9.931270e-01 9.609798e-01

9650 9.931279e-01 9.609720e-01

9651 9.931289e-01 9.609641e-01

9652 9.931299e-01 9.609562e-01

9653 9.931309e-01 9.609482e-01

9654 9.931319e-01 9.609401e-01

9655 9.931329e-01 9.609320e-01

9656 9.931339e-01 9.609237e-01

9657 9.931349e-01 9.609154e-01

9658 9.931359e-01 9.609070e-01

9659 9.931369e-01 9.608986e-01

9660 9.931379e-01 9.608901e-01

9661 9.931389e-01 9.608814e-01

9662 9.931399e-01 9.608728e-01

9663 9.931409e-01 9.608640e-01

9664 9.931419e-01 9.608551e-01

9665 9.931429e-01 9.608462e-01

9666 9.931439e-01 9.608372e-01

9667 9.931449e-01 9.608282e-01

9668 9.931459e-01 9.608190e-01

9669 9.931469e-01 9.608098e-01

9670 9.931479e-01 9.608005e-01

9671 9.931489e-01 9.607911e-01

9672 9.931500e-01 9.607816e-01

9673 9.931510e-01 9.607720e-01

9674 9.931520e-01 9.607624e-01

9675 9.931530e-01 9.607527e-01

9676 9.931541e-01 9.607429e-01

9677 9.931551e-01 9.607330e-01

9678 9.931561e-01 9.607231e-01

9679 9.931572e-01 9.607131e-01

9680 9.931582e-01 9.607030e-01

9681 9.931593e-01 9.606928e-01

9682 9.931603e-01 9.606825e-01

9683 9.931614e-01 9.606722e-01

9684 9.931624e-01 9.606617e-01

9685 9.931635e-01 9.606512e-01

9686 9.931646e-01 9.606406e-01

9687 9.931657e-01 9.606300e-01

9688 9.931667e-01 9.606192e-01

9689 9.931678e-01 9.606084e-01

9690 9.931689e-01 9.605975e-01

9691 9.931700e-01 9.605865e-01

9692 9.931711e-01 9.605754e-01

9693 9.931722e-01 9.605643e-01

9694 9.931733e-01 9.605530e-01

9695 9.931744e-01 9.605417e-01

9696 9.931755e-01 9.605303e-01

9697 9.931767e-01 9.605189e-01

9698 9.931778e-01 9.605073e-01

9699 9.931789e-01 9.604957e-01

9700 9.931801e-01 9.604840e-01

9701 9.931812e-01 9.604722e-01

9702 9.931824e-01 9.604603e-01

9703 9.931835e-01 9.604484e-01

9704 9.931847e-01 9.604364e-01

9705 9.931858e-01 9.604243e-01

9706 9.931870e-01 9.604121e-01

9707 9.931882e-01 9.603999e-01

9708 9.931894e-01 9.603875e-01

9709 9.931906e-01 9.603751e-01

9710 9.931918e-01 9.603626e-01

9711 9.931930e-01 9.603501e-01

9712 9.931942e-01 9.603375e-01

9713 9.931954e-01 9.603248e-01

9714 9.931966e-01 9.603120e-01

9715 9.931978e-01 9.602991e-01

9716 9.931991e-01 9.602862e-01

9717 9.932003e-01 9.602732e-01

9718 9.932015e-01 9.602601e-01

9719 9.932028e-01 9.602470e-01

9720 9.932040e-01 9.602338e-01

9721 9.932053e-01 9.602205e-01

9722 9.932087e-01 9.602181e-01

9723 9.932121e-01 9.602158e-01

9724 9.932154e-01 9.602134e-01

9725 9.932186e-01 9.602109e-01

9726 9.932217e-01 9.602085e-01

9727 9.932248e-01 9.602060e-01

9728 9.932278e-01 9.602035e-01

9729 9.932307e-01 9.602009e-01

9730 9.932336e-01 9.601983e-01

9731 9.932364e-01 9.601957e-01

9732 9.932392e-01 9.601930e-01

9733 9.932419e-01 9.601903e-01

9734 9.932446e-01 9.601876e-01

9735 9.932472e-01 9.601848e-01

9736 9.932497e-01 9.601820e-01

9737 9.932522e-01 9.601792e-01

9738 9.932546e-01 9.601763e-01

9739 9.932570e-01 9.601734e-01

9740 9.932594e-01 9.601705e-01

9741 9.932617e-01 9.601675e-01

9742 9.932639e-01 9.601645e-01

9743 9.932661e-01 9.601614e-01

9744 9.932683e-01 9.601583e-01

9745 9.932704e-01 9.601552e-01

9746 9.932725e-01 9.601520e-01

9747 9.932745e-01 9.601488e-01

9748 9.932765e-01 9.601455e-01

9749 9.932785e-01 9.601423e-01

9750 9.932804e-01 9.601389e-01

9751 9.932823e-01 9.601355e-01

9752 9.932842e-01 9.601321e-01

9753 9.932860e-01 9.601287e-01

9754 9.932878e-01 9.601252e-01

9755 9.932896e-01 9.601216e-01

9756 9.932913e-01 9.601180e-01

9757 9.932930e-01 9.601144e-01

9758 9.932947e-01 9.601107e-01

9759 9.932963e-01 9.601070e-01

9760 9.932979e-01 9.601033e-01

9761 9.932995e-01 9.600995e-01

9762 9.933011e-01 9.600956e-01

9763 9.933026e-01 9.600917e-01

9764 9.933041e-01 9.600878e-01

9765 9.933056e-01 9.600838e-01

9766 9.933071e-01 9.600797e-01

9767 9.933085e-01 9.600756e-01

9768 9.933099e-01 9.600715e-01

9769 9.933113e-01 9.600673e-01

9770 9.933127e-01 9.600631e-01

9771 9.933141e-01 9.600588e-01

9772 9.933154e-01 9.600545e-01

9773 9.933168e-01 9.600501e-01

9774 9.933181e-01 9.600457e-01

9775 9.933193e-01 9.600412e-01

9776 9.933206e-01 9.600366e-01

9777 9.933219e-01 9.600321e-01

9778 9.933231e-01 9.600274e-01

9779 9.933243e-01 9.600227e-01

9780 9.933255e-01 9.600180e-01

9781 9.933267e-01 9.600132e-01

9782 9.933279e-01 9.600083e-01

9783 9.933291e-01 9.600034e-01

9784 9.933302e-01 9.599985e-01

9785 9.933314e-01 9.599934e-01

9786 9.933325e-01 9.599884e-01

9787 9.933336e-01 9.599832e-01

9788 9.933347e-01 9.599780e-01

9789 9.933358e-01 9.599728e-01

9790 9.933369e-01 9.599675e-01

9791 9.933379e-01 9.599621e-01

9792 9.933390e-01 9.599567e-01

9793 9.933401e-01 9.599512e-01

9794 9.933411e-01 9.599457e-01

9795 9.933421e-01 9.599401e-01

9796 9.933432e-01 9.599344e-01

9797 9.933442e-01 9.599287e-01

9798 9.933452e-01 9.599229e-01

9799 9.933462e-01 9.599171e-01

9800 9.933472e-01 9.599112e-01

9801 9.933482e-01 9.599052e-01

9802 9.933491e-01 9.598992e-01

9803 9.933501e-01 9.598931e-01

9804 9.933511e-01 9.598870e-01

9805 9.933520e-01 9.598808e-01

9806 9.933530e-01 9.598745e-01

9807 9.933539e-01 9.598681e-01

9808 9.933549e-01 9.598617e-01

9809 9.933558e-01 9.598552e-01

9810 9.933568e-01 9.598487e-01

9811 9.933577e-01 9.598421e-01

9812 9.933586e-01 9.598354e-01

9813 9.933596e-01 9.598287e-01

9814 9.933605e-01 9.598218e-01

9815 9.933614e-01 9.598150e-01

9816 9.933623e-01 9.598080e-01

9817 9.933632e-01 9.598010e-01

9818 9.933641e-01 9.597939e-01

9819 9.933650e-01 9.597868e-01

9820 9.933659e-01 9.597795e-01

9821 9.933668e-01 9.597722e-01

9822 9.933677e-01 9.597649e-01

9823 9.933686e-01 9.597574e-01

9824 9.933695e-01 9.597499e-01

9825 9.933704e-01 9.597424e-01

9826 9.933713e-01 9.597347e-01

9827 9.933722e-01 9.597270e-01

9828 9.933731e-01 9.597192e-01

9829 9.933740e-01 9.597113e-01

9830 9.933749e-01 9.597034e-01

9831 9.933758e-01 9.596954e-01

9832 9.933767e-01 9.596873e-01

9833 9.933776e-01 9.596791e-01

9834 9.933785e-01 9.596709e-01

9835 9.933794e-01 9.596626e-01

9836 9.933803e-01 9.596542e-01

9837 9.933812e-01 9.596457e-01

9838 9.933820e-01 9.596372e-01

9839 9.933829e-01 9.596286e-01

9840 9.933838e-01 9.596199e-01

9841 9.933847e-01 9.596112e-01

9842 9.933856e-01 9.596023e-01

9843 9.933865e-01 9.595934e-01

9844 9.933874e-01 9.595844e-01

9845 9.933883e-01 9.595754e-01

9846 9.933892e-01 9.595662e-01

9847 9.933902e-01 9.595570e-01

9848 9.933911e-01 9.595477e-01

9849 9.933920e-01 9.595383e-01

9850 9.933929e-01 9.595289e-01

9851 9.933938e-01 9.595193e-01

9852 9.933947e-01 9.595097e-01

9853 9.933957e-01 9.595001e-01

9854 9.933966e-01 9.594903e-01

9855 9.933975e-01 9.594805e-01

9856 9.933984e-01 9.594705e-01

9857 9.933994e-01 9.594605e-01

9858 9.934003e-01 9.594505e-01

9859 9.934012e-01 9.594403e-01

9860 9.934022e-01 9.594301e-01

9861 9.934031e-01 9.594198e-01

9862 9.934041e-01 9.594094e-01

9863 9.934050e-01 9.593989e-01

9864 9.934060e-01 9.593884e-01

9865 9.934070e-01 9.593778e-01

9866 9.934079e-01 9.593671e-01

9867 9.934089e-01 9.593563e-01

9868 9.934099e-01 9.593454e-01

9869 9.934108e-01 9.593345e-01

9870 9.934118e-01 9.593235e-01

9871 9.934128e-01 9.593124e-01

9872 9.934138e-01 9.593012e-01

9873 9.934148e-01 9.592900e-01

9874 9.934158e-01 9.592787e-01

9875 9.934168e-01 9.592673e-01

9876 9.934178e-01 9.592558e-01

9877 9.934188e-01 9.592443e-01

9878 9.934198e-01 9.592326e-01

9879 9.934208e-01 9.592209e-01

9880 9.934218e-01 9.592091e-01

9881 9.934229e-01 9.591973e-01

9882 9.934239e-01 9.591853e-01

9883 9.934249e-01 9.591733e-01

9884 9.934260e-01 9.591613e-01

9885 9.934270e-01 9.591491e-01

9886 9.934281e-01 9.591369e-01

9887 9.934291e-01 9.591246e-01

9888 9.934302e-01 9.591122e-01

9889 9.934312e-01 9.590997e-01

9890 9.934323e-01 9.590872e-01

9891 9.934334e-01 9.590746e-01

9892 9.934344e-01 9.590619e-01

9893 9.934355e-01 9.590492e-01

9894 9.934366e-01 9.590363e-01

9895 9.934377e-01 9.590235e-01

9896 9.934388e-01 9.590105e-01

9897 9.934399e-01 9.589975e-01

9898 9.934410e-01 9.589844e-01

9899 9.934421e-01 9.589712e-01

9900 9.934432e-01 9.589580e-01

9901 9.934443e-01 9.589446e-01

9902 9.934472e-01 9.589422e-01

9903 9.934501e-01 9.589397e-01

9904 9.934529e-01 9.589372e-01

9905 9.934557e-01 9.589347e-01

9906 9.934584e-01 9.589321e-01

9907 9.934610e-01 9.589295e-01

9908 9.934636e-01 9.589269e-01

9909 9.934661e-01 9.589242e-01

9910 9.934686e-01 9.589215e-01

9911 9.934710e-01 9.589188e-01

9912 9.934734e-01 9.589160e-01

9913 9.934757e-01 9.589132e-01

9914 9.934780e-01 9.589103e-01

9915 9.934802e-01 9.589074e-01

9916 9.934824e-01 9.589045e-01

9917 9.934846e-01 9.589016e-01

9918 9.934867e-01 9.588986e-01

9919 9.934887e-01 9.588956e-01

9920 9.934907e-01 9.588925e-01

9921 9.934927e-01 9.588894e-01

9922 9.934947e-01 9.588863e-01

9923 9.934966e-01 9.588831e-01

9924 9.934985e-01 9.588799e-01

9925 9.935003e-01 9.588766e-01

9926 9.935021e-01 9.588733e-01

9927 9.935039e-01 9.588700e-01

9928 9.935056e-01 9.588666e-01

9929 9.935073e-01 9.588632e-01

9930 9.935090e-01 9.588597e-01

9931 9.935106e-01 9.588562e-01

9932 9.935122e-01 9.588526e-01

9933 9.935138e-01 9.588491e-01

9934 9.935154e-01 9.588454e-01

9935 9.935169e-01 9.588418e-01

9936 9.935184e-01 9.588380e-01

9937 9.935199e-01 9.588343e-01

9938 9.935214e-01 9.588305e-01

9939 9.935228e-01 9.588266e-01

9940 9.935242e-01 9.588227e-01

9941 9.935256e-01 9.588188e-01

9942 9.935270e-01 9.588148e-01

9943 9.935283e-01 9.588107e-01

9944 9.935296e-01 9.588067e-01

9945 9.935310e-01 9.588025e-01

9946 9.935322e-01 9.587983e-01

9947 9.935335e-01 9.587941e-01

9948 9.935348e-01 9.587898e-01

9949 9.935360e-01 9.587855e-01

9950 9.935372e-01 9.587811e-01

9951 9.935384e-01 9.587767e-01

9952 9.935396e-01 9.587722e-01

9953 9.935407e-01 9.587677e-01

9954 9.935419e-01 9.587631e-01

9955 9.935430e-01 9.587585e-01

9956 9.935441e-01 9.587538e-01

9957 9.935452e-01 9.587491e-01

9958 9.935463e-01 9.587443e-01

9959 9.935474e-01 9.587395e-01

9960 9.935485e-01 9.587346e-01

9961 9.935495e-01 9.587296e-01

9962 9.935506e-01 9.587246e-01

9963 9.935516e-01 9.587196e-01

9964 9.935526e-01 9.587144e-01

9965 9.935536e-01 9.587093e-01

9966 9.935546e-01 9.587040e-01

9967 9.935556e-01 9.586988e-01

9968 9.935566e-01 9.586934e-01

9969 9.935576e-01 9.586880e-01

9970 9.935585e-01 9.586826e-01

9971 9.935595e-01 9.586771e-01

9972 9.935604e-01 9.586715e-01

9973 9.935614e-01 9.586659e-01

9974 9.935623e-01 9.586602e-01

9975 9.935632e-01 9.586544e-01

9976 9.935641e-01 9.586486e-01

9977 9.935650e-01 9.586427e-01

9978 9.935659e-01 9.586368e-01

9979 9.935668e-01 9.586308e-01

9980 9.935677e-01 9.586247e-01

9981 9.935686e-01 9.586186e-01

9982 9.935694e-01 9.586124e-01

9983 9.935703e-01 9.586062e-01

9984 9.935712e-01 9.585999e-01

9985 9.935720e-01 9.585935e-01

9986 9.935729e-01 9.585871e-01

9987 9.935737e-01 9.585806e-01

9988 9.935746e-01 9.585740e-01

9989 9.935754e-01 9.585674e-01

9990 9.935763e-01 9.585607e-01

9991 9.935771e-01 9.585539e-01

9992 9.935779e-01 9.585471e-01

9993 9.935788e-01 9.585402e-01

9994 9.935796e-01 9.585332e-01

9995 9.935804e-01 9.585262e-01

9996 9.935812e-01 9.585191e-01

9997 9.935820e-01 9.585119e-01

9998 9.935829e-01 9.585047e-01

9999 9.935837e-01 9.584974e-01

10000 9.935845e-01 9.584900e-01

10001 9.935853e-01 9.584826e-01

10002 9.935861e-01 9.584751e-01

10003 9.935869e-01 9.584675e-01

10004 9.935877e-01 9.584598e-01

10005 9.935885e-01 9.584521e-01

10006 9.935893e-01 9.584443e-01

10007 9.935901e-01 9.584364e-01

10008 9.935909e-01 9.584285e-01

10009 9.935917e-01 9.584205e-01

10010 9.935925e-01 9.584124e-01

10011 9.935933e-01 9.584043e-01

10012 9.935941e-01 9.583960e-01

10013 9.935949e-01 9.583877e-01

10014 9.935957e-01 9.583794e-01

10015 9.935966e-01 9.583709e-01

10016 9.935974e-01 9.583624e-01

10017 9.935982e-01 9.583538e-01

10018 9.935990e-01 9.583451e-01

10019 9.935998e-01 9.583364e-01

10020 9.936006e-01 9.583276e-01

10021 9.936014e-01 9.583187e-01

10022 9.936022e-01 9.583097e-01

10023 9.936030e-01 9.583007e-01

10024 9.936038e-01 9.582916e-01

10025 9.936046e-01 9.582824e-01

10026 9.936054e-01 9.582731e-01

10027 9.936063e-01 9.582638e-01

10028 9.936071e-01 9.582544e-01

10029 9.936079e-01 9.582449e-01

10030 9.936087e-01 9.582353e-01

10031 9.936095e-01 9.582257e-01

10032 9.936104e-01 9.582159e-01

10033 9.936112e-01 9.582061e-01

10034 9.936120e-01 9.581963e-01

10035 9.936129e-01 9.581863e-01

10036 9.936137e-01 9.581763e-01

10037 9.936145e-01 9.581662e-01

10038 9.936154e-01 9.581560e-01

10039 9.936162e-01 9.581458e-01

10040 9.936171e-01 9.581354e-01

10041 9.936179e-01 9.581250e-01

10042 9.936187e-01 9.581146e-01

10043 9.936196e-01 9.581040e-01

10044 9.936205e-01 9.580934e-01

10045 9.936213e-01 9.580827e-01

10046 9.936222e-01 9.580719e-01

10047 9.936230e-01 9.580610e-01

10048 9.936239e-01 9.580501e-01

10049 9.936248e-01 9.580391e-01

10050 9.936257e-01 9.580280e-01

10051 9.936265e-01 9.580168e-01

10052 9.936274e-01 9.580056e-01

10053 9.936283e-01 9.579942e-01

10054 9.936292e-01 9.579828e-01

10055 9.936301e-01 9.579714e-01

10056 9.936310e-01 9.579598e-01

10057 9.936319e-01 9.579482e-01

10058 9.936328e-01 9.579365e-01

10059 9.936337e-01 9.579248e-01

10060 9.936346e-01 9.579129e-01

10061 9.936355e-01 9.579010e-01

10062 9.936364e-01 9.578890e-01

10063 9.936373e-01 9.578770e-01

10064 9.936382e-01 9.578649e-01

10065 9.936392e-01 9.578527e-01

10066 9.936401e-01 9.578404e-01

10067 9.936410e-01 9.578280e-01

10068 9.936420e-01 9.578156e-01

10069 9.936429e-01 9.578031e-01

10070 9.936438e-01 9.577906e-01

10071 9.936448e-01 9.577779e-01

10072 9.936457e-01 9.577652e-01

10073 9.936467e-01 9.577525e-01

10074 9.936476e-01 9.577396e-01

10075 9.936486e-01 9.577267e-01

10076 9.936495e-01 9.577137e-01

10077 9.936505e-01 9.577007e-01

10078 9.936515e-01 9.576876e-01

10079 9.936524e-01 9.576744e-01

10080 9.936534e-01 9.576612e-01

10081 9.936544e-01 9.576478e-01

10082 9.936569e-01 9.576453e-01

10083 9.936594e-01 9.576427e-01

10084 9.936618e-01 9.576401e-01

10085 9.936641e-01 9.576375e-01

10086 9.936665e-01 9.576348e-01

10087 9.936687e-01 9.576321e-01

10088 9.936709e-01 9.576293e-01

10089 9.936731e-01 9.576266e-01

10090 9.936753e-01 9.576237e-01

10091 9.936773e-01 9.576209e-01

10092 9.936794e-01 9.576180e-01

10093 9.936814e-01 9.576151e-01

10094 9.936834e-01 9.576121e-01

10095 9.936853e-01 9.576091e-01

10096 9.936872e-01 9.576061e-01

10097 9.936890e-01 9.576030e-01

10098 9.936909e-01 9.575999e-01

10099 9.936926e-01 9.575968e-01

10100 9.936944e-01 9.575936e-01

10101 9.936961e-01 9.575904e-01

10102 9.936978e-01 9.575871e-01

10103 9.936995e-01 9.575838e-01

10104 9.937011e-01 9.575805e-01

10105 9.937027e-01 9.575771e-01

10106 9.937042e-01 9.575737e-01

10107 9.937058e-01 9.575702e-01

10108 9.937073e-01 9.575667e-01

10109 9.937088e-01 9.575632e-01

10110 9.937102e-01 9.575596e-01

10111 9.937117e-01 9.575560e-01

10112 9.937131e-01 9.575523e-01

10113 9.937145e-01 9.575486e-01

10114 9.937158e-01 9.575448e-01

10115 9.937172e-01 9.575410e-01

10116 9.937185e-01 9.575372e-01

10117 9.937198e-01 9.575333e-01

10118 9.937211e-01 9.575293e-01

10119 9.937223e-01 9.575254e-01

10120 9.937236e-01 9.575213e-01

10121 9.937248e-01 9.575172e-01

10122 9.937260e-01 9.575131e-01

10123 9.937271e-01 9.575090e-01

10124 9.937283e-01 9.575047e-01

10125 9.937295e-01 9.575005e-01

10126 9.937306e-01 9.574962e-01

10127 9.937317e-01 9.574918e-01

10128 9.937328e-01 9.574874e-01

10129 9.937339e-01 9.574829e-01

10130 9.937350e-01 9.574784e-01

10131 9.937360e-01 9.574738e-01

10132 9.937370e-01 9.574692e-01

10133 9.937381e-01 9.574646e-01

10134 9.937391e-01 9.574599e-01

10135 9.937401e-01 9.574551e-01

10136 9.937411e-01 9.574503e-01

10137 9.937421e-01 9.574454e-01

10138 9.937430e-01 9.574405e-01

10139 9.937440e-01 9.574355e-01

10140 9.937449e-01 9.574305e-01

10141 9.937458e-01 9.574254e-01

10142 9.937468e-01 9.574202e-01

10143 9.937477e-01 9.574150e-01

10144 9.937486e-01 9.574098e-01

10145 9.937495e-01 9.574045e-01

10146 9.937504e-01 9.573991e-01

10147 9.937512e-01 9.573937e-01

10148 9.937521e-01 9.573882e-01

10149 9.937530e-01 9.573826e-01

10150 9.937538e-01 9.573770e-01

10151 9.937547e-01 9.573714e-01

10152 9.937555e-01 9.573657e-01

10153 9.937563e-01 9.573599e-01

10154 9.937572e-01 9.573541e-01

10155 9.937580e-01 9.573482e-01

10156 9.937588e-01 9.573422e-01

10157 9.937596e-01 9.573362e-01

10158 9.937604e-01 9.573301e-01

10159 9.937612e-01 9.573240e-01

10160 9.937620e-01 9.573178e-01

10161 9.937628e-01 9.573115e-01

10162 9.937636e-01 9.573052e-01

10163 9.937643e-01 9.572988e-01

10164 9.937651e-01 9.572924e-01

10165 9.937659e-01 9.572859e-01

10166 9.937666e-01 9.572793e-01

10167 9.937674e-01 9.572726e-01

10168 9.937682e-01 9.572659e-01

10169 9.937689e-01 9.572592e-01

10170 9.937697e-01 9.572523e-01

10171 9.937704e-01 9.572454e-01

10172 9.937712e-01 9.572385e-01

10173 9.937719e-01 9.572314e-01

10174 9.937726e-01 9.572243e-01

10175 9.937734e-01 9.572172e-01

10176 9.937741e-01 9.572099e-01

10177 9.937748e-01 9.572026e-01

10178 9.937756e-01 9.571952e-01

10179 9.937763e-01 9.571878e-01

10180 9.937770e-01 9.571803e-01

10181 9.937778e-01 9.571727e-01

10182 9.937785e-01 9.571651e-01

10183 9.937792e-01 9.571574e-01

10184 9.937799e-01 9.571496e-01

10185 9.937807e-01 9.571417e-01

10186 9.937814e-01 9.571338e-01

10187 9.937821e-01 9.571258e-01

10188 9.937828e-01 9.571177e-01

10189 9.937835e-01 9.571096e-01

10190 9.937843e-01 9.571014e-01

10191 9.937850e-01 9.570931e-01

10192 9.937857e-01 9.570848e-01

10193 9.937864e-01 9.570763e-01

10194 9.937871e-01 9.570678e-01

10195 9.937878e-01 9.570593e-01

10196 9.937886e-01 9.570506e-01

10197 9.937893e-01 9.570419e-01

10198 9.937900e-01 9.570331e-01

10199 9.937907e-01 9.570243e-01

10200 9.937915e-01 9.570154e-01

10201 9.937922e-01 9.570064e-01

10202 9.937929e-01 9.569973e-01

10203 9.937936e-01 9.569881e-01

10204 9.937944e-01 9.569789e-01

10205 9.937951e-01 9.569696e-01

10206 9.937958e-01 9.569602e-01

10207 9.937965e-01 9.569508e-01

10208 9.937973e-01 9.569413e-01

10209 9.937980e-01 9.569317e-01

10210 9.937987e-01 9.569220e-01

10211 9.937995e-01 9.569123e-01

10212 9.938002e-01 9.569025e-01

10213 9.938009e-01 9.568926e-01

10214 9.938017e-01 9.568826e-01

10215 9.938024e-01 9.568726e-01

10216 9.938032e-01 9.568625e-01

10217 9.938039e-01 9.568523e-01

10218 9.938047e-01 9.568420e-01

10219 9.938054e-01 9.568317e-01

10220 9.938062e-01 9.568213e-01

10221 9.938069e-01 9.568108e-01

10222 9.938077e-01 9.568002e-01

10223 9.938084e-01 9.567896e-01

10224 9.938092e-01 9.567789e-01

10225 9.938100e-01 9.567681e-01

10226 9.938107e-01 9.567572e-01

10227 9.938115e-01 9.567463e-01

10228 9.938123e-01 9.567353e-01

10229 9.938130e-01 9.567242e-01

10230 9.938138e-01 9.567131e-01

10231 9.938146e-01 9.567018e-01

10232 9.938154e-01 9.566905e-01

10233 9.938162e-01 9.566792e-01

10234 9.938169e-01 9.566677e-01

10235 9.938177e-01 9.566562e-01

10236 9.938185e-01 9.566446e-01

10237 9.938193e-01 9.566330e-01

10238 9.938201e-01 9.566212e-01

10239 9.938209e-01 9.566094e-01

10240 9.938217e-01 9.565976e-01

10241 9.938225e-01 9.565856e-01

10242 9.938233e-01 9.565736e-01

10243 9.938241e-01 9.565615e-01

10244 9.938249e-01 9.565493e-01

10245 9.938257e-01 9.565371e-01

10246 9.938265e-01 9.565248e-01

10247 9.938273e-01 9.565124e-01

10248 9.938282e-01 9.565000e-01

10249 9.938290e-01 9.564875e-01

10250 9.938298e-01 9.564749e-01

10251 9.938306e-01 9.564623e-01

10252 9.938314e-01 9.564496e-01

10253 9.938323e-01 9.564368e-01

10254 9.938331e-01 9.564239e-01

10255 9.938339e-01 9.564110e-01

10256 9.938348e-01 9.563980e-01

10257 9.938356e-01 9.563850e-01

10258 9.938364e-01 9.563719e-01

10259 9.938373e-01 9.563587e-01

10260 9.938381e-01 9.563455e-01

10261 9.938389e-01 9.563322e-01

10262 9.938411e-01 9.563295e-01

10263 9.938432e-01 9.563269e-01

10264 9.938453e-01 9.563241e-01

10265 9.938474e-01 9.563214e-01

10266 9.938493e-01 9.563186e-01

10267 9.938513e-01 9.563158e-01

10268 9.938532e-01 9.563130e-01

10269 9.938551e-01 9.563101e-01

10270 9.938569e-01 9.563071e-01

10271 9.938588e-01 9.563042e-01

10272 9.938605e-01 9.563012e-01

10273 9.938623e-01 9.562982e-01

10274 9.938640e-01 9.562951e-01

10275 9.938656e-01 9.562920e-01

10276 9.938673e-01 9.562889e-01

10277 9.938689e-01 9.562857e-01

10278 9.938705e-01 9.562825e-01

10279 9.938720e-01 9.562792e-01

10280 9.938735e-01 9.562759e-01

10281 9.938750e-01 9.562726e-01

10282 9.938765e-01 9.562692e-01

10283 9.938779e-01 9.562658e-01

10284 9.938794e-01 9.562623e-01

10285 9.938807e-01 9.562588e-01

10286 9.938821e-01 9.562553e-01

10287 9.938835e-01 9.562517e-01

10288 9.938848e-01 9.562481e-01

10289 9.938861e-01 9.562444e-01

10290 9.938873e-01 9.562407e-01

10291 9.938886e-01 9.562370e-01

10292 9.938898e-01 9.562332e-01

10293 9.938910e-01 9.562294e-01

10294 9.938922e-01 9.562255e-01

10295 9.938934e-01 9.562215e-01

10296 9.938946e-01 9.562176e-01

10297 9.938957e-01 9.562136e-01

10298 9.938968e-01 9.562095e-01

10299 9.938979e-01 9.562054e-01

10300 9.938990e-01 9.562012e-01

10301 9.939001e-01 9.561970e-01

10302 9.939011e-01 9.561928e-01

10303 9.939022e-01 9.561885e-01

10304 9.939032e-01 9.561842e-01

10305 9.939042e-01 9.561798e-01

10306 9.939052e-01 9.561753e-01

10307 9.939062e-01 9.561708e-01

10308 9.939071e-01 9.561663e-01

10309 9.939081e-01 9.561617e-01

10310 9.939090e-01 9.561571e-01

10311 9.939100e-01 9.561524e-01

10312 9.939109e-01 9.561476e-01

10313 9.939118e-01 9.561428e-01

10314 9.939127e-01 9.561380e-01

10315 9.939136e-01 9.561331e-01

10316 9.939145e-01 9.561281e-01

10317 9.939153e-01 9.561231e-01

10318 9.939162e-01 9.561181e-01

10319 9.939170e-01 9.561130e-01

10320 9.939179e-01 9.561078e-01

10321 9.939187e-01 9.561026e-01

10322 9.939195e-01 9.560973e-01

10323 9.939203e-01 9.560920e-01

10324 9.939211e-01 9.560866e-01

10325 9.939219e-01 9.560811e-01

10326 9.939227e-01 9.560756e-01

10327 9.939235e-01 9.560701e-01

10328 9.939243e-01 9.560645e-01

10329 9.939250e-01 9.560588e-01

10330 9.939258e-01 9.560531e-01

10331 9.939265e-01 9.560473e-01

10332 9.939273e-01 9.560414e-01

10333 9.939280e-01 9.560355e-01

10334 9.939288e-01 9.560296e-01

10335 9.939295e-01 9.560235e-01

10336 9.939302e-01 9.560175e-01

10337 9.939309e-01 9.560113e-01

10338 9.939316e-01 9.560051e-01

10339 9.939324e-01 9.559988e-01

10340 9.939331e-01 9.559925e-01

10341 9.939338e-01 9.559861e-01

10342 9.939345e-01 9.559797e-01

10343 9.939352e-01 9.559732e-01

10344 9.939358e-01 9.559666e-01

10345 9.939365e-01 9.559599e-01

10346 9.939372e-01 9.559532e-01

10347 9.939379e-01 9.559465e-01

10348 9.939386e-01 9.559396e-01

10349 9.939392e-01 9.559327e-01

10350 9.939399e-01 9.559258e-01

10351 9.939406e-01 9.559188e-01

10352 9.939413e-01 9.559117e-01

10353 9.939419e-01 9.559045e-01

10354 9.939426e-01 9.558973e-01

10355 9.939432e-01 9.558900e-01

10356 9.939439e-01 9.558826e-01

10357 9.939445e-01 9.558752e-01

10358 9.939452e-01 9.558677e-01

10359 9.939459e-01 9.558602e-01

10360 9.939465e-01 9.558525e-01

10361 9.939472e-01 9.558448e-01

10362 9.939478e-01 9.558371e-01

10363 9.939485e-01 9.558292e-01

10364 9.939491e-01 9.558213e-01

10365 9.939497e-01 9.558134e-01

10366 9.939504e-01 9.558053e-01

10367 9.939510e-01 9.557972e-01

10368 9.939517e-01 9.557891e-01

10369 9.939523e-01 9.557808e-01

10370 9.939530e-01 9.557725e-01

10371 9.939536e-01 9.557641e-01

10372 9.939542e-01 9.557556e-01

10373 9.939549e-01 9.557471e-01

10374 9.939555e-01 9.557385e-01

10375 9.939562e-01 9.557299e-01

10376 9.939568e-01 9.557211e-01

10377 9.939575e-01 9.557123e-01

10378 9.939581e-01 9.557034e-01

10379 9.939587e-01 9.556945e-01

10380 9.939594e-01 9.556854e-01

10381 9.939600e-01 9.556763e-01

10382 9.939607e-01 9.556672e-01

10383 9.939613e-01 9.556579e-01

10384 9.939620e-01 9.556486e-01

10385 9.939626e-01 9.556392e-01

10386 9.939633e-01 9.556298e-01

10387 9.939639e-01 9.556202e-01

10388 9.939646e-01 9.556106e-01

10389 9.939652e-01 9.556010e-01

10390 9.939659e-01 9.555912e-01

10391 9.939665e-01 9.555814e-01

10392 9.939672e-01 9.555715e-01

10393 9.939678e-01 9.555615e-01

10394 9.939685e-01 9.555515e-01

10395 9.939692e-01 9.555414e-01

10396 9.939698e-01 9.555312e-01

10397 9.939705e-01 9.555210e-01

10398 9.939711e-01 9.555106e-01

10399 9.939718e-01 9.555002e-01

10400 9.939725e-01 9.554898e-01

10401 9.939731e-01 9.554792e-01

10402 9.939738e-01 9.554686e-01

10403 9.939745e-01 9.554579e-01

10404 9.939751e-01 9.554471e-01

10405 9.939758e-01 9.554363e-01

10406 9.939765e-01 9.554254e-01

10407 9.939772e-01 9.554144e-01

10408 9.939778e-01 9.554034e-01

10409 9.939785e-01 9.553922e-01

10410 9.939792e-01 9.553810e-01

10411 9.939799e-01 9.553698e-01

10412 9.939806e-01 9.553584e-01

10413 9.939813e-01 9.553470e-01

10414 9.939819e-01 9.553356e-01

10415 9.939826e-01 9.553240e-01

10416 9.939833e-01 9.553124e-01

10417 9.939840e-01 9.553007e-01

10418 9.939847e-01 9.552889e-01

10419 9.939854e-01 9.552771e-01

10420 9.939861e-01 9.552652e-01

10421 9.939868e-01 9.552533e-01

10422 9.939875e-01 9.552412e-01

10423 9.939882e-01 9.552291e-01

10424 9.939889e-01 9.552169e-01

10425 9.939896e-01 9.552047e-01

10426 9.939903e-01 9.551924e-01

10427 9.939910e-01 9.551800e-01

10428 9.939917e-01 9.551676e-01

10429 9.939924e-01 9.551551e-01

10430 9.939931e-01 9.551425e-01

10431 9.939939e-01 9.551299e-01

10432 9.939946e-01 9.551172e-01

10433 9.939953e-01 9.551044e-01

10434 9.939960e-01 9.550916e-01

10435 9.939967e-01 9.550787e-01

10436 9.939974e-01 9.550657e-01

10437 9.939982e-01 9.550527e-01

10438 9.939989e-01 9.550396e-01

10439 9.939996e-01 9.550264e-01

10440 9.940003e-01 9.550132e-01

10441 9.940010e-01 9.550000e-01

10442 9.940029e-01 9.549972e-01

10443 9.940047e-01 9.549944e-01

10444 9.940065e-01 9.549916e-01

10445 9.940083e-01 9.549888e-01

10446 9.940100e-01 9.549859e-01

10447 9.940117e-01 9.549830e-01

10448 9.940134e-01 9.549800e-01

10449 9.940150e-01 9.549771e-01

10450 9.940166e-01 9.549740e-01

10451 9.940182e-01 9.549710e-01

10452 9.940197e-01 9.549679e-01

10453 9.940212e-01 9.549647e-01

10454 9.940227e-01 9.549616e-01

10455 9.940242e-01 9.549584e-01

10456 9.940256e-01 9.549551e-01

10457 9.940270e-01 9.549518e-01

10458 9.940284e-01 9.549485e-01

10459 9.940297e-01 9.549451e-01

10460 9.940310e-01 9.549417e-01

10461 9.940323e-01 9.549383e-01

10462 9.940336e-01 9.549348e-01

10463 9.940349e-01 9.549313e-01

10464 9.940361e-01 9.549277e-01

10465 9.940373e-01 9.549241e-01

10466 9.940385e-01 9.549205e-01

10467 9.940397e-01 9.549168e-01

10468 9.940409e-01 9.549130e-01

10469 9.940420e-01 9.549093e-01

10470 9.940431e-01 9.549054e-01

10471 9.940442e-01 9.549016e-01

10472 9.940453e-01 9.548977e-01

10473 9.940463e-01 9.548937e-01

10474 9.940474e-01 9.548897e-01

10475 9.940484e-01 9.548857e-01

10476 9.940494e-01 9.548816e-01

10477 9.940504e-01 9.548775e-01

10478 9.940514e-01 9.548733e-01

10479 9.940524e-01 9.548691e-01

10480 9.940533e-01 9.548648e-01

10481 9.940543e-01 9.548605e-01

10482 9.940552e-01 9.548561e-01

10483 9.940561e-01 9.548517e-01

10484 9.940570e-01 9.548472e-01

10485 9.940579e-01 9.548427e-01

10486 9.940588e-01 9.548382e-01

10487 9.940597e-01 9.548335e-01

10488 9.940605e-01 9.548289e-01

10489 9.940614e-01 9.548242e-01

10490 9.940622e-01 9.548194e-01

10491 9.940630e-01 9.548146e-01

10492 9.940638e-01 9.548097e-01

10493 9.940646e-01 9.548048e-01

10494 9.940654e-01 9.547999e-01

10495 9.940662e-01 9.547948e-01

10496 9.940670e-01 9.547898e-01

10497 9.940678e-01 9.547846e-01

10498 9.940685e-01 9.547795e-01

10499 9.940693e-01 9.547742e-01

10500 9.940700e-01 9.547689e-01

10501 9.940708e-01 9.547636e-01

10502 9.940715e-01 9.547582e-01

10503 9.940722e-01 9.547528e-01

10504 9.940729e-01 9.547473e-01

10505 9.940736e-01 9.547417e-01

10506 9.940743e-01 9.547361e-01

10507 9.940750e-01 9.547304e-01

10508 9.940757e-01 9.547247e-01

10509 9.940764e-01 9.547189e-01

10510 9.940771e-01 9.547130e-01

10511 9.940777e-01 9.547071e-01

10512 9.940784e-01 9.547011e-01

10513 9.940791e-01 9.546951e-01

10514 9.940797e-01 9.546890e-01

10515 9.940804e-01 9.546829e-01

10516 9.940810e-01 9.546767e-01

10517 9.940816e-01 9.546704e-01

10518 9.940823e-01 9.546641e-01

10519 9.940829e-01 9.546577e-01

10520 9.940835e-01 9.546513e-01

10521 9.940842e-01 9.546448e-01

10522 9.940848e-01 9.546382e-01

10523 9.940854e-01 9.546316e-01

10524 9.940860e-01 9.546249e-01

10525 9.940866e-01 9.546181e-01

10526 9.940873e-01 9.546113e-01

10527 9.940879e-01 9.546044e-01

10528 9.940885e-01 9.545975e-01

10529 9.940891e-01 9.545905e-01

10530 9.940897e-01 9.545834e-01

10531 9.940903e-01 9.545763e-01

10532 9.940909e-01 9.545691e-01

10533 9.940914e-01 9.545618e-01

10534 9.940920e-01 9.545545e-01

10535 9.940926e-01 9.545471e-01

10536 9.940932e-01 9.545396e-01

10537 9.940938e-01 9.545321e-01

10538 9.940944e-01 9.545245e-01

10539 9.940950e-01 9.545168e-01

10540 9.940955e-01 9.545091e-01

10541 9.940961e-01 9.545013e-01

10542 9.940967e-01 9.544934e-01

10543 9.940973e-01 9.544855e-01

10544 9.940979e-01 9.544775e-01

10545 9.940984e-01 9.544694e-01

10546 9.940990e-01 9.544613e-01

10547 9.940996e-01 9.544531e-01

10548 9.941002e-01 9.544448e-01

10549 9.941007e-01 9.544365e-01

10550 9.941013e-01 9.544281e-01

10551 9.941019e-01 9.544196e-01

10552 9.941024e-01 9.544110e-01

10553 9.941030e-01 9.544024e-01

10554 9.941036e-01 9.543937e-01

10555 9.941042e-01 9.543850e-01

10556 9.941047e-01 9.543761e-01

10557 9.941053e-01 9.543673e-01

10558 9.941059e-01 9.543583e-01

10559 9.941064e-01 9.543493e-01

10560 9.941070e-01 9.543401e-01

10561 9.941076e-01 9.543310e-01

10562 9.941082e-01 9.543217e-01

10563 9.941087e-01 9.543124e-01

10564 9.941093e-01 9.543030e-01

10565 9.941099e-01 9.542936e-01

10566 9.941105e-01 9.542840e-01

10567 9.941110e-01 9.542744e-01

10568 9.941116e-01 9.542648e-01

10569 9.941122e-01 9.542550e-01

10570 9.941128e-01 9.542452e-01

10571 9.941133e-01 9.542353e-01

10572 9.941139e-01 9.542254e-01

10573 9.941145e-01 9.542154e-01

10574 9.941151e-01 9.542053e-01

10575 9.941157e-01 9.541951e-01

10576 9.941162e-01 9.541849e-01

10577 9.941168e-01 9.541745e-01

10578 9.941174e-01 9.541642e-01

10579 9.941180e-01 9.541537e-01

10580 9.941186e-01 9.541432e-01

10581 9.941192e-01 9.541326e-01

10582 9.941197e-01 9.541220e-01

10583 9.941203e-01 9.541112e-01

10584 9.941209e-01 9.541004e-01

10585 9.941215e-01 9.540895e-01

10586 9.941221e-01 9.540786e-01

10587 9.941227e-01 9.540676e-01

10588 9.941233e-01 9.540565e-01

10589 9.941239e-01 9.540454e-01

10590 9.941245e-01 9.540341e-01

10591 9.941251e-01 9.540229e-01

10592 9.941257e-01 9.540115e-01

10593 9.941263e-01 9.540001e-01

10594 9.941269e-01 9.539886e-01

10595 9.941275e-01 9.539770e-01

10596 9.941281e-01 9.539654e-01

10597 9.941287e-01 9.539537e-01

10598 9.941293e-01 9.539419e-01

10599 9.941299e-01 9.539301e-01

10600 9.941305e-01 9.539182e-01

10601 9.941311e-01 9.539062e-01

10602 9.941317e-01 9.538942e-01

10603 9.941323e-01 9.538821e-01

10604 9.941329e-01 9.538699e-01

10605 9.941335e-01 9.538577e-01

10606 9.941342e-01 9.538454e-01

10607 9.941348e-01 9.538330e-01

10608 9.941354e-01 9.538206e-01

10609 9.941360e-01 9.538081e-01

10610 9.941366e-01 9.537956e-01

10611 9.941372e-01 9.537830e-01

10612 9.941378e-01 9.537703e-01

10613 9.941385e-01 9.537575e-01

10614 9.941391e-01 9.537447e-01

10615 9.941397e-01 9.537319e-01

10616 9.941403e-01 9.537190e-01

10617 9.941409e-01 9.537060e-01

10618 9.941415e-01 9.536929e-01

10619 9.941422e-01 9.536798e-01

10620 9.941428e-01 9.536667e-01

10621 9.941434e-01 9.536534e-01

10622 9.941450e-01 9.536506e-01

10623 9.941466e-01 9.536477e-01

10624 9.941482e-01 9.536448e-01

10625 9.941497e-01 9.536419e-01

10626 9.941512e-01 9.536389e-01

10627 9.941527e-01 9.536359e-01

10628 9.941541e-01 9.536328e-01

10629 9.941555e-01 9.536298e-01

10630 9.941569e-01 9.536266e-01

10631 9.941583e-01 9.536235e-01

10632 9.941596e-01 9.536203e-01

10633 9.941609e-01 9.536170e-01

10634 9.941622e-01 9.536138e-01

10635 9.941635e-01 9.536105e-01

10636 9.941647e-01 9.536071e-01

10637 9.941660e-01 9.536037e-01

10638 9.941672e-01 9.536003e-01

10639 9.941683e-01 9.535968e-01

10640 9.941695e-01 9.535933e-01

10641 9.941706e-01 9.535898e-01

10642 9.941718e-01 9.535862e-01

10643 9.941729e-01 9.535826e-01

10644 9.941739e-01 9.535789e-01

10645 9.941750e-01 9.535752e-01

10646 9.941760e-01 9.535714e-01

10647 9.941771e-01 9.535676e-01

10648 9.941781e-01 9.535638e-01

10649 9.941791e-01 9.535599e-01

10650 9.941801e-01 9.535560e-01

10651 9.941810e-01 9.535520e-01

10652 9.941820e-01 9.535480e-01

10653 9.941829e-01 9.535439e-01

10654 9.941838e-01 9.535398e-01

10655 9.941847e-01 9.535357e-01

10656 9.941856e-01 9.535315e-01

10657 9.941865e-01 9.535272e-01

10658 9.941874e-01 9.535230e-01

10659 9.941882e-01 9.535186e-01

10660 9.941891e-01 9.535142e-01

10661 9.941899e-01 9.535098e-01

10662 9.941907e-01 9.535053e-01

10663 9.941915e-01 9.535008e-01

10664 9.941923e-01 9.534962e-01

10665 9.941931e-01 9.534916e-01

10666 9.941939e-01 9.534869e-01

10667 9.941947e-01 9.534822e-01

10668 9.941954e-01 9.534775e-01

10669 9.941962e-01 9.534726e-01

10670 9.941969e-01 9.534678e-01

10671 9.941976e-01 9.534628e-01

10672 9.941983e-01 9.534579e-01

10673 9.941991e-01 9.534528e-01

10674 9.941998e-01 9.534478e-01

10675 9.942005e-01 9.534426e-01

10676 9.942011e-01 9.534374e-01

10677 9.942018e-01 9.534322e-01

10678 9.942025e-01 9.534269e-01

10679 9.942032e-01 9.534216e-01

10680 9.942038e-01 9.534162e-01

10681 9.942045e-01 9.534107e-01

10682 9.942051e-01 9.534052e-01

10683 9.942058e-01 9.533997e-01

10684 9.942064e-01 9.533940e-01

10685 9.942070e-01 9.533884e-01

10686 9.942076e-01 9.533826e-01

10687 9.942083e-01 9.533769e-01

10688 9.942089e-01 9.533710e-01

10689 9.942095e-01 9.533651e-01

10690 9.942101e-01 9.533592e-01

10691 9.942107e-01 9.533531e-01

10692 9.942113e-01 9.533471e-01

10693 9.942119e-01 9.533409e-01

10694 9.942124e-01 9.533348e-01

10695 9.942130e-01 9.533285e-01

10696 9.942136e-01 9.533222e-01

10697 9.942142e-01 9.533158e-01

10698 9.942147e-01 9.533094e-01

10699 9.942153e-01 9.533029e-01

10700 9.942159e-01 9.532964e-01

10701 9.942164e-01 9.532897e-01

10702 9.942170e-01 9.532831e-01

10703 9.942175e-01 9.532763e-01

10704 9.942181e-01 9.532696e-01

10705 9.942186e-01 9.532627e-01

10706 9.942192e-01 9.532558e-01

10707 9.942197e-01 9.532488e-01

10708 9.942202e-01 9.532418e-01

10709 9.942208e-01 9.532346e-01

10710 9.942213e-01 9.532275e-01

10711 9.942219e-01 9.532202e-01

10712 9.942224e-01 9.532129e-01

10713 9.942229e-01 9.532056e-01

10714 9.942234e-01 9.531982e-01

10715 9.942240e-01 9.531907e-01

10716 9.942245e-01 9.531831e-01

10717 9.942250e-01 9.531755e-01

10718 9.942255e-01 9.531678e-01

10719 9.942260e-01 9.531600e-01

10720 9.942266e-01 9.531522e-01

10721 9.942271e-01 9.531443e-01

10722 9.942276e-01 9.531364e-01

10723 9.942281e-01 9.531284e-01

10724 9.942286e-01 9.531203e-01

10725 9.942291e-01 9.531121e-01

10726 9.942296e-01 9.531039e-01

10727 9.942301e-01 9.530957e-01

10728 9.942307e-01 9.530873e-01

10729 9.942312e-01 9.530789e-01

10730 9.942317e-01 9.530704e-01

10731 9.942322e-01 9.530618e-01

10732 9.942327e-01 9.530532e-01

10733 9.942332e-01 9.530445e-01

10734 9.942337e-01 9.530358e-01

10735 9.942342e-01 9.530270e-01

10736 9.942347e-01 9.530181e-01

10737 9.942352e-01 9.530091e-01

10738 9.942357e-01 9.530001e-01

10739 9.942362e-01 9.529910e-01

10740 9.942367e-01 9.529818e-01

10741 9.942372e-01 9.529726e-01

10742 9.942377e-01 9.529633e-01

10743 9.942383e-01 9.529539e-01

10744 9.942388e-01 9.529444e-01

10745 9.942393e-01 9.529349e-01

10746 9.942398e-01 9.529254e-01

10747 9.942403e-01 9.529157e-01

10748 9.942408e-01 9.529060e-01

10749 9.942413e-01 9.528962e-01

10750 9.942418e-01 9.528863e-01

10751 9.942423e-01 9.528764e-01

10752 9.942428e-01 9.528664e-01

10753 9.942433e-01 9.528564e-01

10754 9.942438e-01 9.528462e-01

10755 9.942444e-01 9.528360e-01

10756 9.942449e-01 9.528257e-01

10757 9.942454e-01 9.528154e-01

10758 9.942459e-01 9.528050e-01

10759 9.942464e-01 9.527945e-01

10760 9.942469e-01 9.527840e-01

10761 9.942474e-01 9.527734e-01

10762 9.942479e-01 9.527627e-01

10763 9.942485e-01 9.527519e-01

10764 9.942490e-01 9.527411e-01

10765 9.942495e-01 9.527302e-01

10766 9.942500e-01 9.527193e-01

10767 9.942505e-01 9.527082e-01

10768 9.942510e-01 9.526971e-01

10769 9.942516e-01 9.526860e-01

10770 9.942521e-01 9.526748e-01

10771 9.942526e-01 9.526635e-01

10772 9.942531e-01 9.526521e-01

10773 9.942536e-01 9.526407e-01

10774 9.942542e-01 9.526292e-01

10775 9.942547e-01 9.526176e-01

10776 9.942552e-01 9.526060e-01

10777 9.942557e-01 9.525943e-01

10778 9.942563e-01 9.525826e-01

10779 9.942568e-01 9.525708e-01

10780 9.942573e-01 9.525589e-01

10781 9.942578e-01 9.525469e-01

10782 9.942584e-01 9.525349e-01

10783 9.942589e-01 9.525228e-01

10784 9.942594e-01 9.525107e-01

10785 9.942599e-01 9.524985e-01

10786 9.942605e-01 9.524862e-01

10787 9.942610e-01 9.524739e-01

10788 9.942615e-01 9.524615e-01

10789 9.942620e-01 9.524491e-01

10790 9.942626e-01 9.524366e-01

10791 9.942631e-01 9.524240e-01

10792 9.942636e-01 9.524114e-01

10793 9.942642e-01 9.523987e-01

10794 9.942647e-01 9.523859e-01

10795 9.942652e-01 9.523731e-01

10796 9.942657e-01 9.523603e-01

10797 9.942663e-01 9.523473e-01

10798 9.942668e-01 9.523344e-01

10799 9.942673e-01 9.523213e-01

10800 9.942679e-01 9.523082e-01

10801 9.942684e-01 9.522951e-01

10802 9.942698e-01 9.522922e-01

10803 9.942712e-01 9.522892e-01

10804 9.942725e-01 9.522862e-01

10805 9.942739e-01 9.522832e-01

10806 9.942752e-01 9.522801e-01

10807 9.942764e-01 9.522770e-01

10808 9.942777e-01 9.522739e-01

10809 9.942789e-01 9.522707e-01

10810 9.942801e-01 9.522675e-01

10811 9.942813e-01 9.522642e-01

10812 9.942825e-01 9.522609e-01

10813 9.942836e-01 9.522576e-01

10814 9.942848e-01 9.522542e-01

10815 9.942859e-01 9.522508e-01

10816 9.942870e-01 9.522474e-01

10817 9.942880e-01 9.522439e-01

10818 9.942891e-01 9.522404e-01

10819 9.942901e-01 9.522368e-01

10820 9.942911e-01 9.522332e-01

10821 9.942921e-01 9.522296e-01

10822 9.942931e-01 9.522259e-01

10823 9.942941e-01 9.522221e-01

10824 9.942950e-01 9.522184e-01

10825 9.942960e-01 9.522146e-01

10826 9.942969e-01 9.522107e-01

10827 9.942978e-01 9.522068e-01

10828 9.942987e-01 9.522029e-01

10829 9.942995e-01 9.521989e-01

10830 9.943004e-01 9.521949e-01

10831 9.943013e-01 9.521908e-01

10832 9.943021e-01 9.521867e-01

10833 9.943029e-01 9.521825e-01

10834 9.943037e-01 9.521783e-01

10835 9.943045e-01 9.521741e-01

10836 9.943053e-01 9.521698e-01

10837 9.943061e-01 9.521654e-01

10838 9.943069e-01 9.521610e-01

10839 9.943076e-01 9.521566e-01

10840 9.943084e-01 9.521521e-01

10841 9.943091e-01 9.521476e-01

10842 9.943098e-01 9.521430e-01

10843 9.943105e-01 9.521384e-01

10844 9.943112e-01 9.521337e-01

10845 9.943119e-01 9.521290e-01

10846 9.943126e-01 9.521242e-01

10847 9.943133e-01 9.521194e-01

10848 9.943140e-01 9.521145e-01

10849 9.943146e-01 9.521096e-01

10850 9.943153e-01 9.521046e-01

10851 9.943159e-01 9.520996e-01

10852 9.943166e-01 9.520945e-01

10853 9.943172e-01 9.520894e-01

10854 9.943178e-01 9.520842e-01

10855 9.943184e-01 9.520790e-01

10856 9.943190e-01 9.520737e-01

10857 9.943196e-01 9.520684e-01

10858 9.943202e-01 9.520630e-01

10859 9.943208e-01 9.520575e-01

10860 9.943214e-01 9.520520e-01

10861 9.943220e-01 9.520465e-01

10862 9.943226e-01 9.520409e-01

10863 9.943231e-01 9.520352e-01

10864 9.943237e-01 9.520295e-01

10865 9.943243e-01 9.520237e-01

10866 9.943248e-01 9.520179e-01

10867 9.943254e-01 9.520120e-01

10868 9.943259e-01 9.520061e-01

10869 9.943265e-01 9.520001e-01

10870 9.943270e-01 9.519940e-01

10871 9.943275e-01 9.519879e-01

10872 9.943280e-01 9.519818e-01

10873 9.943286e-01 9.519755e-01

10874 9.943291e-01 9.519692e-01

10875 9.943296e-01 9.519629e-01

10876 9.943301e-01 9.519565e-01

10877 9.943306e-01 9.519500e-01

10878 9.943311e-01 9.519435e-01

10879 9.943316e-01 9.519369e-01

10880 9.943321e-01 9.519303e-01

10881 9.943326e-01 9.519236e-01

10882 9.943331e-01 9.519168e-01

10883 9.943336e-01 9.519100e-01

10884 9.943341e-01 9.519031e-01

10885 9.943346e-01 9.518962e-01

10886 9.943351e-01 9.518892e-01

10887 9.943356e-01 9.518821e-01

10888 9.943360e-01 9.518750e-01

10889 9.943365e-01 9.518678e-01

10890 9.943370e-01 9.518606e-01

10891 9.943375e-01 9.518532e-01

10892 9.943379e-01 9.518459e-01

10893 9.943384e-01 9.518384e-01

10894 9.943389e-01 9.518309e-01

10895 9.943393e-01 9.518233e-01

10896 9.943398e-01 9.518157e-01

10897 9.943403e-01 9.518080e-01

10898 9.943407e-01 9.518002e-01

10899 9.943412e-01 9.517924e-01

10900 9.943416e-01 9.517845e-01

10901 9.943421e-01 9.517766e-01

10902 9.943426e-01 9.517686e-01

10903 9.943430e-01 9.517605e-01

10904 9.943435e-01 9.517523e-01

10905 9.943439e-01 9.517441e-01

10906 9.943444e-01 9.517358e-01

10907 9.943448e-01 9.517275e-01

10908 9.943453e-01 9.517191e-01

10909 9.943457e-01 9.517106e-01

10910 9.943462e-01 9.517020e-01

10911 9.943466e-01 9.516934e-01

10912 9.943471e-01 9.516847e-01

10913 9.943475e-01 9.516760e-01

10914 9.943480e-01 9.516672e-01

10915 9.943484e-01 9.516583e-01

10916 9.943489e-01 9.516494e-01

10917 9.943493e-01 9.516404e-01

10918 9.943498e-01 9.516313e-01

10919 9.943502e-01 9.516221e-01

10920 9.943507e-01 9.516129e-01

10921 9.943511e-01 9.516036e-01

10922 9.943515e-01 9.515943e-01

10923 9.943520e-01 9.515849e-01

10924 9.943524e-01 9.515754e-01

10925 9.943529e-01 9.515658e-01

10926 9.943533e-01 9.515562e-01

10927 9.943538e-01 9.515465e-01

10928 9.943542e-01 9.515368e-01

10929 9.943547e-01 9.515270e-01

10930 9.943551e-01 9.515171e-01

10931 9.943556e-01 9.515071e-01

10932 9.943560e-01 9.514971e-01

10933 9.943564e-01 9.514870e-01

10934 9.943569e-01 9.514769e-01

10935 9.943573e-01 9.514667e-01

10936 9.943578e-01 9.514564e-01

10937 9.943582e-01 9.514460e-01

10938 9.943587e-01 9.514356e-01

10939 9.943591e-01 9.514251e-01

10940 9.943596e-01 9.514145e-01

10941 9.943600e-01 9.514039e-01

10942 9.943605e-01 9.513932e-01

10943 9.943609e-01 9.513825e-01

10944 9.943614e-01 9.513717e-01

10945 9.943618e-01 9.513608e-01

10946 9.943623e-01 9.513498e-01

10947 9.943627e-01 9.513388e-01

10948 9.943632e-01 9.513277e-01

10949 9.943636e-01 9.513166e-01

10950 9.943641e-01 9.513054e-01

10951 9.943645e-01 9.512941e-01

10952 9.943650e-01 9.512828e-01

10953 9.943654e-01 9.512714e-01

10954 9.943659e-01 9.512599e-01

10955 9.943663e-01 9.512484e-01

10956 9.943668e-01 9.512368e-01

10957 9.943672e-01 9.512251e-01

10958 9.943677e-01 9.512134e-01

10959 9.943681e-01 9.512016e-01

10960 9.943686e-01 9.511898e-01

10961 9.943690e-01 9.511778e-01

10962 9.943695e-01 9.511659e-01

10963 9.943699e-01 9.511539e-01

10964 9.943704e-01 9.511418e-01

10965 9.943708e-01 9.511296e-01

10966 9.943713e-01 9.511174e-01

10967 9.943717e-01 9.511051e-01

10968 9.943722e-01 9.510928e-01

10969 9.943726e-01 9.510804e-01

10970 9.943730e-01 9.510680e-01

10971 9.943735e-01 9.510555e-01

10972 9.943739e-01 9.510429e-01

10973 9.943744e-01 9.510303e-01

10974 9.943748e-01 9.510176e-01

10975 9.943753e-01 9.510049e-01

10976 9.943757e-01 9.509921e-01

10977 9.943762e-01 9.509793e-01

10978 9.943766e-01 9.509664e-01

10979 9.943770e-01 9.509535e-01

10980 9.943775e-01 9.509405e-01

10981 9.943779e-01 9.509274e-01

10982 9.943791e-01 9.509244e-01

10983 9.943803e-01 9.509214e-01

10984 9.943815e-01 9.509183e-01

10985 9.943827e-01 9.509152e-01

10986 9.943838e-01 9.509120e-01

10987 9.943849e-01 9.509088e-01

10988 9.943860e-01 9.509056e-01

10989 9.943871e-01 9.509023e-01

10990 9.943882e-01 9.508990e-01

10991 9.943892e-01 9.508957e-01

10992 9.943902e-01 9.508923e-01

10993 9.943912e-01 9.508889e-01

10994 9.943922e-01 9.508854e-01

10995 9.943932e-01 9.508819e-01

10996 9.943942e-01 9.508784e-01

10997 9.943951e-01 9.508748e-01

10998 9.943960e-01 9.508712e-01

10999 9.943969e-01 9.508676e-01

11000 9.943978e-01 9.508639e-01

11001 9.943987e-01 9.508601e-01

11002 9.943995e-01 9.508564e-01

11003 9.944004e-01 9.508525e-01

11004 9.944012e-01 9.508487e-01

11005 9.944020e-01 9.508448e-01

11006 9.944029e-01 9.508408e-01

11007 9.944037e-01 9.508368e-01

11008 9.944044e-01 9.508328e-01

11009 9.944052e-01 9.508287e-01

11010 9.944060e-01 9.508246e-01

11011 9.944067e-01 9.508204e-01

11012 9.944075e-01 9.508162e-01

11013 9.944082e-01 9.508120e-01

11014 9.944089e-01 9.508077e-01

11015 9.944096e-01 9.508033e-01

11016 9.944103e-01 9.507990e-01

11017 9.944110e-01 9.507945e-01

11018 9.944117e-01 9.507900e-01

11019 9.944123e-01 9.507855e-01

11020 9.944130e-01 9.507809e-01

11021 9.944136e-01 9.507763e-01

11022 9.944143e-01 9.507716e-01

11023 9.944149e-01 9.507669e-01

11024 9.944155e-01 9.507622e-01

11025 9.944161e-01 9.507573e-01

11026 9.944167e-01 9.507525e-01

11027 9.944173e-01 9.507476e-01

11028 9.944179e-01 9.507426e-01

11029 9.944185e-01 9.507376e-01

11030 9.944191e-01 9.507325e-01

11031 9.944197e-01 9.507274e-01

11032 9.944202e-01 9.507222e-01

11033 9.944208e-01 9.507170e-01

11034 9.944214e-01 9.507118e-01

11035 9.944219e-01 9.507064e-01

11036 9.944225e-01 9.507011e-01

11037 9.944230e-01 9.506956e-01

11038 9.944235e-01 9.506902e-01

11039 9.944240e-01 9.506846e-01

11040 9.944246e-01 9.506790e-01

11041 9.944251e-01 9.506734e-01

11042 9.944256e-01 9.506677e-01

11043 9.944261e-01 9.506620e-01

11044 9.944266e-01 9.506562e-01

11045 9.944271e-01 9.506503e-01

11046 9.944276e-01 9.506444e-01

11047 9.944281e-01 9.506384e-01

11048 9.944286e-01 9.506324e-01

11049 9.944290e-01 9.506263e-01

11050 9.944295e-01 9.506202e-01

11051 9.944300e-01 9.506140e-01

11052 9.944305e-01 9.506077e-01

11053 9.944309e-01 9.506014e-01

11054 9.944314e-01 9.505950e-01

11055 9.944318e-01 9.505886e-01

11056 9.944323e-01 9.505821e-01

11057 9.944327e-01 9.505756e-01

11058 9.944332e-01 9.505690e-01

11059 9.944336e-01 9.505623e-01

11060 9.944341e-01 9.505556e-01

11061 9.944345e-01 9.505488e-01

11062 9.944350e-01 9.505420e-01

11063 9.944354e-01 9.505351e-01

11064 9.944358e-01 9.505282e-01

11065 9.944363e-01 9.505211e-01

11066 9.944367e-01 9.505141e-01

11067 9.944371e-01 9.505069e-01

11068 9.944376e-01 9.504997e-01

11069 9.944380e-01 9.504925e-01

11070 9.944384e-01 9.504851e-01

11071 9.944388e-01 9.504778e-01

11072 9.944392e-01 9.504703e-01

11073 9.944397e-01 9.504628e-01

11074 9.944401e-01 9.504552e-01

11075 9.944405e-01 9.504476e-01

11076 9.944409e-01 9.504399e-01

11077 9.944413e-01 9.504321e-01

11078 9.944417e-01 9.504243e-01

11079 9.944421e-01 9.504164e-01

11080 9.944425e-01 9.504085e-01

11081 9.944429e-01 9.504005e-01

11082 9.944433e-01 9.503924e-01

11083 9.944437e-01 9.503843e-01

11084 9.944441e-01 9.503761e-01

11085 9.944445e-01 9.503678e-01

11086 9.944449e-01 9.503595e-01

11087 9.944453e-01 9.503511e-01

11088 9.944457e-01 9.503426e-01

11089 9.944461e-01 9.503341e-01

11090 9.944465e-01 9.503255e-01

11091 9.944469e-01 9.503168e-01

11092 9.944473e-01 9.503081e-01

11093 9.944477e-01 9.502993e-01

11094 9.944481e-01 9.502905e-01

11095 9.944485e-01 9.502816e-01

11096 9.944489e-01 9.502726e-01

11097 9.944493e-01 9.502635e-01

11098 9.944497e-01 9.502544e-01

11099 9.944501e-01 9.502452e-01

11100 9.944505e-01 9.502360e-01

11101 9.944509e-01 9.502267e-01

11102 9.944512e-01 9.502173e-01

11103 9.944516e-01 9.502079e-01

11104 9.944520e-01 9.501984e-01

11105 9.944524e-01 9.501888e-01

11106 9.944528e-01 9.501792e-01

11107 9.944532e-01 9.501695e-01

11108 9.944536e-01 9.501597e-01

11109 9.944540e-01 9.501499e-01

11110 9.944544e-01 9.501400e-01

11111 9.944548e-01 9.501300e-01

11112 9.944551e-01 9.501200e-01

11113 9.944555e-01 9.501099e-01

11114 9.944559e-01 9.500998e-01

11115 9.944563e-01 9.500895e-01

11116 9.944567e-01 9.500792e-01

11117 9.944571e-01 9.500689e-01

11118 9.944575e-01 9.500585e-01

11119 9.944579e-01 9.500480e-01

11120 9.944582e-01 9.500375e-01

11121 9.944586e-01 9.500269e-01

11122 9.944590e-01 9.500162e-01

11123 9.944594e-01 9.500054e-01

11124 9.944598e-01 9.499946e-01

11125 9.944602e-01 9.499838e-01

11126 9.944605e-01 9.499729e-01

11127 9.944609e-01 9.499619e-01

11128 9.944613e-01 9.499508e-01

11129 9.944617e-01 9.499397e-01

11130 9.944621e-01 9.499285e-01

11131 9.944625e-01 9.499173e-01

11132 9.944629e-01 9.499060e-01

11133 9.944632e-01 9.498946e-01

11134 9.944636e-01 9.498832e-01

11135 9.944640e-01 9.498717e-01

11136 9.944644e-01 9.498602e-01

11137 9.944648e-01 9.498486e-01

11138 9.944651e-01 9.498369e-01

11139 9.944655e-01 9.498252e-01

11140 9.944659e-01 9.498134e-01

11141 9.944663e-01 9.498016e-01

11142 9.944666e-01 9.497897e-01

11143 9.944670e-01 9.497777e-01

11144 9.944674e-01 9.497657e-01

11145 9.944678e-01 9.497536e-01

11146 9.944681e-01 9.497415e-01

11147 9.944685e-01 9.497293e-01

11148 9.944689e-01 9.497171e-01

11149 9.944692e-01 9.497048e-01

11150 9.944696e-01 9.496924e-01

11151 9.944700e-01 9.496800e-01

11152 9.944703e-01 9.496676e-01

11153 9.944707e-01 9.496551e-01

11154 9.944711e-01 9.496425e-01

11155 9.944714e-01 9.496299e-01

11156 9.944718e-01 9.496172e-01

11157 9.944721e-01 9.496045e-01

11158 9.944725e-01 9.495917e-01

11159 9.944728e-01 9.495789e-01

11160 9.944732e-01 9.495660e-01

11161 9.944735e-01 9.495531e-01

11162 9.944746e-01 9.495500e-01

11163 9.944757e-01 9.495469e-01

11164 9.944767e-01 9.495437e-01

11165 9.944777e-01 9.495405e-01

11166 9.944787e-01 9.495373e-01

11167 9.944797e-01 9.495340e-01

11168 9.944806e-01 9.495307e-01

11169 9.944816e-01 9.495274e-01

11170 9.944825e-01 9.495240e-01

11171 9.944834e-01 9.495206e-01

11172 9.944843e-01 9.495171e-01

11173 9.944852e-01 9.495136e-01

11174 9.944861e-01 9.495101e-01

11175 9.944869e-01 9.495065e-01

11176 9.944878e-01 9.495029e-01

11177 9.944886e-01 9.494992e-01

11178 9.944894e-01 9.494955e-01

11179 9.944902e-01 9.494918e-01

11180 9.944910e-01 9.494880e-01

11181 9.944917e-01 9.494842e-01

11182 9.944925e-01 9.494803e-01

11183 9.944932e-01 9.494764e-01

11184 9.944940e-01 9.494725e-01

11185 9.944947e-01 9.494685e-01

11186 9.944954e-01 9.494644e-01

11187 9.944961e-01 9.494604e-01

11188 9.944968e-01 9.494563e-01

11189 9.944975e-01 9.494521e-01

11190 9.944981e-01 9.494479e-01

11191 9.944988e-01 9.494436e-01

11192 9.944995e-01 9.494394e-01

11193 9.945001e-01 9.494350e-01

11194 9.945007e-01 9.494306e-01

11195 9.945014e-01 9.494262e-01

11196 9.945020e-01 9.494217e-01

11197 9.945026e-01 9.494172e-01

11198 9.945032e-01 9.494127e-01

11199 9.945038e-01 9.494080e-01

11200 9.945043e-01 9.494034e-01

11201 9.945049e-01 9.493987e-01

11202 9.945055e-01 9.493939e-01

11203 9.945060e-01 9.493891e-01

11204 9.945066e-01 9.493843e-01

11205 9.945071e-01 9.493794e-01

11206 9.945077e-01 9.493744e-01

11207 9.945082e-01 9.493694e-01

11208 9.945087e-01 9.493644e-01

11209 9.945093e-01 9.493593e-01

11210 9.945098e-01 9.493541e-01

11211 9.945103e-01 9.493489e-01

11212 9.945108e-01 9.493437e-01

11213 9.945113e-01 9.493384e-01

11214 9.945118e-01 9.493331e-01

11215 9.945123e-01 9.493276e-01

11216 9.945127e-01 9.493222e-01

11217 9.945132e-01 9.493167e-01

11218 9.945137e-01 9.493111e-01

11219 9.945142e-01 9.493055e-01

11220 9.945146e-01 9.492999e-01

11221 9.945151e-01 9.492941e-01

11222 9.945155e-01 9.492884e-01

11223 9.945160e-01 9.492825e-01

11224 9.945164e-01 9.492767e-01

11225 9.945169e-01 9.492707e-01

11226 9.945173e-01 9.492648e-01

11227 9.945177e-01 9.492587e-01

11228 9.945182e-01 9.492526e-01

11229 9.945186e-01 9.492465e-01

11230 9.945190e-01 9.492402e-01

11231 9.945194e-01 9.492340e-01

11232 9.945199e-01 9.492277e-01

11233 9.945203e-01 9.492213e-01

11234 9.945207e-01 9.492148e-01

11235 9.945211e-01 9.492084e-01

11236 9.945215e-01 9.492018e-01

11237 9.945219e-01 9.491952e-01

11238 9.945223e-01 9.491885e-01

11239 9.945227e-01 9.491818e-01

11240 9.945231e-01 9.491750e-01

11241 9.945235e-01 9.491682e-01

11242 9.945239e-01 9.491613e-01

11243 9.945242e-01 9.491543e-01

11244 9.945246e-01 9.491473e-01

11245 9.945250e-01 9.491402e-01

11246 9.945254e-01 9.491331e-01

11247 9.945258e-01 9.491259e-01

11248 9.945262e-01 9.491187e-01

11249 9.945265e-01 9.491113e-01

11250 9.945269e-01 9.491040e-01

11251 9.945273e-01 9.490965e-01

11252 9.945276e-01 9.490890e-01

11253 9.945280e-01 9.490815e-01

11254 9.945284e-01 9.490738e-01

11255 9.945287e-01 9.490662e-01

11256 9.945291e-01 9.490584e-01

11257 9.945295e-01 9.490506e-01

11258 9.945298e-01 9.490428e-01

11259 9.945302e-01 9.490348e-01

11260 9.945305e-01 9.490268e-01

11261 9.945309e-01 9.490188e-01

11262 9.945313e-01 9.490107e-01

11263 9.945316e-01 9.490025e-01

11264 9.945320e-01 9.489943e-01

11265 9.945323e-01 9.489860e-01

11266 9.945327e-01 9.489776e-01

11267 9.945330e-01 9.489692e-01

11268 9.945334e-01 9.489607e-01

11269 9.945337e-01 9.489521e-01

11270 9.945341e-01 9.489435e-01

11271 9.945344e-01 9.489348e-01

11272 9.945348e-01 9.489261e-01

11273 9.945351e-01 9.489173e-01

11274 9.945354e-01 9.489084e-01

11275 9.945358e-01 9.488995e-01

11276 9.945361e-01 9.488905e-01

11277 9.945365e-01 9.488814e-01

11278 9.945368e-01 9.488723e-01

11279 9.945372e-01 9.488631e-01

11280 9.945375e-01 9.488539e-01

11281 9.945378e-01 9.488445e-01

11282 9.945382e-01 9.488352e-01

11283 9.945385e-01 9.488257e-01

11284 9.945389e-01 9.488162e-01

11285 9.945392e-01 9.488066e-01

11286 9.945395e-01 9.487970e-01

11287 9.945399e-01 9.487873e-01

11288 9.945402e-01 9.487776e-01

11289 9.945405e-01 9.487677e-01

11290 9.945409e-01 9.487579e-01

11291 9.945412e-01 9.487479e-01

11292 9.945415e-01 9.487379e-01

11293 9.945419e-01 9.487278e-01

11294 9.945422e-01 9.487177e-01

11295 9.945425e-01 9.487075e-01

11296 9.945429e-01 9.486972e-01

11297 9.945432e-01 9.486869e-01

11298 9.945435e-01 9.486765e-01

11299 9.945438e-01 9.486661e-01

11300 9.945442e-01 9.486556e-01

11301 9.945445e-01 9.486450e-01

11302 9.945448e-01 9.486344e-01

11303 9.945452e-01 9.486237e-01

11304 9.945455e-01 9.486129e-01

11305 9.945458e-01 9.486021e-01

11306 9.945461e-01 9.485912e-01

11307 9.945464e-01 9.485803e-01

11308 9.945468e-01 9.485693e-01

11309 9.945471e-01 9.485582e-01

11310 9.945474e-01 9.485471e-01

11311 9.945477e-01 9.485360e-01

11312 9.945480e-01 9.485247e-01

11313 9.945484e-01 9.485134e-01

11314 9.945487e-01 9.485021e-01

11315 9.945490e-01 9.484907e-01

11316 9.945493e-01 9.484792e-01

11317 9.945496e-01 9.484677e-01

11318 9.945499e-01 9.484561e-01

11319 9.945502e-01 9.484445e-01

11320 9.945505e-01 9.484328e-01

11321 9.945508e-01 9.484210e-01

11322 9.945511e-01 9.484092e-01

11323 9.945514e-01 9.483974e-01

11324 9.945517e-01 9.483855e-01

11325 9.945520e-01 9.483735e-01

11326 9.945523e-01 9.483615e-01

11327 9.945526e-01 9.483494e-01

11328 9.945529e-01 9.483373e-01

11329 9.945532e-01 9.483251e-01

11330 9.945535e-01 9.483129e-01

11331 9.945538e-01 9.483006e-01

11332 9.945540e-01 9.482883e-01

11333 9.945543e-01 9.482759e-01

11334 9.945546e-01 9.482635e-01

11335 9.945549e-01 9.482510e-01

11336 9.945551e-01 9.482384e-01

11337 9.945554e-01 9.482259e-01

11338 9.945557e-01 9.482133e-01

11339 9.945559e-01 9.482006e-01

11340 9.945562e-01 9.481879e-01

11341 9.945564e-01 9.481751e-01

11342 9.945574e-01 9.481719e-01

11343 9.945583e-01 9.481687e-01

11344 9.945592e-01 9.481655e-01

11345 9.945601e-01 9.481622e-01

11346 9.945610e-01 9.481589e-01

11347 9.945618e-01 9.481556e-01

11348 9.945627e-01 9.481522e-01

11349 9.945635e-01 9.481488e-01

11350 9.945643e-01 9.481453e-01

11351 9.945651e-01 9.481418e-01

11352 9.945659e-01 9.481383e-01

11353 9.945667e-01 9.481347e-01

11354 9.945674e-01 9.481311e-01

11355 9.945682e-01 9.481274e-01

11356 9.945689e-01 9.481237e-01

11357 9.945696e-01 9.481200e-01

11358 9.945703e-01 9.481162e-01

11359 9.945710e-01 9.481124e-01

11360 9.945717e-01 9.481086e-01

11361 9.945724e-01 9.481047e-01

11362 9.945731e-01 9.481007e-01

11363 9.945737e-01 9.480967e-01

11364 9.945744e-01 9.480927e-01

11365 9.945750e-01 9.480887e-01

11366 9.945756e-01 9.480846e-01

11367 9.945763e-01 9.480804e-01

11368 9.945769e-01 9.480762e-01

11369 9.945775e-01 9.480720e-01

11370 9.945781e-01 9.480677e-01

11371 9.945786e-01 9.480634e-01

11372 9.945792e-01 9.480590e-01

11373 9.945798e-01 9.480546e-01

11374 9.945803e-01 9.480501e-01

11375 9.945809e-01 9.480456e-01

11376 9.945814e-01 9.480411e-01

11377 9.945820e-01 9.480365e-01

11378 9.945825e-01 9.480319e-01

11379 9.945830e-01 9.480272e-01

11380 9.945835e-01 9.480224e-01

11381 9.945841e-01 9.480177e-01

11382 9.945846e-01 9.480128e-01

11383 9.945851e-01 9.480080e-01

11384 9.945855e-01 9.480030e-01

11385 9.945860e-01 9.479981e-01

11386 9.945865e-01 9.479930e-01

11387 9.945870e-01 9.479880e-01

11388 9.945874e-01 9.479829e-01

11389 9.945879e-01 9.479777e-01

11390 9.945884e-01 9.479725e-01

11391 9.945888e-01 9.479672e-01

11392 9.945893e-01 9.479619e-01

11393 9.945897e-01 9.479565e-01

11394 9.945901e-01 9.479511e-01

11395 9.945906e-01 9.479456e-01

11396 9.945910e-01 9.479401e-01

11397 9.945914e-01 9.479345e-01

11398 9.945918e-01 9.479289e-01

11399 9.945923e-01 9.479232e-01

11400 9.945927e-01 9.479175e-01

11401 9.945931e-01 9.479117e-01

11402 9.945935e-01 9.479059e-01

11403 9.945939e-01 9.479000e-01

11404 9.945943e-01 9.478941e-01

11405 9.945947e-01 9.478881e-01

11406 9.945950e-01 9.478820e-01

11407 9.945954e-01 9.478759e-01

11408 9.945958e-01 9.478698e-01

11409 9.945962e-01 9.478636e-01

11410 9.945966e-01 9.478573e-01

11411 9.945969e-01 9.478510e-01

11412 9.945973e-01 9.478446e-01

11413 9.945977e-01 9.478382e-01

11414 9.945980e-01 9.478317e-01

11415 9.945984e-01 9.478251e-01

11416 9.945988e-01 9.478185e-01

11417 9.945991e-01 9.478119e-01

11418 9.945995e-01 9.478051e-01

11419 9.945998e-01 9.477984e-01

11420 9.946002e-01 9.477915e-01

11421 9.946005e-01 9.477846e-01

11422 9.946009e-01 9.477777e-01

11423 9.946012e-01 9.477707e-01

11424 9.946015e-01 9.477636e-01

11425 9.946019e-01 9.477565e-01

11426 9.946022e-01 9.477493e-01

11427 9.946025e-01 9.477421e-01

11428 9.946029e-01 9.477348e-01

11429 9.946032e-01 9.477274e-01

11430 9.946035e-01 9.477200e-01

11431 9.946039e-01 9.477126e-01

11432 9.946042e-01 9.477050e-01

11433 9.946045e-01 9.476974e-01

11434 9.946048e-01 9.476898e-01

11435 9.946052e-01 9.476821e-01

11436 9.946055e-01 9.476743e-01

11437 9.946058e-01 9.476665e-01

11438 9.946061e-01 9.476586e-01

11439 9.946064e-01 9.476506e-01

11440 9.946067e-01 9.476426e-01

11441 9.946070e-01 9.476345e-01

11442 9.946074e-01 9.476264e-01

11443 9.946077e-01 9.476182e-01

11444 9.946080e-01 9.476099e-01

11445 9.946083e-01 9.476016e-01

11446 9.946086e-01 9.475932e-01

11447 9.946089e-01 9.475848e-01

11448 9.946092e-01 9.475763e-01

11449 9.946095e-01 9.475677e-01

11450 9.946098e-01 9.475591e-01

11451 9.946101e-01 9.475504e-01

11452 9.946104e-01 9.475417e-01

11453 9.946107e-01 9.475329e-01

11454 9.946110e-01 9.475240e-01

11455 9.946113e-01 9.475151e-01

11456 9.946116e-01 9.475061e-01

11457 9.946119e-01 9.474970e-01

11458 9.946122e-01 9.474879e-01

11459 9.946125e-01 9.474787e-01

11460 9.946128e-01 9.474695e-01

11461 9.946130e-01 9.474602e-01

11462 9.946133e-01 9.474508e-01

11463 9.946136e-01 9.474414e-01

11464 9.946139e-01 9.474319e-01

11465 9.946142e-01 9.474224e-01

11466 9.946145e-01 9.474127e-01

11467 9.946148e-01 9.474031e-01

11468 9.946151e-01 9.473933e-01

11469 9.946153e-01 9.473836e-01

11470 9.946156e-01 9.473737e-01

11471 9.946159e-01 9.473638e-01

11472 9.946162e-01 9.473538e-01

11473 9.946165e-01 9.473438e-01

11474 9.946167e-01 9.473337e-01

11475 9.946170e-01 9.473235e-01

11476 9.946173e-01 9.473133e-01

11477 9.946176e-01 9.473031e-01

11478 9.946178e-01 9.472927e-01

11479 9.946181e-01 9.472823e-01

11480 9.946184e-01 9.472719e-01

11481 9.946186e-01 9.472614e-01

11482 9.946189e-01 9.472508e-01

11483 9.946192e-01 9.472402e-01

11484 9.946194e-01 9.472295e-01

11485 9.946197e-01 9.472187e-01

11486 9.946200e-01 9.472079e-01

11487 9.946202e-01 9.471971e-01

11488 9.946205e-01 9.471862e-01

11489 9.946207e-01 9.471752e-01

11490 9.946210e-01 9.471642e-01

11491 9.946212e-01 9.471531e-01

11492 9.946215e-01 9.471419e-01

11493 9.946217e-01 9.471307e-01

11494 9.946220e-01 9.471195e-01

11495 9.946222e-01 9.471082e-01

11496 9.946225e-01 9.470968e-01

11497 9.946227e-01 9.470854e-01

11498 9.946230e-01 9.470739e-01

11499 9.946232e-01 9.470624e-01

11500 9.946234e-01 9.470508e-01

11501 9.946237e-01 9.470392e-01

11502 9.946239e-01 9.470275e-01

11503 9.946241e-01 9.470158e-01

11504 9.946243e-01 9.470040e-01

11505 9.946246e-01 9.469922e-01

11506 9.946248e-01 9.469803e-01

11507 9.946250e-01 9.469684e-01

11508 9.946252e-01 9.469564e-01

11509 9.946254e-01 9.469443e-01

11510 9.946256e-01 9.469323e-01

11511 9.946258e-01 9.469201e-01

11512 9.946260e-01 9.469080e-01

11513 9.946262e-01 9.468957e-01

11514 9.946264e-01 9.468835e-01

11515 9.946266e-01 9.468712e-01

11516 9.946267e-01 9.468588e-01

11517 9.946269e-01 9.468464e-01

11518 9.946271e-01 9.468340e-01

11519 9.946273e-01 9.468215e-01

11520 9.946274e-01 9.468090e-01

11521 9.946276e-01 9.467964e-01

11522 9.946284e-01 9.467932e-01

11523 9.946292e-01 9.467899e-01

11524 9.946300e-01 9.467866e-01

11525 9.946308e-01 9.467832e-01

11526 9.946315e-01 9.467799e-01

11527 9.946323e-01 9.467764e-01

11528 9.946330e-01 9.467730e-01

11529 9.946338e-01 9.467695e-01

11530 9.946345e-01 9.467660e-01

11531 9.946352e-01 9.467624e-01

11532 9.946359e-01 9.467588e-01

11533 9.946366e-01 9.467552e-01

11534 9.946372e-01 9.467515e-01

11535 9.946379e-01 9.467477e-01

11536 9.946385e-01 9.467440e-01

11537 9.946392e-01 9.467402e-01

11538 9.946398e-01 9.467363e-01

11539 9.946404e-01 9.467325e-01

11540 9.946410e-01 9.467285e-01

11541 9.946416e-01 9.467246e-01

11542 9.946422e-01 9.467206e-01

11543 9.946428e-01 9.467165e-01

11544 9.946434e-01 9.467124e-01

11545 9.946439e-01 9.467083e-01

11546 9.946445e-01 9.467041e-01

11547 9.946450e-01 9.466999e-01

11548 9.946456e-01 9.466957e-01

11549 9.946461e-01 9.466914e-01

11550 9.946466e-01 9.466870e-01

11551 9.946471e-01 9.466826e-01

11552 9.946476e-01 9.466782e-01

11553 9.946482e-01 9.466737e-01

11554 9.946486e-01 9.466692e-01

11555 9.946491e-01 9.466646e-01

11556 9.946496e-01 9.466600e-01

11557 9.946501e-01 9.466554e-01

11558 9.946506e-01 9.466507e-01

11559 9.946510e-01 9.466459e-01

11560 9.946515e-01 9.466411e-01

11561 9.946519e-01 9.466363e-01

11562 9.946524e-01 9.466314e-01

11563 9.946528e-01 9.466264e-01

11564 9.946532e-01 9.466215e-01

11565 9.946537e-01 9.466164e-01

11566 9.946541e-01 9.466113e-01

11567 9.946545e-01 9.466062e-01

11568 9.946549e-01 9.466010e-01

11569 9.946553e-01 9.465958e-01

11570 9.946557e-01 9.465905e-01

11571 9.946561e-01 9.465852e-01

11572 9.946565e-01 9.465798e-01

11573 9.946569e-01 9.465744e-01

11574 9.946573e-01 9.465689e-01

11575 9.946577e-01 9.465634e-01

11576 9.946581e-01 9.465578e-01

11577 9.946585e-01 9.465522e-01

11578 9.946588e-01 9.465465e-01

11579 9.946592e-01 9.465408e-01

11580 9.946596e-01 9.465350e-01

11581 9.946599e-01 9.465292e-01

11582 9.946603e-01 9.465233e-01

11583 9.946606e-01 9.465174e-01

11584 9.946610e-01 9.465114e-01

11585 9.946613e-01 9.465053e-01

11586 9.946617e-01 9.464992e-01

11587 9.946620e-01 9.464931e-01

11588 9.946623e-01 9.464869e-01

11589 9.946627e-01 9.464806e-01

11590 9.946630e-01 9.464743e-01

11591 9.946633e-01 9.464679e-01

11592 9.946637e-01 9.464615e-01

11593 9.946640e-01 9.464550e-01

11594 9.946643e-01 9.464485e-01

11595 9.946646e-01 9.464419e-01

11596 9.946649e-01 9.464353e-01

11597 9.946653e-01 9.464286e-01

11598 9.946656e-01 9.464218e-01

11599 9.946659e-01 9.464150e-01

11600 9.946662e-01 9.464082e-01

11601 9.946665e-01 9.464012e-01

11602 9.946668e-01 9.463943e-01

11603 9.946671e-01 9.463872e-01

11604 9.946674e-01 9.463801e-01

11605 9.946677e-01 9.463730e-01

11606 9.946680e-01 9.463658e-01

11607 9.946683e-01 9.463585e-01

11608 9.946686e-01 9.463512e-01

11609 9.946688e-01 9.463438e-01

11610 9.946691e-01 9.463364e-01

11611 9.946694e-01 9.463289e-01

11612 9.946697e-01 9.463213e-01

11613 9.946700e-01 9.463137e-01

11614 9.946703e-01 9.463061e-01

11615 9.946705e-01 9.462983e-01

11616 9.946708e-01 9.462905e-01

11617 9.946711e-01 9.462827e-01

11618 9.946714e-01 9.462748e-01

11619 9.946716e-01 9.462668e-01

11620 9.946719e-01 9.462588e-01

11621 9.946722e-01 9.462507e-01

11622 9.946725e-01 9.462426e-01

11623 9.946727e-01 9.462344e-01

11624 9.946730e-01 9.462261e-01

11625 9.946733e-01 9.462178e-01

11626 9.946735e-01 9.462094e-01

11627 9.946738e-01 9.462010e-01

11628 9.946740e-01 9.461925e-01

11629 9.946743e-01 9.461840e-01

11630 9.946746e-01 9.461754e-01

11631 9.946748e-01 9.461667e-01

11632 9.946751e-01 9.461579e-01

11633 9.946753e-01 9.461492e-01

11634 9.946756e-01 9.461403e-01

11635 9.946758e-01 9.461314e-01

11636 9.946761e-01 9.461224e-01

11637 9.946763e-01 9.461134e-01

11638 9.946766e-01 9.461043e-01

11639 9.946768e-01 9.460952e-01

11640 9.946771e-01 9.460860e-01

11641 9.946773e-01 9.460767e-01

11642 9.946775e-01 9.460674e-01

11643 9.946778e-01 9.460580e-01

11644 9.946780e-01 9.460485e-01

11645 9.946783e-01 9.460390e-01

11646 9.946785e-01 9.460295e-01

11647 9.946787e-01 9.460199e-01

11648 9.946790e-01 9.460102e-01

11649 9.946792e-01 9.460004e-01

11650 9.946794e-01 9.459906e-01

11651 9.946797e-01 9.459808e-01

11652 9.946799e-01 9.459709e-01

11653 9.946801e-01 9.459609e-01

11654 9.946803e-01 9.459509e-01

11655 9.946806e-01 9.459408e-01

11656 9.946808e-01 9.459307e-01

11657 9.946810e-01 9.459205e-01

11658 9.946812e-01 9.459102e-01

11659 9.946814e-01 9.458999e-01

11660 9.946816e-01 9.458895e-01

11661 9.946819e-01 9.458791e-01

11662 9.946821e-01 9.458686e-01

11663 9.946823e-01 9.458581e-01

11664 9.946825e-01 9.458475e-01

11665 9.946827e-01 9.458368e-01

11666 9.946829e-01 9.458261e-01

11667 9.946831e-01 9.458154e-01

11668 9.946833e-01 9.458046e-01

11669 9.946835e-01 9.457937e-01

11670 9.946837e-01 9.457828e-01

11671 9.946838e-01 9.457718e-01

11672 9.946840e-01 9.457608e-01

11673 9.946842e-01 9.457497e-01

11674 9.946844e-01 9.457386e-01

11675 9.946846e-01 9.457274e-01

11676 9.946847e-01 9.457162e-01

11677 9.946849e-01 9.457049e-01

11678 9.946851e-01 9.456936e-01

11679 9.946853e-01 9.456822e-01

11680 9.946854e-01 9.456708e-01

11681 9.946856e-01 9.456593e-01

11682 9.946857e-01 9.456478e-01

11683 9.946859e-01 9.456362e-01

11684 9.946860e-01 9.456246e-01

11685 9.946862e-01 9.456129e-01

11686 9.946863e-01 9.456012e-01

11687 9.946864e-01 9.455894e-01

11688 9.946866e-01 9.455776e-01

11689 9.946867e-01 9.455657e-01

11690 9.946868e-01 9.455538e-01

11691 9.946869e-01 9.455419e-01

11692 9.946871e-01 9.455299e-01

11693 9.946872e-01 9.455179e-01

11694 9.946873e-01 9.455058e-01

11695 9.946874e-01 9.454937e-01

11696 9.946875e-01 9.454815e-01

11697 9.946875e-01 9.454694e-01

11698 9.946876e-01 9.454571e-01

11699 9.946877e-01 9.454448e-01

11700 9.946878e-01 9.454325e-01

11701 9.946878e-01 9.454202e-01

11702 9.946886e-01 9.454169e-01

11703 9.946893e-01 9.454136e-01

11704 9.946900e-01 9.454102e-01

11705 9.946907e-01 9.454068e-01

11706 9.946913e-01 9.454034e-01

11707 9.946920e-01 9.453999e-01

11708 9.946926e-01 9.453964e-01

11709 9.946933e-01 9.453928e-01

11710 9.946939e-01 9.453892e-01

11711 9.946945e-01 9.453856e-01

11712 9.946951e-01 9.453819e-01

11713 9.946957e-01 9.453782e-01

11714 9.946963e-01 9.453745e-01

11715 9.946969e-01 9.453707e-01

11716 9.946975e-01 9.453669e-01

11717 9.946980e-01 9.453630e-01

11718 9.946986e-01 9.453591e-01

11719 9.946991e-01 9.453552e-01

11720 9.946997e-01 9.453512e-01

11721 9.947002e-01 9.453472e-01

11722 9.947007e-01 9.453431e-01

11723 9.947012e-01 9.453390e-01

11724 9.947018e-01 9.453349e-01

11725 9.947023e-01 9.453307e-01

11726 9.947027e-01 9.453264e-01

11727 9.947032e-01 9.453222e-01

11728 9.947037e-01 9.453178e-01

11729 9.947042e-01 9.453135e-01

11730 9.947046e-01 9.453091e-01

11731 9.947051e-01 9.453046e-01

11732 9.947055e-01 9.453002e-01

11733 9.947060e-01 9.452956e-01

11734 9.947064e-01 9.452910e-01

11735 9.947069e-01 9.452864e-01

11736 9.947073e-01 9.452818e-01

11737 9.947077e-01 9.452770e-01

11738 9.947081e-01 9.452723e-01

11739 9.947085e-01 9.452675e-01

11740 9.947089e-01 9.452626e-01

11741 9.947093e-01 9.452577e-01

11742 9.947097e-01 9.452528e-01

11743 9.947101e-01 9.452478e-01

11744 9.947105e-01 9.452428e-01

11745 9.947109e-01 9.452377e-01

11746 9.947113e-01 9.452326e-01

11747 9.947116e-01 9.452274e-01

11748 9.947120e-01 9.452222e-01

11749 9.947124e-01 9.452169e-01

11750 9.947127e-01 9.452116e-01

11751 9.947131e-01 9.452062e-01

11752 9.947134e-01 9.452008e-01

11753 9.947138e-01 9.451953e-01

11754 9.947141e-01 9.451898e-01

11755 9.947144e-01 9.451842e-01

11756 9.947148e-01 9.451786e-01

11757 9.947151e-01 9.451729e-01

11758 9.947154e-01 9.451672e-01

11759 9.947158e-01 9.451614e-01

11760 9.947161e-01 9.451556e-01

11761 9.947164e-01 9.451497e-01

11762 9.947167e-01 9.451438e-01

11763 9.947170e-01 9.451378e-01

11764 9.947173e-01 9.451318e-01

11765 9.947176e-01 9.451257e-01

11766 9.947179e-01 9.451196e-01

11767 9.947182e-01 9.451134e-01

11768 9.947185e-01 9.451072e-01

11769 9.947188e-01 9.451009e-01

11770 9.947191e-01 9.450945e-01

11771 9.947194e-01 9.450881e-01

11772 9.947197e-01 9.450817e-01

11773 9.947200e-01 9.450752e-01

11774 9.947203e-01 9.450686e-01

11775 9.947205e-01 9.450620e-01

11776 9.947208e-01 9.450554e-01

11777 9.947211e-01 9.450486e-01

11778 9.947214e-01 9.450419e-01

11779 9.947216e-01 9.450350e-01

11780 9.947219e-01 9.450282e-01

11781 9.947222e-01 9.450212e-01

11782 9.947224e-01 9.450142e-01

11783 9.947227e-01 9.450072e-01

11784 9.947230e-01 9.450001e-01

11785 9.947232e-01 9.449929e-01

11786 9.947235e-01 9.449857e-01

11787 9.947237e-01 9.449784e-01

11788 9.947240e-01 9.449711e-01

11789 9.947242e-01 9.449637e-01

11790 9.947245e-01 9.449563e-01

11791 9.947247e-01 9.449488e-01

11792 9.947250e-01 9.449412e-01

11793 9.947252e-01 9.449336e-01

11794 9.947254e-01 9.449259e-01

11795 9.947257e-01 9.449182e-01

11796 9.947259e-01 9.449104e-01

11797 9.947262e-01 9.449026e-01

11798 9.947264e-01 9.448947e-01

11799 9.947266e-01 9.448868e-01

11800 9.947269e-01 9.448788e-01

11801 9.947271e-01 9.448707e-01

11802 9.947273e-01 9.448626e-01

11803 9.947275e-01 9.448544e-01

11804 9.947278e-01 9.448461e-01

11805 9.947280e-01 9.448379e-01

11806 9.947282e-01 9.448295e-01

11807 9.947284e-01 9.448211e-01

11808 9.947287e-01 9.448126e-01

11809 9.947289e-01 9.448041e-01

11810 9.947291e-01 9.447955e-01

11811 9.947293e-01 9.447869e-01

11812 9.947295e-01 9.447782e-01

11813 9.947297e-01 9.447694e-01

11814 9.947299e-01 9.447606e-01

11815 9.947301e-01 9.447518e-01

11816 9.947303e-01 9.447428e-01

11817 9.947306e-01 9.447338e-01

11818 9.947308e-01 9.447248e-01

11819 9.947310e-01 9.447157e-01

11820 9.947312e-01 9.447066e-01

11821 9.947314e-01 9.446973e-01

11822 9.947315e-01 9.446881e-01

11823 9.947317e-01 9.446788e-01

11824 9.947319e-01 9.446694e-01

11825 9.947321e-01 9.446599e-01

11826 9.947323e-01 9.446505e-01

11827 9.947325e-01 9.446409e-01

11828 9.947327e-01 9.446313e-01

11829 9.947329e-01 9.446216e-01

11830 9.947330e-01 9.446119e-01

11831 9.947332e-01 9.446022e-01

11832 9.947334e-01 9.445923e-01

11833 9.947336e-01 9.445824e-01

11834 9.947337e-01 9.445725e-01

11835 9.947339e-01 9.445625e-01

11836 9.947341e-01 9.445525e-01

11837 9.947343e-01 9.445424e-01

11838 9.947344e-01 9.445322e-01

11839 9.947346e-01 9.445220e-01

11840 9.947347e-01 9.445117e-01

11841 9.947349e-01 9.445014e-01

11842 9.947350e-01 9.444911e-01

11843 9.947352e-01 9.444806e-01

11844 9.947353e-01 9.444702e-01

11845 9.947355e-01 9.444597e-01

11846 9.947356e-01 9.444491e-01

11847 9.947358e-01 9.444385e-01

11848 9.947359e-01 9.444278e-01

11849 9.947360e-01 9.444170e-01

11850 9.947362e-01 9.444063e-01

11851 9.947363e-01 9.443954e-01

11852 9.947364e-01 9.443846e-01

11853 9.947365e-01 9.443736e-01

11854 9.947367e-01 9.443627e-01

11855 9.947368e-01 9.443516e-01

11856 9.947369e-01 9.443406e-01

11857 9.947370e-01 9.443294e-01

11858 9.947371e-01 9.443183e-01

11859 9.947372e-01 9.443071e-01

11860 9.947373e-01 9.442958e-01

11861 9.947374e-01 9.442845e-01

11862 9.947374e-01 9.442731e-01

11863 9.947375e-01 9.442618e-01

11864 9.947376e-01 9.442503e-01

11865 9.947377e-01 9.442388e-01

11866 9.947377e-01 9.442273e-01

11867 9.947378e-01 9.442157e-01

11868 9.947379e-01 9.442041e-01

11869 9.947379e-01 9.441925e-01

11870 9.947380e-01 9.441808e-01

11871 9.947380e-01 9.441691e-01

11872 9.947380e-01 9.441573e-01

11873 9.947381e-01 9.441455e-01

11874 9.947381e-01 9.441336e-01

11875 9.947381e-01 9.441217e-01

11876 9.947381e-01 9.441098e-01

11877 9.947381e-01 9.440979e-01

11878 9.947381e-01 9.440859e-01

11879 9.947381e-01 9.440738e-01

11880 9.947381e-01 9.440618e-01

11881 9.947381e-01 9.440497e-01

11882 9.947387e-01 9.440463e-01

11883 9.947393e-01 9.440429e-01

11884 9.947399e-01 9.440395e-01

11885 9.947405e-01 9.440361e-01

11886 9.947411e-01 9.440326e-01

11887 9.947417e-01 9.440290e-01

11888 9.947423e-01 9.440255e-01

11889 9.947429e-01 9.440219e-01

11890 9.947434e-01 9.440182e-01

11891 9.947440e-01 9.440145e-01

11892 9.947445e-01 9.440108e-01

11893 9.947450e-01 9.440071e-01

11894 9.947455e-01 9.440033e-01

11895 9.947461e-01 9.439994e-01

11896 9.947466e-01 9.439956e-01

11897 9.947471e-01 9.439917e-01

11898 9.947475e-01 9.439877e-01

11899 9.947480e-01 9.439837e-01

11900 9.947485e-01 9.439797e-01

11901 9.947490e-01 9.439756e-01

11902 9.947494e-01 9.439715e-01

11903 9.947499e-01 9.439673e-01

11904 9.947503e-01 9.439631e-01

11905 9.947508e-01 9.439589e-01

11906 9.947512e-01 9.439546e-01

11907 9.947516e-01 9.439503e-01

11908 9.947521e-01 9.439459e-01

11909 9.947525e-01 9.439415e-01

11910 9.947529e-01 9.439371e-01

11911 9.947533e-01 9.439326e-01

11912 9.947537e-01 9.439281e-01

11913 9.947541e-01 9.439235e-01

11914 9.947545e-01 9.439189e-01

11915 9.947548e-01 9.439142e-01

11916 9.947552e-01 9.439095e-01

11917 9.947556e-01 9.439047e-01

11918 9.947560e-01 9.438999e-01

11919 9.947563e-01 9.438951e-01

11920 9.947567e-01 9.438902e-01

11921 9.947570e-01 9.438853e-01

11922 9.947574e-01 9.438803e-01

11923 9.947577e-01 9.438752e-01

11924 9.947581e-01 9.438702e-01

11925 9.947584e-01 9.438650e-01

11926 9.947587e-01 9.438599e-01

11927 9.947591e-01 9.438547e-01

11928 9.947594e-01 9.438494e-01

11929 9.947597e-01 9.438441e-01

11930 9.947600e-01 9.438387e-01

11931 9.947603e-01 9.438333e-01

11932 9.947606e-01 9.438279e-01

11933 9.947609e-01 9.438224e-01

11934 9.947612e-01 9.438168e-01

11935 9.947615e-01 9.438112e-01

11936 9.947618e-01 9.438056e-01

11937 9.947621e-01 9.437999e-01

11938 9.947624e-01 9.437941e-01

11939 9.947627e-01 9.437883e-01

11940 9.947630e-01 9.437825e-01

11941 9.947633e-01 9.437766e-01

11942 9.947635e-01 9.437706e-01

11943 9.947638e-01 9.437646e-01

11944 9.947641e-01 9.437586e-01

11945 9.947644e-01 9.437525e-01

11946 9.947646e-01 9.437463e-01

11947 9.947649e-01 9.437401e-01

11948 9.947651e-01 9.437339e-01

11949 9.947654e-01 9.437276e-01

11950 9.947657e-01 9.437212e-01

11951 9.947659e-01 9.437148e-01

11952 9.947662e-01 9.437083e-01

11953 9.947664e-01 9.437018e-01

11954 9.947666e-01 9.436952e-01

11955 9.947669e-01 9.436886e-01

11956 9.947671e-01 9.436820e-01

11957 9.947674e-01 9.436752e-01

11958 9.947676e-01 9.436684e-01

11959 9.947678e-01 9.436616e-01

11960 9.947681e-01 9.436547e-01

11961 9.947683e-01 9.436478e-01

11962 9.947685e-01 9.436408e-01

11963 9.947688e-01 9.436337e-01

11964 9.947690e-01 9.436266e-01

11965 9.947692e-01 9.436195e-01

11966 9.947694e-01 9.436123e-01

11967 9.947696e-01 9.436050e-01

11968 9.947699e-01 9.435977e-01

11969 9.947701e-01 9.435903e-01

11970 9.947703e-01 9.435829e-01

11971 9.947705e-01 9.435754e-01

11972 9.947707e-01 9.435679e-01

11973 9.947709e-01 9.435603e-01

11974 9.947711e-01 9.435526e-01

11975 9.947713e-01 9.435449e-01

11976 9.947715e-01 9.435372e-01

11977 9.947717e-01 9.435294e-01

11978 9.947719e-01 9.435215e-01

11979 9.947721e-01 9.435136e-01

11980 9.947723e-01 9.435056e-01

11981 9.947725e-01 9.434976e-01

11982 9.947727e-01 9.434895e-01

11983 9.947729e-01 9.434813e-01

11984 9.947730e-01 9.434731e-01

11985 9.947732e-01 9.434649e-01

11986 9.947734e-01 9.434566e-01

11987 9.947736e-01 9.434482e-01

11988 9.947738e-01 9.434398e-01

11989 9.947740e-01 9.434313e-01

11990 9.947741e-01 9.434228e-01

11991 9.947743e-01 9.434142e-01

11992 9.947745e-01 9.434056e-01

11993 9.947746e-01 9.433969e-01

11994 9.947748e-01 9.433881e-01

11995 9.947750e-01 9.433793e-01

11996 9.947751e-01 9.433705e-01

11997 9.947753e-01 9.433616e-01

11998 9.947755e-01 9.433526e-01

11999 9.947756e-01 9.433436e-01

12000 9.947758e-01 9.433345e-01

12001 9.947759e-01 9.433254e-01

12002 9.947761e-01 9.433162e-01

12003 9.947762e-01 9.433069e-01

12004 9.947764e-01 9.432976e-01

12005 9.947765e-01 9.432883e-01

12006 9.947767e-01 9.432789e-01

12007 9.947768e-01 9.432694e-01

12008 9.947769e-01 9.432599e-01

12009 9.947771e-01 9.432504e-01

12010 9.947772e-01 9.432408e-01

12011 9.947773e-01 9.432311e-01

12012 9.947775e-01 9.432214e-01

12013 9.947776e-01 9.432116e-01

12014 9.947777e-01 9.432018e-01

12015 9.947778e-01 9.431919e-01

12016 9.947780e-01 9.431820e-01

12017 9.947781e-01 9.431720e-01

12018 9.947782e-01 9.431620e-01

12019 9.947783e-01 9.431519e-01

12020 9.947784e-01 9.431418e-01

12021 9.947785e-01 9.431316e-01

12022 9.947786e-01 9.431214e-01

12023 9.947787e-01 9.431111e-01

12024 9.947788e-01 9.431008e-01

12025 9.947789e-01 9.430904e-01

12026 9.947790e-01 9.430800e-01

12027 9.947790e-01 9.430695e-01

12028 9.947791e-01 9.430590e-01

12029 9.947792e-01 9.430484e-01

12030 9.947793e-01 9.430378e-01

12031 9.947793e-01 9.430271e-01

12032 9.947794e-01 9.430164e-01

12033 9.947795e-01 9.430057e-01

12034 9.947795e-01 9.429949e-01

12035 9.947796e-01 9.429840e-01

12036 9.947796e-01 9.429731e-01

12037 9.947797e-01 9.429622e-01

12038 9.947797e-01 9.429512e-01

12039 9.947797e-01 9.429402e-01

12040 9.947798e-01 9.429291e-01

12041 9.947798e-01 9.429180e-01

12042 9.947798e-01 9.429069e-01

12043 9.947798e-01 9.428957e-01

12044 9.947798e-01 9.428845e-01

12045 9.947798e-01 9.428732e-01

12046 9.947798e-01 9.428619e-01

12047 9.947798e-01 9.428506e-01

12048 9.947798e-01 9.428392e-01

12049 9.947798e-01 9.428278e-01

12050 9.947798e-01 9.428163e-01

12051 9.947797e-01 9.428048e-01

12052 9.947797e-01 9.427933e-01

12053 9.947796e-01 9.427818e-01

12054 9.947796e-01 9.427702e-01

12055 9.947795e-01 9.427585e-01

12056 9.947795e-01 9.427469e-01

12057 9.947794e-01 9.427352e-01

12058 9.947793e-01 9.427234e-01

12059 9.947792e-01 9.427117e-01

12060 9.947791e-01 9.426999e-01

12061 9.947790e-01 9.426881e-01

12062 9.947796e-01 9.426847e-01

12063 9.947801e-01 9.426813e-01

12064 9.947807e-01 9.426778e-01

12065 9.947812e-01 9.426743e-01

12066 9.947817e-01 9.426707e-01

12067 9.947823e-01 9.426672e-01

12068 9.947828e-01 9.426636e-01

12069 9.947833e-01 9.426599e-01

12070 9.947837e-01 9.426562e-01

12071 9.947842e-01 9.426525e-01

12072 9.947847e-01 9.426487e-01

12073 9.947852e-01 9.426449e-01

12074 9.947856e-01 9.426411e-01

12075 9.947861e-01 9.426372e-01

12076 9.947865e-01 9.426333e-01

12077 9.947870e-01 9.426294e-01

12078 9.947874e-01 9.426254e-01

12079 9.947878e-01 9.426213e-01

12080 9.947882e-01 9.426172e-01

12081 9.947887e-01 9.426131e-01

12082 9.947891e-01 9.426090e-01

12083 9.947895e-01 9.426048e-01

12084 9.947899e-01 9.426006e-01

12085 9.947902e-01 9.425963e-01

12086 9.947906e-01 9.425920e-01

12087 9.947910e-01 9.425876e-01

12088 9.947914e-01 9.425832e-01

12089 9.947917e-01 9.425788e-01

12090 9.947921e-01 9.425743e-01

12091 9.947925e-01 9.425697e-01

12092 9.947928e-01 9.425652e-01

12093 9.947932e-01 9.425606e-01

12094 9.947935e-01 9.425559e-01

12095 9.947938e-01 9.425512e-01

12096 9.947942e-01 9.425465e-01

12097 9.947945e-01 9.425417e-01

12098 9.947948e-01 9.425368e-01

12099 9.947951e-01 9.425320e-01

12100 9.947955e-01 9.425270e-01

12101 9.947958e-01 9.425221e-01

12102 9.947961e-01 9.425171e-01

12103 9.947964e-01 9.425120e-01

12104 9.947967e-01 9.425069e-01

12105 9.947970e-01 9.425017e-01

12106 9.947973e-01 9.424966e-01

12107 9.947976e-01 9.424913e-01

12108 9.947978e-01 9.424860e-01

12109 9.947981e-01 9.424807e-01

12110 9.947984e-01 9.424753e-01

12111 9.947987e-01 9.424699e-01

12112 9.947990e-01 9.424644e-01

12113 9.947992e-01 9.424589e-01

12114 9.947995e-01 9.424533e-01

12115 9.947997e-01 9.424477e-01

12116 9.948000e-01 9.424420e-01

12117 9.948003e-01 9.424363e-01

12118 9.948005e-01 9.424306e-01

12119 9.948008e-01 9.424247e-01

12120 9.948010e-01 9.424189e-01

12121 9.948013e-01 9.424130e-01

12122 9.948015e-01 9.424070e-01

12123 9.948017e-01 9.424010e-01

12124 9.948020e-01 9.423949e-01

12125 9.948022e-01 9.423888e-01

12126 9.948024e-01 9.423827e-01

12127 9.948027e-01 9.423765e-01

12128 9.948029e-01 9.423702e-01

12129 9.948031e-01 9.423639e-01

12130 9.948033e-01 9.423575e-01

12131 9.948035e-01 9.423511e-01

12132 9.948038e-01 9.423447e-01

12133 9.948040e-01 9.423382e-01

12134 9.948042e-01 9.423316e-01

12135 9.948044e-01 9.423250e-01

12136 9.948046e-01 9.423183e-01

12137 9.948048e-01 9.423116e-01

12138 9.948050e-01 9.423048e-01

12139 9.948052e-01 9.422980e-01

12140 9.948054e-01 9.422911e-01

12141 9.948056e-01 9.422842e-01

12142 9.948058e-01 9.422772e-01

12143 9.948060e-01 9.422702e-01

12144 9.948062e-01 9.422631e-01

12145 9.948064e-01 9.422560e-01

12146 9.948066e-01 9.422488e-01

12147 9.948067e-01 9.422416e-01

12148 9.948069e-01 9.422343e-01

12149 9.948071e-01 9.422269e-01

12150 9.948073e-01 9.422195e-01

12151 9.948075e-01 9.422121e-01

12152 9.948076e-01 9.422046e-01

12153 9.948078e-01 9.421970e-01

12154 9.948080e-01 9.421894e-01

12155 9.948081e-01 9.421818e-01

12156 9.948083e-01 9.421741e-01

12157 9.948085e-01 9.421663e-01

12158 9.948086e-01 9.421585e-01

12159 9.948088e-01 9.421506e-01

12160 9.948089e-01 9.421427e-01

12161 9.948091e-01 9.421347e-01

12162 9.948093e-01 9.421267e-01

12163 9.948094e-01 9.421186e-01

12164 9.948096e-01 9.421104e-01

12165 9.948097e-01 9.421022e-01

12166 9.948098e-01 9.420940e-01

12167 9.948100e-01 9.420857e-01

12168 9.948101e-01 9.420773e-01

12169 9.948103e-01 9.420689e-01

12170 9.948104e-01 9.420605e-01

12171 9.948105e-01 9.420520e-01

12172 9.948107e-01 9.420434e-01

12173 9.948108e-01 9.420348e-01

12174 9.948109e-01 9.420261e-01

12175 9.948111e-01 9.420174e-01

12176 9.948112e-01 9.420086e-01

12177 9.948113e-01 9.419998e-01

12178 9.948114e-01 9.419909e-01

12179 9.948115e-01 9.419820e-01

12180 9.948117e-01 9.419730e-01

12181 9.948118e-01 9.419640e-01

12182 9.948119e-01 9.419549e-01

12183 9.948120e-01 9.419458e-01

12184 9.948121e-01 9.419366e-01

12185 9.948122e-01 9.419274e-01

12186 9.948123e-01 9.419181e-01

12187 9.948124e-01 9.419088e-01

12188 9.948125e-01 9.418994e-01

12189 9.948126e-01 9.418899e-01

12190 9.948127e-01 9.418804e-01

12191 9.948127e-01 9.418709e-01

12192 9.948128e-01 9.418613e-01

12193 9.948129e-01 9.418517e-01

12194 9.948130e-01 9.418420e-01

12195 9.948130e-01 9.418323e-01

12196 9.948131e-01 9.418225e-01

12197 9.948132e-01 9.418127e-01

12198 9.948132e-01 9.418028e-01

12199 9.948133e-01 9.417929e-01

12200 9.948134e-01 9.417829e-01

12201 9.948134e-01 9.417729e-01

12202 9.948135e-01 9.417628e-01

12203 9.948135e-01 9.417527e-01

12204 9.948136e-01 9.417425e-01

12205 9.948136e-01 9.417323e-01

12206 9.948136e-01 9.417221e-01

12207 9.948137e-01 9.417118e-01

12208 9.948137e-01 9.417015e-01

12209 9.948137e-01 9.416911e-01

12210 9.948137e-01 9.416807e-01

12211 9.948137e-01 9.416702e-01

12212 9.948137e-01 9.416597e-01

12213 9.948137e-01 9.416491e-01

12214 9.948137e-01 9.416385e-01

12215 9.948137e-01 9.416279e-01

12216 9.948137e-01 9.416172e-01

12217 9.948137e-01 9.416065e-01

12218 9.948137e-01 9.415958e-01

12219 9.948137e-01 9.415850e-01

12220 9.948136e-01 9.415741e-01

12221 9.948136e-01 9.415633e-01

12222 9.948135e-01 9.415524e-01

12223 9.948135e-01 9.415414e-01

12224 9.948134e-01 9.415304e-01

12225 9.948134e-01 9.415194e-01

12226 9.948133e-01 9.415084e-01

12227 9.948132e-01 9.414973e-01

12228 9.948132e-01 9.414862e-01

12229 9.948131e-01 9.414750e-01

12230 9.948130e-01 9.414638e-01

12231 9.948129e-01 9.414526e-01

12232 9.948128e-01 9.414414e-01

12233 9.948127e-01 9.414301e-01

12234 9.948125e-01 9.414188e-01

12235 9.948124e-01 9.414074e-01

12236 9.948123e-01 9.413960e-01

12237 9.948121e-01 9.413846e-01

12238 9.948120e-01 9.413732e-01

12239 9.948118e-01 9.413618e-01

12240 9.948116e-01 9.413503e-01

12241 9.948115e-01 9.413388e-01

12242 9.948120e-01 9.413353e-01

12243 9.948124e-01 9.413319e-01

12244 9.948129e-01 9.413284e-01

12245 9.948134e-01 9.413248e-01

12246 9.948138e-01 9.413213e-01

12247 9.948143e-01 9.413176e-01

12248 9.948147e-01 9.413140e-01

12249 9.948152e-01 9.413103e-01

12250 9.948156e-01 9.413066e-01

12251 9.948160e-01 9.413028e-01

12252 9.948165e-01 9.412990e-01

12253 9.948169e-01 9.412952e-01

12254 9.948173e-01 9.412913e-01

12255 9.948177e-01 9.412874e-01

12256 9.948181e-01 9.412835e-01

12257 9.948185e-01 9.412795e-01

12258 9.948188e-01 9.412755e-01

12259 9.948192e-01 9.412714e-01

12260 9.948196e-01 9.412673e-01

12261 9.948200e-01 9.412631e-01

12262 9.948203e-01 9.412590e-01

12263 9.948207e-01 9.412547e-01

12264 9.948210e-01 9.412505e-01

12265 9.948214e-01 9.412462e-01

12266 9.948217e-01 9.412418e-01

12267 9.948220e-01 9.412374e-01

12268 9.948224e-01 9.412330e-01

12269 9.948227e-01 9.412285e-01

12270 9.948230e-01 9.412240e-01

12271 9.948233e-01 9.412195e-01

12272 9.948236e-01 9.412149e-01

12273 9.948239e-01 9.412102e-01

12274 9.948242e-01 9.412056e-01

12275 9.948245e-01 9.412008e-01

12276 9.948248e-01 9.411961e-01

12277 9.948251e-01 9.411913e-01

12278 9.948254e-01 9.411864e-01

12279 9.948257e-01 9.411815e-01

12280 9.948260e-01 9.411766e-01

12281 9.948262e-01 9.411716e-01

12282 9.948265e-01 9.411666e-01

12283 9.948268e-01 9.411615e-01

12284 9.948270e-01 9.411564e-01

12285 9.948273e-01 9.411512e-01

12286 9.948275e-01 9.411460e-01

12287 9.948278e-01 9.411407e-01

12288 9.948281e-01 9.411355e-01

12289 9.948283e-01 9.411301e-01

12290 9.948285e-01 9.411247e-01

12291 9.948288e-01 9.411193e-01

12292 9.948290e-01 9.411138e-01

12293 9.948293e-01 9.411083e-01

12294 9.948295e-01 9.411027e-01

12295 9.948297e-01 9.410971e-01

12296 9.948299e-01 9.410914e-01

12297 9.948302e-01 9.410857e-01

12298 9.948304e-01 9.410799e-01

12299 9.948306e-01 9.410741e-01

12300 9.948308e-01 9.410683e-01

12301 9.948310e-01 9.410624e-01

12302 9.948312e-01 9.410564e-01

12303 9.948314e-01 9.410504e-01

12304 9.948316e-01 9.410443e-01

12305 9.948318e-01 9.410382e-01

12306 9.948320e-01 9.410321e-01

12307 9.948322e-01 9.410259e-01

12308 9.948324e-01 9.410197e-01

12309 9.948326e-01 9.410134e-01

12310 9.948328e-01 9.410070e-01

12311 9.948330e-01 9.410006e-01

12312 9.948332e-01 9.409942e-01

12313 9.948334e-01 9.409877e-01

12314 9.948335e-01 9.409812e-01

12315 9.948337e-01 9.409746e-01

12316 9.948339e-01 9.409679e-01

12317 9.948341e-01 9.409612e-01

12318 9.948342e-01 9.409545e-01

12319 9.948344e-01 9.409477e-01

12320 9.948346e-01 9.409409e-01

12321 9.948347e-01 9.409340e-01

12322 9.948349e-01 9.409270e-01

12323 9.948351e-01 9.409200e-01

12324 9.948352e-01 9.409130e-01

12325 9.948354e-01 9.409059e-01

12326 9.948355e-01 9.408988e-01

12327 9.948357e-01 9.408916e-01

12328 9.948358e-01 9.408843e-01

12329 9.948360e-01 9.408770e-01

12330 9.948361e-01 9.408697e-01

12331 9.948363e-01 9.408623e-01

12332 9.948364e-01 9.408548e-01

12333 9.948365e-01 9.408473e-01

12334 9.948367e-01 9.408398e-01

12335 9.948368e-01 9.408322e-01

12336 9.948369e-01 9.408245e-01

12337 9.948371e-01 9.408168e-01

12338 9.948372e-01 9.408091e-01

12339 9.948373e-01 9.408013e-01

12340 9.948375e-01 9.407934e-01

12341 9.948376e-01 9.407855e-01

12342 9.948377e-01 9.407776e-01

12343 9.948378e-01 9.407696e-01

12344 9.948379e-01 9.407615e-01

12345 9.948380e-01 9.407534e-01

12346 9.948381e-01 9.407452e-01

12347 9.948383e-01 9.407370e-01

12348 9.948384e-01 9.407288e-01

12349 9.948385e-01 9.407205e-01

12350 9.948386e-01 9.407121e-01

12351 9.948387e-01 9.407037e-01

12352 9.948388e-01 9.406952e-01

12353 9.948389e-01 9.406867e-01

12354 9.948389e-01 9.406782e-01

12355 9.948390e-01 9.406695e-01

12356 9.948391e-01 9.406609e-01

12357 9.948392e-01 9.406522e-01

12358 9.948393e-01 9.406434e-01

12359 9.948394e-01 9.406346e-01

12360 9.948394e-01 9.406258e-01

12361 9.948395e-01 9.406169e-01

12362 9.948396e-01 9.406079e-01

12363 9.948396e-01 9.405989e-01

12364 9.948397e-01 9.405899e-01

12365 9.948398e-01 9.405808e-01

12366 9.948398e-01 9.405716e-01

12367 9.948399e-01 9.405624e-01

12368 9.948399e-01 9.405532e-01

12369 9.948400e-01 9.405439e-01

12370 9.948400e-01 9.405346e-01

12371 9.948401e-01 9.405252e-01

12372 9.948401e-01 9.405157e-01

12373 9.948401e-01 9.405063e-01

12374 9.948402e-01 9.404968e-01

12375 9.948402e-01 9.404872e-01

12376 9.948402e-01 9.404776e-01

12377 9.948402e-01 9.404679e-01

12378 9.948403e-01 9.404582e-01

12379 9.948403e-01 9.404485e-01

12380 9.948403e-01 9.404387e-01

12381 9.948403e-01 9.404289e-01

12382 9.948403e-01 9.404190e-01

12383 9.948403e-01 9.404091e-01

12384 9.948403e-01 9.403991e-01

12385 9.948403e-01 9.403891e-01

12386 9.948402e-01 9.403791e-01

12387 9.948402e-01 9.403690e-01

12388 9.948402e-01 9.403589e-01

12389 9.948402e-01 9.403487e-01

12390 9.948401e-01 9.403385e-01

12391 9.948401e-01 9.403283e-01

12392 9.948400e-01 9.403180e-01

12393 9.948400e-01 9.403077e-01

12394 9.948399e-01 9.402973e-01

12395 9.948399e-01 9.402869e-01

12396 9.948398e-01 9.402765e-01

12397 9.948397e-01 9.402660e-01

12398 9.948397e-01 9.402555e-01

12399 9.948396e-01 9.402450e-01

12400 9.948395e-01 9.402344e-01

12401 9.948394e-01 9.402238e-01

12402 9.948393e-01 9.402132e-01

12403 9.948392e-01 9.402025e-01

12404 9.948391e-01 9.401918e-01

12405 9.948389e-01 9.401811e-01

12406 9.948388e-01 9.401703e-01

12407 9.948387e-01 9.401595e-01

12408 9.948385e-01 9.401486e-01

12409 9.948384e-01 9.401378e-01

12410 9.948382e-01 9.401269e-01

12411 9.948380e-01 9.401160e-01

12412 9.948379e-01 9.401050e-01

12413 9.948377e-01 9.400941e-01

12414 9.948375e-01 9.400831e-01

12415 9.948373e-01 9.400720e-01

12416 9.948371e-01 9.400610e-01

12417 9.948369e-01 9.400499e-01

12418 9.948366e-01 9.400388e-01

12419 9.948364e-01 9.400277e-01

12420 9.948362e-01 9.400166e-01

12421 9.948359e-01 9.400054e-01

12422 9.948363e-01 9.400019e-01

12423 9.948368e-01 9.399984e-01

12424 9.948372e-01 9.399949e-01

12425 9.948376e-01 9.399913e-01

12426 9.948380e-01 9.399877e-01

12427 9.948384e-01 9.399841e-01

12428 9.948388e-01 9.399804e-01

12429 9.948392e-01 9.399767e-01

12430 9.948396e-01 9.399729e-01

12431 9.948400e-01 9.399692e-01

12432 9.948403e-01 9.399653e-01

12433 9.948407e-01 9.399615e-01

12434 9.948410e-01 9.399576e-01

12435 9.948414e-01 9.399537e-01

12436 9.948417e-01 9.399497e-01

12437 9.948421e-01 9.399457e-01

12438 9.948424e-01 9.399416e-01

12439 9.948428e-01 9.399375e-01

12440 9.948431e-01 9.399334e-01

12441 9.948434e-01 9.399292e-01

12442 9.948437e-01 9.399250e-01

12443 9.948440e-01 9.399208e-01

12444 9.948443e-01 9.399165e-01

12445 9.948446e-01 9.399122e-01

12446 9.948449e-01 9.399078e-01

12447 9.948452e-01 9.399034e-01

12448 9.948455e-01 9.398990e-01

12449 9.948458e-01 9.398945e-01

12450 9.948461e-01 9.398900e-01

12451 9.948464e-01 9.398854e-01

12452 9.948466e-01 9.398808e-01

12453 9.948469e-01 9.398762e-01

12454 9.948472e-01 9.398715e-01

12455 9.948474e-01 9.398667e-01

12456 9.948477e-01 9.398620e-01

12457 9.948479e-01 9.398571e-01
[truncated: 2,882,978 more chars]
