# Supplementary material for: Purity control of simulated moving bed based on advanced fuzzy controller
Source: Sci Rep. 2024 Apr 20;14:9083. doi: 10.1038/s41598-024-59847-1 (PMC11576947; doi:10.1038/s41598-024-59847-1)
Supplement: Supplementary file 5 — Supplementary Information 5. [file 41598_2024_59847_MOESM5_ESM.docx]

**Figure 6(a):**

1 1.806422e-75 1.806804e-75 1.807207e-75 1.807633e-75 1.808083e-75 2.091410e-80 2.100706e-80 2.110045e-80 2.082156e-80 2.119425e-80

2 1.806422e-75 1.806804e-75 1.807207e-75 1.807633e-75 1.808083e-75 2.091410e-80 2.100706e-80 2.110045e-80 2.082156e-80 2.119425e-80

3 1.806422e-75 1.806804e-75 1.807207e-75 1.807633e-75 1.808083e-75 2.091410e-80 2.100706e-80 2.110045e-80 2.082156e-80 2.119425e-80

4 1.806422e-75 1.806804e-75 1.807207e-75 1.807633e-75 1.808083e-75 2.091410e-80 2.100706e-80 2.110045e-80 2.082156e-80 2.119425e-80

5 1.806422e-75 1.806804e-75 1.807207e-75 1.807633e-75 1.808083e-75 2.091410e-80 2.100706e-80 2.110045e-80 2.082156e-80 2.119425e-80

6 1.806422e-75 1.806804e-75 1.807207e-75 1.807633e-75 1.808083e-75 2.091410e-80 2.100706e-80 2.110045e-80 2.082156e-80 2.119425e-80

7 1.806422e-75 1.806804e-75 1.807207e-75 1.807633e-75 1.808083e-75 2.091410e-80 2.100706e-80 2.110045e-80 2.082156e-80 2.119425e-80

8 1.806422e-75 1.806804e-75 1.807207e-75 1.807633e-75 1.808083e-75 2.091410e-80 2.100706e-80 2.110045e-80 2.082156e-80 2.119425e-80

9 1.806422e-75 1.806804e-75 1.807207e-75 1.807633e-75 1.808083e-75 2.091410e-80 2.100706e-80 2.110045e-80 2.082156e-80 2.119425e-80

10 1.806422e-75 1.806804e-75 1.807207e-75 1.807633e-75 1.808083e-75 2.091410e-80 2.100706e-80 2.110045e-80 2.082156e-80 2.119425e-80

11 1.806422e-75 1.806804e-75 1.807207e-75 1.807633e-75 1.808083e-75 2.091410e-80 2.100706e-80 2.110045e-80 2.082156e-80 2.119425e-80

12 1.806422e-75 1.806804e-75 1.807207e-75 1.807633e-75 1.808083e-75 2.091410e-80 2.100706e-80 2.110045e-80 2.082156e-80 2.119425e-80

13 1.806422e-75 1.806804e-75 1.807207e-75 1.807633e-75 1.808083e-75 2.091410e-80 2.100706e-80 2.110045e-80 2.082156e-80 2.119425e-80

14 1.806422e-75 1.806804e-75 1.807207e-75 1.807633e-75 1.808083e-75 2.091410e-80 2.100706e-80 2.110045e-80 2.082156e-80 2.119425e-80

15 1.806422e-75 1.806804e-75 1.807207e-75 1.807633e-75 1.808083e-75 2.091410e-80 2.100706e-80 2.110045e-80 2.082156e-80 2.119425e-80

16 1.806422e-75 1.806804e-75 1.807207e-75 1.807633e-75 1.808083e-75 2.091410e-80 2.100706e-80 2.110045e-80 2.082156e-80 2.119425e-80

17 1.806422e-75 1.806804e-75 1.807207e-75 1.807633e-75 1.808083e-75 2.091410e-80 2.100706e-80 2.110045e-80 2.082156e-80 2.119425e-80

18 1.806422e-75 1.806804e-75 1.807207e-75 1.807633e-75 1.808083e-75 2.091410e-80 2.100706e-80 2.110045e-80 2.082156e-80 2.119425e-80

19 1.806422e-75 1.806804e-75 1.807207e-75 1.807633e-75 1.808083e-75 2.091410e-80 2.100706e-80 2.110045e-80 2.082156e-80 2.119425e-80

20 1.806422e-75 1.806804e-75 1.807207e-75 1.807633e-75 1.808083e-75 2.091410e-80 2.100706e-80 2.110045e-80 2.082156e-80 2.119425e-80

21 1.806422e-75 1.806804e-75 1.807207e-75 1.807633e-75 1.808083e-75 2.091410e-80 2.100706e-80 2.110045e-80 2.082156e-80 2.119425e-80

22 1.806422e-75 1.806804e-75 1.807207e-75 1.807633e-75 1.808083e-75 2.091410e-80 2.100706e-80 2.110045e-80 2.082156e-80 2.119425e-80

23 1.806422e-75 1.806804e-75 1.807207e-75 1.807633e-75 1.808083e-75 2.091410e-80 2.100706e-80 2.110045e-80 2.082156e-80 2.119425e-80

24 1.806422e-75 1.806804e-75 1.807207e-75 1.807633e-75 1.808083e-75 2.091410e-80 2.100706e-80 2.110045e-80 2.082156e-80 2.119425e-80

25 1.806422e-75 1.806804e-75 1.807207e-75 1.807633e-75 1.808083e-75 2.091410e-80 2.100706e-80 2.110045e-80 2.082156e-80 2.119425e-80

26 1.806422e-75 1.806804e-75 1.807207e-75 1.807633e-75 1.808083e-75 2.091410e-80 2.100706e-80 2.110045e-80 2.082156e-80 2.119425e-80

27 1.806422e-75 1.806804e-75 1.807207e-75 1.807633e-75 1.808083e-75 2.091410e-80 2.100706e-80 2.110045e-80 2.082156e-80 2.119425e-80

28 1.806422e-75 1.806804e-75 1.807207e-75 1.807633e-75 1.808083e-75 2.091410e-80 2.100706e-80 2.110045e-80 2.082156e-80 2.119425e-80

29 1.806422e-75 1.806804e-75 1.807207e-75 1.807633e-75 1.808083e-75 2.091410e-80 2.100706e-80 2.110045e-80 2.082156e-80 2.119425e-80

30 1.806422e-75 1.806804e-75 1.807207e-75 1.807633e-75 1.808083e-75 2.091410e-80 2.100706e-80 2.110045e-80 2.082156e-80 2.119425e-80

31 1.806422e-75 1.806804e-75 1.807207e-75 1.807633e-75 1.808083e-75 2.091410e-80 2.100706e-80 2.110045e-80 2.082156e-80 2.119425e-80

32 1.806422e-75 1.806804e-75 1.807207e-75 1.807633e-75 1.808083e-75 2.091410e-80 2.100706e-80 2.110045e-80 2.082156e-80 2.119425e-80

33 1.806422e-75 1.806804e-75 1.807207e-75 1.807633e-75 1.808083e-75 2.091410e-80 2.100706e-80 2.110045e-80 2.082156e-80 2.119425e-80

34 1.806422e-75 1.806804e-75 1.807207e-75 1.807633e-75 1.808083e-75 2.091410e-80 2.100706e-80 2.110045e-80 2.082156e-80 2.119425e-80

35 1.806422e-75 1.806804e-75 1.807207e-75 1.807633e-75 1.808083e-75 2.091410e-80 2.100706e-80 2.110045e-80 2.082156e-80 2.119425e-80

36 1.806422e-75 1.806804e-75 1.807207e-75 1.807633e-75 1.808083e-75 2.091410e-80 2.100706e-80 2.110045e-80 2.082156e-80 2.119425e-80

37 1.806422e-75 1.806804e-75 1.807207e-75 1.807633e-75 1.808083e-75 2.091410e-80 2.100706e-80 2.110045e-80 2.082156e-80 2.119425e-80

38 1.806422e-75 1.806804e-75 1.807207e-75 1.807633e-75 1.808083e-75 2.091410e-80 2.100706e-80 2.110045e-80 2.082156e-80 2.119425e-80

39 1.806422e-75 1.806804e-75 1.807207e-75 1.807633e-75 1.808083e-75 2.091410e-80 2.100706e-80 2.110045e-80 2.082156e-80 2.119425e-80

40 1.806422e-75 1.806804e-75 1.807207e-75 1.807633e-75 1.808083e-75 2.091410e-80 2.100706e-80 2.110045e-80 2.082156e-80 2.119425e-80

41 1.806422e-75 1.806804e-75 1.807207e-75 1.807633e-75 1.808083e-75 2.091410e-80 2.100706e-80 2.110045e-80 2.082156e-80 2.119425e-80

42 1.806422e-75 1.806804e-75 1.807207e-75 1.807633e-75 1.808083e-75 2.091410e-80 2.100706e-80 2.110045e-80 2.082156e-80 2.119425e-80

43 1.806422e-75 1.806804e-75 1.807207e-75 1.807633e-75 1.808083e-75 2.091410e-80 2.100706e-80 2.110045e-80 2.082156e-80 2.119425e-80

44 1.806422e-75 1.806804e-75 1.807207e-75 1.807633e-75 1.808083e-75 2.091410e-80 2.100706e-80 2.110045e-80 2.082156e-80 2.119425e-80

45 1.806422e-75 1.806804e-75 1.807207e-75 1.807633e-75 1.808083e-75 2.091410e-80 2.100706e-80 2.110045e-80 2.082156e-80 2.119425e-80

46 1.806422e-75 1.806804e-75 1.807207e-75 1.807633e-75 1.808083e-75 2.091410e-80 2.100706e-80 2.110045e-80 2.082156e-80 2.119425e-80

47 1.806422e-75 1.806804e-75 1.807207e-75 1.807633e-75 1.808083e-75 2.091410e-80 2.100706e-80 2.110045e-80 2.082156e-80 2.119425e-80

48 1.806422e-75 1.806804e-75 1.807207e-75 1.807633e-75 1.808083e-75 2.091410e-80 2.100706e-80 2.110045e-80 2.082156e-80 2.119425e-80

49 1.806422e-75 1.806804e-75 1.807207e-75 1.807633e-75 1.808083e-75 2.091410e-80 2.100706e-80 2.110045e-80 2.082157e-80 2.119425e-80

50 1.806422e-75 1.806804e-75 1.807207e-75 1.807633e-75 1.808083e-75 2.091411e-80 2.100707e-80 2.110045e-80 2.082157e-80 2.119425e-80

51 1.806422e-75 1.806804e-75 1.807207e-75 1.807633e-75 1.808083e-75 2.091411e-80 2.100707e-80 2.110045e-80 2.082157e-80 2.119426e-80

52 1.806422e-75 1.806804e-75 1.807208e-75 1.807634e-75 1.808083e-75 2.091411e-80 2.100707e-80 2.110045e-80 2.082157e-80 2.119426e-80

53 1.806423e-75 1.806805e-75 1.807208e-75 1.807634e-75 1.808084e-75 2.091412e-80 2.100708e-80 2.110046e-80 2.082158e-80 2.119427e-80

54 1.806425e-75 1.806807e-75 1.807210e-75 1.807636e-75 1.808086e-75 2.091414e-80 2.100710e-80 2.110048e-80 2.082160e-80 2.119429e-80

55 1.806427e-75 1.806810e-75 1.807213e-75 1.807639e-75 1.808089e-75 2.091417e-80 2.100713e-80 2.110051e-80 2.082163e-80 2.119432e-80

56 1.806433e-75 1.806815e-75 1.807219e-75 1.807645e-75 1.808095e-75 2.091424e-80 2.100720e-80 2.110058e-80 2.082170e-80 2.119439e-80

57 1.806445e-75 1.806827e-75 1.807231e-75 1.807657e-75 1.808106e-75 2.091437e-80 2.100733e-80 2.110071e-80 2.082183e-80 2.119452e-80

58 1.806469e-75 1.806851e-75 1.807254e-75 1.807680e-75 1.808130e-75 2.091463e-80 2.100760e-80 2.110098e-80 2.082209e-80 2.119479e-80

59 1.806516e-75 1.806898e-75 1.807301e-75 1.807727e-75 1.808177e-75 2.091516e-80 2.100813e-80 2.110151e-80 2.082261e-80 2.119533e-80

60 1.806609e-75 1.806991e-75 1.807394e-75 1.807820e-75 1.808270e-75 2.091620e-80 2.100917e-80 2.110256e-80 2.082364e-80 2.119638e-80

61 1.806790e-75 1.807173e-75 1.807576e-75 1.808002e-75 1.808452e-75 2.091823e-80 2.101121e-80 2.110462e-80 2.082567e-80 2.119845e-80

62 1.807146e-75 1.807528e-75 1.807932e-75 1.808358e-75 1.808808e-75 2.092220e-80 2.101521e-80 2.110864e-80 2.082961e-80 2.120250e-80

63 1.807838e-75 1.808220e-75 1.808624e-75 1.809050e-75 1.809501e-75 2.092992e-80 2.102298e-80 2.111646e-80 2.083729e-80 2.121036e-80

64 1.809176e-75 1.809558e-75 1.809963e-75 1.810389e-75 1.810840e-75 2.094485e-80 2.103800e-80 2.113157e-80 2.085212e-80 2.122556e-80

65 1.811752e-75 1.812135e-75 1.812540e-75 1.812968e-75 1.813419e-75 2.097356e-80 2.106688e-80 2.116063e-80 2.088066e-80 2.125481e-80

66 1.816687e-75 1.817072e-75 1.817479e-75 1.817908e-75 1.818361e-75 2.102852e-80 2.112218e-80 2.121627e-80 2.093529e-80 2.131079e-80

67 1.826097e-75 1.826485e-75 1.826894e-75 1.827327e-75 1.827783e-75 2.113322e-80 2.122753e-80 2.132226e-80 2.103935e-80 2.141742e-80

68 1.843956e-75 1.844348e-75 1.844763e-75 1.845201e-75 1.845663e-75 2.133174e-80 2.142725e-80 2.152321e-80 2.123665e-80 2.161961e-80

69 1.877690e-75 1.878093e-75 1.878517e-75 1.878966e-75 1.879440e-75 2.170636e-80 2.180417e-80 2.190243e-80 2.160900e-80 2.200115e-80

70 1.941136e-75 1.941556e-75 1.942000e-75 1.942469e-75 1.942964e-75 2.241016e-80 2.251228e-80 2.261488e-80 2.230852e-80 2.271797e-80

71 2.059954e-75 2.060408e-75 2.060888e-75 2.061394e-75 2.061929e-75 2.372669e-80 2.383687e-80 2.394758e-80 2.361703e-80 2.405882e-80

72 2.281571e-75 2.282088e-75 2.282634e-75 2.283211e-75 2.283821e-75 2.617917e-80 2.630436e-80 2.643017e-80 2.605460e-80 2.655661e-80

73 2.693345e-75 2.693979e-75 2.694650e-75 2.695358e-75 2.696106e-75 3.072981e-80 3.088284e-80 3.103667e-80 3.057757e-80 3.119129e-80

74 3.455692e-75 3.456544e-75 3.457445e-75 3.458396e-75 3.459401e-75 3.914231e-80 3.934680e-80 3.955240e-80 3.893893e-80 3.975912e-80

75 4.862361e-75 4.863614e-75 4.864939e-75 4.866339e-75 4.867818e-75 5.464001e-80 5.493926e-80 5.524021e-80 5.434245e-80 5.554286e-80

76 7.449909e-75 7.451901e-75 7.454007e-75 7.456232e-75 7.458585e-75 8.309818e-80 8.357138e-80 8.404735e-80 8.262774e-80 8.452610e-80

77 1.219625e-74 1.219960e-74 1.220314e-74 1.220688e-74 1.221084e-74 1.352005e-79 1.359920e-79 1.367883e-79 1.344136e-79 1.375893e-79

78 2.088030e-74 2.088614e-74 2.089230e-74 2.089882e-74 2.090571e-74 2.303335e-79 2.317061e-79 2.330870e-79 2.289691e-79 2.344762e-79

79 3.673275e-74 3.674313e-74 3.675409e-74 3.676568e-74 3.677793e-74 4.036125e-79 4.060430e-79 4.084883e-79 4.011967e-79 4.109485e-79

80 6.561282e-74 6.563148e-74 6.565120e-74 6.567203e-74 6.569406e-74 7.185435e-79 7.228959e-79 7.272750e-79 7.142176e-79 7.316808e-79

81 1.181348e-73 1.181685e-73 1.182041e-73 1.182418e-73 1.182816e-73 1.289827e-78 1.297664e-78 1.305549e-78 1.282037e-78 1.313483e-78

82 2.135091e-73 2.135702e-73 2.136349e-73 2.137031e-73 2.137753e-73 2.324400e-78 2.338545e-78 2.352778e-78 2.310341e-78 2.367097e-78

83 3.864791e-73 3.865901e-73 3.867073e-73 3.868312e-73 3.869621e-73 4.195279e-78 4.220827e-78 4.246532e-78 4.169887e-78 4.272395e-78

84 6.998443e-73 7.000457e-73 7.002584e-73 7.004830e-73 7.007204e-73 7.574357e-78 7.620491e-78 7.666909e-78 7.528504e-78 7.713612e-78

85 1.267066e-72 1.267431e-72 1.267817e-72 1.268224e-72 1.268655e-72 1.367117e-77 1.375443e-77 1.383821e-77 1.358842e-77 1.392250e-77

86 2.293065e-72 2.293728e-72 2.294427e-72 2.295165e-72 2.295946e-72 2.466210e-77 2.481226e-77 2.496335e-77 2.451285e-77 2.511537e-77

87 4.147853e-72 4.149053e-72 4.150320e-72 4.151658e-72 4.153072e-72 4.446166e-77 4.473231e-77 4.500462e-77 4.419266e-77 4.527861e-77

88 7.499370e-72 7.501544e-72 7.503839e-72 7.506263e-72 7.508822e-72 8.010840e-77 8.059588e-77 8.108637e-77 7.962388e-77 8.157986e-77

89 1.355314e-71 1.355708e-71 1.356123e-71 1.356562e-71 1.357025e-71 1.442543e-76 1.451318e-76 1.460148e-76 1.433821e-76 1.469032e-76

90 2.448448e-71 2.449161e-71 2.449913e-71 2.450707e-71 2.451545e-71 2.596352e-76 2.612141e-76 2.628028e-76 2.580658e-76 2.644013e-76

91 4.421787e-71 4.423077e-71 4.424438e-71 4.425875e-71 4.427391e-71 4.670967e-76 4.699366e-76 4.727939e-76 4.642741e-76 4.756688e-76

92 7.983166e-71 7.985501e-71 7.987964e-71 7.990562e-71 7.993305e-71 8.399974e-76 8.451032e-76 8.502404e-76 8.349227e-76 8.554092e-76

93 1.440894e-70 1.441316e-70 1.441761e-70 1.442231e-70 1.442727e-70 1.510047e-75 1.519224e-75 1.528457e-75 1.500926e-75 1.537747e-75

94 2.599985e-70 2.600749e-70 2.601555e-70 2.602405e-70 2.603301e-70 2.713641e-75 2.730129e-75 2.746719e-75 2.697253e-75 2.763411e-75

95 4.690186e-70 4.691567e-70 4.693024e-70 4.694560e-70 4.696181e-70 4.874899e-75 4.904516e-75 4.934315e-75 4.845462e-75 4.964297e-75

96 8.458298e-70 8.460795e-70 8.463429e-70 8.466206e-70 8.469135e-70 8.754434e-75 8.807618e-75 8.861128e-75 8.701575e-75 8.914967e-75

97 1.524896e-69 1.525347e-69 1.525823e-69 1.526325e-69 1.526854e-69 1.571571e-74 1.581118e-74 1.590724e-74 1.562082e-74 1.600389e-74

98 2.748208e-69 2.749023e-69 2.749883e-69 2.750790e-69 2.751746e-69 2.820168e-74 2.837301e-74 2.854540e-74 2.803139e-74 2.869184e-74

99 4.951056e-69 4.952530e-69 4.954084e-69 4.955721e-69 4.957447e-69 5.058721e-74 5.089457e-74 5.120382e-74 5.028173e-74 5.134095e-74

100 8.916015e-69 8.918677e-69 8.921482e-69 8.924437e-69 8.927553e-69 9.070265e-74 9.125384e-74 9.172191e-74 9.015483e-74 9.170947e-74

101 1.604921e-68 1.605401e-68 1.605907e-68 1.606441e-68 1.607003e-68 1.625551e-73 1.633885e-73 1.639802e-73 1.615731e-73 1.635874e-73

102 2.887560e-68 2.888427e-68 2.889340e-68 2.890303e-68 2.891316e-68 2.909097e-73 2.919607e-73 2.926364e-73 2.894265e-73 2.914710e-73

103 5.192638e-68 5.194202e-68 5.195849e-68 5.197584e-68 5.199411e-68 5.195567e-73 5.207518e-73 5.214642e-73 5.176905e-73 5.188481e-73

104 9.332736e-68 9.335555e-68 9.338524e-68 9.341650e-68 9.344943e-68 9.261815e-73 9.274310e-73 9.281203e-73 9.240684e-73 9.228713e-73

105 1.676400e-67 1.676908e-67 1.677443e-67 1.678006e-67 1.678599e-67 1.648499e-72 1.649688e-72 1.650278e-72 1.646307e-72 1.640326e-72

106 3.009383e-67 3.010298e-67 3.011260e-67 3.012274e-67 3.013341e-67 2.930457e-72 2.931435e-72 2.931831e-72 2.928406e-72 2.913549e-72

107 5.398710e-67 5.400356e-67 5.402088e-67 5.403911e-67 5.405831e-67 5.203853e-72 5.204427e-72 5.204524e-72 5.202236e-72 5.171550e-72

108 9.678224e-67 9.681184e-67 9.684298e-67 9.687576e-67 9.691027e-67 9.232356e-72 9.232294e-72 9.232006e-72 9.231560e-72 9.173189e-72

109 1.733697e-66 1.734228e-66 1.734788e-66 1.735377e-66 1.735997e-66 1.636540e-71 1.636447e-71 1.636382e-71 1.636591e-71 1.625963e-71

110 3.103121e-66 3.104076e-66 3.105080e-66 3.106137e-66 3.107250e-66 2.898524e-71 2.898336e-71 2.898268e-71 2.898759e-71 2.879895e-71

111 5.549408e-66 5.551121e-66 5.552923e-66 5.554819e-66 5.556813e-66 5.129326e-71 5.129079e-71 5.129122e-71 5.129783e-71 5.096825e-71

112 9.914911e-66 9.917980e-66 9.921209e-66 9.924605e-66 9.928178e-66 9.069102e-71 9.068960e-71 9.069408e-71 9.069752e-71 9.012768e-71

113 1.769679e-65 1.770229e-65 1.770807e-65 1.771415e-65 1.772054e-65 1.602027e-70 1.602069e-70 1.602223e-70 1.602089e-70 1.592309e-70

114 3.155230e-65 3.156213e-65 3.157247e-65 3.158333e-65 3.159477e-65 2.827175e-70 2.827384e-70 2.827799e-70 2.827164e-70 2.810476e-70

115 5.619059e-65 5.620814e-65 5.622660e-65 5.624601e-65 5.626642e-65 4.984084e-70 4.984709e-70 4.985705e-70 4.983821e-70 4.955484e-70

116 9.994371e-65 9.997502e-65 1.000079e-64 1.000426e-64 1.000790e-64 8.776811e-70 8.778385e-70 8.780621e-70 8.775890e-70 8.727985e-70

117 1.775287e-64 1.775845e-64 1.776432e-64 1.777048e-64 1.777696e-64 1.543739e-69 1.544102e-69 1.544582e-69 1.543492e-69 1.535429e-69

118 3.148938e-64 3.149930e-64 3.150973e-64 3.152069e-64 3.153222e-64 2.711826e-69 2.712618e-69 2.713615e-69 2.711238e-69 2.697727e-69

119 5.577020e-64 5.578782e-64 5.580634e-64 5.582580e-64 5.584626e-64 4.757339e-69 4.759004e-69 4.761031e-69 4.756033e-69 4.733500e-69

120 9.861502e-64 9.864626e-64 9.867908e-64 9.871358e-64 9.874984e-64 8.333814e-69 8.337222e-69 8.341261e-69 8.331036e-69 8.293646e-69

121 1.740793e-63 1.741346e-63 1.741927e-63 1.742537e-63 1.743179e-63 1.457684e-68 1.458367e-68 1.459160e-68 1.457111e-68 1.450941e-68

122 3.067436e-63 3.068413e-63 3.069439e-63 3.070517e-63 3.071650e-63 2.545573e-68 2.546919e-68 2.548456e-68 2.544419e-68 2.534301e-68

123 5.394988e-63 5.396709e-63 5.398518e-63 5.400418e-63 5.402415e-63 4.437856e-68 4.440472e-68 4.443422e-68 4.435574e-68 4.419099e-68

124 9.470121e-63 9.473150e-63 9.476331e-63 9.479675e-63 9.483188e-63 7.723078e-68 7.728104e-68 7.733714e-68 7.718632e-68 7.692023e-68

125 1.658961e-62 1.659492e-62 1.660051e-62 1.660638e-62 1.661254e-62 1.341536e-67 1.342492e-67 1.343550e-67 1.340680e-67 1.336425e-67

126 2.900009e-62 2.900941e-62 2.901919e-62 2.902947e-62 2.904027e-62 2.325817e-67 2.327621e-67 2.329605e-67 2.324188e-67 2.317461e-67

127 5.058420e-62 5.060048e-62 5.061758e-62 5.063555e-62 5.065442e-62 4.024192e-67 4.027572e-67 4.031266e-67 4.021119e-67 4.010641e-67

128 8.803481e-62 8.806320e-62 8.809302e-62 8.812434e-62 8.815725e-62 6.948363e-67 6.954654e-67 6.961499e-67 6.942611e-67 6.926590e-67

129 1.528594e-61 1.529088e-61 1.529607e-61 1.530152e-61 1.530724e-61 1.197181e-66 1.198345e-66 1.199608e-66 1.196111e-66 1.193722e-66

130 2.647921e-61 2.648779e-61 2.649679e-61 2.650624e-61 2.651618e-61 2.058191e-66 2.060335e-66 2.062654e-66 2.056214e-66 2.052770e-66

131 4.575854e-61 4.577338e-61 4.578897e-61 4.580534e-61 4.582253e-61 3.530514e-66 3.534445e-66 3.538686e-66 3.526879e-66 3.522160e-66

132 7.888155e-61 7.890717e-61 7.893408e-61 7.896234e-61 7.899203e-61 6.042223e-66 6.049400e-66 6.057128e-66 6.035571e-66 6.029616e-66

133 1.356435e-60 1.356876e-60 1.357340e-60 1.357827e-60 1.358338e-60 1.031680e-65 1.032984e-65 1.034387e-65 1.030468e-65 1.029828e-65

134 2.326643e-60 2.327401e-60 2.328197e-60 2.329033e-60 2.329911e-60 1.757386e-65 1.759749e-65 1.762286e-65 1.755187e-65 1.754763e-65

135 3.980673e-60 3.981972e-60 3.983337e-60 3.984769e-60 3.986274e-60 2.986422e-65 2.990685e-65 2.995253e-65 2.982448e-65 2.982894e-65

136 6.793143e-60 6.795364e-60 6.797695e-60 6.800144e-60 6.802715e-60 5.062748e-65 5.070408e-65 5.078597e-65 5.055598e-65 5.058380e-65

137 1.156288e-59 1.156666e-59 1.157064e-59 1.157481e-59 1.157919e-59 8.561787e-65 8.575488e-65 8.590105e-65 8.548972e-65 8.557163e-65

138 1.963077e-59 1.963721e-59 1.964396e-59 1.965106e-59 1.965851e-59 1.444371e-64 1.446811e-64 1.449407e-64 1.442085e-64 1.444059e-64

139 3.324164e-59 3.325255e-59 3.326401e-59 3.327604e-59 3.328867e-59 2.430665e-64 2.434988e-64 2.439576e-64 2.426603e-64 2.430921e-64

140 5.614361e-59 5.616207e-59 5.618145e-59 5.620179e-59 5.622315e-59 4.080372e-64 4.087995e-64 4.096062e-64 4.073190e-64 4.082093e-64

141 9.457837e-59 9.460951e-59 9.464219e-59 9.467650e-59 9.471253e-59 6.832841e-64 6.846213e-64 6.860318e-64 6.820206e-64 6.837826e-64

142 1.589129e-58 1.589653e-58 1.590202e-58 1.590780e-58 1.591386e-58 1.141378e-63 1.143711e-63 1.146163e-63 1.139166e-63 1.142548e-63

143 2.663213e-58 2.664092e-58 2.665014e-58 2.665983e-58 2.667000e-58 1.901887e-63 1.905935e-63 1.910177e-63 1.898037e-63 1.904374e-63

144 4.451813e-58 4.453284e-58 4.454829e-58 4.456450e-58 4.458152e-58 3.161325e-63 3.168312e-63 3.175608e-63 3.154658e-63 3.166301e-63

145 7.422642e-58 7.425098e-58 7.427675e-58 7.430381e-58 7.433222e-58 5.241863e-63 5.253856e-63 5.266337e-63 5.230382e-63 5.251419e-63

146 1.234462e-57 1.234871e-57 1.235300e-57 1.235750e-57 1.236223e-57 8.670381e-63 8.690856e-63 8.712092e-63 8.650715e-63 8.688188e-63

147 2.047869e-57 2.048548e-57 2.049261e-57 2.050010e-57 2.050795e-57 1.430643e-62 1.434120e-62 1.437716e-62 1.427293e-62 1.433885e-62

148 3.388763e-57 3.389889e-57 3.391070e-57 3.392309e-57 3.393611e-57 2.354891e-62 2.360766e-62 2.366822e-62 2.349213e-62 2.360685e-62

149 5.593765e-57 5.595624e-57 5.597576e-57 5.599625e-57 5.601776e-57 3.866899e-62 3.876776e-62 3.886930e-62 3.857324e-62 3.877099e-62

150 9.210884e-57 9.213949e-57 9.217167e-57 9.220544e-57 9.224089e-57 6.334531e-62 6.351058e-62 6.368009e-62 6.318466e-62 6.352262e-62

151 1.513015e-56 1.513519e-56 1.514048e-56 1.514604e-56 1.515187e-56 1.035222e-61 1.037975e-61 1.040793e-61 1.032539e-61 1.038272e-61

152 2.479369e-56 2.480197e-56 2.481065e-56 2.481976e-56 2.482932e-56 1.687828e-61 1.692396e-61 1.697061e-61 1.683368e-61 1.693028e-61

153 4.053283e-56 4.054637e-56 4.056057e-56 4.057548e-56 4.059114e-56 2.745414e-61 2.752961e-61 2.760658e-61 2.738029e-61 2.754211e-61

154 6.610770e-56 6.612981e-56 6.615301e-56 6.617735e-56 6.620291e-56 4.455349e-61 4.467774e-61 4.480430e-61 4.443171e-61 4.470139e-61

155 1.075694e-55 1.076054e-55 1.076432e-55 1.076829e-55 1.077245e-55 7.213732e-61 7.234118e-61 7.254862e-61 7.193725e-61 7.238462e-61

156 1.746344e-55 1.746929e-55 1.747543e-55 1.748188e-55 1.748864e-55 1.165342e-60 1.168676e-60 1.172066e-60 1.162066e-60 1.169457e-60

157 2.828701e-55 2.829650e-55 2.830646e-55 2.831691e-55 2.832789e-55 1.878331e-60 1.883768e-60 1.889293e-60 1.872985e-60 1.885155e-60

158 4.571654e-55 4.573190e-55 4.574801e-55 4.576492e-55 4.578268e-55 3.020844e-60 3.029685e-60 3.038667e-60 3.012144e-60 3.032118e-60

159 7.372270e-55 7.374750e-55 7.377351e-55 7.380082e-55 7.382948e-55 4.847667e-60 4.862009e-60 4.876576e-60 4.833550e-60 4.866241e-60

160 1.186269e-54 1.186669e-54 1.187088e-54 1.187528e-54 1.187989e-54 7.762427e-60 7.785635e-60 7.809207e-60 7.739578e-60 7.792950e-60

161 1.904724e-54 1.905366e-54 1.906040e-54 1.906747e-54 1.907489e-54 1.240322e-59 1.244069e-59 1.247875e-59 1.236632e-59 1.245326e-59

162 3.051827e-54 3.052857e-54 3.053938e-54 3.055072e-54 3.056263e-54 1.977676e-59 1.983713e-59 1.989848e-59 1.971731e-59 1.985865e-59

163 4.879544e-54 4.881193e-54 4.882923e-54 4.884739e-54 4.886644e-54 3.146819e-59 3.156528e-59 3.166396e-59 3.137263e-59 3.160197e-59

164 7.785782e-54 7.788416e-54 7.791180e-54 7.794080e-54 7.797123e-54 4.996853e-59 5.012435e-59 5.028278e-59 4.981518e-59 5.018666e-59

165 1.239769e-53 1.240189e-53 1.240630e-53 1.241092e-53 1.241577e-53 7.918467e-59 7.943431e-59 7.968822e-59 7.893909e-59 7.953978e-59

166 1.970184e-53 1.970852e-53 1.971554e-53 1.972289e-53 1.973061e-53 1.252326e-58 1.256319e-58 1.260381e-58 1.248400e-58 1.258098e-58

167 3.124726e-53 3.125787e-53 3.126900e-53 3.128068e-53 3.129294e-53 1.976684e-58 1.983058e-58 1.989546e-58 1.970419e-58 1.986052e-58

168 4.946152e-53 4.947834e-53 4.949598e-53 4.951449e-53 4.953391e-53 3.113953e-58 3.124112e-58 3.134458e-58 3.103973e-58 3.129134e-58

169 7.814200e-53 7.816860e-53 7.819651e-53 7.822579e-53 7.825651e-53 4.896134e-58 4.912299e-58 4.928770e-58 4.880260e-58 4.920699e-58

170 1.232180e-52 1.232600e-52 1.233040e-52 1.233502e-52 1.233987e-52 7.683732e-58 7.709411e-58 7.735591e-58 7.658525e-58 7.723422e-58

171 1.939307e-52 1.939969e-52 1.940663e-52 1.941391e-52 1.942156e-52 1.203593e-57 1.207666e-57 1.211821e-57 1.199597e-57 1.209997e-57

172 3.046579e-52 3.047620e-52 3.048712e-52 3.049858e-52 3.051060e-52 1.881856e-57 1.888307e-57 1.894891e-57 1.875530e-57 1.892174e-57

173 4.777292e-52 4.778926e-52 4.780641e-52 4.782439e-52 4.784327e-52 2.936995e-57 2.947196e-57 2.957612e-57 2.926997e-57 2.953596e-57

174 7.477636e-52 7.480197e-52 7.482884e-52 7.485703e-52 7.488660e-52 4.575516e-57 4.591624e-57 4.608079e-57 4.559737e-57 4.602190e-57

175 1.168341e-51 1.168741e-51 1.169162e-51 1.169603e-51 1.170065e-51 7.115531e-57 7.140928e-57 7.166887e-57 7.090664e-57 7.158333e-57

176 1.822244e-51 1.822870e-51 1.823526e-51 1.824215e-51 1.824937e-51 1.104624e-56 1.108623e-56 1.112713e-56 1.100711e-56 1.111484e-56

177 2.837164e-51 2.838139e-51 2.839163e-51 2.840236e-51 2.841362e-51 1.711873e-56 1.718160e-56 1.724594e-56 1.705723e-56 1.722852e-56

178 4.409727e-51 4.411245e-51 4.412837e-51 4.414508e-51 4.416260e-51 2.648423e-56 2.658296e-56 2.668405e-56 2.638773e-56 2.665976e-56

179 6.842215e-51 6.844573e-51 6.847047e-51 6.849642e-51 6.852365e-51 4.090453e-56 4.105936e-56 4.121800e-56 4.075329e-56 4.118482e-56

180 1.059858e-50 1.060224e-50 1.060607e-50 1.061010e-50 1.061432e-50 6.307141e-56 6.331390e-56 6.356254e-56 6.283468e-56 6.351849e-56

181 1.638974e-50 1.639540e-50 1.640134e-50 1.640758e-50 1.641411e-50 9.709111e-56 9.747045e-56 9.785967e-56 9.672103e-56 9.780343e-56

182 1.638974e-50 1.639540e-50 1.640134e-50 1.640758e-50 1.641411e-50 9.709111e-56 9.747045e-56 9.785967e-56 9.672103e-56 9.780343e-56

183 1.638974e-50 1.639540e-50 1.640134e-50 1.640758e-50 1.641411e-50 9.709111e-56 9.747045e-56 9.785967e-56 9.672103e-56 9.780343e-56

184 1.638974e-50 1.639540e-50 1.640134e-50 1.640758e-50 1.641411e-50 9.709111e-56 9.747045e-56 9.785967e-56 9.672103e-56 9.780343e-56

185 1.638974e-50 1.639540e-50 1.640134e-50 1.640758e-50 1.641411e-50 9.709111e-56 9.747045e-56 9.785967e-56 9.672103e-56 9.780343e-56

186 1.638974e-50 1.639540e-50 1.640134e-50 1.640758e-50 1.641411e-50 9.709111e-56 9.747045e-56 9.785967e-56 9.672103e-56 9.780343e-56

187 1.638974e-50 1.639540e-50 1.640134e-50 1.640758e-50 1.641411e-50 9.709111e-56 9.747045e-56 9.785967e-56 9.672103e-56 9.780343e-56

188 1.638974e-50 1.639540e-50 1.640134e-50 1.640758e-50 1.641411e-50 9.709111e-56 9.747045e-56 9.785967e-56 9.672103e-56 9.780343e-56

189 1.638974e-50 1.639540e-50 1.640134e-50 1.640758e-50 1.641411e-50 9.709111e-56 9.747045e-56 9.785967e-56 9.672103e-56 9.780343e-56

190 1.638974e-50 1.639540e-50 1.640134e-50 1.640758e-50 1.641411e-50 9.709111e-56 9.747045e-56 9.785967e-56 9.672103e-56 9.780343e-56

191 1.638974e-50 1.639540e-50 1.640134e-50 1.640758e-50 1.641411e-50 9.709111e-56 9.747045e-56 9.785967e-56 9.672103e-56 9.780343e-56

192 1.638974e-50 1.639540e-50 1.640134e-50 1.640758e-50 1.641411e-50 9.709111e-56 9.747045e-56 9.785967e-56 9.672103e-56 9.780343e-56

193 1.638974e-50 1.639540e-50 1.640134e-50 1.640758e-50 1.641411e-50 9.709111e-56 9.747045e-56 9.785967e-56 9.672103e-56 9.780343e-56

194 1.638974e-50 1.639540e-50 1.640134e-50 1.640758e-50 1.641411e-50 9.709111e-56 9.747045e-56 9.785967e-56 9.672103e-56 9.780343e-56

195 1.638974e-50 1.639540e-50 1.640134e-50 1.640758e-50 1.641411e-50 9.709111e-56 9.747045e-56 9.785967e-56 9.672103e-56 9.780343e-56

196 1.638974e-50 1.639540e-50 1.640134e-50 1.640758e-50 1.641411e-50 9.709111e-56 9.747045e-56 9.785967e-56 9.672103e-56 9.780343e-56

197 1.638974e-50 1.639540e-50 1.640134e-50 1.640758e-50 1.641411e-50 9.709111e-56 9.747045e-56 9.785967e-56 9.672103e-56 9.780343e-56

198 1.638974e-50 1.639540e-50 1.640134e-50 1.640758e-50 1.641411e-50 9.709111e-56 9.747045e-56 9.785967e-56 9.672103e-56 9.780343e-56

199 1.638974e-50 1.639540e-50 1.640134e-50 1.640758e-50 1.641411e-50 9.709111e-56 9.747045e-56 9.785967e-56 9.672103e-56 9.780343e-56

200 1.638974e-50 1.639540e-50 1.640134e-50 1.640758e-50 1.641411e-50 9.709111e-56 9.747045e-56 9.785967e-56 9.672103e-56 9.780343e-56

201 1.638974e-50 1.639540e-50 1.640134e-50 1.640758e-50 1.641411e-50 9.709111e-56 9.747045e-56 9.785967e-56 9.672103e-56 9.780343e-56

202 1.638974e-50 1.639540e-50 1.640134e-50 1.640758e-50 1.641411e-50 9.709111e-56 9.747045e-56 9.785967e-56 9.672103e-56 9.780343e-56

203 1.638974e-50 1.639540e-50 1.640134e-50 1.640758e-50 1.641411e-50 9.709111e-56 9.747045e-56 9.785967e-56 9.672103e-56 9.780343e-56

204 1.638974e-50 1.639540e-50 1.640134e-50 1.640758e-50 1.641411e-50 9.709111e-56 9.747045e-56 9.785967e-56 9.672103e-56 9.780343e-56

205 1.638974e-50 1.639540e-50 1.640134e-50 1.640758e-50 1.641411e-50 9.709111e-56 9.747045e-56 9.785967e-56 9.672103e-56 9.780343e-56

206 1.638974e-50 1.639540e-50 1.640134e-50 1.640758e-50 1.641411e-50 9.709111e-56 9.747045e-56 9.785967e-56 9.672103e-56 9.780343e-56

207 1.638974e-50 1.639540e-50 1.640134e-50 1.640758e-50 1.641411e-50 9.709111e-56 9.747045e-56 9.785967e-56 9.672103e-56 9.780343e-56

208 1.638974e-50 1.639540e-50 1.640134e-50 1.640758e-50 1.641411e-50 9.709111e-56 9.747045e-56 9.785967e-56 9.672103e-56 9.780343e-56

209 1.638974e-50 1.639540e-50 1.640134e-50 1.640758e-50 1.641411e-50 9.709111e-56 9.747045e-56 9.785967e-56 9.672103e-56 9.780343e-56

210 1.638974e-50 1.639540e-50 1.640134e-50 1.640758e-50 1.641411e-50 9.709111e-56 9.747045e-56 9.785967e-56 9.672103e-56 9.780343e-56

211 1.638974e-50 1.639540e-50 1.640134e-50 1.640758e-50 1.641411e-50 9.709111e-56 9.747045e-56 9.785967e-56 9.672103e-56 9.780343e-56

212 1.638974e-50 1.639540e-50 1.640134e-50 1.640758e-50 1.641411e-50 9.709111e-56 9.747045e-56 9.785967e-56 9.672103e-56 9.780343e-56

213 1.638974e-50 1.639540e-50 1.640134e-50 1.640758e-50 1.641411e-50 9.709111e-56 9.747045e-56 9.785967e-56 9.672103e-56 9.780343e-56

214 1.638974e-50 1.639540e-50 1.640134e-50 1.640758e-50 1.641411e-50 9.709111e-56 9.747045e-56 9.785967e-56 9.672103e-56 9.780343e-56

215 1.638974e-50 1.639540e-50 1.640134e-50 1.640758e-50 1.641411e-50 9.709111e-56 9.747045e-56 9.785967e-56 9.672103e-56 9.780343e-56

216 1.638974e-50 1.639540e-50 1.640134e-50 1.640758e-50 1.641411e-50 9.709111e-56 9.747045e-56 9.785967e-56 9.672103e-56 9.780343e-56

217 1.638974e-50 1.639540e-50 1.640134e-50 1.640758e-50 1.641411e-50 9.709111e-56 9.747045e-56 9.785967e-56 9.672103e-56 9.780343e-56

218 1.638974e-50 1.639540e-50 1.640134e-50 1.640758e-50 1.641411e-50 9.709111e-56 9.747045e-56 9.785967e-56 9.672103e-56 9.780343e-56

219 1.638974e-50 1.639540e-50 1.640134e-50 1.640758e-50 1.641411e-50 9.709111e-56 9.747045e-56 9.785967e-56 9.672103e-56 9.780343e-56

220 1.638974e-50 1.639540e-50 1.640134e-50 1.640758e-50 1.641411e-50 9.709111e-56 9.747045e-56 9.785967e-56 9.672103e-56 9.780343e-56

221 1.638974e-50 1.639540e-50 1.640134e-50 1.640758e-50 1.641411e-50 9.709111e-56 9.747045e-56 9.785967e-56 9.672103e-56 9.780343e-56

222 1.638974e-50 1.639540e-50 1.640134e-50 1.640758e-50 1.641411e-50 9.709111e-56 9.747045e-56 9.785967e-56 9.672103e-56 9.780343e-56

223 1.638974e-50 1.639540e-50 1.640134e-50 1.640758e-50 1.641411e-50 9.709111e-56 9.747045e-56 9.785967e-56 9.672103e-56 9.780343e-56

224 1.638974e-50 1.639540e-50 1.640134e-50 1.640758e-50 1.641411e-50 9.709111e-56 9.747045e-56 9.785967e-56 9.672103e-56 9.780343e-56

225 1.638974e-50 1.639540e-50 1.640134e-50 1.640758e-50 1.641411e-50 9.709111e-56 9.747045e-56 9.785967e-56 9.672103e-56 9.780343e-56

226 1.638974e-50 1.639540e-50 1.640134e-50 1.640758e-50 1.641411e-50 9.709111e-56 9.747045e-56 9.785967e-56 9.672103e-56 9.780343e-56

227 1.638974e-50 1.639540e-50 1.640134e-50 1.640758e-50 1.641411e-50 9.709111e-56 9.747045e-56 9.785967e-56 9.672103e-56 9.780343e-56

228 1.638974e-50 1.639540e-50 1.640134e-50 1.640758e-50 1.641411e-50 9.709111e-56 9.747045e-56 9.785967e-56 9.672103e-56 9.780343e-56

229 1.638974e-50 1.639540e-50 1.640134e-50 1.640758e-50 1.641411e-50 9.709111e-56 9.747045e-56 9.785967e-56 9.672103e-56 9.780343e-56

230 1.638974e-50 1.639540e-50 1.640134e-50 1.640758e-50 1.641411e-50 9.709111e-56 9.747045e-56 9.785967e-56 9.672103e-56 9.780343e-56

231 1.638974e-50 1.639540e-50 1.640134e-50 1.640758e-50 1.641411e-50 9.709111e-56 9.747045e-56 9.785967e-56 9.672103e-56 9.780343e-56

232 1.638974e-50 1.639540e-50 1.640134e-50 1.640758e-50 1.641411e-50 9.709111e-56 9.747045e-56 9.785967e-56 9.672103e-56 9.780343e-56

233 1.638974e-50 1.639540e-50 1.640134e-50 1.640758e-50 1.641411e-50 9.709111e-56 9.747045e-56 9.785967e-56 9.672103e-56 9.780343e-56

234 1.638974e-50 1.639540e-50 1.640134e-50 1.640758e-50 1.641411e-50 9.709111e-56 9.747045e-56 9.785967e-56 9.672103e-56 9.780344e-56

235 1.638974e-50 1.639540e-50 1.640134e-50 1.640758e-50 1.641411e-50 9.709111e-56 9.747045e-56 9.785967e-56 9.672103e-56 9.780344e-56

236 1.638974e-50 1.639540e-50 1.640134e-50 1.640758e-50 1.641411e-50 9.709111e-56 9.747045e-56 9.785967e-56 9.672104e-56 9.780344e-56

237 1.638974e-50 1.639540e-50 1.640135e-50 1.640758e-50 1.641411e-50 9.709112e-56 9.747046e-56 9.785968e-56 9.672104e-56 9.780344e-56

238 1.638974e-50 1.639541e-50 1.640135e-50 1.640758e-50 1.641412e-50 9.709112e-56 9.747046e-56 9.785968e-56 9.672104e-56 9.780344e-56

239 1.638974e-50 1.639541e-50 1.640135e-50 1.640758e-50 1.641412e-50 9.709113e-56 9.747046e-56 9.785969e-56 9.672105e-56 9.780345e-56

240 1.638975e-50 1.639541e-50 1.640135e-50 1.640758e-50 1.641412e-50 9.709113e-56 9.747047e-56 9.785969e-56 9.672106e-56 9.780346e-56

241 1.638975e-50 1.639541e-50 1.640135e-50 1.640758e-50 1.641412e-50 9.709115e-56 9.747049e-56 9.785971e-56 9.672107e-56 9.780347e-56

242 1.638975e-50 1.639542e-50 1.640136e-50 1.640759e-50 1.641413e-50 9.709117e-56 9.747051e-56 9.785973e-56 9.672109e-56 9.780349e-56

243 1.638976e-50 1.639542e-50 1.640136e-50 1.640759e-50 1.641413e-50 9.709120e-56 9.747053e-56 9.785976e-56 9.672112e-56 9.780352e-56

244 1.638977e-50 1.639543e-50 1.640137e-50 1.640761e-50 1.641414e-50 9.709124e-56 9.747058e-56 9.785980e-56 9.672116e-56 9.780357e-56

245 1.638978e-50 1.639545e-50 1.640139e-50 1.640762e-50 1.641416e-50 9.709131e-56 9.747065e-56 9.785987e-56 9.672123e-56 9.780363e-56

246 1.638981e-50 1.639547e-50 1.640141e-50 1.640764e-50 1.641418e-50 9.709141e-56 9.747075e-56 9.785997e-56 9.672133e-56 9.780374e-56

247 1.638984e-50 1.639551e-50 1.640145e-50 1.640768e-50 1.641422e-50 9.709156e-56 9.747090e-56 9.786013e-56 9.672148e-56 9.780389e-56

248 1.638990e-50 1.639556e-50 1.640150e-50 1.640773e-50 1.641427e-50 9.709179e-56 9.747113e-56 9.786036e-56 9.672171e-56 9.780413e-56

249 1.638998e-50 1.639564e-50 1.640158e-50 1.640781e-50 1.641435e-50 9.709213e-56 9.747148e-56 9.786071e-56 9.672205e-56 9.780448e-56

250 1.639010e-50 1.639576e-50 1.640170e-50 1.640793e-50 1.641447e-50 9.709265e-56 9.747200e-56 9.786123e-56 9.672256e-56 9.780501e-56

251 1.639028e-50 1.639594e-50 1.640188e-50 1.640811e-50 1.641465e-50 9.709342e-56 9.747278e-56 9.786202e-56 9.672333e-56 9.780580e-56

252 1.639055e-50 1.639621e-50 1.640216e-50 1.640839e-50 1.641493e-50 9.709458e-56 9.747395e-56 9.786320e-56 9.672447e-56 9.780699e-56

253 1.639096e-50 1.639662e-50 1.640256e-50 1.640880e-50 1.641533e-50 9.709632e-56 9.747570e-56 9.786497e-56 9.672619e-56 9.780876e-56

254 1.639157e-50 1.639723e-50 1.640317e-50 1.640941e-50 1.641595e-50 9.709890e-56 9.747831e-56 9.786760e-56 9.672876e-56 9.781141e-56

255 1.639248e-50 1.639814e-50 1.640409e-50 1.641032e-50 1.641686e-50 9.710277e-56 9.748221e-56 9.787153e-56 9.673259e-56 9.781537e-56

256 1.639383e-50 1.639950e-50 1.640545e-50 1.641168e-50 1.641822e-50 9.710852e-56 9.748802e-56 9.787740e-56 9.673829e-56 9.782128e-56

257 1.639586e-50 1.640152e-50 1.640747e-50 1.641371e-50 1.642026e-50 9.711709e-56 9.749666e-56 9.788612e-56 9.674678e-56 9.783006e-56

258 1.639887e-50 1.640454e-50 1.641049e-50 1.641674e-50 1.642328e-50 9.712981e-56 9.750951e-56 9.789909e-56 9.675939e-56 9.784313e-56

259 1.640334e-50 1.640901e-50 1.641497e-50 1.642123e-50 1.642777e-50 9.714871e-56 9.752858e-56 9.791835e-56 9.677811e-56 9.786253e-56

260 1.640998e-50 1.641566e-50 1.642163e-50 1.642790e-50 1.643444e-50 9.717672e-56 9.755686e-56 9.794690e-56 9.680586e-56 9.789130e-56

261 1.641983e-50 1.642550e-50 1.643149e-50 1.643778e-50 1.644433e-50 9.721819e-56 9.759873e-56 9.798919e-56 9.684694e-56 9.793392e-56

262 1.643439e-50 1.644007e-50 1.644609e-50 1.645240e-50 1.645896e-50 9.727952e-56 9.766066e-56 9.805173e-56 9.690768e-56 9.799696e-56

263 1.645592e-50 1.646161e-50 1.646767e-50 1.647401e-50 1.648058e-50 9.737008e-56 9.775211e-56 9.814410e-56 9.699737e-56 9.809009e-56

264 1.648770e-50 1.649340e-50 1.649951e-50 1.650591e-50 1.651250e-50 9.750363e-56 9.788700e-56 9.828036e-56 9.712963e-56 9.822748e-56

265 1.653453e-50 1.654026e-50 1.654645e-50 1.655293e-50 1.655954e-50 9.770032e-56 9.808566e-56 9.848105e-56 9.732438e-56 9.842989e-56

266 1.660346e-50 1.660921e-50 1.661552e-50 1.662212e-50 1.662876e-50 9.798958e-56 9.837787e-56 9.877627e-56 9.761078e-56 9.872768e-56

267 1.670475e-50 1.671054e-50 1.671702e-50 1.672380e-50 1.673049e-50 9.841438e-56 9.880703e-56 9.920991e-56 9.803133e-56 9.916516e-56

268 1.685336e-50 1.685922e-50 1.686595e-50 1.687298e-50 1.687975e-50 9.903734e-56 9.943644e-56 9.984593e-56 9.864800e-56 9.980693e-56

269 1.707108e-50 1.707703e-50 1.708413e-50 1.709154e-50 1.709841e-50 9.994954e-56 1.003582e-55 1.007774e-55 9.955092e-56 1.007470e-55

270 1.738955e-50 1.739563e-50 1.740327e-50 1.741123e-50 1.741826e-50 1.012833e-55 1.017059e-55 1.021396e-55 1.008710e-55 1.021219e-55

271 1.785462e-50 1.786091e-50 1.786933e-50 1.787808e-50 1.788534e-50 1.032303e-55 1.036737e-55 1.041285e-55 1.027978e-55 1.041296e-55

272 1.853270e-50 1.853927e-50 1.854884e-50 1.855875e-50 1.856635e-50 1.060683e-55 1.065420e-55 1.070279e-55 1.056062e-55 1.070570e-55

273 1.951969e-50 1.952668e-50 1.953791e-50 1.954950e-50 1.955758e-50 1.101982e-55 1.107163e-55 1.112478e-55 1.096926e-55 1.113181e-55

274 2.095389e-50 2.096150e-50 2.097512e-50 2.098915e-50 2.099795e-50 1.161983e-55 1.167816e-55 1.173797e-55 1.156292e-55 1.175108e-55

275 2.303435e-50 2.304284e-50 2.305994e-50 2.307750e-50 2.308733e-50 1.249013e-55 1.255796e-55 1.262750e-55 1.242393e-55 1.264953e-55

276 2.604700e-50 2.605678e-50 2.607890e-50 2.610155e-50 2.611288e-50 1.375031e-55 1.383201e-55 1.391572e-55 1.367058e-55 1.395082e-55

277 3.040179e-50 3.041344e-50 3.044278e-50 3.047277e-50 3.048627e-50 1.557193e-55 1.567378e-55 1.577810e-55 1.547249e-55 1.583232e-55

278 3.668531e-50 3.669965e-50 3.673940e-50 3.677995e-50 3.679659e-50 1.820050e-55 1.833161e-55 1.846584e-55 1.807246e-55 1.854795e-55

279 4.573528e-50 4.575351e-50 4.580820e-50 4.586392e-50 4.588508e-50 2.198680e-55 2.216028e-55 2.233782e-55 2.181732e-55 2.246053e-55

280 5.874569e-50 5.876951e-50 5.884561e-50 5.892306e-50 5.895073e-50 2.743095e-55 2.766570e-55 2.790583e-55 2.720154e-55 2.808747e-55

281 7.741489e-50 7.744674e-50 7.755348e-50 7.766203e-50 7.769905e-50 3.524468e-55 3.556781e-55 3.589822e-55 3.492880e-55 3.616522e-55

282 1.041539e-49 1.041973e-49 1.043478e-49 1.045008e-49 1.045512e-49 4.643886e-55 4.688922e-55 4.734955e-55 4.599846e-55 4.773992e-55

283 1.423789e-49 1.424388e-49 1.426516e-49 1.428678e-49 1.429374e-49 6.244644e-55 6.307958e-55 6.372650e-55 6.182710e-55 6.429473e-55

284 1.969201e-49 1.970035e-49 1.973050e-49 1.976113e-49 1.977083e-49 8.529474e-55 8.618990e-55 8.710423e-55 8.441880e-55 8.792829e-55

285 2.745943e-49 2.747113e-49 2.751386e-49 2.755728e-49 2.757089e-49 1.178464e-54 1.191164e-54 1.204132e-54 1.166033e-54 1.216044e-54

286 3.850032e-49 3.851680e-49 3.857737e-49 3.863889e-49 3.865806e-49 1.641361e-54 1.659412e-54 1.677838e-54 1.623686e-54 1.695007e-54

287 5.416448e-49 5.418775e-49 5.427354e-49 5.436068e-49 5.438775e-49 2.298389e-54 2.324065e-54 2.350265e-54 2.273244e-54 2.374943e-54

288 7.634589e-49 7.637878e-49 7.650017e-49 7.662347e-49 7.666173e-49 3.229235e-54 3.265748e-54 3.302999e-54 3.193464e-54 3.338379e-54

289 1.076972e-48 1.077437e-48 1.079152e-48 1.080894e-48 1.081436e-48 4.545570e-54 4.597461e-54 4.650386e-54 4.494721e-54 4.700988e-54

290 1.519266e-48 1.519924e-48 1.522344e-48 1.524803e-48 1.525568e-48 6.403620e-54 6.477284e-54 6.552396e-54 6.331415e-54 6.624599e-54

291 2.142093e-48 2.143022e-48 2.146431e-48 2.149895e-48 2.150976e-48 9.021542e-54 9.125975e-54 9.232431e-54 8.919153e-54 9.335223e-54

292 3.017545e-48 3.018855e-48 3.023650e-48 3.028523e-48 3.030047e-48 1.270346e-53 1.285128e-53 1.300194e-53 1.255849e-53 1.314795e-53

293 4.245885e-48 4.247732e-48 4.254464e-48 4.261306e-48 4.263454e-48 1.787262e-53 1.808152e-53 1.829437e-53 1.766770e-53 1.850134e-53

294 5.966330e-48 5.968929e-48 5.978364e-48 5.987956e-48 5.990978e-48 2.511711e-53 2.541182e-53 2.571203e-53 2.482795e-53 2.600478e-53

295 8.371879e-48 8.375531e-48 8.388732e-48 8.402153e-48 8.406400e-48 3.525275e-53 3.566779e-53 3.609048e-53 3.484543e-53 3.650372e-53

296 1.172969e-47 1.173482e-47 1.175325e-47 1.177200e-47 1.177796e-47 4.940960e-53 4.999308e-53 5.058718e-53 4.883685e-53 5.116933e-53

297 1.640906e-47 1.641624e-47 1.644194e-47 1.646809e-47 1.647643e-47 6.915076e-53 6.996960e-53 7.080315e-53 6.834680e-53 7.162165e-53

298 2.291975e-47 2.292979e-47 2.296557e-47 2.300196e-47 2.301364e-47 9.663530e-53 9.778243e-53 9.894993e-53 9.550876e-53 1.000986e-52

299 3.196454e-47 3.197856e-47 3.202827e-47 3.207885e-47 3.209516e-47 1.348416e-52 1.364459e-52 1.380784e-52 1.332658e-52 1.396874e-52

300 4.451113e-47 4.453069e-47 4.459964e-47 4.466983e-47 4.469257e-47 1.878734e-52 1.901134e-52 1.923922e-52 1.856726e-52 1.946419e-52

301 6.189051e-47 6.191775e-47 6.201326e-47 6.211050e-47 6.214216e-47 2.613780e-52 2.645005e-52 2.676764e-52 2.583096e-52 2.708167e-52

302 8.593143e-47 8.596930e-47 8.610140e-47 8.623591e-47 8.627994e-47 3.631193e-52 3.674650e-52 3.718844e-52 3.588480e-52 3.762604e-52

303 1.191441e-46 1.191967e-46 1.193791e-46 1.195649e-46 1.196261e-46 5.037596e-52 5.097987e-52 5.159390e-52 4.978228e-52 5.220272e-52

304 1.649711e-46 1.650441e-46 1.652957e-46 1.655520e-46 1.656368e-46 6.979290e-52 7.063090e-52 7.148279e-52 6.896893e-52 7.232853e-52

305 2.281300e-46 2.282311e-46 2.285777e-46 2.289309e-46 2.290483e-46 9.656847e-52 9.772968e-52 9.890992e-52 9.542649e-52 1.000830e-51

306 3.150807e-46 3.152206e-46 3.156975e-46 3.161835e-46 3.163460e-46 1.334504e-51 1.350573e-51 1.366902e-51 1.318697e-51 1.383152e-51

307 4.346634e-46 4.348568e-46 4.355123e-46 4.361803e-46 4.364050e-46 1.842001e-51 1.864210e-51 1.886775e-51 1.820152e-51 1.909254e-51

308 5.989689e-46 5.992359e-46 6.001360e-46 6.010534e-46 6.013637e-46 2.539643e-51 2.570300e-51 2.601444e-51 2.509476e-51 2.632501e-51

309 8.245255e-46 8.248940e-46 8.261290e-46 8.273878e-46 8.278157e-46 3.497804e-51 3.540076e-51 3.583011e-51 3.456201e-51 3.625867e-51

310 1.133919e-45 1.134427e-45 1.136120e-45 1.137846e-45 1.138436e-45 4.812675e-51 4.870899e-51 4.930029e-51 4.755362e-51 4.989102e-51

311 1.558001e-45 1.558700e-45 1.561020e-45 1.563385e-45 1.564197e-45 6.615681e-51 6.695799e-51 6.777149e-51 6.536805e-51 6.858492e-51

312 2.138901e-45 2.139864e-45 2.143041e-45 2.146279e-45 2.147397e-45 9.086328e-51 9.196469e-51 9.308288e-51 8.977876e-51 9.420188e-51

313 2.934140e-45 2.935464e-45 2.939813e-45 2.944245e-45 2.945783e-45 1.246973e-50 1.262101e-50 1.277458e-50 1.232074e-50 1.292838e-50

314 4.022236e-45 4.024057e-45 4.030008e-45 4.036071e-45 4.038184e-45 1.710050e-50 1.730814e-50 1.751888e-50 1.689598e-50 1.773009e-50

315 5.510355e-45 5.512857e-45 5.520999e-45 5.529292e-45 5.532196e-45 2.343540e-50 2.372018e-50 2.400917e-50 2.315485e-50 2.429901e-50

316 7.544757e-45 7.548193e-45 7.559329e-45 7.570671e-45 7.574658e-45 3.209777e-50 3.248810e-50 3.288415e-50 3.171320e-50 3.328161e-50

317 1.032504e-44 1.032976e-44 1.034499e-44 1.036050e-44 1.036597e-44 4.393833e-50 4.447300e-50 4.501544e-50 4.341145e-50 4.556015e-50

318 1.412359e-44 1.413006e-44 1.415089e-44 1.417210e-44 1.417961e-44 6.011791e-50 6.084993e-50 6.159249e-50 5.939646e-50 6.233859e-50

319 1.931214e-44 1.932103e-44 1.934951e-44 1.937851e-44 1.938881e-44 8.222059e-50 8.322233e-50 8.423837e-50 8.123319e-50 8.525982e-50

320 2.639801e-44 2.641020e-44 2.644916e-44 2.648881e-44 2.650294e-44 1.124079e-49 1.137782e-49 1.151679e-49 1.110571e-49 1.165657e-49

321 3.607360e-44 3.609031e-44 3.614361e-44 3.619783e-44 3.621721e-44 1.536296e-49 1.555034e-49 1.574034e-49 1.517822e-49 1.593155e-49

322 4.928395e-44 4.930687e-44 4.937978e-44 4.945394e-44 4.948051e-44 2.099108e-49 2.124722e-49 2.150693e-49 2.073851e-49 2.176841e-49

323 6.731903e-44 6.735047e-44 6.745022e-44 6.755167e-44 6.758809e-44 2.867445e-49 2.902450e-49 2.937939e-49 2.832924e-49 2.973685e-49

324 9.193978e-44 9.198288e-44 9.211939e-44 9.225818e-44 9.230812e-44 3.916270e-49 3.964098e-49 4.012584e-49 3.869097e-49 4.061439e-49

325 1.255502e-43 1.256093e-43 1.257961e-43 1.259860e-43 1.260545e-43 5.347892e-49 5.413229e-49 5.479458e-49 5.283443e-49 5.546217e-49

326 1.714322e-43 1.715132e-43 1.717690e-43 1.720289e-43 1.721227e-43 7.301941e-49 7.391183e-49 7.481635e-49 7.213904e-49 7.572843e-49

327 2.340670e-43 2.341782e-43 2.345282e-43 2.348840e-43 2.350127e-43 9.968992e-49 1.009087e-48 1.021439e-48 9.848749e-49 1.033898e-48

328 3.195732e-43 3.197256e-43 3.202049e-43 3.206920e-43 3.208683e-43 1.360916e-48 1.377559e-48 1.394425e-48 1.344494e-48 1.411443e-48

329 4.363055e-43 4.365145e-43 4.371707e-43 4.378376e-43 4.380794e-43 1.857746e-48 1.880472e-48 1.903500e-48 1.835321e-48 1.926742e-48

330 5.956723e-43 5.959589e-43 5.968575e-43 5.977707e-43 5.981022e-43 2.535848e-48 2.566876e-48 2.598316e-48 2.505227e-48 2.630056e-48

331 8.132523e-43 8.136454e-43 8.148760e-43 8.161265e-43 8.165811e-43 3.461358e-48 3.503721e-48 3.546643e-48 3.419548e-48 3.589985e-48

332 1.110319e-42 1.110858e-42 1.112544e-42 1.114256e-42 1.114879e-42 4.724546e-48 4.782383e-48 4.840977e-48 4.667462e-48 4.900160e-48

333 1.515923e-42 1.516663e-42 1.518970e-42 1.521315e-42 1.522170e-42 6.448612e-48 6.527571e-48 6.607559e-48 6.370675e-48 6.688368e-48

334 2.069732e-42 2.070747e-42 2.073907e-42 2.077119e-42 2.078291e-42 8.801693e-48 8.909484e-48 9.018675e-48 8.695289e-48 9.129009e-48

335 2.825916e-42 2.827308e-42 2.831635e-42 2.836033e-42 2.837641e-42 1.201324e-47 1.216039e-47 1.230944e-47 1.186798e-47 1.246008e-47

336 3.858437e-42 3.860347e-42 3.866272e-42 3.872294e-42 3.874500e-42 1.639637e-47 1.659724e-47 1.680070e-47 1.619806e-47 1.700636e-47

337 5.268289e-42 5.270909e-42 5.279020e-42 5.287265e-42 5.290291e-42 2.237831e-47 2.265251e-47 2.293024e-47 2.210759e-47 2.321101e-47

338 7.193357e-42 7.196952e-42 7.208054e-42 7.219343e-42 7.223493e-42 3.054197e-47 3.091626e-47 3.129535e-47 3.017241e-47 3.167865e-47

339 9.821895e-42 9.826828e-42 9.842022e-42 9.857474e-42 9.863167e-42 4.168261e-47 4.219351e-47 4.271094e-47 4.117815e-47 4.323419e-47

340 1.341090e-41 1.341767e-41 1.343846e-41 1.345961e-41 1.346742e-41 5.688504e-47 5.758239e-47 5.828862e-47 5.619645e-47 5.900288e-47

341 1.831120e-41 1.832049e-41 1.834893e-41 1.837787e-41 1.838858e-41 7.762895e-47 7.858075e-47 7.954465e-47 7.668908e-47 8.051960e-47

342 2.500167e-41 2.501441e-41 2.505331e-41 2.509289e-41 2.510759e-41 1.059324e-46 1.072314e-46 1.085470e-46 1.046496e-46 1.098777e-46

343 3.413589e-41 3.415338e-41 3.420656e-41 3.426070e-41 3.428086e-41 1.445474e-46 1.463203e-46 1.481156e-46 1.427967e-46 1.499318e-46

344 4.660589e-41 4.662988e-41 4.670257e-41 4.677659e-41 4.680424e-41 1.972265e-46 1.996459e-46 2.020959e-46 1.948373e-46 2.045746e-46

345 6.362891e-41 6.366182e-41 6.376114e-41 6.386233e-41 6.390025e-41 2.690857e-46 2.723871e-46 2.757303e-46 2.658252e-46 2.791129e-46

346 8.686591e-41 8.691106e-41 8.704672e-41 8.718498e-41 8.723699e-41 3.670989e-46 3.716038e-46 3.761654e-46 3.626499e-46 3.807813e-46

347 1.185830e-40 1.186450e-40 1.188302e-40 1.190190e-40 1.190904e-40 5.007719e-46 5.069184e-46 5.131422e-46 4.947016e-46 5.194405e-46

348 1.618717e-40 1.619567e-40 1.622095e-40 1.624673e-40 1.625651e-40 6.830591e-46 6.914447e-46 6.999359e-46 6.747772e-46 7.085290e-46

349 2.209488e-40 2.210654e-40 2.214102e-40 2.217621e-40 2.218963e-40 9.316123e-46 9.430518e-46 9.546353e-46 9.203140e-46 9.663584e-46

350 3.015656e-40 3.017255e-40 3.021957e-40 3.026757e-40 3.028597e-40 1.270480e-45 1.286084e-45 1.301885e-45 1.255068e-45 1.317877e-45

351 4.115648e-40 4.117841e-40 4.124248e-40 4.130794e-40 4.133317e-40 1.732421e-45 1.753704e-45 1.775255e-45 1.711400e-45 1.797068e-45

352 5.616396e-40 5.619403e-40 5.628130e-40 5.637050e-40 5.640509e-40 2.362048e-45 2.391075e-45 2.420466e-45 2.333379e-45 2.450216e-45

353 7.663671e-40 7.667793e-40 7.679673e-40 7.691823e-40 7.696565e-40 3.220112e-45 3.259694e-45 3.299774e-45 3.181016e-45 3.340343e-45

354 1.045615e-39 1.046180e-39 1.047797e-39 1.049450e-39 1.050100e-39 4.389313e-45 4.443285e-45 4.497933e-45 4.336007e-45 4.553252e-45

355 1.426458e-39 1.427233e-39 1.429430e-39 1.431680e-39 1.432570e-39 5.982218e-45 6.055799e-45 6.130304e-45 5.909542e-45 6.205726e-45

356 1.945779e-39 1.946841e-39 1.949826e-39 1.952885e-39 1.954106e-39 8.151997e-45 8.252301e-45 8.353864e-45 8.052928e-45 8.456679e-45

357 2.653817e-39 2.655272e-39 2.659324e-39 2.663481e-39 2.665153e-39 1.110703e-44 1.124374e-44 1.138217e-44 1.097200e-44 1.152230e-44

358 3.618978e-39 3.620971e-39 3.626469e-39 3.632113e-39 3.634403e-39 1.513070e-44 1.531701e-44 1.550565e-44 1.494669e-44 1.569664e-44

359 4.934381e-39 4.937112e-39 4.944565e-39 4.952221e-39 4.955358e-39 2.060832e-44 2.086218e-44 2.111922e-44 2.035760e-44 2.137945e-44

360 6.726745e-39 6.730485e-39 6.740580e-39 6.750960e-39 6.755256e-39 2.806359e-44 2.840942e-44 2.875959e-44 2.772202e-44 2.911411e-44

361 9.168451e-39 9.173572e-39 9.187234e-39 9.201295e-39 9.207175e-39 3.820805e-44 3.867908e-44 3.915603e-44 3.774282e-44 3.963892e-44

362 5.468469e-03 5.468369e-03 5.468144e-03 5.467913e-03 5.467796e-03 4.486461e-06 4.414185e-06 4.342584e-06 4.559411e-06 4.285707e-06

363 1.091525e-02 1.091503e-02 1.091453e-02 1.091401e-02 1.091376e-02 8.455106e-06 8.318418e-06 8.183013e-06 8.593079e-06 8.075492e-06

364 1.633567e-02 1.633531e-02 1.633446e-02 1.633360e-02 1.633318e-02 1.196311e-05 1.176905e-05 1.157683e-05 1.215900e-05 1.142425e-05

365 2.172446e-02 2.172393e-02 2.172268e-02 2.172139e-02 2.172078e-02 1.506162e-05 1.481650e-05 1.457372e-05 1.530907e-05 1.438107e-05

366 2.707577e-02 2.707505e-02 2.707330e-02 2.707152e-02 2.707067e-02 1.779637e-05 1.750584e-05 1.721809e-05 1.808968e-05 1.698983e-05

367 3.238312e-02 3.238216e-02 3.237983e-02 3.237744e-02 3.237632e-02 2.020821e-05 1.987731e-05 1.954960e-05 2.054229e-05 1.928972e-05

368 3.763933e-02 3.763810e-02 3.763508e-02 3.763199e-02 3.763055e-02 2.233362e-05 2.196687e-05 2.160367e-05 2.270392e-05 2.131573e-05

369 4.283655e-02 4.283501e-02 4.283119e-02 4.282727e-02 4.282546e-02 2.420514e-05 2.380657e-05 2.341187e-05 2.460759e-05 2.309905e-05

370 4.796625e-02 4.796434e-02 4.795958e-02 4.795470e-02 4.795246e-02 2.585178e-05 2.542498e-05 2.500235e-05 2.628275e-05 2.466748e-05

371 5.301916e-02 5.301683e-02 5.301098e-02 5.300499e-02 5.300225e-02 2.729938e-05 2.684756e-05 2.640017e-05 2.775564e-05 2.604578e-05

372 5.798532e-02 5.798250e-02 5.797540e-02 5.796813e-02 5.796483e-02 2.857096e-05 2.809698e-05 2.762766e-05 2.904962e-05 2.725600e-05

373 6.285410e-02 6.285071e-02 6.284219e-02 6.283346e-02 6.282950e-02 2.968698e-05 2.919337e-05 2.870466e-05 3.018547e-05 2.831772e-05

374 6.761422e-02 6.761020e-02 6.760006e-02 6.758968e-02 6.758497e-02 3.066562e-05 3.015467e-05 2.964880e-05 3.118166e-05 2.924836e-05

375 7.225389e-02 7.224914e-02 7.223717e-02 7.222493e-02 7.221936e-02 3.152307e-05 3.099678e-05 3.047575e-05 3.205460e-05 3.006339e-05

376 7.676082e-02 7.675525e-02 7.674124e-02 7.672690e-02 7.672038e-02 3.227365e-05 3.173382e-05 3.119939e-05 3.281888e-05 3.077652e-05

377 8.112247e-02 8.111597e-02 8.109969e-02 8.108302e-02 8.107541e-02 3.293009e-05 3.237831e-05 3.183207e-05 3.348742e-05 3.139993e-05

378 8.532613e-02 8.531860e-02 8.529980e-02 8.528056e-02 8.527175e-02 3.350367e-05 3.294135e-05 3.238470e-05 3.407166e-05 3.194443e-05

379 8.935925e-02 8.935058e-02 8.932902e-02 8.930694e-02 8.929681e-02 3.400438e-05 3.343277e-05 3.286698e-05 3.458176e-05 3.241964e-05

380 9.320965e-02 9.319971e-02 9.317514e-02 9.315000e-02 9.313839e-02 3.444108e-05 3.386130e-05 3.328753e-05 3.502670e-05 3.283403e-05

381 9.686585e-02 9.685453e-02 9.682672e-02 9.679825e-02 9.678505e-02 3.482164e-05 3.423467e-05 3.365394e-05 3.541445e-05 3.319509e-05

382 1.003174e-01 1.003046e-01 1.002734e-01 1.002413e-01 1.002264e-01 3.515301e-05 3.455971e-05 3.397291e-05 3.575209e-05 3.350941e-05

383 1.035554e-01 1.035410e-01 1.035061e-01 1.034703e-01 1.034535e-01 3.544128e-05 3.484242e-05 3.425034e-05 3.604582e-05 3.378280e-05

384 1.065726e-01 1.065565e-01 1.065177e-01 1.064780e-01 1.064592e-01 3.569185e-05 3.508810e-05 3.449142e-05 3.630114e-05 3.402036e-05

385 1.093640e-01 1.093461e-01 1.093033e-01 1.092595e-01 1.092387e-01 3.590943e-05 3.530140e-05 3.470071e-05 3.652286e-05 3.422661e-05

386 1.119269e-01 1.119072e-01 1.118603e-01 1.118123e-01 1.117893e-01 3.609820e-05 3.548640e-05 3.488223e-05 3.671523e-05 3.440549e-05

387 1.142614e-01 1.142398e-01 1.141887e-01 1.141364e-01 1.141113e-01 3.626181e-05 3.564671e-05 3.503951e-05 3.688196e-05 3.456049e-05

388 1.163702e-01 1.163466e-01 1.162914e-01 1.162349e-01 1.162075e-01 3.640346e-05 3.578548e-05 3.517565e-05 3.702633e-05 3.469464e-05

389 1.182587e-01 1.182332e-01 1.181739e-01 1.181131e-01 1.180836e-01 3.652598e-05 3.590547e-05 3.529335e-05 3.715120e-05 3.481063e-05

390 1.199350e-01 1.199076e-01 1.198442e-01 1.197794e-01 1.197477e-01 3.663183e-05 3.600911e-05 3.539501e-05 3.725910e-05 3.491081e-05

391 1.214094e-01 1.213802e-01 1.213129e-01 1.212442e-01 1.212103e-01 3.672318e-05 3.609852e-05 3.548271e-05 3.735222e-05 3.499723e-05

392 1.226943e-01 1.226632e-01 1.225923e-01 1.225197e-01 1.224838e-01 3.680192e-05 3.617557e-05 3.555827e-05 3.743250e-05 3.507169e-05

393 1.238033e-01 1.237705e-01 1.236961e-01 1.236200e-01 1.235821e-01 3.686970e-05 3.624189e-05 3.562330e-05 3.750162e-05 3.513576e-05

394 1.247512e-01 1.247169e-01 1.246392e-01 1.245598e-01 1.245201e-01 3.692799e-05 3.629889e-05 3.567919e-05 3.756105e-05 3.519083e-05

395 1.255534e-01 1.255176e-01 1.254370e-01 1.253545e-01 1.253131e-01 3.697804e-05 3.634782e-05 3.572716e-05 3.761209e-05 3.523809e-05

396 1.262253e-01 1.261882e-01 1.261048e-01 1.260196e-01 1.259767e-01 3.702095e-05 3.638976e-05 3.576827e-05 3.765586e-05 3.527859e-05

397 1.267821e-01 1.267436e-01 1.266579e-01 1.265701e-01 1.265258e-01 3.705769e-05 3.642566e-05 3.580345e-05 3.769335e-05 3.531325e-05

398 1.272380e-01 1.271985e-01 1.271105e-01 1.270206e-01 1.269750e-01 3.708911e-05 3.645634e-05 3.583351e-05 3.772540e-05 3.534285e-05

399 1.276069e-01 1.275663e-01 1.274765e-01 1.273845e-01 1.273378e-01 3.711592e-05 3.648251e-05 3.585915e-05 3.775276e-05 3.536811e-05

400 1.279011e-01 1.278597e-01 1.277681e-01 1.276744e-01 1.276267e-01 3.713876e-05 3.650480e-05 3.588098e-05 3.777608e-05 3.538961e-05

401 1.281321e-01 1.280899e-01 1.279969e-01 1.279017e-01 1.278531e-01 3.715819e-05 3.652375e-05 3.589954e-05 3.779591e-05 3.540789e-05

402 1.283100e-01 1.282672e-01 1.281728e-01 1.280764e-01 1.280270e-01 3.717468e-05 3.653983e-05 3.591528e-05 3.781275e-05 3.542339e-05

403 1.284439e-01 1.284004e-01 1.283050e-01 1.282075e-01 1.281575e-01 3.718865e-05 3.655345e-05 3.592861e-05 3.782702e-05 3.543650e-05

404 1.285415e-01 1.284975e-01 1.284012e-01 1.283027e-01 1.282521e-01 3.720046e-05 3.656495e-05 3.593986e-05 3.783909e-05 3.544758e-05

405 1.286096e-01 1.285652e-01 1.284681e-01 1.283688e-01 1.283178e-01 3.721042e-05 3.657465e-05 3.594935e-05 3.784927e-05 3.545691e-05

406 1.286540e-01 1.286093e-01 1.285115e-01 1.284116e-01 1.283601e-01 3.721880e-05 3.658280e-05 3.595732e-05 3.785784e-05 3.546476e-05

407 1.286796e-01 1.286346e-01 1.285363e-01 1.284358e-01 1.283840e-01 3.722583e-05 3.658963e-05 3.596400e-05 3.786503e-05 3.547133e-05

408 1.286905e-01 1.286452e-01 1.285465e-01 1.284455e-01 1.283935e-01 3.723171e-05 3.659534e-05 3.596958e-05 3.787104e-05 3.547681e-05

409 1.286900e-01 1.286446e-01 1.285455e-01 1.284442e-01 1.283919e-01 3.723662e-05 3.660010e-05 3.597422e-05 3.787606e-05 3.548138e-05

410 1.286811e-01 1.286355e-01 1.285361e-01 1.284345e-01 1.283820e-01 3.724069e-05 3.660405e-05 3.597808e-05 3.788023e-05 3.548517e-05

411 1.286660e-01 1.286203e-01 1.285206e-01 1.284187e-01 1.283662e-01 3.724406e-05 3.660732e-05 3.598126e-05 3.788369e-05 3.548830e-05

412 1.286465e-01 1.286007e-01 1.285009e-01 1.283988e-01 1.283461e-01 3.724684e-05 3.661001e-05 3.598389e-05 3.788654e-05 3.549088e-05

413 1.286242e-01 1.285783e-01 1.284783e-01 1.283761e-01 1.283233e-01 3.724912e-05 3.661221e-05 3.598603e-05 3.788887e-05 3.549298e-05

414 1.286001e-01 1.285542e-01 1.284541e-01 1.283518e-01 1.282989e-01 3.725098e-05 3.661400e-05 3.598778e-05 3.789078e-05 3.549470e-05

415 1.285753e-01 1.285293e-01 1.284291e-01 1.283267e-01 1.282738e-01 3.725248e-05 3.661545e-05 3.598919e-05 3.789233e-05 3.549608e-05

416 1.285504e-01 1.285044e-01 1.284042e-01 1.283017e-01 1.282488e-01 3.725369e-05 3.661662e-05 3.599032e-05 3.789358e-05 3.549719e-05

417 1.285260e-01 1.284799e-01 1.283797e-01 1.282772e-01 1.282242e-01 3.725466e-05 3.661755e-05 3.599122e-05 3.789458e-05 3.549807e-05

418 1.285024e-01 1.284564e-01 1.283561e-01 1.282535e-01 1.282006e-01 3.725543e-05 3.661828e-05 3.599193e-05 3.789536e-05 3.549877e-05

419 1.284799e-01 1.284339e-01 1.283336e-01 1.282310e-01 1.281781e-01 3.725602e-05 3.661885e-05 3.599248e-05 3.789598e-05 3.549930e-05

420 1.284588e-01 1.284127e-01 1.283124e-01 1.282098e-01 1.281569e-01 3.725648e-05 3.661929e-05 3.599290e-05 3.789646e-05 3.549971e-05

421 1.284390e-01 1.283930e-01 1.282927e-01 1.281901e-01 1.281371e-01 3.725683e-05 3.661961e-05 3.599321e-05 3.789682e-05 3.550002e-05

422 1.284207e-01 1.283747e-01 1.282744e-01 1.281718e-01 1.281189e-01 3.725708e-05 3.661985e-05 3.599344e-05 3.789708e-05 3.550024e-05

423 1.284039e-01 1.283579e-01 1.282576e-01 1.281551e-01 1.281021e-01 3.725726e-05 3.662002e-05 3.599360e-05 3.789727e-05 3.550039e-05

424 1.283885e-01 1.283425e-01 1.282422e-01 1.281397e-01 1.280868e-01 3.725738e-05 3.662013e-05 3.599370e-05 3.789740e-05 3.550049e-05

425 1.283746e-01 1.283286e-01 1.282283e-01 1.281258e-01 1.280729e-01 3.725745e-05 3.662019e-05 3.599376e-05 3.789748e-05 3.550054e-05

426 1.283620e-01 1.283160e-01 1.282157e-01 1.281133e-01 1.280604e-01 3.725749e-05 3.662022e-05 3.599378e-05 3.789752e-05 3.550057e-05

427 1.283506e-01 1.283046e-01 1.282044e-01 1.281020e-01 1.280491e-01 3.725750e-05 3.662022e-05 3.599378e-05 3.789753e-05 3.550056e-05

428 1.283405e-01 1.282945e-01 1.281943e-01 1.280919e-01 1.280390e-01 3.725748e-05 3.662020e-05 3.599376e-05 3.789752e-05 3.550054e-05

429 1.283314e-01 1.282854e-01 1.281853e-01 1.280829e-01 1.280300e-01 3.725745e-05 3.662017e-05 3.599372e-05 3.789749e-05 3.550050e-05

430 1.283234e-01 1.282774e-01 1.281773e-01 1.280749e-01 1.280221e-01 3.725741e-05 3.662012e-05 3.599368e-05 3.789745e-05 3.550045e-05

431 1.283163e-01 1.282703e-01 1.281702e-01 1.280678e-01 1.280150e-01 3.725736e-05 3.662007e-05 3.599362e-05 3.789740e-05 3.550040e-05

432 1.283100e-01 1.282641e-01 1.281640e-01 1.280616e-01 1.280088e-01 3.725730e-05 3.662001e-05 3.599357e-05 3.789734e-05 3.550034e-05

433 1.283045e-01 1.282586e-01 1.281585e-01 1.280561e-01 1.280034e-01 3.725724e-05 3.661996e-05 3.599351e-05 3.789729e-05 3.550028e-05

434 1.282997e-01 1.282538e-01 1.281537e-01 1.280514e-01 1.279986e-01 3.725718e-05 3.661990e-05 3.599344e-05 3.789723e-05 3.550022e-05

435 1.282955e-01 1.282496e-01 1.281495e-01 1.280472e-01 1.279945e-01 3.725712e-05 3.661984e-05 3.599339e-05 3.789717e-05 3.550016e-05

436 1.282918e-01 1.282459e-01 1.281459e-01 1.280436e-01 1.279909e-01 3.725707e-05 3.661978e-05 3.599333e-05 3.789711e-05 3.550011e-05

437 1.282887e-01 1.282428e-01 1.281428e-01 1.280405e-01 1.279878e-01 3.725701e-05 3.661972e-05 3.599327e-05 3.789705e-05 3.550005e-05

438 1.282859e-01 1.282401e-01 1.281401e-01 1.280378e-01 1.279851e-01 3.725696e-05 3.661967e-05 3.599322e-05 3.789700e-05 3.550000e-05

439 1.282836e-01 1.282378e-01 1.281377e-01 1.280355e-01 1.279828e-01 3.725691e-05 3.661962e-05 3.599317e-05 3.789695e-05 3.549995e-05

440 1.282816e-01 1.282358e-01 1.281358e-01 1.280335e-01 1.279808e-01 3.725686e-05 3.661957e-05 3.599312e-05 3.789690e-05 3.549990e-05

441 1.282799e-01 1.282341e-01 1.281341e-01 1.280318e-01 1.279791e-01 3.725682e-05 3.661953e-05 3.599308e-05 3.789686e-05 3.549986e-05

442 1.282785e-01 1.282326e-01 1.281326e-01 1.280304e-01 1.279777e-01 3.725678e-05 3.661949e-05 3.599304e-05 3.789682e-05 3.549982e-05

443 1.282772e-01 1.282314e-01 1.281314e-01 1.280292e-01 1.279765e-01 3.725674e-05 3.661946e-05 3.599301e-05 3.789678e-05 3.549979e-05

444 1.282762e-01 1.282304e-01 1.281304e-01 1.280282e-01 1.279755e-01 3.725671e-05 3.661942e-05 3.599298e-05 3.789675e-05 3.549976e-05

445 1.282754e-01 1.282295e-01 1.281296e-01 1.280274e-01 1.279747e-01 3.725668e-05 3.661939e-05 3.599295e-05 3.789672e-05 3.549973e-05

446 1.282746e-01 1.282288e-01 1.281289e-01 1.280267e-01 1.279740e-01 3.725665e-05 3.661937e-05 3.599292e-05 3.789669e-05 3.549970e-05

447 1.282741e-01 1.282282e-01 1.281283e-01 1.280261e-01 1.279734e-01 3.725663e-05 3.661934e-05 3.599290e-05 3.789667e-05 3.549968e-05

448 1.282736e-01 1.282278e-01 1.281278e-01 1.280256e-01 1.279729e-01 3.725661e-05 3.661932e-05 3.599287e-05 3.789665e-05 3.549966e-05

449 1.282732e-01 1.282274e-01 1.281274e-01 1.280252e-01 1.279725e-01 3.725659e-05 3.661930e-05 3.599286e-05 3.789663e-05 3.549964e-05

450 1.282729e-01 1.282271e-01 1.281271e-01 1.280249e-01 1.279722e-01 3.725657e-05 3.661928e-05 3.599284e-05 3.789661e-05 3.549962e-05

451 1.282726e-01 1.282268e-01 1.281268e-01 1.280247e-01 1.279720e-01 3.725655e-05 3.661927e-05 3.599282e-05 3.789659e-05 3.549961e-05

452 1.282724e-01 1.282266e-01 1.281266e-01 1.280245e-01 1.279718e-01 3.725654e-05 3.661926e-05 3.599281e-05 3.789658e-05 3.549959e-05

453 1.282723e-01 1.282264e-01 1.281265e-01 1.280243e-01 1.279716e-01 3.725653e-05 3.661924e-05 3.599280e-05 3.789657e-05 3.549958e-05

454 1.282721e-01 1.282263e-01 1.281264e-01 1.280242e-01 1.279715e-01 3.725652e-05 3.661923e-05 3.599279e-05 3.789656e-05 3.549957e-05

455 1.282720e-01 1.282262e-01 1.281263e-01 1.280241e-01 1.279715e-01 3.725651e-05 3.661922e-05 3.599278e-05 3.789655e-05 3.549956e-05

456 1.282720e-01 1.282262e-01 1.281262e-01 1.280240e-01 1.279714e-01 3.725650e-05 3.661922e-05 3.599277e-05 3.789654e-05 3.549955e-05

457 1.282719e-01 1.282261e-01 1.281262e-01 1.280240e-01 1.279714e-01 3.725649e-05 3.661921e-05 3.599276e-05 3.789653e-05 3.549955e-05

458 1.282719e-01 1.282261e-01 1.281262e-01 1.280240e-01 1.279713e-01 3.725649e-05 3.661920e-05 3.599276e-05 3.789652e-05 3.549954e-05

459 1.282719e-01 1.282261e-01 1.281261e-01 1.280240e-01 1.279713e-01 3.725648e-05 3.661920e-05 3.599275e-05 3.789652e-05 3.549954e-05

460 1.282719e-01 1.282261e-01 1.281261e-01 1.280240e-01 1.279713e-01 3.725648e-05 3.661919e-05 3.599275e-05 3.789651e-05 3.549953e-05

461 1.282719e-01 1.282261e-01 1.281261e-01 1.280240e-01 1.279713e-01 3.725647e-05 3.661919e-05 3.599275e-05 3.789651e-05 3.549953e-05

462 1.282719e-01 1.282261e-01 1.281262e-01 1.280240e-01 1.279713e-01 3.725647e-05 3.661919e-05 3.599274e-05 3.789651e-05 3.549953e-05

463 1.282719e-01 1.282261e-01 1.281262e-01 1.280240e-01 1.279713e-01 3.725647e-05 3.661918e-05 3.599274e-05 3.789650e-05 3.549952e-05

464 1.282719e-01 1.282261e-01 1.281262e-01 1.280240e-01 1.279714e-01 3.725646e-05 3.661918e-05 3.599274e-05 3.789650e-05 3.549952e-05

465 1.282719e-01 1.282261e-01 1.281262e-01 1.280240e-01 1.279714e-01 3.725646e-05 3.661918e-05 3.599274e-05 3.789650e-05 3.549952e-05

466 1.282720e-01 1.282262e-01 1.281262e-01 1.280240e-01 1.279714e-01 3.725646e-05 3.661918e-05 3.599273e-05 3.789650e-05 3.549952e-05

467 1.282720e-01 1.282262e-01 1.281262e-01 1.280241e-01 1.279714e-01 3.725646e-05 3.661918e-05 3.599273e-05 3.789650e-05 3.549952e-05

468 1.282720e-01 1.282262e-01 1.281263e-01 1.280241e-01 1.279714e-01 3.725646e-05 3.661918e-05 3.599273e-05 3.789649e-05 3.549951e-05

469 1.282720e-01 1.282262e-01 1.281263e-01 1.280241e-01 1.279715e-01 3.725646e-05 3.661917e-05 3.599273e-05 3.789649e-05 3.549951e-05

470 1.282720e-01 1.282262e-01 1.281263e-01 1.280241e-01 1.279715e-01 3.725646e-05 3.661917e-05 3.599273e-05 3.789649e-05 3.549951e-05

471 1.282721e-01 1.282263e-01 1.281263e-01 1.280241e-01 1.279715e-01 3.725646e-05 3.661917e-05 3.599273e-05 3.789649e-05 3.549951e-05

472 1.282721e-01 1.282263e-01 1.281263e-01 1.280242e-01 1.279715e-01 3.725645e-05 3.661917e-05 3.599273e-05 3.789649e-05 3.549951e-05

473 1.282721e-01 1.282263e-01 1.281263e-01 1.280242e-01 1.279715e-01 3.725645e-05 3.661917e-05 3.599273e-05 3.789649e-05 3.549951e-05

474 1.282721e-01 1.282263e-01 1.281264e-01 1.280242e-01 1.279715e-01 3.725645e-05 3.661917e-05 3.599273e-05 3.789649e-05 3.549951e-05

475 1.282721e-01 1.282263e-01 1.281264e-01 1.280242e-01 1.279715e-01 3.725645e-05 3.661917e-05 3.599273e-05 3.789649e-05 3.549951e-05

476 1.282721e-01 1.282263e-01 1.281264e-01 1.280242e-01 1.279716e-01 3.725645e-05 3.661917e-05 3.599273e-05 3.789649e-05 3.549951e-05

477 1.282721e-01 1.282263e-01 1.281264e-01 1.280242e-01 1.279716e-01 3.725645e-05 3.661917e-05 3.599273e-05 3.789649e-05 3.549951e-05

478 1.282721e-01 1.282263e-01 1.281264e-01 1.280242e-01 1.279716e-01 3.725645e-05 3.661917e-05 3.599273e-05 3.789649e-05 3.549951e-05

479 1.282722e-01 1.282264e-01 1.281264e-01 1.280242e-01 1.279716e-01 3.725645e-05 3.661917e-05 3.599273e-05 3.789649e-05 3.549951e-05

480 1.282722e-01 1.282264e-01 1.281264e-01 1.280242e-01 1.279716e-01 3.725645e-05 3.661917e-05 3.599273e-05 3.789649e-05 3.549951e-05

481 1.282722e-01 1.282264e-01 1.281264e-01 1.280242e-01 1.279716e-01 3.725645e-05 3.661917e-05 3.599273e-05 3.789649e-05 3.549951e-05

482 1.282722e-01 1.282264e-01 1.281264e-01 1.280243e-01 1.279716e-01 3.725645e-05 3.661917e-05 3.599273e-05 3.789649e-05 3.549951e-05

483 1.282722e-01 1.282264e-01 1.281264e-01 1.280243e-01 1.279716e-01 3.725645e-05 3.661917e-05 3.599273e-05 3.789649e-05 3.549951e-05

484 1.282722e-01 1.282264e-01 1.281264e-01 1.280243e-01 1.279716e-01 3.725645e-05 3.661917e-05 3.599273e-05 3.789649e-05 3.549951e-05

485 1.282722e-01 1.282264e-01 1.281264e-01 1.280243e-01 1.279716e-01 3.725645e-05 3.661917e-05 3.599273e-05 3.789649e-05 3.549951e-05

486 1.282722e-01 1.282264e-01 1.281265e-01 1.280243e-01 1.279716e-01 3.725645e-05 3.661917e-05 3.599273e-05 3.789649e-05 3.549951e-05

487 1.282722e-01 1.282264e-01 1.281265e-01 1.280243e-01 1.279716e-01 3.725645e-05 3.661917e-05 3.599273e-05 3.789649e-05 3.549951e-05

488 1.282722e-01 1.282264e-01 1.281265e-01 1.280243e-01 1.279716e-01 3.725645e-05 3.661917e-05 3.599273e-05 3.789649e-05 3.549951e-05

489 1.282722e-01 1.282264e-01 1.281265e-01 1.280243e-01 1.279716e-01 3.725645e-05 3.661917e-05 3.599273e-05 3.789649e-05 3.549951e-05

490 1.282722e-01 1.282264e-01 1.281265e-01 1.280243e-01 1.279716e-01 3.725645e-05 3.661917e-05 3.599273e-05 3.789649e-05 3.549951e-05

491 1.282722e-01 1.282264e-01 1.281265e-01 1.280243e-01 1.279716e-01 3.725645e-05 3.661917e-05 3.599273e-05 3.789649e-05 3.549951e-05

492 1.282722e-01 1.282264e-01 1.281265e-01 1.280243e-01 1.279716e-01 3.725645e-05 3.661917e-05 3.599273e-05 3.789649e-05 3.549951e-05

493 1.282722e-01 1.282264e-01 1.281265e-01 1.280243e-01 1.279716e-01 3.725645e-05 3.661917e-05 3.599273e-05 3.789649e-05 3.549951e-05

494 1.282722e-01 1.282264e-01 1.281265e-01 1.280243e-01 1.279716e-01 3.725645e-05 3.661917e-05 3.599273e-05 3.789649e-05 3.549951e-05

495 1.282722e-01 1.282264e-01 1.281265e-01 1.280243e-01 1.279716e-01 3.725645e-05 3.661917e-05 3.599273e-05 3.789649e-05 3.549951e-05

496 1.282722e-01 1.282264e-01 1.281265e-01 1.280243e-01 1.279716e-01 3.725645e-05 3.661917e-05 3.599273e-05 3.789649e-05 3.549951e-05

497 1.282722e-01 1.282264e-01 1.281265e-01 1.280243e-01 1.279716e-01 3.725645e-05 3.661917e-05 3.599273e-05 3.789649e-05 3.549951e-05

498 1.282722e-01 1.282264e-01 1.281265e-01 1.280243e-01 1.279716e-01 3.725645e-05 3.661917e-05 3.599273e-05 3.789649e-05 3.549951e-05

499 1.282722e-01 1.282264e-01 1.281265e-01 1.280243e-01 1.279716e-01 3.725645e-05 3.661917e-05 3.599273e-05 3.789649e-05 3.549951e-05

500 1.282722e-01 1.282264e-01 1.281265e-01 1.280243e-01 1.279716e-01 3.725645e-05 3.661917e-05 3.599273e-05 3.789649e-05 3.549951e-05

501 1.282722e-01 1.282264e-01 1.281265e-01 1.280243e-01 1.279716e-01 3.725645e-05 3.661917e-05 3.599273e-05 3.789649e-05 3.549951e-05

502 1.282722e-01 1.282264e-01 1.281265e-01 1.280243e-01 1.279716e-01 3.725645e-05 3.661917e-05 3.599273e-05 3.789649e-05 3.549951e-05

503 1.282722e-01 1.282264e-01 1.281265e-01 1.280243e-01 1.279716e-01 3.725645e-05 3.661917e-05 3.599273e-05 3.789649e-05 3.549951e-05

504 1.282722e-01 1.282264e-01 1.281265e-01 1.280243e-01 1.279716e-01 3.725645e-05 3.661917e-05 3.599273e-05 3.789649e-05 3.549951e-05

505 1.282722e-01 1.282264e-01 1.281265e-01 1.280243e-01 1.279716e-01 3.725645e-05 3.661917e-05 3.599273e-05 3.789649e-05 3.549951e-05

506 1.282722e-01 1.282264e-01 1.281265e-01 1.280243e-01 1.279716e-01 3.725645e-05 3.661917e-05 3.599273e-05 3.789649e-05 3.549951e-05

507 1.282722e-01 1.282264e-01 1.281265e-01 1.280243e-01 1.279716e-01 3.725645e-05 3.661917e-05 3.599273e-05 3.789649e-05 3.549951e-05

508 1.282722e-01 1.282264e-01 1.281265e-01 1.280243e-01 1.279716e-01 3.725645e-05 3.661917e-05 3.599273e-05 3.789649e-05 3.549951e-05

509 1.282722e-01 1.282264e-01 1.281265e-01 1.280243e-01 1.279716e-01 3.725645e-05 3.661917e-05 3.599273e-05 3.789649e-05 3.549951e-05

510 1.282722e-01 1.282264e-01 1.281265e-01 1.280243e-01 1.279716e-01 3.725645e-05 3.661917e-05 3.599273e-05 3.789649e-05 3.549951e-05

511 1.282722e-01 1.282264e-01 1.281265e-01 1.280243e-01 1.279716e-01 3.725645e-05 3.661917e-05 3.599273e-05 3.789649e-05 3.549951e-05

512 1.282722e-01 1.282264e-01 1.281265e-01 1.280243e-01 1.279716e-01 3.725645e-05 3.661917e-05 3.599273e-05 3.789649e-05 3.549951e-05

513 1.282722e-01 1.282264e-01 1.281265e-01 1.280243e-01 1.279716e-01 3.725645e-05 3.661917e-05 3.599273e-05 3.789649e-05 3.549951e-05

514 1.282722e-01 1.282264e-01 1.281265e-01 1.280243e-01 1.279716e-01 3.725645e-05 3.661917e-05 3.599273e-05 3.789649e-05 3.549951e-05

515 1.282722e-01 1.282264e-01 1.281265e-01 1.280243e-01 1.279716e-01 3.725645e-05 3.661917e-05 3.599273e-05 3.789649e-05 3.549951e-05

516 1.282722e-01 1.282264e-01 1.281265e-01 1.280243e-01 1.279716e-01 3.725645e-05 3.661917e-05 3.599273e-05 3.789649e-05 3.549951e-05

517 1.282722e-01 1.282264e-01 1.281265e-01 1.280243e-01 1.279716e-01 3.725645e-05 3.661917e-05 3.599273e-05 3.789649e-05 3.549951e-05

518 1.282722e-01 1.282264e-01 1.281265e-01 1.280243e-01 1.279716e-01 3.725645e-05 3.661917e-05 3.599273e-05 3.789649e-05 3.549951e-05

519 1.282722e-01 1.282264e-01 1.281265e-01 1.280243e-01 1.279716e-01 3.725645e-05 3.661917e-05 3.599273e-05 3.789649e-05 3.549951e-05

520 1.282722e-01 1.282264e-01 1.281265e-01 1.280243e-01 1.279716e-01 3.725645e-05 3.661917e-05 3.599273e-05 3.789649e-05 3.549951e-05

521 1.282722e-01 1.282264e-01 1.281265e-01 1.280243e-01 1.279716e-01 3.725645e-05 3.661917e-05 3.599273e-05 3.789649e-05 3.549951e-05

522 1.282722e-01 1.282264e-01 1.281265e-01 1.280243e-01 1.279716e-01 3.725645e-05 3.661917e-05 3.599273e-05 3.789649e-05 3.549951e-05

523 1.282722e-01 1.282264e-01 1.281265e-01 1.280243e-01 1.279716e-01 3.725645e-05 3.661917e-05 3.599273e-05 3.789649e-05 3.549951e-05

524 1.282722e-01 1.282264e-01 1.281265e-01 1.280243e-01 1.279716e-01 3.725645e-05 3.661917e-05 3.599273e-05 3.789649e-05 3.549951e-05

525 1.282722e-01 1.282264e-01 1.281265e-01 1.280243e-01 1.279716e-01 3.725645e-05 3.661917e-05 3.599273e-05 3.789649e-05 3.549951e-05

526 1.282722e-01 1.282264e-01 1.281265e-01 1.280243e-01 1.279716e-01 3.725645e-05 3.661917e-05 3.599273e-05 3.789649e-05 3.549951e-05

527 1.282722e-01 1.282264e-01 1.281265e-01 1.280243e-01 1.279716e-01 3.725645e-05 3.661917e-05 3.599273e-05 3.789649e-05 3.549951e-05

528 1.282722e-01 1.282264e-01 1.281265e-01 1.280243e-01 1.279716e-01 3.725645e-05 3.661917e-05 3.599273e-05 3.789649e-05 3.549951e-05

529 1.282722e-01 1.282264e-01 1.281265e-01 1.280243e-01 1.279716e-01 3.725645e-05 3.661917e-05 3.599273e-05 3.789649e-05 3.549951e-05

530 1.282722e-01 1.282264e-01 1.281265e-01 1.280243e-01 1.279716e-01 3.725645e-05 3.661917e-05 3.599273e-05 3.789649e-05 3.549951e-05

531 1.282722e-01 1.282264e-01 1.281265e-01 1.280243e-01 1.279716e-01 3.725645e-05 3.661917e-05 3.599273e-05 3.789649e-05 3.549951e-05

532 1.282722e-01 1.282264e-01 1.281265e-01 1.280243e-01 1.279716e-01 3.725645e-05 3.661917e-05 3.599273e-05 3.789649e-05 3.549951e-05

533 1.282722e-01 1.282264e-01 1.281265e-01 1.280243e-01 1.279716e-01 3.725645e-05 3.661917e-05 3.599273e-05 3.789649e-05 3.549951e-05

534 1.282722e-01 1.282264e-01 1.281265e-01 1.280243e-01 1.279716e-01 3.725645e-05 3.661917e-05 3.599273e-05 3.789649e-05 3.549951e-05

535 1.282722e-01 1.282264e-01 1.281265e-01 1.280243e-01 1.279716e-01 3.725645e-05 3.661917e-05 3.599273e-05 3.789649e-05 3.549951e-05

536 1.282722e-01 1.282264e-01 1.281265e-01 1.280243e-01 1.279716e-01 3.725645e-05 3.661917e-05 3.599273e-05 3.789649e-05 3.549951e-05

537 1.282722e-01 1.282264e-01 1.281265e-01 1.280243e-01 1.279716e-01 3.725645e-05 3.661917e-05 3.599273e-05 3.789649e-05 3.549951e-05

538 1.282722e-01 1.282264e-01 1.281265e-01 1.280243e-01 1.279716e-01 3.725645e-05 3.661917e-05 3.599273e-05 3.789649e-05 3.549951e-05

539 1.282722e-01 1.282264e-01 1.281265e-01 1.280243e-01 1.279716e-01 3.725645e-05 3.661917e-05 3.599273e-05 3.789649e-05 3.549951e-05

540 1.282722e-01 1.282264e-01 1.281265e-01 1.280243e-01 1.279716e-01 3.725645e-05 3.661917e-05 3.599273e-05 3.789649e-05 3.549951e-05

541 1.282722e-01 1.282264e-01 1.281265e-01 1.280243e-01 1.279716e-01 3.725645e-05 3.661917e-05 3.599273e-05 3.789649e-05 3.549951e-05

542 1.283595e-01 1.283138e-01 1.282140e-01 1.281121e-01 1.280596e-01 1.318754e-03 1.302951e-03 1.288490e-03 1.333257e-03 1.275424e-03

543 1.284683e-01 1.284228e-01 1.283233e-01 1.282216e-01 1.281692e-01 2.538001e-03 2.507501e-03 2.479640e-03 2.565941e-03 2.454404e-03

544 1.286035e-01 1.285581e-01 1.284590e-01 1.283576e-01 1.283054e-01 3.696902e-03 3.652181e-03 3.611361e-03 3.737848e-03 3.574362e-03

545 1.287702e-01 1.287249e-01 1.286262e-01 1.285253e-01 1.284733e-01 4.797440e-03 4.738980e-03 4.685645e-03 4.850954e-03 4.637293e-03

546 1.289743e-01 1.289293e-01 1.288310e-01 1.287306e-01 1.286788e-01 5.841641e-03 5.769926e-03 5.704521e-03 5.907286e-03 5.645224e-03

547 1.292223e-01 1.291775e-01 1.290798e-01 1.289800e-01 1.289285e-01 6.831542e-03 6.747055e-03 6.670025e-03 6.908879e-03 6.600192e-03

548 1.295214e-01 1.294768e-01 1.293798e-01 1.292807e-01 1.292295e-01 7.769183e-03 7.672407e-03 7.584194e-03 7.857775e-03 7.504231e-03

549 1.298793e-01 1.298350e-01 1.297389e-01 1.296406e-01 1.295897e-01 8.656600e-03 8.548013e-03 8.449057e-03 8.756012e-03 8.359366e-03

550 1.303046e-01 1.302607e-01 1.301655e-01 1.300682e-01 1.300177e-01 9.495815e-03 9.375892e-03 9.266629e-03 9.605616e-03 9.167605e-03

551 1.308067e-01 1.307632e-01 1.306690e-01 1.305728e-01 1.305229e-01 1.028883e-02 1.015804e-02 1.003890e-02 1.040860e-02 9.930935e-03

552 1.313953e-01 1.313523e-01 1.312594e-01 1.311645e-01 1.311151e-01 1.103763e-02 1.089643e-02 1.076783e-02 1.116693e-02 1.065131e-02

553 1.320813e-01 1.320388e-01 1.319474e-01 1.318538e-01 1.318051e-01 1.174415e-02 1.159300e-02 1.145535e-02 1.188259e-02 1.133066e-02

554 1.328757e-01 1.328339e-01 1.327441e-01 1.326522e-01 1.326042e-01 1.241030e-02 1.224964e-02 1.210336e-02 1.255747e-02 1.197085e-02

555 1.337906e-01 1.337495e-01 1.336614e-01 1.335714e-01 1.335243e-01 1.303795e-02 1.286822e-02 1.271370e-02 1.319345e-02 1.257374e-02

556 1.348380e-01 1.347977e-01 1.347117e-01 1.346238e-01 1.345776e-01 1.362893e-02 1.345055e-02 1.328817e-02 1.379238e-02 1.314112e-02

557 1.360306e-01 1.359912e-01 1.359075e-01 1.358219e-01 1.357767e-01 1.418501e-02 1.399839e-02 1.382854e-02 1.435603e-02 1.367472e-02

558 1.373810e-01 1.373426e-01 1.372614e-01 1.371784e-01 1.371345e-01 1.470792e-02 1.451346e-02 1.433649e-02 1.488615e-02 1.417625e-02

559 1.389018e-01 1.388646e-01 1.387861e-01 1.387059e-01 1.386633e-01 1.519933e-02 1.499742e-02 1.481368e-02 1.538441e-02 1.464733e-02

560 1.406052e-01 1.405693e-01 1.404938e-01 1.404167e-01 1.403755e-01 1.566085e-02 1.545186e-02 1.526171e-02 1.585244e-02 1.508956e-02

561 1.425027e-01 1.424681e-01 1.423959e-01 1.423221e-01 1.422825e-01 1.609405e-02 1.587835e-02 1.568210e-02 1.629183e-02 1.550445e-02

562 1.446046e-01 1.445715e-01 1.445027e-01 1.444325e-01 1.443946e-01 1.650043e-02 1.627837e-02 1.607634e-02 1.670407e-02 1.589348e-02

563 1.469200e-01 1.468885e-01 1.468234e-01 1.467568e-01 1.467208e-01 1.688145e-02 1.665335e-02 1.644586e-02 1.709064e-02 1.625805e-02

564 1.494560e-01 1.494261e-01 1.493649e-01 1.493023e-01 1.492682e-01 1.723848e-02 1.700468e-02 1.679201e-02 1.745294e-02 1.659953e-02

565 1.522176e-01 1.521895e-01 1.521323e-01 1.520737e-01 1.520418e-01 1.757287e-02 1.733367e-02 1.711610e-02 1.779230e-02 1.691920e-02

566 1.552075e-01 1.551813e-01 1.551281e-01 1.550737e-01 1.550439e-01 1.788588e-02 1.764158e-02 1.741939e-02 1.811002e-02 1.721831e-02

567 1.584257e-01 1.584013e-01 1.583523e-01 1.583022e-01 1.582745e-01 1.817874e-02 1.792963e-02 1.770306e-02 1.840733e-02 1.749805e-02

568 1.618693e-01 1.618469e-01 1.618020e-01 1.617561e-01 1.617306e-01 1.845261e-02 1.819895e-02 1.796827e-02 1.868540e-02 1.775953e-02

569 1.655330e-01 1.655125e-01 1.654717e-01 1.654300e-01 1.654067e-01 1.870861e-02 1.845066e-02 1.821609e-02 1.894535e-02 1.800384e-02

570 1.694086e-01 1.693900e-01 1.693532e-01 1.693156e-01 1.692944e-01 1.894778e-02 1.868579e-02 1.844756e-02 1.918826e-02 1.823200e-02

571 1.734859e-01 1.734691e-01 1.734361e-01 1.734024e-01 1.733834e-01 1.917114e-02 1.890534e-02 1.866366e-02 1.941513e-02 1.844499e-02

572 1.777524e-01 1.777374e-01 1.777081e-01 1.776782e-01 1.776612e-01 1.937963e-02 1.911026e-02 1.886532e-02 1.962694e-02 1.864372e-02

573 1.821945e-01 1.821812e-01 1.821553e-01 1.821289e-01 1.821138e-01 1.957416e-02 1.930142e-02 1.905343e-02 1.982459e-02 1.882907e-02

574 1.867973e-01 1.867855e-01 1.867629e-01 1.867398e-01 1.867264e-01 1.975560e-02 1.947969e-02 1.922882e-02 2.000896e-02 1.900187e-02

575 1.915455e-01 1.915352e-01 1.915154e-01 1.914953e-01 1.914836e-01 1.992475e-02 1.964586e-02 1.939230e-02 2.018086e-02 1.916291e-02

576 1.964236e-01 1.964146e-01 1.963975e-01 1.963801e-01 1.963699e-01 2.008238e-02 1.980070e-02 1.954460e-02 2.034109e-02 1.931292e-02

577 2.014165e-01 2.014086e-01 2.013939e-01 2.013790e-01 2.013701e-01 2.022922e-02 1.994492e-02 1.968644e-02 2.049036e-02 1.945261e-02

578 2.065096e-01 2.065028e-01 2.064902e-01 2.064774e-01 2.064698e-01 2.036596e-02 2.007919e-02 1.981848e-02 2.062938e-02 1.958264e-02

579 2.116893e-01 2.116834e-01 2.116727e-01 2.116618e-01 2.116552e-01 2.049324e-02 2.020416e-02 1.994136e-02 2.075880e-02 1.970363e-02

580 2.169430e-01 2.169380e-01 2.169289e-01 2.169196e-01 2.169139e-01 2.061167e-02 2.032043e-02 2.005566e-02 2.087925e-02 1.981616e-02

581 2.222594e-01 2.222550e-01 2.222473e-01 2.222394e-01 2.222345e-01 2.072184e-02 2.042857e-02 2.016196e-02 2.099129e-02 1.992080e-02

582 2.276281e-01 2.276243e-01 2.276178e-01 2.276111e-01 2.276068e-01 2.082427e-02 2.052910e-02 2.026077e-02 2.109549e-02 2.001805e-02

583 2.330402e-01 2.330368e-01 2.330312e-01 2.330255e-01 2.330218e-01 2.091948e-02 2.062254e-02 2.035259e-02 2.119235e-02 2.010842e-02

584 2.384877e-01 2.384847e-01 2.384798e-01 2.384749e-01 2.384716e-01 2.100795e-02 2.070934e-02 2.043789e-02 2.128237e-02 2.019236e-02

585 2.439637e-01 2.439609e-01 2.439567e-01 2.439524e-01 2.439495e-01 2.109013e-02 2.078997e-02 2.051710e-02 2.136599e-02 2.027029e-02

586 2.494624e-01 2.494598e-01 2.494561e-01 2.494523e-01 2.494495e-01 2.116644e-02 2.086482e-02 2.059064e-02 2.144365e-02 2.034264e-02

587 2.549788e-01 2.549763e-01 2.549729e-01 2.549694e-01 2.549668e-01 2.123727e-02 2.093429e-02 2.065888e-02 2.151575e-02 2.040976e-02

588 2.605087e-01 2.605063e-01 2.605030e-01 2.604998e-01 2.604971e-01 2.130300e-02 2.099876e-02 2.072219e-02 2.158267e-02 2.047204e-02

589 2.660486e-01 2.660461e-01 2.660429e-01 2.660397e-01 2.660370e-01 2.136398e-02 2.105855e-02 2.078091e-02 2.164475e-02 2.052978e-02

590 2.715955e-01 2.715928e-01 2.715896e-01 2.715864e-01 2.715836e-01 2.142053e-02 2.111399e-02 2.083535e-02 2.170233e-02 2.058331e-02

591 2.771470e-01 2.771441e-01 2.771408e-01 2.771374e-01 2.771343e-01 2.147296e-02 2.116539e-02 2.088580e-02 2.175572e-02 2.063292e-02

592 2.827010e-01 2.826978e-01 2.826943e-01 2.826907e-01 2.826872e-01 2.152155e-02 2.121302e-02 2.093256e-02 2.180521e-02 2.067889e-02

593 2.882559e-01 2.882523e-01 2.882485e-01 2.882445e-01 2.882407e-01 2.156657e-02 2.125714e-02 2.097587e-02 2.185107e-02 2.072146e-02

594 2.938104e-01 2.938063e-01 2.938020e-01 2.937976e-01 2.937933e-01 2.160827e-02 2.129800e-02 2.101597e-02 2.189355e-02 2.076088e-02

595 2.993633e-01 2.993587e-01 2.993538e-01 2.993489e-01 2.993440e-01 2.164689e-02 2.133584e-02 2.105310e-02 2.193290e-02 2.079736e-02

596 3.049136e-01 3.049084e-01 3.049029e-01 3.048972e-01 3.048916e-01 2.168263e-02 2.137086e-02 2.108746e-02 2.196932e-02 2.083113e-02

597 3.104606e-01 3.104546e-01 3.104483e-01 3.104419e-01 3.104355e-01 2.171571e-02 2.140326e-02 2.111925e-02 2.200304e-02 2.086236e-02

598 3.160037e-01 3.159968e-01 3.159896e-01 3.159823e-01 3.159749e-01 2.174632e-02 2.143324e-02 2.114865e-02 2.203423e-02 2.089125e-02

599 3.215421e-01 3.215342e-01 3.215260e-01 3.215176e-01 3.215091e-01 2.177463e-02 2.146096e-02 2.117584e-02 2.206309e-02 2.091796e-02

600 3.270755e-01 3.270664e-01 3.270570e-01 3.270474e-01 3.270377e-01 2.180081e-02 2.148659e-02 2.120098e-02 2.208978e-02 2.094265e-02

601 3.326032e-01 3.325929e-01 3.325821e-01 3.325710e-01 3.325599e-01 2.182500e-02 2.151028e-02 2.122421e-02 2.211445e-02 2.096546e-02

602 3.381250e-01 3.381131e-01 3.381007e-01 3.380881e-01 3.380753e-01 2.184737e-02 2.153217e-02 2.124567e-02 2.213725e-02 2.098653e-02

603 3.436402e-01 3.436266e-01 3.436124e-01 3.435979e-01 3.435833e-01 2.186803e-02 2.155240e-02 2.126549e-02 2.215832e-02 2.100600e-02

604 3.491484e-01 3.491328e-01 3.491167e-01 3.491001e-01 3.490833e-01 2.188711e-02 2.157107e-02 2.128380e-02 2.217779e-02 2.102397e-02

605 3.546491e-01 3.546313e-01 3.546128e-01 3.545939e-01 3.545747e-01 2.190473e-02 2.158831e-02 2.130070e-02 2.219576e-02 2.104055e-02

606 3.601417e-01 3.601215e-01 3.601004e-01 3.600787e-01 3.600568e-01 2.192100e-02 2.160423e-02 2.131629e-02 2.221236e-02 2.105586e-02

607 3.656258e-01 3.656027e-01 3.655786e-01 3.655539e-01 3.655289e-01 2.193601e-02 2.161891e-02 2.133068e-02 2.222768e-02 2.106998e-02

608 3.711006e-01 3.710743e-01 3.710468e-01 3.710186e-01 3.709900e-01 2.194986e-02 2.163246e-02 2.134395e-02 2.224181e-02 2.108300e-02

609 3.765654e-01 3.765355e-01 3.765042e-01 3.764720e-01 3.764394e-01 2.196263e-02 2.164495e-02 2.135619e-02 2.225485e-02 2.109501e-02

610 3.820195e-01 3.819854e-01 3.819498e-01 3.819131e-01 3.818759e-01 2.197441e-02 2.165647e-02 2.136747e-02 2.226688e-02 2.110608e-02

611 3.874620e-01 3.874232e-01 3.873826e-01 3.873408e-01 3.872984e-01 2.198528e-02 2.166709e-02 2.137787e-02 2.227797e-02 2.111628e-02

612 3.928918e-01 3.928478e-01 3.928016e-01 3.927540e-01 3.927057e-01 2.199529e-02 2.167688e-02 2.138745e-02 2.228819e-02 2.112568e-02

613 3.983080e-01 3.982580e-01 3.982056e-01 3.981514e-01 3.980964e-01 2.200451e-02 2.168589e-02 2.139628e-02 2.229761e-02 2.113433e-02

614 4.037094e-01 4.036526e-01 4.035931e-01 4.035315e-01 4.034689e-01 2.201300e-02 2.169420e-02 2.140441e-02 2.230628e-02 2.114230e-02

615 4.090947e-01 4.090303e-01 4.089626e-01 4.088927e-01 4.088214e-01 2.202083e-02 2.170184e-02 2.141189e-02 2.231428e-02 2.114964e-02

616 4.144624e-01 4.143894e-01 4.143126e-01 4.142332e-01 4.141522e-01 2.202803e-02 2.170888e-02 2.141878e-02 2.232164e-02 2.115639e-02

617 4.198110e-01 4.197282e-01 4.196412e-01 4.195511e-01 4.194590e-01 2.203466e-02 2.171536e-02 2.142512e-02 2.232841e-02 2.116261e-02

618 4.251388e-01 4.250450e-01 4.249464e-01 4.248442e-01 4.247397e-01 2.204077e-02 2.172132e-02 2.143095e-02 2.233465e-02 2.116832e-02

619 4.304438e-01 4.303378e-01 4.302261e-01 4.301103e-01 4.299917e-01 2.204638e-02 2.172680e-02 2.143632e-02 2.234038e-02 2.117358e-02

620 4.357242e-01 4.356042e-01 4.354779e-01 4.353468e-01 4.352124e-01 2.205154e-02 2.173185e-02 2.144125e-02 2.234566e-02 2.117841e-02

621 4.409775e-01 4.408420e-01 4.406992e-01 4.405508e-01 4.403986e-01 2.205629e-02 2.173648e-02 2.144578e-02 2.235051e-02 2.118285e-02

622 4.462014e-01 4.460485e-01 4.458873e-01 4.457195e-01 4.455473e-01 2.206065e-02 2.174074e-02 2.144995e-02 2.235498e-02 2.118693e-02

623 4.513934e-01 4.512210e-01 4.510391e-01 4.508496e-01 4.506549e-01 2.206466e-02 2.174466e-02 2.145377e-02 2.235908e-02 2.119068e-02

624 4.565504e-01 4.563564e-01 4.561514e-01 4.559377e-01 4.557178e-01 2.206835e-02 2.174825e-02 2.145729e-02 2.236285e-02 2.119412e-02

625 4.616695e-01 4.614515e-01 4.612206e-01 4.609798e-01 4.607319e-01 2.207173e-02 2.175156e-02 2.146052e-02 2.236631e-02 2.119728e-02

626 4.667474e-01 4.665027e-01 4.662432e-01 4.659722e-01 4.656929e-01 2.207484e-02 2.175459e-02 2.146348e-02 2.236949e-02 2.120018e-02

627 4.717804e-01 4.715062e-01 4.712151e-01 4.709105e-01 4.705963e-01 2.207770e-02 2.175737e-02 2.146620e-02 2.237241e-02 2.120285e-02

628 4.767648e-01 4.764582e-01 4.761320e-01 4.757903e-01 4.754374e-01 2.208032e-02 2.175993e-02 2.146870e-02 2.237509e-02 2.120529e-02

629 4.816966e-01 4.813542e-01 4.809895e-01 4.806068e-01 4.802111e-01 2.208272e-02 2.176227e-02 2.147099e-02 2.237755e-02 2.120753e-02

630 4.865714e-01 4.861898e-01 4.857828e-01 4.853550e-01 4.849121e-01 2.208492e-02 2.176442e-02 2.147309e-02 2.237980e-02 2.120958e-02

631 4.913846e-01 4.909602e-01 4.905070e-01 4.900298e-01 4.895350e-01 2.208695e-02 2.176639e-02 2.147501e-02 2.238187e-02 2.121147e-02

632 4.961315e-01 4.956605e-01 4.951568e-01 4.946257e-01 4.940743e-01 2.208880e-02 2.176820e-02 2.147678e-02 2.238377e-02 2.121319e-02

633 5.008071e-01 5.002853e-01 4.997268e-01 4.991372e-01 4.985241e-01 2.209050e-02 2.176986e-02 2.147840e-02 2.238551e-02 2.121478e-02

634 5.054062e-01 5.048293e-01 5.042116e-01 5.035585e-01 5.028786e-01 2.209206e-02 2.177137e-02 2.147988e-02 2.238711e-02 2.121622e-02

635 5.099233e-01 5.092870e-01 5.086053e-01 5.078839e-01 5.071319e-01 2.209349e-02 2.177277e-02 2.148124e-02 2.238857e-02 2.121755e-02

636 5.143529e-01 5.136527e-01 5.129023e-01 5.121076e-01 5.112782e-01 2.209479e-02 2.177404e-02 2.148248e-02 2.238991e-02 2.121877e-02

637 5.186894e-01 5.179206e-01 5.170967e-01 5.162237e-01 5.153117e-01 2.209599e-02 2.177520e-02 2.148362e-02 2.239114e-02 2.121988e-02

638 5.229270e-01 5.220850e-01 5.211828e-01 5.202266e-01 5.192269e-01 2.209709e-02 2.177627e-02 2.148466e-02 2.239226e-02 2.122090e-02

639 5.270601e-01 5.261403e-01 5.251551e-01 5.241106e-01 5.230182e-01 2.209809e-02 2.177725e-02 2.148561e-02 2.239329e-02 2.122183e-02

640 5.310831e-01 5.300808e-01 5.290080e-01 5.278707e-01 5.266809e-01 2.209901e-02 2.177814e-02 2.148648e-02 2.239423e-02 2.122268e-02

641 5.349905e-01 5.339013e-01 5.327364e-01 5.315019e-01 5.302101e-01 2.209985e-02 2.177896e-02 2.148728e-02 2.239509e-02 2.122346e-02

642 5.387770e-01 5.375965e-01 5.363354e-01 5.349996e-01 5.336020e-01 2.210061e-02 2.177971e-02 2.148801e-02 2.239588e-02 2.122417e-02

643 5.424378e-01 5.411620e-01 5.398007e-01 5.383600e-01 5.368530e-01 2.210132e-02 2.178039e-02 2.148867e-02 2.239660e-02 2.122482e-02

644 5.459682e-01 5.445933e-01 5.431285e-01 5.415796e-01 5.399602e-01 2.210196e-02 2.178101e-02 2.148928e-02 2.239726e-02 2.122541e-02

645 5.493641e-01 5.478869e-01 5.463155e-01 5.446557e-01 5.429215e-01 2.210254e-02 2.178159e-02 2.148984e-02 2.239786e-02 2.122596e-02

646 5.526221e-01 5.510395e-01 5.493591e-01 5.475863e-01 5.457357e-01 2.210308e-02 2.178211e-02 2.149035e-02 2.239841e-02 2.122645e-02

647 5.557391e-01 5.540488e-01 5.522575e-01 5.503703e-01 5.484021e-01 2.210357e-02 2.178258e-02 2.149081e-02 2.239891e-02 2.122691e-02

648 5.587130e-01 5.569131e-01 5.550095e-01 5.530071e-01 5.509211e-01 2.210402e-02 2.178302e-02 2.149123e-02 2.239937e-02 2.122732e-02

649 5.615422e-01 5.596314e-01 5.576149e-01 5.554972e-01 5.532938e-01 2.210442e-02 2.178341e-02 2.149162e-02 2.239979e-02 2.122770e-02

650 5.642260e-01 5.622037e-01 5.600743e-01 5.578419e-01 5.555220e-01 2.210480e-02 2.178378e-02 2.149197e-02 2.240017e-02 2.122804e-02

651 5.667645e-01 5.646306e-01 5.623889e-01 5.600430e-01 5.576085e-01 2.210514e-02 2.178411e-02 2.149229e-02 2.240052e-02 2.122835e-02

652 5.691585e-01 5.669136e-01 5.645609e-01 5.621033e-01 5.595567e-01 2.210545e-02 2.178441e-02 2.149259e-02 2.240084e-02 2.122864e-02

653 5.714096e-01 5.690550e-01 5.665931e-01 5.640263e-01 5.613704e-01 2.210573e-02 2.178468e-02 2.149285e-02 2.240113e-02 2.122890e-02

654 5.735202e-01 5.710577e-01 5.684891e-01 5.658161e-01 5.630544e-01 2.210599e-02 2.178493e-02 2.149310e-02 2.240139e-02 2.122914e-02

655 5.754934e-01 5.729254e-01 5.702529e-01 5.674772e-01 5.646137e-01 2.210622e-02 2.178516e-02 2.149332e-02 2.240163e-02 2.122935e-02

656 5.773330e-01 5.746622e-01 5.718894e-01 5.690149e-01 5.660537e-01 2.210644e-02 2.178537e-02 2.149352e-02 2.240185e-02 2.122955e-02

657 5.790433e-01 5.762730e-01 5.734036e-01 5.704345e-01 5.673803e-01 2.210663e-02 2.178556e-02 2.149371e-02 2.240205e-02 2.122973e-02

658 5.806290e-01 5.777630e-01 5.748011e-01 5.717418e-01 5.685993e-01 2.210681e-02 2.178573e-02 2.149387e-02 2.240224e-02 2.122989e-02

659 5.820954e-01 5.791376e-01 5.760876e-01 5.729428e-01 5.697170e-01 2.210697e-02 2.178589e-02 2.149403e-02 2.240240e-02 2.123004e-02

660 5.834481e-01 5.804028e-01 5.772691e-01 5.740436e-01 5.707394e-01 2.210712e-02 2.178603e-02 2.149417e-02 2.240255e-02 2.123018e-02

661 5.846928e-01 5.815643e-01 5.783517e-01 5.750503e-01 5.716727e-01 2.210725e-02 2.178616e-02 2.149429e-02 2.240269e-02 2.123030e-02

662 5.858355e-01 5.826284e-01 5.793414e-01 5.759690e-01 5.725230e-01 2.210737e-02 2.178628e-02 2.149441e-02 2.240282e-02 2.123041e-02

663 5.868823e-01 5.836010e-01 5.802445e-01 5.768058e-01 5.732961e-01 2.210749e-02 2.178639e-02 2.149451e-02 2.240293e-02 2.123051e-02

664 5.878390e-01 5.844883e-01 5.810668e-01 5.775664e-01 5.739978e-01 2.210759e-02 2.178648e-02 2.149461e-02 2.240304e-02 2.123061e-02

665 5.887117e-01 5.852961e-01 5.818141e-01 5.782567e-01 5.746336e-01 2.210768e-02 2.178657e-02 2.149469e-02 2.240313e-02 2.123069e-02

666 5.895063e-01 5.860301e-01 5.824921e-01 5.788819e-01 5.752088e-01 2.210776e-02 2.178665e-02 2.149477e-02 2.240322e-02 2.123077e-02

667 5.902284e-01 5.866960e-01 5.831061e-01 5.794474e-01 5.757283e-01 2.210784e-02 2.178673e-02 2.149484e-02 2.240329e-02 2.123084e-02

668 5.908835e-01 5.872990e-01 5.836614e-01 5.799581e-01 5.761969e-01 2.210791e-02 2.178679e-02 2.149491e-02 2.240337e-02 2.123090e-02

669 5.914769e-01 5.878443e-01 5.841628e-01 5.804187e-01 5.766189e-01 2.210797e-02 2.178685e-02 2.149497e-02 2.240343e-02 2.123096e-02

670 5.920135e-01 5.883367e-01 5.846148e-01 5.808334e-01 5.769986e-01 2.210803e-02 2.178691e-02 2.149502e-02 2.240349e-02 2.123101e-02

671 5.924981e-01 5.887806e-01 5.850219e-01 5.812065e-01 5.773397e-01 2.210808e-02 2.178696e-02 2.149507e-02 2.240354e-02 2.123106e-02

672 5.929352e-01 5.891803e-01 5.853880e-01 5.815416e-01 5.776458e-01 2.210812e-02 2.178700e-02 2.149511e-02 2.240359e-02 2.123110e-02

673 5.933288e-01 5.895399e-01 5.857168e-01 5.818423e-01 5.779203e-01 2.210817e-02 2.178705e-02 2.149515e-02 2.240363e-02 2.123114e-02

674 5.936829e-01 5.898628e-01 5.860119e-01 5.821118e-01 5.781661e-01 2.210820e-02 2.178708e-02 2.149519e-02 2.240367e-02 2.123117e-02

675 5.940011e-01 5.901526e-01 5.862763e-01 5.823532e-01 5.783860e-01 2.210824e-02 2.178712e-02 2.149522e-02 2.240371e-02 2.123120e-02

676 5.942867e-01 5.904124e-01 5.865132e-01 5.825691e-01 5.785826e-01 2.210827e-02 2.178715e-02 2.149525e-02 2.240374e-02 2.123123e-02

677 5.945428e-01 5.906450e-01 5.867250e-01 5.827621e-01 5.787582e-01 2.210830e-02 2.178717e-02 2.149528e-02 2.240377e-02 2.123126e-02

678 5.947722e-01 5.908531e-01 5.869143e-01 5.829345e-01 5.789149e-01 2.210833e-02 2.178720e-02 2.149530e-02 2.240380e-02 2.123128e-02

679 5.949776e-01 5.910392e-01 5.870834e-01 5.830882e-01 5.790546e-01 2.210835e-02 2.178722e-02 2.149532e-02 2.240382e-02 2.123130e-02

680 5.951612e-01 5.912053e-01 5.872343e-01 5.832253e-01 5.791790e-01 2.210837e-02 2.178724e-02 2.149534e-02 2.240384e-02 2.123132e-02

681 5.953252e-01 5.913536e-01 5.873688e-01 5.833475e-01 5.792898e-01 2.210839e-02 2.178726e-02 2.149536e-02 2.240386e-02 2.123134e-02

682 5.954717e-01 5.914859e-01 5.874886e-01 5.834562e-01 5.793884e-01 2.210841e-02 2.178728e-02 2.149538e-02 2.240388e-02 2.123136e-02

683 5.956023e-01 5.916037e-01 5.875953e-01 5.835529e-01 5.794760e-01 2.210842e-02 2.178729e-02 2.149539e-02 2.240390e-02 2.123137e-02

684 5.957188e-01 5.917086e-01 5.876902e-01 5.836389e-01 5.795539e-01 2.210844e-02 2.178731e-02 2.149541e-02 2.240391e-02 2.123138e-02

685 5.958226e-01 5.918020e-01 5.877745e-01 5.837153e-01 5.796230e-01 2.210845e-02 2.178732e-02 2.149542e-02 2.240393e-02 2.123140e-02

686 5.959149e-01 5.918850e-01 5.878495e-01 5.837832e-01 5.796844e-01 2.210846e-02 2.178733e-02 2.149543e-02 2.240394e-02 2.123141e-02

687 5.959971e-01 5.919587e-01 5.879161e-01 5.838433e-01 5.797387e-01 2.210847e-02 2.178734e-02 2.149544e-02 2.240395e-02 2.123142e-02

688 5.960702e-01 5.920242e-01 5.879751e-01 5.838967e-01 5.797869e-01 2.210848e-02 2.178735e-02 2.149545e-02 2.240396e-02 2.123142e-02

689 5.961351e-01 5.920824e-01 5.880275e-01 5.839440e-01 5.798296e-01 2.210849e-02 2.178736e-02 2.149546e-02 2.240397e-02 2.123143e-02

690 5.961927e-01 5.921340e-01 5.880739e-01 5.839858e-01 5.798674e-01 2.210850e-02 2.178737e-02 2.149546e-02 2.240398e-02 2.123144e-02

691 5.962439e-01 5.921797e-01 5.881150e-01 5.840229e-01 5.799008e-01 2.210851e-02 2.178737e-02 2.149547e-02 2.240398e-02 2.123145e-02

692 5.962893e-01 5.922202e-01 5.881514e-01 5.840557e-01 5.799304e-01 2.210851e-02 2.178738e-02 2.149548e-02 2.240399e-02 2.123145e-02

693 5.963295e-01 5.922561e-01 5.881836e-01 5.840847e-01 5.799565e-01 2.210852e-02 2.178739e-02 2.149548e-02 2.240400e-02 2.123146e-02

694 5.963651e-01 5.922878e-01 5.882120e-01 5.841103e-01 5.799795e-01 2.210852e-02 2.178739e-02 2.149549e-02 2.240400e-02 2.123146e-02

695 5.963967e-01 5.923159e-01 5.882372e-01 5.841329e-01 5.799999e-01 2.210853e-02 2.178739e-02 2.149549e-02 2.240401e-02 2.123146e-02

696 5.964246e-01 5.923407e-01 5.882594e-01 5.841529e-01 5.800178e-01 2.210853e-02 2.178740e-02 2.149549e-02 2.240401e-02 2.123147e-02

697 5.964493e-01 5.923626e-01 5.882790e-01 5.841705e-01 5.800336e-01 2.210853e-02 2.178740e-02 2.149550e-02 2.240401e-02 2.123147e-02

698 5.964711e-01 5.923820e-01 5.882963e-01 5.841860e-01 5.800476e-01 2.210854e-02 2.178741e-02 2.149550e-02 2.240402e-02 2.123147e-02

699 5.964904e-01 5.923990e-01 5.883115e-01 5.841997e-01 5.800599e-01 2.210854e-02 2.178741e-02 2.149550e-02 2.240402e-02 2.123148e-02

700 5.965074e-01 5.924141e-01 5.883250e-01 5.842117e-01 5.800707e-01 2.210854e-02 2.178741e-02 2.149551e-02 2.240402e-02 2.123148e-02

701 5.965225e-01 5.924274e-01 5.883368e-01 5.842223e-01 5.800801e-01 2.210855e-02 2.178741e-02 2.149551e-02 2.240403e-02 2.123148e-02

702 5.965357e-01 5.924391e-01 5.883472e-01 5.842316e-01 5.800885e-01 2.210855e-02 2.178742e-02 2.149551e-02 2.240403e-02 2.123148e-02

703 5.965474e-01 5.924494e-01 5.883564e-01 5.842398e-01 5.800958e-01 2.210855e-02 2.178742e-02 2.149551e-02 2.240403e-02 2.123149e-02

704 5.965577e-01 5.924585e-01 5.883644e-01 5.842470e-01 5.801023e-01 2.210855e-02 2.178742e-02 2.149551e-02 2.240403e-02 2.123149e-02

705 5.965668e-01 5.924664e-01 5.883715e-01 5.842533e-01 5.801079e-01 2.210855e-02 2.178742e-02 2.149552e-02 2.240403e-02 2.123149e-02

706 5.965748e-01 5.924735e-01 5.883777e-01 5.842589e-01 5.801129e-01 2.210856e-02 2.178742e-02 2.149552e-02 2.240404e-02 2.123149e-02

707 5.965818e-01 5.924796e-01 5.883832e-01 5.842637e-01 5.801172e-01 2.210856e-02 2.178742e-02 2.149552e-02 2.240404e-02 2.123149e-02

708 5.965880e-01 5.924850e-01 5.883880e-01 5.842680e-01 5.801210e-01 2.210856e-02 2.178742e-02 2.149552e-02 2.240404e-02 2.123149e-02

709 5.965934e-01 5.924898e-01 5.883922e-01 5.842717e-01 5.801243e-01 2.210856e-02 2.178743e-02 2.149552e-02 2.240404e-02 2.123149e-02

710 5.965982e-01 5.924939e-01 5.883958e-01 5.842750e-01 5.801272e-01 2.210856e-02 2.178743e-02 2.149552e-02 2.240404e-02 2.123149e-02

711 5.966024e-01 5.924976e-01 5.883991e-01 5.842778e-01 5.801298e-01 2.210856e-02 2.178743e-02 2.149552e-02 2.240404e-02 2.123150e-02

712 5.966060e-01 5.925008e-01 5.884019e-01 5.842803e-01 5.801320e-01 2.210856e-02 2.178743e-02 2.149552e-02 2.240404e-02 2.123150e-02

713 5.966092e-01 5.925036e-01 5.884043e-01 5.842825e-01 5.801339e-01 2.210856e-02 2.178743e-02 2.149552e-02 2.240404e-02 2.123150e-02

714 5.966121e-01 5.925060e-01 5.884065e-01 5.842844e-01 5.801356e-01 2.210856e-02 2.178743e-02 2.149552e-02 2.240404e-02 2.123150e-02

715 5.966145e-01 5.925081e-01 5.884084e-01 5.842861e-01 5.801371e-01 2.210856e-02 2.178743e-02 2.149552e-02 2.240404e-02 2.123150e-02

716 5.966167e-01 5.925100e-01 5.884100e-01 5.842875e-01 5.801384e-01 2.210856e-02 2.178743e-02 2.149553e-02 2.240404e-02 2.123150e-02

717 5.966186e-01 5.925116e-01 5.884114e-01 5.842888e-01 5.801395e-01 2.210856e-02 2.178743e-02 2.149553e-02 2.240405e-02 2.123150e-02

718 5.966202e-01 5.925131e-01 5.884127e-01 5.842899e-01 5.801405e-01 2.210857e-02 2.178743e-02 2.149553e-02 2.240405e-02 2.123150e-02

719 5.966217e-01 5.925143e-01 5.884138e-01 5.842908e-01 5.801413e-01 2.210857e-02 2.178743e-02 2.149553e-02 2.240405e-02 2.123150e-02

720 5.966229e-01 5.925154e-01 5.884147e-01 5.842917e-01 5.801421e-01 2.210857e-02 2.178743e-02 2.149553e-02 2.240405e-02 2.123150e-02

721 5.966240e-01 5.925163e-01 5.884155e-01 5.842924e-01 5.801427e-01 2.210857e-02 2.178743e-02 2.149553e-02 2.240405e-02 2.123150e-02

722 5.966237e-01 5.925160e-01 5.884152e-01 5.842920e-01 5.801423e-01 2.515405e-02 2.482913e-02 2.453243e-02 2.545374e-02 2.426256e-02

723 5.966234e-01 5.925157e-01 5.884149e-01 5.842918e-01 5.801421e-01 2.821737e-02 2.788762e-02 2.758516e-02 2.852222e-02 2.730852e-02

724 5.966231e-01 5.925155e-01 5.884147e-01 5.842915e-01 5.801419e-01 3.129513e-02 3.095952e-02 3.065030e-02 3.160612e-02 3.036596e-02

725 5.966230e-01 5.925153e-01 5.884145e-01 5.842914e-01 5.801417e-01 3.438388e-02 3.404136e-02 3.372440e-02 3.470200e-02 3.343141e-02

726 5.966230e-01 5.925153e-01 5.884145e-01 5.842914e-01 5.801417e-01 3.748012e-02 3.712965e-02 3.680395e-02 3.780635e-02 3.650137e-02

727 5.966230e-01 5.925153e-01 5.884145e-01 5.842914e-01 5.801418e-01 4.058037e-02 4.022088e-02 3.988545e-02 4.091570e-02 3.957233e-02

728 5.966231e-01 5.925155e-01 5.884147e-01 5.842916e-01 5.801419e-01 4.368111e-02 4.331157e-02 4.296540e-02 4.402654e-02 4.264081e-02

729 5.966234e-01 5.925157e-01 5.884149e-01 5.842918e-01 5.801422e-01 4.677889e-02 4.639823e-02 4.604033e-02 4.713541e-02 4.570334e-02

730 5.966237e-01 5.925161e-01 5.884153e-01 5.842922e-01 5.801426e-01 4.987026e-02 4.947743e-02 4.910682e-02 5.023886e-02 4.875648e-02

731 5.966242e-01 5.925165e-01 5.884158e-01 5.842927e-01 5.801430e-01 5.295182e-02 5.254578e-02 5.216147e-02 5.333347e-02 5.179687e-02

732 5.966247e-01 5.925171e-01 5.884163e-01 5.842933e-01 5.801436e-01 5.602022e-02 5.559993e-02 5.520096e-02 5.641591e-02 5.482117e-02

733 5.966254e-01 5.925178e-01 5.884171e-01 5.842940e-01 5.801444e-01 5.907218e-02 5.863663e-02 5.822201e-02 5.948288e-02 5.782614e-02

734 5.966262e-01 5.925186e-01 5.884179e-01 5.842948e-01 5.801452e-01 6.210449e-02 6.165266e-02 6.122146e-02 6.253116e-02 6.080860e-02

735 5.966272e-01 5.925195e-01 5.884188e-01 5.842958e-01 5.801462e-01 6.511402e-02 6.464491e-02 6.419620e-02 6.555761e-02 6.376548e-02

736 5.966282e-01 5.925206e-01 5.884199e-01 5.842969e-01 5.801473e-01 6.809775e-02 6.761038e-02 6.714322e-02 6.855918e-02 6.669378e-02

737 5.966294e-01 5.925218e-01 5.884211e-01 5.842981e-01 5.801485e-01 7.105273e-02 7.054615e-02 7.005965e-02 7.153293e-02 6.959064e-02

738 5.966307e-01 5.925232e-01 5.884225e-01 5.842994e-01 5.801499e-01 7.397614e-02 7.344941e-02 7.294268e-02 7.447601e-02 7.245328e-02

739 5.966322e-01 5.925246e-01 5.884239e-01 5.843009e-01 5.801514e-01 7.686528e-02 7.631748e-02 7.578967e-02 7.738569e-02 7.527907e-02

740 5.966338e-01 5.925263e-01 5.884256e-01 5.843026e-01 5.801530e-01 7.971757e-02 7.914781e-02 7.859808e-02 8.025937e-02 7.806550e-02

741 5.966356e-01 5.925280e-01 5.884274e-01 5.843044e-01 5.801548e-01 8.253054e-02 8.193796e-02 8.136550e-02 8.309458e-02 8.081019e-02

742 5.966375e-01 5.925299e-01 5.884293e-01 5.843063e-01 5.801567e-01 8.530189e-02 8.468565e-02 8.408969e-02 8.588896e-02 8.351092e-02

743 5.966395e-01 5.925320e-01 5.884314e-01 5.843084e-01 5.801589e-01 8.802943e-02 8.738873e-02 8.676852e-02 8.864032e-02 8.616559e-02

744 5.966418e-01 5.925343e-01 5.884336e-01 5.843107e-01 5.801611e-01 9.071114e-02 9.004521e-02 8.940002e-02 9.134660e-02 8.877226e-02

745 5.966442e-01 5.925367e-01 5.884361e-01 5.843131e-01 5.801636e-01 9.334513e-02 9.265324e-02 9.198239e-02 9.400587e-02 9.132917e-02

746 5.966468e-01 5.925393e-01 5.884387e-01 5.843157e-01 5.801662e-01 9.592967e-02 9.521112e-02 9.451396e-02 9.661639e-02 9.383468e-02

747 5.966496e-01 5.925421e-01 5.884415e-01 5.843186e-01 5.801691e-01 9.846319e-02 9.771732e-02 9.699324e-02 9.917653e-02 9.628732e-02

748 5.966526e-01 5.925451e-01 5.884445e-01 5.843216e-01 5.801721e-01 1.009443e-01 1.001705e-01 9.941888e-02 1.016848e-01 9.868581e-02

749 5.966558e-01 5.925483e-01 5.884477e-01 5.843248e-01 5.801754e-01 1.033717e-01 1.025693e-01 1.017897e-01 1.041400e-01 1.010290e-01

750 5.966592e-01 5.925518e-01 5.884512e-01 5.843283e-01 5.801789e-01 1.057442e-01 1.049128e-01 1.041047e-01 1.065410e-01 1.033159e-01

751 5.966629e-01 5.925555e-01 5.884549e-01 5.843321e-01 5.801827e-01 1.080611e-01 1.072001e-01 1.063630e-01 1.088867e-01 1.055457e-01

752 5.966669e-01 5.925595e-01 5.884590e-01 5.843361e-01 5.801867e-01 1.103214e-01 1.094304e-01 1.085639e-01 1.111763e-01 1.077177e-01

753 5.966712e-01 5.925638e-01 5.884633e-01 5.843405e-01 5.801911e-01 1.125245e-01 1.116031e-01 1.107069e-01 1.134091e-01 1.098314e-01

754 5.966758e-01 5.925684e-01 5.884679e-01 5.843451e-01 5.801958e-01 1.146700e-01 1.137179e-01 1.127915e-01 1.155847e-01 1.118866e-01

755 5.966807e-01 5.925734e-01 5.884729e-01 5.843502e-01 5.802008e-01 1.167574e-01 1.157743e-01 1.148176e-01 1.177026e-01 1.138829e-01

756 5.966860e-01 5.925787e-01 5.884783e-01 5.843556e-01 5.802063e-01 1.187867e-01 1.177722e-01 1.167850e-01 1.197626e-01 1.158203e-01

757 5.966918e-01 5.925845e-01 5.884842e-01 5.843615e-01 5.802122e-01 1.207578e-01 1.197118e-01 1.186937e-01 1.217645e-01 1.176990e-01

758 5.966980e-01 5.925908e-01 5.884905e-01 5.843678e-01 5.802186e-01 1.226707e-01 1.215930e-01 1.205440e-01 1.237085e-01 1.195191e-01

759 5.967047e-01 5.925975e-01 5.884973e-01 5.843747e-01 5.802256e-01 1.245257e-01 1.234161e-01 1.223362e-01 1.255945e-01 1.212810e-01

760 5.967120e-01 5.926048e-01 5.885047e-01 5.843822e-01 5.802331e-01 1.263230e-01 1.251816e-01 1.240706e-01 1.274228e-01 1.229851e-01

761 5.967198e-01 5.926128e-01 5.885127e-01 5.843903e-01 5.802413e-01 1.280630e-01 1.268899e-01 1.257478e-01 1.291937e-01 1.246321e-01

762 5.967284e-01 5.926214e-01 5.885214e-01 5.843991e-01 5.802502e-01 1.297461e-01 1.285415e-01 1.273685e-01 1.309076e-01 1.262226e-01

763 5.967377e-01 5.926308e-01 5.885309e-01 5.844087e-01 5.802599e-01 1.313731e-01 1.301371e-01 1.289333e-01 1.325650e-01 1.277574e-01

764 5.967478e-01 5.926410e-01 5.885412e-01 5.844191e-01 5.802705e-01 1.329444e-01 1.316774e-01 1.304430e-01 1.341666e-01 1.292373e-01

765 5.967587e-01 5.926521e-01 5.885525e-01 5.844305e-01 5.802820e-01 1.344609e-01 1.331633e-01 1.318985e-01 1.357129e-01 1.306634e-01

766 5.967707e-01 5.926642e-01 5.885647e-01 5.844430e-01 5.802946e-01 1.359234e-01 1.345955e-01 1.333006e-01 1.372048e-01 1.320365e-01

767 5.967837e-01 5.926774e-01 5.885781e-01 5.844566e-01 5.803084e-01 1.373327e-01 1.359750e-01 1.346504e-01 1.386431e-01 1.333577e-01

768 5.967980e-01 5.926919e-01 5.885928e-01 5.844714e-01 5.803235e-01 1.386898e-01 1.373027e-01 1.359489e-01 1.400287e-01 1.346280e-01

769 5.968135e-01 5.927076e-01 5.886088e-01 5.844877e-01 5.803400e-01 1.399956e-01 1.385798e-01 1.371971e-01 1.413626e-01 1.358486e-01

770 5.968305e-01 5.927249e-01 5.886263e-01 5.845055e-01 5.803581e-01 1.412512e-01 1.398073e-01 1.383961e-01 1.426457e-01 1.370206e-01

771 5.968491e-01 5.927438e-01 5.886455e-01 5.845251e-01 5.803780e-01 1.424578e-01 1.409862e-01 1.395471e-01 1.438791e-01 1.381452e-01

772 5.968694e-01 5.927644e-01 5.886666e-01 5.845465e-01 5.803998e-01 1.436164e-01 1.421179e-01 1.406514e-01 1.450639e-01 1.392237e-01

773 5.968916e-01 5.927870e-01 5.886896e-01 5.845700e-01 5.804238e-01 1.447281e-01 1.432034e-01 1.417100e-01 1.462013e-01 1.402572e-01

774 5.969159e-01 5.928118e-01 5.887149e-01 5.845958e-01 5.804501e-01 1.457943e-01 1.442439e-01 1.427243e-01 1.472925e-01 1.412470e-01

775 5.969426e-01 5.928390e-01 5.887427e-01 5.846242e-01 5.804791e-01 1.468160e-01 1.452407e-01 1.436954e-01 1.483385e-01 1.421943e-01

776 5.969717e-01 5.928688e-01 5.887732e-01 5.846553e-01 5.805109e-01 1.477946e-01 1.461951e-01 1.446247e-01 1.493407e-01 1.431005e-01

777 5.970037e-01 5.929015e-01 5.888066e-01 5.846895e-01 5.805459e-01 1.487312e-01 1.471082e-01 1.455134e-01 1.503003e-01 1.439669e-01

778 5.970388e-01 5.929374e-01 5.888434e-01 5.847272e-01 5.805844e-01 1.496271e-01 1.479814e-01 1.463627e-01 1.512186e-01 1.447946e-01

779 5.970773e-01 5.929768e-01 5.888837e-01 5.847686e-01 5.806269e-01 1.504837e-01 1.488159e-01 1.471740e-01 1.520967e-01 1.455850e-01

780 5.971195e-01 5.930201e-01 5.889281e-01 5.848141e-01 5.806736e-01 1.513020e-01 1.496130e-01 1.479486e-01 1.529360e-01 1.463393e-01

781 5.971657e-01 5.930676e-01 5.889769e-01 5.848642e-01 5.807250e-01 1.520835e-01 1.503739e-01 1.486876e-01 1.537376e-01 1.470588e-01

782 5.972165e-01 5.931197e-01 5.890305e-01 5.849194e-01 5.807817e-01 1.528293e-01 1.510998e-01 1.493923e-01 1.545030e-01 1.477447e-01

783 5.972723e-01 5.931771e-01 5.890895e-01 5.849801e-01 5.808442e-01 1.535408e-01 1.517921e-01 1.500640e-01 1.552333e-01 1.483983e-01

784 5.973334e-01 5.932400e-01 5.891544e-01 5.850470e-01 5.809131e-01 1.542190e-01 1.524519e-01 1.507039e-01 1.559297e-01 1.490208e-01

785 5.974006e-01 5.933092e-01 5.892258e-01 5.851206e-01 5.809890e-01 1.548653e-01 1.530804e-01 1.513131e-01 1.565935e-01 1.496133e-01

786 5.974743e-01 5.933853e-01 5.893043e-01 5.852017e-01 5.810727e-01 1.554809e-01 1.536789e-01 1.518930e-01 1.572259e-01 1.501771e-01

787 5.975553e-01 5.934689e-01 5.893908e-01 5.852911e-01 5.811651e-01 1.560668e-01 1.542484e-01 1.524445e-01 1.578280e-01 1.507132e-01

788 5.976442e-01 5.935609e-01 5.894859e-01 5.853896e-01 5.812670e-01 1.566243e-01 1.547901e-01 1.529689e-01 1.584011e-01 1.512228e-01

789 5.977418e-01 5.936620e-01 5.895907e-01 5.854981e-01 5.813793e-01 1.571546e-01 1.553053e-01 1.534673e-01 1.589463e-01 1.517071e-01

790 5.978490e-01 5.937731e-01 5.897059e-01 5.856177e-01 5.815033e-01 1.576586e-01 1.557948e-01 1.539407e-01 1.594647e-01 1.521669e-01

791 5.979667e-01 5.938953e-01 5.898328e-01 5.857495e-01 5.816402e-01 1.581375e-01 1.562599e-01 1.543902e-01 1.599573e-01 1.526035e-01

792 5.980960e-01 5.940296e-01 5.899725e-01 5.858948e-01 5.817911e-01 1.585924e-01 1.567015e-01 1.548169e-01 1.604254e-01 1.530178e-01

793 5.982380e-01 5.941774e-01 5.901263e-01 5.860549e-01 5.819577e-01 1.590243e-01 1.571206e-01 1.552217e-01 1.608699e-01 1.534108e-01

794 5.983940e-01 5.943398e-01 5.902956e-01 5.862313e-01 5.821416e-01 1.594341e-01 1.575183e-01 1.556056e-01 1.612918e-01 1.537834e-01

795 5.985652e-01 5.945184e-01 5.904820e-01 5.864258e-01 5.823444e-01 1.598229e-01 1.578955e-01 1.559695e-01 1.616921e-01 1.541365e-01

796 5.987532e-01 5.947147e-01 5.906871e-01 5.866401e-01 5.825681e-01 1.601916e-01 1.582530e-01 1.563143e-01 1.620718e-01 1.544711e-01

797 5.989596e-01 5.949304e-01 5.909127e-01 5.868761e-01 5.828149e-01 1.605410e-01 1.585919e-01 1.566410e-01 1.624318e-01 1.547880e-01

798 5.991861e-01 5.951675e-01 5.911610e-01 5.871361e-01 5.830871e-01 1.608721e-01 1.589129e-01 1.569503e-01 1.627729e-01 1.550880e-01

799 5.994346e-01 5.954279e-01 5.914341e-01 5.874225e-01 5.833871e-01 1.611858e-01 1.592169e-01 1.572432e-01 1.630961e-01 1.553719e-01

800 5.997072e-01 5.957140e-01 5.917344e-01 5.877377e-01 5.837177e-01 1.614827e-01 1.595047e-01 1.575202e-01 1.634022e-01 1.556406e-01

801 6.000062e-01 5.960280e-01 5.920644e-01 5.880845e-01 5.840819e-01 1.617638e-01 1.597771e-01 1.577823e-01 1.636920e-01 1.558947e-01

802 6.003339e-01 5.963726e-01 5.924270e-01 5.884659e-01 5.844829e-01 1.620298e-01 1.600348e-01 1.580302e-01 1.639663e-01 1.561349e-01

803 6.006930e-01 5.967506e-01 5.928251e-01 5.888852e-01 5.849241e-01 1.622814e-01 1.602784e-01 1.582645e-01 1.642258e-01 1.563619e-01

804 6.010864e-01 5.971650e-01 5.932620e-01 5.893458e-01 5.854093e-01 1.625193e-01 1.605088e-01 1.584859e-01 1.644712e-01 1.565765e-01

805 6.015171e-01 5.976190e-01 5.937412e-01 5.898514e-01 5.859424e-01 1.627442e-01 1.607266e-01 1.586951e-01 1.647033e-01 1.567792e-01

806 6.019885e-01 5.981163e-01 5.942663e-01 5.904060e-01 5.865277e-01 1.629567e-01 1.609323e-01 1.588926e-01 1.649226e-01 1.569705e-01

807 6.025040e-01 5.986604e-01 5.948413e-01 5.910139e-01 5.871696e-01 1.631575e-01 1.611266e-01 1.590792e-01 1.651298e-01 1.571512e-01

808 6.030674e-01 5.992554e-01 5.954705e-01 5.916793e-01 5.878729e-01 1.633471e-01 1.613102e-01 1.592552e-01 1.653256e-01 1.573217e-01

809 6.036827e-01 5.999055e-01 5.961582e-01 5.924071e-01 5.886426e-01 1.635261e-01 1.614834e-01 1.594214e-01 1.655104e-01 1.574826e-01

810 6.043543e-01 6.006151e-01 5.969091e-01 5.932022e-01 5.894839e-01 1.636951e-01 1.616469e-01 1.595781e-01 1.656850e-01 1.576343e-01

811 6.050867e-01 6.013891e-01 5.977282e-01 5.940696e-01 5.904020e-01 1.638546e-01 1.618012e-01 1.597260e-01 1.658497e-01 1.577774e-01

812 6.058845e-01 6.022324e-01 5.986206e-01 5.950147e-01 5.914026e-01 1.640051e-01 1.619467e-01 1.598653e-01 1.660051e-01 1.579123e-01

813 6.067527e-01 6.031500e-01 5.995917e-01 5.960429e-01 5.924912e-01 1.641470e-01 1.620839e-01 1.599967e-01 1.661518e-01 1.580394e-01

814 6.076966e-01 6.041473e-01 6.006468e-01 5.971600e-01 5.936737e-01 1.642808e-01 1.622133e-01 1.601205e-01 1.662900e-01 1.581593e-01

815 6.087213e-01 6.052298e-01 6.017916e-01 5.983716e-01 5.949557e-01 1.644069e-01 1.623352e-01 1.602371e-01 1.664204e-01 1.582721e-01

816 6.098325e-01 6.064030e-01 6.030318e-01 5.996833e-01 5.963430e-01 1.645258e-01 1.624501e-01 1.603470e-01 1.665433e-01 1.583784e-01

817 6.110356e-01 6.076727e-01 6.043730e-01 6.011009e-01 5.978412e-01 1.646378e-01 1.625583e-01 1.604505e-01 1.666591e-01 1.584785e-01

818 6.123363e-01 6.090444e-01 6.058208e-01 6.026298e-01 5.994557e-01 1.647433e-01 1.626603e-01 1.605479e-01 1.667682e-01 1.585727e-01

819 6.137402e-01 6.105236e-01 6.073807e-01 6.042756e-01 6.011918e-01 1.648426e-01 1.627563e-01 1.606395e-01 1.668710e-01 1.586613e-01

820 6.152526e-01 6.121159e-01 6.090580e-01 6.060431e-01 6.030542e-01 1.649362e-01 1.628466e-01 1.607258e-01 1.669677e-01 1.587447e-01

821 6.168791e-01 6.138264e-01 6.108577e-01 6.079373e-01 6.050474e-01 1.650242e-01 1.629317e-01 1.608070e-01 1.670588e-01 1.588232e-01

822 6.186247e-01 6.156601e-01 6.127845e-01 6.099623e-01 6.071750e-01 1.651071e-01 1.630117e-01 1.608833e-01 1.671446e-01 1.588970e-01

823 6.204942e-01 6.176214e-01 6.148425e-01 6.121218e-01 6.094405e-01 1.651851e-01 1.630870e-01 1.609551e-01 1.672253e-01 1.589664e-01

824 6.224921e-01 6.197145e-01 6.170354e-01 6.144191e-01 6.118463e-01 1.652584e-01 1.631578e-01 1.610226e-01 1.673012e-01 1.590316e-01

825 6.246221e-01 6.219428e-01 6.193661e-01 6.168565e-01 6.143943e-01 1.653274e-01 1.632244e-01 1.610860e-01 1.673726e-01 1.590929e-01

826 6.268879e-01 6.243092e-01 6.218370e-01 6.194358e-01 6.170853e-01 1.653923e-01 1.632870e-01 1.611456e-01 1.674398e-01 1.591505e-01

827 6.292919e-01 6.268160e-01 6.244496e-01 6.221579e-01 6.199197e-01 1.654532e-01 1.633458e-01 1.612016e-01 1.675029e-01 1.592047e-01

828 6.318363e-01 6.294644e-01 6.272048e-01 6.250227e-01 6.228967e-01 1.655105e-01 1.634011e-01 1.612543e-01 1.675623e-01 1.592555e-01

829 6.345224e-01 6.322553e-01 6.301025e-01 6.280296e-01 6.260148e-01 1.655644e-01 1.634531e-01 1.613037e-01 1.676181e-01 1.593032e-01

830 6.373506e-01 6.351883e-01 6.331419e-01 6.311770e-01 6.292715e-01 1.656150e-01 1.635019e-01 1.613501e-01 1.676705e-01 1.593481e-01

831 6.403205e-01 6.382626e-01 6.363211e-01 6.344623e-01 6.326637e-01 1.656625e-01 1.635478e-01 1.613936e-01 1.677197e-01 1.593901e-01

832 6.434312e-01 6.414761e-01 6.396376e-01 6.378823e-01 6.361875e-01 1.657071e-01 1.635908e-01 1.614345e-01 1.677660e-01 1.594296e-01

833 6.466805e-01 6.448264e-01 6.430881e-01 6.414330e-01 6.398381e-01 1.657490e-01 1.636312e-01 1.614729e-01 1.678094e-01 1.594667e-01

834 6.500658e-01 6.483099e-01 6.466687e-01 6.451098e-01 6.436103e-01 1.657884e-01 1.636691e-01 1.615089e-01 1.678502e-01 1.595014e-01

835 6.535835e-01 6.519227e-01 6.503745e-01 6.489074e-01 6.474984e-01 1.658253e-01 1.637047e-01 1.615426e-01 1.678885e-01 1.595340e-01

836 6.572296e-01 6.556599e-01 6.542002e-01 6.528200e-01 6.514960e-01 1.658600e-01 1.637381e-01 1.615743e-01 1.679244e-01 1.595646e-01

837 6.609992e-01 6.595162e-01 6.581402e-01 6.568413e-01 6.555964e-01 1.658925e-01 1.637695e-01 1.616040e-01 1.679581e-01 1.595932e-01

838 6.648871e-01 6.634859e-01 6.621881e-01 6.609648e-01 6.597928e-01 1.659229e-01 1.637988e-01 1.616318e-01 1.679897e-01 1.596201e-01

839 6.688876e-01 6.675627e-01 6.663375e-01 6.651836e-01 6.640780e-01 1.659515e-01 1.638264e-01 1.616579e-01 1.680194e-01 1.596453e-01

840 6.729944e-01 6.717403e-01 6.705814e-01 6.694905e-01 6.684447e-01 1.659784e-01 1.638522e-01 1.616824e-01 1.680472e-01 1.596689e-01

841 6.772012e-01 6.760118e-01 6.749130e-01 6.738785e-01 6.728855e-01 1.660035e-01 1.638764e-01 1.617053e-01 1.680733e-01 1.596910e-01

842 6.815014e-01 6.803704e-01 6.793251e-01 6.783402e-01 6.773931e-01 1.660271e-01 1.638991e-01 1.617268e-01 1.680978e-01 1.597117e-01

843 6.858882e-01 6.848091e-01 6.838106e-01 6.828684e-01 6.819600e-01 1.660491e-01 1.639204e-01 1.617469e-01 1.681207e-01 1.597311e-01

844 6.903548e-01 6.893210e-01 6.883624e-01 6.874558e-01 6.865790e-01 1.660698e-01 1.639403e-01 1.617657e-01 1.681422e-01 1.597493e-01

845 6.948945e-01 6.938990e-01 6.929734e-01 6.920954e-01 6.912428e-01 1.660892e-01 1.639590e-01 1.617833e-01 1.681623e-01 1.597663e-01

846 6.995005e-01 6.985364e-01 6.976367e-01 6.967801e-01 6.959444e-01 1.661074e-01 1.639765e-01 1.617998e-01 1.681812e-01 1.597822e-01

847 7.041661e-01 7.032263e-01 7.023454e-01 7.015030e-01 7.006768e-01 1.661244e-01 1.639928e-01 1.618153e-01 1.681988e-01 1.597971e-01

848 7.088847e-01 7.079621e-01 7.070928e-01 7.062572e-01 7.054331e-01 1.661403e-01 1.640082e-01 1.618297e-01 1.682153e-01 1.598110e-01

849 7.136501e-01 7.127374e-01 7.118723e-01 7.110363e-01 7.102068e-01 1.661552e-01 1.640225e-01 1.618432e-01 1.682308e-01 1.598241e-01

850 7.184559e-01 7.175459e-01 7.166776e-01 7.158337e-01 7.149912e-01 1.661691e-01 1.640359e-01 1.618559e-01 1.682453e-01 1.598362e-01

851 7.232962e-01 7.223813e-01 7.215025e-01 7.206431e-01 7.197798e-01 1.661822e-01 1.640485e-01 1.618677e-01 1.682589e-01 1.598476e-01

852 7.281651e-01 7.272378e-01 7.263408e-01 7.254582e-01 7.245664e-01 1.661944e-01 1.640602e-01 1.618788e-01 1.682716e-01 1.598583e-01

853 7.330570e-01 7.321096e-01 7.311867e-01 7.302731e-01 7.293446e-01 1.662058e-01 1.640712e-01 1.618891e-01 1.682835e-01 1.598683e-01

854 7.379664e-01 7.369910e-01 7.360343e-01 7.350817e-01 7.341085e-01 1.662165e-01 1.640815e-01 1.618988e-01 1.682946e-01 1.598776e-01

855 7.428879e-01 7.418766e-01 7.408780e-01 7.398782e-01 7.388518e-01 1.662265e-01 1.640911e-01 1.619078e-01 1.683050e-01 1.598863e-01

856 7.478164e-01 7.467609e-01 7.457121e-01 7.446569e-01 7.435686e-01 1.662359e-01 1.641001e-01 1.619163e-01 1.683147e-01 1.598944e-01

857 7.527468e-01 7.516388e-01 7.505313e-01 7.494119e-01 7.482530e-01 1.662446e-01 1.641085e-01 1.619242e-01 1.683238e-01 1.599020e-01

858 7.576744e-01 7.565051e-01 7.553301e-01 7.541376e-01 7.528990e-01 1.662528e-01 1.641164e-01 1.619315e-01 1.683323e-01 1.599091e-01

859 7.625944e-01 7.613548e-01 7.601033e-01 7.588286e-01 7.575009e-01 1.662604e-01 1.641237e-01 1.619384e-01 1.683402e-01 1.599158e-01

860 7.675020e-01 7.661830e-01 7.648455e-01 7.634791e-01 7.620527e-01 1.662675e-01 1.641305e-01 1.619449e-01 1.683476e-01 1.599220e-01

861 7.723927e-01 7.709846e-01 7.695515e-01 7.680838e-01 7.665489e-01 1.662742e-01 1.641369e-01 1.619509e-01 1.683546e-01 1.599277e-01

862 7.772620e-01 7.757549e-01 7.742163e-01 7.726371e-01 7.709836e-01 1.662804e-01 1.641429e-01 1.619565e-01 1.683610e-01 1.599331e-01

863 7.821053e-01 7.804890e-01 7.788345e-01 7.771337e-01 7.753513e-01 1.662862e-01 1.641485e-01 1.619617e-01 1.683671e-01 1.599382e-01

864 7.869181e-01 7.851822e-01 7.834012e-01 7.815683e-01 7.796465e-01 1.662916e-01 1.641537e-01 1.619666e-01 1.683727e-01 1.599429e-01

865 7.916960e-01 7.898297e-01 7.879114e-01 7.859355e-01 7.838638e-01 1.662967e-01 1.641586e-01 1.619711e-01 1.683780e-01 1.599473e-01

866 7.964346e-01 7.944268e-01 7.923600e-01 7.902302e-01 7.879979e-01 1.663014e-01 1.641631e-01 1.619754e-01 1.683829e-01 1.599513e-01

867 8.011292e-01 7.989687e-01 7.967421e-01 7.944474e-01 7.920437e-01 1.663059e-01 1.641674e-01 1.619793e-01 1.683875e-01 1.599552e-01

868 8.057756e-01 8.034507e-01 8.010528e-01 7.985820e-01 7.959964e-01 1.663100e-01 1.641713e-01 1.619830e-01 1.683918e-01 1.599587e-01

869 8.103691e-01 8.078681e-01 8.052875e-01 8.026295e-01 7.998514e-01 1.663138e-01 1.641750e-01 1.619865e-01 1.683958e-01 1.599620e-01

870 8.149053e-01 8.122162e-01 8.094414e-01 8.065851e-01 8.036044e-01 1.663174e-01 1.641784e-01 1.619897e-01 1.683996e-01 1.599651e-01

871 8.193797e-01 8.164903e-01 8.135100e-01 8.104446e-01 8.072513e-01 1.663207e-01 1.641816e-01 1.619927e-01 1.684031e-01 1.599680e-01

872 8.237877e-01 8.206860e-01 8.174888e-01 8.142039e-01 8.107885e-01 1.663239e-01 1.641846e-01 1.619955e-01 1.684063e-01 1.599707e-01

873 8.281249e-01 8.247986e-01 8.213735e-01 8.178591e-01 8.142128e-01 1.663268e-01 1.641874e-01 1.619981e-01 1.684093e-01 1.599732e-01

874 8.323867e-01 8.288239e-01 8.251602e-01 8.214068e-01 8.175214e-01 1.663295e-01 1.641900e-01 1.620005e-01 1.684122e-01 1.599755e-01

875 8.365688e-01 8.327576e-01 8.288450e-01 8.248436e-01 8.207119e-01 1.663320e-01 1.641924e-01 1.620027e-01 1.684148e-01 1.599777e-01

876 8.406668e-01 8.365955e-01 8.324243e-01 8.281667e-01 8.237824e-01 1.663344e-01 1.641947e-01 1.620048e-01 1.684172e-01 1.599797e-01

877 8.446765e-01 8.403338e-01 8.358949e-01 8.313738e-01 8.267314e-01 1.663365e-01 1.641968e-01 1.620068e-01 1.684195e-01 1.599816e-01

878 8.485938e-01 8.439689e-01 8.392538e-01 8.344628e-01 8.295580e-01 1.663386e-01 1.641988e-01 1.620086e-01 1.684217e-01 1.599833e-01

879 8.524146e-01 8.474974e-01 8.424986e-01 8.374321e-01 8.322618e-01 1.663405e-01 1.642006e-01 1.620103e-01 1.684237e-01 1.599850e-01

880 8.561353e-01 8.509162e-01 8.456271e-01 8.402806e-01 8.348427e-01 1.663423e-01 1.642023e-01 1.620119e-01 1.684255e-01 1.599865e-01

881 8.597522e-01 8.542225e-01 8.486375e-01 8.430078e-01 8.373012e-01 1.663439e-01 1.642039e-01 1.620134e-01 1.684272e-01 1.599879e-01

882 8.632622e-01 8.574140e-01 8.515286e-01 8.456134e-01 8.396385e-01 1.663454e-01 1.642053e-01 1.620147e-01 1.684288e-01 1.599892e-01

883 8.666621e-01 8.604888e-01 8.542995e-01 8.480978e-01 8.418560e-01 1.663469e-01 1.642067e-01 1.620160e-01 1.684303e-01 1.599904e-01

884 8.699495e-01 8.634453e-01 8.569499e-01 8.504618e-01 8.439555e-01 1.663482e-01 1.642080e-01 1.620172e-01 1.684317e-01 1.599916e-01

885 8.731220e-01 8.662824e-01 8.594799e-01 8.527067e-01 8.459395e-01 1.663494e-01 1.642092e-01 1.620183e-01 1.684330e-01 1.599926e-01

886 8.761778e-01 8.689994e-01 8.618900e-01 8.548342e-01 8.478106e-01 1.663506e-01 1.642103e-01 1.620193e-01 1.684342e-01 1.599936e-01

887 8.791153e-01 8.715962e-01 8.641814e-01 8.568465e-01 8.495719e-01 1.663517e-01 1.642113e-01 1.620203e-01 1.684353e-01 1.599945e-01

888 8.819337e-01 8.740731e-01 8.663554e-01 8.587460e-01 8.512267e-01 1.663527e-01 1.642123e-01 1.620211e-01 1.684364e-01 1.599954e-01

889 8.846323e-01 8.764309e-01 8.684141e-01 8.605357e-01 8.527787e-01 1.663536e-01 1.642131e-01 1.620220e-01 1.684373e-01 1.599962e-01

890 8.872110e-01 8.786707e-01 8.703597e-01 8.622186e-01 8.542316e-01 1.663545e-01 1.642140e-01 1.620227e-01 1.684382e-01 1.599969e-01

891 8.896702e-01 8.807942e-01 8.721948e-01 8.637984e-01 8.555895e-01 1.663553e-01 1.642147e-01 1.620234e-01 1.684391e-01 1.599976e-01

892 8.920107e-01 8.828035e-01 8.739226e-01 8.652785e-01 8.568564e-01 1.663560e-01 1.642154e-01 1.620241e-01 1.684399e-01 1.599982e-01

893 8.942338e-01 8.847009e-01 8.755462e-01 8.666630e-01 8.580366e-01 1.663567e-01 1.642161e-01 1.620247e-01 1.684406e-01 1.599988e-01

894 8.963412e-01 8.864894e-01 8.770691e-01 8.679558e-01 8.591343e-01 1.663573e-01 1.642167e-01 1.620253e-01 1.684413e-01 1.599993e-01

895 8.983350e-01 8.881720e-01 8.784952e-01 8.691610e-01 8.601537e-01 1.663579e-01 1.642173e-01 1.620258e-01 1.684419e-01 1.599998e-01

896 9.002177e-01 8.897520e-01 8.798283e-01 8.702829e-01 8.610991e-01 1.663585e-01 1.642178e-01 1.620263e-01 1.684425e-01 1.600003e-01

897 9.019921e-01 8.912331e-01 8.810724e-01 8.713255e-01 8.619747e-01 1.663590e-01 1.642183e-01 1.620267e-01 1.684430e-01 1.600007e-01

898 9.036612e-01 8.926191e-01 8.822317e-01 8.722931e-01 8.627845e-01 1.663595e-01 1.642188e-01 1.620272e-01 1.684435e-01 1.600011e-01

899 9.052286e-01 8.939138e-01 8.833102e-01 8.731899e-01 8.635326e-01 1.663599e-01 1.642192e-01 1.620275e-01 1.684440e-01 1.600015e-01

900 9.066978e-01 8.951214e-01 8.843121e-01 8.740199e-01 8.642229e-01 1.663603e-01 1.642196e-01 1.620279e-01 1.684444e-01 1.600019e-01

901 9.080725e-01 8.962460e-01 8.852416e-01 8.747872e-01 8.648591e-01 1.663607e-01 1.642200e-01 1.620282e-01 1.684448e-01 1.600022e-01

902 9.080728e-01 8.962463e-01 8.852418e-01 8.747874e-01 8.648593e-01 1.672669e-01 1.651374e-01 1.629512e-01 1.693406e-01 1.609357e-01

903 9.080731e-01 8.962465e-01 8.852421e-01 8.747876e-01 8.648594e-01 1.682072e-01 1.660892e-01 1.639087e-01 1.702703e-01 1.619038e-01

904 9.080734e-01 8.962468e-01 8.852423e-01 8.747878e-01 8.648596e-01 1.691826e-01 1.670764e-01 1.649016e-01 1.712349e-01 1.629077e-01

905 9.080736e-01 8.962470e-01 8.852425e-01 8.747880e-01 8.648598e-01 1.701942e-01 1.681001e-01 1.659309e-01 1.722353e-01 1.639482e-01

906 9.080739e-01 8.962472e-01 8.852427e-01 8.747882e-01 8.648599e-01 1.712429e-01 1.691611e-01 1.669975e-01 1.732726e-01 1.650264e-01

907 9.080741e-01 8.962474e-01 8.852429e-01 8.747883e-01 8.648601e-01 1.723296e-01 1.702604e-01 1.681025e-01 1.743477e-01 1.661430e-01

908 9.080743e-01 8.962476e-01 8.852430e-01 8.747885e-01 8.648602e-01 1.734553e-01 1.713990e-01 1.692466e-01 1.754616e-01 1.672990e-01

909 9.080745e-01 8.962478e-01 8.852432e-01 8.747886e-01 8.648603e-01 1.746209e-01 1.725777e-01 1.704308e-01 1.766152e-01 1.684953e-01

910 9.080747e-01 8.962479e-01 8.852433e-01 8.747887e-01 8.648605e-01 1.758273e-01 1.737974e-01 1.716558e-01 1.778093e-01 1.697326e-01

911 9.080749e-01 8.962481e-01 8.852435e-01 8.747889e-01 8.648606e-01 1.770753e-01 1.750590e-01 1.729226e-01 1.790448e-01 1.710119e-01

912 9.080751e-01 8.962482e-01 8.852436e-01 8.747890e-01 8.648607e-01 1.783657e-01 1.763631e-01 1.742318e-01 1.803226e-01 1.723338e-01

913 9.080752e-01 8.962484e-01 8.852437e-01 8.747891e-01 8.648608e-01 1.796993e-01 1.777106e-01 1.755842e-01 1.816434e-01 1.736989e-01

914 9.080754e-01 8.962485e-01 8.852438e-01 8.747892e-01 8.648608e-01 1.810768e-01 1.791021e-01 1.769804e-01 1.830079e-01 1.751080e-01

915 9.080755e-01 8.962486e-01 8.852439e-01 8.747893e-01 8.648609e-01 1.824987e-01 1.805383e-01 1.784210e-01 1.844168e-01 1.765616e-01

916 9.080756e-01 8.962487e-01 8.852440e-01 8.747893e-01 8.648610e-01 1.839657e-01 1.820196e-01 1.799065e-01 1.858706e-01 1.780602e-01

917 9.080757e-01 8.962488e-01 8.852441e-01 8.747894e-01 8.648611e-01 1.854782e-01 1.835466e-01 1.814373e-01 1.873699e-01 1.796042e-01

918 9.080759e-01 8.962489e-01 8.852442e-01 8.747895e-01 8.648611e-01 1.870368e-01 1.851197e-01 1.830138e-01 1.889151e-01 1.811939e-01

919 9.080760e-01 8.962490e-01 8.852443e-01 8.747896e-01 8.648612e-01 1.886417e-01 1.867391e-01 1.846363e-01 1.905067e-01 1.828295e-01

920 9.080760e-01 8.962491e-01 8.852443e-01 8.747896e-01 8.648613e-01 1.902932e-01 1.884052e-01 1.863049e-01 1.921448e-01 1.845113e-01

921 9.080761e-01 8.962492e-01 8.852444e-01 8.747897e-01 8.648613e-01 1.919914e-01 1.901179e-01 1.880196e-01 1.938298e-01 1.862392e-01

922 9.080762e-01 8.962492e-01 8.852445e-01 8.747898e-01 8.648614e-01 1.937364e-01 1.918774e-01 1.897806e-01 1.955615e-01 1.880131e-01

923 9.080763e-01 8.962493e-01 8.852445e-01 8.747898e-01 8.648615e-01 1.955282e-01 1.936835e-01 1.915875e-01 1.973402e-01 1.898329e-01

924 9.080764e-01 8.962494e-01 8.852446e-01 8.747899e-01 8.648615e-01 1.973665e-01 1.955361e-01 1.934403e-01 1.991656e-01 1.916983e-01

925 9.080765e-01 8.962495e-01 8.852447e-01 8.747900e-01 8.648616e-01 1.992512e-01 1.974348e-01 1.953384e-01 2.010375e-01 1.936089e-01

926 9.080765e-01 8.962495e-01 8.852448e-01 8.747901e-01 8.648617e-01 2.011819e-01 1.993792e-01 1.972814e-01 2.029556e-01 1.955641e-01

927 9.080766e-01 8.962496e-01 8.852448e-01 8.747901e-01 8.648617e-01 2.031579e-01 2.013688e-01 1.992687e-01 2.049194e-01 1.975632e-01

928 9.080767e-01 8.962497e-01 8.852449e-01 8.747902e-01 8.648618e-01 2.051788e-01 2.034028e-01 2.012995e-01 2.069284e-01 1.996055e-01

929 9.080768e-01 8.962498e-01 8.852450e-01 8.747903e-01 8.648619e-01 2.072437e-01 2.054805e-01 2.033729e-01 2.089818e-01 2.016901e-01

930 9.080769e-01 8.962499e-01 8.852451e-01 8.747904e-01 8.648620e-01 2.093518e-01 2.076010e-01 2.054880e-01 2.110788e-01 2.038157e-01

931 9.080770e-01 8.962500e-01 8.852452e-01 8.747905e-01 8.648621e-01 2.115021e-01 2.097630e-01 2.076436e-01 2.132185e-01 2.059814e-01

932 9.080771e-01 8.962501e-01 8.852454e-01 8.747906e-01 8.648623e-01 2.136934e-01 2.119656e-01 2.098384e-01 2.153998e-01 2.081856e-01

933 9.080772e-01 8.962502e-01 8.852455e-01 8.747908e-01 8.648624e-01 2.159245e-01 2.142073e-01 2.120710e-01 2.176215e-01 2.104271e-01

934 9.080774e-01 8.962504e-01 8.852456e-01 8.747909e-01 8.648626e-01 2.181940e-01 2.164867e-01 2.143399e-01 2.198823e-01 2.127041e-01

935 9.080775e-01 8.962505e-01 8.852458e-01 8.747911e-01 8.648627e-01 2.205004e-01 2.188022e-01 2.166435e-01 2.221807e-01 2.150150e-01

936 9.080777e-01 8.962507e-01 8.852460e-01 8.747913e-01 8.648629e-01 2.228421e-01 2.211522e-01 2.189800e-01 2.245152e-01 2.173580e-01

937 9.080778e-01 8.962509e-01 8.852461e-01 8.747915e-01 8.648631e-01 2.252173e-01 2.235349e-01 2.213475e-01 2.268842e-01 2.197311e-01

938 9.080780e-01 8.962511e-01 8.852463e-01 8.747917e-01 8.648633e-01 2.276242e-01 2.259483e-01 2.237440e-01 2.292859e-01 2.221323e-01

939 9.080782e-01 8.962513e-01 8.852466e-01 8.747919e-01 8.648636e-01 2.300609e-01 2.283904e-01 2.261675e-01 2.317185e-01 2.245594e-01

940 9.080784e-01 8.962515e-01 8.852468e-01 8.747922e-01 8.648638e-01 2.325254e-01 2.308592e-01 2.286157e-01 2.341801e-01 2.270102e-01

941 9.080787e-01 8.962518e-01 8.852471e-01 8.747924e-01 8.648641e-01 2.350156e-01 2.333524e-01 2.310863e-01 2.366687e-01 2.294823e-01

942 9.080789e-01 8.962520e-01 8.852474e-01 8.747927e-01 8.648644e-01 2.375294e-01 2.358678e-01 2.335772e-01 2.391823e-01 2.319733e-01

943 9.080792e-01 8.962523e-01 8.852477e-01 8.747931e-01 8.648647e-01 2.400646e-01 2.384031e-01 2.360858e-01 2.417188e-01 2.344807e-01

944 9.080795e-01 8.962527e-01 8.852480e-01 8.747934e-01 8.648651e-01 2.426189e-01 2.409560e-01 2.386097e-01 2.442759e-01 2.370021e-01

945 9.080799e-01 8.962530e-01 8.852484e-01 8.747938e-01 8.648655e-01 2.451899e-01 2.435241e-01 2.411465e-01 2.468514e-01 2.395348e-01

946 9.080802e-01 8.962534e-01 8.852488e-01 8.747942e-01 8.648659e-01 2.477754e-01 2.461049e-01 2.436936e-01 2.494431e-01 2.420762e-01

947 9.080806e-01 8.962538e-01 8.852492e-01 8.747946e-01 8.648664e-01 2.503729e-01 2.486960e-01 2.462486e-01 2.520485e-01 2.446239e-01

948 9.080810e-01 8.962543e-01 8.852497e-01 8.747951e-01 8.648669e-01 2.529800e-01 2.512949e-01 2.488089e-01 2.546653e-01 2.471751e-01

949 9.080815e-01 8.962547e-01 8.852502e-01 8.747956e-01 8.648674e-01 2.555942e-01 2.538990e-01 2.513718e-01 2.572912e-01 2.497273e-01

950 9.080820e-01 8.962552e-01 8.852507e-01 8.747962e-01 8.648680e-01 2.582130e-01 2.565059e-01 2.539350e-01 2.599235e-01 2.522778e-01

951 9.080825e-01 8.962558e-01 8.852513e-01 8.747968e-01 8.648686e-01 2.608340e-01 2.591130e-01 2.564957e-01 2.625601e-01 2.548241e-01

952 9.080831e-01 8.962564e-01 8.852519e-01 8.747974e-01 8.648693e-01 2.634547e-01 2.617178e-01 2.590515e-01 2.651982e-01 2.573635e-01

953 9.080837e-01 8.962570e-01 8.852526e-01 8.747981e-01 8.648700e-01 2.660726e-01 2.643178e-01 2.615998e-01 2.678356e-01 2.598935e-01

954 9.080843e-01 8.962577e-01 8.852533e-01 8.747989e-01 8.648707e-01 2.686852e-01 2.669104e-01 2.641381e-01 2.704698e-01 2.624117e-01

955 9.080850e-01 8.962585e-01 8.852541e-01 8.747997e-01 8.648716e-01 2.712901e-01 2.694933e-01 2.666640e-01 2.730982e-01 2.649155e-01

956 9.080858e-01 8.962593e-01 8.852549e-01 8.748006e-01 8.648725e-01 2.738848e-01 2.720640e-01 2.691750e-01 2.757187e-01 2.674025e-01

957 9.080866e-01 8.962601e-01 8.852558e-01 8.748015e-01 8.648734e-01 2.764670e-01 2.746201e-01 2.716688e-01 2.783286e-01 2.698704e-01

958 9.080875e-01 8.962611e-01 8.852568e-01 8.748025e-01 8.648744e-01 2.790344e-01 2.771593e-01 2.741431e-01 2.809258e-01 2.723168e-01

959 9.080884e-01 8.962620e-01 8.852578e-01 8.748036e-01 8.648756e-01 2.815846e-01 2.796793e-01 2.765956e-01 2.835079e-01 2.747396e-01

960 9.080894e-01 8.962631e-01 8.852589e-01 8.748047e-01 8.648767e-01 2.841154e-01 2.821780e-01 2.790242e-01 2.860726e-01 2.771366e-01

961 9.080905e-01 8.962642e-01 8.852601e-01 8.748059e-01 8.648780e-01 2.866247e-01 2.846530e-01 2.814268e-01 2.886179e-01 2.795058e-01

962 9.080916e-01 8.962654e-01 8.852614e-01 8.748073e-01 8.648794e-01 2.891103e-01 2.871025e-01 2.838014e-01 2.911415e-01 2.818451e-01

963 9.080928e-01 8.962668e-01 8.852628e-01 8.748087e-01 8.648809e-01 2.915702e-01 2.895244e-01 2.861460e-01 2.936414e-01 2.841528e-01

964 9.080941e-01 8.962681e-01 8.852642e-01 8.748102e-01 8.648824e-01 2.940026e-01 2.919167e-01 2.884589e-01 2.961157e-01 2.864270e-01

965 9.080956e-01 8.962696e-01 8.852658e-01 8.748119e-01 8.648841e-01 2.964055e-01 2.942778e-01 2.907383e-01 2.985624e-01 2.886661e-01

966 9.080971e-01 8.962713e-01 8.852675e-01 8.748136e-01 8.648860e-01 2.987772e-01 2.966059e-01 2.929827e-01 3.009797e-01 2.908685e-01

967 9.080987e-01 8.962730e-01 8.852693e-01 8.748155e-01 8.648879e-01 3.011161e-01 2.988994e-01 2.951904e-01 3.033660e-01 2.930329e-01

968 9.081004e-01 8.962748e-01 8.852713e-01 8.748176e-01 8.648901e-01 3.034205e-01 3.011568e-01 2.973603e-01 3.057195e-01 2.951578e-01

969 9.081023e-01 8.962768e-01 8.852734e-01 8.748198e-01 8.648924e-01 3.056890e-01 3.033767e-01 2.994908e-01 3.080388e-01 2.972422e-01

970 9.081043e-01 8.962789e-01 8.852757e-01 8.748221e-01 8.648948e-01 3.079202e-01 3.055578e-01 3.015810e-01 3.103225e-01 2.992848e-01

971 9.081064e-01 8.962812e-01 8.852781e-01 8.748247e-01 8.648975e-01 3.101129e-01 3.076989e-01 3.036297e-01 3.125692e-01 3.012848e-01

972 9.081087e-01 8.962837e-01 8.852807e-01 8.748274e-01 8.649003e-01 3.122660e-01 3.097990e-01 3.056361e-01 3.147777e-01 3.032412e-01

973 9.081111e-01 8.962863e-01 8.852835e-01 8.748304e-01 8.649034e-01 3.143784e-01 3.118572e-01 3.075992e-01 3.169469e-01 3.051535e-01

974 9.081138e-01 8.962892e-01 8.852865e-01 8.748335e-01 8.649067e-01 3.164493e-01 3.138725e-01 3.095185e-01 3.190757e-01 3.070209e-01

975 9.081166e-01 8.962922e-01 8.852898e-01 8.748370e-01 8.649103e-01 3.184777e-01 3.158444e-01 3.113934e-01 3.211634e-01 3.088429e-01

976 9.081197e-01 8.962955e-01 8.852933e-01 8.748407e-01 8.649142e-01 3.204630e-01 3.177721e-01 3.132233e-01 3.232091e-01 3.106193e-01

977 9.081229e-01 8.962991e-01 8.852971e-01 8.748446e-01 8.649184e-01 3.224046e-01 3.196552e-01 3.150079e-01 3.252121e-01 3.123498e-01

978 9.081264e-01 8.963029e-01 8.853011e-01 8.748489e-01 8.649229e-01 3.243021e-01 3.214932e-01 3.167470e-01 3.271719e-01 3.140341e-01

979 9.081302e-01 8.963069e-01 8.853055e-01 8.748536e-01 8.649278e-01 3.261550e-01 3.232860e-01 3.184405e-01 3.290879e-01 3.156723e-01

980 9.081343e-01 8.963114e-01 8.853103e-01 8.748586e-01 8.649331e-01 3.279631e-01 3.250333e-01 3.200883e-01 3.309599e-01 3.172644e-01

981 9.081386e-01 8.963161e-01 8.853154e-01 8.748640e-01 8.649388e-01 3.297262e-01 3.267351e-01 3.216905e-01 3.327875e-01 3.188106e-01

982 9.081433e-01 8.963212e-01 8.853209e-01 8.748698e-01 8.649450e-01 3.314442e-01 3.283914e-01 3.232472e-01 3.345706e-01 3.203111e-01

983 9.081483e-01 8.963267e-01 8.853268e-01 8.748762e-01 8.649516e-01 3.331172e-01 3.300022e-01 3.247587e-01 3.363090e-01 3.217663e-01

984 9.081538e-01 8.963327e-01 8.853332e-01 8.748830e-01 8.649589e-01 3.347453e-01 3.315678e-01 3.262254e-01 3.380026e-01 3.231766e-01

985 9.081596e-01 8.963391e-01 8.853401e-01 8.748904e-01 8.649667e-01 3.363286e-01 3.330886e-01 3.276476e-01 3.396516e-01 3.245424e-01

986 9.081659e-01 8.963460e-01 8.853476e-01 8.748984e-01 8.649752e-01 3.378675e-01 3.345648e-01 3.290259e-01 3.412559e-01 3.258645e-01

987 9.081727e-01 8.963534e-01 8.853557e-01 8.749070e-01 8.649844e-01 3.393621e-01 3.359969e-01 3.303608e-01 3.428158e-01 3.271433e-01

988 9.081800e-01 8.963615e-01 8.853645e-01 8.749164e-01 8.649944e-01 3.408129e-01 3.373854e-01 3.316529e-01 3.443315e-01 3.283797e-01

989 9.081879e-01 8.963702e-01 8.853739e-01 8.749265e-01 8.650052e-01 3.422203e-01 3.387308e-01 3.329029e-01 3.458033e-01 3.295744e-01

990 9.081964e-01 8.963796e-01 8.853841e-01 8.749375e-01 8.650169e-01 3.435847e-01 3.400338e-01 3.341117e-01 3.472316e-01 3.307281e-01

991 9.082056e-01 8.963897e-01 8.853952e-01 8.749494e-01 8.650297e-01 3.449067e-01 3.412950e-01 3.352798e-01 3.486166e-01 3.318417e-01

992 9.082154e-01 8.964007e-01 8.854072e-01 8.749623e-01 8.650435e-01 3.461868e-01 3.425149e-01 3.364082e-01 3.499591e-01 3.329162e-01

993 9.082261e-01 8.964125e-01 8.854201e-01 8.749763e-01 8.650585e-01 3.474257e-01 3.436944e-01 3.374976e-01 3.512593e-01 3.339524e-01

994 9.082376e-01 8.964253e-01 8.854342e-01 8.749915e-01 8.650748e-01 3.486238e-01 3.448340e-01 3.385489e-01 3.525179e-01 3.349512e-01

995 9.082500e-01 8.964391e-01 8.854494e-01 8.750080e-01 8.650925e-01 3.497820e-01 3.459347e-01 3.395629e-01 3.537355e-01 3.359136e-01

996 9.082634e-01 8.964541e-01 8.854659e-01 8.750258e-01 8.651117e-01 3.509009e-01 3.469970e-01 3.405405e-01 3.549128e-01 3.368404e-01

997 9.082779e-01 8.964703e-01 8.854837e-01 8.750452e-01 8.651326e-01 3.519812e-01 3.480219e-01 3.414824e-01 3.560503e-01 3.377327e-01

998 9.082935e-01 8.964878e-01 8.855031e-01 8.750663e-01 8.651554e-01 3.530238e-01 3.490100e-01 3.423896e-01 3.571489e-01 3.385912e-01

999 9.083104e-01 8.965068e-01 8.855241e-01 8.750891e-01 8.651801e-01 3.540293e-01 3.499624e-01 3.432630e-01 3.582093e-01 3.394170e-01

1000 9.083287e-01 8.965274e-01 8.855468e-01 8.751139e-01 8.652070e-01 3.549987e-01 3.508797e-01 3.441034e-01 3.592322e-01 3.402109e-01

1001 9.083484e-01 8.965496e-01 8.855715e-01 8.751409e-01 8.652363e-01 3.559327e-01 3.517628e-01 3.449117e-01 3.602185e-01 3.409738e-01

1002 9.083698e-01 8.965737e-01 8.855983e-01 8.751703e-01 8.652682e-01 3.568321e-01 3.526127e-01 3.456888e-01 3.611689e-01 3.417067e-01

1003 9.083929e-01 8.965999e-01 8.856274e-01 8.752022e-01 8.653029e-01 3.576978e-01 3.534301e-01 3.464355e-01 3.620843e-01 3.424104e-01

1004 9.084178e-01 8.966282e-01 8.856590e-01 8.752368e-01 8.653408e-01 3.585307e-01 3.542160e-01 3.471528e-01 3.629656e-01 3.430858e-01

1005 9.084448e-01 8.966588e-01 8.856932e-01 8.752746e-01 8.653820e-01 3.593316e-01 3.549711e-01 3.478414e-01 3.638135e-01 3.437338e-01

1006 9.084741e-01 8.966921e-01 8.857305e-01 8.753156e-01 8.654269e-01 3.601014e-01 3.556964e-01 3.485023e-01 3.646290e-01 3.443553e-01

1007 9.085057e-01 8.967282e-01 8.857709e-01 8.753602e-01 8.654758e-01 3.608409e-01 3.563928e-01 3.491363e-01 3.654128e-01 3.449510e-01

1008 9.085399e-01 8.967673e-01 8.858149e-01 8.754088e-01 8.655291e-01 3.615511e-01 3.570611e-01 3.497443e-01 3.661660e-01 3.455219e-01

1009 9.085770e-01 8.968097e-01 8.858627e-01 8.754618e-01 8.655873e-01 3.622328e-01 3.577021e-01 3.503271e-01 3.668894e-01 3.460688e-01

1010 9.086171e-01 8.968558e-01 8.859146e-01 8.755194e-01 8.656507e-01 3.628868e-01 3.583168e-01 3.508855e-01 3.675838e-01 3.465925e-01

1011 9.086606e-01 8.969058e-01 8.859710e-01 8.755821e-01 8.657199e-01 3.635141e-01 3.589060e-01 3.514204e-01 3.682501e-01 3.470938e-01

1012 9.087077e-01 8.969600e-01 8.860324e-01 8.756504e-01 8.657954e-01 3.641154e-01 3.594704e-01 3.519325e-01 3.688891e-01 3.475735e-01

1013 9.087587e-01 8.970189e-01 8.860991e-01 8.757249e-01 8.658778e-01 3.646916e-01 3.600110e-01 3.524226e-01 3.695018e-01 3.480323e-01

1014 9.088140e-01 8.970828e-01 8.861717e-01 8.758060e-01 8.659676e-01 3.652435e-01 3.605286e-01 3.528916e-01 3.700890e-01 3.484710e-01

1015 9.088739e-01 8.971522e-01 8.862507e-01 8.758943e-01 8.660657e-01 3.657720e-01 3.610239e-01 3.533401e-01 3.706515e-01 3.488904e-01

1016 9.089388e-01 8.972275e-01 8.863366e-01 8.759906e-01 8.661728e-01 3.662779e-01 3.614977e-01 3.537689e-01 3.711902e-01 3.492912e-01

1017 9.090091e-01 8.973093e-01 8.864300e-01 8.760955e-01 8.662896e-01 3.667619e-01 3.619508e-01 3.541788e-01 3.717059e-01 3.496740e-01

1018 9.090853e-01 8.973982e-01 8.865317e-01 8.762099e-01 8.664171e-01 3.672249e-01 3.623840e-01 3.545704e-01 3.721993e-01 3.500396e-01

1019 9.091680e-01 8.974947e-01 8.866423e-01 8.763345e-01 8.665563e-01 3.676676e-01 3.627980e-01 3.549445e-01 3.726712e-01 3.503886e-01

1020 9.092576e-01 8.975996e-01 8.867627e-01 8.764703e-01 8.667083e-01 3.680907e-01 3.631935e-01 3.553016e-01 3.731226e-01 3.507217e-01

1021 9.093547e-01 8.977134e-01 8.868937e-01 8.766183e-01 8.668741e-01 3.684949e-01 3.635712e-01 3.556426e-01 3.735540e-01 3.510395e-01

1022 9.094600e-01 8.978371e-01 8.870362e-01 8.767796e-01 8.670551e-01 3.688811e-01 3.639318e-01 3.559679e-01 3.739662e-01 3.513427e-01

1023 9.095742e-01 8.979715e-01 8.871912e-01 8.769554e-01 8.672525e-01 3.692498e-01 3.642760e-01 3.562783e-01 3.743600e-01 3.516317e-01

1024 9.096979e-01 8.981174e-01 8.873599e-01 8.771469e-01 8.674680e-01 3.696018e-01 3.646044e-01 3.565743e-01 3.747360e-01 3.519073e-01

1025 9.098321e-01 8.982758e-01 8.875433e-01 8.773554e-01 8.677029e-01 3.699377e-01 3.649176e-01 3.568566e-01 3.750950e-01 3.521698e-01

1026 9.099775e-01 8.984479e-01 8.877428e-01 8.775826e-01 8.679592e-01 3.702581e-01 3.652163e-01 3.571256e-01 3.754376e-01 3.524200e-01

1027 9.101351e-01 8.986347e-01 8.879598e-01 8.778300e-01 8.682385e-01 3.705638e-01 3.655011e-01 3.573820e-01 3.757645e-01 3.526583e-01

1028 9.103059e-01 8.988375e-01 8.881957e-01 8.780993e-01 8.685430e-01 3.708552e-01 3.657725e-01 3.576262e-01 3.760763e-01 3.528853e-01

1029 9.104910e-01 8.990576e-01 8.884520e-01 8.783923e-01 8.688746e-01 3.711329e-01 3.660311e-01 3.578588e-01 3.763735e-01 3.531013e-01

1030 9.106915e-01 8.992964e-01 8.887306e-01 8.787110e-01 8.692357e-01 3.713976e-01 3.662774e-01 3.580803e-01 3.766569e-01 3.533069e-01

1031 9.109087e-01 8.995555e-01 8.890331e-01 8.790576e-01 8.696287e-01 3.716498e-01 3.665119e-01 3.582912e-01 3.769270e-01 3.535026e-01

1032 9.111439e-01 8.998365e-01 8.893616e-01 8.794343e-01 8.700561e-01 3.718900e-01 3.667353e-01 3.584919e-01 3.771843e-01 3.536887e-01

1033 9.113986e-01 9.001411e-01 8.897181e-01 8.798435e-01 8.705207e-01 3.721187e-01 3.669478e-01 3.586828e-01 3.774294e-01 3.538657e-01

1034 9.116743e-01 9.004712e-01 8.901049e-01 8.802877e-01 8.710254e-01 3.723364e-01 3.671501e-01 3.588645e-01 3.776628e-01 3.540341e-01

1035 9.119725e-01 9.008288e-01 8.905242e-01 8.807696e-01 8.715732e-01 3.725436e-01 3.673426e-01 3.590372e-01 3.778850e-01 3.541941e-01

1036 9.122951e-01 9.012160e-01 8.909785e-01 8.812921e-01 8.721673e-01 3.727408e-01 3.675256e-01 3.592015e-01 3.780964e-01 3.543462e-01

1037 9.126438e-01 9.016349e-01 8.914705e-01 8.818582e-01 8.728110e-01 3.729284e-01 3.676997e-01 3.593576e-01 3.782977e-01 3.544908e-01

1038 9.130206e-01 9.020880e-01 8.920029e-01 8.824708e-01 8.735078e-01 3.731067e-01 3.678651e-01 3.595060e-01 3.784891e-01 3.546281e-01

1039 9.134277e-01 9.025778e-01 8.925786e-01 8.831334e-01 8.742612e-01 3.732764e-01 3.680224e-01 3.596471e-01 3.786712e-01 3.547585e-01

1040 9.138671e-01 9.031068e-01 8.932005e-01 8.838492e-01 8.750749e-01 3.734376e-01 3.681719e-01 3.597810e-01 3.788444e-01 3.548824e-01

1041 9.143412e-01 9.036777e-01 8.938719e-01 8.846218e-01 8.759527e-01 3.735909e-01 3.683139e-01 3.599083e-01 3.790090e-01 3.550000e-01

1042 9.148524e-01 9.042935e-01 8.945959e-01 8.854547e-01 8.768985e-01 3.737365e-01 3.684488e-01 3.600291e-01 3.791654e-01 3.551116e-01

1043 9.154033e-01 9.049570e-01 8.953759e-01 8.863514e-01 8.779159e-01 3.738748e-01 3.685769e-01 3.601439e-01 3.793141e-01 3.552176e-01

1044 9.159966e-01 9.056713e-01 8.962153e-01 8.873158e-01 8.790089e-01 3.740063e-01 3.686986e-01 3.602528e-01 3.794554e-01 3.553182e-01

1045 9.166351e-01 9.064397e-01 8.971177e-01 8.883515e-01 8.801813e-01 3.741311e-01 3.688141e-01 3.603562e-01 3.795896e-01 3.554135e-01

1046 9.173218e-01 9.072653e-01 8.980864e-01 8.894622e-01 8.814367e-01 3.742496e-01 3.689237e-01 3.604543e-01 3.797170e-01 3.555040e-01

1047 9.180598e-01 9.081516e-01 8.991251e-01 8.906515e-01 8.827787e-01 3.743621e-01 3.690277e-01 3.605473e-01 3.798380e-01 3.555898e-01

1048 9.188522e-01 9.091019e-01 9.002373e-01 8.919230e-01 8.842108e-01 3.744688e-01 3.691264e-01 3.606356e-01 3.799529e-01 3.556712e-01

1049 9.197024e-01 9.101199e-01 9.014266e-01 8.932801e-01 8.857360e-01 3.745701e-01 3.692200e-01 3.607193e-01 3.800619e-01 3.557484e-01

1050 9.206138e-01 9.112090e-01 9.026964e-01 8.947261e-01 8.873573e-01 3.746663e-01 3.693088e-01 3.607987e-01 3.801654e-01 3.558215e-01

1051 9.215897e-01 9.123729e-01 9.040502e-01 8.962640e-01 8.890773e-01 3.747574e-01 3.693930e-01 3.608740e-01 3.802636e-01 3.558908e-01

1052 9.226337e-01 9.136149e-01 9.054912e-01 8.978967e-01 8.908980e-01 3.748439e-01 3.694729e-01 3.609453e-01 3.803567e-01 3.559564e-01

1053 9.237495e-01 9.149387e-01 9.070227e-01 8.996267e-01 8.928213e-01 3.749259e-01 3.695485e-01 3.610129e-01 3.804451e-01 3.560186e-01

1054 9.249405e-01 9.163476e-01 9.086476e-01 9.014564e-01 8.948484e-01 3.750037e-01 3.696203e-01 3.610770e-01 3.805288e-01 3.560776e-01

1055 9.262104e-01 9.178450e-01 9.103687e-01 9.033876e-01 8.969802e-01 3.750774e-01 3.696882e-01 3.611377e-01 3.806083e-01 3.561334e-01

1056 9.275625e-01 9.194339e-01 9.121883e-01 9.054218e-01 8.992169e-01 3.751472e-01 3.697526e-01 3.611952e-01 3.806836e-01 3.561862e-01

1057 9.290005e-01 9.211173e-01 9.141087e-01 9.075601e-01 9.015584e-01 3.752134e-01 3.698136e-01 3.612496e-01 3.807549e-01 3.562363e-01

1058 9.305276e-01 9.228979e-01 9.161317e-01 9.098032e-01 9.040039e-01 3.752761e-01 3.698714e-01 3.613012e-01 3.808225e-01 3.562836e-01

1059 9.321469e-01 9.247782e-01 9.182586e-01 9.121511e-01 9.065520e-01 3.753355e-01 3.699261e-01 3.613500e-01 3.808866e-01 3.563284e-01

1060 9.338615e-01 9.267603e-01 9.204904e-01 9.146034e-01 9.092008e-01 3.753918e-01 3.699779e-01 3.613962e-01 3.809473e-01 3.563709e-01

1061 9.356741e-01 9.288458e-01 9.228276e-01 9.171592e-01 9.119477e-01 3.754450e-01 3.700270e-01 3.614400e-01 3.810048e-01 3.564110e-01

1062 9.375871e-01 9.310361e-01 9.252703e-01 9.198169e-01 9.147897e-01 3.754955e-01 3.700734e-01 3.614814e-01 3.810593e-01 3.564490e-01

1063 9.396028e-01 9.333322e-01 9.278178e-01 9.225745e-01 9.177231e-01 3.755432e-01 3.701173e-01 3.615205e-01 3.811108e-01 3.564849e-01

1064 9.417228e-01 9.357345e-01 9.304692e-01 9.254294e-01 9.207435e-01 3.755884e-01 3.701589e-01 3.615576e-01 3.811596e-01 3.565188e-01

1065 9.439486e-01 9.382429e-01 9.332229e-01 9.283783e-01 9.238464e-01 3.756312e-01 3.701982e-01 3.615926e-01 3.812058e-01 3.565510e-01

1066 9.462812e-01 9.408569e-01 9.360767e-01 9.314174e-01 9.270264e-01 3.756717e-01 3.702354e-01 3.616258e-01 3.812496e-01 3.565813e-01

1067 9.487211e-01 9.435753e-01 9.390280e-01 9.345426e-01 9.302778e-01 3.757100e-01 3.702706e-01 3.616571e-01 3.812910e-01 3.566100e-01

1068 9.512683e-01 9.463967e-01 9.420735e-01 9.377491e-01 9.335944e-01 3.757462e-01 3.703039e-01 3.616868e-01 3.813301e-01 3.566371e-01

1069 9.539223e-01 9.493187e-01 9.452097e-01 9.410317e-01 9.369698e-01 3.757805e-01 3.703354e-01 3.617148e-01 3.813671e-01 3.566628e-01

1070 9.566821e-01 9.523388e-01 9.484322e-01 9.443847e-01 9.403971e-01 3.758129e-01 3.703651e-01 3.617413e-01 3.814022e-01 3.566870e-01

1071 9.595463e-01 9.554537e-01 9.517365e-01 9.478021e-01 9.438692e-01 3.758435e-01 3.703932e-01 3.617663e-01 3.814353e-01 3.567099e-01

1072 9.625128e-01 9.586598e-01 9.551174e-01 9.512775e-01 9.473787e-01 3.758725e-01 3.704198e-01 3.617900e-01 3.814666e-01 3.567315e-01

1073 9.655792e-01 9.619530e-01 9.585696e-01 9.548044e-01 9.509181e-01 3.758999e-01 3.704449e-01 3.618123e-01 3.814963e-01 3.567519e-01

1074 9.687423e-01 9.653285e-01 9.620870e-01 9.583757e-01 9.544797e-01 3.759258e-01 3.704687e-01 3.618334e-01 3.815243e-01 3.567712e-01

1075 9.719986e-01 9.687816e-01 9.656638e-01 9.619845e-01 9.580557e-01 3.759502e-01 3.704911e-01 3.618534e-01 3.815507e-01 3.567894e-01

1076 9.753444e-01 9.723068e-01 9.692933e-01 9.656233e-01 9.616383e-01 3.759733e-01 3.705123e-01 3.618722e-01 3.815758e-01 3.568066e-01

1077 9.787751e-01 9.758984e-01 9.729691e-01 9.692848e-01 9.652198e-01 3.759952e-01 3.705323e-01 3.618900e-01 3.815994e-01 3.568229e-01

1078 9.822860e-01 9.795505e-01 9.766843e-01 9.729617e-01 9.687923e-01 3.760158e-01 3.705511e-01 3.619068e-01 3.816217e-01 3.568382e-01

1079 9.858720e-01 9.832569e-01 9.804320e-01 9.766463e-01 9.723483e-01 3.760353e-01 3.705690e-01 3.619226e-01 3.816428e-01 3.568526e-01

1080 9.895278e-01 9.870111e-01 9.842051e-01 9.803312e-01 9.758802e-01 3.760537e-01 3.705858e-01 3.619376e-01 3.816627e-01 3.568663e-01

1081 9.932477e-01 9.908067e-01 9.879966e-01 9.840090e-01 9.793808e-01 3.760710e-01 3.706017e-01 3.619517e-01 3.816816e-01 3.568791e-01

1082 9.932476e-01 9.908067e-01 9.879966e-01 9.840090e-01 9.793808e-01 3.762638e-01 3.707968e-01 3.621527e-01 3.818713e-01 3.570817e-01

1083 9.932476e-01 9.908067e-01 9.879966e-01 9.840090e-01 9.793809e-01 3.764643e-01 3.709997e-01 3.623617e-01 3.820687e-01 3.572922e-01

1084 9.932475e-01 9.908067e-01 9.879966e-01 9.840090e-01 9.793809e-01 3.766727e-01 3.712107e-01 3.625789e-01 3.822739e-01 3.575111e-01

1085 9.932475e-01 9.908067e-01 9.879966e-01 9.840090e-01 9.793809e-01 3.768894e-01 3.714300e-01 3.628048e-01 3.824873e-01 3.577386e-01

1086 9.932474e-01 9.908067e-01 9.879967e-01 9.840091e-01 9.793809e-01 3.771147e-01 3.716579e-01 3.630396e-01 3.827091e-01 3.579751e-01

1087 9.932474e-01 9.908067e-01 9.879967e-01 9.840091e-01 9.793810e-01 3.773489e-01 3.718948e-01 3.632837e-01 3.829398e-01 3.582209e-01

1088 9.932474e-01 9.908067e-01 9.879967e-01 9.840091e-01 9.793810e-01 3.775923e-01 3.721411e-01 3.635374e-01 3.831795e-01 3.584764e-01

1089 9.932474e-01 9.908067e-01 9.879968e-01 9.840092e-01 9.793811e-01 3.778453e-01 3.723970e-01 3.638010e-01 3.834287e-01 3.587419e-01

1090 9.932474e-01 9.908067e-01 9.879968e-01 9.840092e-01 9.793811e-01 3.781082e-01 3.726629e-01 3.640751e-01 3.836877e-01 3.590178e-01

1091 9.932475e-01 9.908068e-01 9.879968e-01 9.840092e-01 9.793811e-01 3.783814e-01 3.729392e-01 3.643598e-01 3.839569e-01 3.593045e-01

1092 9.932475e-01 9.908068e-01 9.879969e-01 9.840093e-01 9.793812e-01 3.786653e-01 3.732263e-01 3.646557e-01 3.842365e-01 3.596024e-01

1093 9.932475e-01 9.908069e-01 9.879969e-01 9.840093e-01 9.793812e-01 3.789603e-01 3.735246e-01 3.649632e-01 3.845271e-01 3.599119e-01

1094 9.932476e-01 9.908069e-01 9.879970e-01 9.840094e-01 9.793813e-01 3.792667e-01 3.738344e-01 3.652827e-01 3.848290e-01 3.602335e-01

1095 9.932476e-01 9.908070e-01 9.879970e-01 9.840094e-01 9.793813e-01 3.795851e-01 3.741563e-01 3.656147e-01 3.851427e-01 3.605677e-01

1096 9.932477e-01 9.908070e-01 9.879971e-01 9.840095e-01 9.793814e-01 3.799157e-01 3.744907e-01 3.659595e-01 3.854685e-01 3.609147e-01

1097 9.932477e-01 9.908071e-01 9.879971e-01 9.840095e-01 9.793814e-01 3.802592e-01 3.748380e-01 3.663177e-01 3.858068e-01 3.612753e-01

1098 9.932478e-01 9.908071e-01 9.879972e-01 9.840096e-01 9.793814e-01 3.806159e-01 3.751987e-01 3.666898e-01 3.861583e-01 3.616498e-01

1099 9.932478e-01 9.908072e-01 9.879972e-01 9.840096e-01 9.793815e-01 3.809864e-01 3.755732e-01 3.670763e-01 3.865232e-01 3.620387e-01

1100 9.932479e-01 9.908072e-01 9.879973e-01 9.840097e-01 9.793815e-01 3.813711e-01 3.759622e-01 3.674777e-01 3.869022e-01 3.624427e-01

1101 9.932480e-01 9.908073e-01 9.879973e-01 9.840097e-01 9.793816e-01 3.817705e-01 3.763660e-01 3.678945e-01 3.872957e-01 3.628622e-01

1102 9.932480e-01 9.908074e-01 9.879974e-01 9.840097e-01 9.793816e-01 3.821852e-01 3.767853e-01 3.683273e-01 3.877042e-01 3.632977e-01

1103 9.932481e-01 9.908074e-01 9.879974e-01 9.840098e-01 9.793816e-01 3.826156e-01 3.772205e-01 3.687766e-01 3.881282e-01 3.637499e-01

1104 9.932481e-01 9.908075e-01 9.879975e-01 9.840098e-01 9.793817e-01 3.830624e-01 3.776722e-01 3.692431e-01 3.885683e-01 3.642193e-01

1105 9.932482e-01 9.908075e-01 9.879975e-01 9.840099e-01 9.793817e-01 3.835261e-01 3.781410e-01 3.697272e-01 3.890250e-01 3.647065e-01

1106 9.932483e-01 9.908076e-01 9.879976e-01 9.840099e-01 9.793817e-01 3.840073e-01 3.786275e-01 3.702297e-01 3.894990e-01 3.652122e-01

1107 9.932483e-01 9.908076e-01 9.879976e-01 9.840100e-01 9.793818e-01 3.845065e-01 3.791323e-01 3.707512e-01 3.899907e-01 3.657370e-01

1108 9.932484e-01 9.908077e-01 9.879977e-01 9.840100e-01 9.793818e-01 3.850244e-01 3.796560e-01 3.712922e-01 3.905008e-01 3.662814e-01

1109 9.932484e-01 9.908077e-01 9.879977e-01 9.840100e-01 9.793818e-01 3.855616e-01 3.801992e-01 3.718535e-01 3.910298e-01 3.668462e-01

1110 9.932485e-01 9.908078e-01 9.879978e-01 9.840101e-01 9.793819e-01 3.861188e-01 3.807625e-01 3.724356e-01 3.915785e-01 3.674319e-01

1111 9.932486e-01 9.908078e-01 9.879978e-01 9.840101e-01 9.793819e-01 3.866964e-01 3.813466e-01 3.730393e-01 3.921474e-01 3.680394e-01

1112 9.932486e-01 9.908079e-01 9.879979e-01 9.840102e-01 9.793819e-01 3.872953e-01 3.819522e-01 3.736653e-01 3.927371e-01 3.686692e-01

1113 9.932487e-01 9.908079e-01 9.879979e-01 9.840102e-01 9.793820e-01 3.879161e-01 3.825799e-01 3.743141e-01 3.933484e-01 3.693220e-01

1114 9.932487e-01 9.908080e-01 9.879979e-01 9.840102e-01 9.793820e-01 3.885594e-01 3.832304e-01 3.749865e-01 3.939819e-01 3.699985e-01

1115 9.932488e-01 9.908080e-01 9.879980e-01 9.840103e-01 9.793821e-01 3.892259e-01 3.839043e-01 3.756832e-01 3.946382e-01 3.706995e-01

1116 9.932488e-01 9.908081e-01 9.879980e-01 9.840103e-01 9.793821e-01 3.899163e-01 3.846024e-01 3.764050e-01 3.953180e-01 3.714256e-01

1117 9.932489e-01 9.908081e-01 9.879981e-01 9.840104e-01 9.793821e-01 3.906313e-01 3.853253e-01 3.771524e-01 3.960219e-01 3.721775e-01

1118 9.932489e-01 9.908082e-01 9.879981e-01 9.840104e-01 9.793822e-01 3.913716e-01 3.860738e-01 3.779262e-01 3.967508e-01 3.729559e-01

1119 9.932490e-01 9.908082e-01 9.879982e-01 9.840104e-01 9.793822e-01 3.921378e-01 3.868484e-01 3.787271e-01 3.975052e-01 3.737615e-01

1120 9.932490e-01 9.908083e-01 9.879982e-01 9.840105e-01 9.793823e-01 3.929307e-01 3.876500e-01 3.795559e-01 3.982859e-01 3.745950e-01

1121 9.932491e-01 9.908083e-01 9.879983e-01 9.840105e-01 9.793823e-01 3.937509e-01 3.884792e-01 3.804131e-01 3.990935e-01 3.754570e-01

1122 9.932492e-01 9.908084e-01 9.879983e-01 9.840106e-01 9.793824e-01 3.945991e-01 3.893367e-01 3.812995e-01 3.999287e-01 3.763483e-01

1123 9.932492e-01 9.908084e-01 9.879984e-01 9.840106e-01 9.793824e-01 3.954760e-01 3.902231e-01 3.822157e-01 4.007921e-01 3.772695e-01

1124 9.932493e-01 9.908085e-01 9.879984e-01 9.840107e-01 9.793825e-01 3.963823e-01 3.911391e-01 3.831624e-01 4.016846e-01 3.782213e-01

1125 9.932493e-01 9.908085e-01 9.879985e-01 9.840107e-01 9.793825e-01 3.973186e-01 3.920853e-01 3.841403e-01 4.026066e-01 3.792041e-01

1126 9.932494e-01 9.908086e-01 9.879985e-01 9.840108e-01 9.793826e-01 3.982855e-01 3.930624e-01 3.851499e-01 4.035589e-01 3.802187e-01

1127 9.932494e-01 9.908087e-01 9.879986e-01 9.840109e-01 9.793826e-01 3.992837e-01 3.940710e-01 3.861918e-01 4.045420e-01 3.812656e-01

1128 9.932495e-01 9.908087e-01 9.879987e-01 9.840109e-01 9.793827e-01 4.003138e-01 3.951117e-01 3.872666e-01 4.055566e-01 3.823454e-01

1129 9.932496e-01 9.908088e-01 9.879987e-01 9.840110e-01 9.793828e-01 4.013764e-01 3.961850e-01 3.883748e-01 4.066033e-01 3.834585e-01

1130 9.932496e-01 9.908089e-01 9.879988e-01 9.840111e-01 9.793828e-01 4.024719e-01 3.972914e-01 3.895169e-01 4.076827e-01 3.846053e-01

1131 9.932497e-01 9.908089e-01 9.879989e-01 9.840112e-01 9.793829e-01 4.036009e-01 3.984314e-01 3.906934e-01 4.087952e-01 3.857863e-01

1132 9.932498e-01 9.908090e-01 9.879990e-01 9.840112e-01 9.793830e-01 4.047640e-01 3.996055e-01 3.919046e-01 4.099414e-01 3.870019e-01

1133 9.932499e-01 9.908091e-01 9.879991e-01 9.840113e-01 9.793831e-01 4.059614e-01 4.008141e-01 3.931509e-01 4.111217e-01 3.882524e-01

1134 9.932500e-01 9.908092e-01 9.879991e-01 9.840114e-01 9.793832e-01 4.071937e-01 4.020575e-01 3.944327e-01 4.123366e-01 3.895380e-01

1135 9.932500e-01 9.908093e-01 9.879992e-01 9.840115e-01 9.793833e-01 4.084612e-01 4.033361e-01 3.957501e-01 4.135864e-01 3.908589e-01

1136 9.932501e-01 9.908094e-01 9.879993e-01 9.840116e-01 9.793834e-01 4.097642e-01 4.046501e-01 3.971034e-01 4.148716e-01 3.922152e-01

1137 9.932502e-01 9.908095e-01 9.879995e-01 9.840117e-01 9.793835e-01 4.111029e-01 4.059997e-01 3.984926e-01 4.161923e-01 3.936070e-01

1138 9.932503e-01 9.908096e-01 9.879996e-01 9.840119e-01 9.793836e-01 4.124776e-01 4.073851e-01 3.999179e-01 4.175489e-01 3.950343e-01

1139 9.932504e-01 9.908097e-01 9.879997e-01 9.840120e-01 9.793837e-01 4.138883e-01 4.088063e-01 4.013792e-01 4.189415e-01 3.964969e-01

1140 9.932506e-01 9.908098e-01 9.879998e-01 9.840121e-01 9.793839e-01 4.153352e-01 4.102633e-01 4.028763e-01 4.203703e-01 3.979948e-01

1141 9.932507e-01 9.908100e-01 9.880000e-01 9.840123e-01 9.793840e-01 4.168182e-01 4.117561e-01 4.044092e-01 4.218352e-01 3.995276e-01

1142 9.932508e-01 9.908101e-01 9.880001e-01 9.840124e-01 9.793842e-01 4.183372e-01 4.132844e-01 4.059775e-01 4.233364e-01 4.010950e-01

1143 9.932509e-01 9.908103e-01 9.880003e-01 9.840126e-01 9.793844e-01 4.198922e-01 4.148482e-01 4.075809e-01 4.248736e-01 4.026965e-01

1144 9.932511e-01 9.908104e-01 9.880004e-01 9.840128e-01 9.793845e-01 4.214829e-01 4.164470e-01 4.092190e-01 4.264468e-01 4.043317e-01

1145 9.932512e-01 9.908106e-01 9.880006e-01 9.840129e-01 9.793847e-01 4.231089e-01 4.180805e-01 4.108912e-01 4.280558e-01 4.059999e-01

1146 9.932514e-01 9.908108e-01 9.880008e-01 9.840131e-01 9.793849e-01 4.247699e-01 4.197482e-01 4.125970e-01 4.297002e-01 4.077003e-01

1147 9.932516e-01 9.908110e-01 9.880010e-01 9.840133e-01 9.793851e-01 4.264655e-01 4.214494e-01 4.143355e-01 4.313796e-01 4.094322e-01

1148 9.932518e-01 9.908112e-01 9.880012e-01 9.840136e-01 9.793854e-01 4.281950e-01 4.231837e-01 4.161060e-01 4.330936e-01 4.111945e-01

1149 9.932520e-01 9.908114e-01 9.880015e-01 9.840138e-01 9.793856e-01 4.299578e-01 4.249501e-01 4.179076e-01 4.348417e-01 4.129864e-01

1150 9.932522e-01 9.908116e-01 9.880017e-01 9.840141e-01 9.793859e-01 4.317532e-01 4.267479e-01 4.197393e-01 4.366231e-01 4.148067e-01

1151 9.932524e-01 9.908118e-01 9.880020e-01 9.840143e-01 9.793861e-01 4.335803e-01 4.285761e-01 4.215999e-01 4.384372e-01 4.166542e-01

1152 9.932526e-01 9.908121e-01 9.880022e-01 9.840146e-01 9.793864e-01 4.354382e-01 4.304337e-01 4.234882e-01 4.402832e-01 4.185275e-01

1153 9.932529e-01 9.908124e-01 9.880025e-01 9.840149e-01 9.793868e-01 4.373260e-01 4.323195e-01 4.254031e-01 4.421601e-01 4.204253e-01

1154 9.932532e-01 9.908127e-01 9.880028e-01 9.840153e-01 9.793871e-01 4.392424e-01 4.342324e-01 4.273430e-01 4.440670e-01 4.223460e-01

1155 9.932534e-01 9.908130e-01 9.880032e-01 9.840156e-01 9.793874e-01 4.411864e-01 4.361711e-01 4.293066e-01 4.460028e-01 4.242882e-01

1156 9.932537e-01 9.908133e-01 9.880035e-01 9.840160e-01 9.793878e-01 4.431567e-01 4.381341e-01 4.312923e-01 4.479663e-01 4.262501e-01

1157 9.932541e-01 9.908137e-01 9.880039e-01 9.840164e-01 9.793882e-01 4.451518e-01 4.401200e-01 4.332984e-01 4.499564e-01 4.282300e-01

1158 9.932544e-01 9.908140e-01 9.880043e-01 9.840168e-01 9.793887e-01 4.471705e-01 4.421272e-01 4.353233e-01 4.519717e-01 4.302261e-01

1159 9.932548e-01 9.908144e-01 9.880047e-01 9.840173e-01 9.793891e-01 4.492111e-01 4.441542e-01 4.373651e-01 4.540108e-01 4.322366e-01

1160 9.932551e-01 9.908149e-01 9.880052e-01 9.840177e-01 9.793896e-01 4.512722e-01 4.461992e-01 4.394221e-01 4.560722e-01 4.342594e-01

1161 9.932555e-01 9.908153e-01 9.880057e-01 9.840183e-01 9.793902e-01 4.533519e-01 4.482605e-01 4.414924e-01 4.581545e-01 4.362927e-01

1162 9.932560e-01 9.908158e-01 9.880062e-01 9.840188e-01 9.793907e-01 4.554487e-01 4.503363e-01 4.435739e-01 4.602560e-01 4.383344e-01

1163 9.932564e-01 9.908163e-01 9.880067e-01 9.840194e-01 9.793914e-01 4.575608e-01 4.524246e-01 4.456647e-01 4.623752e-01 4.403824e-01

1164 9.932569e-01 9.908169e-01 9.880073e-01 9.840200e-01 9.793920e-01 4.596863e-01 4.545236e-01 4.477628e-01 4.645103e-01 4.424346e-01

1165 9.932575e-01 9.908174e-01 9.880080e-01 9.840207e-01 9.793927e-01 4.618234e-01 4.566313e-01 4.498661e-01 4.666598e-01 4.444890e-01

1166 9.932580e-01 9.908181e-01 9.880086e-01 9.840214e-01 9.793934e-01 4.639702e-01 4.587458e-01 4.519724e-01 4.688217e-01 4.465433e-01

1167 9.932586e-01 9.908187e-01 9.880093e-01 9.840222e-01 9.793942e-01 4.661247e-01 4.608649e-01 4.540798e-01 4.709945e-01 4.485954e-01

1168 9.932592e-01 9.908194e-01 9.880101e-01 9.840230e-01 9.793951e-01 4.682852e-01 4.629866e-01 4.561860e-01 4.731763e-01 4.506433e-01

1169 9.932599e-01 9.908202e-01 9.880109e-01 9.840239e-01 9.793960e-01 4.704496e-01 4.651091e-01 4.582891e-01 4.753652e-01 4.526847e-01

1170 9.932606e-01 9.908210e-01 9.880118e-01 9.840248e-01 9.793970e-01 4.726161e-01 4.672301e-01 4.603867e-01 4.775596e-01 4.547175e-01

1171 9.932614e-01 9.908218e-01 9.880127e-01 9.840258e-01 9.793980e-01 4.747827e-01 4.693477e-01 4.624770e-01 4.797574e-01 4.567398e-01

1172 9.932622e-01 9.908227e-01 9.880137e-01 9.840269e-01 9.793992e-01 4.769475e-01 4.714601e-01 4.645577e-01 4.819570e-01 4.587493e-01

1173 9.932630e-01 9.908237e-01 9.880148e-01 9.840280e-01 9.794004e-01 4.791086e-01 4.735652e-01 4.666271e-01 4.841564e-01 4.607442e-01

1174 9.932639e-01 9.908247e-01 9.880159e-01 9.840292e-01 9.794016e-01 4.812642e-01 4.756610e-01 4.686830e-01 4.863538e-01 4.627225e-01

1175 9.932649e-01 9.908258e-01 9.880171e-01 9.840306e-01 9.794030e-01 4.834124e-01 4.777459e-01 4.707238e-01 4.885474e-01 4.646824e-01

1176 9.932659e-01 9.908270e-01 9.880184e-01 9.840320e-01 9.794045e-01 4.855513e-01 4.798179e-01 4.727475e-01 4.907353e-01 4.666222e-01

1177 9.932670e-01 9.908282e-01 9.880197e-01 9.840335e-01 9.794061e-01 4.876792e-01 4.818753e-01 4.747525e-01 4.929158e-01 4.685400e-01

1178 9.932682e-01 9.908296e-01 9.880212e-01 9.840351e-01 9.794078e-01 4.897943e-01 4.839163e-01 4.767370e-01 4.950871e-01 4.704344e-01

1179 9.932695e-01 9.908310e-01 9.880228e-01 9.840368e-01 9.794096e-01 4.918949e-01 4.859392e-01 4.786994e-01 4.972474e-01 4.723039e-01

1180 9.932708e-01 9.908325e-01 9.880245e-01 9.840386e-01 9.794116e-01 4.939792e-01 4.879426e-01 4.806384e-01 4.993951e-01 4.741470e-01

1181 9.932722e-01 9.908342e-01 9.880263e-01 9.840406e-01 9.794137e-01 4.960458e-01 4.899247e-01 4.825523e-01 5.015283e-01 4.759624e-01

1182 9.932738e-01 9.908359e-01 9.880282e-01 9.840427e-01 9.794160e-01 4.980930e-01 4.918842e-01 4.844399e-01 5.036456e-01 4.777490e-01

1183 9.932754e-01 9.908378e-01 9.880303e-01 9.840450e-01 9.794184e-01 5.001192e-01 4.938196e-01 4.863000e-01 5.057454e-01 4.795055e-01

1184 9.932771e-01 9.908397e-01 9.880325e-01 9.840475e-01 9.794210e-01 5.021232e-01 4.957297e-01 4.881312e-01 5.078260e-01 4.812310e-01

1185 9.932789e-01 9.908419e-01 9.880348e-01 9.840501e-01 9.794239e-01 5.041034e-01 4.976131e-01 4.899326e-01 5.098861e-01 4.829244e-01

1186 9.932809e-01 9.908441e-01 9.880374e-01 9.840529e-01 9.794269e-01 5.060587e-01 4.994687e-01 4.917031e-01 5.119242e-01 4.845851e-01

1187 9.932830e-01 9.908466e-01 9.880401e-01 9.840559e-01 9.794302e-01 5.079877e-01 5.012954e-01 4.934417e-01 5.139390e-01 4.862121e-01

1188 9.932853e-01 9.908492e-01 9.880430e-01 9.840591e-01 9.794337e-01 5.098893e-01 5.030921e-01 4.951478e-01 5.159292e-01 4.878050e-01

1189 9.932877e-01 9.908519e-01 9.880461e-01 9.840626e-01 9.794374e-01 5.117624e-01 5.048581e-01 4.968205e-01 5.178936e-01 4.893631e-01

1190 9.932902e-01 9.908549e-01 9.880494e-01 9.840664e-01 9.794415e-01 5.136060e-01 5.065923e-01 4.984592e-01 5.198311e-01 4.908860e-01

1191 9.932929e-01 9.908581e-01 9.880530e-01 9.840704e-01 9.794459e-01 5.154193e-01 5.082942e-01 5.000634e-01 5.217405e-01 4.923733e-01

1192 9.932959e-01 9.908615e-01 9.880568e-01 9.840747e-01 9.794506e-01 5.172012e-01 5.099629e-01 5.016326e-01 5.236210e-01 4.938247e-01

1193 9.932990e-01 9.908652e-01 9.880610e-01 9.840794e-01 9.794557e-01 5.189512e-01 5.115980e-01 5.031664e-01 5.254715e-01 4.952401e-01

1194 9.933023e-01 9.908691e-01 9.880654e-01 9.840844e-01 9.794611e-01 5.206686e-01 5.131989e-01 5.046645e-01 5.272913e-01 4.966193e-01

1195 9.933059e-01 9.908733e-01 9.880701e-01 9.840898e-01 9.794670e-01 5.223527e-01 5.147653e-01 5.061267e-01 5.290797e-01 4.979623e-01

1196 9.933097e-01 9.908778e-01 9.880752e-01 9.840956e-01 9.794734e-01 5.240030e-01 5.162967e-01 5.075529e-01 5.308358e-01 4.992692e-01

1197 9.933138e-01 9.908826e-01 9.880807e-01 9.841018e-01 9.794803e-01 5.256191e-01 5.177930e-01 5.089431e-01 5.325593e-01 5.005401e-01

1198 9.933181e-01 9.908877e-01 9.880866e-01 9.841085e-01 9.794877e-01 5.272006e-01 5.192540e-01 5.102972e-01 5.342494e-01 5.017752e-01

1199 9.933228e-01 9.908933e-01 9.880929e-01 9.841157e-01 9.794957e-01 5.287474e-01 5.206796e-01 5.116154e-01 5.359058e-01 5.029748e-01

1200 9.933278e-01 9.908992e-01 9.880997e-01 9.841235e-01 9.795043e-01 5.302591e-01 5.220698e-01 5.128979e-01 5.375282e-01 5.041391e-01

1201 9.933332e-01 9.909056e-01 9.881070e-01 9.841319e-01 9.795136e-01 5.317356e-01 5.234245e-01 5.141448e-01 5.391162e-01 5.052687e-01

1202 9.933389e-01 9.909124e-01 9.881149e-01 9.841410e-01 9.795237e-01 5.331770e-01 5.247441e-01 5.153565e-01 5.406696e-01 5.063639e-01

1203 9.933450e-01 9.909198e-01 9.881233e-01 9.841507e-01 9.795346e-01 5.345832e-01 5.260285e-01 5.165333e-01 5.421883e-01 5.074251e-01

1204 9.933516e-01 9.909276e-01 9.881324e-01 9.841612e-01 9.795463e-01 5.359544e-01 5.272781e-01 5.176757e-01 5.436722e-01 5.084531e-01

1205 9.933586e-01 9.909361e-01 9.881422e-01 9.841725e-01 9.795590e-01 5.372905e-01 5.284932e-01 5.187840e-01 5.451213e-01 5.094482e-01

1206 9.933662e-01 9.909452e-01 9.881527e-01 9.841848e-01 9.795728e-01 5.385920e-01 5.296741e-01 5.198588e-01 5.465356e-01 5.104112e-01

1207 9.933743e-01 9.909549e-01 9.881640e-01 9.841980e-01 9.795876e-01 5.398589e-01 5.308212e-01 5.209006e-01 5.479153e-01 5.113427e-01

1208 9.933829e-01 9.909654e-01 9.881762e-01 9.842122e-01 9.796037e-01 5.410918e-01 5.319351e-01 5.219101e-01 5.492604e-01 5.122434e-01

1209 9.933922e-01 9.909767e-01 9.881893e-01 9.842276e-01 9.796210e-01 5.422908e-01 5.330161e-01 5.228877e-01 5.505712e-01 5.131139e-01

1210 9.934022e-01 9.909888e-01 9.882035e-01 9.842442e-01 9.796398e-01 5.434563e-01 5.340648e-01 5.238343e-01 5.518477e-01 5.139550e-01

1211 9.934129e-01 9.910018e-01 9.882187e-01 9.842621e-01 9.796602e-01 5.445889e-01 5.350817e-01 5.247503e-01 5.530903e-01 5.147674e-01

1212 9.934244e-01 9.910158e-01 9.882351e-01 9.842815e-01 9.796822e-01 5.456888e-01 5.360675e-01 5.256366e-01 5.542992e-01 5.155520e-01

1213 9.934367e-01 9.910309e-01 9.882528e-01 9.843024e-01 9.797061e-01 5.467565e-01 5.370227e-01 5.264937e-01 5.554748e-01 5.163093e-01

1214 9.934499e-01 9.910471e-01 9.882719e-01 9.843250e-01 9.797319e-01 5.477926e-01 5.379478e-01 5.273224e-01 5.566173e-01 5.170402e-01

1215 9.934641e-01 9.910645e-01 9.882924e-01 9.843494e-01 9.797599e-01 5.487974e-01 5.388435e-01 5.281233e-01 5.577271e-01 5.177454e-01

1216 9.934793e-01 9.910833e-01 9.883146e-01 9.843758e-01 9.797903e-01 5.497715e-01 5.397103e-01 5.288970e-01 5.588046e-01 5.184256e-01

1217 9.934956e-01 9.911034e-01 9.883385e-01 9.844044e-01 9.798231e-01 5.507153e-01 5.405488e-01 5.296443e-01 5.598503e-01 5.190815e-01

1218 9.935132e-01 9.911252e-01 9.883643e-01 9.844353e-01 9.798588e-01 5.516294e-01 5.413597e-01 5.303658e-01 5.608645e-01 5.197137e-01

1219 9.935320e-01 9.911486e-01 9.883921e-01 9.844687e-01 9.798974e-01 5.525144e-01 5.421434e-01 5.310620e-01 5.618478e-01 5.203229e-01

1220 9.935523e-01 9.911738e-01 9.884222e-01 9.845048e-01 9.799392e-01 5.533708e-01 5.429007e-01 5.317338e-01 5.628007e-01 5.209099e-01

1221 9.935740e-01 9.912009e-01 9.884546e-01 9.845439e-01 9.799846e-01 5.541992e-01 5.436321e-01 5.323816e-01 5.637236e-01 5.214751e-01

1222 9.935974e-01 9.912301e-01 9.884896e-01 9.845862e-01 9.800339e-01 5.550001e-01 5.443382e-01 5.330062e-01 5.646172e-01 5.220193e-01

1223 9.936225e-01 9.912616e-01 9.885274e-01 9.846320e-01 9.800872e-01 5.557741e-01 5.450197e-01 5.336081e-01 5.654818e-01 5.225431e-01

1224 9.936496e-01 9.912956e-01 9.885683e-01 9.846816e-01 9.801451e-01 5.565218e-01 5.456772e-01 5.341880e-01 5.663182e-01 5.230471e-01

1225 9.936786e-01 9.913322e-01 9.886123e-01 9.847353e-01 9.802079e-01 5.572439e-01 5.463113e-01 5.347465e-01 5.671269e-01 5.235318e-01

1226 9.937099e-01 9.913716e-01 9.886600e-01 9.847933e-01 9.802760e-01 5.579409e-01 5.469225e-01 5.352842e-01 5.679084e-01 5.239980e-01

1227 9.937434e-01 9.914141e-01 9.887114e-01 9.848562e-01 9.803499e-01 5.586135e-01 5.475115e-01 5.358017e-01 5.686634e-01 5.244461e-01

1228 9.937796e-01 9.914599e-01 9.887670e-01 9.849243e-01 9.804301e-01 5.592622e-01 5.480789e-01 5.362996e-01 5.693924e-01 5.248767e-01

1229 9.938184e-01 9.915093e-01 9.888270e-01 9.849980e-01 9.805170e-01 5.598876e-01 5.486253e-01 5.367785e-01 5.700961e-01 5.252904e-01

1230 9.938603e-01 9.915626e-01 9.888919e-01 9.850778e-01 9.806112e-01 5.604904e-01 5.491514e-01 5.372390e-01 5.707751e-01 5.256878e-01

1231 9.939053e-01 9.916201e-01 9.889619e-01 9.851643e-01 9.807135e-01 5.610712e-01 5.496576e-01 5.376816e-01 5.714300e-01 5.260693e-01

1232 9.939537e-01 9.916820e-01 9.890377e-01 9.852578e-01 9.808243e-01 5.616305e-01 5.501445e-01 5.381069e-01 5.720614e-01 5.264356e-01

1233 9.940058e-01 9.917489e-01 9.891195e-01 9.853591e-01 9.809445e-01 5.621691e-01 5.506128e-01 5.385155e-01 5.726699e-01 5.267870e-01

1234 9.940618e-01 9.918210e-01 9.892080e-01 9.854688e-01 9.810748e-01 5.626874e-01 5.510631e-01 5.389079e-01 5.732562e-01 5.271242e-01

1235 9.941222e-01 9.918987e-01 9.893035e-01 9.855875e-01 9.812160e-01 5.631861e-01 5.514958e-01 5.392846e-01 5.738208e-01 5.274476e-01

1236 9.941871e-01 9.919826e-01 9.894068e-01 9.857160e-01 9.813690e-01 5.636657e-01 5.519115e-01 5.396461e-01 5.743644e-01 5.277577e-01

1237 9.942571e-01 9.920731e-01 9.895184e-01 9.858550e-01 9.815347e-01 5.641269e-01 5.523109e-01 5.399931e-01 5.748876e-01 5.280549e-01

1238 9.943323e-01 9.921706e-01 9.896389e-01 9.860054e-01 9.817142e-01 5.645702e-01 5.526943e-01 5.403259e-01 5.753909e-01 5.283398e-01

1239 9.944133e-01 9.922759e-01 9.897691e-01 9.861681e-01 9.819085e-01 5.649961e-01 5.530624e-01 5.406450e-01 5.758750e-01 5.286127e-01

1240 9.945005e-01 9.923894e-01 9.899097e-01 9.863441e-01 9.821186e-01 5.654053e-01 5.534156e-01 5.409510e-01 5.763405e-01 5.288741e-01

1241 9.945944e-01 9.925118e-01 9.900616e-01 9.865343e-01 9.823460e-01 5.657982e-01 5.537545e-01 5.412443e-01 5.767879e-01 5.291245e-01

1242 9.946955e-01 9.926438e-01 9.902256e-01 9.867399e-01 9.825917e-01 5.661755e-01 5.540796e-01 5.415253e-01 5.772178e-01 5.293642e-01

1243 9.948042e-01 9.927862e-01 9.904026e-01 9.869620e-01 9.828572e-01 5.665376e-01 5.543913e-01 5.417946e-01 5.776308e-01 5.295936e-01

1244 9.949213e-01 9.929396e-01 9.905937e-01 9.872018e-01 9.831439e-01 5.668850e-01 5.546901e-01 5.420524e-01 5.780274e-01 5.298132e-01

1245 9.950473e-01 9.931050e-01 9.907998e-01 9.874607e-01 9.834532e-01 5.672183e-01 5.549765e-01 5.422994e-01 5.784081e-01 5.300232e-01

1246 9.951829e-01 9.932832e-01 9.910221e-01 9.877401e-01 9.837867e-01 5.675379e-01 5.552508e-01 5.425357e-01 5.787736e-01 5.302242e-01

1247 9.953287e-01 9.934753e-01 9.912618e-01 9.880413e-01 9.841460e-01 5.678443e-01 5.555137e-01 5.427620e-01 5.791242e-01 5.304163e-01

1248 9.954857e-01 9.936821e-01 9.915202e-01 9.883659e-01 9.845327e-01 5.681380e-01 5.557654e-01 5.429785e-01 5.794606e-01 5.306000e-01

1249 9.956545e-01 9.939049e-01 9.917985e-01 9.887155e-01 9.849487e-01 5.684195e-01 5.560064e-01 5.431856e-01 5.797832e-01 5.307757e-01

1250 9.958360e-01 9.941446e-01 9.920982e-01 9.890918e-01 9.853956e-01 5.686892e-01 5.562371e-01 5.433837e-01 5.800925e-01 5.309435e-01

1251 9.960312e-01 9.944027e-01 9.924208e-01 9.894964e-01 9.858753e-01 5.689475e-01 5.564579e-01 5.435731e-01 5.803889e-01 5.311039e-01

1252 9.962411e-01 9.946803e-01 9.927678e-01 9.899312e-01 9.863895e-01 5.691948e-01 5.566692e-01 5.437542e-01 5.806730e-01 5.312571e-01

1253 9.964666e-01 9.949788e-01 9.931409e-01 9.903981e-01 9.869402e-01 5.694316e-01 5.568713e-01 5.439273e-01 5.809452e-01 5.314034e-01

1254 9.967089e-01 9.952996e-01 9.935417e-01 9.908988e-01 9.875290e-01 5.696583e-01 5.570646e-01 5.440928e-01 5.812060e-01 5.315432e-01

1255 9.969691e-01 9.956443e-01 9.939720e-01 9.914354e-01 9.881579e-01 5.698752e-01 5.572495e-01 5.442509e-01 5.814556e-01 5.316766e-01

1256 9.972485e-01 9.960143e-01 9.944337e-01 9.920098e-01 9.888286e-01 5.700827e-01 5.574262e-01 5.444019e-01 5.816947e-01 5.318040e-01

1257 9.975484e-01 9.964115e-01 9.949286e-01 9.926240e-01 9.895427e-01 5.702812e-01 5.575951e-01 5.445461e-01 5.819235e-01 5.319256e-01

1258 9.978701e-01 9.968374e-01 9.954587e-01 9.932799e-01 9.903019e-01 5.704711e-01 5.577565e-01 5.446839e-01 5.821425e-01 5.320416e-01

1259 9.982151e-01 9.972939e-01 9.960259e-01 9.939795e-01 9.911076e-01 5.706526e-01 5.579107e-01 5.448154e-01 5.823520e-01 5.321523e-01

1260 9.985849e-01 9.977829e-01 9.966324e-01 9.947248e-01 9.919612e-01 5.708262e-01 5.580580e-01 5.449409e-01 5.825525e-01 5.322578e-01

1261 9.989811e-01 9.983062e-01 9.972801e-01 9.955175e-01 9.928637e-01 5.709920e-01 5.581987e-01 5.450607e-01 5.827442e-01 5.323585e-01

1262 9.989809e-01 9.983061e-01 9.972801e-01 9.955175e-01 9.928637e-01 5.710422e-01 5.582504e-01 5.451150e-01 5.827925e-01 5.324136e-01

1263 9.989808e-01 9.983060e-01 9.972800e-01 9.955175e-01 9.928637e-01 5.710944e-01 5.583041e-01 5.451714e-01 5.828430e-01 5.324708e-01

1264 9.989806e-01 9.983059e-01 9.972800e-01 9.955174e-01 9.928637e-01 5.711489e-01 5.583601e-01 5.452301e-01 5.828957e-01 5.325303e-01

1265 9.989805e-01 9.983058e-01 9.972799e-01 9.955174e-01 9.928636e-01 5.712057e-01 5.584183e-01 5.452911e-01 5.829508e-01 5.325920e-01

1266 9.989804e-01 9.983058e-01 9.972799e-01 9.955174e-01 9.928636e-01 5.712649e-01 5.584789e-01 5.453544e-01 5.830083e-01 5.326561e-01

1267 9.989802e-01 9.983057e-01 9.972799e-01 9.955174e-01 9.928636e-01 5.713265e-01 5.585419e-01 5.454202e-01 5.830682e-01 5.327226e-01

1268 9.989801e-01 9.983057e-01 9.972798e-01 9.955173e-01 9.928636e-01 5.713906e-01 5.586075e-01 5.454886e-01 5.831306e-01 5.327917e-01

1269 9.989801e-01 9.983056e-01 9.972798e-01 9.955173e-01 9.928636e-01 5.714573e-01 5.586756e-01 5.455596e-01 5.831956e-01 5.328633e-01

1270 9.989800e-01 9.983056e-01 9.972798e-01 9.955173e-01 9.928635e-01 5.715267e-01 5.587463e-01 5.456333e-01 5.832633e-01 5.329377e-01

1271 9.989799e-01 9.983055e-01 9.972797e-01 9.955173e-01 9.928635e-01 5.715989e-01 5.588199e-01 5.457098e-01 5.833338e-01 5.330150e-01

1272 9.989798e-01 9.983055e-01 9.972797e-01 9.955173e-01 9.928635e-01 5.716739e-01 5.588962e-01 5.457893e-01 5.834071e-01 5.330951e-01

1273 9.989798e-01 9.983055e-01 9.972797e-01 9.955172e-01 9.928635e-01 5.717518e-01 5.589756e-01 5.458717e-01 5.834834e-01 5.331782e-01

1274 9.989797e-01 9.983054e-01 9.972797e-01 9.955172e-01 9.928635e-01 5.718328e-01 5.590579e-01 5.459573e-01 5.835627e-01 5.332645e-01

1275 9.989796e-01 9.983054e-01 9.972797e-01 9.955172e-01 9.928635e-01 5.719169e-01 5.591435e-01 5.460461e-01 5.836451e-01 5.333540e-01

1276 9.989796e-01 9.983054e-01 9.972797e-01 9.955172e-01 9.928635e-01 5.720043e-01 5.592323e-01 5.461383e-01 5.837307e-01 5.334469e-01

1277 9.989796e-01 9.983054e-01 9.972796e-01 9.955172e-01 9.928635e-01 5.720950e-01 5.593244e-01 5.462339e-01 5.838197e-01 5.335432e-01

1278 9.989795e-01 9.983054e-01 9.972796e-01 9.955172e-01 9.928635e-01 5.721892e-01 5.594201e-01 5.463331e-01 5.839121e-01 5.336432e-01

1279 9.989795e-01 9.983054e-01 9.972796e-01 9.955172e-01 9.928635e-01 5.722869e-01 5.595193e-01 5.464360e-01 5.840081e-01 5.337468e-01

1280 9.989795e-01 9.983053e-01 9.972796e-01 9.955172e-01 9.928635e-01 5.723884e-01 5.596224e-01 5.465427e-01 5.841077e-01 5.338544e-01

1281 9.989795e-01 9.983053e-01 9.972796e-01 9.955172e-01 9.928635e-01 5.724937e-01 5.597292e-01 5.466534e-01 5.842111e-01 5.339660e-01

1282 9.989794e-01 9.983053e-01 9.972796e-01 9.955172e-01 9.928635e-01 5.726030e-01 5.598401e-01 5.467683e-01 5.843185e-01 5.340817e-01

1283 9.989794e-01 9.983053e-01 9.972796e-01 9.955172e-01 9.928635e-01 5.727163e-01 5.599552e-01 5.468874e-01 5.844299e-01 5.342017e-01

1284 9.989794e-01 9.983053e-01 9.972796e-01 9.955172e-01 9.928635e-01 5.728339e-01 5.600745e-01 5.470110e-01 5.845455e-01 5.343263e-01

1285 9.989794e-01 9.983053e-01 9.972796e-01 9.955172e-01 9.928635e-01 5.729560e-01 5.601983e-01 5.471391e-01 5.846654e-01 5.344554e-01

1286 9.989794e-01 9.983053e-01 9.972796e-01 9.955172e-01 9.928635e-01 5.730825e-01 5.603267e-01 5.472720e-01 5.847899e-01 5.345894e-01

1287 9.989794e-01 9.983053e-01 9.972796e-01 9.955172e-01 9.928635e-01 5.732138e-01 5.604599e-01 5.474098e-01 5.849189e-01 5.347284e-01

1288 9.989794e-01 9.983053e-01 9.972796e-01 9.955172e-01 9.928635e-01 5.733499e-01 5.605981e-01 5.475528e-01 5.850528e-01 5.348725e-01

1289 9.989794e-01 9.983053e-01 9.972796e-01 9.955172e-01 9.928635e-01 5.734911e-01 5.607413e-01 5.477010e-01 5.851916e-01 5.350221e-01

1290 9.989794e-01 9.983053e-01 9.972796e-01 9.955172e-01 9.928635e-01 5.736375e-01 5.608899e-01 5.478548e-01 5.853356e-01 5.351772e-01

1291 9.989794e-01 9.983054e-01 9.972796e-01 9.955172e-01 9.928634e-01 5.737893e-01 5.610440e-01 5.480142e-01 5.854848e-01 5.353380e-01

1292 9.989794e-01 9.983054e-01 9.972796e-01 9.955172e-01 9.928634e-01 5.739467e-01 5.612038e-01 5.481795e-01 5.856396e-01 5.355049e-01

1293 9.989794e-01 9.983054e-01 9.972797e-01 9.955172e-01 9.928634e-01 5.741100e-01 5.613696e-01 5.483509e-01 5.858001e-01 5.356780e-01

1294 9.989794e-01 9.983054e-01 9.972797e-01 9.955172e-01 9.928634e-01 5.742792e-01 5.615414e-01 5.485287e-01 5.859665e-01 5.358575e-01

1295 9.989794e-01 9.983054e-01 9.972797e-01 9.955172e-01 9.928634e-01 5.744547e-01 5.617196e-01 5.487131e-01 5.861390e-01 5.360437e-01

1296 9.989794e-01 9.983054e-01 9.972797e-01 9.955172e-01 9.928634e-01 5.746366e-01 5.619044e-01 5.489042e-01 5.863178e-01 5.362369e-01

1297 9.989795e-01 9.983054e-01 9.972797e-01 9.955172e-01 9.928634e-01 5.748251e-01 5.620960e-01 5.491024e-01 5.865031e-01 5.364372e-01

1298 9.989795e-01 9.983054e-01 9.972797e-01 9.955172e-01 9.928634e-01 5.750206e-01 5.622947e-01 5.493080e-01 5.866952e-01 5.366450e-01

1299 9.989795e-01 9.983054e-01 9.972797e-01 9.955172e-01 9.928634e-01 5.752233e-01 5.625007e-01 5.495211e-01 5.868943e-01 5.368605e-01

1300 9.989795e-01 9.983054e-01 9.972797e-01 9.955172e-01 9.928634e-01 5.754333e-01 5.627143e-01 5.497420e-01 5.871007e-01 5.370840e-01

1301 9.989795e-01 9.983055e-01 9.972797e-01 9.955172e-01 9.928634e-01 5.756511e-01 5.629357e-01 5.499711e-01 5.873146e-01 5.373158e-01

1302 9.989795e-01 9.983055e-01 9.972797e-01 9.955172e-01 9.928634e-01 5.758768e-01 5.631653e-01 5.502087e-01 5.875362e-01 5.375562e-01

1303 9.989795e-01 9.983055e-01 9.972797e-01 9.955172e-01 9.928634e-01 5.761107e-01 5.634033e-01 5.504549e-01 5.877659e-01 5.378055e-01

1304 9.989796e-01 9.983055e-01 9.972797e-01 9.955172e-01 9.928634e-01 5.763532e-01 5.636501e-01 5.507102e-01 5.880039e-01 5.380640e-01

1305 9.989796e-01 9.983055e-01 9.972798e-01 9.955172e-01 9.928633e-01 5.766045e-01 5.639059e-01 5.509749e-01 5.882506e-01 5.383321e-01

1306 9.989796e-01 9.983055e-01 9.972798e-01 9.955172e-01 9.928633e-01 5.768649e-01 5.641710e-01 5.512493e-01 5.885061e-01 5.386101e-01

1307 9.989796e-01 9.983055e-01 9.972798e-01 9.955172e-01 9.928633e-01 5.771347e-01 5.644458e-01 5.515337e-01 5.887709e-01 5.388983e-01

1308 9.989796e-01 9.983056e-01 9.972798e-01 9.955172e-01 9.928633e-01 5.774144e-01 5.647307e-01 5.518285e-01 5.890452e-01 5.391971e-01

1309 9.989797e-01 9.983056e-01 9.972798e-01 9.955172e-01 9.928633e-01 5.777041e-01 5.650260e-01 5.521341e-01 5.893294e-01 5.395069e-01

1310 9.989797e-01 9.983056e-01 9.972798e-01 9.955172e-01 9.928633e-01 5.780044e-01 5.653320e-01 5.524507e-01 5.896238e-01 5.398281e-01

1311 9.989797e-01 9.983056e-01 9.972798e-01 9.955172e-01 9.928632e-01 5.783154e-01 5.656491e-01 5.527789e-01 5.899288e-01 5.401610e-01

1312 9.989797e-01 9.983056e-01 9.972798e-01 9.955172e-01 9.928632e-01 5.786377e-01 5.659777e-01 5.531190e-01 5.902446e-01 5.405060e-01

1313 9.989797e-01 9.983056e-01 9.972798e-01 9.955172e-01 9.928632e-01 5.789715e-01 5.663181e-01 5.534714e-01 5.905718e-01 5.408636e-01

1314 9.989798e-01 9.983057e-01 9.972798e-01 9.955172e-01 9.928632e-01 5.793173e-01 5.666709e-01 5.538364e-01 5.909106e-01 5.412342e-01

1315 9.989798e-01 9.983057e-01 9.972798e-01 9.955172e-01 9.928631e-01 5.796754e-01 5.670363e-01 5.542146e-01 5.912615e-01 5.416181e-01

1316 9.989798e-01 9.983057e-01 9.972798e-01 9.955172e-01 9.928631e-01 5.800463e-01 5.674149e-01 5.546064e-01 5.916248e-01 5.420159e-01

1317 9.989798e-01 9.983057e-01 9.972799e-01 9.955172e-01 9.928631e-01 5.804304e-01 5.678070e-01 5.550121e-01 5.920010e-01 5.424279e-01

1318 9.989799e-01 9.983057e-01 9.972799e-01 9.955172e-01 9.928630e-01 5.808282e-01 5.682130e-01 5.554323e-01 5.923904e-01 5.428546e-01

1319 9.989799e-01 9.983057e-01 9.972799e-01 9.955172e-01 9.928630e-01 5.812399e-01 5.686335e-01 5.558673e-01 5.927935e-01 5.432965e-01

1320 9.989799e-01 9.983058e-01 9.972799e-01 9.955172e-01 9.928630e-01 5.816662e-01 5.690688e-01 5.563177e-01 5.932108e-01 5.437540e-01

1321 9.989799e-01 9.983058e-01 9.972799e-01 9.955172e-01 9.928629e-01 5.821075e-01 5.695195e-01 5.567839e-01 5.936427e-01 5.442277e-01

1322 9.989800e-01 9.983058e-01 9.972799e-01 9.955171e-01 9.928629e-01 5.825641e-01 5.699860e-01 5.572664e-01 5.940895e-01 5.447179e-01

1323 9.989800e-01 9.983058e-01 9.972799e-01 9.955171e-01 9.928628e-01 5.830367e-01 5.704688e-01 5.577657e-01 5.945519e-01 5.452251e-01

1324 9.989800e-01 9.983059e-01 9.972799e-01 9.955171e-01 9.928628e-01 5.835256e-01 5.709683e-01 5.582822e-01 5.950303e-01 5.457499e-01

1325 9.989801e-01 9.983059e-01 9.972799e-01 9.955171e-01 9.928627e-01 5.840313e-01 5.714851e-01 5.588165e-01 5.955251e-01 5.462926e-01

1326 9.989801e-01 9.983059e-01 9.972799e-01 9.955171e-01 9.928626e-01 5.845545e-01 5.720197e-01 5.593690e-01 5.960368e-01 5.468539e-01

1327 9.989801e-01 9.983059e-01 9.972800e-01 9.955171e-01 9.928626e-01 5.850954e-01 5.725725e-01 5.599403e-01 5.965660e-01 5.474341e-01

1328 9.989802e-01 9.983060e-01 9.972800e-01 9.955170e-01 9.928625e-01 5.856547e-01 5.731441e-01 5.605307e-01 5.971131e-01 5.480338e-01

1329 9.989802e-01 9.983060e-01 9.972800e-01 9.955170e-01 9.928624e-01 5.862329e-01 5.737349e-01 5.611409e-01 5.976786e-01 5.486534e-01

1330 9.989802e-01 9.983060e-01 9.972800e-01 9.955170e-01 9.928624e-01 5.868304e-01 5.743455e-01 5.617713e-01 5.982630e-01 5.492934e-01

1331 9.989803e-01 9.983061e-01 9.972800e-01 9.955170e-01 9.928623e-01 5.874479e-01 5.749763e-01 5.624223e-01 5.988669e-01 5.499543e-01

1332 9.989803e-01 9.983061e-01 9.972800e-01 9.955170e-01 9.928622e-01 5.880856e-01 5.756280e-01 5.630946e-01 5.994908e-01 5.506365e-01

1333 9.989804e-01 9.983061e-01 9.972800e-01 9.955169e-01 9.928621e-01 5.887443e-01 5.763008e-01 5.637885e-01 6.001351e-01 5.513404e-01

1334 9.989804e-01 9.983062e-01 9.972800e-01 9.955169e-01 9.928620e-01 5.894244e-01 5.769955e-01 5.645044e-01 6.008005e-01 5.520665e-01

1335 9.989805e-01 9.983062e-01 9.972800e-01 9.955169e-01 9.928619e-01 5.901265e-01 5.777124e-01 5.652430e-01 6.014873e-01 5.528152e-01

1336 9.989805e-01 9.983062e-01 9.972801e-01 9.955168e-01 9.928618e-01 5.908509e-01 5.784520e-01 5.660046e-01 6.021962e-01 5.535868e-01

1337 9.989806e-01 9.983063e-01 9.972801e-01 9.955168e-01 9.928616e-01 5.915982e-01 5.792148e-01 5.667896e-01 6.029276e-01 5.543818e-01

1338 9.989806e-01 9.983063e-01 9.972801e-01 9.955167e-01 9.928615e-01 5.923690e-01 5.800012e-01 5.675984e-01 6.036821e-01 5.552006e-01

1339 9.989807e-01 9.983064e-01 9.972801e-01 9.955167e-01 9.928613e-01 5.931636e-01 5.808117e-01 5.684314e-01 6.044600e-01 5.560433e-01

1340 9.989807e-01 9.983064e-01 9.972801e-01 9.955167e-01 9.928612e-01 5.939825e-01 5.816467e-01 5.692891e-01 6.052620e-01 5.569104e-01

1341 9.989808e-01 9.983065e-01 9.972801e-01 9.955166e-01 9.928610e-01 5.948262e-01 5.825067e-01 5.701716e-01 6.060885e-01 5.578021e-01

1342 9.989809e-01 9.983065e-01 9.972801e-01 9.955165e-01 9.928609e-01 5.956951e-01 5.833919e-01 5.710794e-01 6.069400e-01 5.587186e-01

1343 9.989810e-01 9.983066e-01 9.972802e-01 9.955165e-01 9.928607e-01 5.965896e-01 5.843027e-01 5.720128e-01 6.078169e-01 5.596600e-01

1344 9.989810e-01 9.983067e-01 9.972802e-01 9.955164e-01 9.928605e-01 5.975102e-01 5.852395e-01 5.729718e-01 6.087196e-01 5.606267e-01

1345 9.989811e-01 9.983067e-01 9.972802e-01 9.955164e-01 9.928602e-01 5.984571e-01 5.862026e-01 5.739568e-01 6.096486e-01 5.616186e-01

1346 9.989812e-01 9.983068e-01 9.972802e-01 9.955163e-01 9.928600e-01 5.994307e-01 5.871922e-01 5.749680e-01 6.106043e-01 5.626358e-01

1347 9.989813e-01 9.983069e-01 9.972802e-01 9.955162e-01 9.928598e-01 6.004314e-01 5.882085e-01 5.760054e-01 6.115869e-01 5.636784e-01

1348 9.989814e-01 9.983069e-01 9.972803e-01 9.955161e-01 9.928595e-01 6.014594e-01 5.892518e-01 5.770692e-01 6.125970e-01 5.647462e-01

1349 9.989815e-01 9.983070e-01 9.972803e-01 9.955160e-01 9.928592e-01 6.025149e-01 5.903222e-01 5.781593e-01 6.136347e-01 5.658393e-01

1350 9.989816e-01 9.983071e-01 9.972803e-01 9.955159e-01 9.928589e-01 6.035982e-01 5.914198e-01 5.792758e-01 6.147004e-01 5.669574e-01

1351 9.989817e-01 9.983072e-01 9.972803e-01 9.955158e-01 9.928586e-01 6.047094e-01 5.925447e-01 5.804185e-01 6.157943e-01 5.681003e-01

1352 9.989818e-01 9.983073e-01 9.972803e-01 9.955157e-01 9.928582e-01 6.058486e-01 5.936968e-01 5.815874e-01 6.169167e-01 5.692678e-01

1353 9.989819e-01 9.983074e-01 9.972803e-01 9.955156e-01 9.928578e-01 6.070160e-01 5.948761e-01 5.827823e-01 6.180677e-01 5.704594e-01

1354 9.989821e-01 9.983075e-01 9.972804e-01 9.955154e-01 9.928574e-01 6.082115e-01 5.960826e-01 5.840029e-01 6.192474e-01 5.716749e-01

1355 9.989822e-01 9.983076e-01 9.972804e-01 9.955153e-01 9.928570e-01 6.094352e-01 5.973160e-01 5.852488e-01 6.204559e-01 5.729137e-01

1356 9.989824e-01 9.983077e-01 9.972804e-01 9.955151e-01 9.928565e-01 6.106869e-01 5.985762e-01 5.865198e-01 6.216933e-01 5.741753e-01

1357 9.989825e-01 9.983078e-01 9.972804e-01 9.955150e-01 9.928560e-01 6.119666e-01 5.998629e-01 5.878154e-01 6.229596e-01 5.754590e-01

1358 9.989827e-01 9.983079e-01 9.972804e-01 9.955148e-01 9.928555e-01 6.132741e-01 6.011757e-01 5.891350e-01 6.242547e-01 5.767642e-01

1359 9.989828e-01 9.983081e-01 9.972805e-01 9.955146e-01 9.928549e-01 6.146090e-01 6.025143e-01 5.904781e-01 6.255786e-01 5.780902e-01

1360 9.989830e-01 9.983082e-01 9.972805e-01 9.955144e-01 9.928543e-01 6.159712e-01 6.038781e-01 5.918440e-01 6.269309e-01 5.794360e-01

1361 9.989832e-01 9.983083e-01 9.972805e-01 9.955141e-01 9.928536e-01 6.173602e-01 6.052667e-01 5.932320e-01 6.283116e-01 5.808009e-01

1362 9.989834e-01 9.983085e-01 9.972805e-01 9.955139e-01 9.928529e-01 6.187757e-01 6.066793e-01 5.946414e-01 6.297203e-01 5.821838e-01

1363 9.989836e-01 9.983086e-01 9.972805e-01 9.955136e-01 9.928521e-01 6.202170e-01 6.081154e-01 5.960712e-01 6.311567e-01 5.835837e-01

1364 9.989838e-01 9.983088e-01 9.972805e-01 9.955133e-01 9.928513e-01 6.216837e-01 6.095742e-01 5.975205e-01 6.326204e-01 5.849995e-01

1365 9.989841e-01 9.983090e-01 9.972805e-01 9.955130e-01 9.928504e-01 6.231751e-01 6.110549e-01 5.989884e-01 6.341109e-01 5.864301e-01

1366 9.989843e-01 9.983092e-01 9.972806e-01 9.955127e-01 9.928494e-01 6.246906e-01 6.125565e-01 6.004737e-01 6.356277e-01 5.878742e-01

1367 9.989846e-01 9.983094e-01 9.972806e-01 9.955123e-01 9.928484e-01 6.262293e-01 6.140783e-01 6.019754e-01 6.371702e-01 5.893306e-01

1368 9.989848e-01 9.983096e-01 9.972806e-01 9.955119e-01 9.928473e-01 6.277904e-01 6.156190e-01 6.034923e-01 6.387377e-01 5.907980e-01

1369 9.989851e-01 9.983098e-01 9.972806e-01 9.955115e-01 9.928461e-01 6.293730e-01 6.171777e-01 6.050231e-01 6.403296e-01 5.922749e-01

1370 9.989854e-01 9.983100e-01 9.972806e-01 9.955110e-01 9.928448e-01 6.309762e-01 6.187533e-01 6.065665e-01 6.419449e-01 5.937600e-01

1371 9.989858e-01 9.983103e-01 9.972806e-01 9.955106e-01 9.928434e-01 6.325990e-01 6.203444e-01 6.081212e-01 6.435829e-01 5.952518e-01

1372 9.989861e-01 9.983106e-01 9.972806e-01 9.955100e-01 9.928419e-01 6.342401e-01 6.219499e-01 6.096858e-01 6.452427e-01 5.967488e-01

1373 9.989865e-01 9.983108e-01 9.972806e-01 9.955094e-01 9.928403e-01 6.358986e-01 6.235685e-01 6.112588e-01 6.469231e-01 5.982495e-01

1374 9.989869e-01 9.983111e-01 9.972806e-01 9.955088e-01 9.928385e-01 6.375732e-01 6.251988e-01 6.128389e-01 6.486234e-01 5.997524e-01

1375 9.989873e-01 9.983114e-01 9.972805e-01 9.955082e-01 9.928367e-01 6.392626e-01 6.268393e-01 6.144243e-01 6.503422e-01 6.012559e-01

1376 9.989877e-01 9.983118e-01 9.972805e-01 9.955074e-01 9.928346e-01 6.409655e-01 6.284888e-01 6.160138e-01 6.520784e-01 6.027585e-01

1377 9.989882e-01 9.983121e-01 9.972805e-01 9.955067e-01 9.928324e-01 6.426807e-01 6.301456e-01 6.176056e-01 6.538309e-01 6.042586e-01

1378 9.989886e-01 9.983125e-01 9.972805e-01 9.955058e-01 9.928301e-01 6.444065e-01 6.318082e-01 6.191983e-01 6.555984e-01 6.057546e-01

1379 9.989892e-01 9.983128e-01 9.972804e-01 9.955049e-01 9.928275e-01 6.461417e-01 6.334752e-01 6.207902e-01 6.573796e-01 6.072449e-01

1380 9.989897e-01 9.983132e-01 9.972804e-01 9.955039e-01 9.928248e-01 6.478848e-01 6.351450e-01 6.223797e-01 6.591731e-01 6.087281e-01

1381 9.989903e-01 9.983137e-01 9.972803e-01 9.955028e-01 9.928218e-01 6.496342e-01 6.368159e-01 6.239654e-01 6.609775e-01 6.102027e-01

1382 9.989909e-01 9.983141e-01 9.972802e-01 9.955017e-01 9.928186e-01 6.513885e-01 6.384865e-01 6.255456e-01 6.627914e-01 6.116671e-01

1383 9.989915e-01 9.983146e-01 9.972802e-01 9.955004e-01 9.928151e-01 6.531460e-01 6.401551e-01 6.271187e-01 6.646133e-01 6.131199e-01

1384 9.989922e-01 9.983151e-01 9.972801e-01 9.954991e-01 9.928114e-01 6.549052e-01 6.418202e-01 6.286833e-01 6.664417e-01 6.145597e-01

1385 9.989929e-01 9.983156e-01 9.972800e-01 9.954976e-01 9.928073e-01 6.566646e-01 6.434802e-01 6.302379e-01 6.682752e-01 6.159851e-01

1386 9.989937e-01 9.983162e-01 9.972798e-01 9.954960e-01 9.928029e-01 6.584225e-01 6.451335e-01 6.317809e-01 6.701122e-01 6.173948e-01

1387 9.989945e-01 9.983168e-01 9.972797e-01 9.954943e-01 9.927982e-01 6.601774e-01 6.467786e-01 6.333109e-01 6.719512e-01 6.187875e-01

1388 9.989953e-01 9.983175e-01 9.972795e-01 9.954925e-01 9.927931e-01 6.619278e-01 6.484140e-01 6.348266e-01 6.737906e-01 6.201621e-01

1389 9.989963e-01 9.983181e-01 9.972794e-01 9.954905e-01 9.927875e-01 6.636720e-01 6.500382e-01 6.363266e-01 6.756291e-01 6.215174e-01

1390 9.989972e-01 9.983188e-01 9.972792e-01 9.954883e-01 9.927815e-01 6.654085e-01 6.516497e-01 6.378096e-01 6.774651e-01 6.228523e-01

1391 9.989983e-01 9.983196e-01 9.972789e-01 9.954859e-01 9.927750e-01 6.671360e-01 6.532472e-01 6.392743e-01 6.792971e-01 6.241658e-01

1392 9.989993e-01 9.983204e-01 9.972787e-01 9.954834e-01 9.927680e-01 6.688530e-01 6.548293e-01 6.407197e-01 6.811238e-01 6.254570e-01

1393 9.990005e-01 9.983212e-01 9.972784e-01 9.954806e-01 9.927604e-01 6.705581e-01 6.563947e-01 6.421446e-01 6.829437e-01 6.267251e-01

1394 9.990017e-01 9.983221e-01 9.972781e-01 9.954776e-01 9.927521e-01 6.722500e-01 6.579424e-01 6.435481e-01 6.847555e-01 6.279692e-01

1395 9.990030e-01 9.983231e-01 9.972777e-01 9.954744e-01 9.927432e-01 6.739274e-01 6.594711e-01 6.449292e-01 6.865577e-01 6.291888e-01

1396 9.990044e-01 9.983241e-01 9.972773e-01 9.954709e-01 9.927335e-01 6.755892e-01 6.609799e-01 6.462871e-01 6.883491e-01 6.303833e-01

1397 9.990059e-01 9.983252e-01 9.972769e-01 9.954670e-01 9.927230e-01 6.772341e-01 6.624677e-01 6.476212e-01 6.901285e-01 6.315521e-01

1398 9.990074e-01 9.983263e-01 9.972764e-01 9.954629e-01 9.927116e-01 6.788611e-01 6.639338e-01 6.489307e-01 6.918944e-01 6.326950e-01

1399 9.990091e-01 9.983275e-01 9.972758e-01 9.954584e-01 9.926993e-01 6.804691e-01 6.653771e-01 6.502150e-01 6.936459e-01 6.338116e-01

1400 9.990109e-01 9.983288e-01 9.972752e-01 9.954536e-01 9.926859e-01 6.820571e-01 6.667971e-01 6.514738e-01 6.953816e-01 6.349017e-01

1401 9.990127e-01 9.983301e-01 9.972745e-01 9.954483e-01 9.926714e-01 6.836242e-01 6.681929e-01 6.527065e-01 6.971005e-01 6.359651e-01

1402 9.990147e-01 9.983316e-01 9.972738e-01 9.954425e-01 9.926556e-01 6.851695e-01 6.695641e-01 6.539129e-01 6.988016e-01 6.370018e-01

1403 9.990168e-01 9.983331e-01 9.972729e-01 9.954363e-01 9.926385e-01 6.866923e-01 6.709100e-01 6.550926e-01 7.004838e-01 6.380117e-01

1404 9.990191e-01 9.983347e-01 9.972720e-01 9.954296e-01 9.926200e-01 6.881917e-01 6.722302e-01 6.562454e-01 7.021461e-01 6.389949e-01

1405 9.990215e-01 9.983364e-01 9.972710e-01 9.954222e-01 9.925999e-01 6.896672e-01 6.735243e-01 6.573712e-01 7.037877e-01 6.399515e-01

1406 9.990240e-01 9.983383e-01 9.972698e-01 9.954143e-01 9.925780e-01 6.911181e-01 6.747920e-01 6.584700e-01 7.054077e-01 6.408817e-01

1407 9.990267e-01 9.983402e-01 9.972686e-01 9.954056e-01 9.925543e-01 6.925438e-01 6.760330e-01 6.595417e-01 7.070054e-01 6.417857e-01

1408 9.990296e-01 9.983422e-01 9.972672e-01 9.953962e-01 9.925286e-01 6.939440e-01 6.772470e-01 6.605863e-01 7.085799e-01 6.426638e-01

1409 9.990327e-01 9.983444e-01 9.972656e-01 9.953860e-01 9.925006e-01 6.953180e-01 6.784339e-01 6.616040e-01 7.101306e-01 6.435161e-01

1410 9.990360e-01 9.983467e-01 9.972639e-01 9.953750e-01 9.924703e-01 6.966657e-01 6.795937e-01 6.625950e-01 7.116570e-01 6.443432e-01

1411 9.990395e-01 9.983492e-01 9.972621e-01 9.953629e-01 9.924373e-01 6.979867e-01 6.807263e-01 6.635593e-01 7.131584e-01 6.451454e-01

1412 9.990432e-01 9.983518e-01 9.972600e-01 9.953499e-01 9.924015e-01 6.992807e-01 6.818318e-01 6.644973e-01 7.146343e-01 6.459231e-01

1413 9.990471e-01 9.983546e-01 9.972577e-01 9.953356e-01 9.923626e-01 7.005476e-01 6.829101e-01 6.654092e-01 7.160844e-01 6.466768e-01

1414 9.990513e-01 9.983576e-01 9.972552e-01 9.953202e-01 9.923203e-01 7.017872e-01 6.839615e-01 6.662953e-01 7.175081e-01 6.474068e-01

1415 9.990558e-01 9.983607e-01 9.972525e-01 9.953034e-01 9.922744e-01 7.029995e-01 6.849860e-01 6.671561e-01 7.189053e-01 6.481138e-01

1416 9.990606e-01 9.983640e-01 9.972495e-01 9.952852e-01 9.922246e-01 7.041845e-01 6.859840e-01 6.679919e-01 7.202756e-01 6.487983e-01

1417 9.990657e-01 9.983676e-01 9.972461e-01 9.952653e-01 9.921705e-01 7.053422e-01 6.869557e-01 6.688031e-01 7.216188e-01 6.494607e-01

1418 9.990712e-01 9.983714e-01 9.972424e-01 9.952438e-01 9.921118e-01 7.064726e-01 6.879013e-01 6.695902e-01 7.229348e-01 6.501017e-01

1419 9.990770e-01 9.983754e-01 9.972384e-01 9.952203e-01 9.920480e-01 7.075759e-01 6.888212e-01 6.703537e-01 7.242235e-01 6.507217e-01

1420 9.990831e-01 9.983796e-01 9.972340e-01 9.951949e-01 9.919787e-01 7.086522e-01 6.897157e-01 6.710940e-01 7.254848e-01 6.513213e-01

1421 9.990897e-01 9.983841e-01 9.972291e-01 9.951672e-01 9.919036e-01 7.097018e-01 6.905853e-01 6.718116e-01 7.267186e-01 6.519012e-01

1422 9.990968e-01 9.983890e-01 9.972237e-01 9.951371e-01 9.918221e-01 7.107249e-01 6.914303e-01 6.725071e-01 7.279251e-01 6.524619e-01

1423 9.991043e-01 9.983941e-01 9.972178e-01 9.951044e-01 9.917336e-01 7.117218e-01 6.922512e-01 6.731810e-01 7.291043e-01 6.530039e-01

1424 9.991123e-01 9.983995e-01 9.972114e-01 9.950688e-01 9.916377e-01 7.126928e-01 6.930484e-01 6.738338e-01 7.302563e-01 6.535278e-01

1425 9.991208e-01 9.984053e-01 9.972043e-01 9.950302e-01 9.915337e-01 7.136383e-01 6.938225e-01 6.744662e-01 7.313812e-01 6.540343e-01

1426 9.991300e-01 9.984115e-01 9.971964e-01 9.949882e-01 9.914210e-01 7.145585e-01 6.945739e-01 6.750785e-01 7.324792e-01 6.545237e-01

1427 9.991397e-01 9.984180e-01 9.971879e-01 9.949427e-01 9.912990e-01 7.154539e-01 6.953030e-01 6.756714e-01 7.335504e-01 6.549966e-01

1428 9.991501e-01 9.984249e-01 9.971785e-01 9.948932e-01 9.911668e-01 7.163247e-01 6.960102e-01 6.762452e-01 7.345951e-01 6.554534e-01

1429 9.991612e-01 9.984323e-01 9.971682e-01 9.948394e-01 9.910238e-01 7.171714e-01 6.966961e-01 6.768006e-01 7.356133e-01 6.558947e-01

1430 9.991731e-01 9.984402e-01 9.971569e-01 9.947811e-01 9.908692e-01 7.179942e-01 6.973610e-01 6.773379e-01 7.366053e-01 6.563208e-01

1431 9.991858e-01 9.984485e-01 9.971445e-01 9.947178e-01 9.907020e-01 7.187936e-01 6.980054e-01 6.778576e-01 7.375715e-01 6.567322e-01

1432 9.991993e-01 9.984574e-01 9.971309e-01 9.946491e-01 9.905216e-01 7.195700e-01 6.986298e-01 6.783602e-01 7.385119e-01 6.571294e-01

1433 9.992138e-01 9.984668e-01 9.971160e-01 9.945747e-01 9.903268e-01 7.203237e-01 6.992345e-01 6.788460e-01 7.394270e-01 6.575127e-01

1434 9.992293e-01 9.984768e-01 9.970997e-01 9.944940e-01 9.901168e-01 7.210551e-01 6.998200e-01 6.793156e-01 7.403170e-01 6.578825e-01

1435 9.992458e-01 9.984875e-01 9.970819e-01 9.944065e-01 9.898905e-01 7.217646e-01 7.003868e-01 6.797693e-01 7.411823e-01 6.582393e-01

1436 9.992634e-01 9.984988e-01 9.970624e-01 9.943118e-01 9.896471e-01 7.224526e-01 7.009352e-01 6.802076e-01 7.420231e-01 6.585834e-01

1437 9.992823e-01 9.985108e-01 9.970410e-01 9.942094e-01 9.893853e-01 7.231196e-01 7.014657e-01 6.806309e-01 7.428399e-01 6.589151e-01

1438 9.993024e-01 9.985235e-01 9.970177e-01 9.940985e-01 9.891042e-01 7.237659e-01 7.019786e-01 6.810396e-01 7.436329e-01 6.592349e-01

1439 9.993240e-01 9.985371e-01 9.969922e-01 9.939787e-01 9.888028e-01 7.243920e-01 7.024746e-01 6.814340e-01 7.444026e-01 6.595431e-01

1440 9.993470e-01 9.985514e-01 9.969643e-01 9.938493e-01 9.884798e-01 7.249982e-01 7.029538e-01 6.818146e-01 7.451494e-01 6.598401e-01

1441 9.993716e-01 9.985666e-01 9.969339e-01 9.937097e-01 9.881344e-01 7.255851e-01 7.034168e-01 6.821818e-01 7.458737e-01 6.601262e-01

1442 9.993717e-01 9.985667e-01 9.969339e-01 9.937096e-01 9.881344e-01 7.256019e-01 7.034354e-01 6.822014e-01 7.458881e-01 6.601462e-01

1443 9.993718e-01 9.985667e-01 9.969339e-01 9.937096e-01 9.881344e-01 7.256195e-01 7.034547e-01 6.822217e-01 7.459034e-01 6.601668e-01

1444 9.993719e-01 9.985667e-01 9.969339e-01 9.937096e-01 9.881344e-01 7.256379e-01 7.034747e-01 6.822427e-01 7.459195e-01 6.601881e-01

1445 9.993720e-01 9.985667e-01 9.969339e-01 9.937096e-01 9.881343e-01 7.256571e-01 7.034954e-01 6.822644e-01 7.459365e-01 6.602101e-01

1446 9.993720e-01 9.985667e-01 9.969338e-01 9.937096e-01 9.881343e-01 7.256771e-01 7.035170e-01 6.822868e-01 7.459544e-01 6.602328e-01

1447 9.993721e-01 9.985667e-01 9.969338e-01 9.937095e-01 9.881343e-01 7.256980e-01 7.035393e-01 6.823101e-01 7.459733e-01 6.602563e-01

1448 9.993721e-01 9.985667e-01 9.969338e-01 9.937095e-01 9.881343e-01 7.257197e-01 7.035625e-01 6.823341e-01 7.459930e-01 6.602805e-01

1449 9.993721e-01 9.985667e-01 9.969338e-01 9.937095e-01 9.881342e-01 7.257423e-01 7.035865e-01 6.823590e-01 7.460137e-01 6.603056e-01

1450 9.993722e-01 9.985667e-01 9.969338e-01 9.937095e-01 9.881342e-01 7.257659e-01 7.036114e-01 6.823847e-01 7.460353e-01 6.603315e-01

1451 9.993722e-01 9.985667e-01 9.969338e-01 9.937095e-01 9.881342e-01 7.257904e-01 7.036373e-01 6.824114e-01 7.460580e-01 6.603584e-01

1452 9.993722e-01 9.985667e-01 9.969338e-01 9.937094e-01 9.881342e-01 7.258159e-01 7.036640e-01 6.824390e-01 7.460817e-01 6.603861e-01

1453 9.993722e-01 9.985667e-01 9.969337e-01 9.937094e-01 9.881341e-01 7.258424e-01 7.036918e-01 6.824675e-01 7.461064e-01 6.604148e-01

1454 9.993722e-01 9.985667e-01 9.969337e-01 9.937094e-01 9.881341e-01 7.258700e-01 7.037206e-01 6.824971e-01 7.461322e-01 6.604445e-01

1455 9.993722e-01 9.985667e-01 9.969337e-01 9.937094e-01 9.881341e-01 7.258986e-01 7.037504e-01 6.825277e-01 7.461591e-01 6.604752e-01

1456 9.993722e-01 9.985667e-01 9.969337e-01 9.937093e-01 9.881340e-01 7.259283e-01 7.037813e-01 6.825594e-01 7.461872e-01 6.605071e-01

1457 9.993722e-01 9.985667e-01 9.969337e-01 9.937093e-01 9.881340e-01 7.259592e-01 7.038133e-01 6.825922e-01 7.462164e-01 6.605400e-01

1458 9.993722e-01 9.985667e-01 9.969336e-01 9.937093e-01 9.881339e-01 7.259912e-01 7.038465e-01 6.826261e-01 7.462468e-01 6.605741e-01

1459 9.993722e-01 9.985666e-01 9.969336e-01 9.937093e-01 9.881339e-01 7.260244e-01 7.038809e-01 6.826613e-01 7.462784e-01 6.606094e-01

1460 9.993722e-01 9.985666e-01 9.969336e-01 9.937092e-01 9.881339e-01 7.260589e-01 7.039165e-01 6.826977e-01 7.463113e-01 6.606459e-01

1461 9.993722e-01 9.985666e-01 9.969336e-01 9.937092e-01 9.881338e-01 7.260947e-01 7.039534e-01 6.827354e-01 7.463455e-01 6.606838e-01

1462 9.993722e-01 9.985666e-01 9.969336e-01 9.937092e-01 9.881338e-01 7.261317e-01 7.039916e-01 6.827744e-01 7.463810e-01 6.607230e-01

1463 9.993722e-01 9.985666e-01 9.969335e-01 9.937091e-01 9.881337e-01 7.261702e-01 7.040312e-01 6.828149e-01 7.464178e-01 6.607636e-01

1464 9.993722e-01 9.985666e-01 9.969335e-01 9.937091e-01 9.881337e-01 7.262100e-01 7.040722e-01 6.828567e-01 7.464561e-01 6.608057e-01

1465 9.993722e-01 9.985666e-01 9.969335e-01 9.937091e-01 9.881336e-01 7.262513e-01 7.041146e-01 6.829001e-01 7.464958e-01 6.608493e-01

1466 9.993721e-01 9.985665e-01 9.969335e-01 9.937090e-01 9.881336e-01 7.262941e-01 7.041586e-01 6.829450e-01 7.465371e-01 6.608944e-01

1467 9.993721e-01 9.985665e-01 9.969334e-01 9.937090e-01 9.881335e-01 7.263385e-01 7.042041e-01 6.829915e-01 7.465798e-01 6.609412e-01

1468 9.993721e-01 9.985665e-01 9.969334e-01 9.937090e-01 9.881334e-01 7.263844e-01 7.042513e-01 6.830397e-01 7.466242e-01 6.609896e-01

1469 9.993721e-01 9.985665e-01 9.969334e-01 9.937089e-01 9.881334e-01 7.264320e-01 7.043001e-01 6.830895e-01 7.466701e-01 6.610399e-01

1470 9.993721e-01 9.985665e-01 9.969334e-01 9.937089e-01 9.881333e-01 7.264813e-01 7.043506e-01 6.831412e-01 7.467178e-01 6.610919e-01

1471 9.993721e-01 9.985665e-01 9.969333e-01 9.937088e-01 9.881332e-01 7.265324e-01 7.044030e-01 6.831947e-01 7.467672e-01 6.611459e-01

1472 9.993721e-01 9.985664e-01 9.969333e-01 9.937088e-01 9.881332e-01 7.265853e-01 7.044572e-01 6.832502e-01 7.468183e-01 6.612017e-01

1473 9.993721e-01 9.985664e-01 9.969333e-01 9.937087e-01 9.881331e-01 7.266401e-01 7.045133e-01 6.833076e-01 7.468713e-01 6.612597e-01

1474 9.993721e-01 9.985664e-01 9.969333e-01 9.937087e-01 9.881330e-01 7.266968e-01 7.045715e-01 6.833670e-01 7.469262e-01 6.613197e-01

1475 9.993720e-01 9.985664e-01 9.969332e-01 9.937086e-01 9.881329e-01 7.267555e-01 7.046317e-01 6.834286e-01 7.469831e-01 6.613820e-01

1476 9.993720e-01 9.985664e-01 9.969332e-01 9.937086e-01 9.881328e-01 7.268163e-01 7.046940e-01 6.834925e-01 7.470420e-01 6.614465e-01

1477 9.993720e-01 9.985664e-01 9.969332e-01 9.937085e-01 9.881327e-01 7.268792e-01 7.047585e-01 6.835586e-01 7.471030e-01 6.615134e-01

1478 9.993720e-01 9.985663e-01 9.969331e-01 9.937084e-01 9.881326e-01 7.269444e-01 7.048253e-01 6.836271e-01 7.471661e-01 6.615827e-01

1479 9.993720e-01 9.985663e-01 9.969331e-01 9.937084e-01 9.881325e-01 7.270118e-01 7.048945e-01 6.836980e-01 7.472314e-01 6.616546e-01

1480 9.993720e-01 9.985663e-01 9.969331e-01 9.937083e-01 9.881324e-01 7.270816e-01 7.049661e-01 6.837715e-01 7.472991e-01 6.617291e-01

1481 9.993720e-01 9.985663e-01 9.969330e-01 9.937082e-01 9.881322e-01 7.271539e-01 7.050403e-01 6.838477e-01 7.473691e-01 6.618064e-01

1482 9.993720e-01 9.985663e-01 9.969330e-01 9.937081e-01 9.881321e-01 7.272287e-01 7.051171e-01 6.839266e-01 7.474415e-01 6.618865e-01

1483 9.993720e-01 9.985662e-01 9.969329e-01 9.937081e-01 9.881320e-01 7.273061e-01 7.051966e-01 6.840083e-01 7.475165e-01 6.619696e-01

1484 9.993720e-01 9.985662e-01 9.969329e-01 9.937080e-01 9.881318e-01 7.273863e-01 7.052790e-01 6.840930e-01 7.475941e-01 6.620557e-01

1485 9.993719e-01 9.985662e-01 9.969328e-01 9.937079e-01 9.881316e-01 7.274692e-01 7.053642e-01 6.841808e-01 7.476744e-01 6.621451e-01

1486 9.993719e-01 9.985662e-01 9.969328e-01 9.937078e-01 9.881315e-01 7.275551e-01 7.054525e-01 6.842717e-01 7.477575e-01 6.622377e-01

1487 9.993719e-01 9.985661e-01 9.969327e-01 9.937077e-01 9.881313e-01 7.276440e-01 7.055440e-01 6.843659e-01 7.478435e-01 6.623337e-01

1488 9.993719e-01 9.985661e-01 9.969326e-01 9.937075e-01 9.881311e-01 7.277360e-01 7.056387e-01 6.844636e-01 7.479325e-01 6.624333e-01

1489 9.993719e-01 9.985661e-01 9.969326e-01 9.937074e-01 9.881309e-01 7.278312e-01 7.057367e-01 6.845647e-01 7.480245e-01 6.625366e-01

1490 9.993719e-01 9.985661e-01 9.969325e-01 9.937073e-01 9.881307e-01 7.279297e-01 7.058382e-01 6.846696e-01 7.481197e-01 6.626437e-01

1491 9.993719e-01 9.985660e-01 9.969324e-01 9.937072e-01 9.881304e-01 7.280317e-01 7.059434e-01 6.847782e-01 7.482182e-01 6.627548e-01

1492 9.993719e-01 9.985660e-01 9.969324e-01 9.937070e-01 9.881302e-01 7.281372e-01 7.060523e-01 6.848908e-01 7.483201e-01 6.628700e-01

1493 9.993719e-01 9.985660e-01 9.969323e-01 9.937069e-01 9.881299e-01 7.282465e-01 7.061650e-01 6.850074e-01 7.484255e-01 6.629895e-01

1494 9.993718e-01 9.985659e-01 9.969322e-01 9.937067e-01 9.881297e-01 7.283596e-01 7.062818e-01 6.851283e-01 7.485346e-01 6.631134e-01

1495 9.993718e-01 9.985659e-01 9.969321e-01 9.937065e-01 9.881294e-01 7.284766e-01 7.064027e-01 6.852536e-01 7.486474e-01 6.632419e-01

1496 9.993718e-01 9.985658e-01 9.969320e-01 9.937064e-01 9.881291e-01 7.285977e-01 7.065279e-01 6.853835e-01 7.487641e-01 6.633751e-01

1497 9.993718e-01 9.985658e-01 9.969319e-01 9.937062e-01 9.881287e-01 7.287231e-01 7.066576e-01 6.855180e-01 7.488849e-01 6.635133e-01

1498 9.993718e-01 9.985657e-01 9.969318e-01 9.937060e-01 9.881284e-01 7.288529e-01 7.067919e-01 6.856574e-01 7.490098e-01 6.636567e-01

1499 9.993718e-01 9.985657e-01 9.969317e-01 9.937057e-01 9.881280e-01 7.289871e-01 7.069310e-01 6.858019e-01 7.491390e-01 6.638053e-01

1500 9.993718e-01 9.985656e-01 9.969316e-01 9.937055e-01 9.881276e-01 7.291261e-01 7.070750e-01 6.859517e-01 7.492726e-01 6.639595e-01

1501 9.993717e-01 9.985656e-01 9.969314e-01 9.937053e-01 9.881272e-01 7.292700e-01 7.072242e-01 6.861068e-01 7.494109e-01 6.641193e-01

1502 9.993717e-01 9.985655e-01 9.969313e-01 9.937050e-01 9.881268e-01 7.294189e-01 7.073787e-01 6.862676e-01 7.495538e-01 6.642851e-01

1503 9.993717e-01 9.985655e-01 9.969312e-01 9.937047e-01 9.881263e-01 7.295730e-01 7.075387e-01 6.864343e-01 7.497018e-01 6.644570e-01

1504 9.993717e-01 9.985654e-01 9.969310e-01 9.937044e-01 9.881258e-01 7.297325e-01 7.077044e-01 6.866070e-01 7.498548e-01 6.646352e-01

1505 9.993717e-01 9.985653e-01 9.969308e-01 9.937041e-01 9.881253e-01 7.298975e-01 7.078759e-01 6.867859e-01 7.500130e-01 6.648200e-01

1506 9.993716e-01 9.985653e-01 9.969307e-01 9.937038e-01 9.881247e-01 7.300684e-01 7.080536e-01 6.869713e-01 7.501767e-01 6.650116e-01

1507 9.993716e-01 9.985652e-01 9.969305e-01 9.937034e-01 9.881241e-01 7.302452e-01 7.082376e-01 6.871634e-01 7.503460e-01 6.652103e-01

1508 9.993716e-01 9.985651e-01 9.969303e-01 9.937031e-01 9.881234e-01 7.304282e-01 7.084281e-01 6.873625e-01 7.505212e-01 6.654163e-01

1509 9.993716e-01 9.985650e-01 9.969301e-01 9.937027e-01 9.881228e-01 7.306175e-01 7.086254e-01 6.875687e-01 7.507023e-01 6.656298e-01

1510 9.993715e-01 9.985649e-01 9.969299e-01 9.937022e-01 9.881220e-01 7.308135e-01 7.088296e-01 6.877824e-01 7.508897e-01 6.658511e-01

1511 9.993715e-01 9.985648e-01 9.969296e-01 9.937018e-01 9.881213e-01 7.310163e-01 7.090411e-01 6.880037e-01 7.510835e-01 6.660805e-01

1512 9.993715e-01 9.985647e-01 9.969294e-01 9.937013e-01 9.881204e-01 7.312261e-01 7.092601e-01 6.882330e-01 7.512839e-01 6.663183e-01

1513 9.993714e-01 9.985646e-01 9.969291e-01 9.937008e-01 9.881195e-01 7.314433e-01 7.094868e-01 6.884705e-01 7.514912e-01 6.665647e-01

1514 9.993714e-01 9.985645e-01 9.969289e-01 9.937003e-01 9.881186e-01 7.316680e-01 7.097216e-01 6.887165e-01 7.517055e-01 6.668200e-01

1515 9.993714e-01 9.985643e-01 9.969286e-01 9.936997e-01 9.881176e-01 7.319005e-01 7.099645e-01 6.889713e-01 7.519272e-01 6.670845e-01

1516 9.993713e-01 9.985642e-01 9.969282e-01 9.936991e-01 9.881165e-01 7.321411e-01 7.102161e-01 6.892352e-01 7.521565e-01 6.673585e-01

1517 9.993713e-01 9.985641e-01 9.969279e-01 9.936984e-01 9.881154e-01 7.323900e-01 7.104764e-01 6.895084e-01 7.523936e-01 6.676424e-01

1518 9.993712e-01 9.985639e-01 9.969276e-01 9.936977e-01 9.881142e-01 7.326476e-01 7.107459e-01 6.897913e-01 7.526387e-01 6.679364e-01

1519 9.993712e-01 9.985638e-01 9.969272e-01 9.936970e-01 9.881129e-01 7.329140e-01 7.110247e-01 6.900842e-01 7.528922e-01 6.682409e-01

1520 9.993712e-01 9.985636e-01 9.969268e-01 9.936962e-01 9.881115e-01 7.331895e-01 7.113133e-01 6.903874e-01 7.531544e-01 6.685561e-01

1521 9.993711e-01 9.985634e-01 9.969263e-01 9.936954e-01 9.881100e-01 7.334746e-01 7.116119e-01 6.907012e-01 7.534253e-01 6.688825e-01

1522 9.993710e-01 9.985632e-01 9.969259e-01 9.936945e-01 9.881084e-01 7.337694e-01 7.119209e-01 6.910260e-01 7.537055e-01 6.692202e-01

1523 9.993710e-01 9.985630e-01 9.969254e-01 9.936935e-01 9.881067e-01 7.340743e-01 7.122405e-01 6.913621e-01 7.539952e-01 6.695698e-01

1524 9.993709e-01 9.985628e-01 9.969249e-01 9.936925e-01 9.881049e-01 7.343897e-01 7.125711e-01 6.917098e-01 7.542946e-01 6.699315e-01

1525 9.993709e-01 9.985626e-01 9.969243e-01 9.936914e-01 9.881030e-01 7.347157e-01 7.129131e-01 6.920695e-01 7.546041e-01 6.703056e-01

1526 9.993708e-01 9.985623e-01 9.969237e-01 9.936902e-01 9.881009e-01 7.350529e-01 7.132667e-01 6.924415e-01 7.549239e-01 6.706926e-01

1527 9.993707e-01 9.985621e-01 9.969231e-01 9.936890e-01 9.880987e-01 7.354014e-01 7.136324e-01 6.928262e-01 7.552545e-01 6.710927e-01

1528 9.993706e-01 9.985618e-01 9.969225e-01 9.936876e-01 9.880964e-01 7.357617e-01 7.140104e-01 6.932239e-01 7.555961e-01 6.715063e-01

1529 9.993706e-01 9.985615e-01 9.969217e-01 9.936862e-01 9.880938e-01 7.361340e-01 7.144012e-01 6.936351e-01 7.559491e-01 6.719337e-01

1530 9.993705e-01 9.985612e-01 9.969210e-01 9.936847e-01 9.880911e-01 7.365189e-01 7.148051e-01 6.940600e-01 7.563138e-01 6.723753e-01

1531 9.993704e-01 9.985609e-01 9.969202e-01 9.936831e-01 9.880882e-01 7.369165e-01 7.152224e-01 6.944990e-01 7.566906e-01 6.728315e-01

1532 9.993703e-01 9.985605e-01 9.969193e-01 9.936813e-01 9.880851e-01 7.373274e-01 7.156536e-01 6.949525e-01 7.570798e-01 6.733025e-01

1533 9.993702e-01 9.985602e-01 9.969184e-01 9.936795e-01 9.880818e-01 7.377518e-01 7.160989e-01 6.954209e-01 7.574818e-01 6.737887e-01

1534 9.993701e-01 9.985598e-01 9.969174e-01 9.936775e-01 9.880782e-01 7.381901e-01 7.165589e-01 6.959044e-01 7.578969e-01 6.742905e-01

1535 9.993699e-01 9.985593e-01 9.969164e-01 9.936754e-01 9.880744e-01 7.386428e-01 7.170337e-01 6.964036e-01 7.583256e-01 6.748081e-01

1536 9.993698e-01 9.985589e-01 9.969153e-01 9.936731e-01 9.880703e-01 7.391101e-01 7.175240e-01 6.969186e-01 7.587681e-01 6.753418e-01

1537 9.993697e-01 9.985584e-01 9.969141e-01 9.936707e-01 9.880659e-01 7.395926e-01 7.180299e-01 6.974500e-01 7.592250e-01 6.758920e-01

1538 9.993695e-01 9.985579e-01 9.969128e-01 9.936681e-01 9.880612e-01 7.400905e-01 7.185518e-01 6.979979e-01 7.596965e-01 6.764590e-01

1539 9.993694e-01 9.985574e-01 9.969114e-01 9.936653e-01 9.880562e-01 7.406043e-01 7.190902e-01 6.985628e-01 7.601830e-01 6.770430e-01

1540 9.993692e-01 9.985568e-01 9.969100e-01 9.936623e-01 9.880508e-01 7.411344e-01 7.196454e-01 6.991450e-01 7.606850e-01 6.776442e-01

1541 9.993691e-01 9.985562e-01 9.969084e-01 9.936591e-01 9.880449e-01 7.416811e-01 7.202178e-01 6.997448e-01 7.612029e-01 6.782630e-01

1542 9.993689e-01 9.985555e-01 9.969068e-01 9.936557e-01 9.880387e-01 7.422448e-01 7.208076e-01 7.003624e-01 7.617370e-01 6.788995e-01

1543 9.993687e-01 9.985548e-01 9.969050e-01 9.936520e-01 9.880320e-01 7.428260e-01 7.214153e-01 7.009983e-01 7.622877e-01 6.795539e-01

1544 9.993685e-01 9.985541e-01 9.969031e-01 9.936481e-01 9.880248e-01 7.434250e-01 7.220412e-01 7.016526e-01 7.628554e-01 6.802264e-01

1545 9.993683e-01 9.985533e-01 9.969010e-01 9.936438e-01 9.880170e-01 7.440421e-01 7.226856e-01 7.023256e-01 7.634406e-01 6.809172e-01

1546 9.993680e-01 9.985524e-01 9.968989e-01 9.936393e-01 9.880087e-01 7.446778e-01 7.233488e-01 7.030175e-01 7.640436e-01 6.816264e-01

1547 9.993678e-01 9.985515e-01 9.968965e-01 9.936344e-01 9.879997e-01 7.453323e-01 7.240312e-01 7.037286e-01 7.646648e-01 6.823540e-01

1548 9.993675e-01 9.985506e-01 9.968940e-01 9.936292e-01 9.879901e-01 7.460062e-01 7.247329e-01 7.044591e-01 7.653046e-01 6.831002e-01

1549 9.993673e-01 9.985495e-01 9.968914e-01 9.936236e-01 9.879797e-01 7.466996e-01 7.254542e-01 7.052090e-01 7.659634e-01 6.838649e-01

1550 9.993670e-01 9.985485e-01 9.968885e-01 9.936176e-01 9.879686e-01 7.474130e-01 7.261955e-01 7.059786e-01 7.666416e-01 6.846482e-01

1551 9.993667e-01 9.985473e-01 9.968854e-01 9.936111e-01 9.879566e-01 7.481466e-01 7.269569e-01 7.067679e-01 7.673395e-01 6.854500e-01

1552 9.993663e-01 9.985460e-01 9.968821e-01 9.936042e-01 9.879436e-01 7.489007e-01 7.277386e-01 7.075771e-01 7.680574e-01 6.862701e-01

1553 9.993660e-01 9.985447e-01 9.968786e-01 9.935967e-01 9.879297e-01 7.496757e-01 7.285407e-01 7.084061e-01 7.687958e-01 6.871086e-01

1554 9.993656e-01 9.985433e-01 9.968748e-01 9.935887e-01 9.879147e-01 7.504717e-01 7.293635e-01 7.092550e-01 7.695550e-01 6.879652e-01

1555 9.993652e-01 9.985417e-01 9.968708e-01 9.935800e-01 9.878986e-01 7.512891e-01 7.302070e-01 7.101237e-01 7.703353e-01 6.888396e-01

1556 9.993648e-01 9.985401e-01 9.968664e-01 9.935707e-01 9.878812e-01 7.521281e-01 7.310714e-01 7.110122e-01 7.711369e-01 6.897317e-01

1557 9.993643e-01 9.985384e-01 9.968618e-01 9.935607e-01 9.878624e-01 7.529888e-01 7.319565e-01 7.119203e-01 7.719603e-01 6.906411e-01

1558 9.993638e-01 9.985365e-01 9.968568e-01 9.935500e-01 9.878421e-01 7.538714e-01 7.328626e-01 7.128478e-01 7.728056e-01 6.915675e-01

1559 9.993633e-01 9.985345e-01 9.968514e-01 9.935384e-01 9.878203e-01 7.547760e-01 7.337895e-01 7.137947e-01 7.736731e-01 6.925104e-01

1560 9.993628e-01 9.985324e-01 9.968456e-01 9.935259e-01 9.877967e-01 7.557029e-01 7.347372e-01 7.147606e-01 7.745631e-01 6.934694e-01

1561 9.993622e-01 9.985301e-01 9.968394e-01 9.935125e-01 9.877713e-01 7.566519e-01 7.357056e-01 7.157452e-01 7.754757e-01 6.944440e-01

1562 9.993616e-01 9.985276e-01 9.968327e-01 9.934981e-01 9.877439e-01 7.576233e-01 7.366945e-01 7.167481e-01 7.764111e-01 6.954337e-01

1563 9.993609e-01 9.985250e-01 9.968256e-01 9.934825e-01 9.877143e-01 7.586169e-01 7.377037e-01 7.177691e-01 7.773695e-01 6.964377e-01

1564 9.993602e-01 9.985222e-01 9.968179e-01 9.934657e-01 9.876823e-01 7.596328e-01 7.387330e-01 7.188076e-01 7.783510e-01 6.974555e-01

1565 9.993595e-01 9.985192e-01 9.968096e-01 9.934476e-01 9.876477e-01 7.606708e-01 7.397822e-01 7.198631e-01 7.793557e-01 6.984864e-01

1566 9.993586e-01 9.985160e-01 9.968007e-01 9.934281e-01 9.876104e-01 7.617310e-01 7.408507e-01 7.209351e-01 7.803836e-01 6.995296e-01

1567 9.993578e-01 9.985125e-01 9.967911e-01 9.934070e-01 9.875701e-01 7.628130e-01 7.419384e-01 7.220231e-01 7.814348e-01 7.005842e-01

1568 9.993569e-01 9.985088e-01 9.967808e-01 9.933843e-01 9.875265e-01 7.639168e-01 7.430447e-01 7.231262e-01 7.825093e-01 7.016496e-01

1569 9.993559e-01 9.985049e-01 9.967698e-01 9.933598e-01 9.874795e-01 7.650419e-01 7.441692e-01 7.242439e-01 7.836069e-01 7.027247e-01

1570 9.993549e-01 9.985006e-01 9.967578e-01 9.933334e-01 9.874285e-01 7.661883e-01 7.453113e-01 7.253753e-01 7.847276e-01 7.038087e-01

1571 9.993538e-01 9.984961e-01 9.967450e-01 9.933049e-01 9.873735e-01 7.673555e-01 7.464704e-01 7.265198e-01 7.858713e-01 7.049006e-01

1572 9.993526e-01 9.984912e-01 9.967312e-01 9.932740e-01 9.873140e-01 7.685431e-01 7.476460e-01 7.276764e-01 7.870378e-01 7.059994e-01

1573 9.993513e-01 9.984859e-01 9.967163e-01 9.932408e-01 9.872496e-01 7.697506e-01 7.488372e-01 7.288443e-01 7.882269e-01 7.071040e-01

1574 9.993499e-01 9.984803e-01 9.967002e-01 9.932048e-01 9.871800e-01 7.709777e-01 7.500434e-01 7.300225e-01 7.894382e-01 7.082135e-01

1575 9.993485e-01 9.984743e-01 9.966829e-01 9.931660e-01 9.871047e-01 7.722236e-01 7.512639e-01 7.312101e-01 7.906715e-01 7.093268e-01

1576 9.993470e-01 9.984678e-01 9.966643e-01 9.931241e-01 9.870232e-01 7.734880e-01 7.524976e-01 7.324061e-01 7.919264e-01 7.104427e-01

1577 9.993453e-01 9.984608e-01 9.966442e-01 9.930788e-01 9.869350e-01 7.747700e-01 7.537439e-01 7.336094e-01 7.932025e-01 7.115602e-01

1578 9.993435e-01 9.984534e-01 9.966225e-01 9.930298e-01 9.868397e-01 7.760690e-01 7.550017e-01 7.348189e-01 7.944994e-01 7.126782e-01

1579 9.993417e-01 9.984453e-01 9.965992e-01 9.929769e-01 9.867365e-01 7.773842e-01 7.562702e-01 7.360336e-01 7.958165e-01 7.137954e-01

1580 9.993396e-01 9.984367e-01 9.965740e-01 9.929196e-01 9.866248e-01 7.787149e-01 7.575482e-01 7.372524e-01 7.971532e-01 7.149109e-01

1581 9.993375e-01 9.984274e-01 9.965468e-01 9.928578e-01 9.865040e-01 7.800603e-01 7.588348e-01 7.384741e-01 7.985090e-01 7.160234e-01

1582 9.993352e-01 9.984174e-01 9.965175e-01 9.927908e-01 9.863733e-01 7.814193e-01 7.601289e-01 7.396975e-01 7.998831e-01 7.171319e-01

1583 9.993327e-01 9.984067e-01 9.964858e-01 9.927185e-01 9.862319e-01 7.827912e-01 7.614295e-01 7.409216e-01 8.012750e-01 7.182352e-01

1584 9.993301e-01 9.983952e-01 9.964516e-01 9.926402e-01 9.860789e-01 7.841749e-01 7.627353e-01 7.421451e-01 8.026838e-01 7.193324e-01

1585 9.993272e-01 9.983828e-01 9.964147e-01 9.925556e-01 9.859135e-01 7.855694e-01 7.640452e-01 7.433668e-01 8.041087e-01 7.204222e-01

1586 9.993242e-01 9.983694e-01 9.963749e-01 9.924641e-01 9.857346e-01 7.869737e-01 7.653582e-01 7.445857e-01 8.055489e-01 7.215037e-01

1587 9.993210e-01 9.983551e-01 9.963319e-01 9.923651e-01 9.855412e-01 7.883867e-01 7.666730e-01 7.458004e-01 8.070035e-01 7.225758e-01

1588 9.993175e-01 9.983396e-01 9.962854e-01 9.922580e-01 9.853322e-01 7.898073e-01 7.679883e-01 7.470100e-01 8.084715e-01 7.236376e-01

1589 9.993138e-01 9.983230e-01 9.962352e-01 9.921422e-01 9.851063e-01 7.912343e-01 7.693032e-01 7.482132e-01 8.099520e-01 7.246881e-01

1590 9.993098e-01 9.983051e-01 9.961810e-01 9.920169e-01 9.848622e-01 7.926666e-01 7.706162e-01 7.494090e-01 8.114440e-01 7.257265e-01

1591 9.993055e-01 9.982858e-01 9.961224e-01 9.918815e-01 9.845987e-01 7.941030e-01 7.719263e-01 7.505962e-01 8.129464e-01 7.267518e-01

1592 9.993009e-01 9.982650e-01 9.960591e-01 9.917350e-01 9.843142e-01 7.955423e-01 7.732323e-01 7.517738e-01 8.144582e-01 7.277633e-01

1593 9.992960e-01 9.982426e-01 9.959907e-01 9.915767e-01 9.840072e-01 7.969834e-01 7.745330e-01 7.529408e-01 8.159781e-01 7.287601e-01

1594 9.992907e-01 9.982185e-01 9.959168e-01 9.914055e-01 9.836762e-01 7.984249e-01 7.758272e-01 7.540961e-01 8.175052e-01 7.297416e-01

1595 9.992851e-01 9.981925e-01 9.958369e-01 9.912205e-01 9.833195e-01 7.998656e-01 7.771138e-01 7.552388e-01 8.190382e-01 7.307070e-01

1596 9.992790e-01 9.981645e-01 9.957506e-01 9.910207e-01 9.829352e-01 8.013044e-01 7.783916e-01 7.563681e-01 8.205760e-01 7.316558e-01

1597 9.992725e-01 9.981343e-01 9.956573e-01 9.908048e-01 9.825215e-01 8.027400e-01 7.796597e-01 7.574829e-01 8.221174e-01 7.325874e-01

1598 9.992656e-01 9.981018e-01 9.955564e-01 9.905717e-01 9.820765e-01 8.041713e-01 7.809169e-01 7.585826e-01 8.236610e-01 7.335013e-01

1599 9.992581e-01 9.980667e-01 9.954475e-01 9.903200e-01 9.815983e-01 8.055969e-01 7.821623e-01 7.596663e-01 8.252059e-01 7.343970e-01

1600 9.992501e-01 9.980289e-01 9.953298e-01 9.900485e-01 9.810847e-01 8.070157e-01 7.833948e-01 7.607332e-01 8.267506e-01 7.352741e-01

1601 9.992415e-01 9.979882e-01 9.952026e-01 9.897556e-01 9.805337e-01 8.084266e-01 7.846134e-01 7.617828e-01 8.282940e-01 7.361322e-01

1602 9.992322e-01 9.979442e-01 9.950652e-01 9.894399e-01 9.799431e-01 8.098283e-01 7.858174e-01 7.628145e-01 8.298348e-01 7.369712e-01

1603 9.992223e-01 9.978968e-01 9.949168e-01 9.890997e-01 9.793108e-01 8.112199e-01 7.870058e-01 7.638275e-01 8.313719e-01 7.377906e-01

1604 9.992116e-01 9.978456e-01 9.947565e-01 9.887333e-01 9.786347e-01 8.126001e-01 7.881778e-01 7.648215e-01 8.329040e-01 7.385904e-01

1605 9.992001e-01 9.977904e-01 9.945834e-01 9.883391e-01 9.779124e-01 8.139681e-01 7.893327e-01 7.657960e-01 8.344301e-01 7.393703e-01

1606 9.991878e-01 9.977309e-01 9.943966e-01 9.879152e-01 9.771421e-01 8.153227e-01 7.904697e-01 7.667506e-01 8.359490e-01 7.401305e-01

1607 9.991746e-01 9.976667e-01 9.941950e-01 9.874596e-01 9.763215e-01 8.166631e-01 7.915885e-01 7.676850e-01 8.374596e-01 7.408709e-01

1608 9.991604e-01 9.975975e-01 9.939775e-01 9.869705e-01 9.754487e-01 8.179884e-01 7.926882e-01 7.685990e-01 8.389609e-01 7.415915e-01

1609 9.991451e-01 9.975228e-01 9.937429e-01 9.864458e-01 9.745219e-01 8.192977e-01 7.937686e-01 7.694925e-01 8.404518e-01 7.422926e-01

1610 9.991287e-01 9.974422e-01 9.934901e-01 9.858834e-01 9.735393e-01 8.205905e-01 7.948292e-01 7.703653e-01 8.419315e-01 7.429742e-01

1611 9.991111e-01 9.973553e-01 9.932176e-01 9.852814e-01 9.724994e-01 8.218659e-01 7.958697e-01 7.712175e-01 8.433990e-01 7.436366e-01

1612 9.990921e-01 9.972616e-01 9.929241e-01 9.846375e-01 9.714009e-01 8.231233e-01 7.968898e-01 7.720489e-01 8.448533e-01 7.442800e-01

1613 9.990717e-01 9.971605e-01 9.926081e-01 9.839498e-01 9.702427e-01 8.243622e-01 7.978893e-01 7.728597e-01 8.462937e-01 7.449048e-01

1614 9.990497e-01 9.970515e-01 9.922682e-01 9.832160e-01 9.690241e-01 8.255821e-01 7.988680e-01 7.736500e-01 8.477194e-01 7.455111e-01

1615 9.990261e-01 9.969340e-01 9.919027e-01 9.824343e-01 9.677447e-01 8.267824e-01 7.998257e-01 7.744200e-01 8.491295e-01 7.460995e-01

1616 9.990008e-01 9.968073e-01 9.915100e-01 9.816025e-01 9.664043e-01 8.279627e-01 8.007625e-01 7.751697e-01 8.505234e-01 7.466701e-01

1617 9.989735e-01 9.966708e-01 9.910883e-01 9.807188e-01 9.650033e-01 8.291228e-01 8.016784e-01 7.758995e-01 8.519004e-01 7.472235e-01

1618 9.989441e-01 9.965237e-01 9.906359e-01 9.797815e-01 9.635424e-01 8.302622e-01 8.025732e-01 7.766096e-01 8.532598e-01 7.477600e-01

1619 9.989125e-01 9.963652e-01 9.901509e-01 9.787889e-01 9.620228e-01 8.313807e-01 8.034471e-01 7.773002e-01 8.546012e-01 7.482800e-01

1620 9.988785e-01 9.961945e-01 9.896315e-01 9.777396e-01 9.604461e-01 8.324781e-01 8.043003e-01 7.779718e-01 8.559238e-01 7.487839e-01

1621 9.988420e-01 9.960108e-01 9.890757e-01 9.766323e-01 9.588146e-01 8.335543e-01 8.051327e-01 7.786245e-01 8.572274e-01 7.492722e-01

1622 9.988420e-01 9.960108e-01 9.890756e-01 9.766323e-01 9.588145e-01 8.335625e-01 8.051427e-01 7.786352e-01 8.572327e-01 7.492829e-01

1623 9.988420e-01 9.960108e-01 9.890756e-01 9.766322e-01 9.588144e-01 8.335710e-01 8.051530e-01 7.786463e-01 8.572384e-01 7.492938e-01

1624 9.988421e-01 9.960108e-01 9.890756e-01 9.766322e-01 9.588144e-01 8.335799e-01 8.051636e-01 7.786575e-01 8.572446e-01 7.493049e-01

1625 9.988421e-01 9.960108e-01 9.890755e-01 9.766321e-01 9.588143e-01 8.335892e-01 8.051744e-01 7.786691e-01 8.572513e-01 7.493163e-01

1626 9.988421e-01 9.960107e-01 9.890755e-01 9.766321e-01 9.588142e-01 8.335988e-01 8.051856e-01 7.786809e-01 8.572584e-01 7.493279e-01

1627 9.988421e-01 9.960107e-01 9.890755e-01 9.766320e-01 9.588141e-01 8.336089e-01 8.051971e-01 7.786929e-01 8.572660e-01 7.493398e-01

1628 9.988421e-01 9.960107e-01 9.890754e-01 9.766319e-01 9.588140e-01 8.336193e-01 8.052089e-01 7.787053e-01 8.572740e-01 7.493520e-01

1629 9.988421e-01 9.960107e-01 9.890754e-01 9.766319e-01 9.588139e-01 8.336302e-01 8.052210e-01 7.787180e-01 8.572825e-01 7.493644e-01

1630 9.988421e-01 9.960107e-01 9.890753e-01 9.766318e-01 9.588138e-01 8.336414e-01 8.052335e-01 7.787310e-01 8.572914e-01 7.493771e-01

1631 9.988421e-01 9.960106e-01 9.890753e-01 9.766317e-01 9.588136e-01 8.336530e-01 8.052463e-01 7.787443e-01 8.573009e-01 7.493901e-01

1632 9.988421e-01 9.960106e-01 9.890752e-01 9.766316e-01 9.588135e-01 8.336651e-01 8.052594e-01 7.787579e-01 8.573108e-01 7.494035e-01

1633 9.988421e-01 9.960106e-01 9.890752e-01 9.766315e-01 9.588134e-01 8.336775e-01 8.052729e-01 7.787719e-01 8.573212e-01 7.494172e-01

1634 9.988421e-01 9.960106e-01 9.890751e-01 9.766314e-01 9.588132e-01 8.336904e-01 8.052868e-01 7.787862e-01 8.573320e-01 7.494312e-01

1635 9.988421e-01 9.960105e-01 9.890751e-01 9.766313e-01 9.588131e-01 8.337038e-01 8.053011e-01 7.788009e-01 8.573434e-01 7.494457e-01

1636 9.988421e-01 9.960105e-01 9.890750e-01 9.766312e-01 9.588129e-01 8.337175e-01 8.053158e-01 7.788160e-01 8.573553e-01 7.494605e-01

1637 9.988421e-01 9.960105e-01 9.890749e-01 9.766311e-01 9.588128e-01 8.337318e-01 8.053309e-01 7.788315e-01 8.573677e-01 7.494757e-01

1638 9.988421e-01 9.960104e-01 9.890749e-01 9.766310e-01 9.588126e-01 8.337465e-01 8.053464e-01 7.788474e-01 8.573805e-01 7.494913e-01

1639 9.988421e-01 9.960104e-01 9.890748e-01 9.766309e-01 9.588124e-01 8.337616e-01 8.053623e-01 7.788637e-01 8.573940e-01 7.495074e-01

1640 9.988421e-01 9.960104e-01 9.890747e-01 9.766308e-01 9.588122e-01 8.337773e-01 8.053787e-01 7.788804e-01 8.574079e-01 7.495239e-01

1641 9.988420e-01 9.960103e-01 9.890747e-01 9.766306e-01 9.588120e-01 8.337934e-01 8.053955e-01 7.788976e-01 8.574224e-01 7.495409e-01

1642 9.988420e-01 9.960103e-01 9.890746e-01 9.766305e-01 9.588118e-01 8.338101e-01 8.054128e-01 7.789153e-01 8.574374e-01 7.495583e-01

1643 9.988420e-01 9.960102e-01 9.890745e-01 9.766304e-01 9.588116e-01 8.338272e-01 8.054306e-01 7.789334e-01 8.574530e-01 7.495763e-01

1644 9.988420e-01 9.960102e-01 9.890744e-01 9.766302e-01 9.588114e-01 8.338449e-01 8.054488e-01 7.789521e-01 8.574692e-01 7.495948e-01

1645 9.988420e-01 9.960101e-01 9.890743e-01 9.766300e-01 9.588111e-01 8.338632e-01 8.054676e-01 7.789712e-01 8.574859e-01 7.496139e-01

1646 9.988420e-01 9.960101e-01 9.890742e-01 9.766299e-01 9.588108e-01 8.338820e-01 8.054870e-01 7.789909e-01 8.575033e-01 7.496335e-01

1647 9.988419e-01 9.960100e-01 9.890741e-01 9.766297e-01 9.588106e-01 8.339013e-01 8.055068e-01 7.790112e-01 8.575212e-01 7.496537e-01

1648 9.988419e-01 9.960100e-01 9.890740e-01 9.766295e-01 9.588103e-01 8.339213e-01 8.055272e-01 7.790320e-01 8.575398e-01 7.496746e-01

1649 9.988419e-01 9.960099e-01 9.890739e-01 9.766293e-01 9.588100e-01 8.339418e-01 8.055483e-01 7.790534e-01 8.575590e-01 7.496961e-01

1650 9.988419e-01 9.960099e-01 9.890737e-01 9.766291e-01 9.588096e-01 8.339630e-01 8.055699e-01 7.790755e-01 8.575788e-01 7.497182e-01

1651 9.988418e-01 9.960098e-01 9.890736e-01 9.766289e-01 9.588093e-01 8.339848e-01 8.055921e-01 7.790981e-01 8.575994e-01 7.497410e-01

1652 9.988418e-01 9.960097e-01 9.890735e-01 9.766286e-01 9.588089e-01 8.340072e-01 8.056149e-01 7.791214e-01 8.576205e-01 7.497646e-01

1653 9.988418e-01 9.960097e-01 9.890733e-01 9.766284e-01 9.588086e-01 8.340303e-01 8.056385e-01 7.791454e-01 8.576424e-01 7.497889e-01

1654 9.988418e-01 9.960096e-01 9.890732e-01 9.766281e-01 9.588082e-01 8.340541e-01 8.056626e-01 7.791701e-01 8.576650e-01 7.498139e-01

1655 9.988417e-01 9.960095e-01 9.890730e-01 9.766278e-01 9.588077e-01 8.340786e-01 8.056875e-01 7.791956e-01 8.576884e-01 7.498397e-01

1656 9.988417e-01 9.960094e-01 9.890728e-01 9.766275e-01 9.588073e-01 8.341038e-01 8.057132e-01 7.792217e-01 8.577124e-01 7.498664e-01

1657 9.988417e-01 9.960094e-01 9.890727e-01 9.766272e-01 9.588068e-01 8.341298e-01 8.057395e-01 7.792487e-01 8.577373e-01 7.498940e-01

1658 9.988416e-01 9.960093e-01 9.890725e-01 9.766269e-01 9.588063e-01 8.341566e-01 8.057666e-01 7.792764e-01 8.577629e-01 7.499224e-01

1659 9.988416e-01 9.960092e-01 9.890723e-01 9.766266e-01 9.588058e-01 8.341841e-01 8.057946e-01 7.793050e-01 8.577893e-01 7.499517e-01

1660 9.988416e-01 9.960091e-01 9.890721e-01 9.766262e-01 9.588053e-01 8.342124e-01 8.058233e-01 7.793344e-01 8.578166e-01 7.499820e-01

1661 9.988415e-01 9.960090e-01 9.890718e-01 9.766258e-01 9.588047e-01 8.342416e-01 8.058529e-01 7.793647e-01 8.578447e-01 7.500133e-01

1662 9.988415e-01 9.960089e-01 9.890716e-01 9.766254e-01 9.588041e-01 8.342717e-01 8.058833e-01 7.793960e-01 8.578736e-01 7.500456e-01

1663 9.988414e-01 9.960087e-01 9.890713e-01 9.766250e-01 9.588034e-01 8.343026e-01 8.059147e-01 7.794282e-01 8.579035e-01 7.500790e-01

1664 9.988414e-01 9.960086e-01 9.890711e-01 9.766245e-01 9.588027e-01 8.343344e-01 8.059470e-01 7.794613e-01 8.579343e-01 7.501135e-01

1665 9.988414e-01 9.960085e-01 9.890708e-01 9.766240e-01 9.588020e-01 8.343672e-01 8.059802e-01 7.794955e-01 8.579661e-01 7.501491e-01

1666 9.988413e-01 9.960083e-01 9.890705e-01 9.766235e-01 9.588012e-01 8.344010e-01 8.060145e-01 7.795307e-01 8.579988e-01 7.501859e-01

1667 9.988413e-01 9.960082e-01 9.890702e-01 9.766230e-01 9.588004e-01 8.344357e-01 8.060497e-01 7.795671e-01 8.580325e-01 7.502240e-01

1668 9.988412e-01 9.960080e-01 9.890699e-01 9.766224e-01 9.587995e-01 8.344715e-01 8.060861e-01 7.796045e-01 8.580672e-01 7.502633e-01

1669 9.988411e-01 9.960079e-01 9.890695e-01 9.766218e-01 9.587986e-01 8.345083e-01 8.061235e-01 7.796431e-01 8.581030e-01 7.503039e-01

1670 9.988411e-01 9.960077e-01 9.890691e-01 9.766212e-01 9.587976e-01 8.345463e-01 8.061620e-01 7.796829e-01 8.581399e-01 7.503459e-01

1671 9.988410e-01 9.960075e-01 9.890688e-01 9.766205e-01 9.587966e-01 8.345854e-01 8.062017e-01 7.797240e-01 8.581779e-01 7.503894e-01

1672 9.988410e-01 9.960073e-01 9.890683e-01 9.766198e-01 9.587955e-01 8.346256e-01 8.062426e-01 7.797663e-01 8.582171e-01 7.504343e-01

1673 9.988409e-01 9.960071e-01 9.890679e-01 9.766190e-01 9.587943e-01 8.346670e-01 8.062848e-01 7.798100e-01 8.582575e-01 7.504807e-01

1674 9.988408e-01 9.960069e-01 9.890674e-01 9.766182e-01 9.587931e-01 8.347097e-01 8.063283e-01 7.798550e-01 8.582991e-01 7.505287e-01

1675 9.988407e-01 9.960067e-01 9.890670e-01 9.766174e-01 9.587918e-01 8.347536e-01 8.063730e-01 7.799015e-01 8.583419e-01 7.505784e-01

1676 9.988407e-01 9.960065e-01 9.890664e-01 9.766165e-01 9.587904e-01 8.347989e-01 8.064192e-01 7.799494e-01 8.583860e-01 7.506298e-01

1677 9.988406e-01 9.960062e-01 9.890659e-01 9.766155e-01 9.587890e-01 8.348455e-01 8.064667e-01 7.799989e-01 8.584315e-01 7.506829e-01

1678 9.988405e-01 9.960059e-01 9.890653e-01 9.766145e-01 9.587874e-01 8.348935e-01 8.065158e-01 7.800499e-01 8.584784e-01 7.507378e-01

1679 9.988404e-01 9.960057e-01 9.890647e-01 9.766134e-01 9.587858e-01 8.349430e-01 8.065663e-01 7.801026e-01 8.585267e-01 7.507946e-01

1680 9.988403e-01 9.960054e-01 9.890640e-01 9.766123e-01 9.587840e-01 8.349940e-01 8.066184e-01 7.801569e-01 8.585764e-01 7.508534e-01

1681 9.988402e-01 9.960050e-01 9.890633e-01 9.766110e-01 9.587822e-01 8.350465e-01 8.066722e-01 7.802130e-01 8.586277e-01 7.509143e-01

1682 9.988401e-01 9.960047e-01 9.890626e-01 9.766097e-01 9.587802e-01 8.351005e-01 8.067275e-01 7.802709e-01 8.586805e-01 7.509772e-01

1683 9.988400e-01 9.960043e-01 9.890618e-01 9.766084e-01 9.587781e-01 8.351563e-01 8.067847e-01 7.803307e-01 8.587349e-01 7.510423e-01

1684 9.988398e-01 9.960040e-01 9.890610e-01 9.766069e-01 9.587759e-01 8.352137e-01 8.068436e-01 7.803924e-01 8.587910e-01 7.511097e-01

1685 9.988397e-01 9.960036e-01 9.890601e-01 9.766054e-01 9.587735e-01 8.352728e-01 8.069043e-01 7.804561e-01 8.588488e-01 7.511794e-01

1686 9.988396e-01 9.960031e-01 9.890591e-01 9.766037e-01 9.587710e-01 8.353338e-01 8.069669e-01 7.805218e-01 8.589083e-01 7.512515e-01

1687 9.988394e-01 9.960027e-01 9.890581e-01 9.766020e-01 9.587684e-01 8.353966e-01 8.070316e-01 7.805897e-01 8.589697e-01 7.513261e-01

1688 9.988393e-01 9.960022e-01 9.890571e-01 9.766001e-01 9.587656e-01 8.354614e-01 8.070982e-01 7.806598e-01 8.590329e-01 7.514033e-01

1689 9.988391e-01 9.960017e-01 9.890560e-01 9.765982e-01 9.587626e-01 8.355281e-01 8.071669e-01 7.807322e-01 8.590981e-01 7.514833e-01

1690 9.988389e-01 9.960012e-01 9.890548e-01 9.765961e-01 9.587594e-01 8.355968e-01 8.072378e-01 7.808069e-01 8.591652e-01 7.515660e-01

1691 9.988387e-01 9.960006e-01 9.890535e-01 9.765938e-01 9.587560e-01 8.356677e-01 8.073110e-01 7.808841e-01 8.592344e-01 7.516516e-01

1692 9.988385e-01 9.960000e-01 9.890522e-01 9.765915e-01 9.587523e-01 8.357407e-01 8.073864e-01 7.809637e-01 8.593058e-01 7.517401e-01

1693 9.988383e-01 9.959994e-01 9.890507e-01 9.765890e-01 9.587485e-01 8.358160e-01 8.074643e-01 7.810460e-01 8.593793e-01 7.518318e-01

1694 9.988381e-01 9.959987e-01 9.890492e-01 9.765863e-01 9.587444e-01 8.358936e-01 8.075446e-01 7.811310e-01 8.594550e-01 7.519267e-01

1695 9.988379e-01 9.959980e-01 9.890476e-01 9.765834e-01 9.587400e-01 8.359737e-01 8.076274e-01 7.812188e-01 8.595331e-01 7.520249e-01

1696 9.988376e-01 9.959972e-01 9.890458e-01 9.765804e-01 9.587354e-01 8.360561e-01 8.077129e-01 7.813094e-01 8.596136e-01 7.521265e-01

1697 9.988374e-01 9.959964e-01 9.890440e-01 9.765771e-01 9.587304e-01 8.361412e-01 8.078011e-01 7.814030e-01 8.596966e-01 7.522316e-01

1698 9.988371e-01 9.959955e-01 9.890420e-01 9.765737e-01 9.587251e-01 8.362288e-01 8.078921e-01 7.814997e-01 8.597821e-01 7.523404e-01

1699 9.988368e-01 9.959946e-01 9.890400e-01 9.765700e-01 9.587195e-01 8.363192e-01 8.079860e-01 7.815996e-01 8.598703e-01 7.524530e-01

1700 9.988365e-01 9.959936e-01 9.890377e-01 9.765661e-01 9.587135e-01 8.364124e-01 8.080829e-01 7.817027e-01 8.599611e-01 7.525695e-01

1701 9.988362e-01 9.959926e-01 9.890354e-01 9.765619e-01 9.587071e-01 8.365084e-01 8.081829e-01 7.818092e-01 8.600548e-01 7.526901e-01

1702 9.988359e-01 9.959915e-01 9.890329e-01 9.765574e-01 9.587003e-01 8.366075e-01 8.082861e-01 7.819192e-01 8.601514e-01 7.528148e-01

1703 9.988355e-01 9.959903e-01 9.890302e-01 9.765527e-01 9.586931e-01 8.367096e-01 8.083926e-01 7.820328e-01 8.602510e-01 7.529439e-01

1704 9.988351e-01 9.959890e-01 9.890273e-01 9.765476e-01 9.586853e-01 8.368149e-01 8.085025e-01 7.821502e-01 8.603536e-01 7.530774e-01

1705 9.988347e-01 9.959877e-01 9.890243e-01 9.765422e-01 9.586770e-01 8.369235e-01 8.086158e-01 7.822714e-01 8.604594e-01 7.532156e-01

1706 9.988343e-01 9.959863e-01 9.890210e-01 9.765364e-01 9.586682e-01 8.370354e-01 8.087328e-01 7.823965e-01 8.605685e-01 7.533585e-01

1707 9.988338e-01 9.959848e-01 9.890176e-01 9.765302e-01 9.586588e-01 8.371509e-01 8.088536e-01 7.825257e-01 8.606810e-01 7.535063e-01

1708 9.988333e-01 9.959832e-01 9.890139e-01 9.765236e-01 9.586487e-01 8.372699e-01 8.089781e-01 7.826592e-01 8.607970e-01 7.536592e-01

1709 9.988328e-01 9.959815e-01 9.890099e-01 9.765166e-01 9.586379e-01 8.373927e-01 8.091067e-01 7.827970e-01 8.609165e-01 7.538174e-01

1710 9.988323e-01 9.959797e-01 9.890057e-01 9.765091e-01 9.586264e-01 8.375192e-01 8.092393e-01 7.829393e-01 8.610398e-01 7.539809e-01

1711 9.988317e-01 9.959777e-01 9.890012e-01 9.765011e-01 9.586141e-01 8.376497e-01 8.093762e-01 7.830862e-01 8.611669e-01 7.541500e-01

1712 9.988311e-01 9.959757e-01 9.889964e-01 9.764925e-01 9.586010e-01 8.377843e-01 8.095174e-01 7.832379e-01 8.612979e-01 7.543248e-01

1713 9.988304e-01 9.959735e-01 9.889913e-01 9.764833e-01 9.585870e-01 8.379231e-01 8.096630e-01 7.833944e-01 8.614331e-01 7.545055e-01

1714 9.988297e-01 9.959711e-01 9.889858e-01 9.764735e-01 9.585719e-01 8.380662e-01 8.098133e-01 7.835561e-01 8.615724e-01 7.546923e-01

1715 9.988290e-01 9.959686e-01 9.889799e-01 9.764630e-01 9.585559e-01 8.382137e-01 8.099684e-01 7.837229e-01 8.617160e-01 7.548853e-01

1716 9.988282e-01 9.959659e-01 9.889737e-01 9.764518e-01 9.585387e-01 8.383658e-01 8.101283e-01 7.838951e-01 8.618641e-01 7.550848e-01

1717 9.988274e-01 9.959631e-01 9.889670e-01 9.764398e-01 9.585203e-01 8.385226e-01 8.102933e-01 7.840727e-01 8.620168e-01 7.552909e-01

1718 9.988265e-01 9.959600e-01 9.889598e-01 9.764269e-01 9.585006e-01 8.386843e-01 8.104635e-01 7.842561e-01 8.621742e-01 7.555038e-01

1719 9.988256e-01 9.959568e-01 9.889521e-01 9.764131e-01 9.584795e-01 8.388510e-01 8.106390e-01 7.844452e-01 8.623365e-01 7.557236e-01

1720 9.988246e-01 9.959533e-01 9.889439e-01 9.763984e-01 9.584570e-01 8.390229e-01 8.108200e-01 7.846404e-01 8.625039e-01 7.559507e-01

1721 9.988235e-01 9.959496e-01 9.889352e-01 9.763826e-01 9.584328e-01 8.392000e-01 8.110067e-01 7.848416e-01 8.626764e-01 7.561850e-01

1722 9.988224e-01 9.959457e-01 9.889258e-01 9.763657e-01 9.584069e-01 8.393827e-01 8.111991e-01 7.850492e-01 8.628542e-01 7.564270e-01

1723 9.988212e-01 9.959415e-01 9.889157e-01 9.763476e-01 9.583792e-01 8.395709e-01 8.113975e-01 7.852633e-01 8.630376e-01 7.566766e-01

1724 9.988199e-01 9.959370e-01 9.889050e-01 9.763281e-01 9.583495e-01 8.397649e-01 8.116020e-01 7.854839e-01 8.632266e-01 7.569341e-01

1725 9.988185e-01 9.959321e-01 9.888934e-01 9.763073e-01 9.583176e-01 8.399648e-01 8.118129e-01 7.857114e-01 8.634214e-01 7.571998e-01

1726 9.988171e-01 9.959270e-01 9.888811e-01 9.762850e-01 9.582834e-01 8.401709e-01 8.120301e-01 7.859458e-01 8.636223e-01 7.574737e-01

1727 9.988155e-01 9.959215e-01 9.888679e-01 9.762611e-01 9.582468e-01 8.403832e-01 8.122540e-01 7.861873e-01 8.638293e-01 7.577560e-01

1728 9.988139e-01 9.959156e-01 9.888537e-01 9.762354e-01 9.582075e-01 8.406020e-01 8.124847e-01 7.864362e-01 8.640426e-01 7.580470e-01

1729 9.988122e-01 9.959093e-01 9.888385e-01 9.762078e-01 9.581654e-01 8.408274e-01 8.127223e-01 7.866924e-01 8.642625e-01 7.583468e-01

1730 9.988103e-01 9.959025e-01 9.888222e-01 9.761783e-01 9.581202e-01 8.410595e-01 8.129670e-01 7.869563e-01 8.644891e-01 7.586555e-01

1731 9.988083e-01 9.958953e-01 9.888047e-01 9.761465e-01 9.580717e-01 8.412987e-01 8.132190e-01 7.872280e-01 8.647226e-01 7.589734e-01

1732 9.988062e-01 9.958876e-01 9.887859e-01 9.761125e-01 9.580197e-01 8.415450e-01 8.134785e-01 7.875076e-01 8.649632e-01 7.593006e-01

1733 9.988040e-01 9.958793e-01 9.887658e-01 9.760759e-01 9.579639e-01 8.417986e-01 8.137456e-01 7.877952e-01 8.652111e-01 7.596372e-01

1734 9.988016e-01 9.958705e-01 9.887442e-01 9.760366e-01 9.579040e-01 8.420597e-01 8.140205e-01 7.880911e-01 8.654664e-01 7.599833e-01

1735 9.987990e-01 9.958610e-01 9.887210e-01 9.759945e-01 9.578396e-01 8.423285e-01 8.143034e-01 7.883954e-01 8.657295e-01 7.603392e-01

1736 9.987963e-01 9.958508e-01 9.886960e-01 9.759492e-01 9.577706e-01 8.426052e-01 8.145944e-01 7.887082e-01 8.660005e-01 7.607049e-01

1737 9.987934e-01 9.958399e-01 9.886693e-01 9.759005e-01 9.576965e-01 8.428900e-01 8.148936e-01 7.890296e-01 8.662796e-01 7.610806e-01

1738 9.987903e-01 9.958282e-01 9.886406e-01 9.758482e-01 9.576169e-01 8.431830e-01 8.152013e-01 7.893599e-01 8.665670e-01 7.614662e-01

1739 9.987870e-01 9.958157e-01 9.886097e-01 9.757920e-01 9.575315e-01 8.434845e-01 8.155176e-01 7.896990e-01 8.668629e-01 7.618621e-01

1740 9.987834e-01 9.958023e-01 9.885766e-01 9.757316e-01 9.574397e-01 8.437945e-01 8.158426e-01 7.900471e-01 8.671675e-01 7.622681e-01

1741 9.987797e-01 9.957879e-01 9.885409e-01 9.756667e-01 9.573412e-01 8.441134e-01 8.161766e-01 7.904043e-01 8.674811e-01 7.626843e-01

1742 9.987756e-01 9.957724e-01 9.885026e-01 9.755969e-01 9.572355e-01 8.444412e-01 8.165195e-01 7.907708e-01 8.678039e-01 7.631109e-01

1743 9.987713e-01 9.957559e-01 9.884614e-01 9.755219e-01 9.571219e-01 8.447782e-01 8.168716e-01 7.911465e-01 8.681361e-01 7.635478e-01

1744 9.987667e-01 9.957381e-01 9.884172e-01 9.754412e-01 9.570000e-01 8.451245e-01 8.172330e-01 7.915315e-01 8.684779e-01 7.639950e-01

1745 9.987618e-01 9.957190e-01 9.883695e-01 9.753545e-01 9.568691e-01 8.454804e-01 8.176038e-01 7.919260e-01 8.688295e-01 7.644525e-01

1746 9.987566e-01 9.956985e-01 9.883183e-01 9.752612e-01 9.567287e-01 8.458459e-01 8.179840e-01 7.923299e-01 8.691912e-01 7.649203e-01

1747 9.987510e-01 9.956765e-01 9.882632e-01 9.751609e-01 9.565779e-01 8.462212e-01 8.183739e-01 7.927433e-01 8.695631e-01 7.653984e-01

1748 9.987450e-01 9.956528e-01 9.882040e-01 9.750531e-01 9.564161e-01 8.466065e-01 8.187735e-01 7.931662e-01 8.699455e-01 7.658865e-01

1749 9.987385e-01 9.956274e-01 9.881402e-01 9.749372e-01 9.562426e-01 8.470020e-01 8.191828e-01 7.935985e-01 8.703386e-01 7.663848e-01

1750 9.987317e-01 9.956001e-01 9.880716e-01 9.748125e-01 9.560564e-01 8.474078e-01 8.196020e-01 7.940403e-01 8.707426e-01 7.668929e-01

1751 9.987243e-01 9.955708e-01 9.879977e-01 9.746785e-01 9.558567e-01 8.478239e-01 8.200310e-01 7.944916e-01 8.711578e-01 7.674108e-01

1752 9.987165e-01 9.955393e-01 9.879183e-01 9.745344e-01 9.556426e-01 8.482507e-01 8.204700e-01 7.949522e-01 8.715842e-01 7.679383e-01

1753 9.987081e-01 9.955054e-01 9.878327e-01 9.743795e-01 9.554132e-01 8.486881e-01 8.209188e-01 7.954220e-01 8.720222e-01 7.684752e-01

1754 9.986991e-01 9.954689e-01 9.877407e-01 9.742130e-01 9.551673e-01 8.491363e-01 8.213776e-01 7.959011e-01 8.724720e-01 7.690212e-01

1755 9.986895e-01 9.954297e-01 9.876416e-01 9.740341e-01 9.549041e-01 8.495953e-01 8.218464e-01 7.963892e-01 8.729336e-01 7.695762e-01

1756 9.986792e-01 9.953876e-01 9.875349e-01 9.738419e-01 9.546222e-01 8.500653e-01 8.223250e-01 7.968862e-01 8.734074e-01 7.701397e-01

1757 9.986681e-01 9.953422e-01 9.874201e-01 9.736355e-01 9.543206e-01 8.505464e-01 8.228135e-01 7.973919e-01 8.738934e-01 7.707116e-01

1758 9.986563e-01 9.952934e-01 9.872966e-01 9.734138e-01 9.539981e-01 8.510385e-01 8.233117e-01 7.979062e-01 8.743919e-01 7.712915e-01

1759 9.986436e-01 9.952410e-01 9.871636e-01 9.731757e-01 9.536533e-01 8.515417e-01 8.238196e-01 7.984288e-01 8.749030e-01 7.718790e-01

1760 9.986300e-01 9.951845e-01 9.870204e-01 9.729202e-01 9.532850e-01 8.520562e-01 8.243371e-01 7.989595e-01 8.754269e-01 7.724737e-01

1761 9.986154e-01 9.951237e-01 9.868664e-01 9.726461e-01 9.528918e-01 8.525817e-01 8.248640e-01 7.994980e-01 8.759636e-01 7.730752e-01

1762 9.985998e-01 9.950582e-01 9.867007e-01 9.723521e-01 9.524724e-01 8.531185e-01 8.254001e-01 8.000441e-01 8.765135e-01 7.736831e-01

1763 9.985831e-01 9.949878e-01 9.865224e-01 9.720370e-01 9.520253e-01 8.536663e-01 8.259454e-01 8.005974e-01 8.770764e-01 7.742969e-01

1764 9.985651e-01 9.949120e-01 9.863306e-01 9.716993e-01 9.515492e-01 8.542253e-01 8.264995e-01 8.011576e-01 8.776526e-01 7.749162e-01

1765 9.985459e-01 9.948304e-01 9.861244e-01 9.713377e-01 9.510425e-01 8.547952e-01 8.270624e-01 8.017244e-01 8.782422e-01 7.755403e-01

1766 9.985252e-01 9.947425e-01 9.859026e-01 9.709507e-01 9.505038e-01 8.553761e-01 8.276336e-01 8.022973e-01 8.788451e-01 7.761688e-01

1767 9.985031e-01 9.946479e-01 9.856643e-01 9.705366e-01 9.499317e-01 8.559678e-01 8.282130e-01 8.028759e-01 8.794615e-01 7.768012e-01

1768 9.984793e-01 9.945460e-01 9.854081e-01 9.700941e-01 9.493248e-01 8.565702e-01 8.288002e-01 8.034598e-01 8.800914e-01 7.774368e-01

1769 9.984537e-01 9.944364e-01 9.851330e-01 9.696214e-01 9.486817e-01 8.571831e-01 8.293949e-01 8.040487e-01 8.807348e-01 7.780751e-01

1770 9.984263e-01 9.943183e-01 9.848375e-01 9.691168e-01 9.480011e-01 8.578063e-01 8.299968e-01 8.046419e-01 8.813918e-01 7.787155e-01

1771 9.983969e-01 9.941913e-01 9.845204e-01 9.685788e-01 9.472817e-01 8.584397e-01 8.306054e-01 8.052390e-01 8.820622e-01 7.793574e-01

1772 9.983653e-01 9.940545e-01 9.841801e-01 9.680054e-01 9.465224e-01 8.590829e-01 8.312205e-01 8.058396e-01 8.827460e-01 7.800002e-01

1773 9.983314e-01 9.939072e-01 9.838152e-01 9.673952e-01 9.457220e-01 8.597359e-01 8.318415e-01 8.064430e-01 8.834432e-01 7.806432e-01

1774 9.982949e-01 9.937487e-01 9.834240e-01 9.667462e-01 9.448798e-01 8.603981e-01 8.324681e-01 8.070488e-01 8.841537e-01 7.812859e-01

1775 9.982558e-01 9.935782e-01 9.830051e-01 9.660569e-01 9.439948e-01 8.610694e-01 8.330997e-01 8.076565e-01 8.848773e-01 7.819275e-01

1776 9.982137e-01 9.933947e-01 9.825565e-01 9.653255e-01 9.430666e-01 8.617494e-01 8.337359e-01 8.082654e-01 8.856139e-01 7.825676e-01

1777 9.981685e-01 9.931974e-01 9.820767e-01 9.645504e-01 9.420948e-01 8.624378e-01 8.343762e-01 8.088750e-01 8.863634e-01 7.832054e-01

1778 9.981199e-01 9.929851e-01 9.815637e-01 9.637302e-01 9.410791e-01 8.631341e-01 8.350201e-01 8.094847e-01 8.871255e-01 7.838404e-01

1779 9.980677e-01 9.927569e-01 9.810158e-01 9.628633e-01 9.400198e-01 8.638380e-01 8.356671e-01 8.100940e-01 8.878999e-01 7.844719e-01

1780 9.980116e-01 9.925116e-01 9.804310e-01 9.619484e-01 9.389172e-01 8.645490e-01 8.363165e-01 8.107023e-01 8.886866e-01 7.850993e-01

1781 9.979514e-01 9.922480e-01 9.798074e-01 9.609843e-01 9.377719e-01 8.652667e-01 8.369679e-01 8.113090e-01 8.894851e-01 7.857221e-01

1782 9.978865e-01 9.919649e-01 9.791430e-01 9.599701e-01 9.365850e-01 8.659905e-01 8.376206e-01 8.119135e-01 8.902951e-01 7.863396e-01

1783 9.978169e-01 9.916608e-01 9.784360e-01 9.589048e-01 9.353577e-01 8.667200e-01 8.382741e-01 8.125152e-01 8.911164e-01 7.869514e-01

1784 9.977420e-01 9.913345e-01 9.776844e-01 9.577879e-01 9.340917e-01 8.674546e-01 8.389278e-01 8.131137e-01 8.919485e-01 7.875569e-01

1785 9.976615e-01 9.909844e-01 9.768863e-01 9.566191e-01 9.327890e-01 8.681938e-01 8.395811e-01 8.137083e-01 8.927910e-01 7.881555e-01

1786 9.975750e-01 9.906090e-01 9.760399e-01 9.553983e-01 9.314520e-01 8.689371e-01 8.402335e-01 8.142986e-01 8.936436e-01 7.887469e-01

1787 9.974820e-01 9.902067e-01 9.751434e-01 9.541257e-01 9.300832e-01 8.696838e-01 8.408842e-01 8.148839e-01 8.945057e-01 7.893304e-01

1788 9.973820e-01 9.897757e-01 9.741951e-01 9.528018e-01 9.286858e-01 8.704334e-01 8.415328e-01 8.154637e-01 8.953769e-01 7.899058e-01

1789 9.972745e-01 9.893144e-01 9.731935e-01 9.514276e-01 9.272630e-01 8.711851e-01 8.421786e-01 8.160376e-01 8.962567e-01 7.904725e-01

1790 9.971590e-01 9.888210e-01 9.721371e-01 9.500043e-01 9.258185e-01 8.719386e-01 8.428211e-01 8.166052e-01 8.971444e-01 7.910302e-01

1791 9.970349e-01 9.882936e-01 9.710247e-01 9.485335e-01 9.243561e-01 8.726930e-01 8.434597e-01 8.171658e-01 8.980397e-01 7.915785e-01

1792 9.969016e-01 9.877302e-01 9.698553e-01 9.470174e-01 9.228800e-01 8.734477e-01 8.440938e-01 8.177191e-01 8.989418e-01 7.921172e-01

1793 9.967583e-01 9.871291e-01 9.686280e-01 9.454582e-01 9.213944e-01 8.742022e-01 8.447229e-01 8.182647e-01 8.998501e-01 7.926458e-01

1794 9.966043e-01 9.864881e-01 9.673422e-01 9.438588e-01 9.199037e-01 8.749558e-01 8.453464e-01 8.188022e-01 9.007642e-01 7.931641e-01

1795 9.964390e-01 9.858053e-01 9.659978e-01 9.422224e-01 9.184125e-01 8.757077e-01 8.459639e-01 8.193312e-01 9.016831e-01 7.936719e-01

1796 9.962614e-01 9.850786e-01 9.645946e-01 9.405524e-01 9.169254e-01 8.764574e-01 8.465747e-01 8.198514e-01 9.026064e-01 7.941690e-01

1797 9.960708e-01 9.843062e-01 9.631332e-01 9.388528e-01 9.154468e-01 8.772043e-01 8.471786e-01 8.203625e-01 9.035333e-01 7.946553e-01

1798 9.958661e-01 9.834860e-01 9.616142e-01 9.371277e-01 9.139813e-01 8.779476e-01 8.477748e-01 8.208642e-01 9.044632e-01 7.951304e-01

1799 9.956466e-01 9.826161e-01 9.600386e-01 9.353816e-01 9.125333e-01 8.786869e-01 8.483632e-01 8.213562e-01 9.053952e-01 7.955944e-01

1800 9.954110e-01 9.816946e-01 9.584081e-01 9.336192e-01 9.111068e-01 8.794213e-01 8.489431e-01 8.218382e-01 9.063287e-01 7.960471e-01

1801 9.951584e-01 9.807198e-01 9.567245e-01 9.318454e-01 9.097059e-01 8.801504e-01 8.495143e-01 8.223102e-01 9.072630e-01 7.964885e-01

1802 9.951584e-01 9.807198e-01 9.567244e-01 9.318452e-01 9.097057e-01 8.801578e-01 8.495202e-01 8.223146e-01 9.072702e-01 7.964928e-01

1803 9.951583e-01 9.807197e-01 9.567242e-01 9.318450e-01 9.097055e-01 8.801650e-01 8.495259e-01 8.223189e-01 9.072773e-01 7.964970e-01

1804 9.951583e-01 9.807196e-01 9.567241e-01 9.318449e-01 9.097053e-01 8.801721e-01 8.495314e-01 8.223230e-01 9.072844e-01 7.965010e-01

1805 9.951583e-01 9.807195e-01 9.567240e-01 9.318447e-01 9.097050e-01 8.801791e-01 8.495368e-01 8.223270e-01 9.072915e-01 7.965050e-01

1806 9.951583e-01 9.807195e-01 9.567238e-01 9.318445e-01 9.097048e-01 8.801859e-01 8.495420e-01 8.223307e-01 9.072985e-01 7.965088e-01

1807 9.951582e-01 9.807194e-01 9.567237e-01 9.318443e-01 9.097045e-01 8.801926e-01 8.495471e-01 8.223344e-01 9.073054e-01 7.965126e-01

1808 9.951582e-01 9.807193e-01 9.567235e-01 9.318440e-01 9.097043e-01 8.801991e-01 8.495519e-01 8.223378e-01 9.073124e-01 7.965163e-01

1809 9.951581e-01 9.807192e-01 9.567234e-01 9.318438e-01 9.097040e-01 8.802056e-01 8.495567e-01 8.223412e-01 9.073193e-01 7.965199e-01

1810 9.951581e-01 9.807191e-01 9.567232e-01 9.318436e-01 9.097037e-01 8.802119e-01 8.495613e-01 8.223443e-01 9.073262e-01 7.965234e-01

1811 9.951581e-01 9.807190e-01 9.567230e-01 9.318433e-01 9.097034e-01 8.802181e-01 8.495658e-01 8.223473e-01 9.073331e-01 7.965268e-01

1812 9.951580e-01 9.807189e-01 9.567228e-01 9.318431e-01 9.097031e-01 8.802242e-01 8.495701e-01 8.223502e-01 9.073400e-01 7.965301e-01

1813 9.951580e-01 9.807187e-01 9.567226e-01 9.318428e-01 9.097028e-01 8.802303e-01 8.495743e-01 8.223529e-01 9.073469e-01 7.965333e-01

1814 9.951579e-01 9.807186e-01 9.567224e-01 9.318425e-01 9.097025e-01 8.802362e-01 8.495783e-01 8.223555e-01 9.073538e-01 7.965364e-01

1815 9.951579e-01 9.807185e-01 9.567222e-01 9.318422e-01 9.097021e-01 8.802420e-01 8.495823e-01 8.223580e-01 9.073607e-01 7.965394e-01

1816 9.951578e-01 9.807184e-01 9.567219e-01 9.318419e-01 9.097017e-01 8.802478e-01 8.495861e-01 8.223602e-01 9.073676e-01 7.965423e-01

1817 9.951578e-01 9.807182e-01 9.567217e-01 9.318415e-01 9.097013e-01 8.802535e-01 8.495897e-01 8.223624e-01 9.073746e-01 7.965451e-01

1818 9.951577e-01 9.807181e-01 9.567214e-01 9.318412e-01 9.097009e-01 8.802591e-01 8.495933e-01 8.223644e-01 9.073815e-01 7.965478e-01

1819 9.951577e-01 9.807179e-01 9.567211e-01 9.318408e-01 9.097005e-01 8.802646e-01 8.495967e-01 8.223662e-01 9.073885e-01 7.965504e-01

1820 9.951576e-01 9.807178e-01 9.567209e-01 9.318404e-01 9.097001e-01 8.802701e-01 8.496000e-01 8.223679e-01 9.073955e-01 7.965530e-01

1821 9.951575e-01 9.807176e-01 9.567206e-01 9.318400e-01 9.096996e-01 8.802755e-01 8.496032e-01 8.223695e-01 9.074026e-01 7.965554e-01

1822 9.951575e-01 9.807174e-01 9.567203e-01 9.318396e-01 9.096991e-01 8.802808e-01 8.496063e-01 8.223709e-01 9.074097e-01 7.965576e-01

1823 9.951574e-01 9.807172e-01 9.567199e-01 9.318392e-01 9.096986e-01 8.802861e-01 8.496093e-01 8.223721e-01 9.074168e-01 7.965598e-01

1824 9.951573e-01 9.807170e-01 9.567196e-01 9.318387e-01 9.096981e-01 8.802913e-01 8.496121e-01 8.223732e-01 9.074240e-01 7.965619e-01

1825 9.951572e-01 9.807168e-01 9.567192e-01 9.318382e-01 9.096975e-01 8.802965e-01 8.496149e-01 8.223742e-01 9.074313e-01 7.965638e-01

1826 9.951571e-01 9.807166e-01 9.567188e-01 9.318377e-01 9.096969e-01 8.803017e-01 8.496175e-01 8.223749e-01 9.074386e-01 7.965656e-01

1827 9.951571e-01 9.807164e-01 9.567184e-01 9.318372e-01 9.096963e-01 8.803067e-01 8.496200e-01 8.223755e-01 9.074460e-01 7.965673e-01

1828 9.951570e-01 9.807161e-01 9.567180e-01 9.318366e-01 9.096957e-01 8.803118e-01 8.496223e-01 8.223760e-01 9.074534e-01 7.965689e-01

1829 9.951569e-01 9.807159e-01 9.567176e-01 9.318361e-01 9.096950e-01 8.803168e-01 8.496246e-01 8.223762e-01 9.074609e-01 7.965703e-01

1830 9.951568e-01 9.807156e-01 9.567171e-01 9.318355e-01 9.096943e-01 8.803217e-01 8.496267e-01 8.223763e-01 9.074685e-01 7.965715e-01

1831 9.951567e-01 9.807153e-01 9.567167e-01 9.318348e-01 9.096936e-01 8.803266e-01 8.496287e-01 8.223762e-01 9.074762e-01 7.965727e-01

1832 9.951565e-01 9.807150e-01 9.567161e-01 9.318342e-01 9.096928e-01 8.803315e-01 8.496306e-01 8.223759e-01 9.074840e-01 7.965736e-01

1833 9.951564e-01 9.807147e-01 9.567156e-01 9.318335e-01 9.096921e-01 8.803364e-01 8.496324e-01 8.223755e-01 9.074918e-01 7.965744e-01

1834 9.951563e-01 9.807144e-01 9.567151e-01 9.318327e-01 9.096912e-01 8.803412e-01 8.496341e-01 8.223748e-01 9.074997e-01 7.965751e-01

1835 9.951562e-01 9.807140e-01 9.567145e-01 9.318320e-01 9.096904e-01 8.803460e-01 8.496356e-01 8.223739e-01 9.075078e-01 7.965756e-01

1836 9.951560e-01 9.807137e-01 9.567138e-01 9.318311e-01 9.096894e-01 8.803507e-01 8.496370e-01 8.223729e-01 9.075159e-01 7.965758e-01

1837 9.951559e-01 9.807133e-01 9.567132e-01 9.318303e-01 9.096885e-01 8.803554e-01 8.496382e-01 8.223716e-01 9.075242e-01 7.965760e-01

1838 9.951557e-01 9.807129e-01 9.567125e-01 9.318294e-01 9.096875e-01 8.803601e-01 8.496393e-01 8.223701e-01 9.075325e-01 7.965759e-01

1839 9.951556e-01 9.807125e-01 9.567118e-01 9.318285e-01 9.096865e-01 8.803648e-01 8.496403e-01 8.223683e-01 9.075410e-01 7.965756e-01

1840 9.951554e-01 9.807120e-01 9.567110e-01 9.318275e-01 9.096854e-01 8.803695e-01 8.496411e-01 8.223664e-01 9.075496e-01 7.965751e-01

1841 9.951552e-01 9.807115e-01 9.567102e-01 9.318264e-01 9.096842e-01 8.803741e-01 8.496418e-01 8.223641e-01 9.075583e-01 7.965743e-01

1842 9.951550e-01 9.807110e-01 9.567094e-01 9.318254e-01 9.096830e-01 8.803787e-01 8.496424e-01 8.223616e-01 9.075672e-01 7.965734e-01

1843 9.951548e-01 9.807105e-01 9.567085e-01 9.318242e-01 9.096818e-01 8.803833e-01 8.496427e-01 8.223589e-01 9.075762e-01 7.965722e-01

1844 9.951546e-01 9.807100e-01 9.567075e-01 9.318230e-01 9.096805e-01 8.803879e-01 8.496430e-01 8.223559e-01 9.075853e-01 7.965708e-01

1845 9.951544e-01 9.807094e-01 9.567066e-01 9.318218e-01 9.096791e-01 8.803924e-01 8.496430e-01 8.223526e-01 9.075946e-01 7.965691e-01

1846 9.951541e-01 9.807088e-01 9.567055e-01 9.318204e-01 9.096776e-01 8.803969e-01 8.496429e-01 8.223490e-01 9.076041e-01 7.965671e-01

1847 9.951539e-01 9.807081e-01 9.567044e-01 9.318190e-01 9.096761e-01 8.804014e-01 8.496426e-01 8.223451e-01 9.076136e-01 7.965648e-01

1848 9.951536e-01 9.807074e-01 9.567033e-01 9.318176e-01 9.096746e-01 8.804059e-01 8.496421e-01 8.223409e-01 9.076234e-01 7.965623e-01

1849 9.951534e-01 9.807067e-01 9.567021e-01 9.318160e-01 9.096729e-01 8.804104e-01 8.496414e-01 8.223363e-01 9.076333e-01 7.965594e-01

1850 9.951531e-01 9.807059e-01 9.567008e-01 9.318144e-01 9.096712e-01 8.804148e-01 8.496406e-01 8.223314e-01 9.076434e-01 7.965562e-01

1851 9.951528e-01 9.807051e-01 9.566994e-01 9.318127e-01 9.096693e-01 8.804192e-01 8.496395e-01 8.223261e-01 9.076537e-01 7.965526e-01

1852 9.951524e-01 9.807043e-01 9.566980e-01 9.318109e-01 9.096674e-01 8.804236e-01 8.496382e-01 8.223205e-01 9.076642e-01 7.965488e-01

1853 9.951521e-01 9.807034e-01 9.566965e-01 9.318090e-01 9.096654e-01 8.804279e-01 8.496367e-01 8.223145e-01 9.076748e-01 7.965445e-01

1854 9.951517e-01 9.807024e-01 9.566949e-01 9.318070e-01 9.096633e-01 8.804323e-01 8.496349e-01 8.223080e-01 9.076857e-01 7.965398e-01

1855 9.951514e-01 9.807014e-01 9.566933e-01 9.318050e-01 9.096611e-01 8.804366e-01 8.496329e-01 8.223012e-01 9.076967e-01 7.965348e-01

1856 9.951510e-01 9.807004e-01 9.566915e-01 9.318028e-01 9.096588e-01 8.804409e-01 8.496306e-01 8.222939e-01 9.077080e-01 7.965293e-01

1857 9.951505e-01 9.806992e-01 9.566896e-01 9.318005e-01 9.096564e-01 8.804451e-01 8.496281e-01 8.222862e-01 9.077194e-01 7.965234e-01

1858 9.951501e-01 9.806981e-01 9.566877e-01 9.317980e-01 9.096538e-01 8.804493e-01 8.496253e-01 8.222779e-01 9.077311e-01 7.965170e-01

1859 9.951496e-01 9.806968e-01 9.566856e-01 9.317955e-01 9.096512e-01 8.804535e-01 8.496223e-01 8.222692e-01 9.077430e-01 7.965101e-01

1860 9.951491e-01 9.806955e-01 9.566834e-01 9.317928e-01 9.096484e-01 8.804576e-01 8.496189e-01 8.222600e-01 9.077552e-01 7.965027e-01

1861 9.951486e-01 9.806941e-01 9.566811e-01 9.317899e-01 9.096454e-01 8.804617e-01 8.496152e-01 8.222502e-01 9.077676e-01 7.964948e-01

1862 9.951480e-01 9.806926e-01 9.566786e-01 9.317869e-01 9.096424e-01 8.804658e-01 8.496112e-01 8.222398e-01 9.077802e-01 7.964863e-01

1863 9.951474e-01 9.806910e-01 9.566761e-01 9.317838e-01 9.096391e-01 8.804697e-01 8.496069e-01 8.222289e-01 9.077931e-01 7.964773e-01

1864 9.951468e-01 9.806894e-01 9.566733e-01 9.317805e-01 9.096357e-01 8.804737e-01 8.496021e-01 8.222173e-01 9.078063e-01 7.964677e-01

1865 9.951462e-01 9.806876e-01 9.566705e-01 9.317770e-01 9.096322e-01 8.804776e-01 8.495971e-01 8.222052e-01 9.078197e-01 7.964574e-01

1866 9.951455e-01 9.806858e-01 9.566674e-01 9.317733e-01 9.096284e-01 8.804814e-01 8.495916e-01 8.221923e-01 9.078334e-01 7.964464e-01

1867 9.951447e-01 9.806838e-01 9.566642e-01 9.317694e-01 9.096245e-01 8.804852e-01 8.495857e-01 8.221788e-01 9.078473e-01 7.964348e-01

1868 9.951439e-01 9.806817e-01 9.566608e-01 9.317653e-01 9.096204e-01 8.804889e-01 8.495794e-01 8.221645e-01 9.078616e-01 7.964225e-01

1869 9.951431e-01 9.806795e-01 9.566572e-01 9.317610e-01 9.096161e-01 8.804925e-01 8.495727e-01 8.221495e-01 9.078762e-01 7.964094e-01

1870 9.951422e-01 9.806772e-01 9.566534e-01 9.317564e-01 9.096116e-01 8.804960e-01 8.495655e-01 8.221337e-01 9.078910e-01 7.963955e-01

1871 9.951413e-01 9.806747e-01 9.566494e-01 9.317516e-01 9.096068e-01 8.804995e-01 8.495578e-01 8.221170e-01 9.079062e-01 7.963808e-01

1872 9.951403e-01 9.806721e-01 9.566451e-01 9.317465e-01 9.096018e-01 8.805028e-01 8.495497e-01 8.220995e-01 9.079217e-01 7.963652e-01

1873 9.951393e-01 9.806693e-01 9.566406e-01 9.317412e-01 9.095966e-01 8.805061e-01 8.495410e-01 8.220812e-01 9.079376e-01 7.963488e-01

1874 9.951382e-01 9.806663e-01 9.566358e-01 9.317355e-01 9.095911e-01 8.805092e-01 8.495317e-01 8.220619e-01 9.079537e-01 7.963314e-01

1875 9.951370e-01 9.806632e-01 9.566308e-01 9.317296e-01 9.095853e-01 8.805122e-01 8.495219e-01 8.220416e-01 9.079702e-01 7.963130e-01

1876 9.951358e-01 9.806599e-01 9.566254e-01 9.317233e-01 9.095792e-01 8.805151e-01 8.495114e-01 8.220203e-01 9.079871e-01 7.962936e-01

1877 9.951344e-01 9.806564e-01 9.566198e-01 9.317167e-01 9.095728e-01 8.805179e-01 8.495004e-01 8.219980e-01 9.080044e-01 7.962732e-01

1878 9.951331e-01 9.806526e-01 9.566137e-01 9.317097e-01 9.095661e-01 8.805205e-01 8.494886e-01 8.219745e-01 9.080220e-01 7.962517e-01

1879 9.951316e-01 9.806487e-01 9.566074e-01 9.317023e-01 9.095591e-01 8.805230e-01 8.494762e-01 8.219500e-01 9.080400e-01 7.962290e-01

1880 9.951300e-01 9.806445e-01 9.566007e-01 9.316945e-01 9.095517e-01 8.805253e-01 8.494631e-01 8.219242e-01 9.080583e-01 7.962051e-01

1881 9.951283e-01 9.806400e-01 9.565935e-01 9.316862e-01 9.095440e-01 8.805275e-01 8.494493e-01 8.218972e-01 9.080771e-01 7.961799e-01

1882 9.951266e-01 9.806353e-01 9.565859e-01 9.316775e-01 9.095358e-01 8.805294e-01 8.494346e-01 8.218689e-01 9.080963e-01 7.961535e-01

1883 9.951247e-01 9.806302e-01 9.565779e-01 9.316683e-01 9.095273e-01 8.805312e-01 8.494191e-01 8.218393e-01 9.081159e-01 7.961257e-01

1884 9.951227e-01 9.806249e-01 9.565694e-01 9.316586e-01 9.095183e-01 8.805327e-01 8.494028e-01 8.218082e-01 9.081360e-01 7.960964e-01

1885 9.951206e-01 9.806192e-01 9.565604e-01 9.316483e-01 9.095089e-01 8.805340e-01 8.493856e-01 8.217757e-01 9.081565e-01 7.960657e-01

1886 9.951183e-01 9.806132e-01 9.565508e-01 9.316375e-01 9.094990e-01 8.805351e-01 8.493675e-01 8.217417e-01 9.081774e-01 7.960335e-01

1887 9.951160e-01 9.806067e-01 9.565407e-01 9.316260e-01 9.094886e-01 8.805360e-01 8.493484e-01 8.217062e-01 9.081988e-01 7.959997e-01

1888 9.951134e-01 9.805999e-01 9.565299e-01 9.316139e-01 9.094777e-01 8.805365e-01 8.493283e-01 8.216689e-01 9.082207e-01 7.959642e-01

1889 9.951107e-01 9.805927e-01 9.565185e-01 9.316011e-01 9.094663e-01 8.805368e-01 8.493071e-01 8.216300e-01 9.082431e-01 7.959269e-01

1890 9.951079e-01 9.805849e-01 9.565064e-01 9.315876e-01 9.094542e-01 8.805368e-01 8.492849e-01 8.215893e-01 9.082659e-01 7.958879e-01

1891 9.951048e-01 9.805767e-01 9.564935e-01 9.315733e-01 9.094416e-01 8.805364e-01 8.492614e-01 8.215468e-01 9.082893e-01 7.958470e-01

1892 9.951016e-01 9.805680e-01 9.564798e-01 9.315582e-01 9.094284e-01 8.805357e-01 8.492368e-01 8.215023e-01 9.083132e-01 7.958041e-01

1893 9.950982e-01 9.805587e-01 9.564653e-01 9.315422e-01 9.094145e-01 8.805347e-01 8.492110e-01 8.214559e-01 9.083376e-01 7.957593e-01

1894 9.950945e-01 9.805488e-01 9.564499e-01 9.315253e-01 9.093999e-01 8.805333e-01 8.491838e-01 8.214073e-01 9.083625e-01 7.957123e-01

1895 9.950906e-01 9.805383e-01 9.564335e-01 9.315074e-01 9.093846e-01 8.805314e-01 8.491553e-01 8.213567e-01 9.083880e-01 7.956632e-01

1896 9.950865e-01 9.805271e-01 9.564161e-01 9.314885e-01 9.093686e-01 8.805292e-01 8.491254e-01 8.213038e-01 9.084141e-01 7.956118e-01

1897 9.950820e-01 9.805151e-01 9.563976e-01 9.314685e-01 9.093518e-01 8.805265e-01 8.490940e-01 8.212486e-01 9.084408e-01 7.955580e-01

1898 9.950773e-01 9.805024e-01 9.563780e-01 9.314473e-01 9.093342e-01 8.805233e-01 8.490611e-01 8.211910e-01 9.084680e-01 7.955019e-01

1899 9.950723e-01 9.804889e-01 9.563571e-01 9.314249e-01 9.093157e-01 8.805196e-01 8.490266e-01 8.211308e-01 9.084959e-01 7.954432e-01

1900 9.950670e-01 9.804745e-01 9.563350e-01 9.314013e-01 9.092963e-01 8.805154e-01 8.489904e-01 8.210682e-01 9.085243e-01 7.953819e-01

1901 9.950614e-01 9.804591e-01 9.563114e-01 9.313762e-01 9.092760e-01 8.805106e-01 8.489525e-01 8.210028e-01 9.085534e-01 7.953179e-01

1902 9.950553e-01 9.804427e-01 9.562863e-01 9.313497e-01 9.092548e-01 8.805052e-01 8.489129e-01 8.209347e-01 9.085831e-01 7.952511e-01

1903 9.950489e-01 9.804252e-01 9.562596e-01 9.313217e-01 9.092325e-01 8.804993e-01 8.488713e-01 8.208636e-01 9.086135e-01 7.951814e-01

1904 9.950420e-01 9.804066e-01 9.562313e-01 9.312921e-01 9.092092e-01 8.804926e-01 8.488278e-01 8.207897e-01 9.086446e-01 7.951087e-01

1905 9.950347e-01 9.803867e-01 9.562011e-01 9.312608e-01 9.091848e-01 8.804853e-01 8.487823e-01 8.207126e-01 9.086763e-01 7.950329e-01

1906 9.950269e-01 9.803655e-01 9.561691e-01 9.312276e-01 9.091594e-01 8.804773e-01 8.487347e-01 8.206324e-01 9.087087e-01 7.949540e-01

1907 9.950186e-01 9.803429e-01 9.561350e-01 9.311925e-01 9.091327e-01 8.804685e-01 8.486850e-01 8.205488e-01 9.087419e-01 7.948717e-01

1908 9.950097e-01 9.803188e-01 9.560987e-01 9.311554e-01 9.091049e-01 8.804589e-01 8.486330e-01 8.204619e-01 9.087757e-01 7.947861e-01

1909 9.950002e-01 9.802931e-01 9.560601e-01 9.311162e-01 9.090758e-01 8.804484e-01 8.485786e-01 8.203715e-01 9.088103e-01 7.946970e-01

1910 9.949901e-01 9.802657e-01 9.560190e-01 9.310747e-01 9.090455e-01 8.804371e-01 8.485218e-01 8.202774e-01 9.088456e-01 7.946043e-01

1911 9.949794e-01 9.802363e-01 9.559753e-01 9.310309e-01 9.090139e-01 8.804249e-01 8.484625e-01 8.201796e-01 9.088817e-01 7.945078e-01

1912 9.949679e-01 9.802051e-01 9.559288e-01 9.309845e-01 9.089809e-01 8.804117e-01 8.484006e-01 8.200780e-01 9.089186e-01 7.944076e-01

1913 9.949556e-01 9.801717e-01 9.558793e-01 9.309355e-01 9.089466e-01 8.803975e-01 8.483360e-01 8.199724e-01 9.089563e-01 7.943035e-01

1914 9.949425e-01 9.801360e-01 9.558266e-01 9.308837e-01 9.089109e-01 8.803822e-01 8.482686e-01 8.198627e-01 9.089947e-01 7.941953e-01

1915 9.949285e-01 9.800979e-01 9.557705e-01 9.308290e-01 9.088737e-01 8.803658e-01 8.481984e-01 8.197488e-01 9.090340e-01 7.940830e-01

1916 9.949135e-01 9.800572e-01 9.557109e-01 9.307711e-01 9.088352e-01 8.803482e-01 8.481251e-01 8.196306e-01 9.090741e-01 7.939665e-01

1917 9.948975e-01 9.800137e-01 9.556474e-01 9.307100e-01 9.087951e-01 8.803295e-01 8.480487e-01 8.195080e-01 9.091150e-01 7.938457e-01

1918 9.948804e-01 9.799673e-01 9.555798e-01 9.306455e-01 9.087536e-01 8.803094e-01 8.479692e-01 8.193808e-01 9.091568e-01 7.937205e-01

1919 9.948621e-01 9.799177e-01 9.555080e-01 9.305774e-01 9.087107e-01 8.802880e-01 8.478864e-01 8.192489e-01 9.091994e-01 7.935907e-01

1920 9.948426e-01 9.798647e-01 9.554315e-01 9.305055e-01 9.086663e-01 8.802653e-01 8.478002e-01 8.191123e-01 9.092429e-01 7.934564e-01

1921 9.948217e-01 9.798081e-01 9.553501e-01 9.304297e-01 9.086204e-01 8.802411e-01 8.477104e-01 8.189708e-01 9.092873e-01 7.933174e-01

1922 9.947994e-01 9.797476e-01 9.552635e-01 9.303497e-01 9.085732e-01 8.802153e-01 8.476171e-01 8.188242e-01 9.093326e-01 7.931736e-01

1923 9.947756e-01 9.796829e-01 9.551714e-01 9.302654e-01 9.085246e-01 8.801880e-01 8.475201e-01 8.186726e-01 9.093788e-01 7.930249e-01

1924 9.947500e-01 9.796137e-01 9.550735e-01 9.301765e-01 9.084746e-01 8.801591e-01 8.474192e-01 8.185157e-01 9.094260e-01 7.928713e-01

1925 9.947227e-01 9.795399e-01 9.549693e-01 9.300829e-01 9.084234e-01 8.801284e-01 8.473144e-01 8.183536e-01 9.094740e-01 7.927127e-01

1926 9.946935e-01 9.794609e-01 9.548585e-01 9.299844e-01 9.083710e-01 8.800960e-01 8.472056e-01 8.181860e-01 9.095230e-01 7.925490e-01

1927 9.946622e-01 9.793765e-01 9.547408e-01 9.298807e-01 9.083176e-01 8.800617e-01 8.470927e-01 8.180128e-01 9.095729e-01 7.923803e-01

1928 9.946287e-01 9.792863e-01 9.546157e-01 9.297716e-01 9.082632e-01 8.800255e-01 8.469755e-01 8.178341e-01 9.096238e-01 7.922063e-01

1929 9.945928e-01 9.791899e-01 9.544827e-01 9.296570e-01 9.082080e-01 8.799873e-01 8.468540e-01 8.176497e-01 9.096756e-01 7.920271e-01

1930 9.945544e-01 9.790868e-01 9.543414e-01 9.295366e-01 9.081522e-01 8.799471e-01 8.467280e-01 8.174596e-01 9.097284e-01 7.918427e-01

1931 9.945133e-01 9.789767e-01 9.541914e-01 9.294102e-01 9.080959e-01 8.799046e-01 8.465975e-01 8.172636e-01 9.097822e-01 7.916531e-01

1932 9.944693e-01 9.788590e-01 9.540322e-01 9.292777e-01 9.080394e-01 8.798600e-01 8.464624e-01 8.170618e-01 9.098369e-01 7.914581e-01

1933 9.944222e-01 9.787333e-01 9.538632e-01 9.291388e-01 9.079830e-01 8.798131e-01 8.463225e-01 8.168540e-01 9.098926e-01 7.912579e-01

1934 9.943717e-01 9.785990e-01 9.536839e-01 9.289934e-01 9.079269e-01 8.797637e-01 8.461779e-01 8.166402e-01 9.099493e-01 7.910524e-01

1935 9.943176e-01 9.784555e-01 9.534937e-01 9.288412e-01 9.078715e-01 8.797119e-01 8.460283e-01 8.164204e-01 9.100070e-01 7.908416e-01

1936 9.942596e-01 9.783022e-01 9.532922e-01 9.286822e-01 9.078170e-01 8.796576e-01 8.458737e-01 8.161946e-01 9.100656e-01 7.906257e-01

1937 9.941975e-01 9.781386e-01 9.530787e-01 9.285162e-01 9.077641e-01 8.796006e-01 8.457141e-01 8.159628e-01 9.101252e-01 7.904045e-01

1938 9.941310e-01 9.779638e-01 9.528526e-01 9.283431e-01 9.077129e-01 8.795410e-01 8.455493e-01 8.157249e-01 9.101858e-01 7.901782e-01

1939 9.940597e-01 9.777773e-01 9.526133e-01 9.281627e-01 9.076642e-01 8.794785e-01 8.453793e-01 8.154810e-01 9.102474e-01 7.899469e-01

1940 9.939833e-01 9.775782e-01 9.523603e-01 9.279751e-01 9.076183e-01 8.794132e-01 8.452041e-01 8.152311e-01 9.103099e-01 7.897106e-01

1941 9.939014e-01 9.773659e-01 9.520928e-01 9.277801e-01 9.075760e-01 8.793449e-01 8.450236e-01 8.149752e-01 9.103734e-01 7.894694e-01

1942 9.938136e-01 9.771394e-01 9.518102e-01 9.275778e-01 9.075377e-01 8.792736e-01 8.448378e-01 8.147135e-01 9.104378e-01 7.892235e-01

1943 9.937196e-01 9.768979e-01 9.515120e-01 9.273682e-01 9.075043e-01 8.791992e-01 8.446466e-01 8.144459e-01 9.105032e-01 7.889729e-01

1944 9.936188e-01 9.766405e-01 9.511974e-01 9.271514e-01 9.074764e-01 8.791216e-01 8.444500e-01 8.141725e-01 9.105694e-01 7.887178e-01

1945 9.935108e-01 9.763663e-01 9.508659e-01 9.269275e-01 9.074547e-01 8.790408e-01 8.442480e-01 8.138936e-01 9.106366e-01 7.884585e-01

1946 9.933951e-01 9.760743e-01 9.505168e-01 9.266966e-01 9.074402e-01 8.789566e-01 8.440406e-01 8.136091e-01 9.107047e-01 7.881949e-01

1947 9.932711e-01 9.757634e-01 9.501496e-01 9.264590e-01 9.074337e-01 8.788690e-01 8.438278e-01 8.133192e-01 9.107736e-01 7.879273e-01

1948 9.931382e-01 9.754327e-01 9.497637e-01 9.262150e-01 9.074361e-01 8.787779e-01 8.436097e-01 8.130240e-01 9.108434e-01 7.876560e-01

1949 9.929959e-01 9.750811e-01 9.493586e-01 9.259649e-01 9.074483e-01 8.786834e-01 8.433861e-01 8.127238e-01 9.109140e-01 7.873811e-01

1950 9.928433e-01 9.747074e-01 9.489336e-01 9.257091e-01 9.074712e-01 8.785852e-01 8.431573e-01 8.124187e-01 9.109854e-01 7.871028e-01

1951 9.926800e-01 9.743105e-01 9.484885e-01 9.254481e-01 9.075060e-01 8.784834e-01 8.429233e-01 8.121089e-01 9.110575e-01 7.868214e-01

1952 9.925051e-01 9.738892e-01 9.480228e-01 9.251824e-01 9.075536e-01 8.783778e-01 8.426841e-01 8.117946e-01 9.111304e-01 7.865370e-01

1953 9.923177e-01 9.734424e-01 9.475362e-01 9.249127e-01 9.076149e-01 8.782686e-01 8.424398e-01 8.114760e-01 9.112040e-01 7.862501e-01

1954 9.921172e-01 9.729689e-01 9.470283e-01 9.246397e-01 9.076911e-01 8.781555e-01 8.421905e-01 8.111534e-01 9.112782e-01 7.859608e-01

1955 9.919026e-01 9.724674e-01 9.464991e-01 9.243641e-01 9.077831e-01 8.780387e-01 8.419364e-01 8.108270e-01 9.113531e-01 7.856694e-01

1956 9.916729e-01 9.719369e-01 9.459484e-01 9.240867e-01 9.078920e-01 8.779179e-01 8.416776e-01 8.104972e-01 9.114285e-01 7.853763e-01

1957 9.914272e-01 9.713759e-01 9.453763e-01 9.238085e-01 9.080185e-01 8.777934e-01 8.414141e-01 8.101642e-01 9.115045e-01 7.850816e-01

1958 9.911645e-01 9.707835e-01 9.447828e-01 9.235305e-01 9.081637e-01 8.776649e-01 8.411462e-01 8.098282e-01 9.115809e-01 7.847856e-01

1959 9.908836e-01 9.701585e-01 9.441683e-01 9.232536e-01 9.083283e-01 8.775325e-01 8.408740e-01 8.094896e-01 9.116578e-01 7.844888e-01

1960 9.905834e-01 9.694997e-01 9.435330e-01 9.229790e-01 9.085131e-01 8.773962e-01 8.405977e-01 8.091488e-01 9.117351e-01 7.841913e-01

1961 9.902628e-01 9.688062e-01 9.428775e-01 9.227078e-01 9.087187e-01 8.772561e-01 8.403176e-01 8.088059e-01 9.118127e-01 7.838935e-01

1962 9.899203e-01 9.680768e-01 9.422025e-01 9.224412e-01 9.089457e-01 8.771121e-01 8.400337e-01 8.084615e-01 9.118906e-01 7.835957e-01

1963 9.895549e-01 9.673108e-01 9.415087e-01 9.221803e-01 9.091945e-01 8.769642e-01 8.397464e-01 8.081157e-01 9.119687e-01 7.832982e-01

1964 9.891650e-01 9.665073e-01 9.407971e-01 9.219265e-01 9.094655e-01 8.768126e-01 8.394559e-01 8.077690e-01 9.120469e-01 7.830012e-01

1965 9.887493e-01 9.656656e-01 9.400688e-01 9.216809e-01 9.097587e-01 8.766572e-01 8.391624e-01 8.074217e-01 9.121252e-01 7.827052e-01

1966 9.883063e-01 9.647851e-01 9.393251e-01 9.214448e-01 9.100741e-01 8.764981e-01 8.388662e-01 8.070741e-01 9.122035e-01 7.824104e-01

1967 9.878345e-01 9.638655e-01 9.385675e-01 9.212193e-01 9.104118e-01 8.763353e-01 8.385675e-01 8.067266e-01 9.122818e-01 7.821170e-01

1968 9.873324e-01 9.629064e-01 9.377974e-01 9.210055e-01 9.107712e-01 8.761690e-01 8.382666e-01 8.063796e-01 9.123599e-01 7.818254e-01

1969 9.867984e-01 9.619078e-01 9.370166e-01 9.208047e-01 9.111521e-01 8.759992e-01 8.379638e-01 8.060333e-01 9.124379e-01 7.815358e-01

1970 9.862308e-01 9.608697e-01 9.362270e-01 9.206177e-01 9.115538e-01 8.758260e-01 8.376593e-01 8.056881e-01 9.125155e-01 7.812486e-01

1971 9.856281e-01 9.597926e-01 9.354305e-01 9.204456e-01 9.119755e-01 8.756495e-01 8.373536e-01 8.053445e-01 9.125928e-01 7.809638e-01

1972 9.849886e-01 9.586769e-01 9.346292e-01 9.202891e-01 9.124164e-01 8.754699e-01 8.370468e-01 8.050026e-01 9.126696e-01 7.806819e-01

1973 9.843107e-01 9.575234e-01 9.338253e-01 9.201490e-01 9.128755e-01 8.752872e-01 8.367393e-01 8.046628e-01 9.127460e-01 7.804031e-01

1974 9.835926e-01 9.563330e-01 9.330211e-01 9.200260e-01 9.133515e-01 8.751016e-01 8.364313e-01 8.043255e-01 9.128217e-01 7.801275e-01

1975 9.828327e-01 9.551072e-01 9.322187e-01 9.199204e-01 9.138432e-01 8.749132e-01 8.361232e-01 8.039909e-01 9.128967e-01 7.798554e-01

1976 9.820295e-01 9.538474e-01 9.314206e-01 9.198327e-01 9.143493e-01 8.747222e-01 8.358153e-01 8.036593e-01 9.129710e-01 7.795869e-01

1977 9.811814e-01 9.525554e-01 9.306289e-01 9.197630e-01 9.148684e-01 8.745287e-01 8.355079e-01 8.033311e-01 9.130444e-01 7.793223e-01

1978 9.802868e-01 9.512333e-01 9.298461e-01 9.197115e-01 9.153989e-01 8.743329e-01 8.352012e-01 8.030064e-01 9.131169e-01 7.790618e-01

1979 9.793443e-01 9.498833e-01 9.290743e-01 9.196780e-01 9.159393e-01 8.741350e-01 8.348956e-01 8.026856e-01 9.131884e-01 7.788054e-01

1980 9.783526e-01 9.485081e-01 9.283157e-01 9.196625e-01 9.164880e-01 8.739351e-01 8.345913e-01 8.023688e-01 9.132588e-01 7.785533e-01

1981 9.773104e-01 9.471103e-01 9.275724e-01 9.196644e-01 9.170434e-01 8.737334e-01 8.342887e-01 8.020563e-01 9.133280e-01 7.783057e-01

1982 9.773103e-01 9.471101e-01 9.275722e-01 9.196643e-01 9.170434e-01 8.737346e-01 8.342824e-01 8.020439e-01 9.133367e-01 7.782899e-01

1983 9.773102e-01 9.471099e-01 9.275721e-01 9.196641e-01 9.170433e-01 8.737352e-01 8.342754e-01 8.020308e-01 9.133449e-01 7.782734e-01

1984 9.773101e-01 9.471097e-01 9.275719e-01 9.196640e-01 9.170432e-01 8.737352e-01 8.342677e-01 8.020170e-01 9.133525e-01 7.782561e-01

1985 9.773100e-01 9.471096e-01 9.275717e-01 9.196638e-01 9.170431e-01 8.737346e-01 8.342594e-01 8.020025e-01 9.133596e-01 7.782381e-01

1986 9.773099e-01 9.471093e-01 9.275715e-01 9.196637e-01 9.170431e-01 8.737334e-01 8.342504e-01 8.019872e-01 9.133662e-01 7.782193e-01

1987 9.773097e-01 9.471091e-01 9.275713e-01 9.196635e-01 9.170430e-01 8.737316e-01 8.342407e-01 8.019712e-01 9.133723e-01 7.781998e-01

1988 9.773096e-01 9.471089e-01 9.275711e-01 9.196634e-01 9.170429e-01 8.737292e-01 8.342304e-01 8.019544e-01 9.133779e-01 7.781795e-01

1989 9.773094e-01 9.471087e-01 9.275709e-01 9.196632e-01 9.170428e-01 8.737262e-01 8.342194e-01 8.019369e-01 9.133830e-01 7.781584e-01

1990 9.773093e-01 9.471084e-01 9.275706e-01 9.196631e-01 9.170428e-01 8.737226e-01 8.342078e-01 8.019186e-01 9.133876e-01 7.781365e-01

1991 9.773091e-01 9.471082e-01 9.275704e-01 9.196629e-01 9.170427e-01 8.737185e-01 8.341954e-01 8.018996e-01 9.133918e-01 7.781138e-01

1992 9.773090e-01 9.471079e-01 9.275702e-01 9.196628e-01 9.170426e-01 8.737137e-01 8.341824e-01 8.018798e-01 9.133955e-01 7.780902e-01

1993 9.773088e-01 9.471077e-01 9.275699e-01 9.196626e-01 9.170426e-01 8.737084e-01 8.341687e-01 8.018591e-01 9.133987e-01 7.780658e-01

1994 9.773086e-01 9.471074e-01 9.275697e-01 9.196624e-01 9.170425e-01 8.737025e-01 8.341544e-01 8.018377e-01 9.134015e-01 7.780405e-01

1995 9.773084e-01 9.471071e-01 9.275694e-01 9.196623e-01 9.170425e-01 8.736961e-01 8.341393e-01 8.018154e-01 9.134038e-01 7.780144e-01

1996 9.773082e-01 9.471068e-01 9.275691e-01 9.196621e-01 9.170424e-01 8.736890e-01 8.341235e-01 8.017923e-01 9.134057e-01 7.779873e-01

1997 9.773080e-01 9.471064e-01 9.275688e-01 9.196619e-01 9.170424e-01 8.736814e-01 8.341069e-01 8.017683e-01 9.134072e-01 7.779593e-01

1998 9.773078e-01 9.471061e-01 9.275685e-01 9.196618e-01 9.170423e-01 8.736732e-01 8.340897e-01 8.017435e-01 9.134082e-01 7.779304e-01

1999 9.773076e-01 9.471057e-01 9.275682e-01 9.196616e-01 9.170423e-01 8.736644e-01 8.340717e-01 8.017177e-01 9.134089e-01 7.779005e-01

2000 9.773073e-01 9.471054e-01 9.275679e-01 9.196614e-01 9.170423e-01 8.736550e-01 8.340529e-01 8.016910e-01 9.134090e-01 7.778695e-01

2001 9.773071e-01 9.471050e-01 9.275675e-01 9.196612e-01 9.170422e-01 8.736450e-01 8.340334e-01 8.016634e-01 9.134088e-01 7.778376e-01

2002 9.773068e-01 9.471046e-01 9.275672e-01 9.196610e-01 9.170422e-01 8.736344e-01 8.340130e-01 8.016348e-01 9.134082e-01 7.778046e-01

2003 9.773065e-01 9.471042e-01 9.275668e-01 9.196608e-01 9.170422e-01 8.736232e-01 8.339919e-01 8.016052e-01 9.134072e-01 7.777705e-01

2004 9.773063e-01 9.471037e-01 9.275664e-01 9.196606e-01 9.170422e-01 8.736114e-01 8.339699e-01 8.015746e-01 9.134057e-01 7.777353e-01

2005 9.773059e-01 9.471033e-01 9.275661e-01 9.196604e-01 9.170422e-01 8.735989e-01 8.339471e-01 8.015430e-01 9.134039e-01 7.776989e-01

2006 9.773056e-01 9.471028e-01 9.275656e-01 9.196602e-01 9.170422e-01 8.735858e-01 8.339234e-01 8.015103e-01 9.134016e-01 7.776614e-01

2007 9.773053e-01 9.471023e-01 9.275652e-01 9.196600e-01 9.170422e-01 8.735721e-01 8.338989e-01 8.014765e-01 9.133990e-01 7.776227e-01

2008 9.773050e-01 9.471018e-01 9.275648e-01 9.196598e-01 9.170422e-01 8.735577e-01 8.338734e-01 8.014415e-01 9.133959e-01 7.775827e-01

2009 9.773046e-01 9.471013e-01 9.275643e-01 9.196596e-01 9.170422e-01 8.735427e-01 8.338471e-01 8.014054e-01 9.133925e-01 7.775414e-01

2010 9.773042e-01 9.471007e-01 9.275639e-01 9.196594e-01 9.170423e-01 8.735270e-01 8.338197e-01 8.013681e-01 9.133886e-01 7.774988e-01

2011 9.773038e-01 9.471001e-01 9.275634e-01 9.196591e-01 9.170423e-01 8.735106e-01 8.337914e-01 8.013296e-01 9.133843e-01 7.774548e-01

2012 9.773034e-01 9.470995e-01 9.275629e-01 9.196589e-01 9.170424e-01 8.734935e-01 8.337621e-01 8.012898e-01 9.133797e-01 7.774094e-01

2013 9.773029e-01 9.470989e-01 9.275624e-01 9.196587e-01 9.170424e-01 8.734757e-01 8.337318e-01 8.012487e-01 9.133746e-01 7.773626e-01

2014 9.773025e-01 9.470982e-01 9.275618e-01 9.196585e-01 9.170425e-01 8.734571e-01 8.337004e-01 8.012062e-01 9.133691e-01 7.773143e-01

2015 9.773020e-01 9.470975e-01 9.275613e-01 9.196582e-01 9.170426e-01 8.734378e-01 8.336679e-01 8.011624e-01 9.133632e-01 7.772644e-01

2016 9.773015e-01 9.470968e-01 9.275607e-01 9.196580e-01 9.170427e-01 8.734178e-01 8.336343e-01 8.011171e-01 9.133569e-01 7.772129e-01

2017 9.773010e-01 9.470960e-01 9.275601e-01 9.196578e-01 9.170428e-01 8.733969e-01 8.335996e-01 8.010703e-01 9.133502e-01 7.771598e-01

2018 9.773004e-01 9.470952e-01 9.275595e-01 9.196575e-01 9.170429e-01 8.733753e-01 8.335636e-01 8.010221e-01 9.133430e-01 7.771050e-01

2019 9.772998e-01 9.470944e-01 9.275589e-01 9.196573e-01 9.170431e-01 8.733528e-01 8.335264e-01 8.009722e-01 9.133354e-01 7.770484e-01

2020 9.772992e-01 9.470936e-01 9.275582e-01 9.196571e-01 9.170432e-01 8.733295e-01 8.334880e-01 8.009208e-01 9.133274e-01 7.769900e-01

2021 9.772985e-01 9.470927e-01 9.275575e-01 9.196568e-01 9.170434e-01 8.733053e-01 8.334483e-01 8.008676e-01 9.133190e-01 7.769298e-01

2022 9.772978e-01 9.470917e-01 9.275569e-01 9.196566e-01 9.170436e-01 8.732802e-01 8.334072e-01 8.008128e-01 9.133101e-01 7.768676e-01

2023 9.772971e-01 9.470907e-01 9.275561e-01 9.196564e-01 9.170439e-01 8.732542e-01 8.333648e-01 8.007561e-01 9.133007e-01 7.768034e-01

2024 9.772964e-01 9.470897e-01 9.275554e-01 9.196562e-01 9.170441e-01 8.732272e-01 8.333209e-01 8.006976e-01 9.132909e-01 7.767372e-01

2025 9.772956e-01 9.470886e-01 9.275546e-01 9.196560e-01 9.170444e-01 8.731993e-01 8.332755e-01 8.006373e-01 9.132806e-01 7.766689e-01

2026 9.772947e-01 9.470875e-01 9.275539e-01 9.196558e-01 9.170448e-01 8.731704e-01 8.332287e-01 8.005749e-01 9.132698e-01 7.765983e-01

2027 9.772939e-01 9.470864e-01 9.275530e-01 9.196556e-01 9.170451e-01 8.731404e-01 8.331802e-01 8.005105e-01 9.132585e-01 7.765255e-01

2028 9.772929e-01 9.470851e-01 9.275522e-01 9.196554e-01 9.170455e-01 8.731094e-01 8.331302e-01 8.004441e-01 9.132467e-01 7.764504e-01

2029 9.772920e-01 9.470838e-01 9.275513e-01 9.196552e-01 9.170460e-01 8.730773e-01 8.330784e-01 8.003754e-01 9.132344e-01 7.763728e-01

2030 9.772910e-01 9.470825e-01 9.275505e-01 9.196551e-01 9.170465e-01 8.730441e-01 8.330249e-01 8.003045e-01 9.132216e-01 7.762927e-01

2031 9.772899e-01 9.470811e-01 9.275495e-01 9.196550e-01 9.170470e-01 8.730097e-01 8.329697e-01 8.002314e-01 9.132083e-01 7.762101e-01

2032 9.772888e-01 9.470797e-01 9.275486e-01 9.196548e-01 9.170476e-01 8.729741e-01 8.329126e-01 8.001558e-01 9.131943e-01 7.761247e-01

2033 9.772876e-01 9.470781e-01 9.275476e-01 9.196547e-01 9.170482e-01 8.729372e-01 8.328535e-01 8.000777e-01 9.131799e-01 7.760366e-01

2034 9.772863e-01 9.470765e-01 9.275466e-01 9.196547e-01 9.170489e-01 8.728991e-01 8.327925e-01 7.999971e-01 9.131648e-01 7.759457e-01

2035 9.772850e-01 9.470749e-01 9.275456e-01 9.196546e-01 9.170497e-01 8.728596e-01 8.327295e-01 7.999139e-01 9.131492e-01 7.758518e-01

2036 9.772836e-01 9.470731e-01 9.275445e-01 9.196546e-01 9.170505e-01 8.728188e-01 8.326643e-01 7.998279e-01 9.131329e-01 7.757549e-01

2037 9.772822e-01 9.470713e-01 9.275434e-01 9.196546e-01 9.170514e-01 8.727766e-01 8.325970e-01 7.997391e-01 9.131160e-01 7.756548e-01

2038 9.772806e-01 9.470694e-01 9.275423e-01 9.196546e-01 9.170524e-01 8.727329e-01 8.325274e-01 7.996474e-01 9.130985e-01 7.755515e-01

2039 9.772790e-01 9.470674e-01 9.275412e-01 9.196547e-01 9.170535e-01 8.726876e-01 8.324554e-01 7.995527e-01 9.130802e-01 7.754448e-01

2040 9.772773e-01 9.470653e-01 9.275400e-01 9.196549e-01 9.170547e-01 8.726409e-01 8.323811e-01 7.994548e-01 9.130614e-01 7.753346e-01

2041 9.772755e-01 9.470631e-01 9.275388e-01 9.196550e-01 9.170559e-01 8.725925e-01 8.323042e-01 7.993538e-01 9.130418e-01 7.752209e-01

2042 9.772736e-01 9.470608e-01 9.275375e-01 9.196552e-01 9.170573e-01 8.725425e-01 8.322247e-01 7.992494e-01 9.130214e-01 7.751035e-01

2043 9.772717e-01 9.470584e-01 9.275363e-01 9.196555e-01 9.170588e-01 8.724907e-01 8.321426e-01 7.991415e-01 9.130003e-01 7.749822e-01

2044 9.772696e-01 9.470559e-01 9.275350e-01 9.196559e-01 9.170604e-01 8.724371e-01 8.320577e-01 7.990302e-01 9.129785e-01 7.748570e-01

2045 9.772674e-01 9.470533e-01 9.275336e-01 9.196563e-01 9.170621e-01 8.723817e-01 8.319700e-01 7.989151e-01 9.129558e-01 7.747277e-01

2046 9.772650e-01 9.470506e-01 9.275323e-01 9.196567e-01 9.170640e-01 8.723244e-01 8.318793e-01 7.987962e-01 9.129323e-01 7.745941e-01

2047 9.772626e-01 9.470477e-01 9.275309e-01 9.196573e-01 9.170661e-01 8.722651e-01 8.317855e-01 7.986734e-01 9.129080e-01 7.744563e-01

2048 9.772600e-01 9.470447e-01 9.275295e-01 9.196579e-01 9.170683e-01 8.722038e-01 8.316885e-01 7.985466e-01 9.128828e-01 7.743139e-01

2049 9.772573e-01 9.470416e-01 9.275280e-01 9.196587e-01 9.170706e-01 8.721403e-01 8.315883e-01 7.984155e-01 9.128567e-01 7.741669e-01

2050 9.772544e-01 9.470383e-01 9.275265e-01 9.196595e-01 9.170732e-01 8.720747e-01 8.314847e-01 7.982801e-01 9.128296e-01 7.740151e-01

2051 9.772513e-01 9.470349e-01 9.275251e-01 9.196605e-01 9.170760e-01 8.720068e-01 8.313775e-01 7.981403e-01 9.128016e-01 7.738583e-01

2052 9.772481e-01 9.470313e-01 9.275235e-01 9.196616e-01 9.170790e-01 8.719365e-01 8.312668e-01 7.979958e-01 9.127725e-01 7.736965e-01

2053 9.772447e-01 9.470275e-01 9.275220e-01 9.196628e-01 9.170822e-01 8.718638e-01 8.311523e-01 7.978465e-01 9.127425e-01 7.735294e-01

2054 9.772412e-01 9.470236e-01 9.275205e-01 9.196642e-01 9.170857e-01 8.717886e-01 8.310339e-01 7.976923e-01 9.127113e-01 7.733568e-01

2055 9.772374e-01 9.470194e-01 9.275189e-01 9.196657e-01 9.170894e-01 8.717108e-01 8.309115e-01 7.975331e-01 9.126791e-01 7.731787e-01

2056 9.772334e-01 9.470151e-01 9.275173e-01 9.196674e-01 9.170935e-01 8.716303e-01 8.307849e-01 7.973685e-01 9.126457e-01 7.729948e-01

2057 9.772292e-01 9.470106e-01 9.275158e-01 9.196693e-01 9.170979e-01 8.715470e-01 8.306541e-01 7.971985e-01 9.126111e-01 7.728049e-01

2058 9.772248e-01 9.470059e-01 9.275142e-01 9.196714e-01 9.171026e-01 8.714609e-01 8.305188e-01 7.970230e-01 9.125753e-01 7.726089e-01

2059 9.772201e-01 9.470010e-01 9.275126e-01 9.196738e-01 9.171076e-01 8.713717e-01 8.303789e-01 7.968416e-01 9.125382e-01 7.724066e-01

2060 9.772152e-01 9.469958e-01 9.275110e-01 9.196764e-01 9.171131e-01 8.712794e-01 8.302343e-01 7.966543e-01 9.124998e-01 7.721978e-01

2061 9.772099e-01 9.469904e-01 9.275095e-01 9.196792e-01 9.171190e-01 8.711840e-01 8.300848e-01 7.964608e-01 9.124601e-01 7.719823e-01

2062 9.772044e-01 9.469847e-01 9.275080e-01 9.196823e-01 9.171253e-01 8.710852e-01 8.299303e-01 7.962610e-01 9.124189e-01 7.717598e-01

2063 9.771986e-01 9.469788e-01 9.275065e-01 9.196858e-01 9.171321e-01 8.709830e-01 8.297705e-01 7.960546e-01 9.123763e-01 7.715303e-01

2064 9.771924e-01 9.469726e-01 9.275050e-01 9.196896e-01 9.171394e-01 8.708773e-01 8.296053e-01 7.958415e-01 9.123322e-01 7.712934e-01

2065 9.771858e-01 9.469661e-01 9.275036e-01 9.196937e-01 9.171473e-01 8.707679e-01 8.294345e-01 7.956215e-01 9.122865e-01 7.710491e-01

2066 9.771789e-01 9.469593e-01 9.275022e-01 9.196983e-01 9.171557e-01 8.706546e-01 8.292579e-01 7.953943e-01 9.122392e-01 7.707970e-01

2067 9.771716e-01 9.469522e-01 9.275009e-01 9.197033e-01 9.171649e-01 8.705375e-01 8.290754e-01 7.951597e-01 9.121902e-01 7.705370e-01

2068 9.771639e-01 9.469448e-01 9.274997e-01 9.197087e-01 9.171747e-01 8.704163e-01 8.288868e-01 7.949176e-01 9.121395e-01 7.702688e-01

2069 9.771558e-01 9.469370e-01 9.274986e-01 9.197147e-01 9.171852e-01 8.702909e-01 8.286918e-01 7.946677e-01 9.120870e-01 7.699922e-01

2070 9.771471e-01 9.469288e-01 9.274977e-01 9.197212e-01 9.171966e-01 8.701611e-01 8.284903e-01 7.944097e-01 9.120327e-01 7.697071e-01

2071 9.771380e-01 9.469203e-01 9.274968e-01 9.197283e-01 9.172087e-01 8.700268e-01 8.282821e-01 7.941435e-01 9.119764e-01 7.694131e-01

2072 9.771283e-01 9.469114e-01 9.274961e-01 9.197360e-01 9.172218e-01 8.698879e-01 8.280669e-01 7.938688e-01 9.119181e-01 7.691101e-01

2073 9.771181e-01 9.469021e-01 9.274956e-01 9.197444e-01 9.172359e-01 8.697442e-01 8.278445e-01 7.935853e-01 9.118578e-01 7.687979e-01

2074 9.771072e-01 9.468924e-01 9.274952e-01 9.197536e-01 9.172511e-01 8.695954e-01 8.276148e-01 7.932930e-01 9.117953e-01 7.684761e-01

2075 9.770957e-01 9.468822e-01 9.274951e-01 9.197636e-01 9.172673e-01 8.694416e-01 8.273774e-01 7.929914e-01 9.117307e-01 7.681447e-01

2076 9.770836e-01 9.468715e-01 9.274953e-01 9.197745e-01 9.172848e-01 8.692824e-01 8.271322e-01 7.926804e-01 9.116637e-01 7.678033e-01

2077 9.770707e-01 9.468604e-01 9.274957e-01 9.197863e-01 9.173036e-01 8.691178e-01 8.268789e-01 7.923598e-01 9.115944e-01 7.674518e-01

2078 9.770571e-01 9.468488e-01 9.274964e-01 9.197991e-01 9.173238e-01 8.689475e-01 8.266173e-01 7.920292e-01 9.115226e-01 7.670899e-01

2079 9.770427e-01 9.468366e-01 9.274975e-01 9.198131e-01 9.173455e-01 8.687713e-01 8.263472e-01 7.916885e-01 9.114483e-01 7.667175e-01

2080 9.770274e-01 9.468239e-01 9.274990e-01 9.198282e-01 9.173688e-01 8.685891e-01 8.260683e-01 7.913375e-01 9.113714e-01 7.663343e-01

2081 9.770112e-01 9.468107e-01 9.275009e-01 9.198446e-01 9.173938e-01 8.684006e-01 8.257804e-01 7.909758e-01 9.112917e-01 7.659401e-01

2082 9.769940e-01 9.467968e-01 9.275034e-01 9.198624e-01 9.174207e-01 8.682057e-01 8.254832e-01 7.906032e-01 9.112093e-01 7.655346e-01

2083 9.769758e-01 9.467824e-01 9.275063e-01 9.198817e-01 9.174496e-01 8.680042e-01 8.251765e-01 7.902196e-01 9.111239e-01 7.651179e-01

2084 9.769565e-01 9.467673e-01 9.275099e-01 9.199026e-01 9.174805e-01 8.677958e-01 8.248600e-01 7.898246e-01 9.110355e-01 7.646895e-01

2085 9.769361e-01 9.467515e-01 9.275142e-01 9.199252e-01 9.175138e-01 8.675803e-01 8.245335e-01 7.894181e-01 9.109440e-01 7.642494e-01

2086 9.769144e-01 9.467351e-01 9.275191e-01 9.199498e-01 9.175496e-01 8.673575e-01 8.241967e-01 7.889998e-01 9.108493e-01 7.637974e-01

2087 9.768914e-01 9.467179e-01 9.275249e-01 9.199763e-01 9.175879e-01 8.671273e-01 8.238493e-01 7.885696e-01 9.107513e-01 7.633333e-01

2088 9.768671e-01 9.467001e-01 9.275316e-01 9.200051e-01 9.176291e-01 8.668893e-01 8.234912e-01 7.881272e-01 9.106498e-01 7.628571e-01

2089 9.768412e-01 9.466815e-01 9.275393e-01 9.200362e-01 9.176733e-01 8.666433e-01 8.231221e-01 7.876724e-01 9.105447e-01 7.623685e-01

2090 9.768138e-01 9.466621e-01 9.275481e-01 9.200698e-01 9.177207e-01 8.663892e-01 8.227417e-01 7.872050e-01 9.104360e-01 7.618674e-01

2091 9.767848e-01 9.466419e-01 9.275581e-01 9.201062e-01 9.177716e-01 8.661266e-01 8.223498e-01 7.867249e-01 9.103234e-01 7.613538e-01

2092 9.767540e-01 9.466209e-01 9.275694e-01 9.201455e-01 9.178262e-01 8.658554e-01 8.219461e-01 7.862318e-01 9.102070e-01 7.608275e-01

2093 9.767213e-01 9.465991e-01 9.275821e-01 9.201879e-01 9.178848e-01 8.655752e-01 8.215304e-01 7.857257e-01 9.100864e-01 7.602885e-01

2094 9.766866e-01 9.465763e-01 9.275964e-01 9.202338e-01 9.179476e-01 8.652859e-01 8.211025e-01 7.852064e-01 9.099617e-01 7.597368e-01

2095 9.766498e-01 9.465527e-01 9.276124e-01 9.202834e-01 9.180150e-01 8.649872e-01 8.206621e-01 7.846738e-01 9.098326e-01 7.591724e-01

2096 9.766108e-01 9.465283e-01 9.276303e-01 9.203368e-01 9.180873e-01 8.646788e-01 8.202090e-01 7.841277e-01 9.096990e-01 7.585951e-01

2097 9.765694e-01 9.465028e-01 9.276503e-01 9.203945e-01 9.181647e-01 8.643605e-01 8.197431e-01 7.835681e-01 9.095608e-01 7.580051e-01

2098 9.765254e-01 9.464765e-01 9.276725e-01 9.204568e-01 9.182477e-01 8.640321e-01 8.192641e-01 7.829949e-01 9.094178e-01 7.574024e-01

2099 9.764788e-01 9.464492e-01 9.276972e-01 9.205240e-01 9.183367e-01 8.636933e-01 8.187718e-01 7.824081e-01 9.092699e-01 7.567870e-01

2100 9.764293e-01 9.464210e-01 9.277246e-01 9.205963e-01 9.184321e-01 8.633438e-01 8.182661e-01 7.818077e-01 9.091170e-01 7.561592e-01

2101 9.763768e-01 9.463917e-01 9.277548e-01 9.206744e-01 9.185342e-01 8.629834e-01 8.177468e-01 7.811935e-01 9.089588e-01 7.555189e-01

2102 9.763211e-01 9.463616e-01 9.277883e-01 9.207584e-01 9.186436e-01 8.626119e-01 8.172137e-01 7.805658e-01 9.087951e-01 7.548664e-01

2103 9.762620e-01 9.463304e-01 9.278252e-01 9.208490e-01 9.187607e-01 8.622291e-01 8.166667e-01 7.799245e-01 9.086259e-01 7.542018e-01

2104 9.761993e-01 9.462984e-01 9.278658e-01 9.209465e-01 9.188861e-01 8.618346e-01 8.161058e-01 7.792696e-01 9.084510e-01 7.535254e-01

2105 9.761328e-01 9.462653e-01 9.279105e-01 9.210514e-01 9.190203e-01 8.614282e-01 8.155307e-01 7.786014e-01 9.082702e-01 7.528374e-01

2106 9.760622e-01 9.462314e-01 9.279596e-01 9.211643e-01 9.191638e-01 8.610098e-01 8.149415e-01 7.779200e-01 9.080833e-01 7.521382e-01

2107 9.759873e-01 9.461965e-01 9.280134e-01 9.212857e-01 9.193174e-01 8.605791e-01 8.143382e-01 7.772255e-01 9.078901e-01 7.514279e-01

2108 9.759079e-01 9.461608e-01 9.280725e-01 9.214162e-01 9.194815e-01 8.601359e-01 8.137205e-01 7.765182e-01 9.076905e-01 7.507071e-01

2109 9.758236e-01 9.461243e-01 9.281371e-01 9.215564e-01 9.196570e-01 8.596800e-01 8.130887e-01 7.757982e-01 9.074843e-01 7.499760e-01

2110 9.757343e-01 9.460870e-01 9.282078e-01 9.217071e-01 9.198445e-01 8.592111e-01 8.124426e-01 7.750659e-01 9.072713e-01 7.492351e-01

2111 9.756395e-01 9.460490e-01 9.282850e-01 9.218688e-01 9.200448e-01 8.587292e-01 8.117825e-01 7.743216e-01 9.070513e-01 7.484849e-01

2112 9.755390e-01 9.460104e-01 9.283693e-01 9.220424e-01 9.202586e-01 8.582340e-01 8.111082e-01 7.735656e-01 9.068242e-01 7.477258e-01

2113 9.754325e-01 9.459712e-01 9.284611e-01 9.222285e-01 9.204868e-01 8.577254e-01 8.104200e-01 7.727983e-01 9.065896e-01 7.469583e-01

2114 9.753196e-01 9.459316e-01 9.285611e-01 9.224281e-01 9.207302e-01 8.572033e-01 8.097180e-01 7.720201e-01 9.063476e-01 7.461831e-01

2115 9.751999e-01 9.458917e-01 9.286700e-01 9.226420e-01 9.209898e-01 8.566675e-01 8.090024e-01 7.712315e-01 9.060978e-01 7.454006e-01

2116 9.750731e-01 9.458516e-01 9.287883e-01 9.228711e-01 9.212664e-01 8.561179e-01 8.082734e-01 7.704330e-01 9.058401e-01 7.446116e-01

2117 9.749388e-01 9.458115e-01 9.289167e-01 9.231163e-01 9.215612e-01 8.555544e-01 8.075312e-01 7.696250e-01 9.055742e-01 7.438166e-01

2118 9.747966e-01 9.457715e-01 9.290561e-01 9.233786e-01 9.218750e-01 8.549770e-01 8.067762e-01 7.688083e-01 9.053001e-01 7.430163e-01

2119 9.746460e-01 9.457319e-01 9.292071e-01 9.236590e-01 9.222089e-01 8.543856e-01 8.060086e-01 7.679832e-01 9.050176e-01 7.422115e-01

2120 9.744866e-01 9.456929e-01 9.293706e-01 9.239587e-01 9.225640e-01 8.537802e-01 8.052289e-01 7.671506e-01 9.047263e-01 7.414027e-01

2121 9.743180e-01 9.456547e-01 9.295475e-01 9.242787e-01 9.229415e-01 8.531608e-01 8.044373e-01 7.663110e-01 9.044263e-01 7.405909e-01

2122 9.741396e-01 9.456176e-01 9.297386e-01 9.246201e-01 9.233425e-01 8.525275e-01 8.036344e-01 7.654651e-01 9.041172e-01 7.397766e-01

2123 9.739510e-01 9.455818e-01 9.299448e-01 9.249842e-01 9.237682e-01 8.518802e-01 8.028206e-01 7.646136e-01 9.037989e-01 7.389608e-01

2124 9.737517e-01 9.455478e-01 9.301673e-01 9.253721e-01 9.242198e-01 8.512191e-01 8.019964e-01 7.637574e-01 9.034713e-01 7.381441e-01

2125 9.735411e-01 9.455159e-01 9.304069e-01 9.257852e-01 9.246986e-01 8.505444e-01 8.011624e-01 7.628970e-01 9.031342e-01 7.373274e-01

2126 9.733188e-01 9.454864e-01 9.306647e-01 9.262247e-01 9.252057e-01 8.498561e-01 8.003191e-01 7.620334e-01 9.027875e-01 7.365114e-01

2127 9.730841e-01 9.454599e-01 9.309419e-01 9.266919e-01 9.257426e-01 8.491544e-01 7.994672e-01 7.611674e-01 9.024309e-01 7.356970e-01

2128 9.728365e-01 9.454367e-01 9.312395e-01 9.271881e-01 9.263104e-01 8.484395e-01 7.986072e-01 7.602997e-01 9.020644e-01 7.348850e-01

2129 9.725755e-01 9.454173e-01 9.315587e-01 9.277147e-01 9.269105e-01 8.477118e-01 7.977400e-01 7.594311e-01 9.016878e-01 7.340761e-01

2130 9.723005e-01 9.454024e-01 9.319007e-01 9.282730e-01 9.275442e-01 8.469714e-01 7.968661e-01 7.585626e-01 9.013011e-01 7.332711e-01

2131 9.720108e-01 9.453924e-01 9.322666e-01 9.288644e-01 9.282127e-01 8.462188e-01 7.959863e-01 7.576950e-01 9.009040e-01 7.324709e-01

2132 9.717060e-01 9.453879e-01 9.326576e-01 9.294902e-01 9.289174e-01 8.454541e-01 7.951013e-01 7.568290e-01 9.004966e-01 7.316763e-01

2133 9.713855e-01 9.453897e-01 9.330751e-01 9.301518e-01 9.296594e-01 8.446779e-01 7.942121e-01 7.559656e-01 9.000787e-01 7.308878e-01

2134 9.710486e-01 9.453984e-01 9.335201e-01 9.308505e-01 9.304400e-01 8.438906e-01 7.933192e-01 7.551057e-01 8.996502e-01 7.301064e-01

2135 9.706948e-01 9.454147e-01 9.339939e-01 9.315875e-01 9.312604e-01 8.430926e-01 7.924237e-01 7.542499e-01 8.992112e-01 7.293326e-01

2136 9.703236e-01 9.454394e-01 9.344977e-01 9.323641e-01 9.321217e-01 8.422843e-01 7.915263e-01 7.533993e-01 8.987615e-01 7.285673e-01

2137 9.699345e-01 9.454732e-01 9.350326e-01 9.331815e-01 9.330248e-01 8.414665e-01 7.906278e-01 7.525545e-01 8.983012e-01 7.278110e-01

2138 9.695269e-01 9.455170e-01 9.355998e-01 9.340408e-01 9.339709e-01 8.406395e-01 7.897291e-01 7.517163e-01 8.978303e-01 7.270644e-01

2139 9.691003e-01 9.455715e-01 9.362003e-01 9.349430e-01 9.349609e-01 8.398040e-01 7.888311e-01 7.508856e-01 8.973487e-01 7.263281e-01

2140 9.686544e-01 9.456377e-01 9.368352e-01 9.358892e-01 9.359954e-01 8.389606e-01 7.879347e-01 7.500631e-01 8.968565e-01 7.256026e-01

2141 9.681887e-01 9.457164e-01 9.375054e-01 9.368800e-01 9.370753e-01 8.381100e-01 7.870407e-01 7.492495e-01 8.963538e-01 7.248886e-01

2142 9.677028e-01 9.458084e-01 9.382118e-01 9.379164e-01 9.382011e-01 8.372529e-01 7.861499e-01 7.484456e-01 8.958406e-01 7.241864e-01

2143 9.671965e-01 9.459148e-01 9.389552e-01 9.389988e-01 9.393733e-01 8.363899e-01 7.852633e-01 7.476519e-01 8.953171e-01 7.234966e-01

2144 9.666695e-01 9.460363e-01 9.397362e-01 9.401279e-01 9.405922e-01 8.355217e-01 7.843816e-01 7.468690e-01 8.947834e-01 7.228196e-01

2145 9.661217e-01 9.461738e-01 9.405554e-01 9.413039e-01 9.418580e-01 8.346493e-01 7.835056e-01 7.460977e-01 8.942395e-01 7.221557e-01

2146 9.655530e-01 9.463282e-01 9.414133e-01 9.425269e-01 9.431708e-01 8.337732e-01 7.826363e-01 7.453384e-01 8.936858e-01 7.215053e-01

2147 9.649633e-01 9.465003e-01 9.423100e-01 9.437971e-01 9.445302e-01 8.328943e-01 7.817744e-01 7.445917e-01 8.931224e-01 7.208687e-01

2148 9.643527e-01 9.466909e-01 9.432457e-01 9.451141e-01 9.459362e-01 8.320134e-01 7.809205e-01 7.438581e-01 8.925494e-01 7.202462e-01

2149 9.637214e-01 9.469006e-01 9.442204e-01 9.464777e-01 9.473881e-01 8.311313e-01 7.800756e-01 7.431379e-01 8.919673e-01 7.196379e-01

2150 9.630698e-01 9.471303e-01 9.452340e-01 9.478871e-01 9.488853e-01 8.302489e-01 7.792402e-01 7.424316e-01 8.913761e-01 7.190441e-01

2151 9.623981e-01 9.473804e-01 9.462859e-01 9.493416e-01 9.504269e-01 8.293669e-01 7.784151e-01 7.417396e-01 8.907763e-01 7.184649e-01

2152 9.617069e-01 9.476516e-01 9.473755e-01 9.508402e-01 9.520118e-01 8.284861e-01 7.776008e-01 7.410620e-01 8.901682e-01 7.179005e-01

2153 9.609969e-01 9.479443e-01 9.485022e-01 9.523817e-01 9.536387e-01 8.276075e-01 7.767981e-01 7.403994e-01 8.895521e-01 7.173508e-01

2154 9.602688e-01 9.482588e-01 9.496648e-01 9.539646e-01 9.553061e-01 8.267317e-01 7.760074e-01 7.397517e-01 8.889283e-01 7.168160e-01

2155 9.595234e-01 9.485953e-01 9.508622e-01 9.555872e-01 9.570124e-01 8.258597e-01 7.752294e-01 7.391193e-01 8.882973e-01 7.162960e-01

2156 9.587619e-01 9.489541e-01 9.520929e-01 9.572477e-01 9.587557e-01 8.249922e-01 7.744644e-01 7.385023e-01 8.876595e-01 7.157908e-01

2157 9.579853e-01 9.493352e-01 9.533554e-01 9.589442e-01 9.605340e-01 8.241300e-01 7.737129e-01 7.379008e-01 8.870153e-01 7.153003e-01

2158 9.571949e-01 9.497384e-01 9.546478e-01 9.606743e-01 9.623451e-01 8.232738e-01 7.729754e-01 7.373149e-01 8.863652e-01 7.148245e-01

2159 9.563921e-01 9.501635e-01 9.559682e-01 9.624357e-01 9.641866e-01 8.224245e-01 7.722522e-01 7.367446e-01 8.857097e-01 7.143632e-01

2160 9.555784e-01 9.506102e-01 9.573144e-01 9.642259e-01 9.660559e-01 8.215827e-01 7.715436e-01 7.361899e-01 8.850493e-01 7.139163e-01

2161 9.547555e-01 9.510780e-01 9.586841e-01 9.660421e-01 9.679506e-01 8.207491e-01 7.708500e-01 7.356507e-01 8.843845e-01 7.134836e-01

2162 9.547554e-01 9.510780e-01 9.586842e-01 9.660422e-01 9.679508e-01 8.207286e-01 7.708182e-01 7.356114e-01 8.843796e-01 7.134399e-01

2163 9.547552e-01 9.510779e-01 9.586843e-01 9.660424e-01 9.679509e-01 8.207073e-01 7.707853e-01 7.355709e-01 8.843740e-01 7.133950e-01

2164 9.547550e-01 9.510779e-01 9.586844e-01 9.660426e-01 9.679511e-01 8.206850e-01 7.707515e-01 7.355294e-01 8.843677e-01 7.133489e-01

2165 9.547549e-01 9.510778e-01 9.586845e-01 9.660427e-01 9.679513e-01 8.206619e-01 7.707166e-01 7.354867e-01 8.843607e-01 7.133017e-01

2166 9.547547e-01 9.510778e-01 9.586846e-01 9.660429e-01 9.679515e-01 8.206379e-01 7.706807e-01 7.354429e-01 8.843531e-01 7.132533e-01

2167 9.547545e-01 9.510777e-01 9.586847e-01 9.660431e-01 9.679517e-01 8.206130e-01 7.706437e-01 7.353979e-01 8.843447e-01 7.132037e-01

2168 9.547543e-01 9.510777e-01 9.586848e-01 9.660433e-01 9.679519e-01 8.205872e-01 7.706056e-01 7.353518e-01 8.843356e-01 7.131529e-01

2169 9.547541e-01 9.510776e-01 9.586849e-01 9.660435e-01 9.679521e-01 8.205604e-01 7.705664e-01 7.353045e-01 8.843259e-01 7.131008e-01

2170 9.547539e-01 9.510776e-01 9.586850e-01 9.660437e-01 9.679523e-01 8.205326e-01 7.705261e-01 7.352559e-01 8.843155e-01 7.130475e-01

2171 9.547537e-01 9.510775e-01 9.586851e-01 9.660439e-01 9.679526e-01 8.205039e-01 7.704846e-01 7.352061e-01 8.843044e-01 7.129928e-01

2172 9.547534e-01 9.510775e-01 9.586852e-01 9.660441e-01 9.679528e-01 8.204743e-01 7.704419e-01 7.351550e-01 8.842925e-01 7.129368e-01

2173 9.547532e-01 9.510774e-01 9.586854e-01 9.660443e-01 9.679531e-01 8.204436e-01 7.703981e-01 7.351026e-01 8.842800e-01 7.128795e-01

2174 9.547529e-01 9.510774e-01 9.586855e-01 9.660446e-01 9.679533e-01 8.204119e-01 7.703530e-01 7.350489e-01 8.842668e-01 7.128208e-01

2175 9.547527e-01 9.510774e-01 9.586857e-01 9.660448e-01 9.679536e-01 8.203791e-01 7.703066e-01 7.349938e-01 8.842529e-01 7.127607e-01

2176 9.547524e-01 9.510774e-01 9.586858e-01 9.660451e-01 9.679539e-01 8.203453e-01 7.702589e-01 7.349374e-01 8.842383e-01 7.126992e-01

2177 9.547521e-01 9.510773e-01 9.586860e-01 9.660453e-01 9.679542e-01 8.203104e-01 7.702100e-01 7.348795e-01 8.842229e-01 7.126362e-01

2178 9.547518e-01 9.510773e-01 9.586862e-01 9.660456e-01 9.679545e-01 8.202743e-01 7.701596e-01 7.348201e-01 8.842068e-01 7.125716e-01

2179 9.547516e-01 9.510773e-01 9.586864e-01 9.660459e-01 9.679548e-01 8.202371e-01 7.701079e-01 7.347593e-01 8.841900e-01 7.125056e-01

2180 9.547512e-01 9.510773e-01 9.586866e-01 9.660462e-01 9.679551e-01 8.201988e-01 7.700547e-01 7.346969e-01 8.841724e-01 7.124379e-01

2181 9.547509e-01 9.510773e-01 9.586868e-01 9.660465e-01 9.679554e-01 8.201592e-01 7.700001e-01 7.346330e-01 8.841541e-01 7.123686e-01

2182 9.547506e-01 9.510773e-01 9.586870e-01 9.660469e-01 9.679558e-01 8.201185e-01 7.699440e-01 7.345674e-01 8.841350e-01 7.122977e-01

2183 9.547502e-01 9.510773e-01 9.586873e-01 9.660472e-01 9.679562e-01 8.200764e-01 7.698863e-01 7.345002e-01 8.841151e-01 7.122251e-01

2184 9.547499e-01 9.510773e-01 9.586875e-01 9.660476e-01 9.679566e-01 8.200331e-01 7.698271e-01 7.344314e-01 8.840945e-01 7.121508e-01

2185 9.547495e-01 9.510773e-01 9.586878e-01 9.660480e-01 9.679570e-01 8.199884e-01 7.697662e-01 7.343607e-01 8.840730e-01 7.120747e-01

2186 9.547491e-01 9.510773e-01 9.586881e-01 9.660484e-01 9.679574e-01 8.199424e-01 7.697037e-01 7.342884e-01 8.840507e-01 7.119967e-01

2187 9.547487e-01 9.510773e-01 9.586884e-01 9.660488e-01 9.679578e-01 8.198950e-01 7.696395e-01 7.342142e-01 8.840275e-01 7.119170e-01

2188 9.547483e-01 9.510773e-01 9.586887e-01 9.660493e-01 9.679583e-01 8.198461e-01 7.695735e-01 7.341381e-01 8.840035e-01 7.118353e-01

2189 9.547479e-01 9.510774e-01 9.586891e-01 9.660497e-01 9.679587e-01 8.197958e-01 7.695057e-01 7.340601e-01 8.839787e-01 7.117516e-01

2190 9.547475e-01 9.510774e-01 9.586894e-01 9.660502e-01 9.679592e-01 8.197439e-01 7.694360e-01 7.339802e-01 8.839529e-01 7.116659e-01

2191 9.547470e-01 9.510775e-01 9.586898e-01 9.660507e-01 9.679598e-01 8.196905e-01 7.693644e-01 7.338982e-01 8.839262e-01 7.115782e-01

2192 9.547465e-01 9.510775e-01 9.586902e-01 9.660513e-01 9.679603e-01 8.196355e-01 7.692908e-01 7.338142e-01 8.838986e-01 7.114884e-01

2193 9.547461e-01 9.510776e-01 9.586906e-01 9.660518e-01 9.679609e-01 8.195788e-01 7.692153e-01 7.337280e-01 8.838700e-01 7.113965e-01

2194 9.547456e-01 9.510777e-01 9.586911e-01 9.660524e-01 9.679615e-01 8.195203e-01 7.691376e-01 7.336397e-01 8.838404e-01 7.113023e-01

2195 9.547450e-01 9.510778e-01 9.586915e-01 9.660530e-01 9.679621e-01 8.194602e-01 7.690578e-01 7.335492e-01 8.838099e-01 7.112059e-01

2196 9.547445e-01 9.510779e-01 9.586920e-01 9.660537e-01 9.679627e-01 8.193982e-01 7.689758e-01 7.334563e-01 8.837783e-01 7.111072e-01

2197 9.547439e-01 9.510780e-01 9.586925e-01 9.660544e-01 9.679634e-01 8.193344e-01 7.688916e-01 7.333611e-01 8.837456e-01 7.110061e-01

2198 9.547434e-01 9.510781e-01 9.586931e-01 9.660551e-01 9.679641e-01 8.192686e-01 7.688050e-01 7.332635e-01 8.837119e-01 7.109025e-01

2199 9.547428e-01 9.510783e-01 9.586937e-01 9.660558e-01 9.679649e-01 8.192009e-01 7.687160e-01 7.331634e-01 8.836770e-01 7.107965e-01

2200 9.547422e-01 9.510785e-01 9.586943e-01 9.660566e-01 9.679657e-01 8.191311e-01 7.686246e-01 7.330608e-01 8.836410e-01 7.106880e-01

2201 9.547415e-01 9.510787e-01 9.586949e-01 9.660574e-01 9.679665e-01 8.190592e-01 7.685306e-01 7.329556e-01 8.836038e-01 7.105768e-01

2202 9.547409e-01 9.510789e-01 9.586956e-01 9.660583e-01 9.679673e-01 8.189852e-01 7.684340e-01 7.328477e-01 8.835654e-01 7.104630e-01

2203 9.547402e-01 9.510791e-01 9.586963e-01 9.660592e-01 9.679682e-01 8.189089e-01 7.683347e-01 7.327370e-01 8.835257e-01 7.103464e-01

2204 9.547395e-01 9.510793e-01 9.586971e-01 9.660601e-01 9.679692e-01 8.188303e-01 7.682327e-01 7.326236e-01 8.834847e-01 7.102270e-01

2205 9.547388e-01 9.510796e-01 9.586979e-01 9.660611e-01 9.679701e-01 8.187493e-01 7.681279e-01 7.325072e-01 8.834425e-01 7.101048e-01

2206 9.547380e-01 9.510799e-01 9.586987e-01 9.660621e-01 9.679712e-01 8.186659e-01 7.680201e-01 7.323879e-01 8.833988e-01 7.099796e-01

2207 9.547372e-01 9.510803e-01 9.586996e-01 9.660632e-01 9.679723e-01 8.185800e-01 7.679093e-01 7.322656e-01 8.833538e-01 7.098514e-01

2208 9.547364e-01 9.510806e-01 9.587005e-01 9.660643e-01 9.679734e-01 8.184914e-01 7.677954e-01 7.321402e-01 8.833073e-01 7.097202e-01

2209 9.547356e-01 9.510810e-01 9.587015e-01 9.660655e-01 9.679746e-01 8.184002e-01 7.676784e-01 7.320115e-01 8.832593e-01 7.095858e-01

2210 9.547347e-01 9.510815e-01 9.587026e-01 9.660667e-01 9.679758e-01 8.183062e-01 7.675580e-01 7.318796e-01 8.832097e-01 7.094481e-01

2211 9.547338e-01 9.510819e-01 9.587037e-01 9.660680e-01 9.679771e-01 8.182093e-01 7.674343e-01 7.317443e-01 8.831586e-01 7.093072e-01

2212 9.547329e-01 9.510824e-01 9.587048e-01 9.660694e-01 9.679784e-01 8.181095e-01 7.673072e-01 7.316056e-01 8.831059e-01 7.091629e-01

2213 9.547320e-01 9.510830e-01 9.587060e-01 9.660709e-01 9.679798e-01 8.180066e-01 7.671764e-01 7.314634e-01 8.830514e-01 7.090152e-01

2214 9.547310e-01 9.510836e-01 9.587073e-01 9.660724e-01 9.679813e-01 8.179006e-01 7.670421e-01 7.313175e-01 8.829952e-01 7.088640e-01

2215 9.547300e-01 9.510843e-01 9.587087e-01 9.660739e-01 9.679829e-01 8.177914e-01 7.669039e-01 7.311680e-01 8.829373e-01 7.087091e-01

2216 9.547290e-01 9.510850e-01 9.587102e-01 9.660756e-01 9.679845e-01 8.176788e-01 7.667619e-01 7.310146e-01 8.828774e-01 7.085506e-01

2217 9.547279e-01 9.510857e-01 9.587117e-01 9.660773e-01 9.679862e-01 8.175627e-01 7.666158e-01 7.308574e-01 8.828157e-01 7.083884e-01

2218 9.547268e-01 9.510866e-01 9.587133e-01 9.660791e-01 9.679880e-01 8.174431e-01 7.664657e-01 7.306961e-01 8.827519e-01 7.082223e-01

2219 9.547256e-01 9.510875e-01 9.587150e-01 9.660811e-01 9.679899e-01 8.173199e-01 7.663114e-01 7.305308e-01 8.826861e-01 7.080522e-01

2220 9.547245e-01 9.510884e-01 9.587168e-01 9.660831e-01 9.679918e-01 8.171928e-01 7.661527e-01 7.303614e-01 8.826182e-01 7.078782e-01

2221 9.547232e-01 9.510895e-01 9.587187e-01 9.660852e-01 9.679939e-01 8.170618e-01 7.659896e-01 7.301876e-01 8.825482e-01 7.077001e-01

2222 9.547220e-01 9.510906e-01 9.587207e-01 9.660874e-01 9.679961e-01 8.169268e-01 7.658219e-01 7.300095e-01 8.824759e-01 7.075178e-01

2223 9.547207e-01 9.510918e-01 9.587228e-01 9.660897e-01 9.679983e-01 8.167877e-01 7.656495e-01 7.298269e-01 8.824012e-01 7.073313e-01

2224 9.547194e-01 9.510931e-01 9.587251e-01 9.660922e-01 9.680007e-01 8.166442e-01 7.654722e-01 7.296397e-01 8.823242e-01 7.071404e-01

2225 9.547180e-01 9.510945e-01 9.587274e-01 9.660947e-01 9.680032e-01 8.164963e-01 7.652900e-01 7.294479e-01 8.822446e-01 7.069452e-01

2226 9.547166e-01 9.510960e-01 9.587299e-01 9.660974e-01 9.680058e-01 8.163439e-01 7.651027e-01 7.292512e-01 8.821625e-01 7.067454e-01

2227 9.547152e-01 9.510976e-01 9.587326e-01 9.661003e-01 9.680085e-01 8.161868e-01 7.649102e-01 7.290497e-01 8.820778e-01 7.065410e-01

2228 9.547137e-01 9.510994e-01 9.587354e-01 9.661033e-01 9.680114e-01 8.160248e-01 7.647123e-01 7.288432e-01 8.819903e-01 7.063320e-01

2229 9.547122e-01 9.511012e-01 9.587383e-01 9.661064e-01 9.680144e-01 8.158578e-01 7.645088e-01 7.286315e-01 8.819000e-01 7.061182e-01

2230 9.547106e-01 9.511032e-01 9.587415e-01 9.661097e-01 9.680176e-01 8.156856e-01 7.642997e-01 7.284147e-01 8.818068e-01 7.058997e-01

2231 9.547090e-01 9.511054e-01 9.587448e-01 9.661132e-01 9.680209e-01 8.155081e-01 7.640848e-01 7.281925e-01 8.817105e-01 7.056761e-01

2232 9.547073e-01 9.511077e-01 9.587483e-01 9.661168e-01 9.680244e-01 8.153251e-01 7.638639e-01 7.279650e-01 8.816112e-01 7.054477e-01

2233 9.547057e-01 9.511102e-01 9.587519e-01 9.661206e-01 9.680281e-01 8.151365e-01 7.636369e-01 7.277318e-01 8.815086e-01 7.052141e-01

2234 9.547039e-01 9.511129e-01 9.587559e-01 9.661247e-01 9.680319e-01 8.149421e-01 7.634036e-01 7.274931e-01 8.814027e-01 7.049754e-01

2235 9.547022e-01 9.511157e-01 9.587600e-01 9.661289e-01 9.680360e-01 8.147416e-01 7.631638e-01 7.272486e-01 8.812933e-01 7.047315e-01

2236 9.547004e-01 9.511188e-01 9.587644e-01 9.661334e-01 9.680402e-01 8.145350e-01 7.629175e-01 7.269982e-01 8.811804e-01 7.044823e-01

2237 9.546985e-01 9.511221e-01 9.587690e-01 9.661380e-01 9.680447e-01 8.143220e-01 7.626645e-01 7.267419e-01 8.810637e-01 7.042277e-01

2238 9.546966e-01 9.511256e-01 9.587739e-01 9.661430e-01 9.680494e-01 8.141024e-01 7.624045e-01 7.264795e-01 8.809433e-01 7.039677e-01

2239 9.546947e-01 9.511294e-01 9.587790e-01 9.661482e-01 9.680543e-01 8.138761e-01 7.621374e-01 7.262109e-01 8.808190e-01 7.037022e-01

2240 9.546928e-01 9.511334e-01 9.587845e-01 9.661536e-01 9.680595e-01 8.136428e-01 7.618631e-01 7.259361e-01 8.806906e-01 7.034312e-01

2241 9.546908e-01 9.511378e-01 9.587903e-01 9.661594e-01 9.680649e-01 8.134024e-01 7.615814e-01 7.256549e-01 8.805580e-01 7.031545e-01

2242 9.546887e-01 9.511424e-01 9.587964e-01 9.661655e-01 9.680707e-01 8.131546e-01 7.612921e-01 7.253672e-01 8.804211e-01 7.028722e-01

2243 9.546867e-01 9.511474e-01 9.588029e-01 9.661719e-01 9.680767e-01 8.128992e-01 7.609951e-01 7.250730e-01 8.802797e-01 7.025842e-01

2244 9.546846e-01 9.511527e-01 9.588098e-01 9.661786e-01 9.680830e-01 8.126360e-01 7.606901e-01 7.247722e-01 8.801337e-01 7.022905e-01

2245 9.546825e-01 9.511584e-01 9.588171e-01 9.661857e-01 9.680897e-01 8.123648e-01 7.603771e-01 7.244646e-01 8.799829e-01 7.019910e-01

2246 9.546804e-01 9.511645e-01 9.588248e-01 9.661931e-01 9.680967e-01 8.120854e-01 7.600558e-01 7.241502e-01 8.798271e-01 7.016856e-01

2247 9.546783e-01 9.511710e-01 9.588329e-01 9.662010e-01 9.681041e-01 8.117975e-01 7.597261e-01 7.238289e-01 8.796663e-01 7.013744e-01

2248 9.546761e-01 9.511780e-01 9.588415e-01 9.662093e-01 9.681118e-01 8.115009e-01 7.593879e-01 7.235006e-01 8.795002e-01 7.010574e-01

2249 9.546740e-01 9.511855e-01 9.588507e-01 9.662180e-01 9.681200e-01 8.111954e-01 7.590409e-01 7.231654e-01 8.793287e-01 7.007344e-01

2250 9.546718e-01 9.511934e-01 9.588603e-01 9.662272e-01 9.681286e-01 8.108808e-01 7.586850e-01 7.228230e-01 8.791516e-01 7.004056e-01

2251 9.546697e-01 9.512020e-01 9.588706e-01 9.662369e-01 9.681376e-01 8.105567e-01 7.583201e-01 7.224735e-01 8.789687e-01 7.000710e-01

2252 9.546675e-01 9.512111e-01 9.588814e-01 9.662471e-01 9.681471e-01 8.102229e-01 7.579459e-01 7.221168e-01 8.787798e-01 6.997305e-01

2253 9.546654e-01 9.512209e-01 9.588929e-01 9.662579e-01 9.681572e-01 8.098793e-01 7.575624e-01 7.217528e-01 8.785847e-01 6.993841e-01

2254 9.546634e-01 9.512313e-01 9.589051e-01 9.662692e-01 9.681677e-01 8.095256e-01 7.571695e-01 7.213816e-01 8.783833e-01 6.990320e-01

2255 9.546613e-01 9.512425e-01 9.589180e-01 9.662812e-01 9.681789e-01 8.091614e-01 7.567668e-01 7.210031e-01 8.781753e-01 6.986740e-01

2256 9.546594e-01 9.512544e-01 9.589316e-01 9.662938e-01 9.681906e-01 8.087866e-01 7.563544e-01 7.206174e-01 8.779606e-01 6.983104e-01

2257 9.546575e-01 9.512672e-01 9.589461e-01 9.663071e-01 9.682029e-01 8.084010e-01 7.559321e-01 7.202243e-01 8.777389e-01 6.979412e-01

2258 9.546556e-01 9.512808e-01 9.589614e-01 9.663212e-01 9.682159e-01 8.080042e-01 7.554998e-01 7.198239e-01 8.775099e-01 6.975664e-01

2259 9.546539e-01 9.512954e-01 9.589777e-01 9.663360e-01 9.682296e-01 8.075960e-01 7.550574e-01 7.194163e-01 8.772736e-01 6.971861e-01

2260 9.546523e-01 9.513110e-01 9.589949e-01 9.663517e-01 9.682440e-01 8.071762e-01 7.546047e-01 7.190014e-01 8.770296e-01 6.968005e-01

2261 9.546508e-01 9.513276e-01 9.590131e-01 9.663682e-01 9.682593e-01 8.067445e-01 7.541417e-01 7.185794e-01 8.767777e-01 6.964096e-01

2262 9.546495e-01 9.513454e-01 9.590324e-01 9.663856e-01 9.682753e-01 8.063008e-01 7.536684e-01 7.181502e-01 8.765178e-01 6.960135e-01

2263 9.546484e-01 9.513644e-01 9.590529e-01 9.664040e-01 9.682922e-01 8.058446e-01 7.531845e-01 7.177140e-01 8.762494e-01 6.956125e-01

2264 9.546475e-01 9.513847e-01 9.590746e-01 9.664234e-01 9.683100e-01 8.053759e-01 7.526901e-01 7.172707e-01 8.759725e-01 6.952066e-01

2265 9.546468e-01 9.514064e-01 9.590977e-01 9.664438e-01 9.683288e-01 8.048944e-01 7.521852e-01 7.168207e-01 8.756867e-01 6.947961e-01

2266 9.546464e-01 9.514296e-01 9.591221e-01 9.664654e-01 9.683486e-01 8.043998e-01 7.516696e-01 7.163638e-01 8.753918e-01 6.943811e-01

2267 9.546463e-01 9.514544e-01 9.591479e-01 9.664883e-01 9.683695e-01 8.038920e-01 7.511435e-01 7.159004e-01 8.750876e-01 6.939618e-01

2268 9.546465e-01 9.514808e-01 9.591754e-01 9.665124e-01 9.683916e-01 8.033707e-01 7.506067e-01 7.154304e-01 8.747737e-01 6.935385e-01

2269 9.546471e-01 9.515091e-01 9.592044e-01 9.665378e-01 9.684148e-01 8.028357e-01 7.500594e-01 7.149542e-01 8.744499e-01 6.931113e-01

2270 9.546481e-01 9.515392e-01 9.592353e-01 9.665647e-01 9.684394e-01 8.022870e-01 7.495015e-01 7.144719e-01 8.741160e-01 6.926805e-01

2271 9.546495e-01 9.515714e-01 9.592679e-01 9.665931e-01 9.684652e-01 8.017241e-01 7.489331e-01 7.139836e-01 8.737716e-01 6.922464e-01

2272 9.546515e-01 9.516058e-01 9.593026e-01 9.666230e-01 9.684925e-01 8.011471e-01 7.483543e-01 7.134896e-01 8.734165e-01 6.918093e-01

2273 9.546541e-01 9.516425e-01 9.593393e-01 9.666547e-01 9.685213e-01 8.005558e-01 7.477652e-01 7.129902e-01 8.730505e-01 6.913694e-01

2274 9.546573e-01 9.516817e-01 9.593783e-01 9.666881e-01 9.685517e-01 7.999499e-01 7.471659e-01 7.124856e-01 8.726732e-01 6.909270e-01

2275 9.546613e-01 9.517236e-01 9.594195e-01 9.667235e-01 9.685838e-01 7.993295e-01 7.465565e-01 7.119761e-01 8.722843e-01 6.904824e-01

2276 9.546660e-01 9.517682e-01 9.594633e-01 9.667608e-01 9.686177e-01 7.986944e-01 7.459373e-01 7.114619e-01 8.718836e-01 6.900360e-01

2277 9.546715e-01 9.518159e-01 9.595098e-01 9.668002e-01 9.686534e-01 7.980444e-01 7.453083e-01 7.109435e-01 8.714708e-01 6.895882e-01

2278 9.546781e-01 9.518667e-01 9.595590e-01 9.668418e-01 9.686911e-01 7.973797e-01 7.446699e-01 7.104210e-01 8.710456e-01 6.891392e-01

2279 9.546856e-01 9.519210e-01 9.596112e-01 9.668858e-01 9.687310e-01 7.967000e-01 7.440223e-01 7.098949e-01 8.706078e-01 6.886895e-01

2280 9.546943e-01 9.519789e-01 9.596665e-01 9.669323e-01 9.687730e-01 7.960055e-01 7.433657e-01 7.093655e-01 8.701570e-01 6.882394e-01

2281 9.547043e-01 9.520407e-01 9.597251e-01 9.669814e-01 9.688173e-01 7.952961e-01 7.427004e-01 7.088333e-01 8.696930e-01 6.877893e-01

2282 9.547156e-01 9.521066e-01 9.597873e-01 9.670333e-01 9.688642e-01 7.945718e-01 7.420267e-01 7.082985e-01 8.692155e-01 6.873396e-01

2283 9.547284e-01 9.521769e-01 9.598532e-01 9.670881e-01 9.689136e-01 7.938327e-01 7.413451e-01 7.077616e-01 8.687242e-01 6.868907e-01

2284 9.547429e-01 9.522518e-01 9.599231e-01 9.671460e-01 9.689658e-01 7.930788e-01 7.406558e-01 7.072231e-01 8.682190e-01 6.864431e-01

2285 9.547591e-01 9.523317e-01 9.599972e-01 9.672072e-01 9.690209e-01 7.923104e-01 7.399592e-01 7.066834e-01 8.676995e-01 6.859970e-01

2286 9.547773e-01 9.524169e-01 9.600756e-01 9.672718e-01 9.690791e-01 7.915276e-01 7.392559e-01 7.061429e-01 8.671655e-01 6.855530e-01

2287 9.547976e-01 9.525076e-01 9.601588e-01 9.673401e-01 9.691405e-01 7.907305e-01 7.385461e-01 7.056021e-01 8.666167e-01 6.851115e-01

2288 9.548202e-01 9.526044e-01 9.602470e-01 9.674122e-01 9.692053e-01 7.899194e-01 7.378305e-01 7.050615e-01 8.660530e-01 6.846728e-01

2289 9.548454e-01 9.527074e-01 9.603404e-01 9.674884e-01 9.692737e-01 7.890944e-01 7.371094e-01 7.045216e-01 8.654741e-01 6.842375e-01

2290 9.548733e-01 9.528172e-01 9.604393e-01 9.675689e-01 9.693459e-01 7.882560e-01 7.363835e-01 7.039827e-01 8.648798e-01 6.838059e-01

2291 9.549041e-01 9.529342e-01 9.605441e-01 9.676539e-01 9.694221e-01 7.874043e-01 7.356533e-01 7.034455e-01 8.642699e-01 6.833784e-01

2292 9.549381e-01 9.530587e-01 9.606551e-01 9.677437e-01 9.695026e-01 7.865398e-01 7.349192e-01 7.029105e-01 8.636443e-01 6.829554e-01

2293 9.549757e-01 9.531912e-01 9.607727e-01 9.678385e-01 9.695875e-01 7.856629e-01 7.341820e-01 7.023780e-01 8.630028e-01 6.825374e-01

2294 9.550170e-01 9.533323e-01 9.608972e-01 9.679386e-01 9.696771e-01 7.847740e-01 7.334423e-01 7.018487e-01 8.623452e-01 6.821247e-01

2295 9.550623e-01 9.534824e-01 9.610290e-01 9.680443e-01 9.697716e-01 7.838735e-01 7.327005e-01 7.013230e-01 8.616715e-01 6.817178e-01

2296 9.551121e-01 9.536421e-01 9.611685e-01 9.681558e-01 9.698714e-01 7.829620e-01 7.319575e-01 7.008014e-01 8.609815e-01 6.813169e-01

2297 9.551666e-01 9.538119e-01 9.613161e-01 9.682736e-01 9.699766e-01 7.820400e-01 7.312137e-01 7.002844e-01 8.602752e-01 6.809224e-01

2298 9.552262e-01 9.539925e-01 9.614722e-01 9.683979e-01 9.700876e-01 7.811081e-01 7.304700e-01 6.997725e-01 8.595525e-01 6.805347e-01

2299 9.552914e-01 9.541844e-01 9.616374e-01 9.685290e-01 9.702047e-01 7.801668e-01 7.297269e-01 6.992661e-01 8.588134e-01 6.801541e-01

2300 9.553625e-01 9.543882e-01 9.618122e-01 9.686673e-01 9.703281e-01 7.792169e-01 7.289851e-01 6.987657e-01 8.580579e-01 6.797809e-01

2301 9.554400e-01 9.546048e-01 9.619969e-01 9.688132e-01 9.704583e-01 7.782590e-01 7.282453e-01 6.982717e-01 8.572861e-01 6.794154e-01

2302 9.555243e-01 9.548348e-01 9.621921e-01 9.689671e-01 9.705954e-01 7.772938e-01 7.275082e-01 6.977846e-01 8.564979e-01 6.790578e-01

2303 9.556160e-01 9.550788e-01 9.623985e-01 9.691294e-01 9.707400e-01 7.763221e-01 7.267745e-01 6.973048e-01 8.556935e-01 6.787085e-01

2304 9.557156e-01 9.553378e-01 9.626165e-01 9.693004e-01 9.708924e-01 7.753445e-01 7.260448e-01 6.968327e-01 8.548730e-01 6.783676e-01

2305 9.558237e-01 9.556124e-01 9.628467e-01 9.694807e-01 9.710529e-01 7.743620e-01 7.253198e-01 6.963686e-01 8.540366e-01 6.780353e-01

2306 9.559408e-01 9.559036e-01 9.630897e-01 9.696706e-01 9.712219e-01 7.733753e-01 7.246001e-01 6.959129e-01 8.531844e-01 6.777118e-01

2307 9.560676e-01 9.562122e-01 9.633463e-01 9.698707e-01 9.713999e-01 7.723853e-01 7.238865e-01 6.954659e-01 8.523168e-01 6.773973e-01

2308 9.562048e-01 9.565391e-01 9.636169e-01 9.700813e-01 9.715872e-01 7.713928e-01 7.231795e-01 6.950280e-01 8.514339e-01 6.770919e-01

2309 9.563529e-01 9.568852e-01 9.639024e-01 9.703031e-01 9.717844e-01 7.703987e-01 7.224797e-01 6.945994e-01 8.505360e-01 6.767957e-01

2310 9.565129e-01 9.572515e-01 9.642033e-01 9.705365e-01 9.719918e-01 7.694039e-01 7.217879e-01 6.941803e-01 8.496236e-01 6.765088e-01

2311 9.566852e-01 9.576389e-01 9.645205e-01 9.707820e-01 9.722100e-01 7.684093e-01 7.211044e-01 6.937711e-01 8.486970e-01 6.762313e-01

2312 9.568709e-01 9.580485e-01 9.648546e-01 9.710403e-01 9.724394e-01 7.674158e-01 7.204300e-01 6.933719e-01 8.477565e-01 6.759632e-01

2313 9.570706e-01 9.584813e-01 9.652064e-01 9.713118e-01 9.726805e-01 7.664242e-01 7.197651e-01 6.929829e-01 8.468027e-01 6.757045e-01

2314 9.572853e-01 9.589384e-01 9.655767e-01 9.715971e-01 9.729338e-01 7.654356e-01 7.191102e-01 6.926043e-01 8.458361e-01 6.754553e-01

2315 9.575158e-01 9.594209e-01 9.659662e-01 9.718969e-01 9.731998e-01 7.644507e-01 7.184658e-01 6.922361e-01 8.448572e-01 6.752155e-01

2316 9.577629e-01 9.599298e-01 9.663759e-01 9.722117e-01 9.734791e-01 7.634706e-01 7.178324e-01 6.918785e-01 8.438666e-01 6.749850e-01

2317 9.580276e-01 9.604663e-01 9.668064e-01 9.725421e-01 9.737723e-01 7.624960e-01 7.172104e-01 6.915316e-01 8.428649e-01 6.747638e-01

2318 9.583109e-01 9.610315e-01 9.672587e-01 9.728888e-01 9.740798e-01 7.615279e-01 7.166002e-01 6.911954e-01 8.418527e-01 6.745519e-01

2319 9.586137e-01 9.616266e-01 9.677335e-01 9.732525e-01 9.744022e-01 7.605670e-01 7.160020e-01 6.908699e-01 8.408307e-01 6.743490e-01

2320 9.589370e-01 9.622527e-01 9.682317e-01 9.736336e-01 9.747401e-01 7.596144e-01 7.154163e-01 6.905552e-01 8.397997e-01 6.741552e-01

2321 9.592818e-01 9.629110e-01 9.687543e-01 9.740330e-01 9.750941e-01 7.586707e-01 7.148433e-01 6.902512e-01 8.387603e-01 6.739702e-01

2322 9.596490e-01 9.636027e-01 9.693019e-01 9.744512e-01 9.754647e-01 7.577367e-01 7.142833e-01 6.899578e-01 8.377135e-01 6.737940e-01

2323 9.600396e-01 9.643288e-01 9.698756e-01 9.748890e-01 9.758526e-01 7.568133e-01 7.137364e-01 6.896750e-01 8.366599e-01 6.736263e-01

2324 9.604547e-01 9.650906e-01 9.704761e-01 9.753470e-01 9.762583e-01 7.559011e-01 7.132030e-01 6.894028e-01 8.356004e-01 6.734669e-01

2325 9.608953e-01 9.658890e-01 9.711043e-01 9.758258e-01 9.766824e-01 7.550009e-01 7.126830e-01 6.891409e-01 8.345359e-01 6.733158e-01

2326 9.613622e-01 9.667252e-01 9.717610e-01 9.763261e-01 9.771255e-01 7.541132e-01 7.121767e-01 6.888893e-01 8.334673e-01 6.731727e-01

2327 9.618565e-01 9.676002e-01 9.724470e-01 9.768486e-01 9.775881e-01 7.532388e-01 7.116841e-01 6.886479e-01 8.323954e-01 6.730375e-01

2328 9.623790e-01 9.685150e-01 9.731632e-01 9.773941e-01 9.780709e-01 7.523782e-01 7.112053e-01 6.884164e-01 8.313212e-01 6.729098e-01

2329 9.629307e-01 9.694705e-01 9.739104e-01 9.779630e-01 9.785744e-01 7.515319e-01 7.107403e-01 6.881948e-01 8.302456e-01 6.727895e-01

2330 9.635123e-01 9.704676e-01 9.746892e-01 9.785563e-01 9.790992e-01 7.507006e-01 7.102890e-01 6.879828e-01 8.291696e-01 6.726764e-01

2331 9.641247e-01 9.715071e-01 9.755004e-01 9.791745e-01 9.796459e-01 7.498845e-01 7.098515e-01 6.877802e-01 8.280940e-01 6.725702e-01

2332 9.647685e-01 9.725897e-01 9.763447e-01 9.798183e-01 9.802150e-01 7.490842e-01 7.094277e-01 6.875868e-01 8.270199e-01 6.724707e-01

2333 9.654443e-01 9.737160e-01 9.772229e-01 9.804885e-01 9.808072e-01 7.482999e-01 7.090174e-01 6.874024e-01 8.259481e-01 6.723777e-01

2334 9.661527e-01 9.748866e-01 9.781357e-01 9.811857e-01 9.814230e-01 7.475322e-01 7.086207e-01 6.872268e-01 8.248797e-01 6.722910e-01

2335 9.668941e-01 9.761019e-01 9.790836e-01 9.819106e-01 9.820630e-01 7.467811e-01 7.082373e-01 6.870597e-01 8.238154e-01 6.722104e-01

2336 9.676687e-01 9.773622e-01 9.800672e-01 9.826638e-01 9.827277e-01 7.460471e-01 7.078670e-01 6.869010e-01 8.227564e-01 6.721355e-01

2337 9.684769e-01 9.786678e-01 9.810872e-01 9.834459e-01 9.834176e-01 7.453302e-01 7.075098e-01 6.867503e-01 8.217034e-01 6.720662e-01

2338 9.693185e-01 9.800187e-01 9.821440e-01 9.842577e-01 9.841334e-01 7.446306e-01 7.071653e-01 6.866075e-01 8.206574e-01 6.720022e-01

2339 9.701935e-01 9.814148e-01 9.832381e-01 9.850995e-01 9.848753e-01 7.439485e-01 7.068335e-01 6.864722e-01 8.196193e-01 6.719434e-01

2340 9.711016e-01 9.828561e-01 9.843697e-01 9.859721e-01 9.856440e-01 7.432839e-01 7.065140e-01 6.863443e-01 8.185898e-01 6.718895e-01

2341 9.720426e-01 9.843421e-01 9.855392e-01 9.868759e-01 9.864399e-01 7.426370e-01 7.062066e-01 6.862235e-01 8.175698e-01 6.718403e-01

2342 9.720426e-01 9.843423e-01 9.855393e-01 9.868760e-01 9.864399e-01 7.425942e-01 7.061675e-01 6.861928e-01 8.175414e-01 6.718136e-01

2343 9.720427e-01 9.843424e-01 9.855394e-01 9.868760e-01 9.864400e-01 7.425505e-01 7.061276e-01 6.861616e-01 8.175121e-01 6.717866e-01

2344 9.720427e-01 9.843426e-01 9.855395e-01 9.868761e-01 9.864400e-01 7.425057e-01 7.060870e-01 6.861300e-01 8.174818e-01 6.717594e-01

2345 9.720428e-01 9.843428e-01 9.855396e-01 9.868762e-01 9.864401e-01 7.424598e-01 7.060456e-01 6.860980e-01 8.174506e-01 6.717319e-01

2346 9.720429e-01 9.843430e-01 9.855397e-01 9.868763e-01 9.864402e-01 7.424129e-01 7.060035e-01 6.860656e-01 8.174185e-01 6.717041e-01

2347 9.720429e-01 9.843431e-01 9.855398e-01 9.868764e-01 9.864402e-01 7.423649e-01 7.059607e-01 6.860327e-01 8.173854e-01 6.716760e-01

2348 9.720430e-01 9.843433e-01 9.855400e-01 9.868765e-01 9.864403e-01 7.423158e-01 7.059170e-01 6.859994e-01 8.173513e-01 6.716477e-01

2349 9.720431e-01 9.843435e-01 9.855401e-01 9.868766e-01 9.864404e-01 7.422656e-01 7.058726e-01 6.859658e-01 8.173162e-01 6.716192e-01

2350 9.720432e-01 9.843437e-01 9.855402e-01 9.868767e-01 9.864405e-01 7.422143e-01 7.058275e-01 6.859317e-01 8.172800e-01 6.715904e-01

2351 9.720433e-01 9.843440e-01 9.855404e-01 9.868768e-01 9.864405e-01 7.421617e-01 7.057815e-01 6.858972e-01 8.172429e-01 6.715614e-01

2352 9.720434e-01 9.843442e-01 9.855406e-01 9.868769e-01 9.864406e-01 7.421080e-01 7.057348e-01 6.858624e-01 8.172046e-01 6.715322e-01

2353 9.720435e-01 9.843444e-01 9.855407e-01 9.868770e-01 9.864407e-01 7.420531e-01 7.056872e-01 6.858271e-01 8.171653e-01 6.715027e-01

2354 9.720436e-01 9.843447e-01 9.855409e-01 9.868771e-01 9.864408e-01 7.419970e-01 7.056389e-01 6.857914e-01 8.171248e-01 6.714731e-01

2355 9.720437e-01 9.843449e-01 9.855411e-01 9.868772e-01 9.864409e-01 7.419396e-01 7.055897e-01 6.857554e-01 8.170832e-01 6.714432e-01

2356 9.720438e-01 9.843452e-01 9.855413e-01 9.868774e-01 9.864410e-01 7.418809e-01 7.055397e-01 6.857190e-01 8.170404e-01 6.714132e-01

2357 9.720439e-01 9.843454e-01 9.855415e-01 9.868775e-01 9.864411e-01 7.418209e-01 7.054889e-01 6.856822e-01 8.169964e-01 6.713830e-01

2358 9.720441e-01 9.843457e-01 9.855417e-01 9.868777e-01 9.864413e-01 7.417595e-01 7.054373e-01 6.856451e-01 8.169512e-01 6.713527e-01

2359 9.720442e-01 9.843460e-01 9.855419e-01 9.868778e-01 9.864414e-01 7.416968e-01 7.053848e-01 6.856076e-01 8.169047e-01 6.713222e-01

2360 9.720444e-01 9.843463e-01 9.855421e-01 9.868780e-01 9.864415e-01 7.416327e-01 7.053314e-01 6.855697e-01 8.168569e-01 6.712916e-01

2361 9.720446e-01 9.843466e-01 9.855423e-01 9.868781e-01 9.864416e-01 7.415672e-01 7.052772e-01 6.855315e-01 8.168078e-01 6.712608e-01

2362 9.720447e-01 9.843469e-01 9.855426e-01 9.868783e-01 9.864418e-01 7.415002e-01 7.052221e-01 6.854929e-01 8.167573e-01 6.712300e-01

2363 9.720449e-01 9.843472e-01 9.855428e-01 9.868785e-01 9.864419e-01 7.414317e-01 7.051661e-01 6.854540e-01 8.167054e-01 6.711991e-01

2364 9.720451e-01 9.843476e-01 9.855431e-01 9.868787e-01 9.864420e-01 7.413617e-01 7.051092e-01 6.854148e-01 8.166520e-01 6.711680e-01

2365 9.720453e-01 9.843480e-01 9.855433e-01 9.868788e-01 9.864422e-01 7.412901e-01 7.050515e-01 6.853753e-01 8.165972e-01 6.711370e-01

2366 9.720456e-01 9.843483e-01 9.855436e-01 9.868790e-01 9.864424e-01 7.412170e-01 7.049928e-01 6.853355e-01 8.165409e-01 6.711059e-01

2367 9.720458e-01 9.843487e-01 9.855439e-01 9.868792e-01 9.864425e-01 7.411422e-01 7.049332e-01 6.852954e-01 8.164830e-01 6.710748e-01

2368 9.720460e-01 9.843491e-01 9.855442e-01 9.868795e-01 9.864427e-01 7.410658e-01 7.048727e-01 6.852550e-01 8.164234e-01 6.710436e-01

2369 9.720463e-01 9.843495e-01 9.855445e-01 9.868797e-01 9.864429e-01 7.409876e-01 7.048113e-01 6.852143e-01 8.163623e-01 6.710125e-01

2370 9.720466e-01 9.843500e-01 9.855449e-01 9.868799e-01 9.864430e-01 7.409078e-01 7.047489e-01 6.851734e-01 8.162994e-01 6.709815e-01

2371 9.720469e-01 9.843504e-01 9.855452e-01 9.868802e-01 9.864432e-01 7.408261e-01 7.046856e-01 6.851323e-01 8.162348e-01 6.709505e-01

2372 9.720472e-01 9.843509e-01 9.855456e-01 9.868804e-01 9.864434e-01 7.407427e-01 7.046213e-01 6.850909e-01 8.161684e-01 6.709196e-01

2373 9.720475e-01 9.843514e-01 9.855459e-01 9.868807e-01 9.864436e-01 7.406574e-01 7.045561e-01 6.850494e-01 8.161001e-01 6.708889e-01

2374 9.720479e-01 9.843519e-01 9.855463e-01 9.868809e-01 9.864439e-01 7.405702e-01 7.044899e-01 6.850076e-01 8.160299e-01 6.708582e-01

2375 9.720482e-01 9.843525e-01 9.855467e-01 9.868812e-01 9.864441e-01 7.404811e-01 7.044228e-01 6.849657e-01 8.159578e-01 6.708278e-01

2376 9.720486e-01 9.843530e-01 9.855471e-01 9.868815e-01 9.864443e-01 7.403900e-01 7.043546e-01 6.849237e-01 8.158837e-01 6.707976e-01

2377 9.720490e-01 9.843536e-01 9.855476e-01 9.868818e-01 9.864445e-01 7.402968e-01 7.042855e-01 6.848815e-01 8.158074e-01 6.707676e-01

2378 9.720495e-01 9.843543e-01 9.855480e-01 9.868822e-01 9.864448e-01 7.402017e-01 7.042155e-01 6.848392e-01 8.157291e-01 6.707379e-01

2379 9.720499e-01 9.843549e-01 9.855485e-01 9.868825e-01 9.864451e-01 7.401044e-01 7.041444e-01 6.847969e-01 8.156486e-01 6.707085e-01

2380 9.720504e-01 9.843556e-01 9.855490e-01 9.868829e-01 9.864453e-01 7.400049e-01 7.040724e-01 6.847545e-01 8.155658e-01 6.706794e-01

2381 9.720509e-01 9.843563e-01 9.855495e-01 9.868832e-01 9.864456e-01 7.399033e-01 7.039993e-01 6.847121e-01 8.154807e-01 6.706507e-01

2382 9.720514e-01 9.843570e-01 9.855501e-01 9.868836e-01 9.864459e-01 7.397994e-01 7.039253e-01 6.846697e-01 8.153932e-01 6.706225e-01

2383 9.720520e-01 9.843577e-01 9.855506e-01 9.868840e-01 9.864462e-01 7.396932e-01 7.038503e-01 6.846273e-01 8.153032e-01 6.705948e-01

2384 9.720526e-01 9.843585e-01 9.855512e-01 9.868844e-01 9.864466e-01 7.395847e-01 7.037744e-01 6.845850e-01 8.152107e-01 6.705675e-01

2385 9.720532e-01 9.843593e-01 9.855518e-01 9.868849e-01 9.864469e-01 7.394738e-01 7.036974e-01 6.845429e-01 8.151156e-01 6.705408e-01

2386 9.720539e-01 9.843602e-01 9.855525e-01 9.868853e-01 9.864472e-01 7.393604e-01 7.036195e-01 6.845008e-01 8.150178e-01 6.705148e-01

2387 9.720546e-01 9.843611e-01 9.855531e-01 9.868858e-01 9.864476e-01 7.392446e-01 7.035406e-01 6.844590e-01 8.149172e-01 6.704894e-01

2388 9.720553e-01 9.843620e-01 9.855538e-01 9.868863e-01 9.864480e-01 7.391262e-01 7.034608e-01 6.844174e-01 8.148138e-01 6.704647e-01

2389 9.720561e-01 9.843630e-01 9.855546e-01 9.868868e-01 9.864484e-01 7.390053e-01 7.033800e-01 6.843761e-01 8.147074e-01 6.704408e-01

2390 9.720569e-01 9.843640e-01 9.855553e-01 9.868874e-01 9.864488e-01 7.388816e-01 7.032982e-01 6.843351e-01 8.145981e-01 6.704178e-01

2391 9.720578e-01 9.843651e-01 9.855561e-01 9.868880e-01 9.864493e-01 7.387553e-01 7.032156e-01 6.842944e-01 8.144856e-01 6.703957e-01

2392 9.720587e-01 9.843662e-01 9.855570e-01 9.868886e-01 9.864497e-01 7.386263e-01 7.031320e-01 6.842542e-01 8.143699e-01 6.703745e-01

2393 9.720597e-01 9.843673e-01 9.855578e-01 9.868892e-01 9.864502e-01 7.384944e-01 7.030475e-01 6.842145e-01 8.142510e-01 6.703544e-01

2394 9.720607e-01 9.843685e-01 9.855588e-01 9.868898e-01 9.864507e-01 7.383597e-01 7.029621e-01 6.841753e-01 8.141286e-01 6.703354e-01

2395 9.720618e-01 9.843698e-01 9.855597e-01 9.868905e-01 9.864512e-01 7.382221e-01 7.028758e-01 6.841366e-01 8.140028e-01 6.703176e-01

2396 9.720630e-01 9.843711e-01 9.855607e-01 9.868912e-01 9.864518e-01 7.380815e-01 7.027887e-01 6.840987e-01 8.138733e-01 6.703010e-01

2397 9.720642e-01 9.843725e-01 9.855617e-01 9.868920e-01 9.864523e-01 7.379379e-01 7.027007e-01 6.840614e-01 8.137402e-01 6.702858e-01

2398 9.720655e-01 9.843739e-01 9.855628e-01 9.868928e-01 9.864529e-01 7.377913e-01 7.026119e-01 6.840249e-01 8.136032e-01 6.702720e-01

2399 9.720668e-01 9.843754e-01 9.855640e-01 9.868936e-01 9.864535e-01 7.376415e-01 7.025224e-01 6.839893e-01 8.134624e-01 6.702597e-01

2400 9.720682e-01 9.843769e-01 9.855651e-01 9.868944e-01 9.864542e-01 7.374885e-01 7.024321e-01 6.839546e-01 8.133175e-01 6.702490e-01

2401 9.720697e-01 9.843785e-01 9.855664e-01 9.868953e-01 9.864549e-01 7.373323e-01 7.023411e-01 6.839209e-01 8.131684e-01 6.702401e-01

2402 9.720713e-01 9.843802e-01 9.855677e-01 9.868962e-01 9.864556e-01 7.371728e-01 7.022494e-01 6.838883e-01 8.130151e-01 6.702329e-01

2403 9.720730e-01 9.843820e-01 9.855690e-01 9.868972e-01 9.864563e-01 7.370099e-01 7.021570e-01 6.838569e-01 8.128574e-01 6.702276e-01

2404 9.720748e-01 9.843838e-01 9.855704e-01 9.868982e-01 9.864571e-01 7.368437e-01 7.020640e-01 6.838267e-01 8.126952e-01 6.702244e-01

2405 9.720767e-01 9.843858e-01 9.855719e-01 9.868993e-01 9.864579e-01 7.366740e-01 7.019705e-01 6.837979e-01 8.125283e-01 6.702232e-01

2406 9.720786e-01 9.843878e-01 9.855735e-01 9.869004e-01 9.864587e-01 7.365007e-01 7.018764e-01 6.837706e-01 8.123566e-01 6.702243e-01

2407 9.720807e-01 9.843899e-01 9.855751e-01 9.869016e-01 9.864596e-01 7.363240e-01 7.017818e-01 6.837448e-01 8.121800e-01 6.702277e-01

2408 9.720829e-01 9.843921e-01 9.855768e-01 9.869028e-01 9.864606e-01 7.361436e-01 7.016868e-01 6.837207e-01 8.119983e-01 6.702336e-01

2409 9.720853e-01 9.843944e-01 9.855785e-01 9.869041e-01 9.864615e-01 7.359595e-01 7.015915e-01 6.836983e-01 8.118115e-01 6.702421e-01

2410 9.720877e-01 9.843968e-01 9.855804e-01 9.869054e-01 9.864625e-01 7.357718e-01 7.014958e-01 6.836778e-01 8.116192e-01 6.702533e-01

2411 9.720903e-01 9.843993e-01 9.855823e-01 9.869068e-01 9.864636e-01 7.355803e-01 7.013999e-01 6.836593e-01 8.114215e-01 6.702674e-01

2412 9.720931e-01 9.844019e-01 9.855844e-01 9.869083e-01 9.864647e-01 7.353850e-01 7.013038e-01 6.836429e-01 8.112181e-01 6.702845e-01

2413 9.720960e-01 9.844047e-01 9.855865e-01 9.869098e-01 9.864659e-01 7.351858e-01 7.012076e-01 6.836287e-01 8.110089e-01 6.703047e-01

2414 9.720990e-01 9.844075e-01 9.855887e-01 9.869114e-01 9.864671e-01 7.349827e-01 7.011114e-01 6.836169e-01 8.107937e-01 6.703282e-01

2415 9.721023e-01 9.844105e-01 9.855910e-01 9.869131e-01 9.864684e-01 7.347757e-01 7.010152e-01 6.836076e-01 8.105724e-01 6.703551e-01

2416 9.721057e-01 9.844137e-01 9.855935e-01 9.869149e-01 9.864697e-01 7.345647e-01 7.009191e-01 6.836010e-01 8.103448e-01 6.703857e-01

2417 9.721093e-01 9.844170e-01 9.855960e-01 9.869167e-01 9.864711e-01 7.343498e-01 7.008232e-01 6.835971e-01 8.101106e-01 6.704199e-01

2418 9.721131e-01 9.844204e-01 9.855987e-01 9.869187e-01 9.864725e-01 7.341307e-01 7.007276e-01 6.835962e-01 8.098699e-01 6.704581e-01

2419 9.721171e-01 9.844240e-01 9.856015e-01 9.869207e-01 9.864741e-01 7.339076e-01 7.006325e-01 6.835984e-01 8.096223e-01 6.705004e-01

2420 9.721214e-01 9.844277e-01 9.856044e-01 9.869228e-01 9.864757e-01 7.336804e-01 7.005378e-01 6.836038e-01 8.093677e-01 6.705469e-01

2421 9.721259e-01 9.844317e-01 9.856074e-01 9.869251e-01 9.864773e-01 7.334491e-01 7.004438e-01 6.836126e-01 8.091059e-01 6.705979e-01

2422 9.721306e-01 9.844358e-01 9.856107e-01 9.869274e-01 9.864791e-01 7.332136e-01 7.003505e-01 6.836250e-01 8.088367e-01 6.706535e-01

2423 9.721357e-01 9.844401e-01 9.856140e-01 9.869298e-01 9.864809e-01 7.329739e-01 7.002580e-01 6.836411e-01 8.085600e-01 6.707138e-01

2424 9.721410e-01 9.844446e-01 9.856175e-01 9.869324e-01 9.864829e-01 7.327301e-01 7.001665e-01 6.836611e-01 8.082755e-01 6.707792e-01

2425 9.721466e-01 9.844493e-01 9.856212e-01 9.869351e-01 9.864849e-01 7.324820e-01 7.000760e-01 6.836852e-01 8.079831e-01 6.708496e-01

2426 9.721525e-01 9.844542e-01 9.856251e-01 9.869379e-01 9.864870e-01 7.322298e-01 6.999868e-01 6.837137e-01 8.076825e-01 6.709255e-01

2427 9.721588e-01 9.844594e-01 9.856291e-01 9.869409e-01 9.864892e-01 7.319734e-01 6.998990e-01 6.837465e-01 8.073736e-01 6.710069e-01

2428 9.721654e-01 9.844648e-01 9.856334e-01 9.869439e-01 9.864915e-01 7.317128e-01 6.998126e-01 6.837840e-01 8.070562e-01 6.710940e-01

2429 9.721724e-01 9.844705e-01 9.856378e-01 9.869472e-01 9.864940e-01 7.314480e-01 6.997279e-01 6.838264e-01 8.067300e-01 6.711871e-01

2430 9.721798e-01 9.844764e-01 9.856425e-01 9.869506e-01 9.864965e-01 7.311790e-01 6.996450e-01 6.838738e-01 8.063949e-01 6.712864e-01

2431 9.721876e-01 9.844826e-01 9.856474e-01 9.869542e-01 9.864992e-01 7.309059e-01 6.995641e-01 6.839265e-01 8.060507e-01 6.713920e-01

2432 9.721959e-01 9.844891e-01 9.856525e-01 9.869579e-01 9.865020e-01 7.306287e-01 6.994853e-01 6.839846e-01 8.056971e-01 6.715041e-01

2433 9.722047e-01 9.844960e-01 9.856579e-01 9.869619e-01 9.865049e-01 7.303475e-01 6.994088e-01 6.840483e-01 8.053340e-01 6.716231e-01

2434 9.722139e-01 9.845031e-01 9.856635e-01 9.869660e-01 9.865080e-01 7.300622e-01 6.993348e-01 6.841180e-01 8.049611e-01 6.717489e-01

2435 9.722237e-01 9.845106e-01 9.856694e-01 9.869703e-01 9.865113e-01 7.297729e-01 6.992635e-01 6.841937e-01 8.045783e-01 6.718820e-01

2436 9.722340e-01 9.845185e-01 9.856756e-01 9.869749e-01 9.865147e-01 7.294798e-01 6.991949e-01 6.842756e-01 8.041853e-01 6.720224e-01

2437 9.722449e-01 9.845267e-01 9.856821e-01 9.869796e-01 9.865182e-01 7.291827e-01 6.991295e-01 6.843641e-01 8.037820e-01 6.721704e-01

2438 9.722565e-01 9.845353e-01 9.856890e-01 9.869846e-01 9.865220e-01 7.288820e-01 6.990672e-01 6.844593e-01 8.033682e-01 6.723262e-01

2439 9.722687e-01 9.845443e-01 9.856961e-01 9.869899e-01 9.865259e-01 7.285775e-01 6.990083e-01 6.845613e-01 8.029437e-01 6.724900e-01

2440 9.722816e-01 9.845538e-01 9.857037e-01 9.869954e-01 9.865300e-01 7.282694e-01 6.989531e-01 6.846706e-01 8.025082e-01 6.726619e-01

2441 9.722953e-01 9.845638e-01 9.857115e-01 9.870012e-01 9.865344e-01 7.279579e-01 6.989017e-01 6.847871e-01 8.020616e-01 6.728422e-01

2442 9.723098e-01 9.845742e-01 9.857198e-01 9.870073e-01 9.865389e-01 7.276430e-01 6.988544e-01 6.849112e-01 8.016037e-01 6.730311e-01

2443 9.723250e-01 9.845851e-01 9.857285e-01 9.870137e-01 9.865437e-01 7.273248e-01 6.988112e-01 6.850430e-01 8.011344e-01 6.732287e-01

2444 9.723412e-01 9.845965e-01 9.857377e-01 9.870204e-01 9.865487e-01 7.270036e-01 6.987726e-01 6.851828e-01 8.006534e-01 6.734351e-01

2445 9.723583e-01 9.846086e-01 9.857472e-01 9.870275e-01 9.865540e-01 7.266793e-01 6.987386e-01 6.853308e-01 8.001607e-01 6.736507e-01

2446 9.723764e-01 9.846212e-01 9.857573e-01 9.870349e-01 9.865595e-01 7.263523e-01 6.987095e-01 6.854871e-01 7.996560e-01 6.738755e-01

2447 9.723955e-01 9.846344e-01 9.857679e-01 9.870427e-01 9.865653e-01 7.260226e-01 6.986855e-01 6.856519e-01 7.991392e-01 6.741097e-01

2448 9.724157e-01 9.846483e-01 9.857790e-01 9.870509e-01 9.865714e-01 7.256904e-01 6.986669e-01 6.858255e-01 7.986102e-01 6.743535e-01

2449 9.724371e-01 9.846628e-01 9.857906e-01 9.870595e-01 9.865779e-01 7.253559e-01 6.986537e-01 6.860079e-01 7.980689e-01 6.746069e-01

2450 9.724597e-01 9.846781e-01 9.858029e-01 9.870686e-01 9.865846e-01 7.250193e-01 6.986464e-01 6.861994e-01 7.975150e-01 6.748701e-01

2451 9.724837e-01 9.846941e-01 9.858157e-01 9.870781e-01 9.865917e-01 7.246808e-01 6.986449e-01 6.864001e-01 7.969487e-01 6.751432e-01

2452 9.725090e-01 9.847109e-01 9.858293e-01 9.870881e-01 9.865992e-01 7.243407e-01 6.986497e-01 6.866101e-01 7.963696e-01 6.754263e-01

2453 9.725358e-01 9.847286e-01 9.858435e-01 9.870986e-01 9.866070e-01 7.239991e-01 6.986609e-01 6.868297e-01 7.957778e-01 6.757195e-01

2454 9.725641e-01 9.847471e-01 9.858584e-01 9.871097e-01 9.866152e-01 7.236562e-01 6.986786e-01 6.870589e-01 7.951733e-01 6.760229e-01

2455 9.725940e-01 9.847665e-01 9.858741e-01 9.871213e-01 9.866239e-01 7.233125e-01 6.987032e-01 6.872978e-01 7.945559e-01 6.763364e-01

2456 9.726257e-01 9.847869e-01 9.858905e-01 9.871335e-01 9.866330e-01 7.229680e-01 6.987347e-01 6.875466e-01 7.939256e-01 6.766603e-01

2457 9.726593e-01 9.848084e-01 9.859079e-01 9.871464e-01 9.866426e-01 7.226231e-01 6.987734e-01 6.878053e-01 7.932825e-01 6.769944e-01

2458 9.726947e-01 9.848309e-01 9.859261e-01 9.871599e-01 9.866527e-01 7.222780e-01 6.988194e-01 6.880740e-01 7.926266e-01 6.773387e-01

2459 9.727322e-01 9.848545e-01 9.859452e-01 9.871742e-01 9.866633e-01 7.219330e-01 6.988729e-01 6.883527e-01 7.919578e-01 6.776934e-01

2460 9.727719e-01 9.848793e-01 9.859653e-01 9.871891e-01 9.866744e-01 7.215885e-01 6.989341e-01 6.886416e-01 7.912763e-01 6.780583e-01

2461 9.728139e-01 9.849053e-01 9.859865e-01 9.872049e-01 9.866862e-01 7.212447e-01 6.990032e-01 6.889406e-01 7.905821e-01 6.784334e-01

2462 9.728583e-01 9.849327e-01 9.860087e-01 9.872215e-01 9.866985e-01 7.209018e-01 6.990802e-01 6.892497e-01 7.898753e-01 6.788185e-01

2463 9.729052e-01 9.849614e-01 9.860321e-01 9.872389e-01 9.867115e-01 7.205604e-01 6.991653e-01 6.895689e-01 7.891561e-01 6.792138e-01

2464 9.729549e-01 9.849916e-01 9.860567e-01 9.872573e-01 9.867252e-01 7.202205e-01 6.992586e-01 6.898982e-01 7.884246e-01 6.796189e-01

2465 9.730074e-01 9.850233e-01 9.860826e-01 9.872767e-01 9.867396e-01 7.198826e-01 6.993601e-01 6.902375e-01 7.876809e-01 6.800338e-01

2466 9.730629e-01 9.850565e-01 9.861098e-01 9.872970e-01 9.867548e-01 7.195470e-01 6.994701e-01 6.905867e-01 7.869254e-01 6.804584e-01

2467 9.731216e-01 9.850915e-01 9.861384e-01 9.873184e-01 9.867707e-01 7.192140e-01 6.995886e-01 6.909458e-01 7.861581e-01 6.808924e-01

2468 9.731837e-01 9.851282e-01 9.861685e-01 9.873410e-01 9.867875e-01 7.188840e-01 6.997155e-01 6.913147e-01 7.853795e-01 6.813356e-01

2469 9.732494e-01 9.851668e-01 9.862002e-01 9.873647e-01 9.868052e-01 7.185572e-01 6.998511e-01 6.916932e-01 7.845897e-01 6.817879e-01

2470 9.733188e-01 9.852073e-01 9.862335e-01 9.873898e-01 9.868238e-01 7.182340e-01 6.999952e-01 6.920811e-01 7.837891e-01 6.822491e-01

2471 9.733922e-01 9.852498e-01 9.862685e-01 9.874161e-01 9.868434e-01 7.179147e-01 7.001478e-01 6.924783e-01 7.829780e-01 6.827187e-01

2472 9.734698e-01 9.852945e-01 9.863053e-01 9.874438e-01 9.868641e-01 7.175997e-01 7.003091e-01 6.928846e-01 7.821569e-01 6.831967e-01

2473 9.735518e-01 9.853415e-01 9.863441e-01 9.874730e-01 9.868858e-01 7.172892e-01 7.004788e-01 6.932997e-01 7.813261e-01 6.836826e-01

2474 9.736384e-01 9.853908e-01 9.863849e-01 9.875038e-01 9.869087e-01 7.169835e-01 7.006571e-01 6.937235e-01 7.804860e-01 6.841762e-01

2475 9.737300e-01 9.854426e-01 9.864278e-01 9.875361e-01 9.869329e-01 7.166831e-01 7.008437e-01 6.941556e-01 7.796372e-01 6.846772e-01

2476 9.738268e-01 9.854971e-01 9.864729e-01 9.875702e-01 9.869583e-01 7.163881e-01 7.010387e-01 6.945958e-01 7.787800e-01 6.851851e-01

2477 9.739290e-01 9.855542e-01 9.865204e-01 9.876062e-01 9.869850e-01 7.160988e-01 7.012419e-01 6.950438e-01 7.779151e-01 6.856996e-01

2478 9.740370e-01 9.856143e-01 9.865704e-01 9.876440e-01 9.870132e-01 7.158156e-01 7.014532e-01 6.954993e-01 7.770431e-01 6.862203e-01

2479 9.741510e-01 9.856773e-01 9.866229e-01 9.876838e-01 9.870429e-01 7.155386e-01 7.016724e-01 6.959619e-01 7.761643e-01 6.867469e-01

2480 9.742714e-01 9.857436e-01 9.866782e-01 9.877258e-01 9.870742e-01 7.152682e-01 7.018994e-01 6.964313e-01 7.752796e-01 6.872789e-01

2481 9.743986e-01 9.858132e-01 9.867364e-01 9.877700e-01 9.871071e-01 7.150046e-01 7.021340e-01 6.969071e-01 7.743894e-01 6.878159e-01

2482 9.745328e-01 9.858862e-01 9.867975e-01 9.878165e-01 9.871418e-01 7.147480e-01 7.023760e-01 6.973889e-01 7.734945e-01 6.883575e-01

2483 9.746745e-01 9.859630e-01 9.868619e-01 9.878655e-01 9.871783e-01 7.144986e-01 7.026251e-01 6.978764e-01 7.725954e-01 6.889031e-01

2484 9.748240e-01 9.860435e-01 9.869296e-01 9.879171e-01 9.872168e-01 7.142566e-01 7.028812e-01 6.983691e-01 7.716930e-01 6.894524e-01

2485 9.749818e-01 9.861281e-01 9.870008e-01 9.879715e-01 9.872573e-01 7.140222e-01 7.031440e-01 6.988667e-01 7.707879e-01 6.900050e-01

2486 9.751482e-01 9.862170e-01 9.870757e-01 9.880287e-01 9.873000e-01 7.137955e-01 7.034132e-01 6.993686e-01 7.698808e-01 6.905602e-01

2487 9.753236e-01 9.863102e-01 9.871544e-01 9.880890e-01 9.873449e-01 7.135767e-01 7.036886e-01 6.998745e-01 7.689725e-01 6.911178e-01

2488 9.755086e-01 9.864081e-01 9.872372e-01 9.881525e-01 9.873922e-01 7.133660e-01 7.039698e-01 7.003840e-01 7.680637e-01 6.916771e-01

2489 9.757036e-01 9.865108e-01 9.873243e-01 9.882193e-01 9.874421e-01 7.131633e-01 7.042566e-01 7.008965e-01 7.671552e-01 6.922378e-01

2490 9.759091e-01 9.866186e-01 9.874158e-01 9.882896e-01 9.874945e-01 7.129689e-01 7.045487e-01 7.014116e-01 7.662477e-01 6.927994e-01

2491 9.761256e-01 9.867317e-01 9.875120e-01 9.883637e-01 9.875497e-01 7.127827e-01 7.048456e-01 7.019289e-01 7.653420e-01 6.933613e-01

2492 9.763536e-01 9.868504e-01 9.876132e-01 9.884416e-01 9.876078e-01 7.126048e-01 7.051472e-01 7.024479e-01 7.644389e-01 6.939232e-01

2493 9.765937e-01 9.869749e-01 9.877195e-01 9.885237e-01 9.876690e-01 7.124353e-01 7.054531e-01 7.029682e-01 7.635391e-01 6.944845e-01

2494 9.768464e-01 9.871055e-01 9.878312e-01 9.886100e-01 9.877333e-01 7.122741e-01 7.057629e-01 7.034894e-01 7.626434e-01 6.950448e-01

2495 9.771123e-01 9.872424e-01 9.879485e-01 9.887009e-01 9.878010e-01 7.121213e-01 7.060762e-01 7.040109e-01 7.617525e-01 6.956038e-01

2496 9.773920e-01 9.873860e-01 9.880718e-01 9.887964e-01 9.878722e-01 7.119768e-01 7.063928e-01 7.045323e-01 7.608673e-01 6.961608e-01

2497 9.776861e-01 9.875365e-01 9.882013e-01 9.888969e-01 9.879471e-01 7.118405e-01 7.067123e-01 7.050532e-01 7.599884e-01 6.967156e-01

2498 9.779953e-01 9.876942e-01 9.883373e-01 9.890027e-01 9.880258e-01 7.117125e-01 7.070343e-01 7.055732e-01 7.591166e-01 6.972677e-01

2499 9.783202e-01 9.878595e-01 9.884801e-01 9.891138e-01 9.881086e-01 7.115926e-01 7.073585e-01 7.060918e-01 7.582526e-01 6.978167e-01

2500 9.786615e-01 9.880326e-01 9.886300e-01 9.892307e-01 9.881956e-01 7.114807e-01 7.076846e-01 7.066087e-01 7.573970e-01 6.983622e-01

2501 9.790198e-01 9.882140e-01 9.887873e-01 9.893535e-01 9.882870e-01 7.113768e-01 7.080121e-01 7.071235e-01 7.565505e-01 6.989038e-01

2502 9.793959e-01 9.884039e-01 9.889524e-01 9.894826e-01 9.883831e-01 7.112807e-01 7.083408e-01 7.076357e-01 7.557138e-01 6.994412e-01

2503 9.797905e-01 9.886027e-01 9.891255e-01 9.896183e-01 9.884840e-01 7.111922e-01 7.086703e-01 7.081449e-01 7.548874e-01 6.999741e-01

2504 9.802042e-01 9.888108e-01 9.893071e-01 9.897607e-01 9.885899e-01 7.111113e-01 7.090003e-01 7.086510e-01 7.540720e-01 7.005020e-01

2505 9.806380e-01 9.890286e-01 9.894975e-01 9.899103e-01 9.887011e-01 7.110377e-01 7.093305e-01 7.091534e-01 7.532682e-01 7.010248e-01

2506 9.810924e-01 9.892564e-01 9.896971e-01 9.900674e-01 9.888178e-01 7.109713e-01 7.096605e-01 7.096518e-01 7.524764e-01 7.015420e-01

2507 9.815683e-01 9.894947e-01 9.899062e-01 9.902323e-01 9.889401e-01 7.109120e-01 7.099901e-01 7.101460e-01 7.516971e-01 7.020535e-01

2508 9.820665e-01 9.897439e-01 9.901253e-01 9.904052e-01 9.890685e-01 7.108594e-01 7.103190e-01 7.106356e-01 7.509309e-01 7.025590e-01

2509 9.825876e-01 9.900044e-01 9.903547e-01 9.905867e-01 9.892030e-01 7.108135e-01 7.106468e-01 7.111205e-01 7.501780e-01 7.030582e-01

2510 9.831326e-01 9.902768e-01 9.905950e-01 9.907769e-01 9.893440e-01 7.107741e-01 7.109734e-01 7.116002e-01 7.494390e-01 7.035510e-01

2511 9.837021e-01 9.905616e-01 9.908464e-01 9.909764e-01 9.894917e-01 7.107408e-01 7.112984e-01 7.120745e-01 7.487142e-01 7.040370e-01

2512 9.842969e-01 9.908591e-01 9.911094e-01 9.911854e-01 9.896463e-01 7.107136e-01 7.116216e-01 7.125433e-01 7.480039e-01 7.045162e-01

2513 9.849179e-01 9.911700e-01 9.913845e-01 9.914043e-01 9.898081e-01 7.106922e-01 7.119427e-01 7.130063e-01 7.473084e-01 7.049884e-01

2514 9.855658e-01 9.914948e-01 9.916721e-01 9.916335e-01 9.899774e-01 7.106763e-01 7.122616e-01 7.134634e-01 7.466279e-01 7.054533e-01

2515 9.862414e-01 9.918340e-01 9.919726e-01 9.918734e-01 9.901544e-01 7.106659e-01 7.125780e-01 7.139142e-01 7.459627e-01 7.059110e-01

2516 9.869455e-01 9.921882e-01 9.922866e-01 9.921245e-01 9.903394e-01 7.106606e-01 7.128917e-01 7.143588e-01 7.453129e-01 7.063612e-01

2517 9.876790e-01 9.925581e-01 9.926145e-01 9.923870e-01 9.905327e-01 7.106602e-01 7.132026e-01 7.147968e-01 7.446787e-01 7.068039e-01

2518 9.884424e-01 9.929441e-01 9.929569e-01 9.926615e-01 9.907345e-01 7.106645e-01 7.135104e-01 7.152283e-01 7.440602e-01 7.072390e-01

2519 9.892368e-01 9.933469e-01 9.933141e-01 9.929483e-01 9.909451e-01 7.106734e-01 7.138151e-01 7.156531e-01 7.434574e-01 7.076665e-01

2520 9.900628e-01 9.937673e-01 9.936869e-01 9.932478e-01 9.911648e-01 7.106865e-01 7.141164e-01 7.160711e-01 7.428704e-01 7.080862e-01

2521 9.909211e-01 9.942057e-01 9.940757e-01 9.935606e-01 9.913937e-01 7.107037e-01 7.144142e-01 7.164822e-01 7.422993e-01 7.084981e-01

2522 9.909212e-01 9.942058e-01 9.940757e-01 9.935606e-01 9.913938e-01 7.106891e-01 7.144246e-01 7.165043e-01 7.422575e-01 7.085240e-01

2523 9.909213e-01 9.942058e-01 9.940757e-01 9.935606e-01 9.913938e-01 7.106745e-01 7.144355e-01 7.165270e-01 7.422148e-01 7.085506e-01

2524 9.909215e-01 9.942059e-01 9.940758e-01 9.935607e-01 9.913938e-01 7.106599e-01 7.144469e-01 7.165505e-01 7.421712e-01 7.085780e-01

2525 9.909216e-01 9.942060e-01 9.940759e-01 9.935607e-01 9.913938e-01 7.106453e-01 7.144588e-01 7.165747e-01 7.421268e-01 7.086061e-01

2526 9.909217e-01 9.942060e-01 9.940759e-01 9.935608e-01 9.913938e-01 7.106308e-01 7.144714e-01 7.165996e-01 7.420815e-01 7.086350e-01

2527 9.909219e-01 9.942061e-01 9.940760e-01 9.935608e-01 9.913939e-01 7.106163e-01 7.144845e-01 7.166253e-01 7.420353e-01 7.086647e-01

2528 9.909220e-01 9.942062e-01 9.940760e-01 9.935609e-01 9.913939e-01 7.106019e-01 7.144981e-01 7.166518e-01 7.419881e-01 7.086952e-01

2529 9.909222e-01 9.942062e-01 9.940761e-01 9.935609e-01 9.913939e-01 7.105875e-01 7.145125e-01 7.166790e-01 7.419401e-01 7.087265e-01

2530 9.909223e-01 9.942063e-01 9.940761e-01 9.935610e-01 9.913939e-01 7.105732e-01 7.145274e-01 7.167071e-01 7.418910e-01 7.087587e-01

2531 9.909225e-01 9.942064e-01 9.940762e-01 9.935610e-01 9.913939e-01 7.105590e-01 7.145430e-01 7.167360e-01 7.418411e-01 7.087917e-01

2532 9.909226e-01 9.942065e-01 9.940763e-01 9.935611e-01 9.913939e-01 7.105450e-01 7.145593e-01 7.167657e-01 7.417901e-01 7.088257e-01

2533 9.909228e-01 9.942066e-01 9.940764e-01 9.935611e-01 9.913940e-01 7.105311e-01 7.145763e-01 7.167963e-01 7.417382e-01 7.088605e-01

2534 9.909230e-01 9.942066e-01 9.940764e-01 9.935612e-01 9.913940e-01 7.105173e-01 7.145940e-01 7.168279e-01 7.416852e-01 7.088962e-01

2535 9.909232e-01 9.942067e-01 9.940765e-01 9.935612e-01 9.913940e-01 7.105036e-01 7.146124e-01 7.168603e-01 7.416313e-01 7.089329e-01

2536 9.909234e-01 9.942068e-01 9.940766e-01 9.935613e-01 9.913940e-01 7.104902e-01 7.146317e-01 7.168937e-01 7.415763e-01 7.089706e-01

2537 9.909236e-01 9.942069e-01 9.940767e-01 9.935613e-01 9.913940e-01 7.104769e-01 7.146517e-01 7.169280e-01 7.415202e-01 7.090092e-01

2538 9.909238e-01 9.942070e-01 9.940768e-01 9.935614e-01 9.913941e-01 7.104639e-01 7.146726e-01 7.169634e-01 7.414631e-01 7.090489e-01

2539 9.909240e-01 9.942071e-01 9.940769e-01 9.935615e-01 9.913941e-01 7.104511e-01 7.146943e-01 7.169998e-01 7.414049e-01 7.090896e-01

2540 9.909242e-01 9.942073e-01 9.940769e-01 9.935615e-01 9.913941e-01 7.104385e-01 7.147169e-01 7.170372e-01 7.413456e-01 7.091315e-01

2541 9.909245e-01 9.942074e-01 9.940770e-01 9.935616e-01 9.913941e-01 7.104263e-01 7.147404e-01 7.170758e-01 7.412851e-01 7.091744e-01

2542 9.909247e-01 9.942075e-01 9.940771e-01 9.935617e-01 9.913941e-01 7.104143e-01 7.147649e-01 7.171154e-01 7.412235e-01 7.092184e-01

2543 9.909250e-01 9.942076e-01 9.940773e-01 9.935617e-01 9.913942e-01 7.104026e-01 7.147904e-01 7.171563e-01 7.411608e-01 7.092636e-01

2544 9.909252e-01 9.942078e-01 9.940774e-01 9.935618e-01 9.913942e-01 7.103913e-01 7.148169e-01 7.171983e-01 7.410968e-01 7.093100e-01

2545 9.909255e-01 9.942079e-01 9.940775e-01 9.935619e-01 9.913942e-01 7.103804e-01 7.148444e-01 7.172415e-01 7.410316e-01 7.093577e-01

2546 9.909258e-01 9.942080e-01 9.940776e-01 9.935619e-01 9.913942e-01 7.103698e-01 7.148731e-01 7.172860e-01 7.409653e-01 7.094066e-01

2547 9.909261e-01 9.942082e-01 9.940777e-01 9.935620e-01 9.913942e-01 7.103597e-01 7.149028e-01 7.173317e-01 7.408976e-01 7.094568e-01

2548 9.909264e-01 9.942083e-01 9.940778e-01 9.935621e-01 9.913943e-01 7.103501e-01 7.149338e-01 7.173788e-01 7.408287e-01 7.095083e-01

2549 9.909267e-01 9.942085e-01 9.940780e-01 9.935622e-01 9.913943e-01 7.103409e-01 7.149659e-01 7.174273e-01 7.407585e-01 7.095612e-01

2550 9.909270e-01 9.942087e-01 9.940781e-01 9.935623e-01 9.913943e-01 7.103322e-01 7.149993e-01 7.174772e-01 7.406870e-01 7.096155e-01

2551 9.909274e-01 9.942088e-01 9.940782e-01 9.935624e-01 9.913943e-01 7.103241e-01 7.150340e-01 7.175285e-01 7.406142e-01 7.096712e-01

2552 9.909277e-01 9.942090e-01 9.940784e-01 9.935625e-01 9.913944e-01 7.103166e-01 7.150700e-01 7.175813e-01 7.405400e-01 7.097284e-01

2553 9.909281e-01 9.942092e-01 9.940785e-01 9.935626e-01 9.913944e-01 7.103096e-01 7.151074e-01 7.176357e-01 7.404645e-01 7.097872e-01

2554 9.909285e-01 9.942094e-01 9.940787e-01 9.935627e-01 9.913944e-01 7.103034e-01 7.151463e-01 7.176917e-01 7.403875e-01 7.098475e-01

2555 9.909289e-01 9.942096e-01 9.940789e-01 9.935628e-01 9.913944e-01 7.102978e-01 7.151866e-01 7.177493e-01 7.403092e-01 7.099095e-01

2556 9.909293e-01 9.942098e-01 9.940790e-01 9.935629e-01 9.913945e-01 7.102930e-01 7.152285e-01 7.178085e-01 7.402294e-01 7.099730e-01

2557 9.909298e-01 9.942100e-01 9.940792e-01 9.935630e-01 9.913945e-01 7.102889e-01 7.152720e-01 7.178695e-01 7.401481e-01 7.100383e-01

2558 9.909302e-01 9.942103e-01 9.940794e-01 9.935631e-01 9.913945e-01 7.102857e-01 7.153171e-01 7.179323e-01 7.400654e-01 7.101054e-01

2559 9.909307e-01 9.942105e-01 9.940796e-01 9.935632e-01 9.913945e-01 7.102833e-01 7.153639e-01 7.179969e-01 7.399811e-01 7.101742e-01

2560 9.909312e-01 9.942107e-01 9.940798e-01 9.935634e-01 9.913945e-01 7.102818e-01 7.154125e-01 7.180634e-01 7.398953e-01 7.102449e-01

2561 9.909317e-01 9.942110e-01 9.940800e-01 9.935635e-01 9.913946e-01 7.102813e-01 7.154629e-01 7.181318e-01 7.398080e-01 7.103175e-01

2562 9.909322e-01 9.942113e-01 9.940802e-01 9.935636e-01 9.913946e-01 7.102818e-01 7.155151e-01 7.182023e-01 7.397191e-01 7.103920e-01

2563 9.909328e-01 9.942115e-01 9.940804e-01 9.935637e-01 9.913946e-01 7.102834e-01 7.155693e-01 7.182748e-01 7.396287e-01 7.104686e-01

2564 9.909333e-01 9.942118e-01 9.940806e-01 9.935639e-01 9.913946e-01 7.102861e-01 7.156255e-01 7.183494e-01 7.395366e-01 7.105472e-01

2565 9.909339e-01 9.942121e-01 9.940808e-01 9.935640e-01 9.913946e-01 7.102900e-01 7.156838e-01 7.184262e-01 7.394429e-01 7.106279e-01

2566 9.909346e-01 9.942124e-01 9.940811e-01 9.935642e-01 9.913946e-01 7.102951e-01 7.157443e-01 7.185052e-01 7.393475e-01 7.107108e-01

2567 9.909352e-01 9.942127e-01 9.940813e-01 9.935643e-01 9.913947e-01 7.103015e-01 7.158069e-01 7.185866e-01 7.392505e-01 7.107960e-01

2568 9.909359e-01 9.942131e-01 9.940816e-01 9.935645e-01 9.913947e-01 7.103092e-01 7.158719e-01 7.186703e-01 7.391517e-01 7.108834e-01

2569 9.909366e-01 9.942134e-01 9.940819e-01 9.935646e-01 9.913947e-01 7.103184e-01 7.159392e-01 7.187565e-01 7.390513e-01 7.109732e-01

2570 9.909373e-01 9.942138e-01 9.940821e-01 9.935648e-01 9.913947e-01 7.103291e-01 7.160090e-01 7.188452e-01 7.389491e-01 7.110654e-01

2571 9.909381e-01 9.942141e-01 9.940824e-01 9.935650e-01 9.913947e-01 7.103414e-01 7.160813e-01 7.189365e-01 7.388452e-01 7.111602e-01

2572 9.909389e-01 9.942145e-01 9.940827e-01 9.935652e-01 9.913947e-01 7.103553e-01 7.161563e-01 7.190304e-01 7.387395e-01 7.112574e-01

2573 9.909397e-01 9.942149e-01 9.940830e-01 9.935654e-01 9.913947e-01 7.103709e-01 7.162339e-01 7.191272e-01 7.386320e-01 7.113574e-01

2574 9.909405e-01 9.942154e-01 9.940834e-01 9.935656e-01 9.913947e-01 7.103884e-01 7.163143e-01 7.192267e-01 7.385228e-01 7.114600e-01

2575 9.909414e-01 9.942158e-01 9.940837e-01 9.935658e-01 9.913947e-01 7.104077e-01 7.163976e-01 7.193291e-01 7.384117e-01 7.115654e-01

2576 9.909424e-01 9.942163e-01 9.940841e-01 9.935660e-01 9.913947e-01 7.104290e-01 7.164839e-01 7.194346e-01 7.382988e-01 7.116736e-01

2577 9.909433e-01 9.942167e-01 9.940844e-01 9.935662e-01 9.913947e-01 7.104523e-01 7.165733e-01 7.195431e-01 7.381840e-01 7.117848e-01

2578 9.909444e-01 9.942172e-01 9.940848e-01 9.935664e-01 9.913947e-01 7.104778e-01 7.166658e-01 7.196548e-01 7.380674e-01 7.118990e-01

2579 9.909454e-01 9.942178e-01 9.940852e-01 9.935666e-01 9.913947e-01 7.105056e-01 7.167616e-01 7.197697e-01 7.379489e-01 7.120163e-01

2580 9.909465e-01 9.942183e-01 9.940856e-01 9.935669e-01 9.913947e-01 7.105357e-01 7.168608e-01 7.198880e-01 7.378285e-01 7.121368e-01

2581 9.909477e-01 9.942189e-01 9.940860e-01 9.935671e-01 9.913947e-01 7.105683e-01 7.169634e-01 7.200097e-01 7.377063e-01 7.122605e-01

2582 9.909489e-01 9.942194e-01 9.940865e-01 9.935674e-01 9.913947e-01 7.106034e-01 7.170697e-01 7.201349e-01 7.375821e-01 7.123875e-01

2583 9.909501e-01 9.942201e-01 9.940870e-01 9.935676e-01 9.913947e-01 7.106411e-01 7.171797e-01 7.202638e-01 7.374561e-01 7.125180e-01

2584 9.909514e-01 9.942207e-01 9.940874e-01 9.935679e-01 9.913947e-01 7.106817e-01 7.172935e-01 7.203965e-01 7.373281e-01 7.126520e-01

2585 9.909528e-01 9.942214e-01 9.940879e-01 9.935682e-01 9.913947e-01 7.107251e-01 7.174112e-01 7.205329e-01 7.371982e-01 7.127896e-01

2586 9.909542e-01 9.942220e-01 9.940885e-01 9.935685e-01 9.913947e-01 7.107716e-01 7.175330e-01 7.206733e-01 7.370664e-01 7.129310e-01

2587 9.909556e-01 9.942228e-01 9.940890e-01 9.935688e-01 9.913946e-01 7.108212e-01 7.176591e-01 7.208178e-01 7.369327e-01 7.130761e-01

2588 9.909572e-01 9.942235e-01 9.940896e-01 9.935691e-01 9.913946e-01 7.108740e-01 7.177894e-01 7.209664e-01 7.367971e-01 7.132251e-01

2589 9.909588e-01 9.942243e-01 9.940902e-01 9.935694e-01 9.913946e-01 7.109303e-01 7.179242e-01 7.211193e-01 7.366596e-01 7.133781e-01

2590 9.909604e-01 9.942251e-01 9.940908e-01 9.935698e-01 9.913945e-01 7.109901e-01 7.180636e-01 7.212767e-01 7.365201e-01 7.135353e-01

2591 9.909622e-01 9.942260e-01 9.940914e-01 9.935701e-01 9.913945e-01 7.110535e-01 7.182077e-01 7.214385e-01 7.363788e-01 7.136966e-01

2592 9.909640e-01 9.942268e-01 9.940921e-01 9.935705e-01 9.913944e-01 7.111208e-01 7.183566e-01 7.216049e-01 7.362356e-01 7.138622e-01

2593 9.909659e-01 9.942278e-01 9.940928e-01 9.935709e-01 9.913944e-01 7.111920e-01 7.185106e-01 7.217761e-01 7.360905e-01 7.140322e-01

2594 9.909678e-01 9.942287e-01 9.940935e-01 9.935713e-01 9.913943e-01 7.112673e-01 7.186697e-01 7.219521e-01 7.359436e-01 7.142068e-01

2595 9.909699e-01 9.942297e-01 9.940942e-01 9.935717e-01 9.913943e-01 7.113469e-01 7.188342e-01 7.221332e-01 7.357949e-01 7.143860e-01

2596 9.909721e-01 9.942308e-01 9.940950e-01 9.935721e-01 9.913942e-01 7.114309e-01 7.190041e-01 7.223194e-01 7.356444e-01 7.145699e-01

2597 9.909743e-01 9.942319e-01 9.940958e-01 9.935725e-01 9.913941e-01 7.115194e-01 7.191796e-01 7.225108e-01 7.354920e-01 7.147586e-01

2598 9.909766e-01 9.942330e-01 9.940967e-01 9.935730e-01 9.913940e-01 7.116128e-01 7.193608e-01 7.227076e-01 7.353380e-01 7.149524e-01

2599 9.909791e-01 9.942342e-01 9.940976e-01 9.935735e-01 9.913939e-01 7.117110e-01 7.195480e-01 7.229100e-01 7.351822e-01 7.151511e-01

2600 9.909816e-01 9.942354e-01 9.940985e-01 9.935740e-01 9.913938e-01 7.118143e-01 7.197413e-01 7.231180e-01 7.350247e-01 7.153551e-01

2601 9.909843e-01 9.942367e-01 9.940994e-01 9.935745e-01 9.913937e-01 7.119229e-01 7.199408e-01 7.233317e-01 7.348656e-01 7.155644e-01

2602 9.909871e-01 9.942381e-01 9.941004e-01 9.935750e-01 9.913936e-01 7.120370e-01 7.201468e-01 7.235515e-01 7.347049e-01 7.157791e-01

2603 9.909900e-01 9.942395e-01 9.941015e-01 9.935755e-01 9.913934e-01 7.121567e-01 7.203593e-01 7.237772e-01 7.345427e-01 7.159994e-01

2604 9.909930e-01 9.942409e-01 9.941026e-01 9.935761e-01 9.913933e-01 7.122822e-01 7.205787e-01 7.240092e-01 7.343789e-01 7.162253e-01

2605 9.909961e-01 9.942425e-01 9.941037e-01 9.935767e-01 9.913931e-01 7.124137e-01 7.208049e-01 7.242476e-01 7.342138e-01 7.164570e-01

2606 9.909994e-01 9.942441e-01 9.941049e-01 9.935773e-01 9.913929e-01 7.125515e-01 7.210383e-01 7.244924e-01 7.340472e-01 7.166945e-01

2607 9.910029e-01 9.942457e-01 9.941061e-01 9.935779e-01 9.913928e-01 7.126956e-01 7.212790e-01 7.247439e-01 7.338794e-01 7.169381e-01

2608 9.910065e-01 9.942475e-01 9.941074e-01 9.935786e-01 9.913925e-01 7.128464e-01 7.215271e-01 7.250021e-01 7.337103e-01 7.171878e-01

2609 9.910102e-01 9.942493e-01 9.941087e-01 9.935793e-01 9.913923e-01 7.130040e-01 7.217829e-01 7.252673e-01 7.335401e-01 7.174438e-01

2610 9.910142e-01 9.942512e-01 9.941101e-01 9.935800e-01 9.913921e-01 7.131686e-01 7.220465e-01 7.255395e-01 7.333688e-01 7.177062e-01

2611 9.910183e-01 9.942532e-01 9.941116e-01 9.935807e-01 9.913918e-01 7.133404e-01 7.223181e-01 7.258190e-01 7.331965e-01 7.179750e-01

2612 9.910226e-01 9.942552e-01 9.941131e-01 9.935815e-01 9.913915e-01 7.135197e-01 7.225980e-01 7.261058e-01 7.330234e-01 7.182504e-01

2613 9.910270e-01 9.942574e-01 9.941147e-01 9.935823e-01 9.913912e-01 7.137067e-01 7.228862e-01 7.264001e-01 7.328494e-01 7.185325e-01

2614 9.910317e-01 9.942596e-01 9.941163e-01 9.935831e-01 9.913909e-01 7.139015e-01 7.231830e-01 7.267021e-01 7.326748e-01 7.188215e-01

2615 9.910366e-01 9.942620e-01 9.941180e-01 9.935840e-01 9.913905e-01 7.141044e-01 7.234885e-01 7.270118e-01 7.324996e-01 7.191174e-01

2616 9.910417e-01 9.942645e-01 9.941198e-01 9.935849e-01 9.913902e-01 7.143155e-01 7.238030e-01 7.273295e-01 7.323240e-01 7.194204e-01

2617 9.910471e-01 9.942670e-01 9.941217e-01 9.935858e-01 9.913898e-01 7.145352e-01 7.241265e-01 7.276553e-01 7.321480e-01 7.197305e-01

2618 9.910526e-01 9.942697e-01 9.941237e-01 9.935868e-01 9.913893e-01 7.147637e-01 7.244594e-01 7.279892e-01 7.319718e-01 7.200479e-01

2619 9.910585e-01 9.942725e-01 9.941257e-01 9.935878e-01 9.913888e-01 7.150011e-01 7.248017e-01 7.283315e-01 7.317955e-01 7.203727e-01

2620 9.910646e-01 9.942755e-01 9.941279e-01 9.935888e-01 9.913883e-01 7.152476e-01 7.251537e-01 7.286823e-01 7.316194e-01 7.207049e-01

2621 9.910710e-01 9.942785e-01 9.941301e-01 9.935899e-01 9.913878e-01 7.155036e-01 7.255154e-01 7.290417e-01 7.314434e-01 7.210447e-01

2622 9.910776e-01 9.942817e-01 9.941324e-01 9.935910e-01 9.913872e-01 7.157691e-01 7.258872e-01 7.294098e-01 7.312678e-01 7.213921e-01

2623 9.910846e-01 9.942851e-01 9.941348e-01 9.935922e-01 9.913866e-01 7.160444e-01 7.262691e-01 7.297867e-01 7.310928e-01 7.217472e-01

2624 9.910919e-01 9.942886e-01 9.941374e-01 9.935934e-01 9.913859e-01 7.163297e-01 7.266613e-01 7.301726e-01 7.309184e-01 7.221102e-01

2625 9.910996e-01 9.942923e-01 9.941400e-01 9.935946e-01 9.913852e-01 7.166252e-01 7.270640e-01 7.305676e-01 7.307450e-01 7.224810e-01

2626 9.911075e-01 9.942961e-01 9.941428e-01 9.935959e-01 9.913844e-01 7.169312e-01 7.274773e-01 7.309718e-01 7.305725e-01 7.228598e-01

2627 9.911159e-01 9.943001e-01 9.941457e-01 9.935973e-01 9.913836e-01 7.172477e-01 7.279013e-01 7.313853e-01 7.304013e-01 7.232466e-01

2628 9.911246e-01 9.943043e-01 9.941487e-01 9.935987e-01 9.913827e-01 7.175750e-01 7.283362e-01 7.318081e-01 7.302316e-01 7.236415e-01

2629 9.911338e-01 9.943087e-01 9.941518e-01 9.936002e-01 9.913817e-01 7.179132e-01 7.287822e-01 7.322404e-01 7.300634e-01 7.240446e-01

2630 9.911434e-01 9.943132e-01 9.941551e-01 9.936017e-01 9.913807e-01 7.182626e-01 7.292394e-01 7.326823e-01 7.298970e-01 7.244558e-01

2631 9.911534e-01 9.943180e-01 9.941586e-01 9.936033e-01 9.913796e-01 7.186233e-01 7.297078e-01 7.331338e-01 7.297327e-01 7.248751e-01

2632 9.911638e-01 9.943230e-01 9.941622e-01 9.936049e-01 9.913784e-01 7.189955e-01 7.301876e-01 7.335949e-01 7.295706e-01 7.253028e-01

2633 9.911748e-01 9.943283e-01 9.941659e-01 9.936066e-01 9.913772e-01 7.193792e-01 7.306788e-01 7.340658e-01 7.294109e-01 7.257386e-01

2634 9.911863e-01 9.943338e-01 9.941699e-01 9.936084e-01 9.913759e-01 7.197747e-01 7.311817e-01 7.345465e-01 7.292539e-01 7.261827e-01

2635 9.911983e-01 9.943395e-01 9.941740e-01 9.936103e-01 9.913744e-01 7.201821e-01 7.316961e-01 7.350371e-01 7.290997e-01 7.266351e-01

2636 9.912109e-01 9.943455e-01 9.941783e-01 9.936122e-01 9.913729e-01 7.206014e-01 7.322223e-01 7.355375e-01 7.289486e-01 7.270957e-01

2637 9.912241e-01 9.943518e-01 9.941828e-01 9.936142e-01 9.913713e-01 7.210329e-01 7.327603e-01 7.360477e-01 7.288009e-01 7.275645e-01

2638 9.912378e-01 9.943584e-01 9.941875e-01 9.936162e-01 9.913695e-01 7.214765e-01 7.333100e-01 7.365679e-01 7.286566e-01 7.280415e-01

2639 9.912523e-01 9.943653e-01 9.941924e-01 9.936184e-01 9.913677e-01 7.219323e-01 7.338716e-01 7.370979e-01 7.285161e-01 7.285266e-01

2640 9.912674e-01 9.943725e-01 9.941975e-01 9.936206e-01 9.913657e-01 7.224005e-01 7.344450e-01 7.376377e-01 7.283796e-01 7.290198e-01

2641 9.912832e-01 9.943800e-01 9.942029e-01 9.936230e-01 9.913635e-01 7.228810e-01 7.350303e-01 7.381874e-01 7.282474e-01 7.295210e-01

2642 9.912998e-01 9.943880e-01 9.942086e-01 9.936254e-01 9.913613e-01 7.233739e-01 7.356274e-01 7.387469e-01 7.281195e-01 7.300301e-01

2643 9.913171e-01 9.943962e-01 9.942144e-01 9.936279e-01 9.913588e-01 7.238792e-01 7.362363e-01 7.393160e-01 7.279963e-01 7.305470e-01

2644 9.913353e-01 9.944049e-01 9.942206e-01 9.936305e-01 9.913562e-01 7.243969e-01 7.368569e-01 7.398948e-01 7.278779e-01 7.310717e-01

2645 9.913544e-01 9.944140e-01 9.942270e-01 9.936332e-01 9.913535e-01 7.249270e-01 7.374892e-01 7.404832e-01 7.277647e-01 7.316040e-01

2646 9.913743e-01 9.944235e-01 9.942338e-01 9.936361e-01 9.913505e-01 7.254693e-01 7.381332e-01 7.410810e-01 7.276568e-01 7.321438e-01

2647 9.913952e-01 9.944335e-01 9.942408e-01 9.936390e-01 9.913473e-01 7.260240e-01 7.387886e-01 7.416881e-01 7.275543e-01 7.326909e-01

2648 9.914171e-01 9.944439e-01 9.942482e-01 9.936420e-01 9.913440e-01 7.265908e-01 7.394554e-01 7.423045e-01 7.274576e-01 7.332451e-01

2649 9.914400e-01 9.944548e-01 9.942560e-01 9.936452e-01 9.913404e-01 7.271697e-01 7.401335e-01 7.429299e-01 7.273668e-01 7.338063e-01

2650 9.914640e-01 9.944663e-01 9.942641e-01 9.936485e-01 9.913365e-01 7.277605e-01 7.408227e-01 7.435642e-01 7.272821e-01 7.343744e-01

2651 9.914892e-01 9.944783e-01 9.942725e-01 9.936519e-01 9.913324e-01 7.283632e-01 7.415227e-01 7.442072e-01 7.272037e-01 7.349490e-01

2652 9.915156e-01 9.944909e-01 9.942814e-01 9.936555e-01 9.913280e-01 7.289775e-01 7.422336e-01 7.448587e-01 7.271317e-01 7.355300e-01

2653 9.915432e-01 9.945041e-01 9.942907e-01 9.936592e-01 9.913234e-01 7.296032e-01 7.429549e-01 7.455185e-01 7.270663e-01 7.361171e-01

2654 9.915721e-01 9.945179e-01 9.943004e-01 9.936630e-01 9.913184e-01 7.302402e-01 7.436866e-01 7.461864e-01 7.270077e-01 7.367102e-01

2655 9.916025e-01 9.945324e-01 9.943106e-01 9.936670e-01 9.913131e-01 7.308882e-01 7.444283e-01 7.468621e-01 7.269560e-01 7.373089e-01

2656 9.916343e-01 9.945475e-01 9.943213e-01 9.936712e-01 9.913074e-01 7.315470e-01 7.451798e-01 7.475453e-01 7.269113e-01 7.379130e-01

2657 9.916676e-01 9.945635e-01 9.943325e-01 9.936755e-01 9.913014e-01 7.322163e-01 7.459407e-01 7.482359e-01 7.268738e-01 7.385222e-01

2658 9.917025e-01 9.945801e-01 9.943442e-01 9.936799e-01 9.912949e-01 7.328958e-01 7.467109e-01 7.489335e-01 7.268435e-01 7.391362e-01

2659 9.917391e-01 9.945976e-01 9.943564e-01 9.936846e-01 9.912880e-01 7.335852e-01 7.474899e-01 7.496377e-01 7.268206e-01 7.397547e-01

2660 9.917774e-01 9.946159e-01 9.943693e-01 9.936894e-01 9.912807e-01 7.342842e-01 7.482774e-01 7.503484e-01 7.268050e-01 7.403773e-01

2661 9.918176e-01 9.946352e-01 9.943827e-01 9.936944e-01 9.912729e-01 7.349924e-01 7.490731e-01 7.510650e-01 7.267970e-01 7.410038e-01

2662 9.918597e-01 9.946553e-01 9.943968e-01 9.936996e-01 9.912646e-01 7.357094e-01 7.498766e-01 7.517874e-01 7.267965e-01 7.416339e-01

2663 9.919038e-01 9.946764e-01 9.944116e-01 9.937050e-01 9.912557e-01 7.364349e-01 7.506875e-01 7.525151e-01 7.268035e-01 7.422670e-01

2664 9.919500e-01 9.946986e-01 9.944270e-01 9.937107e-01 9.912463e-01 7.371684e-01 7.515054e-01 7.532477e-01 7.268181e-01 7.429030e-01

2665 9.919984e-01 9.947218e-01 9.944432e-01 9.937165e-01 9.912362e-01 7.379095e-01 7.523298e-01 7.539849e-01 7.268403e-01 7.435414e-01

2666 9.920492e-01 9.947462e-01 9.944602e-01 9.937225e-01 9.912255e-01 7.386579e-01 7.531603e-01 7.547262e-01 7.268700e-01 7.441819e-01

2667 9.921024e-01 9.947717e-01 9.944780e-01 9.937288e-01 9.912140e-01 7.394129e-01 7.539966e-01 7.554713e-01 7.269073e-01 7.448241e-01

2668 9.921581e-01 9.947985e-01 9.944966e-01 9.937353e-01 9.912018e-01 7.401743e-01 7.548380e-01 7.562198e-01 7.269520e-01 7.454676e-01

2669 9.922166e-01 9.948266e-01 9.945161e-01 9.937420e-01 9.911889e-01 7.409414e-01 7.556841e-01 7.569711e-01 7.270041e-01 7.461119e-01

2670 9.922777e-01 9.948561e-01 9.945366e-01 9.937490e-01 9.911751e-01 7.417138e-01 7.565345e-01 7.577249e-01 7.270636e-01 7.467568e-01

2671 9.923419e-01 9.948870e-01 9.945580e-01 9.937563e-01 9.911604e-01 7.424910e-01 7.573887e-01 7.584808e-01 7.271302e-01 7.474019e-01

2672 9.924090e-01 9.949194e-01 9.945805e-01 9.937638e-01 9.911448e-01 7.432725e-01 7.582461e-01 7.592382e-01 7.272040e-01 7.480466e-01

2673 9.924794e-01 9.949534e-01 9.946040e-01 9.937716e-01 9.911281e-01 7.440577e-01 7.591062e-01 7.599968e-01 7.272848e-01 7.486906e-01

2674 9.925531e-01 9.949890e-01 9.946287e-01 9.937797e-01 9.911104e-01 7.448462e-01 7.599686e-01 7.607560e-01 7.273725e-01 7.493336e-01

2675 9.926303e-01 9.950264e-01 9.946545e-01 9.937880e-01 9.910916e-01 7.456375e-01 7.608326e-01 7.615155e-01 7.274669e-01 7.499751e-01

2676 9.927112e-01 9.950656e-01 9.946816e-01 9.937967e-01 9.910715e-01 7.464309e-01 7.616978e-01 7.622747e-01 7.275678e-01 7.506147e-01

2677 9.927959e-01 9.951067e-01 9.947100e-01 9.938057e-01 9.910502e-01 7.472260e-01 7.625637e-01 7.630332e-01 7.276751e-01 7.512521e-01

2678 9.928846e-01 9.951498e-01 9.947397e-01 9.938149e-01 9.910276e-01 7.480222e-01 7.634297e-01 7.637905e-01 7.277886e-01 7.518868e-01

2679 9.929774e-01 9.951950e-01 9.947708e-01 9.938245e-01 9.910035e-01 7.488190e-01 7.642952e-01 7.645462e-01 7.279081e-01 7.525184e-01

2680 9.930746e-01 9.952425e-01 9.948034e-01 9.938344e-01 9.909779e-01 7.496159e-01 7.651599e-01 7.652999e-01 7.280334e-01 7.531467e-01

2681 9.931764e-01 9.952922e-01 9.948376e-01 9.938447e-01 9.909508e-01 7.504123e-01 7.660230e-01 7.660510e-01 7.281642e-01 7.537712e-01

2682 9.932829e-01 9.953443e-01 9.948733e-01 9.938553e-01 9.909219e-01 7.512077e-01 7.668842e-01 7.667991e-01 7.283004e-01 7.543915e-01

2683 9.933944e-01 9.953989e-01 9.949108e-01 9.938662e-01 9.908913e-01 7.520017e-01 7.677430e-01 7.675438e-01 7.284418e-01 7.550073e-01

2684 9.935110e-01 9.954562e-01 9.949500e-01 9.938775e-01 9.908588e-01 7.527937e-01 7.685987e-01 7.682847e-01 7.285880e-01 7.556182e-01

2685 9.936331e-01 9.955162e-01 9.949911e-01 9.938891e-01 9.908243e-01 7.535832e-01 7.694509e-01 7.690213e-01 7.287389e-01 7.562240e-01

2686 9.937607e-01 9.955791e-01 9.950341e-01 9.939011e-01 9.907877e-01 7.543697e-01 7.702992e-01 7.697533e-01 7.288942e-01 7.568243e-01

2687 9.938942e-01 9.956451e-01 9.950791e-01 9.939134e-01 9.907488e-01 7.551528e-01 7.711430e-01 7.704801e-01 7.290536e-01 7.574188e-01

2688 9.940339e-01 9.957142e-01 9.951262e-01 9.939262e-01 9.907077e-01 7.559320e-01 7.719819e-01 7.712015e-01 7.292170e-01 7.580071e-01

2689 9.941798e-01 9.957866e-01 9.951755e-01 9.939393e-01 9.906641e-01 7.567068e-01 7.728154e-01 7.719170e-01 7.293840e-01 7.585891e-01

2690 9.943324e-01 9.958624e-01 9.952271e-01 9.939527e-01 9.906179e-01 7.574769e-01 7.736431e-01 7.726263e-01 7.295545e-01 7.591644e-01

2691 9.944919e-01 9.959418e-01 9.952811e-01 9.939666e-01 9.905690e-01 7.582417e-01 7.744646e-01 7.733290e-01 7.297282e-01 7.597327e-01

2692 9.946586e-01 9.960250e-01 9.953376e-01 9.939808e-01 9.905172e-01 7.590009e-01 7.752794e-01 7.740249e-01 7.299047e-01 7.602939e-01

2693 9.948328e-01 9.961122e-01 9.953966e-01 9.939954e-01 9.904624e-01 7.597541e-01 7.760872e-01 7.747134e-01 7.300840e-01 7.608477e-01

2694 9.950147e-01 9.962034e-01 9.954583e-01 9.940103e-01 9.904044e-01 7.605010e-01 7.768875e-01 7.753945e-01 7.302657e-01 7.613938e-01

2695 9.952048e-01 9.962989e-01 9.955228e-01 9.940256e-01 9.903432e-01 7.612411e-01 7.776800e-01 7.760676e-01 7.304495e-01 7.619322e-01

2696 9.954034e-01 9.963988e-01 9.955903e-01 9.940413e-01 9.902784e-01 7.619742e-01 7.784644e-01 7.767327e-01 7.306354e-01 7.624625e-01

2697 9.956107e-01 9.965034e-01 9.956607e-01 9.940573e-01 9.902101e-01 7.626999e-01 7.792403e-01 7.773894e-01 7.308230e-01 7.629847e-01

2698 9.958273e-01 9.966129e-01 9.957343e-01 9.940737e-01 9.901379e-01 7.634180e-01 7.800075e-01 7.780375e-01 7.310121e-01 7.634986e-01

2699 9.960534e-01 9.967273e-01 9.958112e-01 9.940904e-01 9.900617e-01 7.641280e-01 7.807655e-01 7.786767e-01 7.312025e-01 7.640040e-01

2700 9.962895e-01 9.968470e-01 9.958914e-01 9.941074e-01 9.899813e-01 7.648299e-01 7.815143e-01 7.793069e-01 7.313940e-01 7.645008e-01

2701 9.965360e-01 9.969721e-01 9.959751e-01 9.941247e-01 9.898967e-01 7.655233e-01 7.822534e-01 7.799278e-01 7.315863e-01 7.649890e-01

2702 9.965360e-01 9.969722e-01 9.959752e-01 9.941247e-01 9.898966e-01 7.655537e-01 7.822821e-01 7.799504e-01 7.315861e-01 7.650073e-01

2703 9.965361e-01 9.969722e-01 9.959752e-01 9.941247e-01 9.898965e-01 7.655848e-01 7.823114e-01 7.799735e-01 7.315862e-01 7.650259e-01

2704 9.965362e-01 9.969722e-01 9.959752e-01 9.941247e-01 9.898965e-01 7.656168e-01 7.823413e-01 7.799971e-01 7.315866e-01 7.650449e-01

2705 9.965362e-01 9.969723e-01 9.959753e-01 9.941247e-01 9.898964e-01 7.656496e-01 7.823720e-01 7.800211e-01 7.315874e-01 7.650642e-01

2706 9.965363e-01 9.969723e-01 9.959753e-01 9.941247e-01 9.898963e-01 7.656832e-01 7.824033e-01 7.800457e-01 7.315886e-01 7.650838e-01

2707 9.965363e-01 9.969723e-01 9.959753e-01 9.941247e-01 9.898963e-01 7.657177e-01 7.824353e-01 7.800707e-01 7.315901e-01 7.651038e-01

2708 9.965364e-01 9.969724e-01 9.959754e-01 9.941247e-01 9.898962e-01 7.657531e-01 7.824680e-01 7.800961e-01 7.315921e-01 7.651241e-01

2709 9.965365e-01 9.969724e-01 9.959754e-01 9.941246e-01 9.898962e-01 7.657894e-01 7.825014e-01 7.801221e-01 7.315945e-01 7.651448e-01

2710 9.965365e-01 9.969725e-01 9.959754e-01 9.941246e-01 9.898961e-01 7.658266e-01 7.825355e-01 7.801486e-01 7.315973e-01 7.651658e-01

2711 9.965366e-01 9.969725e-01 9.959754e-01 9.941246e-01 9.898960e-01 7.658648e-01 7.825704e-01 7.801756e-01 7.316005e-01 7.651871e-01

2712 9.965367e-01 9.969725e-01 9.959754e-01 9.941246e-01 9.898960e-01 7.659039e-01 7.826061e-01 7.802031e-01 7.316043e-01 7.652088e-01

2713 9.965367e-01 9.969726e-01 9.959755e-01 9.941246e-01 9.898959e-01 7.659440e-01 7.826425e-01 7.802312e-01 7.316085e-01 7.652309e-01

2714 9.965368e-01 9.969726e-01 9.959755e-01 9.941245e-01 9.898959e-01 7.659851e-01 7.826796e-01 7.802598e-01 7.316133e-01 7.652533e-01

2715 9.965369e-01 9.969727e-01 9.959755e-01 9.941245e-01 9.898958e-01 7.660273e-01 7.827176e-01 7.802889e-01 7.316186e-01 7.652761e-01

2716 9.965370e-01 9.969727e-01 9.959755e-01 9.941245e-01 9.898957e-01 7.660705e-01 7.827565e-01 7.803186e-01 7.316245e-01 7.652992e-01

2717 9.965371e-01 9.969728e-01 9.959755e-01 9.941244e-01 9.898956e-01 7.661148e-01 7.827961e-01 7.803488e-01 7.316310e-01 7.653228e-01

2718 9.965372e-01 9.969728e-01 9.959755e-01 9.941244e-01 9.898956e-01 7.661602e-01 7.828366e-01 7.803797e-01 7.316380e-01 7.653467e-01

2719 9.965372e-01 9.969728e-01 9.959755e-01 9.941244e-01 9.898955e-01 7.662067e-01 7.828780e-01 7.804111e-01 7.316458e-01 7.653710e-01

2720 9.965373e-01 9.969729e-01 9.959756e-01 9.941244e-01 9.898954e-01 7.662544e-01 7.829203e-01 7.804431e-01 7.316542e-01 7.653957e-01

2721 9.965374e-01 9.969729e-01 9.959756e-01 9.941243e-01 9.898953e-01 7.663033e-01 7.829634e-01 7.804758e-01 7.316633e-01 7.654208e-01

2722 9.965375e-01 9.969730e-01 9.959756e-01 9.941243e-01 9.898952e-01 7.663535e-01 7.830076e-01 7.805090e-01 7.316731e-01 7.654463e-01

2723 9.965376e-01 9.969730e-01 9.959756e-01 9.941242e-01 9.898951e-01 7.664049e-01 7.830526e-01 7.805429e-01 7.316837e-01 7.654722e-01

2724 9.965378e-01 9.969731e-01 9.959756e-01 9.941242e-01 9.898949e-01 7.664575e-01 7.830987e-01 7.805774e-01 7.316951e-01 7.654985e-01

2725 9.965379e-01 9.969731e-01 9.959756e-01 9.941242e-01 9.898948e-01 7.665116e-01 7.831457e-01 7.806127e-01 7.317073e-01 7.655252e-01

2726 9.965380e-01 9.969732e-01 9.959756e-01 9.941241e-01 9.898947e-01 7.665669e-01 7.831938e-01 7.806485e-01 7.317203e-01 7.655524e-01

2727 9.965381e-01 9.969732e-01 9.959756e-01 9.941241e-01 9.898945e-01 7.666237e-01 7.832429e-01 7.806851e-01 7.317343e-01 7.655800e-01

2728 9.965382e-01 9.969733e-01 9.959757e-01 9.941240e-01 9.898944e-01 7.666819e-01 7.832930e-01 7.807223e-01 7.317492e-01 7.656080e-01

2729 9.965384e-01 9.969733e-01 9.959757e-01 9.941240e-01 9.898942e-01 7.667415e-01 7.833443e-01 7.807603e-01 7.317650e-01 7.656365e-01

2730 9.965385e-01 9.969734e-01 9.959757e-01 9.941239e-01 9.898940e-01 7.668027e-01 7.833967e-01 7.807990e-01 7.317819e-01 7.656655e-01

2731 9.965387e-01 9.969735e-01 9.959757e-01 9.941239e-01 9.898938e-01 7.668654e-01 7.834502e-01 7.808385e-01 7.317998e-01 7.656949e-01

2732 9.965388e-01 9.969735e-01 9.959757e-01 9.941238e-01 9.898936e-01 7.669297e-01 7.835048e-01 7.808787e-01 7.318188e-01 7.657247e-01

2733 9.965390e-01 9.969736e-01 9.959757e-01 9.941237e-01 9.898934e-01 7.669956e-01 7.835607e-01 7.809196e-01 7.318389e-01 7.657550e-01

2734 9.965391e-01 9.969737e-01 9.959757e-01 9.941237e-01 9.898932e-01 7.670632e-01 7.836178e-01 7.809614e-01 7.318602e-01 7.657858e-01

2735 9.965393e-01 9.969737e-01 9.959757e-01 9.941236e-01 9.898930e-01 7.671325e-01 7.836761e-01 7.810040e-01 7.318827e-01 7.658171e-01

2736 9.965394e-01 9.969738e-01 9.959758e-01 9.941235e-01 9.898927e-01 7.672035e-01 7.837358e-01 7.810474e-01 7.319065e-01 7.658489e-01

2737 9.965396e-01 9.969739e-01 9.959758e-01 9.941234e-01 9.898925e-01 7.672764e-01 7.837967e-01 7.810916e-01 7.319316e-01 7.658812e-01

2738 9.965398e-01 9.969739e-01 9.959758e-01 9.941233e-01 9.898922e-01 7.673511e-01 7.838590e-01 7.811367e-01 7.319581e-01 7.659140e-01

2739 9.965400e-01 9.969740e-01 9.959758e-01 9.941232e-01 9.898919e-01 7.674277e-01 7.839226e-01 7.811826e-01 7.319860e-01 7.659473e-01

2740 9.965402e-01 9.969741e-01 9.959758e-01 9.941231e-01 9.898916e-01 7.675062e-01 7.839876e-01 7.812294e-01 7.320153e-01 7.659811e-01

2741 9.965404e-01 9.969742e-01 9.959758e-01 9.941230e-01 9.898913e-01 7.675868e-01 7.840541e-01 7.812771e-01 7.320463e-01 7.660154e-01

2742 9.965406e-01 9.969742e-01 9.959758e-01 9.941229e-01 9.898910e-01 7.676694e-01 7.841220e-01 7.813258e-01 7.320788e-01 7.660503e-01

2743 9.965408e-01 9.969743e-01 9.959758e-01 9.941227e-01 9.898906e-01 7.677541e-01 7.841915e-01 7.813754e-01 7.321129e-01 7.660857e-01

2744 9.965410e-01 9.969744e-01 9.959758e-01 9.941226e-01 9.898903e-01 7.678409e-01 7.842624e-01 7.814259e-01 7.321488e-01 7.661216e-01

2745 9.965413e-01 9.969745e-01 9.959758e-01 9.941224e-01 9.898899e-01 7.679300e-01 7.843350e-01 7.814775e-01 7.321865e-01 7.661581e-01

2746 9.965415e-01 9.969746e-01 9.959758e-01 9.941223e-01 9.898895e-01 7.680214e-01 7.844091e-01 7.815300e-01 7.322260e-01 7.661951e-01

2747 9.965418e-01 9.969747e-01 9.959758e-01 9.941221e-01 9.898891e-01 7.681151e-01 7.844849e-01 7.815835e-01 7.322674e-01 7.662328e-01

2748 9.965420e-01 9.969748e-01 9.959758e-01 9.941220e-01 9.898887e-01 7.682111e-01 7.845624e-01 7.816381e-01 7.323108e-01 7.662709e-01

2749 9.965423e-01 9.969749e-01 9.959758e-01 9.941218e-01 9.898882e-01 7.683097e-01 7.846416e-01 7.816937e-01 7.323563e-01 7.663097e-01

2750 9.965426e-01 9.969750e-01 9.959758e-01 9.941216e-01 9.898877e-01 7.684107e-01 7.847225e-01 7.817504e-01 7.324039e-01 7.663490e-01

2751 9.965429e-01 9.969751e-01 9.959758e-01 9.941214e-01 9.898872e-01 7.685144e-01 7.848053e-01 7.818082e-01 7.324538e-01 7.663890e-01

2752 9.965432e-01 9.969752e-01 9.959758e-01 9.941212e-01 9.898867e-01 7.686206e-01 7.848899e-01 7.818671e-01 7.325059e-01 7.664295e-01

2753 9.965435e-01 9.969753e-01 9.959758e-01 9.941210e-01 9.898862e-01 7.687297e-01 7.849764e-01 7.819272e-01 7.325604e-01 7.664706e-01

2754 9.965438e-01 9.969754e-01 9.959758e-01 9.941207e-01 9.898856e-01 7.688415e-01 7.850648e-01 7.819884e-01 7.326174e-01 7.665123e-01

2755 9.965442e-01 9.969756e-01 9.959758e-01 9.941205e-01 9.898850e-01 7.689561e-01 7.851551e-01 7.820508e-01 7.326769e-01 7.665547e-01

2756 9.965445e-01 9.969757e-01 9.959757e-01 9.941202e-01 9.898843e-01 7.690737e-01 7.852475e-01 7.821144e-01 7.327391e-01 7.665976e-01

2757 9.965449e-01 9.969758e-01 9.959757e-01 9.941199e-01 9.898837e-01 7.691944e-01 7.853420e-01 7.821792e-01 7.328040e-01 7.666412e-01

2758 9.965453e-01 9.969760e-01 9.959757e-01 9.941197e-01 9.898830e-01 7.693181e-01 7.854386e-01 7.822453e-01 7.328717e-01 7.666854e-01

2759 9.965456e-01 9.969761e-01 9.959757e-01 9.941193e-01 9.898822e-01 7.694450e-01 7.855373e-01 7.823127e-01 7.329424e-01 7.667303e-01

2760 9.965461e-01 9.969762e-01 9.959756e-01 9.941190e-01 9.898815e-01 7.695751e-01 7.856383e-01 7.823814e-01 7.330161e-01 7.667758e-01

2761 9.965465e-01 9.969764e-01 9.959756e-01 9.941187e-01 9.898807e-01 7.697086e-01 7.857415e-01 7.824513e-01 7.330930e-01 7.668219e-01

2762 9.965469e-01 9.969765e-01 9.959756e-01 9.941183e-01 9.898798e-01 7.698455e-01 7.858470e-01 7.825227e-01 7.331731e-01 7.668687e-01

2763 9.965474e-01 9.969767e-01 9.959755e-01 9.941180e-01 9.898789e-01 7.699859e-01 7.859549e-01 7.825954e-01 7.332565e-01 7.669161e-01

2764 9.965479e-01 9.969768e-01 9.959755e-01 9.941176e-01 9.898780e-01 7.701299e-01 7.860651e-01 7.826695e-01 7.333435e-01 7.669642e-01

2765 9.965484e-01 9.969770e-01 9.959754e-01 9.941172e-01 9.898770e-01 7.702775e-01 7.861779e-01 7.827450e-01 7.334340e-01 7.670130e-01

2766 9.965489e-01 9.969772e-01 9.959754e-01 9.941167e-01 9.898759e-01 7.704290e-01 7.862932e-01 7.828220e-01 7.335282e-01 7.670624e-01

2767 9.965494e-01 9.969774e-01 9.959753e-01 9.941163e-01 9.898749e-01 7.705843e-01 7.864111e-01 7.829004e-01 7.336263e-01 7.671125e-01

2768 9.965500e-01 9.969775e-01 9.959753e-01 9.941158e-01 9.898737e-01 7.707436e-01 7.865316e-01 7.829804e-01 7.337283e-01 7.671633e-01

2769 9.965505e-01 9.969777e-01 9.959752e-01 9.941153e-01 9.898725e-01 7.709070e-01 7.866548e-01 7.830618e-01 7.338345e-01 7.672147e-01

2770 9.965511e-01 9.969779e-01 9.959751e-01 9.941147e-01 9.898712e-01 7.710745e-01 7.867807e-01 7.831449e-01 7.339448e-01 7.672668e-01

2771 9.965518e-01 9.969781e-01 9.959751e-01 9.941142e-01 9.898699e-01 7.712463e-01 7.869095e-01 7.832295e-01 7.340595e-01 7.673196e-01

2772 9.965524e-01 9.969783e-01 9.959750e-01 9.941136e-01 9.898685e-01 7.714225e-01 7.870412e-01 7.833157e-01 7.341787e-01 7.673731e-01

2773 9.965531e-01 9.969786e-01 9.959749e-01 9.941130e-01 9.898671e-01 7.716032e-01 7.871757e-01 7.834035e-01 7.343026e-01 7.674273e-01

2774 9.965538e-01 9.969788e-01 9.959748e-01 9.941123e-01 9.898655e-01 7.717884e-01 7.873133e-01 7.834930e-01 7.344312e-01 7.674821e-01

2775 9.965545e-01 9.969790e-01 9.959747e-01 9.941116e-01 9.898639e-01 7.719784e-01 7.874540e-01 7.835842e-01 7.345648e-01 7.675377e-01

2776 9.965553e-01 9.969792e-01 9.959746e-01 9.941109e-01 9.898622e-01 7.721732e-01 7.875978e-01 7.836772e-01 7.347035e-01 7.675939e-01

2777 9.965560e-01 9.969795e-01 9.959744e-01 9.941101e-01 9.898604e-01 7.723729e-01 7.877448e-01 7.837718e-01 7.348474e-01 7.676508e-01

2778 9.965569e-01 9.969797e-01 9.959743e-01 9.941093e-01 9.898586e-01 7.725776e-01 7.878951e-01 7.838683e-01 7.349968e-01 7.677084e-01

2779 9.965577e-01 9.969800e-01 9.959742e-01 9.941085e-01 9.898566e-01 7.727875e-01 7.880487e-01 7.839665e-01 7.351517e-01 7.677666e-01

2780 9.965586e-01 9.969803e-01 9.959740e-01 9.941076e-01 9.898545e-01 7.730027e-01 7.882057e-01 7.840666e-01 7.353124e-01 7.678255e-01

2781 9.965595e-01 9.969806e-01 9.959739e-01 9.941066e-01 9.898523e-01 7.732233e-01 7.883662e-01 7.841685e-01 7.354789e-01 7.678852e-01

2782 9.965605e-01 9.969808e-01 9.959737e-01 9.941056e-01 9.898501e-01 7.734494e-01 7.885303e-01 7.842723e-01 7.356515e-01 7.679454e-01

2783 9.965615e-01 9.969811e-01 9.959735e-01 9.941046e-01 9.898477e-01 7.736812e-01 7.886979e-01 7.843780e-01 7.358304e-01 7.680064e-01

2784 9.965625e-01 9.969815e-01 9.959733e-01 9.941035e-01 9.898451e-01 7.739187e-01 7.888693e-01 7.844857e-01 7.360158e-01 7.680680e-01

2785 9.965636e-01 9.969818e-01 9.959731e-01 9.941023e-01 9.898425e-01 7.741622e-01 7.890444e-01 7.845953e-01 7.362077e-01 7.681302e-01

2786 9.965647e-01 9.969821e-01 9.959729e-01 9.941011e-01 9.898397e-01 7.744117e-01 7.892234e-01 7.847069e-01 7.364064e-01 7.681931e-01

2787 9.965659e-01 9.969825e-01 9.959727e-01 9.940998e-01 9.898367e-01 7.746673e-01 7.894062e-01 7.848206e-01 7.366121e-01 7.682567e-01

2788 9.965671e-01 9.969828e-01 9.959724e-01 9.940985e-01 9.898336e-01 7.749293e-01 7.895931e-01 7.849363e-01 7.368250e-01 7.683208e-01

2789 9.965684e-01 9.969832e-01 9.959721e-01 9.940971e-01 9.898304e-01 7.751977e-01 7.897840e-01 7.850540e-01 7.370453e-01 7.683856e-01

2790 9.965697e-01 9.969836e-01 9.959718e-01 9.940956e-01 9.898270e-01 7.754727e-01 7.899791e-01 7.851739e-01 7.372731e-01 7.684510e-01

2791 9.965711e-01 9.969840e-01 9.959715e-01 9.940940e-01 9.898234e-01 7.757544e-01 7.901783e-01 7.852959e-01 7.375086e-01 7.685170e-01

2792 9.965725e-01 9.969844e-01 9.959712e-01 9.940923e-01 9.898196e-01 7.760429e-01 7.903819e-01 7.854200e-01 7.377521e-01 7.685836e-01

2793 9.965740e-01 9.969848e-01 9.959709e-01 9.940906e-01 9.898156e-01 7.763384e-01 7.905899e-01 7.855464e-01 7.380037e-01 7.686508e-01

2794 9.965755e-01 9.969852e-01 9.959705e-01 9.940887e-01 9.898114e-01 7.766411e-01 7.908022e-01 7.856749e-01 7.382637e-01 7.687185e-01

2795 9.965771e-01 9.969857e-01 9.959701e-01 9.940868e-01 9.898070e-01 7.769510e-01 7.910192e-01 7.858056e-01 7.385322e-01 7.687868e-01

2796 9.965788e-01 9.969862e-01 9.959697e-01 9.940848e-01 9.898023e-01 7.772683e-01 7.912407e-01 7.859386e-01 7.388095e-01 7.688556e-01

2797 9.965806e-01 9.969866e-01 9.959693e-01 9.940826e-01 9.897974e-01 7.775931e-01 7.914668e-01 7.860739e-01 7.390957e-01 7.689248e-01

2798 9.965824e-01 9.969871e-01 9.959688e-01 9.940803e-01 9.897923e-01 7.779257e-01 7.916978e-01 7.862114e-01 7.393910e-01 7.689946e-01

2799 9.965843e-01 9.969877e-01 9.959683e-01 9.940779e-01 9.897868e-01 7.782661e-01 7.919335e-01 7.863512e-01 7.396957e-01 7.690649e-01

2800 9.965863e-01 9.969882e-01 9.959678e-01 9.940754e-01 9.897811e-01 7.786144e-01 7.921742e-01 7.864934e-01 7.400099e-01 7.691356e-01

2801 9.965883e-01 9.969888e-01 9.959672e-01 9.940727e-01 9.897751e-01 7.789708e-01 7.924199e-01 7.866379e-01 7.403339e-01 7.692067e-01

2802 9.965905e-01 9.969893e-01 9.959666e-01 9.940699e-01 9.897688e-01 7.793355e-01 7.926706e-01 7.867848e-01 7.406678e-01 7.692782e-01

2803 9.965927e-01 9.969899e-01 9.959660e-01 9.940670e-01 9.897621e-01 7.797086e-01 7.929264e-01 7.869340e-01 7.410118e-01 7.693501e-01

2804 9.965950e-01 9.969905e-01 9.959653e-01 9.940639e-01 9.897550e-01 7.800902e-01 7.931874e-01 7.870856e-01 7.413662e-01 7.694223e-01

2805 9.965975e-01 9.969912e-01 9.959646e-01 9.940606e-01 9.897476e-01 7.804804e-01 7.934537e-01 7.872396e-01 7.417311e-01 7.694949e-01

2806 9.966000e-01 9.969918e-01 9.959638e-01 9.940571e-01 9.897398e-01 7.808794e-01 7.937253e-01 7.873960e-01 7.421067e-01 7.695677e-01

2807 9.966026e-01 9.969925e-01 9.959630e-01 9.940535e-01 9.897316e-01 7.812874e-01 7.940023e-01 7.875548e-01 7.424933e-01 7.696408e-01

2808 9.966054e-01 9.969932e-01 9.959621e-01 9.940496e-01 9.897229e-01 7.817043e-01 7.942848e-01 7.877161e-01 7.428909e-01 7.697141e-01

2809 9.966083e-01 9.969940e-01 9.959612e-01 9.940455e-01 9.897137e-01 7.821305e-01 7.945728e-01 7.878797e-01 7.432997e-01 7.697875e-01

2810 9.966113e-01 9.969947e-01 9.959603e-01 9.940413e-01 9.897041e-01 7.825659e-01 7.948664e-01 7.880458e-01 7.437200e-01 7.698612e-01

2811 9.966144e-01 9.969955e-01 9.959592e-01 9.940367e-01 9.896939e-01 7.830108e-01 7.951656e-01 7.882143e-01 7.441518e-01 7.699349e-01

2812 9.966176e-01 9.969963e-01 9.959581e-01 9.940320e-01 9.896831e-01 7.834652e-01 7.954706e-01 7.883852e-01 7.445954e-01 7.700087e-01

2813 9.966210e-01 9.969972e-01 9.959570e-01 9.940269e-01 9.896718e-01 7.839292e-01 7.957813e-01 7.885585e-01 7.450509e-01 7.700825e-01

2814 9.966246e-01 9.969980e-01 9.959558e-01 9.940216e-01 9.896599e-01 7.844030e-01 7.960978e-01 7.887342e-01 7.455185e-01 7.701564e-01

2815 9.966282e-01 9.969989e-01 9.959545e-01 9.940160e-01 9.896473e-01 7.848866e-01 7.964201e-01 7.889123e-01 7.459982e-01 7.702301e-01

2816 9.966321e-01 9.969999e-01 9.959531e-01 9.940101e-01 9.896340e-01 7.853802e-01 7.967484e-01 7.890928e-01 7.464903e-01 7.703038e-01

2817 9.966361e-01 9.970008e-01 9.959516e-01 9.940039e-01 9.896200e-01 7.858838e-01 7.970825e-01 7.892757e-01 7.469947e-01 7.703773e-01

2818 9.966403e-01 9.970018e-01 9.959500e-01 9.939973e-01 9.896052e-01 7.863975e-01 7.974227e-01 7.894609e-01 7.475117e-01 7.704506e-01

2819 9.966447e-01 9.970029e-01 9.959484e-01 9.939903e-01 9.895896e-01 7.869214e-01 7.977688e-01 7.896484e-01 7.480414e-01 7.705237e-01

2820 9.966492e-01 9.970039e-01 9.959466e-01 9.939830e-01 9.895732e-01 7.874557e-01 7.981209e-01 7.898382e-01 7.485837e-01 7.705964e-01

2821 9.966540e-01 9.970050e-01 9.959448e-01 9.939752e-01 9.895558e-01 7.880002e-01 7.984791e-01 7.900302e-01 7.491389e-01 7.706688e-01

2822 9.966589e-01 9.970062e-01 9.959428e-01 9.939670e-01 9.895375e-01 7.885552e-01 7.988433e-01 7.902245e-01 7.497069e-01 7.707408e-01

2823 9.966641e-01 9.970074e-01 9.959407e-01 9.939584e-01 9.895181e-01 7.891206e-01 7.992136e-01 7.904210e-01 7.502878e-01 7.708124e-01

2824 9.966695e-01 9.970086e-01 9.959385e-01 9.939492e-01 9.894977e-01 7.896965e-01 7.995900e-01 7.906197e-01 7.508816e-01 7.708834e-01

2825 9.966752e-01 9.970099e-01 9.959361e-01 9.939396e-01 9.894761e-01 7.902829e-01 7.999724e-01 7.908204e-01 7.514884e-01 7.709538e-01

2826 9.966810e-01 9.970112e-01 9.959337e-01 9.939294e-01 9.894534e-01 7.908799e-01 8.003609e-01 7.910233e-01 7.521081e-01 7.710236e-01

2827 9.966872e-01 9.970125e-01 9.959310e-01 9.939187e-01 9.894293e-01 7.914874e-01 8.007554e-01 7.912281e-01 7.527408e-01 7.710927e-01

2828 9.966936e-01 9.970139e-01 9.959282e-01 9.939073e-01 9.894040e-01 7.921055e-01 8.011559e-01 7.914349e-01 7.533865e-01 7.711611e-01

2829 9.967003e-01 9.970154e-01 9.959252e-01 9.938953e-01 9.893772e-01 7.927342e-01 8.015625e-01 7.916436e-01 7.540450e-01 7.712287e-01

2830 9.967073e-01 9.970169e-01 9.959221e-01 9.938826e-01 9.893489e-01 7.933734e-01 8.019749e-01 7.918541e-01 7.547163e-01 7.712953e-01

2831 9.967146e-01 9.970185e-01 9.959187e-01 9.938692e-01 9.893191e-01 7.940231e-01 8.023933e-01 7.920664e-01 7.554004e-01 7.713611e-01

2832 9.967223e-01 9.970201e-01 9.959152e-01 9.938550e-01 9.892875e-01 7.946832e-01 8.028176e-01 7.922804e-01 7.560971e-01 7.714258e-01

2833 9.967302e-01 9.970217e-01 9.959114e-01 9.938401e-01 9.892542e-01 7.953538e-01 8.032476e-01 7.924960e-01 7.568064e-01 7.714895e-01

2834 9.967386e-01 9.970235e-01 9.959074e-01 9.938243e-01 9.892191e-01 7.960347e-01 8.036834e-01 7.927131e-01 7.575280e-01 7.715521e-01

2835 9.967472e-01 9.970252e-01 9.959031e-01 9.938076e-01 9.891819e-01 7.967259e-01 8.041248e-01 7.929318e-01 7.582620e-01 7.716135e-01

2836 9.967563e-01 9.970271e-01 9.958987e-01 9.937899e-01 9.891427e-01 7.974273e-01 8.045718e-01 7.931517e-01 7.590080e-01 7.716736e-01

2837 9.967658e-01 9.970290e-01 9.958939e-01 9.937713e-01 9.891013e-01 7.981387e-01 8.050243e-01 7.933730e-01 7.597659e-01 7.717325e-01

2838 9.967757e-01 9.970309e-01 9.958888e-01 9.937515e-01 9.890576e-01 7.988601e-01 8.054821e-01 7.935954e-01 7.605355e-01 7.717899e-01

2839 9.967861e-01 9.970330e-01 9.958835e-01 9.937307e-01 9.890114e-01 7.995912e-01 8.059452e-01 7.938190e-01 7.613166e-01 7.718460e-01

2840 9.967969e-01 9.970351e-01 9.958778e-01 9.937087e-01 9.889627e-01 8.003321e-01 8.064135e-01 7.940435e-01 7.621089e-01 7.719006e-01

2841 9.968082e-01 9.970372e-01 9.958718e-01 9.936854e-01 9.889112e-01 8.010824e-01 8.068868e-01 7.942689e-01 7.629122e-01 7.719536e-01

2842 9.968200e-01 9.970395e-01 9.958654e-01 9.936608e-01 9.888568e-01 8.018421e-01 8.073649e-01 7.944950e-01 7.637261e-01 7.720051e-01

2843 9.968324e-01 9.970418e-01 9.958586e-01 9.936348e-01 9.887995e-01 8.026109e-01 8.078478e-01 7.947218e-01 7.645504e-01 7.720549e-01

2844 9.968453e-01 9.970442e-01 9.958514e-01 9.936073e-01 9.887389e-01 8.033886e-01 8.083353e-01 7.949492e-01 7.653848e-01 7.721030e-01

2845 9.968588e-01 9.970467e-01 9.958438e-01 9.935782e-01 9.886749e-01 8.041751e-01 8.088272e-01 7.951770e-01 7.662289e-01 7.721494e-01

2846 9.968729e-01 9.970492e-01 9.958357e-01 9.935475e-01 9.886074e-01 8.049700e-01 8.093233e-01 7.954050e-01 7.670823e-01 7.721940e-01

2847 9.968876e-01 9.970518e-01 9.958272e-01 9.935150e-01 9.885362e-01 8.057731e-01 8.098235e-01 7.956333e-01 7.679447e-01 7.722367e-01

2848 9.969030e-01 9.970545e-01 9.958181e-01 9.934807e-01 9.884610e-01 8.065842e-01 8.103275e-01 7.958616e-01 7.688158e-01 7.722776e-01

2849 9.969191e-01 9.970573e-01 9.958085e-01 9.934444e-01 9.883817e-01 8.074030e-01 8.108353e-01 7.960898e-01 7.696950e-01 7.723165e-01

2850 9.969359e-01 9.970602e-01 9.957983e-01 9.934060e-01 9.882980e-01 8.082292e-01 8.113464e-01 7.963178e-01 7.705820e-01 7.723535e-01

2851 9.969535e-01 9.970632e-01 9.957875e-01 9.933655e-01 9.882097e-01 8.090625e-01 8.118608e-01 7.965454e-01 7.714764e-01 7.723885e-01

2852 9.969719e-01 9.970663e-01 9.957760e-01 9.933227e-01 9.881166e-01 8.099025e-01 8.123782e-01 7.967726e-01 7.723776e-01 7.724215e-01

2853 9.969912e-01 9.970695e-01 9.957638e-01 9.932774e-01 9.880184e-01 8.107490e-01 8.128984e-01 7.969991e-01 7.732853e-01 7.724524e-01

2854 9.970113e-01 9.970727e-01 9.957510e-01 9.932296e-01 9.879149e-01 8.116015e-01 8.134211e-01 7.972249e-01 7.741990e-01 7.724813e-01

2855 9.970323e-01 9.970761e-01 9.957373e-01 9.931791e-01 9.878058e-01 8.124598e-01 8.139462e-01 7.974499e-01 7.751181e-01 7.725080e-01

2856 9.970543e-01 9.970795e-01 9.957229e-01 9.931257e-01 9.876909e-01 8.133234e-01 8.144732e-01 7.976738e-01 7.760423e-01 7.725327e-01

2857 9.970773e-01 9.970831e-01 9.957075e-01 9.930693e-01 9.875697e-01 8.141921e-01 8.150021e-01 7.978965e-01 7.769709e-01 7.725552e-01

2858 9.971013e-01 9.970868e-01 9.956913e-01 9.930098e-01 9.874421e-01 8.150653e-01 8.155324e-01 7.981179e-01 7.779036e-01 7.725756e-01

2859 9.971264e-01 9.970906e-01 9.956741e-01 9.929469e-01 9.873078e-01 8.159427e-01 8.160640e-01 7.983380e-01 7.788397e-01 7.725938e-01

2860 9.971527e-01 9.970944e-01 9.956559e-01 9.928805e-01 9.871663e-01 8.168239e-01 8.165966e-01 7.985564e-01 7.797787e-01 7.726099e-01

2861 9.971801e-01 9.970984e-01 9.956366e-01 9.928103e-01 9.870174e-01 8.177085e-01 8.171299e-01 7.987732e-01 7.807202e-01 7.726239e-01

2862 9.972089e-01 9.971025e-01 9.956161e-01 9.927363e-01 9.868608e-01 8.185960e-01 8.176635e-01 7.989882e-01 7.816637e-01 7.726357e-01

2863 9.972389e-01 9.971068e-01 9.955945e-01 9.926582e-01 9.866961e-01 8.194860e-01 8.181973e-01 7.992012e-01 7.826085e-01 7.726454e-01

2864 9.972703e-01 9.971111e-01 9.955716e-01 9.925758e-01 9.865229e-01 8.203781e-01 8.187309e-01 7.994121e-01 7.835541e-01 7.726529e-01

2865 9.973031e-01 9.971155e-01 9.955473e-01 9.924888e-01 9.863408e-01 8.212718e-01 8.192641e-01 7.996209e-01 7.845001e-01 7.726584e-01

2866 9.973374e-01 9.971201e-01 9.955216e-01 9.923970e-01 9.861496e-01 8.221667e-01 8.197965e-01 7.998273e-01 7.854459e-01 7.726618e-01

2867 9.973733e-01 9.971247e-01 9.954944e-01 9.923003e-01 9.859488e-01 8.230623e-01 8.203278e-01 8.000314e-01 7.863910e-01 7.726631e-01

2868 9.974108e-01 9.971295e-01 9.954656e-01 9.921983e-01 9.857379e-01 8.239582e-01 8.208579e-01 8.002328e-01 7.873348e-01 7.726624e-01

2869 9.974500e-01 9.971344e-01 9.954352e-01 9.920908e-01 9.855167e-01 8.248539e-01 8.213863e-01 8.004317e-01 7.882769e-01 7.726597e-01

2870 9.974910e-01 9.971394e-01 9.954030e-01 9.919775e-01 9.852847e-01 8.257489e-01 8.219128e-01 8.006278e-01 7.892168e-01 7.726550e-01

2871 9.975339e-01 9.971446e-01 9.953689e-01 9.918581e-01 9.850415e-01 8.266428e-01 8.224371e-01 8.008210e-01 7.901539e-01 7.726484e-01

2872 9.975787e-01 9.971498e-01 9.953328e-01 9.917324e-01 9.847867e-01 8.275352e-01 8.229589e-01 8.010114e-01 7.910877e-01 7.726399e-01

2873 9.976255e-01 9.971551e-01 9.952947e-01 9.916000e-01 9.845198e-01 8.284256e-01 8.234779e-01 8.011987e-01 7.920179e-01 7.726295e-01

2874 9.976744e-01 9.971606e-01 9.952545e-01 9.914607e-01 9.842405e-01 8.293134e-01 8.239939e-01 8.013829e-01 7.929438e-01 7.726173e-01

2875 9.977255e-01 9.971661e-01 9.952119e-01 9.913141e-01 9.839483e-01 8.301984e-01 8.245065e-01 8.015639e-01 7.938651e-01 7.726034e-01

2876 9.977789e-01 9.971718e-01 9.951669e-01 9.911599e-01 9.836428e-01 8.310800e-01 8.250156e-01 8.017416e-01 7.947813e-01 7.725877e-01

2877 9.978347e-01 9.971775e-01 9.951194e-01 9.909978e-01 9.833236e-01 8.319579e-01 8.255209e-01 8.019160e-01 7.956920e-01 7.725704e-01

2878 9.978930e-01 9.971833e-01 9.950692e-01 9.908274e-01 9.829904e-01 8.328315e-01 8.260220e-01 8.020871e-01 7.965968e-01 7.725514e-01

2879 9.979539e-01 9.971893e-01 9.950162e-01 9.906483e-01 9.826426e-01 8.337004e-01 8.265188e-01 8.022547e-01 7.974952e-01 7.725309e-01

2880 9.980175e-01 9.971953e-01 9.949602e-01 9.904603e-01 9.822800e-01 8.345643e-01 8.270110e-01 8.024188e-01 7.983869e-01 7.725089e-01

2881 9.980839e-01 9.972013e-01 9.949012e-01 9.902630e-01 9.819021e-01 8.354227e-01 8.274984e-01 8.025793e-01 7.992714e-01 7.724854e-01

2882 9.980840e-01 9.972013e-01 9.949011e-01 9.902629e-01 9.819020e-01 8.354450e-01 8.275077e-01 8.025768e-01 7.993043e-01 7.724728e-01

2883 9.980840e-01 9.972014e-01 9.949011e-01 9.902628e-01 9.819020e-01 8.354677e-01 8.275172e-01 8.025742e-01 7.993380e-01 7.724598e-01

2884 9.980840e-01 9.972014e-01 9.949010e-01 9.902628e-01 9.819019e-01 8.354908e-01 8.275269e-01 8.025713e-01 7.993725e-01 7.724463e-01

2885 9.980841e-01 9.972014e-01 9.949010e-01 9.902627e-01 9.819018e-01 8.355144e-01 8.275366e-01 8.025683e-01 7.994078e-01 7.724323e-01

2886 9.980841e-01 9.972014e-01 9.949009e-01 9.902627e-01 9.819016e-01 8.355385e-01 8.275465e-01 8.025651e-01 7.994440e-01 7.724179e-01

2887 9.980841e-01 9.972014e-01 9.949009e-01 9.902626e-01 9.819015e-01 8.355630e-01 8.275566e-01 8.025617e-01 7.994810e-01 7.724030e-01

2888 9.980842e-01 9.972014e-01 9.949008e-01 9.902625e-01 9.819014e-01 8.355880e-01 8.275667e-01 8.025580e-01 7.995188e-01 7.723876e-01

2889 9.980842e-01 9.972013e-01 9.949008e-01 9.902625e-01 9.819012e-01 8.356135e-01 8.275770e-01 8.025542e-01 7.995576e-01 7.723716e-01

2890 9.980843e-01 9.972013e-01 9.949007e-01 9.902624e-01 9.819010e-01 8.356395e-01 8.275874e-01 8.025501e-01 7.995973e-01 7.723552e-01

2891 9.980843e-01 9.972013e-01 9.949007e-01 9.902623e-01 9.819008e-01 8.356660e-01 8.275980e-01 8.025458e-01 7.996378e-01 7.723382e-01

2892 9.980843e-01 9.972013e-01 9.949007e-01 9.902622e-01 9.819006e-01 8.356930e-01 8.276086e-01 8.025413e-01 7.996794e-01 7.723206e-01

2893 9.980844e-01 9.972013e-01 9.949006e-01 9.902621e-01 9.819003e-01 8.357205e-01 8.276194e-01 8.025366e-01 7.997219e-01 7.723025e-01

2894 9.980844e-01 9.972013e-01 9.949006e-01 9.902620e-01 9.819001e-01 8.357486e-01 8.276304e-01 8.025315e-01 7.997654e-01 7.722838e-01

2895 9.980844e-01 9.972013e-01 9.949005e-01 9.902619e-01 9.818998e-01 8.357772e-01 8.276415e-01 8.025263e-01 7.998098e-01 7.722644e-01

2896 9.980845e-01 9.972013e-01 9.949005e-01 9.902617e-01 9.818995e-01 8.358063e-01 8.276527e-01 8.025207e-01 7.998554e-01 7.722444e-01

2897 9.980845e-01 9.972012e-01 9.949004e-01 9.902616e-01 9.818992e-01 8.358360e-01 8.276640e-01 8.025149e-01 7.999019e-01 7.722238e-01

2898 9.980846e-01 9.972012e-01 9.949004e-01 9.902614e-01 9.818989e-01 8.358663e-01 8.276755e-01 8.025087e-01 7.999496e-01 7.722025e-01

2899 9.980846e-01 9.972012e-01 9.949003e-01 9.902613e-01 9.818985e-01 8.358972e-01 8.276871e-01 8.025023e-01 7.999984e-01 7.721805e-01

2900 9.980846e-01 9.972012e-01 9.949003e-01 9.902611e-01 9.818982e-01 8.359287e-01 8.276989e-01 8.024955e-01 8.000483e-01 7.721579e-01

2901 9.980847e-01 9.972012e-01 9.949002e-01 9.902609e-01 9.818978e-01 8.359608e-01 8.277108e-01 8.024885e-01 8.000993e-01 7.721344e-01

2902 9.980847e-01 9.972012e-01 9.949002e-01 9.902607e-01 9.818974e-01 8.359935e-01 8.277228e-01 8.024811e-01 8.001515e-01 7.721103e-01

2903 9.980848e-01 9.972011e-01 9.949001e-01 9.902605e-01 9.818971e-01 8.360269e-01 8.277350e-01 8.024733e-01 8.002050e-01 7.720853e-01

2904 9.980848e-01 9.972011e-01 9.949000e-01 9.902602e-01 9.818967e-01 8.360609e-01 8.277474e-01 8.024652e-01 8.002597e-01 7.720595e-01

2905 9.980849e-01 9.972011e-01 9.948999e-01 9.902600e-01 9.818962e-01 8.360955e-01 8.277598e-01 8.024567e-01 8.003156e-01 7.720330e-01

2906 9.980849e-01 9.972011e-01 9.948998e-01 9.902598e-01 9.818958e-01 8.361309e-01 8.277724e-01 8.024478e-01 8.003729e-01 7.720055e-01

2907 9.980849e-01 9.972011e-01 9.948997e-01 9.902595e-01 9.818954e-01 8.361669e-01 8.277852e-01 8.024386e-01 8.004315e-01 7.719772e-01

2908 9.980850e-01 9.972010e-01 9.948996e-01 9.902592e-01 9.818949e-01 8.362037e-01 8.277981e-01 8.024289e-01 8.004914e-01 7.719480e-01

2909 9.980850e-01 9.972010e-01 9.948995e-01 9.902589e-01 9.818944e-01 8.362411e-01 8.278111e-01 8.024188e-01 8.005528e-01 7.719178e-01

2910 9.980851e-01 9.972010e-01 9.948994e-01 9.902586e-01 9.818939e-01 8.362793e-01 8.278243e-01 8.024082e-01 8.006156e-01 7.718867e-01

2911 9.980851e-01 9.972010e-01 9.948993e-01 9.902583e-01 9.818934e-01 8.363183e-01 8.278377e-01 8.023972e-01 8.006798e-01 7.718546e-01

2912 9.980852e-01 9.972009e-01 9.948991e-01 9.902580e-01 9.818929e-01 8.363580e-01 8.278511e-01 8.023857e-01 8.007455e-01 7.718215e-01

2913 9.980852e-01 9.972009e-01 9.948990e-01 9.902576e-01 9.818923e-01 8.363985e-01 8.278648e-01 8.023737e-01 8.008128e-01 7.717873e-01

2914 9.980853e-01 9.972009e-01 9.948988e-01 9.902573e-01 9.818917e-01 8.364398e-01 8.278785e-01 8.023612e-01 8.008817e-01 7.717520e-01

2915 9.980854e-01 9.972008e-01 9.948987e-01 9.902569e-01 9.818911e-01 8.364819e-01 8.278924e-01 8.023482e-01 8.009522e-01 7.717156e-01

2916 9.980854e-01 9.972008e-01 9.948985e-01 9.902565e-01 9.818905e-01 8.365248e-01 8.279065e-01 8.023346e-01 8.010243e-01 7.716781e-01

2917 9.980855e-01 9.972007e-01 9.948983e-01 9.902561e-01 9.818898e-01 8.365686e-01 8.279207e-01 8.023205e-01 8.010981e-01 7.716394e-01

2918 9.980855e-01 9.972007e-01 9.948981e-01 9.902557e-01 9.818891e-01 8.366133e-01 8.279351e-01 8.023058e-01 8.011736e-01 7.715994e-01

2919 9.980856e-01 9.972006e-01 9.948979e-01 9.902552e-01 9.818883e-01 8.366588e-01 8.279495e-01 8.022905e-01 8.012510e-01 7.715582e-01

2920 9.980857e-01 9.972006e-01 9.948977e-01 9.902547e-01 9.818876e-01 8.367052e-01 8.279642e-01 8.022745e-01 8.013301e-01 7.715157e-01

2921 9.980857e-01 9.972005e-01 9.948975e-01 9.902542e-01 9.818867e-01 8.367526e-01 8.279790e-01 8.022579e-01 8.014111e-01 7.714718e-01

2922 9.980858e-01 9.972005e-01 9.948973e-01 9.902537e-01 9.818859e-01 8.368009e-01 8.279939e-01 8.022406e-01 8.014940e-01 7.714266e-01

2923 9.980859e-01 9.972004e-01 9.948971e-01 9.902532e-01 9.818850e-01 8.368502e-01 8.280089e-01 8.022227e-01 8.015789e-01 7.713799e-01

2924 9.980859e-01 9.972003e-01 9.948968e-01 9.902526e-01 9.818840e-01 8.369004e-01 8.280241e-01 8.022040e-01 8.016657e-01 7.713317e-01

2925 9.980860e-01 9.972003e-01 9.948965e-01 9.902520e-01 9.818831e-01 8.369517e-01 8.280395e-01 8.021846e-01 8.017546e-01 7.712821e-01

2926 9.980861e-01 9.972002e-01 9.948963e-01 9.902514e-01 9.818820e-01 8.370039e-01 8.280549e-01 8.021644e-01 8.018456e-01 7.712309e-01

2927 9.980862e-01 9.972001e-01 9.948960e-01 9.902508e-01 9.818809e-01 8.370572e-01 8.280705e-01 8.021434e-01 8.019388e-01 7.711780e-01

2928 9.980862e-01 9.972000e-01 9.948957e-01 9.902501e-01 9.818798e-01 8.371116e-01 8.280862e-01 8.021216e-01 8.020341e-01 7.711235e-01

2929 9.980863e-01 9.971999e-01 9.948954e-01 9.902494e-01 9.818786e-01 8.371671e-01 8.281021e-01 8.020989e-01 8.021318e-01 7.710673e-01

2930 9.980864e-01 9.971998e-01 9.948950e-01 9.902486e-01 9.818774e-01 8.372237e-01 8.281181e-01 8.020754e-01 8.022317e-01 7.710094e-01

2931 9.980865e-01 9.971997e-01 9.948947e-01 9.902478e-01 9.818761e-01 8.372814e-01 8.281342e-01 8.020509e-01 8.023340e-01 7.709496e-01

2932 9.980866e-01 9.971996e-01 9.948943e-01 9.902470e-01 9.818747e-01 8.373402e-01 8.281504e-01 8.020255e-01 8.024387e-01 7.708879e-01

2933 9.980867e-01 9.971995e-01 9.948939e-01 9.902461e-01 9.818733e-01 8.374002e-01 8.281668e-01 8.019991e-01 8.025459e-01 7.708244e-01

2934 9.980868e-01 9.971993e-01 9.948935e-01 9.902452e-01 9.818718e-01 8.374615e-01 8.281832e-01 8.019717e-01 8.026556e-01 7.707588e-01

2935 9.980869e-01 9.971992e-01 9.948931e-01 9.902442e-01 9.818702e-01 8.375240e-01 8.281998e-01 8.019433e-01 8.027679e-01 7.706912e-01

2936 9.980870e-01 9.971991e-01 9.948926e-01 9.902432e-01 9.818685e-01 8.375877e-01 8.282165e-01 8.019138e-01 8.028829e-01 7.706215e-01

2937 9.980871e-01 9.971989e-01 9.948922e-01 9.902422e-01 9.818668e-01 8.376527e-01 8.282333e-01 8.018832e-01 8.030007e-01 7.705496e-01

2938 9.980872e-01 9.971988e-01 9.948917e-01 9.902411e-01 9.818650e-01 8.377190e-01 8.282501e-01 8.018514e-01 8.031212e-01 7.704755e-01

2939 9.980873e-01 9.971986e-01 9.948912e-01 9.902399e-01 9.818631e-01 8.377866e-01 8.282671e-01 8.018184e-01 8.032446e-01 7.703991e-01

2940 9.980874e-01 9.971984e-01 9.948906e-01 9.902387e-01 9.818612e-01 8.378556e-01 8.282841e-01 8.017842e-01 8.033709e-01 7.703203e-01

2941 9.980875e-01 9.971982e-01 9.948901e-01 9.902374e-01 9.818591e-01 8.379260e-01 8.283013e-01 8.017487e-01 8.035002e-01 7.702391e-01

2942 9.980876e-01 9.971980e-01 9.948895e-01 9.902361e-01 9.818570e-01 8.379978e-01 8.283185e-01 8.017119e-01 8.036326e-01 7.701554e-01

2943 9.980877e-01 9.971978e-01 9.948888e-01 9.902347e-01 9.818547e-01 8.380710e-01 8.283358e-01 8.016738e-01 8.037681e-01 7.700691e-01

2944 9.980878e-01 9.971976e-01 9.948882e-01 9.902332e-01 9.818523e-01 8.381457e-01 8.283531e-01 8.016342e-01 8.039068e-01 7.699801e-01

2945 9.980880e-01 9.971974e-01 9.948875e-01 9.902317e-01 9.818499e-01 8.382219e-01 8.283705e-01 8.015932e-01 8.040489e-01 7.698884e-01

2946 9.980881e-01 9.971972e-01 9.948868e-01 9.902300e-01 9.818473e-01 8.382997e-01 8.283879e-01 8.015507e-01 8.041943e-01 7.697939e-01

2947 9.980882e-01 9.971969e-01 9.948860e-01 9.902283e-01 9.818446e-01 8.383790e-01 8.284054e-01 8.015067e-01 8.043432e-01 7.696965e-01

2948 9.980884e-01 9.971967e-01 9.948852e-01 9.902266e-01 9.818417e-01 8.384599e-01 8.284229e-01 8.014610e-01 8.044956e-01 7.695961e-01

2949 9.980885e-01 9.971964e-01 9.948844e-01 9.902247e-01 9.818388e-01 8.385424e-01 8.284404e-01 8.014137e-01 8.046516e-01 7.694927e-01
[truncated: 10,524,276 more chars]
