# Supplementary material for: Purity control of simulated moving bed based on advanced fuzzy controller
Source: Sci Rep. 2024 Apr 20;14:9083. doi: 10.1038/s41598-024-59847-1 (PMC11576947; doi:10.1038/s41598-024-59847-1)
Supplement: Supplementary file 6 — Supplementary Information 6. [file 41598_2024_59847_MOESM6_ESM.docx]

**Figure 6(b):**

1 8.962715e-04 8.963257e-04 8.963820e-04 8.964405e-04 8.965013e-04 1.324665e-05 8.262710e-06 8.271023e-06 8.279348e-06 8.246112e-06

2 8.962715e-04 8.963257e-04 8.963820e-04 8.964405e-04 8.965013e-04 1.324665e-05 8.262710e-06 8.271023e-06 8.279348e-06 8.246112e-06

3 8.962715e-04 8.963257e-04 8.963820e-04 8.964405e-04 8.965013e-04 1.324665e-05 8.262710e-06 8.271023e-06 8.279348e-06 8.246112e-06

4 8.962715e-04 8.963257e-04 8.963820e-04 8.964405e-04 8.965013e-04 1.324665e-05 8.262710e-06 8.271023e-06 8.279348e-06 8.246112e-06

5 8.962715e-04 8.963257e-04 8.963820e-04 8.964405e-04 8.965013e-04 1.324665e-05 8.262710e-06 8.271023e-06 8.279348e-06 8.246112e-06

6 8.962715e-04 8.963257e-04 8.963820e-04 8.964405e-04 8.965013e-04 1.324665e-05 8.262710e-06 8.271023e-06 8.279348e-06 8.246112e-06

7 8.962715e-04 8.963257e-04 8.963820e-04 8.964405e-04 8.965013e-04 1.324665e-05 8.262710e-06 8.271023e-06 8.279348e-06 8.246112e-06

8 8.962715e-04 8.963257e-04 8.963820e-04 8.964405e-04 8.965013e-04 1.324665e-05 8.262710e-06 8.271023e-06 8.279348e-06 8.246112e-06

9 8.962715e-04 8.963257e-04 8.963820e-04 8.964405e-04 8.965013e-04 1.324665e-05 8.262710e-06 8.271023e-06 8.279348e-06 8.246112e-06

10 8.962715e-04 8.963257e-04 8.963820e-04 8.964405e-04 8.965013e-04 1.324665e-05 8.262710e-06 8.271023e-06 8.279348e-06 8.246112e-06

11 8.962715e-04 8.963257e-04 8.963820e-04 8.964405e-04 8.965013e-04 1.324665e-05 8.262710e-06 8.271023e-06 8.279348e-06 8.246112e-06

12 8.962715e-04 8.963257e-04 8.963820e-04 8.964405e-04 8.965013e-04 1.324665e-05 8.262710e-06 8.271023e-06 8.279348e-06 8.246112e-06

13 8.962715e-04 8.963257e-04 8.963820e-04 8.964405e-04 8.965013e-04 1.324665e-05 8.262710e-06 8.271023e-06 8.279348e-06 8.246112e-06

14 8.962715e-04 8.963257e-04 8.963820e-04 8.964405e-04 8.965013e-04 1.324665e-05 8.262710e-06 8.271023e-06 8.279348e-06 8.246112e-06

15 8.962715e-04 8.963257e-04 8.963820e-04 8.964405e-04 8.965013e-04 1.324665e-05 8.262710e-06 8.271023e-06 8.279348e-06 8.246112e-06

16 8.962715e-04 8.963257e-04 8.963820e-04 8.964405e-04 8.965013e-04 1.324665e-05 8.262710e-06 8.271023e-06 8.279348e-06 8.246112e-06

17 8.962715e-04 8.963257e-04 8.963820e-04 8.964405e-04 8.965013e-04 1.324665e-05 8.262710e-06 8.271023e-06 8.279348e-06 8.246112e-06

18 8.962715e-04 8.963257e-04 8.963820e-04 8.964405e-04 8.965013e-04 1.324665e-05 8.262710e-06 8.271023e-06 8.279348e-06 8.246112e-06

19 8.962715e-04 8.963257e-04 8.963820e-04 8.964405e-04 8.965013e-04 1.324665e-05 8.262710e-06 8.271023e-06 8.279348e-06 8.246112e-06

20 8.962715e-04 8.963257e-04 8.963820e-04 8.964405e-04 8.965013e-04 1.324665e-05 8.262710e-06 8.271023e-06 8.279348e-06 8.246112e-06

21 8.962715e-04 8.963257e-04 8.963820e-04 8.964405e-04 8.965013e-04 1.324665e-05 8.262710e-06 8.271023e-06 8.279348e-06 8.246112e-06

22 8.962715e-04 8.963257e-04 8.963820e-04 8.964405e-04 8.965013e-04 1.324665e-05 8.262710e-06 8.271023e-06 8.279348e-06 8.246112e-06

23 8.962715e-04 8.963257e-04 8.963820e-04 8.964405e-04 8.965013e-04 1.324665e-05 8.262710e-06 8.271023e-06 8.279348e-06 8.246112e-06

24 8.962715e-04 8.963257e-04 8.963820e-04 8.964405e-04 8.965013e-04 1.324665e-05 8.262710e-06 8.271023e-06 8.279348e-06 8.246112e-06

25 8.962715e-04 8.963257e-04 8.963820e-04 8.964405e-04 8.965013e-04 1.324665e-05 8.262710e-06 8.271023e-06 8.279348e-06 8.246112e-06

26 8.962715e-04 8.963257e-04 8.963820e-04 8.964405e-04 8.965013e-04 1.324665e-05 8.262710e-06 8.271023e-06 8.279348e-06 8.246112e-06

27 8.962715e-04 8.963257e-04 8.963820e-04 8.964405e-04 8.965013e-04 1.324665e-05 8.262710e-06 8.271023e-06 8.279348e-06 8.246112e-06

28 8.962715e-04 8.963257e-04 8.963820e-04 8.964405e-04 8.965013e-04 1.324665e-05 8.262710e-06 8.271023e-06 8.279348e-06 8.246112e-06

29 8.962715e-04 8.963257e-04 8.963820e-04 8.964405e-04 8.965013e-04 1.324665e-05 8.262710e-06 8.271023e-06 8.279348e-06 8.246112e-06

30 8.962715e-04 8.963257e-04 8.963820e-04 8.964405e-04 8.965013e-04 1.324665e-05 8.262710e-06 8.271023e-06 8.279348e-06 8.246112e-06

31 8.962715e-04 8.963257e-04 8.963820e-04 8.964405e-04 8.965013e-04 1.324665e-05 8.262710e-06 8.271023e-06 8.279348e-06 8.246112e-06

32 8.962715e-04 8.963257e-04 8.963820e-04 8.964405e-04 8.965013e-04 1.324665e-05 8.262710e-06 8.271023e-06 8.279348e-06 8.246112e-06

33 8.962715e-04 8.963257e-04 8.963820e-04 8.964405e-04 8.965013e-04 1.324665e-05 8.262710e-06 8.271023e-06 8.279348e-06 8.246112e-06

34 8.962715e-04 8.963257e-04 8.963820e-04 8.964405e-04 8.965013e-04 1.324665e-05 8.262710e-06 8.271023e-06 8.279348e-06 8.246112e-06

35 8.962715e-04 8.963257e-04 8.963820e-04 8.964405e-04 8.965013e-04 1.324665e-05 8.262710e-06 8.271023e-06 8.279348e-06 8.246112e-06

36 8.962715e-04 8.963257e-04 8.963820e-04 8.964405e-04 8.965013e-04 1.324665e-05 8.262710e-06 8.271023e-06 8.279348e-06 8.246112e-06

37 8.962715e-04 8.963257e-04 8.963820e-04 8.964405e-04 8.965013e-04 1.324665e-05 8.262710e-06 8.271023e-06 8.279348e-06 8.246112e-06

38 8.962715e-04 8.963257e-04 8.963820e-04 8.964405e-04 8.965013e-04 1.324665e-05 8.262710e-06 8.271023e-06 8.279348e-06 8.246112e-06

39 8.962715e-04 8.963257e-04 8.963820e-04 8.964405e-04 8.965013e-04 1.324665e-05 8.262710e-06 8.271023e-06 8.279348e-06 8.246112e-06

40 8.962715e-04 8.963257e-04 8.963820e-04 8.964405e-04 8.965013e-04 1.324665e-05 8.262710e-06 8.271023e-06 8.279348e-06 8.246112e-06

41 8.962715e-04 8.963257e-04 8.963820e-04 8.964405e-04 8.965013e-04 1.324665e-05 8.262710e-06 8.271023e-06 8.279348e-06 8.246112e-06

42 8.962715e-04 8.963257e-04 8.963820e-04 8.964405e-04 8.965013e-04 1.324665e-05 8.262710e-06 8.271023e-06 8.279348e-06 8.246112e-06

43 8.962715e-04 8.963257e-04 8.963820e-04 8.964406e-04 8.965013e-04 1.324665e-05 8.262710e-06 8.271023e-06 8.279348e-06 8.246112e-06

44 8.962715e-04 8.963257e-04 8.963820e-04 8.964406e-04 8.965014e-04 1.324665e-05 8.262710e-06 8.271023e-06 8.279348e-06 8.246112e-06

45 8.962715e-04 8.963257e-04 8.963820e-04 8.964406e-04 8.965014e-04 1.324665e-05 8.262710e-06 8.271023e-06 8.279348e-06 8.246112e-06

46 8.962715e-04 8.963257e-04 8.963820e-04 8.964406e-04 8.965014e-04 1.324665e-05 8.262710e-06 8.271023e-06 8.279348e-06 8.246112e-06

47 8.962715e-04 8.963257e-04 8.963820e-04 8.964406e-04 8.965014e-04 1.324665e-05 8.262710e-06 8.271023e-06 8.279348e-06 8.246112e-06

48 8.962715e-04 8.963257e-04 8.963820e-04 8.964406e-04 8.965014e-04 1.324665e-05 8.262710e-06 8.271024e-06 8.279348e-06 8.246112e-06

49 8.962715e-04 8.963257e-04 8.963820e-04 8.964406e-04 8.965014e-04 1.324665e-05 8.262710e-06 8.271024e-06 8.279348e-06 8.246112e-06

50 8.962715e-04 8.963257e-04 8.963821e-04 8.964406e-04 8.965014e-04 1.324665e-05 8.262710e-06 8.271024e-06 8.279348e-06 8.246112e-06

51 8.962715e-04 8.963257e-04 8.963821e-04 8.964406e-04 8.965014e-04 1.324665e-05 8.262710e-06 8.271024e-06 8.279348e-06 8.246112e-06

52 8.962716e-04 8.963258e-04 8.963821e-04 8.964406e-04 8.965014e-04 1.324665e-05 8.262710e-06 8.271024e-06 8.279348e-06 8.246112e-06

53 8.962716e-04 8.963258e-04 8.963821e-04 8.964406e-04 8.965014e-04 1.324665e-05 8.262710e-06 8.271024e-06 8.279348e-06 8.246112e-06

54 8.962716e-04 8.963258e-04 8.963821e-04 8.964406e-04 8.965014e-04 1.324665e-05 8.262710e-06 8.271024e-06 8.279348e-06 8.246112e-06

55 8.962716e-04 8.963258e-04 8.963821e-04 8.964406e-04 8.965014e-04 1.324665e-05 8.262710e-06 8.271024e-06 8.279348e-06 8.246112e-06

56 8.962716e-04 8.963258e-04 8.963822e-04 8.964407e-04 8.965015e-04 1.324665e-05 8.262710e-06 8.271024e-06 8.279349e-06 8.246113e-06

57 8.962717e-04 8.963259e-04 8.963822e-04 8.964407e-04 8.965015e-04 1.324665e-05 8.262711e-06 8.271025e-06 8.279349e-06 8.246113e-06

58 8.962717e-04 8.963259e-04 8.963822e-04 8.964408e-04 8.965016e-04 1.324665e-05 8.262711e-06 8.271025e-06 8.279349e-06 8.246113e-06

59 8.962718e-04 8.963260e-04 8.963823e-04 8.964408e-04 8.965016e-04 1.324665e-05 8.262712e-06 8.271026e-06 8.279350e-06 8.246114e-06

60 8.962719e-04 8.963261e-04 8.963824e-04 8.964409e-04 8.965017e-04 1.324665e-05 8.262712e-06 8.271026e-06 8.279350e-06 8.246114e-06

61 8.962720e-04 8.963262e-04 8.963825e-04 8.964410e-04 8.965018e-04 1.324665e-05 8.262713e-06 8.271027e-06 8.279351e-06 8.246115e-06

62 8.962721e-04 8.963263e-04 8.963826e-04 8.964412e-04 8.965020e-04 1.324665e-05 8.262714e-06 8.271028e-06 8.279352e-06 8.246116e-06

63 8.962723e-04 8.963265e-04 8.963828e-04 8.964413e-04 8.965021e-04 1.324666e-05 8.262716e-06 8.271029e-06 8.279354e-06 8.246118e-06

64 8.962725e-04 8.963267e-04 8.963831e-04 8.964416e-04 8.965024e-04 1.324666e-05 8.262717e-06 8.271031e-06 8.279355e-06 8.246119e-06

65 8.962728e-04 8.963270e-04 8.963833e-04 8.964419e-04 8.965027e-04 1.324666e-05 8.262719e-06 8.271033e-06 8.279358e-06 8.246122e-06

66 8.962732e-04 8.963274e-04 8.963837e-04 8.964422e-04 8.965030e-04 1.324667e-05 8.262722e-06 8.271036e-06 8.279360e-06 8.246124e-06

67 8.962737e-04 8.963279e-04 8.963842e-04 8.964427e-04 8.965035e-04 1.324667e-05 8.262726e-06 8.271040e-06 8.279364e-06 8.246128e-06

68 8.962743e-04 8.963285e-04 8.963848e-04 8.964433e-04 8.965041e-04 1.324668e-05 8.262730e-06 8.271044e-06 8.279368e-06 8.246132e-06

69 8.962750e-04 8.963292e-04 8.963856e-04 8.964441e-04 8.965049e-04 1.324669e-05 8.262736e-06 8.271050e-06 8.279374e-06 8.246138e-06

70 8.962760e-04 8.963302e-04 8.963865e-04 8.964450e-04 8.965058e-04 1.324670e-05 8.262743e-06 8.271057e-06 8.279381e-06 8.246145e-06

71 8.962772e-04 8.963314e-04 8.963878e-04 8.964463e-04 8.965071e-04 1.324671e-05 8.262752e-06 8.271066e-06 8.279391e-06 8.246154e-06

72 8.962788e-04 8.963330e-04 8.963893e-04 8.964478e-04 8.965086e-04 1.324673e-05 8.262764e-06 8.271078e-06 8.279402e-06 8.246166e-06

73 8.962807e-04 8.963349e-04 8.963912e-04 8.964498e-04 8.965106e-04 1.324675e-05 8.262778e-06 8.271092e-06 8.279417e-06 8.246180e-06

74 8.962832e-04 8.963374e-04 8.963937e-04 8.964522e-04 8.965130e-04 1.324678e-05 8.262796e-06 8.271111e-06 8.279435e-06 8.246198e-06

75 8.962863e-04 8.963405e-04 8.963968e-04 8.964553e-04 8.965161e-04 1.324682e-05 8.262819e-06 8.271134e-06 8.279458e-06 8.246221e-06

76 8.962901e-04 8.963443e-04 8.964007e-04 8.964592e-04 8.965200e-04 1.324687e-05 8.262848e-06 8.271162e-06 8.279487e-06 8.246250e-06

77 8.962950e-04 8.963492e-04 8.964055e-04 8.964640e-04 8.965248e-04 1.324692e-05 8.262884e-06 8.271198e-06 8.279523e-06 8.246286e-06

78 8.963011e-04 8.963553e-04 8.964116e-04 8.964701e-04 8.965309e-04 1.324700e-05 8.262929e-06 8.271244e-06 8.279568e-06 8.246330e-06

79 8.963086e-04 8.963628e-04 8.964192e-04 8.964777e-04 8.965385e-04 1.324709e-05 8.262985e-06 8.271300e-06 8.279625e-06 8.246386e-06

80 8.963181e-04 8.963723e-04 8.964286e-04 8.964872e-04 8.965480e-04 1.324720e-05 8.263056e-06 8.271370e-06 8.279695e-06 8.246456e-06

81 8.963299e-04 8.963841e-04 8.964404e-04 8.964990e-04 8.965598e-04 1.324734e-05 8.263143e-06 8.271458e-06 8.279783e-06 8.246543e-06

82 8.963446e-04 8.963988e-04 8.964551e-04 8.965137e-04 8.965745e-04 1.324752e-05 8.263252e-06 8.271567e-06 8.279892e-06 8.246651e-06

83 8.963628e-04 8.964171e-04 8.964734e-04 8.965319e-04 8.965927e-04 1.324774e-05 8.263387e-06 8.271702e-06 8.280028e-06 8.246786e-06

84 8.963855e-04 8.964397e-04 8.964960e-04 8.965546e-04 8.966154e-04 1.324802e-05 8.263554e-06 8.271870e-06 8.280196e-06 8.246952e-06

85 8.964135e-04 8.964678e-04 8.965241e-04 8.965826e-04 8.966435e-04 1.324836e-05 8.263761e-06 8.272078e-06 8.280404e-06 8.247159e-06

86 8.964483e-04 8.965025e-04 8.965588e-04 8.966174e-04 8.966782e-04 1.324878e-05 8.264018e-06 8.272335e-06 8.280662e-06 8.247414e-06

87 8.964912e-04 8.965454e-04 8.966017e-04 8.966603e-04 8.967211e-04 1.324931e-05 8.264335e-06 8.272652e-06 8.280980e-06 8.247729e-06

88 8.965441e-04 8.965983e-04 8.966547e-04 8.967133e-04 8.967741e-04 1.324996e-05 8.264725e-06 8.273044e-06 8.281373e-06 8.248118e-06

89 8.966093e-04 8.966636e-04 8.967199e-04 8.967785e-04 8.968394e-04 1.325076e-05 8.265206e-06 8.273526e-06 8.281856e-06 8.248597e-06

90 8.966896e-04 8.967439e-04 8.968003e-04 8.968588e-04 8.969197e-04 1.325175e-05 8.265798e-06 8.274119e-06 8.282451e-06 8.249186e-06

91 8.967883e-04 8.968426e-04 8.968990e-04 8.969576e-04 8.970184e-04 1.325298e-05 8.266525e-06 8.274848e-06 8.283181e-06 8.249910e-06

92 8.969094e-04 8.969637e-04 8.970201e-04 8.970787e-04 8.971396e-04 1.325448e-05 8.267417e-06 8.275742e-06 8.284077e-06 8.250798e-06

93 8.970578e-04 8.971121e-04 8.971686e-04 8.972272e-04 8.972881e-04 1.325633e-05 8.268510e-06 8.276837e-06 8.285175e-06 8.251885e-06

94 8.972395e-04 8.972939e-04 8.973503e-04 8.974090e-04 8.974700e-04 1.325861e-05 8.269847e-06 8.278177e-06 8.286518e-06 8.253216e-06

95 8.974616e-04 8.975160e-04 8.975725e-04 8.976312e-04 8.976922e-04 1.326140e-05 8.271481e-06 8.279815e-06 8.288160e-06 8.254842e-06

96 8.977328e-04 8.977872e-04 8.978438e-04 8.979025e-04 8.979636e-04 1.326481e-05 8.273475e-06 8.281814e-06 8.290163e-06 8.256827e-06

97 8.980635e-04 8.981179e-04 8.981745e-04 8.982333e-04 8.982944e-04 1.326899e-05 8.275905e-06 8.284249e-06 8.292604e-06 8.259245e-06

98 8.984661e-04 8.985206e-04 8.985773e-04 8.986361e-04 8.986973e-04 1.327409e-05 8.278862e-06 8.287214e-06 8.295576e-06 8.262190e-06

99 8.989557e-04 8.990103e-04 8.990671e-04 8.991260e-04 8.991872e-04 1.328032e-05 8.282458e-06 8.290818e-06 8.299189e-06 8.265769e-06

100 8.995505e-04 8.996051e-04 8.996620e-04 8.997210e-04 8.997823e-04 1.328791e-05 8.286824e-06 8.295195e-06 8.303575e-06 8.270114e-06

101 9.002719e-04 9.003267e-04 9.003836e-04 9.004428e-04 9.005042e-04 1.329715e-05 8.292118e-06 8.300501e-06 8.308895e-06 8.275383e-06

102 9.011459e-04 9.012008e-04 9.012579e-04 9.013171e-04 9.013788e-04 1.330837e-05 8.298530e-06 8.306928e-06 8.315337e-06 8.281765e-06

103 9.022034e-04 9.022584e-04 9.023157e-04 9.023751e-04 9.024369e-04 1.332201e-05 8.306286e-06 8.314702e-06 8.323129e-06 8.289484e-06

104 9.034813e-04 9.035365e-04 9.035940e-04 9.036536e-04 9.037156e-04 1.333854e-05 8.315656e-06 8.324095e-06 8.332544e-06 8.298810e-06

105 9.050236e-04 9.050791e-04 9.051368e-04 9.051967e-04 9.052589e-04 1.335856e-05 8.326962e-06 8.335427e-06 8.343903e-06 8.310062e-06

106 9.068828e-04 9.069385e-04 9.069965e-04 9.070567e-04 9.071193e-04 1.338278e-05 8.340587e-06 8.349085e-06 8.357593e-06 8.323623e-06

107 9.091210e-04 9.091771e-04 9.092354e-04 9.092960e-04 9.093589e-04 1.341205e-05 8.356988e-06 8.365525e-06 8.374072e-06 8.339946e-06

108 9.118123e-04 9.118688e-04 9.119275e-04 9.119885e-04 9.120519e-04 1.344737e-05 8.376706e-06 8.385289e-06 8.393884e-06 8.359571e-06

109 9.150443e-04 9.151012e-04 9.151604e-04 9.152220e-04 9.152859e-04 1.348994e-05 8.400383e-06 8.409023e-06 8.417674e-06 8.383135e-06

110 9.189207e-04 9.189783e-04 9.190381e-04 9.191002e-04 9.191648e-04 1.354120e-05 8.428781e-06 8.437488e-06 8.446207e-06 8.411398e-06

111 9.235644e-04 9.236227e-04 9.236832e-04 9.237461e-04 9.238115e-04 1.360284e-05 8.462800e-06 8.471589e-06 8.480389e-06 8.445256e-06

112 9.291203e-04 9.291794e-04 9.292408e-04 9.293046e-04 9.293709e-04 1.367689e-05 8.503506e-06 8.512392e-06 8.521290e-06 8.485767e-06

113 9.357592e-04 9.358193e-04 9.358817e-04 9.359466e-04 9.360140e-04 1.376574e-05 8.552155e-06 8.561157e-06 8.570172e-06 8.534185e-06

114 9.436823e-04 9.437436e-04 9.438072e-04 9.438734e-04 9.439421e-04 1.387221e-05 8.610230e-06 8.619371e-06 8.628525e-06 8.591982e-06

115 9.531261e-04 9.531888e-04 9.532539e-04 9.533215e-04 9.533918e-04 1.399967e-05 8.679477e-06 8.688784e-06 8.698104e-06 8.660899e-06

116 9.643681e-04 9.644324e-04 9.644993e-04 9.645687e-04 9.646409e-04 1.415207e-05 8.761950e-06 8.771455e-06 8.780974e-06 8.742978e-06

117 9.777335e-04 9.777998e-04 9.778687e-04 9.779404e-04 9.780148e-04 1.433410e-05 8.860064e-06 8.869804e-06 8.879559e-06 8.840622e-06

118 9.936028e-04 9.936715e-04 9.937429e-04 9.938171e-04 9.938941e-04 1.455126e-05 8.976650e-06 8.986671e-06 8.996706e-06 8.956650e-06

119 1.012420e-03 1.012492e-03 1.012566e-03 1.012643e-03 1.012724e-03 1.481006e-05 9.115032e-06 9.125385e-06 9.135754e-06 9.094369e-06

120 1.034704e-03 1.034779e-03 1.034856e-03 1.034937e-03 1.035021e-03 1.511812e-05 9.279098e-06 9.289846e-06 9.300611e-06 9.257647e-06

121 1.061056e-03 1.061135e-03 1.061216e-03 1.061302e-03 1.061390e-03 1.548443e-05 9.473397e-06 9.484613e-06 9.495847e-06 9.451014e-06

122 1.092176e-03 1.092259e-03 1.092346e-03 1.092436e-03 1.092530e-03 1.591953e-05 9.703246e-06 9.715016e-06 9.726805e-06 9.679759e-06

123 1.128874e-03 1.128963e-03 1.129055e-03 1.129151e-03 1.129251e-03 1.643575e-05 9.974846e-06 9.987271e-06 9.999717e-06 9.950054e-06

124 1.172087e-03 1.172182e-03 1.172281e-03 1.172384e-03 1.172491e-03 1.704757e-05 1.029543e-05 1.030863e-05 1.032185e-05 1.026910e-05

125 1.222893e-03 1.222995e-03 1.223102e-03 1.223213e-03 1.223329e-03 1.777190e-05 1.067341e-05 1.068753e-05 1.070166e-05 1.064526e-05

126 1.282533e-03 1.282644e-03 1.282760e-03 1.282881e-03 1.283006e-03 1.862852e-05 1.111859e-05 1.113378e-05 1.114900e-05 1.108830e-05

127 1.352429e-03 1.352551e-03 1.352677e-03 1.352809e-03 1.352946e-03 1.964050e-05 1.164234e-05 1.165880e-05 1.167528e-05 1.160952e-05

128 1.434204e-03 1.434338e-03 1.434477e-03 1.434622e-03 1.434772e-03 2.083473e-05 1.225787e-05 1.227581e-05 1.229378e-05 1.222208e-05

129 1.529708e-03 1.529856e-03 1.530010e-03 1.530169e-03 1.530335e-03 2.224256e-05 1.298046e-05 1.300015e-05 1.301988e-05 1.294118e-05

130 1.641035e-03 1.641199e-03 1.641370e-03 1.641547e-03 1.641731e-03 2.390042e-05 1.382785e-05 1.384959e-05 1.387138e-05 1.378448e-05

131 1.770551e-03 1.770734e-03 1.770923e-03 1.771121e-03 1.771326e-03 2.585067e-05 1.482053e-05 1.484467e-05 1.486887e-05 1.477237e-05

132 1.920912e-03 1.921116e-03 1.921328e-03 1.921549e-03 1.921778e-03 2.814247e-05 1.598217e-05 1.600913e-05 1.603615e-05 1.592840e-05

133 2.095087e-03 2.095316e-03 2.095554e-03 2.095801e-03 2.096058e-03 3.083280e-05 1.734010e-05 1.737035e-05 1.740066e-05 1.727976e-05

134 2.296374e-03 2.296631e-03 2.296899e-03 2.297177e-03 2.297465e-03 3.398768e-05 1.892581e-05 1.895991e-05 1.899408e-05 1.885781e-05

135 2.528412e-03 2.528702e-03 2.529003e-03 2.529315e-03 2.529640e-03 3.768346e-05 2.077558e-05 2.081416e-05 2.085283e-05 2.069864e-05

136 2.795188e-03 2.795514e-03 2.795854e-03 2.796206e-03 2.796572e-03 4.200837e-05 2.293112e-05 2.297493e-05 2.301884e-05 2.284375e-05

137 3.101035e-03 3.101403e-03 3.101785e-03 3.102183e-03 3.102595e-03 4.706425e-05 2.544037e-05 2.549026e-05 2.554027e-05 2.534085e-05

138 3.450620e-03 3.451035e-03 3.451466e-03 3.451913e-03 3.452378e-03 5.296849e-05 2.835832e-05 2.841529e-05 2.847240e-05 2.824468e-05

139 3.848919e-03 3.849386e-03 3.849871e-03 3.850375e-03 3.850899e-03 5.985628e-05 3.174803e-05 3.181323e-05 3.187858e-05 3.161799e-05

140 4.301180e-03 4.301705e-03 4.302251e-03 4.302818e-03 4.303407e-03 6.788309e-05 3.568171e-05 3.575646e-05 3.583138e-05 3.553264e-05

141 4.812868e-03 4.813457e-03 4.814069e-03 4.814706e-03 4.815367e-03 7.722751e-05 4.024195e-05 4.032776e-05 4.041377e-05 4.007080e-05

142 5.389594e-03 5.390254e-03 5.390940e-03 5.391653e-03 5.392393e-03 8.809445e-05 4.552309e-05 4.562172e-05 4.572057e-05 4.532639e-05

143 6.037033e-03 6.037771e-03 6.038537e-03 6.039333e-03 6.040161e-03 1.007187e-04 5.163281e-05 5.174626e-05 5.185997e-05 5.140654e-05

144 6.760821e-03 6.761643e-03 6.762496e-03 6.763383e-03 6.764304e-03 1.153689e-04 5.869384e-05 5.882440e-05 5.895528e-05 5.843340e-05

145 7.566443e-03 7.567354e-03 7.568301e-03 7.569285e-03 7.570308e-03 1.323520e-04 6.684588e-05 6.699621e-05 6.714690e-05 6.654600e-05

146 8.459110e-03 8.460118e-03 8.461165e-03 8.462254e-03 8.463384e-03 1.520186e-04 7.624782e-05 7.642093e-05 7.659447e-05 7.590248e-05

147 9.443644e-03 9.444754e-03 9.445907e-03 9.447106e-03 9.448351e-03 1.747678e-04 8.708010e-05 8.727946e-05 8.747929e-05 8.668240e-05

148 1.052435e-02 1.052557e-02 1.052683e-02 1.052815e-02 1.052951e-02 2.010540e-04 9.954741e-05 9.977695e-05 1.000070e-04 9.908948e-05

149 1.170491e-02 1.170624e-02 1.170762e-02 1.170905e-02 1.171055e-02 2.313934e-04 1.138816e-04 1.141459e-04 1.144107e-04 1.133545e-04

150 1.298828e-02 1.298973e-02 1.299123e-02 1.299279e-02 1.299441e-02 2.663714e-04 1.303451e-04 1.306492e-04 1.309539e-04 1.297386e-04

151 1.437666e-02 1.437822e-02 1.437985e-02 1.438154e-02 1.438329e-02 3.066508e-04 1.492344e-04 1.495840e-04 1.499345e-04 1.485367e-04

152 1.587137e-02 1.587305e-02 1.587481e-02 1.587663e-02 1.587852e-02 3.529810e-04 1.708839e-04 1.712858e-04 1.716886e-04 1.700819e-04

153 1.747291e-02 1.747472e-02 1.747660e-02 1.747855e-02 1.748058e-02 4.062069e-04 1.956705e-04 1.961322e-04 1.965949e-04 1.947493e-04

154 1.918093e-02 1.918287e-02 1.918487e-02 1.918696e-02 1.918912e-02 4.672800e-04 2.240185e-04 2.245483e-04 2.250795e-04 2.229611e-04

155 2.099429e-02 2.099635e-02 2.099848e-02 2.100070e-02 2.100300e-02 5.372688e-04 2.564042e-04 2.570119e-04 2.576210e-04 2.551914e-04

156 2.291110e-02 2.291327e-02 2.291553e-02 2.291788e-02 2.292032e-02 6.173708e-04 2.933622e-04 2.940585e-04 2.947565e-04 2.919722e-04

157 2.492880e-02 2.493109e-02 2.493347e-02 2.493595e-02 2.493852e-02 7.089246e-04 3.354908e-04 3.362880e-04 3.370872e-04 3.338993e-04

158 2.704428e-02 2.704669e-02 2.704919e-02 2.705179e-02 2.705450e-02 8.134224e-04 3.834592e-04 3.843711e-04 3.852851e-04 3.816388e-04

159 2.925396e-02 2.925649e-02 2.925911e-02 2.926183e-02 2.926466e-02 9.325229e-04 4.380140e-04 4.390559e-04 4.401004e-04 4.359338e-04

160 3.155390e-02 3.155653e-02 3.155927e-02 3.156211e-02 3.156506e-02 1.068064e-03 4.999868e-04 5.011761e-04 5.023682e-04 4.976122e-04

161 3.393988e-02 3.394262e-02 3.394546e-02 3.394841e-02 3.395148e-02 1.222074e-03 5.703020e-04 5.716579e-04 5.730172e-04 5.675942e-04

162 3.640750e-02 3.641034e-02 3.641329e-02 3.641635e-02 3.641953e-02 1.396787e-03 6.499847e-04 6.515290e-04 6.530769e-04 6.469006e-04

163 3.895227e-02 3.895520e-02 3.895825e-02 3.896142e-02 3.896471e-02 1.594649e-03 7.401698e-04 7.419264e-04 7.436871e-04 7.366613e-04

164 4.156966e-02 4.157269e-02 4.157583e-02 4.157910e-02 4.158250e-02 1.818328e-03 8.421098e-04 8.441054e-04 8.461058e-04 8.381234e-04

165 4.425516e-02 4.425828e-02 4.426152e-02 4.426488e-02 4.426838e-02 2.070720e-03 9.571844e-04 9.594486e-04 9.617182e-04 9.526609e-04

166 4.700433e-02 4.700753e-02 4.701086e-02 4.701432e-02 4.701790e-02 2.354955e-03 1.086909e-03 1.089474e-03 1.092046e-03 1.081782e-03

167 4.981281e-02 4.981609e-02 4.981950e-02 4.982304e-02 4.982672e-02 2.674392e-03 1.232941e-03 1.235844e-03 1.238754e-03 1.227140e-03

168 5.267633e-02 5.267968e-02 5.268317e-02 5.268679e-02 5.269056e-02 3.032617e-03 1.397094e-03 1.400373e-03 1.403661e-03 1.390539e-03

169 5.559073e-02 5.559415e-02 5.559771e-02 5.560142e-02 5.560526e-02 3.433427e-03 1.581336e-03 1.585036e-03 1.588744e-03 1.573940e-03

170 5.855193e-02 5.855542e-02 5.855906e-02 5.856283e-02 5.856675e-02 3.880820e-03 1.787803e-03 1.791971e-03 1.796148e-03 1.779471e-03

171 6.155594e-02 6.155950e-02 6.156320e-02 6.156704e-02 6.157103e-02 4.378963e-03 2.018802e-03 2.023488e-03 2.028185e-03 2.009431e-03

172 6.459878e-02 6.460240e-02 6.460617e-02 6.461008e-02 6.461414e-02 4.932166e-03 2.276811e-03 2.282072e-03 2.287345e-03 2.266291e-03

173 6.767651e-02 6.768019e-02 6.768401e-02 6.768798e-02 6.769211e-02 5.544838e-03 2.564486e-03 2.570381e-03 2.576290e-03 2.552695e-03

174 7.078510e-02 7.078883e-02 7.079271e-02 7.079675e-02 7.080093e-02 6.221441e-03 2.884653e-03 2.891247e-03 2.897855e-03 2.871463e-03

175 7.392046e-02 7.392424e-02 7.392818e-02 7.393226e-02 7.393651e-02 6.966437e-03 3.240307e-03 3.247668e-03 3.255045e-03 3.225580e-03

176 7.707832e-02 7.708215e-02 7.708613e-02 7.709027e-02 7.709457e-02 7.784221e-03 3.634603e-03 3.642803e-03 3.651022e-03 3.618194e-03

177 8.025419e-02 8.025807e-02 8.026210e-02 8.026629e-02 8.027063e-02 8.679059e-03 4.070844e-03 4.079961e-03 4.089098e-03 4.052599e-03

178 8.344330e-02 8.344722e-02 8.345130e-02 8.345553e-02 8.345993e-02 9.655015e-03 4.552466e-03 4.562579e-03 4.572714e-03 4.532223e-03

179 8.664053e-02 8.664449e-02 8.664861e-02 8.665288e-02 8.665732e-02 1.071588e-02 5.083012e-03 5.094205e-03 5.105422e-03 5.060603e-03

180 8.984033e-02 8.984433e-02 8.984848e-02 8.985280e-02 8.985728e-02 1.186510e-02 5.666112e-03 5.678472e-03 5.690857e-03 5.641364e-03

181 9.303670e-02 9.304073e-02 9.304492e-02 9.304927e-02 9.305378e-02 1.310570e-02 6.305449e-03 6.319063e-03 6.332705e-03 6.278183e-03

182 9.303670e-02 9.304073e-02 9.304492e-02 9.304927e-02 9.305378e-02 1.310570e-02 6.305449e-03 6.319063e-03 6.332705e-03 6.278183e-03

183 9.303670e-02 9.304073e-02 9.304492e-02 9.304927e-02 9.305378e-02 1.310570e-02 6.305449e-03 6.319063e-03 6.332705e-03 6.278183e-03

184 9.303670e-02 9.304073e-02 9.304492e-02 9.304927e-02 9.305378e-02 1.310570e-02 6.305449e-03 6.319063e-03 6.332705e-03 6.278183e-03

185 9.303670e-02 9.304073e-02 9.304492e-02 9.304927e-02 9.305378e-02 1.310570e-02 6.305449e-03 6.319063e-03 6.332705e-03 6.278183e-03

186 9.303670e-02 9.304073e-02 9.304492e-02 9.304927e-02 9.305378e-02 1.310570e-02 6.305449e-03 6.319063e-03 6.332705e-03 6.278183e-03

187 9.303670e-02 9.304073e-02 9.304492e-02 9.304927e-02 9.305378e-02 1.310570e-02 6.305449e-03 6.319063e-03 6.332705e-03 6.278183e-03

188 9.303670e-02 9.304073e-02 9.304492e-02 9.304927e-02 9.305378e-02 1.310570e-02 6.305449e-03 6.319063e-03 6.332705e-03 6.278183e-03

189 9.303670e-02 9.304073e-02 9.304492e-02 9.304927e-02 9.305378e-02 1.310570e-02 6.305449e-03 6.319063e-03 6.332705e-03 6.278183e-03

190 9.303670e-02 9.304073e-02 9.304492e-02 9.304927e-02 9.305378e-02 1.310570e-02 6.305449e-03 6.319063e-03 6.332705e-03 6.278183e-03

191 9.303670e-02 9.304073e-02 9.304492e-02 9.304927e-02 9.305378e-02 1.310570e-02 6.305449e-03 6.319063e-03 6.332705e-03 6.278183e-03

192 9.303670e-02 9.304073e-02 9.304492e-02 9.304927e-02 9.305378e-02 1.310570e-02 6.305449e-03 6.319063e-03 6.332705e-03 6.278183e-03

193 9.303671e-02 9.304073e-02 9.304492e-02 9.304927e-02 9.305379e-02 1.310570e-02 6.305449e-03 6.319063e-03 6.332705e-03 6.278183e-03

194 9.303671e-02 9.304073e-02 9.304492e-02 9.304927e-02 9.305379e-02 1.310570e-02 6.305449e-03 6.319063e-03 6.332705e-03 6.278183e-03

195 9.303671e-02 9.304073e-02 9.304492e-02 9.304927e-02 9.305379e-02 1.310570e-02 6.305449e-03 6.319063e-03 6.332705e-03 6.278183e-03

196 9.303671e-02 9.304073e-02 9.304492e-02 9.304927e-02 9.305379e-02 1.310570e-02 6.305449e-03 6.319064e-03 6.332705e-03 6.278183e-03

197 9.303671e-02 9.304073e-02 9.304492e-02 9.304927e-02 9.305379e-02 1.310570e-02 6.305449e-03 6.319064e-03 6.332705e-03 6.278183e-03

198 9.303671e-02 9.304074e-02 9.304492e-02 9.304928e-02 9.305379e-02 1.310570e-02 6.305449e-03 6.319064e-03 6.332705e-03 6.278183e-03

199 9.303671e-02 9.304074e-02 9.304493e-02 9.304928e-02 9.305379e-02 1.310570e-02 6.305449e-03 6.319064e-03 6.332705e-03 6.278183e-03

200 9.303671e-02 9.304074e-02 9.304493e-02 9.304928e-02 9.305380e-02 1.310570e-02 6.305449e-03 6.319064e-03 6.332705e-03 6.278183e-03

201 9.303672e-02 9.304074e-02 9.304493e-02 9.304928e-02 9.305380e-02 1.310570e-02 6.305449e-03 6.319064e-03 6.332705e-03 6.278183e-03

202 9.303672e-02 9.304075e-02 9.304493e-02 9.304929e-02 9.305380e-02 1.310570e-02 6.305449e-03 6.319064e-03 6.332705e-03 6.278183e-03

203 9.303673e-02 9.304075e-02 9.304494e-02 9.304929e-02 9.305381e-02 1.310570e-02 6.305449e-03 6.319064e-03 6.332705e-03 6.278183e-03

204 9.303673e-02 9.304075e-02 9.304494e-02 9.304929e-02 9.305381e-02 1.310570e-02 6.305449e-03 6.319064e-03 6.332705e-03 6.278183e-03

205 9.303674e-02 9.304076e-02 9.304495e-02 9.304930e-02 9.305382e-02 1.310570e-02 6.305449e-03 6.319064e-03 6.332705e-03 6.278183e-03

206 9.303674e-02 9.304077e-02 9.304496e-02 9.304931e-02 9.305382e-02 1.310570e-02 6.305449e-03 6.319064e-03 6.332706e-03 6.278183e-03

207 9.303675e-02 9.304078e-02 9.304496e-02 9.304932e-02 9.305383e-02 1.310570e-02 6.305449e-03 6.319064e-03 6.332706e-03 6.278184e-03

208 9.303676e-02 9.304079e-02 9.304497e-02 9.304933e-02 9.305384e-02 1.310570e-02 6.305449e-03 6.319064e-03 6.332706e-03 6.278184e-03

209 9.303677e-02 9.304080e-02 9.304499e-02 9.304934e-02 9.305385e-02 1.310570e-02 6.305449e-03 6.319064e-03 6.332706e-03 6.278184e-03

210 9.303679e-02 9.304081e-02 9.304500e-02 9.304935e-02 9.305387e-02 1.310570e-02 6.305449e-03 6.319064e-03 6.332706e-03 6.278184e-03

211 9.303680e-02 9.304083e-02 9.304502e-02 9.304937e-02 9.305388e-02 1.310570e-02 6.305449e-03 6.319064e-03 6.332706e-03 6.278184e-03

212 9.303682e-02 9.304085e-02 9.304504e-02 9.304939e-02 9.305390e-02 1.310570e-02 6.305450e-03 6.319064e-03 6.332706e-03 6.278184e-03

213 9.303685e-02 9.304087e-02 9.304506e-02 9.304941e-02 9.305393e-02 1.310570e-02 6.305450e-03 6.319064e-03 6.332706e-03 6.278184e-03

214 9.303688e-02 9.304090e-02 9.304509e-02 9.304944e-02 9.305396e-02 1.310570e-02 6.305450e-03 6.319064e-03 6.332706e-03 6.278184e-03

215 9.303691e-02 9.304094e-02 9.304512e-02 9.304948e-02 9.305399e-02 1.310570e-02 6.305450e-03 6.319065e-03 6.332706e-03 6.278184e-03

216 9.303695e-02 9.304098e-02 9.304517e-02 9.304952e-02 9.305403e-02 1.310570e-02 6.305450e-03 6.319065e-03 6.332707e-03 6.278185e-03

217 9.303700e-02 9.304103e-02 9.304521e-02 9.304957e-02 9.305408e-02 1.310570e-02 6.305451e-03 6.319065e-03 6.332707e-03 6.278185e-03

218 9.303706e-02 9.304108e-02 9.304527e-02 9.304962e-02 9.305414e-02 1.310570e-02 6.305451e-03 6.319066e-03 6.332707e-03 6.278185e-03

219 9.303713e-02 9.304115e-02 9.304534e-02 9.304969e-02 9.305421e-02 1.310570e-02 6.305451e-03 6.319066e-03 6.332708e-03 6.278186e-03

220 9.303721e-02 9.304123e-02 9.304542e-02 9.304977e-02 9.305429e-02 1.310570e-02 6.305452e-03 6.319066e-03 6.332708e-03 6.278186e-03

221 9.303730e-02 9.304133e-02 9.304552e-02 9.304987e-02 9.305438e-02 1.310570e-02 6.305452e-03 6.319067e-03 6.332709e-03 6.278187e-03

222 9.303742e-02 9.304144e-02 9.304563e-02 9.304998e-02 9.305450e-02 1.310571e-02 6.305453e-03 6.319068e-03 6.332709e-03 6.278187e-03

223 9.303755e-02 9.304157e-02 9.304576e-02 9.305011e-02 9.305463e-02 1.310571e-02 6.305454e-03 6.319068e-03 6.332710e-03 6.278188e-03

224 9.303771e-02 9.304173e-02 9.304592e-02 9.305027e-02 9.305479e-02 1.310571e-02 6.305455e-03 6.319069e-03 6.332711e-03 6.278189e-03

225 9.303789e-02 9.304192e-02 9.304611e-02 9.305046e-02 9.305497e-02 1.310571e-02 6.305456e-03 6.319070e-03 6.332712e-03 6.278190e-03

226 9.303811e-02 9.304214e-02 9.304633e-02 9.305068e-02 9.305519e-02 1.310571e-02 6.305457e-03 6.319072e-03 6.332713e-03 6.278191e-03

227 9.303837e-02 9.304240e-02 9.304658e-02 9.305094e-02 9.305545e-02 1.310572e-02 6.305458e-03 6.319073e-03 6.332715e-03 6.278193e-03

228 9.303868e-02 9.304270e-02 9.304689e-02 9.305124e-02 9.305576e-02 1.310572e-02 6.305460e-03 6.319075e-03 6.332717e-03 6.278194e-03

229 9.303903e-02 9.304306e-02 9.304725e-02 9.305160e-02 9.305612e-02 1.310572e-02 6.305462e-03 6.319077e-03 6.332719e-03 6.278196e-03

230 9.303945e-02 9.304348e-02 9.304767e-02 9.305202e-02 9.305654e-02 1.310573e-02 6.305465e-03 6.319079e-03 6.332721e-03 6.278199e-03

231 9.303995e-02 9.304397e-02 9.304816e-02 9.305252e-02 9.305703e-02 1.310573e-02 6.305467e-03 6.319082e-03 6.332724e-03 6.278201e-03

232 9.304053e-02 9.304455e-02 9.304874e-02 9.305310e-02 9.305761e-02 1.310574e-02 6.305471e-03 6.319085e-03 6.332727e-03 6.278205e-03

233 9.304121e-02 9.304523e-02 9.304942e-02 9.305378e-02 9.305829e-02 1.310575e-02 6.305474e-03 6.319089e-03 6.332731e-03 6.278209e-03

234 9.304200e-02 9.304603e-02 9.305022e-02 9.305457e-02 9.305909e-02 1.310576e-02 6.305479e-03 6.319094e-03 6.332736e-03 6.278213e-03

235 9.304294e-02 9.304696e-02 9.305115e-02 9.305551e-02 9.306002e-02 1.310577e-02 6.305484e-03 6.319099e-03 6.332741e-03 6.278218e-03

236 9.304403e-02 9.304805e-02 9.305224e-02 9.305660e-02 9.306112e-02 1.310578e-02 6.305490e-03 6.319105e-03 6.332747e-03 6.278224e-03

237 9.304530e-02 9.304933e-02 9.305352e-02 9.305787e-02 9.306239e-02 1.310580e-02 6.305498e-03 6.319112e-03 6.332754e-03 6.278232e-03

238 9.304679e-02 9.305081e-02 9.305501e-02 9.305936e-02 9.306388e-02 1.310581e-02 6.305506e-03 6.319121e-03 6.332763e-03 6.278240e-03

239 9.304852e-02 9.305255e-02 9.305674e-02 9.306110e-02 9.306562e-02 1.310583e-02 6.305516e-03 6.319131e-03 6.332773e-03 6.278250e-03

240 9.305054e-02 9.305457e-02 9.305877e-02 9.306312e-02 9.306764e-02 1.310586e-02 6.305527e-03 6.319142e-03 6.332784e-03 6.278261e-03

241 9.305290e-02 9.305693e-02 9.306112e-02 9.306548e-02 9.307000e-02 1.310588e-02 6.305541e-03 6.319156e-03 6.332798e-03 6.278274e-03

242 9.305564e-02 9.305967e-02 9.306387e-02 9.306823e-02 9.307275e-02 1.310592e-02 6.305556e-03 6.319171e-03 6.332813e-03 6.278290e-03

243 9.305883e-02 9.306286e-02 9.306706e-02 9.307142e-02 9.307594e-02 1.310595e-02 6.305574e-03 6.319189e-03 6.332832e-03 6.278307e-03

244 9.306254e-02 9.306657e-02 9.307077e-02 9.307513e-02 9.307965e-02 1.310600e-02 6.305595e-03 6.319210e-03 6.332853e-03 6.278328e-03

245 9.306684e-02 9.307088e-02 9.307508e-02 9.307944e-02 9.308396e-02 1.310605e-02 6.305620e-03 6.319235e-03 6.332877e-03 6.278352e-03

246 9.307184e-02 9.307587e-02 9.308007e-02 9.308443e-02 9.308896e-02 1.310611e-02 6.305648e-03 6.319263e-03 6.332906e-03 6.278380e-03

247 9.307763e-02 9.308166e-02 9.308586e-02 9.309023e-02 9.309476e-02 1.310617e-02 6.305681e-03 6.319296e-03 6.332939e-03 6.278413e-03

248 9.308433e-02 9.308837e-02 9.309257e-02 9.309694e-02 9.310147e-02 1.310625e-02 6.305719e-03 6.319334e-03 6.332977e-03 6.278451e-03

249 9.309209e-02 9.309614e-02 9.310034e-02 9.310471e-02 9.310924e-02 1.310635e-02 6.305763e-03 6.319379e-03 6.333022e-03 6.278494e-03

250 9.310107e-02 9.310512e-02 9.310932e-02 9.311370e-02 9.311823e-02 1.310646e-02 6.305813e-03 6.319430e-03 6.333073e-03 6.278545e-03

251 9.311145e-02 9.311549e-02 9.311970e-02 9.312408e-02 9.312862e-02 1.310658e-02 6.305872e-03 6.319489e-03 6.333132e-03 6.278603e-03

252 9.312342e-02 9.312747e-02 9.313169e-02 9.313607e-02 9.314061e-02 1.310673e-02 6.305940e-03 6.319557e-03 6.333201e-03 6.278670e-03

253 9.313724e-02 9.314130e-02 9.314551e-02 9.314990e-02 9.315445e-02 1.310689e-02 6.306019e-03 6.319636e-03 6.333280e-03 6.278748e-03

254 9.315317e-02 9.315723e-02 9.316145e-02 9.316584e-02 9.317040e-02 1.310709e-02 6.306109e-03 6.319727e-03 6.333371e-03 6.278838e-03

255 9.317152e-02 9.317558e-02 9.317981e-02 9.318421e-02 9.318877e-02 1.310731e-02 6.306214e-03 6.319832e-03 6.333477e-03 6.278941e-03

256 9.319263e-02 9.319670e-02 9.320094e-02 9.320534e-02 9.320991e-02 1.310757e-02 6.306334e-03 6.319952e-03 6.333598e-03 6.279060e-03

257 9.321690e-02 9.322098e-02 9.322523e-02 9.322964e-02 9.323421e-02 1.310787e-02 6.306472e-03 6.320091e-03 6.333738e-03 6.279197e-03

258 9.324479e-02 9.324888e-02 9.325313e-02 9.325755e-02 9.326213e-02 1.310822e-02 6.306631e-03 6.320251e-03 6.333898e-03 6.279355e-03

259 9.327680e-02 9.328090e-02 9.328516e-02 9.328959e-02 9.329418e-02 1.310862e-02 6.306814e-03 6.320435e-03 6.334083e-03 6.279536e-03

260 9.331350e-02 9.331761e-02 9.332189e-02 9.332633e-02 9.333093e-02 1.310908e-02 6.307024e-03 6.320646e-03 6.334294e-03 6.279745e-03

261 9.335556e-02 9.335968e-02 9.336396e-02 9.336842e-02 9.337304e-02 1.310961e-02 6.307265e-03 6.320888e-03 6.334538e-03 6.279983e-03

262 9.340369e-02 9.340782e-02 9.341212e-02 9.341659e-02 9.342123e-02 1.311022e-02 6.307542e-03 6.321165e-03 6.334817e-03 6.280257e-03

263 9.345872e-02 9.346287e-02 9.346719e-02 9.347168e-02 9.347634e-02 1.311092e-02 6.307858e-03 6.321484e-03 6.335136e-03 6.280572e-03

264 9.352159e-02 9.352576e-02 9.353010e-02 9.353461e-02 9.353928e-02 1.311173e-02 6.308221e-03 6.321848e-03 6.335502e-03 6.280931e-03

265 9.359333e-02 9.359752e-02 9.360188e-02 9.360641e-02 9.361111e-02 1.311266e-02 6.308637e-03 6.322265e-03 6.335921e-03 6.281343e-03

266 9.367511e-02 9.367933e-02 9.368371e-02 9.368827e-02 9.369299e-02 1.311372e-02 6.309112e-03 6.322742e-03 6.336400e-03 6.281814e-03

267 9.376823e-02 9.377247e-02 9.377688e-02 9.378147e-02 9.378623e-02 1.311494e-02 6.309654e-03 6.323288e-03 6.336948e-03 6.282352e-03

268 9.387413e-02 9.387841e-02 9.388285e-02 9.388747e-02 9.389226e-02 1.311634e-02 6.310274e-03 6.323910e-03 6.337574e-03 6.282966e-03

269 9.399443e-02 9.399874e-02 9.400322e-02 9.400788e-02 9.401271e-02 1.311794e-02 6.310982e-03 6.324621e-03 6.338287e-03 6.283667e-03

270 9.413092e-02 9.413526e-02 9.413979e-02 9.414449e-02 9.414936e-02 1.311978e-02 6.311789e-03 6.325431e-03 6.339101e-03 6.284467e-03

271 9.428556e-02 9.428995e-02 9.429452e-02 9.429926e-02 9.430419e-02 1.312187e-02 6.312708e-03 6.326355e-03 6.340029e-03 6.285379e-03

272 9.446052e-02 9.446496e-02 9.446958e-02 9.447439e-02 9.447937e-02 1.312427e-02 6.313756e-03 6.327407e-03 6.341086e-03 6.286417e-03

273 9.465820e-02 9.466270e-02 9.466738e-02 9.467224e-02 9.467728e-02 1.312701e-02 6.314947e-03 6.328604e-03 6.342288e-03 6.287598e-03

274 9.488120e-02 9.488576e-02 9.489050e-02 9.489543e-02 9.490054e-02 1.313014e-02 6.316303e-03 6.329966e-03 6.343656e-03 6.288942e-03

275 9.513234e-02 9.513697e-02 9.514179e-02 9.514680e-02 9.515199e-02 1.313370e-02 6.317843e-03 6.331513e-03 6.345210e-03 6.290469e-03

276 9.541472e-02 9.541943e-02 9.542433e-02 9.542941e-02 9.543470e-02 1.313777e-02 6.319593e-03 6.333270e-03 6.346975e-03 6.292203e-03

277 9.573163e-02 9.573643e-02 9.574142e-02 9.574660e-02 9.575198e-02 1.314240e-02 6.321579e-03 6.335265e-03 6.348978e-03 6.294171e-03

278 9.608663e-02 9.609152e-02 9.609661e-02 9.610190e-02 9.610738e-02 1.314768e-02 6.323831e-03 6.337527e-03 6.351251e-03 6.296404e-03

279 9.648349e-02 9.648849e-02 9.649369e-02 9.649909e-02 9.650470e-02 1.315368e-02 6.326384e-03 6.340091e-03 6.353826e-03 6.298934e-03

280 9.692622e-02 9.693134e-02 9.693666e-02 9.694219e-02 9.694792e-02 1.316050e-02 6.329275e-03 6.342995e-03 6.356743e-03 6.301800e-03

281 9.741902e-02 9.742426e-02 9.742972e-02 9.743538e-02 9.744126e-02 1.316825e-02 6.332548e-03 6.346282e-03 6.360044e-03 6.305044e-03

282 9.796625e-02 9.797164e-02 9.797724e-02 9.798305e-02 9.798909e-02 1.317704e-02 6.336249e-03 6.350000e-03 6.363778e-03 6.308713e-03

283 9.857243e-02 9.857797e-02 9.858373e-02 9.858971e-02 9.859592e-02 1.318703e-02 6.340433e-03 6.354202e-03 6.367999e-03 6.312860e-03

284 9.924218e-02 9.924788e-02 9.925381e-02 9.925997e-02 9.926636e-02 1.319834e-02 6.345159e-03 6.358949e-03 6.372767e-03 6.317544e-03

285 9.998015e-02 9.998603e-02 9.999214e-02 9.999849e-02 1.000051e-01 1.321117e-02 6.350493e-03 6.364306e-03 6.378148e-03 6.322832e-03

286 1.007910e-01 1.007971e-01 1.008034e-01 1.008099e-01 1.008167e-01 1.322569e-02 6.356510e-03 6.370350e-03 6.384218e-03 6.328796e-03

287 1.016793e-01 1.016855e-01 1.016921e-01 1.016988e-01 1.017059e-01 1.324211e-02 6.363293e-03 6.377163e-03 6.391061e-03 6.335520e-03

288 1.026494e-01 1.026559e-01 1.026627e-01 1.026697e-01 1.026769e-01 1.326069e-02 6.370933e-03 6.384837e-03 6.398769e-03 6.343093e-03

289 1.037057e-01 1.037124e-01 1.037194e-01 1.037266e-01 1.037341e-01 1.328167e-02 6.379534e-03 6.393475e-03 6.407445e-03 6.351619e-03

290 1.048519e-01 1.048589e-01 1.048661e-01 1.048736e-01 1.048814e-01 1.330537e-02 6.389210e-03 6.403193e-03 6.417206e-03 6.361210e-03

291 1.060917e-01 1.060990e-01 1.061064e-01 1.061142e-01 1.061223e-01 1.333210e-02 6.400086e-03 6.414118e-03 6.428178e-03 6.371992e-03

292 1.074282e-01 1.074357e-01 1.074434e-01 1.074515e-01 1.074599e-01 1.336224e-02 6.412305e-03 6.426390e-03 6.440504e-03 6.384104e-03

293 1.088640e-01 1.088717e-01 1.088797e-01 1.088881e-01 1.088967e-01 1.339619e-02 6.426022e-03 6.440166e-03 6.454341e-03 6.397701e-03

294 1.104010e-01 1.104091e-01 1.104174e-01 1.104260e-01 1.104350e-01 1.343442e-02 6.441409e-03 6.455621e-03 6.469863e-03 6.412955e-03

295 1.120409e-01 1.120492e-01 1.120578e-01 1.120667e-01 1.120760e-01 1.347742e-02 6.458660e-03 6.472947e-03 6.487265e-03 6.430055e-03

296 1.137843e-01 1.137928e-01 1.138017e-01 1.138110e-01 1.138205e-01 1.352575e-02 6.477984e-03 6.492356e-03 6.506758e-03 6.449212e-03

297 1.156314e-01 1.156402e-01 1.156494e-01 1.156589e-01 1.156688e-01 1.358004e-02 6.499617e-03 6.514083e-03 6.528580e-03 6.470657e-03

298 1.175816e-01 1.175908e-01 1.176003e-01 1.176101e-01 1.176203e-01 1.364097e-02 6.523818e-03 6.538389e-03 6.552992e-03 6.494648e-03

299 1.196340e-01 1.196434e-01 1.196531e-01 1.196633e-01 1.196738e-01 1.370929e-02 6.550871e-03 6.565559e-03 6.580280e-03 6.521467e-03

300 1.217865e-01 1.217962e-01 1.218063e-01 1.218167e-01 1.218276e-01 1.378583e-02 6.581090e-03 6.595910e-03 6.610763e-03 6.551425e-03

301 1.240368e-01 1.240468e-01 1.240572e-01 1.240679e-01 1.240791e-01 1.387152e-02 6.614823e-03 6.629789e-03 6.644788e-03 6.584866e-03

302 1.263821e-01 1.263923e-01 1.264029e-01 1.264140e-01 1.264254e-01 1.396735e-02 6.652449e-03 6.667577e-03 6.682740e-03 6.622168e-03

303 1.288186e-01 1.288291e-01 1.288400e-01 1.288514e-01 1.288631e-01 1.407445e-02 6.694386e-03 6.709695e-03 6.725040e-03 6.663745e-03

304 1.313426e-01 1.313534e-01 1.313645e-01 1.313761e-01 1.313882e-01 1.419400e-02 6.741093e-03 6.756604e-03 6.772151e-03 6.710051e-03

305 1.339496e-01 1.339606e-01 1.339720e-01 1.339839e-01 1.339962e-01 1.432734e-02 6.793074e-03 6.808807e-03 6.824579e-03 6.761586e-03

306 1.366349e-01 1.366461e-01 1.366578e-01 1.366699e-01 1.366825e-01 1.447590e-02 6.850876e-03 6.866858e-03 6.882878e-03 6.818894e-03

307 1.393932e-01 1.394047e-01 1.394166e-01 1.394290e-01 1.394418e-01 1.464125e-02 6.915103e-03 6.931359e-03 6.947656e-03 6.882573e-03

308 1.422193e-01 1.422310e-01 1.422432e-01 1.422558e-01 1.422688e-01 1.482508e-02 6.986411e-03 7.002971e-03 7.019573e-03 6.953274e-03

309 1.451075e-01 1.451193e-01 1.451317e-01 1.451445e-01 1.451578e-01 1.502923e-02 7.065514e-03 7.082410e-03 7.099350e-03 7.031706e-03

310 1.480517e-01 1.480637e-01 1.480763e-01 1.480893e-01 1.481028e-01 1.525568e-02 7.153191e-03 7.170460e-03 7.187773e-03 7.118642e-03

311 1.510458e-01 1.510580e-01 1.510708e-01 1.510840e-01 1.510977e-01 1.550655e-02 7.250290e-03 7.267968e-03 7.285694e-03 7.214921e-03

312 1.540834e-01 1.540959e-01 1.541088e-01 1.541222e-01 1.541361e-01 1.578413e-02 7.357726e-03 7.375857e-03 7.394037e-03 7.321454e-03

313 1.571580e-01 1.571706e-01 1.571836e-01 1.571972e-01 1.572113e-01 1.609083e-02 7.476494e-03 7.495123e-03 7.513804e-03 7.439227e-03

314 1.602627e-01 1.602754e-01 1.602886e-01 1.603023e-01 1.603165e-01 1.642925e-02 7.607668e-03 7.626845e-03 7.646076e-03 7.569307e-03

315 1.633904e-01 1.634032e-01 1.634166e-01 1.634305e-01 1.634448e-01 1.680211e-02 7.752406e-03 7.772185e-03 7.792021e-03 7.712843e-03

316 1.665340e-01 1.665469e-01 1.665604e-01 1.665744e-01 1.665889e-01 1.721228e-02 7.911955e-03 7.932394e-03 7.952893e-03 7.871073e-03

317 1.696861e-01 1.696991e-01 1.697127e-01 1.697269e-01 1.697415e-01 1.766278e-02 8.087654e-03 8.108816e-03 8.130042e-03 8.045326e-03

318 1.728391e-01 1.728522e-01 1.728659e-01 1.728801e-01 1.728948e-01 1.815673e-02 8.280935e-03 8.302889e-03 8.324909e-03 8.237026e-03

319 1.759853e-01 1.759985e-01 1.760123e-01 1.760266e-01 1.760414e-01 1.869737e-02 8.493333e-03 8.516150e-03 8.539039e-03 8.447695e-03

320 1.791169e-01 1.791301e-01 1.791440e-01 1.791584e-01 1.791732e-01 1.928803e-02 8.726478e-03 8.750238e-03 8.774074e-03 8.678956e-03

321 1.822260e-01 1.822393e-01 1.822532e-01 1.822676e-01 1.822825e-01 1.993210e-02 8.982106e-03 9.006892e-03 9.031758e-03 8.932531e-03

322 1.853047e-01 1.853181e-01 1.853320e-01 1.853465e-01 1.853614e-01 2.063299e-02 9.262052e-03 9.287953e-03 9.313939e-03 9.210245e-03

323 1.883453e-01 1.883587e-01 1.883727e-01 1.883872e-01 1.884022e-01 2.139414e-02 9.568254e-03 9.595365e-03 9.622566e-03 9.514026e-03

324 1.913402e-01 1.913536e-01 1.913676e-01 1.913822e-01 1.913971e-01 2.221894e-02 9.902750e-03 9.931171e-03 9.959688e-03 9.845898e-03

325 1.942821e-01 1.942955e-01 1.943095e-01 1.943241e-01 1.943390e-01 2.311069e-02 1.026767e-02 1.029751e-02 1.032745e-02 1.020799e-02

326 1.971641e-01 1.971774e-01 1.971914e-01 1.972060e-01 1.972210e-01 2.407260e-02 1.066525e-02 1.069661e-02 1.072808e-02 1.060250e-02

327 1.999797e-01 1.999931e-01 2.000071e-01 2.000216e-01 2.000365e-01 2.510769e-02 1.109778e-02 1.113079e-02 1.116391e-02 1.103174e-02

328 2.027233e-01 2.027366e-01 2.027506e-01 2.027651e-01 2.027800e-01 2.621877e-02 1.156766e-02 1.160243e-02 1.163733e-02 1.149808e-02

329 2.053898e-01 2.054030e-01 2.054170e-01 2.054315e-01 2.054463e-01 2.740840e-02 1.207733e-02 1.211399e-02 1.215078e-02 1.200396e-02

330 2.079750e-01 2.079882e-01 2.080021e-01 2.080165e-01 2.080313e-01 2.867881e-02 1.262929e-02 1.266797e-02 1.270679e-02 1.255187e-02

331 2.104755e-01 2.104886e-01 2.105025e-01 2.105169e-01 2.105316e-01 3.003186e-02 1.322607e-02 1.326691e-02 1.330789e-02 1.314434e-02

332 2.128889e-01 2.129020e-01 2.129158e-01 2.129302e-01 2.129448e-01 3.146904e-02 1.387023e-02 1.391335e-02 1.395662e-02 1.378390e-02

333 2.152138e-01 2.152268e-01 2.152406e-01 2.152549e-01 2.152695e-01 3.299135e-02 1.456430e-02 1.460984e-02 1.465555e-02 1.447310e-02

334 2.174496e-01 2.174625e-01 2.174762e-01 2.174904e-01 2.175049e-01 3.459932e-02 1.531080e-02 1.535891e-02 1.540719e-02 1.521445e-02

335 2.195964e-01 2.196093e-01 2.196229e-01 2.196371e-01 2.196515e-01 3.629297e-02 1.611219e-02 1.616300e-02 1.621399e-02 1.601042e-02

336 2.216555e-01 2.216683e-01 2.216819e-01 2.216960e-01 2.217103e-01 3.807177e-02 1.697085e-02 1.702449e-02 1.707833e-02 1.686338e-02

337 2.236286e-01 2.236412e-01 2.236548e-01 2.236688e-01 2.236831e-01 3.993464e-02 1.788905e-02 1.794566e-02 1.800247e-02 1.777560e-02

338 2.255180e-01 2.255306e-01 2.255441e-01 2.255581e-01 2.255722e-01 4.187991e-02 1.886890e-02 1.892861e-02 1.898853e-02 1.874922e-02

339 2.273267e-01 2.273392e-01 2.273526e-01 2.273666e-01 2.273806e-01 4.390537e-02 1.991236e-02 1.997529e-02 2.003844e-02 1.978619e-02

340 2.290579e-01 2.290704e-01 2.290837e-01 2.290976e-01 2.291116e-01 4.600819e-02 2.102115e-02 2.108742e-02 2.115392e-02 2.088827e-02

341 2.307153e-01 2.307277e-01 2.307410e-01 2.307548e-01 2.307687e-01 4.818503e-02 2.219678e-02 2.226649e-02 2.233644e-02 2.205695e-02

342 2.323026e-01 2.323149e-01 2.323281e-01 2.323419e-01 2.323557e-01 5.043197e-02 2.344045e-02 2.351370e-02 2.358720e-02 2.329348e-02

343 2.338237e-01 2.338358e-01 2.338491e-01 2.338628e-01 2.338765e-01 5.274457e-02 2.475306e-02 2.482995e-02 2.490709e-02 2.459877e-02

344 2.352824e-01 2.352945e-01 2.353077e-01 2.353214e-01 2.353350e-01 5.511790e-02 2.613520e-02 2.621579e-02 2.629664e-02 2.597344e-02

345 2.366827e-01 2.366947e-01 2.367078e-01 2.367215e-01 2.367350e-01 5.754656e-02 2.758705e-02 2.767140e-02 2.775602e-02 2.741768e-02

346 2.380282e-01 2.380402e-01 2.380533e-01 2.380669e-01 2.380804e-01 6.002471e-02 2.910841e-02 2.919657e-02 2.928501e-02 2.893136e-02

347 2.393227e-01 2.393346e-01 2.393477e-01 2.393612e-01 2.393746e-01 6.254613e-02 3.069869e-02 3.079069e-02 3.088296e-02 3.051388e-02

348 2.405697e-01 2.405814e-01 2.405945e-01 2.406080e-01 2.406214e-01 6.510422e-02 3.235683e-02 3.245268e-02 3.254881e-02 3.216424e-02

349 2.417723e-01 2.417840e-01 2.417970e-01 2.418105e-01 2.418238e-01 6.769211e-02 3.408136e-02 3.418105e-02 3.428103e-02 3.388099e-02

350 2.429338e-01 2.429454e-01 2.429584e-01 2.429719e-01 2.429851e-01 7.030265e-02 3.587033e-02 3.597384e-02 3.607764e-02 3.566222e-02

351 2.440569e-01 2.440685e-01 2.440815e-01 2.440949e-01 2.441080e-01 7.292848e-02 3.772134e-02 3.782863e-02 3.793622e-02 3.750557e-02

352 2.451445e-01 2.451560e-01 2.451689e-01 2.451824e-01 2.451954e-01 7.556213e-02 3.963154e-02 3.974255e-02 3.985387e-02 3.940822e-02

353 2.461990e-01 2.462104e-01 2.462233e-01 2.462368e-01 2.462498e-01 7.819604e-02 4.159763e-02 4.171229e-02 4.182726e-02 4.136692e-02

354 2.472227e-01 2.472340e-01 2.472469e-01 2.472603e-01 2.472733e-01 8.082261e-02 4.361589e-02 4.373410e-02 4.385262e-02 4.337796e-02

355 2.482177e-01 2.482289e-01 2.482418e-01 2.482552e-01 2.482681e-01 8.343436e-02 4.568216e-02 4.580382e-02 4.592578e-02 4.543723e-02

356 2.491859e-01 2.491971e-01 2.492100e-01 2.492234e-01 2.492361e-01 8.602389e-02 4.779191e-02 4.791689e-02 4.804218e-02 4.754023e-02

357 2.501291e-01 2.501403e-01 2.501531e-01 2.501665e-01 2.501792e-01 8.858407e-02 4.994025e-02 5.006842e-02 5.019688e-02 4.968210e-02

358 2.510490e-01 2.510601e-01 2.510729e-01 2.510863e-01 2.510989e-01 9.110801e-02 5.212198e-02 5.225317e-02 5.238467e-02 5.185766e-02

359 2.519470e-01 2.519579e-01 2.519708e-01 2.519841e-01 2.519967e-01 9.358924e-02 5.433159e-02 5.446565e-02 5.460001e-02 5.406143e-02

360 2.528244e-01 2.528353e-01 2.528481e-01 2.528615e-01 2.528740e-01 9.602172e-02 5.656337e-02 5.670012e-02 5.683717e-02 5.628772e-02

361 2.536824e-01 2.536932e-01 2.537061e-01 2.537194e-01 2.537319e-01 9.839991e-02 5.881141e-02 5.895068e-02 5.909023e-02 5.853064e-02

362 2.536826e-01 2.536934e-01 2.537063e-01 2.537196e-01 2.537321e-01 9.839991e-02 5.881141e-02 5.895068e-02 5.909023e-02 5.853064e-02

363 2.536828e-01 2.536937e-01 2.537065e-01 2.537198e-01 2.537323e-01 9.839991e-02 5.881141e-02 5.895068e-02 5.909023e-02 5.853064e-02

364 2.536831e-01 2.536939e-01 2.537068e-01 2.537201e-01 2.537326e-01 9.839991e-02 5.881141e-02 5.895068e-02 5.909024e-02 5.853064e-02

365 2.536834e-01 2.536942e-01 2.537071e-01 2.537204e-01 2.537329e-01 9.839992e-02 5.881142e-02 5.895068e-02 5.909024e-02 5.853064e-02

366 2.536838e-01 2.536946e-01 2.537074e-01 2.537208e-01 2.537332e-01 9.839992e-02 5.881142e-02 5.895068e-02 5.909024e-02 5.853064e-02

367 2.536842e-01 2.536950e-01 2.537078e-01 2.537212e-01 2.537336e-01 9.839992e-02 5.881142e-02 5.895069e-02 5.909024e-02 5.853064e-02

368 2.536846e-01 2.536955e-01 2.537083e-01 2.537216e-01 2.537341e-01 9.839993e-02 5.881142e-02 5.895069e-02 5.909024e-02 5.853064e-02

369 2.536852e-01 2.536960e-01 2.537088e-01 2.537222e-01 2.537346e-01 9.839993e-02 5.881142e-02 5.895069e-02 5.909024e-02 5.853065e-02

370 2.536858e-01 2.536966e-01 2.537095e-01 2.537228e-01 2.537352e-01 9.839994e-02 5.881143e-02 5.895069e-02 5.909025e-02 5.853065e-02

371 2.536865e-01 2.536973e-01 2.537102e-01 2.537235e-01 2.537359e-01 9.839994e-02 5.881143e-02 5.895070e-02 5.909025e-02 5.853065e-02

372 2.536873e-01 2.536981e-01 2.537110e-01 2.537243e-01 2.537368e-01 9.839995e-02 5.881143e-02 5.895070e-02 5.909025e-02 5.853066e-02

373 2.536882e-01 2.536991e-01 2.537119e-01 2.537252e-01 2.537377e-01 9.839996e-02 5.881144e-02 5.895070e-02 5.909026e-02 5.853066e-02

374 2.536893e-01 2.537001e-01 2.537130e-01 2.537263e-01 2.537388e-01 9.839997e-02 5.881144e-02 5.895071e-02 5.909026e-02 5.853066e-02

375 2.536905e-01 2.537013e-01 2.537142e-01 2.537275e-01 2.537400e-01 9.839998e-02 5.881144e-02 5.895071e-02 5.909027e-02 5.853067e-02

376 2.536919e-01 2.537027e-01 2.537156e-01 2.537289e-01 2.537414e-01 9.839999e-02 5.881145e-02 5.895072e-02 5.909027e-02 5.853067e-02

377 2.536935e-01 2.537043e-01 2.537172e-01 2.537305e-01 2.537430e-01 9.840001e-02 5.881146e-02 5.895072e-02 5.909028e-02 5.853068e-02

378 2.536953e-01 2.537061e-01 2.537190e-01 2.537323e-01 2.537448e-01 9.840003e-02 5.881146e-02 5.895073e-02 5.909029e-02 5.853069e-02

379 2.536974e-01 2.537082e-01 2.537211e-01 2.537344e-01 2.537469e-01 9.840005e-02 5.881147e-02 5.895074e-02 5.909030e-02 5.853070e-02

380 2.536997e-01 2.537106e-01 2.537234e-01 2.537368e-01 2.537492e-01 9.840007e-02 5.881148e-02 5.895075e-02 5.909031e-02 5.853071e-02

381 2.537024e-01 2.537133e-01 2.537261e-01 2.537395e-01 2.537519e-01 9.840009e-02 5.881149e-02 5.895076e-02 5.909032e-02 5.853072e-02

382 2.537055e-01 2.537164e-01 2.537292e-01 2.537426e-01 2.537550e-01 9.840012e-02 5.881151e-02 5.895077e-02 5.909033e-02 5.853073e-02

383 2.537090e-01 2.537199e-01 2.537327e-01 2.537461e-01 2.537585e-01 9.840016e-02 5.881152e-02 5.895079e-02 5.909034e-02 5.853074e-02

384 2.537130e-01 2.537239e-01 2.537367e-01 2.537501e-01 2.537625e-01 9.840020e-02 5.881154e-02 5.895080e-02 5.909036e-02 5.853076e-02

385 2.537176e-01 2.537284e-01 2.537413e-01 2.537546e-01 2.537671e-01 9.840024e-02 5.881156e-02 5.895082e-02 5.909038e-02 5.853078e-02

386 2.537227e-01 2.537336e-01 2.537465e-01 2.537598e-01 2.537723e-01 9.840029e-02 5.881158e-02 5.895085e-02 5.909040e-02 5.853080e-02

387 2.537286e-01 2.537395e-01 2.537523e-01 2.537657e-01 2.537782e-01 9.840035e-02 5.881160e-02 5.895087e-02 5.909043e-02 5.853082e-02

388 2.537353e-01 2.537461e-01 2.537590e-01 2.537724e-01 2.537849e-01 9.840041e-02 5.881163e-02 5.895090e-02 5.909045e-02 5.853085e-02

389 2.537429e-01 2.537537e-01 2.537666e-01 2.537800e-01 2.537925e-01 9.840049e-02 5.881166e-02 5.895093e-02 5.909049e-02 5.853088e-02

390 2.537515e-01 2.537623e-01 2.537752e-01 2.537886e-01 2.538011e-01 9.840057e-02 5.881170e-02 5.895096e-02 5.909052e-02 5.853092e-02

391 2.537612e-01 2.537721e-01 2.537850e-01 2.537984e-01 2.538109e-01 9.840067e-02 5.881174e-02 5.895100e-02 5.909056e-02 5.853096e-02

392 2.537723e-01 2.537832e-01 2.537961e-01 2.538094e-01 2.538219e-01 9.840078e-02 5.881178e-02 5.895105e-02 5.909061e-02 5.853100e-02

393 2.537848e-01 2.537957e-01 2.538086e-01 2.538220e-01 2.538345e-01 9.840090e-02 5.881183e-02 5.895110e-02 5.909066e-02 5.853105e-02

394 2.537990e-01 2.538099e-01 2.538228e-01 2.538362e-01 2.538487e-01 9.840104e-02 5.881189e-02 5.895116e-02 5.909072e-02 5.853111e-02

395 2.538150e-01 2.538259e-01 2.538388e-01 2.538523e-01 2.538648e-01 9.840120e-02 5.881196e-02 5.895123e-02 5.909079e-02 5.853118e-02

396 2.538332e-01 2.538441e-01 2.538570e-01 2.538704e-01 2.538830e-01 9.840139e-02 5.881203e-02 5.895130e-02 5.909086e-02 5.853125e-02

397 2.538536e-01 2.538646e-01 2.538775e-01 2.538910e-01 2.539035e-01 9.840159e-02 5.881212e-02 5.895139e-02 5.909095e-02 5.853133e-02

398 2.538768e-01 2.538877e-01 2.539007e-01 2.539141e-01 2.539267e-01 9.840183e-02 5.881221e-02 5.895149e-02 5.909105e-02 5.853143e-02

399 2.539029e-01 2.539139e-01 2.539268e-01 2.539403e-01 2.539529e-01 9.840209e-02 5.881232e-02 5.895160e-02 5.909116e-02 5.853154e-02

400 2.539324e-01 2.539433e-01 2.539563e-01 2.539698e-01 2.539824e-01 9.840240e-02 5.881245e-02 5.895172e-02 5.909128e-02 5.853166e-02

401 2.539656e-01 2.539766e-01 2.539896e-01 2.540031e-01 2.540157e-01 9.840274e-02 5.881259e-02 5.895186e-02 5.909142e-02 5.853179e-02

402 2.540030e-01 2.540140e-01 2.540270e-01 2.540406e-01 2.540532e-01 9.840313e-02 5.881274e-02 5.895202e-02 5.909158e-02 5.853195e-02

403 2.540451e-01 2.540561e-01 2.540692e-01 2.540827e-01 2.540954e-01 9.840356e-02 5.881292e-02 5.895220e-02 5.909176e-02 5.853212e-02

404 2.540925e-01 2.541035e-01 2.541166e-01 2.541302e-01 2.541429e-01 9.840406e-02 5.881312e-02 5.895240e-02 5.909196e-02 5.853232e-02

405 2.541457e-01 2.541568e-01 2.541699e-01 2.541835e-01 2.541962e-01 9.840462e-02 5.881334e-02 5.895262e-02 5.909219e-02 5.853254e-02

406 2.542055e-01 2.542166e-01 2.542298e-01 2.542434e-01 2.542562e-01 9.840525e-02 5.881359e-02 5.895287e-02 5.909245e-02 5.853279e-02

407 2.542726e-01 2.542838e-01 2.542970e-01 2.543107e-01 2.543235e-01 9.840596e-02 5.881388e-02 5.895316e-02 5.909273e-02 5.853307e-02

408 2.543479e-01 2.543591e-01 2.543724e-01 2.543861e-01 2.543989e-01 9.840677e-02 5.881420e-02 5.895348e-02 5.909306e-02 5.853338e-02

409 2.544323e-01 2.544435e-01 2.544568e-01 2.544706e-01 2.544835e-01 9.840768e-02 5.881455e-02 5.895384e-02 5.909342e-02 5.853373e-02

410 2.545268e-01 2.545381e-01 2.545514e-01 2.545653e-01 2.545782e-01 9.840870e-02 5.881496e-02 5.895425e-02 5.909383e-02 5.853413e-02

411 2.546325e-01 2.546439e-01 2.546573e-01 2.546712e-01 2.546842e-01 9.840986e-02 5.881541e-02 5.895470e-02 5.909429e-02 5.853458e-02

412 2.547507e-01 2.547621e-01 2.547756e-01 2.547896e-01 2.548027e-01 9.841116e-02 5.881592e-02 5.895522e-02 5.909480e-02 5.853508e-02

413 2.548827e-01 2.548942e-01 2.549078e-01 2.549218e-01 2.549350e-01 9.841262e-02 5.881649e-02 5.895579e-02 5.909538e-02 5.853564e-02

414 2.550301e-01 2.550417e-01 2.550553e-01 2.550694e-01 2.550827e-01 9.841427e-02 5.881712e-02 5.895643e-02 5.909603e-02 5.853627e-02

415 2.551943e-01 2.552060e-01 2.552197e-01 2.552339e-01 2.552473e-01 9.841612e-02 5.881784e-02 5.895715e-02 5.909675e-02 5.853697e-02

416 2.553772e-01 2.553890e-01 2.554028e-01 2.554172e-01 2.554307e-01 9.841820e-02 5.881864e-02 5.895796e-02 5.909757e-02 5.853776e-02

417 2.555808e-01 2.555926e-01 2.556066e-01 2.556210e-01 2.556346e-01 9.842054e-02 5.881954e-02 5.895886e-02 5.909848e-02 5.853865e-02

418 2.558069e-01 2.558189e-01 2.558330e-01 2.558476e-01 2.558613e-01 9.842317e-02 5.882054e-02 5.895987e-02 5.909950e-02 5.853964e-02

419 2.560580e-01 2.560701e-01 2.560843e-01 2.560990e-01 2.561129e-01 9.842611e-02 5.882167e-02 5.896101e-02 5.910063e-02 5.854075e-02

420 2.563363e-01 2.563486e-01 2.563629e-01 2.563778e-01 2.563919e-01 9.842942e-02 5.882292e-02 5.896227e-02 5.910191e-02 5.854198e-02

421 2.566445e-01 2.566569e-01 2.566715e-01 2.566865e-01 2.567008e-01 9.843312e-02 5.882432e-02 5.896368e-02 5.910333e-02 5.854336e-02

422 2.569853e-01 2.569979e-01 2.570126e-01 2.570279e-01 2.570423e-01 9.843728e-02 5.882589e-02 5.896526e-02 5.910492e-02 5.854491e-02

423 2.573616e-01 2.573744e-01 2.573893e-01 2.574048e-01 2.574194e-01 9.844193e-02 5.882763e-02 5.896702e-02 5.910669e-02 5.854663e-02

424 2.577765e-01 2.577895e-01 2.578047e-01 2.578204e-01 2.578353e-01 9.844714e-02 5.882958e-02 5.896898e-02 5.910866e-02 5.854855e-02

425 2.582333e-01 2.582466e-01 2.582619e-01 2.582779e-01 2.582930e-01 9.845297e-02 5.883175e-02 5.897117e-02 5.911087e-02 5.855069e-02

426 2.587354e-01 2.587489e-01 2.587645e-01 2.587807e-01 2.587962e-01 9.845949e-02 5.883418e-02 5.897360e-02 5.911332e-02 5.855308e-02

427 2.592862e-01 2.593000e-01 2.593160e-01 2.593325e-01 2.593482e-01 9.846678e-02 5.883687e-02 5.897632e-02 5.911606e-02 5.855574e-02

428 2.598896e-01 2.599037e-01 2.599199e-01 2.599367e-01 2.599528e-01 9.847492e-02 5.883987e-02 5.897934e-02 5.911910e-02 5.855870e-02

429 2.605492e-01 2.605636e-01 2.605802e-01 2.605973e-01 2.606138e-01 9.848402e-02 5.884321e-02 5.898270e-02 5.912249e-02 5.856199e-02

430 2.612688e-01 2.612836e-01 2.613005e-01 2.613181e-01 2.613349e-01 9.849417e-02 5.884692e-02 5.898644e-02 5.912625e-02 5.856565e-02

431 2.620524e-01 2.620676e-01 2.620849e-01 2.621028e-01 2.621200e-01 9.850549e-02 5.885105e-02 5.899060e-02 5.913044e-02 5.856972e-02

432 2.629038e-01 2.629193e-01 2.629370e-01 2.629554e-01 2.629730e-01 9.851812e-02 5.885563e-02 5.899521e-02 5.913509e-02 5.857424e-02

433 2.638268e-01 2.638427e-01 2.638608e-01 2.638796e-01 2.638977e-01 9.853220e-02 5.886072e-02 5.900034e-02 5.914025e-02 5.857926e-02

434 2.648250e-01 2.648414e-01 2.648600e-01 2.648792e-01 2.648978e-01 9.854788e-02 5.886637e-02 5.900603e-02 5.914598e-02 5.858483e-02

435 2.659021e-01 2.659190e-01 2.659380e-01 2.659578e-01 2.659769e-01 9.856534e-02 5.887264e-02 5.901234e-02 5.915233e-02 5.859100e-02

436 2.670615e-01 2.670788e-01 2.670984e-01 2.671187e-01 2.671383e-01 9.858476e-02 5.887958e-02 5.901933e-02 5.915937e-02 5.859785e-02

437 2.683062e-01 2.683241e-01 2.683441e-01 2.683649e-01 2.683851e-01 9.860637e-02 5.888728e-02 5.902708e-02 5.916718e-02 5.860544e-02

438 2.696391e-01 2.696575e-01 2.696781e-01 2.696994e-01 2.697202e-01 9.863039e-02 5.889580e-02 5.903567e-02 5.917582e-02 5.861385e-02

439 2.710626e-01 2.710815e-01 2.711026e-01 2.711246e-01 2.711460e-01 9.865708e-02 5.890524e-02 5.904517e-02 5.918539e-02 5.862315e-02

440 2.725787e-01 2.725981e-01 2.726199e-01 2.726424e-01 2.726644e-01 9.868672e-02 5.891568e-02 5.905568e-02 5.919598e-02 5.863344e-02

441 2.741890e-01 2.742090e-01 2.742313e-01 2.742545e-01 2.742771e-01 9.871962e-02 5.892722e-02 5.906730e-02 5.920768e-02 5.864482e-02

442 2.758945e-01 2.759152e-01 2.759381e-01 2.759619e-01 2.759851e-01 9.875612e-02 5.893998e-02 5.908015e-02 5.922062e-02 5.865740e-02

443 2.776959e-01 2.777171e-01 2.777407e-01 2.777651e-01 2.777890e-01 9.879657e-02 5.895407e-02 5.909434e-02 5.923491e-02 5.867130e-02

444 2.795931e-01 2.796149e-01 2.796391e-01 2.796641e-01 2.796887e-01 9.884140e-02 5.896963e-02 5.911001e-02 5.925069e-02 5.868664e-02

445 2.815854e-01 2.816078e-01 2.816326e-01 2.816582e-01 2.816835e-01 9.889105e-02 5.898679e-02 5.912729e-02 5.926809e-02 5.870357e-02

446 2.836717e-01 2.836947e-01 2.837201e-01 2.837464e-01 2.837723e-01 9.894600e-02 5.900573e-02 5.914636e-02 5.928729e-02 5.872224e-02

447 2.858501e-01 2.858737e-01 2.858997e-01 2.859266e-01 2.859532e-01 9.900678e-02 5.902659e-02 5.916737e-02 5.930845e-02 5.874282e-02

448 2.881182e-01 2.881424e-01 2.881690e-01 2.881966e-01 2.882238e-01 9.907397e-02 5.904959e-02 5.919053e-02 5.933177e-02 5.876549e-02

449 2.904732e-01 2.904979e-01 2.905251e-01 2.905532e-01 2.905811e-01 9.914819e-02 5.907490e-02 5.921602e-02 5.935744e-02 5.879046e-02

450 2.929113e-01 2.929366e-01 2.929643e-01 2.929931e-01 2.930215e-01 9.923015e-02 5.910276e-02 5.924407e-02 5.938568e-02 5.881793e-02

451 2.954285e-01 2.954543e-01 2.954826e-01 2.955120e-01 2.955410e-01 9.932057e-02 5.913340e-02 5.927493e-02 5.941675e-02 5.884815e-02

452 2.980201e-01 2.980465e-01 2.980754e-01 2.981053e-01 2.981349e-01 9.942028e-02 5.916709e-02 5.930884e-02 5.945091e-02 5.888137e-02

453 3.006812e-01 3.007081e-01 3.007375e-01 3.007679e-01 3.007981e-01 9.953014e-02 5.920410e-02 5.934611e-02 5.948843e-02 5.891787e-02

454 3.034061e-01 3.034335e-01 3.034633e-01 3.034943e-01 3.035250e-01 9.965111e-02 5.924473e-02 5.938703e-02 5.952963e-02 5.895795e-02

455 3.061888e-01 3.062166e-01 3.062470e-01 3.062784e-01 3.063096e-01 9.978421e-02 5.928933e-02 5.943193e-02 5.957485e-02 5.900193e-02

456 3.090230e-01 3.090513e-01 3.090821e-01 3.091140e-01 3.091457e-01 9.993054e-02 5.933824e-02 5.948118e-02 5.962443e-02 5.905018e-02

457 3.119020e-01 3.119307e-01 3.119619e-01 3.119943e-01 3.120264e-01 1.000913e-01 5.939185e-02 5.953516e-02 5.967879e-02 5.910306e-02

458 3.148188e-01 3.148479e-01 3.148795e-01 3.149122e-01 3.149448e-01 1.002678e-01 5.945059e-02 5.959430e-02 5.973833e-02 5.916099e-02

459 3.177662e-01 3.177956e-01 3.178276e-01 3.178607e-01 3.178936e-01 1.004614e-01 5.951489e-02 5.965904e-02 5.980352e-02 5.922441e-02

460 3.207366e-01 3.207663e-01 3.207986e-01 3.208321e-01 3.208653e-01 1.006735e-01 5.958524e-02 5.972988e-02 5.987484e-02 5.929381e-02

461 3.237224e-01 3.237524e-01 3.237850e-01 3.238188e-01 3.238524e-01 1.009057e-01 5.966217e-02 5.980733e-02 5.995282e-02 5.936970e-02

462 3.267158e-01 3.267461e-01 3.267790e-01 3.268130e-01 3.268469e-01 1.011598e-01 5.974624e-02 5.989197e-02 6.003804e-02 5.945263e-02

463 3.297091e-01 3.297395e-01 3.297726e-01 3.298069e-01 3.298410e-01 1.014374e-01 5.983805e-02 5.998440e-02 6.013110e-02 5.954320e-02

464 3.326942e-01 3.327249e-01 3.327582e-01 3.327927e-01 3.328270e-01 1.017404e-01 5.993824e-02 6.008527e-02 6.023265e-02 5.964204e-02

465 3.356635e-01 3.356943e-01 3.357278e-01 3.357625e-01 3.357970e-01 1.020709e-01 6.004751e-02 6.019527e-02 6.034340e-02 5.974984e-02

466 3.386093e-01 3.386402e-01 3.386738e-01 3.387086e-01 3.387433e-01 1.024309e-01 6.016659e-02 6.031516e-02 6.046409e-02 5.986733e-02

467 3.415240e-01 3.415550e-01 3.415887e-01 3.416237e-01 3.416584e-01 1.028226e-01 6.029629e-02 6.044572e-02 6.059552e-02 5.999529e-02

468 3.444004e-01 3.444315e-01 3.444653e-01 3.445003e-01 3.445351e-01 1.032482e-01 6.043743e-02 6.058780e-02 6.073856e-02 6.013456e-02

469 3.472317e-01 3.472628e-01 3.472967e-01 3.473317e-01 3.473665e-01 1.037102e-01 6.059091e-02 6.074231e-02 6.089409e-02 6.028601e-02

470 3.500113e-01 3.500424e-01 3.500763e-01 3.501114e-01 3.501462e-01 1.042109e-01 6.075770e-02 6.091020e-02 6.106309e-02 6.045059e-02

471 3.527332e-01 3.527643e-01 3.527982e-01 3.528333e-01 3.528681e-01 1.047528e-01 6.093879e-02 6.109248e-02 6.124657e-02 6.062930e-02

472 3.553921e-01 3.554231e-01 3.554570e-01 3.554921e-01 3.555268e-01 1.053386e-01 6.113525e-02 6.129023e-02 6.144562e-02 6.082320e-02

473 3.579830e-01 3.580139e-01 3.580478e-01 3.580828e-01 3.581175e-01 1.059708e-01 6.134822e-02 6.150459e-02 6.166138e-02 6.103340e-02

474 3.605017e-01 3.605326e-01 3.605664e-01 3.606014e-01 3.606359e-01 1.066520e-01 6.157888e-02 6.173674e-02 6.189504e-02 6.126107e-02

475 3.629448e-01 3.629756e-01 3.630093e-01 3.630442e-01 3.630787e-01 1.073848e-01 6.182847e-02 6.198795e-02 6.214787e-02 6.150745e-02

476 3.653095e-01 3.653402e-01 3.653738e-01 3.654086e-01 3.654429e-01 1.081717e-01 6.209832e-02 6.225952e-02 6.242118e-02 6.177385e-02

477 3.675937e-01 3.676242e-01 3.676577e-01 3.676924e-01 3.677266e-01 1.090154e-01 6.238978e-02 6.255284e-02 6.271637e-02 6.206160e-02

478 3.697960e-01 3.698263e-01 3.698597e-01 3.698943e-01 3.699283e-01 1.099181e-01 6.270428e-02 6.286932e-02 6.303486e-02 6.237213e-02

479 3.719157e-01 3.719459e-01 3.719792e-01 3.720137e-01 3.720475e-01 1.108823e-01 6.304329e-02 6.321047e-02 6.337814e-02 6.270690e-02

480 3.739529e-01 3.739830e-01 3.740161e-01 3.740504e-01 3.740841e-01 1.119100e-01 6.340836e-02 6.357780e-02 6.374776e-02 6.306742e-02

481 3.759081e-01 3.759380e-01 3.759710e-01 3.760052e-01 3.760387e-01 1.130033e-01 6.380105e-02 6.397292e-02 6.414531e-02 6.345527e-02

482 3.777825e-01 3.778122e-01 3.778451e-01 3.778791e-01 3.779124e-01 1.141638e-01 6.422299e-02 6.439743e-02 6.457242e-02 6.387206e-02

483 3.795776e-01 3.796071e-01 3.796399e-01 3.796738e-01 3.797069e-01 1.153931e-01 6.467583e-02 6.485301e-02 6.503075e-02 6.431941e-02

484 3.812955e-01 3.813249e-01 3.813575e-01 3.813913e-01 3.814242e-01 1.166923e-01 6.516124e-02 6.534133e-02 6.552200e-02 6.479901e-02

485 3.829387e-01 3.829678e-01 3.830003e-01 3.830339e-01 3.830667e-01 1.180622e-01 6.568094e-02 6.586410e-02 6.604787e-02 6.531255e-02

486 3.845097e-01 3.845387e-01 3.845710e-01 3.846045e-01 3.846371e-01 1.195035e-01 6.623662e-02 6.642303e-02 6.661007e-02 6.586173e-02

487 3.860114e-01 3.860403e-01 3.860725e-01 3.861059e-01 3.861382e-01 1.210161e-01 6.683000e-02 6.701983e-02 6.721031e-02 6.644824e-02

488 3.874470e-01 3.874757e-01 3.875078e-01 3.875410e-01 3.875732e-01 1.225996e-01 6.746275e-02 6.765619e-02 6.785030e-02 6.707377e-02

489 3.888196e-01 3.888481e-01 3.888801e-01 3.889132e-01 3.889452e-01 1.242535e-01 6.813655e-02 6.833376e-02 6.853167e-02 6.773997e-02

490 3.901323e-01 3.901606e-01 3.901925e-01 3.902255e-01 3.902573e-01 1.259763e-01 6.885298e-02 6.905416e-02 6.925605e-02 6.844846e-02

491 3.913884e-01 3.914165e-01 3.914483e-01 3.914812e-01 3.915129e-01 1.277664e-01 6.961361e-02 6.981892e-02 7.002497e-02 6.920078e-02

492 3.925910e-01 3.926190e-01 3.926507e-01 3.926835e-01 3.927150e-01 1.296216e-01 7.041989e-02 7.062951e-02 7.083989e-02 6.999841e-02

493 3.937433e-01 3.937712e-01 3.938028e-01 3.938355e-01 3.938668e-01 1.315393e-01 7.127319e-02 7.148728e-02 7.170216e-02 7.084271e-02

494 3.948483e-01 3.948760e-01 3.949075e-01 3.949401e-01 3.949713e-01 1.335162e-01 7.217474e-02 7.239347e-02 7.261301e-02 7.173493e-02

495 3.959088e-01 3.959364e-01 3.959678e-01 3.960004e-01 3.960314e-01 1.355488e-01 7.312565e-02 7.334918e-02 7.357353e-02 7.267620e-02

496 3.969278e-01 3.969552e-01 3.969866e-01 3.970190e-01 3.970499e-01 1.376329e-01 7.412686e-02 7.435532e-02 7.458463e-02 7.366747e-02

497 3.979078e-01 3.979351e-01 3.979664e-01 3.979988e-01 3.980295e-01 1.397642e-01 7.517912e-02 7.541265e-02 7.564705e-02 7.470951e-02

498 3.988514e-01 3.988785e-01 3.989098e-01 3.989421e-01 3.989727e-01 1.419377e-01 7.628297e-02 7.652169e-02 7.676131e-02 7.580290e-02

499 3.997609e-01 3.997879e-01 3.998191e-01 3.998514e-01 3.998819e-01 1.441482e-01 7.743876e-02 7.768277e-02 7.792770e-02 7.694799e-02

500 4.006386e-01 4.006655e-01 4.006966e-01 4.007288e-01 4.007592e-01 1.463902e-01 7.864655e-02 7.889594e-02 7.914628e-02 7.814491e-02

501 4.014866e-01 4.015134e-01 4.015445e-01 4.015766e-01 4.016069e-01 1.486579e-01 7.990617e-02 8.016102e-02 8.041684e-02 7.939351e-02

502 4.023068e-01 4.023335e-01 4.023645e-01 4.023966e-01 4.024268e-01 1.509452e-01 8.121718e-02 8.147754e-02 8.173889e-02 8.069337e-02

503 4.031011e-01 4.031277e-01 4.031587e-01 4.031907e-01 4.032207e-01 1.532460e-01 8.257882e-02 8.284473e-02 8.311164e-02 8.204380e-02

504 4.038712e-01 4.038976e-01 4.039285e-01 4.039606e-01 4.039905e-01 1.555540e-01 8.399007e-02 8.426153e-02 8.453401e-02 8.344380e-02

505 4.046185e-01 4.046448e-01 4.046758e-01 4.047077e-01 4.047375e-01 1.578628e-01 8.544958e-02 8.572658e-02 8.600462e-02 8.489207e-02

506 4.053446e-01 4.053708e-01 4.054017e-01 4.054337e-01 4.054633e-01 1.601663e-01 8.695569e-02 8.723820e-02 8.752176e-02 8.638700e-02

507 4.060509e-01 4.060769e-01 4.061078e-01 4.061397e-01 4.061693e-01 1.624582e-01 8.850643e-02 8.879439e-02 8.908342e-02 8.792667e-02

508 4.067384e-01 4.067644e-01 4.067953e-01 4.068272e-01 4.068566e-01 1.647324e-01 9.009953e-02 9.039286e-02 9.068727e-02 8.950884e-02

509 4.074085e-01 4.074344e-01 4.074652e-01 4.074971e-01 4.075264e-01 1.669832e-01 9.173241e-02 9.203101e-02 9.233070e-02 9.113100e-02

510 4.080621e-01 4.080879e-01 4.081187e-01 4.081505e-01 4.081798e-01 1.692049e-01 9.340221e-02 9.370596e-02 9.401080e-02 9.279032e-02

511 4.087003e-01 4.087259e-01 4.087567e-01 4.087885e-01 4.088177e-01 1.713924e-01 9.510579e-02 9.541454e-02 9.572439e-02 9.448372e-02

512 4.093238e-01 4.093494e-01 4.093801e-01 4.094119e-01 4.094410e-01 1.735407e-01 9.683977e-02 9.715336e-02 9.746803e-02 9.620784e-02

513 4.099336e-01 4.099591e-01 4.099898e-01 4.100216e-01 4.100505e-01 1.756455e-01 9.860054e-02 9.891876e-02 9.923809e-02 9.795911e-02

514 4.105304e-01 4.105557e-01 4.105865e-01 4.106182e-01 4.106471e-01 1.777026e-01 1.003843e-01 1.007069e-01 1.010307e-01 9.973375e-02

515 4.111148e-01 4.111401e-01 4.111708e-01 4.112026e-01 4.112313e-01 1.797087e-01 1.021870e-01 1.025139e-01 1.028418e-01 1.015278e-01

516 4.116876e-01 4.117128e-01 4.117435e-01 4.117752e-01 4.118039e-01 1.816607e-01 1.040045e-01 1.043354e-01 1.046674e-01 1.033372e-01

517 4.122493e-01 4.122744e-01 4.123051e-01 4.123368e-01 4.123653e-01 1.835561e-01 1.058327e-01 1.061674e-01 1.065030e-01 1.051577e-01

518 4.128005e-01 4.128254e-01 4.128561e-01 4.128879e-01 4.129163e-01 1.853928e-01 1.076673e-01 1.080054e-01 1.083446e-01 1.069851e-01

519 4.133417e-01 4.133665e-01 4.133972e-01 4.134289e-01 4.134572e-01 1.871694e-01 1.095040e-01 1.098453e-01 1.101876e-01 1.088151e-01

520 4.138732e-01 4.138979e-01 4.139286e-01 4.139603e-01 4.139886e-01 1.888849e-01 1.113384e-01 1.116826e-01 1.120280e-01 1.106435e-01

521 4.143956e-01 4.144202e-01 4.144509e-01 4.144826e-01 4.145107e-01 1.905387e-01 1.131664e-01 1.135133e-01 1.138613e-01 1.124659e-01

522 4.149093e-01 4.149338e-01 4.149645e-01 4.149961e-01 4.150242e-01 1.921307e-01 1.149839e-01 1.153332e-01 1.156836e-01 1.142784e-01

523 4.154146e-01 4.154390e-01 4.154696e-01 4.155013e-01 4.155292e-01 1.936612e-01 1.167868e-01 1.171383e-01 1.174908e-01 1.160768e-01

524 4.159118e-01 4.159361e-01 4.159667e-01 4.159984e-01 4.160262e-01 1.951308e-01 1.185714e-01 1.189248e-01 1.192792e-01 1.178575e-01

525 4.164012e-01 4.164254e-01 4.164561e-01 4.164877e-01 4.165155e-01 1.965405e-01 1.203341e-01 1.206891e-01 1.210451e-01 1.196168e-01

526 4.168833e-01 4.169073e-01 4.169380e-01 4.169696e-01 4.169973e-01 1.978916e-01 1.220714e-01 1.224278e-01 1.227852e-01 1.213512e-01

527 4.173581e-01 4.173820e-01 4.174127e-01 4.174443e-01 4.174719e-01 1.991856e-01 1.237804e-01 1.241379e-01 1.244964e-01 1.230577e-01

528 4.178259e-01 4.178498e-01 4.178804e-01 4.179121e-01 4.179395e-01 2.004241e-01 1.254581e-01 1.258165e-01 1.261760e-01 1.247334e-01

529 4.182871e-01 4.183109e-01 4.183415e-01 4.183731e-01 4.184005e-01 2.016090e-01 1.271020e-01 1.274612e-01 1.278213e-01 1.263758e-01

530 4.187418e-01 4.187654e-01 4.187961e-01 4.188277e-01 4.188549e-01 2.027424e-01 1.287099e-01 1.290696e-01 1.294302e-01 1.279825e-01

531 4.191901e-01 4.192137e-01 4.192443e-01 4.192760e-01 4.193031e-01 2.038264e-01 1.302798e-01 1.306398e-01 1.310008e-01 1.295517e-01

532 4.196324e-01 4.196558e-01 4.196865e-01 4.197181e-01 4.197451e-01 2.048630e-01 1.318101e-01 1.321703e-01 1.325314e-01 1.310816e-01

533 4.200687e-01 4.200921e-01 4.201227e-01 4.201543e-01 4.201812e-01 2.058545e-01 1.332996e-01 1.336598e-01 1.340209e-01 1.325709e-01

534 4.204993e-01 4.205225e-01 4.205531e-01 4.205848e-01 4.206116e-01 2.068030e-01 1.347471e-01 1.351072e-01 1.354682e-01 1.340185e-01

535 4.209242e-01 4.209473e-01 4.209779e-01 4.210096e-01 4.210363e-01 2.077108e-01 1.361520e-01 1.365118e-01 1.368725e-01 1.354238e-01

536 4.213437e-01 4.213667e-01 4.213973e-01 4.214289e-01 4.214555e-01 2.085800e-01 1.375137e-01 1.378733e-01 1.382336e-01 1.367862e-01

537 4.217578e-01 4.217807e-01 4.218113e-01 4.218429e-01 4.218694e-01 2.094127e-01 1.388322e-01 1.391913e-01 1.395512e-01 1.381055e-01

538 4.221667e-01 4.221895e-01 4.222201e-01 4.222517e-01 4.222781e-01 2.102110e-01 1.401074e-01 1.404660e-01 1.408254e-01 1.393816e-01

539 4.225705e-01 4.225932e-01 4.226238e-01 4.226554e-01 4.226817e-01 2.109768e-01 1.413396e-01 1.416976e-01 1.420565e-01 1.406149e-01

540 4.229693e-01 4.229919e-01 4.230225e-01 4.230541e-01 4.230803e-01 2.117120e-01 1.425293e-01 1.428867e-01 1.432449e-01 1.418058e-01

541 4.233633e-01 4.233858e-01 4.234164e-01 4.234480e-01 4.234741e-01 2.124185e-01 1.436770e-01 1.440338e-01 1.443913e-01 1.429548e-01

542 4.233844e-01 4.234069e-01 4.234375e-01 4.234691e-01 4.234952e-01 2.124187e-01 1.436771e-01 1.440338e-01 1.443913e-01 1.429549e-01

543 4.234079e-01 4.234304e-01 4.234610e-01 4.234927e-01 4.235188e-01 2.124189e-01 1.436772e-01 1.440339e-01 1.443914e-01 1.429550e-01

544 4.234341e-01 4.234566e-01 4.234873e-01 4.235190e-01 4.235451e-01 2.124191e-01 1.436772e-01 1.440340e-01 1.443915e-01 1.429550e-01

545 4.234632e-01 4.234858e-01 4.235165e-01 4.235482e-01 4.235744e-01 2.124193e-01 1.436773e-01 1.440341e-01 1.443916e-01 1.429551e-01

546 4.234957e-01 4.235183e-01 4.235490e-01 4.235807e-01 4.236070e-01 2.124196e-01 1.436774e-01 1.440342e-01 1.443917e-01 1.429552e-01

547 4.235318e-01 4.235544e-01 4.235852e-01 4.236170e-01 4.236432e-01 2.124198e-01 1.436775e-01 1.440343e-01 1.443918e-01 1.429553e-01

548 4.235719e-01 4.235946e-01 4.236254e-01 4.236572e-01 4.236835e-01 2.124202e-01 1.436776e-01 1.440344e-01 1.443919e-01 1.429554e-01

549 4.236165e-01 4.236392e-01 4.236701e-01 4.237020e-01 4.237283e-01 2.124205e-01 1.436778e-01 1.440345e-01 1.443920e-01 1.429556e-01

550 4.236661e-01 4.236888e-01 4.237197e-01 4.237517e-01 4.237781e-01 2.124209e-01 1.436779e-01 1.440347e-01 1.443922e-01 1.429557e-01

551 4.237211e-01 4.237439e-01 4.237748e-01 4.238068e-01 4.238333e-01 2.124214e-01 1.436781e-01 1.440348e-01 1.443924e-01 1.429559e-01

552 4.237821e-01 4.238050e-01 4.238360e-01 4.238680e-01 4.238946e-01 2.124219e-01 1.436782e-01 1.440350e-01 1.443925e-01 1.429560e-01

553 4.238497e-01 4.238727e-01 4.239038e-01 4.239359e-01 4.239625e-01 2.124225e-01 1.436784e-01 1.440352e-01 1.443927e-01 1.429562e-01

554 4.239247e-01 4.239477e-01 4.239789e-01 4.240111e-01 4.240378e-01 2.124231e-01 1.436787e-01 1.440354e-01 1.443930e-01 1.429564e-01

555 4.240077e-01 4.240309e-01 4.240621e-01 4.240944e-01 4.241212e-01 2.124238e-01 1.436789e-01 1.440357e-01 1.443932e-01 1.429567e-01

556 4.240997e-01 4.241229e-01 4.241542e-01 4.241866e-01 4.242135e-01 2.124246e-01 1.436792e-01 1.440360e-01 1.443935e-01 1.429570e-01

557 4.242013e-01 4.242246e-01 4.242561e-01 4.242886e-01 4.243156e-01 2.124254e-01 1.436795e-01 1.440363e-01 1.443938e-01 1.429573e-01

558 4.243137e-01 4.243371e-01 4.243687e-01 4.244013e-01 4.244284e-01 2.124264e-01 1.436798e-01 1.440366e-01 1.443942e-01 1.429576e-01

559 4.244378e-01 4.244614e-01 4.244931e-01 4.245258e-01 4.245531e-01 2.124275e-01 1.436802e-01 1.440370e-01 1.443945e-01 1.429579e-01

560 4.245748e-01 4.245985e-01 4.246304e-01 4.246633e-01 4.246907e-01 2.124287e-01 1.436806e-01 1.440374e-01 1.443950e-01 1.429584e-01

561 4.247260e-01 4.247498e-01 4.247818e-01 4.248149e-01 4.248425e-01 2.124300e-01 1.436811e-01 1.440379e-01 1.443954e-01 1.429588e-01

562 4.248926e-01 4.249166e-01 4.249487e-01 4.249820e-01 4.250097e-01 2.124315e-01 1.436816e-01 1.440384e-01 1.443959e-01 1.429593e-01

563 4.250760e-01 4.251002e-01 4.251326e-01 4.251660e-01 4.251940e-01 2.124331e-01 1.436821e-01 1.440389e-01 1.443965e-01 1.429599e-01

564 4.252779e-01 4.253023e-01 4.253348e-01 4.253685e-01 4.253967e-01 2.124350e-01 1.436828e-01 1.440396e-01 1.443972e-01 1.429605e-01

565 4.254999e-01 4.255245e-01 4.255573e-01 4.255911e-01 4.256196e-01 2.124370e-01 1.436835e-01 1.440403e-01 1.443979e-01 1.429611e-01

566 4.257437e-01 4.257686e-01 4.258016e-01 4.258357e-01 4.258644e-01 2.124393e-01 1.436842e-01 1.440410e-01 1.443986e-01 1.429619e-01

567 4.260113e-01 4.260364e-01 4.260697e-01 4.261041e-01 4.261331e-01 2.124418e-01 1.436851e-01 1.440419e-01 1.443995e-01 1.429627e-01

568 4.263047e-01 4.263301e-01 4.263637e-01 4.263984e-01 4.264277e-01 2.124445e-01 1.436860e-01 1.440428e-01 1.444004e-01 1.429636e-01

569 4.266261e-01 4.266518e-01 4.266857e-01 4.267207e-01 4.267503e-01 2.124476e-01 1.436870e-01 1.440439e-01 1.444015e-01 1.429646e-01

570 4.269777e-01 4.270037e-01 4.270379e-01 4.270733e-01 4.271033e-01 2.124510e-01 1.436882e-01 1.440450e-01 1.444027e-01 1.429657e-01

571 4.273619e-01 4.273883e-01 4.274229e-01 4.274587e-01 4.274891e-01 2.124548e-01 1.436894e-01 1.440463e-01 1.444039e-01 1.429670e-01

572 4.277814e-01 4.278082e-01 4.278432e-01 4.278794e-01 4.279102e-01 2.124590e-01 1.436908e-01 1.440477e-01 1.444053e-01 1.429683e-01

573 4.282387e-01 4.282659e-01 4.283014e-01 4.283381e-01 4.283694e-01 2.124636e-01 1.436923e-01 1.440492e-01 1.444069e-01 1.429698e-01

574 4.287367e-01 4.287644e-01 4.288003e-01 4.288375e-01 4.288693e-01 2.124687e-01 1.436940e-01 1.440509e-01 1.444086e-01 1.429714e-01

575 4.292782e-01 4.293064e-01 4.293428e-01 4.293805e-01 4.294129e-01 2.124744e-01 1.436958e-01 1.440528e-01 1.444105e-01 1.429733e-01

576 4.298662e-01 4.298949e-01 4.299319e-01 4.299702e-01 4.300031e-01 2.124806e-01 1.436979e-01 1.440548e-01 1.444126e-01 1.429753e-01

577 4.305038e-01 4.305331e-01 4.305707e-01 4.306095e-01 4.306431e-01 2.124875e-01 1.437001e-01 1.440571e-01 1.444149e-01 1.429775e-01

578 4.311940e-01 4.312240e-01 4.312622e-01 4.313017e-01 4.313359e-01 2.124952e-01 1.437026e-01 1.440596e-01 1.444174e-01 1.429799e-01

579 4.319401e-01 4.319707e-01 4.320096e-01 4.320498e-01 4.320847e-01 2.125036e-01 1.437053e-01 1.440623e-01 1.444202e-01 1.429825e-01

580 4.327452e-01 4.327765e-01 4.328161e-01 4.328570e-01 4.328927e-01 2.125129e-01 1.437083e-01 1.440654e-01 1.444232e-01 1.429854e-01

581 4.336125e-01 4.336445e-01 4.336849e-01 4.337265e-01 4.337631e-01 2.125232e-01 1.437116e-01 1.440687e-01 1.444266e-01 1.429887e-01

582 4.345450e-01 4.345778e-01 4.346190e-01 4.346614e-01 4.346988e-01 2.125345e-01 1.437152e-01 1.440723e-01 1.444302e-01 1.429922e-01

583 4.355458e-01 4.355795e-01 4.356214e-01 4.356648e-01 4.357031e-01 2.125470e-01 1.437191e-01 1.440763e-01 1.444343e-01 1.429961e-01

584 4.366178e-01 4.366524e-01 4.366952e-01 4.367394e-01 4.367786e-01 2.125607e-01 1.437235e-01 1.440807e-01 1.444387e-01 1.430003e-01

585 4.377638e-01 4.377992e-01 4.378430e-01 4.378881e-01 4.379283e-01 2.125759e-01 1.437282e-01 1.440855e-01 1.444436e-01 1.430050e-01

586 4.389863e-01 4.390226e-01 4.390673e-01 4.391135e-01 4.391547e-01 2.125926e-01 1.437335e-01 1.440908e-01 1.444489e-01 1.430101e-01

587 4.402875e-01 4.403249e-01 4.403705e-01 4.404177e-01 4.404600e-01 2.126109e-01 1.437392e-01 1.440966e-01 1.444548e-01 1.430157e-01

588 4.416697e-01 4.417080e-01 4.417547e-01 4.418029e-01 4.418463e-01 2.126311e-01 1.437455e-01 1.441029e-01 1.444612e-01 1.430219e-01

589 4.431343e-01 4.431737e-01 4.432215e-01 4.432708e-01 4.433153e-01 2.126534e-01 1.437523e-01 1.441099e-01 1.444682e-01 1.430286e-01

590 4.446829e-01 4.447234e-01 4.447722e-01 4.448226e-01 4.448682e-01 2.126778e-01 1.437599e-01 1.441175e-01 1.444759e-01 1.430360e-01

591 4.463163e-01 4.463578e-01 4.464078e-01 4.464593e-01 4.465061e-01 2.127047e-01 1.437681e-01 1.441258e-01 1.444843e-01 1.430440e-01

592 4.480351e-01 4.480777e-01 4.481287e-01 4.481814e-01 4.482295e-01 2.127341e-01 1.437771e-01 1.441349e-01 1.444935e-01 1.430529e-01

593 4.498392e-01 4.498830e-01 4.499351e-01 4.499889e-01 4.500382e-01 2.127665e-01 1.437870e-01 1.441449e-01 1.445036e-01 1.430625e-01

594 4.517283e-01 4.517732e-01 4.518264e-01 4.518814e-01 4.519319e-01 2.128021e-01 1.437978e-01 1.441558e-01 1.445146e-01 1.430731e-01

595 4.537013e-01 4.537473e-01 4.538018e-01 4.538579e-01 4.539096e-01 2.128411e-01 1.438096e-01 1.441677e-01 1.445266e-01 1.430846e-01

596 4.557569e-01 4.558040e-01 4.558596e-01 4.559169e-01 4.559698e-01 2.128838e-01 1.438224e-01 1.441807e-01 1.445398e-01 1.430972e-01

597 4.578929e-01 4.579412e-01 4.579978e-01 4.580563e-01 4.581105e-01 2.129307e-01 1.438365e-01 1.441949e-01 1.445541e-01 1.431110e-01

598 4.601069e-01 4.601562e-01 4.602140e-01 4.602736e-01 4.603290e-01 2.129820e-01 1.438518e-01 1.442104e-01 1.445698e-01 1.431260e-01

599 4.623956e-01 4.624461e-01 4.625049e-01 4.625657e-01 4.626222e-01 2.130382e-01 1.438685e-01 1.442273e-01 1.445868e-01 1.431424e-01

600 4.647557e-01 4.648071e-01 4.648671e-01 4.649289e-01 4.649866e-01 2.130997e-01 1.438868e-01 1.442457e-01 1.446055e-01 1.431602e-01

601 4.671828e-01 4.672353e-01 4.672963e-01 4.673592e-01 4.674180e-01 2.131670e-01 1.439067e-01 1.442658e-01 1.446258e-01 1.431797e-01

602 4.696725e-01 4.697260e-01 4.697880e-01 4.698519e-01 4.699118e-01 2.132406e-01 1.439283e-01 1.442877e-01 1.446479e-01 1.432009e-01

603 4.722197e-01 4.722742e-01 4.723371e-01 4.724020e-01 4.724629e-01 2.133210e-01 1.439519e-01 1.443115e-01 1.446719e-01 1.432240e-01

604 4.748189e-01 4.748743e-01 4.749382e-01 4.750041e-01 4.750660e-01 2.134089e-01 1.439776e-01 1.443375e-01 1.446982e-01 1.432492e-01

605 4.774644e-01 4.775207e-01 4.775854e-01 4.776522e-01 4.777150e-01 2.135048e-01 1.440055e-01 1.443657e-01 1.447267e-01 1.432765e-01

606 4.801499e-01 4.802069e-01 4.802725e-01 4.803401e-01 4.804038e-01 2.136094e-01 1.440359e-01 1.443964e-01 1.447577e-01 1.433063e-01

607 4.828688e-01 4.829267e-01 4.829930e-01 4.830614e-01 4.831259e-01 2.137235e-01 1.440689e-01 1.444297e-01 1.447914e-01 1.433387e-01

608 4.856146e-01 4.856732e-01 4.857402e-01 4.858093e-01 4.858746e-01 2.138478e-01 1.441048e-01 1.444660e-01 1.448280e-01 1.433738e-01

609 4.883802e-01 4.884394e-01 4.885071e-01 4.885769e-01 4.886429e-01 2.139833e-01 1.441438e-01 1.445054e-01 1.448678e-01 1.434120e-01

610 4.911586e-01 4.912184e-01 4.912867e-01 4.913571e-01 4.914237e-01 2.141307e-01 1.441861e-01 1.445481e-01 1.449110e-01 1.434534e-01

611 4.939426e-01 4.940029e-01 4.940717e-01 4.941427e-01 4.942099e-01 2.142911e-01 1.442320e-01 1.445945e-01 1.449579e-01 1.434984e-01

612 4.967250e-01 4.967858e-01 4.968551e-01 4.969265e-01 4.969942e-01 2.144655e-01 1.442818e-01 1.446448e-01 1.450087e-01 1.435472e-01

613 4.994987e-01 4.995599e-01 4.996296e-01 4.997014e-01 4.997695e-01 2.146550e-01 1.443358e-01 1.446994e-01 1.450638e-01 1.436001e-01

614 5.022565e-01 5.023181e-01 5.023881e-01 5.024603e-01 5.025288e-01 2.148607e-01 1.443943e-01 1.447584e-01 1.451235e-01 1.436574e-01

615 5.049917e-01 5.050535e-01 5.051238e-01 5.051963e-01 5.052651e-01 2.150838e-01 1.444576e-01 1.448224e-01 1.451881e-01 1.437195e-01

616 5.076975e-01 5.077595e-01 5.078301e-01 5.079028e-01 5.079718e-01 2.153257e-01 1.445262e-01 1.448917e-01 1.452581e-01 1.437866e-01

617 5.103675e-01 5.104297e-01 5.105004e-01 5.105733e-01 5.106425e-01 2.155877e-01 1.446004e-01 1.449666e-01 1.453338e-01 1.438593e-01

618 5.129957e-01 5.130580e-01 5.131288e-01 5.132019e-01 5.132711e-01 2.158712e-01 1.446806e-01 1.450477e-01 1.454157e-01 1.439379e-01

619 5.155764e-01 5.156388e-01 5.157097e-01 5.157828e-01 5.158521e-01 2.161779e-01 1.447673e-01 1.451353e-01 1.455041e-01 1.440229e-01

620 5.181045e-01 5.181668e-01 5.182378e-01 5.183109e-01 5.183802e-01 2.165091e-01 1.448609e-01 1.452299e-01 1.455997e-01 1.441147e-01

621 5.205752e-01 5.206375e-01 5.207085e-01 5.207815e-01 5.208508e-01 2.168667e-01 1.449620e-01 1.453320e-01 1.457028e-01 1.442138e-01

622 5.229844e-01 5.230466e-01 5.231175e-01 5.231905e-01 5.232596e-01 2.172523e-01 1.450711e-01 1.454422e-01 1.458141e-01 1.443207e-01

623 5.253284e-01 5.253905e-01 5.254613e-01 5.255342e-01 5.256032e-01 2.176677e-01 1.451888e-01 1.455610e-01 1.459342e-01 1.444360e-01

624 5.276042e-01 5.276661e-01 5.277367e-01 5.278095e-01 5.278783e-01 2.181148e-01 1.453156e-01 1.456891e-01 1.460635e-01 1.445603e-01

625 5.298093e-01 5.298710e-01 5.299415e-01 5.300140e-01 5.300827e-01 2.185953e-01 1.454522e-01 1.458270e-01 1.462028e-01 1.446941e-01

626 5.319418e-01 5.320033e-01 5.320735e-01 5.321459e-01 5.322143e-01 2.191113e-01 1.455992e-01 1.459755e-01 1.463528e-01 1.448383e-01

627 5.340003e-01 5.340616e-01 5.341316e-01 5.342038e-01 5.342719e-01 2.196646e-01 1.457573e-01 1.461352e-01 1.465141e-01 1.449933e-01

628 5.359842e-01 5.360452e-01 5.361150e-01 5.361869e-01 5.362547e-01 2.202573e-01 1.459274e-01 1.463069e-01 1.466875e-01 1.451600e-01

629 5.378931e-01 5.379538e-01 5.380233e-01 5.380950e-01 5.381625e-01 2.208913e-01 1.461101e-01 1.464914e-01 1.468738e-01 1.453391e-01

630 5.397272e-01 5.397876e-01 5.398569e-01 5.399283e-01 5.399955e-01 2.215687e-01 1.463062e-01 1.466894e-01 1.470738e-01 1.455315e-01

631 5.414873e-01 5.415474e-01 5.416165e-01 5.416876e-01 5.417544e-01 2.222912e-01 1.465166e-01 1.469019e-01 1.472883e-01 1.457379e-01

632 5.431744e-01 5.432342e-01 5.433030e-01 5.433738e-01 5.434403e-01 2.230609e-01 1.467423e-01 1.471297e-01 1.475183e-01 1.459592e-01

633 5.447899e-01 5.448494e-01 5.449179e-01 5.449885e-01 5.450547e-01 2.238795e-01 1.469840e-01 1.473738e-01 1.477648e-01 1.461963e-01

634 5.463357e-01 5.463949e-01 5.464631e-01 5.465334e-01 5.465992e-01 2.247488e-01 1.472428e-01 1.476351e-01 1.480285e-01 1.464501e-01

635 5.478136e-01 5.478725e-01 5.479404e-01 5.480104e-01 5.480759e-01 2.256705e-01 1.475196e-01 1.479145e-01 1.483106e-01 1.467217e-01

636 5.492259e-01 5.492845e-01 5.493522e-01 5.494219e-01 5.494871e-01 2.266460e-01 1.478155e-01 1.482132e-01 1.486121e-01 1.470120e-01

637 5.505751e-01 5.506333e-01 5.507008e-01 5.507702e-01 5.508350e-01 2.276766e-01 1.481315e-01 1.485321e-01 1.489341e-01 1.473221e-01

638 5.518635e-01 5.519215e-01 5.519887e-01 5.520579e-01 5.521224e-01 2.287636e-01 1.484686e-01 1.488724e-01 1.492776e-01 1.476530e-01

639 5.530938e-01 5.531515e-01 5.532185e-01 5.532874e-01 5.533516e-01 2.299078e-01 1.488281e-01 1.492352e-01 1.496437e-01 1.480058e-01

640 5.542687e-01 5.543261e-01 5.543929e-01 5.544616e-01 5.545254e-01 2.311100e-01 1.492109e-01 1.496216e-01 1.500336e-01 1.483816e-01

641 5.553909e-01 5.554480e-01 5.555145e-01 5.555830e-01 5.556465e-01 2.323705e-01 1.496183e-01 1.500326e-01 1.504484e-01 1.487816e-01

642 5.564629e-01 5.565197e-01 5.565860e-01 5.566543e-01 5.567176e-01 2.336896e-01 1.500513e-01 1.504696e-01 1.508893e-01 1.492069e-01

643 5.574874e-01 5.575440e-01 5.576101e-01 5.576781e-01 5.577412e-01 2.350671e-01 1.505112e-01 1.509336e-01 1.513574e-01 1.496586e-01

644 5.584671e-01 5.585234e-01 5.585893e-01 5.586571e-01 5.587199e-01 2.365025e-01 1.509991e-01 1.514258e-01 1.518540e-01 1.501378e-01

645 5.594043e-01 5.594604e-01 5.595262e-01 5.595938e-01 5.596563e-01 2.379949e-01 1.515161e-01 1.519473e-01 1.523801e-01 1.506458e-01

646 5.603016e-01 5.603575e-01 5.604231e-01 5.604905e-01 5.605528e-01 2.395432e-01 1.520634e-01 1.524993e-01 1.529368e-01 1.511836e-01

647 5.611613e-01 5.612170e-01 5.612824e-01 5.613497e-01 5.614117e-01 2.411459e-01 1.526420e-01 1.530828e-01 1.535254e-01 1.517523e-01

648 5.619857e-01 5.620412e-01 5.621064e-01 5.621735e-01 5.622353e-01 2.428009e-01 1.532530e-01 1.536990e-01 1.541467e-01 1.523531e-01

649 5.627768e-01 5.628321e-01 5.628972e-01 5.629642e-01 5.630258e-01 2.445061e-01 1.538975e-01 1.543489e-01 1.548020e-01 1.529868e-01

650 5.635368e-01 5.635919e-01 5.636568e-01 5.637237e-01 5.637851e-01 2.462587e-01 1.545764e-01 1.550333e-01 1.554920e-01 1.536546e-01

651 5.642675e-01 5.643224e-01 5.643872e-01 5.644540e-01 5.645152e-01 2.480557e-01 1.552906e-01 1.557533e-01 1.562178e-01 1.543572e-01

652 5.649708e-01 5.650255e-01 5.650902e-01 5.651569e-01 5.652179e-01 2.498937e-01 1.560409e-01 1.565096e-01 1.569801e-01 1.550956e-01

653 5.656484e-01 5.657030e-01 5.657676e-01 5.658341e-01 5.658949e-01 2.517691e-01 1.568281e-01 1.573029e-01 1.577796e-01 1.558705e-01

654 5.663019e-01 5.663563e-01 5.664208e-01 5.664872e-01 5.665479e-01 2.536779e-01 1.576528e-01 1.581339e-01 1.586170e-01 1.566825e-01

655 5.669329e-01 5.669871e-01 5.670516e-01 5.671179e-01 5.671784e-01 2.556157e-01 1.585155e-01 1.590030e-01 1.594927e-01 1.575321e-01

656 5.675427e-01 5.675968e-01 5.676611e-01 5.677274e-01 5.677877e-01 2.575782e-01 1.594165e-01 1.599108e-01 1.604071e-01 1.584198e-01

657 5.681327e-01 5.681867e-01 5.682509e-01 5.683171e-01 5.683773e-01 2.595606e-01 1.603562e-01 1.608572e-01 1.613604e-01 1.593459e-01

658 5.687042e-01 5.687580e-01 5.688221e-01 5.688882e-01 5.689483e-01 2.615581e-01 1.613347e-01 1.618425e-01 1.623526e-01 1.603104e-01

659 5.692582e-01 5.693119e-01 5.693760e-01 5.694419e-01 5.695019e-01 2.635657e-01 1.623518e-01 1.628666e-01 1.633837e-01 1.613134e-01

660 5.697958e-01 5.698494e-01 5.699134e-01 5.699793e-01 5.700391e-01 2.655784e-01 1.634072e-01 1.639292e-01 1.644534e-01 1.623546e-01

661 5.703182e-01 5.703716e-01 5.704356e-01 5.705014e-01 5.705611e-01 2.675912e-01 1.645007e-01 1.650298e-01 1.655612e-01 1.634336e-01

662 5.708261e-01 5.708794e-01 5.709433e-01 5.710091e-01 5.710686e-01 2.695990e-01 1.656316e-01 1.661678e-01 1.667065e-01 1.645499e-01

663 5.713204e-01 5.713736e-01 5.714375e-01 5.715032e-01 5.715627e-01 2.715971e-01 1.667990e-01 1.673424e-01 1.678884e-01 1.657028e-01

664 5.718020e-01 5.718551e-01 5.719189e-01 5.719846e-01 5.720440e-01 2.735805e-01 1.680019e-01 1.685526e-01 1.691057e-01 1.668911e-01

665 5.722716e-01 5.723246e-01 5.723884e-01 5.724540e-01 5.725132e-01 2.755446e-01 1.692392e-01 1.697971e-01 1.703574e-01 1.681139e-01

666 5.727299e-01 5.727828e-01 5.728465e-01 5.729121e-01 5.729712e-01 2.774851e-01 1.705095e-01 1.710744e-01 1.716419e-01 1.693697e-01

667 5.731775e-01 5.732303e-01 5.732940e-01 5.733596e-01 5.734186e-01 2.793979e-01 1.718111e-01 1.723830e-01 1.729576e-01 1.706570e-01

668 5.736151e-01 5.736678e-01 5.737314e-01 5.737969e-01 5.738558e-01 2.812790e-01 1.731421e-01 1.737210e-01 1.743025e-01 1.719740e-01

669 5.740431e-01 5.740957e-01 5.741593e-01 5.742248e-01 5.742836e-01 2.831249e-01 1.745007e-01 1.750864e-01 1.756747e-01 1.733188e-01

670 5.744621e-01 5.745146e-01 5.745782e-01 5.746436e-01 5.747023e-01 2.849324e-01 1.758847e-01 1.764770e-01 1.770719e-01 1.746892e-01

671 5.748725e-01 5.749249e-01 5.749885e-01 5.750539e-01 5.751125e-01 2.866986e-01 1.772917e-01 1.778904e-01 1.784918e-01 1.760830e-01

672 5.752748e-01 5.753272e-01 5.753907e-01 5.754561e-01 5.755146e-01 2.884212e-01 1.787192e-01 1.793241e-01 1.799318e-01 1.774977e-01

673 5.756695e-01 5.757217e-01 5.757852e-01 5.758506e-01 5.759090e-01 2.900979e-01 1.801646e-01 1.807756e-01 1.813892e-01 1.789308e-01

674 5.760568e-01 5.761089e-01 5.761724e-01 5.762377e-01 5.762961e-01 2.917271e-01 1.816251e-01 1.822419e-01 1.828613e-01 1.803796e-01

675 5.764371e-01 5.764892e-01 5.765526e-01 5.766179e-01 5.766762e-01 2.933074e-01 1.830980e-01 1.837203e-01 1.843452e-01 1.818411e-01

676 5.768108e-01 5.768628e-01 5.769262e-01 5.769915e-01 5.770497e-01 2.948378e-01 1.845804e-01 1.852078e-01 1.858380e-01 1.833127e-01

677 5.771781e-01 5.772301e-01 5.772935e-01 5.773587e-01 5.774168e-01 2.963176e-01 1.860691e-01 1.867015e-01 1.873366e-01 1.847913e-01

678 5.775394e-01 5.775913e-01 5.776546e-01 5.777199e-01 5.777779e-01 2.977465e-01 1.875614e-01 1.881984e-01 1.888381e-01 1.862740e-01

679 5.778949e-01 5.779466e-01 5.780100e-01 5.780752e-01 5.781332e-01 2.991245e-01 1.890541e-01 1.896955e-01 1.903395e-01 1.877578e-01

680 5.782448e-01 5.782965e-01 5.783598e-01 5.784250e-01 5.784829e-01 3.004518e-01 1.905444e-01 1.911897e-01 1.918377e-01 1.892397e-01

681 5.785894e-01 5.786410e-01 5.787043e-01 5.787695e-01 5.788273e-01 3.017291e-01 1.920293e-01 1.926783e-01 1.933300e-01 1.907169e-01

682 5.789288e-01 5.789804e-01 5.790437e-01 5.791088e-01 5.791666e-01 3.029569e-01 1.935059e-01 1.941583e-01 1.948133e-01 1.921865e-01

683 5.792633e-01 5.793148e-01 5.793781e-01 5.794433e-01 5.795009e-01 3.041362e-01 1.949715e-01 1.956269e-01 1.962850e-01 1.936457e-01

684 5.795931e-01 5.796445e-01 5.797078e-01 5.797729e-01 5.798306e-01 3.052682e-01 1.964235e-01 1.970817e-01 1.977424e-01 1.950920e-01

685 5.799183e-01 5.799696e-01 5.800329e-01 5.800980e-01 5.801556e-01 3.063541e-01 1.978593e-01 1.985199e-01 1.991831e-01 1.965226e-01

686 5.802391e-01 5.802904e-01 5.803536e-01 5.804187e-01 5.804762e-01 3.073953e-01 1.992766e-01 1.999393e-01 2.006046e-01 1.979354e-01

687 5.805556e-01 5.806068e-01 5.806701e-01 5.807352e-01 5.807926e-01 3.083933e-01 2.006732e-01 2.013377e-01 2.020048e-01 1.993280e-01

688 5.808680e-01 5.809191e-01 5.809824e-01 5.810475e-01 5.811048e-01 3.093496e-01 2.020469e-01 2.027130e-01 2.033817e-01 2.006984e-01

689 5.811764e-01 5.812275e-01 5.812907e-01 5.813558e-01 5.814130e-01 3.102658e-01 2.033961e-01 2.040635e-01 2.047335e-01 2.020448e-01

690 5.814809e-01 5.815319e-01 5.815951e-01 5.816602e-01 5.817174e-01 3.111436e-01 2.047191e-01 2.053875e-01 2.060585e-01 2.033653e-01

691 5.817817e-01 5.818326e-01 5.818958e-01 5.819609e-01 5.820180e-01 3.119847e-01 2.060142e-01 2.066835e-01 2.073553e-01 2.046587e-01

692 5.820788e-01 5.821297e-01 5.821929e-01 5.822579e-01 5.823150e-01 3.127906e-01 2.072804e-01 2.079503e-01 2.086227e-01 2.059235e-01

693 5.823724e-01 5.824232e-01 5.824864e-01 5.825514e-01 5.826084e-01 3.135631e-01 2.085166e-01 2.091869e-01 2.098596e-01 2.071586e-01

694 5.826625e-01 5.827133e-01 5.827764e-01 5.828414e-01 5.828984e-01 3.143038e-01 2.097218e-01 2.103923e-01 2.110653e-01 2.083632e-01

695 5.829492e-01 5.829999e-01 5.830631e-01 5.831281e-01 5.831850e-01 3.150143e-01 2.108954e-01 2.115659e-01 2.122389e-01 2.095365e-01

696 5.832327e-01 5.832834e-01 5.833465e-01 5.834115e-01 5.834683e-01 3.156961e-01 2.120368e-01 2.127073e-01 2.133802e-01 2.106779e-01

697 5.835130e-01 5.835636e-01 5.836267e-01 5.836917e-01 5.837485e-01 3.163508e-01 2.131457e-01 2.138160e-01 2.144886e-01 2.117872e-01

698 5.837901e-01 5.838407e-01 5.839038e-01 5.839688e-01 5.840255e-01 3.169798e-01 2.142220e-01 2.148919e-01 2.155642e-01 2.128640e-01

699 5.840643e-01 5.841148e-01 5.841779e-01 5.842428e-01 5.842995e-01 3.175846e-01 2.152656e-01 2.159351e-01 2.166069e-01 2.139084e-01

700 5.843354e-01 5.843858e-01 5.844489e-01 5.845139e-01 5.845705e-01 3.181665e-01 2.162767e-01 2.169456e-01 2.176169e-01 2.149204e-01

701 5.846036e-01 5.846540e-01 5.847171e-01 5.847820e-01 5.848386e-01 3.187267e-01 2.172554e-01 2.179237e-01 2.185944e-01 2.159003e-01

702 5.848690e-01 5.849193e-01 5.849824e-01 5.850473e-01 5.851038e-01 3.192666e-01 2.182022e-01 2.188698e-01 2.195398e-01 2.168483e-01

703 5.851316e-01 5.851818e-01 5.852449e-01 5.853098e-01 5.853663e-01 3.197873e-01 2.191174e-01 2.197843e-01 2.204536e-01 2.177649e-01

704 5.853914e-01 5.854416e-01 5.855047e-01 5.855696e-01 5.856260e-01 3.202900e-01 2.200017e-01 2.206679e-01 2.213363e-01 2.186507e-01

705 5.856485e-01 5.856987e-01 5.857618e-01 5.858266e-01 5.858830e-01 3.207756e-01 2.208558e-01 2.215211e-01 2.221887e-01 2.195063e-01

706 5.859030e-01 5.859532e-01 5.860162e-01 5.860811e-01 5.861374e-01 3.212452e-01 2.216802e-01 2.223447e-01 2.230115e-01 2.203323e-01

707 5.861550e-01 5.862051e-01 5.862681e-01 5.863329e-01 5.863892e-01 3.216997e-01 2.224758e-01 2.231394e-01 2.238054e-01 2.211295e-01

708 5.864044e-01 5.864544e-01 5.865174e-01 5.865822e-01 5.866385e-01 3.221401e-01 2.232433e-01 2.239061e-01 2.245712e-01 2.218987e-01

709 5.866512e-01 5.867013e-01 5.867642e-01 5.868291e-01 5.868853e-01 3.225671e-01 2.239837e-01 2.246456e-01 2.253099e-01 2.226407e-01

710 5.868957e-01 5.869457e-01 5.870086e-01 5.870734e-01 5.871296e-01 3.229816e-01 2.246977e-01 2.253588e-01 2.260222e-01 2.233564e-01

711 5.871377e-01 5.871876e-01 5.872506e-01 5.873154e-01 5.873715e-01 3.233843e-01 2.253863e-01 2.260466e-01 2.267092e-01 2.240467e-01

712 5.873773e-01 5.874272e-01 5.874902e-01 5.875550e-01 5.876110e-01 3.237759e-01 2.260504e-01 2.267099e-01 2.273716e-01 2.247124e-01

713 5.876146e-01 5.876645e-01 5.877274e-01 5.877922e-01 5.878482e-01 3.241570e-01 2.266910e-01 2.273496e-01 2.280105e-01 2.253545e-01

714 5.878496e-01 5.878995e-01 5.879624e-01 5.880272e-01 5.880832e-01 3.245283e-01 2.273088e-01 2.279667e-01 2.286267e-01 2.259739e-01

715 5.880824e-01 5.881322e-01 5.881951e-01 5.882598e-01 5.883158e-01 3.248902e-01 2.279048e-01 2.285619e-01 2.292212e-01 2.265715e-01

716 5.883129e-01 5.883627e-01 5.884256e-01 5.884903e-01 5.885462e-01 3.252435e-01 2.284800e-01 2.291363e-01 2.297948e-01 2.271481e-01

717 5.885412e-01 5.885909e-01 5.886538e-01 5.887185e-01 5.887744e-01 3.255885e-01 2.290351e-01 2.296907e-01 2.303485e-01 2.277047e-01

718 5.887673e-01 5.888170e-01 5.888799e-01 5.889446e-01 5.890005e-01 3.259257e-01 2.295711e-01 2.302260e-01 2.308831e-01 2.282421e-01

719 5.889913e-01 5.890410e-01 5.891039e-01 5.891685e-01 5.892244e-01 3.262556e-01 2.300888e-01 2.307430e-01 2.313994e-01 2.287611e-01

720 5.892132e-01 5.892629e-01 5.893257e-01 5.893904e-01 5.894462e-01 3.265785e-01 2.305890e-01 2.312426e-01 2.318983e-01 2.292626e-01

721 5.894330e-01 5.894826e-01 5.895455e-01 5.896101e-01 5.896659e-01 3.268949e-01 2.310726e-01 2.317255e-01 2.323806e-01 2.297474e-01

722 5.898392e-01 5.898895e-01 5.899530e-01 5.900183e-01 5.900748e-01 3.268980e-01 2.310735e-01 2.317265e-01 2.323816e-01 2.297483e-01

723 5.902789e-01 5.903299e-01 5.903941e-01 5.904602e-01 5.905174e-01 3.269014e-01 2.310745e-01 2.317275e-01 2.323826e-01 2.297493e-01

724 5.907544e-01 5.908061e-01 5.908711e-01 5.909380e-01 5.909960e-01 3.269051e-01 2.310755e-01 2.317286e-01 2.323837e-01 2.297503e-01

725 5.912679e-01 5.913204e-01 5.913862e-01 5.914539e-01 5.915129e-01 3.269092e-01 2.310767e-01 2.317298e-01 2.323849e-01 2.297515e-01

726 5.918219e-01 5.918753e-01 5.919420e-01 5.920106e-01 5.920705e-01 3.269137e-01 2.310780e-01 2.317311e-01 2.323863e-01 2.297527e-01

727 5.924188e-01 5.924732e-01 5.925408e-01 5.926104e-01 5.926713e-01 3.269186e-01 2.310794e-01 2.317325e-01 2.323877e-01 2.297541e-01

728 5.930613e-01 5.931167e-01 5.931853e-01 5.932559e-01 5.933179e-01 3.269240e-01 2.310810e-01 2.317341e-01 2.323893e-01 2.297556e-01

729 5.937518e-01 5.938083e-01 5.938780e-01 5.939496e-01 5.940128e-01 3.269299e-01 2.310827e-01 2.317358e-01 2.323910e-01 2.297572e-01

730 5.944930e-01 5.945506e-01 5.946214e-01 5.946942e-01 5.947586e-01 3.269364e-01 2.310845e-01 2.317376e-01 2.323929e-01 2.297590e-01

731 5.952874e-01 5.953462e-01 5.954182e-01 5.954922e-01 5.955579e-01 3.269435e-01 2.310865e-01 2.317397e-01 2.323950e-01 2.297610e-01

732 5.961377e-01 5.961976e-01 5.962709e-01 5.963462e-01 5.964132e-01 3.269513e-01 2.310887e-01 2.317419e-01 2.323972e-01 2.297631e-01

733 5.970462e-01 5.971075e-01 5.971821e-01 5.972588e-01 5.973272e-01 3.269597e-01 2.310911e-01 2.317443e-01 2.323997e-01 2.297654e-01

734 5.980155e-01 5.980782e-01 5.981542e-01 5.982323e-01 5.983022e-01 3.269690e-01 2.310937e-01 2.317470e-01 2.324024e-01 2.297679e-01

735 5.990480e-01 5.991122e-01 5.991895e-01 5.992691e-01 5.993406e-01 3.269792e-01 2.310965e-01 2.317498e-01 2.324053e-01 2.297707e-01

736 6.001459e-01 6.002115e-01 6.002904e-01 6.003716e-01 6.004446e-01 3.269903e-01 2.310996e-01 2.317529e-01 2.324085e-01 2.297736e-01

737 6.013112e-01 6.013784e-01 6.014589e-01 6.015416e-01 6.016163e-01 3.270025e-01 2.311029e-01 2.317563e-01 2.324119e-01 2.297769e-01

738 6.025459e-01 6.026147e-01 6.026968e-01 6.027812e-01 6.028576e-01 3.270157e-01 2.311066e-01 2.317601e-01 2.324157e-01 2.297804e-01

739 6.038516e-01 6.039221e-01 6.040058e-01 6.040919e-01 6.041702e-01 3.270302e-01 2.311105e-01 2.317641e-01 2.324198e-01 2.297843e-01

740 6.052297e-01 6.053019e-01 6.053873e-01 6.054752e-01 6.055553e-01 3.270461e-01 2.311148e-01 2.317685e-01 2.324242e-01 2.297885e-01

741 6.066813e-01 6.067553e-01 6.068425e-01 6.069322e-01 6.070142e-01 3.270633e-01 2.311195e-01 2.317732e-01 2.324290e-01 2.297930e-01

742 6.082074e-01 6.082832e-01 6.083722e-01 6.084637e-01 6.085477e-01 3.270822e-01 2.311246e-01 2.317784e-01 2.324343e-01 2.297980e-01

743 6.098082e-01 6.098859e-01 6.099767e-01 6.100701e-01 6.101560e-01 3.271028e-01 2.311302e-01 2.317840e-01 2.324400e-01 2.298034e-01

744 6.114841e-01 6.115636e-01 6.116562e-01 6.117515e-01 6.118395e-01 3.271252e-01 2.311362e-01 2.317901e-01 2.324462e-01 2.298092e-01

745 6.132345e-01 6.133159e-01 6.134104e-01 6.135076e-01 6.135976e-01 3.271497e-01 2.311427e-01 2.317968e-01 2.324529e-01 2.298155e-01

746 6.150588e-01 6.151421e-01 6.152385e-01 6.153377e-01 6.154296e-01 3.271763e-01 2.311498e-01 2.318039e-01 2.324602e-01 2.298224e-01

747 6.169559e-01 6.170411e-01 6.171394e-01 6.172404e-01 6.173345e-01 3.272053e-01 2.311575e-01 2.318117e-01 2.324681e-01 2.298299e-01

748 6.189242e-01 6.190112e-01 6.191114e-01 6.192144e-01 6.193104e-01 3.272369e-01 2.311658e-01 2.318202e-01 2.324767e-01 2.298380e-01

749 6.209615e-01 6.210505e-01 6.211525e-01 6.212574e-01 6.213554e-01 3.272714e-01 2.311749e-01 2.318294e-01 2.324860e-01 2.298468e-01

750 6.230655e-01 6.231563e-01 6.232602e-01 6.233669e-01 6.234669e-01 3.273088e-01 2.311847e-01 2.318393e-01 2.324961e-01 2.298563e-01

751 6.252332e-01 6.253258e-01 6.254314e-01 6.255400e-01 6.256420e-01 3.273495e-01 2.311953e-01 2.318501e-01 2.325071e-01 2.298666e-01

752 6.274611e-01 6.275555e-01 6.276629e-01 6.277732e-01 6.278771e-01 3.273938e-01 2.312068e-01 2.318618e-01 2.325189e-01 2.298777e-01

753 6.297454e-01 6.298416e-01 6.299507e-01 6.300628e-01 6.301685e-01 3.274419e-01 2.312192e-01 2.318744e-01 2.325317e-01 2.298898e-01

754 6.320820e-01 6.321798e-01 6.322906e-01 6.324044e-01 6.325118e-01 3.274942e-01 2.312327e-01 2.318881e-01 2.325456e-01 2.299029e-01

755 6.344662e-01 6.345657e-01 6.346780e-01 6.347934e-01 6.349026e-01 3.275510e-01 2.312473e-01 2.319029e-01 2.325606e-01 2.299171e-01

756 6.368931e-01 6.369941e-01 6.371079e-01 6.372248e-01 6.373357e-01 3.276127e-01 2.312630e-01 2.319189e-01 2.325768e-01 2.299324e-01

757 6.393573e-01 6.394598e-01 6.395750e-01 6.396934e-01 6.398058e-01 3.276796e-01 2.312801e-01 2.319361e-01 2.325944e-01 2.299489e-01

758 6.418534e-01 6.419572e-01 6.420738e-01 6.421936e-01 6.423074e-01 3.277522e-01 2.312985e-01 2.319548e-01 2.326133e-01 2.299668e-01

759 6.443755e-01 6.444806e-01 6.445984e-01 6.447195e-01 6.448346e-01 3.278309e-01 2.313184e-01 2.319750e-01 2.326338e-01 2.299861e-01

760 6.469176e-01 6.470238e-01 6.471428e-01 6.472651e-01 6.473815e-01 3.279162e-01 2.313399e-01 2.319968e-01 2.326559e-01 2.300070e-01

761 6.494735e-01 6.495809e-01 6.497010e-01 6.498243e-01 6.499419e-01 3.280085e-01 2.313631e-01 2.320203e-01 2.326798e-01 2.300295e-01

762 6.520372e-01 6.521455e-01 6.522666e-01 6.523909e-01 6.525095e-01 3.281086e-01 2.313881e-01 2.320457e-01 2.327056e-01 2.300538e-01

763 6.546022e-01 6.547114e-01 6.548333e-01 6.549585e-01 6.550781e-01 3.282169e-01 2.314151e-01 2.320731e-01 2.327334e-01 2.300800e-01

764 6.571623e-01 6.572723e-01 6.573950e-01 6.575210e-01 6.576414e-01 3.283340e-01 2.314442e-01 2.321027e-01 2.327634e-01 2.301083e-01

765 6.597114e-01 6.598221e-01 6.599454e-01 6.600720e-01 6.601932e-01 3.284607e-01 2.314756e-01 2.321345e-01 2.327957e-01 2.301388e-01

766 6.622433e-01 6.623546e-01 6.624785e-01 6.626057e-01 6.627274e-01 3.285975e-01 2.315094e-01 2.321688e-01 2.328305e-01 2.301716e-01

767 6.647522e-01 6.648640e-01 6.649883e-01 6.651160e-01 6.652382e-01 3.287454e-01 2.315458e-01 2.322058e-01 2.328680e-01 2.302070e-01

768 6.672324e-01 6.673446e-01 6.674693e-01 6.675973e-01 6.677199e-01 3.289050e-01 2.315851e-01 2.322456e-01 2.329084e-01 2.302451e-01

769 6.696785e-01 6.697910e-01 6.699159e-01 6.700442e-01 6.701671e-01 3.290771e-01 2.316273e-01 2.322884e-01 2.329518e-01 2.302861e-01

770 6.720854e-01 6.721981e-01 6.723232e-01 6.724517e-01 6.725747e-01 3.292628e-01 2.316727e-01 2.323345e-01 2.329986e-01 2.303302e-01

771 6.744485e-01 6.745612e-01 6.746864e-01 6.748150e-01 6.749381e-01 3.294629e-01 2.317215e-01 2.323841e-01 2.330489e-01 2.303777e-01

772 6.767632e-01 6.768760e-01 6.770013e-01 6.771298e-01 6.772530e-01 3.296783e-01 2.317740e-01 2.324373e-01 2.331029e-01 2.304287e-01

773 6.790259e-01 6.791386e-01 6.792638e-01 6.793923e-01 6.795153e-01 3.299102e-01 2.318305e-01 2.324946e-01 2.331610e-01 2.304835e-01

774 6.812329e-01 6.813455e-01 6.814706e-01 6.815989e-01 6.817218e-01 3.301596e-01 2.318911e-01 2.325560e-01 2.332233e-01 2.305423e-01

775 6.833813e-01 6.834937e-01 6.836186e-01 6.837467e-01 6.838694e-01 3.304277e-01 2.319561e-01 2.326221e-01 2.332903e-01 2.306056e-01

776 6.854686e-01 6.855807e-01 6.857053e-01 6.858332e-01 6.859555e-01 3.307156e-01 2.320260e-01 2.326929e-01 2.333622e-01 2.306734e-01

777 6.874926e-01 6.876044e-01 6.877287e-01 6.878563e-01 6.879783e-01 3.310246e-01 2.321009e-01 2.327689e-01 2.334393e-01 2.307462e-01

778 6.894517e-01 6.895632e-01 6.896872e-01 6.898144e-01 6.899360e-01 3.313559e-01 2.321812e-01 2.328504e-01 2.335219e-01 2.308242e-01

779 6.913449e-01 6.914560e-01 6.915795e-01 6.917063e-01 6.918275e-01 3.317109e-01 2.322673e-01 2.329377e-01 2.336105e-01 2.309079e-01

780 6.931713e-01 6.932819e-01 6.934051e-01 6.935315e-01 6.936521e-01 3.320909e-01 2.323595e-01 2.330312e-01 2.337054e-01 2.309975e-01

781 6.949307e-01 6.950409e-01 6.951636e-01 6.952895e-01 6.954097e-01 3.324974e-01 2.324582e-01 2.331314e-01 2.338070e-01 2.310935e-01

782 6.966232e-01 6.967328e-01 6.968551e-01 6.969805e-01 6.971002e-01 3.329317e-01 2.325639e-01 2.332386e-01 2.339157e-01 2.311962e-01

783 6.982491e-01 6.983583e-01 6.984801e-01 6.986050e-01 6.987241e-01 3.333955e-01 2.326770e-01 2.333533e-01 2.340320e-01 2.313061e-01

784 6.998093e-01 6.999180e-01 7.000393e-01 7.001637e-01 7.002822e-01 3.338900e-01 2.327979e-01 2.334759e-01 2.341563e-01 2.314236e-01

785 7.013048e-01 7.014130e-01 7.015338e-01 7.016577e-01 7.017756e-01 3.344169e-01 2.329271e-01 2.336069e-01 2.342892e-01 2.315492e-01

786 7.027370e-01 7.028446e-01 7.029649e-01 7.030883e-01 7.032057e-01 3.349777e-01 2.330651e-01 2.337468e-01 2.344311e-01 2.316833e-01

787 7.041074e-01 7.042145e-01 7.043343e-01 7.044572e-01 7.045740e-01 3.355737e-01 2.332124e-01 2.338962e-01 2.345826e-01 2.318266e-01

788 7.054177e-01 7.055242e-01 7.056435e-01 7.057659e-01 7.058822e-01 3.362067e-01 2.333696e-01 2.340556e-01 2.347442e-01 2.319794e-01

789 7.066697e-01 7.067758e-01 7.068946e-01 7.070165e-01 7.071322e-01 3.368779e-01 2.335372e-01 2.342255e-01 2.349166e-01 2.321424e-01

790 7.078656e-01 7.079712e-01 7.080895e-01 7.082109e-01 7.083260e-01 3.375888e-01 2.337158e-01 2.344067e-01 2.351002e-01 2.323161e-01

791 7.090074e-01 7.091125e-01 7.092304e-01 7.093513e-01 7.094659e-01 3.383407e-01 2.339061e-01 2.345996e-01 2.352958e-01 2.325012e-01

792 7.100972e-01 7.102018e-01 7.103193e-01 7.104397e-01 7.105538e-01 3.391350e-01 2.341087e-01 2.348049e-01 2.355040e-01 2.326982e-01

793 7.111374e-01 7.112415e-01 7.113585e-01 7.114785e-01 7.115921e-01 3.399728e-01 2.343241e-01 2.350233e-01 2.357254e-01 2.329079e-01

794 7.121300e-01 7.122336e-01 7.123502e-01 7.124698e-01 7.125829e-01 3.408553e-01 2.345532e-01 2.352555e-01 2.359608e-01 2.331308e-01

795 7.130773e-01 7.131805e-01 7.132967e-01 7.134159e-01 7.135285e-01 3.417833e-01 2.347966e-01 2.355022e-01 2.362108e-01 2.333676e-01

796 7.139816e-01 7.140844e-01 7.142002e-01 7.143190e-01 7.144311e-01 3.427577e-01 2.350550e-01 2.357641e-01 2.364763e-01 2.336191e-01

797 7.148449e-01 7.149473e-01 7.150628e-01 7.151811e-01 7.152928e-01 3.437792e-01 2.353292e-01 2.360420e-01 2.367579e-01 2.338860e-01

798 7.156695e-01 7.157715e-01 7.158866e-01 7.160046e-01 7.161159e-01 3.448482e-01 2.356199e-01 2.363366e-01 2.370563e-01 2.341690e-01

799 7.164573e-01 7.165589e-01 7.166737e-01 7.167914e-01 7.169023e-01 3.459650e-01 2.359278e-01 2.366486e-01 2.373725e-01 2.344689e-01

800 7.172104e-01 7.173117e-01 7.174261e-01 7.175435e-01 7.176540e-01 3.471298e-01 2.362539e-01 2.369789e-01 2.377072e-01 2.347864e-01

801 7.179307e-01 7.180316e-01 7.181458e-01 7.182629e-01 7.183730e-01 3.483423e-01 2.365987e-01 2.373283e-01 2.380611e-01 2.351223e-01

802 7.186201e-01 7.187207e-01 7.188346e-01 7.189514e-01 7.190611e-01 3.496021e-01 2.369632e-01 2.376975e-01 2.384351e-01 2.354774e-01

803 7.192803e-01 7.193806e-01 7.194942e-01 7.196107e-01 7.197202e-01 3.509086e-01 2.373481e-01 2.380873e-01 2.388300e-01 2.358525e-01

804 7.199131e-01 7.200131e-01 7.201265e-01 7.202427e-01 7.203519e-01 3.522609e-01 2.377543e-01 2.384987e-01 2.392466e-01 2.362483e-01

805 7.205200e-01 7.206198e-01 7.207329e-01 7.208489e-01 7.209577e-01 3.536577e-01 2.381824e-01 2.389322e-01 2.396856e-01 2.366657e-01

806 7.211026e-01 7.212021e-01 7.213150e-01 7.214308e-01 7.215394e-01 3.550977e-01 2.386333e-01 2.393888e-01 2.401479e-01 2.371054e-01

807 7.216624e-01 7.217616e-01 7.218743e-01 7.219899e-01 7.220982e-01 3.565790e-01 2.391078e-01 2.398691e-01 2.406342e-01 2.375682e-01

808 7.222007e-01 7.222997e-01 7.224122e-01 7.225275e-01 7.226356e-01 3.580995e-01 2.396066e-01 2.403740e-01 2.411453e-01 2.380548e-01

809 7.227188e-01 7.228176e-01 7.229299e-01 7.230450e-01 7.231529e-01 3.596571e-01 2.401304e-01 2.409041e-01 2.416818e-01 2.385660e-01

810 7.232180e-01 7.233165e-01 7.234287e-01 7.235436e-01 7.236512e-01 3.612492e-01 2.406798e-01 2.414601e-01 2.422445e-01 2.391023e-01

811 7.236993e-01 7.237976e-01 7.239096e-01 7.240244e-01 7.241318e-01 3.628728e-01 2.412556e-01 2.420427e-01 2.428339e-01 2.396645e-01

812 7.241639e-01 7.242621e-01 7.243739e-01 7.244885e-01 7.245957e-01 3.645250e-01 2.418583e-01 2.426524e-01 2.434508e-01 2.402532e-01

813 7.246128e-01 7.247108e-01 7.248225e-01 7.249370e-01 7.250439e-01 3.662025e-01 2.424884e-01 2.432898e-01 2.440955e-01 2.408688e-01

814 7.250469e-01 7.251447e-01 7.252563e-01 7.253706e-01 7.254774e-01 3.679018e-01 2.431465e-01 2.439553e-01 2.447685e-01 2.415120e-01

815 7.254671e-01 7.255648e-01 7.256762e-01 7.257905e-01 7.258971e-01 3.696194e-01 2.438330e-01 2.446494e-01 2.454704e-01 2.421831e-01

816 7.258743e-01 7.259719e-01 7.260832e-01 7.261973e-01 7.263037e-01 3.713515e-01 2.445481e-01 2.453724e-01 2.462012e-01 2.428826e-01

817 7.262693e-01 7.263666e-01 7.264778e-01 7.265918e-01 7.266981e-01 3.730941e-01 2.452921e-01 2.461244e-01 2.469614e-01 2.436105e-01

818 7.266527e-01 7.267499e-01 7.268610e-01 7.269749e-01 7.270810e-01 3.748435e-01 2.460653e-01 2.469058e-01 2.477510e-01 2.443673e-01

819 7.270252e-01 7.271223e-01 7.272333e-01 7.273471e-01 7.274531e-01 3.765957e-01 2.468676e-01 2.477164e-01 2.485700e-01 2.451529e-01

820 7.273875e-01 7.274845e-01 7.275954e-01 7.277091e-01 7.278150e-01 3.783466e-01 2.476990e-01 2.485563e-01 2.494184e-01 2.459673e-01

821 7.277402e-01 7.278370e-01 7.279478e-01 7.280614e-01 7.281672e-01 3.800924e-01 2.485594e-01 2.494252e-01 2.502960e-01 2.468105e-01

822 7.280838e-01 7.281805e-01 7.282912e-01 7.284048e-01 7.285104e-01 3.818293e-01 2.494485e-01 2.503229e-01 2.512024e-01 2.476821e-01

823 7.284188e-01 7.285154e-01 7.286261e-01 7.287395e-01 7.288450e-01 3.835534e-01 2.503658e-01 2.512490e-01 2.521373e-01 2.485819e-01

824 7.287457e-01 7.288422e-01 7.289528e-01 7.290662e-01 7.291716e-01 3.852613e-01 2.513110e-01 2.522029e-01 2.531000e-01 2.495094e-01

825 7.290650e-01 7.291614e-01 7.292719e-01 7.293852e-01 7.294906e-01 3.869494e-01 2.522833e-01 2.531840e-01 2.540899e-01 2.504640e-01

826 7.293771e-01 7.294734e-01 7.295838e-01 7.296971e-01 7.298023e-01 3.886145e-01 2.532819e-01 2.541914e-01 2.551062e-01 2.514449e-01

827 7.296823e-01 7.297785e-01 7.298889e-01 7.300021e-01 7.301072e-01 3.902536e-01 2.543060e-01 2.552242e-01 2.561478e-01 2.524513e-01

828 7.299810e-01 7.300772e-01 7.301875e-01 7.303006e-01 7.304057e-01 3.918639e-01 2.553545e-01 2.562814e-01 2.572137e-01 2.534822e-01

829 7.302736e-01 7.303697e-01 7.304800e-01 7.305930e-01 7.306980e-01 3.934428e-01 2.564263e-01 2.573617e-01 2.583027e-01 2.545364e-01

830 7.305604e-01 7.306564e-01 7.307666e-01 7.308796e-01 7.309845e-01 3.949881e-01 2.575200e-01 2.584639e-01 2.594134e-01 2.556129e-01

831 7.308416e-01 7.309376e-01 7.310478e-01 7.311607e-01 7.312655e-01 3.964978e-01 2.586342e-01 2.595865e-01 2.605444e-01 2.567101e-01

832 7.311176e-01 7.312135e-01 7.313236e-01 7.314365e-01 7.315412e-01 3.979701e-01 2.597674e-01 2.607278e-01 2.616940e-01 2.578266e-01

833 7.313886e-01 7.314844e-01 7.315945e-01 7.317073e-01 7.318120e-01 3.994037e-01 2.609179e-01 2.618864e-01 2.628605e-01 2.589608e-01

834 7.316548e-01 7.317506e-01 7.318606e-01 7.319734e-01 7.320780e-01 4.007972e-01 2.620841e-01 2.630603e-01 2.640422e-01 2.601110e-01

835 7.319165e-01 7.320122e-01 7.321222e-01 7.322349e-01 7.323394e-01 4.021498e-01 2.632640e-01 2.642477e-01 2.652373e-01 2.612755e-01

836 7.321738e-01 7.322694e-01 7.323794e-01 7.324921e-01 7.325966e-01 4.034609e-01 2.644557e-01 2.654468e-01 2.664436e-01 2.624523e-01

837 7.324270e-01 7.325226e-01 7.326325e-01 7.327452e-01 7.328496e-01 4.047300e-01 2.656574e-01 2.666554e-01 2.676593e-01 2.636395e-01

838 7.326762e-01 7.327718e-01 7.328816e-01 7.329943e-01 7.330986e-01 4.059569e-01 2.668668e-01 2.678716e-01 2.688822e-01 2.648351e-01

839 7.329217e-01 7.330171e-01 7.331270e-01 7.332396e-01 7.333439e-01 4.071418e-01 2.680821e-01 2.690933e-01 2.701103e-01 2.660371e-01

840 7.331635e-01 7.332589e-01 7.333687e-01 7.334813e-01 7.335855e-01 4.082848e-01 2.693010e-01 2.703183e-01 2.713413e-01 2.672434e-01

841 7.334018e-01 7.334972e-01 7.336069e-01 7.337195e-01 7.338237e-01 4.093864e-01 2.705215e-01 2.715445e-01 2.725733e-01 2.684520e-01

842 7.336368e-01 7.337321e-01 7.338418e-01 7.339543e-01 7.340585e-01 4.104470e-01 2.717415e-01 2.727699e-01 2.738041e-01 2.696607e-01

843 7.338685e-01 7.339638e-01 7.340735e-01 7.341860e-01 7.342900e-01 4.114676e-01 2.729588e-01 2.739923e-01 2.750315e-01 2.708675e-01

844 7.340972e-01 7.341924e-01 7.343021e-01 7.344145e-01 7.345185e-01 4.124488e-01 2.741715e-01 2.752097e-01 2.762536e-01 2.720703e-01

845 7.343228e-01 7.344180e-01 7.345277e-01 7.346401e-01 7.347441e-01 4.133918e-01 2.753776e-01 2.764200e-01 2.774684e-01 2.732672e-01

846 7.345456e-01 7.346407e-01 7.347504e-01 7.348628e-01 7.349667e-01 4.142974e-01 2.765750e-01 2.776215e-01 2.786738e-01 2.744562e-01

847 7.347656e-01 7.348607e-01 7.349703e-01 7.350827e-01 7.351866e-01 4.151668e-01 2.777620e-01 2.788121e-01 2.798680e-01 2.756354e-01

848 7.349829e-01 7.350780e-01 7.351876e-01 7.352999e-01 7.354038e-01 4.160013e-01 2.789367e-01 2.799902e-01 2.810494e-01 2.768031e-01

849 7.351977e-01 7.352927e-01 7.354023e-01 7.355146e-01 7.356184e-01 4.168020e-01 2.800975e-01 2.811540e-01 2.822161e-01 2.779576e-01

850 7.354099e-01 7.355049e-01 7.356144e-01 7.357267e-01 7.358305e-01 4.175702e-01 2.812429e-01 2.823019e-01 2.833668e-01 2.790972e-01

851 7.356197e-01 7.357146e-01 7.358241e-01 7.359364e-01 7.360401e-01 4.183071e-01 2.823712e-01 2.834327e-01 2.844998e-01 2.802205e-01

852 7.358271e-01 7.359220e-01 7.360315e-01 7.361437e-01 7.362474e-01 4.190141e-01 2.834813e-01 2.845447e-01 2.856139e-01 2.813261e-01

853 7.360322e-01 7.361271e-01 7.362366e-01 7.363488e-01 7.364524e-01 4.196924e-01 2.845718e-01 2.856370e-01 2.867079e-01 2.824128e-01

854 7.362351e-01 7.363300e-01 7.364394e-01 7.365516e-01 7.366552e-01 4.203432e-01 2.856417e-01 2.867084e-01 2.877807e-01 2.834794e-01

855 7.364358e-01 7.365307e-01 7.366401e-01 7.367523e-01 7.368559e-01 4.209679e-01 2.866900e-01 2.877579e-01 2.888314e-01 2.845249e-01

856 7.366345e-01 7.367293e-01 7.368387e-01 7.369508e-01 7.370544e-01 4.215677e-01 2.877158e-01 2.887847e-01 2.898592e-01 2.855484e-01

857 7.368311e-01 7.369259e-01 7.370352e-01 7.371473e-01 7.372509e-01 4.221438e-01 2.887185e-01 2.897881e-01 2.908634e-01 2.865492e-01

858 7.370257e-01 7.371204e-01 7.372298e-01 7.373419e-01 7.374454e-01 4.226973e-01 2.896974e-01 2.907676e-01 2.918433e-01 2.875267e-01

859 7.372183e-01 7.373130e-01 7.374224e-01 7.375344e-01 7.376379e-01 4.232294e-01 2.906520e-01 2.917226e-01 2.927987e-01 2.884804e-01

860 7.374090e-01 7.375038e-01 7.376131e-01 7.377251e-01 7.378286e-01 4.237411e-01 2.915821e-01 2.926528e-01 2.937291e-01 2.894098e-01

861 7.375979e-01 7.376926e-01 7.378019e-01 7.379139e-01 7.380173e-01 4.242336e-01 2.924873e-01 2.935581e-01 2.946344e-01 2.903147e-01

862 7.377850e-01 7.378797e-01 7.379889e-01 7.381009e-01 7.382043e-01 4.247079e-01 2.933675e-01 2.944382e-01 2.955143e-01 2.911949e-01

863 7.379703e-01 7.380649e-01 7.381742e-01 7.382861e-01 7.383895e-01 4.251649e-01 2.942227e-01 2.952932e-01 2.963691e-01 2.920504e-01

864 7.381539e-01 7.382485e-01 7.383577e-01 7.384696e-01 7.385730e-01 4.256056e-01 2.950530e-01 2.961231e-01 2.971986e-01 2.928811e-01

865 7.383357e-01 7.384303e-01 7.385395e-01 7.386514e-01 7.387548e-01 4.260308e-01 2.958584e-01 2.969280e-01 2.980031e-01 2.936872e-01

866 7.385159e-01 7.386105e-01 7.387197e-01 7.388316e-01 7.389349e-01 4.264415e-01 2.966392e-01 2.977083e-01 2.987828e-01 2.944689e-01

867 7.386945e-01 7.387890e-01 7.388982e-01 7.390101e-01 7.391134e-01 4.268383e-01 2.973957e-01 2.984642e-01 2.995381e-01 2.952264e-01

868 7.388714e-01 7.389660e-01 7.390751e-01 7.391870e-01 7.392902e-01 4.272222e-01 2.981282e-01 2.991960e-01 3.002692e-01 2.959601e-01

869 7.390468e-01 7.391414e-01 7.392505e-01 7.393623e-01 7.394655e-01 4.275937e-01 2.988371e-01 2.999042e-01 3.009768e-01 2.966703e-01

870 7.392207e-01 7.393152e-01 7.394243e-01 7.395361e-01 7.396393e-01 4.279537e-01 2.995230e-01 3.005893e-01 3.016611e-01 2.973576e-01

871 7.393930e-01 7.394875e-01 7.395966e-01 7.397084e-01 7.398116e-01 4.283027e-01 3.001862e-01 3.012518e-01 3.023228e-01 2.980223e-01

872 7.395639e-01 7.396584e-01 7.397674e-01 7.398791e-01 7.399823e-01 4.286414e-01 3.008274e-01 3.018922e-01 3.029623e-01 2.986650e-01

873 7.397332e-01 7.398277e-01 7.399367e-01 7.400485e-01 7.401517e-01 4.289703e-01 3.014471e-01 3.025111e-01 3.035804e-01 2.992862e-01

874 7.399012e-01 7.399957e-01 7.401047e-01 7.402164e-01 7.403195e-01 4.292900e-01 3.020459e-01 3.031091e-01 3.041775e-01 2.998866e-01

875 7.400677e-01 7.401622e-01 7.402711e-01 7.403828e-01 7.404860e-01 4.296010e-01 3.026245e-01 3.036868e-01 3.047544e-01 3.004668e-01

876 7.402329e-01 7.403273e-01 7.404362e-01 7.405479e-01 7.406510e-01 4.299039e-01 3.031834e-01 3.042449e-01 3.053116e-01 3.010272e-01

877 7.403966e-01 7.404911e-01 7.406000e-01 7.407116e-01 7.408147e-01 4.301989e-01 3.037233e-01 3.047839e-01 3.058499e-01 3.015687e-01

878 7.405590e-01 7.406535e-01 7.407624e-01 7.408740e-01 7.409771e-01 4.304867e-01 3.042448e-01 3.053046e-01 3.063698e-01 3.020918e-01

879 7.407201e-01 7.408145e-01 7.409234e-01 7.410350e-01 7.411381e-01 4.307676e-01 3.047486e-01 3.058076e-01 3.068719e-01 3.025972e-01

880 7.408799e-01 7.409743e-01 7.410831e-01 7.411947e-01 7.412978e-01 4.310419e-01 3.052354e-01 3.062936e-01 3.073571e-01 3.030855e-01

881 7.410383e-01 7.411328e-01 7.412416e-01 7.413531e-01 7.414562e-01 4.313101e-01 3.057057e-01 3.067631e-01 3.078259e-01 3.035573e-01

882 7.411955e-01 7.412900e-01 7.413987e-01 7.415102e-01 7.416133e-01 4.315724e-01 3.061602e-01 3.072169e-01 3.082789e-01 3.040133e-01

883 7.413515e-01 7.414459e-01 7.415546e-01 7.416661e-01 7.417692e-01 4.318292e-01 3.065996e-01 3.076555e-01 3.087168e-01 3.044540e-01

884 7.415062e-01 7.416006e-01 7.417093e-01 7.418208e-01 7.419238e-01 4.320808e-01 3.070244e-01 3.080796e-01 3.091401e-01 3.048802e-01

885 7.416596e-01 7.417540e-01 7.418627e-01 7.419742e-01 7.420772e-01 4.323275e-01 3.074352e-01 3.084898e-01 3.095496e-01 3.052924e-01

886 7.418119e-01 7.419063e-01 7.420150e-01 7.421264e-01 7.422294e-01 4.325694e-01 3.078327e-01 3.088866e-01 3.099457e-01 3.056912e-01

887 7.419629e-01 7.420573e-01 7.421660e-01 7.422774e-01 7.423804e-01 4.328069e-01 3.082174e-01 3.092706e-01 3.103291e-01 3.060771e-01

888 7.421128e-01 7.422072e-01 7.423158e-01 7.424272e-01 7.425302e-01 4.330402e-01 3.085898e-01 3.096424e-01 3.107003e-01 3.064507e-01

889 7.422615e-01 7.423559e-01 7.424645e-01 7.425758e-01 7.426789e-01 4.332695e-01 3.089505e-01 3.100026e-01 3.110599e-01 3.068126e-01

890 7.424091e-01 7.425035e-01 7.426120e-01 7.427233e-01 7.428264e-01 4.334949e-01 3.093001e-01 3.103516e-01 3.114083e-01 3.071632e-01

891 7.425555e-01 7.426499e-01 7.427584e-01 7.428697e-01 7.429727e-01 4.337168e-01 3.096390e-01 3.106899e-01 3.117461e-01 3.075032e-01

892 7.427007e-01 7.427951e-01 7.429037e-01 7.430149e-01 7.431179e-01 4.339352e-01 3.099676e-01 3.110180e-01 3.120737e-01 3.078329e-01

893 7.428449e-01 7.429393e-01 7.430478e-01 7.431590e-01 7.432620e-01 4.341504e-01 3.102866e-01 3.113365e-01 3.123917e-01 3.081528e-01

894 7.429879e-01 7.430824e-01 7.431908e-01 7.433020e-01 7.434050e-01 4.343624e-01 3.105963e-01 3.116457e-01 3.127004e-01 3.084634e-01

895 7.431299e-01 7.432243e-01 7.433328e-01 7.434439e-01 7.435469e-01 4.345714e-01 3.108971e-01 3.119461e-01 3.130003e-01 3.087651e-01

896 7.432708e-01 7.433652e-01 7.434736e-01 7.435847e-01 7.436877e-01 4.347777e-01 3.111895e-01 3.122380e-01 3.132918e-01 3.090583e-01

897 7.434106e-01 7.435050e-01 7.436134e-01 7.437245e-01 7.438275e-01 4.349812e-01 3.114738e-01 3.125220e-01 3.135753e-01 3.093435e-01

898 7.435493e-01 7.436438e-01 7.437521e-01 7.438631e-01 7.439662e-01 4.351821e-01 3.117505e-01 3.127983e-01 3.138512e-01 3.096209e-01

899 7.436870e-01 7.437815e-01 7.438898e-01 7.440008e-01 7.441038e-01 4.353805e-01 3.120199e-01 3.130673e-01 3.141198e-01 3.098910e-01

900 7.438236e-01 7.439181e-01 7.440264e-01 7.441374e-01 7.442404e-01 4.355766e-01 3.122823e-01 3.133293e-01 3.143816e-01 3.101542e-01

901 7.439593e-01 7.440537e-01 7.441620e-01 7.442729e-01 7.443760e-01 4.357704e-01 3.125382e-01 3.135848e-01 3.146367e-01 3.104107e-01

902 7.458418e-01 7.459391e-01 7.460502e-01 7.461640e-01 7.462700e-01 4.357938e-01 3.125436e-01 3.135903e-01 3.146423e-01 3.104158e-01

903 7.477826e-01 7.478828e-01 7.479966e-01 7.481132e-01 7.482223e-01 4.358191e-01 3.125494e-01 3.135963e-01 3.146484e-01 3.104214e-01

904 7.497793e-01 7.498823e-01 7.499988e-01 7.501182e-01 7.502303e-01 4.358465e-01 3.125557e-01 3.136027e-01 3.146549e-01 3.104275e-01

905 7.518291e-01 7.519348e-01 7.520540e-01 7.521761e-01 7.522910e-01 4.358761e-01 3.125625e-01 3.136096e-01 3.146620e-01 3.104340e-01

906 7.539286e-01 7.540370e-01 7.541588e-01 7.542836e-01 7.544013e-01 4.359082e-01 3.125697e-01 3.136170e-01 3.146696e-01 3.104410e-01

907 7.560745e-01 7.561854e-01 7.563097e-01 7.564370e-01 7.565575e-01 4.359429e-01 3.125776e-01 3.136251e-01 3.146777e-01 3.104485e-01

908 7.582627e-01 7.583762e-01 7.585029e-01 7.586327e-01 7.587557e-01 4.359803e-01 3.125861e-01 3.136337e-01 3.146865e-01 3.104567e-01

909 7.604892e-01 7.606050e-01 7.607340e-01 7.608662e-01 7.609918e-01 4.360208e-01 3.125952e-01 3.136430e-01 3.146960e-01 3.104654e-01

910 7.627494e-01 7.628675e-01 7.629988e-01 7.631331e-01 7.632611e-01 4.360646e-01 3.126050e-01 3.136530e-01 3.147062e-01 3.104748e-01

911 7.650387e-01 7.651590e-01 7.652923e-01 7.654288e-01 7.655590e-01 4.361118e-01 3.126155e-01 3.136637e-01 3.147172e-01 3.104849e-01

912 7.673522e-01 7.674745e-01 7.676098e-01 7.677483e-01 7.678806e-01 4.361628e-01 3.126268e-01 3.136753e-01 3.147290e-01 3.104958e-01

913 7.696847e-01 7.698090e-01 7.699460e-01 7.700864e-01 7.702207e-01 4.362179e-01 3.126390e-01 3.136877e-01 3.147416e-01 3.105075e-01

914 7.720312e-01 7.721571e-01 7.722959e-01 7.724379e-01 7.725740e-01 4.362773e-01 3.126521e-01 3.137010e-01 3.147553e-01 3.105201e-01

915 7.743862e-01 7.745137e-01 7.746540e-01 7.747976e-01 7.749354e-01 4.363413e-01 3.126661e-01 3.137154e-01 3.147699e-01 3.105336e-01

916 7.767443e-01 7.768734e-01 7.770150e-01 7.771600e-01 7.772993e-01 4.364103e-01 3.126812e-01 3.137308e-01 3.147856e-01 3.105481e-01

917 7.791004e-01 7.792307e-01 7.793736e-01 7.795198e-01 7.796604e-01 4.364847e-01 3.126975e-01 3.137473e-01 3.148025e-01 3.105637e-01

918 7.814489e-01 7.815804e-01 7.817243e-01 7.818716e-01 7.820134e-01 4.365649e-01 3.127149e-01 3.137651e-01 3.148206e-01 3.105804e-01

919 7.837847e-01 7.839171e-01 7.840620e-01 7.842102e-01 7.843530e-01 4.366511e-01 3.127335e-01 3.137841e-01 3.148400e-01 3.105983e-01

920 7.861027e-01 7.862359e-01 7.863816e-01 7.865306e-01 7.866742e-01 4.367440e-01 3.127536e-01 3.138046e-01 3.148609e-01 3.106176e-01

921 7.883978e-01 7.885318e-01 7.886780e-01 7.888276e-01 7.889719e-01 4.368439e-01 3.127751e-01 3.138265e-01 3.148832e-01 3.106382e-01

922 7.906655e-01 7.907999e-01 7.909467e-01 7.910968e-01 7.912416e-01 4.369514e-01 3.127981e-01 3.138500e-01 3.149072e-01 3.106604e-01

923 7.929011e-01 7.930359e-01 7.931830e-01 7.933334e-01 7.934786e-01 4.370669e-01 3.128228e-01 3.138752e-01 3.149329e-01 3.106841e-01

924 7.951005e-01 7.952356e-01 7.953828e-01 7.955335e-01 7.956789e-01 4.371911e-01 3.128492e-01 3.139021e-01 3.149604e-01 3.107095e-01

925 7.972597e-01 7.973949e-01 7.975423e-01 7.976929e-01 7.978384e-01 4.373244e-01 3.128775e-01 3.139310e-01 3.149899e-01 3.107367e-01

926 7.993752e-01 7.995104e-01 7.996577e-01 7.998083e-01 7.999538e-01 4.374675e-01 3.129079e-01 3.139619e-01 3.150214e-01 3.107659e-01

927 8.014437e-01 8.015788e-01 8.017259e-01 8.018764e-01 8.020216e-01 4.376211e-01 3.129403e-01 3.139951e-01 3.150552e-01 3.107970e-01

928 8.034623e-01 8.035972e-01 8.037441e-01 8.038943e-01 8.040393e-01 4.377859e-01 3.129750e-01 3.140305e-01 3.150913e-01 3.108304e-01

929 8.054287e-01 8.055632e-01 8.057098e-01 8.058596e-01 8.060042e-01 4.379625e-01 3.130122e-01 3.140684e-01 3.151300e-01 3.108661e-01

930 8.073405e-01 8.074747e-01 8.076208e-01 8.077701e-01 8.079142e-01 4.381516e-01 3.130519e-01 3.141089e-01 3.151713e-01 3.109043e-01

931 8.091962e-01 8.093299e-01 8.094755e-01 8.096243e-01 8.097678e-01 4.383542e-01 3.130944e-01 3.141522e-01 3.152154e-01 3.109451e-01

932 8.109944e-01 8.111274e-01 8.112724e-01 8.114206e-01 8.115636e-01 4.385710e-01 3.131397e-01 3.141984e-01 3.152626e-01 3.109887e-01

933 8.127339e-01 8.128663e-01 8.130107e-01 8.131583e-01 8.133005e-01 4.388029e-01 3.131882e-01 3.142479e-01 3.153130e-01 3.110353e-01

934 8.144142e-01 8.145460e-01 8.146897e-01 8.148365e-01 8.149780e-01 4.390507e-01 3.132399e-01 3.143006e-01 3.153669e-01 3.110850e-01

935 8.160350e-01 8.161660e-01 8.163090e-01 8.164551e-01 8.165957e-01 4.393155e-01 3.132952e-01 3.143570e-01 3.154243e-01 3.111381e-01

936 8.175963e-01 8.177265e-01 8.178687e-01 8.180140e-01 8.181538e-01 4.395981e-01 3.133541e-01 3.144171e-01 3.154856e-01 3.111948e-01

937 8.190982e-01 8.192276e-01 8.193691e-01 8.195136e-01 8.196525e-01 4.398996e-01 3.134170e-01 3.144811e-01 3.155509e-01 3.112552e-01

938 8.205414e-01 8.206700e-01 8.208107e-01 8.209543e-01 8.210924e-01 4.402210e-01 3.134840e-01 3.145495e-01 3.156206e-01 3.113196e-01

939 8.219267e-01 8.220545e-01 8.221943e-01 8.223371e-01 8.224742e-01 4.405634e-01 3.135554e-01 3.146223e-01 3.156949e-01 3.113883e-01

940 8.232550e-01 8.233819e-01 8.235209e-01 8.236629e-01 8.237991e-01 4.409278e-01 3.136315e-01 3.146999e-01 3.157740e-01 3.114615e-01

941 8.245275e-01 8.246536e-01 8.247918e-01 8.249330e-01 8.250683e-01 4.413154e-01 3.137125e-01 3.147825e-01 3.158583e-01 3.115394e-01

942 8.257457e-01 8.258709e-01 8.260083e-01 8.261486e-01 8.262830e-01 4.417274e-01 3.137988e-01 3.148705e-01 3.159480e-01 3.116224e-01

943 8.269109e-01 8.270353e-01 8.271719e-01 8.273113e-01 8.274448e-01 4.421647e-01 3.138906e-01 3.149641e-01 3.160434e-01 3.117107e-01

944 8.280248e-01 8.281484e-01 8.282841e-01 8.284228e-01 8.285554e-01 4.426287e-01 3.139883e-01 3.150637e-01 3.161449e-01 3.118046e-01

945 8.290890e-01 8.292118e-01 8.293468e-01 8.294847e-01 8.296164e-01 4.431204e-01 3.140921e-01 3.151695e-01 3.162528e-01 3.119045e-01

946 8.301054e-01 8.302274e-01 8.303616e-01 8.304987e-01 8.306296e-01 4.436411e-01 3.142025e-01 3.152820e-01 3.163675e-01 3.120107e-01

947 8.310757e-01 8.311970e-01 8.313305e-01 8.314668e-01 8.315968e-01 4.441918e-01 3.143197e-01 3.154015e-01 3.164893e-01 3.121235e-01

948 8.320018e-01 8.321223e-01 8.322551e-01 8.323907e-01 8.325199e-01 4.447738e-01 3.144442e-01 3.155284e-01 3.166186e-01 3.122432e-01

949 8.328855e-01 8.330053e-01 8.331374e-01 8.332723e-01 8.334007e-01 4.453880e-01 3.145763e-01 3.156630e-01 3.167559e-01 3.123704e-01

950 8.337287e-01 8.338478e-01 8.339792e-01 8.341134e-01 8.342411e-01 4.460356e-01 3.147164e-01 3.158059e-01 3.169014e-01 3.125052e-01

951 8.345333e-01 8.346517e-01 8.347824e-01 8.349160e-01 8.350429e-01 4.467176e-01 3.148650e-01 3.159573e-01 3.170558e-01 3.126483e-01

952 8.353009e-01 8.354187e-01 8.355488e-01 8.356817e-01 8.358080e-01 4.474350e-01 3.150225e-01 3.161178e-01 3.172193e-01 3.127999e-01

953 8.360335e-01 8.361507e-01 8.362802e-01 8.364125e-01 8.365381e-01 4.481886e-01 3.151893e-01 3.162877e-01 3.173925e-01 3.129605e-01

954 8.367328e-01 8.368493e-01 8.369784e-01 8.371101e-01 8.372350e-01 4.489792e-01 3.153659e-01 3.164677e-01 3.175759e-01 3.131305e-01

955 8.374005e-01 8.375164e-01 8.376449e-01 8.377761e-01 8.379004e-01 4.498076e-01 3.155528e-01 3.166581e-01 3.177698e-01 3.133105e-01

956 8.380381e-01 8.381536e-01 8.382815e-01 8.384122e-01 8.385359e-01 4.506743e-01 3.157504e-01 3.168594e-01 3.179750e-01 3.135009e-01

957 8.386474e-01 8.387623e-01 8.388898e-01 8.390199e-01 8.391432e-01 4.515798e-01 3.159593e-01 3.170722e-01 3.181917e-01 3.137021e-01

958 8.392299e-01 8.393443e-01 8.394713e-01 8.396009e-01 8.397237e-01 4.525246e-01 3.161800e-01 3.172970e-01 3.184207e-01 3.139147e-01

959 8.397869e-01 8.399008e-01 8.400274e-01 8.401566e-01 8.402788e-01 4.535087e-01 3.164130e-01 3.175342e-01 3.186623e-01 3.141392e-01

960 8.403200e-01 8.404335e-01 8.405596e-01 8.406884e-01 8.408101e-01 4.545322e-01 3.166588e-01 3.177845e-01 3.189172e-01 3.143761e-01

961 8.408304e-01 8.409435e-01 8.410692e-01 8.411976e-01 8.413189e-01 4.555951e-01 3.169179e-01 3.180484e-01 3.191859e-01 3.146259e-01

962 8.413194e-01 8.414321e-01 8.415575e-01 8.416855e-01 8.418064e-01 4.566969e-01 3.171910e-01 3.183265e-01 3.194691e-01 3.148893e-01

963 8.417884e-01 8.419007e-01 8.420257e-01 8.421534e-01 8.422738e-01 4.578372e-01 3.174786e-01 3.186193e-01 3.197671e-01 3.151666e-01

964 8.422383e-01 8.423503e-01 8.424750e-01 8.426023e-01 8.427223e-01 4.590152e-01 3.177812e-01 3.189273e-01 3.200807e-01 3.154585e-01

965 8.426704e-01 8.427820e-01 8.429064e-01 8.430334e-01 8.431531e-01 4.602302e-01 3.180994e-01 3.192512e-01 3.204103e-01 3.157656e-01

966 8.430856e-01 8.431969e-01 8.433210e-01 8.434477e-01 8.435670e-01 4.614810e-01 3.184338e-01 3.195915e-01 3.207566e-01 3.160883e-01

967 8.434849e-01 8.435959e-01 8.437197e-01 8.438461e-01 8.439652e-01 4.627662e-01 3.187849e-01 3.199487e-01 3.211202e-01 3.164272e-01

968 8.438694e-01 8.439800e-01 8.441036e-01 8.442297e-01 8.443484e-01 4.640844e-01 3.191533e-01 3.203235e-01 3.215015e-01 3.167830e-01

969 8.442397e-01 8.443501e-01 8.444734e-01 8.445993e-01 8.447177e-01 4.654337e-01 3.195395e-01 3.207164e-01 3.219011e-01 3.171560e-01

970 8.445968e-01 8.447070e-01 8.448300e-01 8.449557e-01 8.450738e-01 4.668124e-01 3.199441e-01 3.211278e-01 3.223196e-01 3.175469e-01

971 8.449414e-01 8.450513e-01 8.451742e-01 8.452996e-01 8.454175e-01 4.682182e-01 3.203675e-01 3.215584e-01 3.227575e-01 3.179561e-01

972 8.452743e-01 8.453840e-01 8.455067e-01 8.456318e-01 8.457495e-01 4.696489e-01 3.208103e-01 3.220086e-01 3.232152e-01 3.183842e-01

973 8.455961e-01 8.457056e-01 8.458281e-01 8.459531e-01 8.460705e-01 4.711019e-01 3.212729e-01 3.224789e-01 3.236933e-01 3.188316e-01

974 8.459075e-01 8.460168e-01 8.461391e-01 8.462639e-01 8.463811e-01 4.725747e-01 3.217557e-01 3.229696e-01 3.241921e-01 3.192987e-01

975 8.462092e-01 8.463182e-01 8.464403e-01 8.465649e-01 8.466820e-01 4.740644e-01 3.222593e-01 3.234814e-01 3.247121e-01 3.197861e-01

976 8.465015e-01 8.466104e-01 8.467324e-01 8.468568e-01 8.469736e-01 4.755683e-01 3.227839e-01 3.240144e-01 3.252537e-01 3.202940e-01

977 8.467852e-01 8.468939e-01 8.470157e-01 8.471400e-01 8.472566e-01 4.770833e-01 3.233299e-01 3.245690e-01 3.258171e-01 3.208229e-01

978 8.470606e-01 8.471692e-01 8.472908e-01 8.474149e-01 8.475314e-01 4.786063e-01 3.238976e-01 3.251456e-01 3.264026e-01 3.213730e-01

979 8.473283e-01 8.474367e-01 8.475582e-01 8.476822e-01 8.477985e-01 4.801343e-01 3.244873e-01 3.257442e-01 3.270105e-01 3.219446e-01

980 8.475886e-01 8.476969e-01 8.478183e-01 8.479421e-01 8.480583e-01 4.816642e-01 3.250990e-01 3.263652e-01 3.276408e-01 3.225379e-01

981 8.478421e-01 8.479502e-01 8.480715e-01 8.481952e-01 8.483112e-01 4.831928e-01 3.257330e-01 3.270086e-01 3.282938e-01 3.231530e-01

982 8.480890e-01 8.481971e-01 8.483182e-01 8.484418e-01 8.485576e-01 4.847170e-01 3.263893e-01 3.276745e-01 3.289694e-01 3.237901e-01

983 8.483298e-01 8.484377e-01 8.485587e-01 8.486822e-01 8.487979e-01 4.862339e-01 3.270679e-01 3.283629e-01 3.296677e-01 3.244491e-01

984 8.485648e-01 8.486725e-01 8.487934e-01 8.489168e-01 8.490324e-01 4.877404e-01 3.277686e-01 3.290735e-01 3.303884e-01 3.251301e-01

985 8.487942e-01 8.489019e-01 8.490227e-01 8.491460e-01 8.492614e-01 4.892338e-01 3.284915e-01 3.298064e-01 3.311314e-01 3.258328e-01

986 8.490184e-01 8.491260e-01 8.492467e-01 8.493699e-01 8.494853e-01 4.907112e-01 3.292361e-01 3.305612e-01 3.318964e-01 3.265572e-01

987 8.492377e-01 8.493452e-01 8.494658e-01 8.495889e-01 8.497041e-01 4.921701e-01 3.300023e-01 3.313375e-01 3.326830e-01 3.273029e-01

988 8.494523e-01 8.495597e-01 8.496802e-01 8.498032e-01 8.499184e-01 4.936081e-01 3.307896e-01 3.321350e-01 3.334909e-01 3.280695e-01

989 8.496624e-01 8.497697e-01 8.498902e-01 8.500131e-01 8.501282e-01 4.950229e-01 3.315975e-01 3.329532e-01 3.343194e-01 3.288567e-01

990 8.498683e-01 8.499756e-01 8.500959e-01 8.502188e-01 8.503338e-01 4.964123e-01 3.324254e-01 3.337914e-01 3.351680e-01 3.296639e-01

991 8.500703e-01 8.501774e-01 8.502977e-01 8.504205e-01 8.505354e-01 4.977746e-01 3.332728e-01 3.346489e-01 3.360359e-01 3.304906e-01

992 8.502684e-01 8.503754e-01 8.504957e-01 8.506184e-01 8.507332e-01 4.991081e-01 3.341387e-01 3.355251e-01 3.369223e-01 3.313359e-01

993 8.504629e-01 8.505699e-01 8.506900e-01 8.508126e-01 8.509274e-01 5.004112e-01 3.350224e-01 3.364189e-01 3.378264e-01 3.321991e-01

994 8.506539e-01 8.507608e-01 8.508809e-01 8.510035e-01 8.511181e-01 5.016827e-01 3.359230e-01 3.373296e-01 3.387471e-01 3.330794e-01

995 8.508416e-01 8.509485e-01 8.510685e-01 8.511910e-01 8.513056e-01 5.029215e-01 3.368395e-01 3.382559e-01 3.396834e-01 3.339757e-01

996 8.510263e-01 8.511331e-01 8.512530e-01 8.513754e-01 8.514900e-01 5.041267e-01 3.377708e-01 3.391969e-01 3.406342e-01 3.348871e-01

997 8.512079e-01 8.513146e-01 8.514345e-01 8.515569e-01 8.516714e-01 5.052978e-01 3.387156e-01 3.401513e-01 3.415982e-01 3.358125e-01

998 8.513866e-01 8.514933e-01 8.516132e-01 8.517355e-01 8.518499e-01 5.064341e-01 3.396729e-01 3.411179e-01 3.425742e-01 3.367506e-01

999 8.515627e-01 8.516693e-01 8.517891e-01 8.519114e-01 8.520257e-01 5.075355e-01 3.406412e-01 3.420954e-01 3.435609e-01 3.377003e-01

1000 8.517361e-01 8.518427e-01 8.519624e-01 8.520846e-01 8.521989e-01 5.086017e-01 3.416194e-01 3.430823e-01 3.445568e-01 3.386602e-01

1001 8.519070e-01 8.520135e-01 8.521332e-01 8.522554e-01 8.523696e-01 5.096328e-01 3.426058e-01 3.440774e-01 3.455604e-01 3.396290e-01

1002 8.520755e-01 8.521820e-01 8.523016e-01 8.524237e-01 8.525380e-01 5.106290e-01 3.435992e-01 3.450791e-01 3.465705e-01 3.406053e-01

1003 8.522417e-01 8.523482e-01 8.524678e-01 8.525898e-01 8.527040e-01 5.115905e-01 3.445981e-01 3.460859e-01 3.475853e-01 3.415877e-01

1004 8.524057e-01 8.525121e-01 8.526317e-01 8.527537e-01 8.528678e-01 5.125179e-01 3.456009e-01 3.470964e-01 3.486034e-01 3.425746e-01

1005 8.525676e-01 8.526740e-01 8.527935e-01 8.529154e-01 8.530295e-01 5.134116e-01 3.466061e-01 3.481089e-01 3.496233e-01 3.435646e-01

1006 8.527274e-01 8.528338e-01 8.529532e-01 8.530751e-01 8.531892e-01 5.142723e-01 3.476123e-01 3.491221e-01 3.506434e-01 3.445563e-01

1007 8.528853e-01 8.529916e-01 8.531110e-01 8.532329e-01 8.533469e-01 5.151006e-01 3.486179e-01 3.501343e-01 3.516623e-01 3.455480e-01

1008 8.530413e-01 8.531475e-01 8.532669e-01 8.533887e-01 8.535027e-01 5.158975e-01 3.496214e-01 3.511441e-01 3.526783e-01 3.465385e-01

1009 8.531954e-01 8.533016e-01 8.534210e-01 8.535428e-01 8.536567e-01 5.166636e-01 3.506214e-01 3.521499e-01 3.536900e-01 3.475261e-01

1010 8.533478e-01 8.534540e-01 8.535733e-01 8.536950e-01 8.538089e-01 5.174000e-01 3.516163e-01 3.531504e-01 3.546959e-01 3.485095e-01

1011 8.534985e-01 8.536046e-01 8.537239e-01 8.538456e-01 8.539595e-01 5.181076e-01 3.526049e-01 3.541441e-01 3.556948e-01 3.494872e-01

1012 8.536475e-01 8.537536e-01 8.538729e-01 8.539945e-01 8.541083e-01 5.187873e-01 3.535857e-01 3.551297e-01 3.566851e-01 3.504579e-01

1013 8.537949e-01 8.539010e-01 8.540202e-01 8.541419e-01 8.542556e-01 5.194401e-01 3.545575e-01 3.561059e-01 3.576656e-01 3.514203e-01

1014 8.539408e-01 8.540469e-01 8.541661e-01 8.542877e-01 8.544014e-01 5.200670e-01 3.555190e-01 3.570714e-01 3.586351e-01 3.523731e-01

1015 8.540852e-01 8.541913e-01 8.543104e-01 8.544320e-01 8.545457e-01 5.206690e-01 3.564690e-01 3.580250e-01 3.595924e-01 3.533152e-01

1016 8.542282e-01 8.543342e-01 8.544533e-01 8.545748e-01 8.546885e-01 5.212471e-01 3.574064e-01 3.589658e-01 3.605365e-01 3.542454e-01

1017 8.543697e-01 8.544757e-01 8.545948e-01 8.547162e-01 8.548299e-01 5.218024e-01 3.583303e-01 3.598926e-01 3.614663e-01 3.551627e-01

1018 8.545099e-01 8.546159e-01 8.547349e-01 8.548563e-01 8.549700e-01 5.223357e-01 3.592396e-01 3.608046e-01 3.623809e-01 3.560661e-01

1019 8.546487e-01 8.547547e-01 8.548737e-01 8.549951e-01 8.551087e-01 5.228480e-01 3.601335e-01 3.617009e-01 3.632795e-01 3.569547e-01

1020 8.547863e-01 8.548922e-01 8.550112e-01 8.551325e-01 8.552461e-01 5.233404e-01 3.610112e-01 3.625807e-01 3.641614e-01 3.578278e-01

1021 8.549225e-01 8.550285e-01 8.551474e-01 8.552687e-01 8.553823e-01 5.238137e-01 3.618721e-01 3.634434e-01 3.650258e-01 3.586845e-01

1022 8.550576e-01 8.551635e-01 8.552824e-01 8.554037e-01 8.555173e-01 5.242688e-01 3.627155e-01 3.642883e-01 3.658723e-01 3.595244e-01

1023 8.551915e-01 8.552974e-01 8.554162e-01 8.555375e-01 8.556510e-01 5.247066e-01 3.635409e-01 3.651150e-01 3.667002e-01 3.603467e-01

1024 8.553242e-01 8.554301e-01 8.555489e-01 8.556701e-01 8.557836e-01 5.251280e-01 3.643478e-01 3.659231e-01 3.675093e-01 3.611511e-01

1025 8.554557e-01 8.555616e-01 8.556804e-01 8.558016e-01 8.559151e-01 5.255337e-01 3.651360e-01 3.667121e-01 3.682992e-01 3.619372e-01

1026 8.555862e-01 8.556921e-01 8.558108e-01 8.559319e-01 8.560454e-01 5.259246e-01 3.659052e-01 3.674819e-01 3.690696e-01 3.627046e-01

1027 8.557155e-01 8.558214e-01 8.559401e-01 8.560612e-01 8.561747e-01 5.263014e-01 3.666550e-01 3.682322e-01 3.698204e-01 3.634532e-01

1028 8.558438e-01 8.559497e-01 8.560684e-01 8.561894e-01 8.563029e-01 5.266648e-01 3.673855e-01 3.689630e-01 3.705514e-01 3.641826e-01

1029 8.559711e-01 8.560769e-01 8.561956e-01 8.563166e-01 8.564300e-01 5.270155e-01 3.680965e-01 3.696742e-01 3.712627e-01 3.648930e-01

1030 8.560973e-01 8.562032e-01 8.563218e-01 8.564428e-01 8.565562e-01 5.273542e-01 3.687881e-01 3.703658e-01 3.719543e-01 3.655842e-01

1031 8.562226e-01 8.563284e-01 8.564470e-01 8.565679e-01 8.566813e-01 5.276815e-01 3.694603e-01 3.710379e-01 3.726263e-01 3.662563e-01

1032 8.563468e-01 8.564526e-01 8.565712e-01 8.566921e-01 8.568055e-01 5.279980e-01 3.701132e-01 3.716906e-01 3.732788e-01 3.669093e-01

1033 8.564701e-01 8.565759e-01 8.566944e-01 8.568153e-01 8.569287e-01 5.283044e-01 3.707471e-01 3.723242e-01 3.739120e-01 3.675435e-01

1034 8.565925e-01 8.566983e-01 8.568167e-01 8.569376e-01 8.570510e-01 5.286010e-01 3.713621e-01 3.729388e-01 3.745262e-01 3.681590e-01

1035 8.567139e-01 8.568197e-01 8.569381e-01 8.570590e-01 8.571723e-01 5.288885e-01 3.719584e-01 3.735347e-01 3.751217e-01 3.687561e-01

1036 8.568345e-01 8.569402e-01 8.570586e-01 8.571794e-01 8.572928e-01 5.291674e-01 3.725365e-01 3.741123e-01 3.756987e-01 3.693349e-01

1037 8.569541e-01 8.570599e-01 8.571782e-01 8.572990e-01 8.574123e-01 5.294380e-01 3.730966e-01 3.746718e-01 3.762577e-01 3.698960e-01

1038 8.570729e-01 8.571786e-01 8.572969e-01 8.574177e-01 8.575310e-01 5.297009e-01 3.736391e-01 3.752137e-01 3.767989e-01 3.704395e-01

1039 8.571908e-01 8.572965e-01 8.574148e-01 8.575355e-01 8.576488e-01 5.299565e-01 3.741644e-01 3.757383e-01 3.773229e-01 3.709658e-01

1040 8.573078e-01 8.574135e-01 8.575318e-01 8.576525e-01 8.577657e-01 5.302051e-01 3.746728e-01 3.762461e-01 3.778300e-01 3.714755e-01

1041 8.574240e-01 8.575298e-01 8.576480e-01 8.577686e-01 8.578819e-01 5.304472e-01 3.751649e-01 3.767375e-01 3.783207e-01 3.719688e-01

1042 8.575394e-01 8.576451e-01 8.577633e-01 8.578839e-01 8.579972e-01 5.306831e-01 3.756411e-01 3.772129e-01 3.787954e-01 3.724462e-01

1043 8.576540e-01 8.577597e-01 8.578779e-01 8.579984e-01 8.581117e-01 5.309131e-01 3.761017e-01 3.776729e-01 3.792546e-01 3.729081e-01

1044 8.577678e-01 8.578735e-01 8.579916e-01 8.581122e-01 8.582254e-01 5.311375e-01 3.765473e-01 3.781178e-01 3.796988e-01 3.733550e-01

1045 8.578808e-01 8.579865e-01 8.581046e-01 8.582251e-01 8.583383e-01 5.313567e-01 3.769784e-01 3.785481e-01 3.801284e-01 3.737874e-01

1046 8.579930e-01 8.580987e-01 8.582168e-01 8.583372e-01 8.584505e-01 5.315709e-01 3.773954e-01 3.789644e-01 3.805439e-01 3.742057e-01

1047 8.581045e-01 8.582102e-01 8.583282e-01 8.584486e-01 8.585618e-01 5.317803e-01 3.777987e-01 3.793670e-01 3.809459e-01 3.746103e-01

1048 8.582152e-01 8.583209e-01 8.584389e-01 8.585593e-01 8.586725e-01 5.319853e-01 3.781889e-01 3.797565e-01 3.813347e-01 3.750018e-01

1049 8.583252e-01 8.584309e-01 8.585488e-01 8.586691e-01 8.587823e-01 5.321861e-01 3.785665e-01 3.801334e-01 3.817108e-01 3.753806e-01

1050 8.584344e-01 8.585401e-01 8.586580e-01 8.587783e-01 8.588915e-01 5.323828e-01 3.789318e-01 3.804980e-01 3.820748e-01 3.757471e-01

1051 8.585429e-01 8.586486e-01 8.587665e-01 8.588867e-01 8.589999e-01 5.325757e-01 3.792853e-01 3.808509e-01 3.824271e-01 3.761019e-01

1052 8.586507e-01 8.587564e-01 8.588742e-01 8.589944e-01 8.591076e-01 5.327650e-01 3.796275e-01 3.811925e-01 3.827680e-01 3.764452e-01

1053 8.587578e-01 8.588635e-01 8.589813e-01 8.591015e-01 8.592146e-01 5.329509e-01 3.799589e-01 3.815232e-01 3.830981e-01 3.767777e-01

1054 8.588642e-01 8.589699e-01 8.590876e-01 8.592078e-01 8.593209e-01 5.331336e-01 3.802798e-01 3.818435e-01 3.834178e-01 3.770997e-01

1055 8.589699e-01 8.590756e-01 8.591933e-01 8.593134e-01 8.594265e-01 5.333131e-01 3.805907e-01 3.821539e-01 3.837276e-01 3.774117e-01

1056 8.590749e-01 8.591806e-01 8.592983e-01 8.594183e-01 8.595315e-01 5.334897e-01 3.808919e-01 3.824546e-01 3.840277e-01 3.777140e-01

1057 8.591793e-01 8.592849e-01 8.594026e-01 8.595226e-01 8.596357e-01 5.336635e-01 3.811840e-01 3.827461e-01 3.843187e-01 3.780070e-01

1058 8.592829e-01 8.593886e-01 8.595062e-01 8.596262e-01 8.597393e-01 5.338346e-01 3.814672e-01 3.830288e-01 3.846009e-01 3.782912e-01

1059 8.593859e-01 8.594916e-01 8.596092e-01 8.597291e-01 8.598422e-01 5.340032e-01 3.817420e-01 3.833031e-01 3.848747e-01 3.785668e-01

1060 8.594883e-01 8.595940e-01 8.597115e-01 8.598314e-01 8.599445e-01 5.341695e-01 3.820086e-01 3.835693e-01 3.851405e-01 3.788344e-01

1061 8.595900e-01 8.596957e-01 8.598131e-01 8.599330e-01 8.600461e-01 5.343334e-01 3.822676e-01 3.838278e-01 3.853985e-01 3.790941e-01

1062 8.596911e-01 8.597967e-01 8.599142e-01 8.600340e-01 8.601471e-01 5.344951e-01 3.825191e-01 3.840789e-01 3.856492e-01 3.793465e-01

1063 8.597915e-01 8.598972e-01 8.600146e-01 8.601343e-01 8.602475e-01 5.346547e-01 3.827636e-01 3.843229e-01 3.858929e-01 3.795917e-01

1064 8.598913e-01 8.599970e-01 8.601143e-01 8.602341e-01 8.603472e-01 5.348124e-01 3.830013e-01 3.845603e-01 3.861298e-01 3.798301e-01

1065 8.599905e-01 8.600962e-01 8.602135e-01 8.603332e-01 8.604463e-01 5.349681e-01 3.832325e-01 3.847911e-01 3.863603e-01 3.800620e-01

1066 8.600891e-01 8.601948e-01 8.603120e-01 8.604317e-01 8.605448e-01 5.351221e-01 3.834576e-01 3.850159e-01 3.865847e-01 3.802877e-01

1067 8.601871e-01 8.602927e-01 8.604099e-01 8.605295e-01 8.606427e-01 5.352743e-01 3.836768e-01 3.852347e-01 3.868032e-01 3.805076e-01

1068 8.602844e-01 8.603901e-01 8.605073e-01 8.606268e-01 8.607399e-01 5.354248e-01 3.838904e-01 3.854480e-01 3.870162e-01 3.807217e-01

1069 8.603812e-01 8.604869e-01 8.606040e-01 8.607235e-01 8.608366e-01 5.355737e-01 3.840986e-01 3.856559e-01 3.872238e-01 3.809305e-01

1070 8.604774e-01 8.605830e-01 8.607001e-01 8.608196e-01 8.609327e-01 5.357211e-01 3.843017e-01 3.858587e-01 3.874263e-01 3.811341e-01

1071 8.605729e-01 8.606786e-01 8.607957e-01 8.609151e-01 8.610282e-01 5.358671e-01 3.845000e-01 3.860567e-01 3.876240e-01 3.813328e-01

1072 8.606679e-01 8.607736e-01 8.608906e-01 8.610100e-01 8.611231e-01 5.360116e-01 3.846935e-01 3.862500e-01 3.878171e-01 3.815269e-01

1073 8.607624e-01 8.608680e-01 8.609850e-01 8.611043e-01 8.612174e-01 5.361548e-01 3.848827e-01 3.864389e-01 3.880057e-01 3.817164e-01

1074 8.608562e-01 8.609619e-01 8.610788e-01 8.611981e-01 8.613112e-01 5.362967e-01 3.850676e-01 3.866236e-01 3.881902e-01 3.819018e-01

1075 8.609495e-01 8.610552e-01 8.611720e-01 8.612913e-01 8.614044e-01 5.364374e-01 3.852484e-01 3.868042e-01 3.883706e-01 3.820830e-01

1076 8.610422e-01 8.611479e-01 8.612647e-01 8.613839e-01 8.614970e-01 5.365768e-01 3.854255e-01 3.869810e-01 3.885472e-01 3.822604e-01

1077 8.611344e-01 8.612401e-01 8.613568e-01 8.614760e-01 8.615891e-01 5.367151e-01 3.855988e-01 3.871542e-01 3.887202e-01 3.824341e-01

1078 8.612260e-01 8.613317e-01 8.614484e-01 8.615675e-01 8.616806e-01 5.368523e-01 3.857687e-01 3.873238e-01 3.888897e-01 3.826043e-01

1079 8.613170e-01 8.614228e-01 8.615394e-01 8.616584e-01 8.617716e-01 5.369883e-01 3.859352e-01 3.874902e-01 3.890558e-01 3.827711e-01

1080 8.614076e-01 8.615133e-01 8.616299e-01 8.617489e-01 8.618620e-01 5.371234e-01 3.860985e-01 3.876534e-01 3.892188e-01 3.829348e-01

1081 8.614975e-01 8.616033e-01 8.617198e-01 8.618387e-01 8.619519e-01 5.372575e-01 3.862589e-01 3.878135e-01 3.893788e-01 3.830954e-01

1082 8.634152e-01 8.635207e-01 8.636370e-01 8.637557e-01 8.638686e-01 5.373571e-01 3.862772e-01 3.878324e-01 3.893982e-01 3.831128e-01

1083 8.652894e-01 8.653946e-01 8.655106e-01 8.656288e-01 8.657413e-01 5.374637e-01 3.862968e-01 3.878525e-01 3.894189e-01 3.831314e-01

1084 8.671181e-01 8.672228e-01 8.673383e-01 8.674560e-01 8.675679e-01 5.375777e-01 3.863178e-01 3.878740e-01 3.894409e-01 3.831512e-01

1085 8.688993e-01 8.690034e-01 8.691182e-01 8.692352e-01 8.693464e-01 5.376996e-01 3.863400e-01 3.878968e-01 3.894644e-01 3.831724e-01

1086 8.706313e-01 8.707347e-01 8.708487e-01 8.709650e-01 8.710754e-01 5.378299e-01 3.863638e-01 3.879212e-01 3.894894e-01 3.831949e-01

1087 8.723128e-01 8.724154e-01 8.725285e-01 8.726439e-01 8.727534e-01 5.379690e-01 3.863891e-01 3.879472e-01 3.895161e-01 3.832189e-01

1088 8.739428e-01 8.740445e-01 8.741567e-01 8.742711e-01 8.743796e-01 5.381175e-01 3.864161e-01 3.879749e-01 3.895445e-01 3.832445e-01

1089 8.755206e-01 8.756212e-01 8.757325e-01 8.758458e-01 8.759532e-01 5.382760e-01 3.864448e-01 3.880044e-01 3.895748e-01 3.832717e-01

1090 8.770457e-01 8.771452e-01 8.772554e-01 8.773677e-01 8.774739e-01 5.384451e-01 3.864753e-01 3.880357e-01 3.896070e-01 3.833007e-01

1091 8.785178e-01 8.786163e-01 8.787253e-01 8.788364e-01 8.789414e-01 5.386254e-01 3.865079e-01 3.880691e-01 3.896412e-01 3.833316e-01

1092 8.799371e-01 8.800344e-01 8.801423e-01 8.802522e-01 8.803559e-01 5.388175e-01 3.865425e-01 3.881046e-01 3.896777e-01 3.833645e-01

1093 8.813038e-01 8.813999e-01 8.815065e-01 8.816152e-01 8.817176e-01 5.390221e-01 3.865793e-01 3.881424e-01 3.897165e-01 3.833994e-01

1094 8.826184e-01 8.827132e-01 8.828187e-01 8.829261e-01 8.830271e-01 5.392400e-01 3.866185e-01 3.881826e-01 3.897577e-01 3.834365e-01

1095 8.838815e-01 8.839751e-01 8.840793e-01 8.841854e-01 8.842851e-01 5.394718e-01 3.866601e-01 3.882253e-01 3.898016e-01 3.834760e-01

1096 8.850940e-01 8.851863e-01 8.852893e-01 8.853941e-01 8.854925e-01 5.397182e-01 3.867043e-01 3.882707e-01 3.898482e-01 3.835180e-01

1097 8.862569e-01 8.863479e-01 8.864496e-01 8.865532e-01 8.866502e-01 5.399801e-01 3.867513e-01 3.883189e-01 3.898976e-01 3.835626e-01

1098 8.873713e-01 8.874610e-01 8.875615e-01 8.876638e-01 8.877594e-01 5.402583e-01 3.868012e-01 3.883701e-01 3.899502e-01 3.836100e-01

1099 8.884384e-01 8.885269e-01 8.886261e-01 8.887272e-01 8.888214e-01 5.405536e-01 3.868542e-01 3.884245e-01 3.900060e-01 3.836602e-01

1100 8.894595e-01 8.895468e-01 8.896448e-01 8.897446e-01 8.898376e-01 5.408668e-01 3.869104e-01 3.884822e-01 3.900652e-01 3.837136e-01

1101 8.904361e-01 8.905221e-01 8.906190e-01 8.907175e-01 8.908092e-01 5.411988e-01 3.869700e-01 3.885434e-01 3.901280e-01 3.837703e-01

1102 8.913695e-01 8.914544e-01 8.915501e-01 8.916475e-01 8.917378e-01 5.415505e-01 3.870333e-01 3.886084e-01 3.901947e-01 3.838303e-01

1103 8.922614e-01 8.923451e-01 8.924396e-01 8.925358e-01 8.926250e-01 5.419227e-01 3.871004e-01 3.886772e-01 3.902653e-01 3.838940e-01

1104 8.931132e-01 8.931957e-01 8.932892e-01 8.933843e-01 8.934722e-01 5.423164e-01 3.871715e-01 3.887502e-01 3.903402e-01 3.839616e-01

1105 8.939264e-01 8.940079e-01 8.941003e-01 8.941943e-01 8.942810e-01 5.427325e-01 3.872469e-01 3.888275e-01 3.904195e-01 3.840331e-01

1106 8.947027e-01 8.947831e-01 8.948745e-01 8.949674e-01 8.950531e-01 5.431718e-01 3.873267e-01 3.889094e-01 3.905035e-01 3.841089e-01

1107 8.954436e-01 8.955230e-01 8.956133e-01 8.957053e-01 8.957898e-01 5.436354e-01 3.874112e-01 3.889961e-01 3.905925e-01 3.841892e-01

1108 8.961507e-01 8.962290e-01 8.963184e-01 8.964094e-01 8.964928e-01 5.441240e-01 3.875007e-01 3.890878e-01 3.906866e-01 3.842742e-01

1109 8.968253e-01 8.969027e-01 8.969912e-01 8.970812e-01 8.971637e-01 5.446387e-01 3.875953e-01 3.891849e-01 3.907862e-01 3.843641e-01

1110 8.974692e-01 8.975456e-01 8.976332e-01 8.977223e-01 8.978038e-01 5.451801e-01 3.876954e-01 3.892875e-01 3.908915e-01 3.844591e-01

1111 8.980836e-01 8.981592e-01 8.982459e-01 8.983341e-01 8.984147e-01 5.457493e-01 3.878012e-01 3.893961e-01 3.910028e-01 3.845597e-01

1112 8.986700e-01 8.987447e-01 8.988307e-01 8.989181e-01 8.989977e-01 5.463470e-01 3.879130e-01 3.895108e-01 3.911204e-01 3.846659e-01

1113 8.992299e-01 8.993038e-01 8.993889e-01 8.994756e-01 8.995544e-01 5.469739e-01 3.880311e-01 3.896319e-01 3.912446e-01 3.847782e-01

1114 8.997645e-01 8.998376e-01 8.999220e-01 9.000079e-01 9.000859e-01 5.476308e-01 3.881559e-01 3.897598e-01 3.913758e-01 3.848968e-01

1115 9.002751e-01 9.003475e-01 9.004312e-01 9.005164e-01 9.005936e-01 5.483183e-01 3.882875e-01 3.898948e-01 3.915142e-01 3.850219e-01

1116 9.007630e-01 9.008347e-01 9.009178e-01 9.010022e-01 9.010787e-01 5.490370e-01 3.884264e-01 3.900372e-01 3.916602e-01 3.851540e-01

1117 9.012294e-01 9.013004e-01 9.013828e-01 9.014666e-01 9.015424e-01 5.497875e-01 3.885728e-01 3.901873e-01 3.918141e-01 3.852932e-01

1118 9.016754e-01 9.017458e-01 9.018276e-01 9.019108e-01 9.019859e-01 5.505701e-01 3.887272e-01 3.903456e-01 3.919764e-01 3.854400e-01

1119 9.021021e-01 9.021719e-01 9.022531e-01 9.023357e-01 9.024102e-01 5.513851e-01 3.888899e-01 3.905123e-01 3.921473e-01 3.855948e-01

1120 9.025106e-01 9.025798e-01 9.026605e-01 9.027426e-01 9.028164e-01 5.522329e-01 3.890611e-01 3.906878e-01 3.923272e-01 3.857577e-01

1121 9.029018e-01 9.029705e-01 9.030507e-01 9.031322e-01 9.032055e-01 5.531135e-01 3.892414e-01 3.908726e-01 3.925165e-01 3.859292e-01

1122 9.032768e-01 9.033449e-01 9.034246e-01 9.035056e-01 9.035784e-01 5.540269e-01 3.894310e-01 3.910669e-01 3.927157e-01 3.861097e-01

1123 9.036363e-01 9.037040e-01 9.037832e-01 9.038638e-01 9.039360e-01 5.549730e-01 3.896304e-01 3.912712e-01 3.929250e-01 3.862995e-01

1124 9.039813e-01 9.040486e-01 9.041273e-01 9.042074e-01 9.042791e-01 5.559514e-01 3.898400e-01 3.914859e-01 3.931449e-01 3.864991e-01

1125 9.043126e-01 9.043794e-01 9.044578e-01 9.045374e-01 9.046087e-01 5.569618e-01 3.900601e-01 3.917113e-01 3.933759e-01 3.867087e-01

1126 9.046310e-01 9.046973e-01 9.047753e-01 9.048546e-01 9.049254e-01 5.580036e-01 3.902911e-01 3.919479e-01 3.936183e-01 3.869287e-01

1127 9.049371e-01 9.050031e-01 9.050807e-01 9.051595e-01 9.052299e-01 5.590761e-01 3.905335e-01 3.921962e-01 3.938725e-01 3.871597e-01

1128 9.052317e-01 9.052973e-01 9.053745e-01 9.054530e-01 9.055231e-01 5.601783e-01 3.907876e-01 3.924564e-01 3.941390e-01 3.874019e-01

1129 9.055154e-01 9.055806e-01 9.056575e-01 9.057357e-01 9.058054e-01 5.613092e-01 3.910539e-01 3.927290e-01 3.944181e-01 3.876558e-01

1130 9.057888e-01 9.058537e-01 9.059303e-01 9.060082e-01 9.060775e-01 5.624677e-01 3.913328e-01 3.930145e-01 3.947104e-01 3.879217e-01

1131 9.060525e-01 9.061171e-01 9.061934e-01 9.062710e-01 9.063400e-01 5.636524e-01 3.916246e-01 3.933132e-01 3.950161e-01 3.882001e-01

1132 9.063071e-01 9.063714e-01 9.064475e-01 9.065247e-01 9.065934e-01 5.648618e-01 3.919298e-01 3.936256e-01 3.953357e-01 3.884913e-01

1133 9.065531e-01 9.066171e-01 9.066929e-01 9.067699e-01 9.068382e-01 5.660942e-01 3.922488e-01 3.939519e-01 3.956697e-01 3.887958e-01

1134 9.067909e-01 9.068547e-01 9.069302e-01 9.070069e-01 9.070750e-01 5.673477e-01 3.925820e-01 3.942928e-01 3.960183e-01 3.891139e-01

1135 9.070210e-01 9.070846e-01 9.071598e-01 9.072363e-01 9.073041e-01 5.686206e-01 3.929297e-01 3.946484e-01 3.963821e-01 3.894460e-01

1136 9.072439e-01 9.073072e-01 9.073822e-01 9.074585e-01 9.075260e-01 5.699107e-01 3.932923e-01 3.950192e-01 3.967613e-01 3.897925e-01

1137 9.074599e-01 9.075230e-01 9.075978e-01 9.076738e-01 9.077412e-01 5.712159e-01 3.936701e-01 3.954055e-01 3.971562e-01 3.901536e-01

1138 9.076695e-01 9.077323e-01 9.078069e-01 9.078828e-01 9.079499e-01 5.725339e-01 3.940636e-01 3.958076e-01 3.975673e-01 3.905299e-01

1139 9.078729e-01 9.079355e-01 9.080100e-01 9.080856e-01 9.081525e-01 5.738624e-01 3.944729e-01 3.962260e-01 3.979948e-01 3.909215e-01

1140 9.080705e-01 9.081330e-01 9.082072e-01 9.082827e-01 9.083494e-01 5.751990e-01 3.948984e-01 3.966607e-01 3.984391e-01 3.913287e-01

1141 9.082627e-01 9.083250e-01 9.083991e-01 9.084743e-01 9.085408e-01 5.765412e-01 3.953404e-01 3.971122e-01 3.989003e-01 3.917520e-01

1142 9.084497e-01 9.085118e-01 9.085857e-01 9.086608e-01 9.087272e-01 5.778866e-01 3.957991e-01 3.975807e-01 3.993787e-01 3.921914e-01

1143 9.086318e-01 9.086938e-01 9.087675e-01 9.088425e-01 9.089087e-01 5.792327e-01 3.962747e-01 3.980663e-01 3.998744e-01 3.926472e-01

1144 9.088093e-01 9.088712e-01 9.089447e-01 9.090195e-01 9.090855e-01 5.805769e-01 3.967674e-01 3.985691e-01 4.003877e-01 3.931196e-01

1145 9.089825e-01 9.090441e-01 9.091176e-01 9.091922e-01 9.092581e-01 5.819169e-01 3.972772e-01 3.990894e-01 4.009187e-01 3.936088e-01

1146 9.091514e-01 9.092130e-01 9.092863e-01 9.093608e-01 9.094265e-01 5.832502e-01 3.978044e-01 3.996272e-01 4.014673e-01 3.941148e-01

1147 9.093165e-01 9.093779e-01 9.094511e-01 9.095254e-01 9.095910e-01 5.845746e-01 3.983490e-01 4.001826e-01 4.020337e-01 3.946379e-01

1148 9.094778e-01 9.095391e-01 9.096121e-01 9.096864e-01 9.097518e-01 5.858876e-01 3.989108e-01 4.007555e-01 4.026178e-01 3.951778e-01

1149 9.096356e-01 9.096967e-01 9.097697e-01 9.098438e-01 9.099091e-01 5.871872e-01 3.994900e-01 4.013458e-01 4.032195e-01 3.957348e-01

1150 9.097900e-01 9.098510e-01 9.099239e-01 9.099979e-01 9.100631e-01 5.884712e-01 4.000865e-01 4.019535e-01 4.038387e-01 3.963087e-01

1151 9.099412e-01 9.100022e-01 9.100749e-01 9.101488e-01 9.102139e-01 5.897377e-01 4.006999e-01 4.025785e-01 4.044752e-01 3.968993e-01

1152 9.100894e-01 9.101503e-01 9.102229e-01 9.102967e-01 9.103617e-01 5.909850e-01 4.013303e-01 4.032203e-01 4.051288e-01 3.975066e-01

1153 9.102348e-01 9.102955e-01 9.103681e-01 9.104418e-01 9.105067e-01 5.922113e-01 4.019772e-01 4.038789e-01 4.057991e-01 3.981303e-01

1154 9.103774e-01 9.104380e-01 9.105105e-01 9.105841e-01 9.106489e-01 5.934150e-01 4.026405e-01 4.045537e-01 4.064858e-01 3.987701e-01

1155 9.105174e-01 9.105780e-01 9.106503e-01 9.107238e-01 9.107885e-01 5.945948e-01 4.033196e-01 4.052446e-01 4.071885e-01 3.994258e-01

1156 9.106549e-01 9.107154e-01 9.107877e-01 9.108611e-01 9.109257e-01 5.957495e-01 4.040142e-01 4.059509e-01 4.079066e-01 4.000968e-01

1157 9.107900e-01 9.108505e-01 9.109227e-01 9.109960e-01 9.110605e-01 5.968779e-01 4.047237e-01 4.066721e-01 4.086397e-01 4.007828e-01

1158 9.109229e-01 9.109833e-01 9.110554e-01 9.111287e-01 9.111931e-01 5.979792e-01 4.054476e-01 4.074077e-01 4.093871e-01 4.014832e-01

1159 9.110537e-01 9.111140e-01 9.111860e-01 9.112592e-01 9.113236e-01 5.990525e-01 4.061854e-01 4.081569e-01 4.101481e-01 4.021975e-01

1160 9.111824e-01 9.112426e-01 9.113146e-01 9.113877e-01 9.114520e-01 6.000973e-01 4.069362e-01 4.089192e-01 4.109219e-01 4.029251e-01

1161 9.113091e-01 9.113693e-01 9.114412e-01 9.115142e-01 9.115785e-01 6.011131e-01 4.076993e-01 4.096937e-01 4.117079e-01 4.036652e-01

1162 9.114339e-01 9.114941e-01 9.115659e-01 9.116389e-01 9.117031e-01 6.020995e-01 4.084740e-01 4.104797e-01 4.125052e-01 4.044171e-01

1163 9.115570e-01 9.116171e-01 9.116888e-01 9.117617e-01 9.118259e-01 6.030564e-01 4.092595e-01 4.112762e-01 4.133128e-01 4.051800e-01

1164 9.116784e-01 9.117384e-01 9.118100e-01 9.118829e-01 9.119470e-01 6.039837e-01 4.100548e-01 4.120823e-01 4.141299e-01 4.059531e-01

1165 9.117980e-01 9.118580e-01 9.119296e-01 9.120024e-01 9.120664e-01 6.048813e-01 4.108590e-01 4.128971e-01 4.149554e-01 4.067356e-01

1166 9.119162e-01 9.119761e-01 9.120476e-01 9.121204e-01 9.121843e-01 6.057495e-01 4.116710e-01 4.137196e-01 4.157883e-01 4.075264e-01

1167 9.120328e-01 9.120926e-01 9.121641e-01 9.122368e-01 9.123007e-01 6.065886e-01 4.124900e-01 4.145487e-01 4.166276e-01 4.083246e-01

1168 9.121479e-01 9.122077e-01 9.122792e-01 9.123518e-01 9.124156e-01 6.073988e-01 4.133149e-01 4.153834e-01 4.174722e-01 4.091292e-01

1169 9.122617e-01 9.123215e-01 9.123928e-01 9.124654e-01 9.125292e-01 6.081806e-01 4.141446e-01 4.162226e-01 4.183209e-01 4.099392e-01

1170 9.123741e-01 9.124338e-01 9.125052e-01 9.125776e-01 9.126414e-01 6.089344e-01 4.149779e-01 4.170652e-01 4.191727e-01 4.107534e-01

1171 9.124852e-01 9.125449e-01 9.126162e-01 9.126886e-01 9.127523e-01 6.096609e-01 4.158139e-01 4.179100e-01 4.200265e-01 4.115710e-01

1172 9.125951e-01 9.126547e-01 9.127260e-01 9.127983e-01 9.128620e-01 6.103607e-01 4.166513e-01 4.187560e-01 4.208810e-01 4.123907e-01

1173 9.127037e-01 9.127634e-01 9.128345e-01 9.129069e-01 9.129705e-01 6.110343e-01 4.174891e-01 4.196020e-01 4.217351e-01 4.132115e-01

1174 9.128113e-01 9.128708e-01 9.129420e-01 9.130142e-01 9.130778e-01 6.116826e-01 4.183262e-01 4.204469e-01 4.225878e-01 4.140322e-01

1175 9.129177e-01 9.129772e-01 9.130483e-01 9.131205e-01 9.131841e-01 6.123063e-01 4.191615e-01 4.212895e-01 4.234378e-01 4.148519e-01

1176 9.130230e-01 9.130825e-01 9.131535e-01 9.132257e-01 9.132892e-01 6.129060e-01 4.199938e-01 4.221289e-01 4.242842e-01 4.156694e-01

1177 9.131272e-01 9.131867e-01 9.132577e-01 9.133298e-01 9.133933e-01 6.134827e-01 4.208222e-01 4.229639e-01 4.251258e-01 4.164837e-01

1178 9.132305e-01 9.132900e-01 9.133609e-01 9.134330e-01 9.134964e-01 6.140370e-01 4.216455e-01 4.237935e-01 4.259616e-01 4.172937e-01

1179 9.133328e-01 9.133922e-01 9.134631e-01 9.135351e-01 9.135986e-01 6.145698e-01 4.224628e-01 4.246166e-01 4.267906e-01 4.180985e-01

1180 9.134341e-01 9.134935e-01 9.135644e-01 9.136364e-01 9.136998e-01 6.150818e-01 4.232731e-01 4.254325e-01 4.276119e-01 4.188970e-01

1181 9.135346e-01 9.135939e-01 9.136647e-01 9.137367e-01 9.138000e-01 6.155740e-01 4.240755e-01 4.262400e-01 4.284244e-01 4.196884e-01

1182 9.136341e-01 9.136935e-01 9.137642e-01 9.138361e-01 9.138994e-01 6.160469e-01 4.248691e-01 4.270383e-01 4.292275e-01 4.204718e-01

1183 9.137328e-01 9.137921e-01 9.138628e-01 9.139346e-01 9.139979e-01 6.165015e-01 4.256530e-01 4.278267e-01 4.300201e-01 4.212462e-01

1184 9.138306e-01 9.138899e-01 9.139606e-01 9.140324e-01 9.140956e-01 6.169386e-01 4.264266e-01 4.286042e-01 4.308017e-01 4.220110e-01

1185 9.139276e-01 9.139869e-01 9.140575e-01 9.141293e-01 9.141925e-01 6.173587e-01 4.271890e-01 4.293703e-01 4.315714e-01 4.227653e-01

1186 9.140238e-01 9.140831e-01 9.141537e-01 9.142254e-01 9.142886e-01 6.177628e-01 4.279396e-01 4.301243e-01 4.323287e-01 4.235085e-01

1187 9.141193e-01 9.141785e-01 9.142491e-01 9.143207e-01 9.143839e-01 6.181515e-01 4.286778e-01 4.308656e-01 4.330729e-01 4.242400e-01

1188 9.142140e-01 9.142732e-01 9.143437e-01 9.144153e-01 9.144785e-01 6.185255e-01 4.294031e-01 4.315935e-01 4.338035e-01 4.249591e-01

1189 9.143080e-01 9.143672e-01 9.144376e-01 9.145092e-01 9.145723e-01 6.188856e-01 4.301149e-01 4.323078e-01 4.345201e-01 4.256653e-01

1190 9.144012e-01 9.144604e-01 9.145308e-01 9.146023e-01 9.146654e-01 6.192322e-01 4.308128e-01 4.330078e-01 4.352222e-01 4.263582e-01

1191 9.144938e-01 9.145529e-01 9.146233e-01 9.146948e-01 9.147579e-01 6.195662e-01 4.314964e-01 4.336933e-01 4.359096e-01 4.270374e-01

1192 9.145857e-01 9.146448e-01 9.147151e-01 9.147865e-01 9.148496e-01 6.198880e-01 4.321654e-01 4.343640e-01 4.365819e-01 4.277025e-01

1193 9.146769e-01 9.147360e-01 9.148062e-01 9.148776e-01 9.149407e-01 6.201984e-01 4.328196e-01 4.350196e-01 4.372388e-01 4.283533e-01

1194 9.147674e-01 9.148265e-01 9.148967e-01 9.149681e-01 9.150311e-01 6.204978e-01 4.334587e-01 4.356599e-01 4.378803e-01 4.289894e-01

1195 9.148574e-01 9.149164e-01 9.149866e-01 9.150579e-01 9.151209e-01 6.207868e-01 4.340826e-01 4.362848e-01 4.385061e-01 4.296108e-01

1196 9.149467e-01 9.150057e-01 9.150759e-01 9.151471e-01 9.152101e-01 6.210659e-01 4.346912e-01 4.368942e-01 4.391163e-01 4.302172e-01

1197 9.150354e-01 9.150944e-01 9.151645e-01 9.152357e-01 9.152987e-01 6.213356e-01 4.352844e-01 4.374881e-01 4.397108e-01 4.308086e-01

1198 9.151235e-01 9.151825e-01 9.152526e-01 9.153237e-01 9.153867e-01 6.215964e-01 4.358622e-01 4.380665e-01 4.402896e-01 4.313849e-01

1199 9.152110e-01 9.152700e-01 9.153400e-01 9.154111e-01 9.154741e-01 6.218488e-01 4.364247e-01 4.386293e-01 4.408527e-01 4.319462e-01

1200 9.152979e-01 9.153569e-01 9.154269e-01 9.154980e-01 9.155609e-01 6.220931e-01 4.369719e-01 4.391767e-01 4.414004e-01 4.324925e-01

1201 9.153843e-01 9.154433e-01 9.155132e-01 9.155843e-01 9.156472e-01 6.223298e-01 4.375040e-01 4.397089e-01 4.419326e-01 4.330239e-01

1202 9.154702e-01 9.155291e-01 9.155990e-01 9.156700e-01 9.157329e-01 6.225593e-01 4.380210e-01 4.402259e-01 4.424496e-01 4.335405e-01

1203 9.155555e-01 9.156144e-01 9.156843e-01 9.157552e-01 9.158181e-01 6.227819e-01 4.385232e-01 4.407280e-01 4.429516e-01 4.340425e-01

1204 9.156402e-01 9.156992e-01 9.157690e-01 9.158399e-01 9.159027e-01 6.229981e-01 4.390107e-01 4.412154e-01 4.434388e-01 4.345300e-01

1205 9.157245e-01 9.157834e-01 9.158532e-01 9.159240e-01 9.159868e-01 6.232081e-01 4.394838e-01 4.416883e-01 4.439114e-01 4.350032e-01

1206 9.158082e-01 9.158671e-01 9.159368e-01 9.160077e-01 9.160705e-01 6.234122e-01 4.399428e-01 4.421470e-01 4.443698e-01 4.354625e-01

1207 9.158915e-01 9.159504e-01 9.160200e-01 9.160908e-01 9.161536e-01 6.236108e-01 4.403879e-01 4.425918e-01 4.448141e-01 4.359079e-01

1208 9.159742e-01 9.160331e-01 9.161027e-01 9.161734e-01 9.162362e-01 6.238042e-01 4.408194e-01 4.430229e-01 4.452448e-01 4.363399e-01

1209 9.160565e-01 9.161153e-01 9.161849e-01 9.162556e-01 9.163183e-01 6.239926e-01 4.412376e-01 4.434407e-01 4.456622e-01 4.367587e-01

1210 9.161382e-01 9.161971e-01 9.162666e-01 9.163372e-01 9.164000e-01 6.241763e-01 4.416429e-01 4.438454e-01 4.460665e-01 4.371646e-01

1211 9.162195e-01 9.162784e-01 9.163478e-01 9.164184e-01 9.164812e-01 6.243555e-01 4.420355e-01 4.442376e-01 4.464581e-01 4.375579e-01

1212 9.163004e-01 9.163592e-01 9.164286e-01 9.164992e-01 9.165619e-01 6.245305e-01 4.424157e-01 4.446174e-01 4.468374e-01 4.379389e-01

1213 9.163808e-01 9.164396e-01 9.165089e-01 9.165795e-01 9.166421e-01 6.247015e-01 4.427841e-01 4.449852e-01 4.472047e-01 4.383080e-01

1214 9.164607e-01 9.165195e-01 9.165888e-01 9.166593e-01 9.167219e-01 6.248686e-01 4.431408e-01 4.453414e-01 4.475603e-01 4.386656e-01

1215 9.165402e-01 9.165989e-01 9.166682e-01 9.167387e-01 9.168013e-01 6.250322e-01 4.434862e-01 4.456863e-01 4.479047e-01 4.390119e-01

1216 9.166192e-01 9.166780e-01 9.167472e-01 9.168176e-01 9.168802e-01 6.251923e-01 4.438208e-01 4.460203e-01 4.482382e-01 4.393472e-01

1217 9.166978e-01 9.167566e-01 9.168258e-01 9.168961e-01 9.169587e-01 6.253491e-01 4.441448e-01 4.463438e-01 4.485611e-01 4.396721e-01

1218 9.167760e-01 9.168348e-01 9.169039e-01 9.169742e-01 9.170368e-01 6.255029e-01 4.444585e-01 4.466570e-01 4.488739e-01 4.399867e-01

1219 9.168538e-01 9.169125e-01 9.169816e-01 9.170518e-01 9.171144e-01 6.256537e-01 4.447624e-01 4.469604e-01 4.491767e-01 4.402914e-01

1220 9.169311e-01 9.169899e-01 9.170589e-01 9.171291e-01 9.171916e-01 6.258017e-01 4.450568e-01 4.472543e-01 4.494701e-01 4.405867e-01

1221 9.170081e-01 9.170668e-01 9.171358e-01 9.172059e-01 9.172685e-01 6.259471e-01 4.453420e-01 4.475390e-01 4.497543e-01 4.408727e-01

1222 9.170846e-01 9.171433e-01 9.172123e-01 9.172823e-01 9.173449e-01 6.260900e-01 4.456184e-01 4.478149e-01 4.500297e-01 4.411498e-01

1223 9.171608e-01 9.172194e-01 9.172884e-01 9.173584e-01 9.174209e-01 6.262305e-01 4.458862e-01 4.480823e-01 4.502966e-01 4.414184e-01

1224 9.172365e-01 9.172952e-01 9.173640e-01 9.174340e-01 9.174965e-01 6.263687e-01 4.461458e-01 4.483415e-01 4.505553e-01 4.416788e-01

1225 9.173119e-01 9.173705e-01 9.174393e-01 9.175093e-01 9.175717e-01 6.265047e-01 4.463976e-01 4.485928e-01 4.508062e-01 4.419313e-01

1226 9.173868e-01 9.174455e-01 9.175142e-01 9.175841e-01 9.176466e-01 6.266387e-01 4.466417e-01 4.488365e-01 4.510495e-01 4.421762e-01

1227 9.174614e-01 9.175201e-01 9.175888e-01 9.176586e-01 9.177210e-01 6.267708e-01 4.468786e-01 4.490730e-01 4.512856e-01 4.424137e-01

1228 9.175356e-01 9.175943e-01 9.176629e-01 9.177327e-01 9.177951e-01 6.269009e-01 4.471086e-01 4.493026e-01 4.515148e-01 4.426443e-01

1229 9.176095e-01 9.176681e-01 9.177367e-01 9.178064e-01 9.178688e-01 6.270293e-01 4.473318e-01 4.495254e-01 4.517373e-01 4.428681e-01

1230 9.176830e-01 9.177416e-01 9.178101e-01 9.178798e-01 9.179422e-01 6.271561e-01 4.475485e-01 4.497418e-01 4.519533e-01 4.430855e-01

1231 9.177561e-01 9.178147e-01 9.178832e-01 9.179528e-01 9.180151e-01 6.272812e-01 4.477592e-01 4.499521e-01 4.521633e-01 4.432967e-01

1232 9.178288e-01 9.178874e-01 9.179558e-01 9.180254e-01 9.180877e-01 6.274047e-01 4.479639e-01 4.501565e-01 4.523674e-01 4.435019e-01

1233 9.179012e-01 9.179598e-01 9.180282e-01 9.180977e-01 9.181600e-01 6.275268e-01 4.481630e-01 4.503553e-01 4.525659e-01 4.437015e-01

1234 9.179733e-01 9.180318e-01 9.181002e-01 9.181696e-01 9.182319e-01 6.276475e-01 4.483566e-01 4.505487e-01 4.527589e-01 4.438956e-01

1235 9.180450e-01 9.181035e-01 9.181718e-01 9.182412e-01 9.183035e-01 6.277669e-01 4.485451e-01 4.507369e-01 4.529469e-01 4.440846e-01

1236 9.181163e-01 9.181748e-01 9.182431e-01 9.183124e-01 9.183747e-01 6.278850e-01 4.487286e-01 4.509201e-01 4.531299e-01 4.442685e-01

1237 9.181874e-01 9.182458e-01 9.183140e-01 9.183833e-01 9.184456e-01 6.280019e-01 4.489074e-01 4.510987e-01 4.533082e-01 4.444477e-01

1238 9.182580e-01 9.183165e-01 9.183846e-01 9.184538e-01 9.185161e-01 6.281176e-01 4.490817e-01 4.512727e-01 4.534820e-01 4.446223e-01

1239 9.183284e-01 9.183868e-01 9.184549e-01 9.185241e-01 9.185863e-01 6.282322e-01 4.492516e-01 4.514424e-01 4.536515e-01 4.447926e-01

1240 9.183984e-01 9.184568e-01 9.185248e-01 9.185939e-01 9.186561e-01 6.283457e-01 4.494174e-01 4.516080e-01 4.538169e-01 4.449587e-01

1241 9.184681e-01 9.185265e-01 9.185944e-01 9.186635e-01 9.187257e-01 6.284582e-01 4.495792e-01 4.517696e-01 4.539783e-01 4.451208e-01

1242 9.185374e-01 9.185959e-01 9.186637e-01 9.187327e-01 9.187949e-01 6.285697e-01 4.497372e-01 4.519275e-01 4.541360e-01 4.452792e-01

1243 9.186065e-01 9.186649e-01 9.187327e-01 9.188016e-01 9.188638e-01 6.286803e-01 4.498917e-01 4.520817e-01 4.542901e-01 4.454339e-01

1244 9.186752e-01 9.187336e-01 9.188014e-01 9.188702e-01 9.189324e-01 6.287899e-01 4.500427e-01 4.522326e-01 4.544408e-01 4.455851e-01

1245 9.187436e-01 9.188020e-01 9.188697e-01 9.189385e-01 9.190006e-01 6.288987e-01 4.501904e-01 4.523801e-01 4.545882e-01 4.457331e-01

1246 9.188117e-01 9.188701e-01 9.189377e-01 9.190065e-01 9.190686e-01 6.290067e-01 4.503349e-01 4.525245e-01 4.547325e-01 4.458779e-01

1247 9.188796e-01 9.189379e-01 9.190054e-01 9.190741e-01 9.191362e-01 6.291139e-01 4.504765e-01 4.526660e-01 4.548738e-01 4.460196e-01

1248 9.189470e-01 9.190053e-01 9.190729e-01 9.191415e-01 9.192035e-01 6.292202e-01 4.506152e-01 4.528046e-01 4.550123e-01 4.461585e-01

1249 9.190142e-01 9.190725e-01 9.191400e-01 9.192085e-01 9.192705e-01 6.293259e-01 4.507512e-01 4.529404e-01 4.551480e-01 4.462947e-01

1250 9.190811e-01 9.191394e-01 9.192068e-01 9.192753e-01 9.193373e-01 6.294308e-01 4.508846e-01 4.530737e-01 4.552812e-01 4.464283e-01

1251 9.191477e-01 9.192060e-01 9.192733e-01 9.193417e-01 9.194037e-01 6.295351e-01 4.510155e-01 4.532045e-01 4.554119e-01 4.465593e-01

1252 9.192141e-01 9.192723e-01 9.193395e-01 9.194079e-01 9.194698e-01 6.296387e-01 4.511440e-01 4.533329e-01 4.555402e-01 4.466880e-01

1253 9.192801e-01 9.193383e-01 9.194055e-01 9.194738e-01 9.195357e-01 6.297416e-01 4.512703e-01 4.534591e-01 4.556663e-01 4.468144e-01

1254 9.193458e-01 9.194040e-01 9.194711e-01 9.195393e-01 9.196012e-01 6.298439e-01 4.513944e-01 4.535831e-01 4.557903e-01 4.469387e-01

1255 9.194113e-01 9.194694e-01 9.195365e-01 9.196046e-01 9.196665e-01 6.299457e-01 4.515165e-01 4.537051e-01 4.559122e-01 4.470609e-01

1256 9.194765e-01 9.195346e-01 9.196016e-01 9.196697e-01 9.197315e-01 6.300468e-01 4.516366e-01 4.538251e-01 4.560322e-01 4.471812e-01

1257 9.195414e-01 9.195995e-01 9.196664e-01 9.197344e-01 9.197962e-01 6.301474e-01 4.517548e-01 4.539433e-01 4.561503e-01 4.472995e-01

1258 9.196060e-01 9.196641e-01 9.197309e-01 9.197988e-01 9.198606e-01 6.302475e-01 4.518713e-01 4.540597e-01 4.562666e-01 4.474161e-01

1259 9.196703e-01 9.197284e-01 9.197952e-01 9.198630e-01 9.199248e-01 6.303470e-01 4.519861e-01 4.541744e-01 4.563813e-01 4.475311e-01

1260 9.197344e-01 9.197925e-01 9.198591e-01 9.199269e-01 9.199887e-01 6.304460e-01 4.520993e-01 4.542875e-01 4.564943e-01 4.476444e-01

1261 9.197983e-01 9.198563e-01 9.199229e-01 9.199906e-01 9.200523e-01 6.305445e-01 4.522109e-01 4.543990e-01 4.566058e-01 4.477561e-01

1262 9.205600e-01 9.206165e-01 9.206815e-01 9.207477e-01 9.208077e-01 6.308254e-01 4.522542e-01 4.544438e-01 4.566521e-01 4.477966e-01

1263 9.212879e-01 9.213428e-01 9.214063e-01 9.214709e-01 9.215293e-01 6.311220e-01 4.523000e-01 4.544911e-01 4.567010e-01 4.478394e-01

1264 9.219831e-01 9.220365e-01 9.220986e-01 9.221618e-01 9.222185e-01 6.314353e-01 4.523484e-01 4.545412e-01 4.567528e-01 4.478846e-01

1265 9.226470e-01 9.226990e-01 9.227597e-01 9.228214e-01 9.228766e-01 6.317659e-01 4.523995e-01 4.545941e-01 4.568075e-01 4.479325e-01

1266 9.232810e-01 9.233315e-01 9.233909e-01 9.234512e-01 9.235050e-01 6.321146e-01 4.524536e-01 4.546500e-01 4.568653e-01 4.479830e-01

1267 9.238861e-01 9.239353e-01 9.239934e-01 9.240524e-01 9.241047e-01 6.324819e-01 4.525107e-01 4.547090e-01 4.569263e-01 4.480364e-01

1268 9.244639e-01 9.245117e-01 9.245686e-01 9.246263e-01 9.246773e-01 6.328688e-01 4.525710e-01 4.547713e-01 4.569907e-01 4.480928e-01

1269 9.250153e-01 9.250620e-01 9.251176e-01 9.251741e-01 9.252238e-01 6.332759e-01 4.526347e-01 4.548372e-01 4.570588e-01 4.481524e-01

1270 9.255418e-01 9.255872e-01 9.256417e-01 9.256971e-01 9.257454e-01 6.337039e-01 4.527019e-01 4.549066e-01 4.571306e-01 4.482152e-01

1271 9.260444e-01 9.260887e-01 9.261421e-01 9.261963e-01 9.262435e-01 6.341536e-01 4.527727e-01 4.549799e-01 4.572063e-01 4.482816e-01

1272 9.265243e-01 9.265675e-01 9.266198e-01 9.266730e-01 9.267190e-01 6.346256e-01 4.528475e-01 4.550571e-01 4.572862e-01 4.483515e-01

1273 9.269826e-01 9.270247e-01 9.270761e-01 9.271282e-01 9.271731e-01 6.351207e-01 4.529263e-01 4.551386e-01 4.573704e-01 4.484253e-01

1274 9.274203e-01 9.274614e-01 9.275118e-01 9.275629e-01 9.276068e-01 6.356395e-01 4.530094e-01 4.552245e-01 4.574591e-01 4.485030e-01

1275 9.278385e-01 9.278787e-01 9.279282e-01 9.279783e-01 9.280212e-01 6.361826e-01 4.530970e-01 4.553149e-01 4.575526e-01 4.485850e-01

1276 9.282382e-01 9.282774e-01 9.283260e-01 9.283753e-01 9.284172e-01 6.367506e-01 4.531892e-01 4.554102e-01 4.576510e-01 4.486713e-01

1277 9.286202e-01 9.286586e-01 9.287064e-01 9.287549e-01 9.287958e-01 6.373441e-01 4.532863e-01 4.555105e-01 4.577547e-01 4.487622e-01

1278 9.289856e-01 9.290232e-01 9.290702e-01 9.291178e-01 9.291579e-01 6.379637e-01 4.533885e-01 4.556161e-01 4.578638e-01 4.488579e-01

1279 9.293351e-01 9.293719e-01 9.294182e-01 9.294651e-01 9.295043e-01 6.386097e-01 4.534960e-01 4.557272e-01 4.579785e-01 4.489586e-01

1280 9.296697e-01 9.297058e-01 9.297513e-01 9.297974e-01 9.298359e-01 6.392825e-01 4.536091e-01 4.558440e-01 4.580992e-01 4.490645e-01

1281 9.299901e-01 9.300254e-01 9.300703e-01 9.301157e-01 9.301535e-01 6.399826e-01 4.537280e-01 4.559668e-01 4.582260e-01 4.491759e-01

1282 9.302970e-01 9.303317e-01 9.303759e-01 9.304207e-01 9.304577e-01 6.407101e-01 4.538530e-01 4.560958e-01 4.583593e-01 4.492929e-01

1283 9.305912e-01 9.306252e-01 9.306688e-01 9.307130e-01 9.307493e-01 6.414652e-01 4.539842e-01 4.562314e-01 4.584993e-01 4.494159e-01

1284 9.308733e-01 9.309067e-01 9.309498e-01 9.309934e-01 9.310291e-01 6.422480e-01 4.541221e-01 4.563737e-01 4.586462e-01 4.495451e-01

1285 9.311441e-01 9.311769e-01 9.312194e-01 9.312624e-01 9.312975e-01 6.430586e-01 4.542668e-01 4.565231e-01 4.588005e-01 4.496808e-01

1286 9.314040e-01 9.314363e-01 9.314783e-01 9.315208e-01 9.315553e-01 6.438968e-01 4.544185e-01 4.566798e-01 4.589622e-01 4.498231e-01

1287 9.316538e-01 9.316856e-01 9.317271e-01 9.317690e-01 9.318030e-01 6.447624e-01 4.545777e-01 4.568441e-01 4.591318e-01 4.499724e-01

1288 9.318939e-01 9.319252e-01 9.319662e-01 9.320077e-01 9.320412e-01 6.456551e-01 4.547446e-01 4.570163e-01 4.593096e-01 4.501289e-01

1289 9.321249e-01 9.321558e-01 9.321963e-01 9.322374e-01 9.322703e-01 6.465745e-01 4.549195e-01 4.571968e-01 4.594958e-01 4.502930e-01

1290 9.323473e-01 9.323777e-01 9.324178e-01 9.324585e-01 9.324910e-01 6.475200e-01 4.551026e-01 4.573857e-01 4.596907e-01 4.504648e-01

1291 9.325616e-01 9.325915e-01 9.326312e-01 9.326715e-01 9.327035e-01 6.484909e-01 4.552942e-01 4.575834e-01 4.598947e-01 4.506447e-01

1292 9.327681e-01 9.327976e-01 9.328370e-01 9.328768e-01 9.329085e-01 6.494866e-01 4.554948e-01 4.577903e-01 4.601081e-01 4.508330e-01

1293 9.329673e-01 9.329965e-01 9.330355e-01 9.330749e-01 9.331062e-01 6.505059e-01 4.557045e-01 4.580065e-01 4.603311e-01 4.510300e-01

1294 9.331596e-01 9.331885e-01 9.332271e-01 9.332662e-01 9.332971e-01 6.515480e-01 4.559236e-01 4.582325e-01 4.605642e-01 4.512359e-01

1295 9.333454e-01 9.333739e-01 9.334123e-01 9.334511e-01 9.334816e-01 6.526117e-01 4.561526e-01 4.584686e-01 4.608075e-01 4.514510e-01

1296 9.335251e-01 9.335533e-01 9.335913e-01 9.336298e-01 9.336600e-01 6.536957e-01 4.563916e-01 4.587149e-01 4.610615e-01 4.516757e-01

1297 9.336989e-01 9.337268e-01 9.337645e-01 9.338027e-01 9.338326e-01 6.547987e-01 4.566409e-01 4.589719e-01 4.613264e-01 4.519102e-01

1298 9.338672e-01 9.338948e-01 9.339323e-01 9.339701e-01 9.339997e-01 6.559190e-01 4.569010e-01 4.592399e-01 4.616025e-01 4.521549e-01

1299 9.340303e-01 9.340576e-01 9.340948e-01 9.341324e-01 9.341617e-01 6.570553e-01 4.571720e-01 4.595191e-01 4.618902e-01 4.524099e-01

1300 9.341884e-01 9.342155e-01 9.342524e-01 9.342898e-01 9.343188e-01 6.582057e-01 4.574542e-01 4.598098e-01 4.621896e-01 4.526757e-01

1301 9.343419e-01 9.343687e-01 9.344054e-01 9.344425e-01 9.344713e-01 6.593685e-01 4.577480e-01 4.601124e-01 4.625012e-01 4.529524e-01

1302 9.344909e-01 9.345175e-01 9.345540e-01 9.345909e-01 9.346194e-01 6.605418e-01 4.580536e-01 4.604270e-01 4.628251e-01 4.532404e-01

1303 9.346357e-01 9.346621e-01 9.346984e-01 9.347351e-01 9.347634e-01 6.617239e-01 4.583713e-01 4.607540e-01 4.631617e-01 4.535398e-01

1304 9.347766e-01 9.348028e-01 9.348389e-01 9.348753e-01 9.349034e-01 6.629127e-01 4.587013e-01 4.610936e-01 4.635112e-01 4.538510e-01

1305 9.349137e-01 9.349397e-01 9.349756e-01 9.350119e-01 9.350397e-01 6.641062e-01 4.590438e-01 4.614460e-01 4.638738e-01 4.541743e-01

1306 9.350473e-01 9.350730e-01 9.351088e-01 9.351448e-01 9.351725e-01 6.653026e-01 4.593991e-01 4.618115e-01 4.642497e-01 4.545097e-01

1307 9.351774e-01 9.352030e-01 9.352386e-01 9.352745e-01 9.353019e-01 6.664996e-01 4.597674e-01 4.621902e-01 4.646391e-01 4.548576e-01

1308 9.353044e-01 9.353298e-01 9.353652e-01 9.354009e-01 9.354282e-01 6.676954e-01 4.601489e-01 4.625823e-01 4.650422e-01 4.552181e-01

1309 9.354283e-01 9.354536e-01 9.354888e-01 9.355244e-01 9.355515e-01 6.688879e-01 4.605437e-01 4.629881e-01 4.654592e-01 4.555914e-01

1310 9.355493e-01 9.355745e-01 9.356095e-01 9.356449e-01 9.356719e-01 6.700751e-01 4.609520e-01 4.634075e-01 4.658901e-01 4.559776e-01

1311 9.356676e-01 9.356926e-01 9.357275e-01 9.357628e-01 9.357896e-01 6.712552e-01 4.613738e-01 4.638408e-01 4.663351e-01 4.563770e-01

1312 9.357833e-01 9.358082e-01 9.358429e-01 9.358781e-01 9.359047e-01 6.724264e-01 4.618093e-01 4.642880e-01 4.667942e-01 4.567895e-01

1313 9.358965e-01 9.359213e-01 9.359559e-01 9.359909e-01 9.360174e-01 6.735867e-01 4.622586e-01 4.647491e-01 4.672675e-01 4.572153e-01

1314 9.360074e-01 9.360320e-01 9.360665e-01 9.361014e-01 9.361278e-01 6.747344e-01 4.627216e-01 4.652242e-01 4.677549e-01 4.576545e-01

1315 9.361161e-01 9.361405e-01 9.361749e-01 9.362097e-01 9.362359e-01 6.758681e-01 4.631984e-01 4.657132e-01 4.682565e-01 4.581070e-01

1316 9.362226e-01 9.362469e-01 9.362812e-01 9.363158e-01 9.363420e-01 6.769860e-01 4.636888e-01 4.662161e-01 4.687721e-01 4.585728e-01

1317 9.363271e-01 9.363513e-01 9.363855e-01 9.364200e-01 9.364460e-01 6.780868e-01 4.641929e-01 4.667328e-01 4.693017e-01 4.590519e-01

1318 9.364296e-01 9.364538e-01 9.364878e-01 9.365222e-01 9.365482e-01 6.791692e-01 4.647106e-01 4.672632e-01 4.698451e-01 4.595443e-01

1319 9.365304e-01 9.365544e-01 9.365884e-01 9.366227e-01 9.366485e-01 6.802320e-01 4.652416e-01 4.678071e-01 4.704020e-01 4.600497e-01

1320 9.366294e-01 9.366533e-01 9.366872e-01 9.367214e-01 9.367471e-01 6.812740e-01 4.657858e-01 4.683642e-01 4.709723e-01 4.605681e-01

1321 9.367267e-01 9.367506e-01 9.367843e-01 9.368184e-01 9.368440e-01 6.822943e-01 4.663429e-01 4.689343e-01 4.715557e-01 4.610993e-01

1322 9.368224e-01 9.368462e-01 9.368799e-01 9.369139e-01 9.369394e-01 6.832920e-01 4.669127e-01 4.695172e-01 4.721519e-01 4.616429e-01

1323 9.369166e-01 9.369403e-01 9.369739e-01 9.370078e-01 9.370333e-01 6.842665e-01 4.674948e-01 4.701124e-01 4.727604e-01 4.621988e-01

1324 9.370094e-01 9.370330e-01 9.370665e-01 9.371003e-01 9.371257e-01 6.852170e-01 4.680889e-01 4.707196e-01 4.733810e-01 4.627666e-01

1325 9.371007e-01 9.371243e-01 9.371577e-01 9.371914e-01 9.372167e-01 6.861432e-01 4.686946e-01 4.713384e-01 4.740130e-01 4.633460e-01

1326 9.371908e-01 9.372143e-01 9.372476e-01 9.372813e-01 9.373065e-01 6.870445e-01 4.693114e-01 4.719682e-01 4.746561e-01 4.639366e-01

1327 9.372796e-01 9.373030e-01 9.373363e-01 9.373698e-01 9.373950e-01 6.879208e-01 4.699389e-01 4.726087e-01 4.753097e-01 4.645379e-01

1328 9.373672e-01 9.373905e-01 9.374237e-01 9.374572e-01 9.374822e-01 6.887718e-01 4.705764e-01 4.732591e-01 4.759731e-01 4.651494e-01

1329 9.374536e-01 9.374769e-01 9.375100e-01 9.375434e-01 9.375684e-01 6.895975e-01 4.712235e-01 4.739189e-01 4.766458e-01 4.657707e-01

1330 9.375389e-01 9.375621e-01 9.375951e-01 9.376285e-01 9.376534e-01 6.903979e-01 4.718795e-01 4.745875e-01 4.773272e-01 4.664011e-01

1331 9.376231e-01 9.376463e-01 9.376792e-01 9.377125e-01 9.377374e-01 6.911731e-01 4.725438e-01 4.752643e-01 4.780165e-01 4.670401e-01

1332 9.377063e-01 9.377294e-01 9.377623e-01 9.377955e-01 9.378203e-01 6.919233e-01 4.732157e-01 4.759484e-01 4.787130e-01 4.676870e-01

1333 9.377885e-01 9.378116e-01 9.378444e-01 9.378775e-01 9.379023e-01 6.926486e-01 4.738945e-01 4.766392e-01 4.794159e-01 4.683413e-01

1334 9.378698e-01 9.378928e-01 9.379256e-01 9.379586e-01 9.379833e-01 6.933495e-01 4.745794e-01 4.773359e-01 4.801245e-01 4.690021e-01

1335 9.379502e-01 9.379732e-01 9.380058e-01 9.380388e-01 9.380635e-01 6.940264e-01 4.752698e-01 4.780378e-01 4.808379e-01 4.696688e-01

1336 9.380297e-01 9.380526e-01 9.380852e-01 9.381182e-01 9.381428e-01 6.946795e-01 4.759647e-01 4.787440e-01 4.815553e-01 4.703406e-01

1337 9.381083e-01 9.381312e-01 9.381638e-01 9.381967e-01 9.382212e-01 6.953095e-01 4.766635e-01 4.794536e-01 4.822760e-01 4.710168e-01

1338 9.381862e-01 9.382090e-01 9.382415e-01 9.382743e-01 9.382988e-01 6.959168e-01 4.773652e-01 4.801660e-01 4.829989e-01 4.716965e-01

1339 9.382633e-01 9.382861e-01 9.383185e-01 9.383513e-01 9.383757e-01 6.965020e-01 4.780691e-01 4.808801e-01 4.837234e-01 4.723791e-01

1340 9.383396e-01 9.383623e-01 9.383947e-01 9.384274e-01 9.384518e-01 6.970656e-01 4.787743e-01 4.815953e-01 4.844484e-01 4.730636e-01

1341 9.384152e-01 9.384379e-01 9.384702e-01 9.385029e-01 9.385272e-01 6.976082e-01 4.794799e-01 4.823105e-01 4.851732e-01 4.737493e-01

1342 9.384901e-01 9.385128e-01 9.385450e-01 9.385776e-01 9.386019e-01 6.981306e-01 4.801852e-01 4.830250e-01 4.858969e-01 4.744354e-01

1343 9.385643e-01 9.385869e-01 9.386192e-01 9.386517e-01 9.386759e-01 6.986332e-01 4.808894e-01 4.837379e-01 4.866186e-01 4.751210e-01

1344 9.386379e-01 9.386605e-01 9.386926e-01 9.387251e-01 9.387493e-01 6.991168e-01 4.815915e-01 4.844485e-01 4.873375e-01 4.758053e-01

1345 9.387108e-01 9.387334e-01 9.387655e-01 9.387979e-01 9.388221e-01 6.995820e-01 4.822907e-01 4.851558e-01 4.880528e-01 4.764876e-01

1346 9.387831e-01 9.388057e-01 9.388377e-01 9.388701e-01 9.388942e-01 7.000294e-01 4.829864e-01 4.858591e-01 4.887636e-01 4.771670e-01

1347 9.388549e-01 9.388774e-01 9.389094e-01 9.389417e-01 9.389658e-01 7.004596e-01 4.836777e-01 4.865576e-01 4.894693e-01 4.778428e-01

1348 9.389260e-01 9.389485e-01 9.389804e-01 9.390127e-01 9.390368e-01 7.008734e-01 4.843638e-01 4.872506e-01 4.901691e-01 4.785143e-01

1349 9.389966e-01 9.390191e-01 9.390510e-01 9.390832e-01 9.391072e-01 7.012714e-01 4.850441e-01 4.879373e-01 4.908622e-01 4.791806e-01

1350 9.390667e-01 9.390891e-01 9.391210e-01 9.391531e-01 9.391771e-01 7.016541e-01 4.857178e-01 4.886171e-01 4.915479e-01 4.798413e-01

1351 9.391362e-01 9.391586e-01 9.391904e-01 9.392225e-01 9.392465e-01 7.020223e-01 4.863843e-01 4.892893e-01 4.922257e-01 4.804954e-01

1352 9.392053e-01 9.392276e-01 9.392594e-01 9.392914e-01 9.393153e-01 7.023764e-01 4.870430e-01 4.899533e-01 4.928949e-01 4.811425e-01

1353 9.392738e-01 9.392961e-01 9.393278e-01 9.393598e-01 9.393837e-01 7.027171e-01 4.876932e-01 4.906085e-01 4.935550e-01 4.817819e-01

1354 9.393419e-01 9.393642e-01 9.393958e-01 9.394278e-01 9.394516e-01 7.030450e-01 4.883345e-01 4.912544e-01 4.942053e-01 4.824131e-01

1355 9.394095e-01 9.394317e-01 9.394633e-01 9.394952e-01 9.395191e-01 7.033607e-01 4.889664e-01 4.918905e-01 4.948456e-01 4.830355e-01

1356 9.394766e-01 9.394989e-01 9.395304e-01 9.395622e-01 9.395860e-01 7.036646e-01 4.895883e-01 4.925163e-01 4.954751e-01 4.836486e-01

1357 9.395433e-01 9.395655e-01 9.395970e-01 9.396288e-01 9.396526e-01 7.039573e-01 4.901998e-01 4.931314e-01 4.960937e-01 4.842521e-01

1358 9.396096e-01 9.396318e-01 9.396632e-01 9.396950e-01 9.397187e-01 7.042394e-01 4.908006e-01 4.937355e-01 4.967010e-01 4.848454e-01

1359 9.396755e-01 9.396976e-01 9.397290e-01 9.397607e-01 9.397844e-01 7.045112e-01 4.913903e-01 4.943282e-01 4.972965e-01 4.854283e-01

1360 9.397409e-01 9.397630e-01 9.397944e-01 9.398260e-01 9.398497e-01 7.047733e-01 4.919686e-01 4.949092e-01 4.978801e-01 4.860003e-01

1361 9.398059e-01 9.398280e-01 9.398593e-01 9.398909e-01 9.399146e-01 7.050262e-01 4.925352e-01 4.954783e-01 4.984516e-01 4.865613e-01

1362 9.398706e-01 9.398927e-01 9.399239e-01 9.399554e-01 9.399791e-01 7.052702e-01 4.930900e-01 4.960353e-01 4.990106e-01 4.871109e-01

1363 9.399349e-01 9.399569e-01 9.399881e-01 9.400196e-01 9.400432e-01 7.055058e-01 4.936328e-01 4.965800e-01 4.995572e-01 4.876490e-01

1364 9.399988e-01 9.400208e-01 9.400519e-01 9.400834e-01 9.401069e-01 7.057335e-01 4.941634e-01 4.971123e-01 5.000911e-01 4.881754e-01

1365 9.400623e-01 9.400843e-01 9.401154e-01 9.401468e-01 9.401703e-01 7.059535e-01 4.946817e-01 4.976322e-01 5.006124e-01 4.886899e-01

1366 9.401255e-01 9.401475e-01 9.401785e-01 9.402098e-01 9.402333e-01 7.061663e-01 4.951877e-01 4.981395e-01 5.011210e-01 4.891925e-01

1367 9.401883e-01 9.402103e-01 9.402413e-01 9.402725e-01 9.402960e-01 7.063722e-01 4.956813e-01 4.986343e-01 5.016168e-01 4.896831e-01

1368 9.402508e-01 9.402727e-01 9.403037e-01 9.403349e-01 9.403584e-01 7.065716e-01 4.961625e-01 4.991165e-01 5.020999e-01 4.901618e-01

1369 9.403130e-01 9.403349e-01 9.403658e-01 9.403970e-01 9.404204e-01 7.067647e-01 4.966314e-01 4.995862e-01 5.025703e-01 4.906283e-01

1370 9.403748e-01 9.403967e-01 9.404275e-01 9.404587e-01 9.404821e-01 7.069519e-01 4.970880e-01 5.000435e-01 5.030283e-01 4.910830e-01

1371 9.404363e-01 9.404582e-01 9.404890e-01 9.405200e-01 9.405434e-01 7.071335e-01 4.975324e-01 5.004885e-01 5.034737e-01 4.915257e-01

1372 9.404975e-01 9.405193e-01 9.405501e-01 9.405811e-01 9.406045e-01 7.073098e-01 4.979648e-01 5.009213e-01 5.039069e-01 4.919565e-01

1373 9.405584e-01 9.405802e-01 9.406109e-01 9.406419e-01 9.406652e-01 7.074810e-01 4.983852e-01 5.013420e-01 5.043279e-01 4.923757e-01

1374 9.406190e-01 9.406408e-01 9.406714e-01 9.407023e-01 9.407256e-01 7.076474e-01 4.987937e-01 5.017509e-01 5.047369e-01 4.927833e-01

1375 9.406793e-01 9.407010e-01 9.407316e-01 9.407625e-01 9.407858e-01 7.078092e-01 4.991907e-01 5.021480e-01 5.051341e-01 4.931794e-01

1376 9.407393e-01 9.407610e-01 9.407916e-01 9.408224e-01 9.408456e-01 7.079667e-01 4.995762e-01 5.025336e-01 5.055198e-01 4.935642e-01

1377 9.407990e-01 9.408207e-01 9.408512e-01 9.408820e-01 9.409052e-01 7.081200e-01 4.999505e-01 5.029079e-01 5.058940e-01 4.939380e-01

1378 9.408584e-01 9.408801e-01 9.409106e-01 9.409413e-01 9.409644e-01 7.082695e-01 5.003138e-01 5.032712e-01 5.062572e-01 4.943009e-01

1379 9.409176e-01 9.409393e-01 9.409696e-01 9.410003e-01 9.410235e-01 7.084152e-01 5.006663e-01 5.036236e-01 5.066095e-01 4.946532e-01

1380 9.409765e-01 9.409981e-01 9.410284e-01 9.410591e-01 9.410822e-01 7.085574e-01 5.010082e-01 5.039654e-01 5.069511e-01 4.949949e-01

1381 9.410351e-01 9.410567e-01 9.410870e-01 9.411176e-01 9.411406e-01 7.086962e-01 5.013398e-01 5.042969e-01 5.072824e-01 4.953265e-01

1382 9.410935e-01 9.411150e-01 9.411453e-01 9.411758e-01 9.411988e-01 7.088318e-01 5.016614e-01 5.046183e-01 5.076035e-01 4.956481e-01

1383 9.411516e-01 9.411731e-01 9.412033e-01 9.412338e-01 9.412568e-01 7.089644e-01 5.019732e-01 5.049299e-01 5.079149e-01 4.959600e-01

1384 9.412094e-01 9.412310e-01 9.412611e-01 9.412915e-01 9.413145e-01 7.090941e-01 5.022755e-01 5.052319e-01 5.082166e-01 4.962625e-01

1385 9.412670e-01 9.412885e-01 9.413186e-01 9.413489e-01 9.413719e-01 7.092211e-01 5.025685e-01 5.055247e-01 5.085091e-01 4.965557e-01

1386 9.413244e-01 9.413459e-01 9.413759e-01 9.414062e-01 9.414291e-01 7.093455e-01 5.028525e-01 5.058084e-01 5.087926e-01 4.968399e-01

1387 9.413815e-01 9.414029e-01 9.414329e-01 9.414631e-01 9.414860e-01 7.094675e-01 5.031278e-01 5.060835e-01 5.090673e-01 4.971155e-01

1388 9.414384e-01 9.414598e-01 9.414897e-01 9.415199e-01 9.415427e-01 7.095871e-01 5.033947e-01 5.063500e-01 5.093335e-01 4.973826e-01

1389 9.414950e-01 9.415164e-01 9.415462e-01 9.415764e-01 9.415992e-01 7.097044e-01 5.036534e-01 5.066084e-01 5.095916e-01 4.976416e-01

1390 9.415514e-01 9.415728e-01 9.416026e-01 9.416326e-01 9.416555e-01 7.098197e-01 5.039041e-01 5.068589e-01 5.098417e-01 4.978926e-01

1391 9.416076e-01 9.416289e-01 9.416587e-01 9.416887e-01 9.417115e-01 7.099329e-01 5.041472e-01 5.071017e-01 5.100842e-01 4.981360e-01

1392 9.416636e-01 9.416849e-01 9.417145e-01 9.417445e-01 9.417672e-01 7.100442e-01 5.043829e-01 5.073371e-01 5.103193e-01 4.983720e-01

1393 9.417193e-01 9.417406e-01 9.417702e-01 9.418001e-01 9.418228e-01 7.101537e-01 5.046114e-01 5.075653e-01 5.105473e-01 4.986009e-01

1394 9.417749e-01 9.417961e-01 9.418256e-01 9.418555e-01 9.418782e-01 7.102615e-01 5.048330e-01 5.077867e-01 5.107684e-01 4.988229e-01

1395 9.418302e-01 9.418514e-01 9.418808e-01 9.419106e-01 9.419333e-01 7.103676e-01 5.050480e-01 5.080015e-01 5.109829e-01 4.990382e-01

1396 9.418853e-01 9.419064e-01 9.419358e-01 9.419656e-01 9.419882e-01 7.104721e-01 5.052567e-01 5.082098e-01 5.111910e-01 4.992471e-01

1397 9.419401e-01 9.419613e-01 9.419906e-01 9.420203e-01 9.420429e-01 7.105752e-01 5.054591e-01 5.084121e-01 5.113929e-01 4.994499e-01

1398 9.419948e-01 9.420159e-01 9.420452e-01 9.420748e-01 9.420974e-01 7.106768e-01 5.056556e-01 5.086083e-01 5.115890e-01 4.996467e-01

1399 9.420493e-01 9.420704e-01 9.420996e-01 9.421291e-01 9.421517e-01 7.107771e-01 5.058464e-01 5.087989e-01 5.117793e-01 4.998377e-01

1400 9.421036e-01 9.421246e-01 9.421538e-01 9.421833e-01 9.422057e-01 7.108761e-01 5.060318e-01 5.089841e-01 5.119642e-01 5.000233e-01

1401 9.421577e-01 9.421787e-01 9.422078e-01 9.422372e-01 9.422596e-01 7.109738e-01 5.062118e-01 5.091639e-01 5.121439e-01 5.002036e-01

1402 9.422116e-01 9.422325e-01 9.422616e-01 9.422909e-01 9.423133e-01 7.110704e-01 5.063868e-01 5.093387e-01 5.123185e-01 5.003789e-01

1403 9.422653e-01 9.422862e-01 9.423152e-01 9.423444e-01 9.423668e-01 7.111659e-01 5.065569e-01 5.095087e-01 5.124883e-01 5.005492e-01

1404 9.423188e-01 9.423397e-01 9.423686e-01 9.423978e-01 9.424201e-01 7.112603e-01 5.067224e-01 5.096740e-01 5.126535e-01 5.007149e-01

1405 9.423721e-01 9.423930e-01 9.424218e-01 9.424509e-01 9.424732e-01 7.113537e-01 5.068834e-01 5.098349e-01 5.128141e-01 5.008761e-01

1406 9.424252e-01 9.424461e-01 9.424748e-01 9.425039e-01 9.425261e-01 7.114461e-01 5.070402e-01 5.099915e-01 5.129706e-01 5.010330e-01

1407 9.424782e-01 9.424990e-01 9.425277e-01 9.425566e-01 9.425789e-01 7.115375e-01 5.071928e-01 5.101439e-01 5.131229e-01 5.011857e-01

1408 9.425310e-01 9.425517e-01 9.425803e-01 9.426092e-01 9.426314e-01 7.116281e-01 5.073414e-01 5.102925e-01 5.132713e-01 5.013345e-01

1409 9.425836e-01 9.426043e-01 9.426328e-01 9.426617e-01 9.426838e-01 7.117179e-01 5.074863e-01 5.104373e-01 5.134160e-01 5.014796e-01

1410 9.426360e-01 9.426567e-01 9.426851e-01 9.427139e-01 9.427360e-01 7.118068e-01 5.076276e-01 5.105785e-01 5.135571e-01 5.016210e-01

1411 9.426882e-01 9.427089e-01 9.427373e-01 9.427660e-01 9.427880e-01 7.118950e-01 5.077654e-01 5.107162e-01 5.136947e-01 5.017589e-01

1412 9.427403e-01 9.427609e-01 9.427892e-01 9.428179e-01 9.428399e-01 7.119824e-01 5.079000e-01 5.108506e-01 5.138291e-01 5.018935e-01

1413 9.427922e-01 9.428128e-01 9.428410e-01 9.428696e-01 9.428916e-01 7.120691e-01 5.080313e-01 5.109819e-01 5.139603e-01 5.020250e-01

1414 9.428440e-01 9.428645e-01 9.428926e-01 9.429211e-01 9.429431e-01 7.121551e-01 5.081596e-01 5.111101e-01 5.140885e-01 5.021534e-01

1415 9.428956e-01 9.429160e-01 9.429441e-01 9.429725e-01 9.429944e-01 7.122405e-01 5.082851e-01 5.112355e-01 5.142138e-01 5.022789e-01

1416 9.429470e-01 9.429674e-01 9.429954e-01 9.430237e-01 9.430456e-01 7.123253e-01 5.084077e-01 5.113581e-01 5.143363e-01 5.024016e-01

1417 9.429983e-01 9.430186e-01 9.430466e-01 9.430748e-01 9.430966e-01 7.124094e-01 5.085277e-01 5.114781e-01 5.144563e-01 5.025217e-01

1418 9.430494e-01 9.430697e-01 9.430975e-01 9.431257e-01 9.431474e-01 7.124930e-01 5.086452e-01 5.115955e-01 5.145737e-01 5.026392e-01

1419 9.431003e-01 9.431206e-01 9.431484e-01 9.431764e-01 9.431981e-01 7.125761e-01 5.087603e-01 5.117105e-01 5.146886e-01 5.027544e-01

1420 9.431511e-01 9.431714e-01 9.431990e-01 9.432270e-01 9.432486e-01 7.126586e-01 5.088730e-01 5.118232e-01 5.148013e-01 5.028672e-01

1421 9.432018e-01 9.432220e-01 9.432495e-01 9.432774e-01 9.432990e-01 7.127406e-01 5.089836e-01 5.119338e-01 5.149118e-01 5.029778e-01

1422 9.432523e-01 9.432724e-01 9.432999e-01 9.433277e-01 9.433492e-01 7.128221e-01 5.090920e-01 5.120422e-01 5.150202e-01 5.030863e-01

1423 9.433027e-01 9.433227e-01 9.433501e-01 9.433778e-01 9.433993e-01 7.129032e-01 5.091985e-01 5.121486e-01 5.151266e-01 5.031928e-01

1424 9.433529e-01 9.433729e-01 9.434002e-01 9.434278e-01 9.434492e-01 7.129838e-01 5.093030e-01 5.122531e-01 5.152311e-01 5.032974e-01

1425 9.434030e-01 9.434229e-01 9.434501e-01 9.434776e-01 9.434990e-01 7.130640e-01 5.094057e-01 5.123558e-01 5.153338e-01 5.034001e-01

1426 9.434529e-01 9.434728e-01 9.434999e-01 9.435273e-01 9.435486e-01 7.131438e-01 5.095066e-01 5.124567e-01 5.154347e-01 5.035012e-01

1427 9.435027e-01 9.435226e-01 9.435495e-01 9.435769e-01 9.435981e-01 7.132231e-01 5.096059e-01 5.125559e-01 5.155340e-01 5.036005e-01

1428 9.435524e-01 9.435722e-01 9.435990e-01 9.436263e-01 9.436474e-01 7.133021e-01 5.097036e-01 5.126536e-01 5.156317e-01 5.036983e-01

1429 9.436019e-01 9.436216e-01 9.436484e-01 9.436755e-01 9.436966e-01 7.133807e-01 5.097997e-01 5.127498e-01 5.157278e-01 5.037946e-01

1430 9.436513e-01 9.436710e-01 9.436976e-01 9.437247e-01 9.437456e-01 7.134590e-01 5.098945e-01 5.128445e-01 5.158225e-01 5.038894e-01

1431 9.437006e-01 9.437202e-01 9.437467e-01 9.437736e-01 9.437946e-01 7.135369e-01 5.099878e-01 5.129378e-01 5.159159e-01 5.039829e-01

1432 9.437497e-01 9.437693e-01 9.437957e-01 9.438225e-01 9.438433e-01 7.136145e-01 5.100799e-01 5.130298e-01 5.160079e-01 5.040750e-01

1433 9.437988e-01 9.438182e-01 9.438445e-01 9.438712e-01 9.438920e-01 7.136917e-01 5.101706e-01 5.131206e-01 5.160987e-01 5.041659e-01

1434 9.438477e-01 9.438670e-01 9.438933e-01 9.439198e-01 9.439405e-01 7.137687e-01 5.102602e-01 5.132102e-01 5.161883e-01 5.042557e-01

1435 9.438964e-01 9.439157e-01 9.439418e-01 9.439683e-01 9.439889e-01 7.138453e-01 5.103487e-01 5.132987e-01 5.162768e-01 5.043443e-01

1436 9.439451e-01 9.439643e-01 9.439903e-01 9.440166e-01 9.440371e-01 7.139216e-01 5.104361e-01 5.133860e-01 5.163642e-01 5.044319e-01

1437 9.439937e-01 9.440128e-01 9.440386e-01 9.440648e-01 9.440852e-01 7.139977e-01 5.105225e-01 5.134724e-01 5.164505e-01 5.045184e-01

1438 9.440421e-01 9.440611e-01 9.440869e-01 9.441129e-01 9.441332e-01 7.140735e-01 5.106078e-01 5.135577e-01 5.165359e-01 5.046040e-01

1439 9.440904e-01 9.441094e-01 9.441350e-01 9.441609e-01 9.441811e-01 7.141490e-01 5.106923e-01 5.136421e-01 5.166203e-01 5.046887e-01

1440 9.441386e-01 9.441575e-01 9.441829e-01 9.442087e-01 9.442288e-01 7.142243e-01 5.107759e-01 5.137257e-01 5.167039e-01 5.047725e-01

1441 9.441867e-01 9.442055e-01 9.442308e-01 9.442564e-01 9.442764e-01 7.142993e-01 5.108586e-01 5.138084e-01 5.167866e-01 5.048555e-01

1442 9.444528e-01 9.444707e-01 9.444951e-01 9.445199e-01 9.445390e-01 7.148595e-01 5.109380e-01 5.138911e-01 5.168728e-01 5.049285e-01

1443 9.447076e-01 9.447247e-01 9.447483e-01 9.447723e-01 9.447905e-01 7.154423e-01 5.110213e-01 5.139780e-01 5.169634e-01 5.050052e-01

1444 9.449519e-01 9.449681e-01 9.449910e-01 9.450142e-01 9.450316e-01 7.160482e-01 5.111088e-01 5.140692e-01 5.170584e-01 5.050857e-01

1445 9.451860e-01 9.452016e-01 9.452237e-01 9.452462e-01 9.452627e-01 7.166774e-01 5.112006e-01 5.141648e-01 5.171580e-01 5.051701e-01

1446 9.454107e-01 9.454255e-01 9.454470e-01 9.454687e-01 9.454845e-01 7.173301e-01 5.112969e-01 5.142651e-01 5.172625e-01 5.052587e-01

1447 9.456263e-01 9.456404e-01 9.456613e-01 9.456823e-01 9.456974e-01 7.180065e-01 5.113978e-01 5.143702e-01 5.173720e-01 5.053516e-01

1448 9.458333e-01 9.458468e-01 9.458670e-01 9.458875e-01 9.459019e-01 7.187066e-01 5.115035e-01 5.144804e-01 5.174868e-01 5.054490e-01

1449 9.460323e-01 9.460451e-01 9.460647e-01 9.460846e-01 9.460984e-01 7.194306e-01 5.116144e-01 5.145958e-01 5.176070e-01 5.055510e-01

1450 9.462235e-01 9.462358e-01 9.462548e-01 9.462741e-01 9.462872e-01 7.201782e-01 5.117304e-01 5.147167e-01 5.177329e-01 5.056579e-01

1451 9.464074e-01 9.464191e-01 9.464377e-01 9.464564e-01 9.464690e-01 7.209495e-01 5.118519e-01 5.148432e-01 5.178647e-01 5.057698e-01

1452 9.465844e-01 9.465956e-01 9.466137e-01 9.466319e-01 9.466439e-01 7.217441e-01 5.119791e-01 5.149756e-01 5.180025e-01 5.058870e-01

1453 9.467549e-01 9.467656e-01 9.467832e-01 9.468009e-01 9.468124e-01 7.225617e-01 5.121121e-01 5.151141e-01 5.181467e-01 5.060096e-01

1454 9.469192e-01 9.469294e-01 9.469465e-01 9.469638e-01 9.469748e-01 7.234019e-01 5.122512e-01 5.152589e-01 5.182974e-01 5.061378e-01

1455 9.470776e-01 9.470874e-01 9.471040e-01 9.471209e-01 9.471314e-01 7.242643e-01 5.123966e-01 5.154102e-01 5.184549e-01 5.062719e-01

1456 9.472305e-01 9.472398e-01 9.472560e-01 9.472724e-01 9.472825e-01 7.251482e-01 5.125486e-01 5.155683e-01 5.186195e-01 5.064120e-01

1457 9.473780e-01 9.473869e-01 9.474028e-01 9.474188e-01 9.474284e-01 7.260530e-01 5.127072e-01 5.157334e-01 5.187912e-01 5.065584e-01

1458 9.475206e-01 9.475291e-01 9.475446e-01 9.475602e-01 9.475695e-01 7.269778e-01 5.128729e-01 5.159058e-01 5.189705e-01 5.067113e-01

1459 9.476584e-01 9.476666e-01 9.476817e-01 9.476970e-01 9.477058e-01 7.279217e-01 5.130458e-01 5.160855e-01 5.191575e-01 5.068708e-01

1460 9.477918e-01 9.477996e-01 9.478144e-01 9.478293e-01 9.478378e-01 7.288839e-01 5.132260e-01 5.162730e-01 5.193525e-01 5.070373e-01

1461 9.479208e-01 9.479283e-01 9.479428e-01 9.479574e-01 9.479656e-01 7.298632e-01 5.134140e-01 5.164685e-01 5.195557e-01 5.072109e-01

1462 9.480459e-01 9.480531e-01 9.480672e-01 9.480815e-01 9.480893e-01 7.308585e-01 5.136098e-01 5.166721e-01 5.197673e-01 5.073919e-01

1463 9.481671e-01 9.481740e-01 9.481879e-01 9.482019e-01 9.482094e-01 7.318685e-01 5.138138e-01 5.168841e-01 5.199877e-01 5.075805e-01

1464 9.482847e-01 9.482913e-01 9.483049e-01 9.483186e-01 9.483259e-01 7.328919e-01 5.140262e-01 5.171047e-01 5.202169e-01 5.077768e-01

1465 9.483989e-01 9.484052e-01 9.484186e-01 9.484320e-01 9.484389e-01 7.339273e-01 5.142471e-01 5.173342e-01 5.204554e-01 5.079812e-01

1466 9.485098e-01 9.485159e-01 9.485290e-01 9.485422e-01 9.485488e-01 7.349733e-01 5.144768e-01 5.175728e-01 5.207032e-01 5.081938e-01

1467 9.486176e-01 9.486235e-01 9.486363e-01 9.486492e-01 9.486556e-01 7.360284e-01 5.147156e-01 5.178208e-01 5.209607e-01 5.084149e-01

1468 9.487225e-01 9.487281e-01 9.487407e-01 9.487534e-01 9.487596e-01 7.370910e-01 5.149636e-01 5.180782e-01 5.212280e-01 5.086446e-01

1469 9.488246e-01 9.488300e-01 9.488423e-01 9.488549e-01 9.488608e-01 7.381595e-01 5.152210e-01 5.183455e-01 5.215053e-01 5.088832e-01

1470 9.489240e-01 9.489292e-01 9.489414e-01 9.489537e-01 9.489594e-01 7.392322e-01 5.154882e-01 5.186226e-01 5.217929e-01 5.091309e-01

1471 9.490209e-01 9.490259e-01 9.490379e-01 9.490500e-01 9.490555e-01 7.403076e-01 5.157651e-01 5.189100e-01 5.220910e-01 5.093878e-01

1472 9.491155e-01 9.491203e-01 9.491321e-01 9.491440e-01 9.491493e-01 7.413840e-01 5.160522e-01 5.192076e-01 5.223996e-01 5.096542e-01

1473 9.492078e-01 9.492124e-01 9.492240e-01 9.492357e-01 9.492408e-01 7.424597e-01 5.163494e-01 5.195158e-01 5.227191e-01 5.099303e-01

1474 9.492979e-01 9.493023e-01 9.493138e-01 9.493253e-01 9.493303e-01 7.435330e-01 5.166570e-01 5.198346e-01 5.230495e-01 5.102161e-01

1475 9.493860e-01 9.493902e-01 9.494015e-01 9.494129e-01 9.494177e-01 7.446024e-01 5.169752e-01 5.201643e-01 5.233911e-01 5.105119e-01

1476 9.494721e-01 9.494762e-01 9.494873e-01 9.494985e-01 9.495031e-01 7.456662e-01 5.173040e-01 5.205049e-01 5.237438e-01 5.108178e-01

1477 9.495564e-01 9.495603e-01 9.495713e-01 9.495824e-01 9.495868e-01 7.467229e-01 5.176436e-01 5.208565e-01 5.241079e-01 5.111339e-01

1478 9.496389e-01 9.496427e-01 9.496535e-01 9.496644e-01 9.496687e-01 7.477710e-01 5.179941e-01 5.212194e-01 5.244834e-01 5.114604e-01

1479 9.497197e-01 9.497234e-01 9.497341e-01 9.497449e-01 9.497490e-01 7.488090e-01 5.183556e-01 5.215934e-01 5.248704e-01 5.117974e-01

1480 9.497989e-01 9.498025e-01 9.498131e-01 9.498237e-01 9.498277e-01 7.498355e-01 5.187281e-01 5.219787e-01 5.252689e-01 5.121449e-01

1481 9.498767e-01 9.498801e-01 9.498905e-01 9.499010e-01 9.499049e-01 7.508493e-01 5.191117e-01 5.223753e-01 5.256789e-01 5.125030e-01

1482 9.499529e-01 9.499563e-01 9.499665e-01 9.499769e-01 9.499807e-01 7.518490e-01 5.195064e-01 5.227833e-01 5.261005e-01 5.128717e-01

1483 9.500278e-01 9.500310e-01 9.500412e-01 9.500515e-01 9.500551e-01 7.528336e-01 5.199122e-01 5.232026e-01 5.265336e-01 5.132511e-01

1484 9.501014e-01 9.501045e-01 9.501146e-01 9.501247e-01 9.501282e-01 7.538019e-01 5.203291e-01 5.236331e-01 5.269782e-01 5.136412e-01

1485 9.501737e-01 9.501767e-01 9.501867e-01 9.501967e-01 9.502001e-01 7.547530e-01 5.207571e-01 5.240749e-01 5.274341e-01 5.140419e-01

1486 9.502448e-01 9.502477e-01 9.502576e-01 9.502675e-01 9.502708e-01 7.556861e-01 5.211959e-01 5.245277e-01 5.279013e-01 5.144532e-01

1487 9.503148e-01 9.503176e-01 9.503273e-01 9.503372e-01 9.503404e-01 7.566002e-01 5.216456e-01 5.249915e-01 5.283795e-01 5.148750e-01

1488 9.503837e-01 9.503864e-01 9.503960e-01 9.504058e-01 9.504089e-01 7.574948e-01 5.221060e-01 5.254661e-01 5.288687e-01 5.153072e-01

1489 9.504515e-01 9.504541e-01 9.504637e-01 9.504733e-01 9.504763e-01 7.583691e-01 5.225770e-01 5.259514e-01 5.293686e-01 5.157498e-01

1490 9.505184e-01 9.505209e-01 9.505304e-01 9.505399e-01 9.505428e-01 7.592228e-01 5.230582e-01 5.264470e-01 5.298790e-01 5.162024e-01

1491 9.505842e-01 9.505867e-01 9.505961e-01 9.506055e-01 9.506084e-01 7.600554e-01 5.235496e-01 5.269529e-01 5.303995e-01 5.166650e-01

1492 9.506492e-01 9.506516e-01 9.506609e-01 9.506702e-01 9.506730e-01 7.608665e-01 5.240508e-01 5.274685e-01 5.309300e-01 5.171373e-01

1493 9.507133e-01 9.507156e-01 9.507248e-01 9.507341e-01 9.507368e-01 7.616559e-01 5.245615e-01 5.279938e-01 5.314700e-01 5.176191e-01

1494 9.507766e-01 9.507788e-01 9.507879e-01 9.507971e-01 9.507997e-01 7.624235e-01 5.250815e-01 5.285282e-01 5.320192e-01 5.181101e-01

1495 9.508390e-01 9.508412e-01 9.508502e-01 9.508594e-01 9.508619e-01 7.631691e-01 5.256103e-01 5.290715e-01 5.325771e-01 5.186100e-01

1496 9.509007e-01 9.509029e-01 9.509118e-01 9.509208e-01 9.509233e-01 7.638928e-01 5.261477e-01 5.296232e-01 5.331434e-01 5.191184e-01

1497 9.509617e-01 9.509638e-01 9.509726e-01 9.509816e-01 9.509840e-01 7.645946e-01 5.266931e-01 5.301829e-01 5.337176e-01 5.196350e-01

1498 9.510220e-01 9.510240e-01 9.510328e-01 9.510416e-01 9.510440e-01 7.652747e-01 5.272461e-01 5.307501e-01 5.342991e-01 5.201595e-01

1499 9.510816e-01 9.510835e-01 9.510922e-01 9.511010e-01 9.511033e-01 7.659331e-01 5.278063e-01 5.313243e-01 5.348875e-01 5.206913e-01

1500 9.511405e-01 9.511424e-01 9.511510e-01 9.511597e-01 9.511619e-01 7.665702e-01 5.283731e-01 5.319050e-01 5.354822e-01 5.212300e-01

1501 9.511988e-01 9.512006e-01 9.512092e-01 9.512178e-01 9.512200e-01 7.671862e-01 5.289461e-01 5.324917e-01 5.360827e-01 5.217752e-01

1502 9.512565e-01 9.512583e-01 9.512668e-01 9.512754e-01 9.512775e-01 7.677815e-01 5.295247e-01 5.330838e-01 5.366883e-01 5.223263e-01

1503 9.513136e-01 9.513153e-01 9.513238e-01 9.513323e-01 9.513343e-01 7.683564e-01 5.301084e-01 5.336806e-01 5.372985e-01 5.228829e-01

1504 9.513702e-01 9.513719e-01 9.513802e-01 9.513887e-01 9.513907e-01 7.689114e-01 5.306964e-01 5.342817e-01 5.379126e-01 5.234443e-01

1505 9.514263e-01 9.514279e-01 9.514362e-01 9.514446e-01 9.514465e-01 7.694468e-01 5.312883e-01 5.348863e-01 5.385300e-01 5.240101e-01

1506 9.514818e-01 9.514833e-01 9.514916e-01 9.514999e-01 9.515018e-01 7.699631e-01 5.318835e-01 5.354939e-01 5.391500e-01 5.245797e-01

1507 9.515368e-01 9.515383e-01 9.515465e-01 9.515548e-01 9.515566e-01 7.704609e-01 5.324812e-01 5.361037e-01 5.397719e-01 5.251524e-01

1508 9.515914e-01 9.515928e-01 9.516010e-01 9.516092e-01 9.516110e-01 7.709405e-01 5.330810e-01 5.367152e-01 5.403952e-01 5.257278e-01

1509 9.516455e-01 9.516469e-01 9.516550e-01 9.516631e-01 9.516648e-01 7.714025e-01 5.336820e-01 5.373277e-01 5.410190e-01 5.263050e-01

1510 9.516991e-01 9.517005e-01 9.517085e-01 9.517166e-01 9.517183e-01 7.718475e-01 5.342837e-01 5.379405e-01 5.416428e-01 5.268836e-01

1511 9.517524e-01 9.517537e-01 9.517616e-01 9.517697e-01 9.517713e-01 7.722760e-01 5.348855e-01 5.385529e-01 5.422659e-01 5.274630e-01

1512 9.518052e-01 9.518065e-01 9.518144e-01 9.518223e-01 9.518239e-01 7.726884e-01 5.354866e-01 5.391644e-01 5.428876e-01 5.280424e-01

1513 9.518576e-01 9.518588e-01 9.518667e-01 9.518746e-01 9.518761e-01 7.730853e-01 5.360865e-01 5.397742e-01 5.435073e-01 5.286213e-01

1514 9.519096e-01 9.519108e-01 9.519186e-01 9.519264e-01 9.519279e-01 7.734673e-01 5.366845e-01 5.403817e-01 5.441243e-01 5.291991e-01

1515 9.519613e-01 9.519624e-01 9.519702e-01 9.519779e-01 9.519794e-01 7.738349e-01 5.372800e-01 5.409864e-01 5.447379e-01 5.297752e-01

1516 9.520126e-01 9.520137e-01 9.520214e-01 9.520291e-01 9.520305e-01 7.741887e-01 5.378723e-01 5.415875e-01 5.453476e-01 5.303489e-01

1517 9.520635e-01 9.520646e-01 9.520722e-01 9.520799e-01 9.520812e-01 7.745290e-01 5.384610e-01 5.421845e-01 5.459528e-01 5.309197e-01

1518 9.521141e-01 9.521152e-01 9.521227e-01 9.521303e-01 9.521317e-01 7.748565e-01 5.390454e-01 5.427768e-01 5.465530e-01 5.314870e-01

1519 9.521644e-01 9.521654e-01 9.521729e-01 9.521805e-01 9.521817e-01 7.751717e-01 5.396249e-01 5.433639e-01 5.471474e-01 5.320502e-01

1520 9.522143e-01 9.522153e-01 9.522228e-01 9.522303e-01 9.522315e-01 7.754750e-01 5.401991e-01 5.439452e-01 5.477357e-01 5.326089e-01

1521 9.522640e-01 9.522649e-01 9.522723e-01 9.522798e-01 9.522810e-01 7.757670e-01 5.407673e-01 5.445202e-01 5.483173e-01 5.331624e-01

1522 9.523133e-01 9.523142e-01 9.523216e-01 9.523290e-01 9.523301e-01 7.760481e-01 5.413293e-01 5.450885e-01 5.488918e-01 5.337104e-01

1523 9.523624e-01 9.523633e-01 9.523705e-01 9.523779e-01 9.523790e-01 7.763187e-01 5.418843e-01 5.456496e-01 5.494586e-01 5.342523e-01

1524 9.524112e-01 9.524120e-01 9.524192e-01 9.524265e-01 9.524276e-01 7.765793e-01 5.424322e-01 5.462030e-01 5.500175e-01 5.347877e-01

1525 9.524597e-01 9.524605e-01 9.524676e-01 9.524749e-01 9.524759e-01 7.768304e-01 5.429723e-01 5.467484e-01 5.505680e-01 5.353161e-01

1526 9.525079e-01 9.525087e-01 9.525158e-01 9.525230e-01 9.525240e-01 7.770723e-01 5.435045e-01 5.472855e-01 5.511098e-01 5.358372e-01

1527 9.525559e-01 9.525566e-01 9.525637e-01 9.525708e-01 9.525718e-01 7.773055e-01 5.440282e-01 5.478139e-01 5.516425e-01 5.363507e-01

1528 9.526036e-01 9.526043e-01 9.526113e-01 9.526184e-01 9.526193e-01 7.775304e-01 5.445433e-01 5.483332e-01 5.521659e-01 5.368561e-01

1529 9.526511e-01 9.526517e-01 9.526587e-01 9.526657e-01 9.526666e-01 7.777472e-01 5.450495e-01 5.488433e-01 5.526797e-01 5.373533e-01

1530 9.526983e-01 9.526989e-01 9.527058e-01 9.527128e-01 9.527137e-01 7.779565e-01 5.455464e-01 5.493439e-01 5.531837e-01 5.378418e-01

1531 9.527453e-01 9.527459e-01 9.527527e-01 9.527597e-01 9.527605e-01 7.781585e-01 5.460340e-01 5.498347e-01 5.536777e-01 5.383216e-01

1532 9.527921e-01 9.527926e-01 9.527994e-01 9.528063e-01 9.528071e-01 7.783536e-01 5.465119e-01 5.503157e-01 5.541616e-01 5.387923e-01

1533 9.528386e-01 9.528391e-01 9.528459e-01 9.528527e-01 9.528534e-01 7.785420e-01 5.469800e-01 5.507867e-01 5.546352e-01 5.392537e-01

1534 9.528850e-01 9.528855e-01 9.528921e-01 9.528989e-01 9.528996e-01 7.787242e-01 5.474383e-01 5.512476e-01 5.550985e-01 5.397058e-01

1535 9.529311e-01 9.529315e-01 9.529382e-01 9.529449e-01 9.529455e-01 7.789004e-01 5.478866e-01 5.516982e-01 5.555512e-01 5.401484e-01

1536 9.529770e-01 9.529774e-01 9.529840e-01 9.529906e-01 9.529913e-01 7.790709e-01 5.483248e-01 5.521385e-01 5.559935e-01 5.405814e-01

1537 9.530228e-01 9.530231e-01 9.530296e-01 9.530362e-01 9.530368e-01 7.792359e-01 5.487529e-01 5.525685e-01 5.564253e-01 5.410048e-01

1538 9.530683e-01 9.530686e-01 9.530751e-01 9.530816e-01 9.530822e-01 7.793958e-01 5.491709e-01 5.529882e-01 5.568465e-01 5.414184e-01

1539 9.531136e-01 9.531139e-01 9.531203e-01 9.531268e-01 9.531273e-01 7.795507e-01 5.495787e-01 5.533976e-01 5.572573e-01 5.418223e-01

1540 9.531588e-01 9.531590e-01 9.531654e-01 9.531718e-01 9.531723e-01 7.797009e-01 5.499765e-01 5.537968e-01 5.576577e-01 5.422164e-01

1541 9.532037e-01 9.532039e-01 9.532102e-01 9.532166e-01 9.532171e-01 7.798467e-01 5.503642e-01 5.541857e-01 5.580477e-01 5.426008e-01

1542 9.532485e-01 9.532487e-01 9.532549e-01 9.532612e-01 9.532617e-01 7.799882e-01 5.507419e-01 5.545645e-01 5.584274e-01 5.429755e-01

1543 9.532931e-01 9.532933e-01 9.532995e-01 9.533057e-01 9.533061e-01 7.801256e-01 5.511096e-01 5.549332e-01 5.587969e-01 5.433405e-01

1544 9.533376e-01 9.533377e-01 9.533438e-01 9.533500e-01 9.533503e-01 7.802592e-01 5.514676e-01 5.552920e-01 5.591564e-01 5.436961e-01

1545 9.533818e-01 9.533819e-01 9.533880e-01 9.533941e-01 9.533944e-01 7.803892e-01 5.518159e-01 5.556410e-01 5.595060e-01 5.440421e-01

1546 9.534259e-01 9.534260e-01 9.534320e-01 9.534381e-01 9.534383e-01 7.805156e-01 5.521546e-01 5.559803e-01 5.598459e-01 5.443789e-01

1547 9.534699e-01 9.534699e-01 9.534758e-01 9.534819e-01 9.534821e-01 7.806388e-01 5.524838e-01 5.563102e-01 5.601761e-01 5.447064e-01

1548 9.535137e-01 9.535137e-01 9.535195e-01 9.535255e-01 9.535257e-01 7.807587e-01 5.528038e-01 5.566307e-01 5.604970e-01 5.450248e-01

1549 9.535573e-01 9.535573e-01 9.535631e-01 9.535690e-01 9.535691e-01 7.808757e-01 5.531147e-01 5.569420e-01 5.608085e-01 5.453343e-01

1550 9.536008e-01 9.536007e-01 9.536065e-01 9.536123e-01 9.536124e-01 7.809899e-01 5.534167e-01 5.572443e-01 5.611111e-01 5.456350e-01

1551 9.536442e-01 9.536440e-01 9.536497e-01 9.536555e-01 9.536555e-01 7.811013e-01 5.537099e-01 5.575378e-01 5.614047e-01 5.459272e-01

1552 9.536873e-01 9.536872e-01 9.536928e-01 9.536985e-01 9.536985e-01 7.812101e-01 5.539946e-01 5.578227e-01 5.616897e-01 5.462108e-01

1553 9.537304e-01 9.537302e-01 9.537358e-01 9.537414e-01 9.537414e-01 7.813165e-01 5.542709e-01 5.580991e-01 5.619663e-01 5.464863e-01

1554 9.537733e-01 9.537731e-01 9.537786e-01 9.537841e-01 9.537841e-01 7.814206e-01 5.545391e-01 5.583674e-01 5.622346e-01 5.467536e-01

1555 9.538161e-01 9.538158e-01 9.538212e-01 9.538268e-01 9.538266e-01 7.815224e-01 5.547992e-01 5.586277e-01 5.624949e-01 5.470131e-01

1556 9.538587e-01 9.538584e-01 9.538638e-01 9.538692e-01 9.538691e-01 7.816221e-01 5.550517e-01 5.588802e-01 5.627474e-01 5.472649e-01

1557 9.539013e-01 9.539009e-01 9.539062e-01 9.539116e-01 9.539113e-01 7.817198e-01 5.552965e-01 5.591251e-01 5.629923e-01 5.475092e-01

1558 9.539436e-01 9.539432e-01 9.539485e-01 9.539538e-01 9.539535e-01 7.818156e-01 5.555341e-01 5.593627e-01 5.632298e-01 5.477463e-01

1559 9.539859e-01 9.539854e-01 9.539906e-01 9.539959e-01 9.539955e-01 7.819096e-01 5.557645e-01 5.595931e-01 5.634602e-01 5.479762e-01

1560 9.540280e-01 9.540275e-01 9.540326e-01 9.540378e-01 9.540374e-01 7.820018e-01 5.559880e-01 5.598166e-01 5.636836e-01 5.481993e-01

1561 9.540701e-01 9.540695e-01 9.540745e-01 9.540796e-01 9.540792e-01 7.820924e-01 5.562047e-01 5.600334e-01 5.639003e-01 5.484158e-01

1562 9.541119e-01 9.541113e-01 9.541163e-01 9.541213e-01 9.541209e-01 7.821814e-01 5.564150e-01 5.602437e-01 5.641105e-01 5.486257e-01

1563 9.541537e-01 9.541531e-01 9.541580e-01 9.541629e-01 9.541624e-01 7.822690e-01 5.566190e-01 5.604476e-01 5.643144e-01 5.488294e-01

1564 9.541954e-01 9.541947e-01 9.541995e-01 9.542044e-01 9.542038e-01 7.823551e-01 5.568169e-01 5.606455e-01 5.645121e-01 5.490270e-01

1565 9.542369e-01 9.542362e-01 9.542409e-01 9.542457e-01 9.542451e-01 7.824398e-01 5.570089e-01 5.608375e-01 5.647040e-01 5.492187e-01

1566 9.542784e-01 9.542776e-01 9.542823e-01 9.542870e-01 9.542863e-01 7.825233e-01 5.571952e-01 5.610237e-01 5.648902e-01 5.494048e-01

1567 9.543197e-01 9.543189e-01 9.543235e-01 9.543281e-01 9.543274e-01 7.826056e-01 5.573760e-01 5.612045e-01 5.650709e-01 5.495854e-01

1568 9.543609e-01 9.543600e-01 9.543646e-01 9.543691e-01 9.543683e-01 7.826867e-01 5.575515e-01 5.613800e-01 5.652463e-01 5.497607e-01

1569 9.544021e-01 9.544011e-01 9.544056e-01 9.544100e-01 9.544092e-01 7.827666e-01 5.577219e-01 5.615503e-01 5.654166e-01 5.499308e-01

1570 9.544431e-01 9.544421e-01 9.544464e-01 9.544508e-01 9.544499e-01 7.828455e-01 5.578873e-01 5.617158e-01 5.655820e-01 5.500961e-01

1571 9.544840e-01 9.544829e-01 9.544872e-01 9.544915e-01 9.544906e-01 7.829234e-01 5.580480e-01 5.618765e-01 5.657426e-01 5.502566e-01

1572 9.545248e-01 9.545237e-01 9.545279e-01 9.545321e-01 9.545311e-01 7.830004e-01 5.582041e-01 5.620326e-01 5.658986e-01 5.504125e-01

1573 9.545655e-01 9.545644e-01 9.545685e-01 9.545726e-01 9.545715e-01 7.830764e-01 5.583558e-01 5.621843e-01 5.660503e-01 5.505640e-01

1574 9.546062e-01 9.546050e-01 9.546090e-01 9.546130e-01 9.546118e-01 7.831516e-01 5.585033e-01 5.623317e-01 5.661977e-01 5.507113e-01

1575 9.546467e-01 9.546454e-01 9.546494e-01 9.546533e-01 9.546521e-01 7.832259e-01 5.586467e-01 5.624751e-01 5.663411e-01 5.508545e-01

1576 9.546872e-01 9.546858e-01 9.546896e-01 9.546935e-01 9.546922e-01 7.832994e-01 5.587861e-01 5.626146e-01 5.664806e-01 5.509937e-01

1577 9.547275e-01 9.547261e-01 9.547298e-01 9.547336e-01 9.547322e-01 7.833722e-01 5.589218e-01 5.627503e-01 5.666163e-01 5.511292e-01

1578 9.547678e-01 9.547663e-01 9.547700e-01 9.547736e-01 9.547721e-01 7.834442e-01 5.590539e-01 5.628824e-01 5.667484e-01 5.512611e-01

1579 9.548080e-01 9.548064e-01 9.548100e-01 9.548135e-01 9.548120e-01 7.835155e-01 5.591825e-01 5.630111e-01 5.668770e-01 5.513895e-01

1580 9.548481e-01 9.548465e-01 9.548499e-01 9.548534e-01 9.548517e-01 7.835862e-01 5.593078e-01 5.631364e-01 5.670023e-01 5.515146e-01

1581 9.548881e-01 9.548864e-01 9.548897e-01 9.548931e-01 9.548914e-01 7.836563e-01 5.594299e-01 5.632585e-01 5.671244e-01 5.516364e-01

1582 9.549280e-01 9.549263e-01 9.549295e-01 9.549327e-01 9.549309e-01 7.837258e-01 5.595488e-01 5.633775e-01 5.672435e-01 5.517552e-01

1583 9.549679e-01 9.549660e-01 9.549692e-01 9.549723e-01 9.549704e-01 7.837947e-01 5.596649e-01 5.634936e-01 5.673596e-01 5.518711e-01

1584 9.550076e-01 9.550057e-01 9.550088e-01 9.550118e-01 9.550098e-01 7.838630e-01 5.597781e-01 5.636069e-01 5.674729e-01 5.519841e-01

1585 9.550473e-01 9.550454e-01 9.550483e-01 9.550511e-01 9.550490e-01 7.839308e-01 5.598886e-01 5.637175e-01 5.675836e-01 5.520944e-01

1586 9.550869e-01 9.550849e-01 9.550877e-01 9.550904e-01 9.550882e-01 7.839981e-01 5.599966e-01 5.638255e-01 5.676916e-01 5.522022e-01

1587 9.551265e-01 9.551244e-01 9.551271e-01 9.551297e-01 9.551274e-01 7.840650e-01 5.601020e-01 5.639310e-01 5.677972e-01 5.523074e-01

1588 9.551660e-01 9.551638e-01 9.551663e-01 9.551688e-01 9.551664e-01 7.841314e-01 5.602051e-01 5.640341e-01 5.679004e-01 5.524103e-01

1589 9.552054e-01 9.552031e-01 9.552055e-01 9.552079e-01 9.552053e-01 7.841973e-01 5.603059e-01 5.641350e-01 5.680013e-01 5.525109e-01

1590 9.552447e-01 9.552424e-01 9.552447e-01 9.552468e-01 9.552442e-01 7.842629e-01 5.604045e-01 5.642337e-01 5.681001e-01 5.526094e-01

1591 9.552840e-01 9.552815e-01 9.552837e-01 9.552857e-01 9.552830e-01 7.843280e-01 5.605010e-01 5.643303e-01 5.681967e-01 5.527057e-01

1592 9.553232e-01 9.553207e-01 9.553227e-01 9.553246e-01 9.553217e-01 7.843927e-01 5.605956e-01 5.644249e-01 5.682914e-01 5.528001e-01

1593 9.553623e-01 9.553597e-01 9.553616e-01 9.553633e-01 9.553603e-01 7.844571e-01 5.606882e-01 5.645176e-01 5.683842e-01 5.528926e-01

1594 9.554014e-01 9.553987e-01 9.554004e-01 9.554020e-01 9.553988e-01 7.845212e-01 5.607790e-01 5.646085e-01 5.684752e-01 5.529832e-01

1595 9.554404e-01 9.554376e-01 9.554392e-01 9.554406e-01 9.554373e-01 7.845849e-01 5.608681e-01 5.646977e-01 5.685644e-01 5.530722e-01

1596 9.554794e-01 9.554765e-01 9.554779e-01 9.554791e-01 9.554756e-01 7.846482e-01 5.609555e-01 5.647852e-01 5.686520e-01 5.531595e-01

1597 9.555183e-01 9.555153e-01 9.555165e-01 9.555175e-01 9.555139e-01 7.847113e-01 5.610413e-01 5.648711e-01 5.687380e-01 5.532452e-01

1598 9.555571e-01 9.555540e-01 9.555551e-01 9.555559e-01 9.555521e-01 7.847741e-01 5.611256e-01 5.649554e-01 5.688225e-01 5.533293e-01

1599 9.555959e-01 9.555927e-01 9.555936e-01 9.555942e-01 9.555902e-01 7.848366e-01 5.612085e-01 5.650384e-01 5.689055e-01 5.534121e-01

1600 9.556347e-01 9.556313e-01 9.556320e-01 9.556324e-01 9.556283e-01 7.848988e-01 5.612899e-01 5.651199e-01 5.689871e-01 5.534935e-01

1601 9.556733e-01 9.556699e-01 9.556704e-01 9.556706e-01 9.556662e-01 7.849608e-01 5.613701e-01 5.652001e-01 5.690674e-01 5.535736e-01

1602 9.557120e-01 9.557084e-01 9.557087e-01 9.557086e-01 9.557041e-01 7.850225e-01 5.614490e-01 5.652791e-01 5.691465e-01 5.536524e-01

1603 9.557506e-01 9.557468e-01 9.557469e-01 9.557467e-01 9.557419e-01 7.850840e-01 5.615267e-01 5.653569e-01 5.692243e-01 5.537300e-01

1604 9.557891e-01 9.557852e-01 9.557851e-01 9.557846e-01 9.557797e-01 7.851452e-01 5.616032e-01 5.654335e-01 5.693010e-01 5.538066e-01

1605 9.558276e-01 9.558236e-01 9.558232e-01 9.558225e-01 9.558173e-01 7.852062e-01 5.616787e-01 5.655090e-01 5.693766e-01 5.538820e-01

1606 9.558660e-01 9.558619e-01 9.558613e-01 9.558602e-01 9.558548e-01 7.852670e-01 5.617531e-01 5.655835e-01 5.694512e-01 5.539564e-01

1607 9.559044e-01 9.559001e-01 9.558993e-01 9.558980e-01 9.558923e-01 7.853276e-01 5.618265e-01 5.656570e-01 5.695248e-01 5.540298e-01

1608 9.559428e-01 9.559383e-01 9.559372e-01 9.559356e-01 9.559297e-01 7.853881e-01 5.618990e-01 5.657295e-01 5.695974e-01 5.541024e-01

1609 9.559811e-01 9.559765e-01 9.559751e-01 9.559732e-01 9.559670e-01 7.854483e-01 5.619705e-01 5.658012e-01 5.696691e-01 5.541740e-01

1610 9.560194e-01 9.560146e-01 9.560129e-01 9.560107e-01 9.560042e-01 7.855083e-01 5.620413e-01 5.658720e-01 5.697400e-01 5.542448e-01

1611 9.560576e-01 9.560527e-01 9.560507e-01 9.560481e-01 9.560414e-01 7.855682e-01 5.621112e-01 5.659419e-01 5.698100e-01 5.543148e-01

1612 9.560958e-01 9.560907e-01 9.560884e-01 9.560855e-01 9.560784e-01 7.856279e-01 5.621803e-01 5.660111e-01 5.698793e-01 5.543840e-01

1613 9.561340e-01 9.561286e-01 9.561260e-01 9.561227e-01 9.561154e-01 7.856874e-01 5.622487e-01 5.660796e-01 5.699478e-01 5.544526e-01

1614 9.561721e-01 9.561666e-01 9.561636e-01 9.561599e-01 9.561522e-01 7.857468e-01 5.623164e-01 5.661473e-01 5.700156e-01 5.545204e-01

1615 9.562102e-01 9.562045e-01 9.562011e-01 9.561971e-01 9.561890e-01 7.858060e-01 5.623835e-01 5.662144e-01 5.700828e-01 5.545877e-01

1616 9.562483e-01 9.562423e-01 9.562386e-01 9.562341e-01 9.562256e-01 7.858651e-01 5.624499e-01 5.662808e-01 5.701493e-01 5.546543e-01

1617 9.562863e-01 9.562801e-01 9.562760e-01 9.562711e-01 9.562622e-01 7.859240e-01 5.625157e-01 5.663467e-01 5.702152e-01 5.547203e-01

1618 9.563243e-01 9.563179e-01 9.563134e-01 9.563080e-01 9.562987e-01 7.859828e-01 5.625809e-01 5.664120e-01 5.702806e-01 5.547858e-01

1619 9.563623e-01 9.563556e-01 9.563507e-01 9.563448e-01 9.563350e-01 7.860415e-01 5.626457e-01 5.664767e-01 5.703454e-01 5.548508e-01

1620 9.564003e-01 9.563933e-01 9.563879e-01 9.563815e-01 9.563713e-01 7.861001e-01 5.627099e-01 5.665410e-01 5.704097e-01 5.549153e-01

1621 9.564382e-01 9.564310e-01 9.564251e-01 9.564181e-01 9.564074e-01 7.861585e-01 5.627736e-01 5.666047e-01 5.704735e-01 5.549793e-01

1622 9.565487e-01 9.565411e-01 9.565347e-01 9.565273e-01 9.565162e-01 7.869713e-01 5.628956e-01 5.667329e-01 5.706082e-01 5.550896e-01

1623 9.566555e-01 9.566475e-01 9.566407e-01 9.566330e-01 9.566214e-01 7.878019e-01 5.630228e-01 5.668666e-01 5.707487e-01 5.552045e-01

1624 9.567589e-01 9.567505e-01 9.567433e-01 9.567352e-01 9.567233e-01 7.886496e-01 5.631554e-01 5.670059e-01 5.708951e-01 5.553244e-01

1625 9.568589e-01 9.568501e-01 9.568427e-01 9.568342e-01 9.568219e-01 7.895136e-01 5.632936e-01 5.671511e-01 5.710476e-01 5.554494e-01

1626 9.569559e-01 9.569467e-01 9.569389e-01 9.569301e-01 9.569174e-01 7.903931e-01 5.634375e-01 5.673023e-01 5.712063e-01 5.555797e-01

1627 9.570498e-01 9.570403e-01 9.570322e-01 9.570230e-01 9.570100e-01 7.912871e-01 5.635874e-01 5.674597e-01 5.713716e-01 5.557153e-01

1628 9.571409e-01 9.571311e-01 9.571227e-01 9.571132e-01 9.570999e-01 7.921948e-01 5.637434e-01 5.676234e-01 5.715435e-01 5.558565e-01

1629 9.572293e-01 9.572192e-01 9.572105e-01 9.572008e-01 9.571871e-01 7.931149e-01 5.639057e-01 5.677938e-01 5.717223e-01 5.560035e-01

1630 9.573152e-01 9.573048e-01 9.572958e-01 9.572858e-01 9.572719e-01 7.940465e-01 5.640744e-01 5.679709e-01 5.719082e-01 5.561564e-01

1631 9.573987e-01 9.573880e-01 9.573787e-01 9.573685e-01 9.573542e-01 7.949884e-01 5.642498e-01 5.681549e-01 5.721012e-01 5.563154e-01

1632 9.574798e-01 9.574689e-01 9.574594e-01 9.574488e-01 9.574343e-01 7.959392e-01 5.644321e-01 5.683461e-01 5.723017e-01 5.564806e-01

1633 9.575588e-01 9.575476e-01 9.575378e-01 9.575271e-01 9.575123e-01 7.968978e-01 5.646213e-01 5.685446e-01 5.725099e-01 5.566523e-01

1634 9.576357e-01 9.576242e-01 9.576142e-01 9.576032e-01 9.575882e-01 7.978629e-01 5.648178e-01 5.687505e-01 5.727258e-01 5.568306e-01

1635 9.577106e-01 9.576989e-01 9.576887e-01 9.576775e-01 9.576622e-01 7.988330e-01 5.650216e-01 5.689641e-01 5.729497e-01 5.570157e-01

1636 9.577836e-01 9.577717e-01 9.577613e-01 9.577498e-01 9.577344e-01 7.998069e-01 5.652329e-01 5.691856e-01 5.731817e-01 5.572077e-01

1637 9.578548e-01 9.578427e-01 9.578321e-01 9.578204e-01 9.578048e-01 8.007832e-01 5.654519e-01 5.694151e-01 5.734221e-01 5.574068e-01

1638 9.579243e-01 9.579120e-01 9.579012e-01 9.578894e-01 9.578735e-01 8.017605e-01 5.656788e-01 5.696527e-01 5.736709e-01 5.576131e-01

1639 9.579922e-01 9.579797e-01 9.579687e-01 9.579567e-01 9.579407e-01 8.027374e-01 5.659137e-01 5.698987e-01 5.739284e-01 5.578269e-01

1640 9.580585e-01 9.580459e-01 9.580347e-01 9.580225e-01 9.580063e-01 8.037125e-01 5.661568e-01 5.701531e-01 5.741946e-01 5.580482e-01

1641 9.581234e-01 9.581106e-01 9.580993e-01 9.580869e-01 9.580705e-01 8.046844e-01 5.664082e-01 5.704161e-01 5.744698e-01 5.582773e-01

1642 9.581869e-01 9.581739e-01 9.581624e-01 9.581499e-01 9.581333e-01 8.056518e-01 5.666680e-01 5.706879e-01 5.747540e-01 5.585141e-01

1643 9.582490e-01 9.582359e-01 9.582242e-01 9.582115e-01 9.581948e-01 8.066135e-01 5.669364e-01 5.709685e-01 5.750474e-01 5.587590e-01

1644 9.583099e-01 9.582966e-01 9.582848e-01 9.582720e-01 9.582551e-01 8.075680e-01 5.672135e-01 5.712582e-01 5.753501e-01 5.590119e-01

1645 9.583696e-01 9.583562e-01 9.583442e-01 9.583312e-01 9.583141e-01 8.085142e-01 5.674993e-01 5.715568e-01 5.756621e-01 5.592730e-01

1646 9.584281e-01 9.584145e-01 9.584024e-01 9.583893e-01 9.583721e-01 8.094509e-01 5.677941e-01 5.718647e-01 5.759835e-01 5.595425e-01

1647 9.584855e-01 9.584718e-01 9.584595e-01 9.584463e-01 9.584290e-01 8.103770e-01 5.680978e-01 5.721818e-01 5.763145e-01 5.598203e-01

1648 9.585418e-01 9.585280e-01 9.585156e-01 9.585023e-01 9.584848e-01 8.112913e-01 5.684105e-01 5.725082e-01 5.766550e-01 5.601066e-01

1649 9.585972e-01 9.585833e-01 9.585708e-01 9.585572e-01 9.585397e-01 8.121930e-01 5.687323e-01 5.728439e-01 5.770051e-01 5.604015e-01

1650 9.586516e-01 9.586375e-01 9.586249e-01 9.586113e-01 9.585936e-01 8.130809e-01 5.690632e-01 5.731889e-01 5.773648e-01 5.607049e-01

1651 9.587051e-01 9.586909e-01 9.586782e-01 9.586644e-01 9.586466e-01 8.139544e-01 5.694033e-01 5.735434e-01 5.777341e-01 5.610170e-01

1652 9.587577e-01 9.587434e-01 9.587306e-01 9.587167e-01 9.586988e-01 8.148125e-01 5.697525e-01 5.739072e-01 5.781130e-01 5.613377e-01

1653 9.588095e-01 9.587951e-01 9.587821e-01 9.587682e-01 9.587501e-01 8.156546e-01 5.701108e-01 5.742803e-01 5.785014e-01 5.616671e-01

1654 9.588604e-01 9.588460e-01 9.588329e-01 9.588188e-01 9.588007e-01 8.164799e-01 5.704781e-01 5.746626e-01 5.788992e-01 5.620052e-01

1655 9.589107e-01 9.588961e-01 9.588830e-01 9.588688e-01 9.588505e-01 8.172881e-01 5.708545e-01 5.750542e-01 5.793064e-01 5.623519e-01

1656 9.589602e-01 9.589455e-01 9.589323e-01 9.589180e-01 9.588997e-01 8.180784e-01 5.712399e-01 5.754549e-01 5.797228e-01 5.627072e-01

1657 9.590090e-01 9.589942e-01 9.589809e-01 9.589665e-01 9.589481e-01 8.188506e-01 5.716341e-01 5.758645e-01 5.801483e-01 5.630709e-01

1658 9.590571e-01 9.590423e-01 9.590289e-01 9.590144e-01 9.589959e-01 8.196043e-01 5.720370e-01 5.762830e-01 5.805828e-01 5.634432e-01

1659 9.591046e-01 9.590897e-01 9.590762e-01 9.590616e-01 9.590430e-01 8.203391e-01 5.724484e-01 5.767101e-01 5.810260e-01 5.638237e-01

1660 9.591515e-01 9.591365e-01 9.591229e-01 9.591083e-01 9.590896e-01 8.210550e-01 5.728683e-01 5.771458e-01 5.814778e-01 5.642124e-01

1661 9.591978e-01 9.591828e-01 9.591691e-01 9.591543e-01 9.591356e-01 8.217517e-01 5.732964e-01 5.775897e-01 5.819379e-01 5.646092e-01

1662 9.592436e-01 9.592285e-01 9.592147e-01 9.591999e-01 9.591810e-01 8.224292e-01 5.737325e-01 5.780417e-01 5.824061e-01 5.650138e-01

1663 9.592888e-01 9.592736e-01 9.592597e-01 9.592448e-01 9.592259e-01 8.230874e-01 5.741764e-01 5.785014e-01 5.828820e-01 5.654261e-01

1664 9.593335e-01 9.593182e-01 9.593043e-01 9.592893e-01 9.592703e-01 8.237264e-01 5.746278e-01 5.789686e-01 5.833654e-01 5.658459e-01

1665 9.593777e-01 9.593624e-01 9.593484e-01 9.593333e-01 9.593143e-01 8.243463e-01 5.750864e-01 5.794431e-01 5.838559e-01 5.662729e-01

1666 9.594215e-01 9.594061e-01 9.593920e-01 9.593768e-01 9.593577e-01 8.249472e-01 5.755519e-01 5.799244e-01 5.843532e-01 5.667069e-01

1667 9.594648e-01 9.594493e-01 9.594351e-01 9.594199e-01 9.594007e-01 8.255293e-01 5.760241e-01 5.804121e-01 5.848569e-01 5.671475e-01

1668 9.595076e-01 9.594921e-01 9.594778e-01 9.594626e-01 9.594433e-01 8.260928e-01 5.765024e-01 5.809061e-01 5.853666e-01 5.675946e-01

1669 9.595501e-01 9.595345e-01 9.595202e-01 9.595048e-01 9.594855e-01 8.266380e-01 5.769867e-01 5.814057e-01 5.858818e-01 5.680476e-01

1670 9.595921e-01 9.595765e-01 9.595621e-01 9.595467e-01 9.595273e-01 8.271651e-01 5.774764e-01 5.819107e-01 5.864022e-01 5.685064e-01

1671 9.596338e-01 9.596181e-01 9.596036e-01 9.595881e-01 9.595687e-01 8.276746e-01 5.779712e-01 5.824206e-01 5.869273e-01 5.689706e-01

1672 9.596751e-01 9.596593e-01 9.596448e-01 9.596292e-01 9.596097e-01 8.281667e-01 5.784706e-01 5.829349e-01 5.874566e-01 5.694397e-01

1673 9.597160e-01 9.597002e-01 9.596856e-01 9.596700e-01 9.596504e-01 8.286418e-01 5.789742e-01 5.834532e-01 5.879897e-01 5.699134e-01

1674 9.597566e-01 9.597407e-01 9.597260e-01 9.597104e-01 9.596908e-01 8.291003e-01 5.794816e-01 5.839750e-01 5.885259e-01 5.703912e-01

1675 9.597968e-01 9.597809e-01 9.597662e-01 9.597504e-01 9.597308e-01 8.295426e-01 5.799922e-01 5.844998e-01 5.890649e-01 5.708728e-01

1676 9.598368e-01 9.598208e-01 9.598060e-01 9.597902e-01 9.597705e-01 8.299691e-01 5.805057e-01 5.850271e-01 5.896061e-01 5.713577e-01

1677 9.598764e-01 9.598604e-01 9.598455e-01 9.598297e-01 9.598099e-01 8.303803e-01 5.810214e-01 5.855564e-01 5.901489e-01 5.718454e-01

1678 9.599157e-01 9.598997e-01 9.598848e-01 9.598688e-01 9.598490e-01 8.307766e-01 5.815390e-01 5.860872e-01 5.906929e-01 5.723355e-01

1679 9.599548e-01 9.599387e-01 9.599237e-01 9.599077e-01 9.598878e-01 8.311585e-01 5.820578e-01 5.866189e-01 5.912375e-01 5.728275e-01

1680 9.599936e-01 9.599774e-01 9.599624e-01 9.599463e-01 9.599264e-01 8.315263e-01 5.825774e-01 5.871511e-01 5.917821e-01 5.733209e-01

1681 9.600321e-01 9.600159e-01 9.600008e-01 9.599847e-01 9.599647e-01 8.318806e-01 5.830973e-01 5.876832e-01 5.923263e-01 5.738152e-01

1682 9.600704e-01 9.600541e-01 9.600390e-01 9.600228e-01 9.600028e-01 8.322217e-01 5.836170e-01 5.882147e-01 5.928695e-01 5.743101e-01

1683 9.601084e-01 9.600921e-01 9.600769e-01 9.600606e-01 9.600406e-01 8.325502e-01 5.841360e-01 5.887451e-01 5.934111e-01 5.748049e-01

1684 9.601462e-01 9.601298e-01 9.601146e-01 9.600983e-01 9.600781e-01 8.328665e-01 5.846537e-01 5.892738e-01 5.939507e-01 5.752992e-01

1685 9.601837e-01 9.601673e-01 9.601520e-01 9.601357e-01 9.601155e-01 8.331710e-01 5.851696e-01 5.898004e-01 5.944878e-01 5.757925e-01

1686 9.602211e-01 9.602046e-01 9.601892e-01 9.601728e-01 9.601526e-01 8.334641e-01 5.856834e-01 5.903244e-01 5.950218e-01 5.762844e-01

1687 9.602582e-01 9.602417e-01 9.602263e-01 9.602098e-01 9.601895e-01 8.337463e-01 5.861944e-01 5.908453e-01 5.955522e-01 5.767743e-01

1688 9.602951e-01 9.602786e-01 9.602631e-01 9.602465e-01 9.602262e-01 8.340180e-01 5.867023e-01 5.913626e-01 5.960787e-01 5.772619e-01

1689 9.603318e-01 9.603152e-01 9.602997e-01 9.602831e-01 9.602627e-01 8.342796e-01 5.872065e-01 5.918758e-01 5.966007e-01 5.777466e-01

1690 9.603683e-01 9.603517e-01 9.603361e-01 9.603195e-01 9.602990e-01 8.345314e-01 5.877067e-01 5.923846e-01 5.971179e-01 5.782281e-01

1691 9.604047e-01 9.603880e-01 9.603723e-01 9.603556e-01 9.603352e-01 8.347740e-01 5.882024e-01 5.928885e-01 5.976297e-01 5.787058e-01

1692 9.604408e-01 9.604241e-01 9.604084e-01 9.603916e-01 9.603711e-01 8.350076e-01 5.886932e-01 5.933871e-01 5.981358e-01 5.791795e-01

1693 9.604768e-01 9.604601e-01 9.604443e-01 9.604274e-01 9.604069e-01 8.352327e-01 5.891788e-01 5.938801e-01 5.986359e-01 5.796487e-01

1694 9.605126e-01 9.604958e-01 9.604800e-01 9.604631e-01 9.604425e-01 8.354495e-01 5.896587e-01 5.943670e-01 5.991295e-01 5.801130e-01

1695 9.605483e-01 9.605314e-01 9.605155e-01 9.604986e-01 9.604779e-01 8.356585e-01 5.901326e-01 5.948476e-01 5.996164e-01 5.805721e-01

1696 9.605837e-01 9.605669e-01 9.605509e-01 9.605339e-01 9.605132e-01 8.358599e-01 5.906002e-01 5.953214e-01 6.000963e-01 5.810257e-01

1697 9.606191e-01 9.606021e-01 9.605861e-01 9.605691e-01 9.605483e-01 8.360542e-01 5.910612e-01 5.957884e-01 6.005688e-01 5.814733e-01

1698 9.606542e-01 9.606373e-01 9.606212e-01 9.606041e-01 9.605833e-01 8.362415e-01 5.915154e-01 5.962481e-01 6.010337e-01 5.819148e-01

1699 9.606893e-01 9.606723e-01 9.606561e-01 9.606390e-01 9.606181e-01 8.364223e-01 5.919624e-01 5.967003e-01 6.014908e-01 5.823499e-01

1700 9.607242e-01 9.607071e-01 9.606909e-01 9.606737e-01 9.606528e-01 8.365967e-01 5.924020e-01 5.971448e-01 6.019399e-01 5.827783e-01

1701 9.607589e-01 9.607418e-01 9.607256e-01 9.607083e-01 9.606873e-01 8.367652e-01 5.928341e-01 5.975814e-01 6.023808e-01 5.831998e-01

1702 9.607935e-01 9.607764e-01 9.607601e-01 9.607427e-01 9.607218e-01 8.369279e-01 5.932584e-01 5.980100e-01 6.028134e-01 5.836141e-01

1703 9.608280e-01 9.608109e-01 9.607945e-01 9.607771e-01 9.607560e-01 8.370851e-01 5.936748e-01 5.984304e-01 6.032374e-01 5.840212e-01

1704 9.608624e-01 9.608452e-01 9.608287e-01 9.608112e-01 9.607902e-01 8.372370e-01 5.940832e-01 5.988424e-01 6.036528e-01 5.844207e-01

1705 9.608966e-01 9.608794e-01 9.608629e-01 9.608453e-01 9.608242e-01 8.373840e-01 5.944834e-01 5.992460e-01 6.040595e-01 5.848127e-01

1706 9.609308e-01 9.609135e-01 9.608969e-01 9.608793e-01 9.608581e-01 8.375261e-01 5.948753e-01 5.996411e-01 6.044575e-01 5.851969e-01

1707 9.609648e-01 9.609474e-01 9.609308e-01 9.609131e-01 9.608919e-01 8.376638e-01 5.952588e-01 6.000276e-01 6.048467e-01 5.855733e-01

1708 9.609987e-01 9.609813e-01 9.609646e-01 9.609468e-01 9.609256e-01 8.377970e-01 5.956340e-01 6.004055e-01 6.052270e-01 5.859418e-01

1709 9.610325e-01 9.610150e-01 9.609983e-01 9.609804e-01 9.609591e-01 8.379262e-01 5.960008e-01 6.007747e-01 6.055984e-01 5.863023e-01

1710 9.610661e-01 9.610487e-01 9.610318e-01 9.610140e-01 9.609926e-01 8.380514e-01 5.963591e-01 6.011353e-01 6.059611e-01 5.866548e-01

1711 9.610997e-01 9.610822e-01 9.610653e-01 9.610474e-01 9.610259e-01 8.381728e-01 5.967090e-01 6.014873e-01 6.063149e-01 5.869993e-01

1712 9.611332e-01 9.611156e-01 9.610987e-01 9.610806e-01 9.610592e-01 8.382906e-01 5.970504e-01 6.018307e-01 6.066600e-01 5.873357e-01

1713 9.611666e-01 9.611490e-01 9.611319e-01 9.611138e-01 9.610923e-01 8.384050e-01 5.973835e-01 6.021656e-01 6.069963e-01 5.876641e-01

1714 9.611999e-01 9.611822e-01 9.611651e-01 9.611469e-01 9.611253e-01 8.385162e-01 5.977083e-01 6.024919e-01 6.073240e-01 5.879846e-01

1715 9.612331e-01 9.612154e-01 9.611982e-01 9.611799e-01 9.611583e-01 8.386242e-01 5.980248e-01 6.028099e-01 6.076432e-01 5.882970e-01

1716 9.612662e-01 9.612484e-01 9.612312e-01 9.612128e-01 9.611911e-01 8.387293e-01 5.983331e-01 6.031195e-01 6.079540e-01 5.886016e-01

1717 9.612992e-01 9.612814e-01 9.612641e-01 9.612457e-01 9.612239e-01 8.388315e-01 5.986332e-01 6.034209e-01 6.082564e-01 5.888984e-01

1718 9.613321e-01 9.613143e-01 9.612969e-01 9.612784e-01 9.612565e-01 8.389311e-01 5.989254e-01 6.037143e-01 6.085506e-01 5.891874e-01

1719 9.613650e-01 9.613471e-01 9.613296e-01 9.613110e-01 9.612891e-01 8.390281e-01 5.992098e-01 6.039996e-01 6.088367e-01 5.894687e-01

1720 9.613977e-01 9.613798e-01 9.613622e-01 9.613436e-01 9.613216e-01 8.391227e-01 5.994863e-01 6.042770e-01 6.091149e-01 5.897426e-01

1721 9.614304e-01 9.614124e-01 9.613948e-01 9.613761e-01 9.613540e-01 8.392150e-01 5.997552e-01 6.045468e-01 6.093853e-01 5.900089e-01

1722 9.614630e-01 9.614450e-01 9.614273e-01 9.614085e-01 9.613863e-01 8.393050e-01 6.000166e-01 6.048089e-01 6.096480e-01 5.902680e-01

1723 9.614956e-01 9.614774e-01 9.614597e-01 9.614408e-01 9.614186e-01 8.393929e-01 6.002707e-01 6.050637e-01 6.099032e-01 5.905198e-01

1724 9.615280e-01 9.615099e-01 9.614920e-01 9.614730e-01 9.614507e-01 8.394789e-01 6.005175e-01 6.053111e-01 6.101511e-01 5.907646e-01

1725 9.615604e-01 9.615422e-01 9.615242e-01 9.615052e-01 9.614828e-01 8.395629e-01 6.007573e-01 6.055515e-01 6.103919e-01 5.910025e-01

1726 9.615928e-01 9.615745e-01 9.615564e-01 9.615372e-01 9.615148e-01 8.396451e-01 6.009901e-01 6.057849e-01 6.106256e-01 5.912336e-01

1727 9.616250e-01 9.616067e-01 9.615885e-01 9.615693e-01 9.615468e-01 8.397256e-01 6.012163e-01 6.060114e-01 6.108525e-01 5.914581e-01

1728 9.616572e-01 9.616388e-01 9.616206e-01 9.616012e-01 9.615786e-01 8.398044e-01 6.014358e-01 6.062314e-01 6.110728e-01 5.916761e-01

1729 9.616893e-01 9.616709e-01 9.616525e-01 9.616331e-01 9.616104e-01 8.398816e-01 6.016489e-01 6.064450e-01 6.112865e-01 5.918878e-01

1730 9.617214e-01 9.617029e-01 9.616844e-01 9.616649e-01 9.616421e-01 8.399574e-01 6.018558e-01 6.066522e-01 6.114940e-01 5.920933e-01

1731 9.617534e-01 9.617348e-01 9.617163e-01 9.616966e-01 9.616738e-01 8.400317e-01 6.020566e-01 6.068533e-01 6.116953e-01 5.922928e-01

1732 9.617854e-01 9.617667e-01 9.617481e-01 9.617283e-01 9.617053e-01 8.401046e-01 6.022515e-01 6.070485e-01 6.118907e-01 5.924865e-01

1733 9.618173e-01 9.617985e-01 9.617798e-01 9.617599e-01 9.617368e-01 8.401763e-01 6.024406e-01 6.072380e-01 6.120803e-01 5.926745e-01

1734 9.618491e-01 9.618303e-01 9.618114e-01 9.617914e-01 9.617683e-01 8.402467e-01 6.026242e-01 6.074218e-01 6.122643e-01 5.928569e-01

1735 9.618809e-01 9.618620e-01 9.618430e-01 9.618229e-01 9.617997e-01 8.403160e-01 6.028023e-01 6.076002e-01 6.124428e-01 5.930340e-01

1736 9.619126e-01 9.618936e-01 9.618746e-01 9.618543e-01 9.618310e-01 8.403841e-01 6.029752e-01 6.077733e-01 6.126161e-01 5.932059e-01

1737 9.619443e-01 9.619252e-01 9.619060e-01 9.618856e-01 9.618622e-01 8.404512e-01 6.031430e-01 6.079414e-01 6.127842e-01 5.933728e-01

1738 9.619759e-01 9.619568e-01 9.619375e-01 9.619169e-01 9.618934e-01 8.405172e-01 6.033059e-01 6.081045e-01 6.129475e-01 5.935347e-01

1739 9.620075e-01 9.619883e-01 9.619688e-01 9.619482e-01 9.619245e-01 8.405823e-01 6.034640e-01 6.082628e-01 6.131059e-01 5.936920e-01

1740 9.620390e-01 9.620197e-01 9.620002e-01 9.619793e-01 9.619556e-01 8.406465e-01 6.036175e-01 6.084165e-01 6.132597e-01 5.938446e-01

1741 9.620705e-01 9.620511e-01 9.620314e-01 9.620105e-01 9.619865e-01 8.407098e-01 6.037666e-01 6.085658e-01 6.134091e-01 5.939928e-01

1742 9.621020e-01 9.620825e-01 9.620626e-01 9.620415e-01 9.620175e-01 8.407722e-01 6.039113e-01 6.087107e-01 6.135542e-01 5.941367e-01

1743 9.621334e-01 9.621138e-01 9.620938e-01 9.620725e-01 9.620483e-01 8.408339e-01 6.040519e-01 6.088515e-01 6.136951e-01 5.942765e-01

1744 9.621647e-01 9.621451e-01 9.621249e-01 9.621035e-01 9.620792e-01 8.408947e-01 6.041885e-01 6.089883e-01 6.138320e-01 5.944123e-01

1745 9.621961e-01 9.621763e-01 9.621560e-01 9.621344e-01 9.621099e-01 8.409549e-01 6.043212e-01 6.091212e-01 6.139650e-01 5.945443e-01

1746 9.622273e-01 9.622075e-01 9.621870e-01 9.621652e-01 9.621406e-01 8.410144e-01 6.044502e-01 6.092504e-01 6.140943e-01 5.946725e-01

1747 9.622586e-01 9.622386e-01 9.622180e-01 9.621960e-01 9.621712e-01 8.410732e-01 6.045755e-01 6.093760e-01 6.142200e-01 5.947972e-01

1748 9.622898e-01 9.622697e-01 9.622489e-01 9.622267e-01 9.622018e-01 8.411314e-01 6.046975e-01 6.094981e-01 6.143423e-01 5.949184e-01

1749 9.623210e-01 9.623008e-01 9.622798e-01 9.622574e-01 9.622323e-01 8.411889e-01 6.048160e-01 6.096169e-01 6.144612e-01 5.950363e-01

1750 9.623521e-01 9.623318e-01 9.623106e-01 9.622880e-01 9.622627e-01 8.412460e-01 6.049314e-01 6.097325e-01 6.145769e-01 5.951510e-01

1751 9.623832e-01 9.623628e-01 9.623414e-01 9.623186e-01 9.622931e-01 8.413024e-01 6.050437e-01 6.098450e-01 6.146896e-01 5.952626e-01

1752 9.624143e-01 9.623938e-01 9.623721e-01 9.623491e-01 9.623234e-01 8.413584e-01 6.051530e-01 6.099545e-01 6.147992e-01 5.953712e-01

1753 9.624453e-01 9.624247e-01 9.624028e-01 9.623796e-01 9.623537e-01 8.414138e-01 6.052595e-01 6.100612e-01 6.149061e-01 5.954770e-01

1754 9.624764e-01 9.624556e-01 9.624335e-01 9.624100e-01 9.623839e-01 8.414688e-01 6.053632e-01 6.101651e-01 6.150101e-01 5.955801e-01

1755 9.625073e-01 9.624864e-01 9.624641e-01 9.624404e-01 9.624141e-01 8.415233e-01 6.054643e-01 6.102664e-01 6.151116e-01 5.956805e-01

1756 9.625383e-01 9.625172e-01 9.624946e-01 9.624707e-01 9.624441e-01 8.415774e-01 6.055628e-01 6.103652e-01 6.152105e-01 5.957784e-01

1757 9.625692e-01 9.625480e-01 9.625252e-01 9.625009e-01 9.624742e-01 8.416311e-01 6.056589e-01 6.104615e-01 6.153070e-01 5.958739e-01

1758 9.626002e-01 9.625788e-01 9.625556e-01 9.625311e-01 9.625041e-01 8.416844e-01 6.057527e-01 6.105555e-01 6.154012e-01 5.959671e-01

1759 9.626310e-01 9.626095e-01 9.625861e-01 9.625613e-01 9.625340e-01 8.417374e-01 6.058443e-01 6.106473e-01 6.154931e-01 5.960580e-01

1760 9.626619e-01 9.626402e-01 9.626165e-01 9.625914e-01 9.625638e-01 8.417899e-01 6.059337e-01 6.107369e-01 6.155829e-01 5.961469e-01

1761 9.626928e-01 9.626708e-01 9.626468e-01 9.626214e-01 9.625936e-01 8.418422e-01 6.060210e-01 6.108244e-01 6.156706e-01 5.962336e-01

1762 9.627236e-01 9.627015e-01 9.626771e-01 9.626514e-01 9.626232e-01 8.418941e-01 6.061064e-01 6.109100e-01 6.157564e-01 5.963184e-01

1763 9.627544e-01 9.627321e-01 9.627074e-01 9.626813e-01 9.626529e-01 8.419457e-01 6.061898e-01 6.109937e-01 6.158403e-01 5.964013e-01

1764 9.627852e-01 9.627627e-01 9.627376e-01 9.627112e-01 9.626824e-01 8.419970e-01 6.062715e-01 6.110756e-01 6.159223e-01 5.964824e-01

1765 9.628159e-01 9.627932e-01 9.627678e-01 9.627410e-01 9.627119e-01 8.420480e-01 6.063514e-01 6.111557e-01 6.160027e-01 5.965618e-01

1766 9.628467e-01 9.628238e-01 9.627979e-01 9.627707e-01 9.627413e-01 8.420988e-01 6.064297e-01 6.112342e-01 6.160813e-01 5.966396e-01

1767 9.628774e-01 9.628543e-01 9.628280e-01 9.628004e-01 9.627706e-01 8.421493e-01 6.065064e-01 6.113111e-01 6.161584e-01 5.967157e-01

1768 9.629081e-01 9.628847e-01 9.628581e-01 9.628300e-01 9.627998e-01 8.421995e-01 6.065815e-01 6.113864e-01 6.162339e-01 5.967904e-01

1769 9.629389e-01 9.629152e-01 9.628881e-01 9.628596e-01 9.628290e-01 8.422495e-01 6.066552e-01 6.114603e-01 6.163080e-01 5.968636e-01

1770 9.629695e-01 9.629456e-01 9.629180e-01 9.628890e-01 9.628580e-01 8.422993e-01 6.067275e-01 6.115329e-01 6.163807e-01 5.969354e-01

1771 9.630002e-01 9.629760e-01 9.629479e-01 9.629185e-01 9.628870e-01 8.423489e-01 6.067985e-01 6.116040e-01 6.164521e-01 5.970059e-01

1772 9.630309e-01 9.630064e-01 9.629778e-01 9.629478e-01 9.629159e-01 8.423983e-01 6.068682e-01 6.116739e-01 6.165221e-01 5.970752e-01

1773 9.630616e-01 9.630368e-01 9.630076e-01 9.629771e-01 9.629447e-01 8.424475e-01 6.069366e-01 6.117426e-01 6.165910e-01 5.971432e-01

1774 9.630922e-01 9.630671e-01 9.630373e-01 9.630063e-01 9.629734e-01 8.424965e-01 6.070039e-01 6.118101e-01 6.166587e-01 5.972102e-01

1775 9.631229e-01 9.630974e-01 9.630670e-01 9.630354e-01 9.630020e-01 8.425453e-01 6.070701e-01 6.118765e-01 6.167253e-01 5.972760e-01

1776 9.631535e-01 9.631277e-01 9.630967e-01 9.630644e-01 9.630305e-01 8.425940e-01 6.071353e-01 6.119419e-01 6.167908e-01 5.973407e-01

1777 9.631841e-01 9.631579e-01 9.631263e-01 9.630934e-01 9.630589e-01 8.426425e-01 6.071994e-01 6.120062e-01 6.168553e-01 5.974045e-01

1778 9.632148e-01 9.631882e-01 9.631558e-01 9.631222e-01 9.630872e-01 8.426908e-01 6.072626e-01 6.120695e-01 6.169188e-01 5.974673e-01

1779 9.632454e-01 9.632184e-01 9.631853e-01 9.631510e-01 9.631153e-01 8.427390e-01 6.073248e-01 6.121320e-01 6.169814e-01 5.975293e-01

1780 9.632760e-01 9.632486e-01 9.632147e-01 9.631797e-01 9.631434e-01 8.427871e-01 6.073862e-01 6.121935e-01 6.170432e-01 5.975903e-01

1781 9.633066e-01 9.632787e-01 9.632440e-01 9.632083e-01 9.631713e-01 8.428350e-01 6.074468e-01 6.122542e-01 6.171041e-01 5.976506e-01

1782 9.633372e-01 9.633088e-01 9.632733e-01 9.632368e-01 9.631991e-01 8.428828e-01 6.075065e-01 6.123142e-01 6.171641e-01 5.977101e-01

1783 9.633678e-01 9.633390e-01 9.633025e-01 9.632651e-01 9.632267e-01 8.429304e-01 6.075655e-01 6.123733e-01 6.172234e-01 5.977688e-01

1784 9.633984e-01 9.633690e-01 9.633316e-01 9.632934e-01 9.632542e-01 8.429780e-01 6.076238e-01 6.124317e-01 6.172820e-01 5.978268e-01

1785 9.634290e-01 9.633991e-01 9.633606e-01 9.633215e-01 9.632816e-01 8.430254e-01 6.076813e-01 6.124895e-01 6.173399e-01 5.978842e-01

1786 9.634596e-01 9.634291e-01 9.633896e-01 9.633496e-01 9.633088e-01 8.430728e-01 6.077383e-01 6.125466e-01 6.173972e-01 5.979409e-01

1787 9.634902e-01 9.634591e-01 9.634185e-01 9.633775e-01 9.633359e-01 8.431200e-01 6.077946e-01 6.126030e-01 6.174538e-01 5.979971e-01

1788 9.635208e-01 9.634890e-01 9.634473e-01 9.634052e-01 9.633627e-01 8.431672e-01 6.078503e-01 6.126589e-01 6.175098e-01 5.980526e-01

1789 9.635514e-01 9.635190e-01 9.634760e-01 9.634329e-01 9.633895e-01 8.432142e-01 6.079055e-01 6.127142e-01 6.175653e-01 5.981077e-01

1790 9.635821e-01 9.635488e-01 9.635046e-01 9.634604e-01 9.634160e-01 8.432612e-01 6.079601e-01 6.127690e-01 6.176202e-01 5.981622e-01

1791 9.636127e-01 9.635787e-01 9.635331e-01 9.634877e-01 9.634423e-01 8.433080e-01 6.080143e-01 6.128233e-01 6.176746e-01 5.982162e-01

1792 9.636433e-01 9.636085e-01 9.635615e-01 9.635149e-01 9.634684e-01 8.433548e-01 6.080679e-01 6.128771e-01 6.177285e-01 5.982698e-01

1793 9.636739e-01 9.636383e-01 9.635898e-01 9.635419e-01 9.634944e-01 8.434016e-01 6.081212e-01 6.129304e-01 6.177820e-01 5.983229e-01

1794 9.637045e-01 9.636680e-01 9.636180e-01 9.635688e-01 9.635201e-01 8.434482e-01 6.081740e-01 6.129833e-01 6.178350e-01 5.983757e-01

1795 9.637351e-01 9.636977e-01 9.636461e-01 9.635954e-01 9.635456e-01 8.434948e-01 6.082264e-01 6.130358e-01 6.178877e-01 5.984281e-01

1796 9.637657e-01 9.637273e-01 9.636740e-01 9.636219e-01 9.635708e-01 8.435413e-01 6.082784e-01 6.130880e-01 6.179399e-01 5.984801e-01

1797 9.637963e-01 9.637569e-01 9.637018e-01 9.636482e-01 9.635958e-01 8.435877e-01 6.083301e-01 6.131397e-01 6.179918e-01 5.985318e-01

1798 9.638270e-01 9.637864e-01 9.637294e-01 9.636742e-01 9.636205e-01 8.436341e-01 6.083814e-01 6.131912e-01 6.180433e-01 5.985831e-01

1799 9.638576e-01 9.638159e-01 9.637569e-01 9.637001e-01 9.636450e-01 8.436804e-01 6.084325e-01 6.132423e-01 6.180945e-01 5.986342e-01

1800 9.638882e-01 9.638453e-01 9.637843e-01 9.637257e-01 9.636692e-01 8.437267e-01 6.084832e-01 6.132931e-01 6.181455e-01 5.986850e-01

1801 9.639188e-01 9.638747e-01 9.638114e-01 9.637511e-01 9.636931e-01 8.437729e-01 6.085337e-01 6.133437e-01 6.181961e-01 5.987355e-01

1802 9.639743e-01 9.639299e-01 9.638664e-01 9.638059e-01 9.637477e-01 8.446620e-01 6.086983e-01 6.135181e-01 6.183809e-01 5.988816e-01

1803 9.640284e-01 9.639838e-01 9.639201e-01 9.638593e-01 9.638009e-01 8.455541e-01 6.088688e-01 6.136988e-01 6.185723e-01 5.990330e-01

1804 9.640812e-01 9.640364e-01 9.639725e-01 9.639115e-01 9.638529e-01 8.464480e-01 6.090455e-01 6.138860e-01 6.187705e-01 5.991900e-01

1805 9.641328e-01 9.640877e-01 9.640237e-01 9.639625e-01 9.639037e-01 8.473426e-01 6.092284e-01 6.140797e-01 6.189756e-01 5.993526e-01

1806 9.641832e-01 9.641380e-01 9.640737e-01 9.640124e-01 9.639534e-01 8.482368e-01 6.094178e-01 6.142801e-01 6.191877e-01 5.995210e-01

1807 9.642325e-01 9.641871e-01 9.641227e-01 9.640612e-01 9.640020e-01 8.491293e-01 6.096136e-01 6.144874e-01 6.194069e-01 5.996953e-01

1808 9.642807e-01 9.642351e-01 9.641706e-01 9.641089e-01 9.640495e-01 8.500189e-01 6.098161e-01 6.147016e-01 6.196334e-01 5.998756e-01

1809 9.643279e-01 9.642822e-01 9.642175e-01 9.641556e-01 9.640961e-01 8.509046e-01 6.100253e-01 6.149228e-01 6.198673e-01 6.000620e-01

1810 9.643742e-01 9.643283e-01 9.642634e-01 9.642014e-01 9.641417e-01 8.517852e-01 6.102415e-01 6.151513e-01 6.201088e-01 6.002548e-01

1811 9.644195e-01 9.643735e-01 9.643085e-01 9.642463e-01 9.641865e-01 8.526597e-01 6.104646e-01 6.153871e-01 6.203578e-01 6.004538e-01

1812 9.644640e-01 9.644178e-01 9.643526e-01 9.642903e-01 9.642303e-01 8.535269e-01 6.106948e-01 6.156303e-01 6.206146e-01 6.006594e-01

1813 9.645076e-01 9.644613e-01 9.643960e-01 9.643335e-01 9.642734e-01 8.543859e-01 6.109322e-01 6.158810e-01 6.208792e-01 6.008715e-01

1814 9.645504e-01 9.645040e-01 9.644385e-01 9.643760e-01 9.643157e-01 8.552357e-01 6.111769e-01 6.161393e-01 6.211517e-01 6.010903e-01

1815 9.645925e-01 9.645459e-01 9.644803e-01 9.644176e-01 9.643572e-01 8.560754e-01 6.114290e-01 6.164052e-01 6.214322e-01 6.013159e-01

1816 9.646338e-01 9.645871e-01 9.645214e-01 9.644586e-01 9.643980e-01 8.569040e-01 6.116885e-01 6.166789e-01 6.217206e-01 6.015483e-01

1817 9.646744e-01 9.646276e-01 9.645618e-01 9.644988e-01 9.644382e-01 8.577209e-01 6.119555e-01 6.169604e-01 6.220172e-01 6.017877e-01

1818 9.647144e-01 9.646674e-01 9.646015e-01 9.645384e-01 9.644777e-01 8.585251e-01 6.122301e-01 6.172497e-01 6.223218e-01 6.020340e-01

1819 9.647537e-01 9.647066e-01 9.646406e-01 9.645774e-01 9.645165e-01 8.593161e-01 6.125122e-01 6.175468e-01 6.226345e-01 6.022873e-01

1820 9.647924e-01 9.647452e-01 9.646791e-01 9.646158e-01 9.645548e-01 8.600932e-01 6.128020e-01 6.178518e-01 6.229554e-01 6.025478e-01

1821 9.648305e-01 9.647833e-01 9.647170e-01 9.646536e-01 9.645925e-01 8.608558e-01 6.130994e-01 6.181647e-01 6.232844e-01 6.028153e-01

1822 9.648681e-01 9.648207e-01 9.647544e-01 9.646909e-01 9.646297e-01 8.616033e-01 6.134044e-01 6.184855e-01 6.236215e-01 6.030900e-01

1823 9.649051e-01 9.648577e-01 9.647912e-01 9.647277e-01 9.646663e-01 8.623354e-01 6.137171e-01 6.188141e-01 6.239666e-01 6.033718e-01

1824 9.649417e-01 9.648941e-01 9.648276e-01 9.647639e-01 9.647025e-01 8.630516e-01 6.140373e-01 6.191505e-01 6.243197e-01 6.036608e-01

1825 9.649777e-01 9.649301e-01 9.648634e-01 9.647997e-01 9.647382e-01 8.637516e-01 6.143651e-01 6.194946e-01 6.246807e-01 6.039569e-01

1826 9.650133e-01 9.649656e-01 9.648988e-01 9.648350e-01 9.647734e-01 8.644351e-01 6.147004e-01 6.198464e-01 6.250496e-01 6.042600e-01

1827 9.650484e-01 9.650006e-01 9.649338e-01 9.648698e-01 9.648082e-01 8.651019e-01 6.150432e-01 6.202058e-01 6.254261e-01 6.045703e-01

1828 9.650831e-01 9.650352e-01 9.649683e-01 9.649043e-01 9.648425e-01 8.657519e-01 6.153932e-01 6.205726e-01 6.258102e-01 6.048875e-01

1829 9.651174e-01 9.650695e-01 9.650025e-01 9.649383e-01 9.648765e-01 8.663848e-01 6.157505e-01 6.209468e-01 6.262018e-01 6.052116e-01

1830 9.651513e-01 9.651033e-01 9.650362e-01 9.649720e-01 9.649101e-01 8.670008e-01 6.161148e-01 6.213281e-01 6.266006e-01 6.055426e-01

1831 9.651848e-01 9.651367e-01 9.650696e-01 9.650053e-01 9.649433e-01 8.675997e-01 6.164861e-01 6.217165e-01 6.270066e-01 6.058803e-01

1832 9.652180e-01 9.651698e-01 9.651026e-01 9.650382e-01 9.649762e-01 8.681815e-01 6.168642e-01 6.221117e-01 6.274193e-01 6.062246e-01

1833 9.652508e-01 9.652026e-01 9.651353e-01 9.650708e-01 9.650087e-01 8.687465e-01 6.172489e-01 6.225136e-01 6.278387e-01 6.065754e-01

1834 9.652833e-01 9.652350e-01 9.651676e-01 9.651031e-01 9.650409e-01 8.692946e-01 6.176400e-01 6.229219e-01 6.282646e-01 6.069324e-01

1835 9.653155e-01 9.652671e-01 9.651997e-01 9.651351e-01 9.650728e-01 8.698261e-01 6.180373e-01 6.233363e-01 6.286965e-01 6.072956e-01

1836 9.653474e-01 9.652989e-01 9.652314e-01 9.651667e-01 9.651044e-01 8.703410e-01 6.184405e-01 6.237567e-01 6.291343e-01 6.076647e-01

1837 9.653790e-01 9.653304e-01 9.652628e-01 9.651981e-01 9.651357e-01 8.708397e-01 6.188495e-01 6.241827e-01 6.295777e-01 6.080396e-01

1838 9.654102e-01 9.653617e-01 9.652940e-01 9.652292e-01 9.651667e-01 8.713224e-01 6.192639e-01 6.246141e-01 6.300263e-01 6.084200e-01

1839 9.654413e-01 9.653926e-01 9.653249e-01 9.652600e-01 9.651974e-01 8.717893e-01 6.196834e-01 6.250505e-01 6.304798e-01 6.088056e-01

1840 9.654720e-01 9.654234e-01 9.653555e-01 9.652906e-01 9.652279e-01 8.722408e-01 6.201078e-01 6.254917e-01 6.309379e-01 6.091963e-01

1841 9.655026e-01 9.654538e-01 9.653859e-01 9.653209e-01 9.652582e-01 8.726771e-01 6.205368e-01 6.259372e-01 6.314002e-01 6.095917e-01

1842 9.655329e-01 9.654840e-01 9.654161e-01 9.653510e-01 9.652882e-01 8.730985e-01 6.209699e-01 6.263868e-01 6.318663e-01 6.099916e-01

1843 9.655629e-01 9.655140e-01 9.654460e-01 9.653808e-01 9.653180e-01 8.735055e-01 6.214069e-01 6.268400e-01 6.323359e-01 6.103956e-01

1844 9.655927e-01 9.655438e-01 9.654757e-01 9.654105e-01 9.653476e-01 8.738984e-01 6.218475e-01 6.272965e-01 6.328085e-01 6.108035e-01

1845 9.656224e-01 9.655734e-01 9.655052e-01 9.654399e-01 9.653770e-01 8.742775e-01 6.222912e-01 6.277560e-01 6.332837e-01 6.112150e-01

1846 9.656518e-01 9.656028e-01 9.655345e-01 9.654692e-01 9.654062e-01 8.746431e-01 6.227376e-01 6.282179e-01 6.337611e-01 6.116296e-01

1847 9.656810e-01 9.656319e-01 9.655636e-01 9.654982e-01 9.654351e-01 8.749958e-01 6.231864e-01 6.286819e-01 6.342403e-01 6.120471e-01

1848 9.657100e-01 9.656609e-01 9.655926e-01 9.655270e-01 9.654639e-01 8.753358e-01 6.236373e-01 6.291477e-01 6.347209e-01 6.124671e-01

1849 9.657389e-01 9.656897e-01 9.656213e-01 9.655557e-01 9.654926e-01 8.756635e-01 6.240897e-01 6.296147e-01 6.352024e-01 6.128892e-01

1850 9.657676e-01 9.657183e-01 9.656499e-01 9.655842e-01 9.655210e-01 8.759794e-01 6.245433e-01 6.300825e-01 6.356844e-01 6.133131e-01

1851 9.657961e-01 9.657468e-01 9.656783e-01 9.656125e-01 9.655493e-01 8.762837e-01 6.249976e-01 6.305508e-01 6.361664e-01 6.137384e-01

1852 9.658244e-01 9.657751e-01 9.657065e-01 9.656407e-01 9.655774e-01 8.765770e-01 6.254524e-01 6.310191e-01 6.366481e-01 6.141647e-01

1853 9.658526e-01 9.658032e-01 9.657346e-01 9.656687e-01 9.656053e-01 8.768594e-01 6.259071e-01 6.314870e-01 6.371289e-01 6.145916e-01

1854 9.658807e-01 9.658312e-01 9.657625e-01 9.656966e-01 9.656331e-01 8.771315e-01 6.263613e-01 6.319540e-01 6.376085e-01 6.150188e-01

1855 9.659086e-01 9.658591e-01 9.657903e-01 9.657243e-01 9.656608e-01 8.773936e-01 6.268147e-01 6.324198e-01 6.380864e-01 6.154458e-01

1856 9.659363e-01 9.658868e-01 9.658179e-01 9.657519e-01 9.656883e-01 8.776460e-01 6.272669e-01 6.328839e-01 6.385623e-01 6.158723e-01

1857 9.659640e-01 9.659144e-01 9.658454e-01 9.657793e-01 9.657157e-01 8.778892e-01 6.277173e-01 6.333460e-01 6.390357e-01 6.162979e-01

1858 9.659915e-01 9.659418e-01 9.658728e-01 9.658066e-01 9.657430e-01 8.781233e-01 6.281657e-01 6.338056e-01 6.395061e-01 6.167222e-01

1859 9.660188e-01 9.659692e-01 9.659001e-01 9.658338e-01 9.657701e-01 8.783489e-01 6.286117e-01 6.342624e-01 6.399733e-01 6.171448e-01

1860 9.660461e-01 9.659964e-01 9.659272e-01 9.658609e-01 9.657971e-01 8.785662e-01 6.290549e-01 6.347159e-01 6.404369e-01 6.175654e-01

1861 9.660732e-01 9.660234e-01 9.659542e-01 9.658878e-01 9.658240e-01 8.787755e-01 6.294949e-01 6.351659e-01 6.408964e-01 6.179837e-01

1862 9.661003e-01 9.660504e-01 9.659811e-01 9.659147e-01 9.658508e-01 8.789772e-01 6.299314e-01 6.356119e-01 6.413516e-01 6.183992e-01

1863 9.661272e-01 9.660773e-01 9.660079e-01 9.659414e-01 9.658774e-01 8.791716e-01 6.303640e-01 6.360536e-01 6.418020e-01 6.188117e-01

1864 9.661540e-01 9.661041e-01 9.660346e-01 9.659680e-01 9.659040e-01 8.793589e-01 6.307925e-01 6.364908e-01 6.422475e-01 6.192208e-01

1865 9.661807e-01 9.661307e-01 9.660612e-01 9.659945e-01 9.659304e-01 8.795394e-01 6.312164e-01 6.369231e-01 6.426877e-01 6.196262e-01

1866 9.662073e-01 9.661573e-01 9.660877e-01 9.660210e-01 9.659568e-01 8.797135e-01 6.316356e-01 6.373502e-01 6.431223e-01 6.200276e-01

1867 9.662339e-01 9.661838e-01 9.661141e-01 9.660473e-01 9.659830e-01 8.798814e-01 6.320497e-01 6.377718e-01 6.435510e-01 6.204247e-01

1868 9.662603e-01 9.662101e-01 9.661404e-01 9.660735e-01 9.660092e-01 8.800434e-01 6.324585e-01 6.381878e-01 6.439737e-01 6.208172e-01

1869 9.662866e-01 9.662364e-01 9.661667e-01 9.660997e-01 9.660353e-01 8.801996e-01 6.328618e-01 6.385978e-01 6.443900e-01 6.212049e-01

1870 9.663129e-01 9.662626e-01 9.661928e-01 9.661257e-01 9.660613e-01 8.803504e-01 6.332592e-01 6.390016e-01 6.447999e-01 6.215876e-01

1871 9.663391e-01 9.662888e-01 9.662188e-01 9.661517e-01 9.660872e-01 8.804960e-01 6.336507e-01 6.393992e-01 6.452030e-01 6.219650e-01

1872 9.663652e-01 9.663148e-01 9.662448e-01 9.661776e-01 9.661130e-01 8.806366e-01 6.340359e-01 6.397901e-01 6.455992e-01 6.223368e-01

1873 9.663912e-01 9.663408e-01 9.662707e-01 9.662034e-01 9.661387e-01 8.807725e-01 6.344148e-01 6.401744e-01 6.459884e-01 6.227030e-01

1874 9.664172e-01 9.663667e-01 9.662965e-01 9.662291e-01 9.661644e-01 8.809037e-01 6.347872e-01 6.405518e-01 6.463705e-01 6.230634e-01

1875 9.664431e-01 9.663925e-01 9.663222e-01 9.662548e-01 9.661899e-01 8.810307e-01 6.351529e-01 6.409223e-01 6.467453e-01 6.234177e-01

1876 9.664689e-01 9.664183e-01 9.663479e-01 9.662803e-01 9.662154e-01 8.811534e-01 6.355118e-01 6.412858e-01 6.471128e-01 6.237658e-01

1877 9.664946e-01 9.664440e-01 9.663735e-01 9.663058e-01 9.662409e-01 8.812722e-01 6.358639e-01 6.416420e-01 6.474728e-01 6.241077e-01

1878 9.665203e-01 9.664696e-01 9.663991e-01 9.663313e-01 9.662662e-01 8.813871e-01 6.362090e-01 6.419911e-01 6.478254e-01 6.244432e-01

1879 9.665459e-01 9.664952e-01 9.664245e-01 9.663567e-01 9.662915e-01 8.814985e-01 6.365471e-01 6.423328e-01 6.481704e-01 6.247722e-01

1880 9.665715e-01 9.665207e-01 9.664499e-01 9.663820e-01 9.663167e-01 8.816063e-01 6.368781e-01 6.426673e-01 6.485078e-01 6.250946e-01

1881 9.665970e-01 9.665461e-01 9.664753e-01 9.664072e-01 9.663419e-01 8.817109e-01 6.372020e-01 6.429943e-01 6.488377e-01 6.254104e-01

1882 9.666225e-01 9.665715e-01 9.665006e-01 9.664324e-01 9.663670e-01 8.818122e-01 6.375188e-01 6.433141e-01 6.491601e-01 6.257195e-01

1883 9.666479e-01 9.665968e-01 9.665258e-01 9.664575e-01 9.663920e-01 8.819106e-01 6.378284e-01 6.436265e-01 6.494748e-01 6.260220e-01

1884 9.666732e-01 9.666221e-01 9.665510e-01 9.664826e-01 9.664170e-01 8.820061e-01 6.381309e-01 6.439315e-01 6.497821e-01 6.263177e-01

1885 9.666986e-01 9.666474e-01 9.665761e-01 9.665076e-01 9.664419e-01 8.820988e-01 6.384262e-01 6.442293e-01 6.500819e-01 6.266067e-01

1886 9.667238e-01 9.666725e-01 9.666012e-01 9.665325e-01 9.664667e-01 8.821889e-01 6.387145e-01 6.445198e-01 6.503742e-01 6.268890e-01

1887 9.667490e-01 9.666977e-01 9.666262e-01 9.665574e-01 9.664915e-01 8.822765e-01 6.389957e-01 6.448031e-01 6.506593e-01 6.271646e-01

1888 9.667742e-01 9.667228e-01 9.666512e-01 9.665823e-01 9.665163e-01 8.823617e-01 6.392699e-01 6.450792e-01 6.509370e-01 6.274335e-01

1889 9.667993e-01 9.667478e-01 9.666761e-01 9.666071e-01 9.665409e-01 8.824446e-01 6.395372e-01 6.453483e-01 6.512075e-01 6.276959e-01

1890 9.668244e-01 9.667728e-01 9.667009e-01 9.666318e-01 9.665656e-01 8.825253e-01 6.397977e-01 6.456104e-01 6.514710e-01 6.279516e-01

1891 9.668494e-01 9.667978e-01 9.667258e-01 9.666565e-01 9.665901e-01 8.826040e-01 6.400514e-01 6.458657e-01 6.517274e-01 6.282009e-01

1892 9.668744e-01 9.668227e-01 9.667505e-01 9.666811e-01 9.666146e-01 8.826807e-01 6.402984e-01 6.461141e-01 6.519769e-01 6.284438e-01

1893 9.668994e-01 9.668476e-01 9.667753e-01 9.667057e-01 9.666391e-01 8.827554e-01 6.405388e-01 6.463558e-01 6.522197e-01 6.286803e-01

1894 9.669244e-01 9.668724e-01 9.668000e-01 9.667303e-01 9.666635e-01 8.828284e-01 6.407727e-01 6.465910e-01 6.524558e-01 6.289105e-01

1895 9.669493e-01 9.668972e-01 9.668246e-01 9.667548e-01 9.666879e-01 8.828997e-01 6.410003e-01 6.468197e-01 6.526854e-01 6.291346e-01

1896 9.669741e-01 9.669220e-01 9.668492e-01 9.667792e-01 9.667122e-01 8.829693e-01 6.412216e-01 6.470421e-01 6.529085e-01 6.293526e-01

1897 9.669990e-01 9.669467e-01 9.668738e-01 9.668036e-01 9.667364e-01 8.830374e-01 6.414367e-01 6.472582e-01 6.531254e-01 6.295647e-01

1898 9.670238e-01 9.669714e-01 9.668983e-01 9.668280e-01 9.667606e-01 8.831040e-01 6.416459e-01 6.474683e-01 6.533362e-01 6.297709e-01

1899 9.670486e-01 9.669961e-01 9.669228e-01 9.668523e-01 9.667848e-01 8.831691e-01 6.418491e-01 6.476724e-01 6.535409e-01 6.299713e-01

1900 9.670733e-01 9.670208e-01 9.669473e-01 9.668765e-01 9.668089e-01 8.832329e-01 6.420466e-01 6.478707e-01 6.537398e-01 6.301662e-01

1901 9.670980e-01 9.670454e-01 9.669717e-01 9.669008e-01 9.668330e-01 8.832955e-01 6.422385e-01 6.480634e-01 6.539330e-01 6.303555e-01

1902 9.671228e-01 9.670700e-01 9.669961e-01 9.669250e-01 9.668570e-01 8.833567e-01 6.424248e-01 6.482505e-01 6.541205e-01 6.305395e-01

1903 9.671474e-01 9.670945e-01 9.670204e-01 9.669491e-01 9.668809e-01 8.834168e-01 6.426058e-01 6.484321e-01 6.543027e-01 6.307182e-01

1904 9.671721e-01 9.671190e-01 9.670447e-01 9.669732e-01 9.669048e-01 8.834758e-01 6.427816e-01 6.486086e-01 6.544795e-01 6.308918e-01

1905 9.671967e-01 9.671435e-01 9.670690e-01 9.669972e-01 9.669287e-01 8.835337e-01 6.429523e-01 6.487799e-01 6.546513e-01 6.310604e-01

1906 9.672213e-01 9.671680e-01 9.670932e-01 9.670212e-01 9.669525e-01 8.835906e-01 6.431181e-01 6.489462e-01 6.548180e-01 6.312241e-01

1907 9.672459e-01 9.671925e-01 9.671174e-01 9.670452e-01 9.669763e-01 8.836465e-01 6.432790e-01 6.491077e-01 6.549798e-01 6.313831e-01

1908 9.672705e-01 9.672169e-01 9.671416e-01 9.670691e-01 9.670000e-01 8.837015e-01 6.434352e-01 6.492645e-01 6.551369e-01 6.315374e-01

1909 9.672951e-01 9.672413e-01 9.671657e-01 9.670930e-01 9.670236e-01 8.837556e-01 6.435869e-01 6.494167e-01 6.552895e-01 6.316873e-01

1910 9.673196e-01 9.672657e-01 9.671898e-01 9.671168e-01 9.670472e-01 8.838089e-01 6.437342e-01 6.495645e-01 6.554376e-01 6.318329e-01

1911 9.673442e-01 9.672900e-01 9.672139e-01 9.671406e-01 9.670708e-01 8.838614e-01 6.438772e-01 6.497080e-01 6.555814e-01 6.319742e-01

1912 9.673687e-01 9.673144e-01 9.672379e-01 9.671644e-01 9.670943e-01 8.839131e-01 6.440160e-01 6.498473e-01 6.557210e-01 6.321114e-01

1913 9.673932e-01 9.673387e-01 9.672619e-01 9.671881e-01 9.671177e-01 8.839641e-01 6.441509e-01 6.499826e-01 6.558566e-01 6.322446e-01

1914 9.674177e-01 9.673630e-01 9.672859e-01 9.672117e-01 9.671411e-01 8.840143e-01 6.442818e-01 6.501140e-01 6.559883e-01 6.323740e-01

1915 9.674421e-01 9.673872e-01 9.673098e-01 9.672353e-01 9.671644e-01 8.840639e-01 6.444090e-01 6.502416e-01 6.561162e-01 6.324997e-01

1916 9.674666e-01 9.674115e-01 9.673337e-01 9.672589e-01 9.671877e-01 8.841129e-01 6.445325e-01 6.503656e-01 6.562405e-01 6.326217e-01

1917 9.674911e-01 9.674357e-01 9.673576e-01 9.672824e-01 9.672109e-01 8.841613e-01 6.446525e-01 6.504860e-01 6.563612e-01 6.327403e-01

1918 9.675155e-01 9.674599e-01 9.673814e-01 9.673059e-01 9.672341e-01 8.842091e-01 6.447691e-01 6.506030e-01 6.564785e-01 6.328555e-01

1919 9.675399e-01 9.674841e-01 9.674052e-01 9.673293e-01 9.672572e-01 8.842563e-01 6.448825e-01 6.507168e-01 6.565925e-01 6.329674e-01

1920 9.675644e-01 9.675083e-01 9.674289e-01 9.673527e-01 9.672802e-01 8.843031e-01 6.449926e-01 6.508273e-01 6.567033e-01 6.330762e-01

1921 9.675888e-01 9.675325e-01 9.674526e-01 9.673760e-01 9.673032e-01 8.843493e-01 6.450997e-01 6.509348e-01 6.568111e-01 6.331819e-01

1922 9.676132e-01 9.675566e-01 9.674763e-01 9.673992e-01 9.673261e-01 8.843951e-01 6.452038e-01 6.510394e-01 6.569159e-01 6.332847e-01

1923 9.676376e-01 9.675807e-01 9.675000e-01 9.674224e-01 9.673489e-01 8.844404e-01 6.453051e-01 6.511410e-01 6.570179e-01 6.333847e-01

1924 9.676620e-01 9.676048e-01 9.675236e-01 9.674456e-01 9.673717e-01 8.844852e-01 6.454036e-01 6.512400e-01 6.571171e-01 6.334820e-01

1925 9.676864e-01 9.676289e-01 9.675471e-01 9.674687e-01 9.673944e-01 8.845297e-01 6.454995e-01 6.513363e-01 6.572137e-01 6.335766e-01

1926 9.677108e-01 9.676530e-01 9.675706e-01 9.674917e-01 9.674170e-01 8.845738e-01 6.455929e-01 6.514300e-01 6.573077e-01 6.336687e-01

1927 9.677352e-01 9.676770e-01 9.675941e-01 9.675147e-01 9.674395e-01 8.846174e-01 6.456838e-01 6.515213e-01 6.573993e-01 6.337584e-01

1928 9.677596e-01 9.677011e-01 9.676175e-01 9.675376e-01 9.674620e-01 8.846608e-01 6.457723e-01 6.516102e-01 6.574885e-01 6.338458e-01

1929 9.677840e-01 9.677251e-01 9.676409e-01 9.675604e-01 9.674844e-01 8.847038e-01 6.458586e-01 6.516969e-01 6.575754e-01 6.339308e-01

1930 9.678084e-01 9.677491e-01 9.676642e-01 9.675832e-01 9.675067e-01 8.847465e-01 6.459427e-01 6.517813e-01 6.576602e-01 6.340138e-01

1931 9.678328e-01 9.677730e-01 9.676875e-01 9.676059e-01 9.675289e-01 8.847888e-01 6.460246e-01 6.518637e-01 6.577429e-01 6.340946e-01

1932 9.678572e-01 9.677970e-01 9.677108e-01 9.676285e-01 9.675510e-01 8.848309e-01 6.461046e-01 6.519440e-01 6.578235e-01 6.341735e-01

1933 9.678815e-01 9.678209e-01 9.677339e-01 9.676511e-01 9.675730e-01 8.848727e-01 6.461826e-01 6.520225e-01 6.579022e-01 6.342504e-01

1934 9.679059e-01 9.678448e-01 9.677571e-01 9.676735e-01 9.675949e-01 8.849142e-01 6.462588e-01 6.520990e-01 6.579790e-01 6.343254e-01

1935 9.679303e-01 9.678687e-01 9.677801e-01 9.676959e-01 9.676167e-01 8.849555e-01 6.463332e-01 6.521737e-01 6.580541e-01 6.343987e-01

1936 9.679547e-01 9.678926e-01 9.678032e-01 9.677182e-01 9.676384e-01 8.849965e-01 6.464058e-01 6.522468e-01 6.581274e-01 6.344703e-01

1937 9.679791e-01 9.679164e-01 9.678261e-01 9.677404e-01 9.676600e-01 8.850373e-01 6.464768e-01 6.523182e-01 6.581991e-01 6.345403e-01

1938 9.680035e-01 9.679402e-01 9.678490e-01 9.677626e-01 9.676815e-01 8.850779e-01 6.465463e-01 6.523880e-01 6.582692e-01 6.346087e-01

1939 9.680279e-01 9.679640e-01 9.678718e-01 9.677846e-01 9.677029e-01 8.851183e-01 6.466142e-01 6.524562e-01 6.583378e-01 6.346756e-01

1940 9.680522e-01 9.679878e-01 9.678946e-01 9.678065e-01 9.677241e-01 8.851584e-01 6.466807e-01 6.525231e-01 6.584049e-01 6.347411e-01

1941 9.680766e-01 9.680115e-01 9.679173e-01 9.678283e-01 9.677453e-01 8.851984e-01 6.467457e-01 6.525885e-01 6.584706e-01 6.348052e-01

1942 9.681010e-01 9.680352e-01 9.679399e-01 9.678500e-01 9.677662e-01 8.852382e-01 6.468095e-01 6.526526e-01 6.585350e-01 6.348680e-01

1943 9.681254e-01 9.680589e-01 9.679624e-01 9.678716e-01 9.677871e-01 8.852779e-01 6.468719e-01 6.527154e-01 6.585981e-01 6.349295e-01

1944 9.681498e-01 9.680826e-01 9.679848e-01 9.678931e-01 9.678078e-01 8.853173e-01 6.469332e-01 6.527770e-01 6.586600e-01 6.349899e-01

1945 9.681742e-01 9.681062e-01 9.680072e-01 9.679145e-01 9.678284e-01 8.853566e-01 6.469932e-01 6.528374e-01 6.587207e-01 6.350490e-01

1946 9.681986e-01 9.681297e-01 9.680295e-01 9.679357e-01 9.678488e-01 8.853958e-01 6.470522e-01 6.528967e-01 6.587802e-01 6.351071e-01

1947 9.682230e-01 9.681533e-01 9.680516e-01 9.679568e-01 9.678690e-01 8.854348e-01 6.471101e-01 6.529549e-01 6.588387e-01 6.351641e-01

1948 9.682474e-01 9.681768e-01 9.680737e-01 9.679778e-01 9.678891e-01 8.854737e-01 6.471669e-01 6.530121e-01 6.588962e-01 6.352201e-01

1949 9.682718e-01 9.682002e-01 9.680957e-01 9.679986e-01 9.679090e-01 8.855125e-01 6.472228e-01 6.530683e-01 6.589527e-01 6.352752e-01

1950 9.682962e-01 9.682236e-01 9.681175e-01 9.680193e-01 9.679287e-01 8.855512e-01 6.472777e-01 6.531236e-01 6.590082e-01 6.353293e-01

1951 9.683206e-01 9.682470e-01 9.681393e-01 9.680398e-01 9.679483e-01 8.855897e-01 6.473318e-01 6.531779e-01 6.590628e-01 6.353825e-01

1952 9.683450e-01 9.682703e-01 9.681609e-01 9.680601e-01 9.679676e-01 8.856281e-01 6.473850e-01 6.532314e-01 6.591166e-01 6.354349e-01

1953 9.683694e-01 9.682936e-01 9.681824e-01 9.680803e-01 9.679867e-01 8.856665e-01 6.474373e-01 6.532841e-01 6.591695e-01 6.354866e-01

1954 9.683938e-01 9.683168e-01 9.682038e-01 9.681003e-01 9.680057e-01 8.857047e-01 6.474889e-01 6.533360e-01 6.592217e-01 6.355374e-01

1955 9.684182e-01 9.683399e-01 9.682251e-01 9.681202e-01 9.680244e-01 8.857429e-01 6.475398e-01 6.533872e-01 6.592731e-01 6.355876e-01

1956 9.684426e-01 9.683630e-01 9.682462e-01 9.681398e-01 9.680429e-01 8.857809e-01 6.475899e-01 6.534376e-01 6.593238e-01 6.356370e-01

1957 9.684670e-01 9.683860e-01 9.682671e-01 9.681592e-01 9.680612e-01 8.858189e-01 6.476394e-01 6.534874e-01 6.593738e-01 6.356858e-01

1958 9.684914e-01 9.684089e-01 9.682879e-01 9.681784e-01 9.680792e-01 8.858568e-01 6.476883e-01 6.535365e-01 6.594232e-01 6.357340e-01

1959 9.685158e-01 9.684318e-01 9.683086e-01 9.681974e-01 9.680970e-01 8.858946e-01 6.477365e-01 6.535850e-01 6.594719e-01 6.357816e-01

1960 9.685402e-01 9.684546e-01 9.683291e-01 9.682162e-01 9.681145e-01 8.859324e-01 6.477841e-01 6.536329e-01 6.595201e-01 6.358286e-01

1961 9.685645e-01 9.684773e-01 9.683494e-01 9.682348e-01 9.681317e-01 8.859701e-01 6.478312e-01 6.536803e-01 6.595676e-01 6.358751e-01

1962 9.685889e-01 9.684999e-01 9.683695e-01 9.682531e-01 9.681487e-01 8.860077e-01 6.478778e-01 6.537271e-01 6.596147e-01 6.359210e-01

1963 9.686132e-01 9.685225e-01 9.683894e-01 9.682711e-01 9.681654e-01 8.860453e-01 6.479239e-01 6.537734e-01 6.596612e-01 6.359665e-01

1964 9.686376e-01 9.685449e-01 9.684091e-01 9.682889e-01 9.681818e-01 8.860828e-01 6.479695e-01 6.538193e-01 6.597073e-01 6.360116e-01

1965 9.686619e-01 9.685672e-01 9.684286e-01 9.683064e-01 9.681978e-01 8.861202e-01 6.480146e-01 6.538647e-01 6.597529e-01 6.360562e-01

1966 9.686862e-01 9.685895e-01 9.684479e-01 9.683237e-01 9.682136e-01 8.861577e-01 6.480594e-01 6.539096e-01 6.597981e-01 6.361004e-01

1967 9.687104e-01 9.686116e-01 9.684670e-01 9.683406e-01 9.682290e-01 8.861950e-01 6.481037e-01 6.539542e-01 6.598429e-01 6.361442e-01

1968 9.687347e-01 9.686336e-01 9.684858e-01 9.683573e-01 9.682441e-01 8.862324e-01 6.481477e-01 6.539984e-01 6.598872e-01 6.361877e-01

1969 9.687589e-01 9.686554e-01 9.685044e-01 9.683736e-01 9.682588e-01 8.862697e-01 6.481913e-01 6.540422e-01 6.599312e-01 6.362308e-01

1970 9.687831e-01 9.686771e-01 9.685227e-01 9.683896e-01 9.682732e-01 8.863069e-01 6.482346e-01 6.540857e-01 6.599749e-01 6.362737e-01

1971 9.688073e-01 9.686987e-01 9.685408e-01 9.684052e-01 9.682872e-01 8.863441e-01 6.482775e-01 6.541288e-01 6.600182e-01 6.363162e-01

1972 9.688314e-01 9.687202e-01 9.685585e-01 9.684205e-01 9.683008e-01 8.863813e-01 6.483202e-01 6.541717e-01 6.600613e-01 6.363584e-01

1973 9.688555e-01 9.687415e-01 9.685760e-01 9.684355e-01 9.683140e-01 8.864185e-01 6.483625e-01 6.542143e-01 6.601040e-01 6.364004e-01

1974 9.688796e-01 9.687626e-01 9.685932e-01 9.684500e-01 9.683267e-01 8.864556e-01 6.484047e-01 6.542566e-01 6.601465e-01 6.364421e-01

1975 9.689036e-01 9.687835e-01 9.686100e-01 9.684642e-01 9.683391e-01 8.864927e-01 6.484465e-01 6.542986e-01 6.601887e-01 6.364836e-01

1976 9.689276e-01 9.688043e-01 9.686265e-01 9.684780e-01 9.683510e-01 8.865298e-01 6.484882e-01 6.543404e-01 6.602306e-01 6.365249e-01

1977 9.689515e-01 9.688249e-01 9.686427e-01 9.684913e-01 9.683625e-01 8.865669e-01 6.485296e-01 6.543820e-01 6.602724e-01 6.365660e-01

1978 9.689754e-01 9.688452e-01 9.686585e-01 9.685042e-01 9.683735e-01 8.866039e-01 6.485709e-01 6.544234e-01 6.603139e-01 6.366069e-01

1979 9.689992e-01 9.688654e-01 9.686740e-01 9.685167e-01 9.683841e-01 8.866409e-01 6.486119e-01 6.544647e-01 6.603553e-01 6.366476e-01

1980 9.690229e-01 9.688853e-01 9.686891e-01 9.685287e-01 9.683941e-01 8.866779e-01 6.486528e-01 6.545057e-01 6.603964e-01 6.366882e-01

1981 9.690466e-01 9.689050e-01 9.687037e-01 9.685402e-01 9.684037e-01 8.867149e-01 6.486935e-01 6.545466e-01 6.604374e-01 6.367286e-01

1982 9.690788e-01 9.689371e-01 9.687356e-01 9.685720e-01 9.684353e-01 8.874968e-01 6.488949e-01 6.547617e-01 6.606670e-01 6.369042e-01

1983 9.691104e-01 9.689686e-01 9.687670e-01 9.686033e-01 9.684664e-01 8.882695e-01 6.491024e-01 6.549832e-01 6.609033e-01 6.370853e-01

1984 9.691415e-01 9.689995e-01 9.687979e-01 9.686340e-01 9.684970e-01 8.890323e-01 6.493160e-01 6.552112e-01 6.611464e-01 6.372718e-01

1985 9.691721e-01 9.690300e-01 9.688282e-01 9.686642e-01 9.685271e-01 8.897846e-01 6.495358e-01 6.554457e-01 6.613963e-01 6.374640e-01

1986 9.692022e-01 9.690600e-01 9.688581e-01 9.686940e-01 9.685568e-01 8.905256e-01 6.497620e-01 6.556868e-01 6.616532e-01 6.376618e-01

1987 9.692318e-01 9.690895e-01 9.688875e-01 9.687233e-01 9.685859e-01 8.912548e-01 6.499944e-01 6.559345e-01 6.619169e-01 6.378654e-01

1988 9.692610e-01 9.691186e-01 9.689164e-01 9.687521e-01 9.686147e-01 8.919716e-01 6.502332e-01 6.561889e-01 6.621876e-01 6.380747e-01

1989 9.692897e-01 9.691472e-01 9.689450e-01 9.687806e-01 9.686430e-01 8.926755e-01 6.504785e-01 6.564499e-01 6.624653e-01 6.382899e-01

1990 9.693181e-01 9.691755e-01 9.689731e-01 9.688086e-01 9.686710e-01 8.933661e-01 6.507302e-01 6.567178e-01 6.627500e-01 6.385110e-01

1991 9.693460e-01 9.692033e-01 9.690009e-01 9.688363e-01 9.686985e-01 8.940429e-01 6.509884e-01 6.569923e-01 6.630416e-01 6.387379e-01

1992 9.693736e-01 9.692308e-01 9.690283e-01 9.688635e-01 9.687257e-01 8.947056e-01 6.512530e-01 6.572736e-01 6.633403e-01 6.389709e-01

1993 9.694008e-01 9.692580e-01 9.690553e-01 9.688905e-01 9.687526e-01 8.953539e-01 6.515242e-01 6.575616e-01 6.636458e-01 6.392097e-01

1994 9.694277e-01 9.692848e-01 9.690820e-01 9.689171e-01 9.687791e-01 8.959876e-01 6.518018e-01 6.578563e-01 6.639583e-01 6.394546e-01

1995 9.694543e-01 9.693112e-01 9.691084e-01 9.689434e-01 9.688053e-01 8.966064e-01 6.520858e-01 6.581576e-01 6.642776e-01 6.397054e-01

1996 9.694805e-01 9.693374e-01 9.691345e-01 9.689694e-01 9.688311e-01 8.972102e-01 6.523763e-01 6.584656e-01 6.646037e-01 6.399622e-01

1997 9.695065e-01 9.693632e-01 9.691602e-01 9.689950e-01 9.688567e-01 8.977989e-01 6.526731e-01 6.587801e-01 6.649366e-01 6.402249e-01

1998 9.695321e-01 9.693888e-01 9.691857e-01 9.690204e-01 9.688821e-01 8.983724e-01 6.529762e-01 6.591010e-01 6.652760e-01 6.404935e-01

1999 9.695575e-01 9.694141e-01 9.692109e-01 9.690456e-01 9.689071e-01 8.989306e-01 6.532856e-01 6.594283e-01 6.656219e-01 6.407680e-01

2000 9.695826e-01 9.694392e-01 9.692359e-01 9.690704e-01 9.689319e-01 8.994736e-01 6.536011e-01 6.597619e-01 6.659742e-01 6.410483e-01

2001 9.696075e-01 9.694639e-01 9.692606e-01 9.690951e-01 9.689564e-01 9.000015e-01 6.539226e-01 6.601016e-01 6.663327e-01 6.413343e-01

2002 9.696321e-01 9.694885e-01 9.692851e-01 9.691194e-01 9.689807e-01 9.005143e-01 6.542500e-01 6.604474e-01 6.666972e-01 6.416260e-01

2003 9.696565e-01 9.695128e-01 9.693093e-01 9.691436e-01 9.690048e-01 9.010120e-01 6.545833e-01 6.607990e-01 6.670677e-01 6.419232e-01

2004 9.696806e-01 9.695369e-01 9.693333e-01 9.691675e-01 9.690286e-01 9.014950e-01 6.549221e-01 6.611563e-01 6.674439e-01 6.422259e-01

2005 9.697046e-01 9.695608e-01 9.693571e-01 9.691912e-01 9.690523e-01 9.019633e-01 6.552665e-01 6.615191e-01 6.678256e-01 6.425340e-01

2006 9.697283e-01 9.695844e-01 9.693807e-01 9.692147e-01 9.690757e-01 9.024171e-01 6.556162e-01 6.618872e-01 6.682125e-01 6.428473e-01

2007 9.697519e-01 9.696079e-01 9.694041e-01 9.692381e-01 9.690990e-01 9.028566e-01 6.559711e-01 6.622605e-01 6.686046e-01 6.431657e-01

2008 9.697752e-01 9.696312e-01 9.694273e-01 9.692612e-01 9.691220e-01 9.032821e-01 6.563308e-01 6.626386e-01 6.690014e-01 6.434889e-01

2009 9.697984e-01 9.696543e-01 9.694503e-01 9.692842e-01 9.691449e-01 9.036938e-01 6.566953e-01 6.630214e-01 6.694028e-01 6.438169e-01

2010 9.698214e-01 9.696773e-01 9.694732e-01 9.693069e-01 9.691676e-01 9.040920e-01 6.570643e-01 6.634086e-01 6.698085e-01 6.441495e-01

2011 9.698442e-01 9.697000e-01 9.694959e-01 9.693296e-01 9.691902e-01 9.044770e-01 6.574376e-01 6.637999e-01 6.702181e-01 6.444865e-01

2012 9.698669e-01 9.697226e-01 9.695184e-01 9.693520e-01 9.692126e-01 9.048491e-01 6.578148e-01 6.641952e-01 6.706315e-01 6.448276e-01

2013 9.698894e-01 9.697451e-01 9.695408e-01 9.693743e-01 9.692348e-01 9.052085e-01 6.581959e-01 6.645940e-01 6.710483e-01 6.451727e-01

2014 9.699118e-01 9.697674e-01 9.695631e-01 9.693965e-01 9.692569e-01 9.055556e-01 6.585804e-01 6.649961e-01 6.714681e-01 6.455215e-01

2015 9.699340e-01 9.697896e-01 9.695851e-01 9.694185e-01 9.692788e-01 9.058907e-01 6.589680e-01 6.654012e-01 6.718907e-01 6.458738e-01

2016 9.699561e-01 9.698116e-01 9.696071e-01 9.694404e-01 9.693006e-01 9.062141e-01 6.593586e-01 6.658089e-01 6.723156e-01 6.462293e-01

2017 9.699781e-01 9.698335e-01 9.696289e-01 9.694621e-01 9.693223e-01 9.065262e-01 6.597518e-01 6.662191e-01 6.727427e-01 6.465877e-01

2018 9.700000e-01 9.698553e-01 9.696506e-01 9.694837e-01 9.693438e-01 9.068272e-01 6.601473e-01 6.666312e-01 6.731714e-01 6.469489e-01

2019 9.700217e-01 9.698770e-01 9.696722e-01 9.695052e-01 9.693653e-01 9.071175e-01 6.605447e-01 6.670450e-01 6.736016e-01 6.473125e-01

2020 9.700433e-01 9.698985e-01 9.696937e-01 9.695266e-01 9.693866e-01 9.073975e-01 6.609438e-01 6.674602e-01 6.740327e-01 6.476782e-01

2021 9.700648e-01 9.699200e-01 9.697150e-01 9.695479e-01 9.694078e-01 9.076674e-01 6.613442e-01 6.678764e-01 6.744645e-01 6.480458e-01

2022 9.700862e-01 9.699413e-01 9.697363e-01 9.695691e-01 9.694289e-01 9.079276e-01 6.617456e-01 6.682932e-01 6.748965e-01 6.484149e-01

2023 9.701075e-01 9.699625e-01 9.697574e-01 9.695901e-01 9.694499e-01 9.081783e-01 6.621476e-01 6.687103e-01 6.753285e-01 6.487853e-01

2024 9.701287e-01 9.699837e-01 9.697785e-01 9.696111e-01 9.694707e-01 9.084200e-01 6.625499e-01 6.691274e-01 6.757600e-01 6.491566e-01

2025 9.701498e-01 9.700047e-01 9.697994e-01 9.696319e-01 9.694915e-01 9.086529e-01 6.629523e-01 6.695441e-01 6.761908e-01 6.495285e-01

2026 9.701708e-01 9.700256e-01 9.698203e-01 9.696527e-01 9.695122e-01 9.088774e-01 6.633543e-01 6.699601e-01 6.766204e-01 6.499008e-01

2027 9.701917e-01 9.700465e-01 9.698411e-01 9.696734e-01 9.695328e-01 9.090937e-01 6.637556e-01 6.703749e-01 6.770485e-01 6.502731e-01

2028 9.702125e-01 9.700673e-01 9.698617e-01 9.696940e-01 9.695533e-01 9.093021e-01 6.641558e-01 6.707884e-01 6.774747e-01 6.506450e-01

2029 9.702333e-01 9.700880e-01 9.698823e-01 9.697145e-01 9.695737e-01 9.095029e-01 6.645548e-01 6.712001e-01 6.778987e-01 6.510164e-01

2030 9.702540e-01 9.701086e-01 9.699029e-01 9.697349e-01 9.695941e-01 9.096964e-01 6.649520e-01 6.716097e-01 6.783202e-01 6.513869e-01

2031 9.702746e-01 9.701291e-01 9.699233e-01 9.697553e-01 9.696143e-01 9.098829e-01 6.653473e-01 6.720169e-01 6.787389e-01 6.517561e-01

2032 9.702951e-01 9.701496e-01 9.699437e-01 9.697755e-01 9.696345e-01 9.100627e-01 6.657403e-01 6.724214e-01 6.791544e-01 6.521239e-01

2033 9.703156e-01 9.701700e-01 9.699640e-01 9.697957e-01 9.696546e-01 9.102360e-01 6.661307e-01 6.728229e-01 6.795665e-01 6.524898e-01

2034 9.703360e-01 9.701903e-01 9.699842e-01 9.698159e-01 9.696747e-01 9.104030e-01 6.665182e-01 6.732211e-01 6.799748e-01 6.528536e-01

2035 9.703564e-01 9.702106e-01 9.700044e-01 9.698359e-01 9.696946e-01 9.105640e-01 6.669025e-01 6.736156e-01 6.803791e-01 6.532151e-01

2036 9.703766e-01 9.702308e-01 9.700245e-01 9.698559e-01 9.697145e-01 9.107193e-01 6.672834e-01 6.740064e-01 6.807791e-01 6.535739e-01

2037 9.703969e-01 9.702510e-01 9.700445e-01 9.698758e-01 9.697344e-01 9.108691e-01 6.676605e-01 6.743930e-01 6.811746e-01 6.539298e-01

2038 9.704171e-01 9.702711e-01 9.700645e-01 9.698957e-01 9.697541e-01 9.110136e-01 6.680338e-01 6.747752e-01 6.815653e-01 6.542825e-01

2039 9.704372e-01 9.702911e-01 9.700844e-01 9.699155e-01 9.697738e-01 9.111530e-01 6.684028e-01 6.751529e-01 6.819510e-01 6.546318e-01

2040 9.704572e-01 9.703111e-01 9.701043e-01 9.699353e-01 9.697935e-01 9.112875e-01 6.687674e-01 6.755257e-01 6.823315e-01 6.549775e-01

2041 9.704773e-01 9.703311e-01 9.701241e-01 9.699550e-01 9.698131e-01 9.114173e-01 6.691274e-01 6.758935e-01 6.827066e-01 6.553194e-01

2042 9.704972e-01 9.703509e-01 9.701439e-01 9.699746e-01 9.698326e-01 9.115427e-01 6.694826e-01 6.762562e-01 6.830761e-01 6.556571e-01

2043 9.705172e-01 9.703708e-01 9.701636e-01 9.699942e-01 9.698521e-01 9.116638e-01 6.698327e-01 6.766134e-01 6.834398e-01 6.559906e-01

2044 9.705371e-01 9.703906e-01 9.701832e-01 9.700137e-01 9.698715e-01 9.117808e-01 6.701777e-01 6.769652e-01 6.837977e-01 6.563197e-01

2045 9.705569e-01 9.704104e-01 9.702028e-01 9.700332e-01 9.698908e-01 9.118938e-01 6.705174e-01 6.773112e-01 6.841495e-01 6.566441e-01

2046 9.705767e-01 9.704301e-01 9.702224e-01 9.700526e-01 9.699101e-01 9.120031e-01 6.708516e-01 6.776515e-01 6.844952e-01 6.569637e-01

2047 9.705965e-01 9.704497e-01 9.702419e-01 9.700720e-01 9.699294e-01 9.121088e-01 6.711801e-01 6.779858e-01 6.848347e-01 6.572784e-01

2048 9.706162e-01 9.704694e-01 9.702614e-01 9.700913e-01 9.699486e-01 9.122110e-01 6.715030e-01 6.783141e-01 6.851678e-01 6.575881e-01

2049 9.706360e-01 9.704890e-01 9.702808e-01 9.701106e-01 9.699677e-01 9.123100e-01 6.718201e-01 6.786363e-01 6.854945e-01 6.578926e-01

2050 9.706556e-01 9.705086e-01 9.703002e-01 9.701298e-01 9.699868e-01 9.124057e-01 6.721313e-01 6.789523e-01 6.858148e-01 6.581917e-01

2051 9.706753e-01 9.705281e-01 9.703196e-01 9.701490e-01 9.700059e-01 9.124985e-01 6.724365e-01 6.792620e-01 6.861285e-01 6.584855e-01

2052 9.706949e-01 9.705476e-01 9.703389e-01 9.701682e-01 9.700249e-01 9.125884e-01 6.727357e-01 6.795655e-01 6.864357e-01 6.587739e-01

2053 9.707145e-01 9.705670e-01 9.703582e-01 9.701873e-01 9.700439e-01 9.126755e-01 6.730288e-01 6.798627e-01 6.867363e-01 6.590567e-01

2054 9.707340e-01 9.705865e-01 9.703774e-01 9.702063e-01 9.700628e-01 9.127600e-01 6.733158e-01 6.801535e-01 6.870304e-01 6.593339e-01

2055 9.707536e-01 9.706059e-01 9.703966e-01 9.702254e-01 9.700816e-01 9.128420e-01 6.735967e-01 6.804380e-01 6.873178e-01 6.596055e-01

2056 9.707731e-01 9.706253e-01 9.704158e-01 9.702444e-01 9.701004e-01 9.129215e-01 6.738715e-01 6.807161e-01 6.875988e-01 6.598715e-01

2057 9.707926e-01 9.706446e-01 9.704349e-01 9.702633e-01 9.701192e-01 9.129988e-01 6.741401e-01 6.809878e-01 6.878732e-01 6.601317e-01

2058 9.708120e-01 9.706640e-01 9.704540e-01 9.702822e-01 9.701379e-01 9.130738e-01 6.744026e-01 6.812533e-01 6.881411e-01 6.603863e-01

2059 9.708315e-01 9.706833e-01 9.704731e-01 9.703010e-01 9.701566e-01 9.131468e-01 6.746590e-01 6.815125e-01 6.884025e-01 6.606352e-01

2060 9.708509e-01 9.707025e-01 9.704921e-01 9.703199e-01 9.701753e-01 9.132177e-01 6.749094e-01 6.817654e-01 6.886576e-01 6.608784e-01

2061 9.708703e-01 9.707218e-01 9.705111e-01 9.703386e-01 9.701938e-01 9.132867e-01 6.751537e-01 6.820122e-01 6.889063e-01 6.611159e-01

2062 9.708897e-01 9.707410e-01 9.705301e-01 9.703574e-01 9.702124e-01 9.133539e-01 6.753921e-01 6.822528e-01 6.891487e-01 6.613478e-01

2063 9.709091e-01 9.707602e-01 9.705491e-01 9.703761e-01 9.702309e-01 9.134193e-01 6.756245e-01 6.824874e-01 6.893850e-01 6.615741e-01

2064 9.709285e-01 9.707794e-01 9.705680e-01 9.703947e-01 9.702493e-01 9.134831e-01 6.758510e-01 6.827159e-01 6.896151e-01 6.617949e-01

2065 9.709478e-01 9.707986e-01 9.705868e-01 9.704134e-01 9.702677e-01 9.135452e-01 6.760718e-01 6.829386e-01 6.898392e-01 6.620101e-01

2066 9.709671e-01 9.708177e-01 9.706057e-01 9.704319e-01 9.702861e-01 9.136058e-01 6.762869e-01 6.831554e-01 6.900574e-01 6.622199e-01

2067 9.709865e-01 9.708368e-01 9.706245e-01 9.704505e-01 9.703044e-01 9.136650e-01 6.764963e-01 6.833665e-01 6.902697e-01 6.624243e-01

2068 9.710058e-01 9.708559e-01 9.706433e-01 9.704690e-01 9.703227e-01 9.137228e-01 6.767002e-01 6.835720e-01 6.904764e-01 6.626234e-01

2069 9.710251e-01 9.708750e-01 9.706620e-01 9.704874e-01 9.703409e-01 9.137792e-01 6.768986e-01 6.837719e-01 6.906773e-01 6.628173e-01

2070 9.710444e-01 9.708941e-01 9.706807e-01 9.705059e-01 9.703591e-01 9.138344e-01 6.770916e-01 6.839663e-01 6.908728e-01 6.630060e-01

2071 9.710637e-01 9.709132e-01 9.706994e-01 9.705242e-01 9.703772e-01 9.138884e-01 6.772794e-01 6.841555e-01 6.910629e-01 6.631897e-01

2072 9.710829e-01 9.709322e-01 9.707181e-01 9.705426e-01 9.703952e-01 9.139412e-01 6.774621e-01 6.843394e-01 6.912477e-01 6.633684e-01

2073 9.711022e-01 9.709512e-01 9.707367e-01 9.705609e-01 9.704133e-01 9.139929e-01 6.776397e-01 6.845181e-01 6.914273e-01 6.635422e-01

2074 9.711215e-01 9.709702e-01 9.707553e-01 9.705791e-01 9.704312e-01 9.140435e-01 6.778123e-01 6.846919e-01 6.916018e-01 6.637112e-01

2075 9.711407e-01 9.709892e-01 9.707738e-01 9.705973e-01 9.704491e-01 9.140932e-01 6.779801e-01 6.848608e-01 6.917714e-01 6.638755e-01

2076 9.711600e-01 9.710081e-01 9.707924e-01 9.706155e-01 9.704670e-01 9.141418e-01 6.781432e-01 6.850249e-01 6.919362e-01 6.640352e-01

2077 9.711792e-01 9.710271e-01 9.708109e-01 9.706336e-01 9.704848e-01 9.141896e-01 6.783017e-01 6.851843e-01 6.920963e-01 6.641904e-01

2078 9.711984e-01 9.710460e-01 9.708293e-01 9.706517e-01 9.705025e-01 9.142365e-01 6.784556e-01 6.853392e-01 6.922518e-01 6.643413e-01

2079 9.712177e-01 9.710649e-01 9.708477e-01 9.706697e-01 9.705202e-01 9.142825e-01 6.786052e-01 6.854896e-01 6.924029e-01 6.644878e-01

2080 9.712369e-01 9.710838e-01 9.708661e-01 9.706876e-01 9.705379e-01 9.143278e-01 6.787504e-01 6.856358e-01 6.925496e-01 6.646302e-01

2081 9.712561e-01 9.711027e-01 9.708845e-01 9.707056e-01 9.705554e-01 9.143723e-01 6.788916e-01 6.857777e-01 6.926921e-01 6.647685e-01

2082 9.712754e-01 9.711216e-01 9.709028e-01 9.707234e-01 9.705729e-01 9.144160e-01 6.790286e-01 6.859156e-01 6.928304e-01 6.649028e-01

2083 9.712946e-01 9.711404e-01 9.709211e-01 9.707412e-01 9.705904e-01 9.144591e-01 6.791617e-01 6.860495e-01 6.929648e-01 6.650333e-01

2084 9.713138e-01 9.711593e-01 9.709393e-01 9.707590e-01 9.706077e-01 9.145015e-01 6.792910e-01 6.861795e-01 6.930954e-01 6.651600e-01

2085 9.713330e-01 9.711781e-01 9.709575e-01 9.707767e-01 9.706250e-01 9.145433e-01 6.794166e-01 6.863058e-01 6.932221e-01 6.652831e-01

2086 9.713523e-01 9.711969e-01 9.709756e-01 9.707943e-01 9.706423e-01 9.145844e-01 6.795385e-01 6.864285e-01 6.933453e-01 6.654026e-01

2087 9.713715e-01 9.712157e-01 9.709937e-01 9.708119e-01 9.706594e-01 9.146250e-01 6.796570e-01 6.865476e-01 6.934649e-01 6.655187e-01

2088 9.713907e-01 9.712344e-01 9.710118e-01 9.708294e-01 9.706765e-01 9.146651e-01 6.797720e-01 6.866634e-01 6.935811e-01 6.656314e-01

2089 9.714099e-01 9.712532e-01 9.710298e-01 9.708469e-01 9.706935e-01 9.147046e-01 6.798838e-01 6.867758e-01 6.936939e-01 6.657409e-01

2090 9.714291e-01 9.712719e-01 9.710478e-01 9.708643e-01 9.707105e-01 9.147436e-01 6.799923e-01 6.868850e-01 6.938036e-01 6.658473e-01

2091 9.714484e-01 9.712906e-01 9.710657e-01 9.708816e-01 9.707273e-01 9.147822e-01 6.800978e-01 6.869911e-01 6.939101e-01 6.659506e-01

2092 9.714676e-01 9.713092e-01 9.710836e-01 9.708988e-01 9.707441e-01 9.148202e-01 6.802003e-01 6.870943e-01 6.940137e-01 6.660509e-01

2093 9.714868e-01 9.713279e-01 9.711014e-01 9.709160e-01 9.707608e-01 9.148579e-01 6.802999e-01 6.871945e-01 6.941143e-01 6.661485e-01

2094 9.715060e-01 9.713465e-01 9.711191e-01 9.709331e-01 9.707773e-01 9.148951e-01 6.803967e-01 6.872919e-01 6.942122e-01 6.662432e-01

2095 9.715253e-01 9.713651e-01 9.711368e-01 9.709501e-01 9.707938e-01 9.149320e-01 6.804909e-01 6.873866e-01 6.943073e-01 6.663353e-01

2096 9.715445e-01 9.713837e-01 9.711545e-01 9.709671e-01 9.708102e-01 9.149685e-01 6.805824e-01 6.874787e-01 6.943998e-01 6.664248e-01

2097 9.715637e-01 9.714023e-01 9.711721e-01 9.709839e-01 9.708265e-01 9.150046e-01 6.806713e-01 6.875683e-01 6.944898e-01 6.665119e-01

2098 9.715829e-01 9.714208e-01 9.711896e-01 9.710007e-01 9.708427e-01 9.150404e-01 6.807579e-01 6.876555e-01 6.945774e-01 6.665965e-01

2099 9.716022e-01 9.714393e-01 9.712070e-01 9.710174e-01 9.708588e-01 9.150758e-01 6.808421e-01 6.877403e-01 6.946626e-01 6.666788e-01

2100 9.716214e-01 9.714578e-01 9.712244e-01 9.710339e-01 9.708748e-01 9.151109e-01 6.809240e-01 6.878228e-01 6.947455e-01 6.667589e-01

2101 9.716406e-01 9.714762e-01 9.712417e-01 9.710504e-01 9.708907e-01 9.151458e-01 6.810038e-01 6.879031e-01 6.948263e-01 6.668369e-01

2102 9.716599e-01 9.714946e-01 9.712590e-01 9.710668e-01 9.709064e-01 9.151804e-01 6.810815e-01 6.879813e-01 6.949049e-01 6.669127e-01

2103 9.716791e-01 9.715130e-01 9.712761e-01 9.710831e-01 9.709220e-01 9.152146e-01 6.811571e-01 6.880575e-01 6.949815e-01 6.669866e-01

2104 9.716983e-01 9.715313e-01 9.712932e-01 9.710993e-01 9.709375e-01 9.152487e-01 6.812308e-01 6.881318e-01 6.950562e-01 6.670585e-01

2105 9.717175e-01 9.715496e-01 9.713102e-01 9.711153e-01 9.709529e-01 9.152825e-01 6.813026e-01 6.882041e-01 6.951289e-01 6.671286e-01

2106 9.717368e-01 9.715678e-01 9.713271e-01 9.711313e-01 9.709682e-01 9.153160e-01 6.813726e-01 6.882747e-01 6.951999e-01 6.671969e-01

2107 9.717560e-01 9.715860e-01 9.713439e-01 9.711471e-01 9.709833e-01 9.153494e-01 6.814409e-01 6.883435e-01 6.952691e-01 6.672635e-01

2108 9.717752e-01 9.716042e-01 9.713606e-01 9.711628e-01 9.709982e-01 9.153825e-01 6.815074e-01 6.884106e-01 6.953366e-01 6.673285e-01

2109 9.717944e-01 9.716223e-01 9.713772e-01 9.711784e-01 9.710130e-01 9.154155e-01 6.815724e-01 6.884761e-01 6.954025e-01 6.673918e-01

2110 9.718136e-01 9.716404e-01 9.713937e-01 9.711938e-01 9.710277e-01 9.154482e-01 6.816358e-01 6.885401e-01 6.954669e-01 6.674536e-01

2111 9.718328e-01 9.716584e-01 9.714101e-01 9.712091e-01 9.710422e-01 9.154808e-01 6.816978e-01 6.886025e-01 6.955297e-01 6.675140e-01

2112 9.718520e-01 9.716763e-01 9.714264e-01 9.712242e-01 9.710566e-01 9.155132e-01 6.817583e-01 6.886636e-01 6.955912e-01 6.675730e-01

2113 9.718712e-01 9.716942e-01 9.714426e-01 9.712392e-01 9.710708e-01 9.155454e-01 6.818174e-01 6.887232e-01 6.956512e-01 6.676306e-01

2114 9.718904e-01 9.717121e-01 9.714586e-01 9.712541e-01 9.710848e-01 9.155775e-01 6.818753e-01 6.887816e-01 6.957099e-01 6.676869e-01

2115 9.719095e-01 9.717299e-01 9.714745e-01 9.712688e-01 9.710986e-01 9.156094e-01 6.819318e-01 6.888386e-01 6.957674e-01 6.677420e-01

2116 9.719287e-01 9.717476e-01 9.714903e-01 9.712833e-01 9.711123e-01 9.156412e-01 6.819872e-01 6.888945e-01 6.958236e-01 6.677958e-01

2117 9.719479e-01 9.717652e-01 9.715060e-01 9.712977e-01 9.711257e-01 9.156729e-01 6.820413e-01 6.889491e-01 6.958787e-01 6.678486e-01

2118 9.719670e-01 9.717828e-01 9.715215e-01 9.713118e-01 9.711390e-01 9.157044e-01 6.820944e-01 6.890027e-01 6.959326e-01 6.679002e-01

2119 9.719861e-01 9.718002e-01 9.715369e-01 9.713258e-01 9.711521e-01 9.157358e-01 6.821464e-01 6.890552e-01 6.959855e-01 6.679508e-01

2120 9.720052e-01 9.718176e-01 9.715521e-01 9.713397e-01 9.711649e-01 9.157672e-01 6.821974e-01 6.891066e-01 6.960373e-01 6.680004e-01

2121 9.720243e-01 9.718350e-01 9.715672e-01 9.713533e-01 9.711776e-01 9.157984e-01 6.822474e-01 6.891571e-01 6.960881e-01 6.680491e-01

2122 9.720434e-01 9.718522e-01 9.715821e-01 9.713667e-01 9.711900e-01 9.158295e-01 6.822964e-01 6.892066e-01 6.961380e-01 6.680968e-01

2123 9.720624e-01 9.718693e-01 9.715968e-01 9.713799e-01 9.712023e-01 9.158605e-01 6.823446e-01 6.892552e-01 6.961870e-01 6.681436e-01

2124 9.720814e-01 9.718864e-01 9.716114e-01 9.713929e-01 9.712143e-01 9.158914e-01 6.823918e-01 6.893029e-01 6.962351e-01 6.681896e-01

2125 9.721004e-01 9.719033e-01 9.716257e-01 9.714057e-01 9.712260e-01 9.159222e-01 6.824383e-01 6.893498e-01 6.962823e-01 6.682348e-01

2126 9.721194e-01 9.719201e-01 9.716399e-01 9.714182e-01 9.712375e-01 9.159530e-01 6.824840e-01 6.893960e-01 6.963288e-01 6.682792e-01

2127 9.721383e-01 9.719368e-01 9.716539e-01 9.714305e-01 9.712488e-01 9.159837e-01 6.825289e-01 6.894413e-01 6.963745e-01 6.683229e-01

2128 9.721573e-01 9.719534e-01 9.716677e-01 9.714426e-01 9.712598e-01 9.160143e-01 6.825731e-01 6.894859e-01 6.964194e-01 6.683659e-01

2129 9.721761e-01 9.719699e-01 9.716812e-01 9.714544e-01 9.712706e-01 9.160448e-01 6.826166e-01 6.895299e-01 6.964637e-01 6.684083e-01

2130 9.721950e-01 9.719862e-01 9.716946e-01 9.714660e-01 9.712811e-01 9.160753e-01 6.826595e-01 6.895731e-01 6.965073e-01 6.684500e-01

2131 9.722137e-01 9.720024e-01 9.717077e-01 9.714773e-01 9.712913e-01 9.161057e-01 6.827017e-01 6.896158e-01 6.965503e-01 6.684911e-01

2132 9.722325e-01 9.720185e-01 9.717206e-01 9.714883e-01 9.713012e-01 9.161361e-01 6.827433e-01 6.896578e-01 6.965926e-01 6.685316e-01

2133 9.722512e-01 9.720344e-01 9.717332e-01 9.714991e-01 9.713108e-01 9.161664e-01 6.827844e-01 6.896993e-01 6.966344e-01 6.685716e-01

2134 9.722698e-01 9.720502e-01 9.717456e-01 9.715095e-01 9.713202e-01 9.161967e-01 6.828249e-01 6.897402e-01 6.966756e-01 6.686110e-01

2135 9.722884e-01 9.720658e-01 9.717577e-01 9.715197e-01 9.713292e-01 9.162270e-01 6.828649e-01 6.897806e-01 6.967163e-01 6.686500e-01

2136 9.723070e-01 9.720812e-01 9.717696e-01 9.715296e-01 9.713380e-01 9.162572e-01 6.829045e-01 6.898205e-01 6.967565e-01 6.686885e-01

2137 9.723254e-01 9.720965e-01 9.717812e-01 9.715391e-01 9.713464e-01 9.162873e-01 6.829435e-01 6.898599e-01 6.967962e-01 6.687265e-01

2138 9.723438e-01 9.721116e-01 9.717925e-01 9.715483e-01 9.713545e-01 9.163174e-01 6.829821e-01 6.898989e-01 6.968354e-01 6.687641e-01

2139 9.723622e-01 9.721265e-01 9.718035e-01 9.715572e-01 9.713623e-01 9.163475e-01 6.830203e-01 6.899374e-01 6.968743e-01 6.688013e-01

2140 9.723804e-01 9.721412e-01 9.718142e-01 9.715658e-01 9.713697e-01 9.163776e-01 6.830581e-01 6.899755e-01 6.969127e-01 6.688382e-01

2141 9.723986e-01 9.721557e-01 9.718246e-01 9.715740e-01 9.713768e-01 9.164076e-01 6.830955e-01 6.900133e-01 6.969507e-01 6.688746e-01

2142 9.724167e-01 9.721699e-01 9.718347e-01 9.715819e-01 9.713836e-01 9.164376e-01 6.831326e-01 6.900507e-01 6.969883e-01 6.689108e-01

2143 9.724347e-01 9.721840e-01 9.718444e-01 9.715894e-01 9.713900e-01 9.164676e-01 6.831693e-01 6.900877e-01 6.970256e-01 6.689466e-01

2144 9.724527e-01 9.721978e-01 9.718538e-01 9.715966e-01 9.713961e-01 9.164976e-01 6.832057e-01 6.901244e-01 6.970625e-01 6.689821e-01

2145 9.724705e-01 9.722114e-01 9.718629e-01 9.716033e-01 9.714018e-01 9.165275e-01 6.832418e-01 6.901608e-01 6.970991e-01 6.690173e-01

2146 9.724882e-01 9.722248e-01 9.718716e-01 9.716097e-01 9.714071e-01 9.165575e-01 6.832776e-01 6.901969e-01 6.971355e-01 6.690523e-01

2147 9.725058e-01 9.722378e-01 9.718799e-01 9.716157e-01 9.714120e-01 9.165874e-01 6.833131e-01 6.902327e-01 6.971715e-01 6.690870e-01

2148 9.725233e-01 9.722507e-01 9.718879e-01 9.716213e-01 9.714166e-01 9.166173e-01 6.833484e-01 6.902683e-01 6.972073e-01 6.691215e-01

2149 9.725406e-01 9.722632e-01 9.718954e-01 9.716265e-01 9.714208e-01 9.166472e-01 6.833835e-01 6.903036e-01 6.972428e-01 6.691558e-01

2150 9.725579e-01 9.722755e-01 9.719026e-01 9.716313e-01 9.714246e-01 9.166771e-01 6.834183e-01 6.903387e-01 6.972781e-01 6.691898e-01

2151 9.725750e-01 9.722874e-01 9.719093e-01 9.716356e-01 9.714280e-01 9.167070e-01 6.834529e-01 6.903736e-01 6.973132e-01 6.692237e-01

2152 9.725919e-01 9.722991e-01 9.719156e-01 9.716395e-01 9.714310e-01 9.167369e-01 6.834874e-01 6.904083e-01 6.973480e-01 6.692574e-01

2153 9.726087e-01 9.723105e-01 9.719215e-01 9.716430e-01 9.714336e-01 9.167667e-01 6.835216e-01 6.904428e-01 6.973827e-01 6.692909e-01

2154 9.726254e-01 9.723215e-01 9.719270e-01 9.716460e-01 9.714358e-01 9.167966e-01 6.835557e-01 6.904771e-01 6.974172e-01 6.693243e-01

2155 9.726418e-01 9.723322e-01 9.719320e-01 9.716486e-01 9.714376e-01 9.168265e-01 6.835897e-01 6.905113e-01 6.974515e-01 6.693575e-01

2156 9.726581e-01 9.723425e-01 9.719365e-01 9.716507e-01 9.714389e-01 9.168564e-01 6.836235e-01 6.905453e-01 6.974856e-01 6.693907e-01

2157 9.726743e-01 9.723525e-01 9.719406e-01 9.716524e-01 9.714399e-01 9.168862e-01 6.836571e-01 6.905792e-01 6.975196e-01 6.694237e-01

2158 9.726902e-01 9.723621e-01 9.719442e-01 9.716536e-01 9.714405e-01 9.169161e-01 6.836907e-01 6.906130e-01 6.975535e-01 6.694566e-01

2159 9.727059e-01 9.723713e-01 9.719473e-01 9.716543e-01 9.714406e-01 9.169460e-01 6.837241e-01 6.906466e-01 6.975872e-01 6.694894e-01

2160 9.727214e-01 9.723802e-01 9.719500e-01 9.716545e-01 9.714404e-01 9.169759e-01 6.837575e-01 6.906801e-01 6.976208e-01 6.695221e-01

2161 9.727367e-01 9.723886e-01 9.719521e-01 9.716543e-01 9.714397e-01 9.170058e-01 6.837907e-01 6.907136e-01 6.976544e-01 6.695548e-01

2162 9.727574e-01 9.724092e-01 9.719726e-01 9.716747e-01 9.714600e-01 9.176035e-01 6.840195e-01 6.909596e-01 6.979185e-01 6.697509e-01

2163 9.727779e-01 9.724296e-01 9.719929e-01 9.716949e-01 9.714801e-01 9.181881e-01 6.842537e-01 6.912114e-01 6.981888e-01 6.699520e-01

2164 9.727981e-01 9.724497e-01 9.720129e-01 9.717148e-01 9.714999e-01 9.187592e-01 6.844936e-01 6.914691e-01 6.984651e-01 6.701582e-01

2165 9.728181e-01 9.724696e-01 9.720327e-01 9.717345e-01 9.715195e-01 9.193169e-01 6.847390e-01 6.917326e-01 6.987475e-01 6.703694e-01

2166 9.728379e-01 9.724893e-01 9.720523e-01 9.717540e-01 9.715389e-01 9.198609e-01 6.849900e-01 6.920018e-01 6.990359e-01 6.705856e-01

2167 9.728574e-01 9.725088e-01 9.720717e-01 9.717733e-01 9.715581e-01 9.203913e-01 6.852466e-01 6.922768e-01 6.993302e-01 6.708069e-01

2168 9.728768e-01 9.725281e-01 9.720908e-01 9.717924e-01 9.715771e-01 9.209079e-01 6.855086e-01 6.925575e-01 6.996303e-01 6.710333e-01

2169 9.728959e-01 9.725471e-01 9.721098e-01 9.718113e-01 9.715959e-01 9.214109e-01 6.857761e-01 6.928439e-01 6.999363e-01 6.712646e-01

2170 9.729149e-01 9.725660e-01 9.721286e-01 9.718300e-01 9.716145e-01 9.219002e-01 6.860490e-01 6.931358e-01 7.002480e-01 6.715010e-01

2171 9.729337e-01 9.725848e-01 9.721472e-01 9.718485e-01 9.716330e-01 9.223759e-01 6.863272e-01 6.934331e-01 7.005652e-01 6.717423e-01

2172 9.729523e-01 9.726033e-01 9.721657e-01 9.718669e-01 9.716512e-01 9.228381e-01 6.866107e-01 6.937359e-01 7.008880e-01 6.719885e-01

2173 9.729708e-01 9.726217e-01 9.721840e-01 9.718851e-01 9.716693e-01 9.232869e-01 6.868993e-01 6.940440e-01 7.012161e-01 6.722396e-01

2174 9.729891e-01 9.726399e-01 9.722021e-01 9.719031e-01 9.716873e-01 9.237225e-01 6.871931e-01 6.943572e-01 7.015494e-01 6.724955e-01

2175 9.730073e-01 9.726580e-01 9.722201e-01 9.719210e-01 9.717051e-01 9.241450e-01 6.874918e-01 6.946755e-01 7.018878e-01 6.727561e-01

2176 9.730253e-01 9.726759e-01 9.722380e-01 9.719388e-01 9.717228e-01 9.245547e-01 6.877953e-01 6.949986e-01 7.022311e-01 6.730214e-01

2177 9.730432e-01 9.726937e-01 9.722557e-01 9.719564e-01 9.717403e-01 9.249516e-01 6.881036e-01 6.953265e-01 7.025791e-01 6.732912e-01

2178 9.730609e-01 9.727114e-01 9.722732e-01 9.719738e-01 9.717577e-01 9.253360e-01 6.884164e-01 6.956590e-01 7.029317e-01 6.735654e-01

2179 9.730785e-01 9.727290e-01 9.722907e-01 9.719912e-01 9.717749e-01 9.257082e-01 6.887337e-01 6.959959e-01 7.032886e-01 6.738440e-01

2180 9.730960e-01 9.727464e-01 9.723080e-01 9.720084e-01 9.717921e-01 9.260684e-01 6.890552e-01 6.963370e-01 7.036497e-01 6.741268e-01

2181 9.731134e-01 9.727637e-01 9.723252e-01 9.720255e-01 9.718091e-01 9.264168e-01 6.893808e-01 6.966821e-01 7.040147e-01 6.744136e-01

2182 9.731307e-01 9.727809e-01 9.723423e-01 9.720425e-01 9.718260e-01 9.267538e-01 6.897103e-01 6.970310e-01 7.043833e-01 6.747044e-01

2183 9.731479e-01 9.727980e-01 9.723593e-01 9.720594e-01 9.718428e-01 9.270794e-01 6.900435e-01 6.973835e-01 7.047554e-01 6.749989e-01

2184 9.731649e-01 9.728150e-01 9.723762e-01 9.720762e-01 9.718595e-01 9.273941e-01 6.903802e-01 6.977394e-01 7.051306e-01 6.752971e-01

2185 9.731819e-01 9.728318e-01 9.723929e-01 9.720929e-01 9.718761e-01 9.276982e-01 6.907202e-01 6.980984e-01 7.055088e-01 6.755987e-01

2186 9.731988e-01 9.728486e-01 9.724096e-01 9.721095e-01 9.718926e-01 9.279918e-01 6.910632e-01 6.984603e-01 7.058896e-01 6.759036e-01

2187 9.732155e-01 9.728653e-01 9.724262e-01 9.721259e-01 9.719090e-01 9.282753e-01 6.914090e-01 6.988248e-01 7.062729e-01 6.762115e-01

2188 9.732322e-01 9.728819e-01 9.724427e-01 9.721423e-01 9.719253e-01 9.285489e-01 6.917574e-01 6.991916e-01 7.066582e-01 6.765223e-01

2189 9.732488e-01 9.728985e-01 9.724591e-01 9.721586e-01 9.719415e-01 9.288130e-01 6.921081e-01 6.995606e-01 7.070453e-01 6.768358e-01

2190 9.732654e-01 9.729149e-01 9.724755e-01 9.721749e-01 9.719576e-01 9.290678e-01 6.924609e-01 6.999313e-01 7.074339e-01 6.771517e-01

2191 9.732818e-01 9.729313e-01 9.724917e-01 9.721910e-01 9.719736e-01 9.293136e-01 6.928155e-01 7.003036e-01 7.078237e-01 6.774698e-01

2192 9.732982e-01 9.729476e-01 9.725079e-01 9.722071e-01 9.719896e-01 9.295507e-01 6.931717e-01 7.006772e-01 7.082145e-01 6.777899e-01

2193 9.733145e-01 9.729638e-01 9.725240e-01 9.722231e-01 9.720055e-01 9.297794e-01 6.935291e-01 7.010517e-01 7.086058e-01 6.781118e-01

2194 9.733308e-01 9.729800e-01 9.725400e-01 9.722390e-01 9.720213e-01 9.300000e-01 6.938876e-01 7.014269e-01 7.089975e-01 6.784352e-01

2195 9.733470e-01 9.729961e-01 9.725560e-01 9.722548e-01 9.720371e-01 9.302126e-01 6.942467e-01 7.018024e-01 7.093891e-01 6.787598e-01

2196 9.733631e-01 9.730121e-01 9.725719e-01 9.722706e-01 9.720527e-01 9.304176e-01 6.946063e-01 7.021781e-01 7.097804e-01 6.790855e-01

2197 9.733792e-01 9.730281e-01 9.725877e-01 9.722863e-01 9.720683e-01 9.306153e-01 6.949661e-01 7.025535e-01 7.101712e-01 6.794120e-01

2198 9.733952e-01 9.730440e-01 9.726035e-01 9.723020e-01 9.720839e-01 9.308059e-01 6.953257e-01 7.029285e-01 7.105610e-01 6.797390e-01

2199 9.734112e-01 9.730599e-01 9.726192e-01 9.723176e-01 9.720994e-01 9.309896e-01 6.956850e-01 7.033026e-01 7.109496e-01 6.800663e-01

2200 9.734271e-01 9.730757e-01 9.726349e-01 9.723331e-01 9.721148e-01 9.311667e-01 6.960435e-01 7.036757e-01 7.113367e-01 6.803936e-01

2201 9.734429e-01 9.730914e-01 9.726505e-01 9.723486e-01 9.721301e-01 9.313375e-01 6.964012e-01 7.040474e-01 7.117220e-01 6.807207e-01

2202 9.734587e-01 9.731071e-01 9.726660e-01 9.723640e-01 9.721454e-01 9.315021e-01 6.967576e-01 7.044175e-01 7.121052e-01 6.810473e-01

2203 9.734745e-01 9.731228e-01 9.726815e-01 9.723793e-01 9.721606e-01 9.316608e-01 6.971125e-01 7.047857e-01 7.124860e-01 6.813732e-01

2204 9.734902e-01 9.731384e-01 9.726970e-01 9.723946e-01 9.721758e-01 9.318139e-01 6.974657e-01 7.051517e-01 7.128643e-01 6.816980e-01

2205 9.735059e-01 9.731540e-01 9.727124e-01 9.724099e-01 9.721909e-01 9.319615e-01 6.978168e-01 7.055153e-01 7.132396e-01 6.820216e-01

2206 9.735216e-01 9.731695e-01 9.727278e-01 9.724251e-01 9.722060e-01 9.321039e-01 6.981657e-01 7.058762e-01 7.136118e-01 6.823438e-01

2207 9.735372e-01 9.731850e-01 9.727431e-01 9.724402e-01 9.722210e-01 9.322412e-01 6.985121e-01 7.062342e-01 7.139807e-01 6.826642e-01

2208 9.735528e-01 9.732005e-01 9.727583e-01 9.724553e-01 9.722360e-01 9.323736e-01 6.988557e-01 7.065890e-01 7.143459e-01 6.829827e-01

2209 9.735683e-01 9.732159e-01 9.727736e-01 9.724704e-01 9.722509e-01 9.325014e-01 6.991964e-01 7.069404e-01 7.147073e-01 6.832990e-01

2210 9.735839e-01 9.732313e-01 9.727887e-01 9.724854e-01 9.722658e-01 9.326247e-01 6.995339e-01 7.072882e-01 7.150646e-01 6.836129e-01

2211 9.735993e-01 9.732466e-01 9.728039e-01 9.725004e-01 9.722806e-01 9.327438e-01 6.998680e-01 7.076321e-01 7.154178e-01 6.839242e-01

2212 9.736148e-01 9.732619e-01 9.728190e-01 9.725153e-01 9.722954e-01 9.328587e-01 7.001984e-01 7.079721e-01 7.157665e-01 6.842327e-01

2213 9.736302e-01 9.732772e-01 9.728340e-01 9.725302e-01 9.723101e-01 9.329696e-01 7.005251e-01 7.083079e-01 7.161105e-01 6.845382e-01

2214 9.736456e-01 9.732925e-01 9.728491e-01 9.725450e-01 9.723248e-01 9.330768e-01 7.008478e-01 7.086393e-01 7.164499e-01 6.848405e-01

2215 9.736610e-01 9.733077e-01 9.728641e-01 9.725598e-01 9.723394e-01 9.331803e-01 7.011664e-01 7.089662e-01 7.167843e-01 6.851394e-01

2216 9.736764e-01 9.733229e-01 9.728790e-01 9.725746e-01 9.723540e-01 9.332803e-01 7.014807e-01 7.092884e-01 7.171136e-01 6.854348e-01

2217 9.736917e-01 9.733381e-01 9.728939e-01 9.725893e-01 9.723686e-01 9.333770e-01 7.017906e-01 7.096058e-01 7.174378e-01 6.857264e-01

2218 9.737070e-01 9.733532e-01 9.729088e-01 9.726040e-01 9.723831e-01 9.334705e-01 7.020958e-01 7.099183e-01 7.177567e-01 6.860142e-01

2219 9.737223e-01 9.733683e-01 9.729237e-01 9.726186e-01 9.723975e-01 9.335609e-01 7.023964e-01 7.102257e-01 7.180702e-01 6.862981e-01

2220 9.737376e-01 9.733834e-01 9.729385e-01 9.726332e-01 9.724119e-01 9.336484e-01 7.026922e-01 7.105280e-01 7.183782e-01 6.865778e-01

2221 9.737529e-01 9.733985e-01 9.729533e-01 9.726477e-01 9.724263e-01 9.337330e-01 7.029830e-01 7.108250e-01 7.186806e-01 6.868532e-01

2222 9.737681e-01 9.734135e-01 9.729680e-01 9.726623e-01 9.724406e-01 9.338149e-01 7.032689e-01 7.111167e-01 7.189774e-01 6.871244e-01

2223 9.737834e-01 9.734286e-01 9.729828e-01 9.726767e-01 9.724549e-01 9.338943e-01 7.035496e-01 7.114031e-01 7.192686e-01 6.873910e-01

2224 9.737986e-01 9.734436e-01 9.729974e-01 9.726912e-01 9.724691e-01 9.339712e-01 7.038253e-01 7.116840e-01 7.195540e-01 6.876532e-01

2225 9.738138e-01 9.734585e-01 9.730121e-01 9.727056e-01 9.724833e-01 9.340457e-01 7.040957e-01 7.119594e-01 7.198337e-01 6.879107e-01

2226 9.738290e-01 9.734735e-01 9.730267e-01 9.727199e-01 9.724974e-01 9.341179e-01 7.043609e-01 7.122293e-01 7.201076e-01 6.881636e-01

2227 9.738442e-01 9.734885e-01 9.730413e-01 9.727342e-01 9.725115e-01 9.341879e-01 7.046208e-01 7.124937e-01 7.203757e-01 6.884117e-01

2228 9.738593e-01 9.735034e-01 9.730559e-01 9.727485e-01 9.725256e-01 9.342559e-01 7.048753e-01 7.127525e-01 7.206380e-01 6.886550e-01

2229 9.738745e-01 9.735183e-01 9.730704e-01 9.727628e-01 9.725396e-01 9.343219e-01 7.051246e-01 7.130057e-01 7.208945e-01 6.888936e-01

2230 9.738896e-01 9.735332e-01 9.730849e-01 9.727770e-01 9.725536e-01 9.343860e-01 7.053685e-01 7.132534e-01 7.211453e-01 6.891272e-01

2231 9.739048e-01 9.735480e-01 9.730994e-01 9.727911e-01 9.725675e-01 9.344482e-01 7.056071e-01 7.134956e-01 7.213903e-01 6.893561e-01

2232 9.739199e-01 9.735629e-01 9.731138e-01 9.728052e-01 9.725814e-01 9.345087e-01 7.058404e-01 7.137322e-01 7.216297e-01 6.895800e-01

2233 9.739350e-01 9.735777e-01 9.731282e-01 9.728193e-01 9.725952e-01 9.345676e-01 7.060684e-01 7.139634e-01 7.218634e-01 6.897991e-01

2234 9.739502e-01 9.735925e-01 9.731426e-01 9.728334e-01 9.726090e-01 9.346248e-01 7.062911e-01 7.141891e-01 7.220914e-01 6.900132e-01

2235 9.739653e-01 9.736073e-01 9.731569e-01 9.728474e-01 9.726227e-01 9.346805e-01 7.065085e-01 7.144094e-01 7.223139e-01 6.902226e-01

2236 9.739804e-01 9.736221e-01 9.731712e-01 9.728613e-01 9.726364e-01 9.347348e-01 7.067208e-01 7.146243e-01 7.225309e-01 6.904271e-01

2237 9.739955e-01 9.736368e-01 9.731855e-01 9.728752e-01 9.726500e-01 9.347877e-01 7.069279e-01 7.148340e-01 7.227425e-01 6.906267e-01

2238 9.740106e-01 9.736516e-01 9.731997e-01 9.728891e-01 9.726636e-01 9.348392e-01 7.071299e-01 7.150383e-01 7.229487e-01 6.908216e-01

2239 9.740257e-01 9.736663e-01 9.732140e-01 9.729029e-01 9.726771e-01 9.348894e-01 7.073268e-01 7.152375e-01 7.231495e-01 6.910118e-01

2240 9.740408e-01 9.736810e-01 9.732281e-01 9.729167e-01 9.726906e-01 9.349384e-01 7.075188e-01 7.154316e-01 7.233452e-01 6.911972e-01

2241 9.740558e-01 9.736957e-01 9.732423e-01 9.729304e-01 9.727040e-01 9.349863e-01 7.077058e-01 7.156207e-01 7.235358e-01 6.913781e-01

2242 9.740709e-01 9.737104e-01 9.732563e-01 9.729441e-01 9.727173e-01 9.350330e-01 7.078880e-01 7.158048e-01 7.237213e-01 6.915543e-01

2243 9.740860e-01 9.737250e-01 9.732704e-01 9.729577e-01 9.727306e-01 9.350787e-01 7.080654e-01 7.159841e-01 7.239019e-01 6.917260e-01

2244 9.741011e-01 9.737397e-01 9.732844e-01 9.729713e-01 9.727439e-01 9.351234e-01 7.082382e-01 7.161586e-01 7.240776e-01 6.918933e-01

2245 9.741162e-01 9.737543e-01 9.732984e-01 9.729848e-01 9.727571e-01 9.351670e-01 7.084063e-01 7.163284e-01 7.242485e-01 6.920561e-01

2246 9.741312e-01 9.737689e-01 9.733123e-01 9.729983e-01 9.727702e-01 9.352098e-01 7.085699e-01 7.164935e-01 7.244147e-01 6.922146e-01

2247 9.741463e-01 9.737834e-01 9.733262e-01 9.730117e-01 9.727832e-01 9.352516e-01 7.087291e-01 7.166542e-01 7.245764e-01 6.923689e-01

2248 9.741614e-01 9.737980e-01 9.733401e-01 9.730250e-01 9.727962e-01 9.352926e-01 7.088839e-01 7.168104e-01 7.247337e-01 6.925190e-01

2249 9.741764e-01 9.738125e-01 9.733539e-01 9.730383e-01 9.728092e-01 9.353328e-01 7.090344e-01 7.169624e-01 7.248865e-01 6.926650e-01

2250 9.741915e-01 9.738270e-01 9.733677e-01 9.730516e-01 9.728220e-01 9.353722e-01 7.091808e-01 7.171101e-01 7.250351e-01 6.928070e-01

2251 9.742066e-01 9.738415e-01 9.733814e-01 9.730648e-01 9.728348e-01 9.354109e-01 7.093231e-01 7.172536e-01 7.251795e-01 6.929451e-01

2252 9.742216e-01 9.738560e-01 9.733951e-01 9.730779e-01 9.728475e-01 9.354488e-01 7.094614e-01 7.173932e-01 7.253198e-01 6.930793e-01

2253 9.742367e-01 9.738704e-01 9.734087e-01 9.730909e-01 9.728602e-01 9.354861e-01 7.095959e-01 7.175288e-01 7.254562e-01 6.932098e-01

2254 9.742518e-01 9.738848e-01 9.734222e-01 9.731039e-01 9.728728e-01 9.355227e-01 7.097265e-01 7.176606e-01 7.255887e-01 6.933366e-01

2255 9.742668e-01 9.738992e-01 9.734358e-01 9.731168e-01 9.728852e-01 9.355587e-01 7.098535e-01 7.177886e-01 7.257175e-01 6.934598e-01

2256 9.742819e-01 9.739136e-01 9.734492e-01 9.731297e-01 9.728977e-01 9.355941e-01 7.099769e-01 7.179130e-01 7.258426e-01 6.935796e-01

2257 9.742969e-01 9.739279e-01 9.734626e-01 9.731425e-01 9.729100e-01 9.356290e-01 7.100967e-01 7.180339e-01 7.259641e-01 6.936959e-01

2258 9.743120e-01 9.739422e-01 9.734760e-01 9.731552e-01 9.729222e-01 9.356633e-01 7.102131e-01 7.181513e-01 7.260822e-01 6.938088e-01

2259 9.743270e-01 9.739565e-01 9.734893e-01 9.731678e-01 9.729344e-01 9.356970e-01 7.103262e-01 7.182654e-01 7.261969e-01 6.939186e-01

2260 9.743421e-01 9.739708e-01 9.735025e-01 9.731803e-01 9.729465e-01 9.357303e-01 7.104361e-01 7.183763e-01 7.263083e-01 6.940252e-01

2261 9.743571e-01 9.739850e-01 9.735156e-01 9.731928e-01 9.729585e-01 9.357632e-01 7.105429e-01 7.184839e-01 7.264166e-01 6.941288e-01

2262 9.743721e-01 9.739992e-01 9.735287e-01 9.732052e-01 9.729704e-01 9.357955e-01 7.106466e-01 7.185885e-01 7.265218e-01 6.942293e-01

2263 9.743872e-01 9.740133e-01 9.735417e-01 9.732174e-01 9.729822e-01 9.358275e-01 7.107473e-01 7.186902e-01 7.266240e-01 6.943270e-01

2264 9.744022e-01 9.740274e-01 9.735547e-01 9.732296e-01 9.729939e-01 9.358590e-01 7.108452e-01 7.187889e-01 7.267233e-01 6.944219e-01

2265 9.744172e-01 9.740415e-01 9.735676e-01 9.732417e-01 9.730055e-01 9.358902e-01 7.109403e-01 7.188849e-01 7.268198e-01 6.945141e-01

2266 9.744322e-01 9.740555e-01 9.735804e-01 9.732538e-01 9.730169e-01 9.359209e-01 7.110327e-01 7.189781e-01 7.269136e-01 6.946036e-01

2267 9.744473e-01 9.740695e-01 9.735931e-01 9.732657e-01 9.730283e-01 9.359514e-01 7.111225e-01 7.190687e-01 7.270048e-01 6.946906e-01

2268 9.744623e-01 9.740834e-01 9.736057e-01 9.732775e-01 9.730396e-01 9.359814e-01 7.112098e-01 7.191568e-01 7.270934e-01 6.947751e-01

2269 9.744773e-01 9.740973e-01 9.736183e-01 9.732892e-01 9.730508e-01 9.360112e-01 7.112946e-01 7.192425e-01 7.271795e-01 6.948572e-01

2270 9.744922e-01 9.741112e-01 9.736307e-01 9.733008e-01 9.730618e-01 9.360406e-01 7.113771e-01 7.193257e-01 7.272633e-01 6.949370e-01

2271 9.745072e-01 9.741250e-01 9.736431e-01 9.733123e-01 9.730727e-01 9.360698e-01 7.114572e-01 7.194066e-01 7.273448e-01 6.950145e-01

2272 9.745222e-01 9.741387e-01 9.736554e-01 9.733237e-01 9.730836e-01 9.360987e-01 7.115352e-01 7.194854e-01 7.274240e-01 6.950899e-01

2273 9.745371e-01 9.741524e-01 9.736675e-01 9.733349e-01 9.730942e-01 9.361273e-01 7.116110e-01 7.195619e-01 7.275011e-01 6.951632e-01

2274 9.745521e-01 9.741661e-01 9.736796e-01 9.733461e-01 9.731048e-01 9.361556e-01 7.116847e-01 7.196364e-01 7.275761e-01 6.952344e-01

2275 9.745670e-01 9.741797e-01 9.736916e-01 9.733571e-01 9.731152e-01 9.361837e-01 7.117565e-01 7.197089e-01 7.276492e-01 6.953037e-01

2276 9.745819e-01 9.741932e-01 9.737035e-01 9.733680e-01 9.731255e-01 9.362116e-01 7.118263e-01 7.197795e-01 7.277202e-01 6.953711e-01

2277 9.745968e-01 9.742066e-01 9.737152e-01 9.733787e-01 9.731357e-01 9.362392e-01 7.118943e-01 7.198482e-01 7.277894e-01 6.954367e-01

2278 9.746117e-01 9.742200e-01 9.737269e-01 9.733893e-01 9.731457e-01 9.362667e-01 7.119605e-01 7.199151e-01 7.278568e-01 6.955005e-01

2279 9.746266e-01 9.742334e-01 9.737384e-01 9.733998e-01 9.731555e-01 9.362939e-01 7.120249e-01 7.199803e-01 7.279225e-01 6.955626e-01

2280 9.746414e-01 9.742466e-01 9.737498e-01 9.734102e-01 9.731653e-01 9.363210e-01 7.120877e-01 7.200438e-01 7.279865e-01 6.956231e-01

2281 9.746562e-01 9.742598e-01 9.737611e-01 9.734203e-01 9.731748e-01 9.363478e-01 7.121489e-01 7.201056e-01 7.280488e-01 6.956820e-01

2282 9.746710e-01 9.742729e-01 9.737722e-01 9.734304e-01 9.731843e-01 9.363745e-01 7.122085e-01 7.201659e-01 7.281097e-01 6.957394e-01

2283 9.746858e-01 9.742859e-01 9.737832e-01 9.734403e-01 9.731935e-01 9.364011e-01 7.122666e-01 7.202248e-01 7.281690e-01 6.957953e-01

2284 9.747005e-01 9.742988e-01 9.737941e-01 9.734500e-01 9.732026e-01 9.364275e-01 7.123233e-01 7.202821e-01 7.282268e-01 6.958499e-01

2285 9.747153e-01 9.743117e-01 9.738048e-01 9.734596e-01 9.732115e-01 9.364537e-01 7.123786e-01 7.203381e-01 7.282833e-01 6.959031e-01

2286 9.747299e-01 9.743244e-01 9.738154e-01 9.734690e-01 9.732203e-01 9.364798e-01 7.124326e-01 7.203928e-01 7.283384e-01 6.959549e-01

2287 9.747446e-01 9.743371e-01 9.738258e-01 9.734782e-01 9.732289e-01 9.365058e-01 7.124853e-01 7.204461e-01 7.283923e-01 6.960056e-01

2288 9.747592e-01 9.743496e-01 9.738361e-01 9.734873e-01 9.732373e-01 9.365316e-01 7.125368e-01 7.204982e-01 7.284449e-01 6.960550e-01

2289 9.747738e-01 9.743621e-01 9.738462e-01 9.734961e-01 9.732455e-01 9.365573e-01 7.125871e-01 7.205492e-01 7.284963e-01 6.961033e-01

2290 9.747883e-01 9.743744e-01 9.738561e-01 9.735048e-01 9.732536e-01 9.365829e-01 7.126362e-01 7.205990e-01 7.285465e-01 6.961505e-01

2291 9.748028e-01 9.743867e-01 9.738659e-01 9.735133e-01 9.732614e-01 9.366084e-01 7.126843e-01 7.206477e-01 7.285957e-01 6.961966e-01

2292 9.748173e-01 9.743988e-01 9.738755e-01 9.735216e-01 9.732691e-01 9.366338e-01 7.127313e-01 7.206953e-01 7.286438e-01 6.962417e-01

2293 9.748317e-01 9.744108e-01 9.738849e-01 9.735297e-01 9.732766e-01 9.366591e-01 7.127773e-01 7.207419e-01 7.286908e-01 6.962858e-01

2294 9.748461e-01 9.744227e-01 9.738942e-01 9.735376e-01 9.732839e-01 9.366843e-01 7.128223e-01 7.207876e-01 7.287369e-01 6.963289e-01

2295 9.748604e-01 9.744344e-01 9.739032e-01 9.735454e-01 9.732910e-01 9.367095e-01 7.128664e-01 7.208323e-01 7.287821e-01 6.963712e-01

2296 9.748746e-01 9.744461e-01 9.739121e-01 9.735528e-01 9.732979e-01 9.367345e-01 7.129096e-01 7.208761e-01 7.288263e-01 6.964126e-01

2297 9.748888e-01 9.744576e-01 9.739207e-01 9.735601e-01 9.733045e-01 9.367595e-01 7.129520e-01 7.209190e-01 7.288697e-01 6.964532e-01

2298 9.749030e-01 9.744689e-01 9.739292e-01 9.735672e-01 9.733110e-01 9.367844e-01 7.129935e-01 7.209611e-01 7.289123e-01 6.964929e-01

2299 9.749170e-01 9.744801e-01 9.739374e-01 9.735740e-01 9.733173e-01 9.368092e-01 7.130343e-01 7.210025e-01 7.289540e-01 6.965320e-01

2300 9.749310e-01 9.744912e-01 9.739454e-01 9.735806e-01 9.733233e-01 9.368340e-01 7.130743e-01 7.210430e-01 7.289950e-01 6.965703e-01

2301 9.749450e-01 9.745021e-01 9.739532e-01 9.735870e-01 9.733291e-01 9.368587e-01 7.131136e-01 7.210829e-01 7.290352e-01 6.966079e-01

2302 9.749588e-01 9.745128e-01 9.739608e-01 9.735932e-01 9.733347e-01 9.368834e-01 7.131522e-01 7.211220e-01 7.290748e-01 6.966448e-01

2303 9.749726e-01 9.745234e-01 9.739681e-01 9.735991e-01 9.733401e-01 9.369080e-01 7.131901e-01 7.211605e-01 7.291137e-01 6.966811e-01

2304 9.749863e-01 9.745338e-01 9.739752e-01 9.736047e-01 9.733452e-01 9.369326e-01 7.132274e-01 7.211983e-01 7.291519e-01 6.967168e-01

2305 9.749999e-01 9.745440e-01 9.739821e-01 9.736101e-01 9.733502e-01 9.369571e-01 7.132641e-01 7.212355e-01 7.291895e-01 6.967520e-01

2306 9.750134e-01 9.745541e-01 9.739887e-01 9.736153e-01 9.733549e-01 9.369816e-01 7.133003e-01 7.212722e-01 7.292265e-01 6.967866e-01

2307 9.750268e-01 9.745639e-01 9.739951e-01 9.736202e-01 9.733593e-01 9.370061e-01 7.133358e-01 7.213083e-01 7.292629e-01 6.968206e-01

2308 9.750402e-01 9.745736e-01 9.740012e-01 9.736248e-01 9.733635e-01 9.370305e-01 7.133709e-01 7.213438e-01 7.292988e-01 6.968542e-01

2309 9.750534e-01 9.745830e-01 9.740070e-01 9.736292e-01 9.733675e-01 9.370549e-01 7.134054e-01 7.213788e-01 7.293342e-01 6.968872e-01

2310 9.750665e-01 9.745923e-01 9.740126e-01 9.736333e-01 9.733713e-01 9.370792e-01 7.134395e-01 7.214134e-01 7.293691e-01 6.969199e-01

2311 9.750795e-01 9.746014e-01 9.740179e-01 9.736372e-01 9.733748e-01 9.371035e-01 7.134731e-01 7.214475e-01 7.294035e-01 6.969521e-01

2312 9.750924e-01 9.746102e-01 9.740229e-01 9.736407e-01 9.733781e-01 9.371278e-01 7.135063e-01 7.214811e-01 7.294375e-01 6.969838e-01

2313 9.751051e-01 9.746188e-01 9.740277e-01 9.736440e-01 9.733811e-01 9.371521e-01 7.135391e-01 7.215143e-01 7.294710e-01 6.970152e-01

2314 9.751177e-01 9.746272e-01 9.740321e-01 9.736471e-01 9.733839e-01 9.371764e-01 7.135715e-01 7.215472e-01 7.295041e-01 6.970463e-01

2315 9.751302e-01 9.746354e-01 9.740363e-01 9.736498e-01 9.733864e-01 9.372006e-01 7.136035e-01 7.215796e-01 7.295369e-01 6.970769e-01

2316 9.751426e-01 9.746433e-01 9.740402e-01 9.736523e-01 9.733888e-01 9.372249e-01 7.136351e-01 7.216117e-01 7.295692e-01 6.971073e-01

2317 9.751548e-01 9.746510e-01 9.740438e-01 9.736545e-01 9.733908e-01 9.372491e-01 7.136664e-01 7.216434e-01 7.296012e-01 6.971373e-01

2318 9.751668e-01 9.746584e-01 9.740470e-01 9.736564e-01 9.733927e-01 9.372733e-01 7.136974e-01 7.216748e-01 7.296329e-01 6.971671e-01

2319 9.751787e-01 9.746656e-01 9.740500e-01 9.736580e-01 9.733943e-01 9.372975e-01 7.137281e-01 7.217059e-01 7.296642e-01 6.971965e-01

2320 9.751904e-01 9.746725e-01 9.740527e-01 9.736593e-01 9.733956e-01 9.373217e-01 7.137585e-01 7.217367e-01 7.296952e-01 6.972257e-01

2321 9.752020e-01 9.746792e-01 9.740550e-01 9.736603e-01 9.733968e-01 9.373459e-01 7.137887e-01 7.217672e-01 7.297260e-01 6.972546e-01

2322 9.752134e-01 9.746856e-01 9.740570e-01 9.736611e-01 9.733977e-01 9.373701e-01 7.138185e-01 7.217974e-01 7.297564e-01 6.972834e-01

2323 9.752246e-01 9.746917e-01 9.740587e-01 9.736616e-01 9.733983e-01 9.373943e-01 7.138482e-01 7.218274e-01 7.297866e-01 6.973118e-01

2324 9.752356e-01 9.746975e-01 9.740601e-01 9.736618e-01 9.733988e-01 9.374185e-01 7.138776e-01 7.218571e-01 7.298166e-01 6.973401e-01

2325 9.752464e-01 9.747030e-01 9.740612e-01 9.736616e-01 9.733990e-01 9.374427e-01 7.139068e-01 7.218867e-01 7.298463e-01 6.973682e-01

2326 9.752570e-01 9.747083e-01 9.740619e-01 9.736613e-01 9.733990e-01 9.374669e-01 7.139358e-01 7.219160e-01 7.298758e-01 6.973961e-01

2327 9.752675e-01 9.747132e-01 9.740623e-01 9.736606e-01 9.733988e-01 9.374911e-01 7.139646e-01 7.219451e-01 7.299050e-01 6.974239e-01

2328 9.752776e-01 9.747178e-01 9.740624e-01 9.736597e-01 9.733984e-01 9.375153e-01 7.139932e-01 7.219741e-01 7.299341e-01 6.974515e-01

2329 9.752876e-01 9.747222e-01 9.740622e-01 9.736584e-01 9.733978e-01 9.375395e-01 7.140217e-01 7.220028e-01 7.299630e-01 6.974789e-01

2330 9.752973e-01 9.747262e-01 9.740616e-01 9.736570e-01 9.733970e-01 9.375637e-01 7.140500e-01 7.220314e-01 7.299917e-01 6.975063e-01

2331 9.753068e-01 9.747299e-01 9.740607e-01 9.736552e-01 9.733960e-01 9.375879e-01 7.140782e-01 7.220599e-01 7.300203e-01 6.975334e-01

2332 9.753161e-01 9.747333e-01 9.740595e-01 9.736532e-01 9.733948e-01 9.376121e-01 7.141062e-01 7.220882e-01 7.300487e-01 6.975605e-01

2333 9.753251e-01 9.747363e-01 9.740580e-01 9.736509e-01 9.733934e-01 9.376364e-01 7.141342e-01 7.221164e-01 7.300769e-01 6.975875e-01

2334 9.753338e-01 9.747390e-01 9.740561e-01 9.736484e-01 9.733919e-01 9.376606e-01 7.141620e-01 7.221444e-01 7.301050e-01 6.976144e-01

2335 9.753423e-01 9.747414e-01 9.740539e-01 9.736456e-01 9.733903e-01 9.376849e-01 7.141897e-01 7.221724e-01 7.301330e-01 6.976412e-01

2336 9.753505e-01 9.747435e-01 9.740514e-01 9.736426e-01 9.733884e-01 9.377092e-01 7.142173e-01 7.222002e-01 7.301609e-01 6.976680e-01

2337 9.753584e-01 9.747452e-01 9.740486e-01 9.736394e-01 9.733865e-01 9.377335e-01 7.142449e-01 7.222279e-01 7.301886e-01 6.976946e-01

2338 9.753660e-01 9.747466e-01 9.740455e-01 9.736359e-01 9.733844e-01 9.377578e-01 7.142723e-01 7.222556e-01 7.302163e-01 6.977213e-01

2339 9.753733e-01 9.747476e-01 9.740421e-01 9.736323e-01 9.733822e-01 9.377821e-01 7.142997e-01 7.222832e-01 7.302438e-01 6.977478e-01

2340 9.753803e-01 9.747483e-01 9.740383e-01 9.736284e-01 9.733799e-01 9.378065e-01 7.143271e-01 7.223107e-01 7.302713e-01 6.977744e-01

2341 9.753869e-01 9.747487e-01 9.740343e-01 9.736243e-01 9.733775e-01 9.378308e-01 7.143544e-01 7.223382e-01 7.302987e-01 6.978009e-01

2342 9.754013e-01 9.747629e-01 9.740485e-01 9.736383e-01 9.733914e-01 9.382557e-01 7.145997e-01 7.226036e-01 7.305850e-01 6.980080e-01

2343 9.754155e-01 9.747770e-01 9.740624e-01 9.736522e-01 9.734052e-01 9.386685e-01 7.148497e-01 7.228738e-01 7.308763e-01 6.982193e-01

2344 9.754296e-01 9.747910e-01 9.740763e-01 9.736660e-01 9.734189e-01 9.390693e-01 7.151042e-01 7.231487e-01 7.311724e-01 6.984349e-01

2345 9.754436e-01 9.748049e-01 9.740901e-01 9.736796e-01 9.734324e-01 9.394583e-01 7.153634e-01 7.234283e-01 7.314733e-01 6.986547e-01

2346 9.754574e-01 9.748187e-01 9.741037e-01 9.736932e-01 9.734459e-01 9.398358e-01 7.156269e-01 7.237125e-01 7.317788e-01 6.988786e-01

2347 9.754712e-01 9.748324e-01 9.741173e-01 9.737066e-01 9.734592e-01 9.402017e-01 7.158949e-01 7.240011e-01 7.320887e-01 6.991066e-01

2348 9.754849e-01 9.748460e-01 9.741308e-01 9.737200e-01 9.734724e-01 9.405564e-01 7.161671e-01 7.242940e-01 7.324030e-01 6.993386e-01

2349 9.754985e-01 9.748595e-01 9.741441e-01 9.737332e-01 9.734856e-01 9.409000e-01 7.164434e-01 7.245911e-01 7.327215e-01 6.995745e-01

2350 9.755120e-01 9.748729e-01 9.741574e-01 9.737464e-01 9.734986e-01 9.412328e-01 7.167238e-01 7.248923e-01 7.330440e-01 6.998144e-01

2351 9.755254e-01 9.748862e-01 9.741706e-01 9.737594e-01 9.735116e-01 9.415549e-01 7.170082e-01 7.251974e-01 7.333704e-01 7.000580e-01

2352 9.755388e-01 9.748994e-01 9.741837e-01 9.737724e-01 9.735245e-01 9.418666e-01 7.172963e-01 7.255063e-01 7.337005e-01 7.003053e-01

2353 9.755520e-01 9.749126e-01 9.741967e-01 9.737853e-01 9.735372e-01 9.421682e-01 7.175881e-01 7.258188e-01 7.340340e-01 7.005562e-01

2354 9.755652e-01 9.749256e-01 9.742096e-01 9.737981e-01 9.735499e-01 9.424598e-01 7.178833e-01 7.261347e-01 7.343709e-01 7.008105e-01

2355 9.755783e-01 9.749386e-01 9.742225e-01 9.738108e-01 9.735626e-01 9.427417e-01 7.181819e-01 7.264538e-01 7.347108e-01 7.010683e-01

2356 9.755914e-01 9.749516e-01 9.742353e-01 9.738235e-01 9.735751e-01 9.430142e-01 7.184836e-01 7.267760e-01 7.350537e-01 7.013292e-01

2357 9.756044e-01 9.749645e-01 9.742480e-01 9.738361e-01 9.735876e-01 9.432775e-01 7.187884e-01 7.271010e-01 7.353992e-01 7.015933e-01

2358 9.756173e-01 9.749773e-01 9.742606e-01 9.738486e-01 9.736000e-01 9.435318e-01 7.190959e-01 7.274287e-01 7.357471e-01 7.018603e-01

2359 9.756302e-01 9.749900e-01 9.742732e-01 9.738610e-01 9.736123e-01 9.437775e-01 7.194061e-01 7.277588e-01 7.360972e-01 7.021301e-01

2360 9.756430e-01 9.750027e-01 9.742857e-01 9.738734e-01 9.736246e-01 9.440147e-01 7.197186e-01 7.280911e-01 7.364493e-01 7.024025e-01

2361 9.756557e-01 9.750153e-01 9.742982e-01 9.738857e-01 9.736368e-01 9.442436e-01 7.200334e-01 7.284254e-01 7.368031e-01 7.026774e-01

2362 9.756684e-01 9.750279e-01 9.743106e-01 9.738980e-01 9.736489e-01 9.444647e-01 7.203502e-01 7.287615e-01 7.371584e-01 7.029547e-01

2363 9.756811e-01 9.750404e-01 9.743229e-01 9.739102e-01 9.736610e-01 9.446780e-01 7.206688e-01 7.290991e-01 7.375148e-01 7.032341e-01

2364 9.756937e-01 9.750529e-01 9.743352e-01 9.739223e-01 9.736730e-01 9.448838e-01 7.209889e-01 7.294380e-01 7.378722e-01 7.035154e-01

2365 9.757062e-01 9.750653e-01 9.743474e-01 9.739344e-01 9.736850e-01 9.450824e-01 7.213103e-01 7.297779e-01 7.382303e-01 7.037985e-01

2366 9.757187e-01 9.750777e-01 9.743596e-01 9.739465e-01 9.736969e-01 9.452740e-01 7.216329e-01 7.301186e-01 7.385889e-01 7.040831e-01

2367 9.757312e-01 9.750900e-01 9.743718e-01 9.739584e-01 9.737087e-01 9.454589e-01 7.219564e-01 7.304599e-01 7.389476e-01 7.043692e-01

2368 9.757436e-01 9.751023e-01 9.743839e-01 9.739704e-01 9.737205e-01 9.456372e-01 7.222804e-01 7.308015e-01 7.393062e-01 7.046564e-01

2369 9.757560e-01 9.751145e-01 9.743959e-01 9.739822e-01 9.737323e-01 9.458092e-01 7.226049e-01 7.311431e-01 7.396644e-01 7.049446e-01

2370 9.757684e-01 9.751267e-01 9.744079e-01 9.739940e-01 9.737439e-01 9.459751e-01 7.229296e-01 7.314845e-01 7.400221e-01 7.052336e-01

2371 9.757807e-01 9.751389e-01 9.744198e-01 9.740058e-01 9.737556e-01 9.461351e-01 7.232542e-01 7.318255e-01 7.403789e-01 7.055231e-01

2372 9.757930e-01 9.751510e-01 9.744317e-01 9.740176e-01 9.737672e-01 9.462894e-01 7.235785e-01 7.321658e-01 7.407345e-01 7.058130e-01

2373 9.758053e-01 9.751631e-01 9.744436e-01 9.740292e-01 9.737787e-01 9.464383e-01 7.239023e-01 7.325051e-01 7.410888e-01 7.061030e-01

2374 9.758175e-01 9.751752e-01 9.744554e-01 9.740409e-01 9.737902e-01 9.465818e-01 7.242253e-01 7.328433e-01 7.414414e-01 7.063929e-01

2375 9.758297e-01 9.751872e-01 9.744672e-01 9.740525e-01 9.738016e-01 9.467204e-01 7.245473e-01 7.331801e-01 7.417922e-01 7.066826e-01

2376 9.758419e-01 9.751992e-01 9.744790e-01 9.740640e-01 9.738130e-01 9.468540e-01 7.248681e-01 7.335152e-01 7.421408e-01 7.069717e-01

2377 9.758541e-01 9.752112e-01 9.744907e-01 9.740755e-01 9.738244e-01 9.469829e-01 7.251874e-01 7.338484e-01 7.424871e-01 7.072602e-01

2378 9.758662e-01 9.752231e-01 9.745023e-01 9.740870e-01 9.738357e-01 9.471072e-01 7.255050e-01 7.341795e-01 7.428309e-01 7.075477e-01

2379 9.758783e-01 9.752350e-01 9.745140e-01 9.740984e-01 9.738470e-01 9.472272e-01 7.258208e-01 7.345083e-01 7.431719e-01 7.078341e-01

2380 9.758904e-01 9.752469e-01 9.745256e-01 9.741098e-01 9.738582e-01 9.473430e-01 7.261344e-01 7.348346e-01 7.435099e-01 7.081192e-01

2381 9.759024e-01 9.752588e-01 9.745371e-01 9.741211e-01 9.738694e-01 9.474548e-01 7.264458e-01 7.351581e-01 7.438447e-01 7.084028e-01

2382 9.759145e-01 9.752706e-01 9.745486e-01 9.741325e-01 9.738805e-01 9.475627e-01 7.267546e-01 7.354787e-01 7.441761e-01 7.086847e-01

2383 9.759265e-01 9.752824e-01 9.745601e-01 9.741437e-01 9.738916e-01 9.476669e-01 7.270608e-01 7.357962e-01 7.445040e-01 7.089647e-01

2384 9.759385e-01 9.752942e-01 9.745716e-01 9.741549e-01 9.739026e-01 9.477674e-01 7.273641e-01 7.361104e-01 7.448281e-01 7.092426e-01

2385 9.759505e-01 9.753059e-01 9.745830e-01 9.741661e-01 9.739136e-01 9.478646e-01 7.276643e-01 7.364211e-01 7.451484e-01 7.095183e-01

2386 9.759625e-01 9.753176e-01 9.745944e-01 9.741773e-01 9.739246e-01 9.479584e-01 7.279613e-01 7.367282e-01 7.454646e-01 7.097915e-01

2387 9.759745e-01 9.753294e-01 9.746058e-01 9.741884e-01 9.739355e-01 9.480491e-01 7.282550e-01 7.370315e-01 7.457766e-01 7.100621e-01

2388 9.759864e-01 9.753410e-01 9.746171e-01 9.741995e-01 9.739464e-01 9.481367e-01 7.285451e-01 7.373309e-01 7.460842e-01 7.103300e-01

2389 9.759984e-01 9.753527e-01 9.746284e-01 9.742105e-01 9.739572e-01 9.482214e-01 7.288316e-01 7.376263e-01 7.463875e-01 7.105950e-01

2390 9.760103e-01 9.753643e-01 9.746397e-01 9.742215e-01 9.739680e-01 9.483033e-01 7.291142e-01 7.379174e-01 7.466861e-01 7.108570e-01

2391 9.760222e-01 9.753760e-01 9.746509e-01 9.742324e-01 9.739787e-01 9.483825e-01 7.293930e-01 7.382043e-01 7.469801e-01 7.111157e-01

2392 9.760341e-01 9.753876e-01 9.746621e-01 9.742433e-01 9.739895e-01 9.484591e-01 7.296677e-01 7.384868e-01 7.472694e-01 7.113712e-01

2393 9.760460e-01 9.753991e-01 9.746733e-01 9.742542e-01 9.740001e-01 9.485332e-01 7.299383e-01 7.387648e-01 7.475538e-01 7.116233e-01

2394 9.760579e-01 9.754107e-01 9.746844e-01 9.742651e-01 9.740107e-01 9.486050e-01 7.302046e-01 7.390382e-01 7.478332e-01 7.118718e-01

2395 9.760698e-01 9.754222e-01 9.746955e-01 9.742758e-01 9.740213e-01 9.486745e-01 7.304667e-01 7.393070e-01 7.481078e-01 7.121167e-01

2396 9.760816e-01 9.754338e-01 9.747066e-01 9.742866e-01 9.740318e-01 9.487418e-01 7.307243e-01 7.395710e-01 7.483772e-01 7.123579e-01

2397 9.760935e-01 9.754453e-01 9.747176e-01 9.742973e-01 9.740423e-01 9.488070e-01 7.309775e-01 7.398303e-01 7.486417e-01 7.125952e-01

2398 9.761053e-01 9.754567e-01 9.747286e-01 9.743080e-01 9.740528e-01 9.488702e-01 7.312261e-01 7.400848e-01 7.489010e-01 7.128287e-01

2399 9.761172e-01 9.754682e-01 9.747396e-01 9.743186e-01 9.740632e-01 9.489315e-01 7.314702e-01 7.403344e-01 7.491551e-01 7.130582e-01

2400 9.761290e-01 9.754796e-01 9.747505e-01 9.743292e-01 9.740735e-01 9.489909e-01 7.317097e-01 7.405791e-01 7.494042e-01 7.132837e-01

2401 9.761408e-01 9.754911e-01 9.747614e-01 9.743398e-01 9.740838e-01 9.490486e-01 7.319446e-01 7.408189e-01 7.496481e-01 7.135051e-01

2402 9.761527e-01 9.755025e-01 9.747723e-01 9.743503e-01 9.740941e-01 9.491045e-01 7.321748e-01 7.410538e-01 7.498868e-01 7.137224e-01

2403 9.761645e-01 9.755138e-01 9.747831e-01 9.743607e-01 9.741043e-01 9.491589e-01 7.324003e-01 7.412838e-01 7.501204e-01 7.139355e-01

2404 9.761763e-01 9.755252e-01 9.747939e-01 9.743712e-01 9.741144e-01 9.492117e-01 7.326211e-01 7.415089e-01 7.503488e-01 7.141445e-01

2405 9.761881e-01 9.755366e-01 9.748047e-01 9.743815e-01 9.741245e-01 9.492630e-01 7.328372e-01 7.417291e-01 7.505721e-01 7.143493e-01

2406 9.761999e-01 9.755479e-01 9.748154e-01 9.743918e-01 9.741346e-01 9.493128e-01 7.330487e-01 7.419444e-01 7.507904e-01 7.145498e-01

2407 9.762117e-01 9.755592e-01 9.748261e-01 9.744021e-01 9.741446e-01 9.493614e-01 7.332555e-01 7.421548e-01 7.510036e-01 7.147462e-01

2408 9.762235e-01 9.755705e-01 9.748367e-01 9.744124e-01 9.741545e-01 9.494086e-01 7.334576e-01 7.423604e-01 7.512118e-01 7.149383e-01

2409 9.762353e-01 9.755817e-01 9.748473e-01 9.744225e-01 9.741644e-01 9.494545e-01 7.336551e-01 7.425611e-01 7.514151e-01 7.151262e-01

2410 9.762471e-01 9.755930e-01 9.748579e-01 9.744327e-01 9.741743e-01 9.494993e-01 7.338480e-01 7.427571e-01 7.516134e-01 7.153099e-01

2411 9.762588e-01 9.756042e-01 9.748684e-01 9.744427e-01 9.741840e-01 9.495429e-01 7.340364e-01 7.429485e-01 7.518069e-01 7.154894e-01

2412 9.762706e-01 9.756154e-01 9.748789e-01 9.744528e-01 9.741938e-01 9.495854e-01 7.342202e-01 7.431351e-01 7.519955e-01 7.156647e-01

2413 9.762824e-01 9.756265e-01 9.748893e-01 9.744627e-01 9.742034e-01 9.496268e-01 7.343996e-01 7.433171e-01 7.521795e-01 7.158359e-01

2414 9.762941e-01 9.756377e-01 9.748997e-01 9.744727e-01 9.742131e-01 9.496673e-01 7.345746e-01 7.434946e-01 7.523588e-01 7.160030e-01

2415 9.763059e-01 9.756488e-01 9.749100e-01 9.744825e-01 9.742226e-01 9.497067e-01 7.347452e-01 7.436676e-01 7.525335e-01 7.161660e-01

2416 9.763176e-01 9.756599e-01 9.749203e-01 9.744923e-01 9.742321e-01 9.497453e-01 7.349114e-01 7.438362e-01 7.527037e-01 7.163251e-01

2417 9.763294e-01 9.756710e-01 9.749306e-01 9.745021e-01 9.742415e-01 9.497830e-01 7.350735e-01 7.440004e-01 7.528694e-01 7.164801e-01

2418 9.763411e-01 9.756820e-01 9.749408e-01 9.745118e-01 9.742509e-01 9.498198e-01 7.352313e-01 7.441603e-01 7.530308e-01 7.166312e-01

2419 9.763529e-01 9.756930e-01 9.749509e-01 9.745214e-01 9.742602e-01 9.498558e-01 7.353851e-01 7.443161e-01 7.531878e-01 7.167785e-01

2420 9.763646e-01 9.757040e-01 9.749610e-01 9.745309e-01 9.742694e-01 9.498910e-01 7.355348e-01 7.444677e-01 7.533407e-01 7.169219e-01

2421 9.763763e-01 9.757149e-01 9.749711e-01 9.745404e-01 9.742786e-01 9.499255e-01 7.356805e-01 7.446152e-01 7.534895e-01 7.170616e-01

2422 9.763880e-01 9.757259e-01 9.749811e-01 9.745499e-01 9.742876e-01 9.499593e-01 7.358224e-01 7.447588e-01 7.536342e-01 7.171976e-01

2423 9.763998e-01 9.757368e-01 9.749910e-01 9.745592e-01 9.742967e-01 9.499924e-01 7.359604e-01 7.448985e-01 7.537750e-01 7.173299e-01

2424 9.764115e-01 9.757476e-01 9.750009e-01 9.745685e-01 9.743056e-01 9.500249e-01 7.360946e-01 7.450344e-01 7.539119e-01 7.174587e-01

2425 9.764232e-01 9.757584e-01 9.750107e-01 9.745778e-01 9.743145e-01 9.500567e-01 7.362253e-01 7.451666e-01 7.540451e-01 7.175840e-01

2426 9.764348e-01 9.757692e-01 9.750204e-01 9.745869e-01 9.743233e-01 9.500879e-01 7.363523e-01 7.452951e-01 7.541745e-01 7.177059e-01

2427 9.764465e-01 9.757800e-01 9.750301e-01 9.745960e-01 9.743320e-01 9.501186e-01 7.364758e-01 7.454200e-01 7.543004e-01 7.178244e-01

2428 9.764582e-01 9.757907e-01 9.750397e-01 9.746050e-01 9.743406e-01 9.501487e-01 7.365959e-01 7.455415e-01 7.544227e-01 7.179396e-01

2429 9.764698e-01 9.758014e-01 9.750493e-01 9.746139e-01 9.743492e-01 9.501783e-01 7.367126e-01 7.456596e-01 7.545417e-01 7.180517e-01

2430 9.764815e-01 9.758120e-01 9.750588e-01 9.746227e-01 9.743576e-01 9.502074e-01 7.368261e-01 7.457744e-01 7.546573e-01 7.181605e-01

2431 9.764931e-01 9.758226e-01 9.750682e-01 9.746315e-01 9.743660e-01 9.502361e-01 7.369364e-01 7.458859e-01 7.547696e-01 7.182663e-01

2432 9.765048e-01 9.758332e-01 9.750775e-01 9.746401e-01 9.743743e-01 9.502642e-01 7.370436e-01 7.459944e-01 7.548788e-01 7.183692e-01

2433 9.765164e-01 9.758437e-01 9.750868e-01 9.746487e-01 9.743825e-01 9.502920e-01 7.371477e-01 7.460997e-01 7.549849e-01 7.184691e-01

2434 9.765280e-01 9.758541e-01 9.750960e-01 9.746572e-01 9.743906e-01 9.503193e-01 7.372489e-01 7.462021e-01 7.550881e-01 7.185661e-01

2435 9.765396e-01 9.758646e-01 9.751051e-01 9.746656e-01 9.743986e-01 9.503462e-01 7.373473e-01 7.463016e-01 7.551883e-01 7.186604e-01

2436 9.765511e-01 9.758749e-01 9.751141e-01 9.746739e-01 9.744066e-01 9.503728e-01 7.374428e-01 7.463983e-01 7.552856e-01 7.187520e-01

2437 9.765627e-01 9.758852e-01 9.751230e-01 9.746821e-01 9.744144e-01 9.503990e-01 7.375357e-01 7.464922e-01 7.553803e-01 7.188410e-01

2438 9.765742e-01 9.758955e-01 9.751319e-01 9.746903e-01 9.744221e-01 9.504248e-01 7.376259e-01 7.465835e-01 7.554722e-01 7.189274e-01

2439 9.765857e-01 9.759057e-01 9.751407e-01 9.746983e-01 9.744298e-01 9.504503e-01 7.377136e-01 7.466723e-01 7.555616e-01 7.190114e-01

2440 9.765972e-01 9.759158e-01 9.751493e-01 9.747062e-01 9.744373e-01 9.504755e-01 7.377988e-01 7.467585e-01 7.556485e-01 7.190929e-01

2441 9.766087e-01 9.759259e-01 9.751579e-01 9.747140e-01 9.744447e-01 9.505004e-01 7.378816e-01 7.468423e-01 7.557329e-01 7.191721e-01

2442 9.766202e-01 9.759360e-01 9.751664e-01 9.747217e-01 9.744521e-01 9.505250e-01 7.379620e-01 7.469237e-01 7.558150e-01 7.192490e-01

2443 9.766316e-01 9.759459e-01 9.751748e-01 9.747293e-01 9.744593e-01 9.505494e-01 7.380402e-01 7.470029e-01 7.558948e-01 7.193237e-01

2444 9.766430e-01 9.759558e-01 9.751831e-01 9.747368e-01 9.744664e-01 9.505734e-01 7.381162e-01 7.470798e-01 7.559724e-01 7.193963e-01

2445 9.766544e-01 9.759657e-01 9.751913e-01 9.747442e-01 9.744734e-01 9.505972e-01 7.381900e-01 7.471547e-01 7.560478e-01 7.194668e-01

2446 9.766657e-01 9.759754e-01 9.751994e-01 9.747514e-01 9.744803e-01 9.506208e-01 7.382618e-01 7.472274e-01 7.561212e-01 7.195354e-01

2447 9.766771e-01 9.759851e-01 9.752073e-01 9.747586e-01 9.744870e-01 9.506442e-01 7.383316e-01 7.472981e-01 7.561925e-01 7.196019e-01

2448 9.766883e-01 9.759947e-01 9.752152e-01 9.747656e-01 9.744937e-01 9.506673e-01 7.383995e-01 7.473669e-01 7.562619e-01 7.196666e-01

2449 9.766996e-01 9.760043e-01 9.752229e-01 9.747725e-01 9.745002e-01 9.506902e-01 7.384655e-01 7.474339e-01 7.563294e-01 7.197295e-01

2450 9.767108e-01 9.760137e-01 9.752306e-01 9.747792e-01 9.745066e-01 9.507129e-01 7.385297e-01 7.474990e-01 7.563951e-01 7.197907e-01

2451 9.767220e-01 9.760231e-01 9.752381e-01 9.747859e-01 9.745129e-01 9.507355e-01 7.385922e-01 7.475623e-01 7.564590e-01 7.198501e-01

2452 9.767332e-01 9.760324e-01 9.752455e-01 9.747924e-01 9.745191e-01 9.507578e-01 7.386530e-01 7.476240e-01 7.565213e-01 7.199079e-01

2453 9.767443e-01 9.760416e-01 9.752527e-01 9.747988e-01 9.745251e-01 9.507800e-01 7.387121e-01 7.476840e-01 7.565819e-01 7.199641e-01

2454 9.767554e-01 9.760508e-01 9.752599e-01 9.748050e-01 9.745311e-01 9.508021e-01 7.387697e-01 7.477424e-01 7.566409e-01 7.200188e-01

2455 9.767664e-01 9.760598e-01 9.752669e-01 9.748111e-01 9.745369e-01 9.508239e-01 7.388257e-01 7.477994e-01 7.566984e-01 7.200720e-01

2456 9.767774e-01 9.760687e-01 9.752737e-01 9.748171e-01 9.745425e-01 9.508457e-01 7.388803e-01 7.478548e-01 7.567544e-01 7.201238e-01

2457 9.767883e-01 9.760776e-01 9.752805e-01 9.748229e-01 9.745480e-01 9.508673e-01 7.389335e-01 7.479088e-01 7.568090e-01 7.201742e-01

2458 9.767992e-01 9.760863e-01 9.752871e-01 9.748286e-01 9.745534e-01 9.508887e-01 7.389854e-01 7.479615e-01 7.568622e-01 7.202233e-01

2459 9.768100e-01 9.760949e-01 9.752935e-01 9.748341e-01 9.745587e-01 9.509100e-01 7.390359e-01 7.480128e-01 7.569140e-01 7.202711e-01

2460 9.768208e-01 9.761035e-01 9.752998e-01 9.748395e-01 9.745638e-01 9.509313e-01 7.390851e-01 7.480628e-01 7.569646e-01 7.203177e-01

2461 9.768315e-01 9.761119e-01 9.753060e-01 9.748448e-01 9.745688e-01 9.509524e-01 7.391331e-01 7.481116e-01 7.570140e-01 7.203631e-01

2462 9.768422e-01 9.761202e-01 9.753120e-01 9.748499e-01 9.745737e-01 9.509733e-01 7.391800e-01 7.481593e-01 7.570622e-01 7.204074e-01

2463 9.768528e-01 9.761284e-01 9.753179e-01 9.748548e-01 9.745784e-01 9.509942e-01 7.392257e-01 7.482058e-01 7.571092e-01 7.204506e-01

2464 9.768634e-01 9.761365e-01 9.753236e-01 9.748596e-01 9.745829e-01 9.510150e-01 7.392703e-01 7.482511e-01 7.571551e-01 7.204927e-01

2465 9.768738e-01 9.761444e-01 9.753291e-01 9.748642e-01 9.745874e-01 9.510357e-01 7.393139e-01 7.482955e-01 7.572000e-01 7.205338e-01

2466 9.768842e-01 9.761523e-01 9.753345e-01 9.748687e-01 9.745917e-01 9.510564e-01 7.393564e-01 7.483388e-01 7.572438e-01 7.205739e-01

2467 9.768946e-01 9.761600e-01 9.753397e-01 9.748730e-01 9.745958e-01 9.510769e-01 7.393980e-01 7.483811e-01 7.572866e-01 7.206131e-01

2468 9.769048e-01 9.761676e-01 9.753448e-01 9.748771e-01 9.745998e-01 9.510974e-01 7.394387e-01 7.484225e-01 7.573285e-01 7.206514e-01

2469 9.769150e-01 9.761750e-01 9.753496e-01 9.748811e-01 9.746037e-01 9.511177e-01 7.394784e-01 7.484630e-01 7.573695e-01 7.206888e-01

2470 9.769251e-01 9.761823e-01 9.753544e-01 9.748849e-01 9.746074e-01 9.511381e-01 7.395173e-01 7.485026e-01 7.574096e-01 7.207254e-01

2471 9.769351e-01 9.761895e-01 9.753589e-01 9.748886e-01 9.746110e-01 9.511583e-01 7.395554e-01 7.485414e-01 7.574489e-01 7.207613e-01

2472 9.769450e-01 9.761965e-01 9.753633e-01 9.748921e-01 9.746144e-01 9.511785e-01 7.395927e-01 7.485793e-01 7.574873e-01 7.207963e-01

2473 9.769549e-01 9.762034e-01 9.753675e-01 9.748954e-01 9.746177e-01 9.511987e-01 7.396292e-01 7.486165e-01 7.575250e-01 7.208306e-01

2474 9.769646e-01 9.762102e-01 9.753715e-01 9.748985e-01 9.746209e-01 9.512188e-01 7.396649e-01 7.486530e-01 7.575619e-01 7.208642e-01

2475 9.769742e-01 9.762168e-01 9.753753e-01 9.749015e-01 9.746239e-01 9.512388e-01 7.397000e-01 7.486887e-01 7.575981e-01 7.208971e-01

2476 9.769838e-01 9.762232e-01 9.753790e-01 9.749043e-01 9.746268e-01 9.512588e-01 7.397344e-01 7.487237e-01 7.576336e-01 7.209294e-01

2477 9.769932e-01 9.762295e-01 9.753825e-01 9.749070e-01 9.746296e-01 9.512788e-01 7.397681e-01 7.487581e-01 7.576684e-01 7.209611e-01

2478 9.770025e-01 9.762356e-01 9.753857e-01 9.749095e-01 9.746322e-01 9.512987e-01 7.398012e-01 7.487919e-01 7.577026e-01 7.209922e-01

2479 9.770118e-01 9.762415e-01 9.753888e-01 9.749118e-01 9.746347e-01 9.513186e-01 7.398338e-01 7.488250e-01 7.577361e-01 7.210227e-01

2480 9.770209e-01 9.762473e-01 9.753918e-01 9.749139e-01 9.746370e-01 9.513385e-01 7.398657e-01 7.488576e-01 7.577691e-01 7.210527e-01

2481 9.770298e-01 9.762529e-01 9.753945e-01 9.749159e-01 9.746392e-01 9.513583e-01 7.398971e-01 7.488896e-01 7.578015e-01 7.210822e-01

2482 9.770387e-01 9.762584e-01 9.753970e-01 9.749177e-01 9.746413e-01 9.513781e-01 7.399280e-01 7.489211e-01 7.578334e-01 7.211111e-01

2483 9.770474e-01 9.762637e-01 9.753994e-01 9.749194e-01 9.746433e-01 9.513979e-01 7.399584e-01 7.489520e-01 7.578647e-01 7.211396e-01

2484 9.770560e-01 9.762688e-01 9.754015e-01 9.749209e-01 9.746451e-01 9.514176e-01 7.399883e-01 7.489825e-01 7.578956e-01 7.211677e-01

2485 9.770645e-01 9.762737e-01 9.754035e-01 9.749222e-01 9.746469e-01 9.514373e-01 7.400177e-01 7.490125e-01 7.579259e-01 7.211953e-01

2486 9.770728e-01 9.762784e-01 9.754053e-01 9.749234e-01 9.746485e-01 9.514571e-01 7.400467e-01 7.490421e-01 7.579559e-01 7.212225e-01

2487 9.770810e-01 9.762830e-01 9.754069e-01 9.749244e-01 9.746500e-01 9.514768e-01 7.400753e-01 7.490712e-01 7.579853e-01 7.212494e-01

2488 9.770890e-01 9.762874e-01 9.754083e-01 9.749253e-01 9.746513e-01 9.514965e-01 7.401035e-01 7.490999e-01 7.580144e-01 7.212758e-01

2489 9.770969e-01 9.762915e-01 9.754095e-01 9.749260e-01 9.746526e-01 9.515161e-01 7.401313e-01 7.491283e-01 7.580430e-01 7.213019e-01

2490 9.771046e-01 9.762955e-01 9.754105e-01 9.749265e-01 9.746538e-01 9.515358e-01 7.401588e-01 7.491562e-01 7.580713e-01 7.213277e-01

2491 9.771122e-01 9.762993e-01 9.754114e-01 9.749270e-01 9.746549e-01 9.515555e-01 7.401859e-01 7.491839e-01 7.580992e-01 7.213532e-01

2492 9.771196e-01 9.763030e-01 9.754120e-01 9.749273e-01 9.746559e-01 9.515751e-01 7.402127e-01 7.492111e-01 7.581267e-01 7.213783e-01

2493 9.771268e-01 9.763064e-01 9.754125e-01 9.749274e-01 9.746568e-01 9.515948e-01 7.402391e-01 7.492381e-01 7.581539e-01 7.214032e-01

2494 9.771339e-01 9.763096e-01 9.754128e-01 9.749274e-01 9.746577e-01 9.516144e-01 7.402653e-01 7.492647e-01 7.581808e-01 7.214278e-01

2495 9.771407e-01 9.763126e-01 9.754129e-01 9.749273e-01 9.746585e-01 9.516341e-01 7.402912e-01 7.492910e-01 7.582074e-01 7.214522e-01

2496 9.771474e-01 9.763154e-01 9.754129e-01 9.749271e-01 9.746592e-01 9.516537e-01 7.403168e-01 7.493171e-01 7.582337e-01 7.214763e-01

2497 9.771540e-01 9.763181e-01 9.754127e-01 9.749268e-01 9.746598e-01 9.516734e-01 7.403422e-01 7.493429e-01 7.582597e-01 7.215002e-01

2498 9.771603e-01 9.763205e-01 9.754123e-01 9.749263e-01 9.746604e-01 9.516930e-01 7.403673e-01 7.493685e-01 7.582854e-01 7.215238e-01

2499 9.771664e-01 9.763227e-01 9.754118e-01 9.749258e-01 9.746610e-01 9.517127e-01 7.403922e-01 7.493938e-01 7.583109e-01 7.215473e-01

2500 9.771723e-01 9.763248e-01 9.754111e-01 9.749252e-01 9.746615e-01 9.517323e-01 7.404169e-01 7.494189e-01 7.583361e-01 7.215706e-01

2501 9.771781e-01 9.763266e-01 9.754103e-01 9.749245e-01 9.746620e-01 9.517520e-01 7.404414e-01 7.494437e-01 7.583611e-01 7.215937e-01

2502 9.771836e-01 9.763283e-01 9.754093e-01 9.749237e-01 9.746625e-01 9.517717e-01 7.404657e-01 7.494684e-01 7.583859e-01 7.216166e-01

2503 9.771889e-01 9.763297e-01 9.754082e-01 9.749228e-01 9.746630e-01 9.517914e-01 7.404898e-01 7.494929e-01 7.584105e-01 7.216394e-01

2504 9.771940e-01 9.763310e-01 9.754070e-01 9.749219e-01 9.746635e-01 9.518111e-01 7.405138e-01 7.495172e-01 7.584349e-01 7.216620e-01

2505 9.771989e-01 9.763320e-01 9.754056e-01 9.749209e-01 9.746640e-01 9.518308e-01 7.405376e-01 7.495413e-01 7.584591e-01 7.216846e-01

2506 9.772036e-01 9.763329e-01 9.754041e-01 9.749199e-01 9.746645e-01 9.518505e-01 7.405612e-01 7.495653e-01 7.584831e-01 7.217069e-01

2507 9.772080e-01 9.763336e-01 9.754026e-01 9.749189e-01 9.746650e-01 9.518703e-01 7.405847e-01 7.495891e-01 7.585070e-01 7.217292e-01

2508 9.772122e-01 9.763341e-01 9.754009e-01 9.749178e-01 9.746656e-01 9.518900e-01 7.406081e-01 7.496128e-01 7.585306e-01 7.217514e-01

2509 9.772162e-01 9.763344e-01 9.753991e-01 9.749167e-01 9.746662e-01 9.519098e-01 7.406313e-01 7.496363e-01 7.585542e-01 7.217734e-01

2510 9.772199e-01 9.763346e-01 9.753972e-01 9.749156e-01 9.746669e-01 9.519296e-01 7.406545e-01 7.496597e-01 7.585776e-01 7.217954e-01

2511 9.772234e-01 9.763345e-01 9.753953e-01 9.749146e-01 9.746677e-01 9.519494e-01 7.406775e-01 7.496830e-01 7.586009e-01 7.218173e-01

2512 9.772267e-01 9.763343e-01 9.753933e-01 9.749136e-01 9.746686e-01 9.519692e-01 7.407005e-01 7.497062e-01 7.586240e-01 7.218391e-01

2513 9.772297e-01 9.763339e-01 9.753913e-01 9.749126e-01 9.746696e-01 9.519890e-01 7.407233e-01 7.497293e-01 7.586470e-01 7.218609e-01

2514 9.772325e-01 9.763334e-01 9.753892e-01 9.749116e-01 9.746706e-01 9.520089e-01 7.407461e-01 7.497523e-01 7.586699e-01 7.218826e-01

2515 9.772350e-01 9.763327e-01 9.753871e-01 9.749107e-01 9.746719e-01 9.520288e-01 7.407688e-01 7.497753e-01 7.586927e-01 7.219042e-01

2516 9.772372e-01 9.763319e-01 9.753850e-01 9.749099e-01 9.746732e-01 9.520487e-01 7.407915e-01 7.497981e-01 7.587154e-01 7.219258e-01

2517 9.772393e-01 9.763309e-01 9.753829e-01 9.749092e-01 9.746747e-01 9.520686e-01 7.408141e-01 7.498209e-01 7.587380e-01 7.219474e-01

2518 9.772410e-01 9.763298e-01 9.753808e-01 9.749086e-01 9.746764e-01 9.520885e-01 7.408366e-01 7.498436e-01 7.587606e-01 7.219690e-01

2519 9.772425e-01 9.763285e-01 9.753787e-01 9.749082e-01 9.746783e-01 9.521085e-01 7.408591e-01 7.498663e-01 7.587830e-01 7.219905e-01

2520 9.772438e-01 9.763272e-01 9.753767e-01 9.749078e-01 9.746803e-01 9.521285e-01 7.408816e-01 7.498889e-01 7.588054e-01 7.220120e-01

2521 9.772448e-01 9.763257e-01 9.753747e-01 9.749076e-01 9.746826e-01 9.521485e-01 7.409040e-01 7.499115e-01 7.588277e-01 7.220335e-01

2522 9.772552e-01 9.763360e-01 9.753848e-01 9.749176e-01 9.746924e-01 9.524422e-01 7.411559e-01 7.501853e-01 7.591240e-01 7.222431e-01

2523 9.772655e-01 9.763462e-01 9.753948e-01 9.749275e-01 9.747022e-01 9.527265e-01 7.414114e-01 7.504626e-01 7.594238e-01 7.224560e-01

2524 9.772758e-01 9.763563e-01 9.754048e-01 9.749373e-01 9.747119e-01 9.530017e-01 7.416703e-01 7.507434e-01 7.597271e-01 7.226723e-01

2525 9.772861e-01 9.763664e-01 9.754147e-01 9.749471e-01 9.747216e-01 9.532680e-01 7.419326e-01 7.510275e-01 7.600335e-01 7.228918e-01

2526 9.772963e-01 9.763765e-01 9.754245e-01 9.749568e-01 9.747311e-01 9.535256e-01 7.421980e-01 7.513148e-01 7.603430e-01 7.231144e-01

2527 9.773064e-01 9.763865e-01 9.754343e-01 9.749664e-01 9.747407e-01 9.537747e-01 7.424666e-01 7.516051e-01 7.606554e-01 7.233400e-01

2528 9.773165e-01 9.763964e-01 9.754441e-01 9.749760e-01 9.747501e-01 9.540155e-01 7.427381e-01 7.518983e-01 7.609705e-01 7.235686e-01

2529 9.773266e-01 9.764063e-01 9.754538e-01 9.749856e-01 9.747595e-01 9.542483e-01 7.430124e-01 7.521941e-01 7.612881e-01 7.238001e-01

2530 9.773366e-01 9.764161e-01 9.754634e-01 9.749950e-01 9.747689e-01 9.544733e-01 7.432893e-01 7.524925e-01 7.616080e-01 7.240342e-01

2531 9.773466e-01 9.764259e-01 9.754730e-01 9.750045e-01 9.747782e-01 9.546906e-01 7.435688e-01 7.527932e-01 7.619301e-01 7.242710e-01

2532 9.773565e-01 9.764357e-01 9.754825e-01 9.750138e-01 9.747874e-01 9.549006e-01 7.438506e-01 7.530960e-01 7.622541e-01 7.245103e-01

2533 9.773664e-01 9.764454e-01 9.754920e-01 9.750231e-01 9.747966e-01 9.551033e-01 7.441345e-01 7.534009e-01 7.625798e-01 7.247519e-01

2534 9.773762e-01 9.764551e-01 9.755014e-01 9.750324e-01 9.748058e-01 9.552992e-01 7.444205e-01 7.537075e-01 7.629069e-01 7.249958e-01

2535 9.773860e-01 9.764647e-01 9.755108e-01 9.750416e-01 9.748149e-01 9.554883e-01 7.447083e-01 7.540157e-01 7.632354e-01 7.252417e-01

2536 9.773958e-01 9.764743e-01 9.755202e-01 9.750508e-01 9.748239e-01 9.556708e-01 7.449977e-01 7.543253e-01 7.635649e-01 7.254896e-01

2537 9.774056e-01 9.764839e-01 9.755295e-01 9.750600e-01 9.748329e-01 9.558470e-01 7.452885e-01 7.546360e-01 7.638953e-01 7.257394e-01

2538 9.774153e-01 9.764934e-01 9.755388e-01 9.750690e-01 9.748419e-01 9.560171e-01 7.455806e-01 7.549477e-01 7.642262e-01 7.259907e-01

2539 9.774250e-01 9.765029e-01 9.755480e-01 9.750781e-01 9.748508e-01 9.561813e-01 7.458738e-01 7.552602e-01 7.645576e-01 7.262436e-01

2540 9.774347e-01 9.765123e-01 9.755572e-01 9.750871e-01 9.748596e-01 9.563398e-01 7.461678e-01 7.555732e-01 7.648891e-01 7.264977e-01

2541 9.774443e-01 9.765218e-01 9.755663e-01 9.750960e-01 9.748684e-01 9.564927e-01 7.464625e-01 7.558866e-01 7.652205e-01 7.267531e-01

2542 9.774539e-01 9.765312e-01 9.755754e-01 9.751049e-01 9.748772e-01 9.566402e-01 7.467577e-01 7.562000e-01 7.655516e-01 7.270094e-01

2543 9.774635e-01 9.765405e-01 9.755845e-01 9.751138e-01 9.748859e-01 9.567826e-01 7.470531e-01 7.565134e-01 7.658822e-01 7.272666e-01

2544 9.774731e-01 9.765498e-01 9.755935e-01 9.751227e-01 9.748946e-01 9.569200e-01 7.473486e-01 7.568264e-01 7.662121e-01 7.275244e-01

2545 9.774826e-01 9.765592e-01 9.756025e-01 9.751314e-01 9.749033e-01 9.570526e-01 7.476440e-01 7.571389e-01 7.665410e-01 7.277827e-01

2546 9.774922e-01 9.765684e-01 9.756115e-01 9.751402e-01 9.749119e-01 9.571806e-01 7.479390e-01 7.574507e-01 7.668687e-01 7.280413e-01

2547 9.775017e-01 9.765777e-01 9.756204e-01 9.751489e-01 9.749204e-01 9.573041e-01 7.482334e-01 7.577615e-01 7.671949e-01 7.283000e-01

2548 9.775112e-01 9.765869e-01 9.756293e-01 9.751576e-01 9.749289e-01 9.574233e-01 7.485272e-01 7.580711e-01 7.675196e-01 7.285586e-01

2549 9.775206e-01 9.765961e-01 9.756382e-01 9.751662e-01 9.749374e-01 9.575383e-01 7.488199e-01 7.583794e-01 7.678424e-01 7.288170e-01

2550 9.775301e-01 9.766053e-01 9.756470e-01 9.751748e-01 9.749458e-01 9.576493e-01 7.491116e-01 7.586861e-01 7.681632e-01 7.290750e-01

2551 9.775395e-01 9.766144e-01 9.756558e-01 9.751834e-01 9.749542e-01 9.577565e-01 7.494019e-01 7.589910e-01 7.684818e-01 7.293324e-01

2552 9.775489e-01 9.766236e-01 9.756646e-01 9.751919e-01 9.749626e-01 9.578599e-01 7.496907e-01 7.592940e-01 7.687980e-01 7.295891e-01

2553 9.775584e-01 9.766327e-01 9.756733e-01 9.752004e-01 9.749709e-01 9.579598e-01 7.499778e-01 7.595949e-01 7.691115e-01 7.298448e-01

2554 9.775677e-01 9.766417e-01 9.756820e-01 9.752089e-01 9.749792e-01 9.580562e-01 7.502630e-01 7.598934e-01 7.694223e-01 7.300994e-01

2555 9.775771e-01 9.766508e-01 9.756907e-01 9.752173e-01 9.749874e-01 9.581494e-01 7.505461e-01 7.601895e-01 7.697301e-01 7.303528e-01

2556 9.775865e-01 9.766598e-01 9.756993e-01 9.752256e-01 9.749956e-01 9.582393e-01 7.508270e-01 7.604829e-01 7.700348e-01 7.306047e-01

2557 9.775958e-01 9.766689e-01 9.757079e-01 9.752340e-01 9.750038e-01 9.583262e-01 7.511056e-01 7.607735e-01 7.703363e-01 7.308550e-01

2558 9.776052e-01 9.766779e-01 9.757165e-01 9.752423e-01 9.750119e-01 9.584102e-01 7.513816e-01 7.610611e-01 7.706343e-01 7.311036e-01

2559 9.776145e-01 9.766868e-01 9.757250e-01 9.752505e-01 9.750200e-01 9.584913e-01 7.516549e-01 7.613456e-01 7.709288e-01 7.313502e-01

2560 9.776238e-01 9.766958e-01 9.757335e-01 9.752588e-01 9.750280e-01 9.585697e-01 7.519253e-01 7.616268e-01 7.712196e-01 7.315948e-01

2561 9.776331e-01 9.767047e-01 9.757420e-01 9.752670e-01 9.750360e-01 9.586455e-01 7.521928e-01 7.619046e-01 7.715065e-01 7.318373e-01

2562 9.776424e-01 9.767136e-01 9.757505e-01 9.752751e-01 9.750440e-01 9.587188e-01 7.524572e-01 7.621790e-01 7.717896e-01 7.320774e-01

2563 9.776517e-01 9.767225e-01 9.757589e-01 9.752832e-01 9.750519e-01 9.587896e-01 7.527184e-01 7.624497e-01 7.720686e-01 7.323150e-01

2564 9.776610e-01 9.767314e-01 9.757672e-01 9.752913e-01 9.750598e-01 9.588582e-01 7.529762e-01 7.627167e-01 7.723435e-01 7.325501e-01

2565 9.776703e-01 9.767402e-01 9.757756e-01 9.752993e-01 9.750676e-01 9.589245e-01 7.532306e-01 7.629798e-01 7.726142e-01 7.327824e-01

2566 9.776796e-01 9.767490e-01 9.757839e-01 9.753073e-01 9.750754e-01 9.589887e-01 7.534814e-01 7.632390e-01 7.728806e-01 7.330120e-01

2567 9.776888e-01 9.767578e-01 9.757922e-01 9.753153e-01 9.750832e-01 9.590509e-01 7.537285e-01 7.634942e-01 7.731426e-01 7.332386e-01

2568 9.776981e-01 9.767666e-01 9.758004e-01 9.753232e-01 9.750909e-01 9.591111e-01 7.539720e-01 7.637454e-01 7.734002e-01 7.334623e-01

2569 9.777073e-01 9.767754e-01 9.758086e-01 9.753310e-01 9.750985e-01 9.591694e-01 7.542116e-01 7.639924e-01 7.736534e-01 7.336828e-01

2570 9.777165e-01 9.767841e-01 9.758168e-01 9.753389e-01 9.751062e-01 9.592258e-01 7.544474e-01 7.642352e-01 7.739020e-01 7.339001e-01

2571 9.777257e-01 9.767929e-01 9.758249e-01 9.753467e-01 9.751137e-01 9.592806e-01 7.546792e-01 7.644737e-01 7.741460e-01 7.341142e-01

2572 9.777350e-01 9.768016e-01 9.758330e-01 9.753544e-01 9.751213e-01 9.593337e-01 7.549071e-01 7.647079e-01 7.743855e-01 7.343249e-01

2573 9.777442e-01 9.768102e-01 9.758411e-01 9.753621e-01 9.751287e-01 9.593851e-01 7.551309e-01 7.649379e-01 7.746203e-01 7.345323e-01

2574 9.777534e-01 9.768189e-01 9.758491e-01 9.753698e-01 9.751362e-01 9.594351e-01 7.553506e-01 7.651634e-01 7.748505e-01 7.347361e-01

2575 9.777626e-01 9.768275e-01 9.758571e-01 9.753774e-01 9.751436e-01 9.594835e-01 7.555663e-01 7.653846e-01 7.750761e-01 7.349365e-01

2576 9.777717e-01 9.768361e-01 9.758650e-01 9.753849e-01 9.751509e-01 9.595305e-01 7.557778e-01 7.656014e-01 7.752970e-01 7.351333e-01

2577 9.777809e-01 9.768447e-01 9.758729e-01 9.753925e-01 9.751582e-01 9.595762e-01 7.559852e-01 7.658138e-01 7.755133e-01 7.353265e-01

2578 9.777901e-01 9.768533e-01 9.758808e-01 9.753999e-01 9.751655e-01 9.596206e-01 7.561884e-01 7.660218e-01 7.757250e-01 7.355161e-01

2579 9.777993e-01 9.768618e-01 9.758886e-01 9.754074e-01 9.751727e-01 9.596637e-01 7.563874e-01 7.662254e-01 7.759321e-01 7.357020e-01

2580 9.778084e-01 9.768703e-01 9.758964e-01 9.754147e-01 9.751798e-01 9.597057e-01 7.565823e-01 7.664246e-01 7.761345e-01 7.358843e-01

2581 9.778176e-01 9.768788e-01 9.759041e-01 9.754221e-01 9.751869e-01 9.597465e-01 7.567731e-01 7.666194e-01 7.763325e-01 7.360629e-01

2582 9.778267e-01 9.768873e-01 9.759118e-01 9.754294e-01 9.751940e-01 9.597862e-01 7.569596e-01 7.668099e-01 7.765259e-01 7.362378e-01

2583 9.778358e-01 9.768957e-01 9.759195e-01 9.754366e-01 9.752010e-01 9.598248e-01 7.571421e-01 7.669961e-01 7.767148e-01 7.364091e-01

2584 9.778449e-01 9.769041e-01 9.759271e-01 9.754438e-01 9.752079e-01 9.598624e-01 7.573205e-01 7.671780e-01 7.768993e-01 7.365766e-01

2585 9.778541e-01 9.769125e-01 9.759347e-01 9.754509e-01 9.752148e-01 9.598991e-01 7.574947e-01 7.673557e-01 7.770794e-01 7.367405e-01

2586 9.778632e-01 9.769208e-01 9.759422e-01 9.754579e-01 9.752216e-01 9.599348e-01 7.576650e-01 7.675292e-01 7.772551e-01 7.369007e-01

2587 9.778723e-01 9.769291e-01 9.759496e-01 9.754650e-01 9.752284e-01 9.599696e-01 7.578312e-01 7.676985e-01 7.774266e-01 7.370572e-01

2588 9.778813e-01 9.769374e-01 9.759570e-01 9.754719e-01 9.752351e-01 9.600036e-01 7.579935e-01 7.678637e-01 7.775938e-01 7.372102e-01

2589 9.778904e-01 9.769457e-01 9.759644e-01 9.754788e-01 9.752418e-01 9.600368e-01 7.581518e-01 7.680248e-01 7.777568e-01 7.373595e-01

2590 9.778995e-01 9.769539e-01 9.759717e-01 9.754856e-01 9.752484e-01 9.600691e-01 7.583063e-01 7.681819e-01 7.779158e-01 7.375053e-01

2591 9.779085e-01 9.769621e-01 9.759790e-01 9.754924e-01 9.752549e-01 9.601008e-01 7.584569e-01 7.683351e-01 7.780706e-01 7.376476e-01

2592 9.779176e-01 9.769703e-01 9.759862e-01 9.754991e-01 9.752614e-01 9.601317e-01 7.586037e-01 7.684844e-01 7.782216e-01 7.377863e-01

2593 9.779266e-01 9.769784e-01 9.759933e-01 9.755058e-01 9.752678e-01 9.601619e-01 7.587469e-01 7.686299e-01 7.783685e-01 7.379216e-01

2594 9.779356e-01 9.769865e-01 9.760004e-01 9.755124e-01 9.752742e-01 9.601914e-01 7.588863e-01 7.687716e-01 7.785117e-01 7.380535e-01

2595 9.779446e-01 9.769945e-01 9.760075e-01 9.755189e-01 9.752805e-01 9.602203e-01 7.590222e-01 7.689096e-01 7.786511e-01 7.381821e-01

2596 9.779536e-01 9.770025e-01 9.760144e-01 9.755254e-01 9.752867e-01 9.602487e-01 7.591545e-01 7.690440e-01 7.787868e-01 7.383073e-01

2597 9.779625e-01 9.770105e-01 9.760213e-01 9.755318e-01 9.752928e-01 9.602764e-01 7.592834e-01 7.691748e-01 7.789188e-01 7.384293e-01

2598 9.779715e-01 9.770184e-01 9.760282e-01 9.755381e-01 9.752989e-01 9.603036e-01 7.594088e-01 7.693021e-01 7.790474e-01 7.385481e-01

2599 9.779804e-01 9.770263e-01 9.760350e-01 9.755444e-01 9.753050e-01 9.603302e-01 7.595309e-01 7.694260e-01 7.791724e-01 7.386637e-01

2600 9.779894e-01 9.770342e-01 9.760417e-01 9.755506e-01 9.753109e-01 9.603563e-01 7.596496e-01 7.695466e-01 7.792941e-01 7.387762e-01

2601 9.779983e-01 9.770420e-01 9.760484e-01 9.755567e-01 9.753168e-01 9.603820e-01 7.597652e-01 7.696639e-01 7.794124e-01 7.388857e-01

2602 9.780072e-01 9.770498e-01 9.760550e-01 9.755627e-01 9.753226e-01 9.604071e-01 7.598777e-01 7.697780e-01 7.795275e-01 7.389922e-01

2603 9.780160e-01 9.770575e-01 9.760615e-01 9.755687e-01 9.753284e-01 9.604319e-01 7.599870e-01 7.698889e-01 7.796394e-01 7.390958e-01

2604 9.780249e-01 9.770651e-01 9.760679e-01 9.755746e-01 9.753341e-01 9.604562e-01 7.600934e-01 7.699969e-01 7.797483e-01 7.391966e-01

2605 9.780337e-01 9.770728e-01 9.760743e-01 9.755804e-01 9.753397e-01 9.604800e-01 7.601968e-01 7.701018e-01 7.798541e-01 7.392945e-01

2606 9.780425e-01 9.770803e-01 9.760806e-01 9.755862e-01 9.753452e-01 9.605035e-01 7.602973e-01 7.702038e-01 7.799570e-01 7.393897e-01

2607 9.780513e-01 9.770879e-01 9.760869e-01 9.755918e-01 9.753507e-01 9.605266e-01 7.603951e-01 7.703030e-01 7.800570e-01 7.394823e-01

2608 9.780600e-01 9.770953e-01 9.760930e-01 9.755974e-01 9.753560e-01 9.605494e-01 7.604901e-01 7.703994e-01 7.801543e-01 7.395722e-01

2609 9.780688e-01 9.771027e-01 9.760991e-01 9.756029e-01 9.753613e-01 9.605718e-01 7.605824e-01 7.704931e-01 7.802488e-01 7.396596e-01

2610 9.780775e-01 9.771101e-01 9.761051e-01 9.756083e-01 9.753666e-01 9.605939e-01 7.606722e-01 7.705842e-01 7.803407e-01 7.397445e-01

2611 9.780861e-01 9.771174e-01 9.761110e-01 9.756137e-01 9.753717e-01 9.606156e-01 7.607595e-01 7.706728e-01 7.804300e-01 7.398270e-01

2612 9.780948e-01 9.771247e-01 9.761169e-01 9.756189e-01 9.753768e-01 9.606371e-01 7.608442e-01 7.707588e-01 7.805168e-01 7.399071e-01

2613 9.781034e-01 9.771318e-01 9.761226e-01 9.756241e-01 9.753818e-01 9.606583e-01 7.609266e-01 7.708425e-01 7.806012e-01 7.399850e-01

2614 9.781120e-01 9.771390e-01 9.761283e-01 9.756291e-01 9.753867e-01 9.606792e-01 7.610067e-01 7.709238e-01 7.806833e-01 7.400606e-01

2615 9.781205e-01 9.771460e-01 9.761339e-01 9.756341e-01 9.753915e-01 9.606998e-01 7.610845e-01 7.710028e-01 7.807630e-01 7.401340e-01

2616 9.781290e-01 9.771530e-01 9.761394e-01 9.756390e-01 9.753963e-01 9.607202e-01 7.611601e-01 7.710796e-01 7.808405e-01 7.402053e-01

2617 9.781375e-01 9.771599e-01 9.761448e-01 9.756438e-01 9.754009e-01 9.607404e-01 7.612336e-01 7.711543e-01 7.809159e-01 7.402746e-01

2618 9.781460e-01 9.771668e-01 9.761501e-01 9.756486e-01 9.754055e-01 9.607603e-01 7.613051e-01 7.712268e-01 7.809891e-01 7.403419e-01

2619 9.781544e-01 9.771736e-01 9.761553e-01 9.756532e-01 9.754100e-01 9.607800e-01 7.613745e-01 7.712974e-01 7.810604e-01 7.404072e-01

2620 9.781627e-01 9.771803e-01 9.761604e-01 9.756577e-01 9.754144e-01 9.607995e-01 7.614419e-01 7.713659e-01 7.811296e-01 7.404707e-01

2621 9.781711e-01 9.771870e-01 9.761655e-01 9.756621e-01 9.754188e-01 9.608187e-01 7.615075e-01 7.714326e-01 7.811970e-01 7.405323e-01

2622 9.781794e-01 9.771935e-01 9.761704e-01 9.756665e-01 9.754230e-01 9.608378e-01 7.615713e-01 7.714974e-01 7.812625e-01 7.405922e-01

2623 9.781876e-01 9.772000e-01 9.761753e-01 9.756707e-01 9.754272e-01 9.608567e-01 7.616332e-01 7.715605e-01 7.813262e-01 7.406504e-01

2624 9.781958e-01 9.772065e-01 9.761800e-01 9.756749e-01 9.754313e-01 9.608755e-01 7.616935e-01 7.716218e-01 7.813881e-01 7.407069e-01

2625 9.782039e-01 9.772128e-01 9.761846e-01 9.756789e-01 9.754353e-01 9.608941e-01 7.617521e-01 7.716814e-01 7.814484e-01 7.407618e-01

2626 9.782120e-01 9.772191e-01 9.761892e-01 9.756829e-01 9.754392e-01 9.609125e-01 7.618091e-01 7.717395e-01 7.815071e-01 7.408151e-01

2627 9.782201e-01 9.772253e-01 9.761936e-01 9.756868e-01 9.754430e-01 9.609308e-01 7.618645e-01 7.717959e-01 7.815642e-01 7.408670e-01

2628 9.782281e-01 9.772313e-01 9.761980e-01 9.756905e-01 9.754468e-01 9.609489e-01 7.619184e-01 7.718508e-01 7.816197e-01 7.409174e-01

2629 9.782360e-01 9.772374e-01 9.762022e-01 9.756942e-01 9.754504e-01 9.609669e-01 7.619709e-01 7.719043e-01 7.816738e-01 7.409664e-01

2630 9.782439e-01 9.772433e-01 9.762063e-01 9.756977e-01 9.754540e-01 9.609847e-01 7.620219e-01 7.719563e-01 7.817265e-01 7.410140e-01

2631 9.782517e-01 9.772491e-01 9.762103e-01 9.757012e-01 9.754575e-01 9.610025e-01 7.620716e-01 7.720070e-01 7.817778e-01 7.410604e-01

2632 9.782595e-01 9.772549e-01 9.762143e-01 9.757046e-01 9.754609e-01 9.610201e-01 7.621200e-01 7.720563e-01 7.818277e-01 7.411054e-01

2633 9.782672e-01 9.772605e-01 9.762181e-01 9.757078e-01 9.754643e-01 9.610376e-01 7.621671e-01 7.721044e-01 7.818764e-01 7.411493e-01

2634 9.782748e-01 9.772661e-01 9.762218e-01 9.757110e-01 9.754675e-01 9.610550e-01 7.622130e-01 7.721512e-01 7.819238e-01 7.411919e-01

2635 9.782823e-01 9.772715e-01 9.762253e-01 9.757141e-01 9.754707e-01 9.610723e-01 7.622577e-01 7.721968e-01 7.819700e-01 7.412335e-01

2636 9.782898e-01 9.772769e-01 9.762288e-01 9.757170e-01 9.754738e-01 9.610895e-01 7.623012e-01 7.722413e-01 7.820151e-01 7.412739e-01

2637 9.782973e-01 9.772822e-01 9.762322e-01 9.757199e-01 9.754768e-01 9.611066e-01 7.623437e-01 7.722847e-01 7.820590e-01 7.413132e-01

2638 9.783046e-01 9.772873e-01 9.762354e-01 9.757227e-01 9.754798e-01 9.611237e-01 7.623851e-01 7.723269e-01 7.821019e-01 7.413516e-01

2639 9.783119e-01 9.772924e-01 9.762386e-01 9.757254e-01 9.754827e-01 9.611406e-01 7.624254e-01 7.723682e-01 7.821437e-01 7.413890e-01

2640 9.783191e-01 9.772974e-01 9.762416e-01 9.757280e-01 9.754855e-01 9.611575e-01 7.624648e-01 7.724084e-01 7.821846e-01 7.414254e-01

2641 9.783262e-01 9.773022e-01 9.762446e-01 9.757305e-01 9.754882e-01 9.611743e-01 7.625032e-01 7.724477e-01 7.822244e-01 7.414609e-01

2642 9.783333e-01 9.773070e-01 9.762474e-01 9.757329e-01 9.754909e-01 9.611910e-01 7.625407e-01 7.724861e-01 7.822633e-01 7.414955e-01

2643 9.783402e-01 9.773116e-01 9.762501e-01 9.757352e-01 9.754935e-01 9.612077e-01 7.625774e-01 7.725235e-01 7.823013e-01 7.415293e-01

2644 9.783471e-01 9.773162e-01 9.762527e-01 9.757374e-01 9.754960e-01 9.612243e-01 7.626131e-01 7.725602e-01 7.823385e-01 7.415623e-01

2645 9.783538e-01 9.773206e-01 9.762552e-01 9.757395e-01 9.754985e-01 9.612409e-01 7.626481e-01 7.725959e-01 7.823748e-01 7.415945e-01

2646 9.783605e-01 9.773249e-01 9.762575e-01 9.757416e-01 9.755009e-01 9.612574e-01 7.626822e-01 7.726309e-01 7.824103e-01 7.416260e-01

2647 9.783671e-01 9.773291e-01 9.762598e-01 9.757435e-01 9.755033e-01 9.612739e-01 7.627156e-01 7.726651e-01 7.824450e-01 7.416567e-01

2648 9.783736e-01 9.773332e-01 9.762620e-01 9.757454e-01 9.755056e-01 9.612903e-01 7.627483e-01 7.726986e-01 7.824790e-01 7.416867e-01

2649 9.783800e-01 9.773372e-01 9.762641e-01 9.757472e-01 9.755078e-01 9.613067e-01 7.627803e-01 7.727313e-01 7.825122e-01 7.417161e-01

2650 9.783863e-01 9.773411e-01 9.762660e-01 9.757490e-01 9.755100e-01 9.613230e-01 7.628116e-01 7.727634e-01 7.825448e-01 7.417448e-01

2651 9.783925e-01 9.773448e-01 9.762679e-01 9.757506e-01 9.755122e-01 9.613393e-01 7.628422e-01 7.727948e-01 7.825766e-01 7.417730e-01

2652 9.783986e-01 9.773485e-01 9.762696e-01 9.757522e-01 9.755143e-01 9.613556e-01 7.628722e-01 7.728255e-01 7.826079e-01 7.418005e-01

2653 9.784046e-01 9.773520e-01 9.762713e-01 9.757537e-01 9.755164e-01 9.613718e-01 7.629016e-01 7.728557e-01 7.826385e-01 7.418275e-01

2654 9.784105e-01 9.773554e-01 9.762729e-01 9.757552e-01 9.755185e-01 9.613880e-01 7.629305e-01 7.728853e-01 7.826685e-01 7.418540e-01

2655 9.784162e-01 9.773587e-01 9.762743e-01 9.757566e-01 9.755205e-01 9.614042e-01 7.629588e-01 7.729143e-01 7.826980e-01 7.418799e-01

2656 9.784219e-01 9.773619e-01 9.762757e-01 9.757579e-01 9.755225e-01 9.614203e-01 7.629866e-01 7.729427e-01 7.827269e-01 7.419053e-01

2657 9.784274e-01 9.773650e-01 9.762770e-01 9.757592e-01 9.755245e-01 9.614365e-01 7.630138e-01 7.729707e-01 7.827553e-01 7.419303e-01

2658 9.784328e-01 9.773680e-01 9.762782e-01 9.757605e-01 9.755265e-01 9.614526e-01 7.630406e-01 7.729981e-01 7.827831e-01 7.419548e-01

2659 9.784381e-01 9.773708e-01 9.762794e-01 9.757617e-01 9.755285e-01 9.614687e-01 7.630669e-01 7.730251e-01 7.828105e-01 7.419789e-01

2660 9.784432e-01 9.773736e-01 9.762804e-01 9.757628e-01 9.755305e-01 9.614848e-01 7.630928e-01 7.730516e-01 7.828374e-01 7.420026e-01

2661 9.784483e-01 9.773762e-01 9.762814e-01 9.757640e-01 9.755325e-01 9.615008e-01 7.631182e-01 7.730777e-01 7.828638e-01 7.420258e-01

2662 9.784532e-01 9.773787e-01 9.762823e-01 9.757651e-01 9.755344e-01 9.615169e-01 7.631432e-01 7.731034e-01 7.828899e-01 7.420487e-01

2663 9.784580e-01 9.773811e-01 9.762831e-01 9.757661e-01 9.755364e-01 9.615330e-01 7.631678e-01 7.731286e-01 7.829155e-01 7.420713e-01

2664 9.784626e-01 9.773834e-01 9.762839e-01 9.757672e-01 9.755385e-01 9.615490e-01 7.631921e-01 7.731535e-01 7.829407e-01 7.420935e-01

2665 9.784671e-01 9.773856e-01 9.762847e-01 9.757683e-01 9.755405e-01 9.615650e-01 7.632160e-01 7.731780e-01 7.829655e-01 7.421154e-01

2666 9.784715e-01 9.773877e-01 9.762853e-01 9.757693e-01 9.755426e-01 9.615811e-01 7.632396e-01 7.732021e-01 7.829899e-01 7.421369e-01

2667 9.784758e-01 9.773896e-01 9.762860e-01 9.757704e-01 9.755447e-01 9.615971e-01 7.632628e-01 7.732259e-01 7.830140e-01 7.421582e-01

2668 9.784799e-01 9.773915e-01 9.762866e-01 9.757715e-01 9.755469e-01 9.616131e-01 7.632857e-01 7.732494e-01 7.830378e-01 7.421792e-01

2669 9.784838e-01 9.773933e-01 9.762871e-01 9.757726e-01 9.755491e-01 9.616292e-01 7.633083e-01 7.732726e-01 7.830612e-01 7.421999e-01

2670 9.784876e-01 9.773950e-01 9.762876e-01 9.757737e-01 9.755514e-01 9.616452e-01 7.633307e-01 7.732955e-01 7.830844e-01 7.422204e-01

2671 9.784913e-01 9.773966e-01 9.762882e-01 9.757748e-01 9.755538e-01 9.616612e-01 7.633527e-01 7.733181e-01 7.831072e-01 7.422407e-01

2672 9.784949e-01 9.773981e-01 9.762887e-01 9.757760e-01 9.755562e-01 9.616773e-01 7.633746e-01 7.733404e-01 7.831297e-01 7.422607e-01

2673 9.784982e-01 9.773995e-01 9.762891e-01 9.757772e-01 9.755587e-01 9.616933e-01 7.633961e-01 7.733625e-01 7.831520e-01 7.422805e-01

2674 9.785015e-01 9.774008e-01 9.762896e-01 9.757785e-01 9.755613e-01 9.617094e-01 7.634175e-01 7.733843e-01 7.831740e-01 7.423001e-01

2675 9.785046e-01 9.774021e-01 9.762901e-01 9.757799e-01 9.755640e-01 9.617254e-01 7.634386e-01 7.734059e-01 7.831958e-01 7.423195e-01

2676 9.785075e-01 9.774032e-01 9.762906e-01 9.757813e-01 9.755668e-01 9.617415e-01 7.634595e-01 7.734273e-01 7.832173e-01 7.423387e-01

2677 9.785103e-01 9.774043e-01 9.762912e-01 9.757828e-01 9.755698e-01 9.617576e-01 7.634802e-01 7.734484e-01 7.832386e-01 7.423578e-01

2678 9.785129e-01 9.774054e-01 9.762917e-01 9.757844e-01 9.755728e-01 9.617737e-01 7.635007e-01 7.734694e-01 7.832597e-01 7.423767e-01

2679 9.785154e-01 9.774063e-01 9.762924e-01 9.757862e-01 9.755760e-01 9.617898e-01 7.635211e-01 7.734902e-01 7.832806e-01 7.423954e-01

2680 9.785178e-01 9.774073e-01 9.762930e-01 9.757880e-01 9.755794e-01 9.618059e-01 7.635412e-01 7.735108e-01 7.833013e-01 7.424140e-01

2681 9.785200e-01 9.774081e-01 9.762937e-01 9.757899e-01 9.755828e-01 9.618221e-01 7.635613e-01 7.735312e-01 7.833218e-01 7.424325e-01

2682 9.785220e-01 9.774089e-01 9.762945e-01 9.757920e-01 9.755865e-01 9.618382e-01 7.635811e-01 7.735515e-01 7.833421e-01 7.424509e-01

2683 9.785239e-01 9.774097e-01 9.762954e-01 9.757942e-01 9.755903e-01 9.618544e-01 7.636009e-01 7.735716e-01 7.833623e-01 7.424691e-01

2684 9.785257e-01 9.774105e-01 9.762963e-01 9.757966e-01 9.755943e-01 9.618706e-01 7.636205e-01 7.735916e-01 7.833823e-01 7.424873e-01

2685 9.785273e-01 9.774112e-01 9.762974e-01 9.757991e-01 9.755985e-01 9.618868e-01 7.636400e-01 7.736114e-01 7.834021e-01 7.425053e-01

2686 9.785287e-01 9.774119e-01 9.762986e-01 9.758018e-01 9.756029e-01 9.619030e-01 7.636593e-01 7.736311e-01 7.834218e-01 7.425233e-01

2687 9.785300e-01 9.774126e-01 9.762998e-01 9.758047e-01 9.756075e-01 9.619192e-01 7.636786e-01 7.736507e-01 7.834413e-01 7.425412e-01

2688 9.785312e-01 9.774133e-01 9.763012e-01 9.758077e-01 9.756123e-01 9.619355e-01 7.636977e-01 7.736702e-01 7.834608e-01 7.425590e-01

2689 9.785322e-01 9.774140e-01 9.763028e-01 9.758110e-01 9.756173e-01 9.619518e-01 7.637168e-01 7.736896e-01 7.834801e-01 7.425767e-01

2690 9.785331e-01 9.774147e-01 9.763045e-01 9.758145e-01 9.756226e-01 9.619681e-01 7.637358e-01 7.737088e-01 7.834993e-01 7.425944e-01

2691 9.785339e-01 9.774154e-01 9.763064e-01 9.758182e-01 9.756281e-01 9.619844e-01 7.637547e-01 7.737280e-01 7.835183e-01 7.426120e-01

2692 9.785345e-01 9.774161e-01 9.763084e-01 9.758222e-01 9.756338e-01 9.620007e-01 7.637735e-01 7.737471e-01 7.835373e-01 7.426296e-01

2693 9.785350e-01 9.774169e-01 9.763107e-01 9.758264e-01 9.756398e-01 9.620171e-01 7.637923e-01 7.737661e-01 7.835562e-01 7.426471e-01

2694 9.785353e-01 9.774177e-01 9.763131e-01 9.758308e-01 9.756461e-01 9.620335e-01 7.638110e-01 7.737851e-01 7.835749e-01 7.426646e-01

2695 9.785356e-01 9.774186e-01 9.763158e-01 9.758356e-01 9.756527e-01 9.620499e-01 7.638297e-01 7.738040e-01 7.835936e-01 7.426821e-01

2696 9.785357e-01 9.774196e-01 9.763186e-01 9.758406e-01 9.756595e-01 9.620663e-01 7.638483e-01 7.738228e-01 7.836122e-01 7.426996e-01

2697 9.785357e-01 9.774206e-01 9.763218e-01 9.758459e-01 9.756667e-01 9.620828e-01 7.638668e-01 7.738416e-01 7.836308e-01 7.427170e-01

2698 9.785356e-01 9.774218e-01 9.763251e-01 9.758515e-01 9.756742e-01 9.620993e-01 7.638854e-01 7.738603e-01 7.836493e-01 7.427344e-01

2699 9.785354e-01 9.774230e-01 9.763287e-01 9.758574e-01 9.756819e-01 9.621158e-01 7.639039e-01 7.738790e-01 7.836677e-01 7.427518e-01

2700 9.785351e-01 9.774243e-01 9.763326e-01 9.758636e-01 9.756900e-01 9.621323e-01 7.639223e-01 7.738977e-01 7.836860e-01 7.427692e-01

2701 9.785347e-01 9.774257e-01 9.763368e-01 9.758702e-01 9.756985e-01 9.621489e-01 7.639408e-01 7.739163e-01 7.837043e-01 7.427866e-01

2702 9.785425e-01 9.774333e-01 9.763442e-01 9.758774e-01 9.757055e-01 9.623515e-01 7.641911e-01 7.741892e-01 7.840001e-01 7.429921e-01

2703 9.785503e-01 9.774409e-01 9.763515e-01 9.758845e-01 9.757125e-01 9.625474e-01 7.644438e-01 7.744645e-01 7.842981e-01 7.432001e-01

2704 9.785580e-01 9.774484e-01 9.763587e-01 9.758916e-01 9.757195e-01 9.627367e-01 7.646989e-01 7.747419e-01 7.845980e-01 7.434104e-01

2705 9.785658e-01 9.774559e-01 9.763660e-01 9.758987e-01 9.757264e-01 9.629196e-01 7.649561e-01 7.750213e-01 7.848997e-01 7.436230e-01

2706 9.785735e-01 9.774634e-01 9.763732e-01 9.759057e-01 9.757333e-01 9.630963e-01 7.652153e-01 7.753026e-01 7.852029e-01 7.438377e-01

2707 9.785811e-01 9.774708e-01 9.763803e-01 9.759127e-01 9.757402e-01 9.632671e-01 7.654764e-01 7.755855e-01 7.855075e-01 7.440546e-01

2708 9.785888e-01 9.774782e-01 9.763874e-01 9.759196e-01 9.757470e-01 9.634320e-01 7.657392e-01 7.758699e-01 7.858133e-01 7.442733e-01

2709 9.785964e-01 9.774856e-01 9.763945e-01 9.759265e-01 9.757537e-01 9.635913e-01 7.660036e-01 7.761557e-01 7.861201e-01 7.444939e-01

2710 9.786040e-01 9.774929e-01 9.764015e-01 9.759333e-01 9.757605e-01 9.637452e-01 7.662694e-01 7.764426e-01 7.864278e-01 7.447163e-01

2711 9.786116e-01 9.775002e-01 9.764085e-01 9.759401e-01 9.757671e-01 9.638937e-01 7.665365e-01 7.767304e-01 7.867360e-01 7.449402e-01

2712 9.786191e-01 9.775075e-01 9.764155e-01 9.759469e-01 9.757738e-01 9.640372e-01 7.668046e-01 7.770190e-01 7.870446e-01 7.451655e-01

2713 9.786266e-01 9.775148e-01 9.764224e-01 9.759537e-01 9.757804e-01 9.641757e-01 7.670736e-01 7.773083e-01 7.873534e-01 7.453922e-01

2714 9.786341e-01 9.775220e-01 9.764294e-01 9.759604e-01 9.757870e-01 9.643094e-01 7.673434e-01 7.775979e-01 7.876623e-01 7.456201e-01

2715 9.786416e-01 9.775292e-01 9.764362e-01 9.759670e-01 9.757935e-01 9.644386e-01 7.676137e-01 7.778877e-01 7.879710e-01 7.458490e-01

2716 9.786491e-01 9.775364e-01 9.764431e-01 9.759737e-01 9.758000e-01 9.645632e-01 7.678844e-01 7.781776e-01 7.882792e-01 7.460788e-01

2717 9.786566e-01 9.775436e-01 9.764499e-01 9.759802e-01 9.758065e-01 9.646836e-01 7.681554e-01 7.784674e-01 7.885869e-01 7.463094e-01

2718 9.786640e-01 9.775507e-01 9.764566e-01 9.759868e-01 9.758129e-01 9.647998e-01 7.684263e-01 7.787567e-01 7.888938e-01 7.465406e-01

2719 9.786714e-01 9.775578e-01 9.764634e-01 9.759933e-01 9.758193e-01 9.649120e-01 7.686972e-01 7.790456e-01 7.891997e-01 7.467723e-01

2720 9.786788e-01 9.775649e-01 9.764701e-01 9.759998e-01 9.758256e-01 9.650203e-01 7.689677e-01 7.793337e-01 7.895045e-01 7.470042e-01

2721 9.786862e-01 9.775719e-01 9.764768e-01 9.760063e-01 9.758319e-01 9.651249e-01 7.692378e-01 7.796210e-01 7.898078e-01 7.472364e-01

2722 9.786936e-01 9.775790e-01 9.764834e-01 9.760127e-01 9.758382e-01 9.652259e-01 7.695072e-01 7.799072e-01 7.901097e-01 7.474686e-01

2723 9.787010e-01 9.775860e-01 9.764900e-01 9.760191e-01 9.758444e-01 9.653234e-01 7.697758e-01 7.801921e-01 7.904098e-01 7.477006e-01

2724 9.787083e-01 9.775930e-01 9.764966e-01 9.760254e-01 9.758507e-01 9.654176e-01 7.700433e-01 7.804756e-01 7.907080e-01 7.479324e-01

2725 9.787157e-01 9.776000e-01 9.765032e-01 9.760317e-01 9.758568e-01 9.655085e-01 7.703098e-01 7.807575e-01 7.910041e-01 7.481637e-01

2726 9.787230e-01 9.776069e-01 9.765097e-01 9.760380e-01 9.758630e-01 9.655964e-01 7.705749e-01 7.810376e-01 7.912980e-01 7.483944e-01

2727 9.787303e-01 9.776139e-01 9.765162e-01 9.760443e-01 9.758691e-01 9.656812e-01 7.708385e-01 7.813158e-01 7.915895e-01 7.486244e-01

2728 9.787376e-01 9.776208e-01 9.765227e-01 9.760505e-01 9.758751e-01 9.657632e-01 7.711004e-01 7.815920e-01 7.918784e-01 7.488536e-01

2729 9.787449e-01 9.776276e-01 9.765291e-01 9.760567e-01 9.758812e-01 9.658424e-01 7.713606e-01 7.818658e-01 7.921647e-01 7.490817e-01

2730 9.787522e-01 9.776345e-01 9.765355e-01 9.760628e-01 9.758872e-01 9.659190e-01 7.716188e-01 7.821373e-01 7.924480e-01 7.493087e-01

2731 9.787595e-01 9.776414e-01 9.765419e-01 9.760689e-01 9.758931e-01 9.659929e-01 7.718750e-01 7.824063e-01 7.927284e-01 7.495343e-01

2732 9.787667e-01 9.776482e-01 9.765482e-01 9.760750e-01 9.758990e-01 9.660644e-01 7.721289e-01 7.826726e-01 7.930057e-01 7.497585e-01

2733 9.787740e-01 9.776550e-01 9.765545e-01 9.760810e-01 9.759049e-01 9.661336e-01 7.723804e-01 7.829362e-01 7.932798e-01 7.499812e-01

2734 9.787812e-01 9.776618e-01 9.765608e-01 9.760870e-01 9.759108e-01 9.662004e-01 7.726295e-01 7.831968e-01 7.935504e-01 7.502021e-01

2735 9.787885e-01 9.776685e-01 9.765670e-01 9.760930e-01 9.759166e-01 9.662650e-01 7.728760e-01 7.834544e-01 7.938177e-01 7.504213e-01

2736 9.787957e-01 9.776753e-01 9.765733e-01 9.760989e-01 9.759224e-01 9.663276e-01 7.731197e-01 7.837088e-01 7.940814e-01 7.506385e-01

2737 9.788029e-01 9.776820e-01 9.765794e-01 9.761048e-01 9.759281e-01 9.663881e-01 7.733606e-01 7.839600e-01 7.943414e-01 7.508536e-01

2738 9.788101e-01 9.776887e-01 9.765856e-01 9.761107e-01 9.759338e-01 9.664466e-01 7.735986e-01 7.842079e-01 7.945977e-01 7.510666e-01

2739 9.788173e-01 9.776954e-01 9.765917e-01 9.761165e-01 9.759395e-01 9.665033e-01 7.738335e-01 7.844523e-01 7.948501e-01 7.512773e-01

2740 9.788245e-01 9.777020e-01 9.765978e-01 9.761223e-01 9.759451e-01 9.665581e-01 7.740653e-01 7.846933e-01 7.950987e-01 7.514857e-01

2741 9.788316e-01 9.777087e-01 9.766038e-01 9.761280e-01 9.759507e-01 9.666113e-01 7.742939e-01 7.849306e-01 7.953434e-01 7.516916e-01

2742 9.788388e-01 9.777153e-01 9.766098e-01 9.761337e-01 9.759562e-01 9.666627e-01 7.745192e-01 7.851644e-01 7.955840e-01 7.518949e-01

2743 9.788459e-01 9.777219e-01 9.766158e-01 9.761394e-01 9.759618e-01 9.667126e-01 7.747412e-01 7.853944e-01 7.958206e-01 7.520955e-01

2744 9.788531e-01 9.777284e-01 9.766218e-01 9.761450e-01 9.759672e-01 9.667609e-01 7.749597e-01 7.856206e-01 7.960530e-01 7.522935e-01

2745 9.788602e-01 9.777350e-01 9.766277e-01 9.761506e-01 9.759727e-01 9.668077e-01 7.751748e-01 7.858430e-01 7.962814e-01 7.524887e-01

2746 9.788673e-01 9.777415e-01 9.766335e-01 9.761561e-01 9.759781e-01 9.668531e-01 7.753863e-01 7.860616e-01 7.965056e-01 7.526810e-01

2747 9.788745e-01 9.777480e-01 9.766394e-01 9.761616e-01 9.759834e-01 9.668972e-01 7.755942e-01 7.862763e-01 7.967256e-01 7.528704e-01

2748 9.788816e-01 9.777545e-01 9.766452e-01 9.761671e-01 9.759887e-01 9.669399e-01 7.757985e-01 7.864870e-01 7.969414e-01 7.530568e-01

2749 9.788886e-01 9.777609e-01 9.766509e-01 9.761725e-01 9.759940e-01 9.669814e-01 7.759992e-01 7.866938e-01 7.971529e-01 7.532401e-01

2750 9.788957e-01 9.777673e-01 9.766567e-01 9.761779e-01 9.759992e-01 9.670217e-01 7.761962e-01 7.868967e-01 7.973603e-01 7.534204e-01

2751 9.789028e-01 9.777737e-01 9.766623e-01 9.761833e-01 9.760044e-01 9.670608e-01 7.763895e-01 7.870956e-01 7.975635e-01 7.535976e-01

2752 9.789099e-01 9.777801e-01 9.766680e-01 9.761886e-01 9.760095e-01 9.670988e-01 7.765791e-01 7.872905e-01 7.977624e-01 7.537716e-01

2753 9.789169e-01 9.777865e-01 9.766736e-01 9.761938e-01 9.760147e-01 9.671357e-01 7.767650e-01 7.874815e-01 7.979572e-01 7.539425e-01

2754 9.789239e-01 9.777928e-01 9.766791e-01 9.761990e-01 9.760197e-01 9.671716e-01 7.769471e-01 7.876685e-01 7.981477e-01 7.541101e-01

2755 9.789310e-01 9.777991e-01 9.766847e-01 9.762042e-01 9.760247e-01 9.672065e-01 7.771255e-01 7.878515e-01 7.983342e-01 7.542746e-01

2756 9.789380e-01 9.778053e-01 9.766901e-01 9.762093e-01 9.760297e-01 9.672405e-01 7.773002e-01 7.880307e-01 7.985165e-01 7.544358e-01

2757 9.789450e-01 9.778116e-01 9.766956e-01 9.762144e-01 9.760346e-01 9.672736e-01 7.774711e-01 7.882058e-01 7.986947e-01 7.545937e-01

2758 9.789520e-01 9.778178e-01 9.767010e-01 9.762194e-01 9.760395e-01 9.673057e-01 7.776384e-01 7.883771e-01 7.988688e-01 7.547484e-01

2759 9.789589e-01 9.778239e-01 9.767063e-01 9.762244e-01 9.760443e-01 9.673371e-01 7.778020e-01 7.885446e-01 7.990389e-01 7.548999e-01

2760 9.789659e-01 9.778301e-01 9.767116e-01 9.762293e-01 9.760491e-01 9.673676e-01 7.779619e-01 7.887082e-01 7.992051e-01 7.550481e-01

2761 9.789728e-01 9.778362e-01 9.767169e-01 9.762342e-01 9.760539e-01 9.673974e-01 7.781182e-01 7.888680e-01 7.993673e-01 7.551931e-01

2762 9.789798e-01 9.778423e-01 9.767221e-01 9.762390e-01 9.760586e-01 9.674265e-01 7.782709e-01 7.890241e-01 7.995256e-01 7.553349e-01

2763 9.789867e-01 9.778483e-01 9.767272e-01 9.762438e-01 9.760632e-01 9.674548e-01 7.784200e-01 7.891764e-01 7.996800e-01 7.554735e-01

2764 9.789936e-01 9.778543e-01 9.767324e-01 9.762485e-01 9.760678e-01 9.674824e-01 7.785656e-01 7.893251e-01 7.998307e-01 7.556089e-01

2765 9.790005e-01 9.778603e-01 9.767374e-01 9.762532e-01 9.760724e-01 9.675094e-01 7.787077e-01 7.894701e-01 7.999776e-01 7.557411e-01

2766 9.790073e-01 9.778662e-01 9.767424e-01 9.762578e-01 9.760769e-01 9.675358e-01 7.788464e-01 7.896116e-01 8.001209e-01 7.558703e-01

2767 9.790142e-01 9.778722e-01 9.767474e-01 9.762624e-01 9.760814e-01 9.675616e-01 7.789816e-01 7.897495e-01 8.002606e-01 7.559963e-01

2768 9.790210e-01 9.778780e-01 9.767523e-01 9.762669e-01 9.760858e-01 9.675868e-01 7.791135e-01 7.898840e-01 8.003967e-01 7.561192e-01

2769 9.790278e-01 9.778839e-01 9.767572e-01 9.762714e-01 9.760902e-01 9.676114e-01 7.792421e-01 7.900151e-01 8.005293e-01 7.562391e-01

2770 9.790346e-01 9.778897e-01 9.767620e-01 9.762758e-01 9.760945e-01 9.676355e-01 7.793674e-01 7.901428e-01 8.006585e-01 7.563561e-01

2771 9.790414e-01 9.778954e-01 9.767668e-01 9.762802e-01 9.760987e-01 9.676591e-01 7.794896e-01 7.902672e-01 8.007843e-01 7.564700e-01

2772 9.790481e-01 9.779011e-01 9.767715e-01 9.762845e-01 9.761030e-01 9.676822e-01 7.796085e-01 7.903884e-01 8.009068e-01 7.565811e-01

2773 9.790548e-01 9.779068e-01 9.767761e-01 9.762887e-01 9.761071e-01 9.677049e-01 7.797244e-01 7.905064e-01 8.010261e-01 7.566893e-01

2774 9.790615e-01 9.779125e-01 9.767807e-01 9.762929e-01 9.761112e-01 9.677270e-01 7.798373e-01 7.906213e-01 8.011422e-01 7.567947e-01

2775 9.790682e-01 9.779181e-01 9.767853e-01 9.762971e-01 9.761153e-01 9.677488e-01 7.799471e-01 7.907332e-01 8.012552e-01 7.568973e-01

2776 9.790749e-01 9.779236e-01 9.767897e-01 9.763012e-01 9.761193e-01 9.677702e-01 7.800540e-01 7.908421e-01 8.013652e-01 7.569971e-01

2777 9.790815e-01 9.779291e-01 9.767942e-01 9.763052e-01 9.761233e-01 9.677911e-01 7.801581e-01 7.909480e-01 8.014723e-01 7.570943e-01

2778 9.790881e-01 9.779346e-01 9.767985e-01 9.763092e-01 9.761272e-01 9.678117e-01 7.802594e-01 7.910511e-01 8.015764e-01 7.571889e-01

2779 9.790947e-01 9.779400e-01 9.768028e-01 9.763131e-01 9.761311e-01 9.678319e-01 7.803579e-01 7.911514e-01 8.016777e-01 7.572809e-01

2780 9.791012e-01 9.779454e-01 9.768071e-01 9.763169e-01 9.761349e-01 9.678518e-01 7.804538e-01 7.912489e-01 8.017762e-01 7.573704e-01

2781 9.791078e-01 9.779507e-01 9.768113e-01 9.763207e-01 9.761386e-01 9.678713e-01 7.805470e-01 7.913438e-01 8.018720e-01 7.574574e-01

2782 9.791142e-01 9.779560e-01 9.768154e-01 9.763245e-01 9.761424e-01 9.678905e-01 7.806377e-01 7.914361e-01 8.019652e-01 7.575420e-01

2783 9.791207e-01 9.779612e-01 9.768195e-01 9.763281e-01 9.761460e-01 9.679095e-01 7.807258e-01 7.915258e-01 8.020559e-01 7.576242e-01

2784 9.791271e-01 9.779664e-01 9.768235e-01 9.763318e-01 9.761496e-01 9.679281e-01 7.808115e-01 7.916131e-01 8.021440e-01 7.577041e-01

2785 9.791335e-01 9.779715e-01 9.768274e-01 9.763353e-01 9.761532e-01 9.679464e-01 7.808949e-01 7.916980e-01 8.022297e-01 7.577818e-01

2786 9.791399e-01 9.779766e-01 9.768313e-01 9.763388e-01 9.761567e-01 9.679645e-01 7.809759e-01 7.917805e-01 8.023130e-01 7.578572e-01

2787 9.791462e-01 9.779816e-01 9.768351e-01 9.763423e-01 9.761602e-01 9.679823e-01 7.810547e-01 7.918607e-01 8.023941e-01 7.579305e-01

2788 9.791525e-01 9.779866e-01 9.768389e-01 9.763457e-01 9.761636e-01 9.679999e-01 7.811312e-01 7.919386e-01 8.024728e-01 7.580018e-01

2789 9.791588e-01 9.779915e-01 9.768426e-01 9.763490e-01 9.761670e-01 9.680172e-01 7.812056e-01 7.920144e-01 8.025494e-01 7.580709e-01

2790 9.791650e-01 9.779964e-01 9.768462e-01 9.763523e-01 9.761703e-01 9.680343e-01 7.812779e-01 7.920881e-01 8.026239e-01 7.581381e-01

2791 9.791712e-01 9.780012e-01 9.768498e-01 9.763555e-01 9.761736e-01 9.680512e-01 7.813482e-01 7.921598e-01 8.026963e-01 7.582034e-01

2792 9.791773e-01 9.780059e-01 9.768533e-01 9.763587e-01 9.761768e-01 9.680679e-01 7.814166e-01 7.922294e-01 8.027667e-01 7.582668e-01

2793 9.791834e-01 9.780106e-01 9.768567e-01 9.763618e-01 9.761800e-01 9.680844e-01 7.814829e-01 7.922971e-01 8.028351e-01 7.583283e-01

2794 9.791894e-01 9.780152e-01 9.768601e-01 9.763648e-01 9.761831e-01 9.681007e-01 7.815475e-01 7.923629e-01 8.029017e-01 7.583881e-01

2795 9.791954e-01 9.780198e-01 9.768634e-01 9.763678e-01 9.761862e-01 9.681168e-01 7.816102e-01 7.924269e-01 8.029664e-01 7.584461e-01

2796 9.792014e-01 9.780243e-01 9.768666e-01 9.763707e-01 9.761893e-01 9.681328e-01 7.816712e-01 7.924891e-01 8.030293e-01 7.585025e-01

2797 9.792073e-01 9.780288e-01 9.768698e-01 9.763736e-01 9.761923e-01 9.681486e-01 7.817304e-01 7.925496e-01 8.030905e-01 7.585572e-01

2798 9.792132e-01 9.780332e-01 9.768729e-01 9.763764e-01 9.761953e-01 9.681642e-01 7.817880e-01 7.926084e-01 8.031501e-01 7.586103e-01

2799 9.792190e-01 9.780375e-01 9.768759e-01 9.763792e-01 9.761982e-01 9.681797e-01 7.818440e-01 7.926656e-01 8.032080e-01 7.586620e-01

2800 9.792248e-01 9.780418e-01 9.768789e-01 9.763819e-01 9.762011e-01 9.681950e-01 7.818985e-01 7.927212e-01 8.032643e-01 7.587121e-01

2801 9.792305e-01 9.780460e-01 9.768818e-01 9.763846e-01 9.762040e-01 9.682102e-01 7.819514e-01 7.927753e-01 8.033191e-01 7.587608e-01

2802 9.792362e-01 9.780501e-01 9.768847e-01 9.763872e-01 9.762068e-01 9.682253e-01 7.820028e-01 7.928279e-01 8.033724e-01 7.588080e-01

2803 9.792418e-01 9.780542e-01 9.768875e-01 9.763897e-01 9.762096e-01 9.682403e-01 7.820529e-01 7.928791e-01 8.034242e-01 7.588540e-01

2804 9.792473e-01 9.780582e-01 9.768902e-01 9.763923e-01 9.762124e-01 9.682551e-01 7.821016e-01 7.929289e-01 8.034747e-01 7.588986e-01

2805 9.792528e-01 9.780621e-01 9.768929e-01 9.763947e-01 9.762151e-01 9.682698e-01 7.821489e-01 7.929773e-01 8.035239e-01 7.589420e-01

2806 9.792583e-01 9.780660e-01 9.768955e-01 9.763972e-01 9.762178e-01 9.682844e-01 7.821950e-01 7.930245e-01 8.035717e-01 7.589841e-01

2807 9.792636e-01 9.780698e-01 9.768981e-01 9.763995e-01 9.762205e-01 9.682990e-01 7.822398e-01 7.930704e-01 8.036183e-01 7.590250e-01

2808 9.792690e-01 9.780736e-01 9.769006e-01 9.764019e-01 9.762231e-01 9.683134e-01 7.822834e-01 7.931151e-01 8.036636e-01 7.590648e-01

2809 9.792742e-01 9.780773e-01 9.769030e-01 9.764042e-01 9.762258e-01 9.683277e-01 7.823259e-01 7.931586e-01 8.037078e-01 7.591035e-01

2810 9.792794e-01 9.780809e-01 9.769054e-01 9.764065e-01 9.762284e-01 9.683420e-01 7.823672e-01 7.932010e-01 8.037508e-01 7.591411e-01

2811 9.792846e-01 9.780844e-01 9.769077e-01 9.764087e-01 9.762310e-01 9.683561e-01 7.824074e-01 7.932423e-01 8.037927e-01 7.591777e-01

2812 9.792896e-01 9.780879e-01 9.769100e-01 9.764109e-01 9.762336e-01 9.683702e-01 7.824466e-01 7.932825e-01 8.038336e-01 7.592133e-01

2813 9.792946e-01 9.780914e-01 9.769122e-01 9.764131e-01 9.762361e-01 9.683842e-01 7.824848e-01 7.933217e-01 8.038734e-01 7.592479e-01

2814 9.792995e-01 9.780947e-01 9.769144e-01 9.764152e-01 9.762387e-01 9.683982e-01 7.825220e-01 7.933599e-01 8.039122e-01 7.592816e-01

2815 9.793044e-01 9.780980e-01 9.769165e-01 9.764173e-01 9.762413e-01 9.684120e-01 7.825582e-01 7.933971e-01 8.039501e-01 7.593144e-01

2816 9.793092e-01 9.781012e-01 9.769186e-01 9.764194e-01 9.762438e-01 9.684259e-01 7.825936e-01 7.934334e-01 8.039870e-01 7.593463e-01

2817 9.793139e-01 9.781044e-01 9.769206e-01 9.764215e-01 9.762464e-01 9.684396e-01 7.826280e-01 7.934689e-01 8.040231e-01 7.593775e-01

2818 9.793186e-01 9.781075e-01 9.769226e-01 9.764235e-01 9.762489e-01 9.684533e-01 7.826616e-01 7.935034e-01 8.040582e-01 7.594078e-01

2819 9.793231e-01 9.781105e-01 9.769246e-01 9.764256e-01 9.762515e-01 9.684670e-01 7.826944e-01 7.935371e-01 8.040926e-01 7.594373e-01

2820 9.793276e-01 9.781135e-01 9.769265e-01 9.764276e-01 9.762541e-01 9.684806e-01 7.827264e-01 7.935701e-01 8.041261e-01 7.594661e-01

2821 9.793320e-01 9.781164e-01 9.769284e-01 9.764296e-01 9.762567e-01 9.684941e-01 7.827576e-01 7.936022e-01 8.041588e-01 7.594941e-01

2822 9.793364e-01 9.781192e-01 9.769302e-01 9.764317e-01 9.762593e-01 9.685076e-01 7.827881e-01 7.936336e-01 8.041908e-01 7.595215e-01

2823 9.793406e-01 9.781220e-01 9.769321e-01 9.764337e-01 9.762619e-01 9.685211e-01 7.828179e-01 7.936643e-01 8.042221e-01 7.595482e-01

2824 9.793448e-01 9.781247e-01 9.769339e-01 9.764357e-01 9.762646e-01 9.685345e-01 7.828470e-01 7.936943e-01 8.042526e-01 7.595743e-01

2825 9.793489e-01 9.781274e-01 9.769357e-01 9.764378e-01 9.762672e-01 9.685479e-01 7.828755e-01 7.937237e-01 8.042825e-01 7.595998e-01

2826 9.793529e-01 9.781300e-01 9.769374e-01 9.764398e-01 9.762700e-01 9.685613e-01 7.829033e-01 7.937524e-01 8.043117e-01 7.596247e-01

2827 9.793569e-01 9.781326e-01 9.769392e-01 9.764419e-01 9.762727e-01 9.685747e-01 7.829305e-01 7.937804e-01 8.043403e-01 7.596490e-01

2828 9.793607e-01 9.781351e-01 9.769409e-01 9.764440e-01 9.762755e-01 9.685880e-01 7.829571e-01 7.938079e-01 8.043683e-01 7.596728e-01

2829 9.793645e-01 9.781375e-01 9.769427e-01 9.764461e-01 9.762784e-01 9.686013e-01 7.829832e-01 7.938348e-01 8.043957e-01 7.596960e-01

2830 9.793682e-01 9.781399e-01 9.769444e-01 9.764482e-01 9.762812e-01 9.686145e-01 7.830087e-01 7.938611e-01 8.044226e-01 7.597188e-01

2831 9.793718e-01 9.781422e-01 9.769461e-01 9.764504e-01 9.762842e-01 9.686278e-01 7.830337e-01 7.938870e-01 8.044489e-01 7.597411e-01

2832 9.793753e-01 9.781445e-01 9.769479e-01 9.764526e-01 9.762872e-01 9.686410e-01 7.830582e-01 7.939123e-01 8.044746e-01 7.597629e-01

2833 9.793787e-01 9.781468e-01 9.769496e-01 9.764549e-01 9.762903e-01 9.686542e-01 7.830823e-01 7.939371e-01 8.044999e-01 7.597843e-01

2834 9.793820e-01 9.781490e-01 9.769514e-01 9.764572e-01 9.762934e-01 9.686674e-01 7.831058e-01 7.939614e-01 8.045247e-01 7.598052e-01

2835 9.793853e-01 9.781512e-01 9.769532e-01 9.764596e-01 9.762966e-01 9.686806e-01 7.831290e-01 7.939853e-01 8.045490e-01 7.598258e-01

2836 9.793884e-01 9.781533e-01 9.769550e-01 9.764620e-01 9.762999e-01 9.686938e-01 7.831517e-01 7.940087e-01 8.045729e-01 7.598460e-01

2837 9.793915e-01 9.781554e-01 9.769568e-01 9.764645e-01 9.763033e-01 9.687070e-01 7.831739e-01 7.940318e-01 8.045964e-01 7.598658e-01

2838 9.793945e-01 9.781575e-01 9.769587e-01 9.764671e-01 9.763068e-01 9.687201e-01 7.831958e-01 7.940544e-01 8.046194e-01 7.598852e-01

2839 9.793974e-01 9.781596e-01 9.769606e-01 9.764697e-01 9.763103e-01 9.687333e-01 7.832174e-01 7.940766e-01 8.046420e-01 7.599043e-01

2840 9.794002e-01 9.781616e-01 9.769626e-01 9.764724e-01 9.763140e-01 9.687464e-01 7.832385e-01 7.940985e-01 8.046643e-01 7.599231e-01

2841 9.794030e-01 9.781636e-01 9.769646e-01 9.764753e-01 9.763178e-01 9.687596e-01 7.832593e-01 7.941200e-01 8.046862e-01 7.599416e-01

2842 9.794056e-01 9.781656e-01 9.769667e-01 9.764782e-01 9.763216e-01 9.687727e-01 7.832798e-01 7.941411e-01 8.047077e-01 7.599598e-01

2843 9.794082e-01 9.781676e-01 9.769689e-01 9.764812e-01 9.763256e-01 9.687859e-01 7.833000e-01 7.941620e-01 8.047289e-01 7.599777e-01

2844 9.794106e-01 9.781695e-01 9.769711e-01 9.764843e-01 9.763298e-01 9.687990e-01 7.833199e-01 7.941825e-01 8.047497e-01 7.599953e-01

2845 9.794130e-01 9.781715e-01 9.769734e-01 9.764876e-01 9.763340e-01 9.688122e-01 7.833395e-01 7.942027e-01 8.047703e-01 7.600127e-01

2846 9.794153e-01 9.781735e-01 9.769758e-01 9.764910e-01 9.763384e-01 9.688254e-01 7.833588e-01 7.942227e-01 8.047905e-01 7.600299e-01

2847 9.794175e-01 9.781754e-01 9.769782e-01 9.764945e-01 9.763429e-01 9.688385e-01 7.833778e-01 7.942423e-01 8.048105e-01 7.600468e-01

2848 9.794197e-01 9.781774e-01 9.769808e-01 9.764981e-01 9.763476e-01 9.688517e-01 7.833966e-01 7.942617e-01 8.048301e-01 7.600635e-01

2849 9.794217e-01 9.781794e-01 9.769835e-01 9.765019e-01 9.763524e-01 9.688649e-01 7.834151e-01 7.942808e-01 8.048495e-01 7.600800e-01

2850 9.794237e-01 9.781814e-01 9.769863e-01 9.765058e-01 9.763573e-01 9.688781e-01 7.834335e-01 7.942998e-01 8.048687e-01 7.600964e-01

2851 9.794256e-01 9.781835e-01 9.769892e-01 9.765099e-01 9.763625e-01 9.688912e-01 7.834516e-01 7.943184e-01 8.048876e-01 7.601125e-01

2852 9.794275e-01 9.781855e-01 9.769922e-01 9.765141e-01 9.763678e-01 9.689045e-01 7.834695e-01 7.943369e-01 8.049062e-01 7.601284e-01

2853 9.794292e-01 9.781876e-01 9.769954e-01 9.765185e-01 9.763732e-01 9.689177e-01 7.834872e-01 7.943551e-01 8.049247e-01 7.601442e-01

2854 9.794309e-01 9.781898e-01 9.769987e-01 9.765231e-01 9.763789e-01 9.689309e-01 7.835047e-01 7.943732e-01 8.049429e-01 7.601599e-01

2855 9.794326e-01 9.781920e-01 9.770022e-01 9.765279e-01 9.763847e-01 9.689441e-01 7.835220e-01 7.943910e-01 8.049609e-01 7.601753e-01

2856 9.794341e-01 9.781942e-01 9.770058e-01 9.765328e-01 9.763907e-01 9.689574e-01 7.835392e-01 7.944087e-01 8.049787e-01 7.601907e-01

2857 9.794356e-01 9.781965e-01 9.770096e-01 9.765380e-01 9.763969e-01 9.689707e-01 7.835562e-01 7.944262e-01 8.049964e-01 7.602059e-01

2858 9.794371e-01 9.781989e-01 9.770136e-01 9.765433e-01 9.764033e-01 9.689840e-01 7.835731e-01 7.944435e-01 8.050138e-01 7.602210e-01

2859 9.794385e-01 9.782014e-01 9.770177e-01 9.765489e-01 9.764099e-01 9.689973e-01 7.835898e-01 7.944607e-01 8.050311e-01 7.602360e-01

2860 9.794398e-01 9.782039e-01 9.770220e-01 9.765546e-01 9.764167e-01 9.690106e-01 7.836063e-01 7.944777e-01 8.050482e-01 7.602509e-01

2861 9.794411e-01 9.782065e-01 9.770266e-01 9.765606e-01 9.764237e-01 9.690239e-01 7.836228e-01 7.944946e-01 8.050652e-01 7.602657e-01

2862 9.794424e-01 9.782092e-01 9.770313e-01 9.765669e-01 9.764309e-01 9.690373e-01 7.836391e-01 7.945113e-01 8.050820e-01 7.602804e-01

2863 9.794436e-01 9.782120e-01 9.770362e-01 9.765733e-01 9.764384e-01 9.690507e-01 7.836553e-01 7.945280e-01 8.050986e-01 7.602950e-01

2864 9.794447e-01 9.782149e-01 9.770414e-01 9.765800e-01 9.764460e-01 9.690641e-01 7.836714e-01 7.945445e-01 8.051151e-01 7.603096e-01

2865 9.794459e-01 9.782180e-01 9.770468e-01 9.765869e-01 9.764540e-01 9.690775e-01 7.836875e-01 7.945609e-01 8.051315e-01 7.603240e-01

2866 9.794470e-01 9.782211e-01 9.770524e-01 9.765941e-01 9.764621e-01 9.690909e-01 7.837034e-01 7.945772e-01 8.051478e-01 7.603384e-01

2867 9.794481e-01 9.782244e-01 9.770583e-01 9.766016e-01 9.764705e-01 9.691044e-01 7.837192e-01 7.945934e-01 8.051639e-01 7.603528e-01

2868 9.794492e-01 9.782278e-01 9.770644e-01 9.766093e-01 9.764791e-01 9.691179e-01 7.837350e-01 7.946095e-01 8.051799e-01 7.603671e-01

2869 9.794503e-01 9.782314e-01 9.770708e-01 9.766173e-01 9.764879e-01 9.691314e-01 7.837506e-01 7.946255e-01 8.051958e-01 7.603813e-01

2870 9.794513e-01 9.782351e-01 9.770774e-01 9.766256e-01 9.764970e-01 9.691449e-01 7.837662e-01 7.946414e-01 8.052116e-01 7.603955e-01

2871 9.794524e-01 9.782390e-01 9.770843e-01 9.766341e-01 9.765064e-01 9.691584e-01 7.837818e-01 7.946573e-01 8.052273e-01 7.604097e-01

2872 9.794535e-01 9.782430e-01 9.770915e-01 9.766429e-01 9.765160e-01 9.691720e-01 7.837973e-01 7.946730e-01 8.052429e-01 7.604239e-01

2873 9.794546e-01 9.782473e-01 9.770990e-01 9.766520e-01 9.765258e-01 9.691856e-01 7.838127e-01 7.946888e-01 8.052585e-01 7.604380e-01

2874 9.794557e-01 9.782517e-01 9.771068e-01 9.766614e-01 9.765359e-01 9.691992e-01 7.838281e-01 7.947044e-01 8.052739e-01 7.604521e-01

2875 9.794569e-01 9.782563e-01 9.771148e-01 9.766711e-01 9.765463e-01 9.692129e-01 7.838434e-01 7.947200e-01 8.052892e-01 7.604662e-01

2876 9.794580e-01 9.782611e-01 9.771232e-01 9.766812e-01 9.765569e-01 9.692265e-01 7.838587e-01 7.947356e-01 8.053045e-01 7.604802e-01

2877 9.794593e-01 9.782661e-01 9.771319e-01 9.766915e-01 9.765678e-01 9.692402e-01 7.838740e-01 7.947510e-01 8.053197e-01 7.604943e-01

2878 9.794606e-01 9.782713e-01 9.771410e-01 9.767021e-01 9.765790e-01 9.692540e-01 7.838892e-01 7.947665e-01 8.053348e-01 7.605083e-01

2879 9.794619e-01 9.782768e-01 9.771503e-01 9.767130e-01 9.765904e-01 9.692677e-01 7.839044e-01 7.947819e-01 8.053499e-01 7.605224e-01

2880 9.794633e-01 9.782825e-01 9.771600e-01 9.767243e-01 9.766021e-01 9.692815e-01 7.839196e-01 7.947973e-01 8.053648e-01 7.605365e-01

2881 9.794648e-01 9.782884e-01 9.771700e-01 9.767358e-01 9.766140e-01 9.692953e-01 7.839348e-01 7.948126e-01 8.053797e-01 7.605505e-01

2882 9.794707e-01 9.782941e-01 9.771754e-01 9.767410e-01 9.766191e-01 9.694367e-01 7.841775e-01 7.950779e-01 8.056673e-01 7.607472e-01

2883 9.794767e-01 9.782997e-01 9.771807e-01 9.767461e-01 9.766241e-01 9.695733e-01 7.844216e-01 7.953443e-01 8.059556e-01 7.609456e-01

2884 9.794826e-01 9.783053e-01 9.771860e-01 9.767513e-01 9.766291e-01 9.697053e-01 7.846670e-01 7.956118e-01 8.062446e-01 7.611455e-01

2885 9.794885e-01 9.783109e-01 9.771912e-01 9.767563e-01 9.766341e-01 9.698329e-01 7.849135e-01 7.958800e-01 8.065341e-01 7.613469e-01

2886 9.794944e-01 9.783165e-01 9.771964e-01 9.767614e-01 9.766390e-01 9.699561e-01 7.851610e-01 7.961490e-01 8.068239e-01 7.615496e-01

2887 9.795002e-01 9.783220e-01 9.772016e-01 9.767664e-01 9.766439e-01 9.700751e-01 7.854093e-01 7.964184e-01 8.071138e-01 7.617535e-01

2888 9.795061e-01 9.783276e-01 9.772068e-01 9.767714e-01 9.766488e-01 9.701900e-01 7.856583e-01 7.966883e-01 8.074036e-01 7.619585e-01

2889 9.795119e-01 9.783331e-01 9.772120e-01 9.767763e-01 9.766537e-01 9.703011e-01 7.859078e-01 7.969583e-01 8.076932e-01 7.621645e-01

2890 9.795177e-01 9.783385e-01 9.772171e-01 9.767812e-01 9.766585e-01 9.704084e-01 7.861578e-01 7.972283e-01 8.079824e-01 7.623714e-01

2891 9.795235e-01 9.783440e-01 9.772221e-01 9.767861e-01 9.766632e-01 9.705120e-01 7.864079e-01 7.974982e-01 8.082710e-01 7.625790e-01

2892 9.795293e-01 9.783494e-01 9.772272e-01 9.767910e-01 9.766680e-01 9.706120e-01 7.866582e-01 7.977678e-01 8.085588e-01 7.627873e-01

2893 9.795351e-01 9.783549e-01 9.772322e-01 9.767958e-01 9.766727e-01 9.707087e-01 7.869083e-01 7.980369e-01 8.088457e-01 7.629961e-01

2894 9.795408e-01 9.783603e-01 9.772372e-01 9.768006e-01 9.766774e-01 9.708021e-01 7.871583e-01 7.983053e-01 8.091315e-01 7.632052e-01

2895 9.795466e-01 9.783656e-01 9.772422e-01 9.768053e-01 9.766821e-01 9.708923e-01 7.874078e-01 7.985730e-01 8.094160e-01 7.634146e-01

2896 9.795523e-01 9.783710e-01 9.772471e-01 9.768101e-01 9.766867e-01 9.709795e-01 7.876568e-01 7.988397e-01 8.096991e-01 7.636241e-01

2897 9.795581e-01 9.783763e-01 9.772521e-01 9.768148e-01 9.766913e-01 9.710637e-01 7.879052e-01 7.991053e-01 8.099806e-01 7.638336e-01

2898 9.795638e-01 9.783816e-01 9.772570e-01 9.768194e-01 9.766959e-01 9.711450e-01 7.881527e-01 7.993697e-01 8.102604e-01 7.640430e-01

2899 9.795695e-01 9.783869e-01 9.772618e-01 9.768241e-01 9.767004e-01 9.712236e-01 7.883992e-01 7.996326e-01 8.105382e-01 7.642520e-01

2900 9.795752e-01 9.783922e-01 9.772666e-01 9.768287e-01 9.767049e-01 9.712996e-01 7.886446e-01 7.998939e-01 8.108140e-01 7.644607e-01

2901 9.795809e-01 9.783975e-01 9.772715e-01 9.768333e-01 9.767094e-01 9.713730e-01 7.888887e-01 8.001536e-01 8.110876e-01 7.646689e-01

2902 9.795865e-01 9.784027e-01 9.772762e-01 9.768378e-01 9.767138e-01 9.714440e-01 7.891314e-01 8.004114e-01 8.113588e-01 7.648764e-01

2903 9.795922e-01 9.784079e-01 9.772810e-01 9.768424e-01 9.767183e-01 9.715126e-01 7.893726e-01 8.006672e-01 8.116276e-01 7.650831e-01

2904 9.795978e-01 9.784131e-01 9.772857e-01 9.768469e-01 9.767226e-01 9.715789e-01 7.896121e-01 8.009208e-01 8.118938e-01 7.652890e-01

2905 9.796035e-01 9.784183e-01 9.772904e-01 9.768513e-01 9.767270e-01 9.716430e-01 7.898498e-01 8.011723e-01 8.121573e-01 7.654938e-01

2906 9.796091e-01 9.784235e-01 9.772951e-01 9.768558e-01 9.767313e-01 9.717050e-01 7.900856e-01 8.014213e-01 8.124179e-01 7.656974e-01

2907 9.796147e-01 9.784286e-01 9.772997e-01 9.768602e-01 9.767356e-01 9.717649e-01 7.903193e-01 8.016679e-01 8.126756e-01 7.658998e-01

2908 9.796203e-01 9.784337e-01 9.773043e-01 9.768645e-01 9.767399e-01 9.718230e-01 7.905509e-01 8.019119e-01 8.129303e-01 7.661009e-01

2909 9.796259e-01 9.784388e-01 9.773089e-01 9.768689e-01 9.767441e-01 9.718791e-01 7.907802e-01 8.021532e-01 8.131818e-01 7.663004e-01

2910 9.796315e-01 9.784439e-01 9.773134e-01 9.768732e-01 9.767484e-01 9.719334e-01 7.910071e-01 8.023917e-01 8.134301e-01 7.664984e-01

2911 9.796370e-01 9.784489e-01 9.773180e-01 9.768774e-01 9.767525e-01 9.719859e-01 7.912316e-01 8.026273e-01 8.136751e-01 7.666947e-01

2912 9.796426e-01 9.784540e-01 9.773225e-01 9.768817e-01 9.767567e-01 9.720368e-01 7.914535e-01 8.028599e-01 8.139166e-01 7.668891e-01

2913 9.796482e-01 9.784590e-01 9.773269e-01 9.768859e-01 9.767608e-01 9.720861e-01 7.916727e-01 8.030894e-01 8.141547e-01 7.670817e-01

2914 9.796537e-01 9.784640e-01 9.773313e-01 9.768901e-01 9.767649e-01 9.721338e-01 7.918891e-01 8.033158e-01 8.143893e-01 7.672723e-01

2915 9.796592e-01 9.784690e-01 9.773358e-01 9.768942e-01 9.767690e-01 9.721800e-01 7.921028e-01 8.035390e-01 8.146203e-01 7.674609e-01

2916 9.796647e-01 9.784739e-01 9.773401e-01 9.768984e-01 9.767730e-01 9.722247e-01 7.923135e-01 8.037589e-01 8.148476e-01 7.676473e-01

2917 9.796702e-01 9.784788e-01 9.773445e-01 9.769024e-01 9.767770e-01 9.722681e-01 7.925213e-01 8.039755e-01 8.150712e-01 7.678314e-01

2918 9.796757e-01 9.784838e-01 9.773488e-01 9.769065e-01 9.767809e-01 9.723101e-01 7.927260e-01 8.041886e-01 8.152911e-01 7.680133e-01

2919 9.796812e-01 9.784886e-01 9.773531e-01 9.769105e-01 9.767849e-01 9.723509e-01 7.929277e-01 8.043984e-01 8.155073e-01 7.681927e-01

2920 9.796867e-01 9.784935e-01 9.773573e-01 9.769145e-01 9.767888e-01 9.723904e-01 7.931262e-01 8.046047e-01 8.157196e-01 7.683697e-01

2921 9.796922e-01 9.784983e-01 9.773615e-01 9.769184e-01 9.767927e-01 9.724288e-01 7.933215e-01 8.048074e-01 8.159281e-01 7.685443e-01

2922 9.796976e-01 9.785032e-01 9.773657e-01 9.769224e-01 9.767965e-01 9.724660e-01 7.935136e-01 8.050066e-01 8.161328e-01 7.687162e-01

2923 9.797030e-01 9.785080e-01 9.773698e-01 9.769263e-01 9.768003e-01 9.725021e-01 7.937024e-01 8.052023e-01 8.163336e-01 7.688856e-01

2924 9.797085e-01 9.785127e-01 9.773740e-01 9.769301e-01 9.768041e-01 9.725372e-01 7.938880e-01 8.053944e-01 8.165306e-01 7.690523e-01

2925 9.797139e-01 9.785175e-01 9.773780e-01 9.769339e-01 9.768078e-01 9.725712e-01 7.940702e-01 8.055828e-01 8.167237e-01 7.692162e-01

2926 9.797193e-01 9.785222e-01 9.773821e-01 9.769377e-01 9.768116e-01 9.726043e-01 7.942491e-01 8.057677e-01 8.169129e-01 7.693775e-01

2927 9.797246e-01 9.785269e-01 9.773861e-01 9.769415e-01 9.768152e-01 9.726364e-01 7.944247e-01 8.059490e-01 8.170983e-01 7.695360e-01

2928 9.797300e-01 9.785316e-01 9.773901e-01 9.769452e-01 9.768189e-01 9.726677e-01 7.945969e-01 8.061266e-01 8.172799e-01 7.696917e-01

2929 9.797354e-01 9.785362e-01 9.773940e-01 9.769489e-01 9.768225e-01 9.726981e-01 7.947657e-01 8.063006e-01 8.174576e-01 7.698445e-01

2930 9.797407e-01 9.785408e-01 9.773980e-01 9.769525e-01 9.768261e-01 9.727276e-01 7.949311e-01 8.064711e-01 8.176315e-01 7.699945e-01

2931 9.797460e-01 9.785454e-01 9.774018e-01 9.769561e-01 9.768297e-01 9.727564e-01 7.950932e-01 8.066379e-01 8.178017e-01 7.701417e-01

2932 9.797513e-01 9.785500e-01 9.774057e-01 9.769597e-01 9.768332e-01 9.727844e-01 7.952520e-01 8.068012e-01 8.179681e-01 7.702860e-01

2933 9.797566e-01 9.785545e-01 9.774095e-01 9.769632e-01 9.768367e-01 9.728116e-01 7.954073e-01 8.069609e-01 8.181308e-01 7.704274e-01

2934 9.797619e-01 9.785590e-01 9.774133e-01 9.769667e-01 9.768402e-01 9.728382e-01 7.955594e-01 8.071172e-01 8.182898e-01 7.705660e-01

2935 9.797672e-01 9.785635e-01 9.774170e-01 9.769702e-01 9.768436e-01 9.728640e-01 7.957081e-01 8.072699e-01 8.184452e-01 7.707016e-01

2936 9.797724e-01 9.785680e-01 9.774207e-01 9.769736e-01 9.768470e-01 9.728892e-01 7.958536e-01 8.074192e-01 8.185969e-01 7.708344e-01

2937 9.797776e-01 9.785724e-01 9.774244e-01 9.769770e-01 9.768504e-01 9.729138e-01 7.959958e-01 8.075650e-01 8.187451e-01 7.709644e-01

2938 9.797828e-01 9.785768e-01 9.774280e-01 9.769804e-01 9.768537e-01 9.729378e-01 7.961347e-01 8.077074e-01 8.188898e-01 7.710915e-01

2939 9.797880e-01 9.785812e-01 9.774316e-01 9.769837e-01 9.768570e-01 9.729612e-01 7.962704e-01 8.078465e-01 8.190309e-01 7.712157e-01

2940 9.797932e-01 9.785855e-01 9.774351e-01 9.769870e-01 9.768603e-01 9.729841e-01 7.964030e-01 8.079823e-01 8.191687e-01 7.713371e-01

2941 9.797983e-01 9.785898e-01 9.774386e-01 9.769903e-01 9.768636e-01 9.730064e-01 7.965324e-01 8.081148e-01 8.193031e-01 7.714558e-01

2942 9.798035e-01 9.785941e-01 9.774421e-01 9.769935e-01 9.768668e-01 9.730282e-01 7.966587e-01 8.082440e-01 8.194342e-01 7.715716e-01

2943 9.798086e-01 9.785983e-01 9.774456e-01 9.769967e-01 9.768700e-01 9.730495e-01 7.967819e-01 8.083701e-01 8.195620e-01 7.716847e-01

2944 9.798137e-01 9.786026e-01 9.774490e-01 9.769998e-01 9.768731e-01 9.730704e-01 7.969022e-01 8.084931e-01 8.196866e-01 7.717951e-01

2945 9.798187e-01 9.786067e-01 9.774523e-01 9.770030e-01 9.768763e-01 9.730908e-01 7.970194e-01 8.086130e-01 8.198080e-01 7.719028e-01

2946 9.798238e-01 9.786109e-01 9.774557e-01 9.770060e-01 9.768794e-01 9.731107e-01 7.971337e-01 8.087299e-01 8.199263e-01 7.720079e-01

2947 9.798288e-01 9.786150e-01 9.774589e-01 9.770091e-01 9.768825e-01 9.731303e-01 7.972452e-01 8.088437e-01 8.200416e-01 7.721103e-01

2948 9.798338e-01 9.786191e-01 9.774622e-01 9.770121e-01 9.768855e-01 9.731494e-01 7.973538e-01 8.089547e-01 8.201539e-01 7.722101e-01

2949 9.798387e-01 9.786231e-01 9.774654e-01 9.770151e-01 9.768885e-01 9.731682e-01 7.974596e-01 8.090628e-01 8.202633e-01 7.723074e-01
[truncated: 10,524,276 more chars]
