# Supplementary material for: Purity control of simulated moving bed based on advanced fuzzy controller
Source: Sci Rep. 2024 Apr 20;14:9083. doi: 10.1038/s41598-024-59847-1 (PMC11576947; doi:10.1038/s41598-024-59847-1)
Supplement: Supplementary file 7 — Supplementary Information 7. [file 41598_2024_59847_MOESM7_ESM.docx]

**Figure 7(a):**

1 1.886238e-75 1.885839e-75 1.886659e-75 1.887104e-75 1.887574e-75 2.173498e-80 2.091410e-80 2.100706e-80 2.110045e-80 2.212409e-80

2 1.886238e-75 1.885839e-75 1.886659e-75 1.887104e-75 1.887574e-75 2.173498e-80 2.091410e-80 2.100706e-80 2.110045e-80 2.212409e-80

3 1.886238e-75 1.885839e-75 1.886659e-75 1.887104e-75 1.887574e-75 2.173498e-80 2.091410e-80 2.100706e-80 2.110045e-80 2.212409e-80

4 1.886238e-75 1.885839e-75 1.886659e-75 1.887104e-75 1.887574e-75 2.173498e-80 2.091410e-80 2.100706e-80 2.110045e-80 2.212409e-80

5 1.886238e-75 1.885839e-75 1.886659e-75 1.887104e-75 1.887574e-75 2.173498e-80 2.091410e-80 2.100706e-80 2.110045e-80 2.212409e-80

6 1.886238e-75 1.885839e-75 1.886659e-75 1.887104e-75 1.887574e-75 2.173498e-80 2.091410e-80 2.100706e-80 2.110045e-80 2.212409e-80

7 1.886238e-75 1.885839e-75 1.886659e-75 1.887104e-75 1.887574e-75 2.173498e-80 2.091410e-80 2.100706e-80 2.110045e-80 2.212409e-80

8 1.886238e-75 1.885839e-75 1.886659e-75 1.887104e-75 1.887574e-75 2.173498e-80 2.091410e-80 2.100706e-80 2.110045e-80 2.212409e-80

9 1.886238e-75 1.885839e-75 1.886659e-75 1.887104e-75 1.887574e-75 2.173498e-80 2.091410e-80 2.100706e-80 2.110045e-80 2.212409e-80

10 1.886238e-75 1.885839e-75 1.886659e-75 1.887104e-75 1.887574e-75 2.173498e-80 2.091410e-80 2.100706e-80 2.110045e-80 2.212409e-80

11 1.886238e-75 1.885839e-75 1.886659e-75 1.887104e-75 1.887574e-75 2.173498e-80 2.091410e-80 2.100706e-80 2.110045e-80 2.212409e-80

12 1.886238e-75 1.885839e-75 1.886659e-75 1.887104e-75 1.887574e-75 2.173498e-80 2.091410e-80 2.100706e-80 2.110045e-80 2.212409e-80

13 1.886238e-75 1.885839e-75 1.886659e-75 1.887104e-75 1.887574e-75 2.173498e-80 2.091410e-80 2.100706e-80 2.110045e-80 2.212409e-80

14 1.886238e-75 1.885839e-75 1.886659e-75 1.887104e-75 1.887574e-75 2.173498e-80 2.091410e-80 2.100706e-80 2.110045e-80 2.212409e-80

15 1.886238e-75 1.885839e-75 1.886659e-75 1.887104e-75 1.887574e-75 2.173498e-80 2.091410e-80 2.100706e-80 2.110045e-80 2.212409e-80

16 1.886238e-75 1.885839e-75 1.886659e-75 1.887104e-75 1.887574e-75 2.173498e-80 2.091410e-80 2.100706e-80 2.110045e-80 2.212409e-80

17 1.886238e-75 1.885839e-75 1.886659e-75 1.887104e-75 1.887574e-75 2.173498e-80 2.091410e-80 2.100706e-80 2.110045e-80 2.212409e-80

18 1.886238e-75 1.885839e-75 1.886659e-75 1.887104e-75 1.887574e-75 2.173498e-80 2.091410e-80 2.100706e-80 2.110045e-80 2.212409e-80

19 1.886238e-75 1.885839e-75 1.886659e-75 1.887104e-75 1.887574e-75 2.173498e-80 2.091410e-80 2.100706e-80 2.110045e-80 2.212409e-80

20 1.886238e-75 1.885839e-75 1.886659e-75 1.887104e-75 1.887574e-75 2.173498e-80 2.091410e-80 2.100706e-80 2.110045e-80 2.212409e-80

21 1.886238e-75 1.885839e-75 1.886659e-75 1.887104e-75 1.887574e-75 2.173498e-80 2.091410e-80 2.100706e-80 2.110045e-80 2.212409e-80

22 1.886238e-75 1.885839e-75 1.886659e-75 1.887104e-75 1.887574e-75 2.173498e-80 2.091410e-80 2.100706e-80 2.110045e-80 2.212409e-80

23 1.886238e-75 1.885839e-75 1.886659e-75 1.887104e-75 1.887574e-75 2.173498e-80 2.091410e-80 2.100706e-80 2.110045e-80 2.212409e-80

24 1.886238e-75 1.885839e-75 1.886659e-75 1.887104e-75 1.887574e-75 2.173498e-80 2.091410e-80 2.100706e-80 2.110045e-80 2.212409e-80

25 1.886238e-75 1.885839e-75 1.886659e-75 1.887104e-75 1.887574e-75 2.173498e-80 2.091410e-80 2.100706e-80 2.110045e-80 2.212409e-80

26 1.886238e-75 1.885839e-75 1.886659e-75 1.887104e-75 1.887574e-75 2.173498e-80 2.091410e-80 2.100706e-80 2.110045e-80 2.212409e-80

27 1.886238e-75 1.885839e-75 1.886659e-75 1.887104e-75 1.887574e-75 2.173498e-80 2.091410e-80 2.100706e-80 2.110045e-80 2.212409e-80

28 1.886238e-75 1.885839e-75 1.886659e-75 1.887104e-75 1.887574e-75 2.173498e-80 2.091410e-80 2.100706e-80 2.110045e-80 2.212409e-80

29 1.886238e-75 1.885839e-75 1.886659e-75 1.887104e-75 1.887574e-75 2.173498e-80 2.091410e-80 2.100706e-80 2.110045e-80 2.212409e-80

30 1.886238e-75 1.885839e-75 1.886659e-75 1.887104e-75 1.887574e-75 2.173498e-80 2.091410e-80 2.100706e-80 2.110045e-80 2.212409e-80

31 1.886238e-75 1.885839e-75 1.886659e-75 1.887104e-75 1.887574e-75 2.173498e-80 2.091410e-80 2.100706e-80 2.110045e-80 2.212409e-80

32 1.886238e-75 1.885839e-75 1.886659e-75 1.887104e-75 1.887574e-75 2.173498e-80 2.091410e-80 2.100706e-80 2.110045e-80 2.212409e-80

33 1.886238e-75 1.885839e-75 1.886659e-75 1.887104e-75 1.887574e-75 2.173498e-80 2.091410e-80 2.100706e-80 2.110045e-80 2.212409e-80

34 1.886238e-75 1.885839e-75 1.886659e-75 1.887104e-75 1.887574e-75 2.173498e-80 2.091410e-80 2.100706e-80 2.110045e-80 2.212409e-80

35 1.886238e-75 1.885839e-75 1.886659e-75 1.887104e-75 1.887574e-75 2.173498e-80 2.091410e-80 2.100706e-80 2.110045e-80 2.212409e-80

36 1.886238e-75 1.885839e-75 1.886659e-75 1.887104e-75 1.887574e-75 2.173498e-80 2.091410e-80 2.100706e-80 2.110045e-80 2.212409e-80

37 1.886238e-75 1.885839e-75 1.886659e-75 1.887104e-75 1.887574e-75 2.173498e-80 2.091410e-80 2.100706e-80 2.110045e-80 2.212409e-80

38 1.886238e-75 1.885839e-75 1.886659e-75 1.887104e-75 1.887574e-75 2.173498e-80 2.091410e-80 2.100706e-80 2.110045e-80 2.212409e-80

39 1.886238e-75 1.885839e-75 1.886659e-75 1.887104e-75 1.887574e-75 2.173498e-80 2.091410e-80 2.100706e-80 2.110045e-80 2.212409e-80

40 1.886238e-75 1.885839e-75 1.886659e-75 1.887104e-75 1.887574e-75 2.173498e-80 2.091410e-80 2.100706e-80 2.110045e-80 2.212409e-80

41 1.886238e-75 1.885839e-75 1.886659e-75 1.887104e-75 1.887574e-75 2.173498e-80 2.091410e-80 2.100706e-80 2.110045e-80 2.212409e-80

42 1.886238e-75 1.885839e-75 1.886659e-75 1.887104e-75 1.887574e-75 2.173498e-80 2.091410e-80 2.100706e-80 2.110045e-80 2.212409e-80

43 1.886238e-75 1.885839e-75 1.886659e-75 1.887104e-75 1.887574e-75 2.173498e-80 2.091410e-80 2.100706e-80 2.110045e-80 2.212409e-80

44 1.886238e-75 1.885839e-75 1.886659e-75 1.887104e-75 1.887574e-75 2.173498e-80 2.091410e-80 2.100706e-80 2.110045e-80 2.212409e-80

45 1.886238e-75 1.885839e-75 1.886659e-75 1.887104e-75 1.887574e-75 2.173498e-80 2.091410e-80 2.100706e-80 2.110045e-80 2.212409e-80

46 1.886238e-75 1.885839e-75 1.886659e-75 1.887104e-75 1.887574e-75 2.173498e-80 2.091410e-80 2.100706e-80 2.110045e-80 2.212409e-80

47 1.886238e-75 1.885839e-75 1.886659e-75 1.887104e-75 1.887574e-75 2.173499e-80 2.091410e-80 2.100706e-80 2.110045e-80 2.212409e-80

48 1.886238e-75 1.885839e-75 1.886659e-75 1.887104e-75 1.887574e-75 2.173499e-80 2.091410e-80 2.100706e-80 2.110045e-80 2.212409e-80

49 1.886238e-75 1.885839e-75 1.886659e-75 1.887104e-75 1.887574e-75 2.173499e-80 2.091410e-80 2.100706e-80 2.110045e-80 2.212409e-80

50 1.886238e-75 1.885839e-75 1.886660e-75 1.887104e-75 1.887574e-75 2.173499e-80 2.091411e-80 2.100707e-80 2.110045e-80 2.212409e-80

51 1.886238e-75 1.885840e-75 1.886660e-75 1.887104e-75 1.887574e-75 2.173499e-80 2.091411e-80 2.100707e-80 2.110045e-80 2.212409e-80

52 1.886239e-75 1.885840e-75 1.886660e-75 1.887105e-75 1.887574e-75 2.173499e-80 2.091411e-80 2.100707e-80 2.110045e-80 2.212409e-80

53 1.886240e-75 1.885841e-75 1.886661e-75 1.887106e-75 1.887575e-75 2.173500e-80 2.091412e-80 2.100708e-80 2.110046e-80 2.212410e-80

54 1.886241e-75 1.885842e-75 1.886662e-75 1.887107e-75 1.887577e-75 2.173502e-80 2.091414e-80 2.100710e-80 2.110048e-80 2.212412e-80

55 1.886244e-75 1.885845e-75 1.886665e-75 1.887110e-75 1.887580e-75 2.173505e-80 2.091417e-80 2.100713e-80 2.110051e-80 2.212416e-80

56 1.886250e-75 1.885851e-75 1.886672e-75 1.887116e-75 1.887586e-75 2.173512e-80 2.091424e-80 2.100720e-80 2.110058e-80 2.212423e-80

57 1.886263e-75 1.885864e-75 1.886684e-75 1.887129e-75 1.887598e-75 2.173526e-80 2.091437e-80 2.100733e-80 2.110071e-80 2.212437e-80

58 1.886288e-75 1.885889e-75 1.886709e-75 1.887154e-75 1.887623e-75 2.173554e-80 2.091463e-80 2.100760e-80 2.110098e-80 2.212465e-80

59 1.886337e-75 1.885938e-75 1.886758e-75 1.887203e-75 1.887673e-75 2.173608e-80 2.091516e-80 2.100813e-80 2.110151e-80 2.212521e-80

60 1.886434e-75 1.886035e-75 1.886855e-75 1.887300e-75 1.887770e-75 2.173716e-80 2.091620e-80 2.100917e-80 2.110256e-80 2.212632e-80

61 1.886625e-75 1.886226e-75 1.887046e-75 1.887491e-75 1.887961e-75 2.173928e-80 2.091823e-80 2.101121e-80 2.110462e-80 2.212849e-80

62 1.886998e-75 1.886599e-75 1.887419e-75 1.887864e-75 1.888334e-75 2.174342e-80 2.092220e-80 2.101521e-80 2.110864e-80 2.213273e-80

63 1.887723e-75 1.887324e-75 1.888145e-75 1.888590e-75 1.889060e-75 2.175147e-80 2.092992e-80 2.102298e-80 2.111646e-80 2.214098e-80

64 1.889127e-75 1.888727e-75 1.889549e-75 1.889995e-75 1.890465e-75 2.176703e-80 2.094485e-80 2.103800e-80 2.113157e-80 2.215692e-80

65 1.891830e-75 1.891429e-75 1.892253e-75 1.892699e-75 1.893171e-75 2.179695e-80 2.097356e-80 2.106688e-80 2.116063e-80 2.218759e-80

66 1.897009e-75 1.896607e-75 1.897433e-75 1.897881e-75 1.898354e-75 2.185425e-80 2.102852e-80 2.112218e-80 2.121627e-80 2.224630e-80

67 1.906884e-75 1.906479e-75 1.907311e-75 1.907763e-75 1.908239e-75 2.196342e-80 2.113322e-80 2.122753e-80 2.132226e-80 2.235817e-80

68 1.925626e-75 1.925216e-75 1.926059e-75 1.926516e-75 1.926999e-75 2.217041e-80 2.133174e-80 2.142725e-80 2.152321e-80 2.257028e-80

69 1.961035e-75 1.960615e-75 1.961478e-75 1.961947e-75 1.962442e-75 2.256107e-80 2.170636e-80 2.180417e-80 2.190243e-80 2.297060e-80

70 2.027635e-75 2.027196e-75 2.028099e-75 2.028588e-75 2.029106e-75 2.329508e-80 2.241016e-80 2.251228e-80 2.261488e-80 2.372276e-80

71 2.152374e-75 2.151899e-75 2.152875e-75 2.153404e-75 2.153963e-75 2.466825e-80 2.372669e-80 2.383687e-80 2.394758e-80 2.512987e-80

72 2.385058e-75 2.384517e-75 2.385630e-75 2.386233e-75 2.386871e-75 2.722653e-80 2.617917e-80 2.630436e-80 2.643017e-80 2.775135e-80

73 2.817444e-75 2.816780e-75 2.818146e-75 2.818887e-75 2.819670e-75 3.197397e-80 3.072981e-80 3.088284e-80 3.103667e-80 3.261608e-80

74 3.618041e-75 3.617148e-75 3.618984e-75 3.619980e-75 3.621033e-75 4.075127e-80 3.914231e-80 3.934680e-80 3.955240e-80 4.161013e-80

75 5.095456e-75 5.094143e-75 5.096845e-75 5.098312e-75 5.099863e-75 5.692284e-80 5.464001e-80 5.493926e-80 5.524021e-80 5.818093e-80

76 7.813467e-75 7.811377e-75 7.815676e-75 7.818011e-75 7.820479e-75 8.662193e-80 8.309818e-80 8.357138e-80 8.404735e-80 8.861295e-80

77 1.279974e-74 1.279622e-74 1.280345e-74 1.280738e-74 1.281153e-74 1.410028e-79 1.352005e-79 1.359920e-79 1.367883e-79 1.443353e-79

78 2.192393e-74 2.191781e-74 2.193041e-74 2.193725e-74 2.194448e-74 2.403088e-79 2.303335e-79 2.317061e-79 2.330870e-79 2.460901e-79

79 3.858208e-74 3.857119e-74 3.859360e-74 3.860577e-74 3.861864e-74 4.212124e-79 4.036125e-79 4.060430e-79 4.084883e-79 4.314529e-79

80 6.893417e-74 6.891457e-74 6.895488e-74 6.897677e-74 6.899991e-74 7.500458e-79 7.185435e-79 7.228959e-79 7.272750e-79 7.683882e-79

81 1.241410e-73 1.241056e-73 1.241785e-73 1.242181e-73 1.242599e-73 1.346632e-78 1.289827e-78 1.297664e-78 1.305549e-78 1.379667e-78

82 2.244057e-73 2.243415e-73 2.244737e-73 2.245454e-73 2.246213e-73 2.427184e-78 2.324400e-78 2.338545e-78 2.352778e-78 2.486820e-78

83 4.062727e-73 4.061560e-73 4.063959e-73 4.065261e-73 4.066637e-73 4.381504e-78 4.195279e-78 4.220827e-78 4.246532e-78 4.489228e-78

84 7.358077e-73 7.355960e-73 7.360313e-73 7.362675e-73 7.365171e-73 7.911830e-78 7.574357e-78 7.620491e-78 7.666909e-78 8.106388e-78

85 1.332394e-72 1.332010e-72 1.332799e-72 1.333228e-72 1.333680e-72 1.428255e-77 1.367117e-77 1.375443e-77 1.383821e-77 1.463374e-77

86 2.411686e-72 2.410989e-72 2.412421e-72 2.413198e-72 2.414018e-72 2.576909e-77 2.466210e-77 2.481226e-77 2.496335e-77 2.640258e-77

87 4.363141e-72 4.361878e-72 4.364474e-72 4.365881e-72 4.367368e-72 4.646490e-77 4.446166e-77 4.473231e-77 4.500462e-77 4.760682e-77

88 7.889929e-72 7.887642e-72 7.892343e-72 7.894893e-72 7.897585e-72 8.373142e-77 8.010840e-77 8.059588e-77 8.108637e-77 8.578856e-77

89 1.426138e-71 1.425724e-71 1.426575e-71 1.427037e-71 1.427524e-71 1.508034e-76 1.442543e-76 1.451318e-76 1.460148e-76 1.545072e-76

90 2.576835e-71 2.576085e-71 2.577627e-71 2.578463e-71 2.579345e-71 2.714681e-76 2.596352e-76 2.612141e-76 2.628028e-76 2.781335e-76

91 4.654450e-71 4.653092e-71 4.655883e-71 4.657395e-71 4.658991e-71 4.884674e-76 4.670967e-76 4.699366e-76 4.727939e-76 5.004574e-76

92 8.404678e-71 8.402220e-71 8.407271e-71 8.410006e-71 8.412894e-71 8.785789e-76 8.399974e-76 8.451032e-76 8.502404e-76 9.001394e-76

93 1.517237e-70 1.516793e-70 1.517706e-70 1.518201e-70 1.518723e-70 1.579675e-75 1.510047e-75 1.519224e-75 1.528457e-75 1.618433e-75

94 2.738221e-70 2.737416e-70 2.739069e-70 2.739964e-70 2.740908e-70 2.839253e-75 2.713641e-75 2.730129e-75 2.746719e-75 2.908903e-75

95 4.940418e-70 4.938963e-70 4.941952e-70 4.943571e-70 4.945278e-70 5.101432e-75 4.874899e-75 4.904516e-75 4.934315e-75 5.226561e-75

96 8.911130e-70 8.908498e-70 8.913904e-70 8.916830e-70 8.919915e-70 9.162822e-75 8.754434e-75 8.807618e-75 8.861128e-75 9.387555e-75

97 1.606815e-69 1.606340e-69 1.607317e-69 1.607846e-69 1.608403e-69 1.645166e-74 1.571571e-74 1.581118e-74 1.590724e-74 1.685516e-74

98 2.896353e-69 2.895493e-69 2.897259e-69 2.898215e-69 2.899222e-69 2.952739e-74 2.820168e-74 2.837301e-74 2.854540e-74 3.022316e-74

99 5.218861e-69 5.217307e-69 5.220498e-69 5.222224e-69 5.224043e-69 5.297428e-74 5.058721e-74 5.089457e-74 5.120382e-74 5.409028e-74

100 9.399925e-69 9.397119e-69 9.402882e-69 9.405998e-69 9.409282e-69 9.499881e-74 9.070265e-74 9.125384e-74 9.172191e-74 9.663689e-74

101 1.692321e-68 1.691814e-68 1.692855e-68 1.693417e-68 1.694010e-68 1.702835e-73 1.625551e-73 1.633885e-73 1.639802e-73 1.724058e-73

102 3.045338e-68 3.044424e-68 3.046301e-68 3.047316e-68 3.048385e-68 3.050814e-73 2.909097e-73 2.919607e-73 2.926364e-73 3.072349e-73

103 5.477314e-68 5.475665e-68 5.479051e-68 5.480881e-68 5.482809e-68 5.457846e-73 5.195567e-73 5.207518e-73 5.214642e-73 5.470014e-73

104 9.846081e-68 9.843107e-68 9.849213e-68 9.852511e-68 9.855985e-68 9.743797e-73 9.261815e-73 9.274310e-73 9.281203e-73 9.731105e-73

105 1.768914e-67 1.768378e-67 1.769478e-67 1.770072e-67 1.770698e-67 1.736231e-72 1.648499e-72 1.649688e-72 1.650278e-72 1.729911e-72

106 3.176000e-67 3.175035e-67 3.177016e-67 3.178086e-67 3.179212e-67 3.088874e-72 2.930457e-72 2.931435e-72 2.931831e-72 3.073183e-72

107 5.698581e-67 5.696844e-67 5.700409e-67 5.702334e-67 5.704360e-67 5.488213e-72 5.203853e-72 5.204427e-72 5.204524e-72 5.455805e-72

108 1.021752e-66 1.021440e-66 1.022081e-66 1.022427e-66 1.022791e-66 9.740641e-72 9.232356e-72 9.232294e-72 9.232006e-72 9.678994e-72

109 1.830608e-66 1.830047e-66 1.831199e-66 1.831821e-66 1.832475e-66 1.727125e-71 1.636540e-71 1.636447e-71 1.636382e-71 1.715899e-71

110 3.277123e-66 3.276115e-66 3.278184e-66 3.279301e-66 3.280475e-66 3.059611e-71 2.898524e-71 2.898336e-71 2.898268e-71 3.039685e-71

111 5.861539e-66 5.859730e-66 5.863442e-66 5.865444e-66 5.867551e-66 5.415307e-71 5.129326e-71 5.129079e-71 5.129122e-71 5.380488e-71

112 1.047427e-65 1.047103e-65 1.047768e-65 1.048127e-65 1.048504e-65 9.576099e-71 9.069102e-71 9.068960e-71 9.069408e-71 9.515890e-71

113 1.869813e-65 1.869233e-65 1.870424e-65 1.871066e-65 1.871742e-65 1.691796e-70 1.602027e-70 1.602069e-70 1.602223e-70 1.681462e-70

114 3.334283e-65 3.333245e-65 3.335375e-65 3.336523e-65 3.337731e-65 2.985931e-70 2.827175e-70 2.827384e-70 2.827799e-70 2.968294e-70

115 5.938836e-65 5.936981e-65 5.940786e-65 5.942837e-65 5.944995e-65 5.264504e-70 4.984084e-70 4.984709e-70 4.985705e-70 5.234551e-70

116 1.056472e-64 1.056141e-64 1.056820e-64 1.057186e-64 1.057571e-64 9.271525e-70 8.776811e-70 8.778385e-70 8.780621e-70 9.220881e-70

117 1.876873e-64 1.876283e-64 1.877493e-64 1.878144e-64 1.878829e-64 1.630903e-69 1.543739e-69 1.544102e-69 1.544582e-69 1.622378e-69

118 3.329601e-64 3.328552e-64 3.330704e-64 3.331863e-64 3.333081e-64 2.865192e-69 2.711826e-69 2.712618e-69 2.713615e-69 2.850905e-69

119 5.897807e-64 5.895944e-64 5.899765e-64 5.901824e-64 5.903987e-64 5.026802e-69 4.757339e-69 4.759004e-69 4.761031e-69 5.002971e-69

120 1.043014e-63 1.042684e-63 1.043361e-63 1.043726e-63 1.044109e-63 8.806538e-69 8.333814e-69 8.337222e-69 8.341261e-69 8.766990e-69

121 1.841413e-63 1.840828e-63 1.842027e-63 1.842673e-63 1.843352e-63 1.540482e-68 1.457684e-68 1.458367e-68 1.459160e-68 1.533956e-68

122 3.245151e-63 3.244118e-63 3.246236e-63 3.247377e-63 3.248576e-63 2.690350e-68 2.545573e-68 2.546919e-68 2.548456e-68 2.679646e-68

123 5.708257e-63 5.706436e-63 5.710171e-63 5.712181e-63 5.714294e-63 4.690560e-68 4.437856e-68 4.440472e-68 4.443422e-68 4.673128e-68

124 1.002122e-62 1.001802e-62 1.002459e-62 1.002813e-62 1.003184e-62 8.163345e-68 7.723078e-68 7.728104e-68 7.733714e-68 8.135189e-68

125 1.755705e-62 1.755143e-62 1.756296e-62 1.756917e-62 1.757570e-62 1.418092e-67 1.341536e-67 1.342492e-67 1.343550e-67 1.413589e-67

126 3.069473e-62 3.068487e-62 3.070508e-62 3.071596e-62 3.072739e-62 2.458672e-67 2.325817e-67 2.327621e-67 2.329605e-67 2.451552e-67

127 5.354595e-62 5.352872e-62 5.356406e-62 5.358307e-62 5.360305e-62 4.254266e-67 4.024192e-67 4.027572e-67 4.031266e-67 4.243175e-67

128 9.319919e-62 9.316913e-62 9.323076e-62 9.326392e-62 9.329875e-62 7.345941e-67 6.948363e-67 6.954654e-67 6.961499e-67 7.328983e-67

129 1.618432e-61 1.617909e-61 1.618981e-61 1.619558e-61 1.620164e-61 1.265732e-66 1.197181e-66 1.198345e-66 1.199608e-66 1.263203e-66

130 2.803823e-61 2.802915e-61 2.804776e-61 2.805777e-61 2.806829e-61 2.176119e-66 2.058191e-66 2.060335e-66 2.062654e-66 2.172474e-66

131 4.845736e-61 4.844165e-61 4.847386e-61 4.849119e-61 4.850940e-61 3.732913e-66 3.530514e-66 3.534445e-66 3.538686e-66 3.727918e-66

132 8.354180e-61 8.351467e-61 8.357030e-61 8.360023e-61 8.363166e-61 6.388774e-66 6.042223e-66 6.049400e-66 6.057128e-66 6.382471e-66

133 1.436704e-60 1.436236e-60 1.437195e-60 1.437710e-60 1.438252e-60 1.090873e-65 1.031680e-65 1.032984e-65 1.034387e-65 1.090196e-65

134 2.464545e-60 2.463742e-60 2.465388e-60 2.466274e-60 2.467204e-60 1.858244e-65 1.757386e-65 1.759749e-65 1.762286e-65 1.857796e-65

135 4.216980e-60 4.215603e-60 4.218425e-60 4.219942e-60 4.221536e-60 3.157846e-65 2.986422e-65 2.990685e-65 2.995253e-65 3.158321e-65

136 7.197022e-60 7.194670e-60 7.199492e-60 7.202086e-60 7.204810e-60 5.353383e-65 5.062748e-65 5.070408e-65 5.078597e-65 5.356335e-65

137 1.225136e-59 1.224735e-59 1.225557e-59 1.225999e-59 1.226464e-59 9.053294e-65 8.561787e-65 8.575488e-65 8.590105e-65 9.061979e-65

138 2.080135e-59 2.079453e-59 2.080851e-59 2.081602e-59 2.082392e-59 1.527284e-64 1.444371e-64 1.446811e-64 1.449407e-64 1.529377e-64

139 3.522667e-59 3.521511e-59 3.523881e-59 3.525156e-59 3.526494e-59 2.570177e-64 2.430665e-64 2.434988e-64 2.439576e-64 2.574756e-64

140 5.950097e-59 5.948141e-59 5.952151e-59 5.954306e-59 5.956570e-59 4.314535e-64 4.080372e-64 4.087995e-64 4.096062e-64 4.323973e-64

141 1.002420e-58 1.002090e-58 1.002766e-58 1.003130e-58 1.003511e-58 7.224889e-64 6.832841e-64 6.846213e-64 6.860318e-64 7.243567e-64

142 1.684420e-58 1.683865e-58 1.685003e-58 1.685615e-58 1.686257e-58 1.206854e-63 1.141378e-63 1.143711e-63 1.146163e-63 1.210439e-63

143 2.823127e-58 2.822196e-58 2.824105e-58 2.825132e-58 2.826210e-58 2.010970e-63 1.901887e-63 1.905935e-63 1.910177e-63 2.017688e-63

144 4.719484e-58 4.717924e-58 4.721121e-58 4.722839e-58 4.724643e-58 3.342616e-63 3.161325e-63 3.168312e-63 3.175608e-63 3.354958e-63

145 7.869530e-58 7.866926e-58 7.872262e-58 7.875131e-58 7.878143e-58 5.542431e-63 5.241863e-63 5.253856e-63 5.266337e-63 5.564732e-63

146 1.308882e-57 1.308448e-57 1.309337e-57 1.309814e-57 1.310316e-57 9.167510e-63 8.670381e-63 8.690856e-63 8.712092e-63 9.207235e-63

147 2.171487e-57 2.170767e-57 2.172243e-57 2.173037e-57 2.173870e-57 1.512672e-62 1.430643e-62 1.434120e-62 1.437716e-62 1.519661e-62

148 3.593590e-57 3.592397e-57 3.594842e-57 3.596157e-57 3.597537e-57 2.489924e-62 2.354891e-62 2.360766e-62 2.366822e-62 2.502087e-62

149 5.932306e-57 5.930334e-57 5.934376e-57 5.936549e-57 5.938829e-57 4.088667e-62 3.866899e-62 3.876776e-62 3.886930e-62 4.109633e-62

150 9.769059e-57 9.765807e-57 9.772471e-57 9.776052e-57 9.779812e-57 6.697906e-62 6.334531e-62 6.351058e-62 6.368009e-62 6.733738e-62

151 1.604821e-56 1.604287e-56 1.605383e-56 1.605972e-56 1.606590e-56 1.094625e-61 1.035222e-61 1.037975e-61 1.040793e-61 1.100704e-61

152 2.630006e-56 2.629128e-56 2.630927e-56 2.631893e-56 2.632907e-56 1.784718e-61 1.687828e-61 1.692396e-61 1.697061e-61 1.794961e-61

153 4.299861e-56 4.298425e-56 4.301368e-56 4.302949e-56 4.304610e-56 2.903089e-61 2.745414e-61 2.752961e-61 2.760658e-61 2.920249e-61

154 7.013449e-56 7.011104e-56 7.015910e-56 7.018493e-56 7.021204e-56 4.711367e-61 4.455349e-61 4.467774e-61 4.480430e-61 4.739966e-61

155 1.141302e-55 1.140920e-55 1.141703e-55 1.142124e-55 1.142565e-55 7.628504e-61 7.213732e-61 7.234118e-61 7.254862e-61 7.675950e-61

156 1.852993e-55 1.852372e-55 1.853644e-55 1.854328e-55 1.855046e-55 1.232389e-60 1.165342e-60 1.168676e-60 1.172066e-60 1.240229e-60

157 3.001672e-55 3.000665e-55 3.002729e-55 3.003838e-55 3.005003e-55 1.986476e-60 1.878331e-60 1.883768e-60 1.889293e-60 1.999384e-60

158 4.851567e-55 4.849937e-55 4.853277e-55 4.855072e-55 4.856956e-55 3.194894e-60 3.020844e-60 3.029685e-60 3.038667e-60 3.216081e-60

159 7.824246e-55 7.821615e-55 7.827007e-55 7.829905e-55 7.832946e-55 5.127183e-60 4.847667e-60 4.862009e-60 4.876576e-60 5.161863e-60

160 1.259091e-54 1.258667e-54 1.259536e-54 1.260003e-54 1.260493e-54 8.210352e-60 7.762427e-60 7.785635e-60 7.809207e-60 8.266976e-60

161 2.021803e-54 2.021121e-54 2.022518e-54 2.023269e-54 2.024056e-54 1.311949e-59 1.240322e-59 1.244069e-59 1.247875e-59 1.321174e-59

162 3.239662e-54 3.238568e-54 3.240809e-54 3.242013e-54 3.243276e-54 2.091975e-59 1.977676e-59 1.983713e-59 1.989848e-59 2.106973e-59

163 5.180266e-54 5.178516e-54 5.182103e-54 5.184030e-54 5.186053e-54 3.328833e-59 3.146819e-59 3.156528e-59 3.166396e-59 3.353170e-59

164 8.266245e-54 8.263449e-54 8.269180e-54 8.272258e-54 8.275490e-54 5.286099e-59 4.996853e-59 5.012435e-59 5.028278e-59 5.325522e-59

165 1.316377e-53 1.315931e-53 1.316845e-53 1.317336e-53 1.317851e-53 8.377188e-59 7.918467e-59 7.943431e-59 7.968822e-59 8.440942e-59

166 2.092088e-53 2.091378e-53 2.092832e-53 2.093613e-53 2.094433e-53 1.324929e-58 1.252326e-58 1.256319e-58 1.260381e-58 1.335223e-58

167 3.318323e-53 3.317196e-53 3.319505e-53 3.320745e-53 3.322047e-53 2.091367e-58 1.976684e-58 1.983058e-58 1.989546e-58 2.107962e-58

168 5.253007e-53 5.251221e-53 5.254880e-53 5.256846e-53 5.258909e-53 3.294750e-58 3.113953e-58 3.124112e-58 3.134458e-58 3.321462e-58

169 8.299635e-53 8.296810e-53 8.302598e-53 8.305708e-53 8.308971e-53 5.180608e-58 4.896134e-58 4.912299e-58 4.928770e-58 5.223542e-58

170 1.308828e-52 1.308382e-52 1.309296e-52 1.309787e-52 1.310302e-52 8.130479e-58 7.683732e-58 7.709411e-58 7.735591e-58 8.199387e-58

171 2.060105e-52 2.059402e-52 2.060842e-52 2.061616e-52 2.062428e-52 1.273619e-57 1.203593e-57 1.207666e-57 1.211821e-57 1.284663e-57

172 3.236604e-52 3.235498e-52 3.237764e-52 3.238981e-52 3.240258e-52 1.991416e-57 1.881856e-57 1.888307e-57 1.894891e-57 2.009092e-57

173 5.075669e-52 5.073933e-52 5.077491e-52 5.079401e-52 5.081406e-52 3.108091e-57 2.936995e-57 2.947196e-57 2.957612e-57 3.136342e-57

174 7.945302e-52 7.942581e-52 7.948156e-52 7.951151e-52 7.954293e-52 4.842225e-57 4.575516e-57 4.591624e-57 4.608079e-57 4.887319e-57

175 1.241510e-51 1.241084e-51 1.241957e-51 1.242425e-51 1.242917e-51 7.530537e-57 7.115531e-57 7.140928e-57 7.166887e-57 7.602422e-57

176 1.936520e-51 1.935855e-51 1.937218e-51 1.937949e-51 1.938717e-51 1.169086e-56 1.104624e-56 1.108623e-56 1.112713e-56 1.180530e-56

177 3.015329e-51 3.014293e-51 3.016417e-51 3.017557e-51 3.018754e-51 1.811822e-56 1.711873e-56 1.718160e-56 1.724594e-56 1.830022e-56

178 4.687021e-51 4.685408e-51 4.688713e-51 4.690489e-51 4.692351e-51 2.803129e-56 2.648423e-56 2.658296e-56 2.668405e-56 2.832036e-56

179 7.273056e-51 7.270549e-51 7.275685e-51 7.278443e-51 7.281337e-51 4.329503e-56 4.090453e-56 4.105936e-56 4.121800e-56 4.375363e-56

180 1.126686e-50 1.126297e-50 1.127094e-50 1.127521e-50 1.127970e-50 6.675889e-56 6.307141e-56 6.331390e-56 6.356254e-56 6.748566e-56

181 1.742458e-50 1.741856e-50 1.743090e-50 1.743752e-50 1.744447e-50 1.027697e-55 9.709111e-56 9.747045e-56 9.785967e-56 1.039202e-55

182 1.742458e-50 1.741856e-50 1.743090e-50 1.743752e-50 1.744447e-50 1.027697e-55 9.709111e-56 9.747045e-56 9.785967e-56 1.039202e-55

183 1.742458e-50 1.741856e-50 1.743090e-50 1.743752e-50 1.744447e-50 1.027697e-55 9.709111e-56 9.747045e-56 9.785967e-56 1.039202e-55

184 1.742458e-50 1.741856e-50 1.743090e-50 1.743752e-50 1.744447e-50 1.027697e-55 9.709111e-56 9.747045e-56 9.785967e-56 1.039202e-55

185 1.742458e-50 1.741856e-50 1.743090e-50 1.743752e-50 1.744447e-50 1.027697e-55 9.709111e-56 9.747045e-56 9.785967e-56 1.039202e-55

186 1.742458e-50 1.741856e-50 1.743090e-50 1.743752e-50 1.744447e-50 1.027697e-55 9.709111e-56 9.747045e-56 9.785967e-56 1.039202e-55

187 1.742458e-50 1.741856e-50 1.743090e-50 1.743752e-50 1.744447e-50 1.027697e-55 9.709111e-56 9.747045e-56 9.785967e-56 1.039202e-55

188 1.742458e-50 1.741856e-50 1.743090e-50 1.743752e-50 1.744447e-50 1.027697e-55 9.709111e-56 9.747045e-56 9.785967e-56 1.039202e-55

189 1.742458e-50 1.741856e-50 1.743090e-50 1.743752e-50 1.744447e-50 1.027697e-55 9.709111e-56 9.747045e-56 9.785967e-56 1.039202e-55

190 1.742458e-50 1.741856e-50 1.743090e-50 1.743752e-50 1.744447e-50 1.027697e-55 9.709111e-56 9.747045e-56 9.785967e-56 1.039202e-55

191 1.742458e-50 1.741856e-50 1.743090e-50 1.743752e-50 1.744447e-50 1.027697e-55 9.709111e-56 9.747045e-56 9.785967e-56 1.039202e-55

192 1.742458e-50 1.741856e-50 1.743090e-50 1.743752e-50 1.744447e-50 1.027697e-55 9.709111e-56 9.747045e-56 9.785967e-56 1.039202e-55

193 1.742458e-50 1.741856e-50 1.743090e-50 1.743752e-50 1.744447e-50 1.027697e-55 9.709111e-56 9.747045e-56 9.785967e-56 1.039202e-55

194 1.742458e-50 1.741856e-50 1.743090e-50 1.743752e-50 1.744447e-50 1.027697e-55 9.709111e-56 9.747045e-56 9.785967e-56 1.039202e-55

195 1.742458e-50 1.741856e-50 1.743090e-50 1.743752e-50 1.744447e-50 1.027697e-55 9.709111e-56 9.747045e-56 9.785967e-56 1.039202e-55

196 1.742458e-50 1.741856e-50 1.743090e-50 1.743752e-50 1.744447e-50 1.027697e-55 9.709111e-56 9.747045e-56 9.785967e-56 1.039202e-55

197 1.742458e-50 1.741856e-50 1.743090e-50 1.743752e-50 1.744447e-50 1.027697e-55 9.709111e-56 9.747045e-56 9.785967e-56 1.039202e-55

198 1.742458e-50 1.741856e-50 1.743090e-50 1.743752e-50 1.744447e-50 1.027697e-55 9.709111e-56 9.747045e-56 9.785967e-56 1.039202e-55

199 1.742458e-50 1.741856e-50 1.743090e-50 1.743752e-50 1.744447e-50 1.027697e-55 9.709111e-56 9.747045e-56 9.785967e-56 1.039202e-55

200 1.742458e-50 1.741856e-50 1.743090e-50 1.743752e-50 1.744447e-50 1.027697e-55 9.709111e-56 9.747045e-56 9.785967e-56 1.039202e-55

201 1.742458e-50 1.741856e-50 1.743090e-50 1.743752e-50 1.744447e-50 1.027697e-55 9.709111e-56 9.747045e-56 9.785967e-56 1.039202e-55

202 1.742458e-50 1.741856e-50 1.743090e-50 1.743752e-50 1.744447e-50 1.027697e-55 9.709111e-56 9.747045e-56 9.785967e-56 1.039202e-55

203 1.742458e-50 1.741856e-50 1.743090e-50 1.743752e-50 1.744447e-50 1.027697e-55 9.709111e-56 9.747045e-56 9.785967e-56 1.039202e-55

204 1.742458e-50 1.741856e-50 1.743090e-50 1.743752e-50 1.744447e-50 1.027697e-55 9.709111e-56 9.747045e-56 9.785967e-56 1.039202e-55

205 1.742458e-50 1.741856e-50 1.743090e-50 1.743752e-50 1.744447e-50 1.027697e-55 9.709111e-56 9.747045e-56 9.785967e-56 1.039202e-55

206 1.742458e-50 1.741856e-50 1.743090e-50 1.743752e-50 1.744447e-50 1.027697e-55 9.709111e-56 9.747045e-56 9.785967e-56 1.039202e-55

207 1.742458e-50 1.741856e-50 1.743090e-50 1.743752e-50 1.744447e-50 1.027697e-55 9.709111e-56 9.747045e-56 9.785967e-56 1.039202e-55

208 1.742458e-50 1.741856e-50 1.743090e-50 1.743752e-50 1.744447e-50 1.027697e-55 9.709111e-56 9.747045e-56 9.785967e-56 1.039202e-55

209 1.742458e-50 1.741856e-50 1.743090e-50 1.743752e-50 1.744447e-50 1.027697e-55 9.709111e-56 9.747045e-56 9.785967e-56 1.039202e-55

210 1.742458e-50 1.741856e-50 1.743090e-50 1.743752e-50 1.744447e-50 1.027697e-55 9.709111e-56 9.747045e-56 9.785967e-56 1.039202e-55

211 1.742458e-50 1.741856e-50 1.743090e-50 1.743752e-50 1.744447e-50 1.027697e-55 9.709111e-56 9.747045e-56 9.785967e-56 1.039202e-55

212 1.742458e-50 1.741856e-50 1.743090e-50 1.743752e-50 1.744447e-50 1.027697e-55 9.709111e-56 9.747045e-56 9.785967e-56 1.039202e-55

213 1.742458e-50 1.741856e-50 1.743090e-50 1.743752e-50 1.744447e-50 1.027697e-55 9.709111e-56 9.747045e-56 9.785967e-56 1.039202e-55

214 1.742458e-50 1.741856e-50 1.743090e-50 1.743752e-50 1.744447e-50 1.027697e-55 9.709111e-56 9.747045e-56 9.785967e-56 1.039202e-55

215 1.742458e-50 1.741856e-50 1.743090e-50 1.743752e-50 1.744447e-50 1.027697e-55 9.709111e-56 9.747045e-56 9.785967e-56 1.039202e-55

216 1.742458e-50 1.741856e-50 1.743090e-50 1.743752e-50 1.744447e-50 1.027697e-55 9.709111e-56 9.747045e-56 9.785967e-56 1.039202e-55

217 1.742458e-50 1.741856e-50 1.743090e-50 1.743752e-50 1.744447e-50 1.027697e-55 9.709111e-56 9.747045e-56 9.785967e-56 1.039202e-55

218 1.742458e-50 1.741856e-50 1.743090e-50 1.743752e-50 1.744447e-50 1.027697e-55 9.709111e-56 9.747045e-56 9.785967e-56 1.039202e-55

219 1.742458e-50 1.741856e-50 1.743090e-50 1.743752e-50 1.744447e-50 1.027697e-55 9.709111e-56 9.747045e-56 9.785967e-56 1.039202e-55

220 1.742458e-50 1.741856e-50 1.743090e-50 1.743752e-50 1.744447e-50 1.027697e-55 9.709111e-56 9.747045e-56 9.785967e-56 1.039202e-55

221 1.742458e-50 1.741856e-50 1.743090e-50 1.743752e-50 1.744447e-50 1.027697e-55 9.709111e-56 9.747045e-56 9.785967e-56 1.039202e-55

222 1.742458e-50 1.741856e-50 1.743090e-50 1.743752e-50 1.744447e-50 1.027697e-55 9.709111e-56 9.747045e-56 9.785967e-56 1.039202e-55

223 1.742458e-50 1.741856e-50 1.743090e-50 1.743752e-50 1.744447e-50 1.027697e-55 9.709111e-56 9.747045e-56 9.785967e-56 1.039202e-55

224 1.742458e-50 1.741856e-50 1.743090e-50 1.743752e-50 1.744447e-50 1.027697e-55 9.709111e-56 9.747045e-56 9.785967e-56 1.039202e-55

225 1.742458e-50 1.741856e-50 1.743090e-50 1.743752e-50 1.744447e-50 1.027697e-55 9.709111e-56 9.747045e-56 9.785967e-56 1.039202e-55

226 1.742458e-50 1.741856e-50 1.743090e-50 1.743752e-50 1.744447e-50 1.027697e-55 9.709111e-56 9.747045e-56 9.785967e-56 1.039202e-55

227 1.742458e-50 1.741856e-50 1.743090e-50 1.743752e-50 1.744447e-50 1.027697e-55 9.709111e-56 9.747045e-56 9.785967e-56 1.039202e-55

228 1.742458e-50 1.741856e-50 1.743090e-50 1.743752e-50 1.744447e-50 1.027697e-55 9.709111e-56 9.747045e-56 9.785967e-56 1.039202e-55

229 1.742458e-50 1.741856e-50 1.743090e-50 1.743752e-50 1.744447e-50 1.027697e-55 9.709111e-56 9.747045e-56 9.785967e-56 1.039202e-55

230 1.742458e-50 1.741856e-50 1.743090e-50 1.743752e-50 1.744447e-50 1.027697e-55 9.709111e-56 9.747045e-56 9.785967e-56 1.039202e-55

231 1.742458e-50 1.741856e-50 1.743090e-50 1.743752e-50 1.744447e-50 1.027697e-55 9.709111e-56 9.747045e-56 9.785967e-56 1.039202e-55

232 1.742458e-50 1.741856e-50 1.743090e-50 1.743752e-50 1.744447e-50 1.027697e-55 9.709111e-56 9.747045e-56 9.785967e-56 1.039202e-55

233 1.742458e-50 1.741856e-50 1.743090e-50 1.743752e-50 1.744447e-50 1.027697e-55 9.709111e-56 9.747045e-56 9.785967e-56 1.039202e-55

234 1.742458e-50 1.741856e-50 1.743090e-50 1.743752e-50 1.744447e-50 1.027697e-55 9.709111e-56 9.747045e-56 9.785967e-56 1.039202e-55

235 1.742458e-50 1.741856e-50 1.743090e-50 1.743752e-50 1.744447e-50 1.027697e-55 9.709111e-56 9.747045e-56 9.785967e-56 1.039202e-55

236 1.742458e-50 1.741856e-50 1.743090e-50 1.743752e-50 1.744447e-50 1.027697e-55 9.709111e-56 9.747045e-56 9.785967e-56 1.039202e-55

237 1.742458e-50 1.741856e-50 1.743090e-50 1.743752e-50 1.744447e-50 1.027697e-55 9.709112e-56 9.747046e-56 9.785968e-56 1.039202e-55

238 1.742458e-50 1.741856e-50 1.743090e-50 1.743752e-50 1.744447e-50 1.027697e-55 9.709112e-56 9.747046e-56 9.785968e-56 1.039202e-55

239 1.742459e-50 1.741857e-50 1.743090e-50 1.743753e-50 1.744448e-50 1.027697e-55 9.709113e-56 9.747046e-56 9.785969e-56 1.039202e-55

240 1.742459e-50 1.741857e-50 1.743090e-50 1.743753e-50 1.744448e-50 1.027697e-55 9.709113e-56 9.747047e-56 9.785969e-56 1.039202e-55

241 1.742459e-50 1.741857e-50 1.743091e-50 1.743753e-50 1.744448e-50 1.027697e-55 9.709115e-56 9.747049e-56 9.785971e-56 1.039202e-55

242 1.742460e-50 1.741858e-50 1.743091e-50 1.743754e-50 1.744449e-50 1.027697e-55 9.709117e-56 9.747051e-56 9.785973e-56 1.039203e-55

243 1.742460e-50 1.741858e-50 1.743092e-50 1.743754e-50 1.744449e-50 1.027698e-55 9.709120e-56 9.747053e-56 9.785976e-56 1.039203e-55

244 1.742461e-50 1.741859e-50 1.743093e-50 1.743755e-50 1.744450e-50 1.027698e-55 9.709124e-56 9.747058e-56 9.785980e-56 1.039203e-55

245 1.742463e-50 1.741861e-50 1.743094e-50 1.743757e-50 1.744452e-50 1.027699e-55 9.709131e-56 9.747065e-56 9.785987e-56 1.039204e-55

246 1.742465e-50 1.741863e-50 1.743097e-50 1.743759e-50 1.744454e-50 1.027700e-55 9.709141e-56 9.747075e-56 9.785997e-56 1.039205e-55

247 1.742469e-50 1.741867e-50 1.743101e-50 1.743763e-50 1.744458e-50 1.027702e-55 9.709156e-56 9.747090e-56 9.786013e-56 1.039207e-55

248 1.742475e-50 1.741873e-50 1.743106e-50 1.743769e-50 1.744464e-50 1.027704e-55 9.709179e-56 9.747113e-56 9.786036e-56 1.039209e-55

249 1.742483e-50 1.741881e-50 1.743115e-50 1.743777e-50 1.744472e-50 1.027708e-55 9.709213e-56 9.747148e-56 9.786071e-56 1.039213e-55

250 1.742496e-50 1.741894e-50 1.743128e-50 1.743790e-50 1.744485e-50 1.027713e-55 9.709265e-56 9.747200e-56 9.786123e-56 1.039219e-55

251 1.742516e-50 1.741913e-50 1.743147e-50 1.743810e-50 1.744505e-50 1.027721e-55 9.709342e-56 9.747278e-56 9.786202e-56 1.039227e-55

252 1.742545e-50 1.741942e-50 1.743176e-50 1.743839e-50 1.744534e-50 1.027733e-55 9.709458e-56 9.747395e-56 9.786320e-56 1.039240e-55

253 1.742588e-50 1.741986e-50 1.743220e-50 1.743882e-50 1.744577e-50 1.027752e-55 9.709632e-56 9.747570e-56 9.786497e-56 1.039259e-55

254 1.742653e-50 1.742051e-50 1.743285e-50 1.743947e-50 1.744642e-50 1.027779e-55 9.709890e-56 9.747831e-56 9.786760e-56 1.039287e-55

255 1.742750e-50 1.742147e-50 1.743382e-50 1.744044e-50 1.744740e-50 1.027820e-55 9.710277e-56 9.748221e-56 9.787153e-56 1.039329e-55

256 1.742895e-50 1.742292e-50 1.743527e-50 1.744190e-50 1.744885e-50 1.027880e-55 9.710852e-56 9.748802e-56 9.787740e-56 1.039392e-55

257 1.743111e-50 1.742507e-50 1.743743e-50 1.744406e-50 1.745102e-50 1.027971e-55 9.711709e-56 9.749666e-56 9.788612e-56 1.039485e-55

258 1.743432e-50 1.742828e-50 1.744065e-50 1.744728e-50 1.745424e-50 1.028105e-55 9.712981e-56 9.750951e-56 9.789909e-56 1.039625e-55

259 1.743909e-50 1.743305e-50 1.744543e-50 1.745206e-50 1.745903e-50 1.028304e-55 9.714871e-56 9.752858e-56 9.791835e-56 1.039831e-55

260 1.744617e-50 1.744012e-50 1.745252e-50 1.745916e-50 1.746615e-50 1.028600e-55 9.717672e-56 9.755686e-56 9.794690e-56 1.040137e-55

261 1.745667e-50 1.745060e-50 1.746304e-50 1.746968e-50 1.747669e-50 1.029037e-55 9.721819e-56 9.759873e-56 9.798919e-56 1.040591e-55

262 1.747222e-50 1.746612e-50 1.747861e-50 1.748526e-50 1.749229e-50 1.029684e-55 9.727952e-56 9.766066e-56 9.805173e-56 1.041263e-55

263 1.749519e-50 1.748905e-50 1.750162e-50 1.750828e-50 1.751535e-50 1.030639e-55 9.737008e-56 9.775211e-56 9.814410e-56 1.042254e-55

264 1.752910e-50 1.752290e-50 1.753559e-50 1.754226e-50 1.754940e-50 1.032048e-55 9.750363e-56 9.788700e-56 9.828036e-56 1.043718e-55

265 1.757908e-50 1.757280e-50 1.758565e-50 1.759235e-50 1.759957e-50 1.034122e-55 9.770032e-56 9.808566e-56 9.848105e-56 1.045873e-55

266 1.765263e-50 1.764623e-50 1.765934e-50 1.766607e-50 1.767342e-50 1.037173e-55 9.798958e-56 9.837787e-56 9.877627e-56 1.049045e-55

267 1.776073e-50 1.775414e-50 1.776762e-50 1.777440e-50 1.778194e-50 1.041652e-55 9.841438e-56 9.880703e-56 9.920991e-56 1.053706e-55

268 1.791935e-50 1.791250e-50 1.792651e-50 1.793336e-50 1.794118e-50 1.048222e-55 9.903734e-56 9.943644e-56 9.984593e-56 1.060542e-55

269 1.815174e-50 1.814450e-50 1.815929e-50 1.816626e-50 1.817448e-50 1.057841e-55 9.994954e-56 1.003582e-55 1.007774e-55 1.070557e-55

270 1.849168e-50 1.848387e-50 1.849981e-50 1.850693e-50 1.851574e-50 1.071905e-55 1.012833e-55 1.017059e-55 1.021396e-55 1.085206e-55

271 1.898815e-50 1.897951e-50 1.899711e-50 1.900447e-50 1.901414e-50 1.092435e-55 1.032303e-55 1.036737e-55 1.041285e-55 1.106599e-55

272 1.971203e-50 1.970220e-50 1.972221e-50 1.972991e-50 1.974084e-50 1.122359e-55 1.060683e-55 1.065420e-55 1.070279e-55 1.137791e-55

273 2.076574e-50 2.075417e-50 2.077769e-50 2.078589e-50 2.079864e-50 1.165904e-55 1.101982e-55 1.107163e-55 1.112478e-55 1.183199e-55

274 2.229698e-50 2.228288e-50 2.231149e-50 2.232041e-50 2.233580e-50 1.229168e-55 1.161983e-55 1.167816e-55 1.173797e-55 1.249192e-55

275 2.451832e-50 2.450056e-50 2.453654e-50 2.454650e-50 2.456571e-50 1.320928e-55 1.249013e-55 1.255796e-55 1.262750e-55 1.344944e-55

276 2.773512e-50 2.771209e-50 2.775870e-50 2.777018e-50 2.779490e-50 1.453793e-55 1.375031e-55 1.383201e-55 1.391572e-55 1.483636e-55

277 3.238524e-50 3.235460e-50 3.241654e-50 3.243022e-50 3.246289e-50 1.645849e-55 1.557193e-55 1.567378e-55 1.577810e-55 1.684177e-55

278 3.909522e-50 3.905363e-50 3.913761e-50 3.915448e-50 3.919859e-50 1.922979e-55 1.820050e-55 1.833161e-55 1.846584e-55 1.973640e-55

279 4.875985e-50 4.870255e-50 4.881819e-50 4.883964e-50 4.890018e-50 2.322164e-55 2.198680e-55 2.216028e-55 2.233782e-55 2.390710e-55

280 6.265452e-50 6.257470e-50 6.273572e-50 6.276377e-50 6.284786e-50 2.896127e-55 2.743095e-55 2.766570e-55 2.790583e-55 2.990557e-55

281 8.259350e-50 8.248145e-50 8.270740e-50 8.274494e-50 8.286271e-50 3.719903e-55 3.524468e-55 3.556781e-55 3.589822e-55 3.851711e-55

282 1.111525e-49 1.109944e-49 1.113131e-49 1.113642e-49 1.115301e-49 4.900061e-55 4.643886e-55 4.688922e-55 4.734955e-55 5.085731e-55

283 1.519808e-49 1.517572e-49 1.522080e-49 1.522786e-49 1.525130e-49 6.587667e-55 6.244644e-55 6.307958e-55 6.372650e-55 6.850784e-55

284 2.102392e-49 2.099223e-49 2.105610e-49 2.106594e-49 2.109914e-49 8.996450e-55 8.529474e-55 8.618990e-55 8.710423e-55 9.370689e-55

285 2.932106e-49 2.927613e-49 2.936668e-49 2.938049e-49 2.942754e-49 1.242821e-54 1.178464e-54 1.191164e-54 1.204132e-54 1.296155e-54

286 4.111541e-49 4.105172e-49 4.118007e-49 4.119952e-49 4.126621e-49 1.730829e-54 1.641361e-54 1.659412e-54 1.677838e-54 1.806893e-54

287 5.784921e-49 5.775900e-49 5.794081e-49 5.796827e-49 5.806273e-49 2.423503e-54 2.298389e-54 2.324065e-54 2.350265e-54 2.531972e-54

288 8.154627e-49 8.141861e-49 8.167588e-49 8.171470e-49 8.184837e-49 3.404853e-54 3.229235e-54 3.265748e-54 3.302999e-54 3.559423e-54

289 1.150411e-48 1.148607e-48 1.152243e-48 1.152792e-48 1.154681e-48 4.792616e-54 4.545570e-54 4.597461e-54 4.650386e-54 5.012635e-54

290 1.622965e-48 1.620420e-48 1.625550e-48 1.626326e-48 1.628992e-48 6.751504e-54 6.403620e-54 6.477284e-54 6.552396e-54 7.064249e-54

291 2.288429e-48 2.284843e-48 2.292071e-48 2.293167e-48 2.296924e-48 9.511530e-54 9.021542e-54 9.125975e-54 9.232431e-54 9.955380e-54

292 3.223849e-48 3.218806e-48 3.228972e-48 3.230519e-48 3.235804e-48 1.339335e-53 1.270346e-53 1.285128e-53 1.300194e-53 1.402220e-53

293 4.536383e-48 4.529302e-48 4.543576e-48 4.545756e-48 4.553178e-48 1.884324e-53 1.787262e-53 1.808152e-53 1.829437e-53 1.973261e-53

294 6.374824e-48 6.364900e-48 6.384907e-48 6.387975e-48 6.398382e-48 2.648127e-53 2.511711e-53 2.541182e-53 2.571203e-53 2.773684e-53

295 8.945460e-48 8.931575e-48 8.959569e-48 8.963879e-48 8.978445e-48 3.716770e-53 3.525275e-53 3.566779e-53 3.609048e-53 3.893701e-53

296 1.253385e-47 1.251446e-47 1.255356e-47 1.255961e-47 1.257996e-47 5.209415e-53 4.940960e-53 4.999308e-53 5.058718e-53 5.458288e-53

297 1.753476e-47 1.750772e-47 1.756224e-47 1.757071e-47 1.759910e-47 7.290892e-53 6.915076e-53 6.996960e-53 7.080315e-53 7.640330e-53

298 2.449310e-47 2.445548e-47 2.453136e-47 2.454321e-47 2.458274e-47 1.018889e-52 9.663530e-53 9.778243e-53 9.894993e-53 1.067866e-52

299 3.416018e-47 3.410790e-47 3.421336e-47 3.422991e-47 3.428486e-47 1.421750e-52 1.348416e-52 1.364459e-52 1.380784e-52 1.490276e-52

300 4.757056e-47 4.749804e-47 4.764434e-47 4.766743e-47 4.774370e-47 1.980950e-52 1.878734e-52 1.901134e-52 1.923922e-52 2.076668e-52

301 6.614725e-47 6.604680e-47 6.624946e-47 6.628162e-47 6.638732e-47 2.756053e-52 2.613780e-52 2.645005e-52 2.676764e-52 2.889531e-52

302 9.184554e-47 9.170662e-47 9.198693e-47 9.203164e-47 9.217790e-47 3.828943e-52 3.631193e-52 3.674650e-52 3.718844e-52 4.014781e-52

303 1.273495e-46 1.271576e-46 1.275448e-46 1.276069e-46 1.278090e-46 5.312085e-52 5.037596e-52 5.097987e-52 5.159390e-52 5.570424e-52

304 1.763403e-46 1.760757e-46 1.766097e-46 1.766958e-46 1.769747e-46 7.359798e-52 6.979290e-52 7.063090e-52 7.148279e-52 7.718393e-52

305 2.438627e-46 2.434982e-46 2.442339e-46 2.443533e-46 2.447376e-46 1.018366e-51 9.656847e-52 9.772968e-52 9.890992e-52 1.068071e-51

306 3.368253e-46 3.363238e-46 3.373361e-46 3.375013e-46 3.380302e-46 1.407353e-51 1.334504e-51 1.350573e-51 1.366902e-51 1.476158e-51

307 4.646827e-46 4.639933e-46 4.653848e-46 4.656131e-46 4.663404e-46 1.942626e-51 1.842001e-51 1.864210e-51 1.886775e-51 2.037745e-51

308 6.403667e-46 6.394201e-46 6.413309e-46 6.416462e-46 6.426451e-46 2.678481e-51 2.539643e-51 2.570300e-51 2.601444e-51 2.809821e-51

309 8.815569e-46 8.802582e-46 8.828799e-46 8.833149e-46 8.846857e-46 3.689172e-51 3.497804e-51 3.540076e-51 3.583011e-51 3.870317e-51

310 1.212413e-45 1.210633e-45 1.214227e-45 1.214827e-45 1.216707e-45 5.076199e-51 4.812675e-51 4.870899e-51 4.930029e-51 5.325767e-51

311 1.665941e-45 1.663501e-45 1.668426e-45 1.669252e-45 1.671827e-45 6.978245e-51 6.615681e-51 6.695799e-51 6.777149e-51 7.321736e-51

312 2.287213e-45 2.283872e-45 2.290616e-45 2.291753e-45 2.295280e-45 9.584746e-51 9.086328e-51 9.196469e-51 9.308288e-51 1.005707e-50

313 3.137774e-45 3.133200e-45 3.142432e-45 3.143995e-45 3.148823e-45 1.315439e-50 1.246973e-50 1.262101e-50 1.277458e-50 1.380330e-50

314 4.301639e-45 4.295381e-45 4.308013e-45 4.310162e-45 4.316767e-45 1.804035e-50 1.710050e-50 1.730814e-50 1.751888e-50 1.893116e-50

315 5.893491e-45 5.884929e-45 5.902209e-45 5.905162e-45 5.914195e-45 2.472474e-50 2.343540e-50 2.372018e-50 2.400917e-50 2.594675e-50

316 8.069853e-45 8.058141e-45 8.081776e-45 8.085832e-45 8.098185e-45 3.386558e-50 3.209777e-50 3.248810e-50 3.288415e-50 3.554083e-50

317 1.104435e-44 1.102833e-44 1.106066e-44 1.106623e-44 1.108312e-44 4.636094e-50 4.393833e-50 4.447300e-50 4.501544e-50 4.865617e-50

318 1.510855e-44 1.508664e-44 1.513084e-44 1.513849e-44 1.516158e-44 6.343641e-50 6.011791e-50 6.084993e-50 6.159249e-50 6.657939e-50

319 2.066037e-44 2.063040e-44 2.069085e-44 2.070134e-44 2.073291e-44 8.676451e-50 8.222059e-50 8.322233e-50 8.423837e-50 9.106636e-50

320 2.824292e-44 2.820193e-44 2.828460e-44 2.829899e-44 2.834215e-44 1.186277e-49 1.124079e-49 1.137782e-49 1.151679e-49 1.245133e-49

321 3.859750e-44 3.854143e-44 3.865452e-44 3.867425e-44 3.873327e-44 1.621409e-49 1.536296e-49 1.555034e-49 1.574034e-49 1.701903e-49

322 5.273602e-44 5.265931e-44 5.281401e-44 5.284107e-44 5.292179e-44 2.215550e-49 2.099108e-49 2.124722e-49 2.150693e-49 2.325603e-49

323 7.203981e-44 7.193484e-44 7.214650e-44 7.218361e-44 7.229401e-44 3.026716e-49 2.867445e-49 2.902450e-49 2.937939e-49 3.177143e-49

324 9.839467e-44 9.825102e-44 9.854065e-44 9.859154e-44 9.874257e-44 4.134088e-49 3.916270e-49 3.964098e-49 4.012584e-49 4.339654e-49

325 1.343753e-43 1.341786e-43 1.345750e-43 1.346448e-43 1.348515e-43 5.645738e-49 5.347892e-49 5.413229e-49 5.479458e-49 5.926602e-49

326 1.834970e-43 1.832278e-43 1.837704e-43 1.838661e-43 1.841489e-43 7.709178e-49 7.301941e-49 7.391183e-49 7.481635e-49 8.092860e-49

327 2.505600e-43 2.501915e-43 2.509343e-43 2.510655e-43 2.514526e-43 1.052575e-48 9.968992e-49 1.009087e-48 1.021439e-48 1.104982e-48

328 3.421189e-43 3.416143e-43 3.426314e-43 3.428113e-43 3.433412e-43 1.437030e-48 1.360916e-48 1.377559e-48 1.394425e-48 1.508605e-48

329 4.671248e-43 4.664338e-43 4.678266e-43 4.680733e-43 4.687988e-43 1.961796e-48 1.857746e-48 1.880472e-48 1.903500e-48 2.059543e-48

330 6.378014e-43 6.368551e-43 6.387624e-43 6.391007e-43 6.400942e-43 2.678082e-48 2.535848e-48 2.566876e-48 2.598316e-48 2.811561e-48

331 8.708420e-43 8.695460e-43 8.721581e-43 8.726222e-43 8.739828e-43 3.655787e-48 3.461358e-48 3.503721e-48 3.546643e-48 3.838051e-48

332 1.189044e-42 1.187269e-42 1.190847e-42 1.191483e-42 1.193347e-42 4.990320e-48 4.724546e-48 4.782383e-48 4.840977e-48 5.239188e-48

333 1.623542e-42 1.621111e-42 1.626011e-42 1.626884e-42 1.629436e-42 6.811907e-48 6.448612e-48 6.527571e-48 6.607559e-48 7.151705e-48

334 2.216853e-42 2.213523e-42 2.220234e-42 2.221432e-42 2.224927e-42 9.298287e-48 8.801693e-48 8.909484e-48 9.018675e-48 9.762226e-48

335 3.027039e-42 3.022480e-42 3.031670e-42 3.033314e-42 3.038101e-42 1.269204e-47 1.201324e-47 1.216039e-47 1.230944e-47 1.332545e-47

336 4.133391e-42 4.127149e-42 4.139733e-42 4.141988e-42 4.148545e-42 1.732421e-47 1.639637e-47 1.659724e-47 1.680070e-47 1.818899e-47

337 5.644179e-42 5.635631e-42 5.652863e-42 5.655956e-42 5.664936e-42 2.364654e-47 2.237831e-47 2.265251e-47 2.293024e-47 2.482716e-47

338 7.707239e-42 7.695538e-42 7.719129e-42 7.723373e-42 7.735670e-42 3.227544e-47 3.054197e-47 3.091626e-47 3.129535e-47 3.388720e-47

339 1.052442e-41 1.050841e-41 1.054070e-41 1.054652e-41 1.056336e-41 4.405191e-47 4.168261e-47 4.219351e-47 4.271094e-47 4.625219e-47

340 1.437132e-41 1.434941e-41 1.439360e-41 1.440159e-41 1.442464e-41 6.012328e-47 5.688504e-47 5.758239e-47 5.828862e-47 6.312685e-47

341 1.962417e-41 1.959419e-41 1.965466e-41 1.966562e-41 1.969717e-41 8.205462e-47 7.762895e-47 7.858075e-47 7.954465e-47 8.615460e-47

342 2.679654e-41 2.675553e-41 2.683825e-41 2.685329e-41 2.689646e-41 1.119806e-46 1.059324e-46 1.072314e-46 1.085470e-46 1.175770e-46

343 3.658947e-41 3.653340e-41 3.664652e-41 3.666716e-41 3.672623e-41 1.528126e-46 1.445474e-46 1.463203e-46 1.481156e-46 1.604511e-46

344 4.995979e-41 4.988314e-41 5.003780e-41 5.006611e-41 5.014691e-41 2.085205e-46 1.972265e-46 1.996459e-46 2.020959e-46 2.189456e-46

345 6.821328e-41 6.810854e-41 6.831992e-41 6.835876e-41 6.846924e-41 2.845172e-46 2.690857e-46 2.723871e-46 2.757303e-46 2.987448e-46

346 9.313184e-41 9.298878e-41 9.327756e-41 9.333085e-41 9.348188e-41 3.881821e-46 3.670989e-46 3.716038e-46 3.761654e-46 4.075978e-46

347 1.271468e-40 1.269515e-40 1.273458e-40 1.274190e-40 1.276253e-40 5.295742e-46 5.007719e-46 5.069184e-46 5.131422e-46 5.560678e-46

348 1.735752e-40 1.733086e-40 1.738470e-40 1.739473e-40 1.742292e-40 7.224029e-46 6.830591e-46 6.914447e-46 6.999359e-46 7.585519e-46

349 2.369419e-40 2.365782e-40 2.373129e-40 2.374505e-40 2.378354e-40 9.853502e-46 9.316123e-46 9.430518e-46 9.546353e-46 1.034669e-45

350 3.234188e-40 3.229229e-40 3.239248e-40 3.241135e-40 3.246390e-40 1.343870e-45 1.270480e-45 1.286084e-45 1.301885e-45 1.411152e-45

351 4.414225e-40 4.407466e-40 4.421125e-40 4.423713e-40 4.430882e-40 1.832639e-45 1.732421e-45 1.753704e-45 1.775255e-45 1.924417e-45

352 6.024296e-40 6.015091e-40 6.033700e-40 6.037249e-40 6.047026e-40 2.498884e-45 2.362048e-45 2.391075e-45 2.420466e-45 2.624064e-45

353 8.220863e-40 8.208332e-40 8.233672e-40 8.238538e-40 8.251864e-40 3.406920e-45 3.220112e-45 3.259694e-45 3.299774e-45 3.577641e-45

354 1.121719e-39 1.120014e-39 1.123463e-39 1.124130e-39 1.125945e-39 4.644310e-45 4.389313e-45 4.443285e-45 4.497933e-45 4.877112e-45

355 1.530390e-39 1.528072e-39 1.532762e-39 1.533677e-39 1.536148e-39 6.330240e-45 5.982218e-45 6.055799e-45 6.130304e-45 6.647661e-45

356 2.087696e-39 2.084547e-39 2.090921e-39 2.092175e-39 2.095538e-39 8.626907e-45 8.151997e-45 8.252301e-45 8.353864e-45 9.059647e-45

357 2.847572e-39 2.843297e-39 2.851954e-39 2.853672e-39 2.858244e-39 1.175498e-44 1.110703e-44 1.124374e-44 1.138217e-44 1.234485e-44

358 3.883463e-39 3.877664e-39 3.889412e-39 3.891766e-39 3.897980e-39 1.601459e-44 1.513070e-44 1.531701e-44 1.550565e-44 1.681853e-44

359 5.295352e-39 5.287492e-39 5.303424e-39 5.306647e-39 5.315086e-39 2.181383e-44 2.060832e-44 2.086218e-44 2.111922e-44 2.290934e-44

360 7.219306e-39 7.208660e-39 7.230248e-39 7.234663e-39 7.246114e-39 2.970740e-44 2.806359e-44 2.840942e-44 2.875959e-44 3.119997e-44

361 9.840430e-39 9.826024e-39 9.855253e-39 9.861297e-39 9.876824e-39 4.044904e-44 3.820805e-44 3.867908e-44 3.915603e-44 4.248219e-44

362 5.480112e-03 5.480313e-03 5.479905e-03 5.479804e-03 5.479587e-03 5.014925e-06 4.486461e-06 4.414185e-06 4.342584e-06 4.713898e-06

363 1.093994e-02 1.094040e-02 1.093948e-02 1.093926e-02 1.093877e-02 9.451624e-06 8.455106e-06 8.318418e-06 8.183013e-06 8.882364e-06

364 1.637510e-02 1.637585e-02 1.637432e-02 1.637395e-02 1.637314e-02 1.337387e-05 1.196311e-05 1.176905e-05 1.157683e-05 1.256576e-05

365 2.178064e-02 2.178177e-02 2.177948e-02 2.177894e-02 2.177773e-02 1.683874e-05 1.506162e-05 1.481650e-05 1.457372e-05 1.581808e-05

366 2.715108e-02 2.715266e-02 2.714947e-02 2.714873e-02 2.714703e-02 1.989725e-05 1.779637e-05 1.750584e-05 1.721809e-05 1.868757e-05

367 3.248034e-02 3.248244e-02 3.247818e-02 3.247720e-02 3.247494e-02 2.259499e-05 2.020821e-05 1.987731e-05 1.954960e-05 2.121734e-05

368 3.776169e-02 3.776443e-02 3.775889e-02 3.775764e-02 3.775470e-02 2.497268e-05 2.233362e-05 2.196687e-05 2.160367e-05 2.344586e-05

369 4.298779e-02 4.299126e-02 4.298424e-02 4.298265e-02 4.297893e-02 2.706664e-05 2.420514e-05 2.380657e-05 2.341187e-05 2.540744e-05

370 4.815060e-02 4.815493e-02 4.814617e-02 4.814420e-02 4.813956e-02 2.890926e-05 2.585178e-05 2.542498e-05 2.500235e-05 2.713266e-05

371 5.324139e-02 5.324671e-02 5.323594e-02 5.323354e-02 5.322783e-02 3.052939e-05 2.729938e-05 2.684756e-05 2.640017e-05 2.864875e-05

372 5.825076e-02 5.825723e-02 5.824415e-02 5.824123e-02 5.823430e-02 3.195272e-05 2.857096e-05 2.809698e-05 2.762766e-05 2.997995e-05

373 6.316863e-02 6.317640e-02 6.316067e-02 6.315717e-02 6.314883e-02 3.320213e-05 2.968698e-05 2.919337e-05 2.870466e-05 3.114781e-05

374 6.798423e-02 6.799350e-02 6.797475e-02 6.797058e-02 6.796064e-02 3.429791e-05 3.066562e-05 3.015467e-05 2.964880e-05 3.217150e-05

375 7.268623e-02 7.269719e-02 7.267502e-02 7.267008e-02 7.265834e-02 3.525814e-05 3.152307e-05 3.099678e-05 3.047575e-05 3.306801e-05

376 7.726274e-02 7.727560e-02 7.724959e-02 7.724379e-02 7.723001e-02 3.609883e-05 3.227365e-05 3.173382e-05 3.119939e-05 3.385245e-05

377 8.170147e-02 8.171645e-02 8.168615e-02 8.167937e-02 8.166332e-02 3.683420e-05 3.293009e-05 3.237831e-05 3.183207e-05 3.453819e-05

378 8.598986e-02 8.600719e-02 8.597213e-02 8.596424e-02 8.594567e-02 3.747686e-05 3.350367e-05 3.294135e-05 3.238470e-05 3.513709e-05

379 9.011526e-02 9.013520e-02 9.009487e-02 9.008576e-02 9.006441e-02 3.803796e-05 3.400438e-05 3.343277e-05 3.286698e-05 3.565964e-05

380 9.406522e-02 9.408801e-02 9.404192e-02 9.403145e-02 9.400705e-02 3.852739e-05 3.444108e-05 3.386130e-05 3.328753e-05 3.611518e-05

381 9.782773e-02 9.785361e-02 9.780127e-02 9.778931e-02 9.776159e-02 3.895388e-05 3.482164e-05 3.423467e-05 3.365394e-05 3.651195e-05

382 1.013916e-01 1.014208e-01 1.013617e-01 1.013481e-01 1.013168e-01 3.932515e-05 3.515301e-05 3.455971e-05 3.397291e-05 3.685724e-05

383 1.047467e-01 1.047795e-01 1.047132e-01 1.046979e-01 1.046628e-01 3.964804e-05 3.544128e-05 3.484242e-05 3.425034e-05 3.715747e-05

384 1.078846e-01 1.079211e-01 1.078473e-01 1.078301e-01 1.077910e-01 3.992861e-05 3.569185e-05 3.508810e-05 3.449142e-05 3.741827e-05

385 1.107987e-01 1.108392e-01 1.107574e-01 1.107382e-01 1.106949e-01 4.017217e-05 3.590943e-05 3.530140e-05 3.470071e-05 3.764462e-05

386 1.134847e-01 1.135292e-01 1.134392e-01 1.134179e-01 1.133703e-01 4.038341e-05 3.609820e-05 3.548640e-05 3.488223e-05 3.784086e-05

387 1.159409e-01 1.159895e-01 1.158911e-01 1.158677e-01 1.158156e-01 4.056644e-05 3.626181e-05 3.564671e-05 3.503951e-05 3.801084e-05

388 1.181681e-01 1.182210e-01 1.181141e-01 1.180884e-01 1.180319e-01 4.072487e-05 3.640346e-05 3.578548e-05 3.517565e-05 3.815791e-05

389 1.201703e-01 1.202273e-01 1.201120e-01 1.200841e-01 1.200231e-01 4.086185e-05 3.652598e-05 3.590547e-05 3.529335e-05 3.828503e-05

390 1.219539e-01 1.220150e-01 1.218914e-01 1.218613e-01 1.217959e-01 4.098017e-05 3.663183e-05 3.600911e-05 3.539501e-05 3.839478e-05

391 1.235281e-01 1.235932e-01 1.234615e-01 1.234292e-01 1.233595e-01 4.108224e-05 3.672318e-05 3.609852e-05 3.548271e-05 3.848942e-05

392 1.249042e-01 1.249732e-01 1.248336e-01 1.247993e-01 1.247255e-01 4.117020e-05 3.680192e-05 3.617557e-05 3.555827e-05 3.857093e-05

393 1.260954e-01 1.261680e-01 1.260211e-01 1.259848e-01 1.259071e-01 4.124591e-05 3.686970e-05 3.624189e-05 3.562330e-05 3.864105e-05

394 1.271162e-01 1.271922e-01 1.270384e-01 1.270002e-01 1.269188e-01 4.131099e-05 3.692799e-05 3.629889e-05 3.567919e-05 3.870129e-05

395 1.279820e-01 1.280612e-01 1.279010e-01 1.278610e-01 1.277763e-01 4.136685e-05 3.697804e-05 3.634782e-05 3.572716e-05 3.875297e-05

396 1.287086e-01 1.287907e-01 1.286246e-01 1.285830e-01 1.284952e-01 4.141474e-05 3.702095e-05 3.638976e-05 3.576827e-05 3.879724e-05

397 1.293115e-01 1.293962e-01 1.292249e-01 1.291817e-01 1.290911e-01 4.145573e-05 3.705769e-05 3.642566e-05 3.580345e-05 3.883511e-05

398 1.298059e-01 1.298929e-01 1.297169e-01 1.296723e-01 1.295792e-01 4.149077e-05 3.708911e-05 3.645634e-05 3.583351e-05 3.886745e-05

399 1.302061e-01 1.302952e-01 1.301150e-01 1.300692e-01 1.299739e-01 4.152067e-05 3.711592e-05 3.648251e-05 3.585915e-05 3.889502e-05

400 1.305255e-01 1.306164e-01 1.304325e-01 1.303857e-01 1.302884e-01 4.154613e-05 3.713876e-05 3.650480e-05 3.588098e-05 3.891849e-05

401 1.307762e-01 1.308688e-01 1.306816e-01 1.306339e-01 1.305349e-01 4.156779e-05 3.715819e-05 3.652375e-05 3.589954e-05 3.893842e-05

402 1.309693e-01 1.310632e-01 1.308733e-01 1.308247e-01 1.307243e-01 4.158617e-05 3.717468e-05 3.653983e-05 3.591528e-05 3.895532e-05

403 1.311144e-01 1.312095e-01 1.310171e-01 1.309679e-01 1.308662e-01 4.160173e-05 3.718865e-05 3.655345e-05 3.592861e-05 3.896961e-05

404 1.312200e-01 1.313161e-01 1.311217e-01 1.310718e-01 1.309691e-01 4.161489e-05 3.720046e-05 3.656495e-05 3.593986e-05 3.898168e-05

405 1.312934e-01 1.313905e-01 1.311943e-01 1.311439e-01 1.310403e-01 4.162598e-05 3.721042e-05 3.657465e-05 3.594935e-05 3.899184e-05

406 1.313411e-01 1.314388e-01 1.312412e-01 1.311904e-01 1.310860e-01 4.163531e-05 3.721880e-05 3.658280e-05 3.595732e-05 3.900037e-05

407 1.313682e-01 1.314666e-01 1.312678e-01 1.312166e-01 1.311116e-01 4.164313e-05 3.722583e-05 3.658963e-05 3.596400e-05 3.900752e-05

408 1.313794e-01 1.314782e-01 1.312784e-01 1.312269e-01 1.311214e-01 4.164968e-05 3.723171e-05 3.659534e-05 3.596958e-05 3.901348e-05

409 1.313782e-01 1.314774e-01 1.312769e-01 1.312251e-01 1.311191e-01 4.165513e-05 3.723662e-05 3.660010e-05 3.597422e-05 3.901845e-05

410 1.313678e-01 1.314674e-01 1.312661e-01 1.312142e-01 1.311079e-01 4.165966e-05 3.724069e-05 3.660405e-05 3.597808e-05 3.902256e-05

411 1.313507e-01 1.314505e-01 1.312488e-01 1.311967e-01 1.310901e-01 4.166341e-05 3.724406e-05 3.660732e-05 3.598126e-05 3.902596e-05

412 1.313289e-01 1.314289e-01 1.312268e-01 1.311745e-01 1.310677e-01 4.166650e-05 3.724684e-05 3.661001e-05 3.598389e-05 3.902874e-05

413 1.313041e-01 1.314042e-01 1.312017e-01 1.311494e-01 1.310424e-01 4.166904e-05 3.724912e-05 3.661221e-05 3.598603e-05 3.903102e-05

414 1.312774e-01 1.313777e-01 1.311749e-01 1.311225e-01 1.310154e-01 4.167110e-05 3.725098e-05 3.661400e-05 3.598778e-05 3.903288e-05

415 1.312499e-01 1.313503e-01 1.311473e-01 1.310949e-01 1.309877e-01 4.167278e-05 3.725248e-05 3.661545e-05 3.598919e-05 3.903437e-05

416 1.312224e-01 1.313228e-01 1.311198e-01 1.310673e-01 1.309600e-01 4.167412e-05 3.725369e-05 3.661662e-05 3.599032e-05 3.903557e-05

417 1.311954e-01 1.312959e-01 1.310927e-01 1.310403e-01 1.309329e-01 4.167520e-05 3.725466e-05 3.661755e-05 3.599122e-05 3.903652e-05

418 1.311694e-01 1.312699e-01 1.310667e-01 1.310142e-01 1.309069e-01 4.167605e-05 3.725543e-05 3.661828e-05 3.599193e-05 3.903726e-05

419 1.311446e-01 1.312452e-01 1.310419e-01 1.309894e-01 1.308821e-01 4.167671e-05 3.725602e-05 3.661885e-05 3.599248e-05 3.903784e-05

420 1.311213e-01 1.312219e-01 1.310186e-01 1.309661e-01 1.308588e-01 4.167722e-05 3.725648e-05 3.661929e-05 3.599290e-05 3.903828e-05

421 1.310996e-01 1.312001e-01 1.309969e-01 1.309444e-01 1.308370e-01 4.167760e-05 3.725683e-05 3.661961e-05 3.599321e-05 3.903860e-05

422 1.310795e-01 1.311800e-01 1.309768e-01 1.309243e-01 1.308170e-01 4.167788e-05 3.725708e-05 3.661985e-05 3.599344e-05 3.903884e-05

423 1.310610e-01 1.311615e-01 1.309583e-01 1.309059e-01 1.307985e-01 4.167808e-05 3.725726e-05 3.662002e-05 3.599360e-05 3.903900e-05

424 1.310441e-01 1.311446e-01 1.309415e-01 1.308890e-01 1.307817e-01 4.167822e-05 3.725738e-05 3.662013e-05 3.599370e-05 3.903910e-05

425 1.310288e-01 1.311293e-01 1.309262e-01 1.308737e-01 1.307665e-01 4.167830e-05 3.725745e-05 3.662019e-05 3.599376e-05 3.903915e-05

426 1.310150e-01 1.311154e-01 1.309124e-01 1.308599e-01 1.307527e-01 4.167834e-05 3.725749e-05 3.662022e-05 3.599378e-05 3.903917e-05

427 1.310025e-01 1.311030e-01 1.308999e-01 1.308475e-01 1.307403e-01 4.167835e-05 3.725750e-05 3.662022e-05 3.599378e-05 3.903916e-05

428 1.309914e-01 1.310918e-01 1.308888e-01 1.308364e-01 1.307292e-01 4.167833e-05 3.725748e-05 3.662020e-05 3.599376e-05 3.903914e-05

429 1.309815e-01 1.310819e-01 1.308789e-01 1.308266e-01 1.307194e-01 4.167829e-05 3.725745e-05 3.662017e-05 3.599372e-05 3.903909e-05

430 1.309727e-01 1.310730e-01 1.308701e-01 1.308178e-01 1.307106e-01 4.167825e-05 3.725741e-05 3.662012e-05 3.599368e-05 3.903904e-05

431 1.309649e-01 1.310652e-01 1.308624e-01 1.308100e-01 1.307029e-01 4.167819e-05 3.725736e-05 3.662007e-05 3.599362e-05 3.903898e-05

432 1.309580e-01 1.310583e-01 1.308555e-01 1.308032e-01 1.306961e-01 4.167813e-05 3.725730e-05 3.662001e-05 3.599357e-05 3.903891e-05

433 1.309520e-01 1.310523e-01 1.308495e-01 1.307972e-01 1.306901e-01 4.167806e-05 3.725724e-05 3.661996e-05 3.599351e-05 3.903884e-05

434 1.309467e-01 1.310470e-01 1.308443e-01 1.307920e-01 1.306849e-01 4.167800e-05 3.725718e-05 3.661990e-05 3.599344e-05 3.903878e-05

435 1.309422e-01 1.310424e-01 1.308397e-01 1.307875e-01 1.306804e-01 4.167793e-05 3.725712e-05 3.661984e-05 3.599339e-05 3.903871e-05

436 1.309382e-01 1.310384e-01 1.308358e-01 1.307835e-01 1.306765e-01 4.167787e-05 3.725707e-05 3.661978e-05 3.599333e-05 3.903865e-05

437 1.309347e-01 1.310350e-01 1.308323e-01 1.307801e-01 1.306730e-01 4.167781e-05 3.725701e-05 3.661972e-05 3.599327e-05 3.903859e-05

438 1.309318e-01 1.310320e-01 1.308294e-01 1.307771e-01 1.306701e-01 4.167775e-05 3.725696e-05 3.661967e-05 3.599322e-05 3.903853e-05

439 1.309292e-01 1.310294e-01 1.308268e-01 1.307746e-01 1.306676e-01 4.167769e-05 3.725691e-05 3.661962e-05 3.599317e-05 3.903848e-05

440 1.309270e-01 1.310272e-01 1.308247e-01 1.307724e-01 1.306655e-01 4.167764e-05 3.725686e-05 3.661957e-05 3.599312e-05 3.903843e-05

441 1.309252e-01 1.310254e-01 1.308228e-01 1.307706e-01 1.306636e-01 4.167759e-05 3.725682e-05 3.661953e-05 3.599308e-05 3.903838e-05

442 1.309236e-01 1.310238e-01 1.308213e-01 1.307691e-01 1.306621e-01 4.167755e-05 3.725678e-05 3.661949e-05 3.599304e-05 3.903834e-05

443 1.309223e-01 1.310225e-01 1.308199e-01 1.307677e-01 1.306608e-01 4.167751e-05 3.725674e-05 3.661946e-05 3.599301e-05 3.903830e-05

444 1.309212e-01 1.310213e-01 1.308188e-01 1.307666e-01 1.306597e-01 4.167747e-05 3.725671e-05 3.661942e-05 3.599298e-05 3.903826e-05

445 1.309202e-01 1.310204e-01 1.308179e-01 1.307657e-01 1.306588e-01 4.167744e-05 3.725668e-05 3.661939e-05 3.599295e-05 3.903823e-05

446 1.309195e-01 1.310196e-01 1.308171e-01 1.307649e-01 1.306580e-01 4.167741e-05 3.725665e-05 3.661937e-05 3.599292e-05 3.903820e-05

447 1.309188e-01 1.310190e-01 1.308165e-01 1.307643e-01 1.306574e-01 4.167738e-05 3.725663e-05 3.661934e-05 3.599290e-05 3.903818e-05

448 1.309183e-01 1.310185e-01 1.308160e-01 1.307638e-01 1.306569e-01 4.167736e-05 3.725661e-05 3.661932e-05 3.599287e-05 3.903815e-05

449 1.309179e-01 1.310180e-01 1.308156e-01 1.307634e-01 1.306564e-01 4.167734e-05 3.725659e-05 3.661930e-05 3.599286e-05 3.903813e-05

450 1.309175e-01 1.310177e-01 1.308152e-01 1.307630e-01 1.306561e-01 4.167732e-05 3.725657e-05 3.661928e-05 3.599284e-05 3.903811e-05

451 1.309173e-01 1.310174e-01 1.308149e-01 1.307628e-01 1.306558e-01 4.167730e-05 3.725655e-05 3.661927e-05 3.599282e-05 3.903810e-05

452 1.309170e-01 1.310172e-01 1.308147e-01 1.307626e-01 1.306556e-01 4.167728e-05 3.725654e-05 3.661926e-05 3.599281e-05 3.903808e-05

453 1.309169e-01 1.310170e-01 1.308146e-01 1.307624e-01 1.306555e-01 4.167727e-05 3.725653e-05 3.661924e-05 3.599280e-05 3.903807e-05

454 1.309167e-01 1.310169e-01 1.308144e-01 1.307623e-01 1.306553e-01 4.167726e-05 3.725652e-05 3.661923e-05 3.599279e-05 3.903806e-05

455 1.309167e-01 1.310168e-01 1.308143e-01 1.307622e-01 1.306553e-01 4.167725e-05 3.725651e-05 3.661922e-05 3.599278e-05 3.903805e-05

456 1.309166e-01 1.310167e-01 1.308143e-01 1.307621e-01 1.306552e-01 4.167724e-05 3.725650e-05 3.661922e-05 3.599277e-05 3.903804e-05

457 1.309165e-01 1.310167e-01 1.308142e-01 1.307621e-01 1.306551e-01 4.167723e-05 3.725649e-05 3.661921e-05 3.599276e-05 3.903804e-05

458 1.309165e-01 1.310166e-01 1.308142e-01 1.307620e-01 1.306551e-01 4.167722e-05 3.725649e-05 3.661920e-05 3.599276e-05 3.903803e-05

459 1.309165e-01 1.310166e-01 1.308142e-01 1.307620e-01 1.306551e-01 4.167722e-05 3.725648e-05 3.661920e-05 3.599275e-05 3.903802e-05

460 1.309165e-01 1.310166e-01 1.308142e-01 1.307620e-01 1.306551e-01 4.167721e-05 3.725648e-05 3.661919e-05 3.599275e-05 3.903802e-05

461 1.309165e-01 1.310166e-01 1.308142e-01 1.307620e-01 1.306551e-01 4.167721e-05 3.725647e-05 3.661919e-05 3.599275e-05 3.903801e-05

462 1.309165e-01 1.310166e-01 1.308142e-01 1.307620e-01 1.306551e-01 4.167721e-05 3.725647e-05 3.661919e-05 3.599274e-05 3.903801e-05

463 1.309165e-01 1.310167e-01 1.308142e-01 1.307621e-01 1.306551e-01 4.167720e-05 3.725647e-05 3.661918e-05 3.599274e-05 3.903801e-05

464 1.309165e-01 1.310167e-01 1.308142e-01 1.307621e-01 1.306552e-01 4.167720e-05 3.725646e-05 3.661918e-05 3.599274e-05 3.903801e-05

465 1.309166e-01 1.310167e-01 1.308143e-01 1.307621e-01 1.306552e-01 4.167720e-05 3.725646e-05 3.661918e-05 3.599274e-05 3.903800e-05

466 1.309166e-01 1.310167e-01 1.308143e-01 1.307621e-01 1.306552e-01 4.167720e-05 3.725646e-05 3.661918e-05 3.599273e-05 3.903800e-05

467 1.309166e-01 1.310167e-01 1.308143e-01 1.307621e-01 1.306552e-01 4.167719e-05 3.725646e-05 3.661918e-05 3.599273e-05 3.903800e-05

468 1.309166e-01 1.310168e-01 1.308143e-01 1.307622e-01 1.306552e-01 4.167719e-05 3.725646e-05 3.661918e-05 3.599273e-05 3.903800e-05

469 1.309166e-01 1.310168e-01 1.308143e-01 1.307622e-01 1.306553e-01 4.167719e-05 3.725646e-05 3.661917e-05 3.599273e-05 3.903800e-05

470 1.309167e-01 1.310168e-01 1.308144e-01 1.307622e-01 1.306553e-01 4.167719e-05 3.725646e-05 3.661917e-05 3.599273e-05 3.903800e-05

471 1.309167e-01 1.310168e-01 1.308144e-01 1.307622e-01 1.306553e-01 4.167719e-05 3.725646e-05 3.661917e-05 3.599273e-05 3.903800e-05

472 1.309167e-01 1.310168e-01 1.308144e-01 1.307622e-01 1.306553e-01 4.167719e-05 3.725645e-05 3.661917e-05 3.599273e-05 3.903800e-05

473 1.309167e-01 1.310168e-01 1.308144e-01 1.307623e-01 1.306553e-01 4.167719e-05 3.725645e-05 3.661917e-05 3.599273e-05 3.903800e-05

474 1.309167e-01 1.310169e-01 1.308144e-01 1.307623e-01 1.306553e-01 4.167719e-05 3.725645e-05 3.661917e-05 3.599273e-05 3.903800e-05

475 1.309167e-01 1.310169e-01 1.308144e-01 1.307623e-01 1.306554e-01 4.167719e-05 3.725645e-05 3.661917e-05 3.599273e-05 3.903799e-05

476 1.309168e-01 1.310169e-01 1.308145e-01 1.307623e-01 1.306554e-01 4.167719e-05 3.725645e-05 3.661917e-05 3.599273e-05 3.903799e-05

477 1.309168e-01 1.310169e-01 1.308145e-01 1.307623e-01 1.306554e-01 4.167719e-05 3.725645e-05 3.661917e-05 3.599273e-05 3.903799e-05

478 1.309168e-01 1.310169e-01 1.308145e-01 1.307623e-01 1.306554e-01 4.167719e-05 3.725645e-05 3.661917e-05 3.599273e-05 3.903799e-05

479 1.309168e-01 1.310169e-01 1.308145e-01 1.307623e-01 1.306554e-01 4.167719e-05 3.725645e-05 3.661917e-05 3.599273e-05 3.903799e-05

480 1.309168e-01 1.310169e-01 1.308145e-01 1.307623e-01 1.306554e-01 4.167719e-05 3.725645e-05 3.661917e-05 3.599273e-05 3.903799e-05

481 1.309168e-01 1.310169e-01 1.308145e-01 1.307623e-01 1.306554e-01 4.167719e-05 3.725645e-05 3.661917e-05 3.599273e-05 3.903799e-05

482 1.309168e-01 1.310169e-01 1.308145e-01 1.307623e-01 1.306554e-01 4.167719e-05 3.725645e-05 3.661917e-05 3.599273e-05 3.903799e-05

483 1.309168e-01 1.310169e-01 1.308145e-01 1.307624e-01 1.306554e-01 4.167719e-05 3.725645e-05 3.661917e-05 3.599273e-05 3.903799e-05

484 1.309168e-01 1.310170e-01 1.308145e-01 1.307624e-01 1.306554e-01 4.167719e-05 3.725645e-05 3.661917e-05 3.599273e-05 3.903799e-05

485 1.309168e-01 1.310170e-01 1.308145e-01 1.307624e-01 1.306554e-01 4.167719e-05 3.725645e-05 3.661917e-05 3.599273e-05 3.903799e-05

486 1.309168e-01 1.310170e-01 1.308145e-01 1.307624e-01 1.306554e-01 4.167719e-05 3.725645e-05 3.661917e-05 3.599273e-05 3.903799e-05

487 1.309168e-01 1.310170e-01 1.308145e-01 1.307624e-01 1.306554e-01 4.167719e-05 3.725645e-05 3.661917e-05 3.599273e-05 3.903799e-05

488 1.309168e-01 1.310170e-01 1.308145e-01 1.307624e-01 1.306555e-01 4.167719e-05 3.725645e-05 3.661917e-05 3.599273e-05 3.903799e-05

489 1.309168e-01 1.310170e-01 1.308145e-01 1.307624e-01 1.306555e-01 4.167719e-05 3.725645e-05 3.661917e-05 3.599273e-05 3.903799e-05

490 1.309168e-01 1.310170e-01 1.308145e-01 1.307624e-01 1.306555e-01 4.167719e-05 3.725645e-05 3.661917e-05 3.599273e-05 3.903799e-05

491 1.309168e-01 1.310170e-01 1.308145e-01 1.307624e-01 1.306555e-01 4.167719e-05 3.725645e-05 3.661917e-05 3.599273e-05 3.903799e-05

492 1.309168e-01 1.310170e-01 1.308145e-01 1.307624e-01 1.306555e-01 4.167719e-05 3.725645e-05 3.661917e-05 3.599273e-05 3.903799e-05

493 1.309168e-01 1.310170e-01 1.308145e-01 1.307624e-01 1.306555e-01 4.167719e-05 3.725645e-05 3.661917e-05 3.599273e-05 3.903799e-05

494 1.309168e-01 1.310170e-01 1.308145e-01 1.307624e-01 1.306555e-01 4.167719e-05 3.725645e-05 3.661917e-05 3.599273e-05 3.903799e-05

495 1.309168e-01 1.310170e-01 1.308145e-01 1.307624e-01 1.306555e-01 4.167719e-05 3.725645e-05 3.661917e-05 3.599273e-05 3.903799e-05

496 1.309168e-01 1.310170e-01 1.308145e-01 1.307624e-01 1.306555e-01 4.167719e-05 3.725645e-05 3.661917e-05 3.599273e-05 3.903799e-05

497 1.309168e-01 1.310170e-01 1.308145e-01 1.307624e-01 1.306555e-01 4.167719e-05 3.725645e-05 3.661917e-05 3.599273e-05 3.903799e-05

498 1.309168e-01 1.310170e-01 1.308145e-01 1.307624e-01 1.306555e-01 4.167719e-05 3.725645e-05 3.661917e-05 3.599273e-05 3.903799e-05

499 1.309168e-01 1.310170e-01 1.308145e-01 1.307624e-01 1.306555e-01 4.167719e-05 3.725645e-05 3.661917e-05 3.599273e-05 3.903799e-05

500 1.309168e-01 1.310170e-01 1.308145e-01 1.307624e-01 1.306555e-01 4.167719e-05 3.725645e-05 3.661917e-05 3.599273e-05 3.903799e-05

501 1.309168e-01 1.310170e-01 1.308145e-01 1.307624e-01 1.306555e-01 4.167719e-05 3.725645e-05 3.661917e-05 3.599273e-05 3.903799e-05

502 1.309168e-01 1.310170e-01 1.308145e-01 1.307624e-01 1.306555e-01 4.167719e-05 3.725645e-05 3.661917e-05 3.599273e-05 3.903799e-05

503 1.309168e-01 1.310170e-01 1.308145e-01 1.307624e-01 1.306555e-01 4.167719e-05 3.725645e-05 3.661917e-05 3.599273e-05 3.903799e-05

504 1.309168e-01 1.310170e-01 1.308145e-01 1.307624e-01 1.306555e-01 4.167719e-05 3.725645e-05 3.661917e-05 3.599273e-05 3.903799e-05

505 1.309168e-01 1.310170e-01 1.308145e-01 1.307624e-01 1.306555e-01 4.167719e-05 3.725645e-05 3.661917e-05 3.599273e-05 3.903799e-05

506 1.309168e-01 1.310170e-01 1.308145e-01 1.307624e-01 1.306555e-01 4.167719e-05 3.725645e-05 3.661917e-05 3.599273e-05 3.903799e-05

507 1.309168e-01 1.310170e-01 1.308145e-01 1.307624e-01 1.306555e-01 4.167719e-05 3.725645e-05 3.661917e-05 3.599273e-05 3.903799e-05

508 1.309168e-01 1.310170e-01 1.308145e-01 1.307624e-01 1.306555e-01 4.167719e-05 3.725645e-05 3.661917e-05 3.599273e-05 3.903799e-05

509 1.309168e-01 1.310170e-01 1.308145e-01 1.307624e-01 1.306555e-01 4.167719e-05 3.725645e-05 3.661917e-05 3.599273e-05 3.903799e-05

510 1.309168e-01 1.310170e-01 1.308145e-01 1.307624e-01 1.306555e-01 4.167719e-05 3.725645e-05 3.661917e-05 3.599273e-05 3.903799e-05

511 1.309168e-01 1.310170e-01 1.308145e-01 1.307624e-01 1.306555e-01 4.167719e-05 3.725645e-05 3.661917e-05 3.599273e-05 3.903799e-05

512 1.309168e-01 1.310170e-01 1.308145e-01 1.307624e-01 1.306555e-01 4.167719e-05 3.725645e-05 3.661917e-05 3.599273e-05 3.903799e-05

513 1.309168e-01 1.310170e-01 1.308145e-01 1.307624e-01 1.306555e-01 4.167719e-05 3.725645e-05 3.661917e-05 3.599273e-05 3.903799e-05

514 1.309168e-01 1.310170e-01 1.308145e-01 1.307624e-01 1.306555e-01 4.167719e-05 3.725645e-05 3.661917e-05 3.599273e-05 3.903799e-05

515 1.309168e-01 1.310170e-01 1.308145e-01 1.307624e-01 1.306555e-01 4.167719e-05 3.725645e-05 3.661917e-05 3.599273e-05 3.903799e-05

516 1.309168e-01 1.310170e-01 1.308145e-01 1.307624e-01 1.306555e-01 4.167719e-05 3.725645e-05 3.661917e-05 3.599273e-05 3.903799e-05

517 1.309168e-01 1.310170e-01 1.308145e-01 1.307624e-01 1.306555e-01 4.167719e-05 3.725645e-05 3.661917e-05 3.599273e-05 3.903799e-05

518 1.309168e-01 1.310170e-01 1.308145e-01 1.307624e-01 1.306555e-01 4.167719e-05 3.725645e-05 3.661917e-05 3.599273e-05 3.903799e-05

519 1.309168e-01 1.310170e-01 1.308145e-01 1.307624e-01 1.306555e-01 4.167719e-05 3.725645e-05 3.661917e-05 3.599273e-05 3.903799e-05

520 1.309168e-01 1.310170e-01 1.308145e-01 1.307624e-01 1.306555e-01 4.167719e-05 3.725645e-05 3.661917e-05 3.599273e-05 3.903799e-05

521 1.309168e-01 1.310170e-01 1.308145e-01 1.307624e-01 1.306555e-01 4.167719e-05 3.725645e-05 3.661917e-05 3.599273e-05 3.903799e-05

522 1.309168e-01 1.310170e-01 1.308145e-01 1.307624e-01 1.306555e-01 4.167719e-05 3.725645e-05 3.661917e-05 3.599273e-05 3.903799e-05

523 1.309168e-01 1.310170e-01 1.308145e-01 1.307624e-01 1.306555e-01 4.167719e-05 3.725645e-05 3.661917e-05 3.599273e-05 3.903799e-05

524 1.309168e-01 1.310170e-01 1.308145e-01 1.307624e-01 1.306555e-01 4.167719e-05 3.725645e-05 3.661917e-05 3.599273e-05 3.903799e-05

525 1.309168e-01 1.310170e-01 1.308145e-01 1.307624e-01 1.306555e-01 4.167719e-05 3.725645e-05 3.661917e-05 3.599273e-05 3.903799e-05

526 1.309168e-01 1.310170e-01 1.308145e-01 1.307624e-01 1.306555e-01 4.167719e-05 3.725645e-05 3.661917e-05 3.599273e-05 3.903799e-05

527 1.309168e-01 1.310170e-01 1.308145e-01 1.307624e-01 1.306555e-01 4.167719e-05 3.725645e-05 3.661917e-05 3.599273e-05 3.903799e-05

528 1.309168e-01 1.310170e-01 1.308145e-01 1.307624e-01 1.306555e-01 4.167719e-05 3.725645e-05 3.661917e-05 3.599273e-05 3.903799e-05

529 1.309168e-01 1.310170e-01 1.308145e-01 1.307624e-01 1.306555e-01 4.167719e-05 3.725645e-05 3.661917e-05 3.599273e-05 3.903799e-05

530 1.309168e-01 1.310170e-01 1.308145e-01 1.307624e-01 1.306555e-01 4.167719e-05 3.725645e-05 3.661917e-05 3.599273e-05 3.903799e-05

531 1.309168e-01 1.310170e-01 1.308145e-01 1.307624e-01 1.306555e-01 4.167719e-05 3.725645e-05 3.661917e-05 3.599273e-05 3.903799e-05

532 1.309168e-01 1.310170e-01 1.308145e-01 1.307624e-01 1.306555e-01 4.167719e-05 3.725645e-05 3.661917e-05 3.599273e-05 3.903799e-05

533 1.309168e-01 1.310170e-01 1.308145e-01 1.307624e-01 1.306555e-01 4.167719e-05 3.725645e-05 3.661917e-05 3.599273e-05 3.903799e-05

534 1.309168e-01 1.310170e-01 1.308145e-01 1.307624e-01 1.306555e-01 4.167719e-05 3.725645e-05 3.661917e-05 3.599273e-05 3.903799e-05

535 1.309168e-01 1.310170e-01 1.308145e-01 1.307624e-01 1.306555e-01 4.167719e-05 3.725645e-05 3.661917e-05 3.599273e-05 3.903799e-05

536 1.309168e-01 1.310170e-01 1.308145e-01 1.307624e-01 1.306555e-01 4.167719e-05 3.725645e-05 3.661917e-05 3.599273e-05 3.903799e-05

537 1.309168e-01 1.310170e-01 1.308145e-01 1.307624e-01 1.306555e-01 4.167719e-05 3.725645e-05 3.661917e-05 3.599273e-05 3.903799e-05

538 1.309168e-01 1.310170e-01 1.308145e-01 1.307624e-01 1.306555e-01 4.167719e-05 3.725645e-05 3.661917e-05 3.599273e-05 3.903799e-05

539 1.309168e-01 1.310170e-01 1.308145e-01 1.307624e-01 1.306555e-01 4.167719e-05 3.725645e-05 3.661917e-05 3.599273e-05 3.903799e-05

540 1.309168e-01 1.310170e-01 1.308145e-01 1.307624e-01 1.306555e-01 4.167719e-05 3.725645e-05 3.661917e-05 3.599273e-05 3.903799e-05

541 1.309168e-01 1.310170e-01 1.308145e-01 1.307624e-01 1.306555e-01 4.167719e-05 3.725645e-05 3.661917e-05 3.599273e-05 3.903799e-05

542 1.309924e-01 1.310924e-01 1.308904e-01 1.308383e-01 1.307316e-01 1.431034e-03 1.318754e-03 1.302951e-03 1.288490e-03 1.370272e-03

543 1.310883e-01 1.311879e-01 1.309864e-01 1.309345e-01 1.308280e-01 2.754592e-03 2.538001e-03 2.507501e-03 2.479640e-03 2.637399e-03

544 1.312087e-01 1.313081e-01 1.311072e-01 1.310554e-01 1.309492e-01 4.014198e-03 3.696902e-03 3.652181e-03 3.611361e-03 3.842308e-03

545 1.313587e-01 1.314577e-01 1.312575e-01 1.312059e-01 1.311001e-01 5.211796e-03 4.797440e-03 4.738980e-03 4.685645e-03 4.986968e-03

546 1.315436e-01 1.316422e-01 1.314429e-01 1.313915e-01 1.312862e-01 6.349392e-03 5.841641e-03 5.769926e-03 5.704521e-03 6.073417e-03

547 1.317697e-01 1.318678e-01 1.316696e-01 1.316183e-01 1.315137e-01 7.429018e-03 6.831542e-03 6.747055e-03 6.670025e-03 7.103729e-03

548 1.320436e-01 1.321410e-01 1.319441e-01 1.318932e-01 1.317891e-01 8.452726e-03 7.769183e-03 7.672407e-03 7.584194e-03 8.079983e-03

549 1.323727e-01 1.324694e-01 1.322739e-01 1.322233e-01 1.321201e-01 9.422578e-03 8.656600e-03 8.548013e-03 8.449057e-03 9.004256e-03

550 1.327649e-01 1.328608e-01 1.326670e-01 1.326168e-01 1.325145e-01 1.034063e-02 9.495815e-03 9.375892e-03 9.266629e-03 9.878615e-03

551 1.332291e-01 1.333239e-01 1.331322e-01 1.330824e-01 1.329812e-01 1.120895e-02 1.028883e-02 1.015804e-02 1.003890e-02 1.070511e-02

552 1.337746e-01 1.338683e-01 1.336788e-01 1.336296e-01 1.335295e-01 1.202956e-02 1.103763e-02 1.089643e-02 1.076783e-02 1.148576e-02

553 1.344115e-01 1.345039e-01 1.343171e-01 1.342684e-01 1.341697e-01 1.280448e-02 1.174415e-02 1.159300e-02 1.145535e-02 1.222257e-02

554 1.351505e-01 1.352414e-01 1.350576e-01 1.350096e-01 1.349125e-01 1.353571e-02 1.241030e-02 1.224964e-02 1.210336e-02 1.291749e-02

555 1.360030e-01 1.360923e-01 1.359119e-01 1.358646e-01 1.357694e-01 1.422519e-02 1.303795e-02 1.286822e-02 1.271370e-02 1.357245e-02

556 1.369809e-01 1.370683e-01 1.368917e-01 1.368453e-01 1.367521e-01 1.487484e-02 1.362893e-02 1.345055e-02 1.328817e-02 1.418932e-02

557 1.380965e-01 1.381817e-01 1.380095e-01 1.379640e-01 1.378731e-01 1.548654e-02 1.418501e-02 1.399839e-02 1.382854e-02 1.476992e-02

558 1.393623e-01 1.394452e-01 1.392777e-01 1.392333e-01 1.391449e-01 1.606211e-02 1.470792e-02 1.451346e-02 1.433649e-02 1.531604e-02

559 1.407910e-01 1.408712e-01 1.407090e-01 1.406658e-01 1.405802e-01 1.660333e-02 1.519933e-02 1.499742e-02 1.481368e-02 1.582941e-02

560 1.423949e-01 1.424724e-01 1.423158e-01 1.422740e-01 1.421914e-01 1.711192e-02 1.566085e-02 1.545186e-02 1.526171e-02 1.631168e-02

561 1.441862e-01 1.442606e-01 1.441103e-01 1.440700e-01 1.439907e-01 1.758955e-02 1.609405e-02 1.587835e-02 1.568210e-02 1.676447e-02

562 1.461760e-01 1.462471e-01 1.461035e-01 1.460648e-01 1.459890e-01 1.803783e-02 1.650043e-02 1.627837e-02 1.607634e-02 1.718934e-02

563 1.483744e-01 1.484419e-01 1.483055e-01 1.482685e-01 1.481966e-01 1.845831e-02 1.688145e-02 1.665335e-02 1.644586e-02 1.758778e-02

564 1.507898e-01 1.508536e-01 1.507248e-01 1.506897e-01 1.506217e-01 1.885250e-02 1.723848e-02 1.700468e-02 1.679201e-02 1.796124e-02

565 1.534289e-01 1.534888e-01 1.533678e-01 1.533347e-01 1.532710e-01 1.922184e-02 1.757287e-02 1.733367e-02 1.711610e-02 1.831108e-02

566 1.562960e-01 1.563517e-01 1.562390e-01 1.562080e-01 1.561485e-01 1.956770e-02 1.788588e-02 1.764158e-02 1.741939e-02 1.863865e-02

567 1.593927e-01 1.594443e-01 1.593400e-01 1.593111e-01 1.592561e-01 1.989140e-02 1.817874e-02 1.792963e-02 1.770306e-02 1.894519e-02

568 1.627181e-01 1.627656e-01 1.626696e-01 1.626429e-01 1.625924e-01 2.019421e-02 1.845261e-02 1.819895e-02 1.796827e-02 1.923192e-02

569 1.662684e-01 1.663117e-01 1.662242e-01 1.661997e-01 1.661536e-01 2.047733e-02 1.870861e-02 1.845066e-02 1.821609e-02 1.949999e-02

570 1.700370e-01 1.700762e-01 1.699970e-01 1.699747e-01 1.699330e-01 2.074193e-02 1.894778e-02 1.868579e-02 1.844756e-02 1.975050e-02

571 1.740148e-01 1.740501e-01 1.739789e-01 1.739587e-01 1.739212e-01 2.098908e-02 1.917114e-02 1.890534e-02 1.866366e-02 1.998449e-02

572 1.781905e-01 1.782219e-01 1.781584e-01 1.781403e-01 1.781069e-01 2.121985e-02 1.937963e-02 1.911026e-02 1.886532e-02 2.020295e-02

573 1.825507e-01 1.825785e-01 1.825224e-01 1.825062e-01 1.824767e-01 2.143521e-02 1.957416e-02 1.930142e-02 1.905343e-02 2.040683e-02

574 1.870810e-01 1.871055e-01 1.870561e-01 1.870419e-01 1.870159e-01 2.163610e-02 1.975560e-02 1.947969e-02 1.922882e-02 2.059701e-02

575 1.917660e-01 1.917873e-01 1.917443e-01 1.917318e-01 1.917092e-01 2.182343e-02 1.992475e-02 1.964586e-02 1.939230e-02 2.077435e-02

576 1.965899e-01 1.966084e-01 1.965711e-01 1.965602e-01 1.965406e-01 2.199802e-02 2.008238e-02 1.980070e-02 1.954460e-02 2.093964e-02

577 2.015370e-01 2.015529e-01 2.015209e-01 2.015114e-01 2.014946e-01 2.216069e-02 2.022922e-02 1.994492e-02 1.968644e-02 2.109365e-02

578 2.065923e-01 2.066059e-01 2.065784e-01 2.065702e-01 2.065558e-01 2.231219e-02 2.036596e-02 2.007919e-02 1.981848e-02 2.123709e-02

579 2.117411e-01 2.117527e-01 2.117293e-01 2.117223e-01 2.117101e-01 2.245322e-02 2.049324e-02 2.020416e-02 1.994136e-02 2.137063e-02

580 2.169703e-01 2.169801e-01 2.169603e-01 2.169543e-01 2.169439e-01 2.258446e-02 2.061167e-02 2.032043e-02 2.005566e-02 2.149490e-02

581 2.222675e-01 2.222759e-01 2.222591e-01 2.222539e-01 2.222451e-01 2.270654e-02 2.072184e-02 2.042857e-02 2.016196e-02 2.161052e-02

582 2.276218e-01 2.276289e-01 2.276147e-01 2.276102e-01 2.276028e-01 2.282007e-02 2.082427e-02 2.052910e-02 2.026077e-02 2.171805e-02

583 2.330234e-01 2.330293e-01 2.330173e-01 2.330134e-01 2.330072e-01 2.292560e-02 2.091948e-02 2.062254e-02 2.035259e-02 2.181801e-02

584 2.384636e-01 2.384687e-01 2.384584e-01 2.384550e-01 2.384497e-01 2.302367e-02 2.100795e-02 2.070934e-02 2.043789e-02 2.191091e-02

585 2.439351e-01 2.439395e-01 2.439307e-01 2.439276e-01 2.439230e-01 2.311476e-02 2.109013e-02 2.078997e-02 2.051710e-02 2.199721e-02

586 2.494315e-01 2.494353e-01 2.494276e-01 2.494248e-01 2.494208e-01 2.319935e-02 2.116644e-02 2.086482e-02 2.059064e-02 2.207736e-02

587 2.549473e-01 2.549508e-01 2.549438e-01 2.549412e-01 2.549376e-01 2.327787e-02 2.123727e-02 2.093429e-02 2.065888e-02 2.215178e-02

588 2.604780e-01 2.604812e-01 2.604748e-01 2.604722e-01 2.604689e-01 2.335074e-02 2.130300e-02 2.099876e-02 2.072219e-02 2.222085e-02

589 2.660197e-01 2.660228e-01 2.660167e-01 2.660141e-01 2.660109e-01 2.341834e-02 2.136398e-02 2.105855e-02 2.078091e-02 2.228493e-02

590 2.715693e-01 2.715723e-01 2.715662e-01 2.715636e-01 2.715604e-01 2.348102e-02 2.142053e-02 2.111399e-02 2.083535e-02 2.234436e-02

591 2.771240e-01 2.771271e-01 2.771209e-01 2.771181e-01 2.771148e-01 2.353914e-02 2.147296e-02 2.116539e-02 2.088580e-02 2.239948e-02

592 2.826818e-01 2.826850e-01 2.826785e-01 2.826754e-01 2.826719e-01 2.359301e-02 2.152155e-02 2.121302e-02 2.093256e-02 2.245056e-02

593 2.882408e-01 2.882443e-01 2.882372e-01 2.882337e-01 2.882300e-01 2.364291e-02 2.156657e-02 2.125714e-02 2.097587e-02 2.249790e-02

594 2.937995e-01 2.938034e-01 2.937955e-01 2.937916e-01 2.937875e-01 2.368914e-02 2.160827e-02 2.129800e-02 2.101597e-02 2.254176e-02

595 2.993567e-01 2.993611e-01 2.993523e-01 2.993478e-01 2.993432e-01 2.373194e-02 2.164689e-02 2.133584e-02 2.105310e-02 2.258238e-02

596 3.049116e-01 3.049165e-01 3.049065e-01 3.049015e-01 3.048962e-01 2.377156e-02 2.168263e-02 2.137086e-02 2.108746e-02 2.261998e-02

597 3.104631e-01 3.104688e-01 3.104574e-01 3.104516e-01 3.104456e-01 2.380823e-02 2.171571e-02 2.140326e-02 2.111925e-02 2.265479e-02

598 3.160108e-01 3.160172e-01 3.160042e-01 3.159975e-01 3.159906e-01 2.384215e-02 2.174632e-02 2.143324e-02 2.114865e-02 2.268700e-02

599 3.215538e-01 3.215612e-01 3.215462e-01 3.215386e-01 3.215307e-01 2.387352e-02 2.177463e-02 2.146096e-02 2.117584e-02 2.271680e-02

600 3.270918e-01 3.271003e-01 3.270831e-01 3.270743e-01 3.270653e-01 2.390253e-02 2.180081e-02 2.148659e-02 2.120098e-02 2.274435e-02

601 3.326242e-01 3.326339e-01 3.326143e-01 3.326042e-01 3.325938e-01 2.392934e-02 2.182500e-02 2.151028e-02 2.122421e-02 2.276983e-02

602 3.381506e-01 3.381618e-01 3.381392e-01 3.381277e-01 3.381157e-01 2.395412e-02 2.184737e-02 2.153217e-02 2.124567e-02 2.279337e-02

603 3.436706e-01 3.436833e-01 3.436575e-01 3.436443e-01 3.436305e-01 2.397701e-02 2.186803e-02 2.155240e-02 2.126549e-02 2.281513e-02

604 3.491836e-01 3.491982e-01 3.491686e-01 3.491535e-01 3.491377e-01 2.399815e-02 2.188711e-02 2.157107e-02 2.128380e-02 2.283523e-02

605 3.546892e-01 3.547059e-01 3.546721e-01 3.546547e-01 3.546367e-01 2.401767e-02 2.190473e-02 2.158831e-02 2.130070e-02 2.285379e-02

606 3.601869e-01 3.602059e-01 3.601673e-01 3.601475e-01 3.601269e-01 2.403569e-02 2.192100e-02 2.160423e-02 2.131629e-02 2.287093e-02

607 3.656761e-01 3.656978e-01 3.656537e-01 3.656311e-01 3.656075e-01 2.405232e-02 2.193601e-02 2.161891e-02 2.133068e-02 2.288675e-02

608 3.711562e-01 3.711809e-01 3.711307e-01 3.711048e-01 3.710779e-01 2.406766e-02 2.194986e-02 2.163246e-02 2.134395e-02 2.290135e-02

609 3.766264e-01 3.766546e-01 3.765974e-01 3.765679e-01 3.765372e-01 2.408180e-02 2.196263e-02 2.164495e-02 2.135619e-02 2.291482e-02

610 3.820862e-01 3.821182e-01 3.820531e-01 3.820195e-01 3.819844e-01 2.409485e-02 2.197441e-02 2.165647e-02 2.136747e-02 2.292724e-02

611 3.875345e-01 3.875710e-01 3.874968e-01 3.874585e-01 3.874185e-01 2.410687e-02 2.198528e-02 2.166709e-02 2.137787e-02 2.293869e-02

612 3.929704e-01 3.930119e-01 3.929275e-01 3.928839e-01 3.928383e-01 2.411796e-02 2.199529e-02 2.167688e-02 2.138745e-02 2.294925e-02

613 3.983929e-01 3.984401e-01 3.983442e-01 3.982945e-01 3.982426e-01 2.412817e-02 2.200451e-02 2.168589e-02 2.139628e-02 2.295898e-02

614 4.038009e-01 4.038544e-01 4.037455e-01 4.036890e-01 4.036299e-01 2.413757e-02 2.201300e-02 2.169420e-02 2.140441e-02 2.296794e-02

615 4.091929e-01 4.092537e-01 4.091300e-01 4.090658e-01 4.089985e-01 2.414623e-02 2.202083e-02 2.170184e-02 2.141189e-02 2.297620e-02

616 4.145677e-01 4.146366e-01 4.144962e-01 4.144232e-01 4.143468e-01 2.415420e-02 2.202803e-02 2.170888e-02 2.141878e-02 2.298380e-02

617 4.199236e-01 4.200016e-01 4.198425e-01 4.197596e-01 4.196727e-01 2.416154e-02 2.203466e-02 2.171536e-02 2.142512e-02 2.299080e-02

618 4.252589e-01 4.253473e-01 4.251670e-01 4.250729e-01 4.249742e-01 2.416829e-02 2.204077e-02 2.172132e-02 2.143095e-02 2.299724e-02

619 4.305717e-01 4.306718e-01 4.304676e-01 4.303608e-01 4.302488e-01 2.417450e-02 2.204638e-02 2.172680e-02 2.143632e-02 2.300317e-02

620 4.358599e-01 4.359731e-01 4.357421e-01 4.356211e-01 4.354941e-01 2.418021e-02 2.205154e-02 2.173185e-02 2.144125e-02 2.300862e-02

621 4.411213e-01 4.412493e-01 4.409880e-01 4.408510e-01 4.407071e-01 2.418546e-02 2.205629e-02 2.173648e-02 2.144578e-02 2.301363e-02

622 4.463535e-01 4.464980e-01 4.462028e-01 4.460477e-01 4.458848e-01 2.419029e-02 2.206065e-02 2.174074e-02 2.144995e-02 2.301824e-02

623 4.515537e-01 4.517167e-01 4.513836e-01 4.512082e-01 4.510239e-01 2.419473e-02 2.206466e-02 2.174466e-02 2.145377e-02 2.302248e-02

624 4.567192e-01 4.569028e-01 4.565272e-01 4.563292e-01 4.561209e-01 2.419880e-02 2.206835e-02 2.174825e-02 2.145729e-02 2.302638e-02

625 4.618468e-01 4.620534e-01 4.616304e-01 4.614069e-01 4.611718e-01 2.420254e-02 2.207173e-02 2.175156e-02 2.146052e-02 2.302995e-02

626 4.669331e-01 4.671652e-01 4.666895e-01 4.664377e-01 4.661726e-01 2.420598e-02 2.207484e-02 2.175459e-02 2.146348e-02 2.303324e-02

627 4.719745e-01 4.722350e-01 4.717008e-01 4.714174e-01 4.711189e-01 2.420913e-02 2.207770e-02 2.175737e-02 2.146620e-02 2.303626e-02

628 4.769673e-01 4.772591e-01 4.766602e-01 4.763416e-01 4.760060e-01 2.421203e-02 2.208032e-02 2.175993e-02 2.146870e-02 2.303903e-02

629 4.819073e-01 4.822335e-01 4.815633e-01 4.812058e-01 4.808290e-01 2.421468e-02 2.208272e-02 2.176227e-02 2.147099e-02 2.304157e-02

630 4.867901e-01 4.871542e-01 4.864055e-01 4.860050e-01 4.855827e-01 2.421712e-02 2.208492e-02 2.176442e-02 2.147309e-02 2.304390e-02

631 4.916111e-01 4.920167e-01 4.911819e-01 4.907343e-01 4.902619e-01 2.421935e-02 2.208695e-02 2.176639e-02 2.147501e-02 2.304604e-02

632 4.963654e-01 4.968165e-01 4.958875e-01 4.953883e-01 4.948608e-01 2.422140e-02 2.208880e-02 2.176820e-02 2.147678e-02 2.304800e-02

633 5.010480e-01 5.015485e-01 5.005170e-01 4.999614e-01 4.993738e-01 2.422328e-02 2.209050e-02 2.176986e-02 2.147840e-02 2.304980e-02

634 5.056535e-01 5.062078e-01 5.050649e-01 5.044481e-01 5.037951e-01 2.422500e-02 2.209206e-02 2.177137e-02 2.147988e-02 2.305145e-02

635 5.101764e-01 5.107890e-01 5.095254e-01 5.088424e-01 5.081186e-01 2.422658e-02 2.209349e-02 2.177277e-02 2.148124e-02 2.305296e-02

636 5.146112e-01 5.152866e-01 5.138930e-01 5.131386e-01 5.123386e-01 2.422802e-02 2.209479e-02 2.177404e-02 2.148248e-02 2.305435e-02

637 5.189520e-01 5.196950e-01 5.181616e-01 5.173308e-01 5.164490e-01 2.422934e-02 2.209599e-02 2.177520e-02 2.148362e-02 2.305562e-02

638 5.231930e-01 5.240085e-01 5.223256e-01 5.214131e-01 5.204441e-01 2.423055e-02 2.209709e-02 2.177627e-02 2.148466e-02 2.305678e-02

639 5.273286e-01 5.282214e-01 5.263790e-01 5.253798e-01 5.243183e-01 2.423166e-02 2.209809e-02 2.177725e-02 2.148561e-02 2.305784e-02

640 5.313529e-01 5.323278e-01 5.303164e-01 5.292254e-01 5.280664e-01 2.423267e-02 2.209901e-02 2.177814e-02 2.148648e-02 2.305882e-02

641 5.352604e-01 5.363223e-01 5.341322e-01 5.329448e-01 5.316833e-01 2.423360e-02 2.209985e-02 2.177896e-02 2.148728e-02 2.305971e-02

642 5.390459e-01 5.401992e-01 5.378214e-01 5.365331e-01 5.351646e-01 2.423444e-02 2.210061e-02 2.177971e-02 2.148801e-02 2.306052e-02

643 5.427041e-01 5.439535e-01 5.413792e-01 5.399858e-01 5.385065e-01 2.423522e-02 2.210132e-02 2.178039e-02 2.148867e-02 2.306126e-02

644 5.462307e-01 5.475802e-01 5.448014e-01 5.432993e-01 5.417055e-01 2.423593e-02 2.210196e-02 2.178101e-02 2.148928e-02 2.306195e-02

645 5.496214e-01 5.510747e-01 5.480842e-01 5.464704e-01 5.447592e-01 2.423657e-02 2.210254e-02 2.178159e-02 2.148984e-02 2.306257e-02

646 5.528726e-01 5.544332e-01 5.512247e-01 5.494964e-01 5.476657e-01 2.423716e-02 2.210308e-02 2.178211e-02 2.149035e-02 2.306314e-02

647 5.559815e-01 5.576523e-01 5.542204e-01 5.523757e-01 5.504238e-01 2.423770e-02 2.210357e-02 2.178258e-02 2.149081e-02 2.306366e-02

648 5.589457e-01 5.607291e-01 5.570697e-01 5.551074e-01 5.530335e-01 2.423820e-02 2.210402e-02 2.178302e-02 2.149123e-02 2.306413e-02

649 5.617640e-01 5.636615e-01 5.597718e-01 5.576913e-01 5.554954e-01 2.423864e-02 2.210442e-02 2.178341e-02 2.149162e-02 2.306456e-02

650 5.644355e-01 5.664484e-01 5.623268e-01 5.601281e-01 5.578108e-01 2.423906e-02 2.210480e-02 2.178378e-02 2.149197e-02 2.306496e-02

651 5.669604e-01 5.690892e-01 5.647353e-01 5.624193e-01 5.599820e-01 2.423943e-02 2.210514e-02 2.178411e-02 2.149229e-02 2.306532e-02

652 5.693397e-01 5.715842e-01 5.669991e-01 5.645672e-01 5.620120e-01 2.423977e-02 2.210545e-02 2.178441e-02 2.149259e-02 2.306565e-02

653 5.715751e-01 5.739344e-01 5.691205e-01 5.665749e-01 5.639045e-01 2.424008e-02 2.210573e-02 2.178468e-02 2.149285e-02 2.306595e-02

654 5.736692e-01 5.761419e-01 5.711027e-01 5.684462e-01 5.656638e-01 2.424037e-02 2.210599e-02 2.178493e-02 2.149310e-02 2.306623e-02

655 5.756251e-01 5.782091e-01 5.729495e-01 5.701852e-01 5.672948e-01 2.424063e-02 2.210622e-02 2.178516e-02 2.149332e-02 2.306648e-02

656 5.774468e-01 5.801394e-01 5.746653e-01 5.717971e-01 5.688027e-01 2.424086e-02 2.210644e-02 2.178537e-02 2.149352e-02 2.306670e-02

657 5.791387e-01 5.819369e-01 5.762550e-01 5.732869e-01 5.701932e-01 2.424108e-02 2.210663e-02 2.178556e-02 2.149371e-02 2.306691e-02

658 5.807058e-01 5.836059e-01 5.777240e-01 5.746604e-01 5.714723e-01 2.424127e-02 2.210681e-02 2.178573e-02 2.149387e-02 2.306710e-02

659 5.821535e-01 5.851516e-01 5.790778e-01 5.759236e-01 5.726460e-01 2.424145e-02 2.210697e-02 2.178589e-02 2.149403e-02 2.306727e-02

660 5.834875e-01 5.865793e-01 5.803225e-01 5.770824e-01 5.737205e-01 2.424161e-02 2.210712e-02 2.178603e-02 2.149417e-02 2.306743e-02

661 5.847136e-01 5.878947e-01 5.814641e-01 5.781431e-01 5.747021e-01 2.424176e-02 2.210725e-02 2.178616e-02 2.149429e-02 2.306757e-02

662 5.858380e-01 5.891037e-01 5.825088e-01 5.791119e-01 5.755969e-01 2.424189e-02 2.210737e-02 2.178628e-02 2.149441e-02 2.306770e-02

663 5.868668e-01 5.902123e-01 5.834628e-01 5.799948e-01 5.764110e-01 2.424201e-02 2.210749e-02 2.178639e-02 2.149451e-02 2.306782e-02

664 5.878060e-01 5.912266e-01 5.843320e-01 5.807980e-01 5.771502e-01 2.424213e-02 2.210759e-02 2.178648e-02 2.149461e-02 2.306793e-02

665 5.886617e-01 5.921527e-01 5.851226e-01 5.815271e-01 5.778203e-01 2.424223e-02 2.210768e-02 2.178657e-02 2.149469e-02 2.306803e-02

666 5.894399e-01 5.929965e-01 5.858402e-01 5.821880e-01 5.784268e-01 2.424232e-02 2.210776e-02 2.178665e-02 2.149477e-02 2.306811e-02

667 5.901463e-01 5.937640e-01 5.864905e-01 5.827860e-01 5.789747e-01 2.424240e-02 2.210784e-02 2.178673e-02 2.149484e-02 2.306820e-02

668 5.907864e-01 5.944607e-01 5.870788e-01 5.833262e-01 5.794690e-01 2.424248e-02 2.210791e-02 2.178679e-02 2.149491e-02 2.306827e-02

669 5.913654e-01 5.950921e-01 5.876102e-01 5.838134e-01 5.799143e-01 2.424255e-02 2.210797e-02 2.178685e-02 2.149497e-02 2.306834e-02

670 5.918884e-01 5.956634e-01 5.880895e-01 5.842523e-01 5.803149e-01 2.424261e-02 2.210803e-02 2.178691e-02 2.149502e-02 2.306840e-02

671 5.923602e-01 5.961796e-01 5.885211e-01 5.846472e-01 5.806749e-01 2.424266e-02 2.210808e-02 2.178696e-02 2.149507e-02 2.306845e-02

672 5.927851e-01 5.966452e-01 5.889094e-01 5.850019e-01 5.809980e-01 2.424272e-02 2.210812e-02 2.178700e-02 2.149511e-02 2.306850e-02

673 5.931673e-01 5.970648e-01 5.892583e-01 5.853203e-01 5.812876e-01 2.424276e-02 2.210817e-02 2.178705e-02 2.149515e-02 2.306855e-02

674 5.935108e-01 5.974423e-01 5.895713e-01 5.856056e-01 5.815470e-01 2.424280e-02 2.210820e-02 2.178708e-02 2.149519e-02 2.306859e-02

675 5.938190e-01 5.977816e-01 5.898519e-01 5.858612e-01 5.817791e-01 2.424284e-02 2.210824e-02 2.178712e-02 2.149522e-02 2.306863e-02

676 5.940953e-01 5.980862e-01 5.901032e-01 5.860898e-01 5.819865e-01 2.424288e-02 2.210827e-02 2.178715e-02 2.149525e-02 2.306866e-02

677 5.943427e-01 5.983593e-01 5.903280e-01 5.862941e-01 5.821717e-01 2.424291e-02 2.210830e-02 2.178717e-02 2.149528e-02 2.306869e-02

678 5.945641e-01 5.986041e-01 5.905288e-01 5.864765e-01 5.823370e-01 2.424294e-02 2.210833e-02 2.178720e-02 2.149530e-02 2.306872e-02

679 5.947620e-01 5.988231e-01 5.907082e-01 5.866392e-01 5.824843e-01 2.424296e-02 2.210835e-02 2.178722e-02 2.149532e-02 2.306874e-02

680 5.949387e-01 5.990190e-01 5.908682e-01 5.867843e-01 5.826155e-01 2.424299e-02 2.210837e-02 2.178724e-02 2.149534e-02 2.306877e-02

681 5.950965e-01 5.991940e-01 5.910109e-01 5.869135e-01 5.827323e-01 2.424301e-02 2.210839e-02 2.178726e-02 2.149536e-02 2.306879e-02

682 5.952371e-01 5.993502e-01 5.911380e-01 5.870285e-01 5.828361e-01 2.424303e-02 2.210841e-02 2.178728e-02 2.149538e-02 2.306880e-02

683 5.953623e-01 5.994895e-01 5.912511e-01 5.871308e-01 5.829285e-01 2.424304e-02 2.210842e-02 2.178729e-02 2.149539e-02 2.306882e-02

684 5.954739e-01 5.996138e-01 5.913517e-01 5.872217e-01 5.830105e-01 2.424306e-02 2.210844e-02 2.178731e-02 2.149541e-02 2.306884e-02

685 5.955731e-01 5.997244e-01 5.914411e-01 5.873024e-01 5.830832e-01 2.424307e-02 2.210845e-02 2.178732e-02 2.149542e-02 2.306885e-02

686 5.956613e-01 5.998229e-01 5.915205e-01 5.873741e-01 5.831478e-01 2.424309e-02 2.210846e-02 2.178733e-02 2.149543e-02 2.306886e-02

687 5.957397e-01 5.999105e-01 5.915910e-01 5.874376e-01 5.832050e-01 2.424310e-02 2.210847e-02 2.178734e-02 2.149544e-02 2.306887e-02

688 5.958093e-01 5.999883e-01 5.916535e-01 5.874940e-01 5.832557e-01 2.424311e-02 2.210848e-02 2.178735e-02 2.149545e-02 2.306888e-02

689 5.958710e-01 6.000575e-01 5.917089e-01 5.875439e-01 5.833006e-01 2.424312e-02 2.210849e-02 2.178736e-02 2.149546e-02 2.306889e-02

690 5.959258e-01 6.001189e-01 5.917581e-01 5.875881e-01 5.833403e-01 2.424313e-02 2.210850e-02 2.178737e-02 2.149546e-02 2.306890e-02

691 5.959743e-01 6.001734e-01 5.918015e-01 5.876271e-01 5.833754e-01 2.424313e-02 2.210851e-02 2.178737e-02 2.149547e-02 2.306891e-02

692 5.960173e-01 6.002217e-01 5.918400e-01 5.876617e-01 5.834064e-01 2.424314e-02 2.210851e-02 2.178738e-02 2.149548e-02 2.306892e-02

693 5.960553e-01 6.002645e-01 5.918741e-01 5.876923e-01 5.834338e-01 2.424315e-02 2.210852e-02 2.178739e-02 2.149548e-02 2.306892e-02

694 5.960890e-01 6.003024e-01 5.919041e-01 5.877192e-01 5.834580e-01 2.424315e-02 2.210852e-02 2.178739e-02 2.149549e-02 2.306893e-02

695 5.961187e-01 6.003360e-01 5.919307e-01 5.877431e-01 5.834793e-01 2.424316e-02 2.210853e-02 2.178739e-02 2.149549e-02 2.306893e-02

696 5.961450e-01 6.003657e-01 5.919542e-01 5.877641e-01 5.834981e-01 2.424316e-02 2.210853e-02 2.178740e-02 2.149549e-02 2.306894e-02

697 5.961682e-01 6.003919e-01 5.919749e-01 5.877826e-01 5.835147e-01 2.424317e-02 2.210853e-02 2.178740e-02 2.149550e-02 2.306894e-02

698 5.961887e-01 6.004151e-01 5.919931e-01 5.877989e-01 5.835293e-01 2.424317e-02 2.210854e-02 2.178741e-02 2.149550e-02 2.306895e-02

699 5.962068e-01 6.004356e-01 5.920092e-01 5.878133e-01 5.835421e-01 2.424317e-02 2.210854e-02 2.178741e-02 2.149550e-02 2.306895e-02

700 5.962227e-01 6.004537e-01 5.920234e-01 5.878259e-01 5.835534e-01 2.424318e-02 2.210854e-02 2.178741e-02 2.149551e-02 2.306895e-02

701 5.962368e-01 6.004697e-01 5.920358e-01 5.878371e-01 5.835633e-01 2.424318e-02 2.210855e-02 2.178741e-02 2.149551e-02 2.306895e-02

702 5.962492e-01 6.004838e-01 5.920468e-01 5.878468e-01 5.835721e-01 2.424318e-02 2.210855e-02 2.178742e-02 2.149551e-02 2.306896e-02

703 5.962601e-01 6.004962e-01 5.920564e-01 5.878554e-01 5.835797e-01 2.424318e-02 2.210855e-02 2.178742e-02 2.149551e-02 2.306896e-02

704 5.962696e-01 6.005071e-01 5.920649e-01 5.878630e-01 5.835864e-01 2.424319e-02 2.210855e-02 2.178742e-02 2.149551e-02 2.306896e-02

705 5.962780e-01 6.005167e-01 5.920724e-01 5.878696e-01 5.835923e-01 2.424319e-02 2.210855e-02 2.178742e-02 2.149552e-02 2.306896e-02

706 5.962854e-01 6.005252e-01 5.920789e-01 5.878754e-01 5.835975e-01 2.424319e-02 2.210856e-02 2.178742e-02 2.149552e-02 2.306896e-02

707 5.962919e-01 6.005326e-01 5.920846e-01 5.878805e-01 5.836020e-01 2.424319e-02 2.210856e-02 2.178742e-02 2.149552e-02 2.306897e-02

708 5.962976e-01 6.005392e-01 5.920897e-01 5.878850e-01 5.836060e-01 2.424319e-02 2.210856e-02 2.178742e-02 2.149552e-02 2.306897e-02

709 5.963026e-01 6.005449e-01 5.920941e-01 5.878889e-01 5.836094e-01 2.424319e-02 2.210856e-02 2.178743e-02 2.149552e-02 2.306897e-02

710 5.963070e-01 6.005499e-01 5.920979e-01 5.878923e-01 5.836125e-01 2.424319e-02 2.210856e-02 2.178743e-02 2.149552e-02 2.306897e-02

711 5.963109e-01 6.005544e-01 5.921013e-01 5.878953e-01 5.836151e-01 2.424320e-02 2.210856e-02 2.178743e-02 2.149552e-02 2.306897e-02

712 5.963142e-01 6.005582e-01 5.921043e-01 5.878979e-01 5.836174e-01 2.424320e-02 2.210856e-02 2.178743e-02 2.149552e-02 2.306897e-02

713 5.963172e-01 6.005616e-01 5.921068e-01 5.879002e-01 5.836194e-01 2.424320e-02 2.210856e-02 2.178743e-02 2.149552e-02 2.306897e-02

714 5.963197e-01 6.005646e-01 5.921091e-01 5.879021e-01 5.836212e-01 2.424320e-02 2.210856e-02 2.178743e-02 2.149552e-02 2.306897e-02

715 5.963220e-01 6.005672e-01 5.921110e-01 5.879039e-01 5.836227e-01 2.424320e-02 2.210856e-02 2.178743e-02 2.149552e-02 2.306897e-02

716 5.963240e-01 6.005695e-01 5.921128e-01 5.879054e-01 5.836240e-01 2.424320e-02 2.210856e-02 2.178743e-02 2.149553e-02 2.306897e-02

717 5.963257e-01 6.005715e-01 5.921143e-01 5.879067e-01 5.836252e-01 2.424320e-02 2.210856e-02 2.178743e-02 2.149553e-02 2.306897e-02

718 5.963272e-01 6.005732e-01 5.921156e-01 5.879078e-01 5.836262e-01 2.424320e-02 2.210857e-02 2.178743e-02 2.149553e-02 2.306897e-02

719 5.963285e-01 6.005747e-01 5.921167e-01 5.879088e-01 5.836271e-01 2.424320e-02 2.210857e-02 2.178743e-02 2.149553e-02 2.306898e-02

720 5.963296e-01 6.005761e-01 5.921177e-01 5.879097e-01 5.836278e-01 2.424320e-02 2.210857e-02 2.178743e-02 2.149553e-02 2.306898e-02

721 5.963306e-01 6.005772e-01 5.921185e-01 5.879104e-01 5.836285e-01 2.424320e-02 2.210857e-02 2.178743e-02 2.149553e-02 2.306898e-02

722 5.963303e-01 6.005769e-01 5.921182e-01 5.879101e-01 5.836282e-01 2.727100e-02 2.515405e-02 2.482913e-02 2.453243e-02 2.613137e-02

723 5.963300e-01 6.005766e-01 5.921179e-01 5.879098e-01 5.836279e-01 3.032257e-02 2.821737e-02 2.788762e-02 2.758516e-02 2.921544e-02

724 5.963298e-01 6.005764e-01 5.921177e-01 5.879097e-01 5.836277e-01 3.339458e-02 3.129513e-02 3.095952e-02 3.065030e-02 3.231781e-02

725 5.963297e-01 6.005763e-01 5.921176e-01 5.879096e-01 5.836276e-01 3.648362e-02 3.438388e-02 3.404136e-02 3.372440e-02 3.543507e-02

726 5.963296e-01 6.005762e-01 5.921176e-01 5.879095e-01 5.836276e-01 3.958620e-02 3.748012e-02 3.712965e-02 3.680395e-02 3.856368e-02

727 5.963297e-01 6.005763e-01 5.921177e-01 5.879096e-01 5.836277e-01 4.269879e-02 4.058037e-02 4.022088e-02 3.988545e-02 4.170011e-02

728 5.963298e-01 6.005764e-01 5.921178e-01 5.879098e-01 5.836278e-01 4.581788e-02 4.368111e-02 4.331157e-02 4.296540e-02 4.484079e-02

729 5.963301e-01 6.005767e-01 5.921181e-01 5.879100e-01 5.836281e-01 4.893992e-02 4.677889e-02 4.639823e-02 4.604033e-02 4.798219e-02

730 5.963304e-01 6.005770e-01 5.921184e-01 5.879104e-01 5.836285e-01 5.206140e-02 4.987026e-02 4.947743e-02 4.910682e-02 5.112076e-02

731 5.963309e-01 6.005775e-01 5.921189e-01 5.879109e-01 5.836290e-01 5.517881e-02 5.295182e-02 5.254578e-02 5.216147e-02 5.425300e-02

732 5.963315e-01 6.005780e-01 5.921195e-01 5.879114e-01 5.836295e-01 5.828871e-02 5.602022e-02 5.559993e-02 5.520096e-02 5.737547e-02

733 5.963321e-01 6.005787e-01 5.921201e-01 5.879121e-01 5.836302e-01 6.138765e-02 5.907218e-02 5.863663e-02 5.822201e-02 6.048475e-02

734 5.963329e-01 6.005794e-01 5.921209e-01 5.879129e-01 5.836310e-01 6.447229e-02 6.210449e-02 6.165266e-02 6.122146e-02 6.357749e-02

735 5.963338e-01 6.005803e-01 5.921218e-01 5.879138e-01 5.836320e-01 6.753934e-02 6.511402e-02 6.464491e-02 6.419620e-02 6.665042e-02

736 5.963348e-01 6.005813e-01 5.921229e-01 5.879149e-01 5.836330e-01 7.058557e-02 6.809775e-02 6.761038e-02 6.714322e-02 6.970035e-02

737 5.963359e-01 6.005825e-01 5.921240e-01 5.879160e-01 5.836342e-01 7.360786e-02 7.105273e-02 7.054615e-02 7.005965e-02 7.272417e-02

738 5.963372e-01 6.005837e-01 5.921253e-01 5.879173e-01 5.836354e-01 7.660318e-02 7.397614e-02 7.344941e-02 7.294268e-02 7.571889e-02

739 5.963386e-01 6.005851e-01 5.921267e-01 5.879187e-01 5.836369e-01 7.956860e-02 7.686528e-02 7.631748e-02 7.578967e-02 7.868159e-02

740 5.963401e-01 6.005866e-01 5.921282e-01 5.879202e-01 5.836384e-01 8.250131e-02 7.971757e-02 7.914781e-02 7.859808e-02 8.160950e-02

741 5.963417e-01 6.005882e-01 5.921298e-01 5.879219e-01 5.836401e-01 8.539862e-02 8.253054e-02 8.193796e-02 8.136550e-02 8.449995e-02

742 5.963435e-01 6.005900e-01 5.921316e-01 5.879237e-01 5.836419e-01 8.825796e-02 8.530189e-02 8.468565e-02 8.408969e-02 8.735042e-02

743 5.963455e-01 6.005919e-01 5.921336e-01 5.879256e-01 5.836438e-01 9.107691e-02 8.802943e-02 8.738873e-02 8.676852e-02 9.015850e-02

744 5.963475e-01 6.005940e-01 5.921356e-01 5.879277e-01 5.836459e-01 9.385318e-02 9.071114e-02 9.004521e-02 8.940002e-02 9.292193e-02

745 5.963498e-01 6.005962e-01 5.921379e-01 5.879300e-01 5.836482e-01 9.658461e-02 9.334513e-02 9.265324e-02 9.198239e-02 9.563861e-02

746 5.963522e-01 6.005986e-01 5.921403e-01 5.879324e-01 5.836506e-01 9.926922e-02 9.592967e-02 9.521112e-02 9.451396e-02 9.830656e-02

747 5.963547e-01 6.006012e-01 5.921429e-01 5.879350e-01 5.836532e-01 1.019052e-01 9.846319e-02 9.771732e-02 9.699324e-02 1.009240e-01

748 5.963575e-01 6.006039e-01 5.921457e-01 5.879378e-01 5.836560e-01 1.044907e-01 1.009443e-01 1.001705e-01 9.941888e-02 1.034892e-01

749 5.963605e-01 6.006069e-01 5.921486e-01 5.879407e-01 5.836590e-01 1.070244e-01 1.033717e-01 1.025693e-01 1.017897e-01 1.060007e-01

750 5.963636e-01 6.006100e-01 5.921518e-01 5.879439e-01 5.836623e-01 1.095048e-01 1.057442e-01 1.049128e-01 1.041047e-01 1.084572e-01

751 5.963670e-01 6.006134e-01 5.921552e-01 5.879474e-01 5.836657e-01 1.119308e-01 1.080611e-01 1.072001e-01 1.063630e-01 1.108576e-01

752 5.963707e-01 6.006170e-01 5.921589e-01 5.879511e-01 5.836694e-01 1.143012e-01 1.103214e-01 1.094304e-01 1.085639e-01 1.132007e-01

753 5.963746e-01 6.006209e-01 5.921628e-01 5.879550e-01 5.836734e-01 1.166152e-01 1.125245e-01 1.116031e-01 1.107069e-01 1.154857e-01

754 5.963788e-01 6.006251e-01 5.921671e-01 5.879593e-01 5.836777e-01 1.188720e-01 1.146700e-01 1.137179e-01 1.127915e-01 1.177120e-01

755 5.963834e-01 6.006296e-01 5.921717e-01 5.879639e-01 5.836823e-01 1.210711e-01 1.167574e-01 1.157743e-01 1.148176e-01 1.198789e-01

756 5.963882e-01 6.006345e-01 5.921766e-01 5.879689e-01 5.836873e-01 1.232121e-01 1.187867e-01 1.177722e-01 1.167850e-01 1.219862e-01

757 5.963935e-01 6.006397e-01 5.921819e-01 5.879742e-01 5.836927e-01 1.252945e-01 1.207578e-01 1.197118e-01 1.186937e-01 1.240336e-01

758 5.963992e-01 6.006454e-01 5.921876e-01 5.879800e-01 5.836986e-01 1.273184e-01 1.226707e-01 1.215930e-01 1.205440e-01 1.260210e-01

759 5.964054e-01 6.006515e-01 5.921939e-01 5.879863e-01 5.837049e-01 1.292837e-01 1.245257e-01 1.234161e-01 1.223362e-01 1.279484e-01

760 5.964121e-01 6.006581e-01 5.922006e-01 5.879931e-01 5.837118e-01 1.311904e-01 1.263230e-01 1.251816e-01 1.240706e-01 1.298161e-01

761 5.964193e-01 6.006653e-01 5.922079e-01 5.880005e-01 5.837192e-01 1.330388e-01 1.280630e-01 1.268899e-01 1.257478e-01 1.316243e-01

762 5.964272e-01 6.006731e-01 5.922159e-01 5.880085e-01 5.837274e-01 1.348292e-01 1.297461e-01 1.285415e-01 1.273685e-01 1.333736e-01

763 5.964358e-01 6.006816e-01 5.922245e-01 5.880173e-01 5.837362e-01 1.365619e-01 1.313731e-01 1.301371e-01 1.289333e-01 1.350645e-01

764 5.964451e-01 6.006908e-01 5.922340e-01 5.880268e-01 5.837459e-01 1.382375e-01 1.329444e-01 1.316774e-01 1.304430e-01 1.366977e-01

765 5.964552e-01 6.007008e-01 5.922443e-01 5.880372e-01 5.837564e-01 1.398566e-01 1.344609e-01 1.331633e-01 1.318985e-01 1.382739e-01

766 5.964663e-01 6.007118e-01 5.922555e-01 5.880486e-01 5.837680e-01 1.414198e-01 1.359234e-01 1.345955e-01 1.333006e-01 1.397940e-01

767 5.964784e-01 6.007237e-01 5.922677e-01 5.880610e-01 5.837806e-01 1.429279e-01 1.373327e-01 1.359750e-01 1.346504e-01 1.412589e-01

768 5.964916e-01 6.007367e-01 5.922811e-01 5.880746e-01 5.837944e-01 1.443817e-01 1.386898e-01 1.373027e-01 1.359489e-01 1.426696e-01

769 5.965060e-01 6.007509e-01 5.922958e-01 5.880895e-01 5.838095e-01 1.457822e-01 1.399956e-01 1.385798e-01 1.371971e-01 1.440271e-01

770 5.965218e-01 6.007664e-01 5.923118e-01 5.881058e-01 5.838261e-01 1.471303e-01 1.412512e-01 1.398073e-01 1.383961e-01 1.453324e-01

771 5.965391e-01 6.007834e-01 5.923294e-01 5.881237e-01 5.838443e-01 1.484270e-01 1.424578e-01 1.409862e-01 1.395471e-01 1.465868e-01

772 5.965580e-01 6.008020e-01 5.923487e-01 5.881433e-01 5.838643e-01 1.496734e-01 1.436164e-01 1.421179e-01 1.406514e-01 1.477913e-01

773 5.965787e-01 6.008223e-01 5.923698e-01 5.881649e-01 5.838863e-01 1.508706e-01 1.447281e-01 1.432034e-01 1.417100e-01 1.489472e-01

774 5.966014e-01 6.008446e-01 5.923930e-01 5.881885e-01 5.839104e-01 1.520197e-01 1.457943e-01 1.442439e-01 1.427243e-01 1.500557e-01

775 5.966264e-01 6.008690e-01 5.924184e-01 5.882145e-01 5.839370e-01 1.531220e-01 1.468160e-01 1.452407e-01 1.436954e-01 1.511180e-01

776 5.966537e-01 6.008958e-01 5.924464e-01 5.882431e-01 5.839662e-01 1.541786e-01 1.477946e-01 1.461951e-01 1.446247e-01 1.521355e-01

777 5.966837e-01 6.009251e-01 5.924771e-01 5.882745e-01 5.839983e-01 1.551908e-01 1.487312e-01 1.471082e-01 1.455134e-01 1.531094e-01

778 5.967166e-01 6.009572e-01 5.925108e-01 5.883090e-01 5.840337e-01 1.561598e-01 1.496271e-01 1.479814e-01 1.463627e-01 1.540410e-01

779 5.967528e-01 6.009925e-01 5.925479e-01 5.883470e-01 5.840726e-01 1.570869e-01 1.504837e-01 1.488159e-01 1.471740e-01 1.549316e-01

780 5.967925e-01 6.010312e-01 5.925886e-01 5.883888e-01 5.841155e-01 1.579734e-01 1.513020e-01 1.496130e-01 1.479486e-01 1.557825e-01

781 5.968361e-01 6.010736e-01 5.926334e-01 5.884348e-01 5.841628e-01 1.588206e-01 1.520835e-01 1.503739e-01 1.486876e-01 1.565950e-01

782 5.968840e-01 6.011203e-01 5.926827e-01 5.884854e-01 5.842149e-01 1.596296e-01 1.528293e-01 1.510998e-01 1.493923e-01 1.573704e-01

783 5.969366e-01 6.011714e-01 5.927369e-01 5.885412e-01 5.842723e-01 1.604019e-01 1.535408e-01 1.517921e-01 1.500640e-01 1.581100e-01

784 5.969945e-01 6.012276e-01 5.927965e-01 5.886027e-01 5.843357e-01 1.611387e-01 1.542190e-01 1.524519e-01 1.507039e-01 1.588151e-01

785 5.970581e-01 6.012893e-01 5.928622e-01 5.886704e-01 5.844055e-01 1.618411e-01 1.548653e-01 1.530804e-01 1.513131e-01 1.594870e-01

786 5.971281e-01 6.013570e-01 5.929344e-01 5.887450e-01 5.844826e-01 1.625106e-01 1.554809e-01 1.536789e-01 1.518930e-01 1.601268e-01

787 5.972050e-01 6.014314e-01 5.930139e-01 5.888272e-01 5.845676e-01 1.631482e-01 1.560668e-01 1.542484e-01 1.524445e-01 1.607358e-01

788 5.972896e-01 6.015131e-01 5.931015e-01 5.889178e-01 5.846614e-01 1.637552e-01 1.566243e-01 1.547901e-01 1.529689e-01 1.613151e-01

789 5.973826e-01 6.016029e-01 5.931979e-01 5.890177e-01 5.847650e-01 1.643329e-01 1.571546e-01 1.553053e-01 1.534673e-01 1.618661e-01

790 5.974849e-01 6.017015e-01 5.933041e-01 5.891278e-01 5.848792e-01 1.648822e-01 1.576586e-01 1.557948e-01 1.539407e-01 1.623898e-01

791 5.975974e-01 6.018098e-01 5.934210e-01 5.892492e-01 5.850054e-01 1.654045e-01 1.581375e-01 1.562599e-01 1.543902e-01 1.628873e-01

792 5.977212e-01 6.019289e-01 5.935498e-01 5.893831e-01 5.851447e-01 1.659008e-01 1.585924e-01 1.567015e-01 1.548169e-01 1.633598e-01

793 5.978574e-01 6.020597e-01 5.936916e-01 5.895308e-01 5.852984e-01 1.663721e-01 1.590243e-01 1.571206e-01 1.552217e-01 1.638083e-01

794 5.980072e-01 6.022034e-01 5.938478e-01 5.896936e-01 5.854682e-01 1.668196e-01 1.594341e-01 1.575183e-01 1.556056e-01 1.642339e-01

795 5.981720e-01 6.023612e-01 5.940199e-01 5.898731e-01 5.856556e-01 1.672443e-01 1.598229e-01 1.578955e-01 1.559695e-01 1.646375e-01

796 5.983533e-01 6.025347e-01 5.942093e-01 5.900710e-01 5.858625e-01 1.676472e-01 1.601916e-01 1.582530e-01 1.563143e-01 1.650201e-01

797 5.985526e-01 6.027251e-01 5.944179e-01 5.902893e-01 5.860908e-01 1.680292e-01 1.605410e-01 1.585919e-01 1.566410e-01 1.653827e-01

798 5.987717e-01 6.029343e-01 5.946475e-01 5.905298e-01 5.863428e-01 1.683912e-01 1.608721e-01 1.589129e-01 1.569503e-01 1.657263e-01

799 5.990127e-01 6.031640e-01 5.949003e-01 5.907948e-01 5.866208e-01 1.687343e-01 1.611858e-01 1.592169e-01 1.572432e-01 1.660516e-01

800 5.992775e-01 6.034161e-01 5.951784e-01 5.910868e-01 5.869274e-01 1.690593e-01 1.614827e-01 1.595047e-01 1.575202e-01 1.663596e-01

801 5.995684e-01 6.036927e-01 5.954844e-01 5.914084e-01 5.872655e-01 1.693670e-01 1.617638e-01 1.597771e-01 1.577823e-01 1.666510e-01

802 5.998879e-01 6.039962e-01 5.958208e-01 5.917624e-01 5.876380e-01 1.696582e-01 1.620298e-01 1.600348e-01 1.580302e-01 1.669267e-01

803 6.002387e-01 6.043290e-01 5.961905e-01 5.921518e-01 5.880484e-01 1.699337e-01 1.622814e-01 1.602784e-01 1.582645e-01 1.671874e-01

804 6.006236e-01 6.046939e-01 5.965967e-01 5.925802e-01 5.885001e-01 1.701943e-01 1.625193e-01 1.605088e-01 1.584859e-01 1.674338e-01

805 6.010458e-01 6.050937e-01 5.970426e-01 5.930509e-01 5.889970e-01 1.704407e-01 1.627442e-01 1.607266e-01 1.586951e-01 1.676667e-01

806 6.015085e-01 6.055316e-01 5.975318e-01 5.935678e-01 5.895432e-01 1.706737e-01 1.629567e-01 1.609323e-01 1.588926e-01 1.678868e-01

807 6.020153e-01 6.060110e-01 5.980681e-01 5.941350e-01 5.901431e-01 1.708938e-01 1.631575e-01 1.611266e-01 1.590792e-01 1.680946e-01

808 6.025702e-01 6.065354e-01 5.986555e-01 5.947567e-01 5.908012e-01 1.711017e-01 1.633471e-01 1.613102e-01 1.592552e-01 1.682908e-01

809 6.031770e-01 6.071087e-01 5.992984e-01 5.954376e-01 5.915224e-01 1.712980e-01 1.635261e-01 1.614834e-01 1.594214e-01 1.684760e-01

810 6.038403e-01 6.077351e-01 6.000013e-01 5.961825e-01 5.923118e-01 1.714834e-01 1.636951e-01 1.616469e-01 1.595781e-01 1.686508e-01

811 6.045645e-01 6.084189e-01 6.007690e-01 5.969963e-01 5.931747e-01 1.716584e-01 1.638546e-01 1.618012e-01 1.597260e-01 1.688156e-01

812 6.053545e-01 6.091647e-01 6.016065e-01 5.978843e-01 5.941166e-01 1.718235e-01 1.640051e-01 1.619467e-01 1.598653e-01 1.689711e-01

813 6.062153e-01 6.099774e-01 6.025191e-01 5.988520e-01 5.951432e-01 1.719792e-01 1.641470e-01 1.620839e-01 1.599967e-01 1.691177e-01

814 6.071522e-01 6.108619e-01 6.035122e-01 5.999049e-01 5.962601e-01 1.721261e-01 1.642808e-01 1.622133e-01 1.601205e-01 1.692559e-01

815 6.081705e-01 6.118236e-01 6.045913e-01 6.010487e-01 5.974731e-01 1.722645e-01 1.644069e-01 1.623352e-01 1.602371e-01 1.693861e-01

816 6.092758e-01 6.128678e-01 6.057622e-01 6.022892e-01 5.987882e-01 1.723951e-01 1.645258e-01 1.624501e-01 1.603470e-01 1.695088e-01

817 6.104738e-01 6.140001e-01 6.070304e-01 6.036322e-01 6.002110e-01 1.725180e-01 1.646378e-01 1.625583e-01 1.604505e-01 1.696243e-01

818 6.117702e-01 6.152261e-01 6.084019e-01 6.050833e-01 6.017471e-01 1.726339e-01 1.647433e-01 1.626603e-01 1.605479e-01 1.697331e-01

819 6.131706e-01 6.165515e-01 6.098821e-01 6.066480e-01 6.034021e-01 1.727430e-01 1.648426e-01 1.627563e-01 1.606395e-01 1.698355e-01

820 6.146806e-01 6.179819e-01 6.114767e-01 6.083318e-01 6.051810e-01 1.728458e-01 1.649362e-01 1.628466e-01 1.607258e-01 1.699319e-01

821 6.163057e-01 6.195228e-01 6.131907e-01 6.101397e-01 6.070886e-01 1.729425e-01 1.650242e-01 1.629317e-01 1.608070e-01 1.700226e-01

822 6.180510e-01 6.211796e-01 6.150293e-01 6.120762e-01 6.091290e-01 1.730336e-01 1.651071e-01 1.630117e-01 1.608833e-01 1.701079e-01

823 6.199214e-01 6.229574e-01 6.169969e-01 6.141456e-01 6.113059e-01 1.731193e-01 1.651851e-01 1.630870e-01 1.609551e-01 1.701881e-01

824 6.219214e-01 6.248608e-01 6.190977e-01 6.163514e-01 6.136224e-01 1.731999e-01 1.652584e-01 1.631578e-01 1.610226e-01 1.702636e-01

825 6.240548e-01 6.268943e-01 6.213349e-01 6.186965e-01 6.160809e-01 1.732757e-01 1.653274e-01 1.632244e-01 1.610860e-01 1.703345e-01

826 6.263250e-01 6.290616e-01 6.237116e-01 6.211832e-01 6.186827e-01 1.733470e-01 1.653923e-01 1.632870e-01 1.611456e-01 1.704012e-01

827 6.287348e-01 6.313660e-01 6.262298e-01 6.238130e-01 6.214286e-01 1.734140e-01 1.654532e-01 1.633458e-01 1.612016e-01 1.704639e-01

828 6.312861e-01 6.338101e-01 6.288908e-01 6.265863e-01 6.243184e-01 1.734770e-01 1.655105e-01 1.634011e-01 1.612543e-01 1.705227e-01

829 6.339801e-01 6.363956e-01 6.316952e-01 6.295031e-01 6.273512e-01 1.735362e-01 1.655644e-01 1.634531e-01 1.613037e-01 1.705780e-01

830 6.368172e-01 6.391236e-01 6.346425e-01 6.325621e-01 6.305249e-01 1.735918e-01 1.656150e-01 1.635019e-01 1.613501e-01 1.706299e-01

831 6.397970e-01 6.419944e-01 6.377317e-01 6.357615e-01 6.338369e-01 1.736441e-01 1.656625e-01 1.635478e-01 1.613936e-01 1.706787e-01

832 6.429181e-01 6.450075e-01 6.409606e-01 6.390985e-01 6.372836e-01 1.736931e-01 1.657071e-01 1.635908e-01 1.614345e-01 1.707244e-01

833 6.461784e-01 6.481613e-01 6.443265e-01 6.425694e-01 6.408608e-01 1.737392e-01 1.657490e-01 1.636312e-01 1.614729e-01 1.707674e-01

834 6.495751e-01 6.514537e-01 6.478257e-01 6.461701e-01 6.445634e-01 1.737825e-01 1.657884e-01 1.636691e-01 1.615089e-01 1.708077e-01

835 6.531043e-01 6.548818e-01 6.514539e-01 6.498955e-01 6.483860e-01 1.738231e-01 1.658253e-01 1.637047e-01 1.615426e-01 1.708455e-01

836 6.567619e-01 6.584417e-01 6.552061e-01 6.537401e-01 6.523224e-01 1.738612e-01 1.658600e-01 1.637381e-01 1.615743e-01 1.708809e-01

837 6.605427e-01 6.621291e-01 6.590767e-01 6.576979e-01 6.563661e-01 1.738969e-01 1.658925e-01 1.637695e-01 1.616040e-01 1.709142e-01

838 6.644412e-01 6.659390e-01 6.630599e-01 6.617625e-01 6.605103e-01 1.739305e-01 1.659229e-01 1.637988e-01 1.616318e-01 1.709454e-01

839 6.684515e-01 6.698659e-01 6.671491e-01 6.659269e-01 6.647479e-01 1.739619e-01 1.659515e-01 1.638264e-01 1.616579e-01 1.709746e-01

840 6.725673e-01 6.739039e-01 6.713376e-01 6.701843e-01 6.690716e-01 1.739914e-01 1.659784e-01 1.638522e-01 1.616824e-01 1.710020e-01

841 6.767818e-01 6.780467e-01 6.756186e-01 6.745274e-01 6.734740e-01 1.740191e-01 1.660035e-01 1.638764e-01 1.617053e-01 1.710276e-01

842 6.810882e-01 6.822878e-01 6.799848e-01 6.789490e-01 6.779477e-01 1.740450e-01 1.660271e-01 1.638991e-01 1.617268e-01 1.710517e-01

843 6.854797e-01 6.866204e-01 6.844293e-01 6.834417e-01 6.824851e-01 1.740693e-01 1.660491e-01 1.639204e-01 1.617469e-01 1.710742e-01

844 6.899490e-01 6.910378e-01 6.889446e-01 6.879981e-01 6.870789e-01 1.740920e-01 1.660698e-01 1.639403e-01 1.617657e-01 1.710953e-01

845 6.944893e-01 6.955331e-01 6.935238e-01 6.926111e-01 6.917218e-01 1.741134e-01 1.660892e-01 1.639590e-01 1.617833e-01 1.711151e-01

846 6.990935e-01 7.000994e-01 6.981596e-01 6.972735e-01 6.964065e-01 1.741333e-01 1.661074e-01 1.639765e-01 1.617998e-01 1.711335e-01

847 7.037547e-01 7.047300e-01 7.028453e-01 7.019783e-01 7.011260e-01 1.741520e-01 1.661244e-01 1.639928e-01 1.618153e-01 1.711509e-01

848 7.084662e-01 7.094182e-01 7.075738e-01 7.067185e-01 7.058733e-01 1.741696e-01 1.661403e-01 1.640082e-01 1.618297e-01 1.711671e-01

849 7.132214e-01 7.141575e-01 7.123385e-01 7.114874e-01 7.106415e-01 1.741859e-01 1.661552e-01 1.640225e-01 1.618432e-01 1.711822e-01

850 7.180138e-01 7.189415e-01 7.171330e-01 7.162784e-01 7.154241e-01 1.742013e-01 1.661691e-01 1.640359e-01 1.618559e-01 1.711964e-01

851 7.228372e-01 7.237640e-01 7.219508e-01 7.210850e-01 7.202143e-01 1.742157e-01 1.661822e-01 1.640485e-01 1.618677e-01 1.712097e-01

852 7.276853e-01 7.286190e-01 7.267856e-01 7.259009e-01 7.250059e-01 1.742291e-01 1.661944e-01 1.640602e-01 1.618788e-01 1.712221e-01

853 7.325524e-01 7.335008e-01 7.316316e-01 7.307200e-01 7.297924e-01 1.742417e-01 1.662058e-01 1.640712e-01 1.618891e-01 1.712337e-01

854 7.374327e-01 7.384037e-01 7.364827e-01 7.355361e-01 7.345676e-01 1.742534e-01 1.662165e-01 1.640815e-01 1.618988e-01 1.712445e-01

855 7.423205e-01 7.433222e-01 7.413332e-01 7.403433e-01 7.393254e-01 1.742644e-01 1.662265e-01 1.640911e-01 1.619078e-01 1.712546e-01

856 7.472104e-01 7.482510e-01 7.461774e-01 7.451357e-01 7.440596e-01 1.742747e-01 1.662359e-01 1.641001e-01 1.619163e-01 1.712641e-01

857 7.520970e-01 7.531851e-01 7.510098e-01 7.499076e-01 7.487643e-01 1.742843e-01 1.662446e-01 1.641085e-01 1.619242e-01 1.712730e-01

858 7.569751e-01 7.581195e-01 7.558248e-01 7.546531e-01 7.534335e-01 1.742933e-01 1.662528e-01 1.641164e-01 1.619315e-01 1.712812e-01

859 7.618396e-01 7.630492e-01 7.606171e-01 7.593667e-01 7.580613e-01 1.743017e-01 1.662604e-01 1.641237e-01 1.619384e-01 1.712889e-01

860 7.666855e-01 7.679696e-01 7.653814e-01 7.640427e-01 7.626417e-01 1.743096e-01 1.662675e-01 1.641305e-01 1.619449e-01 1.712962e-01

861 7.715078e-01 7.728760e-01 7.701123e-01 7.686756e-01 7.671689e-01 1.743169e-01 1.662742e-01 1.641369e-01 1.619509e-01 1.713029e-01

862 7.763015e-01 7.777638e-01 7.748047e-01 7.732598e-01 7.716373e-01 1.743237e-01 1.662804e-01 1.641429e-01 1.619565e-01 1.713092e-01

863 7.810618e-01 7.826284e-01 7.794533e-01 7.777898e-01 7.760410e-01 1.743301e-01 1.662862e-01 1.641485e-01 1.619617e-01 1.713150e-01

864 7.857839e-01 7.874654e-01 7.840530e-01 7.822602e-01 7.803744e-01 1.743361e-01 1.662916e-01 1.641537e-01 1.619666e-01 1.713205e-01

865 7.904630e-01 7.922704e-01 7.885987e-01 7.866657e-01 7.846321e-01 1.743417e-01 1.662967e-01 1.641586e-01 1.619711e-01 1.713256e-01

866 7.950944e-01 7.970387e-01 7.930852e-01 7.910009e-01 7.888086e-01 1.743469e-01 1.663014e-01 1.641631e-01 1.619754e-01 1.713304e-01

867 7.996732e-01 8.017661e-01 7.975077e-01 7.952608e-01 7.928987e-01 1.743517e-01 1.663059e-01 1.641674e-01 1.619793e-01 1.713348e-01

868 8.041947e-01 8.064479e-01 8.018612e-01 7.994401e-01 7.968973e-01 1.743563e-01 1.663100e-01 1.641713e-01 1.619830e-01 1.713390e-01

869 8.086542e-01 8.110798e-01 8.061408e-01 8.035341e-01 8.007998e-01 1.743605e-01 1.663138e-01 1.641750e-01 1.619865e-01 1.713428e-01

870 8.130470e-01 8.156571e-01 8.103417e-01 8.075381e-01 8.046015e-01 1.743645e-01 1.663174e-01 1.641784e-01 1.619897e-01 1.713465e-01

871 8.173684e-01 8.201755e-01 8.144593e-01 8.114474e-01 8.082983e-01 1.743681e-01 1.663207e-01 1.641816e-01 1.619927e-01 1.713498e-01

872 8.216139e-01 8.246304e-01 8.184890e-01 8.152579e-01 8.118862e-01 1.743716e-01 1.663239e-01 1.641846e-01 1.619955e-01 1.713529e-01

873 8.257789e-01 8.290173e-01 8.224265e-01 8.189656e-01 8.153619e-01 1.743748e-01 1.663268e-01 1.641874e-01 1.619981e-01 1.713559e-01

874 8.298588e-01 8.333317e-01 8.262675e-01 8.225667e-01 8.187223e-01 1.743777e-01 1.663295e-01 1.641900e-01 1.620005e-01 1.713586e-01

875 8.338495e-01 8.375692e-01 8.300081e-01 8.260579e-01 8.219648e-01 1.743805e-01 1.663320e-01 1.641924e-01 1.620027e-01 1.713611e-01

876 8.377467e-01 8.417254e-01 8.336446e-01 8.294360e-01 8.250873e-01 1.743831e-01 1.663344e-01 1.641947e-01 1.620048e-01 1.713635e-01

877 8.415464e-01 8.457959e-01 8.371734e-01 8.326985e-01 8.280882e-01 1.743855e-01 1.663365e-01 1.641968e-01 1.620068e-01 1.713657e-01

878 8.452449e-01 8.497766e-01 8.405915e-01 8.358432e-01 8.309662e-01 1.743878e-01 1.663386e-01 1.641988e-01 1.620086e-01 1.713677e-01

879 8.488385e-01 8.536633e-01 8.438961e-01 8.388682e-01 8.337207e-01 1.743899e-01 1.663405e-01 1.642006e-01 1.620103e-01 1.713696e-01

880 8.523242e-01 8.574523e-01 8.470849e-01 8.417722e-01 8.363517e-01 1.743918e-01 1.663423e-01 1.642023e-01 1.620119e-01 1.713714e-01

881 8.556988e-01 8.611398e-01 8.501559e-01 8.445544e-01 8.388593e-01 1.743936e-01 1.663439e-01 1.642039e-01 1.620134e-01 1.713730e-01

882 8.589599e-01 8.647224e-01 8.531075e-01 8.472144e-01 8.412445e-01 1.743953e-01 1.663454e-01 1.642053e-01 1.620147e-01 1.713746e-01

883 8.621053e-01 8.681969e-01 8.559388e-01 8.497525e-01 8.435086e-01 1.743969e-01 1.663469e-01 1.642067e-01 1.620160e-01 1.713760e-01

884 8.651332e-01 8.715604e-01 8.586490e-01 8.521691e-01 8.456533e-01 1.743984e-01 1.663482e-01 1.642080e-01 1.620172e-01 1.713773e-01

885 8.680422e-01 8.748104e-01 8.612382e-01 8.544654e-01 8.476810e-01 1.743997e-01 1.663494e-01 1.642092e-01 1.620183e-01 1.713786e-01

886 8.708314e-01 8.779449e-01 8.637066e-01 8.566429e-01 8.495941e-01 1.744010e-01 1.663506e-01 1.642103e-01 1.620193e-01 1.713797e-01

887 8.735003e-01 8.809620e-01 8.660552e-01 8.587037e-01 8.513957e-01 1.744022e-01 1.663517e-01 1.642113e-01 1.620203e-01 1.713808e-01

888 8.760492e-01 8.838605e-01 8.682851e-01 8.606501e-01 8.530890e-01 1.744033e-01 1.663527e-01 1.642123e-01 1.620211e-01 1.713818e-01

889 8.784783e-01 8.866395e-01 8.703982e-01 8.624849e-01 8.546777e-01 1.744043e-01 1.663536e-01 1.642131e-01 1.620220e-01 1.713827e-01

890 8.807887e-01 8.892986e-01 8.723965e-01 8.642111e-01 8.561654e-01 1.744052e-01 1.663545e-01 1.642140e-01 1.620227e-01 1.713835e-01

891 8.829817e-01 8.918378e-01 8.742827e-01 8.658322e-01 8.575562e-01 1.744061e-01 1.663553e-01 1.642147e-01 1.620234e-01 1.713843e-01

892 8.850593e-01 8.942578e-01 8.760595e-01 8.673518e-01 8.588542e-01 1.744069e-01 1.663560e-01 1.642154e-01 1.620241e-01 1.713851e-01

893 8.870235e-01 8.965595e-01 8.777302e-01 8.687737e-01 8.600636e-01 1.744077e-01 1.663567e-01 1.642161e-01 1.620247e-01 1.713858e-01

894 8.888771e-01 8.987443e-01 8.792982e-01 8.701020e-01 8.611887e-01 1.744084e-01 1.663573e-01 1.642167e-01 1.620253e-01 1.713864e-01

895 8.906229e-01 9.008140e-01 8.807672e-01 8.713406e-01 8.622338e-01 1.744091e-01 1.663579e-01 1.642173e-01 1.620258e-01 1.713870e-01

896 8.922641e-01 9.027709e-01 8.821411e-01 8.724939e-01 8.632031e-01 1.744097e-01 1.663585e-01 1.642178e-01 1.620263e-01 1.713875e-01

897 8.938043e-01 9.046175e-01 8.834238e-01 8.735661e-01 8.641009e-01 1.744103e-01 1.663590e-01 1.642183e-01 1.620267e-01 1.713881e-01

898 8.952470e-01 9.063569e-01 8.846195e-01 8.745613e-01 8.649314e-01 1.744108e-01 1.663595e-01 1.642188e-01 1.620272e-01 1.713885e-01

899 8.965963e-01 9.079922e-01 8.857323e-01 8.754838e-01 8.656987e-01 1.744113e-01 1.663599e-01 1.642192e-01 1.620275e-01 1.713890e-01

900 8.978559e-01 9.095268e-01 8.867664e-01 8.763378e-01 8.664066e-01 1.744117e-01 1.663603e-01 1.642196e-01 1.620279e-01 1.713894e-01

901 8.990301e-01 9.109645e-01 8.877260e-01 8.771273e-01 8.670591e-01 1.744121e-01 1.663607e-01 1.642200e-01 1.620282e-01 1.713898e-01

902 8.990303e-01 9.109648e-01 8.877262e-01 8.771275e-01 8.670592e-01 1.752571e-01 1.672669e-01 1.651374e-01 1.629512e-01 1.722371e-01

903 8.990305e-01 9.109650e-01 8.877264e-01 8.771277e-01 8.670594e-01 1.761348e-01 1.682072e-01 1.660892e-01 1.639087e-01 1.731167e-01

904 8.990307e-01 9.109653e-01 8.877266e-01 8.771279e-01 8.670596e-01 1.770463e-01 1.691826e-01 1.670764e-01 1.649016e-01 1.740298e-01

905 8.990309e-01 9.109655e-01 8.877268e-01 8.771280e-01 8.670597e-01 1.779925e-01 1.701942e-01 1.681001e-01 1.659309e-01 1.749772e-01

906 8.990311e-01 9.109657e-01 8.877269e-01 8.771282e-01 8.670598e-01 1.789746e-01 1.712429e-01 1.691611e-01 1.669975e-01 1.759599e-01

907 8.990313e-01 9.109659e-01 8.877271e-01 8.771283e-01 8.670599e-01 1.799935e-01 1.723296e-01 1.702604e-01 1.681025e-01 1.769789e-01

908 8.990315e-01 9.109661e-01 8.877272e-01 8.771284e-01 8.670601e-01 1.810502e-01 1.734553e-01 1.713990e-01 1.692466e-01 1.780352e-01

909 8.990316e-01 9.109663e-01 8.877274e-01 8.771286e-01 8.670602e-01 1.821456e-01 1.746209e-01 1.725777e-01 1.704308e-01 1.791296e-01

910 8.990318e-01 9.109665e-01 8.877275e-01 8.771287e-01 8.670603e-01 1.832808e-01 1.758273e-01 1.737974e-01 1.716558e-01 1.802632e-01

911 8.990319e-01 9.109666e-01 8.877276e-01 8.771288e-01 8.670603e-01 1.844566e-01 1.770753e-01 1.750590e-01 1.729226e-01 1.814367e-01

912 8.990320e-01 9.109668e-01 8.877277e-01 8.771289e-01 8.670604e-01 1.856740e-01 1.783657e-01 1.763631e-01 1.742318e-01 1.826512e-01

913 8.990321e-01 9.109669e-01 8.877278e-01 8.771289e-01 8.670605e-01 1.869338e-01 1.796993e-01 1.777106e-01 1.755842e-01 1.839073e-01

914 8.990322e-01 9.109670e-01 8.877279e-01 8.771290e-01 8.670606e-01 1.882368e-01 1.810768e-01 1.791021e-01 1.769804e-01 1.852058e-01

915 8.990323e-01 9.109671e-01 8.877280e-01 8.771291e-01 8.670606e-01 1.895838e-01 1.824987e-01 1.805383e-01 1.784210e-01 1.865475e-01

916 8.990324e-01 9.109672e-01 8.877280e-01 8.771292e-01 8.670607e-01 1.909755e-01 1.839657e-01 1.820196e-01 1.799065e-01 1.879331e-01

917 8.990325e-01 9.109673e-01 8.877281e-01 8.771292e-01 8.670607e-01 1.924125e-01 1.854782e-01 1.835466e-01 1.814373e-01 1.893631e-01

918 8.990326e-01 9.109674e-01 8.877282e-01 8.771293e-01 8.670608e-01 1.938953e-01 1.870368e-01 1.851197e-01 1.830138e-01 1.908381e-01

919 8.990326e-01 9.109675e-01 8.877282e-01 8.771293e-01 8.670608e-01 1.954246e-01 1.886417e-01 1.867391e-01 1.846363e-01 1.923586e-01

920 8.990327e-01 9.109676e-01 8.877283e-01 8.771294e-01 8.670609e-01 1.970007e-01 1.902932e-01 1.884052e-01 1.863049e-01 1.939249e-01

921 8.990328e-01 9.109676e-01 8.877283e-01 8.771294e-01 8.670610e-01 1.986239e-01 1.919914e-01 1.901179e-01 1.880196e-01 1.955373e-01

922 8.990328e-01 9.109677e-01 8.877284e-01 8.771295e-01 8.670610e-01 2.002946e-01 1.937364e-01 1.918774e-01 1.897806e-01 1.971960e-01

923 8.990329e-01 9.109678e-01 8.877285e-01 8.771296e-01 8.670611e-01 2.020127e-01 1.955282e-01 1.936835e-01 1.915875e-01 1.989012e-01

924 8.990330e-01 9.109678e-01 8.877285e-01 8.771296e-01 8.670611e-01 2.037785e-01 1.973665e-01 1.955361e-01 1.934403e-01 2.006528e-01

925 8.990330e-01 9.109679e-01 8.877286e-01 8.771297e-01 8.670612e-01 2.055918e-01 1.992512e-01 1.974348e-01 1.953384e-01 2.024507e-01

926 8.990331e-01 9.109680e-01 8.877286e-01 8.771297e-01 8.670612e-01 2.074525e-01 2.011819e-01 1.993792e-01 1.972814e-01 2.042946e-01

927 8.990332e-01 9.109680e-01 8.877287e-01 8.771298e-01 8.670613e-01 2.093602e-01 2.031579e-01 2.013688e-01 1.992687e-01 2.061843e-01

928 8.990332e-01 9.109681e-01 8.877288e-01 8.771299e-01 8.670614e-01 2.113147e-01 2.051788e-01 2.034028e-01 2.012995e-01 2.081192e-01

929 8.990333e-01 9.109682e-01 8.877289e-01 8.771300e-01 8.670615e-01 2.133152e-01 2.072437e-01 2.054805e-01 2.033729e-01 2.100988e-01

930 8.990334e-01 9.109683e-01 8.877290e-01 8.771301e-01 8.670616e-01 2.153612e-01 2.093518e-01 2.076010e-01 2.054880e-01 2.121223e-01

931 8.990335e-01 9.109684e-01 8.877291e-01 8.771302e-01 8.670617e-01 2.174520e-01 2.115021e-01 2.097630e-01 2.076436e-01 2.141888e-01

932 8.990336e-01 9.109685e-01 8.877292e-01 8.771303e-01 8.670618e-01 2.195865e-01 2.136934e-01 2.119656e-01 2.098384e-01 2.162974e-01

933 8.990337e-01 9.109686e-01 8.877293e-01 8.771304e-01 8.670619e-01 2.217638e-01 2.159245e-01 2.142073e-01 2.120710e-01 2.184469e-01

934 8.990338e-01 9.109687e-01 8.877294e-01 8.771305e-01 8.670621e-01 2.239827e-01 2.181940e-01 2.164867e-01 2.143399e-01 2.206360e-01

935 8.990340e-01 9.109688e-01 8.877296e-01 8.771307e-01 8.670622e-01 2.262419e-01 2.205004e-01 2.188022e-01 2.166435e-01 2.228635e-01

936 8.990341e-01 9.109690e-01 8.877297e-01 8.771308e-01 8.670624e-01 2.285400e-01 2.228421e-01 2.211522e-01 2.189800e-01 2.251276e-01

937 8.990343e-01 9.109691e-01 8.877299e-01 8.771310e-01 8.670626e-01 2.308755e-01 2.252173e-01 2.235349e-01 2.213475e-01 2.274268e-01

938 8.990345e-01 9.109693e-01 8.877301e-01 8.771312e-01 8.670628e-01 2.332467e-01 2.276242e-01 2.259483e-01 2.237440e-01 2.297593e-01

939 8.990347e-01 9.109695e-01 8.877303e-01 8.771314e-01 8.670630e-01 2.356519e-01 2.300609e-01 2.283904e-01 2.261675e-01 2.321232e-01

940 8.990349e-01 9.109697e-01 8.877305e-01 8.771317e-01 8.670632e-01 2.380893e-01 2.325254e-01 2.308592e-01 2.286157e-01 2.345165e-01

941 8.990351e-01 9.109699e-01 8.877308e-01 8.771319e-01 8.670635e-01 2.405569e-01 2.350156e-01 2.333524e-01 2.310863e-01 2.369371e-01

942 8.990354e-01 9.109701e-01 8.877310e-01 8.771322e-01 8.670638e-01 2.430528e-01 2.375294e-01 2.358678e-01 2.335772e-01 2.393827e-01

943 8.990357e-01 9.109704e-01 8.877313e-01 8.771325e-01 8.670641e-01 2.455749e-01 2.400646e-01 2.384031e-01 2.360858e-01 2.418510e-01

944 8.990360e-01 9.109707e-01 8.877316e-01 8.771328e-01 8.670644e-01 2.481210e-01 2.426189e-01 2.409560e-01 2.386097e-01 2.443397e-01

945 8.990363e-01 9.109710e-01 8.877320e-01 8.771332e-01 8.670648e-01 2.506890e-01 2.451899e-01 2.435241e-01 2.411465e-01 2.468461e-01

946 8.990366e-01 9.109713e-01 8.877324e-01 8.771336e-01 8.670652e-01 2.532766e-01 2.477754e-01 2.461049e-01 2.436936e-01 2.493680e-01

947 8.990370e-01 9.109717e-01 8.877328e-01 8.771340e-01 8.670656e-01 2.558815e-01 2.503729e-01 2.486960e-01 2.462486e-01 2.519026e-01

948 8.990374e-01 9.109721e-01 8.877332e-01 8.771344e-01 8.670661e-01 2.585012e-01 2.529800e-01 2.512949e-01 2.488089e-01 2.544473e-01

949 8.990379e-01 9.109725e-01 8.877337e-01 8.771349e-01 8.670666e-01 2.611334e-01 2.555942e-01 2.538990e-01 2.513718e-01 2.569996e-01

950 8.990384e-01 9.109730e-01 8.877342e-01 8.771354e-01 8.670671e-01 2.637756e-01 2.582130e-01 2.565059e-01 2.539350e-01 2.595569e-01

951 8.990389e-01 9.109735e-01 8.877347e-01 8.771360e-01 8.670677e-01 2.664254e-01 2.608340e-01 2.591130e-01 2.564957e-01 2.621164e-01

952 8.990395e-01 9.109740e-01 8.877353e-01 8.771366e-01 8.670683e-01 2.690803e-01 2.634547e-01 2.617178e-01 2.590515e-01 2.646756e-01

953 8.990401e-01 9.109746e-01 8.877359e-01 8.771372e-01 8.670690e-01 2.717378e-01 2.660726e-01 2.643178e-01 2.615998e-01 2.672318e-01

954 8.990407e-01 9.109752e-01 8.877366e-01 8.771379e-01 8.670697e-01 2.743954e-01 2.686852e-01 2.669104e-01 2.641381e-01 2.697823e-01

955 8.990414e-01 9.109758e-01 8.877373e-01 8.771387e-01 8.670705e-01 2.770505e-01 2.712901e-01 2.694933e-01 2.666640e-01 2.723247e-01

956 8.990421e-01 9.109765e-01 8.877381e-01 8.771395e-01 8.670713e-01 2.797008e-01 2.738848e-01 2.720640e-01 2.691750e-01 2.748562e-01

957 8.990429e-01 9.109773e-01 8.877389e-01 8.771404e-01 8.670722e-01 2.823437e-01 2.764670e-01 2.746201e-01 2.716688e-01 2.773744e-01

958 8.990438e-01 9.109781e-01 8.877398e-01 8.771413e-01 8.670731e-01 2.849769e-01 2.790344e-01 2.771593e-01 2.741431e-01 2.798768e-01

959 8.990447e-01 9.109789e-01 8.877408e-01 8.771423e-01 8.670742e-01 2.875978e-01 2.815846e-01 2.796793e-01 2.765956e-01 2.823610e-01

960 8.990457e-01 9.109799e-01 8.877418e-01 8.771433e-01 8.670753e-01 2.902043e-01 2.841154e-01 2.821780e-01 2.790242e-01 2.848245e-01

961 8.990467e-01 9.109809e-01 8.877429e-01 8.771445e-01 8.670764e-01 2.927939e-01 2.866247e-01 2.846530e-01 2.814268e-01 2.872652e-01

962 8.990479e-01 9.109819e-01 8.877441e-01 8.771457e-01 8.670777e-01 2.953644e-01 2.891103e-01 2.871025e-01 2.838014e-01 2.896808e-01

963 8.990491e-01 9.109831e-01 8.877453e-01 8.771470e-01 8.670791e-01 2.979137e-01 2.915702e-01 2.895244e-01 2.861460e-01 2.920692e-01

964 8.990504e-01 9.109843e-01 8.877467e-01 8.771485e-01 8.670806e-01 3.004396e-01 2.940026e-01 2.919167e-01 2.884589e-01 2.944282e-01

965 8.990518e-01 9.109856e-01 8.877482e-01 8.771500e-01 8.670821e-01 3.029401e-01 2.964055e-01 2.942778e-01 2.907383e-01 2.967561e-01

966 8.990533e-01 9.109870e-01 8.877497e-01 8.771516e-01 8.670838e-01 3.054133e-01 2.987772e-01 2.966059e-01 2.929827e-01 2.990509e-01

967 8.990549e-01 9.109885e-01 8.877514e-01 8.771534e-01 8.670857e-01 3.078572e-01 3.011161e-01 2.988994e-01 2.951904e-01 3.013109e-01

968 8.990566e-01 9.109901e-01 8.877532e-01 8.771553e-01 8.670876e-01 3.102702e-01 3.034205e-01 3.011568e-01 2.973603e-01 3.035345e-01

969 8.990584e-01 9.109918e-01 8.877552e-01 8.771573e-01 8.670898e-01 3.126504e-01 3.056890e-01 3.033767e-01 2.994908e-01 3.057201e-01

970 8.990604e-01 9.109937e-01 8.877573e-01 8.771595e-01 8.670921e-01 3.149964e-01 3.079202e-01 3.055578e-01 3.015810e-01 3.078665e-01

971 8.990625e-01 9.109956e-01 8.877595e-01 8.771619e-01 8.670945e-01 3.173067e-01 3.101129e-01 3.076989e-01 3.036297e-01 3.099722e-01

972 8.990648e-01 9.109978e-01 8.877620e-01 8.771644e-01 8.670972e-01 3.195798e-01 3.122660e-01 3.097990e-01 3.056361e-01 3.120361e-01

973 8.990673e-01 9.110000e-01 8.877646e-01 8.771672e-01 8.671001e-01 3.218145e-01 3.143784e-01 3.118572e-01 3.075992e-01 3.140573e-01

974 8.990699e-01 9.110025e-01 8.877674e-01 8.771701e-01 8.671031e-01 3.240097e-01 3.164493e-01 3.138725e-01 3.095185e-01 3.160348e-01

975 8.990728e-01 9.110051e-01 8.877704e-01 8.771733e-01 8.671065e-01 3.261643e-01 3.184777e-01 3.158444e-01 3.113934e-01 3.179678e-01

976 8.990758e-01 9.110079e-01 8.877736e-01 8.771767e-01 8.671101e-01 3.282773e-01 3.204630e-01 3.177721e-01 3.132233e-01 3.198556e-01

977 8.990791e-01 9.110110e-01 8.877771e-01 8.771804e-01 8.671140e-01 3.303479e-01 3.224046e-01 3.196552e-01 3.150079e-01 3.216978e-01

978 8.990826e-01 9.110142e-01 8.877809e-01 8.771844e-01 8.671182e-01 3.323755e-01 3.243021e-01 3.214932e-01 3.167470e-01 3.234939e-01

979 8.990864e-01 9.110177e-01 8.877850e-01 8.771887e-01 8.671228e-01 3.343593e-01 3.261550e-01 3.232860e-01 3.184405e-01 3.252435e-01

980 8.990905e-01 9.110215e-01 8.877894e-01 8.771934e-01 8.671277e-01 3.362989e-01 3.279631e-01 3.250333e-01 3.200883e-01 3.269466e-01

981 8.990950e-01 9.110256e-01 8.877941e-01 8.771984e-01 8.671330e-01 3.381939e-01 3.297262e-01 3.267351e-01 3.216905e-01 3.286030e-01

982 8.990997e-01 9.110299e-01 8.877992e-01 8.772039e-01 8.671388e-01 3.400440e-01 3.314442e-01 3.283914e-01 3.232472e-01 3.302128e-01

983 8.991049e-01 9.110346e-01 8.878048e-01 8.772098e-01 8.671450e-01 3.418489e-01 3.331172e-01 3.300022e-01 3.247587e-01 3.317760e-01

984 8.991104e-01 9.110397e-01 8.878107e-01 8.772161e-01 8.671517e-01 3.436086e-01 3.347453e-01 3.315678e-01 3.262254e-01 3.332930e-01

985 8.991164e-01 9.110451e-01 8.878172e-01 8.772230e-01 8.671591e-01 3.453230e-01 3.363286e-01 3.330886e-01 3.276476e-01 3.347639e-01

986 8.991228e-01 9.110509e-01 8.878241e-01 8.772304e-01 8.671670e-01 3.469921e-01 3.378675e-01 3.345648e-01 3.290259e-01 3.361894e-01

987 8.991297e-01 9.110573e-01 8.878317e-01 8.772385e-01 8.671756e-01 3.486160e-01 3.393621e-01 3.359969e-01 3.303608e-01 3.375697e-01

988 8.991372e-01 9.110641e-01 8.878398e-01 8.772472e-01 8.671849e-01 3.501949e-01 3.408129e-01 3.373854e-01 3.316529e-01 3.389055e-01

989 8.991453e-01 9.110714e-01 8.878486e-01 8.772567e-01 8.671950e-01 3.517291e-01 3.422203e-01 3.387308e-01 3.329029e-01 3.401975e-01

990 8.991541e-01 9.110793e-01 8.878581e-01 8.772669e-01 8.672060e-01 3.532187e-01 3.435847e-01 3.400338e-01 3.341117e-01 3.414463e-01

991 8.991636e-01 9.110878e-01 8.878684e-01 8.772780e-01 8.672179e-01 3.546641e-01 3.449067e-01 3.412950e-01 3.352798e-01 3.426526e-01

992 8.991738e-01 9.110970e-01 8.878796e-01 8.772901e-01 8.672308e-01 3.560658e-01 3.461868e-01 3.425149e-01 3.364082e-01 3.438174e-01

993 8.991848e-01 9.111069e-01 8.878916e-01 8.773031e-01 8.672448e-01 3.574243e-01 3.474257e-01 3.436944e-01 3.374976e-01 3.449414e-01

994 8.991968e-01 9.111176e-01 8.879047e-01 8.773173e-01 8.672601e-01 3.587400e-01 3.486238e-01 3.448340e-01 3.385489e-01 3.460256e-01

995 8.992097e-01 9.111292e-01 8.879189e-01 8.773327e-01 8.672766e-01 3.600135e-01 3.497820e-01 3.459347e-01 3.395629e-01 3.470709e-01

996 8.992237e-01 9.111417e-01 8.879342e-01 8.773494e-01 8.672946e-01 3.612454e-01 3.509009e-01 3.469970e-01 3.405405e-01 3.480781e-01

997 8.992388e-01 9.111552e-01 8.879509e-01 8.773675e-01 8.673142e-01 3.624364e-01 3.519812e-01 3.480219e-01 3.414824e-01 3.490484e-01

998 8.992552e-01 9.111698e-01 8.879689e-01 8.773872e-01 8.673355e-01 3.635871e-01 3.530238e-01 3.490100e-01 3.423896e-01 3.499826e-01

999 8.992729e-01 9.111855e-01 8.879885e-01 8.774085e-01 8.673587e-01 3.646984e-01 3.540293e-01 3.499624e-01 3.432630e-01 3.508817e-01

1000 8.992921e-01 9.112026e-01 8.880098e-01 8.774317e-01 8.673839e-01 3.657709e-01 3.549987e-01 3.508797e-01 3.441034e-01 3.517467e-01

1001 8.993129e-01 9.112210e-01 8.880328e-01 8.774570e-01 8.674114e-01 3.668054e-01 3.559327e-01 3.517628e-01 3.449117e-01 3.525784e-01

1002 8.993354e-01 9.112409e-01 8.880578e-01 8.774844e-01 8.674413e-01 3.678027e-01 3.568321e-01 3.526127e-01 3.456888e-01 3.533778e-01

1003 8.993599e-01 9.112624e-01 8.880850e-01 8.775143e-01 8.674738e-01 3.687637e-01 3.576978e-01 3.534301e-01 3.464355e-01 3.541459e-01

1004 8.993863e-01 9.112857e-01 8.881145e-01 8.775467e-01 8.675093e-01 3.696891e-01 3.585307e-01 3.542160e-01 3.471528e-01 3.548835e-01

1005 8.994150e-01 9.113109e-01 8.881465e-01 8.775821e-01 8.675480e-01 3.705800e-01 3.593316e-01 3.549711e-01 3.478414e-01 3.555915e-01

1006 8.994462e-01 9.113382e-01 8.881813e-01 8.776205e-01 8.675902e-01 3.714370e-01 3.601014e-01 3.556964e-01 3.485023e-01 3.562710e-01

1007 8.994799e-01 9.113677e-01 8.882192e-01 8.776624e-01 8.676361e-01 3.722611e-01 3.608409e-01 3.563928e-01 3.491363e-01 3.569227e-01

1008 8.995165e-01 9.113997e-01 8.882603e-01 8.777079e-01 8.676862e-01 3.730532e-01 3.615511e-01 3.570611e-01 3.497443e-01 3.575476e-01

1009 8.995563e-01 9.114343e-01 8.883050e-01 8.777575e-01 8.677409e-01 3.738142e-01 3.622328e-01 3.577021e-01 3.503271e-01 3.581466e-01

1010 8.995994e-01 9.114719e-01 8.883536e-01 8.778116e-01 8.678006e-01 3.745449e-01 3.628868e-01 3.583168e-01 3.508855e-01 3.587204e-01

1011 8.996462e-01 9.115125e-01 8.884064e-01 8.778704e-01 8.678656e-01 3.752462e-01 3.635141e-01 3.589060e-01 3.514204e-01 3.592699e-01

1012 8.996971e-01 9.115565e-01 8.884639e-01 8.779346e-01 8.679367e-01 3.759191e-01 3.641154e-01 3.594704e-01 3.519325e-01 3.597960e-01

1013 8.997523e-01 9.116042e-01 8.885264e-01 8.780045e-01 8.680142e-01 3.765644e-01 3.646916e-01 3.600110e-01 3.524226e-01 3.602995e-01

1014 8.998122e-01 9.116559e-01 8.885944e-01 8.780807e-01 8.680989e-01 3.771830e-01 3.652435e-01 3.605286e-01 3.528916e-01 3.607812e-01

1015 8.998773e-01 9.117120e-01 8.886685e-01 8.781637e-01 8.681913e-01 3.777757e-01 3.657720e-01 3.610239e-01 3.533401e-01 3.612418e-01

1016 8.999480e-01 9.117727e-01 8.887490e-01 8.782543e-01 8.682922e-01 3.783434e-01 3.662779e-01 3.614977e-01 3.537689e-01 3.616822e-01

1017 9.000248e-01 9.118386e-01 8.888367e-01 8.783530e-01 8.684024e-01 3.788869e-01 3.667619e-01 3.619508e-01 3.541788e-01 3.621030e-01

1018 9.001083e-01 9.119100e-01 8.889322e-01 8.784606e-01 8.685227e-01 3.794072e-01 3.672249e-01 3.623840e-01 3.545704e-01 3.625050e-01

1019 9.001990e-01 9.119874e-01 8.890361e-01 8.785779e-01 8.686541e-01 3.799049e-01 3.676676e-01 3.627980e-01 3.549445e-01 3.628890e-01

1020 9.002975e-01 9.120714e-01 8.891491e-01 8.787059e-01 8.687977e-01 3.803808e-01 3.680907e-01 3.631935e-01 3.553016e-01 3.632556e-01

1021 9.004046e-01 9.121625e-01 8.892723e-01 8.788454e-01 8.689544e-01 3.808359e-01 3.684949e-01 3.635712e-01 3.556426e-01 3.636055e-01

1022 9.005210e-01 9.122613e-01 8.894063e-01 8.789975e-01 8.691255e-01 3.812708e-01 3.688811e-01 3.639318e-01 3.559679e-01 3.639393e-01

1023 9.006474e-01 9.123684e-01 8.895522e-01 8.791634e-01 8.693124e-01 3.816863e-01 3.692498e-01 3.642760e-01 3.562783e-01 3.642578e-01

1024 9.007848e-01 9.124846e-01 8.897109e-01 8.793442e-01 8.695164e-01 3.820831e-01 3.696018e-01 3.646044e-01 3.565743e-01 3.645614e-01

1025 9.009341e-01 9.126106e-01 8.898838e-01 8.795412e-01 8.697390e-01 3.824620e-01 3.699377e-01 3.649176e-01 3.568566e-01 3.648509e-01

1026 9.010962e-01 9.127473e-01 8.900718e-01 8.797560e-01 8.699820e-01 3.828236e-01 3.702581e-01 3.652163e-01 3.571256e-01 3.651268e-01

1027 9.012724e-01 9.128955e-01 8.902765e-01 8.799900e-01 8.702470e-01 3.831687e-01 3.705638e-01 3.655011e-01 3.573820e-01 3.653896e-01

1028 9.014638e-01 9.130562e-01 8.904990e-01 8.802449e-01 8.705361e-01 3.834978e-01 3.708552e-01 3.657725e-01 3.576262e-01 3.656400e-01

1029 9.016716e-01 9.132304e-01 8.907411e-01 8.805225e-01 8.708512e-01 3.838117e-01 3.711329e-01 3.660311e-01 3.578588e-01 3.658784e-01

1030 9.018973e-01 9.134193e-01 8.910044e-01 8.808247e-01 8.711946e-01 3.841109e-01 3.713976e-01 3.662774e-01 3.580803e-01 3.661054e-01

1031 9.021422e-01 9.136240e-01 8.912905e-01 8.811535e-01 8.715687e-01 3.843960e-01 3.716498e-01 3.665119e-01 3.582912e-01 3.663214e-01

1032 9.024080e-01 9.138458e-01 8.916014e-01 8.815112e-01 8.719758e-01 3.846678e-01 3.718900e-01 3.667353e-01 3.584919e-01 3.665270e-01

1033 9.026964e-01 9.140860e-01 8.919390e-01 8.819001e-01 8.724188e-01 3.849266e-01 3.721187e-01 3.669478e-01 3.586828e-01 3.667225e-01

1034 9.030092e-01 9.143463e-01 8.923056e-01 8.823226e-01 8.729005e-01 3.851730e-01 3.723364e-01 3.671501e-01 3.588645e-01 3.669085e-01

1035 9.033482e-01 9.146280e-01 8.927034e-01 8.827813e-01 8.734237e-01 3.854077e-01 3.725436e-01 3.673426e-01 3.590372e-01 3.670854e-01

1036 9.037155e-01 9.149330e-01 8.931347e-01 8.832792e-01 8.739918e-01 3.856310e-01 3.727408e-01 3.675256e-01 3.592015e-01 3.672535e-01

1037 9.041134e-01 9.152629e-01 8.936023e-01 8.838190e-01 8.746079e-01 3.858435e-01 3.729284e-01 3.676997e-01 3.593576e-01 3.674132e-01

1038 9.045440e-01 9.156197e-01 8.941087e-01 8.844040e-01 8.752755e-01 3.860457e-01 3.731067e-01 3.678651e-01 3.595060e-01 3.675651e-01

1039 9.050099e-01 9.160054e-01 8.946568e-01 8.850372e-01 8.759981e-01 3.862380e-01 3.732764e-01 3.680224e-01 3.596471e-01 3.677093e-01

1040 9.055135e-01 9.164221e-01 8.952495e-01 8.857220e-01 8.767795e-01 3.864209e-01 3.734376e-01 3.681719e-01 3.597810e-01 3.678463e-01

1041 9.060577e-01 9.168721e-01 8.958900e-01 8.864619e-01 8.776234e-01 3.865948e-01 3.735909e-01 3.683139e-01 3.599083e-01 3.679763e-01

1042 9.066451e-01 9.173577e-01 8.965814e-01 8.872604e-01 8.785336e-01 3.867600e-01 3.737365e-01 3.684488e-01 3.600291e-01 3.680998e-01

1043 9.072787e-01 9.178814e-01 8.973271e-01 8.881212e-01 8.795140e-01 3.869170e-01 3.738748e-01 3.685769e-01 3.601439e-01 3.682171e-01

1044 9.079615e-01 9.184458e-01 8.981305e-01 8.890480e-01 8.805685e-01 3.870662e-01 3.740063e-01 3.686986e-01 3.602528e-01 3.683283e-01

1045 9.086967e-01 9.190538e-01 8.989950e-01 8.900446e-01 8.817011e-01 3.872079e-01 3.741311e-01 3.688141e-01 3.603562e-01 3.684339e-01

1046 9.094876e-01 9.197081e-01 8.999243e-01 8.911145e-01 8.829154e-01 3.873425e-01 3.742496e-01 3.689237e-01 3.604543e-01 3.685340e-01

1047 9.103374e-01 9.204118e-01 9.009219e-01 8.922617e-01 8.842151e-01 3.874703e-01 3.743621e-01 3.690277e-01 3.605473e-01 3.686290e-01

1048 9.112496e-01 9.211681e-01 9.019915e-01 8.934898e-01 8.856039e-01 3.875916e-01 3.744688e-01 3.691264e-01 3.606356e-01 3.687190e-01

1049 9.122277e-01 9.219801e-01 9.031365e-01 8.948022e-01 8.870851e-01 3.877067e-01 3.745701e-01 3.692200e-01 3.607193e-01 3.688044e-01

1050 9.132752e-01 9.228512e-01 9.043606e-01 8.962024e-01 8.886617e-01 3.878160e-01 3.746663e-01 3.693088e-01 3.607987e-01 3.688854e-01

1051 9.143956e-01 9.237849e-01 9.056672e-01 8.976937e-01 8.903365e-01 3.879196e-01 3.747574e-01 3.693930e-01 3.608740e-01 3.689621e-01

1052 9.155927e-01 9.247846e-01 9.070597e-01 8.992789e-01 8.921118e-01 3.880179e-01 3.748439e-01 3.694729e-01 3.609453e-01 3.690348e-01

1053 9.168698e-01 9.258539e-01 9.085416e-01 9.009609e-01 8.939898e-01 3.881112e-01 3.749259e-01 3.695485e-01 3.610129e-01 3.691037e-01

1054 9.182306e-01 9.269964e-01 9.101157e-01 9.027421e-01 8.959718e-01 3.881996e-01 3.750037e-01 3.696203e-01 3.610770e-01 3.691689e-01

1055 9.196783e-01 9.282156e-01 9.117851e-01 9.046245e-01 8.980590e-01 3.882834e-01 3.750774e-01 3.696882e-01 3.611377e-01 3.692307e-01

1056 9.212162e-01 9.295152e-01 9.135524e-01 9.066100e-01 9.002519e-01 3.883629e-01 3.751472e-01 3.697526e-01 3.611952e-01 3.692892e-01

1057 9.228474e-01 9.308986e-01 9.154199e-01 9.086998e-01 9.025505e-01 3.884382e-01 3.752134e-01 3.698136e-01 3.612496e-01 3.693446e-01

1058 9.245749e-01 9.323692e-01 9.173896e-01 9.108947e-01 9.049541e-01 3.885096e-01 3.752761e-01 3.698714e-01 3.613012e-01 3.693971e-01

1059 9.264011e-01 9.339304e-01 9.194631e-01 9.131951e-01 9.074618e-01 3.885772e-01 3.753355e-01 3.699261e-01 3.613500e-01 3.694467e-01

1060 9.283285e-01 9.355853e-01 9.216417e-01 9.156008e-01 9.100718e-01 3.886413e-01 3.753918e-01 3.699779e-01 3.613962e-01 3.694937e-01

1061 9.303590e-01 9.373368e-01 9.239260e-01 9.181111e-01 9.127817e-01 3.887019e-01 3.754450e-01 3.700270e-01 3.614400e-01 3.695381e-01

1062 9.324943e-01 9.391876e-01 9.263164e-01 9.207248e-01 9.155887e-01 3.887594e-01 3.754955e-01 3.700734e-01 3.614814e-01 3.695802e-01

1063 9.347354e-01 9.411401e-01 9.288126e-01 9.234399e-01 9.184892e-01 3.888137e-01 3.755432e-01 3.701173e-01 3.615205e-01 3.696200e-01

1064 9.370833e-01 9.431963e-01 9.314138e-01 9.262540e-01 9.214791e-01 3.888652e-01 3.755884e-01 3.701589e-01 3.615576e-01 3.696576e-01

1065 9.395381e-01 9.453578e-01 9.341186e-01 9.291642e-01 9.245540e-01 3.889140e-01 3.756312e-01 3.701982e-01 3.615926e-01 3.696931e-01

1066 9.420996e-01 9.476261e-01 9.369252e-01 9.321669e-01 9.277086e-01 3.889601e-01 3.756717e-01 3.702354e-01 3.616258e-01 3.697268e-01

1067 9.447672e-01 9.500019e-01 9.398312e-01 9.352580e-01 9.309373e-01 3.890037e-01 3.757100e-01 3.702706e-01 3.616571e-01 3.697586e-01

1068 9.475395e-01 9.524857e-01 9.428335e-01 9.384329e-01 9.342341e-01 3.890450e-01 3.757462e-01 3.703039e-01 3.616868e-01 3.697886e-01

1069 9.504147e-01 9.550772e-01 9.459288e-01 9.416866e-01 9.375925e-01 3.890841e-01 3.757805e-01 3.703354e-01 3.617148e-01 3.698170e-01

1070 9.533906e-01 9.577760e-01 9.491129e-01 9.450135e-01 9.410058e-01 3.891210e-01 3.758129e-01 3.703651e-01 3.617413e-01 3.698438e-01

1071 9.564643e-01 9.605808e-01 9.523815e-01 9.484077e-01 9.444669e-01 3.891559e-01 3.758435e-01 3.703932e-01 3.617663e-01 3.698692e-01

1072 9.596325e-01 9.634902e-01 9.557295e-01 9.518629e-01 9.479683e-01 3.891889e-01 3.758725e-01 3.704198e-01 3.617900e-01 3.698931e-01

1073 9.628913e-01 9.665018e-01 9.591516e-01 9.553726e-01 9.515025e-01 3.892202e-01 3.758999e-01 3.704449e-01 3.618123e-01 3.699157e-01

1074 9.662365e-01 9.696130e-01 9.626421e-01 9.589298e-01 9.550619e-01 3.892497e-01 3.759258e-01 3.704687e-01 3.618334e-01 3.699371e-01

1075 9.696634e-01 9.728208e-01 9.661951e-01 9.625274e-01 9.586386e-01 3.892776e-01 3.759502e-01 3.704911e-01 3.618534e-01 3.699573e-01

1076 9.731669e-01 9.761215e-01 9.698040e-01 9.661582e-01 9.622247e-01 3.893039e-01 3.759733e-01 3.705123e-01 3.618722e-01 3.699763e-01

1077 9.767417e-01 9.795111e-01 9.734624e-01 9.698147e-01 9.658123e-01 3.893288e-01 3.759952e-01 3.705323e-01 3.618900e-01 3.699943e-01

1078 9.803819e-01 9.829851e-01 9.771634e-01 9.734894e-01 9.693937e-01 3.893523e-01 3.760158e-01 3.705511e-01 3.619068e-01 3.700112e-01

1079 9.840816e-01 9.865387e-01 9.809002e-01 9.771748e-01 9.729610e-01 3.893746e-01 3.760353e-01 3.705690e-01 3.619226e-01 3.700272e-01

1080 9.878345e-01 9.901668e-01 9.846656e-01 9.808633e-01 9.765066e-01 3.893956e-01 3.760537e-01 3.705858e-01 3.619376e-01 3.700423e-01

1081 9.916343e-01 9.938640e-01 9.884525e-01 9.845474e-01 9.800231e-01 3.894154e-01 3.760710e-01 3.706017e-01 3.619517e-01 3.700566e-01

1082 9.916343e-01 9.938639e-01 9.884525e-01 9.845474e-01 9.800231e-01 3.895922e-01 3.762638e-01 3.707968e-01 3.621527e-01 3.702352e-01

1083 9.916343e-01 9.938638e-01 9.884525e-01 9.845475e-01 9.800231e-01 3.897762e-01 3.764643e-01 3.709997e-01 3.623617e-01 3.704209e-01

1084 9.916343e-01 9.938638e-01 9.884525e-01 9.845475e-01 9.800232e-01 3.899676e-01 3.766727e-01 3.712107e-01 3.625789e-01 3.706140e-01

1085 9.916343e-01 9.938638e-01 9.884525e-01 9.845475e-01 9.800232e-01 3.901666e-01 3.768894e-01 3.714300e-01 3.628048e-01 3.708149e-01

1086 9.916343e-01 9.938638e-01 9.884525e-01 9.845475e-01 9.800232e-01 3.903736e-01 3.771147e-01 3.716579e-01 3.630396e-01 3.710237e-01

1087 9.916343e-01 9.938638e-01 9.884526e-01 9.845476e-01 9.800233e-01 3.905888e-01 3.773489e-01 3.718948e-01 3.632837e-01 3.712408e-01

1088 9.916344e-01 9.938638e-01 9.884526e-01 9.845476e-01 9.800233e-01 3.908125e-01 3.775923e-01 3.721411e-01 3.635374e-01 3.714666e-01

1089 9.916344e-01 9.938638e-01 9.884527e-01 9.845477e-01 9.800233e-01 3.910451e-01 3.778453e-01 3.723970e-01 3.638010e-01 3.717013e-01

1090 9.916344e-01 9.938638e-01 9.884527e-01 9.845477e-01 9.800234e-01 3.912869e-01 3.781082e-01 3.726629e-01 3.640751e-01 3.719453e-01

1091 9.916345e-01 9.938639e-01 9.884527e-01 9.845477e-01 9.800234e-01 3.915382e-01 3.783814e-01 3.729392e-01 3.643598e-01 3.721990e-01

1092 9.916345e-01 9.938639e-01 9.884528e-01 9.845478e-01 9.800235e-01 3.917995e-01 3.786653e-01 3.732263e-01 3.646557e-01 3.724627e-01

1093 9.916346e-01 9.938639e-01 9.884528e-01 9.845478e-01 9.800235e-01 3.920710e-01 3.789603e-01 3.735246e-01 3.649632e-01 3.727368e-01

1094 9.916346e-01 9.938640e-01 9.884529e-01 9.845479e-01 9.800235e-01 3.923532e-01 3.792667e-01 3.738344e-01 3.652827e-01 3.730218e-01

1095 9.916347e-01 9.938640e-01 9.884529e-01 9.845479e-01 9.800236e-01 3.926464e-01 3.795851e-01 3.741563e-01 3.656147e-01 3.733180e-01

1096 9.916347e-01 9.938641e-01 9.884530e-01 9.845480e-01 9.800236e-01 3.929511e-01 3.799157e-01 3.744907e-01 3.659595e-01 3.736258e-01

1097 9.916348e-01 9.938641e-01 9.884530e-01 9.845480e-01 9.800237e-01 3.932677e-01 3.802592e-01 3.748380e-01 3.663177e-01 3.739457e-01

1098 9.916348e-01 9.938642e-01 9.884531e-01 9.845480e-01 9.800237e-01 3.935966e-01 3.806159e-01 3.751987e-01 3.666898e-01 3.742783e-01

1099 9.916349e-01 9.938642e-01 9.884531e-01 9.845481e-01 9.800237e-01 3.939384e-01 3.809864e-01 3.755732e-01 3.670763e-01 3.746238e-01

1100 9.916349e-01 9.938643e-01 9.884532e-01 9.845481e-01 9.800238e-01 3.942933e-01 3.813711e-01 3.759622e-01 3.674777e-01 3.749829e-01

1101 9.916350e-01 9.938644e-01 9.884532e-01 9.845482e-01 9.800238e-01 3.946620e-01 3.817705e-01 3.763660e-01 3.678945e-01 3.753559e-01

1102 9.916350e-01 9.938644e-01 9.884532e-01 9.845482e-01 9.800238e-01 3.950449e-01 3.821852e-01 3.767853e-01 3.683273e-01 3.757436e-01

1103 9.916351e-01 9.938645e-01 9.884533e-01 9.845482e-01 9.800239e-01 3.954426e-01 3.826156e-01 3.772205e-01 3.687766e-01 3.761463e-01

1104 9.916351e-01 9.938645e-01 9.884533e-01 9.845483e-01 9.800239e-01 3.958555e-01 3.830624e-01 3.776722e-01 3.692431e-01 3.765647e-01

1105 9.916352e-01 9.938646e-01 9.884534e-01 9.845483e-01 9.800239e-01 3.962842e-01 3.835261e-01 3.781410e-01 3.697272e-01 3.769992e-01

1106 9.916352e-01 9.938646e-01 9.884534e-01 9.845483e-01 9.800240e-01 3.967293e-01 3.840073e-01 3.786275e-01 3.702297e-01 3.774505e-01

1107 9.916353e-01 9.938647e-01 9.884535e-01 9.845484e-01 9.800240e-01 3.971913e-01 3.845065e-01 3.791323e-01 3.707512e-01 3.779192e-01

1108 9.916353e-01 9.938647e-01 9.884535e-01 9.845484e-01 9.800240e-01 3.976708e-01 3.850244e-01 3.796560e-01 3.712922e-01 3.784059e-01

1109 9.916354e-01 9.938648e-01 9.884535e-01 9.845485e-01 9.800240e-01 3.981685e-01 3.855616e-01 3.801992e-01 3.718535e-01 3.789111e-01

1110 9.916354e-01 9.938649e-01 9.884536e-01 9.845485e-01 9.800241e-01 3.986848e-01 3.861188e-01 3.807625e-01 3.724356e-01 3.794356e-01

1111 9.916355e-01 9.938649e-01 9.884536e-01 9.845485e-01 9.800241e-01 3.992205e-01 3.866964e-01 3.813466e-01 3.730393e-01 3.799800e-01

1112 9.916355e-01 9.938650e-01 9.884537e-01 9.845486e-01 9.800241e-01 3.997761e-01 3.872953e-01 3.819522e-01 3.736653e-01 3.805449e-01

1113 9.916356e-01 9.938650e-01 9.884537e-01 9.845486e-01 9.800242e-01 4.003524e-01 3.879161e-01 3.825799e-01 3.743141e-01 3.811311e-01

1114 9.916356e-01 9.938651e-01 9.884537e-01 9.845486e-01 9.800242e-01 4.009500e-01 3.885594e-01 3.832304e-01 3.749865e-01 3.817391e-01

1115 9.916356e-01 9.938651e-01 9.884538e-01 9.845487e-01 9.800242e-01 4.015694e-01 3.892259e-01 3.839043e-01 3.756832e-01 3.823697e-01

1116 9.916357e-01 9.938652e-01 9.884538e-01 9.845487e-01 9.800243e-01 4.022115e-01 3.899163e-01 3.846024e-01 3.764050e-01 3.830236e-01

1117 9.916357e-01 9.938652e-01 9.884539e-01 9.845487e-01 9.800243e-01 4.028769e-01 3.906313e-01 3.853253e-01 3.771524e-01 3.837015e-01

1118 9.916358e-01 9.938652e-01 9.884539e-01 9.845488e-01 9.800243e-01 4.035663e-01 3.913716e-01 3.860738e-01 3.779262e-01 3.844041e-01

1119 9.916358e-01 9.938653e-01 9.884539e-01 9.845488e-01 9.800244e-01 4.042804e-01 3.921378e-01 3.868484e-01 3.787271e-01 3.851321e-01

1120 9.916359e-01 9.938653e-01 9.884540e-01 9.845488e-01 9.800244e-01 4.050198e-01 3.929307e-01 3.876500e-01 3.795559e-01 3.858862e-01

1121 9.916359e-01 9.938654e-01 9.884540e-01 9.845489e-01 9.800245e-01 4.057854e-01 3.937509e-01 3.884792e-01 3.804131e-01 3.866671e-01

1122 9.916360e-01 9.938654e-01 9.884541e-01 9.845489e-01 9.800245e-01 4.065777e-01 3.945991e-01 3.893367e-01 3.812995e-01 3.874755e-01

1123 9.916360e-01 9.938655e-01 9.884541e-01 9.845490e-01 9.800245e-01 4.073974e-01 3.954760e-01 3.902231e-01 3.822157e-01 3.883122e-01

1124 9.916361e-01 9.938655e-01 9.884542e-01 9.845490e-01 9.800246e-01 4.082454e-01 3.963823e-01 3.911391e-01 3.831624e-01 3.891777e-01

1125 9.916361e-01 9.938656e-01 9.884542e-01 9.845491e-01 9.800246e-01 4.091222e-01 3.973186e-01 3.920853e-01 3.841403e-01 3.900728e-01

1126 9.916362e-01 9.938656e-01 9.884543e-01 9.845491e-01 9.800247e-01 4.100286e-01 3.982855e-01 3.930624e-01 3.851499e-01 3.909982e-01

1127 9.916362e-01 9.938657e-01 9.884543e-01 9.845492e-01 9.800248e-01 4.109651e-01 3.992837e-01 3.940710e-01 3.861918e-01 3.919545e-01

1128 9.916363e-01 9.938658e-01 9.884544e-01 9.845492e-01 9.800248e-01 4.119326e-01 4.003138e-01 3.951117e-01 3.872666e-01 3.929422e-01

1129 9.916363e-01 9.938658e-01 9.884544e-01 9.845493e-01 9.800249e-01 4.129315e-01 4.013764e-01 3.961850e-01 3.883748e-01 3.939620e-01

1130 9.916364e-01 9.938659e-01 9.884545e-01 9.845494e-01 9.800249e-01 4.139625e-01 4.024719e-01 3.972914e-01 3.895169e-01 3.950145e-01

1131 9.916365e-01 9.938659e-01 9.884546e-01 9.845495e-01 9.800250e-01 4.150263e-01 4.036009e-01 3.984314e-01 3.906934e-01 3.961001e-01

1132 9.916365e-01 9.938660e-01 9.884547e-01 9.845495e-01 9.800251e-01 4.161233e-01 4.047640e-01 3.996055e-01 3.919046e-01 3.972194e-01

1133 9.916366e-01 9.938661e-01 9.884547e-01 9.845496e-01 9.800252e-01 4.172541e-01 4.059614e-01 4.008141e-01 3.931509e-01 3.983728e-01

1134 9.916367e-01 9.938662e-01 9.884548e-01 9.845497e-01 9.800253e-01 4.184192e-01 4.071937e-01 4.020575e-01 3.944327e-01 3.995607e-01

1135 9.916368e-01 9.938662e-01 9.884549e-01 9.845498e-01 9.800254e-01 4.196191e-01 4.084612e-01 4.033361e-01 3.957501e-01 4.007834e-01

1136 9.916369e-01 9.938663e-01 9.884550e-01 9.845499e-01 9.800255e-01 4.208543e-01 4.097642e-01 4.046501e-01 3.971034e-01 4.020413e-01

1137 9.916370e-01 9.938664e-01 9.884551e-01 9.845500e-01 9.800256e-01 4.221250e-01 4.111029e-01 4.059997e-01 3.984926e-01 4.033346e-01

1138 9.916371e-01 9.938665e-01 9.884552e-01 9.845501e-01 9.800257e-01 4.234316e-01 4.124776e-01 4.073851e-01 3.999179e-01 4.046634e-01

1139 9.916372e-01 9.938666e-01 9.884553e-01 9.845502e-01 9.800258e-01 4.247744e-01 4.138883e-01 4.088063e-01 4.013792e-01 4.060278e-01

1140 9.916373e-01 9.938667e-01 9.884554e-01 9.845503e-01 9.800259e-01 4.261536e-01 4.153352e-01 4.102633e-01 4.028763e-01 4.074278e-01

1141 9.916374e-01 9.938668e-01 9.884556e-01 9.845505e-01 9.800261e-01 4.275694e-01 4.168182e-01 4.117561e-01 4.044092e-01 4.088635e-01

1142 9.916375e-01 9.938669e-01 9.884557e-01 9.845506e-01 9.800262e-01 4.290219e-01 4.183372e-01 4.132844e-01 4.059775e-01 4.103346e-01

1143 9.916377e-01 9.938671e-01 9.884559e-01 9.845508e-01 9.800263e-01 4.305111e-01 4.198922e-01 4.148482e-01 4.075809e-01 4.118409e-01

1144 9.916378e-01 9.938672e-01 9.884560e-01 9.845509e-01 9.800265e-01 4.320370e-01 4.214829e-01 4.164470e-01 4.092190e-01 4.133822e-01

1145 9.916380e-01 9.938673e-01 9.884562e-01 9.845511e-01 9.800267e-01 4.335994e-01 4.231089e-01 4.180805e-01 4.108912e-01 4.149580e-01

1146 9.916381e-01 9.938675e-01 9.884564e-01 9.845513e-01 9.800269e-01 4.351983e-01 4.247699e-01 4.197482e-01 4.125970e-01 4.165678e-01

1147 9.916383e-01 9.938677e-01 9.884565e-01 9.845515e-01 9.800271e-01 4.368333e-01 4.264655e-01 4.214494e-01 4.143355e-01 4.182110e-01

1148 9.916385e-01 9.938678e-01 9.884567e-01 9.845517e-01 9.800273e-01 4.385040e-01 4.281950e-01 4.231837e-01 4.161060e-01 4.198870e-01

1149 9.916387e-01 9.938680e-01 9.884570e-01 9.845519e-01 9.800275e-01 4.402101e-01 4.299578e-01 4.249501e-01 4.179076e-01 4.215949e-01

1150 9.916389e-01 9.938682e-01 9.884572e-01 9.845522e-01 9.800278e-01 4.419510e-01 4.317532e-01 4.267479e-01 4.197393e-01 4.233339e-01

1151 9.916391e-01 9.938684e-01 9.884574e-01 9.845524e-01 9.800280e-01 4.437262e-01 4.335803e-01 4.285761e-01 4.215999e-01 4.251029e-01

1152 9.916394e-01 9.938686e-01 9.884577e-01 9.845527e-01 9.800283e-01 4.455349e-01 4.354382e-01 4.304337e-01 4.234882e-01 4.269009e-01

1153 9.916396e-01 9.938688e-01 9.884580e-01 9.845530e-01 9.800286e-01 4.473763e-01 4.373260e-01 4.323195e-01 4.254031e-01 4.287267e-01

1154 9.916399e-01 9.938691e-01 9.884582e-01 9.845533e-01 9.800289e-01 4.492497e-01 4.392424e-01 4.342324e-01 4.273430e-01 4.305789e-01

1155 9.916402e-01 9.938694e-01 9.884586e-01 9.845536e-01 9.800292e-01 4.511540e-01 4.411864e-01 4.361711e-01 4.293066e-01 4.324563e-01

1156 9.916405e-01 9.938696e-01 9.884589e-01 9.845539e-01 9.800296e-01 4.530881e-01 4.431567e-01 4.381341e-01 4.312923e-01 4.343572e-01

1157 9.916408e-01 9.938699e-01 9.884592e-01 9.845543e-01 9.800300e-01 4.550511e-01 4.451518e-01 4.401200e-01 4.332984e-01 4.362802e-01

1158 9.916411e-01 9.938702e-01 9.884596e-01 9.845547e-01 9.800304e-01 4.570416e-01 4.471705e-01 4.421272e-01 4.353233e-01 4.382236e-01

1159 9.916415e-01 9.938706e-01 9.884600e-01 9.845551e-01 9.800308e-01 4.590583e-01 4.492111e-01 4.441542e-01 4.373651e-01 4.401856e-01

1160 9.916419e-01 9.938709e-01 9.884604e-01 9.845556e-01 9.800313e-01 4.610999e-01 4.512722e-01 4.461992e-01 4.394221e-01 4.421644e-01

1161 9.916423e-01 9.938713e-01 9.884609e-01 9.845561e-01 9.800317e-01 4.631649e-01 4.533519e-01 4.482605e-01 4.414924e-01 4.441582e-01

1162 9.916428e-01 9.938717e-01 9.884614e-01 9.845566e-01 9.800323e-01 4.652518e-01 4.554487e-01 4.503363e-01 4.435739e-01 4.461651e-01

1163 9.916432e-01 9.938721e-01 9.884619e-01 9.845571e-01 9.800328e-01 4.673590e-01 4.575608e-01 4.524246e-01 4.456647e-01 4.481829e-01

1164 9.916437e-01 9.938725e-01 9.884624e-01 9.845577e-01 9.800334e-01 4.694848e-01 4.596863e-01 4.545236e-01 4.477628e-01 4.502097e-01

1165 9.916442e-01 9.938730e-01 9.884630e-01 9.845583e-01 9.800341e-01 4.716276e-01 4.618234e-01 4.566313e-01 4.498661e-01 4.522434e-01

1166 9.916448e-01 9.938735e-01 9.884636e-01 9.845590e-01 9.800348e-01 4.737856e-01 4.639702e-01 4.587458e-01 4.519724e-01 4.542818e-01

1167 9.916454e-01 9.938741e-01 9.884643e-01 9.845597e-01 9.800355e-01 4.759572e-01 4.661247e-01 4.608649e-01 4.540798e-01 4.563228e-01

1168 9.916461e-01 9.938746e-01 9.884650e-01 9.845605e-01 9.800363e-01 4.781404e-01 4.682852e-01 4.629866e-01 4.561860e-01 4.583643e-01

1169 9.916467e-01 9.938752e-01 9.884658e-01 9.845613e-01 9.800372e-01 4.803335e-01 4.704496e-01 4.651091e-01 4.582891e-01 4.604040e-01

1170 9.916475e-01 9.938759e-01 9.884666e-01 9.845621e-01 9.800381e-01 4.825347e-01 4.726161e-01 4.672301e-01 4.603867e-01 4.624398e-01

1171 9.916482e-01 9.938766e-01 9.884674e-01 9.845631e-01 9.800390e-01 4.847421e-01 4.747827e-01 4.693477e-01 4.624770e-01 4.644696e-01

1172 9.916491e-01 9.938773e-01 9.884683e-01 9.845641e-01 9.800401e-01 4.869538e-01 4.769475e-01 4.714601e-01 4.645577e-01 4.664911e-01

1173 9.916499e-01 9.938781e-01 9.884693e-01 9.845651e-01 9.800412e-01 4.891680e-01 4.791086e-01 4.735652e-01 4.666271e-01 4.685022e-01

1174 9.916509e-01 9.938789e-01 9.884704e-01 9.845663e-01 9.800424e-01 4.913827e-01 4.812642e-01 4.756610e-01 4.686830e-01 4.705010e-01

1175 9.916519e-01 9.938798e-01 9.884715e-01 9.845675e-01 9.800437e-01 4.935962e-01 4.834124e-01 4.777459e-01 4.707238e-01 4.724853e-01

1176 9.916529e-01 9.938808e-01 9.884727e-01 9.845688e-01 9.800451e-01 4.958064e-01 4.855513e-01 4.798179e-01 4.727475e-01 4.744531e-01

1177 9.916541e-01 9.938818e-01 9.884740e-01 9.845702e-01 9.800465e-01 4.980116e-01 4.876792e-01 4.818753e-01 4.747525e-01 4.764025e-01

1178 9.916553e-01 9.938828e-01 9.884753e-01 9.845717e-01 9.800481e-01 5.002099e-01 4.897943e-01 4.839163e-01 4.767370e-01 4.783319e-01

1179 9.916566e-01 9.938840e-01 9.884768e-01 9.845733e-01 9.800498e-01 5.023995e-01 4.918949e-01 4.859392e-01 4.786994e-01 4.802393e-01

1180 9.916580e-01 9.938852e-01 9.884784e-01 9.845750e-01 9.800517e-01 5.045786e-01 4.939792e-01 4.879426e-01 4.806384e-01 4.821232e-01

1181 9.916595e-01 9.938865e-01 9.884800e-01 9.845769e-01 9.800536e-01 5.067454e-01 4.960458e-01 4.899247e-01 4.825523e-01 4.839821e-01

1182 9.916610e-01 9.938879e-01 9.884818e-01 9.845788e-01 9.800557e-01 5.088982e-01 4.980930e-01 4.918842e-01 4.844399e-01 4.858144e-01

1183 9.916627e-01 9.938893e-01 9.884838e-01 9.845810e-01 9.800580e-01 5.110354e-01 5.001192e-01 4.938196e-01 4.863000e-01 4.876189e-01

1184 9.916645e-01 9.938909e-01 9.884858e-01 9.845832e-01 9.800604e-01 5.131553e-01 5.021232e-01 4.957297e-01 4.881312e-01 4.893943e-01

1185 9.916665e-01 9.938926e-01 9.884880e-01 9.845857e-01 9.800631e-01 5.152564e-01 5.041034e-01 4.976131e-01 4.899326e-01 4.911395e-01

1186 9.916685e-01 9.938944e-01 9.884904e-01 9.845883e-01 9.800659e-01 5.173370e-01 5.060587e-01 4.994687e-01 4.917031e-01 4.928534e-01

1187 9.916707e-01 9.938963e-01 9.884929e-01 9.845911e-01 9.800689e-01 5.193959e-01 5.079877e-01 5.012954e-01 4.934417e-01 4.945350e-01

1188 9.916731e-01 9.938983e-01 9.884956e-01 9.845941e-01 9.800722e-01 5.214315e-01 5.098893e-01 5.030921e-01 4.951478e-01 4.961836e-01

1189 9.916756e-01 9.939005e-01 9.884985e-01 9.845974e-01 9.800757e-01 5.234425e-01 5.117624e-01 5.048581e-01 4.968205e-01 4.977983e-01

1190 9.916783e-01 9.939028e-01 9.885017e-01 9.846009e-01 9.800795e-01 5.254278e-01 5.136060e-01 5.065923e-01 4.984592e-01 4.993785e-01

1191 9.916812e-01 9.939053e-01 9.885050e-01 9.846046e-01 9.800836e-01 5.273860e-01 5.154193e-01 5.082942e-01 5.000634e-01 5.009237e-01

1192 9.916843e-01 9.939079e-01 9.885086e-01 9.846087e-01 9.800880e-01 5.293161e-01 5.172012e-01 5.099629e-01 5.016326e-01 5.024334e-01

1193 9.916876e-01 9.939107e-01 9.885124e-01 9.846130e-01 9.800927e-01 5.312172e-01 5.189512e-01 5.115980e-01 5.031664e-01 5.039073e-01

1194 9.916911e-01 9.939138e-01 9.885166e-01 9.846177e-01 9.800978e-01 5.330881e-01 5.206686e-01 5.131989e-01 5.046645e-01 5.053450e-01

1195 9.916949e-01 9.939170e-01 9.885210e-01 9.846227e-01 9.801033e-01 5.349281e-01 5.223527e-01 5.147653e-01 5.061267e-01 5.067464e-01

1196 9.916990e-01 9.939204e-01 9.885258e-01 9.846282e-01 9.801093e-01 5.367364e-01 5.240030e-01 5.162967e-01 5.075529e-01 5.081115e-01

1197 9.917033e-01 9.939241e-01 9.885309e-01 9.846340e-01 9.801157e-01 5.385122e-01 5.256191e-01 5.177930e-01 5.089431e-01 5.094401e-01

1198 9.917080e-01 9.939281e-01 9.885364e-01 9.846403e-01 9.801226e-01 5.402549e-01 5.272006e-01 5.192540e-01 5.102972e-01 5.107324e-01

1199 9.917130e-01 9.939323e-01 9.885423e-01 9.846470e-01 9.801300e-01 5.419640e-01 5.287474e-01 5.206796e-01 5.116154e-01 5.119885e-01

1200 9.917184e-01 9.939368e-01 9.885487e-01 9.846543e-01 9.801381e-01 5.436390e-01 5.302591e-01 5.220698e-01 5.128979e-01 5.132087e-01

1201 9.917241e-01 9.939417e-01 9.885555e-01 9.846622e-01 9.801468e-01 5.452796e-01 5.317356e-01 5.234245e-01 5.141448e-01 5.143931e-01

1202 9.917303e-01 9.939469e-01 9.885628e-01 9.846706e-01 9.801562e-01 5.468853e-01 5.331770e-01 5.247441e-01 5.153565e-01 5.155423e-01

1203 9.917369e-01 9.939524e-01 9.885707e-01 9.846797e-01 9.801664e-01 5.484561e-01 5.345832e-01 5.260285e-01 5.165333e-01 5.166565e-01

1204 9.917441e-01 9.939583e-01 9.885792e-01 9.846896e-01 9.801774e-01 5.499916e-01 5.359544e-01 5.272781e-01 5.176757e-01 5.177362e-01

1205 9.917517e-01 9.939647e-01 9.885884e-01 9.847002e-01 9.801892e-01 5.514919e-01 5.372905e-01 5.284932e-01 5.187840e-01 5.187820e-01

1206 9.917599e-01 9.939715e-01 9.885983e-01 9.847117e-01 9.802021e-01 5.529568e-01 5.385920e-01 5.296741e-01 5.198588e-01 5.197944e-01

1207 9.917687e-01 9.939788e-01 9.886089e-01 9.847240e-01 9.802160e-01 5.543865e-01 5.398589e-01 5.308212e-01 5.209006e-01 5.207740e-01

1208 9.917782e-01 9.939867e-01 9.886203e-01 9.847374e-01 9.802310e-01 5.557810e-01 5.410918e-01 5.319351e-01 5.219101e-01 5.217214e-01

1209 9.917883e-01 9.939951e-01 9.886326e-01 9.847518e-01 9.802472e-01 5.571404e-01 5.422908e-01 5.330161e-01 5.228877e-01 5.226374e-01

1210 9.917992e-01 9.940041e-01 9.886458e-01 9.847674e-01 9.802648e-01 5.584649e-01 5.434563e-01 5.340648e-01 5.238343e-01 5.235225e-01

1211 9.918110e-01 9.940137e-01 9.886601e-01 9.847842e-01 9.802839e-01 5.597547e-01 5.445889e-01 5.350817e-01 5.247503e-01 5.243776e-01

1212 9.918236e-01 9.940241e-01 9.886755e-01 9.848023e-01 9.803045e-01 5.610099e-01 5.456888e-01 5.360675e-01 5.256366e-01 5.252033e-01

1213 9.918372e-01 9.940352e-01 9.886921e-01 9.848220e-01 9.803269e-01 5.622310e-01 5.467565e-01 5.370227e-01 5.264937e-01 5.260004e-01

1214 9.918518e-01 9.940471e-01 9.887100e-01 9.848432e-01 9.803511e-01 5.634181e-01 5.477926e-01 5.379478e-01 5.273224e-01 5.267698e-01

1215 9.918675e-01 9.940599e-01 9.887293e-01 9.848662e-01 9.803774e-01 5.645716e-01 5.487974e-01 5.388435e-01 5.281233e-01 5.275121e-01

1216 9.918844e-01 9.940736e-01 9.887502e-01 9.848910e-01 9.804058e-01 5.656919e-01 5.497715e-01 5.397103e-01 5.288970e-01 5.282282e-01

1217 9.919025e-01 9.940883e-01 9.887726e-01 9.849178e-01 9.804366e-01 5.667794e-01 5.507153e-01 5.405488e-01 5.296443e-01 5.289187e-01

1218 9.919221e-01 9.941042e-01 9.887969e-01 9.849469e-01 9.804700e-01 5.678345e-01 5.516294e-01 5.413597e-01 5.303658e-01 5.295845e-01

1219 9.919432e-01 9.941212e-01 9.888231e-01 9.849783e-01 9.805063e-01 5.688577e-01 5.525144e-01 5.421434e-01 5.310620e-01 5.302262e-01

1220 9.919658e-01 9.941394e-01 9.888513e-01 9.850123e-01 9.805456e-01 5.698494e-01 5.533708e-01 5.429007e-01 5.317338e-01 5.308446e-01

1221 9.919903e-01 9.941590e-01 9.888818e-01 9.850491e-01 9.805882e-01 5.708102e-01 5.541992e-01 5.436321e-01 5.323816e-01 5.314403e-01

1222 9.920166e-01 9.941801e-01 9.889148e-01 9.850889e-01 9.806345e-01 5.717405e-01 5.550001e-01 5.443382e-01 5.330062e-01 5.320140e-01

1223 9.920449e-01 9.942028e-01 9.889503e-01 9.851320e-01 9.806846e-01 5.726410e-01 5.557741e-01 5.450197e-01 5.336081e-01 5.325663e-01

1224 9.920754e-01 9.942271e-01 9.889888e-01 9.851787e-01 9.807391e-01 5.735121e-01 5.565218e-01 5.456772e-01 5.341880e-01 5.330979e-01

1225 9.921083e-01 9.942533e-01 9.890303e-01 9.852293e-01 9.807981e-01 5.743545e-01 5.572439e-01 5.463113e-01 5.347465e-01 5.336095e-01

1226 9.921438e-01 9.942814e-01 9.890752e-01 9.852841e-01 9.808622e-01 5.751688e-01 5.579409e-01 5.469225e-01 5.352842e-01 5.341016e-01

1227 9.921820e-01 9.943117e-01 9.891236e-01 9.853434e-01 9.809318e-01 5.759555e-01 5.586135e-01 5.475115e-01 5.358017e-01 5.345748e-01

1228 9.922232e-01 9.943442e-01 9.891760e-01 9.854076e-01 9.810072e-01 5.767152e-01 5.592622e-01 5.480789e-01 5.362996e-01 5.350297e-01

1229 9.922677e-01 9.943792e-01 9.892327e-01 9.854772e-01 9.810891e-01 5.774486e-01 5.598876e-01 5.486253e-01 5.367785e-01 5.354670e-01

1230 9.923156e-01 9.944169e-01 9.892939e-01 9.855525e-01 9.811779e-01 5.781563e-01 5.604904e-01 5.491514e-01 5.372390e-01 5.358871e-01

1231 9.923672e-01 9.944574e-01 9.893600e-01 9.856342e-01 9.812743e-01 5.788390e-01 5.610712e-01 5.496576e-01 5.376816e-01 5.362907e-01

1232 9.924229e-01 9.945010e-01 9.894315e-01 9.857226e-01 9.813789e-01 5.794971e-01 5.616305e-01 5.501445e-01 5.381069e-01 5.366782e-01

1233 9.924830e-01 9.945479e-01 9.895089e-01 9.858184e-01 9.814923e-01 5.801315e-01 5.621691e-01 5.506128e-01 5.385155e-01 5.370503e-01

1234 9.925478e-01 9.945984e-01 9.895924e-01 9.859221e-01 9.816153e-01 5.807427e-01 5.626874e-01 5.510631e-01 5.389079e-01 5.374073e-01

1235 9.926177e-01 9.946527e-01 9.896828e-01 9.860344e-01 9.817487e-01 5.813313e-01 5.631861e-01 5.514958e-01 5.392846e-01 5.377499e-01

1236 9.926931e-01 9.947112e-01 9.897805e-01 9.861561e-01 9.818933e-01 5.818980e-01 5.636657e-01 5.519115e-01 5.396461e-01 5.380786e-01

1237 9.927744e-01 9.947742e-01 9.898860e-01 9.862877e-01 9.820500e-01 5.824433e-01 5.641269e-01 5.523109e-01 5.399931e-01 5.383938e-01

1238 9.928622e-01 9.948420e-01 9.900001e-01 9.864303e-01 9.822198e-01 5.829680e-01 5.645702e-01 5.526943e-01 5.403259e-01 5.386959e-01

1239 9.929568e-01 9.949149e-01 9.901235e-01 9.865846e-01 9.824036e-01 5.834727e-01 5.649961e-01 5.530624e-01 5.406450e-01 5.389855e-01

1240 9.930589e-01 9.949934e-01 9.902567e-01 9.867515e-01 9.826027e-01 5.839578e-01 5.654053e-01 5.534156e-01 5.409510e-01 5.392631e-01

1241 9.931690e-01 9.950780e-01 9.904007e-01 9.869321e-01 9.828181e-01 5.844241e-01 5.657982e-01 5.537545e-01 5.412443e-01 5.395289e-01

1242 9.932877e-01 9.951690e-01 9.905563e-01 9.871273e-01 9.830511e-01 5.848721e-01 5.661755e-01 5.540796e-01 5.415253e-01 5.397836e-01

1243 9.934158e-01 9.952670e-01 9.907243e-01 9.873384e-01 9.833030e-01 5.853025e-01 5.665376e-01 5.543913e-01 5.417946e-01 5.400274e-01

1244 9.935539e-01 9.953725e-01 9.909057e-01 9.875664e-01 9.835752e-01 5.857157e-01 5.668850e-01 5.546901e-01 5.420524e-01 5.402608e-01

1245 9.937028e-01 9.954860e-01 9.911015e-01 9.878127e-01 9.838690e-01 5.861124e-01 5.672183e-01 5.549765e-01 5.422994e-01 5.404842e-01

1246 9.938633e-01 9.956082e-01 9.913128e-01 9.880786e-01 9.841859e-01 5.864931e-01 5.675379e-01 5.552508e-01 5.425357e-01 5.406980e-01

1247 9.940363e-01 9.957398e-01 9.915408e-01 9.883655e-01 9.845277e-01 5.868583e-01 5.678443e-01 5.555137e-01 5.427620e-01 5.409025e-01

1248 9.942227e-01 9.958813e-01 9.917867e-01 9.886749e-01 9.848958e-01 5.872086e-01 5.681380e-01 5.557654e-01 5.429785e-01 5.410981e-01

1249 9.944235e-01 9.960336e-01 9.920517e-01 9.890084e-01 9.852919e-01 5.875445e-01 5.684195e-01 5.560064e-01 5.431856e-01 5.412851e-01

1250 9.946397e-01 9.961974e-01 9.923373e-01 9.893674e-01 9.857179e-01 5.878665e-01 5.686892e-01 5.562371e-01 5.433837e-01 5.414640e-01

1251 9.948726e-01 9.963736e-01 9.926449e-01 9.897539e-01 9.861753e-01 5.881751e-01 5.689475e-01 5.564579e-01 5.435731e-01 5.416349e-01

1252 9.951232e-01 9.965631e-01 9.929759e-01 9.901694e-01 9.866661e-01 5.884708e-01 5.691948e-01 5.566692e-01 5.437542e-01 5.417982e-01

1253 9.953928e-01 9.967668e-01 9.933321e-01 9.906158e-01 9.871919e-01 5.887540e-01 5.694316e-01 5.568713e-01 5.439273e-01 5.419543e-01

1254 9.956827e-01 9.969858e-01 9.937149e-01 9.910950e-01 9.877547e-01 5.890252e-01 5.696583e-01 5.570646e-01 5.440928e-01 5.421034e-01

1255 9.959944e-01 9.972211e-01 9.941263e-01 9.916088e-01 9.883562e-01 5.892849e-01 5.698752e-01 5.572495e-01 5.442509e-01 5.422458e-01

1256 9.963293e-01 9.974739e-01 9.945679e-01 9.921593e-01 9.889981e-01 5.895335e-01 5.700827e-01 5.574262e-01 5.444019e-01 5.423817e-01

1257 9.966890e-01 9.977453e-01 9.950417e-01 9.927483e-01 9.896820e-01 5.897715e-01 5.702812e-01 5.575951e-01 5.445461e-01 5.425115e-01

1258 9.970751e-01 9.980367e-01 9.955495e-01 9.933778e-01 9.904097e-01 5.899991e-01 5.704711e-01 5.577565e-01 5.446839e-01 5.426354e-01

1259 9.974892e-01 9.983494e-01 9.960932e-01 9.940497e-01 9.911825e-01 5.902168e-01 5.706526e-01 5.579107e-01 5.448154e-01 5.427537e-01

1260 9.979331e-01 9.986848e-01 9.966750e-01 9.947660e-01 9.920018e-01 5.904251e-01 5.708262e-01 5.580580e-01 5.449409e-01 5.428665e-01

1261 9.984086e-01 9.990443e-01 9.972969e-01 9.955285e-01 9.928688e-01 5.906242e-01 5.709920e-01 5.581987e-01 5.450607e-01 5.429742e-01

1262 9.984085e-01 9.990442e-01 9.972968e-01 9.955285e-01 9.928687e-01 5.906693e-01 5.710422e-01 5.582504e-01 5.451150e-01 5.430235e-01

1263 9.984085e-01 9.990440e-01 9.972968e-01 9.955285e-01 9.928687e-01 5.907164e-01 5.710944e-01 5.583041e-01 5.451714e-01 5.430747e-01

1264 9.984084e-01 9.990439e-01 9.972968e-01 9.955284e-01 9.928687e-01 5.907656e-01 5.711489e-01 5.583601e-01 5.452301e-01 5.431279e-01

1265 9.984083e-01 9.990438e-01 9.972967e-01 9.955284e-01 9.928687e-01 5.908169e-01 5.712057e-01 5.584183e-01 5.452911e-01 5.431831e-01

1266 9.984083e-01 9.990437e-01 9.972967e-01 9.955284e-01 9.928687e-01 5.908704e-01 5.712649e-01 5.584789e-01 5.453544e-01 5.432405e-01

1267 9.984082e-01 9.990436e-01 9.972967e-01 9.955284e-01 9.928686e-01 5.909261e-01 5.713265e-01 5.585419e-01 5.454202e-01 5.433001e-01

1268 9.984082e-01 9.990435e-01 9.972966e-01 9.955284e-01 9.928686e-01 5.909842e-01 5.713906e-01 5.586075e-01 5.454886e-01 5.433620e-01

1269 9.984082e-01 9.990435e-01 9.972966e-01 9.955283e-01 9.928686e-01 5.910447e-01 5.714573e-01 5.586756e-01 5.455596e-01 5.434263e-01

1270 9.984081e-01 9.990434e-01 9.972966e-01 9.955283e-01 9.928686e-01 5.911076e-01 5.715267e-01 5.587463e-01 5.456333e-01 5.434930e-01

1271 9.984081e-01 9.990433e-01 9.972966e-01 9.955283e-01 9.928686e-01 5.911731e-01 5.715989e-01 5.588199e-01 5.457098e-01 5.435622e-01

1272 9.984081e-01 9.990433e-01 9.972966e-01 9.955283e-01 9.928686e-01 5.912412e-01 5.716739e-01 5.588962e-01 5.457893e-01 5.436341e-01

1273 9.984080e-01 9.990432e-01 9.972965e-01 9.955283e-01 9.928686e-01 5.913121e-01 5.717518e-01 5.589756e-01 5.458717e-01 5.437087e-01

1274 9.984080e-01 9.990432e-01 9.972965e-01 9.955283e-01 9.928686e-01 5.913857e-01 5.718328e-01 5.590579e-01 5.459573e-01 5.437861e-01

1275 9.984080e-01 9.990431e-01 9.972965e-01 9.955283e-01 9.928686e-01 5.914622e-01 5.719169e-01 5.591435e-01 5.460461e-01 5.438665e-01

1276 9.984080e-01 9.990431e-01 9.972965e-01 9.955283e-01 9.928686e-01 5.915417e-01 5.720043e-01 5.592323e-01 5.461383e-01 5.439499e-01

1277 9.984080e-01 9.990431e-01 9.972965e-01 9.955283e-01 9.928686e-01 5.916243e-01 5.720950e-01 5.593244e-01 5.462339e-01 5.440365e-01

1278 9.984079e-01 9.990430e-01 9.972965e-01 9.955283e-01 9.928686e-01 5.917100e-01 5.721892e-01 5.594201e-01 5.463331e-01 5.441263e-01

1279 9.984079e-01 9.990430e-01 9.972965e-01 9.955283e-01 9.928686e-01 5.917991e-01 5.722869e-01 5.595193e-01 5.464360e-01 5.442196e-01

1280 9.984079e-01 9.990430e-01 9.972965e-01 9.955283e-01 9.928685e-01 5.918915e-01 5.723884e-01 5.596224e-01 5.465427e-01 5.443163e-01

1281 9.984079e-01 9.990430e-01 9.972965e-01 9.955283e-01 9.928685e-01 5.919874e-01 5.724937e-01 5.597292e-01 5.466534e-01 5.444167e-01

1282 9.984079e-01 9.990430e-01 9.972965e-01 9.955283e-01 9.928685e-01 5.920870e-01 5.726030e-01 5.598401e-01 5.467683e-01 5.445209e-01

1283 9.984079e-01 9.990430e-01 9.972965e-01 9.955283e-01 9.928685e-01 5.921904e-01 5.727163e-01 5.599552e-01 5.468874e-01 5.446291e-01

1284 9.984079e-01 9.990429e-01 9.972965e-01 9.955283e-01 9.928685e-01 5.922976e-01 5.728339e-01 5.600745e-01 5.470110e-01 5.447413e-01

1285 9.984079e-01 9.990429e-01 9.972965e-01 9.955283e-01 9.928685e-01 5.924088e-01 5.729560e-01 5.601983e-01 5.471391e-01 5.448577e-01

1286 9.984079e-01 9.990429e-01 9.972965e-01 9.955283e-01 9.928685e-01 5.925242e-01 5.730825e-01 5.603267e-01 5.472720e-01 5.449786e-01

1287 9.984079e-01 9.990429e-01 9.972965e-01 9.955283e-01 9.928685e-01 5.926439e-01 5.732138e-01 5.604599e-01 5.474098e-01 5.451040e-01

1288 9.984079e-01 9.990429e-01 9.972965e-01 9.955283e-01 9.928685e-01 5.927681e-01 5.733499e-01 5.605981e-01 5.475528e-01 5.452341e-01

1289 9.984079e-01 9.990429e-01 9.972965e-01 9.955283e-01 9.928685e-01 5.928969e-01 5.734911e-01 5.607413e-01 5.477010e-01 5.453692e-01

1290 9.984079e-01 9.990429e-01 9.972965e-01 9.955283e-01 9.928685e-01 5.930304e-01 5.736375e-01 5.608899e-01 5.478548e-01 5.455093e-01

1291 9.984079e-01 9.990429e-01 9.972965e-01 9.955283e-01 9.928685e-01 5.931689e-01 5.737893e-01 5.610440e-01 5.480142e-01 5.456548e-01

1292 9.984079e-01 9.990430e-01 9.972965e-01 9.955283e-01 9.928685e-01 5.933125e-01 5.739467e-01 5.612038e-01 5.481795e-01 5.458057e-01

1293 9.984080e-01 9.990430e-01 9.972965e-01 9.955283e-01 9.928685e-01 5.934614e-01 5.741100e-01 5.613696e-01 5.483509e-01 5.459623e-01

1294 9.984080e-01 9.990430e-01 9.972965e-01 9.955283e-01 9.928685e-01 5.936158e-01 5.742792e-01 5.615414e-01 5.485287e-01 5.461249e-01

1295 9.984080e-01 9.990430e-01 9.972965e-01 9.955283e-01 9.928685e-01 5.937759e-01 5.744547e-01 5.617196e-01 5.487131e-01 5.462936e-01

1296 9.984080e-01 9.990430e-01 9.972966e-01 9.955283e-01 9.928685e-01 5.939418e-01 5.746366e-01 5.619044e-01 5.489042e-01 5.464687e-01

1297 9.984080e-01 9.990430e-01 9.972966e-01 9.955283e-01 9.928685e-01 5.941139e-01 5.748251e-01 5.620960e-01 5.491024e-01 5.466503e-01

1298 9.984080e-01 9.990430e-01 9.972966e-01 9.955283e-01 9.928685e-01 5.942923e-01 5.750206e-01 5.622947e-01 5.493080e-01 5.468389e-01

1299 9.984080e-01 9.990430e-01 9.972966e-01 9.955283e-01 9.928685e-01 5.944772e-01 5.752233e-01 5.625007e-01 5.495211e-01 5.470345e-01

1300 9.984080e-01 9.990430e-01 9.972966e-01 9.955283e-01 9.928685e-01 5.946689e-01 5.754333e-01 5.627143e-01 5.497420e-01 5.472376e-01

1301 9.984080e-01 9.990430e-01 9.972966e-01 9.955283e-01 9.928685e-01 5.948676e-01 5.756511e-01 5.629357e-01 5.499711e-01 5.474483e-01

1302 9.984080e-01 9.990431e-01 9.972966e-01 9.955283e-01 9.928684e-01 5.950735e-01 5.758768e-01 5.631653e-01 5.502087e-01 5.476669e-01

1303 9.984081e-01 9.990431e-01 9.972966e-01 9.955283e-01 9.928684e-01 5.952870e-01 5.761107e-01 5.634033e-01 5.504549e-01 5.478938e-01

1304 9.984081e-01 9.990431e-01 9.972966e-01 9.955283e-01 9.928684e-01 5.955083e-01 5.763532e-01 5.636501e-01 5.507102e-01 5.481293e-01

1305 9.984081e-01 9.990431e-01 9.972966e-01 9.955283e-01 9.928684e-01 5.957376e-01 5.766045e-01 5.639059e-01 5.509749e-01 5.483736e-01

1306 9.984081e-01 9.990431e-01 9.972966e-01 9.955283e-01 9.928684e-01 5.959753e-01 5.768649e-01 5.641710e-01 5.512493e-01 5.486271e-01

1307 9.984081e-01 9.990431e-01 9.972966e-01 9.955283e-01 9.928684e-01 5.962216e-01 5.771347e-01 5.644458e-01 5.515337e-01 5.488901e-01

1308 9.984081e-01 9.990432e-01 9.972966e-01 9.955283e-01 9.928684e-01 5.964769e-01 5.774144e-01 5.647307e-01 5.518285e-01 5.491629e-01

1309 9.984081e-01 9.990432e-01 9.972966e-01 9.955283e-01 9.928683e-01 5.967414e-01 5.777041e-01 5.650260e-01 5.521341e-01 5.494460e-01

1310 9.984081e-01 9.990432e-01 9.972966e-01 9.955283e-01 9.928683e-01 5.970155e-01 5.780044e-01 5.653320e-01 5.524507e-01 5.497397e-01

1311 9.984082e-01 9.990432e-01 9.972967e-01 9.955283e-01 9.928683e-01 5.972996e-01 5.783154e-01 5.656491e-01 5.527789e-01 5.500444e-01

1312 9.984082e-01 9.990432e-01 9.972967e-01 9.955283e-01 9.928683e-01 5.975939e-01 5.786377e-01 5.659777e-01 5.531190e-01 5.503604e-01

1313 9.984082e-01 9.990432e-01 9.972967e-01 9.955282e-01 9.928682e-01 5.978988e-01 5.789715e-01 5.663181e-01 5.534714e-01 5.506881e-01

1314 9.984082e-01 9.990433e-01 9.972967e-01 9.955282e-01 9.928682e-01 5.982146e-01 5.793173e-01 5.666709e-01 5.538364e-01 5.510280e-01

1315 9.984082e-01 9.990433e-01 9.972967e-01 9.955282e-01 9.928682e-01 5.985419e-01 5.796754e-01 5.670363e-01 5.542146e-01 5.513805e-01

1316 9.984082e-01 9.990433e-01 9.972967e-01 9.955282e-01 9.928681e-01 5.988808e-01 5.800463e-01 5.674149e-01 5.546064e-01 5.517459e-01

1317 9.984082e-01 9.990433e-01 9.972967e-01 9.955282e-01 9.928681e-01 5.992319e-01 5.804304e-01 5.678070e-01 5.550121e-01 5.521248e-01

1318 9.984083e-01 9.990433e-01 9.972967e-01 9.955282e-01 9.928681e-01 5.995955e-01 5.808282e-01 5.682130e-01 5.554323e-01 5.525175e-01

1319 9.984083e-01 9.990434e-01 9.972967e-01 9.955282e-01 9.928680e-01 5.999721e-01 5.812399e-01 5.686335e-01 5.558673e-01 5.529246e-01

1320 9.984083e-01 9.990434e-01 9.972967e-01 9.955282e-01 9.928680e-01 6.003620e-01 5.816662e-01 5.690688e-01 5.563177e-01 5.533464e-01

1321 9.984083e-01 9.990434e-01 9.972967e-01 9.955282e-01 9.928679e-01 6.007657e-01 5.821075e-01 5.695195e-01 5.567839e-01 5.537835e-01

1322 9.984083e-01 9.990434e-01 9.972967e-01 9.955281e-01 9.928679e-01 6.011836e-01 5.825641e-01 5.699860e-01 5.572664e-01 5.542363e-01

1323 9.984083e-01 9.990435e-01 9.972967e-01 9.955281e-01 9.928678e-01 6.016162e-01 5.830367e-01 5.704688e-01 5.577657e-01 5.547054e-01

1324 9.984084e-01 9.990435e-01 9.972967e-01 9.955281e-01 9.928678e-01 6.020640e-01 5.835256e-01 5.709683e-01 5.582822e-01 5.551911e-01

1325 9.984084e-01 9.990435e-01 9.972967e-01 9.955281e-01 9.928677e-01 6.025274e-01 5.840313e-01 5.714851e-01 5.588165e-01 5.556940e-01

1326 9.984084e-01 9.990435e-01 9.972967e-01 9.955281e-01 9.928676e-01 6.030069e-01 5.845545e-01 5.720197e-01 5.593690e-01 5.562146e-01

1327 9.984084e-01 9.990436e-01 9.972967e-01 9.955280e-01 9.928676e-01 6.035030e-01 5.850954e-01 5.725725e-01 5.599403e-01 5.567534e-01

1328 9.984085e-01 9.990436e-01 9.972968e-01 9.955280e-01 9.928675e-01 6.040161e-01 5.856547e-01 5.731441e-01 5.605307e-01 5.573108e-01

1329 9.984085e-01 9.990436e-01 9.972968e-01 9.955280e-01 9.928674e-01 6.045468e-01 5.862329e-01 5.737349e-01 5.611409e-01 5.578875e-01

1330 9.984085e-01 9.990437e-01 9.972968e-01 9.955280e-01 9.928673e-01 6.050956e-01 5.868304e-01 5.743455e-01 5.617713e-01 5.584838e-01

1331 9.984085e-01 9.990437e-01 9.972968e-01 9.955279e-01 9.928672e-01 6.056629e-01 5.874479e-01 5.749763e-01 5.624223e-01 5.591003e-01

1332 9.984086e-01 9.990438e-01 9.972968e-01 9.955279e-01 9.928671e-01 6.062494e-01 5.880856e-01 5.756280e-01 5.630946e-01 5.597374e-01

1333 9.984086e-01 9.990438e-01 9.972968e-01 9.955279e-01 9.928670e-01 6.068554e-01 5.887443e-01 5.763008e-01 5.637885e-01 5.603956e-01

1334 9.984086e-01 9.990438e-01 9.972968e-01 9.955278e-01 9.928669e-01 6.074816e-01 5.894244e-01 5.769955e-01 5.645044e-01 5.610755e-01

1335 9.984086e-01 9.990439e-01 9.972968e-01 9.955278e-01 9.928668e-01 6.081284e-01 5.901265e-01 5.777124e-01 5.652430e-01 5.617774e-01

1336 9.984087e-01 9.990439e-01 9.972968e-01 9.955277e-01 9.928667e-01 6.087964e-01 5.908509e-01 5.784520e-01 5.660046e-01 5.625019e-01

1337 9.984087e-01 9.990440e-01 9.972968e-01 9.955277e-01 9.928665e-01 6.094861e-01 5.915982e-01 5.792148e-01 5.667896e-01 5.632493e-01

1338 9.984088e-01 9.990440e-01 9.972968e-01 9.955276e-01 9.928664e-01 6.101980e-01 5.923690e-01 5.800012e-01 5.675984e-01 5.640201e-01

1339 9.984088e-01 9.990441e-01 9.972968e-01 9.955276e-01 9.928662e-01 6.109326e-01 5.931636e-01 5.808117e-01 5.684314e-01 5.648146e-01

1340 9.984088e-01 9.990441e-01 9.972968e-01 9.955275e-01 9.928661e-01 6.116903e-01 5.939825e-01 5.816467e-01 5.692891e-01 5.656333e-01

1341 9.984089e-01 9.990442e-01 9.972968e-01 9.955275e-01 9.928659e-01 6.124718e-01 5.948262e-01 5.825067e-01 5.701716e-01 5.664765e-01

1342 9.984089e-01 9.990442e-01 9.972968e-01 9.955274e-01 9.928657e-01 6.132775e-01 5.956951e-01 5.833919e-01 5.710794e-01 5.673444e-01

1343 9.984090e-01 9.990443e-01 9.972969e-01 9.955273e-01 9.928655e-01 6.141079e-01 5.965896e-01 5.843027e-01 5.720128e-01 5.682374e-01

1344 9.984090e-01 9.990444e-01 9.972969e-01 9.955273e-01 9.928653e-01 6.149633e-01 5.975102e-01 5.852395e-01 5.729718e-01 5.691557e-01

1345 9.984091e-01 9.990444e-01 9.972969e-01 9.955272e-01 9.928651e-01 6.158444e-01 5.984571e-01 5.862026e-01 5.739568e-01 5.700996e-01

1346 9.984091e-01 9.990445e-01 9.972969e-01 9.955271e-01 9.928648e-01 6.167514e-01 5.994307e-01 5.871922e-01 5.749680e-01 5.710692e-01

1347 9.984092e-01 9.990446e-01 9.972969e-01 9.955270e-01 9.928645e-01 6.176848e-01 6.004314e-01 5.882085e-01 5.760054e-01 5.720646e-01

1348 9.984092e-01 9.990447e-01 9.972969e-01 9.955269e-01 9.928643e-01 6.186451e-01 6.014594e-01 5.892518e-01 5.770692e-01 5.730859e-01

1349 9.984093e-01 9.990448e-01 9.972969e-01 9.955268e-01 9.928639e-01 6.196324e-01 6.025149e-01 5.903222e-01 5.781593e-01 5.741331e-01

1350 9.984094e-01 9.990449e-01 9.972969e-01 9.955267e-01 9.928636e-01 6.206473e-01 6.035982e-01 5.914198e-01 5.792758e-01 5.752062e-01

1351 9.984094e-01 9.990450e-01 9.972969e-01 9.955265e-01 9.928633e-01 6.216899e-01 6.047094e-01 5.925447e-01 5.804185e-01 5.763051e-01

1352 9.984095e-01 9.990451e-01 9.972969e-01 9.955264e-01 9.928629e-01 6.227605e-01 6.058486e-01 5.936968e-01 5.815874e-01 5.774297e-01

1353 9.984096e-01 9.990452e-01 9.972969e-01 9.955262e-01 9.928625e-01 6.238594e-01 6.070160e-01 5.948761e-01 5.827823e-01 5.785798e-01

1354 9.984097e-01 9.990453e-01 9.972969e-01 9.955261e-01 9.928620e-01 6.249868e-01 6.082115e-01 5.960826e-01 5.840029e-01 5.797550e-01

1355 9.984097e-01 9.990454e-01 9.972969e-01 9.955259e-01 9.928616e-01 6.261428e-01 6.094352e-01 5.973160e-01 5.852488e-01 5.809551e-01

1356 9.984098e-01 9.990455e-01 9.972969e-01 9.955257e-01 9.928611e-01 6.273275e-01 6.106869e-01 5.985762e-01 5.865198e-01 5.821797e-01

1357 9.984099e-01 9.990457e-01 9.972969e-01 9.955255e-01 9.928605e-01 6.285410e-01 6.119666e-01 5.998629e-01 5.878154e-01 5.834282e-01

1358 9.984100e-01 9.990458e-01 9.972969e-01 9.955253e-01 9.928599e-01 6.297833e-01 6.132741e-01 6.011757e-01 5.891350e-01 5.847002e-01

1359 9.984101e-01 9.990459e-01 9.972969e-01 9.955251e-01 9.928593e-01 6.310544e-01 6.146090e-01 6.025143e-01 5.904781e-01 5.859949e-01

1360 9.984102e-01 9.990461e-01 9.972969e-01 9.955248e-01 9.928586e-01 6.323541e-01 6.159712e-01 6.038781e-01 5.918440e-01 5.873118e-01

1361 9.984103e-01 9.990463e-01 9.972968e-01 9.955246e-01 9.928579e-01 6.336823e-01 6.173602e-01 6.052667e-01 5.932320e-01 5.886500e-01

1362 9.984105e-01 9.990464e-01 9.972968e-01 9.955243e-01 9.928572e-01 6.350389e-01 6.187757e-01 6.066793e-01 5.946414e-01 5.900087e-01

1363 9.984106e-01 9.990466e-01 9.972968e-01 9.955240e-01 9.928563e-01 6.364235e-01 6.202170e-01 6.081154e-01 5.960712e-01 5.913871e-01

1364 9.984107e-01 9.990468e-01 9.972968e-01 9.955236e-01 9.928554e-01 6.378358e-01 6.216837e-01 6.095742e-01 5.975205e-01 5.927840e-01

1365 9.984109e-01 9.990470e-01 9.972968e-01 9.955233e-01 9.928545e-01 6.392755e-01 6.231751e-01 6.110549e-01 5.989884e-01 5.941986e-01

1366 9.984110e-01 9.990472e-01 9.972967e-01 9.955229e-01 9.928534e-01 6.407421e-01 6.246906e-01 6.125565e-01 6.004737e-01 5.956295e-01

1367 9.984112e-01 9.990475e-01 9.972967e-01 9.955224e-01 9.928523e-01 6.422350e-01 6.262293e-01 6.140783e-01 6.019754e-01 5.970758e-01

1368 9.984113e-01 9.990477e-01 9.972967e-01 9.955220e-01 9.928511e-01 6.437537e-01 6.277904e-01 6.156190e-01 6.034923e-01 5.985360e-01

1369 9.984115e-01 9.990479e-01 9.972966e-01 9.955215e-01 9.928498e-01 6.452976e-01 6.293730e-01 6.171777e-01 6.050231e-01 6.000090e-01

1370 9.984117e-01 9.990482e-01 9.972966e-01 9.955210e-01 9.928484e-01 6.468658e-01 6.309762e-01 6.187533e-01 6.065665e-01 6.014934e-01

1371 9.984119e-01 9.990485e-01 9.972965e-01 9.955204e-01 9.928469e-01 6.484578e-01 6.325990e-01 6.203444e-01 6.081212e-01 6.029877e-01

1372 9.984121e-01 9.990488e-01 9.972965e-01 9.955198e-01 9.928453e-01 6.500725e-01 6.342401e-01 6.219499e-01 6.096858e-01 6.044905e-01

1373 9.984123e-01 9.990491e-01 9.972964e-01 9.955191e-01 9.928435e-01 6.517090e-01 6.358986e-01 6.235685e-01 6.112588e-01 6.060003e-01

1374 9.984125e-01 9.990494e-01 9.972963e-01 9.955184e-01 9.928416e-01 6.533665e-01 6.375732e-01 6.251988e-01 6.128389e-01 6.075156e-01

1375 9.984128e-01 9.990498e-01 9.972963e-01 9.955177e-01 9.928396e-01 6.550439e-01 6.392626e-01 6.268393e-01 6.144243e-01 6.090348e-01

1376 9.984130e-01 9.990502e-01 9.972962e-01 9.955168e-01 9.928374e-01 6.567400e-01 6.409655e-01 6.284888e-01 6.160138e-01 6.105564e-01

1377 9.984133e-01 9.990506e-01 9.972961e-01 9.955159e-01 9.928351e-01 6.584537e-01 6.426807e-01 6.301456e-01 6.176056e-01 6.120788e-01

1378 9.984135e-01 9.990510e-01 9.972959e-01 9.955150e-01 9.928325e-01 6.601838e-01 6.444065e-01 6.318082e-01 6.191983e-01 6.136003e-01

1379 9.984138e-01 9.990514e-01 9.972958e-01 9.955139e-01 9.928298e-01 6.619291e-01 6.461417e-01 6.334752e-01 6.207902e-01 6.151194e-01

1380 9.984141e-01 9.990519e-01 9.972957e-01 9.955128e-01 9.928268e-01 6.636882e-01 6.478848e-01 6.351450e-01 6.223797e-01 6.166344e-01

1381 9.984145e-01 9.990524e-01 9.972955e-01 9.955115e-01 9.928236e-01 6.654597e-01 6.496342e-01 6.368159e-01 6.239654e-01 6.181437e-01

1382 9.984148e-01 9.990529e-01 9.972953e-01 9.955102e-01 9.928201e-01 6.672424e-01 6.513885e-01 6.384865e-01 6.255456e-01 6.196459e-01

1383 9.984152e-01 9.990535e-01 9.972951e-01 9.955088e-01 9.928163e-01 6.690346e-01 6.531460e-01 6.401551e-01 6.271187e-01 6.211393e-01

1384 9.984156e-01 9.990541e-01 9.972949e-01 9.955073e-01 9.928123e-01 6.708350e-01 6.549052e-01 6.418202e-01 6.286833e-01 6.226224e-01

1385 9.984160e-01 9.990547e-01 9.972947e-01 9.955056e-01 9.928079e-01 6.726420e-01 6.566646e-01 6.434802e-01 6.302379e-01 6.240937e-01

1386 9.984164e-01 9.990554e-01 9.972944e-01 9.955038e-01 9.928032e-01 6.744542e-01 6.584225e-01 6.451335e-01 6.317809e-01 6.255518e-01

1387 9.984169e-01 9.990561e-01 9.972941e-01 9.955018e-01 9.927980e-01 6.762699e-01 6.601774e-01 6.467786e-01 6.333109e-01 6.269952e-01

1388 9.984173e-01 9.990568e-01 9.972938e-01 9.954997e-01 9.927925e-01 6.780877e-01 6.619278e-01 6.484140e-01 6.348266e-01 6.284227e-01

1389 9.984178e-01 9.990576e-01 9.972934e-01 9.954974e-01 9.927865e-01 6.799061e-01 6.636720e-01 6.500382e-01 6.363266e-01 6.298329e-01

1390 9.984184e-01 9.990585e-01 9.972930e-01 9.954949e-01 9.927800e-01 6.817235e-01 6.654085e-01 6.516497e-01 6.378096e-01 6.312246e-01

1391 9.984189e-01 9.990594e-01 9.972926e-01 9.954922e-01 9.927729e-01 6.835385e-01 6.671360e-01 6.532472e-01 6.392743e-01 6.325966e-01

1392 9.984195e-01 9.990603e-01 9.972921e-01 9.954893e-01 9.927653e-01 6.853495e-01 6.688530e-01 6.548293e-01 6.407197e-01 6.339479e-01

1393 9.984202e-01 9.990613e-01 9.972916e-01 9.954862e-01 9.927571e-01 6.871552e-01 6.705581e-01 6.563947e-01 6.421446e-01 6.352773e-01

1394 9.984208e-01 9.990624e-01 9.972911e-01 9.954828e-01 9.927481e-01 6.889541e-01 6.722500e-01 6.579424e-01 6.435481e-01 6.365841e-01

1395 9.984215e-01 9.990635e-01 9.972904e-01 9.954791e-01 9.927384e-01 6.907448e-01 6.739274e-01 6.594711e-01 6.449292e-01 6.378672e-01

1396 9.984223e-01 9.990647e-01 9.972897e-01 9.954751e-01 9.927279e-01 6.925259e-01 6.755892e-01 6.609799e-01 6.462871e-01 6.391259e-01

1397 9.984231e-01 9.990660e-01 9.972890e-01 9.954707e-01 9.927165e-01 6.942962e-01 6.772341e-01 6.624677e-01 6.476212e-01 6.403595e-01

1398 9.984239e-01 9.990674e-01 9.972882e-01 9.954660e-01 9.927042e-01 6.960543e-01 6.788611e-01 6.639338e-01 6.489307e-01 6.415674e-01

1399 9.984248e-01 9.990688e-01 9.972873e-01 9.954609e-01 9.926908e-01 6.977990e-01 6.804691e-01 6.653771e-01 6.502150e-01 6.427491e-01

1400 9.984257e-01 9.990703e-01 9.972863e-01 9.954554e-01 9.926762e-01 6.995290e-01 6.820571e-01 6.667971e-01 6.514738e-01 6.439043e-01

1401 9.984267e-01 9.990720e-01 9.972852e-01 9.954494e-01 9.926605e-01 7.012433e-01 6.836242e-01 6.681929e-01 6.527065e-01 6.450326e-01

1402 9.984278e-01 9.990737e-01 9.972840e-01 9.954429e-01 9.926434e-01 7.029406e-01 6.851695e-01 6.695641e-01 6.539129e-01 6.461338e-01

1403 9.984289e-01 9.990755e-01 9.972827e-01 9.954358e-01 9.926248e-01 7.046199e-01 6.866923e-01 6.709100e-01 6.550926e-01 6.472077e-01

1404 9.984300e-01 9.990775e-01 9.972812e-01 9.954282e-01 9.926046e-01 7.062803e-01 6.881917e-01 6.722302e-01 6.562454e-01 6.482543e-01

1405 9.984313e-01 9.990796e-01 9.972796e-01 9.954198e-01 9.925827e-01 7.079207e-01 6.896672e-01 6.735243e-01 6.573712e-01 6.492735e-01

1406 9.984326e-01 9.990818e-01 9.972779e-01 9.954108e-01 9.925590e-01 7.095402e-01 6.911181e-01 6.747920e-01 6.584700e-01 6.502655e-01

1407 9.984340e-01 9.990842e-01 9.972760e-01 9.954010e-01 9.925331e-01 7.111380e-01 6.925438e-01 6.760330e-01 6.595417e-01 6.512302e-01

1408 9.984354e-01 9.990867e-01 9.972739e-01 9.953903e-01 9.925051e-01 7.127133e-01 6.939440e-01 6.772470e-01 6.605863e-01 6.521680e-01

1409 9.984370e-01 9.990894e-01 9.972717e-01 9.953788e-01 9.924746e-01 7.142653e-01 6.953180e-01 6.784339e-01 6.616040e-01 6.530790e-01

1410 9.984387e-01 9.990922e-01 9.972692e-01 9.953662e-01 9.924416e-01 7.157935e-01 6.966657e-01 6.795937e-01 6.625950e-01 6.539635e-01

1411 9.984404e-01 9.990952e-01 9.972665e-01 9.953526e-01 9.924056e-01 7.172971e-01 6.979867e-01 6.807263e-01 6.635593e-01 6.548218e-01

1412 9.984422e-01 9.990985e-01 9.972635e-01 9.953377e-01 9.923666e-01 7.187757e-01 6.992807e-01 6.818318e-01 6.644973e-01 6.556544e-01

1413 9.984442e-01 9.991019e-01 9.972602e-01 9.953216e-01 9.923241e-01 7.202287e-01 7.005476e-01 6.829101e-01 6.654092e-01 6.564615e-01

1414 9.984463e-01 9.991056e-01 9.972566e-01 9.953041e-01 9.922781e-01 7.216557e-01 7.017872e-01 6.839615e-01 6.662953e-01 6.572437e-01

1415 9.984485e-01 9.991095e-01 9.972527e-01 9.952851e-01 9.922280e-01 7.230563e-01 7.029995e-01 6.849860e-01 6.671561e-01 6.580014e-01

1416 9.984508e-01 9.991137e-01 9.972484e-01 9.952644e-01 9.921736e-01 7.244303e-01 7.041845e-01 6.859840e-01 6.679919e-01 6.587351e-01

1417 9.984532e-01 9.991181e-01 9.972437e-01 9.952419e-01 9.921145e-01 7.257774e-01 7.053422e-01 6.869557e-01 6.688031e-01 6.594453e-01

1418 9.984558e-01 9.991228e-01 9.972386e-01 9.952175e-01 9.920503e-01 7.270973e-01 7.064726e-01 6.879013e-01 6.695902e-01 6.601325e-01

1419 9.984586e-01 9.991279e-01 9.972330e-01 9.951909e-01 9.919806e-01 7.283900e-01 7.075759e-01 6.888212e-01 6.703537e-01 6.607974e-01

1420 9.984615e-01 9.991333e-01 9.972269e-01 9.951621e-01 9.919050e-01 7.296553e-01 7.086522e-01 6.897157e-01 6.710940e-01 6.614404e-01

1421 9.984646e-01 9.991390e-01 9.972201e-01 9.951307e-01 9.918228e-01 7.308931e-01 7.097018e-01 6.905853e-01 6.718116e-01 6.620622e-01

1422 9.984678e-01 9.991452e-01 9.972128e-01 9.950966e-01 9.917336e-01 7.321036e-01 7.107249e-01 6.914303e-01 6.725071e-01 6.626633e-01

1423 9.984713e-01 9.991517e-01 9.972048e-01 9.950596e-01 9.916369e-01 7.332868e-01 7.117218e-01 6.922512e-01 6.731810e-01 6.632444e-01

1424 9.984750e-01 9.991587e-01 9.971960e-01 9.950193e-01 9.915320e-01 7.344427e-01 7.126928e-01 6.930484e-01 6.738338e-01 6.638059e-01

1425 9.984788e-01 9.991661e-01 9.971863e-01 9.949756e-01 9.914182e-01 7.355715e-01 7.136383e-01 6.938225e-01 6.744662e-01 6.643485e-01

1426 9.984829e-01 9.991741e-01 9.971758e-01 9.949281e-01 9.912948e-01 7.366734e-01 7.145585e-01 6.945739e-01 6.750785e-01 6.648729e-01

1427 9.984873e-01 9.991826e-01 9.971643e-01 9.948765e-01 9.911612e-01 7.377484e-01 7.154539e-01 6.953030e-01 6.756714e-01 6.653795e-01

1428 9.984918e-01 9.991916e-01 9.971518e-01 9.948204e-01 9.910165e-01 7.387969e-01 7.163247e-01 6.960102e-01 6.762452e-01 6.658688e-01

1429 9.984967e-01 9.992013e-01 9.971381e-01 9.947596e-01 9.908600e-01 7.398190e-01 7.171714e-01 6.966961e-01 6.768006e-01 6.663414e-01

1430 9.985018e-01 9.992117e-01 9.971231e-01 9.946936e-01 9.906906e-01 7.408150e-01 7.179942e-01 6.973610e-01 6.773379e-01 6.667978e-01

1431 9.985072e-01 9.992227e-01 9.971067e-01 9.946219e-01 9.905076e-01 7.417850e-01 7.187936e-01 6.980054e-01 6.778576e-01 6.672385e-01

1432 9.985129e-01 9.992345e-01 9.970888e-01 9.945442e-01 9.903099e-01 7.427293e-01 7.195700e-01 6.986298e-01 6.783602e-01 6.676639e-01

1433 9.985189e-01 9.992472e-01 9.970692e-01 9.944600e-01 9.900966e-01 7.436483e-01 7.203237e-01 6.992345e-01 6.788460e-01 6.680744e-01

1434 9.985253e-01 9.992606e-01 9.970479e-01 9.943687e-01 9.898666e-01 7.445422e-01 7.210551e-01 6.998200e-01 6.793156e-01 6.684705e-01

1435 9.985320e-01 9.992750e-01 9.970246e-01 9.942698e-01 9.896188e-01 7.454114e-01 7.217646e-01 7.003868e-01 6.797693e-01 6.688527e-01

1436 9.985391e-01 9.992904e-01 9.969992e-01 9.941628e-01 9.893522e-01 7.462561e-01 7.224526e-01 7.009352e-01 6.802076e-01 6.692213e-01

1437 9.985466e-01 9.993069e-01 9.969715e-01 9.940469e-01 9.890656e-01 7.470767e-01 7.231196e-01 7.014657e-01 6.806309e-01 6.695767e-01

1438 9.985545e-01 9.993245e-01 9.969413e-01 9.939217e-01 9.887579e-01 7.478737e-01 7.237659e-01 7.019786e-01 6.810396e-01 6.699193e-01

1439 9.985628e-01 9.993433e-01 9.969083e-01 9.937863e-01 9.884279e-01 7.486472e-01 7.243920e-01 7.024746e-01 6.814340e-01 6.702495e-01

1440 9.985715e-01 9.993634e-01 9.968725e-01 9.936402e-01 9.880746e-01 7.493978e-01 7.249982e-01 7.029538e-01 6.818146e-01 6.705677e-01

1441 9.985807e-01 9.993848e-01 9.968334e-01 9.934825e-01 9.876968e-01 7.501258e-01 7.255851e-01 7.034168e-01 6.821818e-01 6.708743e-01

1442 9.985808e-01 9.993849e-01 9.968334e-01 9.934825e-01 9.876968e-01 7.501398e-01 7.256019e-01 7.034354e-01 6.822014e-01 6.708927e-01

1443 9.985808e-01 9.993850e-01 9.968334e-01 9.934825e-01 9.876968e-01 7.501546e-01 7.256195e-01 7.034547e-01 6.822217e-01 6.709117e-01

1444 9.985808e-01 9.993850e-01 9.968334e-01 9.934825e-01 9.876968e-01 7.501701e-01 7.256379e-01 7.034747e-01 6.822427e-01 6.709313e-01

1445 9.985808e-01 9.993851e-01 9.968334e-01 9.934825e-01 9.876968e-01 7.501864e-01 7.256571e-01 7.034954e-01 6.822644e-01 6.709515e-01

1446 9.985808e-01 9.993851e-01 9.968334e-01 9.934824e-01 9.876967e-01 7.502035e-01 7.256771e-01 7.035170e-01 6.822868e-01 6.709725e-01

1447 9.985808e-01 9.993852e-01 9.968333e-01 9.934824e-01 9.876967e-01 7.502215e-01 7.256980e-01 7.035393e-01 6.823101e-01 6.709941e-01

1448 9.985808e-01 9.993852e-01 9.968333e-01 9.934824e-01 9.876967e-01 7.502402e-01 7.257197e-01 7.035625e-01 6.823341e-01 6.710164e-01

1449 9.985808e-01 9.993852e-01 9.968333e-01 9.934824e-01 9.876967e-01 7.502599e-01 7.257423e-01 7.035865e-01 6.823590e-01 6.710395e-01

1450 9.985808e-01 9.993852e-01 9.968333e-01 9.934824e-01 9.876966e-01 7.502804e-01 7.257659e-01 7.036114e-01 6.823847e-01 6.710634e-01

1451 9.985808e-01 9.993852e-01 9.968333e-01 9.934823e-01 9.876966e-01 7.503018e-01 7.257904e-01 7.036373e-01 6.824114e-01 6.710880e-01

1452 9.985808e-01 9.993853e-01 9.968333e-01 9.934823e-01 9.876966e-01 7.503242e-01 7.258159e-01 7.036640e-01 6.824390e-01 6.711135e-01

1453 9.985808e-01 9.993853e-01 9.968332e-01 9.934823e-01 9.876965e-01 7.503475e-01 7.258424e-01 7.036918e-01 6.824675e-01 6.711399e-01

1454 9.985808e-01 9.993853e-01 9.968332e-01 9.934823e-01 9.876965e-01 7.503718e-01 7.258700e-01 7.037206e-01 6.824971e-01 6.711672e-01

1455 9.985807e-01 9.993853e-01 9.968332e-01 9.934822e-01 9.876965e-01 7.503971e-01 7.258986e-01 7.037504e-01 6.825277e-01 6.711955e-01

1456 9.985807e-01 9.993853e-01 9.968332e-01 9.934822e-01 9.876964e-01 7.504234e-01 7.259283e-01 7.037813e-01 6.825594e-01 6.712247e-01

1457 9.985807e-01 9.993853e-01 9.968332e-01 9.934822e-01 9.876964e-01 7.504509e-01 7.259592e-01 7.038133e-01 6.825922e-01 6.712550e-01

1458 9.985807e-01 9.993853e-01 9.968332e-01 9.934822e-01 9.876964e-01 7.504794e-01 7.259912e-01 7.038465e-01 6.826261e-01 6.712863e-01

1459 9.985807e-01 9.993852e-01 9.968331e-01 9.934821e-01 9.876963e-01 7.505090e-01 7.260244e-01 7.038809e-01 6.826613e-01 6.713187e-01

1460 9.985807e-01 9.993852e-01 9.968331e-01 9.934821e-01 9.876963e-01 7.505398e-01 7.260589e-01 7.039165e-01 6.826977e-01 6.713523e-01

1461 9.985807e-01 9.993852e-01 9.968331e-01 9.934821e-01 9.876962e-01 7.505718e-01 7.260947e-01 7.039534e-01 6.827354e-01 6.713870e-01

1462 9.985806e-01 9.993852e-01 9.968331e-01 9.934821e-01 9.876962e-01 7.506050e-01 7.261317e-01 7.039916e-01 6.827744e-01 6.714230e-01

1463 9.985806e-01 9.993852e-01 9.968331e-01 9.934820e-01 9.876962e-01 7.506395e-01 7.261702e-01 7.040312e-01 6.828149e-01 6.714603e-01

1464 9.985806e-01 9.993852e-01 9.968330e-01 9.934820e-01 9.876961e-01 7.506753e-01 7.262100e-01 7.040722e-01 6.828567e-01 6.714989e-01

1465 9.985806e-01 9.993852e-01 9.968330e-01 9.934820e-01 9.876961e-01 7.507125e-01 7.262513e-01 7.041146e-01 6.829001e-01 6.715388e-01

1466 9.985806e-01 9.993852e-01 9.968330e-01 9.934819e-01 9.876960e-01 7.507510e-01 7.262941e-01 7.041586e-01 6.829450e-01 6.715802e-01

1467 9.985806e-01 9.993852e-01 9.968330e-01 9.934819e-01 9.876959e-01 7.507910e-01 7.263385e-01 7.042041e-01 6.829915e-01 6.716231e-01

1468 9.985806e-01 9.993852e-01 9.968330e-01 9.934819e-01 9.876959e-01 7.508324e-01 7.263844e-01 7.042513e-01 6.830397e-01 6.716675e-01

1469 9.985805e-01 9.993852e-01 9.968329e-01 9.934818e-01 9.876958e-01 7.508754e-01 7.264320e-01 7.043001e-01 6.830895e-01 6.717136e-01

1470 9.985805e-01 9.993851e-01 9.968329e-01 9.934818e-01 9.876957e-01 7.509199e-01 7.264813e-01 7.043506e-01 6.831412e-01 6.717613e-01

1471 9.985805e-01 9.993851e-01 9.968329e-01 9.934817e-01 9.876957e-01 7.509660e-01 7.265324e-01 7.044030e-01 6.831947e-01 6.718107e-01

1472 9.985805e-01 9.993851e-01 9.968329e-01 9.934817e-01 9.876956e-01 7.510139e-01 7.265853e-01 7.044572e-01 6.832502e-01 6.718619e-01

1473 9.985805e-01 9.993851e-01 9.968328e-01 9.934816e-01 9.876955e-01 7.510634e-01 7.266401e-01 7.045133e-01 6.833076e-01 6.719149e-01

1474 9.985805e-01 9.993851e-01 9.968328e-01 9.934816e-01 9.876954e-01 7.511147e-01 7.266968e-01 7.045715e-01 6.833670e-01 6.719699e-01

1475 9.985805e-01 9.993851e-01 9.968328e-01 9.934815e-01 9.876953e-01 7.511679e-01 7.267555e-01 7.046317e-01 6.834286e-01 6.720269e-01

1476 9.985804e-01 9.993851e-01 9.968327e-01 9.934815e-01 9.876952e-01 7.512229e-01 7.268163e-01 7.046940e-01 6.834925e-01 6.720860e-01

1477 9.985804e-01 9.993851e-01 9.968327e-01 9.934814e-01 9.876951e-01 7.512799e-01 7.268792e-01 7.047585e-01 6.835586e-01 6.721473e-01

1478 9.985804e-01 9.993851e-01 9.968327e-01 9.934813e-01 9.876950e-01 7.513390e-01 7.269444e-01 7.048253e-01 6.836271e-01 6.722107e-01

1479 9.985804e-01 9.993851e-01 9.968326e-01 9.934813e-01 9.876949e-01 7.514001e-01 7.270118e-01 7.048945e-01 6.836980e-01 6.722766e-01

1480 9.985804e-01 9.993850e-01 9.968326e-01 9.934812e-01 9.876948e-01 7.514634e-01 7.270816e-01 7.049661e-01 6.837715e-01 6.723448e-01

1481 9.985803e-01 9.993850e-01 9.968325e-01 9.934811e-01 9.876946e-01 7.515289e-01 7.271539e-01 7.050403e-01 6.838477e-01 6.724155e-01

1482 9.985803e-01 9.993850e-01 9.968325e-01 9.934810e-01 9.876945e-01 7.515967e-01 7.272287e-01 7.051171e-01 6.839266e-01 6.724888e-01

1483 9.985803e-01 9.993850e-01 9.968325e-01 9.934810e-01 9.876944e-01 7.516669e-01 7.273061e-01 7.051966e-01 6.840083e-01 6.725649e-01

1484 9.985803e-01 9.993850e-01 9.968324e-01 9.934809e-01 9.876942e-01 7.517396e-01 7.273863e-01 7.052790e-01 6.840930e-01 6.726437e-01

1485 9.985803e-01 9.993850e-01 9.968323e-01 9.934808e-01 9.876940e-01 7.518148e-01 7.274692e-01 7.053642e-01 6.841808e-01 6.727254e-01

1486 9.985802e-01 9.993850e-01 9.968323e-01 9.934807e-01 9.876939e-01 7.518927e-01 7.275551e-01 7.054525e-01 6.842717e-01 6.728102e-01

1487 9.985802e-01 9.993850e-01 9.968322e-01 9.934805e-01 9.876937e-01 7.519733e-01 7.276440e-01 7.055440e-01 6.843659e-01 6.728981e-01

1488 9.985802e-01 9.993850e-01 9.968322e-01 9.934804e-01 9.876935e-01 7.520567e-01 7.277360e-01 7.056387e-01 6.844636e-01 6.729892e-01

1489 9.985801e-01 9.993850e-01 9.968321e-01 9.934803e-01 9.876933e-01 7.521431e-01 7.278312e-01 7.057367e-01 6.845647e-01 6.730837e-01

1490 9.985801e-01 9.993849e-01 9.968320e-01 9.934802e-01 9.876931e-01 7.522324e-01 7.279297e-01 7.058382e-01 6.846696e-01 6.731817e-01

1491 9.985801e-01 9.993849e-01 9.968320e-01 9.934800e-01 9.876928e-01 7.523249e-01 7.280317e-01 7.059434e-01 6.847782e-01 6.732834e-01

1492 9.985800e-01 9.993849e-01 9.968319e-01 9.934799e-01 9.876926e-01 7.524206e-01 7.281372e-01 7.060523e-01 6.848908e-01 6.733888e-01

1493 9.985800e-01 9.993849e-01 9.968318e-01 9.934797e-01 9.876923e-01 7.525197e-01 7.282465e-01 7.061650e-01 6.850074e-01 6.734982e-01

1494 9.985800e-01 9.993849e-01 9.968317e-01 9.934796e-01 9.876920e-01 7.526222e-01 7.283596e-01 7.062818e-01 6.851283e-01 6.736116e-01

1495 9.985799e-01 9.993849e-01 9.968316e-01 9.934794e-01 9.876917e-01 7.527283e-01 7.284766e-01 7.064027e-01 6.852536e-01 6.737292e-01

1496 9.985799e-01 9.993849e-01 9.968315e-01 9.934792e-01 9.876914e-01 7.528381e-01 7.285977e-01 7.065279e-01 6.853835e-01 6.738512e-01

1497 9.985798e-01 9.993849e-01 9.968314e-01 9.934790e-01 9.876911e-01 7.529517e-01 7.287231e-01 7.066576e-01 6.855180e-01 6.739777e-01

1498 9.985798e-01 9.993848e-01 9.968313e-01 9.934788e-01 9.876907e-01 7.530693e-01 7.288529e-01 7.067919e-01 6.856574e-01 6.741089e-01

1499 9.985797e-01 9.993848e-01 9.968312e-01 9.934786e-01 9.876904e-01 7.531910e-01 7.289871e-01 7.069310e-01 6.858019e-01 6.742451e-01

1500 9.985797e-01 9.993848e-01 9.968311e-01 9.934784e-01 9.876900e-01 7.533169e-01 7.291261e-01 7.070750e-01 6.859517e-01 6.743862e-01

1501 9.985796e-01 9.993848e-01 9.968309e-01 9.934781e-01 9.876895e-01 7.534473e-01 7.292700e-01 7.072242e-01 6.861068e-01 6.745326e-01

1502 9.985796e-01 9.993848e-01 9.968308e-01 9.934778e-01 9.876891e-01 7.535821e-01 7.294189e-01 7.073787e-01 6.862676e-01 6.746845e-01

1503 9.985795e-01 9.993848e-01 9.968307e-01 9.934776e-01 9.876886e-01 7.537217e-01 7.295730e-01 7.075387e-01 6.864343e-01 6.748420e-01

1504 9.985794e-01 9.993847e-01 9.968305e-01 9.934773e-01 9.876881e-01 7.538662e-01 7.297325e-01 7.077044e-01 6.866070e-01 6.750053e-01

1505 9.985794e-01 9.993847e-01 9.968303e-01 9.934769e-01 9.876876e-01 7.540157e-01 7.298975e-01 7.078759e-01 6.867859e-01 6.751747e-01

1506 9.985793e-01 9.993847e-01 9.968302e-01 9.934766e-01 9.876870e-01 7.541704e-01 7.300684e-01 7.080536e-01 6.869713e-01 6.753504e-01

1507 9.985792e-01 9.993847e-01 9.968300e-01 9.934762e-01 9.876864e-01 7.543305e-01 7.302452e-01 7.082376e-01 6.871634e-01 6.755326e-01

1508 9.985791e-01 9.993846e-01 9.968298e-01 9.934759e-01 9.876857e-01 7.544962e-01 7.304282e-01 7.084281e-01 6.873625e-01 6.757215e-01

1509 9.985790e-01 9.993846e-01 9.968296e-01 9.934755e-01 9.876850e-01 7.546676e-01 7.306175e-01 7.086254e-01 6.875687e-01 6.759174e-01

1510 9.985789e-01 9.993846e-01 9.968293e-01 9.934750e-01 9.876843e-01 7.548451e-01 7.308135e-01 7.088296e-01 6.877824e-01 6.761205e-01

1511 9.985788e-01 9.993845e-01 9.968291e-01 9.934746e-01 9.876835e-01 7.550287e-01 7.310163e-01 7.090411e-01 6.880037e-01 6.763311e-01

1512 9.985787e-01 9.993845e-01 9.968288e-01 9.934741e-01 9.876826e-01 7.552187e-01 7.312261e-01 7.092601e-01 6.882330e-01 6.765494e-01

1513 9.985786e-01 9.993845e-01 9.968286e-01 9.934736e-01 9.876817e-01 7.554153e-01 7.314433e-01 7.094868e-01 6.884705e-01 6.767757e-01

1514 9.985785e-01 9.993844e-01 9.968283e-01 9.934730e-01 9.876808e-01 7.556187e-01 7.316680e-01 7.097216e-01 6.887165e-01 6.770103e-01

1515 9.985784e-01 9.993844e-01 9.968280e-01 9.934724e-01 9.876797e-01 7.558292e-01 7.319005e-01 7.099645e-01 6.889713e-01 6.772534e-01

1516 9.985782e-01 9.993844e-01 9.968277e-01 9.934718e-01 9.876786e-01 7.560471e-01 7.321411e-01 7.102161e-01 6.892352e-01 6.775054e-01

1517 9.985781e-01 9.993843e-01 9.968273e-01 9.934711e-01 9.876775e-01 7.562724e-01 7.323900e-01 7.104764e-01 6.895084e-01 6.777666e-01

1518 9.985779e-01 9.993843e-01 9.968269e-01 9.934704e-01 9.876762e-01 7.565056e-01 7.326476e-01 7.107459e-01 6.897913e-01 6.780371e-01

1519 9.985777e-01 9.993842e-01 9.968266e-01 9.934696e-01 9.876749e-01 7.567468e-01 7.329140e-01 7.110247e-01 6.900842e-01 6.783174e-01

1520 9.985776e-01 9.993842e-01 9.968261e-01 9.934688e-01 9.876735e-01 7.569964e-01 7.331895e-01 7.113133e-01 6.903874e-01 6.786078e-01

1521 9.985774e-01 9.993841e-01 9.968257e-01 9.934680e-01 9.876720e-01 7.572546e-01 7.334746e-01 7.116119e-01 6.907012e-01 6.789086e-01

1522 9.985772e-01 9.993840e-01 9.968252e-01 9.934670e-01 9.876703e-01 7.575217e-01 7.337694e-01 7.119209e-01 6.910260e-01 6.792200e-01

1523 9.985770e-01 9.993840e-01 9.968247e-01 9.934661e-01 9.876686e-01 7.577980e-01 7.340743e-01 7.122405e-01 6.913621e-01 6.795425e-01

1524 9.985768e-01 9.993839e-01 9.968242e-01 9.934650e-01 9.876668e-01 7.580837e-01 7.343897e-01 7.125711e-01 6.917098e-01 6.798763e-01

1525 9.985765e-01 9.993839e-01 9.968236e-01 9.934639e-01 9.876648e-01 7.583793e-01 7.347157e-01 7.129131e-01 6.920695e-01 6.802218e-01

1526 9.985763e-01 9.993838e-01 9.968230e-01 9.934627e-01 9.876627e-01 7.586849e-01 7.350529e-01 7.132667e-01 6.924415e-01 6.805794e-01

1527 9.985760e-01 9.993837e-01 9.968224e-01 9.934614e-01 9.876604e-01 7.590010e-01 7.354014e-01 7.136324e-01 6.928262e-01 6.809493e-01

1528 9.985757e-01 9.993836e-01 9.968217e-01 9.934600e-01 9.876580e-01 7.593278e-01 7.357617e-01 7.140104e-01 6.932239e-01 6.813320e-01

1529 9.985754e-01 9.993835e-01 9.968210e-01 9.934586e-01 9.876554e-01 7.596658e-01 7.361340e-01 7.144012e-01 6.936351e-01 6.817277e-01

1530 9.985751e-01 9.993834e-01 9.968202e-01 9.934570e-01 9.876526e-01 7.600151e-01 7.365189e-01 7.148051e-01 6.940600e-01 6.821369e-01

1531 9.985748e-01 9.993833e-01 9.968193e-01 9.934553e-01 9.876496e-01 7.603763e-01 7.369165e-01 7.152224e-01 6.944990e-01 6.825598e-01

1532 9.985744e-01 9.993832e-01 9.968185e-01 9.934536e-01 9.876464e-01 7.607496e-01 7.373274e-01 7.156536e-01 6.949525e-01 6.829969e-01

1533 9.985740e-01 9.993831e-01 9.968175e-01 9.934516e-01 9.876430e-01 7.611354e-01 7.377518e-01 7.160989e-01 6.954209e-01 6.834483e-01

1534 9.985736e-01 9.993830e-01 9.968165e-01 9.934496e-01 9.876393e-01 7.615341e-01 7.381901e-01 7.165589e-01 6.959044e-01 6.839146e-01

1535 9.985732e-01 9.993829e-01 9.968154e-01 9.934474e-01 9.876354e-01 7.619460e-01 7.386428e-01 7.170337e-01 6.964036e-01 6.843961e-01

1536 9.985727e-01 9.993827e-01 9.968142e-01 9.934451e-01 9.876311e-01 7.623716e-01 7.391101e-01 7.175240e-01 6.969186e-01 6.848929e-01

1537 9.985722e-01 9.993826e-01 9.968130e-01 9.934426e-01 9.876266e-01 7.628112e-01 7.395926e-01 7.180299e-01 6.974500e-01 6.854056e-01

1538 9.985717e-01 9.993825e-01 9.968117e-01 9.934399e-01 9.876217e-01 7.632652e-01 7.400905e-01 7.185518e-01 6.979979e-01 6.859343e-01

1539 9.985711e-01 9.993823e-01 9.968103e-01 9.934370e-01 9.876165e-01 7.637340e-01 7.406043e-01 7.190902e-01 6.985628e-01 6.864794e-01

1540 9.985705e-01 9.993821e-01 9.968088e-01 9.934339e-01 9.876109e-01 7.642181e-01 7.411344e-01 7.196454e-01 6.991450e-01 6.870412e-01

1541 9.985699e-01 9.993819e-01 9.968072e-01 9.934306e-01 9.876049e-01 7.647177e-01 7.416811e-01 7.202178e-01 6.997448e-01 6.876200e-01

1542 9.985692e-01 9.993818e-01 9.968054e-01 9.934270e-01 9.875984e-01 7.652334e-01 7.422448e-01 7.208076e-01 7.003624e-01 6.882160e-01

1543 9.985685e-01 9.993816e-01 9.968036e-01 9.934232e-01 9.875915e-01 7.657656e-01 7.428260e-01 7.214153e-01 7.009983e-01 6.888294e-01

1544 9.985677e-01 9.993813e-01 9.968016e-01 9.934192e-01 9.875840e-01 7.663145e-01 7.434250e-01 7.220412e-01 7.016526e-01 6.894605e-01

1545 9.985669e-01 9.993811e-01 9.967995e-01 9.934148e-01 9.875760e-01 7.668808e-01 7.440421e-01 7.226856e-01 7.023256e-01 6.901095e-01

1546 9.985660e-01 9.993809e-01 9.967972e-01 9.934101e-01 9.875674e-01 7.674647e-01 7.446778e-01 7.233488e-01 7.030175e-01 6.907765e-01

1547 9.985650e-01 9.993806e-01 9.967948e-01 9.934050e-01 9.875581e-01 7.680667e-01 7.453323e-01 7.240312e-01 7.037286e-01 6.914617e-01

1548 9.985640e-01 9.993803e-01 9.967922e-01 9.933996e-01 9.875481e-01 7.686871e-01 7.460062e-01 7.247329e-01 7.044591e-01 6.921652e-01

1549 9.985630e-01 9.993800e-01 9.967894e-01 9.933938e-01 9.875373e-01 7.693265e-01 7.466996e-01 7.254542e-01 7.052090e-01 6.928871e-01

1550 9.985618e-01 9.993797e-01 9.967864e-01 9.933875e-01 9.875257e-01 7.699851e-01 7.474130e-01 7.261955e-01 7.059786e-01 6.936275e-01

1551 9.985606e-01 9.993794e-01 9.967832e-01 9.933808e-01 9.875133e-01 7.706634e-01 7.481466e-01 7.269569e-01 7.067679e-01 6.943864e-01

1552 9.985593e-01 9.993791e-01 9.967798e-01 9.933736e-01 9.874998e-01 7.713617e-01 7.489007e-01 7.277386e-01 7.075771e-01 6.951638e-01

1553 9.985579e-01 9.993787e-01 9.967761e-01 9.933658e-01 9.874853e-01 7.720805e-01 7.496757e-01 7.285407e-01 7.084061e-01 6.959596e-01

1554 9.985564e-01 9.993783e-01 9.967721e-01 9.933574e-01 9.874697e-01 7.728200e-01 7.504717e-01 7.293635e-01 7.092550e-01 6.967738e-01

1555 9.985548e-01 9.993779e-01 9.967679e-01 9.933484e-01 9.874529e-01 7.735807e-01 7.512891e-01 7.302070e-01 7.101237e-01 6.976061e-01

1556 9.985531e-01 9.993774e-01 9.967634e-01 9.933387e-01 9.874348e-01 7.743629e-01 7.521281e-01 7.310714e-01 7.110122e-01 6.984565e-01

1557 9.985512e-01 9.993769e-01 9.967585e-01 9.933283e-01 9.874152e-01 7.751668e-01 7.529888e-01 7.319565e-01 7.119203e-01 6.993248e-01

1558 9.985493e-01 9.993764e-01 9.967532e-01 9.933171e-01 9.873941e-01 7.759929e-01 7.538714e-01 7.328626e-01 7.128478e-01 7.002105e-01

1559 9.985472e-01 9.993759e-01 9.967476e-01 9.933050e-01 9.873713e-01 7.768413e-01 7.547760e-01 7.337895e-01 7.137947e-01 7.011135e-01

1560 9.985449e-01 9.993753e-01 9.967415e-01 9.932920e-01 9.873467e-01 7.777124e-01 7.557029e-01 7.347372e-01 7.147606e-01 7.020334e-01

1561 9.985425e-01 9.993747e-01 9.967350e-01 9.932780e-01 9.873202e-01 7.786064e-01 7.566519e-01 7.357056e-01 7.157452e-01 7.029697e-01

1562 9.985399e-01 9.993740e-01 9.967280e-01 9.932629e-01 9.872915e-01 7.795234e-01 7.576233e-01 7.366945e-01 7.167481e-01 7.039220e-01

1563 9.985372e-01 9.993733e-01 9.967205e-01 9.932466e-01 9.872606e-01 7.804638e-01 7.586169e-01 7.377037e-01 7.177691e-01 7.048898e-01

1564 9.985342e-01 9.993726e-01 9.967124e-01 9.932290e-01 9.872271e-01 7.814276e-01 7.596328e-01 7.387330e-01 7.188076e-01 7.058725e-01

1565 9.985311e-01 9.993718e-01 9.967038e-01 9.932101e-01 9.871910e-01 7.824150e-01 7.606708e-01 7.397822e-01 7.198631e-01 7.068695e-01

1566 9.985277e-01 9.993709e-01 9.966944e-01 9.931897e-01 9.871520e-01 7.834261e-01 7.617310e-01 7.408507e-01 7.209351e-01 7.078801e-01

1567 9.985240e-01 9.993700e-01 9.966843e-01 9.931676e-01 9.871097e-01 7.844609e-01 7.628130e-01 7.419384e-01 7.220231e-01 7.089036e-01

1568 9.985201e-01 9.993690e-01 9.966735e-01 9.931438e-01 9.870641e-01 7.855195e-01 7.639168e-01 7.430447e-01 7.231262e-01 7.099393e-01

1569 9.985159e-01 9.993680e-01 9.966618e-01 9.931181e-01 9.870147e-01 7.866018e-01 7.650419e-01 7.441692e-01 7.242439e-01 7.109864e-01

1570 9.985115e-01 9.993669e-01 9.966493e-01 9.930904e-01 9.869613e-01 7.877079e-01 7.661883e-01 7.453113e-01 7.253753e-01 7.120439e-01

1571 9.985066e-01 9.993657e-01 9.966358e-01 9.930604e-01 9.869036e-01 7.888375e-01 7.673555e-01 7.464704e-01 7.265198e-01 7.131110e-01

1572 9.985015e-01 9.993644e-01 9.966212e-01 9.930281e-01 9.868411e-01 7.899907e-01 7.685431e-01 7.476460e-01 7.276764e-01 7.141868e-01

1573 9.984959e-01 9.993631e-01 9.966055e-01 9.929931e-01 9.867735e-01 7.911671e-01 7.697506e-01 7.488372e-01 7.288443e-01 7.152703e-01

1574 9.984900e-01 9.993616e-01 9.965885e-01 9.929554e-01 9.867004e-01 7.923666e-01 7.709777e-01 7.500434e-01 7.300225e-01 7.163605e-01

1575 9.984836e-01 9.993601e-01 9.965703e-01 9.929145e-01 9.866212e-01 7.935888e-01 7.722236e-01 7.512639e-01 7.312101e-01 7.174563e-01

1576 9.984767e-01 9.993584e-01 9.965506e-01 9.928704e-01 9.865355e-01 7.948336e-01 7.734880e-01 7.524976e-01 7.324061e-01 7.185568e-01

1577 9.984693e-01 9.993567e-01 9.965294e-01 9.928227e-01 9.864428e-01 7.961004e-01 7.747700e-01 7.537439e-01 7.336094e-01 7.196608e-01

1578 9.984614e-01 9.993548e-01 9.965065e-01 9.927711e-01 9.863424e-01 7.973889e-01 7.760690e-01 7.550017e-01 7.348189e-01 7.207671e-01

1579 9.984528e-01 9.993528e-01 9.964817e-01 9.927153e-01 9.862338e-01 7.986986e-01 7.773842e-01 7.562702e-01 7.360336e-01 7.218748e-01

1580 9.984436e-01 9.993506e-01 9.964551e-01 9.926550e-01 9.861162e-01 8.000289e-01 7.787149e-01 7.575482e-01 7.372524e-01 7.229827e-01

1581 9.984338e-01 9.993483e-01 9.964263e-01 9.925897e-01 9.859889e-01 8.013794e-01 7.800603e-01 7.588348e-01 7.384741e-01 7.240896e-01

1582 9.984232e-01 9.993458e-01 9.963952e-01 9.925191e-01 9.858511e-01 8.027493e-01 7.814193e-01 7.601289e-01 7.396975e-01 7.251945e-01

1583 9.984117e-01 9.993432e-01 9.963616e-01 9.924428e-01 9.857020e-01 8.041379e-01 7.827912e-01 7.614295e-01 7.409216e-01 7.262961e-01

1584 9.983995e-01 9.993404e-01 9.963254e-01 9.923601e-01 9.855407e-01 8.055447e-01 7.841749e-01 7.627353e-01 7.421451e-01 7.273935e-01

1585 9.983862e-01 9.993373e-01 9.962862e-01 9.922707e-01 9.853662e-01 8.069687e-01 7.855694e-01 7.640452e-01 7.433668e-01 7.284854e-01

1586 9.983720e-01 9.993341e-01 9.962439e-01 9.921740e-01 9.851774e-01 8.084091e-01 7.869737e-01 7.653582e-01 7.445857e-01 7.295708e-01

1587 9.983567e-01 9.993306e-01 9.961982e-01 9.920693e-01 9.849732e-01 8.098651e-01 7.883867e-01 7.666730e-01 7.458004e-01 7.306486e-01

1588 9.983402e-01 9.993269e-01 9.961488e-01 9.919560e-01 9.847525e-01 8.113358e-01 7.898073e-01 7.679883e-01 7.470100e-01 7.317178e-01

1589 9.983224e-01 9.993229e-01 9.960955e-01 9.918335e-01 9.845139e-01 8.128201e-01 7.912343e-01 7.693032e-01 7.482132e-01 7.327773e-01

1590 9.983033e-01 9.993186e-01 9.960378e-01 9.917009e-01 9.842561e-01 8.143171e-01 7.926666e-01 7.706162e-01 7.494090e-01 7.338263e-01

1591 9.982826e-01 9.993140e-01 9.959754e-01 9.915575e-01 9.839777e-01 8.158258e-01 7.941030e-01 7.719263e-01 7.505962e-01 7.348637e-01

1592 9.982604e-01 9.993091e-01 9.959080e-01 9.914023e-01 9.836771e-01 8.173450e-01 7.955423e-01 7.732323e-01 7.517738e-01 7.358887e-01

1593 9.982364e-01 9.993038e-01 9.958351e-01 9.912346e-01 9.833527e-01 8.188737e-01 7.969834e-01 7.745330e-01 7.529408e-01 7.369004e-01

1594 9.982106e-01 9.992982e-01 9.957563e-01 9.910532e-01 9.830029e-01 8.204107e-01 7.984249e-01 7.758272e-01 7.540961e-01 7.378979e-01

1595 9.981827e-01 9.992921e-01 9.956712e-01 9.908571e-01 9.826258e-01 8.219548e-01 7.998656e-01 7.771138e-01 7.552388e-01 7.388807e-01

1596 9.981527e-01 9.992856e-01 9.955791e-01 9.906452e-01 9.822197e-01 8.235049e-01 8.013044e-01 7.783916e-01 7.563681e-01 7.398478e-01

1597 9.981203e-01 9.992786e-01 9.954795e-01 9.904162e-01 9.817825e-01 8.250597e-01 8.027400e-01 7.796597e-01 7.574829e-01 7.407987e-01

1598 9.980854e-01 9.992711e-01 9.953719e-01 9.901689e-01 9.813123e-01 8.266180e-01 8.041713e-01 7.809169e-01 7.585826e-01 7.417328e-01

1599 9.980477e-01 9.992630e-01 9.952555e-01 9.899019e-01 9.808070e-01 8.281786e-01 8.055969e-01 7.821623e-01 7.596663e-01 7.426495e-01

1600 9.980070e-01 9.992544e-01 9.951297e-01 9.896138e-01 9.802646e-01 8.297401e-01 8.070157e-01 7.833948e-01 7.607332e-01 7.435483e-01

1601 9.979631e-01 9.992451e-01 9.949938e-01 9.893030e-01 9.796827e-01 8.313014e-01 8.084266e-01 7.846134e-01 7.617828e-01 7.444288e-01

1602 9.979158e-01 9.992351e-01 9.948469e-01 9.889679e-01 9.790592e-01 8.328612e-01 8.098283e-01 7.858174e-01 7.628145e-01 7.452906e-01

1603 9.978647e-01 9.992244e-01 9.946882e-01 9.886068e-01 9.783920e-01 8.344181e-01 8.112199e-01 7.870058e-01 7.638275e-01 7.461333e-01

1604 9.978096e-01 9.992129e-01 9.945167e-01 9.882181e-01 9.776787e-01 8.359711e-01 8.126001e-01 7.881778e-01 7.648215e-01 7.469567e-01

1605 9.977501e-01 9.992005e-01 9.943315e-01 9.877997e-01 9.769173e-01 8.375188e-01 8.139681e-01 7.893327e-01 7.657960e-01 7.477605e-01

1606 9.976859e-01 9.991872e-01 9.941316e-01 9.873499e-01 9.761055e-01 8.390601e-01 8.153227e-01 7.904697e-01 7.667506e-01 7.485446e-01

1607 9.976166e-01 9.991729e-01 9.939157e-01 9.868666e-01 9.752414e-01 8.405938e-01 8.166631e-01 7.915885e-01 7.676850e-01 7.493090e-01

1608 9.975417e-01 9.991576e-01 9.936829e-01 9.863478e-01 9.743230e-01 8.421188e-01 8.179884e-01 7.926882e-01 7.685990e-01 7.500535e-01

1609 9.974610e-01 9.991410e-01 9.934317e-01 9.857914e-01 9.733485e-01 8.436340e-01 8.192977e-01 7.937686e-01 7.694925e-01 7.507781e-01

1610 9.973739e-01 9.991233e-01 9.931608e-01 9.851954e-01 9.723163e-01 8.451383e-01 8.205905e-01 7.948292e-01 7.703653e-01 7.514831e-01

1611 9.972798e-01 9.991041e-01 9.928690e-01 9.845575e-01 9.712249e-01 8.466309e-01 8.218659e-01 7.958697e-01 7.712175e-01 7.521686e-01

1612 9.971784e-01 9.990836e-01 9.925546e-01 9.838755e-01 9.700732e-01 8.481107e-01 8.231233e-01 7.968898e-01 7.720489e-01 7.528347e-01

1613 9.970689e-01 9.990615e-01 9.922162e-01 9.831475e-01 9.688603e-01 8.495768e-01 8.243622e-01 7.978893e-01 7.728597e-01 7.534816e-01

1614 9.969508e-01 9.990377e-01 9.918521e-01 9.823713e-01 9.675856e-01 8.510284e-01 8.255821e-01 7.988680e-01 7.736500e-01 7.541097e-01

1615 9.968234e-01 9.990121e-01 9.914607e-01 9.815447e-01 9.662491e-01 8.524647e-01 8.267824e-01 7.998257e-01 7.744200e-01 7.547192e-01

1616 9.966860e-01 9.989845e-01 9.910401e-01 9.806660e-01 9.648507e-01 8.538849e-01 8.279627e-01 8.007625e-01 7.751697e-01 7.553104e-01

1617 9.965378e-01 9.989549e-01 9.905886e-01 9.797332e-01 9.633913e-01 8.552882e-01 8.291228e-01 8.016784e-01 7.758995e-01 7.558838e-01

1618 9.963781e-01 9.989230e-01 9.901044e-01 9.787446e-01 9.618718e-01 8.566740e-01 8.302622e-01 8.025732e-01 7.766096e-01 7.564396e-01

1619 9.962060e-01 9.988887e-01 9.895854e-01 9.776987e-01 9.602939e-01 8.580416e-01 8.313807e-01 8.034471e-01 7.773002e-01 7.569784e-01

1620 9.960206e-01 9.988517e-01 9.890297e-01 9.765943e-01 9.586595e-01 8.593906e-01 8.324781e-01 8.043003e-01 7.779718e-01 7.575004e-01

1621 9.958209e-01 9.988120e-01 9.884354e-01 9.754302e-01 9.569712e-01 8.607202e-01 8.335543e-01 8.051327e-01 7.786245e-01 7.580061e-01

1622 9.958209e-01 9.988120e-01 9.884353e-01 9.754301e-01 9.569711e-01 8.607257e-01 8.335625e-01 8.051427e-01 7.786352e-01 7.580156e-01

1623 9.958209e-01 9.988120e-01 9.884353e-01 9.754301e-01 9.569710e-01 8.607315e-01 8.335710e-01 8.051530e-01 7.786463e-01 7.580253e-01

1624 9.958209e-01 9.988120e-01 9.884353e-01 9.754300e-01 9.569709e-01 8.607378e-01 8.335799e-01 8.051636e-01 7.786575e-01 7.580352e-01

1625 9.958209e-01 9.988121e-01 9.884352e-01 9.754300e-01 9.569708e-01 8.607445e-01 8.335892e-01 8.051744e-01 7.786691e-01 7.580453e-01

1626 9.958208e-01 9.988121e-01 9.884352e-01 9.754299e-01 9.569708e-01 8.607516e-01 8.335988e-01 8.051856e-01 7.786809e-01 7.580556e-01

1627 9.958208e-01 9.988121e-01 9.884352e-01 9.754298e-01 9.569707e-01 8.607592e-01 8.336089e-01 8.051971e-01 7.786929e-01 7.580661e-01

1628 9.958208e-01 9.988121e-01 9.884351e-01 9.754298e-01 9.569706e-01 8.607672e-01 8.336193e-01 8.052089e-01 7.787053e-01 7.580768e-01

1629 9.958208e-01 9.988121e-01 9.884351e-01 9.754297e-01 9.569704e-01 8.607756e-01 8.336302e-01 8.052210e-01 7.787180e-01 7.580877e-01

1630 9.958207e-01 9.988121e-01 9.884350e-01 9.754296e-01 9.569703e-01 8.607845e-01 8.336414e-01 8.052335e-01 7.787310e-01 7.580989e-01

1631 9.958207e-01 9.988121e-01 9.884350e-01 9.754295e-01 9.569702e-01 8.607938e-01 8.336530e-01 8.052463e-01 7.787443e-01 7.581103e-01

1632 9.958207e-01 9.988121e-01 9.884349e-01 9.754295e-01 9.569701e-01 8.608036e-01 8.336651e-01 8.052594e-01 7.787579e-01 7.581220e-01

1633 9.958207e-01 9.988121e-01 9.884349e-01 9.754294e-01 9.569700e-01 8.608139e-01 8.336775e-01 8.052729e-01 7.787719e-01 7.581340e-01

1634 9.958206e-01 9.988120e-01 9.884348e-01 9.754293e-01 9.569698e-01 8.608246e-01 8.336904e-01 8.052868e-01 7.787862e-01 7.581462e-01

1635 9.958206e-01 9.988120e-01 9.884348e-01 9.754292e-01 9.569697e-01 8.608357e-01 8.337038e-01 8.053011e-01 7.788009e-01 7.581588e-01

1636 9.958206e-01 9.988120e-01 9.884347e-01 9.754291e-01 9.569695e-01 8.608474e-01 8.337175e-01 8.053158e-01 7.788160e-01 7.581717e-01

1637 9.958205e-01 9.988120e-01 9.884346e-01 9.754290e-01 9.569693e-01 8.608595e-01 8.337318e-01 8.053309e-01 7.788315e-01 7.581849e-01

1638 9.958205e-01 9.988120e-01 9.884346e-01 9.754289e-01 9.569692e-01 8.608721e-01 8.337465e-01 8.053464e-01 7.788474e-01 7.581984e-01

1639 9.958205e-01 9.988120e-01 9.884345e-01 9.754287e-01 9.569690e-01 8.608852e-01 8.337616e-01 8.053623e-01 7.788637e-01 7.582124e-01

1640 9.958204e-01 9.988120e-01 9.884344e-01 9.754286e-01 9.569688e-01 8.608988e-01 8.337773e-01 8.053787e-01 7.788804e-01 7.582266e-01

1641 9.958204e-01 9.988120e-01 9.884343e-01 9.754285e-01 9.569686e-01 8.609129e-01 8.337934e-01 8.053955e-01 7.788976e-01 7.582413e-01

1642 9.958204e-01 9.988119e-01 9.884343e-01 9.754283e-01 9.569684e-01 8.609276e-01 8.338101e-01 8.054128e-01 7.789153e-01 7.582563e-01

1643 9.958203e-01 9.988119e-01 9.884342e-01 9.754282e-01 9.569682e-01 8.609428e-01 8.338272e-01 8.054306e-01 7.789334e-01 7.582718e-01

1644 9.958203e-01 9.988119e-01 9.884341e-01 9.754280e-01 9.569679e-01 8.609585e-01 8.338449e-01 8.054488e-01 7.789521e-01 7.582877e-01

1645 9.958202e-01 9.988119e-01 9.884340e-01 9.754279e-01 9.569677e-01 8.609748e-01 8.338632e-01 8.054676e-01 7.789712e-01 7.583041e-01

1646 9.958202e-01 9.988119e-01 9.884339e-01 9.754277e-01 9.569674e-01 8.609916e-01 8.338820e-01 8.054870e-01 7.789909e-01 7.583209e-01

1647 9.958201e-01 9.988118e-01 9.884338e-01 9.754275e-01 9.569671e-01 8.610090e-01 8.339013e-01 8.055068e-01 7.790112e-01 7.583382e-01

1648 9.958201e-01 9.988118e-01 9.884337e-01 9.754273e-01 9.569668e-01 8.610270e-01 8.339213e-01 8.055272e-01 7.790320e-01 7.583560e-01

1649 9.958200e-01 9.988118e-01 9.884335e-01 9.754271e-01 9.569665e-01 8.610456e-01 8.339418e-01 8.055483e-01 7.790534e-01 7.583743e-01

1650 9.958199e-01 9.988118e-01 9.884334e-01 9.754269e-01 9.569662e-01 8.610648e-01 8.339630e-01 8.055699e-01 7.790755e-01 7.583932e-01

1651 9.958199e-01 9.988118e-01 9.884333e-01 9.754267e-01 9.569659e-01 8.610846e-01 8.339848e-01 8.055921e-01 7.790981e-01 7.584126e-01

1652 9.958198e-01 9.988117e-01 9.884331e-01 9.754264e-01 9.569655e-01 8.611051e-01 8.340072e-01 8.056149e-01 7.791214e-01 7.584326e-01

1653 9.958197e-01 9.988117e-01 9.884330e-01 9.754262e-01 9.569651e-01 8.611263e-01 8.340303e-01 8.056385e-01 7.791454e-01 7.584532e-01

1654 9.958197e-01 9.988117e-01 9.884328e-01 9.754259e-01 9.569647e-01 8.611481e-01 8.340541e-01 8.056626e-01 7.791701e-01 7.584744e-01

1655 9.958196e-01 9.988116e-01 9.884327e-01 9.754256e-01 9.569643e-01 8.611706e-01 8.340786e-01 8.056875e-01 7.791956e-01 7.584962e-01

1656 9.958195e-01 9.988116e-01 9.884325e-01 9.754254e-01 9.569638e-01 8.611938e-01 8.341038e-01 8.057132e-01 7.792217e-01 7.585188e-01

1657 9.958194e-01 9.988116e-01 9.884323e-01 9.754250e-01 9.569634e-01 8.612177e-01 8.341298e-01 8.057395e-01 7.792487e-01 7.585420e-01

1658 9.958193e-01 9.988116e-01 9.884321e-01 9.754247e-01 9.569629e-01 8.612424e-01 8.341566e-01 8.057666e-01 7.792764e-01 7.585660e-01

1659 9.958192e-01 9.988115e-01 9.884319e-01 9.754244e-01 9.569623e-01 8.612679e-01 8.341841e-01 8.057946e-01 7.793050e-01 7.585907e-01

1660 9.958191e-01 9.988115e-01 9.884317e-01 9.754240e-01 9.569618e-01 8.612941e-01 8.342124e-01 8.058233e-01 7.793344e-01 7.586161e-01

1661 9.958190e-01 9.988114e-01 9.884315e-01 9.754236e-01 9.569612e-01 8.613211e-01 8.342416e-01 8.058529e-01 7.793647e-01 7.586424e-01

1662 9.958189e-01 9.988114e-01 9.884312e-01 9.754232e-01 9.569606e-01 8.613490e-01 8.342717e-01 8.058833e-01 7.793960e-01 7.586695e-01

1663 9.958188e-01 9.988114e-01 9.884310e-01 9.754228e-01 9.569599e-01 8.613777e-01 8.343026e-01 8.059147e-01 7.794282e-01 7.586974e-01

1664 9.958187e-01 9.988113e-01 9.884307e-01 9.754223e-01 9.569592e-01 8.614073e-01 8.343344e-01 8.059470e-01 7.794613e-01 7.587263e-01

1665 9.958185e-01 9.988113e-01 9.884304e-01 9.754218e-01 9.569585e-01 8.614378e-01 8.343672e-01 8.059802e-01 7.794955e-01 7.587561e-01

1666 9.958184e-01 9.988112e-01 9.884301e-01 9.754213e-01 9.569577e-01 8.614693e-01 8.344010e-01 8.060145e-01 7.795307e-01 7.587868e-01

1667 9.958182e-01 9.988112e-01 9.884298e-01 9.754208e-01 9.569568e-01 8.615016e-01 8.344357e-01 8.060497e-01 7.795671e-01 7.588185e-01

1668 9.958181e-01 9.988111e-01 9.884295e-01 9.754202e-01 9.569560e-01 8.615350e-01 8.344715e-01 8.060861e-01 7.796045e-01 7.588513e-01

1669 9.958179e-01 9.988111e-01 9.884291e-01 9.754196e-01 9.569550e-01 8.615693e-01 8.345083e-01 8.061235e-01 7.796431e-01 7.588851e-01

1670 9.958177e-01 9.988110e-01 9.884288e-01 9.754189e-01 9.569541e-01 8.616047e-01 8.345463e-01 8.061620e-01 7.796829e-01 7.589201e-01

1671 9.958176e-01 9.988109e-01 9.884284e-01 9.754182e-01 9.569530e-01 8.616412e-01 8.345854e-01 8.062017e-01 7.797240e-01 7.589561e-01

1672 9.958174e-01 9.988109e-01 9.884279e-01 9.754175e-01 9.569519e-01 8.616788e-01 8.346256e-01 8.062426e-01 7.797663e-01 7.589934e-01

1673 9.958171e-01 9.988108e-01 9.884275e-01 9.754167e-01 9.569507e-01 8.617175e-01 8.346670e-01 8.062848e-01 7.798100e-01 7.590319e-01

1674 9.958169e-01 9.988107e-01 9.884270e-01 9.754159e-01 9.569495e-01 8.617573e-01 8.347097e-01 8.063283e-01 7.798550e-01 7.590717e-01

1675 9.958167e-01 9.988106e-01 9.884265e-01 9.754151e-01 9.569482e-01 8.617984e-01 8.347536e-01 8.063730e-01 7.799015e-01 7.591128e-01

1676 9.958164e-01 9.988106e-01 9.884260e-01 9.754141e-01 9.569468e-01 8.618407e-01 8.347989e-01 8.064192e-01 7.799494e-01 7.591552e-01

1677 9.958162e-01 9.988105e-01 9.884254e-01 9.754132e-01 9.569453e-01 8.618843e-01 8.348455e-01 8.064667e-01 7.799989e-01 7.591991e-01

1678 9.958159e-01 9.988104e-01 9.884248e-01 9.754121e-01 9.569437e-01 8.619292e-01 8.348935e-01 8.065158e-01 7.800499e-01 7.592444e-01

1679 9.958156e-01 9.988103e-01 9.884242e-01 9.754110e-01 9.569421e-01 8.619754e-01 8.349430e-01 8.065663e-01 7.801026e-01 7.592913e-01

1680 9.958153e-01 9.988102e-01 9.884235e-01 9.754099e-01 9.569403e-01 8.620231e-01 8.349940e-01 8.066184e-01 7.801569e-01 7.593397e-01

1681 9.958150e-01 9.988101e-01 9.884228e-01 9.754086e-01 9.569384e-01 8.620722e-01 8.350465e-01 8.066722e-01 7.802130e-01 7.593898e-01

1682 9.958146e-01 9.988100e-01 9.884221e-01 9.754073e-01 9.569364e-01 8.621227e-01 8.351005e-01 8.067275e-01 7.802709e-01 7.594415e-01

1683 9.958143e-01 9.988098e-01 9.884213e-01 9.754059e-01 9.569343e-01 8.621748e-01 8.351563e-01 8.067847e-01 7.803307e-01 7.594951e-01

1684 9.958139e-01 9.988097e-01 9.884204e-01 9.754045e-01 9.569321e-01 8.622285e-01 8.352137e-01 8.068436e-01 7.803924e-01 7.595504e-01

1685 9.958135e-01 9.988096e-01 9.884195e-01 9.754029e-01 9.569297e-01 8.622838e-01 8.352728e-01 8.069043e-01 7.804561e-01 7.596076e-01

1686 9.958130e-01 9.988094e-01 9.884185e-01 9.754012e-01 9.569271e-01 8.623408e-01 8.353338e-01 8.069669e-01 7.805218e-01 7.596667e-01

1687 9.958125e-01 9.988093e-01 9.884175e-01 9.753994e-01 9.569244e-01 8.623995e-01 8.353966e-01 8.070316e-01 7.805897e-01 7.597279e-01

1688 9.958121e-01 9.988091e-01 9.884164e-01 9.753976e-01 9.569216e-01 8.624600e-01 8.354614e-01 8.070982e-01 7.806598e-01 7.597911e-01

1689 9.958115e-01 9.988089e-01 9.884153e-01 9.753956e-01 9.569185e-01 8.625224e-01 8.355281e-01 8.071669e-01 7.807322e-01 7.598565e-01

1690 9.958110e-01 9.988088e-01 9.884141e-01 9.753934e-01 9.569153e-01 8.625866e-01 8.355968e-01 8.072378e-01 7.808069e-01 7.599242e-01

1691 9.958104e-01 9.988086e-01 9.884128e-01 9.753911e-01 9.569118e-01 8.626528e-01 8.356677e-01 8.073110e-01 7.808841e-01 7.599941e-01

1692 9.958098e-01 9.988084e-01 9.884114e-01 9.753887e-01 9.569081e-01 8.627211e-01 8.357407e-01 8.073864e-01 7.809637e-01 7.600665e-01

1693 9.958091e-01 9.988082e-01 9.884099e-01 9.753862e-01 9.569042e-01 8.627914e-01 8.358160e-01 8.074643e-01 7.810460e-01 7.601413e-01

1694 9.958084e-01 9.988079e-01 9.884084e-01 9.753834e-01 9.569001e-01 8.628639e-01 8.358936e-01 8.075446e-01 7.811310e-01 7.602187e-01

1695 9.958076e-01 9.988077e-01 9.884067e-01 9.753805e-01 9.568956e-01 8.629386e-01 8.359737e-01 8.076274e-01 7.812188e-01 7.602988e-01

1696 9.958068e-01 9.988075e-01 9.884049e-01 9.753774e-01 9.568909e-01 8.630155e-01 8.360561e-01 8.077129e-01 7.813094e-01 7.603816e-01

1697 9.958060e-01 9.988072e-01 9.884030e-01 9.753741e-01 9.568859e-01 8.630949e-01 8.361412e-01 8.078011e-01 7.814030e-01 7.604672e-01

1698 9.958051e-01 9.988069e-01 9.884010e-01 9.753706e-01 9.568805e-01 8.631767e-01 8.362288e-01 8.078921e-01 7.814997e-01 7.605558e-01

1699 9.958041e-01 9.988066e-01 9.883989e-01 9.753668e-01 9.568748e-01 8.632610e-01 8.363192e-01 8.079860e-01 7.815996e-01 7.606474e-01

1700 9.958031e-01 9.988063e-01 9.883966e-01 9.753628e-01 9.568687e-01 8.633480e-01 8.364124e-01 8.080829e-01 7.817027e-01 7.607422e-01

1701 9.958020e-01 9.988060e-01 9.883942e-01 9.753585e-01 9.568622e-01 8.634376e-01 8.365084e-01 8.081829e-01 7.818092e-01 7.608402e-01

1702 9.958009e-01 9.988056e-01 9.883916e-01 9.753540e-01 9.568552e-01 8.635299e-01 8.366075e-01 8.082861e-01 7.819192e-01 7.609415e-01

1703 9.957996e-01 9.988052e-01 9.883889e-01 9.753491e-01 9.568478e-01 8.636252e-01 8.367096e-01 8.083926e-01 7.820328e-01 7.610463e-01

1704 9.957983e-01 9.988048e-01 9.883859e-01 9.753439e-01 9.568399e-01 8.637233e-01 8.368149e-01 8.085025e-01 7.821502e-01 7.611547e-01

1705 9.957970e-01 9.988044e-01 9.883828e-01 9.753384e-01 9.568315e-01 8.638246e-01 8.369235e-01 8.086158e-01 7.822714e-01 7.612669e-01

1706 9.957955e-01 9.988040e-01 9.883794e-01 9.753325e-01 9.568225e-01 8.639289e-01 8.370354e-01 8.087328e-01 7.823965e-01 7.613828e-01

1707 9.957939e-01 9.988035e-01 9.883759e-01 9.753262e-01 9.568128e-01 8.640365e-01 8.371509e-01 8.088536e-01 7.825257e-01 7.615026e-01

1708 9.957922e-01 9.988030e-01 9.883721e-01 9.753194e-01 9.568026e-01 8.641475e-01 8.372699e-01 8.089781e-01 7.826592e-01 7.616266e-01

1709 9.957904e-01 9.988025e-01 9.883680e-01 9.753122e-01 9.567916e-01 8.642619e-01 8.373927e-01 8.091067e-01 7.827970e-01 7.617547e-01

1710 9.957885e-01 9.988019e-01 9.883637e-01 9.753045e-01 9.567799e-01 8.643798e-01 8.375192e-01 8.092393e-01 7.829393e-01 7.618872e-01

1711 9.957865e-01 9.988013e-01 9.883590e-01 9.752963e-01 9.567673e-01 8.645014e-01 8.376497e-01 8.093762e-01 7.830862e-01 7.620241e-01

1712 9.957843e-01 9.988006e-01 9.883541e-01 9.752875e-01 9.567539e-01 8.646268e-01 8.377843e-01 8.095174e-01 7.832379e-01 7.621656e-01

1713 9.957820e-01 9.988000e-01 9.883488e-01 9.752781e-01 9.567396e-01 8.647561e-01 8.379231e-01 8.096630e-01 7.833944e-01 7.623119e-01

1714 9.957795e-01 9.987992e-01 9.883431e-01 9.752681e-01 9.567242e-01 8.648894e-01 8.380662e-01 8.098133e-01 7.835561e-01 7.624630e-01

1715 9.957769e-01 9.987985e-01 9.883371e-01 9.752573e-01 9.567078e-01 8.650269e-01 8.382137e-01 8.099684e-01 7.837229e-01 7.626192e-01

1716 9.957741e-01 9.987977e-01 9.883306e-01 9.752458e-01 9.566903e-01 8.651687e-01 8.383658e-01 8.101283e-01 7.838951e-01 7.627805e-01

1717 9.957711e-01 9.987968e-01 9.883237e-01 9.752335e-01 9.566715e-01 8.653148e-01 8.385226e-01 8.102933e-01 7.840727e-01 7.629471e-01

1718 9.957679e-01 9.987959e-01 9.883163e-01 9.752203e-01 9.566514e-01 8.654655e-01 8.386843e-01 8.104635e-01 7.842561e-01 7.631192e-01

1719 9.957645e-01 9.987949e-01 9.883084e-01 9.752061e-01 9.566298e-01 8.656209e-01 8.388510e-01 8.106390e-01 7.844452e-01 7.632969e-01

1720 9.957608e-01 9.987939e-01 9.882999e-01 9.751910e-01 9.566067e-01 8.657811e-01 8.390229e-01 8.108200e-01 7.846404e-01 7.634804e-01

1721 9.957569e-01 9.987928e-01 9.882908e-01 9.751748e-01 9.565820e-01 8.659463e-01 8.392000e-01 8.110067e-01 7.848416e-01 7.636698e-01

1722 9.957527e-01 9.987916e-01 9.882811e-01 9.751574e-01 9.565555e-01 8.661167e-01 8.393827e-01 8.111991e-01 7.850492e-01 7.638652e-01

1723 9.957483e-01 9.987903e-01 9.882707e-01 9.751388e-01 9.565272e-01 8.662923e-01 8.395709e-01 8.113975e-01 7.852633e-01 7.640669e-01

1724 9.957435e-01 9.987890e-01 9.882596e-01 9.751188e-01 9.564967e-01 8.664733e-01 8.397649e-01 8.116020e-01 7.854839e-01 7.642749e-01

1725 9.957384e-01 9.987876e-01 9.882476e-01 9.750974e-01 9.564641e-01 8.666600e-01 8.399648e-01 8.118129e-01 7.857114e-01 7.644895e-01

1726 9.957330e-01 9.987861e-01 9.882349e-01 9.750744e-01 9.564291e-01 8.668524e-01 8.401709e-01 8.120301e-01 7.859458e-01 7.647107e-01

1727 9.957271e-01 9.987844e-01 9.882211e-01 9.750498e-01 9.563916e-01 8.670508e-01 8.403832e-01 8.122540e-01 7.861873e-01 7.649387e-01

1728 9.957209e-01 9.987827e-01 9.882064e-01 9.750234e-01 9.563514e-01 8.672552e-01 8.406020e-01 8.124847e-01 7.864362e-01 7.651737e-01

1729 9.957142e-01 9.987809e-01 9.881907e-01 9.749950e-01 9.563082e-01 8.674660e-01 8.408274e-01 8.127223e-01 7.866924e-01 7.654158e-01

1730 9.957071e-01 9.987789e-01 9.881737e-01 9.749645e-01 9.562619e-01 8.676832e-01 8.410595e-01 8.129670e-01 7.869563e-01 7.656651e-01

1731 9.956994e-01 9.987769e-01 9.881556e-01 9.749319e-01 9.562122e-01 8.679071e-01 8.412987e-01 8.132190e-01 7.872280e-01 7.659218e-01

1732 9.956912e-01 9.987746e-01 9.881361e-01 9.748968e-01 9.561589e-01 8.681378e-01 8.415450e-01 8.134785e-01 7.875076e-01 7.661860e-01

1733 9.956824e-01 9.987723e-01 9.881152e-01 9.748591e-01 9.561016e-01 8.683756e-01 8.417986e-01 8.137456e-01 7.877952e-01 7.664579e-01

1734 9.956730e-01 9.987698e-01 9.880928e-01 9.748186e-01 9.560402e-01 8.686206e-01 8.420597e-01 8.140205e-01 7.880911e-01 7.667375e-01

1735 9.956629e-01 9.987671e-01 9.880687e-01 9.747751e-01 9.559742e-01 8.688730e-01 8.423285e-01 8.143034e-01 7.883954e-01 7.670249e-01

1736 9.956521e-01 9.987642e-01 9.880428e-01 9.747284e-01 9.559033e-01 8.691330e-01 8.426052e-01 8.145944e-01 7.887082e-01 7.673203e-01

1737 9.956405e-01 9.987611e-01 9.880150e-01 9.746781e-01 9.558273e-01 8.694009e-01 8.428900e-01 8.148936e-01 7.890296e-01 7.676237e-01

1738 9.956281e-01 9.987579e-01 9.879851e-01 9.746242e-01 9.557456e-01 8.696767e-01 8.431830e-01 8.152013e-01 7.893599e-01 7.679353e-01

1739 9.956147e-01 9.987544e-01 9.879530e-01 9.745661e-01 9.556579e-01 8.699609e-01 8.434845e-01 8.155176e-01 7.896990e-01 7.682552e-01

1740 9.956004e-01 9.987506e-01 9.879185e-01 9.745038e-01 9.555637e-01 8.702534e-01 8.437945e-01 8.158426e-01 7.900471e-01 7.685832e-01

1741 9.955850e-01 9.987467e-01 9.878814e-01 9.744367e-01 9.554626e-01 8.705547e-01 8.441134e-01 8.161766e-01 7.904043e-01 7.689197e-01

1742 9.955685e-01 9.987424e-01 9.878415e-01 9.743646e-01 9.553539e-01 8.708648e-01 8.444412e-01 8.165195e-01 7.907708e-01 7.692645e-01

1743 9.955508e-01 9.987379e-01 9.877986e-01 9.742871e-01 9.552373e-01 8.711840e-01 8.447782e-01 8.168716e-01 7.911465e-01 7.696176e-01

1744 9.955318e-01 9.987330e-01 9.877524e-01 9.742037e-01 9.551121e-01 8.715125e-01 8.451245e-01 8.172330e-01 7.915315e-01 7.699792e-01

1745 9.955113e-01 9.987278e-01 9.877028e-01 9.741140e-01 9.549777e-01 8.718505e-01 8.454804e-01 8.176038e-01 7.919260e-01 7.703492e-01

1746 9.954894e-01 9.987222e-01 9.876494e-01 9.740176e-01 9.548334e-01 8.721983e-01 8.458459e-01 8.179840e-01 7.923299e-01 7.707276e-01

1747 9.954658e-01 9.987163e-01 9.875919e-01 9.739139e-01 9.546785e-01 8.725560e-01 8.462212e-01 8.183739e-01 7.927433e-01 7.711143e-01

1748 9.954405e-01 9.987099e-01 9.875300e-01 9.738023e-01 9.545122e-01 8.729239e-01 8.466065e-01 8.187735e-01 7.931662e-01 7.715092e-01

1749 9.954132e-01 9.987031e-01 9.874635e-01 9.736824e-01 9.543339e-01 8.733022e-01 8.470020e-01 8.191828e-01 7.935985e-01 7.719124e-01

1750 9.953839e-01 9.986959e-01 9.873918e-01 9.735534e-01 9.541426e-01 8.736911e-01 8.474078e-01 8.196020e-01 7.940403e-01 7.723237e-01

1751 9.953524e-01 9.986881e-01 9.873147e-01 9.734147e-01 9.539374e-01 8.740908e-01 8.478239e-01 8.200310e-01 7.944916e-01 7.727429e-01

1752 9.953185e-01 9.986797e-01 9.872317e-01 9.732655e-01 9.537174e-01 8.745015e-01 8.482507e-01 8.204700e-01 7.949522e-01 7.731700e-01

1753 9.952821e-01 9.986708e-01 9.871423e-01 9.731052e-01 9.534817e-01 8.749234e-01 8.486881e-01 8.209188e-01 7.954220e-01 7.736048e-01

1754 9.952429e-01 9.986612e-01 9.870461e-01 9.729328e-01 9.532291e-01 8.753568e-01 8.491363e-01 8.213776e-01 7.959011e-01 7.740470e-01

1755 9.952007e-01 9.986510e-01 9.869425e-01 9.727476e-01 9.529586e-01 8.758017e-01 8.495953e-01 8.218464e-01 7.963892e-01 7.744966e-01

1756 9.951553e-01 9.986400e-01 9.868309e-01 9.725486e-01 9.526691e-01 8.762585e-01 8.500653e-01 8.223250e-01 7.968862e-01 7.749532e-01

1757 9.951064e-01 9.986282e-01 9.867108e-01 9.723348e-01 9.523594e-01 8.767272e-01 8.505464e-01 8.228135e-01 7.973919e-01 7.754166e-01

1758 9.950538e-01 9.986156e-01 9.865816e-01 9.721052e-01 9.520282e-01 8.772081e-01 8.510385e-01 8.233117e-01 7.979062e-01 7.758866e-01

1759 9.949972e-01 9.986020e-01 9.864424e-01 9.718586e-01 9.516742e-01 8.777013e-01 8.515417e-01 8.238196e-01 7.984288e-01 7.763628e-01

1760 9.949362e-01 9.985875e-01 9.862926e-01 9.715940e-01 9.512962e-01 8.782070e-01 8.520562e-01 8.243371e-01 7.989595e-01 7.768449e-01

1761 9.948706e-01 9.985720e-01 9.861314e-01 9.713102e-01 9.508927e-01 8.787253e-01 8.525817e-01 8.248640e-01 7.994980e-01 7.773327e-01

1762 9.947999e-01 9.985553e-01 9.859579e-01 9.710058e-01 9.504625e-01 8.792564e-01 8.531185e-01 8.254001e-01 8.000441e-01 7.778256e-01

1763 9.947237e-01 9.985374e-01 9.857712e-01 9.706795e-01 9.500040e-01 8.798003e-01 8.536663e-01 8.259454e-01 8.005974e-01 7.783234e-01

1764 9.946417e-01 9.985182e-01 9.855704e-01 9.703299e-01 9.495158e-01 8.803573e-01 8.542253e-01 8.264995e-01 8.011576e-01 7.788256e-01

1765 9.945533e-01 9.984975e-01 9.853544e-01 9.699555e-01 9.489966e-01 8.809273e-01 8.547952e-01 8.270624e-01 8.017244e-01 7.793318e-01

1766 9.944582e-01 9.984754e-01 9.851222e-01 9.695549e-01 9.484449e-01 8.815104e-01 8.553761e-01 8.276336e-01 8.022973e-01 7.798416e-01

1767 9.943557e-01 9.984516e-01 9.848725e-01 9.691265e-01 9.478593e-01 8.821068e-01 8.559678e-01 8.282130e-01 8.028759e-01 7.803545e-01

1768 9.942453e-01 9.984261e-01 9.846042e-01 9.686687e-01 9.472383e-01 8.827165e-01 8.565702e-01 8.288002e-01 8.034598e-01 7.808701e-01

1769 9.941264e-01 9.983987e-01 9.843161e-01 9.681798e-01 9.465807e-01 8.833395e-01 8.571831e-01 8.293949e-01 8.040487e-01 7.813878e-01

1770 9.939983e-01 9.983692e-01 9.840066e-01 9.676582e-01 9.458851e-01 8.839757e-01 8.578063e-01 8.299968e-01 8.046419e-01 7.819073e-01

1771 9.938604e-01 9.983376e-01 9.836745e-01 9.671021e-01 9.451505e-01 8.846253e-01 8.584397e-01 8.306054e-01 8.052390e-01 7.824279e-01

1772 9.937119e-01 9.983036e-01 9.833182e-01 9.665098e-01 9.443756e-01 8.852881e-01 8.590829e-01 8.312205e-01 8.058396e-01 7.829491e-01

1773 9.935520e-01 9.982671e-01 9.829361e-01 9.658796e-01 9.435596e-01 8.859641e-01 8.597359e-01 8.318415e-01 8.064430e-01 7.834705e-01

1774 9.933798e-01 9.982278e-01 9.825266e-01 9.652099e-01 9.427016e-01 8.866532e-01 8.603981e-01 8.324681e-01 8.070488e-01 7.839915e-01

1775 9.931945e-01 9.981856e-01 9.820881e-01 9.644988e-01 9.418009e-01 8.873553e-01 8.610694e-01 8.330997e-01 8.076565e-01 7.845117e-01

1776 9.929950e-01 9.981403e-01 9.816188e-01 9.637448e-01 9.408572e-01 8.880703e-01 8.617494e-01 8.337359e-01 8.082654e-01 7.850304e-01

1777 9.927804e-01 9.980915e-01 9.811169e-01 9.629463e-01 9.398702e-01 8.887980e-01 8.624378e-01 8.343762e-01 8.088750e-01 7.855471e-01

1778 9.925495e-01 9.980391e-01 9.805805e-01 9.621018e-01 9.388398e-01 8.895383e-01 8.631341e-01 8.350201e-01 8.094847e-01 7.860614e-01

1779 9.923013e-01 9.979827e-01 9.800077e-01 9.612100e-01 9.377664e-01 8.902909e-01 8.638380e-01 8.356671e-01 8.100940e-01 7.865727e-01

1780 9.920344e-01 9.979221e-01 9.793966e-01 9.602697e-01 9.366506e-01 8.910556e-01 8.645490e-01 8.363165e-01 8.107023e-01 7.870806e-01

1781 9.917475e-01 9.978569e-01 9.787454e-01 9.592796e-01 9.354931e-01 8.918321e-01 8.652667e-01 8.369679e-01 8.113090e-01 7.875845e-01

1782 9.914394e-01 9.977868e-01 9.780519e-01 9.582390e-01 9.342953e-01 8.926201e-01 8.659905e-01 8.376206e-01 8.119135e-01 7.880840e-01

1783 9.911084e-01 9.977114e-01 9.773144e-01 9.571473e-01 9.330586e-01 8.934194e-01 8.667200e-01 8.382741e-01 8.125152e-01 7.885785e-01

1784 9.907533e-01 9.976303e-01 9.765308e-01 9.560038e-01 9.317849e-01 8.942294e-01 8.674546e-01 8.389278e-01 8.131137e-01 7.890678e-01

1785 9.903722e-01 9.975431e-01 9.756994e-01 9.548086e-01 9.304764e-01 8.950500e-01 8.681938e-01 8.395811e-01 8.137083e-01 7.895512e-01

1786 9.899637e-01 9.974493e-01 9.748182e-01 9.535617e-01 9.291357e-01 8.958806e-01 8.689371e-01 8.402335e-01 8.142986e-01 7.900285e-01

1787 9.895259e-01 9.973484e-01 9.738857e-01 9.522636e-01 9.277657e-01 8.967208e-01 8.696838e-01 8.408842e-01 8.148839e-01 7.904993e-01

1788 9.890571e-01 9.972399e-01 9.729002e-01 9.509150e-01 9.263696e-01 8.975702e-01 8.704334e-01 8.415328e-01 8.154637e-01 7.909631e-01

1789 9.885554e-01 9.971233e-01 9.718602e-01 9.495172e-01 9.249510e-01 8.984282e-01 8.711851e-01 8.421786e-01 8.160376e-01 7.914196e-01

1790 9.880189e-01 9.969978e-01 9.707645e-01 9.480717e-01 9.235136e-01 8.992944e-01 8.719386e-01 8.428211e-01 8.166052e-01 7.918686e-01

1791 9.874456e-01 9.968630e-01 9.696120e-01 9.465803e-01 9.220614e-01 9.001681e-01 8.726930e-01 8.434597e-01 8.171658e-01 7.923096e-01

1792 9.868336e-01 9.967180e-01 9.684017e-01 9.450454e-01 9.205987e-01 9.010488e-01 8.734477e-01 8.440938e-01 8.177191e-01 7.927425e-01

1793 9.861807e-01 9.965622e-01 9.671332e-01 9.434698e-01 9.191299e-01 9.019359e-01 8.742022e-01 8.447229e-01 8.182647e-01 7.931670e-01

1794 9.854851e-01 9.963947e-01 9.658059e-01 9.418564e-01 9.176595e-01 9.028289e-01 8.749558e-01 8.453464e-01 8.188022e-01 7.935829e-01

1795 9.847445e-01 9.962148e-01 9.644200e-01 9.402086e-01 9.161919e-01 9.037269e-01 8.757077e-01 8.459639e-01 8.193312e-01 7.939900e-01

1796 9.839570e-01 9.960215e-01 9.629757e-01 9.385305e-01 9.147319e-01 9.046295e-01 8.764574e-01 8.465747e-01 8.198514e-01 7.943881e-01

1797 9.831205e-01 9.958140e-01 9.614737e-01 9.368259e-01 9.132838e-01 9.055359e-01 8.772043e-01 8.471786e-01 8.203625e-01 7.947771e-01

1798 9.822331e-01 9.955911e-01 9.599149e-01 9.350993e-01 9.118520e-01 9.064454e-01 8.779476e-01 8.477748e-01 8.208642e-01 7.951569e-01

1799 9.812927e-01 9.953520e-01 9.583010e-01 9.333555e-01 9.104409e-01 9.073573e-01 8.786869e-01 8.483632e-01 8.213562e-01 7.955274e-01

1800 9.802977e-01 9.950953e-01 9.566335e-01 9.315992e-01 9.090543e-01 9.082709e-01 8.794213e-01 8.489431e-01 8.218382e-01 7.958884e-01

1801 9.792463e-01 9.948201e-01 9.549150e-01 9.298355e-01 9.076960e-01 9.091854e-01 8.801504e-01 8.495143e-01 8.223102e-01 7.962400e-01

1802 9.792463e-01 9.948201e-01 9.549148e-01 9.298353e-01 9.076958e-01 9.091923e-01 8.801578e-01 8.495202e-01 8.223146e-01 7.962418e-01

1803 9.792462e-01 9.948200e-01 9.549147e-01 9.298351e-01 9.076956e-01 9.091990e-01 8.801650e-01 8.495259e-01 8.223189e-01 7.962433e-01

1804 9.792461e-01 9.948200e-01 9.549146e-01 9.298350e-01 9.076954e-01 9.092057e-01 8.801721e-01 8.495314e-01 8.223230e-01 7.962447e-01

1805 9.792460e-01 9.948200e-01 9.549144e-01 9.298348e-01 9.076952e-01 9.092123e-01 8.801791e-01 8.495368e-01 8.223270e-01 7.962459e-01

1806 9.792459e-01 9.948199e-01 9.549143e-01 9.298346e-01 9.076950e-01 9.092189e-01 8.801859e-01 8.495420e-01 8.223307e-01 7.962469e-01

1807 9.792459e-01 9.948199e-01 9.549142e-01 9.298344e-01 9.076947e-01 9.092255e-01 8.801926e-01 8.495471e-01 8.223344e-01 7.962477e-01

1808 9.792458e-01 9.948199e-01 9.549140e-01 9.298342e-01 9.076945e-01 9.092320e-01 8.801991e-01 8.495519e-01 8.223378e-01 7.962483e-01

1809 9.792457e-01 9.948198e-01 9.549138e-01 9.298339e-01 9.076942e-01 9.092384e-01 8.802056e-01 8.495567e-01 8.223412e-01 7.962488e-01

1810 9.792456e-01 9.948198e-01 9.549136e-01 9.298337e-01 9.076939e-01 9.092448e-01 8.802119e-01 8.495613e-01 8.223443e-01 7.962490e-01

1811 9.792455e-01 9.948197e-01 9.549135e-01 9.298334e-01 9.076936e-01 9.092513e-01 8.802181e-01 8.495658e-01 8.223473e-01 7.962491e-01

1812 9.792453e-01 9.948197e-01 9.549133e-01 9.298332e-01 9.076933e-01 9.092576e-01 8.802242e-01 8.495701e-01 8.223502e-01 7.962490e-01

1813 9.792452e-01 9.948197e-01 9.549131e-01 9.298329e-01 9.076930e-01 9.092640e-01 8.802303e-01 8.495743e-01 8.223529e-01 7.962487e-01

1814 9.792451e-01 9.948196e-01 9.549128e-01 9.298326e-01 9.076927e-01 9.092704e-01 8.802362e-01 8.495783e-01 8.223555e-01 7.962482e-01

1815 9.792450e-01 9.948196e-01 9.549126e-01 9.298323e-01 9.076923e-01 9.092768e-01 8.802420e-01 8.495823e-01 8.223580e-01 7.962475e-01

1816 9.792448e-01 9.948195e-01 9.549124e-01 9.298320e-01 9.076920e-01 9.092831e-01 8.802478e-01 8.495861e-01 8.223602e-01 7.962467e-01

1817 9.792447e-01 9.948194e-01 9.549121e-01 9.298317e-01 9.076916e-01 9.092895e-01 8.802535e-01 8.495897e-01 8.223624e-01 7.962456e-01

1818 9.792445e-01 9.948194e-01 9.549119e-01 9.298313e-01 9.076912e-01 9.092959e-01 8.802591e-01 8.495933e-01 8.223644e-01 7.962443e-01

1819 9.792444e-01 9.948193e-01 9.549116e-01 9.298309e-01 9.076908e-01 9.093023e-01 8.802646e-01 8.495967e-01 8.223662e-01 7.962428e-01

1820 9.792442e-01 9.948193e-01 9.549113e-01 9.298306e-01 9.076903e-01 9.093087e-01 8.802701e-01 8.496000e-01 8.223679e-01 7.962411e-01

1821 9.792440e-01 9.948192e-01 9.549110e-01 9.298302e-01 9.076899e-01 9.093151e-01 8.802755e-01 8.496032e-01 8.223695e-01 7.962392e-01

1822 9.792438e-01 9.948191e-01 9.549107e-01 9.298298e-01 9.076894e-01 9.093216e-01 8.802808e-01 8.496063e-01 8.223709e-01 7.962370e-01

1823 9.792436e-01 9.948190e-01 9.549104e-01 9.298293e-01 9.076889e-01 9.093281e-01 8.802861e-01 8.496093e-01 8.223721e-01 7.962347e-01

1824 9.792434e-01 9.948190e-01 9.549101e-01 9.298289e-01 9.076884e-01 9.093346e-01 8.802913e-01 8.496121e-01 8.223732e-01 7.962320e-01

1825 9.792432e-01 9.948189e-01 9.549097e-01 9.298284e-01 9.076878e-01 9.093411e-01 8.802965e-01 8.496149e-01 8.223742e-01 7.962292e-01

1826 9.792430e-01 9.948188e-01 9.549093e-01 9.298279e-01 9.076872e-01 9.093478e-01 8.803017e-01 8.496175e-01 8.223749e-01 7.962261e-01

1827 9.792428e-01 9.948187e-01 9.549089e-01 9.298274e-01 9.076867e-01 9.093544e-01 8.803067e-01 8.496200e-01 8.223755e-01 7.962227e-01

1828 9.792425e-01 9.948186e-01 9.549085e-01 9.298268e-01 9.076860e-01 9.093611e-01 8.803118e-01 8.496223e-01 8.223760e-01 7.962191e-01

1829 9.792422e-01 9.948185e-01 9.549081e-01 9.298263e-01 9.076854e-01 9.093679e-01 8.803168e-01 8.496246e-01 8.223762e-01 7.962152e-01

1830 9.792420e-01 9.948184e-01 9.549076e-01 9.298257e-01 9.076847e-01 9.093747e-01 8.803217e-01 8.496267e-01 8.223763e-01 7.962110e-01

1831 9.792417e-01 9.948183e-01 9.549071e-01 9.298250e-01 9.076840e-01 9.093816e-01 8.803266e-01 8.496287e-01 8.223762e-01 7.962065e-01

1832 9.792414e-01 9.948182e-01 9.549066e-01 9.298244e-01 9.076832e-01 9.093885e-01 8.803315e-01 8.496306e-01 8.223759e-01 7.962017e-01

1833 9.792411e-01 9.948181e-01 9.549061e-01 9.298237e-01 9.076825e-01 9.093956e-01 8.803364e-01 8.496324e-01 8.223755e-01 7.961967e-01

1834 9.792407e-01 9.948179e-01 9.549055e-01 9.298230e-01 9.076817e-01 9.094027e-01 8.803412e-01 8.496341e-01 8.223748e-01 7.961913e-01

1835 9.792404e-01 9.948178e-01 9.549050e-01 9.298222e-01 9.076808e-01 9.094098e-01 8.803460e-01 8.496356e-01 8.223739e-01 7.961855e-01

1836 9.792400e-01 9.948176e-01 9.549043e-01 9.298214e-01 9.076799e-01 9.094171e-01 8.803507e-01 8.496370e-01 8.223729e-01 7.961794e-01

1837 9.792396e-01 9.948175e-01 9.549037e-01 9.298206e-01 9.076790e-01 9.094245e-01 8.803554e-01 8.496382e-01 8.223716e-01 7.961730e-01

1838 9.792392e-01 9.948173e-01 9.549030e-01 9.298197e-01 9.076780e-01 9.094319e-01 8.803601e-01 8.496393e-01 8.223701e-01 7.961662e-01

1839 9.792387e-01 9.948172e-01 9.549023e-01 9.298187e-01 9.076770e-01 9.094395e-01 8.803648e-01 8.496403e-01 8.223683e-01 7.961590e-01

1840 9.792383e-01 9.948170e-01 9.549015e-01 9.298178e-01 9.076759e-01 9.094471e-01 8.803695e-01 8.496411e-01 8.223664e-01 7.961514e-01

1841 9.792378e-01 9.948168e-01 9.549007e-01 9.298167e-01 9.076748e-01 9.094548e-01 8.803741e-01 8.496418e-01 8.223641e-01 7.961434e-01

1842 9.792373e-01 9.948166e-01 9.548999e-01 9.298157e-01 9.076736e-01 9.094627e-01 8.803787e-01 8.496424e-01 8.223616e-01 7.961349e-01

1843 9.792367e-01 9.948164e-01 9.548990e-01 9.298145e-01 9.076724e-01 9.094707e-01 8.803833e-01 8.496427e-01 8.223589e-01 7.961260e-01

1844 9.792361e-01 9.948162e-01 9.548981e-01 9.298134e-01 9.076711e-01 9.094788e-01 8.803879e-01 8.496430e-01 8.223559e-01 7.961167e-01

1845 9.792355e-01 9.948160e-01 9.548971e-01 9.298121e-01 9.076698e-01 9.094870e-01 8.803924e-01 8.496430e-01 8.223526e-01 7.961069e-01

1846 9.792349e-01 9.948157e-01 9.548960e-01 9.298108e-01 9.076684e-01 9.094953e-01 8.803969e-01 8.496429e-01 8.223490e-01 7.960966e-01

1847 9.792342e-01 9.948155e-01 9.548949e-01 9.298094e-01 9.076669e-01 9.095038e-01 8.804014e-01 8.496426e-01 8.223451e-01 7.960857e-01

1848 9.792335e-01 9.948152e-01 9.548938e-01 9.298080e-01 9.076654e-01 9.095124e-01 8.804059e-01 8.496421e-01 8.223409e-01 7.960743e-01

1849 9.792328e-01 9.948149e-01 9.548926e-01 9.298065e-01 9.076637e-01 9.095211e-01 8.804104e-01 8.496414e-01 8.223363e-01 7.960624e-01

1850 9.792320e-01 9.948146e-01 9.548913e-01 9.298049e-01 9.076620e-01 9.095300e-01 8.804148e-01 8.496406e-01 8.223314e-01 7.960499e-01

1851 9.792311e-01 9.948143e-01 9.548900e-01 9.298032e-01 9.076603e-01 9.095391e-01 8.804192e-01 8.496395e-01 8.223261e-01 7.960368e-01

1852 9.792303e-01 9.948140e-01 9.548885e-01 9.298014e-01 9.076584e-01 9.095483e-01 8.804236e-01 8.496382e-01 8.223205e-01 7.960230e-01

1853 9.792293e-01 9.948136e-01 9.548870e-01 9.297995e-01 9.076564e-01 9.095576e-01 8.804279e-01 8.496367e-01 8.223145e-01 7.960086e-01

1854 9.792283e-01 9.948132e-01 9.548855e-01 9.297976e-01 9.076544e-01 9.095672e-01 8.804323e-01 8.496349e-01 8.223080e-01 7.959936e-01

1855 9.792273e-01 9.948128e-01 9.548838e-01 9.297955e-01 9.076522e-01 9.095769e-01 8.804366e-01 8.496329e-01 8.223012e-01 7.959778e-01

1856 9.792262e-01 9.948124e-01 9.548820e-01 9.297934e-01 9.076500e-01 9.095867e-01 8.804409e-01 8.496306e-01 8.222939e-01 7.959613e-01

1857 9.792250e-01 9.948120e-01 9.548802e-01 9.297911e-01 9.076476e-01 9.095968e-01 8.804451e-01 8.496281e-01 8.222862e-01 7.959440e-01

1858 9.792238e-01 9.948115e-01 9.548782e-01 9.297887e-01 9.076451e-01 9.096070e-01 8.804493e-01 8.496253e-01 8.222779e-01 7.959259e-01

1859 9.792225e-01 9.948110e-01 9.548761e-01 9.297862e-01 9.076425e-01 9.096174e-01 8.804535e-01 8.496223e-01 8.222692e-01 7.959070e-01

1860 9.792211e-01 9.948105e-01 9.548740e-01 9.297835e-01 9.076398e-01 9.096280e-01 8.804576e-01 8.496189e-01 8.222600e-01 7.958873e-01

1861 9.792197e-01 9.948100e-01 9.548717e-01 9.297807e-01 9.076370e-01 9.096389e-01 8.804617e-01 8.496152e-01 8.222502e-01 7.958666e-01

1862 9.792181e-01 9.948094e-01 9.548692e-01 9.297777e-01 9.076340e-01 9.096499e-01 8.804658e-01 8.496112e-01 8.222398e-01 7.958451e-01

1863 9.792165e-01 9.948088e-01 9.548666e-01 9.297746e-01 9.076308e-01 9.096611e-01 8.804697e-01 8.496069e-01 8.222289e-01 7.958225e-01

1864 9.792148e-01 9.948081e-01 9.548639e-01 9.297714e-01 9.076275e-01 9.096726e-01 8.804737e-01 8.496021e-01 8.222173e-01 7.957990e-01

1865 9.792130e-01 9.948074e-01 9.548610e-01 9.297679e-01 9.076241e-01 9.096843e-01 8.804776e-01 8.495971e-01 8.222052e-01 7.957744e-01

1866 9.792110e-01 9.948067e-01 9.548580e-01 9.297643e-01 9.076205e-01 9.096962e-01 8.804814e-01 8.495916e-01 8.221923e-01 7.957487e-01

1867 9.792090e-01 9.948059e-01 9.548548e-01 9.297604e-01 9.076166e-01 9.097083e-01 8.804852e-01 8.495857e-01 8.221788e-01 7.957219e-01

1868 9.792068e-01 9.948051e-01 9.548514e-01 9.297564e-01 9.076127e-01 9.097207e-01 8.804889e-01 8.495794e-01 8.221645e-01 7.956939e-01

1869 9.792045e-01 9.948043e-01 9.548478e-01 9.297522e-01 9.076085e-01 9.097333e-01 8.804925e-01 8.495727e-01 8.221495e-01 7.956647e-01

1870 9.792021e-01 9.948033e-01 9.548440e-01 9.297477e-01 9.076041e-01 9.097462e-01 8.804960e-01 8.495655e-01 8.221337e-01 7.956342e-01

1871 9.791995e-01 9.948024e-01 9.548400e-01 9.297429e-01 9.075995e-01 9.097594e-01 8.804995e-01 8.495578e-01 8.221170e-01 7.956025e-01

1872 9.791967e-01 9.948013e-01 9.548357e-01 9.297379e-01 9.075946e-01 9.097728e-01 8.805028e-01 8.495497e-01 8.220995e-01 7.955693e-01

1873 9.791938e-01 9.948003e-01 9.548312e-01 9.297327e-01 9.075895e-01 9.097864e-01 8.805061e-01 8.495410e-01 8.220812e-01 7.955347e-01

1874 9.791908e-01 9.947991e-01 9.548265e-01 9.297271e-01 9.075842e-01 9.098004e-01 8.805092e-01 8.495317e-01 8.220619e-01 7.954986e-01

1875 9.791875e-01 9.947979e-01 9.548214e-01 9.297213e-01 9.075786e-01 9.098147e-01 8.805122e-01 8.495219e-01 8.220416e-01 7.954610e-01

1876 9.791840e-01 9.947966e-01 9.548161e-01 9.297151e-01 9.075728e-01 9.098292e-01 8.805151e-01 8.495114e-01 8.220203e-01 7.954218e-01

1877 9.791804e-01 9.947952e-01 9.548105e-01 9.297086e-01 9.075666e-01 9.098441e-01 8.805179e-01 8.495004e-01 8.219980e-01 7.953809e-01

1878 9.791765e-01 9.947938e-01 9.548045e-01 9.297017e-01 9.075601e-01 9.098592e-01 8.805205e-01 8.494886e-01 8.219745e-01 7.953382e-01

1879 9.791723e-01 9.947922e-01 9.547981e-01 9.296944e-01 9.075534e-01 9.098747e-01 8.805230e-01 8.494762e-01 8.219500e-01 7.952938e-01

1880 9.791679e-01 9.947906e-01 9.547914e-01 9.296868e-01 9.075463e-01 9.098905e-01 8.805253e-01 8.494631e-01 8.219242e-01 7.952475e-01

1881 9.791633e-01 9.947888e-01 9.547843e-01 9.296787e-01 9.075388e-01 9.099066e-01 8.805275e-01 8.494493e-01 8.218972e-01 7.951993e-01

1882 9.791583e-01 9.947870e-01 9.547768e-01 9.296701e-01 9.075310e-01 9.099230e-01 8.805294e-01 8.494346e-01 8.218689e-01 7.951490e-01

1883 9.791530e-01 9.947850e-01 9.547688e-01 9.296611e-01 9.075227e-01 9.099398e-01 8.805312e-01 8.494191e-01 8.218393e-01 7.950967e-01

1884 9.791474e-01 9.947829e-01 9.547603e-01 9.296516e-01 9.075141e-01 9.099570e-01 8.805327e-01 8.494028e-01 8.218082e-01 7.950422e-01

1885 9.791415e-01 9.947807e-01 9.547513e-01 9.296415e-01 9.075051e-01 9.099745e-01 8.805340e-01 8.493856e-01 8.217757e-01 7.949855e-01

1886 9.791351e-01 9.947783e-01 9.547417e-01 9.296308e-01 9.074956e-01 9.099923e-01 8.805351e-01 8.493675e-01 8.217417e-01 7.949264e-01

1887 9.791284e-01 9.947758e-01 9.547316e-01 9.296196e-01 9.074856e-01 9.100106e-01 8.805360e-01 8.493484e-01 8.217062e-01 7.948649e-01

1888 9.791213e-01 9.947732e-01 9.547209e-01 9.296077e-01 9.074752e-01 9.100292e-01 8.805365e-01 8.493283e-01 8.216689e-01 7.948008e-01

1889 9.791137e-01 9.947704e-01 9.547095e-01 9.295952e-01 9.074642e-01 9.100482e-01 8.805368e-01 8.493071e-01 8.216300e-01 7.947342e-01

1890 9.791056e-01 9.947673e-01 9.546974e-01 9.295819e-01 9.074527e-01 9.100677e-01 8.805368e-01 8.492849e-01 8.215893e-01 7.946649e-01

1891 9.790970e-01 9.947641e-01 9.546846e-01 9.295679e-01 9.074407e-01 9.100875e-01 8.805364e-01 8.492614e-01 8.215468e-01 7.945928e-01

1892 9.790878e-01 9.947607e-01 9.546710e-01 9.295531e-01 9.074280e-01 9.101077e-01 8.805357e-01 8.492368e-01 8.215023e-01 7.945178e-01

1893 9.790781e-01 9.947571e-01 9.546565e-01 9.295374e-01 9.074148e-01 9.101284e-01 8.805347e-01 8.492110e-01 8.214559e-01 7.944398e-01

1894 9.790677e-01 9.947533e-01 9.546411e-01 9.295209e-01 9.074009e-01 9.101495e-01 8.805333e-01 8.491838e-01 8.214073e-01 7.943587e-01

1895 9.790566e-01 9.947492e-01 9.546248e-01 9.295034e-01 9.073864e-01 9.101710e-01 8.805314e-01 8.491553e-01 8.213567e-01 7.942744e-01

1896 9.790449e-01 9.947448e-01 9.546075e-01 9.294849e-01 9.073711e-01 9.101930e-01 8.805292e-01 8.491254e-01 8.213038e-01 7.941867e-01

1897 9.790323e-01 9.947402e-01 9.545891e-01 9.294654e-01 9.073552e-01 9.102155e-01 8.805265e-01 8.490940e-01 8.212486e-01 7.940957e-01

1898 9.790190e-01 9.947352e-01 9.545695e-01 9.294447e-01 9.073385e-01 9.102384e-01 8.805233e-01 8.490611e-01 8.211910e-01 7.940011e-01

1899 9.790047e-01 9.947299e-01 9.545488e-01 9.294228e-01 9.073210e-01 9.102618e-01 8.805196e-01 8.490266e-01 8.211308e-01 7.939028e-01

1900 9.789895e-01 9.947243e-01 9.545267e-01 9.293997e-01 9.073027e-01 9.102857e-01 8.805154e-01 8.489904e-01 8.210682e-01 7.938008e-01

1901 9.789734e-01 9.947183e-01 9.545032e-01 9.293753e-01 9.072835e-01 9.103100e-01 8.805106e-01 8.489525e-01 8.210028e-01 7.936949e-01

1902 9.789561e-01 9.947119e-01 9.544782e-01 9.293495e-01 9.072635e-01 9.103349e-01 8.805052e-01 8.489129e-01 8.209347e-01 7.935849e-01

1903 9.789377e-01 9.947051e-01 9.544517e-01 9.293222e-01 9.072426e-01 9.103603e-01 8.804993e-01 8.488713e-01 8.208636e-01 7.934709e-01

1904 9.789181e-01 9.946979e-01 9.544235e-01 9.292933e-01 9.072207e-01 9.103862e-01 8.804926e-01 8.488278e-01 8.207897e-01 7.933525e-01

1905 9.788972e-01 9.946902e-01 9.543935e-01 9.292628e-01 9.071979e-01 9.104126e-01 8.804853e-01 8.487823e-01 8.207126e-01 7.932298e-01

1906 9.788749e-01 9.946819e-01 9.543615e-01 9.292305e-01 9.071740e-01 9.104396e-01 8.804773e-01 8.487347e-01 8.206324e-01 7.931025e-01

1907 9.788511e-01 9.946731e-01 9.543276e-01 9.291964e-01 9.071492e-01 9.104671e-01 8.804685e-01 8.486850e-01 8.205488e-01 7.929706e-01

1908 9.788257e-01 9.946637e-01 9.542915e-01 9.291603e-01 9.071233e-01 9.104951e-01 8.804589e-01 8.486330e-01 8.204619e-01 7.928339e-01

1909 9.787985e-01 9.946537e-01 9.542531e-01 9.291222e-01 9.070963e-01 9.105237e-01 8.804484e-01 8.485786e-01 8.203715e-01 7.926923e-01

1910 9.787696e-01 9.946430e-01 9.542122e-01 9.290819e-01 9.070681e-01 9.105529e-01 8.804371e-01 8.485218e-01 8.202774e-01 7.925457e-01

1911 9.787387e-01 9.946315e-01 9.541688e-01 9.290394e-01 9.070389e-01 9.105827e-01 8.804249e-01 8.484625e-01 8.201796e-01 7.923940e-01

1912 9.787057e-01 9.946193e-01 9.541225e-01 9.289944e-01 9.070085e-01 9.106130e-01 8.804117e-01 8.484006e-01 8.200780e-01 7.922370e-01

1913 9.786704e-01 9.946063e-01 9.540733e-01 9.289469e-01 9.069769e-01 9.106440e-01 8.803975e-01 8.483360e-01 8.199724e-01 7.920746e-01

1914 9.786327e-01 9.945924e-01 9.540209e-01 9.288967e-01 9.069442e-01 9.106755e-01 8.803822e-01 8.482686e-01 8.198627e-01 7.919066e-01

1915 9.785925e-01 9.945775e-01 9.539652e-01 9.288437e-01 9.069103e-01 9.107077e-01 8.803658e-01 8.481984e-01 8.197488e-01 7.917330e-01

1916 9.785495e-01 9.945616e-01 9.539060e-01 9.287878e-01 9.068751e-01 9.107404e-01 8.803482e-01 8.481251e-01 8.196306e-01 7.915537e-01

1917 9.785036e-01 9.945446e-01 9.538429e-01 9.287287e-01 9.068388e-01 9.107738e-01 8.803295e-01 8.480487e-01 8.195080e-01 7.913685e-01

1918 9.784546e-01 9.945264e-01 9.537758e-01 9.286664e-01 9.068013e-01 9.108078e-01 8.803094e-01 8.479692e-01 8.193808e-01 7.911773e-01

1919 9.784021e-01 9.945070e-01 9.537045e-01 9.286007e-01 9.067627e-01 9.108424e-01 8.802880e-01 8.478864e-01 8.192489e-01 7.909801e-01

1920 9.783461e-01 9.944862e-01 9.536286e-01 9.285314e-01 9.067229e-01 9.108777e-01 8.802653e-01 8.478002e-01 8.191123e-01 7.907767e-01

1921 9.782862e-01 9.944639e-01 9.535478e-01 9.284584e-01 9.066821e-01 9.109136e-01 8.802411e-01 8.477104e-01 8.189708e-01 7.905671e-01

1922 9.782222e-01 9.944401e-01 9.534619e-01 9.283814e-01 9.066402e-01 9.109501e-01 8.802153e-01 8.476171e-01 8.188242e-01 7.903511e-01

1923 9.781538e-01 9.944147e-01 9.533706e-01 9.283004e-01 9.065974e-01 9.109873e-01 8.801880e-01 8.475201e-01 8.186726e-01 7.901287e-01

1924 9.780806e-01 9.943874e-01 9.532734e-01 9.282150e-01 9.065537e-01 9.110251e-01 8.801591e-01 8.474192e-01 8.185157e-01 7.898998e-01

1925 9.780024e-01 9.943583e-01 9.531702e-01 9.281253e-01 9.065092e-01 9.110636e-01 8.801284e-01 8.473144e-01 8.183536e-01 7.896644e-01

1926 9.779188e-01 9.943271e-01 9.530604e-01 9.280308e-01 9.064641e-01 9.111028e-01 8.800960e-01 8.472056e-01 8.181860e-01 7.894224e-01

1927 9.778295e-01 9.942937e-01 9.529438e-01 9.279316e-01 9.064185e-01 9.111426e-01 8.800617e-01 8.470927e-01 8.180128e-01 7.891738e-01

1928 9.777340e-01 9.942579e-01 9.528199e-01 9.278274e-01 9.063724e-01 9.111830e-01 8.800255e-01 8.469755e-01 8.178341e-01 7.889186e-01

1929 9.776319e-01 9.942195e-01 9.526883e-01 9.277180e-01 9.063263e-01 9.112241e-01 8.799873e-01 8.468540e-01 8.176497e-01 7.886567e-01

1930 9.775228e-01 9.941785e-01 9.525485e-01 9.276033e-01 9.062801e-01 9.112658e-01 8.799471e-01 8.467280e-01 8.174596e-01 7.883882e-01

1931 9.774062e-01 9.941345e-01 9.524002e-01 9.274830e-01 9.062342e-01 9.113082e-01 8.799046e-01 8.465975e-01 8.172636e-01 7.881130e-01

1932 9.772816e-01 9.940874e-01 9.522427e-01 9.273571e-01 9.061889e-01 9.113512e-01 8.798600e-01 8.464624e-01 8.170618e-01 7.878313e-01

1933 9.771484e-01 9.940369e-01 9.520757e-01 9.272253e-01 9.061445e-01 9.113949e-01 8.798131e-01 8.463225e-01 8.168540e-01 7.875430e-01

1934 9.770062e-01 9.939829e-01 9.518986e-01 9.270876e-01 9.061013e-01 9.114392e-01 8.797637e-01 8.461779e-01 8.166402e-01 7.872482e-01

1935 9.768542e-01 9.939249e-01 9.517109e-01 9.269438e-01 9.060596e-01 9.114841e-01 8.797119e-01 8.460283e-01 8.164204e-01 7.869470e-01

1936 9.766919e-01 9.938628e-01 9.515121e-01 9.267938e-01 9.060200e-01 9.115296e-01 8.796576e-01 8.458737e-01 8.161946e-01 7.866395e-01

1937 9.765186e-01 9.937962e-01 9.513015e-01 9.266375e-01 9.059829e-01 9.115758e-01 8.796006e-01 8.457141e-01 8.159628e-01 7.863258e-01

1938 9.763335e-01 9.937248e-01 9.510787e-01 9.264748e-01 9.059487e-01 9.116225e-01 8.795410e-01 8.455493e-01 8.157249e-01 7.860061e-01

1939 9.761361e-01 9.936483e-01 9.508430e-01 9.263057e-01 9.059181e-01 9.116698e-01 8.794785e-01 8.453793e-01 8.154810e-01 7.856804e-01

1940 9.759253e-01 9.935663e-01 9.505939e-01 9.261302e-01 9.058916e-01 9.117176e-01 8.794132e-01 8.452041e-01 8.152311e-01 7.853489e-01

1941 9.757005e-01 9.934784e-01 9.503307e-01 9.259483e-01 9.058699e-01 9.117660e-01 8.793449e-01 8.450236e-01 8.149752e-01 7.850119e-01

1942 9.754608e-01 9.933841e-01 9.500530e-01 9.257601e-01 9.058537e-01 9.118149e-01 8.792736e-01 8.448378e-01 8.147135e-01 7.846696e-01

1943 9.752053e-01 9.932831e-01 9.497600e-01 9.255656e-01 9.058438e-01 9.118644e-01 8.791992e-01 8.446466e-01 8.144459e-01 7.843221e-01

1944 9.749330e-01 9.931748e-01 9.494512e-01 9.253650e-01 9.058408e-01 9.119143e-01 8.791216e-01 8.444500e-01 8.141725e-01 7.839697e-01

1945 9.746431e-01 9.930587e-01 9.491261e-01 9.251585e-01 9.058457e-01 9.119647e-01 8.790408e-01 8.442480e-01 8.138936e-01 7.836126e-01

1946 9.743343e-01 9.929343e-01 9.487840e-01 9.249463e-01 9.058594e-01 9.120155e-01 8.789566e-01 8.440406e-01 8.136091e-01 7.832512e-01

1947 9.740059e-01 9.928009e-01 9.484245e-01 9.247287e-01 9.058827e-01 9.120667e-01 8.788690e-01 8.438278e-01 8.133192e-01 7.828857e-01

1948 9.736565e-01 9.926579e-01 9.480470e-01 9.245061e-01 9.059167e-01 9.121183e-01 8.787779e-01 8.436097e-01 8.130240e-01 7.825164e-01

1949 9.732852e-01 9.925048e-01 9.476511e-01 9.242789e-01 9.059622e-01 9.121702e-01 8.786834e-01 8.433861e-01 8.127238e-01 7.821437e-01

1950 9.728908e-01 9.923407e-01 9.472363e-01 9.240476e-01 9.060204e-01 9.122225e-01 8.785852e-01 8.431573e-01 8.124187e-01 7.817678e-01

1951 9.724722e-01 9.921649e-01 9.468022e-01 9.238126e-01 9.060923e-01 9.122750e-01 8.784834e-01 8.429233e-01 8.121089e-01 7.813892e-01

1952 9.720281e-01 9.919766e-01 9.463485e-01 9.235747e-01 9.061788e-01 9.123277e-01 8.783778e-01 8.426841e-01 8.117946e-01 7.810081e-01

1953 9.715575e-01 9.917749e-01 9.458750e-01 9.233345e-01 9.062810e-01 9.123807e-01 8.782686e-01 8.424398e-01 8.114760e-01 7.806251e-01

1954 9.710590e-01 9.915590e-01 9.453815e-01 9.230928e-01 9.064001e-01 9.124338e-01 8.781555e-01 8.421905e-01 8.111534e-01 7.802403e-01

1955 9.705314e-01 9.913280e-01 9.448679e-01 9.228504e-01 9.065368e-01 9.124870e-01 8.780387e-01 8.419364e-01 8.108270e-01 7.798543e-01

1956 9.699737e-01 9.910807e-01 9.443342e-01 9.226082e-01 9.066923e-01 9.125403e-01 8.779179e-01 8.416776e-01 8.104972e-01 7.794674e-01

1957 9.693847e-01 9.908162e-01 9.437804e-01 9.223670e-01 9.068673e-01 9.125936e-01 8.777934e-01 8.414141e-01 8.101642e-01 7.790800e-01

1958 9.687631e-01 9.905333e-01 9.432068e-01 9.221281e-01 9.070629e-01 9.126469e-01 8.776649e-01 8.411462e-01 8.098282e-01 7.786925e-01

1959 9.681080e-01 9.902309e-01 9.426138e-01 9.218923e-01 9.072796e-01 9.127000e-01 8.775325e-01 8.408740e-01 8.094896e-01 7.783054e-01

1960 9.674182e-01 9.899078e-01 9.420018e-01 9.216608e-01 9.075183e-01 9.127531e-01 8.773962e-01 8.405977e-01 8.091488e-01 7.779189e-01

1961 9.666928e-01 9.895627e-01 9.413714e-01 9.214348e-01 9.077794e-01 9.128059e-01 8.772561e-01 8.403176e-01 8.088059e-01 7.775335e-01

1962 9.659309e-01 9.891942e-01 9.407233e-01 9.212153e-01 9.080634e-01 9.128585e-01 8.771121e-01 8.400337e-01 8.084615e-01 7.771497e-01

1963 9.651317e-01 9.888011e-01 9.400584e-01 9.210037e-01 9.083707e-01 9.129107e-01 8.769642e-01 8.397464e-01 8.081157e-01 7.767677e-01

1964 9.642946e-01 9.883817e-01 9.393779e-01 9.208010e-01 9.087013e-01 9.129626e-01 8.768126e-01 8.394559e-01 8.077690e-01 7.763879e-01

1965 9.634189e-01 9.879348e-01 9.386827e-01 9.206084e-01 9.090553e-01 9.130140e-01 8.766572e-01 8.391624e-01 8.074217e-01 7.760108e-01

1966 9.625043e-01 9.874587e-01 9.379744e-01 9.204271e-01 9.094326e-01 9.130648e-01 8.764981e-01 8.388662e-01 8.070741e-01 7.756367e-01

1967 9.615505e-01 9.869519e-01 9.372543e-01 9.202581e-01 9.098329e-01 9.131152e-01 8.763353e-01 8.385675e-01 8.067266e-01 7.752659e-01

1968 9.605575e-01 9.864127e-01 9.365241e-01 9.201025e-01 9.102557e-01 9.131648e-01 8.761690e-01 8.382666e-01 8.063796e-01 7.748988e-01

1969 9.595254e-01 9.858396e-01 9.357855e-01 9.199613e-01 9.107003e-01 9.132138e-01 8.759992e-01 8.379638e-01 8.060333e-01 7.745357e-01

1970 9.584545e-01 9.852308e-01 9.350404e-01 9.198353e-01 9.111661e-01 9.132619e-01 8.758260e-01 8.376593e-01 8.056881e-01 7.741769e-01

1971 9.573455e-01 9.845848e-01 9.342908e-01 9.197253e-01 9.116520e-01 9.133093e-01 8.756495e-01 8.373536e-01 8.053445e-01 7.738227e-01

1972 9.561991e-01 9.838997e-01 9.335387e-01 9.196320e-01 9.121570e-01 9.133556e-01 8.754699e-01 8.370468e-01 8.050026e-01 7.734734e-01

1973 9.550164e-01 9.831740e-01 9.327863e-01 9.195560e-01 9.126799e-01 9.134010e-01 8.752872e-01 8.367393e-01 8.046628e-01 7.731292e-01

1974 9.537987e-01 9.824060e-01 9.320357e-01 9.194976e-01 9.132194e-01 9.134454e-01 8.751016e-01 8.364313e-01 8.043255e-01 7.727903e-01

1975 9.525476e-01 9.815941e-01 9.312892e-01 9.194571e-01 9.137739e-01 9.134886e-01 8.749132e-01 8.361232e-01 8.039909e-01 7.724570e-01

1976 9.512649e-01 9.807366e-01 9.305489e-01 9.194348e-01 9.143421e-01 9.135306e-01 8.747222e-01 8.358153e-01 8.036593e-01 7.721295e-01

1977 9.499526e-01 9.798321e-01 9.298171e-01 9.194306e-01 9.149223e-01 9.135713e-01 8.745287e-01 8.355079e-01 8.033311e-01 7.718079e-01

1978 9.486133e-01 9.788792e-01 9.290959e-01 9.194444e-01 9.155130e-01 9.136107e-01 8.743329e-01 8.352012e-01 8.030064e-01 7.714925e-01

1979 9.472494e-01 9.778764e-01 9.283873e-01 9.194759e-01 9.161123e-01 9.136487e-01 8.741350e-01 8.348956e-01 8.026856e-01 7.711833e-01

1980 9.458638e-01 9.768225e-01 9.276934e-01 9.195248e-01 9.167186e-01 9.136852e-01 8.739351e-01 8.345913e-01 8.023688e-01 7.708804e-01

1981 9.444596e-01 9.757165e-01 9.270160e-01 9.195906e-01 9.173301e-01 9.137202e-01 8.737334e-01 8.342887e-01 8.020563e-01 7.705841e-01

1982 9.444594e-01 9.757164e-01 9.270158e-01 9.195904e-01 9.173301e-01 9.137272e-01 8.737346e-01 8.342824e-01 8.020439e-01 7.705652e-01

1983 9.444592e-01 9.757163e-01 9.270156e-01 9.195903e-01 9.173300e-01 9.137337e-01 8.737352e-01 8.342754e-01 8.020308e-01 7.705456e-01

1984 9.444590e-01 9.757162e-01 9.270155e-01 9.195901e-01 9.173299e-01 9.137397e-01 8.737352e-01 8.342677e-01 8.020170e-01 7.705252e-01

1985 9.444588e-01 9.757161e-01 9.270153e-01 9.195900e-01 9.173299e-01 9.137451e-01 8.737346e-01 8.342594e-01 8.020025e-01 7.705041e-01

1986 9.444586e-01 9.757159e-01 9.270151e-01 9.195898e-01 9.173298e-01 9.137502e-01 8.737334e-01 8.342504e-01 8.019872e-01 7.704822e-01

1987 9.444584e-01 9.757158e-01 9.270149e-01 9.195897e-01 9.173298e-01 9.137547e-01 8.737316e-01 8.342407e-01 8.019712e-01 7.704594e-01

1988 9.444582e-01 9.757156e-01 9.270147e-01 9.195895e-01 9.173297e-01 9.137588e-01 8.737292e-01 8.342304e-01 8.019544e-01 7.704359e-01

1989 9.444580e-01 9.757155e-01 9.270145e-01 9.195894e-01 9.173296e-01 9.137624e-01 8.737262e-01 8.342194e-01 8.019369e-01 7.704115e-01

1990 9.444577e-01 9.757153e-01 9.270143e-01 9.195893e-01 9.173296e-01 9.137655e-01 8.737226e-01 8.342078e-01 8.019186e-01 7.703863e-01

1991 9.444575e-01 9.757152e-01 9.270141e-01 9.195891e-01 9.173295e-01 9.137683e-01 8.737185e-01 8.341954e-01 8.018996e-01 7.703603e-01

1992 9.444572e-01 9.757150e-01 9.270139e-01 9.195890e-01 9.173295e-01 9.137705e-01 8.737137e-01 8.341824e-01 8.018798e-01 7.703333e-01

1993 9.444569e-01 9.757148e-01 9.270136e-01 9.195888e-01 9.173294e-01 9.137724e-01 8.737084e-01 8.341687e-01 8.018591e-01 7.703055e-01

1994 9.444566e-01 9.757146e-01 9.270134e-01 9.195887e-01 9.173294e-01 9.137738e-01 8.737025e-01 8.341544e-01 8.018377e-01 7.702768e-01

1995 9.444563e-01 9.757145e-01 9.270132e-01 9.195885e-01 9.173293e-01 9.137749e-01 8.736961e-01 8.341393e-01 8.018154e-01 7.702471e-01

1996 9.444560e-01 9.757143e-01 9.270129e-01 9.195884e-01 9.173293e-01 9.137755e-01 8.736890e-01 8.341235e-01 8.017923e-01 7.702164e-01

1997 9.444557e-01 9.757140e-01 9.270126e-01 9.195882e-01 9.173293e-01 9.137757e-01 8.736814e-01 8.341069e-01 8.017683e-01 7.701848e-01

1998 9.444554e-01 9.757138e-01 9.270123e-01 9.195881e-01 9.173292e-01 9.137755e-01 8.736732e-01 8.340897e-01 8.017435e-01 7.701522e-01

1999 9.444550e-01 9.757136e-01 9.270120e-01 9.195879e-01 9.173292e-01 9.137749e-01 8.736644e-01 8.340717e-01 8.017177e-01 7.701185e-01

2000 9.444546e-01 9.757133e-01 9.270117e-01 9.195878e-01 9.173292e-01 9.137739e-01 8.736550e-01 8.340529e-01 8.016910e-01 7.700838e-01

2001 9.444543e-01 9.757131e-01 9.270114e-01 9.195876e-01 9.173292e-01 9.137725e-01 8.736450e-01 8.340334e-01 8.016634e-01 7.700479e-01

2002 9.444539e-01 9.757128e-01 9.270111e-01 9.195874e-01 9.173292e-01 9.137707e-01 8.736344e-01 8.340130e-01 8.016348e-01 7.700110e-01

2003 9.444534e-01 9.757125e-01 9.270107e-01 9.195873e-01 9.173292e-01 9.137685e-01 8.736232e-01 8.339919e-01 8.016052e-01 7.699729e-01

2004 9.444530e-01 9.757122e-01 9.270104e-01 9.195871e-01 9.173292e-01 9.137660e-01 8.736114e-01 8.339699e-01 8.015746e-01 7.699336e-01

2005 9.444525e-01 9.757119e-01 9.270100e-01 9.195869e-01 9.173292e-01 9.137630e-01 8.735989e-01 8.339471e-01 8.015430e-01 7.698931e-01

2006 9.444521e-01 9.757116e-01 9.270096e-01 9.195867e-01 9.173292e-01 9.137597e-01 8.735858e-01 8.339234e-01 8.015103e-01 7.698513e-01

2007 9.444516e-01 9.757113e-01 9.270092e-01 9.195866e-01 9.173293e-01 9.137559e-01 8.735721e-01 8.338989e-01 8.014765e-01 7.698082e-01

2008 9.444511e-01 9.757109e-01 9.270088e-01 9.195864e-01 9.173293e-01 9.137518e-01 8.735577e-01 8.338734e-01 8.014415e-01 7.697638e-01

2009 9.444505e-01 9.757106e-01 9.270084e-01 9.195862e-01 9.173293e-01 9.137473e-01 8.735427e-01 8.338471e-01 8.014054e-01 7.697181e-01

2010 9.444500e-01 9.757102e-01 9.270080e-01 9.195860e-01 9.173294e-01 9.137424e-01 8.735270e-01 8.338197e-01 8.013681e-01 7.696709e-01

2011 9.444494e-01 9.757098e-01 9.270075e-01 9.195858e-01 9.173295e-01 9.137371e-01 8.735106e-01 8.337914e-01 8.013296e-01 7.696222e-01

2012 9.444488e-01 9.757093e-01 9.270070e-01 9.195856e-01 9.173295e-01 9.137314e-01 8.734935e-01 8.337621e-01 8.012898e-01 7.695721e-01

2013 9.444481e-01 9.757089e-01 9.270066e-01 9.195854e-01 9.173296e-01 9.137253e-01 8.734757e-01 8.337318e-01 8.012487e-01 7.695204e-01

2014 9.444475e-01 9.757084e-01 9.270061e-01 9.195852e-01 9.173297e-01 9.137188e-01 8.734571e-01 8.337004e-01 8.012062e-01 7.694671e-01

2015 9.444468e-01 9.757079e-01 9.270056e-01 9.195851e-01 9.173298e-01 9.137119e-01 8.734378e-01 8.336679e-01 8.011624e-01 7.694121e-01

2016 9.444461e-01 9.757074e-01 9.270050e-01 9.195849e-01 9.173300e-01 9.137046e-01 8.734178e-01 8.336343e-01 8.011171e-01 7.693554e-01

2017 9.444453e-01 9.757068e-01 9.270045e-01 9.195847e-01 9.173301e-01 9.136969e-01 8.733969e-01 8.335996e-01 8.010703e-01 7.692970e-01

2018 9.444445e-01 9.757063e-01 9.270039e-01 9.195845e-01 9.173303e-01 9.136887e-01 8.733753e-01 8.335636e-01 8.010221e-01 7.692368e-01

2019 9.444437e-01 9.757057e-01 9.270033e-01 9.195843e-01 9.173304e-01 9.136801e-01 8.733528e-01 8.335264e-01 8.009722e-01 7.691747e-01

2020 9.444429e-01 9.757050e-01 9.270027e-01 9.195841e-01 9.173306e-01 9.136711e-01 8.733295e-01 8.334880e-01 8.009208e-01 7.691107e-01

2021 9.444420e-01 9.757044e-01 9.270021e-01 9.195839e-01 9.173309e-01 9.136616e-01 8.733053e-01 8.334483e-01 8.008676e-01 7.690446e-01

2022 9.444410e-01 9.757037e-01 9.270015e-01 9.195838e-01 9.173311e-01 9.136517e-01 8.732802e-01 8.334072e-01 8.008128e-01 7.689766e-01

2023 9.444401e-01 9.757029e-01 9.270008e-01 9.195836e-01 9.173314e-01 9.136413e-01 8.732542e-01 8.333648e-01 8.007561e-01 7.689064e-01

2024 9.444390e-01 9.757022e-01 9.270001e-01 9.195835e-01 9.173317e-01 9.136304e-01 8.732272e-01 8.333209e-01 8.006976e-01 7.688340e-01

2025 9.444380e-01 9.757014e-01 9.269994e-01 9.195833e-01 9.173320e-01 9.136190e-01 8.731993e-01 8.332755e-01 8.006373e-01 7.687593e-01

2026 9.444369e-01 9.757005e-01 9.269987e-01 9.195832e-01 9.173324e-01 9.136072e-01 8.731704e-01 8.332287e-01 8.005749e-01 7.686823e-01

2027 9.444357e-01 9.756996e-01 9.269980e-01 9.195830e-01 9.173328e-01 9.135949e-01 8.731404e-01 8.331802e-01 8.005105e-01 7.686029e-01

2028 9.444345e-01 9.756987e-01 9.269972e-01 9.195829e-01 9.173332e-01 9.135820e-01 8.731094e-01 8.331302e-01 8.004441e-01 7.685210e-01

2029 9.444332e-01 9.756977e-01 9.269964e-01 9.195828e-01 9.173337e-01 9.135686e-01 8.730773e-01 8.330784e-01 8.003754e-01 7.684365e-01

2030 9.444319e-01 9.756966e-01 9.269956e-01 9.195828e-01 9.173342e-01 9.135547e-01 8.730441e-01 8.330249e-01 8.003045e-01 7.683493e-01

2031 9.444305e-01 9.756955e-01 9.269948e-01 9.195827e-01 9.173348e-01 9.135402e-01 8.730097e-01 8.329697e-01 8.002314e-01 7.682594e-01

2032 9.444290e-01 9.756944e-01 9.269939e-01 9.195827e-01 9.173355e-01 9.135252e-01 8.729741e-01 8.329126e-01 8.001558e-01 7.681667e-01

2033 9.444275e-01 9.756932e-01 9.269931e-01 9.195827e-01 9.173361e-01 9.135095e-01 8.729372e-01 8.328535e-01 8.000777e-01 7.680710e-01

2034 9.444260e-01 9.756919e-01 9.269922e-01 9.195827e-01 9.173369e-01 9.134933e-01 8.728991e-01 8.327925e-01 7.999971e-01 7.679724e-01

2035 9.444243e-01 9.756906e-01 9.269912e-01 9.195827e-01 9.173377e-01 9.134764e-01 8.728596e-01 8.327295e-01 7.999139e-01 7.678705e-01

2036 9.444226e-01 9.756891e-01 9.269903e-01 9.195828e-01 9.173386e-01 9.134589e-01 8.728188e-01 8.326643e-01 7.998279e-01 7.677655e-01

2037 9.444208e-01 9.756876e-01 9.269893e-01 9.195829e-01 9.173395e-01 9.134408e-01 8.727766e-01 8.325970e-01 7.997391e-01 7.676571e-01

2038 9.444189e-01 9.756861e-01 9.269883e-01 9.195831e-01 9.173406e-01 9.134219e-01 8.727329e-01 8.325274e-01 7.996474e-01 7.675453e-01

2039 9.444169e-01 9.756844e-01 9.269873e-01 9.195833e-01 9.173417e-01 9.134024e-01 8.726876e-01 8.324554e-01 7.995527e-01 7.674299e-01

2040 9.444148e-01 9.756827e-01 9.269862e-01 9.195835e-01 9.173429e-01 9.133821e-01 8.726409e-01 8.323811e-01 7.994548e-01 7.673109e-01

2041 9.444127e-01 9.756809e-01 9.269852e-01 9.195838e-01 9.173442e-01 9.133612e-01 8.725925e-01 8.323042e-01 7.993538e-01 7.671881e-01

2042 9.444104e-01 9.756789e-01 9.269841e-01 9.195842e-01 9.173457e-01 9.133394e-01 8.725425e-01 8.322247e-01 7.992494e-01 7.670613e-01

2043 9.444081e-01 9.756769e-01 9.269830e-01 9.195846e-01 9.173472e-01 9.133169e-01 8.724907e-01 8.321426e-01 7.991415e-01 7.669306e-01

2044 9.444056e-01 9.756748e-01 9.269818e-01 9.195851e-01 9.173489e-01 9.132935e-01 8.724371e-01 8.320577e-01 7.990302e-01 7.667956e-01

2045 9.444030e-01 9.756725e-01 9.269807e-01 9.195856e-01 9.173507e-01 9.132693e-01 8.723817e-01 8.319700e-01 7.989151e-01 7.666564e-01

2046 9.444003e-01 9.756701e-01 9.269795e-01 9.195863e-01 9.173526e-01 9.132442e-01 8.723244e-01 8.318793e-01 7.987962e-01 7.665127e-01

2047 9.443975e-01 9.756676e-01 9.269783e-01 9.195870e-01 9.173547e-01 9.132183e-01 8.722651e-01 8.317855e-01 7.986734e-01 7.663644e-01

2048 9.443946e-01 9.756650e-01 9.269771e-01 9.195878e-01 9.173570e-01 9.131914e-01 8.722038e-01 8.316885e-01 7.985466e-01 7.662114e-01

2049 9.443915e-01 9.756622e-01 9.269758e-01 9.195887e-01 9.173594e-01 9.131635e-01 8.721403e-01 8.315883e-01 7.984155e-01 7.660536e-01

2050 9.443883e-01 9.756592e-01 9.269746e-01 9.195898e-01 9.173621e-01 9.131347e-01 8.720747e-01 8.314847e-01 7.982801e-01 7.658907e-01

2051 9.443849e-01 9.756561e-01 9.269733e-01 9.195910e-01 9.173649e-01 9.131048e-01 8.720068e-01 8.313775e-01 7.981403e-01 7.657226e-01

2052 9.443814e-01 9.756528e-01 9.269721e-01 9.195923e-01 9.173680e-01 9.130739e-01 8.719365e-01 8.312668e-01 7.979958e-01 7.655492e-01

2053 9.443777e-01 9.756494e-01 9.269708e-01 9.195937e-01 9.173713e-01 9.130418e-01 8.718638e-01 8.311523e-01 7.978465e-01 7.653703e-01

2054 9.443739e-01 9.756457e-01 9.269696e-01 9.195953e-01 9.173748e-01 9.130087e-01 8.717886e-01 8.310339e-01 7.976923e-01 7.651857e-01

2055 9.443698e-01 9.756419e-01 9.269683e-01 9.195971e-01 9.173787e-01 9.129743e-01 8.717108e-01 8.309115e-01 7.975331e-01 7.649952e-01

2056 9.443656e-01 9.756378e-01 9.269670e-01 9.195991e-01 9.173828e-01 9.129388e-01 8.716303e-01 8.307849e-01 7.973685e-01 7.647987e-01

2057 9.443612e-01 9.756335e-01 9.269658e-01 9.196013e-01 9.173872e-01 9.129020e-01 8.715470e-01 8.306541e-01 7.971985e-01 7.645961e-01

2058 9.443566e-01 9.756289e-01 9.269646e-01 9.196036e-01 9.173920e-01 9.128638e-01 8.714609e-01 8.305188e-01 7.970230e-01 7.643870e-01

2059 9.443518e-01 9.756241e-01 9.269633e-01 9.196063e-01 9.173971e-01 9.128244e-01 8.713717e-01 8.303789e-01 7.968416e-01 7.641713e-01

2060 9.443468e-01 9.756191e-01 9.269622e-01 9.196092e-01 9.174027e-01 9.127835e-01 8.712794e-01 8.302343e-01 7.966543e-01 7.639489e-01

2061 9.443415e-01 9.756137e-01 9.269610e-01 9.196124e-01 9.174086e-01 9.127412e-01 8.711840e-01 8.300848e-01 7.964608e-01 7.637196e-01

2062 9.443360e-01 9.756081e-01 9.269599e-01 9.196159e-01 9.174150e-01 9.126974e-01 8.710852e-01 8.299303e-01 7.962610e-01 7.634830e-01

2063 9.443303e-01 9.756021e-01 9.269589e-01 9.196197e-01 9.174219e-01 9.126521e-01 8.709830e-01 8.297705e-01 7.960546e-01 7.632392e-01

2064 9.443243e-01 9.755958e-01 9.269579e-01 9.196238e-01 9.174293e-01 9.126051e-01 8.708773e-01 8.296053e-01 7.958415e-01 7.629877e-01

2065 9.443180e-01 9.755891e-01 9.269570e-01 9.196284e-01 9.174372e-01 9.125565e-01 8.707679e-01 8.294345e-01 7.956215e-01 7.627285e-01

2066 9.443114e-01 9.755820e-01 9.269562e-01 9.196334e-01 9.174458e-01 9.125061e-01 8.706546e-01 8.292579e-01 7.953943e-01 7.624614e-01

2067 9.443046e-01 9.755745e-01 9.269555e-01 9.196388e-01 9.174550e-01 9.124540e-01 8.705375e-01 8.290754e-01 7.951597e-01 7.621860e-01

2068 9.442974e-01 9.755666e-01 9.269549e-01 9.196448e-01 9.174648e-01 9.124000e-01 8.704163e-01 8.288868e-01 7.949176e-01 7.619023e-01

2069 9.442899e-01 9.755583e-01 9.269544e-01 9.196512e-01 9.174754e-01 9.123442e-01 8.702909e-01 8.286918e-01 7.946677e-01 7.616100e-01

2070 9.442821e-01 9.755494e-01 9.269541e-01 9.196582e-01 9.174869e-01 9.122863e-01 8.701611e-01 8.284903e-01 7.944097e-01 7.613088e-01

2071 9.442739e-01 9.755401e-01 9.269540e-01 9.196659e-01 9.174991e-01 9.122264e-01 8.700268e-01 8.282821e-01 7.941435e-01 7.609987e-01

2072 9.442653e-01 9.755302e-01 9.269541e-01 9.196742e-01 9.175123e-01 9.121643e-01 8.698879e-01 8.280669e-01 7.938688e-01 7.606793e-01

2073 9.442564e-01 9.755197e-01 9.269543e-01 9.196833e-01 9.175264e-01 9.121001e-01 8.697442e-01 8.278445e-01 7.935853e-01 7.603504e-01

2074 9.442471e-01 9.755086e-01 9.269548e-01 9.196931e-01 9.175416e-01 9.120336e-01 8.695954e-01 8.276148e-01 7.932930e-01 7.600119e-01

2075 9.442374e-01 9.754968e-01 9.269556e-01 9.197038e-01 9.175579e-01 9.119647e-01 8.694416e-01 8.273774e-01 7.929914e-01 7.596635e-01

2076 9.442272e-01 9.754844e-01 9.269567e-01 9.197154e-01 9.175755e-01 9.118934e-01 8.692824e-01 8.271322e-01 7.926804e-01 7.593051e-01

2077 9.442166e-01 9.754712e-01 9.269582e-01 9.197279e-01 9.175943e-01 9.118195e-01 8.691178e-01 8.268789e-01 7.923598e-01 7.589364e-01

2078 9.442056e-01 9.754573e-01 9.269600e-01 9.197416e-01 9.176145e-01 9.117431e-01 8.689475e-01 8.266173e-01 7.920292e-01 7.585572e-01

2079 9.441940e-01 9.754425e-01 9.269622e-01 9.197563e-01 9.176362e-01 9.116639e-01 8.687713e-01 8.263472e-01 7.916885e-01 7.581674e-01

2080 9.441820e-01 9.754268e-01 9.269649e-01 9.197724e-01 9.176596e-01 9.115819e-01 8.685891e-01 8.260683e-01 7.913375e-01 7.577667e-01

2081 9.441695e-01 9.754102e-01 9.269682e-01 9.197897e-01 9.176846e-01 9.114970e-01 8.684006e-01 8.257804e-01 7.909758e-01 7.573550e-01

2082 9.441564e-01 9.753927e-01 9.269719e-01 9.198085e-01 9.177115e-01 9.114091e-01 8.682057e-01 8.254832e-01 7.906032e-01 7.569320e-01

2083 9.441428e-01 9.753740e-01 9.269764e-01 9.198288e-01 9.177404e-01 9.113181e-01 8.680042e-01 8.251765e-01 7.902196e-01 7.564977e-01

2084 9.441286e-01 9.753543e-01 9.269815e-01 9.198509e-01 9.177713e-01 9.112238e-01 8.677958e-01 8.248600e-01 7.898246e-01 7.560519e-01

2085 9.441139e-01 9.753334e-01 9.269873e-01 9.198747e-01 9.178046e-01 9.111263e-01 8.675803e-01 8.245335e-01 7.894181e-01 7.555945e-01

2086 9.440985e-01 9.753112e-01 9.269940e-01 9.199004e-01 9.178403e-01 9.110252e-01 8.673575e-01 8.241967e-01 7.889998e-01 7.551252e-01

2087 9.440826e-01 9.752877e-01 9.270017e-01 9.199283e-01 9.178786e-01 9.109206e-01 8.671273e-01 8.238493e-01 7.885696e-01 7.546440e-01

2088 9.440660e-01 9.752627e-01 9.270103e-01 9.199584e-01 9.179197e-01 9.108123e-01 8.668893e-01 8.234912e-01 7.881272e-01 7.541508e-01

2089 9.440487e-01 9.752363e-01 9.270201e-01 9.199910e-01 9.179638e-01 9.107002e-01 8.666433e-01 8.231221e-01 7.876724e-01 7.536455e-01

2090 9.440308e-01 9.752083e-01 9.270310e-01 9.200261e-01 9.180111e-01 9.105842e-01 8.663892e-01 8.227417e-01 7.872050e-01 7.531280e-01

2091 9.440122e-01 9.751785e-01 9.270433e-01 9.200641e-01 9.180619e-01 9.104640e-01 8.661266e-01 8.223498e-01 7.867249e-01 7.525983e-01

2092 9.439930e-01 9.751470e-01 9.270571e-01 9.201051e-01 9.181164e-01 9.103397e-01 8.658554e-01 8.219461e-01 7.862318e-01 7.520563e-01

2093 9.439730e-01 9.751136e-01 9.270724e-01 9.201494e-01 9.181748e-01 9.102109e-01 8.655752e-01 8.215304e-01 7.857257e-01 7.515020e-01

2094 9.439523e-01 9.750781e-01 9.270895e-01 9.201972e-01 9.182374e-01 9.100777e-01 8.652859e-01 8.211025e-01 7.852064e-01 7.509355e-01

2095 9.439309e-01 9.750404e-01 9.271085e-01 9.202487e-01 9.183045e-01 9.099398e-01 8.649872e-01 8.206621e-01 7.846738e-01 7.503567e-01

2096 9.439088e-01 9.750005e-01 9.271295e-01 9.203043e-01 9.183765e-01 9.097971e-01 8.646788e-01 8.202090e-01 7.841277e-01 7.497658e-01

2097 9.438859e-01 9.749582e-01 9.271528e-01 9.203643e-01 9.184537e-01 9.096495e-01 8.643605e-01 8.197431e-01 7.835681e-01 7.491628e-01

2098 9.438623e-01 9.749132e-01 9.271785e-01 9.204289e-01 9.185364e-01 9.094967e-01 8.640321e-01 8.192641e-01 7.829949e-01 7.485477e-01

2099 9.438380e-01 9.748656e-01 9.272070e-01 9.204986e-01 9.186250e-01 9.093386e-01 8.636933e-01 8.187718e-01 7.824081e-01 7.479209e-01

2100 9.438130e-01 9.748150e-01 9.272383e-01 9.205736e-01 9.187200e-01 9.091751e-01 8.633438e-01 8.182661e-01 7.818077e-01 7.472824e-01

2101 9.437873e-01 9.747613e-01 9.272728e-01 9.206544e-01 9.188216e-01 9.090059e-01 8.629834e-01 8.177468e-01 7.811935e-01 7.466323e-01

2102 9.437609e-01 9.747044e-01 9.273107e-01 9.207415e-01 9.189305e-01 9.088310e-01 8.626119e-01 8.172137e-01 7.805658e-01 7.459711e-01

2103 9.437338e-01 9.746440e-01 9.273523e-01 9.208352e-01 9.190471e-01 9.086500e-01 8.622291e-01 8.166667e-01 7.799245e-01 7.452988e-01

2104 9.437061e-01 9.745800e-01 9.273980e-01 9.209359e-01 9.191718e-01 9.084629e-01 8.618346e-01 8.161058e-01 7.792696e-01 7.446159e-01

2105 9.436779e-01 9.745120e-01 9.274481e-01 9.210444e-01 9.193053e-01 9.082695e-01 8.614282e-01 8.155307e-01 7.786014e-01 7.439226e-01

2106 9.436491e-01 9.744399e-01 9.275028e-01 9.211609e-01 9.194482e-01 9.080695e-01 8.610098e-01 8.149415e-01 7.779200e-01 7.432192e-01

2107 9.436198e-01 9.743635e-01 9.275627e-01 9.212862e-01 9.196009e-01 9.078628e-01 8.605791e-01 8.143382e-01 7.772255e-01 7.425063e-01

2108 9.435900e-01 9.742824e-01 9.276282e-01 9.214209e-01 9.197642e-01 9.076492e-01 8.601359e-01 8.137205e-01 7.765182e-01 7.417841e-01

2109 9.435600e-01 9.741964e-01 9.276996e-01 9.215655e-01 9.199387e-01 9.074285e-01 8.596800e-01 8.130887e-01 7.757982e-01 7.410532e-01

2110 9.435296e-01 9.741053e-01 9.277775e-01 9.217207e-01 9.201251e-01 9.072005e-01 8.592111e-01 8.124426e-01 7.750659e-01 7.403140e-01

2111 9.434992e-01 9.740086e-01 9.278623e-01 9.218873e-01 9.203242e-01 9.069650e-01 8.587292e-01 8.117825e-01 7.743216e-01 7.395671e-01

2112 9.434686e-01 9.739062e-01 9.279547e-01 9.220660e-01 9.205368e-01 9.067218e-01 8.582340e-01 8.111082e-01 7.735656e-01 7.388131e-01

2113 9.434382e-01 9.737976e-01 9.280551e-01 9.222576e-01 9.207636e-01 9.064707e-01 8.577254e-01 8.104200e-01 7.727983e-01 7.380524e-01

2114 9.434080e-01 9.736826e-01 9.281643e-01 9.224629e-01 9.210056e-01 9.062114e-01 8.572033e-01 8.097180e-01 7.720201e-01 7.372857e-01

2115 9.433783e-01 9.735608e-01 9.282827e-01 9.226828e-01 9.212636e-01 9.059439e-01 8.566675e-01 8.090024e-01 7.712315e-01 7.365137e-01

2116 9.433491e-01 9.734317e-01 9.284112e-01 9.229182e-01 9.215385e-01 9.056679e-01 8.561179e-01 8.082734e-01 7.704330e-01 7.357370e-01

2117 9.433208e-01 9.732951e-01 9.285504e-01 9.231701e-01 9.218314e-01 9.053832e-01 8.555544e-01 8.075312e-01 7.696250e-01 7.349563e-01

2118 9.432935e-01 9.731504e-01 9.287012e-01 9.234395e-01 9.221432e-01 9.050895e-01 8.549770e-01 8.067762e-01 7.688083e-01 7.341723e-01

2119 9.432675e-01 9.729974e-01 9.288643e-01 9.237274e-01 9.224749e-01 9.047868e-01 8.543856e-01 8.060086e-01 7.679832e-01 7.333858e-01

2120 9.432430e-01 9.728354e-01 9.290405e-01 9.240349e-01 9.228278e-01 9.044748e-01 8.537802e-01 8.052289e-01 7.671506e-01 7.325974e-01

2121 9.432205e-01 9.726642e-01 9.292309e-01 9.243632e-01 9.232028e-01 9.041533e-01 8.531608e-01 8.044373e-01 7.663110e-01 7.318079e-01

2122 9.432003e-01 9.724832e-01 9.294362e-01 9.247133e-01 9.236011e-01 9.038221e-01 8.525275e-01 8.036344e-01 7.654651e-01 7.310182e-01

2123 9.431826e-01 9.722919e-01 9.296575e-01 9.250866e-01 9.240239e-01 9.034811e-01 8.518802e-01 8.028206e-01 7.646136e-01 7.302289e-01

2124 9.431679e-01 9.720899e-01 9.298958e-01 9.254841e-01 9.244725e-01 9.031301e-01 8.512191e-01 8.019964e-01 7.637574e-01 7.294409e-01

2125 9.431568e-01 9.718766e-01 9.301521e-01 9.259073e-01 9.249480e-01 9.027689e-01 8.505444e-01 8.011624e-01 7.628970e-01 7.286550e-01

2126 9.431495e-01 9.716515e-01 9.304275e-01 9.263575e-01 9.254516e-01 9.023974e-01 8.498561e-01 8.003191e-01 7.620334e-01 7.278719e-01

2127 9.431467e-01 9.714141e-01 9.307232e-01 9.268358e-01 9.259848e-01 9.020154e-01 8.491544e-01 7.994672e-01 7.611674e-01 7.270923e-01

2128 9.431488e-01 9.711639e-01 9.310403e-01 9.273438e-01 9.265486e-01 9.016227e-01 8.484395e-01 7.986072e-01 7.602997e-01 7.263172e-01

2129 9.431566e-01 9.709003e-01 9.313800e-01 9.278827e-01 9.271445e-01 9.012193e-01 8.477118e-01 7.977400e-01 7.594311e-01 7.255472e-01

2130 9.431705e-01 9.706228e-01 9.317435e-01 9.284540e-01 9.277737e-01 9.008050e-01 8.469714e-01 7.968661e-01 7.585626e-01 7.247831e-01

2131 9.431913e-01 9.703308e-01 9.321320e-01 9.290589e-01 9.284374e-01 9.003797e-01 8.462188e-01 7.959863e-01 7.576950e-01 7.240256e-01

2132 9.432196e-01 9.700237e-01 9.325467e-01 9.296990e-01 9.291370e-01 8.999433e-01 8.454541e-01 7.951013e-01 7.568290e-01 7.232755e-01

2133 9.432563e-01 9.697012e-01 9.329890e-01 9.303754e-01 9.298736e-01 8.994957e-01 8.446779e-01 7.942121e-01 7.559656e-01 7.225334e-01

2134 9.433019e-01 9.693625e-01 9.334600e-01 9.310896e-01 9.306485e-01 8.990369e-01 8.438906e-01 7.933192e-01 7.551057e-01 7.217999e-01

2135 9.433574e-01 9.690073e-01 9.339609e-01 9.318429e-01 9.314628e-01 8.985669e-01 8.430926e-01 7.924237e-01 7.542499e-01 7.210758e-01

2136 9.434236e-01 9.686350e-01 9.344931e-01 9.326365e-01 9.323177e-01 8.980855e-01 8.422843e-01 7.915263e-01 7.533993e-01 7.203616e-01

2137 9.435014e-01 9.682451e-01 9.350577e-01 9.334716e-01 9.332141e-01 8.975929e-01 8.414665e-01 7.906278e-01 7.525545e-01 7.196579e-01

2138 9.435916e-01 9.678372e-01 9.356558e-01 9.343494e-01 9.341531e-01 8.970889e-01 8.406395e-01 7.897291e-01 7.517163e-01 7.189652e-01

2139 9.436951e-01 9.674109e-01 9.362885e-01 9.352709e-01 9.351355e-01 8.965736e-01 8.398040e-01 7.888311e-01 7.508856e-01 7.182841e-01

2140 9.438128e-01 9.669659e-01 9.369569e-01 9.362371e-01 9.361622e-01 8.960472e-01 8.389606e-01 7.879347e-01 7.500631e-01 7.176149e-01

2141 9.439457e-01 9.665017e-01 9.376619e-01 9.372488e-01 9.372338e-01 8.955096e-01 8.381100e-01 7.870407e-01 7.492495e-01 7.169582e-01

2142 9.440947e-01 9.660182e-01 9.384044e-01 9.383069e-01 9.383509e-01 8.949610e-01 8.372529e-01 7.861499e-01 7.484456e-01 7.163144e-01

2143 9.442606e-01 9.655151e-01 9.391852e-01 9.394119e-01 9.395139e-01 8.944015e-01 8.363899e-01 7.852633e-01 7.476519e-01 7.156837e-01

2144 9.444445e-01 9.649923e-01 9.400049e-01 9.405644e-01 9.407232e-01 8.938312e-01 8.355217e-01 7.843816e-01 7.468690e-01 7.150665e-01

2145 9.446471e-01 9.644498e-01 9.408641e-01 9.417646e-01 9.419790e-01 8.932503e-01 8.346493e-01 7.835056e-01 7.460977e-01 7.144631e-01

2146 9.448694e-01 9.638874e-01 9.417633e-01 9.430128e-01 9.432812e-01 8.926591e-01 8.337732e-01 7.826363e-01 7.453384e-01 7.138737e-01

2147 9.451120e-01 9.633055e-01 9.427026e-01 9.443090e-01 9.446296e-01 8.920577e-01 8.328943e-01 7.817744e-01 7.445917e-01 7.132985e-01

2148 9.453758e-01 9.627040e-01 9.436822e-01 9.456530e-01 9.460241e-01 8.914464e-01 8.320134e-01 7.809205e-01 7.438581e-01 7.127376e-01

2149 9.456614e-01 9.620835e-01 9.447020e-01 9.470443e-01 9.474641e-01 8.908255e-01 8.311313e-01 7.800756e-01 7.431379e-01 7.121912e-01

2150 9.459694e-01 9.614441e-01 9.457618e-01 9.484824e-01 9.489488e-01 8.901953e-01 8.302489e-01 7.792402e-01 7.424316e-01 7.116593e-01

2151 9.463003e-01 9.607866e-01 9.468611e-01 9.499665e-01 9.504775e-01 8.895561e-01 8.293669e-01 7.784151e-01 7.417396e-01 7.111420e-01

2152 9.466546e-01 9.601115e-01 9.479994e-01 9.514956e-01 9.520490e-01 8.889084e-01 8.284861e-01 7.776008e-01 7.410620e-01 7.106393e-01

2153 9.470326e-01 9.594196e-01 9.491756e-01 9.530684e-01 9.536621e-01 8.882525e-01 8.276075e-01 7.767981e-01 7.403994e-01 7.101512e-01

2154 9.474344e-01 9.587117e-01 9.503889e-01 9.546835e-01 9.553152e-01 8.875887e-01 8.267317e-01 7.760074e-01 7.397517e-01 7.096776e-01

2155 9.478602e-01 9.579890e-01 9.516380e-01 9.563392e-01 9.570068e-01 8.869177e-01 8.258597e-01 7.752294e-01 7.391193e-01 7.092185e-01

2156 9.483099e-01 9.572524e-01 9.529212e-01 9.580337e-01 9.587349e-01 8.862398e-01 8.249922e-01 7.744644e-01 7.385023e-01 7.087736e-01

2157 9.487834e-01 9.565034e-01 9.542371e-01 9.597649e-01 9.604976e-01 8.855555e-01 8.241300e-01 7.737129e-01 7.379008e-01 7.083430e-01

2158 9.492803e-01 9.557431e-01 9.555837e-01 9.615306e-01 9.622927e-01 8.848654e-01 8.232738e-01 7.729754e-01 7.373149e-01 7.079264e-01

2159 9.498003e-01 9.549732e-01 9.569590e-01 9.633285e-01 9.641177e-01 8.841699e-01 8.224245e-01 7.722522e-01 7.367446e-01 7.075236e-01

2160 9.503427e-01 9.541953e-01 9.583607e-01 9.651558e-01 9.659703e-01 8.834697e-01 8.215827e-01 7.715436e-01 7.361899e-01 7.071345e-01

2161 9.509067e-01 9.534109e-01 9.597866e-01 9.670099e-01 9.678479e-01 8.827652e-01 8.207491e-01 7.708500e-01 7.356507e-01 7.067588e-01

2162 9.509067e-01 9.534107e-01 9.597867e-01 9.670101e-01 9.678481e-01 8.827586e-01 8.207286e-01 7.708182e-01 7.356114e-01 7.067162e-01

2163 9.509067e-01 9.534106e-01 9.597868e-01 9.670103e-01 9.678482e-01 8.827513e-01 8.207073e-01 7.707853e-01 7.355709e-01 7.066725e-01

2164 9.509066e-01 9.534104e-01 9.597869e-01 9.670104e-01 9.678484e-01 8.827433e-01 8.206850e-01 7.707515e-01 7.355294e-01 7.066278e-01

2165 9.509066e-01 9.534102e-01 9.597870e-01 9.670106e-01 9.678486e-01 8.827347e-01 8.206619e-01 7.707166e-01 7.354867e-01 7.065821e-01

2166 9.509065e-01 9.534101e-01 9.597871e-01 9.670108e-01 9.678488e-01 8.827255e-01 8.206379e-01 7.706807e-01 7.354429e-01 7.065352e-01

2167 9.509065e-01 9.534099e-01 9.597872e-01 9.670110e-01 9.678490e-01 8.827156e-01 8.206130e-01 7.706437e-01 7.353979e-01 7.064873e-01

2168 9.509065e-01 9.534097e-01 9.597873e-01 9.670112e-01 9.678492e-01 8.827050e-01 8.205872e-01 7.706056e-01 7.353518e-01 7.064382e-01

2169 9.509064e-01 9.534095e-01 9.597874e-01 9.670114e-01 9.678494e-01 8.826939e-01 8.205604e-01 7.705664e-01 7.353045e-01 7.063880e-01

2170 9.509064e-01 9.534093e-01 9.597876e-01 9.670116e-01 9.678496e-01 8.826820e-01 8.205326e-01 7.705261e-01 7.352559e-01 7.063366e-01

2171 9.509064e-01 9.534091e-01 9.597877e-01 9.670118e-01 9.678498e-01 8.826695e-01 8.205039e-01 7.704846e-01 7.352061e-01 7.062840e-01

2172 9.509064e-01 9.534089e-01 9.597878e-01 9.670120e-01 9.678500e-01 8.826563e-01 8.204743e-01 7.704419e-01 7.351550e-01 7.062303e-01

2173 9.509063e-01 9.534086e-01 9.597880e-01 9.670123e-01 9.678503e-01 8.826425e-01 8.204436e-01 7.703981e-01 7.351026e-01 7.061753e-01

2174 9.509063e-01 9.534084e-01 9.597881e-01 9.670125e-01 9.678505e-01 8.826280e-01 8.204119e-01 7.703530e-01 7.350489e-01 7.061190e-01

2175 9.509063e-01 9.534081e-01 9.597883e-01 9.670128e-01 9.678508e-01 8.826128e-01 8.203791e-01 7.703066e-01 7.349938e-01 7.060615e-01

2176 9.509063e-01 9.534079e-01 9.597885e-01 9.670130e-01 9.678511e-01 8.825969e-01 8.203453e-01 7.702589e-01 7.349374e-01 7.060027e-01

2177 9.509063e-01 9.534076e-01 9.597886e-01 9.670133e-01 9.678514e-01 8.825803e-01 8.203104e-01 7.702100e-01 7.348795e-01 7.059425e-01

2178 9.509063e-01 9.534073e-01 9.597888e-01 9.670136e-01 9.678517e-01 8.825630e-01 8.202743e-01 7.701596e-01 7.348201e-01 7.058810e-01

2179 9.509063e-01 9.534071e-01 9.597890e-01 9.670139e-01 9.678520e-01 8.825450e-01 8.202371e-01 7.701079e-01 7.347593e-01 7.058181e-01

2180 9.509063e-01 9.534068e-01 9.597892e-01 9.670142e-01 9.678523e-01 8.825262e-01 8.201988e-01 7.700547e-01 7.346969e-01 7.057537e-01

2181 9.509063e-01 9.534065e-01 9.597895e-01 9.670145e-01 9.678526e-01 8.825067e-01 8.201592e-01 7.700001e-01 7.346330e-01 7.056879e-01

2182 9.509063e-01 9.534061e-01 9.597897e-01 9.670148e-01 9.678529e-01 8.824865e-01 8.201185e-01 7.699440e-01 7.345674e-01 7.056207e-01

2183 9.509063e-01 9.534058e-01 9.597899e-01 9.670152e-01 9.678533e-01 8.824654e-01 8.200764e-01 7.698863e-01 7.345002e-01 7.055519e-01

2184 9.509064e-01 9.534055e-01 9.597902e-01 9.670155e-01 9.678537e-01 8.824436e-01 8.200331e-01 7.698271e-01 7.344314e-01 7.054815e-01

2185 9.509064e-01 9.534051e-01 9.597905e-01 9.670159e-01 9.678540e-01 8.824210e-01 8.199884e-01 7.697662e-01 7.343607e-01 7.054096e-01

2186 9.509065e-01 9.534047e-01 9.597908e-01 9.670163e-01 9.678544e-01 8.823976e-01 8.199424e-01 7.697037e-01 7.342884e-01 7.053360e-01

2187 9.509065e-01 9.534044e-01 9.597911e-01 9.670167e-01 9.678549e-01 8.823733e-01 8.198950e-01 7.696395e-01 7.342142e-01 7.052608e-01

2188 9.509066e-01 9.534040e-01 9.597914e-01 9.670172e-01 9.678553e-01 8.823482e-01 8.198461e-01 7.695735e-01 7.341381e-01 7.051839e-01

2189 9.509066e-01 9.534036e-01 9.597918e-01 9.670176e-01 9.678558e-01 8.823222e-01 8.197958e-01 7.695057e-01 7.340601e-01 7.051052e-01

2190 9.509067e-01 9.534032e-01 9.597921e-01 9.670181e-01 9.678562e-01 8.822952e-01 8.197439e-01 7.694360e-01 7.339802e-01 7.050248e-01

2191 9.509068e-01 9.534027e-01 9.597925e-01 9.670186e-01 9.678567e-01 8.822674e-01 8.196905e-01 7.693644e-01 7.338982e-01 7.049426e-01

2192 9.509069e-01 9.534023e-01 9.597929e-01 9.670191e-01 9.678573e-01 8.822386e-01 8.196355e-01 7.692908e-01 7.338142e-01 7.048585e-01

2193 9.509070e-01 9.534018e-01 9.597933e-01 9.670197e-01 9.678578e-01 8.822089e-01 8.195788e-01 7.692153e-01 7.337280e-01 7.047725e-01

2194 9.509071e-01 9.534013e-01 9.597938e-01 9.670203e-01 9.678584e-01 8.821782e-01 8.195203e-01 7.691376e-01 7.336397e-01 7.046845e-01

2195 9.509072e-01 9.534009e-01 9.597943e-01 9.670209e-01 9.678590e-01 8.821464e-01 8.194602e-01 7.690578e-01 7.335492e-01 7.045946e-01

2196 9.509074e-01 9.534003e-01 9.597948e-01 9.670215e-01 9.678596e-01 8.821136e-01 8.193982e-01 7.689758e-01 7.334563e-01 7.045026e-01

2197 9.509075e-01 9.533998e-01 9.597953e-01 9.670222e-01 9.678602e-01 8.820797e-01 8.193344e-01 7.688916e-01 7.333611e-01 7.044086e-01

2198 9.509077e-01 9.533993e-01 9.597959e-01 9.670229e-01 9.678609e-01 8.820447e-01 8.192686e-01 7.688050e-01 7.332635e-01 7.043125e-01

2199 9.509079e-01 9.533987e-01 9.597964e-01 9.670236e-01 9.678616e-01 8.820086e-01 8.192009e-01 7.687160e-01 7.331634e-01 7.042141e-01

2200 9.509081e-01 9.533981e-01 9.597971e-01 9.670244e-01 9.678624e-01 8.819713e-01 8.191311e-01 7.686246e-01 7.330608e-01 7.041136e-01

2201 9.509084e-01 9.533975e-01 9.597977e-01 9.670252e-01 9.678632e-01 8.819328e-01 8.190592e-01 7.685306e-01 7.329556e-01 7.040108e-01

2202 9.509086e-01 9.533969e-01 9.597984e-01 9.670260e-01 9.678640e-01 8.818930e-01 8.189852e-01 7.684340e-01 7.328477e-01 7.039057e-01

2203 9.509089e-01 9.533963e-01 9.597991e-01 9.670269e-01 9.678649e-01 8.818520e-01 8.189089e-01 7.683347e-01 7.327370e-01 7.037982e-01

2204 9.509092e-01 9.533956e-01 9.597999e-01 9.670278e-01 9.678658e-01 8.818096e-01 8.188303e-01 7.682327e-01 7.326236e-01 7.036883e-01

2205 9.509095e-01 9.533950e-01 9.598007e-01 9.670288e-01 9.678667e-01 8.817659e-01 8.187493e-01 7.681279e-01 7.325072e-01 7.035760e-01

2206 9.509099e-01 9.533943e-01 9.598015e-01 9.670298e-01 9.678677e-01 8.817207e-01 8.186659e-01 7.680201e-01 7.323879e-01 7.034611e-01

2207 9.509103e-01 9.533935e-01 9.598024e-01 9.670309e-01 9.678687e-01 8.816742e-01 8.185800e-01 7.679093e-01 7.322656e-01 7.033437e-01

2208 9.509107e-01 9.533928e-01 9.598034e-01 9.670320e-01 9.678698e-01 8.816261e-01 8.184914e-01 7.677954e-01 7.321402e-01 7.032236e-01

2209 9.509112e-01 9.533920e-01 9.598044e-01 9.670331e-01 9.678710e-01 8.815765e-01 8.184002e-01 7.676784e-01 7.320115e-01 7.031009e-01

2210 9.509116e-01 9.533912e-01 9.598054e-01 9.670344e-01 9.678722e-01 8.815253e-01 8.183062e-01 7.675580e-01 7.318796e-01 7.029754e-01

2211 9.509122e-01 9.533904e-01 9.598065e-01 9.670356e-01 9.678734e-01 8.814725e-01 8.182093e-01 7.674343e-01 7.317443e-01 7.028472e-01

2212 9.509128e-01 9.533896e-01 9.598077e-01 9.670370e-01 9.678747e-01 8.814180e-01 8.181095e-01 7.673072e-01 7.316056e-01 7.027161e-01

2213 9.509134e-01 9.533887e-01 9.598089e-01 9.670384e-01 9.678761e-01 8.813617e-01 8.180066e-01 7.671764e-01 7.314634e-01 7.025821e-01

2214 9.509141e-01 9.533878e-01 9.598102e-01 9.670399e-01 9.678775e-01 8.813036e-01 8.179006e-01 7.670421e-01 7.313175e-01 7.024451e-01

2215 9.509148e-01 9.533869e-01 9.598116e-01 9.670414e-01 9.678790e-01 8.812437e-01 8.177914e-01 7.669039e-01 7.311680e-01 7.023052e-01

2216 9.509156e-01 9.533859e-01 9.598130e-01 9.670430e-01 9.678806e-01 8.811818e-01 8.176788e-01 7.667619e-01 7.310146e-01 7.021622e-01

2217 9.509164e-01 9.533849e-01 9.598145e-01 9.670448e-01 9.678822e-01 8.811180e-01 8.175627e-01 7.666158e-01 7.308574e-01 7.020160e-01

2218 9.509173e-01 9.533839e-01 9.598162e-01 9.670465e-01 9.678840e-01 8.810521e-01 8.174431e-01 7.664657e-01 7.306961e-01 7.018667e-01

2219 9.509183e-01 9.533829e-01 9.598179e-01 9.670484e-01 9.678858e-01 8.809841e-01 8.173199e-01 7.663114e-01 7.305308e-01 7.017141e-01

2220 9.509194e-01 9.533818e-01 9.598197e-01 9.670504e-01 9.678877e-01 8.809138e-01 8.171928e-01 7.661527e-01 7.303614e-01 7.015583e-01

2221 9.509205e-01 9.533807e-01 9.598216e-01 9.670525e-01 9.678897e-01 8.808414e-01 8.170618e-01 7.659896e-01 7.301876e-01 7.013991e-01

2222 9.509217e-01 9.533796e-01 9.598236e-01 9.670546e-01 9.678918e-01 8.807666e-01 8.169268e-01 7.658219e-01 7.300095e-01 7.012364e-01

2223 9.509230e-01 9.533784e-01 9.598257e-01 9.670569e-01 9.678940e-01 8.806893e-01 8.167877e-01 7.656495e-01 7.298269e-01 7.010704e-01

2224 9.509244e-01 9.533772e-01 9.598279e-01 9.670593e-01 9.678963e-01 8.806096e-01 8.166442e-01 7.654722e-01 7.296397e-01 7.009008e-01

2225 9.509259e-01 9.533760e-01 9.598303e-01 9.670618e-01 9.678987e-01 8.805273e-01 8.164963e-01 7.652900e-01 7.294479e-01 7.007276e-01

2226 9.509275e-01 9.533748e-01 9.598328e-01 9.670645e-01 9.679013e-01 8.804423e-01 8.163439e-01 7.651027e-01 7.292512e-01 7.005508e-01

2227 9.509293e-01 9.533735e-01 9.598354e-01 9.670672e-01 9.679039e-01 8.803546e-01 8.161868e-01 7.649102e-01 7.290497e-01 7.003704e-01

2228 9.509311e-01 9.533722e-01 9.598382e-01 9.670702e-01 9.679067e-01 8.802640e-01 8.160248e-01 7.647123e-01 7.288432e-01 7.001862e-01

2229 9.509331e-01 9.533708e-01 9.598411e-01 9.670732e-01 9.679097e-01 8.801705e-01 8.158578e-01 7.645088e-01 7.286315e-01 6.999983e-01

2230 9.509352e-01 9.533694e-01 9.598442e-01 9.670764e-01 9.679127e-01 8.800739e-01 8.156856e-01 7.642997e-01 7.284147e-01 6.998065e-01

2231 9.509375e-01 9.533680e-01 9.598475e-01 9.670798e-01 9.679160e-01 8.799742e-01 8.155081e-01 7.640848e-01 7.281925e-01 6.996108e-01

2232 9.509400e-01 9.533666e-01 9.598510e-01 9.670834e-01 9.679194e-01 8.798712e-01 8.153251e-01 7.638639e-01 7.279650e-01 6.994113e-01

2233 9.509426e-01 9.533651e-01 9.598546e-01 9.670871e-01 9.679230e-01 8.797648e-01 8.151365e-01 7.636369e-01 7.277318e-01 6.992078e-01

2234 9.509454e-01 9.533637e-01 9.598585e-01 9.670911e-01 9.679267e-01 8.796550e-01 8.149421e-01 7.634036e-01 7.274931e-01 6.990003e-01

2235 9.509484e-01 9.533621e-01 9.598626e-01 9.670952e-01 9.679307e-01 8.795416e-01 8.147416e-01 7.631638e-01 7.272486e-01 6.987888e-01

2236 9.509516e-01 9.533606e-01 9.598669e-01 9.670996e-01 9.679348e-01 8.794244e-01 8.145350e-01 7.629175e-01 7.269982e-01 6.985732e-01

2237 9.509551e-01 9.533591e-01 9.598715e-01 9.671041e-01 9.679392e-01 8.793035e-01 8.143220e-01 7.626645e-01 7.267419e-01 6.983535e-01

2238 9.509588e-01 9.533575e-01 9.598764e-01 9.671090e-01 9.679438e-01 8.791785e-01 8.141024e-01 7.624045e-01 7.264795e-01 6.981297e-01

2239 9.509627e-01 9.533559e-01 9.598815e-01 9.671140e-01 9.679486e-01 8.790494e-01 8.138761e-01 7.621374e-01 7.262109e-01 6.979018e-01

2240 9.509670e-01 9.533543e-01 9.598869e-01 9.671194e-01 9.679536e-01 8.789161e-01 8.136428e-01 7.618631e-01 7.259361e-01 6.976697e-01

2241 9.509715e-01 9.533526e-01 9.598926e-01 9.671250e-01 9.679590e-01 8.787784e-01 8.134024e-01 7.615814e-01 7.256549e-01 6.974334e-01

2242 9.509763e-01 9.533510e-01 9.598987e-01 9.671309e-01 9.679646e-01 8.786362e-01 8.131546e-01 7.612921e-01 7.253672e-01 6.971929e-01

2243 9.509815e-01 9.533493e-01 9.599051e-01 9.671371e-01 9.679705e-01 8.784893e-01 8.128992e-01 7.609951e-01 7.250730e-01 6.969483e-01

2244 9.509870e-01 9.533477e-01 9.599119e-01 9.671437e-01 9.679767e-01 8.783375e-01 8.126360e-01 7.606901e-01 7.247722e-01 6.966995e-01

2245 9.509929e-01 9.533460e-01 9.599191e-01 9.671506e-01 9.679832e-01 8.781808e-01 8.123648e-01 7.603771e-01 7.244646e-01 6.964465e-01

2246 9.509992e-01 9.533444e-01 9.599267e-01 9.671578e-01 9.679901e-01 8.780189e-01 8.120854e-01 7.600558e-01 7.241502e-01 6.961893e-01

2247 9.510060e-01 9.533428e-01 9.599347e-01 9.671655e-01 9.679973e-01 8.778516e-01 8.117975e-01 7.597261e-01 7.238289e-01 6.959280e-01

2248 9.510132e-01 9.533412e-01 9.599432e-01 9.671736e-01 9.680049e-01 8.776789e-01 8.115009e-01 7.593879e-01 7.235006e-01 6.956625e-01

2249 9.510209e-01 9.533396e-01 9.599522e-01 9.671821e-01 9.680129e-01 8.775004e-01 8.111954e-01 7.590409e-01 7.231654e-01 6.953930e-01

2250 9.510291e-01 9.533381e-01 9.599618e-01 9.671910e-01 9.680214e-01 8.773161e-01 8.108808e-01 7.586850e-01 7.228230e-01 6.951194e-01

2251 9.510379e-01 9.533366e-01 9.599718e-01 9.672005e-01 9.680303e-01 8.771257e-01 8.105567e-01 7.583201e-01 7.224735e-01 6.948419e-01

2252 9.510474e-01 9.533352e-01 9.599825e-01 9.672104e-01 9.680396e-01 8.769291e-01 8.102229e-01 7.579459e-01 7.221168e-01 6.945604e-01

2253 9.510574e-01 9.533338e-01 9.599938e-01 9.672209e-01 9.680495e-01 8.767259e-01 8.098793e-01 7.575624e-01 7.217528e-01 6.942750e-01

2254 9.510682e-01 9.533325e-01 9.600058e-01 9.672319e-01 9.680599e-01 8.765161e-01 8.095256e-01 7.571695e-01 7.213816e-01 6.939858e-01

2255 9.510796e-01 9.533313e-01 9.600185e-01 9.672436e-01 9.680708e-01 8.762995e-01 8.091614e-01 7.567668e-01 7.210031e-01 6.936929e-01

2256 9.510919e-01 9.533302e-01 9.600319e-01 9.672559e-01 9.680823e-01 8.760757e-01 8.087866e-01 7.563544e-01 7.206174e-01 6.933964e-01

2257 9.511050e-01 9.533293e-01 9.600462e-01 9.672688e-01 9.680945e-01 8.758446e-01 8.084010e-01 7.559321e-01 7.202243e-01 6.930963e-01

2258 9.511190e-01 9.533284e-01 9.600612e-01 9.672825e-01 9.681073e-01 8.756060e-01 8.080042e-01 7.554998e-01 7.198239e-01 6.927929e-01

2259 9.511339e-01 9.533278e-01 9.600772e-01 9.672969e-01 9.681208e-01 8.753596e-01 8.075960e-01 7.550574e-01 7.194163e-01 6.924861e-01

2260 9.511499e-01 9.533273e-01 9.600941e-01 9.673121e-01 9.681350e-01 8.751051e-01 8.071762e-01 7.546047e-01 7.190014e-01 6.921762e-01

2261 9.511669e-01 9.533271e-01 9.601120e-01 9.673281e-01 9.681500e-01 8.748424e-01 8.067445e-01 7.541417e-01 7.185794e-01 6.918634e-01

2262 9.511851e-01 9.533270e-01 9.601310e-01 9.673450e-01 9.681658e-01 8.745712e-01 8.063008e-01 7.536684e-01 7.181502e-01 6.915476e-01

2263 9.512045e-01 9.533273e-01 9.601511e-01 9.673629e-01 9.681825e-01 8.742912e-01 8.058446e-01 7.531845e-01 7.177140e-01 6.912292e-01

2264 9.512252e-01 9.533278e-01 9.601725e-01 9.673817e-01 9.682001e-01 8.740022e-01 8.053759e-01 7.526901e-01 7.172707e-01 6.909083e-01

2265 9.512474e-01 9.533286e-01 9.601950e-01 9.674016e-01 9.682187e-01 8.737039e-01 8.048944e-01 7.521852e-01 7.168207e-01 6.905851e-01

2266 9.512710e-01 9.533298e-01 9.602190e-01 9.674226e-01 9.682382e-01 8.733961e-01 8.043998e-01 7.516696e-01 7.163638e-01 6.902598e-01

2267 9.512963e-01 9.533314e-01 9.602443e-01 9.674448e-01 9.682589e-01 8.730784e-01 8.038920e-01 7.511435e-01 7.159004e-01 6.899326e-01

2268 9.513232e-01 9.533335e-01 9.602712e-01 9.674682e-01 9.682807e-01 8.727506e-01 8.033707e-01 7.506067e-01 7.154304e-01 6.896038e-01

2269 9.513520e-01 9.533361e-01 9.602997e-01 9.674929e-01 9.683037e-01 8.724125e-01 8.028357e-01 7.500594e-01 7.149542e-01 6.892735e-01

2270 9.513827e-01 9.533392e-01 9.603299e-01 9.675189e-01 9.683280e-01 8.720637e-01 8.022870e-01 7.495015e-01 7.144719e-01 6.889420e-01

2271 9.514154e-01 9.533429e-01 9.603619e-01 9.675465e-01 9.683536e-01 8.717040e-01 8.017241e-01 7.489331e-01 7.139836e-01 6.886096e-01

2272 9.514504e-01 9.533472e-01 9.603959e-01 9.675756e-01 9.683806e-01 8.713330e-01 8.011471e-01 7.483543e-01 7.134896e-01 6.882765e-01

2273 9.514877e-01 9.533523e-01 9.604319e-01 9.676063e-01 9.684092e-01 8.709505e-01 8.005558e-01 7.477652e-01 7.129902e-01 6.879431e-01

2274 9.515275e-01 9.533582e-01 9.604700e-01 9.676387e-01 9.684393e-01 8.705563e-01 7.999499e-01 7.471659e-01 7.124856e-01 6.876095e-01

2275 9.515700e-01 9.533650e-01 9.605104e-01 9.676730e-01 9.684711e-01 8.701499e-01 7.993295e-01 7.465565e-01 7.119761e-01 6.872761e-01

2276 9.516154e-01 9.533728e-01 9.605533e-01 9.677091e-01 9.685047e-01 8.697311e-01 7.986944e-01 7.459373e-01 7.114619e-01 6.869433e-01

2277 9.516637e-01 9.533816e-01 9.605987e-01 9.677474e-01 9.685402e-01 8.692997e-01 7.980444e-01 7.453083e-01 7.109435e-01 6.866112e-01

2278 9.517153e-01 9.533915e-01 9.606468e-01 9.677877e-01 9.685776e-01 8.688553e-01 7.973797e-01 7.446699e-01 7.104210e-01 6.862803e-01

2279 9.517703e-01 9.534028e-01 9.606979e-01 9.678304e-01 9.686172e-01 8.683976e-01 7.967000e-01 7.440223e-01 7.098949e-01 6.859508e-01

2280 9.518290e-01 9.534154e-01 9.607520e-01 9.678755e-01 9.686589e-01 8.679264e-01 7.960055e-01 7.433657e-01 7.093655e-01 6.856231e-01

2281 9.518916e-01 9.534294e-01 9.608093e-01 9.679231e-01 9.687030e-01 8.674414e-01 7.952961e-01 7.427004e-01 7.088333e-01 6.852975e-01

2282 9.519583e-01 9.534452e-01 9.608701e-01 9.679733e-01 9.687496e-01 8.669423e-01 7.945718e-01 7.420267e-01 7.082985e-01 6.849743e-01

2283 9.520294e-01 9.534627e-01 9.609346e-01 9.680265e-01 9.687988e-01 8.664288e-01 7.938327e-01 7.413451e-01 7.077616e-01 6.846538e-01

2284 9.521052e-01 9.534822e-01 9.610028e-01 9.680826e-01 9.688507e-01 8.659007e-01 7.930788e-01 7.406558e-01 7.072231e-01 6.843365e-01

2285 9.521860e-01 9.535037e-01 9.610752e-01 9.681419e-01 9.689056e-01 8.653578e-01 7.923104e-01 7.399592e-01 7.066834e-01 6.840226e-01

2286 9.522721e-01 9.535276e-01 9.611519e-01 9.682045e-01 9.689636e-01 8.647996e-01 7.915276e-01 7.392559e-01 7.061429e-01 6.837124e-01

2287 9.523638e-01 9.535539e-01 9.612332e-01 9.682707e-01 9.690247e-01 8.642262e-01 7.907305e-01 7.385461e-01 7.056021e-01 6.834063e-01

2288 9.524616e-01 9.535829e-01 9.613193e-01 9.683406e-01 9.690894e-01 8.636371e-01 7.899194e-01 7.378305e-01 7.050615e-01 6.831046e-01

2289 9.525657e-01 9.536149e-01 9.614105e-01 9.684144e-01 9.691576e-01 8.630323e-01 7.890944e-01 7.371094e-01 7.045216e-01 6.828077e-01

2290 9.526765e-01 9.536500e-01 9.615072e-01 9.684924e-01 9.692297e-01 8.624114e-01 7.882560e-01 7.363835e-01 7.039827e-01 6.825157e-01

2291 9.527945e-01 9.536885e-01 9.616095e-01 9.685747e-01 9.693058e-01 8.617744e-01 7.874043e-01 7.356533e-01 7.034455e-01 6.822291e-01

2292 9.529201e-01 9.537308e-01 9.617179e-01 9.686617e-01 9.693861e-01 8.611210e-01 7.865398e-01 7.349192e-01 7.029105e-01 6.819480e-01

2293 9.530538e-01 9.537770e-01 9.618327e-01 9.687535e-01 9.694710e-01 8.604512e-01 7.856629e-01 7.341820e-01 7.023780e-01 6.816729e-01

2294 9.531960e-01 9.538275e-01 9.619543e-01 9.688505e-01 9.695605e-01 8.597647e-01 7.847740e-01 7.334423e-01 7.018487e-01 6.814039e-01

2295 9.533473e-01 9.538827e-01 9.620829e-01 9.689529e-01 9.696551e-01 8.590616e-01 7.838735e-01 7.327005e-01 7.013230e-01 6.811413e-01

2296 9.535082e-01 9.539428e-01 9.622191e-01 9.690610e-01 9.697549e-01 8.583416e-01 7.829620e-01 7.319575e-01 7.008014e-01 6.808853e-01

2297 9.536793e-01 9.540084e-01 9.623632e-01 9.691751e-01 9.698602e-01 8.576048e-01 7.820400e-01 7.312137e-01 7.002844e-01 6.806362e-01

2298 9.538610e-01 9.540798e-01 9.625157e-01 9.692955e-01 9.699713e-01 8.568511e-01 7.811081e-01 7.304700e-01 6.997725e-01 6.803941e-01

2299 9.540542e-01 9.541574e-01 9.626770e-01 9.694226e-01 9.700886e-01 8.560806e-01 7.801668e-01 7.297269e-01 6.992661e-01 6.801592e-01

2300 9.542594e-01 9.542417e-01 9.628475e-01 9.695566e-01 9.702123e-01 8.552932e-01 7.792169e-01 7.289851e-01 6.987657e-01 6.799318e-01

2301 9.544772e-01 9.543332e-01 9.630279e-01 9.696981e-01 9.703428e-01 8.544891e-01 7.782590e-01 7.282453e-01 6.982717e-01 6.797118e-01

2302 9.547084e-01 9.544324e-01 9.632185e-01 9.698472e-01 9.704804e-01 8.536682e-01 7.772938e-01 7.275082e-01 6.977846e-01 6.794996e-01

2303 9.549538e-01 9.545398e-01 9.634199e-01 9.700045e-01 9.706255e-01 8.528308e-01 7.763221e-01 7.267745e-01 6.973048e-01 6.792951e-01

2304 9.552141e-01 9.546561e-01 9.636328e-01 9.701703e-01 9.707784e-01 8.519771e-01 7.753445e-01 7.260448e-01 6.968327e-01 6.790985e-01

2305 9.554900e-01 9.547818e-01 9.638575e-01 9.703450e-01 9.709396e-01 8.511071e-01 7.743620e-01 7.253198e-01 6.963686e-01 6.789099e-01

2306 9.557825e-01 9.549176e-01 9.640948e-01 9.705291e-01 9.711095e-01 8.502212e-01 7.733753e-01 7.246001e-01 6.959129e-01 6.787292e-01

2307 9.560923e-01 9.550641e-01 9.643453e-01 9.707231e-01 9.712884e-01 8.493196e-01 7.723853e-01 7.238865e-01 6.954659e-01 6.785565e-01

2308 9.564204e-01 9.552221e-01 9.646096e-01 9.709274e-01 9.714768e-01 8.484027e-01 7.713928e-01 7.231795e-01 6.950280e-01 6.783919e-01

2309 9.567677e-01 9.553923e-01 9.648884e-01 9.711425e-01 9.716751e-01 8.474707e-01 7.703987e-01 7.224797e-01 6.945994e-01 6.782352e-01

2310 9.571351e-01 9.555755e-01 9.651823e-01 9.713689e-01 9.718839e-01 8.465242e-01 7.694039e-01 7.217879e-01 6.941803e-01 6.780865e-01

2311 9.575236e-01 9.557724e-01 9.654921e-01 9.716071e-01 9.721036e-01 8.455635e-01 7.684093e-01 7.211044e-01 6.937711e-01 6.779458e-01

2312 9.579343e-01 9.559839e-01 9.658185e-01 9.718576e-01 9.723347e-01 8.445891e-01 7.674158e-01 7.204300e-01 6.933719e-01 6.778129e-01

2313 9.583680e-01 9.562109e-01 9.661623e-01 9.721211e-01 9.725777e-01 8.436015e-01 7.664242e-01 7.197651e-01 6.929829e-01 6.776878e-01

2314 9.588260e-01 9.564543e-01 9.665241e-01 9.723981e-01 9.728331e-01 8.426013e-01 7.654356e-01 7.191102e-01 6.926043e-01 6.775703e-01

2315 9.593092e-01 9.567150e-01 9.669048e-01 9.726891e-01 9.731014e-01 8.415891e-01 7.644507e-01 7.184658e-01 6.922361e-01 6.774604e-01

2316 9.598188e-01 9.569939e-01 9.673052e-01 9.729947e-01 9.733833e-01 8.405655e-01 7.634706e-01 7.178324e-01 6.918785e-01 6.773579e-01

2317 9.603558e-01 9.572920e-01 9.677261e-01 9.733156e-01 9.736792e-01 8.395312e-01 7.624960e-01 7.172104e-01 6.915316e-01 6.772628e-01

2318 9.609214e-01 9.576103e-01 9.681683e-01 9.736524e-01 9.739898e-01 8.384869e-01 7.615279e-01 7.166002e-01 6.911954e-01 6.771747e-01

2319 9.615167e-01 9.579498e-01 9.686327e-01 9.740056e-01 9.743155e-01 8.374334e-01 7.605670e-01 7.160020e-01 6.908699e-01 6.770936e-01

2320 9.621428e-01 9.583115e-01 9.691201e-01 9.743760e-01 9.746570e-01 8.363714e-01 7.596144e-01 7.154163e-01 6.905552e-01 6.770193e-01

2321 9.628009e-01 9.586965e-01 9.696313e-01 9.747642e-01 9.750150e-01 8.353017e-01 7.586707e-01 7.148433e-01 6.902512e-01 6.769516e-01

2322 9.634922e-01 9.591058e-01 9.701673e-01 9.751708e-01 9.753899e-01 8.342253e-01 7.577367e-01 7.142833e-01 6.899578e-01 6.768902e-01

2323 9.642176e-01 9.595405e-01 9.707288e-01 9.755965e-01 9.757824e-01 8.331429e-01 7.568133e-01 7.137364e-01 6.896750e-01 6.768351e-01

2324 9.649784e-01 9.600014e-01 9.713168e-01 9.760419e-01 9.761931e-01 8.320555e-01 7.559011e-01 7.132030e-01 6.894028e-01 6.767860e-01

2325 9.657756e-01 9.604898e-01 9.719321e-01 9.765078e-01 9.766226e-01 8.309639e-01 7.550009e-01 7.126830e-01 6.891409e-01 6.767427e-01

2326 9.666103e-01 9.610065e-01 9.725754e-01 9.769947e-01 9.770715e-01 8.298692e-01 7.541132e-01 7.121767e-01 6.888893e-01 6.767049e-01

2327 9.674835e-01 9.615526e-01 9.732478e-01 9.775034e-01 9.775404e-01 8.287722e-01 7.532388e-01 7.116841e-01 6.886479e-01 6.766725e-01

2328 9.683960e-01 9.621289e-01 9.739498e-01 9.780345e-01 9.780299e-01 8.276740e-01 7.523782e-01 7.112053e-01 6.884164e-01 6.766452e-01

2329 9.693490e-01 9.627363e-01 9.746825e-01 9.785888e-01 9.785405e-01 8.265754e-01 7.515319e-01 7.107403e-01 6.881948e-01 6.766228e-01

2330 9.703431e-01 9.633757e-01 9.754464e-01 9.791668e-01 9.790729e-01 8.254775e-01 7.507006e-01 7.102890e-01 6.879828e-01 6.766052e-01

2331 9.713791e-01 9.640479e-01 9.762424e-01 9.797694e-01 9.796276e-01 8.243813e-01 7.498845e-01 7.098515e-01 6.877802e-01 6.765920e-01

2332 9.724578e-01 9.647534e-01 9.770713e-01 9.803972e-01 9.802053e-01 8.232877e-01 7.490842e-01 7.094277e-01 6.875868e-01 6.765831e-01

2333 9.735797e-01 9.654929e-01 9.779337e-01 9.810509e-01 9.808065e-01 8.221977e-01 7.482999e-01 7.090174e-01 6.874024e-01 6.765782e-01

2334 9.747454e-01 9.662668e-01 9.788303e-01 9.817311e-01 9.814317e-01 8.211123e-01 7.475322e-01 7.086207e-01 6.872268e-01 6.765772e-01

2335 9.759552e-01 9.670756e-01 9.797619e-01 9.824387e-01 9.820816e-01 8.200324e-01 7.467811e-01 7.082373e-01 6.870597e-01 6.765798e-01

2336 9.772095e-01 9.679194e-01 9.807291e-01 9.831742e-01 9.827567e-01 8.189590e-01 7.460471e-01 7.078670e-01 6.869010e-01 6.765859e-01

2337 9.785085e-01 9.687983e-01 9.817324e-01 9.839382e-01 9.834575e-01 8.178929e-01 7.453302e-01 7.075098e-01 6.867503e-01 6.765952e-01

2338 9.798521e-01 9.697124e-01 9.827723e-01 9.847315e-01 9.841845e-01 8.168352e-01 7.446306e-01 7.071653e-01 6.866075e-01 6.766075e-01

2339 9.812403e-01 9.706614e-01 9.838494e-01 9.855545e-01 9.849382e-01 8.157866e-01 7.439485e-01 7.068335e-01 6.864722e-01 6.766227e-01

2340 9.826730e-01 9.716450e-01 9.849640e-01 9.864079e-01 9.857192e-01 8.147481e-01 7.432839e-01 7.065140e-01 6.863443e-01 6.766405e-01

2341 9.841497e-01 9.726627e-01 9.861165e-01 9.872922e-01 9.865277e-01 8.137204e-01 7.426370e-01 7.062066e-01 6.862235e-01 6.766609e-01

2342 9.841499e-01 9.726628e-01 9.861166e-01 9.872923e-01 9.865277e-01 8.136910e-01 7.425942e-01 7.061675e-01 6.861928e-01 6.766434e-01

2343 9.841500e-01 9.726628e-01 9.861167e-01 9.872924e-01 9.865278e-01 8.136608e-01 7.425505e-01 7.061276e-01 6.861616e-01 6.766257e-01

2344 9.841502e-01 9.726629e-01 9.861168e-01 9.872925e-01 9.865278e-01 8.136296e-01 7.425057e-01 7.060870e-01 6.861300e-01 6.766080e-01

2345 9.841503e-01 9.726630e-01 9.861169e-01 9.872925e-01 9.865279e-01 8.135976e-01 7.424598e-01 7.060456e-01 6.860980e-01 6.765902e-01

2346 9.841505e-01 9.726631e-01 9.861170e-01 9.872926e-01 9.865280e-01 8.135646e-01 7.424129e-01 7.060035e-01 6.860656e-01 6.765724e-01

2347 9.841507e-01 9.726631e-01 9.861171e-01 9.872927e-01 9.865281e-01 8.135306e-01 7.423649e-01 7.059607e-01 6.860327e-01 6.765545e-01

2348 9.841509e-01 9.726632e-01 9.861172e-01 9.872928e-01 9.865281e-01 8.134957e-01 7.423158e-01 7.059170e-01 6.859994e-01 6.765365e-01

2349 9.841511e-01 9.726633e-01 9.861174e-01 9.872929e-01 9.865282e-01 8.134598e-01 7.422656e-01 7.058726e-01 6.859658e-01 6.765185e-01

2350 9.841513e-01 9.726634e-01 9.861175e-01 9.872930e-01 9.865283e-01 8.134228e-01 7.422143e-01 7.058275e-01 6.859317e-01 6.765005e-01

2351 9.841515e-01 9.726635e-01 9.861176e-01 9.872931e-01 9.865284e-01 8.133848e-01 7.421617e-01 7.057815e-01 6.858972e-01 6.764825e-01

2352 9.841517e-01 9.726636e-01 9.861178e-01 9.872932e-01 9.865285e-01 8.133458e-01 7.421080e-01 7.057348e-01 6.858624e-01 6.764645e-01

2353 9.841519e-01 9.726637e-01 9.861179e-01 9.872933e-01 9.865286e-01 8.133056e-01 7.420531e-01 7.056872e-01 6.858271e-01 6.764464e-01

2354 9.841521e-01 9.726639e-01 9.861181e-01 9.872934e-01 9.865287e-01 8.132643e-01 7.419970e-01 7.056389e-01 6.857914e-01 6.764284e-01

2355 9.841524e-01 9.726640e-01 9.861183e-01 9.872935e-01 9.865288e-01 8.132219e-01 7.419396e-01 7.055897e-01 6.857554e-01 6.764105e-01

2356 9.841526e-01 9.726641e-01 9.861184e-01 9.872936e-01 9.865289e-01 8.131783e-01 7.418809e-01 7.055397e-01 6.857190e-01 6.763926e-01

2357 9.841529e-01 9.726643e-01 9.861186e-01 9.872938e-01 9.865290e-01 8.131334e-01 7.418209e-01 7.054889e-01 6.856822e-01 6.763747e-01

2358 9.841531e-01 9.726644e-01 9.861188e-01 9.872939e-01 9.865291e-01 8.130874e-01 7.417595e-01 7.054373e-01 6.856451e-01 6.763570e-01

2359 9.841534e-01 9.726646e-01 9.861190e-01 9.872940e-01 9.865292e-01 8.130400e-01 7.416968e-01 7.053848e-01 6.856076e-01 6.763393e-01

2360 9.841537e-01 9.726648e-01 9.861192e-01 9.872942e-01 9.865294e-01 8.129914e-01 7.416327e-01 7.053314e-01 6.855697e-01 6.763217e-01

2361 9.841540e-01 9.726649e-01 9.861194e-01 9.872943e-01 9.865295e-01 8.129414e-01 7.415672e-01 7.052772e-01 6.855315e-01 6.763043e-01

2362 9.841543e-01 9.726651e-01 9.861197e-01 9.872945e-01 9.865296e-01 8.128901e-01 7.415002e-01 7.052221e-01 6.854929e-01 6.762871e-01

2363 9.841546e-01 9.726653e-01 9.861199e-01 9.872947e-01 9.865298e-01 8.128373e-01 7.414317e-01 7.051661e-01 6.854540e-01 6.762700e-01

2364 9.841550e-01 9.726656e-01 9.861202e-01 9.872948e-01 9.865299e-01 8.127831e-01 7.413617e-01 7.051092e-01 6.854148e-01 6.762531e-01

2365 9.841553e-01 9.726658e-01 9.861204e-01 9.872950e-01 9.865300e-01 8.127273e-01 7.412901e-01 7.050515e-01 6.853753e-01 6.762364e-01

2366 9.841557e-01 9.726660e-01 9.861207e-01 9.872952e-01 9.865302e-01 8.126701e-01 7.412170e-01 7.049928e-01 6.853355e-01 6.762199e-01

2367 9.841561e-01 9.726663e-01 9.861210e-01 9.872954e-01 9.865304e-01 8.126112e-01 7.411422e-01 7.049332e-01 6.852954e-01 6.762037e-01

2368 9.841564e-01 9.726666e-01 9.861212e-01 9.872956e-01 9.865305e-01 8.125508e-01 7.410658e-01 7.048727e-01 6.852550e-01 6.761878e-01

2369 9.841569e-01 9.726668e-01 9.861215e-01 9.872958e-01 9.865307e-01 8.124886e-01 7.409876e-01 7.048113e-01 6.852143e-01 6.761722e-01

2370 9.841573e-01 9.726671e-01 9.861219e-01 9.872960e-01 9.865309e-01 8.124248e-01 7.409078e-01 7.047489e-01 6.851734e-01 6.761570e-01

2371 9.841577e-01 9.726675e-01 9.861222e-01 9.872962e-01 9.865311e-01 8.123591e-01 7.408261e-01 7.046856e-01 6.851323e-01 6.761421e-01

2372 9.841582e-01 9.726678e-01 9.861225e-01 9.872965e-01 9.865313e-01 8.122917e-01 7.407427e-01 7.046213e-01 6.850909e-01 6.761276e-01

2373 9.841587e-01 9.726681e-01 9.861229e-01 9.872967e-01 9.865315e-01 8.122224e-01 7.406574e-01 7.045561e-01 6.850494e-01 6.761135e-01

2374 9.841592e-01 9.726685e-01 9.861232e-01 9.872970e-01 9.865317e-01 8.121511e-01 7.405702e-01 7.044899e-01 6.850076e-01 6.761000e-01

2375 9.841597e-01 9.726689e-01 9.861236e-01 9.872973e-01 9.865319e-01 8.120779e-01 7.404811e-01 7.044228e-01 6.849657e-01 6.760869e-01

2376 9.841602e-01 9.726693e-01 9.861240e-01 9.872975e-01 9.865321e-01 8.120027e-01 7.403900e-01 7.043546e-01 6.849237e-01 6.760743e-01

2377 9.841608e-01 9.726697e-01 9.861244e-01 9.872978e-01 9.865324e-01 8.119253e-01 7.402968e-01 7.042855e-01 6.848815e-01 6.760623e-01

2378 9.841614e-01 9.726702e-01 9.861249e-01 9.872981e-01 9.865326e-01 8.118458e-01 7.402017e-01 7.042155e-01 6.848392e-01 6.760509e-01

2379 9.841620e-01 9.726707e-01 9.861253e-01 9.872985e-01 9.865329e-01 8.117640e-01 7.401044e-01 7.041444e-01 6.847969e-01 6.760402e-01

2380 9.841626e-01 9.726712e-01 9.861258e-01 9.872988e-01 9.865331e-01 8.116800e-01 7.400049e-01 7.040724e-01 6.847545e-01 6.760302e-01

2381 9.841633e-01 9.726717e-01 9.861263e-01 9.872991e-01 9.865334e-01 8.115936e-01 7.399033e-01 7.039993e-01 6.847121e-01 6.760209e-01

2382 9.841640e-01 9.726723e-01 9.861268e-01 9.872995e-01 9.865337e-01 8.115048e-01 7.397994e-01 7.039253e-01 6.846697e-01 6.760124e-01

2383 9.841648e-01 9.726729e-01 9.861273e-01 9.872999e-01 9.865340e-01 8.114135e-01 7.396932e-01 7.038503e-01 6.846273e-01 6.760048e-01

2384 9.841655e-01 9.726735e-01 9.861279e-01 9.873003e-01 9.865343e-01 8.113196e-01 7.395847e-01 7.037744e-01 6.845850e-01 6.759980e-01

2385 9.841663e-01 9.726742e-01 9.861285e-01 9.873007e-01 9.865347e-01 8.112231e-01 7.394738e-01 7.036974e-01 6.845429e-01 6.759922e-01

2386 9.841671e-01 9.726749e-01 9.861291e-01 9.873011e-01 9.865350e-01 8.111238e-01 7.393604e-01 7.036195e-01 6.845008e-01 6.759874e-01

2387 9.841680e-01 9.726756e-01 9.861297e-01 9.873016e-01 9.865354e-01 8.110218e-01 7.392446e-01 7.035406e-01 6.844590e-01 6.759836e-01

2388 9.841689e-01 9.726764e-01 9.861304e-01 9.873020e-01 9.865358e-01 8.109168e-01 7.391262e-01 7.034608e-01 6.844174e-01 6.759809e-01

2389 9.841699e-01 9.726772e-01 9.861311e-01 9.873025e-01 9.865362e-01 8.108089e-01 7.390053e-01 7.033800e-01 6.843761e-01 6.759795e-01

2390 9.841708e-01 9.726780e-01 9.861318e-01 9.873030e-01 9.865366e-01 8.106979e-01 7.388816e-01 7.032982e-01 6.843351e-01 6.759793e-01

2391 9.841719e-01 9.726790e-01 9.861326e-01 9.873036e-01 9.865370e-01 8.105837e-01 7.387553e-01 7.032156e-01 6.842944e-01 6.759804e-01

2392 9.841729e-01 9.726799e-01 9.861333e-01 9.873041e-01 9.865375e-01 8.104664e-01 7.386263e-01 7.031320e-01 6.842542e-01 6.759829e-01

2393 9.841741e-01 9.726809e-01 9.861342e-01 9.873047e-01 9.865379e-01 8.103456e-01 7.384944e-01 7.030475e-01 6.842145e-01 6.759868e-01

2394 9.841752e-01 9.726820e-01 9.861350e-01 9.873053e-01 9.865384e-01 8.102215e-01 7.383597e-01 7.029621e-01 6.841753e-01 6.759923e-01

2395 9.841765e-01 9.726831e-01 9.861359e-01 9.873060e-01 9.865389e-01 8.100938e-01 7.382221e-01 7.028758e-01 6.841366e-01 6.759994e-01

2396 9.841777e-01 9.726843e-01 9.861369e-01 9.873066e-01 9.865395e-01 8.099624e-01 7.380815e-01 7.027887e-01 6.840987e-01 6.760082e-01

2397 9.841791e-01 9.726856e-01 9.861378e-01 9.873073e-01 9.865400e-01 8.098273e-01 7.379379e-01 7.027007e-01 6.840614e-01 6.760188e-01

2398 9.841805e-01 9.726869e-01 9.861389e-01 9.873081e-01 9.865406e-01 8.096883e-01 7.377913e-01 7.026119e-01 6.840249e-01 6.760312e-01

2399 9.841819e-01 9.726883e-01 9.861399e-01 9.873088e-01 9.865412e-01 8.095454e-01 7.376415e-01 7.025224e-01 6.839893e-01 6.760457e-01

2400 9.841834e-01 9.726897e-01 9.861411e-01 9.873096e-01 9.865419e-01 8.093983e-01 7.374885e-01 7.024321e-01 6.839546e-01 6.760622e-01

2401 9.841850e-01 9.726913e-01 9.861422e-01 9.873105e-01 9.865426e-01 8.092471e-01 7.373323e-01 7.023411e-01 6.839209e-01 6.760808e-01

2402 9.841867e-01 9.726929e-01 9.861434e-01 9.873114e-01 9.865433e-01 8.090915e-01 7.371728e-01 7.022494e-01 6.838883e-01 6.761018e-01

2403 9.841884e-01 9.726946e-01 9.861447e-01 9.873123e-01 9.865440e-01 8.089315e-01 7.370099e-01 7.021570e-01 6.838569e-01 6.761251e-01

2404 9.841902e-01 9.726965e-01 9.861461e-01 9.873132e-01 9.865448e-01 8.087669e-01 7.368437e-01 7.020640e-01 6.838267e-01 6.761508e-01

2405 9.841921e-01 9.726984e-01 9.861475e-01 9.873142e-01 9.865456e-01 8.085975e-01 7.366740e-01 7.019705e-01 6.837979e-01 6.761792e-01

2406 9.841940e-01 9.727004e-01 9.861489e-01 9.873153e-01 9.865464e-01 8.084233e-01 7.365007e-01 7.018764e-01 6.837706e-01 6.762103e-01

2407 9.841961e-01 9.727025e-01 9.861505e-01 9.873164e-01 9.865473e-01 8.082441e-01 7.363240e-01 7.017818e-01 6.837448e-01 6.762443e-01

2408 9.841983e-01 9.727048e-01 9.861520e-01 9.873175e-01 9.865482e-01 8.080598e-01 7.361436e-01 7.016868e-01 6.837207e-01 6.762811e-01

2409 9.842005e-01 9.727072e-01 9.861537e-01 9.873187e-01 9.865491e-01 8.078702e-01 7.359595e-01 7.015915e-01 6.836983e-01 6.763211e-01

2410 9.842029e-01 9.727097e-01 9.861555e-01 9.873200e-01 9.865501e-01 8.076752e-01 7.357718e-01 7.014958e-01 6.836778e-01 6.763643e-01

2411 9.842053e-01 9.727123e-01 9.861573e-01 9.873213e-01 9.865512e-01 8.074746e-01 7.355803e-01 7.013999e-01 6.836593e-01 6.764109e-01

2412 9.842079e-01 9.727151e-01 9.861592e-01 9.873227e-01 9.865523e-01 8.072682e-01 7.353850e-01 7.013038e-01 6.836429e-01 6.764610e-01

2413 9.842106e-01 9.727181e-01 9.861612e-01 9.873241e-01 9.865534e-01 8.070560e-01 7.351858e-01 7.012076e-01 6.836287e-01 6.765147e-01

2414 9.842134e-01 9.727212e-01 9.861633e-01 9.873256e-01 9.865546e-01 8.068377e-01 7.349827e-01 7.011114e-01 6.836169e-01 6.765722e-01

2415 9.842164e-01 9.727245e-01 9.861655e-01 9.873272e-01 9.865559e-01 8.066132e-01 7.347757e-01 7.010152e-01 6.836076e-01 6.766336e-01

2416 9.842194e-01 9.727280e-01 9.861678e-01 9.873288e-01 9.865572e-01 8.063823e-01 7.345647e-01 7.009191e-01 6.836010e-01 6.766992e-01

2417 9.842227e-01 9.727316e-01 9.861702e-01 9.873306e-01 9.865586e-01 8.061449e-01 7.343498e-01 7.008232e-01 6.835971e-01 6.767690e-01

2418 9.842260e-01 9.727355e-01 9.861727e-01 9.873324e-01 9.865600e-01 8.059007e-01 7.341307e-01 7.007276e-01 6.835962e-01 6.768432e-01

2419 9.842296e-01 9.727396e-01 9.861753e-01 9.873343e-01 9.865615e-01 8.056496e-01 7.339076e-01 7.006325e-01 6.835984e-01 6.769221e-01

2420 9.842333e-01 9.727439e-01 9.861781e-01 9.873363e-01 9.865631e-01 8.053915e-01 7.336804e-01 7.005378e-01 6.836038e-01 6.770056e-01

2421 9.842371e-01 9.727484e-01 9.861810e-01 9.873384e-01 9.865648e-01 8.051261e-01 7.334491e-01 7.004438e-01 6.836126e-01 6.770942e-01

2422 9.842412e-01 9.727532e-01 9.861840e-01 9.873405e-01 9.865665e-01 8.048532e-01 7.332136e-01 7.003505e-01 6.836250e-01 6.771878e-01

2423 9.842454e-01 9.727583e-01 9.861872e-01 9.873428e-01 9.865683e-01 8.045727e-01 7.329739e-01 7.002580e-01 6.836411e-01 6.772868e-01

2424 9.842499e-01 9.727637e-01 9.861905e-01 9.873452e-01 9.865702e-01 8.042843e-01 7.327301e-01 7.001665e-01 6.836611e-01 6.773912e-01

2425 9.842545e-01 9.727694e-01 9.861940e-01 9.873478e-01 9.865722e-01 8.039879e-01 7.324820e-01 7.000760e-01 6.836852e-01 6.775013e-01

2426 9.842594e-01 9.727753e-01 9.861976e-01 9.873504e-01 9.865743e-01 8.036834e-01 7.322298e-01 6.999868e-01 6.837137e-01 6.776172e-01

2427 9.842645e-01 9.727817e-01 9.862014e-01 9.873532e-01 9.865765e-01 8.033704e-01 7.319734e-01 6.998990e-01 6.837465e-01 6.777392e-01

2428 9.842698e-01 9.727883e-01 9.862054e-01 9.873561e-01 9.865788e-01 8.030488e-01 7.317128e-01 6.998126e-01 6.837840e-01 6.778675e-01

2429 9.842754e-01 9.727954e-01 9.862096e-01 9.873591e-01 9.865812e-01 8.027184e-01 7.314480e-01 6.997279e-01 6.838264e-01 6.780021e-01

2430 9.842813e-01 9.728028e-01 9.862140e-01 9.873623e-01 9.865837e-01 8.023790e-01 7.311790e-01 6.996450e-01 6.838738e-01 6.781435e-01

2431 9.842874e-01 9.728107e-01 9.862186e-01 9.873657e-01 9.865864e-01 8.020304e-01 7.309059e-01 6.995641e-01 6.839265e-01 6.782916e-01

2432 9.842939e-01 9.728190e-01 9.862235e-01 9.873692e-01 9.865892e-01 8.016724e-01 7.306287e-01 6.994853e-01 6.839846e-01 6.784468e-01

2433 9.843006e-01 9.728278e-01 9.862285e-01 9.873729e-01 9.865921e-01 8.013048e-01 7.303475e-01 6.994088e-01 6.840483e-01 6.786091e-01

2434 9.843077e-01 9.728371e-01 9.862338e-01 9.873767e-01 9.865951e-01 8.009274e-01 7.300622e-01 6.993348e-01 6.841180e-01 6.787790e-01

2435 9.843151e-01 9.728469e-01 9.862394e-01 9.873808e-01 9.865983e-01 8.005400e-01 7.297729e-01 6.992635e-01 6.841937e-01 6.789564e-01

2436 9.843229e-01 9.728572e-01 9.862453e-01 9.873851e-01 9.866017e-01 8.001424e-01 7.294798e-01 6.991949e-01 6.842756e-01 6.791417e-01

2437 9.843311e-01 9.728682e-01 9.862514e-01 9.873895e-01 9.866052e-01 7.997345e-01 7.291827e-01 6.991295e-01 6.843641e-01 6.793349e-01

2438 9.843396e-01 9.728798e-01 9.862578e-01 9.873942e-01 9.866089e-01 7.993160e-01 7.288820e-01 6.990672e-01 6.844593e-01 6.795364e-01

2439 9.843486e-01 9.728920e-01 9.862646e-01 9.873992e-01 9.866128e-01 7.988867e-01 7.285775e-01 6.990083e-01 6.845613e-01 6.797463e-01

2440 9.843580e-01 9.729049e-01 9.862717e-01 9.874043e-01 9.866169e-01 7.984465e-01 7.282694e-01 6.989531e-01 6.846706e-01 6.799647e-01

2441 9.843679e-01 9.729186e-01 9.862791e-01 9.874098e-01 9.866212e-01 7.979952e-01 7.279579e-01 6.989017e-01 6.847871e-01 6.801920e-01

2442 9.843782e-01 9.729330e-01 9.862869e-01 9.874155e-01 9.866257e-01 7.975326e-01 7.276430e-01 6.988544e-01 6.849112e-01 6.804281e-01

2443 9.843891e-01 9.729483e-01 9.862951e-01 9.874215e-01 9.866304e-01 7.970586e-01 7.273248e-01 6.988112e-01 6.850430e-01 6.806734e-01

2444 9.844005e-01 9.729644e-01 9.863037e-01 9.874278e-01 9.866354e-01 7.965730e-01 7.270036e-01 6.987726e-01 6.851828e-01 6.809279e-01

2445 9.844124e-01 9.729815e-01 9.863127e-01 9.874344e-01 9.866406e-01 7.960756e-01 7.266793e-01 6.987386e-01 6.853308e-01 6.811919e-01

2446 9.844250e-01 9.729995e-01 9.863222e-01 9.874414e-01 9.866461e-01 7.955663e-01 7.263523e-01 6.987095e-01 6.854871e-01 6.814655e-01

2447 9.844381e-01 9.730186e-01 9.863321e-01 9.874487e-01 9.866518e-01 7.950450e-01 7.260226e-01 6.986855e-01 6.856519e-01 6.817487e-01

2448 9.844520e-01 9.730387e-01 9.863426e-01 9.874563e-01 9.866579e-01 7.945115e-01 7.256904e-01 6.986669e-01 6.858255e-01 6.820419e-01

2449 9.844665e-01 9.730600e-01 9.863535e-01 9.874644e-01 9.866642e-01 7.939658e-01 7.253559e-01 6.986537e-01 6.860079e-01 6.823450e-01

2450 9.844817e-01 9.730826e-01 9.863651e-01 9.874729e-01 9.866709e-01 7.934077e-01 7.250193e-01 6.986464e-01 6.861994e-01 6.826582e-01

2451 9.844977e-01 9.731064e-01 9.863772e-01 9.874818e-01 9.866779e-01 7.928371e-01 7.246808e-01 6.986449e-01 6.864001e-01 6.829816e-01

2452 9.845144e-01 9.731316e-01 9.863899e-01 9.874912e-01 9.866853e-01 7.922541e-01 7.243407e-01 6.986497e-01 6.866101e-01 6.833152e-01

2453 9.845321e-01 9.731582e-01 9.864033e-01 9.875011e-01 9.866931e-01 7.916585e-01 7.239991e-01 6.986609e-01 6.868297e-01 6.836592e-01

2454 9.845506e-01 9.731863e-01 9.864173e-01 9.875114e-01 9.867012e-01 7.910503e-01 7.236562e-01 6.986786e-01 6.870589e-01 6.840136e-01

2455 9.845700e-01 9.732161e-01 9.864321e-01 9.875223e-01 9.867098e-01 7.904294e-01 7.233125e-01 6.987032e-01 6.872978e-01 6.843785e-01

2456 9.845904e-01 9.732476e-01 9.864476e-01 9.875338e-01 9.867189e-01 7.897959e-01 7.229680e-01 6.987347e-01 6.875466e-01 6.847538e-01

2457 9.846118e-01 9.732808e-01 9.864639e-01 9.875459e-01 9.867283e-01 7.891498e-01 7.226231e-01 6.987734e-01 6.878053e-01 6.851395e-01

2458 9.846343e-01 9.733160e-01 9.864810e-01 9.875586e-01 9.867383e-01 7.884912e-01 7.222780e-01 6.988194e-01 6.880740e-01 6.855358e-01

2459 9.846580e-01 9.733532e-01 9.864990e-01 9.875719e-01 9.867488e-01 7.878200e-01 7.219330e-01 6.988729e-01 6.883527e-01 6.859425e-01

2460 9.846828e-01 9.733926e-01 9.865180e-01 9.875860e-01 9.867599e-01 7.871364e-01 7.215885e-01 6.989341e-01 6.886416e-01 6.863596e-01

2461 9.847089e-01 9.734342e-01 9.865379e-01 9.876008e-01 9.867715e-01 7.864404e-01 7.212447e-01 6.990032e-01 6.889406e-01 6.867870e-01

2462 9.847363e-01 9.734781e-01 9.865588e-01 9.876163e-01 9.867838e-01 7.857323e-01 7.209018e-01 6.990802e-01 6.892497e-01 6.872246e-01

2463 9.847651e-01 9.735246e-01 9.865808e-01 9.876327e-01 9.867967e-01 7.850121e-01 7.205604e-01 6.991653e-01 6.895689e-01 6.876724e-01

2464 9.847954e-01 9.735738e-01 9.866040e-01 9.876499e-01 9.868102e-01 7.842801e-01 7.202205e-01 6.992586e-01 6.898982e-01 6.881303e-01

2465 9.848272e-01 9.736257e-01 9.866283e-01 9.876681e-01 9.868245e-01 7.835364e-01 7.198826e-01 6.993601e-01 6.902375e-01 6.885980e-01

2466 9.848606e-01 9.736807e-01 9.866539e-01 9.876872e-01 9.868395e-01 7.827813e-01 7.195470e-01 6.994701e-01 6.905867e-01 6.890754e-01

2467 9.848957e-01 9.737387e-01 9.866809e-01 9.877073e-01 9.868553e-01 7.820150e-01 7.192140e-01 6.995886e-01 6.909458e-01 6.895623e-01

2468 9.849326e-01 9.738001e-01 9.867092e-01 9.877285e-01 9.868720e-01 7.812379e-01 7.188840e-01 6.997155e-01 6.913147e-01 6.900586e-01

2469 9.849714e-01 9.738650e-01 9.867390e-01 9.877507e-01 9.868895e-01 7.804502e-01 7.185572e-01 6.998511e-01 6.916932e-01 6.905639e-01

2470 9.850121e-01 9.739336e-01 9.867703e-01 9.877742e-01 9.869080e-01 7.796524e-01 7.182340e-01 6.999952e-01 6.920811e-01 6.910780e-01

2471 9.850549e-01 9.740060e-01 9.868033e-01 9.877989e-01 9.869274e-01 7.788447e-01 7.179147e-01 7.001478e-01 6.924783e-01 6.916008e-01

2472 9.850999e-01 9.740826e-01 9.868380e-01 9.878250e-01 9.869479e-01 7.780277e-01 7.175997e-01 7.003091e-01 6.928846e-01 6.921317e-01

2473 9.851472e-01 9.741636e-01 9.868745e-01 9.878524e-01 9.869695e-01 7.772016e-01 7.172892e-01 7.004788e-01 6.932997e-01 6.926707e-01

2474 9.851969e-01 9.742491e-01 9.869129e-01 9.878812e-01 9.869922e-01 7.763671e-01 7.169835e-01 7.006571e-01 6.937235e-01 6.932173e-01

2475 9.852491e-01 9.743394e-01 9.869533e-01 9.879117e-01 9.870161e-01 7.755246e-01 7.166831e-01 7.008437e-01 6.941556e-01 6.937711e-01

2476 9.853040e-01 9.744348e-01 9.869957e-01 9.879437e-01 9.870414e-01 7.746746e-01 7.163881e-01 7.010387e-01 6.945958e-01 6.943319e-01

2477 9.853617e-01 9.745356e-01 9.870405e-01 9.879774e-01 9.870679e-01 7.738177e-01 7.160988e-01 7.012419e-01 6.950438e-01 6.948991e-01

2478 9.854223e-01 9.746420e-01 9.870875e-01 9.880129e-01 9.870959e-01 7.729545e-01 7.158156e-01 7.014532e-01 6.954993e-01 6.954725e-01

2479 9.854859e-01 9.747543e-01 9.871370e-01 9.880504e-01 9.871253e-01 7.720855e-01 7.155386e-01 7.016724e-01 6.959619e-01 6.960517e-01

2480 9.855529e-01 9.748730e-01 9.871890e-01 9.880898e-01 9.871564e-01 7.712114e-01 7.152682e-01 7.018994e-01 6.964313e-01 6.966361e-01

2481 9.856232e-01 9.749982e-01 9.872438e-01 9.881313e-01 9.871891e-01 7.703328e-01 7.150046e-01 7.021340e-01 6.969071e-01 6.972253e-01

2482 9.856970e-01 9.751304e-01 9.873014e-01 9.881751e-01 9.872235e-01 7.694504e-01 7.147480e-01 7.023760e-01 6.973889e-01 6.978190e-01

2483 9.857746e-01 9.752698e-01 9.873620e-01 9.882211e-01 9.872598e-01 7.685649e-01 7.144986e-01 7.026251e-01 6.978764e-01 6.984165e-01

2484 9.858562e-01 9.754170e-01 9.874258e-01 9.882697e-01 9.872980e-01 7.676770e-01 7.142566e-01 7.028812e-01 6.983691e-01 6.990176e-01

2485 9.859418e-01 9.755721e-01 9.874929e-01 9.883208e-01 9.873382e-01 7.667874e-01 7.140222e-01 7.031440e-01 6.988667e-01 6.996216e-01

2486 9.860318e-01 9.757358e-01 9.875634e-01 9.883746e-01 9.873806e-01 7.658969e-01 7.137955e-01 7.034132e-01 6.993686e-01 7.002281e-01

2487 9.861262e-01 9.759084e-01 9.876376e-01 9.884313e-01 9.874252e-01 7.650062e-01 7.135767e-01 7.036886e-01 6.998745e-01 7.008366e-01

2488 9.862254e-01 9.760903e-01 9.877157e-01 9.884910e-01 9.874722e-01 7.641160e-01 7.133660e-01 7.039698e-01 7.003840e-01 7.014467e-01

2489 9.863296e-01 9.762820e-01 9.877978e-01 9.885539e-01 9.875217e-01 7.632271e-01 7.131633e-01 7.042566e-01 7.008965e-01 7.020578e-01

2490 9.864389e-01 9.764839e-01 9.878841e-01 9.886201e-01 9.875738e-01 7.623403e-01 7.129689e-01 7.045487e-01 7.014116e-01 7.026694e-01

2491 9.865537e-01 9.766967e-01 9.879748e-01 9.886898e-01 9.876286e-01 7.614563e-01 7.127827e-01 7.048456e-01 7.019289e-01 7.032811e-01

2492 9.866742e-01 9.769207e-01 9.880702e-01 9.887631e-01 9.876864e-01 7.605759e-01 7.126048e-01 7.051472e-01 7.024479e-01 7.038923e-01

2493 9.868007e-01 9.771565e-01 9.881704e-01 9.888404e-01 9.877471e-01 7.596998e-01 7.124353e-01 7.054531e-01 7.029682e-01 7.045026e-01

2494 9.869333e-01 9.774047e-01 9.882758e-01 9.889217e-01 9.878111e-01 7.588288e-01 7.122741e-01 7.057629e-01 7.034894e-01 7.051116e-01

2495 9.870725e-01 9.776658e-01 9.883866e-01 9.890072e-01 9.878784e-01 7.579637e-01 7.121213e-01 7.060762e-01 7.040109e-01 7.057187e-01

2496 9.872185e-01 9.779405e-01 9.885029e-01 9.890973e-01 9.879492e-01 7.571052e-01 7.119768e-01 7.063928e-01 7.045323e-01 7.063235e-01

2497 9.873716e-01 9.782292e-01 9.886251e-01 9.891920e-01 9.880236e-01 7.562539e-01 7.118405e-01 7.067123e-01 7.050532e-01 7.069255e-01

2498 9.875321e-01 9.785327e-01 9.887535e-01 9.892916e-01 9.881019e-01 7.554106e-01 7.117125e-01 7.070343e-01 7.055732e-01 7.075244e-01

2499 9.877004e-01 9.788516e-01 9.888884e-01 9.893964e-01 9.881843e-01 7.545759e-01 7.115926e-01 7.073585e-01 7.060918e-01 7.081197e-01

2500 9.878768e-01 9.791865e-01 9.890299e-01 9.895066e-01 9.882708e-01 7.537506e-01 7.114807e-01 7.076846e-01 7.066087e-01 7.087109e-01

2501 9.880615e-01 9.795382e-01 9.891785e-01 9.896225e-01 9.883617e-01 7.529351e-01 7.113768e-01 7.080121e-01 7.071235e-01 7.092977e-01

2502 9.882551e-01 9.799072e-01 9.893345e-01 9.897443e-01 9.884573e-01 7.521302e-01 7.112807e-01 7.083408e-01 7.076357e-01 7.098798e-01

2503 9.884578e-01 9.802943e-01 9.894981e-01 9.898722e-01 9.885577e-01 7.513364e-01 7.111922e-01 7.086703e-01 7.081449e-01 7.104566e-01

2504 9.886701e-01 9.807003e-01 9.896698e-01 9.900067e-01 9.886631e-01 7.505543e-01 7.111113e-01 7.090003e-01 7.086510e-01 7.110280e-01

2505 9.888922e-01 9.811257e-01 9.898498e-01 9.901479e-01 9.887737e-01 7.497843e-01 7.110377e-01 7.093305e-01 7.091534e-01 7.115936e-01

2506 9.891247e-01 9.815715e-01 9.900386e-01 9.902962e-01 9.888898e-01 7.490269e-01 7.109713e-01 7.096605e-01 7.096518e-01 7.121530e-01

2507 9.893680e-01 9.820383e-01 9.902364e-01 9.904519e-01 9.890116e-01 7.482827e-01 7.109120e-01 7.099901e-01 7.101460e-01 7.127060e-01

2508 9.896225e-01 9.825269e-01 9.904438e-01 9.906153e-01 9.891394e-01 7.475519e-01 7.108594e-01 7.103190e-01 7.106356e-01 7.132524e-01

2509 9.898886e-01 9.830381e-01 9.906609e-01 9.907868e-01 9.892733e-01 7.468350e-01 7.108135e-01 7.106468e-01 7.111205e-01 7.137917e-01

2510 9.901669e-01 9.835726e-01 9.908884e-01 9.909666e-01 9.894137e-01 7.461323e-01 7.107741e-01 7.109734e-01 7.116002e-01 7.143239e-01

2511 9.904578e-01 9.841312e-01 9.911265e-01 9.911552e-01 9.895607e-01 7.454442e-01 7.107408e-01 7.112984e-01 7.120745e-01 7.148487e-01

2512 9.907619e-01 9.847146e-01 9.913757e-01 9.913528e-01 9.897147e-01 7.447708e-01 7.107136e-01 7.116216e-01 7.125433e-01 7.153659e-01

2513 9.910796e-01 9.853238e-01 9.916363e-01 9.915599e-01 9.898759e-01 7.441125e-01 7.106922e-01 7.119427e-01 7.130063e-01 7.158753e-01

2514 9.914117e-01 9.859594e-01 9.919090e-01 9.917768e-01 9.900445e-01 7.434694e-01 7.106763e-01 7.122616e-01 7.134634e-01 7.163768e-01

2515 9.917585e-01 9.866224e-01 9.921939e-01 9.920039e-01 9.902209e-01 7.428416e-01 7.106659e-01 7.125780e-01 7.139142e-01 7.168702e-01

2516 9.921207e-01 9.873134e-01 9.924918e-01 9.922415e-01 9.904052e-01 7.422294e-01 7.106606e-01 7.128917e-01 7.143588e-01 7.173554e-01

2517 9.924988e-01 9.880333e-01 9.928029e-01 9.924902e-01 9.905978e-01 7.416327e-01 7.106602e-01 7.132026e-01 7.147968e-01 7.178323e-01

2518 9.928936e-01 9.887830e-01 9.931278e-01 9.927502e-01 9.907989e-01 7.410517e-01 7.106645e-01 7.135104e-01 7.152283e-01 7.183009e-01

2519 9.933057e-01 9.895632e-01 9.934670e-01 9.930219e-01 9.910088e-01 7.404864e-01 7.106734e-01 7.138151e-01 7.156531e-01 7.187609e-01

2520 9.937356e-01 9.903746e-01 9.938210e-01 9.933058e-01 9.912277e-01 7.399367e-01 7.106865e-01 7.141164e-01 7.160711e-01 7.192125e-01

2521 9.941841e-01 9.912182e-01 9.941904e-01 9.936023e-01 9.914560e-01 7.394027e-01 7.107037e-01 7.144142e-01 7.164822e-01 7.196555e-01

2522 9.941842e-01 9.912183e-01 9.941904e-01 9.936023e-01 9.914560e-01 7.393623e-01 7.106891e-01 7.144246e-01 7.165043e-01 7.196806e-01

2523 9.941842e-01 9.912184e-01 9.941905e-01 9.936024e-01 9.914560e-01 7.393210e-01 7.106745e-01 7.144355e-01 7.165270e-01 7.197064e-01

2524 9.941843e-01 9.912185e-01 9.941905e-01 9.936024e-01 9.914561e-01 7.392790e-01 7.106599e-01 7.144469e-01 7.165505e-01 7.197328e-01

2525 9.941843e-01 9.912186e-01 9.941906e-01 9.936024e-01 9.914561e-01 7.392361e-01 7.106453e-01 7.144588e-01 7.165747e-01 7.197600e-01

2526 9.941844e-01 9.912187e-01 9.941906e-01 9.936025e-01 9.914561e-01 7.391923e-01 7.106308e-01 7.144714e-01 7.165996e-01 7.197878e-01

2527 9.941845e-01 9.912189e-01 9.941907e-01 9.936025e-01 9.914561e-01 7.391477e-01 7.106163e-01 7.144845e-01 7.166253e-01 7.198163e-01

2528 9.941845e-01 9.912190e-01 9.941907e-01 9.936026e-01 9.914561e-01 7.391023e-01 7.106019e-01 7.144981e-01 7.166518e-01 7.198456e-01

2529 9.941846e-01 9.912191e-01 9.941908e-01 9.936026e-01 9.914561e-01 7.390560e-01 7.105875e-01 7.145125e-01 7.166790e-01 7.198756e-01

2530 9.941847e-01 9.912193e-01 9.941908e-01 9.936027e-01 9.914561e-01 7.390088e-01 7.105732e-01 7.145274e-01 7.167071e-01 7.199064e-01

2531 9.941848e-01 9.912194e-01 9.941909e-01 9.936027e-01 9.914562e-01 7.389607e-01 7.105590e-01 7.145430e-01 7.167360e-01 7.199380e-01

2532 9.941848e-01 9.912196e-01 9.941910e-01 9.936027e-01 9.914562e-01 7.389116e-01 7.105450e-01 7.145593e-01 7.167657e-01 7.199703e-01

2533 9.941849e-01 9.912198e-01 9.941910e-01 9.936028e-01 9.914562e-01 7.388617e-01 7.105311e-01 7.145763e-01 7.167963e-01 7.200035e-01

2534 9.941850e-01 9.912199e-01 9.941911e-01 9.936028e-01 9.914562e-01 7.388108e-01 7.105173e-01 7.145940e-01 7.168279e-01 7.200375e-01

2535 9.941851e-01 9.912201e-01 9.941912e-01 9.936029e-01 9.914562e-01 7.387590e-01 7.105036e-01 7.146124e-01 7.168603e-01 7.200724e-01

2536 9.941852e-01 9.912203e-01 9.941913e-01 9.936029e-01 9.914562e-01 7.387062e-01 7.104902e-01 7.146317e-01 7.168937e-01 7.201081e-01

2537 9.941853e-01 9.912205e-01 9.941913e-01 9.936030e-01 9.914562e-01 7.386524e-01 7.104769e-01 7.146517e-01 7.169280e-01 7.201447e-01

2538 9.941854e-01 9.912207e-01 9.941914e-01 9.936030e-01 9.914562e-01 7.385976e-01 7.104639e-01 7.146726e-01 7.169634e-01 7.201822e-01

2539 9.941855e-01 9.912209e-01 9.941915e-01 9.936031e-01 9.914563e-01 7.385418e-01 7.104511e-01 7.146943e-01 7.169998e-01 7.202206e-01

2540 9.941856e-01 9.912211e-01 9.941916e-01 9.936031e-01 9.914563e-01 7.384850e-01 7.104385e-01 7.147169e-01 7.170372e-01 7.202601e-01

2541 9.941858e-01 9.912214e-01 9.941917e-01 9.936032e-01 9.914563e-01 7.384271e-01 7.104263e-01 7.147404e-01 7.170758e-01 7.203005e-01

2542 9.941859e-01 9.912216e-01 9.941918e-01 9.936032e-01 9.914563e-01 7.383682e-01 7.104143e-01 7.147649e-01 7.171154e-01 7.203419e-01

2543 9.941860e-01 9.912218e-01 9.941919e-01 9.936033e-01 9.914563e-01 7.383082e-01 7.104026e-01 7.147904e-01 7.171563e-01 7.203843e-01

2544 9.941861e-01 9.912221e-01 9.941920e-01 9.936034e-01 9.914563e-01 7.382471e-01 7.103913e-01 7.148169e-01 7.171983e-01 7.204278e-01

2545 9.941863e-01 9.912223e-01 9.941921e-01 9.936034e-01 9.914564e-01 7.381849e-01 7.103804e-01 7.148444e-01 7.172415e-01 7.204724e-01

2546 9.941864e-01 9.912226e-01 9.941922e-01 9.936035e-01 9.914564e-01 7.381215e-01 7.103698e-01 7.148731e-01 7.172860e-01 7.205181e-01

2547 9.941866e-01 9.912229e-01 9.941923e-01 9.936036e-01 9.914564e-01 7.380570e-01 7.103597e-01 7.149028e-01 7.173317e-01 7.205649e-01

2548 9.941867e-01 9.912232e-01 9.941924e-01 9.936037e-01 9.914564e-01 7.379913e-01 7.103501e-01 7.149338e-01 7.173788e-01 7.206129e-01

2549 9.941869e-01 9.912235e-01 9.941925e-01 9.936037e-01 9.914565e-01 7.379245e-01 7.103409e-01 7.149659e-01 7.174273e-01 7.206622e-01

2550 9.941870e-01 9.912238e-01 9.941926e-01 9.936038e-01 9.914565e-01 7.378564e-01 7.103322e-01 7.149993e-01 7.174772e-01 7.207126e-01

2551 9.941872e-01 9.912241e-01 9.941928e-01 9.936039e-01 9.914565e-01 7.377872e-01 7.103241e-01 7.150340e-01 7.175285e-01 7.207644e-01

2552 9.941874e-01 9.912245e-01 9.941929e-01 9.936040e-01 9.914565e-01 7.377167e-01 7.103166e-01 7.150700e-01 7.175813e-01 7.208174e-01

2553 9.941876e-01 9.912248e-01 9.941930e-01 9.936041e-01 9.914566e-01 7.376449e-01 7.103096e-01 7.151074e-01 7.176357e-01 7.208718e-01

2554 9.941878e-01 9.912252e-01 9.941932e-01 9.936041e-01 9.914566e-01 7.375719e-01 7.103034e-01 7.151463e-01 7.176917e-01 7.209275e-01

2555 9.941880e-01 9.912256e-01 9.941933e-01 9.936042e-01 9.914566e-01 7.374976e-01 7.102978e-01 7.151866e-01 7.177493e-01 7.209847e-01

2556 9.941882e-01 9.912260e-01 9.941935e-01 9.936043e-01 9.914566e-01 7.374220e-01 7.102930e-01 7.152285e-01 7.178085e-01 7.210433e-01

2557 9.941884e-01 9.912264e-01 9.941936e-01 9.936044e-01 9.914567e-01 7.373450e-01 7.102889e-01 7.152720e-01 7.178695e-01 7.211034e-01

2558 9.941886e-01 9.912268e-01 9.941938e-01 9.936045e-01 9.914567e-01 7.372668e-01 7.102857e-01 7.153171e-01 7.179323e-01 7.211651e-01

2559 9.941889e-01 9.912273e-01 9.941940e-01 9.936046e-01 9.914567e-01 7.371871e-01 7.102833e-01 7.153639e-01 7.179969e-01 7.212283e-01

2560 9.941891e-01 9.912278e-01 9.941941e-01 9.936047e-01 9.914567e-01 7.371061e-01 7.102818e-01 7.154125e-01 7.180634e-01 7.212931e-01

2561 9.941893e-01 9.912282e-01 9.941943e-01 9.936049e-01 9.914567e-01 7.370237e-01 7.102813e-01 7.154629e-01 7.181318e-01 7.213595e-01

2562 9.941896e-01 9.912288e-01 9.941945e-01 9.936050e-01 9.914568e-01 7.369399e-01 7.102818e-01 7.155151e-01 7.182023e-01 7.214277e-01

2563 9.941899e-01 9.912293e-01 9.941947e-01 9.936051e-01 9.914568e-01 7.368547e-01 7.102834e-01 7.155693e-01 7.182748e-01 7.214976e-01

2564 9.941902e-01 9.912298e-01 9.941949e-01 9.936052e-01 9.914568e-01 7.367681e-01 7.102861e-01 7.156255e-01 7.183494e-01 7.215693e-01

2565 9.941905e-01 9.912304e-01 9.941951e-01 9.936053e-01 9.914568e-01 7.366800e-01 7.102900e-01 7.156838e-01 7.184262e-01 7.216428e-01

2566 9.941908e-01 9.912310e-01 9.941953e-01 9.936055e-01 9.914568e-01 7.365904e-01 7.102951e-01 7.157443e-01 7.185052e-01 7.217182e-01

2567 9.941911e-01 9.912316e-01 9.941956e-01 9.936056e-01 9.914568e-01 7.364994e-01 7.103015e-01 7.158069e-01 7.185866e-01 7.217956e-01

2568 9.941914e-01 9.912323e-01 9.941958e-01 9.936057e-01 9.914568e-01 7.364068e-01 7.103092e-01 7.158719e-01 7.186703e-01 7.218749e-01

2569 9.941918e-01 9.912329e-01 9.941960e-01 9.936059e-01 9.914569e-01 7.363128e-01 7.103184e-01 7.159392e-01 7.187565e-01 7.219563e-01

2570 9.941921e-01 9.912336e-01 9.941963e-01 9.936060e-01 9.914569e-01 7.362172e-01 7.103291e-01 7.160090e-01 7.188452e-01 7.220398e-01

2571 9.941925e-01 9.912344e-01 9.941966e-01 9.936062e-01 9.914569e-01 7.361201e-01 7.103414e-01 7.160813e-01 7.189365e-01 7.221255e-01

2572 9.941929e-01 9.912351e-01 9.941968e-01 9.936063e-01 9.914569e-01 7.360215e-01 7.103553e-01 7.161563e-01 7.190304e-01 7.222133e-01

2573 9.941933e-01 9.912359e-01 9.941971e-01 9.936065e-01 9.914569e-01 7.359213e-01 7.103709e-01 7.162339e-01 7.191272e-01 7.223034e-01

2574 9.941937e-01 9.912367e-01 9.941974e-01 9.936067e-01 9.914569e-01 7.358196e-01 7.103884e-01 7.163143e-01 7.192267e-01 7.223959e-01

2575 9.941941e-01 9.912376e-01 9.941977e-01 9.936068e-01 9.914569e-01 7.357163e-01 7.104077e-01 7.163976e-01 7.193291e-01 7.224907e-01

2576 9.941946e-01 9.912385e-01 9.941980e-01 9.936070e-01 9.914569e-01 7.356114e-01 7.104290e-01 7.164839e-01 7.194346e-01 7.225880e-01

2577 9.941951e-01 9.912394e-01 9.941984e-01 9.936072e-01 9.914569e-01 7.355050e-01 7.104523e-01 7.165733e-01 7.195431e-01 7.226878e-01

2578 9.941956e-01 9.912404e-01 9.941987e-01 9.936074e-01 9.914569e-01 7.353970e-01 7.104778e-01 7.166658e-01 7.196548e-01 7.227902e-01

2579 9.941961e-01 9.912414e-01 9.941991e-01 9.936076e-01 9.914569e-01 7.352874e-01 7.105056e-01 7.167616e-01 7.197697e-01 7.228952e-01

2580 9.941966e-01 9.912425e-01 9.941995e-01 9.936078e-01 9.914569e-01 7.351762e-01 7.105357e-01 7.168608e-01 7.198880e-01 7.230030e-01

2581 9.941972e-01 9.912436e-01 9.941998e-01 9.936080e-01 9.914569e-01 7.350634e-01 7.105683e-01 7.169634e-01 7.200097e-01 7.231136e-01

2582 9.941978e-01 9.912447e-01 9.942003e-01 9.936082e-01 9.914569e-01 7.349491e-01 7.106034e-01 7.170697e-01 7.201349e-01 7.232270e-01

2583 9.941984e-01 9.912459e-01 9.942007e-01 9.936085e-01 9.914568e-01 7.348332e-01 7.106411e-01 7.171797e-01 7.202638e-01 7.233433e-01

2584 9.941990e-01 9.912471e-01 9.942011e-01 9.936087e-01 9.914568e-01 7.347157e-01 7.106817e-01 7.172935e-01 7.203965e-01 7.234627e-01

2585 9.941997e-01 9.912484e-01 9.942016e-01 9.936089e-01 9.914568e-01 7.345966e-01 7.107251e-01 7.174112e-01 7.205329e-01 7.235851e-01

2586 9.942004e-01 9.912498e-01 9.942020e-01 9.936092e-01 9.914568e-01 7.344760e-01 7.107716e-01 7.175330e-01 7.206733e-01 7.237107e-01

2587 9.942011e-01 9.912512e-01 9.942025e-01 9.936094e-01 9.914567e-01 7.343538e-01 7.108212e-01 7.176591e-01 7.208178e-01 7.238396e-01

2588 9.942018e-01 9.912527e-01 9.942030e-01 9.936097e-01 9.914567e-01 7.342301e-01 7.108740e-01 7.177894e-01 7.209664e-01 7.239718e-01

2589 9.942026e-01 9.912542e-01 9.942036e-01 9.936100e-01 9.914567e-01 7.341049e-01 7.109303e-01 7.179242e-01 7.211193e-01 7.241074e-01

2590 9.942034e-01 9.912558e-01 9.942041e-01 9.936103e-01 9.914566e-01 7.339782e-01 7.109901e-01 7.180636e-01 7.212767e-01 7.242465e-01

2591 9.942043e-01 9.912575e-01 9.942047e-01 9.936106e-01 9.914566e-01 7.338501e-01 7.110535e-01 7.182077e-01 7.214385e-01 7.243892e-01

2592 9.942051e-01 9.912592e-01 9.942053e-01 9.936109e-01 9.914565e-01 7.337204e-01 7.111208e-01 7.183566e-01 7.216049e-01 7.245355e-01

2593 9.942061e-01 9.912610e-01 9.942059e-01 9.936112e-01 9.914564e-01 7.335894e-01 7.111920e-01 7.185106e-01 7.217761e-01 7.246856e-01

2594 9.942070e-01 9.912629e-01 9.942066e-01 9.936116e-01 9.914564e-01 7.334570e-01 7.112673e-01 7.186697e-01 7.219521e-01 7.248395e-01

2595 9.942080e-01 9.912649e-01 9.942073e-01 9.936119e-01 9.914563e-01 7.333232e-01 7.113469e-01 7.188342e-01 7.221332e-01 7.249974e-01

2596 9.942091e-01 9.912670e-01 9.942080e-01 9.936123e-01 9.914562e-01 7.331881e-01 7.114309e-01 7.190041e-01 7.223194e-01 7.251593e-01

2597 9.942102e-01 9.912691e-01 9.942087e-01 9.936126e-01 9.914561e-01 7.330517e-01 7.115194e-01 7.191796e-01 7.225108e-01 7.253253e-01

2598 9.942113e-01 9.912714e-01 9.942095e-01 9.936130e-01 9.914560e-01 7.329141e-01 7.116128e-01 7.193608e-01 7.227076e-01 7.254955e-01

2599 9.942125e-01 9.912737e-01 9.942103e-01 9.936134e-01 9.914559e-01 7.327753e-01 7.117110e-01 7.195480e-01 7.229100e-01 7.256700e-01

2600 9.942137e-01 9.912762e-01 9.942111e-01 9.936138e-01 9.914558e-01 7.326354e-01 7.118143e-01 7.197413e-01 7.231180e-01 7.258490e-01

2601 9.942150e-01 9.912787e-01 9.942120e-01 9.936143e-01 9.914557e-01 7.324944e-01 7.119229e-01 7.199408e-01 7.233317e-01 7.260324e-01

2602 9.942163e-01 9.912814e-01 9.942129e-01 9.936147e-01 9.914555e-01 7.323524e-01 7.120370e-01 7.201468e-01 7.235515e-01 7.262204e-01

2603 9.942178e-01 9.912842e-01 9.942138e-01 9.936152e-01 9.914554e-01 7.322094e-01 7.121567e-01 7.203593e-01 7.237772e-01 7.264132e-01

2604 9.942192e-01 9.912871e-01 9.942148e-01 9.936156e-01 9.914552e-01 7.320656e-01 7.122822e-01 7.205787e-01 7.240092e-01 7.266107e-01

2605 9.942207e-01 9.912901e-01 9.942158e-01 9.936161e-01 9.914550e-01 7.319209e-01 7.124137e-01 7.208049e-01 7.242476e-01 7.268132e-01

2606 9.942223e-01 9.912933e-01 9.942169e-01 9.936167e-01 9.914548e-01 7.317756e-01 7.125515e-01 7.210383e-01 7.244924e-01 7.270206e-01

2607 9.942240e-01 9.912966e-01 9.942180e-01 9.936172e-01 9.914546e-01 7.316295e-01 7.126956e-01 7.212790e-01 7.247439e-01 7.272331e-01

2608 9.942258e-01 9.913000e-01 9.942192e-01 9.936177e-01 9.914544e-01 7.314830e-01 7.128464e-01 7.215271e-01 7.250021e-01 7.274508e-01

2609 9.942276e-01 9.913037e-01 9.942204e-01 9.936183e-01 9.914542e-01 7.313359e-01 7.130040e-01 7.217829e-01 7.252673e-01 7.276739e-01

2610 9.942295e-01 9.913074e-01 9.942217e-01 9.936189e-01 9.914539e-01 7.311885e-01 7.131686e-01 7.220465e-01 7.255395e-01 7.279023e-01

2611 9.942315e-01 9.913114e-01 9.942230e-01 9.936195e-01 9.914536e-01 7.310409e-01 7.133404e-01 7.223181e-01 7.258190e-01 7.281362e-01

2612 9.942335e-01 9.913155e-01 9.942244e-01 9.936202e-01 9.914533e-01 7.308931e-01 7.135197e-01 7.225980e-01 7.261058e-01 7.283757e-01

2613 9.942357e-01 9.913198e-01 9.942258e-01 9.936208e-01 9.914530e-01 7.307453e-01 7.137067e-01 7.228862e-01 7.264001e-01 7.286209e-01

2614 9.942379e-01 9.913243e-01 9.942273e-01 9.936215e-01 9.914526e-01 7.305976e-01 7.139015e-01 7.231830e-01 7.267021e-01 7.288719e-01

2615 9.942403e-01 9.913290e-01 9.942289e-01 9.936222e-01 9.914522e-01 7.304501e-01 7.141044e-01 7.234885e-01 7.270118e-01 7.291288e-01

2616 9.942428e-01 9.913339e-01 9.942305e-01 9.936230e-01 9.914518e-01 7.303030e-01 7.143155e-01 7.238030e-01 7.273295e-01 7.293916e-01

2617 9.942453e-01 9.913391e-01 9.942322e-01 9.936237e-01 9.914514e-01 7.301563e-01 7.145352e-01 7.241265e-01 7.276553e-01 7.296605e-01

2618 9.942480e-01 9.913444e-01 9.942340e-01 9.936245e-01 9.914509e-01 7.300103e-01 7.147637e-01 7.244594e-01 7.279892e-01 7.299356e-01

2619 9.942508e-01 9.913500e-01 9.942358e-01 9.936254e-01 9.914504e-01 7.298651e-01 7.150011e-01 7.248017e-01 7.283315e-01 7.302169e-01

2620 9.942538e-01 9.913559e-01 9.942377e-01 9.936262e-01 9.914498e-01 7.297209e-01 7.152476e-01 7.251537e-01 7.286823e-01 7.305045e-01

2621 9.942569e-01 9.913621e-01 9.942398e-01 9.936271e-01 9.914492e-01 7.295777e-01 7.155036e-01 7.255154e-01 7.290417e-01 7.307985e-01

2622 9.942601e-01 9.913685e-01 9.942419e-01 9.936280e-01 9.914486e-01 7.294359e-01 7.157691e-01 7.258872e-01 7.294098e-01 7.310990e-01

2623 9.942634e-01 9.913752e-01 9.942441e-01 9.936290e-01 9.914479e-01 7.292955e-01 7.160444e-01 7.262691e-01 7.297867e-01 7.314061e-01

2624 9.942669e-01 9.913822e-01 9.942464e-01 9.936300e-01 9.914472e-01 7.291567e-01 7.163297e-01 7.266613e-01 7.301726e-01 7.317198e-01

2625 9.942706e-01 9.913896e-01 9.942488e-01 9.936310e-01 9.914464e-01 7.290198e-01 7.166252e-01 7.270640e-01 7.305676e-01 7.320401e-01

2626 9.942745e-01 9.913973e-01 9.942513e-01 9.936321e-01 9.914456e-01 7.288848e-01 7.169312e-01 7.274773e-01 7.309718e-01 7.323673e-01

2627 9.942785e-01 9.914053e-01 9.942539e-01 9.936332e-01 9.914447e-01 7.287521e-01 7.172477e-01 7.279013e-01 7.313853e-01 7.327012e-01

2628 9.942827e-01 9.914137e-01 9.942566e-01 9.936343e-01 9.914438e-01 7.286218e-01 7.175750e-01 7.283362e-01 7.318081e-01 7.330420e-01

2629 9.942871e-01 9.914225e-01 9.942595e-01 9.936355e-01 9.914427e-01 7.284940e-01 7.179132e-01 7.287822e-01 7.322404e-01 7.333896e-01

2630 9.942917e-01 9.914317e-01 9.942625e-01 9.936367e-01 9.914416e-01 7.283691e-01 7.182626e-01 7.292394e-01 7.326823e-01 7.337442e-01

2631 9.942965e-01 9.914414e-01 9.942656e-01 9.936380e-01 9.914405e-01 7.282472e-01 7.186233e-01 7.297078e-01 7.331338e-01 7.341058e-01

2632 9.943015e-01 9.914515e-01 9.942689e-01 9.936393e-01 9.914392e-01 7.281286e-01 7.189955e-01 7.301876e-01 7.335949e-01 7.344744e-01

2633 9.943068e-01 9.914620e-01 9.942723e-01 9.936407e-01 9.914379e-01 7.280134e-01 7.193792e-01 7.306788e-01 7.340658e-01 7.348500e-01

2634 9.943123e-01 9.914731e-01 9.942758e-01 9.936421e-01 9.914365e-01 7.279018e-01 7.197747e-01 7.311817e-01 7.345465e-01 7.352325e-01

2635 9.943181e-01 9.914847e-01 9.942796e-01 9.936436e-01 9.914349e-01 7.277942e-01 7.201821e-01 7.316961e-01 7.350371e-01 7.356221e-01

2636 9.943241e-01 9.914968e-01 9.942835e-01 9.936452e-01 9.914333e-01 7.276907e-01 7.206014e-01 7.322223e-01 7.355375e-01 7.360187e-01

2637 9.943304e-01 9.915095e-01 9.942875e-01 9.936468e-01 9.914316e-01 7.275915e-01 7.210329e-01 7.327603e-01 7.360477e-01 7.364223e-01

2638 9.943370e-01 9.915228e-01 9.942918e-01 9.936484e-01 9.914297e-01 7.274969e-01 7.214765e-01 7.333100e-01 7.365679e-01 7.368329e-01

2639 9.943440e-01 9.915367e-01 9.942963e-01 9.936501e-01 9.914277e-01 7.274070e-01 7.219323e-01 7.338716e-01 7.370979e-01 7.372504e-01

2640 9.943512e-01 9.915512e-01 9.943009e-01 9.936519e-01 9.914256e-01 7.273222e-01 7.224005e-01 7.344450e-01 7.376377e-01 7.376748e-01

2641 9.943588e-01 9.915665e-01 9.943058e-01 9.936537e-01 9.914233e-01 7.272425e-01 7.228810e-01 7.350303e-01 7.381874e-01 7.381060e-01

2642 9.943668e-01 9.915825e-01 9.943109e-01 9.936556e-01 9.914209e-01 7.271683e-01 7.233739e-01 7.356274e-01 7.387469e-01 7.385439e-01

2643 9.943751e-01 9.915992e-01 9.943162e-01 9.936576e-01 9.914183e-01 7.270998e-01 7.238792e-01 7.362363e-01 7.393160e-01 7.389886e-01

2644 9.943838e-01 9.916167e-01 9.943218e-01 9.936597e-01 9.914156e-01 7.270370e-01 7.243969e-01 7.368569e-01 7.398948e-01 7.394398e-01

2645 9.943930e-01 9.916351e-01 9.943277e-01 9.936618e-01 9.914126e-01 7.269804e-01 7.249270e-01 7.374892e-01 7.404832e-01 7.398976e-01

2646 9.944025e-01 9.916543e-01 9.943338e-01 9.936640e-01 9.914095e-01 7.269299e-01 7.254693e-01 7.381332e-01 7.410810e-01 7.403617e-01

2647 9.944126e-01 9.916745e-01 9.943402e-01 9.936663e-01 9.914061e-01 7.268859e-01 7.260240e-01 7.387886e-01 7.416881e-01 7.408321e-01

2648 9.944231e-01 9.916956e-01 9.943469e-01 9.936687e-01 9.914026e-01 7.268486e-01 7.265908e-01 7.394554e-01 7.423045e-01 7.413086e-01

2649 9.944341e-01 9.917177e-01 9.943539e-01 9.936711e-01 9.913987e-01 7.268180e-01 7.271697e-01 7.401335e-01 7.429299e-01 7.417910e-01

2650 9.944456e-01 9.917409e-01 9.943612e-01 9.936737e-01 9.913947e-01 7.267943e-01 7.277605e-01 7.408227e-01 7.435642e-01 7.422793e-01

2651 9.944577e-01 9.917652e-01 9.943689e-01 9.936763e-01 9.913903e-01 7.267778e-01 7.283632e-01 7.415227e-01 7.442072e-01 7.427733e-01

2652 9.944704e-01 9.917906e-01 9.943770e-01 9.936790e-01 9.913857e-01 7.267685e-01 7.289775e-01 7.422336e-01 7.448587e-01 7.432727e-01

2653 9.944837e-01 9.918173e-01 9.943854e-01 9.936819e-01 9.913807e-01 7.267665e-01 7.296032e-01 7.429549e-01 7.455185e-01 7.437774e-01

2654 9.944976e-01 9.918452e-01 9.943942e-01 9.936848e-01 9.913755e-01 7.267721e-01 7.302402e-01 7.436866e-01 7.461864e-01 7.442871e-01

2655 9.945122e-01 9.918745e-01 9.944034e-01 9.936878e-01 9.913698e-01 7.267852e-01 7.308882e-01 7.444283e-01 7.468621e-01 7.448017e-01

2656 9.945275e-01 9.919052e-01 9.944131e-01 9.936910e-01 9.913638e-01 7.268060e-01 7.315470e-01 7.451798e-01 7.475453e-01 7.453209e-01

2657 9.945436e-01 9.919374e-01 9.944232e-01 9.936942e-01 9.913574e-01 7.268346e-01 7.322163e-01 7.459407e-01 7.482359e-01 7.458445e-01

2658 9.945604e-01 9.919711e-01 9.944339e-01 9.936976e-01 9.913506e-01 7.268710e-01 7.328958e-01 7.467109e-01 7.489335e-01 7.463722e-01

2659 9.945780e-01 9.920064e-01 9.944450e-01 9.937010e-01 9.913433e-01 7.269153e-01 7.335852e-01 7.474899e-01 7.496377e-01 7.469038e-01

2660 9.945965e-01 9.920435e-01 9.944566e-01 9.937046e-01 9.913356e-01 7.269675e-01 7.342842e-01 7.482774e-01 7.503484e-01 7.474389e-01

2661 9.946159e-01 9.920823e-01 9.944688e-01 9.937084e-01 9.913273e-01 7.270276e-01 7.349924e-01 7.490731e-01 7.510650e-01 7.479773e-01

2662 9.946362e-01 9.921230e-01 9.944816e-01 9.937122e-01 9.913185e-01 7.270956e-01 7.357094e-01 7.498766e-01 7.517874e-01 7.485188e-01

2663 9.946576e-01 9.921656e-01 9.944950e-01 9.937162e-01 9.913091e-01 7.271714e-01 7.364349e-01 7.506875e-01 7.525151e-01 7.490629e-01

2664 9.946799e-01 9.922103e-01 9.945090e-01 9.937203e-01 9.912991e-01 7.272552e-01 7.371684e-01 7.515054e-01 7.532477e-01 7.496095e-01

2665 9.947034e-01 9.922571e-01 9.945237e-01 9.937245e-01 9.912885e-01 7.273467e-01 7.379095e-01 7.523298e-01 7.539849e-01 7.501581e-01

2666 9.947280e-01 9.923062e-01 9.945391e-01 9.937289e-01 9.912771e-01 7.274460e-01 7.386579e-01 7.531603e-01 7.547262e-01 7.507085e-01

2667 9.947538e-01 9.923577e-01 9.945552e-01 9.937334e-01 9.912650e-01 7.275530e-01 7.394129e-01 7.539966e-01 7.554713e-01 7.512603e-01

2668 9.947809e-01 9.924116e-01 9.945721e-01 9.937381e-01 9.912522e-01 7.276674e-01 7.401743e-01 7.548380e-01 7.562198e-01 7.518132e-01

2669 9.948093e-01 9.924681e-01 9.945898e-01 9.937429e-01 9.912385e-01 7.277894e-01 7.409414e-01 7.556841e-01 7.569711e-01 7.523668e-01

2670 9.948391e-01 9.925274e-01 9.946084e-01 9.937478e-01 9.912239e-01 7.279186e-01 7.417138e-01 7.565345e-01 7.577249e-01 7.529209e-01

2671 9.948703e-01 9.925894e-01 9.946278e-01 9.937530e-01 9.912083e-01 7.280550e-01 7.424910e-01 7.573887e-01 7.584808e-01 7.534750e-01

2672 9.949031e-01 9.926545e-01 9.946482e-01 9.937582e-01 9.911918e-01 7.281984e-01 7.432725e-01 7.582461e-01 7.592382e-01 7.540288e-01

2673 9.949375e-01 9.927226e-01 9.946695e-01 9.937636e-01 9.911742e-01 7.283486e-01 7.440577e-01 7.591062e-01 7.599968e-01 7.545820e-01

2674 9.949735e-01 9.927940e-01 9.946919e-01 9.937692e-01 9.911555e-01 7.285054e-01 7.448462e-01 7.599686e-01 7.607560e-01 7.551342e-01

2675 9.950113e-01 9.928688e-01 9.947153e-01 9.937749e-01 9.911356e-01 7.286686e-01 7.456375e-01 7.608326e-01 7.615155e-01 7.556851e-01

2676 9.950510e-01 9.929472e-01 9.947399e-01 9.937808e-01 9.911144e-01 7.288381e-01 7.464309e-01 7.616978e-01 7.622747e-01 7.562343e-01

2677 9.950926e-01 9.930293e-01 9.947656e-01 9.937869e-01 9.910919e-01 7.290135e-01 7.472260e-01 7.625637e-01 7.630332e-01 7.567815e-01

2678 9.951363e-01 9.931153e-01 9.947926e-01 9.937931e-01 9.910680e-01 7.291948e-01 7.480222e-01 7.634297e-01 7.637905e-01 7.573263e-01

2679 9.951820e-01 9.932053e-01 9.948208e-01 9.937995e-01 9.910425e-01 7.293815e-01 7.488190e-01 7.642952e-01 7.645462e-01 7.578684e-01

2680 9.952300e-01 9.932996e-01 9.948504e-01 9.938060e-01 9.910155e-01 7.295735e-01 7.496159e-01 7.651599e-01 7.652999e-01 7.584075e-01

2681 9.952804e-01 9.933984e-01 9.948814e-01 9.938127e-01 9.909868e-01 7.297705e-01 7.504123e-01 7.660230e-01 7.660510e-01 7.589432e-01

2682 9.953331e-01 9.935017e-01 9.949138e-01 9.938195e-01 9.909563e-01 7.299722e-01 7.512077e-01 7.668842e-01 7.667991e-01 7.594752e-01

2683 9.953885e-01 9.936099e-01 9.949478e-01 9.938265e-01 9.909239e-01 7.301785e-01 7.520017e-01 7.677430e-01 7.675438e-01 7.600032e-01

2684 9.954465e-01 9.937232e-01 9.949834e-01 9.938336e-01 9.908895e-01 7.303889e-01 7.527937e-01 7.685987e-01 7.682847e-01 7.605269e-01

2685 9.955074e-01 9.938417e-01 9.950206e-01 9.938409e-01 9.908531e-01 7.306033e-01 7.535832e-01 7.694509e-01 7.690213e-01 7.610461e-01

2686 9.955711e-01 9.939658e-01 9.950596e-01 9.938483e-01 9.908144e-01 7.308213e-01 7.543697e-01 7.702992e-01 7.697533e-01 7.615603e-01

2687 9.956380e-01 9.940955e-01 9.951004e-01 9.938559e-01 9.907734e-01 7.310427e-01 7.551528e-01 7.711430e-01 7.704801e-01 7.620694e-01

2688 9.957081e-01 9.942312e-01 9.951432e-01 9.938635e-01 9.907299e-01 7.312672e-01 7.559320e-01 7.719819e-01 7.712015e-01 7.625731e-01

2689 9.957815e-01 9.943732e-01 9.951879e-01 9.938713e-01 9.906838e-01 7.314945e-01 7.567068e-01 7.728154e-01 7.719170e-01 7.630711e-01

2690 9.958584e-01 9.945216e-01 9.952347e-01 9.938792e-01 9.906350e-01 7.317244e-01 7.574769e-01 7.736431e-01 7.726263e-01 7.635633e-01

2691 9.959390e-01 9.946768e-01 9.952836e-01 9.938871e-01 9.905832e-01 7.319565e-01 7.582417e-01 7.744646e-01 7.733290e-01 7.640493e-01

2692 9.960234e-01 9.948390e-01 9.953348e-01 9.938952e-01 9.905285e-01 7.321907e-01 7.590009e-01 7.752794e-01 7.740249e-01 7.645289e-01

2693 9.961118e-01 9.950085e-01 9.953883e-01 9.939033e-01 9.904706e-01 7.324266e-01 7.597541e-01 7.760872e-01 7.747134e-01 7.650020e-01

2694 9.962044e-01 9.951856e-01 9.954443e-01 9.939114e-01 9.904093e-01 7.326640e-01 7.605010e-01 7.768875e-01 7.753945e-01 7.654684e-01

2695 9.963014e-01 9.953707e-01 9.955028e-01 9.939196e-01 9.903446e-01 7.329026e-01 7.612411e-01 7.776800e-01 7.760676e-01 7.659278e-01

2696 9.964029e-01 9.955641e-01 9.955640e-01 9.939278e-01 9.902761e-01 7.331422e-01 7.619742e-01 7.784644e-01 7.767327e-01 7.663802e-01

2697 9.965091e-01 9.957661e-01 9.956278e-01 9.939359e-01 9.902038e-01 7.333826e-01 7.626999e-01 7.792403e-01 7.773894e-01 7.668254e-01

2698 9.966202e-01 9.959771e-01 9.956945e-01 9.939440e-01 9.901275e-01 7.336235e-01 7.634180e-01 7.800075e-01 7.780375e-01 7.672632e-01

2699 9.967364e-01 9.961975e-01 9.957642e-01 9.939521e-01 9.900470e-01 7.338646e-01 7.641280e-01 7.807655e-01 7.786767e-01 7.676935e-01

2700 9.968580e-01 9.964276e-01 9.958369e-01 9.939600e-01 9.899621e-01 7.341058e-01 7.648299e-01 7.815143e-01 7.793069e-01 7.681163e-01

2701 9.969852e-01 9.966678e-01 9.959128e-01 9.939678e-01 9.898725e-01 7.343469e-01 7.655233e-01 7.822534e-01 7.799278e-01 7.685314e-01

2702 9.969852e-01 9.966679e-01 9.959129e-01 9.939678e-01 9.898725e-01 7.343502e-01 7.655537e-01 7.822821e-01 7.799504e-01 7.685438e-01

2703 9.969852e-01 9.966680e-01 9.959129e-01 9.939677e-01 9.898724e-01 7.343539e-01 7.655848e-01 7.823114e-01 7.799735e-01 7.685564e-01

2704 9.969853e-01 9.966680e-01 9.959129e-01 9.939677e-01 9.898723e-01 7.343581e-01 7.656168e-01 7.823413e-01 7.799971e-01 7.685692e-01

2705 9.969853e-01 9.966681e-01 9.959130e-01 9.939677e-01 9.898723e-01 7.343626e-01 7.656496e-01 7.823720e-01 7.800211e-01 7.685823e-01

2706 9.969854e-01 9.966681e-01 9.959130e-01 9.939677e-01 9.898722e-01 7.343676e-01 7.656832e-01 7.824033e-01 7.800457e-01 7.685955e-01

2707 9.969854e-01 9.966682e-01 9.959130e-01 9.939677e-01 9.898722e-01 7.343730e-01 7.657177e-01 7.824353e-01 7.800707e-01 7.686089e-01

2708 9.969854e-01 9.966682e-01 9.959130e-01 9.939677e-01 9.898721e-01 7.343789e-01 7.657531e-01 7.824680e-01 7.800961e-01 7.686224e-01

2709 9.969855e-01 9.966683e-01 9.959131e-01 9.939676e-01 9.898721e-01 7.343853e-01 7.657894e-01 7.825014e-01 7.801221e-01 7.686362e-01

2710 9.969855e-01 9.966684e-01 9.959131e-01 9.939676e-01 9.898720e-01 7.343922e-01 7.658266e-01 7.825355e-01 7.801486e-01 7.686502e-01

2711 9.969856e-01 9.966684e-01 9.959131e-01 9.939676e-01 9.898720e-01 7.343997e-01 7.658648e-01 7.825704e-01 7.801756e-01 7.686643e-01

2712 9.969856e-01 9.966685e-01 9.959131e-01 9.939676e-01 9.898719e-01 7.344077e-01 7.659039e-01 7.826061e-01 7.802031e-01 7.686787e-01

2713 9.969857e-01 9.966686e-01 9.959131e-01 9.939675e-01 9.898718e-01 7.344162e-01 7.659440e-01 7.826425e-01 7.802312e-01 7.686932e-01

2714 9.969857e-01 9.966686e-01 9.959131e-01 9.939675e-01 9.898718e-01 7.344254e-01 7.659851e-01 7.826796e-01 7.802598e-01 7.687080e-01

2715 9.969857e-01 9.966687e-01 9.959132e-01 9.939675e-01 9.898717e-01 7.344352e-01 7.660273e-01 7.827176e-01 7.802889e-01 7.687229e-01

2716 9.969858e-01 9.966688e-01 9.959132e-01 9.939674e-01 9.898717e-01 7.344456e-01 7.660705e-01 7.827565e-01 7.803186e-01 7.687381e-01

2717 9.969858e-01 9.966689e-01 9.959132e-01 9.939674e-01 9.898716e-01 7.344567e-01 7.661148e-01 7.827961e-01 7.803488e-01 7.687534e-01

2718 9.969859e-01 9.966690e-01 9.959132e-01 9.939674e-01 9.898715e-01 7.344685e-01 7.661602e-01 7.828366e-01 7.803797e-01 7.687689e-01

2719 9.969859e-01 9.966691e-01 9.959132e-01 9.939673e-01 9.898714e-01 7.344811e-01 7.662067e-01 7.828780e-01 7.804111e-01 7.687847e-01

2720 9.969860e-01 9.966692e-01 9.959132e-01 9.939673e-01 9.898714e-01 7.344943e-01 7.662544e-01 7.829203e-01 7.804431e-01 7.688006e-01

2721 9.969860e-01 9.966692e-01 9.959132e-01 9.939673e-01 9.898713e-01 7.345084e-01 7.663033e-01 7.829634e-01 7.804758e-01 7.688167e-01

2722 9.969861e-01 9.966693e-01 9.959132e-01 9.939672e-01 9.898712e-01 7.345233e-01 7.663535e-01 7.830076e-01 7.805090e-01 7.688331e-01

2723 9.969861e-01 9.966694e-01 9.959132e-01 9.939672e-01 9.898711e-01 7.345390e-01 7.664049e-01 7.830526e-01 7.805429e-01 7.688496e-01

2724 9.969862e-01 9.966696e-01 9.959132e-01 9.939672e-01 9.898709e-01 7.345556e-01 7.664575e-01 7.830987e-01 7.805774e-01 7.688664e-01

2725 9.969862e-01 9.966697e-01 9.959132e-01 9.939671e-01 9.898708e-01 7.345732e-01 7.665116e-01 7.831457e-01 7.806127e-01 7.688834e-01

2726 9.969863e-01 9.966698e-01 9.959132e-01 9.939671e-01 9.898707e-01 7.345916e-01 7.665669e-01 7.831938e-01 7.806485e-01 7.689005e-01

2727 9.969863e-01 9.966699e-01 9.959133e-01 9.939670e-01 9.898705e-01 7.346111e-01 7.666237e-01 7.832429e-01 7.806851e-01 7.689179e-01

2728 9.969864e-01 9.966700e-01 9.959133e-01 9.939670e-01 9.898704e-01 7.346316e-01 7.666819e-01 7.832930e-01 7.807223e-01 7.689355e-01

2729 9.969864e-01 9.966701e-01 9.959133e-01 9.939669e-01 9.898702e-01 7.346531e-01 7.667415e-01 7.833443e-01 7.807603e-01 7.689533e-01

2730 9.969865e-01 9.966703e-01 9.959133e-01 9.939669e-01 9.898700e-01 7.346758e-01 7.668027e-01 7.833967e-01 7.807990e-01 7.689713e-01

2731 9.969865e-01 9.966704e-01 9.959133e-01 9.939668e-01 9.898699e-01 7.346996e-01 7.668654e-01 7.834502e-01 7.808385e-01 7.689895e-01

2732 9.969866e-01 9.966705e-01 9.959133e-01 9.939667e-01 9.898697e-01 7.347246e-01 7.669297e-01 7.835048e-01 7.808787e-01 7.690080e-01

2733 9.969867e-01 9.966707e-01 9.959133e-01 9.939667e-01 9.898694e-01 7.347508e-01 7.669956e-01 7.835607e-01 7.809196e-01 7.690266e-01

2734 9.969867e-01 9.966708e-01 9.959133e-01 9.939666e-01 9.898692e-01 7.347783e-01 7.670632e-01 7.836178e-01 7.809614e-01 7.690455e-01

2735 9.969868e-01 9.966710e-01 9.959133e-01 9.939665e-01 9.898690e-01 7.348071e-01 7.671325e-01 7.836761e-01 7.810040e-01 7.690646e-01

2736 9.969869e-01 9.966712e-01 9.959133e-01 9.939664e-01 9.898688e-01 7.348373e-01 7.672035e-01 7.837358e-01 7.810474e-01 7.690839e-01

2737 9.969869e-01 9.966713e-01 9.959133e-01 9.939663e-01 9.898685e-01 7.348689e-01 7.672764e-01 7.837967e-01 7.810916e-01 7.691034e-01

2738 9.969870e-01 9.966715e-01 9.959133e-01 9.939662e-01 9.898682e-01 7.349021e-01 7.673511e-01 7.838590e-01 7.811367e-01 7.691231e-01

2739 9.969871e-01 9.966717e-01 9.959133e-01 9.939661e-01 9.898680e-01 7.349367e-01 7.674277e-01 7.839226e-01 7.811826e-01 7.691430e-01

2740 9.969872e-01 9.966719e-01 9.959133e-01 9.939660e-01 9.898677e-01 7.349730e-01 7.675062e-01 7.839876e-01 7.812294e-01 7.691631e-01

2741 9.969873e-01 9.966721e-01 9.959133e-01 9.939658e-01 9.898674e-01 7.350109e-01 7.675868e-01 7.840541e-01 7.812771e-01 7.691835e-01

2742 9.969873e-01 9.966723e-01 9.959133e-01 9.939657e-01 9.898670e-01 7.350505e-01 7.676694e-01 7.841220e-01 7.813258e-01 7.692040e-01

2743 9.969874e-01 9.966725e-01 9.959133e-01 9.939656e-01 9.898667e-01 7.350919e-01 7.677541e-01 7.841915e-01 7.813754e-01 7.692248e-01

2744 9.969875e-01 9.966727e-01 9.959133e-01 9.939654e-01 9.898663e-01 7.351351e-01 7.678409e-01 7.842624e-01 7.814259e-01 7.692458e-01

2745 9.969876e-01 9.966729e-01 9.959133e-01 9.939653e-01 9.898660e-01 7.351802e-01 7.679300e-01 7.843350e-01 7.814775e-01 7.692669e-01

2746 9.969877e-01 9.966731e-01 9.959133e-01 9.939651e-01 9.898656e-01 7.352273e-01 7.680214e-01 7.844091e-01 7.815300e-01 7.692883e-01

2747 9.969878e-01 9.966734e-01 9.959133e-01 9.939649e-01 9.898652e-01 7.352765e-01 7.681151e-01 7.844849e-01 7.815835e-01 7.693099e-01

2748 9.969879e-01 9.966736e-01 9.959133e-01 9.939647e-01 9.898647e-01 7.353278e-01 7.682111e-01 7.845624e-01 7.816381e-01 7.693317e-01

2749 9.969880e-01 9.966739e-01 9.959133e-01 9.939645e-01 9.898643e-01 7.353812e-01 7.683097e-01 7.846416e-01 7.816937e-01 7.693537e-01

2750 9.969881e-01 9.966741e-01 9.959132e-01 9.939643e-01 9.898638e-01 7.354370e-01 7.684107e-01 7.847225e-01 7.817504e-01 7.693758e-01

2751 9.969882e-01 9.966744e-01 9.959132e-01 9.939641e-01 9.898633e-01 7.354950e-01 7.685144e-01 7.848053e-01 7.818082e-01 7.693982e-01

2752 9.969883e-01 9.966747e-01 9.959132e-01 9.939639e-01 9.898628e-01 7.355556e-01 7.686206e-01 7.848899e-01 7.818671e-01 7.694207e-01

2753 9.969884e-01 9.966750e-01 9.959132e-01 9.939636e-01 9.898623e-01 7.356186e-01 7.687297e-01 7.849764e-01 7.819272e-01 7.694434e-01

2754 9.969886e-01 9.966753e-01 9.959131e-01 9.939634e-01 9.898617e-01 7.356843e-01 7.688415e-01 7.850648e-01 7.819884e-01 7.694663e-01

2755 9.969887e-01 9.966756e-01 9.959131e-01 9.939631e-01 9.898611e-01 7.357526e-01 7.689561e-01 7.851551e-01 7.820508e-01 7.694894e-01

2756 9.969888e-01 9.966760e-01 9.959131e-01 9.939628e-01 9.898605e-01 7.358237e-01 7.690737e-01 7.852475e-01 7.821144e-01 7.695127e-01

2757 9.969889e-01 9.966763e-01 9.959130e-01 9.939625e-01 9.898598e-01 7.358978e-01 7.691944e-01 7.853420e-01 7.821792e-01 7.695361e-01

2758 9.969891e-01 9.966767e-01 9.959130e-01 9.939622e-01 9.898591e-01 7.359748e-01 7.693181e-01 7.854386e-01 7.822453e-01 7.695596e-01

2759 9.969892e-01 9.966771e-01 9.959129e-01 9.939619e-01 9.898584e-01 7.360549e-01 7.694450e-01 7.855373e-01 7.823127e-01 7.695833e-01

2760 9.969894e-01 9.966774e-01 9.959129e-01 9.939616e-01 9.898576e-01 7.361381e-01 7.695751e-01 7.856383e-01 7.823814e-01 7.696072e-01

2761 9.969895e-01 9.966778e-01 9.959128e-01 9.939612e-01 9.898568e-01 7.362247e-01 7.697086e-01 7.857415e-01 7.824513e-01 7.696312e-01

2762 9.969897e-01 9.966783e-01 9.959128e-01 9.939608e-01 9.898559e-01 7.363147e-01 7.698455e-01 7.858470e-01 7.825227e-01 7.696553e-01

2763 9.969898e-01 9.966787e-01 9.959127e-01 9.939604e-01 9.898550e-01 7.364082e-01 7.699859e-01 7.859549e-01 7.825954e-01 7.696795e-01

2764 9.969900e-01 9.966792e-01 9.959126e-01 9.939600e-01 9.898541e-01 7.365054e-01 7.701299e-01 7.860651e-01 7.826695e-01 7.697039e-01

2765 9.969901e-01 9.966796e-01 9.959125e-01 9.939595e-01 9.898531e-01 7.366063e-01 7.702775e-01 7.861779e-01 7.827450e-01 7.697284e-01

2766 9.969903e-01 9.966801e-01 9.959125e-01 9.939591e-01 9.898520e-01 7.367110e-01 7.704290e-01 7.862932e-01 7.828220e-01 7.697529e-01

2767 9.969905e-01 9.966806e-01 9.959124e-01 9.939586e-01 9.898510e-01 7.368198e-01 7.705843e-01 7.864111e-01 7.829004e-01 7.697775e-01

2768 9.969907e-01 9.966811e-01 9.959123e-01 9.939581e-01 9.898498e-01 7.369327e-01 7.707436e-01 7.865316e-01 7.829804e-01 7.698022e-01

2769 9.969909e-01 9.966817e-01 9.959122e-01 9.939575e-01 9.898486e-01 7.370499e-01 7.709070e-01 7.866548e-01 7.830618e-01 7.698270e-01

2770 9.969911e-01 9.966823e-01 9.959121e-01 9.939569e-01 9.898473e-01 7.371715e-01 7.710745e-01 7.867807e-01 7.831449e-01 7.698518e-01

2771 9.969913e-01 9.966829e-01 9.959120e-01 9.939563e-01 9.898460e-01 7.372976e-01 7.712463e-01 7.869095e-01 7.832295e-01 7.698767e-01

2772 9.969915e-01 9.966835e-01 9.959118e-01 9.939557e-01 9.898446e-01 7.374284e-01 7.714225e-01 7.870412e-01 7.833157e-01 7.699015e-01

2773 9.969917e-01 9.966841e-01 9.959117e-01 9.939550e-01 9.898431e-01 7.375641e-01 7.716032e-01 7.871757e-01 7.834035e-01 7.699264e-01

2774 9.969919e-01 9.966848e-01 9.959116e-01 9.939543e-01 9.898416e-01 7.377047e-01 7.717884e-01 7.873133e-01 7.834930e-01 7.699513e-01

2775 9.969921e-01 9.966855e-01 9.959114e-01 9.939536e-01 9.898400e-01 7.378504e-01 7.719784e-01 7.874540e-01 7.835842e-01 7.699761e-01

2776 9.969924e-01 9.966862e-01 9.959113e-01 9.939528e-01 9.898382e-01 7.380015e-01 7.721732e-01 7.875978e-01 7.836772e-01 7.700010e-01

2777 9.969926e-01 9.966869e-01 9.959111e-01 9.939519e-01 9.898365e-01 7.381579e-01 7.723729e-01 7.877448e-01 7.837718e-01 7.700257e-01

2778 9.969929e-01 9.966877e-01 9.959109e-01 9.939511e-01 9.898346e-01 7.383200e-01 7.725776e-01 7.878951e-01 7.838683e-01 7.700504e-01

2779 9.969932e-01 9.966885e-01 9.959107e-01 9.939502e-01 9.898326e-01 7.384878e-01 7.727875e-01 7.880487e-01 7.839665e-01 7.700750e-01

2780 9.969934e-01 9.966893e-01 9.959105e-01 9.939492e-01 9.898305e-01 7.386616e-01 7.730027e-01 7.882057e-01 7.840666e-01 7.700995e-01

2781 9.969937e-01 9.966902e-01 9.959103e-01 9.939482e-01 9.898283e-01 7.388415e-01 7.732233e-01 7.883662e-01 7.841685e-01 7.701238e-01

2782 9.969940e-01 9.966911e-01 9.959101e-01 9.939471e-01 9.898260e-01 7.390277e-01 7.734494e-01 7.885303e-01 7.842723e-01 7.701480e-01

2783 9.969943e-01 9.966921e-01 9.959098e-01 9.939460e-01 9.898236e-01 7.392203e-01 7.736812e-01 7.886979e-01 7.843780e-01 7.701720e-01

2784 9.969946e-01 9.966931e-01 9.959096e-01 9.939448e-01 9.898210e-01 7.394196e-01 7.739187e-01 7.888693e-01 7.844857e-01 7.701958e-01

2785 9.969949e-01 9.966941e-01 9.959093e-01 9.939436e-01 9.898183e-01 7.396256e-01 7.741622e-01 7.890444e-01 7.845953e-01 7.702194e-01

2786 9.969953e-01 9.966951e-01 9.959090e-01 9.939422e-01 9.898155e-01 7.398387e-01 7.744117e-01 7.892234e-01 7.847069e-01 7.702428e-01

2787 9.969956e-01 9.966963e-01 9.959087e-01 9.939409e-01 9.898125e-01 7.400590e-01 7.746673e-01 7.894062e-01 7.848206e-01 7.702659e-01

2788 9.969960e-01 9.966974e-01 9.959084e-01 9.939394e-01 9.898094e-01 7.402867e-01 7.749293e-01 7.895931e-01 7.849363e-01 7.702886e-01

2789 9.969964e-01 9.966986e-01 9.959080e-01 9.939379e-01 9.898061e-01 7.405220e-01 7.751977e-01 7.897840e-01 7.850540e-01 7.703111e-01

2790 9.969967e-01 9.966999e-01 9.959076e-01 9.939363e-01 9.898027e-01 7.407651e-01 7.754727e-01 7.899791e-01 7.851739e-01 7.703332e-01

2791 9.969971e-01 9.967012e-01 9.959072e-01 9.939346e-01 9.897990e-01 7.410161e-01 7.757544e-01 7.901783e-01 7.852959e-01 7.703549e-01

2792 9.969975e-01 9.967025e-01 9.959068e-01 9.939328e-01 9.897952e-01 7.412753e-01 7.760429e-01 7.903819e-01 7.854200e-01 7.703761e-01

2793 9.969980e-01 9.967039e-01 9.959064e-01 9.939309e-01 9.897912e-01 7.415428e-01 7.763384e-01 7.905899e-01 7.855464e-01 7.703970e-01

2794 9.969984e-01 9.967054e-01 9.959059e-01 9.939289e-01 9.897869e-01 7.418190e-01 7.766411e-01 7.908022e-01 7.856749e-01 7.704173e-01

2795 9.969989e-01 9.967069e-01 9.959054e-01 9.939268e-01 9.897824e-01 7.421039e-01 7.769510e-01 7.910192e-01 7.858056e-01 7.704372e-01

2796 9.969993e-01 9.967085e-01 9.959049e-01 9.939246e-01 9.897777e-01 7.423978e-01 7.772683e-01 7.912407e-01 7.859386e-01 7.704564e-01

2797 9.969998e-01 9.967102e-01 9.959043e-01 9.939223e-01 9.897728e-01 7.427009e-01 7.775931e-01 7.914668e-01 7.860739e-01 7.704751e-01

2798 9.970003e-01 9.967119e-01 9.959037e-01 9.939198e-01 9.897675e-01 7.430133e-01 7.779257e-01 7.916978e-01 7.862114e-01 7.704932e-01

2799 9.970009e-01 9.967137e-01 9.959031e-01 9.939173e-01 9.897620e-01 7.433354e-01 7.782661e-01 7.919335e-01 7.863512e-01 7.705106e-01

2800 9.970014e-01 9.967156e-01 9.959024e-01 9.939145e-01 9.897562e-01 7.436673e-01 7.786144e-01 7.921742e-01 7.864934e-01 7.705273e-01

2801 9.970020e-01 9.967175e-01 9.959017e-01 9.939117e-01 9.897501e-01 7.440091e-01 7.789708e-01 7.924199e-01 7.866379e-01 7.705433e-01

2802 9.970025e-01 9.967195e-01 9.959010e-01 9.939087e-01 9.897437e-01 7.443612e-01 7.793355e-01 7.926706e-01 7.867848e-01 7.705584e-01

2803 9.970031e-01 9.967217e-01 9.959002e-01 9.939055e-01 9.897369e-01 7.447236e-01 7.797086e-01 7.929264e-01 7.869340e-01 7.705728e-01

2804 9.970038e-01 9.967239e-01 9.958994e-01 9.939021e-01 9.897298e-01 7.450966e-01 7.800902e-01 7.931874e-01 7.870856e-01 7.705863e-01

2805 9.970044e-01 9.967262e-01 9.958985e-01 9.938986e-01 9.897222e-01 7.454805e-01 7.804804e-01 7.934537e-01 7.872396e-01 7.705988e-01

2806 9.970051e-01 9.967286e-01 9.958975e-01 9.938948e-01 9.897143e-01 7.458753e-01 7.808794e-01 7.937253e-01 7.873960e-01 7.706104e-01

2807 9.970058e-01 9.967311e-01 9.958965e-01 9.938909e-01 9.897059e-01 7.462813e-01 7.812874e-01 7.940023e-01 7.875548e-01 7.706210e-01

2808 9.970065e-01 9.967337e-01 9.958955e-01 9.938868e-01 9.896971e-01 7.466986e-01 7.817043e-01 7.942848e-01 7.877161e-01 7.706306e-01

2809 9.970072e-01 9.967364e-01 9.958943e-01 9.938824e-01 9.896878e-01 7.471274e-01 7.821305e-01 7.945728e-01 7.878797e-01 7.706390e-01

2810 9.970080e-01 9.967392e-01 9.958932e-01 9.938778e-01 9.896779e-01 7.475680e-01 7.825659e-01 7.948664e-01 7.880458e-01 7.706463e-01

2811 9.970088e-01 9.967422e-01 9.958919e-01 9.938729e-01 9.896676e-01 7.480204e-01 7.830108e-01 7.951656e-01 7.882143e-01 7.706525e-01

2812 9.970096e-01 9.967453e-01 9.958906e-01 9.938678e-01 9.896567e-01 7.484849e-01 7.834652e-01 7.954706e-01 7.883852e-01 7.706573e-01

2813 9.970104e-01 9.967485e-01 9.958892e-01 9.938623e-01 9.896451e-01 7.489616e-01 7.839292e-01 7.957813e-01 7.885585e-01 7.706609e-01

2814 9.970113e-01 9.967518e-01 9.958877e-01 9.938566e-01 9.896330e-01 7.494506e-01 7.844030e-01 7.960978e-01 7.887342e-01 7.706632e-01

2815 9.970122e-01 9.967553e-01 9.958861e-01 9.938506e-01 9.896202e-01 7.499521e-01 7.848866e-01 7.964201e-01 7.889123e-01 7.706640e-01

2816 9.970132e-01 9.967589e-01 9.958844e-01 9.938442e-01 9.896066e-01 7.504661e-01 7.853802e-01 7.967484e-01 7.890928e-01 7.706634e-01

2817 9.970141e-01 9.967627e-01 9.958826e-01 9.938375e-01 9.895924e-01 7.509930e-01 7.858838e-01 7.970825e-01 7.892757e-01 7.706613e-01

2818 9.970152e-01 9.967667e-01 9.958807e-01 9.938304e-01 9.895773e-01 7.515326e-01 7.863975e-01 7.974227e-01 7.894609e-01 7.706577e-01

2819 9.970162e-01 9.967708e-01 9.958788e-01 9.938229e-01 9.895614e-01 7.520852e-01 7.869214e-01 7.977688e-01 7.896484e-01 7.706525e-01

2820 9.970173e-01 9.967752e-01 9.958766e-01 9.938150e-01 9.895446e-01 7.526509e-01 7.874557e-01 7.981209e-01 7.898382e-01 7.706456e-01

2821 9.970184e-01 9.967797e-01 9.958744e-01 9.938066e-01 9.895269e-01 7.532296e-01 7.880002e-01 7.984791e-01 7.900302e-01 7.706371e-01

2822 9.970196e-01 9.967843e-01 9.958720e-01 9.937978e-01 9.895082e-01 7.538215e-01 7.885552e-01 7.988433e-01 7.902245e-01 7.706268e-01

2823 9.970208e-01 9.967893e-01 9.958695e-01 9.937885e-01 9.894884e-01 7.544267e-01 7.891206e-01 7.992136e-01 7.904210e-01 7.706147e-01

2824 9.970220e-01 9.967944e-01 9.958669e-01 9.937786e-01 9.894676e-01 7.550451e-01 7.896965e-01 7.995900e-01 7.906197e-01 7.706007e-01

2825 9.970233e-01 9.967997e-01 9.958641e-01 9.937682e-01 9.894455e-01 7.556768e-01 7.902829e-01 7.999724e-01 7.908204e-01 7.705849e-01

2826 9.970246e-01 9.968053e-01 9.958611e-01 9.937573e-01 9.894223e-01 7.563218e-01 7.908799e-01 8.003609e-01 7.910233e-01 7.705672e-01

2827 9.970260e-01 9.968111e-01 9.958579e-01 9.937457e-01 9.893977e-01 7.569801e-01 7.914874e-01 8.007554e-01 7.912281e-01 7.705475e-01

2828 9.970274e-01 9.968172e-01 9.958546e-01 9.937334e-01 9.893718e-01 7.576517e-01 7.921055e-01 8.011559e-01 7.914349e-01 7.705258e-01

2829 9.970289e-01 9.968235e-01 9.958510e-01 9.937205e-01 9.893444e-01 7.583365e-01 7.927342e-01 8.015625e-01 7.916436e-01 7.705020e-01

2830 9.970304e-01 9.968301e-01 9.958473e-01 9.937068e-01 9.893155e-01 7.590345e-01 7.933734e-01 8.019749e-01 7.918541e-01 7.704761e-01

2831 9.970320e-01 9.968371e-01 9.958433e-01 9.936924e-01 9.892850e-01 7.597455e-01 7.940231e-01 8.023933e-01 7.920664e-01 7.704481e-01

2832 9.970336e-01 9.968443e-01 9.958391e-01 9.936771e-01 9.892527e-01 7.604696e-01 7.946832e-01 8.028176e-01 7.922804e-01 7.704179e-01

2833 9.970353e-01 9.968518e-01 9.958346e-01 9.936610e-01 9.892186e-01 7.612066e-01 7.953538e-01 8.032476e-01 7.924960e-01 7.703855e-01

2834 9.970371e-01 9.968597e-01 9.958299e-01 9.936439e-01 9.891826e-01 7.619563e-01 7.960347e-01 8.036834e-01 7.927131e-01 7.703509e-01

2835 9.970389e-01 9.968679e-01 9.958249e-01 9.936259e-01 9.891446e-01 7.627187e-01 7.967259e-01 8.041248e-01 7.929318e-01 7.703140e-01

2836 9.970407e-01 9.968765e-01 9.958196e-01 9.936069e-01 9.891044e-01 7.634935e-01 7.974273e-01 8.045718e-01 7.931517e-01 7.702749e-01

2837 9.970427e-01 9.968855e-01 9.958140e-01 9.935867e-01 9.890620e-01 7.642806e-01 7.981387e-01 8.050243e-01 7.933730e-01 7.702334e-01

2838 9.970447e-01 9.968949e-01 9.958080e-01 9.935655e-01 9.890172e-01 7.650797e-01 7.988601e-01 8.054821e-01 7.935954e-01 7.701896e-01

2839 9.970467e-01 9.969047e-01 9.958017e-01 9.935430e-01 9.889698e-01 7.658907e-01 7.995912e-01 8.059452e-01 7.938190e-01 7.701435e-01

2840 9.970488e-01 9.969149e-01 9.957950e-01 9.935192e-01 9.889198e-01 7.667133e-01 8.003321e-01 8.064135e-01 7.940435e-01 7.700950e-01

2841 9.970511e-01 9.969256e-01 9.957880e-01 9.934941e-01 9.888670e-01 7.675471e-01 8.010824e-01 8.068868e-01 7.942689e-01 7.700442e-01

2842 9.970533e-01 9.969368e-01 9.957805e-01 9.934675e-01 9.888112e-01 7.683921e-01 8.018421e-01 8.073649e-01 7.944950e-01 7.699910e-01

2843 9.970557e-01 9.969485e-01 9.957725e-01 9.934395e-01 9.887523e-01 7.692477e-01 8.026109e-01 8.078478e-01 7.947218e-01 7.699354e-01

2844 9.970581e-01 9.969607e-01 9.957641e-01 9.934098e-01 9.886901e-01 7.701138e-01 8.033886e-01 8.083353e-01 7.949492e-01 7.698775e-01

2845 9.970606e-01 9.969734e-01 9.957552e-01 9.933784e-01 9.886244e-01 7.709900e-01 8.041751e-01 8.088272e-01 7.951770e-01 7.698172e-01

2846 9.970632e-01 9.969868e-01 9.957458e-01 9.933453e-01 9.885551e-01 7.718759e-01 8.049700e-01 8.093233e-01 7.954050e-01 7.697546e-01

2847 9.970659e-01 9.970007e-01 9.957357e-01 9.933102e-01 9.884819e-01 7.727711e-01 8.057731e-01 8.098235e-01 7.956333e-01 7.696897e-01

2848 9.970687e-01 9.970153e-01 9.957252e-01 9.932732e-01 9.884046e-01 7.736753e-01 8.065842e-01 8.103275e-01 7.958616e-01 7.696224e-01

2849 9.970715e-01 9.970305e-01 9.957139e-01 9.932340e-01 9.883231e-01 7.745880e-01 8.074030e-01 8.108353e-01 7.960898e-01 7.695529e-01

2850 9.970745e-01 9.970464e-01 9.957020e-01 9.931926e-01 9.882370e-01 7.755088e-01 8.082292e-01 8.113464e-01 7.963178e-01 7.694811e-01

2851 9.970775e-01 9.970631e-01 9.956894e-01 9.931489e-01 9.881462e-01 7.764373e-01 8.090625e-01 8.118608e-01 7.965454e-01 7.694072e-01

2852 9.970807e-01 9.970805e-01 9.956761e-01 9.931026e-01 9.880504e-01 7.773731e-01 8.099025e-01 8.123782e-01 7.967726e-01 7.693310e-01

2853 9.970839e-01 9.970987e-01 9.956620e-01 9.930538e-01 9.879494e-01 7.783155e-01 8.107490e-01 8.128984e-01 7.969991e-01 7.692527e-01

2854 9.970872e-01 9.971177e-01 9.956470e-01 9.930021e-01 9.878428e-01 7.792643e-01 8.116015e-01 8.134211e-01 7.972249e-01 7.691723e-01

2855 9.970907e-01 9.971376e-01 9.956312e-01 9.929476e-01 9.877305e-01 7.802189e-01 8.124598e-01 8.139462e-01 7.974499e-01 7.690899e-01

2856 9.970942e-01 9.971584e-01 9.956144e-01 9.928900e-01 9.876120e-01 7.811787e-01 8.133234e-01 8.144732e-01 7.976738e-01 7.690054e-01

2857 9.970978e-01 9.971801e-01 9.955966e-01 9.928291e-01 9.874872e-01 7.821433e-01 8.141921e-01 8.150021e-01 7.978965e-01 7.689191e-01

2858 9.971016e-01 9.972028e-01 9.955778e-01 9.927648e-01 9.873558e-01 7.831122e-01 8.150653e-01 8.155324e-01 7.981179e-01 7.688309e-01

2859 9.971055e-01 9.972266e-01 9.955579e-01 9.926969e-01 9.872173e-01 7.840848e-01 8.159427e-01 8.160640e-01 7.983380e-01 7.687409e-01

2860 9.971094e-01 9.972514e-01 9.955368e-01 9.926252e-01 9.870714e-01 7.850606e-01 8.168239e-01 8.165966e-01 7.985564e-01 7.686492e-01

2861 9.971135e-01 9.972774e-01 9.955144e-01 9.925494e-01 9.869179e-01 7.860391e-01 8.177085e-01 8.171299e-01 7.987732e-01 7.685558e-01

2862 9.971177e-01 9.973046e-01 9.954908e-01 9.924695e-01 9.867564e-01 7.870197e-01 8.185960e-01 8.176635e-01 7.989882e-01 7.684608e-01

2863 9.971221e-01 9.973330e-01 9.954658e-01 9.923852e-01 9.865865e-01 7.880018e-01 8.194860e-01 8.181973e-01 7.992012e-01 7.683644e-01

2864 9.971265e-01 9.973627e-01 9.954393e-01 9.922962e-01 9.864077e-01 7.889850e-01 8.203781e-01 8.187309e-01 7.994121e-01 7.682665e-01

2865 9.971311e-01 9.973938e-01 9.954113e-01 9.922023e-01 9.862199e-01 7.899687e-01 8.212718e-01 8.192641e-01 7.996209e-01 7.681673e-01

2866 9.971358e-01 9.974263e-01 9.953817e-01 9.921033e-01 9.860225e-01 7.909524e-01 8.221667e-01 8.197965e-01 7.998273e-01 7.680668e-01

2867 9.971406e-01 9.974602e-01 9.953503e-01 9.919988e-01 9.858151e-01 7.919354e-01 8.230623e-01 8.203278e-01 8.000314e-01 7.679652e-01

2868 9.971455e-01 9.974957e-01 9.953172e-01 9.918887e-01 9.855974e-01 7.929173e-01 8.239582e-01 8.208579e-01 8.002328e-01 7.678625e-01

2869 9.971505e-01 9.975328e-01 9.952821e-01 9.917727e-01 9.853689e-01 7.938976e-01 8.248539e-01 8.213863e-01 8.004317e-01 7.677589e-01

2870 9.971557e-01 9.975716e-01 9.952450e-01 9.916504e-01 9.851293e-01 7.948757e-01 8.257489e-01 8.219128e-01 8.006278e-01 7.676543e-01

2871 9.971609e-01 9.976122e-01 9.952058e-01 9.915216e-01 9.848780e-01 7.958511e-01 8.266428e-01 8.224371e-01 8.008210e-01 7.675490e-01

2872 9.971663e-01 9.976546e-01 9.951644e-01 9.913860e-01 9.846146e-01 7.968233e-01 8.275352e-01 8.229589e-01 8.010114e-01 7.674429e-01

2873 9.971719e-01 9.976989e-01 9.951206e-01 9.912432e-01 9.843388e-01 7.977917e-01 8.284256e-01 8.234779e-01 8.011987e-01 7.673362e-01

2874 9.971775e-01 9.977452e-01 9.950743e-01 9.910929e-01 9.840501e-01 7.987560e-01 8.293134e-01 8.239939e-01 8.013829e-01 7.672291e-01

2875 9.971832e-01 9.977936e-01 9.950255e-01 9.909348e-01 9.837480e-01 7.997156e-01 8.301984e-01 8.245065e-01 8.015639e-01 7.671215e-01

2876 9.971891e-01 9.978442e-01 9.949739e-01 9.907686e-01 9.834322e-01 8.006700e-01 8.310800e-01 8.250156e-01 8.017416e-01 7.670135e-01

2877 9.971950e-01 9.978970e-01 9.949194e-01 9.905938e-01 9.831022e-01 8.016189e-01 8.319579e-01 8.255209e-01 8.019160e-01 7.669054e-01

2878 9.972011e-01 9.979522e-01 9.948619e-01 9.904102e-01 9.827575e-01 8.025617e-01 8.328315e-01 8.260220e-01 8.020871e-01 7.667970e-01

2879 9.972072e-01 9.980099e-01 9.948012e-01 9.902173e-01 9.823978e-01 8.034980e-01 8.337004e-01 8.265188e-01 8.022547e-01 7.666886e-01

2880 9.972134e-01 9.980701e-01 9.947372e-01 9.900147e-01 9.820227e-01 8.044275e-01 8.345643e-01 8.270110e-01 8.024188e-01 7.665803e-01

2881 9.972197e-01 9.981331e-01 9.946696e-01 9.898022e-01 9.816318e-01 8.053496e-01 8.354227e-01 8.274984e-01 8.025793e-01 7.664720e-01

2882 9.972197e-01 9.981331e-01 9.946696e-01 9.898021e-01 9.816318e-01 8.053821e-01 8.354450e-01 8.275077e-01 8.025768e-01 7.664543e-01

2883 9.972197e-01 9.981331e-01 9.946695e-01 9.898021e-01 9.816317e-01 8.054154e-01 8.354677e-01 8.275172e-01 8.025742e-01 7.664361e-01

2884 9.972197e-01 9.981332e-01 9.946695e-01 9.898021e-01 9.816316e-01 8.054494e-01 8.354908e-01 8.275269e-01 8.025713e-01 7.664173e-01

2885 9.972197e-01 9.981332e-01 9.946694e-01 9.898020e-01 9.816315e-01 8.054842e-01 8.355144e-01 8.275366e-01 8.025683e-01 7.663979e-01

2886 9.972197e-01 9.981332e-01 9.946694e-01 9.898020e-01 9.816314e-01 8.055199e-01 8.355385e-01 8.275465e-01 8.025651e-01 7.663780e-01

2887 9.972197e-01 9.981333e-01 9.946693e-01 9.898019e-01 9.816313e-01 8.055563e-01 8.355630e-01 8.275566e-01 8.025617e-01 7.663574e-01

2888 9.972197e-01 9.981333e-01 9.946693e-01 9.898018e-01 9.816311e-01 8.055936e-01 8.355880e-01 8.275667e-01 8.025580e-01 7.663362e-01

2889 9.972197e-01 9.981333e-01 9.946693e-01 9.898018e-01 9.816309e-01 8.056318e-01 8.356135e-01 8.275770e-01 8.025542e-01 7.663144e-01

2890 9.972197e-01 9.981334e-01 9.946692e-01 9.898017e-01 9.816308e-01 8.056709e-01 8.356395e-01 8.275874e-01 8.025501e-01 7.662919e-01

2891 9.972197e-01 9.981334e-01 9.946692e-01 9.898016e-01 9.816305e-01 8.057108e-01 8.356660e-01 8.275980e-01 8.025458e-01 7.662687e-01

2892 9.972197e-01 9.981335e-01 9.946692e-01 9.898015e-01 9.816303e-01 8.057517e-01 8.356930e-01 8.276086e-01 8.025413e-01 7.662449e-01

2893 9.972197e-01 9.981335e-01 9.946691e-01 9.898014e-01 9.816301e-01 8.057935e-01 8.357205e-01 8.276194e-01 8.025366e-01 7.662203e-01

2894 9.972197e-01 9.981335e-01 9.946691e-01 9.898013e-01 9.816298e-01 8.058363e-01 8.357486e-01 8.276304e-01 8.025315e-01 7.661950e-01

2895 9.972197e-01 9.981336e-01 9.946690e-01 9.898012e-01 9.816295e-01 8.058800e-01 8.357772e-01 8.276415e-01 8.025263e-01 7.661689e-01

2896 9.972197e-01 9.981336e-01 9.946690e-01 9.898010e-01 9.816293e-01 8.059247e-01 8.358063e-01 8.276527e-01 8.025207e-01 7.661421e-01

2897 9.972197e-01 9.981336e-01 9.946689e-01 9.898009e-01 9.816290e-01 8.059705e-01 8.358360e-01 8.276640e-01 8.025149e-01 7.661144e-01

2898 9.972197e-01 9.981337e-01 9.946689e-01 9.898007e-01 9.816286e-01 8.060173e-01 8.358663e-01 8.276755e-01 8.025087e-01 7.660859e-01

2899 9.972196e-01 9.981337e-01 9.946688e-01 9.898005e-01 9.816283e-01 8.060652e-01 8.358972e-01 8.276871e-01 8.025023e-01 7.660566e-01

2900 9.972196e-01 9.981338e-01 9.946688e-01 9.898004e-01 9.816280e-01 8.061142e-01 8.359287e-01 8.276989e-01 8.024955e-01 7.660264e-01

2901 9.972196e-01 9.981338e-01 9.946687e-01 9.898002e-01 9.816276e-01 8.061643e-01 8.359608e-01 8.277108e-01 8.024885e-01 7.659953e-01

2902 9.972196e-01 9.981338e-01 9.946687e-01 9.897999e-01 9.816273e-01 8.062155e-01 8.359935e-01 8.277228e-01 8.024811e-01 7.659633e-01

2903 9.972196e-01 9.981339e-01 9.946686e-01 9.897997e-01 9.816269e-01 8.062679e-01 8.360269e-01 8.277350e-01 8.024733e-01 7.659303e-01

2904 9.972196e-01 9.981339e-01 9.946685e-01 9.897995e-01 9.816265e-01 8.063215e-01 8.360609e-01 8.277474e-01 8.024652e-01 7.658964e-01

2905 9.972196e-01 9.981340e-01 9.946684e-01 9.897992e-01 9.816261e-01 8.063764e-01 8.360955e-01 8.277598e-01 8.024567e-01 7.658614e-01

2906 9.972196e-01 9.981340e-01 9.946683e-01 9.897990e-01 9.816257e-01 8.064325e-01 8.361309e-01 8.277724e-01 8.024478e-01 7.658255e-01

2907 9.972195e-01 9.981340e-01 9.946682e-01 9.897987e-01 9.816252e-01 8.064899e-01 8.361669e-01 8.277852e-01 8.024386e-01 7.657884e-01

2908 9.972195e-01 9.981341e-01 9.946681e-01 9.897984e-01 9.816248e-01 8.065486e-01 8.362037e-01 8.277981e-01 8.024289e-01 7.657502e-01

2909 9.972195e-01 9.981341e-01 9.946680e-01 9.897981e-01 9.816243e-01 8.066087e-01 8.362411e-01 8.278111e-01 8.024188e-01 7.657109e-01

2910 9.972195e-01 9.981342e-01 9.946679e-01 9.897978e-01 9.816238e-01 8.066702e-01 8.362793e-01 8.278243e-01 8.024082e-01 7.656705e-01

2911 9.972195e-01 9.981342e-01 9.946677e-01 9.897975e-01 9.816233e-01 8.067330e-01 8.363183e-01 8.278377e-01 8.023972e-01 7.656288e-01

2912 9.972194e-01 9.981343e-01 9.946676e-01 9.897972e-01 9.816228e-01 8.067974e-01 8.363580e-01 8.278511e-01 8.023857e-01 7.655860e-01

2913 9.972194e-01 9.981343e-01 9.946674e-01 9.897968e-01 9.816223e-01 8.068632e-01 8.363985e-01 8.278648e-01 8.023737e-01 7.655418e-01

2914 9.972194e-01 9.981344e-01 9.946673e-01 9.897965e-01 9.816217e-01 8.069305e-01 8.364398e-01 8.278785e-01 8.023612e-01 7.654963e-01

2915 9.972193e-01 9.981344e-01 9.946671e-01 9.897961e-01 9.816211e-01 8.069994e-01 8.364819e-01 8.278924e-01 8.023482e-01 7.654495e-01

2916 9.972193e-01 9.981345e-01 9.946669e-01 9.897957e-01 9.816204e-01 8.070699e-01 8.365248e-01 8.279065e-01 8.023346e-01 7.654013e-01

2917 9.972193e-01 9.981345e-01 9.946667e-01 9.897953e-01 9.816198e-01 8.071420e-01 8.365686e-01 8.279207e-01 8.023205e-01 7.653517e-01

2918 9.972192e-01 9.981346e-01 9.946665e-01 9.897948e-01 9.816191e-01 8.072158e-01 8.366133e-01 8.279351e-01 8.023058e-01 7.653006e-01

2919 9.972192e-01 9.981347e-01 9.946663e-01 9.897944e-01 9.816183e-01 8.072913e-01 8.366588e-01 8.279495e-01 8.022905e-01 7.652479e-01

2920 9.972191e-01 9.981347e-01 9.946661e-01 9.897939e-01 9.816176e-01 8.073686e-01 8.367052e-01 8.279642e-01 8.022745e-01 7.651937e-01

2921 9.972191e-01 9.981348e-01 9.946659e-01 9.897934e-01 9.816168e-01 8.074476e-01 8.367526e-01 8.279790e-01 8.022579e-01 7.651380e-01

2922 9.972190e-01 9.981348e-01 9.946657e-01 9.897929e-01 9.816159e-01 8.075286e-01 8.368009e-01 8.279939e-01 8.022406e-01 7.650805e-01

2923 9.972190e-01 9.981349e-01 9.946654e-01 9.897923e-01 9.816150e-01 8.076113e-01 8.368502e-01 8.280089e-01 8.022227e-01 7.650214e-01

2924 9.972189e-01 9.981350e-01 9.946652e-01 9.897918e-01 9.816141e-01 8.076961e-01 8.369004e-01 8.280241e-01 8.022040e-01 7.649605e-01

2925 9.972188e-01 9.981350e-01 9.946649e-01 9.897912e-01 9.816131e-01 8.077828e-01 8.369517e-01 8.280395e-01 8.021846e-01 7.648978e-01

2926 9.972188e-01 9.981351e-01 9.946646e-01 9.897905e-01 9.816121e-01 8.078715e-01 8.370039e-01 8.280549e-01 8.021644e-01 7.648332e-01

2927 9.972187e-01 9.981352e-01 9.946643e-01 9.897899e-01 9.816110e-01 8.079623e-01 8.370572e-01 8.280705e-01 8.021434e-01 7.647668e-01

2928 9.972186e-01 9.981353e-01 9.946640e-01 9.897891e-01 9.816098e-01 8.080552e-01 8.371116e-01 8.280862e-01 8.021216e-01 7.646983e-01

2929 9.972185e-01 9.981353e-01 9.946637e-01 9.897884e-01 9.816087e-01 8.081503e-01 8.371671e-01 8.281021e-01 8.020989e-01 7.646279e-01

2930 9.972184e-01 9.981354e-01 9.946633e-01 9.897876e-01 9.816074e-01 8.082476e-01 8.372237e-01 8.281181e-01 8.020754e-01 7.645553e-01

2931 9.972183e-01 9.981355e-01 9.946629e-01 9.897868e-01 9.816061e-01 8.083472e-01 8.372814e-01 8.281342e-01 8.020509e-01 7.644806e-01

2932 9.972182e-01 9.981356e-01 9.946626e-01 9.897860e-01 9.816047e-01 8.084491e-01 8.373402e-01 8.281504e-01 8.020255e-01 7.644037e-01

2933 9.972181e-01 9.981357e-01 9.946622e-01 9.897851e-01 9.816033e-01 8.085535e-01 8.374002e-01 8.281668e-01 8.019991e-01 7.643246e-01

2934 9.972180e-01 9.981357e-01 9.946617e-01 9.897841e-01 9.816018e-01 8.086603e-01 8.374615e-01 8.281832e-01 8.019717e-01 7.642431e-01

2935 9.972178e-01 9.981358e-01 9.946613e-01 9.897832e-01 9.816002e-01 8.087695e-01 8.375240e-01 8.281998e-01 8.019433e-01 7.641592e-01

2936 9.972177e-01 9.981359e-01 9.946608e-01 9.897821e-01 9.815986e-01 8.088814e-01 8.375877e-01 8.282165e-01 8.019138e-01 7.640728e-01

2937 9.972176e-01 9.981360e-01 9.946603e-01 9.897811e-01 9.815969e-01 8.089959e-01 8.376527e-01 8.282333e-01 8.018832e-01 7.639839e-01

2938 9.972174e-01 9.981361e-01 9.946598e-01 9.897799e-01 9.815951e-01 8.091130e-01 8.377190e-01 8.282501e-01 8.018514e-01 7.638923e-01

2939 9.972173e-01 9.981362e-01 9.946593e-01 9.897787e-01 9.815932e-01 8.092330e-01 8.377866e-01 8.282671e-01 8.018184e-01 7.637981e-01

2940 9.972171e-01 9.981363e-01 9.946587e-01 9.897775e-01 9.815912e-01 8.093557e-01 8.378556e-01 8.282841e-01 8.017842e-01 7.637011e-01

2941 9.972169e-01 9.981364e-01 9.946581e-01 9.897762e-01 9.815892e-01 8.094814e-01 8.379260e-01 8.283013e-01 8.017487e-01 7.636012e-01

2942 9.972167e-01 9.981365e-01 9.946575e-01 9.897748e-01 9.815870e-01 8.096100e-01 8.379978e-01 8.283185e-01 8.017119e-01 7.634985e-01

2943 9.972166e-01 9.981366e-01 9.946569e-01 9.897734e-01 9.815847e-01 8.097416e-01 8.380710e-01 8.283358e-01 8.016738e-01 7.633927e-01

2944 9.972164e-01 9.981367e-01 9.946562e-01 9.897718e-01 9.815824e-01 8.098763e-01 8.381457e-01 8.283531e-01 8.016342e-01 7.632838e-01

2945 9.972161e-01 9.981368e-01 9.946554e-01 9.897703e-01 9.815799e-01 8.100142e-01 8.382219e-01 8.283705e-01 8.015932e-01 7.631718e-01

2946 9.972159e-01 9.981370e-01 9.946547e-01 9.897686e-01 9.815773e-01 8.101553e-01 8.382997e-01 8.283879e-01 8.015507e-01 7.630565e-01

2947 9.972157e-01 9.981371e-01 9.946539e-01 9.897669e-01 9.815745e-01 8.102998e-01 8.383790e-01 8.284054e-01 8.015067e-01 7.629378e-01

2948 9.972154e-01 9.981372e-01 9.946531e-01 9.897650e-01 9.815717e-01 8.104477e-01 8.384599e-01 8.284229e-01 8.014610e-01 7.628157e-01

2949 9.972152e-01 9.981373e-01 9.946522e-01 9.897631e-01 9.815687e-01 8.105990e-01 8.385424e-01 8.284404e-01 8.014137e-01 7.626901e-01
[truncated: 10,524,276 more chars]
