# Supplementary material for: Purity control of simulated moving bed based on advanced fuzzy controller
Source: Sci Rep. 2024 Apr 20;14:9083. doi: 10.1038/s41598-024-59847-1 (PMC11576947; doi:10.1038/s41598-024-59847-1)
Supplement: Supplementary file 8 — Supplementary Information 8. [file 41598_2024_59847_MOESM8_ESM.docx]

**Figure 7(b):**

1 8.962715e-04 8.963257e-04 8.963820e-04 8.964405e-04 1.001853e-03 9.257608e-06 8.254405e-06 8.262710e-06 9.285570e-06 9.294913e-06

2 8.962715e-04 8.963257e-04 8.963820e-04 8.964405e-04 1.001853e-03 9.257608e-06 8.254405e-06 8.262710e-06 9.285570e-06 9.294913e-06

3 8.962715e-04 8.963257e-04 8.963820e-04 8.964405e-04 1.001853e-03 9.257608e-06 8.254405e-06 8.262710e-06 9.285570e-06 9.294913e-06

4 8.962715e-04 8.963257e-04 8.963820e-04 8.964405e-04 1.001853e-03 9.257608e-06 8.254405e-06 8.262710e-06 9.285570e-06 9.294913e-06

5 8.962715e-04 8.963257e-04 8.963820e-04 8.964405e-04 1.001853e-03 9.257608e-06 8.254405e-06 8.262710e-06 9.285570e-06 9.294913e-06

6 8.962715e-04 8.963257e-04 8.963820e-04 8.964405e-04 1.001853e-03 9.257608e-06 8.254405e-06 8.262710e-06 9.285570e-06 9.294913e-06

7 8.962715e-04 8.963257e-04 8.963820e-04 8.964405e-04 1.001853e-03 9.257608e-06 8.254405e-06 8.262710e-06 9.285570e-06 9.294913e-06

8 8.962715e-04 8.963257e-04 8.963820e-04 8.964405e-04 1.001853e-03 9.257608e-06 8.254405e-06 8.262710e-06 9.285570e-06 9.294913e-06

9 8.962715e-04 8.963257e-04 8.963820e-04 8.964405e-04 1.001853e-03 9.257608e-06 8.254405e-06 8.262710e-06 9.285570e-06 9.294913e-06

10 8.962715e-04 8.963257e-04 8.963820e-04 8.964405e-04 1.001853e-03 9.257608e-06 8.254405e-06 8.262710e-06 9.285570e-06 9.294913e-06

11 8.962715e-04 8.963257e-04 8.963820e-04 8.964405e-04 1.001853e-03 9.257608e-06 8.254405e-06 8.262710e-06 9.285570e-06 9.294913e-06

12 8.962715e-04 8.963257e-04 8.963820e-04 8.964405e-04 1.001853e-03 9.257608e-06 8.254405e-06 8.262710e-06 9.285570e-06 9.294913e-06

13 8.962715e-04 8.963257e-04 8.963820e-04 8.964405e-04 1.001853e-03 9.257608e-06 8.254405e-06 8.262710e-06 9.285570e-06 9.294913e-06

14 8.962715e-04 8.963257e-04 8.963820e-04 8.964405e-04 1.001853e-03 9.257608e-06 8.254405e-06 8.262710e-06 9.285570e-06 9.294913e-06

15 8.962715e-04 8.963257e-04 8.963820e-04 8.964405e-04 1.001853e-03 9.257608e-06 8.254405e-06 8.262710e-06 9.285570e-06 9.294913e-06

16 8.962715e-04 8.963257e-04 8.963820e-04 8.964405e-04 1.001853e-03 9.257608e-06 8.254405e-06 8.262710e-06 9.285570e-06 9.294913e-06

17 8.962715e-04 8.963257e-04 8.963820e-04 8.964405e-04 1.001853e-03 9.257608e-06 8.254405e-06 8.262710e-06 9.285570e-06 9.294913e-06

18 8.962715e-04 8.963257e-04 8.963820e-04 8.964405e-04 1.001853e-03 9.257608e-06 8.254405e-06 8.262710e-06 9.285570e-06 9.294913e-06

19 8.962715e-04 8.963257e-04 8.963820e-04 8.964405e-04 1.001853e-03 9.257608e-06 8.254405e-06 8.262710e-06 9.285570e-06 9.294913e-06

20 8.962715e-04 8.963257e-04 8.963820e-04 8.964405e-04 1.001853e-03 9.257608e-06 8.254405e-06 8.262710e-06 9.285570e-06 9.294913e-06

21 8.962715e-04 8.963257e-04 8.963820e-04 8.964405e-04 1.001853e-03 9.257608e-06 8.254405e-06 8.262710e-06 9.285570e-06 9.294913e-06

22 8.962715e-04 8.963257e-04 8.963820e-04 8.964405e-04 1.001853e-03 9.257608e-06 8.254405e-06 8.262710e-06 9.285570e-06 9.294913e-06

23 8.962715e-04 8.963257e-04 8.963820e-04 8.964405e-04 1.001853e-03 9.257608e-06 8.254405e-06 8.262710e-06 9.285570e-06 9.294913e-06

24 8.962715e-04 8.963257e-04 8.963820e-04 8.964405e-04 1.001853e-03 9.257608e-06 8.254405e-06 8.262710e-06 9.285570e-06 9.294913e-06

25 8.962715e-04 8.963257e-04 8.963820e-04 8.964405e-04 1.001853e-03 9.257608e-06 8.254405e-06 8.262710e-06 9.285570e-06 9.294913e-06

26 8.962715e-04 8.963257e-04 8.963820e-04 8.964405e-04 1.001853e-03 9.257608e-06 8.254405e-06 8.262710e-06 9.285570e-06 9.294913e-06

27 8.962715e-04 8.963257e-04 8.963820e-04 8.964405e-04 1.001853e-03 9.257608e-06 8.254405e-06 8.262710e-06 9.285570e-06 9.294913e-06

28 8.962715e-04 8.963257e-04 8.963820e-04 8.964405e-04 1.001853e-03 9.257608e-06 8.254405e-06 8.262710e-06 9.285570e-06 9.294913e-06

29 8.962715e-04 8.963257e-04 8.963820e-04 8.964405e-04 1.001853e-03 9.257608e-06 8.254405e-06 8.262710e-06 9.285570e-06 9.294913e-06

30 8.962715e-04 8.963257e-04 8.963820e-04 8.964405e-04 1.001853e-03 9.257608e-06 8.254405e-06 8.262710e-06 9.285570e-06 9.294913e-06

31 8.962715e-04 8.963257e-04 8.963820e-04 8.964405e-04 1.001853e-03 9.257608e-06 8.254405e-06 8.262710e-06 9.285570e-06 9.294913e-06

32 8.962715e-04 8.963257e-04 8.963820e-04 8.964405e-04 1.001853e-03 9.257608e-06 8.254405e-06 8.262710e-06 9.285570e-06 9.294913e-06

33 8.962715e-04 8.963257e-04 8.963820e-04 8.964405e-04 1.001853e-03 9.257608e-06 8.254405e-06 8.262710e-06 9.285570e-06 9.294913e-06

34 8.962715e-04 8.963257e-04 8.963820e-04 8.964405e-04 1.001853e-03 9.257608e-06 8.254405e-06 8.262710e-06 9.285570e-06 9.294913e-06

35 8.962715e-04 8.963257e-04 8.963820e-04 8.964405e-04 1.001853e-03 9.257608e-06 8.254405e-06 8.262710e-06 9.285570e-06 9.294913e-06

36 8.962715e-04 8.963257e-04 8.963820e-04 8.964405e-04 1.001853e-03 9.257608e-06 8.254405e-06 8.262710e-06 9.285570e-06 9.294913e-06

37 8.962715e-04 8.963257e-04 8.963820e-04 8.964405e-04 1.001853e-03 9.257608e-06 8.254405e-06 8.262710e-06 9.285570e-06 9.294913e-06

38 8.962715e-04 8.963257e-04 8.963820e-04 8.964405e-04 1.001853e-03 9.257608e-06 8.254405e-06 8.262710e-06 9.285570e-06 9.294913e-06

39 8.962715e-04 8.963257e-04 8.963820e-04 8.964405e-04 1.001853e-03 9.257608e-06 8.254405e-06 8.262710e-06 9.285570e-06 9.294913e-06

40 8.962715e-04 8.963257e-04 8.963820e-04 8.964405e-04 1.001853e-03 9.257608e-06 8.254405e-06 8.262710e-06 9.285570e-06 9.294913e-06

41 8.962715e-04 8.963257e-04 8.963820e-04 8.964405e-04 1.001853e-03 9.257608e-06 8.254405e-06 8.262710e-06 9.285570e-06 9.294913e-06

42 8.962715e-04 8.963257e-04 8.963820e-04 8.964405e-04 1.001853e-03 9.257608e-06 8.254405e-06 8.262710e-06 9.285570e-06 9.294913e-06

43 8.962715e-04 8.963257e-04 8.963820e-04 8.964406e-04 1.001853e-03 9.257608e-06 8.254405e-06 8.262710e-06 9.285570e-06 9.294913e-06

44 8.962715e-04 8.963257e-04 8.963820e-04 8.964406e-04 1.001853e-03 9.257608e-06 8.254405e-06 8.262710e-06 9.285570e-06 9.294913e-06

45 8.962715e-04 8.963257e-04 8.963820e-04 8.964406e-04 1.001853e-03 9.257608e-06 8.254405e-06 8.262710e-06 9.285570e-06 9.294913e-06

46 8.962715e-04 8.963257e-04 8.963820e-04 8.964406e-04 1.001853e-03 9.257608e-06 8.254405e-06 8.262710e-06 9.285570e-06 9.294914e-06

47 8.962715e-04 8.963257e-04 8.963820e-04 8.964406e-04 1.001853e-03 9.257608e-06 8.254405e-06 8.262710e-06 9.285570e-06 9.294914e-06

48 8.962715e-04 8.963257e-04 8.963820e-04 8.964406e-04 1.001853e-03 9.257608e-06 8.254405e-06 8.262710e-06 9.285570e-06 9.294914e-06

49 8.962715e-04 8.963257e-04 8.963820e-04 8.964406e-04 1.001853e-03 9.257608e-06 8.254405e-06 8.262710e-06 9.285570e-06 9.294914e-06

50 8.962715e-04 8.963257e-04 8.963821e-04 8.964406e-04 1.001853e-03 9.257608e-06 8.254405e-06 8.262710e-06 9.285570e-06 9.294914e-06

51 8.962715e-04 8.963257e-04 8.963821e-04 8.964406e-04 1.001853e-03 9.257608e-06 8.254405e-06 8.262710e-06 9.285570e-06 9.294914e-06

52 8.962716e-04 8.963258e-04 8.963821e-04 8.964406e-04 1.001853e-03 9.257608e-06 8.254405e-06 8.262710e-06 9.285570e-06 9.294914e-06

53 8.962716e-04 8.963258e-04 8.963821e-04 8.964406e-04 1.001853e-03 9.257609e-06 8.254405e-06 8.262710e-06 9.285571e-06 9.294914e-06

54 8.962716e-04 8.963258e-04 8.963821e-04 8.964406e-04 1.001853e-03 9.257609e-06 8.254406e-06 8.262710e-06 9.285571e-06 9.294914e-06

55 8.962716e-04 8.963258e-04 8.963821e-04 8.964406e-04 1.001853e-03 9.257609e-06 8.254406e-06 8.262710e-06 9.285571e-06 9.294914e-06

56 8.962716e-04 8.963258e-04 8.963822e-04 8.964407e-04 1.001853e-03 9.257609e-06 8.254406e-06 8.262710e-06 9.285571e-06 9.294915e-06

57 8.962717e-04 8.963259e-04 8.963822e-04 8.964407e-04 1.001853e-03 9.257610e-06 8.254406e-06 8.262711e-06 9.285571e-06 9.294915e-06

58 8.962717e-04 8.963259e-04 8.963822e-04 8.964408e-04 1.001853e-03 9.257610e-06 8.254407e-06 8.262711e-06 9.285572e-06 9.294915e-06

59 8.962718e-04 8.963260e-04 8.963823e-04 8.964408e-04 1.001854e-03 9.257611e-06 8.254407e-06 8.262712e-06 9.285572e-06 9.294916e-06

60 8.962719e-04 8.963261e-04 8.963824e-04 8.964409e-04 1.001854e-03 9.257611e-06 8.254408e-06 8.262712e-06 9.285573e-06 9.294917e-06

61 8.962720e-04 8.963262e-04 8.963825e-04 8.964410e-04 1.001854e-03 9.257612e-06 8.254409e-06 8.262713e-06 9.285574e-06 9.294918e-06

62 8.962721e-04 8.963263e-04 8.963826e-04 8.964412e-04 1.001854e-03 9.257613e-06 8.254410e-06 8.262714e-06 9.285575e-06 9.294919e-06

63 8.962723e-04 8.963265e-04 8.963828e-04 8.964413e-04 1.001854e-03 9.257615e-06 8.254411e-06 8.262716e-06 9.285577e-06 9.294920e-06

64 8.962725e-04 8.963267e-04 8.963831e-04 8.964416e-04 1.001854e-03 9.257617e-06 8.254413e-06 8.262717e-06 9.285579e-06 9.294922e-06

65 8.962728e-04 8.963270e-04 8.963833e-04 8.964419e-04 1.001855e-03 9.257619e-06 8.254415e-06 8.262719e-06 9.285581e-06 9.294925e-06

66 8.962732e-04 8.963274e-04 8.963837e-04 8.964422e-04 1.001855e-03 9.257623e-06 8.254418e-06 8.262722e-06 9.285585e-06 9.294928e-06

67 8.962737e-04 8.963279e-04 8.963842e-04 8.964427e-04 1.001856e-03 9.257627e-06 8.254421e-06 8.262726e-06 9.285589e-06 9.294932e-06

68 8.962743e-04 8.963285e-04 8.963848e-04 8.964433e-04 1.001856e-03 9.257632e-06 8.254426e-06 8.262730e-06 9.285594e-06 9.294937e-06

69 8.962750e-04 8.963292e-04 8.963856e-04 8.964441e-04 1.001857e-03 9.257638e-06 8.254431e-06 8.262736e-06 9.285600e-06 9.294944e-06

70 8.962760e-04 8.963302e-04 8.963865e-04 8.964450e-04 1.001858e-03 9.257646e-06 8.254439e-06 8.262743e-06 9.285609e-06 9.294952e-06

71 8.962772e-04 8.963314e-04 8.963878e-04 8.964463e-04 1.001860e-03 9.257657e-06 8.254448e-06 8.262752e-06 9.285619e-06 9.294963e-06

72 8.962788e-04 8.963330e-04 8.963893e-04 8.964478e-04 1.001862e-03 9.257670e-06 8.254459e-06 8.262764e-06 9.285632e-06 9.294976e-06

73 8.962807e-04 8.963349e-04 8.963912e-04 8.964498e-04 1.001864e-03 9.257687e-06 8.254474e-06 8.262778e-06 9.285649e-06 9.294993e-06

74 8.962832e-04 8.963374e-04 8.963937e-04 8.964522e-04 1.001867e-03 9.257707e-06 8.254492e-06 8.262796e-06 9.285670e-06 9.295014e-06

75 8.962863e-04 8.963405e-04 8.963968e-04 8.964553e-04 1.001870e-03 9.257734e-06 8.254515e-06 8.262819e-06 9.285696e-06 9.295040e-06

76 8.962901e-04 8.963443e-04 8.964007e-04 8.964592e-04 1.001875e-03 9.257766e-06 8.254543e-06 8.262848e-06 9.285729e-06 9.295073e-06

77 8.962950e-04 8.963492e-04 8.964055e-04 8.964640e-04 1.001880e-03 9.257807e-06 8.254579e-06 8.262884e-06 9.285771e-06 9.295115e-06

78 8.963011e-04 8.963553e-04 8.964116e-04 8.964701e-04 1.001887e-03 9.257859e-06 8.254624e-06 8.262929e-06 9.285823e-06 9.295167e-06

79 8.963086e-04 8.963628e-04 8.964192e-04 8.964777e-04 1.001896e-03 9.257923e-06 8.254680e-06 8.262985e-06 9.285887e-06 9.295231e-06

80 8.963181e-04 8.963723e-04 8.964286e-04 8.964872e-04 1.001907e-03 9.258003e-06 8.254750e-06 8.263056e-06 9.285968e-06 9.295312e-06

81 8.963299e-04 8.963841e-04 8.964404e-04 8.964990e-04 1.001920e-03 9.258103e-06 8.254838e-06 8.263143e-06 9.286068e-06 9.295413e-06

82 8.963446e-04 8.963988e-04 8.964551e-04 8.965137e-04 1.001937e-03 9.258227e-06 8.254946e-06 8.263252e-06 9.286193e-06 9.295538e-06

83 8.963628e-04 8.964171e-04 8.964734e-04 8.965319e-04 1.001958e-03 9.258381e-06 8.255081e-06 8.263387e-06 9.286348e-06 9.295693e-06

84 8.963855e-04 8.964397e-04 8.964960e-04 8.965546e-04 1.001984e-03 9.258572e-06 8.255248e-06 8.263554e-06 9.286541e-06 9.295886e-06

85 8.964135e-04 8.964678e-04 8.965241e-04 8.965826e-04 1.002016e-03 9.258809e-06 8.255455e-06 8.263761e-06 9.286779e-06 9.296125e-06

86 8.964483e-04 8.965025e-04 8.965588e-04 8.966174e-04 1.002056e-03 9.259101e-06 8.255710e-06 8.264018e-06 9.287073e-06 9.296420e-06

87 8.964912e-04 8.965454e-04 8.966017e-04 8.966603e-04 1.002105e-03 9.259462e-06 8.256026e-06 8.264335e-06 9.287437e-06 9.296785e-06

88 8.965441e-04 8.965983e-04 8.966547e-04 8.967133e-04 1.002166e-03 9.259908e-06 8.256416e-06 8.264725e-06 9.287886e-06 9.297234e-06

89 8.966093e-04 8.966636e-04 8.967199e-04 8.967785e-04 1.002241e-03 9.260457e-06 8.256896e-06 8.265206e-06 9.288438e-06 9.297788e-06

90 8.966896e-04 8.967439e-04 8.968003e-04 8.968588e-04 1.002333e-03 9.261131e-06 8.257487e-06 8.265798e-06 9.289118e-06 9.298469e-06

91 8.967883e-04 8.968426e-04 8.968990e-04 8.969576e-04 1.002446e-03 9.261960e-06 8.258212e-06 8.266525e-06 9.289953e-06 9.299306e-06

92 8.969094e-04 8.969637e-04 8.970201e-04 8.970787e-04 1.002584e-03 9.262977e-06 8.259102e-06 8.267417e-06 9.290977e-06 9.300332e-06

93 8.970578e-04 8.971121e-04 8.971686e-04 8.972272e-04 1.002755e-03 9.264223e-06 8.260192e-06 8.268510e-06 9.292231e-06 9.301590e-06

94 8.972395e-04 8.972939e-04 8.973503e-04 8.974090e-04 1.002963e-03 9.265747e-06 8.261526e-06 8.269847e-06 9.293766e-06 9.303128e-06

95 8.974616e-04 8.975160e-04 8.975725e-04 8.976312e-04 1.003217e-03 9.267609e-06 8.263156e-06 8.271481e-06 9.295641e-06 9.305008e-06

96 8.977328e-04 8.977872e-04 8.978438e-04 8.979025e-04 1.003528e-03 9.269882e-06 8.265145e-06 8.273475e-06 9.297929e-06 9.307301e-06

97 8.980635e-04 8.981179e-04 8.981745e-04 8.982333e-04 1.003907e-03 9.272651e-06 8.267569e-06 8.275905e-06 9.300718e-06 9.310097e-06

98 8.984661e-04 8.985206e-04 8.985773e-04 8.986361e-04 1.004368e-03 9.276021e-06 8.270520e-06 8.278862e-06 9.304112e-06 9.313499e-06

99 8.989557e-04 8.990103e-04 8.990671e-04 8.991260e-04 1.004929e-03 9.280118e-06 8.274108e-06 8.282458e-06 9.308238e-06 9.317635e-06

100 8.995505e-04 8.996051e-04 8.996620e-04 8.997210e-04 1.005610e-03 9.285092e-06 8.278463e-06 8.286824e-06 9.313247e-06 9.322656e-06

101 9.002719e-04 9.003267e-04 9.003836e-04 9.004428e-04 1.006436e-03 9.291123e-06 8.283745e-06 8.292118e-06 9.319321e-06 9.328744e-06

102 9.011459e-04 9.012008e-04 9.012579e-04 9.013171e-04 1.007436e-03 9.298426e-06 8.290142e-06 8.298530e-06 9.326676e-06 9.336116e-06

103 9.022034e-04 9.022584e-04 9.023157e-04 9.023751e-04 1.008647e-03 9.307260e-06 8.297879e-06 8.306286e-06 9.335572e-06 9.345033e-06

104 9.034813e-04 9.035365e-04 9.035940e-04 9.036536e-04 1.010110e-03 9.317930e-06 8.307227e-06 8.315656e-06 9.346319e-06 9.355805e-06

105 9.050236e-04 9.050791e-04 9.051368e-04 9.051967e-04 1.011875e-03 9.330804e-06 8.318506e-06 8.326962e-06 9.359285e-06 9.368802e-06

106 9.068828e-04 9.069385e-04 9.069965e-04 9.070567e-04 1.014003e-03 9.346318e-06 8.332099e-06 8.340587e-06 9.374909e-06 9.384463e-06

107 9.091210e-04 9.091771e-04 9.092354e-04 9.092960e-04 1.016564e-03 9.364990e-06 8.348461e-06 8.356988e-06 9.393715e-06 9.403313e-06

108 9.118123e-04 9.118688e-04 9.119275e-04 9.119885e-04 1.019644e-03 9.387436e-06 8.368132e-06 8.376706e-06 9.416321e-06 9.425974e-06

109 9.150443e-04 9.151012e-04 9.151604e-04 9.152220e-04 1.023341e-03 9.414386e-06 8.391753e-06 8.400383e-06 9.443464e-06 9.453182e-06

110 9.189207e-04 9.189783e-04 9.190381e-04 9.191002e-04 1.027776e-03 9.446707e-06 8.420083e-06 8.428781e-06 9.476017e-06 9.485811e-06

111 9.235644e-04 9.236227e-04 9.236832e-04 9.237461e-04 1.033087e-03 9.485421e-06 8.454022e-06 8.462800e-06 9.515009e-06 9.524897e-06

112 9.291203e-04 9.291794e-04 9.292408e-04 9.293046e-04 1.039441e-03 9.531740e-06 8.494630e-06 8.503506e-06 9.561660e-06 9.571660e-06

113 9.357592e-04 9.358193e-04 9.358817e-04 9.359466e-04 1.047032e-03 9.587092e-06 8.543163e-06 8.552155e-06 9.617410e-06 9.627543e-06

114 9.436823e-04 9.437436e-04 9.438072e-04 9.438734e-04 1.056090e-03 9.653162e-06 8.601099e-06 8.610230e-06 9.683955e-06 9.694247e-06

115 9.531261e-04 9.531888e-04 9.532539e-04 9.533215e-04 1.066884e-03 9.731933e-06 8.670181e-06 8.679477e-06 9.763294e-06 9.773775e-06

116 9.643681e-04 9.644324e-04 9.644993e-04 9.645687e-04 1.079732e-03 9.825739e-06 8.752457e-06 8.761950e-06 9.857776e-06 9.868485e-06

117 9.777335e-04 9.777998e-04 9.778687e-04 9.779404e-04 1.095003e-03 9.937323e-06 8.850335e-06 8.860064e-06 9.970166e-06 9.981144e-06

118 9.936028e-04 9.936715e-04 9.937429e-04 9.938171e-04 1.113131e-03 1.006990e-05 8.966642e-06 8.976650e-06 1.010370e-05 1.011500e-05

119 1.012420e-03 1.012492e-03 1.012566e-03 1.012643e-03 1.134622e-03 1.022725e-05 9.104691e-06 9.115032e-06 1.026219e-05 1.027387e-05

120 1.034704e-03 1.034779e-03 1.034856e-03 1.034937e-03 1.160064e-03 1.041378e-05 9.268363e-06 9.279098e-06 1.045007e-05 1.046220e-05

121 1.061056e-03 1.061135e-03 1.061216e-03 1.061302e-03 1.190144e-03 1.063466e-05 9.462196e-06 9.473397e-06 1.067254e-05 1.068521e-05

122 1.092176e-03 1.092259e-03 1.092346e-03 1.092436e-03 1.225654e-03 1.089592e-05 9.691491e-06 9.703246e-06 1.093570e-05 1.094900e-05

123 1.128874e-03 1.128963e-03 1.129055e-03 1.129151e-03 1.267514e-03 1.120461e-05 9.962438e-06 9.974846e-06 1.124662e-05 1.126067e-05

124 1.172087e-03 1.172182e-03 1.172281e-03 1.172384e-03 1.316786e-03 1.156893e-05 1.028225e-05 1.029543e-05 1.161358e-05 1.162852e-05

125 1.222893e-03 1.222995e-03 1.223102e-03 1.223213e-03 1.374691e-03 1.199843e-05 1.065932e-05 1.067341e-05 1.204620e-05 1.206218e-05

126 1.282533e-03 1.282644e-03 1.282760e-03 1.282881e-03 1.442632e-03 1.250422e-05 1.110343e-05 1.111859e-05 1.255567e-05 1.257288e-05

127 1.352429e-03 1.352551e-03 1.352677e-03 1.352809e-03 1.522212e-03 1.309921e-05 1.162591e-05 1.164234e-05 1.315499e-05 1.317364e-05

128 1.434204e-03 1.434338e-03 1.434477e-03 1.434622e-03 1.615260e-03 1.379838e-05 1.223995e-05 1.225787e-05 1.385924e-05 1.387960e-05

129 1.529708e-03 1.529856e-03 1.530010e-03 1.530169e-03 1.723854e-03 1.461907e-05 1.296080e-05 1.298046e-05 1.468590e-05 1.470826e-05

130 1.641035e-03 1.641199e-03 1.641370e-03 1.641547e-03 1.850343e-03 1.558139e-05 1.380614e-05 1.382785e-05 1.565522e-05 1.567992e-05

131 1.770551e-03 1.770734e-03 1.770923e-03 1.771121e-03 1.997370e-03 1.670856e-05 1.479641e-05 1.482053e-05 1.679060e-05 1.681805e-05

132 1.920912e-03 1.921116e-03 1.921328e-03 1.921549e-03 2.167897e-03 1.802742e-05 1.595524e-05 1.598217e-05 1.811907e-05 1.814974e-05

133 2.095087e-03 2.095316e-03 2.095554e-03 2.095801e-03 2.365218e-03 1.956895e-05 1.730988e-05 1.734010e-05 1.967183e-05 1.970626e-05

134 2.296374e-03 2.296631e-03 2.296899e-03 2.297177e-03 2.592978e-03 2.136884e-05 1.889176e-05 1.892581e-05 2.148484e-05 2.152366e-05

135 2.528412e-03 2.528702e-03 2.529003e-03 2.529315e-03 2.855182e-03 2.346818e-05 2.073705e-05 2.077558e-05 2.359948e-05 2.364342e-05

136 2.795188e-03 2.795514e-03 2.795854e-03 2.796206e-03 3.156189e-03 2.591422e-05 2.288736e-05 2.293112e-05 2.606336e-05 2.611328e-05

137 3.101035e-03 3.101403e-03 3.101785e-03 3.102183e-03 3.500712e-03 2.876126e-05 2.539052e-05 2.544037e-05 2.893116e-05 2.898803e-05

138 3.450620e-03 3.451035e-03 3.451466e-03 3.451913e-03 3.893786e-03 3.207158e-05 2.830140e-05 2.835832e-05 3.226563e-05 3.233058e-05

139 3.848919e-03 3.849386e-03 3.849871e-03 3.850375e-03 4.340741e-03 3.591657e-05 3.168289e-05 3.174803e-05 3.613867e-05 3.621301e-05

140 4.301180e-03 4.301705e-03 4.302251e-03 4.302818e-03 4.847142e-03 4.037796e-05 3.560703e-05 3.568171e-05 4.063261e-05 4.071785e-05

141 4.812868e-03 4.813457e-03 4.814069e-03 4.814706e-03 5.418726e-03 4.554921e-05 4.015621e-05 4.024195e-05 4.584158e-05 4.593945e-05

142 5.389594e-03 5.390254e-03 5.390940e-03 5.391653e-03 6.061312e-03 5.153707e-05 4.542454e-05 4.552309e-05 5.187312e-05 5.198561e-05

143 6.037033e-03 6.037771e-03 6.038537e-03 6.039333e-03 6.780705e-03 5.846332e-05 5.151944e-05 5.163281e-05 5.884989e-05 5.897929e-05

144 6.760821e-03 6.761643e-03 6.762496e-03 6.763383e-03 7.582576e-03 6.646676e-05 5.856334e-05 5.869384e-05 6.691168e-05 6.706061e-05

145 7.566443e-03 7.567354e-03 7.568301e-03 7.569285e-03 8.472342e-03 7.570531e-05 6.669561e-05 6.684588e-05 7.621758e-05 7.638905e-05

146 8.459110e-03 8.460118e-03 8.461165e-03 8.462254e-03 9.455035e-03 8.635855e-05 7.607476e-05 7.624782e-05 8.694845e-05 8.714589e-05

147 9.443644e-03 9.444754e-03 9.445907e-03 9.447106e-03 1.053518e-02 9.863034e-05 8.688079e-05 8.708010e-05 9.930960e-05 9.953695e-05

148 1.052435e-02 1.052557e-02 1.052683e-02 1.052815e-02 1.171667e-02 1.127518e-04 9.931790e-05 9.954741e-05 1.135339e-04 1.137956e-04

149 1.170491e-02 1.170624e-02 1.170762e-02 1.170905e-02 1.300269e-02 1.289848e-04 1.136174e-04 1.138816e-04 1.298849e-04 1.301862e-04

150 1.298828e-02 1.298973e-02 1.299123e-02 1.299279e-02 1.439558e-02 1.476254e-04 1.300411e-04 1.303451e-04 1.486610e-04 1.490075e-04

151 1.437666e-02 1.437822e-02 1.437985e-02 1.438154e-02 1.589687e-02 1.690079e-04 1.488847e-04 1.492344e-04 1.701987e-04 1.705972e-04

152 1.587137e-02 1.587305e-02 1.587481e-02 1.587663e-02 1.750718e-02 1.935094e-04 1.704818e-04 1.708839e-04 1.948780e-04 1.953360e-04

153 1.747291e-02 1.747472e-02 1.747660e-02 1.747855e-02 1.922626e-02 2.215548e-04 1.952087e-04 1.956705e-04 2.231265e-04 2.236525e-04

154 1.918093e-02 1.918287e-02 1.918487e-02 1.918696e-02 2.105302e-02 2.536216e-04 2.234883e-04 2.240185e-04 2.554253e-04 2.560288e-04

155 2.099429e-02 2.099635e-02 2.099848e-02 2.100070e-02 2.298561e-02 2.902459e-04 2.557961e-04 2.564042e-04 2.923141e-04 2.930061e-04

156 2.291110e-02 2.291327e-02 2.291553e-02 2.291788e-02 2.502146e-02 3.320289e-04 2.926652e-04 2.933622e-04 3.343983e-04 3.351911e-04

157 2.492880e-02 2.493109e-02 2.493347e-02 2.493595e-02 2.715742e-02 3.796429e-04 3.346927e-04 3.354908e-04 3.823550e-04 3.832623e-04

158 2.704428e-02 2.704669e-02 2.704919e-02 2.705179e-02 2.938984e-02 4.338392e-04 3.825462e-04 3.834592e-04 4.369403e-04 4.379777e-04

159 2.925396e-02 2.925649e-02 2.925911e-02 2.926183e-02 3.171469e-02 4.954551e-04 4.369707e-04 4.380140e-04 4.989975e-04 5.001824e-04

160 3.155390e-02 3.155653e-02 3.155927e-02 3.156211e-02 3.412762e-02 5.654224e-04 4.987957e-04 4.999868e-04 5.694643e-04 5.708162e-04

161 3.393988e-02 3.394262e-02 3.394546e-02 3.394841e-02 3.662414e-02 6.447753e-04 5.689437e-04 5.703020e-04 6.493820e-04 6.509228e-04

162 3.640750e-02 3.641034e-02 3.641329e-02 3.641635e-02 3.919960e-02 7.346597e-04 6.484375e-04 6.499847e-04 7.399039e-04 7.416578e-04

163 3.895227e-02 3.895520e-02 3.895825e-02 3.896142e-02 4.184936e-02 8.363417e-04 7.384095e-04 7.401698e-04 8.423043e-04 8.442983e-04

164 4.156966e-02 4.157269e-02 4.157583e-02 4.157910e-02 4.456878e-02 9.512170e-04 8.401096e-04 8.421098e-04 9.579877e-04 9.602517e-04

165 4.425516e-02 4.425828e-02 4.426152e-02 4.426488e-02 4.735329e-02 1.080820e-03 9.549144e-04 9.571844e-04 1.088498e-03 1.091065e-03

166 4.700433e-02 4.700753e-02 4.701086e-02 4.701432e-02 5.019843e-02 1.226832e-03 1.084336e-03 1.086909e-03 1.235526e-03 1.238433e-03

167 4.981281e-02 4.981609e-02 4.981950e-02 4.982304e-02 5.309982e-02 1.391090e-03 1.230030e-03 1.232941e-03 1.400921e-03 1.404208e-03

168 5.267633e-02 5.267968e-02 5.268317e-02 5.268679e-02 5.605323e-02 1.575594e-03 1.393803e-03 1.397094e-03 1.586695e-03 1.590405e-03

169 5.559073e-02 5.559415e-02 5.559771e-02 5.560142e-02 5.905452e-02 1.782517e-03 1.577623e-03 1.581336e-03 1.795030e-03 1.799212e-03

170 5.855193e-02 5.855542e-02 5.855906e-02 5.856283e-02 6.209965e-02 2.014202e-03 1.783620e-03 1.787803e-03 2.028285e-03 2.032991e-03

171 6.155594e-02 6.155950e-02 6.156320e-02 6.156704e-02 6.518465e-02 2.273176e-03 2.014097e-03 2.018802e-03 2.288997e-03 2.294283e-03

172 6.459878e-02 6.460240e-02 6.460617e-02 6.461008e-02 6.830553e-02 2.562142e-03 2.271528e-03 2.276811e-03 2.579885e-03 2.585812e-03

173 6.767651e-02 6.768019e-02 6.768401e-02 6.768798e-02 7.145834e-02 2.883984e-03 2.558565e-03 2.564486e-03 2.903844e-03 2.910479e-03

174 7.078510e-02 7.078883e-02 7.079271e-02 7.079675e-02 7.463902e-02 3.241759e-03 2.878028e-03 2.884653e-03 3.263947e-03 3.271358e-03

175 7.392046e-02 7.392424e-02 7.392818e-02 7.393226e-02 7.784338e-02 3.638691e-03 3.232909e-03 3.240307e-03 3.663428e-03 3.671689e-03

176 7.707832e-02 7.708215e-02 7.708613e-02 7.709027e-02 8.106705e-02 4.078155e-03 3.626359e-03 3.634603e-03 4.105677e-03 4.114867e-03

177 8.025419e-02 8.025807e-02 8.026210e-02 8.026629e-02 8.430537e-02 4.563667e-03 4.061677e-03 4.070844e-03 4.594218e-03 4.604418e-03

178 8.344330e-02 8.344722e-02 8.345130e-02 8.345553e-02 8.755337e-02 5.098853e-03 4.542294e-03 4.552466e-03 5.132691e-03 5.143987e-03

179 8.664053e-02 8.664449e-02 8.664861e-02 8.665288e-02 9.080566e-02 5.687433e-03 5.071750e-03 5.083012e-03 5.724822e-03 5.737301e-03

180 8.984033e-02 8.984433e-02 8.984848e-02 8.985280e-02 9.405641e-02 6.333180e-03 5.653673e-03 5.666112e-03 6.374390e-03 6.388143e-03

181 9.303670e-02 9.304073e-02 9.304492e-02 9.304927e-02 9.729928e-02 7.039888e-03 6.291744e-03 6.305449e-03 7.085196e-03 7.100314e-03

182 9.303670e-02 9.304073e-02 9.304492e-02 9.304927e-02 9.729928e-02 7.039888e-03 6.291744e-03 6.305449e-03 7.085196e-03 7.100314e-03

183 9.303670e-02 9.304073e-02 9.304492e-02 9.304927e-02 9.729928e-02 7.039888e-03 6.291744e-03 6.305449e-03 7.085196e-03 7.100314e-03

184 9.303670e-02 9.304073e-02 9.304492e-02 9.304927e-02 9.729928e-02 7.039888e-03 6.291744e-03 6.305449e-03 7.085196e-03 7.100314e-03

185 9.303670e-02 9.304073e-02 9.304492e-02 9.304927e-02 9.729928e-02 7.039888e-03 6.291744e-03 6.305449e-03 7.085196e-03 7.100314e-03

186 9.303670e-02 9.304073e-02 9.304492e-02 9.304927e-02 9.729928e-02 7.039888e-03 6.291744e-03 6.305449e-03 7.085196e-03 7.100314e-03

187 9.303670e-02 9.304073e-02 9.304492e-02 9.304927e-02 9.729928e-02 7.039888e-03 6.291744e-03 6.305449e-03 7.085196e-03 7.100314e-03

188 9.303670e-02 9.304073e-02 9.304492e-02 9.304927e-02 9.729928e-02 7.039888e-03 6.291744e-03 6.305449e-03 7.085196e-03 7.100314e-03

189 9.303670e-02 9.304073e-02 9.304492e-02 9.304927e-02 9.729928e-02 7.039888e-03 6.291744e-03 6.305449e-03 7.085196e-03 7.100314e-03

190 9.303670e-02 9.304073e-02 9.304492e-02 9.304927e-02 9.729928e-02 7.039888e-03 6.291744e-03 6.305449e-03 7.085196e-03 7.100314e-03

191 9.303670e-02 9.304073e-02 9.304492e-02 9.304927e-02 9.729928e-02 7.039888e-03 6.291744e-03 6.305449e-03 7.085196e-03 7.100314e-03

192 9.303670e-02 9.304073e-02 9.304492e-02 9.304927e-02 9.729928e-02 7.039888e-03 6.291744e-03 6.305449e-03 7.085196e-03 7.100314e-03

193 9.303671e-02 9.304073e-02 9.304492e-02 9.304927e-02 9.729928e-02 7.039888e-03 6.291744e-03 6.305449e-03 7.085196e-03 7.100314e-03

194 9.303671e-02 9.304073e-02 9.304492e-02 9.304927e-02 9.729928e-02 7.039888e-03 6.291744e-03 6.305449e-03 7.085196e-03 7.100314e-03

195 9.303671e-02 9.304073e-02 9.304492e-02 9.304927e-02 9.729928e-02 7.039888e-03 6.291744e-03 6.305449e-03 7.085196e-03 7.100314e-03

196 9.303671e-02 9.304073e-02 9.304492e-02 9.304927e-02 9.729928e-02 7.039888e-03 6.291744e-03 6.305449e-03 7.085196e-03 7.100314e-03

197 9.303671e-02 9.304073e-02 9.304492e-02 9.304927e-02 9.729929e-02 7.039888e-03 6.291744e-03 6.305449e-03 7.085196e-03 7.100314e-03

198 9.303671e-02 9.304074e-02 9.304492e-02 9.304928e-02 9.729929e-02 7.039888e-03 6.291744e-03 6.305449e-03 7.085196e-03 7.100314e-03

199 9.303671e-02 9.304074e-02 9.304493e-02 9.304928e-02 9.729929e-02 7.039888e-03 6.291744e-03 6.305449e-03 7.085196e-03 7.100314e-03

200 9.303671e-02 9.304074e-02 9.304493e-02 9.304928e-02 9.729929e-02 7.039888e-03 6.291744e-03 6.305449e-03 7.085196e-03 7.100314e-03

201 9.303672e-02 9.304074e-02 9.304493e-02 9.304928e-02 9.729930e-02 7.039888e-03 6.291744e-03 6.305449e-03 7.085196e-03 7.100314e-03

202 9.303672e-02 9.304075e-02 9.304493e-02 9.304929e-02 9.729930e-02 7.039888e-03 6.291744e-03 6.305449e-03 7.085196e-03 7.100314e-03

203 9.303673e-02 9.304075e-02 9.304494e-02 9.304929e-02 9.729930e-02 7.039888e-03 6.291744e-03 6.305449e-03 7.085196e-03 7.100314e-03

204 9.303673e-02 9.304075e-02 9.304494e-02 9.304929e-02 9.729931e-02 7.039888e-03 6.291744e-03 6.305449e-03 7.085196e-03 7.100314e-03

205 9.303674e-02 9.304076e-02 9.304495e-02 9.304930e-02 9.729932e-02 7.039888e-03 6.291744e-03 6.305449e-03 7.085196e-03 7.100314e-03

206 9.303674e-02 9.304077e-02 9.304496e-02 9.304931e-02 9.729932e-02 7.039888e-03 6.291744e-03 6.305449e-03 7.085196e-03 7.100314e-03

207 9.303675e-02 9.304078e-02 9.304496e-02 9.304932e-02 9.729933e-02 7.039888e-03 6.291744e-03 6.305449e-03 7.085196e-03 7.100314e-03

208 9.303676e-02 9.304079e-02 9.304497e-02 9.304933e-02 9.729935e-02 7.039888e-03 6.291744e-03 6.305449e-03 7.085197e-03 7.100314e-03

209 9.303677e-02 9.304080e-02 9.304499e-02 9.304934e-02 9.729936e-02 7.039888e-03 6.291744e-03 6.305449e-03 7.085197e-03 7.100314e-03

210 9.303679e-02 9.304081e-02 9.304500e-02 9.304935e-02 9.729938e-02 7.039889e-03 6.291744e-03 6.305449e-03 7.085197e-03 7.100314e-03

211 9.303680e-02 9.304083e-02 9.304502e-02 9.304937e-02 9.729940e-02 7.039889e-03 6.291744e-03 6.305449e-03 7.085197e-03 7.100314e-03

212 9.303682e-02 9.304085e-02 9.304504e-02 9.304939e-02 9.729942e-02 7.039889e-03 6.291744e-03 6.305450e-03 7.085197e-03 7.100314e-03

213 9.303685e-02 9.304087e-02 9.304506e-02 9.304941e-02 9.729945e-02 7.039889e-03 6.291744e-03 6.305450e-03 7.085197e-03 7.100315e-03

214 9.303688e-02 9.304090e-02 9.304509e-02 9.304944e-02 9.729948e-02 7.039889e-03 6.291745e-03 6.305450e-03 7.085197e-03 7.100315e-03

215 9.303691e-02 9.304094e-02 9.304512e-02 9.304948e-02 9.729952e-02 7.039889e-03 6.291745e-03 6.305450e-03 7.085198e-03 7.100315e-03

216 9.303695e-02 9.304098e-02 9.304517e-02 9.304952e-02 9.729957e-02 7.039890e-03 6.291745e-03 6.305450e-03 7.085198e-03 7.100315e-03

217 9.303700e-02 9.304103e-02 9.304521e-02 9.304957e-02 9.729962e-02 7.039890e-03 6.291745e-03 6.305451e-03 7.085198e-03 7.100316e-03

218 9.303706e-02 9.304108e-02 9.304527e-02 9.304962e-02 9.729969e-02 7.039890e-03 6.291746e-03 6.305451e-03 7.085198e-03 7.100316e-03

219 9.303713e-02 9.304115e-02 9.304534e-02 9.304969e-02 9.729977e-02 7.039891e-03 6.291746e-03 6.305451e-03 7.085199e-03 7.100316e-03

220 9.303721e-02 9.304123e-02 9.304542e-02 9.304977e-02 9.729987e-02 7.039891e-03 6.291746e-03 6.305452e-03 7.085199e-03 7.100317e-03

221 9.303730e-02 9.304133e-02 9.304552e-02 9.304987e-02 9.729998e-02 7.039892e-03 6.291747e-03 6.305452e-03 7.085200e-03 7.100318e-03

222 9.303742e-02 9.304144e-02 9.304563e-02 9.304998e-02 9.730011e-02 7.039893e-03 6.291748e-03 6.305453e-03 7.085201e-03 7.100318e-03

223 9.303755e-02 9.304157e-02 9.304576e-02 9.305011e-02 9.730026e-02 7.039894e-03 6.291748e-03 6.305454e-03 7.085202e-03 7.100319e-03

224 9.303771e-02 9.304173e-02 9.304592e-02 9.305027e-02 9.730045e-02 7.039895e-03 6.291749e-03 6.305455e-03 7.085203e-03 7.100320e-03

225 9.303789e-02 9.304192e-02 9.304611e-02 9.305046e-02 9.730066e-02 7.039896e-03 6.291750e-03 6.305456e-03 7.085204e-03 7.100322e-03

226 9.303811e-02 9.304214e-02 9.304633e-02 9.305068e-02 9.730092e-02 7.039897e-03 6.291752e-03 6.305457e-03 7.085205e-03 7.100323e-03

227 9.303837e-02 9.304240e-02 9.304658e-02 9.305094e-02 9.730122e-02 7.039899e-03 6.291753e-03 6.305458e-03 7.085207e-03 7.100325e-03

228 9.303868e-02 9.304270e-02 9.304689e-02 9.305124e-02 9.730157e-02 7.039901e-03 6.291755e-03 6.305460e-03 7.085209e-03 7.100327e-03

229 9.303903e-02 9.304306e-02 9.304725e-02 9.305160e-02 9.730199e-02 7.039903e-03 6.291757e-03 6.305462e-03 7.085212e-03 7.100329e-03

230 9.303945e-02 9.304348e-02 9.304767e-02 9.305202e-02 9.730248e-02 7.039906e-03 6.291759e-03 6.305465e-03 7.085214e-03 7.100332e-03

231 9.303995e-02 9.304397e-02 9.304816e-02 9.305252e-02 9.730306e-02 7.039909e-03 6.291762e-03 6.305467e-03 7.085218e-03 7.100335e-03

232 9.304053e-02 9.304455e-02 9.304874e-02 9.305310e-02 9.730373e-02 7.039913e-03 6.291765e-03 6.305471e-03 7.085221e-03 7.100339e-03

233 9.304121e-02 9.304523e-02 9.304942e-02 9.305378e-02 9.730452e-02 7.039917e-03 6.291769e-03 6.305474e-03 7.085226e-03 7.100344e-03

234 9.304200e-02 9.304603e-02 9.305022e-02 9.305457e-02 9.730545e-02 7.039923e-03 6.291773e-03 6.305479e-03 7.085231e-03 7.100349e-03

235 9.304294e-02 9.304696e-02 9.305115e-02 9.305551e-02 9.730653e-02 7.039929e-03 6.291779e-03 6.305484e-03 7.085237e-03 7.100355e-03

236 9.304403e-02 9.304805e-02 9.305224e-02 9.305660e-02 9.730780e-02 7.039936e-03 6.291785e-03 6.305490e-03 7.085245e-03 7.100362e-03

237 9.304530e-02 9.304933e-02 9.305352e-02 9.305787e-02 9.730928e-02 7.039944e-03 6.291792e-03 6.305498e-03 7.085253e-03 7.100371e-03

238 9.304679e-02 9.305081e-02 9.305501e-02 9.305936e-02 9.731101e-02 7.039954e-03 6.291800e-03 6.305506e-03 7.085263e-03 7.100381e-03

239 9.304852e-02 9.305255e-02 9.305674e-02 9.306110e-02 9.731302e-02 7.039965e-03 6.291810e-03 6.305516e-03 7.085274e-03 7.100392e-03

240 9.305054e-02 9.305457e-02 9.305877e-02 9.306312e-02 9.731537e-02 7.039978e-03 6.291822e-03 6.305527e-03 7.085288e-03 7.100406e-03

241 9.305290e-02 9.305693e-02 9.306112e-02 9.306548e-02 9.731811e-02 7.039994e-03 6.291835e-03 6.305541e-03 7.085303e-03 7.100421e-03

242 9.305564e-02 9.305967e-02 9.306387e-02 9.306823e-02 9.732129e-02 7.040011e-03 6.291850e-03 6.305556e-03 7.085321e-03 7.100439e-03

243 9.305883e-02 9.306286e-02 9.306706e-02 9.307142e-02 9.732500e-02 7.040032e-03 6.291868e-03 6.305574e-03 7.085342e-03 7.100460e-03

244 9.306254e-02 9.306657e-02 9.307077e-02 9.307513e-02 9.732930e-02 7.040056e-03 6.291889e-03 6.305595e-03 7.085367e-03 7.100485e-03

245 9.306684e-02 9.307088e-02 9.307508e-02 9.307944e-02 9.733430e-02 7.040084e-03 6.291913e-03 6.305620e-03 7.085395e-03 7.100514e-03

246 9.307184e-02 9.307587e-02 9.308007e-02 9.308443e-02 9.734009e-02 7.040117e-03 6.291942e-03 6.305648e-03 7.085428e-03 7.100547e-03

247 9.307763e-02 9.308166e-02 9.308586e-02 9.309023e-02 9.734681e-02 7.040155e-03 6.291974e-03 6.305681e-03 7.085466e-03 7.100585e-03

248 9.308433e-02 9.308837e-02 9.309257e-02 9.309694e-02 9.735459e-02 7.040198e-03 6.292012e-03 6.305719e-03 7.085511e-03 7.100629e-03

249 9.309209e-02 9.309614e-02 9.310034e-02 9.310471e-02 9.736360e-02 7.040249e-03 6.292056e-03 6.305763e-03 7.085562e-03 7.100681e-03

250 9.310107e-02 9.310512e-02 9.310932e-02 9.311370e-02 9.737401e-02 7.040307e-03 6.292106e-03 6.305813e-03 7.085621e-03 7.100740e-03

251 9.311145e-02 9.311549e-02 9.311970e-02 9.312408e-02 9.738604e-02 7.040375e-03 6.292165e-03 6.305872e-03 7.085690e-03 7.100809e-03

252 9.312342e-02 9.312747e-02 9.313169e-02 9.313607e-02 9.739992e-02 7.040453e-03 6.292233e-03 6.305940e-03 7.085769e-03 7.100889e-03

253 9.313724e-02 9.314130e-02 9.314551e-02 9.314990e-02 9.741594e-02 7.040543e-03 6.292311e-03 6.306019e-03 7.085860e-03 7.100981e-03

254 9.315317e-02 9.315723e-02 9.316145e-02 9.316584e-02 9.743440e-02 7.040647e-03 6.292401e-03 6.306109e-03 7.085965e-03 7.101086e-03

255 9.317152e-02 9.317558e-02 9.317981e-02 9.318421e-02 9.745566e-02 7.040767e-03 6.292505e-03 6.306214e-03 7.086087e-03 7.101208e-03

256 9.319263e-02 9.319670e-02 9.320094e-02 9.320534e-02 9.748011e-02 7.040905e-03 6.292624e-03 6.306334e-03 7.086227e-03 7.101349e-03

257 9.321690e-02 9.322098e-02 9.322523e-02 9.322964e-02 9.750822e-02 7.041064e-03 6.292762e-03 6.306472e-03 7.086388e-03 7.101510e-03

258 9.324479e-02 9.324888e-02 9.325313e-02 9.325755e-02 9.754050e-02 7.041247e-03 6.292921e-03 6.306631e-03 7.086573e-03 7.101696e-03

259 9.327680e-02 9.328090e-02 9.328516e-02 9.328959e-02 9.757754e-02 7.041456e-03 6.293103e-03 6.306814e-03 7.086785e-03 7.101910e-03

260 9.331350e-02 9.331761e-02 9.332189e-02 9.332633e-02 9.762000e-02 7.041698e-03 6.293312e-03 6.307024e-03 7.087030e-03 7.102155e-03

261 9.335556e-02 9.335968e-02 9.336396e-02 9.336842e-02 9.766863e-02 7.041974e-03 6.293552e-03 6.307265e-03 7.087310e-03 7.102437e-03

262 9.340369e-02 9.340782e-02 9.341212e-02 9.341659e-02 9.772427e-02 7.042291e-03 6.293827e-03 6.307542e-03 7.087632e-03 7.102760e-03

263 9.345872e-02 9.346287e-02 9.346719e-02 9.347168e-02 9.778787e-02 7.042655e-03 6.294142e-03 6.307858e-03 7.088000e-03 7.103130e-03

264 9.352159e-02 9.352576e-02 9.353010e-02 9.353461e-02 9.786048e-02 7.043071e-03 6.294504e-03 6.308221e-03 7.088422e-03 7.103554e-03

265 9.359333e-02 9.359752e-02 9.360188e-02 9.360641e-02 9.794330e-02 7.043548e-03 6.294917e-03 6.308637e-03 7.088905e-03 7.104038e-03

266 9.367511e-02 9.367933e-02 9.368371e-02 9.368827e-02 9.803765e-02 7.044092e-03 6.295390e-03 6.309112e-03 7.089456e-03 7.104593e-03

267 9.376823e-02 9.377247e-02 9.377688e-02 9.378147e-02 9.814503e-02 7.044715e-03 6.295930e-03 6.309654e-03 7.090087e-03 7.105226e-03

268 9.387413e-02 9.387841e-02 9.388285e-02 9.388747e-02 9.826706e-02 7.045425e-03 6.296548e-03 6.310274e-03 7.090807e-03 7.105950e-03

269 9.399443e-02 9.399874e-02 9.400322e-02 9.400788e-02 9.840560e-02 7.046236e-03 6.297252e-03 6.310982e-03 7.091629e-03 7.106775e-03

270 9.413092e-02 9.413526e-02 9.413979e-02 9.414449e-02 9.856264e-02 7.047161e-03 6.298055e-03 6.311789e-03 7.092566e-03 7.107717e-03

271 9.428556e-02 9.428995e-02 9.429452e-02 9.429926e-02 9.874043e-02 7.048215e-03 6.298971e-03 6.312708e-03 7.093634e-03 7.108789e-03

272 9.446052e-02 9.446496e-02 9.446958e-02 9.447439e-02 9.894140e-02 7.049415e-03 6.300013e-03 6.313756e-03 7.094850e-03 7.110010e-03

273 9.465820e-02 9.466270e-02 9.466738e-02 9.467224e-02 9.916823e-02 7.050780e-03 6.301200e-03 6.314947e-03 7.096234e-03 7.111400e-03

274 9.488120e-02 9.488576e-02 9.489050e-02 9.489543e-02 9.942383e-02 7.052333e-03 6.302550e-03 6.316303e-03 7.097807e-03 7.112981e-03

275 9.513234e-02 9.513697e-02 9.514179e-02 9.514680e-02 9.971136e-02 7.054097e-03 6.304083e-03 6.317843e-03 7.099595e-03 7.114776e-03

276 9.541472e-02 9.541943e-02 9.542433e-02 9.542941e-02 1.000342e-01 7.056101e-03 6.305825e-03 6.319593e-03 7.101625e-03 7.116816e-03

277 9.573163e-02 9.573643e-02 9.574142e-02 9.574660e-02 1.003960e-01 7.058374e-03 6.307802e-03 6.321579e-03 7.103929e-03 7.119130e-03

278 9.608663e-02 9.609152e-02 9.609661e-02 9.610190e-02 1.008007e-01 7.060953e-03 6.310045e-03 6.323831e-03 7.106542e-03 7.121754e-03

279 9.648349e-02 9.648849e-02 9.649369e-02 9.649909e-02 1.012523e-01 7.063874e-03 6.312586e-03 6.326384e-03 7.109502e-03 7.124727e-03

280 9.692622e-02 9.693134e-02 9.693666e-02 9.694219e-02 1.017553e-01 7.067183e-03 6.315465e-03 6.329275e-03 7.112855e-03 7.128095e-03

281 9.741902e-02 9.742426e-02 9.742972e-02 9.743538e-02 1.023140e-01 7.070927e-03 6.318723e-03 6.332548e-03 7.116649e-03 7.131906e-03

282 9.796625e-02 9.797164e-02 9.797724e-02 9.798305e-02 1.029331e-01 7.075162e-03 6.322408e-03 6.336249e-03 7.120940e-03 7.136216e-03

283 9.857243e-02 9.857797e-02 9.858373e-02 9.858971e-02 1.036173e-01 7.079947e-03 6.326573e-03 6.340433e-03 7.125789e-03 7.141086e-03

284 9.924218e-02 9.924788e-02 9.925381e-02 9.925997e-02 1.043715e-01 7.085352e-03 6.331278e-03 6.345159e-03 7.131265e-03 7.146587e-03

285 9.998015e-02 9.998603e-02 9.999214e-02 9.999849e-02 1.052004e-01 7.091451e-03 6.336589e-03 6.350493e-03 7.137446e-03 7.152794e-03

286 1.007910e-01 1.007971e-01 1.008034e-01 1.008099e-01 1.061087e-01 7.098330e-03 6.342580e-03 6.356510e-03 7.144416e-03 7.159795e-03

287 1.016793e-01 1.016855e-01 1.016921e-01 1.016988e-01 1.071009e-01 7.106083e-03 6.349333e-03 6.363293e-03 7.152272e-03 7.167686e-03

288 1.026494e-01 1.026559e-01 1.026627e-01 1.026697e-01 1.081813e-01 7.114815e-03 6.356939e-03 6.370933e-03 7.161120e-03 7.176572e-03

289 1.037057e-01 1.037124e-01 1.037194e-01 1.037266e-01 1.093539e-01 7.124643e-03 6.365503e-03 6.379534e-03 7.171077e-03 7.186574e-03

290 1.048519e-01 1.048589e-01 1.048661e-01 1.048736e-01 1.106224e-01 7.135696e-03 6.375136e-03 6.389210e-03 7.182277e-03 7.197822e-03

291 1.060917e-01 1.060990e-01 1.061064e-01 1.061142e-01 1.119898e-01 7.148119e-03 6.385965e-03 6.400086e-03 7.194865e-03 7.210465e-03

292 1.074282e-01 1.074357e-01 1.074434e-01 1.074515e-01 1.134588e-01 7.162073e-03 6.398130e-03 6.412305e-03 7.209002e-03 7.224665e-03

293 1.088640e-01 1.088717e-01 1.088797e-01 1.088881e-01 1.150314e-01 7.177734e-03 6.411786e-03 6.426022e-03 7.224870e-03 7.240602e-03

294 1.104010e-01 1.104091e-01 1.104174e-01 1.104260e-01 1.167091e-01 7.195299e-03 6.427106e-03 6.441409e-03 7.242667e-03 7.258477e-03

295 1.120409e-01 1.120492e-01 1.120578e-01 1.120667e-01 1.184925e-01 7.214986e-03 6.444281e-03 6.458660e-03 7.262613e-03 7.278510e-03

296 1.137843e-01 1.137928e-01 1.138017e-01 1.138110e-01 1.203818e-01 7.237036e-03 6.463521e-03 6.477984e-03 7.284953e-03 7.300947e-03

297 1.156314e-01 1.156402e-01 1.156494e-01 1.156589e-01 1.223763e-01 7.261714e-03 6.485060e-03 6.499617e-03 7.309955e-03 7.326059e-03

298 1.175816e-01 1.175908e-01 1.176003e-01 1.176101e-01 1.244749e-01 7.289314e-03 6.509155e-03 6.523818e-03 7.337918e-03 7.354143e-03

299 1.196340e-01 1.196434e-01 1.196531e-01 1.196633e-01 1.266754e-01 7.320160e-03 6.536090e-03 6.550871e-03 7.369168e-03 7.385529e-03

300 1.217865e-01 1.217962e-01 1.218063e-01 1.218167e-01 1.289755e-01 7.354608e-03 6.566179e-03 6.581090e-03 7.404067e-03 7.420578e-03

301 1.240368e-01 1.240468e-01 1.240572e-01 1.240679e-01 1.313720e-01 7.393050e-03 6.599765e-03 6.614823e-03 7.443011e-03 7.459691e-03

302 1.263821e-01 1.263923e-01 1.264029e-01 1.264140e-01 1.338612e-01 7.435917e-03 6.637227e-03 6.652449e-03 7.486436e-03 7.503304e-03

303 1.288186e-01 1.288291e-01 1.288400e-01 1.288514e-01 1.364392e-01 7.483683e-03 6.678983e-03 6.694386e-03 7.534823e-03 7.551899e-03

304 1.313426e-01 1.313534e-01 1.313645e-01 1.313761e-01 1.391012e-01 7.536864e-03 6.725489e-03 6.741093e-03 7.588695e-03 7.606003e-03

305 1.339496e-01 1.339606e-01 1.339720e-01 1.339839e-01 1.418426e-01 7.596030e-03 6.777244e-03 6.793074e-03 7.648627e-03 7.666193e-03

306 1.366349e-01 1.366461e-01 1.366578e-01 1.366699e-01 1.446579e-01 7.661801e-03 6.834798e-03 6.850876e-03 7.715248e-03 7.733099e-03

307 1.393932e-01 1.394047e-01 1.394166e-01 1.394290e-01 1.475418e-01 7.734856e-03 6.898750e-03 6.915103e-03 7.789245e-03 7.807411e-03

308 1.422193e-01 1.422310e-01 1.422432e-01 1.422558e-01 1.504883e-01 7.815933e-03 6.969751e-03 6.986411e-03 7.871364e-03 7.889880e-03

309 1.451075e-01 1.451193e-01 1.451317e-01 1.451445e-01 1.534915e-01 7.905838e-03 7.048517e-03 7.065514e-03 7.962421e-03 7.981324e-03

310 1.480517e-01 1.480637e-01 1.480763e-01 1.480893e-01 1.565450e-01 8.005446e-03 7.135821e-03 7.153191e-03 8.063301e-03 8.082630e-03

311 1.510458e-01 1.510580e-01 1.510708e-01 1.510840e-01 1.596424e-01 8.115705e-03 7.232506e-03 7.250290e-03 8.174964e-03 8.194763e-03

312 1.540834e-01 1.540959e-01 1.541088e-01 1.541222e-01 1.627771e-01 8.237646e-03 7.339487e-03 7.357726e-03 8.298449e-03 8.318767e-03

313 1.571580e-01 1.571706e-01 1.571836e-01 1.571972e-01 1.659421e-01 8.372378e-03 7.457754e-03 7.476494e-03 8.434883e-03 8.455771e-03

314 1.602627e-01 1.602754e-01 1.602886e-01 1.603023e-01 1.691304e-01 8.521102e-03 7.588377e-03 7.607668e-03 8.585476e-03 8.606990e-03

315 1.633904e-01 1.634032e-01 1.634166e-01 1.634305e-01 1.723346e-01 8.685108e-03 7.732509e-03 7.752406e-03 8.751534e-03 8.773736e-03

316 1.665340e-01 1.665469e-01 1.665604e-01 1.665744e-01 1.755473e-01 8.865784e-03 7.891393e-03 7.911955e-03 8.934458e-03 8.957413e-03

317 1.696861e-01 1.696991e-01 1.697127e-01 1.697269e-01 1.787609e-01 9.064616e-03 8.066363e-03 8.087654e-03 9.135749e-03 9.159528e-03

318 1.728391e-01 1.728522e-01 1.728659e-01 1.728801e-01 1.819675e-01 9.283190e-03 8.258847e-03 8.280935e-03 9.357011e-03 9.381690e-03

319 1.759853e-01 1.759985e-01 1.760123e-01 1.760266e-01 1.851592e-01 9.523198e-03 8.470373e-03 8.493333e-03 9.599951e-03 9.625613e-03

320 1.791169e-01 1.791301e-01 1.791440e-01 1.791584e-01 1.883280e-01 9.786438e-03 8.702568e-03 8.726478e-03 9.866384e-03 9.893115e-03

321 1.822260e-01 1.822393e-01 1.822532e-01 1.822676e-01 1.914660e-01 1.007481e-02 8.957160e-03 8.982106e-03 1.015823e-02 1.018612e-02

322 1.853047e-01 1.853181e-01 1.853320e-01 1.853465e-01 1.945651e-01 1.039033e-02 9.235980e-03 9.262052e-03 1.047751e-02 1.050667e-02

323 1.883453e-01 1.883587e-01 1.883727e-01 1.883872e-01 1.976176e-01 1.073510e-02 9.540960e-03 9.568254e-03 1.082636e-02 1.085688e-02

324 1.913402e-01 1.913536e-01 1.913676e-01 1.913822e-01 2.006160e-01 1.111133e-02 9.874132e-03 9.902750e-03 1.120700e-02 1.123899e-02

325 1.942821e-01 1.942955e-01 1.943095e-01 1.943241e-01 2.035533e-01 1.152132e-02 1.023762e-02 1.026767e-02 1.162175e-02 1.165533e-02

326 1.971641e-01 1.971774e-01 1.971914e-01 1.972060e-01 2.064227e-01 1.196747e-02 1.063365e-02 1.066525e-02 1.207302e-02 1.210831e-02

327 1.999797e-01 1.999931e-01 2.000071e-01 2.000216e-01 2.092183e-01 1.245224e-02 1.106453e-02 1.109778e-02 1.256328e-02 1.260041e-02

328 2.027233e-01 2.027366e-01 2.027506e-01 2.027651e-01 2.119349e-01 1.297815e-02 1.153262e-02 1.156766e-02 1.309509e-02 1.313418e-02

329 2.053898e-01 2.054030e-01 2.054170e-01 2.054315e-01 2.145681e-01 1.354780e-02 1.204037e-02 1.207733e-02 1.367102e-02 1.371222e-02

330 2.079750e-01 2.079882e-01 2.080021e-01 2.080165e-01 2.171143e-01 1.416378e-02 1.259029e-02 1.262929e-02 1.429371e-02 1.433714e-02

331 2.104755e-01 2.104886e-01 2.105025e-01 2.105169e-01 2.195710e-01 1.482874e-02 1.318489e-02 1.322607e-02 1.496578e-02 1.501159e-02

332 2.128889e-01 2.129020e-01 2.129158e-01 2.129302e-01 2.219364e-01 1.554527e-02 1.382672e-02 1.387023e-02 1.568986e-02 1.573819e-02

333 2.152138e-01 2.152268e-01 2.152406e-01 2.152549e-01 2.242101e-01 1.631597e-02 1.451833e-02 1.456430e-02 1.646854e-02 1.651952e-02

334 2.174496e-01 2.174625e-01 2.174762e-01 2.174904e-01 2.263921e-01 1.714336e-02 1.526223e-02 1.531080e-02 1.730432e-02 1.735811e-02

335 2.195964e-01 2.196093e-01 2.196229e-01 2.196371e-01 2.284836e-01 1.802988e-02 1.606088e-02 1.611219e-02 1.819965e-02 1.825637e-02

336 2.216555e-01 2.216683e-01 2.216819e-01 2.216960e-01 2.304863e-01 1.897782e-02 1.691666e-02 1.697085e-02 1.915682e-02 1.921661e-02

337 2.236286e-01 2.236412e-01 2.236548e-01 2.236688e-01 2.324026e-01 1.998935e-02 1.783183e-02 1.788905e-02 2.017796e-02 2.024096e-02

338 2.255180e-01 2.255306e-01 2.255441e-01 2.255581e-01 2.342356e-01 2.106643e-02 1.880853e-02 1.886890e-02 2.126504e-02 2.133136e-02

339 2.273267e-01 2.273392e-01 2.273526e-01 2.273666e-01 2.359887e-01 2.221081e-02 1.984871e-02 1.991236e-02 2.241976e-02 2.248953e-02

340 2.290579e-01 2.290704e-01 2.290837e-01 2.290976e-01 2.376655e-01 2.342396e-02 2.095410e-02 2.102115e-02 2.364359e-02 2.371691e-02

341 2.307153e-01 2.307277e-01 2.307410e-01 2.307548e-01 2.392700e-01 2.470709e-02 2.212621e-02 2.219678e-02 2.493769e-02 2.501465e-02

342 2.323026e-01 2.323149e-01 2.323281e-01 2.323419e-01 2.408062e-01 2.606107e-02 2.336627e-02 2.344045e-02 2.630289e-02 2.638358e-02

343 2.338237e-01 2.338358e-01 2.338491e-01 2.338628e-01 2.422781e-01 2.748639e-02 2.467518e-02 2.475306e-02 2.773965e-02 2.782414e-02

344 2.352824e-01 2.352945e-01 2.353077e-01 2.353214e-01 2.436897e-01 2.898320e-02 2.605353e-02 2.613520e-02 2.924807e-02 2.933642e-02

345 2.366827e-01 2.366947e-01 2.367078e-01 2.367215e-01 2.450451e-01 3.055121e-02 2.750153e-02 2.758705e-02 3.082782e-02 3.092006e-02

346 2.380282e-01 2.380402e-01 2.380533e-01 2.380669e-01 2.463479e-01 3.218970e-02 2.901900e-02 2.910841e-02 3.247813e-02 3.257428e-02

347 2.393227e-01 2.393346e-01 2.393477e-01 2.393612e-01 2.476018e-01 3.389753e-02 3.060535e-02 3.069869e-02 3.419780e-02 3.429787e-02

348 2.405697e-01 2.405814e-01 2.405945e-01 2.406080e-01 2.488102e-01 3.567307e-02 3.225955e-02 3.235683e-02 3.598514e-02 3.608912e-02

349 2.417723e-01 2.417840e-01 2.417970e-01 2.418105e-01 2.499764e-01 3.751423e-02 3.398013e-02 3.408136e-02 3.783802e-02 3.794588e-02

350 2.429338e-01 2.429454e-01 2.429584e-01 2.429719e-01 2.511033e-01 3.941846e-02 3.576518e-02 3.587033e-02 3.975383e-02 3.986552e-02

351 2.440569e-01 2.440685e-01 2.440815e-01 2.440949e-01 2.521938e-01 4.138274e-02 3.761230e-02 3.772134e-02 4.172950e-02 4.184495e-02

352 2.451445e-01 2.451560e-01 2.451689e-01 2.451824e-01 2.532504e-01 4.340360e-02 3.951868e-02 3.963154e-02 4.376149e-02 4.388062e-02

353 2.461990e-01 2.462104e-01 2.462233e-01 2.462368e-01 2.542756e-01 4.547711e-02 4.148102e-02 4.159763e-02 4.584583e-02 4.596853e-02

354 2.472227e-01 2.472340e-01 2.472469e-01 2.472603e-01 2.552715e-01 4.759893e-02 4.349561e-02 4.361589e-02 4.797814e-02 4.810430e-02

355 2.482177e-01 2.482289e-01 2.482418e-01 2.482552e-01 2.562401e-01 4.976435e-02 4.555833e-02 4.568216e-02 5.015363e-02 5.028312e-02

356 2.491859e-01 2.491971e-01 2.492100e-01 2.492234e-01 2.571833e-01 5.196826e-02 4.766465e-02 4.779191e-02 5.236719e-02 5.249985e-02

357 2.501291e-01 2.501403e-01 2.501531e-01 2.501665e-01 2.581028e-01 5.420525e-02 4.980971e-02 4.994025e-02 5.461334e-02 5.474902e-02

358 2.510490e-01 2.510601e-01 2.510729e-01 2.510863e-01 2.589999e-01 5.646964e-02 5.198830e-02 5.212198e-02 5.688637e-02 5.702490e-02

359 2.519470e-01 2.519579e-01 2.519708e-01 2.519841e-01 2.598763e-01 5.875550e-02 5.419494e-02 5.433159e-02 5.918033e-02 5.932152e-02

360 2.528244e-01 2.528353e-01 2.528481e-01 2.528615e-01 2.607330e-01 6.105673e-02 5.642393e-02 5.656337e-02 6.148910e-02 6.163275e-02

361 2.536824e-01 2.536932e-01 2.537061e-01 2.537194e-01 2.615712e-01 6.336713e-02 5.866936e-02 5.881141e-02 6.380643e-02 6.395236e-02

362 2.536826e-01 2.536934e-01 2.537063e-01 2.537196e-01 2.615715e-01 6.336713e-02 5.866936e-02 5.881141e-02 6.380643e-02 6.395236e-02

363 2.536828e-01 2.536937e-01 2.537065e-01 2.537198e-01 2.615718e-01 6.336713e-02 5.866936e-02 5.881141e-02 6.380643e-02 6.395236e-02

364 2.536831e-01 2.536939e-01 2.537068e-01 2.537201e-01 2.615721e-01 6.336713e-02 5.866937e-02 5.881141e-02 6.380643e-02 6.395236e-02

365 2.536834e-01 2.536942e-01 2.537071e-01 2.537204e-01 2.615724e-01 6.336713e-02 5.866937e-02 5.881142e-02 6.380643e-02 6.395236e-02

366 2.536838e-01 2.536946e-01 2.537074e-01 2.537208e-01 2.615728e-01 6.336713e-02 5.866937e-02 5.881142e-02 6.380643e-02 6.395236e-02

367 2.536842e-01 2.536950e-01 2.537078e-01 2.537212e-01 2.615733e-01 6.336713e-02 5.866937e-02 5.881142e-02 6.380644e-02 6.395237e-02

368 2.536846e-01 2.536955e-01 2.537083e-01 2.537216e-01 2.615739e-01 6.336714e-02 5.866937e-02 5.881142e-02 6.380644e-02 6.395237e-02

369 2.536852e-01 2.536960e-01 2.537088e-01 2.537222e-01 2.615745e-01 6.336714e-02 5.866937e-02 5.881142e-02 6.380644e-02 6.395237e-02

370 2.536858e-01 2.536966e-01 2.537095e-01 2.537228e-01 2.615752e-01 6.336714e-02 5.866938e-02 5.881143e-02 6.380644e-02 6.395237e-02

371 2.536865e-01 2.536973e-01 2.537102e-01 2.537235e-01 2.615761e-01 6.336715e-02 5.866938e-02 5.881143e-02 6.380645e-02 6.395238e-02

372 2.536873e-01 2.536981e-01 2.537110e-01 2.537243e-01 2.615770e-01 6.336715e-02 5.866938e-02 5.881143e-02 6.380645e-02 6.395238e-02

373 2.536882e-01 2.536991e-01 2.537119e-01 2.537252e-01 2.615781e-01 6.336715e-02 5.866939e-02 5.881144e-02 6.380645e-02 6.395239e-02

374 2.536893e-01 2.537001e-01 2.537130e-01 2.537263e-01 2.615794e-01 6.336716e-02 5.866939e-02 5.881144e-02 6.380646e-02 6.395239e-02

375 2.536905e-01 2.537013e-01 2.537142e-01 2.537275e-01 2.615808e-01 6.336716e-02 5.866940e-02 5.881144e-02 6.380647e-02 6.395240e-02

376 2.536919e-01 2.537027e-01 2.537156e-01 2.537289e-01 2.615824e-01 6.336717e-02 5.866940e-02 5.881145e-02 6.380647e-02 6.395240e-02

377 2.536935e-01 2.537043e-01 2.537172e-01 2.537305e-01 2.615843e-01 6.336718e-02 5.866941e-02 5.881146e-02 6.380648e-02 6.395241e-02

378 2.536953e-01 2.537061e-01 2.537190e-01 2.537323e-01 2.615864e-01 6.336719e-02 5.866942e-02 5.881146e-02 6.380649e-02 6.395242e-02

379 2.536974e-01 2.537082e-01 2.537211e-01 2.537344e-01 2.615889e-01 6.336720e-02 5.866942e-02 5.881147e-02 6.380650e-02 6.395243e-02

380 2.536997e-01 2.537106e-01 2.537234e-01 2.537368e-01 2.615917e-01 6.336721e-02 5.866943e-02 5.881148e-02 6.380651e-02 6.395244e-02

381 2.537024e-01 2.537133e-01 2.537261e-01 2.537395e-01 2.615948e-01 6.336722e-02 5.866944e-02 5.881149e-02 6.380652e-02 6.395246e-02

382 2.537055e-01 2.537164e-01 2.537292e-01 2.537426e-01 2.615985e-01 6.336724e-02 5.866946e-02 5.881151e-02 6.380654e-02 6.395247e-02

383 2.537090e-01 2.537199e-01 2.537327e-01 2.537461e-01 2.616026e-01 6.336725e-02 5.866947e-02 5.881152e-02 6.380656e-02 6.395249e-02

384 2.537130e-01 2.537239e-01 2.537367e-01 2.537501e-01 2.616073e-01 6.336727e-02 5.866949e-02 5.881154e-02 6.380658e-02 6.395251e-02

385 2.537176e-01 2.537284e-01 2.537413e-01 2.537546e-01 2.616126e-01 6.336729e-02 5.866951e-02 5.881156e-02 6.380660e-02 6.395253e-02

386 2.537227e-01 2.537336e-01 2.537465e-01 2.537598e-01 2.616187e-01 6.336732e-02 5.866953e-02 5.881158e-02 6.380662e-02 6.395256e-02

387 2.537286e-01 2.537395e-01 2.537523e-01 2.537657e-01 2.616256e-01 6.336735e-02 5.866955e-02 5.881160e-02 6.380665e-02 6.395258e-02

388 2.537353e-01 2.537461e-01 2.537590e-01 2.537724e-01 2.616335e-01 6.336738e-02 5.866958e-02 5.881163e-02 6.380668e-02 6.395262e-02

389 2.537429e-01 2.537537e-01 2.537666e-01 2.537800e-01 2.616424e-01 6.336741e-02 5.866961e-02 5.881166e-02 6.380672e-02 6.395265e-02

390 2.537515e-01 2.537623e-01 2.537752e-01 2.537886e-01 2.616525e-01 6.336746e-02 5.866965e-02 5.881170e-02 6.380676e-02 6.395270e-02

391 2.537612e-01 2.537721e-01 2.537850e-01 2.537984e-01 2.616640e-01 6.336750e-02 5.866969e-02 5.881174e-02 6.380681e-02 6.395274e-02

392 2.537723e-01 2.537832e-01 2.537961e-01 2.538094e-01 2.616770e-01 6.336756e-02 5.866973e-02 5.881178e-02 6.380686e-02 6.395280e-02

393 2.537848e-01 2.537957e-01 2.538086e-01 2.538220e-01 2.616917e-01 6.336762e-02 5.866978e-02 5.881183e-02 6.380693e-02 6.395286e-02

394 2.537990e-01 2.538099e-01 2.538228e-01 2.538362e-01 2.617083e-01 6.336768e-02 5.866984e-02 5.881189e-02 6.380700e-02 6.395293e-02

395 2.538150e-01 2.538259e-01 2.538388e-01 2.538523e-01 2.617271e-01 6.336776e-02 5.866991e-02 5.881196e-02 6.380707e-02 6.395301e-02

396 2.538332e-01 2.538441e-01 2.538570e-01 2.538704e-01 2.617484e-01 6.336785e-02 5.866998e-02 5.881203e-02 6.380716e-02 6.395310e-02

397 2.538536e-01 2.538646e-01 2.538775e-01 2.538910e-01 2.617725e-01 6.336795e-02 5.867007e-02 5.881212e-02 6.380726e-02 6.395320e-02

398 2.538768e-01 2.538877e-01 2.539007e-01 2.539141e-01 2.617996e-01 6.336806e-02 5.867016e-02 5.881221e-02 6.380738e-02 6.395332e-02

399 2.539029e-01 2.539139e-01 2.539268e-01 2.539403e-01 2.618303e-01 6.336818e-02 5.867027e-02 5.881232e-02 6.380751e-02 6.395344e-02

400 2.539324e-01 2.539433e-01 2.539563e-01 2.539698e-01 2.618648e-01 6.336832e-02 5.867039e-02 5.881245e-02 6.380765e-02 6.395359e-02

401 2.539656e-01 2.539766e-01 2.539896e-01 2.540031e-01 2.619037e-01 6.336849e-02 5.867053e-02 5.881259e-02 6.380781e-02 6.395376e-02

402 2.540030e-01 2.540140e-01 2.540270e-01 2.540406e-01 2.619475e-01 6.336867e-02 5.867068e-02 5.881274e-02 6.380800e-02 6.395394e-02

403 2.540451e-01 2.540561e-01 2.540692e-01 2.540827e-01 2.619969e-01 6.336887e-02 5.867086e-02 5.881292e-02 6.380821e-02 6.395415e-02

404 2.540925e-01 2.541035e-01 2.541166e-01 2.541302e-01 2.620523e-01 6.336910e-02 5.867106e-02 5.881312e-02 6.380844e-02 6.395439e-02

405 2.541457e-01 2.541568e-01 2.541699e-01 2.541835e-01 2.621146e-01 6.336936e-02 5.867128e-02 5.881334e-02 6.380871e-02 6.395465e-02

406 2.542055e-01 2.542166e-01 2.542298e-01 2.542434e-01 2.621846e-01 6.336965e-02 5.867153e-02 5.881359e-02 6.380900e-02 6.395495e-02

407 2.542726e-01 2.542838e-01 2.542970e-01 2.543107e-01 2.622631e-01 6.336998e-02 5.867181e-02 5.881388e-02 6.380934e-02 6.395529e-02

408 2.543479e-01 2.543591e-01 2.543724e-01 2.543861e-01 2.623511e-01 6.337035e-02 5.867213e-02 5.881420e-02 6.380972e-02 6.395567e-02

409 2.544323e-01 2.544435e-01 2.544568e-01 2.544706e-01 2.624496e-01 6.337076e-02 5.867248e-02 5.881455e-02 6.381014e-02 6.395610e-02

410 2.545268e-01 2.545381e-01 2.545514e-01 2.545653e-01 2.625600e-01 6.337123e-02 5.867288e-02 5.881496e-02 6.381061e-02 6.395658e-02

411 2.546325e-01 2.546439e-01 2.546573e-01 2.546712e-01 2.626833e-01 6.337175e-02 5.867333e-02 5.881541e-02 6.381115e-02 6.395711e-02

412 2.547507e-01 2.547621e-01 2.547756e-01 2.547896e-01 2.628212e-01 6.337233e-02 5.867384e-02 5.881592e-02 6.381175e-02 6.395772e-02

413 2.548827e-01 2.548942e-01 2.549078e-01 2.549218e-01 2.629750e-01 6.337299e-02 5.867440e-02 5.881649e-02 6.381242e-02 6.395839e-02

414 2.550301e-01 2.550417e-01 2.550553e-01 2.550694e-01 2.631465e-01 6.337373e-02 5.867504e-02 5.881712e-02 6.381317e-02 6.395915e-02

415 2.551943e-01 2.552060e-01 2.552197e-01 2.552339e-01 2.633376e-01 6.337455e-02 5.867575e-02 5.881784e-02 6.381401e-02 6.396000e-02

416 2.553772e-01 2.553890e-01 2.554028e-01 2.554172e-01 2.635503e-01 6.337548e-02 5.867654e-02 5.881864e-02 6.381496e-02 6.396095e-02

417 2.555808e-01 2.555926e-01 2.556066e-01 2.556210e-01 2.637866e-01 6.337651e-02 5.867743e-02 5.881954e-02 6.381602e-02 6.396202e-02

418 2.558069e-01 2.558189e-01 2.558330e-01 2.558476e-01 2.640490e-01 6.337767e-02 5.867843e-02 5.882054e-02 6.381720e-02 6.396321e-02

419 2.560580e-01 2.560701e-01 2.560843e-01 2.560990e-01 2.643400e-01 6.337897e-02 5.867954e-02 5.882167e-02 6.381852e-02 6.396454e-02

420 2.563363e-01 2.563486e-01 2.563629e-01 2.563778e-01 2.646621e-01 6.338041e-02 5.868079e-02 5.882292e-02 6.382000e-02 6.396603e-02

421 2.566445e-01 2.566569e-01 2.566715e-01 2.566865e-01 2.650184e-01 6.338203e-02 5.868218e-02 5.882432e-02 6.382165e-02 6.396769e-02

422 2.569853e-01 2.569979e-01 2.570126e-01 2.570279e-01 2.654119e-01 6.338383e-02 5.868374e-02 5.882589e-02 6.382349e-02 6.396955e-02

423 2.573616e-01 2.573744e-01 2.573893e-01 2.574048e-01 2.658457e-01 6.338585e-02 5.868547e-02 5.882763e-02 6.382555e-02 6.397162e-02

424 2.577765e-01 2.577895e-01 2.578047e-01 2.578204e-01 2.663232e-01 6.338809e-02 5.868741e-02 5.882958e-02 6.382784e-02 6.397393e-02

425 2.582333e-01 2.582466e-01 2.582619e-01 2.582779e-01 2.668481e-01 6.339060e-02 5.868956e-02 5.883175e-02 6.383040e-02 6.397650e-02

426 2.587354e-01 2.587489e-01 2.587645e-01 2.587807e-01 2.674239e-01 6.339338e-02 5.869197e-02 5.883418e-02 6.383325e-02 6.397937e-02

427 2.592862e-01 2.593000e-01 2.593160e-01 2.593325e-01 2.680545e-01 6.339649e-02 5.869464e-02 5.883687e-02 6.383642e-02 6.398257e-02

428 2.598896e-01 2.599037e-01 2.599199e-01 2.599367e-01 2.687438e-01 6.339995e-02 5.869762e-02 5.883987e-02 6.383995e-02 6.398612e-02

429 2.605492e-01 2.605636e-01 2.605802e-01 2.605973e-01 2.694956e-01 6.340379e-02 5.870094e-02 5.884321e-02 6.384388e-02 6.399007e-02

430 2.612688e-01 2.612836e-01 2.613005e-01 2.613181e-01 2.703140e-01 6.340806e-02 5.870462e-02 5.884692e-02 6.384824e-02 6.399447e-02

431 2.620524e-01 2.620676e-01 2.620849e-01 2.621028e-01 2.712029e-01 6.341281e-02 5.870872e-02 5.885105e-02 6.385309e-02 6.399936e-02

432 2.629038e-01 2.629193e-01 2.629370e-01 2.629554e-01 2.721661e-01 6.341809e-02 5.871327e-02 5.885563e-02 6.385848e-02 6.400478e-02

433 2.638268e-01 2.638427e-01 2.638608e-01 2.638796e-01 2.732076e-01 6.342395e-02 5.871833e-02 5.886072e-02 6.386446e-02 6.401081e-02

434 2.648250e-01 2.648414e-01 2.648600e-01 2.648792e-01 2.743307e-01 6.343044e-02 5.872394e-02 5.886637e-02 6.387110e-02 6.401749e-02

435 2.659021e-01 2.659190e-01 2.659380e-01 2.659578e-01 2.755390e-01 6.343765e-02 5.873016e-02 5.887264e-02 6.387846e-02 6.402490e-02

436 2.670615e-01 2.670788e-01 2.670984e-01 2.671187e-01 2.768355e-01 6.344564e-02 5.873705e-02 5.887958e-02 6.388662e-02 6.403312e-02

437 2.683062e-01 2.683241e-01 2.683441e-01 2.683649e-01 2.782230e-01 6.345449e-02 5.874470e-02 5.888728e-02 6.389566e-02 6.404222e-02

438 2.696391e-01 2.696575e-01 2.696781e-01 2.696994e-01 2.797038e-01 6.346429e-02 5.875316e-02 5.889580e-02 6.390567e-02 6.405230e-02

439 2.710626e-01 2.710815e-01 2.711026e-01 2.711246e-01 2.812799e-01 6.347513e-02 5.876253e-02 5.890524e-02 6.391674e-02 6.406345e-02

440 2.725787e-01 2.725981e-01 2.726199e-01 2.726424e-01 2.829527e-01 6.348713e-02 5.877289e-02 5.891568e-02 6.392899e-02 6.407579e-02

441 2.741890e-01 2.742090e-01 2.742313e-01 2.742545e-01 2.847231e-01 6.350039e-02 5.878436e-02 5.892722e-02 6.394253e-02 6.408943e-02

442 2.758945e-01 2.759152e-01 2.759381e-01 2.759619e-01 2.865915e-01 6.351504e-02 5.879702e-02 5.893998e-02 6.395750e-02 6.410450e-02

443 2.776959e-01 2.777171e-01 2.777407e-01 2.777651e-01 2.885576e-01 6.353122e-02 5.881102e-02 5.895407e-02 6.397402e-02 6.412114e-02

444 2.795931e-01 2.796149e-01 2.796391e-01 2.796641e-01 2.906206e-01 6.354908e-02 5.882646e-02 5.896963e-02 6.399227e-02 6.413951e-02

445 2.815854e-01 2.816078e-01 2.816326e-01 2.816582e-01 2.927790e-01 6.356879e-02 5.884351e-02 5.898679e-02 6.401239e-02 6.415977e-02

446 2.836717e-01 2.836947e-01 2.837201e-01 2.837464e-01 2.950308e-01 6.359052e-02 5.886231e-02 5.900573e-02 6.403458e-02 6.418212e-02

447 2.858501e-01 2.858737e-01 2.858997e-01 2.859266e-01 2.973733e-01 6.361447e-02 5.888303e-02 5.902659e-02 6.405903e-02 6.420674e-02

448 2.881182e-01 2.881424e-01 2.881690e-01 2.881966e-01 2.998032e-01 6.364084e-02 5.890587e-02 5.904959e-02 6.408596e-02 6.423386e-02

449 2.904732e-01 2.904979e-01 2.905251e-01 2.905532e-01 3.023167e-01 6.366987e-02 5.893100e-02 5.907490e-02 6.411561e-02 6.426371e-02

450 2.929113e-01 2.929366e-01 2.929643e-01 2.929931e-01 3.049095e-01 6.370182e-02 5.895867e-02 5.910276e-02 6.414822e-02 6.429655e-02

451 2.954285e-01 2.954543e-01 2.954826e-01 2.955120e-01 3.075766e-01 6.373694e-02 5.898910e-02 5.913340e-02 6.418409e-02 6.433267e-02

452 2.980201e-01 2.980465e-01 2.980754e-01 2.981053e-01 3.103128e-01 6.377555e-02 5.902255e-02 5.916709e-02 6.422350e-02 6.437236e-02

453 3.006812e-01 3.007081e-01 3.007375e-01 3.007679e-01 3.131121e-01 6.381795e-02 5.905930e-02 5.920410e-02 6.426679e-02 6.441595e-02

454 3.034061e-01 3.034335e-01 3.034633e-01 3.034943e-01 3.159683e-01 6.386449e-02 5.909966e-02 5.924473e-02 6.431431e-02 6.446379e-02

455 3.061888e-01 3.062166e-01 3.062470e-01 3.062784e-01 3.188750e-01 6.391556e-02 5.914394e-02 5.928933e-02 6.436644e-02 6.451628e-02

456 3.090230e-01 3.090513e-01 3.090821e-01 3.091140e-01 3.218251e-01 6.397155e-02 5.919252e-02 5.933824e-02 6.442360e-02 6.457383e-02

457 3.119020e-01 3.119307e-01 3.119619e-01 3.119943e-01 3.248115e-01 6.403290e-02 5.924576e-02 5.939185e-02 6.448623e-02 6.463689e-02

458 3.148188e-01 3.148479e-01 3.148795e-01 3.149122e-01 3.278267e-01 6.410008e-02 5.930409e-02 5.945059e-02 6.455481e-02 6.470595e-02

459 3.177662e-01 3.177956e-01 3.178276e-01 3.178607e-01 3.308630e-01 6.417362e-02 5.936795e-02 5.951489e-02 6.462986e-02 6.478152e-02

460 3.207366e-01 3.207663e-01 3.207986e-01 3.208321e-01 3.339125e-01 6.425404e-02 5.943782e-02 5.958524e-02 6.471195e-02 6.486417e-02

461 3.237224e-01 3.237524e-01 3.237850e-01 3.238188e-01 3.369674e-01 6.434195e-02 5.951422e-02 5.966217e-02 6.480167e-02 6.495450e-02

462 3.267158e-01 3.267461e-01 3.267790e-01 3.268130e-01 3.400196e-01 6.443797e-02 5.959772e-02 5.974624e-02 6.489967e-02 6.505316e-02

463 3.297091e-01 3.297395e-01 3.297726e-01 3.298069e-01 3.430612e-01 6.454279e-02 5.968890e-02 5.983805e-02 6.500664e-02 6.516086e-02

464 3.326942e-01 3.327249e-01 3.327582e-01 3.327927e-01 3.460840e-01 6.465714e-02 5.978841e-02 5.993824e-02 6.512332e-02 6.527833e-02

465 3.356635e-01 3.356943e-01 3.357278e-01 3.357625e-01 3.490805e-01 6.478178e-02 5.989693e-02 6.004751e-02 6.525051e-02 6.540637e-02

466 3.386093e-01 3.386402e-01 3.386738e-01 3.387086e-01 3.520429e-01 6.491755e-02 6.001521e-02 6.016659e-02 6.538904e-02 6.554583e-02

467 3.415240e-01 3.415550e-01 3.415887e-01 3.416237e-01 3.549639e-01 6.506534e-02 6.014403e-02 6.029629e-02 6.553982e-02 6.569761e-02

468 3.444004e-01 3.444315e-01 3.444653e-01 3.445003e-01 3.578366e-01 6.522609e-02 6.028422e-02 6.043743e-02 6.570381e-02 6.586269e-02

469 3.472317e-01 3.472628e-01 3.472967e-01 3.473317e-01 3.606544e-01 6.540080e-02 6.043668e-02 6.059091e-02 6.588202e-02 6.604208e-02

470 3.500113e-01 3.500424e-01 3.500763e-01 3.501114e-01 3.634113e-01 6.559052e-02 6.060235e-02 6.075770e-02 6.607553e-02 6.623687e-02

471 3.527332e-01 3.527643e-01 3.527982e-01 3.528333e-01 3.661018e-01 6.579639e-02 6.078224e-02 6.093879e-02 6.628549e-02 6.644820e-02

472 3.553921e-01 3.554231e-01 3.554570e-01 3.554921e-01 3.687211e-01 6.601958e-02 6.097740e-02 6.113525e-02 6.651309e-02 6.667729e-02

473 3.579830e-01 3.580139e-01 3.580478e-01 3.580828e-01 3.712650e-01 6.626134e-02 6.118897e-02 6.134822e-02 6.675960e-02 6.692540e-02

474 3.605017e-01 3.605326e-01 3.605664e-01 3.606014e-01 3.737301e-01 6.652298e-02 6.141812e-02 6.157888e-02 6.702635e-02 6.719387e-02

475 3.629448e-01 3.629756e-01 3.630093e-01 3.630442e-01 3.761137e-01 6.680587e-02 6.166609e-02 6.182847e-02 6.731474e-02 6.748410e-02

476 3.653095e-01 3.653402e-01 3.653738e-01 3.654086e-01 3.784139e-01 6.711145e-02 6.193419e-02 6.209832e-02 6.762620e-02 6.779755e-02

477 3.675937e-01 3.676242e-01 3.676577e-01 3.676924e-01 3.806294e-01 6.744120e-02 6.222377e-02 6.238978e-02 6.796226e-02 6.813573e-02

478 3.697960e-01 3.698263e-01 3.698597e-01 3.698943e-01 3.827597e-01 6.779667e-02 6.253626e-02 6.270428e-02 6.832448e-02 6.850021e-02

479 3.719157e-01 3.719459e-01 3.719792e-01 3.720137e-01 3.848049e-01 6.817946e-02 6.287313e-02 6.304329e-02 6.871447e-02 6.889262e-02

480 3.739529e-01 3.739830e-01 3.740161e-01 3.740504e-01 3.867657e-01 6.859121e-02 6.323590e-02 6.340836e-02 6.913391e-02 6.931463e-02

481 3.759081e-01 3.759380e-01 3.759710e-01 3.760052e-01 3.886436e-01 6.903361e-02 6.362614e-02 6.380105e-02 6.958449e-02 6.976795e-02

482 3.777825e-01 3.778122e-01 3.778451e-01 3.778791e-01 3.904402e-01 6.950839e-02 6.404546e-02 6.422299e-02 7.006794e-02 7.025432e-02

483 3.795776e-01 3.796071e-01 3.796399e-01 3.796738e-01 3.921579e-01 7.001728e-02 6.449552e-02 6.467583e-02 7.058603e-02 7.077550e-02

484 3.812955e-01 3.813249e-01 3.813575e-01 3.813913e-01 3.937991e-01 7.056205e-02 6.497800e-02 6.516124e-02 7.114054e-02 7.133327e-02

485 3.829387e-01 3.829678e-01 3.830003e-01 3.830339e-01 3.953668e-01 7.114446e-02 6.549457e-02 6.568094e-02 7.173323e-02 7.192941e-02

486 3.845097e-01 3.845387e-01 3.845710e-01 3.846045e-01 3.968640e-01 7.176627e-02 6.604696e-02 6.623662e-02 7.236587e-02 7.256567e-02

487 3.860114e-01 3.860403e-01 3.860725e-01 3.861059e-01 3.982939e-01 7.242921e-02 6.663686e-02 6.683000e-02 7.304020e-02 7.324381e-02

488 3.874470e-01 3.874757e-01 3.875078e-01 3.875410e-01 3.996597e-01 7.313498e-02 6.726595e-02 6.746275e-02 7.375791e-02 7.396552e-02

489 3.888196e-01 3.888481e-01 3.888801e-01 3.889132e-01 4.009649e-01 7.388522e-02 6.793590e-02 6.813655e-02 7.452064e-02 7.473242e-02

490 3.901323e-01 3.901606e-01 3.901925e-01 3.902255e-01 4.022128e-01 7.468149e-02 6.864831e-02 6.885298e-02 7.532995e-02 7.554609e-02

491 3.913884e-01 3.914165e-01 3.914483e-01 3.914812e-01 4.034066e-01 7.552528e-02 6.940472e-02 6.961361e-02 7.618730e-02 7.640797e-02

492 3.925910e-01 3.926190e-01 3.926507e-01 3.926835e-01 4.045496e-01 7.641793e-02 7.020662e-02 7.041989e-02 7.709403e-02 7.731940e-02

493 3.937433e-01 3.937712e-01 3.938028e-01 3.938355e-01 4.056448e-01 7.736068e-02 7.105535e-02 7.127319e-02 7.805135e-02 7.828159e-02

494 3.948483e-01 3.948760e-01 3.949075e-01 3.949401e-01 4.066954e-01 7.835460e-02 7.195218e-02 7.217474e-02 7.906031e-02 7.929556e-02

495 3.959088e-01 3.959364e-01 3.959678e-01 3.960004e-01 4.077040e-01 7.940059e-02 7.289820e-02 7.312565e-02 8.012177e-02 8.036217e-02

496 3.969278e-01 3.969552e-01 3.969866e-01 3.970190e-01 4.086736e-01 8.049934e-02 7.389436e-02 7.412686e-02 8.123638e-02 8.148207e-02

497 3.979078e-01 3.979351e-01 3.979664e-01 3.979988e-01 4.096065e-01 8.165132e-02 7.494143e-02 7.517912e-02 8.240457e-02 8.265566e-02

498 3.988514e-01 3.988785e-01 3.989098e-01 3.989421e-01 4.105054e-01 8.285677e-02 7.603998e-02 7.628297e-02 8.362655e-02 8.388313e-02

499 3.997609e-01 3.997879e-01 3.998191e-01 3.998514e-01 4.113723e-01 8.411567e-02 7.719034e-02 7.743876e-02 8.490222e-02 8.516437e-02

500 4.006386e-01 4.006655e-01 4.006966e-01 4.007288e-01 4.122096e-01 8.542772e-02 7.839261e-02 7.864655e-02 8.623123e-02 8.649902e-02

501 4.014866e-01 4.015134e-01 4.015445e-01 4.015766e-01 4.130191e-01 8.679232e-02 7.964664e-02 7.990617e-02 8.761292e-02 8.788639e-02

502 4.023068e-01 4.023335e-01 4.023645e-01 4.023966e-01 4.138026e-01 8.820858e-02 8.095199e-02 8.121718e-02 8.904635e-02 8.932551e-02

503 4.031011e-01 4.031277e-01 4.031587e-01 4.031907e-01 4.145620e-01 8.967529e-02 8.230794e-02 8.257882e-02 9.053022e-02 9.081506e-02

504 4.038712e-01 4.038976e-01 4.039285e-01 4.039606e-01 4.152988e-01 9.119092e-02 8.371348e-02 8.399007e-02 9.206293e-02 9.235344e-02

505 4.046185e-01 4.046448e-01 4.046758e-01 4.047077e-01 4.160145e-01 9.275361e-02 8.516728e-02 8.544958e-02 9.364257e-02 9.393869e-02

506 4.053446e-01 4.053708e-01 4.054017e-01 4.054337e-01 4.167103e-01 9.436120e-02 8.666770e-02 8.695569e-02 9.526689e-02 9.556853e-02

507 4.060509e-01 4.060769e-01 4.061078e-01 4.061397e-01 4.173877e-01 9.601119e-02 8.821282e-02 8.850643e-02 9.693332e-02 9.724040e-02

508 4.067384e-01 4.067644e-01 4.067953e-01 4.068272e-01 4.180476e-01 9.770078e-02 8.980036e-02 9.009953e-02 9.863900e-02 9.895138e-02

509 4.074085e-01 4.074344e-01 4.074652e-01 4.074971e-01 4.186912e-01 9.942690e-02 9.142779e-02 9.173241e-02 1.003808e-01 1.006983e-01

510 4.080621e-01 4.080879e-01 4.081187e-01 4.081505e-01 4.193195e-01 1.011862e-01 9.309227e-02 9.340221e-02 1.021552e-01 1.024777e-01

511 4.087003e-01 4.087259e-01 4.087567e-01 4.087885e-01 4.199333e-01 1.029750e-01 9.479067e-02 9.510579e-02 1.039586e-01 1.042860e-01

512 4.093238e-01 4.093494e-01 4.093801e-01 4.094119e-01 4.205335e-01 1.047895e-01 9.651963e-02 9.683977e-02 1.057871e-01 1.061191e-01

513 4.099336e-01 4.099591e-01 4.099898e-01 4.100216e-01 4.211208e-01 1.066257e-01 9.827557e-02 9.860054e-02 1.076367e-01 1.079730e-01

514 4.105304e-01 4.105557e-01 4.105865e-01 4.106182e-01 4.216959e-01 1.084794e-01 1.000547e-01 1.003843e-01 1.095030e-01 1.098434e-01

515 4.111148e-01 4.111401e-01 4.111708e-01 4.112026e-01 4.222595e-01 1.103463e-01 1.018530e-01 1.021870e-01 1.113817e-01 1.117260e-01

516 4.116876e-01 4.117128e-01 4.117435e-01 4.117752e-01 4.228121e-01 1.122219e-01 1.036664e-01 1.040045e-01 1.132684e-01 1.136163e-01

517 4.122493e-01 4.122744e-01 4.123051e-01 4.123368e-01 4.233543e-01 1.141019e-01 1.054907e-01 1.058327e-01 1.151587e-01 1.155099e-01

518 4.128005e-01 4.128254e-01 4.128561e-01 4.128879e-01 4.238867e-01 1.159818e-01 1.073216e-01 1.076673e-01 1.170480e-01 1.174023e-01

519 4.133417e-01 4.133665e-01 4.133972e-01 4.134289e-01 4.244096e-01 1.178572e-01 1.091548e-01 1.095040e-01 1.189319e-01 1.192890e-01

520 4.138732e-01 4.138979e-01 4.139286e-01 4.139603e-01 4.249234e-01 1.197237e-01 1.109862e-01 1.113384e-01 1.208062e-01 1.211658e-01

521 4.143956e-01 4.144202e-01 4.144509e-01 4.144826e-01 4.254287e-01 1.215773e-01 1.128113e-01 1.131664e-01 1.226666e-01 1.230284e-01

522 4.149093e-01 4.149338e-01 4.149645e-01 4.149961e-01 4.259257e-01 1.234137e-01 1.146262e-01 1.149839e-01 1.245091e-01 1.248729e-01

523 4.154146e-01 4.154390e-01 4.154696e-01 4.155013e-01 4.264147e-01 1.252292e-01 1.164269e-01 1.167868e-01 1.263299e-01 1.266954e-01

524 4.159118e-01 4.159361e-01 4.159667e-01 4.159984e-01 4.268961e-01 1.270202e-01 1.182095e-01 1.185714e-01 1.281254e-01 1.284923e-01

525 4.164012e-01 4.164254e-01 4.164561e-01 4.164877e-01 4.273702e-01 1.287834e-01 1.199704e-01 1.203341e-01 1.298922e-01 1.302603e-01

526 4.168833e-01 4.169073e-01 4.169380e-01 4.169696e-01 4.278372e-01 1.305155e-01 1.217062e-01 1.220714e-01 1.316273e-01 1.319963e-01

527 4.173581e-01 4.173820e-01 4.174127e-01 4.174443e-01 4.282974e-01 1.322138e-01 1.234139e-01 1.237804e-01 1.333280e-01 1.336977e-01

528 4.178259e-01 4.178498e-01 4.178804e-01 4.179121e-01 4.287510e-01 1.338759e-01 1.250906e-01 1.254581e-01 1.349918e-01 1.353620e-01

529 4.182871e-01 4.183109e-01 4.183415e-01 4.183731e-01 4.291982e-01 1.354996e-01 1.267337e-01 1.271020e-01 1.366166e-01 1.369872e-01

530 4.187418e-01 4.187654e-01 4.187961e-01 4.188277e-01 4.296393e-01 1.370831e-01 1.283410e-01 1.287099e-01 1.382007e-01 1.385713e-01

531 4.191901e-01 4.192137e-01 4.192443e-01 4.192760e-01 4.300743e-01 1.386248e-01 1.299105e-01 1.302798e-01 1.397424e-01 1.401131e-01

532 4.196324e-01 4.196558e-01 4.196865e-01 4.197181e-01 4.305035e-01 1.401235e-01 1.314406e-01 1.318101e-01 1.412408e-01 1.416113e-01

533 4.200687e-01 4.200921e-01 4.201227e-01 4.201543e-01 4.309270e-01 1.415784e-01 1.329299e-01 1.332996e-01 1.426950e-01 1.430652e-01

534 4.204993e-01 4.205225e-01 4.205531e-01 4.205848e-01 4.313450e-01 1.429889e-01 1.343775e-01 1.347471e-01 1.441043e-01 1.444741e-01

535 4.209242e-01 4.209473e-01 4.209779e-01 4.210096e-01 4.317577e-01 1.443545e-01 1.357826e-01 1.361520e-01 1.454686e-01 1.458379e-01

536 4.213437e-01 4.213667e-01 4.213973e-01 4.214289e-01 4.321651e-01 1.456754e-01 1.371446e-01 1.375137e-01 1.467878e-01 1.471566e-01

537 4.217578e-01 4.217807e-01 4.218113e-01 4.218429e-01 4.325674e-01 1.469516e-01 1.384635e-01 1.388322e-01 1.480621e-01 1.484302e-01

538 4.221667e-01 4.221895e-01 4.222201e-01 4.222517e-01 4.329647e-01 1.481835e-01 1.397391e-01 1.401074e-01 1.492920e-01 1.496594e-01

539 4.225705e-01 4.225932e-01 4.226238e-01 4.226554e-01 4.333571e-01 1.493717e-01 1.409719e-01 1.413396e-01 1.504780e-01 1.508447e-01

540 4.229693e-01 4.229919e-01 4.230225e-01 4.230541e-01 4.337448e-01 1.505169e-01 1.421621e-01 1.425293e-01 1.516210e-01 1.519869e-01

541 4.233633e-01 4.233858e-01 4.234164e-01 4.234480e-01 4.341277e-01 1.516202e-01 1.433105e-01 1.436770e-01 1.527219e-01 1.530870e-01

542 4.233844e-01 4.234069e-01 4.234375e-01 4.234691e-01 4.341528e-01 1.516202e-01 1.433106e-01 1.436771e-01 1.527220e-01 1.530871e-01

543 4.234079e-01 4.234304e-01 4.234610e-01 4.234927e-01 4.341807e-01 1.516203e-01 1.433107e-01 1.436772e-01 1.527221e-01 1.530872e-01

544 4.234341e-01 4.234566e-01 4.234873e-01 4.235190e-01 4.342118e-01 1.516204e-01 1.433107e-01 1.436772e-01 1.527222e-01 1.530873e-01

545 4.234632e-01 4.234858e-01 4.235165e-01 4.235482e-01 4.342464e-01 1.516205e-01 1.433108e-01 1.436773e-01 1.527223e-01 1.530874e-01

546 4.234957e-01 4.235183e-01 4.235490e-01 4.235807e-01 4.342849e-01 1.516206e-01 1.433109e-01 1.436774e-01 1.527224e-01 1.530875e-01

547 4.235318e-01 4.235544e-01 4.235852e-01 4.236170e-01 4.343277e-01 1.516207e-01 1.433110e-01 1.436775e-01 1.527225e-01 1.530876e-01

548 4.235719e-01 4.235946e-01 4.236254e-01 4.236572e-01 4.343753e-01 1.516209e-01 1.433111e-01 1.436776e-01 1.527226e-01 1.530877e-01

549 4.236165e-01 4.236392e-01 4.236701e-01 4.237020e-01 4.344282e-01 1.516210e-01 1.433113e-01 1.436778e-01 1.527228e-01 1.530879e-01

550 4.236661e-01 4.236888e-01 4.237197e-01 4.237517e-01 4.344869e-01 1.516212e-01 1.433114e-01 1.436779e-01 1.527230e-01 1.530881e-01

551 4.237211e-01 4.237439e-01 4.237748e-01 4.238068e-01 4.345521e-01 1.516214e-01 1.433116e-01 1.436781e-01 1.527232e-01 1.530883e-01

552 4.237821e-01 4.238050e-01 4.238360e-01 4.238680e-01 4.346244e-01 1.516216e-01 1.433117e-01 1.436782e-01 1.527234e-01 1.530885e-01

553 4.238497e-01 4.238727e-01 4.239038e-01 4.239359e-01 4.347044e-01 1.516218e-01 1.433119e-01 1.436784e-01 1.527236e-01 1.530887e-01

554 4.239247e-01 4.239477e-01 4.239789e-01 4.240111e-01 4.347932e-01 1.516221e-01 1.433121e-01 1.436787e-01 1.527239e-01 1.530890e-01

555 4.240077e-01 4.240309e-01 4.240621e-01 4.240944e-01 4.348914e-01 1.516224e-01 1.433124e-01 1.436789e-01 1.527242e-01 1.530893e-01

556 4.240997e-01 4.241229e-01 4.241542e-01 4.241866e-01 4.350000e-01 1.516227e-01 1.433127e-01 1.436792e-01 1.527245e-01 1.530896e-01

557 4.242013e-01 4.242246e-01 4.242561e-01 4.242886e-01 4.351201e-01 1.516230e-01 1.433130e-01 1.436795e-01 1.527249e-01 1.530900e-01

558 4.243137e-01 4.243371e-01 4.243687e-01 4.244013e-01 4.352527e-01 1.516234e-01 1.433133e-01 1.436798e-01 1.527253e-01 1.530904e-01

559 4.244378e-01 4.244614e-01 4.244931e-01 4.245258e-01 4.353992e-01 1.516239e-01 1.433137e-01 1.436802e-01 1.527257e-01 1.530909e-01

560 4.245748e-01 4.245985e-01 4.246304e-01 4.246633e-01 4.355607e-01 1.516244e-01 1.433141e-01 1.436806e-01 1.527262e-01 1.530914e-01

561 4.247260e-01 4.247498e-01 4.247818e-01 4.248149e-01 4.357388e-01 1.516249e-01 1.433145e-01 1.436811e-01 1.527268e-01 1.530919e-01

562 4.248926e-01 4.249166e-01 4.249487e-01 4.249820e-01 4.359349e-01 1.516255e-01 1.433150e-01 1.436816e-01 1.527274e-01 1.530925e-01

563 4.250760e-01 4.251002e-01 4.251326e-01 4.251660e-01 4.361507e-01 1.516261e-01 1.433156e-01 1.436821e-01 1.527281e-01 1.530932e-01

564 4.252779e-01 4.253023e-01 4.253348e-01 4.253685e-01 4.363880e-01 1.516269e-01 1.433162e-01 1.436828e-01 1.527288e-01 1.530940e-01

565 4.254999e-01 4.255245e-01 4.255573e-01 4.255911e-01 4.366486e-01 1.516277e-01 1.433169e-01 1.436835e-01 1.527296e-01 1.530948e-01

566 4.257437e-01 4.257686e-01 4.258016e-01 4.258357e-01 4.369346e-01 1.516285e-01 1.433176e-01 1.436842e-01 1.527305e-01 1.530957e-01

567 4.260113e-01 4.260364e-01 4.260697e-01 4.261041e-01 4.372481e-01 1.516295e-01 1.433185e-01 1.436851e-01 1.527316e-01 1.530967e-01

568 4.263047e-01 4.263301e-01 4.263637e-01 4.263984e-01 4.375914e-01 1.516306e-01 1.433194e-01 1.436860e-01 1.527327e-01 1.530979e-01

569 4.266261e-01 4.266518e-01 4.266857e-01 4.267207e-01 4.379670e-01 1.516318e-01 1.433204e-01 1.436870e-01 1.527339e-01 1.530991e-01

570 4.269777e-01 4.270037e-01 4.270379e-01 4.270733e-01 4.383773e-01 1.516331e-01 1.433215e-01 1.436882e-01 1.527353e-01 1.531005e-01

571 4.273619e-01 4.273883e-01 4.274229e-01 4.274587e-01 4.388252e-01 1.516345e-01 1.433228e-01 1.436894e-01 1.527368e-01 1.531020e-01

572 4.277814e-01 4.278082e-01 4.278432e-01 4.278794e-01 4.393133e-01 1.516361e-01 1.433241e-01 1.436908e-01 1.527384e-01 1.531037e-01

573 4.282387e-01 4.282659e-01 4.283014e-01 4.283381e-01 4.398446e-01 1.516379e-01 1.433257e-01 1.436923e-01 1.527402e-01 1.531055e-01

574 4.287367e-01 4.287644e-01 4.288003e-01 4.288375e-01 4.404221e-01 1.516399e-01 1.433273e-01 1.436940e-01 1.527422e-01 1.531075e-01

575 4.292782e-01 4.293064e-01 4.293428e-01 4.293805e-01 4.410489e-01 1.516420e-01 1.433291e-01 1.436958e-01 1.527444e-01 1.531098e-01

576 4.298662e-01 4.298949e-01 4.299319e-01 4.299702e-01 4.417282e-01 1.516444e-01 1.433312e-01 1.436979e-01 1.527469e-01 1.531122e-01

577 4.305038e-01 4.305331e-01 4.305707e-01 4.306095e-01 4.424633e-01 1.516470e-01 1.433334e-01 1.437001e-01 1.527496e-01 1.531149e-01

578 4.311940e-01 4.312240e-01 4.312622e-01 4.313017e-01 4.432572e-01 1.516498e-01 1.433358e-01 1.437026e-01 1.527525e-01 1.531179e-01

579 4.319401e-01 4.319707e-01 4.320096e-01 4.320498e-01 4.441135e-01 1.516529e-01 1.433385e-01 1.437053e-01 1.527557e-01 1.531212e-01

580 4.327452e-01 4.327765e-01 4.328161e-01 4.328570e-01 4.450351e-01 1.516564e-01 1.433415e-01 1.437083e-01 1.527593e-01 1.531248e-01

581 4.336125e-01 4.336445e-01 4.336849e-01 4.337265e-01 4.460253e-01 1.516602e-01 1.433447e-01 1.437116e-01 1.527632e-01 1.531287e-01

582 4.345450e-01 4.345778e-01 4.346190e-01 4.346614e-01 4.470871e-01 1.516644e-01 1.433483e-01 1.437152e-01 1.527675e-01 1.531331e-01

583 4.355458e-01 4.355795e-01 4.356214e-01 4.356648e-01 4.482235e-01 1.516689e-01 1.433522e-01 1.437191e-01 1.527722e-01 1.531379e-01

584 4.366178e-01 4.366524e-01 4.366952e-01 4.367394e-01 4.494370e-01 1.516740e-01 1.433565e-01 1.437235e-01 1.527774e-01 1.531431e-01

585 4.377638e-01 4.377992e-01 4.378430e-01 4.378881e-01 4.507303e-01 1.516795e-01 1.433612e-01 1.437282e-01 1.527831e-01 1.531488e-01

586 4.389863e-01 4.390226e-01 4.390673e-01 4.391135e-01 4.521054e-01 1.516855e-01 1.433664e-01 1.437335e-01 1.527893e-01 1.531551e-01

587 4.402875e-01 4.403249e-01 4.403705e-01 4.404177e-01 4.535644e-01 1.516921e-01 1.433720e-01 1.437392e-01 1.527962e-01 1.531620e-01

588 4.416697e-01 4.417080e-01 4.417547e-01 4.418029e-01 4.551086e-01 1.516994e-01 1.433782e-01 1.437455e-01 1.528037e-01 1.531696e-01

589 4.431343e-01 4.431737e-01 4.432215e-01 4.432708e-01 4.567393e-01 1.517073e-01 1.433850e-01 1.437523e-01 1.528119e-01 1.531779e-01

590 4.446829e-01 4.447234e-01 4.447722e-01 4.448226e-01 4.584572e-01 1.517160e-01 1.433925e-01 1.437599e-01 1.528208e-01 1.531870e-01

591 4.463163e-01 4.463578e-01 4.464078e-01 4.464593e-01 4.602624e-01 1.517255e-01 1.434007e-01 1.437681e-01 1.528307e-01 1.531969e-01

592 4.480351e-01 4.480777e-01 4.481287e-01 4.481814e-01 4.621548e-01 1.517360e-01 1.434096e-01 1.437771e-01 1.528414e-01 1.532078e-01

593 4.498392e-01 4.498830e-01 4.499351e-01 4.499889e-01 4.641335e-01 1.517473e-01 1.434193e-01 1.437870e-01 1.528532e-01 1.532196e-01

594 4.517283e-01 4.517732e-01 4.518264e-01 4.518814e-01 4.661972e-01 1.517598e-01 1.434300e-01 1.437978e-01 1.528660e-01 1.532326e-01

595 4.537013e-01 4.537473e-01 4.538018e-01 4.538579e-01 4.683441e-01 1.517734e-01 1.434417e-01 1.438096e-01 1.528800e-01 1.532468e-01

596 4.557569e-01 4.558040e-01 4.558596e-01 4.559169e-01 4.705717e-01 1.517882e-01 1.434544e-01 1.438224e-01 1.528953e-01 1.532623e-01

597 4.578929e-01 4.579412e-01 4.579978e-01 4.580563e-01 4.728772e-01 1.518044e-01 1.434683e-01 1.438365e-01 1.529121e-01 1.532791e-01

598 4.601069e-01 4.601562e-01 4.602140e-01 4.602736e-01 4.752571e-01 1.518221e-01 1.434835e-01 1.438518e-01 1.529303e-01 1.532976e-01

599 4.623956e-01 4.624461e-01 4.625049e-01 4.625657e-01 4.777072e-01 1.518414e-01 1.435000e-01 1.438685e-01 1.529502e-01 1.533177e-01

600 4.647557e-01 4.648071e-01 4.648671e-01 4.649289e-01 4.802232e-01 1.518624e-01 1.435181e-01 1.438868e-01 1.529719e-01 1.533396e-01

601 4.671828e-01 4.672353e-01 4.672963e-01 4.673592e-01 4.828001e-01 1.518853e-01 1.435378e-01 1.439067e-01 1.529955e-01 1.533635e-01

602 4.696725e-01 4.697260e-01 4.697880e-01 4.698519e-01 4.854324e-01 1.519103e-01 1.435592e-01 1.439283e-01 1.530213e-01 1.533895e-01

603 4.722197e-01 4.722742e-01 4.723371e-01 4.724020e-01 4.881143e-01 1.519375e-01 1.435825e-01 1.439519e-01 1.530493e-01 1.534178e-01

604 4.748189e-01 4.748743e-01 4.749382e-01 4.750041e-01 4.908397e-01 1.519670e-01 1.436079e-01 1.439776e-01 1.530798e-01 1.534486e-01

605 4.774644e-01 4.775207e-01 4.775854e-01 4.776522e-01 4.936020e-01 1.519992e-01 1.436356e-01 1.440055e-01 1.531130e-01 1.534822e-01

606 4.801499e-01 4.802069e-01 4.802725e-01 4.803401e-01 4.963944e-01 1.520342e-01 1.436657e-01 1.440359e-01 1.531491e-01 1.535186e-01

607 4.828688e-01 4.829267e-01 4.829930e-01 4.830614e-01 4.992099e-01 1.520722e-01 1.436983e-01 1.440689e-01 1.531883e-01 1.535582e-01

608 4.856146e-01 4.856732e-01 4.857402e-01 4.858093e-01 5.020413e-01 1.521135e-01 1.437339e-01 1.441048e-01 1.532308e-01 1.536012e-01

609 4.883802e-01 4.884394e-01 4.885071e-01 4.885769e-01 5.048814e-01 1.521583e-01 1.437724e-01 1.441438e-01 1.532771e-01 1.536480e-01

610 4.911586e-01 4.912184e-01 4.912867e-01 4.913571e-01 5.077227e-01 1.522070e-01 1.438143e-01 1.441861e-01 1.533272e-01 1.536986e-01

611 4.939426e-01 4.940029e-01 4.940717e-01 4.941427e-01 5.105579e-01 1.522597e-01 1.438597e-01 1.442320e-01 1.533817e-01 1.537536e-01

612 4.967250e-01 4.967858e-01 4.968551e-01 4.969265e-01 5.133797e-01 1.523169e-01 1.439090e-01 1.442818e-01 1.534407e-01 1.538132e-01

613 4.994987e-01 4.995599e-01 4.996296e-01 4.997014e-01 5.161810e-01 1.523790e-01 1.439625e-01 1.443358e-01 1.535046e-01 1.538778e-01

614 5.022565e-01 5.023181e-01 5.023881e-01 5.024603e-01 5.189548e-01 1.524461e-01 1.440203e-01 1.443943e-01 1.535739e-01 1.539478e-01

615 5.049917e-01 5.050535e-01 5.051238e-01 5.051963e-01 5.216945e-01 1.525188e-01 1.440830e-01 1.444576e-01 1.536489e-01 1.540235e-01

616 5.076975e-01 5.077595e-01 5.078301e-01 5.079028e-01 5.243936e-01 1.525975e-01 1.441509e-01 1.445262e-01 1.537300e-01 1.541055e-01

617 5.103675e-01 5.104297e-01 5.105004e-01 5.105733e-01 5.270462e-01 1.526826e-01 1.442243e-01 1.446004e-01 1.538177e-01 1.541941e-01

618 5.129957e-01 5.130580e-01 5.131288e-01 5.132019e-01 5.296467e-01 1.527746e-01 1.443037e-01 1.446806e-01 1.539125e-01 1.542899e-01

619 5.155764e-01 5.156388e-01 5.157097e-01 5.157828e-01 5.321901e-01 1.528740e-01 1.443896e-01 1.447673e-01 1.540150e-01 1.543934e-01

620 5.181045e-01 5.181668e-01 5.182378e-01 5.183109e-01 5.346717e-01 1.529813e-01 1.444823e-01 1.448609e-01 1.541255e-01 1.545051e-01

621 5.205752e-01 5.206375e-01 5.207085e-01 5.207815e-01 5.370877e-01 1.530971e-01 1.445823e-01 1.449620e-01 1.542449e-01 1.546256e-01

622 5.229844e-01 5.230466e-01 5.231175e-01 5.231905e-01 5.394346e-01 1.532219e-01 1.446903e-01 1.450711e-01 1.543736e-01 1.547556e-01

623 5.253284e-01 5.253905e-01 5.254613e-01 5.255342e-01 5.417096e-01 1.533565e-01 1.448068e-01 1.451888e-01 1.545122e-01 1.548956e-01

624 5.276042e-01 5.276661e-01 5.277367e-01 5.278095e-01 5.439105e-01 1.535015e-01 1.449323e-01 1.453156e-01 1.546616e-01 1.550465e-01

625 5.298093e-01 5.298710e-01 5.299415e-01 5.300140e-01 5.460356e-01 1.536576e-01 1.450675e-01 1.454522e-01 1.548224e-01 1.552089e-01

626 5.319418e-01 5.320033e-01 5.320735e-01 5.321459e-01 5.480840e-01 1.538255e-01 1.452131e-01 1.455992e-01 1.549954e-01 1.553836e-01

627 5.340003e-01 5.340616e-01 5.341316e-01 5.342038e-01 5.500550e-01 1.540061e-01 1.453697e-01 1.457573e-01 1.551813e-01 1.555714e-01

628 5.359842e-01 5.360452e-01 5.361150e-01 5.361869e-01 5.519488e-01 1.542001e-01 1.455380e-01 1.459274e-01 1.553811e-01 1.557731e-01

629 5.378931e-01 5.379538e-01 5.380233e-01 5.380950e-01 5.537659e-01 1.544083e-01 1.457189e-01 1.461101e-01 1.555956e-01 1.559897e-01

630 5.397272e-01 5.397876e-01 5.398569e-01 5.399283e-01 5.555072e-01 1.546318e-01 1.459131e-01 1.463062e-01 1.558256e-01 1.562220e-01

631 5.414873e-01 5.415474e-01 5.416165e-01 5.416876e-01 5.571740e-01 1.548714e-01 1.461215e-01 1.465166e-01 1.560722e-01 1.564710e-01

632 5.431744e-01 5.432342e-01 5.433030e-01 5.433738e-01 5.587680e-01 1.551280e-01 1.463449e-01 1.467423e-01 1.563364e-01 1.567376e-01

633 5.447899e-01 5.448494e-01 5.449179e-01 5.449885e-01 5.602913e-01 1.554028e-01 1.465843e-01 1.469840e-01 1.566191e-01 1.570231e-01

634 5.463357e-01 5.463949e-01 5.464631e-01 5.465334e-01 5.617460e-01 1.556966e-01 1.468406e-01 1.472428e-01 1.569215e-01 1.573283e-01

635 5.478136e-01 5.478725e-01 5.479404e-01 5.480104e-01 5.631345e-01 1.560107e-01 1.471148e-01 1.475196e-01 1.572446e-01 1.576544e-01

636 5.492259e-01 5.492845e-01 5.493522e-01 5.494219e-01 5.644594e-01 1.563461e-01 1.474078e-01 1.478155e-01 1.575895e-01 1.580026e-01

637 5.505751e-01 5.506333e-01 5.507008e-01 5.507702e-01 5.657234e-01 1.567039e-01 1.477208e-01 1.481315e-01 1.579574e-01 1.583739e-01

638 5.518635e-01 5.519215e-01 5.519887e-01 5.520579e-01 5.669293e-01 1.570853e-01 1.480548e-01 1.484686e-01 1.583495e-01 1.587696e-01

639 5.530938e-01 5.531515e-01 5.532185e-01 5.532874e-01 5.680797e-01 1.574914e-01 1.484109e-01 1.488281e-01 1.587669e-01 1.591908e-01

640 5.542687e-01 5.543261e-01 5.543929e-01 5.544616e-01 5.691774e-01 1.579234e-01 1.487901e-01 1.492109e-01 1.592109e-01 1.596388e-01

641 5.553909e-01 5.554480e-01 5.555145e-01 5.555830e-01 5.702253e-01 1.583826e-01 1.491938e-01 1.496183e-01 1.596826e-01 1.601147e-01

642 5.564629e-01 5.565197e-01 5.565860e-01 5.566543e-01 5.712260e-01 1.588701e-01 1.496229e-01 1.500513e-01 1.601832e-01 1.606198e-01

643 5.574874e-01 5.575440e-01 5.576101e-01 5.576781e-01 5.721822e-01 1.593871e-01 1.500786e-01 1.505112e-01 1.607141e-01 1.611553e-01

644 5.584671e-01 5.585234e-01 5.585893e-01 5.586571e-01 5.730964e-01 1.599348e-01 1.505621e-01 1.509991e-01 1.612762e-01 1.617223e-01

645 5.594043e-01 5.594604e-01 5.595262e-01 5.595938e-01 5.739711e-01 1.605143e-01 1.510745e-01 1.515161e-01 1.618708e-01 1.623220e-01

646 5.603016e-01 5.603575e-01 5.604231e-01 5.604905e-01 5.748086e-01 1.611267e-01 1.516170e-01 1.520634e-01 1.624991e-01 1.629556e-01

647 5.611613e-01 5.612170e-01 5.612824e-01 5.613497e-01 5.756113e-01 1.617732e-01 1.521906e-01 1.526420e-01 1.631620e-01 1.636240e-01

648 5.619857e-01 5.620412e-01 5.621064e-01 5.621735e-01 5.763813e-01 1.624547e-01 1.527964e-01 1.532530e-01 1.638606e-01 1.643284e-01

649 5.627768e-01 5.628321e-01 5.628972e-01 5.629642e-01 5.771207e-01 1.631723e-01 1.534354e-01 1.538975e-01 1.645958e-01 1.650695e-01

650 5.635368e-01 5.635919e-01 5.636568e-01 5.637237e-01 5.778313e-01 1.639267e-01 1.541087e-01 1.545764e-01 1.653686e-01 1.658484e-01

651 5.642675e-01 5.643224e-01 5.643872e-01 5.644540e-01 5.785150e-01 1.647189e-01 1.548170e-01 1.552906e-01 1.661796e-01 1.666658e-01

652 5.649708e-01 5.650255e-01 5.650902e-01 5.651569e-01 5.791736e-01 1.655494e-01 1.555613e-01 1.560409e-01 1.670296e-01 1.675223e-01

653 5.656484e-01 5.657030e-01 5.657676e-01 5.658341e-01 5.798086e-01 1.664188e-01 1.563422e-01 1.568281e-01 1.679190e-01 1.684184e-01

654 5.663019e-01 5.663563e-01 5.664208e-01 5.664872e-01 5.804215e-01 1.673277e-01 1.571605e-01 1.576528e-01 1.688484e-01 1.693546e-01

655 5.669329e-01 5.669871e-01 5.670516e-01 5.671179e-01 5.810137e-01 1.682763e-01 1.580165e-01 1.585155e-01 1.698179e-01 1.703311e-01

656 5.675427e-01 5.675968e-01 5.676611e-01 5.677274e-01 5.815866e-01 1.692648e-01 1.589108e-01 1.594165e-01 1.708276e-01 1.713480e-01

657 5.681327e-01 5.681867e-01 5.682509e-01 5.683171e-01 5.821414e-01 1.702931e-01 1.598436e-01 1.603562e-01 1.718776e-01 1.724051e-01

658 5.687042e-01 5.687580e-01 5.688221e-01 5.688882e-01 5.826792e-01 1.713611e-01 1.608150e-01 1.613347e-01 1.729675e-01 1.735023e-01

659 5.692582e-01 5.693119e-01 5.693760e-01 5.694419e-01 5.832011e-01 1.724684e-01 1.618249e-01 1.623518e-01 1.740969e-01 1.746391e-01

660 5.697958e-01 5.698494e-01 5.699134e-01 5.699793e-01 5.837081e-01 1.736144e-01 1.628731e-01 1.634072e-01 1.752651e-01 1.758148e-01

661 5.703182e-01 5.703716e-01 5.704356e-01 5.705014e-01 5.842010e-01 1.747983e-01 1.639593e-01 1.645007e-01 1.764714e-01 1.770285e-01

662 5.708261e-01 5.708794e-01 5.709433e-01 5.710091e-01 5.846808e-01 1.760193e-01 1.650827e-01 1.656316e-01 1.777147e-01 1.782792e-01

663 5.713204e-01 5.713736e-01 5.714375e-01 5.715032e-01 5.851482e-01 1.772760e-01 1.662428e-01 1.667990e-01 1.789937e-01 1.795655e-01

664 5.718020e-01 5.718551e-01 5.719189e-01 5.719846e-01 5.856039e-01 1.785671e-01 1.674383e-01 1.680019e-01 1.803069e-01 1.808861e-01

665 5.722716e-01 5.723246e-01 5.723884e-01 5.724540e-01 5.860486e-01 1.798910e-01 1.686682e-01 1.692392e-01 1.816526e-01 1.822390e-01

666 5.727299e-01 5.727828e-01 5.728465e-01 5.729121e-01 5.864830e-01 1.812459e-01 1.699312e-01 1.705095e-01 1.830290e-01 1.836226e-01

667 5.731775e-01 5.732303e-01 5.732940e-01 5.733596e-01 5.869077e-01 1.826298e-01 1.712255e-01 1.718111e-01 1.844340e-01 1.850345e-01

668 5.736151e-01 5.736678e-01 5.737314e-01 5.737969e-01 5.873231e-01 1.840405e-01 1.725494e-01 1.731421e-01 1.858653e-01 1.864726e-01

669 5.740431e-01 5.740957e-01 5.741593e-01 5.742248e-01 5.877297e-01 1.854756e-01 1.739010e-01 1.745007e-01 1.873204e-01 1.879343e-01

670 5.744621e-01 5.745146e-01 5.745782e-01 5.746436e-01 5.881281e-01 1.869325e-01 1.752781e-01 1.758847e-01 1.887968e-01 1.894171e-01

671 5.748725e-01 5.749249e-01 5.749885e-01 5.750539e-01 5.885187e-01 1.884087e-01 1.766784e-01 1.772917e-01 1.902917e-01 1.909181e-01

672 5.752748e-01 5.753272e-01 5.753907e-01 5.754561e-01 5.889018e-01 1.899012e-01 1.780994e-01 1.787192e-01 1.918022e-01 1.924345e-01

673 5.756695e-01 5.757217e-01 5.757852e-01 5.758506e-01 5.892778e-01 1.914073e-01 1.795385e-01 1.801646e-01 1.933253e-01 1.939633e-01

674 5.760568e-01 5.761089e-01 5.761724e-01 5.762377e-01 5.896470e-01 1.929238e-01 1.809931e-01 1.816251e-01 1.948581e-01 1.955013e-01

675 5.764371e-01 5.764892e-01 5.765526e-01 5.766179e-01 5.900099e-01 1.944477e-01 1.824602e-01 1.830980e-01 1.963974e-01 1.970456e-01

676 5.768108e-01 5.768628e-01 5.769262e-01 5.769915e-01 5.903666e-01 1.959759e-01 1.839371e-01 1.845804e-01 1.979400e-01 1.985929e-01

677 5.771781e-01 5.772301e-01 5.772935e-01 5.773587e-01 5.907175e-01 1.975054e-01 1.854207e-01 1.860691e-01 1.994829e-01 2.001402e-01

678 5.775394e-01 5.775913e-01 5.776546e-01 5.777199e-01 5.910627e-01 1.990330e-01 1.869081e-01 1.875614e-01 2.010229e-01 2.016843e-01

679 5.778949e-01 5.779466e-01 5.780100e-01 5.780752e-01 5.914026e-01 2.005557e-01 1.883963e-01 1.890541e-01 2.025571e-01 2.032221e-01

680 5.782448e-01 5.782965e-01 5.783598e-01 5.784250e-01 5.917373e-01 2.020705e-01 1.898823e-01 1.905444e-01 2.040823e-01 2.047507e-01

681 5.785894e-01 5.786410e-01 5.787043e-01 5.787695e-01 5.920671e-01 2.035745e-01 1.913633e-01 1.920293e-01 2.055958e-01 2.062672e-01

682 5.789288e-01 5.789804e-01 5.790437e-01 5.791088e-01 5.923921e-01 2.050650e-01 1.928364e-01 1.935059e-01 2.070947e-01 2.077688e-01

683 5.792633e-01 5.793148e-01 5.793781e-01 5.794433e-01 5.927126e-01 2.065393e-01 1.942987e-01 1.949715e-01 2.085765e-01 2.092529e-01

684 5.795931e-01 5.796445e-01 5.797078e-01 5.797729e-01 5.930286e-01 2.079949e-01 1.957478e-01 1.964235e-01 2.100386e-01 2.107171e-01

685 5.799183e-01 5.799696e-01 5.800329e-01 5.800980e-01 5.933404e-01 2.094295e-01 1.971810e-01 1.978593e-01 2.114788e-01 2.121591e-01

686 5.802391e-01 5.802904e-01 5.803536e-01 5.804187e-01 5.936481e-01 2.108409e-01 1.985960e-01 1.992766e-01 2.128950e-01 2.135768e-01

687 5.805556e-01 5.806068e-01 5.806701e-01 5.807352e-01 5.939518e-01 2.122272e-01 1.999905e-01 2.006732e-01 2.142852e-01 2.149682e-01

688 5.808680e-01 5.809191e-01 5.809824e-01 5.810475e-01 5.942516e-01 2.135865e-01 2.013626e-01 2.020469e-01 2.156477e-01 2.163316e-01

689 5.811764e-01 5.812275e-01 5.812907e-01 5.813558e-01 5.945477e-01 2.149175e-01 2.027103e-01 2.033961e-01 2.169810e-01 2.176657e-01

690 5.814809e-01 5.815319e-01 5.815951e-01 5.816602e-01 5.948402e-01 2.162186e-01 2.040321e-01 2.047191e-01 2.182838e-01 2.189690e-01

691 5.817817e-01 5.818326e-01 5.818958e-01 5.819609e-01 5.951292e-01 2.174887e-01 2.053263e-01 2.060142e-01 2.195551e-01 2.202405e-01

692 5.820788e-01 5.821297e-01 5.821929e-01 5.822579e-01 5.954147e-01 2.187269e-01 2.065918e-01 2.072804e-01 2.207938e-01 2.214793e-01

693 5.823724e-01 5.824232e-01 5.824864e-01 5.825514e-01 5.956969e-01 2.199325e-01 2.078274e-01 2.085166e-01 2.219993e-01 2.226848e-01

694 5.826625e-01 5.827133e-01 5.827764e-01 5.828414e-01 5.959759e-01 2.211048e-01 2.090323e-01 2.097218e-01 2.231712e-01 2.238564e-01

695 5.829492e-01 5.829999e-01 5.830631e-01 5.831281e-01 5.962518e-01 2.222435e-01 2.102057e-01 2.108954e-01 2.243090e-01 2.249938e-01

696 5.832327e-01 5.832834e-01 5.833465e-01 5.834115e-01 5.965245e-01 2.233484e-01 2.113472e-01 2.120368e-01 2.254125e-01 2.260969e-01

697 5.835130e-01 5.835636e-01 5.836267e-01 5.836917e-01 5.967942e-01 2.244193e-01 2.124563e-01 2.131457e-01 2.264819e-01 2.271657e-01

698 5.837901e-01 5.838407e-01 5.839038e-01 5.839688e-01 5.970610e-01 2.254566e-01 2.135328e-01 2.142220e-01 2.275173e-01 2.282004e-01

699 5.840643e-01 5.841148e-01 5.841779e-01 5.842428e-01 5.973250e-01 2.264602e-01 2.145768e-01 2.152656e-01 2.285188e-01 2.292012e-01

700 5.843354e-01 5.843858e-01 5.844489e-01 5.845139e-01 5.975861e-01 2.274307e-01 2.155883e-01 2.162767e-01 2.294870e-01 2.301686e-01

701 5.846036e-01 5.846540e-01 5.847171e-01 5.847820e-01 5.978445e-01 2.283685e-01 2.165676e-01 2.172554e-01 2.304223e-01 2.311030e-01

702 5.848690e-01 5.849193e-01 5.849824e-01 5.850473e-01 5.981002e-01 2.292742e-01 2.175150e-01 2.182022e-01 2.313254e-01 2.320052e-01

703 5.851316e-01 5.851818e-01 5.852449e-01 5.853098e-01 5.983532e-01 2.301483e-01 2.184310e-01 2.191174e-01 2.321968e-01 2.328757e-01

704 5.853914e-01 5.854416e-01 5.855047e-01 5.855696e-01 5.986037e-01 2.309917e-01 2.193160e-01 2.200017e-01 2.330375e-01 2.337154e-01

705 5.856485e-01 5.856987e-01 5.857618e-01 5.858266e-01 5.988516e-01 2.318052e-01 2.201708e-01 2.208558e-01 2.338481e-01 2.345251e-01

706 5.859030e-01 5.859532e-01 5.860162e-01 5.860811e-01 5.990971e-01 2.325895e-01 2.209961e-01 2.216802e-01 2.346296e-01 2.353056e-01

707 5.861550e-01 5.862051e-01 5.862681e-01 5.863329e-01 5.993401e-01 2.333456e-01 2.217924e-01 2.224758e-01 2.353829e-01 2.360579e-01

708 5.864044e-01 5.864544e-01 5.865174e-01 5.865822e-01 5.995807e-01 2.340743e-01 2.225608e-01 2.232433e-01 2.361088e-01 2.367829e-01

709 5.866512e-01 5.867013e-01 5.867642e-01 5.868291e-01 5.998189e-01 2.347767e-01 2.233020e-01 2.239837e-01 2.368084e-01 2.374815e-01

710 5.868957e-01 5.869457e-01 5.870086e-01 5.870734e-01 6.000548e-01 2.354536e-01 2.240169e-01 2.246977e-01 2.374825e-01 2.381548e-01

711 5.871377e-01 5.871876e-01 5.872506e-01 5.873154e-01 6.002884e-01 2.361060e-01 2.247063e-01 2.253863e-01 2.381322e-01 2.388035e-01

712 5.873773e-01 5.874272e-01 5.874902e-01 5.875550e-01 6.005198e-01 2.367348e-01 2.253713e-01 2.260504e-01 2.387584e-01 2.394289e-01

713 5.876146e-01 5.876645e-01 5.877274e-01 5.877922e-01 6.007489e-01 2.373410e-01 2.260126e-01 2.266910e-01 2.393621e-01 2.400317e-01

714 5.878496e-01 5.878995e-01 5.879624e-01 5.880272e-01 6.009759e-01 2.379256e-01 2.266312e-01 2.273088e-01 2.399442e-01 2.406129e-01

715 5.880824e-01 5.881322e-01 5.881951e-01 5.882598e-01 6.012007e-01 2.384894e-01 2.272280e-01 2.279048e-01 2.405056e-01 2.411736e-01

716 5.883129e-01 5.883627e-01 5.884256e-01 5.884903e-01 6.014234e-01 2.390334e-01 2.278039e-01 2.284800e-01 2.410473e-01 2.417145e-01

717 5.885412e-01 5.885909e-01 5.886538e-01 5.887185e-01 6.016441e-01 2.395585e-01 2.283597e-01 2.290351e-01 2.415701e-01 2.422365e-01

718 5.887673e-01 5.888170e-01 5.888799e-01 5.889446e-01 6.018626e-01 2.400654e-01 2.288964e-01 2.295711e-01 2.420749e-01 2.427406e-01

719 5.889913e-01 5.890410e-01 5.891039e-01 5.891685e-01 6.020791e-01 2.405551e-01 2.294148e-01 2.300888e-01 2.425625e-01 2.432275e-01

720 5.892132e-01 5.892629e-01 5.893257e-01 5.893904e-01 6.022937e-01 2.410284e-01 2.299157e-01 2.305890e-01 2.430338e-01 2.436981e-01

721 5.894330e-01 5.894826e-01 5.895455e-01 5.896101e-01 6.025062e-01 2.414860e-01 2.303998e-01 2.310726e-01 2.434895e-01 2.441532e-01

722 5.898392e-01 5.898895e-01 5.899530e-01 5.900183e-01 6.029844e-01 2.414870e-01 2.304007e-01 2.310735e-01 2.434906e-01 2.441543e-01

723 5.902789e-01 5.903299e-01 5.903941e-01 5.904602e-01 6.035013e-01 2.414882e-01 2.304017e-01 2.310745e-01 2.434918e-01 2.441555e-01

724 5.907544e-01 5.908061e-01 5.908711e-01 5.909380e-01 6.040591e-01 2.414895e-01 2.304028e-01 2.310755e-01 2.434931e-01 2.441568e-01

725 5.912679e-01 5.913204e-01 5.913862e-01 5.914539e-01 6.046606e-01 2.414908e-01 2.304039e-01 2.310767e-01 2.434946e-01 2.441583e-01

726 5.918219e-01 5.918753e-01 5.919420e-01 5.920106e-01 6.053082e-01 2.414923e-01 2.304052e-01 2.310780e-01 2.434961e-01 2.441599e-01

727 5.924188e-01 5.924732e-01 5.925408e-01 5.926104e-01 6.060046e-01 2.414940e-01 2.304066e-01 2.310794e-01 2.434978e-01 2.441616e-01

728 5.930613e-01 5.931167e-01 5.931853e-01 5.932559e-01 6.067525e-01 2.414958e-01 2.304081e-01 2.310810e-01 2.434997e-01 2.441635e-01

729 5.937518e-01 5.938083e-01 5.938780e-01 5.939496e-01 6.075546e-01 2.414977e-01 2.304098e-01 2.310827e-01 2.435018e-01 2.441656e-01

730 5.944930e-01 5.945506e-01 5.946214e-01 5.946942e-01 6.084134e-01 2.414999e-01 2.304116e-01 2.310845e-01 2.435040e-01 2.441679e-01

731 5.952874e-01 5.953462e-01 5.954182e-01 5.954922e-01 6.093316e-01 2.415022e-01 2.304136e-01 2.310865e-01 2.435064e-01 2.441704e-01

732 5.961377e-01 5.961976e-01 5.962709e-01 5.963462e-01 6.103117e-01 2.415047e-01 2.304157e-01 2.310887e-01 2.435091e-01 2.441731e-01

733 5.970462e-01 5.971075e-01 5.971821e-01 5.972588e-01 6.113561e-01 2.415075e-01 2.304181e-01 2.310911e-01 2.435120e-01 2.441760e-01

734 5.980155e-01 5.980782e-01 5.981542e-01 5.982323e-01 6.124672e-01 2.415105e-01 2.304206e-01 2.310937e-01 2.435151e-01 2.441792e-01

735 5.990480e-01 5.991122e-01 5.991895e-01 5.992691e-01 6.136472e-01 2.415138e-01 2.304234e-01 2.310965e-01 2.435186e-01 2.441827e-01

736 6.001459e-01 6.002115e-01 6.002904e-01 6.003716e-01 6.148979e-01 2.415174e-01 2.304265e-01 2.310996e-01 2.435223e-01 2.441865e-01

737 6.013112e-01 6.013784e-01 6.014589e-01 6.015416e-01 6.162211e-01 2.415213e-01 2.304298e-01 2.311029e-01 2.435264e-01 2.441906e-01

738 6.025459e-01 6.026147e-01 6.026968e-01 6.027812e-01 6.176184e-01 2.415255e-01 2.304333e-01 2.311066e-01 2.435308e-01 2.441951e-01

739 6.038516e-01 6.039221e-01 6.040058e-01 6.040919e-01 6.190908e-01 2.415301e-01 2.304373e-01 2.311105e-01 2.435356e-01 2.442000e-01

740 6.052297e-01 6.053019e-01 6.053873e-01 6.054752e-01 6.206393e-01 2.415351e-01 2.304415e-01 2.311148e-01 2.435409e-01 2.442053e-01

741 6.066813e-01 6.067553e-01 6.068425e-01 6.069322e-01 6.222644e-01 2.415406e-01 2.304461e-01 2.311195e-01 2.435466e-01 2.442111e-01

742 6.082074e-01 6.082832e-01 6.083722e-01 6.084637e-01 6.239662e-01 2.415465e-01 2.304512e-01 2.311246e-01 2.435527e-01 2.442174e-01

743 6.098082e-01 6.098859e-01 6.099767e-01 6.100701e-01 6.257445e-01 2.415529e-01 2.304566e-01 2.311302e-01 2.435595e-01 2.442242e-01

744 6.114841e-01 6.115636e-01 6.116562e-01 6.117515e-01 6.275985e-01 2.415599e-01 2.304625e-01 2.311362e-01 2.435668e-01 2.442316e-01

745 6.132345e-01 6.133159e-01 6.134104e-01 6.135076e-01 6.295271e-01 2.415675e-01 2.304690e-01 2.311427e-01 2.435747e-01 2.442396e-01

746 6.150588e-01 6.151421e-01 6.152385e-01 6.153377e-01 6.315288e-01 2.415757e-01 2.304760e-01 2.311498e-01 2.435833e-01 2.442483e-01

747 6.169559e-01 6.170411e-01 6.171394e-01 6.172404e-01 6.336014e-01 2.415846e-01 2.304835e-01 2.311575e-01 2.435926e-01 2.442578e-01

748 6.189242e-01 6.190112e-01 6.191114e-01 6.192144e-01 6.357425e-01 2.415943e-01 2.304918e-01 2.311658e-01 2.436027e-01 2.442680e-01

749 6.209615e-01 6.210505e-01 6.211525e-01 6.212574e-01 6.379491e-01 2.416047e-01 2.305007e-01 2.311749e-01 2.436136e-01 2.442791e-01

750 6.230655e-01 6.231563e-01 6.232602e-01 6.233669e-01 6.402177e-01 2.416161e-01 2.305103e-01 2.311847e-01 2.436255e-01 2.442912e-01

751 6.252332e-01 6.253258e-01 6.254314e-01 6.255400e-01 6.425444e-01 2.416284e-01 2.305208e-01 2.311953e-01 2.436383e-01 2.443042e-01

752 6.274611e-01 6.275555e-01 6.276629e-01 6.277732e-01 6.449250e-01 2.416417e-01 2.305321e-01 2.312068e-01 2.436523e-01 2.443183e-01

753 6.297454e-01 6.298416e-01 6.299507e-01 6.300628e-01 6.473548e-01 2.416561e-01 2.305444e-01 2.312192e-01 2.436673e-01 2.443336e-01

754 6.320820e-01 6.321798e-01 6.322906e-01 6.324044e-01 6.498288e-01 2.416717e-01 2.305576e-01 2.312327e-01 2.436836e-01 2.443502e-01

755 6.344662e-01 6.345657e-01 6.346780e-01 6.347934e-01 6.523414e-01 2.416885e-01 2.305720e-01 2.312473e-01 2.437012e-01 2.443681e-01

756 6.368931e-01 6.369941e-01 6.371079e-01 6.372248e-01 6.548872e-01 2.417068e-01 2.305875e-01 2.312630e-01 2.437203e-01 2.443874e-01

757 6.393573e-01 6.394598e-01 6.395750e-01 6.396934e-01 6.574601e-01 2.417265e-01 2.306043e-01 2.312801e-01 2.437409e-01 2.444083e-01

758 6.418534e-01 6.419572e-01 6.420738e-01 6.421936e-01 6.600540e-01 2.417478e-01 2.306225e-01 2.312985e-01 2.437631e-01 2.444309e-01

759 6.443755e-01 6.444806e-01 6.445984e-01 6.447195e-01 6.626627e-01 2.417708e-01 2.306421e-01 2.313184e-01 2.437872e-01 2.444553e-01

760 6.469176e-01 6.470238e-01 6.471428e-01 6.472651e-01 6.652797e-01 2.417956e-01 2.306632e-01 2.313399e-01 2.438131e-01 2.444816e-01

761 6.494735e-01 6.495809e-01 6.497010e-01 6.498243e-01 6.678987e-01 2.418224e-01 2.306861e-01 2.313631e-01 2.438411e-01 2.445100e-01

762 6.520372e-01 6.521455e-01 6.522666e-01 6.523909e-01 6.705132e-01 2.418513e-01 2.307107e-01 2.313881e-01 2.438713e-01 2.445407e-01

763 6.546022e-01 6.547114e-01 6.548333e-01 6.549585e-01 6.731168e-01 2.418825e-01 2.307373e-01 2.314151e-01 2.439039e-01 2.445737e-01

764 6.571623e-01 6.572723e-01 6.573950e-01 6.575210e-01 6.757032e-01 2.419161e-01 2.307660e-01 2.314442e-01 2.439391e-01 2.446094e-01

765 6.597114e-01 6.598221e-01 6.599454e-01 6.600720e-01 6.782665e-01 2.419524e-01 2.307970e-01 2.314756e-01 2.439769e-01 2.446478e-01

766 6.622433e-01 6.623546e-01 6.624785e-01 6.626057e-01 6.808008e-01 2.419914e-01 2.308303e-01 2.315094e-01 2.440177e-01 2.446892e-01

767 6.647522e-01 6.648640e-01 6.649883e-01 6.651160e-01 6.833004e-01 2.420334e-01 2.308662e-01 2.315458e-01 2.440616e-01 2.447337e-01

768 6.672324e-01 6.673446e-01 6.674693e-01 6.675973e-01 6.857601e-01 2.420787e-01 2.309048e-01 2.315851e-01 2.441088e-01 2.447817e-01

769 6.696785e-01 6.697910e-01 6.699159e-01 6.700442e-01 6.881751e-01 2.421273e-01 2.309464e-01 2.316273e-01 2.441597e-01 2.448333e-01

770 6.720854e-01 6.721981e-01 6.723232e-01 6.724517e-01 6.905407e-01 2.421797e-01 2.309912e-01 2.316727e-01 2.442144e-01 2.448887e-01

771 6.744485e-01 6.745612e-01 6.746864e-01 6.748150e-01 6.928529e-01 2.422360e-01 2.310393e-01 2.317215e-01 2.442732e-01 2.449484e-01

772 6.767632e-01 6.768760e-01 6.770013e-01 6.771298e-01 6.951081e-01 2.422965e-01 2.310911e-01 2.317740e-01 2.443363e-01 2.450125e-01

773 6.790259e-01 6.791386e-01 6.792638e-01 6.793923e-01 6.973032e-01 2.423614e-01 2.311467e-01 2.318305e-01 2.444042e-01 2.450813e-01

774 6.812329e-01 6.813455e-01 6.814706e-01 6.815989e-01 6.994354e-01 2.424312e-01 2.312064e-01 2.318911e-01 2.444770e-01 2.451552e-01

775 6.833813e-01 6.834937e-01 6.836186e-01 6.837467e-01 7.015026e-01 2.425061e-01 2.312705e-01 2.319561e-01 2.445552e-01 2.452345e-01

776 6.854686e-01 6.855807e-01 6.857053e-01 6.858332e-01 7.035031e-01 2.425864e-01 2.313393e-01 2.320260e-01 2.446391e-01 2.453196e-01

777 6.874926e-01 6.876044e-01 6.877287e-01 6.878563e-01 7.054356e-01 2.426726e-01 2.314132e-01 2.321009e-01 2.447290e-01 2.454108e-01

778 6.894517e-01 6.895632e-01 6.896872e-01 6.898144e-01 7.072993e-01 2.427649e-01 2.314923e-01 2.321812e-01 2.448254e-01 2.455086e-01

779 6.913449e-01 6.914560e-01 6.915795e-01 6.917063e-01 7.090939e-01 2.428638e-01 2.315772e-01 2.322673e-01 2.449286e-01 2.456133e-01

780 6.931713e-01 6.932819e-01 6.934051e-01 6.935315e-01 7.108195e-01 2.429697e-01 2.316681e-01 2.323595e-01 2.450392e-01 2.457254e-01

781 6.949307e-01 6.950409e-01 6.951636e-01 6.952895e-01 7.124764e-01 2.430831e-01 2.317654e-01 2.324582e-01 2.451575e-01 2.458454e-01

782 6.966232e-01 6.967328e-01 6.968551e-01 6.969805e-01 7.140654e-01 2.432044e-01 2.318696e-01 2.325639e-01 2.452840e-01 2.459738e-01

783 6.982491e-01 6.983583e-01 6.984801e-01 6.986050e-01 7.155877e-01 2.433340e-01 2.319811e-01 2.326770e-01 2.454193e-01 2.461110e-01

784 6.998093e-01 6.999180e-01 7.000393e-01 7.001637e-01 7.170445e-01 2.434726e-01 2.321002e-01 2.327979e-01 2.455638e-01 2.462576e-01

785 7.013048e-01 7.014130e-01 7.015338e-01 7.016577e-01 7.184375e-01 2.436206e-01 2.322276e-01 2.329271e-01 2.457182e-01 2.464141e-01

786 7.027370e-01 7.028446e-01 7.029649e-01 7.030883e-01 7.197684e-01 2.437786e-01 2.323637e-01 2.330651e-01 2.458830e-01 2.465812e-01

787 7.041074e-01 7.042145e-01 7.043343e-01 7.044572e-01 7.210391e-01 2.439472e-01 2.325089e-01 2.332124e-01 2.460588e-01 2.467594e-01

788 7.054177e-01 7.055242e-01 7.056435e-01 7.057659e-01 7.222519e-01 2.441269e-01 2.326639e-01 2.333696e-01 2.462462e-01 2.469494e-01

789 7.066697e-01 7.067758e-01 7.068946e-01 7.070165e-01 7.234087e-01 2.443184e-01 2.328292e-01 2.335372e-01 2.464458e-01 2.471519e-01

790 7.078656e-01 7.079712e-01 7.080895e-01 7.082109e-01 7.245119e-01 2.445225e-01 2.330053e-01 2.337158e-01 2.466584e-01 2.473674e-01

791 7.090074e-01 7.091125e-01 7.092304e-01 7.093513e-01 7.255638e-01 2.447396e-01 2.331929e-01 2.339061e-01 2.468847e-01 2.475968e-01

792 7.100972e-01 7.102018e-01 7.103193e-01 7.104397e-01 7.265667e-01 2.449706e-01 2.333927e-01 2.341087e-01 2.471253e-01 2.478407e-01

793 7.111374e-01 7.112415e-01 7.113585e-01 7.114785e-01 7.275228e-01 2.452161e-01 2.336052e-01 2.343241e-01 2.473810e-01 2.480999e-01

794 7.121300e-01 7.122336e-01 7.123502e-01 7.124698e-01 7.284345e-01 2.454769e-01 2.338311e-01 2.345532e-01 2.476526e-01 2.483751e-01

795 7.130773e-01 7.131805e-01 7.132967e-01 7.134159e-01 7.293040e-01 2.457537e-01 2.340712e-01 2.347966e-01 2.479408e-01 2.486672e-01

796 7.139816e-01 7.140844e-01 7.142002e-01 7.143190e-01 7.301335e-01 2.460474e-01 2.343261e-01 2.350550e-01 2.482465e-01 2.489770e-01

797 7.148449e-01 7.149473e-01 7.150628e-01 7.151811e-01 7.309253e-01 2.463586e-01 2.345966e-01 2.353292e-01 2.485704e-01 2.493052e-01

798 7.156695e-01 7.157715e-01 7.158866e-01 7.160046e-01 7.316813e-01 2.466883e-01 2.348834e-01 2.356199e-01 2.489134e-01 2.496527e-01

799 7.164573e-01 7.165589e-01 7.166737e-01 7.167914e-01 7.324035e-01 2.470372e-01 2.351872e-01 2.359278e-01 2.492762e-01 2.500203e-01

800 7.172104e-01 7.173117e-01 7.174261e-01 7.175435e-01 7.330940e-01 2.474062e-01 2.355089e-01 2.362539e-01 2.496598e-01 2.504088e-01

801 7.179307e-01 7.180316e-01 7.181458e-01 7.182629e-01 7.337545e-01 2.477960e-01 2.358492e-01 2.365987e-01 2.500650e-01 2.508192e-01

802 7.186201e-01 7.187207e-01 7.188346e-01 7.189514e-01 7.343868e-01 2.482075e-01 2.362090e-01 2.369632e-01 2.504925e-01 2.512522e-01

803 7.192803e-01 7.193806e-01 7.194942e-01 7.196107e-01 7.349926e-01 2.486415e-01 2.365889e-01 2.373481e-01 2.509433e-01 2.517087e-01

804 7.199131e-01 7.200131e-01 7.201265e-01 7.202427e-01 7.355735e-01 2.490989e-01 2.369898e-01 2.377543e-01 2.514182e-01 2.521895e-01

805 7.205200e-01 7.206198e-01 7.207329e-01 7.208489e-01 7.361309e-01 2.495803e-01 2.374125e-01 2.381824e-01 2.519179e-01 2.526954e-01

806 7.211026e-01 7.212021e-01 7.213150e-01 7.214308e-01 7.366664e-01 2.500867e-01 2.378577e-01 2.386333e-01 2.524432e-01 2.532271e-01

807 7.216624e-01 7.217616e-01 7.218743e-01 7.219899e-01 7.371813e-01 2.506186e-01 2.383263e-01 2.391078e-01 2.529948e-01 2.537854e-01

808 7.222007e-01 7.222997e-01 7.224122e-01 7.225275e-01 7.376767e-01 2.511769e-01 2.388189e-01 2.396066e-01 2.535736e-01 2.543710e-01

809 7.227188e-01 7.228176e-01 7.229299e-01 7.230450e-01 7.381540e-01 2.517623e-01 2.393362e-01 2.401304e-01 2.541800e-01 2.549846e-01

810 7.232180e-01 7.233165e-01 7.234287e-01 7.235436e-01 7.386142e-01 2.523752e-01 2.398790e-01 2.406798e-01 2.548148e-01 2.556268e-01

811 7.236993e-01 7.237976e-01 7.239096e-01 7.240244e-01 7.390585e-01 2.530164e-01 2.404479e-01 2.412556e-01 2.554785e-01 2.562981e-01

812 7.241639e-01 7.242621e-01 7.243739e-01 7.244885e-01 7.394876e-01 2.536863e-01 2.410435e-01 2.418583e-01 2.561715e-01 2.569989e-01

813 7.246128e-01 7.247108e-01 7.248225e-01 7.249370e-01 7.399027e-01 2.543853e-01 2.416663e-01 2.424884e-01 2.568944e-01 2.577298e-01

814 7.250469e-01 7.251447e-01 7.252563e-01 7.253706e-01 7.403045e-01 2.551139e-01 2.423168e-01 2.431465e-01 2.576473e-01 2.584909e-01

815 7.254671e-01 7.255648e-01 7.256762e-01 7.257905e-01 7.406939e-01 2.558722e-01 2.429955e-01 2.438330e-01 2.584306e-01 2.592826e-01

816 7.258743e-01 7.259719e-01 7.260832e-01 7.261973e-01 7.410716e-01 2.566606e-01 2.437026e-01 2.445481e-01 2.592443e-01 2.601048e-01

817 7.262693e-01 7.263666e-01 7.264778e-01 7.265918e-01 7.414382e-01 2.574789e-01 2.444385e-01 2.452921e-01 2.600886e-01 2.609578e-01

818 7.266527e-01 7.267499e-01 7.268610e-01 7.269749e-01 7.417945e-01 2.583273e-01 2.452033e-01 2.460653e-01 2.609632e-01 2.618412e-01

819 7.270252e-01 7.271223e-01 7.272333e-01 7.273471e-01 7.421411e-01 2.592056e-01 2.459972e-01 2.468676e-01 2.618681e-01 2.627550e-01

820 7.273875e-01 7.274845e-01 7.275954e-01 7.277091e-01 7.424785e-01 2.601135e-01 2.468200e-01 2.476990e-01 2.628029e-01 2.636988e-01

821 7.277402e-01 7.278370e-01 7.279478e-01 7.280614e-01 7.428073e-01 2.610506e-01 2.476716e-01 2.485594e-01 2.637670e-01 2.646720e-01

822 7.280838e-01 7.281805e-01 7.282912e-01 7.284048e-01 7.431279e-01 2.620164e-01 2.485518e-01 2.494485e-01 2.647600e-01 2.656741e-01

823 7.284188e-01 7.285154e-01 7.286261e-01 7.287395e-01 7.434408e-01 2.630102e-01 2.494603e-01 2.503658e-01 2.657811e-01 2.667043e-01

824 7.287457e-01 7.288422e-01 7.289528e-01 7.290662e-01 7.437465e-01 2.640313e-01 2.503965e-01 2.513110e-01 2.668295e-01 2.677617e-01

825 7.290650e-01 7.291614e-01 7.292719e-01 7.293852e-01 7.440453e-01 2.650788e-01 2.513598e-01 2.522833e-01 2.679040e-01 2.688452e-01

826 7.293771e-01 7.294734e-01 7.295838e-01 7.296971e-01 7.443376e-01 2.661514e-01 2.523494e-01 2.532819e-01 2.690036e-01 2.699538e-01

827 7.296823e-01 7.297785e-01 7.298889e-01 7.300021e-01 7.446238e-01 2.672481e-01 2.533646e-01 2.543060e-01 2.701269e-01 2.710860e-01

828 7.299810e-01 7.300772e-01 7.301875e-01 7.303006e-01 7.449041e-01 2.683676e-01 2.544042e-01 2.553545e-01 2.712726e-01 2.722404e-01

829 7.302736e-01 7.303697e-01 7.304800e-01 7.305930e-01 7.451789e-01 2.695082e-01 2.554671e-01 2.564263e-01 2.724392e-01 2.734154e-01

830 7.305604e-01 7.306564e-01 7.307666e-01 7.308796e-01 7.454485e-01 2.706686e-01 2.565520e-01 2.575200e-01 2.736248e-01 2.746094e-01

831 7.308416e-01 7.309376e-01 7.310478e-01 7.311607e-01 7.457130e-01 2.718469e-01 2.576576e-01 2.586342e-01 2.748278e-01 2.758206e-01

832 7.311176e-01 7.312135e-01 7.313236e-01 7.314365e-01 7.459729e-01 2.730414e-01 2.587824e-01 2.597674e-01 2.760463e-01 2.770470e-01

833 7.313886e-01 7.314844e-01 7.315945e-01 7.317073e-01 7.462282e-01 2.742501e-01 2.599246e-01 2.609179e-01 2.772783e-01 2.782867e-01

834 7.316548e-01 7.317506e-01 7.318606e-01 7.319734e-01 7.464792e-01 2.754712e-01 2.610827e-01 2.620841e-01 2.785218e-01 2.795375e-01

835 7.319165e-01 7.320122e-01 7.321222e-01 7.322349e-01 7.467261e-01 2.767024e-01 2.622548e-01 2.632640e-01 2.797746e-01 2.807974e-01

836 7.321738e-01 7.322694e-01 7.323794e-01 7.324921e-01 7.469690e-01 2.779418e-01 2.634390e-01 2.644557e-01 2.810346e-01 2.820641e-01

837 7.324270e-01 7.325226e-01 7.326325e-01 7.327452e-01 7.472083e-01 2.791871e-01 2.646333e-01 2.656574e-01 2.822996e-01 2.833355e-01

838 7.326762e-01 7.327718e-01 7.328816e-01 7.329943e-01 7.474439e-01 2.804363e-01 2.658358e-01 2.668668e-01 2.835674e-01 2.846093e-01

839 7.329217e-01 7.330171e-01 7.331270e-01 7.332396e-01 7.476761e-01 2.816870e-01 2.670444e-01 2.680821e-01 2.848357e-01 2.858833e-01

840 7.331635e-01 7.332589e-01 7.333687e-01 7.334813e-01 7.479049e-01 2.829372e-01 2.682569e-01 2.693010e-01 2.861023e-01 2.871553e-01

841 7.334018e-01 7.334972e-01 7.336069e-01 7.337195e-01 7.481306e-01 2.841847e-01 2.694714e-01 2.705215e-01 2.873651e-01 2.884230e-01

842 7.336368e-01 7.337321e-01 7.338418e-01 7.339543e-01 7.483533e-01 2.854272e-01 2.706857e-01 2.717415e-01 2.886220e-01 2.896845e-01

843 7.338685e-01 7.339638e-01 7.340735e-01 7.341860e-01 7.485730e-01 2.866629e-01 2.718977e-01 2.729588e-01 2.898708e-01 2.909375e-01

844 7.340972e-01 7.341924e-01 7.343021e-01 7.344145e-01 7.487899e-01 2.878895e-01 2.731054e-01 2.741715e-01 2.911095e-01 2.921800e-01

845 7.343228e-01 7.344180e-01 7.345277e-01 7.346401e-01 7.490040e-01 2.891053e-01 2.743068e-01 2.753776e-01 2.923362e-01 2.934102e-01

846 7.345456e-01 7.346407e-01 7.347504e-01 7.348628e-01 7.492156e-01 2.903083e-01 2.755000e-01 2.765750e-01 2.935491e-01 2.946262e-01

847 7.347656e-01 7.348607e-01 7.349703e-01 7.350827e-01 7.494246e-01 2.914969e-01 2.766831e-01 2.777620e-01 2.947465e-01 2.958264e-01

848 7.349829e-01 7.350780e-01 7.351876e-01 7.352999e-01 7.496311e-01 2.926693e-01 2.778543e-01 2.789367e-01 2.959267e-01 2.970090e-01

849 7.351977e-01 7.352927e-01 7.354023e-01 7.355146e-01 7.498353e-01 2.938241e-01 2.790119e-01 2.800975e-01 2.970883e-01 2.981727e-01

850 7.354099e-01 7.355049e-01 7.356144e-01 7.357267e-01 7.500371e-01 2.949598e-01 2.801544e-01 2.812429e-01 2.982299e-01 2.993162e-01

851 7.356197e-01 7.357146e-01 7.358241e-01 7.359364e-01 7.502367e-01 2.960752e-01 2.812802e-01 2.823712e-01 2.993503e-01 3.004381e-01

852 7.358271e-01 7.359220e-01 7.360315e-01 7.361437e-01 7.504342e-01 2.971692e-01 2.823880e-01 2.834813e-01 3.004484e-01 3.015374e-01

853 7.360322e-01 7.361271e-01 7.362366e-01 7.363488e-01 7.506295e-01 2.982407e-01 2.834766e-01 2.845718e-01 3.015232e-01 3.026132e-01

854 7.362351e-01 7.363300e-01 7.364394e-01 7.365516e-01 7.508228e-01 2.992888e-01 2.845449e-01 2.856417e-01 3.025740e-01 3.036647e-01

855 7.364358e-01 7.365307e-01 7.366401e-01 7.367523e-01 7.510141e-01 3.003129e-01 2.855917e-01 2.866900e-01 3.036000e-01 3.046912e-01

856 7.366345e-01 7.367293e-01 7.368387e-01 7.369508e-01 7.512035e-01 3.013123e-01 2.866164e-01 2.877158e-01 3.046006e-01 3.056922e-01

857 7.368311e-01 7.369259e-01 7.370352e-01 7.371473e-01 7.513909e-01 3.022866e-01 2.876182e-01 2.887185e-01 3.055756e-01 3.066672e-01

858 7.370257e-01 7.371204e-01 7.372298e-01 7.373419e-01 7.515766e-01 3.032353e-01 2.885964e-01 2.896974e-01 3.065244e-01 3.076160e-01

859 7.372183e-01 7.373130e-01 7.374224e-01 7.375344e-01 7.517604e-01 3.041582e-01 2.895506e-01 2.906520e-01 3.074470e-01 3.085384e-01

860 7.374090e-01 7.375038e-01 7.376131e-01 7.377251e-01 7.519425e-01 3.050552e-01 2.904803e-01 2.915821e-01 3.083433e-01 3.094343e-01

861 7.375979e-01 7.376926e-01 7.378019e-01 7.379139e-01 7.521228e-01 3.059263e-01 2.913854e-01 2.924873e-01 3.092132e-01 3.103038e-01

862 7.377850e-01 7.378797e-01 7.379889e-01 7.381009e-01 7.523015e-01 3.067715e-01 2.922656e-01 2.933675e-01 3.100570e-01 3.111470e-01

863 7.379703e-01 7.380649e-01 7.381742e-01 7.382861e-01 7.524785e-01 3.075910e-01 2.931210e-01 2.942227e-01 3.108747e-01 3.119640e-01

864 7.381539e-01 7.382485e-01 7.383577e-01 7.384696e-01 7.526539e-01 3.083850e-01 2.939515e-01 2.950530e-01 3.116667e-01 3.127553e-01

865 7.383357e-01 7.384303e-01 7.385395e-01 7.386514e-01 7.528277e-01 3.091538e-01 2.947573e-01 2.958584e-01 3.124333e-01 3.135211e-01

866 7.385159e-01 7.386105e-01 7.387197e-01 7.388316e-01 7.530000e-01 3.098979e-01 2.955386e-01 2.966392e-01 3.131749e-01 3.142619e-01

867 7.386945e-01 7.387890e-01 7.388982e-01 7.390101e-01 7.531708e-01 3.106176e-01 2.962956e-01 2.973957e-01 3.138921e-01 3.149781e-01

868 7.388714e-01 7.389660e-01 7.390751e-01 7.391870e-01 7.533401e-01 3.113134e-01 2.970287e-01 2.981282e-01 3.145852e-01 3.156703e-01

869 7.390468e-01 7.391414e-01 7.392505e-01 7.393623e-01 7.535079e-01 3.119858e-01 2.977383e-01 2.988371e-01 3.152549e-01 3.163391e-01

870 7.392207e-01 7.393152e-01 7.394243e-01 7.395361e-01 7.536743e-01 3.126355e-01 2.984249e-01 2.995230e-01 3.159018e-01 3.169850e-01

871 7.393930e-01 7.394875e-01 7.395966e-01 7.397084e-01 7.538392e-01 3.132631e-01 2.990889e-01 3.001862e-01 3.165265e-01 3.176087e-01

872 7.395639e-01 7.396584e-01 7.397674e-01 7.398791e-01 7.540028e-01 3.138690e-01 2.997308e-01 3.008274e-01 3.171296e-01 3.182108e-01

873 7.397332e-01 7.398277e-01 7.399367e-01 7.400485e-01 7.541651e-01 3.144541e-01 3.003514e-01 3.014471e-01 3.177119e-01 3.187921e-01

874 7.399012e-01 7.399957e-01 7.401047e-01 7.402164e-01 7.543259e-01 3.150190e-01 3.009510e-01 3.020459e-01 3.182739e-01 3.193531e-01

875 7.400677e-01 7.401622e-01 7.402711e-01 7.403828e-01 7.544855e-01 3.155642e-01 3.015303e-01 3.026245e-01 3.188163e-01 3.198946e-01

876 7.402329e-01 7.403273e-01 7.404362e-01 7.405479e-01 7.546438e-01 3.160906e-01 3.020901e-01 3.031834e-01 3.193399e-01 3.204173e-01

877 7.403966e-01 7.404911e-01 7.406000e-01 7.407116e-01 7.548007e-01 3.165988e-01 3.026308e-01 3.037233e-01 3.198454e-01 3.209218e-01

878 7.405590e-01 7.406535e-01 7.407624e-01 7.408740e-01 7.549565e-01 3.170894e-01 3.031531e-01 3.042448e-01 3.203333e-01 3.214088e-01

879 7.407201e-01 7.408145e-01 7.409234e-01 7.410350e-01 7.551109e-01 3.175631e-01 3.036577e-01 3.047486e-01 3.208045e-01 3.218791e-01

880 7.408799e-01 7.409743e-01 7.410831e-01 7.411947e-01 7.552642e-01 3.180207e-01 3.041453e-01 3.052354e-01 3.212595e-01 3.223332e-01

881 7.410383e-01 7.411328e-01 7.412416e-01 7.413531e-01 7.554162e-01 3.184626e-01 3.046163e-01 3.057057e-01 3.216990e-01 3.227719e-01

882 7.411955e-01 7.412900e-01 7.413987e-01 7.415102e-01 7.555671e-01 3.188897e-01 3.050716e-01 3.061602e-01 3.221236e-01 3.231957e-01

883 7.413515e-01 7.414459e-01 7.415546e-01 7.416661e-01 7.557167e-01 3.193024e-01 3.055117e-01 3.065996e-01 3.225340e-01 3.236054e-01

884 7.415062e-01 7.416006e-01 7.417093e-01 7.418208e-01 7.558652e-01 3.197014e-01 3.059372e-01 3.070244e-01 3.229309e-01 3.240015e-01

885 7.416596e-01 7.417540e-01 7.418627e-01 7.419742e-01 7.560126e-01 3.200874e-01 3.063487e-01 3.074352e-01 3.233147e-01 3.243845e-01

886 7.418119e-01 7.419063e-01 7.420150e-01 7.421264e-01 7.561588e-01 3.204608e-01 3.067469e-01 3.078327e-01 3.236860e-01 3.247552e-01

887 7.419629e-01 7.420573e-01 7.421660e-01 7.422774e-01 7.563039e-01 3.208222e-01 3.071322e-01 3.082174e-01 3.240455e-01 3.251140e-01

888 7.421128e-01 7.422072e-01 7.423158e-01 7.424272e-01 7.564479e-01 3.211722e-01 3.075052e-01 3.085898e-01 3.243937e-01 3.254615e-01

889 7.422615e-01 7.423559e-01 7.424645e-01 7.425758e-01 7.565907e-01 3.215114e-01 3.078665e-01 3.089505e-01 3.247310e-01 3.257983e-01

890 7.424091e-01 7.425035e-01 7.426120e-01 7.427233e-01 7.567325e-01 3.218401e-01 3.082166e-01 3.093001e-01 3.250580e-01 3.261247e-01

891 7.425555e-01 7.426499e-01 7.427584e-01 7.428697e-01 7.568733e-01 3.221589e-01 3.085561e-01 3.096390e-01 3.253751e-01 3.264412e-01

892 7.427007e-01 7.427951e-01 7.429037e-01 7.430149e-01 7.570129e-01 3.224682e-01 3.088853e-01 3.099676e-01 3.256829e-01 3.267485e-01

893 7.428449e-01 7.429393e-01 7.430478e-01 7.431590e-01 7.571516e-01 3.227685e-01 3.092047e-01 3.102866e-01 3.259817e-01 3.270468e-01

894 7.429879e-01 7.430824e-01 7.431908e-01 7.433020e-01 7.572891e-01 3.230603e-01 3.095148e-01 3.105963e-01 3.262720e-01 3.273366e-01

895 7.431299e-01 7.432243e-01 7.433328e-01 7.434439e-01 7.574257e-01 3.233438e-01 3.098161e-01 3.108971e-01 3.265541e-01 3.276183e-01

896 7.432708e-01 7.433652e-01 7.434736e-01 7.435847e-01 7.575612e-01 3.236196e-01 3.101089e-01 3.111895e-01 3.268286e-01 3.278924e-01

897 7.434106e-01 7.435050e-01 7.436134e-01 7.437245e-01 7.576958e-01 3.238880e-01 3.103937e-01 3.114738e-01 3.270957e-01 3.281590e-01

898 7.435493e-01 7.436438e-01 7.437521e-01 7.438631e-01 7.578293e-01 3.241493e-01 3.106708e-01 3.117505e-01 3.273558e-01 3.284187e-01

899 7.436870e-01 7.437815e-01 7.438898e-01 7.440008e-01 7.579618e-01 3.244038e-01 3.109405e-01 3.120199e-01 3.276093e-01 3.286718e-01

900 7.438236e-01 7.439181e-01 7.440264e-01 7.441374e-01 7.580934e-01 3.246520e-01 3.112033e-01 3.122823e-01 3.278563e-01 3.289185e-01

901 7.439593e-01 7.440537e-01 7.441620e-01 7.442729e-01 7.582240e-01 3.248941e-01 3.114595e-01 3.125382e-01 3.280974e-01 3.291593e-01

902 7.458418e-01 7.459391e-01 7.460502e-01 7.461640e-01 7.602761e-01 3.249004e-01 3.114648e-01 3.125436e-01 3.281041e-01 3.291661e-01

903 7.477826e-01 7.478828e-01 7.479966e-01 7.481132e-01 7.623818e-01 3.249072e-01 3.114705e-01 3.125494e-01 3.281113e-01 3.291734e-01

904 7.497793e-01 7.498823e-01 7.499988e-01 7.501182e-01 7.645377e-01 3.249145e-01 3.114766e-01 3.125557e-01 3.281190e-01 3.291813e-01

905 7.518291e-01 7.519348e-01 7.520540e-01 7.521761e-01 7.667402e-01 3.249223e-01 3.114833e-01 3.125625e-01 3.281274e-01 3.291899e-01

906 7.539286e-01 7.540370e-01 7.541588e-01 7.542836e-01 7.689852e-01 3.249308e-01 3.114904e-01 3.125697e-01 3.281364e-01 3.291991e-01

907 7.560745e-01 7.561854e-01 7.563097e-01 7.564370e-01 7.712683e-01 3.249399e-01 3.114981e-01 3.125776e-01 3.281461e-01 3.292089e-01

908 7.582627e-01 7.583762e-01 7.585029e-01 7.586327e-01 7.735849e-01 3.249498e-01 3.115064e-01 3.125861e-01 3.281565e-01 3.292196e-01

909 7.604892e-01 7.606050e-01 7.607340e-01 7.608662e-01 7.759301e-01 3.249603e-01 3.115153e-01 3.125952e-01 3.281678e-01 3.292310e-01

910 7.627494e-01 7.628675e-01 7.629988e-01 7.631331e-01 7.782988e-01 3.249717e-01 3.115249e-01 3.126050e-01 3.281798e-01 3.292434e-01

911 7.650387e-01 7.651590e-01 7.652923e-01 7.654288e-01 7.806858e-01 3.249839e-01 3.115353e-01 3.126155e-01 3.281928e-01 3.292566e-01

912 7.673522e-01 7.674745e-01 7.676098e-01 7.677483e-01 7.830857e-01 3.249971e-01 3.115464e-01 3.126268e-01 3.282068e-01 3.292709e-01

913 7.696847e-01 7.698090e-01 7.699460e-01 7.700864e-01 7.854930e-01 3.250112e-01 3.115583e-01 3.126390e-01 3.282218e-01 3.292862e-01

914 7.720312e-01 7.721571e-01 7.722959e-01 7.724379e-01 7.879023e-01 3.250264e-01 3.115711e-01 3.126521e-01 3.282379e-01 3.293026e-01

915 7.743862e-01 7.745137e-01 7.746540e-01 7.747976e-01 7.903079e-01 3.250427e-01 3.115849e-01 3.126661e-01 3.282552e-01 3.293203e-01

916 7.767443e-01 7.768734e-01 7.770150e-01 7.771600e-01 7.927045e-01 3.250602e-01 3.115997e-01 3.126812e-01 3.282738e-01 3.293393e-01

917 7.791004e-01 7.792307e-01 7.793736e-01 7.795198e-01 7.950866e-01 3.250790e-01 3.116156e-01 3.126975e-01 3.282938e-01 3.293596e-01

918 7.814489e-01 7.815804e-01 7.817243e-01 7.818716e-01 7.974490e-01 3.250992e-01 3.116326e-01 3.127149e-01 3.283152e-01 3.293815e-01

919 7.837847e-01 7.839171e-01 7.840620e-01 7.842102e-01 7.997868e-01 3.251208e-01 3.116509e-01 3.127335e-01 3.283382e-01 3.294049e-01

920 7.861027e-01 7.862359e-01 7.863816e-01 7.865306e-01 8.020951e-01 3.251441e-01 3.116706e-01 3.127536e-01 3.283629e-01 3.294301e-01

921 7.883978e-01 7.885318e-01 7.886780e-01 7.888276e-01 8.043692e-01 3.251689e-01 3.116916e-01 3.127751e-01 3.283893e-01 3.294570e-01

922 7.906655e-01 7.907999e-01 7.909467e-01 7.910968e-01 8.066050e-01 3.251956e-01 3.117142e-01 3.127981e-01 3.284176e-01 3.294859e-01

923 7.929011e-01 7.930359e-01 7.931830e-01 7.933334e-01 8.087984e-01 3.252242e-01 3.117384e-01 3.128228e-01 3.284479e-01 3.295169e-01

924 7.951005e-01 7.952356e-01 7.953828e-01 7.955335e-01 8.109459e-01 3.252548e-01 3.117643e-01 3.128492e-01 3.284804e-01 3.295500e-01

925 7.972597e-01 7.973949e-01 7.975423e-01 7.976929e-01 8.130443e-01 3.252876e-01 3.117921e-01 3.128775e-01 3.285152e-01 3.295855e-01

926 7.993752e-01 7.995104e-01 7.996577e-01 7.998083e-01 8.150905e-01 3.253227e-01 3.118218e-01 3.129079e-01 3.285525e-01 3.296235e-01

927 8.014437e-01 8.015788e-01 8.017259e-01 8.018764e-01 8.170823e-01 3.253602e-01 3.118536e-01 3.129403e-01 3.285923e-01 3.296641e-01

928 8.034623e-01 8.035972e-01 8.037441e-01 8.038943e-01 8.190174e-01 3.254004e-01 3.118877e-01 3.129750e-01 3.286349e-01 3.297076e-01

929 8.054287e-01 8.055632e-01 8.057098e-01 8.058596e-01 8.208941e-01 3.254433e-01 3.119241e-01 3.130122e-01 3.286805e-01 3.297541e-01

930 8.073405e-01 8.074747e-01 8.076208e-01 8.077701e-01 8.227112e-01 3.254892e-01 3.119630e-01 3.130519e-01 3.287292e-01 3.298038e-01

931 8.091962e-01 8.093299e-01 8.094755e-01 8.096243e-01 8.244677e-01 3.255383e-01 3.120046e-01 3.130944e-01 3.287813e-01 3.298569e-01

932 8.109944e-01 8.111274e-01 8.112724e-01 8.114206e-01 8.261631e-01 3.255907e-01 3.120491e-01 3.131397e-01 3.288368e-01 3.299136e-01

933 8.127339e-01 8.128663e-01 8.130107e-01 8.131583e-01 8.277969e-01 3.256466e-01 3.120966e-01 3.131882e-01 3.288962e-01 3.299741e-01

934 8.144142e-01 8.145460e-01 8.146897e-01 8.148365e-01 8.293694e-01 3.257063e-01 3.121473e-01 3.132399e-01 3.289596e-01 3.300387e-01

935 8.160350e-01 8.161660e-01 8.163090e-01 8.164551e-01 8.308809e-01 3.257701e-01 3.122015e-01 3.132952e-01 3.290272e-01 3.301076e-01

936 8.175963e-01 8.177265e-01 8.178687e-01 8.180140e-01 8.323321e-01 3.258380e-01 3.122592e-01 3.133541e-01 3.290992e-01 3.301812e-01

937 8.190982e-01 8.192276e-01 8.193691e-01 8.195136e-01 8.337237e-01 3.259105e-01 3.123209e-01 3.134170e-01 3.291761e-01 3.302595e-01

938 8.205414e-01 8.206700e-01 8.208107e-01 8.209543e-01 8.350569e-01 3.259877e-01 3.123866e-01 3.134840e-01 3.292580e-01 3.303430e-01

939 8.219267e-01 8.220545e-01 8.221943e-01 8.223371e-01 8.363330e-01 3.260700e-01 3.124566e-01 3.135554e-01 3.293453e-01 3.304320e-01

940 8.232550e-01 8.233819e-01 8.235209e-01 8.236629e-01 8.375534e-01 3.261577e-01 3.125312e-01 3.136315e-01 3.294382e-01 3.305267e-01

941 8.245275e-01 8.246536e-01 8.247918e-01 8.249330e-01 8.387197e-01 3.262510e-01 3.126107e-01 3.137125e-01 3.295371e-01 3.306276e-01

942 8.257457e-01 8.258709e-01 8.260083e-01 8.261486e-01 8.398336e-01 3.263502e-01 3.126953e-01 3.137988e-01 3.296423e-01 3.307348e-01

943 8.269109e-01 8.270353e-01 8.271719e-01 8.273113e-01 8.408968e-01 3.264558e-01 3.127853e-01 3.138906e-01 3.297542e-01 3.308489e-01

944 8.280248e-01 8.281484e-01 8.282841e-01 8.284228e-01 8.419113e-01 3.265681e-01 3.128811e-01 3.139883e-01 3.298731e-01 3.309702e-01

945 8.290890e-01 8.292118e-01 8.293468e-01 8.294847e-01 8.428789e-01 3.266874e-01 3.129829e-01 3.140921e-01 3.299995e-01 3.310990e-01

946 8.301054e-01 8.302274e-01 8.303616e-01 8.304987e-01 8.438015e-01 3.268141e-01 3.130911e-01 3.142025e-01 3.301337e-01 3.312358e-01

947 8.310757e-01 8.311970e-01 8.313305e-01 8.314668e-01 8.446810e-01 3.269486e-01 3.132061e-01 3.143197e-01 3.302762e-01 3.313811e-01

948 8.320018e-01 8.321223e-01 8.322551e-01 8.323907e-01 8.455194e-01 3.270914e-01 3.133282e-01 3.144442e-01 3.304274e-01 3.315352e-01

949 8.328855e-01 8.330053e-01 8.331374e-01 8.332723e-01 8.463186e-01 3.272428e-01 3.134577e-01 3.145763e-01 3.305878e-01 3.316986e-01

950 8.337287e-01 8.338478e-01 8.339792e-01 8.341134e-01 8.470804e-01 3.274034e-01 3.135952e-01 3.147164e-01 3.307577e-01 3.318718e-01

951 8.345333e-01 8.346517e-01 8.347824e-01 8.349160e-01 8.478067e-01 3.275735e-01 3.137410e-01 3.148650e-01 3.309378e-01 3.320552e-01

952 8.353009e-01 8.354187e-01 8.355488e-01 8.356817e-01 8.484993e-01 3.277537e-01 3.138955e-01 3.150225e-01 3.311284e-01 3.322495e-01

953 8.360335e-01 8.361507e-01 8.362802e-01 8.364125e-01 8.491599e-01 3.279444e-01 3.140591e-01 3.151893e-01 3.313302e-01 3.324550e-01

954 8.367328e-01 8.368493e-01 8.369784e-01 8.371101e-01 8.497903e-01 3.281462e-01 3.142324e-01 3.153659e-01 3.315436e-01 3.326724e-01

955 8.374005e-01 8.375164e-01 8.376449e-01 8.377761e-01 8.503921e-01 3.283596e-01 3.144158e-01 3.155528e-01 3.317692e-01 3.329022e-01

956 8.380381e-01 8.381536e-01 8.382815e-01 8.384122e-01 8.509667e-01 3.285850e-01 3.146097e-01 3.157504e-01 3.320075e-01 3.331449e-01

957 8.386474e-01 8.387623e-01 8.388898e-01 8.390199e-01 8.515158e-01 3.288231e-01 3.148147e-01 3.159593e-01 3.322590e-01 3.334012e-01

958 8.392299e-01 8.393443e-01 8.394713e-01 8.396009e-01 8.520408e-01 3.290744e-01 3.150313e-01 3.161800e-01 3.325245e-01 3.336715e-01

959 8.397869e-01 8.399008e-01 8.400274e-01 8.401566e-01 8.525430e-01 3.293394e-01 3.152599e-01 3.164130e-01 3.328044e-01 3.339565e-01

960 8.403200e-01 8.404335e-01 8.405596e-01 8.406884e-01 8.530237e-01 3.296188e-01 3.155012e-01 3.166588e-01 3.330994e-01 3.342568e-01

961 8.408304e-01 8.409435e-01 8.410692e-01 8.411976e-01 8.534842e-01 3.299131e-01 3.157556e-01 3.169179e-01 3.334099e-01 3.345730e-01

962 8.413194e-01 8.414321e-01 8.415575e-01 8.416855e-01 8.539257e-01 3.302228e-01 3.160238e-01 3.171910e-01 3.337368e-01 3.349057e-01

963 8.417884e-01 8.419007e-01 8.420257e-01 8.421534e-01 8.543493e-01 3.305487e-01 3.163061e-01 3.174786e-01 3.340804e-01 3.352554e-01

964 8.422383e-01 8.423503e-01 8.424750e-01 8.426023e-01 8.547560e-01 3.308912e-01 3.166033e-01 3.177812e-01 3.344415e-01 3.356228e-01

965 8.426704e-01 8.427820e-01 8.429064e-01 8.430334e-01 8.551468e-01 3.312509e-01 3.169159e-01 3.180994e-01 3.348205e-01 3.360085e-01

966 8.430856e-01 8.431969e-01 8.433210e-01 8.434477e-01 8.555227e-01 3.316284e-01 3.172443e-01 3.184338e-01 3.352182e-01 3.364130e-01

967 8.434849e-01 8.435959e-01 8.437197e-01 8.438461e-01 8.558846e-01 3.320243e-01 3.175893e-01 3.187849e-01 3.356350e-01 3.368370e-01

968 8.438694e-01 8.439800e-01 8.441036e-01 8.442297e-01 8.562332e-01 3.324391e-01 3.179512e-01 3.191533e-01 3.360715e-01 3.372809e-01

969 8.442397e-01 8.443501e-01 8.444734e-01 8.445993e-01 8.565694e-01 3.328734e-01 3.183307e-01 3.195395e-01 3.365283e-01 3.377454e-01

970 8.445968e-01 8.447070e-01 8.448300e-01 8.449557e-01 8.568939e-01 3.333276e-01 3.187283e-01 3.199441e-01 3.370058e-01 3.382308e-01

971 8.449414e-01 8.450513e-01 8.451742e-01 8.452996e-01 8.572073e-01 3.338023e-01 3.191445e-01 3.203675e-01 3.375046e-01 3.387378e-01

972 8.452743e-01 8.453840e-01 8.455067e-01 8.456318e-01 8.575105e-01 3.342979e-01 3.195798e-01 3.208103e-01 3.380250e-01 3.392667e-01

973 8.455961e-01 8.457056e-01 8.458281e-01 8.459531e-01 8.578038e-01 3.348148e-01 3.200347e-01 3.212729e-01 3.385675e-01 3.398179e-01

974 8.459075e-01 8.460168e-01 8.461391e-01 8.462639e-01 8.580880e-01 3.353535e-01 3.205096e-01 3.217557e-01 3.391325e-01 3.403919e-01

975 8.462092e-01 8.463182e-01 8.464403e-01 8.465649e-01 8.583636e-01 3.359143e-01 3.210049e-01 3.222593e-01 3.397203e-01 3.409889e-01

976 8.465015e-01 8.466104e-01 8.467324e-01 8.468568e-01 8.586310e-01 3.364974e-01 3.215211e-01 3.227839e-01 3.403312e-01 3.416091e-01

977 8.467852e-01 8.468939e-01 8.470157e-01 8.471400e-01 8.588907e-01 3.371031e-01 3.220584e-01 3.233299e-01 3.409653e-01 3.422528e-01

978 8.470606e-01 8.471692e-01 8.472908e-01 8.474149e-01 8.591432e-01 3.377317e-01 3.226172e-01 3.238976e-01 3.416228e-01 3.429202e-01

979 8.473283e-01 8.474367e-01 8.475582e-01 8.476822e-01 8.593888e-01 3.383831e-01 3.231976e-01 3.244873e-01 3.423038e-01 3.436112e-01

980 8.475886e-01 8.476969e-01 8.478183e-01 8.479421e-01 8.596280e-01 3.390576e-01 3.238000e-01 3.250990e-01 3.430083e-01 3.443258e-01

981 8.478421e-01 8.479502e-01 8.480715e-01 8.481952e-01 8.598612e-01 3.397551e-01 3.244244e-01 3.257330e-01 3.437363e-01 3.450641e-01

982 8.480890e-01 8.481971e-01 8.483182e-01 8.484418e-01 8.600885e-01 3.404754e-01 3.250710e-01 3.263893e-01 3.444876e-01 3.458258e-01

983 8.483298e-01 8.484377e-01 8.485587e-01 8.486822e-01 8.603105e-01 3.412185e-01 3.257396e-01 3.270679e-01 3.452619e-01 3.466107e-01

984 8.485648e-01 8.486725e-01 8.487934e-01 8.489168e-01 8.605273e-01 3.419840e-01 3.264304e-01 3.277686e-01 3.460590e-01 3.474184e-01

985 8.487942e-01 8.489019e-01 8.490227e-01 8.491460e-01 8.607392e-01 3.427717e-01 3.271430e-01 3.284915e-01 3.468784e-01 3.482485e-01

986 8.490184e-01 8.491260e-01 8.492467e-01 8.493699e-01 8.609466e-01 3.435811e-01 3.278774e-01 3.292361e-01 3.477197e-01 3.491004e-01

987 8.492377e-01 8.493452e-01 8.494658e-01 8.495889e-01 8.611496e-01 3.444117e-01 3.286332e-01 3.300023e-01 3.485823e-01 3.499736e-01

988 8.494523e-01 8.495597e-01 8.496802e-01 8.498032e-01 8.613484e-01 3.452629e-01 3.294100e-01 3.307896e-01 3.494653e-01 3.508674e-01

989 8.496624e-01 8.497697e-01 8.498902e-01 8.500131e-01 8.615433e-01 3.461340e-01 3.302075e-01 3.315975e-01 3.503682e-01 3.517809e-01

990 8.498683e-01 8.499756e-01 8.500959e-01 8.502188e-01 8.617345e-01 3.470242e-01 3.310249e-01 3.324254e-01 3.512900e-01 3.527132e-01

991 8.500703e-01 8.501774e-01 8.502977e-01 8.504205e-01 8.619221e-01 3.479326e-01 3.318617e-01 3.332728e-01 3.522297e-01 3.536633e-01

992 8.502684e-01 8.503754e-01 8.504957e-01 8.506184e-01 8.621063e-01 3.488582e-01 3.327173e-01 3.341387e-01 3.531863e-01 3.546302e-01

993 8.504629e-01 8.505699e-01 8.506900e-01 8.508126e-01 8.622874e-01 3.498000e-01 3.335906e-01 3.350224e-01 3.541587e-01 3.556127e-01

994 8.506539e-01 8.507608e-01 8.508809e-01 8.510035e-01 8.624654e-01 3.507569e-01 3.344809e-01 3.359230e-01 3.551456e-01 3.566095e-01

995 8.508416e-01 8.509485e-01 8.510685e-01 8.511910e-01 8.626404e-01 3.517276e-01 3.353872e-01 3.368395e-01 3.561457e-01 3.576193e-01

996 8.510263e-01 8.511331e-01 8.512530e-01 8.513754e-01 8.628127e-01 3.527109e-01 3.363085e-01 3.377708e-01 3.571577e-01 3.586407e-01

997 8.512079e-01 8.513146e-01 8.514345e-01 8.515569e-01 8.629823e-01 3.537054e-01 3.372435e-01 3.387156e-01 3.581801e-01 3.596723e-01

998 8.513866e-01 8.514933e-01 8.516132e-01 8.517355e-01 8.631494e-01 3.547097e-01 3.381911e-01 3.396729e-01 3.592115e-01 3.607126e-01

999 8.515627e-01 8.516693e-01 8.517891e-01 8.519114e-01 8.633141e-01 3.557223e-01 3.391500e-01 3.406412e-01 3.602503e-01 3.617601e-01

1000 8.517361e-01 8.518427e-01 8.519624e-01 8.520846e-01 8.634764e-01 3.567418e-01 3.401190e-01 3.416194e-01 3.612950e-01 3.628130e-01

1001 8.519070e-01 8.520135e-01 8.521332e-01 8.522554e-01 8.636365e-01 3.577665e-01 3.410965e-01 3.426058e-01 3.623441e-01 3.638699e-01

1002 8.520755e-01 8.521820e-01 8.523016e-01 8.524237e-01 8.637944e-01 3.587950e-01 3.420813e-01 3.435992e-01 3.633958e-01 3.649292e-01

1003 8.522417e-01 8.523482e-01 8.524678e-01 8.525898e-01 8.639503e-01 3.598257e-01 3.430718e-01 3.445981e-01 3.644486e-01 3.659891e-01

1004 8.524057e-01 8.525121e-01 8.526317e-01 8.527537e-01 8.641042e-01 3.608570e-01 3.440666e-01 3.456009e-01 3.655008e-01 3.670482e-01

1005 8.525676e-01 8.526740e-01 8.527935e-01 8.529154e-01 8.642562e-01 3.618873e-01 3.450642e-01 3.466061e-01 3.665510e-01 3.681047e-01

1006 8.527274e-01 8.528338e-01 8.529532e-01 8.530751e-01 8.644064e-01 3.629151e-01 3.460631e-01 3.476123e-01 3.675974e-01 3.691572e-01

1007 8.528853e-01 8.529916e-01 8.531110e-01 8.532329e-01 8.645548e-01 3.639388e-01 3.470617e-01 3.486179e-01 3.686387e-01 3.702040e-01

1008 8.530413e-01 8.531475e-01 8.532669e-01 8.533887e-01 8.647015e-01 3.649570e-01 3.480587e-01 3.496214e-01 3.696732e-01 3.712437e-01

1009 8.531954e-01 8.533016e-01 8.534210e-01 8.535428e-01 8.648466e-01 3.659682e-01 3.490525e-01 3.506214e-01 3.706995e-01 3.722749e-01

1010 8.533478e-01 8.534540e-01 8.535733e-01 8.536950e-01 8.649901e-01 3.669710e-01 3.500416e-01 3.516163e-01 3.717163e-01 3.732961e-01

1011 8.534985e-01 8.536046e-01 8.537239e-01 8.538456e-01 8.651320e-01 3.679641e-01 3.510248e-01 3.526049e-01 3.727222e-01 3.743060e-01

1012 8.536475e-01 8.537536e-01 8.538729e-01 8.539945e-01 8.652725e-01 3.689462e-01 3.520005e-01 3.535857e-01 3.737160e-01 3.753034e-01

1013 8.537949e-01 8.539010e-01 8.540202e-01 8.541419e-01 8.654115e-01 3.699161e-01 3.529676e-01 3.545575e-01 3.746965e-01 3.762871e-01

1014 8.539408e-01 8.540469e-01 8.541661e-01 8.542877e-01 8.655492e-01 3.708727e-01 3.539248e-01 3.555190e-01 3.756625e-01 3.772561e-01

1015 8.540852e-01 8.541913e-01 8.543104e-01 8.544320e-01 8.656854e-01 3.718149e-01 3.548708e-01 3.564690e-01 3.766132e-01 3.782094e-01

1016 8.542282e-01 8.543342e-01 8.544533e-01 8.545748e-01 8.658204e-01 3.727418e-01 3.558046e-01 3.574064e-01 3.775475e-01 3.791460e-01

1017 8.543697e-01 8.544757e-01 8.545948e-01 8.547162e-01 8.659541e-01 3.736524e-01 3.567252e-01 3.583303e-01 3.784647e-01 3.800651e-01

1018 8.545099e-01 8.546159e-01 8.547349e-01 8.548563e-01 8.660866e-01 3.745461e-01 3.576316e-01 3.592396e-01 3.793640e-01 3.809661e-01

1019 8.546487e-01 8.547547e-01 8.548737e-01 8.549951e-01 8.662178e-01 3.754221e-01 3.585229e-01 3.601335e-01 3.802448e-01 3.818483e-01

1020 8.547863e-01 8.548922e-01 8.550112e-01 8.551325e-01 8.663479e-01 3.762798e-01 3.593983e-01 3.610112e-01 3.811065e-01 3.827111e-01

1021 8.549225e-01 8.550285e-01 8.551474e-01 8.552687e-01 8.664769e-01 3.771187e-01 3.602572e-01 3.618721e-01 3.819487e-01 3.835541e-01

1022 8.550576e-01 8.551635e-01 8.552824e-01 8.554037e-01 8.666047e-01 3.779384e-01 3.610988e-01 3.627155e-01 3.827709e-01 3.843770e-01

1023 8.551915e-01 8.552974e-01 8.554162e-01 8.555375e-01 8.667314e-01 3.787385e-01 3.619227e-01 3.635409e-01 3.835729e-01 3.851794e-01

1024 8.553242e-01 8.554301e-01 8.555489e-01 8.556701e-01 8.668571e-01 3.795187e-01 3.627285e-01 3.643478e-01 3.843545e-01 3.859613e-01

1025 8.554557e-01 8.555616e-01 8.556804e-01 8.558016e-01 8.669818e-01 3.802790e-01 3.635156e-01 3.651360e-01 3.851155e-01 3.867224e-01

1026 8.555862e-01 8.556921e-01 8.558108e-01 8.559319e-01 8.671054e-01 3.810191e-01 3.642839e-01 3.659052e-01 3.858558e-01 3.874627e-01

1027 8.557155e-01 8.558214e-01 8.559401e-01 8.560612e-01 8.672280e-01 3.817390e-01 3.650332e-01 3.666550e-01 3.865756e-01 3.881823e-01

1028 8.558438e-01 8.559497e-01 8.560684e-01 8.561894e-01 8.673497e-01 3.824388e-01 3.657632e-01 3.673855e-01 3.872748e-01 3.888812e-01

1029 8.559711e-01 8.560769e-01 8.561956e-01 8.563166e-01 8.674704e-01 3.831185e-01 3.664739e-01 3.680965e-01 3.879536e-01 3.895595e-01

1030 8.560973e-01 8.562032e-01 8.563218e-01 8.564428e-01 8.675902e-01 3.837783e-01 3.671654e-01 3.687881e-01 3.886122e-01 3.902176e-01

1031 8.562226e-01 8.563284e-01 8.564470e-01 8.565679e-01 8.677091e-01 3.844184e-01 3.678376e-01 3.694603e-01 3.892508e-01 3.908556e-01

1032 8.563468e-01 8.564526e-01 8.565712e-01 8.566921e-01 8.678271e-01 3.850391e-01 3.684906e-01 3.701132e-01 3.898697e-01 3.914738e-01

1033 8.564701e-01 8.565759e-01 8.566944e-01 8.568153e-01 8.679442e-01 3.856405e-01 3.691246e-01 3.707471e-01 3.904693e-01 3.920726e-01

1034 8.565925e-01 8.566983e-01 8.568167e-01 8.569376e-01 8.680604e-01 3.862231e-01 3.697399e-01 3.713621e-01 3.910498e-01 3.926523e-01

1035 8.567139e-01 8.568197e-01 8.569381e-01 8.570590e-01 8.681758e-01 3.867872e-01 3.703367e-01 3.719584e-01 3.916117e-01 3.932133e-01

1036 8.568345e-01 8.569402e-01 8.570586e-01 8.571794e-01 8.682904e-01 3.873333e-01 3.709152e-01 3.725365e-01 3.921553e-01 3.937561e-01

1037 8.569541e-01 8.570599e-01 8.571782e-01 8.572990e-01 8.684041e-01 3.878616e-01 3.714758e-01 3.730966e-01 3.926812e-01 3.942812e-01

1038 8.570729e-01 8.571786e-01 8.572969e-01 8.574177e-01 8.685170e-01 3.883727e-01 3.720188e-01 3.736391e-01 3.931898e-01 3.947888e-01

1039 8.571908e-01 8.572965e-01 8.574148e-01 8.575355e-01 8.686292e-01 3.888669e-01 3.725447e-01 3.741644e-01 3.936815e-01 3.952797e-01

1040 8.573078e-01 8.574135e-01 8.575318e-01 8.576525e-01 8.687405e-01 3.893449e-01 3.730538e-01 3.746728e-01 3.941569e-01 3.957541e-01

1041 8.574240e-01 8.575298e-01 8.576480e-01 8.577686e-01 8.688511e-01 3.898070e-01 3.735465e-01 3.751649e-01 3.946164e-01 3.962127e-01

1042 8.575394e-01 8.576451e-01 8.577633e-01 8.578839e-01 8.689610e-01 3.902537e-01 3.740233e-01 3.756411e-01 3.950606e-01 3.966560e-01

1043 8.576540e-01 8.577597e-01 8.578779e-01 8.579984e-01 8.690701e-01 3.906855e-01 3.744847e-01 3.761017e-01 3.954899e-01 3.970844e-01

1044 8.577678e-01 8.578735e-01 8.579916e-01 8.581122e-01 8.691784e-01 3.911030e-01 3.749310e-01 3.765473e-01 3.959048e-01 3.974984e-01

1045 8.578808e-01 8.579865e-01 8.581046e-01 8.582251e-01 8.692861e-01 3.915065e-01 3.753627e-01 3.769784e-01 3.963059e-01 3.978986e-01

1046 8.579930e-01 8.580987e-01 8.582168e-01 8.583372e-01 8.693930e-01 3.918966e-01 3.757804e-01 3.773954e-01 3.966936e-01 3.982855e-01

1047 8.581045e-01 8.582102e-01 8.583282e-01 8.584486e-01 8.694992e-01 3.922738e-01 3.761844e-01 3.777987e-01 3.970685e-01 3.986595e-01

1048 8.582152e-01 8.583209e-01 8.584389e-01 8.585593e-01 8.696048e-01 3.926386e-01 3.765753e-01 3.781889e-01 3.974309e-01 3.990212e-01

1049 8.583252e-01 8.584309e-01 8.585488e-01 8.586691e-01 8.697096e-01 3.929914e-01 3.769535e-01 3.785665e-01 3.977815e-01 3.993710e-01

1050 8.584344e-01 8.585401e-01 8.586580e-01 8.587783e-01 8.698138e-01 3.933327e-01 3.773194e-01 3.789318e-01 3.981206e-01 3.997093e-01

1051 8.585429e-01 8.586486e-01 8.587665e-01 8.588867e-01 8.699173e-01 3.936629e-01 3.776736e-01 3.792853e-01 3.984487e-01 4.000367e-01

1052 8.586507e-01 8.587564e-01 8.588742e-01 8.589944e-01 8.700201e-01 3.939825e-01 3.780164e-01 3.796275e-01 3.987663e-01 4.003536e-01

1053 8.587578e-01 8.588635e-01 8.589813e-01 8.591015e-01 8.701223e-01 3.942920e-01 3.783484e-01 3.799589e-01 3.990738e-01 4.006604e-01

1054 8.588642e-01 8.589699e-01 8.590876e-01 8.592078e-01 8.702238e-01 3.945917e-01 3.786698e-01 3.802798e-01 3.993716e-01 4.009576e-01

1055 8.589699e-01 8.590756e-01 8.591933e-01 8.593134e-01 8.703247e-01 3.948820e-01 3.789813e-01 3.805907e-01 3.996602e-01 4.012455e-01

1056 8.590749e-01 8.591806e-01 8.592983e-01 8.594183e-01 8.704250e-01 3.951635e-01 3.792831e-01 3.808919e-01 3.999398e-01 4.015246e-01

1057 8.591793e-01 8.592849e-01 8.594026e-01 8.595226e-01 8.705246e-01 3.954363e-01 3.795756e-01 3.811840e-01 4.002110e-01 4.017952e-01

1058 8.592829e-01 8.593886e-01 8.595062e-01 8.596262e-01 8.706236e-01 3.957010e-01 3.798594e-01 3.814672e-01 4.004741e-01 4.020577e-01

1059 8.593859e-01 8.594916e-01 8.596092e-01 8.597291e-01 8.707220e-01 3.959578e-01 3.801346e-01 3.817420e-01 4.007294e-01 4.023125e-01

1060 8.594883e-01 8.595940e-01 8.597115e-01 8.598314e-01 8.708198e-01 3.962072e-01 3.804017e-01 3.820086e-01 4.009774e-01 4.025600e-01

1061 8.595900e-01 8.596957e-01 8.598131e-01 8.599330e-01 8.709170e-01 3.964494e-01 3.806611e-01 3.822676e-01 4.012182e-01 4.028003e-01

1062 8.596911e-01 8.597967e-01 8.599142e-01 8.600340e-01 8.710137e-01 3.966848e-01 3.809130e-01 3.825191e-01 4.014523e-01 4.030340e-01

1063 8.597915e-01 8.598972e-01 8.600146e-01 8.601343e-01 8.711097e-01 3.969137e-01 3.811579e-01 3.827636e-01 4.016799e-01 4.032612e-01

1064 8.598913e-01 8.599970e-01 8.601143e-01 8.602341e-01 8.712051e-01 3.971364e-01 3.813960e-01 3.830013e-01 4.019014e-01 4.034823e-01

1065 8.599905e-01 8.600962e-01 8.602135e-01 8.603332e-01 8.713000e-01 3.973531e-01 3.816276e-01 3.832325e-01 4.021170e-01 4.036975e-01

1066 8.600891e-01 8.601948e-01 8.603120e-01 8.604317e-01 8.713943e-01 3.975642e-01 3.818530e-01 3.834576e-01 4.023271e-01 4.039071e-01

1067 8.601871e-01 8.602927e-01 8.604099e-01 8.605295e-01 8.714880e-01 3.977699e-01 3.820725e-01 3.836768e-01 4.025317e-01 4.041115e-01

1068 8.602844e-01 8.603901e-01 8.605073e-01 8.606268e-01 8.715812e-01 3.979705e-01 3.822864e-01 3.838904e-01 4.027313e-01 4.043107e-01

1069 8.603812e-01 8.604869e-01 8.606040e-01 8.607235e-01 8.716738e-01 3.981661e-01 3.824949e-01 3.840986e-01 4.029260e-01 4.045051e-01

1070 8.604774e-01 8.605830e-01 8.607001e-01 8.608196e-01 8.717659e-01 3.983571e-01 3.826983e-01 3.843017e-01 4.031161e-01 4.046949e-01

1071 8.605729e-01 8.606786e-01 8.607957e-01 8.609151e-01 8.718574e-01 3.985436e-01 3.828968e-01 3.845000e-01 4.033018e-01 4.048803e-01

1072 8.606679e-01 8.607736e-01 8.608906e-01 8.610100e-01 8.719483e-01 3.987259e-01 3.830906e-01 3.846935e-01 4.034833e-01 4.050615e-01

1073 8.607624e-01 8.608680e-01 8.609850e-01 8.611043e-01 8.720388e-01 3.989041e-01 3.832800e-01 3.848827e-01 4.036608e-01 4.052387e-01

1074 8.608562e-01 8.609619e-01 8.610788e-01 8.611981e-01 8.721287e-01 3.990785e-01 3.834651e-01 3.850676e-01 4.038344e-01 4.054121e-01

1075 8.609495e-01 8.610552e-01 8.611720e-01 8.612913e-01 8.722180e-01 3.992491e-01 3.836462e-01 3.852484e-01 4.040044e-01 4.055819e-01

1076 8.610422e-01 8.611479e-01 8.612647e-01 8.613839e-01 8.723069e-01 3.994163e-01 3.838234e-01 3.854255e-01 4.041710e-01 4.057482e-01

1077 8.611344e-01 8.612401e-01 8.613568e-01 8.614760e-01 8.723952e-01 3.995802e-01 3.839969e-01 3.855988e-01 4.043342e-01 4.059113e-01

1078 8.612260e-01 8.613317e-01 8.614484e-01 8.615675e-01 8.724830e-01 3.997408e-01 3.841670e-01 3.857687e-01 4.044943e-01 4.060712e-01

1079 8.613170e-01 8.614228e-01 8.615394e-01 8.616584e-01 8.725704e-01 3.998985e-01 3.843337e-01 3.859352e-01 4.046514e-01 4.062281e-01

1080 8.614076e-01 8.615133e-01 8.616299e-01 8.617489e-01 8.726571e-01 4.000532e-01 3.844972e-01 3.860985e-01 4.048056e-01 4.063821e-01

1081 8.614975e-01 8.616033e-01 8.617198e-01 8.618387e-01 8.727434e-01 4.002052e-01 3.846576e-01 3.862589e-01 4.049571e-01 4.065335e-01

1082 8.634152e-01 8.635207e-01 8.636370e-01 8.637557e-01 8.745813e-01 4.002266e-01 3.846755e-01 3.862772e-01 4.049802e-01 4.065572e-01

1083 8.652894e-01 8.653946e-01 8.655106e-01 8.656288e-01 8.763692e-01 4.002493e-01 3.846946e-01 3.862968e-01 4.050048e-01 4.065824e-01

1084 8.671181e-01 8.672228e-01 8.673383e-01 8.674560e-01 8.781054e-01 4.002736e-01 3.847150e-01 3.863178e-01 4.050311e-01 4.066094e-01

1085 8.688993e-01 8.690034e-01 8.691182e-01 8.692352e-01 8.797889e-01 4.002994e-01 3.847367e-01 3.863400e-01 4.050591e-01 4.066381e-01

1086 8.706313e-01 8.707347e-01 8.708487e-01 8.709650e-01 8.814187e-01 4.003270e-01 3.847598e-01 3.863638e-01 4.050889e-01 4.066687e-01

1087 8.723128e-01 8.724154e-01 8.725285e-01 8.726439e-01 8.829943e-01 4.003563e-01 3.847845e-01 3.863891e-01 4.051206e-01 4.067013e-01

1088 8.739428e-01 8.740445e-01 8.741567e-01 8.742711e-01 8.845154e-01 4.003876e-01 3.848107e-01 3.864161e-01 4.051544e-01 4.067360e-01

1089 8.755206e-01 8.756212e-01 8.757325e-01 8.758458e-01 8.859819e-01 4.004208e-01 3.848387e-01 3.864448e-01 4.051904e-01 4.067730e-01

1090 8.770457e-01 8.771452e-01 8.772554e-01 8.773677e-01 8.873940e-01 4.004563e-01 3.848684e-01 3.864753e-01 4.052287e-01 4.068123e-01

1091 8.785178e-01 8.786163e-01 8.787253e-01 8.788364e-01 8.887521e-01 4.004939e-01 3.849001e-01 3.865079e-01 4.052695e-01 4.068541e-01

1092 8.799371e-01 8.800344e-01 8.801423e-01 8.802522e-01 8.900568e-01 4.005340e-01 3.849338e-01 3.865425e-01 4.053128e-01 4.068986e-01

1093 8.813038e-01 8.813999e-01 8.815065e-01 8.816152e-01 8.913091e-01 4.005766e-01 3.849697e-01 3.865793e-01 4.053589e-01 4.069459e-01

1094 8.826184e-01 8.827132e-01 8.828187e-01 8.829261e-01 8.925097e-01 4.006219e-01 3.850078e-01 3.866185e-01 4.054079e-01 4.069962e-01

1095 8.838815e-01 8.839751e-01 8.840793e-01 8.841854e-01 8.936599e-01 4.006701e-01 3.850484e-01 3.866601e-01 4.054600e-01 4.070496e-01

1096 8.850940e-01 8.851863e-01 8.852893e-01 8.853941e-01 8.947609e-01 4.007212e-01 3.850914e-01 3.867043e-01 4.055152e-01 4.071064e-01

1097 8.862569e-01 8.863479e-01 8.864496e-01 8.865532e-01 8.958140e-01 4.007755e-01 3.851372e-01 3.867513e-01 4.055740e-01 4.071666e-01

1098 8.873713e-01 8.874610e-01 8.875615e-01 8.876638e-01 8.968206e-01 4.008332e-01 3.851858e-01 3.868012e-01 4.056363e-01 4.072306e-01

1099 8.884384e-01 8.885269e-01 8.886261e-01 8.887272e-01 8.977823e-01 4.008944e-01 3.852374e-01 3.868542e-01 4.057025e-01 4.072985e-01

1100 8.894595e-01 8.895468e-01 8.896448e-01 8.897446e-01 8.987005e-01 4.009593e-01 3.852922e-01 3.869104e-01 4.057726e-01 4.073705e-01

1101 8.904361e-01 8.905221e-01 8.906190e-01 8.907175e-01 8.995769e-01 4.010282e-01 3.853503e-01 3.869700e-01 4.058471e-01 4.074469e-01

1102 8.913695e-01 8.914544e-01 8.915501e-01 8.916475e-01 9.004131e-01 4.011012e-01 3.854120e-01 3.870333e-01 4.059260e-01 4.075278e-01

1103 8.922614e-01 8.923451e-01 8.924396e-01 8.925358e-01 9.012107e-01 4.011786e-01 3.854773e-01 3.871004e-01 4.060096e-01 4.076136e-01

1104 8.931132e-01 8.931957e-01 8.932892e-01 8.933843e-01 9.019713e-01 4.012606e-01 3.855466e-01 3.871715e-01 4.060981e-01 4.077045e-01

1105 8.939264e-01 8.940079e-01 8.941003e-01 8.941943e-01 9.026965e-01 4.013474e-01 3.856200e-01 3.872469e-01 4.061919e-01 4.078007e-01

1106 8.947027e-01 8.947831e-01 8.948745e-01 8.949674e-01 9.033879e-01 4.014394e-01 3.856978e-01 3.873267e-01 4.062912e-01 4.079025e-01

1107 8.954436e-01 8.955230e-01 8.956133e-01 8.957053e-01 9.040470e-01 4.015367e-01 3.857802e-01 3.874112e-01 4.063963e-01 4.080103e-01

1108 8.961507e-01 8.962290e-01 8.963184e-01 8.964094e-01 9.046755e-01 4.016396e-01 3.858673e-01 3.875007e-01 4.065074e-01 4.081243e-01

1109 8.968253e-01 8.969027e-01 8.969912e-01 8.970812e-01 9.052747e-01 4.017485e-01 3.859595e-01 3.875953e-01 4.066249e-01 4.082448e-01

1110 8.974692e-01 8.975456e-01 8.976332e-01 8.977223e-01 9.058461e-01 4.018635e-01 3.860571e-01 3.876954e-01 4.067490e-01 4.083721e-01

1111 8.980836e-01 8.981592e-01 8.982459e-01 8.983341e-01 9.063911e-01 4.019851e-01 3.861602e-01 3.878012e-01 4.068802e-01 4.085066e-01

1112 8.986700e-01 8.987447e-01 8.988307e-01 8.989181e-01 9.069111e-01 4.021135e-01 3.862692e-01 3.879130e-01 4.070187e-01 4.086487e-01

1113 8.992299e-01 8.993038e-01 8.993889e-01 8.994756e-01 9.074074e-01 4.022491e-01 3.863843e-01 3.880311e-01 4.071649e-01 4.087986e-01

1114 8.997645e-01 8.998376e-01 8.999220e-01 9.000079e-01 9.078812e-01 4.023922e-01 3.865059e-01 3.881559e-01 4.073192e-01 4.089567e-01

1115 9.002751e-01 9.003475e-01 9.004312e-01 9.005164e-01 9.083337e-01 4.025431e-01 3.866343e-01 3.882875e-01 4.074818e-01 4.091235e-01

1116 9.007630e-01 9.008347e-01 9.009178e-01 9.010022e-01 9.087662e-01 4.027022e-01 3.867697e-01 3.884264e-01 4.076533e-01 4.092992e-01

1117 9.012294e-01 9.013004e-01 9.013828e-01 9.014666e-01 9.091796e-01 4.028698e-01 3.869125e-01 3.885728e-01 4.078339e-01 4.094843e-01

1118 9.016754e-01 9.017458e-01 9.018276e-01 9.019108e-01 9.095750e-01 4.030464e-01 3.870630e-01 3.887272e-01 4.080241e-01 4.096793e-01

1119 9.021021e-01 9.021719e-01 9.022531e-01 9.023357e-01 9.099535e-01 4.032323e-01 3.872216e-01 3.888899e-01 4.082242e-01 4.098844e-01

1120 9.025106e-01 9.025798e-01 9.026605e-01 9.027426e-01 9.103160e-01 4.034279e-01 3.873886e-01 3.890611e-01 4.084348e-01 4.101001e-01

1121 9.029018e-01 9.029705e-01 9.030507e-01 9.031322e-01 9.106633e-01 4.036337e-01 3.875644e-01 3.892414e-01 4.086561e-01 4.103269e-01

1122 9.032768e-01 9.033449e-01 9.034246e-01 9.035056e-01 9.109964e-01 4.038499e-01 3.877494e-01 3.894310e-01 4.088887e-01 4.105652e-01

1123 9.036363e-01 9.037040e-01 9.037832e-01 9.038638e-01 9.113160e-01 4.040771e-01 3.879439e-01 3.896304e-01 4.091330e-01 4.108153e-01

1124 9.039813e-01 9.040486e-01 9.041273e-01 9.042074e-01 9.116229e-01 4.043156e-01 3.881484e-01 3.898400e-01 4.093893e-01 4.110779e-01

1125 9.043126e-01 9.043794e-01 9.044578e-01 9.045374e-01 9.119179e-01 4.045659e-01 3.883631e-01 3.900601e-01 4.096582e-01 4.113532e-01

1126 9.046310e-01 9.046973e-01 9.047753e-01 9.048546e-01 9.122015e-01 4.048283e-01 3.885886e-01 3.902911e-01 4.099400e-01 4.116418e-01

1127 9.049371e-01 9.050031e-01 9.050807e-01 9.051595e-01 9.124745e-01 4.051034e-01 3.888251e-01 3.905335e-01 4.102353e-01 4.119440e-01

1128 9.052317e-01 9.052973e-01 9.053745e-01 9.054530e-01 9.127375e-01 4.053915e-01 3.890732e-01 3.907876e-01 4.105444e-01 4.122604e-01

1129 9.055154e-01 9.055806e-01 9.056575e-01 9.057357e-01 9.129910e-01 4.056930e-01 3.893332e-01 3.910539e-01 4.108677e-01 4.125913e-01

1130 9.057888e-01 9.058537e-01 9.059303e-01 9.060082e-01 9.132356e-01 4.060084e-01 3.896055e-01 3.913328e-01 4.112058e-01 4.129372e-01

1131 9.060525e-01 9.061171e-01 9.061934e-01 9.062710e-01 9.134718e-01 4.063381e-01 3.898904e-01 3.916246e-01 4.115590e-01 4.132985e-01

1132 9.063071e-01 9.063714e-01 9.064475e-01 9.065247e-01 9.137000e-01 4.066825e-01 3.901885e-01 3.919298e-01 4.119277e-01 4.136756e-01

1133 9.065531e-01 9.066171e-01 9.066929e-01 9.067699e-01 9.139208e-01 4.070419e-01 3.905002e-01 3.922488e-01 4.123123e-01 4.140688e-01

1134 9.067909e-01 9.068547e-01 9.069302e-01 9.070069e-01 9.141345e-01 4.074168e-01 3.908257e-01 3.925820e-01 4.127132e-01 4.144787e-01

1135 9.070210e-01 9.070846e-01 9.071598e-01 9.072363e-01 9.143415e-01 4.078074e-01 3.911654e-01 3.929297e-01 4.131307e-01 4.149054e-01

1136 9.072439e-01 9.073072e-01 9.073822e-01 9.074585e-01 9.145422e-01 4.082142e-01 3.915198e-01 3.932923e-01 4.135652e-01 4.153495e-01

1137 9.074599e-01 9.075230e-01 9.075978e-01 9.076738e-01 9.147370e-01 4.086375e-01 3.918892e-01 3.936701e-01 4.140170e-01 4.158110e-01

1138 9.076695e-01 9.077323e-01 9.078069e-01 9.078828e-01 9.149262e-01 4.090776e-01 3.922739e-01 3.940636e-01 4.144863e-01 4.162904e-01

1139 9.078729e-01 9.079355e-01 9.080100e-01 9.080856e-01 9.151101e-01 4.095346e-01 3.926742e-01 3.944729e-01 4.149735e-01 4.167879e-01

1140 9.080705e-01 9.081330e-01 9.082072e-01 9.082827e-01 9.152890e-01 4.100090e-01 3.930904e-01 3.948984e-01 4.154786e-01 4.173036e-01

1141 9.082627e-01 9.083250e-01 9.083991e-01 9.084743e-01 9.154631e-01 4.105008e-01 3.935229e-01 3.953404e-01 4.160020e-01 4.178378e-01

1142 9.084497e-01 9.085118e-01 9.085857e-01 9.086608e-01 9.156328e-01 4.110103e-01 3.939718e-01 3.957991e-01 4.165437e-01 4.183905e-01

1143 9.086318e-01 9.086938e-01 9.087675e-01 9.088425e-01 9.157982e-01 4.115375e-01 3.944373e-01 3.962747e-01 4.171039e-01 4.189619e-01

1144 9.088093e-01 9.088712e-01 9.089447e-01 9.090195e-01 9.159596e-01 4.120826e-01 3.949197e-01 3.967674e-01 4.176825e-01 4.195519e-01

1145 9.089825e-01 9.090441e-01 9.091176e-01 9.091922e-01 9.161173e-01 4.126456e-01 3.954191e-01 3.972772e-01 4.182796e-01 4.201606e-01

1146 9.091514e-01 9.092130e-01 9.092863e-01 9.093608e-01 9.162713e-01 4.132265e-01 3.959356e-01 3.978044e-01 4.188951e-01 4.207878e-01

1147 9.093165e-01 9.093779e-01 9.094511e-01 9.095254e-01 9.164219e-01 4.138252e-01 3.964692e-01 3.983490e-01 4.195288e-01 4.214335e-01

1148 9.094778e-01 9.095391e-01 9.096121e-01 9.096864e-01 9.165693e-01 4.144417e-01 3.970199e-01 3.989108e-01 4.201807e-01 4.220974e-01

1149 9.096356e-01 9.096967e-01 9.097697e-01 9.098438e-01 9.167136e-01 4.150756e-01 3.975879e-01 3.994900e-01 4.208505e-01 4.227793e-01

1150 9.097900e-01 9.098510e-01 9.099239e-01 9.099979e-01 9.168550e-01 4.157269e-01 3.981728e-01 4.000865e-01 4.215379e-01 4.234788e-01

1151 9.099412e-01 9.100022e-01 9.100749e-01 9.101488e-01 9.169936e-01 4.163953e-01 3.987748e-01 4.006999e-01 4.222425e-01 4.241957e-01

1152 9.100894e-01 9.101503e-01 9.102229e-01 9.102967e-01 9.171296e-01 4.170803e-01 3.993934e-01 4.013303e-01 4.229639e-01 4.249293e-01

1153 9.102348e-01 9.102955e-01 9.103681e-01 9.104418e-01 9.172631e-01 4.177816e-01 4.000286e-01 4.019772e-01 4.237016e-01 4.256792e-01

1154 9.103774e-01 9.104380e-01 9.105105e-01 9.105841e-01 9.173942e-01 4.184986e-01 4.006800e-01 4.026405e-01 4.244550e-01 4.264449e-01

1155 9.105174e-01 9.105780e-01 9.106503e-01 9.107238e-01 9.175231e-01 4.192309e-01 4.013472e-01 4.033196e-01 4.252236e-01 4.272256e-01

1156 9.106549e-01 9.107154e-01 9.107877e-01 9.108611e-01 9.176497e-01 4.199778e-01 4.020299e-01 4.040142e-01 4.260067e-01 4.280207e-01

1157 9.107900e-01 9.108505e-01 9.109227e-01 9.109960e-01 9.177744e-01 4.207387e-01 4.027275e-01 4.047237e-01 4.268034e-01 4.288294e-01

1158 9.109229e-01 9.109833e-01 9.110554e-01 9.111287e-01 9.178970e-01 4.215129e-01 4.034396e-01 4.054476e-01 4.276130e-01 4.296508e-01

1159 9.110537e-01 9.111140e-01 9.111860e-01 9.112592e-01 9.180178e-01 4.222995e-01 4.041654e-01 4.061854e-01 4.284346e-01 4.304840e-01

1160 9.111824e-01 9.112426e-01 9.113146e-01 9.113877e-01 9.181368e-01 4.230977e-01 4.049045e-01 4.069362e-01 4.292673e-01 4.313281e-01

1161 9.113091e-01 9.113693e-01 9.114412e-01 9.115142e-01 9.182541e-01 4.239067e-01 4.056560e-01 4.076993e-01 4.301102e-01 4.321822e-01

1162 9.114339e-01 9.114941e-01 9.115659e-01 9.116389e-01 9.183697e-01 4.247255e-01 4.064192e-01 4.084740e-01 4.309621e-01 4.330451e-01

1163 9.115570e-01 9.116171e-01 9.116888e-01 9.117617e-01 9.184838e-01 4.255530e-01 4.071933e-01 4.092595e-01 4.318221e-01 4.339157e-01

1164 9.116784e-01 9.117384e-01 9.118100e-01 9.118829e-01 9.185963e-01 4.263883e-01 4.079774e-01 4.100548e-01 4.326890e-01 4.347930e-01

1165 9.117980e-01 9.118580e-01 9.119296e-01 9.120024e-01 9.187074e-01 4.272303e-01 4.087707e-01 4.108590e-01 4.335617e-01 4.356758e-01

1166 9.119162e-01 9.119761e-01 9.120476e-01 9.121204e-01 9.188171e-01 4.280780e-01 4.095720e-01 4.116710e-01 4.344391e-01 4.365629e-01

1167 9.120328e-01 9.120926e-01 9.121641e-01 9.122368e-01 9.189255e-01 4.289301e-01 4.103806e-01 4.124900e-01 4.353200e-01 4.374531e-01

1168 9.121479e-01 9.122077e-01 9.122792e-01 9.123518e-01 9.190327e-01 4.297855e-01 4.111952e-01 4.133149e-01 4.362031e-01 4.383452e-01

1169 9.122617e-01 9.123215e-01 9.123928e-01 9.124654e-01 9.191385e-01 4.306432e-01 4.120150e-01 4.141446e-01 4.370874e-01 4.392381e-01

1170 9.123741e-01 9.124338e-01 9.125052e-01 9.125776e-01 9.192432e-01 4.315019e-01 4.128388e-01 4.149779e-01 4.379715e-01 4.401304e-01

1171 9.124852e-01 9.125449e-01 9.126162e-01 9.126886e-01 9.193468e-01 4.323604e-01 4.136655e-01 4.158139e-01 4.388544e-01 4.410210e-01

1172 9.125951e-01 9.126547e-01 9.127260e-01 9.127983e-01 9.194493e-01 4.332178e-01 4.144940e-01 4.166513e-01 4.397348e-01 4.419088e-01

1173 9.127037e-01 9.127634e-01 9.128345e-01 9.129069e-01 9.195507e-01 4.340726e-01 4.153233e-01 4.174891e-01 4.406115e-01 4.427925e-01

1174 9.128113e-01 9.128708e-01 9.129420e-01 9.130142e-01 9.196511e-01 4.349240e-01 4.161522e-01 4.183262e-01 4.414835e-01 4.436711e-01

1175 9.129177e-01 9.129772e-01 9.130483e-01 9.131205e-01 9.197505e-01 4.357708e-01 4.169797e-01 4.191615e-01 4.423497e-01 4.445434e-01

1176 9.130230e-01 9.130825e-01 9.131535e-01 9.132257e-01 9.198489e-01 4.366118e-01 4.178046e-01 4.199938e-01 4.432090e-01 4.454083e-01

1177 9.131272e-01 9.131867e-01 9.132577e-01 9.133298e-01 9.199464e-01 4.374462e-01 4.186259e-01 4.208222e-01 4.440603e-01 4.462650e-01

1178 9.132305e-01 9.132900e-01 9.133609e-01 9.134330e-01 9.200430e-01 4.382728e-01 4.194426e-01 4.216455e-01 4.449027e-01 4.471123e-01

1179 9.133328e-01 9.133922e-01 9.134631e-01 9.135351e-01 9.201388e-01 4.390909e-01 4.202537e-01 4.224628e-01 4.457353e-01 4.479494e-01

1180 9.134341e-01 9.134935e-01 9.135644e-01 9.136364e-01 9.202337e-01 4.398993e-01 4.210581e-01 4.232731e-01 4.465572e-01 4.487754e-01

1181 9.135346e-01 9.135939e-01 9.136647e-01 9.137367e-01 9.203278e-01 4.406974e-01 4.218551e-01 4.240755e-01 4.473676e-01 4.495895e-01

1182 9.136341e-01 9.136935e-01 9.137642e-01 9.138361e-01 9.204211e-01 4.414844e-01 4.226436e-01 4.248691e-01 4.481657e-01 4.503910e-01

1183 9.137328e-01 9.137921e-01 9.138628e-01 9.139346e-01 9.205136e-01 4.422594e-01 4.234228e-01 4.256530e-01 4.489509e-01 4.511793e-01

1184 9.138306e-01 9.138899e-01 9.139606e-01 9.140324e-01 9.206054e-01 4.430219e-01 4.241920e-01 4.264266e-01 4.497225e-01 4.519536e-01

1185 9.139276e-01 9.139869e-01 9.140575e-01 9.141293e-01 9.206965e-01 4.437713e-01 4.249504e-01 4.271890e-01 4.504800e-01 4.527134e-01

1186 9.140238e-01 9.140831e-01 9.141537e-01 9.142254e-01 9.207868e-01 4.445069e-01 4.256974e-01 4.279396e-01 4.512228e-01 4.534583e-01

1187 9.141193e-01 9.141785e-01 9.142491e-01 9.143207e-01 9.208765e-01 4.452284e-01 4.264323e-01 4.286778e-01 4.519506e-01 4.541879e-01

1188 9.142140e-01 9.142732e-01 9.143437e-01 9.144153e-01 9.209655e-01 4.459352e-01 4.271545e-01 4.294031e-01 4.526629e-01 4.549018e-01

1189 9.143080e-01 9.143672e-01 9.144376e-01 9.145092e-01 9.210538e-01 4.466271e-01 4.278636e-01 4.301149e-01 4.533595e-01 4.555996e-01

1190 9.144012e-01 9.144604e-01 9.145308e-01 9.146023e-01 9.211415e-01 4.473036e-01 4.285591e-01 4.308128e-01 4.540401e-01 4.562812e-01

1191 9.144938e-01 9.145529e-01 9.146233e-01 9.146948e-01 9.212286e-01 4.479647e-01 4.292405e-01 4.314964e-01 4.547044e-01 4.569464e-01

1192 9.145857e-01 9.146448e-01 9.147151e-01 9.147865e-01 9.213150e-01 4.486101e-01 4.299077e-01 4.321654e-01 4.553525e-01 4.575951e-01

1193 9.146769e-01 9.147360e-01 9.148062e-01 9.148776e-01 9.214009e-01 4.492396e-01 4.305602e-01 4.328196e-01 4.559841e-01 4.582272e-01

1194 9.147674e-01 9.148265e-01 9.148967e-01 9.149681e-01 9.214862e-01 4.498532e-01 4.311979e-01 4.334587e-01 4.565993e-01 4.588426e-01

1195 9.148574e-01 9.149164e-01 9.149866e-01 9.150579e-01 9.215709e-01 4.504509e-01 4.318206e-01 4.340826e-01 4.571980e-01 4.594415e-01

1196 9.149467e-01 9.150057e-01 9.150759e-01 9.151471e-01 9.216551e-01 4.510327e-01 4.324282e-01 4.346912e-01 4.577804e-01 4.600238e-01

1197 9.150354e-01 9.150944e-01 9.151645e-01 9.152357e-01 9.217387e-01 4.515985e-01 4.330205e-01 4.352844e-01 4.583464e-01 4.605898e-01

1198 9.151235e-01 9.151825e-01 9.152526e-01 9.153237e-01 9.218217e-01 4.521486e-01 4.335977e-01 4.358622e-01 4.588964e-01 4.611395e-01

1199 9.152110e-01 9.152700e-01 9.153400e-01 9.154111e-01 9.219043e-01 4.526831e-01 4.341597e-01 4.364247e-01 4.594304e-01 4.616731e-01

1200 9.152979e-01 9.153569e-01 9.154269e-01 9.154980e-01 9.219863e-01 4.532021e-01 4.347065e-01 4.369719e-01 4.599486e-01 4.621909e-01

1201 9.153843e-01 9.154433e-01 9.155132e-01 9.155843e-01 9.220679e-01 4.537058e-01 4.352383e-01 4.375040e-01 4.604513e-01 4.626931e-01

1202 9.154702e-01 9.155291e-01 9.155990e-01 9.156700e-01 9.221489e-01 4.541945e-01 4.357551e-01 4.380210e-01 4.609388e-01 4.631800e-01

1203 9.155555e-01 9.156144e-01 9.156843e-01 9.157552e-01 9.222294e-01 4.546683e-01 4.362573e-01 4.385232e-01 4.614113e-01 4.636519e-01

1204 9.156402e-01 9.156992e-01 9.157690e-01 9.158399e-01 9.223095e-01 4.551277e-01 4.367448e-01 4.390107e-01 4.618691e-01 4.641091e-01

1205 9.157245e-01 9.157834e-01 9.158532e-01 9.159240e-01 9.223891e-01 4.555729e-01 4.372181e-01 4.394838e-01 4.623126e-01 4.645518e-01

1206 9.158082e-01 9.158671e-01 9.159368e-01 9.160077e-01 9.224682e-01 4.560042e-01 4.376773e-01 4.399428e-01 4.627421e-01 4.649806e-01

1207 9.158915e-01 9.159504e-01 9.160200e-01 9.160908e-01 9.225469e-01 4.564219e-01 4.381226e-01 4.403879e-01 4.631579e-01 4.653957e-01

1208 9.159742e-01 9.160331e-01 9.161027e-01 9.161734e-01 9.226251e-01 4.568264e-01 4.385544e-01 4.408194e-01 4.635605e-01 4.657974e-01

1209 9.160565e-01 9.161153e-01 9.161849e-01 9.162556e-01 9.227029e-01 4.572179e-01 4.389729e-01 4.412376e-01 4.639501e-01 4.661863e-01

1210 9.161382e-01 9.161971e-01 9.162666e-01 9.163372e-01 9.227803e-01 4.575970e-01 4.393786e-01 4.416429e-01 4.643271e-01 4.665625e-01

1211 9.162195e-01 9.162784e-01 9.163478e-01 9.164184e-01 9.228572e-01 4.579639e-01 4.397716e-01 4.420355e-01 4.646919e-01 4.669266e-01

1212 9.163004e-01 9.163592e-01 9.164286e-01 9.164992e-01 9.229337e-01 4.583189e-01 4.401523e-01 4.424157e-01 4.650450e-01 4.672788e-01

1213 9.163808e-01 9.164396e-01 9.165089e-01 9.165795e-01 9.230098e-01 4.586626e-01 4.405211e-01 4.427841e-01 4.653866e-01 4.676197e-01

1214 9.164607e-01 9.165195e-01 9.165888e-01 9.166593e-01 9.230855e-01 4.589951e-01 4.408782e-01 4.431408e-01 4.657171e-01 4.679495e-01

1215 9.165402e-01 9.165989e-01 9.166682e-01 9.167387e-01 9.231607e-01 4.593170e-01 4.412241e-01 4.434862e-01 4.660370e-01 4.682686e-01

1216 9.166192e-01 9.166780e-01 9.167472e-01 9.168176e-01 9.232356e-01 4.596285e-01 4.415592e-01 4.438208e-01 4.663466e-01 4.685774e-01

1217 9.166978e-01 9.167566e-01 9.168258e-01 9.168961e-01 9.233101e-01 4.599300e-01 4.418836e-01 4.441448e-01 4.666462e-01 4.688763e-01

1218 9.167760e-01 9.168348e-01 9.169039e-01 9.169742e-01 9.233842e-01 4.602219e-01 4.421978e-01 4.444585e-01 4.669362e-01 4.691657e-01

1219 9.168538e-01 9.169125e-01 9.169816e-01 9.170518e-01 9.234579e-01 4.605045e-01 4.425022e-01 4.447624e-01 4.672170e-01 4.694458e-01

1220 9.169311e-01 9.169899e-01 9.170589e-01 9.171291e-01 9.235312e-01 4.607782e-01 4.427970e-01 4.450568e-01 4.674889e-01 4.697171e-01

1221 9.170081e-01 9.170668e-01 9.171358e-01 9.172059e-01 9.236042e-01 4.610433e-01 4.430827e-01 4.453420e-01 4.677523e-01 4.699798e-01

1222 9.170846e-01 9.171433e-01 9.172123e-01 9.172823e-01 9.236768e-01 4.613001e-01 4.433595e-01 4.456184e-01 4.680075e-01 4.702344e-01

1223 9.171608e-01 9.172194e-01 9.172884e-01 9.173584e-01 9.237490e-01 4.615490e-01 4.436277e-01 4.458862e-01 4.682548e-01 4.704811e-01

1224 9.172365e-01 9.172952e-01 9.173640e-01 9.174340e-01 9.238209e-01 4.617903e-01 4.438878e-01 4.461458e-01 4.684945e-01 4.707203e-01

1225 9.173119e-01 9.173705e-01 9.174393e-01 9.175093e-01 9.238924e-01 4.620242e-01 4.441399e-01 4.463976e-01 4.687270e-01 4.709522e-01

1226 9.173868e-01 9.174455e-01 9.175142e-01 9.175841e-01 9.239636e-01 4.622511e-01 4.443845e-01 4.466417e-01 4.689525e-01 4.711772e-01

1227 9.174614e-01 9.175201e-01 9.175888e-01 9.176586e-01 9.240344e-01 4.624713e-01 4.446218e-01 4.468786e-01 4.691713e-01 4.713956e-01

1228 9.175356e-01 9.175943e-01 9.176629e-01 9.177327e-01 9.241049e-01 4.626850e-01 4.448520e-01 4.471086e-01 4.693838e-01 4.716075e-01

1229 9.176095e-01 9.176681e-01 9.177367e-01 9.178064e-01 9.241750e-01 4.628925e-01 4.450756e-01 4.473318e-01 4.695901e-01 4.718134e-01

1230 9.176830e-01 9.177416e-01 9.178101e-01 9.178798e-01 9.242448e-01 4.630941e-01 4.452927e-01 4.475485e-01 4.697906e-01 4.720135e-01

1231 9.177561e-01 9.178147e-01 9.178832e-01 9.179528e-01 9.243143e-01 4.632901e-01 4.455036e-01 4.477592e-01 4.699854e-01 4.722079e-01

1232 9.178288e-01 9.178874e-01 9.179558e-01 9.180254e-01 9.243834e-01 4.634806e-01 4.457086e-01 4.479639e-01 4.701749e-01 4.723970e-01

1233 9.179012e-01 9.179598e-01 9.180282e-01 9.180977e-01 9.244522e-01 4.636660e-01 4.459080e-01 4.481630e-01 4.703592e-01 4.725810e-01

1234 9.179733e-01 9.180318e-01 9.181002e-01 9.181696e-01 9.245207e-01 4.638463e-01 4.461019e-01 4.483566e-01 4.705387e-01 4.727601e-01

1235 9.180450e-01 9.181035e-01 9.181718e-01 9.182412e-01 9.245889e-01 4.640220e-01 4.462906e-01 4.485451e-01 4.707134e-01 4.729345e-01

1236 9.181163e-01 9.181748e-01 9.182431e-01 9.183124e-01 9.246567e-01 4.641931e-01 4.464744e-01 4.487286e-01 4.708837e-01 4.731045e-01

1237 9.181874e-01 9.182458e-01 9.183140e-01 9.183833e-01 9.247243e-01 4.643599e-01 4.466534e-01 4.489074e-01 4.710497e-01 4.732702e-01

1238 9.182580e-01 9.183165e-01 9.183846e-01 9.184538e-01 9.247915e-01 4.645225e-01 4.468279e-01 4.490817e-01 4.712116e-01 4.734318e-01

1239 9.183284e-01 9.183868e-01 9.184549e-01 9.185241e-01 9.248584e-01 4.646812e-01 4.469980e-01 4.492516e-01 4.713696e-01 4.735895e-01

1240 9.183984e-01 9.184568e-01 9.185248e-01 9.185939e-01 9.249251e-01 4.648361e-01 4.471640e-01 4.494174e-01 4.715239e-01 4.737435e-01

1241 9.184681e-01 9.185265e-01 9.185944e-01 9.186635e-01 9.249914e-01 4.649875e-01 4.473260e-01 4.495792e-01 4.716746e-01 4.738940e-01

1242 9.185374e-01 9.185959e-01 9.186637e-01 9.187327e-01 9.250574e-01 4.651354e-01 4.474842e-01 4.497372e-01 4.718219e-01 4.740411e-01

1243 9.186065e-01 9.186649e-01 9.187327e-01 9.188016e-01 9.251232e-01 4.652800e-01 4.476388e-01 4.498917e-01 4.719660e-01 4.741850e-01

1244 9.186752e-01 9.187336e-01 9.188014e-01 9.188702e-01 9.251886e-01 4.654215e-01 4.477900e-01 4.500427e-01 4.721069e-01 4.743258e-01

1245 9.187436e-01 9.188020e-01 9.188697e-01 9.189385e-01 9.252538e-01 4.655600e-01 4.479378e-01 4.501904e-01 4.722450e-01 4.744636e-01

1246 9.188117e-01 9.188701e-01 9.189377e-01 9.190065e-01 9.253186e-01 4.656957e-01 4.480825e-01 4.503349e-01 4.723802e-01 4.745986e-01

1247 9.188796e-01 9.189379e-01 9.190054e-01 9.190741e-01 9.253832e-01 4.658286e-01 4.482242e-01 4.504765e-01 4.725127e-01 4.747310e-01

1248 9.189470e-01 9.190053e-01 9.190729e-01 9.191415e-01 9.254475e-01 4.659590e-01 4.483631e-01 4.506152e-01 4.726427e-01 4.748608e-01

1249 9.190142e-01 9.190725e-01 9.191400e-01 9.192085e-01 9.255116e-01 4.660869e-01 4.484992e-01 4.507512e-01 4.727702e-01 4.749882e-01

1250 9.190811e-01 9.191394e-01 9.192068e-01 9.192753e-01 9.255753e-01 4.662125e-01 4.486327e-01 4.508846e-01 4.728954e-01 4.751133e-01

1251 9.191477e-01 9.192060e-01 9.192733e-01 9.193417e-01 9.256388e-01 4.663358e-01 4.487637e-01 4.510155e-01 4.730184e-01 4.752361e-01

1252 9.192141e-01 9.192723e-01 9.193395e-01 9.194079e-01 9.257020e-01 4.664570e-01 4.488923e-01 4.511440e-01 4.731393e-01 4.753569e-01

1253 9.192801e-01 9.193383e-01 9.194055e-01 9.194738e-01 9.257650e-01 4.665761e-01 4.490187e-01 4.512703e-01 4.732581e-01 4.754756e-01

1254 9.193458e-01 9.194040e-01 9.194711e-01 9.195393e-01 9.258277e-01 4.666933e-01 4.491429e-01 4.513944e-01 4.733751e-01 4.755924e-01

1255 9.194113e-01 9.194694e-01 9.195365e-01 9.196046e-01 9.258901e-01 4.668087e-01 4.492651e-01 4.515165e-01 4.734902e-01 4.757074e-01

1256 9.194765e-01 9.195346e-01 9.196016e-01 9.196697e-01 9.259523e-01 4.669223e-01 4.493853e-01 4.516366e-01 4.736035e-01 4.758206e-01

1257 9.195414e-01 9.195995e-01 9.196664e-01 9.197344e-01 9.260142e-01 4.670342e-01 4.495037e-01 4.517548e-01 4.737152e-01 4.759322e-01

1258 9.196060e-01 9.196641e-01 9.197309e-01 9.197988e-01 9.260758e-01 4.671446e-01 4.496203e-01 4.518713e-01 4.738253e-01 4.760422e-01

1259 9.196703e-01 9.197284e-01 9.197952e-01 9.198630e-01 9.261372e-01 4.672534e-01 4.497352e-01 4.519861e-01 4.739339e-01 4.761508e-01

1260 9.197344e-01 9.197925e-01 9.198591e-01 9.199269e-01 9.261984e-01 4.673608e-01 4.498485e-01 4.520993e-01 4.740411e-01 4.762578e-01

1261 9.197983e-01 9.198563e-01 9.199229e-01 9.199906e-01 9.262593e-01 4.674668e-01 4.499602e-01 4.522109e-01 4.741468e-01 4.763635e-01

1262 9.205600e-01 9.206165e-01 9.206815e-01 9.207477e-01 9.269298e-01 4.675168e-01 4.500021e-01 4.522542e-01 4.742022e-01 4.764208e-01

1263 9.212879e-01 9.213428e-01 9.214063e-01 9.214709e-01 9.275695e-01 4.675698e-01 4.500463e-01 4.523000e-01 4.742608e-01 4.764813e-01

1264 9.219831e-01 9.220365e-01 9.220986e-01 9.221618e-01 9.281796e-01 4.676258e-01 4.500931e-01 4.523484e-01 4.743226e-01 4.765453e-01

1265 9.226470e-01 9.226990e-01 9.227597e-01 9.228214e-01 9.287616e-01 4.676849e-01 4.501426e-01 4.523995e-01 4.743880e-01 4.766128e-01

1266 9.232810e-01 9.233315e-01 9.233909e-01 9.234512e-01 9.293167e-01 4.677473e-01 4.501948e-01 4.524536e-01 4.744570e-01 4.766842e-01

1267 9.238861e-01 9.239353e-01 9.239934e-01 9.240524e-01 9.298461e-01 4.678133e-01 4.502501e-01 4.525107e-01 4.745299e-01 4.767595e-01

1268 9.244639e-01 9.245117e-01 9.245686e-01 9.246263e-01 9.303510e-01 4.678829e-01 4.503084e-01 4.525710e-01 4.746068e-01 4.768390e-01

1269 9.250153e-01 9.250620e-01 9.251176e-01 9.251741e-01 9.308327e-01 4.679564e-01 4.503699e-01 4.526347e-01 4.746879e-01 4.769228e-01

1270 9.255418e-01 9.255872e-01 9.256417e-01 9.256971e-01 9.312922e-01 4.680339e-01 4.504349e-01 4.527019e-01 4.747735e-01 4.770113e-01

1271 9.260444e-01 9.260887e-01 9.261421e-01 9.261963e-01 9.317308e-01 4.681156e-01 4.505035e-01 4.527727e-01 4.748637e-01 4.771046e-01

1272 9.265243e-01 9.265675e-01 9.266198e-01 9.266730e-01 9.321493e-01 4.682018e-01 4.505758e-01 4.528475e-01 4.749589e-01 4.772028e-01

1273 9.269826e-01 9.270247e-01 9.270761e-01 9.271282e-01 9.325489e-01 4.682926e-01 4.506520e-01 4.529263e-01 4.750591e-01 4.773064e-01

1274 9.274203e-01 9.274614e-01 9.275118e-01 9.275629e-01 9.329305e-01 4.683883e-01 4.507324e-01 4.530094e-01 4.751646e-01 4.774155e-01

1275 9.278385e-01 9.278787e-01 9.279282e-01 9.279783e-01 9.332951e-01 4.684890e-01 4.508171e-01 4.530970e-01 4.752758e-01 4.775303e-01

1276 9.282382e-01 9.282774e-01 9.283260e-01 9.283753e-01 9.336435e-01 4.685951e-01 4.509063e-01 4.531892e-01 4.753928e-01 4.776511e-01

1277 9.286202e-01 9.286586e-01 9.287064e-01 9.287549e-01 9.339766e-01 4.687067e-01 4.510002e-01 4.532863e-01 4.755158e-01 4.777782e-01

1278 9.289856e-01 9.290232e-01 9.290702e-01 9.291178e-01 9.342953e-01 4.688241e-01 4.510991e-01 4.533885e-01 4.756452e-01 4.779119e-01

1279 9.293351e-01 9.293719e-01 9.294182e-01 9.294651e-01 9.346002e-01 4.689475e-01 4.512031e-01 4.534960e-01 4.757813e-01 4.780524e-01

1280 9.296697e-01 9.297058e-01 9.297513e-01 9.297974e-01 9.348922e-01 4.690773e-01 4.513125e-01 4.536091e-01 4.759243e-01 4.782000e-01

1281 9.299901e-01 9.300254e-01 9.300703e-01 9.301157e-01 9.351720e-01 4.692136e-01 4.514276e-01 4.537280e-01 4.760744e-01 4.783551e-01

1282 9.302970e-01 9.303317e-01 9.303759e-01 9.304207e-01 9.354401e-01 4.693568e-01 4.515485e-01 4.538530e-01 4.762321e-01 4.785179e-01

1283 9.305912e-01 9.306252e-01 9.306688e-01 9.307130e-01 9.356973e-01 4.695071e-01 4.516756e-01 4.539842e-01 4.763976e-01 4.786887e-01

1284 9.308733e-01 9.309067e-01 9.309498e-01 9.309934e-01 9.359441e-01 4.696649e-01 4.518090e-01 4.541221e-01 4.765711e-01 4.788678e-01

1285 9.311441e-01 9.311769e-01 9.312194e-01 9.312624e-01 9.361811e-01 4.698303e-01 4.519490e-01 4.542668e-01 4.767531e-01 4.790556e-01

1286 9.314040e-01 9.314363e-01 9.314783e-01 9.315208e-01 9.364089e-01 4.700038e-01 4.520960e-01 4.544185e-01 4.769438e-01 4.792524e-01

1287 9.316538e-01 9.316856e-01 9.317271e-01 9.317690e-01 9.366280e-01 4.701855e-01 4.522501e-01 4.545777e-01 4.771436e-01 4.794585e-01

1288 9.318939e-01 9.319252e-01 9.319662e-01 9.320077e-01 9.368387e-01 4.703759e-01 4.524117e-01 4.547446e-01 4.773527e-01 4.796742e-01

1289 9.321249e-01 9.321558e-01 9.321963e-01 9.322374e-01 9.370417e-01 4.705752e-01 4.525811e-01 4.549195e-01 4.775716e-01 4.798999e-01

1290 9.323473e-01 9.323777e-01 9.324178e-01 9.324585e-01 9.372373e-01 4.707837e-01 4.527584e-01 4.551026e-01 4.778004e-01 4.801359e-01

1291 9.325616e-01 9.325915e-01 9.326312e-01 9.326715e-01 9.374259e-01 4.710018e-01 4.529441e-01 4.552942e-01 4.780397e-01 4.803826e-01

1292 9.327681e-01 9.327976e-01 9.328370e-01 9.328768e-01 9.376079e-01 4.712297e-01 4.531384e-01 4.554948e-01 4.782896e-01 4.806402e-01

1293 9.329673e-01 9.329965e-01 9.330355e-01 9.330749e-01 9.377837e-01 4.714678e-01 4.533416e-01 4.557045e-01 4.785505e-01 4.809092e-01

1294 9.331596e-01 9.331885e-01 9.332271e-01 9.332662e-01 9.379536e-01 4.717164e-01 4.535540e-01 4.559236e-01 4.788228e-01 4.811898e-01

1295 9.333454e-01 9.333739e-01 9.334123e-01 9.334511e-01 9.381180e-01 4.719758e-01 4.537759e-01 4.561526e-01 4.791068e-01 4.814824e-01

1296 9.335251e-01 9.335533e-01 9.335913e-01 9.336298e-01 9.382771e-01 4.722463e-01 4.540076e-01 4.563916e-01 4.794028e-01 4.817872e-01

1297 9.336989e-01 9.337268e-01 9.337645e-01 9.338027e-01 9.384312e-01 4.725282e-01 4.542494e-01 4.566409e-01 4.797110e-01 4.821046e-01

1298 9.338672e-01 9.338948e-01 9.339323e-01 9.339701e-01 9.385806e-01 4.728218e-01 4.545016e-01 4.569010e-01 4.800318e-01 4.824349e-01

1299 9.340303e-01 9.340576e-01 9.340948e-01 9.341324e-01 9.387256e-01 4.731275e-01 4.547645e-01 4.571720e-01 4.803655e-01 4.827784e-01

1300 9.341884e-01 9.342155e-01 9.342524e-01 9.342898e-01 9.388663e-01 4.734453e-01 4.550383e-01 4.574542e-01 4.807124e-01 4.831354e-01

1301 9.343419e-01 9.343687e-01 9.344054e-01 9.344425e-01 9.390031e-01 4.737758e-01 4.553234e-01 4.577480e-01 4.810727e-01 4.835061e-01

1302 9.344909e-01 9.345175e-01 9.345540e-01 9.345909e-01 9.391361e-01 4.741190e-01 4.556200e-01 4.580536e-01 4.814467e-01 4.838907e-01

1303 9.346357e-01 9.346621e-01 9.346984e-01 9.347351e-01 9.392655e-01 4.744753e-01 4.559284e-01 4.583713e-01 4.818346e-01 4.842896e-01

1304 9.347766e-01 9.348028e-01 9.348389e-01 9.348753e-01 9.393915e-01 4.748448e-01 4.562488e-01 4.587013e-01 4.822365e-01 4.847028e-01

1305 9.349137e-01 9.349397e-01 9.349756e-01 9.350119e-01 9.395143e-01 4.752278e-01 4.565815e-01 4.590438e-01 4.826528e-01 4.851306e-01

1306 9.350473e-01 9.350730e-01 9.351088e-01 9.351448e-01 9.396341e-01 4.756244e-01 4.569267e-01 4.593991e-01 4.830835e-01 4.855731e-01

1307 9.351774e-01 9.352030e-01 9.352386e-01 9.352745e-01 9.397510e-01 4.760349e-01 4.572846e-01 4.597674e-01 4.835288e-01 4.860304e-01

1308 9.353044e-01 9.353298e-01 9.353652e-01 9.354009e-01 9.398652e-01 4.764593e-01 4.576554e-01 4.601489e-01 4.839888e-01 4.865027e-01

1309 9.354283e-01 9.354536e-01 9.354888e-01 9.355244e-01 9.399767e-01 4.768977e-01 4.580393e-01 4.605437e-01 4.844636e-01 4.869900e-01

1310 9.355493e-01 9.355745e-01 9.356095e-01 9.356449e-01 9.400859e-01 4.773503e-01 4.584364e-01 4.609520e-01 4.849532e-01 4.874923e-01

1311 9.356676e-01 9.356926e-01 9.357275e-01 9.357628e-01 9.401926e-01 4.778170e-01 4.588468e-01 4.613738e-01 4.854576e-01 4.880096e-01

1312 9.357833e-01 9.358082e-01 9.358429e-01 9.358781e-01 9.402972e-01 4.782980e-01 4.592706e-01 4.618093e-01 4.859768e-01 4.885419e-01

1313 9.358965e-01 9.359213e-01 9.359559e-01 9.359909e-01 9.403996e-01 4.787931e-01 4.597080e-01 4.622586e-01 4.865106e-01 4.890890e-01

1314 9.360074e-01 9.360320e-01 9.360665e-01 9.361014e-01 9.405000e-01 4.793023e-01 4.601589e-01 4.627216e-01 4.870591e-01 4.896509e-01

1315 9.361161e-01 9.361405e-01 9.361749e-01 9.362097e-01 9.405986e-01 4.798255e-01 4.606233e-01 4.631984e-01 4.876220e-01 4.902273e-01

1316 9.362226e-01 9.362469e-01 9.362812e-01 9.363158e-01 9.406953e-01 4.803626e-01 4.611013e-01 4.636888e-01 4.881992e-01 4.908181e-01

1317 9.363271e-01 9.363513e-01 9.363855e-01 9.364200e-01 9.407902e-01 4.809134e-01 4.615927e-01 4.641929e-01 4.887903e-01 4.914230e-01

1318 9.364296e-01 9.364538e-01 9.364878e-01 9.365222e-01 9.408836e-01 4.814777e-01 4.620975e-01 4.647106e-01 4.893952e-01 4.920416e-01

1319 9.365304e-01 9.365544e-01 9.365884e-01 9.366227e-01 9.409753e-01 4.820551e-01 4.626156e-01 4.652416e-01 4.900134e-01 4.926736e-01

1320 9.366294e-01 9.366533e-01 9.366872e-01 9.367214e-01 9.410656e-01 4.826455e-01 4.631467e-01 4.657858e-01 4.906446e-01 4.933186e-01

1321 9.367267e-01 9.367506e-01 9.367843e-01 9.368184e-01 9.411544e-01 4.832484e-01 4.636906e-01 4.663429e-01 4.912884e-01 4.939761e-01

1322 9.368224e-01 9.368462e-01 9.368799e-01 9.369139e-01 9.412418e-01 4.838635e-01 4.642472e-01 4.669127e-01 4.919442e-01 4.946457e-01

1323 9.369166e-01 9.369403e-01 9.369739e-01 9.370078e-01 9.413280e-01 4.844903e-01 4.648161e-01 4.674948e-01 4.926116e-01 4.953267e-01

1324 9.370094e-01 9.370330e-01 9.370665e-01 9.371003e-01 9.414129e-01 4.851283e-01 4.653969e-01 4.680889e-01 4.932900e-01 4.960185e-01

1325 9.371007e-01 9.371243e-01 9.371577e-01 9.371914e-01 9.414966e-01 4.857770e-01 4.659893e-01 4.686946e-01 4.939787e-01 4.967206e-01

1326 9.371908e-01 9.372143e-01 9.372476e-01 9.372813e-01 9.415792e-01 4.864358e-01 4.665928e-01 4.693114e-01 4.946772e-01 4.974323e-01

1327 9.372796e-01 9.373030e-01 9.373363e-01 9.373698e-01 9.416607e-01 4.871041e-01 4.672070e-01 4.699389e-01 4.953846e-01 4.981528e-01

1328 9.373672e-01 9.373905e-01 9.374237e-01 9.374572e-01 9.417411e-01 4.877812e-01 4.678315e-01 4.705764e-01 4.961004e-01 4.988813e-01

1329 9.374536e-01 9.374769e-01 9.375100e-01 9.375434e-01 9.418206e-01 4.884665e-01 4.684655e-01 4.712235e-01 4.968237e-01 4.996172e-01

1330 9.375389e-01 9.375621e-01 9.375951e-01 9.376285e-01 9.418991e-01 4.891592e-01 4.691086e-01 4.718795e-01 4.975537e-01 5.003595e-01

1331 9.376231e-01 9.376463e-01 9.376792e-01 9.377125e-01 9.419766e-01 4.898586e-01 4.697602e-01 4.725438e-01 4.982896e-01 5.011074e-01

1332 9.377063e-01 9.377294e-01 9.377623e-01 9.377955e-01 9.420533e-01 4.905639e-01 4.704195e-01 4.732157e-01 4.990306e-01 5.018600e-01

1333 9.377885e-01 9.378116e-01 9.378444e-01 9.378775e-01 9.421292e-01 4.912743e-01 4.710860e-01 4.738945e-01 4.997757e-01 5.026165e-01

1334 9.378698e-01 9.378928e-01 9.379256e-01 9.379586e-01 9.422042e-01 4.919890e-01 4.717588e-01 4.745794e-01 5.005242e-01 5.033759e-01

1335 9.379502e-01 9.379732e-01 9.380058e-01 9.380388e-01 9.422784e-01 4.927070e-01 4.724372e-01 4.752698e-01 5.012750e-01 5.041373e-01

1336 9.380297e-01 9.380526e-01 9.380852e-01 9.381182e-01 9.423519e-01 4.934277e-01 4.731205e-01 4.759647e-01 5.020273e-01 5.048999e-01

1337 9.381083e-01 9.381312e-01 9.381638e-01 9.381967e-01 9.424246e-01 4.941500e-01 4.738080e-01 4.766635e-01 5.027802e-01 5.056626e-01

1338 9.381862e-01 9.382090e-01 9.382415e-01 9.382743e-01 9.424967e-01 4.948731e-01 4.744987e-01 4.773652e-01 5.035327e-01 5.064246e-01

1339 9.382633e-01 9.382861e-01 9.383185e-01 9.383513e-01 9.425680e-01 4.955962e-01 4.751919e-01 4.780691e-01 5.042840e-01 5.071849e-01

1340 9.383396e-01 9.383623e-01 9.383947e-01 9.384274e-01 9.426387e-01 4.963184e-01 4.758867e-01 4.787743e-01 5.050332e-01 5.079426e-01

1341 9.384152e-01 9.384379e-01 9.384702e-01 9.385029e-01 9.427088e-01 4.970388e-01 4.765824e-01 4.794799e-01 5.057794e-01 5.086969e-01

1342 9.384901e-01 9.385128e-01 9.385450e-01 9.385776e-01 9.427783e-01 4.977566e-01 4.772781e-01 4.801852e-01 5.065216e-01 5.094469e-01

1343 9.385643e-01 9.385869e-01 9.386192e-01 9.386517e-01 9.428472e-01 4.984709e-01 4.779730e-01 4.808894e-01 5.072592e-01 5.101918e-01

1344 9.386379e-01 9.386605e-01 9.386926e-01 9.387251e-01 9.429155e-01 4.991810e-01 4.786662e-01 4.815915e-01 5.079912e-01 5.109306e-01

1345 9.387108e-01 9.387334e-01 9.387655e-01 9.387979e-01 9.429833e-01 4.998860e-01 4.793570e-01 4.822907e-01 5.087169e-01 5.116628e-01

1346 9.387831e-01 9.388057e-01 9.388377e-01 9.388701e-01 9.430505e-01 5.005852e-01 4.800446e-01 4.829864e-01 5.094356e-01 5.123874e-01

1347 9.388549e-01 9.388774e-01 9.389094e-01 9.389417e-01 9.431172e-01 5.012778e-01 4.807282e-01 4.836777e-01 5.101465e-01 5.131039e-01

1348 9.389260e-01 9.389485e-01 9.389804e-01 9.390127e-01 9.431835e-01 5.019632e-01 4.814070e-01 4.843638e-01 5.108489e-01 5.138115e-01

1349 9.389966e-01 9.390191e-01 9.390510e-01 9.390832e-01 9.432492e-01 5.026407e-01 4.820804e-01 4.850441e-01 5.115422e-01 5.145096e-01

1350 9.390667e-01 9.390891e-01 9.391210e-01 9.391531e-01 9.433144e-01 5.033097e-01 4.827477e-01 4.857178e-01 5.122258e-01 5.151976e-01

1351 9.391362e-01 9.391586e-01 9.391904e-01 9.392225e-01 9.433793e-01 5.039696e-01 4.834081e-01 4.863843e-01 5.128992e-01 5.158750e-01

1352 9.392053e-01 9.392276e-01 9.392594e-01 9.392914e-01 9.434436e-01 5.046198e-01 4.840610e-01 4.870430e-01 5.135619e-01 5.165413e-01

1353 9.392738e-01 9.392961e-01 9.393278e-01 9.393598e-01 9.435075e-01 5.052598e-01 4.847059e-01 4.876932e-01 5.142133e-01 5.171961e-01

1354 9.393419e-01 9.393642e-01 9.393958e-01 9.394278e-01 9.435710e-01 5.058893e-01 4.853423e-01 4.883345e-01 5.148531e-01 5.178389e-01

1355 9.394095e-01 9.394317e-01 9.394633e-01 9.394952e-01 9.436341e-01 5.065077e-01 4.859695e-01 4.889664e-01 5.154810e-01 5.184694e-01

1356 9.394766e-01 9.394989e-01 9.395304e-01 9.395622e-01 9.436968e-01 5.071148e-01 4.865871e-01 4.895883e-01 5.160965e-01 5.190873e-01

1357 9.395433e-01 9.395655e-01 9.395970e-01 9.396288e-01 9.437591e-01 5.077101e-01 4.871946e-01 4.901998e-01 5.166994e-01 5.196923e-01

1358 9.396096e-01 9.396318e-01 9.396632e-01 9.396950e-01 9.438211e-01 5.082935e-01 4.877918e-01 4.908006e-01 5.172895e-01 5.202842e-01

1359 9.396755e-01 9.396976e-01 9.397290e-01 9.397607e-01 9.438826e-01 5.088646e-01 4.883782e-01 4.913903e-01 5.178666e-01 5.208629e-01

1360 9.397409e-01 9.397630e-01 9.397944e-01 9.398260e-01 9.439438e-01 5.094233e-01 4.889534e-01 4.919686e-01 5.184306e-01 5.214282e-01

1361 9.398059e-01 9.398280e-01 9.398593e-01 9.398909e-01 9.440046e-01 5.099694e-01 4.895173e-01 4.925352e-01 5.189812e-01 5.219800e-01

1362 9.398706e-01 9.398927e-01 9.399239e-01 9.399554e-01 9.440651e-01 5.105028e-01 4.900697e-01 4.930900e-01 5.195186e-01 5.225182e-01

1363 9.399349e-01 9.399569e-01 9.399881e-01 9.400196e-01 9.441253e-01 5.110235e-01 4.906102e-01 4.936328e-01 5.200425e-01 5.230429e-01

1364 9.399988e-01 9.400208e-01 9.400519e-01 9.400834e-01 9.441851e-01 5.115313e-01 4.911388e-01 4.941634e-01 5.205531e-01 5.235541e-01

1365 9.400623e-01 9.400843e-01 9.401154e-01 9.401468e-01 9.442447e-01 5.120264e-01 4.916553e-01 4.946817e-01 5.210504e-01 5.240518e-01

1366 9.401255e-01 9.401475e-01 9.401785e-01 9.402098e-01 9.443039e-01 5.125086e-01 4.921597e-01 4.951877e-01 5.215344e-01 5.245360e-01

1367 9.401883e-01 9.402103e-01 9.402413e-01 9.402725e-01 9.443627e-01 5.129781e-01 4.926519e-01 4.956813e-01 5.220053e-01 5.250070e-01

1368 9.402508e-01 9.402727e-01 9.403037e-01 9.403349e-01 9.444213e-01 5.134349e-01 4.931319e-01 4.961625e-01 5.224631e-01 5.254648e-01

1369 9.403130e-01 9.403349e-01 9.403658e-01 9.403970e-01 9.444796e-01 5.138791e-01 4.935998e-01 4.966314e-01 5.229080e-01 5.259097e-01

1370 9.403748e-01 9.403967e-01 9.404275e-01 9.404587e-01 9.445376e-01 5.143110e-01 4.940555e-01 4.970880e-01 5.233401e-01 5.263417e-01

1371 9.404363e-01 9.404582e-01 9.404890e-01 9.405200e-01 9.445953e-01 5.147306e-01 4.944991e-01 4.975324e-01 5.237598e-01 5.267610e-01

1372 9.404975e-01 9.405193e-01 9.405501e-01 9.405811e-01 9.446528e-01 5.151381e-01 4.949308e-01 4.979648e-01 5.241670e-01 5.271680e-01

1373 9.405584e-01 9.405802e-01 9.406109e-01 9.406419e-01 9.447099e-01 5.155337e-01 4.953507e-01 4.983852e-01 5.245622e-01 5.275628e-01

1374 9.406190e-01 9.406408e-01 9.406714e-01 9.407023e-01 9.447668e-01 5.159176e-01 4.957588e-01 4.987937e-01 5.249455e-01 5.279457e-01

1375 9.406793e-01 9.407010e-01 9.407316e-01 9.407625e-01 9.448235e-01 5.162901e-01 4.961555e-01 4.991907e-01 5.253172e-01 5.283169e-01

1376 9.407393e-01 9.407610e-01 9.407916e-01 9.408224e-01 9.448798e-01 5.166513e-01 4.965407e-01 4.995762e-01 5.256776e-01 5.286767e-01

1377 9.407990e-01 9.408207e-01 9.408512e-01 9.408820e-01 9.449360e-01 5.170016e-01 4.969149e-01 4.999505e-01 5.260268e-01 5.290254e-01

1378 9.408584e-01 9.408801e-01 9.409106e-01 9.409413e-01 9.449918e-01 5.173411e-01 4.972780e-01 5.003138e-01 5.263652e-01 5.293632e-01

1379 9.409176e-01 9.409393e-01 9.409696e-01 9.410003e-01 9.450475e-01 5.176702e-01 4.976305e-01 5.006663e-01 5.266931e-01 5.296905e-01

1380 9.409765e-01 9.409981e-01 9.410284e-01 9.410591e-01 9.451028e-01 5.179891e-01 4.979724e-01 5.010082e-01 5.270107e-01 5.300075e-01

1381 9.410351e-01 9.410567e-01 9.410870e-01 9.411176e-01 9.451580e-01 5.182980e-01 4.983041e-01 5.013398e-01 5.273184e-01 5.303146e-01

1382 9.410935e-01 9.411150e-01 9.411453e-01 9.411758e-01 9.452129e-01 5.185974e-01 4.986258e-01 5.016614e-01 5.276163e-01 5.306120e-01

1383 9.411516e-01 9.411731e-01 9.412033e-01 9.412338e-01 9.452676e-01 5.188873e-01 4.989377e-01 5.019732e-01 5.279049e-01 5.309000e-01

1384 9.412094e-01 9.412310e-01 9.412611e-01 9.412915e-01 9.453220e-01 5.191682e-01 4.992401e-01 5.022755e-01 5.281845e-01 5.311789e-01

1385 9.412670e-01 9.412885e-01 9.413186e-01 9.413489e-01 9.453762e-01 5.194403e-01 4.995333e-01 5.025685e-01 5.284552e-01 5.314490e-01

1386 9.413244e-01 9.413459e-01 9.413759e-01 9.414062e-01 9.454303e-01 5.197039e-01 4.998175e-01 5.028525e-01 5.287174e-01 5.317106e-01

1387 9.413815e-01 9.414029e-01 9.414329e-01 9.414631e-01 9.454840e-01 5.199592e-01 5.000930e-01 5.031278e-01 5.289713e-01 5.319640e-01

1388 9.414384e-01 9.414598e-01 9.414897e-01 9.415199e-01 9.455376e-01 5.202065e-01 5.003601e-01 5.033947e-01 5.292173e-01 5.322094e-01

1389 9.414950e-01 9.415164e-01 9.415462e-01 9.415764e-01 9.455910e-01 5.204462e-01 5.006189e-01 5.036534e-01 5.294557e-01 5.324472e-01

1390 9.415514e-01 9.415728e-01 9.416026e-01 9.416326e-01 9.456442e-01 5.206784e-01 5.008699e-01 5.039041e-01 5.296866e-01 5.326776e-01

1391 9.416076e-01 9.416289e-01 9.416587e-01 9.416887e-01 9.456971e-01 5.209034e-01 5.011132e-01 5.041472e-01 5.299104e-01 5.329009e-01

1392 9.416636e-01 9.416849e-01 9.417145e-01 9.417445e-01 9.457499e-01 5.211215e-01 5.013491e-01 5.043829e-01 5.301273e-01 5.331173e-01

1393 9.417193e-01 9.417406e-01 9.417702e-01 9.418001e-01 9.458024e-01 5.213330e-01 5.015779e-01 5.046114e-01 5.303377e-01 5.333271e-01

1394 9.417749e-01 9.417961e-01 9.418256e-01 9.418555e-01 9.458548e-01 5.215381e-01 5.017997e-01 5.048330e-01 5.305416e-01 5.335306e-01

1395 9.418302e-01 9.418514e-01 9.418808e-01 9.419106e-01 9.459070e-01 5.217370e-01 5.020150e-01 5.050480e-01 5.307394e-01 5.337280e-01

1396 9.418853e-01 9.419064e-01 9.419358e-01 9.419656e-01 9.459589e-01 5.219299e-01 5.022238e-01 5.052567e-01 5.309314e-01 5.339195e-01

1397 9.419401e-01 9.419613e-01 9.419906e-01 9.420203e-01 9.460107e-01 5.221172e-01 5.024264e-01 5.054591e-01 5.311177e-01 5.341054e-01

1398 9.419948e-01 9.420159e-01 9.420452e-01 9.420748e-01 9.460623e-01 5.222990e-01 5.026232e-01 5.056556e-01 5.312985e-01 5.342859e-01

1399 9.420493e-01 9.420704e-01 9.420996e-01 9.421291e-01 9.461138e-01 5.224755e-01 5.028142e-01 5.058464e-01 5.314742e-01 5.344611e-01

1400 9.421036e-01 9.421246e-01 9.421538e-01 9.421833e-01 9.461650e-01 5.226470e-01 5.029997e-01 5.060318e-01 5.316448e-01 5.346314e-01

1401 9.421577e-01 9.421787e-01 9.422078e-01 9.422372e-01 9.462161e-01 5.228136e-01 5.031799e-01 5.062118e-01 5.318107e-01 5.347970e-01

1402 9.422116e-01 9.422325e-01 9.422616e-01 9.422909e-01 9.462669e-01 5.229756e-01 5.033551e-01 5.063868e-01 5.319719e-01 5.349579e-01

1403 9.422653e-01 9.422862e-01 9.423152e-01 9.423444e-01 9.463176e-01 5.231331e-01 5.035254e-01 5.065569e-01 5.321288e-01 5.351144e-01

1404 9.423188e-01 9.423397e-01 9.423686e-01 9.423978e-01 9.463682e-01 5.232864e-01 5.036910e-01 5.067224e-01 5.322814e-01 5.352668e-01

1405 9.423721e-01 9.423930e-01 9.424218e-01 9.424509e-01 9.464186e-01 5.234355e-01 5.038522e-01 5.068834e-01 5.324300e-01 5.354151e-01

1406 9.424252e-01 9.424461e-01 9.424748e-01 9.425039e-01 9.464688e-01 5.235808e-01 5.040091e-01 5.070402e-01 5.325747e-01 5.355595e-01

1407 9.424782e-01 9.424990e-01 9.425277e-01 9.425566e-01 9.465188e-01 5.237223e-01 5.041618e-01 5.071928e-01 5.327157e-01 5.357003e-01

1408 9.425310e-01 9.425517e-01 9.425803e-01 9.426092e-01 9.465687e-01 5.238603e-01 5.043106e-01 5.073414e-01 5.328531e-01 5.358375e-01

1409 9.425836e-01 9.426043e-01 9.426328e-01 9.426617e-01 9.466184e-01 5.239948e-01 5.044556e-01 5.074863e-01 5.329871e-01 5.359713e-01

1410 9.426360e-01 9.426567e-01 9.426851e-01 9.427139e-01 9.466679e-01 5.241260e-01 5.045971e-01 5.076276e-01 5.331179e-01 5.361019e-01

1411 9.426882e-01 9.427089e-01 9.427373e-01 9.427660e-01 9.467173e-01 5.242540e-01 5.047350e-01 5.077654e-01 5.332456e-01 5.362294e-01

1412 9.427403e-01 9.427609e-01 9.427892e-01 9.428179e-01 9.467665e-01 5.243791e-01 5.048696e-01 5.079000e-01 5.333703e-01 5.363540e-01

1413 9.427922e-01 9.428128e-01 9.428410e-01 9.428696e-01 9.468156e-01 5.245013e-01 5.050011e-01 5.080313e-01 5.334922e-01 5.364757e-01

1414 9.428440e-01 9.428645e-01 9.428926e-01 9.429211e-01 9.468646e-01 5.246208e-01 5.051296e-01 5.081596e-01 5.336114e-01 5.365947e-01

1415 9.428956e-01 9.429160e-01 9.429441e-01 9.429725e-01 9.469133e-01 5.247376e-01 5.052551e-01 5.082851e-01 5.337279e-01 5.367111e-01

1416 9.429470e-01 9.429674e-01 9.429954e-01 9.430237e-01 9.469620e-01 5.248520e-01 5.053779e-01 5.084077e-01 5.338420e-01 5.368251e-01

1417 9.429983e-01 9.430186e-01 9.430466e-01 9.430748e-01 9.470105e-01 5.249639e-01 5.054980e-01 5.085277e-01 5.339538e-01 5.369367e-01

1418 9.430494e-01 9.430697e-01 9.430975e-01 9.431257e-01 9.470588e-01 5.250736e-01 5.056156e-01 5.086452e-01 5.340632e-01 5.370460e-01

1419 9.431003e-01 9.431206e-01 9.431484e-01 9.431764e-01 9.471070e-01 5.251811e-01 5.057308e-01 5.087603e-01 5.341705e-01 5.371532e-01

1420 9.431511e-01 9.431714e-01 9.431990e-01 9.432270e-01 9.471550e-01 5.252865e-01 5.058436e-01 5.088730e-01 5.342758e-01 5.372584e-01

1421 9.432018e-01 9.432220e-01 9.432495e-01 9.432774e-01 9.472029e-01 5.253899e-01 5.059543e-01 5.089836e-01 5.343791e-01 5.373616e-01

1422 9.432523e-01 9.432724e-01 9.432999e-01 9.433277e-01 9.472507e-01 5.254915e-01 5.060629e-01 5.090920e-01 5.344805e-01 5.374629e-01

1423 9.433027e-01 9.433227e-01 9.433501e-01 9.433778e-01 9.472983e-01 5.255912e-01 5.061694e-01 5.091985e-01 5.345801e-01 5.375624e-01

1424 9.433529e-01 9.433729e-01 9.434002e-01 9.434278e-01 9.473458e-01 5.256892e-01 5.062741e-01 5.093030e-01 5.346780e-01 5.376602e-01

1425 9.434030e-01 9.434229e-01 9.434501e-01 9.434776e-01 9.473932e-01 5.257856e-01 5.063769e-01 5.094057e-01 5.347742e-01 5.377564e-01

1426 9.434529e-01 9.434728e-01 9.434999e-01 9.435273e-01 9.474404e-01 5.258804e-01 5.064780e-01 5.095066e-01 5.348690e-01 5.378511e-01

1427 9.435027e-01 9.435226e-01 9.435495e-01 9.435769e-01 9.474875e-01 5.259737e-01 5.065774e-01 5.096059e-01 5.349622e-01 5.379443e-01

1428 9.435524e-01 9.435722e-01 9.435990e-01 9.436263e-01 9.475345e-01 5.260656e-01 5.066753e-01 5.097036e-01 5.350540e-01 5.380360e-01

1429 9.436019e-01 9.436216e-01 9.436484e-01 9.436755e-01 9.475813e-01 5.261562e-01 5.067716e-01 5.097997e-01 5.351445e-01 5.381264e-01

1430 9.436513e-01 9.436710e-01 9.436976e-01 9.437247e-01 9.476281e-01 5.262454e-01 5.068665e-01 5.098945e-01 5.352337e-01 5.382156e-01

1431 9.437006e-01 9.437202e-01 9.437467e-01 9.437736e-01 9.476746e-01 5.263335e-01 5.069601e-01 5.099878e-01 5.353217e-01 5.383035e-01

1432 9.437497e-01 9.437693e-01 9.437957e-01 9.438225e-01 9.477211e-01 5.264203e-01 5.070523e-01 5.100799e-01 5.354085e-01 5.383903e-01

1433 9.437988e-01 9.438182e-01 9.438445e-01 9.438712e-01 9.477674e-01 5.265061e-01 5.071433e-01 5.101706e-01 5.354942e-01 5.384759e-01

1434 9.438477e-01 9.438670e-01 9.438933e-01 9.439198e-01 9.478136e-01 5.265908e-01 5.072331e-01 5.102602e-01 5.355788e-01 5.385605e-01

1435 9.438964e-01 9.439157e-01 9.439418e-01 9.439683e-01 9.478597e-01 5.266745e-01 5.073218e-01 5.103487e-01 5.356624e-01 5.386441e-01

1436 9.439451e-01 9.439643e-01 9.439903e-01 9.440166e-01 9.479057e-01 5.267572e-01 5.074095e-01 5.104361e-01 5.357451e-01 5.387267e-01

1437 9.439937e-01 9.440128e-01 9.440386e-01 9.440648e-01 9.479516e-01 5.268390e-01 5.074961e-01 5.105225e-01 5.358269e-01 5.388084e-01

1438 9.440421e-01 9.440611e-01 9.440869e-01 9.441129e-01 9.479973e-01 5.269200e-01 5.075818e-01 5.106078e-01 5.359078e-01 5.388893e-01

1439 9.440904e-01 9.441094e-01 9.441350e-01 9.441609e-01 9.480429e-01 5.270001e-01 5.076665e-01 5.106923e-01 5.359879e-01 5.389693e-01

1440 9.441386e-01 9.441575e-01 9.441829e-01 9.442087e-01 9.480884e-01 5.270795e-01 5.077504e-01 5.107759e-01 5.360671e-01 5.390485e-01

1441 9.441867e-01 9.442055e-01 9.442308e-01 9.442564e-01 9.481338e-01 5.271581e-01 5.078334e-01 5.108586e-01 5.361457e-01 5.391270e-01

1442 9.444528e-01 9.444707e-01 9.444951e-01 9.445199e-01 9.483622e-01 5.272495e-01 5.079096e-01 5.109380e-01 5.362491e-01 5.392348e-01

1443 9.447076e-01 9.447247e-01 9.447483e-01 9.447723e-01 9.485812e-01 5.273453e-01 5.079895e-01 5.110213e-01 5.363576e-01 5.393478e-01

1444 9.449519e-01 9.449681e-01 9.449910e-01 9.450142e-01 9.487912e-01 5.274459e-01 5.080734e-01 5.111088e-01 5.364713e-01 5.394662e-01

1445 9.451860e-01 9.452016e-01 9.452237e-01 9.452462e-01 9.489927e-01 5.275513e-01 5.081614e-01 5.112006e-01 5.365905e-01 5.395904e-01

1446 9.454107e-01 9.454255e-01 9.454470e-01 9.454687e-01 9.491861e-01 5.276618e-01 5.082537e-01 5.112969e-01 5.367155e-01 5.397205e-01

1447 9.456263e-01 9.456404e-01 9.456613e-01 9.456823e-01 9.493719e-01 5.277776e-01 5.083505e-01 5.113978e-01 5.368463e-01 5.398568e-01

1448 9.458333e-01 9.458468e-01 9.458670e-01 9.458875e-01 9.495504e-01 5.278989e-01 5.084520e-01 5.115035e-01 5.369833e-01 5.399994e-01

1449 9.460323e-01 9.460451e-01 9.460647e-01 9.460846e-01 9.497221e-01 5.280259e-01 5.085583e-01 5.116144e-01 5.371267e-01 5.401487e-01

1450 9.462235e-01 9.462358e-01 9.462548e-01 9.462741e-01 9.498873e-01 5.281588e-01 5.086697e-01 5.117304e-01 5.372768e-01 5.403048e-01

1451 9.464074e-01 9.464191e-01 9.464377e-01 9.464564e-01 9.500464e-01 5.282979e-01 5.087863e-01 5.118519e-01 5.374337e-01 5.404681e-01

1452 9.465844e-01 9.465956e-01 9.466137e-01 9.466319e-01 9.501996e-01 5.284433e-01 5.089083e-01 5.119791e-01 5.375977e-01 5.406387e-01

1453 9.467549e-01 9.467656e-01 9.467832e-01 9.468009e-01 9.503473e-01 5.285953e-01 5.090360e-01 5.121121e-01 5.377690e-01 5.408170e-01

1454 9.469192e-01 9.469294e-01 9.469465e-01 9.469638e-01 9.504898e-01 5.287541e-01 5.091696e-01 5.122512e-01 5.379480e-01 5.410032e-01

1455 9.470776e-01 9.470874e-01 9.471040e-01 9.471209e-01 9.506274e-01 5.289200e-01 5.093092e-01 5.123966e-01 5.381349e-01 5.411975e-01

1456 9.472305e-01 9.472398e-01 9.472560e-01 9.472724e-01 9.507603e-01 5.290932e-01 5.094551e-01 5.125486e-01 5.383298e-01 5.414002e-01

1457 9.473780e-01 9.473869e-01 9.474028e-01 9.474188e-01 9.508888e-01 5.292739e-01 5.096074e-01 5.127072e-01 5.385331e-01 5.416116e-01

1458 9.475206e-01 9.475291e-01 9.475446e-01 9.475602e-01 9.510130e-01 5.294624e-01 5.097666e-01 5.128729e-01 5.387451e-01 5.418319e-01

1459 9.476584e-01 9.476666e-01 9.476817e-01 9.476970e-01 9.511333e-01 5.296590e-01 5.099326e-01 5.130458e-01 5.389659e-01 5.420614e-01

1460 9.477918e-01 9.477996e-01 9.478144e-01 9.478293e-01 9.512499e-01 5.298637e-01 5.101058e-01 5.132260e-01 5.391959e-01 5.423003e-01

1461 9.479208e-01 9.479283e-01 9.479428e-01 9.479574e-01 9.513628e-01 5.300770e-01 5.102865e-01 5.134140e-01 5.394353e-01 5.425489e-01

1462 9.480459e-01 9.480531e-01 9.480672e-01 9.480815e-01 9.514724e-01 5.302990e-01 5.104747e-01 5.136098e-01 5.396843e-01 5.428075e-01

1463 9.481671e-01 9.481740e-01 9.481879e-01 9.482019e-01 9.515787e-01 5.305300e-01 5.106708e-01 5.138138e-01 5.399432e-01 5.430763e-01

1464 9.482847e-01 9.482913e-01 9.483049e-01 9.483186e-01 9.516821e-01 5.307701e-01 5.108749e-01 5.140262e-01 5.402122e-01 5.433554e-01

1465 9.483989e-01 9.484052e-01 9.484186e-01 9.484320e-01 9.517825e-01 5.310197e-01 5.110874e-01 5.142471e-01 5.404915e-01 5.436453e-01

1466 9.485098e-01 9.485159e-01 9.485290e-01 9.485422e-01 9.518802e-01 5.312789e-01 5.113084e-01 5.144768e-01 5.407814e-01 5.439460e-01

1467 9.486176e-01 9.486235e-01 9.486363e-01 9.486492e-01 9.519753e-01 5.315480e-01 5.115381e-01 5.147156e-01 5.410821e-01 5.442579e-01

1468 9.487225e-01 9.487281e-01 9.487407e-01 9.487534e-01 9.520679e-01 5.318272e-01 5.117768e-01 5.149636e-01 5.413938e-01 5.445810e-01

1469 9.488246e-01 9.488300e-01 9.488423e-01 9.488549e-01 9.521582e-01 5.321166e-01 5.120246e-01 5.152210e-01 5.417167e-01 5.449156e-01

1470 9.489240e-01 9.489292e-01 9.489414e-01 9.489537e-01 9.522463e-01 5.324164e-01 5.122818e-01 5.154882e-01 5.420509e-01 5.452619e-01

1471 9.490209e-01 9.490259e-01 9.490379e-01 9.490500e-01 9.523322e-01 5.327269e-01 5.125486e-01 5.157651e-01 5.423966e-01 5.456200e-01

1472 9.491155e-01 9.491203e-01 9.491321e-01 9.491440e-01 9.524162e-01 5.330482e-01 5.128251e-01 5.160522e-01 5.427540e-01 5.459901e-01

1473 9.492078e-01 9.492124e-01 9.492240e-01 9.492357e-01 9.524983e-01 5.333804e-01 5.131115e-01 5.163494e-01 5.431232e-01 5.463722e-01

1474 9.492979e-01 9.493023e-01 9.493138e-01 9.493253e-01 9.525785e-01 5.337237e-01 5.134080e-01 5.166570e-01 5.435044e-01 5.467665e-01

1475 9.493860e-01 9.493902e-01 9.494015e-01 9.494129e-01 9.526571e-01 5.340781e-01 5.137147e-01 5.169752e-01 5.438975e-01 5.471731e-01

1476 9.494721e-01 9.494762e-01 9.494873e-01 9.494985e-01 9.527339e-01 5.344439e-01 5.140319e-01 5.173040e-01 5.443026e-01 5.475920e-01

1477 9.495564e-01 9.495603e-01 9.495713e-01 9.495824e-01 9.528093e-01 5.348210e-01 5.143595e-01 5.176436e-01 5.447199e-01 5.480232e-01

1478 9.496389e-01 9.496427e-01 9.496535e-01 9.496644e-01 9.528831e-01 5.352095e-01 5.146978e-01 5.179941e-01 5.451493e-01 5.484668e-01

1479 9.497197e-01 9.497234e-01 9.497341e-01 9.497449e-01 9.529556e-01 5.356094e-01 5.150468e-01 5.183556e-01 5.455908e-01 5.489227e-01

1480 9.497989e-01 9.498025e-01 9.498131e-01 9.498237e-01 9.530267e-01 5.360208e-01 5.154066e-01 5.187281e-01 5.460444e-01 5.493909e-01

1481 9.498767e-01 9.498801e-01 9.498905e-01 9.499010e-01 9.530965e-01 5.364437e-01 5.157772e-01 5.191117e-01 5.465100e-01 5.498712e-01

1482 9.499529e-01 9.499563e-01 9.499665e-01 9.499769e-01 9.531651e-01 5.368779e-01 5.161587e-01 5.195064e-01 5.469875e-01 5.503636e-01

1483 9.500278e-01 9.500310e-01 9.500412e-01 9.500515e-01 9.532325e-01 5.373234e-01 5.165511e-01 5.199122e-01 5.474768e-01 5.508679e-01

1484 9.501014e-01 9.501045e-01 9.501146e-01 9.501247e-01 9.532989e-01 5.377802e-01 5.169544e-01 5.203291e-01 5.479777e-01 5.513840e-01

1485 9.501737e-01 9.501767e-01 9.501867e-01 9.501967e-01 9.533641e-01 5.382480e-01 5.173685e-01 5.207571e-01 5.484901e-01 5.519115e-01

1486 9.502448e-01 9.502477e-01 9.502576e-01 9.502675e-01 9.534284e-01 5.387268e-01 5.177933e-01 5.211959e-01 5.490136e-01 5.524503e-01

1487 9.503148e-01 9.503176e-01 9.503273e-01 9.503372e-01 9.534917e-01 5.392162e-01 5.182289e-01 5.216456e-01 5.495481e-01 5.530001e-01

1488 9.503837e-01 9.503864e-01 9.503960e-01 9.504058e-01 9.535540e-01 5.397162e-01 5.186750e-01 5.221060e-01 5.500932e-01 5.535605e-01

1489 9.504515e-01 9.504541e-01 9.504637e-01 9.504733e-01 9.536155e-01 5.402264e-01 5.191315e-01 5.225770e-01 5.506485e-01 5.541312e-01

1490 9.505184e-01 9.505209e-01 9.505304e-01 9.505399e-01 9.536762e-01 5.407465e-01 5.195983e-01 5.230582e-01 5.512139e-01 5.547118e-01

1491 9.505842e-01 9.505867e-01 9.505961e-01 9.506055e-01 9.537360e-01 5.412763e-01 5.200751e-01 5.235496e-01 5.517887e-01 5.553018e-01

1492 9.506492e-01 9.506516e-01 9.506609e-01 9.506702e-01 9.537951e-01 5.418153e-01 5.205616e-01 5.240508e-01 5.523727e-01 5.559008e-01

1493 9.507133e-01 9.507156e-01 9.507248e-01 9.507341e-01 9.538534e-01 5.423632e-01 5.210577e-01 5.245615e-01 5.529652e-01 5.565083e-01

1494 9.507766e-01 9.507788e-01 9.507879e-01 9.507971e-01 9.539110e-01 5.429196e-01 5.215630e-01 5.250815e-01 5.535659e-01 5.571238e-01

1495 9.508390e-01 9.508412e-01 9.508502e-01 9.508594e-01 9.539680e-01 5.434839e-01 5.220772e-01 5.256103e-01 5.541742e-01 5.577468e-01

1496 9.509007e-01 9.509029e-01 9.509118e-01 9.509208e-01 9.540243e-01 5.440558e-01 5.226000e-01 5.261477e-01 5.547895e-01 5.583765e-01

1497 9.509617e-01 9.509638e-01 9.509726e-01 9.509816e-01 9.540799e-01 5.446347e-01 5.231309e-01 5.266931e-01 5.554113e-01 5.590125e-01

1498 9.510220e-01 9.510240e-01 9.510328e-01 9.510416e-01 9.541350e-01 5.452201e-01 5.236695e-01 5.272461e-01 5.560389e-01 5.596540e-01

1499 9.510816e-01 9.510835e-01 9.510922e-01 9.511010e-01 9.541895e-01 5.458114e-01 5.242153e-01 5.278063e-01 5.566717e-01 5.603005e-01

1500 9.511405e-01 9.511424e-01 9.511510e-01 9.511597e-01 9.542434e-01 5.464080e-01 5.247680e-01 5.283731e-01 5.573091e-01 5.609512e-01

1501 9.511988e-01 9.512006e-01 9.512092e-01 9.512178e-01 9.542968e-01 5.470094e-01 5.253270e-01 5.289461e-01 5.579504e-01 5.616055e-01

1502 9.512565e-01 9.512583e-01 9.512668e-01 9.512754e-01 9.543497e-01 5.476149e-01 5.258918e-01 5.295247e-01 5.585949e-01 5.622627e-01

1503 9.513136e-01 9.513153e-01 9.513238e-01 9.513323e-01 9.544022e-01 5.482239e-01 5.264618e-01 5.301084e-01 5.592419e-01 5.629220e-01

1504 9.513702e-01 9.513719e-01 9.513802e-01 9.513887e-01 9.544541e-01 5.488357e-01 5.270366e-01 5.306964e-01 5.598907e-01 5.635828e-01

1505 9.514263e-01 9.514279e-01 9.514362e-01 9.514446e-01 9.545056e-01 5.494497e-01 5.276154e-01 5.312883e-01 5.605406e-01 5.642442e-01

1506 9.514818e-01 9.514833e-01 9.514916e-01 9.514999e-01 9.545566e-01 5.500652e-01 5.281977e-01 5.318835e-01 5.611909e-01 5.649056e-01

1507 9.515368e-01 9.515383e-01 9.515465e-01 9.515548e-01 9.546072e-01 5.506815e-01 5.287829e-01 5.324812e-01 5.618408e-01 5.655663e-01

1508 9.515914e-01 9.515928e-01 9.516010e-01 9.516092e-01 9.546575e-01 5.512980e-01 5.293704e-01 5.330810e-01 5.624898e-01 5.662255e-01

1509 9.516455e-01 9.516469e-01 9.516550e-01 9.516631e-01 9.547073e-01 5.519141e-01 5.299596e-01 5.336820e-01 5.631370e-01 5.668826e-01

1510 9.516991e-01 9.517005e-01 9.517085e-01 9.517166e-01 9.547567e-01 5.525289e-01 5.305498e-01 5.342837e-01 5.637818e-01 5.675368e-01

1511 9.517524e-01 9.517537e-01 9.517616e-01 9.517697e-01 9.548058e-01 5.531419e-01 5.311403e-01 5.348855e-01 5.644235e-01 5.681874e-01

1512 9.518052e-01 9.518065e-01 9.518144e-01 9.518223e-01 9.548545e-01 5.537525e-01 5.317307e-01 5.354866e-01 5.650614e-01 5.688339e-01

1513 9.518576e-01 9.518588e-01 9.518667e-01 9.518746e-01 9.549029e-01 5.543599e-01 5.323201e-01 5.360865e-01 5.656949e-01 5.694755e-01

1514 9.519096e-01 9.519108e-01 9.519186e-01 9.519264e-01 9.549509e-01 5.549636e-01 5.329081e-01 5.366845e-01 5.663234e-01 5.701116e-01

1515 9.519613e-01 9.519624e-01 9.519702e-01 9.519779e-01 9.549987e-01 5.555630e-01 5.334939e-01 5.372800e-01 5.669463e-01 5.707417e-01

1516 9.520126e-01 9.520137e-01 9.520214e-01 9.520291e-01 9.550461e-01 5.561574e-01 5.340770e-01 5.378723e-01 5.675631e-01 5.713652e-01

1517 9.520635e-01 9.520646e-01 9.520722e-01 9.520799e-01 9.550932e-01 5.567464e-01 5.346568e-01 5.384610e-01 5.681730e-01 5.719815e-01

1518 9.521141e-01 9.521152e-01 9.521227e-01 9.521303e-01 9.551400e-01 5.573294e-01 5.352327e-01 5.390454e-01 5.687757e-01 5.725901e-01

1519 9.521644e-01 9.521654e-01 9.521729e-01 9.521805e-01 9.551865e-01 5.579059e-01 5.358042e-01 5.396249e-01 5.693707e-01 5.731905e-01

1520 9.522143e-01 9.522153e-01 9.522228e-01 9.522303e-01 9.552328e-01 5.584753e-01 5.363707e-01 5.401991e-01 5.699575e-01 5.737824e-01

1521 9.522640e-01 9.522649e-01 9.522723e-01 9.522798e-01 9.552788e-01 5.590374e-01 5.369318e-01 5.407673e-01 5.705357e-01 5.743653e-01

1522 9.523133e-01 9.523142e-01 9.523216e-01 9.523290e-01 9.553246e-01 5.595915e-01 5.374868e-01 5.413293e-01 5.711049e-01 5.749388e-01

1523 9.523624e-01 9.523633e-01 9.523705e-01 9.523779e-01 9.553700e-01 5.601374e-01 5.380354e-01 5.418843e-01 5.716647e-01 5.755026e-01

1524 9.524112e-01 9.524120e-01 9.524192e-01 9.524265e-01 9.554153e-01 5.606747e-01 5.385771e-01 5.424322e-01 5.722149e-01 5.760564e-01

1525 9.524597e-01 9.524605e-01 9.524676e-01 9.524749e-01 9.554603e-01 5.612030e-01 5.391116e-01 5.429723e-01 5.727551e-01 5.765999e-01

1526 9.525079e-01 9.525087e-01 9.525158e-01 9.525230e-01 9.555051e-01 5.617221e-01 5.396383e-01 5.435045e-01 5.732851e-01 5.771329e-01

1527 9.525559e-01 9.525566e-01 9.525637e-01 9.525708e-01 9.555496e-01 5.622318e-01 5.401571e-01 5.440282e-01 5.738046e-01 5.776552e-01

1528 9.526036e-01 9.526043e-01 9.526113e-01 9.526184e-01 9.555940e-01 5.627316e-01 5.406675e-01 5.445433e-01 5.743136e-01 5.781665e-01

1529 9.526511e-01 9.526517e-01 9.526587e-01 9.526657e-01 9.556381e-01 5.632216e-01 5.411693e-01 5.450495e-01 5.748118e-01 5.786669e-01

1530 9.526983e-01 9.526989e-01 9.527058e-01 9.527128e-01 9.556820e-01 5.637015e-01 5.416622e-01 5.455464e-01 5.752991e-01 5.791561e-01

1531 9.527453e-01 9.527459e-01 9.527527e-01 9.527597e-01 9.557257e-01 5.641711e-01 5.421459e-01 5.460340e-01 5.757754e-01 5.796341e-01

1532 9.527921e-01 9.527926e-01 9.527994e-01 9.528063e-01 9.557692e-01 5.646305e-01 5.426204e-01 5.465119e-01 5.762407e-01 5.801008e-01

1533 9.528386e-01 9.528391e-01 9.528459e-01 9.528527e-01 9.558125e-01 5.650794e-01 5.430853e-01 5.469800e-01 5.766949e-01 5.805562e-01

1534 9.528850e-01 9.528855e-01 9.528921e-01 9.528989e-01 9.558557e-01 5.655178e-01 5.435407e-01 5.474383e-01 5.771381e-01 5.810004e-01

1535 9.529311e-01 9.529315e-01 9.529382e-01 9.529449e-01 9.558986e-01 5.659458e-01 5.439863e-01 5.478866e-01 5.775702e-01 5.814334e-01

1536 9.529770e-01 9.529774e-01 9.529840e-01 9.529906e-01 9.559414e-01 5.663632e-01 5.444220e-01 5.483248e-01 5.779912e-01 5.818552e-01

1537 9.530228e-01 9.530231e-01 9.530296e-01 9.530362e-01 9.559840e-01 5.667702e-01 5.448479e-01 5.487529e-01 5.784014e-01 5.822658e-01

1538 9.530683e-01 9.530686e-01 9.530751e-01 9.530816e-01 9.560264e-01 5.671668e-01 5.452638e-01 5.491709e-01 5.788006e-01 5.826655e-01

1539 9.531136e-01 9.531139e-01 9.531203e-01 9.531268e-01 9.560687e-01 5.675530e-01 5.456698e-01 5.495787e-01 5.791891e-01 5.830543e-01

1540 9.531588e-01 9.531590e-01 9.531654e-01 9.531718e-01 9.561108e-01 5.679290e-01 5.460658e-01 5.499765e-01 5.795669e-01 5.834323e-01

1541 9.532037e-01 9.532039e-01 9.532102e-01 9.532166e-01 9.561527e-01 5.682948e-01 5.464520e-01 5.503642e-01 5.799342e-01 5.837998e-01

1542 9.532485e-01 9.532487e-01 9.532549e-01 9.532612e-01 9.561945e-01 5.686505e-01 5.468283e-01 5.507419e-01 5.802912e-01 5.841567e-01

1543 9.532931e-01 9.532933e-01 9.532995e-01 9.533057e-01 9.562361e-01 5.689964e-01 5.471949e-01 5.511096e-01 5.806380e-01 5.845035e-01

1544 9.533376e-01 9.533377e-01 9.533438e-01 9.533500e-01 9.562776e-01 5.693325e-01 5.475518e-01 5.514676e-01 5.809748e-01 5.848401e-01

1545 9.533818e-01 9.533819e-01 9.533880e-01 9.533941e-01 9.563189e-01 5.696590e-01 5.478990e-01 5.518159e-01 5.813017e-01 5.851668e-01

1546 9.534259e-01 9.534260e-01 9.534320e-01 9.534381e-01 9.563601e-01 5.699760e-01 5.482369e-01 5.521546e-01 5.816190e-01 5.854839e-01

1547 9.534699e-01 9.534699e-01 9.534758e-01 9.534819e-01 9.564011e-01 5.702838e-01 5.485654e-01 5.524838e-01 5.819269e-01 5.857915e-01

1548 9.535137e-01 9.535137e-01 9.535195e-01 9.535255e-01 9.564420e-01 5.705825e-01 5.488847e-01 5.528038e-01 5.822256e-01 5.860899e-01

1549 9.535573e-01 9.535573e-01 9.535631e-01 9.535690e-01 9.564828e-01 5.708724e-01 5.491950e-01 5.531147e-01 5.825153e-01 5.863792e-01

1550 9.536008e-01 9.536007e-01 9.536065e-01 9.536123e-01 9.565234e-01 5.711536e-01 5.494965e-01 5.534167e-01 5.827962e-01 5.866597e-01

1551 9.536442e-01 9.536440e-01 9.536497e-01 9.536555e-01 9.565639e-01 5.714264e-01 5.497893e-01 5.537099e-01 5.830686e-01 5.869317e-01

1552 9.536873e-01 9.536872e-01 9.536928e-01 9.536985e-01 9.566043e-01 5.716909e-01 5.500736e-01 5.539946e-01 5.833326e-01 5.871953e-01

1553 9.537304e-01 9.537302e-01 9.537358e-01 9.537414e-01 9.566446e-01 5.719474e-01 5.503495e-01 5.542709e-01 5.835886e-01 5.874508e-01

1554 9.537733e-01 9.537731e-01 9.537786e-01 9.537841e-01 9.566847e-01 5.721961e-01 5.506174e-01 5.545391e-01 5.838367e-01 5.876985e-01

1555 9.538161e-01 9.538158e-01 9.538212e-01 9.538268e-01 9.567247e-01 5.724372e-01 5.508773e-01 5.547992e-01 5.840771e-01 5.879385e-01

1556 9.538587e-01 9.538584e-01 9.538638e-01 9.538692e-01 9.567646e-01 5.726709e-01 5.511295e-01 5.550517e-01 5.843102e-01 5.881711e-01

1557 9.539013e-01 9.539009e-01 9.539062e-01 9.539116e-01 9.568044e-01 5.728974e-01 5.513742e-01 5.552965e-01 5.845360e-01 5.883965e-01

1558 9.539436e-01 9.539432e-01 9.539485e-01 9.539538e-01 9.568440e-01 5.731170e-01 5.516116e-01 5.555341e-01 5.847550e-01 5.886150e-01

1559 9.539859e-01 9.539854e-01 9.539906e-01 9.539959e-01 9.568836e-01 5.733299e-01 5.518419e-01 5.557645e-01 5.849672e-01 5.888267e-01

1560 9.540280e-01 9.540275e-01 9.540326e-01 9.540378e-01 9.569230e-01 5.735363e-01 5.520653e-01 5.559880e-01 5.851728e-01 5.890320e-01

1561 9.540701e-01 9.540695e-01 9.540745e-01 9.540796e-01 9.569623e-01 5.737363e-01 5.522820e-01 5.562047e-01 5.853722e-01 5.892310e-01

1562 9.541119e-01 9.541113e-01 9.541163e-01 9.541213e-01 9.570015e-01 5.739303e-01 5.524922e-01 5.564150e-01 5.855655e-01 5.894239e-01

1563 9.541537e-01 9.541531e-01 9.541580e-01 9.541629e-01 9.570406e-01 5.741184e-01 5.526961e-01 5.566190e-01 5.857530e-01 5.896109e-01

1564 9.541954e-01 9.541947e-01 9.541995e-01 9.542044e-01 9.570796e-01 5.743008e-01 5.528940e-01 5.568169e-01 5.859348e-01 5.897924e-01

1565 9.542369e-01 9.542362e-01 9.542409e-01 9.542457e-01 9.571185e-01 5.744778e-01 5.530859e-01 5.570089e-01 5.861111e-01 5.899683e-01

1566 9.542784e-01 9.542776e-01 9.542823e-01 9.542870e-01 9.571573e-01 5.746494e-01 5.532722e-01 5.571952e-01 5.862822e-01 5.901391e-01

1567 9.543197e-01 9.543189e-01 9.543235e-01 9.543281e-01 9.571960e-01 5.748160e-01 5.534530e-01 5.573760e-01 5.864482e-01 5.903047e-01

1568 9.543609e-01 9.543600e-01 9.543646e-01 9.543691e-01 9.572346e-01 5.749776e-01 5.536285e-01 5.575515e-01 5.866094e-01 5.904656e-01

1569 9.544021e-01 9.544011e-01 9.544056e-01 9.544100e-01 9.572731e-01 5.751345e-01 5.537989e-01 5.577219e-01 5.867658e-01 5.906217e-01

1570 9.544431e-01 9.544421e-01 9.544464e-01 9.544508e-01 9.573115e-01 5.752869e-01 5.539643e-01 5.578873e-01 5.869177e-01 5.907733e-01

1571 9.544840e-01 9.544829e-01 9.544872e-01 9.544915e-01 9.573498e-01 5.754349e-01 5.541250e-01 5.580480e-01 5.870653e-01 5.909207e-01

1572 9.545248e-01 9.545237e-01 9.545279e-01 9.545321e-01 9.573881e-01 5.755787e-01 5.542811e-01 5.582041e-01 5.872087e-01 5.910638e-01

1573 9.545655e-01 9.545644e-01 9.545685e-01 9.545726e-01 9.574262e-01 5.757184e-01 5.544328e-01 5.583558e-01 5.873481e-01 5.912030e-01

1574 9.546062e-01 9.546050e-01 9.546090e-01 9.546130e-01 9.574642e-01 5.758543e-01 5.545803e-01 5.585033e-01 5.874836e-01 5.913383e-01

1575 9.546467e-01 9.546454e-01 9.546494e-01 9.546533e-01 9.575022e-01 5.759864e-01 5.547236e-01 5.586467e-01 5.876155e-01 5.914699e-01

1576 9.546872e-01 9.546858e-01 9.546896e-01 9.546935e-01 9.575400e-01 5.761150e-01 5.548631e-01 5.587861e-01 5.877438e-01 5.915980e-01

1577 9.547275e-01 9.547261e-01 9.547298e-01 9.547336e-01 9.575778e-01 5.762401e-01 5.549988e-01 5.589218e-01 5.878687e-01 5.917227e-01

1578 9.547678e-01 9.547663e-01 9.547700e-01 9.547736e-01 9.576155e-01 5.763619e-01 5.551309e-01 5.590539e-01 5.879903e-01 5.918441e-01

1579 9.548080e-01 9.548064e-01 9.548100e-01 9.548135e-01 9.576531e-01 5.764805e-01 5.552595e-01 5.591825e-01 5.881088e-01 5.919625e-01

1580 9.548481e-01 9.548465e-01 9.548499e-01 9.548534e-01 9.576906e-01 5.765961e-01 5.553848e-01 5.593078e-01 5.882242e-01 5.920778e-01

1581 9.548881e-01 9.548864e-01 9.548897e-01 9.548931e-01 9.577280e-01 5.767088e-01 5.555068e-01 5.594299e-01 5.883368e-01 5.921903e-01

1582 9.549280e-01 9.549263e-01 9.549295e-01 9.549327e-01 9.577654e-01 5.768187e-01 5.556258e-01 5.595488e-01 5.884467e-01 5.923000e-01

1583 9.549679e-01 9.549660e-01 9.549692e-01 9.549723e-01 9.578027e-01 5.769260e-01 5.557419e-01 5.596649e-01 5.885539e-01 5.924070e-01

1584 9.550076e-01 9.550057e-01 9.550088e-01 9.550118e-01 9.578398e-01 5.770307e-01 5.558551e-01 5.597781e-01 5.886585e-01 5.925116e-01

1585 9.550473e-01 9.550454e-01 9.550483e-01 9.550511e-01 9.578770e-01 5.771329e-01 5.559656e-01 5.598886e-01 5.887607e-01 5.926137e-01

1586 9.550869e-01 9.550849e-01 9.550877e-01 9.550904e-01 9.579140e-01 5.772329e-01 5.560736e-01 5.599966e-01 5.888607e-01 5.927136e-01

1587 9.551265e-01 9.551244e-01 9.551271e-01 9.551297e-01 9.579509e-01 5.773305e-01 5.561791e-01 5.601020e-01 5.889584e-01 5.928112e-01

1588 9.551660e-01 9.551638e-01 9.551663e-01 9.551688e-01 9.579878e-01 5.774261e-01 5.562822e-01 5.602051e-01 5.890539e-01 5.929067e-01

1589 9.552054e-01 9.552031e-01 9.552055e-01 9.552079e-01 9.580246e-01 5.775195e-01 5.563830e-01 5.603059e-01 5.891475e-01 5.930002e-01

1590 9.552447e-01 9.552424e-01 9.552447e-01 9.552468e-01 9.580613e-01 5.776111e-01 5.564816e-01 5.604045e-01 5.892391e-01 5.930917e-01

1591 9.552840e-01 9.552815e-01 9.552837e-01 9.552857e-01 9.580979e-01 5.777007e-01 5.565782e-01 5.605010e-01 5.893288e-01 5.931814e-01

1592 9.553232e-01 9.553207e-01 9.553227e-01 9.553246e-01 9.581345e-01 5.777886e-01 5.566728e-01 5.605956e-01 5.894167e-01 5.932693e-01

1593 9.553623e-01 9.553597e-01 9.553616e-01 9.553633e-01 9.581709e-01 5.778747e-01 5.567655e-01 5.606882e-01 5.895030e-01 5.933555e-01

1594 9.554014e-01 9.553987e-01 9.554004e-01 9.554020e-01 9.582073e-01 5.779593e-01 5.568564e-01 5.607790e-01 5.895876e-01 5.934401e-01

1595 9.554404e-01 9.554376e-01 9.554392e-01 9.554406e-01 9.582436e-01 5.780422e-01 5.569455e-01 5.608681e-01 5.896707e-01 5.935232e-01

1596 9.554794e-01 9.554765e-01 9.554779e-01 9.554791e-01 9.582799e-01 5.781237e-01 5.570330e-01 5.609555e-01 5.897523e-01 5.936048e-01

1597 9.555183e-01 9.555153e-01 9.555165e-01 9.555175e-01 9.583160e-01 5.782038e-01 5.571189e-01 5.610413e-01 5.898325e-01 5.936849e-01

1598 9.555571e-01 9.555540e-01 9.555551e-01 9.555559e-01 9.583521e-01 5.782825e-01 5.572033e-01 5.611256e-01 5.899114e-01 5.937638e-01

1599 9.555959e-01 9.555927e-01 9.555936e-01 9.555942e-01 9.583881e-01 5.783599e-01 5.572863e-01 5.612085e-01 5.899889e-01 5.938413e-01

1600 9.556347e-01 9.556313e-01 9.556320e-01 9.556324e-01 9.584240e-01 5.784361e-01 5.573678e-01 5.612899e-01 5.900652e-01 5.939176e-01

1601 9.556733e-01 9.556699e-01 9.556704e-01 9.556706e-01 9.584598e-01 5.785111e-01 5.574481e-01 5.613701e-01 5.901404e-01 5.939928e-01

1602 9.557120e-01 9.557084e-01 9.557087e-01 9.557086e-01 9.584956e-01 5.785849e-01 5.575272e-01 5.614490e-01 5.902144e-01 5.940668e-01

1603 9.557506e-01 9.557468e-01 9.557469e-01 9.557467e-01 9.585312e-01 5.786578e-01 5.576050e-01 5.615267e-01 5.902874e-01 5.941398e-01

1604 9.557891e-01 9.557852e-01 9.557851e-01 9.557846e-01 9.585668e-01 5.787296e-01 5.576817e-01 5.616032e-01 5.903593e-01 5.942117e-01

1605 9.558276e-01 9.558236e-01 9.558232e-01 9.558225e-01 9.586023e-01 5.788004e-01 5.577573e-01 5.616787e-01 5.904303e-01 5.942827e-01

1606 9.558660e-01 9.558619e-01 9.558613e-01 9.558602e-01 9.586377e-01 5.788703e-01 5.578319e-01 5.617531e-01 5.905004e-01 5.943527e-01

1607 9.559044e-01 9.559001e-01 9.558993e-01 9.558980e-01 9.586730e-01 5.789394e-01 5.579056e-01 5.618265e-01 5.905696e-01 5.944219e-01

1608 9.559428e-01 9.559383e-01 9.559372e-01 9.559356e-01 9.587083e-01 5.790076e-01 5.579783e-01 5.618990e-01 5.906379e-01 5.944902e-01

1609 9.559811e-01 9.559765e-01 9.559751e-01 9.559732e-01 9.587434e-01 5.790750e-01 5.580501e-01 5.619705e-01 5.907055e-01 5.945578e-01

1610 9.560194e-01 9.560146e-01 9.560129e-01 9.560107e-01 9.587784e-01 5.791417e-01 5.581210e-01 5.620413e-01 5.907722e-01 5.946246e-01

1611 9.560576e-01 9.560527e-01 9.560507e-01 9.560481e-01 9.588134e-01 5.792076e-01 5.581912e-01 5.621112e-01 5.908383e-01 5.946906e-01

1612 9.560958e-01 9.560907e-01 9.560884e-01 9.560855e-01 9.588482e-01 5.792729e-01 5.582606e-01 5.621803e-01 5.909037e-01 5.947560e-01

1613 9.561340e-01 9.561286e-01 9.561260e-01 9.561227e-01 9.588829e-01 5.793375e-01 5.583293e-01 5.622487e-01 5.909684e-01 5.948208e-01

1614 9.561721e-01 9.561666e-01 9.561636e-01 9.561599e-01 9.589176e-01 5.794015e-01 5.583974e-01 5.623164e-01 5.910326e-01 5.948849e-01

1615 9.562102e-01 9.562045e-01 9.562011e-01 9.561971e-01 9.589521e-01 5.794650e-01 5.584647e-01 5.623835e-01 5.910961e-01 5.949484e-01

1616 9.562483e-01 9.562423e-01 9.562386e-01 9.562341e-01 9.589865e-01 5.795279e-01 5.585315e-01 5.624499e-01 5.911591e-01 5.950114e-01

1617 9.562863e-01 9.562801e-01 9.562760e-01 9.562711e-01 9.590208e-01 5.795902e-01 5.585977e-01 5.625157e-01 5.912215e-01 5.950738e-01

1618 9.563243e-01 9.563179e-01 9.563134e-01 9.563080e-01 9.590549e-01 5.796521e-01 5.586633e-01 5.625809e-01 5.912835e-01 5.951358e-01

1619 9.563623e-01 9.563556e-01 9.563507e-01 9.563448e-01 9.590890e-01 5.797136e-01 5.587284e-01 5.626457e-01 5.913450e-01 5.951972e-01

1620 9.564003e-01 9.563933e-01 9.563879e-01 9.563815e-01 9.591229e-01 5.797746e-01 5.587931e-01 5.627099e-01 5.914060e-01 5.952582e-01

1621 9.564382e-01 9.564310e-01 9.564251e-01 9.564181e-01 9.591567e-01 5.798351e-01 5.588572e-01 5.627736e-01 5.914667e-01 5.953188e-01

1622 9.565487e-01 9.565411e-01 9.565347e-01 9.565273e-01 9.592516e-01 5.799743e-01 5.589732e-01 5.628956e-01 5.916280e-01 5.954883e-01

1623 9.566555e-01 9.566475e-01 9.566407e-01 9.566330e-01 9.593435e-01 5.801194e-01 5.590941e-01 5.630228e-01 5.917961e-01 5.956647e-01

1624 9.567589e-01 9.567505e-01 9.567433e-01 9.567352e-01 9.594325e-01 5.802705e-01 5.592202e-01 5.631554e-01 5.919711e-01 5.958483e-01

1625 9.568589e-01 9.568501e-01 9.568427e-01 9.568342e-01 9.595188e-01 5.804278e-01 5.593516e-01 5.632936e-01 5.921531e-01 5.960392e-01

1626 9.569559e-01 9.569467e-01 9.569389e-01 9.569301e-01 9.596025e-01 5.805915e-01 5.594885e-01 5.634375e-01 5.923425e-01 5.962378e-01

1627 9.570498e-01 9.570403e-01 9.570322e-01 9.570230e-01 9.596837e-01 5.807618e-01 5.596311e-01 5.635874e-01 5.925393e-01 5.964442e-01

1628 9.571409e-01 9.571311e-01 9.571227e-01 9.571132e-01 9.597626e-01 5.809389e-01 5.597795e-01 5.637434e-01 5.927439e-01 5.966587e-01

1629 9.572293e-01 9.572192e-01 9.572105e-01 9.572008e-01 9.598393e-01 5.811230e-01 5.599339e-01 5.639057e-01 5.929563e-01 5.968813e-01

1630 9.573152e-01 9.573048e-01 9.572958e-01 9.572858e-01 9.599139e-01 5.813142e-01 5.600945e-01 5.640744e-01 5.931768e-01 5.971124e-01

1631 9.573987e-01 9.573880e-01 9.573787e-01 9.573685e-01 9.599865e-01 5.815128e-01 5.602615e-01 5.642498e-01 5.934057e-01 5.973520e-01

1632 9.574798e-01 9.574689e-01 9.574594e-01 9.574488e-01 9.600572e-01 5.817188e-01 5.604350e-01 5.644321e-01 5.936429e-01 5.976005e-01

1633 9.575588e-01 9.575476e-01 9.575378e-01 9.575271e-01 9.601260e-01 5.819326e-01 5.606153e-01 5.646213e-01 5.938889e-01 5.978579e-01

1634 9.576357e-01 9.576242e-01 9.576142e-01 9.576032e-01 9.601932e-01 5.821542e-01 5.608024e-01 5.648178e-01 5.941436e-01 5.981246e-01

1635 9.577106e-01 9.576989e-01 9.576887e-01 9.576775e-01 9.602587e-01 5.823839e-01 5.609966e-01 5.650216e-01 5.944074e-01 5.984005e-01

1636 9.577836e-01 9.577717e-01 9.577613e-01 9.577498e-01 9.603227e-01 5.826218e-01 5.611980e-01 5.652329e-01 5.946803e-01 5.986859e-01

1637 9.578548e-01 9.578427e-01 9.578321e-01 9.578204e-01 9.603851e-01 5.828680e-01 5.614069e-01 5.654519e-01 5.949626e-01 5.989810e-01

1638 9.579243e-01 9.579120e-01 9.579012e-01 9.578894e-01 9.604462e-01 5.831228e-01 5.616232e-01 5.656788e-01 5.952543e-01 5.992858e-01

1639 9.579922e-01 9.579797e-01 9.579687e-01 9.579567e-01 9.605059e-01 5.833862e-01 5.618473e-01 5.659137e-01 5.955556e-01 5.996005e-01

1640 9.580585e-01 9.580459e-01 9.580347e-01 9.580225e-01 9.605644e-01 5.836584e-01 5.620793e-01 5.661568e-01 5.958666e-01 5.999253e-01

1641 9.581234e-01 9.581106e-01 9.580993e-01 9.580869e-01 9.606216e-01 5.839395e-01 5.623192e-01 5.664082e-01 5.961874e-01 6.002602e-01

1642 9.581869e-01 9.581739e-01 9.581624e-01 9.581499e-01 9.606777e-01 5.842296e-01 5.625673e-01 5.666680e-01 5.965181e-01 6.006052e-01

1643 9.582490e-01 9.582359e-01 9.582242e-01 9.582115e-01 9.607326e-01 5.845288e-01 5.628237e-01 5.669364e-01 5.968588e-01 6.009605e-01

1644 9.583099e-01 9.582966e-01 9.582848e-01 9.582720e-01 9.607865e-01 5.848373e-01 5.630884e-01 5.672135e-01 5.972096e-01 6.013262e-01

1645 9.583696e-01 9.583562e-01 9.583442e-01 9.583312e-01 9.608395e-01 5.851550e-01 5.633616e-01 5.674993e-01 5.975704e-01 6.017022e-01

1646 9.584281e-01 9.584145e-01 9.584024e-01 9.583893e-01 9.608914e-01 5.854820e-01 5.636434e-01 5.677941e-01 5.979414e-01 6.020885e-01

1647 9.584855e-01 9.584718e-01 9.584595e-01 9.584463e-01 9.609425e-01 5.858184e-01 5.639339e-01 5.680978e-01 5.983225e-01 6.024852e-01

1648 9.585418e-01 9.585280e-01 9.585156e-01 9.585023e-01 9.609926e-01 5.861643e-01 5.642332e-01 5.684105e-01 5.987136e-01 6.028922e-01

1649 9.585972e-01 9.585833e-01 9.585708e-01 9.585572e-01 9.610420e-01 5.865195e-01 5.645412e-01 5.687323e-01 5.991149e-01 6.033095e-01

1650 9.586516e-01 9.586375e-01 9.586249e-01 9.586113e-01 9.610905e-01 5.868841e-01 5.648581e-01 5.690632e-01 5.995261e-01 6.037369e-01

1651 9.587051e-01 9.586909e-01 9.586782e-01 9.586644e-01 9.611383e-01 5.872581e-01 5.651839e-01 5.694033e-01 5.999473e-01 6.041744e-01

1652 9.587577e-01 9.587434e-01 9.587306e-01 9.587167e-01 9.611854e-01 5.876413e-01 5.655186e-01 5.697525e-01 6.003782e-01 6.046219e-01

1653 9.588095e-01 9.587951e-01 9.587821e-01 9.587682e-01 9.612318e-01 5.880338e-01 5.658622e-01 5.701108e-01 6.008188e-01 6.050791e-01

1654 9.588604e-01 9.588460e-01 9.588329e-01 9.588188e-01 9.612775e-01 5.884354e-01 5.662147e-01 5.704781e-01 6.012690e-01 6.055459e-01

1655 9.589107e-01 9.588961e-01 9.588830e-01 9.588688e-01 9.613226e-01 5.888461e-01 5.665760e-01 5.708545e-01 6.017285e-01 6.060222e-01

1656 9.589602e-01 9.589455e-01 9.589323e-01 9.589180e-01 9.613670e-01 5.892656e-01 5.669460e-01 5.712399e-01 6.021971e-01 6.065076e-01

1657 9.590090e-01 9.589942e-01 9.589809e-01 9.589665e-01 9.614109e-01 5.896938e-01 5.673247e-01 5.716341e-01 6.026745e-01 6.070018e-01

1658 9.590571e-01 9.590423e-01 9.590289e-01 9.590144e-01 9.614543e-01 5.901304e-01 5.677121e-01 5.720370e-01 6.031606e-01 6.075047e-01

1659 9.591046e-01 9.590897e-01 9.590762e-01 9.590616e-01 9.614971e-01 5.905754e-01 5.681078e-01 5.724484e-01 6.036550e-01 6.080159e-01

1660 9.591515e-01 9.591365e-01 9.591229e-01 9.591083e-01 9.615394e-01 5.910284e-01 5.685119e-01 5.728683e-01 6.041575e-01 6.085351e-01

1661 9.591978e-01 9.591828e-01 9.591691e-01 9.591543e-01 9.615813e-01 5.914892e-01 5.689241e-01 5.732964e-01 6.046675e-01 6.090618e-01

1662 9.592436e-01 9.592285e-01 9.592147e-01 9.591999e-01 9.616226e-01 5.919575e-01 5.693443e-01 5.737325e-01 6.051849e-01 6.095957e-01

1663 9.592888e-01 9.592736e-01 9.592597e-01 9.592448e-01 9.616635e-01 5.924330e-01 5.697722e-01 5.741764e-01 6.057092e-01 6.101364e-01

1664 9.593335e-01 9.593182e-01 9.593043e-01 9.592893e-01 9.617040e-01 5.929153e-01 5.702076e-01 5.746278e-01 6.062400e-01 6.106833e-01

1665 9.593777e-01 9.593624e-01 9.593484e-01 9.593333e-01 9.617441e-01 5.934040e-01 5.706502e-01 5.750864e-01 6.067769e-01 6.112362e-01

1666 9.594215e-01 9.594061e-01 9.593920e-01 9.593768e-01 9.617838e-01 5.938989e-01 5.710998e-01 5.755519e-01 6.073194e-01 6.117944e-01

1667 9.594648e-01 9.594493e-01 9.594351e-01 9.594199e-01 9.618231e-01 5.943995e-01 5.715560e-01 5.760241e-01 6.078669e-01 6.123576e-01

1668 9.595076e-01 9.594921e-01 9.594778e-01 9.594626e-01 9.618621e-01 5.949053e-01 5.720186e-01 5.765024e-01 6.084191e-01 6.129250e-01

1669 9.595501e-01 9.595345e-01 9.595202e-01 9.595048e-01 9.619007e-01 5.954160e-01 5.724871e-01 5.769867e-01 6.089755e-01 6.134963e-01

1670 9.595921e-01 9.595765e-01 9.595621e-01 9.595467e-01 9.619389e-01 5.959310e-01 5.729613e-01 5.774764e-01 6.095354e-01 6.140709e-01

1671 9.596338e-01 9.596181e-01 9.596036e-01 9.595881e-01 9.619769e-01 5.964500e-01 5.734406e-01 5.779712e-01 6.100984e-01 6.146482e-01

1672 9.596751e-01 9.596593e-01 9.596448e-01 9.596292e-01 9.620145e-01 5.969724e-01 5.739248e-01 5.784706e-01 6.106638e-01 6.152276e-01

1673 9.597160e-01 9.597002e-01 9.596856e-01 9.596700e-01 9.620518e-01 5.974977e-01 5.744134e-01 5.789742e-01 6.112313e-01 6.158086e-01

1674 9.597566e-01 9.597407e-01 9.597260e-01 9.597104e-01 9.620889e-01 5.980254e-01 5.749060e-01 5.794816e-01 6.118001e-01 6.163906e-01

1675 9.597968e-01 9.597809e-01 9.597662e-01 9.597504e-01 9.621256e-01 5.985550e-01 5.754020e-01 5.799922e-01 6.123697e-01 6.169730e-01

1676 9.598368e-01 9.598208e-01 9.598060e-01 9.597902e-01 9.621621e-01 5.990859e-01 5.759012e-01 5.805057e-01 6.129396e-01 6.175552e-01

1677 9.598764e-01 9.598604e-01 9.598455e-01 9.598297e-01 9.621984e-01 5.996178e-01 5.764029e-01 5.810214e-01 6.135091e-01 6.181367e-01

1678 9.599157e-01 9.598997e-01 9.598848e-01 9.598688e-01 9.622344e-01 6.001499e-01 5.769067e-01 5.815390e-01 6.140778e-01 6.187168e-01

1679 9.599548e-01 9.599387e-01 9.599237e-01 9.599077e-01 9.622701e-01 6.006818e-01 5.774121e-01 5.820578e-01 6.146450e-01 6.192951e-01

1680 9.599936e-01 9.599774e-01 9.599624e-01 9.599463e-01 9.623056e-01 6.012130e-01 5.779187e-01 5.825774e-01 6.152102e-01 6.198709e-01

1681 9.600321e-01 9.600159e-01 9.600008e-01 9.599847e-01 9.623409e-01 6.017429e-01 5.784258e-01 5.830973e-01 6.157729e-01 6.204437e-01

1682 9.600704e-01 9.600541e-01 9.600390e-01 9.600228e-01 9.623760e-01 6.022711e-01 5.789331e-01 5.836170e-01 6.163325e-01 6.210130e-01

1683 9.601084e-01 9.600921e-01 9.600769e-01 9.600606e-01 9.624109e-01 6.027969e-01 5.794401e-01 5.841360e-01 6.168885e-01 6.215782e-01

1684 9.601462e-01 9.601298e-01 9.601146e-01 9.600983e-01 9.624456e-01 6.033200e-01 5.799462e-01 5.846537e-01 6.174404e-01 6.221388e-01

1685 9.601837e-01 9.601673e-01 9.601520e-01 9.601357e-01 9.624801e-01 6.038398e-01 5.804509e-01 5.851696e-01 6.179877e-01 6.226944e-01

1686 9.602211e-01 9.602046e-01 9.601892e-01 9.601728e-01 9.625144e-01 6.043559e-01 5.809538e-01 5.856834e-01 6.185300e-01 6.232445e-01

1687 9.602582e-01 9.602417e-01 9.602263e-01 9.602098e-01 9.625485e-01 6.048678e-01 5.814544e-01 5.861944e-01 6.190667e-01 6.237887e-01

1688 9.602951e-01 9.602786e-01 9.602631e-01 9.602465e-01 9.625825e-01 6.053750e-01 5.819522e-01 5.867023e-01 6.195976e-01 6.243265e-01

1689 9.603318e-01 9.603152e-01 9.602997e-01 9.602831e-01 9.626162e-01 6.058772e-01 5.824469e-01 5.872065e-01 6.201221e-01 6.248576e-01

1690 9.603683e-01 9.603517e-01 9.603361e-01 9.603195e-01 9.626498e-01 6.063740e-01 5.829378e-01 5.877067e-01 6.206400e-01 6.253816e-01

1691 9.604047e-01 9.603880e-01 9.603723e-01 9.603556e-01 9.626833e-01 6.068649e-01 5.834247e-01 5.882024e-01 6.211508e-01 6.258980e-01

1692 9.604408e-01 9.604241e-01 9.604084e-01 9.603916e-01 9.627166e-01 6.073497e-01 5.839071e-01 5.886932e-01 6.216542e-01 6.264068e-01

1693 9.604768e-01 9.604601e-01 9.604443e-01 9.604274e-01 9.627498e-01 6.078279e-01 5.843846e-01 5.891788e-01 6.221499e-01 6.269074e-01

1694 9.605126e-01 9.604958e-01 9.604800e-01 9.604631e-01 9.627828e-01 6.082993e-01 5.848569e-01 5.896587e-01 6.226377e-01 6.273997e-01

1695 9.605483e-01 9.605314e-01 9.605155e-01 9.604986e-01 9.628156e-01 6.087636e-01 5.853236e-01 5.901326e-01 6.231173e-01 6.278835e-01

1696 9.605837e-01 9.605669e-01 9.605509e-01 9.605339e-01 9.628484e-01 6.092205e-01 5.857843e-01 5.906002e-01 6.235884e-01 6.283585e-01

1697 9.606191e-01 9.606021e-01 9.605861e-01 9.605691e-01 9.628809e-01 6.096698e-01 5.862388e-01 5.910612e-01 6.240509e-01 6.288245e-01

1698 9.606542e-01 9.606373e-01 9.606212e-01 9.606041e-01 9.629134e-01 6.101112e-01 5.866868e-01 5.915154e-01 6.245046e-01 6.292814e-01

1699 9.606893e-01 9.606723e-01 9.606561e-01 9.606390e-01 9.629458e-01 6.105447e-01 5.871281e-01 5.919624e-01 6.249493e-01 6.297290e-01

1700 9.607242e-01 9.607071e-01 9.606909e-01 9.606737e-01 9.629780e-01 6.109699e-01 5.875623e-01 5.924020e-01 6.253849e-01 6.301673e-01

1701 9.607589e-01 9.607418e-01 9.607256e-01 9.607083e-01 9.630101e-01 6.113868e-01 5.879892e-01 5.928341e-01 6.258114e-01 6.305962e-01

1702 9.607935e-01 9.607764e-01 9.607601e-01 9.607427e-01 9.630421e-01 6.117953e-01 5.884087e-01 5.932584e-01 6.262286e-01 6.310155e-01

1703 9.608280e-01 9.608109e-01 9.607945e-01 9.607771e-01 9.630739e-01 6.121952e-01 5.888206e-01 5.936748e-01 6.266365e-01 6.314253e-01

1704 9.608624e-01 9.608452e-01 9.608287e-01 9.608112e-01 9.631057e-01 6.125864e-01 5.892248e-01 5.940832e-01 6.270351e-01 6.318256e-01

1705 9.608966e-01 9.608794e-01 9.608629e-01 9.608453e-01 9.631374e-01 6.129690e-01 5.896210e-01 5.944834e-01 6.274244e-01 6.322163e-01

1706 9.609308e-01 9.609135e-01 9.608969e-01 9.608793e-01 9.631689e-01 6.133429e-01 5.900093e-01 5.948753e-01 6.278042e-01 6.325974e-01

1707 9.609648e-01 9.609474e-01 9.609308e-01 9.609131e-01 9.632004e-01 6.137081e-01 5.903894e-01 5.952588e-01 6.281748e-01 6.329691e-01

1708 9.609987e-01 9.609813e-01 9.609646e-01 9.609468e-01 9.632317e-01 6.140645e-01 5.907615e-01 5.956340e-01 6.285361e-01 6.333313e-01

1709 9.610325e-01 9.610150e-01 9.609983e-01 9.609804e-01 9.632630e-01 6.144123e-01 5.911253e-01 5.960008e-01 6.288882e-01 6.336842e-01

1710 9.610661e-01 9.610487e-01 9.610318e-01 9.610140e-01 9.632942e-01 6.147514e-01 5.914809e-01 5.963591e-01 6.292312e-01 6.340278e-01

1711 9.610997e-01 9.610822e-01 9.610653e-01 9.610474e-01 9.633253e-01 6.150818e-01 5.918282e-01 5.967090e-01 6.295651e-01 6.343623e-01

1712 9.611332e-01 9.611156e-01 9.610987e-01 9.610806e-01 9.633563e-01 6.154038e-01 5.921673e-01 5.970504e-01 6.298901e-01 6.346877e-01

1713 9.611666e-01 9.611490e-01 9.611319e-01 9.611138e-01 9.633872e-01 6.157172e-01 5.924982e-01 5.973835e-01 6.302062e-01 6.350042e-01

1714 9.611999e-01 9.611822e-01 9.611651e-01 9.611469e-01 9.634180e-01 6.160224e-01 5.928210e-01 5.977083e-01 6.305137e-01 6.353118e-01

1715 9.612331e-01 9.612154e-01 9.611982e-01 9.611799e-01 9.634487e-01 6.163192e-01 5.931356e-01 5.980248e-01 6.308126e-01 6.356109e-01

1716 9.612662e-01 9.612484e-01 9.612312e-01 9.612128e-01 9.634794e-01 6.166079e-01 5.934422e-01 5.983331e-01 6.311030e-01 6.359014e-01

1717 9.612992e-01 9.612814e-01 9.612641e-01 9.612457e-01 9.635099e-01 6.168886e-01 5.937409e-01 5.986332e-01 6.313853e-01 6.361836e-01

1718 9.613321e-01 9.613143e-01 9.612969e-01 9.612784e-01 9.635404e-01 6.171615e-01 5.940316e-01 5.989254e-01 6.316594e-01 6.364577e-01

1719 9.613650e-01 9.613471e-01 9.613296e-01 9.613110e-01 9.635709e-01 6.174266e-01 5.943146e-01 5.992098e-01 6.319255e-01 6.367238e-01

1720 9.613977e-01 9.613798e-01 9.613622e-01 9.613436e-01 9.636012e-01 6.176841e-01 5.945899e-01 5.994863e-01 6.321839e-01 6.369820e-01

1721 9.614304e-01 9.614124e-01 9.613948e-01 9.613761e-01 9.636315e-01 6.179341e-01 5.948577e-01 5.997552e-01 6.324348e-01 6.372326e-01

1722 9.614630e-01 9.614450e-01 9.614273e-01 9.614085e-01 9.636617e-01 6.181769e-01 5.951181e-01 6.000166e-01 6.326781e-01 6.374758e-01

1723 9.614956e-01 9.614774e-01 9.614597e-01 9.614408e-01 9.636918e-01 6.184126e-01 5.953712e-01 6.002707e-01 6.329143e-01 6.377117e-01

1724 9.615280e-01 9.615099e-01 9.614920e-01 9.614730e-01 9.637219e-01 6.186413e-01 5.956171e-01 6.005175e-01 6.331434e-01 6.379406e-01

1725 9.615604e-01 9.615422e-01 9.615242e-01 9.615052e-01 9.637519e-01 6.188633e-01 5.958561e-01 6.007573e-01 6.333656e-01 6.381625e-01

1726 9.615928e-01 9.615745e-01 9.615564e-01 9.615372e-01 9.637818e-01 6.190786e-01 5.960882e-01 6.009901e-01 6.335812e-01 6.383778e-01

1727 9.616250e-01 9.616067e-01 9.615885e-01 9.615693e-01 9.638116e-01 6.192876e-01 5.963136e-01 6.012163e-01 6.337902e-01 6.385865e-01

1728 9.616572e-01 9.616388e-01 9.616206e-01 9.616012e-01 9.638414e-01 6.194902e-01 5.965325e-01 6.014358e-01 6.339930e-01 6.387890e-01

1729 9.616893e-01 9.616709e-01 9.616525e-01 9.616331e-01 9.638712e-01 6.196868e-01 5.967451e-01 6.016489e-01 6.341896e-01 6.389853e-01

1730 9.617214e-01 9.617029e-01 9.616844e-01 9.616649e-01 9.639008e-01 6.198775e-01 5.969514e-01 6.018558e-01 6.343803e-01 6.391756e-01

1731 9.617534e-01 9.617348e-01 9.617163e-01 9.616966e-01 9.639304e-01 6.200624e-01 5.971517e-01 6.020566e-01 6.345652e-01 6.393602e-01

1732 9.617854e-01 9.617667e-01 9.617481e-01 9.617283e-01 9.639600e-01 6.202418e-01 5.973461e-01 6.022515e-01 6.347445e-01 6.395392e-01

1733 9.618173e-01 9.617985e-01 9.617798e-01 9.617599e-01 9.639895e-01 6.204157e-01 5.975347e-01 6.024406e-01 6.349184e-01 6.397128e-01

1734 9.618491e-01 9.618303e-01 9.618114e-01 9.617914e-01 9.640189e-01 6.205845e-01 5.977179e-01 6.026242e-01 6.350871e-01 6.398812e-01

1735 9.618809e-01 9.618620e-01 9.618430e-01 9.618229e-01 9.640482e-01 6.207481e-01 5.978956e-01 6.028023e-01 6.352507e-01 6.400446e-01

1736 9.619126e-01 9.618936e-01 9.618746e-01 9.618543e-01 9.640775e-01 6.209069e-01 5.980681e-01 6.029752e-01 6.354095e-01 6.402030e-01

1737 9.619443e-01 9.619252e-01 9.619060e-01 9.618856e-01 9.641068e-01 6.210610e-01 5.982355e-01 6.031430e-01 6.355635e-01 6.403568e-01

1738 9.619759e-01 9.619568e-01 9.619375e-01 9.619169e-01 9.641360e-01 6.212105e-01 5.983981e-01 6.033059e-01 6.357129e-01 6.405059e-01

1739 9.620075e-01 9.619883e-01 9.619688e-01 9.619482e-01 9.641651e-01 6.213555e-01 5.985558e-01 6.034640e-01 6.358579e-01 6.406507e-01

1740 9.620390e-01 9.620197e-01 9.620002e-01 9.619793e-01 9.641942e-01 6.214963e-01 5.987090e-01 6.036175e-01 6.359987e-01 6.407913e-01

1741 9.620705e-01 9.620511e-01 9.620314e-01 9.620105e-01 9.642232e-01 6.216330e-01 5.988578e-01 6.037666e-01 6.361354e-01 6.409278e-01

1742 9.621020e-01 9.620825e-01 9.620626e-01 9.620415e-01 9.642521e-01 6.217658e-01 5.990022e-01 6.039113e-01 6.362682e-01 6.410603e-01

1743 9.621334e-01 9.621138e-01 9.620938e-01 9.620725e-01 9.642810e-01 6.218947e-01 5.991425e-01 6.040519e-01 6.363971e-01 6.411891e-01

1744 9.621647e-01 9.621451e-01 9.621249e-01 9.621035e-01 9.643098e-01 6.220199e-01 5.992788e-01 6.041885e-01 6.365224e-01 6.413142e-01

1745 9.621961e-01 9.621763e-01 9.621560e-01 9.621344e-01 9.643386e-01 6.221415e-01 5.994112e-01 6.043212e-01 6.366441e-01 6.414358e-01

1746 9.622273e-01 9.622075e-01 9.621870e-01 9.621652e-01 9.643673e-01 6.222598e-01 5.995400e-01 6.044502e-01 6.367625e-01 6.415540e-01

1747 9.622586e-01 9.622386e-01 9.622180e-01 9.621960e-01 9.643960e-01 6.223748e-01 5.996651e-01 6.045755e-01 6.368776e-01 6.416689e-01

1748 9.622898e-01 9.622697e-01 9.622489e-01 9.622267e-01 9.644246e-01 6.224866e-01 5.997868e-01 6.046975e-01 6.369895e-01 6.417808e-01

1749 9.623210e-01 9.623008e-01 9.622798e-01 9.622574e-01 9.644531e-01 6.225953e-01 5.999051e-01 6.048160e-01 6.370985e-01 6.418896e-01

1750 9.623521e-01 9.623318e-01 9.623106e-01 9.622880e-01 9.644816e-01 6.227012e-01 6.000202e-01 6.049314e-01 6.372045e-01 6.419955e-01

1751 9.623832e-01 9.623628e-01 9.623414e-01 9.623186e-01 9.645100e-01 6.228042e-01 6.001323e-01 6.050437e-01 6.373077e-01 6.420987e-01

1752 9.624143e-01 9.623938e-01 9.623721e-01 9.623491e-01 9.645384e-01 6.229045e-01 6.002414e-01 6.051530e-01 6.374083e-01 6.421991e-01

1753 9.624453e-01 9.624247e-01 9.624028e-01 9.623796e-01 9.645667e-01 6.230023e-01 6.003476e-01 6.052595e-01 6.375063e-01 6.422971e-01

1754 9.624764e-01 9.624556e-01 9.624335e-01 9.624100e-01 9.645949e-01 6.230975e-01 6.004511e-01 6.053632e-01 6.376018e-01 6.423925e-01

1755 9.625073e-01 9.624864e-01 9.624641e-01 9.624404e-01 9.646231e-01 6.231904e-01 6.005520e-01 6.054643e-01 6.376950e-01 6.424856e-01

1756 9.625383e-01 9.625172e-01 9.624946e-01 9.624707e-01 9.646512e-01 6.232810e-01 6.006503e-01 6.055628e-01 6.377859e-01 6.425765e-01

1757 9.625692e-01 9.625480e-01 9.625252e-01 9.625009e-01 9.646792e-01 6.233694e-01 6.007463e-01 6.056589e-01 6.378746e-01 6.426652e-01

1758 9.626002e-01 9.625788e-01 9.625556e-01 9.625311e-01 9.647072e-01 6.234557e-01 6.008399e-01 6.057527e-01 6.379612e-01 6.427518e-01

1759 9.626310e-01 9.626095e-01 9.625861e-01 9.625613e-01 9.647351e-01 6.235399e-01 6.009312e-01 6.058443e-01 6.380458e-01 6.428363e-01

1760 9.626619e-01 9.626402e-01 9.626165e-01 9.625914e-01 9.647629e-01 6.236223e-01 6.010205e-01 6.059337e-01 6.381285e-01 6.429190e-01

1761 9.626928e-01 9.626708e-01 9.626468e-01 9.626214e-01 9.647906e-01 6.237027e-01 6.011076e-01 6.060210e-01 6.382093e-01 6.429999e-01

1762 9.627236e-01 9.627015e-01 9.626771e-01 9.626514e-01 9.648183e-01 6.237815e-01 6.011928e-01 6.061064e-01 6.382884e-01 6.430790e-01

1763 9.627544e-01 9.627321e-01 9.627074e-01 9.626813e-01 9.648459e-01 6.238585e-01 6.012761e-01 6.061898e-01 6.383658e-01 6.431564e-01

1764 9.627852e-01 9.627627e-01 9.627376e-01 9.627112e-01 9.648734e-01 6.239339e-01 6.013577e-01 6.062715e-01 6.384416e-01 6.432322e-01

1765 9.628159e-01 9.627932e-01 9.627678e-01 9.627410e-01 9.649008e-01 6.240077e-01 6.014374e-01 6.063514e-01 6.385159e-01 6.433064e-01

1766 9.628467e-01 9.628238e-01 9.627979e-01 9.627707e-01 9.649281e-01 6.240800e-01 6.015156e-01 6.064297e-01 6.385886e-01 6.433792e-01

1767 9.628774e-01 9.628543e-01 9.628280e-01 9.628004e-01 9.649554e-01 6.241510e-01 6.015921e-01 6.065064e-01 6.386599e-01 6.434506e-01

1768 9.629081e-01 9.628847e-01 9.628581e-01 9.628300e-01 9.649825e-01 6.242206e-01 6.016672e-01 6.065815e-01 6.387299e-01 6.435206e-01

1769 9.629389e-01 9.629152e-01 9.628881e-01 9.628596e-01 9.650096e-01 6.242888e-01 6.017407e-01 6.066552e-01 6.387986e-01 6.435893e-01

1770 9.629695e-01 9.629456e-01 9.629180e-01 9.628890e-01 9.650366e-01 6.243559e-01 6.018130e-01 6.067275e-01 6.388661e-01 6.436568e-01

1771 9.630002e-01 9.629760e-01 9.629479e-01 9.629185e-01 9.650634e-01 6.244217e-01 6.018838e-01 6.067985e-01 6.389323e-01 6.437231e-01

1772 9.630309e-01 9.630064e-01 9.629778e-01 9.629478e-01 9.650902e-01 6.244864e-01 6.019535e-01 6.068682e-01 6.389975e-01 6.437883e-01

1773 9.630616e-01 9.630368e-01 9.630076e-01 9.629771e-01 9.651168e-01 6.245501e-01 6.020219e-01 6.069366e-01 6.390615e-01 6.438524e-01

1774 9.630922e-01 9.630671e-01 9.630373e-01 9.630063e-01 9.651433e-01 6.246127e-01 6.020891e-01 6.070039e-01 6.391245e-01 6.439155e-01

1775 9.631229e-01 9.630974e-01 9.630670e-01 9.630354e-01 9.651697e-01 6.246743e-01 6.021553e-01 6.070701e-01 6.391866e-01 6.439776e-01

1776 9.631535e-01 9.631277e-01 9.630967e-01 9.630644e-01 9.651960e-01 6.247350e-01 6.022204e-01 6.071353e-01 6.392477e-01 6.440387e-01

1777 9.631841e-01 9.631579e-01 9.631263e-01 9.630934e-01 9.652222e-01 6.247948e-01 6.022845e-01 6.071994e-01 6.393079e-01 6.440989e-01

1778 9.632148e-01 9.631882e-01 9.631558e-01 9.631222e-01 9.652482e-01 6.248537e-01 6.023477e-01 6.072626e-01 6.393672e-01 6.441583e-01

1779 9.632454e-01 9.632184e-01 9.631853e-01 9.631510e-01 9.652740e-01 6.249118e-01 6.024099e-01 6.073248e-01 6.394257e-01 6.442169e-01

1780 9.632760e-01 9.632486e-01 9.632147e-01 9.631797e-01 9.652998e-01 6.249692e-01 6.024713e-01 6.073862e-01 6.394835e-01 6.442747e-01

1781 9.633066e-01 9.632787e-01 9.632440e-01 9.632083e-01 9.653253e-01 6.250258e-01 6.025319e-01 6.074468e-01 6.395405e-01 6.443318e-01

1782 9.633372e-01 9.633088e-01 9.632733e-01 9.632368e-01 9.653507e-01 6.250818e-01 6.025917e-01 6.075065e-01 6.395968e-01 6.443881e-01

1783 9.633678e-01 9.633390e-01 9.633025e-01 9.632651e-01 9.653760e-01 6.251370e-01 6.026507e-01 6.075655e-01 6.396524e-01 6.444438e-01

1784 9.633984e-01 9.633690e-01 9.633316e-01 9.632934e-01 9.654011e-01 6.251917e-01 6.027090e-01 6.076238e-01 6.397074e-01 6.444989e-01

1785 9.634290e-01 9.633991e-01 9.633606e-01 9.633215e-01 9.654260e-01 6.252457e-01 6.027667e-01 6.076813e-01 6.397618e-01 6.445533e-01

1786 9.634596e-01 9.634291e-01 9.633896e-01 9.633496e-01 9.654507e-01 6.252992e-01 6.028237e-01 6.077383e-01 6.398156e-01 6.446072e-01

1787 9.634902e-01 9.634591e-01 9.634185e-01 9.633775e-01 9.654752e-01 6.253521e-01 6.028801e-01 6.077946e-01 6.398689e-01 6.446605e-01

1788 9.635208e-01 9.634890e-01 9.634473e-01 9.634052e-01 9.654995e-01 6.254046e-01 6.029359e-01 6.078503e-01 6.399216e-01 6.447133e-01

1789 9.635514e-01 9.635190e-01 9.634760e-01 9.634329e-01 9.655235e-01 6.254565e-01 6.029912e-01 6.079055e-01 6.399739e-01 6.447656e-01

1790 9.635821e-01 9.635488e-01 9.635046e-01 9.634604e-01 9.655474e-01 6.255080e-01 6.030460e-01 6.079601e-01 6.400257e-01 6.448174e-01

1791 9.636127e-01 9.635787e-01 9.635331e-01 9.634877e-01 9.655710e-01 6.255590e-01 6.031003e-01 6.080143e-01 6.400770e-01 6.448688e-01

1792 9.636433e-01 9.636085e-01 9.635615e-01 9.635149e-01 9.655944e-01 6.256097e-01 6.031541e-01 6.080679e-01 6.401280e-01 6.449197e-01

1793 9.636739e-01 9.636383e-01 9.635898e-01 9.635419e-01 9.656175e-01 6.256600e-01 6.032075e-01 6.081212e-01 6.401785e-01 6.449703e-01

1794 9.637045e-01 9.636680e-01 9.636180e-01 9.635688e-01 9.656404e-01 6.257099e-01 6.032605e-01 6.081740e-01 6.402287e-01 6.450205e-01

1795 9.637351e-01 9.636977e-01 9.636461e-01 9.635954e-01 9.656629e-01 6.257595e-01 6.033131e-01 6.082264e-01 6.402785e-01 6.450703e-01

1796 9.637657e-01 9.637273e-01 9.636740e-01 9.636219e-01 9.656852e-01 6.258087e-01 6.033653e-01 6.082784e-01 6.403280e-01 6.451198e-01

1797 9.637963e-01 9.637569e-01 9.637018e-01 9.636482e-01 9.657072e-01 6.258577e-01 6.034172e-01 6.083301e-01 6.403771e-01 6.451690e-01

1798 9.638270e-01 9.637864e-01 9.637294e-01 9.636742e-01 9.657289e-01 6.259063e-01 6.034688e-01 6.083814e-01 6.404260e-01 6.452179e-01

1799 9.638576e-01 9.638159e-01 9.637569e-01 9.637001e-01 9.657502e-01 6.259548e-01 6.035201e-01 6.084325e-01 6.404746e-01 6.452666e-01

1800 9.638882e-01 9.638453e-01 9.637843e-01 9.637257e-01 9.657712e-01 6.260029e-01 6.035711e-01 6.084832e-01 6.405230e-01 6.453149e-01

1801 9.639188e-01 9.638747e-01 9.638114e-01 9.637511e-01 9.657918e-01 6.260509e-01 6.036218e-01 6.085337e-01 6.405711e-01 6.453631e-01

1802 9.639743e-01 9.639299e-01 9.638664e-01 9.638059e-01 9.658398e-01 6.262366e-01 6.037768e-01 6.086983e-01 6.407914e-01 6.455958e-01

1803 9.640284e-01 9.639838e-01 9.639201e-01 9.638593e-01 9.658867e-01 6.264288e-01 6.039376e-01 6.088688e-01 6.410191e-01 6.458365e-01

1804 9.640812e-01 9.640364e-01 9.639725e-01 9.639115e-01 9.659325e-01 6.266277e-01 6.041041e-01 6.090455e-01 6.412545e-01 6.460851e-01

1805 9.641328e-01 9.640877e-01 9.640237e-01 9.639625e-01 9.659773e-01 6.268334e-01 6.042766e-01 6.092284e-01 6.414978e-01 6.463419e-01

1806 9.641832e-01 9.641380e-01 9.640737e-01 9.640124e-01 9.660212e-01 6.270460e-01 6.044552e-01 6.094178e-01 6.417489e-01 6.466070e-01

1807 9.642325e-01 9.641871e-01 9.641227e-01 9.640612e-01 9.660642e-01 6.272656e-01 6.046399e-01 6.096136e-01 6.420081e-01 6.468804e-01

1808 9.642807e-01 9.642351e-01 9.641706e-01 9.641089e-01 9.661063e-01 6.274925e-01 6.048310e-01 6.098161e-01 6.422754e-01 6.471622e-01

1809 9.643279e-01 9.642822e-01 9.642175e-01 9.641556e-01 9.661476e-01 6.277266e-01 6.050286e-01 6.100253e-01 6.425510e-01 6.474527e-01

1810 9.643742e-01 9.643283e-01 9.642634e-01 9.642014e-01 9.661881e-01 6.279681e-01 6.052327e-01 6.102415e-01 6.428349e-01 6.477517e-01

1811 9.644195e-01 9.643735e-01 9.643085e-01 9.642463e-01 9.662278e-01 6.282171e-01 6.054435e-01 6.104646e-01 6.431271e-01 6.480595e-01

1812 9.644640e-01 9.644178e-01 9.643526e-01 9.642903e-01 9.662669e-01 6.284736e-01 6.056610e-01 6.106948e-01 6.434279e-01 6.483760e-01

1813 9.645076e-01 9.644613e-01 9.643960e-01 9.643335e-01 9.663052e-01 6.287378e-01 6.058855e-01 6.109322e-01 6.437372e-01 6.487014e-01

1814 9.645504e-01 9.645040e-01 9.644385e-01 9.643760e-01 9.663429e-01 6.290096e-01 6.061169e-01 6.111769e-01 6.440550e-01 6.490355e-01

1815 9.645925e-01 9.645459e-01 9.644803e-01 9.644176e-01 9.663800e-01 6.292893e-01 6.063554e-01 6.114290e-01 6.443814e-01 6.493786e-01

1816 9.646338e-01 9.645871e-01 9.645214e-01 9.644586e-01 9.664164e-01 6.295767e-01 6.066010e-01 6.116885e-01 6.447165e-01 6.497304e-01

1817 9.646744e-01 9.646276e-01 9.645618e-01 9.644988e-01 9.664523e-01 6.298719e-01 6.068539e-01 6.119555e-01 6.450601e-01 6.500911e-01

1818 9.647144e-01 9.646674e-01 9.646015e-01 9.645384e-01 9.664877e-01 6.301750e-01 6.071140e-01 6.122301e-01 6.454123e-01 6.504605e-01

1819 9.647537e-01 9.647066e-01 9.646406e-01 9.645774e-01 9.665225e-01 6.304859e-01 6.073814e-01 6.125122e-01 6.457730e-01 6.508387e-01

1820 9.647924e-01 9.647452e-01 9.646791e-01 9.646158e-01 9.665569e-01 6.308047e-01 6.076562e-01 6.128020e-01 6.461421e-01 6.512255e-01

1821 9.648305e-01 9.647833e-01 9.647170e-01 9.646536e-01 9.665907e-01 6.311313e-01 6.079383e-01 6.130994e-01 6.465197e-01 6.516209e-01

1822 9.648681e-01 9.648207e-01 9.647544e-01 9.646909e-01 9.666241e-01 6.314656e-01 6.082279e-01 6.134044e-01 6.469055e-01 6.520247e-01

1823 9.649051e-01 9.648577e-01 9.647912e-01 9.647277e-01 9.666571e-01 6.318077e-01 6.085248e-01 6.137171e-01 6.472996e-01 6.524368e-01

1824 9.649417e-01 9.648941e-01 9.648276e-01 9.647639e-01 9.666897e-01 6.321574e-01 6.088291e-01 6.140373e-01 6.477016e-01 6.528570e-01

1825 9.649777e-01 9.649301e-01 9.648634e-01 9.647997e-01 9.667218e-01 6.325146e-01 6.091407e-01 6.143651e-01 6.481116e-01 6.532851e-01

1826 9.650133e-01 9.649656e-01 9.648988e-01 9.648350e-01 9.667536e-01 6.328792e-01 6.094596e-01 6.147004e-01 6.485293e-01 6.537211e-01

1827 9.650484e-01 9.650006e-01 9.649338e-01 9.648698e-01 9.667850e-01 6.332512e-01 6.097858e-01 6.150432e-01 6.489545e-01 6.541645e-01

1828 9.650831e-01 9.650352e-01 9.649683e-01 9.649043e-01 9.668161e-01 6.336302e-01 6.101192e-01 6.153932e-01 6.493870e-01 6.546153e-01

1829 9.651174e-01 9.650695e-01 9.650025e-01 9.649383e-01 9.668468e-01 6.340163e-01 6.104596e-01 6.157505e-01 6.498265e-01 6.550731e-01

1830 9.651513e-01 9.651033e-01 9.650362e-01 9.649720e-01 9.668772e-01 6.344092e-01 6.108070e-01 6.161148e-01 6.502729e-01 6.555376e-01

1831 9.651848e-01 9.651367e-01 9.650696e-01 9.650053e-01 9.669073e-01 6.348086e-01 6.111612e-01 6.164861e-01 6.507257e-01 6.560085e-01

1832 9.652180e-01 9.651698e-01 9.651026e-01 9.650382e-01 9.669371e-01 6.352145e-01 6.115221e-01 6.168642e-01 6.511848e-01 6.564856e-01

1833 9.652508e-01 9.652026e-01 9.651353e-01 9.650708e-01 9.669666e-01 6.356264e-01 6.118896e-01 6.172489e-01 6.516499e-01 6.569684e-01

1834 9.652833e-01 9.652350e-01 9.651676e-01 9.651031e-01 9.669958e-01 6.360443e-01 6.122634e-01 6.176400e-01 6.521204e-01 6.574567e-01

1835 9.653155e-01 9.652671e-01 9.651997e-01 9.651351e-01 9.670248e-01 6.364677e-01 6.126435e-01 6.180373e-01 6.525963e-01 6.579499e-01

1836 9.653474e-01 9.652989e-01 9.652314e-01 9.651667e-01 9.670535e-01 6.368964e-01 6.130294e-01 6.184405e-01 6.530769e-01 6.584479e-01

1837 9.653790e-01 9.653304e-01 9.652628e-01 9.651981e-01 9.670820e-01 6.373301e-01 6.134212e-01 6.188495e-01 6.535621e-01 6.589500e-01

1838 9.654102e-01 9.653617e-01 9.652940e-01 9.652292e-01 9.671103e-01 6.377685e-01 6.138184e-01 6.192639e-01 6.540513e-01 6.594559e-01

1839 9.654413e-01 9.653926e-01 9.653249e-01 9.652600e-01 9.671383e-01 6.382112e-01 6.142208e-01 6.196834e-01 6.545441e-01 6.599652e-01

1840 9.654720e-01 9.654234e-01 9.653555e-01 9.652906e-01 9.671661e-01 6.386579e-01 6.146282e-01 6.201078e-01 6.550402e-01 6.604774e-01

1841 9.655026e-01 9.654538e-01 9.653859e-01 9.653209e-01 9.671937e-01 6.391081e-01 6.150402e-01 6.205368e-01 6.555391e-01 6.609921e-01

1842 9.655329e-01 9.654840e-01 9.654161e-01 9.653510e-01 9.672211e-01 6.395616e-01 6.154566e-01 6.209699e-01 6.560403e-01 6.615088e-01

1843 9.655629e-01 9.655140e-01 9.654460e-01 9.653808e-01 9.672484e-01 6.400179e-01 6.158771e-01 6.214069e-01 6.565435e-01 6.620270e-01

1844 9.655927e-01 9.655438e-01 9.654757e-01 9.654105e-01 9.672754e-01 6.404767e-01 6.163012e-01 6.218475e-01 6.570481e-01 6.625463e-01

1845 9.656224e-01 9.655734e-01 9.655052e-01 9.654399e-01 9.673023e-01 6.409374e-01 6.167287e-01 6.222912e-01 6.575537e-01 6.630661e-01

1846 9.656518e-01 9.656028e-01 9.655345e-01 9.654692e-01 9.673290e-01 6.413998e-01 6.171592e-01 6.227376e-01 6.580597e-01 6.635860e-01

1847 9.656810e-01 9.656319e-01 9.655636e-01 9.654982e-01 9.673555e-01 6.418634e-01 6.175923e-01 6.231864e-01 6.585659e-01 6.641056e-01

1848 9.657100e-01 9.656609e-01 9.655926e-01 9.655270e-01 9.673819e-01 6.423277e-01 6.180277e-01 6.236373e-01 6.590716e-01 6.646243e-01

1849 9.657389e-01 9.656897e-01 9.656213e-01 9.655557e-01 9.674081e-01 6.427924e-01 6.184650e-01 6.240897e-01 6.595765e-01 6.651417e-01

1850 9.657676e-01 9.657183e-01 9.656499e-01 9.655842e-01 9.674342e-01 6.432570e-01 6.189038e-01 6.245433e-01 6.600800e-01 6.656572e-01

1851 9.657961e-01 9.657468e-01 9.656783e-01 9.656125e-01 9.674601e-01 6.437211e-01 6.193436e-01 6.249976e-01 6.605818e-01 6.661706e-01

1852 9.658244e-01 9.657751e-01 9.657065e-01 9.656407e-01 9.674859e-01 6.441842e-01 6.197842e-01 6.254524e-01 6.610813e-01 6.666812e-01

1853 9.658526e-01 9.658032e-01 9.657346e-01 9.656687e-01 9.675116e-01 6.446460e-01 6.202250e-01 6.259071e-01 6.615782e-01 6.671887e-01

1854 9.658807e-01 9.658312e-01 9.657625e-01 9.656966e-01 9.675372e-01 6.451061e-01 6.206658e-01 6.263613e-01 6.620720e-01 6.676926e-01

1855 9.659086e-01 9.658591e-01 9.657903e-01 9.657243e-01 9.675626e-01 6.455640e-01 6.211061e-01 6.268147e-01 6.625623e-01 6.681926e-01

1856 9.659363e-01 9.658868e-01 9.658179e-01 9.657519e-01 9.675879e-01 6.460194e-01 6.215455e-01 6.272669e-01 6.630487e-01 6.686882e-01

1857 9.659640e-01 9.659144e-01 9.658454e-01 9.657793e-01 9.676131e-01 6.464718e-01 6.219837e-01 6.277173e-01 6.635308e-01 6.691791e-01

1858 9.659915e-01 9.659418e-01 9.658728e-01 9.658066e-01 9.676382e-01 6.469209e-01 6.224202e-01 6.281657e-01 6.640083e-01 6.696649e-01

1859 9.660188e-01 9.659692e-01 9.659001e-01 9.658338e-01 9.676632e-01 6.473663e-01 6.228546e-01 6.286117e-01 6.644808e-01 6.701452e-01

1860 9.660461e-01 9.659964e-01 9.659272e-01 9.658609e-01 9.676881e-01 6.478077e-01 6.232867e-01 6.290549e-01 6.649479e-01 6.706197e-01

1861 9.660732e-01 9.660234e-01 9.659542e-01 9.658878e-01 9.677128e-01 6.482447e-01 6.237160e-01 6.294949e-01 6.654094e-01 6.710882e-01

1862 9.661003e-01 9.660504e-01 9.659811e-01 9.659147e-01 9.677375e-01 6.486771e-01 6.241422e-01 6.299314e-01 6.658650e-01 6.715503e-01

1863 9.661272e-01 9.660773e-01 9.660079e-01 9.659414e-01 9.677621e-01 6.491045e-01 6.245649e-01 6.303640e-01 6.663143e-01 6.720057e-01

1864 9.661540e-01 9.661041e-01 9.660346e-01 9.659680e-01 9.677866e-01 6.495266e-01 6.249839e-01 6.307925e-01 6.667572e-01 6.724543e-01

1865 9.661807e-01 9.661307e-01 9.660612e-01 9.659945e-01 9.678110e-01 6.499433e-01 6.253987e-01 6.312164e-01 6.671933e-01 6.728958e-01

1866 9.662073e-01 9.661573e-01 9.660877e-01 9.660210e-01 9.678353e-01 6.503541e-01 6.258092e-01 6.316356e-01 6.676226e-01 6.733300e-01

1867 9.662339e-01 9.661838e-01 9.661141e-01 9.660473e-01 9.678596e-01 6.507589e-01 6.262150e-01 6.320497e-01 6.680447e-01 6.737566e-01

1868 9.662603e-01 9.662101e-01 9.661404e-01 9.660735e-01 9.678838e-01 6.511575e-01 6.266159e-01 6.324585e-01 6.684595e-01 6.741756e-01

1869 9.662866e-01 9.662364e-01 9.661667e-01 9.660997e-01 9.679078e-01 6.515497e-01 6.270116e-01 6.328618e-01 6.688668e-01 6.745869e-01

1870 9.663129e-01 9.662626e-01 9.661928e-01 9.661257e-01 9.679319e-01 6.519353e-01 6.274018e-01 6.332592e-01 6.692665e-01 6.749902e-01

1871 9.663391e-01 9.662888e-01 9.662188e-01 9.661517e-01 9.679558e-01 6.523141e-01 6.277865e-01 6.336507e-01 6.696586e-01 6.753855e-01

1872 9.663652e-01 9.663148e-01 9.662448e-01 9.661776e-01 9.679797e-01 6.526860e-01 6.281652e-01 6.340359e-01 6.700428e-01 6.757727e-01

1873 9.663912e-01 9.663408e-01 9.662707e-01 9.662034e-01 9.680035e-01 6.530510e-01 6.285380e-01 6.344148e-01 6.704191e-01 6.761518e-01

1874 9.664172e-01 9.663667e-01 9.662965e-01 9.662291e-01 9.680272e-01 6.534088e-01 6.289046e-01 6.347872e-01 6.707875e-01 6.765226e-01

1875 9.664431e-01 9.663925e-01 9.663222e-01 9.662548e-01 9.680509e-01 6.537595e-01 6.292648e-01 6.351529e-01 6.711479e-01 6.768852e-01

1876 9.664689e-01 9.664183e-01 9.663479e-01 9.662803e-01 9.680745e-01 6.541028e-01 6.296186e-01 6.355118e-01 6.715003e-01 6.772396e-01

1877 9.664946e-01 9.664440e-01 9.663735e-01 9.663058e-01 9.680980e-01 6.544389e-01 6.299658e-01 6.358639e-01 6.718446e-01 6.775858e-01

1878 9.665203e-01 9.664696e-01 9.663991e-01 9.663313e-01 9.681215e-01 6.547676e-01 6.303063e-01 6.362090e-01 6.721810e-01 6.779237e-01

1879 9.665459e-01 9.664952e-01 9.664245e-01 9.663567e-01 9.681449e-01 6.550890e-01 6.306400e-01 6.365471e-01 6.725093e-01 6.782534e-01

1880 9.665715e-01 9.665207e-01 9.664499e-01 9.663820e-01 9.681683e-01 6.554029e-01 6.309670e-01 6.368781e-01 6.728297e-01 6.785751e-01

1881 9.665970e-01 9.665461e-01 9.664753e-01 9.664072e-01 9.681916e-01 6.557096e-01 6.312870e-01 6.372020e-01 6.731422e-01 6.788886e-01

1882 9.666225e-01 9.665715e-01 9.665006e-01 9.664324e-01 9.682148e-01 6.560089e-01 6.316002e-01 6.375188e-01 6.734469e-01 6.791942e-01

1883 9.666479e-01 9.665968e-01 9.665258e-01 9.664575e-01 9.682380e-01 6.563008e-01 6.319064e-01 6.378284e-01 6.737437e-01 6.794918e-01

1884 9.666732e-01 9.666221e-01 9.665510e-01 9.664826e-01 9.682612e-01 6.565856e-01 6.322057e-01 6.381309e-01 6.740328e-01 6.797816e-01

1885 9.666986e-01 9.666474e-01 9.665761e-01 9.665076e-01 9.682842e-01 6.568631e-01 6.324981e-01 6.384262e-01 6.743144e-01 6.800637e-01

1886 9.667238e-01 9.666725e-01 9.666012e-01 9.665325e-01 9.683073e-01 6.571335e-01 6.327835e-01 6.387145e-01 6.745884e-01 6.803382e-01

1887 9.667490e-01 9.666977e-01 9.666262e-01 9.665574e-01 9.683303e-01 6.573968e-01 6.330621e-01 6.389957e-01 6.748551e-01 6.806052e-01

1888 9.667742e-01 9.667228e-01 9.666512e-01 9.665823e-01 9.683532e-01 6.576532e-01 6.333339e-01 6.392699e-01 6.751144e-01 6.808648e-01

1889 9.667993e-01 9.667478e-01 9.666761e-01 9.666071e-01 9.683761e-01 6.579027e-01 6.335989e-01 6.395372e-01 6.753666e-01 6.811172e-01

1890 9.668244e-01 9.667728e-01 9.667009e-01 9.666318e-01 9.683989e-01 6.581455e-01 6.338572e-01 6.397977e-01 6.756117e-01 6.813624e-01

1891 9.668494e-01 9.667978e-01 9.667258e-01 9.666565e-01 9.684217e-01 6.583816e-01 6.341089e-01 6.400514e-01 6.758500e-01 6.816008e-01

1892 9.668744e-01 9.668227e-01 9.667505e-01 9.666811e-01 9.684444e-01 6.586112e-01 6.343540e-01 6.402984e-01 6.760815e-01 6.818323e-01

1893 9.668994e-01 9.668476e-01 9.667753e-01 9.667057e-01 9.684671e-01 6.588343e-01 6.345926e-01 6.405388e-01 6.763064e-01 6.820571e-01

1894 9.669244e-01 9.668724e-01 9.668000e-01 9.667303e-01 9.684897e-01 6.590511e-01 6.348248e-01 6.407727e-01 6.765248e-01 6.822755e-01

1895 9.669493e-01 9.668972e-01 9.668246e-01 9.667548e-01 9.685123e-01 6.592618e-01 6.350508e-01 6.410003e-01 6.767368e-01 6.824874e-01

1896 9.669741e-01 9.669220e-01 9.668492e-01 9.667792e-01 9.685349e-01 6.594664e-01 6.352706e-01 6.412216e-01 6.769427e-01 6.826932e-01

1897 9.669990e-01 9.669467e-01 9.668738e-01 9.668036e-01 9.685573e-01 6.596651e-01 6.354844e-01 6.414367e-01 6.771426e-01 6.828929e-01

1898 9.670238e-01 9.669714e-01 9.668983e-01 9.668280e-01 9.685798e-01 6.598581e-01 6.356922e-01 6.416459e-01 6.773366e-01 6.830867e-01

1899 9.670486e-01 9.669961e-01 9.669228e-01 9.668523e-01 9.686022e-01 6.600454e-01 6.358942e-01 6.418491e-01 6.775248e-01 6.832748e-01

1900 9.670733e-01 9.670208e-01 9.669473e-01 9.668765e-01 9.686245e-01 6.602273e-01 6.360905e-01 6.420466e-01 6.777075e-01 6.834573e-01

1901 9.670980e-01 9.670454e-01 9.669717e-01 9.669008e-01 9.686468e-01 6.604037e-01 6.362812e-01 6.422385e-01 6.778848e-01 6.836343e-01

1902 9.671228e-01 9.670700e-01 9.669961e-01 9.669250e-01 9.686691e-01 6.605750e-01 6.364665e-01 6.424248e-01 6.780568e-01 6.838061e-01

1903 9.671474e-01 9.670945e-01 9.670204e-01 9.669491e-01 9.686913e-01 6.607412e-01 6.366465e-01 6.426058e-01 6.782236e-01 6.839728e-01

1904 9.671721e-01 9.671190e-01 9.670447e-01 9.669732e-01 9.687134e-01 6.609025e-01 6.368213e-01 6.427816e-01 6.783856e-01 6.841345e-01

1905 9.671967e-01 9.671435e-01 9.670690e-01 9.669972e-01 9.687355e-01 6.610591e-01 6.369911e-01 6.429523e-01 6.785426e-01 6.842914e-01

1906 9.672213e-01 9.671680e-01 9.670932e-01 9.670212e-01 9.687576e-01 6.612109e-01 6.371560e-01 6.431181e-01 6.786951e-01 6.844436e-01

1907 9.672459e-01 9.671925e-01 9.671174e-01 9.670452e-01 9.687796e-01 6.613583e-01 6.373160e-01 6.432790e-01 6.788430e-01 6.845913e-01

1908 9.672705e-01 9.672169e-01 9.671416e-01 9.670691e-01 9.688015e-01 6.615013e-01 6.374714e-01 6.434352e-01 6.789865e-01 6.847346e-01

1909 9.672951e-01 9.672413e-01 9.671657e-01 9.670930e-01 9.688234e-01 6.616400e-01 6.376223e-01 6.435869e-01 6.791257e-01 6.848736e-01

1910 9.673196e-01 9.672657e-01 9.671898e-01 9.671168e-01 9.688452e-01 6.617747e-01 6.377689e-01 6.437342e-01 6.792609e-01 6.850086e-01

1911 9.673442e-01 9.672900e-01 9.672139e-01 9.671406e-01 9.688670e-01 6.619054e-01 6.379111e-01 6.438772e-01 6.793920e-01 6.851396e-01

1912 9.673687e-01 9.673144e-01 9.672379e-01 9.671644e-01 9.688887e-01 6.620322e-01 6.380493e-01 6.440160e-01 6.795194e-01 6.852668e-01

1913 9.673932e-01 9.673387e-01 9.672619e-01 9.671881e-01 9.689104e-01 6.621554e-01 6.381834e-01 6.441509e-01 6.796430e-01 6.853902e-01

1914 9.674177e-01 9.673630e-01 9.672859e-01 9.672117e-01 9.689320e-01 6.622749e-01 6.383137e-01 6.442818e-01 6.797630e-01 6.855101e-01

1915 9.674421e-01 9.673872e-01 9.673098e-01 9.672353e-01 9.689535e-01 6.623910e-01 6.384402e-01 6.444090e-01 6.798796e-01 6.856266e-01

1916 9.674666e-01 9.674115e-01 9.673337e-01 9.672589e-01 9.689750e-01 6.625037e-01 6.385631e-01 6.445325e-01 6.799929e-01 6.857397e-01

1917 9.674911e-01 9.674357e-01 9.673576e-01 9.672824e-01 9.689964e-01 6.626132e-01 6.386825e-01 6.446525e-01 6.801029e-01 6.858496e-01

1918 9.675155e-01 9.674599e-01 9.673814e-01 9.673059e-01 9.690177e-01 6.627196e-01 6.387985e-01 6.447691e-01 6.802098e-01 6.859564e-01

1919 9.675399e-01 9.674841e-01 9.674052e-01 9.673293e-01 9.690390e-01 6.628230e-01 6.389112e-01 6.448825e-01 6.803137e-01 6.860603e-01

1920 9.675644e-01 9.675083e-01 9.674289e-01 9.673527e-01 9.690602e-01 6.629235e-01 6.390207e-01 6.449926e-01 6.804147e-01 6.861612e-01

1921 9.675888e-01 9.675325e-01 9.674526e-01 9.673760e-01 9.690813e-01 6.630211e-01 6.391272e-01 6.450997e-01 6.805130e-01 6.862594e-01

1922 9.676132e-01 9.675566e-01 9.674763e-01 9.673992e-01 9.691024e-01 6.631161e-01 6.392308e-01 6.452038e-01 6.806086e-01 6.863550e-01

1923 9.676376e-01 9.675807e-01 9.675000e-01 9.674224e-01 9.691233e-01 6.632085e-01 6.393315e-01 6.453051e-01 6.807016e-01 6.864479e-01

1924 9.676620e-01 9.676048e-01 9.675236e-01 9.674456e-01 9.691442e-01 6.632985e-01 6.394295e-01 6.454036e-01 6.807921e-01 6.865384e-01

1925 9.676864e-01 9.676289e-01 9.675471e-01 9.674687e-01 9.691650e-01 6.633860e-01 6.395249e-01 6.454995e-01 6.808802e-01 6.866265e-01

1926 9.677108e-01 9.676530e-01 9.675706e-01 9.674917e-01 9.691858e-01 6.634712e-01 6.396177e-01 6.455929e-01 6.809661e-01 6.867124e-01

1927 9.677352e-01 9.676770e-01 9.675941e-01 9.675147e-01 9.692064e-01 6.635542e-01 6.397081e-01 6.456838e-01 6.810497e-01 6.867960e-01

1928 9.677596e-01 9.677011e-01 9.676175e-01 9.675376e-01 9.692269e-01 6.636350e-01 6.397961e-01 6.457723e-01 6.811312e-01 6.868775e-01

1929 9.677840e-01 9.677251e-01 9.676409e-01 9.675604e-01 9.692474e-01 6.637139e-01 6.398819e-01 6.458586e-01 6.812107e-01 6.869570e-01

1930 9.678084e-01 9.677491e-01 9.676642e-01 9.675832e-01 9.692677e-01 6.637907e-01 6.399655e-01 6.459427e-01 6.812882e-01 6.870346e-01

1931 9.678328e-01 9.677730e-01 9.676875e-01 9.676059e-01 9.692879e-01 6.638657e-01 6.400470e-01 6.460246e-01 6.813639e-01 6.871102e-01

1932 9.678572e-01 9.677970e-01 9.677108e-01 9.676285e-01 9.693081e-01 6.639388e-01 6.401265e-01 6.461046e-01 6.814377e-01 6.871841e-01

1933 9.678815e-01 9.678209e-01 9.677339e-01 9.676511e-01 9.693281e-01 6.640102e-01 6.402041e-01 6.461826e-01 6.815098e-01 6.872563e-01

1934 9.679059e-01 9.678448e-01 9.677571e-01 9.676735e-01 9.693480e-01 6.640799e-01 6.402798e-01 6.462588e-01 6.815802e-01 6.873267e-01

1935 9.679303e-01 9.678687e-01 9.677801e-01 9.676959e-01 9.693677e-01 6.641481e-01 6.403537e-01 6.463332e-01 6.816490e-01 6.873956e-01

1936 9.679547e-01 9.678926e-01 9.678032e-01 9.677182e-01 9.693874e-01 6.642147e-01 6.404259e-01 6.464058e-01 6.817163e-01 6.874630e-01

1937 9.679791e-01 9.679164e-01 9.678261e-01 9.677404e-01 9.694069e-01 6.642798e-01 6.404965e-01 6.464768e-01 6.817822e-01 6.875289e-01

1938 9.680035e-01 9.679402e-01 9.678490e-01 9.677626e-01 9.694263e-01 6.643435e-01 6.405655e-01 6.465463e-01 6.818466e-01 6.875934e-01

1939 9.680279e-01 9.679640e-01 9.678718e-01 9.677846e-01 9.694455e-01 6.644058e-01 6.406330e-01 6.466142e-01 6.819097e-01 6.876566e-01

1940 9.680522e-01 9.679878e-01 9.678946e-01 9.678065e-01 9.694646e-01 6.644669e-01 6.406991e-01 6.466807e-01 6.819715e-01 6.877185e-01

1941 9.680766e-01 9.680115e-01 9.679173e-01 9.678283e-01 9.694835e-01 6.645267e-01 6.407638e-01 6.467457e-01 6.820320e-01 6.877791e-01

1942 9.681010e-01 9.680352e-01 9.679399e-01 9.678500e-01 9.695023e-01 6.645854e-01 6.408271e-01 6.468095e-01 6.820914e-01 6.878385e-01

1943 9.681254e-01 9.680589e-01 9.679624e-01 9.678716e-01 9.695209e-01 6.646429e-01 6.408892e-01 6.468719e-01 6.821496e-01 6.878969e-01

1944 9.681498e-01 9.680826e-01 9.679848e-01 9.678931e-01 9.695394e-01 6.646993e-01 6.409501e-01 6.469332e-01 6.822067e-01 6.879541e-01

1945 9.681742e-01 9.681062e-01 9.680072e-01 9.679145e-01 9.695576e-01 6.647547e-01 6.410098e-01 6.469932e-01 6.822628e-01 6.880103e-01

1946 9.681986e-01 9.681297e-01 9.680295e-01 9.679357e-01 9.695757e-01 6.648091e-01 6.410684e-01 6.470522e-01 6.823179e-01 6.880655e-01

1947 9.682230e-01 9.681533e-01 9.680516e-01 9.679568e-01 9.695936e-01 6.648625e-01 6.411260e-01 6.471101e-01 6.823720e-01 6.881197e-01

1948 9.682474e-01 9.681768e-01 9.680737e-01 9.679778e-01 9.696113e-01 6.649150e-01 6.411825e-01 6.471669e-01 6.824253e-01 6.881731e-01

1949 9.682718e-01 9.682002e-01 9.680957e-01 9.679986e-01 9.696288e-01 6.649667e-01 6.412380e-01 6.472228e-01 6.824776e-01 6.882256e-01

1950 9.682962e-01 9.682236e-01 9.681175e-01 9.680193e-01 9.696461e-01 6.650176e-01 6.412927e-01 6.472777e-01 6.825292e-01 6.882772e-01

1951 9.683206e-01 9.682470e-01 9.681393e-01 9.680398e-01 9.696631e-01 6.650676e-01 6.413464e-01 6.473318e-01 6.825799e-01 6.883280e-01

1952 9.683450e-01 9.682703e-01 9.681609e-01 9.680601e-01 9.696799e-01 6.651169e-01 6.413993e-01 6.473850e-01 6.826299e-01 6.883781e-01

1953 9.683694e-01 9.682936e-01 9.681824e-01 9.680803e-01 9.696965e-01 6.651655e-01 6.414514e-01 6.474373e-01 6.826791e-01 6.884275e-01

1954 9.683938e-01 9.683168e-01 9.682038e-01 9.681003e-01 9.697129e-01 6.652135e-01 6.415027e-01 6.474889e-01 6.827277e-01 6.884762e-01

1955 9.684182e-01 9.683399e-01 9.682251e-01 9.681202e-01 9.697289e-01 6.652607e-01 6.415533e-01 6.475398e-01 6.827756e-01 6.885242e-01

1956 9.684426e-01 9.683630e-01 9.682462e-01 9.681398e-01 9.697447e-01 6.653074e-01 6.416032e-01 6.475899e-01 6.828229e-01 6.885716e-01

1957 9.684670e-01 9.683860e-01 9.682671e-01 9.681592e-01 9.697603e-01 6.653534e-01 6.416525e-01 6.476394e-01 6.828696e-01 6.886184e-01

1958 9.684914e-01 9.684089e-01 9.682879e-01 9.681784e-01 9.697755e-01 6.653989e-01 6.417011e-01 6.476883e-01 6.829157e-01 6.886646e-01

1959 9.685158e-01 9.684318e-01 9.683086e-01 9.681974e-01 9.697905e-01 6.654439e-01 6.417491e-01 6.477365e-01 6.829613e-01 6.887103e-01

1960 9.685402e-01 9.684546e-01 9.683291e-01 9.682162e-01 9.698051e-01 6.654884e-01 6.417965e-01 6.477841e-01 6.830063e-01 6.887554e-01

1961 9.685645e-01 9.684773e-01 9.683494e-01 9.682348e-01 9.698194e-01 6.655324e-01 6.418434e-01 6.478312e-01 6.830509e-01 6.888001e-01

1962 9.685889e-01 9.684999e-01 9.683695e-01 9.682531e-01 9.698334e-01 6.655759e-01 6.418898e-01 6.478778e-01 6.830950e-01 6.888443e-01

1963 9.686132e-01 9.685225e-01 9.683894e-01 9.682711e-01 9.698471e-01 6.656190e-01 6.419357e-01 6.479239e-01 6.831387e-01 6.888881e-01

1964 9.686376e-01 9.685449e-01 9.684091e-01 9.682889e-01 9.698604e-01 6.656618e-01 6.419811e-01 6.479695e-01 6.831819e-01 6.889314e-01

1965 9.686619e-01 9.685672e-01 9.684286e-01 9.683064e-01 9.698734e-01 6.657041e-01 6.420261e-01 6.480146e-01 6.832248e-01 6.889744e-01

1966 9.686862e-01 9.685895e-01 9.684479e-01 9.683237e-01 9.698859e-01 6.657460e-01 6.420707e-01 6.480594e-01 6.832673e-01 6.890169e-01

1967 9.687104e-01 9.686116e-01 9.684670e-01 9.683406e-01 9.698981e-01 6.657877e-01 6.421149e-01 6.481037e-01 6.833094e-01 6.890591e-01

1968 9.687347e-01 9.686336e-01 9.684858e-01 9.683573e-01 9.699099e-01 6.658290e-01 6.421587e-01 6.481477e-01 6.833512e-01 6.891010e-01

1969 9.687589e-01 9.686554e-01 9.685044e-01 9.683736e-01 9.699213e-01 6.658700e-01 6.422022e-01 6.481913e-01 6.833927e-01 6.891426e-01

1970 9.687831e-01 9.686771e-01 9.685227e-01 9.683896e-01 9.699323e-01 6.659107e-01 6.422454e-01 6.482346e-01 6.834339e-01 6.891838e-01

1971 9.688073e-01 9.686987e-01 9.685408e-01 9.684052e-01 9.699428e-01 6.659512e-01 6.422882e-01 6.482775e-01 6.834748e-01 6.892248e-01

1972 9.688314e-01 9.687202e-01 9.685585e-01 9.684205e-01 9.699529e-01 6.659914e-01 6.423308e-01 6.483202e-01 6.835154e-01 6.892655e-01

1973 9.688555e-01 9.687415e-01 9.685760e-01 9.684355e-01 9.699625e-01 6.660314e-01 6.423730e-01 6.483625e-01 6.835558e-01 6.893060e-01

1974 9.688796e-01 9.687626e-01 9.685932e-01 9.684500e-01 9.699716e-01 6.660711e-01 6.424151e-01 6.484047e-01 6.835960e-01 6.893462e-01

1975 9.689036e-01 9.687835e-01 9.686100e-01 9.684642e-01 9.699803e-01 6.661107e-01 6.424569e-01 6.484465e-01 6.836359e-01 6.893862e-01

1976 9.689276e-01 9.688043e-01 9.686265e-01 9.684780e-01 9.699884e-01 6.661501e-01 6.424984e-01 6.484882e-01 6.836757e-01 6.894260e-01

1977 9.689515e-01 9.688249e-01 9.686427e-01 9.684913e-01 9.699961e-01 6.661893e-01 6.425398e-01 6.485296e-01 6.837152e-01 6.894656e-01

1978 9.689754e-01 9.688452e-01 9.686585e-01 9.685042e-01 9.700032e-01 6.662283e-01 6.425810e-01 6.485709e-01 6.837546e-01 6.895050e-01

1979 9.689992e-01 9.688654e-01 9.686740e-01 9.685167e-01 9.700098e-01 6.662672e-01 6.426220e-01 6.486119e-01 6.837939e-01 6.895443e-01

1980 9.690229e-01 9.688853e-01 9.686891e-01 9.685287e-01 9.700158e-01 6.663060e-01 6.426628e-01 6.486528e-01 6.838329e-01 6.895834e-01

1981 9.690466e-01 9.689050e-01 9.687037e-01 9.685402e-01 9.700213e-01 6.663447e-01 6.427035e-01 6.486935e-01 6.838719e-01 6.896224e-01

1982 9.690788e-01 9.689371e-01 9.687356e-01 9.685720e-01 9.700493e-01 6.665686e-01 6.428916e-01 6.488949e-01 6.841429e-01 6.899104e-01

1983 9.691104e-01 9.689686e-01 9.687670e-01 9.686033e-01 9.700769e-01 6.667989e-01 6.430855e-01 6.491024e-01 6.844212e-01 6.902060e-01

1984 9.691415e-01 9.689995e-01 9.687979e-01 9.686340e-01 9.701041e-01 6.670358e-01 6.432852e-01 6.493160e-01 6.847069e-01 6.905092e-01

1985 9.691721e-01 9.690300e-01 9.688282e-01 9.686642e-01 9.701309e-01 6.672792e-01 6.434908e-01 6.495358e-01 6.850000e-01 6.908202e-01

1986 9.692022e-01 9.690600e-01 9.688581e-01 9.686940e-01 9.701572e-01 6.675292e-01 6.437024e-01 6.497620e-01 6.853005e-01 6.911388e-01

1987 9.692318e-01 9.690895e-01 9.688875e-01 9.687233e-01 9.701832e-01 6.677857e-01 6.439200e-01 6.499944e-01 6.856085e-01 6.914651e-01

1988 9.692610e-01 9.691186e-01 9.689164e-01 9.687521e-01 9.702088e-01 6.680490e-01 6.441437e-01 6.502332e-01 6.859238e-01 6.917990e-01

1989 9.692897e-01 9.691472e-01 9.689450e-01 9.687806e-01 9.702341e-01 6.683188e-01 6.443735e-01 6.504785e-01 6.862465e-01 6.921404e-01

1990 9.693181e-01 9.691755e-01 9.689731e-01 9.688086e-01 9.702591e-01 6.685953e-01 6.446095e-01 6.507302e-01 6.865766e-01 6.924894e-01

1991 9.693460e-01 9.692033e-01 9.690009e-01 9.688363e-01 9.702837e-01 6.688784e-01 6.448516e-01 6.509884e-01 6.869139e-01 6.928458e-01

1992 9.693736e-01 9.692308e-01 9.690283e-01 9.688635e-01 9.703081e-01 6.691682e-01 6.451000e-01 6.512530e-01 6.872584e-01 6.932096e-01

1993 9.694008e-01 9.692580e-01 9.690553e-01 9.688905e-01 9.703321e-01 6.694645e-01 6.453546e-01 6.515242e-01 6.876100e-01 6.935805e-01

1994 9.694277e-01 9.692848e-01 9.690820e-01 9.689171e-01 9.703559e-01 6.697673e-01 6.456155e-01 6.518018e-01 6.879685e-01 6.939586e-01

1995 9.694543e-01 9.693112e-01 9.691084e-01 9.689434e-01 9.703794e-01 6.700765e-01 6.458825e-01 6.520858e-01 6.883340e-01 6.943436e-01

1996 9.694805e-01 9.693374e-01 9.691345e-01 9.689694e-01 9.704027e-01 6.703922e-01 6.461558e-01 6.523763e-01 6.887062e-01 6.947354e-01

1997 9.695065e-01 9.693632e-01 9.691602e-01 9.689950e-01 9.704257e-01 6.707141e-01 6.464352e-01 6.526731e-01 6.890849e-01 6.951338e-01

1998 9.695321e-01 9.693888e-01 9.691857e-01 9.690204e-01 9.704485e-01 6.710422e-01 6.467207e-01 6.529762e-01 6.894701e-01 6.955387e-01

1999 9.695575e-01 9.694141e-01 9.692109e-01 9.690456e-01 9.704710e-01 6.713764e-01 6.470122e-01 6.532856e-01 6.898615e-01 6.959497e-01

2000 9.695826e-01 9.694392e-01 9.692359e-01 9.690704e-01 9.704933e-01 6.717165e-01 6.473098e-01 6.536011e-01 6.902589e-01 6.963667e-01

2001 9.696075e-01 9.694639e-01 9.692606e-01 9.690951e-01 9.705155e-01 6.720624e-01 6.476132e-01 6.539226e-01 6.906621e-01 6.967895e-01

2002 9.696321e-01 9.694885e-01 9.692851e-01 9.691194e-01 9.705374e-01 6.724139e-01 6.479224e-01 6.542500e-01 6.910709e-01 6.972177e-01

2003 9.696565e-01 9.695128e-01 9.693093e-01 9.691436e-01 9.705591e-01 6.727708e-01 6.482374e-01 6.545833e-01 6.914850e-01 6.976510e-01

2004 9.696806e-01 9.695369e-01 9.693333e-01 9.691675e-01 9.705807e-01 6.731330e-01 6.485579e-01 6.549221e-01 6.919041e-01 6.980893e-01

2005 9.697046e-01 9.695608e-01 9.693571e-01 9.691912e-01 9.706021e-01 6.735002e-01 6.488838e-01 6.552665e-01 6.923280e-01 6.985322e-01

2006 9.697283e-01 9.695844e-01 9.693807e-01 9.692147e-01 9.706233e-01 6.738722e-01 6.492150e-01 6.556162e-01 6.927563e-01 6.989793e-01

2007 9.697519e-01 9.696079e-01 9.694041e-01 9.692381e-01 9.706444e-01 6.742488e-01 6.495513e-01 6.559711e-01 6.931889e-01 6.994304e-01

2008 9.697752e-01 9.696312e-01 9.694273e-01 9.692612e-01 9.706653e-01 6.746298e-01 6.498926e-01 6.563308e-01 6.936252e-01 6.998851e-01

2009 9.697984e-01 9.696543e-01 9.694503e-01 9.692842e-01 9.706860e-01 6.750148e-01 6.502386e-01 6.566953e-01 6.940651e-01 7.003430e-01

2010 9.698214e-01 9.696773e-01 9.694732e-01 9.693069e-01 9.707066e-01 6.754036e-01 6.505892e-01 6.570643e-01 6.945081e-01 7.008038e-01

2011 9.698442e-01 9.697000e-01 9.694959e-01 9.693296e-01 9.707271e-01 6.757960e-01 6.509441e-01 6.574376e-01 6.949540e-01 7.012670e-01

2012 9.698669e-01 9.697226e-01 9.695184e-01 9.693520e-01 9.707474e-01 6.761916e-01 6.513032e-01 6.578148e-01 6.954023e-01 7.017324e-01

2013 9.698894e-01 9.697451e-01 9.695408e-01 9.693743e-01 9.707676e-01 6.765901e-01 6.516660e-01 6.581959e-01 6.958527e-01 7.021995e-01

2014 9.699118e-01 9.697674e-01 9.695631e-01 9.693965e-01 9.707877e-01 6.769912e-01 6.520325e-01 6.585804e-01 6.963048e-01 7.026679e-01

2015 9.699340e-01 9.697896e-01 9.695851e-01 9.694185e-01 9.708077e-01 6.773946e-01 6.524024e-01 6.589680e-01 6.967582e-01 7.031373e-01

2016 9.699561e-01 9.698116e-01 9.696071e-01 9.694404e-01 9.708275e-01 6.778000e-01 6.527753e-01 6.593586e-01 6.972125e-01 7.036071e-01

2017 9.699781e-01 9.698335e-01 9.696289e-01 9.694621e-01 9.708473e-01 6.782070e-01 6.531511e-01 6.597518e-01 6.976675e-01 7.040772e-01

2018 9.700000e-01 9.698553e-01 9.696506e-01 9.694837e-01 9.708669e-01 6.786153e-01 6.535293e-01 6.601473e-01 6.981226e-01 7.045469e-01

2019 9.700217e-01 9.698770e-01 9.696722e-01 9.695052e-01 9.708865e-01 6.790245e-01 6.539098e-01 6.605447e-01 6.985775e-01 7.050160e-01

2020 9.700433e-01 9.698985e-01 9.696937e-01 9.695266e-01 9.709059e-01 6.794344e-01 6.542922e-01 6.609438e-01 6.990318e-01 7.054840e-01

2021 9.700648e-01 9.699200e-01 9.697150e-01 9.695479e-01 9.709252e-01 6.798445e-01 6.546761e-01 6.613442e-01 6.994851e-01 7.059506e-01

2022 9.700862e-01 9.699413e-01 9.697363e-01 9.695691e-01 9.709445e-01 6.802545e-01 6.550614e-01 6.617456e-01 6.999370e-01 7.064153e-01

2023 9.701075e-01 9.699625e-01 9.697574e-01 9.695901e-01 9.709637e-01 6.806640e-01 6.554476e-01 6.621476e-01 7.003873e-01 7.068778e-01

2024 9.701287e-01 9.699837e-01 9.697785e-01 9.696111e-01 9.709827e-01 6.810728e-01 6.558345e-01 6.625499e-01 7.008354e-01 7.073378e-01

2025 9.701498e-01 9.700047e-01 9.697994e-01 9.696319e-01 9.710017e-01 6.814805e-01 6.562217e-01 6.629523e-01 7.012810e-01 7.077948e-01

2026 9.701708e-01 9.700256e-01 9.698203e-01 9.696527e-01 9.710207e-01 6.818867e-01 6.566089e-01 6.633543e-01 7.017239e-01 7.082485e-01

2027 9.701917e-01 9.700465e-01 9.698411e-01 9.696734e-01 9.710395e-01 6.822911e-01 6.569958e-01 6.637556e-01 7.021636e-01 7.086985e-01

2028 9.702125e-01 9.700673e-01 9.698617e-01 9.696940e-01 9.710583e-01 6.826934e-01 6.573820e-01 6.641558e-01 7.025998e-01 7.091446e-01

2029 9.702333e-01 9.700880e-01 9.698823e-01 9.697145e-01 9.710770e-01 6.830933e-01 6.577673e-01 6.645548e-01 7.030323e-01 7.095864e-01

2030 9.702540e-01 9.701086e-01 9.699029e-01 9.697349e-01 9.710956e-01 6.834904e-01 6.581513e-01 6.649520e-01 7.034606e-01 7.100237e-01

2031 9.702746e-01 9.701291e-01 9.699233e-01 9.697553e-01 9.711141e-01 6.838844e-01 6.585337e-01 6.653473e-01 7.038846e-01 7.104561e-01

2032 9.702951e-01 9.701496e-01 9.699437e-01 9.697755e-01 9.711326e-01 6.842752e-01 6.589143e-01 6.657403e-01 7.043038e-01 7.108834e-01

2033 9.703156e-01 9.701700e-01 9.699640e-01 9.697957e-01 9.711511e-01 6.846623e-01 6.592926e-01 6.661307e-01 7.047182e-01 7.113053e-01

2034 9.703360e-01 9.701903e-01 9.699842e-01 9.698159e-01 9.711694e-01 6.850456e-01 6.596684e-01 6.665182e-01 7.051274e-01 7.117215e-01

2035 9.703564e-01 9.702106e-01 9.700044e-01 9.698359e-01 9.711877e-01 6.854247e-01 6.600415e-01 6.669025e-01 7.055311e-01 7.121320e-01

2036 9.703766e-01 9.702308e-01 9.700245e-01 9.698559e-01 9.712060e-01 6.857994e-01 6.604116e-01 6.672834e-01 7.059293e-01 7.125364e-01

2037 9.703969e-01 9.702510e-01 9.700445e-01 9.698758e-01 9.712242e-01 6.861696e-01 6.607783e-01 6.676605e-01 7.063216e-01 7.129346e-01

2038 9.704171e-01 9.702711e-01 9.700645e-01 9.698957e-01 9.712423e-01 6.865349e-01 6.611415e-01 6.680338e-01 7.067079e-01 7.133263e-01

2039 9.704372e-01 9.702911e-01 9.700844e-01 9.699155e-01 9.712604e-01 6.868952e-01 6.615009e-01 6.684028e-01 7.070880e-01 7.137116e-01

2040 9.704572e-01 9.703111e-01 9.701043e-01 9.699353e-01 9.712784e-01 6.872503e-01 6.618563e-01 6.687674e-01 7.074618e-01 7.140901e-01

2041 9.704773e-01 9.703311e-01 9.701241e-01 9.699550e-01 9.712964e-01 6.876001e-01 6.622075e-01 6.691274e-01 7.078292e-01 7.144619e-01

2042 9.704972e-01 9.703509e-01 9.701439e-01 9.699746e-01 9.713143e-01 6.879443e-01 6.625542e-01 6.694826e-01 7.081900e-01 7.148267e-01

2043 9.705172e-01 9.703708e-01 9.701636e-01 9.699942e-01 9.713321e-01 6.882829e-01 6.628963e-01 6.698327e-01 7.085441e-01 7.151845e-01

2044 9.705371e-01 9.703906e-01 9.701832e-01 9.700137e-01 9.713500e-01 6.886156e-01 6.632335e-01 6.701777e-01 7.088914e-01 7.155353e-01

2045 9.705569e-01 9.704104e-01 9.702028e-01 9.700332e-01 9.713677e-01 6.889425e-01 6.635658e-01 6.705174e-01 7.092319e-01 7.158789e-01

2046 9.705767e-01 9.704301e-01 9.702224e-01 9.700526e-01 9.713855e-01 6.892633e-01 6.638930e-01 6.708516e-01 7.095655e-01 7.162154e-01

2047 9.705965e-01 9.704497e-01 9.702419e-01 9.700720e-01 9.714031e-01 6.895781e-01 6.642149e-01 6.711801e-01 7.098921e-01 7.165447e-01

2048 9.706162e-01 9.704694e-01 9.702614e-01 9.700913e-01 9.714208e-01 6.898867e-01 6.645314e-01 6.715030e-01 7.102118e-01 7.168667e-01

2049 9.706360e-01 9.704890e-01 9.702808e-01 9.701106e-01 9.714384e-01 6.901891e-01 6.648424e-01 6.718201e-01 7.105245e-01 7.171816e-01

2050 9.706556e-01 9.705086e-01 9.703002e-01 9.701298e-01 9.714559e-01 6.904852e-01 6.651478e-01 6.721313e-01 7.108303e-01 7.174893e-01

2051 9.706753e-01 9.705281e-01 9.703196e-01 9.701490e-01 9.714734e-01 6.907751e-01 6.654476e-01 6.724365e-01 7.111290e-01 7.177897e-01

2052 9.706949e-01 9.705476e-01 9.703389e-01 9.701682e-01 9.714908e-01 6.910586e-01 6.657416e-01 6.727357e-01 7.114208e-01 7.180831e-01

2053 9.707145e-01 9.705670e-01 9.703582e-01 9.701873e-01 9.715082e-01 6.913359e-01 6.660298e-01 6.730288e-01 7.117057e-01 7.183693e-01

2054 9.707340e-01 9.705865e-01 9.703774e-01 9.702063e-01 9.715256e-01 6.916068e-01 6.663122e-01 6.733158e-01 7.119837e-01 7.186486e-01

2055 9.707536e-01 9.706059e-01 9.703966e-01 9.702254e-01 9.715429e-01 6.918715e-01 6.665886e-01 6.735967e-01 7.122549e-01 7.189208e-01

2056 9.707731e-01 9.706253e-01 9.704158e-01 9.702444e-01 9.715602e-01 6.921299e-01 6.668592e-01 6.738715e-01 7.125193e-01 7.191861e-01

2057 9.707926e-01 9.706446e-01 9.704349e-01 9.702633e-01 9.715774e-01 6.923820e-01 6.671239e-01 6.741401e-01 7.127770e-01 7.194446e-01

2058 9.708120e-01 9.706640e-01 9.704540e-01 9.702822e-01 9.715946e-01 6.926280e-01 6.673826e-01 6.744026e-01 7.130281e-01 7.196964e-01

2059 9.708315e-01 9.706833e-01 9.704731e-01 9.703010e-01 9.716117e-01 6.928679e-01 6.676355e-01 6.746590e-01 7.132726e-01 7.199415e-01

2060 9.708509e-01 9.707025e-01 9.704921e-01 9.703199e-01 9.716288e-01 6.931016e-01 6.678825e-01 6.749094e-01 7.135107e-01 7.201801e-01

2061 9.708703e-01 9.707218e-01 9.705111e-01 9.703386e-01 9.716458e-01 6.933294e-01 6.681236e-01 6.751537e-01 7.137425e-01 7.204123e-01

2062 9.708897e-01 9.707410e-01 9.705301e-01 9.703574e-01 9.716628e-01 6.935513e-01 6.683589e-01 6.753921e-01 7.139680e-01 7.206381e-01

2063 9.709091e-01 9.707602e-01 9.705491e-01 9.703761e-01 9.716798e-01 6.937673e-01 6.685885e-01 6.756245e-01 7.141874e-01 7.208577e-01

2064 9.709285e-01 9.707794e-01 9.705680e-01 9.703947e-01 9.716967e-01 6.939775e-01 6.688123e-01 6.758510e-01 7.144007e-01 7.210713e-01

2065 9.709478e-01 9.707986e-01 9.705868e-01 9.704134e-01 9.717135e-01 6.941821e-01 6.690305e-01 6.760718e-01 7.146082e-01 7.212788e-01

2066 9.709671e-01 9.708177e-01 9.706057e-01 9.704319e-01 9.717303e-01 6.943811e-01 6.692431e-01 6.762869e-01 7.148098e-01 7.214805e-01

2067 9.709865e-01 9.708368e-01 9.706245e-01 9.704505e-01 9.717471e-01 6.945747e-01 6.694502e-01 6.764963e-01 7.150057e-01 7.216765e-01

2068 9.710058e-01 9.708559e-01 9.706433e-01 9.704690e-01 9.717638e-01 6.947628e-01 6.696519e-01 6.767002e-01 7.151962e-01 7.218669e-01

2069 9.710251e-01 9.708750e-01 9.706620e-01 9.704874e-01 9.717805e-01 6.949458e-01 6.698482e-01 6.768986e-01 7.153811e-01 7.220518e-01

2070 9.710444e-01 9.708941e-01 9.706807e-01 9.705059e-01 9.717971e-01 6.951235e-01 6.700393e-01 6.770916e-01 7.155608e-01 7.222315e-01

2071 9.710637e-01 9.709132e-01 9.706994e-01 9.705242e-01 9.718136e-01 6.952962e-01 6.702251e-01 6.772794e-01 7.157353e-01 7.224059e-01

2072 9.710829e-01 9.709322e-01 9.707181e-01 9.705426e-01 9.718301e-01 6.954640e-01 6.704060e-01 6.774621e-01 7.159047e-01 7.225752e-01

2073 9.711022e-01 9.709512e-01 9.707367e-01 9.705609e-01 9.718465e-01 6.956270e-01 6.705818e-01 6.776397e-01 7.160693e-01 7.227396e-01

2074 9.711215e-01 9.709702e-01 9.707553e-01 9.705791e-01 9.718629e-01 6.957852e-01 6.707528e-01 6.778123e-01 7.162290e-01 7.228992e-01

2075 9.711407e-01 9.709892e-01 9.707738e-01 9.705973e-01 9.718792e-01 6.959389e-01 6.709189e-01 6.779801e-01 7.163840e-01 7.230541e-01

2076 9.711600e-01 9.710081e-01 9.707924e-01 9.706155e-01 9.718955e-01 6.960882e-01 6.710805e-01 6.781432e-01 7.165346e-01 7.232045e-01

2077 9.711792e-01 9.710271e-01 9.708109e-01 9.706336e-01 9.719117e-01 6.962330e-01 6.712374e-01 6.783017e-01 7.166807e-01 7.233504e-01

2078 9.711984e-01 9.710460e-01 9.708293e-01 9.706517e-01 9.719278e-01 6.963737e-01 6.713900e-01 6.784556e-01 7.168225e-01 7.234921e-01

2079 9.712177e-01 9.710649e-01 9.708477e-01 9.706697e-01 9.719439e-01 6.965102e-01 6.715381e-01 6.786052e-01 7.169601e-01 7.236296e-01

2080 9.712369e-01 9.710838e-01 9.708661e-01 9.706876e-01 9.719599e-01 6.966427e-01 6.716821e-01 6.787504e-01 7.170938e-01 7.237631e-01

2081 9.712561e-01 9.711027e-01 9.708845e-01 9.707056e-01 9.719758e-01 6.967713e-01 6.718219e-01 6.788916e-01 7.172234e-01 7.238926e-01

2082 9.712754e-01 9.711216e-01 9.709028e-01 9.707234e-01 9.719917e-01 6.968962e-01 6.719577e-01 6.790286e-01 7.173493e-01 7.240184e-01

2083 9.712946e-01 9.711404e-01 9.709211e-01 9.707412e-01 9.720075e-01 6.970173e-01 6.720896e-01 6.791617e-01 7.174715e-01 7.241404e-01

2084 9.713138e-01 9.711593e-01 9.709393e-01 9.707590e-01 9.720232e-01 6.971350e-01 6.722177e-01 6.792910e-01 7.175902e-01 7.242589e-01

2085 9.713330e-01 9.711781e-01 9.709575e-01 9.707767e-01 9.720388e-01 6.972492e-01 6.723421e-01 6.794166e-01 7.177053e-01 7.243740e-01

2086 9.713523e-01 9.711969e-01 9.709756e-01 9.707943e-01 9.720544e-01 6.973600e-01 6.724629e-01 6.795385e-01 7.178172e-01 7.244857e-01

2087 9.713715e-01 9.712157e-01 9.709937e-01 9.708119e-01 9.720698e-01 6.974677e-01 6.725803e-01 6.796570e-01 7.179258e-01 7.245942e-01

2088 9.713907e-01 9.712344e-01 9.710118e-01 9.708294e-01 9.720852e-01 6.975722e-01 6.726943e-01 6.797720e-01 7.180312e-01 7.246996e-01

2089 9.714099e-01 9.712532e-01 9.710298e-01 9.708469e-01 9.721005e-01 6.976736e-01 6.728050e-01 6.798838e-01 7.181337e-01 7.248020e-01

2090 9.714291e-01 9.712719e-01 9.710478e-01 9.708643e-01 9.721157e-01 6.977722e-01 6.729125e-01 6.799923e-01 7.182332e-01 7.249014e-01

2091 9.714484e-01 9.712906e-01 9.710657e-01 9.708816e-01 9.721308e-01 6.978679e-01 6.730170e-01 6.800978e-01 7.183299e-01 7.249980e-01

2092 9.714676e-01 9.713092e-01 9.710836e-01 9.708988e-01 9.721458e-01 6.979609e-01 6.731185e-01 6.802003e-01 7.184238e-01 7.250920e-01

2093 9.714868e-01 9.713279e-01 9.711014e-01 9.709160e-01 9.721607e-01 6.980513e-01 6.732171e-01 6.802999e-01 7.185152e-01 7.251833e-01

2094 9.715060e-01 9.713465e-01 9.711191e-01 9.709331e-01 9.721755e-01 6.981391e-01 6.733130e-01 6.803967e-01 7.186039e-01 7.252720e-01

2095 9.715253e-01 9.713651e-01 9.711368e-01 9.709501e-01 9.721902e-01 6.982245e-01 6.734062e-01 6.804909e-01 7.186903e-01 7.253584e-01

2096 9.715445e-01 9.713837e-01 9.711545e-01 9.709671e-01 9.722047e-01 6.983075e-01 6.734968e-01 6.805824e-01 7.187743e-01 7.254423e-01

2097 9.715637e-01 9.714023e-01 9.711721e-01 9.709839e-01 9.722192e-01 6.983882e-01 6.735849e-01 6.806713e-01 7.188560e-01 7.255241e-01

2098 9.715829e-01 9.714208e-01 9.711896e-01 9.710007e-01 9.722335e-01 6.984667e-01 6.736705e-01 6.807579e-01 7.189355e-01 7.256036e-01

2099 9.716022e-01 9.714393e-01 9.712070e-01 9.710174e-01 9.722477e-01 6.985431e-01 6.737539e-01 6.808421e-01 7.190128e-01 7.256810e-01

2100 9.716214e-01 9.714578e-01 9.712244e-01 9.710339e-01 9.722618e-01 6.986174e-01 6.738349e-01 6.809240e-01 7.190882e-01 7.257564e-01

2101 9.716406e-01 9.714762e-01 9.712417e-01 9.710504e-01 9.722758e-01 6.986898e-01 6.739139e-01 6.810038e-01 7.191616e-01 7.258298e-01

2102 9.716599e-01 9.714946e-01 9.712590e-01 9.710668e-01 9.722896e-01 6.987603e-01 6.739907e-01 6.810815e-01 7.192331e-01 7.259014e-01

2103 9.716791e-01 9.715130e-01 9.712761e-01 9.710831e-01 9.723033e-01 6.988289e-01 6.740655e-01 6.811571e-01 7.193027e-01 7.259711e-01

2104 9.716983e-01 9.715313e-01 9.712932e-01 9.710993e-01 9.723168e-01 6.988959e-01 6.741384e-01 6.812308e-01 7.193707e-01 7.260391e-01

2105 9.717175e-01 9.715496e-01 9.713102e-01 9.711153e-01 9.723302e-01 6.989611e-01 6.742094e-01 6.813026e-01 7.194369e-01 7.261055e-01

2106 9.717368e-01 9.715678e-01 9.713271e-01 9.711313e-01 9.723434e-01 6.990247e-01 6.742786e-01 6.813726e-01 7.195015e-01 7.261702e-01

2107 9.717560e-01 9.715860e-01 9.713439e-01 9.711471e-01 9.723564e-01 6.990867e-01 6.743461e-01 6.814409e-01 7.195646e-01 7.262334e-01

2108 9.717752e-01 9.716042e-01 9.713606e-01 9.711628e-01 9.723693e-01 6.991473e-01 6.744119e-01 6.815074e-01 7.196262e-01 7.262950e-01

2109 9.717944e-01 9.716223e-01 9.713772e-01 9.711784e-01 9.723820e-01 6.992064e-01 6.744761e-01 6.815724e-01 7.196863e-01 7.263553e-01

2110 9.718136e-01 9.716404e-01 9.713937e-01 9.711938e-01 9.723946e-01 6.992641e-01 6.745388e-01 6.816358e-01 7.197451e-01 7.264142e-01

2111 9.718328e-01 9.716584e-01 9.714101e-01 9.712091e-01 9.724070e-01 6.993205e-01 6.746000e-01 6.816978e-01 7.198025e-01 7.264717e-01

2112 9.718520e-01 9.716763e-01 9.714264e-01 9.712242e-01 9.724191e-01 6.993756e-01 6.746598e-01 6.817583e-01 7.198587e-01 7.265280e-01

2113 9.718712e-01 9.716942e-01 9.714426e-01 9.712392e-01 9.724311e-01 6.994295e-01 6.747182e-01 6.818174e-01 7.199136e-01 7.265831e-01

2114 9.718904e-01 9.717121e-01 9.714586e-01 9.712541e-01 9.724429e-01 6.994822e-01 6.747753e-01 6.818753e-01 7.199674e-01 7.266370e-01

2115 9.719095e-01 9.717299e-01 9.714745e-01 9.712688e-01 9.724545e-01 6.995339e-01 6.748312e-01 6.819318e-01 7.200200e-01 7.266898e-01

2116 9.719287e-01 9.717476e-01 9.714903e-01 9.712833e-01 9.724658e-01 6.995844e-01 6.748858e-01 6.819872e-01 7.200716e-01 7.267415e-01

2117 9.719479e-01 9.717652e-01 9.715060e-01 9.712977e-01 9.724770e-01 6.996339e-01 6.749393e-01 6.820413e-01 7.201221e-01 7.267921e-01

2118 9.719670e-01 9.717828e-01 9.715215e-01 9.713118e-01 9.724879e-01 6.996824e-01 6.749917e-01 6.820944e-01 7.201716e-01 7.268418e-01

2119 9.719861e-01 9.718002e-01 9.715369e-01 9.713258e-01 9.724986e-01 6.997300e-01 6.750431e-01 6.821464e-01 7.202202e-01 7.268905e-01

2120 9.720052e-01 9.718176e-01 9.715521e-01 9.713397e-01 9.725091e-01 6.997767e-01 6.750934e-01 6.821974e-01 7.202679e-01 7.269383e-01

2121 9.720243e-01 9.718350e-01 9.715672e-01 9.713533e-01 9.725193e-01 6.998225e-01 6.751428e-01 6.822474e-01 7.203146e-01 7.269853e-01

2122 9.720434e-01 9.718522e-01 9.715821e-01 9.713667e-01 9.725293e-01 6.998674e-01 6.751912e-01 6.822964e-01 7.203606e-01 7.270314e-01

2123 9.720624e-01 9.718693e-01 9.715968e-01 9.713799e-01 9.725390e-01 6.999116e-01 6.752387e-01 6.823446e-01 7.204058e-01 7.270767e-01

2124 9.720814e-01 9.718864e-01 9.716114e-01 9.713929e-01 9.725484e-01 6.999551e-01 6.752854e-01 6.823918e-01 7.204501e-01 7.271213e-01

2125 9.721004e-01 9.719033e-01 9.716257e-01 9.714057e-01 9.725576e-01 6.999978e-01 6.753313e-01 6.824383e-01 7.204938e-01 7.271651e-01

2126 9.721194e-01 9.719201e-01 9.716399e-01 9.714182e-01 9.725665e-01 7.000398e-01 6.753764e-01 6.824840e-01 7.205368e-01 7.272082e-01

2127 9.721383e-01 9.719368e-01 9.716539e-01 9.714305e-01 9.725752e-01 7.000811e-01 6.754207e-01 6.825289e-01 7.205790e-01 7.272506e-01

2128 9.721573e-01 9.719534e-01 9.716677e-01 9.714426e-01 9.725835e-01 7.001219e-01 6.754644e-01 6.825731e-01 7.206207e-01 7.272924e-01

2129 9.721761e-01 9.719699e-01 9.716812e-01 9.714544e-01 9.725915e-01 7.001620e-01 6.755074e-01 6.826166e-01 7.206617e-01 7.273336e-01

2130 9.721950e-01 9.719862e-01 9.716946e-01 9.714660e-01 9.725993e-01 7.002016e-01 6.755497e-01 6.826595e-01 7.207022e-01 7.273742e-01

2131 9.722137e-01 9.720024e-01 9.717077e-01 9.714773e-01 9.726067e-01 7.002406e-01 6.755914e-01 6.827017e-01 7.207421e-01 7.274143e-01

2132 9.722325e-01 9.720185e-01 9.717206e-01 9.714883e-01 9.726138e-01 7.002791e-01 6.756325e-01 6.827433e-01 7.207814e-01 7.274538e-01

2133 9.722512e-01 9.720344e-01 9.717332e-01 9.714991e-01 9.726206e-01 7.003171e-01 6.756731e-01 6.827844e-01 7.208203e-01 7.274928e-01

2134 9.722698e-01 9.720502e-01 9.717456e-01 9.715095e-01 9.726271e-01 7.003546e-01 6.757131e-01 6.828249e-01 7.208587e-01 7.275313e-01

2135 9.722884e-01 9.720658e-01 9.717577e-01 9.715197e-01 9.726332e-01 7.003917e-01 6.757526e-01 6.828649e-01 7.208966e-01 7.275694e-01

2136 9.723070e-01 9.720812e-01 9.717696e-01 9.715296e-01 9.726390e-01 7.004284e-01 6.757916e-01 6.829045e-01 7.209341e-01 7.276070e-01

2137 9.723254e-01 9.720965e-01 9.717812e-01 9.715391e-01 9.726445e-01 7.004646e-01 6.758302e-01 6.829435e-01 7.209711e-01 7.276442e-01

2138 9.723438e-01 9.721116e-01 9.717925e-01 9.715483e-01 9.726495e-01 7.005005e-01 6.758684e-01 6.829821e-01 7.210078e-01 7.276810e-01

2139 9.723622e-01 9.721265e-01 9.718035e-01 9.715572e-01 9.726543e-01 7.005361e-01 6.759061e-01 6.830203e-01 7.210441e-01 7.277174e-01

2140 9.723804e-01 9.721412e-01 9.718142e-01 9.715658e-01 9.726586e-01 7.005713e-01 6.759434e-01 6.830581e-01 7.210800e-01 7.277534e-01

2141 9.723986e-01 9.721557e-01 9.718246e-01 9.715740e-01 9.726626e-01 7.006061e-01 6.759804e-01 6.830955e-01 7.211156e-01 7.277892e-01

2142 9.724167e-01 9.721699e-01 9.718347e-01 9.715819e-01 9.726662e-01 7.006407e-01 6.760170e-01 6.831326e-01 7.211509e-01 7.278246e-01

2143 9.724347e-01 9.721840e-01 9.718444e-01 9.715894e-01 9.726695e-01 7.006750e-01 6.760533e-01 6.831693e-01 7.211859e-01 7.278596e-01

2144 9.724527e-01 9.721978e-01 9.718538e-01 9.715966e-01 9.726723e-01 7.007090e-01 6.760893e-01 6.832057e-01 7.212205e-01 7.278944e-01

2145 9.724705e-01 9.722114e-01 9.718629e-01 9.716033e-01 9.726747e-01 7.007428e-01 6.761250e-01 6.832418e-01 7.212550e-01 7.279290e-01

2146 9.724882e-01 9.722248e-01 9.718716e-01 9.716097e-01 9.726768e-01 7.007763e-01 6.761604e-01 6.832776e-01 7.212891e-01 7.279632e-01

2147 9.725058e-01 9.722378e-01 9.718799e-01 9.716157e-01 9.726784e-01 7.008096e-01 6.761956e-01 6.833131e-01 7.213230e-01 7.279972e-01

2148 9.725233e-01 9.722507e-01 9.718879e-01 9.716213e-01 9.726797e-01 7.008427e-01 6.762305e-01 6.833484e-01 7.213567e-01 7.280310e-01

2149 9.725406e-01 9.722632e-01 9.718954e-01 9.716265e-01 9.726805e-01 7.008756e-01 6.762651e-01 6.833835e-01 7.213902e-01 7.280646e-01

2150 9.725579e-01 9.722755e-01 9.719026e-01 9.716313e-01 9.726810e-01 7.009083e-01 6.762996e-01 6.834183e-01 7.214235e-01 7.280979e-01

2151 9.725750e-01 9.722874e-01 9.719093e-01 9.716356e-01 9.726810e-01 7.009409e-01 6.763339e-01 6.834529e-01 7.214566e-01 7.281311e-01

2152 9.725919e-01 9.722991e-01 9.719156e-01 9.716395e-01 9.726806e-01 7.009733e-01 6.763680e-01 6.834874e-01 7.214895e-01 7.281641e-01

2153 9.726087e-01 9.723105e-01 9.719215e-01 9.716430e-01 9.726798e-01 7.010055e-01 6.764019e-01 6.835216e-01 7.215223e-01 7.281969e-01

2154 9.726254e-01 9.723215e-01 9.719270e-01 9.716460e-01 9.726786e-01 7.010377e-01 6.764356e-01 6.835557e-01 7.215549e-01 7.282296e-01

2155 9.726418e-01 9.723322e-01 9.719320e-01 9.716486e-01 9.726769e-01 7.010697e-01 6.764692e-01 6.835897e-01 7.215874e-01 7.282621e-01

2156 9.726581e-01 9.723425e-01 9.719365e-01 9.716507e-01 9.726749e-01 7.011016e-01 6.765027e-01 6.836235e-01 7.216197e-01 7.282945e-01

2157 9.726743e-01 9.723525e-01 9.719406e-01 9.716524e-01 9.726724e-01 7.011334e-01 6.765360e-01 6.836571e-01 7.216519e-01 7.283267e-01

2158 9.726902e-01 9.723621e-01 9.719442e-01 9.716536e-01 9.726695e-01 7.011651e-01 6.765692e-01 6.836907e-01 7.216841e-01 7.283588e-01

2159 9.727059e-01 9.723713e-01 9.719473e-01 9.716543e-01 9.726662e-01 7.011968e-01 6.766024e-01 6.837241e-01 7.217161e-01 7.283909e-01

2160 9.727214e-01 9.723802e-01 9.719500e-01 9.716545e-01 9.726625e-01 7.012283e-01 6.766354e-01 6.837575e-01 7.217480e-01 7.284228e-01

2161 9.727367e-01 9.723886e-01 9.719521e-01 9.716543e-01 9.726585e-01 7.012599e-01 6.766684e-01 6.837907e-01 7.217799e-01 7.284546e-01

2162 9.727574e-01 9.724092e-01 9.719726e-01 9.716747e-01 9.726765e-01 7.015098e-01 6.768803e-01 6.840195e-01 7.220872e-01 7.287825e-01

2163 9.727779e-01 9.724296e-01 9.719929e-01 9.716949e-01 9.726944e-01 7.017655e-01 6.770975e-01 6.842537e-01 7.224009e-01 7.291167e-01

2164 9.727981e-01 9.724497e-01 9.720129e-01 9.717148e-01 9.727121e-01 7.020268e-01 6.773201e-01 6.844936e-01 7.227208e-01 7.294573e-01

2165 9.728181e-01 9.724696e-01 9.720327e-01 9.717345e-01 9.727296e-01 7.022937e-01 6.775479e-01 6.847390e-01 7.230468e-01 7.298041e-01

2166 9.728379e-01 9.724893e-01 9.720523e-01 9.717540e-01 9.727470e-01 7.025661e-01 6.777811e-01 6.849900e-01 7.233788e-01 7.301570e-01

2167 9.728574e-01 9.725088e-01 9.720717e-01 9.717733e-01 9.727641e-01 7.028441e-01 6.780195e-01 6.852466e-01 7.237167e-01 7.305158e-01

2168 9.728768e-01 9.725281e-01 9.720908e-01 9.717924e-01 9.727811e-01 7.031275e-01 6.782632e-01 6.855086e-01 7.240604e-01 7.308805e-01

2169 9.728959e-01 9.725471e-01 9.721098e-01 9.718113e-01 9.727980e-01 7.034163e-01 6.785122e-01 6.857761e-01 7.244097e-01 7.312508e-01

2170 9.729149e-01 9.725660e-01 9.721286e-01 9.718300e-01 9.728147e-01 7.037103e-01 6.787664e-01 6.860490e-01 7.247645e-01 7.316266e-01

2171 9.729337e-01 9.725848e-01 9.721472e-01 9.718485e-01 9.728312e-01 7.040095e-01 6.790257e-01 6.863272e-01 7.251246e-01 7.320076e-01

2172 9.729523e-01 9.726033e-01 9.721657e-01 9.718669e-01 9.728476e-01 7.043137e-01 6.792902e-01 6.866107e-01 7.254898e-01 7.323937e-01

2173 9.729708e-01 9.726217e-01 9.721840e-01 9.718851e-01 9.728639e-01 7.046229e-01 6.795596e-01 6.868993e-01 7.258599e-01 7.327846e-01

2174 9.729891e-01 9.726399e-01 9.722021e-01 9.719031e-01 9.728800e-01 7.049369e-01 6.798340e-01 6.871931e-01 7.262348e-01 7.331802e-01

2175 9.730073e-01 9.726580e-01 9.722201e-01 9.719210e-01 9.728960e-01 7.052555e-01 6.801133e-01 6.874918e-01 7.266141e-01 7.335800e-01

2176 9.730253e-01 9.726759e-01 9.722380e-01 9.719388e-01 9.729119e-01 7.055786e-01 6.803973e-01 6.877953e-01 7.269977e-01 7.339840e-01

2177 9.730432e-01 9.726937e-01 9.722557e-01 9.719564e-01 9.729277e-01 7.059060e-01 6.806860e-01 6.881036e-01 7.273854e-01 7.343918e-01

2178 9.730609e-01 9.727114e-01 9.722732e-01 9.719738e-01 9.729433e-01 7.062375e-01 6.809792e-01 6.884164e-01 7.277768e-01 7.348031e-01

2179 9.730785e-01 9.727290e-01 9.722907e-01 9.719912e-01 9.729589e-01 7.065730e-01 6.812768e-01 6.887337e-01 7.281717e-01 7.352177e-01

2180 9.730960e-01 9.727464e-01 9.723080e-01 9.720084e-01 9.729744e-01 7.069122e-01 6.815786e-01 6.890552e-01 7.285698e-01 7.356353e-01

2181 9.731134e-01 9.727637e-01 9.723252e-01 9.720255e-01 9.729897e-01 7.072549e-01 6.818846e-01 6.893808e-01 7.289709e-01 7.360555e-01

2182 9.731307e-01 9.727809e-01 9.723423e-01 9.720425e-01 9.730050e-01 7.076009e-01 6.821944e-01 6.897103e-01 7.293746e-01 7.364780e-01

2183 9.731479e-01 9.727980e-01 9.723593e-01 9.720594e-01 9.730202e-01 7.079499e-01 6.825081e-01 6.900435e-01 7.297807e-01 7.369025e-01

2184 9.731649e-01 9.728150e-01 9.723762e-01 9.720762e-01 9.730352e-01 7.083018e-01 6.828253e-01 6.903802e-01 7.301888e-01 7.373287e-01

2185 9.731819e-01 9.728318e-01 9.723929e-01 9.720929e-01 9.730502e-01 7.086562e-01 6.831458e-01 6.907202e-01 7.305986e-01 7.377563e-01

2186 9.731988e-01 9.728486e-01 9.724096e-01 9.721095e-01 9.730651e-01 7.090130e-01 6.834696e-01 6.910632e-01 7.310098e-01 7.381848e-01

2187 9.732155e-01 9.728653e-01 9.724262e-01 9.721259e-01 9.730800e-01 7.093718e-01 6.837963e-01 6.914090e-01 7.314221e-01 7.386141e-01

2188 9.732322e-01 9.728819e-01 9.724427e-01 9.721423e-01 9.730947e-01 7.097324e-01 6.841257e-01 6.917574e-01 7.318352e-01 7.390436e-01

2189 9.732488e-01 9.728985e-01 9.724591e-01 9.721586e-01 9.731094e-01 7.100944e-01 6.844577e-01 6.921081e-01 7.322487e-01 7.394732e-01

2190 9.732654e-01 9.729149e-01 9.724755e-01 9.721749e-01 9.731240e-01 7.104577e-01 6.847919e-01 6.924609e-01 7.326623e-01 7.399024e-01

2191 9.732818e-01 9.729313e-01 9.724917e-01 9.721910e-01 9.731386e-01 7.108220e-01 6.851282e-01 6.928155e-01 7.330757e-01 7.403309e-01

2192 9.732982e-01 9.729476e-01 9.725079e-01 9.722071e-01 9.731530e-01 7.111869e-01 6.854663e-01 6.931717e-01 7.334886e-01 7.407584e-01

2193 9.733145e-01 9.729638e-01 9.725240e-01 9.722231e-01 9.731674e-01 7.115521e-01 6.858059e-01 6.935291e-01 7.339006e-01 7.411846e-01

2194 9.733308e-01 9.729800e-01 9.725400e-01 9.722390e-01 9.731818e-01 7.119174e-01 6.861468e-01 6.938876e-01 7.343113e-01 7.416090e-01

2195 9.733470e-01 9.729961e-01 9.725560e-01 9.722548e-01 9.731961e-01 7.122826e-01 6.864887e-01 6.942467e-01 7.347206e-01 7.420315e-01

2196 9.733631e-01 9.730121e-01 9.725719e-01 9.722706e-01 9.732103e-01 7.126472e-01 6.868314e-01 6.946063e-01 7.351281e-01 7.424516e-01

2197 9.733792e-01 9.730281e-01 9.725877e-01 9.722863e-01 9.732244e-01 7.130111e-01 6.871746e-01 6.949661e-01 7.355334e-01 7.428691e-01

2198 9.733952e-01 9.730440e-01 9.726035e-01 9.723020e-01 9.732386e-01 7.133738e-01 6.875179e-01 6.953257e-01 7.359363e-01 7.432837e-01

2199 9.734112e-01 9.730599e-01 9.726192e-01 9.723176e-01 9.732526e-01 7.137353e-01 6.878613e-01 6.956850e-01 7.363365e-01 7.436951e-01

2200 9.734271e-01 9.730757e-01 9.726349e-01 9.723331e-01 9.732666e-01 7.140951e-01 6.882043e-01 6.960435e-01 7.367337e-01 7.441030e-01

2201 9.734429e-01 9.730914e-01 9.726505e-01 9.723486e-01 9.732805e-01 7.144531e-01 6.885468e-01 6.964012e-01 7.371277e-01 7.445071e-01

2202 9.734587e-01 9.731071e-01 9.726660e-01 9.723640e-01 9.732944e-01 7.148088e-01 6.888884e-01 6.967576e-01 7.375181e-01 7.449072e-01

2203 9.734745e-01 9.731228e-01 9.726815e-01 9.723793e-01 9.733083e-01 7.151622e-01 6.892290e-01 6.971125e-01 7.379047e-01 7.453031e-01

2204 9.734902e-01 9.731384e-01 9.726970e-01 9.723946e-01 9.733220e-01 7.155130e-01 6.895681e-01 6.974657e-01 7.382873e-01 7.456945e-01

2205 9.735059e-01 9.731540e-01 9.727124e-01 9.724099e-01 9.733358e-01 7.158608e-01 6.899057e-01 6.978168e-01 7.386657e-01 7.460811e-01

2206 9.735216e-01 9.731695e-01 9.727278e-01 9.724251e-01 9.733495e-01 7.162055e-01 6.902414e-01 6.981657e-01 7.390396e-01 7.464629e-01

2207 9.735372e-01 9.731850e-01 9.727431e-01 9.724402e-01 9.733631e-01 7.165469e-01 6.905750e-01 6.985121e-01 7.394089e-01 7.468395e-01

2208 9.735528e-01 9.732005e-01 9.727583e-01 9.724553e-01 9.733767e-01 7.168847e-01 6.909063e-01 6.988557e-01 7.397733e-01 7.472108e-01

2209 9.735683e-01 9.732159e-01 9.727736e-01 9.724704e-01 9.733903e-01 7.172188e-01 6.912350e-01 6.991964e-01 7.401326e-01 7.475767e-01

2210 9.735839e-01 9.732313e-01 9.727887e-01 9.724854e-01 9.734038e-01 7.175489e-01 6.915609e-01 6.995339e-01 7.404868e-01 7.479370e-01

2211 9.735993e-01 9.732466e-01 9.728039e-01 9.725004e-01 9.734172e-01 7.178748e-01 6.918839e-01 6.998680e-01 7.408356e-01 7.482916e-01

2212 9.736148e-01 9.732619e-01 9.728190e-01 9.725153e-01 9.734307e-01 7.181965e-01 6.922036e-01 7.001984e-01 7.411790e-01 7.486402e-01

2213 9.736302e-01 9.732772e-01 9.728340e-01 9.725302e-01 9.734440e-01 7.185137e-01 6.925199e-01 7.005251e-01 7.415167e-01 7.489829e-01

2214 9.736456e-01 9.732925e-01 9.728491e-01 9.725450e-01 9.734574e-01 7.188263e-01 6.928327e-01 7.008478e-01 7.418487e-01 7.493196e-01

2215 9.736610e-01 9.733077e-01 9.728641e-01 9.725598e-01 9.734706e-01 7.191341e-01 6.931417e-01 7.011664e-01 7.421749e-01 7.496501e-01

2216 9.736764e-01 9.733229e-01 9.728790e-01 9.725746e-01 9.734839e-01 7.194371e-01 6.934468e-01 7.014807e-01 7.424952e-01 7.499743e-01

2217 9.736917e-01 9.733381e-01 9.728939e-01 9.725893e-01 9.734971e-01 7.197351e-01 6.937479e-01 7.017906e-01 7.428095e-01 7.502923e-01

2218 9.737070e-01 9.733532e-01 9.729088e-01 9.726040e-01 9.735102e-01 7.200281e-01 6.940447e-01 7.020958e-01 7.431178e-01 7.506039e-01

2219 9.737223e-01 9.733683e-01 9.729237e-01 9.726186e-01 9.735233e-01 7.203159e-01 6.943372e-01 7.023964e-01 7.434200e-01 7.509092e-01

2220 9.737376e-01 9.733834e-01 9.729385e-01 9.726332e-01 9.735364e-01 7.205984e-01 6.946252e-01 7.026922e-01 7.437161e-01 7.512081e-01

2221 9.737529e-01 9.733985e-01 9.729533e-01 9.726477e-01 9.735494e-01 7.208757e-01 6.949086e-01 7.029830e-01 7.440060e-01 7.515006e-01

2222 9.737681e-01 9.734135e-01 9.729680e-01 9.726623e-01 9.735624e-01 7.211476e-01 6.951874e-01 7.032689e-01 7.442898e-01 7.517868e-01

2223 9.737834e-01 9.734286e-01 9.729828e-01 9.726767e-01 9.735753e-01 7.214141e-01 6.954614e-01 7.035496e-01 7.445674e-01 7.520665e-01

2224 9.737986e-01 9.734436e-01 9.729974e-01 9.726912e-01 9.735882e-01 7.216752e-01 6.957305e-01 7.038253e-01 7.448389e-01 7.523399e-01

2225 9.738138e-01 9.734585e-01 9.730121e-01 9.727056e-01 9.736011e-01 7.219308e-01 6.959948e-01 7.040957e-01 7.451042e-01 7.526069e-01

2226 9.738290e-01 9.734735e-01 9.730267e-01 9.727199e-01 9.736139e-01 7.221810e-01 6.962540e-01 7.043609e-01 7.453634e-01 7.528677e-01

2227 9.738442e-01 9.734885e-01 9.730413e-01 9.727342e-01 9.736266e-01 7.224257e-01 6.965083e-01 7.046208e-01 7.456166e-01 7.531222e-01

2228 9.738593e-01 9.735034e-01 9.730559e-01 9.727485e-01 9.736393e-01 7.226650e-01 6.967575e-01 7.048753e-01 7.458637e-01 7.533706e-01

2229 9.738745e-01 9.735183e-01 9.730704e-01 9.727628e-01 9.736520e-01 7.228988e-01 6.970017e-01 7.051246e-01 7.461048e-01 7.536128e-01

2230 9.738896e-01 9.735332e-01 9.730849e-01 9.727770e-01 9.736646e-01 7.231272e-01 6.972407e-01 7.053685e-01 7.463400e-01 7.538489e-01

2231 9.739048e-01 9.735480e-01 9.730994e-01 9.727911e-01 9.736771e-01 7.233502e-01 6.974747e-01 7.056071e-01 7.465694e-01 7.540790e-01

2232 9.739199e-01 9.735629e-01 9.731138e-01 9.728052e-01 9.736896e-01 7.235678e-01 6.977035e-01 7.058404e-01 7.467929e-01 7.543033e-01

2233 9.739350e-01 9.735777e-01 9.731282e-01 9.728193e-01 9.737021e-01 7.237802e-01 6.979273e-01 7.060684e-01 7.470107e-01 7.545217e-01

2234 9.739502e-01 9.735925e-01 9.731426e-01 9.728334e-01 9.737145e-01 7.239872e-01 6.981459e-01 7.062911e-01 7.472229e-01 7.547343e-01

2235 9.739653e-01 9.736073e-01 9.731569e-01 9.728474e-01 9.737268e-01 7.241891e-01 6.983596e-01 7.065085e-01 7.474295e-01 7.549414e-01

2236 9.739804e-01 9.736221e-01 9.731712e-01 9.728613e-01 9.737391e-01 7.243859e-01 6.985681e-01 7.067208e-01 7.476306e-01 7.551428e-01

2237 9.739955e-01 9.736368e-01 9.731855e-01 9.728752e-01 9.737514e-01 7.245775e-01 6.987717e-01 7.069279e-01 7.478264e-01 7.553389e-01

2238 9.740106e-01 9.736516e-01 9.731997e-01 9.728891e-01 9.737636e-01 7.247642e-01 6.989704e-01 7.071299e-01 7.480169e-01 7.555296e-01

2239 9.740257e-01 9.736663e-01 9.732140e-01 9.729029e-01 9.737757e-01 7.249459e-01 6.991641e-01 7.073268e-01 7.482022e-01 7.557150e-01

2240 9.740408e-01 9.736810e-01 9.732281e-01 9.729167e-01 9.737877e-01 7.251228e-01 6.993530e-01 7.075188e-01 7.483824e-01 7.558953e-01

2241 9.740558e-01 9.736957e-01 9.732423e-01 9.729304e-01 9.737997e-01 7.252949e-01 6.995372e-01 7.077058e-01 7.485576e-01 7.560706e-01

2242 9.740709e-01 9.737104e-01 9.732563e-01 9.729441e-01 9.738117e-01 7.254624e-01 6.997166e-01 7.078880e-01 7.487279e-01 7.562410e-01

2243 9.740860e-01 9.737250e-01 9.732704e-01 9.729577e-01 9.738236e-01 7.256252e-01 6.998913e-01 7.080654e-01 7.488935e-01 7.564065e-01

2244 9.741011e-01 9.737397e-01 9.732844e-01 9.729713e-01 9.738354e-01 7.257836e-01 7.000615e-01 7.082382e-01 7.490544e-01 7.565674e-01

2245 9.741162e-01 9.737543e-01 9.732984e-01 9.729848e-01 9.738471e-01 7.259375e-01 7.002271e-01 7.084063e-01 7.492108e-01 7.567237e-01

2246 9.741312e-01 9.737689e-01 9.733123e-01 9.729983e-01 9.738588e-01 7.260872e-01 7.003883e-01 7.085699e-01 7.493627e-01 7.568756e-01

2247 9.741463e-01 9.737834e-01 9.733262e-01 9.730117e-01 9.738704e-01 7.262326e-01 7.005452e-01 7.087291e-01 7.495102e-01 7.570230e-01

2248 9.741614e-01 9.737980e-01 9.733401e-01 9.730250e-01 9.738819e-01 7.263739e-01 7.006978e-01 7.088839e-01 7.496536e-01 7.571663e-01

2249 9.741764e-01 9.738125e-01 9.733539e-01 9.730383e-01 9.738934e-01 7.265111e-01 7.008462e-01 7.090344e-01 7.497928e-01 7.573054e-01

2250 9.741915e-01 9.738270e-01 9.733677e-01 9.730516e-01 9.739047e-01 7.266445e-01 7.009906e-01 7.091808e-01 7.499280e-01 7.574404e-01

2251 9.742066e-01 9.738415e-01 9.733814e-01 9.730648e-01 9.739160e-01 7.267740e-01 7.011309e-01 7.093231e-01 7.500593e-01 7.575716e-01

2252 9.742216e-01 9.738560e-01 9.733951e-01 9.730779e-01 9.739272e-01 7.268997e-01 7.012673e-01 7.094614e-01 7.501868e-01 7.576990e-01

2253 9.742367e-01 9.738704e-01 9.734087e-01 9.730909e-01 9.739384e-01 7.270219e-01 7.013999e-01 7.095959e-01 7.503106e-01 7.578226e-01

2254 9.742518e-01 9.738848e-01 9.734222e-01 9.731039e-01 9.739494e-01 7.271405e-01 7.015287e-01 7.097265e-01 7.504308e-01 7.579427e-01

2255 9.742668e-01 9.738992e-01 9.734358e-01 9.731168e-01 9.739604e-01 7.272556e-01 7.016539e-01 7.098535e-01 7.505475e-01 7.580593e-01

2256 9.742819e-01 9.739136e-01 9.734492e-01 9.731297e-01 9.739712e-01 7.273674e-01 7.017756e-01 7.099769e-01 7.506608e-01 7.581725e-01

2257 9.742969e-01 9.739279e-01 9.734626e-01 9.731425e-01 9.739820e-01 7.274760e-01 7.018937e-01 7.100967e-01 7.507708e-01 7.582825e-01

2258 9.743120e-01 9.739422e-01 9.734760e-01 9.731552e-01 9.739927e-01 7.275814e-01 7.020085e-01 7.102131e-01 7.508777e-01 7.583892e-01

2259 9.743270e-01 9.739565e-01 9.734893e-01 9.731678e-01 9.740032e-01 7.276837e-01 7.021201e-01 7.103262e-01 7.509815e-01 7.584929e-01

2260 9.743421e-01 9.739708e-01 9.735025e-01 9.731803e-01 9.740137e-01 7.277831e-01 7.022284e-01 7.104361e-01 7.510822e-01 7.585936e-01

2261 9.743571e-01 9.739850e-01 9.735156e-01 9.731928e-01 9.740241e-01 7.278795e-01 7.023337e-01 7.105429e-01 7.511801e-01 7.586914e-01

2262 9.743721e-01 9.739992e-01 9.735287e-01 9.732052e-01 9.740343e-01 7.279732e-01 7.024359e-01 7.106466e-01 7.512752e-01 7.587864e-01

2263 9.743872e-01 9.740133e-01 9.735417e-01 9.732174e-01 9.740445e-01 7.280642e-01 7.025352e-01 7.107473e-01 7.513675e-01 7.588787e-01

2264 9.744022e-01 9.740274e-01 9.735547e-01 9.732296e-01 9.740545e-01 7.281525e-01 7.026316e-01 7.108452e-01 7.514572e-01 7.589683e-01

2265 9.744172e-01 9.740415e-01 9.735676e-01 9.732417e-01 9.740644e-01 7.282383e-01 7.027253e-01 7.109403e-01 7.515444e-01 7.590555e-01

2266 9.744322e-01 9.740555e-01 9.735804e-01 9.732538e-01 9.740742e-01 7.283217e-01 7.028164e-01 7.110327e-01 7.516291e-01 7.591402e-01

2267 9.744473e-01 9.740695e-01 9.735931e-01 9.732657e-01 9.740839e-01 7.284026e-01 7.029048e-01 7.111225e-01 7.517114e-01 7.592225e-01

2268 9.744623e-01 9.740834e-01 9.736057e-01 9.732775e-01 9.740935e-01 7.284813e-01 7.029908e-01 7.112098e-01 7.517914e-01 7.593025e-01

2269 9.744773e-01 9.740973e-01 9.736183e-01 9.732892e-01 9.741029e-01 7.285578e-01 7.030743e-01 7.112946e-01 7.518692e-01 7.593803e-01

2270 9.744922e-01 9.741112e-01 9.736307e-01 9.733008e-01 9.741122e-01 7.286321e-01 7.031555e-01 7.113771e-01 7.519449e-01 7.594560e-01

2271 9.745072e-01 9.741250e-01 9.736431e-01 9.733123e-01 9.741214e-01 7.287043e-01 7.032344e-01 7.114572e-01 7.520185e-01 7.595296e-01

2272 9.745222e-01 9.741387e-01 9.736554e-01 9.733237e-01 9.741304e-01 7.287746e-01 7.033111e-01 7.115352e-01 7.520901e-01 7.596013e-01

2273 9.745371e-01 9.741524e-01 9.736675e-01 9.733349e-01 9.741393e-01 7.288429e-01 7.033857e-01 7.116110e-01 7.521597e-01 7.596710e-01

2274 9.745521e-01 9.741661e-01 9.736796e-01 9.733461e-01 9.741480e-01 7.289093e-01 7.034583e-01 7.116847e-01 7.522275e-01 7.597388e-01

2275 9.745670e-01 9.741797e-01 9.736916e-01 9.733571e-01 9.741566e-01 7.289740e-01 7.035288e-01 7.117565e-01 7.522935e-01 7.598049e-01

2276 9.745819e-01 9.741932e-01 9.737035e-01 9.733680e-01 9.741650e-01 7.290369e-01 7.035975e-01 7.118263e-01 7.523578e-01 7.598693e-01

2277 9.745968e-01 9.742066e-01 9.737152e-01 9.733787e-01 9.741733e-01 7.290981e-01 7.036643e-01 7.118943e-01 7.524204e-01 7.599320e-01

2278 9.746117e-01 9.742200e-01 9.737269e-01 9.733893e-01 9.741814e-01 7.291578e-01 7.037293e-01 7.119605e-01 7.524814e-01 7.599931e-01

2279 9.746266e-01 9.742334e-01 9.737384e-01 9.733998e-01 9.741894e-01 7.292159e-01 7.037926e-01 7.120249e-01 7.525408e-01 7.600526e-01

2280 9.746414e-01 9.742466e-01 9.737498e-01 9.734102e-01 9.741971e-01 7.292725e-01 7.038543e-01 7.120877e-01 7.525988e-01 7.601107e-01

2281 9.746562e-01 9.742598e-01 9.737611e-01 9.734203e-01 9.742048e-01 7.293277e-01 7.039144e-01 7.121489e-01 7.526553e-01 7.601673e-01

2282 9.746710e-01 9.742729e-01 9.737722e-01 9.734304e-01 9.742122e-01 7.293815e-01 7.039729e-01 7.122085e-01 7.527104e-01 7.602226e-01

2283 9.746858e-01 9.742859e-01 9.737832e-01 9.734403e-01 9.742195e-01 7.294339e-01 7.040300e-01 7.122666e-01 7.527642e-01 7.602765e-01

2284 9.747005e-01 9.742988e-01 9.737941e-01 9.734500e-01 9.742266e-01 7.294851e-01 7.040856e-01 7.123233e-01 7.528167e-01 7.603292e-01

2285 9.747153e-01 9.743117e-01 9.738048e-01 9.734596e-01 9.742335e-01 7.295351e-01 7.041399e-01 7.123786e-01 7.528680e-01 7.603806e-01

2286 9.747299e-01 9.743244e-01 9.738154e-01 9.734690e-01 9.742402e-01 7.295838e-01 7.041929e-01 7.124326e-01 7.529181e-01 7.604309e-01

2287 9.747446e-01 9.743371e-01 9.738258e-01 9.734782e-01 9.742467e-01 7.296315e-01 7.042446e-01 7.124853e-01 7.529671e-01 7.604800e-01

2288 9.747592e-01 9.743496e-01 9.738361e-01 9.734873e-01 9.742530e-01 7.296780e-01 7.042950e-01 7.125368e-01 7.530149e-01 7.605280e-01

2289 9.747738e-01 9.743621e-01 9.738462e-01 9.734961e-01 9.742592e-01 7.297235e-01 7.043444e-01 7.125871e-01 7.530617e-01 7.605750e-01

2290 9.747883e-01 9.743744e-01 9.738561e-01 9.735048e-01 9.742651e-01 7.297680e-01 7.043925e-01 7.126362e-01 7.531075e-01 7.606210e-01

2291 9.748028e-01 9.743867e-01 9.738659e-01 9.735133e-01 9.742708e-01 7.298115e-01 7.044396e-01 7.126843e-01 7.531524e-01 7.606660e-01

2292 9.748173e-01 9.743988e-01 9.738755e-01 9.735216e-01 9.742764e-01 7.298542e-01 7.044857e-01 7.127313e-01 7.531963e-01 7.607100e-01

2293 9.748317e-01 9.744108e-01 9.738849e-01 9.735297e-01 9.742817e-01 7.298959e-01 7.045308e-01 7.127773e-01 7.532393e-01 7.607532e-01

2294 9.748461e-01 9.744227e-01 9.738942e-01 9.735376e-01 9.742868e-01 7.299367e-01 7.045749e-01 7.128223e-01 7.532814e-01 7.607955e-01

2295 9.748604e-01 9.744344e-01 9.739032e-01 9.735454e-01 9.742916e-01 7.299768e-01 7.046181e-01 7.128664e-01 7.533227e-01 7.608370e-01

2296 9.748746e-01 9.744461e-01 9.739121e-01 9.735528e-01 9.742963e-01 7.300161e-01 7.046604e-01 7.129096e-01 7.533632e-01 7.608777e-01

2297 9.748888e-01 9.744576e-01 9.739207e-01 9.735601e-01 9.743007e-01 7.300546e-01 7.047019e-01 7.129520e-01 7.534030e-01 7.609176e-01

2298 9.749030e-01 9.744689e-01 9.739292e-01 9.735672e-01 9.743050e-01 7.300924e-01 7.047425e-01 7.129935e-01 7.534420e-01 7.609568e-01

2299 9.749170e-01 9.744801e-01 9.739374e-01 9.735740e-01 9.743089e-01 7.301295e-01 7.047824e-01 7.130343e-01 7.534803e-01 7.609953e-01

2300 9.749310e-01 9.744912e-01 9.739454e-01 9.735806e-01 9.743127e-01 7.301660e-01 7.048216e-01 7.130743e-01 7.535179e-01 7.610331e-01

2301 9.749450e-01 9.745021e-01 9.739532e-01 9.735870e-01 9.743162e-01 7.302018e-01 7.048600e-01 7.131136e-01 7.535550e-01 7.610703e-01

2302 9.749588e-01 9.745128e-01 9.739608e-01 9.735932e-01 9.743195e-01 7.302371e-01 7.048978e-01 7.131522e-01 7.535914e-01 7.611069e-01

2303 9.749726e-01 9.745234e-01 9.739681e-01 9.735991e-01 9.743226e-01 7.302718e-01 7.049349e-01 7.131901e-01 7.536272e-01 7.611428e-01

2304 9.749863e-01 9.745338e-01 9.739752e-01 9.736047e-01 9.743254e-01 7.303059e-01 7.049714e-01 7.132274e-01 7.536624e-01 7.611782e-01

2305 9.749999e-01 9.745440e-01 9.739821e-01 9.736101e-01 9.743280e-01 7.303395e-01 7.050074e-01 7.132641e-01 7.536971e-01 7.612131e-01

2306 9.750134e-01 9.745541e-01 9.739887e-01 9.736153e-01 9.743304e-01 7.303726e-01 7.050427e-01 7.133003e-01 7.537313e-01 7.612475e-01

2307 9.750268e-01 9.745639e-01 9.739951e-01 9.736202e-01 9.743325e-01 7.304052e-01 7.050775e-01 7.133358e-01 7.537650e-01 7.612813e-01

2308 9.750402e-01 9.745736e-01 9.740012e-01 9.736248e-01 9.743343e-01 7.304374e-01 7.051118e-01 7.133709e-01 7.537982e-01 7.613147e-01

2309 9.750534e-01 9.745830e-01 9.740070e-01 9.736292e-01 9.743360e-01 7.304691e-01 7.051456e-01 7.134054e-01 7.538310e-01 7.613476e-01

2310 9.750665e-01 9.745923e-01 9.740126e-01 9.736333e-01 9.743374e-01 7.305005e-01 7.051790e-01 7.134395e-01 7.538633e-01 7.613801e-01

2311 9.750795e-01 9.746014e-01 9.740179e-01 9.736372e-01 9.743385e-01 7.305314e-01 7.052119e-01 7.134731e-01 7.538953e-01 7.614122e-01

2312 9.750924e-01 9.746102e-01 9.740229e-01 9.736407e-01 9.743395e-01 7.305620e-01 7.052443e-01 7.135063e-01 7.539268e-01 7.614438e-01

2313 9.751051e-01 9.746188e-01 9.740277e-01 9.736440e-01 9.743402e-01 7.305922e-01 7.052764e-01 7.135391e-01 7.539580e-01 7.614751e-01

2314 9.751177e-01 9.746272e-01 9.740321e-01 9.736471e-01 9.743406e-01 7.306221e-01 7.053081e-01 7.135715e-01 7.539888e-01 7.615061e-01

2315 9.751302e-01 9.746354e-01 9.740363e-01 9.736498e-01 9.743408e-01 7.306517e-01 7.053394e-01 7.136035e-01 7.540193e-01 7.615366e-01

2316 9.751426e-01 9.746433e-01 9.740402e-01 9.736523e-01 9.743408e-01 7.306809e-01 7.053704e-01 7.136351e-01 7.540494e-01 7.615669e-01

2317 9.751548e-01 9.746510e-01 9.740438e-01 9.736545e-01 9.743406e-01 7.307099e-01 7.054010e-01 7.136664e-01 7.540793e-01 7.615968e-01

2318 9.751668e-01 9.746584e-01 9.740470e-01 9.736564e-01 9.743401e-01 7.307387e-01 7.054314e-01 7.136974e-01 7.541088e-01 7.616265e-01

2319 9.751787e-01 9.746656e-01 9.740500e-01 9.736580e-01 9.743395e-01 7.307671e-01 7.054614e-01 7.137281e-01 7.541381e-01 7.616558e-01

2320 9.751904e-01 9.746725e-01 9.740527e-01 9.736593e-01 9.743386e-01 7.307954e-01 7.054912e-01 7.137585e-01 7.541671e-01 7.616849e-01

2321 9.752020e-01 9.746792e-01 9.740550e-01 9.736603e-01 9.743375e-01 7.308234e-01 7.055207e-01 7.137887e-01 7.541959e-01 7.617138e-01

2322 9.752134e-01 9.746856e-01 9.740570e-01 9.736611e-01 9.743362e-01 7.308512e-01 7.055499e-01 7.138185e-01 7.542244e-01 7.617423e-01

2323 9.752246e-01 9.746917e-01 9.740587e-01 9.736616e-01 9.743347e-01 7.308788e-01 7.055790e-01 7.138482e-01 7.542528e-01 7.617707e-01

2324 9.752356e-01 9.746975e-01 9.740601e-01 9.736618e-01 9.743330e-01 7.309062e-01 7.056078e-01 7.138776e-01 7.542809e-01 7.617988e-01

2325 9.752464e-01 9.747030e-01 9.740612e-01 9.736616e-01 9.743311e-01 7.309334e-01 7.056364e-01 7.139068e-01 7.543088e-01 7.618267e-01

2326 9.752570e-01 9.747083e-01 9.740619e-01 9.736613e-01 9.743290e-01 7.309605e-01 7.056648e-01 7.139358e-01 7.543365e-01 7.618545e-01

2327 9.752675e-01 9.747132e-01 9.740623e-01 9.736606e-01 9.743268e-01 7.309874e-01 7.056930e-01 7.139646e-01 7.543640e-01 7.618820e-01

2328 9.752776e-01 9.747178e-01 9.740624e-01 9.736597e-01 9.743244e-01 7.310142e-01 7.057211e-01 7.139932e-01 7.543914e-01 7.619094e-01

2329 9.752876e-01 9.747222e-01 9.740622e-01 9.736584e-01 9.743219e-01 7.310409e-01 7.057490e-01 7.140217e-01 7.544186e-01 7.619365e-01

2330 9.752973e-01 9.747262e-01 9.740616e-01 9.736570e-01 9.743192e-01 7.310674e-01 7.057767e-01 7.140500e-01 7.544457e-01 7.619636e-01

2331 9.753068e-01 9.747299e-01 9.740607e-01 9.736552e-01 9.743164e-01 7.310939e-01 7.058044e-01 7.140782e-01 7.544726e-01 7.619904e-01

2332 9.753161e-01 9.747333e-01 9.740595e-01 9.736532e-01 9.743134e-01 7.311202e-01 7.058319e-01 7.141062e-01 7.544994e-01 7.620172e-01

2333 9.753251e-01 9.747363e-01 9.740580e-01 9.736509e-01 9.743104e-01 7.311465e-01 7.058593e-01 7.141342e-01 7.545261e-01 7.620438e-01

2334 9.753338e-01 9.747390e-01 9.740561e-01 9.736484e-01 9.743072e-01 7.311727e-01 7.058865e-01 7.141620e-01 7.545527e-01 7.620702e-01

2335 9.753423e-01 9.747414e-01 9.740539e-01 9.736456e-01 9.743040e-01 7.311988e-01 7.059137e-01 7.141897e-01 7.545792e-01 7.620966e-01

2336 9.753505e-01 9.747435e-01 9.740514e-01 9.736426e-01 9.743007e-01 7.312248e-01 7.059408e-01 7.142173e-01 7.546055e-01 7.621228e-01

2337 9.753584e-01 9.747452e-01 9.740486e-01 9.736394e-01 9.742974e-01 7.312508e-01 7.059679e-01 7.142449e-01 7.546318e-01 7.621490e-01

2338 9.753660e-01 9.747466e-01 9.740455e-01 9.736359e-01 9.742940e-01 7.312767e-01 7.059948e-01 7.142723e-01 7.546581e-01 7.621750e-01

2339 9.753733e-01 9.747476e-01 9.740421e-01 9.736323e-01 9.742906e-01 7.313026e-01 7.060217e-01 7.142997e-01 7.546842e-01 7.622009e-01

2340 9.753803e-01 9.747483e-01 9.740383e-01 9.736284e-01 9.742871e-01 7.313285e-01 7.060486e-01 7.143271e-01 7.547103e-01 7.622268e-01

2341 9.753869e-01 9.747487e-01 9.740343e-01 9.736243e-01 9.742837e-01 7.313543e-01 7.060754e-01 7.143544e-01 7.547363e-01 7.622526e-01

2342 9.754013e-01 9.747629e-01 9.740485e-01 9.736383e-01 9.742961e-01 7.316174e-01 7.063010e-01 7.145997e-01 7.550635e-01 7.626020e-01

2343 9.754155e-01 9.747770e-01 9.740624e-01 9.736522e-01 9.743084e-01 7.318849e-01 7.065312e-01 7.148497e-01 7.553954e-01 7.629561e-01

2344 9.754296e-01 9.747910e-01 9.740763e-01 9.736660e-01 9.743206e-01 7.321569e-01 7.067657e-01 7.151042e-01 7.557317e-01 7.633146e-01

2345 9.754436e-01 9.748049e-01 9.740901e-01 9.736796e-01 9.743327e-01 7.324332e-01 7.070047e-01 7.153634e-01 7.560724e-01 7.636773e-01

2346 9.754574e-01 9.748187e-01 9.741037e-01 9.736932e-01 9.743447e-01 7.327137e-01 7.072479e-01 7.156269e-01 7.564172e-01 7.640440e-01

2347 9.754712e-01 9.748324e-01 9.741173e-01 9.737066e-01 9.743566e-01 7.329982e-01 7.074954e-01 7.158949e-01 7.567660e-01 7.644145e-01

2348 9.754849e-01 9.748460e-01 9.741308e-01 9.737200e-01 9.743684e-01 7.332867e-01 7.077470e-01 7.161671e-01 7.571185e-01 7.647886e-01

2349 9.754985e-01 9.748595e-01 9.741441e-01 9.737332e-01 9.743802e-01 7.335790e-01 7.080027e-01 7.164434e-01 7.574746e-01 7.651660e-01

2350 9.755120e-01 9.748729e-01 9.741574e-01 9.737464e-01 9.743919e-01 7.338750e-01 7.082624e-01 7.167238e-01 7.578340e-01 7.655465e-01

2351 9.755254e-01 9.748862e-01 9.741706e-01 9.737594e-01 9.744035e-01 7.341744e-01 7.085260e-01 7.170082e-01 7.581964e-01 7.659298e-01

2352 9.755388e-01 9.748994e-01 9.741837e-01 9.737724e-01 9.744150e-01 7.344772e-01 7.087933e-01 7.172963e-01 7.585618e-01 7.663157e-01

2353 9.755520e-01 9.749126e-01 9.741967e-01 9.737853e-01 9.744265e-01 7.347832e-01 7.090642e-01 7.175881e-01 7.589297e-01 7.667039e-01

2354 9.755652e-01 9.749256e-01 9.742096e-01 9.737981e-01 9.744378e-01 7.350921e-01 7.093387e-01 7.178833e-01 7.593000e-01 7.670942e-01

2355 9.755783e-01 9.749386e-01 9.742225e-01 9.738108e-01 9.744492e-01 7.354038e-01 7.096165e-01 7.181819e-01 7.596724e-01 7.674862e-01

2356 9.755914e-01 9.749516e-01 9.742353e-01 9.738235e-01 9.744604e-01 7.357181e-01 7.098975e-01 7.184836e-01 7.600466e-01 7.678796e-01

2357 9.756044e-01 9.749645e-01 9.742480e-01 9.738361e-01 9.744716e-01 7.360348e-01 7.101816e-01 7.187884e-01 7.604224e-01 7.682743e-01

2358 9.756173e-01 9.749773e-01 9.742606e-01 9.738486e-01 9.744827e-01 7.363537e-01 7.104686e-01 7.190959e-01 7.607995e-01 7.686698e-01

2359 9.756302e-01 9.749900e-01 9.742732e-01 9.738610e-01 9.744938e-01 7.366745e-01 7.107583e-01 7.194061e-01 7.611776e-01 7.690660e-01

2360 9.756430e-01 9.750027e-01 9.742857e-01 9.738734e-01 9.745048e-01 7.369971e-01 7.110506e-01 7.197186e-01 7.615565e-01 7.694624e-01

2361 9.756557e-01 9.750153e-01 9.742982e-01 9.738857e-01 9.745158e-01 7.373212e-01 7.113453e-01 7.200334e-01 7.619358e-01 7.698589e-01

2362 9.756684e-01 9.750279e-01 9.743106e-01 9.738980e-01 9.745267e-01 7.376465e-01 7.116421e-01 7.203502e-01 7.623153e-01 7.702550e-01

2363 9.756811e-01 9.750404e-01 9.743229e-01 9.739102e-01 9.745375e-01 7.379729e-01 7.119409e-01 7.206688e-01 7.626948e-01 7.706507e-01

2364 9.756937e-01 9.750529e-01 9.743352e-01 9.739223e-01 9.745483e-01 7.383001e-01 7.122415e-01 7.209889e-01 7.630738e-01 7.710454e-01

2365 9.757062e-01 9.750653e-01 9.743474e-01 9.739344e-01 9.745590e-01 7.386279e-01 7.125437e-01 7.213103e-01 7.634522e-01 7.714390e-01

2366 9.757187e-01 9.750777e-01 9.743596e-01 9.739465e-01 9.745697e-01 7.389560e-01 7.128472e-01 7.216329e-01 7.638296e-01 7.718311e-01

2367 9.757312e-01 9.750900e-01 9.743718e-01 9.739584e-01 9.745804e-01 7.392842e-01 7.131519e-01 7.219564e-01 7.642059e-01 7.722216e-01

2368 9.757436e-01 9.751023e-01 9.743839e-01 9.739704e-01 9.745910e-01 7.396122e-01 7.134575e-01 7.222804e-01 7.645806e-01 7.726100e-01

2369 9.757560e-01 9.751145e-01 9.743959e-01 9.739822e-01 9.746015e-01 7.399398e-01 7.137638e-01 7.226049e-01 7.649536e-01 7.729962e-01

2370 9.757684e-01 9.751267e-01 9.744079e-01 9.739940e-01 9.746120e-01 7.402667e-01 7.140706e-01 7.229296e-01 7.653247e-01 7.733799e-01

2371 9.757807e-01 9.751389e-01 9.744198e-01 9.740058e-01 9.746224e-01 7.405928e-01 7.143777e-01 7.232542e-01 7.656934e-01 7.737608e-01

2372 9.757930e-01 9.751510e-01 9.744317e-01 9.740176e-01 9.746329e-01 7.409178e-01 7.146849e-01 7.235785e-01 7.660597e-01 7.741387e-01

2373 9.758053e-01 9.751631e-01 9.744436e-01 9.740292e-01 9.746432e-01 7.412414e-01 7.149918e-01 7.239023e-01 7.664232e-01 7.745134e-01

2374 9.758175e-01 9.751752e-01 9.744554e-01 9.740409e-01 9.746535e-01 7.415634e-01 7.152984e-01 7.242253e-01 7.667837e-01 7.748845e-01

2375 9.758297e-01 9.751872e-01 9.744672e-01 9.740525e-01 9.746638e-01 7.418837e-01 7.156043e-01 7.245473e-01 7.671411e-01 7.752520e-01

2376 9.758419e-01 9.751992e-01 9.744790e-01 9.740640e-01 9.746740e-01 7.422019e-01 7.159094e-01 7.248681e-01 7.674950e-01 7.756156e-01

2377 9.758541e-01 9.752112e-01 9.744907e-01 9.740755e-01 9.746842e-01 7.425179e-01 7.162134e-01 7.251874e-01 7.678454e-01 7.759751e-01

2378 9.758662e-01 9.752231e-01 9.745023e-01 9.740870e-01 9.746944e-01 7.428314e-01 7.165162e-01 7.255050e-01 7.681919e-01 7.763302e-01

2379 9.758783e-01 9.752350e-01 9.745140e-01 9.740984e-01 9.747045e-01 7.431423e-01 7.168175e-01 7.258208e-01 7.685344e-01 7.766810e-01

2380 9.758904e-01 9.752469e-01 9.745256e-01 9.741098e-01 9.747145e-01 7.434504e-01 7.171170e-01 7.261344e-01 7.688727e-01 7.770271e-01

2381 9.759024e-01 9.752588e-01 9.745371e-01 9.741211e-01 9.747245e-01 7.437555e-01 7.174147e-01 7.264458e-01 7.692068e-01 7.773684e-01

2382 9.759145e-01 9.752706e-01 9.745486e-01 9.741325e-01 9.747345e-01 7.440574e-01 7.177103e-01 7.267546e-01 7.695363e-01 7.777048e-01

2383 9.759265e-01 9.752824e-01 9.745601e-01 9.741437e-01 9.747444e-01 7.443560e-01 7.180036e-01 7.270608e-01 7.698612e-01 7.780362e-01

2384 9.759385e-01 9.752942e-01 9.745716e-01 9.741549e-01 9.747543e-01 7.446510e-01 7.182944e-01 7.273641e-01 7.701814e-01 7.783624e-01

2385 9.759505e-01 9.753059e-01 9.745830e-01 9.741661e-01 9.747642e-01 7.449424e-01 7.185826e-01 7.276643e-01 7.704967e-01 7.786834e-01

2386 9.759625e-01 9.753176e-01 9.745944e-01 9.741773e-01 9.747740e-01 7.452299e-01 7.188680e-01 7.279613e-01 7.708070e-01 7.789990e-01

2387 9.759745e-01 9.753294e-01 9.746058e-01 9.741884e-01 9.747837e-01 7.455136e-01 7.191504e-01 7.282550e-01 7.711122e-01 7.793091e-01

2388 9.759864e-01 9.753410e-01 9.746171e-01 9.741995e-01 9.747934e-01 7.457932e-01 7.194297e-01 7.285451e-01 7.714122e-01 7.796138e-01

2389 9.759984e-01 9.753527e-01 9.746284e-01 9.742105e-01 9.748031e-01 7.460686e-01 7.197057e-01 7.288316e-01 7.717070e-01 7.799128e-01

2390 9.760103e-01 9.753643e-01 9.746397e-01 9.742215e-01 9.748127e-01 7.463398e-01 7.199783e-01 7.291142e-01 7.719965e-01 7.802062e-01

2391 9.760222e-01 9.753760e-01 9.746509e-01 9.742324e-01 9.748223e-01 7.466066e-01 7.202473e-01 7.293930e-01 7.722806e-01 7.804940e-01

2392 9.760341e-01 9.753876e-01 9.746621e-01 9.742433e-01 9.748318e-01 7.468689e-01 7.205127e-01 7.296677e-01 7.725594e-01 7.807761e-01

2393 9.760460e-01 9.753991e-01 9.746733e-01 9.742542e-01 9.748413e-01 7.471268e-01 7.207743e-01 7.299383e-01 7.728326e-01 7.810524e-01

2394 9.760579e-01 9.754107e-01 9.746844e-01 9.742651e-01 9.748508e-01 7.473800e-01 7.210320e-01 7.302046e-01 7.731004e-01 7.813230e-01

2395 9.760698e-01 9.754222e-01 9.746955e-01 9.742758e-01 9.748602e-01 7.476287e-01 7.212858e-01 7.304667e-01 7.733628e-01 7.815879e-01

2396 9.760816e-01 9.754338e-01 9.747066e-01 9.742866e-01 9.748695e-01 7.478726e-01 7.215355e-01 7.307243e-01 7.736196e-01 7.818471e-01

2397 9.760935e-01 9.754453e-01 9.747176e-01 9.742973e-01 9.748788e-01 7.481119e-01 7.217810e-01 7.309775e-01 7.738709e-01 7.821005e-01

2398 9.761053e-01 9.754567e-01 9.747286e-01 9.743080e-01 9.748881e-01 7.483464e-01 7.220224e-01 7.312261e-01 7.741168e-01 7.823483e-01

2399 9.761172e-01 9.754682e-01 9.747396e-01 9.743186e-01 9.748973e-01 7.485761e-01 7.222595e-01 7.314702e-01 7.743572e-01 7.825904e-01

2400 9.761290e-01 9.754796e-01 9.747505e-01 9.743292e-01 9.749065e-01 7.488010e-01 7.224922e-01 7.317097e-01 7.745922e-01 7.828269e-01

2401 9.761408e-01 9.754911e-01 9.747614e-01 9.743398e-01 9.749156e-01 7.490212e-01 7.227206e-01 7.319446e-01 7.748217e-01 7.830578e-01

2402 9.761527e-01 9.755025e-01 9.747723e-01 9.743503e-01 9.749246e-01 7.492366e-01 7.229447e-01 7.321748e-01 7.750459e-01 7.832832e-01

2403 9.761645e-01 9.755138e-01 9.747831e-01 9.743607e-01 9.749336e-01 7.494472e-01 7.231643e-01 7.324003e-01 7.752647e-01 7.835031e-01

2404 9.761763e-01 9.755252e-01 9.747939e-01 9.743712e-01 9.749426e-01 7.496531e-01 7.233794e-01 7.326211e-01 7.754783e-01 7.837176e-01

2405 9.761881e-01 9.755366e-01 9.748047e-01 9.743815e-01 9.749515e-01 7.498543e-01 7.235901e-01 7.328372e-01 7.756867e-01 7.839268e-01

2406 9.761999e-01 9.755479e-01 9.748154e-01 9.743918e-01 9.749603e-01 7.500507e-01 7.237964e-01 7.330487e-01 7.758899e-01 7.841307e-01

2407 9.762117e-01 9.755592e-01 9.748261e-01 9.744021e-01 9.749691e-01 7.502425e-01 7.239982e-01 7.332555e-01 7.760880e-01 7.843294e-01

2408 9.762235e-01 9.755705e-01 9.748367e-01 9.744124e-01 9.749778e-01 7.504297e-01 7.241955e-01 7.334576e-01 7.762810e-01 7.845230e-01

2409 9.762353e-01 9.755817e-01 9.748473e-01 9.744225e-01 9.749864e-01 7.506123e-01 7.243885e-01 7.336551e-01 7.764692e-01 7.847115e-01

2410 9.762471e-01 9.755930e-01 9.748579e-01 9.744327e-01 9.749950e-01 7.507903e-01 7.245770e-01 7.338480e-01 7.766524e-01 7.848951e-01

2411 9.762588e-01 9.756042e-01 9.748684e-01 9.744427e-01 9.750036e-01 7.509639e-01 7.247612e-01 7.340364e-01 7.768308e-01 7.850738e-01

2412 9.762706e-01 9.756154e-01 9.748789e-01 9.744528e-01 9.750120e-01 7.511331e-01 7.249410e-01 7.342202e-01 7.770046e-01 7.852477e-01

2413 9.762824e-01 9.756265e-01 9.748893e-01 9.744627e-01 9.750205e-01 7.512979e-01 7.251165e-01 7.343996e-01 7.771737e-01 7.854170e-01

2414 9.762941e-01 9.756377e-01 9.748997e-01 9.744727e-01 9.750288e-01 7.514584e-01 7.252877e-01 7.345746e-01 7.773382e-01 7.855816e-01

2415 9.763059e-01 9.756488e-01 9.749100e-01 9.744825e-01 9.750371e-01 7.516147e-01 7.254547e-01 7.347452e-01 7.774983e-01 7.857418e-01

2416 9.763176e-01 9.756599e-01 9.749203e-01 9.744923e-01 9.750453e-01 7.517669e-01 7.256176e-01 7.349114e-01 7.776540e-01 7.858975e-01

2417 9.763294e-01 9.756710e-01 9.749306e-01 9.745021e-01 9.750534e-01 7.519149e-01 7.257763e-01 7.350735e-01 7.778055e-01 7.860490e-01

2418 9.763411e-01 9.756820e-01 9.749408e-01 9.745118e-01 9.750615e-01 7.520590e-01 7.259309e-01 7.352313e-01 7.779527e-01 7.861962e-01

2419 9.763529e-01 9.756930e-01 9.749509e-01 9.745214e-01 9.750694e-01 7.521991e-01 7.260816e-01 7.353851e-01 7.780959e-01 7.863393e-01

2420 9.763646e-01 9.757040e-01 9.749610e-01 9.745309e-01 9.750774e-01 7.523354e-01 7.262283e-01 7.355348e-01 7.782351e-01 7.864784e-01

2421 9.763763e-01 9.757149e-01 9.749711e-01 9.745404e-01 9.750852e-01 7.524680e-01 7.263712e-01 7.356805e-01 7.783703e-01 7.866136e-01

2422 9.763880e-01 9.757259e-01 9.749811e-01 9.745499e-01 9.750929e-01 7.525968e-01 7.265103e-01 7.358224e-01 7.785018e-01 7.867449e-01

2423 9.763998e-01 9.757368e-01 9.749910e-01 9.745592e-01 9.751006e-01 7.527220e-01 7.266456e-01 7.359604e-01 7.786295e-01 7.868725e-01

2424 9.764115e-01 9.757476e-01 9.750009e-01 9.745685e-01 9.751082e-01 7.528437e-01 7.267773e-01 7.360946e-01 7.787536e-01 7.869965e-01

2425 9.764232e-01 9.757584e-01 9.750107e-01 9.745778e-01 9.751157e-01 7.529620e-01 7.269054e-01 7.362253e-01 7.788742e-01 7.871170e-01

2426 9.764348e-01 9.757692e-01 9.750204e-01 9.745869e-01 9.751231e-01 7.530769e-01 7.270299e-01 7.363523e-01 7.789913e-01 7.872340e-01

2427 9.764465e-01 9.757800e-01 9.750301e-01 9.745960e-01 9.751305e-01 7.531886e-01 7.271511e-01 7.364758e-01 7.791051e-01 7.873476e-01

2428 9.764582e-01 9.757907e-01 9.750397e-01 9.746050e-01 9.751377e-01 7.532970e-01 7.272688e-01 7.365959e-01 7.792156e-01 7.874580e-01

2429 9.764698e-01 9.758014e-01 9.750493e-01 9.746139e-01 9.751449e-01 7.534023e-01 7.273833e-01 7.367126e-01 7.793230e-01 7.875652e-01

2430 9.764815e-01 9.758120e-01 9.750588e-01 9.746227e-01 9.751519e-01 7.535046e-01 7.274946e-01 7.368261e-01 7.794273e-01 7.876694e-01

2431 9.764931e-01 9.758226e-01 9.750682e-01 9.746315e-01 9.751589e-01 7.536040e-01 7.276028e-01 7.369364e-01 7.795286e-01 7.877706e-01

2432 9.765048e-01 9.758332e-01 9.750775e-01 9.746401e-01 9.751658e-01 7.537005e-01 7.277079e-01 7.370436e-01 7.796270e-01 7.878688e-01

2433 9.765164e-01 9.758437e-01 9.750868e-01 9.746487e-01 9.751725e-01 7.537942e-01 7.278100e-01 7.371477e-01 7.797225e-01 7.879643e-01

2434 9.765280e-01 9.758541e-01 9.750960e-01 9.746572e-01 9.751792e-01 7.538852e-01 7.279092e-01 7.372489e-01 7.798153e-01 7.880570e-01

2435 9.765396e-01 9.758646e-01 9.751051e-01 9.746656e-01 9.751858e-01 7.539735e-01 7.280056e-01 7.373473e-01 7.799055e-01 7.881471e-01

2436 9.765511e-01 9.758749e-01 9.751141e-01 9.746739e-01 9.751922e-01 7.540593e-01 7.280993e-01 7.374428e-01 7.799931e-01 7.882346e-01

2437 9.765627e-01 9.758852e-01 9.751230e-01 9.746821e-01 9.751986e-01 7.541427e-01 7.281903e-01 7.375357e-01 7.800782e-01 7.883196e-01

2438 9.765742e-01 9.758955e-01 9.751319e-01 9.746903e-01 9.752048e-01 7.542236e-01 7.282787e-01 7.376259e-01 7.801608e-01 7.884023e-01

2439 9.765857e-01 9.759057e-01 9.751407e-01 9.746983e-01 9.752110e-01 7.543022e-01 7.283645e-01 7.377136e-01 7.802412e-01 7.884825e-01

2440 9.765972e-01 9.759158e-01 9.751493e-01 9.747062e-01 9.752170e-01 7.543785e-01 7.284480e-01 7.377988e-01 7.803192e-01 7.885606e-01

2441 9.766087e-01 9.759259e-01 9.751579e-01 9.747140e-01 9.752229e-01 7.544527e-01 7.285290e-01 7.378816e-01 7.803951e-01 7.886364e-01

2442 9.766202e-01 9.759360e-01 9.751664e-01 9.747217e-01 9.752287e-01 7.545247e-01 7.286078e-01 7.379620e-01 7.804688e-01 7.887101e-01

2443 9.766316e-01 9.759459e-01 9.751748e-01 9.747293e-01 9.752344e-01 7.545947e-01 7.286843e-01 7.380402e-01 7.805405e-01 7.887818e-01

2444 9.766430e-01 9.759558e-01 9.751831e-01 9.747368e-01 9.752400e-01 7.546627e-01 7.287586e-01 7.381162e-01 7.806102e-01 7.888515e-01

2445 9.766544e-01 9.759657e-01 9.751913e-01 9.747442e-01 9.752454e-01 7.547288e-01 7.288308e-01 7.381900e-01 7.806779e-01 7.889193e-01

2446 9.766657e-01 9.759754e-01 9.751994e-01 9.747514e-01 9.752507e-01 7.547930e-01 7.289010e-01 7.382618e-01 7.807438e-01 7.889852e-01

2447 9.766771e-01 9.759851e-01 9.752073e-01 9.747586e-01 9.752559e-01 7.548554e-01 7.289693e-01 7.383316e-01 7.808079e-01 7.890494e-01

2448 9.766883e-01 9.759947e-01 9.752152e-01 9.747656e-01 9.752610e-01 7.549161e-01 7.290356e-01 7.383995e-01 7.808702e-01 7.891118e-01

2449 9.766996e-01 9.760043e-01 9.752229e-01 9.747725e-01 9.752659e-01 7.549752e-01 7.291001e-01 7.384655e-01 7.809309e-01 7.891725e-01

2450 9.767108e-01 9.760137e-01 9.752306e-01 9.747792e-01 9.752707e-01 7.550326e-01 7.291628e-01 7.385297e-01 7.809900e-01 7.892317e-01

2451 9.767220e-01 9.760231e-01 9.752381e-01 9.747859e-01 9.752754e-01 7.550884e-01 7.292238e-01 7.385922e-01 7.810475e-01 7.892892e-01

2452 9.767332e-01 9.760324e-01 9.752455e-01 9.747924e-01 9.752800e-01 7.551427e-01 7.292831e-01 7.386530e-01 7.811035e-01 7.893453e-01

2453 9.767443e-01 9.760416e-01 9.752527e-01 9.747988e-01 9.752844e-01 7.551956e-01 7.293408e-01 7.387121e-01 7.811580e-01 7.893999e-01

2454 9.767554e-01 9.760508e-01 9.752599e-01 9.748050e-01 9.752887e-01 7.552471e-01 7.293970e-01 7.387697e-01 7.812111e-01 7.894532e-01

2455 9.767664e-01 9.760598e-01 9.752669e-01 9.748111e-01 9.752929e-01 7.552973e-01 7.294516e-01 7.388257e-01 7.812629e-01 7.895051e-01

2456 9.767774e-01 9.760687e-01 9.752737e-01 9.748171e-01 9.752969e-01 7.553461e-01 7.295048e-01 7.388803e-01 7.813133e-01 7.895556e-01

2457 9.767883e-01 9.760776e-01 9.752805e-01 9.748229e-01 9.753008e-01 7.553937e-01 7.295566e-01 7.389335e-01 7.813625e-01 7.896049e-01

2458 9.767992e-01 9.760863e-01 9.752871e-01 9.748286e-01 9.753045e-01 7.554400e-01 7.296071e-01 7.389854e-01 7.814105e-01 7.896531e-01

2459 9.768100e-01 9.760949e-01 9.752935e-01 9.748341e-01 9.753081e-01 7.554852e-01 7.296563e-01 7.390359e-01 7.814573e-01 7.897000e-01

2460 9.768208e-01 9.761035e-01 9.752998e-01 9.748395e-01 9.753116e-01 7.555293e-01 7.297042e-01 7.390851e-01 7.815029e-01 7.897458e-01

2461 9.768315e-01 9.761119e-01 9.753060e-01 9.748448e-01 9.753149e-01 7.555723e-01 7.297509e-01 7.391331e-01 7.815475e-01 7.897906e-01

2462 9.768422e-01 9.761202e-01 9.753120e-01 9.748499e-01 9.753181e-01 7.556143e-01 7.297965e-01 7.391800e-01 7.815911e-01 7.898342e-01

2463 9.768528e-01 9.761284e-01 9.753179e-01 9.748548e-01 9.753212e-01 7.556553e-01 7.298409e-01 7.392257e-01 7.816336e-01 7.898769e-01

2464 9.768634e-01 9.761365e-01 9.753236e-01 9.748596e-01 9.753241e-01 7.556953e-01 7.298843e-01 7.392703e-01 7.816751e-01 7.899186e-01

2465 9.768738e-01 9.761444e-01 9.753291e-01 9.748642e-01 9.753269e-01 7.557343e-01 7.299266e-01 7.393139e-01 7.817158e-01 7.899594e-01

2466 9.768842e-01 9.761523e-01 9.753345e-01 9.748687e-01 9.753295e-01 7.557725e-01 7.299680e-01 7.393564e-01 7.817555e-01 7.899993e-01

2467 9.768946e-01 9.761600e-01 9.753397e-01 9.748730e-01 9.753320e-01 7.558099e-01 7.300084e-01 7.393980e-01 7.817943e-01 7.900383e-01

2468 9.769048e-01 9.761676e-01 9.753448e-01 9.748771e-01 9.753344e-01 7.558464e-01 7.300478e-01 7.394387e-01 7.818324e-01 7.900765e-01

2469 9.769150e-01 9.761750e-01 9.753496e-01 9.748811e-01 9.753367e-01 7.558821e-01 7.300864e-01 7.394784e-01 7.818696e-01 7.901139e-01

2470 9.769251e-01 9.761823e-01 9.753544e-01 9.748849e-01 9.753388e-01 7.559171e-01 7.301242e-01 7.395173e-01 7.819061e-01 7.901505e-01

2471 9.769351e-01 9.761895e-01 9.753589e-01 9.748886e-01 9.753407e-01 7.559514e-01 7.301611e-01 7.395554e-01 7.819418e-01 7.901864e-01

2472 9.769450e-01 9.761965e-01 9.753633e-01 9.748921e-01 9.753426e-01 7.559850e-01 7.301972e-01 7.395927e-01 7.819768e-01 7.902216e-01

2473 9.769549e-01 9.762034e-01 9.753675e-01 9.748954e-01 9.753443e-01 7.560179e-01 7.302326e-01 7.396292e-01 7.820111e-01 7.902560e-01

2474 9.769646e-01 9.762102e-01 9.753715e-01 9.748985e-01 9.753458e-01 7.560501e-01 7.302673e-01 7.396649e-01 7.820448e-01 7.902899e-01

2475 9.769742e-01 9.762168e-01 9.753753e-01 9.749015e-01 9.753473e-01 7.560818e-01 7.303013e-01 7.397000e-01 7.820778e-01 7.903230e-01

2476 9.769838e-01 9.762232e-01 9.753790e-01 9.749043e-01 9.753486e-01 7.561128e-01 7.303346e-01 7.397344e-01 7.821102e-01 7.903556e-01

2477 9.769932e-01 9.762295e-01 9.753825e-01 9.749070e-01 9.753498e-01 7.561433e-01 7.303673e-01 7.397681e-01 7.821421e-01 7.903876e-01

2478 9.770025e-01 9.762356e-01 9.753857e-01 9.749095e-01 9.753509e-01 7.561733e-01 7.303993e-01 7.398012e-01 7.821734e-01 7.904190e-01

2479 9.770118e-01 9.762415e-01 9.753888e-01 9.749118e-01 9.753519e-01 7.562027e-01 7.304308e-01 7.398338e-01 7.822041e-01 7.904499e-01

2480 9.770209e-01 9.762473e-01 9.753918e-01 9.749139e-01 9.753528e-01 7.562317e-01 7.304618e-01 7.398657e-01 7.822344e-01 7.904803e-01

2481 9.770298e-01 9.762529e-01 9.753945e-01 9.749159e-01 9.753535e-01 7.562602e-01 7.304922e-01 7.398971e-01 7.822641e-01 7.905102e-01

2482 9.770387e-01 9.762584e-01 9.753970e-01 9.749177e-01 9.753542e-01 7.562882e-01 7.305221e-01 7.399280e-01 7.822934e-01 7.905396e-01

2483 9.770474e-01 9.762637e-01 9.753994e-01 9.749194e-01 9.753547e-01 7.563158e-01 7.305515e-01 7.399584e-01 7.823222e-01 7.905685e-01

2484 9.770560e-01 9.762688e-01 9.754015e-01 9.749209e-01 9.753552e-01 7.563430e-01 7.305804e-01 7.399883e-01 7.823506e-01 7.905970e-01

2485 9.770645e-01 9.762737e-01 9.754035e-01 9.749222e-01 9.753556e-01 7.563699e-01 7.306089e-01 7.400177e-01 7.823786e-01 7.906251e-01

2486 9.770728e-01 9.762784e-01 9.754053e-01 9.749234e-01 9.753559e-01 7.563963e-01 7.306370e-01 7.400467e-01 7.824062e-01 7.906528e-01

2487 9.770810e-01 9.762830e-01 9.754069e-01 9.749244e-01 9.753561e-01 7.564224e-01 7.306646e-01 7.400753e-01 7.824334e-01 7.906801e-01

2488 9.770890e-01 9.762874e-01 9.754083e-01 9.749253e-01 9.753563e-01 7.564481e-01 7.306919e-01 7.401035e-01 7.824603e-01 7.907070e-01

2489 9.770969e-01 9.762915e-01 9.754095e-01 9.749260e-01 9.753564e-01 7.564736e-01 7.307188e-01 7.401313e-01 7.824868e-01 7.907335e-01

2490 9.771046e-01 9.762955e-01 9.754105e-01 9.749265e-01 9.753564e-01 7.564987e-01 7.307454e-01 7.401588e-01 7.825129e-01 7.907597e-01

2491 9.771122e-01 9.762993e-01 9.754114e-01 9.749270e-01 9.753564e-01 7.565235e-01 7.307716e-01 7.401859e-01 7.825388e-01 7.907856e-01

2492 9.771196e-01 9.763030e-01 9.754120e-01 9.749273e-01 9.753564e-01 7.565481e-01 7.307976e-01 7.402127e-01 7.825643e-01 7.908112e-01

2493 9.771268e-01 9.763064e-01 9.754125e-01 9.749274e-01 9.753563e-01 7.565724e-01 7.308232e-01 7.402391e-01 7.825896e-01 7.908365e-01

2494 9.771339e-01 9.763096e-01 9.754128e-01 9.749274e-01 9.753562e-01 7.565964e-01 7.308485e-01 7.402653e-01 7.826146e-01 7.908614e-01

2495 9.771407e-01 9.763126e-01 9.754129e-01 9.749273e-01 9.753560e-01 7.566202e-01 7.308736e-01 7.402912e-01 7.826393e-01 7.908862e-01

2496 9.771474e-01 9.763154e-01 9.754129e-01 9.749271e-01 9.753559e-01 7.566438e-01 7.308984e-01 7.403168e-01 7.826638e-01 7.909106e-01

2497 9.771540e-01 9.763181e-01 9.754127e-01 9.749268e-01 9.753558e-01 7.566672e-01 7.309229e-01 7.403422e-01 7.826880e-01 7.909348e-01

2498 9.771603e-01 9.763205e-01 9.754123e-01 9.749263e-01 9.753556e-01 7.566904e-01 7.309473e-01 7.403673e-01 7.827121e-01 7.909587e-01

2499 9.771664e-01 9.763227e-01 9.754118e-01 9.749258e-01 9.753555e-01 7.567134e-01 7.309714e-01 7.403922e-01 7.827359e-01 7.909825e-01

2500 9.771723e-01 9.763248e-01 9.754111e-01 9.749252e-01 9.753555e-01 7.567362e-01 7.309953e-01 7.404169e-01 7.827595e-01 7.910060e-01

2501 9.771781e-01 9.763266e-01 9.754103e-01 9.749245e-01 9.753554e-01 7.567589e-01 7.310190e-01 7.404414e-01 7.827829e-01 7.910293e-01

2502 9.771836e-01 9.763283e-01 9.754093e-01 9.749237e-01 9.753555e-01 7.567814e-01 7.310425e-01 7.404657e-01 7.828061e-01 7.910524e-01

2503 9.771889e-01 9.763297e-01 9.754082e-01 9.749228e-01 9.753556e-01 7.568038e-01 7.310659e-01 7.404898e-01 7.828291e-01 7.910753e-01

2504 9.771940e-01 9.763310e-01 9.754070e-01 9.749219e-01 9.753557e-01 7.568260e-01 7.310891e-01 7.405138e-01 7.828520e-01 7.910980e-01

2505 9.771989e-01 9.763320e-01 9.754056e-01 9.749209e-01 9.753560e-01 7.568481e-01 7.311122e-01 7.405376e-01 7.828747e-01 7.911205e-01

2506 9.772036e-01 9.763329e-01 9.754041e-01 9.749199e-01 9.753563e-01 7.568701e-01 7.311351e-01 7.405612e-01 7.828973e-01 7.911429e-01

2507 9.772080e-01 9.763336e-01 9.754026e-01 9.749189e-01 9.753568e-01 7.568920e-01 7.311579e-01 7.405847e-01 7.829198e-01 7.911651e-01

2508 9.772122e-01 9.763341e-01 9.754009e-01 9.749178e-01 9.753574e-01 7.569138e-01 7.311806e-01 7.406081e-01 7.829421e-01 7.911872e-01

2509 9.772162e-01 9.763344e-01 9.753991e-01 9.749167e-01 9.753581e-01 7.569356e-01 7.312031e-01 7.406313e-01 7.829643e-01 7.912091e-01

2510 9.772199e-01 9.763346e-01 9.753972e-01 9.749156e-01 9.753590e-01 7.569572e-01 7.312256e-01 7.406545e-01 7.829863e-01 7.912309e-01

2511 9.772234e-01 9.763345e-01 9.753953e-01 9.749146e-01 9.753601e-01 7.569787e-01 7.312480e-01 7.406775e-01 7.830083e-01 7.912525e-01

2512 9.772267e-01 9.763343e-01 9.753933e-01 9.749136e-01 9.753613e-01 7.570002e-01 7.312702e-01 7.407005e-01 7.830302e-01 7.912740e-01

2513 9.772297e-01 9.763339e-01 9.753913e-01 9.749126e-01 9.753627e-01 7.570217e-01 7.312925e-01 7.407233e-01 7.830519e-01 7.912954e-01

2514 9.772325e-01 9.763334e-01 9.753892e-01 9.749116e-01 9.753644e-01 7.570430e-01 7.313146e-01 7.407461e-01 7.830736e-01 7.913167e-01

2515 9.772350e-01 9.763327e-01 9.753871e-01 9.749107e-01 9.753662e-01 7.570644e-01 7.313367e-01 7.407688e-01 7.830952e-01 7.913379e-01

2516 9.772372e-01 9.763319e-01 9.753850e-01 9.749099e-01 9.753683e-01 7.570857e-01 7.313587e-01 7.407915e-01 7.831168e-01 7.913590e-01

2517 9.772393e-01 9.763309e-01 9.753829e-01 9.749092e-01 9.753707e-01 7.571069e-01 7.313806e-01 7.408141e-01 7.831382e-01 7.913800e-01

2518 9.772410e-01 9.763298e-01 9.753808e-01 9.749086e-01 9.753733e-01 7.571282e-01 7.314026e-01 7.408366e-01 7.831596e-01 7.914009e-01

2519 9.772425e-01 9.763285e-01 9.753787e-01 9.749082e-01 9.753762e-01 7.571494e-01 7.314244e-01 7.408591e-01 7.831810e-01 7.914217e-01

2520 9.772438e-01 9.763272e-01 9.753767e-01 9.749078e-01 9.753794e-01 7.571706e-01 7.314463e-01 7.408816e-01 7.832022e-01 7.914424e-01

2521 9.772448e-01 9.763257e-01 9.753747e-01 9.749076e-01 9.753829e-01 7.571918e-01 7.314681e-01 7.409040e-01 7.832235e-01 7.914630e-01

2522 9.772552e-01 9.763360e-01 9.753848e-01 9.749176e-01 9.753916e-01 7.574565e-01 7.316983e-01 7.411559e-01 7.835551e-01 7.918170e-01

2523 9.772655e-01 9.763462e-01 9.753948e-01 9.749275e-01 9.754003e-01 7.577243e-01 7.319319e-01 7.414114e-01 7.838895e-01 7.921735e-01

2524 9.772758e-01 9.763563e-01 9.754048e-01 9.749373e-01 9.754089e-01 7.579953e-01 7.321689e-01 7.416703e-01 7.842266e-01 7.925323e-01

2525 9.772861e-01 9.763664e-01 9.754147e-01 9.749471e-01 9.754174e-01 7.582692e-01 7.324092e-01 7.419326e-01 7.845661e-01 7.928933e-01

2526 9.772963e-01 9.763765e-01 9.754245e-01 9.749568e-01 9.754260e-01 7.585458e-01 7.326527e-01 7.421980e-01 7.849078e-01 7.932562e-01

2527 9.773064e-01 9.763865e-01 9.754343e-01 9.749664e-01 9.754344e-01 7.588251e-01 7.328993e-01 7.424666e-01 7.852515e-01 7.936207e-01

2528 9.773165e-01 9.763964e-01 9.754441e-01 9.749760e-01 9.754428e-01 7.591069e-01 7.331489e-01 7.427381e-01 7.855970e-01 7.939866e-01

2529 9.773266e-01 9.764063e-01 9.754538e-01 9.749856e-01 9.754512e-01 7.593909e-01 7.334013e-01 7.430124e-01 7.859440e-01 7.943537e-01

2530 9.773366e-01 9.764161e-01 9.754634e-01 9.749950e-01 9.754595e-01 7.596771e-01 7.336564e-01 7.432893e-01 7.862923e-01 7.947216e-01

2531 9.773466e-01 9.764259e-01 9.754730e-01 9.750045e-01 9.754677e-01 7.599652e-01 7.339141e-01 7.435688e-01 7.866416e-01 7.950902e-01

2532 9.773565e-01 9.764357e-01 9.754825e-01 9.750138e-01 9.754759e-01 7.602551e-01 7.341742e-01 7.438506e-01 7.869918e-01 7.954592e-01

2533 9.773664e-01 9.764454e-01 9.754920e-01 9.750231e-01 9.754841e-01 7.605465e-01 7.344366e-01 7.441345e-01 7.873425e-01 7.958282e-01

2534 9.773762e-01 9.764551e-01 9.755014e-01 9.750324e-01 9.754922e-01 7.608394e-01 7.347012e-01 7.444205e-01 7.876936e-01 7.961972e-01

2535 9.773860e-01 9.764647e-01 9.755108e-01 9.750416e-01 9.755003e-01 7.611334e-01 7.349678e-01 7.447083e-01 7.880448e-01 7.965657e-01

2536 9.773958e-01 9.764743e-01 9.755202e-01 9.750508e-01 9.755083e-01 7.614284e-01 7.352361e-01 7.449977e-01 7.883958e-01 7.969336e-01

2537 9.774056e-01 9.764839e-01 9.755295e-01 9.750600e-01 9.755163e-01 7.617242e-01 7.355062e-01 7.452885e-01 7.887464e-01 7.973006e-01

2538 9.774153e-01 9.764934e-01 9.755388e-01 9.750690e-01 9.755242e-01 7.620206e-01 7.357777e-01 7.455806e-01 7.890964e-01 7.976664e-01

2539 9.774250e-01 9.765029e-01 9.755480e-01 9.750781e-01 9.755321e-01 7.623173e-01 7.360505e-01 7.458738e-01 7.894455e-01 7.980309e-01

2540 9.774347e-01 9.765123e-01 9.755572e-01 9.750871e-01 9.755399e-01 7.626143e-01 7.363244e-01 7.461678e-01 7.897935e-01 7.983937e-01

2541 9.774443e-01 9.765218e-01 9.755663e-01 9.750960e-01 9.755478e-01 7.629112e-01 7.365993e-01 7.464625e-01 7.901401e-01 7.987546e-01

2542 9.774539e-01 9.765312e-01 9.755754e-01 9.751049e-01 9.755555e-01 7.632078e-01 7.368749e-01 7.467577e-01 7.904851e-01 7.991134e-01

2543 9.774635e-01 9.765405e-01 9.755845e-01 9.751138e-01 9.755632e-01 7.635041e-01 7.371511e-01 7.470531e-01 7.908283e-01 7.994699e-01

2544 9.774731e-01 9.765498e-01 9.755935e-01 9.751227e-01 9.755709e-01 7.637996e-01 7.374276e-01 7.473486e-01 7.911695e-01 7.998238e-01

2545 9.774826e-01 9.765592e-01 9.756025e-01 9.751314e-01 9.755786e-01 7.640944e-01 7.377044e-01 7.476440e-01 7.915085e-01 8.001750e-01

2546 9.774922e-01 9.765684e-01 9.756115e-01 9.751402e-01 9.755862e-01 7.643880e-01 7.379812e-01 7.479390e-01 7.918450e-01 8.005231e-01

2547 9.775017e-01 9.765777e-01 9.756204e-01 9.751489e-01 9.755938e-01 7.646805e-01 7.382578e-01 7.482334e-01 7.921788e-01 8.008681e-01

2548 9.775112e-01 9.765869e-01 9.756293e-01 9.751576e-01 9.756013e-01 7.649715e-01 7.385340e-01 7.485272e-01 7.925098e-01 8.012098e-01

2549 9.775206e-01 9.765961e-01 9.756382e-01 9.751662e-01 9.756088e-01 7.652608e-01 7.388096e-01 7.488199e-01 7.928377e-01 8.015479e-01

2550 9.775301e-01 9.766053e-01 9.756470e-01 9.751748e-01 9.756162e-01 7.655484e-01 7.390845e-01 7.491116e-01 7.931625e-01 8.018822e-01

2551 9.775395e-01 9.766144e-01 9.756558e-01 9.751834e-01 9.756236e-01 7.658340e-01 7.393584e-01 7.494019e-01 7.934838e-01 8.022127e-01

2552 9.775489e-01 9.766236e-01 9.756646e-01 9.751919e-01 9.756310e-01 7.661174e-01 7.396313e-01 7.496907e-01 7.938016e-01 8.025392e-01

2553 9.775584e-01 9.766327e-01 9.756733e-01 9.752004e-01 9.756383e-01 7.663984e-01 7.399028e-01 7.499778e-01 7.941157e-01 8.028615e-01

2554 9.775677e-01 9.766417e-01 9.756820e-01 9.752089e-01 9.756456e-01 7.666770e-01 7.401729e-01 7.502630e-01 7.944259e-01 8.031795e-01

2555 9.775771e-01 9.766508e-01 9.756907e-01 9.752173e-01 9.756528e-01 7.669529e-01 7.404413e-01 7.505461e-01 7.947321e-01 8.034930e-01

2556 9.775865e-01 9.766598e-01 9.756993e-01 9.752256e-01 9.756601e-01 7.672260e-01 7.407079e-01 7.508270e-01 7.950342e-01 8.038020e-01

2557 9.775958e-01 9.766689e-01 9.757079e-01 9.752340e-01 9.756672e-01 7.674961e-01 7.409725e-01 7.511056e-01 7.953321e-01 8.041064e-01

2558 9.776052e-01 9.766779e-01 9.757165e-01 9.752423e-01 9.756743e-01 7.677631e-01 7.412350e-01 7.513816e-01 7.956257e-01 8.044060e-01

2559 9.776145e-01 9.766868e-01 9.757250e-01 9.752505e-01 9.756814e-01 7.680270e-01 7.414952e-01 7.516549e-01 7.959148e-01 8.047008e-01

2560 9.776238e-01 9.766958e-01 9.757335e-01 9.752588e-01 9.756885e-01 7.682875e-01 7.417530e-01 7.519253e-01 7.961993e-01 8.049906e-01

2561 9.776331e-01 9.767047e-01 9.757420e-01 9.752670e-01 9.756955e-01 7.685445e-01 7.420082e-01 7.521928e-01 7.964793e-01 8.052755e-01

2562 9.776424e-01 9.767136e-01 9.757505e-01 9.752751e-01 9.757024e-01 7.687981e-01 7.422606e-01 7.524572e-01 7.967546e-01 8.055554e-01

2563 9.776517e-01 9.767225e-01 9.757589e-01 9.752832e-01 9.757093e-01 7.690479e-01 7.425103e-01 7.527184e-01 7.970251e-01 8.058302e-01

2564 9.776610e-01 9.767314e-01 9.757672e-01 9.752913e-01 9.757162e-01 7.692940e-01 7.427570e-01 7.529762e-01 7.972908e-01 8.060998e-01

2565 9.776703e-01 9.767402e-01 9.757756e-01 9.752993e-01 9.757230e-01 7.695363e-01 7.430006e-01 7.532306e-01 7.975517e-01 8.063644e-01

2566 9.776796e-01 9.767490e-01 9.757839e-01 9.753073e-01 9.757298e-01 7.697747e-01 7.432411e-01 7.534814e-01 7.978077e-01 8.066237e-01

2567 9.776888e-01 9.767578e-01 9.757922e-01 9.753153e-01 9.757365e-01 7.700091e-01 7.434783e-01 7.537285e-01 7.980588e-01 8.068779e-01

2568 9.776981e-01 9.767666e-01 9.758004e-01 9.753232e-01 9.757432e-01 7.702395e-01 7.437121e-01 7.539720e-01 7.983050e-01 8.071268e-01

2569 9.777073e-01 9.767754e-01 9.758086e-01 9.753310e-01 9.757499e-01 7.704659e-01 7.439425e-01 7.542116e-01 7.985462e-01 8.073706e-01

2570 9.777165e-01 9.767841e-01 9.758168e-01 9.753389e-01 9.757565e-01 7.706881e-01 7.441693e-01 7.544474e-01 7.987825e-01 8.076092e-01

2571 9.777257e-01 9.767929e-01 9.758249e-01 9.753467e-01 9.757630e-01 7.709062e-01 7.443926e-01 7.546792e-01 7.990138e-01 8.078427e-01

2572 9.777350e-01 9.768016e-01 9.758330e-01 9.753544e-01 9.757695e-01 7.711201e-01 7.446121e-01 7.549071e-01 7.992402e-01 8.080710e-01

2573 9.777442e-01 9.768102e-01 9.758411e-01 9.753621e-01 9.757760e-01 7.713298e-01 7.448280e-01 7.551309e-01 7.994617e-01 8.082942e-01

2574 9.777534e-01 9.768189e-01 9.758491e-01 9.753698e-01 9.757824e-01 7.715353e-01 7.450401e-01 7.553506e-01 7.996783e-01 8.085123e-01

2575 9.777626e-01 9.768275e-01 9.758571e-01 9.753774e-01 9.757887e-01 7.717366e-01 7.452484e-01 7.555663e-01 7.998900e-01 8.087254e-01

2576 9.777717e-01 9.768361e-01 9.758650e-01 9.753849e-01 9.757950e-01 7.719337e-01 7.454528e-01 7.557778e-01 8.000969e-01 8.089335e-01

2577 9.777809e-01 9.768447e-01 9.758729e-01 9.753925e-01 9.758013e-01 7.721265e-01 7.456534e-01 7.559852e-01 8.002990e-01 8.091366e-01

2578 9.777901e-01 9.768533e-01 9.758808e-01 9.753999e-01 9.758075e-01 7.723151e-01 7.458501e-01 7.561884e-01 8.004963e-01 8.093349e-01

2579 9.777993e-01 9.768618e-01 9.758886e-01 9.754074e-01 9.758136e-01 7.724996e-01 7.460428e-01 7.563874e-01 8.006889e-01 8.095283e-01

2580 9.778084e-01 9.768703e-01 9.758964e-01 9.754147e-01 9.758197e-01 7.726799e-01 7.462317e-01 7.565823e-01 8.008769e-01 8.097169e-01

2581 9.778176e-01 9.768788e-01 9.759041e-01 9.754221e-01 9.758257e-01 7.728560e-01 7.464166e-01 7.567731e-01 8.010602e-01 8.099009e-01

2582 9.778267e-01 9.768873e-01 9.759118e-01 9.754294e-01 9.758317e-01 7.730280e-01 7.465976e-01 7.569596e-01 8.012390e-01 8.100802e-01

2583 9.778358e-01 9.768957e-01 9.759195e-01 9.754366e-01 9.758376e-01 7.731960e-01 7.467747e-01 7.571421e-01 8.014134e-01 8.102549e-01

2584 9.778449e-01 9.769041e-01 9.759271e-01 9.754438e-01 9.758435e-01 7.733599e-01 7.469479e-01 7.573205e-01 8.015833e-01 8.104251e-01

2585 9.778541e-01 9.769125e-01 9.759347e-01 9.754509e-01 9.758493e-01 7.735198e-01 7.471172e-01 7.574947e-01 8.017488e-01 8.105909e-01

2586 9.778632e-01 9.769208e-01 9.759422e-01 9.754579e-01 9.758550e-01 7.736757e-01 7.472827e-01 7.576650e-01 8.019101e-01 8.107524e-01

2587 9.778723e-01 9.769291e-01 9.759496e-01 9.754650e-01 9.758607e-01 7.738278e-01 7.474443e-01 7.578312e-01 8.020672e-01 8.109097e-01

2588 9.778813e-01 9.769374e-01 9.759570e-01 9.754719e-01 9.758663e-01 7.739760e-01 7.476021e-01 7.579935e-01 8.022202e-01 8.110627e-01

2589 9.778904e-01 9.769457e-01 9.759644e-01 9.754788e-01 9.758719e-01 7.741204e-01 7.477562e-01 7.581518e-01 8.023691e-01 8.112116e-01

2590 9.778995e-01 9.769539e-01 9.759717e-01 9.754856e-01 9.758773e-01 7.742611e-01 7.479065e-01 7.583063e-01 8.025140e-01 8.113566e-01

2591 9.779085e-01 9.769621e-01 9.759790e-01 9.754924e-01 9.758828e-01 7.743981e-01 7.480532e-01 7.584569e-01 8.026551e-01 8.114976e-01

2592 9.779176e-01 9.769703e-01 9.759862e-01 9.754991e-01 9.758881e-01 7.745315e-01 7.481962e-01 7.586037e-01 8.027923e-01 8.116347e-01

2593 9.779266e-01 9.769784e-01 9.759933e-01 9.755058e-01 9.758934e-01 7.746613e-01 7.483356e-01 7.587469e-01 8.029258e-01 8.117681e-01

2594 9.779356e-01 9.769865e-01 9.760004e-01 9.755124e-01 9.758986e-01 7.747877e-01 7.484715e-01 7.588863e-01 8.030556e-01 8.118978e-01

2595 9.779446e-01 9.769945e-01 9.760075e-01 9.755189e-01 9.759038e-01 7.749106e-01 7.486039e-01 7.590222e-01 8.031819e-01 8.120240e-01

2596 9.779536e-01 9.770025e-01 9.760144e-01 9.755254e-01 9.759089e-01 7.750302e-01 7.487328e-01 7.591545e-01 8.033047e-01 8.121466e-01

2597 9.779625e-01 9.770105e-01 9.760213e-01 9.755318e-01 9.759139e-01 7.751465e-01 7.488584e-01 7.592834e-01 8.034241e-01 8.122658e-01

2598 9.779715e-01 9.770184e-01 9.760282e-01 9.755381e-01 9.759189e-01 7.752596e-01 7.489807e-01 7.594088e-01 8.035401e-01 8.123817e-01

2599 9.779804e-01 9.770263e-01 9.760350e-01 9.755444e-01 9.759237e-01 7.753696e-01 7.490997e-01 7.595309e-01 8.036529e-01 8.124943e-01

2600 9.779894e-01 9.770342e-01 9.760417e-01 9.755506e-01 9.759285e-01 7.754765e-01 7.492155e-01 7.596496e-01 8.037625e-01 8.126037e-01

2601 9.779983e-01 9.770420e-01 9.760484e-01 9.755567e-01 9.759333e-01 7.755804e-01 7.493282e-01 7.597652e-01 8.038690e-01 8.127101e-01

2602 9.780072e-01 9.770498e-01 9.760550e-01 9.755627e-01 9.759379e-01 7.756814e-01 7.494378e-01 7.598777e-01 8.039726e-01 8.128134e-01

2603 9.780160e-01 9.770575e-01 9.760615e-01 9.755687e-01 9.759425e-01 7.757795e-01 7.495444e-01 7.599870e-01 8.040732e-01 8.129139e-01

2604 9.780249e-01 9.770651e-01 9.760679e-01 9.755746e-01 9.759470e-01 7.758749e-01 7.496481e-01 7.600934e-01 8.041709e-01 8.130115e-01

2605 9.780337e-01 9.770728e-01 9.760743e-01 9.755804e-01 9.759514e-01 7.759675e-01 7.497489e-01 7.601968e-01 8.042659e-01 8.131063e-01

2606 9.780425e-01 9.770803e-01 9.760806e-01 9.755862e-01 9.759558e-01 7.760575e-01 7.498469e-01 7.602973e-01 8.043582e-01 8.131984e-01

2607 9.780513e-01 9.770879e-01 9.760869e-01 9.755918e-01 9.759601e-01 7.761449e-01 7.499422e-01 7.603951e-01 8.044479e-01 8.132879e-01

2608 9.780600e-01 9.770953e-01 9.760930e-01 9.755974e-01 9.759642e-01 7.762298e-01 7.500348e-01 7.604901e-01 8.045350e-01 8.133749e-01

2609 9.780688e-01 9.771027e-01 9.760991e-01 9.756029e-01 9.759684e-01 7.763122e-01 7.501247e-01 7.605824e-01 8.046196e-01 8.134594e-01

2610 9.780775e-01 9.771101e-01 9.761051e-01 9.756083e-01 9.759724e-01 7.763923e-01 7.502122e-01 7.606722e-01 8.047018e-01 8.135415e-01

2611 9.780861e-01 9.771174e-01 9.761110e-01 9.756137e-01 9.759764e-01 7.764701e-01 7.502971e-01 7.607595e-01 8.047818e-01 8.136213e-01

2612 9.780948e-01 9.771247e-01 9.761169e-01 9.756189e-01 9.759802e-01 7.765457e-01 7.503797e-01 7.608442e-01 8.048594e-01 8.136989e-01

2613 9.781034e-01 9.771318e-01 9.761226e-01 9.756241e-01 9.759840e-01 7.766190e-01 7.504599e-01 7.609266e-01 8.049348e-01 8.137742e-01

2614 9.781120e-01 9.771390e-01 9.761283e-01 9.756291e-01 9.759877e-01 7.766903e-01 7.505378e-01 7.610067e-01 8.050082e-01 8.138475e-01

2615 9.781205e-01 9.771460e-01 9.761339e-01 9.756341e-01 9.759914e-01 7.767595e-01 7.506135e-01 7.610845e-01 8.050794e-01 8.139187e-01

2616 9.781290e-01 9.771530e-01 9.761394e-01 9.756390e-01 9.759949e-01 7.768267e-01 7.506871e-01 7.611601e-01 8.051486e-01 8.139879e-01

2617 9.781375e-01 9.771599e-01 9.761448e-01 9.756438e-01 9.759984e-01 7.768920e-01 7.507585e-01 7.612336e-01 8.052160e-01 8.140552e-01

2618 9.781460e-01 9.771668e-01 9.761501e-01 9.756486e-01 9.760018e-01 7.769555e-01 7.508279e-01 7.613051e-01 8.052814e-01 8.141206e-01

2619 9.781544e-01 9.771736e-01 9.761553e-01 9.756532e-01 9.760051e-01 7.770171e-01 7.508954e-01 7.613745e-01 8.053450e-01 8.141842e-01

2620 9.781627e-01 9.771803e-01 9.761604e-01 9.756577e-01 9.760083e-01 7.770770e-01 7.509609e-01 7.614419e-01 8.054068e-01 8.142460e-01

2621 9.781711e-01 9.771870e-01 9.761655e-01 9.756621e-01 9.760115e-01 7.771352e-01 7.510246e-01 7.615075e-01 8.054670e-01 8.143062e-01

2622 9.781794e-01 9.771935e-01 9.761704e-01 9.756665e-01 9.760145e-01 7.771917e-01 7.510865e-01 7.615713e-01 8.055255e-01 8.143647e-01

2623 9.781876e-01 9.772000e-01 9.761753e-01 9.756707e-01 9.760175e-01 7.772467e-01 7.511466e-01 7.616332e-01 8.055824e-01 8.144216e-01

2624 9.781958e-01 9.772065e-01 9.761800e-01 9.756749e-01 9.760204e-01 7.773001e-01 7.512050e-01 7.616935e-01 8.056377e-01 8.144770e-01

2625 9.782039e-01 9.772128e-01 9.761846e-01 9.756789e-01 9.760232e-01 7.773520e-01 7.512618e-01 7.617521e-01 8.056916e-01 8.145309e-01

2626 9.782120e-01 9.772191e-01 9.761892e-01 9.756829e-01 9.760260e-01 7.774025e-01 7.513170e-01 7.618091e-01 8.057440e-01 8.145834e-01

2627 9.782201e-01 9.772253e-01 9.761936e-01 9.756868e-01 9.760287e-01 7.774517e-01 7.513707e-01 7.618645e-01 8.057950e-01 8.146345e-01

2628 9.782281e-01 9.772313e-01 9.761980e-01 9.756905e-01 9.760313e-01 7.774994e-01 7.514229e-01 7.619184e-01 8.058447e-01 8.146842e-01

2629 9.782360e-01 9.772374e-01 9.762022e-01 9.756942e-01 9.760338e-01 7.775459e-01 7.514737e-01 7.619709e-01 8.058931e-01 8.147327e-01

2630 9.782439e-01 9.772433e-01 9.762063e-01 9.756977e-01 9.760362e-01 7.775912e-01 7.515231e-01 7.620219e-01 8.059402e-01 8.147799e-01

2631 9.782517e-01 9.772491e-01 9.762103e-01 9.757012e-01 9.760386e-01 7.776352e-01 7.515711e-01 7.620716e-01 8.059861e-01 8.148259e-01

2632 9.782595e-01 9.772549e-01 9.762143e-01 9.757046e-01 9.760409e-01 7.776780e-01 7.516179e-01 7.621200e-01 8.060308e-01 8.148707e-01

2633 9.782672e-01 9.772605e-01 9.762181e-01 9.757078e-01 9.760431e-01 7.777198e-01 7.516634e-01 7.621671e-01 8.060744e-01 8.149144e-01

2634 9.782748e-01 9.772661e-01 9.762218e-01 9.757110e-01 9.760453e-01 7.777604e-01 7.517076e-01 7.622130e-01 8.061169e-01 8.149571e-01

2635 9.782823e-01 9.772715e-01 9.762253e-01 9.757141e-01 9.760474e-01 7.778001e-01 7.517508e-01 7.622577e-01 8.061584e-01 8.149986e-01

2636 9.782898e-01 9.772769e-01 9.762288e-01 9.757170e-01 9.760495e-01 7.778387e-01 7.517928e-01 7.623012e-01 8.061988e-01 8.150392e-01

2637 9.782973e-01 9.772822e-01 9.762322e-01 9.757199e-01 9.760514e-01 7.778763e-01 7.518337e-01 7.623437e-01 8.062383e-01 8.150788e-01

2638 9.783046e-01 9.772873e-01 9.762354e-01 9.757227e-01 9.760534e-01 7.779130e-01 7.518736e-01 7.623851e-01 8.062768e-01 8.151174e-01

2639 9.783119e-01 9.772924e-01 9.762386e-01 9.757254e-01 9.760552e-01 7.779488e-01 7.519125e-01 7.624254e-01 8.063144e-01 8.151551e-01

2640 9.783191e-01 9.772974e-01 9.762416e-01 9.757280e-01 9.760570e-01 7.779838e-01 7.519504e-01 7.624648e-01 8.063511e-01 8.151920e-01

2641 9.783262e-01 9.773022e-01 9.762446e-01 9.757305e-01 9.760588e-01 7.780179e-01 7.519873e-01 7.625032e-01 8.063869e-01 8.152279e-01

2642 9.783333e-01 9.773070e-01 9.762474e-01 9.757329e-01 9.760605e-01 7.780512e-01 7.520234e-01 7.625407e-01 8.064220e-01 8.152631e-01

2643 9.783402e-01 9.773116e-01 9.762501e-01 9.757352e-01 9.760622e-01 7.780837e-01 7.520586e-01 7.625774e-01 8.064562e-01 8.152975e-01

2644 9.783471e-01 9.773162e-01 9.762527e-01 9.757374e-01 9.760638e-01 7.781155e-01 7.520930e-01 7.626131e-01 8.064897e-01 8.153311e-01

2645 9.783538e-01 9.773206e-01 9.762552e-01 9.757395e-01 9.760654e-01 7.781466e-01 7.521266e-01 7.626481e-01 8.065225e-01 8.153641e-01

2646 9.783605e-01 9.773249e-01 9.762575e-01 9.757416e-01 9.760670e-01 7.781770e-01 7.521594e-01 7.626822e-01 8.065546e-01 8.153962e-01

2647 9.783671e-01 9.773291e-01 9.762598e-01 9.757435e-01 9.760685e-01 7.782067e-01 7.521914e-01 7.627156e-01 8.065860e-01 8.154278e-01

2648 9.783736e-01 9.773332e-01 9.762620e-01 9.757454e-01 9.760700e-01 7.782358e-01 7.522228e-01 7.627483e-01 8.066167e-01 8.154586e-01

2649 9.783800e-01 9.773372e-01 9.762641e-01 9.757472e-01 9.760715e-01 7.782643e-01 7.522534e-01 7.627803e-01 8.066468e-01 8.154889e-01

2650 9.783863e-01 9.773411e-01 9.762660e-01 9.757490e-01 9.760730e-01 7.782923e-01 7.522834e-01 7.628116e-01 8.066763e-01 8.155185e-01

2651 9.783925e-01 9.773448e-01 9.762679e-01 9.757506e-01 9.760745e-01 7.783196e-01 7.523128e-01 7.628422e-01 8.067053e-01 8.155475e-01

2652 9.783986e-01 9.773485e-01 9.762696e-01 9.757522e-01 9.760760e-01 7.783464e-01 7.523416e-01 7.628722e-01 8.067336e-01 8.155760e-01

2653 9.784046e-01 9.773520e-01 9.762713e-01 9.757537e-01 9.760774e-01 7.783727e-01 7.523697e-01 7.629016e-01 8.067615e-01 8.156039e-01

2654 9.784105e-01 9.773554e-01 9.762729e-01 9.757552e-01 9.760789e-01 7.783985e-01 7.523974e-01 7.629305e-01 8.067888e-01 8.156313e-01

2655 9.784162e-01 9.773587e-01 9.762743e-01 9.757566e-01 9.760804e-01 7.784239e-01 7.524244e-01 7.629588e-01 8.068156e-01 8.156582e-01

2656 9.784219e-01 9.773619e-01 9.762757e-01 9.757579e-01 9.760819e-01 7.784488e-01 7.524510e-01 7.629866e-01 8.068420e-01 8.156847e-01

2657 9.784274e-01 9.773650e-01 9.762770e-01 9.757592e-01 9.760834e-01 7.784732e-01 7.524771e-01 7.630138e-01 8.068679e-01 8.157106e-01

2658 9.784328e-01 9.773680e-01 9.762782e-01 9.757605e-01 9.760850e-01 7.784972e-01 7.525027e-01 7.630406e-01 8.068933e-01 8.157361e-01

2659 9.784381e-01 9.773708e-01 9.762794e-01 9.757617e-01 9.760865e-01 7.785209e-01 7.525279e-01 7.630669e-01 8.069183e-01 8.157612e-01

2660 9.784432e-01 9.773736e-01 9.762804e-01 9.757628e-01 9.760882e-01 7.785441e-01 7.525526e-01 7.630928e-01 8.069430e-01 8.157859e-01

2661 9.784483e-01 9.773762e-01 9.762814e-01 9.757640e-01 9.760899e-01 7.785670e-01 7.525769e-01 7.631182e-01 8.069672e-01 8.158102e-01

2662 9.784532e-01 9.773787e-01 9.762823e-01 9.757651e-01 9.760916e-01 7.785896e-01 7.526009e-01 7.631432e-01 8.069911e-01 8.158340e-01

2663 9.784580e-01 9.773811e-01 9.762831e-01 9.757661e-01 9.760934e-01 7.786118e-01 7.526244e-01 7.631678e-01 8.070146e-01 8.158576e-01

2664 9.784626e-01 9.773834e-01 9.762839e-01 9.757672e-01 9.760953e-01 7.786337e-01 7.526476e-01 7.631921e-01 8.070377e-01 8.158807e-01

2665 9.784671e-01 9.773856e-01 9.762847e-01 9.757683e-01 9.760973e-01 7.786553e-01 7.526704e-01 7.632160e-01 8.070606e-01 8.159035e-01

2666 9.784715e-01 9.773877e-01 9.762853e-01 9.757693e-01 9.760993e-01 7.786766e-01 7.526930e-01 7.632396e-01 8.070831e-01 8.159260e-01

2667 9.784758e-01 9.773896e-01 9.762860e-01 9.757704e-01 9.761014e-01 7.786977e-01 7.527152e-01 7.632628e-01 8.071053e-01 8.159482e-01

2668 9.784799e-01 9.773915e-01 9.762866e-01 9.757715e-01 9.761037e-01 7.787185e-01 7.527371e-01 7.632857e-01 8.071273e-01 8.159701e-01

2669 9.784838e-01 9.773933e-01 9.762871e-01 9.757726e-01 9.761060e-01 7.787390e-01 7.527587e-01 7.633083e-01 8.071489e-01 8.159917e-01

2670 9.784876e-01 9.773950e-01 9.762876e-01 9.757737e-01 9.761085e-01 7.787593e-01 7.527800e-01 7.633307e-01 8.071703e-01 8.160130e-01

2671 9.784913e-01 9.773966e-01 9.762882e-01 9.757748e-01 9.761111e-01 7.787794e-01 7.528011e-01 7.633527e-01 8.071915e-01 8.160341e-01

2672 9.784949e-01 9.773981e-01 9.762887e-01 9.757760e-01 9.761138e-01 7.787992e-01 7.528220e-01 7.633746e-01 8.072124e-01 8.160548e-01

2673 9.784982e-01 9.773995e-01 9.762891e-01 9.757772e-01 9.761167e-01 7.788189e-01 7.528426e-01 7.633961e-01 8.072330e-01 8.160754e-01

2674 9.785015e-01 9.774008e-01 9.762896e-01 9.757785e-01 9.761198e-01 7.788384e-01 7.528630e-01 7.634175e-01 8.072535e-01 8.160957e-01

2675 9.785046e-01 9.774021e-01 9.762901e-01 9.757799e-01 9.761230e-01 7.788577e-01 7.528832e-01 7.634386e-01 8.072737e-01 8.161158e-01

2676 9.785075e-01 9.774032e-01 9.762906e-01 9.757813e-01 9.761263e-01 7.788768e-01 7.529032e-01 7.634595e-01 8.072938e-01 8.161357e-01

2677 9.785103e-01 9.774043e-01 9.762912e-01 9.757828e-01 9.761299e-01 7.788958e-01 7.529230e-01 7.634802e-01 8.073136e-01 8.161553e-01

2678 9.785129e-01 9.774054e-01 9.762917e-01 9.757844e-01 9.761336e-01 7.789146e-01 7.529427e-01 7.635007e-01 8.073333e-01 8.161748e-01

2679 9.785154e-01 9.774063e-01 9.762924e-01 9.757862e-01 9.761376e-01 7.789333e-01 7.529622e-01 7.635211e-01 8.073528e-01 8.161940e-01

2680 9.785178e-01 9.774073e-01 9.762930e-01 9.757880e-01 9.761417e-01 7.789518e-01 7.529815e-01 7.635412e-01 8.073722e-01 8.162131e-01

2681 9.785200e-01 9.774081e-01 9.762937e-01 9.757899e-01 9.761461e-01 7.789703e-01 7.530007e-01 7.635613e-01 8.073913e-01 8.162320e-01

2682 9.785220e-01 9.774089e-01 9.762945e-01 9.757920e-01 9.761507e-01 7.789886e-01 7.530197e-01 7.635811e-01 8.074104e-01 8.162508e-01

2683 9.785239e-01 9.774097e-01 9.762954e-01 9.757942e-01 9.761555e-01 7.790068e-01 7.530386e-01 7.636009e-01 8.074293e-01 8.162694e-01

2684 9.785257e-01 9.774105e-01 9.762963e-01 9.757966e-01 9.761606e-01 7.790249e-01 7.530574e-01 7.636205e-01 8.074481e-01 8.162878e-01

2685 9.785273e-01 9.774112e-01 9.762974e-01 9.757991e-01 9.761659e-01 7.790429e-01 7.530761e-01 7.636400e-01 8.074667e-01 8.163061e-01

2686 9.785287e-01 9.774119e-01 9.762986e-01 9.758018e-01 9.761715e-01 7.790608e-01 7.530946e-01 7.636593e-01 8.074852e-01 8.163242e-01

2687 9.785300e-01 9.774126e-01 9.762998e-01 9.758047e-01 9.761774e-01 7.790787e-01 7.531131e-01 7.636786e-01 8.075036e-01 8.163422e-01

2688 9.785312e-01 9.774133e-01 9.763012e-01 9.758077e-01 9.761835e-01 7.790964e-01 7.531315e-01 7.636977e-01 8.075219e-01 8.163601e-01

2689 9.785322e-01 9.774140e-01 9.763028e-01 9.758110e-01 9.761900e-01 7.791141e-01 7.531498e-01 7.637168e-01 8.075401e-01 8.163778e-01

2690 9.785331e-01 9.774147e-01 9.763045e-01 9.758145e-01 9.761967e-01 7.791318e-01 7.531681e-01 7.637358e-01 8.075583e-01 8.163955e-01

2691 9.785339e-01 9.774154e-01 9.763064e-01 9.758182e-01 9.762037e-01 7.791494e-01 7.531862e-01 7.637547e-01 8.075763e-01 8.164130e-01

2692 9.785345e-01 9.774161e-01 9.763084e-01 9.758222e-01 9.762111e-01 7.791669e-01 7.532043e-01 7.637735e-01 8.075942e-01 8.164304e-01

2693 9.785350e-01 9.774169e-01 9.763107e-01 9.758264e-01 9.762188e-01 7.791845e-01 7.532224e-01 7.637923e-01 8.076121e-01 8.164477e-01

2694 9.785353e-01 9.774177e-01 9.763131e-01 9.758308e-01 9.762268e-01 7.792019e-01 7.532404e-01 7.638110e-01 8.076299e-01 8.164650e-01

2695 9.785356e-01 9.774186e-01 9.763158e-01 9.758356e-01 9.762352e-01 7.792194e-01 7.532584e-01 7.638297e-01 8.076477e-01 8.164821e-01

2696 9.785357e-01 9.774196e-01 9.763186e-01 9.758406e-01 9.762439e-01 7.792368e-01 7.532763e-01 7.638483e-01 8.076653e-01 8.164991e-01

2697 9.785357e-01 9.774206e-01 9.763218e-01 9.758459e-01 9.762530e-01 7.792542e-01 7.532942e-01 7.638668e-01 8.076830e-01 8.165161e-01

2698 9.785356e-01 9.774218e-01 9.763251e-01 9.758515e-01 9.762624e-01 7.792716e-01 7.533120e-01 7.638854e-01 8.077005e-01 8.165329e-01

2699 9.785354e-01 9.774230e-01 9.763287e-01 9.758574e-01 9.762722e-01 7.792890e-01 7.533299e-01 7.639039e-01 8.077181e-01 8.165497e-01

2700 9.785351e-01 9.774243e-01 9.763326e-01 9.758636e-01 9.762824e-01 7.793063e-01 7.533477e-01 7.639223e-01 8.077355e-01 8.165665e-01

2701 9.785347e-01 9.774257e-01 9.763368e-01 9.758702e-01 9.762930e-01 7.793237e-01 7.533655e-01 7.639408e-01 8.077530e-01 8.165831e-01

2702 9.785425e-01 9.774333e-01 9.763442e-01 9.758774e-01 9.762992e-01 7.795812e-01 7.535928e-01 7.641911e-01 8.080767e-01 8.169277e-01

2703 9.785503e-01 9.774409e-01 9.763515e-01 9.758845e-01 9.763053e-01 7.798408e-01 7.538226e-01 7.644438e-01 8.084015e-01 8.172731e-01

2704 9.785580e-01 9.774484e-01 9.763587e-01 9.758916e-01 9.763114e-01 7.801021e-01 7.540548e-01 7.646989e-01 8.087273e-01 8.176189e-01

2705 9.785658e-01 9.774559e-01 9.763660e-01 9.758987e-01 9.763175e-01 7.803651e-01 7.542892e-01 7.649561e-01 8.090537e-01 8.179650e-01

2706 9.785735e-01 9.774634e-01 9.763732e-01 9.759057e-01 9.763235e-01 7.806296e-01 7.545257e-01 7.652153e-01 8.093807e-01 8.183111e-01

2707 9.785811e-01 9.774708e-01 9.763803e-01 9.759127e-01 9.763295e-01 7.808955e-01 7.547643e-01 7.654764e-01 8.097081e-01 8.186571e-01

2708 9.785888e-01 9.774782e-01 9.763874e-01 9.759196e-01 9.763354e-01 7.811625e-01 7.550047e-01 7.657392e-01 8.100354e-01 8.190027e-01

2709 9.785964e-01 9.774856e-01 9.763945e-01 9.759265e-01 9.763413e-01 7.814305e-01 7.552468e-01 7.660036e-01 8.103627e-01 8.193476e-01

2710 9.786040e-01 9.774929e-01 9.764015e-01 9.759333e-01 9.763472e-01 7.816994e-01 7.554905e-01 7.662694e-01 8.106896e-01 8.196916e-01

2711 9.786116e-01 9.775002e-01 9.764085e-01 9.759401e-01 9.763531e-01 7.819689e-01 7.557356e-01 7.665365e-01 8.110160e-01 8.200346e-01

2712 9.786191e-01 9.775075e-01 9.764155e-01 9.759469e-01 9.763589e-01 7.822389e-01 7.559821e-01 7.668046e-01 8.113416e-01 8.203763e-01

2713 9.786266e-01 9.775148e-01 9.764224e-01 9.759537e-01 9.763646e-01 7.825092e-01 7.562297e-01 7.670736e-01 8.116662e-01 8.207164e-01

2714 9.786341e-01 9.775220e-01 9.764294e-01 9.759604e-01 9.763704e-01 7.827797e-01 7.564783e-01 7.673434e-01 8.119896e-01 8.210549e-01

2715 9.786416e-01 9.775292e-01 9.764362e-01 9.759670e-01 9.763761e-01 7.830501e-01 7.567277e-01 7.676137e-01 8.123116e-01 8.213914e-01

2716 9.786491e-01 9.775364e-01 9.764431e-01 9.759737e-01 9.763817e-01 7.833203e-01 7.569778e-01 7.678844e-01 8.126321e-01 8.217258e-01

2717 9.786566e-01 9.775436e-01 9.764499e-01 9.759802e-01 9.763873e-01 7.835900e-01 7.572285e-01 7.681554e-01 8.129508e-01 8.220579e-01

2718 9.786640e-01 9.775507e-01 9.764566e-01 9.759868e-01 9.763929e-01 7.838593e-01 7.574795e-01 7.684263e-01 8.132674e-01 8.223874e-01

2719 9.786714e-01 9.775578e-01 9.764634e-01 9.759933e-01 9.763985e-01 7.841277e-01 7.577307e-01 7.686972e-01 8.135820e-01 8.227143e-01

2720 9.786788e-01 9.775649e-01 9.764701e-01 9.759998e-01 9.764040e-01 7.843953e-01 7.579819e-01 7.689677e-01 8.138942e-01 8.230382e-01

2721 9.786862e-01 9.775719e-01 9.764768e-01 9.760063e-01 9.764095e-01 7.846617e-01 7.582330e-01 7.692378e-01 8.142038e-01 8.233592e-01

2722 9.786936e-01 9.775790e-01 9.764834e-01 9.760127e-01 9.764149e-01 7.849269e-01 7.584838e-01 7.695072e-01 8.145108e-01 8.236769e-01

2723 9.787010e-01 9.775860e-01 9.764900e-01 9.760191e-01 9.764203e-01 7.851907e-01 7.587342e-01 7.697758e-01 8.148150e-01 8.239913e-01

2724 9.787083e-01 9.775930e-01 9.764966e-01 9.760254e-01 9.764257e-01 7.854529e-01 7.589839e-01 7.700433e-01 8.151161e-01 8.243022e-01

2725 9.787157e-01 9.776000e-01 9.765032e-01 9.760317e-01 9.764310e-01 7.857134e-01 7.592329e-01 7.703098e-01 8.154141e-01 8.246094e-01

2726 9.787230e-01 9.776069e-01 9.765097e-01 9.760380e-01 9.764363e-01 7.859720e-01 7.594809e-01 7.705749e-01 8.157088e-01 8.249129e-01

2727 9.787303e-01 9.776139e-01 9.765162e-01 9.760443e-01 9.764416e-01 7.862285e-01 7.597279e-01 7.708385e-01 8.160001e-01 8.252125e-01

2728 9.787376e-01 9.776208e-01 9.765227e-01 9.760505e-01 9.764468e-01 7.864829e-01 7.599736e-01 7.711004e-01 8.162879e-01 8.255080e-01

2729 9.787449e-01 9.776276e-01 9.765291e-01 9.760567e-01 9.764520e-01 7.867349e-01 7.602179e-01 7.713606e-01 8.165720e-01 8.257995e-01

2730 9.787522e-01 9.776345e-01 9.765355e-01 9.760628e-01 9.764572e-01 7.869845e-01 7.604607e-01 7.716188e-01 8.168523e-01 8.260868e-01

2731 9.787595e-01 9.776414e-01 9.765419e-01 9.760689e-01 9.764623e-01 7.872316e-01 7.607018e-01 7.718750e-01 8.171287e-01 8.263697e-01

2732 9.787667e-01 9.776482e-01 9.765482e-01 9.760750e-01 9.764673e-01 7.874759e-01 7.609411e-01 7.721289e-01 8.174012e-01 8.266483e-01

2733 9.787740e-01 9.776550e-01 9.765545e-01 9.760810e-01 9.764724e-01 7.877175e-01 7.611785e-01 7.723804e-01 8.176696e-01 8.269225e-01

2734 9.787812e-01 9.776618e-01 9.765608e-01 9.760870e-01 9.764774e-01 7.879561e-01 7.614138e-01 7.726295e-01 8.179339e-01 8.271921e-01

2735 9.787885e-01 9.776685e-01 9.765670e-01 9.760930e-01 9.764824e-01 7.881917e-01 7.616468e-01 7.728760e-01 8.181940e-01 8.274572e-01

2736 9.787957e-01 9.776753e-01 9.765733e-01 9.760989e-01 9.764873e-01 7.884242e-01 7.618776e-01 7.731197e-01 8.184499e-01 8.277176e-01

2737 9.788029e-01 9.776820e-01 9.765794e-01 9.761048e-01 9.764922e-01 7.886535e-01 7.621059e-01 7.733606e-01 8.187014e-01 8.279734e-01

2738 9.788101e-01 9.776887e-01 9.765856e-01 9.761107e-01 9.764970e-01 7.888795e-01 7.623317e-01 7.735986e-01 8.189485e-01 8.282246e-01

2739 9.788173e-01 9.776954e-01 9.765917e-01 9.761165e-01 9.765018e-01 7.891022e-01 7.625549e-01 7.738335e-01 8.191913e-01 8.284710e-01

2740 9.788245e-01 9.777020e-01 9.765978e-01 9.761223e-01 9.765066e-01 7.893215e-01 7.627753e-01 7.740653e-01 8.194296e-01 8.287127e-01

2741 9.788316e-01 9.777087e-01 9.766038e-01 9.761280e-01 9.765114e-01 7.895372e-01 7.629929e-01 7.742939e-01 8.196635e-01 8.289496e-01

2742 9.788388e-01 9.777153e-01 9.766098e-01 9.761337e-01 9.765160e-01 7.897495e-01 7.632075e-01 7.745192e-01 8.198929e-01 8.291818e-01

2743 9.788459e-01 9.777219e-01 9.766158e-01 9.761394e-01 9.765207e-01 7.899581e-01 7.634192e-01 7.747412e-01 8.201178e-01 8.294093e-01

2744 9.788531e-01 9.777284e-01 9.766218e-01 9.761450e-01 9.765253e-01 7.901631e-01 7.636278e-01 7.749597e-01 8.203382e-01 8.296321e-01

2745 9.788602e-01 9.777350e-01 9.766277e-01 9.761506e-01 9.765299e-01 7.903645e-01 7.638333e-01 7.751748e-01 8.205541e-01 8.298501e-01

2746 9.788673e-01 9.777415e-01 9.766335e-01 9.761561e-01 9.765344e-01 7.905621e-01 7.640355e-01 7.753863e-01 8.207656e-01 8.300634e-01

2747 9.788745e-01 9.777480e-01 9.766394e-01 9.761616e-01 9.765389e-01 7.907561e-01 7.642346e-01 7.755942e-01 8.209725e-01 8.302721e-01

2748 9.788816e-01 9.777545e-01 9.766452e-01 9.761671e-01 9.765433e-01 7.909463e-01 7.644303e-01 7.757985e-01 8.211750e-01 8.304761e-01

2749 9.788886e-01 9.777609e-01 9.766509e-01 9.761725e-01 9.765477e-01 7.911327e-01 7.646227e-01 7.759992e-01 8.213731e-01 8.306756e-01

2750 9.788957e-01 9.777673e-01 9.766567e-01 9.761779e-01 9.765521e-01 7.913154e-01 7.648117e-01 7.761962e-01 8.215668e-01 8.308704e-01

2751 9.789028e-01 9.777737e-01 9.766623e-01 9.761833e-01 9.765564e-01 7.914944e-01 7.649973e-01 7.763895e-01 8.217561e-01 8.310608e-01

2752 9.789099e-01 9.777801e-01 9.766680e-01 9.761886e-01 9.765607e-01 7.916696e-01 7.651794e-01 7.765791e-01 8.219410e-01 8.312466e-01

2753 9.789169e-01 9.777865e-01 9.766736e-01 9.761938e-01 9.765649e-01 7.918410e-01 7.653582e-01 7.767650e-01 8.221217e-01 8.314281e-01

2754 9.789239e-01 9.777928e-01 9.766791e-01 9.761990e-01 9.765691e-01 7.920087e-01 7.655334e-01 7.769471e-01 8.222981e-01 8.316052e-01

2755 9.789310e-01 9.777991e-01 9.766847e-01 9.762042e-01 9.765732e-01 7.921727e-01 7.657052e-01 7.771255e-01 8.224703e-01 8.317779e-01

2756 9.789380e-01 9.778053e-01 9.766901e-01 9.762093e-01 9.765773e-01 7.923330e-01 7.658734e-01 7.773002e-01 8.226384e-01 8.319464e-01

2757 9.789450e-01 9.778116e-01 9.766956e-01 9.762144e-01 9.765814e-01 7.924897e-01 7.660382e-01 7.774711e-01 8.228023e-01 8.321108e-01

2758 9.789520e-01 9.778178e-01 9.767010e-01 9.762194e-01 9.765854e-01 7.926427e-01 7.661995e-01 7.776384e-01 8.229622e-01 8.322710e-01

2759 9.789589e-01 9.778239e-01 9.767063e-01 9.762244e-01 9.765893e-01 7.927920e-01 7.663574e-01 7.778020e-01 8.231181e-01 8.324271e-01

2760 9.789659e-01 9.778301e-01 9.767116e-01 9.762293e-01 9.765932e-01 7.929379e-01 7.665118e-01 7.779619e-01 8.232701e-01 8.325792e-01

2761 9.789728e-01 9.778362e-01 9.767169e-01 9.762342e-01 9.765971e-01 7.930802e-01 7.666628e-01 7.781182e-01 8.234183e-01 8.327274e-01

2762 9.789798e-01 9.778423e-01 9.767221e-01 9.762390e-01 9.766009e-01 7.932190e-01 7.668103e-01 7.782709e-01 8.235626e-01 8.328718e-01

2763 9.789867e-01 9.778483e-01 9.767272e-01 9.762438e-01 9.766046e-01 7.933543e-01 7.669545e-01 7.784200e-01 8.237032e-01 8.330124e-01

2764 9.789936e-01 9.778543e-01 9.767324e-01 9.762485e-01 9.766084e-01 7.934863e-01 7.670952e-01 7.785656e-01 8.238402e-01 8.331493e-01

2765 9.790005e-01 9.778603e-01 9.767374e-01 9.762532e-01 9.766120e-01 7.936149e-01 7.672327e-01 7.787077e-01 8.239736e-01 8.332826e-01

2766 9.790073e-01 9.778662e-01 9.767424e-01 9.762578e-01 9.766156e-01 7.937403e-01 7.673669e-01 7.788464e-01 8.241034e-01 8.334123e-01

2767 9.790142e-01 9.778722e-01 9.767474e-01 9.762624e-01 9.766192e-01 7.938624e-01 7.674978e-01 7.789816e-01 8.242298e-01 8.335385e-01

2768 9.790210e-01 9.778780e-01 9.767523e-01 9.762669e-01 9.766227e-01 7.939812e-01 7.676255e-01 7.791135e-01 8.243528e-01 8.336613e-01

2769 9.790278e-01 9.778839e-01 9.767572e-01 9.762714e-01 9.766262e-01 7.940970e-01 7.677500e-01 7.792421e-01 8.244725e-01 8.337808e-01

2770 9.790346e-01 9.778897e-01 9.767620e-01 9.762758e-01 9.766296e-01 7.942097e-01 7.678714e-01 7.793674e-01 8.245889e-01 8.338970e-01

2771 9.790414e-01 9.778954e-01 9.767668e-01 9.762802e-01 9.766330e-01 7.943194e-01 7.679897e-01 7.794896e-01 8.247022e-01 8.340101e-01

2772 9.790481e-01 9.779011e-01 9.767715e-01 9.762845e-01 9.766363e-01 7.944261e-01 7.681049e-01 7.796085e-01 8.248124e-01 8.341200e-01

2773 9.790548e-01 9.779068e-01 9.767761e-01 9.762887e-01 9.766396e-01 7.945299e-01 7.682172e-01 7.797244e-01 8.249195e-01 8.342269e-01

2774 9.790615e-01 9.779125e-01 9.767807e-01 9.762929e-01 9.766428e-01 7.946309e-01 7.683265e-01 7.798373e-01 8.250237e-01 8.343309e-01

2775 9.790682e-01 9.779181e-01 9.767853e-01 9.762971e-01 9.766460e-01 7.947291e-01 7.684330e-01 7.799471e-01 8.251250e-01 8.344320e-01

2776 9.790749e-01 9.779236e-01 9.767897e-01 9.763012e-01 9.766492e-01 7.948246e-01 7.685366e-01 7.800540e-01 8.252235e-01 8.345302e-01

2777 9.790815e-01 9.779291e-01 9.767942e-01 9.763052e-01 9.766523e-01 7.949174e-01 7.686374e-01 7.801581e-01 8.253193e-01 8.346258e-01

2778 9.790881e-01 9.779346e-01 9.767985e-01 9.763092e-01 9.766553e-01 7.950077e-01 7.687356e-01 7.802594e-01 8.254124e-01 8.347186e-01

2779 9.790947e-01 9.779400e-01 9.768028e-01 9.763131e-01 9.766583e-01 7.950954e-01 7.688310e-01 7.803579e-01 8.255029e-01 8.348089e-01

2780 9.791012e-01 9.779454e-01 9.768071e-01 9.763169e-01 9.766612e-01 7.951806e-01 7.689239e-01 7.804538e-01 8.255909e-01 8.348966e-01

2781 9.791078e-01 9.779507e-01 9.768113e-01 9.763207e-01 9.766641e-01 7.952635e-01 7.690142e-01 7.805470e-01 8.256764e-01 8.349819e-01

2782 9.791142e-01 9.779560e-01 9.768154e-01 9.763245e-01 9.766670e-01 7.953440e-01 7.691020e-01 7.806377e-01 8.257594e-01 8.350648e-01

2783 9.791207e-01 9.779612e-01 9.768195e-01 9.763281e-01 9.766698e-01 7.954222e-01 7.691874e-01 7.807258e-01 8.258402e-01 8.351453e-01

2784 9.791271e-01 9.779664e-01 9.768235e-01 9.763318e-01 9.766726e-01 7.954981e-01 7.692703e-01 7.808115e-01 8.259187e-01 8.352236e-01

2785 9.791335e-01 9.779715e-01 9.768274e-01 9.763353e-01 9.766753e-01 7.955719e-01 7.693510e-01 7.808949e-01 8.259950e-01 8.352997e-01

2786 9.791399e-01 9.779766e-01 9.768313e-01 9.763388e-01 9.766780e-01 7.956436e-01 7.694294e-01 7.809759e-01 8.260691e-01 8.353737e-01

2787 9.791462e-01 9.779816e-01 9.768351e-01 9.763423e-01 9.766806e-01 7.957133e-01 7.695056e-01 7.810547e-01 8.261411e-01 8.354456e-01

2788 9.791525e-01 9.779866e-01 9.768389e-01 9.763457e-01 9.766832e-01 7.957809e-01 7.695796e-01 7.811312e-01 8.262112e-01 8.355155e-01

2789 9.791588e-01 9.779915e-01 9.768426e-01 9.763490e-01 9.766858e-01 7.958466e-01 7.696516e-01 7.812056e-01 8.262792e-01 8.355834e-01

2790 9.791650e-01 9.779964e-01 9.768462e-01 9.763523e-01 9.766883e-01 7.959104e-01 7.697215e-01 7.812779e-01 8.263454e-01 8.356495e-01

2791 9.791712e-01 9.780012e-01 9.768498e-01 9.763555e-01 9.766908e-01 7.959724e-01 7.697894e-01 7.813482e-01 8.264097e-01 8.357137e-01

2792 9.791773e-01 9.780059e-01 9.768533e-01 9.763587e-01 9.766932e-01 7.960326e-01 7.698553e-01 7.814166e-01 8.264722e-01 8.357761e-01

2793 9.791834e-01 9.780106e-01 9.768567e-01 9.763618e-01 9.766956e-01 7.960911e-01 7.699194e-01 7.814829e-01 8.265329e-01 8.358368e-01

2794 9.791894e-01 9.780152e-01 9.768601e-01 9.763648e-01 9.766980e-01 7.961479e-01 7.699817e-01 7.815475e-01 8.265920e-01 8.358958e-01

2795 9.791954e-01 9.780198e-01 9.768634e-01 9.763678e-01 9.767004e-01 7.962031e-01 7.700422e-01 7.816102e-01 8.266494e-01 8.359532e-01

2796 9.792014e-01 9.780243e-01 9.768666e-01 9.763707e-01 9.767027e-01 7.962567e-01 7.701010e-01 7.816712e-01 8.267053e-01 8.360090e-01

2797 9.792073e-01 9.780288e-01 9.768698e-01 9.763736e-01 9.767050e-01 7.963088e-01 7.701581e-01 7.817304e-01 8.267596e-01 8.360633e-01

2798 9.792132e-01 9.780332e-01 9.768729e-01 9.763764e-01 9.767072e-01 7.963594e-01 7.702135e-01 7.817880e-01 8.268124e-01 8.361161e-01

2799 9.792190e-01 9.780375e-01 9.768759e-01 9.763792e-01 9.767095e-01 7.964086e-01 7.702674e-01 7.818440e-01 8.268638e-01 8.361675e-01

2800 9.792248e-01 9.780418e-01 9.768789e-01 9.763819e-01 9.767117e-01 7.964564e-01 7.703198e-01 7.818985e-01 8.269138e-01 8.362175e-01

2801 9.792305e-01 9.780460e-01 9.768818e-01 9.763846e-01 9.767139e-01 7.965028e-01 7.703707e-01 7.819514e-01 8.269624e-01 8.362662e-01

2802 9.792362e-01 9.780501e-01 9.768847e-01 9.763872e-01 9.767161e-01 7.965480e-01 7.704202e-01 7.820028e-01 8.270097e-01 8.363135e-01

2803 9.792418e-01 9.780542e-01 9.768875e-01 9.763897e-01 9.767182e-01 7.965919e-01 7.704682e-01 7.820529e-01 8.270558e-01 8.363596e-01

2804 9.792473e-01 9.780582e-01 9.768902e-01 9.763923e-01 9.767204e-01 7.966346e-01 7.705150e-01 7.821016e-01 8.271006e-01 8.364045e-01

2805 9.792528e-01 9.780621e-01 9.768929e-01 9.763947e-01 9.767225e-01 7.966761e-01 7.705604e-01 7.821489e-01 8.271443e-01 8.364482e-01

2806 9.792583e-01 9.780660e-01 9.768955e-01 9.763972e-01 9.767246e-01 7.967165e-01 7.706046e-01 7.821950e-01 8.271868e-01 8.364908e-01

2807 9.792636e-01 9.780698e-01 9.768981e-01 9.763995e-01 9.767268e-01 7.967558e-01 7.706475e-01 7.822398e-01 8.272282e-01 8.365323e-01

2808 9.792690e-01 9.780736e-01 9.769006e-01 9.764019e-01 9.767289e-01 7.967940e-01 7.706893e-01 7.822834e-01 8.272686e-01 8.365727e-01

2809 9.792742e-01 9.780773e-01 9.769030e-01 9.764042e-01 9.767310e-01 7.968312e-01 7.707299e-01 7.823259e-01 8.273079e-01 8.366121e-01

2810 9.792794e-01 9.780809e-01 9.769054e-01 9.764065e-01 9.767331e-01 7.968674e-01 7.707694e-01 7.823672e-01 8.273462e-01 8.366505e-01

2811 9.792846e-01 9.780844e-01 9.769077e-01 9.764087e-01 9.767353e-01 7.969027e-01 7.708079e-01 7.824074e-01 8.273835e-01 8.366879e-01

2812 9.792896e-01 9.780879e-01 9.769100e-01 9.764109e-01 9.767374e-01 7.969370e-01 7.708453e-01 7.824466e-01 8.274199e-01 8.367244e-01

2813 9.792946e-01 9.780914e-01 9.769122e-01 9.764131e-01 9.767396e-01 7.969705e-01 7.708818e-01 7.824848e-01 8.274554e-01 8.367601e-01

2814 9.792995e-01 9.780947e-01 9.769144e-01 9.764152e-01 9.767417e-01 7.970031e-01 7.709173e-01 7.825220e-01 8.274901e-01 8.367948e-01

2815 9.793044e-01 9.780980e-01 9.769165e-01 9.764173e-01 9.767439e-01 7.970349e-01 7.709519e-01 7.825582e-01 8.275238e-01 8.368287e-01

2816 9.793092e-01 9.781012e-01 9.769186e-01 9.764194e-01 9.767462e-01 7.970659e-01 7.709855e-01 7.825936e-01 8.275568e-01 8.368618e-01

2817 9.793139e-01 9.781044e-01 9.769206e-01 9.764215e-01 9.767484e-01 7.970961e-01 7.710183e-01 7.826280e-01 8.275890e-01 8.368941e-01

2818 9.793186e-01 9.781075e-01 9.769226e-01 9.764235e-01 9.767507e-01 7.971256e-01 7.710503e-01 7.826616e-01 8.276205e-01 8.369257e-01

2819 9.793231e-01 9.781105e-01 9.769246e-01 9.764256e-01 9.767530e-01 7.971544e-01 7.710815e-01 7.826944e-01 8.276512e-01 8.369565e-01

2820 9.793276e-01 9.781135e-01 9.769265e-01 9.764276e-01 9.767554e-01 7.971825e-01 7.711119e-01 7.827264e-01 8.276812e-01 8.369867e-01

2821 9.793320e-01 9.781164e-01 9.769284e-01 9.764296e-01 9.767578e-01 7.972099e-01 7.711416e-01 7.827576e-01 8.277106e-01 8.370161e-01

2822 9.793364e-01 9.781192e-01 9.769302e-01 9.764317e-01 9.767602e-01 7.972367e-01 7.711706e-01 7.827881e-01 8.277393e-01 8.370449e-01

2823 9.793406e-01 9.781220e-01 9.769321e-01 9.764337e-01 9.767628e-01 7.972629e-01 7.711989e-01 7.828179e-01 8.277673e-01 8.370731e-01

2824 9.793448e-01 9.781247e-01 9.769339e-01 9.764357e-01 9.767653e-01 7.972885e-01 7.712265e-01 7.828470e-01 8.277948e-01 8.371006e-01

2825 9.793489e-01 9.781274e-01 9.769357e-01 9.764378e-01 9.767680e-01 7.973135e-01 7.712535e-01 7.828755e-01 8.278217e-01 8.371276e-01

2826 9.793529e-01 9.781300e-01 9.769374e-01 9.764398e-01 9.767707e-01 7.973380e-01 7.712798e-01 7.829033e-01 8.278480e-01 8.371540e-01

2827 9.793569e-01 9.781326e-01 9.769392e-01 9.764419e-01 9.767735e-01 7.973620e-01 7.713056e-01 7.829305e-01 8.278737e-01 8.371799e-01

2828 9.793607e-01 9.781351e-01 9.769409e-01 9.764440e-01 9.767763e-01 7.973855e-01 7.713308e-01 7.829571e-01 8.278990e-01 8.372052e-01

2829 9.793645e-01 9.781375e-01 9.769427e-01 9.764461e-01 9.767793e-01 7.974085e-01 7.713555e-01 7.829832e-01 8.279237e-01 8.372300e-01

2830 9.793682e-01 9.781399e-01 9.769444e-01 9.764482e-01 9.767823e-01 7.974310e-01 7.713796e-01 7.830087e-01 8.279480e-01 8.372543e-01

2831 9.793718e-01 9.781422e-01 9.769461e-01 9.764504e-01 9.767854e-01 7.974531e-01 7.714033e-01 7.830337e-01 8.279718e-01 8.372782e-01

2832 9.793753e-01 9.781445e-01 9.769479e-01 9.764526e-01 9.767887e-01 7.974748e-01 7.714264e-01 7.830582e-01 8.279951e-01 8.373016e-01

2833 9.793787e-01 9.781468e-01 9.769496e-01 9.764549e-01 9.767920e-01 7.974960e-01 7.714491e-01 7.830823e-01 8.280180e-01 8.373245e-01

2834 9.793820e-01 9.781490e-01 9.769514e-01 9.764572e-01 9.767954e-01 7.975169e-01 7.714714e-01 7.831058e-01 8.280405e-01 8.373471e-01

2835 9.793853e-01 9.781512e-01 9.769532e-01 9.764596e-01 9.767990e-01 7.975374e-01 7.714932e-01 7.831290e-01 8.280626e-01 8.373692e-01

2836 9.793884e-01 9.781533e-01 9.769550e-01 9.764620e-01 9.768027e-01 7.975575e-01 7.715147e-01 7.831517e-01 8.280843e-01 8.373909e-01

2837 9.793915e-01 9.781554e-01 9.769568e-01 9.764645e-01 9.768065e-01 7.975773e-01 7.715357e-01 7.831739e-01 8.281056e-01 8.374123e-01

2838 9.793945e-01 9.781575e-01 9.769587e-01 9.764671e-01 9.768104e-01 7.975967e-01 7.715564e-01 7.831958e-01 8.281266e-01 8.374333e-01

2839 9.793974e-01 9.781596e-01 9.769606e-01 9.764697e-01 9.768145e-01 7.976159e-01 7.715767e-01 7.832174e-01 8.281472e-01 8.374539e-01

2840 9.794002e-01 9.781616e-01 9.769626e-01 9.764724e-01 9.768187e-01 7.976347e-01 7.715966e-01 7.832385e-01 8.281676e-01 8.374742e-01

2841 9.794030e-01 9.781636e-01 9.769646e-01 9.764753e-01 9.768231e-01 7.976533e-01 7.716162e-01 7.832593e-01 8.281876e-01 8.374942e-01

2842 9.794056e-01 9.781656e-01 9.769667e-01 9.764782e-01 9.768276e-01 7.976716e-01 7.716356e-01 7.832798e-01 8.282072e-01 8.375138e-01

2843 9.794082e-01 9.781676e-01 9.769689e-01 9.764812e-01 9.768323e-01 7.976896e-01 7.716546e-01 7.833000e-01 8.282267e-01 8.375332e-01

2844 9.794106e-01 9.781695e-01 9.769711e-01 9.764843e-01 9.768372e-01 7.977074e-01 7.716733e-01 7.833199e-01 8.282458e-01 8.375523e-01

2845 9.794130e-01 9.781715e-01 9.769734e-01 9.764876e-01 9.768422e-01 7.977249e-01 7.716918e-01 7.833395e-01 8.282646e-01 8.375710e-01

2846 9.794153e-01 9.781735e-01 9.769758e-01 9.764910e-01 9.768474e-01 7.977422e-01 7.717100e-01 7.833588e-01 8.282832e-01 8.375896e-01

2847 9.794175e-01 9.781754e-01 9.769782e-01 9.764945e-01 9.768528e-01 7.977593e-01 7.717279e-01 7.833778e-01 8.283016e-01 8.376078e-01

2848 9.794197e-01 9.781774e-01 9.769808e-01 9.764981e-01 9.768584e-01 7.977762e-01 7.717457e-01 7.833966e-01 8.283197e-01 8.376258e-01

2849 9.794217e-01 9.781794e-01 9.769835e-01 9.765019e-01 9.768642e-01 7.977928e-01 7.717632e-01 7.834151e-01 8.283376e-01 8.376436e-01

2850 9.794237e-01 9.781814e-01 9.769863e-01 9.765058e-01 9.768701e-01 7.978093e-01 7.717804e-01 7.834335e-01 8.283553e-01 8.376611e-01

2851 9.794256e-01 9.781835e-01 9.769892e-01 9.765099e-01 9.768763e-01 7.978257e-01 7.717975e-01 7.834516e-01 8.283728e-01 8.376784e-01

2852 9.794275e-01 9.781855e-01 9.769922e-01 9.765141e-01 9.768827e-01 7.978418e-01 7.718144e-01 7.834695e-01 8.283901e-01 8.376955e-01

2853 9.794292e-01 9.781876e-01 9.769954e-01 9.765185e-01 9.768893e-01 7.978578e-01 7.718311e-01 7.834872e-01 8.284071e-01 8.377124e-01

2854 9.794309e-01 9.781898e-01 9.769987e-01 9.765231e-01 9.768961e-01 7.978737e-01 7.718477e-01 7.835047e-01 8.284241e-01 8.377291e-01

2855 9.794326e-01 9.781920e-01 9.770022e-01 9.765279e-01 9.769031e-01 7.978894e-01 7.718640e-01 7.835220e-01 8.284408e-01 8.377456e-01

2856 9.794341e-01 9.781942e-01 9.770058e-01 9.765328e-01 9.769104e-01 7.979050e-01 7.718802e-01 7.835392e-01 8.284574e-01 8.377619e-01

2857 9.794356e-01 9.781965e-01 9.770096e-01 9.765380e-01 9.769179e-01 7.979204e-01 7.718963e-01 7.835562e-01 8.284738e-01 8.377780e-01

2858 9.794371e-01 9.781989e-01 9.770136e-01 9.765433e-01 9.769257e-01 7.979357e-01 7.719122e-01 7.835731e-01 8.284900e-01 8.377940e-01

2859 9.794385e-01 9.782014e-01 9.770177e-01 9.765489e-01 9.769336e-01 7.979510e-01 7.719280e-01 7.835898e-01 8.285062e-01 8.378098e-01

2860 9.794398e-01 9.782039e-01 9.770220e-01 9.765546e-01 9.769419e-01 7.979661e-01 7.719437e-01 7.836063e-01 8.285221e-01 8.378254e-01

2861 9.794411e-01 9.782065e-01 9.770266e-01 9.765606e-01 9.769504e-01 7.979811e-01 7.719593e-01 7.836228e-01 8.285380e-01 8.378409e-01

2862 9.794424e-01 9.782092e-01 9.770313e-01 9.765669e-01 9.769591e-01 7.979960e-01 7.719747e-01 7.836391e-01 8.285537e-01 8.378562e-01

2863 9.794436e-01 9.782120e-01 9.770362e-01 9.765733e-01 9.769681e-01 7.980109e-01 7.719901e-01 7.836553e-01 8.285693e-01 8.378714e-01

2864 9.794447e-01 9.782149e-01 9.770414e-01 9.765800e-01 9.769773e-01 7.980256e-01 7.720054e-01 7.836714e-01 8.285848e-01 8.378865e-01

2865 9.794459e-01 9.782180e-01 9.770468e-01 9.765869e-01 9.769869e-01 7.980403e-01 7.720206e-01 7.836875e-01 8.286002e-01 8.379014e-01

2866 9.794470e-01 9.782211e-01 9.770524e-01 9.765941e-01 9.769966e-01 7.980550e-01 7.720357e-01 7.837034e-01 8.286155e-01 8.379162e-01

2867 9.794481e-01 9.782244e-01 9.770583e-01 9.766016e-01 9.770067e-01 7.980695e-01 7.720507e-01 7.837192e-01 8.286307e-01 8.379309e-01

2868 9.794492e-01 9.782278e-01 9.770644e-01 9.766093e-01 9.770170e-01 7.980841e-01 7.720657e-01 7.837350e-01 8.286458e-01 8.379454e-01

2869 9.794503e-01 9.782314e-01 9.770708e-01 9.766173e-01 9.770276e-01 7.980985e-01 7.720806e-01 7.837506e-01 8.286609e-01 8.379599e-01

2870 9.794513e-01 9.782351e-01 9.770774e-01 9.766256e-01 9.770385e-01 7.981130e-01 7.720954e-01 7.837662e-01 8.286758e-01 8.379742e-01

2871 9.794524e-01 9.782390e-01 9.770843e-01 9.766341e-01 9.770497e-01 7.981273e-01 7.721102e-01 7.837818e-01 8.286907e-01 8.379884e-01

2872 9.794535e-01 9.782430e-01 9.770915e-01 9.766429e-01 9.770611e-01 7.981417e-01 7.721249e-01 7.837973e-01 8.287054e-01 8.380025e-01

2873 9.794546e-01 9.782473e-01 9.770990e-01 9.766520e-01 9.770729e-01 7.981560e-01 7.721396e-01 7.838127e-01 8.287202e-01 8.380165e-01

2874 9.794557e-01 9.782517e-01 9.771068e-01 9.766614e-01 9.770849e-01 7.981703e-01 7.721543e-01 7.838281e-01 8.287348e-01 8.380305e-01

2875 9.794569e-01 9.782563e-01 9.771148e-01 9.766711e-01 9.770972e-01 7.981846e-01 7.721690e-01 7.838434e-01 8.287494e-01 8.380443e-01

2876 9.794580e-01 9.782611e-01 9.771232e-01 9.766812e-01 9.771097e-01 7.981988e-01 7.721836e-01 7.838587e-01 8.287639e-01 8.380580e-01

2877 9.794593e-01 9.782661e-01 9.771319e-01 9.766915e-01 9.771226e-01 7.982131e-01 7.721982e-01 7.838740e-01 8.287784e-01 8.380716e-01

2878 9.794606e-01 9.782713e-01 9.771410e-01 9.767021e-01 9.771357e-01 7.982273e-01 7.722127e-01 7.838892e-01 8.287929e-01 8.380851e-01

2879 9.794619e-01 9.782768e-01 9.771503e-01 9.767130e-01 9.771492e-01 7.982415e-01 7.722273e-01 7.839044e-01 8.288072e-01 8.380985e-01

2880 9.794633e-01 9.782825e-01 9.771600e-01 9.767243e-01 9.771629e-01 7.982557e-01 7.722419e-01 7.839196e-01 8.288216e-01 8.381119e-01

2881 9.794648e-01 9.782884e-01 9.771700e-01 9.767358e-01 9.771769e-01 7.982699e-01 7.722564e-01 7.839348e-01 8.288358e-01 8.381251e-01

2882 9.794707e-01 9.782941e-01 9.771754e-01 9.767410e-01 9.771812e-01 7.985145e-01 7.724756e-01 7.841775e-01 8.291431e-01 8.384509e-01

2883 9.794767e-01 9.782997e-01 9.771807e-01 9.767461e-01 9.771856e-01 7.987599e-01 7.726964e-01 7.844216e-01 8.294500e-01 8.387758e-01

2884 9.794826e-01 9.783053e-01 9.771860e-01 9.767513e-01 9.771899e-01 7.990062e-01 7.729187e-01 7.846670e-01 8.297565e-01 8.390998e-01

2885 9.794885e-01 9.783109e-01 9.771912e-01 9.767563e-01 9.771941e-01 7.992530e-01 7.731422e-01 7.849135e-01 8.300625e-01 8.394226e-01

2886 9.794944e-01 9.783165e-01 9.771964e-01 9.767614e-01 9.771984e-01 7.995003e-01 7.733670e-01 7.851610e-01 8.303675e-01 8.397441e-01

2887 9.795002e-01 9.783220e-01 9.772016e-01 9.767664e-01 9.772026e-01 7.997479e-01 7.735928e-01 7.854093e-01 8.306716e-01 8.400640e-01

2888 9.795061e-01 9.783276e-01 9.772068e-01 9.767714e-01 9.772068e-01 7.999956e-01 7.738195e-01 7.856583e-01 8.309746e-01 8.403822e-01

2889 9.795119e-01 9.783331e-01 9.772120e-01 9.767763e-01 9.772109e-01 8.002433e-01 7.740471e-01 7.859078e-01 8.312761e-01 8.406985e-01

2890 9.795177e-01 9.783385e-01 9.772171e-01 9.767812e-01 9.772150e-01 8.004909e-01 7.742753e-01 7.861578e-01 8.315761e-01 8.410126e-01

2891 9.795235e-01 9.783440e-01 9.772221e-01 9.767861e-01 9.772191e-01 8.007382e-01 7.745040e-01 7.864079e-01 8.318745e-01 8.413246e-01

2892 9.795293e-01 9.783494e-01 9.772272e-01 9.767910e-01 9.772232e-01 8.009851e-01 7.747331e-01 7.866582e-01 8.321709e-01 8.416341e-01

2893 9.795351e-01 9.783549e-01 9.772322e-01 9.767958e-01 9.772272e-01 8.012313e-01 7.749625e-01 7.869083e-01 8.324653e-01 8.419410e-01

2894 9.795408e-01 9.783603e-01 9.772372e-01 9.768006e-01 9.772312e-01 8.014767e-01 7.751920e-01 7.871583e-01 8.327575e-01 8.422452e-01

2895 9.795466e-01 9.783656e-01 9.772422e-01 9.768053e-01 9.772352e-01 8.017213e-01 7.754214e-01 7.874078e-01 8.330474e-01 8.425465e-01

2896 9.795523e-01 9.783710e-01 9.772471e-01 9.768101e-01 9.772391e-01 8.019648e-01 7.756507e-01 7.876568e-01 8.333347e-01 8.428447e-01

2897 9.795581e-01 9.783763e-01 9.772521e-01 9.768148e-01 9.772430e-01 8.022071e-01 7.758796e-01 7.879052e-01 8.336195e-01 8.431399e-01

2898 9.795638e-01 9.783816e-01 9.772570e-01 9.768194e-01 9.772469e-01 8.024480e-01 7.761081e-01 7.881527e-01 8.339014e-01 8.434317e-01

2899 9.795695e-01 9.783869e-01 9.772618e-01 9.768241e-01 9.772507e-01 8.026875e-01 7.763360e-01 7.883992e-01 8.341805e-01 8.437201e-01

2900 9.795752e-01 9.783922e-01 9.772666e-01 9.768287e-01 9.772546e-01 8.029253e-01 7.765632e-01 7.886446e-01 8.344565e-01 8.440050e-01

2901 9.795809e-01 9.783975e-01 9.772715e-01 9.768333e-01 9.772583e-01 8.031615e-01 7.767895e-01 7.888887e-01 8.347294e-01 8.442863e-01

2902 9.795865e-01 9.784027e-01 9.772762e-01 9.768378e-01 9.772621e-01 8.033957e-01 7.770148e-01 7.891314e-01 8.349990e-01 8.445639e-01

2903 9.795922e-01 9.784079e-01 9.772810e-01 9.768424e-01 9.772658e-01 8.036280e-01 7.772390e-01 7.893726e-01 8.352652e-01 8.448376e-01

2904 9.795978e-01 9.784131e-01 9.772857e-01 9.768469e-01 9.772695e-01 8.038581e-01 7.774619e-01 7.896121e-01 8.355280e-01 8.451075e-01

2905 9.796035e-01 9.784183e-01 9.772904e-01 9.768513e-01 9.772732e-01 8.040860e-01 7.776834e-01 7.898498e-01 8.357873e-01 8.453733e-01

2906 9.796091e-01 9.784235e-01 9.772951e-01 9.768558e-01 9.772768e-01 8.043116e-01 7.779034e-01 7.900856e-01 8.360429e-01 8.456352e-01

2907 9.796147e-01 9.784286e-01 9.772997e-01 9.768602e-01 9.772805e-01 8.045348e-01 7.781217e-01 7.903193e-01 8.362948e-01 8.458929e-01

2908 9.796203e-01 9.784337e-01 9.773043e-01 9.768645e-01 9.772840e-01 8.047554e-01 7.783384e-01 7.905509e-01 8.365430e-01 8.461465e-01

2909 9.796259e-01 9.784388e-01 9.773089e-01 9.768689e-01 9.772876e-01 8.049734e-01 7.785531e-01 7.907802e-01 8.367873e-01 8.463958e-01

2910 9.796315e-01 9.784439e-01 9.773134e-01 9.768732e-01 9.772911e-01 8.051887e-01 7.787659e-01 7.910071e-01 8.370277e-01 8.466409e-01

2911 9.796370e-01 9.784489e-01 9.773180e-01 9.768774e-01 9.772946e-01 8.054012e-01 7.789766e-01 7.912316e-01 8.372642e-01 8.468818e-01

2912 9.796426e-01 9.784540e-01 9.773225e-01 9.768817e-01 9.772981e-01 8.056108e-01 7.791852e-01 7.914535e-01 8.374967e-01 8.471183e-01

2913 9.796482e-01 9.784590e-01 9.773269e-01 9.768859e-01 9.773015e-01 8.058175e-01 7.793915e-01 7.916727e-01 8.377252e-01 8.473504e-01

2914 9.796537e-01 9.784640e-01 9.773313e-01 9.768901e-01 9.773049e-01 8.060212e-01 7.795954e-01 7.918891e-01 8.379496e-01 8.475783e-01

2915 9.796592e-01 9.784690e-01 9.773358e-01 9.768942e-01 9.773083e-01 8.062218e-01 7.797969e-01 7.921028e-01 8.381699e-01 8.478017e-01

2916 9.796647e-01 9.784739e-01 9.773401e-01 9.768984e-01 9.773116e-01 8.064193e-01 7.799959e-01 7.923135e-01 8.383862e-01 8.480208e-01

2917 9.796702e-01 9.784788e-01 9.773445e-01 9.769024e-01 9.773149e-01 8.066136e-01 7.801923e-01 7.925213e-01 8.385983e-01 8.482356e-01

2918 9.796757e-01 9.784838e-01 9.773488e-01 9.769065e-01 9.773182e-01 8.068048e-01 7.803860e-01 7.927260e-01 8.388063e-01 8.484460e-01

2919 9.796812e-01 9.784886e-01 9.773531e-01 9.769105e-01 9.773215e-01 8.069926e-01 7.805770e-01 7.929277e-01 8.390103e-01 8.486520e-01

2920 9.796867e-01 9.784935e-01 9.773573e-01 9.769145e-01 9.773247e-01 8.071772e-01 7.807652e-01 7.931262e-01 8.392101e-01 8.488537e-01

2921 9.796922e-01 9.784983e-01 9.773615e-01 9.769184e-01 9.773279e-01 8.073585e-01 7.809506e-01 7.933215e-01 8.394058e-01 8.490512e-01

2922 9.796976e-01 9.785032e-01 9.773657e-01 9.769224e-01 9.773310e-01 8.075364e-01 7.811331e-01 7.935136e-01 8.395974e-01 8.492443e-01

2923 9.797030e-01 9.785080e-01 9.773698e-01 9.769263e-01 9.773342e-01 8.077111e-01 7.813126e-01 7.937024e-01 8.397849e-01 8.494332e-01

2924 9.797085e-01 9.785127e-01 9.773740e-01 9.769301e-01 9.773373e-01 8.078823e-01 7.814892e-01 7.938880e-01 8.399685e-01 8.496179e-01

2925 9.797139e-01 9.785175e-01 9.773780e-01 9.769339e-01 9.773403e-01 8.080502e-01 7.816628e-01 7.940702e-01 8.401479e-01 8.497984e-01

2926 9.797193e-01 9.785222e-01 9.773821e-01 9.769377e-01 9.773434e-01 8.082147e-01 7.818333e-01 7.942491e-01 8.403234e-01 8.499748e-01

2927 9.797246e-01 9.785269e-01 9.773861e-01 9.769415e-01 9.773464e-01 8.083759e-01 7.820008e-01 7.944247e-01 8.404950e-01 8.501471e-01

2928 9.797300e-01 9.785316e-01 9.773901e-01 9.769452e-01 9.773493e-01 8.085337e-01 7.821652e-01 7.945969e-01 8.406626e-01 8.503154e-01

2929 9.797354e-01 9.785362e-01 9.773940e-01 9.769489e-01 9.773523e-01 8.086881e-01 7.823264e-01 7.947657e-01 8.408264e-01 8.504796e-01

2930 9.797407e-01 9.785408e-01 9.773980e-01 9.769525e-01 9.773552e-01 8.088392e-01 7.824846e-01 7.949311e-01 8.409863e-01 8.506400e-01

2931 9.797460e-01 9.785454e-01 9.774018e-01 9.769561e-01 9.773581e-01 8.089870e-01 7.826397e-01 7.950932e-01 8.411424e-01 8.507964e-01

2932 9.797513e-01 9.785500e-01 9.774057e-01 9.769597e-01 9.773610e-01 8.091315e-01 7.827916e-01 7.952520e-01 8.412948e-01 8.509490e-01

2933 9.797566e-01 9.785545e-01 9.774095e-01 9.769632e-01 9.773638e-01 8.092727e-01 7.829405e-01 7.954073e-01 8.414434e-01 8.510978e-01

2934 9.797619e-01 9.785590e-01 9.774133e-01 9.769667e-01 9.773666e-01 8.094106e-01 7.830862e-01 7.955594e-01 8.415885e-01 8.512430e-01

2935 9.797672e-01 9.785635e-01 9.774170e-01 9.769702e-01 9.773694e-01 8.095454e-01 7.832288e-01 7.957081e-01 8.417300e-01 8.513845e-01

2936 9.797724e-01 9.785680e-01 9.774207e-01 9.769736e-01 9.773721e-01 8.096770e-01 7.833684e-01 7.958536e-01 8.418679e-01 8.515224e-01

2937 9.797776e-01 9.785724e-01 9.774244e-01 9.769770e-01 9.773748e-01 8.098054e-01 7.835048e-01 7.959958e-01 8.420024e-01 8.516568e-01

2938 9.797828e-01 9.785768e-01 9.774280e-01 9.769804e-01 9.773775e-01 8.099307e-01 7.836382e-01 7.961347e-01 8.421334e-01 8.517877e-01

2939 9.797880e-01 9.785812e-01 9.774316e-01 9.769837e-01 9.773802e-01 8.100529e-01 7.837686e-01 7.962704e-01 8.422611e-01 8.519152e-01

2940 9.797932e-01 9.785855e-01 9.774351e-01 9.769870e-01 9.773828e-01 8.101721e-01 7.838960e-01 7.964030e-01 8.423856e-01 8.520394e-01

2941 9.797983e-01 9.785898e-01 9.774386e-01 9.769903e-01 9.773855e-01 8.102883e-01 7.840204e-01 7.965324e-01 8.425067e-01 8.521604e-01

2942 9.798035e-01 9.785941e-01 9.774421e-01 9.769935e-01 9.773880e-01 8.104015e-01 7.841419e-01 7.966587e-01 8.426248e-01 8.522782e-01

2943 9.798086e-01 9.785983e-01 9.774456e-01 9.769967e-01 9.773906e-01 8.105119e-01 7.842604e-01 7.967819e-01 8.427397e-01 8.523928e-01

2944 9.798137e-01 9.786026e-01 9.774490e-01 9.769998e-01 9.773932e-01 8.106194e-01 7.843761e-01 7.969022e-01 8.428516e-01 8.525044e-01

2945 9.798187e-01 9.786067e-01 9.774523e-01 9.770030e-01 9.773957e-01 8.107241e-01 7.844889e-01 7.970194e-01 8.429605e-01 8.526130e-01

2946 9.798238e-01 9.786109e-01 9.774557e-01 9.770060e-01 9.773982e-01 8.108261e-01 7.845989e-01 7.971337e-01 8.430665e-01 8.527187e-01

2947 9.798288e-01 9.786150e-01 9.774589e-01 9.770091e-01 9.774006e-01 8.109254e-01 7.847062e-01 7.972452e-01 8.431696e-01 8.528215e-01

2948 9.798338e-01 9.786191e-01 9.774622e-01 9.770121e-01 9.774031e-01 8.110220e-01 7.848107e-01 7.973538e-01 8.432700e-01 8.529215e-01

2949 9.798387e-01 9.786231e-01 9.774654e-01 9.770151e-01 9.774055e-01 8.111160e-01 7.849126e-01 7.974596e-01 8.433677e-01 8.530189e-01
[truncated: 10,524,276 more chars]
