# Supplementary material for: Purity control of simulated moving bed based on advanced fuzzy controller
Source: Sci Rep. 2024 Apr 20;14:9083. doi: 10.1038/s41598-024-59847-1 (PMC11576947; doi:10.1038/s41598-024-59847-1)
Supplement: Supplementary file 9 — Supplementary Information 9. [file 41598_2024_59847_MOESM9_ESM.docx]

**Figure 8(a):**

1 8.114365e-76 8.116078e-76 8.117886e-76 8.119796e-76 8.121812e-76 6.670775e-81

2 8.114365e-76 8.116078e-76 8.117886e-76 8.119796e-76 8.121812e-76 6.670775e-81

3 8.114365e-76 8.116078e-76 8.117886e-76 8.119796e-76 8.121812e-76 6.670775e-81

4 8.114365e-76 8.116078e-76 8.117886e-76 8.119796e-76 8.121812e-76 6.670775e-81

5 8.114365e-76 8.116078e-76 8.117886e-76 8.119796e-76 8.121812e-76 6.670775e-81

6 8.114365e-76 8.116078e-76 8.117886e-76 8.119796e-76 8.121812e-76 6.670775e-81

7 8.114365e-76 8.116078e-76 8.117886e-76 8.119796e-76 8.121812e-76 6.670775e-81

8 8.114365e-76 8.116078e-76 8.117886e-76 8.119796e-76 8.121812e-76 6.670775e-81

9 8.114365e-76 8.116078e-76 8.117886e-76 8.119796e-76 8.121812e-76 6.670775e-81

10 8.114365e-76 8.116078e-76 8.117886e-76 8.119796e-76 8.121812e-76 6.670775e-81

11 8.114365e-76 8.116078e-76 8.117886e-76 8.119796e-76 8.121812e-76 6.670775e-81

12 8.114365e-76 8.116078e-76 8.117886e-76 8.119796e-76 8.121812e-76 6.670775e-81

13 8.114365e-76 8.116078e-76 8.117886e-76 8.119796e-76 8.121812e-76 6.670775e-81

14 8.114365e-76 8.116078e-76 8.117886e-76 8.119796e-76 8.121812e-76 6.670775e-81

15 8.114365e-76 8.116078e-76 8.117886e-76 8.119796e-76 8.121812e-76 6.670775e-81

16 8.114365e-76 8.116078e-76 8.117886e-76 8.119796e-76 8.121812e-76 6.670775e-81

17 8.114365e-76 8.116078e-76 8.117886e-76 8.119796e-76 8.121812e-76 6.670775e-81

18 8.114365e-76 8.116078e-76 8.117886e-76 8.119796e-76 8.121812e-76 6.670775e-81

19 8.114365e-76 8.116078e-76 8.117886e-76 8.119796e-76 8.121812e-76 6.670775e-81

20 8.114365e-76 8.116078e-76 8.117886e-76 8.119796e-76 8.121812e-76 6.670775e-81

21 8.114365e-76 8.116078e-76 8.117886e-76 8.119796e-76 8.121812e-76 6.670775e-81

22 8.114365e-76 8.116078e-76 8.117886e-76 8.119796e-76 8.121812e-76 6.670775e-81

23 8.114365e-76 8.116078e-76 8.117886e-76 8.119796e-76 8.121812e-76 6.670775e-81

24 8.114365e-76 8.116078e-76 8.117886e-76 8.119796e-76 8.121812e-76 6.670775e-81

25 8.114365e-76 8.116078e-76 8.117886e-76 8.119796e-76 8.121812e-76 6.670775e-81

26 8.114365e-76 8.116078e-76 8.117886e-76 8.119796e-76 8.121812e-76 6.670775e-81

27 8.114365e-76 8.116078e-76 8.117886e-76 8.119796e-76 8.121812e-76 6.670775e-81

28 8.114365e-76 8.116078e-76 8.117886e-76 8.119796e-76 8.121812e-76 6.670775e-81

29 8.114365e-76 8.116078e-76 8.117886e-76 8.119796e-76 8.121812e-76 6.670775e-81

30 8.114365e-76 8.116078e-76 8.117886e-76 8.119796e-76 8.121812e-76 6.670775e-81

31 8.114365e-76 8.116078e-76 8.117886e-76 8.119796e-76 8.121812e-76 6.670775e-81

32 8.114365e-76 8.116078e-76 8.117886e-76 8.119796e-76 8.121812e-76 6.670775e-81

33 8.114365e-76 8.116078e-76 8.117886e-76 8.119796e-76 8.121812e-76 6.670775e-81

34 8.114365e-76 8.116078e-76 8.117886e-76 8.119796e-76 8.121812e-76 6.670775e-81

35 8.114365e-76 8.116078e-76 8.117886e-76 8.119796e-76 8.121812e-76 6.670775e-81

36 8.114365e-76 8.116078e-76 8.117886e-76 8.119796e-76 8.121812e-76 6.670775e-81

37 8.114365e-76 8.116078e-76 8.117886e-76 8.119796e-76 8.121812e-76 6.670775e-81

38 8.114365e-76 8.116078e-76 8.117886e-76 8.119796e-76 8.121812e-76 6.670775e-81

39 8.114365e-76 8.116078e-76 8.117886e-76 8.119796e-76 8.121812e-76 6.670775e-81

40 8.114365e-76 8.116078e-76 8.117886e-76 8.119796e-76 8.121812e-76 6.670775e-81

41 8.114365e-76 8.116078e-76 8.117886e-76 8.119796e-76 8.121812e-76 6.670775e-81

42 8.114365e-76 8.116078e-76 8.117886e-76 8.119796e-76 8.121812e-76 6.670775e-81

43 8.114365e-76 8.116078e-76 8.117886e-76 8.119796e-76 8.121812e-76 6.670775e-81

44 8.114365e-76 8.116078e-76 8.117886e-76 8.119796e-76 8.121812e-76 6.670775e-81

45 8.114365e-76 8.116078e-76 8.117886e-76 8.119796e-76 8.121812e-76 6.670775e-81

46 8.114365e-76 8.116078e-76 8.117886e-76 8.119796e-76 8.121812e-76 6.670775e-81

47 8.114365e-76 8.116078e-76 8.117887e-76 8.119796e-76 8.121813e-76 6.670775e-81

48 8.114366e-76 8.116078e-76 8.117887e-76 8.119796e-76 8.121813e-76 6.670776e-81

49 8.114366e-76 8.116079e-76 8.117887e-76 8.119797e-76 8.121813e-76 6.670776e-81

50 8.114367e-76 8.116080e-76 8.117888e-76 8.119797e-76 8.121814e-76 6.670777e-81

51 8.114369e-76 8.116081e-76 8.117890e-76 8.119799e-76 8.121816e-76 6.670778e-81

52 8.114372e-76 8.116085e-76 8.117893e-76 8.119803e-76 8.121819e-76 6.670781e-81

53 8.114379e-76 8.116092e-76 8.117900e-76 8.119810e-76 8.121826e-76 6.670786e-81

54 8.114394e-76 8.116106e-76 8.117915e-76 8.119824e-76 8.121841e-76 6.670798e-81

55 8.114423e-76 8.116135e-76 8.117944e-76 8.119853e-76 8.121870e-76 6.670821e-81

56 8.114481e-76 8.116194e-76 8.118002e-76 8.119912e-76 8.121928e-76 6.670868e-81

57 8.114598e-76 8.116310e-76 8.118119e-76 8.120028e-76 8.122045e-76 6.670961e-81

58 8.114830e-76 8.116542e-76 8.118351e-76 8.120261e-76 8.122277e-76 6.671146e-81

59 8.115288e-76 8.117001e-76 8.118810e-76 8.120720e-76 8.122737e-76 6.671513e-81

60 8.116192e-76 8.117905e-76 8.119714e-76 8.121624e-76 8.123641e-76 6.672233e-81

61 8.117962e-76 8.119675e-76 8.121485e-76 8.123395e-76 8.125413e-76 6.673644e-81

62 8.121411e-76 8.123126e-76 8.124936e-76 8.126848e-76 8.128867e-76 6.676391e-81

63 8.128099e-76 8.129816e-76 8.131628e-76 8.133542e-76 8.135563e-76 6.681715e-81

64 8.141005e-76 8.142725e-76 8.144541e-76 8.146459e-76 8.148485e-76 6.691978e-81

65 8.165784e-76 8.167511e-76 8.169335e-76 8.171261e-76 8.173294e-76 6.711667e-81

66 8.213132e-76 8.214872e-76 8.216710e-76 8.218651e-76 8.220701e-76 6.749255e-81

67 8.303180e-76 8.304947e-76 8.306812e-76 8.308781e-76 8.310861e-76 6.820673e-81

68 8.473659e-76 8.475474e-76 8.477390e-76 8.479413e-76 8.481551e-76 6.955740e-81

69 8.794991e-76 8.796897e-76 8.798909e-76 8.801035e-76 8.803280e-76 7.210044e-81

70 9.398117e-76 9.400194e-76 9.402388e-76 9.404706e-76 9.407153e-76 7.686791e-81

71 1.052564e-75 1.052804e-75 1.053057e-75 1.053325e-75 1.053607e-75 8.576895e-81

72 1.262555e-75 1.262855e-75 1.263171e-75 1.263506e-75 1.263859e-75 1.023230e-80

73 1.652268e-75 1.652679e-75 1.653113e-75 1.653571e-75 1.654055e-75 1.329977e-80

74 2.373151e-75 2.373767e-75 2.374418e-75 2.375106e-75 2.375833e-75 1.896447e-80

75 3.702621e-75 3.703616e-75 3.704668e-75 3.705780e-75 3.706955e-75 2.939254e-80

76 6.147766e-75 6.149459e-75 6.151249e-75 6.153141e-75 6.155140e-75 4.853409e-80

77 1.063381e-74 1.063679e-74 1.063993e-74 1.064326e-74 1.064677e-74 8.357811e-80

78 1.884631e-74 1.885164e-74 1.885726e-74 1.886321e-74 1.886950e-74 1.475856e-79

79 3.385202e-74 3.386165e-74 3.387181e-74 3.388256e-74 3.389392e-74 2.642513e-79

80 6.122486e-74 6.124234e-74 6.126080e-74 6.128031e-74 6.130093e-74 4.765087e-79

81 1.110871e-73 1.111189e-73 1.111524e-73 1.111879e-73 1.112255e-73 8.620723e-79

82 2.018084e-73 2.018663e-73 2.019274e-73 2.019920e-73 2.020603e-73 1.561498e-78

83 3.667090e-73 3.668144e-73 3.669258e-73 3.670434e-73 3.671677e-73 2.828831e-78

84 6.662033e-73 6.663951e-73 6.665977e-73 6.668118e-73 6.670378e-73 5.122994e-78

85 1.209797e-72 1.210146e-72 1.210514e-72 1.210904e-72 1.211315e-72 9.272656e-78

86 2.195928e-72 2.196563e-72 2.197233e-72 2.197941e-72 2.198689e-72 1.677360e-77

87 3.984119e-72 3.985273e-72 3.986491e-72 3.987777e-72 3.989135e-72 3.032492e-77

88 7.225597e-72 7.227694e-72 7.229907e-72 7.232244e-72 7.234711e-72 5.479571e-77

89 1.309975e-71 1.310356e-71 1.310758e-71 1.311182e-71 1.311630e-71 9.896725e-77

90 2.374197e-71 2.374889e-71 2.375620e-71 2.376390e-71 2.377204e-71 1.786718e-76

91 4.301769e-71 4.303026e-71 4.304352e-71 4.305751e-71 4.307227e-71 3.224458e-76

92 7.792188e-71 7.794470e-71 7.796878e-71 7.799417e-71 7.802097e-71 5.817067e-76

93 1.411088e-70 1.411502e-70 1.411939e-70 1.412400e-70 1.412886e-70 1.049067e-75

94 2.554623e-70 2.555375e-70 2.556167e-70 2.557004e-70 2.557886e-70 1.891270e-75

95 4.623480e-70 4.624844e-70 4.626283e-70 4.627800e-70 4.629399e-70 3.408407e-75

96 8.365075e-70 8.367550e-70 8.370159e-70 8.372909e-70 8.375810e-70 6.140295e-75

97 1.512921e-69 1.513370e-69 1.513843e-69 1.514342e-69 1.514868e-69 1.105751e-74

98 2.735232e-69 2.736046e-69 2.736904e-69 2.737807e-69 2.738760e-69 1.988526e-74

99 4.942979e-69 4.944453e-69 4.946007e-69 4.947644e-69 4.949370e-69 3.569059e-74

100 8.928625e-69 8.931296e-69 8.934111e-69 8.937076e-69 8.940200e-69 6.394380e-74

101 1.612005e-68 1.612488e-68 1.612998e-68 1.613535e-68 1.614100e-68 1.143952e-73

102 2.908828e-68 2.909703e-68 2.910625e-68 2.911596e-68 2.912619e-68 2.044124e-73

103 5.245944e-68 5.247527e-68 5.249195e-68 5.250951e-68 5.252800e-68 3.649103e-73

104 9.455100e-68 9.457963e-68 9.460977e-68 9.464150e-68 9.467493e-68 6.508791e-73

105 1.703050e-67 1.703568e-67 1.704112e-67 1.704685e-67 1.705289e-67 1.160060e-72

106 3.065408e-67 3.066342e-67 3.067325e-67 3.068359e-67 3.069449e-67 2.066044e-72

107 5.513500e-67 5.515185e-67 5.516958e-67 5.518824e-67 5.520788e-67 3.676866e-72

108 9.908809e-67 9.911847e-67 9.915043e-67 9.918406e-67 9.921946e-67 6.538635e-72

109 1.779284e-66 1.779831e-66 1.780406e-66 1.781012e-66 1.781649e-66 1.161859e-71

110 3.192057e-66 3.193042e-66 3.194077e-66 3.195167e-66 3.196313e-66 2.062800e-71

111 5.720943e-66 5.722713e-66 5.724574e-66 5.726533e-66 5.728593e-66 3.659113e-71

112 1.024244e-65 1.024562e-65 1.024896e-65 1.025248e-65 1.025618e-65 6.484599e-71

113 1.831654e-65 1.832224e-65 1.832824e-65 1.833454e-65 1.834117e-65 1.148021e-70

114 3.271531e-65 3.272552e-65 3.273626e-65 3.274755e-65 3.275942e-65 2.030227e-70

115 5.835632e-65 5.837459e-65 5.839379e-65 5.841399e-65 5.843522e-65 3.586193e-70

116 1.039477e-64 1.039803e-64 1.040146e-64 1.040506e-64 1.040886e-64 6.326762e-70

117 1.848806e-64 1.849388e-64 1.849999e-64 1.850642e-64 1.851318e-64 1.114683e-69

118 3.283047e-64 3.284083e-64 3.285172e-64 3.286317e-64 3.287520e-64 1.961128e-69

119 5.820121e-64 5.821963e-64 5.823898e-64 5.825932e-64 5.828070e-64 3.445138e-69

120 1.029950e-63 1.030277e-63 1.030620e-63 1.030981e-63 1.031360e-63 6.042485e-69

121 1.819243e-63 1.819822e-63 1.820429e-63 1.821068e-63 1.821739e-63 1.058023e-68

122 3.207144e-63 3.208166e-63 3.209240e-63 3.210368e-63 3.211554e-63 1.849303e-68

123 5.642393e-63 5.644195e-63 5.646088e-63 5.648078e-63 5.650168e-63 3.226407e-68

124 9.905848e-63 9.909019e-63 9.912350e-63 9.915849e-63 9.919526e-63 5.618161e-68

125 1.735289e-62 1.735845e-62 1.736430e-62 1.737044e-62 1.737690e-62 9.763395e-68

126 3.033013e-62 3.033988e-62 3.035012e-62 3.036088e-62 3.037218e-62 1.693203e-67

127 5.288986e-62 5.290689e-62 5.292478e-62 5.294357e-62 5.296332e-62 2.930159e-67

128 9.201143e-62 9.204111e-62 9.207229e-62 9.210504e-62 9.213944e-62 5.059662e-67

129 1.596837e-61 1.597353e-61 1.597895e-61 1.598465e-61 1.599063e-61 8.717201e-67

130 2.764456e-61 2.765352e-61 2.766292e-61 2.767279e-61 2.768316e-61 1.498433e-66

131 4.773892e-61 4.775440e-61 4.777066e-61 4.778774e-61 4.780567e-61 2.569710e-66

132 8.223089e-61 8.225761e-61 8.228566e-61 8.231511e-61 8.234605e-61 4.396445e-66

133 1.412815e-60 1.413275e-60 1.413757e-60 1.414264e-60 1.414796e-60 7.503712e-66

134 2.421107e-60 2.421896e-60 2.422724e-60 2.423594e-60 2.424508e-60 1.277604e-65

135 4.138223e-60 4.139574e-60 4.140992e-60 4.142481e-60 4.144044e-60 2.169962e-65

136 7.054724e-60 7.057030e-60 7.059450e-60 7.061992e-60 7.064661e-60 3.676516e-65

137 1.199525e-59 1.199917e-59 1.200329e-59 1.200762e-59 1.201216e-59 6.213595e-65

138 2.034230e-59 2.034896e-59 2.035596e-59 2.036331e-59 2.037103e-59 1.047532e-64

139 3.440748e-59 3.441877e-59 3.443063e-59 3.444307e-59 3.445614e-59 1.761596e-64

140 5.804568e-59 5.806475e-59 5.808477e-59 5.810579e-59 5.812786e-59 2.955007e-64

141 9.766846e-59 9.770059e-59 9.773433e-59 9.776974e-59 9.780692e-59 4.944518e-64

142 1.639121e-58 1.639661e-58 1.640228e-58 1.640823e-58 1.641447e-58 8.252842e-64

143 2.743758e-58 2.744663e-58 2.745613e-58 2.746610e-58 2.747657e-58 1.374040e-63

144 4.581056e-58 4.582569e-58 4.584157e-58 4.585824e-58 4.587574e-58 2.281995e-63

145 7.629192e-58 7.631715e-58 7.634362e-58 7.637142e-58 7.640059e-58 3.780543e-63

146 1.267341e-57 1.267760e-57 1.268200e-57 1.268663e-57 1.269148e-57 6.247742e-63

147 2.100001e-57 2.100697e-57 2.101428e-57 2.102195e-57 2.103000e-57 1.029978e-62

148 3.471103e-57 3.472255e-57 3.473463e-57 3.474732e-57 3.476064e-57 1.693853e-62

149 5.723317e-57 5.725219e-57 5.727214e-57 5.729309e-57 5.731507e-57 2.778911e-62

150 9.413951e-57 9.417082e-57 9.420368e-57 9.423817e-57 9.427437e-57 4.548140e-62

151 1.544726e-56 1.545240e-56 1.545780e-56 1.546347e-56 1.546941e-56 7.426129e-62

152 2.528706e-56 2.529549e-56 2.530434e-56 2.531363e-56 2.532337e-56 1.209681e-61

153 4.129763e-56 4.131141e-56 4.132588e-56 4.134106e-56 4.135699e-56 1.965938e-61

154 6.728900e-56 6.731148e-56 6.733508e-56 6.735984e-56 6.738583e-56 3.187646e-61

155 1.093875e-55 1.094241e-55 1.094625e-55 1.095028e-55 1.095451e-55 5.156840e-61

156 1.774227e-55 1.774821e-55 1.775445e-55 1.776099e-55 1.776786e-55 8.323809e-61

157 2.871316e-55 2.872278e-55 2.873289e-55 2.874349e-55 2.875462e-55 1.340595e-60

158 4.636558e-55 4.638115e-55 4.639748e-55 4.641462e-55 4.643262e-55 2.154380e-60

159 7.470787e-55 7.473298e-55 7.475933e-55 7.478698e-55 7.481600e-55 3.454686e-60

160 1.201173e-54 1.201577e-54 1.202001e-54 1.202446e-54 1.202913e-54 5.528010e-60

161 1.927195e-54 1.927844e-54 1.928525e-54 1.929240e-54 1.929990e-54 8.827047e-60

162 3.085595e-54 3.086636e-54 3.087728e-54 3.088874e-54 3.090077e-54 1.406568e-59

163 4.930125e-54 4.931790e-54 4.933537e-54 4.935371e-54 4.937295e-54 2.236749e-59

164 7.861303e-54 7.863961e-54 7.866750e-54 7.869677e-54 7.872748e-54 3.549743e-59

165 1.251009e-53 1.251433e-53 1.251877e-53 1.252343e-53 1.252833e-53 5.622270e-59

166 1.986860e-53 1.987534e-53 1.988240e-53 1.988982e-53 1.989760e-53 8.887375e-59

167 3.149390e-53 3.150459e-53 3.151580e-53 3.152757e-53 3.153992e-53 1.402149e-58

168 4.982518e-53 4.984211e-53 4.985987e-53 4.987851e-53 4.989807e-53 2.207927e-58

169 7.867657e-53 7.870333e-53 7.873142e-53 7.876088e-53 7.879180e-53 3.470217e-58

170 1.240014e-52 1.240436e-52 1.240879e-52 1.241344e-52 1.241832e-52 5.444036e-58

171 1.950754e-52 1.951419e-52 1.952117e-52 1.952849e-52 1.953618e-52 8.524885e-58

172 3.063255e-52 3.064301e-52 3.065399e-52 3.066550e-52 3.067758e-52 1.332508e-57

173 4.801515e-52 4.803156e-52 4.804879e-52 4.806686e-52 4.808582e-52 2.079099e-57

174 7.512718e-52 7.515290e-52 7.517988e-52 7.520819e-52 7.523790e-52 3.238281e-57

175 1.173407e-51 1.173809e-51 1.174231e-51 1.174674e-51 1.175139e-51 5.034984e-57

176 1.829540e-51 1.830168e-51 1.830827e-51 1.831518e-51 1.832243e-51 7.815115e-57

177 2.847641e-51 2.848620e-51 2.849647e-51 2.850724e-51 2.851854e-51 1.210977e-56

178 4.424731e-51 4.426253e-51 4.427850e-51 4.429526e-51 4.431284e-51 1.873305e-56

179 6.863641e-51 6.866006e-51 6.868486e-51 6.871089e-51 6.873819e-51 2.893095e-56

180 6.863641e-51 6.866006e-51 6.868486e-51 6.871089e-51 6.873819e-51 2.893095e-56

181 6.863641e-51 6.866006e-51 6.868486e-51 6.871089e-51 6.873819e-51 2.893095e-56

182 6.863641e-51 6.866006e-51 6.868486e-51 6.871089e-51 6.873819e-51 2.893095e-56

183 6.863641e-51 6.866006e-51 6.868486e-51 6.871089e-51 6.873819e-51 2.893095e-56

184 6.863641e-51 6.866006e-51 6.868486e-51 6.871089e-51 6.873819e-51 2.893095e-56

185 6.863641e-51 6.866006e-51 6.868486e-51 6.871089e-51 6.873819e-51 2.893095e-56

186 6.863641e-51 6.866006e-51 6.868486e-51 6.871089e-51 6.873819e-51 2.893095e-56

187 6.863641e-51 6.866006e-51 6.868486e-51 6.871089e-51 6.873819e-51 2.893095e-56

188 6.863641e-51 6.866006e-51 6.868486e-51 6.871089e-51 6.873819e-51 2.893095e-56

189 6.863641e-51 6.866006e-51 6.868486e-51 6.871089e-51 6.873819e-51 2.893095e-56

190 6.863641e-51 6.866006e-51 6.868486e-51 6.871089e-51 6.873819e-51 2.893095e-56

191 6.863641e-51 6.866006e-51 6.868486e-51 6.871089e-51 6.873819e-51 2.893095e-56

192 6.863641e-51 6.866006e-51 6.868486e-51 6.871089e-51 6.873819e-51 2.893095e-56

193 6.863641e-51 6.866006e-51 6.868486e-51 6.871089e-51 6.873819e-51 2.893095e-56

194 6.863641e-51 6.866006e-51 6.868486e-51 6.871089e-51 6.873819e-51 2.893095e-56

195 6.863641e-51 6.866006e-51 6.868486e-51 6.871089e-51 6.873819e-51 2.893095e-56

196 6.863641e-51 6.866006e-51 6.868486e-51 6.871089e-51 6.873819e-51 2.893095e-56

197 6.863641e-51 6.866006e-51 6.868486e-51 6.871089e-51 6.873819e-51 2.893095e-56

198 6.863641e-51 6.866006e-51 6.868486e-51 6.871089e-51 6.873819e-51 2.893095e-56

199 6.863641e-51 6.866006e-51 6.868486e-51 6.871089e-51 6.873819e-51 2.893095e-56

200 6.863641e-51 6.866006e-51 6.868486e-51 6.871089e-51 6.873819e-51 2.893095e-56

201 6.863641e-51 6.866006e-51 6.868486e-51 6.871089e-51 6.873819e-51 2.893095e-56

202 6.863641e-51 6.866006e-51 6.868486e-51 6.871089e-51 6.873819e-51 2.893095e-56

203 6.863641e-51 6.866006e-51 6.868486e-51 6.871089e-51 6.873819e-51 2.893095e-56

204 6.863641e-51 6.866006e-51 6.868486e-51 6.871089e-51 6.873819e-51 2.893095e-56

205 6.863641e-51 6.866006e-51 6.868486e-51 6.871089e-51 6.873819e-51 2.893095e-56

206 6.863641e-51 6.866006e-51 6.868486e-51 6.871089e-51 6.873819e-51 2.893095e-56

207 6.863641e-51 6.866006e-51 6.868486e-51 6.871089e-51 6.873819e-51 2.893095e-56

208 6.863641e-51 6.866006e-51 6.868486e-51 6.871089e-51 6.873819e-51 2.893095e-56

209 6.863641e-51 6.866006e-51 6.868486e-51 6.871089e-51 6.873819e-51 2.893095e-56

210 6.863641e-51 6.866006e-51 6.868486e-51 6.871089e-51 6.873819e-51 2.893095e-56

211 6.863641e-51 6.866006e-51 6.868486e-51 6.871089e-51 6.873819e-51 2.893095e-56

212 6.863641e-51 6.866006e-51 6.868486e-51 6.871089e-51 6.873819e-51 2.893095e-56

213 6.863641e-51 6.866006e-51 6.868486e-51 6.871089e-51 6.873819e-51 2.893095e-56

214 6.863641e-51 6.866006e-51 6.868486e-51 6.871089e-51 6.873819e-51 2.893095e-56

215 6.863641e-51 6.866006e-51 6.868486e-51 6.871089e-51 6.873819e-51 2.893095e-56

216 6.863641e-51 6.866006e-51 6.868486e-51 6.871089e-51 6.873819e-51 2.893095e-56

217 6.863641e-51 6.866006e-51 6.868486e-51 6.871089e-51 6.873819e-51 2.893095e-56

218 6.863641e-51 6.866006e-51 6.868486e-51 6.871089e-51 6.873819e-51 2.893095e-56

219 6.863641e-51 6.866006e-51 6.868486e-51 6.871089e-51 6.873819e-51 2.893095e-56

220 6.863641e-51 6.866006e-51 6.868486e-51 6.871089e-51 6.873819e-51 2.893095e-56

221 6.863641e-51 6.866006e-51 6.868486e-51 6.871089e-51 6.873819e-51 2.893095e-56

222 6.863641e-51 6.866006e-51 6.868486e-51 6.871089e-51 6.873819e-51 2.893095e-56

223 6.863641e-51 6.866006e-51 6.868486e-51 6.871089e-51 6.873819e-51 2.893095e-56

224 6.863641e-51 6.866006e-51 6.868486e-51 6.871089e-51 6.873819e-51 2.893095e-56

225 6.863641e-51 6.866006e-51 6.868486e-51 6.871089e-51 6.873819e-51 2.893095e-56

226 6.863641e-51 6.866006e-51 6.868486e-51 6.871089e-51 6.873819e-51 2.893095e-56

227 6.863641e-51 6.866006e-51 6.868486e-51 6.871089e-51 6.873819e-51 2.893095e-56

228 6.863641e-51 6.866006e-51 6.868486e-51 6.871089e-51 6.873819e-51 2.893095e-56

229 6.863641e-51 6.866006e-51 6.868486e-51 6.871089e-51 6.873819e-51 2.893095e-56

230 6.863641e-51 6.866006e-51 6.868486e-51 6.871089e-51 6.873819e-51 2.893095e-56

231 6.863641e-51 6.866006e-51 6.868486e-51 6.871089e-51 6.873819e-51 2.893095e-56

232 6.863641e-51 6.866006e-51 6.868487e-51 6.871089e-51 6.873819e-51 2.893095e-56

233 6.863641e-51 6.866006e-51 6.868487e-51 6.871089e-51 6.873819e-51 2.893096e-56

234 6.863641e-51 6.866006e-51 6.868487e-51 6.871089e-51 6.873820e-51 2.893096e-56

235 6.863641e-51 6.866006e-51 6.868487e-51 6.871089e-51 6.873820e-51 2.893096e-56

236 6.863642e-51 6.866007e-51 6.868487e-51 6.871090e-51 6.873820e-51 2.893096e-56

237 6.863642e-51 6.866007e-51 6.868488e-51 6.871090e-51 6.873821e-51 2.893096e-56

238 6.863643e-51 6.866008e-51 6.868489e-51 6.871091e-51 6.873821e-51 2.893096e-56

239 6.863644e-51 6.866009e-51 6.868490e-51 6.871092e-51 6.873823e-51 2.893097e-56

240 6.863646e-51 6.866011e-51 6.868492e-51 6.871094e-51 6.873825e-51 2.893097e-56

241 6.863649e-51 6.866014e-51 6.868495e-51 6.871097e-51 6.873828e-51 2.893098e-56

242 6.863654e-51 6.866019e-51 6.868499e-51 6.871102e-51 6.873832e-51 2.893099e-56

243 6.863660e-51 6.866025e-51 6.868506e-51 6.871108e-51 6.873839e-51 2.893102e-56

244 6.863671e-51 6.866035e-51 6.868516e-51 6.871118e-51 6.873849e-51 2.893105e-56

245 6.863686e-51 6.866051e-51 6.868531e-51 6.871134e-51 6.873864e-51 2.893110e-56

246 6.863709e-51 6.866074e-51 6.868555e-51 6.871157e-51 6.873888e-51 2.893117e-56

247 6.863744e-51 6.866109e-51 6.868590e-51 6.871192e-51 6.873923e-51 2.893128e-56

248 6.863798e-51 6.866163e-51 6.868643e-51 6.871246e-51 6.873976e-51 2.893144e-56

249 6.863878e-51 6.866243e-51 6.868724e-51 6.871326e-51 6.874057e-51 2.893169e-56

250 6.863998e-51 6.866363e-51 6.868844e-51 6.871447e-51 6.874177e-51 2.893206e-56

251 6.864179e-51 6.866545e-51 6.869026e-51 6.871628e-51 6.874359e-51 2.893261e-56

252 6.864451e-51 6.866817e-51 6.869298e-51 6.871901e-51 6.874632e-51 2.893344e-56

253 6.864858e-51 6.867224e-51 6.869706e-51 6.872309e-51 6.875041e-51 2.893469e-56

254 6.865466e-51 6.867833e-51 6.870316e-51 6.872920e-51 6.875652e-51 2.893654e-56

255 6.866374e-51 6.868743e-51 6.871228e-51 6.873831e-51 6.876566e-51 2.893931e-56

256 6.867729e-51 6.870100e-51 6.872587e-51 6.875192e-51 6.877928e-51 2.894344e-56

257 6.869747e-51 6.872122e-51 6.874613e-51 6.877218e-51 6.879958e-51 2.894959e-56

258 6.872750e-51 6.875130e-51 6.877625e-51 6.880232e-51 6.882977e-51 2.895872e-56

259 6.877210e-51 6.879597e-51 6.882101e-51 6.884709e-51 6.887462e-51 2.897228e-56

260 6.883826e-51 6.886225e-51 6.888739e-51 6.891351e-51 6.894116e-51 2.899238e-56

261 6.893627e-51 6.896042e-51 6.898574e-51 6.901190e-51 6.903972e-51 2.902213e-56

262 6.908124e-51 6.910564e-51 6.913120e-51 6.915743e-51 6.918551e-51 2.906612e-56

263 6.929537e-51 6.932013e-51 6.934606e-51 6.937239e-51 6.940085e-51 2.913106e-56

264 6.961118e-51 6.963648e-51 6.966295e-51 6.968943e-51 6.971844e-51 2.922680e-56

265 7.007625e-51 7.010233e-51 7.012960e-51 7.015629e-51 7.018613e-51 2.936774e-56

266 7.076006e-51 7.078730e-51 7.081573e-51 7.084275e-51 7.087378e-51 2.957491e-56

267 7.176393e-51 7.179285e-51 7.182300e-51 7.185048e-51 7.188327e-51 2.987896e-56

268 7.323529e-51 7.326667e-51 7.329932e-51 7.332749e-51 7.336285e-51 3.032454e-56

269 7.538832e-51 7.542330e-51 7.545959e-51 7.548877e-51 7.552789e-51 3.097647e-56

270 7.853359e-51 7.857381e-51 7.861542e-51 7.864608e-51 7.869067e-51 3.192879e-56

271 8.312061e-51 8.316844e-51 8.321778e-51 8.325060e-51 8.330314e-51 3.331763e-56

272 8.979869e-51 8.985760e-51 8.991816e-51 8.995412e-51 9.001822e-51 3.533970e-56

273 9.950407e-51 9.957901e-51 9.965583e-51 9.969637e-51 9.977722e-51 3.827871e-56

274 1.135839e-50 1.136821e-50 1.137824e-50 1.138296e-50 1.139347e-50 4.254308e-56

275 1.339731e-50 1.341047e-50 1.342390e-50 1.342958e-50 1.344359e-50 4.871959e-56

276 1.634447e-50 1.636245e-50 1.638078e-50 1.638786e-50 1.640691e-50 5.764969e-56

277 2.059656e-50 2.062149e-50 2.064686e-50 2.065596e-50 2.068226e-50 7.053767e-56

278 2.671992e-50 2.675481e-50 2.679030e-50 2.680229e-50 2.683901e-50 8.910367e-56

279 3.552138e-50 3.557056e-50 3.562053e-50 3.563671e-50 3.568836e-50 1.157998e-55

280 4.814822e-50 4.821781e-50 4.828851e-50 4.831069e-50 4.838369e-50 1.541150e-55

281 6.622844e-50 6.632718e-50 6.642745e-50 6.645825e-50 6.656173e-50 2.090031e-55

282 9.206770e-50 9.220796e-50 9.235037e-50 9.239348e-50 9.254039e-50 2.874845e-55

283 1.289249e-49 1.291242e-49 1.293265e-49 1.293872e-49 1.295959e-49 3.994889e-55

284 1.813973e-49 1.816804e-49 1.819677e-49 1.820535e-49 1.823498e-49 5.590340e-55

285 2.559578e-49 2.563595e-49 2.567673e-49 2.568888e-49 2.573092e-49 7.858701e-55

286 3.617029e-49 3.622723e-49 3.628504e-49 3.630224e-49 3.636185e-49 1.107773e-54

287 5.113921e-49 5.121982e-49 5.130164e-49 5.132603e-49 5.141041e-49 1.563732e-54

288 7.228911e-49 7.240303e-49 7.251869e-49 7.255323e-49 7.267250e-49 2.208379e-54

289 1.021170e-48 1.022778e-48 1.024410e-48 1.024899e-48 1.026582e-48 3.118131e-54

290 1.441070e-48 1.443335e-48 1.445634e-48 1.446325e-48 1.448697e-48 4.399688e-54

291 2.031123e-48 2.034308e-48 2.037542e-48 2.038517e-48 2.041853e-48 6.201792e-54

292 2.858825e-48 2.863295e-48 2.867835e-48 2.869210e-48 2.873895e-48 8.731454e-54

293 4.017899e-48 4.024163e-48 4.030526e-48 4.032460e-48 4.039028e-48 1.227634e-53

294 5.638299e-48 5.647060e-48 5.655964e-48 5.658682e-48 5.667873e-48 1.723559e-53

295 7.899959e-48 7.912194e-48 7.924629e-48 7.928443e-48 7.941284e-48 2.416227e-53

296 1.105169e-47 1.106875e-47 1.108609e-47 1.109143e-47 1.110934e-47 3.382163e-53

297 1.543711e-47 1.546085e-47 1.548499e-47 1.549247e-47 1.551741e-47 4.727124e-53

298 2.153024e-47 2.156323e-47 2.159678e-47 2.160722e-47 2.164189e-47 6.597088e-53

299 2.998423e-47 3.002999e-47 3.007655e-47 3.009111e-47 3.013925e-47 9.193326e-53

300 4.169819e-47 4.176159e-47 4.182611e-47 4.184639e-47 4.191311e-47 1.279306e-52

301 5.790868e-47 5.799639e-47 5.808567e-47 5.811388e-47 5.820622e-47 1.777775e-52

302 8.031490e-47 8.043609e-47 8.055946e-47 8.059866e-47 8.072630e-47 2.467186e-52

303 1.112501e-46 1.114173e-46 1.115876e-46 1.116420e-46 1.118182e-46 3.419585e-52

304 1.539158e-46 1.541463e-46 1.543810e-46 1.544564e-46 1.546994e-46 4.733877e-52

305 2.127026e-46 2.130200e-46 2.133432e-46 2.134476e-46 2.137823e-46 6.545749e-52

306 2.936286e-46 2.940652e-46 2.945099e-46 2.946543e-46 2.951149e-46 9.041248e-52

307 4.049388e-46 4.055389e-46 4.061502e-46 4.063498e-46 4.069830e-46 1.247534e-51

308 5.579252e-46 5.587495e-46 5.595891e-46 5.598649e-46 5.607347e-46 1.719733e-51

309 7.680483e-46 7.691798e-46 7.703323e-46 7.707128e-46 7.719069e-46 2.368552e-51

310 1.056470e-45 1.058022e-45 1.059603e-45 1.060128e-45 1.061767e-45 3.259475e-51

311 1.452151e-45 1.454280e-45 1.456448e-45 1.457171e-45 1.459418e-45 4.482133e-51

312 1.994719e-45 1.997639e-45 2.000611e-45 2.001607e-45 2.004687e-45 6.159178e-51

313 2.738396e-45 2.742398e-45 2.746472e-45 2.747843e-45 2.752064e-45 8.458435e-51

314 3.757362e-45 3.762846e-45 3.768429e-45 3.770317e-45 3.776100e-45 1.160951e-50

315 5.153106e-45 5.160622e-45 5.168271e-45 5.170868e-45 5.178792e-45 1.592654e-50

316 7.064474e-45 7.074775e-45 7.085256e-45 7.088828e-45 7.099683e-45 2.183923e-50

317 9.681435e-45 9.695553e-45 9.709915e-45 9.714827e-45 9.729698e-45 2.993545e-50

318 1.326390e-44 1.328326e-44 1.330294e-44 1.330969e-44 1.333007e-44 4.101943e-50

319 1.816753e-44 1.819406e-44 1.822103e-44 1.823032e-44 1.825824e-44 5.619136e-50

320 2.487897e-44 2.491535e-44 2.495232e-44 2.496508e-44 2.500335e-44 7.695647e-50

321 3.406423e-44 3.411411e-44 3.416481e-44 3.418235e-44 3.423480e-44 1.053741e-49

322 4.663477e-44 4.670318e-44 4.677271e-44 4.679682e-44 4.686874e-44 1.442617e-49

323 6.383810e-44 6.393196e-44 6.402733e-44 6.406045e-44 6.415909e-44 1.974746e-49

324 8.738176e-44 8.751055e-44 8.764138e-44 8.768691e-44 8.782220e-44 2.702877e-49

325 1.196031e-43 1.197798e-43 1.199594e-43 1.200219e-43 1.202076e-43 3.699188e-49

326 1.637017e-43 1.639443e-43 1.641906e-43 1.642766e-43 1.645313e-43 5.062443e-49

327 2.240576e-43 2.243905e-43 2.247287e-43 2.248469e-43 2.251965e-43 6.927786e-49

328 3.066669e-43 3.071240e-43 3.075881e-43 3.077507e-43 3.082305e-43 9.480140e-49

329 4.197383e-43 4.203658e-43 4.210030e-43 4.212265e-43 4.218851e-43 1.297253e-48

330 5.745093e-43 5.753709e-43 5.762458e-43 5.765529e-43 5.774572e-43 1.775119e-48

331 7.863640e-43 7.875469e-43 7.887481e-43 7.891704e-43 7.904119e-43 2.428979e-48

332 1.076362e-42 1.077986e-42 1.079635e-42 1.080216e-42 1.081921e-42 3.323645e-48

333 1.473333e-42 1.475563e-42 1.477827e-42 1.478626e-42 1.480966e-42 4.547777e-48

334 2.016740e-42 2.019800e-42 2.022909e-42 2.024007e-42 2.027220e-42 6.222664e-48

335 2.760598e-42 2.764799e-42 2.769066e-42 2.770576e-42 2.774987e-42 8.514211e-48

336 3.778842e-42 3.784606e-42 3.790463e-42 3.792540e-42 3.798595e-42 1.164933e-47

337 5.172657e-42 5.180566e-42 5.188604e-42 5.191460e-42 5.199772e-42 1.593836e-47

338 7.080523e-42 7.091372e-42 7.102401e-42 7.106329e-42 7.117735e-42 2.180567e-47

339 9.691936e-42 9.706817e-42 9.721946e-42 9.727348e-42 9.742999e-42 2.983150e-47

340 1.326619e-41 1.328660e-41 1.330735e-41 1.331478e-41 1.333625e-41 4.080919e-47

341 1.815806e-41 1.818603e-41 1.821448e-41 1.822470e-41 1.825415e-41 5.582318e-47

342 2.485290e-41 2.489123e-41 2.493023e-41 2.494428e-41 2.498466e-41 7.635581e-47

343 3.401465e-41 3.406715e-41 3.412059e-41 3.413992e-41 3.419527e-41 1.044329e-46

344 4.655144e-41 4.662333e-41 4.669655e-41 4.672311e-41 4.679896e-41 1.428227e-46

345 6.370528e-41 6.380370e-41 6.390395e-41 6.394048e-41 6.404439e-41 1.953076e-46

346 8.717461e-41 8.730927e-41 8.744650e-41 8.749673e-41 8.763903e-41 2.670544e-46

347 1.192817e-40 1.194658e-40 1.196536e-40 1.197227e-40 1.199175e-40 3.651207e-46

348 1.632012e-40 1.634530e-40 1.637098e-40 1.638047e-40 1.640712e-40 4.991443e-46

349 2.232727e-40 2.236167e-40 2.239677e-40 2.240982e-40 2.244628e-40 6.822851e-46

350 3.054268e-40 3.058965e-40 3.063761e-40 3.065554e-40 3.070538e-40 9.325077e-46

351 4.177669e-40 4.184078e-40 4.190628e-40 4.193092e-40 4.199902e-40 1.274332e-45

352 5.713630e-40 5.722372e-40 5.731311e-40 5.734696e-40 5.743997e-40 1.741215e-45

353 7.813346e-40 7.825261e-40 7.837453e-40 7.842104e-40 7.854798e-40 2.378803e-45

354 1.068326e-39 1.069949e-39 1.071611e-39 1.072250e-39 1.073981e-39 3.249347e-45

355 1.460520e-39 1.462729e-39 1.464992e-39 1.465869e-39 1.468230e-39 4.437730e-45

356 1.996373e-39 1.999377e-39 2.002458e-39 2.003662e-39 2.006878e-39 6.059651e-45

357 2.728351e-39 2.732433e-39 2.736623e-39 2.738276e-39 2.742654e-39 8.272764e-45

358 3.728001e-39 3.733544e-39 3.739238e-39 3.741507e-39 3.747463e-39 1.129182e-44

359 5.497750e-03 5.497606e-03 5.497459e-03 5.497380e-03 5.497225e-03 3.786705e-06

360 1.098098e-02 1.098065e-02 1.098032e-02 1.098015e-02 1.097980e-02 7.139928e-06

361 1.644604e-02 1.644549e-02 1.644492e-02 1.644464e-02 1.644404e-02 1.010714e-05

362 2.188881e-02 2.188797e-02 2.188712e-02 2.188670e-02 2.188580e-02 1.273084e-05

363 2.730461e-02 2.730344e-02 2.730223e-02 2.730165e-02 2.730038e-02 1.504909e-05

364 3.268824e-02 3.268665e-02 3.268503e-02 3.268425e-02 3.268254e-02 1.709591e-05

365 3.803389e-02 3.803181e-02 3.802968e-02 3.802868e-02 3.802644e-02 1.890169e-05

366 4.333512e-02 4.333245e-02 4.332972e-02 4.332845e-02 4.332559e-02 2.049360e-05

367 4.858485e-02 4.858150e-02 4.857806e-02 4.857648e-02 4.857287e-02 2.189586e-05

368 5.377533e-02 5.377116e-02 5.376690e-02 5.376495e-02 5.376048e-02 2.313009e-05

369 5.889810e-02 5.889299e-02 5.888776e-02 5.888539e-02 5.887990e-02 2.421555e-05

370 6.394400e-02 6.393780e-02 6.393146e-02 6.392859e-02 6.392194e-02 2.516938e-05

371 6.890319e-02 6.889574e-02 6.888811e-02 6.888467e-02 6.887668e-02 2.600685e-05

372 7.376514e-02 7.375626e-02 7.374717e-02 7.374306e-02 7.373354e-02 2.674152e-05

373 7.851869e-02 7.850818e-02 7.849743e-02 7.849257e-02 7.848131e-02 2.738547e-05

374 8.315213e-02 8.313978e-02 8.312715e-02 8.312143e-02 8.310820e-02 2.794939e-05

375 8.765327e-02 8.763886e-02 8.762412e-02 8.761742e-02 8.760198e-02 2.844279e-05

376 9.200961e-02 9.199290e-02 9.197580e-02 9.196800e-02 9.195009e-02 2.887412e-05

377 9.620850e-02 9.618924e-02 9.616954e-02 9.616050e-02 9.613987e-02 2.925090e-05

378 1.002374e-01 1.002153e-01 1.001928e-01 1.001823e-01 1.001587e-01 2.957977e-05

379 1.040841e-01 1.040590e-01 1.040333e-01 1.040213e-01 1.039944e-01 2.986658e-05

380 1.077371e-01 1.077086e-01 1.076795e-01 1.076659e-01 1.076355e-01 3.011651e-05

381 1.111858e-01 1.111538e-01 1.111211e-01 1.111057e-01 1.110715e-01 3.033411e-05

382 1.144213e-01 1.143855e-01 1.143490e-01 1.143316e-01 1.142933e-01 3.052340e-05

383 1.174362e-01 1.173965e-01 1.173559e-01 1.173364e-01 1.172939e-01 3.068790e-05

384 1.202253e-01 1.201815e-01 1.201367e-01 1.201150e-01 1.200681e-01 3.083073e-05

385 1.227860e-01 1.227379e-01 1.226887e-01 1.226648e-01 1.226134e-01 3.095463e-05

386 1.251180e-01 1.250656e-01 1.250121e-01 1.249859e-01 1.249298e-01 3.106199e-05

387 1.272242e-01 1.271675e-01 1.271095e-01 1.270809e-01 1.270203e-01 3.115493e-05

388 1.291099e-01 1.290489e-01 1.289865e-01 1.289556e-01 1.288904e-01 3.123529e-05

389 1.307831e-01 1.307179e-01 1.306513e-01 1.306180e-01 1.305483e-01 3.130470e-05

390 1.322542e-01 1.321850e-01 1.321142e-01 1.320786e-01 1.320046e-01 3.136459e-05

391 1.335355e-01 1.334624e-01 1.333877e-01 1.333499e-01 1.332718e-01 3.141619e-05

392 1.346408e-01 1.345641e-01 1.344856e-01 1.344457e-01 1.343638e-01 3.146061e-05

393 1.355850e-01 1.355049e-01 1.354230e-01 1.353811e-01 1.352955e-01 3.149878e-05

394 1.363835e-01 1.363002e-01 1.362151e-01 1.361714e-01 1.360825e-01 3.153154e-05

395 1.370517e-01 1.369656e-01 1.368776e-01 1.368322e-01 1.367403e-01 3.155962e-05

396 1.376049e-01 1.375162e-01 1.374256e-01 1.373787e-01 1.372840e-01 3.158366e-05

397 1.380576e-01 1.379666e-01 1.378736e-01 1.378253e-01 1.377282e-01 3.160419e-05

398 1.384233e-01 1.383303e-01 1.382353e-01 1.381857e-01 1.380865e-01 3.162171e-05

399 1.387147e-01 1.386199e-01 1.385231e-01 1.384724e-01 1.383713e-01 3.163662e-05

400 1.389432e-01 1.388468e-01 1.387484e-01 1.386967e-01 1.385940e-01 3.164930e-05

401 1.391188e-01 1.390211e-01 1.389213e-01 1.388689e-01 1.387647e-01 3.166005e-05

402 1.392507e-01 1.391518e-01 1.390509e-01 1.389977e-01 1.388923e-01 3.166915e-05

403 1.393465e-01 1.392467e-01 1.391447e-01 1.390909e-01 1.389845e-01 3.167684e-05

404 1.394131e-01 1.393125e-01 1.392097e-01 1.391554e-01 1.390480e-01 3.168332e-05

405 1.394563e-01 1.393549e-01 1.392514e-01 1.391967e-01 1.390886e-01 3.168876e-05

406 1.394808e-01 1.393789e-01 1.392748e-01 1.392197e-01 1.391110e-01 3.169332e-05

407 1.394908e-01 1.393884e-01 1.392838e-01 1.392284e-01 1.391192e-01 3.169713e-05

408 1.394896e-01 1.393868e-01 1.392819e-01 1.392262e-01 1.391166e-01 3.170030e-05

409 1.394801e-01 1.393770e-01 1.392717e-01 1.392158e-01 1.391059e-01 3.170293e-05

410 1.394645e-01 1.393611e-01 1.392556e-01 1.391996e-01 1.390894e-01 3.170511e-05

411 1.394446e-01 1.393411e-01 1.392353e-01 1.391792e-01 1.390688e-01 3.170689e-05

412 1.394220e-01 1.393183e-01 1.392124e-01 1.391561e-01 1.390456e-01 3.170836e-05

413 1.393978e-01 1.392939e-01 1.391879e-01 1.391316e-01 1.390209e-01 3.170955e-05

414 1.393728e-01 1.392689e-01 1.391627e-01 1.391063e-01 1.389956e-01 3.171051e-05

415 1.393478e-01 1.392438e-01 1.391376e-01 1.390812e-01 1.389704e-01 3.171128e-05

416 1.393233e-01 1.392192e-01 1.391130e-01 1.390566e-01 1.389457e-01 3.171189e-05

417 1.392997e-01 1.391956e-01 1.390893e-01 1.390329e-01 1.389220e-01 3.171237e-05

418 1.392772e-01 1.391731e-01 1.390668e-01 1.390104e-01 1.388995e-01 3.171275e-05

419 1.392560e-01 1.391519e-01 1.390457e-01 1.389892e-01 1.388783e-01 3.171303e-05

420 1.392363e-01 1.391322e-01 1.390259e-01 1.389695e-01 1.388586e-01 3.171325e-05

421 1.392180e-01 1.391140e-01 1.390077e-01 1.389513e-01 1.388404e-01 3.171340e-05

422 1.392013e-01 1.390972e-01 1.389910e-01 1.389345e-01 1.388237e-01 3.171350e-05

423 1.391859e-01 1.390819e-01 1.389757e-01 1.389193e-01 1.388084e-01 3.171357e-05

424 1.391720e-01 1.390680e-01 1.389618e-01 1.389054e-01 1.387946e-01 3.171361e-05

425 1.391595e-01 1.390555e-01 1.389493e-01 1.388929e-01 1.387821e-01 3.171363e-05

426 1.391482e-01 1.390442e-01 1.389381e-01 1.388817e-01 1.387709e-01 3.171363e-05

427 1.391381e-01 1.390342e-01 1.389280e-01 1.388717e-01 1.387609e-01 3.171361e-05

428 1.391291e-01 1.390252e-01 1.389191e-01 1.388627e-01 1.387520e-01 3.171358e-05

429 1.391211e-01 1.390172e-01 1.389111e-01 1.388548e-01 1.387441e-01 3.171355e-05

430 1.391141e-01 1.390102e-01 1.389041e-01 1.388478e-01 1.387371e-01 3.171351e-05

431 1.391078e-01 1.390040e-01 1.388979e-01 1.388416e-01 1.387310e-01 3.171347e-05

432 1.391024e-01 1.389985e-01 1.388925e-01 1.388362e-01 1.387256e-01 3.171343e-05

433 1.390976e-01 1.389938e-01 1.388878e-01 1.388315e-01 1.387209e-01 3.171339e-05

434 1.390935e-01 1.389897e-01 1.388837e-01 1.388274e-01 1.387168e-01 3.171335e-05

435 1.390899e-01 1.389861e-01 1.388801e-01 1.388239e-01 1.387133e-01 3.171331e-05

436 1.390868e-01 1.389830e-01 1.388770e-01 1.388208e-01 1.387102e-01 3.171327e-05

437 1.390841e-01 1.389803e-01 1.388743e-01 1.388181e-01 1.387075e-01 3.171323e-05

438 1.390818e-01 1.389780e-01 1.388720e-01 1.388158e-01 1.387053e-01 3.171320e-05

439 1.390798e-01 1.389760e-01 1.388701e-01 1.388139e-01 1.387034e-01 3.171317e-05

440 1.390781e-01 1.389744e-01 1.388684e-01 1.388123e-01 1.387017e-01 3.171314e-05

441 1.390767e-01 1.389730e-01 1.388670e-01 1.388109e-01 1.387003e-01 3.171311e-05

442 1.390755e-01 1.389718e-01 1.388658e-01 1.388097e-01 1.386991e-01 3.171309e-05

443 1.390745e-01 1.389708e-01 1.388649e-01 1.388087e-01 1.386982e-01 3.171307e-05

444 1.390736e-01 1.389699e-01 1.388640e-01 1.388079e-01 1.386973e-01 3.171305e-05

445 1.390729e-01 1.389692e-01 1.388633e-01 1.388072e-01 1.386967e-01 3.171303e-05

446 1.390724e-01 1.389687e-01 1.388628e-01 1.388066e-01 1.386961e-01 3.171301e-05

447 1.390719e-01 1.389682e-01 1.388623e-01 1.388061e-01 1.386956e-01 3.171300e-05

448 1.390715e-01 1.389678e-01 1.388619e-01 1.388058e-01 1.386953e-01 3.171298e-05

449 1.390712e-01 1.389675e-01 1.388616e-01 1.388055e-01 1.386950e-01 3.171297e-05

450 1.390710e-01 1.389673e-01 1.388614e-01 1.388052e-01 1.386947e-01 3.171296e-05

451 1.390708e-01 1.389671e-01 1.388612e-01 1.388050e-01 1.386946e-01 3.171295e-05

452 1.390706e-01 1.389669e-01 1.388610e-01 1.388049e-01 1.386944e-01 3.171294e-05

453 1.390705e-01 1.389668e-01 1.388609e-01 1.388048e-01 1.386943e-01 3.171294e-05

454 1.390704e-01 1.389667e-01 1.388608e-01 1.388047e-01 1.386942e-01 3.171293e-05

455 1.390703e-01 1.389667e-01 1.388608e-01 1.388046e-01 1.386942e-01 3.171293e-05

456 1.390703e-01 1.389666e-01 1.388607e-01 1.388046e-01 1.386941e-01 3.171292e-05

457 1.390703e-01 1.389666e-01 1.388607e-01 1.388046e-01 1.386941e-01 3.171292e-05

458 1.390703e-01 1.389666e-01 1.388607e-01 1.388046e-01 1.386941e-01 3.171291e-05

459 1.390703e-01 1.389666e-01 1.388607e-01 1.388046e-01 1.386941e-01 3.171291e-05

460 1.390703e-01 1.389666e-01 1.388607e-01 1.388046e-01 1.386941e-01 3.171291e-05

461 1.390703e-01 1.389666e-01 1.388607e-01 1.388046e-01 1.386941e-01 3.171291e-05

462 1.390703e-01 1.389666e-01 1.388607e-01 1.388046e-01 1.386941e-01 3.171290e-05

463 1.390703e-01 1.389666e-01 1.388608e-01 1.388046e-01 1.386941e-01 3.171290e-05

464 1.390703e-01 1.389666e-01 1.388608e-01 1.388046e-01 1.386942e-01 3.171290e-05

465 1.390704e-01 1.389667e-01 1.388608e-01 1.388047e-01 1.386942e-01 3.171290e-05

466 1.390704e-01 1.389667e-01 1.388608e-01 1.388047e-01 1.386942e-01 3.171290e-05

467 1.390704e-01 1.389667e-01 1.388608e-01 1.388047e-01 1.386942e-01 3.171290e-05

468 1.390704e-01 1.389667e-01 1.388609e-01 1.388047e-01 1.386942e-01 3.171290e-05

469 1.390704e-01 1.389667e-01 1.388609e-01 1.388047e-01 1.386943e-01 3.171290e-05

470 1.390704e-01 1.389668e-01 1.388609e-01 1.388048e-01 1.386943e-01 3.171290e-05

471 1.390705e-01 1.389668e-01 1.388609e-01 1.388048e-01 1.386943e-01 3.171290e-05

472 1.390705e-01 1.389668e-01 1.388609e-01 1.388048e-01 1.386943e-01 3.171290e-05

473 1.390705e-01 1.389668e-01 1.388609e-01 1.388048e-01 1.386943e-01 3.171290e-05

474 1.390705e-01 1.389668e-01 1.388609e-01 1.388048e-01 1.386943e-01 3.171290e-05

475 1.390705e-01 1.389668e-01 1.388610e-01 1.388048e-01 1.386943e-01 3.171290e-05

476 1.390705e-01 1.389668e-01 1.388610e-01 1.388048e-01 1.386944e-01 3.171290e-05

477 1.390705e-01 1.389668e-01 1.388610e-01 1.388048e-01 1.386944e-01 3.171290e-05

478 1.390705e-01 1.389669e-01 1.388610e-01 1.388049e-01 1.386944e-01 3.171290e-05

479 1.390705e-01 1.389669e-01 1.388610e-01 1.388049e-01 1.386944e-01 3.171290e-05

480 1.390706e-01 1.389669e-01 1.388610e-01 1.388049e-01 1.386944e-01 3.171290e-05

481 1.390706e-01 1.389669e-01 1.388610e-01 1.388049e-01 1.386944e-01 3.171290e-05

482 1.390706e-01 1.389669e-01 1.388610e-01 1.388049e-01 1.386944e-01 3.171290e-05

483 1.390706e-01 1.389669e-01 1.388610e-01 1.388049e-01 1.386944e-01 3.171290e-05

484 1.390706e-01 1.389669e-01 1.388610e-01 1.388049e-01 1.386944e-01 3.171290e-05

485 1.390706e-01 1.389669e-01 1.388610e-01 1.388049e-01 1.386944e-01 3.171290e-05

486 1.390706e-01 1.389669e-01 1.388610e-01 1.388049e-01 1.386944e-01 3.171290e-05

487 1.390706e-01 1.389669e-01 1.388610e-01 1.388049e-01 1.386944e-01 3.171290e-05

488 1.390706e-01 1.389669e-01 1.388610e-01 1.388049e-01 1.386944e-01 3.171290e-05

489 1.390706e-01 1.389669e-01 1.388610e-01 1.388049e-01 1.386944e-01 3.171290e-05

490 1.390706e-01 1.389669e-01 1.388610e-01 1.388049e-01 1.386944e-01 3.171290e-05

491 1.390706e-01 1.389669e-01 1.388610e-01 1.388049e-01 1.386944e-01 3.171290e-05

492 1.390706e-01 1.389669e-01 1.388610e-01 1.388049e-01 1.386944e-01 3.171290e-05

493 1.390706e-01 1.389669e-01 1.388610e-01 1.388049e-01 1.386944e-01 3.171290e-05

494 1.390706e-01 1.389669e-01 1.388610e-01 1.388049e-01 1.386944e-01 3.171290e-05

495 1.390706e-01 1.389669e-01 1.388610e-01 1.388049e-01 1.386944e-01 3.171290e-05

496 1.390706e-01 1.389669e-01 1.388610e-01 1.388049e-01 1.386944e-01 3.171290e-05

497 1.390706e-01 1.389669e-01 1.388610e-01 1.388049e-01 1.386944e-01 3.171290e-05

498 1.390706e-01 1.389669e-01 1.388610e-01 1.388049e-01 1.386944e-01 3.171290e-05

499 1.390706e-01 1.389669e-01 1.388610e-01 1.388049e-01 1.386944e-01 3.171290e-05

500 1.390706e-01 1.389669e-01 1.388610e-01 1.388049e-01 1.386944e-01 3.171290e-05

501 1.390706e-01 1.389669e-01 1.388610e-01 1.388049e-01 1.386944e-01 3.171290e-05

502 1.390706e-01 1.389669e-01 1.388610e-01 1.388049e-01 1.386944e-01 3.171290e-05

503 1.390706e-01 1.389669e-01 1.388610e-01 1.388049e-01 1.386944e-01 3.171290e-05

504 1.390706e-01 1.389669e-01 1.388610e-01 1.388049e-01 1.386944e-01 3.171290e-05

505 1.390706e-01 1.389669e-01 1.388610e-01 1.388049e-01 1.386944e-01 3.171290e-05

506 1.390706e-01 1.389669e-01 1.388610e-01 1.388049e-01 1.386944e-01 3.171290e-05

507 1.390706e-01 1.389669e-01 1.388610e-01 1.388049e-01 1.386944e-01 3.171290e-05

508 1.390706e-01 1.389669e-01 1.388610e-01 1.388049e-01 1.386944e-01 3.171290e-05

509 1.390706e-01 1.389669e-01 1.388610e-01 1.388049e-01 1.386944e-01 3.171290e-05

510 1.390706e-01 1.389669e-01 1.388610e-01 1.388049e-01 1.386944e-01 3.171290e-05

511 1.390706e-01 1.389669e-01 1.388610e-01 1.388049e-01 1.386944e-01 3.171290e-05

512 1.390706e-01 1.389669e-01 1.388610e-01 1.388049e-01 1.386944e-01 3.171290e-05

513 1.390706e-01 1.389669e-01 1.388610e-01 1.388049e-01 1.386944e-01 3.171290e-05

514 1.390706e-01 1.389669e-01 1.388610e-01 1.388049e-01 1.386944e-01 3.171290e-05

515 1.390706e-01 1.389669e-01 1.388610e-01 1.388049e-01 1.386944e-01 3.171290e-05

516 1.390706e-01 1.389669e-01 1.388610e-01 1.388049e-01 1.386944e-01 3.171290e-05

517 1.390706e-01 1.389669e-01 1.388610e-01 1.388049e-01 1.386944e-01 3.171290e-05

518 1.390706e-01 1.389669e-01 1.388610e-01 1.388049e-01 1.386944e-01 3.171290e-05

519 1.390706e-01 1.389669e-01 1.388610e-01 1.388049e-01 1.386944e-01 3.171290e-05

520 1.390706e-01 1.389669e-01 1.388610e-01 1.388049e-01 1.386944e-01 3.171290e-05

521 1.390706e-01 1.389669e-01 1.388610e-01 1.388049e-01 1.386944e-01 3.171290e-05

522 1.390706e-01 1.389669e-01 1.388610e-01 1.388049e-01 1.386944e-01 3.171290e-05

523 1.390706e-01 1.389669e-01 1.388610e-01 1.388049e-01 1.386944e-01 3.171290e-05

524 1.390706e-01 1.389669e-01 1.388610e-01 1.388049e-01 1.386944e-01 3.171290e-05

525 1.390706e-01 1.389669e-01 1.388610e-01 1.388049e-01 1.386944e-01 3.171290e-05

526 1.390706e-01 1.389669e-01 1.388610e-01 1.388049e-01 1.386944e-01 3.171290e-05

527 1.390706e-01 1.389669e-01 1.388610e-01 1.388049e-01 1.386944e-01 3.171290e-05

528 1.390706e-01 1.389669e-01 1.388610e-01 1.388049e-01 1.386944e-01 3.171290e-05

529 1.390706e-01 1.389669e-01 1.388610e-01 1.388049e-01 1.386944e-01 3.171290e-05

530 1.390706e-01 1.389669e-01 1.388610e-01 1.388049e-01 1.386944e-01 3.171290e-05

531 1.390706e-01 1.389669e-01 1.388610e-01 1.388049e-01 1.386944e-01 3.171290e-05

532 1.390706e-01 1.389669e-01 1.388610e-01 1.388049e-01 1.386944e-01 3.171290e-05

533 1.390706e-01 1.389669e-01 1.388610e-01 1.388049e-01 1.386944e-01 3.171290e-05

534 1.390706e-01 1.389669e-01 1.388610e-01 1.388049e-01 1.386944e-01 3.171290e-05

535 1.390706e-01 1.389669e-01 1.388610e-01 1.388049e-01 1.386944e-01 3.171290e-05

536 1.390706e-01 1.389669e-01 1.388610e-01 1.388049e-01 1.386944e-01 3.171290e-05

537 1.390706e-01 1.389669e-01 1.388610e-01 1.388049e-01 1.386944e-01 3.171290e-05

538 1.446264e-01 1.445227e-01 1.444169e-01 1.443607e-01 1.442503e-01 1.116835e-03

539 1.446845e-01 1.445809e-01 1.444752e-01 1.444191e-01 1.443088e-01 2.143380e-03

540 1.447570e-01 1.446536e-01 1.445481e-01 1.444921e-01 1.443820e-01 3.117481e-03

541 1.448476e-01 1.447444e-01 1.446391e-01 1.445833e-01 1.444734e-01 4.041038e-03

542 1.449604e-01 1.448575e-01 1.447525e-01 1.446968e-01 1.445872e-01 4.915980e-03

543 1.451001e-01 1.449976e-01 1.448929e-01 1.448373e-01 1.447281e-01 5.744235e-03

544 1.452719e-01 1.451698e-01 1.450656e-01 1.450102e-01 1.449014e-01 6.527715e-03

545 1.454817e-01 1.453800e-01 1.452763e-01 1.452211e-01 1.451129e-01 7.268304e-03

546 1.457357e-01 1.456347e-01 1.455315e-01 1.454766e-01 1.453690e-01 7.967856e-03

547 1.460411e-01 1.459408e-01 1.458383e-01 1.457837e-01 1.456768e-01 8.628192e-03

548 1.464058e-01 1.463062e-01 1.462046e-01 1.461503e-01 1.460443e-01 9.251093e-03

549 1.468380e-01 1.467394e-01 1.466387e-01 1.465849e-01 1.464798e-01 9.838297e-03

550 1.473469e-01 1.472494e-01 1.471498e-01 1.470965e-01 1.469926e-01 1.039150e-02

551 1.479425e-01 1.478462e-01 1.477479e-01 1.476952e-01 1.475926e-01 1.091234e-02

552 1.486352e-01 1.485404e-01 1.484435e-01 1.483914e-01 1.482904e-01 1.140243e-02

553 1.494362e-01 1.493430e-01 1.492478e-01 1.491965e-01 1.490972e-01 1.186330e-02

554 1.503572e-01 1.502658e-01 1.501725e-01 1.501220e-01 1.500247e-01 1.229645e-02

555 1.514104e-01 1.513210e-01 1.512298e-01 1.511803e-01 1.510852e-01 1.270332e-02

556 1.526082e-01 1.525211e-01 1.524323e-01 1.523839e-01 1.522912e-01 1.308530e-02

557 1.539634e-01 1.538788e-01 1.537926e-01 1.537454e-01 1.536554e-01 1.344372e-02

558 1.554884e-01 1.554067e-01 1.553232e-01 1.552774e-01 1.551905e-01 1.377987e-02

559 1.571955e-01 1.571168e-01 1.570365e-01 1.569922e-01 1.569085e-01 1.409495e-02

560 1.590961e-01 1.590207e-01 1.589438e-01 1.589012e-01 1.588210e-01 1.439016e-02

561 1.612008e-01 1.611290e-01 1.610557e-01 1.610148e-01 1.609385e-01 1.466662e-02

562 1.635187e-01 1.634506e-01 1.633811e-01 1.633422e-01 1.632699e-01 1.492538e-02

563 1.660569e-01 1.659928e-01 1.659274e-01 1.658905e-01 1.658224e-01 1.516749e-02

564 1.688208e-01 1.687608e-01 1.686995e-01 1.686648e-01 1.686011e-01 1.539390e-02

565 1.718129e-01 1.717571e-01 1.717002e-01 1.716678e-01 1.716085e-01 1.560555e-02

566 1.750335e-01 1.749819e-01 1.749294e-01 1.748993e-01 1.748446e-01 1.580330e-02

567 1.784797e-01 1.784325e-01 1.783843e-01 1.783565e-01 1.783064e-01 1.598801e-02

568 1.821462e-01 1.821032e-01 1.820594e-01 1.820340e-01 1.819884e-01 1.616046e-02

569 1.860249e-01 1.859860e-01 1.859465e-01 1.859234e-01 1.858822e-01 1.632139e-02

570 1.901054e-01 1.900706e-01 1.900351e-01 1.900143e-01 1.899774e-01 1.647153e-02

571 1.943754e-01 1.943445e-01 1.943129e-01 1.942943e-01 1.942615e-01 1.661153e-02

572 1.988212e-01 1.987938e-01 1.987659e-01 1.987493e-01 1.987204e-01 1.674204e-02

573 2.034276e-01 2.034036e-01 2.033792e-01 2.033645e-01 2.033391e-01 1.686366e-02

574 2.081795e-01 2.081585e-01 2.081372e-01 2.081244e-01 2.081022e-01 1.697694e-02

575 2.130611e-01 2.130430e-01 2.130245e-01 2.130133e-01 2.129941e-01 1.708243e-02

576 2.180574e-01 2.180418e-01 2.180259e-01 2.180161e-01 2.179996e-01 1.718063e-02

577 2.231537e-01 2.231403e-01 2.231266e-01 2.231182e-01 2.231041e-01 1.727200e-02

578 2.283364e-01 2.283249e-01 2.283132e-01 2.283059e-01 2.282939e-01 1.735700e-02

579 2.335928e-01 2.335830e-01 2.335731e-01 2.335667e-01 2.335565e-01 1.743605e-02

580 2.389115e-01 2.389032e-01 2.388947e-01 2.388892e-01 2.388805e-01 1.750952e-02

581 2.442823e-01 2.442752e-01 2.442679e-01 2.442632e-01 2.442557e-01 1.757781e-02

582 2.496961e-01 2.496900e-01 2.496838e-01 2.496796e-01 2.496731e-01 1.764125e-02

583 2.551450e-01 2.551397e-01 2.551343e-01 2.551306e-01 2.551250e-01 1.770016e-02

584 2.606223e-01 2.606176e-01 2.606128e-01 2.606094e-01 2.606045e-01 1.775487e-02

585 2.661219e-01 2.661177e-01 2.661134e-01 2.661102e-01 2.661058e-01 1.780564e-02

586 2.716390e-01 2.716351e-01 2.716312e-01 2.716281e-01 2.716240e-01 1.785275e-02

587 2.771694e-01 2.771657e-01 2.771619e-01 2.771588e-01 2.771549e-01 1.789645e-02

588 2.827095e-01 2.827059e-01 2.827022e-01 2.826990e-01 2.826951e-01 1.793697e-02

589 2.882565e-01 2.882528e-01 2.882490e-01 2.882456e-01 2.882417e-01 1.797454e-02

590 2.938078e-01 2.938039e-01 2.938000e-01 2.937963e-01 2.937922e-01 1.800935e-02

591 2.993616e-01 2.993574e-01 2.993532e-01 2.993491e-01 2.993447e-01 1.804161e-02

592 3.049161e-01 3.049115e-01 3.049068e-01 3.049023e-01 3.048975e-01 1.807149e-02

593 3.104699e-01 3.104648e-01 3.104597e-01 3.104545e-01 3.104491e-01 1.809916e-02

594 3.160220e-01 3.160163e-01 3.160105e-01 3.160047e-01 3.159986e-01 1.812477e-02

595 3.215715e-01 3.215650e-01 3.215584e-01 3.215517e-01 3.215448e-01 1.814848e-02

596 3.271174e-01 3.271101e-01 3.271025e-01 3.270950e-01 3.270871e-01 1.817041e-02

597 3.326593e-01 3.326509e-01 3.326422e-01 3.326336e-01 3.326246e-01 1.819070e-02

598 3.381963e-01 3.381867e-01 3.381769e-01 3.381670e-01 3.381567e-01 1.820946e-02

599 3.437281e-01 3.437172e-01 3.437059e-01 3.436946e-01 3.436828e-01 1.822680e-02

600 3.492542e-01 3.492416e-01 3.492288e-01 3.492158e-01 3.492023e-01 1.824283e-02

601 3.547740e-01 3.547596e-01 3.547449e-01 3.547301e-01 3.547146e-01 1.825763e-02

602 3.602870e-01 3.602707e-01 3.602539e-01 3.602369e-01 3.602192e-01 1.827131e-02

603 3.657928e-01 3.657742e-01 3.657550e-01 3.657356e-01 3.657154e-01 1.828394e-02

604 3.712909e-01 3.712697e-01 3.712478e-01 3.712256e-01 3.712025e-01 1.829561e-02

605 3.767807e-01 3.767565e-01 3.767316e-01 3.767063e-01 3.766799e-01 1.830637e-02

606 3.822615e-01 3.822340e-01 3.822056e-01 3.821767e-01 3.821467e-01 1.831630e-02

607 3.877328e-01 3.877014e-01 3.876691e-01 3.876362e-01 3.876019e-01 1.832547e-02

608 3.931937e-01 3.931580e-01 3.931212e-01 3.930838e-01 3.930447e-01 1.833392e-02

609 3.986434e-01 3.986029e-01 3.985611e-01 3.985185e-01 3.984739e-01 1.834171e-02

610 4.040810e-01 4.040351e-01 4.039875e-01 4.039390e-01 4.038883e-01 1.834889e-02

611 4.095056e-01 4.094534e-01 4.093994e-01 4.093443e-01 4.092866e-01 1.835550e-02

612 4.149159e-01 4.148568e-01 4.147955e-01 4.147328e-01 4.146672e-01 1.836160e-02

613 4.203108e-01 4.202438e-01 4.201742e-01 4.201030e-01 4.200285e-01 1.836722e-02

614 4.256889e-01 4.256130e-01 4.255341e-01 4.254533e-01 4.253686e-01 1.837239e-02

615 4.310487e-01 4.309627e-01 4.308734e-01 4.307817e-01 4.306855e-01 1.837715e-02

616 4.363885e-01 4.362913e-01 4.361901e-01 4.360861e-01 4.359769e-01 1.838153e-02

617 4.417066e-01 4.415967e-01 4.414821e-01 4.413643e-01 4.412406e-01 1.838556e-02

618 4.470011e-01 4.468768e-01 4.467472e-01 4.466138e-01 4.464736e-01 1.838927e-02

619 4.522696e-01 4.521294e-01 4.519829e-01 4.518320e-01 4.516733e-01 1.839268e-02

620 4.575100e-01 4.573518e-01 4.571865e-01 4.570158e-01 4.568363e-01 1.839581e-02

621 4.627197e-01 4.625414e-01 4.623549e-01 4.621622e-01 4.619593e-01 1.839869e-02

622 4.678960e-01 4.676953e-01 4.674851e-01 4.672677e-01 4.670386e-01 1.840134e-02

623 4.730359e-01 4.728103e-01 4.725736e-01 4.723285e-01 4.720703e-01 1.840377e-02

624 4.781361e-01 4.778830e-01 4.776168e-01 4.773409e-01 4.770500e-01 1.840601e-02

625 4.831933e-01 4.829097e-01 4.826107e-01 4.823005e-01 4.819734e-01 1.840806e-02

626 4.882037e-01 4.878865e-01 4.875513e-01 4.872030e-01 4.868356e-01 1.840994e-02

627 4.931634e-01 4.928093e-01 4.924341e-01 4.920436e-01 4.916316e-01 1.841167e-02

628 4.980683e-01 4.976737e-01 4.972544e-01 4.968174e-01 4.963562e-01 1.841325e-02

629 5.029138e-01 5.024750e-01 5.020074e-01 5.015193e-01 5.010038e-01 1.841471e-02

630 5.076952e-01 5.072082e-01 5.066879e-01 5.061439e-01 5.055689e-01 1.841604e-02

631 5.124076e-01 5.118684e-01 5.112906e-01 5.106856e-01 5.100457e-01 1.841727e-02

632 5.170459e-01 5.164501e-01 5.158100e-01 5.151388e-01 5.144283e-01 1.841839e-02

633 5.216048e-01 5.209480e-01 5.202404e-01 5.194977e-01 5.187108e-01 1.841941e-02

634 5.260786e-01 5.253563e-01 5.245762e-01 5.237564e-01 5.228872e-01 1.842035e-02

635 5.304618e-01 5.296694e-01 5.288115e-01 5.279092e-01 5.269516e-01 1.842122e-02

636 5.347487e-01 5.338815e-01 5.329405e-01 5.319504e-01 5.308985e-01 1.842201e-02

637 5.389336e-01 5.379870e-01 5.369577e-01 5.358742e-01 5.347222e-01 1.842273e-02

638 5.430107e-01 5.419802e-01 5.408575e-01 5.396754e-01 5.384176e-01 1.842339e-02

639 5.469744e-01 5.458556e-01 5.446346e-01 5.433490e-01 5.419800e-01 1.842399e-02

640 5.508195e-01 5.496082e-01 5.482840e-01 5.468903e-01 5.454052e-01 1.842455e-02

641 5.545407e-01 5.532330e-01 5.518013e-01 5.502952e-01 5.486894e-01 1.842505e-02

642 5.581333e-01 5.567256e-01 5.551824e-01 5.535602e-01 5.518297e-01 1.842551e-02

643 5.615929e-01 5.600820e-01 5.584239e-01 5.566823e-01 5.548238e-01 1.842594e-02

644 5.649156e-01 5.632989e-01 5.615228e-01 5.596594e-01 5.576701e-01 1.842632e-02

645 5.680984e-01 5.663736e-01 5.644772e-01 5.624900e-01 5.603680e-01 1.842668e-02

646 5.711384e-01 5.693040e-01 5.672856e-01 5.651734e-01 5.629175e-01 1.842700e-02

647 5.740340e-01 5.720888e-01 5.699474e-01 5.677098e-01 5.653197e-01 1.842729e-02

648 5.767840e-01 5.747277e-01 5.724629e-01 5.701002e-01 5.675763e-01 1.842756e-02

649 5.793881e-01 5.772210e-01 5.748331e-01 5.723464e-01 5.696898e-01 1.842781e-02

650 5.818468e-01 5.795696e-01 5.770599e-01 5.744508e-01 5.716636e-01 1.842803e-02

651 5.841613e-01 5.817757e-01 5.791458e-01 5.764168e-01 5.735015e-01 1.842823e-02

652 5.863338e-01 5.838417e-01 5.810942e-01 5.782482e-01 5.752082e-01 1.842842e-02

653 5.883670e-01 5.857712e-01 5.829088e-01 5.799495e-01 5.767886e-01 1.842859e-02

654 5.902645e-01 5.875679e-01 5.845943e-01 5.815257e-01 5.782483e-01 1.842874e-02

655 5.920302e-01 5.892365e-01 5.861557e-01 5.829822e-01 5.795930e-01 1.842889e-02

656 5.936690e-01 5.907819e-01 5.875982e-01 5.843245e-01 5.808286e-01 1.842901e-02

657 5.951858e-01 5.922095e-01 5.889275e-01 5.855588e-01 5.819615e-01 1.842913e-02

658 5.965860e-01 5.935250e-01 5.901496e-01 5.866909e-01 5.829977e-01 1.842924e-02

659 5.978755e-01 5.947343e-01 5.912706e-01 5.877271e-01 5.839434e-01 1.842933e-02

660 5.990602e-01 5.958434e-01 5.922964e-01 5.886734e-01 5.848049e-01 1.842942e-02

661 6.001460e-01 5.968583e-01 5.932331e-01 5.895359e-01 5.855880e-01 1.842950e-02

662 6.011391e-01 5.977851e-01 5.940869e-01 5.903205e-01 5.862987e-01 1.842957e-02

663 6.020455e-01 5.986298e-01 5.948635e-01 5.910329e-01 5.869425e-01 1.842964e-02

664 6.028711e-01 5.993981e-01 5.955686e-01 5.916787e-01 5.875246e-01 1.842970e-02

665 6.036217e-01 6.000957e-01 5.962078e-01 5.922631e-01 5.880503e-01 1.842976e-02

666 6.043030e-01 6.007281e-01 5.967862e-01 5.927912e-01 5.885243e-01 1.842981e-02

667 6.049202e-01 6.013005e-01 5.973088e-01 5.932677e-01 5.889511e-01 1.842985e-02

668 6.054785e-01 6.018176e-01 5.977804e-01 5.936970e-01 5.893349e-01 1.842989e-02

669 6.059829e-01 6.022843e-01 5.982053e-01 5.940834e-01 5.896795e-01 1.842993e-02

670 6.064378e-01 6.027049e-01 5.985877e-01 5.944307e-01 5.899887e-01 1.842996e-02

671 6.068476e-01 6.030834e-01 5.989314e-01 5.947424e-01 5.902658e-01 1.842999e-02

672 6.072162e-01 6.034236e-01 5.992399e-01 5.950220e-01 5.905138e-01 1.843002e-02

673 6.075476e-01 6.037292e-01 5.995167e-01 5.952725e-01 5.907357e-01 1.843005e-02

674 6.078449e-01 6.040032e-01 5.997646e-01 5.954967e-01 5.909338e-01 1.843007e-02

675 6.081116e-01 6.042488e-01 5.999865e-01 5.956971e-01 5.911107e-01 1.843009e-02

676 6.083505e-01 6.044686e-01 6.001850e-01 5.958762e-01 5.912685e-01 1.843011e-02

677 6.085643e-01 6.046653e-01 6.003623e-01 5.960360e-01 5.914091e-01 1.843013e-02

678 6.087554e-01 6.048410e-01 6.005205e-01 5.961786e-01 5.915343e-01 1.843014e-02

679 6.089262e-01 6.049979e-01 6.006617e-01 5.963057e-01 5.916457e-01 1.843016e-02

680 6.090787e-01 6.051378e-01 6.007875e-01 5.964188e-01 5.917448e-01 1.843017e-02

681 6.092146e-01 6.052626e-01 6.008996e-01 5.965195e-01 5.918328e-01 1.843018e-02

682 6.093358e-01 6.053738e-01 6.009994e-01 5.966091e-01 5.919109e-01 1.843019e-02

683 6.094438e-01 6.054728e-01 6.010881e-01 5.966887e-01 5.919802e-01 1.843020e-02

684 6.095399e-01 6.055608e-01 6.011669e-01 5.967594e-01 5.920417e-01 1.843021e-02

685 6.096253e-01 6.056391e-01 6.012370e-01 5.968221e-01 5.920962e-01 1.843022e-02

686 6.097013e-01 6.057087e-01 6.012991e-01 5.968777e-01 5.921444e-01 1.843022e-02

687 6.097688e-01 6.057705e-01 6.013543e-01 5.969271e-01 5.921872e-01 1.843023e-02

688 6.098287e-01 6.058253e-01 6.014032e-01 5.969708e-01 5.922249e-01 1.843023e-02

689 6.098818e-01 6.058739e-01 6.014465e-01 5.970095e-01 5.922583e-01 1.843024e-02

690 6.099290e-01 6.059170e-01 6.014849e-01 5.970437e-01 5.922878e-01 1.843024e-02

691 6.099707e-01 6.059551e-01 6.015189e-01 5.970740e-01 5.923138e-01 1.843025e-02

692 6.100077e-01 6.059889e-01 6.015489e-01 5.971007e-01 5.923368e-01 1.843025e-02

693 6.100405e-01 6.060188e-01 6.015755e-01 5.971244e-01 5.923571e-01 1.843026e-02

694 6.100695e-01 6.060452e-01 6.015989e-01 5.971452e-01 5.923750e-01 1.843026e-02

695 6.100951e-01 6.060686e-01 6.016196e-01 5.971637e-01 5.923907e-01 1.843026e-02

696 6.101177e-01 6.060893e-01 6.016379e-01 5.971799e-01 5.924045e-01 1.843026e-02

697 6.101377e-01 6.061075e-01 6.016540e-01 5.971942e-01 5.924167e-01 1.843027e-02

698 6.101554e-01 6.061236e-01 6.016683e-01 5.972068e-01 5.924274e-01 1.843027e-02

699 6.101709e-01 6.061378e-01 6.016808e-01 5.972179e-01 5.924368e-01 1.843027e-02

700 6.101847e-01 6.061503e-01 6.016918e-01 5.972277e-01 5.924451e-01 1.843027e-02

701 6.101968e-01 6.061613e-01 6.017015e-01 5.972363e-01 5.924524e-01 1.843027e-02

702 6.102074e-01 6.061710e-01 6.017101e-01 5.972438e-01 5.924587e-01 1.843027e-02

703 6.102168e-01 6.061795e-01 6.017176e-01 5.972504e-01 5.924643e-01 1.843028e-02

704 6.102250e-01 6.061870e-01 6.017242e-01 5.972562e-01 5.924692e-01 1.843028e-02

705 6.102323e-01 6.061936e-01 6.017299e-01 5.972613e-01 5.924735e-01 1.843028e-02

706 6.102387e-01 6.061994e-01 6.017350e-01 5.972658e-01 5.924772e-01 1.843028e-02

707 6.102443e-01 6.062045e-01 6.017395e-01 5.972697e-01 5.924805e-01 1.843028e-02

708 6.102492e-01 6.062089e-01 6.017434e-01 5.972732e-01 5.924834e-01 1.843028e-02

709 6.102535e-01 6.062129e-01 6.017468e-01 5.972762e-01 5.924859e-01 1.843028e-02

710 6.102573e-01 6.062163e-01 6.017498e-01 5.972788e-01 5.924880e-01 1.843028e-02

711 6.102606e-01 6.062193e-01 6.017524e-01 5.972811e-01 5.924899e-01 1.843028e-02

712 6.102635e-01 6.062219e-01 6.017547e-01 5.972831e-01 5.924916e-01 1.843028e-02

713 6.102661e-01 6.062242e-01 6.017567e-01 5.972848e-01 5.924930e-01 1.843028e-02

714 6.102683e-01 6.062262e-01 6.017584e-01 5.972864e-01 5.924943e-01 1.843028e-02

715 6.102702e-01 6.062280e-01 6.017600e-01 5.972877e-01 5.924954e-01 1.843028e-02

716 6.102719e-01 6.062295e-01 6.017613e-01 5.972888e-01 5.924963e-01 1.843028e-02

717 6.158273e-01 6.117849e-01 6.073167e-01 6.028442e-01 5.980517e-01 2.252869e-02

718 6.158268e-01 6.117844e-01 6.073162e-01 6.028437e-01 5.980512e-01 2.549234e-02

719 6.158264e-01 6.117840e-01 6.073158e-01 6.028433e-01 5.980508e-01 2.845979e-02

720 6.158260e-01 6.117836e-01 6.073154e-01 6.028430e-01 5.980505e-01 3.142779e-02

721 6.158258e-01 6.117833e-01 6.073151e-01 6.028427e-01 5.980502e-01 3.439306e-02

722 6.158255e-01 6.117831e-01 6.073149e-01 6.028425e-01 5.980500e-01 3.735232e-02

723 6.158254e-01 6.117830e-01 6.073148e-01 6.028423e-01 5.980498e-01 4.030230e-02

724 6.158253e-01 6.117829e-01 6.073147e-01 6.028423e-01 5.980498e-01 4.323979e-02

725 6.158253e-01 6.117829e-01 6.073147e-01 6.028423e-01 5.980498e-01 4.616162e-02

726 6.158254e-01 6.117830e-01 6.073149e-01 6.028424e-01 5.980500e-01 4.906471e-02

727 6.158256e-01 6.117833e-01 6.073151e-01 6.028427e-01 5.980502e-01 5.194605e-02

728 6.158259e-01 6.117836e-01 6.073154e-01 6.028430e-01 5.980505e-01 5.480269e-02

729 6.158264e-01 6.117840e-01 6.073158e-01 6.028434e-01 5.980510e-01 5.763181e-02

730 6.158269e-01 6.117845e-01 6.073164e-01 6.028440e-01 5.980516e-01 6.043068e-02

731 6.158275e-01 6.117852e-01 6.073170e-01 6.028446e-01 5.980522e-01 6.319667e-02

732 6.158283e-01 6.117859e-01 6.073178e-01 6.028454e-01 5.980530e-01 6.592727e-02

733 6.158292e-01 6.117868e-01 6.073187e-01 6.028463e-01 5.980539e-01 6.862009e-02

734 6.158301e-01 6.117878e-01 6.073197e-01 6.028474e-01 5.980550e-01 7.127288e-02

735 6.158313e-01 6.117890e-01 6.073209e-01 6.028485e-01 5.980561e-01 7.388351e-02

736 6.158325e-01 6.117902e-01 6.073221e-01 6.028498e-01 5.980574e-01 7.645000e-02

737 6.158339e-01 6.117916e-01 6.073235e-01 6.028512e-01 5.980588e-01 7.897049e-02

738 6.158354e-01 6.117931e-01 6.073251e-01 6.028527e-01 5.980604e-01 8.144328e-02

739 6.158371e-01 6.117948e-01 6.073267e-01 6.028544e-01 5.980621e-01 8.386681e-02

740 6.158389e-01 6.117966e-01 6.073286e-01 6.028563e-01 5.980639e-01 8.623969e-02

741 6.158408e-01 6.117986e-01 6.073305e-01 6.028582e-01 5.980659e-01 8.856065e-02

742 6.158429e-01 6.118007e-01 6.073327e-01 6.028604e-01 5.980681e-01 9.082858e-02

743 6.158452e-01 6.118030e-01 6.073350e-01 6.028627e-01 5.980704e-01 9.304254e-02

744 6.158476e-01 6.118054e-01 6.073374e-01 6.028652e-01 5.980729e-01 9.520172e-02

745 6.158503e-01 6.118081e-01 6.073401e-01 6.028678e-01 5.980756e-01 9.730546e-02

746 6.158531e-01 6.118109e-01 6.073429e-01 6.028707e-01 5.980785e-01 9.935326e-02

747 6.158561e-01 6.118140e-01 6.073460e-01 6.028738e-01 5.980815e-01 1.013447e-01

748 6.158594e-01 6.118172e-01 6.073493e-01 6.028771e-01 5.980849e-01 1.032797e-01

749 6.158629e-01 6.118207e-01 6.073528e-01 6.028806e-01 5.980884e-01 1.051580e-01

750 6.158666e-01 6.118245e-01 6.073566e-01 6.028845e-01 5.980923e-01 1.069797e-01

751 6.158706e-01 6.118286e-01 6.073607e-01 6.028886e-01 5.980964e-01 1.087451e-01

752 6.158749e-01 6.118329e-01 6.073651e-01 6.028930e-01 5.981008e-01 1.104542e-01

753 6.158796e-01 6.118376e-01 6.073698e-01 6.028977e-01 5.981056e-01 1.121077e-01

754 6.158846e-01 6.118427e-01 6.073749e-01 6.029029e-01 5.981108e-01 1.137060e-01

755 6.158900e-01 6.118481e-01 6.073804e-01 6.029084e-01 5.981164e-01 1.152497e-01

756 6.158959e-01 6.118540e-01 6.073864e-01 6.029144e-01 5.981224e-01 1.167395e-01

757 6.159022e-01 6.118604e-01 6.073928e-01 6.029210e-01 5.981290e-01 1.181762e-01

758 6.159090e-01 6.118673e-01 6.073998e-01 6.029280e-01 5.981361e-01 1.195608e-01

759 6.159164e-01 6.118748e-01 6.074074e-01 6.029357e-01 5.981439e-01 1.208941e-01

760 6.159245e-01 6.118830e-01 6.074156e-01 6.029440e-01 5.981523e-01 1.221773e-01

761 6.159332e-01 6.118918e-01 6.074246e-01 6.029531e-01 5.981615e-01 1.234113e-01

762 6.159427e-01 6.119015e-01 6.074344e-01 6.029630e-01 5.981715e-01 1.245973e-01

763 6.159531e-01 6.119120e-01 6.074450e-01 6.029738e-01 5.981824e-01 1.257365e-01

764 6.159643e-01 6.119234e-01 6.074566e-01 6.029855e-01 5.981943e-01 1.268300e-01

765 6.159766e-01 6.119359e-01 6.074693e-01 6.029984e-01 5.982074e-01 1.278792e-01

766 6.159900e-01 6.119495e-01 6.074831e-01 6.030125e-01 5.982217e-01 1.288851e-01

767 6.160046e-01 6.119644e-01 6.074983e-01 6.030279e-01 5.982374e-01 1.298491e-01

768 6.160206e-01 6.119807e-01 6.075149e-01 6.030448e-01 5.982546e-01 1.307723e-01

769 6.160381e-01 6.119985e-01 6.075331e-01 6.030634e-01 5.982734e-01 1.316561e-01

770 6.160572e-01 6.120181e-01 6.075530e-01 6.030837e-01 5.982941e-01 1.325015e-01

771 6.160782e-01 6.120395e-01 6.075749e-01 6.031060e-01 5.983169e-01 1.333099e-01

772 6.161011e-01 6.120629e-01 6.075988e-01 6.031305e-01 5.983419e-01 1.340825e-01

773 6.161262e-01 6.120886e-01 6.076251e-01 6.031574e-01 5.983694e-01 1.348204e-01

774 6.161537e-01 6.121168e-01 6.076540e-01 6.031869e-01 5.983996e-01 1.355249e-01

775 6.161839e-01 6.121478e-01 6.076857e-01 6.032195e-01 5.984329e-01 1.361972e-01

776 6.162169e-01 6.121817e-01 6.077206e-01 6.032552e-01 5.984696e-01 1.368383e-01

777 6.162532e-01 6.122190e-01 6.077589e-01 6.032945e-01 5.985099e-01 1.374496e-01

778 6.162930e-01 6.122599e-01 6.078010e-01 6.033378e-01 5.985544e-01 1.380320e-01

779 6.163366e-01 6.123049e-01 6.078473e-01 6.033855e-01 5.986034e-01 1.385867e-01

780 6.163846e-01 6.123543e-01 6.078982e-01 6.034380e-01 5.986574e-01 1.391149e-01

781 6.164371e-01 6.124086e-01 6.079542e-01 6.034958e-01 5.987170e-01 1.396174e-01

782 6.164949e-01 6.124683e-01 6.080159e-01 6.035594e-01 5.987827e-01 1.400955e-01

783 6.165583e-01 6.125339e-01 6.080837e-01 6.036295e-01 5.988551e-01 1.405500e-01

784 6.166279e-01 6.126061e-01 6.081584e-01 6.037068e-01 5.989351e-01 1.409820e-01

785 6.167044e-01 6.126854e-01 6.082406e-01 6.037920e-01 5.990233e-01 1.413923e-01

786 6.167884e-01 6.127726e-01 6.083312e-01 6.038859e-01 5.991206e-01 1.417821e-01

787 6.168807e-01 6.128685e-01 6.084308e-01 6.039894e-01 5.992280e-01 1.421521e-01

788 6.169820e-01 6.129741e-01 6.085406e-01 6.041036e-01 5.993467e-01 1.425032e-01

789 6.170934e-01 6.130901e-01 6.086615e-01 6.042294e-01 5.994776e-01 1.428362e-01

790 6.172157e-01 6.132178e-01 6.087947e-01 6.043682e-01 5.996222e-01 1.431520e-01

791 6.173502e-01 6.133582e-01 6.089413e-01 6.045213e-01 5.997818e-01 1.434514e-01

792 6.174978e-01 6.135127e-01 6.091028e-01 6.046900e-01 5.999580e-01 1.437351e-01

793 6.176600e-01 6.136826e-01 6.092806e-01 6.048761e-01 6.001526e-01 1.440038e-01

794 6.178382e-01 6.138694e-01 6.094765e-01 6.050813e-01 6.003674e-01 1.442583e-01

795 6.180338e-01 6.140749e-01 6.096921e-01 6.053075e-01 6.006044e-01 1.444993e-01

796 6.182487e-01 6.143008e-01 6.099295e-01 6.055567e-01 6.008661e-01 1.447273e-01

797 6.184845e-01 6.145491e-01 6.101907e-01 6.058314e-01 6.011547e-01 1.449430e-01

798 6.187434e-01 6.148220e-01 6.104781e-01 6.061340e-01 6.014730e-01 1.451470e-01

799 6.190274e-01 6.151217e-01 6.107943e-01 6.064672e-01 6.018239e-01 1.453400e-01

800 6.193390e-01 6.154509e-01 6.111418e-01 6.068339e-01 6.022105e-01 1.455223e-01

801 6.196806e-01 6.158122e-01 6.115237e-01 6.072374e-01 6.026364e-01 1.456947e-01

802 6.200550e-01 6.162086e-01 6.119432e-01 6.076809e-01 6.031050e-01 1.458575e-01

803 6.204651e-01 6.166431e-01 6.124036e-01 6.081683e-01 6.036204e-01 1.460113e-01

804 6.209142e-01 6.171193e-01 6.129085e-01 6.087033e-01 6.041868e-01 1.461564e-01

805 6.214057e-01 6.176408e-01 6.134619e-01 6.092902e-01 6.048086e-01 1.462935e-01

806 6.219432e-01 6.182113e-01 6.140679e-01 6.099334e-01 6.054906e-01 1.464229e-01

807 6.225308e-01 6.188352e-01 6.147308e-01 6.106374e-01 6.062376e-01 1.465449e-01

808 6.231724e-01 6.195166e-01 6.154552e-01 6.114073e-01 6.070550e-01 1.466600e-01

809 6.238726e-01 6.202602e-01 6.162460e-01 6.122481e-01 6.079480e-01 1.467686e-01

810 6.246360e-01 6.210710e-01 6.171082e-01 6.131650e-01 6.089222e-01 1.468709e-01

811 6.254675e-01 6.219538e-01 6.180472e-01 6.141635e-01 6.099832e-01 1.469674e-01

812 6.263722e-01 6.229141e-01 6.190682e-01 6.152492e-01 6.111370e-01 1.470583e-01

813 6.273553e-01 6.239571e-01 6.201770e-01 6.164278e-01 6.123891e-01 1.471440e-01

814 6.284223e-01 6.250884e-01 6.213790e-01 6.177050e-01 6.137456e-01 1.472246e-01

815 6.295787e-01 6.263137e-01 6.226801e-01 6.190865e-01 6.152120e-01 1.473006e-01

816 6.308302e-01 6.276385e-01 6.240858e-01 6.205780e-01 6.167939e-01 1.473722e-01

817 6.321823e-01 6.290684e-01 6.256016e-01 6.221847e-01 6.184966e-01 1.474395e-01

818 6.336407e-01 6.306090e-01 6.272330e-01 6.239119e-01 6.203250e-01 1.475029e-01

819 6.352108e-01 6.322656e-01 6.289850e-01 6.257645e-01 6.222837e-01 1.475625e-01

820 6.368979e-01 6.340431e-01 6.308624e-01 6.277468e-01 6.243766e-01 1.476186e-01

821 6.387070e-01 6.359462e-01 6.328696e-01 6.298627e-01 6.266072e-01 1.476713e-01

822 6.406426e-01 6.379794e-01 6.350104e-01 6.321157e-01 6.289781e-01 1.477209e-01

823 6.427091e-01 6.401462e-01 6.372880e-01 6.345082e-01 6.314914e-01 1.477676e-01

824 6.449099e-01 6.424499e-01 6.397050e-01 6.370423e-01 6.341481e-01 1.478114e-01

825 6.472483e-01 6.448929e-01 6.422632e-01 6.397191e-01 6.369487e-01 1.478526e-01

826 6.497264e-01 6.474771e-01 6.449638e-01 6.425388e-01 6.398925e-01 1.478913e-01

827 6.523460e-01 6.502034e-01 6.478070e-01 6.455010e-01 6.429781e-01 1.479276e-01

828 6.551080e-01 6.530719e-01 6.507922e-01 6.486042e-01 6.462032e-01 1.479618e-01

829 6.580122e-01 6.560821e-01 6.539179e-01 6.518463e-01 6.495648e-01 1.479938e-01

830 6.610580e-01 6.592325e-01 6.571820e-01 6.552242e-01 6.530589e-01 1.480239e-01

831 6.642437e-01 6.625207e-01 6.605814e-01 6.587341e-01 6.566810e-01 1.480522e-01

832 6.675669e-01 6.659438e-01 6.641123e-01 6.623715e-01 6.604256e-01 1.480787e-01

833 6.710244e-01 6.694979e-01 6.677703e-01 6.661315e-01 6.642872e-01 1.481036e-01

834 6.746123e-01 6.731786e-01 6.715503e-01 6.700082e-01 6.682592e-01 1.481269e-01

835 6.783260e-01 6.769808e-01 6.754467e-01 6.739956e-01 6.723350e-01 1.481488e-01

836 6.821605e-01 6.808991e-01 6.794534e-01 6.780871e-01 6.765075e-01 1.481693e-01

837 6.861100e-01 6.849272e-01 6.835640e-01 6.822760e-01 6.807695e-01 1.481886e-01

838 6.901687e-01 6.890591e-01 6.877718e-01 6.865553e-01 6.851136e-01 1.482066e-01

839 6.943300e-01 6.932879e-01 6.920699e-01 6.909177e-01 6.895322e-01 1.482235e-01

840 6.985875e-01 6.976070e-01 6.964512e-01 6.953561e-01 6.940178e-01 1.482394e-01

841 7.029343e-01 7.020093e-01 7.009086e-01 6.998632e-01 6.985628e-01 1.482542e-01

842 7.073636e-01 7.064880e-01 7.054350e-01 7.044316e-01 7.031598e-01 1.482681e-01

843 7.118685e-01 7.110361e-01 7.100233e-01 7.090543e-01 7.078014e-01 1.482812e-01

844 7.164421e-01 7.156466e-01 7.146665e-01 7.137241e-01 7.124802e-01 1.482934e-01

845 7.210777e-01 7.203128e-01 7.193578e-01 7.184342e-01 7.171891e-01 1.483048e-01

846 7.257686e-01 7.250280e-01 7.240903e-01 7.231776e-01 7.219210e-01 1.483155e-01

847 7.305082e-01 7.297856e-01 7.288574e-01 7.279478e-01 7.266691e-01 1.483255e-01

848 7.352902e-01 7.345793e-01 7.336528e-01 7.327381e-01 7.314266e-01 1.483349e-01

849 7.401084e-01 7.394028e-01 7.384701e-01 7.375422e-01 7.361868e-01 1.483436e-01

850 7.449568e-01 7.442502e-01 7.433032e-01 7.423538e-01 7.409430e-01 1.483518e-01

851 7.498295e-01 7.491156e-01 7.481460e-01 7.471668e-01 7.456890e-01 1.483595e-01

852 7.547209e-01 7.539932e-01 7.529928e-01 7.519752e-01 7.504183e-01 1.483666e-01

853 7.596256e-01 7.588775e-01 7.578378e-01 7.567730e-01 7.551245e-01 1.483733e-01

854 7.645381e-01 7.637631e-01 7.626753e-01 7.615544e-01 7.598016e-01 1.483796e-01

855 7.694534e-01 7.686447e-01 7.674999e-01 7.663137e-01 7.644433e-01 1.483855e-01

856 7.743664e-01 7.735171e-01 7.723062e-01 7.710451e-01 7.690435e-01 1.483909e-01

857 7.792722e-01 7.783752e-01 7.770886e-01 7.757430e-01 7.735963e-01 1.483961e-01

858 7.841660e-01 7.832141e-01 7.818421e-01 7.804018e-01 7.780955e-01 1.484008e-01

859 7.890431e-01 7.880287e-01 7.865612e-01 7.850160e-01 7.825353e-01 1.484053e-01

860 7.938987e-01 7.928142e-01 7.912409e-01 7.895800e-01 7.869099e-01 1.484095e-01

861 7.987284e-01 7.975657e-01 7.958758e-01 7.940885e-01 7.912135e-01 1.484133e-01

862 8.035274e-01 8.022784e-01 8.004610e-01 7.985359e-01 7.954405e-01 1.484170e-01

863 8.082912e-01 8.069477e-01 8.049912e-01 8.029171e-01 7.995854e-01 1.484204e-01

864 8.130153e-01 8.115686e-01 8.094616e-01 8.072267e-01 8.036430e-01 1.484235e-01

865 8.176952e-01 8.161365e-01 8.138670e-01 8.114596e-01 8.076082e-01 1.484265e-01

866 8.223261e-01 8.206466e-01 8.182027e-01 8.156108e-01 8.114762e-01 1.484292e-01

867 8.269036e-01 8.250942e-01 8.224637e-01 8.196755e-01 8.152424e-01 1.484318e-01

868 8.314231e-01 8.294745e-01 8.266455e-01 8.236491e-01 8.189026e-01 1.484342e-01

869 8.358800e-01 8.337830e-01 8.307432e-01 8.275271e-01 8.224531e-01 1.484364e-01

870 8.402698e-01 8.380150e-01 8.347526e-01 8.313053e-01 8.258904e-01 1.484385e-01

871 8.445879e-01 8.421660e-01 8.386692e-01 8.349799e-01 8.292116e-01 1.484405e-01

872 8.488298e-01 8.462316e-01 8.424889e-01 8.385473e-01 8.324143e-01 1.484423e-01

873 8.529910e-01 8.502074e-01 8.462078e-01 8.420042e-01 8.354963e-01 1.484439e-01

874 8.570673e-01 8.540892e-01 8.498223e-01 8.453477e-01 8.384563e-01 1.484455e-01

875 8.610543e-01 8.578731e-01 8.533290e-01 8.485753e-01 8.412933e-01 1.484470e-01

876 8.649479e-01 8.615554e-01 8.567248e-01 8.516849e-01 8.440067e-01 1.484483e-01

877 8.687442e-01 8.651325e-01 8.600072e-01 8.546748e-01 8.465966e-01 1.484496e-01

878 8.724394e-01 8.686011e-01 8.631737e-01 8.575439e-01 8.490636e-01 1.484508e-01

879 8.760300e-01 8.719585e-01 8.662226e-01 8.602915e-01 8.514086e-01 1.484519e-01

880 8.795129e-01 8.752021e-01 8.691525e-01 8.629174e-01 8.536332e-01 1.484529e-01

881 8.828851e-01 8.783297e-01 8.719623e-01 8.654218e-01 8.557393e-01 1.484539e-01

882 8.861440e-01 8.813396e-01 8.746515e-01 8.678055e-01 8.577292e-01 1.484548e-01

883 8.892874e-01 8.842304e-01 8.772202e-01 8.700697e-01 8.596057e-01 1.484556e-01

884 8.923136e-01 8.870015e-01 8.796687e-01 8.722160e-01 8.613718e-01 1.484563e-01

885 8.952213e-01 8.896523e-01 8.819979e-01 8.742466e-01 8.630310e-01 1.484571e-01

886 8.980094e-01 8.921829e-01 8.842093e-01 8.761640e-01 8.645869e-01 1.484577e-01

887 9.006776e-01 8.945940e-01 8.863045e-01 8.779708e-01 8.660433e-01 1.484583e-01

888 9.032258e-01 8.968865e-01 8.882858e-01 8.796704e-01 8.674043e-01 1.484589e-01

889 9.056546e-01 8.990619e-01 8.901558e-01 8.812661e-01 8.686740e-01 1.484594e-01

890 9.079649e-01 9.011220e-01 8.919173e-01 8.827615e-01 8.698566e-01 1.484599e-01

891 9.101580e-01 9.030692e-01 8.935735e-01 8.841606e-01 8.709565e-01 1.484604e-01

892 9.122359e-01 9.049061e-01 8.951280e-01 8.854673e-01 8.719778e-01 1.484608e-01

893 9.142006e-01 9.066356e-01 8.965843e-01 8.866857e-01 8.729248e-01 1.484612e-01

894 9.160547e-01 9.082610e-01 8.979463e-01 8.878201e-01 8.738019e-01 1.484616e-01

895 9.178013e-01 9.097858e-01 8.992180e-01 8.888745e-01 8.746130e-01 1.484619e-01

896 9.233553e-01 9.153400e-01 9.047723e-01 8.944289e-01 8.801676e-01 1.536051e-01

897 9.233556e-01 9.153403e-01 9.047726e-01 8.944292e-01 8.801678e-01 1.546364e-01

898 9.233559e-01 9.153405e-01 9.047728e-01 8.944294e-01 8.801680e-01 1.557034e-01

899 9.233562e-01 9.153408e-01 9.047731e-01 8.944296e-01 8.801682e-01 1.568068e-01

900 9.233564e-01 9.153411e-01 9.047733e-01 8.944298e-01 8.801684e-01 1.579475e-01

901 9.233567e-01 9.153413e-01 9.047735e-01 8.944300e-01 8.801685e-01 1.591263e-01

902 9.233570e-01 9.153415e-01 9.047737e-01 8.944302e-01 8.801687e-01 1.603439e-01

903 9.233572e-01 9.153418e-01 9.047739e-01 8.944304e-01 8.801688e-01 1.616010e-01

904 9.233574e-01 9.153420e-01 9.047741e-01 8.944306e-01 8.801690e-01 1.628982e-01

905 9.233576e-01 9.153422e-01 9.047743e-01 8.944307e-01 8.801691e-01 1.642363e-01

906 9.233578e-01 9.153423e-01 9.047744e-01 8.944308e-01 8.801692e-01 1.656157e-01

907 9.233580e-01 9.153425e-01 9.047746e-01 8.944310e-01 8.801693e-01 1.670370e-01

908 9.233582e-01 9.153427e-01 9.047747e-01 8.944311e-01 8.801694e-01 1.685005e-01

909 9.233584e-01 9.153428e-01 9.047749e-01 8.944312e-01 8.801695e-01 1.700068e-01

910 9.233585e-01 9.153429e-01 9.047750e-01 8.944313e-01 8.801696e-01 1.715559e-01

911 9.233587e-01 9.153431e-01 9.047751e-01 8.944314e-01 8.801697e-01 1.731483e-01

912 9.233588e-01 9.153432e-01 9.047752e-01 8.944315e-01 8.801698e-01 1.747839e-01

913 9.233589e-01 9.153433e-01 9.047753e-01 8.944316e-01 8.801699e-01 1.764629e-01

914 9.233590e-01 9.153434e-01 9.047754e-01 8.944317e-01 8.801699e-01 1.781851e-01

915 9.233591e-01 9.153435e-01 9.047754e-01 8.944317e-01 8.801700e-01 1.799504e-01

916 9.233592e-01 9.153436e-01 9.047755e-01 8.944318e-01 8.801700e-01 1.817585e-01

917 9.233593e-01 9.153437e-01 9.047756e-01 8.944319e-01 8.801701e-01 1.836091e-01

918 9.233594e-01 9.153437e-01 9.047757e-01 8.944319e-01 8.801702e-01 1.855017e-01

919 9.233595e-01 9.153438e-01 9.047757e-01 8.944320e-01 8.801702e-01 1.874356e-01

920 9.233595e-01 9.153439e-01 9.047758e-01 8.944321e-01 8.801703e-01 1.894102e-01

921 9.233596e-01 9.153439e-01 9.047759e-01 8.944321e-01 8.801703e-01 1.914246e-01

922 9.233597e-01 9.153440e-01 9.047759e-01 8.944322e-01 8.801704e-01 1.934778e-01

923 9.233598e-01 9.153441e-01 9.047760e-01 8.944322e-01 8.801705e-01 1.955688e-01

924 9.233598e-01 9.153442e-01 9.047761e-01 8.944323e-01 8.801705e-01 1.976964e-01

925 9.233599e-01 9.153442e-01 9.047761e-01 8.944324e-01 8.801706e-01 1.998592e-01

926 9.233600e-01 9.153443e-01 9.047762e-01 8.944325e-01 8.801707e-01 2.020559e-01

927 9.233601e-01 9.153444e-01 9.047763e-01 8.944326e-01 8.801708e-01 2.042848e-01

928 9.233602e-01 9.153445e-01 9.047764e-01 8.944327e-01 8.801709e-01 2.065444e-01

929 9.233603e-01 9.153446e-01 9.047765e-01 8.944328e-01 8.801710e-01 2.088328e-01

930 9.233604e-01 9.153447e-01 9.047766e-01 8.944329e-01 8.801711e-01 2.111481e-01

931 9.233605e-01 9.153448e-01 9.047768e-01 8.944330e-01 8.801713e-01 2.134885e-01

932 9.233606e-01 9.153450e-01 9.047769e-01 8.944332e-01 8.801714e-01 2.158518e-01

933 9.233608e-01 9.153451e-01 9.047770e-01 8.944333e-01 8.801716e-01 2.182359e-01

934 9.233609e-01 9.153453e-01 9.047772e-01 8.944335e-01 8.801718e-01 2.206385e-01

935 9.233611e-01 9.153454e-01 9.047774e-01 8.944337e-01 8.801720e-01 2.230575e-01

936 9.233613e-01 9.153456e-01 9.047776e-01 8.944339e-01 8.801722e-01 2.254903e-01

937 9.233615e-01 9.153458e-01 9.047778e-01 8.944341e-01 8.801724e-01 2.279346e-01

938 9.233617e-01 9.153460e-01 9.047780e-01 8.944343e-01 8.801727e-01 2.303880e-01

939 9.233619e-01 9.153463e-01 9.047783e-01 8.944346e-01 8.801730e-01 2.328479e-01

940 9.233622e-01 9.153465e-01 9.047786e-01 8.944349e-01 8.801733e-01 2.353118e-01

941 9.233625e-01 9.153468e-01 9.047789e-01 8.944352e-01 8.801736e-01 2.377772e-01

942 9.233628e-01 9.153471e-01 9.047792e-01 8.944355e-01 8.801740e-01 2.402414e-01

943 9.233631e-01 9.153475e-01 9.047795e-01 8.944359e-01 8.801744e-01 2.427021e-01

944 9.233635e-01 9.153478e-01 9.047799e-01 8.944363e-01 8.801748e-01 2.451565e-01

945 9.233638e-01 9.153482e-01 9.047803e-01 8.944367e-01 8.801753e-01 2.476023e-01

946 9.233643e-01 9.153487e-01 9.047808e-01 8.944372e-01 8.801758e-01 2.500371e-01

947 9.233647e-01 9.153491e-01 9.047813e-01 8.944377e-01 8.801763e-01 2.524583e-01

948 9.233652e-01 9.153496e-01 9.047818e-01 8.944382e-01 8.801769e-01 2.548638e-01

949 9.233657e-01 9.153502e-01 9.047823e-01 8.944388e-01 8.801775e-01 2.572511e-01

950 9.233663e-01 9.153507e-01 9.047829e-01 8.944394e-01 8.801782e-01 2.596182e-01

951 9.233669e-01 9.153513e-01 9.047836e-01 8.944401e-01 8.801789e-01 2.619628e-01

952 9.233675e-01 9.153520e-01 9.047843e-01 8.944408e-01 8.801796e-01 2.642828e-01

953 9.233682e-01 9.153527e-01 9.047850e-01 8.944416e-01 8.801805e-01 2.665764e-01

954 9.233689e-01 9.153535e-01 9.047858e-01 8.944424e-01 8.801814e-01 2.688416e-01

955 9.233697e-01 9.153543e-01 9.047867e-01 8.944433e-01 8.801823e-01 2.710765e-01

956 9.233706e-01 9.153552e-01 9.047876e-01 8.944443e-01 8.801834e-01 2.732796e-01

957 9.233715e-01 9.153561e-01 9.047886e-01 8.944453e-01 8.801845e-01 2.754492e-01

958 9.233725e-01 9.153571e-01 9.047897e-01 8.944464e-01 8.801857e-01 2.775837e-01

959 9.233735e-01 9.153582e-01 9.047908e-01 8.944476e-01 8.801869e-01 2.796819e-01

960 9.233747e-01 9.153594e-01 9.047920e-01 8.944489e-01 8.801883e-01 2.817423e-01

961 9.233759e-01 9.153606e-01 9.047933e-01 8.944503e-01 8.801898e-01 2.837639e-01

962 9.233772e-01 9.153620e-01 9.047948e-01 8.944517e-01 8.801914e-01 2.857455e-01

963 9.233785e-01 9.153634e-01 9.047963e-01 8.944533e-01 8.801931e-01 2.876862e-01

964 9.233800e-01 9.153649e-01 9.047979e-01 8.944550e-01 8.801950e-01 2.895851e-01

965 9.233816e-01 9.153666e-01 9.047996e-01 8.944569e-01 8.801969e-01 2.914416e-01

966 9.233833e-01 9.153683e-01 9.048015e-01 8.944588e-01 8.801991e-01 2.932550e-01

967 9.233852e-01 9.153702e-01 9.048035e-01 8.944609e-01 8.802014e-01 2.950248e-01

968 9.233871e-01 9.153723e-01 9.048057e-01 8.944632e-01 8.802039e-01 2.967505e-01

969 9.233892e-01 9.153745e-01 9.048080e-01 8.944657e-01 8.802066e-01 2.984320e-01

970 9.233915e-01 9.153768e-01 9.048105e-01 8.944683e-01 8.802095e-01 3.000690e-01

971 9.233939e-01 9.153793e-01 9.048132e-01 8.944712e-01 8.802126e-01 3.016614e-01

972 9.233965e-01 9.153820e-01 9.048161e-01 8.944742e-01 8.802160e-01 3.032092e-01

973 9.233993e-01 9.153850e-01 9.048192e-01 8.944775e-01 8.802196e-01 3.047126e-01

974 9.234023e-01 9.153881e-01 9.048226e-01 8.944811e-01 8.802235e-01 3.061717e-01

975 9.234055e-01 9.153915e-01 9.048262e-01 8.944849e-01 8.802278e-01 3.075868e-01

976 9.234090e-01 9.153951e-01 9.048301e-01 8.944891e-01 8.802324e-01 3.089583e-01

977 9.234127e-01 9.153990e-01 9.048343e-01 8.944935e-01 8.802374e-01 3.102865e-01

978 9.234167e-01 9.154032e-01 9.048389e-01 8.944984e-01 8.802427e-01 3.115721e-01

979 9.234210e-01 9.154077e-01 9.048438e-01 8.945036e-01 8.802486e-01 3.128155e-01

980 9.234257e-01 9.154126e-01 9.048490e-01 8.945092e-01 8.802549e-01 3.140174e-01

981 9.234307e-01 9.154179e-01 9.048547e-01 8.945153e-01 8.802617e-01 3.151786e-01

982 9.234361e-01 9.154236e-01 9.048609e-01 8.945219e-01 8.802691e-01 3.162996e-01

983 9.234419e-01 9.154297e-01 9.048675e-01 8.945291e-01 8.802771e-01 3.173814e-01

984 9.234481e-01 9.154363e-01 9.048747e-01 8.945368e-01 8.802858e-01 3.184247e-01

985 9.234548e-01 9.154434e-01 9.048825e-01 8.945451e-01 8.802952e-01 3.194304e-01

986 9.234621e-01 9.154511e-01 9.048909e-01 8.945542e-01 8.803054e-01 3.203994e-01

987 9.234699e-01 9.154594e-01 9.048999e-01 8.945640e-01 8.803165e-01 3.213326e-01

988 9.234783e-01 9.154683e-01 9.049097e-01 8.945746e-01 8.803286e-01 3.222309e-01

989 9.234874e-01 9.154780e-01 9.049204e-01 8.945861e-01 8.803417e-01 3.230953e-01

990 9.234972e-01 9.154885e-01 9.049318e-01 8.945986e-01 8.803559e-01 3.239267e-01

991 9.235078e-01 9.154998e-01 9.049443e-01 8.946121e-01 8.803713e-01 3.247262e-01

992 9.235193e-01 9.155120e-01 9.049578e-01 8.946267e-01 8.803881e-01 3.254947e-01

993 9.235316e-01 9.155252e-01 9.049723e-01 8.946427e-01 8.804064e-01 3.262331e-01

994 9.235450e-01 9.155395e-01 9.049882e-01 8.946599e-01 8.804262e-01 3.269424e-01

995 9.235594e-01 9.155550e-01 9.050053e-01 8.946787e-01 8.804478e-01 3.276235e-01

996 9.235749e-01 9.155717e-01 9.050239e-01 8.946990e-01 8.804713e-01 3.282773e-01

997 9.235918e-01 9.155898e-01 9.050440e-01 8.947212e-01 8.804969e-01 3.289046e-01

998 9.236100e-01 9.156095e-01 9.050659e-01 8.947452e-01 8.805248e-01 3.295064e-01

999 9.236297e-01 9.156307e-01 9.050896e-01 8.947713e-01 8.805551e-01 3.300835e-01

1000 9.236509e-01 9.156537e-01 9.051153e-01 8.947997e-01 8.805882e-01 3.306367e-01

1001 9.236740e-01 9.156787e-01 9.051433e-01 8.948306e-01 8.806242e-01 3.311668e-01

1002 9.236989e-01 9.157057e-01 9.051736e-01 8.948641e-01 8.806635e-01 3.316746e-01

1003 9.237258e-01 9.157350e-01 9.052065e-01 8.949007e-01 8.807063e-01 3.321610e-01

1004 9.237550e-01 9.157668e-01 9.052423e-01 8.949404e-01 8.807529e-01 3.326266e-01

1005 9.237866e-01 9.158012e-01 9.052812e-01 8.949837e-01 8.808039e-01 3.330722e-01

1006 9.238209e-01 9.158386e-01 9.053234e-01 8.950308e-01 8.808594e-01 3.334985e-01

1007 9.238579e-01 9.158791e-01 9.053693e-01 8.950820e-01 8.809200e-01 3.339062e-01

1008 9.238981e-01 9.159231e-01 9.054192e-01 8.951379e-01 8.809862e-01 3.342961e-01

1009 9.239416e-01 9.159708e-01 9.054734e-01 8.951987e-01 8.810584e-01 3.346687e-01

1010 9.239887e-01 9.160226e-01 9.055324e-01 8.952649e-01 8.811373e-01 3.350248e-01

1011 9.240398e-01 9.160789e-01 9.055966e-01 8.953371e-01 8.812234e-01 3.353649e-01

1012 9.240952e-01 9.161399e-01 9.056664e-01 8.954158e-01 8.813174e-01 3.356898e-01

1013 9.241552e-01 9.162062e-01 9.057424e-01 8.955015e-01 8.814201e-01 3.360000e-01

1014 9.242203e-01 9.162782e-01 9.058250e-01 8.955950e-01 8.815322e-01 3.362960e-01

1015 9.242909e-01 9.163564e-01 9.059149e-01 8.956969e-01 8.816547e-01 3.365785e-01

1016 9.243674e-01 9.164414e-01 9.060127e-01 8.958079e-01 8.817885e-01 3.368479e-01

1017 9.244504e-01 9.165337e-01 9.061192e-01 8.959289e-01 8.819347e-01 3.371049e-01

1018 9.245404e-01 9.166339e-01 9.062351e-01 8.960609e-01 8.820943e-01 3.373499e-01

1019 9.246380e-01 9.167428e-01 9.063612e-01 8.962047e-01 8.822686e-01 3.375835e-01

1020 9.247439e-01 9.168611e-01 9.064984e-01 8.963615e-01 8.824589e-01 3.378061e-01

1021 9.248587e-01 9.169896e-01 9.066477e-01 8.965324e-01 8.826667e-01 3.380181e-01

1022 9.249832e-01 9.171292e-01 9.068102e-01 8.967187e-01 8.828936e-01 3.382201e-01

1023 9.251182e-01 9.172808e-01 9.069870e-01 8.969217e-01 8.831412e-01 3.384124e-01

1024 9.252647e-01 9.174455e-01 9.071794e-01 8.971428e-01 8.834113e-01 3.385955e-01

1025 9.254234e-01 9.176243e-01 9.073886e-01 8.973837e-01 8.837059e-01 3.387698e-01

1026 9.255956e-01 9.178185e-01 9.076161e-01 8.976459e-01 8.840271e-01 3.389356e-01

1027 9.257822e-01 9.180294e-01 9.078635e-01 8.979315e-01 8.843771e-01 3.390934e-01

1028 9.259845e-01 9.182582e-01 9.081323e-01 8.982422e-01 8.847584e-01 3.392434e-01

1029 9.262037e-01 9.185065e-01 9.084244e-01 8.985801e-01 8.851734e-01 3.393861e-01

1030 9.264411e-01 9.187759e-01 9.087417e-01 8.989476e-01 8.856250e-01 3.395218e-01

1031 9.266983e-01 9.190681e-01 9.090862e-01 8.993469e-01 8.861160e-01 3.396508e-01

1032 9.269768e-01 9.193848e-01 9.094601e-01 8.997806e-01 8.866493e-01 3.397734e-01

1033 9.272782e-01 9.197280e-01 9.098656e-01 9.002513e-01 8.872283e-01 3.398899e-01

1034 9.276043e-01 9.200997e-01 9.103052e-01 9.007618e-01 8.878563e-01 3.400005e-01

1035 9.279570e-01 9.205021e-01 9.107814e-01 9.013152e-01 8.885366e-01 3.401056e-01

1036 9.283383e-01 9.209376e-01 9.112969e-01 9.019144e-01 8.892730e-01 3.402054e-01

1037 9.287503e-01 9.214084e-01 9.118547e-01 9.025627e-01 8.900690e-01 3.403001e-01

1038 9.291952e-01 9.219172e-01 9.124576e-01 9.032635e-01 8.909286e-01 3.403901e-01

1039 9.296753e-01 9.224667e-01 9.131087e-01 9.040202e-01 8.918556e-01 3.404754e-01

1040 9.301931e-01 9.230596e-01 9.138114e-01 9.048364e-01 8.928539e-01 3.405564e-01

1041 9.307513e-01 9.236989e-01 9.145687e-01 9.057157e-01 8.939273e-01 3.406333e-01

1042 9.313525e-01 9.243875e-01 9.153843e-01 9.066617e-01 8.950798e-01 3.407061e-01

1043 9.319997e-01 9.251287e-01 9.162614e-01 9.076782e-01 8.963151e-01 3.407753e-01

1044 9.326958e-01 9.259255e-01 9.172038e-01 9.087689e-01 8.976369e-01 3.408408e-01

1045 9.334440e-01 9.267814e-01 9.182148e-01 9.099374e-01 8.990486e-01 3.409030e-01

1046 9.342473e-01 9.276997e-01 9.192981e-01 9.111874e-01 9.005536e-01 3.409619e-01

1047 9.351093e-01 9.286840e-01 9.204572e-01 9.125222e-01 9.021549e-01 3.410177e-01

1048 9.360332e-01 9.297377e-01 9.216956e-01 9.139452e-01 9.038550e-01 3.410706e-01

1049 9.370226e-01 9.308643e-01 9.230167e-01 9.154595e-01 9.056562e-01 3.411207e-01

1050 9.380810e-01 9.320675e-01 9.244238e-01 9.170679e-01 9.075605e-01 3.411682e-01

1051 9.392120e-01 9.333506e-01 9.259202e-01 9.187730e-01 9.095692e-01 3.412132e-01

1052 9.404191e-01 9.347173e-01 9.275089e-01 9.205771e-01 9.116830e-01 3.412558e-01

1053 9.417058e-01 9.361706e-01 9.291925e-01 9.224821e-01 9.139024e-01 3.412961e-01

1054 9.430758e-01 9.377140e-01 9.309738e-01 9.244895e-01 9.162271e-01 3.413343e-01

1055 9.445324e-01 9.393503e-01 9.328548e-01 9.266005e-01 9.186562e-01 3.413704e-01

1056 9.460788e-01 9.410824e-01 9.348374e-01 9.288156e-01 9.211883e-01 3.414046e-01

1057 9.477183e-01 9.429128e-01 9.369232e-01 9.311351e-01 9.238216e-01 3.414369e-01

1058 9.494537e-01 9.448437e-01 9.391132e-01 9.335584e-01 9.265532e-01 3.414675e-01

1059 9.512876e-01 9.468770e-01 9.414080e-01 9.360847e-01 9.293802e-01 3.414965e-01

1060 9.532225e-01 9.490142e-01 9.438077e-01 9.387124e-01 9.322987e-01 3.415239e-01

1061 9.552605e-01 9.512564e-01 9.463119e-01 9.414395e-01 9.353045e-01 3.415498e-01

1062 9.574031e-01 9.536042e-01 9.489197e-01 9.442633e-01 9.383926e-01 3.415743e-01

1063 9.596517e-01 9.560577e-01 9.516296e-01 9.471806e-01 9.415577e-01 3.415974e-01

1064 9.620071e-01 9.586165e-01 9.544395e-01 9.501877e-01 9.447940e-01 3.416193e-01

1065 9.644697e-01 9.612797e-01 9.573470e-01 9.532803e-01 9.480953e-01 3.416400e-01

1066 9.670393e-01 9.640460e-01 9.603489e-01 9.564535e-01 9.514549e-01 3.416595e-01

1067 9.697154e-01 9.669134e-01 9.634415e-01 9.597021e-01 9.548659e-01 3.416780e-01

1068 9.724968e-01 9.698792e-01 9.666208e-01 9.630205e-01 9.583211e-01 3.416955e-01

1069 9.753818e-01 9.729407e-01 9.698821e-01 9.664026e-01 9.618131e-01 3.417119e-01

1070 9.783683e-01 9.760941e-01 9.732205e-01 9.698419e-01 9.653341e-01 3.417275e-01

1071 9.814535e-01 9.793355e-01 9.766304e-01 9.733317e-01 9.688764e-01 3.417422e-01

1072 9.846342e-01 9.826606e-01 9.801061e-01 9.768651e-01 9.724321e-01 3.417561e-01

1073 9.879069e-01 9.860642e-01 9.836415e-01 9.804348e-01 9.759935e-01 3.417692e-01

1074 9.912674e-01 9.895413e-01 9.872301e-01 9.840334e-01 9.795525e-01 3.417815e-01

1075 9.950745e-01 9.935703e-01 9.915125e-01 9.885333e-01 9.842959e-01 3.471574e-01

1076 9.950743e-01 9.935702e-01 9.915124e-01 9.885333e-01 9.842959e-01 3.473858e-01

1077 9.950742e-01 9.935702e-01 9.915124e-01 9.885332e-01 9.842959e-01 3.476230e-01

1078 9.950741e-01 9.935701e-01 9.915123e-01 9.885332e-01 9.842959e-01 3.478691e-01

1079 9.950740e-01 9.935701e-01 9.915123e-01 9.885332e-01 9.842960e-01 3.481247e-01

1080 9.950739e-01 9.935700e-01 9.915123e-01 9.885332e-01 9.842960e-01 3.483899e-01

1081 9.950739e-01 9.935700e-01 9.915123e-01 9.885333e-01 9.842960e-01 3.486652e-01

1082 9.950739e-01 9.935700e-01 9.915123e-01 9.885333e-01 9.842960e-01 3.489508e-01

1083 9.950738e-01 9.935700e-01 9.915124e-01 9.885333e-01 9.842961e-01 3.492473e-01

1084 9.950738e-01 9.935700e-01 9.915124e-01 9.885333e-01 9.842961e-01 3.495549e-01

1085 9.950738e-01 9.935700e-01 9.915124e-01 9.885334e-01 9.842961e-01 3.498741e-01

1086 9.950738e-01 9.935701e-01 9.915124e-01 9.885334e-01 9.842962e-01 3.502052e-01

1087 9.950739e-01 9.935701e-01 9.915125e-01 9.885334e-01 9.842962e-01 3.505487e-01

1088 9.950739e-01 9.935701e-01 9.915125e-01 9.885335e-01 9.842963e-01 3.509051e-01

1089 9.950739e-01 9.935702e-01 9.915125e-01 9.885335e-01 9.842963e-01 3.512748e-01

1090 9.950739e-01 9.935702e-01 9.915126e-01 9.885336e-01 9.842963e-01 3.516581e-01

1091 9.950740e-01 9.935702e-01 9.915126e-01 9.885336e-01 9.842964e-01 3.520557e-01

1092 9.950740e-01 9.935703e-01 9.915127e-01 9.885336e-01 9.842964e-01 3.524680e-01

1093 9.950741e-01 9.935703e-01 9.915127e-01 9.885337e-01 9.842965e-01 3.528955e-01

1094 9.950741e-01 9.935704e-01 9.915128e-01 9.885337e-01 9.842965e-01 3.533387e-01

1095 9.950742e-01 9.935704e-01 9.915128e-01 9.885338e-01 9.842965e-01 3.537982e-01

1096 9.950742e-01 9.935705e-01 9.915129e-01 9.885338e-01 9.842966e-01 3.542744e-01

1097 9.950743e-01 9.935705e-01 9.915129e-01 9.885339e-01 9.842966e-01 3.547679e-01

1098 9.950743e-01 9.935706e-01 9.915130e-01 9.885339e-01 9.842966e-01 3.552793e-01

1099 9.950744e-01 9.935706e-01 9.915130e-01 9.885339e-01 9.842967e-01 3.558092e-01

1100 9.950745e-01 9.935707e-01 9.915130e-01 9.885340e-01 9.842967e-01 3.563580e-01

1101 9.950745e-01 9.935707e-01 9.915131e-01 9.885340e-01 9.842967e-01 3.569265e-01

1102 9.950746e-01 9.935708e-01 9.915131e-01 9.885340e-01 9.842968e-01 3.575152e-01

1103 9.950746e-01 9.935708e-01 9.915132e-01 9.885341e-01 9.842968e-01 3.581246e-01

1104 9.950747e-01 9.935709e-01 9.915132e-01 9.885341e-01 9.842968e-01 3.587555e-01

1105 9.950747e-01 9.935709e-01 9.915133e-01 9.885342e-01 9.842969e-01 3.594084e-01

1106 9.950748e-01 9.935710e-01 9.915133e-01 9.885342e-01 9.842969e-01 3.600839e-01

1107 9.950748e-01 9.935710e-01 9.915133e-01 9.885342e-01 9.842969e-01 3.607826e-01

1108 9.950749e-01 9.935711e-01 9.915134e-01 9.885343e-01 9.842970e-01 3.615052e-01

1109 9.950750e-01 9.935711e-01 9.915134e-01 9.885343e-01 9.842970e-01 3.622522e-01

1110 9.950750e-01 9.935711e-01 9.915135e-01 9.885343e-01 9.842970e-01 3.630244e-01

1111 9.950751e-01 9.935712e-01 9.915135e-01 9.885344e-01 9.842971e-01 3.638222e-01

1112 9.950751e-01 9.935712e-01 9.915135e-01 9.885344e-01 9.842971e-01 3.646462e-01

1113 9.950752e-01 9.935713e-01 9.915136e-01 9.885344e-01 9.842971e-01 3.654971e-01

1114 9.950752e-01 9.935713e-01 9.915136e-01 9.885345e-01 9.842972e-01 3.663755e-01

1115 9.950752e-01 9.935714e-01 9.915137e-01 9.885345e-01 9.842972e-01 3.672817e-01

1116 9.950753e-01 9.935714e-01 9.915137e-01 9.885345e-01 9.842972e-01 3.682165e-01

1117 9.950753e-01 9.935715e-01 9.915137e-01 9.885346e-01 9.842973e-01 3.691803e-01

1118 9.950754e-01 9.935715e-01 9.915138e-01 9.885346e-01 9.842973e-01 3.701736e-01

1119 9.950754e-01 9.935715e-01 9.915138e-01 9.885347e-01 9.842974e-01 3.711968e-01

1120 9.950755e-01 9.935716e-01 9.915139e-01 9.885347e-01 9.842974e-01 3.722505e-01

1121 9.950755e-01 9.935716e-01 9.915139e-01 9.885348e-01 9.842974e-01 3.733348e-01

1122 9.950756e-01 9.935717e-01 9.915140e-01 9.885348e-01 9.842975e-01 3.744503e-01

1123 9.950757e-01 9.935717e-01 9.915140e-01 9.885349e-01 9.842976e-01 3.755972e-01

1124 9.950757e-01 9.935718e-01 9.915141e-01 9.885349e-01 9.842976e-01 3.767758e-01

1125 9.950758e-01 9.935719e-01 9.915141e-01 9.885350e-01 9.842977e-01 3.779862e-01

1126 9.950758e-01 9.935719e-01 9.915142e-01 9.885350e-01 9.842977e-01 3.792287e-01

1127 9.950759e-01 9.935720e-01 9.915143e-01 9.885351e-01 9.842978e-01 3.805033e-01

1128 9.950760e-01 9.935720e-01 9.915143e-01 9.885352e-01 9.842979e-01 3.818101e-01

1129 9.950760e-01 9.935721e-01 9.915144e-01 9.885352e-01 9.842979e-01 3.831491e-01

1130 9.950761e-01 9.935722e-01 9.915145e-01 9.885353e-01 9.842980e-01 3.845200e-01

1131 9.950762e-01 9.935723e-01 9.915146e-01 9.885354e-01 9.842981e-01 3.859229e-01

1132 9.950763e-01 9.935723e-01 9.915146e-01 9.885355e-01 9.842982e-01 3.873573e-01

1133 9.950763e-01 9.935724e-01 9.915147e-01 9.885356e-01 9.842983e-01 3.888231e-01

1134 9.950764e-01 9.935725e-01 9.915148e-01 9.885357e-01 9.842984e-01 3.903198e-01

1135 9.950765e-01 9.935726e-01 9.915149e-01 9.885358e-01 9.842985e-01 3.918469e-01

1136 9.950766e-01 9.935727e-01 9.915150e-01 9.885359e-01 9.842986e-01 3.934038e-01

1137 9.950767e-01 9.935728e-01 9.915151e-01 9.885360e-01 9.842987e-01 3.949899e-01

1138 9.950769e-01 9.935729e-01 9.915153e-01 9.885361e-01 9.842989e-01 3.966045e-01

1139 9.950770e-01 9.935731e-01 9.915154e-01 9.885362e-01 9.842990e-01 3.982467e-01

1140 9.950771e-01 9.935732e-01 9.915155e-01 9.885364e-01 9.842992e-01 3.999156e-01

1141 9.950772e-01 9.935733e-01 9.915157e-01 9.885365e-01 9.842993e-01 4.016103e-01

1142 9.950774e-01 9.935735e-01 9.915158e-01 9.885367e-01 9.842995e-01 4.033295e-01

1143 9.950775e-01 9.935736e-01 9.915160e-01 9.885369e-01 9.842997e-01 4.050722e-01

1144 9.950777e-01 9.935738e-01 9.915162e-01 9.885370e-01 9.842999e-01 4.068371e-01

1145 9.950779e-01 9.935740e-01 9.915164e-01 9.885372e-01 9.843001e-01 4.086228e-01

1146 9.950780e-01 9.935742e-01 9.915166e-01 9.885374e-01 9.843003e-01 4.104279e-01

1147 9.950782e-01 9.935744e-01 9.915168e-01 9.885376e-01 9.843005e-01 4.122510e-01

1148 9.950784e-01 9.935746e-01 9.915170e-01 9.885379e-01 9.843008e-01 4.140905e-01

1149 9.950787e-01 9.935748e-01 9.915172e-01 9.885381e-01 9.843010e-01 4.159448e-01

1150 9.950789e-01 9.935751e-01 9.915175e-01 9.885384e-01 9.843013e-01 4.178121e-01

1151 9.950791e-01 9.935753e-01 9.915178e-01 9.885387e-01 9.843016e-01 4.196907e-01

1152 9.950794e-01 9.935756e-01 9.915180e-01 9.885390e-01 9.843019e-01 4.215789e-01

1153 9.950797e-01 9.935759e-01 9.915183e-01 9.885393e-01 9.843023e-01 4.234748e-01

1154 9.950800e-01 9.935762e-01 9.915187e-01 9.885396e-01 9.843026e-01 4.253765e-01

1155 9.950803e-01 9.935765e-01 9.915190e-01 9.885400e-01 9.843030e-01 4.272821e-01

1156 9.950806e-01 9.935768e-01 9.915194e-01 9.885404e-01 9.843034e-01 4.291897e-01

1157 9.950810e-01 9.935772e-01 9.915198e-01 9.885408e-01 9.843039e-01 4.310974e-01

1158 9.950813e-01 9.935776e-01 9.915202e-01 9.885412e-01 9.843044e-01 4.330032e-01

1159 9.950817e-01 9.935780e-01 9.915206e-01 9.885417e-01 9.843049e-01 4.349052e-01

1160 9.950822e-01 9.935785e-01 9.915211e-01 9.885422e-01 9.843054e-01 4.368013e-01

1161 9.950826e-01 9.935789e-01 9.915216e-01 9.885427e-01 9.843060e-01 4.386898e-01

1162 9.950831e-01 9.935795e-01 9.915222e-01 9.885433e-01 9.843066e-01 4.405687e-01

1163 9.950836e-01 9.935800e-01 9.915227e-01 9.885439e-01 9.843073e-01 4.424360e-01

1164 9.950841e-01 9.935806e-01 9.915233e-01 9.885445e-01 9.843080e-01 4.442900e-01

1165 9.950847e-01 9.935812e-01 9.915240e-01 9.885452e-01 9.843087e-01 4.461288e-01

1166 9.950853e-01 9.935818e-01 9.915247e-01 9.885459e-01 9.843095e-01 4.479508e-01

1167 9.950860e-01 9.935825e-01 9.915254e-01 9.885467e-01 9.843104e-01 4.497542e-01

1168 9.950867e-01 9.935832e-01 9.915262e-01 9.885476e-01 9.843113e-01 4.515373e-01

1169 9.950874e-01 9.935840e-01 9.915271e-01 9.885485e-01 9.843123e-01 4.532987e-01

1170 9.950882e-01 9.935849e-01 9.915280e-01 9.885494e-01 9.843134e-01 4.550367e-01

1171 9.950891e-01 9.935858e-01 9.915289e-01 9.885504e-01 9.843145e-01 4.567501e-01

1172 9.950899e-01 9.935867e-01 9.915300e-01 9.885515e-01 9.843158e-01 4.584374e-01

1173 9.950909e-01 9.935877e-01 9.915311e-01 9.885527e-01 9.843171e-01 4.600975e-01

1174 9.950919e-01 9.935888e-01 9.915323e-01 9.885540e-01 9.843185e-01 4.617292e-01

1175 9.950930e-01 9.935900e-01 9.915335e-01 9.885553e-01 9.843200e-01 4.633315e-01

1176 9.950941e-01 9.935912e-01 9.915349e-01 9.885568e-01 9.843216e-01 4.649034e-01

1177 9.950954e-01 9.935925e-01 9.915363e-01 9.885583e-01 9.843234e-01 4.664443e-01

1178 9.950967e-01 9.935939e-01 9.915378e-01 9.885600e-01 9.843252e-01 4.679533e-01

1179 9.950981e-01 9.935954e-01 9.915395e-01 9.885618e-01 9.843272e-01 4.694298e-01

1180 9.950996e-01 9.935971e-01 9.915412e-01 9.885637e-01 9.843294e-01 4.708733e-01

1181 9.951012e-01 9.935988e-01 9.915431e-01 9.885658e-01 9.843317e-01 4.722833e-01

1182 9.951029e-01 9.936006e-01 9.915452e-01 9.885680e-01 9.843342e-01 4.736596e-01

1183 9.951047e-01 9.936026e-01 9.915473e-01 9.885703e-01 9.843369e-01 4.750018e-01

1184 9.951066e-01 9.936047e-01 9.915496e-01 9.885728e-01 9.843398e-01 4.763099e-01

1185 9.951087e-01 9.936069e-01 9.915521e-01 9.885756e-01 9.843429e-01 4.775836e-01

1186 9.951109e-01 9.936093e-01 9.915548e-01 9.885785e-01 9.843462e-01 4.788229e-01

1187 9.951132e-01 9.936119e-01 9.915576e-01 9.885816e-01 9.843498e-01 4.800280e-01

1188 9.951157e-01 9.936147e-01 9.915607e-01 9.885850e-01 9.843537e-01 4.811990e-01

1189 9.951184e-01 9.936176e-01 9.915640e-01 9.885886e-01 9.843579e-01 4.823360e-01

1190 9.951213e-01 9.936208e-01 9.915675e-01 9.885925e-01 9.843624e-01 4.834394e-01

1191 9.951244e-01 9.936241e-01 9.915713e-01 9.885967e-01 9.843672e-01 4.845094e-01

1192 9.951276e-01 9.936278e-01 9.915754e-01 9.886013e-01 9.843725e-01 4.855464e-01

1193 9.951311e-01 9.936317e-01 9.915797e-01 9.886061e-01 9.843781e-01 4.865509e-01

1194 9.951349e-01 9.936358e-01 9.915844e-01 9.886113e-01 9.843842e-01 4.875233e-01

1195 9.951389e-01 9.936403e-01 9.915894e-01 9.886170e-01 9.843907e-01 4.884640e-01

1196 9.951432e-01 9.936451e-01 9.915948e-01 9.886230e-01 9.843978e-01 4.893738e-01

1197 9.951478e-01 9.936502e-01 9.916006e-01 9.886296e-01 9.844055e-01 4.902532e-01

1198 9.951527e-01 9.936557e-01 9.916069e-01 9.886366e-01 9.844137e-01 4.911027e-01

1199 9.951580e-01 9.936616e-01 9.916136e-01 9.886442e-01 9.844226e-01 4.919231e-01

1200 9.951637e-01 9.936680e-01 9.916208e-01 9.886524e-01 9.844323e-01 4.927149e-01

1201 9.951697e-01 9.936748e-01 9.916285e-01 9.886612e-01 9.844427e-01 4.934790e-01

1202 9.951762e-01 9.936821e-01 9.916369e-01 9.886707e-01 9.844539e-01 4.942159e-01

1203 9.951832e-01 9.936899e-01 9.916459e-01 9.886809e-01 9.844661e-01 4.949265e-01

1204 9.951906e-01 9.936984e-01 9.916556e-01 9.886920e-01 9.844793e-01 4.956114e-01

1205 9.951986e-01 9.937074e-01 9.916660e-01 9.887040e-01 9.844935e-01 4.962714e-01

1206 9.952072e-01 9.937172e-01 9.916772e-01 9.887168e-01 9.845089e-01 4.969071e-01

1207 9.952164e-01 9.937277e-01 9.916893e-01 9.887308e-01 9.845256e-01 4.975195e-01

1208 9.952262e-01 9.937389e-01 9.917023e-01 9.887458e-01 9.845436e-01 4.981091e-01

1209 9.952368e-01 9.937510e-01 9.917163e-01 9.887621e-01 9.845632e-01 4.986767e-01

1210 9.952482e-01 9.937641e-01 9.917314e-01 9.887796e-01 9.845843e-01 4.992231e-01

1211 9.952603e-01 9.937781e-01 9.917477e-01 9.887986e-01 9.846073e-01 4.997490e-01

1212 9.952734e-01 9.937931e-01 9.917653e-01 9.888191e-01 9.846321e-01 5.002551e-01

1213 9.952875e-01 9.938094e-01 9.917843e-01 9.888413e-01 9.846590e-01 5.007420e-01

1214 9.953025e-01 9.938268e-01 9.918047e-01 9.888652e-01 9.846882e-01 5.012104e-01

1215 9.953187e-01 9.938456e-01 9.918268e-01 9.888912e-01 9.847198e-01 5.016610e-01

1216 9.953361e-01 9.938659e-01 9.918506e-01 9.889192e-01 9.847541e-01 5.020943e-01

1217 9.953549e-01 9.938877e-01 9.918763e-01 9.889496e-01 9.847912e-01 5.025110e-01

1218 9.953750e-01 9.939112e-01 9.919041e-01 9.889824e-01 9.848315e-01 5.029115e-01

1219 9.953966e-01 9.939365e-01 9.919341e-01 9.890180e-01 9.848752e-01 5.032965e-01

1220 9.954198e-01 9.939638e-01 9.919664e-01 9.890565e-01 9.849226e-01 5.036665e-01

1221 9.954448e-01 9.939931e-01 9.920014e-01 9.890981e-01 9.849740e-01 5.040219e-01

1222 9.954716e-01 9.940248e-01 9.920391e-01 9.891432e-01 9.850298e-01 5.043633e-01

1223 9.955005e-01 9.940590e-01 9.920799e-01 9.891920e-01 9.850903e-01 5.046912e-01

1224 9.955316e-01 9.940958e-01 9.921239e-01 9.892449e-01 9.851559e-01 5.050060e-01

1225 9.955650e-01 9.941355e-01 9.921716e-01 9.893022e-01 9.852270e-01 5.053081e-01

1226 9.956010e-01 9.941783e-01 9.922230e-01 9.893642e-01 9.853042e-01 5.055981e-01

1227 9.956397e-01 9.942245e-01 9.922786e-01 9.894313e-01 9.853879e-01 5.058764e-01

1228 9.956813e-01 9.942743e-01 9.923387e-01 9.895040e-01 9.854787e-01 5.061433e-01

1229 9.957261e-01 9.943280e-01 9.924036e-01 9.895828e-01 9.855771e-01 5.063992e-01

1230 9.957744e-01 9.943860e-01 9.924738e-01 9.896680e-01 9.856838e-01 5.066447e-01

1231 9.958263e-01 9.944485e-01 9.925497e-01 9.897603e-01 9.857995e-01 5.068800e-01

1232 9.958823e-01 9.945160e-01 9.926317e-01 9.898603e-01 9.859248e-01 5.071055e-01

1233 9.959425e-01 9.945888e-01 9.927203e-01 9.899684e-01 9.860605e-01 5.073216e-01

1234 9.960073e-01 9.946673e-01 9.928161e-01 9.900855e-01 9.862075e-01 5.075286e-01

1235 9.960771e-01 9.947521e-01 9.929196e-01 9.902122e-01 9.863666e-01 5.077269e-01

1236 9.961522e-01 9.948435e-01 9.930314e-01 9.903492e-01 9.865388e-01 5.079168e-01

1237 9.962332e-01 9.949421e-01 9.931522e-01 9.904975e-01 9.867250e-01 5.080986e-01

1238 9.963203e-01 9.950485e-01 9.932827e-01 9.906577e-01 9.869263e-01 5.082726e-01

1239 9.964141e-01 9.951633e-01 9.934236e-01 9.908310e-01 9.871437e-01 5.084392e-01

1240 9.965151e-01 9.952871e-01 9.935758e-01 9.910181e-01 9.873785e-01 5.085985e-01

1241 9.966239e-01 9.954206e-01 9.937401e-01 9.912202e-01 9.876319e-01 5.087510e-01

1242 9.967409e-01 9.955645e-01 9.939174e-01 9.914384e-01 9.879050e-01 5.088968e-01

1243 9.968669e-01 9.957197e-01 9.941087e-01 9.916738e-01 9.881993e-01 5.090363e-01

1244 9.970026e-01 9.958869e-01 9.943150e-01 9.919276e-01 9.885160e-01 5.091696e-01

1245 9.971485e-01 9.960671e-01 9.945374e-01 9.922011e-01 9.888565e-01 5.092970e-01

1246 9.973056e-01 9.962612e-01 9.947770e-01 9.924956e-01 9.892224e-01 5.094188e-01

1247 9.974745e-01 9.964703e-01 9.950351e-01 9.928126e-01 9.896149e-01 5.095352e-01

1248 9.976562e-01 9.966954e-01 9.953130e-01 9.931534e-01 9.900356e-01 5.096464e-01

1249 9.978516e-01 9.969376e-01 9.956120e-01 9.935195e-01 9.904860e-01 5.097527e-01

1250 9.980617e-01 9.971981e-01 9.959334e-01 9.939124e-01 9.909675e-01 5.098541e-01

1251 9.982874e-01 9.974783e-01 9.962789e-01 9.943338e-01 9.914814e-01 5.099510e-01

1252 9.985299e-01 9.977794e-01 9.966498e-01 9.947852e-01 9.920292e-01 5.100435e-01

1253 9.987904e-01 9.981028e-01 9.970478e-01 9.952681e-01 9.926122e-01 5.101318e-01

1254 9.991289e-01 9.985538e-01 9.976509e-01 9.960891e-01 9.937305e-01 5.155608e-01

1255 9.991288e-01 9.985537e-01 9.976509e-01 9.960891e-01 9.937304e-01 5.156242e-01

1256 9.991287e-01 9.985536e-01 9.976509e-01 9.960890e-01 9.937304e-01 5.156900e-01

1257 9.991285e-01 9.985536e-01 9.976508e-01 9.960890e-01 9.937304e-01 5.157582e-01

1258 9.991284e-01 9.985535e-01 9.976508e-01 9.960889e-01 9.937303e-01 5.158290e-01

1259 9.991283e-01 9.985535e-01 9.976507e-01 9.960889e-01 9.937303e-01 5.159023e-01

1260 9.991282e-01 9.985534e-01 9.976507e-01 9.960889e-01 9.937303e-01 5.159785e-01

1261 9.991281e-01 9.985534e-01 9.976506e-01 9.960888e-01 9.937303e-01 5.160574e-01

1262 9.991281e-01 9.985533e-01 9.976506e-01 9.960888e-01 9.937302e-01 5.161392e-01

1263 9.991280e-01 9.985533e-01 9.976506e-01 9.960888e-01 9.937302e-01 5.162241e-01

1264 9.991279e-01 9.985532e-01 9.976505e-01 9.960888e-01 9.937302e-01 5.163121e-01

1265 9.991278e-01 9.985532e-01 9.976505e-01 9.960887e-01 9.937302e-01 5.164034e-01

1266 9.991278e-01 9.985531e-01 9.976505e-01 9.960887e-01 9.937301e-01 5.164980e-01

1267 9.991277e-01 9.985531e-01 9.976505e-01 9.960887e-01 9.937301e-01 5.165961e-01

1268 9.991277e-01 9.985531e-01 9.976504e-01 9.960887e-01 9.937301e-01 5.166979e-01

1269 9.991276e-01 9.985530e-01 9.976504e-01 9.960887e-01 9.937301e-01 5.168033e-01

1270 9.991276e-01 9.985530e-01 9.976504e-01 9.960886e-01 9.937301e-01 5.169127e-01

1271 9.991275e-01 9.985530e-01 9.976504e-01 9.960886e-01 9.937301e-01 5.170260e-01

1272 9.991275e-01 9.985530e-01 9.976503e-01 9.960886e-01 9.937301e-01 5.171435e-01

1273 9.991275e-01 9.985529e-01 9.976503e-01 9.960886e-01 9.937301e-01 5.172654e-01

1274 9.991274e-01 9.985529e-01 9.976503e-01 9.960886e-01 9.937301e-01 5.173917e-01

1275 9.991274e-01 9.985529e-01 9.976503e-01 9.960886e-01 9.937300e-01 5.175226e-01

1276 9.991274e-01 9.985529e-01 9.976503e-01 9.960886e-01 9.937300e-01 5.176583e-01

1277 9.991274e-01 9.985529e-01 9.976503e-01 9.960886e-01 9.937300e-01 5.177990e-01

1278 9.991274e-01 9.985529e-01 9.976503e-01 9.960886e-01 9.937300e-01 5.179449e-01

1279 9.991273e-01 9.985529e-01 9.976503e-01 9.960886e-01 9.937300e-01 5.180961e-01

1280 9.991273e-01 9.985529e-01 9.976503e-01 9.960885e-01 9.937300e-01 5.182528e-01

1281 9.991273e-01 9.985529e-01 9.976503e-01 9.960885e-01 9.937300e-01 5.184153e-01

1282 9.991273e-01 9.985529e-01 9.976503e-01 9.960885e-01 9.937300e-01 5.185837e-01

1283 9.991273e-01 9.985529e-01 9.976503e-01 9.960885e-01 9.937300e-01 5.187583e-01

1284 9.991273e-01 9.985529e-01 9.976503e-01 9.960885e-01 9.937300e-01 5.189392e-01

1285 9.991273e-01 9.985529e-01 9.976503e-01 9.960885e-01 9.937300e-01 5.191268e-01

1286 9.991273e-01 9.985529e-01 9.976503e-01 9.960885e-01 9.937300e-01 5.193212e-01

1287 9.991273e-01 9.985529e-01 9.976503e-01 9.960885e-01 9.937300e-01 5.195227e-01

1288 9.991273e-01 9.985529e-01 9.976503e-01 9.960885e-01 9.937299e-01 5.197315e-01

1289 9.991273e-01 9.985529e-01 9.976503e-01 9.960885e-01 9.937299e-01 5.199479e-01

1290 9.991273e-01 9.985529e-01 9.976503e-01 9.960885e-01 9.937299e-01 5.201722e-01

1291 9.991273e-01 9.985529e-01 9.976503e-01 9.960885e-01 9.937299e-01 5.204047e-01

1292 9.991273e-01 9.985529e-01 9.976503e-01 9.960885e-01 9.937299e-01 5.206456e-01

1293 9.991273e-01 9.985529e-01 9.976503e-01 9.960885e-01 9.937299e-01 5.208952e-01

1294 9.991274e-01 9.985529e-01 9.976503e-01 9.960885e-01 9.937299e-01 5.211539e-01

1295 9.991274e-01 9.985529e-01 9.976503e-01 9.960885e-01 9.937298e-01 5.214219e-01

1296 9.991274e-01 9.985529e-01 9.976503e-01 9.960885e-01 9.937298e-01 5.216995e-01

1297 9.991274e-01 9.985529e-01 9.976503e-01 9.960885e-01 9.937298e-01 5.219872e-01

1298 9.991274e-01 9.985529e-01 9.976503e-01 9.960884e-01 9.937298e-01 5.222852e-01

1299 9.991274e-01 9.985529e-01 9.976503e-01 9.960884e-01 9.937297e-01 5.225938e-01

1300 9.991274e-01 9.985529e-01 9.976503e-01 9.960884e-01 9.937297e-01 5.229135e-01

1301 9.991274e-01 9.985529e-01 9.976503e-01 9.960884e-01 9.937297e-01 5.232446e-01

1302 9.991274e-01 9.985529e-01 9.976503e-01 9.960884e-01 9.937297e-01 5.235875e-01

1303 9.991275e-01 9.985529e-01 9.976503e-01 9.960884e-01 9.937296e-01 5.239425e-01

1304 9.991275e-01 9.985529e-01 9.976503e-01 9.960884e-01 9.937296e-01 5.243101e-01

1305 9.991275e-01 9.985530e-01 9.976503e-01 9.960883e-01 9.937295e-01 5.246905e-01

1306 9.991275e-01 9.985530e-01 9.976502e-01 9.960883e-01 9.937295e-01 5.250843e-01

1307 9.991275e-01 9.985530e-01 9.976502e-01 9.960883e-01 9.937294e-01 5.254918e-01

1308 9.991275e-01 9.985530e-01 9.976502e-01 9.960883e-01 9.937294e-01 5.259135e-01

1309 9.991275e-01 9.985530e-01 9.976502e-01 9.960882e-01 9.937293e-01 5.263498e-01

1310 9.991276e-01 9.985530e-01 9.976502e-01 9.960882e-01 9.937293e-01 5.268010e-01

1311 9.991276e-01 9.985530e-01 9.976502e-01 9.960882e-01 9.937292e-01 5.272676e-01

1312 9.991276e-01 9.985530e-01 9.976502e-01 9.960881e-01 9.937292e-01 5.277501e-01

1313 9.991276e-01 9.985530e-01 9.976502e-01 9.960881e-01 9.937291e-01 5.282489e-01

1314 9.991276e-01 9.985530e-01 9.976502e-01 9.960881e-01 9.937290e-01 5.287644e-01

1315 9.991276e-01 9.985530e-01 9.976502e-01 9.960880e-01 9.937289e-01 5.292970e-01

1316 9.991277e-01 9.985530e-01 9.976502e-01 9.960880e-01 9.937289e-01 5.298473e-01

1317 9.991277e-01 9.985530e-01 9.976502e-01 9.960879e-01 9.937288e-01 5.304155e-01

1318 9.991277e-01 9.985530e-01 9.976501e-01 9.960879e-01 9.937287e-01 5.310022e-01

1319 9.991277e-01 9.985531e-01 9.976501e-01 9.960878e-01 9.937286e-01 5.316077e-01

1320 9.991277e-01 9.985531e-01 9.976501e-01 9.960878e-01 9.937285e-01 5.322326e-01

1321 9.991278e-01 9.985531e-01 9.976501e-01 9.960877e-01 9.937283e-01 5.328771e-01

1322 9.991278e-01 9.985531e-01 9.976501e-01 9.960877e-01 9.937282e-01 5.335417e-01

1323 9.991278e-01 9.985531e-01 9.976501e-01 9.960876e-01 9.937281e-01 5.342268e-01

1324 9.991278e-01 9.985531e-01 9.976500e-01 9.960875e-01 9.937279e-01 5.349327e-01

1325 9.991279e-01 9.985531e-01 9.976500e-01 9.960874e-01 9.937278e-01 5.356599e-01

1326 9.991279e-01 9.985531e-01 9.976500e-01 9.960874e-01 9.937276e-01 5.364086e-01

1327 9.991279e-01 9.985531e-01 9.976500e-01 9.960873e-01 9.937275e-01 5.371791e-01

1328 9.991280e-01 9.985531e-01 9.976499e-01 9.960872e-01 9.937273e-01 5.379718e-01

1329 9.991280e-01 9.985531e-01 9.976499e-01 9.960871e-01 9.937271e-01 5.387869e-01

1330 9.991280e-01 9.985531e-01 9.976499e-01 9.960870e-01 9.937269e-01 5.396247e-01

1331 9.991280e-01 9.985531e-01 9.976498e-01 9.960869e-01 9.937267e-01 5.404854e-01

1332 9.991281e-01 9.985532e-01 9.976498e-01 9.960867e-01 9.937264e-01 5.413692e-01

1333 9.991281e-01 9.985532e-01 9.976497e-01 9.960866e-01 9.937262e-01 5.422761e-01

1334 9.991282e-01 9.985532e-01 9.976497e-01 9.960865e-01 9.937259e-01 5.432064e-01

1335 9.991282e-01 9.985532e-01 9.976496e-01 9.960863e-01 9.937256e-01 5.441600e-01

1336 9.991282e-01 9.985532e-01 9.976496e-01 9.960862e-01 9.937253e-01 5.451370e-01

1337 9.991283e-01 9.985532e-01 9.976495e-01 9.960860e-01 9.937250e-01 5.461375e-01

1338 9.991283e-01 9.985532e-01 9.976495e-01 9.960858e-01 9.937246e-01 5.471612e-01

1339 9.991284e-01 9.985532e-01 9.976494e-01 9.960856e-01 9.937242e-01 5.482081e-01

1340 9.991284e-01 9.985532e-01 9.976493e-01 9.960854e-01 9.937238e-01 5.492780e-01

1341 9.991285e-01 9.985532e-01 9.976493e-01 9.960852e-01 9.937234e-01 5.503707e-01

1342 9.991286e-01 9.985532e-01 9.976492e-01 9.960849e-01 9.937229e-01 5.514860e-01

1343 9.991286e-01 9.985533e-01 9.976491e-01 9.960847e-01 9.937224e-01 5.526233e-01

1344 9.991287e-01 9.985533e-01 9.976490e-01 9.960844e-01 9.937219e-01 5.537825e-01

1345 9.991288e-01 9.985533e-01 9.976489e-01 9.960841e-01 9.937213e-01 5.549629e-01

1346 9.991288e-01 9.985533e-01 9.976488e-01 9.960838e-01 9.937207e-01 5.561641e-01

1347 9.991289e-01 9.985533e-01 9.976487e-01 9.960834e-01 9.937200e-01 5.573854e-01

1348 9.991290e-01 9.985533e-01 9.976486e-01 9.960831e-01 9.937193e-01 5.586263e-01

1349 9.991291e-01 9.985533e-01 9.976484e-01 9.960827e-01 9.937185e-01 5.598861e-01

1350 9.991292e-01 9.985533e-01 9.976483e-01 9.960823e-01 9.937177e-01 5.611638e-01

1351 9.991293e-01 9.985533e-01 9.976481e-01 9.960818e-01 9.937168e-01 5.624588e-01

1352 9.991294e-01 9.985534e-01 9.976480e-01 9.960813e-01 9.937159e-01 5.637701e-01

1353 9.991295e-01 9.985534e-01 9.976478e-01 9.960808e-01 9.937148e-01 5.650967e-01

1354 9.991296e-01 9.985534e-01 9.976476e-01 9.960803e-01 9.937137e-01 5.664377e-01

1355 9.991297e-01 9.985534e-01 9.976474e-01 9.960797e-01 9.937126e-01 5.677919e-01

1356 9.991298e-01 9.985534e-01 9.976472e-01 9.960790e-01 9.937113e-01 5.691583e-01

1357 9.991299e-01 9.985534e-01 9.976469e-01 9.960783e-01 9.937099e-01 5.705357e-01

1358 9.991301e-01 9.985534e-01 9.976467e-01 9.960776e-01 9.937085e-01 5.719229e-01

1359 9.991302e-01 9.985534e-01 9.976464e-01 9.960768e-01 9.937069e-01 5.733186e-01

1360 9.991304e-01 9.985534e-01 9.976461e-01 9.960759e-01 9.937052e-01 5.747214e-01

1361 9.991305e-01 9.985534e-01 9.976458e-01 9.960750e-01 9.937034e-01 5.761302e-01

1362 9.991307e-01 9.985534e-01 9.976455e-01 9.960740e-01 9.937014e-01 5.775435e-01

1363 9.991309e-01 9.985534e-01 9.976451e-01 9.960730e-01 9.936993e-01 5.789600e-01

1364 9.991311e-01 9.985534e-01 9.976447e-01 9.960718e-01 9.936970e-01 5.803782e-01

1365 9.991313e-01 9.985534e-01 9.976443e-01 9.960706e-01 9.936945e-01 5.817967e-01

1366 9.991315e-01 9.985534e-01 9.976438e-01 9.960692e-01 9.936918e-01 5.832142e-01

1367 9.991317e-01 9.985534e-01 9.976434e-01 9.960678e-01 9.936890e-01 5.846291e-01

1368 9.991320e-01 9.985534e-01 9.976428e-01 9.960663e-01 9.936859e-01 5.860402e-01

1369 9.991322e-01 9.985534e-01 9.976423e-01 9.960646e-01 9.936825e-01 5.874459e-01

1370 9.991325e-01 9.985534e-01 9.976416e-01 9.960628e-01 9.936789e-01 5.888448e-01

1371 9.991328e-01 9.985534e-01 9.976410e-01 9.960609e-01 9.936751e-01 5.902356e-01

1372 9.991331e-01 9.985534e-01 9.976403e-01 9.960588e-01 9.936709e-01 5.916170e-01

1373 9.991334e-01 9.985534e-01 9.976395e-01 9.960566e-01 9.936663e-01 5.929876e-01

1374 9.991337e-01 9.985533e-01 9.976387e-01 9.960542e-01 9.936614e-01 5.943460e-01

1375 9.991341e-01 9.985533e-01 9.976378e-01 9.960516e-01 9.936561e-01 5.956912e-01

1376 9.991344e-01 9.985533e-01 9.976368e-01 9.960487e-01 9.936504e-01 5.970218e-01

1377 9.991348e-01 9.985532e-01 9.976358e-01 9.960457e-01 9.936442e-01 5.983367e-01

1378 9.991353e-01 9.985532e-01 9.976346e-01 9.960424e-01 9.936375e-01 5.996349e-01

1379 9.991357e-01 9.985531e-01 9.976334e-01 9.960389e-01 9.936303e-01 6.009152e-01

1380 9.991362e-01 9.985530e-01 9.976321e-01 9.960351e-01 9.936224e-01 6.021766e-01

1381 9.991367e-01 9.985529e-01 9.976307e-01 9.960309e-01 9.936139e-01 6.034183e-01

1382 9.991372e-01 9.985529e-01 9.976292e-01 9.960265e-01 9.936047e-01 6.046394e-01

1383 9.991378e-01 9.985528e-01 9.976275e-01 9.960216e-01 9.935948e-01 6.058390e-01

1384 9.991384e-01 9.985526e-01 9.976257e-01 9.960164e-01 9.935840e-01 6.070165e-01

1385 9.991390e-01 9.985525e-01 9.976238e-01 9.960108e-01 9.935723e-01 6.081712e-01

1386 9.991397e-01 9.985524e-01 9.976217e-01 9.960047e-01 9.935597e-01 6.093024e-01

1387 9.991404e-01 9.985522e-01 9.976195e-01 9.959980e-01 9.935460e-01 6.104097e-01

1388 9.991411e-01 9.985520e-01 9.976170e-01 9.959909e-01 9.935311e-01 6.114925e-01

1389 9.991419e-01 9.985518e-01 9.976144e-01 9.959832e-01 9.935150e-01 6.125505e-01

1390 9.991428e-01 9.985516e-01 9.976116e-01 9.959748e-01 9.934975e-01 6.135834e-01

1391 9.991437e-01 9.985513e-01 9.976085e-01 9.959657e-01 9.934785e-01 6.145909e-01

1392 9.991447e-01 9.985511e-01 9.976052e-01 9.959559e-01 9.934579e-01 6.155728e-01

1393 9.991457e-01 9.985508e-01 9.976015e-01 9.959452e-01 9.934355e-01 6.165290e-01

1394 9.991468e-01 9.985504e-01 9.975976e-01 9.959337e-01 9.934113e-01 6.174595e-01

1395 9.991479e-01 9.985500e-01 9.975934e-01 9.959212e-01 9.933849e-01 6.183643e-01

1396 9.991491e-01 9.985496e-01 9.975888e-01 9.959076e-01 9.933563e-01 6.192436e-01

1397 9.991504e-01 9.985491e-01 9.975839e-01 9.958929e-01 9.933252e-01 6.200974e-01

1398 9.991518e-01 9.985486e-01 9.975785e-01 9.958770e-01 9.932914e-01 6.209260e-01

1399 9.991533e-01 9.985480e-01 9.975727e-01 9.958597e-01 9.932548e-01 6.217296e-01

1400 9.991549e-01 9.985474e-01 9.975664e-01 9.958410e-01 9.932149e-01 6.225087e-01

1401 9.991565e-01 9.985467e-01 9.975595e-01 9.958206e-01 9.931716e-01 6.232634e-01

1402 9.991583e-01 9.985460e-01 9.975521e-01 9.957985e-01 9.931245e-01 6.239943e-01

1403 9.991602e-01 9.985451e-01 9.975441e-01 9.957745e-01 9.930733e-01 6.247017e-01

1404 9.991622e-01 9.985442e-01 9.975354e-01 9.957485e-01 9.930177e-01 6.253860e-01

1405 9.991643e-01 9.985432e-01 9.975260e-01 9.957203e-01 9.929572e-01 6.260478e-01

1406 9.991666e-01 9.985420e-01 9.975157e-01 9.956896e-01 9.928915e-01 6.266876e-01

1407 9.991690e-01 9.985408e-01 9.975046e-01 9.956563e-01 9.928201e-01 6.273058e-01

1408 9.991716e-01 9.985394e-01 9.974925e-01 9.956201e-01 9.927425e-01 6.279030e-01

1409 9.991744e-01 9.985379e-01 9.974795e-01 9.955809e-01 9.926581e-01 6.284797e-01

1410 9.991773e-01 9.985363e-01 9.974653e-01 9.955382e-01 9.925664e-01 6.290365e-01

1411 9.991804e-01 9.985345e-01 9.974499e-01 9.954919e-01 9.924668e-01 6.295739e-01

1412 9.991837e-01 9.985325e-01 9.974331e-01 9.954416e-01 9.923586e-01 6.300925e-01

1413 9.991873e-01 9.985303e-01 9.974150e-01 9.953869e-01 9.922411e-01 6.305929e-01

1414 9.991911e-01 9.985279e-01 9.973953e-01 9.953276e-01 9.921135e-01 6.310756e-01

1415 9.991951e-01 9.985253e-01 9.973739e-01 9.952631e-01 9.919750e-01 6.315411e-01

1416 9.991994e-01 9.985224e-01 9.973506e-01 9.951931e-01 9.918246e-01 6.319901e-01

1417 9.992040e-01 9.985192e-01 9.973254e-01 9.951172e-01 9.916616e-01 6.324231e-01

1418 9.992089e-01 9.985157e-01 9.972980e-01 9.950347e-01 9.914848e-01 6.328406e-01

1419 9.992141e-01 9.985119e-01 9.972683e-01 9.949452e-01 9.912932e-01 6.332432e-01

1420 9.992197e-01 9.985078e-01 9.972360e-01 9.948480e-01 9.910857e-01 6.336314e-01

1421 9.992256e-01 9.985032e-01 9.972009e-01 9.947426e-01 9.908610e-01 6.340058e-01

1422 9.992319e-01 9.984982e-01 9.971629e-01 9.946283e-01 9.906180e-01 6.343667e-01

1423 9.992387e-01 9.984927e-01 9.971216e-01 9.945044e-01 9.903552e-01 6.347148e-01

1424 9.992459e-01 9.984866e-01 9.970767e-01 9.943701e-01 9.900714e-01 6.350503e-01

1425 9.992536e-01 9.984800e-01 9.970281e-01 9.942247e-01 9.897651e-01 6.353738e-01

1426 9.992619e-01 9.984728e-01 9.969752e-01 9.940671e-01 9.894348e-01 6.356857e-01

1427 9.992707e-01 9.984649e-01 9.969179e-01 9.938966e-01 9.890790e-01 6.359862e-01

1428 9.992801e-01 9.984562e-01 9.968558e-01 9.937122e-01 9.886961e-01 6.362759e-01

1429 9.992901e-01 9.984468e-01 9.967884e-01 9.935129e-01 9.882846e-01 6.365550e-01

1430 9.993008e-01 9.984364e-01 9.967153e-01 9.932975e-01 9.878429e-01 6.368240e-01

1431 9.993122e-01 9.984251e-01 9.966361e-01 9.930651e-01 9.873694e-01 6.370830e-01

1432 9.993245e-01 9.984127e-01 9.965503e-01 9.928144e-01 9.868625e-01 6.373326e-01

1433 9.993796e-01 9.985093e-01 9.967142e-01 9.930971e-01 9.873252e-01 6.426980e-01

1434 9.993797e-01 9.985093e-01 9.967142e-01 9.930971e-01 9.873252e-01 6.427223e-01

1435 9.993798e-01 9.985094e-01 9.967142e-01 9.930971e-01 9.873252e-01 6.427473e-01

1436 9.993799e-01 9.985094e-01 9.967142e-01 9.930971e-01 9.873252e-01 6.427730e-01

1437 9.993800e-01 9.985094e-01 9.967142e-01 9.930970e-01 9.873251e-01 6.427995e-01

1438 9.993801e-01 9.985094e-01 9.967142e-01 9.930970e-01 9.873251e-01 6.428269e-01

1439 9.993801e-01 9.985094e-01 9.967142e-01 9.930970e-01 9.873251e-01 6.428551e-01

1440 9.993802e-01 9.985095e-01 9.967141e-01 9.930970e-01 9.873250e-01 6.428842e-01

1441 9.993802e-01 9.985095e-01 9.967141e-01 9.930969e-01 9.873250e-01 6.429142e-01

1442 9.993803e-01 9.985095e-01 9.967141e-01 9.930969e-01 9.873250e-01 6.429452e-01

1443 9.993803e-01 9.985094e-01 9.967141e-01 9.930969e-01 9.873249e-01 6.429772e-01

1444 9.993803e-01 9.985094e-01 9.967141e-01 9.930969e-01 9.873249e-01 6.430103e-01

1445 9.993803e-01 9.985094e-01 9.967140e-01 9.930968e-01 9.873249e-01 6.430444e-01

1446 9.993804e-01 9.985094e-01 9.967140e-01 9.930968e-01 9.873248e-01 6.430797e-01

1447 9.993804e-01 9.985094e-01 9.967140e-01 9.930968e-01 9.873248e-01 6.431162e-01

1448 9.993804e-01 9.985094e-01 9.967140e-01 9.930967e-01 9.873248e-01 6.431539e-01

1449 9.993804e-01 9.985094e-01 9.967139e-01 9.930967e-01 9.873247e-01 6.431929e-01

1450 9.993804e-01 9.985094e-01 9.967139e-01 9.930967e-01 9.873247e-01 6.432332e-01

1451 9.993804e-01 9.985093e-01 9.967139e-01 9.930966e-01 9.873246e-01 6.432748e-01

1452 9.993804e-01 9.985093e-01 9.967139e-01 9.930966e-01 9.873246e-01 6.433179e-01

1453 9.993804e-01 9.985093e-01 9.967139e-01 9.930966e-01 9.873245e-01 6.433625e-01

1454 9.993803e-01 9.985093e-01 9.967138e-01 9.930965e-01 9.873245e-01 6.434086e-01

1455 9.993803e-01 9.985093e-01 9.967138e-01 9.930965e-01 9.873244e-01 6.434563e-01

1456 9.993803e-01 9.985093e-01 9.967138e-01 9.930964e-01 9.873243e-01 6.435056e-01

1457 9.993803e-01 9.985092e-01 9.967137e-01 9.930964e-01 9.873243e-01 6.435567e-01

1458 9.993803e-01 9.985092e-01 9.967137e-01 9.930964e-01 9.873242e-01 6.436096e-01

1459 9.993803e-01 9.985092e-01 9.967137e-01 9.930963e-01 9.873241e-01 6.436642e-01

1460 9.993803e-01 9.985092e-01 9.967137e-01 9.930963e-01 9.873241e-01 6.437209e-01

1461 9.993803e-01 9.985092e-01 9.967136e-01 9.930962e-01 9.873240e-01 6.437795e-01

1462 9.993803e-01 9.985091e-01 9.967136e-01 9.930962e-01 9.873239e-01 6.438401e-01

1463 9.993802e-01 9.985091e-01 9.967136e-01 9.930961e-01 9.873238e-01 6.439029e-01

1464 9.993802e-01 9.985091e-01 9.967135e-01 9.930960e-01 9.873237e-01 6.439679e-01

1465 9.993802e-01 9.985091e-01 9.967135e-01 9.930960e-01 9.873236e-01 6.440352e-01

1466 9.993802e-01 9.985091e-01 9.967135e-01 9.930959e-01 9.873235e-01 6.441049e-01

1467 9.993802e-01 9.985090e-01 9.967134e-01 9.930958e-01 9.873234e-01 6.441771e-01

1468 9.993802e-01 9.985090e-01 9.967134e-01 9.930958e-01 9.873233e-01 6.442518e-01

1469 9.993802e-01 9.985090e-01 9.967133e-01 9.930957e-01 9.873231e-01 6.443292e-01

1470 9.993801e-01 9.985090e-01 9.967133e-01 9.930956e-01 9.873230e-01 6.444094e-01

1471 9.993801e-01 9.985089e-01 9.967132e-01 9.930955e-01 9.873229e-01 6.444924e-01

1472 9.993801e-01 9.985089e-01 9.967132e-01 9.930954e-01 9.873227e-01 6.445783e-01

1473 9.993801e-01 9.985089e-01 9.967131e-01 9.930953e-01 9.873225e-01 6.446673e-01

1474 9.993801e-01 9.985089e-01 9.967131e-01 9.930952e-01 9.873224e-01 6.447595e-01

1475 9.993801e-01 9.985088e-01 9.967130e-01 9.930951e-01 9.873222e-01 6.448551e-01

1476 9.993801e-01 9.985088e-01 9.967129e-01 9.930950e-01 9.873220e-01 6.449540e-01

1477 9.993801e-01 9.985088e-01 9.967129e-01 9.930949e-01 9.873218e-01 6.450564e-01

1478 9.993800e-01 9.985087e-01 9.967128e-01 9.930947e-01 9.873216e-01 6.451626e-01

1479 9.993800e-01 9.985087e-01 9.967127e-01 9.930946e-01 9.873214e-01 6.452725e-01

1480 9.993800e-01 9.985087e-01 9.967126e-01 9.930944e-01 9.873211e-01 6.453864e-01

1481 9.993800e-01 9.985086e-01 9.967126e-01 9.930943e-01 9.873209e-01 6.455043e-01

1482 9.993800e-01 9.985086e-01 9.967125e-01 9.930941e-01 9.873206e-01 6.456265e-01

1483 9.993800e-01 9.985085e-01 9.967124e-01 9.930939e-01 9.873203e-01 6.457531e-01

1484 9.993799e-01 9.985085e-01 9.967123e-01 9.930937e-01 9.873200e-01 6.458842e-01

1485 9.993799e-01 9.985084e-01 9.967122e-01 9.930935e-01 9.873197e-01 6.460200e-01

1486 9.993799e-01 9.985084e-01 9.967121e-01 9.930933e-01 9.873193e-01 6.461607e-01

1487 9.993799e-01 9.985083e-01 9.967119e-01 9.930931e-01 9.873190e-01 6.463064e-01

1488 9.993799e-01 9.985083e-01 9.967118e-01 9.930929e-01 9.873186e-01 6.464573e-01

1489 9.993799e-01 9.985082e-01 9.967117e-01 9.930926e-01 9.873181e-01 6.466136e-01

1490 9.993798e-01 9.985081e-01 9.967115e-01 9.930923e-01 9.873177e-01 6.467755e-01

1491 9.993798e-01 9.985081e-01 9.967114e-01 9.930920e-01 9.873172e-01 6.469431e-01

1492 9.993798e-01 9.985080e-01 9.967112e-01 9.930917e-01 9.873167e-01 6.471168e-01

1493 9.993798e-01 9.985079e-01 9.967110e-01 9.930914e-01 9.873162e-01 6.472966e-01

1494 9.993797e-01 9.985078e-01 9.967109e-01 9.930911e-01 9.873156e-01 6.474828e-01

1495 9.993797e-01 9.985078e-01 9.967107e-01 9.930907e-01 9.873150e-01 6.476756e-01

1496 9.993797e-01 9.985077e-01 9.967105e-01 9.930903e-01 9.873144e-01 6.478753e-01

1497 9.993796e-01 9.985076e-01 9.967102e-01 9.930899e-01 9.873137e-01 6.480820e-01

1498 9.993796e-01 9.985075e-01 9.967100e-01 9.930894e-01 9.873130e-01 6.482960e-01

1499 9.993796e-01 9.985073e-01 9.967098e-01 9.930890e-01 9.873122e-01 6.485176e-01

1500 9.993795e-01 9.985072e-01 9.967095e-01 9.930885e-01 9.873114e-01 6.487469e-01

1501 9.993795e-01 9.985071e-01 9.967092e-01 9.930879e-01 9.873105e-01 6.489842e-01

1502 9.993795e-01 9.985070e-01 9.967089e-01 9.930874e-01 9.873095e-01 6.492298e-01

1503 9.993794e-01 9.985068e-01 9.967086e-01 9.930868e-01 9.873085e-01 6.494840e-01

1504 9.993794e-01 9.985067e-01 9.967083e-01 9.930861e-01 9.873075e-01 6.497470e-01

1505 9.993793e-01 9.985065e-01 9.967079e-01 9.930854e-01 9.873063e-01 6.500190e-01

1506 9.993793e-01 9.985064e-01 9.967075e-01 9.930847e-01 9.873051e-01 6.503005e-01

1507 9.993792e-01 9.985062e-01 9.967071e-01 9.930839e-01 9.873038e-01 6.505915e-01

1508 9.993792e-01 9.985060e-01 9.967067e-01 9.930831e-01 9.873024e-01 6.508925e-01

1509 9.993791e-01 9.985058e-01 9.967063e-01 9.930822e-01 9.873009e-01 6.512037e-01

1510 9.993790e-01 9.985056e-01 9.967058e-01 9.930812e-01 9.872993e-01 6.515254e-01

1511 9.993790e-01 9.985054e-01 9.967053e-01 9.930802e-01 9.872976e-01 6.518579e-01

1512 9.993789e-01 9.985051e-01 9.967047e-01 9.930791e-01 9.872958e-01 6.522014e-01

1513 9.993788e-01 9.985049e-01 9.967041e-01 9.930780e-01 9.872939e-01 6.525564e-01

1514 9.993787e-01 9.985046e-01 9.967035e-01 9.930768e-01 9.872918e-01 6.529231e-01

1515 9.993786e-01 9.985043e-01 9.967028e-01 9.930754e-01 9.872896e-01 6.533017e-01

1516 9.993785e-01 9.985040e-01 9.967021e-01 9.930740e-01 9.872872e-01 6.536927e-01

1517 9.993785e-01 9.985037e-01 9.967014e-01 9.930725e-01 9.872846e-01 6.540962e-01

1518 9.993783e-01 9.985034e-01 9.967006e-01 9.930709e-01 9.872819e-01 6.545126e-01

1519 9.993782e-01 9.985030e-01 9.966997e-01 9.930692e-01 9.872789e-01 6.549422e-01

1520 9.993781e-01 9.985026e-01 9.966988e-01 9.930674e-01 9.872758e-01 6.553853e-01

1521 9.993780e-01 9.985022e-01 9.966978e-01 9.930654e-01 9.872724e-01 6.558422e-01

1522 9.993779e-01 9.985018e-01 9.966967e-01 9.930633e-01 9.872688e-01 6.563130e-01

1523 9.993777e-01 9.985013e-01 9.966956e-01 9.930610e-01 9.872650e-01 6.567982e-01

1524 9.993776e-01 9.985008e-01 9.966944e-01 9.930586e-01 9.872608e-01 6.572980e-01

1525 9.993774e-01 9.985003e-01 9.966932e-01 9.930560e-01 9.872563e-01 6.578126e-01

1526 9.993773e-01 9.984997e-01 9.966918e-01 9.930533e-01 9.872516e-01 6.583423e-01

1527 9.993771e-01 9.984991e-01 9.966903e-01 9.930503e-01 9.872464e-01 6.588872e-01

1528 9.993769e-01 9.984985e-01 9.966888e-01 9.930471e-01 9.872409e-01 6.594477e-01

1529 9.993767e-01 9.984978e-01 9.966871e-01 9.930437e-01 9.872350e-01 6.600240e-01

1530 9.993765e-01 9.984971e-01 9.966853e-01 9.930400e-01 9.872286e-01 6.606161e-01

1531 9.993763e-01 9.984963e-01 9.966834e-01 9.930361e-01 9.872218e-01 6.612244e-01

1532 9.993761e-01 9.984955e-01 9.966814e-01 9.930319e-01 9.872144e-01 6.618488e-01

1533 9.993758e-01 9.984946e-01 9.966792e-01 9.930273e-01 9.872065e-01 6.624896e-01

1534 9.993755e-01 9.984937e-01 9.966768e-01 9.930225e-01 9.871979e-01 6.631469e-01

1535 9.993753e-01 9.984927e-01 9.966743e-01 9.930173e-01 9.871888e-01 6.638207e-01

1536 9.993750e-01 9.984916e-01 9.966716e-01 9.930117e-01 9.871789e-01 6.645110e-01

1537 9.993746e-01 9.984905e-01 9.966687e-01 9.930056e-01 9.871683e-01 6.652180e-01

1538 9.993743e-01 9.984893e-01 9.966656e-01 9.929991e-01 9.871568e-01 6.659415e-01

1539 9.993739e-01 9.984880e-01 9.966623e-01 9.929922e-01 9.871445e-01 6.666816e-01

1540 9.993736e-01 9.984866e-01 9.966588e-01 9.929847e-01 9.871312e-01 6.674381e-01

1541 9.993732e-01 9.984851e-01 9.966550e-01 9.929766e-01 9.871169e-01 6.682110e-01

1542 9.993727e-01 9.984835e-01 9.966509e-01 9.929680e-01 9.871014e-01 6.690001e-01

1543 9.993723e-01 9.984818e-01 9.966465e-01 9.929586e-01 9.870848e-01 6.698052e-01

1544 9.993718e-01 9.984800e-01 9.966418e-01 9.929486e-01 9.870668e-01 6.706261e-01

1545 9.993713e-01 9.984781e-01 9.966367e-01 9.929378e-01 9.870474e-01 6.714626e-01

1546 9.993707e-01 9.984760e-01 9.966312e-01 9.929261e-01 9.870265e-01 6.723142e-01

1547 9.993701e-01 9.984738e-01 9.966254e-01 9.929135e-01 9.870039e-01 6.731807e-01

1548 9.993695e-01 9.984714e-01 9.966191e-01 9.929000e-01 9.869795e-01 6.740617e-01

1549 9.993688e-01 9.984689e-01 9.966124e-01 9.928854e-01 9.869531e-01 6.749567e-01

1550 9.993681e-01 9.984661e-01 9.966051e-01 9.928697e-01 9.869247e-01 6.758653e-01

1551 9.993673e-01 9.984632e-01 9.965973e-01 9.928527e-01 9.868939e-01 6.767870e-01

1552 9.993665e-01 9.984601e-01 9.965889e-01 9.928344e-01 9.868606e-01 6.777212e-01

1553 9.993657e-01 9.984567e-01 9.965798e-01 9.928146e-01 9.868246e-01 6.786672e-01

1554 9.993647e-01 9.984531e-01 9.965701e-01 9.927933e-01 9.867857e-01 6.796245e-01

1555 9.993638e-01 9.984493e-01 9.965596e-01 9.927703e-01 9.867437e-01 6.805924e-01

1556 9.993627e-01 9.984451e-01 9.965484e-01 9.927454e-01 9.866981e-01 6.815701e-01

1557 9.993616e-01 9.984407e-01 9.965362e-01 9.927185e-01 9.866489e-01 6.825569e-01

1558 9.993604e-01 9.984360e-01 9.965231e-01 9.926895e-01 9.865956e-01 6.835520e-01

1559 9.993591e-01 9.984308e-01 9.965090e-01 9.926582e-01 9.865378e-01 6.845546e-01

1560 9.993578e-01 9.984254e-01 9.964938e-01 9.926243e-01 9.864754e-01 6.855638e-01

1561 9.993563e-01 9.984195e-01 9.964774e-01 9.925876e-01 9.864077e-01 6.865787e-01

1562 9.993548e-01 9.984132e-01 9.964597e-01 9.925480e-01 9.863344e-01 6.875985e-01

1563 9.993531e-01 9.984064e-01 9.964406e-01 9.925051e-01 9.862551e-01 6.886222e-01

1564 9.993513e-01 9.983991e-01 9.964200e-01 9.924588e-01 9.861691e-01 6.896488e-01

1565 9.993494e-01 9.983912e-01 9.963978e-01 9.924086e-01 9.860760e-01 6.906774e-01

1566 9.993474e-01 9.983828e-01 9.963739e-01 9.923543e-01 9.859751e-01 6.917070e-01

1567 9.993453e-01 9.983738e-01 9.963480e-01 9.922956e-01 9.858658e-01 6.927367e-01

1568 9.993429e-01 9.983640e-01 9.963200e-01 9.922320e-01 9.857474e-01 6.937654e-01

1569 9.993405e-01 9.983535e-01 9.962898e-01 9.921632e-01 9.856191e-01 6.947922e-01

1570 9.993378e-01 9.983423e-01 9.962572e-01 9.920887e-01 9.854800e-01 6.958161e-01

1571 9.993350e-01 9.983301e-01 9.962220e-01 9.920080e-01 9.853295e-01 6.968360e-01

1572 9.993319e-01 9.983171e-01 9.961839e-01 9.919206e-01 9.851663e-01 6.978511e-01

1573 9.993287e-01 9.983030e-01 9.961428e-01 9.918259e-01 9.849896e-01 6.988603e-01

1574 9.993252e-01 9.982879e-01 9.960983e-01 9.917234e-01 9.847983e-01 6.998628e-01

1575 9.993215e-01 9.982716e-01 9.960501e-01 9.916125e-01 9.845911e-01 7.008576e-01

1576 9.993175e-01 9.982540e-01 9.959981e-01 9.914922e-01 9.843669e-01 7.018438e-01

1577 9.993132e-01 9.982351e-01 9.959419e-01 9.913621e-01 9.841241e-01 7.028206e-01

1578 9.993087e-01 9.982147e-01 9.958810e-01 9.912211e-01 9.838615e-01 7.037871e-01

1579 9.993037e-01 9.981927e-01 9.958152e-01 9.910684e-01 9.835775e-01 7.047426e-01

1580 9.992985e-01 9.981690e-01 9.957439e-01 9.909031e-01 9.832705e-01 7.056862e-01

1581 9.992928e-01 9.981434e-01 9.956669e-01 9.907242e-01 9.829386e-01 7.066173e-01

1582 9.992868e-01 9.981159e-01 9.955835e-01 9.905305e-01 9.825802e-01 7.075351e-01

1583 9.992803e-01 9.980861e-01 9.954933e-01 9.903209e-01 9.821934e-01 7.084390e-01

1584 9.992733e-01 9.980541e-01 9.953956e-01 9.900942e-01 9.817760e-01 7.093284e-01

1585 9.992659e-01 9.980195e-01 9.952900e-01 9.898489e-01 9.813259e-01 7.102028e-01

1586 9.992579e-01 9.979821e-01 9.951756e-01 9.895837e-01 9.808411e-01 7.110616e-01

1587 9.992492e-01 9.979418e-01 9.950520e-01 9.892971e-01 9.803192e-01 7.119044e-01

1588 9.992400e-01 9.978983e-01 9.949181e-01 9.889874e-01 9.797579e-01 7.127307e-01

1589 9.992301e-01 9.978514e-01 9.947733e-01 9.886530e-01 9.791547e-01 7.135403e-01

1590 9.992194e-01 9.978007e-01 9.946167e-01 9.882920e-01 9.785072e-01 7.143326e-01

1591 9.992080e-01 9.977459e-01 9.944473e-01 9.879026e-01 9.778129e-01 7.151076e-01

1592 9.991957e-01 9.976868e-01 9.942641e-01 9.874828e-01 9.770692e-01 7.158649e-01

1593 9.991825e-01 9.976230e-01 9.940661e-01 9.870306e-01 9.762737e-01 7.166043e-01

1594 9.991682e-01 9.975540e-01 9.938520e-01 9.865437e-01 9.754239e-01 7.173258e-01

1595 9.991530e-01 9.974796e-01 9.936207e-01 9.860200e-01 9.745174e-01 7.180291e-01

1596 9.991366e-01 9.973992e-01 9.933709e-01 9.854571e-01 9.735518e-01 7.187143e-01

1597 9.991189e-01 9.973123e-01 9.931011e-01 9.848527e-01 9.725250e-01 7.193813e-01

1598 9.990999e-01 9.972185e-01 9.928099e-01 9.842044e-01 9.714348e-01 7.200302e-01

1599 9.990795e-01 9.971173e-01 9.924957e-01 9.835098e-01 9.702796e-01 7.206609e-01

1600 9.990575e-01 9.970079e-01 9.921569e-01 9.827664e-01 9.690576e-01 7.212736e-01

1601 9.990339e-01 9.968898e-01 9.917918e-01 9.819717e-01 9.677676e-01 7.218683e-01

1602 9.990085e-01 9.967623e-01 9.913985e-01 9.811235e-01 9.664086e-01 7.224454e-01

1603 9.989811e-01 9.966247e-01 9.909751e-01 9.802192e-01 9.649800e-01 7.230049e-01

1604 9.989517e-01 9.964762e-01 9.905197e-01 9.792566e-01 9.634816e-01 7.235472e-01

1605 9.989200e-01 9.963159e-01 9.900303e-01 9.782337e-01 9.619136e-01 7.240725e-01

1606 9.988859e-01 9.961430e-01 9.895046e-01 9.771484e-01 9.602769e-01 7.245811e-01

1607 9.988491e-01 9.959565e-01 9.889406e-01 9.759988e-01 9.585726e-01 7.250734e-01

1608 9.988096e-01 9.957554e-01 9.883359e-01 9.747836e-01 9.568025e-01 7.255498e-01

1609 9.987671e-01 9.955387e-01 9.876885e-01 9.735013e-01 9.549689e-01 7.260105e-01

1610 9.987212e-01 9.953051e-01 9.869959e-01 9.721510e-01 9.530748e-01 7.264561e-01

1611 9.986719e-01 9.950535e-01 9.862559e-01 9.707320e-01 9.511236e-01 7.268869e-01

1612 9.987112e-01 9.951558e-01 9.864938e-01 9.712448e-01 9.520593e-01 7.320117e-01

1613 9.987112e-01 9.951558e-01 9.864938e-01 9.712448e-01 9.520593e-01 7.320245e-01

1614 9.987113e-01 9.951558e-01 9.864938e-01 9.712447e-01 9.520592e-01 7.320375e-01

1615 9.987113e-01 9.951558e-01 9.864937e-01 9.712446e-01 9.520591e-01 7.320507e-01

1616 9.987113e-01 9.951558e-01 9.864937e-01 9.712445e-01 9.520589e-01 7.320642e-01

1617 9.987113e-01 9.951557e-01 9.864936e-01 9.712445e-01 9.520588e-01 7.320778e-01

1618 9.987113e-01 9.951557e-01 9.864936e-01 9.712444e-01 9.520587e-01 7.320916e-01

1619 9.987113e-01 9.951557e-01 9.864935e-01 9.712443e-01 9.520586e-01 7.321057e-01

1620 9.987113e-01 9.951557e-01 9.864935e-01 9.712442e-01 9.520585e-01 7.321200e-01

1621 9.987113e-01 9.951556e-01 9.864934e-01 9.712441e-01 9.520583e-01 7.321346e-01

1622 9.987113e-01 9.951556e-01 9.864934e-01 9.712440e-01 9.520582e-01 7.321495e-01

1623 9.987113e-01 9.951556e-01 9.864933e-01 9.712439e-01 9.520580e-01 7.321646e-01

1624 9.987113e-01 9.951555e-01 9.864932e-01 9.712438e-01 9.520578e-01 7.321800e-01

1625 9.987113e-01 9.951555e-01 9.864932e-01 9.712437e-01 9.520577e-01 7.321958e-01

1626 9.987113e-01 9.951555e-01 9.864931e-01 9.712436e-01 9.520575e-01 7.322118e-01

1627 9.987113e-01 9.951554e-01 9.864930e-01 9.712434e-01 9.520573e-01 7.322281e-01

1628 9.987113e-01 9.951554e-01 9.864929e-01 9.712433e-01 9.520571e-01 7.322448e-01

1629 9.987112e-01 9.951553e-01 9.864928e-01 9.712431e-01 9.520569e-01 7.322619e-01

1630 9.987112e-01 9.951553e-01 9.864928e-01 9.712430e-01 9.520567e-01 7.322793e-01

1631 9.987112e-01 9.951553e-01 9.864927e-01 9.712428e-01 9.520564e-01 7.322970e-01

1632 9.987112e-01 9.951552e-01 9.864926e-01 9.712427e-01 9.520562e-01 7.323152e-01

1633 9.987112e-01 9.951552e-01 9.864925e-01 9.712425e-01 9.520559e-01 7.323337e-01

1634 9.987111e-01 9.951551e-01 9.864923e-01 9.712423e-01 9.520556e-01 7.323527e-01

1635 9.987111e-01 9.951550e-01 9.864922e-01 9.712421e-01 9.520553e-01 7.323721e-01

1636 9.987111e-01 9.951550e-01 9.864921e-01 9.712419e-01 9.520550e-01 7.323919e-01

1637 9.987111e-01 9.951549e-01 9.864920e-01 9.712417e-01 9.520547e-01 7.324122e-01

1638 9.987111e-01 9.951549e-01 9.864918e-01 9.712414e-01 9.520544e-01 7.324329e-01

1639 9.987110e-01 9.951548e-01 9.864917e-01 9.712412e-01 9.520540e-01 7.324542e-01

1640 9.987110e-01 9.951547e-01 9.864916e-01 9.712409e-01 9.520536e-01 7.324759e-01

1641 9.987110e-01 9.951546e-01 9.864914e-01 9.712407e-01 9.520532e-01 7.324982e-01

1642 9.987109e-01 9.951546e-01 9.864912e-01 9.712404e-01 9.520528e-01 7.325210e-01

1643 9.987109e-01 9.951545e-01 9.864911e-01 9.712401e-01 9.520523e-01 7.325444e-01

1644 9.987109e-01 9.951544e-01 9.864909e-01 9.712398e-01 9.520519e-01 7.325683e-01

1645 9.987108e-01 9.951543e-01 9.864907e-01 9.712394e-01 9.520514e-01 7.325928e-01

1646 9.987108e-01 9.951542e-01 9.864905e-01 9.712391e-01 9.520508e-01 7.326180e-01

1647 9.987108e-01 9.951541e-01 9.864902e-01 9.712387e-01 9.520503e-01 7.326437e-01

1648 9.987107e-01 9.951540e-01 9.864900e-01 9.712383e-01 9.520497e-01 7.326702e-01

1649 9.987107e-01 9.951539e-01 9.864898e-01 9.712379e-01 9.520491e-01 7.326973e-01

1650 9.987106e-01 9.951537e-01 9.864895e-01 9.712374e-01 9.520484e-01 7.327251e-01

1651 9.987106e-01 9.951536e-01 9.864892e-01 9.712369e-01 9.520477e-01 7.327536e-01

1652 9.987106e-01 9.951535e-01 9.864889e-01 9.712364e-01 9.520470e-01 7.327828e-01

1653 9.987105e-01 9.951533e-01 9.864886e-01 9.712359e-01 9.520462e-01 7.328129e-01

1654 9.987105e-01 9.951532e-01 9.864883e-01 9.712354e-01 9.520454e-01 7.328437e-01

1655 9.987104e-01 9.951530e-01 9.864880e-01 9.712348e-01 9.520445e-01 7.328753e-01

1656 9.987103e-01 9.951529e-01 9.864876e-01 9.712341e-01 9.520436e-01 7.329078e-01

1657 9.987103e-01 9.951527e-01 9.864872e-01 9.712335e-01 9.520426e-01 7.329411e-01

1658 9.987102e-01 9.951525e-01 9.864868e-01 9.712328e-01 9.520415e-01 7.329754e-01

1659 9.987102e-01 9.951523e-01 9.864864e-01 9.712320e-01 9.520404e-01 7.330105e-01

1660 9.987101e-01 9.951521e-01 9.864859e-01 9.712312e-01 9.520393e-01 7.330466e-01

1661 9.987100e-01 9.951519e-01 9.864855e-01 9.712304e-01 9.520380e-01 7.330837e-01

1662 9.987099e-01 9.951516e-01 9.864849e-01 9.712295e-01 9.520367e-01 7.331218e-01

1663 9.987098e-01 9.951514e-01 9.864844e-01 9.712286e-01 9.520353e-01 7.331610e-01

1664 9.987098e-01 9.951511e-01 9.864838e-01 9.712276e-01 9.520338e-01 7.332012e-01

1665 9.987097e-01 9.951508e-01 9.864832e-01 9.712265e-01 9.520323e-01 7.332426e-01

1666 9.987096e-01 9.951505e-01 9.864826e-01 9.712254e-01 9.520306e-01 7.332851e-01

1667 9.987095e-01 9.951502e-01 9.864819e-01 9.712242e-01 9.520289e-01 7.333288e-01

1668 9.987094e-01 9.951499e-01 9.864812e-01 9.712229e-01 9.520270e-01 7.333737e-01

1669 9.987092e-01 9.951495e-01 9.864804e-01 9.712216e-01 9.520250e-01 7.334199e-01

1670 9.987091e-01 9.951492e-01 9.864796e-01 9.712202e-01 9.520229e-01 7.334674e-01

1671 9.987090e-01 9.951488e-01 9.864787e-01 9.712187e-01 9.520206e-01 7.335162e-01

1672 9.987089e-01 9.951483e-01 9.864778e-01 9.712171e-01 9.520183e-01 7.335664e-01

1673 9.987087e-01 9.951479e-01 9.864768e-01 9.712153e-01 9.520157e-01 7.336180e-01

1674 9.987086e-01 9.951474e-01 9.864758e-01 9.712135e-01 9.520130e-01 7.336711e-01

1675 9.987084e-01 9.951469e-01 9.864747e-01 9.712116e-01 9.520102e-01 7.337257e-01

1676 9.987082e-01 9.951464e-01 9.864735e-01 9.712095e-01 9.520071e-01 7.337818e-01

1677 9.987081e-01 9.951458e-01 9.864722e-01 9.712074e-01 9.520039e-01 7.338395e-01

1678 9.987079e-01 9.951452e-01 9.864709e-01 9.712050e-01 9.520004e-01 7.338989e-01

1679 9.987077e-01 9.951446e-01 9.864695e-01 9.712026e-01 9.519968e-01 7.339600e-01

1680 9.987075e-01 9.951439e-01 9.864680e-01 9.711999e-01 9.519929e-01 7.340229e-01

1681 9.987073e-01 9.951432e-01 9.864664e-01 9.711971e-01 9.519887e-01 7.340875e-01

1682 9.987070e-01 9.951425e-01 9.864647e-01 9.711941e-01 9.519843e-01 7.341540e-01

1683 9.987068e-01 9.951417e-01 9.864629e-01 9.711909e-01 9.519796e-01 7.342224e-01

1684 9.987065e-01 9.951408e-01 9.864610e-01 9.711876e-01 9.519745e-01 7.342928e-01

1685 9.987062e-01 9.951399e-01 9.864589e-01 9.711839e-01 9.519692e-01 7.343652e-01

1686 9.987059e-01 9.951389e-01 9.864568e-01 9.711801e-01 9.519635e-01 7.344397e-01

1687 9.987056e-01 9.951379e-01 9.864544e-01 9.711760e-01 9.519574e-01 7.345162e-01

1688 9.987053e-01 9.951368e-01 9.864520e-01 9.711716e-01 9.519509e-01 7.345950e-01

1689 9.987049e-01 9.951356e-01 9.864493e-01 9.711669e-01 9.519439e-01 7.346761e-01

1690 9.987046e-01 9.951344e-01 9.864465e-01 9.711620e-01 9.519366e-01 7.347594e-01

1691 9.987042e-01 9.951331e-01 9.864435e-01 9.711566e-01 9.519287e-01 7.348452e-01

1692 9.987038e-01 9.951317e-01 9.864403e-01 9.711510e-01 9.519202e-01 7.349333e-01

1693 9.987033e-01 9.951302e-01 9.864369e-01 9.711449e-01 9.519112e-01 7.350240e-01

1694 9.987028e-01 9.951286e-01 9.864332e-01 9.711384e-01 9.519016e-01 7.351173e-01

1695 9.987024e-01 9.951269e-01 9.864294e-01 9.711315e-01 9.518914e-01 7.352131e-01

1696 9.987018e-01 9.951251e-01 9.864252e-01 9.711241e-01 9.518804e-01 7.353117e-01

1697 9.987013e-01 9.951232e-01 9.864208e-01 9.711162e-01 9.518687e-01 7.354131e-01

1698 9.987007e-01 9.951211e-01 9.864160e-01 9.711078e-01 9.518561e-01 7.355172e-01

1699 9.987000e-01 9.951189e-01 9.864110e-01 9.710988e-01 9.518427e-01 7.356243e-01

1700 9.986994e-01 9.951166e-01 9.864056e-01 9.710891e-01 9.518284e-01 7.357344e-01

1701 9.986986e-01 9.951141e-01 9.863998e-01 9.710788e-01 9.518131e-01 7.358474e-01

1702 9.986979e-01 9.951114e-01 9.863936e-01 9.710678e-01 9.517967e-01 7.359636e-01

1703 9.986971e-01 9.951086e-01 9.863870e-01 9.710559e-01 9.517791e-01 7.360830e-01

1704 9.986962e-01 9.951056e-01 9.863799e-01 9.710433e-01 9.517603e-01 7.362056e-01

1705 9.986953e-01 9.951024e-01 9.863723e-01 9.710297e-01 9.517402e-01 7.363315e-01

1706 9.986944e-01 9.950989e-01 9.863642e-01 9.710152e-01 9.517186e-01 7.364608e-01

1707 9.986933e-01 9.950952e-01 9.863556e-01 9.709997e-01 9.516955e-01 7.365935e-01

1708 9.986922e-01 9.950913e-01 9.863463e-01 9.709830e-01 9.516708e-01 7.367297e-01

1709 9.986911e-01 9.950871e-01 9.863364e-01 9.709652e-01 9.516442e-01 7.368695e-01

1710 9.986898e-01 9.950826e-01 9.863257e-01 9.709460e-01 9.516158e-01 7.370129e-01

1711 9.986885e-01 9.950778e-01 9.863143e-01 9.709255e-01 9.515853e-01 7.371599e-01

1712 9.986871e-01 9.950726e-01 9.863021e-01 9.709035e-01 9.515526e-01 7.373107e-01

1713 9.986856e-01 9.950671e-01 9.862890e-01 9.708799e-01 9.515176e-01 7.374653e-01

1714 9.986840e-01 9.950612e-01 9.862749e-01 9.708546e-01 9.514800e-01 7.376237e-01

1715 9.986823e-01 9.950549e-01 9.862598e-01 9.708274e-01 9.514396e-01 7.377860e-01

1716 9.986805e-01 9.950482e-01 9.862437e-01 9.707982e-01 9.513963e-01 7.379522e-01

1717 9.986786e-01 9.950410e-01 9.862263e-01 9.707669e-01 9.513499e-01 7.381224e-01

1718 9.986765e-01 9.950332e-01 9.862077e-01 9.707332e-01 9.513000e-01 7.382966e-01

1719 9.986743e-01 9.950250e-01 9.861877e-01 9.706971e-01 9.512464e-01 7.384748e-01

1720 9.986720e-01 9.950161e-01 9.861663e-01 9.706583e-01 9.511889e-01 7.386571e-01

1721 9.986695e-01 9.950066e-01 9.861432e-01 9.706166e-01 9.511271e-01 7.388434e-01

1722 9.986668e-01 9.949964e-01 9.861185e-01 9.705718e-01 9.510608e-01 7.390338e-01

1723 9.986640e-01 9.949855e-01 9.860919e-01 9.705236e-01 9.509896e-01 7.392284e-01

1724 9.986610e-01 9.949737e-01 9.860633e-01 9.704719e-01 9.509131e-01 7.394270e-01

1725 9.986577e-01 9.949612e-01 9.860326e-01 9.704162e-01 9.508309e-01 7.396297e-01

1726 9.986543e-01 9.949477e-01 9.859996e-01 9.703564e-01 9.507426e-01 7.398365e-01

1727 9.986506e-01 9.949332e-01 9.859641e-01 9.702921e-01 9.506477e-01 7.400473e-01

1728 9.986466e-01 9.949177e-01 9.859260e-01 9.702229e-01 9.505458e-01 7.402622e-01

1729 9.986424e-01 9.949010e-01 9.858849e-01 9.701485e-01 9.504363e-01 7.404811e-01

1730 9.986379e-01 9.948831e-01 9.858408e-01 9.700684e-01 9.503187e-01 7.407039e-01

1731 9.986331e-01 9.948639e-01 9.857933e-01 9.699823e-01 9.501923e-01 7.409305e-01

1732 9.986280e-01 9.948432e-01 9.857421e-01 9.698897e-01 9.500566e-01 7.411610e-01

1733 9.986225e-01 9.948210e-01 9.856871e-01 9.697901e-01 9.499108e-01 7.413953e-01

1734 9.986166e-01 9.947971e-01 9.856279e-01 9.696829e-01 9.497543e-01 7.416332e-01

1735 9.986103e-01 9.947715e-01 9.855641e-01 9.695675e-01 9.495861e-01 7.418746e-01

1736 9.986036e-01 9.947439e-01 9.854955e-01 9.694435e-01 9.494056e-01 7.421196e-01

1737 9.985964e-01 9.947143e-01 9.854216e-01 9.693100e-01 9.492119e-01 7.423678e-01

1738 9.985886e-01 9.946824e-01 9.853420e-01 9.691664e-01 9.490040e-01 7.426193e-01

1739 9.985804e-01 9.946481e-01 9.852562e-01 9.690120e-01 9.487810e-01 7.428738e-01

1740 9.985715e-01 9.946112e-01 9.851639e-01 9.688460e-01 9.485417e-01 7.431313e-01

1741 9.985620e-01 9.945714e-01 9.850645e-01 9.686674e-01 9.482853e-01 7.433915e-01

1742 9.985519e-01 9.945287e-01 9.849574e-01 9.684754e-01 9.480104e-01 7.436543e-01

1743 9.985410e-01 9.944827e-01 9.848421e-01 9.682690e-01 9.477159e-01 7.439195e-01

1744 9.985293e-01 9.944332e-01 9.847178e-01 9.680473e-01 9.474006e-01 7.441870e-01

1745 9.985168e-01 9.943798e-01 9.845840e-01 9.678090e-01 9.470632e-01 7.444565e-01

1746 9.985034e-01 9.943224e-01 9.844399e-01 9.675531e-01 9.467022e-01 7.447278e-01

1747 9.984890e-01 9.942605e-01 9.842848e-01 9.672783e-01 9.463164e-01 7.450007e-01

1748 9.984736e-01 9.941939e-01 9.841177e-01 9.669834e-01 9.459042e-01 7.452750e-01

1749 9.984570e-01 9.941221e-01 9.839378e-01 9.666670e-01 9.454642e-01 7.455504e-01

1750 9.984392e-01 9.940448e-01 9.837442e-01 9.663278e-01 9.449949e-01 7.458267e-01

1751 9.984202e-01 9.939615e-01 9.835358e-01 9.659641e-01 9.444947e-01 7.461037e-01

1752 9.983997e-01 9.938717e-01 9.833115e-01 9.655746e-01 9.439620e-01 7.463811e-01

1753 9.983777e-01 9.937750e-01 9.830703e-01 9.651575e-01 9.433954e-01 7.466587e-01

1754 9.983541e-01 9.936708e-01 9.828108e-01 9.647113e-01 9.427932e-01 7.469361e-01

1755 9.983287e-01 9.935585e-01 9.825319e-01 9.642343e-01 9.421540e-01 7.472132e-01

1756 9.983015e-01 9.934376e-01 9.822321e-01 9.637247e-01 9.414762e-01 7.474897e-01

1757 9.982722e-01 9.933072e-01 9.819101e-01 9.631807e-01 9.407584e-01 7.477653e-01

1758 9.982407e-01 9.931668e-01 9.815643e-01 9.626005e-01 9.399992e-01 7.480397e-01

1759 9.982069e-01 9.930156e-01 9.811931e-01 9.619823e-01 9.391974e-01 7.483127e-01

1760 9.981705e-01 9.928526e-01 9.807948e-01 9.613243e-01 9.383518e-01 7.485841e-01

1761 9.981315e-01 9.926771e-01 9.803679e-01 9.606246e-01 9.374615e-01 7.488535e-01

1762 9.980894e-01 9.924881e-01 9.799104e-01 9.598815e-01 9.365255e-01 7.491208e-01

1763 9.980442e-01 9.922847e-01 9.794205e-01 9.590932e-01 9.355433e-01 7.493856e-01

1764 9.979956e-01 9.920656e-01 9.788964e-01 9.582580e-01 9.345144e-01 7.496478e-01

1765 9.979434e-01 9.918299e-01 9.783359e-01 9.573744e-01 9.334387e-01 7.499070e-01

1766 9.978871e-01 9.915763e-01 9.777372e-01 9.564409e-01 9.323162e-01 7.501632e-01

1767 9.978266e-01 9.913036e-01 9.770982e-01 9.554561e-01 9.311475e-01 7.504160e-01

1768 9.977616e-01 9.910103e-01 9.764168e-01 9.544190e-01 9.299331e-01 7.506653e-01

1769 9.976915e-01 9.906951e-01 9.756909e-01 9.533285e-01 9.286741e-01 7.509109e-01

1770 9.976162e-01 9.903564e-01 9.749186e-01 9.521840e-01 9.273721e-01 7.511525e-01

1771 9.975351e-01 9.899928e-01 9.740976e-01 9.509849e-01 9.260287e-01 7.513901e-01

1772 9.974479e-01 9.896025e-01 9.732262e-01 9.497310e-01 9.246461e-01 7.516234e-01

1773 9.973540e-01 9.891838e-01 9.723023e-01 9.484226e-01 9.232269e-01 7.518523e-01

1774 9.972530e-01 9.887350e-01 9.713241e-01 9.470600e-01 9.217740e-01 7.520767e-01

1775 9.971444e-01 9.882540e-01 9.702900e-01 9.456441e-01 9.202907e-01 7.522965e-01

1776 9.970274e-01 9.877391e-01 9.691983e-01 9.441762e-01 9.187805e-01 7.525114e-01

1777 9.969017e-01 9.871882e-01 9.680478e-01 9.426577e-01 9.172475e-01 7.527215e-01

1778 9.967663e-01 9.865993e-01 9.668371e-01 9.410907e-01 9.156958e-01 7.529267e-01

1779 9.966208e-01 9.859702e-01 9.655655e-01 9.394778e-01 9.141299e-01 7.531269e-01

1780 9.964642e-01 9.852989e-01 9.642322e-01 9.378216e-01 9.125545e-01 7.533219e-01

1781 9.962959e-01 9.845832e-01 9.628370e-01 9.361256e-01 9.109744e-01 7.535119e-01

1782 9.961149e-01 9.838209e-01 9.613796e-01 9.343933e-01 9.093946e-01 7.536967e-01

1783 9.959204e-01 9.830100e-01 9.598606e-01 9.326288e-01 9.078199e-01 7.538764e-01

1784 9.957113e-01 9.821481e-01 9.582805e-01 9.308365e-01 9.062553e-01 7.540509e-01

1785 9.954867e-01 9.812334e-01 9.566406e-01 9.290210e-01 9.047057e-01 7.542202e-01

1786 9.952455e-01 9.802637e-01 9.549423e-01 9.271873e-01 9.031758e-01 7.543843e-01

1787 9.949865e-01 9.792372e-01 9.531876e-01 9.253406e-01 9.016702e-01 7.545434e-01

1788 9.947084e-01 9.781520e-01 9.513788e-01 9.234863e-01 9.001930e-01 7.546973e-01

1789 9.944101e-01 9.770065e-01 9.495189e-01 9.216297e-01 8.987483e-01 7.548461e-01

1790 9.940901e-01 9.757991e-01 9.476111e-01 9.197765e-01 8.973397e-01 7.549900e-01

1791 9.941761e-01 9.761457e-01 9.485714e-01 9.216706e-01 9.001617e-01 7.596672e-01

1792 9.941761e-01 9.761456e-01 9.485713e-01 9.216704e-01 9.001615e-01 7.596636e-01

1793 9.941761e-01 9.761455e-01 9.485711e-01 9.216702e-01 9.001613e-01 7.596595e-01

1794 9.941760e-01 9.761455e-01 9.485710e-01 9.216700e-01 9.001610e-01 7.596549e-01

1795 9.941760e-01 9.761454e-01 9.485708e-01 9.216698e-01 9.001608e-01 7.596497e-01

1796 9.941760e-01 9.761453e-01 9.485706e-01 9.216696e-01 9.001605e-01 7.596441e-01

1797 9.941759e-01 9.761451e-01 9.485705e-01 9.216693e-01 9.001602e-01 7.596380e-01

1798 9.941759e-01 9.761450e-01 9.485703e-01 9.216690e-01 9.001599e-01 7.596313e-01

1799 9.941758e-01 9.761449e-01 9.485701e-01 9.216688e-01 9.001596e-01 7.596241e-01

1800 9.941758e-01 9.761448e-01 9.485698e-01 9.216685e-01 9.001593e-01 7.596164e-01

1801 9.941757e-01 9.761447e-01 9.485696e-01 9.216682e-01 9.001589e-01 7.596082e-01

1802 9.941757e-01 9.761445e-01 9.485694e-01 9.216679e-01 9.001585e-01 7.595994e-01

1803 9.941756e-01 9.761444e-01 9.485691e-01 9.216675e-01 9.001582e-01 7.595901e-01

1804 9.941756e-01 9.761442e-01 9.485689e-01 9.216672e-01 9.001578e-01 7.595802e-01

1805 9.941755e-01 9.761441e-01 9.485686e-01 9.216668e-01 9.001574e-01 7.595697e-01

1806 9.941755e-01 9.761439e-01 9.485683e-01 9.216665e-01 9.001569e-01 7.595586e-01

1807 9.941754e-01 9.761437e-01 9.485680e-01 9.216661e-01 9.001565e-01 7.595469e-01

1808 9.941753e-01 9.761436e-01 9.485677e-01 9.216656e-01 9.001560e-01 7.595347e-01

1809 9.941752e-01 9.761434e-01 9.485674e-01 9.216652e-01 9.001555e-01 7.595218e-01

1810 9.941752e-01 9.761432e-01 9.485671e-01 9.216648e-01 9.001550e-01 7.595082e-01

1811 9.941751e-01 9.761430e-01 9.485667e-01 9.216643e-01 9.001545e-01 7.594940e-01

1812 9.941750e-01 9.761427e-01 9.485663e-01 9.216638e-01 9.001539e-01 7.594791e-01

1813 9.941749e-01 9.761425e-01 9.485659e-01 9.216633e-01 9.001533e-01 7.594636e-01

1814 9.941748e-01 9.761423e-01 9.485655e-01 9.216627e-01 9.001527e-01 7.594473e-01

1815 9.941747e-01 9.761420e-01 9.485651e-01 9.216622e-01 9.001520e-01 7.594303e-01

1816 9.941746e-01 9.761417e-01 9.485646e-01 9.216616e-01 9.001514e-01 7.594125e-01

1817 9.941745e-01 9.761414e-01 9.485642e-01 9.216610e-01 9.001507e-01 7.593940e-01

1818 9.941744e-01 9.761411e-01 9.485636e-01 9.216603e-01 9.001500e-01 7.593747e-01

1819 9.941743e-01 9.761408e-01 9.485631e-01 9.216596e-01 9.001492e-01 7.593545e-01

1820 9.941741e-01 9.761405e-01 9.485626e-01 9.216589e-01 9.001484e-01 7.593336e-01

1821 9.941740e-01 9.761402e-01 9.485620e-01 9.216582e-01 9.001476e-01 7.593117e-01

1822 9.941739e-01 9.761398e-01 9.485614e-01 9.216574e-01 9.001467e-01 7.592889e-01

1823 9.941737e-01 9.761394e-01 9.485607e-01 9.216566e-01 9.001458e-01 7.592653e-01

1824 9.941736e-01 9.761390e-01 9.485600e-01 9.216557e-01 9.001448e-01 7.592406e-01

1825 9.941734e-01 9.761386e-01 9.485593e-01 9.216548e-01 9.001438e-01 7.592150e-01

1826 9.941732e-01 9.761381e-01 9.485586e-01 9.216538e-01 9.001428e-01 7.591884e-01

1827 9.941731e-01 9.761376e-01 9.485578e-01 9.216528e-01 9.001417e-01 7.591607e-01

1828 9.941729e-01 9.761371e-01 9.485570e-01 9.216518e-01 9.001405e-01 7.591319e-01

1829 9.941727e-01 9.761366e-01 9.485561e-01 9.216507e-01 9.001393e-01 7.591020e-01

1830 9.941725e-01 9.761360e-01 9.485552e-01 9.216495e-01 9.001381e-01 7.590710e-01

1831 9.941722e-01 9.761355e-01 9.485542e-01 9.216483e-01 9.001368e-01 7.590387e-01

1832 9.941720e-01 9.761348e-01 9.485532e-01 9.216470e-01 9.001354e-01 7.590052e-01

1833 9.941717e-01 9.761342e-01 9.485521e-01 9.216457e-01 9.001339e-01 7.589704e-01

1834 9.941715e-01 9.761335e-01 9.485510e-01 9.216443e-01 9.001324e-01 7.589343e-01

1835 9.941712e-01 9.761328e-01 9.485498e-01 9.216428e-01 9.001309e-01 7.588968e-01

1836 9.941709e-01 9.761320e-01 9.485485e-01 9.216412e-01 9.001292e-01 7.588578e-01

1837 9.941706e-01 9.761312e-01 9.485472e-01 9.216396e-01 9.001275e-01 7.588174e-01

1838 9.941703e-01 9.761303e-01 9.485458e-01 9.216379e-01 9.001256e-01 7.587755e-01

1839 9.941700e-01 9.761294e-01 9.485443e-01 9.216361e-01 9.001237e-01 7.587320e-01

1840 9.941696e-01 9.761285e-01 9.485428e-01 9.216342e-01 9.001217e-01 7.586868e-01

1841 9.941692e-01 9.761275e-01 9.485411e-01 9.216322e-01 9.001197e-01 7.586400e-01

1842 9.941688e-01 9.761264e-01 9.485394e-01 9.216301e-01 9.001175e-01 7.585914e-01

1843 9.941684e-01 9.761253e-01 9.485376e-01 9.216279e-01 9.001152e-01 7.585410e-01

1844 9.941679e-01 9.761241e-01 9.485357e-01 9.216255e-01 9.001128e-01 7.584887e-01

1845 9.941675e-01 9.761228e-01 9.485337e-01 9.216231e-01 9.001102e-01 7.584344e-01

1846 9.941670e-01 9.761215e-01 9.485315e-01 9.216205e-01 9.001076e-01 7.583781e-01

1847 9.941664e-01 9.761201e-01 9.485293e-01 9.216178e-01 9.001048e-01 7.583197e-01

1848 9.941659e-01 9.761186e-01 9.485269e-01 9.216150e-01 9.001019e-01 7.582592e-01

1849 9.941653e-01 9.761171e-01 9.485244e-01 9.216120e-01 9.000988e-01 7.581964e-01

1850 9.941646e-01 9.761154e-01 9.485217e-01 9.216088e-01 9.000956e-01 7.581313e-01

1851 9.941640e-01 9.761136e-01 9.485189e-01 9.216055e-01 9.000923e-01 7.580638e-01

1852 9.941633e-01 9.761118e-01 9.485160e-01 9.216020e-01 9.000888e-01 7.579937e-01

1853 9.941625e-01 9.761098e-01 9.485128e-01 9.215983e-01 9.000851e-01 7.579211e-01

1854 9.941618e-01 9.761077e-01 9.485095e-01 9.215944e-01 9.000812e-01 7.578459e-01

1855 9.941609e-01 9.761055e-01 9.485060e-01 9.215904e-01 9.000772e-01 7.577678e-01

1856 9.941600e-01 9.761032e-01 9.485024e-01 9.215861e-01 9.000729e-01 7.576869e-01

1857 9.941591e-01 9.761007e-01 9.484984e-01 9.215815e-01 9.000685e-01 7.576031e-01

1858 9.941581e-01 9.760981e-01 9.484943e-01 9.215767e-01 9.000638e-01 7.575161e-01

1859 9.941571e-01 9.760953e-01 9.484900e-01 9.215717e-01 9.000589e-01 7.574260e-01

1860 9.941560e-01 9.760923e-01 9.484853e-01 9.215664e-01 9.000537e-01 7.573326e-01

1861 9.941548e-01 9.760892e-01 9.484805e-01 9.215608e-01 9.000483e-01 7.572358e-01

1862 9.941535e-01 9.760859e-01 9.484753e-01 9.215549e-01 9.000427e-01 7.571355e-01

1863 9.941522e-01 9.760824e-01 9.484698e-01 9.215487e-01 9.000367e-01 7.570315e-01

1864 9.941508e-01 9.760786e-01 9.484640e-01 9.215422e-01 9.000305e-01 7.569238e-01

1865 9.941493e-01 9.760747e-01 9.484579e-01 9.215352e-01 9.000240e-01 7.568122e-01

1866 9.941477e-01 9.760705e-01 9.484514e-01 9.215280e-01 9.000171e-01 7.566966e-01

1867 9.941461e-01 9.760660e-01 9.484445e-01 9.215203e-01 9.000099e-01 7.565768e-01

1868 9.941443e-01 9.760613e-01 9.484372e-01 9.215122e-01 9.000024e-01 7.564528e-01

1869 9.941424e-01 9.760563e-01 9.484295e-01 9.215036e-01 8.999944e-01 7.563243e-01

1870 9.941404e-01 9.760509e-01 9.484214e-01 9.214946e-01 8.999861e-01 7.561913e-01

1871 9.941383e-01 9.760452e-01 9.484127e-01 9.214850e-01 8.999774e-01 7.560535e-01

1872 9.941360e-01 9.760392e-01 9.484035e-01 9.214750e-01 8.999683e-01 7.559109e-01

1873 9.941336e-01 9.760328e-01 9.483938e-01 9.214644e-01 8.999587e-01 7.557633e-01

1874 9.941311e-01 9.760260e-01 9.483835e-01 9.214532e-01 8.999487e-01 7.556105e-01

1875 9.941284e-01 9.760188e-01 9.483725e-01 9.214414e-01 8.999382e-01 7.554523e-01

1876 9.941255e-01 9.760111e-01 9.483609e-01 9.214289e-01 8.999271e-01 7.552887e-01

1877 9.941224e-01 9.760029e-01 9.483486e-01 9.214157e-01 8.999155e-01 7.551194e-01

1878 9.941191e-01 9.759942e-01 9.483355e-01 9.214018e-01 8.999034e-01 7.549443e-01

1879 9.941157e-01 9.759850e-01 9.483217e-01 9.213871e-01 8.998907e-01 7.547632e-01

1880 9.941120e-01 9.759751e-01 9.483069e-01 9.213716e-01 8.998774e-01 7.545759e-01

1881 9.941081e-01 9.759646e-01 9.482913e-01 9.213552e-01 8.998635e-01 7.543824e-01

1882 9.941039e-01 9.759535e-01 9.482747e-01 9.213379e-01 8.998489e-01 7.541823e-01

1883 9.940994e-01 9.759416e-01 9.482571e-01 9.213196e-01 8.998336e-01 7.539755e-01

1884 9.940947e-01 9.759289e-01 9.482384e-01 9.213003e-01 8.998176e-01 7.537619e-01

1885 9.940896e-01 9.759154e-01 9.482186e-01 9.212799e-01 8.998008e-01 7.535412e-01

1886 9.940843e-01 9.759011e-01 9.481975e-01 9.212584e-01 8.997833e-01 7.533134e-01

1887 9.940785e-01 9.758858e-01 9.481751e-01 9.212357e-01 8.997650e-01 7.530782e-01

1888 9.940724e-01 9.758695e-01 9.481513e-01 9.212117e-01 8.997458e-01 7.528354e-01

1889 9.940659e-01 9.758521e-01 9.481260e-01 9.211863e-01 8.997258e-01 7.525850e-01

1890 9.940590e-01 9.758336e-01 9.480991e-01 9.211595e-01 8.997049e-01 7.523266e-01

1891 9.940516e-01 9.758139e-01 9.480706e-01 9.211312e-01 8.996830e-01 7.520602e-01

1892 9.940437e-01 9.757928e-01 9.480402e-01 9.211014e-01 8.996602e-01 7.517856e-01

1893 9.940353e-01 9.757703e-01 9.480080e-01 9.210698e-01 8.996365e-01 7.515026e-01

1894 9.940263e-01 9.757464e-01 9.479737e-01 9.210365e-01 8.996117e-01 7.512111e-01

1895 9.940167e-01 9.757208e-01 9.479372e-01 9.210014e-01 8.995859e-01 7.509109e-01

1896 9.940065e-01 9.756935e-01 9.478984e-01 9.209643e-01 8.995590e-01 7.506020e-01

1897 9.939956e-01 9.756644e-01 9.478572e-01 9.209251e-01 8.995311e-01 7.502840e-01

1898 9.939840e-01 9.756334e-01 9.478134e-01 9.208838e-01 8.995021e-01 7.499571e-01

1899 9.939715e-01 9.756002e-01 9.477668e-01 9.208402e-01 8.994719e-01 7.496209e-01

1900 9.939582e-01 9.755648e-01 9.477172e-01 9.207942e-01 8.994406e-01 7.492755e-01

1901 9.939440e-01 9.755270e-01 9.476645e-01 9.207457e-01 8.994082e-01 7.489207e-01

1902 9.939288e-01 9.754866e-01 9.476085e-01 9.206945e-01 8.993747e-01 7.485564e-01

1903 9.939126e-01 9.754435e-01 9.475489e-01 9.206406e-01 8.993400e-01 7.481826e-01

1904 9.938953e-01 9.753974e-01 9.474855e-01 9.205838e-01 8.993042e-01 7.477992e-01

1905 9.938767e-01 9.753482e-01 9.474182e-01 9.205240e-01 8.992673e-01 7.474062e-01

1906 9.938569e-01 9.752956e-01 9.473465e-01 9.204610e-01 8.992293e-01 7.470036e-01

1907 9.938357e-01 9.752395e-01 9.472704e-01 9.203946e-01 8.991903e-01 7.465913e-01

1908 9.938130e-01 9.751795e-01 9.471894e-01 9.203248e-01 8.991502e-01 7.461694e-01

1909 9.937888e-01 9.751153e-01 9.471034e-01 9.202514e-01 8.991093e-01 7.457379e-01

1910 9.937628e-01 9.750468e-01 9.470119e-01 9.201743e-01 8.990675e-01 7.452969e-01

1911 9.937350e-01 9.749736e-01 9.469148e-01 9.200932e-01 8.990250e-01 7.448464e-01

1912 9.937053e-01 9.748953e-01 9.468115e-01 9.200081e-01 8.989818e-01 7.443865e-01

1913 9.936734e-01 9.748117e-01 9.467019e-01 9.199187e-01 8.989381e-01 7.439173e-01

1914 9.936393e-01 9.747223e-01 9.465854e-01 9.198251e-01 8.988940e-01 7.434390e-01

1915 9.936028e-01 9.746268e-01 9.464618e-01 9.197269e-01 8.988498e-01 7.429518e-01

1916 9.935637e-01 9.745247e-01 9.463305e-01 9.196241e-01 8.988056e-01 7.424558e-01

1917 9.935218e-01 9.744157e-01 9.461913e-01 9.195166e-01 8.987616e-01 7.419512e-01

1918 9.934769e-01 9.742992e-01 9.460436e-01 9.194042e-01 8.987181e-01 7.414384e-01

1919 9.934288e-01 9.741747e-01 9.458869e-01 9.192868e-01 8.986755e-01 7.409175e-01

1920 9.933772e-01 9.740417e-01 9.457209e-01 9.191643e-01 8.986340e-01 7.403888e-01

1921 9.933220e-01 9.738996e-01 9.455450e-01 9.190367e-01 8.985940e-01 7.398527e-01

1922 9.932628e-01 9.737480e-01 9.453587e-01 9.189038e-01 8.985559e-01 7.393095e-01

1923 9.931993e-01 9.735860e-01 9.451615e-01 9.187656e-01 8.985202e-01 7.387595e-01

1924 9.931313e-01 9.734131e-01 9.449529e-01 9.186222e-01 8.984873e-01 7.382032e-01

1925 9.930584e-01 9.732285e-01 9.447323e-01 9.184734e-01 8.984579e-01 7.376410e-01

1926 9.929802e-01 9.730316e-01 9.444991e-01 9.183194e-01 8.984325e-01 7.370732e-01

1927 9.928964e-01 9.728216e-01 9.442529e-01 9.181602e-01 8.984117e-01 7.365004e-01

1928 9.928066e-01 9.725975e-01 9.439931e-01 9.179959e-01 8.983962e-01 7.359231e-01

1929 9.927102e-01 9.723587e-01 9.437192e-01 9.178266e-01 8.983868e-01 7.353416e-01

1930 9.926069e-01 9.721042e-01 9.434305e-01 9.176526e-01 8.983843e-01 7.347566e-01

1931 9.924962e-01 9.718331e-01 9.431265e-01 9.174740e-01 8.983894e-01 7.341686e-01

1932 9.923775e-01 9.715445e-01 9.428068e-01 9.172912e-01 8.984032e-01 7.335781e-01

1933 9.922502e-01 9.712373e-01 9.424708e-01 9.171045e-01 8.984265e-01 7.329856e-01

1934 9.921138e-01 9.709104e-01 9.421180e-01 9.169143e-01 8.984604e-01 7.323918e-01

1935 9.919676e-01 9.705630e-01 9.417480e-01 9.167210e-01 8.985058e-01 7.317971e-01

1936 9.918108e-01 9.701938e-01 9.413604e-01 9.165252e-01 8.985638e-01 7.312022e-01

1937 9.916429e-01 9.698017e-01 9.409548e-01 9.163274e-01 8.986354e-01 7.306077e-01

1938 9.914630e-01 9.693856e-01 9.405309e-01 9.161282e-01 8.987219e-01 7.300142e-01

1939 9.912702e-01 9.689444e-01 9.400884e-01 9.159285e-01 8.988242e-01 7.294221e-01

1940 9.910637e-01 9.684769e-01 9.396273e-01 9.157290e-01 8.989435e-01 7.288322e-01

1941 9.908427e-01 9.679818e-01 9.391473e-01 9.155306e-01 8.990809e-01 7.282449e-01

1942 9.906060e-01 9.674581e-01 9.386484e-01 9.153340e-01 8.992374e-01 7.276609e-01

1943 9.903527e-01 9.669046e-01 9.381308e-01 9.151404e-01 8.994141e-01 7.270807e-01

1944 9.900818e-01 9.663202e-01 9.375947e-01 9.149507e-01 8.996120e-01 7.265048e-01

1945 9.897919e-01 9.657036e-01 9.370403e-01 9.147661e-01 8.998320e-01 7.259338e-01

1946 9.894820e-01 9.650539e-01 9.364680e-01 9.145875e-01 9.000748e-01 7.253682e-01

1947 9.891509e-01 9.643701e-01 9.358784e-01 9.144163e-01 9.003413e-01 7.248085e-01

1948 9.887971e-01 9.636512e-01 9.352723e-01 9.142535e-01 9.006320e-01 7.242551e-01

1949 9.884193e-01 9.628964e-01 9.346504e-01 9.141002e-01 9.009474e-01 7.237086e-01

1950 9.880162e-01 9.621048e-01 9.340136e-01 9.139578e-01 9.012879e-01 7.231693e-01

1951 9.875861e-01 9.612760e-01 9.333632e-01 9.138273e-01 9.016536e-01 7.226376e-01

1952 9.871277e-01 9.604092e-01 9.327003e-01 9.137099e-01 9.020446e-01 7.221140e-01

1953 9.866393e-01 9.595043e-01 9.320264e-01 9.136066e-01 9.024608e-01 7.215988e-01

1954 9.861194e-01 9.585609e-01 9.313430e-01 9.135185e-01 9.029018e-01 7.210923e-01

1955 9.855661e-01 9.575791e-01 9.306517e-01 9.134464e-01 9.033671e-01 7.205949e-01

1956 9.849780e-01 9.565590e-01 9.299543e-01 9.133913e-01 9.038560e-01 7.201067e-01

1957 9.843533e-01 9.555010e-01 9.292528e-01 9.133538e-01 9.043678e-01 7.196281e-01

1958 9.836902e-01 9.544057e-01 9.285491e-01 9.133346e-01 9.049014e-01 7.191592e-01

1959 9.829870e-01 9.532741e-01 9.278453e-01 9.133342e-01 9.054557e-01 7.187004e-01

1960 9.822421e-01 9.521071e-01 9.271434e-01 9.133529e-01 9.060294e-01 7.182516e-01

1961 9.814537e-01 9.509062e-01 9.264457e-01 9.133910e-01 9.066209e-01 7.178131e-01

1962 9.806202e-01 9.496729e-01 9.257542e-01 9.134484e-01 9.072289e-01 7.173849e-01

1963 9.797399e-01 9.484091e-01 9.250711e-01 9.135252e-01 9.078516e-01 7.169672e-01

1964 9.788113e-01 9.471171e-01 9.243985e-01 9.136211e-01 9.084873e-01 7.165600e-01

1965 9.778329e-01 9.457990e-01 9.237384e-01 9.137357e-01 9.091343e-01 7.161633e-01

1966 9.768034e-01 9.444577e-01 9.230928e-01 9.138685e-01 9.097907e-01 7.157772e-01

1967 9.757215e-01 9.430959e-01 9.224634e-01 9.140188e-01 9.104546e-01 7.154016e-01

1968 9.745861e-01 9.417168e-01 9.218521e-01 9.141860e-01 9.111242e-01 7.150365e-01

1969 9.733963e-01 9.403235e-01 9.212604e-01 9.143692e-01 9.117975e-01 7.146818e-01

1970 9.737962e-01 9.418524e-01 9.240648e-01 9.180129e-01 9.159098e-01 7.191495e-01

1971 9.737961e-01 9.418522e-01 9.240646e-01 9.180128e-01 9.159097e-01 7.191111e-01

1972 9.737960e-01 9.418520e-01 9.240644e-01 9.180126e-01 9.159097e-01 7.190715e-01

1973 9.737958e-01 9.418518e-01 9.240643e-01 9.180125e-01 9.159096e-01 7.190305e-01

1974 9.737957e-01 9.418516e-01 9.240641e-01 9.180124e-01 9.159096e-01 7.189882e-01

1975 9.737955e-01 9.418514e-01 9.240639e-01 9.180122e-01 9.159095e-01 7.189445e-01

1976 9.737954e-01 9.418512e-01 9.240637e-01 9.180121e-01 9.159095e-01 7.188994e-01

1977 9.737952e-01 9.418510e-01 9.240635e-01 9.180120e-01 9.159094e-01 7.188529e-01

1978 9.737951e-01 9.418507e-01 9.240633e-01 9.180119e-01 9.159094e-01 7.188049e-01

1979 9.737949e-01 9.418504e-01 9.240630e-01 9.180117e-01 9.159093e-01 7.187554e-01

1980 9.737947e-01 9.418502e-01 9.240628e-01 9.180116e-01 9.159093e-01 7.187044e-01

1981 9.737945e-01 9.418499e-01 9.240626e-01 9.180115e-01 9.159093e-01 7.186518e-01

1982 9.737943e-01 9.418496e-01 9.240624e-01 9.180113e-01 9.159092e-01 7.185976e-01

1983 9.737941e-01 9.418493e-01 9.240621e-01 9.180112e-01 9.159092e-01 7.185418e-01

1984 9.737939e-01 9.418490e-01 9.240618e-01 9.180111e-01 9.159092e-01 7.184843e-01

1985 9.737937e-01 9.418486e-01 9.240616e-01 9.180110e-01 9.159092e-01 7.184251e-01

1986 9.737934e-01 9.418483e-01 9.240613e-01 9.180108e-01 9.159092e-01 7.183641e-01

1987 9.737932e-01 9.418479e-01 9.240610e-01 9.180107e-01 9.159092e-01 7.183013e-01

1988 9.737929e-01 9.418475e-01 9.240607e-01 9.180106e-01 9.159092e-01 7.182366e-01

1989 9.737926e-01 9.418471e-01 9.240604e-01 9.180104e-01 9.159092e-01 7.181701e-01

1990 9.737923e-01 9.418467e-01 9.240601e-01 9.180103e-01 9.159092e-01 7.181016e-01

1991 9.737920e-01 9.418463e-01 9.240597e-01 9.180102e-01 9.159093e-01 7.180310e-01

1992 9.737917e-01 9.418458e-01 9.240594e-01 9.180100e-01 9.159093e-01 7.179584e-01

1993 9.737914e-01 9.418454e-01 9.240590e-01 9.180099e-01 9.159093e-01 7.178837e-01

1994 9.737910e-01 9.418449e-01 9.240587e-01 9.180097e-01 9.159094e-01 7.178067e-01

1995 9.737907e-01 9.418444e-01 9.240583e-01 9.180096e-01 9.159095e-01 7.177275e-01

1996 9.737903e-01 9.418438e-01 9.240579e-01 9.180095e-01 9.159095e-01 7.176460e-01

1997 9.737899e-01 9.418433e-01 9.240575e-01 9.180093e-01 9.159096e-01 7.175621e-01

1998 9.737895e-01 9.418427e-01 9.240570e-01 9.180092e-01 9.159097e-01 7.174757e-01

1999 9.737890e-01 9.418421e-01 9.240566e-01 9.180091e-01 9.159098e-01 7.173868e-01

2000 9.737886e-01 9.418415e-01 9.240561e-01 9.180089e-01 9.159099e-01 7.172953e-01

2001 9.737881e-01 9.418409e-01 9.240557e-01 9.180088e-01 9.159100e-01 7.172011e-01

2002 9.737876e-01 9.418402e-01 9.240552e-01 9.180087e-01 9.159102e-01 7.171042e-01

2003 9.737870e-01 9.418395e-01 9.240547e-01 9.180086e-01 9.159103e-01 7.170044e-01

2004 9.737865e-01 9.418387e-01 9.240542e-01 9.180084e-01 9.159105e-01 7.169017e-01

2005 9.737859e-01 9.418380e-01 9.240537e-01 9.180083e-01 9.159107e-01 7.167960e-01

2006 9.737853e-01 9.418372e-01 9.240531e-01 9.180082e-01 9.159109e-01 7.166871e-01

2007 9.737846e-01 9.418363e-01 9.240526e-01 9.180081e-01 9.159112e-01 7.165751e-01

2008 9.737839e-01 9.418355e-01 9.240520e-01 9.180080e-01 9.159114e-01 7.164597e-01

2009 9.737832e-01 9.418346e-01 9.240514e-01 9.180079e-01 9.159117e-01 7.163410e-01

2010 9.737825e-01 9.418336e-01 9.240508e-01 9.180079e-01 9.159120e-01 7.162187e-01

2011 9.737817e-01 9.418326e-01 9.240502e-01 9.180078e-01 9.159124e-01 7.160929e-01

2012 9.737808e-01 9.418316e-01 9.240496e-01 9.180078e-01 9.159128e-01 7.159633e-01

2013 9.737800e-01 9.418305e-01 9.240489e-01 9.180078e-01 9.159132e-01 7.158299e-01

2014 9.737790e-01 9.418294e-01 9.240483e-01 9.180078e-01 9.159136e-01 7.156926e-01

2015 9.737781e-01 9.418282e-01 9.240476e-01 9.180078e-01 9.159141e-01 7.155512e-01

2016 9.737771e-01 9.418270e-01 9.240469e-01 9.180078e-01 9.159147e-01 7.154057e-01

2017 9.737760e-01 9.418257e-01 9.240462e-01 9.180079e-01 9.159152e-01 7.152558e-01

2018 9.737749e-01 9.418243e-01 9.240454e-01 9.180079e-01 9.159159e-01 7.151015e-01

2019 9.737737e-01 9.418229e-01 9.240447e-01 9.180081e-01 9.159166e-01 7.149426e-01

2020 9.737724e-01 9.418215e-01 9.240439e-01 9.180082e-01 9.159173e-01 7.147790e-01

2021 9.737711e-01 9.418200e-01 9.240431e-01 9.180084e-01 9.159181e-01 7.146106e-01

2022 9.737698e-01 9.418184e-01 9.240423e-01 9.180086e-01 9.159190e-01 7.144372e-01

2023 9.737683e-01 9.418167e-01 9.240415e-01 9.180089e-01 9.159199e-01 7.142587e-01

2024 9.737668e-01 9.418150e-01 9.240407e-01 9.180092e-01 9.159210e-01 7.140749e-01

2025 9.737652e-01 9.418132e-01 9.240398e-01 9.180095e-01 9.159221e-01 7.138856e-01

2026 9.737635e-01 9.418113e-01 9.240390e-01 9.180099e-01 9.159233e-01 7.136908e-01

2027 9.737617e-01 9.418093e-01 9.240381e-01 9.180104e-01 9.159246e-01 7.134902e-01

2028 9.737598e-01 9.418072e-01 9.240372e-01 9.180109e-01 9.159260e-01 7.132837e-01

2029 9.737579e-01 9.418051e-01 9.240363e-01 9.180115e-01 9.159275e-01 7.130711e-01

2030 9.737558e-01 9.418028e-01 9.240354e-01 9.180122e-01 9.159291e-01 7.128523e-01

2031 9.737536e-01 9.418005e-01 9.240344e-01 9.180129e-01 9.159309e-01 7.126270e-01

2032 9.737513e-01 9.417980e-01 9.240335e-01 9.180138e-01 9.159328e-01 7.123951e-01

2033 9.737489e-01 9.417955e-01 9.240325e-01 9.180147e-01 9.159348e-01 7.121564e-01

2034 9.737463e-01 9.417928e-01 9.240316e-01 9.180158e-01 9.159370e-01 7.119108e-01

2035 9.737436e-01 9.417900e-01 9.240306e-01 9.180169e-01 9.159394e-01 7.116580e-01

2036 9.737407e-01 9.417871e-01 9.240297e-01 9.180182e-01 9.159419e-01 7.113978e-01

2037 9.737377e-01 9.417840e-01 9.240288e-01 9.180196e-01 9.159447e-01 7.111301e-01

2038 9.737346e-01 9.417808e-01 9.240278e-01 9.180212e-01 9.159476e-01 7.108547e-01

2039 9.737312e-01 9.417775e-01 9.240269e-01 9.180229e-01 9.159508e-01 7.105713e-01

2040 9.737277e-01 9.417741e-01 9.240260e-01 9.180247e-01 9.159542e-01 7.102798e-01

2041 9.737240e-01 9.417704e-01 9.240251e-01 9.180268e-01 9.159579e-01 7.099799e-01

2042 9.737200e-01 9.417666e-01 9.240242e-01 9.180291e-01 9.159618e-01 7.096715e-01

2043 9.737159e-01 9.417627e-01 9.240234e-01 9.180315e-01 9.159660e-01 7.093543e-01

2044 9.737115e-01 9.417586e-01 9.240226e-01 9.180342e-01 9.159706e-01 7.090282e-01

2045 9.737069e-01 9.417542e-01 9.240219e-01 9.180372e-01 9.159755e-01 7.086929e-01

2046 9.737020e-01 9.417497e-01 9.240212e-01 9.180404e-01 9.159808e-01 7.083483e-01

2047 9.736968e-01 9.417450e-01 9.240205e-01 9.180439e-01 9.159864e-01 7.079941e-01

2048 9.736914e-01 9.417401e-01 9.240200e-01 9.180477e-01 9.159925e-01 7.076301e-01

2049 9.736856e-01 9.417350e-01 9.240195e-01 9.180518e-01 9.159990e-01 7.072561e-01

2050 9.736795e-01 9.417297e-01 9.240191e-01 9.180563e-01 9.160060e-01 7.068720e-01

2051 9.736731e-01 9.417241e-01 9.240188e-01 9.180613e-01 9.160136e-01 7.064775e-01

2052 9.736663e-01 9.417183e-01 9.240186e-01 9.180666e-01 9.160217e-01 7.060724e-01

2053 9.736591e-01 9.417122e-01 9.240186e-01 9.180724e-01 9.160303e-01 7.056565e-01

2054 9.736515e-01 9.417059e-01 9.240187e-01 9.180787e-01 9.160397e-01 7.052297e-01

2055 9.736435e-01 9.416993e-01 9.240190e-01 9.180855e-01 9.160497e-01 7.047918e-01

2056 9.736350e-01 9.416924e-01 9.240194e-01 9.180929e-01 9.160604e-01 7.043426e-01

2057 9.736260e-01 9.416852e-01 9.240201e-01 9.181009e-01 9.160720e-01 7.038819e-01

2058 9.736165e-01 9.416777e-01 9.240210e-01 9.181096e-01 9.160843e-01 7.034095e-01

2059 9.736064e-01 9.416699e-01 9.240222e-01 9.181190e-01 9.160976e-01 7.029254e-01

2060 9.735958e-01 9.416618e-01 9.240236e-01 9.181292e-01 9.161119e-01 7.024294e-01

2061 9.735845e-01 9.416533e-01 9.240254e-01 9.181402e-01 9.161272e-01 7.019213e-01

2062 9.735726e-01 9.416444e-01 9.240275e-01 9.181521e-01 9.161436e-01 7.014011e-01

2063 9.735599e-01 9.416352e-01 9.240300e-01 9.181650e-01 9.161613e-01 7.008685e-01

2064 9.735466e-01 9.416257e-01 9.240330e-01 9.181790e-01 9.161802e-01 7.003237e-01

2065 9.735324e-01 9.416157e-01 9.240364e-01 9.181941e-01 9.162005e-01 6.997664e-01

2066 9.735174e-01 9.416053e-01 9.240403e-01 9.182104e-01 9.162223e-01 6.991966e-01

2067 9.735016e-01 9.415946e-01 9.240448e-01 9.182280e-01 9.162457e-01 6.986143e-01

2068 9.734848e-01 9.415834e-01 9.240499e-01 9.182470e-01 9.162707e-01 6.980194e-01

2069 9.734670e-01 9.415717e-01 9.240557e-01 9.182676e-01 9.162977e-01 6.974120e-01

2070 9.734481e-01 9.415596e-01 9.240622e-01 9.182897e-01 9.163265e-01 6.967921e-01

2071 9.734281e-01 9.415471e-01 9.240696e-01 9.183137e-01 9.163575e-01 6.961597e-01

2072 9.734069e-01 9.415341e-01 9.240779e-01 9.183395e-01 9.163907e-01 6.955148e-01

2073 9.733845e-01 9.415206e-01 9.240872e-01 9.183674e-01 9.164263e-01 6.948577e-01

2074 9.733607e-01 9.415066e-01 9.240975e-01 9.183975e-01 9.164645e-01 6.941884e-01

2075 9.733354e-01 9.414921e-01 9.241090e-01 9.184299e-01 9.165054e-01 6.935070e-01

2076 9.733087e-01 9.414771e-01 9.241218e-01 9.184649e-01 9.165494e-01 6.928137e-01

2077 9.732803e-01 9.414616e-01 9.241360e-01 9.185026e-01 9.165964e-01 6.921088e-01

2078 9.732503e-01 9.414456e-01 9.241518e-01 9.185432e-01 9.166469e-01 6.913924e-01

2079 9.732184e-01 9.414291e-01 9.241691e-01 9.185870e-01 9.167010e-01 6.906648e-01

2080 9.731846e-01 9.414120e-01 9.241884e-01 9.186342e-01 9.167591e-01 6.899263e-01

2081 9.731488e-01 9.413944e-01 9.242095e-01 9.186850e-01 9.168212e-01 6.891773e-01

2082 9.731108e-01 9.413764e-01 9.242329e-01 9.187397e-01 9.168878e-01 6.884180e-01

2083 9.730705e-01 9.413578e-01 9.242585e-01 9.187986e-01 9.169592e-01 6.876490e-01

2084 9.730277e-01 9.413387e-01 9.242867e-01 9.188621e-01 9.170357e-01 6.868705e-01

2085 9.729824e-01 9.413192e-01 9.243177e-01 9.189303e-01 9.171176e-01 6.860831e-01

2086 9.729343e-01 9.412992e-01 9.243516e-01 9.190037e-01 9.172053e-01 6.852872e-01

2087 9.728833e-01 9.412788e-01 9.243888e-01 9.190827e-01 9.172992e-01 6.844833e-01

2088 9.728292e-01 9.412580e-01 9.244294e-01 9.191676e-01 9.173998e-01 6.836721e-01

2089 9.727718e-01 9.412368e-01 9.244739e-01 9.192589e-01 9.175074e-01 6.828539e-01

2090 9.727110e-01 9.412153e-01 9.245225e-01 9.193570e-01 9.176226e-01 6.820296e-01

2091 9.726464e-01 9.411935e-01 9.245755e-01 9.194625e-01 9.177458e-01 6.811996e-01

2092 9.725780e-01 9.411716e-01 9.246333e-01 9.195757e-01 9.178776e-01 6.803647e-01

2093 9.725054e-01 9.411495e-01 9.246964e-01 9.196973e-01 9.180185e-01 6.795255e-01

2094 9.724285e-01 9.411274e-01 9.247650e-01 9.198278e-01 9.181691e-01 6.786827e-01

2095 9.723469e-01 9.411053e-01 9.248397e-01 9.199678e-01 9.183301e-01 6.778371e-01

2096 9.722604e-01 9.410833e-01 9.249209e-01 9.201181e-01 9.185021e-01 6.769893e-01

2097 9.721688e-01 9.410616e-01 9.250091e-01 9.202791e-01 9.186858e-01 6.761401e-01

2098 9.720716e-01 9.410404e-01 9.251048e-01 9.204518e-01 9.188819e-01 6.752904e-01

2099 9.719687e-01 9.410196e-01 9.252088e-01 9.206368e-01 9.190913e-01 6.744408e-01

2100 9.718596e-01 9.409996e-01 9.253214e-01 9.208349e-01 9.193146e-01 6.735922e-01

2101 9.717441e-01 9.409805e-01 9.254434e-01 9.210470e-01 9.195527e-01 6.727454e-01

2102 9.716218e-01 9.409624e-01 9.255755e-01 9.212739e-01 9.198066e-01 6.719010e-01

2103 9.714923e-01 9.409457e-01 9.257184e-01 9.215166e-01 9.200770e-01 6.710601e-01

2104 9.713552e-01 9.409306e-01 9.258728e-01 9.217761e-01 9.203651e-01 6.702232e-01

2105 9.712102e-01 9.409173e-01 9.260396e-01 9.220533e-01 9.206717e-01 6.693913e-01

2106 9.710567e-01 9.409062e-01 9.262196e-01 9.223493e-01 9.209979e-01 6.685650e-01

2107 9.708945e-01 9.408975e-01 9.264136e-01 9.226652e-01 9.213448e-01 6.677452e-01

2108 9.707229e-01 9.408917e-01 9.266227e-01 9.230022e-01 9.217134e-01 6.669325e-01

2109 9.705417e-01 9.408891e-01 9.268479e-01 9.233614e-01 9.221050e-01 6.661277e-01

2110 9.703502e-01 9.408902e-01 9.270900e-01 9.237440e-01 9.225205e-01 6.653316e-01

2111 9.701481e-01 9.408954e-01 9.273502e-01 9.241513e-01 9.229614e-01 6.645447e-01

2112 9.699347e-01 9.409051e-01 9.276296e-01 9.245846e-01 9.234287e-01 6.637678e-01

2113 9.697097e-01 9.409200e-01 9.279293e-01 9.250452e-01 9.239237e-01 6.630014e-01

2114 9.694725e-01 9.409406e-01 9.282506e-01 9.255344e-01 9.244478e-01 6.622462e-01

2115 9.692226e-01 9.409675e-01 9.285945e-01 9.260537e-01 9.250020e-01 6.615027e-01

2116 9.689594e-01 9.410013e-01 9.289624e-01 9.266045e-01 9.255878e-01 6.607714e-01

2117 9.686824e-01 9.410427e-01 9.293555e-01 9.271881e-01 9.262064e-01 6.600528e-01

2118 9.683912e-01 9.410924e-01 9.297751e-01 9.278059e-01 9.268591e-01 6.593475e-01

2119 9.680851e-01 9.411512e-01 9.302225e-01 9.284594e-01 9.275472e-01 6.586557e-01

2120 9.677637e-01 9.412199e-01 9.306990e-01 9.291500e-01 9.282719e-01 6.579779e-01

2121 9.674264e-01 9.412993e-01 9.312059e-01 9.298790e-01 9.290345e-01 6.573145e-01

2122 9.670728e-01 9.413902e-01 9.317445e-01 9.306479e-01 9.298360e-01 6.566656e-01

2123 9.667023e-01 9.414936e-01 9.323161e-01 9.314579e-01 9.306778e-01 6.560316e-01

2124 9.663147e-01 9.416103e-01 9.329219e-01 9.323103e-01 9.315608e-01 6.554127e-01

2125 9.659093e-01 9.417414e-01 9.335631e-01 9.332064e-01 9.324860e-01 6.548091e-01

2126 9.654860e-01 9.418876e-01 9.342410e-01 9.341472e-01 9.334545e-01 6.542209e-01

2127 9.650442e-01 9.420501e-01 9.349565e-01 9.351340e-01 9.344671e-01 6.536482e-01

2128 9.645838e-01 9.422298e-01 9.357108e-01 9.361675e-01 9.355245e-01 6.530911e-01

2129 9.641046e-01 9.424277e-01 9.365049e-01 9.372488e-01 9.366274e-01 6.525496e-01

2130 9.636062e-01 9.426446e-01 9.373394e-01 9.383786e-01 9.377764e-01 6.520237e-01

2131 9.630887e-01 9.428816e-01 9.382152e-01 9.395575e-01 9.389717e-01 6.515134e-01

2132 9.625520e-01 9.431395e-01 9.391329e-01 9.407859e-01 9.402137e-01 6.510186e-01

2133 9.619962e-01 9.434192e-01 9.400930e-01 9.420642e-01 9.415025e-01 6.505392e-01

2134 9.614215e-01 9.437216e-01 9.410958e-01 9.433926e-01 9.428381e-01 6.500751e-01

2135 9.608280e-01 9.440474e-01 9.421414e-01 9.447709e-01 9.442202e-01 6.496261e-01

2136 9.602161e-01 9.443972e-01 9.432299e-01 9.461991e-01 9.456485e-01 6.491921e-01

2137 9.595863e-01 9.447717e-01 9.443611e-01 9.476766e-01 9.471224e-01 6.487729e-01

2138 9.589392e-01 9.451714e-01 9.455345e-01 9.492028e-01 9.486412e-01 6.483681e-01

2139 9.582753e-01 9.455967e-01 9.467495e-01 9.507770e-01 9.502038e-01 6.479777e-01

2140 9.575957e-01 9.460479e-01 9.480053e-01 9.523979e-01 9.518092e-01 6.476014e-01

2141 9.569011e-01 9.465252e-01 9.493010e-01 9.540644e-01 9.534560e-01 6.472387e-01

2142 9.561926e-01 9.470286e-01 9.506351e-01 9.557749e-01 9.551426e-01 6.468896e-01

2143 9.554714e-01 9.475580e-01 9.520063e-01 9.575278e-01 9.568673e-01 6.465537e-01

2144 9.547388e-01 9.481132e-01 9.534129e-01 9.593212e-01 9.586283e-01 6.462306e-01

2145 9.539962e-01 9.486939e-01 9.548530e-01 9.611530e-01 9.604233e-01 6.459201e-01

2146 9.532451e-01 9.492995e-01 9.563246e-01 9.630209e-01 9.622503e-01 6.456218e-01

2147 9.524871e-01 9.499293e-01 9.578254e-01 9.649224e-01 9.641068e-01 6.453354e-01

2148 9.517239e-01 9.505826e-01 9.593530e-01 9.668551e-01 9.659903e-01 6.450606e-01

2149 9.532863e-01 9.534200e-01 9.626411e-01 9.702186e-01 9.693476e-01 6.497834e-01

2150 9.532861e-01 9.534200e-01 9.626413e-01 9.702187e-01 9.693477e-01 6.497158e-01

2151 9.532860e-01 9.534200e-01 9.626414e-01 9.702189e-01 9.693479e-01 6.496468e-01

2152 9.532858e-01 9.534199e-01 9.626415e-01 9.702191e-01 9.693481e-01 6.495762e-01

2153 9.532856e-01 9.534199e-01 9.626416e-01 9.702193e-01 9.693483e-01 6.495042e-01

2154 9.532854e-01 9.534199e-01 9.626417e-01 9.702195e-01 9.693484e-01 6.494306e-01

2155 9.532853e-01 9.534199e-01 9.626418e-01 9.702197e-01 9.693486e-01 6.493554e-01

2156 9.532851e-01 9.534198e-01 9.626420e-01 9.702199e-01 9.693488e-01 6.492786e-01

2157 9.532849e-01 9.534198e-01 9.626421e-01 9.702201e-01 9.693491e-01 6.492003e-01

2158 9.532846e-01 9.534198e-01 9.626423e-01 9.702204e-01 9.693493e-01 6.491203e-01

2159 9.532844e-01 9.534198e-01 9.626424e-01 9.702206e-01 9.693495e-01 6.490386e-01

2160 9.532842e-01 9.534198e-01 9.626426e-01 9.702208e-01 9.693498e-01 6.489553e-01

2161 9.532840e-01 9.534198e-01 9.626427e-01 9.702211e-01 9.693500e-01 6.488702e-01

2162 9.532837e-01 9.534198e-01 9.626429e-01 9.702213e-01 9.693503e-01 6.487834e-01

2163 9.532835e-01 9.534198e-01 9.626431e-01 9.702216e-01 9.693505e-01 6.486948e-01

2164 9.532832e-01 9.534198e-01 9.626433e-01 9.702219e-01 9.693508e-01 6.486044e-01

2165 9.532829e-01 9.534198e-01 9.626435e-01 9.702222e-01 9.693511e-01 6.485121e-01

2166 9.532826e-01 9.534199e-01 9.626437e-01 9.702225e-01 9.693514e-01 6.484180e-01

2167 9.532823e-01 9.534199e-01 9.626440e-01 9.702228e-01 9.693517e-01 6.483220e-01

2168 9.532820e-01 9.534199e-01 9.626442e-01 9.702231e-01 9.693520e-01 6.482240e-01

2169 9.532817e-01 9.534200e-01 9.626445e-01 9.702235e-01 9.693524e-01 6.481240e-01

2170 9.532814e-01 9.534200e-01 9.626447e-01 9.702238e-01 9.693527e-01 6.480220e-01

2171 9.532811e-01 9.534201e-01 9.626450e-01 9.702242e-01 9.693531e-01 6.479180e-01

2172 9.532807e-01 9.534201e-01 9.626453e-01 9.702246e-01 9.693534e-01 6.478118e-01

2173 9.532803e-01 9.534202e-01 9.626456e-01 9.702250e-01 9.693538e-01 6.477036e-01

2174 9.532800e-01 9.534203e-01 9.626460e-01 9.702254e-01 9.693543e-01 6.475931e-01

2175 9.532796e-01 9.534204e-01 9.626463e-01 9.702259e-01 9.693547e-01 6.474805e-01

2176 9.532792e-01 9.534205e-01 9.626467e-01 9.702264e-01 9.693551e-01 6.473656e-01

2177 9.532788e-01 9.534206e-01 9.626471e-01 9.702269e-01 9.693556e-01 6.472484e-01

2178 9.532783e-01 9.534207e-01 9.626475e-01 9.702274e-01 9.693561e-01 6.471289e-01

2179 9.532779e-01 9.534208e-01 9.626479e-01 9.702279e-01 9.693566e-01 6.470070e-01

2180 9.532774e-01 9.534210e-01 9.626484e-01 9.702285e-01 9.693571e-01 6.468827e-01

2181 9.532770e-01 9.534211e-01 9.626489e-01 9.702291e-01 9.693577e-01 6.467560e-01

2182 9.532765e-01 9.534213e-01 9.626494e-01 9.702297e-01 9.693583e-01 6.466267e-01

2183 9.532760e-01 9.534215e-01 9.626499e-01 9.702303e-01 9.693589e-01 6.464949e-01

2184 9.532755e-01 9.534216e-01 9.626504e-01 9.702310e-01 9.693595e-01 6.463606e-01

2185 9.532749e-01 9.534219e-01 9.626510e-01 9.702317e-01 9.693602e-01 6.462236e-01

2186 9.532744e-01 9.534221e-01 9.626516e-01 9.702324e-01 9.693609e-01 6.460840e-01

2187 9.532738e-01 9.534223e-01 9.626523e-01 9.702332e-01 9.693616e-01 6.459416e-01

2188 9.532732e-01 9.534226e-01 9.626530e-01 9.702340e-01 9.693624e-01 6.457966e-01

2189 9.532726e-01 9.534229e-01 9.626537e-01 9.702349e-01 9.693632e-01 6.456487e-01

2190 9.532720e-01 9.534232e-01 9.626545e-01 9.702358e-01 9.693640e-01 6.454980e-01

2191 9.532713e-01 9.534236e-01 9.626552e-01 9.702367e-01 9.693649e-01 6.453445e-01

2192 9.532707e-01 9.534240e-01 9.626561e-01 9.702377e-01 9.693658e-01 6.451880e-01

2193 9.532700e-01 9.534244e-01 9.626570e-01 9.702387e-01 9.693668e-01 6.450286e-01

2194 9.532693e-01 9.534248e-01 9.626579e-01 9.702397e-01 9.693678e-01 6.448662e-01

2195 9.532685e-01 9.534253e-01 9.626589e-01 9.702409e-01 9.693689e-01 6.447008e-01

2196 9.532678e-01 9.534258e-01 9.626599e-01 9.702420e-01 9.693700e-01 6.445323e-01

2197 9.532670e-01 9.534263e-01 9.626610e-01 9.702432e-01 9.693711e-01 6.443608e-01

2198 9.532662e-01 9.534269e-01 9.626621e-01 9.702445e-01 9.693724e-01 6.441861e-01

2199 9.532654e-01 9.534276e-01 9.626633e-01 9.702459e-01 9.693736e-01 6.440082e-01

2200 9.532645e-01 9.534283e-01 9.626646e-01 9.702473e-01 9.693750e-01 6.438272e-01

2201 9.532636e-01 9.534290e-01 9.626659e-01 9.702487e-01 9.693764e-01 6.436429e-01

2202 9.532627e-01 9.534298e-01 9.626673e-01 9.702503e-01 9.693778e-01 6.434553e-01

2203 9.532618e-01 9.534306e-01 9.626688e-01 9.702519e-01 9.693794e-01 6.432645e-01

2204 9.532608e-01 9.534315e-01 9.626703e-01 9.702536e-01 9.693810e-01 6.430704e-01

2205 9.532599e-01 9.534325e-01 9.626720e-01 9.702553e-01 9.693826e-01 6.428729e-01

2206 9.532589e-01 9.534335e-01 9.626737e-01 9.702572e-01 9.693844e-01 6.426721e-01

2207 9.532578e-01 9.534347e-01 9.626755e-01 9.702591e-01 9.693862e-01 6.424679e-01

2208 9.532568e-01 9.534359e-01 9.626774e-01 9.702612e-01 9.693882e-01 6.422603e-01

2209 9.532557e-01 9.534371e-01 9.626794e-01 9.702633e-01 9.693902e-01 6.420494e-01

2210 9.532545e-01 9.534385e-01 9.626816e-01 9.702655e-01 9.693923e-01 6.418350e-01

2211 9.532534e-01 9.534400e-01 9.626838e-01 9.702679e-01 9.693946e-01 6.416172e-01

2212 9.532522e-01 9.534416e-01 9.626862e-01 9.702704e-01 9.693969e-01 6.413960e-01

2213 9.532510e-01 9.534433e-01 9.626887e-01 9.702729e-01 9.693994e-01 6.411714e-01

2214 9.532498e-01 9.534451e-01 9.626913e-01 9.702757e-01 9.694019e-01 6.409433e-01

2215 9.532485e-01 9.534470e-01 9.626941e-01 9.702785e-01 9.694046e-01 6.407119e-01

2216 9.532472e-01 9.534491e-01 9.626971e-01 9.702815e-01 9.694075e-01 6.404771e-01

2217 9.532459e-01 9.534513e-01 9.627002e-01 9.702846e-01 9.694104e-01 6.402389e-01

2218 9.532446e-01 9.534536e-01 9.627034e-01 9.702879e-01 9.694136e-01 6.399974e-01

2219 9.532432e-01 9.534562e-01 9.627069e-01 9.702914e-01 9.694168e-01 6.397525e-01

2220 9.532418e-01 9.534589e-01 9.627105e-01 9.702950e-01 9.694203e-01 6.395044e-01

2221 9.532404e-01 9.534617e-01 9.627144e-01 9.702988e-01 9.694239e-01 6.392531e-01

2222 9.532390e-01 9.534648e-01 9.627184e-01 9.703028e-01 9.694277e-01 6.389985e-01

2223 9.532375e-01 9.534681e-01 9.627227e-01 9.703071e-01 9.694317e-01 6.387409e-01

2224 9.532361e-01 9.534716e-01 9.627273e-01 9.703115e-01 9.694359e-01 6.384801e-01

2225 9.532346e-01 9.534754e-01 9.627320e-01 9.703161e-01 9.694403e-01 6.382164e-01

2226 9.532331e-01 9.534794e-01 9.627371e-01 9.703210e-01 9.694449e-01 6.379497e-01

2227 9.532316e-01 9.534837e-01 9.627424e-01 9.703261e-01 9.694498e-01 6.376802e-01

2228 9.532301e-01 9.534882e-01 9.627480e-01 9.703315e-01 9.694549e-01 6.374080e-01

2229 9.532286e-01 9.534931e-01 9.627540e-01 9.703372e-01 9.694602e-01 6.371330e-01

2230 9.532271e-01 9.534983e-01 9.627603e-01 9.703432e-01 9.694659e-01 6.368555e-01

2231 9.532256e-01 9.535039e-01 9.627669e-01 9.703495e-01 9.694718e-01 6.365756e-01

2232 9.532241e-01 9.535099e-01 9.627739e-01 9.703561e-01 9.694781e-01 6.362933e-01

2233 9.532226e-01 9.535162e-01 9.627814e-01 9.703630e-01 9.694847e-01 6.360089e-01

2234 9.532211e-01 9.535230e-01 9.627892e-01 9.703703e-01 9.694916e-01 6.357224e-01

2235 9.532197e-01 9.535302e-01 9.627975e-01 9.703780e-01 9.694989e-01 6.354340e-01

2236 9.532183e-01 9.535379e-01 9.628062e-01 9.703861e-01 9.695065e-01 6.351439e-01

2237 9.532170e-01 9.535461e-01 9.628155e-01 9.703946e-01 9.695146e-01 6.348522e-01

2238 9.532157e-01 9.535549e-01 9.628253e-01 9.704035e-01 9.695231e-01 6.345591e-01

2239 9.532145e-01 9.535643e-01 9.628357e-01 9.704130e-01 9.695320e-01 6.342648e-01

2240 9.532134e-01 9.535743e-01 9.628466e-01 9.704229e-01 9.695414e-01 6.339695e-01

2241 9.532124e-01 9.535849e-01 9.628582e-01 9.704334e-01 9.695513e-01 6.336733e-01

2242 9.532115e-01 9.535963e-01 9.628704e-01 9.704444e-01 9.695617e-01 6.333766e-01

2243 9.532107e-01 9.536085e-01 9.628834e-01 9.704560e-01 9.695727e-01 6.330795e-01

2244 9.532101e-01 9.536214e-01 9.628971e-01 9.704682e-01 9.695843e-01 6.327822e-01

2245 9.532096e-01 9.536352e-01 9.629116e-01 9.704810e-01 9.695964e-01 6.324850e-01

2246 9.532093e-01 9.536500e-01 9.629269e-01 9.704946e-01 9.696093e-01 6.321881e-01

2247 9.532092e-01 9.536657e-01 9.629432e-01 9.705089e-01 9.696228e-01 6.318918e-01

2248 9.532093e-01 9.536825e-01 9.629604e-01 9.705239e-01 9.696371e-01 6.315963e-01

2249 9.532097e-01 9.537004e-01 9.629786e-01 9.705398e-01 9.696521e-01 6.313019e-01

2250 9.532104e-01 9.537194e-01 9.629978e-01 9.705565e-01 9.696680e-01 6.310089e-01

2251 9.532114e-01 9.537398e-01 9.630182e-01 9.705741e-01 9.696847e-01 6.307175e-01

2252 9.532128e-01 9.537615e-01 9.630397e-01 9.705927e-01 9.697023e-01 6.304281e-01

2253 9.532145e-01 9.537846e-01 9.630626e-01 9.706123e-01 9.697209e-01 6.301409e-01

2254 9.532167e-01 9.538093e-01 9.630868e-01 9.706330e-01 9.697404e-01 6.298562e-01

2255 9.532194e-01 9.538356e-01 9.631123e-01 9.706548e-01 9.697611e-01 6.295742e-01

2256 9.532226e-01 9.538637e-01 9.631394e-01 9.706777e-01 9.697829e-01 6.292954e-01

2257 9.532264e-01 9.538937e-01 9.631681e-01 9.707020e-01 9.698059e-01 6.290199e-01

2258 9.532308e-01 9.539256e-01 9.631985e-01 9.707275e-01 9.698301e-01 6.287481e-01

2259 9.532359e-01 9.539596e-01 9.632307e-01 9.707545e-01 9.698557e-01 6.284802e-01

2260 9.532419e-01 9.539959e-01 9.632647e-01 9.707829e-01 9.698827e-01 6.282166e-01

2261 9.532486e-01 9.540346e-01 9.633008e-01 9.708129e-01 9.699112e-01 6.279574e-01

2262 9.532563e-01 9.540758e-01 9.633390e-01 9.708446e-01 9.699413e-01 6.277031e-01

2263 9.532651e-01 9.541198e-01 9.633795e-01 9.708780e-01 9.699731e-01 6.274539e-01

2264 9.532749e-01 9.541666e-01 9.634223e-01 9.709133e-01 9.700066e-01 6.272100e-01

2265 9.532859e-01 9.542165e-01 9.634677e-01 9.709505e-01 9.700419e-01 6.269717e-01

2266 9.532983e-01 9.542698e-01 9.635158e-01 9.709898e-01 9.700792e-01 6.267392e-01

2267 9.533122e-01 9.543265e-01 9.635667e-01 9.710312e-01 9.701187e-01 6.265129e-01

2268 9.533276e-01 9.543869e-01 9.636206e-01 9.710749e-01 9.701603e-01 6.262928e-01

2269 9.533447e-01 9.544513e-01 9.636776e-01 9.711211e-01 9.702042e-01 6.260793e-01

2270 9.533637e-01 9.545198e-01 9.637381e-01 9.711698e-01 9.702506e-01 6.258725e-01

2271 9.533847e-01 9.545929e-01 9.638021e-01 9.712213e-01 9.702995e-01 6.256726e-01

2272 9.534079e-01 9.546707e-01 9.638699e-01 9.712755e-01 9.703512e-01 6.254798e-01

2273 9.534335e-01 9.547535e-01 9.639417e-01 9.713328e-01 9.704058e-01 6.252942e-01

2274 9.534617e-01 9.548417e-01 9.640177e-01 9.713933e-01 9.704634e-01 6.251161e-01

2275 9.534926e-01 9.549356e-01 9.640981e-01 9.714571e-01 9.705242e-01 6.249455e-01

2276 9.535266e-01 9.550356e-01 9.641833e-01 9.715245e-01 9.705885e-01 6.247825e-01

2277 9.535639e-01 9.551419e-01 9.642735e-01 9.715956e-01 9.706563e-01 6.246272e-01

2278 9.536048e-01 9.552551e-01 9.643689e-01 9.716706e-01 9.707279e-01 6.244797e-01

2279 9.536494e-01 9.553755e-01 9.644699e-01 9.717498e-01 9.708034e-01 6.243400e-01

2280 9.536982e-01 9.555036e-01 9.645768e-01 9.718334e-01 9.708832e-01 6.242083e-01

2281 9.537515e-01 9.556398e-01 9.646899e-01 9.719216e-01 9.709674e-01 6.240844e-01

2282 9.538095e-01 9.557845e-01 9.648095e-01 9.720146e-01 9.710562e-01 6.239684e-01

2283 9.538728e-01 9.559384e-01 9.649361e-01 9.721127e-01 9.711500e-01 6.238603e-01

2284 9.539415e-01 9.561019e-01 9.650699e-01 9.722162e-01 9.712490e-01 6.237600e-01

2285 9.540163e-01 9.562756e-01 9.652114e-01 9.723254e-01 9.713534e-01 6.236675e-01

2286 9.540975e-01 9.564601e-01 9.653611e-01 9.724406e-01 9.714635e-01 6.235827e-01

2287 9.541855e-01 9.566560e-01 9.655192e-01 9.725620e-01 9.715797e-01 6.235055e-01

2288 9.542810e-01 9.568639e-01 9.656863e-01 9.726900e-01 9.717022e-01 6.234358e-01

2289 9.543844e-01 9.570845e-01 9.658629e-01 9.728250e-01 9.718314e-01 6.233735e-01

2290 9.544962e-01 9.573186e-01 9.660494e-01 9.729672e-01 9.719676e-01 6.233184e-01

2291 9.546171e-01 9.575667e-01 9.662463e-01 9.731171e-01 9.721111e-01 6.232705e-01

2292 9.547477e-01 9.578297e-01 9.664542e-01 9.732750e-01 9.722624e-01 6.232295e-01

2293 9.548887e-01 9.581084e-01 9.666737e-01 9.734413e-01 9.724218e-01 6.231952e-01

2294 9.550406e-01 9.584036e-01 9.669052e-01 9.736164e-01 9.725897e-01 6.231676e-01

2295 9.552043e-01 9.587162e-01 9.671494e-01 9.738008e-01 9.727666e-01 6.231463e-01

2296 9.553805e-01 9.590469e-01 9.674069e-01 9.739949e-01 9.729527e-01 6.231312e-01

2297 9.555699e-01 9.593968e-01 9.676784e-01 9.741992e-01 9.731487e-01 6.231221e-01

2298 9.557734e-01 9.597667e-01 9.679644e-01 9.744140e-01 9.733548e-01 6.231187e-01

2299 9.559919e-01 9.601577e-01 9.682657e-01 9.746400e-01 9.735717e-01 6.231209e-01

2300 9.562261e-01 9.605707e-01 9.685829e-01 9.748776e-01 9.737997e-01 6.231284e-01

2301 9.564771e-01 9.610067e-01 9.689168e-01 9.751273e-01 9.740395e-01 6.231409e-01

2302 9.567457e-01 9.614667e-01 9.692680e-01 9.753896e-01 9.742914e-01 6.231583e-01

2303 9.570329e-01 9.619519e-01 9.696374e-01 9.756652e-01 9.745559e-01 6.231803e-01

2304 9.573398e-01 9.624632e-01 9.700257e-01 9.759545e-01 9.748338e-01 6.232067e-01

2305 9.576672e-01 9.630018e-01 9.704337e-01 9.762581e-01 9.751253e-01 6.232372e-01

2306 9.580162e-01 9.635689e-01 9.708621e-01 9.765766e-01 9.754313e-01 6.232716e-01

2307 9.583879e-01 9.641654e-01 9.713117e-01 9.769106e-01 9.757521e-01 6.233097e-01

2308 9.587833e-01 9.647925e-01 9.717834e-01 9.772607e-01 9.760883e-01 6.233512e-01

2309 9.592035e-01 9.654514e-01 9.722780e-01 9.776275e-01 9.764406e-01 6.233960e-01

2310 9.596495e-01 9.661431e-01 9.727962e-01 9.780116e-01 9.768095e-01 6.234437e-01

2311 9.601222e-01 9.668688e-01 9.733390e-01 9.784137e-01 9.771955e-01 6.234942e-01

2312 9.606229e-01 9.676296e-01 9.739071e-01 9.788343e-01 9.775994e-01 6.235473e-01

2313 9.611524e-01 9.684265e-01 9.745013e-01 9.792742e-01 9.780216e-01 6.236027e-01

2314 9.617118e-01 9.692605e-01 9.751225e-01 9.797339e-01 9.784627e-01 6.236603e-01

2315 9.623019e-01 9.701327e-01 9.757714e-01 9.802141e-01 9.789234e-01 6.237199e-01

2316 9.629237e-01 9.710441e-01 9.764489e-01 9.807155e-01 9.794041e-01 6.237812e-01

2317 9.635779e-01 9.719954e-01 9.771557e-01 9.812388e-01 9.799055e-01 6.238441e-01

2318 9.642655e-01 9.729876e-01 9.778926e-01 9.817847e-01 9.804281e-01 6.239085e-01

2319 9.649870e-01 9.740214e-01 9.786604e-01 9.823537e-01 9.809725e-01 6.239741e-01

2320 9.657431e-01 9.750976e-01 9.794599e-01 9.829468e-01 9.815393e-01 6.240409e-01

2321 9.665342e-01 9.762167e-01 9.802917e-01 9.835644e-01 9.821291e-01 6.241086e-01

2322 9.673607e-01 9.773793e-01 9.811567e-01 9.842074e-01 9.827425e-01 6.241770e-01

2323 9.682228e-01 9.785857e-01 9.820555e-01 9.848765e-01 9.833799e-01 6.242462e-01

2324 9.691207e-01 9.798364e-01 9.829889e-01 9.855722e-01 9.840419e-01 6.243159e-01

2325 9.700543e-01 9.811314e-01 9.839574e-01 9.862953e-01 9.847292e-01 6.243861e-01

2326 9.710234e-01 9.824710e-01 9.849616e-01 9.870464e-01 9.854420e-01 6.244566e-01

2327 9.720278e-01 9.838550e-01 9.860022e-01 9.878262e-01 9.861810e-01 6.245272e-01

2328 9.742736e-01 9.859433e-01 9.876744e-01 9.891736e-01 9.875737e-01 6.294900e-01

2329 9.742737e-01 9.859435e-01 9.876745e-01 9.891737e-01 9.875737e-01 6.294781e-01

2330 9.742737e-01 9.859436e-01 9.876746e-01 9.891738e-01 9.875738e-01 6.294664e-01

2331 9.742738e-01 9.859437e-01 9.876747e-01 9.891738e-01 9.875738e-01 6.294548e-01

2332 9.742739e-01 9.859439e-01 9.876748e-01 9.891739e-01 9.875739e-01 6.294436e-01

2333 9.742740e-01 9.859441e-01 9.876749e-01 9.891740e-01 9.875739e-01 6.294325e-01

2334 9.742741e-01 9.859442e-01 9.876750e-01 9.891740e-01 9.875740e-01 6.294217e-01

2335 9.742741e-01 9.859444e-01 9.876751e-01 9.891741e-01 9.875741e-01 6.294113e-01

2336 9.742742e-01 9.859446e-01 9.876752e-01 9.891742e-01 9.875741e-01 6.294011e-01

2337 9.742743e-01 9.859448e-01 9.876753e-01 9.891743e-01 9.875742e-01 6.293912e-01

2338 9.742744e-01 9.859450e-01 9.876755e-01 9.891744e-01 9.875743e-01 6.293817e-01

2339 9.742746e-01 9.859452e-01 9.876756e-01 9.891745e-01 9.875744e-01 6.293725e-01

2340 9.742747e-01 9.859454e-01 9.876757e-01 9.891746e-01 9.875745e-01 6.293637e-01

2341 9.742748e-01 9.859457e-01 9.876759e-01 9.891747e-01 9.875746e-01 6.293553e-01

2342 9.742749e-01 9.859459e-01 9.876760e-01 9.891748e-01 9.875747e-01 6.293473e-01

2343 9.742751e-01 9.859461e-01 9.876762e-01 9.891749e-01 9.875748e-01 6.293398e-01

2344 9.742752e-01 9.859464e-01 9.876764e-01 9.891750e-01 9.875749e-01 6.293328e-01

2345 9.742754e-01 9.859466e-01 9.876766e-01 9.891751e-01 9.875750e-01 6.293263e-01

2346 9.742756e-01 9.859469e-01 9.876768e-01 9.891753e-01 9.875751e-01 6.293203e-01

2347 9.742757e-01 9.859472e-01 9.876769e-01 9.891754e-01 9.875752e-01 6.293149e-01

2348 9.742759e-01 9.859475e-01 9.876771e-01 9.891755e-01 9.875753e-01 6.293101e-01

2349 9.742761e-01 9.859478e-01 9.876774e-01 9.891757e-01 9.875754e-01 6.293059e-01

2350 9.742763e-01 9.859481e-01 9.876776e-01 9.891758e-01 9.875756e-01 6.293024e-01

2351 9.742766e-01 9.859484e-01 9.876778e-01 9.891760e-01 9.875757e-01 6.292996e-01

2352 9.742768e-01 9.859487e-01 9.876780e-01 9.891761e-01 9.875758e-01 6.292976e-01

2353 9.742771e-01 9.859491e-01 9.876783e-01 9.891763e-01 9.875760e-01 6.292963e-01

2354 9.742773e-01 9.859494e-01 9.876785e-01 9.891765e-01 9.875761e-01 6.292959e-01

2355 9.742776e-01 9.859498e-01 9.876788e-01 9.891767e-01 9.875763e-01 6.292963e-01

2356 9.742779e-01 9.859502e-01 9.876791e-01 9.891769e-01 9.875764e-01 6.292976e-01

2357 9.742782e-01 9.859506e-01 9.876794e-01 9.891771e-01 9.875766e-01 6.292999e-01

2358 9.742785e-01 9.859510e-01 9.876797e-01 9.891773e-01 9.875768e-01 6.293032e-01

2359 9.742789e-01 9.859515e-01 9.876800e-01 9.891775e-01 9.875769e-01 6.293076e-01

2360 9.742792e-01 9.859519e-01 9.876803e-01 9.891777e-01 9.875771e-01 6.293131e-01

2361 9.742796e-01 9.859524e-01 9.876807e-01 9.891779e-01 9.875773e-01 6.293197e-01

2362 9.742800e-01 9.859529e-01 9.876810e-01 9.891782e-01 9.875775e-01 6.293275e-01

2363 9.742804e-01 9.859534e-01 9.876814e-01 9.891784e-01 9.875777e-01 6.293367e-01

2364 9.742809e-01 9.859540e-01 9.876818e-01 9.891787e-01 9.875779e-01 6.293471e-01

2365 9.742813e-01 9.859546e-01 9.876822e-01 9.891789e-01 9.875782e-01 6.293590e-01

2366 9.742818e-01 9.859551e-01 9.876826e-01 9.891792e-01 9.875784e-01 6.293723e-01

2367 9.742823e-01 9.859558e-01 9.876830e-01 9.891795e-01 9.875787e-01 6.293872e-01

2368 9.742829e-01 9.859564e-01 9.876835e-01 9.891798e-01 9.875789e-01 6.294036e-01

2369 9.742835e-01 9.859571e-01 9.876840e-01 9.891801e-01 9.875792e-01 6.294218e-01

2370 9.742841e-01 9.859578e-01 9.876845e-01 9.891805e-01 9.875795e-01 6.294416e-01

2371 9.742847e-01 9.859585e-01 9.876850e-01 9.891808e-01 9.875798e-01 6.294633e-01

2372 9.742854e-01 9.859593e-01 9.876855e-01 9.891812e-01 9.875801e-01 6.294869e-01

2373 9.742861e-01 9.859601e-01 9.876861e-01 9.891816e-01 9.875804e-01 6.295125e-01

2374 9.742868e-01 9.859609e-01 9.876867e-01 9.891820e-01 9.875807e-01 6.295401e-01

2375 9.742876e-01 9.859618e-01 9.876873e-01 9.891824e-01 9.875810e-01 6.295699e-01

2376 9.742885e-01 9.859627e-01 9.876880e-01 9.891828e-01 9.875814e-01 6.296020e-01

2377 9.742893e-01 9.859636e-01 9.876886e-01 9.891832e-01 9.875818e-01 6.296364e-01

2378 9.742903e-01 9.859646e-01 9.876893e-01 9.891837e-01 9.875822e-01 6.296732e-01

2379 9.742912e-01 9.859656e-01 9.876901e-01 9.891842e-01 9.875826e-01 6.297126e-01

2380 9.742923e-01 9.859667e-01 9.876908e-01 9.891847e-01 9.875830e-01 6.297545e-01

2381 9.742934e-01 9.859678e-01 9.876916e-01 9.891852e-01 9.875835e-01 6.297993e-01

2382 9.742945e-01 9.859689e-01 9.876924e-01 9.891858e-01 9.875839e-01 6.298469e-01

2383 9.742957e-01 9.859701e-01 9.876933e-01 9.891864e-01 9.875844e-01 6.298974e-01

2384 9.742970e-01 9.859714e-01 9.876942e-01 9.891870e-01 9.875849e-01 6.299510e-01

2385 9.742983e-01 9.859727e-01 9.876952e-01 9.891876e-01 9.875854e-01 6.300079e-01

2386 9.742997e-01 9.859741e-01 9.876962e-01 9.891883e-01 9.875860e-01 6.300680e-01

2387 9.743012e-01 9.859755e-01 9.876972e-01 9.891890e-01 9.875866e-01 6.301316e-01

2388 9.743028e-01 9.859770e-01 9.876983e-01 9.891897e-01 9.875872e-01 6.301987e-01

2389 9.743044e-01 9.859786e-01 9.876994e-01 9.891905e-01 9.875878e-01 6.302695e-01

2390 9.743062e-01 9.859803e-01 9.877006e-01 9.891912e-01 9.875885e-01 6.303442e-01

2391 9.743080e-01 9.859820e-01 9.877018e-01 9.891921e-01 9.875891e-01 6.304228e-01

2392 9.743100e-01 9.859837e-01 9.877031e-01 9.891929e-01 9.875899e-01 6.305055e-01

2393 9.743120e-01 9.859856e-01 9.877045e-01 9.891938e-01 9.875906e-01 6.305925e-01

2394 9.743142e-01 9.859876e-01 9.877059e-01 9.891948e-01 9.875914e-01 6.306839e-01

2395 9.743164e-01 9.859896e-01 9.877074e-01 9.891958e-01 9.875922e-01 6.307798e-01

2396 9.743188e-01 9.859917e-01 9.877089e-01 9.891968e-01 9.875931e-01 6.308803e-01

2397 9.743214e-01 9.859940e-01 9.877106e-01 9.891979e-01 9.875940e-01 6.309857e-01

2398 9.743240e-01 9.859963e-01 9.877122e-01 9.891990e-01 9.875949e-01 6.310962e-01

2399 9.743269e-01 9.859987e-01 9.877140e-01 9.892002e-01 9.875959e-01 6.312117e-01

2400 9.743298e-01 9.860013e-01 9.877159e-01 9.892014e-01 9.875969e-01 6.313326e-01

2401 9.743330e-01 9.860039e-01 9.877178e-01 9.892027e-01 9.875980e-01 6.314589e-01

2402 9.743363e-01 9.860067e-01 9.877198e-01 9.892040e-01 9.875991e-01 6.315909e-01

2403 9.743398e-01 9.860096e-01 9.877220e-01 9.892054e-01 9.876002e-01 6.317287e-01

2404 9.743435e-01 9.860126e-01 9.877242e-01 9.892069e-01 9.876015e-01 6.318724e-01

2405 9.743474e-01 9.860158e-01 9.877265e-01 9.892084e-01 9.876028e-01 6.320223e-01

2406 9.743515e-01 9.860191e-01 9.877289e-01 9.892100e-01 9.876041e-01 6.321784e-01

2407 9.743558e-01 9.860226e-01 9.877315e-01 9.892117e-01 9.876055e-01 6.323411e-01

2408 9.743604e-01 9.860263e-01 9.877342e-01 9.892135e-01 9.876070e-01 6.325104e-01

2409 9.743652e-01 9.860301e-01 9.877369e-01 9.892153e-01 9.876085e-01 6.326864e-01

2410 9.743703e-01 9.860341e-01 9.877399e-01 9.892173e-01 9.876101e-01 6.328695e-01

2411 9.743757e-01 9.860382e-01 9.877429e-01 9.892193e-01 9.876118e-01 6.330597e-01

2412 9.743814e-01 9.860426e-01 9.877461e-01 9.892214e-01 9.876136e-01 6.332571e-01

2413 9.743874e-01 9.860472e-01 9.877495e-01 9.892237e-01 9.876154e-01 6.334621e-01

2414 9.743937e-01 9.860520e-01 9.877530e-01 9.892260e-01 9.876173e-01 6.336747e-01

2415 9.744004e-01 9.860570e-01 9.877567e-01 9.892284e-01 9.876194e-01 6.338950e-01

2416 9.744075e-01 9.860622e-01 9.877606e-01 9.892310e-01 9.876215e-01 6.341233e-01

2417 9.744150e-01 9.860677e-01 9.877646e-01 9.892337e-01 9.876237e-01 6.343597e-01

2418 9.744229e-01 9.860735e-01 9.877689e-01 9.892365e-01 9.876260e-01 6.346044e-01

2419 9.744312e-01 9.860795e-01 9.877734e-01 9.892394e-01 9.876285e-01 6.348574e-01

2420 9.744400e-01 9.860858e-01 9.877780e-01 9.892425e-01 9.876310e-01 6.351189e-01

2421 9.744493e-01 9.860924e-01 9.877829e-01 9.892457e-01 9.876337e-01 6.353891e-01

2422 9.744591e-01 9.860994e-01 9.877881e-01 9.892491e-01 9.876365e-01 6.356681e-01

2423 9.744694e-01 9.861066e-01 9.877934e-01 9.892527e-01 9.876395e-01 6.359560e-01

2424 9.744804e-01 9.861143e-01 9.877991e-01 9.892564e-01 9.876426e-01 6.362529e-01

2425 9.744919e-01 9.861223e-01 9.878050e-01 9.892603e-01 9.876458e-01 6.365589e-01

2426 9.745042e-01 9.861306e-01 9.878112e-01 9.892644e-01 9.876492e-01 6.368740e-01

2427 9.745171e-01 9.861394e-01 9.878177e-01 9.892687e-01 9.876528e-01 6.371985e-01

2428 9.745307e-01 9.861486e-01 9.878246e-01 9.892732e-01 9.876566e-01 6.375323e-01

2429 9.745451e-01 9.861582e-01 9.878318e-01 9.892779e-01 9.876605e-01 6.378756e-01

2430 9.745604e-01 9.861684e-01 9.878393e-01 9.892829e-01 9.876646e-01 6.382283e-01

2431 9.745765e-01 9.861790e-01 9.878472e-01 9.892881e-01 9.876690e-01 6.385905e-01

2432 9.745935e-01 9.861901e-01 9.878555e-01 9.892936e-01 9.876735e-01 6.389622e-01

2433 9.746115e-01 9.862018e-01 9.878642e-01 9.892993e-01 9.876783e-01 6.393435e-01

2434 9.746305e-01 9.862140e-01 9.878734e-01 9.893053e-01 9.876833e-01 6.397344e-01

2435 9.746505e-01 9.862269e-01 9.878830e-01 9.893117e-01 9.876886e-01 6.401347e-01

2436 9.746718e-01 9.862404e-01 9.878931e-01 9.893183e-01 9.876941e-01 6.405446e-01

2437 9.746942e-01 9.862545e-01 9.879037e-01 9.893253e-01 9.876999e-01 6.409638e-01

2438 9.747179e-01 9.862693e-01 9.879148e-01 9.893326e-01 9.877060e-01 6.413925e-01

2439 9.747430e-01 9.862849e-01 9.879265e-01 9.893403e-01 9.877124e-01 6.418305e-01

2440 9.747695e-01 9.863013e-01 9.879388e-01 9.893484e-01 9.877192e-01 6.422777e-01

2441 9.747975e-01 9.863184e-01 9.879517e-01 9.893569e-01 9.877262e-01 6.427339e-01

2442 9.748271e-01 9.863365e-01 9.879652e-01 9.893658e-01 9.877337e-01 6.431992e-01

2443 9.748583e-01 9.863554e-01 9.879794e-01 9.893752e-01 9.877415e-01 6.436732e-01

2444 9.748914e-01 9.863752e-01 9.879944e-01 9.893850e-01 9.877497e-01 6.441559e-01

2445 9.749264e-01 9.863961e-01 9.880101e-01 9.893954e-01 9.877583e-01 6.446471e-01

2446 9.749633e-01 9.864180e-01 9.880267e-01 9.894062e-01 9.877674e-01 6.451465e-01

2447 9.750024e-01 9.864410e-01 9.880440e-01 9.894177e-01 9.877770e-01 6.456540e-01

2448 9.750437e-01 9.864651e-01 9.880623e-01 9.894297e-01 9.877870e-01 6.461692e-01

2449 9.750873e-01 9.864904e-01 9.880815e-01 9.894424e-01 9.877976e-01 6.466921e-01

2450 9.751335e-01 9.865171e-01 9.881017e-01 9.894557e-01 9.878087e-01 6.472222e-01

2451 9.751822e-01 9.865450e-01 9.881229e-01 9.894697e-01 9.878204e-01 6.477594e-01

2452 9.752338e-01 9.865744e-01 9.881453e-01 9.894844e-01 9.878327e-01 6.483033e-01

2453 9.752883e-01 9.866053e-01 9.881687e-01 9.894998e-01 9.878456e-01 6.488535e-01

2454 9.753458e-01 9.866377e-01 9.881934e-01 9.895161e-01 9.878592e-01 6.494098e-01

2455 9.754067e-01 9.866718e-01 9.882194e-01 9.895332e-01 9.878736e-01 6.499719e-01

2456 9.754710e-01 9.867075e-01 9.882467e-01 9.895512e-01 9.878886e-01 6.505393e-01

2457 9.755389e-01 9.867451e-01 9.882754e-01 9.895702e-01 9.879045e-01 6.511117e-01

2458 9.756107e-01 9.867846e-01 9.883056e-01 9.895901e-01 9.879212e-01 6.516887e-01

2459 9.756866e-01 9.868261e-01 9.883374e-01 9.896111e-01 9.879387e-01 6.522699e-01

2460 9.757667e-01 9.868697e-01 9.883708e-01 9.896332e-01 9.879572e-01 6.528549e-01

2461 9.758513e-01 9.869155e-01 9.884060e-01 9.896564e-01 9.879767e-01 6.534434e-01

2462 9.759407e-01 9.869636e-01 9.884430e-01 9.896809e-01 9.879972e-01 6.540348e-01

2463 9.760351e-01 9.870141e-01 9.884819e-01 9.897066e-01 9.880188e-01 6.546288e-01

2464 9.761348e-01 9.870672e-01 9.885229e-01 9.897337e-01 9.880415e-01 6.552249e-01

2465 9.762401e-01 9.871230e-01 9.885660e-01 9.897622e-01 9.880654e-01 6.558227e-01

2466 9.763512e-01 9.871816e-01 9.886113e-01 9.897922e-01 9.880906e-01 6.564218e-01

2467 9.764685e-01 9.872432e-01 9.886590e-01 9.898238e-01 9.881171e-01 6.570218e-01

2468 9.765923e-01 9.873079e-01 9.887091e-01 9.898570e-01 9.881450e-01 6.576221e-01

2469 9.767229e-01 9.873758e-01 9.887619e-01 9.898920e-01 9.881743e-01 6.582224e-01

2470 9.768607e-01 9.874472e-01 9.888175e-01 9.899289e-01 9.882052e-01 6.588222e-01

2471 9.770061e-01 9.875222e-01 9.888759e-01 9.899676e-01 9.882378e-01 6.594211e-01

2472 9.771594e-01 9.876009e-01 9.889373e-01 9.900085e-01 9.882721e-01 6.600186e-01

2473 9.773210e-01 9.876836e-01 9.890019e-01 9.900514e-01 9.883081e-01 6.606145e-01

2474 9.774914e-01 9.877705e-01 9.890699e-01 9.900966e-01 9.883461e-01 6.612081e-01

2475 9.776710e-01 9.878616e-01 9.891414e-01 9.901442e-01 9.883860e-01 6.617991e-01

2476 9.778603e-01 9.879574e-01 9.892166e-01 9.901943e-01 9.884281e-01 6.623872e-01

2477 9.780596e-01 9.880579e-01 9.892957e-01 9.902470e-01 9.884723e-01 6.629720e-01

2478 9.782696e-01 9.881634e-01 9.893789e-01 9.903025e-01 9.885189e-01 6.635529e-01

2479 9.784907e-01 9.882741e-01 9.894663e-01 9.903608e-01 9.885678e-01 6.641298e-01

2480 9.787234e-01 9.883903e-01 9.895583e-01 9.904222e-01 9.886193e-01 6.647022e-01

2481 9.789683e-01 9.885122e-01 9.896549e-01 9.904868e-01 9.886735e-01 6.652698e-01

2482 9.792259e-01 9.886401e-01 9.897565e-01 9.905548e-01 9.887305e-01 6.658323e-01

2483 9.794968e-01 9.887742e-01 9.898632e-01 9.906262e-01 9.887904e-01 6.663894e-01

2484 9.797816e-01 9.889149e-01 9.899754e-01 9.907014e-01 9.888534e-01 6.669407e-01

2485 9.800809e-01 9.890624e-01 9.900932e-01 9.907804e-01 9.889196e-01 6.674860e-01

2486 9.803954e-01 9.892171e-01 9.902170e-01 9.908635e-01 9.889891e-01 6.680250e-01

2487 9.807256e-01 9.893792e-01 9.903470e-01 9.909509e-01 9.890622e-01 6.685574e-01

2488 9.810723e-01 9.895490e-01 9.904835e-01 9.910426e-01 9.891390e-01 6.690831e-01

2489 9.814362e-01 9.897270e-01 9.906268e-01 9.911391e-01 9.892196e-01 6.696018e-01

2490 9.818179e-01 9.899134e-01 9.907772e-01 9.912404e-01 9.893042e-01 6.701133e-01

2491 9.822181e-01 9.901086e-01 9.909350e-01 9.913468e-01 9.893930e-01 6.706174e-01

2492 9.826375e-01 9.903129e-01 9.911006e-01 9.914586e-01 9.894861e-01 6.711140e-01

2493 9.830769e-01 9.905268e-01 9.912743e-01 9.915759e-01 9.895839e-01 6.716028e-01

2494 9.835371e-01 9.907506e-01 9.914564e-01 9.916990e-01 9.896863e-01 6.720838e-01

2495 9.840187e-01 9.909847e-01 9.916473e-01 9.918282e-01 9.897937e-01 6.725569e-01

2496 9.845226e-01 9.912296e-01 9.918473e-01 9.919638e-01 9.899062e-01 6.730219e-01

2497 9.850495e-01 9.914858e-01 9.920570e-01 9.921059e-01 9.900241e-01 6.734787e-01

2498 9.856001e-01 9.917536e-01 9.922765e-01 9.922549e-01 9.901475e-01 6.739273e-01

2499 9.861753e-01 9.920335e-01 9.925064e-01 9.924110e-01 9.902766e-01 6.743676e-01

2500 9.867758e-01 9.923261e-01 9.927470e-01 9.925746e-01 9.904117e-01 6.747996e-01

2501 9.874024e-01 9.926319e-01 9.929987e-01 9.927459e-01 9.905529e-01 6.752232e-01

2502 9.880560e-01 9.929514e-01 9.932620e-01 9.929253e-01 9.907005e-01 6.756384e-01

2503 9.887373e-01 9.932851e-01 9.935374e-01 9.931130e-01 9.908547e-01 6.760452e-01

2504 9.894472e-01 9.936336e-01 9.938251e-01 9.933093e-01 9.910157e-01 6.764436e-01

2505 9.901865e-01 9.939975e-01 9.941257e-01 9.935146e-01 9.911837e-01 6.768336e-01

2506 9.909558e-01 9.943774e-01 9.944397e-01 9.937292e-01 9.913589e-01 6.772154e-01

2507 9.921149e-01 9.950091e-01 9.950088e-01 9.942432e-01 9.919574e-01 6.821306e-01

2508 9.921150e-01 9.950092e-01 9.950088e-01 9.942433e-01 9.919574e-01 6.821669e-01

2509 9.921151e-01 9.950092e-01 9.950089e-01 9.942433e-01 9.919575e-01 6.822040e-01

2510 9.921153e-01 9.950093e-01 9.950089e-01 9.942433e-01 9.919575e-01 6.822421e-01

2511 9.921154e-01 9.950093e-01 9.950090e-01 9.942434e-01 9.919575e-01 6.822810e-01

2512 9.921155e-01 9.950094e-01 9.950090e-01 9.942434e-01 9.919575e-01 6.823209e-01

2513 9.921156e-01 9.950094e-01 9.950091e-01 9.942434e-01 9.919575e-01 6.823617e-01

2514 9.921158e-01 9.950095e-01 9.950091e-01 9.942435e-01 9.919575e-01 6.824034e-01

2515 9.921159e-01 9.950096e-01 9.950092e-01 9.942435e-01 9.919575e-01 6.824461e-01

2516 9.921160e-01 9.950096e-01 9.950092e-01 9.942435e-01 9.919575e-01 6.824897e-01

2517 9.921162e-01 9.950097e-01 9.950093e-01 9.942435e-01 9.919575e-01 6.825344e-01

2518 9.921163e-01 9.950098e-01 9.950093e-01 9.942436e-01 9.919575e-01 6.825800e-01

2519 9.921165e-01 9.950098e-01 9.950094e-01 9.942436e-01 9.919575e-01 6.826266e-01

2520 9.921167e-01 9.950099e-01 9.950095e-01 9.942436e-01 9.919575e-01 6.826743e-01

2521 9.921168e-01 9.950100e-01 9.950095e-01 9.942437e-01 9.919575e-01 6.827230e-01

2522 9.921170e-01 9.950101e-01 9.950096e-01 9.942437e-01 9.919575e-01 6.827728e-01

2523 9.921172e-01 9.950102e-01 9.950097e-01 9.942437e-01 9.919575e-01 6.828237e-01

2524 9.921174e-01 9.950103e-01 9.950097e-01 9.942438e-01 9.919575e-01 6.828758e-01

2525 9.921176e-01 9.950104e-01 9.950098e-01 9.942438e-01 9.919575e-01 6.829289e-01

2526 9.921178e-01 9.950105e-01 9.950099e-01 9.942438e-01 9.919575e-01 6.829833e-01

2527 9.921180e-01 9.950106e-01 9.950100e-01 9.942439e-01 9.919575e-01 6.830388e-01

2528 9.921182e-01 9.950107e-01 9.950101e-01 9.942439e-01 9.919575e-01 6.830956e-01

2529 9.921185e-01 9.950108e-01 9.950101e-01 9.942439e-01 9.919575e-01 6.831536e-01

2530 9.921187e-01 9.950109e-01 9.950102e-01 9.942440e-01 9.919575e-01 6.832129e-01

2531 9.921190e-01 9.950110e-01 9.950103e-01 9.942440e-01 9.919575e-01 6.832735e-01

2532 9.921192e-01 9.950112e-01 9.950104e-01 9.942440e-01 9.919575e-01 6.833354e-01

2533 9.921195e-01 9.950113e-01 9.950105e-01 9.942441e-01 9.919575e-01 6.833987e-01

2534 9.921198e-01 9.950114e-01 9.950106e-01 9.942441e-01 9.919575e-01 6.834634e-01

2535 9.921201e-01 9.950116e-01 9.950107e-01 9.942442e-01 9.919575e-01 6.835295e-01

2536 9.921204e-01 9.950117e-01 9.950108e-01 9.942442e-01 9.919575e-01 6.835971e-01

2537 9.921207e-01 9.950119e-01 9.950109e-01 9.942443e-01 9.919575e-01 6.836661e-01

2538 9.921210e-01 9.950120e-01 9.950111e-01 9.942443e-01 9.919575e-01 6.837367e-01

2539 9.921213e-01 9.950122e-01 9.950112e-01 9.942444e-01 9.919575e-01 6.838088e-01

2540 9.921217e-01 9.950124e-01 9.950113e-01 9.942444e-01 9.919575e-01 6.838826e-01

2541 9.921221e-01 9.950126e-01 9.950114e-01 9.942445e-01 9.919575e-01 6.839579e-01

2542 9.921224e-01 9.950127e-01 9.950116e-01 9.942445e-01 9.919575e-01 6.840350e-01

2543 9.921228e-01 9.950129e-01 9.950117e-01 9.942446e-01 9.919574e-01 6.841137e-01

2544 9.921233e-01 9.950131e-01 9.950118e-01 9.942446e-01 9.919574e-01 6.841942e-01

2545 9.921237e-01 9.950133e-01 9.950120e-01 9.942447e-01 9.919574e-01 6.842764e-01

2546 9.921241e-01 9.950135e-01 9.950122e-01 9.942447e-01 9.919574e-01 6.843605e-01

2547 9.921246e-01 9.950138e-01 9.950123e-01 9.942448e-01 9.919574e-01 6.844465e-01

2548 9.921251e-01 9.950140e-01 9.950125e-01 9.942448e-01 9.919573e-01 6.845343e-01

2549 9.921256e-01 9.950142e-01 9.950126e-01 9.942449e-01 9.919573e-01 6.846241e-01

2550 9.921261e-01 9.950145e-01 9.950128e-01 9.942450e-01 9.919573e-01 6.847159e-01

2551 9.921267e-01 9.950147e-01 9.950130e-01 9.942450e-01 9.919573e-01 6.848098e-01

2552 9.921272e-01 9.950150e-01 9.950132e-01 9.942451e-01 9.919572e-01 6.849057e-01

2553 9.921278e-01 9.950153e-01 9.950134e-01 9.942452e-01 9.919572e-01 6.850037e-01

2554 9.921284e-01 9.950156e-01 9.950136e-01 9.942452e-01 9.919571e-01 6.851039e-01

2555 9.921291e-01 9.950159e-01 9.950138e-01 9.942453e-01 9.919571e-01 6.852064e-01

2556 9.921297e-01 9.950162e-01 9.950140e-01 9.942454e-01 9.919570e-01 6.853111e-01

2557 9.921304e-01 9.950165e-01 9.950142e-01 9.942454e-01 9.919570e-01 6.854182e-01

2558 9.921312e-01 9.950169e-01 9.950145e-01 9.942455e-01 9.919569e-01 6.855276e-01

2559 9.921319e-01 9.950172e-01 9.950147e-01 9.942456e-01 9.919568e-01 6.856395e-01

2560 9.921327e-01 9.950176e-01 9.950150e-01 9.942457e-01 9.919568e-01 6.857538e-01

2561 9.921335e-01 9.950180e-01 9.950152e-01 9.942457e-01 9.919567e-01 6.858707e-01

2562 9.921344e-01 9.950184e-01 9.950155e-01 9.942458e-01 9.919566e-01 6.859901e-01

2563 9.921353e-01 9.950188e-01 9.950158e-01 9.942459e-01 9.919565e-01 6.861122e-01

2564 9.921362e-01 9.950192e-01 9.950161e-01 9.942460e-01 9.919564e-01 6.862370e-01

2565 9.921371e-01 9.950196e-01 9.950164e-01 9.942461e-01 9.919563e-01 6.863646e-01

2566 9.921381e-01 9.950201e-01 9.950167e-01 9.942461e-01 9.919562e-01 6.864949e-01

2567 9.921392e-01 9.950206e-01 9.950170e-01 9.942462e-01 9.919561e-01 6.866282e-01

2568 9.921403e-01 9.950211e-01 9.950174e-01 9.942463e-01 9.919560e-01 6.867643e-01

2569 9.921414e-01 9.950216e-01 9.950177e-01 9.942464e-01 9.919559e-01 6.869035e-01

2570 9.921426e-01 9.950222e-01 9.950181e-01 9.942465e-01 9.919558e-01 6.870457e-01

2571 9.921438e-01 9.950227e-01 9.950185e-01 9.942466e-01 9.919556e-01 6.871910e-01

2572 9.921451e-01 9.950233e-01 9.950189e-01 9.942467e-01 9.919555e-01 6.873395e-01

2573 9.921464e-01 9.950239e-01 9.950193e-01 9.942468e-01 9.919553e-01 6.874912e-01

2574 9.921478e-01 9.950246e-01 9.950197e-01 9.942469e-01 9.919551e-01 6.876462e-01

2575 9.921493e-01 9.950253e-01 9.950202e-01 9.942470e-01 9.919549e-01 6.878046e-01

2576 9.921508e-01 9.950260e-01 9.950206e-01 9.942471e-01 9.919547e-01 6.879663e-01

2577 9.921524e-01 9.950267e-01 9.950211e-01 9.942472e-01 9.919545e-01 6.881316e-01

2578 9.921540e-01 9.950274e-01 9.950216e-01 9.942473e-01 9.919543e-01 6.883004e-01

2579 9.921557e-01 9.950282e-01 9.950221e-01 9.942474e-01 9.919540e-01 6.884728e-01

2580 9.921575e-01 9.950290e-01 9.950227e-01 9.942475e-01 9.919538e-01 6.886489e-01

2581 9.921594e-01 9.950299e-01 9.950232e-01 9.942477e-01 9.919535e-01 6.888287e-01

2582 9.921614e-01 9.950308e-01 9.950238e-01 9.942478e-01 9.919532e-01 6.890123e-01

2583 9.921634e-01 9.950317e-01 9.950244e-01 9.942479e-01 9.919529e-01 6.891998e-01

2584 9.921655e-01 9.950327e-01 9.950251e-01 9.942480e-01 9.919526e-01 6.893912e-01

2585 9.921677e-01 9.950337e-01 9.950257e-01 9.942481e-01 9.919522e-01 6.895866e-01

2586 9.921700e-01 9.950348e-01 9.950264e-01 9.942483e-01 9.919519e-01 6.897860e-01

2587 9.921724e-01 9.950359e-01 9.950271e-01 9.942484e-01 9.919515e-01 6.899895e-01

2588 9.921750e-01 9.950370e-01 9.950279e-01 9.942485e-01 9.919511e-01 6.901971e-01

2589 9.921776e-01 9.950382e-01 9.950286e-01 9.942486e-01 9.919506e-01 6.904090e-01

2590 9.921803e-01 9.950394e-01 9.950294e-01 9.942487e-01 9.919501e-01 6.906252e-01

2591 9.921832e-01 9.950407e-01 9.950303e-01 9.942489e-01 9.919496e-01 6.908457e-01

2592 9.921862e-01 9.950421e-01 9.950311e-01 9.942490e-01 9.919491e-01 6.910705e-01

2593 9.921893e-01 9.950435e-01 9.950320e-01 9.942491e-01 9.919485e-01 6.912998e-01

2594 9.921926e-01 9.950450e-01 9.950330e-01 9.942493e-01 9.919479e-01 6.915336e-01

2595 9.921960e-01 9.950465e-01 9.950339e-01 9.942494e-01 9.919473e-01 6.917720e-01

2596 9.921995e-01 9.950481e-01 9.950350e-01 9.942495e-01 9.919466e-01 6.920148e-01

2597 9.922032e-01 9.950498e-01 9.950360e-01 9.942496e-01 9.919459e-01 6.922624e-01

2598 9.922071e-01 9.950515e-01 9.950371e-01 9.942498e-01 9.919451e-01 6.925145e-01

2599 9.922112e-01 9.950533e-01 9.950383e-01 9.942499e-01 9.919443e-01 6.927714e-01

2600 9.922154e-01 9.950552e-01 9.950395e-01 9.942500e-01 9.919435e-01 6.930330e-01

2601 9.922198e-01 9.950572e-01 9.950407e-01 9.942501e-01 9.919425e-01 6.932993e-01

2602 9.922244e-01 9.950593e-01 9.950420e-01 9.942502e-01 9.919416e-01 6.935705e-01

2603 9.922293e-01 9.950615e-01 9.950434e-01 9.942503e-01 9.919405e-01 6.938464e-01

2604 9.922343e-01 9.950637e-01 9.950448e-01 9.942505e-01 9.919394e-01 6.941272e-01

2605 9.922396e-01 9.950661e-01 9.950463e-01 9.942506e-01 9.919383e-01 6.944129e-01

2606 9.922451e-01 9.950685e-01 9.950478e-01 9.942507e-01 9.919370e-01 6.947033e-01

2607 9.922509e-01 9.950711e-01 9.950494e-01 9.942508e-01 9.919357e-01 6.949987e-01

2608 9.922569e-01 9.950738e-01 9.950511e-01 9.942508e-01 9.919343e-01 6.952989e-01

2609 9.922632e-01 9.950766e-01 9.950528e-01 9.942509e-01 9.919328e-01 6.956039e-01

2610 9.922698e-01 9.950795e-01 9.950546e-01 9.942510e-01 9.919313e-01 6.959138e-01

2611 9.922767e-01 9.950826e-01 9.950565e-01 9.942510e-01 9.919296e-01 6.962286e-01

2612 9.922839e-01 9.950858e-01 9.950585e-01 9.942511e-01 9.919278e-01 6.965481e-01

2613 9.922914e-01 9.950891e-01 9.950605e-01 9.942511e-01 9.919259e-01 6.968724e-01

2614 9.922993e-01 9.950926e-01 9.950626e-01 9.942512e-01 9.919239e-01 6.972015e-01

2615 9.923075e-01 9.950963e-01 9.950649e-01 9.942512e-01 9.919218e-01 6.975353e-01

2616 9.923162e-01 9.951001e-01 9.950672e-01 9.942512e-01 9.919196e-01 6.978737e-01

2617 9.923252e-01 9.951041e-01 9.950696e-01 9.942511e-01 9.919172e-01 6.982167e-01

2618 9.923346e-01 9.951083e-01 9.950721e-01 9.942511e-01 9.919146e-01 6.985643e-01

2619 9.923445e-01 9.951126e-01 9.950748e-01 9.942510e-01 9.919119e-01 6.989164e-01

2620 9.923548e-01 9.951172e-01 9.950775e-01 9.942510e-01 9.919090e-01 6.992728e-01

2621 9.923657e-01 9.951220e-01 9.950804e-01 9.942508e-01 9.919060e-01 6.996336e-01

2622 9.923770e-01 9.951269e-01 9.950834e-01 9.942507e-01 9.919028e-01 6.999985e-01

2623 9.923888e-01 9.951322e-01 9.950865e-01 9.942505e-01 9.918993e-01 7.003676e-01

2624 9.924012e-01 9.951376e-01 9.950898e-01 9.942503e-01 9.918957e-01 7.007407e-01

2625 9.924142e-01 9.951433e-01 9.950932e-01 9.942501e-01 9.918918e-01 7.011178e-01

2626 9.924278e-01 9.951493e-01 9.950968e-01 9.942498e-01 9.918877e-01 7.014985e-01

2627 9.924421e-01 9.951556e-01 9.951005e-01 9.942495e-01 9.918833e-01 7.018830e-01

2628 9.924570e-01 9.951621e-01 9.951044e-01 9.942491e-01 9.918787e-01 7.022709e-01

2629 9.924726e-01 9.951689e-01 9.951084e-01 9.942487e-01 9.918737e-01 7.026622e-01

2630 9.924889e-01 9.951761e-01 9.951126e-01 9.942482e-01 9.918685e-01 7.030567e-01

2631 9.925061e-01 9.951836e-01 9.951171e-01 9.942477e-01 9.918629e-01 7.034543e-01

2632 9.925240e-01 9.951915e-01 9.951217e-01 9.942471e-01 9.918570e-01 7.038547e-01

2633 9.925428e-01 9.951997e-01 9.951265e-01 9.942465e-01 9.918507e-01 7.042579e-01

2634 9.925624e-01 9.952083e-01 9.951315e-01 9.942457e-01 9.918441e-01 7.046635e-01

2635 9.925830e-01 9.952173e-01 9.951368e-01 9.942449e-01 9.918370e-01 7.050715e-01

2636 9.926046e-01 9.952267e-01 9.951423e-01 9.942440e-01 9.918294e-01 7.054816e-01

2637 9.926272e-01 9.952366e-01 9.951480e-01 9.942430e-01 9.918214e-01 7.058937e-01

2638 9.926509e-01 9.952470e-01 9.951540e-01 9.942419e-01 9.918130e-01 7.063074e-01

2639 9.926758e-01 9.952578e-01 9.951602e-01 9.942407e-01 9.918039e-01 7.067227e-01

2640 9.927018e-01 9.952692e-01 9.951668e-01 9.942394e-01 9.917943e-01 7.071393e-01

2641 9.927290e-01 9.952810e-01 9.951736e-01 9.942380e-01 9.917842e-01 7.075569e-01

2642 9.927576e-01 9.952935e-01 9.951808e-01 9.942364e-01 9.917733e-01 7.079753e-01

2643 9.927875e-01 9.953066e-01 9.951882e-01 9.942347e-01 9.917619e-01 7.083944e-01

2644 9.928188e-01 9.953202e-01 9.951960e-01 9.942328e-01 9.917496e-01 7.088138e-01

2645 9.928517e-01 9.953346e-01 9.952042e-01 9.942308e-01 9.917367e-01 7.092334e-01

2646 9.928861e-01 9.953496e-01 9.952127e-01 9.942285e-01 9.917229e-01 7.096528e-01

2647 9.929222e-01 9.953653e-01 9.952216e-01 9.942261e-01 9.917083e-01 7.100719e-01

2648 9.929600e-01 9.953818e-01 9.952309e-01 9.942235e-01 9.916927e-01 7.104903e-01

2649 9.929996e-01 9.953991e-01 9.952407e-01 9.942206e-01 9.916762e-01 7.109080e-01

2650 9.930411e-01 9.954172e-01 9.952508e-01 9.942175e-01 9.916587e-01 7.113245e-01

2651 9.930846e-01 9.954361e-01 9.952615e-01 9.942142e-01 9.916401e-01 7.117397e-01

2652 9.931302e-01 9.954560e-01 9.952726e-01 9.942105e-01 9.916204e-01 7.121534e-01

2653 9.931780e-01 9.954769e-01 9.952842e-01 9.942066e-01 9.915994e-01 7.125652e-01

2654 9.932280e-01 9.954987e-01 9.952964e-01 9.942023e-01 9.915771e-01 7.129749e-01

2655 9.932805e-01 9.955217e-01 9.953091e-01 9.941977e-01 9.915535e-01 7.133824e-01

2656 9.933355e-01 9.955457e-01 9.953224e-01 9.941928e-01 9.915284e-01 7.137873e-01

2657 9.933931e-01 9.955709e-01 9.953363e-01 9.941874e-01 9.915017e-01 7.141895e-01

2658 9.934534e-01 9.955972e-01 9.953508e-01 9.941817e-01 9.914735e-01 7.145887e-01

2659 9.935166e-01 9.956249e-01 9.953660e-01 9.941754e-01 9.914435e-01 7.149847e-01

2660 9.935829e-01 9.956539e-01 9.953819e-01 9.941688e-01 9.914116e-01 7.153773e-01

2661 9.936523e-01 9.956843e-01 9.953984e-01 9.941615e-01 9.913779e-01 7.157663e-01

2662 9.937250e-01 9.957162e-01 9.954158e-01 9.941538e-01 9.913421e-01 7.161514e-01

2663 9.938012e-01 9.957496e-01 9.954339e-01 9.941454e-01 9.913041e-01 7.165325e-01

2664 9.938809e-01 9.957846e-01 9.954529e-01 9.941365e-01 9.912638e-01 7.169095e-01

2665 9.939644e-01 9.958214e-01 9.954727e-01 9.941268e-01 9.912212e-01 7.172820e-01

2666 9.940519e-01 9.958599e-01 9.954934e-01 9.941165e-01 9.911759e-01 7.176500e-01

2667 9.941435e-01 9.959002e-01 9.955150e-01 9.941054e-01 9.911280e-01 7.180133e-01

2668 9.942394e-01 9.959425e-01 9.955376e-01 9.940935e-01 9.910773e-01 7.183718e-01

2669 9.943398e-01 9.959868e-01 9.955612e-01 9.940807e-01 9.910235e-01 7.187252e-01

2670 9.944449e-01 9.960332e-01 9.955859e-01 9.940670e-01 9.909666e-01 7.190735e-01

2671 9.945548e-01 9.960819e-01 9.956116e-01 9.940523e-01 9.909063e-01 7.194165e-01

2672 9.946699e-01 9.961329e-01 9.956385e-01 9.940366e-01 9.908426e-01 7.197541e-01

2673 9.947903e-01 9.961863e-01 9.956666e-01 9.940197e-01 9.907752e-01 7.200862e-01

2674 9.949162e-01 9.962423e-01 9.956960e-01 9.940017e-01 9.907038e-01 7.204128e-01

2675 9.950480e-01 9.963009e-01 9.957266e-01 9.939825e-01 9.906284e-01 7.207336e-01

2676 9.951857e-01 9.963624e-01 9.957585e-01 9.939619e-01 9.905488e-01 7.210487e-01

2677 9.953298e-01 9.964267e-01 9.957919e-01 9.939399e-01 9.904646e-01 7.213580e-01

2678 9.954803e-01 9.964940e-01 9.958267e-01 9.939164e-01 9.903757e-01 7.216614e-01

2679 9.956377e-01 9.965645e-01 9.958630e-01 9.938913e-01 9.902819e-01 7.219588e-01

2680 9.958022e-01 9.966384e-01 9.959009e-01 9.938645e-01 9.901829e-01 7.222503e-01

2681 9.959740e-01 9.967156e-01 9.959403e-01 9.938360e-01 9.900786e-01 7.225358e-01

2682 9.961536e-01 9.967965e-01 9.959815e-01 9.938056e-01 9.899685e-01 7.228152e-01

2683 9.963412e-01 9.968810e-01 9.960244e-01 9.937732e-01 9.898526e-01 7.230886e-01

2684 9.965371e-01 9.969695e-01 9.960690e-01 9.937387e-01 9.897304e-01 7.233560e-01

2685 9.967418e-01 9.970621e-01 9.961156e-01 9.937019e-01 9.896019e-01 7.236173e-01

2686 9.970853e-01 9.972868e-01 9.963457e-01 9.939780e-01 9.899977e-01 7.281450e-01

2687 9.970853e-01 9.972869e-01 9.963458e-01 9.939779e-01 9.899976e-01 7.281530e-01

2688 9.970854e-01 9.972869e-01 9.963458e-01 9.939779e-01 9.899975e-01 7.281610e-01

2689 9.970854e-01 9.972869e-01 9.963458e-01 9.939779e-01 9.899974e-01 7.281690e-01

2690 9.970855e-01 9.972870e-01 9.963458e-01 9.939778e-01 9.899974e-01 7.281771e-01

2691 9.970855e-01 9.972870e-01 9.963458e-01 9.939778e-01 9.899973e-01 7.281852e-01

2692 9.970856e-01 9.972870e-01 9.963459e-01 9.939777e-01 9.899972e-01 7.281933e-01

2693 9.970856e-01 9.972871e-01 9.963459e-01 9.939777e-01 9.899972e-01 7.282014e-01

2694 9.970857e-01 9.972871e-01 9.963459e-01 9.939776e-01 9.899971e-01 7.282094e-01

2695 9.970857e-01 9.972871e-01 9.963459e-01 9.939776e-01 9.899970e-01 7.282175e-01

2696 9.970858e-01 9.972872e-01 9.963459e-01 9.939775e-01 9.899970e-01 7.282255e-01

2697 9.970859e-01 9.972872e-01 9.963459e-01 9.939775e-01 9.899969e-01 7.282336e-01

2698 9.970859e-01 9.972872e-01 9.963459e-01 9.939774e-01 9.899968e-01 7.282416e-01

2699 9.970860e-01 9.972873e-01 9.963459e-01 9.939774e-01 9.899967e-01 7.282495e-01

2700 9.970861e-01 9.972873e-01 9.963459e-01 9.939773e-01 9.899967e-01 7.282574e-01

2701 9.970861e-01 9.972873e-01 9.963459e-01 9.939772e-01 9.899966e-01 7.282653e-01

2702 9.970862e-01 9.972874e-01 9.963459e-01 9.939772e-01 9.899965e-01 7.282730e-01

2703 9.970863e-01 9.972874e-01 9.963459e-01 9.939771e-01 9.899964e-01 7.282808e-01

2704 9.970864e-01 9.972874e-01 9.963459e-01 9.939771e-01 9.899963e-01 7.282884e-01

2705 9.970865e-01 9.972875e-01 9.963459e-01 9.939770e-01 9.899961e-01 7.282960e-01

2706 9.970865e-01 9.972875e-01 9.963459e-01 9.939770e-01 9.899960e-01 7.283035e-01

2707 9.970866e-01 9.972875e-01 9.963459e-01 9.939769e-01 9.899959e-01 7.283108e-01

2708 9.970867e-01 9.972876e-01 9.963459e-01 9.939768e-01 9.899957e-01 7.283181e-01

2709 9.970868e-01 9.972876e-01 9.963459e-01 9.939768e-01 9.899956e-01 7.283252e-01

2710 9.970869e-01 9.972876e-01 9.963459e-01 9.939767e-01 9.899954e-01 7.283322e-01

2711 9.970870e-01 9.972877e-01 9.963458e-01 9.939766e-01 9.899952e-01 7.283391e-01

2712 9.970871e-01 9.972877e-01 9.963458e-01 9.939765e-01 9.899950e-01 7.283458e-01

2713 9.970872e-01 9.972877e-01 9.963458e-01 9.939764e-01 9.899948e-01 7.283524e-01

2714 9.970874e-01 9.972878e-01 9.963458e-01 9.939763e-01 9.899946e-01 7.283588e-01

2715 9.970875e-01 9.972878e-01 9.963458e-01 9.939762e-01 9.899944e-01 7.283650e-01

2716 9.970876e-01 9.972879e-01 9.963458e-01 9.939761e-01 9.899941e-01 7.283710e-01

2717 9.970877e-01 9.972879e-01 9.963458e-01 9.939760e-01 9.899939e-01 7.283769e-01

2718 9.970879e-01 9.972879e-01 9.963458e-01 9.939758e-01 9.899936e-01 7.283824e-01

2719 9.970880e-01 9.972880e-01 9.963457e-01 9.939757e-01 9.899933e-01 7.283878e-01

2720 9.970881e-01 9.972880e-01 9.963457e-01 9.939755e-01 9.899930e-01 7.283929e-01

2721 9.970883e-01 9.972881e-01 9.963457e-01 9.939754e-01 9.899927e-01 7.283978e-01

2722 9.970884e-01 9.972881e-01 9.963457e-01 9.939752e-01 9.899924e-01 7.284023e-01

2723 9.970886e-01 9.972882e-01 9.963456e-01 9.939750e-01 9.899921e-01 7.284066e-01

2724 9.970888e-01 9.972882e-01 9.963456e-01 9.939749e-01 9.899917e-01 7.284106e-01

2725 9.970889e-01 9.972882e-01 9.963456e-01 9.939747e-01 9.899913e-01 7.284142e-01

2726 9.970891e-01 9.972883e-01 9.963455e-01 9.939745e-01 9.899909e-01 7.284175e-01

2727 9.970893e-01 9.972883e-01 9.963455e-01 9.939742e-01 9.899905e-01 7.284204e-01

2728 9.970895e-01 9.972884e-01 9.963455e-01 9.939740e-01 9.899901e-01 7.284230e-01

2729 9.970897e-01 9.972885e-01 9.963454e-01 9.939738e-01 9.899897e-01 7.284251e-01

2730 9.970899e-01 9.972885e-01 9.963454e-01 9.939735e-01 9.899892e-01 7.284269e-01

2731 9.970901e-01 9.972886e-01 9.963453e-01 9.939733e-01 9.899887e-01 7.284281e-01

2732 9.970903e-01 9.972886e-01 9.963453e-01 9.939730e-01 9.899882e-01 7.284290e-01

2733 9.970905e-01 9.972887e-01 9.963452e-01 9.939727e-01 9.899877e-01 7.284293e-01

2734 9.970907e-01 9.972887e-01 9.963451e-01 9.939724e-01 9.899871e-01 7.284291e-01

2735 9.970910e-01 9.972888e-01 9.963451e-01 9.939721e-01 9.899865e-01 7.284284e-01

2736 9.970912e-01 9.972889e-01 9.963450e-01 9.939718e-01 9.899859e-01 7.284271e-01

2737 9.970915e-01 9.972889e-01 9.963449e-01 9.939714e-01 9.899852e-01 7.284252e-01

2738 9.970917e-01 9.972890e-01 9.963448e-01 9.939710e-01 9.899846e-01 7.284227e-01

2739 9.970920e-01 9.972891e-01 9.963448e-01 9.939707e-01 9.899838e-01 7.284195e-01

2740 9.970923e-01 9.972891e-01 9.963447e-01 9.939703e-01 9.899831e-01 7.284157e-01

2741 9.970926e-01 9.972892e-01 9.963446e-01 9.939698e-01 9.899823e-01 7.284112e-01

2742 9.970929e-01 9.972893e-01 9.963445e-01 9.939694e-01 9.899815e-01 7.284059e-01

2743 9.970932e-01 9.972893e-01 9.963443e-01 9.939689e-01 9.899806e-01 7.283999e-01

2744 9.970936e-01 9.972894e-01 9.963442e-01 9.939684e-01 9.899797e-01 7.283930e-01

2745 9.970939e-01 9.972895e-01 9.963441e-01 9.939679e-01 9.899787e-01 7.283854e-01

2746 9.970943e-01 9.972896e-01 9.963440e-01 9.939674e-01 9.899777e-01 7.283768e-01

2747 9.970946e-01 9.972897e-01 9.963438e-01 9.939668e-01 9.899766e-01 7.283674e-01

2748 9.970950e-01 9.972897e-01 9.963437e-01 9.939662e-01 9.899755e-01 7.283570e-01

2749 9.970954e-01 9.972898e-01 9.963435e-01 9.939655e-01 9.899743e-01 7.283456e-01

2750 9.970958e-01 9.972899e-01 9.963434e-01 9.939649e-01 9.899731e-01 7.283332e-01

2751 9.970963e-01 9.972900e-01 9.963432e-01 9.939642e-01 9.899718e-01 7.283197e-01

2752 9.970967e-01 9.972901e-01 9.963430e-01 9.939634e-01 9.899704e-01 7.283051e-01

2753 9.970972e-01 9.972902e-01 9.963428e-01 9.939627e-01 9.899690e-01 7.282893e-01

2754 9.970977e-01 9.972903e-01 9.963426e-01 9.939619e-01 9.899675e-01 7.282724e-01

2755 9.970982e-01 9.972904e-01 9.963424e-01 9.939610e-01 9.899659e-01 7.282542e-01

2756 9.970987e-01 9.972905e-01 9.963421e-01 9.939601e-01 9.899643e-01 7.282348e-01

2757 9.970992e-01 9.972906e-01 9.963419e-01 9.939592e-01 9.899625e-01 7.282140e-01

2758 9.970998e-01 9.972907e-01 9.963416e-01 9.939582e-01 9.899607e-01 7.281918e-01

2759 9.971004e-01 9.972908e-01 9.963413e-01 9.939571e-01 9.899588e-01 7.281682e-01

2760 9.971010e-01 9.972909e-01 9.963410e-01 9.939560e-01 9.899568e-01 7.281430e-01

2761 9.971016e-01 9.972910e-01 9.963407e-01 9.939549e-01 9.899546e-01 7.281164e-01

2762 9.971023e-01 9.972911e-01 9.963404e-01 9.939537e-01 9.899524e-01 7.280882e-01

2763 9.971029e-01 9.972912e-01 9.963401e-01 9.939524e-01 9.899500e-01 7.280583e-01

2764 9.971037e-01 9.972914e-01 9.963397e-01 9.939510e-01 9.899476e-01 7.280267e-01

2765 9.971044e-01 9.972915e-01 9.963393e-01 9.939496e-01 9.899450e-01 7.279933e-01

2766 9.971052e-01 9.972916e-01 9.963389e-01 9.939481e-01 9.899422e-01 7.279582e-01

2767 9.971059e-01 9.972917e-01 9.963385e-01 9.939466e-01 9.899394e-01 7.279211e-01

2768 9.971068e-01 9.972919e-01 9.963380e-01 9.939449e-01 9.899364e-01 7.278821e-01

2769 9.971076e-01 9.972920e-01 9.963375e-01 9.939432e-01 9.899332e-01 7.278412e-01

2770 9.971085e-01 9.972921e-01 9.963370e-01 9.939414e-01 9.899298e-01 7.277981e-01

2771 9.971095e-01 9.972923e-01 9.963365e-01 9.939395e-01 9.899263e-01 7.277530e-01

2772 9.971104e-01 9.972924e-01 9.963359e-01 9.939375e-01 9.899226e-01 7.277057e-01

2773 9.971114e-01 9.972926e-01 9.963353e-01 9.939353e-01 9.899187e-01 7.276561e-01

2774 9.971125e-01 9.972927e-01 9.963347e-01 9.939331e-01 9.899146e-01 7.276042e-01

2775 9.971136e-01 9.972929e-01 9.963341e-01 9.939308e-01 9.899103e-01 7.275499e-01

2776 9.971147e-01 9.972930e-01 9.963334e-01 9.939283e-01 9.899057e-01 7.274932e-01

2777 9.971159e-01 9.972932e-01 9.963326e-01 9.939257e-01 9.899010e-01 7.274340e-01

2778 9.971171e-01 9.972934e-01 9.963318e-01 9.939230e-01 9.898959e-01 7.273723e-01

2779 9.971184e-01 9.972935e-01 9.963310e-01 9.939201e-01 9.898906e-01 7.273079e-01

2780 9.971197e-01 9.972937e-01 9.963301e-01 9.939170e-01 9.898850e-01 7.272408e-01

2781 9.971211e-01 9.972939e-01 9.963292e-01 9.939138e-01 9.898791e-01 7.271709e-01

2782 9.971225e-01 9.972940e-01 9.963283e-01 9.939105e-01 9.898729e-01 7.270982e-01

2783 9.971240e-01 9.972942e-01 9.963272e-01 9.939069e-01 9.898664e-01 7.270226e-01

2784 9.971256e-01 9.972944e-01 9.963262e-01 9.939032e-01 9.898595e-01 7.269441e-01

2785 9.971272e-01 9.972946e-01 9.963250e-01 9.938992e-01 9.898522e-01 7.268625e-01

2786 9.971289e-01 9.972948e-01 9.963238e-01 9.938951e-01 9.898446e-01 7.267779e-01

2787 9.971306e-01 9.972949e-01 9.963225e-01 9.938907e-01 9.898365e-01 7.266901e-01

2788 9.971324e-01 9.972951e-01 9.963212e-01 9.938861e-01 9.898280e-01 7.265991e-01

2789 9.971343e-01 9.972953e-01 9.963198e-01 9.938812e-01 9.898191e-01 7.265049e-01

2790 9.971363e-01 9.972955e-01 9.963183e-01 9.938761e-01 9.898096e-01 7.264073e-01

2791 9.971384e-01 9.972957e-01 9.963167e-01 9.938707e-01 9.897997e-01 7.263064e-01

2792 9.971405e-01 9.972959e-01 9.963150e-01 9.938650e-01 9.897892e-01 7.262021e-01

2793 9.971428e-01 9.972961e-01 9.963133e-01 9.938590e-01 9.897781e-01 7.260942e-01

2794 9.971451e-01 9.972963e-01 9.963114e-01 9.938526e-01 9.897664e-01 7.259829e-01

2795 9.971475e-01 9.972965e-01 9.963094e-01 9.938459e-01 9.897540e-01 7.258680e-01

2796 9.971501e-01 9.972967e-01 9.963074e-01 9.938389e-01 9.897410e-01 7.257494e-01

2797 9.971527e-01 9.972969e-01 9.963052e-01 9.938315e-01 9.897273e-01 7.256272e-01

2798 9.971555e-01 9.972971e-01 9.963029e-01 9.938236e-01 9.897128e-01 7.255014e-01

2799 9.971583e-01 9.972973e-01 9.963004e-01 9.938153e-01 9.896975e-01 7.253718e-01

2800 9.971613e-01 9.972975e-01 9.962978e-01 9.938066e-01 9.896814e-01 7.252385e-01

2801 9.971644e-01 9.972977e-01 9.962951e-01 9.937974e-01 9.896643e-01 7.251014e-01

2802 9.971677e-01 9.972979e-01 9.962922e-01 9.937876e-01 9.896464e-01 7.249605e-01

2803 9.971711e-01 9.972981e-01 9.962891e-01 9.937773e-01 9.896274e-01 7.248158e-01

2804 9.971746e-01 9.972983e-01 9.962859e-01 9.937665e-01 9.896073e-01 7.246673e-01

2805 9.971783e-01 9.972985e-01 9.962825e-01 9.937550e-01 9.895862e-01 7.245149e-01

2806 9.971821e-01 9.972986e-01 9.962789e-01 9.937430e-01 9.895638e-01 7.243588e-01

2807 9.971861e-01 9.972988e-01 9.962751e-01 9.937302e-01 9.895402e-01 7.241988e-01

2808 9.971903e-01 9.972990e-01 9.962711e-01 9.937167e-01 9.895153e-01 7.240350e-01

2809 9.971946e-01 9.972991e-01 9.962668e-01 9.937025e-01 9.894889e-01 7.238674e-01

2810 9.971992e-01 9.972993e-01 9.962623e-01 9.936874e-01 9.894611e-01 7.236960e-01

2811 9.972039e-01 9.972994e-01 9.962576e-01 9.936715e-01 9.894317e-01 7.235209e-01

2812 9.972088e-01 9.972995e-01 9.962526e-01 9.936548e-01 9.894007e-01 7.233421e-01

2813 9.972140e-01 9.972996e-01 9.962472e-01 9.936370e-01 9.893679e-01 7.231596e-01

2814 9.972194e-01 9.972997e-01 9.962416e-01 9.936183e-01 9.893332e-01 7.229734e-01

2815 9.972250e-01 9.972998e-01 9.962357e-01 9.935985e-01 9.892966e-01 7.227838e-01

2816 9.972308e-01 9.972999e-01 9.962294e-01 9.935776e-01 9.892579e-01 7.225905e-01

2817 9.972369e-01 9.972999e-01 9.962228e-01 9.935555e-01 9.892171e-01 7.223939e-01

2818 9.972433e-01 9.973000e-01 9.962158e-01 9.935322e-01 9.891739e-01 7.221939e-01

2819 9.972500e-01 9.973000e-01 9.962083e-01 9.935075e-01 9.891283e-01 7.219905e-01

2820 9.972569e-01 9.972999e-01 9.962005e-01 9.934814e-01 9.890801e-01 7.217840e-01

2821 9.972642e-01 9.972999e-01 9.961922e-01 9.934538e-01 9.890291e-01 7.215743e-01

2822 9.972717e-01 9.972998e-01 9.961834e-01 9.934247e-01 9.889753e-01 7.213617e-01

2823 9.972796e-01 9.972997e-01 9.961741e-01 9.933939e-01 9.889184e-01 7.211461e-01

2824 9.972879e-01 9.972995e-01 9.961643e-01 9.933613e-01 9.888584e-01 7.209277e-01

2825 9.972965e-01 9.972993e-01 9.961539e-01 9.933269e-01 9.887949e-01 7.207066e-01

2826 9.973055e-01 9.972991e-01 9.961429e-01 9.932906e-01 9.887279e-01 7.204830e-01

2827 9.973149e-01 9.972988e-01 9.961313e-01 9.932521e-01 9.886570e-01 7.202569e-01

2828 9.973247e-01 9.972984e-01 9.961190e-01 9.932115e-01 9.885822e-01 7.200285e-01

2829 9.973350e-01 9.972980e-01 9.961059e-01 9.931685e-01 9.885032e-01 7.197980e-01

2830 9.973457e-01 9.972976e-01 9.960922e-01 9.931231e-01 9.884198e-01 7.195655e-01

2831 9.973568e-01 9.972970e-01 9.960776e-01 9.930751e-01 9.883317e-01 7.193311e-01

2832 9.973685e-01 9.972964e-01 9.960622e-01 9.930244e-01 9.882387e-01 7.190949e-01

2833 9.973807e-01 9.972957e-01 9.960459e-01 9.929707e-01 9.881405e-01 7.188573e-01

2834 9.973935e-01 9.972950e-01 9.960287e-01 9.929141e-01 9.880369e-01 7.186182e-01

2835 9.974068e-01 9.972941e-01 9.960104e-01 9.928542e-01 9.879275e-01 7.183779e-01

2836 9.974207e-01 9.972931e-01 9.959911e-01 9.927909e-01 9.878121e-01 7.181365e-01

2837 9.974352e-01 9.972921e-01 9.959707e-01 9.927240e-01 9.876904e-01 7.178943e-01

2838 9.974504e-01 9.972909e-01 9.959491e-01 9.926534e-01 9.875620e-01 7.176513e-01

2839 9.974663e-01 9.972896e-01 9.959262e-01 9.925787e-01 9.874266e-01 7.174077e-01

2840 9.974829e-01 9.972881e-01 9.959021e-01 9.924999e-01 9.872839e-01 7.171638e-01

2841 9.975002e-01 9.972865e-01 9.958765e-01 9.924166e-01 9.871335e-01 7.169196e-01

2842 9.975183e-01 9.972848e-01 9.958494e-01 9.923287e-01 9.869751e-01 7.166754e-01

2843 9.975372e-01 9.972829e-01 9.958208e-01 9.922358e-01 9.868082e-01 7.164313e-01

2844 9.975570e-01 9.972808e-01 9.957906e-01 9.921378e-01 9.866324e-01 7.161876e-01

2845 9.975776e-01 9.972785e-01 9.957586e-01 9.920343e-01 9.864474e-01 7.159443e-01

2846 9.975992e-01 9.972760e-01 9.957247e-01 9.919251e-01 9.862527e-01 7.157016e-01

2847 9.976218e-01 9.972733e-01 9.956889e-01 9.918099e-01 9.860479e-01 7.154597e-01

2848 9.976454e-01 9.972704e-01 9.956511e-01 9.916884e-01 9.858325e-01 7.152188e-01

2849 9.976700e-01 9.972672e-01 9.956111e-01 9.915602e-01 9.856061e-01 7.149790e-01

2850 9.976958e-01 9.972637e-01 9.955688e-01 9.914251e-01 9.853683e-01 7.147405e-01

2851 9.977227e-01 9.972600e-01 9.955242e-01 9.912826e-01 9.851185e-01 7.145034e-01

2852 9.977508e-01 9.972559e-01 9.954769e-01 9.911325e-01 9.848562e-01 7.142679e-01

2853 9.977802e-01 9.972515e-01 9.954270e-01 9.909744e-01 9.845811e-01 7.140341e-01

2854 9.978109e-01 9.972468e-01 9.953743e-01 9.908078e-01 9.842925e-01 7.138022e-01

2855 9.978430e-01 9.972417e-01 9.953186e-01 9.906325e-01 9.839901e-01 7.135722e-01

2856 9.978765e-01 9.972361e-01 9.952598e-01 9.904479e-01 9.836732e-01 7.133444e-01

2857 9.979115e-01 9.972302e-01 9.951977e-01 9.902538e-01 9.833414e-01 7.131187e-01

2858 9.979481e-01 9.972238e-01 9.951322e-01 9.900495e-01 9.829943e-01 7.128954e-01

2859 9.979864e-01 9.972169e-01 9.950629e-01 9.898348e-01 9.826312e-01 7.126746e-01

2860 9.980263e-01 9.972095e-01 9.949899e-01 9.896092e-01 9.822517e-01 7.124563e-01

2861 9.980680e-01 9.972015e-01 9.949129e-01 9.893722e-01 9.818554e-01 7.122407e-01

2862 9.981116e-01 9.971929e-01 9.948316e-01 9.891233e-01 9.814417e-01 7.120278e-01

2863 9.981571e-01 9.971837e-01 9.947459e-01 9.888622e-01 9.810102e-01 7.118178e-01

2864 9.982046e-01 9.971738e-01 9.946555e-01 9.885882e-01 9.805606e-01 7.116106e-01

2865 9.983287e-01 9.972959e-01 9.948284e-01 9.888877e-01 9.810657e-01 7.158169e-01

2866 9.983288e-01 9.972959e-01 9.948284e-01 9.888877e-01 9.810656e-01 7.157785e-01

2867 9.983288e-01 9.972959e-01 9.948283e-01 9.888876e-01 9.810654e-01 7.157391e-01

2868 9.983288e-01 9.972959e-01 9.948282e-01 9.888875e-01 9.810653e-01 7.156986e-01

2869 9.983289e-01 9.972959e-01 9.948282e-01 9.888875e-01 9.810651e-01 7.156570e-01

2870 9.983289e-01 9.972959e-01 9.948281e-01 9.888874e-01 9.810649e-01 7.156143e-01

2871 9.983289e-01 9.972958e-01 9.948281e-01 9.888873e-01 9.810647e-01 7.155704e-01

2872 9.983290e-01 9.972958e-01 9.948280e-01 9.888872e-01 9.810645e-01 7.155252e-01

2873 9.983290e-01 9.972958e-01 9.948280e-01 9.888870e-01 9.810643e-01 7.154789e-01

2874 9.983290e-01 9.972958e-01 9.948279e-01 9.888869e-01 9.810640e-01 7.154313e-01

2875 9.983291e-01 9.972957e-01 9.948279e-01 9.888868e-01 9.810637e-01 7.153823e-01

2876 9.983291e-01 9.972957e-01 9.948278e-01 9.888866e-01 9.810634e-01 7.153321e-01

2877 9.983291e-01 9.972957e-01 9.948278e-01 9.888864e-01 9.810631e-01 7.152805e-01

2878 9.983292e-01 9.972957e-01 9.948277e-01 9.888862e-01 9.810628e-01 7.152274e-01

2879 9.983292e-01 9.972956e-01 9.948276e-01 9.888860e-01 9.810624e-01 7.151730e-01

2880 9.983292e-01 9.972956e-01 9.948276e-01 9.888858e-01 9.810620e-01 7.151170e-01

2881 9.983293e-01 9.972956e-01 9.948275e-01 9.888856e-01 9.810617e-01 7.150595e-01

2882 9.983293e-01 9.972956e-01 9.948274e-01 9.888853e-01 9.810613e-01 7.150005e-01

2883 9.983293e-01 9.972955e-01 9.948273e-01 9.888851e-01 9.810609e-01 7.149398e-01

2884 9.983294e-01 9.972955e-01 9.948272e-01 9.888848e-01 9.810605e-01 7.148775e-01

2885 9.983294e-01 9.972955e-01 9.948272e-01 9.888845e-01 9.810601e-01 7.148135e-01

2886 9.983294e-01 9.972955e-01 9.948270e-01 9.888842e-01 9.810596e-01 7.147477e-01

2887 9.983294e-01 9.972954e-01 9.948269e-01 9.888839e-01 9.810592e-01 7.146801e-01

2888 9.983295e-01 9.972954e-01 9.948268e-01 9.888836e-01 9.810587e-01 7.146107e-01

2889 9.983295e-01 9.972954e-01 9.948267e-01 9.888833e-01 9.810582e-01 7.145394e-01

2890 9.983295e-01 9.972953e-01 9.948266e-01 9.888829e-01 9.810577e-01 7.144662e-01

2891 9.983296e-01 9.972953e-01 9.948264e-01 9.888826e-01 9.810572e-01 7.143909e-01

2892 9.983296e-01 9.972953e-01 9.948263e-01 9.888822e-01 9.810567e-01 7.143136e-01

2893 9.983296e-01 9.972952e-01 9.948261e-01 9.888818e-01 9.810561e-01 7.142342e-01

2894 9.983297e-01 9.972952e-01 9.948259e-01 9.888814e-01 9.810555e-01 7.141526e-01

2895 9.983297e-01 9.972951e-01 9.948257e-01 9.888810e-01 9.810549e-01 7.140688e-01

2896 9.983297e-01 9.972951e-01 9.948255e-01 9.888805e-01 9.810542e-01 7.139826e-01

2897 9.983298e-01 9.972950e-01 9.948253e-01 9.888801e-01 9.810536e-01 7.138942e-01

2898 9.983298e-01 9.972950e-01 9.948251e-01 9.888796e-01 9.810528e-01 7.138033e-01

2899 9.983298e-01 9.972949e-01 9.948249e-01 9.888791e-01 9.810521e-01 7.137099e-01

2900 9.983299e-01 9.972948e-01 9.948247e-01 9.888786e-01 9.810513e-01 7.136140e-01

2901 9.983299e-01 9.972948e-01 9.948244e-01 9.888780e-01 9.810505e-01 7.135155e-01

2902 9.983300e-01 9.972947e-01 9.948242e-01 9.888774e-01 9.810496e-01 7.134142e-01

2903 9.983300e-01 9.972946e-01 9.948239e-01 9.888768e-01 9.810487e-01 7.133103e-01

2904 9.983300e-01 9.972945e-01 9.948236e-01 9.888762e-01 9.810478e-01 7.132034e-01

2905 9.983301e-01 9.972944e-01 9.948233e-01 9.888755e-01 9.810468e-01 7.130937e-01

2906 9.983301e-01 9.972943e-01 9.948230e-01 9.888748e-01 9.810457e-01 7.129810e-01

2907 9.983302e-01 9.972942e-01 9.948227e-01 9.888741e-01 9.810446e-01 7.128652e-01

2908 9.983302e-01 9.972941e-01 9.948224e-01 9.888733e-01 9.810435e-01 7.127462e-01

2909 9.983303e-01 9.972940e-01 9.948220e-01 9.888725e-01 9.810423e-01 7.126241e-01

2910 9.983303e-01 9.972939e-01 9.948216e-01 9.888717e-01 9.810410e-01 7.124986e-01

2911 9.983304e-01 9.972937e-01 9.948213e-01 9.888708e-01 9.810397e-01 7.123697e-01

2912 9.983304e-01 9.972936e-01 9.948209e-01 9.888699e-01 9.810383e-01 7.122373e-01

2913 9.983305e-01 9.972934e-01 9.948204e-01 9.888689e-01 9.810369e-01 7.121013e-01

2914 9.983305e-01 9.972933e-01 9.948200e-01 9.888679e-01 9.810354e-01 7.119616e-01

2915 9.983306e-01 9.972931e-01 9.948195e-01 9.888668e-01 9.810338e-01 7.118182e-01

2916 9.983306e-01 9.972930e-01 9.948190e-01 9.888657e-01 9.810321e-01 7.116709e-01

2917 9.983307e-01 9.972928e-01 9.948185e-01 9.888645e-01 9.810304e-01 7.115196e-01

2918 9.983307e-01 9.972926e-01 9.948180e-01 9.888632e-01 9.810285e-01 7.113643e-01

2919 9.983308e-01 9.972924e-01 9.948174e-01 9.888620e-01 9.810266e-01 7.112048e-01

2920 9.983308e-01 9.972922e-01 9.948168e-01 9.888606e-01 9.810246e-01 7.110411e-01

2921 9.983309e-01 9.972920e-01 9.948162e-01 9.888592e-01 9.810225e-01 7.108730e-01

2922 9.983309e-01 9.972918e-01 9.948155e-01 9.888577e-01 9.810204e-01 7.107003e-01

2923 9.983310e-01 9.972915e-01 9.948148e-01 9.888561e-01 9.810181e-01 7.105231e-01

2924 9.983311e-01 9.972913e-01 9.948141e-01 9.888545e-01 9.810157e-01 7.103412e-01

2925 9.983311e-01 9.972910e-01 9.948133e-01 9.888528e-01 9.810132e-01 7.101545e-01

2926 9.983312e-01 9.972907e-01 9.948125e-01 9.888510e-01 9.810105e-01 7.099629e-01

2927 9.983313e-01 9.972905e-01 9.948117e-01 9.888491e-01 9.810078e-01 7.097663e-01

2928 9.983313e-01 9.972902e-01 9.948108e-01 9.888471e-01 9.810049e-01 7.095644e-01

2929 9.983314e-01 9.972898e-01 9.948099e-01 9.888451e-01 9.810019e-01 7.093574e-01

2930 9.983315e-01 9.972895e-01 9.948089e-01 9.888429e-01 9.809987e-01 7.091449e-01

2931 9.983315e-01 9.972892e-01 9.948079e-01 9.888406e-01 9.809954e-01 7.089269e-01

2932 9.983316e-01 9.972888e-01 9.948068e-01 9.888382e-01 9.809920e-01 7.087033e-01

2933 9.983317e-01 9.972884e-01 9.948057e-01 9.888357e-01 9.809883e-01 7.084740e-01

2934 9.983318e-01 9.972880e-01 9.948045e-01 9.888331e-01 9.809845e-01 7.082388e-01

2935 9.983318e-01 9.972876e-01 9.948033e-01 9.888304e-01 9.809805e-01 7.079976e-01

2936 9.983319e-01 9.972871e-01 9.948020e-01 9.888275e-01 9.809763e-01 7.077503e-01

2937 9.983320e-01 9.972867e-01 9.948006e-01 9.888244e-01 9.809719e-01 7.074968e-01

2938 9.983321e-01 9.972862e-01 9.947992e-01 9.888213e-01 9.809673e-01 7.072369e-01

2939 9.983322e-01 9.972856e-01 9.947977e-01 9.888179e-01 9.809625e-01 7.069705e-01

2940 9.983323e-01 9.972851e-01 9.947961e-01 9.888144e-01 9.809574e-01 7.066976e-01

2941 9.983323e-01 9.972845e-01 9.947945e-01 9.888108e-01 9.809521e-01 7.064179e-01

2942 9.983324e-01 9.972839e-01 9.947927e-01 9.888069e-01 9.809466e-01 7.061314e-01

2943 9.983325e-01 9.972833e-01 9.947909e-01 9.888029e-01 9.809407e-01 7.058380e-01

2944 9.983326e-01 9.972826e-01 9.947890e-01 9.887986e-01 9.809346e-01 7.055375e-01

2945 9.983327e-01 9.972819e-01 9.947870e-01 9.887942e-01 9.809282e-01 7.052298e-01

2946 9.983328e-01 9.972812e-01 9.947849e-01 9.887895e-01 9.809214e-01 7.049148e-01

2947 9.983329e-01 9.972804e-01 9.947827e-01 9.887846e-01 9.809144e-01 7.045924e-01

2948 9.983330e-01 9.972796e-01 9.947804e-01 9.887794e-01 9.809070e-01 7.042625e-01

2949 9.983331e-01 9.972788e-01 9.947779e-01 9.887740e-01 9.808992e-01 7.039250e-01

2950 9.983332e-01 9.972779e-01 9.947754e-01 9.887683e-01 9.808910e-01 7.035797e-01

2951 9.983333e-01 9.972770e-01 9.947727e-01 9.887624e-01 9.808824e-01 7.032267e-01

2952 9.983334e-01 9.972760e-01 9.947699e-01 9.887561e-01 9.808734e-01 7.028657e-01

2953 9.983336e-01 9.972750e-01 9.947669e-01 9.887495e-01 9.808640e-01 7.024968e-01

2954 9.983337e-01 9.972739e-01 9.947638e-01 9.887426e-01 9.808541e-01 7.021197e-01

2955 9.983338e-01 9.972727e-01 9.947605e-01 9.887353e-01 9.808437e-01 7.017345e-01

2956 9.983339e-01 9.972715e-01 9.947571e-01 9.887276e-01 9.808327e-01 7.013411e-01

2957 9.983340e-01 9.972703e-01 9.947534e-01 9.887196e-01 9.808213e-01 7.009394e-01

2958 9.983342e-01 9.972690e-01 9.947496e-01 9.887112e-01 9.808092e-01 7.005293e-01

2959 9.983343e-01 9.972676e-01 9.947456e-01 9.887023e-01 9.807965e-01 7.001108e-01

2960 9.983344e-01 9.972661e-01 9.947414e-01 9.886929e-01 9.807832e-01 6.996839e-01

2961 9.983346e-01 9.972646e-01 9.947370e-01 9.886831e-01 9.807693e-01 6.992485e-01

2962 9.983347e-01 9.972630e-01 9.947324e-01 9.886728e-01 9.807546e-01 6.988046e-01

2963 9.983348e-01 9.972613e-01 9.947275e-01 9.886620e-01 9.807392e-01 6.983521e-01

2964 9.983350e-01 9.972596e-01 9.947224e-01 9.886505e-01 9.807230e-01 6.978911e-01

2965 9.983351e-01 9.972577e-01 9.947170e-01 9.886385e-01 9.807060e-01 6.974216e-01

2966 9.983353e-01 9.972558e-01 9.947113e-01 9.886259e-01 9.806881e-01 6.969436e-01

2967 9.983354e-01 9.972537e-01 9.947053e-01 9.886126e-01 9.806693e-01 6.964570e-01

2968 9.983355e-01 9.972515e-01 9.946990e-01 9.885987e-01 9.806495e-01 6.959620e-01

2969 9.983357e-01 9.972493e-01 9.946924e-01 9.885840e-01 9.806287e-01 6.954585e-01

2970 9.983358e-01 9.972469e-01 9.946855e-01 9.885685e-01 9.806069e-01 6.949467e-01

2971 9.983360e-01 9.972444e-01 9.946781e-01 9.885522e-01 9.805839e-01 6.944266e-01

2972 9.983362e-01 9.972417e-01 9.946704e-01 9.885351e-01 9.805598e-01 6.938983e-01

2973 9.983363e-01 9.972390e-01 9.946623e-01 9.885170e-01 9.805344e-01 6.933619e-01

2974 9.983365e-01 9.972361e-01 9.946538e-01 9.884980e-01 9.805077e-01 6.928174e-01

2975 9.983366e-01 9.972330e-01 9.946448e-01 9.884781e-01 9.804796e-01 6.922651e-01

2976 9.983368e-01 9.972298e-01 9.946353e-01 9.884570e-01 9.804501e-01 6.917051e-01

2977 9.983370e-01 9.972264e-01 9.946254e-01 9.884348e-01 9.804190e-01 6.911375e-01

2978 9.983371e-01 9.972228e-01 9.946149e-01 9.884115e-01 9.803864e-01 6.905624e-01

2979 9.983373e-01 9.972191e-01 9.946038e-01 9.883869e-01 9.803520e-01 6.899802e-01

2980 9.983375e-01 9.972151e-01 9.945922e-01 9.883611e-01 9.803159e-01 6.893909e-01

2981 9.983376e-01 9.972110e-01 9.945800e-01 9.883338e-01 9.802779e-01 6.887947e-01

2982 9.983378e-01 9.972066e-01 9.945671e-01 9.883051e-01 9.802380e-01 6.881920e-01

2983 9.983379e-01 9.972020e-01 9.945535e-01 9.882748e-01 9.801959e-01 6.875829e-01

2984 9.983381e-01 9.971972e-01 9.945391e-01 9.882430e-01 9.801517e-01 6.869678e-01

2985 9.983383e-01 9.971921e-01 9.945240e-01 9.882094e-01 9.801052e-01 6.863468e-01

2986 9.983384e-01 9.971867e-01 9.945081e-01 9.881740e-01 9.800562e-01 6.857203e-01

2987 9.983386e-01 9.971811e-01 9.944914e-01 9.881368e-01 9.800048e-01 6.850885e-01

2988 9.983388e-01 9.971751e-01 9.944737e-01 9.880975e-01 9.799506e-01 6.844518e-01

2989 9.983389e-01 9.971689e-01 9.944551e-01 9.880562e-01 9.798937e-01 6.838105e-01

2990 9.983391e-01 9.971623e-01 9.944355e-01 9.880126e-01 9.798338e-01 6.831650e-01

2991 9.983392e-01 9.971553e-01 9.944148e-01 9.879667e-01 9.797708e-01 6.825155e-01

2992 9.983394e-01 9.971480e-01 9.943930e-01 9.879183e-01 9.797045e-01 6.818625e-01

2993 9.983395e-01 9.971403e-01 9.943700e-01 9.878673e-01 9.796348e-01 6.812064e-01

2994 9.983396e-01 9.971322e-01 9.943458e-01 9.878135e-01 9.795616e-01 6.805474e-01

2995 9.983397e-01 9.971237e-01 9.943202e-01 9.877569e-01 9.794845e-01 6.798860e-01

2996 9.983399e-01 9.971147e-01 9.942933e-01 9.876972e-01 9.794035e-01 6.792227e-01

2997 9.983400e-01 9.971053e-01 9.942648e-01 9.876344e-01 9.793184e-01 6.785578e-01

2998 9.983401e-01 9.970953e-01 9.942349e-01 9.875681e-01 9.792289e-01 6.778916e-01

2999 9.983402e-01 9.970848e-01 9.942033e-01 9.874983e-01 9.791348e-01 6.772248e-01

3000 9.983402e-01 9.970737e-01 9.941699e-01 9.874248e-01 9.790360e-01 6.765576e-01

3001 9.983403e-01 9.970620e-01 9.941348e-01 9.873473e-01 9.789322e-01 6.758905e-01

3002 9.983403e-01 9.970497e-01 9.940977e-01 9.872657e-01 9.788231e-01 6.752239e-01

3003 9.983404e-01 9.970368e-01 9.940586e-01 9.871798e-01 9.787085e-01 6.745583e-01

3004 9.983404e-01 9.970231e-01 9.940173e-01 9.870892e-01 9.785882e-01 6.738942e-01

3005 9.983404e-01 9.970087e-01 9.939738e-01 9.869939e-01 9.784619e-01 6.732318e-01

3006 9.983404e-01 9.969935e-01 9.939279e-01 9.868935e-01 9.783293e-01 6.725717e-01

3007 9.983403e-01 9.969775e-01 9.938795e-01 9.867878e-01 9.781902e-01 6.719143e-01

3008 9.983402e-01 9.969607e-01 9.938285e-01 9.866765e-01 9.780443e-01 6.712600e-01

3009 9.983401e-01 9.969429e-01 9.937746e-01 9.865593e-01 9.778912e-01 6.706092e-01

3010 9.983400e-01 9.969242e-01 9.937178e-01 9.864361e-01 9.777307e-01 6.699624e-01

3011 9.983398e-01 9.969044e-01 9.936579e-01 9.863063e-01 9.775625e-01 6.693199e-01

3012 9.983396e-01 9.968836e-01 9.935947e-01 9.861699e-01 9.773863e-01 6.686821e-01

3013 9.983394e-01 9.968616e-01 9.935280e-01 9.860263e-01 9.772016e-01 6.680495e-01

3014 9.983391e-01 9.968385e-01 9.934577e-01 9.858754e-01 9.770083e-01 6.674223e-01

3015 9.983388e-01 9.968141e-01 9.933836e-01 9.857166e-01 9.768059e-01 6.668010e-01

3016 9.983384e-01 9.967883e-01 9.933055e-01 9.855498e-01 9.765942e-01 6.661859e-01

3017 9.983380e-01 9.967612e-01 9.932231e-01 9.853745e-01 9.763727e-01 6.655774e-01

3018 9.983375e-01 9.967326e-01 9.931363e-01 9.851904e-01 9.761412e-01 6.649757e-01

3019 9.983370e-01 9.967024e-01 9.930447e-01 9.849970e-01 9.758992e-01 6.643812e-01

3020 9.983364e-01 9.966706e-01 9.929482e-01 9.847939e-01 9.756465e-01 6.637941e-01

3021 9.983357e-01 9.966370e-01 9.928465e-01 9.845808e-01 9.753827e-01 6.632148e-01

3022 9.983350e-01 9.966017e-01 9.927394e-01 9.843571e-01 9.751074e-01 6.626435e-01

3023 9.983341e-01 9.965644e-01 9.926265e-01 9.841226e-01 9.748203e-01 6.620805e-01

3024 9.983332e-01 9.965251e-01 9.925076e-01 9.838767e-01 9.745210e-01 6.615259e-01

3025 9.983322e-01 9.964836e-01 9.923824e-01 9.836190e-01 9.742093e-01 6.609800e-01

3026 9.983311e-01 9.964399e-01 9.922505e-01 9.833490e-01 9.738847e-01 6.604430e-01

3027 9.983299e-01 9.963938e-01 9.921117e-01 9.830662e-01 9.735470e-01 6.599151e-01

3028 9.983286e-01 9.963453e-01 9.919656e-01 9.827703e-01 9.731958e-01 6.593963e-01

3029 9.983271e-01 9.962941e-01 9.918118e-01 9.824606e-01 9.728309e-01 6.588870e-01

3030 9.983255e-01 9.962401e-01 9.916501e-01 9.821368e-01 9.724520e-01 6.583870e-01

3031 9.983238e-01 9.961833e-01 9.914799e-01 9.817984e-01 9.720589e-01 6.578967e-01

3032 9.983219e-01 9.961234e-01 9.913010e-01 9.814449e-01 9.716512e-01 6.574161e-01

3033 9.983199e-01 9.960602e-01 9.911130e-01 9.810758e-01 9.712288e-01 6.569451e-01

3034 9.983177e-01 9.959937e-01 9.909153e-01 9.806906e-01 9.707915e-01 6.564840e-01

3035 9.983153e-01 9.959237e-01 9.907077e-01 9.802889e-01 9.703391e-01 6.560327e-01

3036 9.983127e-01 9.958499e-01 9.904896e-01 9.798703e-01 9.698715e-01 6.555913e-01

3037 9.983099e-01 9.957722e-01 9.902607e-01 9.794342e-01 9.693886e-01 6.551597e-01

3038 9.983068e-01 9.956904e-01 9.900204e-01 9.789803e-01 9.688904e-01 6.547380e-01

3039 9.983036e-01 9.956042e-01 9.897683e-01 9.785081e-01 9.683769e-01 6.543261e-01

3040 9.983000e-01 9.955136e-01 9.895039e-01 9.780173e-01 9.678480e-01 6.539241e-01

3041 9.982963e-01 9.954181e-01 9.892267e-01 9.775075e-01 9.673039e-01 6.535318e-01

3042 9.982922e-01 9.953177e-01 9.889363e-01 9.769784e-01 9.667446e-01 6.531492e-01

3043 9.982878e-01 9.952121e-01 9.886321e-01 9.764295e-01 9.661704e-01 6.527762e-01

3044 9.983591e-01 9.953384e-01 9.888870e-01 9.769880e-01 9.671003e-01 6.570744e-01

3045 9.983591e-01 9.953384e-01 9.888869e-01 9.769876e-01 9.671000e-01 6.569857e-01

3046 9.983591e-01 9.953383e-01 9.888868e-01 9.769873e-01 9.670996e-01 6.568950e-01

3047 9.983591e-01 9.953383e-01 9.888866e-01 9.769870e-01 9.670993e-01 6.568024e-01

3048 9.983591e-01 9.953383e-01 9.888865e-01 9.769867e-01 9.670990e-01 6.567077e-01

3049 9.983590e-01 9.953382e-01 9.888863e-01 9.769863e-01 9.670986e-01 6.566110e-01

3050 9.983590e-01 9.953382e-01 9.888861e-01 9.769860e-01 9.670982e-01 6.565122e-01

3051 9.983590e-01 9.953381e-01 9.888859e-01 9.769856e-01 9.670979e-01 6.564113e-01

3052 9.983590e-01 9.953381e-01 9.888857e-01 9.769852e-01 9.670975e-01 6.563082e-01

3053 9.983590e-01 9.953380e-01 9.888855e-01 9.769849e-01 9.670971e-01 6.562030e-01

3054 9.983590e-01 9.953379e-01 9.888852e-01 9.769845e-01 9.670967e-01 6.560954e-01

3055 9.983590e-01 9.953379e-01 9.888850e-01 9.769841e-01 9.670963e-01 6.559856e-01

3056 9.983589e-01 9.953378e-01 9.888848e-01 9.769838e-01 9.670959e-01 6.558734e-01

3057 9.983589e-01 9.953377e-01 9.888845e-01 9.769834e-01 9.670955e-01 6.557589e-01

3058 9.983589e-01 9.953376e-01 9.888842e-01 9.769830e-01 9.670950e-01 6.556419e-01

3059 9.983589e-01 9.953375e-01 9.888840e-01 9.769826e-01 9.670946e-01 6.555224e-01

3060 9.983589e-01 9.953374e-01 9.888837e-01 9.769822e-01 9.670941e-01 6.554004e-01

3061 9.983589e-01 9.953373e-01 9.888834e-01 9.769817e-01 9.670937e-01 6.552758e-01

3062 9.983588e-01 9.953372e-01 9.888831e-01 9.769813e-01 9.670932e-01 6.551486e-01

3063 9.983588e-01 9.953370e-01 9.888828e-01 9.769808e-01 9.670927e-01 6.550187e-01

3064 9.983588e-01 9.953369e-01 9.888825e-01 9.769803e-01 9.670922e-01 6.548860e-01

3065 9.983588e-01 9.953368e-01 9.888821e-01 9.769798e-01 9.670916e-01 6.547505e-01

3066 9.983588e-01 9.953366e-01 9.888818e-01 9.769793e-01 9.670910e-01 6.546122e-01

3067 9.983588e-01 9.953364e-01 9.888814e-01 9.769787e-01 9.670905e-01 6.544710e-01

3068 9.983588e-01 9.953363e-01 9.888810e-01 9.769781e-01 9.670898e-01 6.543267e-01

3069 9.983587e-01 9.953361e-01 9.888806e-01 9.769775e-01 9.670892e-01 6.541795e-01

3070 9.983587e-01 9.953359e-01 9.888802e-01 9.769768e-01 9.670885e-01 6.540291e-01

3071 9.983587e-01 9.953357e-01 9.888798e-01 9.769761e-01 9.670878e-01 6.538756e-01

3072 9.983587e-01 9.953355e-01 9.888794e-01 9.769754e-01 9.670871e-01 6.537188e-01

3073 9.983587e-01 9.953353e-01 9.888789e-01 9.769746e-01 9.670863e-01 6.535588e-01

3074 9.983586e-01 9.953351e-01 9.888784e-01 9.769738e-01 9.670855e-01 6.533954e-01

3075 9.983586e-01 9.953348e-01 9.888779e-01 9.769730e-01 9.670846e-01 6.532286e-01

3076 9.983586e-01 9.953346e-01 9.888773e-01 9.769721e-01 9.670838e-01 6.530583e-01

3077 9.983586e-01 9.953343e-01 9.888768e-01 9.769711e-01 9.670828e-01 6.528845e-01

3078 9.983585e-01 9.953341e-01 9.888762e-01 9.769702e-01 9.670819e-01 6.527071e-01

3079 9.983585e-01 9.953338e-01 9.888755e-01 9.769691e-01 9.670809e-01 6.525259e-01

3080 9.983584e-01 9.953335e-01 9.888749e-01 9.769681e-01 9.670798e-01 6.523410e-01

3081 9.983584e-01 9.953332e-01 9.888742e-01 9.769670e-01 9.670787e-01 6.521523e-01

3082 9.983584e-01 9.953329e-01 9.888734e-01 9.769658e-01 9.670776e-01 6.519598e-01

3083 9.983583e-01 9.953326e-01 9.888727e-01 9.769646e-01 9.670764e-01 6.517632e-01

3084 9.983583e-01 9.953322e-01 9.888719e-01 9.769633e-01 9.670751e-01 6.515626e-01

3085 9.983582e-01 9.953319e-01 9.888710e-01 9.769620e-01 9.670738e-01 6.513579e-01

3086 9.983581e-01 9.953315e-01 9.888702e-01 9.769607e-01 9.670725e-01 6.511490e-01

3087 9.983581e-01 9.953311e-01 9.888692e-01 9.769592e-01 9.670711e-01 6.509359e-01

3088 9.983580e-01 9.953307e-01 9.888683e-01 9.769577e-01 9.670696e-01 6.507185e-01

3089 9.983579e-01 9.953302e-01 9.888673e-01 9.769562e-01 9.670681e-01 6.504967e-01

3090 9.983579e-01 9.953298e-01 9.888662e-01 9.769546e-01 9.670665e-01 6.502704e-01

3091 9.983578e-01 9.953293e-01 9.888651e-01 9.769529e-01 9.670648e-01 6.500395e-01

3092 9.983577e-01 9.953288e-01 9.888640e-01 9.769511e-01 9.670631e-01 6.498041e-01

3093 9.983576e-01 9.953282e-01 9.888628e-01 9.769493e-01 9.670613e-01 6.495640e-01

3094 9.983575e-01 9.953277e-01 9.888615e-01 9.769474e-01 9.670595e-01 6.493191e-01

3095 9.983574e-01 9.953271e-01 9.888602e-01 9.769454e-01 9.670576e-01 6.490694e-01

3096 9.983573e-01 9.953265e-01 9.888588e-01 9.769433e-01 9.670555e-01 6.488149e-01

3097 9.983572e-01 9.953258e-01 9.888574e-01 9.769411e-01 9.670535e-01 6.485553e-01

3098 9.983571e-01 9.953252e-01 9.888558e-01 9.769389e-01 9.670513e-01 6.482907e-01

3099 9.983569e-01 9.953245e-01 9.888543e-01 9.769365e-01 9.670490e-01 6.480211e-01

3100 9.983568e-01 9.953237e-01 9.888526e-01 9.769340e-01 9.670467e-01 6.477462e-01

3101 9.983567e-01 9.953229e-01 9.888509e-01 9.769315e-01 9.670442e-01 6.474662e-01

3102 9.983565e-01 9.953221e-01 9.888491e-01 9.769288e-01 9.670416e-01 6.471808e-01

3103 9.983564e-01 9.953213e-01 9.888472e-01 9.769260e-01 9.670390e-01 6.468901e-01

3104 9.983562e-01 9.953204e-01 9.888452e-01 9.769230e-01 9.670362e-01 6.465940e-01

3105 9.983561e-01 9.953195e-01 9.888431e-01 9.769200e-01 9.670333e-01 6.462924e-01

3106 9.983559e-01 9.953185e-01 9.888410e-01 9.769168e-01 9.670304e-01 6.459853e-01

3107 9.983557e-01 9.953175e-01 9.888387e-01 9.769134e-01 9.670272e-01 6.456726e-01

3108 9.983555e-01 9.953164e-01 9.888363e-01 9.769099e-01 9.670240e-01 6.453543e-01

3109 9.983553e-01 9.953153e-01 9.888338e-01 9.769063e-01 9.670206e-01 6.450303e-01

3110 9.983551e-01 9.953141e-01 9.888312e-01 9.769025e-01 9.670171e-01 6.447005e-01

3111 9.983549e-01 9.953129e-01 9.888285e-01 9.768985e-01 9.670135e-01 6.443650e-01

3112 9.983546e-01 9.953116e-01 9.888256e-01 9.768944e-01 9.670096e-01 6.440237e-01

3113 9.983544e-01 9.953102e-01 9.888226e-01 9.768900e-01 9.670057e-01 6.436766e-01

3114 9.983541e-01 9.953088e-01 9.888195e-01 9.768855e-01 9.670016e-01 6.433236e-01

3115 9.983539e-01 9.953073e-01 9.888162e-01 9.768808e-01 9.669973e-01 6.429647e-01

3116 9.983536e-01 9.953058e-01 9.888128e-01 9.768759e-01 9.669928e-01 6.425999e-01

3117 9.983533e-01 9.953042e-01 9.888092e-01 9.768707e-01 9.669882e-01 6.422292e-01

3118 9.983529e-01 9.953025e-01 9.888054e-01 9.768653e-01 9.669834e-01 6.418525e-01

3119 9.983526e-01 9.953007e-01 9.888015e-01 9.768597e-01 9.669783e-01 6.414699e-01

3120 9.983523e-01 9.952988e-01 9.887973e-01 9.768538e-01 9.669731e-01 6.410813e-01

3121 9.983519e-01 9.952968e-01 9.887930e-01 9.768477e-01 9.669677e-01 6.406868e-01

3122 9.983515e-01 9.952948e-01 9.887885e-01 9.768413e-01 9.669620e-01 6.402864e-01

3123 9.983511e-01 9.952926e-01 9.887837e-01 9.768346e-01 9.669561e-01 6.398801e-01

3124 9.983507e-01 9.952904e-01 9.887787e-01 9.768276e-01 9.669500e-01 6.394679e-01

3125 9.983502e-01 9.952880e-01 9.887735e-01 9.768203e-01 9.669436e-01 6.390498e-01

3126 9.983498e-01 9.952855e-01 9.887680e-01 9.768127e-01 9.669370e-01 6.386260e-01

3127 9.983493e-01 9.952829e-01 9.887623e-01 9.768047e-01 9.669301e-01 6.381964e-01

3128 9.983488e-01 9.952802e-01 9.887563e-01 9.767964e-01 9.669230e-01 6.377611e-01

3129 9.983482e-01 9.952773e-01 9.887500e-01 9.767877e-01 9.669155e-01 6.373201e-01

3130 9.983477e-01 9.952743e-01 9.887434e-01 9.767786e-01 9.669078e-01 6.368736e-01

3131 9.983471e-01 9.952712e-01 9.887365e-01 9.767691e-01 9.668997e-01 6.364216e-01

3132 9.983465e-01 9.952679e-01 9.887292e-01 9.767592e-01 9.668913e-01 6.359642e-01

3133 9.983458e-01 9.952644e-01 9.887216e-01 9.767488e-01 9.668826e-01 6.355015e-01

3134 9.983451e-01 9.952608e-01 9.887136e-01 9.767380e-01 9.668735e-01 6.350336e-01

3135 9.983444e-01 9.952570e-01 9.887053e-01 9.767266e-01 9.668641e-01 6.345606e-01

3136 9.983437e-01 9.952530e-01 9.886965e-01 9.767148e-01 9.668543e-01 6.340827e-01

3137 9.983429e-01 9.952488e-01 9.886873e-01 9.767024e-01 9.668441e-01 6.336000e-01

3138 9.983421e-01 9.952444e-01 9.886776e-01 9.766895e-01 9.668335e-01 6.331127e-01

3139 9.983412e-01 9.952398e-01 9.886675e-01 9.766759e-01 9.668225e-01 6.326208e-01

3140 9.983403e-01 9.952349e-01 9.886569e-01 9.766618e-01 9.668110e-01 6.321246e-01

3141 9.983393e-01 9.952298e-01 9.886458e-01 9.766470e-01 9.667991e-01 6.316242e-01

3142 9.983383e-01 9.952245e-01 9.886341e-01 9.766315e-01 9.667867e-01 6.311198e-01

3143 9.983373e-01 9.952189e-01 9.886218e-01 9.766153e-01 9.667739e-01 6.306117e-01

3144 9.983362e-01 9.952130e-01 9.886090e-01 9.765984e-01 9.667605e-01 6.301000e-01

3145 9.983350e-01 9.952069e-01 9.885955e-01 9.765807e-01 9.667466e-01 6.295849e-01

3146 9.983338e-01 9.952004e-01 9.885813e-01 9.765622e-01 9.667322e-01 6.290667e-01

3147 9.983326e-01 9.951936e-01 9.885664e-01 9.765429e-01 9.667172e-01 6.285456e-01

3148 9.983312e-01 9.951865e-01 9.885508e-01 9.765226e-01 9.667016e-01 6.280218e-01

3149 9.983298e-01 9.951790e-01 9.885344e-01 9.765015e-01 9.666854e-01 6.274956e-01

3150 9.983284e-01 9.951711e-01 9.885172e-01 9.764794e-01 9.666686e-01 6.269672e-01

3151 9.983268e-01 9.951628e-01 9.884991e-01 9.764562e-01 9.666511e-01 6.264370e-01

3152 9.983252e-01 9.951541e-01 9.884802e-01 9.764320e-01 9.666330e-01 6.259051e-01

3153 9.983235e-01 9.951450e-01 9.884602e-01 9.764067e-01 9.666141e-01 6.253719e-01

3154 9.983218e-01 9.951354e-01 9.884393e-01 9.763802e-01 9.665946e-01 6.248377e-01

3155 9.983199e-01 9.951253e-01 9.884173e-01 9.763525e-01 9.665743e-01 6.243028e-01

3156 9.983179e-01 9.951147e-01 9.883943e-01 9.763236e-01 9.665533e-01 6.237674e-01

3157 9.983159e-01 9.951036e-01 9.883700e-01 9.762933e-01 9.665314e-01 6.232318e-01

3158 9.983137e-01 9.950919e-01 9.883446e-01 9.762616e-01 9.665088e-01 6.226965e-01

3159 9.983115e-01 9.950796e-01 9.883178e-01 9.762285e-01 9.664853e-01 6.221616e-01

3160 9.983091e-01 9.950667e-01 9.882897e-01 9.761938e-01 9.664609e-01 6.216275e-01

3161 9.983066e-01 9.950531e-01 9.882602e-01 9.761576e-01 9.664357e-01 6.210946e-01

3162 9.983040e-01 9.950389e-01 9.882292e-01 9.761197e-01 9.664095e-01 6.205631e-01

3163 9.983013e-01 9.950238e-01 9.881966e-01 9.760801e-01 9.663824e-01 6.200333e-01

3164 9.982984e-01 9.950081e-01 9.881623e-01 9.760387e-01 9.663543e-01 6.195057e-01

3165 9.982953e-01 9.949915e-01 9.881264e-01 9.759954e-01 9.663252e-01 6.189805e-01

3166 9.982922e-01 9.949740e-01 9.880886e-01 9.759501e-01 9.662951e-01 6.184580e-01

3167 9.982888e-01 9.949557e-01 9.880488e-01 9.759028e-01 9.662640e-01 6.179386e-01

3168 9.982853e-01 9.949364e-01 9.880071e-01 9.758533e-01 9.662318e-01 6.174226e-01

3169 9.982816e-01 9.949161e-01 9.879632e-01 9.758016e-01 9.661985e-01 6.169102e-01

3170 9.982778e-01 9.948947e-01 9.879171e-01 9.757476e-01 9.661640e-01 6.164019e-01

3171 9.982737e-01 9.948722e-01 9.878686e-01 9.756911e-01 9.661285e-01 6.158978e-01

3172 9.982694e-01 9.948486e-01 9.878177e-01 9.756321e-01 9.660917e-01 6.153983e-01

3173 9.982649e-01 9.948237e-01 9.877642e-01 9.755705e-01 9.660538e-01 6.149038e-01

3174 9.982602e-01 9.947976e-01 9.877080e-01 9.755061e-01 9.660147e-01 6.144143e-01

3175 9.982553e-01 9.947700e-01 9.876489e-01 9.754389e-01 9.659744e-01 6.139303e-01

3176 9.982501e-01 9.947411e-01 9.875868e-01 9.753687e-01 9.659328e-01 6.134520e-01

3177 9.982446e-01 9.947106e-01 9.875216e-01 9.752955e-01 9.658899e-01 6.129797e-01

3178 9.982389e-01 9.946785e-01 9.874530e-01 9.752190e-01 9.658459e-01 6.125135e-01

3179 9.982328e-01 9.946447e-01 9.873810e-01 9.751392e-01 9.658005e-01 6.120538e-01

3180 9.982265e-01 9.946092e-01 9.873053e-01 9.750560e-01 9.657538e-01 6.116008e-01

3181 9.982198e-01 9.945718e-01 9.872259e-01 9.749692e-01 9.657059e-01 6.111546e-01

3182 9.982128e-01 9.945324e-01 9.871424e-01 9.748786e-01 9.656567e-01 6.107154e-01

3183 9.982054e-01 9.944910e-01 9.870547e-01 9.747842e-01 9.656062e-01 6.102835e-01

3184 9.981977e-01 9.944474e-01 9.869626e-01 9.746859e-01 9.655545e-01 6.098590e-01

3185 9.981895e-01 9.944015e-01 9.868659e-01 9.745834e-01 9.655015e-01 6.094421e-01

3186 9.981810e-01 9.943532e-01 9.867643e-01 9.744767e-01 9.654473e-01 6.090329e-01

3187 9.981719e-01 9.943024e-01 9.866577e-01 9.743655e-01 9.653918e-01 6.086315e-01

3188 9.981625e-01 9.942488e-01 9.865458e-01 9.742498e-01 9.653352e-01 6.082381e-01

3189 9.981525e-01 9.941925e-01 9.864284e-01 9.741295e-01 9.652774e-01 6.078528e-01

3190 9.981420e-01 9.941332e-01 9.863051e-01 9.740043e-01 9.652184e-01 6.074757e-01

3191 9.981310e-01 9.940708e-01 9.861758e-01 9.738741e-01 9.651584e-01 6.071067e-01

3192 9.981194e-01 9.940052e-01 9.860402e-01 9.737388e-01 9.650974e-01 6.067461e-01

3193 9.981072e-01 9.939361e-01 9.858980e-01 9.735983e-01 9.650355e-01 6.063938e-01

3194 9.980944e-01 9.938634e-01 9.857488e-01 9.734523e-01 9.649726e-01 6.060499e-01

3195 9.980809e-01 9.937869e-01 9.855925e-01 9.733008e-01 9.649089e-01 6.057144e-01

3196 9.980667e-01 9.937064e-01 9.854287e-01 9.731437e-01 9.648445e-01 6.053873e-01

3197 9.980518e-01 9.936217e-01 9.852570e-01 9.729807e-01 9.647795e-01 6.050686e-01

3198 9.980361e-01 9.935326e-01 9.850772e-01 9.728118e-01 9.647139e-01 6.047583e-01

3199 9.980196e-01 9.934388e-01 9.848888e-01 9.726368e-01 9.646479e-01 6.044564e-01

3200 9.980022e-01 9.933403e-01 9.846917e-01 9.724557e-01 9.645815e-01 6.041628e-01

3201 9.979839e-01 9.932366e-01 9.844854e-01 9.722682e-01 9.645150e-01 6.038775e-01

3202 9.979647e-01 9.931276e-01 9.842695e-01 9.720744e-01 9.644485e-01 6.036003e-01

3203 9.979444e-01 9.930130e-01 9.840437e-01 9.718740e-01 9.643820e-01 6.033314e-01

3204 9.979231e-01 9.928925e-01 9.838077e-01 9.716671e-01 9.643159e-01 6.030705e-01

3205 9.979007e-01 9.927659e-01 9.835610e-01 9.714535e-01 9.642502e-01 6.028176e-01

3206 9.978772e-01 9.926328e-01 9.833032e-01 9.712331e-01 9.641851e-01 6.025726e-01

3207 9.978523e-01 9.924930e-01 9.830341e-01 9.710060e-01 9.641208e-01 6.023354e-01

3208 9.978262e-01 9.923461e-01 9.827531e-01 9.707720e-01 9.640575e-01 6.021059e-01

3209 9.977988e-01 9.921918e-01 9.824599e-01 9.705312e-01 9.639954e-01 6.018839e-01

3210 9.977699e-01 9.920298e-01 9.821542e-01 9.702835e-01 9.639348e-01 6.016693e-01

3211 9.977395e-01 9.918598e-01 9.818354e-01 9.700289e-01 9.638759e-01 6.014621e-01

3212 9.977075e-01 9.916813e-01 9.815033e-01 9.697675e-01 9.638188e-01 6.012621e-01

3213 9.976738e-01 9.914940e-01 9.811574e-01 9.694993e-01 9.637640e-01 6.010691e-01

3214 9.976384e-01 9.912975e-01 9.807974e-01 9.692244e-01 9.637115e-01 6.008829e-01

3215 9.976012e-01 9.910915e-01 9.804229e-01 9.689427e-01 9.636617e-01 6.007036e-01

3216 9.975620e-01 9.908755e-01 9.800334e-01 9.686546e-01 9.636148e-01 6.005308e-01

3217 9.975208e-01 9.906490e-01 9.796287e-01 9.683599e-01 9.635711e-01 6.003645e-01

3218 9.974775e-01 9.904117e-01 9.792085e-01 9.680590e-01 9.635308e-01 6.002045e-01

3219 9.974319e-01 9.901632e-01 9.787723e-01 9.677520e-01 9.634943e-01 6.000507e-01

3220 9.973839e-01 9.899030e-01 9.783199e-01 9.674391e-01 9.634618e-01 5.999028e-01

3221 9.973335e-01 9.896305e-01 9.778510e-01 9.671204e-01 9.634336e-01 5.997608e-01

3222 9.972805e-01 9.893455e-01 9.773653e-01 9.667963e-01 9.634099e-01 5.996245e-01

3223 9.973530e-01 9.895713e-01 9.779115e-01 9.678766e-01 9.648723e-01 6.042018e-01

3224 9.973530e-01 9.895711e-01 9.779113e-01 9.678766e-01 9.648724e-01 6.041314e-01

3225 9.973529e-01 9.895709e-01 9.779110e-01 9.678766e-01 9.648726e-01 6.040602e-01

3226 9.973529e-01 9.895707e-01 9.779107e-01 9.678766e-01 9.648728e-01 6.039881e-01

3227 9.973529e-01 9.895705e-01 9.779104e-01 9.678765e-01 9.648731e-01 6.039151e-01

3228 9.973528e-01 9.895703e-01 9.779101e-01 9.678764e-01 9.648733e-01 6.038413e-01

3229 9.973528e-01 9.895701e-01 9.779098e-01 9.678763e-01 9.648736e-01 6.037666e-01

3230 9.973528e-01 9.895699e-01 9.779095e-01 9.678761e-01 9.648738e-01 6.036910e-01

3231 9.973528e-01 9.895696e-01 9.779092e-01 9.678760e-01 9.648741e-01 6.036147e-01

3232 9.973527e-01 9.895694e-01 9.779089e-01 9.678758e-01 9.648743e-01 6.035374e-01

3233 9.973527e-01 9.895692e-01 9.779086e-01 9.678756e-01 9.648746e-01 6.034593e-01

3234 9.973526e-01 9.895689e-01 9.779083e-01 9.678753e-01 9.648748e-01 6.033804e-01

3235 9.973526e-01 9.895687e-01 9.779079e-01 9.678751e-01 9.648750e-01 6.033007e-01

3236 9.973526e-01 9.895684e-01 9.779076e-01 9.678748e-01 9.648752e-01 6.032201e-01

3237 9.973525e-01 9.895682e-01 9.779072e-01 9.678746e-01 9.648753e-01 6.031387e-01

3238 9.973524e-01 9.895679e-01 9.779068e-01 9.678743e-01 9.648755e-01 6.030565e-01

3239 9.973524e-01 9.895677e-01 9.779065e-01 9.678740e-01 9.648756e-01 6.029735e-01

3240 9.973523e-01 9.895674e-01 9.779060e-01 9.678736e-01 9.648757e-01 6.028897e-01

3241 9.973522e-01 9.895671e-01 9.779056e-01 9.678733e-01 9.648758e-01 6.028052e-01

3242 9.973522e-01 9.895668e-01 9.779052e-01 9.678730e-01 9.648759e-01 6.027198e-01

3243 9.973521e-01 9.895665e-01 9.779047e-01 9.678727e-01 9.648759e-01 6.026337e-01

3244 9.973520e-01 9.895661e-01 9.779042e-01 9.678723e-01 9.648760e-01 6.025468e-01

3245 9.973519e-01 9.895658e-01 9.779037e-01 9.678720e-01 9.648760e-01 6.024592e-01

3246 9.973518e-01 9.895654e-01 9.779032e-01 9.678717e-01 9.648760e-01 6.023708e-01

3247 9.973517e-01 9.895650e-01 9.779026e-01 9.678713e-01 9.648761e-01 6.022818e-01

3248 9.973516e-01 9.895646e-01 9.779021e-01 9.678710e-01 9.648761e-01 6.021920e-01

3249 9.973515e-01 9.895642e-01 9.779014e-01 9.678706e-01 9.648761e-01 6.021016e-01

3250 9.973513e-01 9.895638e-01 9.779008e-01 9.678703e-01 9.648761e-01 6.020105e-01

3251 9.973512e-01 9.895633e-01 9.779001e-01 9.678699e-01 9.648762e-01 6.019187e-01

3252 9.973511e-01 9.895628e-01 9.778994e-01 9.678696e-01 9.648762e-01 6.018263e-01

3253 9.973509e-01 9.895623e-01 9.778987e-01 9.678692e-01 9.648763e-01 6.017333e-01

3254 9.973508e-01 9.895618e-01 9.778979e-01 9.678689e-01 9.648763e-01 6.016398e-01

3255 9.973506e-01 9.895612e-01 9.778971e-01 9.678685e-01 9.648764e-01 6.015456e-01

3256 9.973505e-01 9.895606e-01 9.778963e-01 9.678681e-01 9.648765e-01 6.014510e-01

3257 9.973503e-01 9.895600e-01 9.778954e-01 9.678677e-01 9.648767e-01 6.013558e-01

3258 9.973501e-01 9.895593e-01 9.778945e-01 9.678673e-01 9.648768e-01 6.012602e-01

3259 9.973500e-01 9.895586e-01 9.778936e-01 9.678669e-01 9.648770e-01 6.011641e-01

3260 9.973498e-01 9.895579e-01 9.778926e-01 9.678665e-01 9.648772e-01 6.010676e-01

3261 9.973496e-01 9.895572e-01 9.778916e-01 9.678661e-01 9.648774e-01 6.009707e-01

3262 9.973494e-01 9.895564e-01 9.778905e-01 9.678656e-01 9.648776e-01 6.008734e-01

3263 9.973491e-01 9.895556e-01 9.778894e-01 9.678651e-01 9.648779e-01 6.007759e-01

3264 9.973489e-01 9.895547e-01 9.778883e-01 9.678646e-01 9.648782e-01 6.006780e-01

3265 9.973487e-01 9.895538e-01 9.778871e-01 9.678641e-01 9.648785e-01 6.005800e-01

3266 9.973484e-01 9.895529e-01 9.778858e-01 9.678636e-01 9.648788e-01 6.004817e-01

3267 9.973482e-01 9.895519e-01 9.778845e-01 9.678630e-01 9.648792e-01 6.003833e-01

3268 9.973479e-01 9.895509e-01 9.778832e-01 9.678625e-01 9.648796e-01 6.002848e-01

3269 9.973476e-01 9.895499e-01 9.778818e-01 9.678619e-01 9.648800e-01 6.001863e-01

3270 9.973473e-01 9.895488e-01 9.778803e-01 9.678613e-01 9.648804e-01 6.000877e-01

3271 9.973470e-01 9.895476e-01 9.778788e-01 9.678606e-01 9.648808e-01 5.999892e-01

3272 9.973466e-01 9.895464e-01 9.778772e-01 9.678600e-01 9.648813e-01 5.998908e-01

3273 9.973463e-01 9.895452e-01 9.778756e-01 9.678593e-01 9.648818e-01 5.997926e-01

3274 9.973459e-01 9.895439e-01 9.778739e-01 9.678586e-01 9.648823e-01 5.996945e-01

3275 9.973455e-01 9.895425e-01 9.778721e-01 9.678579e-01 9.648829e-01 5.995968e-01

3276 9.973451e-01 9.895411e-01 9.778702e-01 9.678572e-01 9.648835e-01 5.994994e-01

3277 9.973447e-01 9.895396e-01 9.778683e-01 9.678564e-01 9.648841e-01 5.994025e-01

3278 9.973443e-01 9.895380e-01 9.778663e-01 9.678557e-01 9.648847e-01 5.993060e-01

3279 9.973438e-01 9.895364e-01 9.778642e-01 9.678549e-01 9.648854e-01 5.992100e-01

3280 9.973433e-01 9.895347e-01 9.778620e-01 9.678541e-01 9.648861e-01 5.991147e-01

3281 9.973428e-01 9.895329e-01 9.778598e-01 9.678532e-01 9.648869e-01 5.990201e-01

3282 9.973423e-01 9.895311e-01 9.778574e-01 9.678524e-01 9.648877e-01 5.989263e-01

3283 9.973417e-01 9.895291e-01 9.778549e-01 9.678515e-01 9.648886e-01 5.988333e-01

3284 9.973411e-01 9.895271e-01 9.778524e-01 9.678507e-01 9.648895e-01 5.987413e-01

3285 9.973405e-01 9.895250e-01 9.778497e-01 9.678498e-01 9.648904e-01 5.986503e-01

3286 9.973399e-01 9.895228e-01 9.778469e-01 9.678489e-01 9.648915e-01 5.985604e-01

3287 9.973392e-01 9.895205e-01 9.778441e-01 9.678479e-01 9.648925e-01 5.984717e-01

3288 9.973385e-01 9.895181e-01 9.778411e-01 9.678470e-01 9.648937e-01 5.983843e-01

3289 9.973377e-01 9.895156e-01 9.778379e-01 9.678460e-01 9.648949e-01 5.982983e-01

3290 9.973370e-01 9.895129e-01 9.778347e-01 9.678451e-01 9.648962e-01 5.982138e-01

3291 9.973362e-01 9.895102e-01 9.778313e-01 9.678441e-01 9.648976e-01 5.981309e-01

3292 9.973353e-01 9.895073e-01 9.778278e-01 9.678431e-01 9.648991e-01 5.980497e-01

3293 9.973344e-01 9.895043e-01 9.778241e-01 9.678421e-01 9.649007e-01 5.979703e-01

3294 9.973335e-01 9.895011e-01 9.778203e-01 9.678411e-01 9.649023e-01 5.978927e-01

3295 9.973325e-01 9.894979e-01 9.778163e-01 9.678401e-01 9.649041e-01 5.978172e-01

3296 9.973315e-01 9.894944e-01 9.778122e-01 9.678391e-01 9.649060e-01 5.977437e-01

3297 9.973304e-01 9.894908e-01 9.778079e-01 9.678381e-01 9.649080e-01 5.976725e-01

3298 9.973293e-01 9.894871e-01 9.778034e-01 9.678370e-01 9.649101e-01 5.976036e-01

3299 9.973282e-01 9.894832e-01 9.777988e-01 9.678360e-01 9.649124e-01 5.975372e-01

3300 9.973269e-01 9.894791e-01 9.777940e-01 9.678349e-01 9.649148e-01 5.974733e-01

3301 9.973257e-01 9.894748e-01 9.777889e-01 9.678339e-01 9.649174e-01 5.974121e-01

3302 9.973243e-01 9.894703e-01 9.777837e-01 9.678329e-01 9.649201e-01 5.973537e-01

3303 9.973229e-01 9.894656e-01 9.777782e-01 9.678319e-01 9.649230e-01 5.972982e-01

3304 9.973214e-01 9.894607e-01 9.777725e-01 9.678309e-01 9.649261e-01 5.972457e-01

3305 9.973199e-01 9.894556e-01 9.777666e-01 9.678299e-01 9.649294e-01 5.971964e-01

3306 9.973183e-01 9.894503e-01 9.777605e-01 9.678289e-01 9.649329e-01 5.971503e-01

3307 9.973166e-01 9.894447e-01 9.777541e-01 9.678279e-01 9.649366e-01 5.971076e-01

3308 9.973148e-01 9.894389e-01 9.777475e-01 9.678270e-01 9.649405e-01 5.970684e-01

3309 9.973130e-01 9.894327e-01 9.777405e-01 9.678261e-01 9.649447e-01 5.970328e-01

3310 9.973110e-01 9.894263e-01 9.777333e-01 9.678252e-01 9.649492e-01 5.970009e-01

3311 9.973090e-01 9.894197e-01 9.777258e-01 9.678244e-01 9.649539e-01 5.969729e-01

3312 9.973069e-01 9.894127e-01 9.777180e-01 9.678236e-01 9.649589e-01 5.969489e-01

3313 9.973047e-01 9.894054e-01 9.777099e-01 9.678229e-01 9.649642e-01 5.969289e-01

3314 9.973023e-01 9.893977e-01 9.777015e-01 9.678223e-01 9.649699e-01 5.969131e-01

3315 9.972999e-01 9.893897e-01 9.776927e-01 9.678217e-01 9.649759e-01 5.969016e-01

3316 9.972973e-01 9.893813e-01 9.776835e-01 9.678212e-01 9.649823e-01 5.968944e-01

3317 9.972946e-01 9.893726e-01 9.776740e-01 9.678208e-01 9.649891e-01 5.968918e-01

3318 9.972918e-01 9.893634e-01 9.776641e-01 9.678205e-01 9.649963e-01 5.968938e-01

3319 9.972889e-01 9.893538e-01 9.776538e-01 9.678203e-01 9.650040e-01 5.969004e-01

3320 9.972858e-01 9.893437e-01 9.776431e-01 9.678203e-01 9.650121e-01 5.969119e-01

3321 9.972826e-01 9.893332e-01 9.776320e-01 9.678203e-01 9.650207e-01 5.969282e-01

3322 9.972792e-01 9.893222e-01 9.776204e-01 9.678205e-01 9.650299e-01 5.969495e-01

3323 9.972756e-01 9.893107e-01 9.776083e-01 9.678209e-01 9.650396e-01 5.969758e-01

3324 9.972719e-01 9.892987e-01 9.775957e-01 9.678215e-01 9.650499e-01 5.970073e-01

3325 9.972680e-01 9.892860e-01 9.775827e-01 9.678222e-01 9.650609e-01 5.970439e-01

3326 9.972639e-01 9.892728e-01 9.775691e-01 9.678232e-01 9.650725e-01 5.970858e-01

3327 9.972596e-01 9.892590e-01 9.775550e-01 9.678244e-01 9.650848e-01 5.971330e-01

3328 9.972551e-01 9.892445e-01 9.775402e-01 9.678259e-01 9.650979e-01 5.971855e-01

3329 9.972504e-01 9.892293e-01 9.775249e-01 9.678276e-01 9.651119e-01 5.972434e-01

3330 9.972455e-01 9.892135e-01 9.775090e-01 9.678297e-01 9.651266e-01 5.973068e-01

3331 9.972403e-01 9.891968e-01 9.774925e-01 9.678320e-01 9.651423e-01 5.973756e-01

3332 9.972348e-01 9.891794e-01 9.774752e-01 9.678347e-01 9.651589e-01 5.974499e-01

3333 9.972291e-01 9.891611e-01 9.774573e-01 9.678378e-01 9.651765e-01 5.975298e-01

3334 9.972232e-01 9.891420e-01 9.774387e-01 9.678413e-01 9.651951e-01 5.976152e-01

3335 9.972169e-01 9.891220e-01 9.774193e-01 9.678453e-01 9.652149e-01 5.977060e-01

3336 9.972103e-01 9.891010e-01 9.773992e-01 9.678497e-01 9.652360e-01 5.978024e-01

3337 9.972034e-01 9.890790e-01 9.773782e-01 9.678547e-01 9.652582e-01 5.979043e-01

3338 9.971962e-01 9.890560e-01 9.773564e-01 9.678602e-01 9.652818e-01 5.980117e-01

3339 9.971886e-01 9.890319e-01 9.773338e-01 9.678663e-01 9.653069e-01 5.981245e-01

3340 9.971806e-01 9.890066e-01 9.773102e-01 9.678730e-01 9.653334e-01 5.982427e-01

3341 9.971722e-01 9.889801e-01 9.772858e-01 9.678805e-01 9.653615e-01 5.983663e-01

3342 9.971634e-01 9.889523e-01 9.772603e-01 9.678887e-01 9.653913e-01 5.984952e-01

3343 9.971542e-01 9.889233e-01 9.772339e-01 9.678977e-01 9.654228e-01 5.986294e-01

3344 9.971446e-01 9.888928e-01 9.772064e-01 9.679075e-01 9.654563e-01 5.987687e-01

3345 9.971344e-01 9.888608e-01 9.771779e-01 9.679183e-01 9.654916e-01 5.989130e-01

3346 9.971238e-01 9.888273e-01 9.771482e-01 9.679301e-01 9.655291e-01 5.990624e-01

3347 9.971126e-01 9.887922e-01 9.771174e-01 9.679429e-01 9.655688e-01 5.992167e-01

3348 9.971008e-01 9.887554e-01 9.770854e-01 9.679568e-01 9.656108e-01 5.993758e-01

3349 9.970885e-01 9.887168e-01 9.770522e-01 9.679720e-01 9.656553e-01 5.995396e-01

3350 9.970755e-01 9.886764e-01 9.770177e-01 9.679884e-01 9.657023e-01 5.997079e-01

3351 9.970619e-01 9.886340e-01 9.769819e-01 9.680062e-01 9.657521e-01 5.998807e-01

3352 9.970477e-01 9.885896e-01 9.769447e-01 9.680255e-01 9.658047e-01 6.000578e-01

3353 9.970327e-01 9.885430e-01 9.769061e-01 9.680464e-01 9.658603e-01 6.002390e-01

3354 9.970169e-01 9.884942e-01 9.768661e-01 9.680689e-01 9.659192e-01 6.004243e-01

3355 9.970003e-01 9.884431e-01 9.768246e-01 9.680932e-01 9.659814e-01 6.006135e-01

3356 9.969830e-01 9.883894e-01 9.767816e-01 9.681194e-01 9.660471e-01 6.008064e-01

3357 9.969647e-01 9.883332e-01 9.767370e-01 9.681476e-01 9.661165e-01 6.010029e-01

3358 9.969455e-01 9.882743e-01 9.766907e-01 9.681780e-01 9.661898e-01 6.012027e-01

3359 9.969253e-01 9.882126e-01 9.766428e-01 9.682106e-01 9.662673e-01 6.014058e-01

3360 9.969041e-01 9.881478e-01 9.765931e-01 9.682456e-01 9.663490e-01 6.016119e-01

3361 9.968819e-01 9.880800e-01 9.765417e-01 9.682832e-01 9.664353e-01 6.018210e-01

3362 9.968585e-01 9.880089e-01 9.764885e-01 9.683235e-01 9.665263e-01 6.020327e-01

3363 9.968339e-01 9.879344e-01 9.764334e-01 9.683667e-01 9.666223e-01 6.022469e-01

3364 9.968080e-01 9.878564e-01 9.763764e-01 9.684129e-01 9.667236e-01 6.024635e-01

3365 9.967809e-01 9.877746e-01 9.763175e-01 9.684624e-01 9.668303e-01 6.026823e-01

3366 9.967523e-01 9.876890e-01 9.762565e-01 9.685153e-01 9.669427e-01 6.029030e-01

3367 9.967223e-01 9.875993e-01 9.761936e-01 9.685717e-01 9.670612e-01 6.031255e-01

3368 9.966908e-01 9.875053e-01 9.761285e-01 9.686320e-01 9.671859e-01 6.033496e-01

3369 9.966576e-01 9.874068e-01 9.760613e-01 9.686963e-01 9.673173e-01 6.035751e-01

3370 9.966227e-01 9.873038e-01 9.759920e-01 9.687648e-01 9.674555e-01 6.038018e-01

3371 9.965861e-01 9.871959e-01 9.759204e-01 9.688378e-01 9.676008e-01 6.040296e-01

3372 9.965475e-01 9.870829e-01 9.758467e-01 9.689155e-01 9.677537e-01 6.042582e-01

3373 9.965070e-01 9.869646e-01 9.757706e-01 9.689981e-01 9.679144e-01 6.044876e-01

3374 9.964644e-01 9.868409e-01 9.756923e-01 9.690859e-01 9.680832e-01 6.047174e-01

3375 9.964196e-01 9.867114e-01 9.756116e-01 9.691792e-01 9.682605e-01 6.049476e-01

3376 9.963725e-01 9.865759e-01 9.755286e-01 9.692782e-01 9.684467e-01 6.051780e-01

3377 9.963230e-01 9.864342e-01 9.754432e-01 9.693833e-01 9.686420e-01 6.054083e-01

3378 9.962709e-01 9.862861e-01 9.753555e-01 9.694946e-01 9.688469e-01 6.056385e-01

3379 9.962162e-01 9.861312e-01 9.752653e-01 9.696125e-01 9.690618e-01 6.058684e-01

3380 9.961586e-01 9.859692e-01 9.751727e-01 9.697373e-01 9.692869e-01 6.060978e-01

3381 9.960981e-01 9.858000e-01 9.750778e-01 9.698693e-01 9.695227e-01 6.063266e-01

3382 9.960345e-01 9.856232e-01 9.749804e-01 9.700088e-01 9.697696e-01 6.065546e-01

3383 9.959676e-01 9.854386e-01 9.748807e-01 9.701562e-01 9.700279e-01 6.067817e-01

3384 9.958973e-01 9.852458e-01 9.747785e-01 9.703117e-01 9.702980e-01 6.070077e-01

3385 9.958234e-01 9.850445e-01 9.746741e-01 9.704757e-01 9.705804e-01 6.072326e-01

3386 9.957457e-01 9.848345e-01 9.745673e-01 9.706486e-01 9.708754e-01 6.074562e-01

3387 9.956641e-01 9.846154e-01 9.744582e-01 9.708306e-01 9.711834e-01 6.076784e-01

3388 9.955783e-01 9.843868e-01 9.743469e-01 9.710221e-01 9.715049e-01 6.078990e-01

3389 9.954881e-01 9.841486e-01 9.742333e-01 9.712235e-01 9.718400e-01 6.081180e-01

3390 9.953933e-01 9.839004e-01 9.741177e-01 9.714351e-01 9.721894e-01 6.083353e-01

3391 9.952937e-01 9.836418e-01 9.740000e-01 9.716573e-01 9.725533e-01 6.085508e-01

3392 9.951890e-01 9.833724e-01 9.738804e-01 9.718903e-01 9.729321e-01 6.087643e-01

3393 9.950791e-01 9.830921e-01 9.737588e-01 9.721345e-01 9.733261e-01 6.089759e-01

3394 9.949636e-01 9.828005e-01 9.736355e-01 9.723902e-01 9.737357e-01 6.091854e-01

3395 9.948423e-01 9.824972e-01 9.735105e-01 9.726578e-01 9.741613e-01 6.093927e-01

3396 9.947149e-01 9.821819e-01 9.733840e-01 9.729375e-01 9.746030e-01 6.095978e-01

3397 9.945811e-01 9.818543e-01 9.732560e-01 9.732297e-01 9.750612e-01 6.098006e-01

3398 9.944406e-01 9.815141e-01 9.731268e-01 9.735346e-01 9.755361e-01 6.100011e-01

3399 9.942932e-01 9.811609e-01 9.729965e-01 9.738525e-01 9.760279e-01 6.101992e-01

3400 9.941384e-01 9.807946e-01 9.728652e-01 9.741835e-01 9.765367e-01 6.103949e-01

3401 9.939760e-01 9.804148e-01 9.727331e-01 9.745278e-01 9.770627e-01 6.105882e-01

3402 9.940981e-01 9.809145e-01 9.737395e-01 9.758867e-01 9.784938e-01 6.153245e-01

3403 9.940980e-01 9.809143e-01 9.737397e-01 9.758866e-01 9.784942e-01 6.153632e-01

3404 9.940979e-01 9.809141e-01 9.737398e-01 9.758865e-01 9.784945e-01 6.154030e-01

3405 9.940978e-01 9.809139e-01 9.737400e-01 9.758865e-01 9.784948e-01 6.154439e-01

3406 9.940977e-01 9.809137e-01 9.737402e-01 9.758864e-01 9.784950e-01 6.154859e-01

3407 9.940975e-01 9.809135e-01 9.737403e-01 9.758864e-01 9.784953e-01 6.155291e-01

3408 9.940974e-01 9.809132e-01 9.737405e-01 9.758865e-01 9.784955e-01 6.155735e-01

3409 9.940972e-01 9.809130e-01 9.737406e-01 9.758866e-01 9.784957e-01 6.156191e-01

3410 9.940971e-01 9.809127e-01 9.737407e-01 9.758867e-01 9.784959e-01 6.156660e-01

3411 9.940969e-01 9.809125e-01 9.737408e-01 9.758869e-01 9.784960e-01 6.157141e-01

3412 9.940968e-01 9.809122e-01 9.737409e-01 9.758871e-01 9.784962e-01 6.157635e-01

3413 9.940966e-01 9.809119e-01 9.737410e-01 9.758874e-01 9.784964e-01 6.158142e-01

3414 9.940964e-01 9.809116e-01 9.737410e-01 9.758877e-01 9.784966e-01 6.158662e-01

3415 9.940962e-01 9.809113e-01 9.737410e-01 9.758880e-01 9.784968e-01 6.159196e-01

3416 9.940961e-01 9.809110e-01 9.737410e-01 9.758884e-01 9.784971e-01 6.159744e-01

3417 9.940959e-01 9.809107e-01 9.737410e-01 9.758888e-01 9.784974e-01 6.160306e-01

3418 9.940957e-01 9.809104e-01 9.737410e-01 9.758893e-01 9.784977e-01 6.160883e-01

3419 9.940955e-01 9.809101e-01 9.737409e-01 9.758898e-01 9.784980e-01 6.161476e-01

3420 9.940953e-01 9.809097e-01 9.737409e-01 9.758903e-01 9.784984e-01 6.162083e-01

3421 9.940951e-01 9.809094e-01 9.737408e-01 9.758909e-01 9.784989e-01 6.162706e-01

3422 9.940948e-01 9.809090e-01 9.737408e-01 9.758914e-01 9.784993e-01 6.163346e-01

3423 9.940946e-01 9.809086e-01 9.737407e-01 9.758920e-01 9.784999e-01 6.164002e-01

3424 9.940944e-01 9.809083e-01 9.737407e-01 9.758927e-01 9.785004e-01 6.164675e-01

3425 9.940941e-01 9.809079e-01 9.737406e-01 9.758933e-01 9.785010e-01 6.165365e-01

3426 9.940938e-01 9.809074e-01 9.737405e-01 9.758939e-01 9.785017e-01 6.166074e-01

3427 9.940936e-01 9.809070e-01 9.737405e-01 9.758946e-01 9.785024e-01 6.166800e-01

3428 9.940933e-01 9.809066e-01 9.737405e-01 9.758953e-01 9.785031e-01 6.167546e-01

3429 9.940929e-01 9.809061e-01 9.737404e-01 9.758960e-01 9.785039e-01 6.168310e-01

3430 9.940926e-01 9.809056e-01 9.737404e-01 9.758967e-01 9.785048e-01 6.169094e-01

3431 9.940923e-01 9.809051e-01 9.737404e-01 9.758975e-01 9.785056e-01 6.169899e-01

3432 9.940919e-01 9.809046e-01 9.737404e-01 9.758983e-01 9.785065e-01 6.170724e-01

3433 9.940915e-01 9.809040e-01 9.737404e-01 9.758990e-01 9.785075e-01 6.171570e-01

3434 9.940911e-01 9.809034e-01 9.737405e-01 9.758998e-01 9.785085e-01 6.172438e-01

3435 9.940907e-01 9.809029e-01 9.737405e-01 9.759007e-01 9.785095e-01 6.173329e-01

3436 9.940903e-01 9.809022e-01 9.737406e-01 9.759015e-01 9.785106e-01 6.174242e-01

3437 9.940898e-01 9.809016e-01 9.737406e-01 9.759024e-01 9.785117e-01 6.175178e-01

3438 9.940894e-01 9.809009e-01 9.737407e-01 9.759033e-01 9.785129e-01 6.176139e-01

3439 9.940889e-01 9.809002e-01 9.737408e-01 9.759042e-01 9.785141e-01 6.177124e-01

3440 9.940883e-01 9.808995e-01 9.737409e-01 9.759052e-01 9.785153e-01 6.178133e-01

3441 9.940878e-01 9.808987e-01 9.737411e-01 9.759062e-01 9.785166e-01 6.179169e-01

3442 9.940872e-01 9.808979e-01 9.737412e-01 9.759072e-01 9.785180e-01 6.180231e-01

3443 9.940866e-01 9.808971e-01 9.737413e-01 9.759083e-01 9.785193e-01 6.181320e-01

3444 9.940860e-01 9.808963e-01 9.737415e-01 9.759095e-01 9.785207e-01 6.182436e-01

3445 9.940853e-01 9.808954e-01 9.737417e-01 9.759106e-01 9.785222e-01 6.183580e-01

3446 9.940846e-01 9.808944e-01 9.737419e-01 9.759119e-01 9.785237e-01 6.184754e-01

3447 9.940839e-01 9.808935e-01 9.737421e-01 9.759131e-01 9.785253e-01 6.185956e-01

3448 9.940832e-01 9.808925e-01 9.737423e-01 9.759145e-01 9.785269e-01 6.187189e-01

3449 9.940824e-01 9.808915e-01 9.737425e-01 9.759159e-01 9.785285e-01 6.188453e-01

3450 9.940816e-01 9.808904e-01 9.737427e-01 9.759173e-01 9.785303e-01 6.189748e-01

3451 9.940807e-01 9.808893e-01 9.737430e-01 9.759188e-01 9.785321e-01 6.191075e-01

3452 9.940798e-01 9.808881e-01 9.737432e-01 9.759204e-01 9.785339e-01 6.192436e-01

3453 9.940789e-01 9.808869e-01 9.737435e-01 9.759221e-01 9.785359e-01 6.193830e-01

3454 9.940779e-01 9.808857e-01 9.737438e-01 9.759238e-01 9.785379e-01 6.195258e-01

3455 9.940769e-01 9.808844e-01 9.737441e-01 9.759256e-01 9.785399e-01 6.196721e-01

3456 9.940759e-01 9.808831e-01 9.737445e-01 9.759275e-01 9.785421e-01 6.198220e-01

3457 9.940748e-01 9.808817e-01 9.737448e-01 9.759294e-01 9.785444e-01 6.199755e-01

3458 9.940736e-01 9.808803e-01 9.737452e-01 9.759315e-01 9.785467e-01 6.201328e-01

3459 9.940724e-01 9.808788e-01 9.737456e-01 9.759336e-01 9.785491e-01 6.202939e-01

3460 9.940711e-01 9.808772e-01 9.737460e-01 9.759358e-01 9.785516e-01 6.204588e-01

3461 9.940698e-01 9.808756e-01 9.737465e-01 9.759381e-01 9.785543e-01 6.206277e-01

3462 9.940684e-01 9.808740e-01 9.737470e-01 9.759405e-01 9.785570e-01 6.208006e-01

3463 9.940670e-01 9.808722e-01 9.737475e-01 9.759431e-01 9.785598e-01 6.209776e-01

3464 9.940655e-01 9.808705e-01 9.737481e-01 9.759457e-01 9.785628e-01 6.211588e-01

3465 9.940639e-01 9.808686e-01 9.737487e-01 9.759484e-01 9.785658e-01 6.213443e-01

3466 9.940623e-01 9.808667e-01 9.737494e-01 9.759512e-01 9.785690e-01 6.215341e-01

3467 9.940606e-01 9.808647e-01 9.737501e-01 9.759542e-01 9.785723e-01 6.217283e-01

3468 9.940588e-01 9.808626e-01 9.737508e-01 9.759573e-01 9.785758e-01 6.219269e-01

3469 9.940569e-01 9.808605e-01 9.737516e-01 9.759605e-01 9.785793e-01 6.221301e-01

3470 9.940549e-01 9.808583e-01 9.737525e-01 9.759638e-01 9.785831e-01 6.223380e-01

3471 9.940529e-01 9.808560e-01 9.737534e-01 9.759673e-01 9.785869e-01 6.225505e-01

3472 9.940507e-01 9.808536e-01 9.737544e-01 9.759710e-01 9.785910e-01 6.227678e-01

3473 9.940485e-01 9.808511e-01 9.737555e-01 9.759748e-01 9.785951e-01 6.229900e-01

3474 9.940461e-01 9.808486e-01 9.737566e-01 9.759787e-01 9.785995e-01 6.232171e-01

3475 9.940437e-01 9.808459e-01 9.737578e-01 9.759828e-01 9.786040e-01 6.234492e-01

3476 9.940411e-01 9.808432e-01 9.737591e-01 9.759871e-01 9.786087e-01 6.236863e-01

3477 9.940384e-01 9.808403e-01 9.737604e-01 9.759916e-01 9.786136e-01 6.239285e-01

3478 9.940356e-01 9.808373e-01 9.737619e-01 9.759963e-01 9.786186e-01 6.241760e-01

3479 9.940327e-01 9.808343e-01 9.737635e-01 9.760011e-01 9.786239e-01 6.244287e-01

3480 9.940297e-01 9.808311e-01 9.737651e-01 9.760062e-01 9.786294e-01 6.246867e-01

3481 9.940265e-01 9.808278e-01 9.737669e-01 9.760115e-01 9.786351e-01 6.249500e-01

3482 9.940231e-01 9.808244e-01 9.737688e-01 9.760171e-01 9.786410e-01 6.252188e-01

3483 9.940196e-01 9.808209e-01 9.737708e-01 9.760228e-01 9.786472e-01 6.254931e-01

3484 9.940160e-01 9.808172e-01 9.737730e-01 9.760289e-01 9.786536e-01 6.257729e-01

3485 9.940121e-01 9.808134e-01 9.737752e-01 9.760352e-01 9.786602e-01 6.260583e-01

3486 9.940082e-01 9.808095e-01 9.737777e-01 9.760417e-01 9.786671e-01 6.263493e-01

3487 9.940040e-01 9.808054e-01 9.737803e-01 9.760486e-01 9.786743e-01 6.266459e-01

3488 9.939996e-01 9.808012e-01 9.737830e-01 9.760558e-01 9.786818e-01 6.269483e-01

3489 9.939950e-01 9.807969e-01 9.737860e-01 9.760633e-01 9.786896e-01 6.272564e-01

3490 9.939903e-01 9.807924e-01 9.737891e-01 9.760711e-01 9.786977e-01 6.275703e-01

3491 9.939853e-01 9.807877e-01 9.737924e-01 9.760793e-01 9.787062e-01 6.278900e-01

3492 9.939800e-01 9.807828e-01 9.737959e-01 9.760878e-01 9.787149e-01 6.282155e-01

3493 9.939746e-01 9.807778e-01 9.737997e-01 9.760967e-01 9.787241e-01 6.285468e-01

3494 9.939688e-01 9.807726e-01 9.738037e-01 9.761061e-01 9.787336e-01 6.288840e-01

3495 9.939629e-01 9.807673e-01 9.738079e-01 9.761158e-01 9.787435e-01 6.292270e-01

3496 9.939566e-01 9.807617e-01 9.738125e-01 9.761260e-01 9.787538e-01 6.295759e-01

3497 9.939500e-01 9.807560e-01 9.738173e-01 9.761367e-01 9.787645e-01 6.299307e-01

3498 9.939432e-01 9.807500e-01 9.738224e-01 9.761479e-01 9.787757e-01 6.302912e-01

3499 9.939360e-01 9.807439e-01 9.738278e-01 9.761595e-01 9.787873e-01 6.306577e-01

3500 9.939285e-01 9.807375e-01 9.738336e-01 9.761717e-01 9.787994e-01 6.310299e-01

3501 9.939206e-01 9.807309e-01 9.738397e-01 9.761845e-01 9.788120e-01 6.314079e-01

3502 9.939124e-01 9.807241e-01 9.738462e-01 9.761978e-01 9.788252e-01 6.317917e-01

3503 9.939038e-01 9.807170e-01 9.738531e-01 9.762118e-01 9.788389e-01 6.321812e-01

3504 9.938948e-01 9.807097e-01 9.738604e-01 9.762264e-01 9.788531e-01 6.325764e-01

3505 9.938853e-01 9.807022e-01 9.738682e-01 9.762417e-01 9.788680e-01 6.329772e-01

3506 9.938754e-01 9.806944e-01 9.738765e-01 9.762577e-01 9.788835e-01 6.333835e-01

3507 9.938651e-01 9.806863e-01 9.738853e-01 9.762745e-01 9.788997e-01 6.337954e-01

3508 9.938542e-01 9.806779e-01 9.738946e-01 9.762920e-01 9.789165e-01 6.342126e-01

3509 9.938428e-01 9.806693e-01 9.739045e-01 9.763103e-01 9.789340e-01 6.346352e-01

3510 9.938309e-01 9.806604e-01 9.739150e-01 9.763295e-01 9.789524e-01 6.350631e-01

3511 9.938185e-01 9.806512e-01 9.739261e-01 9.763496e-01 9.789714e-01 6.354962e-01

3512 9.938054e-01 9.806416e-01 9.739380e-01 9.763707e-01 9.789913e-01 6.359343e-01

3513 9.937917e-01 9.806318e-01 9.739505e-01 9.763927e-01 9.790121e-01 6.363774e-01

3514 9.937774e-01 9.806216e-01 9.739638e-01 9.764158e-01 9.790337e-01 6.368253e-01

3515 9.937624e-01 9.806111e-01 9.739779e-01 9.764400e-01 9.790563e-01 6.372779e-01

3516 9.937466e-01 9.806003e-01 9.739928e-01 9.764653e-01 9.790798e-01 6.377351e-01

3517 9.937301e-01 9.805891e-01 9.740087e-01 9.764918e-01 9.791044e-01 6.381968e-01

3518 9.937128e-01 9.805775e-01 9.740255e-01 9.765196e-01 9.791300e-01 6.386628e-01

3519 9.936947e-01 9.805655e-01 9.740433e-01 9.765487e-01 9.791567e-01 6.391330e-01

3520 9.936757e-01 9.805532e-01 9.740622e-01 9.765791e-01 9.791846e-01 6.396072e-01

3521 9.936558e-01 9.805405e-01 9.740822e-01 9.766110e-01 9.792137e-01 6.400852e-01

3522 9.936349e-01 9.805273e-01 9.741034e-01 9.766444e-01 9.792441e-01 6.405669e-01

3523 9.936129e-01 9.805138e-01 9.741258e-01 9.766795e-01 9.792757e-01 6.410521e-01

3524 9.935900e-01 9.804998e-01 9.741496e-01 9.767161e-01 9.793088e-01 6.415407e-01

3525 9.935659e-01 9.804854e-01 9.741748e-01 9.767545e-01 9.793433e-01 6.420323e-01

3526 9.935406e-01 9.804705e-01 9.742014e-01 9.767948e-01 9.793793e-01 6.425269e-01

3527 9.935141e-01 9.804552e-01 9.742296e-01 9.768369e-01 9.794168e-01 6.430242e-01

3528 9.934862e-01 9.804394e-01 9.742595e-01 9.768811e-01 9.794561e-01 6.435241e-01

3529 9.934571e-01 9.804231e-01 9.742911e-01 9.769273e-01 9.794970e-01 6.440263e-01

3530 9.934264e-01 9.804064e-01 9.743245e-01 9.769758e-01 9.795397e-01 6.445307e-01

3531 9.933943e-01 9.803891e-01 9.743599e-01 9.770265e-01 9.795842e-01 6.450369e-01

3532 9.933606e-01 9.803713e-01 9.743973e-01 9.770796e-01 9.796307e-01 6.455448e-01

3533 9.933252e-01 9.803530e-01 9.744369e-01 9.771353e-01 9.796793e-01 6.460542e-01

3534 9.932880e-01 9.803342e-01 9.744787e-01 9.771936e-01 9.797299e-01 6.465648e-01

3535 9.932491e-01 9.803149e-01 9.745229e-01 9.772546e-01 9.797828e-01 6.470764e-01

3536 9.932081e-01 9.802950e-01 9.745696e-01 9.773185e-01 9.798379e-01 6.475887e-01

3537 9.931652e-01 9.802746e-01 9.746190e-01 9.773854e-01 9.798955e-01 6.481017e-01

3538 9.931201e-01 9.802536e-01 9.746711e-01 9.774555e-01 9.799556e-01 6.486149e-01

3539 9.930727e-01 9.802320e-01 9.747262e-01 9.775289e-01 9.800182e-01 6.491282e-01

3540 9.930230e-01 9.802099e-01 9.747844e-01 9.776056e-01 9.800836e-01 6.496413e-01

3541 9.929708e-01 9.801872e-01 9.748458e-01 9.776860e-01 9.801518e-01 6.501540e-01

3542 9.929160e-01 9.801639e-01 9.749106e-01 9.777701e-01 9.802229e-01 6.506661e-01

3543 9.928585e-01 9.801400e-01 9.749789e-01 9.778581e-01 9.802971e-01 6.511773e-01

3544 9.927980e-01 9.801156e-01 9.750510e-01 9.779501e-01 9.803744e-01 6.516874e-01

3545 9.927346e-01 9.800905e-01 9.751271e-01 9.780464e-01 9.804551e-01 6.521962e-01

3546 9.926679e-01 9.800649e-01 9.752073e-01 9.781472e-01 9.805392e-01 6.527034e-01

3547 9.925979e-01 9.800387e-01 9.752918e-01 9.782525e-01 9.806268e-01 6.532088e-01

3548 9.925244e-01 9.800119e-01 9.753809e-01 9.783627e-01 9.807182e-01 6.537123e-01

3549 9.924472e-01 9.799846e-01 9.754747e-01 9.784778e-01 9.808135e-01 6.542134e-01

3550 9.923662e-01 9.799566e-01 9.755735e-01 9.785981e-01 9.809127e-01 6.547121e-01

3551 9.922810e-01 9.799282e-01 9.756775e-01 9.787239e-01 9.810161e-01 6.552082e-01

3552 9.921916e-01 9.798991e-01 9.757870e-01 9.788553e-01 9.811237e-01 6.557014e-01

3553 9.920977e-01 9.798696e-01 9.759021e-01 9.789925e-01 9.812359e-01 6.561915e-01

3554 9.919991e-01 9.798395e-01 9.760233e-01 9.791359e-01 9.813526e-01 6.566783e-01

3555 9.918955e-01 9.798090e-01 9.761507e-01 9.792855e-01 9.814742e-01 6.571616e-01

3556 9.917868e-01 9.797779e-01 9.762846e-01 9.794417e-01 9.816007e-01 6.576413e-01

3557 9.916727e-01 9.797465e-01 9.764253e-01 9.796047e-01 9.817323e-01 6.581172e-01

3558 9.915528e-01 9.797146e-01 9.765730e-01 9.797748e-01 9.818692e-01 6.585890e-01

3559 9.914269e-01 9.796823e-01 9.767282e-01 9.799521e-01 9.820115e-01 6.590567e-01

3560 9.912948e-01 9.796497e-01 9.768910e-01 9.801370e-01 9.821596e-01 6.595200e-01

3561 9.911561e-01 9.796167e-01 9.770618e-01 9.803298e-01 9.823134e-01 6.599788e-01

3562 9.910106e-01 9.795835e-01 9.772410e-01 9.805307e-01 9.824733e-01 6.604329e-01

3563 9.908578e-01 9.795501e-01 9.774288e-01 9.807400e-01 9.826394e-01 6.608823e-01

3564 9.906975e-01 9.795165e-01 9.776256e-01 9.809579e-01 9.828119e-01 6.613267e-01

3565 9.905293e-01 9.794828e-01 9.778317e-01 9.811847e-01 9.829909e-01 6.617661e-01

3566 9.903529e-01 9.794490e-01 9.780475e-01 9.814208e-01 9.831767e-01 6.622004e-01

3567 9.901678e-01 9.794153e-01 9.782734e-01 9.816665e-01 9.833695e-01 6.626293e-01

3568 9.899737e-01 9.793816e-01 9.785096e-01 9.819219e-01 9.835694e-01 6.630528e-01

3569 9.897702e-01 9.793482e-01 9.787565e-01 9.821874e-01 9.837767e-01 6.634709e-01

3570 9.895568e-01 9.793150e-01 9.790145e-01 9.824633e-01 9.839915e-01 6.638834e-01

3571 9.893331e-01 9.792821e-01 9.792841e-01 9.827499e-01 9.842140e-01 6.642903e-01

3572 9.890987e-01 9.792496e-01 9.795654e-01 9.830475e-01 9.844444e-01 6.646914e-01

3573 9.888532e-01 9.792177e-01 9.798589e-01 9.833563e-01 9.846829e-01 6.650867e-01

3574 9.885960e-01 9.791865e-01 9.801649e-01 9.836766e-01 9.849296e-01 6.654762e-01

3575 9.883266e-01 9.791560e-01 9.804838e-01 9.840088e-01 9.851848e-01 6.658598e-01

3576 9.880446e-01 9.791263e-01 9.808160e-01 9.843531e-01 9.854486e-01 6.662375e-01

3577 9.877495e-01 9.790977e-01 9.811616e-01 9.847097e-01 9.857212e-01 6.666092e-01

3578 9.874408e-01 9.790702e-01 9.815210e-01 9.850791e-01 9.860028e-01 6.669748e-01

3579 9.871178e-01 9.790440e-01 9.818944e-01 9.854613e-01 9.862937e-01 6.673345e-01

3580 9.867801e-01 9.790192e-01 9.822822e-01 9.858568e-01 9.865939e-01 6.676880e-01

3581 9.870587e-01 9.798757e-01 9.833795e-01 9.868222e-01 9.874495e-01 6.722789e-01

3582 9.870585e-01 9.798758e-01 9.833797e-01 9.868226e-01 9.874493e-01 6.723384e-01

3583 9.870583e-01 9.798759e-01 9.833800e-01 9.868230e-01 9.874492e-01 6.723987e-01

3584 9.870581e-01 9.798760e-01 9.833802e-01 9.868235e-01 9.874492e-01 6.724600e-01

3585 9.870579e-01 9.798761e-01 9.833804e-01 9.868240e-01 9.874492e-01 6.725221e-01

3586 9.870577e-01 9.798763e-01 9.833806e-01 9.868245e-01 9.874493e-01 6.725851e-01

3587 9.870575e-01 9.798765e-01 9.833808e-01 9.868251e-01 9.874494e-01 6.726491e-01

3588 9.870573e-01 9.798767e-01 9.833810e-01 9.868256e-01 9.874496e-01 6.727139e-01

3589 9.870571e-01 9.798769e-01 9.833811e-01 9.868262e-01 9.874498e-01 6.727797e-01

3590 9.870568e-01 9.798771e-01 9.833813e-01 9.868267e-01 9.874500e-01 6.728465e-01

3591 9.870566e-01 9.798774e-01 9.833815e-01 9.868273e-01 9.874503e-01 6.729142e-01

3592 9.870563e-01 9.798776e-01 9.833818e-01 9.868278e-01 9.874506e-01 6.729829e-01

3593 9.870561e-01 9.798778e-01 9.833820e-01 9.868283e-01 9.874510e-01 6.730526e-01

3594 9.870558e-01 9.798780e-01 9.833822e-01 9.868288e-01 9.874514e-01 6.731232e-01

3595 9.870555e-01 9.798783e-01 9.833825e-01 9.868293e-01 9.874517e-01 6.731949e-01

3596 9.870552e-01 9.798785e-01 9.833828e-01 9.868298e-01 9.874521e-01 6.732677e-01

3597 9.870549e-01 9.798787e-01 9.833831e-01 9.868302e-01 9.874526e-01 6.733414e-01

3598 9.870546e-01 9.798789e-01 9.833835e-01 9.868307e-01 9.874530e-01 6.734163e-01

3599 9.870543e-01 9.798791e-01 9.833839e-01 9.868311e-01 9.874534e-01 6.734922e-01

3600 9.870540e-01 9.798793e-01 9.833843e-01 9.868315e-01 9.874538e-01 6.735692e-01

3601 9.870536e-01 9.798794e-01 9.833847e-01 9.868319e-01 9.874542e-01 6.736473e-01

3602 9.870532e-01 9.798796e-01 9.833852e-01 9.868323e-01 9.874547e-01 6.737266e-01

3603 9.870528e-01 9.798798e-01 9.833857e-01 9.868327e-01 9.874551e-01 6.738070e-01

3604 9.870524e-01 9.798800e-01 9.833863e-01 9.868331e-01 9.874555e-01 6.738886e-01

3605 9.870520e-01 9.798801e-01 9.833868e-01 9.868335e-01 9.874559e-01 6.739713e-01

3606 9.870516e-01 9.798803e-01 9.833874e-01 9.868339e-01 9.874563e-01 6.740553e-01

3607 9.870511e-01 9.798805e-01 9.833881e-01 9.868344e-01 9.874567e-01 6.741405e-01

3608 9.870506e-01 9.798807e-01 9.833888e-01 9.868348e-01 9.874571e-01 6.742269e-01

3609 9.870501e-01 9.798809e-01 9.833895e-01 9.868352e-01 9.874575e-01 6.743146e-01

3610 9.870496e-01 9.798811e-01 9.833902e-01 9.868357e-01 9.874579e-01 6.744035e-01

3611 9.870490e-01 9.798813e-01 9.833910e-01 9.868362e-01 9.874583e-01 6.744938e-01

3612 9.870485e-01 9.798815e-01 9.833918e-01 9.868367e-01 9.874588e-01 6.745853e-01

3613 9.870479e-01 9.798818e-01 9.833926e-01 9.868373e-01 9.874592e-01 6.746783e-01

3614 9.870472e-01 9.798821e-01 9.833935e-01 9.868379e-01 9.874596e-01 6.747725e-01

3615 9.870466e-01 9.798823e-01 9.833944e-01 9.868385e-01 9.874601e-01 6.748682e-01

3616 9.870459e-01 9.798827e-01 9.833953e-01 9.868392e-01 9.874605e-01 6.749652e-01

3617 9.870452e-01 9.798830e-01 9.833963e-01 9.868399e-01 9.874610e-01 6.750637e-01

3618 9.870445e-01 9.798833e-01 9.833973e-01 9.868406e-01 9.874615e-01 6.751636e-01

3619 9.870437e-01 9.798837e-01 9.833983e-01 9.868414e-01 9.874620e-01 6.752650e-01

3620 9.870429e-01 9.798841e-01 9.833994e-01 9.868422e-01 9.874626e-01 6.753678e-01

3621 9.870421e-01 9.798845e-01 9.834005e-01 9.868431e-01 9.874631e-01 6.754721e-01

3622 9.870412e-01 9.798850e-01 9.834017e-01 9.868440e-01 9.874638e-01 6.755780e-01

3623 9.870404e-01 9.798854e-01 9.834029e-01 9.868450e-01 9.874644e-01 6.756854e-01

3624 9.870394e-01 9.798859e-01 9.834042e-01 9.868460e-01 9.874651e-01 6.757944e-01

3625 9.870385e-01 9.798865e-01 9.834054e-01 9.868471e-01 9.874658e-01 6.759050e-01

3626 9.870375e-01 9.798870e-01 9.834068e-01 9.868482e-01 9.874665e-01 6.760172e-01

3627 9.870364e-01 9.798876e-01 9.834082e-01 9.868493e-01 9.874673e-01 6.761310e-01

3628 9.870353e-01 9.798882e-01 9.834096e-01 9.868506e-01 9.874681e-01 6.762464e-01

3629 9.870342e-01 9.798889e-01 9.834111e-01 9.868518e-01 9.874690e-01 6.763636e-01

3630 9.870330e-01 9.798895e-01 9.834127e-01 9.868531e-01 9.874699e-01 6.764824e-01

3631 9.870318e-01 9.798902e-01 9.834143e-01 9.868545e-01 9.874709e-01 6.766029e-01

3632 9.870305e-01 9.798910e-01 9.834159e-01 9.868559e-01 9.874718e-01 6.767252e-01

3633 9.870292e-01 9.798918e-01 9.834177e-01 9.868574e-01 9.874729e-01 6.768493e-01

3634 9.870278e-01 9.798926e-01 9.834195e-01 9.868590e-01 9.874740e-01 6.769751e-01

3635 9.870264e-01 9.798934e-01 9.834214e-01 9.868606e-01 9.874751e-01 6.771027e-01

3636 9.870249e-01 9.798943e-01 9.834233e-01 9.868622e-01 9.874763e-01 6.772321e-01

3637 9.870233e-01 9.798953e-01 9.834253e-01 9.868639e-01 9.874775e-01 6.773634e-01

3638 9.870217e-01 9.798963e-01 9.834275e-01 9.868657e-01 9.874788e-01 6.774965e-01

3639 9.870200e-01 9.798973e-01 9.834296e-01 9.868676e-01 9.874801e-01 6.776315e-01

3640 9.870183e-01 9.798984e-01 9.834319e-01 9.868695e-01 9.874815e-01 6.777685e-01

3641 9.870164e-01 9.798995e-01 9.834343e-01 9.868715e-01 9.874829e-01 6.779073e-01

3642 9.870145e-01 9.799007e-01 9.834367e-01 9.868735e-01 9.874844e-01 6.780481e-01

3643 9.870126e-01 9.799019e-01 9.834393e-01 9.868757e-01 9.874860e-01 6.781908e-01

3644 9.870105e-01 9.799032e-01 9.834420e-01 9.868779e-01 9.874876e-01 6.783355e-01

3645 9.870084e-01 9.799046e-01 9.834447e-01 9.868802e-01 9.874892e-01 6.784822e-01

3646 9.870062e-01 9.799060e-01 9.834476e-01 9.868826e-01 9.874910e-01 6.786309e-01

3647 9.870038e-01 9.799075e-01 9.834506e-01 9.868851e-01 9.874928e-01 6.787816e-01

3648 9.870014e-01 9.799090e-01 9.834537e-01 9.868876e-01 9.874946e-01 6.789344e-01

3649 9.869989e-01 9.799107e-01 9.834569e-01 9.868903e-01 9.874966e-01 6.790892e-01

3650 9.869963e-01 9.799124e-01 9.834603e-01 9.868931e-01 9.874986e-01 6.792461e-01

3651 9.869936e-01 9.799142e-01 9.834637e-01 9.868960e-01 9.875007e-01 6.794051e-01

3652 9.869908e-01 9.799161e-01 9.834674e-01 9.868990e-01 9.875029e-01 6.795662e-01

3653 9.869878e-01 9.799181e-01 9.834711e-01 9.869021e-01 9.875051e-01 6.797295e-01

3654 9.869848e-01 9.799202e-01 9.834750e-01 9.869053e-01 9.875074e-01 6.798948e-01

3655 9.869816e-01 9.799224e-01 9.834791e-01 9.869087e-01 9.875099e-01 6.800623e-01

3656 9.869783e-01 9.799247e-01 9.834833e-01 9.869122e-01 9.875124e-01 6.802319e-01

3657 9.869748e-01 9.799271e-01 9.834877e-01 9.869158e-01 9.875150e-01 6.804037e-01

3658 9.869712e-01 9.799296e-01 9.834922e-01 9.869196e-01 9.875177e-01 6.805776e-01

3659 9.869675e-01 9.799323e-01 9.834970e-01 9.869235e-01 9.875206e-01 6.807537e-01

3660 9.869636e-01 9.799351e-01 9.835019e-01 9.869276e-01 9.875235e-01 6.809320e-01

3661 9.869595e-01 9.799380e-01 9.835070e-01 9.869318e-01 9.875265e-01 6.811125e-01

3662 9.869553e-01 9.799411e-01 9.835123e-01 9.869362e-01 9.875297e-01 6.812951e-01

3663 9.869509e-01 9.799443e-01 9.835178e-01 9.869408e-01 9.875330e-01 6.814799e-01

3664 9.869463e-01 9.799477e-01 9.835235e-01 9.869456e-01 9.875364e-01 6.816669e-01

3665 9.869416e-01 9.799513e-01 9.835295e-01 9.869505e-01 9.875399e-01 6.818561e-01

3666 9.869366e-01 9.799550e-01 9.835357e-01 9.869556e-01 9.875436e-01 6.820475e-01

3667 9.869314e-01 9.799589e-01 9.835422e-01 9.869610e-01 9.875474e-01 6.822411e-01

3668 9.869260e-01 9.799631e-01 9.835489e-01 9.869665e-01 9.875514e-01 6.824368e-01

3669 9.869204e-01 9.799674e-01 9.835558e-01 9.869722e-01 9.875555e-01 6.826347e-01

3670 9.869146e-01 9.799720e-01 9.835631e-01 9.869782e-01 9.875597e-01 6.828347e-01

3671 9.869085e-01 9.799768e-01 9.835706e-01 9.869844e-01 9.875641e-01 6.830369e-01

3672 9.869022e-01 9.799818e-01 9.835785e-01 9.869908e-01 9.875687e-01 6.832412e-01

3673 9.868956e-01 9.799871e-01 9.835866e-01 9.869975e-01 9.875735e-01 6.834476e-01

3674 9.868887e-01 9.799926e-01 9.835951e-01 9.870045e-01 9.875784e-01 6.836561e-01

3675 9.868816e-01 9.799985e-01 9.836039e-01 9.870117e-01 9.875835e-01 6.838667e-01

3676 9.868741e-01 9.800046e-01 9.836131e-01 9.870192e-01 9.875889e-01 6.840794e-01

3677 9.868663e-01 9.800111e-01 9.836227e-01 9.870269e-01 9.875944e-01 6.842940e-01

3678 9.868582e-01 9.800178e-01 9.836326e-01 9.870350e-01 9.876001e-01 6.845107e-01

3679 9.868498e-01 9.800249e-01 9.836429e-01 9.870434e-01 9.876060e-01 6.847294e-01

3680 9.868409e-01 9.800324e-01 9.836537e-01 9.870521e-01 9.876121e-01 6.849500e-01

3681 9.868318e-01 9.800403e-01 9.836649e-01 9.870612e-01 9.876185e-01 6.851725e-01

3682 9.868222e-01 9.800485e-01 9.836766e-01 9.870706e-01 9.876251e-01 6.853969e-01

3683 9.868122e-01 9.800572e-01 9.836887e-01 9.870803e-01 9.876320e-01 6.856231e-01

3684 9.868018e-01 9.800664e-01 9.837014e-01 9.870905e-01 9.876391e-01 6.858512e-01

3685 9.867909e-01 9.800760e-01 9.837145e-01 9.871010e-01 9.876464e-01 6.860810e-01

3686 9.867796e-01 9.800861e-01 9.837282e-01 9.871120e-01 9.876540e-01 6.863125e-01

3687 9.867678e-01 9.800967e-01 9.837425e-01 9.871234e-01 9.876620e-01 6.865456e-01

3688 9.867555e-01 9.801078e-01 9.837574e-01 9.871352e-01 9.876702e-01 6.867804e-01

3689 9.867426e-01 9.801195e-01 9.837728e-01 9.871475e-01 9.876787e-01 6.870167e-01

3690 9.867292e-01 9.801319e-01 9.837890e-01 9.871603e-01 9.876875e-01 6.872546e-01

3691 9.867152e-01 9.801448e-01 9.838058e-01 9.871736e-01 9.876966e-01 6.874938e-01

3692 9.867006e-01 9.801585e-01 9.838233e-01 9.871874e-01 9.877061e-01 6.877345e-01

3693 9.866853e-01 9.801728e-01 9.838415e-01 9.872017e-01 9.877159e-01 6.879765e-01

3694 9.866694e-01 9.801879e-01 9.838605e-01 9.872166e-01 9.877261e-01 6.882197e-01

3695 9.866527e-01 9.802037e-01 9.838803e-01 9.872321e-01 9.877366e-01 6.884641e-01

3696 9.866354e-01 9.802204e-01 9.839009e-01 9.872483e-01 9.877475e-01 6.887097e-01

3697 9.866172e-01 9.802380e-01 9.839224e-01 9.872650e-01 9.877589e-01 6.889563e-01

3698 9.865983e-01 9.802564e-01 9.839448e-01 9.872824e-01 9.877706e-01 6.892038e-01

3699 9.865785e-01 9.802758e-01 9.839681e-01 9.873005e-01 9.877828e-01 6.894522e-01

3700 9.865579e-01 9.802962e-01 9.839925e-01 9.873194e-01 9.877954e-01 6.897014e-01

3701 9.865363e-01 9.803177e-01 9.840178e-01 9.873390e-01 9.878084e-01 6.899514e-01

3702 9.865137e-01 9.803402e-01 9.840443e-01 9.873593e-01 9.878220e-01 6.902020e-01

3703 9.864901e-01 9.803640e-01 9.840718e-01 9.873805e-01 9.878360e-01 6.904531e-01

3704 9.864655e-01 9.803889e-01 9.841006e-01 9.874025e-01 9.878506e-01 6.907047e-01

3705 9.864397e-01 9.804152e-01 9.841305e-01 9.874254e-01 9.878657e-01 6.909566e-01

3706 9.864128e-01 9.804428e-01 9.841618e-01 9.874492e-01 9.878813e-01 6.912089e-01

3707 9.863847e-01 9.804718e-01 9.841943e-01 9.874740e-01 9.878975e-01 6.914613e-01

3708 9.863552e-01 9.805024e-01 9.842283e-01 9.874998e-01 9.879143e-01 6.917138e-01

3709 9.863245e-01 9.805345e-01 9.842637e-01 9.875266e-01 9.879317e-01 6.919662e-01

3710 9.862923e-01 9.805683e-01 9.843006e-01 9.875545e-01 9.879497e-01 6.922186e-01

3711 9.862586e-01 9.806038e-01 9.843392e-01 9.875835e-01 9.879684e-01 6.924708e-01

3712 9.862234e-01 9.806411e-01 9.843793e-01 9.876137e-01 9.879878e-01 6.927226e-01

3713 9.861866e-01 9.806804e-01 9.844212e-01 9.876451e-01 9.880078e-01 6.929740e-01

3714 9.861481e-01 9.807217e-01 9.844649e-01 9.876777e-01 9.880286e-01 6.932249e-01

3715 9.861078e-01 9.807651e-01 9.845105e-01 9.877117e-01 9.880502e-01 6.934752e-01

3716 9.860657e-01 9.808107e-01 9.845580e-01 9.877471e-01 9.880725e-01 6.937248e-01

3717 9.860216e-01 9.808587e-01 9.846075e-01 9.877838e-01 9.880956e-01 6.939735e-01

3718 9.859754e-01 9.809092e-01 9.846592e-01 9.878221e-01 9.881195e-01 6.942213e-01

3719 9.859272e-01 9.809622e-01 9.847131e-01 9.878619e-01 9.881443e-01 6.944681e-01

3720 9.858767e-01 9.810179e-01 9.847693e-01 9.879033e-01 9.881700e-01 6.947137e-01

3721 9.858238e-01 9.810764e-01 9.848279e-01 9.879464e-01 9.881966e-01 6.949581e-01

3722 9.857685e-01 9.811379e-01 9.848890e-01 9.879912e-01 9.882241e-01 6.952012e-01

3723 9.857107e-01 9.812026e-01 9.849527e-01 9.880379e-01 9.882527e-01 6.954428e-01

3724 9.856502e-01 9.812705e-01 9.850191e-01 9.880864e-01 9.882822e-01 6.956829e-01

3725 9.855868e-01 9.813418e-01 9.850883e-01 9.881368e-01 9.883128e-01 6.959213e-01

3726 9.855206e-01 9.814167e-01 9.851605e-01 9.881893e-01 9.883444e-01 6.961580e-01

3727 9.854512e-01 9.814954e-01 9.852358e-01 9.882439e-01 9.883772e-01 6.963929e-01

3728 9.853787e-01 9.815780e-01 9.853142e-01 9.883007e-01 9.884111e-01 6.966259e-01

3729 9.853028e-01 9.816647e-01 9.853960e-01 9.883598e-01 9.884461e-01 6.968568e-01

3730 9.852235e-01 9.817557e-01 9.854812e-01 9.884212e-01 9.884824e-01 6.970856e-01

3731 9.851405e-01 9.818512e-01 9.855699e-01 9.884850e-01 9.885199e-01 6.973123e-01

3732 9.850536e-01 9.819515e-01 9.856624e-01 9.885514e-01 9.885588e-01 6.975367e-01

3733 9.849629e-01 9.820566e-01 9.857588e-01 9.886205e-01 9.885989e-01 6.977587e-01

3734 9.848680e-01 9.821669e-01 9.858591e-01 9.886922e-01 9.886404e-01 6.979783e-01

3735 9.847688e-01 9.822826e-01 9.859636e-01 9.887668e-01 9.886833e-01 6.981954e-01

3736 9.846651e-01 9.824038e-01 9.860725e-01 9.888443e-01 9.887276e-01 6.984099e-01

3737 9.845567e-01 9.825310e-01 9.861858e-01 9.889249e-01 9.887733e-01 6.986218e-01

3738 9.844435e-01 9.826642e-01 9.863037e-01 9.890086e-01 9.888206e-01 6.988310e-01

3739 9.843252e-01 9.828038e-01 9.864265e-01 9.890955e-01 9.888694e-01 6.990373e-01

3740 9.842017e-01 9.829500e-01 9.865542e-01 9.891858e-01 9.889198e-01 6.992409e-01

3741 9.840727e-01 9.831031e-01 9.866871e-01 9.892796e-01 9.889718e-01 6.994415e-01

3742 9.839381e-01 9.832634e-01 9.868253e-01 9.893770e-01 9.890255e-01 6.996392e-01

3743 9.837976e-01 9.834312e-01 9.869690e-01 9.894781e-01 9.890808e-01 6.998339e-01

3744 9.836511e-01 9.836068e-01 9.871185e-01 9.895830e-01 9.891378e-01 7.000256e-01

3745 9.834982e-01 9.837904e-01 9.872738e-01 9.896919e-01 9.891966e-01 7.002142e-01

3746 9.833389e-01 9.839825e-01 9.874352e-01 9.898048e-01 9.892572e-01 7.003997e-01

3747 9.831729e-01 9.841833e-01 9.876029e-01 9.899220e-01 9.893196e-01 7.005820e-01

3748 9.829999e-01 9.843931e-01 9.877771e-01 9.900435e-01 9.893838e-01 7.007611e-01

3749 9.828198e-01 9.846122e-01 9.879579e-01 9.901694e-01 9.894499e-01 7.009371e-01

3750 9.826324e-01 9.848411e-01 9.881457e-01 9.902999e-01 9.895179e-01 7.011097e-01

3751 9.824374e-01 9.850801e-01 9.883405e-01 9.904352e-01 9.895878e-01 7.012792e-01

3752 9.822346e-01 9.853295e-01 9.885426e-01 9.905753e-01 9.896596e-01 7.014454e-01

3753 9.820238e-01 9.855896e-01 9.887522e-01 9.907203e-01 9.897334e-01 7.016083e-01

3754 9.818049e-01 9.858608e-01 9.889695e-01 9.908705e-01 9.898091e-01 7.017679e-01

3755 9.815776e-01 9.861434e-01 9.891947e-01 9.910259e-01 9.898869e-01 7.019242e-01

3756 9.813417e-01 9.864378e-01 9.894281e-01 9.911867e-01 9.899666e-01 7.020772e-01

3757 9.810971e-01 9.867444e-01 9.896699e-01 9.913530e-01 9.900483e-01 7.022269e-01

3758 9.808437e-01 9.870633e-01 9.899204e-01 9.915248e-01 9.901321e-01 7.023734e-01

3759 9.805812e-01 9.873950e-01 9.901799e-01 9.917025e-01 9.902178e-01 7.025165e-01

3760 9.811844e-01 9.882410e-01 9.908484e-01 9.922375e-01 9.907420e-01 7.067583e-01

3761 9.811845e-01 9.882414e-01 9.908482e-01 9.922377e-01 9.907421e-01 7.067772e-01

3762 9.811845e-01 9.882417e-01 9.908481e-01 9.922378e-01 9.907423e-01 7.067959e-01

3763 9.811846e-01 9.882421e-01 9.908480e-01 9.922378e-01 9.907424e-01 7.068144e-01

3764 9.811847e-01 9.882425e-01 9.908480e-01 9.922379e-01 9.907425e-01 7.068325e-01

3765 9.811847e-01 9.882428e-01 9.908480e-01 9.922380e-01 9.907426e-01 7.068504e-01

3766 9.811848e-01 9.882431e-01 9.908480e-01 9.922380e-01 9.907427e-01 7.068679e-01

3767 9.811848e-01 9.882434e-01 9.908481e-01 9.922380e-01 9.907427e-01 7.068851e-01

3768 9.811848e-01 9.882437e-01 9.908483e-01 9.922381e-01 9.907428e-01 7.069020e-01

3769 9.811848e-01 9.882440e-01 9.908485e-01 9.922381e-01 9.907428e-01 7.069186e-01

3770 9.811848e-01 9.882443e-01 9.908487e-01 9.922382e-01 9.907428e-01 7.069347e-01

3771 9.811847e-01 9.882446e-01 9.908489e-01 9.922382e-01 9.907429e-01 7.069506e-01

3772 9.811847e-01 9.882449e-01 9.908492e-01 9.922382e-01 9.907429e-01 7.069660e-01

3773 9.811846e-01 9.882452e-01 9.908495e-01 9.922383e-01 9.907429e-01 7.069811e-01

3774 9.811846e-01 9.882455e-01 9.908498e-01 9.922383e-01 9.907429e-01 7.069958e-01

3775 9.811845e-01 9.882458e-01 9.908502e-01 9.922384e-01 9.907429e-01 7.070100e-01

3776 9.811844e-01 9.882461e-01 9.908505e-01 9.922385e-01 9.907430e-01 7.070239e-01

3777 9.811843e-01 9.882464e-01 9.908509e-01 9.922385e-01 9.907430e-01 7.070373e-01

3778 9.811842e-01 9.882467e-01 9.908512e-01 9.922386e-01 9.907430e-01 7.070502e-01

3779 9.811841e-01 9.882470e-01 9.908516e-01 9.922387e-01 9.907430e-01 7.070627e-01

3780 9.811840e-01 9.882473e-01 9.908520e-01 9.922389e-01 9.907431e-01 7.070747e-01

3781 9.811839e-01 9.882477e-01 9.908524e-01 9.922390e-01 9.907431e-01 7.070862e-01

3782 9.811838e-01 9.882481e-01 9.908528e-01 9.922391e-01 9.907432e-01 7.070972e-01

3783 9.811837e-01 9.882485e-01 9.908532e-01 9.922393e-01 9.907433e-01 7.071077e-01

3784 9.811836e-01 9.882489e-01 9.908535e-01 9.922395e-01 9.907434e-01 7.071176e-01

3785 9.811835e-01 9.882494e-01 9.908539e-01 9.922396e-01 9.907435e-01 7.071270e-01

3786 9.811834e-01 9.882498e-01 9.908543e-01 9.922398e-01 9.907436e-01 7.071359e-01

3787 9.811833e-01 9.882504e-01 9.908547e-01 9.922400e-01 9.907437e-01 7.071441e-01

3788 9.811832e-01 9.882509e-01 9.908551e-01 9.922403e-01 9.907438e-01 7.071517e-01

3789 9.811831e-01 9.882514e-01 9.908555e-01 9.922405e-01 9.907440e-01 7.071587e-01

3790 9.811831e-01 9.882520e-01 9.908559e-01 9.922407e-01 9.907441e-01 7.071651e-01

3791 9.811830e-01 9.882527e-01 9.908562e-01 9.922410e-01 9.907443e-01 7.071708e-01

3792 9.811829e-01 9.882533e-01 9.908566e-01 9.922413e-01 9.907445e-01 7.071758e-01

3793 9.811829e-01 9.882540e-01 9.908571e-01 9.922415e-01 9.907447e-01 7.071801e-01

3794 9.811828e-01 9.882547e-01 9.908575e-01 9.922418e-01 9.907449e-01 7.071837e-01

3795 9.811828e-01 9.882555e-01 9.908579e-01 9.922421e-01 9.907451e-01 7.071866e-01

3796 9.811827e-01 9.882562e-01 9.908583e-01 9.922424e-01 9.907453e-01 7.071887e-01

3797 9.811827e-01 9.882570e-01 9.908588e-01 9.922428e-01 9.907455e-01 7.071900e-01

3798 9.811827e-01 9.882579e-01 9.908593e-01 9.922431e-01 9.907457e-01 7.071905e-01

3799 9.811826e-01 9.882588e-01 9.908598e-01 9.922435e-01 9.907460e-01 7.071901e-01

3800 9.811826e-01 9.882597e-01 9.908603e-01 9.922438e-01 9.907462e-01 7.071889e-01

3801 9.811826e-01 9.882607e-01 9.908609e-01 9.922442e-01 9.907464e-01 7.071868e-01

3802 9.811825e-01 9.882616e-01 9.908614e-01 9.922446e-01 9.907467e-01 7.071838e-01

3803 9.811825e-01 9.882627e-01 9.908620e-01 9.922450e-01 9.907470e-01 7.071799e-01

3804 9.811825e-01 9.882637e-01 9.908627e-01 9.922454e-01 9.907472e-01 7.071750e-01

3805 9.811825e-01 9.882649e-01 9.908633e-01 9.922458e-01 9.907475e-01 7.071691e-01

3806 9.811824e-01 9.882660e-01 9.908641e-01 9.922463e-01 9.907478e-01 7.071622e-01

3807 9.811824e-01 9.882672e-01 9.908648e-01 9.922467e-01 9.907480e-01 7.071542e-01

3808 9.811824e-01 9.882684e-01 9.908656e-01 9.922472e-01 9.907483e-01 7.071451e-01

3809 9.811824e-01 9.882697e-01 9.908664e-01 9.922477e-01 9.907486e-01 7.071349e-01

3810 9.811824e-01 9.882711e-01 9.908672e-01 9.922482e-01 9.907489e-01 7.071236e-01

3811 9.811824e-01 9.882724e-01 9.908681e-01 9.922487e-01 9.907492e-01 7.071111e-01

3812 9.811823e-01 9.882739e-01 9.908690e-01 9.922493e-01 9.907496e-01 7.070974e-01

3813 9.811823e-01 9.882754e-01 9.908700e-01 9.922499e-01 9.907499e-01 7.070824e-01

3814 9.811823e-01 9.882769e-01 9.908710e-01 9.922504e-01 9.907502e-01 7.070662e-01

3815 9.811823e-01 9.882785e-01 9.908721e-01 9.922511e-01 9.907506e-01 7.070486e-01

3816 9.811823e-01 9.882802e-01 9.908732e-01 9.922517e-01 9.907509e-01 7.070297e-01

3817 9.811824e-01 9.882819e-01 9.908744e-01 9.922524e-01 9.907513e-01 7.070094e-01

3818 9.811824e-01 9.882837e-01 9.908756e-01 9.922530e-01 9.907517e-01 7.069876e-01

3819 9.811824e-01 9.882855e-01 9.908768e-01 9.922538e-01 9.907521e-01 7.069644e-01

3820 9.811825e-01 9.882874e-01 9.908781e-01 9.922545e-01 9.907525e-01 7.069397e-01

3821 9.811825e-01 9.882894e-01 9.908795e-01 9.922553e-01 9.907529e-01 7.069134e-01

3822 9.811826e-01 9.882915e-01 9.908809e-01 9.922561e-01 9.907533e-01 7.068855e-01

3823 9.811827e-01 9.882937e-01 9.908823e-01 9.922569e-01 9.907537e-01 7.068560e-01

3824 9.811827e-01 9.882959e-01 9.908838e-01 9.922578e-01 9.907542e-01 7.068248e-01

3825 9.811828e-01 9.882982e-01 9.908854e-01 9.922587e-01 9.907547e-01 7.067919e-01

3826 9.811830e-01 9.883006e-01 9.908871e-01 9.922596e-01 9.907552e-01 7.067572e-01

3827 9.811831e-01 9.883031e-01 9.908888e-01 9.922606e-01 9.907557e-01 7.067207e-01

3828 9.811832e-01 9.883057e-01 9.908905e-01 9.922616e-01 9.907562e-01 7.066823e-01

3829 9.811834e-01 9.883084e-01 9.908924e-01 9.922626e-01 9.907567e-01 7.066420e-01

3830 9.811836e-01 9.883112e-01 9.908943e-01 9.922637e-01 9.907573e-01 7.065998e-01

3831 9.811838e-01 9.883142e-01 9.908963e-01 9.922648e-01 9.907578e-01 7.065556e-01

3832 9.811840e-01 9.883172e-01 9.908984e-01 9.922660e-01 9.907584e-01 7.065093e-01

3833 9.811843e-01 9.883203e-01 9.909005e-01 9.922672e-01 9.907590e-01 7.064610e-01

3834 9.811846e-01 9.883236e-01 9.909027e-01 9.922685e-01 9.907596e-01 7.064105e-01

3835 9.811849e-01 9.883269e-01 9.909050e-01 9.922698e-01 9.907602e-01 7.063578e-01

3836 9.811852e-01 9.883304e-01 9.909074e-01 9.922711e-01 9.907609e-01 7.063029e-01

3837 9.811856e-01 9.883341e-01 9.909099e-01 9.922725e-01 9.907615e-01 7.062456e-01

3838 9.811860e-01 9.883379e-01 9.909125e-01 9.922740e-01 9.907622e-01 7.061861e-01

3839 9.811864e-01 9.883418e-01 9.909152e-01 9.922755e-01 9.907629e-01 7.061241e-01

3840 9.811869e-01 9.883459e-01 9.909180e-01 9.922770e-01 9.907635e-01 7.060597e-01

3841 9.811874e-01 9.883501e-01 9.909209e-01 9.922787e-01 9.907642e-01 7.059928e-01

3842 9.811880e-01 9.883545e-01 9.909240e-01 9.922803e-01 9.907650e-01 7.059234e-01

3843 9.811886e-01 9.883590e-01 9.909271e-01 9.922821e-01 9.907657e-01 7.058514e-01

3844 9.811892e-01 9.883638e-01 9.909304e-01 9.922838e-01 9.907664e-01 7.057767e-01

3845 9.811899e-01 9.883687e-01 9.909338e-01 9.922857e-01 9.907671e-01 7.056993e-01

3846 9.811907e-01 9.883738e-01 9.909373e-01 9.922876e-01 9.907679e-01 7.056192e-01

3847 9.811915e-01 9.883790e-01 9.909410e-01 9.922896e-01 9.907686e-01 7.055363e-01

3848 9.811923e-01 9.883845e-01 9.909448e-01 9.922917e-01 9.907694e-01 7.054506e-01

3849 9.811932e-01 9.883902e-01 9.909487e-01 9.922938e-01 9.907702e-01 7.053619e-01

3850 9.811942e-01 9.883961e-01 9.909528e-01 9.922960e-01 9.907709e-01 7.052704e-01

3851 9.811953e-01 9.884023e-01 9.909571e-01 9.922983e-01 9.907717e-01 7.051758e-01

3852 9.811965e-01 9.884086e-01 9.909615e-01 9.923006e-01 9.907725e-01 7.050782e-01

3853 9.811977e-01 9.884153e-01 9.909661e-01 9.923031e-01 9.907732e-01 7.049776e-01

3854 9.811990e-01 9.884221e-01 9.909709e-01 9.923056e-01 9.907740e-01 7.048738e-01

3855 9.812004e-01 9.884293e-01 9.909758e-01 9.923082e-01 9.907747e-01 7.047669e-01

3856 9.812019e-01 9.884367e-01 9.909810e-01 9.923109e-01 9.907754e-01 7.046567e-01

3857 9.812035e-01 9.884444e-01 9.909863e-01 9.923136e-01 9.907762e-01 7.045434e-01

3858 9.812052e-01 9.884524e-01 9.909919e-01 9.923165e-01 9.907769e-01 7.044267e-01

3859 9.812070e-01 9.884607e-01 9.909977e-01 9.923194e-01 9.907776e-01 7.043067e-01

3860 9.812090e-01 9.884693e-01 9.910036e-01 9.923225e-01 9.907782e-01 7.041834e-01

3861 9.812111e-01 9.884782e-01 9.910099e-01 9.923256e-01 9.907788e-01 7.040567e-01

3862 9.812133e-01 9.884875e-01 9.910163e-01 9.923289e-01 9.907794e-01 7.039266e-01

3863 9.812157e-01 9.884972e-01 9.910230e-01 9.923322e-01 9.907800e-01 7.037931e-01

3864 9.812182e-01 9.885072e-01 9.910300e-01 9.923357e-01 9.907805e-01 7.036560e-01

3865 9.812209e-01 9.885176e-01 9.910372e-01 9.923392e-01 9.907810e-01 7.035155e-01

3866 9.812238e-01 9.885285e-01 9.910447e-01 9.923429e-01 9.907814e-01 7.033715e-01

3867 9.812269e-01 9.885397e-01 9.910524e-01 9.923466e-01 9.907817e-01 7.032240e-01

3868 9.812301e-01 9.885514e-01 9.910605e-01 9.923505e-01 9.907820e-01 7.030729e-01

3869 9.812336e-01 9.885636e-01 9.910689e-01 9.923545e-01 9.907822e-01 7.029182e-01

3870 9.812373e-01 9.885762e-01 9.910776e-01 9.923586e-01 9.907823e-01 7.027600e-01

3871 9.812413e-01 9.885893e-01 9.910866e-01 9.923628e-01 9.907823e-01 7.025983e-01

3872 9.812455e-01 9.886030e-01 9.910959e-01 9.923672e-01 9.907822e-01 7.024329e-01

3873 9.812499e-01 9.886172e-01 9.911057e-01 9.923717e-01 9.907820e-01 7.022640e-01

3874 9.812547e-01 9.886319e-01 9.911158e-01 9.923763e-01 9.907816e-01 7.020915e-01

3875 9.812597e-01 9.886473e-01 9.911262e-01 9.923810e-01 9.907811e-01 7.019154e-01

3876 9.812651e-01 9.886632e-01 9.911371e-01 9.923858e-01 9.907804e-01 7.017359e-01

3877 9.812708e-01 9.886798e-01 9.911484e-01 9.923908e-01 9.907796e-01 7.015527e-01

3878 9.812768e-01 9.886970e-01 9.911601e-01 9.923959e-01 9.907786e-01 7.013661e-01

3879 9.812833e-01 9.887150e-01 9.911722e-01 9.924011e-01 9.907773e-01 7.011760e-01

3880 9.812901e-01 9.887336e-01 9.911849e-01 9.924065e-01 9.907759e-01 7.009824e-01

3881 9.812974e-01 9.887530e-01 9.911980e-01 9.924120e-01 9.907742e-01 7.007855e-01

3882 9.813051e-01 9.887732e-01 9.912116e-01 9.924176e-01 9.907722e-01 7.005851e-01

3883 9.813133e-01 9.887942e-01 9.912257e-01 9.924234e-01 9.907699e-01 7.003814e-01

3884 9.813221e-01 9.888160e-01 9.912403e-01 9.924293e-01 9.907673e-01 7.001744e-01

3885 9.813313e-01 9.888387e-01 9.912555e-01 9.924353e-01 9.907644e-01 6.999641e-01

3886 9.813411e-01 9.888623e-01 9.912713e-01 9.924415e-01 9.907611e-01 6.997507e-01

3887 9.813516e-01 9.888869e-01 9.912877e-01 9.924478e-01 9.907575e-01 6.995341e-01

3888 9.813627e-01 9.889125e-01 9.913047e-01 9.924542e-01 9.907534e-01 6.993145e-01

3889 9.813744e-01 9.889391e-01 9.913224e-01 9.924608e-01 9.907488e-01 6.990919e-01

3890 9.813869e-01 9.889669e-01 9.913407e-01 9.924675e-01 9.907438e-01 6.988663e-01

3891 9.814001e-01 9.889957e-01 9.913598e-01 9.924743e-01 9.907382e-01 6.986379e-01

3892 9.814142e-01 9.890257e-01 9.913795e-01 9.924812e-01 9.907320e-01 6.984068e-01

3893 9.814291e-01 9.890570e-01 9.914000e-01 9.924882e-01 9.907252e-01 6.981729e-01

3894 9.814449e-01 9.890895e-01 9.914213e-01 9.924954e-01 9.907178e-01 6.979365e-01

3895 9.814617e-01 9.891234e-01 9.914435e-01 9.925027e-01 9.907096e-01 6.976976e-01

3896 9.814795e-01 9.891586e-01 9.914664e-01 9.925100e-01 9.907007e-01 6.974563e-01

3897 9.814984e-01 9.891953e-01 9.914903e-01 9.925175e-01 9.906909e-01 6.972128e-01

3898 9.815184e-01 9.892336e-01 9.915150e-01 9.925251e-01 9.906803e-01 6.969670e-01

3899 9.815396e-01 9.892733e-01 9.915407e-01 9.925327e-01 9.906688e-01 6.967192e-01

3900 9.815621e-01 9.893148e-01 9.915674e-01 9.925405e-01 9.906562e-01 6.964694e-01

3901 9.815860e-01 9.893579e-01 9.915951e-01 9.925483e-01 9.906426e-01 6.962178e-01

3902 9.816113e-01 9.894028e-01 9.916239e-01 9.925561e-01 9.906278e-01 6.959644e-01

3903 9.816381e-01 9.894496e-01 9.916537e-01 9.925640e-01 9.906117e-01 6.957095e-01

3904 9.816665e-01 9.894983e-01 9.916847e-01 9.925719e-01 9.905944e-01 6.954530e-01

3905 9.816966e-01 9.895490e-01 9.917169e-01 9.925798e-01 9.905757e-01 6.951953e-01

3906 9.817285e-01 9.896018e-01 9.917504e-01 9.925878e-01 9.905555e-01 6.949363e-01

3907 9.817623e-01 9.896567e-01 9.917850e-01 9.925957e-01 9.905336e-01 6.946763e-01

3908 9.817980e-01 9.897140e-01 9.918211e-01 9.926036e-01 9.905101e-01 6.944153e-01

3909 9.818359e-01 9.897736e-01 9.918584e-01 9.926114e-01 9.904848e-01 6.941535e-01

3910 9.818760e-01 9.898356e-01 9.918972e-01 9.926192e-01 9.904576e-01 6.938910e-01

3911 9.819185e-01 9.899002e-01 9.919375e-01 9.926268e-01 9.904283e-01 6.936280e-01

3912 9.819634e-01 9.899674e-01 9.919793e-01 9.926344e-01 9.903969e-01 6.933646e-01

3913 9.820110e-01 9.900374e-01 9.920227e-01 9.926417e-01 9.903631e-01 6.931009e-01

3914 9.820613e-01 9.901103e-01 9.920677e-01 9.926489e-01 9.903270e-01 6.928371e-01

3915 9.821145e-01 9.901862e-01 9.921145e-01 9.926559e-01 9.902882e-01 6.925733e-01

3916 9.821708e-01 9.902651e-01 9.921630e-01 9.926626e-01 9.902466e-01 6.923097e-01

3917 9.822304e-01 9.903473e-01 9.922133e-01 9.926691e-01 9.902022e-01 6.920464e-01

3918 9.822933e-01 9.904328e-01 9.922654e-01 9.926752e-01 9.901547e-01 6.917835e-01

3919 9.823598e-01 9.905217e-01 9.923196e-01 9.926809e-01 9.901039e-01 6.915212e-01

3920 9.824301e-01 9.906143e-01 9.923757e-01 9.926862e-01 9.900496e-01 6.912596e-01

3921 9.825043e-01 9.907105e-01 9.924340e-01 9.926911e-01 9.899918e-01 6.909988e-01

3922 9.825827e-01 9.908107e-01 9.924943e-01 9.926954e-01 9.899300e-01 6.907389e-01

3923 9.826655e-01 9.909148e-01 9.925569e-01 9.926992e-01 9.898642e-01 6.904802e-01

3924 9.827529e-01 9.910231e-01 9.926218e-01 9.927023e-01 9.897941e-01 6.902227e-01

3925 9.828450e-01 9.911357e-01 9.926891e-01 9.927048e-01 9.897196e-01 6.899665e-01

3926 9.829423e-01 9.912527e-01 9.927588e-01 9.927065e-01 9.896402e-01 6.897117e-01

3927 9.830448e-01 9.913744e-01 9.928310e-01 9.927073e-01 9.895559e-01 6.894586e-01

3928 9.831528e-01 9.915007e-01 9.929058e-01 9.927072e-01 9.894663e-01 6.892071e-01

3929 9.832666e-01 9.916320e-01 9.929832e-01 9.927062e-01 9.893712e-01 6.889574e-01

3930 9.833865e-01 9.917684e-01 9.930634e-01 9.927040e-01 9.892703e-01 6.887096e-01

3931 9.835128e-01 9.919100e-01 9.931465e-01 9.927007e-01 9.891633e-01 6.884638e-01

3932 9.836456e-01 9.920571e-01 9.932324e-01 9.926962e-01 9.890500e-01 6.882201e-01

3933 9.837853e-01 9.922097e-01 9.933213e-01 9.926903e-01 9.889300e-01 6.879785e-01

3934 9.839322e-01 9.923680e-01 9.934133e-01 9.926829e-01 9.888030e-01 6.877393e-01

3935 9.840865e-01 9.925324e-01 9.935085e-01 9.926740e-01 9.886688e-01 6.875024e-01

3936 9.842486e-01 9.927028e-01 9.936068e-01 9.926634e-01 9.885270e-01 6.872679e-01

3937 9.844187e-01 9.928796e-01 9.937085e-01 9.926510e-01 9.883772e-01 6.870360e-01

3938 9.845971e-01 9.930630e-01 9.938135e-01 9.926367e-01 9.882192e-01 6.868066e-01

3939 9.854358e-01 9.935442e-01 9.941972e-01 9.929734e-01 9.886367e-01 6.908495e-01

3940 9.854359e-01 9.935441e-01 9.941973e-01 9.929734e-01 9.886366e-01 6.908135e-01

3941 9.854360e-01 9.935440e-01 9.941974e-01 9.929735e-01 9.886365e-01 6.907760e-01

3942 9.854362e-01 9.935439e-01 9.941976e-01 9.929735e-01 9.886365e-01 6.907370e-01

3943 9.854363e-01 9.935438e-01 9.941977e-01 9.929735e-01 9.886364e-01 6.906964e-01

3944 9.854364e-01 9.935437e-01 9.941979e-01 9.929735e-01 9.886363e-01 6.906543e-01

3945 9.854365e-01 9.935436e-01 9.941980e-01 9.929735e-01 9.886362e-01 6.906106e-01

3946 9.854366e-01 9.935436e-01 9.941981e-01 9.929735e-01 9.886362e-01 6.905652e-01

3947 9.854367e-01 9.935435e-01 9.941982e-01 9.929735e-01 9.886361e-01 6.905181e-01

3948 9.854369e-01 9.935435e-01 9.941983e-01 9.929735e-01 9.886361e-01 6.904693e-01

3949 9.854370e-01 9.935435e-01 9.941984e-01 9.929734e-01 9.886360e-01 6.904188e-01

3950 9.854371e-01 9.935435e-01 9.941985e-01 9.929734e-01 9.886360e-01 6.903664e-01

3951 9.854373e-01 9.935435e-01 9.941986e-01 9.929734e-01 9.886360e-01 6.903122e-01

3952 9.854375e-01 9.935436e-01 9.941987e-01 9.929734e-01 9.886359e-01 6.902561e-01

3953 9.854377e-01 9.935437e-01 9.941988e-01 9.929733e-01 9.886359e-01 6.901982e-01

3954 9.854379e-01 9.935438e-01 9.941989e-01 9.929733e-01 9.886359e-01 6.901382e-01

3955 9.854382e-01 9.935439e-01 9.941989e-01 9.929733e-01 9.886359e-01 6.900763e-01

3956 9.854384e-01 9.935441e-01 9.941990e-01 9.929733e-01 9.886360e-01 6.900122e-01

3957 9.854387e-01 9.935442e-01 9.941991e-01 9.929733e-01 9.886360e-01 6.899461e-01

3958 9.854390e-01 9.935444e-01 9.941992e-01 9.929733e-01 9.886360e-01 6.898779e-01

3959 9.854393e-01 9.935446e-01 9.941992e-01 9.929733e-01 9.886361e-01 6.898075e-01

3960 9.854397e-01 9.935449e-01 9.941993e-01 9.929733e-01 9.886361e-01 6.897348e-01

3961 9.854401e-01 9.935451e-01 9.941994e-01 9.929733e-01 9.886361e-01 6.896599e-01

3962 9.854405e-01 9.935454e-01 9.941995e-01 9.929734e-01 9.886362e-01 6.895826e-01

3963 9.854409e-01 9.935456e-01 9.941996e-01 9.929734e-01 9.886362e-01 6.895029e-01

3964 9.854413e-01 9.935459e-01 9.941997e-01 9.929735e-01 9.886363e-01 6.894208e-01

3965 9.854418e-01 9.935462e-01 9.941998e-01 9.929735e-01 9.886363e-01 6.893363e-01

3966 9.854423e-01 9.935465e-01 9.941999e-01 9.929736e-01 9.886363e-01 6.892492e-01

3967 9.854428e-01 9.935468e-01 9.942000e-01 9.929736e-01 9.886364e-01 6.891595e-01

3968 9.854433e-01 9.935471e-01 9.942001e-01 9.929737e-01 9.886364e-01 6.890672e-01

3969 9.854439e-01 9.935474e-01 9.942002e-01 9.929738e-01 9.886364e-01 6.889722e-01

3970 9.854445e-01 9.935477e-01 9.942004e-01 9.929739e-01 9.886364e-01 6.888744e-01

3971 9.854451e-01 9.935480e-01 9.942005e-01 9.929740e-01 9.886364e-01 6.887738e-01

3972 9.854457e-01 9.935483e-01 9.942007e-01 9.929740e-01 9.886364e-01 6.886704e-01

3973 9.854463e-01 9.935487e-01 9.942008e-01 9.929741e-01 9.886364e-01 6.885640e-01

3974 9.854470e-01 9.935490e-01 9.942010e-01 9.929742e-01 9.886364e-01 6.884547e-01

3975 9.854477e-01 9.935493e-01 9.942012e-01 9.929743e-01 9.886364e-01 6.883423e-01

3976 9.854484e-01 9.935497e-01 9.942014e-01 9.929744e-01 9.886364e-01 6.882269e-01

3977 9.854492e-01 9.935501e-01 9.942016e-01 9.929745e-01 9.886363e-01 6.881082e-01

3978 9.854499e-01 9.935504e-01 9.942018e-01 9.929746e-01 9.886363e-01 6.879864e-01

3979 9.854508e-01 9.935508e-01 9.942020e-01 9.929747e-01 9.886362e-01 6.878613e-01

3980 9.854516e-01 9.935512e-01 9.942022e-01 9.929748e-01 9.886362e-01 6.877328e-01

3981 9.854525e-01 9.935516e-01 9.942025e-01 9.929749e-01 9.886361e-01 6.876009e-01

3982 9.854534e-01 9.935520e-01 9.942027e-01 9.929750e-01 9.886360e-01 6.874655e-01

3983 9.854543e-01 9.935525e-01 9.942030e-01 9.929751e-01 9.886359e-01 6.873266e-01

3984 9.854553e-01 9.935529e-01 9.942032e-01 9.929752e-01 9.886359e-01 6.871840e-01

3985 9.854563e-01 9.935534e-01 9.942035e-01 9.929753e-01 9.886358e-01 6.870378e-01

3986 9.854573e-01 9.935539e-01 9.942038e-01 9.929753e-01 9.886357e-01 6.868878e-01

3987 9.854584e-01 9.935544e-01 9.942041e-01 9.929754e-01 9.886356e-01 6.867340e-01

3988 9.854595e-01 9.935549e-01 9.942044e-01 9.929755e-01 9.886354e-01 6.865764e-01

3989 9.854607e-01 9.935555e-01 9.942047e-01 9.929756e-01 9.886353e-01 6.864147e-01

3990 9.854620e-01 9.935561e-01 9.942050e-01 9.929756e-01 9.886352e-01 6.862491e-01

3991 9.854632e-01 9.935567e-01 9.942053e-01 9.929757e-01 9.886350e-01 6.860793e-01

3992 9.854645e-01 9.935573e-01 9.942057e-01 9.929758e-01 9.886349e-01 6.859054e-01

3993 9.854659e-01 9.935580e-01 9.942060e-01 9.929759e-01 9.886347e-01 6.857272e-01

3994 9.854674e-01 9.935586e-01 9.942064e-01 9.929759e-01 9.886345e-01 6.855447e-01

3995 9.854689e-01 9.935594e-01 9.942068e-01 9.929760e-01 9.886343e-01 6.853578e-01

3996 9.854704e-01 9.935601e-01 9.942072e-01 9.929761e-01 9.886341e-01 6.851664e-01

3997 9.854720e-01 9.935609e-01 9.942076e-01 9.929761e-01 9.886338e-01 6.849705e-01

3998 9.854737e-01 9.935617e-01 9.942080e-01 9.929762e-01 9.886336e-01 6.847700e-01

3999 9.854754e-01 9.935626e-01 9.942084e-01 9.929763e-01 9.886333e-01 6.845648e-01

4000 9.854772e-01 9.935634e-01 9.942089e-01 9.929763e-01 9.886330e-01 6.843549e-01

4001 9.854791e-01 9.935644e-01 9.942094e-01 9.929764e-01 9.886327e-01 6.841401e-01

4002 9.854811e-01 9.935653e-01 9.942099e-01 9.929764e-01 9.886323e-01 6.839204e-01

4003 9.854831e-01 9.935663e-01 9.942104e-01 9.929765e-01 9.886319e-01 6.836957e-01

4004 9.854853e-01 9.935674e-01 9.942109e-01 9.929766e-01 9.886315e-01 6.834660e-01

4005 9.854875e-01 9.935684e-01 9.942114e-01 9.929766e-01 9.886310e-01 6.832311e-01

4006 9.854898e-01 9.935696e-01 9.942120e-01 9.929766e-01 9.886305e-01 6.829911e-01

4007 9.854921e-01 9.935707e-01 9.942126e-01 9.929767e-01 9.886299e-01 6.827458e-01

4008 9.854946e-01 9.935719e-01 9.942132e-01 9.929767e-01 9.886293e-01 6.824952e-01

4009 9.854972e-01 9.935732e-01 9.942138e-01 9.929767e-01 9.886286e-01 6.822392e-01

4010 9.854999e-01 9.935745e-01 9.942144e-01 9.929767e-01 9.886279e-01 6.819777e-01

4011 9.855027e-01 9.935759e-01 9.942151e-01 9.929767e-01 9.886271e-01 6.817107e-01

4012 9.855056e-01 9.935773e-01 9.942158e-01 9.929767e-01 9.886263e-01 6.814381e-01

4013 9.855086e-01 9.935787e-01 9.942165e-01 9.929766e-01 9.886254e-01 6.811599e-01

4014 9.855117e-01 9.935803e-01 9.942172e-01 9.929766e-01 9.886244e-01 6.808759e-01

4015 9.855150e-01 9.935818e-01 9.942180e-01 9.929765e-01 9.886234e-01 6.805862e-01

4016 9.855184e-01 9.935835e-01 9.942188e-01 9.929764e-01 9.886222e-01 6.802906e-01

4017 9.855219e-01 9.935852e-01 9.942196e-01 9.929763e-01 9.886210e-01 6.799892e-01

4018 9.855256e-01 9.935869e-01 9.942205e-01 9.929762e-01 9.886197e-01 6.796819e-01

4019 9.855294e-01 9.935888e-01 9.942213e-01 9.929760e-01 9.886183e-01 6.793686e-01

4020 9.855333e-01 9.935907e-01 9.942222e-01 9.929758e-01 9.886167e-01 6.790492e-01

4021 9.855375e-01 9.935926e-01 9.942232e-01 9.929756e-01 9.886151e-01 6.787239e-01

4022 9.855417e-01 9.935947e-01 9.942241e-01 9.929753e-01 9.886133e-01 6.783924e-01

4023 9.855462e-01 9.935968e-01 9.942251e-01 9.929750e-01 9.886114e-01 6.780548e-01

4024 9.855508e-01 9.935990e-01 9.942262e-01 9.929746e-01 9.886093e-01 6.777111e-01

4025 9.855557e-01 9.936013e-01 9.942272e-01 9.929742e-01 9.886071e-01 6.773612e-01

4026 9.855607e-01 9.936037e-01 9.942283e-01 9.929738e-01 9.886048e-01 6.770051e-01

4027 9.855659e-01 9.936062e-01 9.942295e-01 9.929733e-01 9.886022e-01 6.766427e-01

4028 9.855713e-01 9.936088e-01 9.942306e-01 9.929728e-01 9.885995e-01 6.762742e-01

4029 9.855770e-01 9.936114e-01 9.942318e-01 9.929721e-01 9.885965e-01 6.758994e-01

4030 9.855829e-01 9.936142e-01 9.942331e-01 9.929715e-01 9.885934e-01 6.755184e-01

4031 9.855890e-01 9.936170e-01 9.942343e-01 9.929707e-01 9.885900e-01 6.751312e-01

4032 9.855954e-01 9.936200e-01 9.942357e-01 9.929699e-01 9.885864e-01 6.747378e-01

4033 9.856020e-01 9.936231e-01 9.942370e-01 9.929690e-01 9.885825e-01 6.743381e-01

4034 9.856089e-01 9.936263e-01 9.942384e-01 9.929680e-01 9.885783e-01 6.739323e-01

4035 9.856161e-01 9.936296e-01 9.942398e-01 9.929669e-01 9.885739e-01 6.735203e-01

4036 9.856236e-01 9.936331e-01 9.942413e-01 9.929657e-01 9.885691e-01 6.731023e-01

4037 9.856314e-01 9.936367e-01 9.942428e-01 9.929644e-01 9.885640e-01 6.726781e-01

4038 9.856395e-01 9.936404e-01 9.942443e-01 9.929629e-01 9.885585e-01 6.722480e-01

4039 9.856480e-01 9.936442e-01 9.942459e-01 9.929614e-01 9.885526e-01 6.718118e-01

4040 9.856568e-01 9.936482e-01 9.942475e-01 9.929597e-01 9.885463e-01 6.713698e-01

4041 9.856659e-01 9.936524e-01 9.942492e-01 9.929578e-01 9.885396e-01 6.709219e-01

4042 9.856755e-01 9.936566e-01 9.942509e-01 9.929558e-01 9.885324e-01 6.704683e-01

4043 9.856855e-01 9.936611e-01 9.942527e-01 9.929536e-01 9.885247e-01 6.700090e-01

4044 9.856958e-01 9.936657e-01 9.942545e-01 9.929513e-01 9.885164e-01 6.695441e-01

4045 9.857066e-01 9.936705e-01 9.942563e-01 9.929487e-01 9.885076e-01 6.690738e-01

4046 9.857179e-01 9.936754e-01 9.942581e-01 9.929459e-01 9.884981e-01 6.685981e-01

4047 9.857297e-01 9.936806e-01 9.942601e-01 9.929429e-01 9.884880e-01 6.681171e-01

4048 9.857419e-01 9.936859e-01 9.942620e-01 9.929397e-01 9.884772e-01 6.676310e-01

4049 9.857547e-01 9.936914e-01 9.942640e-01 9.929361e-01 9.884657e-01 6.671399e-01

4050 9.857680e-01 9.936972e-01 9.942660e-01 9.929323e-01 9.884534e-01 6.666439e-01

4051 9.857819e-01 9.937031e-01 9.942680e-01 9.929282e-01 9.884402e-01 6.661433e-01

4052 9.857964e-01 9.937092e-01 9.942701e-01 9.929238e-01 9.884261e-01 6.656380e-01

4053 9.858115e-01 9.937156e-01 9.942722e-01 9.929190e-01 9.884111e-01 6.651284e-01

4054 9.858273e-01 9.937222e-01 9.942744e-01 9.929139e-01 9.883950e-01 6.646146e-01

4055 9.858438e-01 9.937291e-01 9.942765e-01 9.929084e-01 9.883779e-01 6.640967e-01

4056 9.858609e-01 9.937362e-01 9.942787e-01 9.929024e-01 9.883596e-01 6.635750e-01

4057 9.858788e-01 9.937435e-01 9.942809e-01 9.928960e-01 9.883401e-01 6.630497e-01

4058 9.858976e-01 9.937511e-01 9.942832e-01 9.928890e-01 9.883192e-01 6.625208e-01

4059 9.859171e-01 9.937590e-01 9.942854e-01 9.928816e-01 9.882970e-01 6.619887e-01

4060 9.859375e-01 9.937672e-01 9.942876e-01 9.928736e-01 9.882732e-01 6.614536e-01

4061 9.859588e-01 9.937756e-01 9.942899e-01 9.928650e-01 9.882479e-01 6.609157e-01

4062 9.859810e-01 9.937844e-01 9.942921e-01 9.928558e-01 9.882209e-01 6.603752e-01

4063 9.860043e-01 9.937935e-01 9.942944e-01 9.928459e-01 9.881921e-01 6.598323e-01

4064 9.860285e-01 9.938029e-01 9.942966e-01 9.928353e-01 9.881614e-01 6.592873e-01

4065 9.860539e-01 9.938126e-01 9.942988e-01 9.928239e-01 9.881287e-01 6.587405e-01

4066 9.860804e-01 9.938227e-01 9.943010e-01 9.928117e-01 9.880938e-01 6.581920e-01

4067 9.861080e-01 9.938332e-01 9.943031e-01 9.927986e-01 9.880566e-01 6.576421e-01

4068 9.861370e-01 9.938440e-01 9.943052e-01 9.927846e-01 9.880171e-01 6.570911e-01

4069 9.861672e-01 9.938552e-01 9.943072e-01 9.927695e-01 9.879749e-01 6.565393e-01

4070 9.861988e-01 9.938668e-01 9.943092e-01 9.927534e-01 9.879300e-01 6.559869e-01

4071 9.862318e-01 9.938789e-01 9.943111e-01 9.927362e-01 9.878822e-01 6.554341e-01

4072 9.862664e-01 9.938913e-01 9.943129e-01 9.927177e-01 9.878313e-01 6.548813e-01

4073 9.863025e-01 9.939042e-01 9.943146e-01 9.926980e-01 9.877771e-01 6.543287e-01

4074 9.863402e-01 9.939176e-01 9.943161e-01 9.926769e-01 9.877194e-01 6.537765e-01

4075 9.863797e-01 9.939314e-01 9.943176e-01 9.926543e-01 9.876581e-01 6.532252e-01

4076 9.864210e-01 9.939457e-01 9.943188e-01 9.926302e-01 9.875929e-01 6.526748e-01

4077 9.864642e-01 9.939606e-01 9.943200e-01 9.926044e-01 9.875235e-01 6.521257e-01

4078 9.865094e-01 9.939759e-01 9.943209e-01 9.925769e-01 9.874498e-01 6.515782e-01

4079 9.865567e-01 9.939918e-01 9.943216e-01 9.925475e-01 9.873715e-01 6.510325e-01

4080 9.866061e-01 9.940083e-01 9.943221e-01 9.925161e-01 9.872882e-01 6.504889e-01

4081 9.866578e-01 9.940253e-01 9.943223e-01 9.924826e-01 9.871998e-01 6.499476e-01

4082 9.867119e-01 9.940430e-01 9.943222e-01 9.924469e-01 9.871059e-01 6.494090e-01

4083 9.867686e-01 9.940612e-01 9.943218e-01 9.924088e-01 9.870062e-01 6.488732e-01

4084 9.868278e-01 9.940801e-01 9.943211e-01 9.923682e-01 9.869004e-01 6.483406e-01

4085 9.868898e-01 9.940997e-01 9.943200e-01 9.923249e-01 9.867881e-01 6.478113e-01

4086 9.869546e-01 9.941200e-01 9.943185e-01 9.922787e-01 9.866691e-01 6.472856e-01

4087 9.870224e-01 9.941409e-01 9.943165e-01 9.922296e-01 9.865429e-01 6.467637e-01

4088 9.870934e-01 9.941626e-01 9.943141e-01 9.921772e-01 9.864091e-01 6.462459e-01

4089 9.871677e-01 9.941851e-01 9.943111e-01 9.921215e-01 9.862673e-01 6.457325e-01

4090 9.872453e-01 9.942083e-01 9.943076e-01 9.920622e-01 9.861172e-01 6.452235e-01

4091 9.873266e-01 9.942323e-01 9.943034e-01 9.919992e-01 9.859582e-01 6.447192e-01

4092 9.874116e-01 9.942572e-01 9.942986e-01 9.919321e-01 9.857900e-01 6.442199e-01

4093 9.875006e-01 9.942830e-01 9.942931e-01 9.918608e-01 9.856120e-01 6.437257e-01

4094 9.875936e-01 9.943096e-01 9.942868e-01 9.917851e-01 9.854238e-01 6.432368e-01

4095 9.876909e-01 9.943371e-01 9.942796e-01 9.917046e-01 9.852248e-01 6.427533e-01

4096 9.877926e-01 9.943656e-01 9.942716e-01 9.916192e-01 9.850147e-01 6.422755e-01

4097 9.878990e-01 9.943951e-01 9.942625e-01 9.915285e-01 9.847927e-01 6.418035e-01

4098 9.880102e-01 9.944256e-01 9.942525e-01 9.914323e-01 9.845584e-01 6.413375e-01

4099 9.881265e-01 9.944571e-01 9.942413e-01 9.913303e-01 9.843112e-01 6.408775e-01

4100 9.882480e-01 9.944896e-01 9.942290e-01 9.912221e-01 9.840505e-01 6.404238e-01

4101 9.883750e-01 9.945233e-01 9.942153e-01 9.911075e-01 9.837757e-01 6.399764e-01

4102 9.885077e-01 9.945581e-01 9.942003e-01 9.909861e-01 9.834863e-01 6.395355e-01

4103 9.886464e-01 9.945941e-01 9.941839e-01 9.908575e-01 9.831816e-01 6.391011e-01

4104 9.887912e-01 9.946312e-01 9.941659e-01 9.907215e-01 9.828609e-01 6.386734e-01

4105 9.889425e-01 9.946696e-01 9.941462e-01 9.905775e-01 9.825237e-01 6.382524e-01

4106 9.891004e-01 9.947093e-01 9.941247e-01 9.904254e-01 9.821693e-01 6.378382e-01

4107 9.892652e-01 9.947502e-01 9.941014e-01 9.902646e-01 9.817971e-01 6.374309e-01

4108 9.894372e-01 9.947925e-01 9.940761e-01 9.900947e-01 9.814063e-01 6.370306e-01

4109 9.896166e-01 9.948362e-01 9.940487e-01 9.899154e-01 9.809964e-01 6.366372e-01

4110 9.898038e-01 9.948812e-01 9.940190e-01 9.897261e-01 9.805667e-01 6.362508e-01

4111 9.899989e-01 9.949277e-01 9.939869e-01 9.895265e-01 9.801166e-01 6.358714e-01

4112 9.902023e-01 9.949757e-01 9.939523e-01 9.893161e-01 9.796453e-01 6.354992e-01

4113 9.904143e-01 9.950251e-01 9.939150e-01 9.890945e-01 9.791524e-01 6.351339e-01

4114 9.906350e-01 9.950761e-01 9.938749e-01 9.888611e-01 9.786371e-01 6.347758e-01

4115 9.908649e-01 9.951287e-01 9.938318e-01 9.886156e-01 9.780988e-01 6.344247e-01

4116 9.911043e-01 9.951829e-01 9.937855e-01 9.883573e-01 9.775371e-01 6.340807e-01

4117 9.913533e-01 9.952387e-01 9.937359e-01 9.880859e-01 9.769513e-01 6.337438e-01

4118 9.919783e-01 9.955174e-01 9.939988e-01 9.884226e-01 9.775086e-01 6.379091e-01

4119 9.919782e-01 9.955175e-01 9.939989e-01 9.884226e-01 9.775086e-01 6.378236e-01

4120 9.919782e-01 9.955175e-01 9.939989e-01 9.884225e-01 9.775086e-01 6.377359e-01

4121 9.919782e-01 9.955176e-01 9.939989e-01 9.884225e-01 9.775086e-01 6.376460e-01

4122 9.919782e-01 9.955176e-01 9.939989e-01 9.884224e-01 9.775086e-01 6.375538e-01

4123 9.919782e-01 9.955177e-01 9.939989e-01 9.884224e-01 9.775086e-01 6.374594e-01

4124 9.919783e-01 9.955178e-01 9.939989e-01 9.884224e-01 9.775086e-01 6.373627e-01

4125 9.919784e-01 9.955179e-01 9.939988e-01 9.884224e-01 9.775086e-01 6.372636e-01

4126 9.919785e-01 9.955179e-01 9.939988e-01 9.884224e-01 9.775086e-01 6.371622e-01

4127 9.919787e-01 9.955180e-01 9.939988e-01 9.884223e-01 9.775086e-01 6.370583e-01

4128 9.919789e-01 9.955181e-01 9.939988e-01 9.884223e-01 9.775086e-01 6.369519e-01

4129 9.919791e-01 9.955182e-01 9.939987e-01 9.884223e-01 9.775085e-01 6.368431e-01

4130 9.919793e-01 9.955182e-01 9.939987e-01 9.884223e-01 9.775085e-01 6.367317e-01

4131 9.919796e-01 9.955183e-01 9.939987e-01 9.884223e-01 9.775085e-01 6.366178e-01

4132 9.919799e-01 9.955184e-01 9.939986e-01 9.884223e-01 9.775084e-01 6.365012e-01

4133 9.919801e-01 9.955184e-01 9.939986e-01 9.884223e-01 9.775084e-01 6.363819e-01

4134 9.919804e-01 9.955185e-01 9.939986e-01 9.884223e-01 9.775083e-01 6.362599e-01

4135 9.919807e-01 9.955186e-01 9.939986e-01 9.884223e-01 9.775082e-01 6.361352e-01

4136 9.919810e-01 9.955186e-01 9.939985e-01 9.884223e-01 9.775081e-01 6.360077e-01

4137 9.919813e-01 9.955187e-01 9.939985e-01 9.884223e-01 9.775080e-01 6.358773e-01

4138 9.919817e-01 9.955188e-01 9.939985e-01 9.884223e-01 9.775079e-01 6.357441e-01

4139 9.919820e-01 9.955188e-01 9.939985e-01 9.884223e-01 9.775078e-01 6.356079e-01

4140 9.919823e-01 9.955189e-01 9.939985e-01 9.884222e-01 9.775077e-01 6.354687e-01

4141 9.919826e-01 9.955189e-01 9.939985e-01 9.884222e-01 9.775076e-01 6.353265e-01

4142 9.919830e-01 9.955190e-01 9.939985e-01 9.884222e-01 9.775075e-01 6.351812e-01

4143 9.919833e-01 9.955191e-01 9.939986e-01 9.884222e-01 9.775074e-01 6.350329e-01

4144 9.919837e-01 9.955191e-01 9.939986e-01 9.884221e-01 9.775072e-01 6.348813e-01

4145 9.919840e-01 9.955192e-01 9.939986e-01 9.884221e-01 9.775071e-01 6.347266e-01

4146 9.919843e-01 9.955193e-01 9.939987e-01 9.884220e-01 9.775070e-01 6.345686e-01

4147 9.919847e-01 9.955194e-01 9.939987e-01 9.884220e-01 9.775068e-01 6.344073e-01

4148 9.919850e-01 9.955195e-01 9.939987e-01 9.884219e-01 9.775066e-01 6.342426e-01

4149 9.919854e-01 9.955196e-01 9.939988e-01 9.884219e-01 9.775065e-01 6.340746e-01

4150 9.919858e-01 9.955196e-01 9.939988e-01 9.884218e-01 9.775063e-01 6.339032e-01

4151 9.919862e-01 9.955197e-01 9.939989e-01 9.884217e-01 9.775061e-01 6.337282e-01

4152 9.919866e-01 9.955199e-01 9.939989e-01 9.884216e-01 9.775059e-01 6.335497e-01

4153 9.919870e-01 9.955200e-01 9.939990e-01 9.884215e-01 9.775057e-01 6.333677e-01

4154 9.919874e-01 9.955201e-01 9.939990e-01 9.884214e-01 9.775055e-01 6.331821e-01

4155 9.919878e-01 9.955202e-01 9.939991e-01 9.884213e-01 9.775052e-01 6.329928e-01

4156 9.919883e-01 9.955203e-01 9.939991e-01 9.884212e-01 9.775050e-01 6.327998e-01

4157 9.919887e-01 9.955205e-01 9.939992e-01 9.884211e-01 9.775047e-01 6.326030e-01

4158 9.919892e-01 9.955206e-01 9.939992e-01 9.884210e-01 9.775044e-01 6.324025e-01

4159 9.919897e-01 9.955207e-01 9.939992e-01 9.884208e-01 9.775041e-01 6.321981e-01

4160 9.919903e-01 9.955209e-01 9.939993e-01 9.884207e-01 9.775037e-01 6.319899e-01

4161 9.919908e-01 9.955210e-01 9.939993e-01 9.884205e-01 9.775034e-01 6.317778e-01

4162 9.919914e-01 9.955212e-01 9.939994e-01 9.884203e-01 9.775030e-01 6.315617e-01

4163 9.919920e-01 9.955214e-01 9.939994e-01 9.884202e-01 9.775025e-01 6.313417e-01

4164 9.919927e-01 9.955215e-01 9.939994e-01 9.884200e-01 9.775021e-01 6.311176e-01

4165 9.919933e-01 9.955217e-01 9.939994e-01 9.884198e-01 9.775016e-01 6.308895e-01

4166 9.919940e-01 9.955219e-01 9.939995e-01 9.884195e-01 9.775011e-01 6.306573e-01

4167 9.919948e-01 9.955221e-01 9.939995e-01 9.884193e-01 9.775005e-01 6.304209e-01

4168 9.919955e-01 9.955223e-01 9.939995e-01 9.884190e-01 9.774999e-01 6.301804e-01

4169 9.919963e-01 9.955225e-01 9.939995e-01 9.884188e-01 9.774992e-01 6.299357e-01

4170 9.919971e-01 9.955227e-01 9.939995e-01 9.884185e-01 9.774985e-01 6.296868e-01

4171 9.919980e-01 9.955229e-01 9.939995e-01 9.884181e-01 9.774978e-01 6.294337e-01

4172 9.919989e-01 9.955231e-01 9.939995e-01 9.884178e-01 9.774970e-01 6.291763e-01

4173 9.919999e-01 9.955234e-01 9.939995e-01 9.884174e-01 9.774961e-01 6.289145e-01

4174 9.920008e-01 9.955236e-01 9.939995e-01 9.884170e-01 9.774951e-01 6.286485e-01

4175 9.920019e-01 9.955238e-01 9.939995e-01 9.884166e-01 9.774941e-01 6.283781e-01

4176 9.920029e-01 9.955241e-01 9.939995e-01 9.884161e-01 9.774931e-01 6.281034e-01

4177 9.920040e-01 9.955243e-01 9.939994e-01 9.884156e-01 9.774919e-01 6.278243e-01

4178 9.920052e-01 9.955246e-01 9.939994e-01 9.884150e-01 9.774907e-01 6.275408e-01

4179 9.920064e-01 9.955249e-01 9.939994e-01 9.884144e-01 9.774894e-01 6.272529e-01

4180 9.920076e-01 9.955252e-01 9.939993e-01 9.884138e-01 9.774880e-01 6.269607e-01

4181 9.920089e-01 9.955255e-01 9.939993e-01 9.884131e-01 9.774864e-01 6.266640e-01

4182 9.920102e-01 9.955258e-01 9.939992e-01 9.884124e-01 9.774848e-01 6.263629e-01

4183 9.920116e-01 9.955261e-01 9.939992e-01 9.884116e-01 9.774831e-01 6.260573e-01

4184 9.920131e-01 9.955264e-01 9.939991e-01 9.884107e-01 9.774812e-01 6.257474e-01

4185 9.920146e-01 9.955267e-01 9.939990e-01 9.884098e-01 9.774793e-01 6.254331e-01

4186 9.920161e-01 9.955270e-01 9.939989e-01 9.884088e-01 9.774771e-01 6.251143e-01

4187 9.920178e-01 9.955274e-01 9.939988e-01 9.884078e-01 9.774749e-01 6.247912e-01

4188 9.920195e-01 9.955277e-01 9.939987e-01 9.884066e-01 9.774724e-01 6.244638e-01

4189 9.920212e-01 9.955281e-01 9.939985e-01 9.884054e-01 9.774698e-01 6.241320e-01

4190 9.920230e-01 9.955285e-01 9.939984e-01 9.884041e-01 9.774670e-01 6.237958e-01

4191 9.920249e-01 9.955289e-01 9.939982e-01 9.884026e-01 9.774640e-01 6.234554e-01

4192 9.920269e-01 9.955293e-01 9.939980e-01 9.884011e-01 9.774608e-01 6.231107e-01

4193 9.920289e-01 9.955297e-01 9.939977e-01 9.883995e-01 9.774574e-01 6.227618e-01

4194 9.920310e-01 9.955301e-01 9.939975e-01 9.883977e-01 9.774538e-01 6.224087e-01

4195 9.920332e-01 9.955306e-01 9.939972e-01 9.883958e-01 9.774498e-01 6.220515e-01

4196 9.920355e-01 9.955310e-01 9.939969e-01 9.883938e-01 9.774456e-01 6.216902e-01

4197 9.920379e-01 9.955315e-01 9.939965e-01 9.883916e-01 9.774412e-01 6.213249e-01

4198 9.920404e-01 9.955320e-01 9.939961e-01 9.883893e-01 9.774364e-01 6.209556e-01

4199 9.920430e-01 9.955324e-01 9.939957e-01 9.883868e-01 9.774312e-01 6.205824e-01

4200 9.920456e-01 9.955329e-01 9.939952e-01 9.883841e-01 9.774257e-01 6.202053e-01

4201 9.920484e-01 9.955335e-01 9.939947e-01 9.883813e-01 9.774199e-01 6.198245e-01

4202 9.920513e-01 9.955340e-01 9.939941e-01 9.883782e-01 9.774136e-01 6.194400e-01

4203 9.920543e-01 9.955345e-01 9.939935e-01 9.883749e-01 9.774069e-01 6.190519e-01

4204 9.920574e-01 9.955351e-01 9.939928e-01 9.883714e-01 9.773997e-01 6.186603e-01

4205 9.920607e-01 9.955356e-01 9.939921e-01 9.883676e-01 9.773920e-01 6.182653e-01

4206 9.920640e-01 9.955362e-01 9.939913e-01 9.883635e-01 9.773838e-01 6.178670e-01

4207 9.920675e-01 9.955368e-01 9.939904e-01 9.883592e-01 9.773750e-01 6.174654e-01

4208 9.920712e-01 9.955374e-01 9.939894e-01 9.883545e-01 9.773656e-01 6.170608e-01

4209 9.920750e-01 9.955380e-01 9.939884e-01 9.883495e-01 9.773556e-01 6.166531e-01

4210 9.920789e-01 9.955386e-01 9.939873e-01 9.883442e-01 9.773449e-01 6.162426e-01

4211 9.920830e-01 9.955392e-01 9.939861e-01 9.883385e-01 9.773335e-01 6.158294e-01

4212 9.920872e-01 9.955399e-01 9.939847e-01 9.883324e-01 9.773213e-01 6.154135e-01

4213 9.920917e-01 9.955405e-01 9.939833e-01 9.883259e-01 9.773083e-01 6.149952e-01

4214 9.920962e-01 9.955412e-01 9.939818e-01 9.883189e-01 9.772943e-01 6.145745e-01

4215 9.921010e-01 9.955419e-01 9.939801e-01 9.883114e-01 9.772795e-01 6.141517e-01

4216 9.921060e-01 9.955425e-01 9.939783e-01 9.883034e-01 9.772636e-01 6.137268e-01

4217 9.921111e-01 9.955432e-01 9.939763e-01 9.882948e-01 9.772466e-01 6.133000e-01

4218 9.921165e-01 9.955439e-01 9.939742e-01 9.882857e-01 9.772286e-01 6.128715e-01

4219 9.921221e-01 9.955446e-01 9.939719e-01 9.882759e-01 9.772093e-01 6.124415e-01

4220 9.921279e-01 9.955453e-01 9.939695e-01 9.882654e-01 9.771887e-01 6.120101e-01

4221 9.921339e-01 9.955460e-01 9.939668e-01 9.882542e-01 9.771667e-01 6.115774e-01

4222 9.921402e-01 9.955467e-01 9.939640e-01 9.882423e-01 9.771432e-01 6.111437e-01

4223 9.921467e-01 9.955474e-01 9.939609e-01 9.882295e-01 9.771182e-01 6.107092e-01

4224 9.921535e-01 9.955481e-01 9.939576e-01 9.882159e-01 9.770915e-01 6.102740e-01

4225 9.921605e-01 9.955487e-01 9.939540e-01 9.882013e-01 9.770630e-01 6.098383e-01

4226 9.921678e-01 9.955494e-01 9.939502e-01 9.881858e-01 9.770327e-01 6.094023e-01

4227 9.921755e-01 9.955501e-01 9.939461e-01 9.881692e-01 9.770003e-01 6.089662e-01

4228 9.921834e-01 9.955507e-01 9.939416e-01 9.881515e-01 9.769658e-01 6.085301e-01

4229 9.921916e-01 9.955514e-01 9.939369e-01 9.881326e-01 9.769290e-01 6.080944e-01

4230 9.922002e-01 9.955520e-01 9.939318e-01 9.881124e-01 9.768898e-01 6.076591e-01

4231 9.922091e-01 9.955526e-01 9.939263e-01 9.880909e-01 9.768480e-01 6.072246e-01

4232 9.922184e-01 9.955531e-01 9.939204e-01 9.880679e-01 9.768035e-01 6.067909e-01

4233 9.922280e-01 9.955537e-01 9.939141e-01 9.880434e-01 9.767561e-01 6.063583e-01

4234 9.922381e-01 9.955542e-01 9.939073e-01 9.880172e-01 9.767055e-01 6.059269e-01

4235 9.922485e-01 9.955546e-01 9.939000e-01 9.879893e-01 9.766517e-01 6.054971e-01

4236 9.922594e-01 9.955550e-01 9.938923e-01 9.879596e-01 9.765944e-01 6.050689e-01

4237 9.922707e-01 9.955554e-01 9.938839e-01 9.879279e-01 9.765333e-01 6.046427e-01

4238 9.922824e-01 9.955556e-01 9.938749e-01 9.878941e-01 9.764683e-01 6.042185e-01

4239 9.922947e-01 9.955559e-01 9.938654e-01 9.878581e-01 9.763992e-01 6.037966e-01

4240 9.923074e-01 9.955560e-01 9.938551e-01 9.878197e-01 9.763255e-01 6.033772e-01

4241 9.923207e-01 9.955561e-01 9.938441e-01 9.877789e-01 9.762472e-01 6.029604e-01

4242 9.923345e-01 9.955561e-01 9.938323e-01 9.877353e-01 9.761639e-01 6.025465e-01

4243 9.923489e-01 9.955559e-01 9.938197e-01 9.876890e-01 9.760752e-01 6.021357e-01

4244 9.923638e-01 9.955557e-01 9.938063e-01 9.876396e-01 9.759809e-01 6.017281e-01

4245 9.923794e-01 9.955553e-01 9.937919e-01 9.875870e-01 9.758807e-01 6.013240e-01

4246 9.923956e-01 9.955548e-01 9.937764e-01 9.875310e-01 9.757741e-01 6.009234e-01

4247 9.924125e-01 9.955542e-01 9.937600e-01 9.874715e-01 9.756608e-01 6.005265e-01

4248 9.924301e-01 9.955534e-01 9.937424e-01 9.874081e-01 9.755404e-01 6.001337e-01

4249 9.924484e-01 9.955524e-01 9.937236e-01 9.873407e-01 9.754126e-01 5.997449e-01

4250 9.924674e-01 9.955512e-01 9.937035e-01 9.872690e-01 9.752768e-01 5.993603e-01

4251 9.924873e-01 9.955499e-01 9.936820e-01 9.871927e-01 9.751326e-01 5.989802e-01

4252 9.925080e-01 9.955483e-01 9.936591e-01 9.871117e-01 9.749795e-01 5.986046e-01

4253 9.925295e-01 9.955464e-01 9.936346e-01 9.870255e-01 9.748171e-01 5.982337e-01

4254 9.925520e-01 9.955443e-01 9.936085e-01 9.869339e-01 9.746448e-01 5.978676e-01

4255 9.925754e-01 9.955419e-01 9.935807e-01 9.868367e-01 9.744621e-01 5.975065e-01

4256 9.925998e-01 9.955392e-01 9.935510e-01 9.867333e-01 9.742683e-01 5.971504e-01

4257 9.926252e-01 9.955362e-01 9.935193e-01 9.866236e-01 9.740630e-01 5.967996e-01

4258 9.926516e-01 9.955328e-01 9.934855e-01 9.865071e-01 9.738454e-01 5.964540e-01

4259 9.926792e-01 9.955290e-01 9.934495e-01 9.863835e-01 9.736150e-01 5.961137e-01

4260 9.927080e-01 9.955248e-01 9.934111e-01 9.862523e-01 9.733710e-01 5.957790e-01

4261 9.927380e-01 9.955201e-01 9.933703e-01 9.861131e-01 9.731128e-01 5.954498e-01

4262 9.927692e-01 9.955150e-01 9.933267e-01 9.859655e-01 9.728397e-01 5.951263e-01

4263 9.928018e-01 9.955093e-01 9.932804e-01 9.858091e-01 9.725509e-01 5.948084e-01

4264 9.928357e-01 9.955031e-01 9.932310e-01 9.856433e-01 9.722456e-01 5.944963e-01

4265 9.928711e-01 9.954963e-01 9.931785e-01 9.854677e-01 9.719232e-01 5.941901e-01

4266 9.929080e-01 9.954888e-01 9.931227e-01 9.852817e-01 9.715827e-01 5.938897e-01

4267 9.929464e-01 9.954807e-01 9.930633e-01 9.850847e-01 9.712233e-01 5.935952e-01

4268 9.929865e-01 9.954718e-01 9.930001e-01 9.848763e-01 9.708443e-01 5.933066e-01

4269 9.930283e-01 9.954621e-01 9.929330e-01 9.846558e-01 9.704447e-01 5.930240e-01

4270 9.930719e-01 9.954516e-01 9.928617e-01 9.844227e-01 9.700237e-01 5.927474e-01

4271 9.931174e-01 9.954402e-01 9.927859e-01 9.841762e-01 9.695804e-01 5.924767e-01

4272 9.931648e-01 9.954278e-01 9.927054e-01 9.839158e-01 9.691139e-01 5.922121e-01

4273 9.932142e-01 9.954144e-01 9.926200e-01 9.836408e-01 9.686233e-01 5.919534e-01

4274 9.932657e-01 9.953999e-01 9.925294e-01 9.833504e-01 9.681078e-01 5.917007e-01

4275 9.933194e-01 9.953842e-01 9.924332e-01 9.830441e-01 9.675664e-01 5.914540e-01

4276 9.933754e-01 9.953673e-01 9.923312e-01 9.827209e-01 9.669982e-01 5.912132e-01

4277 9.934337e-01 9.953491e-01 9.922231e-01 9.823803e-01 9.664024e-01 5.909783e-01

4278 9.934946e-01 9.953295e-01 9.921086e-01 9.820214e-01 9.657782e-01 5.907493e-01

4279 9.935580e-01 9.953084e-01 9.919873e-01 9.816435e-01 9.651246e-01 5.905262e-01

4280 9.936242e-01 9.952857e-01 9.918588e-01 9.812457e-01 9.644411e-01 5.903088e-01

4281 9.936931e-01 9.952613e-01 9.917228e-01 9.808273e-01 9.637266e-01 5.900972e-01

4282 9.937649e-01 9.952351e-01 9.915790e-01 9.803874e-01 9.629807e-01 5.898912e-01

4283 9.938398e-01 9.952070e-01 9.914269e-01 9.799252e-01 9.622025e-01 5.896909e-01

4284 9.939178e-01 9.951769e-01 9.912661e-01 9.794399e-01 9.613916e-01 5.894962e-01

4285 9.939991e-01 9.951447e-01 9.910962e-01 9.789307e-01 9.605473e-01 5.893069e-01

4286 9.940837e-01 9.951102e-01 9.909168e-01 9.783967e-01 9.596693e-01 5.891231e-01

4287 9.941719e-01 9.950734e-01 9.907274e-01 9.778371e-01 9.587571e-01 5.889447e-01

4288 9.942638e-01 9.950340e-01 9.905276e-01 9.772511e-01 9.578104e-01 5.887715e-01

4289 9.943594e-01 9.949920e-01 9.903168e-01 9.766379e-01 9.568291e-01 5.886036e-01

4290 9.944590e-01 9.949471e-01 9.900947e-01 9.759968e-01 9.558132e-01 5.884407e-01

4291 9.945627e-01 9.948993e-01 9.898608e-01 9.753268e-01 9.547625e-01 5.882829e-01

4292 9.946706e-01 9.948484e-01 9.896144e-01 9.746274e-01 9.536773e-01 5.881301e-01

4293 9.947829e-01 9.947942e-01 9.893552e-01 9.738978e-01 9.525578e-01 5.879821e-01

4294 9.948997e-01 9.947366e-01 9.890826e-01 9.731373e-01 9.514044e-01 5.878389e-01

4295 9.950212e-01 9.946753e-01 9.887960e-01 9.723453e-01 9.502176e-01 5.877004e-01

4296 9.951475e-01 9.946102e-01 9.884950e-01 9.715213e-01 9.489981e-01 5.875664e-01

4297 9.954978e-01 9.948211e-01 9.887958e-01 9.721139e-01 9.501156e-01 5.920095e-01

4298 9.954979e-01 9.948212e-01 9.887958e-01 9.721139e-01 9.501155e-01 5.919689e-01

4299 9.954980e-01 9.948212e-01 9.887958e-01 9.721138e-01 9.501155e-01 5.919277e-01

4300 9.954981e-01 9.948212e-01 9.887958e-01 9.721138e-01 9.501154e-01 5.918858e-01

4301 9.954982e-01 9.948212e-01 9.887958e-01 9.721138e-01 9.501153e-01 5.918433e-01

4302 9.954983e-01 9.948212e-01 9.887957e-01 9.721137e-01 9.501152e-01 5.918001e-01

4303 9.954984e-01 9.948212e-01 9.887957e-01 9.721137e-01 9.501151e-01 5.917562e-01

4304 9.954985e-01 9.948212e-01 9.887957e-01 9.721136e-01 9.501150e-01 5.917116e-01

4305 9.954986e-01 9.948212e-01 9.887957e-01 9.721136e-01 9.501149e-01 5.916664e-01

4306 9.954987e-01 9.948212e-01 9.887957e-01 9.721135e-01 9.501148e-01 5.916205e-01

4307 9.954988e-01 9.948212e-01 9.887957e-01 9.721134e-01 9.501147e-01 5.915739e-01

4308 9.954989e-01 9.948211e-01 9.887957e-01 9.721133e-01 9.501146e-01 5.915266e-01

4309 9.954990e-01 9.948211e-01 9.887957e-01 9.721133e-01 9.501145e-01 5.914786e-01

4310 9.954991e-01 9.948211e-01 9.887957e-01 9.721132e-01 9.501144e-01 5.914300e-01

4311 9.954992e-01 9.948211e-01 9.887957e-01 9.721131e-01 9.501143e-01 5.913807e-01

4312 9.954993e-01 9.948211e-01 9.887957e-01 9.721130e-01 9.501142e-01 5.913307e-01

4313 9.954994e-01 9.948210e-01 9.887956e-01 9.721129e-01 9.501140e-01 5.912800e-01

4314 9.954994e-01 9.948210e-01 9.887956e-01 9.721128e-01 9.501139e-01 5.912287e-01

4315 9.954995e-01 9.948210e-01 9.887956e-01 9.721127e-01 9.501138e-01 5.911767e-01

4316 9.954996e-01 9.948210e-01 9.887956e-01 9.721126e-01 9.501136e-01 5.911240e-01

4317 9.954997e-01 9.948210e-01 9.887956e-01 9.721124e-01 9.501135e-01 5.910706e-01

4318 9.954998e-01 9.948210e-01 9.887955e-01 9.721123e-01 9.501133e-01 5.910166e-01

4319 9.954999e-01 9.948210e-01 9.887955e-01 9.721122e-01 9.501132e-01 5.909620e-01

4320 9.955000e-01 9.948210e-01 9.887955e-01 9.721121e-01 9.501130e-01 5.909067e-01

4321 9.955001e-01 9.948210e-01 9.887954e-01 9.721119e-01 9.501128e-01 5.908508e-01

4322 9.955003e-01 9.948210e-01 9.887954e-01 9.721118e-01 9.501127e-01 5.907942e-01

4323 9.955004e-01 9.948210e-01 9.887953e-01 9.721116e-01 9.501125e-01 5.907370e-01

4324 9.955005e-01 9.948210e-01 9.887953e-01 9.721114e-01 9.501122e-01 5.906793e-01

4325 9.955006e-01 9.948210e-01 9.887952e-01 9.721113e-01 9.501120e-01 5.906209e-01

4326 9.955008e-01 9.948210e-01 9.887951e-01 9.721111e-01 9.501117e-01 5.905619e-01

4327 9.955009e-01 9.948210e-01 9.887951e-01 9.721109e-01 9.501115e-01 5.905024e-01

4328 9.955010e-01 9.948211e-01 9.887950e-01 9.721106e-01 9.501112e-01 5.904424e-01

4329 9.955012e-01 9.948211e-01 9.887949e-01 9.721104e-01 9.501109e-01 5.903818e-01

4330 9.955013e-01 9.948211e-01 9.887948e-01 9.721102e-01 9.501105e-01 5.903207e-01

4331 9.955015e-01 9.948211e-01 9.887947e-01 9.721099e-01 9.501102e-01 5.902591e-01

4332 9.955017e-01 9.948211e-01 9.887946e-01 9.721096e-01 9.501098e-01 5.901970e-01

4333 9.955018e-01 9.948212e-01 9.887945e-01 9.721093e-01 9.501094e-01 5.901345e-01

4334 9.955020e-01 9.948212e-01 9.887944e-01 9.721089e-01 9.501090e-01 5.900716e-01

4335 9.955022e-01 9.948212e-01 9.887943e-01 9.721086e-01 9.501085e-01 5.900082e-01

4336 9.955024e-01 9.948212e-01 9.887942e-01 9.721082e-01 9.501080e-01 5.899445e-01

4337 9.955026e-01 9.948212e-01 9.887940e-01 9.721078e-01 9.501075e-01 5.898805e-01

4338 9.955028e-01 9.948213e-01 9.887939e-01 9.721073e-01 9.501069e-01 5.898161e-01

4339 9.955030e-01 9.948213e-01 9.887937e-01 9.721068e-01 9.501063e-01 5.897515e-01

4340 9.955033e-01 9.948213e-01 9.887936e-01 9.721063e-01 9.501056e-01 5.896866e-01

4341 9.955035e-01 9.948213e-01 9.887934e-01 9.721058e-01 9.501049e-01 5.896215e-01

4342 9.955037e-01 9.948213e-01 9.887932e-01 9.721052e-01 9.501042e-01 5.895562e-01

4343 9.955040e-01 9.948213e-01 9.887930e-01 9.721045e-01 9.501034e-01 5.894908e-01

4344 9.955042e-01 9.948213e-01 9.887928e-01 9.721038e-01 9.501026e-01 5.894253e-01

4345 9.955045e-01 9.948213e-01 9.887925e-01 9.721031e-01 9.501017e-01 5.893598e-01

4346 9.955048e-01 9.948213e-01 9.887923e-01 9.721023e-01 9.501007e-01 5.892942e-01

4347 9.955051e-01 9.948213e-01 9.887920e-01 9.721015e-01 9.500997e-01 5.892287e-01

4348 9.955054e-01 9.948213e-01 9.887917e-01 9.721006e-01 9.500986e-01 5.891632e-01

4349 9.955057e-01 9.948213e-01 9.887914e-01 9.720996e-01 9.500975e-01 5.890979e-01

4350 9.955060e-01 9.948212e-01 9.887910e-01 9.720986e-01 9.500963e-01 5.890328e-01

4351 9.955064e-01 9.948212e-01 9.887906e-01 9.720975e-01 9.500950e-01 5.889678e-01

4352 9.955067e-01 9.948212e-01 9.887902e-01 9.720963e-01 9.500936e-01 5.889032e-01

4353 9.955071e-01 9.948212e-01 9.887898e-01 9.720950e-01 9.500921e-01 5.888389e-01

4354 9.955075e-01 9.948211e-01 9.887893e-01 9.720937e-01 9.500905e-01 5.887750e-01

4355 9.955079e-01 9.948211e-01 9.887888e-01 9.720922e-01 9.500888e-01 5.887116e-01

4356 9.955083e-01 9.948210e-01 9.887883e-01 9.720907e-01 9.500870e-01 5.886486e-01

4357 9.955087e-01 9.948210e-01 9.887877e-01 9.720890e-01 9.500851e-01 5.885863e-01

4358 9.955091e-01 9.948209e-01 9.887871e-01 9.720872e-01 9.500830e-01 5.885246e-01

4359 9.955096e-01 9.948208e-01 9.887864e-01 9.720853e-01 9.500808e-01 5.884635e-01

4360 9.955101e-01 9.948207e-01 9.887857e-01 9.720833e-01 9.500785e-01 5.884033e-01

4361 9.955105e-01 9.948206e-01 9.887849e-01 9.720811e-01 9.500759e-01 5.883438e-01

4362 9.955111e-01 9.948205e-01 9.887840e-01 9.720788e-01 9.500733e-01 5.882853e-01

4363 9.955116e-01 9.948204e-01 9.887831e-01 9.720763e-01 9.500704e-01 5.882277e-01

4364 9.955121e-01 9.948203e-01 9.887821e-01 9.720736e-01 9.500673e-01 5.881712e-01

4365 9.955127e-01 9.948201e-01 9.887811e-01 9.720708e-01 9.500641e-01 5.881158e-01

4366 9.955133e-01 9.948200e-01 9.887800e-01 9.720677e-01 9.500606e-01 5.880616e-01

4367 9.955139e-01 9.948198e-01 9.887787e-01 9.720644e-01 9.500569e-01 5.880086e-01

4368 9.955145e-01 9.948196e-01 9.887774e-01 9.720609e-01 9.500529e-01 5.879570e-01

4369 9.955152e-01 9.948194e-01 9.887760e-01 9.720572e-01 9.500486e-01 5.879067e-01

4370 9.955159e-01 9.948191e-01 9.887745e-01 9.720532e-01 9.500441e-01 5.878580e-01

4371 9.955166e-01 9.948189e-01 9.887729e-01 9.720488e-01 9.500392e-01 5.878108e-01

4372 9.955173e-01 9.948186e-01 9.887712e-01 9.720442e-01 9.500341e-01 5.877652e-01

4373 9.955181e-01 9.948183e-01 9.887693e-01 9.720393e-01 9.500285e-01 5.877214e-01

4374 9.955189e-01 9.948179e-01 9.887673e-01 9.720341e-01 9.500226e-01 5.876793e-01

4375 9.955197e-01 9.948175e-01 9.887652e-01 9.720284e-01 9.500163e-01 5.876391e-01

4376 9.955206e-01 9.948171e-01 9.887629e-01 9.720224e-01 9.500096e-01 5.876009e-01

4377 9.955215e-01 9.948167e-01 9.887604e-01 9.720159e-01 9.500024e-01 5.875647e-01

4378 9.955224e-01 9.948162e-01 9.887577e-01 9.720091e-01 9.499947e-01 5.875306e-01

4379 9.955233e-01 9.948156e-01 9.887549e-01 9.720017e-01 9.499865e-01 5.874986e-01

4380 9.955243e-01 9.948150e-01 9.887518e-01 9.719938e-01 9.499777e-01 5.874690e-01

4381 9.955254e-01 9.948144e-01 9.887486e-01 9.719854e-01 9.499683e-01 5.874416e-01

4382 9.955264e-01 9.948137e-01 9.887451e-01 9.719764e-01 9.499583e-01 5.874167e-01

4383 9.955275e-01 9.948129e-01 9.887413e-01 9.719667e-01 9.499476e-01 5.873942e-01

4384 9.955287e-01 9.948121e-01 9.887373e-01 9.719565e-01 9.499362e-01 5.873743e-01

4385 9.955299e-01 9.948112e-01 9.887329e-01 9.719455e-01 9.499240e-01 5.873571e-01

4386 9.955311e-01 9.948103e-01 9.887283e-01 9.719337e-01 9.499109e-01 5.873425e-01

4387 9.955324e-01 9.948092e-01 9.887234e-01 9.719212e-01 9.498970e-01 5.873307e-01

4388 9.955337e-01 9.948081e-01 9.887180e-01 9.719078e-01 9.498821e-01 5.873218e-01

4389 9.955351e-01 9.948068e-01 9.887123e-01 9.718934e-01 9.498663e-01 5.873157e-01

4390 9.955365e-01 9.948055e-01 9.887062e-01 9.718781e-01 9.498493e-01 5.873126e-01

4391 9.955380e-01 9.948041e-01 9.886997e-01 9.718618e-01 9.498312e-01 5.873126e-01

4392 9.955395e-01 9.948025e-01 9.886927e-01 9.718444e-01 9.498118e-01 5.873157e-01

4393 9.955411e-01 9.948009e-01 9.886853e-01 9.718257e-01 9.497912e-01 5.873219e-01

4394 9.955428e-01 9.947991e-01 9.886773e-01 9.718058e-01 9.497691e-01 5.873313e-01

4395 9.955445e-01 9.947971e-01 9.886687e-01 9.717846e-01 9.497455e-01 5.873440e-01

4396 9.955463e-01 9.947951e-01 9.886595e-01 9.717620e-01 9.497203e-01 5.873600e-01

4397 9.955481e-01 9.947928e-01 9.886498e-01 9.717378e-01 9.496934e-01 5.873793e-01

4398 9.955500e-01 9.947904e-01 9.886393e-01 9.717120e-01 9.496647e-01 5.874021e-01

4399 9.955520e-01 9.947878e-01 9.886281e-01 9.716844e-01 9.496340e-01 5.874283e-01

4400 9.955540e-01 9.947850e-01 9.886161e-01 9.716550e-01 9.496013e-01 5.874579e-01

4401 9.955561e-01 9.947820e-01 9.886033e-01 9.716237e-01 9.495663e-01 5.874911e-01

4402 9.955583e-01 9.947788e-01 9.885896e-01 9.715903e-01 9.495290e-01 5.875278e-01

4403 9.955606e-01 9.947753e-01 9.885750e-01 9.715546e-01 9.494891e-01 5.875681e-01

4404 9.955630e-01 9.947715e-01 9.885594e-01 9.715165e-01 9.494465e-01 5.876120e-01

4405 9.955654e-01 9.947675e-01 9.885428e-01 9.714760e-01 9.494010e-01 5.876595e-01

4406 9.955679e-01 9.947632e-01 9.885250e-01 9.714327e-01 9.493525e-01 5.877106e-01

4407 9.955706e-01 9.947586e-01 9.885059e-01 9.713866e-01 9.493006e-01 5.877653e-01

4408 9.955733e-01 9.947537e-01 9.884856e-01 9.713375e-01 9.492453e-01 5.878237e-01

4409 9.955761e-01 9.947484e-01 9.884640e-01 9.712851e-01 9.491863e-01 5.878857e-01

4410 9.955790e-01 9.947427e-01 9.884409e-01 9.712292e-01 9.491232e-01 5.879513e-01

4411 9.955820e-01 9.947366e-01 9.884162e-01 9.711697e-01 9.490560e-01 5.880206e-01

4412 9.955851e-01 9.947301e-01 9.883899e-01 9.711064e-01 9.489842e-01 5.880935e-01

4413 9.955883e-01 9.947231e-01 9.883618e-01 9.710388e-01 9.489076e-01 5.881700e-01

4414 9.955917e-01 9.947156e-01 9.883319e-01 9.709669e-01 9.488259e-01 5.882500e-01

4415 9.955951e-01 9.947076e-01 9.882999e-01 9.708903e-01 9.487387e-01 5.883337e-01

4416 9.955987e-01 9.946990e-01 9.882659e-01 9.708087e-01 9.486458e-01 5.884208e-01

4417 9.956024e-01 9.946898e-01 9.882296e-01 9.707218e-01 9.485467e-01 5.885114e-01

4418 9.956062e-01 9.946800e-01 9.881910e-01 9.706293e-01 9.484410e-01 5.886055e-01

4419 9.956101e-01 9.946696e-01 9.881497e-01 9.705309e-01 9.483283e-01 5.887030e-01

4420 9.956142e-01 9.946583e-01 9.881059e-01 9.704261e-01 9.482082e-01 5.888038e-01

4421 9.956184e-01 9.946464e-01 9.880591e-01 9.703146e-01 9.480803e-01 5.889079e-01

4422 9.956228e-01 9.946336e-01 9.880093e-01 9.701960e-01 9.479440e-01 5.890153e-01

4423 9.956273e-01 9.946199e-01 9.879563e-01 9.700698e-01 9.477989e-01 5.891259e-01

4424 9.956320e-01 9.946053e-01 9.878998e-01 9.699357e-01 9.476443e-01 5.892396e-01

4425 9.956368e-01 9.945897e-01 9.878398e-01 9.697930e-01 9.474799e-01 5.893564e-01

4426 9.956417e-01 9.945731e-01 9.877758e-01 9.696414e-01 9.473049e-01 5.894761e-01

4427 9.956468e-01 9.945553e-01 9.877078e-01 9.694802e-01 9.471188e-01 5.895988e-01

4428 9.956521e-01 9.945364e-01 9.876354e-01 9.693090e-01 9.469209e-01 5.897243e-01

4429 9.956576e-01 9.945162e-01 9.875585e-01 9.691270e-01 9.467106e-01 5.898526e-01

4430 9.956632e-01 9.944946e-01 9.874767e-01 9.689339e-01 9.464872e-01 5.899835e-01

4431 9.956690e-01 9.944717e-01 9.873897e-01 9.687287e-01 9.462500e-01 5.901170e-01

4432 9.956750e-01 9.944472e-01 9.872973e-01 9.685110e-01 9.459983e-01 5.902530e-01

4433 9.956812e-01 9.944211e-01 9.871990e-01 9.682801e-01 9.457312e-01 5.903914e-01

4434 9.956876e-01 9.943933e-01 9.870947e-01 9.680350e-01 9.454482e-01 5.905321e-01

4435 9.956941e-01 9.943637e-01 9.869839e-01 9.677753e-01 9.451483e-01 5.906750e-01

4436 9.957009e-01 9.943321e-01 9.868662e-01 9.674999e-01 9.448308e-01 5.908200e-01

4437 9.957079e-01 9.942986e-01 9.867413e-01 9.672081e-01 9.444948e-01 5.909671e-01

4438 9.957151e-01 9.942629e-01 9.866088e-01 9.668991e-01 9.441396e-01 5.911160e-01

4439 9.957224e-01 9.942248e-01 9.864681e-01 9.665720e-01 9.437643e-01 5.912667e-01

4440 9.957301e-01 9.941844e-01 9.863190e-01 9.662259e-01 9.433681e-01 5.914192e-01

4441 9.957379e-01 9.941414e-01 9.861608e-01 9.658598e-01 9.429501e-01 5.915732e-01

4442 9.957459e-01 9.940957e-01 9.859932e-01 9.654728e-01 9.425096e-01 5.917287e-01

4443 9.957542e-01 9.940471e-01 9.858155e-01 9.650640e-01 9.420457e-01 5.918856e-01

4444 9.957627e-01 9.939954e-01 9.856273e-01 9.646322e-01 9.415578e-01 5.920437e-01

4445 9.957715e-01 9.939406e-01 9.854279e-01 9.641766e-01 9.410450e-01 5.922031e-01

4446 9.957805e-01 9.938823e-01 9.852169e-01 9.636961e-01 9.405066e-01 5.923634e-01

4447 9.957897e-01 9.938204e-01 9.849936e-01 9.631896e-01 9.399420e-01 5.925248e-01

4448 9.957992e-01 9.937547e-01 9.847574e-01 9.626561e-01 9.393505e-01 5.926870e-01

4449 9.958089e-01 9.936850e-01 9.845075e-01 9.620947e-01 9.387317e-01 5.928499e-01

4450 9.958189e-01 9.936111e-01 9.842434e-01 9.615042e-01 9.380850e-01 5.930135e-01

4451 9.958292e-01 9.935327e-01 9.839644e-01 9.608837e-01 9.374101e-01 5.931776e-01

4452 9.958397e-01 9.934495e-01 9.836696e-01 9.602321e-01 9.367066e-01 5.933421e-01

4453 9.958504e-01 9.933614e-01 9.833584e-01 9.595486e-01 9.359743e-01 5.935070e-01

4454 9.958615e-01 9.932680e-01 9.830301e-01 9.588321e-01 9.352131e-01 5.936721e-01

4455 9.958727e-01 9.931691e-01 9.826837e-01 9.580819e-01 9.344230e-01 5.938374e-01

4456 9.958843e-01 9.930644e-01 9.823185e-01 9.572970e-01 9.336041e-01 5.940028e-01

4457 9.958961e-01 9.929536e-01 9.819337e-01 9.564767e-01 9.327567e-01 5.941680e-01

4458 9.959082e-01 9.928363e-01 9.815284e-01 9.556203e-01 9.318811e-01 5.943332e-01

4459 9.959205e-01 9.927123e-01 9.811017e-01 9.547272e-01 9.309779e-01 5.944981e-01

4460 9.959331e-01 9.925812e-01 9.806529e-01 9.537968e-01 9.300476e-01 5.946628e-01

4461 9.959459e-01 9.924426e-01 9.801809e-01 9.528289e-01 9.290912e-01 5.948270e-01

4462 9.959590e-01 9.922962e-01 9.796850e-01 9.518230e-01 9.281095e-01 5.949908e-01

4463 9.959724e-01 9.921416e-01 9.791641e-01 9.507789e-01 9.271036e-01 5.951541e-01

4464 9.959860e-01 9.919784e-01 9.786175e-01 9.496967e-01 9.260748e-01 5.953167e-01

4465 9.959999e-01 9.918062e-01 9.780442e-01 9.485763e-01 9.250244e-01 5.954787e-01

4466 9.960139e-01 9.916246e-01 9.774433e-01 9.474181e-01 9.239539e-01 5.956399e-01

4467 9.960283e-01 9.914332e-01 9.768139e-01 9.462224e-01 9.228650e-01 5.958003e-01

4468 9.960428e-01 9.912314e-01 9.761552e-01 9.449897e-01 9.217593e-01 5.959598e-01

4469 9.960575e-01 9.910190e-01 9.754663e-01 9.437208e-01 9.206387e-01 5.961184e-01

4470 9.960725e-01 9.907953e-01 9.747464e-01 9.424165e-01 9.195051e-01 5.962760e-01

4471 9.960876e-01 9.905600e-01 9.739947e-01 9.410778e-01 9.183606e-01 5.964325e-01

4472 9.961029e-01 9.903125e-01 9.732103e-01 9.397061e-01 9.172072e-01 5.965879e-01

4473 9.961184e-01 9.900524e-01 9.723928e-01 9.383026e-01 9.160470e-01 5.967422e-01

4474 9.961341e-01 9.897792e-01 9.715412e-01 9.368689e-01 9.148823e-01 5.968953e-01

4475 9.961498e-01 9.894923e-01 9.706551e-01 9.354067e-01 9.137153e-01 5.970472e-01

4476 9.963589e-01 9.897585e-01 9.712424e-01 9.367951e-01 9.160035e-01 6.017016e-01

4477 9.963589e-01 9.897585e-01 9.712423e-01 9.367950e-01 9.160035e-01 6.017934e-01

4478 9.963590e-01 9.897585e-01 9.712423e-01 9.367949e-01 9.160035e-01 6.018872e-01

4479 9.963590e-01 9.897585e-01 9.712423e-01 9.367949e-01 9.160035e-01 6.019832e-01

4480 9.963591e-01 9.897585e-01 9.712422e-01 9.367948e-01 9.160035e-01 6.020813e-01

4481 9.963591e-01 9.897585e-01 9.712422e-01 9.367947e-01 9.160035e-01 6.021815e-01

4482 9.963592e-01 9.897585e-01 9.712421e-01 9.367946e-01 9.160035e-01 6.022838e-01

4483 9.963592e-01 9.897585e-01 9.712420e-01 9.367945e-01 9.160035e-01 6.023882e-01

4484 9.963592e-01 9.897585e-01 9.712420e-01 9.367945e-01 9.160035e-01 6.024948e-01

4485 9.963593e-01 9.897585e-01 9.712419e-01 9.367944e-01 9.160035e-01 6.026036e-01

4486 9.963593e-01 9.897585e-01 9.712418e-01 9.367943e-01 9.160035e-01 6.027145e-01

4487 9.963593e-01 9.897585e-01 9.712418e-01 9.367942e-01 9.160035e-01 6.028276e-01

4488 9.963593e-01 9.897585e-01 9.712417e-01 9.367941e-01 9.160035e-01 6.029429e-01

4489 9.963594e-01 9.897584e-01 9.712416e-01 9.367939e-01 9.160034e-01 6.030604e-01

4490 9.963594e-01 9.897584e-01 9.712415e-01 9.367938e-01 9.160034e-01 6.031802e-01

4491 9.963594e-01 9.897584e-01 9.712414e-01 9.367937e-01 9.160034e-01 6.033022e-01

4492 9.963594e-01 9.897584e-01 9.712413e-01 9.367936e-01 9.160033e-01 6.034265e-01

4493 9.963594e-01 9.897584e-01 9.712412e-01 9.367934e-01 9.160033e-01 6.035530e-01

4494 9.963594e-01 9.897584e-01 9.712411e-01 9.367933e-01 9.160032e-01 6.036819e-01

4495 9.963594e-01 9.897583e-01 9.712410e-01 9.367932e-01 9.160031e-01 6.038131e-01

4496 9.963594e-01 9.897583e-01 9.712409e-01 9.367930e-01 9.160031e-01 6.039466e-01

4497 9.963594e-01 9.897583e-01 9.712408e-01 9.367928e-01 9.160030e-01 6.040825e-01

4498 9.963594e-01 9.897583e-01 9.712406e-01 9.367927e-01 9.160029e-01 6.042207e-01

4499 9.963595e-01 9.897582e-01 9.712405e-01 9.367925e-01 9.160029e-01 6.043614e-01

4500 9.963595e-01 9.897582e-01 9.712404e-01 9.367923e-01 9.160028e-01 6.045044e-01

4501 9.963595e-01 9.897581e-01 9.712402e-01 9.367921e-01 9.160027e-01 6.046499e-01

4502 9.963595e-01 9.897581e-01 9.712400e-01 9.367918e-01 9.160026e-01 6.047978e-01

4503 9.963595e-01 9.897580e-01 9.712399e-01 9.367916e-01 9.160026e-01 6.049482e-01

4504 9.963595e-01 9.897580e-01 9.712397e-01 9.367913e-01 9.160025e-01 6.051011e-01

4505 9.963595e-01 9.897579e-01 9.712395e-01 9.367911e-01 9.160024e-01 6.052565e-01

4506 9.963596e-01 9.897579e-01 9.712393e-01 9.367908e-01 9.160023e-01 6.054144e-01

4507 9.963596e-01 9.897578e-01 9.712390e-01 9.367905e-01 9.160022e-01 6.055748e-01

4508 9.963596e-01 9.897577e-01 9.712388e-01 9.367901e-01 9.160022e-01 6.057378e-01

4509 9.963597e-01 9.897576e-01 9.712385e-01 9.367898e-01 9.160021e-01 6.059034e-01

4510 9.963597e-01 9.897576e-01 9.712382e-01 9.367894e-01 9.160020e-01 6.060716e-01

4511 9.963597e-01 9.897575e-01 9.712379e-01 9.367890e-01 9.160019e-01 6.062425e-01

4512 9.963598e-01 9.897574e-01 9.712376e-01 9.367886e-01 9.160019e-01 6.064160e-01

4513 9.963598e-01 9.897573e-01 9.712372e-01 9.367881e-01 9.160018e-01 6.065921e-01

4514 9.963599e-01 9.897572e-01 9.712368e-01 9.367877e-01 9.160017e-01 6.067710e-01

4515 9.963600e-01 9.897570e-01 9.712364e-01 9.367872e-01 9.160016e-01 6.069525e-01

4516 9.963600e-01 9.897569e-01 9.712359e-01 9.367866e-01 9.160016e-01 6.071368e-01

4517 9.963601e-01 9.897568e-01 9.712355e-01 9.367860e-01 9.160015e-01 6.073239e-01

4518 9.963602e-01 9.897566e-01 9.712350e-01 9.367854e-01 9.160014e-01 6.075138e-01

4519 9.963602e-01 9.897565e-01 9.712344e-01 9.367848e-01 9.160013e-01 6.077064e-01

4520 9.963603e-01 9.897563e-01 9.712338e-01 9.367841e-01 9.160012e-01 6.079019e-01

4521 9.963604e-01 9.897561e-01 9.712332e-01 9.367834e-01 9.160012e-01 6.081003e-01

4522 9.963605e-01 9.897559e-01 9.712325e-01 9.367826e-01 9.160011e-01 6.083015e-01

4523 9.963605e-01 9.897557e-01 9.712318e-01 9.367818e-01 9.160010e-01 6.085056e-01

4524 9.963606e-01 9.897555e-01 9.712310e-01 9.367809e-01 9.160009e-01 6.087127e-01

4525 9.963607e-01 9.897553e-01 9.712302e-01 9.367800e-01 9.160008e-01 6.089227e-01

4526 9.963608e-01 9.897550e-01 9.712293e-01 9.367790e-01 9.160007e-01 6.091358e-01

4527 9.963609e-01 9.897547e-01 9.712283e-01 9.367779e-01 9.160006e-01 6.093518e-01

4528 9.963610e-01 9.897544e-01 9.712273e-01 9.367768e-01 9.160006e-01 6.095709e-01

4529 9.963611e-01 9.897541e-01 9.712262e-01 9.367756e-01 9.160005e-01 6.097930e-01

4530 9.963612e-01 9.897538e-01 9.712251e-01 9.367743e-01 9.160004e-01 6.100183e-01

4531 9.963613e-01 9.897534e-01 9.712238e-01 9.367730e-01 9.160003e-01 6.102466e-01

4532 9.963614e-01 9.897530e-01 9.712225e-01 9.367715e-01 9.160002e-01 6.104782e-01

4533 9.963615e-01 9.897525e-01 9.712211e-01 9.367700e-01 9.160000e-01 6.107129e-01

4534 9.963616e-01 9.897521e-01 9.712195e-01 9.367684e-01 9.159999e-01 6.109508e-01

4535 9.963617e-01 9.897516e-01 9.712179e-01 9.367667e-01 9.159998e-01 6.111919e-01

4536 9.963618e-01 9.897510e-01 9.712162e-01 9.367648e-01 9.159997e-01 6.114363e-01

4537 9.963619e-01 9.897504e-01 9.712143e-01 9.367628e-01 9.159996e-01 6.116840e-01

4538 9.963621e-01 9.897498e-01 9.712123e-01 9.367608e-01 9.159995e-01 6.119350e-01

4539 9.963622e-01 9.897491e-01 9.712101e-01 9.367585e-01 9.159994e-01 6.121894e-01

4540 9.963623e-01 9.897484e-01 9.712078e-01 9.367562e-01 9.159993e-01 6.124472e-01

4541 9.963624e-01 9.897476e-01 9.712054e-01 9.367536e-01 9.159992e-01 6.127083e-01

4542 9.963625e-01 9.897467e-01 9.712027e-01 9.367509e-01 9.159991e-01 6.129729e-01

4543 9.963627e-01 9.897458e-01 9.711999e-01 9.367481e-01 9.159990e-01 6.132410e-01

4544 9.963628e-01 9.897448e-01 9.711969e-01 9.367450e-01 9.159989e-01 6.135125e-01

4545 9.963629e-01 9.897438e-01 9.711936e-01 9.367418e-01 9.159988e-01 6.137875e-01

4546 9.963630e-01 9.897426e-01 9.711902e-01 9.367383e-01 9.159986e-01 6.140661e-01

4547 9.963632e-01 9.897414e-01 9.711865e-01 9.367346e-01 9.159985e-01 6.143482e-01

4548 9.963633e-01 9.897401e-01 9.711825e-01 9.367307e-01 9.159984e-01 6.146339e-01

4549 9.963634e-01 9.897387e-01 9.711782e-01 9.367265e-01 9.159983e-01 6.149232e-01

4550 9.963635e-01 9.897371e-01 9.711737e-01 9.367220e-01 9.159982e-01 6.152162e-01

4551 9.963637e-01 9.897355e-01 9.711688e-01 9.367172e-01 9.159981e-01 6.155127e-01

4552 9.963638e-01 9.897338e-01 9.711636e-01 9.367121e-01 9.159979e-01 6.158130e-01

4553 9.963639e-01 9.897319e-01 9.711581e-01 9.367067e-01 9.159978e-01 6.161169e-01

4554 9.963641e-01 9.897298e-01 9.711521e-01 9.367009e-01 9.159976e-01 6.164245e-01

4555 9.963642e-01 9.897277e-01 9.711458e-01 9.366947e-01 9.159975e-01 6.167358e-01

4556 9.963643e-01 9.897253e-01 9.711390e-01 9.366881e-01 9.159973e-01 6.170508e-01

4557 9.963644e-01 9.897228e-01 9.711317e-01 9.366811e-01 9.159971e-01 6.173695e-01

4558 9.963645e-01 9.897201e-01 9.711239e-01 9.366736e-01 9.159968e-01 6.176920e-01

4559 9.963647e-01 9.897173e-01 9.711156e-01 9.366655e-01 9.159966e-01 6.180182e-01

4560 9.963648e-01 9.897142e-01 9.711067e-01 9.366570e-01 9.159963e-01 6.183482e-01

4561 9.963649e-01 9.897108e-01 9.710972e-01 9.366478e-01 9.159959e-01 6.186819e-01

4562 9.963650e-01 9.897073e-01 9.710871e-01 9.366381e-01 9.159955e-01 6.190193e-01

4563 9.963650e-01 9.897034e-01 9.710763e-01 9.366276e-01 9.159951e-01 6.193605e-01

4564 9.963651e-01 9.896994e-01 9.710647e-01 9.366165e-01 9.159946e-01 6.197054e-01

4565 9.963652e-01 9.896950e-01 9.710523e-01 9.366046e-01 9.159940e-01 6.200541e-01

4566 9.963652e-01 9.896903e-01 9.710391e-01 9.365919e-01 9.159934e-01 6.204064e-01

4567 9.963653e-01 9.896852e-01 9.710250e-01 9.365783e-01 9.159927e-01 6.207624e-01

4568 9.963653e-01 9.896798e-01 9.710099e-01 9.365638e-01 9.159918e-01 6.211222e-01

4569 9.963653e-01 9.896740e-01 9.709938e-01 9.365483e-01 9.159909e-01 6.214855e-01

4570 9.963653e-01 9.896678e-01 9.709766e-01 9.365317e-01 9.159898e-01 6.218525e-01

4571 9.963652e-01 9.896612e-01 9.709582e-01 9.365139e-01 9.159885e-01 6.222231e-01

4572 9.963652e-01 9.896541e-01 9.709386e-01 9.364950e-01 9.159871e-01 6.225973e-01

4573 9.963651e-01 9.896465e-01 9.709177e-01 9.364747e-01 9.159855e-01 6.229750e-01

4574 9.963650e-01 9.896383e-01 9.708953e-01 9.364530e-01 9.159837e-01 6.233562e-01

4575 9.963648e-01 9.896296e-01 9.708715e-01 9.364298e-01 9.159817e-01 6.237408e-01

4576 9.963647e-01 9.896203e-01 9.708461e-01 9.364050e-01 9.159793e-01 6.241289e-01

4577 9.963645e-01 9.896104e-01 9.708189e-01 9.363785e-01 9.159767e-01 6.245203e-01

4578 9.963642e-01 9.895997e-01 9.707899e-01 9.363501e-01 9.159738e-01 6.249150e-01

4579 9.963639e-01 9.895884e-01 9.707590e-01 9.363197e-01 9.159705e-01 6.253129e-01

4580 9.963636e-01 9.895762e-01 9.707261e-01 9.362872e-01 9.159668e-01 6.257140e-01

4581 9.963632e-01 9.895632e-01 9.706909e-01 9.362524e-01 9.159626e-01 6.261183e-01

4582 9.963627e-01 9.895493e-01 9.706534e-01 9.362152e-01 9.159579e-01 6.265256e-01

4583 9.963622e-01 9.895345e-01 9.706134e-01 9.361754e-01 9.159527e-01 6.269359e-01

4584 9.963616e-01 9.895187e-01 9.705707e-01 9.361328e-01 9.159468e-01 6.273490e-01

4585 9.963610e-01 9.895018e-01 9.705252e-01 9.360872e-01 9.159403e-01 6.277650e-01

4586 9.963603e-01 9.894837e-01 9.704767e-01 9.360384e-01 9.159330e-01 6.281837e-01

4587 9.963595e-01 9.894645e-01 9.704250e-01 9.359862e-01 9.159249e-01 6.286051e-01

4588 9.963586e-01 9.894439e-01 9.703699e-01 9.359303e-01 9.159159e-01 6.290290e-01

4589 9.963576e-01 9.894220e-01 9.703111e-01 9.358705e-01 9.159059e-01 6.294553e-01

4590 9.963565e-01 9.893986e-01 9.702485e-01 9.358065e-01 9.158948e-01 6.298841e-01

4591 9.963553e-01 9.893737e-01 9.701818e-01 9.357381e-01 9.158825e-01 6.303150e-01

4592 9.963540e-01 9.893471e-01 9.701107e-01 9.356648e-01 9.158690e-01 6.307481e-01

4593 9.963526e-01 9.893188e-01 9.700349e-01 9.355865e-01 9.158540e-01 6.311832e-01

4594 9.963510e-01 9.892886e-01 9.699542e-01 9.355027e-01 9.158375e-01 6.316203e-01

4595 9.963493e-01 9.892564e-01 9.698683e-01 9.354131e-01 9.158193e-01 6.320591e-01

4596 9.963474e-01 9.892222e-01 9.697767e-01 9.353174e-01 9.157993e-01 6.324996e-01

4597 9.963453e-01 9.891857e-01 9.696792e-01 9.352150e-01 9.157773e-01 6.329417e-01

4598 9.963431e-01 9.891468e-01 9.695754e-01 9.351056e-01 9.157532e-01 6.333852e-01

4599 9.963407e-01 9.891054e-01 9.694649e-01 9.349887e-01 9.157268e-01 6.338299e-01

4600 9.963380e-01 9.890614e-01 9.693473e-01 9.348639e-01 9.156979e-01 6.342759e-01

4601 9.963352e-01 9.890145e-01 9.692220e-01 9.347306e-01 9.156663e-01 6.347229e-01

4602 9.963321e-01 9.889646e-01 9.690888e-01 9.345883e-01 9.156318e-01 6.351707e-01

4603 9.963287e-01 9.889115e-01 9.689470e-01 9.344365e-01 9.155942e-01 6.356193e-01

4604 9.963251e-01 9.888551e-01 9.687962e-01 9.342746e-01 9.155533e-01 6.360686e-01

4605 9.963212e-01 9.887951e-01 9.686359e-01 9.341021e-01 9.155089e-01 6.365183e-01

4606 9.963169e-01 9.887313e-01 9.684653e-01 9.339183e-01 9.154606e-01 6.369683e-01

4607 9.963123e-01 9.886635e-01 9.682840e-01 9.337225e-01 9.154083e-01 6.374185e-01

4608 9.963074e-01 9.885914e-01 9.680913e-01 9.335142e-01 9.153518e-01 6.378687e-01

4609 9.963021e-01 9.885148e-01 9.678865e-01 9.332926e-01 9.152906e-01 6.383188e-01

4610 9.962963e-01 9.884335e-01 9.676690e-01 9.330571e-01 9.152246e-01 6.387687e-01

4611 9.962902e-01 9.883471e-01 9.674380e-01 9.328069e-01 9.151535e-01 6.392181e-01

4612 9.962835e-01 9.882554e-01 9.671928e-01 9.325414e-01 9.150771e-01 6.396670e-01

4613 9.962764e-01 9.881581e-01 9.669325e-01 9.322598e-01 9.149950e-01 6.401151e-01

4614 9.962687e-01 9.880548e-01 9.666563e-01 9.319614e-01 9.149069e-01 6.405624e-01

4615 9.962605e-01 9.879452e-01 9.663634e-01 9.316455e-01 9.148127e-01 6.410087e-01

4616 9.962517e-01 9.878289e-01 9.660529e-01 9.313114e-01 9.147119e-01 6.414539e-01

4617 9.962422e-01 9.877056e-01 9.657238e-01 9.309583e-01 9.146045e-01 6.418977e-01

4618 9.962321e-01 9.875749e-01 9.653753e-01 9.305855e-01 9.144901e-01 6.423401e-01

4619 9.962212e-01 9.874363e-01 9.650063e-01 9.301924e-01 9.143685e-01 6.427809e-01

4620 9.962096e-01 9.872895e-01 9.646158e-01 9.297784e-01 9.142396e-01 6.432199e-01

4621 9.961971e-01 9.871340e-01 9.642027e-01 9.293428e-01 9.141030e-01 6.436571e-01

4622 9.961838e-01 9.869693e-01 9.637662e-01 9.288851e-01 9.139587e-01 6.440923e-01

4623 9.961695e-01 9.867948e-01 9.633049e-01 9.284048e-01 9.138064e-01 6.445253e-01

4624 9.961543e-01 9.866102e-01 9.628180e-01 9.279013e-01 9.136462e-01 6.449560e-01

4625 9.961380e-01 9.864149e-01 9.623042e-01 9.273745e-01 9.134780e-01 6.453843e-01

4626 9.961206e-01 9.862083e-01 9.617626e-01 9.268238e-01 9.133016e-01 6.458100e-01

4627 9.961021e-01 9.859897e-01 9.611919e-01 9.262491e-01 9.131172e-01 6.462331e-01

4628 9.960823e-01 9.857587e-01 9.605911e-01 9.256504e-01 9.129248e-01 6.466534e-01

4629 9.960612e-01 9.855146e-01 9.599591e-01 9.250275e-01 9.127244e-01 6.470707e-01

4630 9.960387e-01 9.852567e-01 9.592949e-01 9.243805e-01 9.125163e-01 6.474850e-01

4631 9.960147e-01 9.849844e-01 9.585975e-01 9.237097e-01 9.123006e-01 6.478961e-01

4632 9.959891e-01 9.846969e-01 9.578659e-01 9.230153e-01 9.120777e-01 6.483040e-01

4633 9.959619e-01 9.843936e-01 9.570990e-01 9.222978e-01 9.118477e-01 6.487085e-01

4634 9.959329e-01 9.840736e-01 9.562962e-01 9.215578e-01 9.116111e-01 6.491095e-01

4635 9.959021e-01 9.837363e-01 9.554565e-01 9.207960e-01 9.113682e-01 6.495069e-01

4636 9.958693e-01 9.833808e-01 9.545792e-01 9.200131e-01 9.111195e-01 6.499007e-01

4637 9.958344e-01 9.830063e-01 9.536638e-01 9.192103e-01 9.108656e-01 6.502907e-01

4638 9.957974e-01 9.826120e-01 9.527096e-01 9.183885e-01 9.106069e-01 6.506769e-01

4639 9.957580e-01 9.821971e-01 9.517163e-01 9.175489e-01 9.103441e-01 6.510591e-01

4640 9.957163e-01 9.817606e-01 9.506836e-01 9.166930e-01 9.100777e-01 6.514374e-01

4641 9.956719e-01 9.813018e-01 9.496112e-01 9.158222e-01 9.098083e-01 6.518115e-01

4642 9.956249e-01 9.808197e-01 9.484992e-01 9.149380e-01 9.095367e-01 6.521815e-01

4643 9.955750e-01 9.803134e-01 9.473477e-01 9.140421e-01 9.092636e-01 6.525473e-01

4644 9.955221e-01 9.797820e-01 9.461570e-01 9.131362e-01 9.089895e-01 6.529089e-01

4645 9.954660e-01 9.792247e-01 9.449276e-01 9.122221e-01 9.087152e-01 6.532661e-01

4646 9.954066e-01 9.786404e-01 9.436601e-01 9.113016e-01 9.084415e-01 6.536189e-01

4647 9.953438e-01 9.780284e-01 9.423552e-01 9.103768e-01 9.081689e-01 6.539673e-01

4648 9.952773e-01 9.773878e-01 9.410141e-01 9.094495e-01 9.078981e-01 6.543112e-01

4649 9.952069e-01 9.767175e-01 9.396378e-01 9.085215e-01 9.076299e-01 6.546506e-01

4650 9.951325e-01 9.760169e-01 9.382277e-01 9.075950e-01 9.073647e-01 6.549855e-01

4651 9.950539e-01 9.752851e-01 9.367854e-01 9.066718e-01 9.071033e-01 6.553158e-01

4652 9.949709e-01 9.745212e-01 9.353126e-01 9.057537e-01 9.068462e-01 6.556416e-01

4653 9.948832e-01 9.737245e-01 9.338110e-01 9.048426e-01 9.065938e-01 6.559627e-01

4654 9.947907e-01 9.728943e-01 9.322828e-01 9.039402e-01 9.063467e-01 6.562792e-01

4655 9.949744e-01 9.734343e-01 9.337278e-01 9.067318e-01 9.097221e-01 6.608679e-01

4656 9.949744e-01 9.734343e-01 9.337277e-01 9.067318e-01 9.097220e-01 6.609668e-01

4657 9.949744e-01 9.734342e-01 9.337276e-01 9.067318e-01 9.097220e-01 6.610680e-01

4658 9.949744e-01 9.734342e-01 9.337276e-01 9.067318e-01 9.097219e-01 6.611717e-01

4659 9.949744e-01 9.734342e-01 9.337275e-01 9.067319e-01 9.097219e-01 6.612777e-01

4660 9.949744e-01 9.734341e-01 9.337274e-01 9.067319e-01 9.097218e-01 6.613861e-01

4661 9.949744e-01 9.734341e-01 9.337273e-01 9.067320e-01 9.097218e-01 6.614969e-01

4662 9.949744e-01 9.734340e-01 9.337272e-01 9.067320e-01 9.097218e-01 6.616102e-01

4663 9.949744e-01 9.734340e-01 9.337271e-01 9.067320e-01 9.097218e-01 6.617259e-01

4664 9.949744e-01 9.734339e-01 9.337270e-01 9.067321e-01 9.097218e-01 6.618442e-01

4665 9.949744e-01 9.734338e-01 9.337269e-01 9.067321e-01 9.097218e-01 6.619649e-01

4666 9.949744e-01 9.734338e-01 9.337268e-01 9.067322e-01 9.097219e-01 6.620882e-01

4667 9.949744e-01 9.734337e-01 9.337267e-01 9.067322e-01 9.097219e-01 6.622141e-01

4668 9.949744e-01 9.734336e-01 9.337266e-01 9.067322e-01 9.097220e-01 6.623426e-01

4669 9.949744e-01 9.734336e-01 9.337265e-01 9.067323e-01 9.097221e-01 6.624737e-01

4670 9.949744e-01 9.734335e-01 9.337263e-01 9.067323e-01 9.097222e-01 6.626074e-01

4671 9.949744e-01 9.734334e-01 9.337262e-01 9.067323e-01 9.097223e-01 6.627438e-01

4672 9.949744e-01 9.734333e-01 9.337260e-01 9.067323e-01 9.097224e-01 6.628829e-01

4673 9.949744e-01 9.734332e-01 9.337259e-01 9.067324e-01 9.097225e-01 6.630247e-01

4674 9.949744e-01 9.734331e-01 9.337257e-01 9.067324e-01 9.097227e-01 6.631692e-01

4675 9.949744e-01 9.734330e-01 9.337255e-01 9.067324e-01 9.097228e-01 6.633165e-01

4676 9.949744e-01 9.734329e-01 9.337254e-01 9.067324e-01 9.097230e-01 6.634665e-01

4677 9.949744e-01 9.734328e-01 9.337252e-01 9.067324e-01 9.097232e-01 6.636194e-01

4678 9.949744e-01 9.734326e-01 9.337250e-01 9.067324e-01 9.097234e-01 6.637750e-01

4679 9.949744e-01 9.734325e-01 9.337247e-01 9.067324e-01 9.097236e-01 6.639335e-01

4680 9.949744e-01 9.734324e-01 9.337245e-01 9.067324e-01 9.097238e-01 6.640949e-01

4681 9.949744e-01 9.734322e-01 9.337243e-01 9.067325e-01 9.097240e-01 6.642591e-01

4682 9.949744e-01 9.734320e-01 9.337240e-01 9.067325e-01 9.097242e-01 6.644262e-01

4683 9.949744e-01 9.734319e-01 9.337237e-01 9.067325e-01 9.097244e-01 6.645962e-01

4684 9.949744e-01 9.734317e-01 9.337234e-01 9.067325e-01 9.097247e-01 6.647691e-01

4685 9.949744e-01 9.734315e-01 9.337231e-01 9.067326e-01 9.097249e-01 6.649449e-01

4686 9.949743e-01 9.734312e-01 9.337228e-01 9.067326e-01 9.097252e-01 6.651237e-01

4687 9.949743e-01 9.734310e-01 9.337224e-01 9.067327e-01 9.097255e-01 6.653053e-01

4688 9.949743e-01 9.734307e-01 9.337220e-01 9.067327e-01 9.097258e-01 6.654900e-01

4689 9.949743e-01 9.734305e-01 9.337216e-01 9.067328e-01 9.097261e-01 6.656776e-01

4690 9.949743e-01 9.734302e-01 9.337212e-01 9.067329e-01 9.097264e-01 6.658681e-01

4691 9.949743e-01 9.734299e-01 9.337208e-01 9.067330e-01 9.097267e-01 6.660616e-01

4692 9.949743e-01 9.734295e-01 9.337203e-01 9.067331e-01 9.097271e-01 6.662581e-01

4693 9.949743e-01 9.734292e-01 9.337198e-01 9.067333e-01 9.097275e-01 6.664575e-01

4694 9.949743e-01 9.734288e-01 9.337192e-01 9.067334e-01 9.097279e-01 6.666599e-01

4695 9.949742e-01 9.734284e-01 9.337186e-01 9.067336e-01 9.097283e-01 6.668652e-01

4696 9.949742e-01 9.734279e-01 9.337180e-01 9.067338e-01 9.097288e-01 6.670736e-01

4697 9.949742e-01 9.734274e-01 9.337174e-01 9.067340e-01 9.097293e-01 6.672848e-01

4698 9.949742e-01 9.734269e-01 9.337167e-01 9.067342e-01 9.097298e-01 6.674990e-01

4699 9.949741e-01 9.734264e-01 9.337160e-01 9.067344e-01 9.097303e-01 6.677161e-01

4700 9.949741e-01 9.734258e-01 9.337152e-01 9.067347e-01 9.097310e-01 6.679362e-01

4701 9.949741e-01 9.734251e-01 9.337144e-01 9.067350e-01 9.097316e-01 6.681592e-01

4702 9.949740e-01 9.734245e-01 9.337135e-01 9.067353e-01 9.097323e-01 6.683850e-01

4703 9.949740e-01 9.734237e-01 9.337125e-01 9.067356e-01 9.097330e-01 6.686138e-01

4704 9.949740e-01 9.734230e-01 9.337116e-01 9.067360e-01 9.097338e-01 6.688454e-01

4705 9.949739e-01 9.734221e-01 9.337105e-01 9.067364e-01 9.097347e-01 6.690798e-01

4706 9.949738e-01 9.734212e-01 9.337094e-01 9.067368e-01 9.097356e-01 6.693171e-01

4707 9.949738e-01 9.734203e-01 9.337082e-01 9.067373e-01 9.097366e-01 6.695572e-01

4708 9.949737e-01 9.734193e-01 9.337069e-01 9.067378e-01 9.097376e-01 6.698001e-01

4709 9.949737e-01 9.734182e-01 9.337056e-01 9.067383e-01 9.097387e-01 6.700457e-01

4710 9.949736e-01 9.734170e-01 9.337042e-01 9.067389e-01 9.097399e-01 6.702941e-01

4711 9.949735e-01 9.734157e-01 9.337027e-01 9.067395e-01 9.097412e-01 6.705451e-01

4712 9.949734e-01 9.734144e-01 9.337011e-01 9.067402e-01 9.097425e-01 6.707989e-01

4713 9.949733e-01 9.734129e-01 9.336993e-01 9.067409e-01 9.097440e-01 6.710553e-01

4714 9.949732e-01 9.734114e-01 9.336975e-01 9.067417e-01 9.097455e-01 6.713143e-01

4715 9.949731e-01 9.734098e-01 9.336956e-01 9.067425e-01 9.097472e-01 6.715759e-01

4716 9.949730e-01 9.734080e-01 9.336935e-01 9.067434e-01 9.097490e-01 6.718400e-01

4717 9.949728e-01 9.734061e-01 9.336914e-01 9.067444e-01 9.097508e-01 6.721066e-01

4718 9.949727e-01 9.734041e-01 9.336891e-01 9.067454e-01 9.097529e-01 6.723757e-01

4719 9.949725e-01 9.734019e-01 9.336866e-01 9.067465e-01 9.097550e-01 6.726473e-01

4720 9.949723e-01 9.733996e-01 9.336840e-01 9.067478e-01 9.097573e-01 6.729212e-01

4721 9.949721e-01 9.733971e-01 9.336812e-01 9.067491e-01 9.097597e-01 6.731975e-01

4722 9.949719e-01 9.733944e-01 9.336782e-01 9.067505e-01 9.097623e-01 6.734761e-01

4723 9.949717e-01 9.733916e-01 9.336750e-01 9.067520e-01 9.097651e-01 6.737570e-01

4724 9.949714e-01 9.733885e-01 9.336717e-01 9.067536e-01 9.097681e-01 6.740401e-01

4725 9.949712e-01 9.733853e-01 9.336681e-01 9.067553e-01 9.097712e-01 6.743254e-01

4726 9.949709e-01 9.733818e-01 9.336643e-01 9.067572e-01 9.097746e-01 6.746128e-01

4727 9.949705e-01 9.733780e-01 9.336603e-01 9.067592e-01 9.097782e-01 6.749023e-01

4728 9.949702e-01 9.733741e-01 9.336559e-01 9.067613e-01 9.097820e-01 6.751938e-01

4729 9.949698e-01 9.733698e-01 9.336514e-01 9.067636e-01 9.097861e-01 6.754873e-01

4730 9.949694e-01 9.733652e-01 9.336465e-01 9.067661e-01 9.097905e-01 6.757827e-01

4731 9.949689e-01 9.733603e-01 9.336413e-01 9.067687e-01 9.097951e-01 6.760801e-01

4732 9.949685e-01 9.733551e-01 9.336357e-01 9.067716e-01 9.098001e-01 6.763792e-01

4733 9.949679e-01 9.733495e-01 9.336298e-01 9.067746e-01 9.098054e-01 6.766801e-01

4734 9.949673e-01 9.733435e-01 9.336235e-01 9.067778e-01 9.098110e-01 6.769828e-01

4735 9.949667e-01 9.733371e-01 9.336168e-01 9.067813e-01 9.098170e-01 6.772871e-01

4736 9.949661e-01 9.733303e-01 9.336096e-01 9.067850e-01 9.098234e-01 6.775930e-01

4737 9.949653e-01 9.733230e-01 9.336020e-01 9.067889e-01 9.098302e-01 6.779004e-01

4738 9.949645e-01 9.733152e-01 9.335938e-01 9.067931e-01 9.098375e-01 6.782093e-01

4739 9.949637e-01 9.733068e-01 9.335852e-01 9.067976e-01 9.098453e-01 6.785197e-01

4740 9.949628e-01 9.732979e-01 9.335759e-01 9.068024e-01 9.098535e-01 6.788314e-01

4741 9.949618e-01 9.732884e-01 9.335660e-01 9.068075e-01 9.098623e-01 6.791445e-01

4742 9.949607e-01 9.732782e-01 9.335555e-01 9.068129e-01 9.098717e-01 6.794588e-01

4743 9.949595e-01 9.732673e-01 9.335442e-01 9.068187e-01 9.098818e-01 6.797742e-01

4744 9.949583e-01 9.732557e-01 9.335322e-01 9.068248e-01 9.098924e-01 6.800908e-01

4745 9.949569e-01 9.732433e-01 9.335194e-01 9.068314e-01 9.099038e-01 6.804085e-01

4746 9.949555e-01 9.732301e-01 9.335057e-01 9.068383e-01 9.099159e-01 6.807271e-01

4747 9.949539e-01 9.732160e-01 9.334910e-01 9.068458e-01 9.099288e-01 6.810466e-01

4748 9.949522e-01 9.732009e-01 9.334754e-01 9.068536e-01 9.099426e-01 6.813671e-01

4749 9.949504e-01 9.731848e-01 9.334587e-01 9.068620e-01 9.099573e-01 6.816883e-01

4750 9.949484e-01 9.731676e-01 9.334408e-01 9.068709e-01 9.099729e-01 6.820102e-01

4751 9.949463e-01 9.731493e-01 9.334217e-01 9.068803e-01 9.099895e-01 6.823328e-01

4752 9.949440e-01 9.731297e-01 9.334013e-01 9.068903e-01 9.100073e-01 6.826559e-01

4753 9.949415e-01 9.731088e-01 9.333795e-01 9.069010e-01 9.100262e-01 6.829796e-01

4754 9.949389e-01 9.730865e-01 9.333562e-01 9.069122e-01 9.100463e-01 6.833037e-01

4755 9.949360e-01 9.730627e-01 9.333313e-01 9.069242e-01 9.100677e-01 6.836282e-01

4756 9.949330e-01 9.730374e-01 9.333046e-01 9.069368e-01 9.100905e-01 6.839530e-01

4757 9.949297e-01 9.730103e-01 9.332761e-01 9.069502e-01 9.101148e-01 6.842780e-01

4758 9.949261e-01 9.729815e-01 9.332456e-01 9.069644e-01 9.101406e-01 6.846032e-01

4759 9.949223e-01 9.729507e-01 9.332129e-01 9.069794e-01 9.101681e-01 6.849284e-01

4760 9.949183e-01 9.729179e-01 9.331779e-01 9.069953e-01 9.101974e-01 6.852537e-01

4761 9.949139e-01 9.728830e-01 9.331405e-01 9.070121e-01 9.102286e-01 6.855788e-01

4762 9.949092e-01 9.728457e-01 9.331005e-01 9.070299e-01 9.102617e-01 6.859039e-01

4763 9.949041e-01 9.728060e-01 9.330577e-01 9.070486e-01 9.102970e-01 6.862287e-01

4764 9.948987e-01 9.727636e-01 9.330118e-01 9.070685e-01 9.103345e-01 6.865532e-01

4765 9.948929e-01 9.727185e-01 9.329627e-01 9.070894e-01 9.103745e-01 6.868773e-01

4766 9.948867e-01 9.726704e-01 9.329102e-01 9.071115e-01 9.104169e-01 6.872010e-01

4767 9.948801e-01 9.726191e-01 9.328540e-01 9.071348e-01 9.104620e-01 6.875241e-01

4768 9.948729e-01 9.725645e-01 9.327938e-01 9.071594e-01 9.105100e-01 6.878467e-01

4769 9.948653e-01 9.725063e-01 9.327294e-01 9.071853e-01 9.105610e-01 6.881685e-01

4770 9.948571e-01 9.724443e-01 9.326604e-01 9.072126e-01 9.106152e-01 6.884896e-01

4771 9.948484e-01 9.723783e-01 9.325867e-01 9.072414e-01 9.106728e-01 6.888098e-01

4772 9.948390e-01 9.723079e-01 9.325077e-01 9.072716e-01 9.107340e-01 6.891291e-01

4773 9.948290e-01 9.722330e-01 9.324233e-01 9.073035e-01 9.107989e-01 6.894474e-01

4774 9.948183e-01 9.721532e-01 9.323330e-01 9.073370e-01 9.108678e-01 6.897647e-01
[truncated: 6,411,605 more chars]
